# Analytic Thinking and Political Orientation in the Corona Crisis

**DOI:** 10.3389/fpsyg.2021.631800

**Published:** 2021-07-22

**Authors:** Marina Maglić, Tomislav Pavlović, Renata Franc

**Affiliations:** Institute of Social Sciences Ivo Pilar (IPI), Zagreb, Croatia

**Keywords:** COVID-19, open-minded thinking, cognitive reflection, political orientation, preventive behavior, policy support, conspiracy beliefs, cross-national

## Abstract

With much unknown about the new coronavirus, the scientific consensus is that human hosts are crucial to its spread and reproduction—the more people behave like regular socializing beings they are, the more likely it is that the virus will propagate. Hence, many nations worldwide have mandated physical-distancing measures. In the current preregistered research, we focus on examining two factors that may help explain differences in adherence to COVID-19 preventive behaviors and policy support across different countries—political orientation and analytic thinking. We positioned our research within the dual-process framework of human reasoning and investigated the role of cognitive reflection, open-minded thinking, and political ideology in determining COVID-19 responsible behavior (physical distancing and maintaining hygiene) and support for restrictive COVID-19 policies on a sample of 12,490 participants from 17 countries. We have not been able to detect substantial relationships of political orientation with preventive behaviors and policy support, and overall found no reliable evidence of politicization, nor polarization regarding the issue. The results of structural equation modeling showed that the inclination towards COVID-19 preventive measures and their endorsement were defined primarily by the tendency of open-minded thinking. Specifically, open-minded thinking was shown to be a predictor of all three criteria—avoiding physical contact, maintaining physical hygiene, and supporting COVID-19 restrictive mitigation policies. Cognitive reflection was predictive of lesser adherence to stricter hygiene and only very weakly predictive of lesser policy support. Furthermore, there was no evidence of these effects varying across political contexts. The mediation analysis suggested a partial mediation effect of COVID-19 conspiracy beliefs on the relationships of open-mindedness and cognitive reflection with physical distancing (but not adherence to stricter hygiene) and COVID-19 policy support, albeit very small and significant primarily due to sample size. There was also no evidence of these effects varying across political contexts. Finally, we have not been able to find strong evidence of political orientation modifying the relationship between analytical thinking and COVID-19 behaviors and policy support, although we explored the pattern of these effects in the US and Canadian samples for exploratory purposes and comparison with other similar studies.

## Introduction

### Public Response to the COVID-19 Pandemic—Preventive Behaviors and Policy Support

The COVID-19 pandemic is a global health crisis affecting all major aspects of human life—political, social, economic, and psychological. Given the lack of any clinically approved antiviral drugs or vaccines at the time when our survey was conducted, the only way of mitigating and controlling the spread of the novel coronavirus was to break the chain of infection. Thus, responsible preventive behaviors guided by reliable information were paramount in combating COVID-19. However, public health response is not uniform, and preventive measures, such as physical distancing, self-isolating, and maintaining good hygiene, can hardly be implemented by coercion alone. Citizens need to understand what is required of them and realize the importance of complying.

Various preventive behaviors against COVID-19 have been identified and advised, such as those summarized by the World Health Organization (2021). Many have been promoted by relevant public health officials and bodies in most countries and incorporated in their COVID-19 policies. Generally, preventive behaviors can be broadly categorized into two types: spatial distancing and stricter hygiene. Although many studies (e.g., Alper et al., 2020; Harper et al., 2020; Plohl and Musil, 2020; Qian and Yahara, 2020; Raude et al., 2020) used composite measures of multiple types of preventive behaviors, several studies demonstrated that spatial distancing and stricter hygiene represent distinct types of preventive behaviors with different correlates (Wismans et al., 2020; van Mulukom et al., 2021).

In addition to these universally advocated preventive behaviors, almost all countries have implemented some type of COVID-19 restrictive policy, ranging from advising work from home to governments enacting full lockdowns. Investigating public support for imposing different restrictions that limit some of the fundamental civil rights for the collective good should provide valuable information. A better understanding of public response is vital for modeling the course of a pandemic and appropriate public health communication. Indeed, epidemiological research has acknowledged the weakness of many traditional mathematical models of infectious diseases in that they generally do not allow for behavioral heterogeneity, which inevitably limits their accuracy and predictive validity (Weston et al., 2018).

Like any behavior, such behavior change is presumed to be influenced by numerous individual, interpersonal, societal, and ecological factors. Research conducted both before (Bish and Michie, 2010; Lunn et al., 2020) and during (e.g., Clark et al., 2020; Earnshaw et al., 2020; Harper et al., 2020; Sabat et al., 2020) this pandemic has explored various sociodemographic, psychological, and contextual determinants of engaging in preventive behavior and support for official public health policies. Although the current crisis sparked the proliferation of social and behavioral science research, much is still unknown about how people respond to the COVID-19 pandemic, including the causes and motives of engaging in health-protective behaviors.

The COVID-19 pandemic is a public health and policy issue, but also a scientific issue, and there is a wide range of factors at play in reasoning about it. Communicating complex medical and scientific concepts to the public is difficult enough without conflicting or unclear messages from government officials and public health advisors, with an abundance of misinformation in the media adding to this complexity. Thus, reasoning and judgment are done in the highly uncertain context of a global pandemic and infodemic, bearing significant psychological loads on individuals.

With regard to the aforementioned, we sought to contribute to social and behavioral science efforts by taking the cognitive science lens to investigate psychological determinants of COVID-19 preventive behavior and policy support, focusing on the role of reasoning and political ideology. Specifically, we positioned our research within the dual-process framework of human reasoning, examining the postulates of classical reasoning account and identity-protective cognition account.

### Theoretical Framework—Analytic Thinking Within the Dual-Process Framework of Human Reasoning

The fundamental idea within the influential dual-process framework is that there are two qualitatively different types of processing—autonomous, intuitive (Type 1) processing and typically deliberative and computationally demanding (Type 2) processing achieved by some form of deliberative control (Evans and Stanovich, 2013; Pennycook et al., 2015c). With heavy loading on working memory resources, Type 2 processing is computationally expensive. Consequently, humans often act as “cognitive misers,” typically seeking to avoid resource-demanding processes and defaulting to processing mechanisms of low computational expense (e.g., Kahneman, 2011; Stanovich, 2015). In fact, processing outcomes of both types are often consistent, and, in such cases, heuristic mechanisms of low computational cost are very efficient (Kahneman, 2011; Stanovich et al., 2016). However, they can also result in very different and conflicting outcomes. Because Type 1 processing has not evolved for the fine-grained, deep analysis required by many situations of the modern world, in such cases, a propensity for analytical, computationally demanding thinking may be crucial (Stanovich, 2012). Indeed, in the dual-process literature, conflict detection, and an override of incorrect autonomous responses are deemed as fundamental functions of analytic processing (Evans and Frankish, 2009; Pennycook et al., 2015c; Stanovich et al., 2016).

#### Classical Reasoning Perspective

From the “classical reasoning” or “reflectionist” perspective (see Pennycook, 2018) deliberative, analytic thinking is viewed to support rational thinking, reasoning, and decision making by overriding incorrect intuitive responses (Pennycook et al., 2015c; Stanovich et al., 2016; for a review of conflict detection in reasoning, see De Neys, 2014). Moreover, a crucial finding within the dual-process framework is that, to think rationally, one has to have the adequate *computational capacity* (i.e., cognitive ability, intelligence) to respond to the processing requirements and the *willingness to engage deliberative reasoning processes* (i.e., thinking dispositions that foster thorough and prudent, unbiased thought, and knowledge acquisition) (Stanovich and West, 2000; Stanovich, 2011; Pennycook et al., 2015c).

Within the dual-process framework, numerous measures have been used as indicators of analytic or rational thinking (see for example, Stanovich et al., 2016). Some of the more commonly used are different versions of the cognitive reflection test and open-minded thinking scale (for a review, see Stanovich et al., 2016).

The *cognitive reflection test* (CRT) was originally designed to measure the ability or disposition to override a predominant intuitive but incorrect response and to engage in further reflection, leading to the correct response (Frederick, 2005). As such, it is assumed to represent a prime measure of overcoming miserly processing, proposed by authors, most prominently by Stanovich et al. (2016). It is one of the most widely used measures of the propensity to engage in analytic thinking and has proved to be a potent predictor of performance on various kinds of reasoning (e.g., Lesage et al., 2013; Sirota et al., 2014; Pennycook et al., 2017) and decision-making tasks (e.g., Frederick, 2005; Cokely and Kelley, 2009; Oechssler et al., 2009; Koehler and James, 2010; Hoppe and Kusterer, 2011), with its predictive effect proven stronger than intelligence and executive functioning measures on a wide range of these tasks (Toplak et al., 2011, 2014; see also Trippas et al., 2015). Moreover, cognitive reflection has been associated with a broad range of beliefs and behaviors in everyday life, such as paranormal disbelief, utilitarian moral judgment, science understanding, and smartphone use, to name a few (see Pennycook et al., 2015b for a review). In fact, its predictive potency may derive from the fact that it happens to tap both aspects of Type 2 processing—the ability and disposition to engage in analytic thinking (Toplak et al., 2011; Campitelli and Gerrans, 2014; Pennycook and Ross, 2016; but see also Szaszi et al., 2017; Erceg et al., 2020a for a further discussion).

The dispositional tendency of actively open-minded thinking is one of the thinking dispositions deemed specifically relevant to rational thinking (Baron, 1985, 2019; Stanovich et al., 2016). Unlike the CRT, a primarily maximal performance measure, *open-minded thinking* is a self-reported measure of the tendency of recognizing the limitations of one's own knowledge (especially in relation to others) and openness to new information and knowledge as opposed to arrogance about one's own knowledge and intellectual abilities (Alfano et al., 2017). Thus, it is a (typical performance) indicator of the willingness to initiate an override and engage deliberative reasoning processes. It should be mentioned that there are different versions of the scales intended to measure the tendency of open-minded thinking (comprising different dimensions of the construct), which vary between six, seven (e.g., Haran et al., 2013; Alfano et al., 2017), up to 41 items (Stanovich and West, 2007).

Research shows that the two indicators of analytic thinking, CRT, and open-mindedness, are positively correlated, typically in the range 0.2–0.3 (Haran et al., 2013; Toplak et al., 2014; Szaszi et al., 2017; Svedholm-Häkkinen and Lindeman, 2018; Bronstein et al., 2019; McPhetres et al., 2021). Similar to CRT, open-mindedness is associated with lower susceptibility to biases in reasoning and decision-making tasks (e.g., Sá et al., 1999; West et al., 2008; Toplak et al., 2011; Heijltjes et al., 2014; Svedholm-Häkkinen and Lindeman, 2018).

Moreover, a growing body of evidence has linked these two as well as other indicators of analytic reasoning with various beneficial psychological and behavioral outcomes. For example, analytic, cognitively sophisticated individuals exhibit more discerning social media use (Mosleh et al., 2021); they are less prone to various unfounded, epistemically suspect beliefs (Pennycook et al., 2015b), the so-called pseudo-profound bullshit (Pennycook et al., 2015a), and fake news (Bronstein et al., 2019; Pennycook and Rand, 2019), as well as religious beliefs (Pennycook et al., 2014, 2020a). There is also some evidence suggesting that activation of analytic thinking can lead to a higher endorsement of (some domains of) secular belief (Hudiyana et al., 2019). In addition, recent research has indicated that individuals more prone to analytic thinking are also more likely to form or adhere to scientifically founded beliefs (Pennycook et al., 2020a; McPhetres et al., 2021).

Individual differences in analytic thinking are also reflected in the health domain, specifically health-related attitudes and behaviors. Analytically sophisticated individuals (i.e., those characterized by higher cognitive reflection and open-mindedness) are generally less inclined to complementary and alternative forms of medical treatment and to believe in their effectiveness (Browne et al., 2015; Svedholm-Häkkinen and Lindeman, 2018; McPhetres et al., 2021).

Initial findings on the relationship between different indicators of analytic thinking and responsible behaviors in the context of COVID-19 are somewhat mixed.

Regarding the role of cognitive reflection, while some researchers found a negative predictive effect of cognitive reflection on preventive behavior (Thoma et al., 2021), others found a negative effect of cognitive intuition on responsible behavior (Teovanović et al., 2021), yet others did not detect an effect (although it negatively predicted conspiracy beliefs; Alper et al., 2020) or showed that the effect of cognitive reflection is fully mediated by unfounded beliefs (Erceg et al., 2020b; Stanley et al., 2020).

As for open-minded thinking, Thoma et al. (2021) found it did not predict reported preventive behavior, but the results of Erceg et al. (2020b) indicate that the effect of actively open-minded thinking on responsible behavior is mediated by unfounded beliefs.

Here, we present the results in more detail.

Erceg et al. (2020b) used several indicators of analytic thinking, i.e., the CRT and three thinking dispositions—actively open-minded thinking, faith in intuition, and science curiosity. Zero-order correlations showed that cognitive reflection, actively open-minded thinking, and science curiosity were associated with less unfounded COVID-19 beliefs and higher knowledge. Conversely, faith in intuition was related to more unfounded beliefs and worse COVID-19 knowledge. Furthermore, out of the aforementioned variables, only actively open-minded thinking and science curiosity were associated with responsible behavior (avoiding physical contact, washing hands, avoiding going out, and coughing and sneezing in the elbow). Within an SEM model, among those variables, only science curiosity emerged as a direct predictor of COVID-19 responsible behavior once other measures were controlled for. Additionally, the authors found that faith in intuition positively and cognitive reflection and actively open-minded thinking negatively predicted COVID-19 unfounded beliefs, while the effect of science curiosity was non-significant. Moreover, unfounded beliefs predicted less responsible behavior and mediated the effects of cognitive reflection, actively open-minded thinking, and faith in intuition on responsible behavior.

Alper et al. (2020) found that higher faith in intuition, generic conspiracy beliefs, and a lower level of cognitive reflection predicted COVID-19 conspiracy beliefs. On the other hand, they did not detect the predictive effects of any of these variables on adherence to preventive measures.

Thoma et al. (2021) found that tendency toward cognitive failures (a self-report measure of the tendency of lapses of attention, memory, and cognition in everyday life) and actively open-minded thinking did not predict reported preventive behavior. Lower understanding of the infection and transmission mechanism of COVID-19, a higher risk-taking tendency and higher cognitive reflection predicted adopting fewer preventive behaviors while more concern predicted adopting more of the preventive behaviors.

Teovanović et al. (2021) found that, in addition to COVID-19 conspiracy beliefs predicting engaging in pseudoscientific practices, lower adherence to COVID-19 guidelines, and unwillingness to get vaccinated, cognitive intuition (calculated as a mean of intuitive responses on the CRT) predicted only lesser adherence to COVID-19 guidelines. Furthermore, overestimation of COVID-19-related knowledge predicted lesser adherence to COVID-19 guidelines but also lesser engagement in pseudoscientific practices, while cognitive biases predicted greater use of pseudoscientific practices but also greater adherence to COVID-19 guidelines and willingness to get vaccinated.

Stanley et al. (2020) found significant indirect effects of CRT performance on distancing and hand-washing behaviors, with cognitively reflective individuals being more likely to believe the pandemic was a hoax and consequently less likely to engage in distancing and hand-washing behaviors. In addition, CRT did not exhibit any direct effects on physical distancing and handwashing.

Swami and Barron (2020) also tested and confirmed a mediation model in which conspiracy beliefs mediated the relationship between analytic thinking (indexed by scores on the analytic thinking subscale of the Rational/Experiential Multimodal Inventory) and compliance with mandated distancing measures. Specifically, they found that greater analytic thinking was directly associated with physical distancing behavior that was mandated in the UK in early April 2020, as well as indirectly *via* lower COVID-19 conspiracy beliefs.

When considering these results, one has to keep in mind that the studies were conducted in March and April 2020 in different countries during the first wave of the COVID-19 pandemic. Thus, some of the differences in the findings between studies may be due to differences in the specific policies and restrictions in place at the time of data collection. To sum up, despite somewhat mixed results, a few of the studies provide evidence that the relationship between analytical thinking and COVID-19 preventive behavior or intentions could be explained by COVID-19 conspiracy beliefs (Stanley et al., 2020; Swami and Barron, 2020) and similar unfounded beliefs (Erceg et al., 2020b). Such links between COVID-19 conspiracy beliefs and cognitive processing are in line with previous findings on the importance of cognitive factors in explaining conspiracy beliefs in general, with different indicators of cognitive proficiency being generally associated with reduced conspiratorial ideation (Oliver and Wood, 2014a,b; Swami et al., 2014, 2017; Stanovich et al., 2016; Van Prooijen, 2017; Georgiou et al., 2019). We further outline the role of conspiracy beliefs within the dual-process framework in the following section.

##### Conspiracy Beliefs as Contaminated Mindware

In addition to adequate computational power and willingness to engage deliberative reasoning processes, Stanovich et al. (2016) stress that procedural and declarative knowledge is required for successful Type 2 override. Stanovich (e.g., Stanovich, 2011; Stanovich et al., 2016) adopted the term “mindware” to refer to these knowledge structures, strategies, rules, and belief bases. However, various thinking problems can arise related to mindware—even if the first two prerequisites are satisfied, lack, or inaccessibility of appropriate mindware, or having one that is contaminated can inhibit reasoning processes and hinder rational thought (Stanovich et al., 2016).

The tendency toward conspiracy beliefs generally reflects the inclination of an individual to attribute the causes of various events or phenomena to conspiracies secretly plotted by individuals or groups of powerful people with predominantly sinister intentions (Douglas and Sutton, 2008; Bruder et al., 2013). Although some conspiracies may turn out to be true, they generally lack evidential support and resist falsification (Sutton and Douglas, 2014). Quintessentially, conspiracy beliefs bear the “unnecessary assumption of conspiracy when other explanations are more probable” (Aaronovitch, 2009, p. 5) and represent an important domain of *contaminated mindware* (Stanovich et al., 2016; Rizeq et al., 2021).

It has been well-documented that endorsement of specific conspiracy theories is associated with greater beliefs in other conspiracy theories (Goertzel, 1994; Swami et al., 2010; Lewandowsky et al., 2013; Majima, 2015), even when conspiracy theories themselves are contradictory (Wood et al., 2012). This speaks to the notion of a general disposition toward conspiracist ideation, i.e., a conspiracy mentality (Imhoff and Bruder, 2014). As an explanation of the pervasiveness of conspiratorial thinking and the allure of various conspiracy theories, Oliver and Wood (2014a) postulated two psychological predispositions underlying conspiratorial ideation – attributing intentionality to unseen others and the tendency for melodramatic narratives when faced with important events that require explanation. These resonate well with the proposed “fundamental computational biases” of Stanovich in human cognition (Stanovich, 2003), specifically with the human proclivity to infer intentionality and to rely on a narrative mode of thought.

Not surprisingly, the rapid spread of the COVID-19 and the global crisis produced laid fertile ground for the mass proliferation of various COVID-19-related conspiracies. In the state of global emergency, adverse outcomes beyond an individual, such as vaccination resistance that can lead to devastating collective consequences, are particularly worrying. Indeed, previous research suggested that conspiracy beliefs are related to unwarranted health behavior, such as vaccination refusal, medical treatment non-adherence, and alternative medicine use (Bogart et al., 2010; Grebe and Nattrass, 2012; Jolley and Douglas, 2014; Oliver and Wood, 2014b). Furthermore, initial evidence in the context of the COVID-19 pandemic suggests that belief in conspiracy theories undermines engagement in preventive behaviors and support for public health policies (Erceg et al., 2020b; Imhoff and Lamberty, 2020; Plohl and Musil, 2020; Stanley et al., 2020; Swami and Barron, 2020; Pavela Banai et al., 2021). However, Alper et al. (2020) did not find any evidence of the association between COVID-19 conspiracy beliefs and preventive measures. In fact, a recent systematic review (van Mulukom et al., 2021) has revealed that the type of preventive behavior measure matters. In the case of general measures of preventive behavior (measures that combine hygiene, distancing, and/or mask-wearing), COVID conspiracy beliefs were negatively associated with self-reported adherence to behavioral guidelines in most studies and across different countries. However, in the case of separate measures of hygiene and distancing, studies from the USA and Europe mainly (although not all) indicate a negative association of conspiracy beliefs (general or COVID-19) with distancing but not with hygiene guidelines (van Mulukom et al., 2021). Longitudinal studies also point to similar findings. Bierwiaczonek et al. (2020) found that overall conspiracy beliefs generally decreased, and distancing behavior increased over time, with individuals endorsing more conspiracy beliefs at the beginning of the crisis, exhibiting the lowest increase of distancing behavior. Pummerer et al. (2021) detected the adverse effect of conspiracy beliefs on distancing, but not hygiene behaviors. Inevitably, this issue warrants further investigation.

Theoretically and empirically, computational power, willingness to engage deliberative reasoning processes, and mindware are unavoidably intertwined (Stanovich et al., 2016). With regard to the indicators of analytic thinking, the presence of contaminated conspiratorial mindware has been shown to correlate negatively (weak to moderate correlations) with cognitive reflection (Stanovich et al., 2016; Van Prooijen, 2017; Pennycook et al., 2020a) and open-mindedness (Swami et al., 2014; Stanovich et al., 2016; Pennycook et al., 2020a). In the context of the coronavirus pandemic, initial findings confirm that analytically sophisticated individuals are less prone to believe various misinformation and pseudoscientific practices regarding coronavirus prevention and treatment, including fake news and conspiracy theories about its nature and origin (Alper et al., 2020; Čavojová et al., 2020; Erceg et al., 2020b; Pennycook et al., 2020b, 2021; Stanley et al., 2020; Teovanović et al., 2021). Furthermore, indicators of analytic thinking have shown to be significant and relatively strong negative predictors of various misperceptions and unfounded beliefs and knowledge about COVID-19 (Čavojová et al., 2020; Erceg et al., 2020b; Stanley et al., 2020; Swami and Barron, 2020; Pennycook et al., 2021). Overall, described patterns of associations between analytic thinking, conspiracy beliefs, and COVID-19 protective behavior further suggest the possibility of endorsement of conspiracy beliefs mediating the negative relation between analytic thinking and responsible behavior. However, such a hypothesis has been investigated and confirmed only in a few studies and warrants further research (Erceg et al., 2020b; Stanley et al., 2020; Swami and Barron, 2020).

### Motivated Reasoning—A Case for Politicization of the Crisis

In addition to being cognitive misers, humans are also “motivated reasoners” in the sense that they perceive and process information directed by certain motives or goals (Kunda, 1990; Taber and Lodge, 2006; Leeper and Slothuus, 2014). More often than not, our reasoning is directed by some goals other than accuracy (Kunda, 1990; Taber and Lodge, 2006). Namely, we are often motivated to maintain and support our existing conceptions and beliefs using any of the many processes by which we explain new inconsistent information we encounter, which is in contrast to the classical notions of rational updating (Kunda, 1990; Taber and Lodge, 2006).

Some of the most common sources of directional motivated reasoning are political ideology and partisanship, and issue-related prior opinions (Taber and Lodge, 2006; Bolsen et al., 2014; Leeper and Slothuus, 2014). Indeed, in Western societies (primarily in the US and Europe), there is an ideological polarization of the public on a number of political as well as scientific issues, such as climate change, gun policy, nuclear power, and immigration (Pew Research Center, 2018a,b; Simmons et al., 2018), and it persists despite scientific consensus on many of these contentious issues (Kahan et al., 2011; Lewandowsky et al., 2012). Moreover, research has shown that ideology and partisanship influence information processing and reasoning and judgment of information on some contested issues, e.g., embryonic stem cell research, affirmative action, gun control, capital punishment, climate change (Lord et al., 1979; Nisbet, 2005; Taber and Lodge, 2006; Ho et al., 2008; Hart and Nisbet, 2012; Bolsen and Druckman, 2018).

Since the beginning of the current crisis, political ideology and partisanship have been some of the most salient apparent sources of disagreement on COVID-19 issues. Probably, the most prominent examples of conservative and right-leaning leaders downplaying the severity of the outbreak, attacking experts, and resisting physical distancing are Donald Trump and Jair Bolsonaro. There is some initial evidence of the politicization of the crisis—for example, a Pew poll from March 2020 found that 59% of Democrats vs. 33% of Republicans perceived COVID-19 to be a major threat to the health of the U.S. population (see also Pew Research Center, 2020a,b; Saad, 2020). Being a global crisis and requiring action from political leaders around the world, opinions, policies, and actions regarding COVID-19 may have, indeed, become linked to political identities, thus acting as an important identity marker or symbol, differentiating right-leaning individuals from the left-leaning ones, at least in some countries. This would be in line with the findings suggesting that individuals are more persuaded by policy experts perceived to hold congenial values and cultural outlooks to their own (Kahan et al., 2010a, 2011).

On the other hand, society generally accepts scientific findings, and, in the absence of cultural or social divisions, citizens generally do form beliefs in accordance with the best available evidence (Kahan et al., 2017). Thus, for example, the public is not polarized about the usefulness of antibiotics in treating bacterial infections, the health risks associated with obesity, etc. The same may be true of attitudes and behaviors regarding the COVID-19 coronavirus pandemic, at least in some countries. Namely, in health crises, people are more likely to trust medical experts than politicians (Albertson and Gadarian, 2015). Also, it is possible that a situation in which individuals feel that they are jointly faced with the same risk may trigger a sense of shared destiny (Van Bavel et al., 2020). The consequent common identity in a global catastrophe situation could, in some way, put ideological differences in the background (Gaertner and Dovidio, 2012; Vezzali et al., 2015; Schellhaas and Dovidio, 2016).

The findings so far regarding the role of political identity in the context of COVID-19 preventive behavior and policy support are mixed. It seems they differ by country (political context) and the stage of the pandemic, whether political identity is operationalized by party affiliation, last voting preference, or political orientation, as well as concrete COVID-19 psychological reaction or behavior.

For example, Harper et al. (2020) conducted a study in the UK at the end of March 2020 and did not find a self-reported measure of political orientation to correlate with behavior change in response to the pandemic (i.e., engaging with WHO-recommended behaviors) or with fear of the novel coronavirus, despite a generally polarized nature of the UK political landscape. Moreover, political orientation did not predict engagement with WHO-recommended behaviors after controlling for fear of the virus (Harper et al., 2020).

However, studies conducted primarily in the US and Canada (regardless of the operationalization of political identity) point to the politicization and public polarization. For example, Pennycook et al. (2021) found that conservativism (a mean of social and economic dimensions), at the end of March 2020, was associated with COVID-19 misperceptions in the US, Canada, and the UK, and the association was greater in the US than in the UK. This pattern was evident for perceptions of COVID-19 risk and behavior change intentions as well. Kerr et al. (2021) investigated the extent of the polarization among the US public across two national studies. The first study conducted in March showed that liberals (compared with conservatives) perceived higher risk, exhibited less trust in politicians to effectively handle the pandemic and more trust of medical experts, such as the WHO, and reported engaging in more health-protective actions. Results of the following study in April 2020 replicated these results when considering partisanship, rather than political ideology.

Overall, increasing evidence suggests that, in the US, Republicans and conservatives tend to express less concern or perceive a lower risk of coronavirus, and are less prone to increased responsible behavior (hygiene and physical distancing) than Democrats and liberals (Allcott et al., 2020; Calvillo et al., 2020; Pickup et al., 2020; Rothgerber et al., 2020; Conway et al., 2021; Kushner Gadarian et al., 2021; Pennycook et al., 2021). In addition, Republicans and conservatives are less accurate at discerning between real and fake news and less likely to share the news with accurate coronavirus content than Democrats or liberals (Calvillo et al., 2020; Pennycook et al., 2020b). In line with this, US studies examining objective indicators, such as GPS location data, Google searches, debit card transactions, provide additional support by showing a higher reduction in mobility in counties and states with lower Republican vote shares (Allcott et al., 2020; Andersen, 2020; Barrios and Hochberg, 2020; Engle et al., 2020; Gollwitzer et al., 2020; Painter and Qiu, 2021).

#### Identity—Protective Cognition Account of Motivated Reasoning

Following the outlined theoretical review, the question of the role of analytic thinking in motivated reasoning arises. One account that has gained significant traction is the *identity-protective cognition* account (also called “Motivated System 2 Reasoning”), and it postulates that engaging analytic thinking exacerbates motivated reasoning (Kahan, 2013, 2017b). Namely, individuals engage in deliberation and use their cognitive capacities to secure, protect, and defend their (often political) identities and their preexisting beliefs (Kahan et al., 2007; Drummond and Fischhoff, 2017). This can, in turn, lead individuals to become further entrenched in what they already believe, and, consequently, to polarization over contested issues that convey special meaning for opposing groups (i.e., have particular significance for their interests, status, or commitments) to which they belong or have an affinity to (Kahan, 2017a). By this account, individuals equipped with the most proficient Type 2 reasoning capacities end up most polarized. Thus, the identity-protective cognition account is in direct contrast to the classical reasoning account, which presumes that deliberation facilitates accurate belief formation and not ideological or partisan bias.

There is evidence that political polarization about contentious scientific issues and other facts that admit of empirical inquiry is actually greater among individuals who are more reflective (Kahan, 2013), numerical (Kahan et al., 2017), actively open-minded (Kahan and Corbin, 2016; Baron, 2017), and scientifically literate (Hamilton et al., 2012; Kahan et al., 2012; Bolsen et al., 2015; Drummond and Fischhoff, 2017; Motta, 2018; Sarathchandra et al., 2018). On the other hand, some recent findings have supported the classical reasoning account over the identity-protective account by showing that more analytical individuals (indexed by CRT) are less susceptible to false news, whether or not they are consistent with their political ideology (Pennycook and Rand, 2019; Bago et al., 2020).

In the context of the current crisis, a few studies investigated the effect of analytic thinking together with the effect of political ideology on COVID-19 preventive behaviors (Alper et al., 2020; Erceg et al., 2020b; Stanley et al., 2020; Pennycook et al., 2021; Thoma et al., 2021). Most did not particularly focus on the role of political ideology. Alper et al. (2020), Erceg et al. (2020b), Thoma et al. (2021), and Stanley et al. (2020), measured indicators of analytic thinking (actively opened-minded thinking, faith in intuition, science curiosity, cognitive failures tendency, and CRT) along with a left-right and liberal-conservative political orientation, but only briefly reported on the results (and the latter only in their Supplementary Materials). Thoma et al. (2021) found that political leaning was practically uncorrelated with all of their predictor (except actively open-minded thinking, *r* = 0.26) or outcome (preventive behaviors) measures and did not investigate it any further. Stanley et al. (2020) showed that political and economic conservativism was significantly positively correlated with COVID-19 hoax belief and negatively with distancing behavior but unrelated to handwashing and a number of helping behaviors (also, CRT performance was negatively associated with both political measures). Erceg et al. (2020b) treated political orientation as a control variable and found that right/conservative leaning was predictive of COVID-19 unfounded beliefs, but not of COVID-19 responsible behavior, while Alper et al. (2020) found it was not predictive in either case.

Crucially, only Pennycook et al. (2021) aimed to examine the interactions of these variables in a two-wave study conducted in March and December 2020 in the US, Canada, and the UK. Firstly, they found that polarization was greater in the US than in Canada and the UK, with political conservatism in the US strongly related to weaker mitigation behaviors (regarding hygiene and physical distancing), lower COVID-19 risk perceptions, and greater misperceptions (and stronger vaccination hesitancy, measured only in the second wave). Overall, cognitive sophistication [composite of the CRT performance, numeracy, bullshit receptivity (reverse-scored), and basic science knowledge] was consistently negatively correlated with misperceptions across time, countries, and political lines (whether political ideology as a combined social and fiscal conservatism or partisan identification). On the other hand, cognitive sophistication was not a strong or consistent predictor of COVID-19 risk perceptions or behavior change intentions.

But, moreover, they focused on the interaction of political ideology and cognitive sophistication, which they tested in both waves. In the first wave, they found no evidence for an interaction between ideology and cognitive sophistication in predicting COVID-19 misperceptions, COVID-19 risk perceptions, and behavior change intentions. Thus, contrary to the identity-protective cognition account, the result showed that cognitive sophistication was a better predictor of misperceptions than political ideology in all three countries, with the absence of any interaction. On the other hand, in the US and Canada, only political ideology, i.e., conservativism, significantly predicted weaker engagement in mitigation behaviors.

In the second wave, in the US sample, interestingly, they detected significant interactions of political partisanship and cognitive sophistication for COVID-19 misperceptions, risk perceptions, and behavior change intentions (but not for vaccination intentions). When they zoomed in on the correlation between cognitive sophistication and these measures separately for strong Democrats and strong Republicans, they found that, although cognitive sophistication was associated with decreased misperceptions for both groups, this association was notably weaker for Republicans compared with Democrats. What is more, risk perceptions and behavior intentions were positively correlated with cognitive sophistication among strong Democrats but nominally negatively (albeit not significantly) correlated with cognitive sophistication among strong Republicans, indicating that polarization seems to widen between partisans with the rise of their reasoning skills. Although this is in line with the identity-protective cognition account, when the authors controlled for liberal and conservative media trust (and their interactions with cognitive sophistication), partisan identification no longer interacted with cognitive sophistication in predicting misperceptions, vaccination intentions, or mitigation behaviors, although it did remain significant for risk perceptions.

Given these initial findings, the question of the role of analytic thinking in motivated reasoning remains open to debate.

### The Present Study

We aim to explore the psychological determinants of COVID-19 (self-reported) responsible behavior and policy support, focusing on cognitive and sociopolitical factors, thus seeking to contribute to the emerging discussion with insights within the dual-process framework of human reasoning, and testing the postulates of the identity-protective cognition account and the classical reasoning account.

Specifically, in this study, we examine^1^:

(RQ1) whether political orientation predicts adherence to COVID-19 preventive behaviors and policy support and whether this relationship varies across countries.

Since previous results regarding the relationship between political identity and COVID-19 preventive behavior differed for different countries, we expected to detect a degree of variability in this relationship across countries. Based on some initial findings, we tested the hypothesis that COVID-19 has become a politically divisive topic in some countries, which is reflected in relation between political ideology and COVID-19 preventive behaviors and policy support. Moreover, since political ideology can have different meanings in different countries, we expected to be able to differentiate between three groups of countries—countries where a positive correlation between right-leaning orientation and COVID-19 policy support would emerge; countries where this relationship would be in the opposite (negative) direction, and countries where these phenomena would be uncorrelated (suggesting the COVID-19 issue is not politicized).

(RQ2) whether analytic thinking predicts adherence to COVID-19 preventive behaviors and policy support and whether this relationship varies across political contexts.

Here, we wanted to first test the main effect of analytical thinking on adherence to preventive measures and policy support, the hypothesis being that analytical thinking (cognitive reflection and open-mindedness) should aid deliberation about COVID-19 and lead to scientifically backed reasoning and adherence to preventive measures. Also, we wanted to explore whether this relationship differs across political contexts. Specifically, we wanted to investigate if the effect of analytical thinking on the three outcome measures would be different in countries where a positive correlation between right-leaning orientation and COVID-19 policy support would have emerged vs. countries where this relationship would have been in the opposite (negative) direction, as well as countries where no such relationship existed (indicating that the pandemic has not been politicized).

(RQ3) whether the effect of analytic thinking on adherence to COVID-19 preventive behaviors and policy support is mediated by conspiracy beliefs and whether this relationship varies across political contexts.

Contaminated mindware, i.e., endorsement of COVID-19 conspiracy theories, is hypothesized to mediate the relationship between analytic thinking and adherence to preventive behaviors and policy support. Additionally, this effect could vary across political contexts, being more pronounced in countries where COVID-19 has been politicized—i.e., a group of countries where right-leaning orientation would be associated with COVID-19 policy support and countries where left-leaning orientation would be associated with COVID-19 policy support vs. countries where no such link existed (indicating that the pandemic has not been politicized).

(RQ4) whether political orientation moderates the relationship between analytic thinking and adherence to COVID-19 preventive behaviors and policy support and whether this relationship varies across political contexts.

Put differently, we aimed to investigate whether analytic thinking leads to scientifically recommended preventive behavior overall (in line with the *classical reasoning approach*), or whether it is primarily used to support motivated reasoning, leading to politically polarized behavior (in line with the *identity-protective cognition account*). Here, we first planned to test the potential interaction between analytic thinking and individual-level political orientation. In addition, we wanted to investigate if the possible interaction effects differ on the contextual level—whether the direction of the interaction would be different in countries where a positive correlation between right-leaning orientation and COVID-19 policy support would have emerged vs. countries where this relation would have been in the opposite (negative) direction.

## Materials and Methods

### Participants and Data Collection

Data used in this study were collected within the scope of the “International Collaboration on Social and Moral Psychology of COVID-19,”^2^ whose initiators launched an open call for international collaborators *via* social media in April 2020. They asked each interested team to collect data from at least 500 participants, representative with respect to age and gender, in their own country. The core team of the project created a survey in English approved by the University of Kent ethics committee.

Data collection was conducted online in 67 countries/regions during April and May 2020, with national teams of each country, including the authors of the present study, translating the questionnaire (a forward–backward method), and administering it to the participants, in most cases with the help of local paneling companies. Such data were gathered to create an overarching database that was used as a source for this study. Overall, the initial sample included 51,717 participants from countries from all the continents (except for Antarctica), with some overrepresented (e.g., from the Americas and Europe) while others underrepresented (e.g., from the Middle East and Africa).

Data cleaning (described in detail in Supplementary Material) resulted with a final sample of 12,490 participants from 17 countries with acceptable variability in all of the relevant variables: Australia, Belgium, Canada, Switzerland, Germany, Greece, Iraq, Israel, Japan, South Korea, Nigeria, New Zealand, Pakistan, Poland, Singapore, Slovakia, and the USA. The sex ratio was balanced (51% women), while the average participant was 45.1 years old (*SD* = 17.1).

### Measures and Instruments

#### Outcome Variables

We employed three outcome variables^3^:

1) *avoiding physical contact*, i.e., physical distancing [e.g., during the days of the coronavirus (COVID-19) pandemic, I have been staying at home as much as practically possible]
2) *maintaining physical hygiene* [e.g., During the days of the coronavirus (COVID-19) pandemic, I have been washing my hands longer than usual]
3) *COVID-19 policy support* [e.g., During the days of the coronavirus (COVID-19) pandemic, I have been in favor of closing all schools and universities].

The first two measures are indicators of adherence to COVID-19 preventive behaviors, while the latter is an indicator of endorsement of COVID-19 preventive measures.

Each construct was operationalized by five items measured on a 0–10 scale with higher values indicating higher levels of the measured construct (with item 2 of the contact subscale being reverse-coded). Due to insufficient variation in multiple countries, item 5 of the contact subscale was excluded from further analyses. The three factors extracted from their respective items exhibited acceptable internal consistency (ω_contact_ = 0.69, ω_hygiene_ = 0.74, ω_support_ = 0.86) and were moderately correlated, implying the existence of a general factor of attitudes and behaviors related to COVID-19 (for further details, see Supplementary Material).

#### Indicators of Analytic Thinking

*Open-mindedness* was operationalized using the open-mindedness subscale from the multidimensional measure of intellectual humility (Alfano et al., 2017). The scale consists of six items, three positively (e.g., If I do not know much about some topic, I don't mind being taught about it, even if I know about other topics.) and three negatively worded (e.g., I think that paying attention to people who disagree with me is a waste of time.). In this study, a unitary latent factor of open-mindedness was extracted with the overall CFA model displaying a very good fit, although the scale exhibited a lower level of reliability (ω = 0.53; for further details, see Supplementary Material). Higher scores indicate higher levels of the measured concept.

Our performance-based measure of the disposition and ability to engage in analytic and reflective thinking was *the cognitive reflection test* (Frederick, 2005)—a slightly adapted three-item version, with the structure of the tasks intact, but the numbers and particular subjects, objects, and predicates slightly changed (e.g., A postcard and a pen cost 150 cents in total. The postcard costs 100 cents more than the pen. How many cents does the pen cost?). Due to the low number of included items (*k* = 3), in this study, we were focused only on correct answers (coded as 1), while incorrect and intuitive answers were coded as 0. The sum of scores on these three items represented the total score on cognitive reflection (i.e., the most commonly used scoring technique; but see Erceg and Bubić, 2017 for different scoring procedures).

#### Hypothesized Moderator and Mediator Variables

A four-item measure of *support for conspiracy theories related to COVID-19* (i.e., COVID-19 conspiracy beliefs) was developed for the purpose of this study. Items presented statements that claimed COVID-19 is a bioweapon or a scam to implement totalitarian regimes, hide the fall of global economy, or allow certain individuals to get economic benefits. The participants rated their agreement with the statements using a 0–10 scale with higher values indicating higher agreement with conspiracy theories. A single factor was extracted from these items, with a very good internal consistency (ω = 0.91; for further details, see Supplementary Material).

*Political orientation* was measured using an 11-point scale, with values lower than the midpoint, indicating political left, and values higher than the midpoint, indicating political right.

*Political context* represents a complex phenomenon that can be operationalized in multiple ways. In our study, however, the political context simply refers to the overall pattern of the relationship between political ideology and COVID-19 policy support, the logic being that the direction of the relationship should speak to a general left or right political outlook toward restrictive mitigation policies across countries. Thus, we operationalized the COVID-19-related political context *via* correlations between support for COVID-19 policy decisions and political orientation. This context would have reflected whether the COVID-19 pandemic was politicized and the pattern of the polarization on the issue. Specifically, we preregistered that countries where the correlation between political orientation and support for policy decisions would be significantly below −0.10, i.e., where the upper bound of 95% confidence intervals of the correlation between political orientation and support for policy decisions would be below −0.10, would represent one group. Countries where this correlation would be significantly above 0.10, i.e., where the lower bound of 95% confidence intervals of the correlation between political orientation and support for restrictive COVID-19 mitigation measures would be above 0.10, would represent another group. The third in-between group would denote the absence of the aforementioned association. Our preregistered operationalization of political context assumed that this grouping achieved at least partial strong invariance. Unfortunately, as the variation of the relationship between political orientation and policy support was not substantial (see Results section), the grouping of countries based on confidence intervals, as preregistered, would have resulted in the US comprising the first group, with all the remaining countries in the second group. Hence, we decided to follow the basic logic of our preregistered grouping and form the groups based simply on correlations, not confidence intervals, which, of course, presents a deviation from our preregistration.

### Analytic Strategy

Our analytic strategy was based on structural equation modeling and accompanying multivariate analyses on a wide cross-cultural data set. We opted for SEM over traditional multivariate techniques because of its advantages, the major ones being: explicit assessment of measurement error, estimation of latent constructs *via* manifest indicators and of the relations among constructs, and providing measures of a global fit of the model to the data. Moreover, the specific reason for using SEM was to be able to first establish invariances (see Vandenberg and Lance, 2000) that allow for the treatment of constructs as identical across different groups and then to examine the relationships between constructs with a verified similar meaning.

We performed all analyses using R version 4.0.3 (R Core Team, 2020), specifically the semTools (Jorgensen et al., 2020) and the lavaan (Rosseel, 2012) packages for structural equation modeling. The full reproducible code with the results is available in Supplementary Material.

Our preregistration can be accessed at https://aspredicted.org/xj83u.pdf. All non-preregistered analyses are noted as such.

## Results

This section summarizes the results of conducted analyses and reflects the order of posed research questions, while the results of confirmatory factor analyses, indicating construct validity of our variables, are presented in Supplementary Material, as well as descriptive data and intercorrelations among latent factor scores of outcome variables, open-mindedness and conspiracy beliefs, and manifest variables: simple sum scores of CRT, political ideology and sex and age. Here, we first present the relationships between outcome variables and political orientation, followed by regression, mediation, and moderation analyses. The complete output of all the analyses is available in Supplementary Material.

Overall, the results presented in Table 1 suggest the absence of a consistent and practically meaningful relationship between political orientation and COVID-19 preventive behaviors and policy support as they generally shared <2% of the variance. For all three outcome measures, the correlations in a majority of the countries did not exceed 0.10, and, where they did, they were below 0.20 in magnitude (only in the US for policy support exactly 0.20), i.e., relatively small (Gignac and Szodorai, 2016). This suggests that, although there appeared to be some variation across countries, COVID-19 preventive behaviors and policy support were largely unpoliticized at the time when the survey was conducted. The strongest association with political ideology was observed in the case of the USA: −0.2 for policy support and −0.13 physical distancing, then −0.14 in both Austria and New Zealand for policy support, and in the case of Singapore: 0.14 for physical hygiene and 0.12 for policy support.

**Table 1:** Correlations of political orientation with COVID-19 preventive behaviors and policy support across the 17 different countries (*N* = 12,490).

	**Political orientation**																
	**AUS**	**BEL**	**CAN**	**CHE**	**DEU**	**GRC**	**IRQ**	**ISR**	**JPN**	**KOR**	**NGA**	**NZL**	**PAK**	**POL**	**SGP**	**SVK**	**USA**
Physical contact	−0.13	−0.07	−0.1	−0.09	−0.04	0.01	−0.08	−0.02	−0.01	−0.08	0.01	−0.08	−0.03	0.06	0.05	0.03	−0.13
Physical hygiene	−0.02	0	−0.04	−0.04	0.02	0	0	−0.03	0.05	−0.06	0.12	−0.04	0.01	0.06	0.14	0.01	0.05
Policy support	−0.14	−0.12	−0.12	−0.09	−0.05	0.08	−0.07	0.09	0.02	−0.04	0.06	−0.14	−0.08	0.11	0.12	0.06	−0.2

*ISO 3166-1 alpha-3 country codes: AUS, Australia; BEL, Belgium; CAN, Canada; CHE, Switzerland; DEU, Germany; GRC, Greece; IRQ, Iraq; ISR, Israel; JPN, Japan; KOR, South Korea; NGA, Nigeria; NZL, New Zealand; PAK, Pakistan; POL, Poland; SGP, Singapore; SVK, Slovakia; USA, United States of America*.

**Figure 1:**
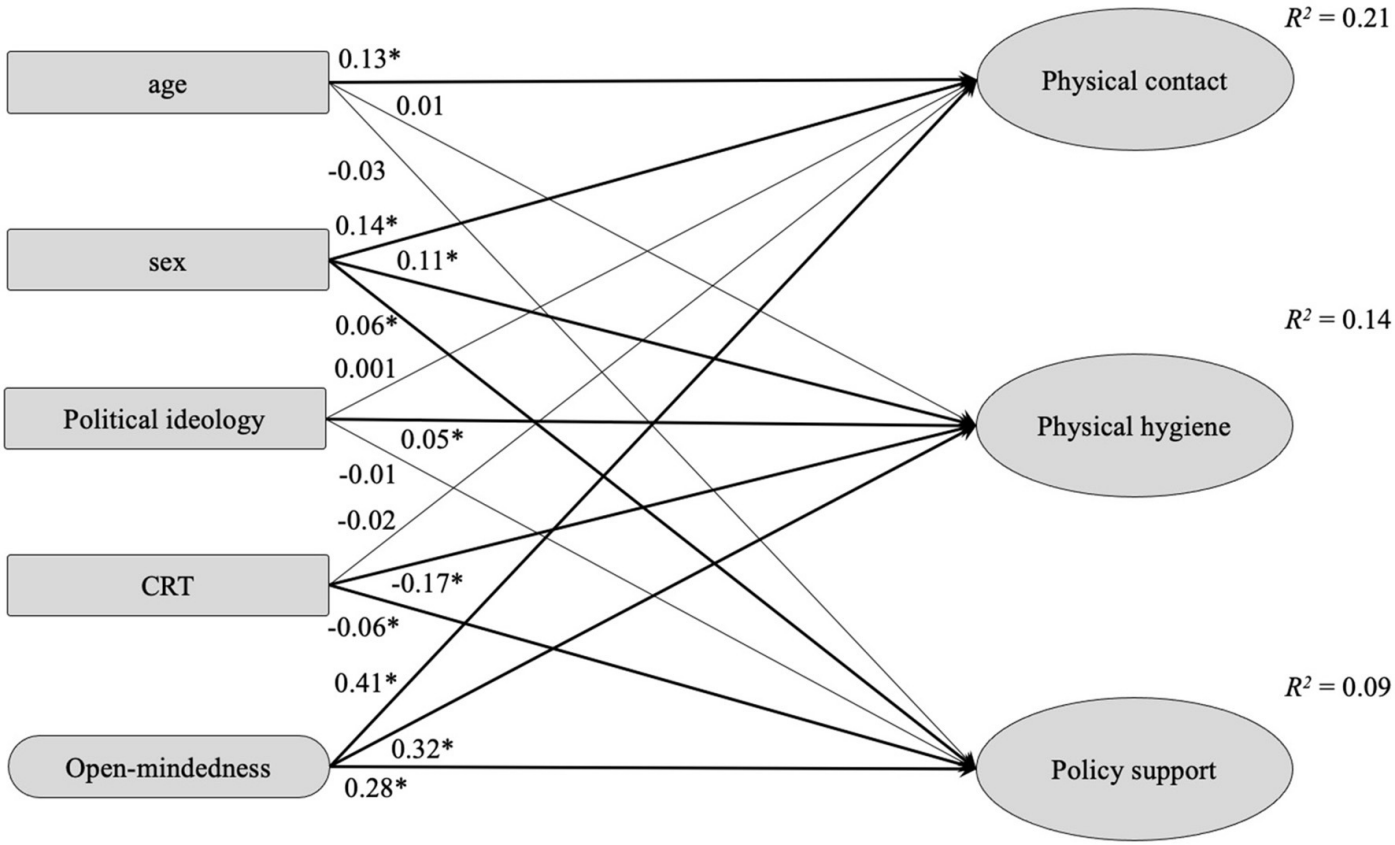
Structural equation modeling of prediction of adherence to COVID-19 preventive behaviors (avoiding physical contact and maintaining physical hygiene) and COVID-19 policy support (*N* = 12,490). Values shown are standardized regression coefficients. Latent variables are denoted as ellipse nodes, and observed variables are denoted as rectangle nodes. Sex coded as males = 1 and females = 2; CRT, cognitive reflection. *p < 0.001.

Secondly, we tested whether analytic thinking predicted COVID-19 responses (Figure 1). The model achieved an adequate data fit (robust CFI = 0.944, robust RMSEA = 0.048, SRMR = 0.044). After controlling for age and sex, open-mindedness emerged as a positive predictor of avoiding physical contact, stricter physical hygiene, and policy support related to the COVID-19 pandemic. Higher CRT scores predicted slightly decreased physical hygiene and policy support. As expected from the zero-order correlations, political ideology did not exhibit a relevant predictive effect.

Furthermore, we tested if these relationships varied across political contexts. Following the principal logic of our preregistered operationalization of political context based simply on correlations between political orientation and policy support but diverging from our preregistration as we did not take into account the confidence intervals, we grouped Australia, Belgium, Canada, New Zealand, and the US together as countries where right-leaning orientation was correlated (*r* ≥ 0.10) with COVID-19 policy support. Poland and Singapore formed a group of countries where left-leaning orientation was correlated (*r* ≤ −0.10) with COVID-19 policy support, with the remaining countries forming a “neutral” group where no such link (−0.10 < *r* < 0.10) was detected, thus indicating that the pandemic has not been politicized. Based on the notion that mediation cannot vary if regression slopes forming it do not vary, we tested the variations in regression slopes of the new models and found no significant differences (Table 2).

**Table 2:** Invariance of analytic thinking in prediction of COVID-19 preventive behaviors and policy support across the three political contexts (*N* = 12,490).

**Level of invariance**	**Robust CFI**	**Robust RMSEA**	**SRMR**
Configural	0.934	0.052	0.05
Metric	0.932	0.052	0.052
Scalar	0.921	0.055	0.054
Regressions	0.919	0.055	0.056

**Figure 2:**
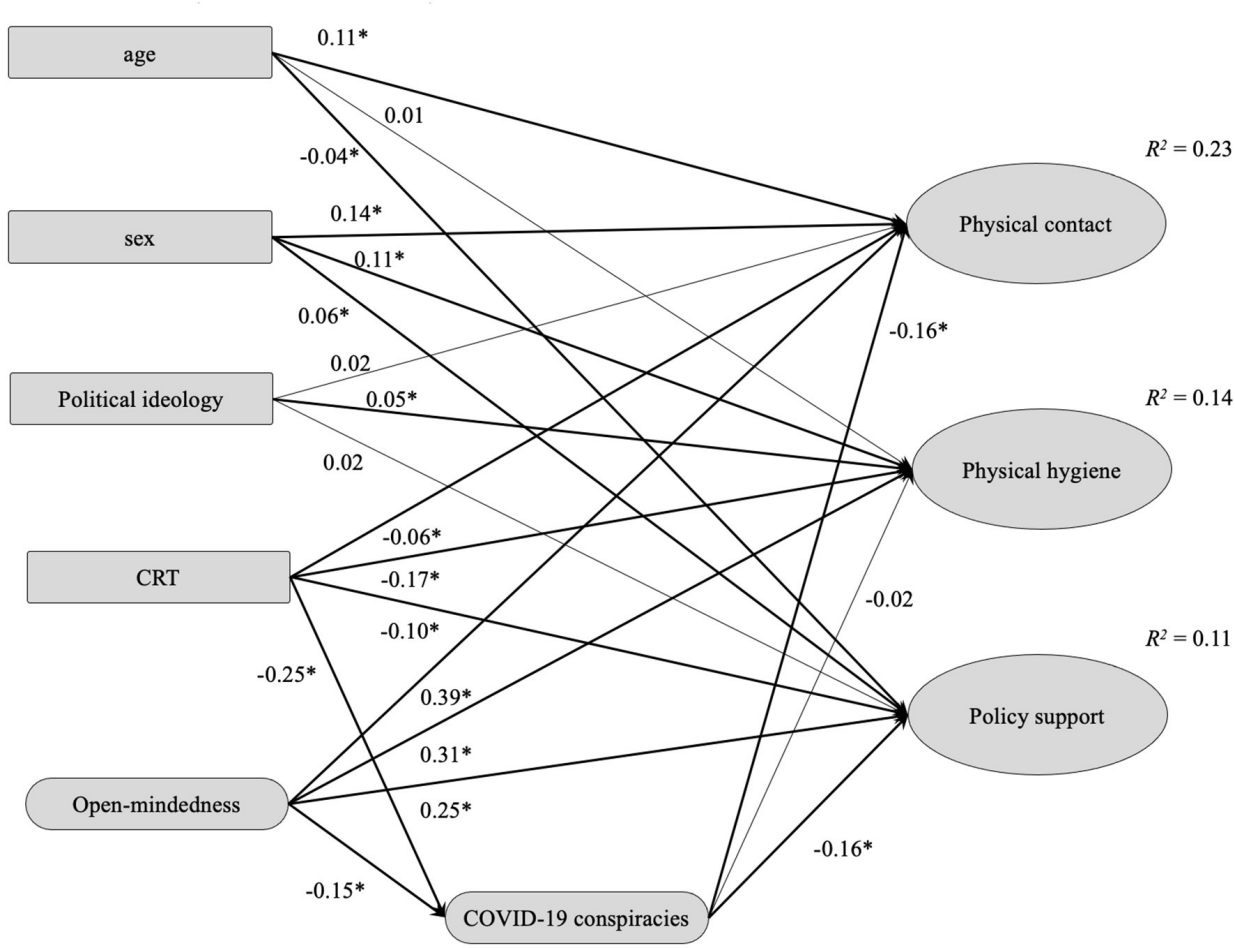
Structural equation modeling of belief in COVID-19 conspiracies as a mediator of the relationship of open-mindedness and CRT with COVID-19 preventive behaviors and policy support (*N* = 12,490). Values shown are standardized regression coefficients. Latent variables are denoted as ellipse nodes, and observed variables are denoted as rectangle nodes. Sex coded as males = 1 and females = 2; CRT, cognitive reflection. *p < 0.001.

In the next step, we tested if COVID-19 conspiracy beliefs mediated the relationship between analytic thinking and COVID-preventive behaviors and policy support (Figure 2). The model achieved an adequate fit (robust CFI = 0.943, robust RMSEA = 0.048, SRMR = 0.060) and demonstrated that support for conspiracy theories was negative, albeit a relatively weak predictor of physical contact and policy support. It mediated only a minor portion of the relationships between open-mindedness (indirect β = 0.03, *p* < 0.001 for physical contact, indirect β = 0.03, *p* < 0.001 for policy support, and indirect β = 0.01, *p* = 0.112 for physical hygiene) and CRT (indirect β = 0.04, *p* < 0.001 for physical contact, indirect β = 0.04, *p* < 0.001 for policy support and indirect β = 0.01, *p* = 0.114 for physical hygiene) with COVID-19 behaviors and attitudes, with the effects being significant mainly due to sample size. Only 9.4% of the variance of COVID-19-related conspiracy beliefs was explained in this model.

In the following step, we tested if the detected mediation varied across political contexts defined by our country grouping (based on correlations between political orientation and policy support as before) by employing invariance testing which (as in the former case) indicated that no significant variation across the context existed (Table 3).

**Table 3:** Invariance of belief in COVID-19 conspiracies as a mediator in the prediction of COVID-19 preventive behaviors and policy support on analytic thinking across the three political contexts (*N* = 12,490).

**Level of invariance**	**Robust CFI**	**Robust RMSEA**	**SRMR**
Configural	0.93	0.054	0.068
Metric	0.928	0.053	0.069
Scalar	0.919	0.056	0.07
Regressions	0.918	0.055	0.073

Finally, we tested if political orientation moderated the relationship between analytic thinking and COVID-19 preventive behaviors and policy support. To compute these analyses, we extracted the factor scores from the model that achieved strong invariance before including conspiracy beliefs (Figure 1), using the Ten Berge method (see Ten Berge, 1977), and multiplied their scaled version with a scaled version of political orientation.

As evident from Table 4, the interactions of political ideology with CRT and open-mindedness in the prediction of our three dependent variables were all practically negligible.

**Table 4:** Multiple regression analyses interacting political orientation with open-mindedness and cognitive reflection in the prediction of our three dependent variables (*N* = 12,490).

	***b* (*SE*)**	**β**	***b* (*SE*)**	**β**
**Physical contact**				
Age	0.004 (<0.001) ^***^	0.07 ^***^	0.004 (<0.001) ^***^	0.07 ^***^
Sex^a^	0.21 (0.02) ^***^	0.1 ^***^	0.21 (0.02) ^***^	0.1 ^***^
Political orientation	−0.002 (0.004)	−0.003	−0.003 (0.004)	−0.01
Open-mindedness	0.43 (0.01) ^***^	0.43 ^***^	0.43 (0.01) ^***^	0.43 ^***^
CRT	−0.04 (0.01) ^***^	−0.04 ^***^	−0.04 (0.01) ^***^	−0.04 ^***^
Open-mindedness × Political orientation	−0.02 (0.01) ^*^	−0.02 ^*^		
CRT × Political orientation			−0.02 (0.01) ^*^	−0.02 ^*^
*R*^2^	0.21		0.21	
**Physical hygiene**				
Age	−0.001 (<0.001)	−0.01	−0.001 (<0.001)	−0.01
Sex^a^	0.19 (0.02) ^***^	0.1 ^***^	0.19 (0.02) ^***^	0.1 ^***^
Political orientation	0.02 (0.004) ^***^	0.04 ^***^	0.02 (0.004) ^***^	0.04 ^***^
Open-mindedness	0.32 (0.01) ^***^	0.32 ^***^	0.32 (0.01) ^***^	0.32 ^***^
CRT	−0.14 (0.01) ^***^	−0.15 ^***^	−0.15 (0.01) ^***^	−0.16 ^***^
Open-mindedness × Political orientation	−0.02 (0.01) ^*^	−0.02 ^*^		
CRT × Political orientation			−0.03 (0.01) ^**^	−0.03 ^**^
*R*^2^	0.14		0.14	
**Policy support**				
Age	−0.003 (<0.001) ^***^	−0.05 ^***^	−0.003 (<0.001) ^***^	−0.05 ^***^
Sex^a^	0.08 (0.02) ^***^	0.04 ^***^	0.08 (0.02) ^***^	0.04 ^***^
Political orientation	−0.004 (0.004)	−0.01	−0.01 (0.004)	−0.01
Open-mindedness	0.28 (0.01) ^***^	0.28 ^***^	0.28 (0.01) ^***^	0.28 ^***^
CRT	−0.07 (0.01) ^***^	−0.08 ^***^	−0.07 (0.01) ^***^	−0.08 ^***^
Open-mindedness × Political orientation	−0.02 (0.01) ^*^	−0.02 ^*^		
CRT × Political orientation			−0.02 (0.01) ^*^	−0.02 ^*^
*R*^2^	0.09		0.09	

*** *p < 0.001*,

** *p < 0.01*,

* *p < 0.05*.

a *Sex coded as males = 1 and females = 2*.

Additionally, results of invariance testing did not indicate significant differences in these relationships across political contexts defined by our country grouping based on correlations between political orientation and policy support (Table 5).

**Table 5:** Invariance of political orientation as a moderator of the relationship between analytic thinking and COVID-19 preventive behaviors and policy support across the three political contexts (*N* = 12,490).

**IVs**	**Constraints**	**Robust CFI**	**Robust RMSEA**	**SRMR**
CRT	Scalar invariance	0.998	0.034	0.005
	Scalar invariance + constrained interactions	0.997	0.027	0.006
Open-mindedness	Scalar invariance	0.998	0.035	0.005
	Scalar invariance + constrained interactions	0.998	0.027	0.006

Although the interactions of political ideology and the two indicators of analytic thinking were negligible and did not vary across political contexts, i.e., the three country groups, for exploratory purposes of comparing our results with the results of Pennycook et al. (2021), we decided to focus on these relationships in the US and Canadian sample.

Hence, we conducted path analyses. Separate models were formed to test the contribution of each interaction (open-mindedness and the three outcome variables and CRT and the three outcome variables) in the US and Canadian samples (Table 6). None of the interactions emerged as significant, following the preset criteria of *p* < 0.001. Yet it is reasonable to assume that interaction effects are smaller, and detecting them requires more power (McClelland and Judd, 1993; see Gelman, 2018). Thus, we report and explore the effects at less stringent significance thresholds.

**Table 6:** Multiple regression analyses interacting political orientation with open-mindedness and cognitive reflection in the prediction of our three dependent variables on separate samples from Canada (*n* = 740) and the USA (*n* = 905).

	**Canada (*****n*** **= 740)**				**US (*****n*** **= 905)**			
	***b* (*SE*)**	**β**	***b* (*SE*)**	**β**	***b* (*SE*)**	**β**	***b* (*SE*)**	**β**
**Physical contact**								
Age	0.01 (0.002)^***^	0.12^***^	0.01 (0.002)^***^	0.11^***^	0.01 (0.002)^**^	0.09^**^	0.01 (0.002)^**^	0.09^**^
Sex^a^	0.19 (0.06)^**^	0.09^**^	0.19 (0.06)^**^	0.09^**^	0.15 (0.05)^**^	0.07^**^	0.14 (0.05)^**^	0.07^**^
Political orientation	−0.05 (0.02)^*^	−0.09^*^	−0.05 (0.02)^*^	−0.09^*^	−0.02 (0.01)	−0.04	−0.03 (0.01)^*^	−0.07^*^
Open-mindedness	0.36 (0.04)^***^	0.36^***^	0.36 (0.04)^***^	0.36^***^	0.52 (0.04)^***^	0.52^***^	0.52 (0.03)^***^	0.52^***^
CRT	−0.04 (0.03)	−0.04	−0.07 (0.04)	−0.08	−0.06 (0.03)	−0.05	−0.07 (0.03)^*^	−0.06^*^
Open-mindedness × political orientation	−0.02 (0.05)	−0.01			−0.02 (0.03)	−0.02		
CRT × political orientation			−0.12 (0.05)^**^	−0.1^**^			−0.06 (0.03)^*^	−0.07^*^
*R*^2^	0.16		0.17		0.31		0.32	
**Physical hygiene**								
Age	−0.001 (0.002)	−0.02	−0.001 (0.002)	−0.02	−0.001 (0.002)	−0.01	−0.001 (0.002)	−0.02
Sex^a^	0.24 (0.06)^***^	0.12^***^	0.24 (0.06)^***^	0.12^***^	0.16 (0.06)^**^	0.08^**^	0.15 (0.06)^**^	0.08^**^
Political orientation	−0.002 (0.02)	−0.003	−0.002 (0.02)	−0.004	0.03 (0.01)^**^	0.09^**^	0.03 (0.02)^*^	0.08^*^
Open-mindedness	0.34 (0.04)^***^	0.34^***^	0.34 (0.04)^***^	0.34^***^	0.39 (0.04)^***^	0.4^***^	0.39 (0.04)^***^	0.39^***^
CRT	−0.11 (0.03)^**^	−0.11^**^	−0.13 (0.04)^**^	−0.13^**^	−0.21 (0.04)^***^	−0.19^***^	−0.21 (0.04)^***^	−0.19^***^
Open-mindedness × political orientation	−0.04 (0.04)	−0.03			−0.01 (0.03)	−0.01		
CRT × political orientation			−0.1 (0.05)^*^	−0.08^*^			−0.002 (0.04)	−0.002
*R*^2^	0.15		0.16		0.2		0.2	
**Policy support**								
Age	0.01 (0.002)^**^	0.09^**^	0.01 (0.002)^**^	0.08^**^	0.003 (0.002)	0.06	0.004 (0.002)^*^	0.06^*^
Sex^a^	0.06 (0.06)	0.03	0.06 (0.06)	0.03	0.07 (0.06)	0.03	0.07 (0.06)	0.03
Political orientation	−0.07 (0.02)^***^	−0.13^***^	−0.08 (0.02)^***^	−0.14^***^	−0.05 (0.01)^***^	−0.13^***^	−0.06 (0.01)^***^	−0.17^***^
Open-mindedness	0.24 (0.04)^***^	0.24^***^	0.25 (0.04)^***^	0.25^***^	0.38 (0.03)^***^	0.38^***^	0.38 (0.03)^***^	0.38^***^
CRT	−0.04 (0.03)	−0.04	−0.06 (0.04)	−0.06	−0.06 (0.04)	−0.05	−0.06 (0.04)	−0.06
Open-mindedness × political orientation	−0.06 (0.05)	−0.05			−0.01 (0.02)	−0.01		
CRT × political orientation			−0.07 (0.04)	−0.06			−0.09 (0.03)^**^	−0.1^**^
*R*^2^	0.09		0.09		0.19		0.19	

*** *p < 0.001*,

** *p ≤ 0.01*,

* *p < 0.05*.

a *Sex coded as males = 1 and females = 2*.

Overall, all the models showed weak, both main and interaction, effects with the exception of the main effect of open-mindedness, which was expected based on our previous overall results. But the goal was to focus on the interactions, and some interesting trends emerged.

In the case of Canada, a weak interaction between CRT and ideology was observed in predicting reduced physical contact and stricter physical hygiene. To clarify these interactions, we plotted them.

**Figure 3:**
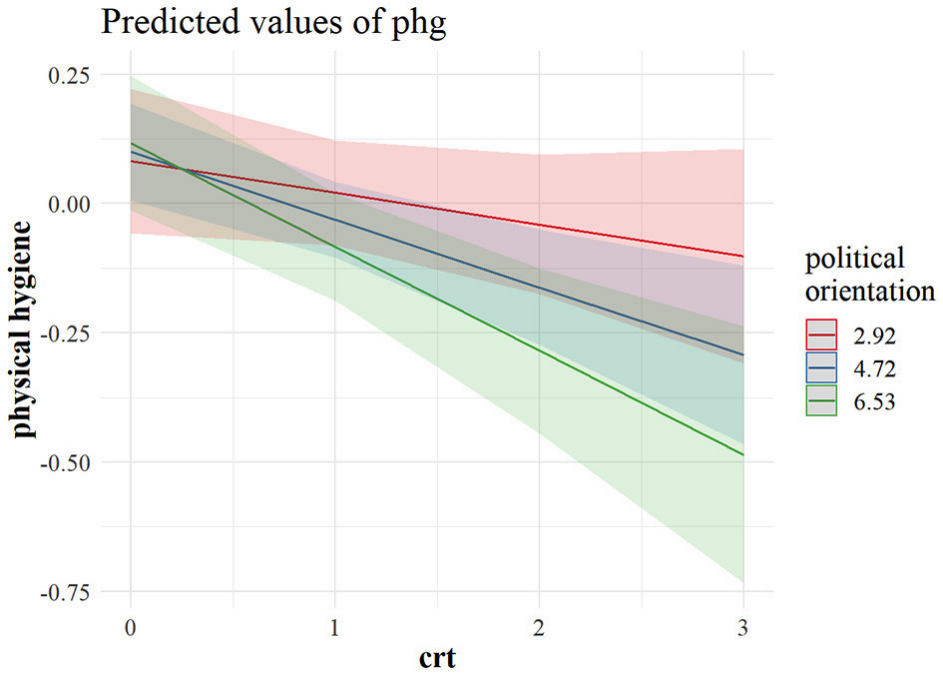
Marginal predicted values for stricter physical hygiene maintenance from a model interacting CRT and political orientation in the Canadian sample (*n* = 740). The predictor values for left (2.29), centrist (4.72), and right (6.53) political orientation are ± 1 *SD*. CRT values (0–3) indicate the number of correct responses. phg, stricter hygiene maintenance; crt, cognitive reflection.

**Figure 4:**
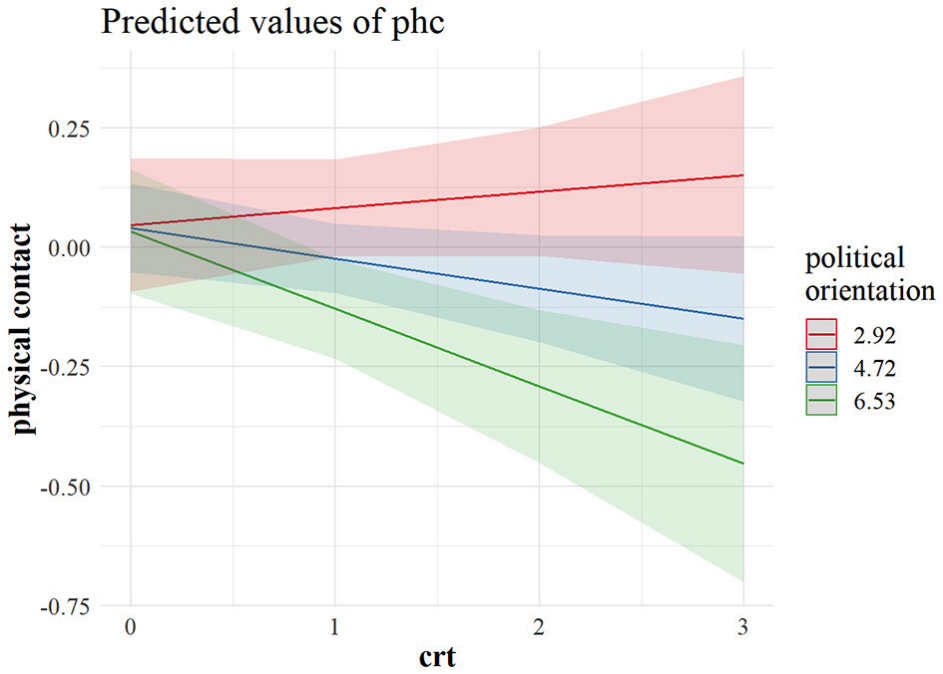
Marginal predicted values for avoiding physical contact from a model interacting CRT and political orientation in the Canadian sample (*n* = 740). The predictor values for left (2.29), centrist (4.72), and right (6.53) political orientation are ± 1 *SD*. CRT values (0–3) indicate the number of correct responses. phc, avoiding physical contact; crt, cognitive reflection.

**Figure 5:**
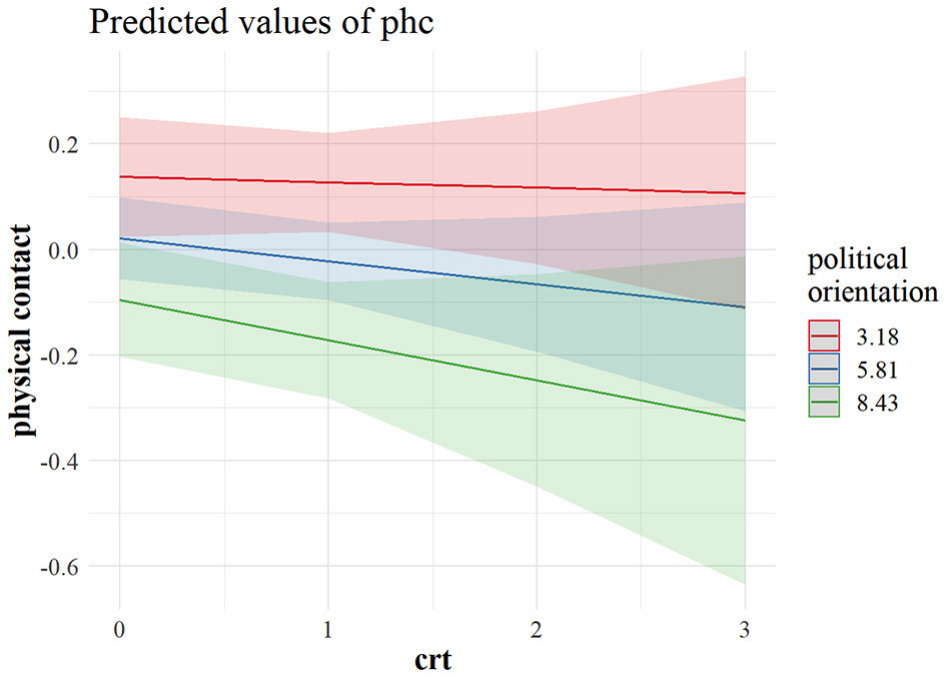
Marginal predicted values for avoiding physical contact from a model interacting CRT and political orientation in the US sample (*n* = 905). The predictor values for left (3.18), centrist (5.81), and right (8.43) political orientation are ± 1 *SD*. CRT values (0–3) indicate the number of correct responses. phc, avoiding physical contact; crt, cognitive reflection.

**Figure 6:**
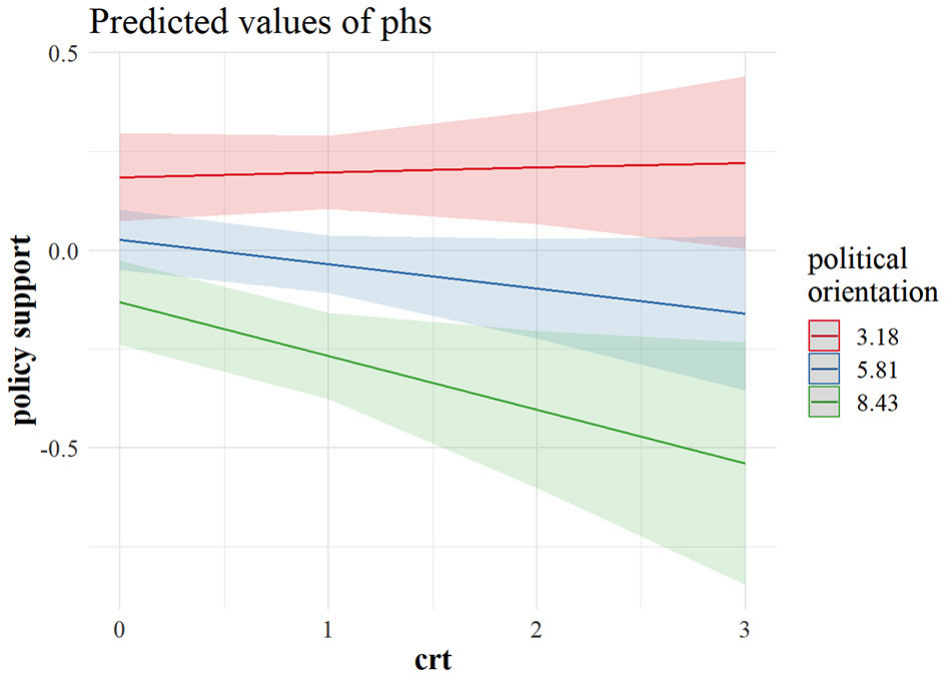
Marginal predicted values for policy support from a model interacting CRT and political orientation in the US sample (*n* = 905). The predictor values for left (3.18), centrist (5.81), and right (8.43) political orientation are ± 1 *SD*. CRT values (0–3) indicate the number of correct responses. phs, support for restrictive COVID-19 policies; crt, cognitive reflection.

Figure 3 shows that a weak negative effect of CRT on hygiene maintenance was driven primarily by right leaning reflective individuals who were more likely not to adhere to stricter hygiene practices. This was even more evident regarding physical distancing. As can be observed in Figure 4, the most reflective individuals were the ones differing the most in their tendency to physical distancing, depending on their political outlook. It seems that, although cognitive reflection does not lead left-leaning individuals to engage in more distancing behavior, with an increase in cognitive reflection, the right-leaning ones are less prone to engage in physical distancing, and the same pattern is visible in the US sample when it comes to predicting physical distancing (Figure 5), as well as policy support (Figure 6).

This broadly supplies some evidence consistent with the identity-protective cognition account but, of course, has to be treated/interpreted with caution since our analysis was exploratory and deviated from our preregistration. In addition, we did not measure any other potentially relevant variables, such as liberal and conservative media trust for which Pennycook et al. (2021) showed that, when controlled for, leads to political identity no longer interacting with cognitive sophistication.

Finally, models with moderation were also tested for variation across the two countries, and no indications in favor of such variation were found (Table 7).

**Table 7:** Invariance of interactions of political orientation with CRT and open-mindedness across the two countries: Canada and the USA.

**IVs**	**Constraints**	**Robust CFI**	**Robust RMSEA**	**SRMR**
CRT	Scalar invariance	0.999	0.029	0.005
	Scalar invariance + constrained interactions	0.998	0.027	0.007
Open-mindedness	Scalar invariance	0.999	0.024	0.005
	Scalar invariance + constrained interactions	1	0	0.006

## Discussion

Dealing with this global health crisis exhorts large-scale behavior change, the so-called new normal, and poses a considerable psychological load on individuals. Therefore, we tried to contribute to a collaborative effort in social and behavioral sciences in providing valuable insights from within the dual-process framework regarding the determinants of COVID-19 preventive behavior and policy support. In particular, we focused on the role of analytic propensity and political ideology, factors that have been shown to affect reasoning and decisions-making regarding many contested issues. We wanted to investigate how it may translate to the issue of preventive behavior and policy support in the context of the COVID-19 pandemic.

Regarding the question of the relationship between political orientation and COVID-19 preventive behaviors and policy support, i.e., whether it varies across countries (RQ1), political orientation, generally, was not substantially related to COVID-19 self-reported behaviors and opinions, with it generally explaining <2% of their variance across different countries. This result was in line with the other studies conducted in March and April 2020 (Alper et al., 2020; Erceg et al., 2020b; Thoma et al., 2021), showing no or relatively weak associations of political ideology and COVID-19 preventive behaviors.

On the other hand, based on some evidence outlined in the introduction, which suggested the possibility of politization and, consequently, polarization of COVID-19 issues, we expected to find some degree of variability in the relationship between political ideology and COVID-19 policy support and, possibly, preventive behaviors across countries. Indeed, in line with previous research, the strongest correlations were observed in the case of the USA for physical distancing and policy support (see also Choma et al., 2021).

If, in fact, a strong link between these phenomena exists, at least in some countries, several reasons may explain our results. Firstly, there is an obvious weakness of the used measure of political orientation—a single item likely connoting different meanings across the 17 different countries. We did not have any other individual-level measure of political ideology, such as party affiliation at our disposal. Namely, in the US, party identification or leaning is commonly used, often yielding stronger polarization effects (e.g., Kahan et al., 2012; McPhetres et al., 2021), this being the case in the pandemic context as well (Pennycook et al., 2021). Also, ideology and partisanship are not the only basis on which the public is divided on many issues regarding decision-relevant science. Other ideological factors, such as “cultural worldviews” proposed by Kahan (e.g., Kahan et al., 2010b, 2012) could also be implicated in motivated reasoning.

Furthermore, there is evidence that, in March 2020, there were partisan differences, both in the US and Canada, regarding COVID-19 concern, government reaction assessments, confidence in government ability to deal with the pandemic, and self-reported behavior change (e.g., Pickup et al., 2020; Pennycook et al., 2021), but the same pattern was not evident in the UK (Pennycook et al., 2021). Moreover, these differences might have widened as the pandemic progressed. Pennycook et al. (2021) found that political polarization seemed to have increased between their first study conducted in March and the second one in December, indicated by a noticeable increase in the correlation between political ideology and risk perceptions (*r*_1_ = −0.36 in Study 1, *r*_2_ = −0.54 in Study 2) and misperceptions (*r*_1_ = 0.31, *r*_2_ = 0.51), as well as mitigation behavior (*r*_1_ = −0.15, *r*_2_ = −0.36) in the US, but this noticeable increase was not apparent or less so (mitigation behavior: *r*_1_ = 0.07, *r*_2_ = −0.02; risk perceptions: *r*_1_ = −0.02, *r*_2_ = −0.18; misperceptions: *r*_1_ = 0.14, *r*_2_ = 0.21) in the UK (unfortunately, they did not include a Canadian sample in their second study). Thus, the point of time in the progression of the pandemic might matter and for a clearer and more nuanced look at this question, the need for longitudinal studies is evident and essential.

Taking this together, there is evidence for the current crisis evoking both ideological differences in motivated reasoning in some countries, or conversely a sense of shared humanity and destiny, putting the common ideological differences aside in others (Gaertner and Dovidio, 2012; Vezzali et al., 2015; Schellhaas and Dovidio, 2016; Van Bavel et al., 2020). Still, in view of the dynamic aspect of the many cognitive processes operating in the background, this question begets further investigation, especially in light of observed deepening political debates about crisis management and mitigation behaviors, following our research.

Regarding the role of analytic thinking, political orientation and conspiracy beliefs in predicting the adherence to preventive behaviors and policy support (RQ2, RQ3), the SEM models we tested (both simple and mediation models), show that endorsement of and adherence to COVID-19 preventive measures follow primarily (considering investigated variables), from an open-minded outlook, over and above political ideology and cognitive reflection. While open-mindedness was a considerable predictor of inclination to all three outcome measures, CRT was predictive of lower adherence to stricter hygiene maintenance and lower support of restrictive COVID-19 policies, albeit these effects were weak and possibly significant due to the sample size. Political ideology, practically, did not exhibit any effects (as expected due to its generally low correlations observed across countries), although a very weak positive relationship of a right-leaning outlook and stricter hygiene managed to reach the threshold for statistical significance, again mainly due to the sample size. In addition, there was a partial mediation effect of COVID-19 conspiracy beliefs on the relationship of open-mindedness and CRT with two out of the three dependent variables, which indicates that less open-minded and more reflective individuals engaged in less physical distancing and were less supportive of restrictive policies partly due to their support for COVID-19 conspiracy theories as well (although noting again that these effects are small in size). All of these predictive effects stood controlling for sex and age.

In sum, as expected, we found that the indicators of analytic thinking, especially the propensity for open-minded thinking, were relevant determinates of preventive behavior and policy support. In fact, they were stronger predictors than political ideology. Overall, the two indicators of analytic thinking, together with political ideology, sex, and age explained between 9 and 21% of the variance of the three dependent variables (whereas the mediation model which included COVID-19 conspiracy beliefs explained 11–23% of the outcome variables). This might not appear to be a sizable amount, but considering only two short cognitive measures (especially the three-item CRT) were used, and, for example, compared with the theory of planned behavior, a prominent social cognition theory, which has been shown to account for around 14–40% of the variance in behavior and behavior intentions (Armitage and Conner, 2001; McEachan et al., 2011), it presents a relevant result and a notable avenue worth pursuing in further research.

Moreover, from the dual-process perspective, our results resonate with the notion that the ability and disposition to engage analytic thinking is not the same as having a general open-minded stance (Baron, 1985; Stanovich and West, 1997), suggesting that, for COVID-19 mitigation behavior, the latter seems more important. As expected, individuals more open to new information and knowledge, unconstrained by prior or favored beliefs, were more likely to engage in and support preventive measures. This finding is convergent with recent evidence, suggesting that actively open-minded thinking (about evidence) is robustly associated with acceptance of science and (negatively) with a range of unfounded beliefs (e.g., paranormal and conspiracy beliefs) and more strongly and over and above cognitive reflection (Pennycook et al., 2020a).

What was not expected is for cognitively reflective individuals to be somewhat less likely to adhere to stricter hygiene maintenance and support restrictive policies. However, CRT did correlate positively, albeit weakly, with open-mindedness (0.09, *p* < 0.001), meaning that the two indicators of analytic thinking were related (albeit weakly) in the direction expected within the dual-process framework (e.g., Stanovich et al., 2016; also Baron et al., 2015).

The results regarding the CRT are comparable to the findings of Thoma et al. (2021). Interestingly, they also found that cognitively reflective individuals adopted fewer preventive behaviors (open-mindedness in their case was not predictive at all). What is more, they found that the only factor of the underlying individual responses referring to COVID-19 prevention measures that were positively correlated with CRT was cleanliness (wash, soap, face, disinfect), conspicuously similar to our physical hygiene maintenance measure, which, in our case, also exhibited the strongest relationship with CRT. Guided by classical reasoning account within the dual-process framework, both Thoma et al. (2021) and we expected the reflective individuals, ones more able to detect and overcome their automatic, intuitive responses (previous behavior), to be more likely to engage in the recommended distancing and hygiene behaviors deemed relevant for controlling and mitigating the spread of COVID-19. Namely, these demanding behavior changes should be easier for them to appreciate as rational in the current situation, as well as adhere to. Indeed, there is some evidence that cognitive intuition (calculated as a mean of intuitive responses on the CRT, as opposed to cognitive reflection) predicts lesser adherence to COVID-19 guidelines (Teovanović et al., 2021).

So, what could account for our results? There are several potential reasons some of them also considered by Thoma et al. (2021). Firstly, as Thoma et al. (2021) also noted, previous research showed CRT to be related to numeracy (e.g., Cokely and Kelley, 2009; Campitelli and Gerrans, 2014; Thomson and Oppenheimer, 2016; Szaszi et al., 2017), which is generally higher among men. Although this may explain the correlation of CRT (especially the classical three items) and gender (Baron et al., 2015), we have controlled for the effects of sex in our SEM models. In addition, we observed a negative correlation of sex and CRT, with females likely to score lower (*r* = −0.17, *p* < 0.001), while higher open-mindedness was weakly associated with females (*r* = 0.08, *p* < 0.001, see data output in Supplementary Material).

Furthermore, Baron argued (Baron et al., 2015; Baron, 2017, 2019) that CRT is, primarily, a measure of a reflection/impulsivity trait, i.e., the amount (but see Raoelison et al., 2020 for a “logical intuitions” perspective) opposed to the direction (fairness of the direction to both sides vs. my side bias) of thinking, which is better tapped by open-minded thinking. The two are related because being actively open to new information and knowledge will result in increased search, he suggested (Baron, 2019). Additionally, Baron (2019) and Thoma et al. (2021), referring to arguments made by other researchers, pointed that, in well-structured laboratory settings where normative responses are clearly defined, more search leads to better normative judgment, while this might not be the case in real-world situations. When it comes to important and controversial questions, we might engage reflective capacities in motivated reasoning (e.g., Kahan, 2013, 2017a,b; Baron, 2017), or as Stanovich (2004, pp. 228–243) proposes successful Type-2 override outcomes may be rejected to achieve rational integration of preferences, or as Risen (2016) suggests “acquiescence” is a possible Type 2 response (detecting an error, but choosing not to correct it).

Another possibility is that, during the first wave of the pandemic, at a time when almost all countries had some kind of restrictive policies in place (Hale et al., 2021), cognitively reflective individuals were reflecting on various, sometimes even miscommunicated or seemingly contradictory, guidelines and measures, dissected them and their consequences rather than simply complying. In such uncertain circumstances, with generally high levels of compliance observed around the world, what was cognitively or behaviorally more effortful and rational or irrational may be open to some debate.

And, finally, also mentioned by Thoma et al. (2021), a negative relationship of CRT and cooperation and prosociality (e.g., Rand et al., 2012; Capraro et al., 2017) may contribute to the negative predictive effect of CRT. Namely, theoretical and empirical work suggests that, in social environments where cooperation, on average, leads to better individual outcomes, intuition leads to prosociality (Rand et al., 2014; Rand, 2016; Everett et al., 2017; but see Chen et al., 2013; Verkoeijen and Bouwmeester, 2014). Unfortunately, the design of our research did not permit us to test these different possibilities. Thus, these speculative arguments are in need of clear empirical testing. However, our last question (RQ4) is aimed at exploring the possibility that cognitive reflection and open-mindedness may be utilized in motivated reasoning, which we intended to investigate by interacting political orientation with CRT and open-mindedness in predicting preventive behaviors and policy support (discussed further down).

The partial mediation effects we found are in line with theoretical expectations and consistent with a growing body of evidence, suggesting a general detrimental effect of inclination toward conspiracist thinking on reasoning and decision-making, but, moreover, with evidence, thus far, on the role of different COVID-19 unfounded beliefs, most prominently conspiracy beliefs in the current crisis (Erceg et al., 2020b; Imhoff and Lamberty, 2020; Pennycook et al., 2020b; Stanley et al., 2020; Swami and Barron, 2020; Pavela Banai et al., 2021). In fact, our results (albeit weak in size) are generally consistent with other studies that specifically tested and provided evidence for mediating effects of various misperceptions and unfounded beliefs and knowledge about COVID-19 on the relationship between indicators of analytic thinking and preventive behavior (Erceg et al., 2020b; Stanley et al., 2020; Swami and Barron, 2020). Taken together, these results broadly speak to the importance of having the right, uncontaminated mindware—in addition to having an efficient analytic processor and a tendency to engage it; unhindered rational reasoning and judgment requires mindware that is not contaminated with epistemically suspect beliefs and attitudes not founded in evidence (Stanovich et al., 2016; Rizeq et al., 2021).

Our next question (RQ4) on whether political orientation moderates the relationship between analytic thinking and COVID-19 preventive behaviors and policy support required analyzing the interaction of individual-level political orientation and analytic thinking in the prediction of the three outcome variables. On an overall sample, the interaction effects were negligible.

We also wanted to investigate whether the relationships between political orientation, analytic thinking, and COVID-19 conspiracy beliefs vary across political contexts defined *via* the direction of the relationship of political orientation and policy support. We found no evidence that the SEM model, including only direct effects of political orientation and analytic thinking, neither the SEM model with the mediation effects of conspiracy beliefs included, nor the modeled interactions of political orientation with the two indicators of analytic thinking varied across the three country groups (Tables 2, 3, 5). Put differently, we did not observe the effect of analytical thinking on the three outcome measures being different in countries where a relatively positive correlation between right-leaning orientation and COVID-19 policy emerged vs. countries where this relationship was in the opposite (negative) direction, or countries where no such relationship existed (indicating that the pandemic has not been politicized). This was an expected result of political orientation, generally weakly correlating with policy support (admittedly, a somewhat circular operationalization of political context *via* the association of individual-level variables).

Finally, our last exploratory analyses were not preregistered as we wanted to seize the opportunity and directly compare our results in the two countries included in the research of ours and Pennycook et al. (2021) and gain some insight into whether analytic thinking leads to universally advocated preventive behaviors and policy support, or whether it is primarily co-opted to support motivated reasoning, thus leading to increased political polarization. Although on the level of the entire sample, the moderating effects of political orientation proved to be negligible (and did not vary across political contexts), we focused on running the analyses on the Canadian and US samples separately. The results point to a possibility of interactions between CRT and political ideology in predicting reduced physical contact and stricter physical hygiene. The findings, which we interpret only as indicative trends, show that, in the case of Canada, a weak negative effect of CRT was driven primarily by right-leaning reflective individuals who were more likely *not to adhere* to physical distancing and stricter hygiene practice, with the same pattern being visible in the US sample when it comes to predicting physical distancing and policy support. In fact, the moderations models did not seem to vary across the two countries as indicated by our results of invariance testing. The overall pattern was that the most reflective individuals seemed to be the ones differing the most in their tendency to adhere to preventive behaviors and support restrictive policies conditional on their political outlook. Specifically, a trend we observed was: although cognitive reflection may not lead left-leaning individuals to engage in more preventive behavior and exhibit stronger policy support, with an increase in cognitive reflection, the right-leaning ones seem less prone to engage in physical distancing in both countries, and in the US they seem less likely to support restrictive policies, while in Canada less likely to adhere to stricter hygiene. This would broadly be in line with the identity-protective cognition account, even in the early stages of the pandemic. As we have already stressed, in addition to not being preregistered, our analysis yielded minor interaction effects. We have to note that, unlike Pennycook et al. (2021), we did not have data on partisan identification, which may be deemed a stronger measure of political identity, especially in the US. In addition, Pennycook et al. (2021) also used a composite measure of cognitive sophistication (science knowledge, CRT, numeracy, and bushtit receptivity). Also, we were unable to control for other potentially relevant variables which could attenuate or diminish these effects, such as liberal and conservative media trust which Pennycook et al. (2021) controlled for. By presenting these results, our desire is to encourage further research, providing more evidence for the debate of the two accounts of the role of analytic thinking in motivated reasoning and behavior.

Finally, several limitations of our study should be taken into account. The first and most notable one is the questionable variability in the dependent variables, which implies that only a small portion of the possible specter of physical distancing, hygiene maintenance, and COVID-19 policy support was measured in this study. This is not unusual as the data were collected during the full-blown first wave of the COVID-19 pandemic, with lockdowns and financial penalties for violating imposed restrictions, defining everyday life and behavior. Also, although, in most cases, at least some of the restrictive measures were in force, specific policies varied across countries and regions. We tried to counter this problem by eliminating countries with insufficient variability from the analyses, which resulted in a greatly reduced number of countries, primarily WEIRD societies (Henrich et al., 2010). However, this was the only way to protect ecological validity (i.e., that our results reflect real-life phenomena as they occur) without annulling the validity of the applied statistical procedures. Even considering the full sample, comprising of 67 countries/regions, it was simply not possible to ensure representativeness and balanced representation of countries (African and Middle Eastern countries), having in mind the circumstances of a developing pandemic. This obviously presented a crucial obstacle to our intention to examine the relations of the investigated factors in a cross-cultural context.

Once again, we have to note that we deviated from our preregistration regarding the operationalization of political context by not taking into account the confidence intervals when forming the three country groups, although following the principal logic of country grouping based on the correlation between political orientation and policy support. Additionally, we did not preregister our final analyses, exploring the interactions of political ideology and the two indicators of analytic thinking in predicting the three outcome variables in the US and Canadian sample, following Pennycook et al. (2021). The results stemming from these analyses are purely exploratory and have to be treated with caution.

Furthermore, the use of brief versions of instruments may undermine construct validity as even broad phenomena are measured using only several items. This is most notable in the application of CRT, which can, in general, yield two scores: one for the number of correct answers and one for the number of intuitive answers (Frederick, 2005). However, on a set of three items, that we were bound to due to project limitations, it was impossible to extract both results without multicollinearity. Therefore, in the future studies, we would recommend the use of longer measures of cognitive reflection. Consequently, incorporating additional potentially relevant variables, such as trust in science (see for example, Plohl and Musil, 2020), risk perception, attitudes toward vaccination, and other indicators of cognitive capacity and motivation of critical thinking (e.g., scientific reasoning, see Čavojová et al., 2020, science curiosity, see Erceg et al., 2020b), as well as taking into account the dynamic factors, which fluctuate with respect to the time and the phase of the crisis when collecting data, may provide a broader picture in understanding psychological and behavioral responses to the pandemic. This, of course, implies careful and theory-informed development of potential models.

Since the literature on the effects of social desirability on reporting risk behaviors remains inconclusive (Crutzen and Göritz, 2010; Davis et al., 2010), we would also recommend future researchers to use some measure of overclaiming or social desirability to ensure the robustness of findings.

Ultimately, this was a correlational cross-sectional study, so no causal conclusions should be drawn. As already stated, a longitudinal study that would allow monitoring of public responses to the pandemic during different phases would be of great value, as well as experimental and meta-analytical studies informed by previous work.

## Conclusions

In the current study, within the dual-process framework of human reasoning, we focused on examining political orientation and analytic thinking (cognitive reflection, open-minded thinking) as possible sources of differences in adherence to COVID-19 preventive behaviors (physical distancing and maintaining hygiene) and support for restrictive COVID-19 policies across different countries.

We have not been able to detect substantial relationships of political orientation with preventive behaviors and policy support, and overall found no reliable evidence of politicization nor polarization regarding the issue. The SEM results showed that the inclination toward and endorsement of COVID-19 preventive measures was defined primarily by the tendency of open-minded thinking. Specifically, it was shown to be a predictor of all three criteria: avoiding physical contact, maintaining physical hygiene, and policy support. Cognitive reflection was predictive of lesser adherence to stricter hygiene and weakly to lesser policy support. Furthermore, there was no evidence of these effects varying across political contexts. The mediation analysis suggested a partial mediation effect of COVID-19 conspiracy beliefs on the relationships of open-mindedness and CRT with physical distancing (but not adherence to stricter hygiene) and COVID-19 policy support, albeit very small and significant primarily due to the sample size. There was also no evidence of these effects varying across political contexts. Finally, we have not been able to find strong evidence of political orientation modifying the relationship between analytical thinking and COVID-19 behaviors and policy support, although we explored the pattern of these effects in the US and Canadian sample for exploratory purposes and comparison with findings of Pennycook et al. (2021).

## Data Availability Statement

The original contributions presented in the study are included in the article/Supplementary Material, further inquiries can be directed to the corresponding author/s.

## Ethics Statement

The studies involving human participants were reviewed and approved by Research Ethics and Governance Committee, University of Kent. The patients/participants provided their written informed consent to participate in this study.

## Author Contributions

All the authors made substantial contributions to the theoretical framework, design, data collection, interpretation of this study, contributed to this article and approved its publication, and agreed to be accountable for the accuracy and integrity of the project.

## Conflict of Interest

The authors declare that the research was conducted in the absence of any commercial or financial relationships that could be construed as a potential conflict of interest.

## Footnotes

^1^RQ = research questions outlined in our preregistration (Available online at: https://aspredicted.org/xj83u.pdf) and further explained here.

^2^https://icsmp-covid19.netlify.app/.

^3^Items were devised for the purposes of the International Collaboration on the Social and Moral Psychology (ICSMP) of COVID-19 Project.
