# Supplementary material for: Analytic Thinking and Political Orientation in the Corona Crisis
Source: Front Psychol. 2021 Jul 22;12:631800. doi: 10.3389/fpsyg.2021.631800 (PMC8341110; doi:10.3389/fpsyg.2021.631800)
Supplement: Supplementary file 1 [file Data_Sheet_1.zip › Supplementary Materials/Main analyses.html]

Data analyses - Analytic Thinking and Political Orientation in the Corona Crisis


# Data analyses - Analytic Thinking and Political Orientation in the Corona Crisis

#### Deadpool

#### 15 05 2021

## Preparations

```
lapply(c("readr",  "dplyr", "caret", "psych", "lavaan", "semTools", "semPlot", "psych", "MASS", "xfun", "mime", "htmltools", "seminr", "ggplot2", "sjPlot"), library, character.only = T)
```

## Preparing functions

```
"%^%" <- function(S, power) with(eigen(S), vectors %*% (values ^ power * t(vectors)))

# ten Berge factor score calculator
calc_ten_berge_scores <- function(X, Lambda, Phi, i.means, i.sds) {
  if (any(is.na(X))) {
    # if any missing, impute using person average
    p.means <- rowMeans(X, na.rm = TRUE)
    missings <- which(is.na(X), arr.ind = TRUE)
    X[is.na(X)] <- p.means[missings[, 1]]
    X <- scale(X)
  } else {
    X <- t((t(X) - i.means) / i.sds)
  }
  R <- stats::cor(X, use = "pairwise")
  R.sqrt.i <- R %^% -0.5
  Phi.sqrt <- Phi %^% 0.5
  L <- Lambda %*% Phi.sqrt
  C <- R.sqrt.i %*% L %*% ((t(L) %*% chol2inv(chol(R)) %*% L) %^% -0.5)
  W <- R.sqrt.i %*% C %*% Phi.sqrt
  colnames(W) <- colnames(Lambda)
  rownames(W) <- rownames(Lambda)
  scores <- X %*% W
  colnames(scores) <- colnames(Lambda)
  list(scores = scores)
}

#### original function ####
estimate_lavaan_ten_berge <- function (fit) {
  X <- lavaan::lavInspect(fit, "data")
  i.means <- fit@SampleStats@mean[[1]]
  i.sds <- sqrt(fit@SampleStats@var[[1]])
  Lambda_mat <- lavaan::lavInspect(fit, what = "std.lv")$lambda
  Phi_mat <- matrix(lavaan::lavInspect(fit, what = "cor.lv"), ncol(Lambda_mat))
  calc_ten_berge_scores(X, Lambda_mat, Phi_mat, i.means, i.sds)
}

#### modified function ####
estimate_lavaan_ten_berge_per_group <- function (fit) {
  Lambda_mats <- function(fit){
    longlist <- lavaan::lavInspect(fit, "std.lv")
    outputs <- list()
    for(i in seq_along(names(longlist))){
      outputs[[i]] <- eval(parse(text = paste0("longlist$", names(longlist)[i], "$lambda")))
    }
    outputs
  }
  Phi_mats <- function(fit){
    longlist2 <- lavaan::lavInspect(fit, what = "cor.lv")
    outputs <- list()
    for(i in seq_along(names(longlist2))){
      outputs[[i]] <- eval(parse(text = paste0("matrix(longlist2$", names(longlist2)[i], ", ncol(lambda_mat[[1]]))")))
    }
    outputs
  }
  X <- lavaan::lavInspect(fit, "data")
  i.means <- fit@SampleStats@mean
  i.sds <- lapply(fit@SampleStats@var, sqrt)
  lambda_mat <- Lambda_mats(fit)
  phi_mat <- Phi_mats(fit)
  ouut <- list()
  for(i in seq_along(X)){
    ouut[[i]] <- calc_ten_berge_scores(X[[i]], lambda_mat[[i]], phi_mat[[i]], i.means[[i]], i.sds[[i]])
  } 
  ouut
}
```

## Data manipulations

```
c19 <- read_csv("finaldata_marina.csv")
```

```
## Warning: Missing column names filled in: 'X1' [1]
```

```
## 
## -- Column specification --------------------------------------------------------
## cols(
##   .default = col_double(),
##   country = col_character()
## )
## i Use `spec()` for the full column specifications.
```

```
pls2 <- c19[, c("country", "contact1", "contact2", "contact3", "contact4", "contact5", "hygiene1", "hygiene2", "hygiene3", "hygiene4", "hygiene5", "psupport1", "psupport2", "psupport3", "psupport4","psupport5", "political_ideology", "omind1", "omind2", "omind3","omind4", "omind5", "omind6", "CRT1", "CRT2", "CRT3", "age", "sex1", "ctheory1", "ctheory2", "ctheory3", "ctheory4", "employ_status1")]
table(pls2$country)
```

```
## 
##   ARE   ARG   AUS   AUT   BEL   BGD   BGR   BRA   CAN CENAM   CHE   CHN   COL 
##   183   715  1819  1368  1140   323   631  1666   941   238   969  1030  1200 
##   DEU   DNK   ESP   FIN   FRA   GBR   GHA   GRC   HRV   HUN   IND   IRL   IRQ 
##  1416   453  1081   662   800   543   193   614   493   479   607   712   414 
##   ISR   ITA   JPN   KOR LATAM   LVA   MAR   MEX mixed   MKD   NGA   NLD   NOR 
##  1174  1137   891   308   524   921   561  1237   183   678   509  1289   505 
##   NPL   NZL   PAK   PHL   POL   ROU   RUS   SEN   SGP   SRB   SVK   SWE   TUR 
##   338   448   451   482  1677   932   498   301   454   712  1010  1546  1256 
##   TWN   UKR   USA   ZAF 
##   786   546  1147   602
```

```
#excluding countries with too low variability
plsx <- split.data.frame(pls2, pls2$country)

nzv2 <- list()
for(i in 1:length(plsx)){
  nam <- names(plsx)
  df <-  plsx[[i]]
  nzv2[[i]] <- colnames(pls2)[nearZeroVar(df, freqCut = 6)]
  names(nzv2)[i] <- nam[i]
}

nzv2
```

```
## $ARE
## [1] "country"
## 
## $ARG
##  [1] "country"   "contact2"  "contact5"  "hygiene1"  "hygiene2"  "hygiene3" 
##  [7] "psupport1" "psupport2" "psupport3" "psupport4" "psupport5" "omind2"   
## [13] "omind4"    "omind5"    "omind6"   
## 
## $AUS
## [1] "country"
## 
## $AUT
## [1] "country"  "contact5" "ctheory2" "ctheory3" "ctheory4"
## 
## $BEL
## [1] "country"  "contact5"
## 
## $BGD
## [1] "country"   "contact5"  "hygiene3"  "psupport1" "psupport2" "psupport3"
## [7] "psupport4" "psupport5"
## 
## $BGR
##  [1] "country"   "contact5"  "hygiene1"  "hygiene3"  "psupport1" "psupport2"
##  [7] "psupport4" "psupport5" "omind2"    "omind3"    "omind5"   
## 
## $BRA
##  [1] "country"   "contact5"  "hygiene3"  "psupport1" "psupport2" "psupport3"
##  [7] "psupport4" "psupport5" "omind5"    "ctheory2"  "ctheory3" 
## 
## $CAN
## [1] "country"  "contact5"
## 
## $CENAM
##  [1] "country"            "contact5"           "hygiene3"          
##  [4] "hygiene5"           "psupport1"          "psupport2"         
##  [7] "psupport3"          "psupport4"          "psupport5"         
## [10] "political_ideology" "omind2"             "omind3"            
## [13] "omind4"             "omind5"             "CRT1"              
## 
## $CHE
## [1] "country"  "contact5"
## 
## $CHN
## [1] "country" "CRT2"   
## 
## $COL
##  [1] "country"   "contact2"  "contact5"  "hygiene3"  "psupport1" "psupport2"
##  [7] "psupport3" "psupport4" "psupport5" "omind2"    "omind3"    "omind4"   
## [13] "omind5"   
## 
## $DEU
## [1] "country"  "contact5"
## 
## $DNK
## [1] "country"   "contact5"  "psupport2" "psupport5"
## 
## $ESP
## [1] "country"   "contact2"  "contact5"  "psupport4" "psupport5"
## 
## $FIN
## [1] "country"  "contact5" "omind5"   "ctheory2" "ctheory3" "ctheory4"
## 
## $FRA
## [1] "country"  "contact2" "contact5"
## 
## $GBR
## [1] "country"  "contact5" "hygiene3"
## 
## $GHA
## [1] "country"   "psupport1" "omind3"    "omind4"   
## 
## $GRC
## [1] "country"
## 
## $HRV
## [1] "country"   "contact5"  "hygiene3"  "hygiene5"  "psupport5" "omind2"   
## [7] "omind4"   
## 
## $HUN
## [1] "country"   "contact5"  "psupport4"
## 
## $IND
## [1] "country"   "contact5"  "psupport1" "psupport2" "psupport3" "psupport4"
## [7] "psupport5"
## 
## $IRL
## [1] "country"   "contact5"  "psupport2" "ctheory3"  "ctheory4" 
## 
## $IRQ
## [1] "country"
## 
## $ISR
## [1] "country"  "contact5"
## 
## $ITA
## [1] "country"  "contact2" "contact5"
## 
## $JPN
## [1] "country"
## 
## $KOR
## [1] "country"
## 
## $LATAM
##  [1] "country"   "contact5"  "hygiene1"  "hygiene2"  "hygiene3"  "hygiene5" 
##  [7] "psupport1" "psupport2" "psupport3" "psupport4" "psupport5" "omind2"   
## [13] "omind3"    "omind4"    "omind5"   
## 
## $LVA
## [1] "country"        "contact5"       "hygiene3"       "psupport5"     
## [5] "employ_status1"
## 
## $MAR
## [1] "country"   "contact5"  "hygiene3"  "psupport1" "psupport2" "psupport3"
## [7] "psupport4" "psupport5" "omind5"   
## 
## $MEX
## [1] "country"   "contact5"  "hygiene3"  "psupport1" "psupport2" "psupport4"
## [7] "psupport5" "omind2"    "omind5"   
## 
## $mixed
## [1] "country"
## 
## $MKD
## [1] "country"        "contact5"       "hygiene3"       "psupport2"     
## [5] "psupport4"      "psupport5"      "employ_status1"
## 
## $NGA
## [1] "country"
## 
## $NLD
## [1] "country"  "contact5" "ctheory3"
## 
## $NOR
## [1] "country"  "contact5" "ctheory2" "ctheory3" "ctheory4"
## 
## $NPL
## [1] "country"   "contact5"  "psupport5"
## 
## $NZL
## [1] "country"  "contact5"
## 
## $PAK
## [1] "country"
## 
## $PHL
## [1] "country"   "contact5"  "hygiene3"  "psupport1" "psupport2" "psupport4"
## [7] "psupport5"
## 
## $POL
## [1] "country"
## 
## $ROU
## [1] "country"   "contact5"  "hygiene3"  "hygiene5"  "psupport2" "psupport4"
## [7] "psupport5"
## 
## $RUS
## [1] "country"   "contact2"  "contact5"  "hygiene3"  "psupport2" "psupport4"
## 
## $SEN
## [1] "country"   "psupport4" "psupport5"
## 
## $SGP
## [1] "country"        "employ_status1"
## 
## $SRB
##  [1] "country"        "contact5"       "hygiene3"       "psupport1"     
##  [5] "psupport2"      "psupport4"      "psupport5"      "omind2"        
##  [9] "omind3"         "omind4"         "omind5"         "employ_status1"
## 
## $SVK
## [1] "country"  "contact5"
## 
## $SWE
## [1] "country"  "contact5" "ctheory3" "ctheory4"
## 
## $TUR
## [1] "country"   "contact5"  "hygiene3"  "psupport2" "psupport4" "psupport5"
## 
## $TWN
## [1] "country"            "political_ideology"
## 
## $UKR
## [1] "country"            "hygiene3"           "political_ideology"
## 
## $USA
## [1] "country"
## 
## $ZAF
## [1] "country"   "contact5"  "hygiene3"  "psupport4"
```

```
pls3 <- subset(pls2, country %in% c("ARE", "AUS", "BEL", "CAN", "CHE", "DEU", "GRC", "IRQ", "ISR", "JPN", "KOR", "NGA", "NZL", "PAK", "POL", "SGP", "SVK", "USA"))
pls3$contact2r <- 10-pls3$contact2
pls3$omind1 <- 10 - pls3$omind1
pls3$omind5 <- 10 - pls3$omind5
pls3$omind6 <- 10 - pls3$omind6

pls3x <- split.data.frame(pls3, pls3$country)

lyst <- list()
n_excl <- list()
for(i in 1:length(pls3x)){
  nam <- names(pls3x)
  print(i)
  df <- pls3x[[i]]
  set.seed(i)
  df75 <- cov.mcd(df[ ,c(-1, -3, -6, -17, -24, -25, -26, -27, -28)], quantile.used = nrow(df)*.75) # variable no. 3 is excluded as its recoded version was included in the calculations 
  set.seed(i)
  df75m <- mahalanobis(df[ ,c(-1, -3, -6, -17, -24, -25, -26, -27, -28)], df75$center, df75$cov) #calculating Mahalanobis distance using the robust estimates
  (cutoff <- (qchisq(p = 1 - .001, df = ncol(df[ ,c(-1, -3, -6, -17, -24, -25, -26, -27, -28)]))))
  outcat <- which(df75m > cutoff)
  n_excl[[i]] <- length(outcat)
  names(n_excl)[i] <- nam[i]
  lyst[[i]] <- df[-outcat, ]
  names(lyst)[i] <- nam[i]
}
```

```
## [1] 1
## [1] 2
## [1] 3
## [1] 4
## [1] 5
## [1] 6
## [1] 7
## [1] 8
## [1] 9
## [1] 10
## [1] 11
## [1] 12
## [1] 13
## [1] 14
## [1] 15
## [1] 16
## [1] 17
## [1] 18
```

```
n_excl #number of excluded participants per country
```

```
## $ARE
## [1] 30
## 
## $AUS
## [1] 331
## 
## $BEL
## [1] 189
## 
## $CAN
## [1] 196
## 
## $CHE
## [1] 168
## 
## $DEU
## [1] 296
## 
## $GRC
## [1] 134
## 
## $IRQ
## [1] 90
## 
## $ISR
## [1] 180
## 
## $JPN
## [1] 142
## 
## $KOR
## [1] 54
## 
## $NGA
## [1] 106
## 
## $NZL
## [1] 114
## 
## $PAK
## [1] 84
## 
## $POL
## [1] 281
## 
## $SGP
## [1] 80
## 
## $SVK
## [1] 176
## 
## $USA
## [1] 240
```

```
pls4 <- bind_rows(lyst)
pls4$CRT1 <- ifelse(pls4$CRT1 == 1, 1, 0)
pls4$CRT2 <- ifelse(pls4$CRT2 == 1, 1, 0)
pls4$CRT3 <- ifelse(pls4$CRT3 == 1, 1, 0)
pls4$ccrt <- rowSums(pls4[, c("CRT1", "CRT2", "CRT3")])
table(pls4$country)
```

```
## 
##  ARE  AUS  BEL  CAN  CHE  DEU  GRC  IRQ  ISR  JPN  KOR  NGA  NZL  PAK  POL  SGP 
##  153 1488  951  745  801 1120  480  324  994  749  254  403  334  367 1396  374 
##  SVK  USA 
##  834  907
```

```
table(pls4$sex1)
```

```
## 
##    1    2    3 
## 6216 6426   32
```

```
pls4 <- subset(pls4, country != "ARE") #due to the number of participants
pls4 <- subset(pls4, sex1 != 3) #due to the number of participants
describe(pls4)
```

```
##                    vars     n  mean    sd median trimmed   mad min max range
## country*              1 12490  8.31  5.40      8    8.17  7.41   1  17    16
## contact1              2 12490  8.65  1.77      9    8.99  1.48   0  10    10
## contact2              3 12490  2.14  2.76      1    1.63  1.48   0  10    10
## contact3              4 12490  8.13  2.22      9    8.52  1.48   0  10    10
## contact4              5 12490  8.63  1.78      9    8.97  1.48   0  10    10
## contact5              6 12490  9.22  1.68     10    9.66  0.00   0  10    10
## hygiene1              7 12490  8.10  2.04      9    8.40  1.48   0  10    10
## hygiene2              8 12490  8.23  1.99      9    8.54  1.48   0  10    10
## hygiene3              9 12490  8.69  1.88     10    9.06  0.00   0  10    10
## hygiene4             10 12490  6.19  3.12      7    6.45  2.97   0  10    10
## hygiene5             11 12490  7.93  2.46      9    8.35  1.48   0  10    10
## psupport1            12 12490  7.77  2.53      8    8.20  2.97   0  10    10
## psupport2            13 12490  8.24  2.28      9    8.67  1.48   0  10    10
## psupport3            14 12490  6.35  3.21      7    6.65  4.45   0  10    10
## psupport4            15 12490  8.56  2.07     10    8.99  0.00   0  10    10
## psupport5            16 12490  8.32  2.33      9    8.79  1.48   0  10    10
## political_ideology   17 12490  5.19  2.22      5    5.19  1.48   0  10    10
## omind1               18 12490  6.12  2.71      6    6.27  2.97   0  10    10
## omind2               19 12490  8.69  1.67      9    8.99  1.48   0  10    10
## omind3               20 12490  8.61  1.50      9    8.83  1.48   1  10     9
## omind4               21 12490  8.51  1.63      9    8.76  1.48   0  10    10
## omind5               22 12490  8.29  2.52      9    8.85  1.48   0  10    10
## omind6               23 12490  7.33  2.58      8    7.67  2.97   0  10    10
## CRT1                 24 12490  0.28  0.45      0    0.23  0.00   0   1     1
## CRT2                 25 12490  0.43  0.50      0    0.41  0.00   0   1     1
## CRT3                 26 12490  0.30  0.46      0    0.25  0.00   0   1     1
## age                  27 12490 45.14 17.09     44   44.78 22.24  18 100    82
## sex1                 28 12490  1.51  0.50      2    1.51  0.00   1   2     1
## ctheory1             29 12490  4.02  3.30      4    3.81  4.45   0  10    10
## ctheory2             30 12490  2.92  3.07      2    2.53  2.97   0  10    10
## ctheory3             31 12490  2.47  2.96      1    1.99  1.48   0  10    10
## ctheory4             32 12490  2.81  3.02      2    2.40  2.97   0  10    10
## employ_status1       33 12490  2.91  1.84      3    2.77  2.97   1   6     5
## contact2r            34 12490  7.86  2.76      9    8.37  1.48   0  10    10
## ccrt                 35 12490  1.01  1.07      1    0.89  1.48   0   3     3
##                     skew kurtosis   se
## country*            0.19    -1.37 0.05
## contact1           -1.71     3.24 0.02
## contact2            1.28     0.56 0.02
## contact3           -1.38     1.52 0.02
## contact4           -1.70     3.26 0.02
## contact5           -2.97     9.97 0.01
## hygiene1           -1.23     1.58 0.02
## hygiene2           -1.33     1.94 0.02
## hygiene3           -1.74     3.18 0.02
## hygiene4           -0.50    -0.81 0.03
## hygiene5           -1.32     1.25 0.02
## psupport1          -1.18     0.70 0.02
## psupport2          -1.47     1.69 0.02
## psupport3          -0.51    -0.94 0.03
## psupport4          -1.78     3.18 0.02
## psupport5          -1.59     2.05 0.02
## political_ideology -0.01     0.01 0.02
## omind1             -0.39    -0.63 0.02
## omind2             -1.96     5.40 0.01
## omind3             -1.11     1.13 0.01
## omind4             -1.20     1.42 0.01
## omind5             -1.68     2.01 0.02
## omind6             -0.86    -0.10 0.02
## CRT1                0.97    -1.06 0.00
## CRT2                0.28    -1.92 0.00
## CRT3                0.89    -1.20 0.00
## age                 0.16    -1.09 0.15
## sex1               -0.03    -2.00 0.00
## ctheory1            0.25    -1.20 0.03
## ctheory2            0.72    -0.69 0.03
## ctheory3            1.00    -0.18 0.03
## ctheory4            0.78    -0.59 0.03
## employ_status1      0.32    -1.45 0.02
## contact2r          -1.28     0.56 0.02
## ccrt                0.66    -0.88 0.01
```

```
describeBy(pls4, pls4$country)
```

```
## 
##  Descriptive statistics by group 
## group: AUS
##                    vars    n  mean    sd median trimmed   mad min max range
## country*              1 1480  1.00  0.00      1    1.00  0.00   1   1     0
## contact1              2 1480  8.78  1.51      9    9.04  1.48   1  10     9
## contact2              3 1480  2.07  2.67      1    1.58  1.48   0  10    10
## contact3              4 1480  8.04  2.12      9    8.35  1.48   0  10    10
## contact4              5 1480  8.86  1.46      9    9.11  1.48   2  10     8
## contact5              6 1480  9.22  1.54     10    9.60  0.00   0  10    10
## hygiene1              7 1480  8.28  1.80      9    8.54  1.48   0  10    10
## hygiene2              8 1480  8.35  1.81      9    8.62  1.48   0  10    10
## hygiene3              9 1480  8.39  1.94      9    8.72  1.48   0  10    10
## hygiene4             10 1480  6.52  2.88      7    6.80  2.97   0  10    10
## hygiene5             11 1480  7.95  2.25      9    8.27  1.48   0  10    10
## psupport1            12 1480  7.02  2.70      8    7.35  2.97   0  10    10
## psupport2            13 1480  8.29  2.05      9    8.66  1.48   0  10    10
## psupport3            14 1480  6.61  2.84      7    6.88  2.97   0  10    10
## psupport4            15 1480  8.52  1.91      9    8.88  1.48   0  10    10
## psupport5            16 1480  8.45  2.00      9    8.83  1.48   0  10    10
## political_ideology   17 1480  5.31  2.01      5    5.32  1.48   0  10    10
## omind1               18 1480  5.70  2.58      6    5.78  2.97   0  10    10
## omind2               19 1480  8.52  1.52      9    8.73  1.48   2  10     8
## omind3               20 1480  8.43  1.46      9    8.61  1.48   2  10     8
## omind4               21 1480  8.30  1.56      8    8.49  1.48   2  10     8
## omind5               22 1480  8.20  2.60      9    8.75  1.48   0  10    10
## omind6               23 1480  7.26  2.64      8    7.58  2.97   0  10    10
## CRT1                 24 1480  0.20  0.40      0    0.13  0.00   0   1     1
## CRT2                 25 1480  0.41  0.49      0    0.39  0.00   0   1     1
## CRT3                 26 1480  0.26  0.44      0    0.20  0.00   0   1     1
## age                  27 1480 50.01 17.09     51   50.21 22.24  18  89    71
## sex1                 28 1480  1.53  0.50      2    1.54  0.00   1   2     1
## ctheory1             29 1480  4.10  3.17      5    3.94  4.45   0  10    10
## ctheory2             30 1480  2.97  3.05      2    2.60  2.97   0  10    10
## ctheory3             31 1480  2.06  2.78      1    1.54  1.48   0  10    10
## ctheory4             32 1480  2.54  2.91      1    2.11  1.48   0  10    10
## employ_status1       33 1480  2.96  1.80      2    2.86  1.48   1   6     5
## contact2r            34 1480  7.93  2.67      9    8.42  1.48   0  10    10
## ccrt                 35 1480  0.87  1.00      1    0.72  1.48   0   3     3
##                     skew kurtosis   se
## country*             NaN      NaN 0.00
## contact1           -1.53     2.66 0.04
## contact2            1.26     0.51 0.07
## contact3           -1.15     0.92 0.05
## contact4           -1.53     2.42 0.04
## contact5           -2.85     9.95 0.04
## hygiene1           -1.07     0.97 0.05
## hygiene2           -1.18     1.26 0.05
## hygiene3           -1.35     1.69 0.05
## hygiene4           -0.60    -0.52 0.07
## hygiene5           -1.22     1.25 0.06
## psupport1          -0.78    -0.22 0.07
## psupport2          -1.35     1.39 0.05
## psupport3          -0.56    -0.63 0.07
## psupport4          -1.50     2.00 0.05
## psupport5          -1.52     2.09 0.05
## political_ideology -0.01     0.40 0.05
## omind1             -0.28    -0.61 0.07
## omind2             -1.19     1.67 0.04
## omind3             -0.80     0.28 0.04
## omind4             -0.75     0.02 0.04
## omind5             -1.58     1.47 0.07
## omind6             -0.79    -0.38 0.07
## CRT1                1.49     0.23 0.01
## CRT2                0.36    -1.87 0.01
## CRT3                1.10    -0.80 0.01
## age                -0.08    -1.13 0.44
## sex1               -0.14    -1.98 0.01
## ctheory1            0.15    -1.16 0.08
## ctheory2            0.68    -0.77 0.08
## ctheory3            1.25     0.37 0.07
## ctheory4            0.90    -0.38 0.08
## employ_status1      0.29    -1.52 0.05
## contact2r          -1.26     0.51 0.07
## ccrt                0.84    -0.49 0.03
## ------------------------------------------------------------ 
## group: BEL
##                    vars   n  mean    sd median trimmed   mad min max range
## country*              1 950  1.00  0.00      1    1.00  0.00   1   1     0
## contact1              2 950  8.73  1.74      9    9.10  1.48   0  10    10
## contact2              3 950  1.86  2.68      1    1.32  1.48   0  10    10
## contact3              4 950  8.25  2.32      9    8.73  1.48   0  10    10
## contact4              5 950  9.02  1.32      9    9.27  1.48   2  10     8
## contact5              6 950  9.73  1.00     10    9.96  0.00   0  10    10
## hygiene1              7 950  7.56  2.36      8    7.91  2.97   0  10    10
## hygiene2              8 950  7.84  2.21      8    8.18  2.97   0  10    10
## hygiene3              9 950  7.94  2.39      9    8.37  1.48   0  10    10
## hygiene4             10 950  4.48  3.21      5    4.37  4.45   0  10    10
## hygiene5             11 950  7.41  2.65      8    7.81  2.97   0  10    10
## psupport1            12 950  7.19  2.88      8    7.59  2.97   0  10    10
## psupport2            13 950  8.46  2.14      9    8.92  1.48   0  10    10
## psupport3            14 950  5.10  3.27      5    5.13  4.45   0  10    10
## psupport4            15 950  8.77  1.82     10    9.16  0.00   0  10    10
## psupport5            16 950  8.21  2.42      9    8.70  1.48   0  10    10
## political_ideology   17 950  5.01  2.67      5    5.07  2.97   0  10    10
## omind1               18 950  6.35  2.40      7    6.47  2.97   0  10    10
## omind2               19 950  8.91  1.17      9    9.07  1.48   4  10     6
## omind3               20 950  8.91  1.13      9    9.07  1.48   4  10     6
## omind4               21 950  8.84  1.22      9    9.01  1.48   4  10     6
## omind5               22 950  8.91  2.11     10    9.46  0.00   0  10    10
## omind6               23 950  7.88  2.14      8    8.18  2.97   0  10    10
## CRT1                 24 950  0.45  0.50      0    0.44  0.00   0   1     1
## CRT2                 25 950  0.64  0.48      1    0.67  0.00   0   1     1
## CRT3                 26 950  0.59  0.49      1    0.61  0.00   0   1     1
## age                  27 950 46.22 18.91     48   45.96 26.69  18  87    69
## sex1                 28 950  1.43  0.49      1    1.41  0.00   1   2     1
## ctheory1             29 950  2.49  2.84      1    2.08  1.48   0  10    10
## ctheory2             30 950  1.57  2.34      0    1.11  0.00   0  10    10
## ctheory3             31 950  1.35  2.26      0    0.84  0.00   0  10    10
## ctheory4             32 950  1.61  2.42      0    1.11  0.00   0  10    10
## employ_status1       33 950  3.61  1.81      4    3.63  1.48   1   6     5
## contact2r            34 950  8.14  2.68      9    8.68  1.48   0  10    10
## ccrt                 35 950  1.68  1.12      2    1.72  1.48   0   3     3
##                     skew kurtosis   se
## country*             NaN      NaN 0.00
## contact1           -2.06     4.76 0.06
## contact2            1.48     1.10 0.09
## contact3           -1.63     2.13 0.08
## contact4           -1.78     3.80 0.04
## contact5           -6.04    44.95 0.03
## hygiene1           -1.12     0.98 0.08
## hygiene2           -1.34     1.91 0.07
## hygiene3           -1.34     1.37 0.08
## hygiene4            0.12    -1.14 0.10
## hygiene5           -1.06     0.49 0.09
## psupport1          -0.92    -0.19 0.09
## psupport2          -1.74     2.68 0.07
## psupport3           0.02    -1.27 0.11
## psupport4          -1.95     4.23 0.06
## psupport5          -1.50     1.54 0.08
## political_ideology -0.12    -1.00 0.09
## omind1             -0.51    -0.30 0.08
## omind2             -0.95     0.57 0.04
## omind3             -0.93     0.62 0.04
## omind4             -1.03     0.89 0.04
## omind5             -2.63     6.74 0.07
## omind6             -1.06     0.70 0.07
## CRT1                0.19    -1.97 0.02
## CRT2               -0.56    -1.68 0.02
## CRT3               -0.37    -1.87 0.02
## age                -0.02    -1.42 0.61
## sex1                0.29    -1.92 0.02
## ctheory1            0.90    -0.38 0.09
## ctheory2            1.57     1.70 0.08
## ctheory3            1.86     2.83 0.07
## ctheory4            1.53     1.43 0.08
## employ_status1     -0.41    -1.31 0.06
## contact2r          -1.48     1.10 0.09
## ccrt               -0.22    -1.32 0.04
## ------------------------------------------------------------ 
## group: CAN
##                    vars   n  mean    sd median trimmed   mad min max range
## country*              1 740  1.00  0.00      1    1.00  0.00   1   1     0
## contact1              2 740  9.20  1.17     10    9.44  0.00   3  10     7
## contact2              3 740  1.27  2.10      0    0.76  0.00   0  10    10
## contact3              4 740  8.59  1.94      9    9.00  1.48   0  10    10
## contact4              5 740  9.30  1.07     10    9.52  0.00   4  10     6
## contact5              6 740  9.68  1.03     10    9.92  0.00   0  10    10
## hygiene1              7 740  8.66  1.64      9    8.95  1.48   1  10     9
## hygiene2              8 740  8.78  1.59      9    9.07  1.48   2  10     8
## hygiene3              9 740  8.79  1.84     10    9.18  0.00   0  10    10
## hygiene4             10 740  7.59  2.57      8    8.00  2.97   0  10    10
## hygiene5             11 740  8.75  1.81     10    9.12  0.00   0  10    10
## psupport1            12 740  9.03  1.44     10    9.32  0.00   2  10     8
## psupport2            13 740  9.01  1.51     10    9.33  0.00   3  10     7
## psupport3            14 740  7.95  2.45      9    8.35  1.48   0  10    10
## psupport4            15 740  9.17  1.43     10    9.51  0.00   2  10     8
## psupport5            16 740  9.13  1.51     10    9.49  0.00   1  10     9
## political_ideology   17 740  4.72  1.80      5    4.74  1.48   0  10    10
## omind1               18 740  6.49  2.54      7    6.68  2.97   0  10    10
## omind2               19 740  8.79  1.38      9    8.99  1.48   2  10     8
## omind3               20 740  8.65  1.38      9    8.82  1.48   2  10     8
## omind4               21 740  8.52  1.56      9    8.76  1.48   2  10     8
## omind5               22 740  8.95  1.81     10    9.38  0.00   0  10    10
## omind6               23 740  8.07  2.17      9    8.44  1.48   0  10    10
## CRT1                 24 740  0.21  0.41      0    0.14  0.00   0   1     1
## CRT2                 25 740  0.35  0.48      0    0.31  0.00   0   1     1
## CRT3                 26 740  0.27  0.44      0    0.21  0.00   0   1     1
## age                  27 740 44.05 17.55     42   43.43 22.24  18  99    81
## sex1                 28 740  1.64  0.48      2    1.67  0.00   1   2     1
## ctheory1             29 740  2.83  2.98      2    2.45  2.97   0  10    10
## ctheory2             30 740  1.88  2.54      1    1.42  1.48   0  10    10
## ctheory3             31 740  1.37  2.17      0    0.90  0.00   0  10    10
## ctheory4             32 740  1.77  2.42      0    1.32  0.00   0  10    10
## employ_status1       33 740  2.98  1.79      3    2.86  2.97   1   6     5
## contact2r            34 740  8.73  2.10     10    9.24  0.00   0  10    10
## ccrt                 35 740  0.83  1.01      0    0.66  0.00   0   3     3
##                     skew kurtosis   se
## country*             NaN      NaN 0.00
## contact1           -1.78     3.43 0.04
## contact2            2.09     4.13 0.08
## contact3           -1.85     3.59 0.07
## contact4           -1.79     3.24 0.04
## contact5           -5.64    41.16 0.04
## hygiene1           -1.46     2.29 0.06
## hygiene2           -1.44     1.94 0.06
## hygiene3           -1.98     4.44 0.07
## hygiene4           -1.13     0.68 0.09
## hygiene5           -1.82     3.66 0.07
## psupport1          -1.63     2.32 0.05
## psupport2          -1.70     2.47 0.06
## psupport3          -1.13     0.42 0.09
## psupport4          -2.05     4.12 0.05
## psupport5          -2.08     4.14 0.06
## political_ideology -0.08     0.51 0.07
## omind1             -0.59    -0.30 0.09
## omind2             -1.60     4.12 0.05
## omind3             -1.01     1.10 0.05
## omind4             -1.19     1.62 0.06
## omind5             -2.56     7.29 0.07
## omind6             -1.36     1.61 0.08
## CRT1                1.44     0.06 0.01
## CRT2                0.63    -1.60 0.02
## CRT3                1.03    -0.94 0.02
## age                 0.26    -1.02 0.65
## sex1               -0.57    -1.67 0.02
## ctheory1            0.74    -0.66 0.11
## ctheory2            1.28     0.56 0.09
## ctheory3            1.72     2.19 0.08
## ctheory4            1.30     0.69 0.09
## employ_status1      0.26    -1.40 0.07
## contact2r          -2.09     4.13 0.08
## ccrt                0.96    -0.28 0.04
## ------------------------------------------------------------ 
## group: CHE
##                    vars   n  mean    sd median trimmed   mad min max range
## country*              1 801  1.00  0.00      1    1.00  0.00   1   1     0
## contact1              2 801  8.34  2.16      9    8.75  1.48   0  10    10
## contact2              3 801  2.39  2.94      1    1.90  1.48   0  10    10
## contact3              4 801  8.27  2.19      9    8.67  1.48   0  10    10
## contact4              5 801  8.83  1.62     10    9.16  0.00   1  10     9
## contact5              6 801  9.57  1.17     10    9.88  0.00   0  10    10
## hygiene1              7 801  7.93  2.42      9    8.32  1.48   0  10    10
## hygiene2              8 801  7.97  2.35      9    8.34  1.48   0  10    10
## hygiene3              9 801  8.55  2.16     10    9.00  0.00   0  10    10
## hygiene4             10 801  5.30  3.11      5    5.37  2.97   0  10    10
## hygiene5             11 801  8.44  2.20      9    8.86  1.48   0  10    10
## psupport1            12 801  7.37  2.82      8    7.81  2.97   0  10    10
## psupport2            13 801  7.92  2.63      9    8.40  1.48   0  10    10
## psupport3            14 801  6.30  3.17      7    6.60  2.97   0  10    10
## psupport4            15 801  8.51  2.34     10    9.05  0.00   0  10    10
## psupport5            16 801  8.29  2.54     10    8.83  0.00   0  10    10
## political_ideology   17 801  5.04  2.11      5    5.03  1.48   0  10    10
## omind1               18 801  6.82  2.73      7    7.09  2.97   0  10    10
## omind2               19 801  9.06  1.34     10    9.31  0.00   2  10     8
## omind3               20 801  8.84  1.41      9    9.06  1.48   2  10     8
## omind4               21 801  8.70  1.60      9    8.99  1.48   1  10     9
## omind5               22 801  8.33  2.78     10    9.00  0.00   0  10    10
## omind6               23 801  7.63  2.54      8    7.97  2.97   0  10    10
## CRT1                 24 801  0.26  0.44      0    0.20  0.00   0   1     1
## CRT2                 25 801  0.54  0.50      1    0.55  0.00   0   1     1
## CRT3                 26 801  0.31  0.46      0    0.27  0.00   0   1     1
## age                  27 801 49.33 16.72     51   49.45 19.27  18 100    82
## sex1                 28 801  1.52  0.50      2    1.52  0.00   1   2     1
## ctheory1             29 801  3.05  3.23      2    2.66  2.97   0  10    10
## ctheory2             30 801  2.24  2.91      0    1.75  0.00   0  10    10
## ctheory3             31 801  1.80  2.71      0    1.27  0.00   0  10    10
## ctheory4             32 801  2.22  2.94      0    1.71  0.00   0  10    10
## employ_status1       33 801  2.94  1.89      2    2.80  1.48   1   6     5
## contact2r            34 801  7.61  2.94      9    8.10  1.48   0  10    10
## ccrt                 35 801  1.12  1.06      1    1.02  1.48   0   3     3
##                     skew kurtosis   se
## country*             NaN      NaN 0.00
## contact1           -1.62     2.55 0.08
## contact2            1.10     0.03 0.10
## contact3           -1.45     1.67 0.08
## contact4           -1.75     3.27 0.06
## contact5           -4.01    20.31 0.04
## hygiene1           -1.32     1.42 0.09
## hygiene2           -1.31     1.50 0.08
## hygiene3           -1.83     3.24 0.08
## hygiene4           -0.23    -0.96 0.11
## hygiene5           -1.69     2.72 0.08
## psupport1          -1.01     0.20 0.10
## psupport2          -1.37     1.22 0.09
## psupport3          -0.54    -0.83 0.11
## psupport4          -1.98     3.60 0.08
## psupport5          -1.73     2.42 0.09
## political_ideology  0.03     0.20 0.07
## omind1             -0.55    -0.49 0.10
## omind2             -1.68     3.11 0.05
## omind3             -1.45     2.50 0.05
## omind4             -1.44     2.16 0.06
## omind5             -1.77     1.98 0.10
## omind6             -0.94     0.04 0.09
## CRT1                1.09    -0.82 0.02
## CRT2               -0.16    -1.98 0.02
## CRT3                0.80    -1.37 0.02
## age                -0.04    -0.87 0.59
## sex1               -0.07    -2.00 0.02
## ctheory1            0.63    -0.93 0.11
## ctheory2            1.02    -0.21 0.10
## ctheory3            1.35     0.62 0.10
## ctheory4            1.06    -0.13 0.10
## employ_status1      0.36    -1.51 0.07
## contact2r          -1.10     0.03 0.10
## ccrt                0.52    -0.99 0.04
## ------------------------------------------------------------ 
## group: DEU
##                    vars    n  mean    sd median trimmed   mad min max range
## country*              1 1117  1.00  0.00      1    1.00  0.00   1   1     0
## contact1              2 1117  8.41  1.96      9    8.77  1.48   0  10    10
## contact2              3 1117  2.24  2.77      1    1.76  1.48   0  10    10
## contact3              4 1117  8.03  2.20      9    8.37  1.48   0  10    10
## contact4              5 1117  8.72  1.66      9    9.04  1.48   1  10     9
## contact5              6 1117  9.64  1.12     10    9.93  0.00   0  10    10
## hygiene1              7 1117  8.20  2.15      9    8.54  1.48   0  10    10
## hygiene2              8 1117  8.40  2.07      9    8.77  1.48   0  10    10
## hygiene3              9 1117  8.90  1.84     10    9.33  0.00   0  10    10
## hygiene4             10 1117  4.52  3.28      5    4.40  4.45   0  10    10
## hygiene5             11 1117  8.58  1.94      9    8.95  1.48   0  10    10
## psupport1            12 1117  7.06  2.68      8    7.38  2.97   0  10    10
## psupport2            13 1117  7.48  2.69      8    7.90  2.97   0  10    10
## psupport3            14 1117  4.54  3.27      5    4.44  4.45   0  10    10
## psupport4            15 1117  8.58  2.10     10    9.03  0.00   0  10    10
## psupport5            16 1117  8.27  2.40      9    8.75  1.48   0  10    10
## political_ideology   17 1117  4.69  1.73      5    4.70  1.48   0  10    10
## omind1               18 1117  7.99  2.17      9    8.29  1.48   0  10    10
## omind2               19 1117  9.21  1.19     10    9.45  0.00   2  10     8
## omind3               20 1117  8.30  1.65      9    8.51  1.48   1  10     9
## omind4               21 1117  8.29  1.84      9    8.58  1.48   0  10    10
## omind5               22 1117  8.85  2.02     10    9.36  0.00   0  10    10
## omind6               23 1117  7.51  2.31      8    7.77  2.97   0  10    10
## CRT1                 24 1117  0.26  0.44      0    0.20  0.00   0   1     1
## CRT2                 25 1117  0.60  0.49      1    0.62  0.00   0   1     1
## CRT3                 26 1117  0.39  0.49      0    0.36  0.00   0   1     1
## age                  27 1117 50.07 16.15     52   50.77 20.76  18  83    65
## sex1                 28 1117  1.49  0.50      1    1.49  0.00   1   2     1
## ctheory1             29 1117  2.35  2.88      1    1.88  1.48   0  10    10
## ctheory2             30 1117  1.41  2.44      0    0.85  0.00   0  10    10
## ctheory3             31 1117  1.22  2.20      0    0.71  0.00   0  10    10
## ctheory4             32 1117  1.67  2.59      0    1.12  0.00   0  10    10
## employ_status1       33 1117  3.04  1.89      3    2.96  2.97   1   6     5
## contact2r            34 1117  7.76  2.77      9    8.24  1.48   0  10    10
## ccrt                 35 1117  1.25  1.07      1    1.19  1.48   0   3     3
##                     skew kurtosis   se
## country*             NaN      NaN 0.00
## contact1           -1.50     2.18 0.06
## contact2            1.19     0.32 0.08
## contact3           -1.25     1.22 0.07
## contact4           -1.68     3.03 0.05
## contact5           -4.77    28.05 0.03
## hygiene1           -1.39     1.94 0.06
## hygiene2           -1.62     2.86 0.06
## hygiene3           -2.15     4.87 0.06
## hygiene4            0.06    -1.17 0.10
## hygiene5           -1.62     2.57 0.06
## psupport1          -0.77    -0.14 0.08
## psupport2          -1.06     0.31 0.08
## psupport3           0.12    -1.23 0.10
## psupport4          -1.84     3.34 0.06
## psupport5          -1.58     1.92 0.07
## political_ideology -0.02     0.60 0.05
## omind1             -1.04     0.39 0.07
## omind2             -2.09     5.69 0.04
## omind3             -1.03     1.07 0.05
## omind4             -1.29     1.78 0.06
## omind5             -2.29     5.14 0.06
## omind6             -0.76    -0.23 0.07
## CRT1                1.07    -0.85 0.01
## CRT2               -0.40    -1.84 0.01
## CRT3                0.46    -1.79 0.01
## age                -0.31    -1.09 0.48
## sex1                0.04    -2.00 0.01
## ctheory1            1.05    -0.03 0.09
## ctheory2            1.81     2.34 0.07
## ctheory3            1.94     2.94 0.07
## ctheory4            1.57     1.44 0.08
## employ_status1      0.15    -1.68 0.06
## contact2r          -1.19     0.32 0.08
## ccrt                0.36    -1.13 0.03
## ------------------------------------------------------------ 
## group: GRC
##                    vars   n  mean    sd median trimmed  mad min max range  skew
## country*              1 479  1.00  0.00      1    1.00 0.00   1   1     0   NaN
## contact1              2 479  9.01  1.35      9    9.26 1.48   2  10     8 -1.78
## contact2              3 479  1.80  2.26      1    1.39 1.48   0  10    10  1.33
## contact3              4 479  8.17  2.12      9    8.54 1.48   0  10    10 -1.52
## contact4              5 479  8.50  1.96      9    8.88 1.48   1  10     9 -1.64
## contact5              6 479  9.23  1.58     10    9.61 0.00   0  10    10 -3.12
## hygiene1              7 479  7.81  2.08      8    8.09 1.48   0  10    10 -1.30
## hygiene2              8 479  8.01  2.00      8    8.30 1.48   0  10    10 -1.35
## hygiene3              9 479  9.45  1.08     10    9.70 0.00   4  10     6 -2.20
## hygiene4             10 479  7.08  2.87      8    7.48 2.97   0  10    10 -0.93
## hygiene5             11 479  8.83  1.82     10    9.23 0.00   0  10    10 -2.16
## psupport1            12 479  9.18  1.21     10    9.43 0.00   3  10     7 -1.67
## psupport2            13 479  9.36  1.04     10    9.58 0.00   4  10     6 -1.76
## psupport3            14 479  6.87  2.94      8    7.16 2.97   0  10    10 -0.61
## psupport4            15 479  9.01  1.46     10    9.31 0.00   3  10     7 -1.75
## psupport5            16 479  9.11  1.58     10    9.48 0.00   2  10     8 -2.19
## political_ideology   17 479  4.30  1.95      4    4.31 1.48   0  10    10  0.00
## omind1               18 479  6.56  2.29      7    6.71 1.48   0  10    10 -0.65
## omind2               19 479  8.68  1.97      9    9.10 1.48   0  10    10 -2.64
## omind3               20 479  9.09  1.05      9    9.24 1.48   5  10     5 -1.00
## omind4               21 479  8.97  1.23      9    9.16 1.48   3  10     7 -1.31
## omind5               22 479  9.21  1.16     10    9.44 0.00   3  10     7 -1.83
## omind6               23 479  8.13  1.84      8    8.39 1.48   1  10     9 -1.17
## CRT1                 24 479  0.58  0.49      1    0.60 0.00   0   1     1 -0.33
## CRT2                 25 479  0.62  0.49      1    0.64 0.00   0   1     1 -0.47
## CRT3                 26 479  0.62  0.49      1    0.65 0.00   0   1     1 -0.49
## age                  27 479 30.05 11.72     26   27.83 5.93  18  80    62  1.92
## sex1                 28 479  1.38  0.49      1    1.35 0.00   1   2     1  0.49
## ctheory1             29 479  2.52  2.72      1    2.16 1.48   0  10    10  0.84
## ctheory2             30 479  1.76  2.33      1    1.34 1.48   0  10    10  1.39
## ctheory3             31 479  1.67  2.35      0    1.21 0.00   0  10    10  1.49
## ctheory4             32 479  2.17  2.54      1    1.79 1.48   0  10    10  1.00
## employ_status1       33 479  2.71  1.45      3    2.65 1.48   1   6     5  0.10
## contact2r            34 479  8.20  2.26      9    8.61 1.48   0  10    10 -1.33
## ccrt                 35 479  1.82  1.18      2    1.90 1.48   0   3     3 -0.43
##                    kurtosis   se
## country*                NaN 0.00
## contact1               3.74 0.06
## contact2               0.82 0.10
## contact3               2.40 0.10
## contact4               2.46 0.09
## contact5              11.95 0.07
## hygiene1               2.14 0.09
## hygiene2               2.22 0.09
## hygiene3               4.61 0.05
## hygiene4              -0.05 0.13
## hygiene5               5.25 0.08
## psupport1              2.85 0.06
## psupport2              2.85 0.05
## psupport3             -0.78 0.13
## psupport4              2.89 0.07
## psupport5              4.70 0.07
## political_ideology    -0.21 0.09
## omind1                 0.00 0.10
## omind2                 7.88 0.09
## omind3                 0.33 0.05
## omind4                 1.78 0.06
## omind5                 4.02 0.05
## omind6                 1.35 0.08
## CRT1                  -1.89 0.02
## CRT2                  -1.78 0.02
## CRT3                  -1.76 0.02
## age                    3.84 0.54
## sex1                  -1.76 0.02
## ctheory1              -0.37 0.12
## ctheory2               1.35 0.11
## ctheory3               1.50 0.11
## ctheory4              -0.03 0.12
## employ_status1        -1.26 0.07
## contact2r              0.82 0.10
## ccrt                  -1.34 0.05
## ------------------------------------------------------------ 
## group: IRQ
##                    vars   n  mean    sd median trimmed   mad min max range
## country*              1 320  1.00  0.00    1.0    1.00  0.00   1   1     0
## contact1              2 320  8.53  1.96    9.0    8.93  1.48   2  10     8
## contact2              3 320  5.44  3.48    5.5    5.55  5.19   0  10    10
## contact3              4 320  7.98  2.75    9.0    8.57  1.48   0  10    10
## contact4              5 320  7.53  2.86    9.0    8.03  1.48   0  10    10
## contact5              6 320  8.17  2.53    9.0    8.68  1.48   0  10    10
## hygiene1              7 320  7.87  2.15    8.0    8.16  2.97   0  10    10
## hygiene2              8 320  8.05  2.12    9.0    8.37  1.48   0  10    10
## hygiene3              9 320  8.59  2.03   10.0    9.01  0.00   1  10     9
## hygiene4             10 320  6.65  3.08    7.0    6.97  4.45   0  10    10
## hygiene5             11 320  8.26  2.28    9.0    8.68  1.48   0  10    10
## psupport1            12 320  8.49  2.26   10.0    8.96  0.00   0  10    10
## psupport2            13 320  9.07  1.84   10.0    9.55  0.00   0  10    10
## psupport3            14 320  7.76  2.90    9.0    8.29  1.48   0  10    10
## psupport4            15 320  8.88  1.88   10.0    9.31  0.00   0  10    10
## psupport5            16 320  8.72  2.13   10.0    9.21  0.00   0  10    10
## political_ideology   17 320  5.26  2.74    5.0    5.30  1.48   0  10    10
## omind1               18 320  5.53  3.23    5.0    5.64  4.45   0  10    10
## omind2               19 320  8.86  1.75   10.0    9.24  0.00   1  10     9
## omind3               20 320  8.88  1.54    9.0    9.18  1.48   2  10     8
## omind4               21 320  8.72  1.78    9.0    9.09  1.48   1  10     9
## omind5               22 320  7.48  3.31    9.0    8.04  1.48   0  10    10
## omind6               23 320  6.11  3.29    7.0    6.34  4.45   0  10    10
## CRT1                 24 320  0.12  0.33    0.0    0.03  0.00   0   1     1
## CRT2                 25 320  0.17  0.38    0.0    0.09  0.00   0   1     1
## CRT3                 26 320  0.06  0.24    0.0    0.00  0.00   0   1     1
## age                  27 320 32.40 13.78   28.0   30.53 11.12  18 100    82
## sex1                 28 320  1.47  0.50    1.0    1.46  0.00   1   2     1
## ctheory1             29 320  5.01  3.31    5.0    5.02  4.45   0  10    10
## ctheory2             30 320  4.35  3.39    5.0    4.20  4.45   0  10    10
## ctheory3             31 320  4.76  3.47    5.0    4.70  4.45   0  10    10
## ctheory4             32 320  4.59  3.39    5.0    4.50  4.45   0  10    10
## employ_status1       33 320  3.01  1.58    3.0    2.89  1.48   1   6     5
## contact2r            34 320  4.56  3.48    4.5    4.45  5.19   0  10    10
## ccrt                 35 320  0.36  0.70    0.0    0.20  0.00   0   3     3
##                     skew kurtosis   se
## country*             NaN      NaN 0.00
## contact1           -1.60     2.00 0.11
## contact2           -0.15    -1.38 0.19
## contact3           -1.55     1.41 0.15
## contact4           -1.19     0.45 0.16
## contact5           -1.49     1.33 0.14
## hygiene1           -1.09     1.06 0.12
## hygiene2           -1.08     0.65 0.12
## hygiene3           -1.64     2.18 0.11
## hygiene4           -0.61    -0.74 0.17
## hygiene5           -1.58     2.25 0.13
## psupport1          -1.73     2.56 0.13
## psupport2          -2.57     6.95 0.10
## psupport3          -1.20     0.37 0.16
## psupport4          -2.09     4.46 0.11
## psupport5          -2.01     3.82 0.12
## political_ideology -0.10    -0.38 0.15
## omind1             -0.16    -1.23 0.18
## omind2             -2.05     4.69 0.10
## omind3             -1.98     4.49 0.09
## omind4             -1.78     3.02 0.10
## omind5             -1.11    -0.25 0.19
## omind6             -0.38    -1.15 0.18
## CRT1                2.26     3.10 0.02
## CRT2                1.70     0.90 0.02
## CRT3                3.60    10.98 0.01
## age                 1.83     5.44 0.77
## sex1                0.14    -1.99 0.03
## ctheory1           -0.13    -1.22 0.19
## ctheory2            0.15    -1.23 0.19
## ctheory3           -0.05    -1.35 0.19
## ctheory4            0.00    -1.29 0.19
## employ_status1      0.25    -0.94 0.09
## contact2r           0.15    -1.38 0.19
## ccrt                2.00     3.48 0.04
## ------------------------------------------------------------ 
## group: ISR
##                    vars   n  mean    sd median trimmed   mad min max range
## country*              1 994  1.00  0.00      1    1.00  0.00   1   1     0
## contact1              2 994  8.75  1.69      9    9.08  1.48   1  10     9
## contact2              3 994  1.89  2.61      1    1.34  1.48   0  10    10
## contact3              4 994  8.00  2.59      9    8.54  1.48   0  10    10
## contact4              5 994  8.77  1.66      9    9.10  1.48   1  10     9
## contact5              6 994  9.52  1.38     10    9.89  0.00   0  10    10
## hygiene1              7 994  8.55  1.73      9    8.85  1.48   0  10    10
## hygiene2              8 994  8.53  1.73      9    8.81  1.48   0  10    10
## hygiene3              9 994  9.05  1.58     10    9.41  0.00   1  10     9
## hygiene4             10 994  6.30  3.06      7    6.55  2.97   0  10    10
## hygiene5             11 994  8.04  2.40      9    8.46  1.48   0  10    10
## psupport1            12 994  6.99  2.97      8    7.34  2.97   0  10    10
## psupport2            13 994  7.88  2.60      9    8.34  1.48   0  10    10
## psupport3            14 994  6.43  3.23      7    6.73  4.45   0  10    10
## psupport4            15 994  8.16  2.43      9    8.64  1.48   0  10    10
## psupport5            16 994  6.78  3.12      8    7.11  2.97   0  10    10
## political_ideology   17 994  5.65  2.81      6    5.74  2.97   0  10    10
## omind1               18 994  7.17  2.39      8    7.44  2.22   0  10    10
## omind2               19 994  8.00  2.93      9    8.68  1.48   0  10    10
## omind3               20 994  9.01  1.25      9    9.23  1.48   3  10     7
## omind4               21 994  8.95  1.34      9    9.19  1.48   3  10     7
## omind5               22 994  8.89  2.12     10    9.45  0.00   0  10    10
## omind6               23 994  8.03  2.45      9    8.49  1.48   0  10    10
## CRT1                 24 994  0.42  0.49      0    0.40  0.00   0   1     1
## CRT2                 25 994  0.44  0.50      0    0.43  0.00   0   1     1
## CRT3                 26 994  0.38  0.49      0    0.35  0.00   0   1     1
## age                  27 994 41.27 15.05     40   40.65 17.79  18  74    56
## sex1                 28 994  1.52  0.50      2    1.52  0.00   1   2     1
## ctheory1             29 994  4.23  3.14      5    4.09  4.45   0  10    10
## ctheory2             30 994  2.11  2.52      1    1.72  1.48   0  10    10
## ctheory3             31 994  1.77  2.50      0    1.28  0.00   0  10    10
## ctheory4             32 994  1.96  2.53      1    1.51  1.48   0  10    10
## employ_status1       33 994  2.86  1.92      2    2.70  1.48   1   6     5
## contact2r            34 994  8.11  2.61      9    8.66  1.48   0  10    10
## ccrt                 35 994  1.24  1.13      1    1.17  1.48   0   3     3
##                     skew kurtosis   se
## country*             NaN      NaN 0.00
## contact1           -1.73     3.17 0.05
## contact2            1.59     1.66 0.08
## contact3           -1.53     1.49 0.08
## contact4           -1.73     3.17 0.05
## contact5           -4.13    19.61 0.04
## hygiene1           -1.45     2.38 0.05
## hygiene2           -1.36     1.99 0.05
## hygiene3           -1.99     3.94 0.05
## hygiene4           -0.47    -0.85 0.10
## hygiene5           -1.28     0.93 0.08
## psupport1          -0.71    -0.65 0.09
## psupport2          -1.24     0.61 0.08
## psupport3          -0.48    -1.03 0.10
## psupport4          -1.52     1.79 0.08
## psupport5          -0.64    -0.84 0.10
## political_ideology -0.24    -0.91 0.09
## omind1             -0.85     0.17 0.08
## omind2             -1.74     1.80 0.09
## omind3             -1.42     2.30 0.04
## omind4             -1.50     2.48 0.04
## omind5             -2.49     5.88 0.07
## omind6             -1.46     1.47 0.08
## CRT1                0.34    -1.89 0.02
## CRT2                0.23    -1.95 0.02
## CRT3                0.50    -1.75 0.02
## age                 0.31    -1.06 0.48
## sex1               -0.08    -2.00 0.02
## ctheory1            0.14    -1.15 0.10
## ctheory2            1.06     0.11 0.08
## ctheory3            1.47     1.36 0.08
## ctheory4            1.23     0.54 0.08
## employ_status1      0.54    -1.25 0.06
## contact2r          -1.59     1.66 0.08
## ccrt                0.31    -1.33 0.04
## ------------------------------------------------------------ 
## group: JPN
##                    vars   n  mean    sd median trimmed   mad min max range
## country*              1 747  1.00  0.00      1    1.00  0.00   1   1     0
## contact1              2 747  8.18  1.81      8    8.43  1.48   0  10    10
## contact2              3 747  1.43  1.97      1    1.04  1.48   0  10    10
## contact3              4 747  7.28  2.17      7    7.44  2.97   0  10    10
## contact4              5 747  7.18  1.87      7    7.23  1.48   0  10    10
## contact5              6 747  8.56  2.11     10    8.96  0.00   0  10    10
## hygiene1              7 747  7.04  2.10      7    7.17  1.48   0  10    10
## hygiene2              8 747  7.16  2.18      7    7.34  1.48   0  10    10
## hygiene3              9 747  8.12  2.09      9    8.42  1.48   0  10    10
## hygiene4             10 747  4.57  2.82      5    4.51  2.97   0  10    10
## hygiene5             11 747  6.76  2.59      7    7.00  2.97   0  10    10
## psupport1            12 747  7.34  2.12      7    7.47  2.97   0  10    10
## psupport2            13 747  7.17  2.17      7    7.28  2.97   0  10    10
## psupport3            14 747  6.09  2.41      6    6.14  2.97   0  10    10
## psupport4            15 747  8.22  1.90      9    8.47  1.48   0  10    10
## psupport5            16 747  8.35  1.90      9    8.63  1.48   0  10    10
## political_ideology   17 747  5.14  1.20      5    5.13  0.00   0  10    10
## omind1               18 747  5.47  2.12      5    5.46  1.48   0  10    10
## omind2               19 747  7.92  1.69      8    8.04  1.48   2  10     8
## omind3               20 747  7.61  1.79      8    7.70  1.48   2  10     8
## omind4               21 747  7.43  1.87      7    7.52  1.48   2  10     8
## omind5               22 747  7.70  2.17      8    7.95  2.97   0  10    10
## omind6               23 747  5.88  2.22      6    5.91  1.48   0  10    10
## CRT1                 24 747  0.40  0.49      0    0.38  0.00   0   1     1
## CRT2                 25 747  0.30  0.46      0    0.25  0.00   0   1     1
## CRT3                 26 747  0.31  0.46      0    0.27  0.00   0   1     1
## age                  27 747 50.43 14.61     52   51.16 16.31  18  74    56
## sex1                 28 747  1.52  0.50      2    1.53  0.00   1   2     1
## ctheory1             29 747  4.69  2.70      5    4.69  2.97   0  10    10
## ctheory2             30 747  3.66  2.53      4    3.56  2.97   0  10    10
## ctheory3             31 747  2.33  2.17      2    2.15  2.97   0  10    10
## ctheory4             32 747  2.83  2.33      3    2.66  2.97   0  10    10
## employ_status1       33 747  2.52  1.69      2    2.31  1.48   1   6     5
## contact2r            34 747  8.57  1.97      9    8.96  1.48   0  10    10
## ccrt                 35 747  1.02  0.89      1    0.96  1.48   0   3     3
##                     skew kurtosis   se
## country*             NaN      NaN 0.00
## contact1           -1.20     1.76 0.07
## contact2            1.67     2.73 0.07
## contact3           -0.51    -0.29 0.08
## contact4           -0.29    -0.29 0.07
## contact5           -1.61     2.37 0.08
## hygiene1           -0.54     0.47 0.08
## hygiene2           -0.67     0.52 0.08
## hygiene3           -1.21     1.58 0.08
## hygiene4            0.06    -0.70 0.10
## hygiene5           -0.58    -0.18 0.09
## psupport1          -0.40    -0.49 0.08
## psupport2          -0.25    -0.85 0.08
## psupport3          -0.17    -0.55 0.09
## psupport4          -0.95     0.50 0.07
## psupport5          -1.02     0.33 0.07
## political_ideology  0.04     3.20 0.04
## omind1             -0.04     0.02 0.08
## omind2             -0.46    -0.33 0.06
## omind3             -0.34    -0.57 0.07
## omind4             -0.30    -0.61 0.07
## omind5             -0.77     0.03 0.08
## omind6             -0.10    -0.22 0.08
## CRT1                0.39    -1.85 0.02
## CRT2                0.86    -1.27 0.02
## CRT3                0.80    -1.37 0.02
## age                -0.36    -0.93 0.53
## sex1               -0.08    -2.00 0.02
## ctheory1           -0.03    -0.58 0.10
## ctheory2            0.18    -0.62 0.09
## ctheory3            0.52    -0.66 0.08
## ctheory4            0.36    -0.73 0.09
## employ_status1      0.77    -0.75 0.06
## contact2r          -1.67     2.73 0.07
## ccrt                0.42    -0.76 0.03
## ------------------------------------------------------------ 
## group: KOR
##                    vars   n  mean    sd median trimmed   mad min max range
## country*              1 253  1.00  0.00      1    1.00  0.00   1   1     0
## contact1              2 253  8.23  1.61      8    8.40  1.48   4  10     6
## contact2              3 253  3.21  2.42      3    3.01  2.97   0   9     9
## contact3              4 253  7.48  2.05      8    7.68  1.48   1  10     9
## contact4              5 253  7.78  1.88      8    7.96  1.48   1  10     9
## contact5              6 253  7.90  2.23      9    8.24  1.48   0  10    10
## hygiene1              7 253  8.08  1.67      8    8.21  1.48   4  10     6
## hygiene2              8 253  8.32  1.63      9    8.49  1.48   4  10     6
## hygiene3              9 253  8.62  1.53      9    8.85  1.48   4  10     6
## hygiene4             10 253  6.96  2.15      7    7.04  2.97   0  10    10
## hygiene5             11 253  8.36  1.68      9    8.58  1.48   3  10     7
## psupport1            12 253  7.91  1.80      8    8.06  1.48   0  10    10
## psupport2            13 253  7.08  2.08      7    7.21  2.97   0  10    10
## psupport3            14 253  6.37  2.37      7    6.41  2.97   0  10    10
## psupport4            15 253  7.70  2.02      8    7.88  2.97   2  10     8
## psupport5            16 253  8.32  1.77      9    8.55  1.48   2  10     8
## political_ideology   17 253  5.03  2.16      5    5.05  1.48   0  10    10
## omind1               18 253  5.38  2.16      5    5.43  2.97   0  10    10
## omind2               19 253  7.87  1.70      8    8.01  1.48   1  10     9
## omind3               20 253  7.97  1.61      8    8.11  1.48   2  10     8
## omind4               21 253  7.98  1.64      8    8.11  1.48   2  10     8
## omind5               22 253  7.90  2.27      8    8.29  2.97   0  10    10
## omind6               23 253  6.49  2.21      7    6.54  2.97   0  10    10
## CRT1                 24 253  0.28  0.45      0    0.22  0.00   0   1     1
## CRT2                 25 253  0.45  0.50      0    0.43  0.00   0   1     1
## CRT3                 26 253  0.19  0.40      0    0.12  0.00   0   1     1
## age                  27 253 46.16 12.87     47   46.73 14.83  18  72    54
## sex1                 28 253  1.47  0.50      1    1.46  0.00   1   2     1
## ctheory1             29 253  4.01  2.92      4    3.89  4.45   0  10    10
## ctheory2             30 253  2.63  2.40      2    2.36  2.97   0  10    10
## ctheory3             31 253  2.59  2.45      2    2.29  2.97   0  10    10
## ctheory4             32 253  2.62  2.48      2    2.31  2.97   0  10    10
## employ_status1       33 253  2.49  1.93      1    2.24  0.00   1   6     5
## contact2r            34 253  6.79  2.42      7    6.99  2.97   1  10     9
## ccrt                 35 253  0.92  1.02      1    0.77  1.48   0   3     3
##                     skew kurtosis   se
## country*             NaN      NaN 0.00
## contact1           -0.66    -0.49 0.10
## contact2            0.60    -0.39 0.15
## contact3           -0.78     0.03 0.13
## contact4           -0.76     0.17 0.12
## contact5           -1.19     1.02 0.14
## hygiene1           -0.39    -0.95 0.11
## hygiene2           -0.64    -0.64 0.10
## hygiene3           -0.93    -0.03 0.10
## hygiene4           -0.27    -0.68 0.13
## hygiene5           -0.84    -0.20 0.11
## psupport1          -0.74     0.51 0.11
## psupport2          -0.55     0.01 0.13
## psupport3          -0.18    -0.94 0.15
## psupport4          -0.60    -0.51 0.13
## psupport5          -0.91     0.13 0.11
## political_ideology -0.08     0.02 0.14
## omind1             -0.19    -0.25 0.14
## omind2             -0.78     0.75 0.11
## omind3             -0.85     1.03 0.10
## omind4             -0.66     0.17 0.10
## omind5             -1.29     1.18 0.14
## omind6             -0.18    -0.58 0.14
## CRT1                0.99    -1.02 0.03
## CRT2                0.21    -1.96 0.03
## CRT3                1.54     0.38 0.02
## age                -0.33    -0.71 0.81
## sex1                0.12    -1.99 0.03
## ctheory1            0.14    -1.03 0.18
## ctheory2            0.70    -0.26 0.15
## ctheory3            0.85     0.18 0.15
## ctheory4            0.89     0.25 0.16
## employ_status1      0.85    -0.96 0.12
## contact2r          -0.60    -0.39 0.15
## ccrt                0.79    -0.61 0.06
## ------------------------------------------------------------ 
## group: NGA
##                    vars   n  mean    sd median trimmed   mad min max range
## country*              1 403  1.00  0.00      1    1.00  0.00   1   1     0
## contact1              2 403  9.02  1.49     10    9.33  0.00   1  10     9
## contact2              3 403  2.05  2.54      1    1.56  1.48   0  10    10
## contact3              4 403  7.96  2.30      9    8.35  1.48   0  10    10
## contact4              5 403  8.42  2.02      9    8.81  1.48   0  10    10
## contact5              6 403  8.80  2.20     10    9.36  0.00   0  10    10
## hygiene1              7 403  8.67  1.67      9    8.98  1.48   3  10     7
## hygiene2              8 403  8.78  1.56      9    9.08  1.48   3  10     7
## hygiene3              9 403  9.06  1.47     10    9.38  0.00   2  10     8
## hygiene4             10 403  7.38  2.61      8    7.71  2.97   0  10    10
## hygiene5             11 403  7.96  2.42      9    8.37  1.48   0  10    10
## psupport1            12 403  8.24  2.50      9    8.77  1.48   0  10    10
## psupport2            13 403  8.69  2.16     10    9.21  0.00   0  10    10
## psupport3            14 403  8.28  2.38      9    8.76  1.48   0  10    10
## psupport4            15 403  8.57  2.24     10    9.09  0.00   0  10    10
## psupport5            16 403  8.80  2.05     10    9.28  0.00   0  10    10
## political_ideology   17 403  6.16  2.16      6    6.20  1.48   0  10    10
## omind1               18 403  5.11  3.12      5    5.13  4.45   0  10    10
## omind2               19 403  9.45  0.93     10    9.63  0.00   5  10     5
## omind3               20 403  9.37  0.99     10    9.56  0.00   4  10     6
## omind4               21 403  9.32  1.03     10    9.51  0.00   5  10     5
## omind5               22 403  7.41  3.00      9    7.89  1.48   0  10    10
## omind6               23 403  7.30  2.77      8    7.70  2.97   0  10    10
## CRT1                 24 403  0.18  0.39      0    0.11  0.00   0   1     1
## CRT2                 25 403  0.27  0.44      0    0.21  0.00   0   1     1
## CRT3                 26 403  0.09  0.28      0    0.00  0.00   0   1     1
## age                  27 403 32.63 11.37     30   31.31 10.38  18 100    82
## sex1                 28 403  1.49  0.50      1    1.48  0.00   1   2     1
## ctheory1             29 403  6.00  3.11      6    6.21  2.97   0  10    10
## ctheory2             30 403  4.54  3.19      5    4.43  4.45   0  10    10
## ctheory3             31 403  4.59  3.30      5    4.49  4.45   0  10    10
## ctheory4             32 403  4.13  3.08      5    3.97  4.45   0  10    10
## employ_status1       33 403  2.56  1.51      2    2.38  1.48   1   6     5
## contact2r            34 403  7.95  2.54      9    8.44  1.48   0  10    10
## ccrt                 35 403  0.54  0.80      0    0.38  0.00   0   3     3
##                     skew kurtosis   se
## country*             NaN      NaN 0.00
## contact1           -2.03     4.90 0.07
## contact2            1.45     1.36 0.13
## contact3           -1.31     1.16 0.11
## contact4           -1.66     2.72 0.10
## contact5           -2.30     4.99 0.11
## hygiene1           -1.29     0.98 0.08
## hygiene2           -1.38     1.28 0.08
## hygiene3           -2.02     4.51 0.07
## hygiene4           -0.82    -0.19 0.13
## hygiene5           -1.28     1.14 0.12
## psupport1          -1.56     1.55 0.12
## psupport2          -2.04     3.72 0.11
## psupport3          -1.55     1.71 0.12
## psupport4          -1.92     3.25 0.11
## psupport5          -2.17     4.74 0.10
## political_ideology -0.16     0.07 0.11
## omind1             -0.02    -1.11 0.16
## omind2             -1.83     3.32 0.05
## omind3             -1.77     3.57 0.05
## omind4             -1.45     1.50 0.05
## omind5             -1.03    -0.11 0.15
## omind6             -0.93    -0.15 0.14
## CRT1                1.63     0.65 0.02
## CRT2                1.03    -0.94 0.02
## CRT3                2.92     6.56 0.01
## age                 1.29     2.58 0.57
## sex1                0.05    -2.00 0.02
## ctheory1           -0.37    -0.91 0.16
## ctheory2            0.15    -1.05 0.16
## ctheory3            0.10    -1.20 0.16
## ctheory4            0.24    -1.02 0.15
## employ_status1      0.66    -0.46 0.08
## contact2r          -1.45     1.36 0.13
## ccrt                1.47     1.48 0.04
## ------------------------------------------------------------ 
## group: NZL
##                    vars   n  mean    sd median trimmed   mad min max range
## country*              1 333  1.00  0.00      1    1.00  0.00   1   1     0
## contact1              2 333  9.44  0.94     10    9.64  0.00   5  10     5
## contact2              3 333  0.80  1.89      0    0.28  0.00   0  10    10
## contact3              4 333  8.83  1.84     10    9.24  0.00   2  10     8
## contact4              5 333  9.39  1.03     10    9.60  0.00   5  10     5
## contact5              6 333  9.63  1.08     10    9.90  0.00   1  10     9
## hygiene1              7 333  7.95  2.04      8    8.21  2.97   1  10     9
## hygiene2              8 333  8.16  1.88      9    8.42  1.48   1  10     9
## hygiene3              9 333  8.45  2.01      9    8.82  1.48   0  10    10
## hygiene4             10 333  6.00  3.08      6    6.22  2.97   0  10    10
## hygiene5             11 333  8.05  2.29      9    8.44  1.48   0  10    10
## psupport1            12 333  9.24  1.19     10    9.49  0.00   3  10     7
## psupport2            13 333  9.48  0.97     10    9.70  0.00   5  10     5
## psupport3            14 333  8.22  2.45      9    8.71  1.48   0  10    10
## psupport4            15 333  9.48  0.96     10    9.70  0.00   5  10     5
## psupport5            16 333  9.43  1.00     10    9.66  0.00   5  10     5
## political_ideology   17 333  5.04  1.99      5    5.07  1.48   0  10    10
## omind1               18 333  5.88  2.70      6    6.03  2.97   0  10    10
## omind2               19 333  8.89  1.25      9    9.08  1.48   4  10     6
## omind3               20 333  8.75  1.34      9    8.94  1.48   4  10     6
## omind4               21 333  8.70  1.40      9    8.91  1.48   3  10     7
## omind5               22 333  8.61  2.33     10    9.18  0.00   0  10    10
## omind6               23 333  7.56  2.70      9    8.02  1.48   0  10    10
## CRT1                 24 333  0.22  0.42      0    0.15  0.00   0   1     1
## CRT2                 25 333  0.42  0.49      0    0.40  0.00   0   1     1
## CRT3                 26 333  0.30  0.46      0    0.25  0.00   0   1     1
## age                  27 333 49.52 16.96     48   49.49 19.27  18  89    71
## sex1                 28 333  1.46  0.50      1    1.45  0.00   1   2     1
## ctheory1             29 333  3.12  3.12      2    2.77  2.97   0  10    10
## ctheory2             30 333  2.10  2.76      1    1.62  1.48   0  10    10
## ctheory3             31 333  1.73  2.58      0    1.18  0.00   0  10    10
## ctheory4             32 333  2.01  2.74      1    1.51  1.48   0  10    10
## employ_status1       33 333  2.92  1.96      2    2.78  1.48   1   6     5
## contact2r            34 333  9.20  1.89     10    9.72  0.00   0  10    10
## ccrt                 35 333  0.95  1.03      1    0.81  1.48   0   3     3
##                     skew kurtosis   se
## country*             NaN      NaN 0.00
## contact1           -1.90     3.76 0.05
## contact2            3.04     9.17 0.10
## contact3           -1.86     3.15 0.10
## contact4           -1.80     3.00 0.06
## contact5           -4.44    23.70 0.06
## hygiene1           -1.01     0.61 0.11
## hygiene2           -1.08     0.86 0.10
## hygiene3           -1.60     2.68 0.11
## hygiene4           -0.44    -0.82 0.17
## hygiene5           -1.41     1.80 0.13
## psupport1          -1.79     3.40 0.07
## psupport2          -1.97     3.45 0.05
## psupport3          -1.53     1.69 0.13
## psupport4          -1.90     2.99 0.05
## psupport5          -1.92     3.24 0.05
## political_ideology -0.09     0.54 0.11
## omind1             -0.41    -0.60 0.15
## omind2             -1.21     1.34 0.07
## omind3             -1.01     0.58 0.07
## omind4             -1.14     1.20 0.08
## omind5             -2.07     3.62 0.13
## omind6             -1.20     0.60 0.15
## CRT1                1.33    -0.23 0.02
## CRT2                0.32    -1.90 0.03
## CRT3                0.85    -1.28 0.03
## age                 0.07    -1.00 0.93
## sex1                0.15    -1.98 0.03
## ctheory1            0.67    -0.81 0.17
## ctheory2            1.19     0.21 0.15
## ctheory3            1.59     1.52 0.14
## ctheory4            1.25     0.37 0.15
## employ_status1      0.41    -1.51 0.11
## contact2r          -3.04     9.17 0.10
## ccrt                0.71    -0.74 0.06
## ------------------------------------------------------------ 
## group: PAK
##                    vars   n  mean   sd median trimmed  mad min max range  skew
## country*              1 367  1.00 0.00      1    1.00 0.00   1   1     0   NaN
## contact1              2 367  9.00 1.41     10    9.29 0.00   4  10     6 -1.62
## contact2              3 367  2.38 2.86      1    1.96 1.48   0  10    10  1.01
## contact3              4 367  8.23 2.38      9    8.72 1.48   0  10    10 -1.63
## contact4              5 367  8.61 2.08      9    9.06 1.48   0  10    10 -2.08
## contact5              6 367  9.00 2.04     10    9.53 0.00   0  10    10 -2.62
## hygiene1              7 367  8.23 1.96      9    8.55 1.48   0  10    10 -1.45
## hygiene2              8 367  8.52 1.72      9    8.81 1.48   0  10    10 -1.55
## hygiene3              9 367  9.27 1.28     10    9.57 0.00   3  10     7 -2.03
## hygiene4             10 367  7.09 2.68      8    7.42 2.97   0  10    10 -0.82
## hygiene5             11 367  8.23 2.22      9    8.63 1.48   0  10    10 -1.48
## psupport1            12 367  8.92 1.63     10    9.25 0.00   2  10     8 -1.84
## psupport2            13 367  9.32 1.15     10    9.56 0.00   4  10     6 -1.95
## psupport3            14 367  9.03 1.61     10    9.39 0.00   0  10    10 -2.07
## psupport4            15 367  9.42 1.06     10    9.66 0.00   4  10     6 -2.20
## psupport5            16 367  9.37 1.18     10    9.65 0.00   4  10     6 -2.30
## political_ideology   17 367  5.41 2.18      5    5.48 1.48   0  10    10 -0.27
## omind1               18 367  5.29 3.19      5    5.37 4.45   0  10    10 -0.18
## omind2               19 367  9.19 1.20     10    9.42 0.00   3  10     7 -1.93
## omind3               20 367  8.94 1.42     10    9.20 0.00   3  10     7 -1.52
## omind4               21 367  9.15 1.22     10    9.39 0.00   4  10     6 -1.61
## omind5               22 367  6.66 3.13      7    6.94 4.45   0  10    10 -0.44
## omind6               23 367  6.43 3.11      7    6.69 2.97   0  10    10 -0.57
## CRT1                 24 367  0.25 0.43      0    0.19 0.00   0   1     1  1.16
## CRT2                 25 367  0.44 0.50      0    0.43 0.00   0   1     1  0.22
## CRT3                 26 367  0.25 0.43      0    0.19 0.00   0   1     1  1.16
## age                  27 367 26.97 8.41     24   25.52 4.45  18 100    82  3.15
## sex1                 28 367  1.56 0.50      2    1.57 0.00   1   2     1 -0.23
## ctheory1             29 367  5.34 3.18      6    5.42 2.97   0  10    10 -0.29
## ctheory2             30 367  4.01 3.03      4    3.86 4.45   0  10    10  0.25
## ctheory3             31 367  4.31 3.24      5    4.19 4.45   0  10    10  0.10
## ctheory4             32 367  4.68 3.16      5    4.65 4.45   0  10    10 -0.05
## employ_status1       33 367  3.04 1.49      4    3.03 1.48   1   6     5 -0.18
## contact2r            34 367  7.62 2.86      9    8.04 1.48   0  10    10 -1.01
## ccrt                 35 367  0.94 1.01      1    0.80 1.48   0   3     3  0.76
##                    kurtosis   se
## country*                NaN 0.00
## contact1               2.14 0.07
## contact2              -0.29 0.15
## contact3               2.02 0.12
## contact4               4.52 0.11
## contact5               6.85 0.11
## hygiene1               2.33 0.10
## hygiene2               3.05 0.09
## hygiene3               3.89 0.07
## hygiene4              -0.07 0.14
## hygiene5               1.74 0.12
## psupport1              3.56 0.09
## psupport2              3.98 0.06
## psupport3              4.89 0.08
## psupport4              5.07 0.06
## psupport5              5.37 0.06
## political_ideology     0.15 0.11
## omind1                -1.30 0.17
## omind2                 4.55 0.06
## omind3                 2.02 0.07
## omind4                 2.34 0.06
## omind5                -1.12 0.16
## omind6                -0.97 0.16
## CRT1                  -0.65 0.02
## CRT2                  -1.96 0.03
## CRT3                  -0.65 0.02
## age                   17.50 0.44
## sex1                  -1.95 0.03
## ctheory1              -1.05 0.17
## ctheory2              -1.07 0.16
## ctheory3              -1.24 0.17
## ctheory4              -1.17 0.16
## employ_status1        -1.03 0.08
## contact2r             -0.29 0.15
## ccrt                  -0.58 0.05
## ------------------------------------------------------------ 
## group: POL
##                    vars    n  mean    sd median trimmed   mad min max range
## country*              1 1394  1.00  0.00      1    1.00  0.00   1   1     0
## contact1              2 1394  8.23  2.17      9    8.62  1.48   0  10    10
## contact2              3 1394  2.58  2.94      1    2.14  1.48   0  10    10
## contact3              4 1394  8.23  2.18      9    8.63  1.48   0  10    10
## contact4              5 1394  8.57  1.85      9    8.92  1.48   0  10    10
## contact5              6 1394  8.88  2.07     10    9.39  0.00   0  10    10
## hygiene1              7 1394  7.98  2.18      9    8.29  1.48   0  10    10
## hygiene2              8 1394  8.05  2.16      9    8.38  1.48   0  10    10
## hygiene3              9 1394  8.72  1.86     10    9.09  0.00   0  10    10
## hygiene4             10 1394  6.35  3.15      7    6.65  2.97   0  10    10
## hygiene5             11 1394  7.48  2.78      8    7.92  2.97   0  10    10
## psupport1            12 1394  7.95  2.52      9    8.41  1.48   0  10    10
## psupport2            13 1394  7.92  2.60      9    8.39  1.48   0  10    10
## psupport3            14 1394  5.21  3.63      5    5.27  4.45   0  10    10
## psupport4            15 1394  8.26  2.45     10    8.76  0.00   0  10    10
## psupport5            16 1394  8.01  2.57      9    8.49  1.48   0  10    10
## political_ideology   17 1394  5.07  2.43      5    5.04  1.48   0  10    10
## omind1               18 1394  5.73  2.62      6    5.80  2.97   0  10    10
## omind2               19 1394  8.54  1.60      9    8.78  1.48   1  10     9
## omind3               20 1394  8.47  1.58      9    8.68  1.48   1  10     9
## omind4               21 1394  8.46  1.65      9    8.69  1.48   2  10     8
## omind5               22 1394  8.20  2.48      9    8.72  1.48   0  10    10
## omind6               23 1394  7.55  2.53      8    7.91  2.97   0  10    10
## CRT1                 24 1394  0.22  0.41      0    0.14  0.00   0   1     1
## CRT2                 25 1394  0.41  0.49      0    0.39  0.00   0   1     1
## CRT3                 26 1394  0.18  0.39      0    0.10  0.00   0   1     1
## age                  27 1394 47.61 16.99     47   47.89 20.76  18 100    82
## sex1                 28 1394  1.50  0.50      2    1.51  0.00   1   2     1
## ctheory1             29 1394  5.66  3.17      6    5.82  2.97   0  10    10
## ctheory2             30 1394  4.67  3.20      5    4.60  4.45   0  10    10
## ctheory3             31 1394  4.24  3.21      5    4.08  4.45   0  10    10
## ctheory4             32 1394  4.41  3.08      5    4.30  4.45   0  10    10
## employ_status1       33 1394  3.13  1.92      3    3.04  2.97   1   6     5
## contact2r            34 1394  7.42  2.94      9    7.86  1.48   0  10    10
## ccrt                 35 1394  0.81  0.97      1    0.65  1.48   0   3     3
##                     skew kurtosis   se
## country*             NaN      NaN 0.00
## contact1           -1.40     1.56 0.06
## contact2            0.96    -0.32 0.08
## contact3           -1.44     1.72 0.06
## contact4           -1.64     2.81 0.05
## contact5           -2.36     5.59 0.06
## hygiene1           -1.20     1.32 0.06
## hygiene2           -1.30     1.68 0.06
## hygiene3           -1.65     2.55 0.05
## hygiene4           -0.56    -0.80 0.08
## hygiene5           -1.03     0.25 0.07
## psupport1          -1.30     0.99 0.07
## psupport2          -1.26     0.79 0.07
## psupport3          -0.07    -1.41 0.10
## psupport4          -1.58     1.99 0.07
## psupport5          -1.35     1.11 0.07
## political_ideology  0.09    -0.25 0.07
## omind1             -0.25    -0.60 0.07
## omind2             -1.06     0.76 0.04
## omind3             -0.91     0.37 0.04
## omind4             -0.99     0.46 0.04
## omind5             -1.59     1.79 0.07
## omind6             -0.95     0.09 0.07
## CRT1                1.38    -0.08 0.01
## CRT2                0.37    -1.86 0.01
## CRT3                1.64     0.71 0.01
## age                -0.02    -0.99 0.45
## sex1               -0.02    -2.00 0.01
## ctheory1           -0.33    -0.95 0.08
## ctheory2            0.00    -1.12 0.09
## ctheory3            0.19    -1.12 0.09
## ctheory4            0.11    -1.01 0.08
## employ_status1      0.09    -1.64 0.05
## contact2r          -0.96    -0.32 0.08
## ccrt                0.98    -0.16 0.03
## ------------------------------------------------------------ 
## group: SGP
##                    vars   n  mean    sd median trimmed   mad min max range
## country*              1 373  1.00  0.00      1    1.00  0.00   1   1     0
## contact1              2 373  8.92  1.22      9    9.10  1.48   4  10     6
## contact2              3 373  1.34  2.30      0    0.76  0.00   0  10    10
## contact3              4 373  7.97  2.03      8    8.24  2.97   2  10     8
## contact4              5 373  8.79  1.36      9    8.99  1.48   3  10     7
## contact5              6 373  9.24  1.44     10    9.58  0.00   0  10    10
## hygiene1              7 373  7.89  1.74      8    8.03  1.48   2  10     8
## hygiene2              8 373  8.13  1.61      8    8.28  1.48   2  10     8
## hygiene3              9 373  8.51  1.71      9    8.77  1.48   2  10     8
## hygiene4             10 373  6.90  2.43      7    7.13  2.97   0  10    10
## hygiene5             11 373  6.01  3.11      7    6.24  2.97   0  10    10
## psupport1            12 373  7.80  2.08      8    8.06  1.48   0  10    10
## psupport2            13 373  8.42  1.86      9    8.72  1.48   0  10    10
## psupport3            14 373  6.81  2.79      7    7.09  2.97   0  10    10
## psupport4            15 373  8.60  1.77      9    8.91  1.48   0  10    10
## psupport5            16 373  8.78  1.57      9    9.06  1.48   2  10     8
## political_ideology   17 373  5.53  1.55      5    5.53  1.48   0  10    10
## omind1               18 373  5.00  2.61      5    5.06  2.97   0  10    10
## omind2               19 373  8.41  1.40      8    8.54  1.48   4  10     6
## omind3               20 373  8.29  1.37      8    8.40  1.48   4  10     6
## omind4               21 373  8.40  1.45      9    8.57  1.48   3  10     7
## omind5               22 373  7.55  2.61      8    7.95  2.97   0  10    10
## omind6               23 373  6.64  2.57      7    6.82  2.97   0  10    10
## CRT1                 24 373  0.46  0.50      0    0.45  0.00   0   1     1
## CRT2                 25 373  0.50  0.50      0    0.50  0.00   0   1     1
## CRT3                 26 373  0.34  0.48      0    0.30  0.00   0   1     1
## age                  27 373 43.43 13.59     46   43.35 16.31  18  73    55
## sex1                 28 373  1.51  0.50      2    1.52  0.00   1   2     1
## ctheory1             29 373  3.69  2.87      4    3.53  4.45   0  10    10
## ctheory2             30 373  3.05  2.76      2    2.79  2.97   0  10    10
## ctheory3             31 373  2.71  2.62      2    2.40  2.97   0  10    10
## ctheory4             32 373  2.92  2.68      2    2.66  2.97   0  10    10
## employ_status1       33 373  1.99  1.62      1    1.66  0.00   1   6     5
## contact2r            34 373  8.66  2.30     10    9.24  0.00   0  10    10
## ccrt                 35 373  1.30  1.18      1    1.25  1.48   0   3     3
##                     skew kurtosis   se
## country*             NaN      NaN 0.00
## contact1           -1.09     0.79 0.06
## contact2            1.95     2.82 0.12
## contact3           -0.95     0.16 0.11
## contact4           -1.14     1.13 0.07
## contact5           -2.97    11.75 0.07
## hygiene1           -0.52    -0.51 0.09
## hygiene2           -0.71     0.13 0.08
## hygiene3           -1.05     0.32 0.09
## hygiene4           -0.72     0.02 0.13
## hygiene5           -0.49    -0.88 0.16
## psupport1          -0.99     0.53 0.11
## psupport2          -1.24     1.28 0.10
## psupport3          -0.65    -0.56 0.14
## psupport4          -1.59     3.05 0.09
## psupport5          -1.46     2.06 0.08
## political_ideology -0.20     1.50 0.08
## omind1             -0.15    -0.83 0.14
## omind2             -0.62    -0.08 0.07
## omind3             -0.51    -0.30 0.07
## omind4             -0.83     0.46 0.08
## omind5             -1.05     0.30 0.14
## omind6             -0.47    -0.64 0.13
## CRT1                0.17    -1.98 0.03
## CRT2                0.01    -2.01 0.03
## CRT3                0.66    -1.57 0.02
## age                -0.01    -0.88 0.70
## sex1               -0.05    -2.00 0.03
## ctheory1            0.25    -1.04 0.15
## ctheory2            0.52    -0.85 0.14
## ctheory3            0.71    -0.49 0.14
## ctheory4            0.54    -0.77 0.14
## employ_status1      1.39     0.50 0.08
## contact2r          -1.95     2.82 0.12
## ccrt                0.29    -1.41 0.06
## ------------------------------------------------------------ 
## group: SVK
##                    vars   n  mean    sd median trimmed   mad min max range
## country*              1 834  1.00  0.00      1    1.00  0.00   1   1     0
## contact1              2 834  8.38  2.09      9    8.77  1.48   0  10    10
## contact2              3 834  2.15  2.67      1    1.69  1.48   0  10    10
## contact3              4 834  8.42  2.11      9    8.81  1.48   0  10    10
## contact4              5 834  8.31  2.22      9    8.75  1.48   0  10    10
## contact5              6 834  9.20  1.74     10    9.65  0.00   0  10    10
## hygiene1              7 834  8.18  2.04      9    8.46  1.48   0  10    10
## hygiene2              8 834  8.32  1.97      9    8.62  1.48   0  10    10
## hygiene3              9 834  9.13  1.49     10    9.48  0.00   0  10    10
## hygiene4             10 834  6.50  2.97      7    6.79  2.97   0  10    10
## hygiene5             11 834  7.59  2.87      9    8.10  1.48   0  10    10
## psupport1            12 834  7.89  2.55      9    8.35  1.48   0  10    10
## psupport2            13 834  8.32  2.31      9    8.78  1.48   0  10    10
## psupport3            14 834  5.37  3.26      5    5.46  4.45   0  10    10
## psupport4            15 834  8.29  2.38     10    8.75  0.00   0  10    10
## psupport5            16 834  8.11  2.51      9    8.58  1.48   0  10    10
## political_ideology   17 834  5.25  1.93      5    5.25  1.48   0  10    10
## omind1               18 834  5.17  2.66      5    5.19  2.97   0  10    10
## omind2               19 834  9.15  1.28     10    9.41  0.00   3  10     7
## omind3               20 834  8.85  1.41      9    9.08  1.48   3  10     7
## omind4               21 834  8.26  1.95      9    8.56  1.48   0  10    10
## omind5               22 834  8.13  2.67      9    8.72  1.48   0  10    10
## omind6               23 834  6.78  2.57      7    6.99  2.97   0  10    10
## CRT1                 24 834  0.23  0.42      0    0.16  0.00   0   1     1
## CRT2                 25 834  0.32  0.47      0    0.27  0.00   0   1     1
## CRT3                 26 834  0.16  0.37      0    0.08  0.00   0   1     1
## age                  27 834 44.31 15.91     43   44.10 20.76  18  88    70
## sex1                 28 834  1.52  0.50      2    1.52  0.00   1   2     1
## ctheory1             29 834  5.35  3.45      5    5.44  4.45   0  10    10
## ctheory2             30 834  4.14  3.36      5    3.94  4.45   0  10    10
## ctheory3             31 834  3.44  3.32      3    3.08  4.45   0  10    10
## ctheory4             32 834  4.24  3.38      5    4.06  4.45   0  10    10
## employ_status1       33 834  2.79  1.93      2    2.64  1.48   1   6     5
## contact2r            34 834  7.85  2.67      9    8.31  1.48   0  10    10
## ccrt                 35 834  0.71  0.93      0    0.54  0.00   0   3     3
##                     skew kurtosis   se
## country*             NaN      NaN 0.00
## contact1           -1.45     1.51 0.07
## contact2            1.20     0.38 0.09
## contact3           -1.49     1.69 0.07
## contact4           -1.71     2.76 0.08
## contact5           -2.93     9.58 0.06
## hygiene1           -1.15     1.18 0.07
## hygiene2           -1.28     1.56 0.07
## hygiene3           -1.94     3.62 0.05
## hygiene4           -0.55    -0.64 0.10
## hygiene5           -1.21     0.61 0.10
## psupport1          -1.29     0.99 0.09
## psupport2          -1.53     1.74 0.08
## psupport3          -0.09    -1.15 0.11
## psupport4          -1.53     1.77 0.08
## psupport5          -1.39     1.24 0.09
## political_ideology -0.03     0.89 0.07
## omind1             -0.09    -0.64 0.09
## omind2             -1.71     2.89 0.04
## omind3             -1.24     1.09 0.05
## omind4             -1.29     1.80 0.07
## omind5             -1.65     1.90 0.09
## omind6             -0.50    -0.45 0.09
## CRT1                1.30    -0.30 0.01
## CRT2                0.79    -1.38 0.02
## CRT3                1.81     1.27 0.01
## age                 0.13    -1.12 0.55
## sex1               -0.08    -2.00 0.02
## ctheory1           -0.23    -1.24 0.12
## ctheory2            0.22    -1.21 0.12
## ctheory3            0.55    -0.98 0.12
## ctheory4            0.17    -1.24 0.12
## employ_status1      0.37    -1.59 0.07
## contact2r          -1.20     0.38 0.09
## ccrt                1.19     0.41 0.03
## ------------------------------------------------------------ 
## group: USA
##                    vars   n  mean    sd median trimmed   mad min max range
## country*              1 905  1.00  0.00      1    1.00  0.00   1   1     0
## contact1              2 905  8.88  1.40      9    9.11  1.48   3  10     7
## contact2              3 905  2.51  3.07      1    2.02  1.48   0  10    10
## contact3              4 905  8.28  1.97      9    8.63  1.48   0  10    10
## contact4              5 905  8.88  1.46      9    9.14  1.48   2  10     8
## contact5              6 905  9.14  1.58     10    9.50  0.00   0  10    10
## hygiene1              7 905  8.58  1.61      9    8.82  1.48   0  10    10
## hygiene2              8 905  8.63  1.65      9    8.92  1.48   0  10    10
## hygiene3              9 905  8.73  1.74     10    9.07  0.00   0  10    10
## hygiene4             10 905  7.80  2.42      8    8.20  2.97   0  10    10
## hygiene5             11 905  8.29  2.16      9    8.69  1.48   0  10    10
## psupport1            12 905  8.42  2.09      9    8.83  1.48   0  10    10
## psupport2            13 905  8.51  2.07      9    8.93  1.48   0  10    10
## psupport3            14 905  7.41  2.83      8    7.85  2.97   0  10    10
## psupport4            15 905  8.53  2.07      9    8.95  1.48   0  10    10
## psupport5            16 905  8.18  2.37      9    8.63  1.48   0  10    10
## political_ideology   17 905  5.81  2.63      5    5.90  2.97   0  10    10
## omind1               18 905  5.69  2.93      6    5.81  2.97   0  10    10
## omind2               19 905  8.55  1.64      9    8.82  1.48   1  10     9
## omind3               20 905  8.50  1.59      9    8.74  1.48   1  10     9
## omind4               21 905  8.43  1.65      9    8.68  1.48   1  10     9
## omind5               22 905  7.80  3.00      9    8.35  1.48   0  10    10
## omind6               23 905  7.25  2.91      8    7.64  2.97   0  10    10
## CRT1                 24 905  0.14  0.35      0    0.06  0.00   0   1     1
## CRT2                 25 905  0.29  0.45      0    0.23  0.00   0   1     1
## CRT3                 26 905  0.16  0.37      0    0.08  0.00   0   1     1
## age                  27 905 48.29 16.39     48   48.20 20.76  18 100    82
## sex1                 28 905  1.53  0.50      2    1.54  0.00   1   2     1
## ctheory1             29 905  4.30  3.41      5    4.15  4.45   0  10    10
## ctheory2             30 905  3.31  3.32      2    2.97  2.97   0  10    10
## ctheory3             31 905  2.64  3.13      1    2.18  1.48   0  10    10
## ctheory4             32 905  3.04  3.28      2    2.63  2.97   0  10    10
## employ_status1       33 905  2.71  1.85      2    2.56  1.48   1   6     5
## contact2r            34 905  7.49  3.07      9    7.98  1.48   0  10    10
## ccrt                 35 905  0.59  0.89      0    0.41  0.00   0   3     3
##                     skew kurtosis   se
## country*             NaN      NaN 0.00
## contact1           -1.34     1.48 0.05
## contact2            1.03    -0.28 0.10
## contact3           -1.36     1.59 0.07
## contact4           -1.52     2.23 0.05
## contact5           -2.81    10.24 0.05
## hygiene1           -1.16     1.31 0.05
## hygiene2           -1.38     1.88 0.05
## hygiene3           -1.60     2.53 0.06
## hygiene4           -1.26     1.23 0.08
## hygiene5           -1.67     3.04 0.07
## psupport1          -1.70     2.97 0.07
## psupport2          -1.79     3.25 0.07
## psupport3          -1.06     0.19 0.09
## psupport4          -1.79     3.29 0.07
## psupport5          -1.48     1.69 0.08
## political_ideology -0.25    -0.55 0.09
## omind1             -0.31    -0.95 0.10
## omind2             -1.40     2.14 0.05
## omind3             -1.22     1.54 0.05
## omind4             -1.18     1.38 0.05
## omind5             -1.24     0.15 0.10
## omind6             -0.90    -0.43 0.10
## CRT1                2.03     2.12 0.01
## CRT2                0.94    -1.11 0.02
## CRT3                1.84     1.38 0.01
## age                 0.11    -0.95 0.54
## sex1               -0.11    -1.99 0.02
## ctheory1            0.15    -1.33 0.11
## ctheory2            0.56    -1.08 0.11
## ctheory3            0.92    -0.49 0.10
## ctheory4            0.72    -0.84 0.11
## employ_status1      0.49    -1.42 0.06
## contact2r          -1.03    -0.28 0.10
## ccrt                1.41     0.98 0.03
```

```
pls5 <- split.data.frame(pls4, pls4$country)

for(i in seq_along(pls5)){
  df <- pls5[[i]]
  print(names(pls5)[i])
  print(prop.table(table(df$sex1)))
}
```

```
## [1] "AUS"
## 
##         1         2 
## 0.4662162 0.5337838 
## [1] "BEL"
## 
##         1         2 
## 0.5726316 0.4273684 
## [1] "CAN"
## 
##         1         2 
## 0.3621622 0.6378378 
## [1] "CHE"
## 
##         1         2 
## 0.4818976 0.5181024 
## [1] "DEU"
## 
##         1         2 
## 0.5111907 0.4888093 
## [1] "GRC"
## 
##         1         2 
## 0.6200418 0.3799582 
## [1] "IRQ"
## 
##        1        2 
## 0.534375 0.465625 
## [1] "ISR"
## 
##         1         2 
## 0.4808853 0.5191147 
## [1] "JPN"
## 
##         1         2 
## 0.4792503 0.5207497 
## [1] "KOR"
## 
##         1         2 
## 0.5296443 0.4703557 
## [1] "NGA"
## 
##         1         2 
## 0.5136476 0.4863524 
## [1] "NZL"
## 
##         1         2 
## 0.5375375 0.4624625 
## [1] "PAK"
## 
##         1         2 
## 0.4414169 0.5585831 
## [1] "POL"
## 
##         1         2 
## 0.4956958 0.5043042 
## [1] "SGP"
## 
##         1         2 
## 0.4879357 0.5120643 
## [1] "SVK"
## 
##         1         2 
## 0.4808153 0.5191847 
## [1] "USA"
## 
##         1         2 
## 0.4718232 0.5281768
```

```
for(i in seq_along(pls5)){
  df <- pls5[[i]]
  print(names(pls5)[i])
  print(prop.table(table(df$employ_status1)))
}
```

```
## [1] "AUS"
## 
##          1          2          3          4          5          6 
## 0.32635135 0.18783784 0.09797297 0.04527027 0.27297297 0.06959459 
## [1] "BEL"
## 
##          1          2          3          4          5          6 
## 0.27157895 0.04105263 0.02842105 0.26210526 0.26315789 0.13368421 
## [1] "CAN"
## 
##          1          2          3          4          5          6 
## 0.34729730 0.09459459 0.17567568 0.08918919 0.20135135 0.09189189 
## [1] "CHE"
## 
##          1          2          3          4          5          6 
## 0.35705368 0.18851436 0.05617978 0.06491885 0.22097378 0.11235955 
## [1] "DEU"
## 
##          1          2          3          4          5          6 
## 0.37242614 0.12175470 0.04923903 0.08146822 0.29722471 0.07788720 
## [1] "GRC"
## 
##          1          2          3          4          5          6 
## 0.34237996 0.10438413 0.14196242 0.35073069 0.03757829 0.02296451 
## [1] "IRQ"
## 
##        1        2        3        4        5        6 
## 0.246875 0.168750 0.140625 0.309375 0.037500 0.096875 
## [1] "ISR"
## 
##          1          2          3          4          5          6 
## 0.38832998 0.13782696 0.14386318 0.06036217 0.09255533 0.17706237 
## [1] "JPN"
## 
##          1          2          3          4          5          6 
## 0.42704150 0.14190094 0.19277108 0.03212851 0.13119143 0.07496653 
## [1] "KOR"
## 
##          1          2          3          4          5          6 
## 0.53359684 0.11857708 0.05533597 0.05138340 0.09881423 0.14229249 
## [1] "NGA"
## 
##          1          2          3          4          5          6 
## 0.35732010 0.16625310 0.19354839 0.19851117 0.01488834 0.06947891 
## [1] "NZL"
## 
##          1          2          3          4          5          6 
## 0.39339339 0.15015015 0.08408408 0.02702703 0.20720721 0.13813814 
## [1] "PAK"
## 
##           1           2           3           4           5           6 
## 0.286103542 0.059945504 0.095367847 0.495912807 0.008174387 0.054495913 
## [1] "POL"
## 
##          1          2          3          4          5          6 
## 0.37230990 0.06743185 0.10760402 0.06599713 0.28192253 0.10473458 
## [1] "SGP"
## 
##          1          2          3          4          5          6 
## 0.66219839 0.06434316 0.08847185 0.05361930 0.06434316 0.06702413 
## [1] "SVK"
## 
##          1          2          3          4          5          6 
## 0.48201439 0.05275779 0.06354916 0.07673861 0.24220624 0.08273381 
## [1] "USA"
## 
##          1          2          3          4          5          6 
## 0.44972376 0.09944751 0.11712707 0.02430939 0.24088398 0.06850829
```

```
write.csv(pls4, "Data_c2.csv")
```

## CFA - model variables

### Dependent variables

```
phc <- 'phc =~ contact1 + contact2r + contact3 + contact4
        phs =~ psupport1 + psupport2 + psupport3 + psupport4 + psupport5
        phg =~ hygiene1 + hygiene2 + hygiene3 + hygiene4 + hygiene5'

phymod <- cfa(phc, estimator = "MLM", data = pls4)
fitmeasures(phymod, fit.measures = c("chisq.scaled", "df.scaled", "cfi.robust", "rmsea.robust", "srmr"))
```

```
## chisq.scaled    df.scaled   cfi.robust rmsea.robust         srmr 
##     3695.302       74.000        0.948        0.068        0.054
```

```
phc <- 'phc =~ contact1 + contact2r + contact3 + contact4
        phs =~ psupport1 + psupport2 + psupport3 + psupport4 + psupport5
        phg =~ hygiene1 + hygiene2 + hygiene3 + hygiene4 + hygiene5
        hygiene1 ~~ hygiene2
        psupport4 ~~ psupport5'

phymod <- cfa(phc, estimator = "MLM", data = pls4)
fitmeasures(phymod, fit.measures = c("chisq.scaled", "df.scaled", "cfi.robust", "rmsea.robust", "srmr"))
```

```
## chisq.scaled    df.scaled   cfi.robust rmsea.robust         srmr 
##     1842.877       72.000        0.975        0.048        0.030
```

```
semPaths(phymod, what = "std", edge.label.cex = 1.2, label.cex = 1.2)
```

```
reliability(phymod)
```

```
##              phc       phs       phg
## alpha  0.6867700 0.8693538 0.7880084
## omega  0.6879093 0.8593874 0.7408419
## omega2 0.6879093 0.8593874 0.7408419
## omega3 0.6867099 0.8568363 0.7373203
## avevar 0.3575329 0.5662227 0.4022453
```

### Open-mindedness

```
phc <- 'om1 =~ omind2 + omind3 + omind4 + omind1 + omind5 + omind6'

phymod <- cfa(phc, estimator = "MLM", data = pls4)
fitmeasures(phymod, fit.measures = c("chisq.scaled", "df.scaled", "cfi.robust", "rmsea.robust", "srmr"))
```

```
## chisq.scaled    df.scaled   cfi.robust rmsea.robust         srmr 
##     3365.564        9.000        0.819        0.197        0.114
```

```
phc <- 'om1 =~ omind2 + omind3 + omind4 + omind1 + omind5 + omind6
        omind1 ~~ omind5
        omind5 ~~ omind6
        omind1 ~~ omind6'

phymod <- cfa(phc, estimator = "MLM", data = pls4)
fitmeasures(phymod, fit.measures = c("chisq.scaled", "df.scaled", "cfi.robust", "rmsea.robust", "srmr"))
```

```
## chisq.scaled    df.scaled   cfi.robust rmsea.robust         srmr 
##       61.978        6.000        0.998        0.028        0.009
```

```
semPaths(phymod, what = "std", edge.label.cex = 1.2, label.cex = 1.2)
```

```
reliability(phymod)
```

```
##              om1
## alpha  0.7401993
## omega  0.5293343
## omega2 0.5293343
## omega3 0.5273229
## avevar 0.2448879
```

## CFA - altogether

```
pls4$cpol <- ifelse(pls4$country %in% c("AUS", "BEL", "CAN", "NZL", "USA"), "negative", ifelse(pls4$country %in% c("POL", "SGP"), "positive", "neutral"))

phc <- 'phc =~ contact1 + contact2r + contact3 + contact4
        phs =~ psupport1 + psupport2 + psupport3 + psupport4 + psupport5
        phg =~ hygiene1 + hygiene2 + hygiene3 + hygiene4 + hygiene5
        hygiene1 ~~ hygiene2
        psupport4 ~~ psupport5
        om1 =~ omind2 + omind3 + omind4 + omind1 + omind5 + omind6
        omind1 ~~ omind5
        omind5 ~~ omind6
        omind1 ~~ omind6'

phymod <- cfa(phc, estimator = "MLM", data = pls4)
fitmeasures(phymod, fit.measures = c("chisq.scaled", "df.scaled", "cfi.robust", "rmsea.robust", "srmr"))
```

```
## chisq.scaled    df.scaled   cfi.robust rmsea.robust         srmr 
##     3903.328      159.000        0.963        0.045        0.041
```

```
semPaths(phymod, what = "std", edge.label.cex = 1.2, label.cex = 1.2)
```

```
reliability(phymod)
```

```
##              phc       phs       phg       om1
## alpha  0.6867700 0.8693538 0.7880084 0.7401993
## omega  0.6887037 0.8592015 0.7397524 0.5305899
## omega2 0.6887037 0.8592015 0.7397524 0.5305899
## omega3 0.6885468 0.8561371 0.7352640 0.5291870
## avevar 0.3581956 0.5656993 0.4007822 0.2455824
```

```
phymodcc <- cfa(phc, estimator = "MLM", data = pls4, group = "cpol")
summary(phymodcc, standardized = T)
```

```
## lavaan 0.6-8 ended normally after 199 iterations
## 
##   Estimator                                         ML
##   Optimization method                           NLMINB
##   Number of model parameters                       213
##                                                       
##   Number of observations per group:                   
##     negative                                      4408
##     neutral                                       6315
##     positive                                      1767
##                                                       
## Model Test User Model:
##                                               Standard      Robust
##   Test Statistic                              5475.363    5153.892
##   Degrees of freedom                               477         477
##   P-value (Chi-square)                           0.000       0.000
##   Scaling correction factor                                  1.062
##        Satorra-Bentler correction                                 
##   Test statistic for each group:
##     negative                                  2500.950    2354.114
##     neutral                                   2093.124    1970.232
##     positive                                   881.288     829.546
## 
## Parameter Estimates:
## 
##   Standard errors                           Robust.sem
##   Information                                 Expected
##   Information saturated (h1) model          Structured
## 
## 
## Group 1 [negative]:
## 
## Latent Variables:
##                    Estimate  Std.Err  z-value  P(>|z|)   Std.lv  Std.all
##   phc =~                                                                
##     contact1          1.000                               1.122    0.764
##     contact2r         1.026    0.041   24.808    0.000    1.152    0.432
##     contact3          1.126    0.032   35.210    0.000    1.263    0.602
##     contact4          0.953    0.024   39.294    0.000    1.069    0.789
##   phs =~                                                                
##     psupport1         1.000                               1.764    0.699
##     psupport2         0.985    0.019   52.961    0.000    1.737    0.885
##     psupport3         1.032    0.021   48.081    0.000    1.820    0.600
##     psupport4         0.880    0.021   42.381    0.000    1.552    0.851
##     psupport5         0.874    0.023   38.644    0.000    1.541    0.736
##   phg =~                                                                
##     hygiene1          1.000                               1.437    0.743
##     hygiene2          0.979    0.011   85.763    0.000    1.407    0.752
##     hygiene3          1.061    0.028   37.426    0.000    1.525    0.756
##     hygiene4          1.262    0.039   32.590    0.000    1.814    0.590
##     hygiene5          0.921    0.032   28.501    0.000    1.324    0.574
##   om1 =~                                                                
##     omind2            1.000                               1.237    0.856
##     omind3            1.018    0.017   60.511    0.000    1.260    0.893
##     omind4            0.971    0.019   52.229    0.000    1.201    0.794
##     omind1            0.511    0.033   15.287    0.000    0.632    0.239
##     omind5            0.787    0.030   26.081    0.000    0.973    0.390
##     omind6            0.777    0.031   24.811    0.000    0.962    0.377
## 
## Covariances:
##                    Estimate  Std.Err  z-value  P(>|z|)   Std.lv  Std.all
##  .hygiene1 ~~                                                           
##    .hygiene2          1.206    0.078   15.558    0.000    1.206    0.754
##  .psupport4 ~~                                                          
##    .psupport5         0.228    0.044    5.233    0.000    0.228    0.168
##  .omind1 ~~                                                             
##    .omind5            2.048    0.125   16.446    0.000    2.048    0.348
##  .omind5 ~~                                                             
##    .omind6            2.699    0.142   18.970    0.000    2.699    0.497
##  .omind1 ~~                                                             
##    .omind6            2.480    0.119   20.756    0.000    2.480    0.409
##   phc ~~                                                                
##     phs               1.182    0.054   22.048    0.000    0.598    0.598
##     phg               0.835    0.042   20.114    0.000    0.518    0.518
##     om1               0.731    0.036   20.078    0.000    0.527    0.527
##   phs ~~                                                                
##     phg               0.974    0.058   16.812    0.000    0.384    0.384
##     om1               0.741    0.041   17.860    0.000    0.340    0.340
##   phg ~~                                                                
##     om1               0.509    0.035   14.633    0.000    0.286    0.286
## 
## Intercepts:
##                    Estimate  Std.Err  z-value  P(>|z|)   Std.lv  Std.all
##    .contact1          8.910    0.022  402.869    0.000    8.910    6.068
##    .contact2r         8.116    0.040  202.165    0.000    8.116    3.045
##    .contact3          8.288    0.032  262.127    0.000    8.288    3.948
##    .contact4          9.010    0.020  441.722    0.000    9.010    6.653
##    .psupport1         7.850    0.038  206.680    0.000    7.850    3.113
##    .psupport2         8.584    0.030  290.384    0.000    8.584    4.374
##    .psupport3         6.795    0.046  148.778    0.000    6.795    2.241
##    .psupport4         8.758    0.027  318.892    0.000    8.758    4.803
##    .psupport5         8.529    0.032  270.376    0.000    8.529    4.072
##    .hygiene1          8.227    0.029  282.302    0.000    8.227    4.252
##    .hygiene2          8.353    0.028  296.356    0.000    8.353    4.464
##    .hygiene3          8.437    0.030  277.465    0.000    8.437    4.179
##    .hygiene4          6.485    0.046  139.971    0.000    6.485    2.108
##    .hygiene5          8.045    0.035  231.746    0.000    8.045    3.491
##    .omind2            8.680    0.022  398.678    0.000    8.680    6.005
##    .omind3            8.610    0.021  405.088    0.000    8.610    6.101
##    .omind4            8.513    0.023  373.650    0.000    8.513    5.628
##    .omind1            5.986    0.040  150.521    0.000    5.986    2.267
##    .omind5            8.426    0.038  224.212    0.000    8.426    3.377
##    .omind6            7.550    0.038  196.408    0.000    7.550    2.958
##     phc               0.000                               0.000    0.000
##     phs               0.000                               0.000    0.000
##     phg               0.000                               0.000    0.000
##     om1               0.000                               0.000    0.000
## 
## Variances:
##                    Estimate  Std.Err  z-value  P(>|z|)   Std.lv  Std.all
##    .contact1          0.898    0.054   16.745    0.000    0.898    0.416
##    .contact2r         5.778    0.199   28.998    0.000    5.778    0.813
##    .contact3          2.811    0.132   21.265    0.000    2.811    0.638
##    .contact4          0.691    0.039   17.847    0.000    0.691    0.377
##    .psupport1         3.248    0.135   23.980    0.000    3.248    0.511
##    .psupport2         0.833    0.049   17.067    0.000    0.833    0.216
##    .psupport3         5.884    0.163   36.161    0.000    5.884    0.640
##    .psupport4         0.915    0.045   20.338    0.000    0.915    0.275
##    .psupport5         2.012    0.088   22.810    0.000    2.012    0.459
##    .hygiene1          1.679    0.085   19.688    0.000    1.679    0.448
##    .hygiene2          1.524    0.080   19.001    0.000    1.524    0.435
##    .hygiene3          1.749    0.090   19.390    0.000    1.749    0.429
##    .hygiene4          6.171    0.180   34.258    0.000    6.171    0.652
##    .hygiene5          3.560    0.149   23.867    0.000    3.560    0.670
##    .omind2            0.559    0.033   16.982    0.000    0.559    0.268
##    .omind3            0.405    0.027   14.922    0.000    0.405    0.203
##    .omind4            0.845    0.047   17.960    0.000    0.845    0.370
##    .omind1            6.572    0.134   49.060    0.000    6.572    0.943
##    .omind5            5.279    0.204   25.841    0.000    5.279    0.848
##    .omind6            5.589    0.163   34.261    0.000    5.589    0.858
##     phc               1.259    0.060   20.943    0.000    1.000    1.000
##     phs               3.110    0.131   23.825    0.000    1.000    1.000
##     phg               2.065    0.100   20.551    0.000    1.000    1.000
##     om1               1.530    0.057   26.676    0.000    1.000    1.000
## 
## 
## Group 2 [neutral]:
## 
## Latent Variables:
##                    Estimate  Std.Err  z-value  P(>|z|)   Std.lv  Std.all
##   phc =~                                                                
##     contact1          1.000                               1.376    0.740
##     contact2r         0.719    0.029   24.525    0.000    0.990    0.356
##     contact3          1.079    0.028   38.761    0.000    1.484    0.643
##     contact4          0.966    0.026   37.054    0.000    1.329    0.667
##   phs =~                                                                
##     psupport1         1.000                               2.080    0.810
##     psupport2         1.018    0.013   77.632    0.000    2.117    0.880
##     psupport3         0.996    0.016   61.752    0.000    2.072    0.649
##     psupport4         0.817    0.015   54.385    0.000    1.698    0.792
##     psupport5         0.850    0.016   54.861    0.000    1.769    0.721
##   phg =~                                                                
##     hygiene1          1.000                               1.437    0.687
##     hygiene2          1.012    0.011   89.863    0.000    1.453    0.713
##     hygiene3          0.941    0.023   41.227    0.000    1.352    0.761
##     hygiene4          1.100    0.032   34.747    0.000    1.581    0.502
##     hygiene5          0.855    0.029   29.116    0.000    1.228    0.515
##   om1 =~                                                                
##     omind2            1.000                               1.089    0.591
##     omind3            1.236    0.028   44.791    0.000    1.346    0.876
##     omind4            1.262    0.029   42.916    0.000    1.375    0.802
##     omind1            0.457    0.033   14.020    0.000    0.498    0.181
##     omind5            0.781    0.032   24.759    0.000    0.851    0.337
##     omind6            0.775    0.033   23.601    0.000    0.844    0.325
## 
## Covariances:
##                    Estimate  Std.Err  z-value  P(>|z|)   Std.lv  Std.all
##  .hygiene1 ~~                                                           
##    .hygiene2          1.597    0.087   18.413    0.000    1.597    0.735
##  .psupport4 ~~                                                          
##    .psupport5         0.512    0.050   10.293    0.000    0.512    0.230
##  .omind1 ~~                                                             
##    .omind5            1.775    0.104   17.089    0.000    1.775    0.276
##  .omind5 ~~                                                             
##    .omind6            1.851    0.104   17.859    0.000    1.851    0.317
##  .omind1 ~~                                                             
##    .omind6            2.641    0.104   25.389    0.000    2.641    0.396
##   phc ~~                                                                
##     phs               1.428    0.064   22.405    0.000    0.499    0.499
##     phg               1.190    0.051   23.134    0.000    0.602    0.602
##     om1               0.536    0.029   18.535    0.000    0.358    0.358
##   phs ~~                                                                
##     phg               1.198    0.059   20.131    0.000    0.401    0.401
##     om1               0.551    0.035   15.625    0.000    0.243    0.243
##   phg ~~                                                                
##     om1               0.511    0.031   16.535    0.000    0.327    0.327
## 
## Intercepts:
##                    Estimate  Std.Err  z-value  P(>|z|)   Std.lv  Std.all
##    .contact1          8.541    0.023  365.072    0.000    8.541    4.594
##    .contact2r         7.741    0.035  221.455    0.000    7.741    2.787
##    .contact3          8.012    0.029  275.958    0.000    8.012    3.473
##    .contact4          8.366    0.025  333.764    0.000    8.366    4.200
##    .psupport1         7.682    0.032  237.857    0.000    7.682    2.993
##    .psupport2         8.063    0.030  266.316    0.000    8.063    3.351
##    .psupport3         6.267    0.040  156.003    0.000    6.267    1.963
##    .psupport4         8.484    0.027  314.295    0.000    8.484    3.955
##    .psupport5         8.213    0.031  266.041    0.000    8.213    3.348
##    .hygiene1          8.060    0.026  306.295    0.000    8.060    3.854
##    .hygiene2          8.190    0.026  319.242    0.000    8.190    4.017
##    .hygiene3          8.865    0.022  396.654    0.000    8.865    4.991
##    .hygiene4          5.899    0.040  148.965    0.000    5.899    1.875
##    .hygiene5          8.063    0.030  268.566    0.000    8.063    3.380
##    .omind2            8.743    0.023  376.978    0.000    8.743    4.744
##    .omind3            8.652    0.019  447.324    0.000    8.652    5.629
##    .omind4            8.516    0.022  394.797    0.000    8.516    4.968
##    .omind1            6.363    0.035  183.608    0.000    6.363    2.310
##    .omind5            8.260    0.032  259.996    0.000    8.260    3.272
##    .omind6            7.178    0.033  219.387    0.000    7.178    2.761
##     phc               0.000                               0.000    0.000
##     phs               0.000                               0.000    0.000
##     phg               0.000                               0.000    0.000
##     om1               0.000                               0.000    0.000
## 
## Variances:
##                    Estimate  Std.Err  z-value  P(>|z|)   Std.lv  Std.all
##    .contact1          1.563    0.071   22.016    0.000    1.563    0.452
##    .contact2r         6.736    0.174   38.641    0.000    6.736    0.873
##    .contact3          3.119    0.124   25.124    0.000    3.119    0.586
##    .contact4          2.202    0.093   23.636    0.000    2.202    0.555
##    .psupport1         2.263    0.086   26.183    0.000    2.263    0.344
##    .psupport2         1.308    0.060   21.950    0.000    1.308    0.226
##    .psupport3         5.901    0.134   43.984    0.000    5.901    0.579
##    .psupport4         1.718    0.060   28.824    0.000    1.718    0.373
##    .psupport5         2.891    0.095   30.319    0.000    2.891    0.480
##    .hygiene1          2.308    0.096   24.093    0.000    2.308    0.528
##    .hygiene2          2.044    0.092   22.250    0.000    2.044    0.492
##    .hygiene3          1.327    0.053   24.928    0.000    1.327    0.421
##    .hygiene4          7.403    0.144   51.374    0.000    7.403    0.748
##    .hygiene5          4.184    0.131   32.057    0.000    4.184    0.735
##    .omind2            2.210    0.139   15.954    0.000    2.210    0.651
##    .omind3            0.551    0.037   14.851    0.000    0.551    0.233
##    .omind4            1.048    0.058   18.014    0.000    1.048    0.357
##    .omind1            7.338    0.122   60.238    0.000    7.338    0.967
##    .omind5            5.650    0.172   32.874    0.000    5.650    0.886
##    .omind6            6.047    0.128   47.083    0.000    6.047    0.895
##     phc               1.894    0.078   24.183    0.000    1.000    1.000
##     phs               4.324    0.127   34.055    0.000    1.000    1.000
##     phg               2.064    0.091   22.787    0.000    1.000    1.000
##     om1               1.187    0.056   21.050    0.000    1.000    1.000
## 
## 
## Group 3 [positive]:
## 
## Latent Variables:
##                    Estimate  Std.Err  z-value  P(>|z|)   Std.lv  Std.all
##   phc =~                                                                
##     contact1          1.000                               1.567    0.774
##     contact2r         0.814    0.047   17.272    0.000    1.275    0.445
##     contact3          1.049    0.034   30.403    0.000    1.643    0.763
##     contact4          0.963    0.031   31.158    0.000    1.510    0.857
##   phs =~                                                                
##     psupport1         1.000                               2.108    0.865
##     psupport2         1.083    0.021   51.708    0.000    2.283    0.925
##     psupport3         0.975    0.033   29.565    0.000    2.056    0.583
##     psupport4         0.922    0.026   35.673    0.000    1.943    0.837
##     psupport5         0.918    0.029   32.122    0.000    1.935    0.802
##   phg =~                                                                
##     hygiene1          1.000                               1.651    0.787
##     hygiene2          1.001    0.012   85.732    0.000    1.652    0.803
##     hygiene3          0.875    0.032   27.544    0.000    1.444    0.790
##     hygiene4          1.108    0.042   26.470    0.000    1.828    0.605
##     hygiene5          0.950    0.041   23.102    0.000    1.568    0.538
##   om1 =~                                                                
##     omind2            1.000                               1.321    0.846
##     omind3            1.060    0.024   44.503    0.000    1.400    0.912
##     omind4            1.045    0.026   40.484    0.000    1.381    0.858
##     omind1            0.398    0.048    8.330    0.000    0.525    0.200
##     omind5            0.864    0.046   18.836    0.000    1.142    0.453
##     omind6            0.832    0.046   17.915    0.000    1.099    0.428
## 
## Covariances:
##                    Estimate  Std.Err  z-value  P(>|z|)   Std.lv  Std.all
##  .hygiene1 ~~                                                           
##    .hygiene2          1.267    0.127    9.963    0.000    1.267    0.799
##  .psupport4 ~~                                                          
##    .psupport5         0.593    0.089    6.660    0.000    0.593    0.323
##  .omind1 ~~                                                             
##    .omind5            1.516    0.160    9.497    0.000    1.516    0.262
##  .omind5 ~~                                                             
##    .omind6            2.117    0.190   11.163    0.000    2.117    0.406
##  .omind1 ~~                                                             
##    .omind6            2.358    0.172   13.743    0.000    2.358    0.394
##   phc ~~                                                                
##     phs               2.068    0.158   13.109    0.000    0.626    0.626
##     phg               1.684    0.136   12.426    0.000    0.651    0.651
##     om1               0.979    0.071   13.801    0.000    0.473    0.473
##   phs ~~                                                                
##     phg               1.799    0.149   12.083    0.000    0.517    0.517
##     om1               0.766    0.080    9.619    0.000    0.275    0.275
##   phg ~~                                                                
##     om1               0.657    0.070    9.423    0.000    0.301    0.301
## 
## Intercepts:
##                    Estimate  Std.Err  z-value  P(>|z|)   Std.lv  Std.all
##    .contact1          8.379    0.048  173.912    0.000    8.379    4.137
##    .contact2r         7.680    0.068  112.739    0.000    7.680    2.682
##    .contact3          8.179    0.051  159.718    0.000    8.179    3.800
##    .contact4          8.617    0.042  205.576    0.000    8.617    4.891
##    .psupport1         7.916    0.058  136.600    0.000    7.916    3.250
##    .psupport2         8.027    0.059  136.697    0.000    8.027    3.252
##    .psupport3         5.551    0.084   66.137    0.000    5.551    1.573
##    .psupport4         8.334    0.055  150.879    0.000    8.334    3.589
##    .psupport5         8.171    0.057  142.320    0.000    8.171    3.386
##    .hygiene1          7.962    0.050  159.541    0.000    7.962    3.795
##    .hygiene2          8.067    0.049  164.854    0.000    8.067    3.922
##    .hygiene3          8.678    0.043  199.534    0.000    8.678    4.747
##    .hygiene4          6.467    0.072   89.985    0.000    6.467    2.141
##    .hygiene5          7.169    0.069  103.495    0.000    7.169    2.462
##    .omind2            8.511    0.037  229.195    0.000    8.511    5.452
##    .omind3            8.432    0.037  230.957    0.000    8.432    5.494
##    .omind4            8.450    0.038  220.690    0.000    8.450    5.250
##    .omind1            5.576    0.063   89.051    0.000    5.576    2.118
##    .omind5            8.060    0.060  134.408    0.000    8.060    3.197
##    .omind6            7.359    0.061  120.445    0.000    7.359    2.865
##     phc               0.000                               0.000    0.000
##     phs               0.000                               0.000    0.000
##     phg               0.000                               0.000    0.000
##     om1               0.000                               0.000    0.000
## 
## Variances:
##                    Estimate  Std.Err  z-value  P(>|z|)   Std.lv  Std.all
##    .contact1          1.645    0.121   13.609    0.000    1.645    0.401
##    .contact2r         6.574    0.311   21.113    0.000    6.574    0.802
##    .contact3          1.933    0.145   13.302    0.000    1.933    0.417
##    .contact4          0.825    0.059   14.103    0.000    0.825    0.266
##    .psupport1         1.491    0.117   12.777    0.000    1.491    0.251
##    .psupport2         0.881    0.076   11.584    0.000    0.881    0.145
##    .psupport3         8.223    0.274   29.966    0.000    8.223    0.661
##    .psupport4         1.618    0.115   14.027    0.000    1.618    0.300
##    .psupport5         2.082    0.139   14.942    0.000    2.082    0.357
##    .hygiene1          1.675    0.130   12.889    0.000    1.675    0.381
##    .hygiene2          1.500    0.133   11.269    0.000    1.500    0.355
##    .hygiene3          1.256    0.082   15.363    0.000    1.256    0.376
##    .hygiene4          5.783    0.264   21.930    0.000    5.783    0.634
##    .hygiene5          6.022    0.297   20.274    0.000    6.022    0.710
##    .omind2            0.692    0.056   12.378    0.000    0.692    0.284
##    .omind3            0.396    0.041    9.703    0.000    0.396    0.168
##    .omind4            0.683    0.058   11.736    0.000    0.683    0.264
##    .omind1            6.651    0.202   32.877    0.000    6.651    0.960
##    .omind5            5.051    0.304   16.612    0.000    5.051    0.795
##    .omind6            5.389    0.247   21.791    0.000    5.389    0.817
##     phc               2.457    0.177   13.900    0.000    1.000    1.000
##     phs               4.443    0.248   17.888    0.000    1.000    1.000
##     phg               2.725    0.178   15.342    0.000    1.000    1.000
##     om1               1.745    0.089   19.592    0.000    1.000    1.000
```

```
phymodcw <- cfa(phc, estimator = "MLM", data = pls4, group = "cpol", group.equal = "loadings")
summary(phymodcw, standardized = T)
```

```
## lavaan 0.6-8 ended normally after 200 iterations
## 
##   Estimator                                         ML
##   Optimization method                           NLMINB
##   Number of model parameters                       213
##   Number of equality constraints                    32
##                                                       
##   Number of observations per group:                   
##     negative                                      4408
##     neutral                                       6315
##     positive                                      1767
##                                                       
## Model Test User Model:
##                                               Standard      Robust
##   Test Statistic                              5761.073    5402.417
##   Degrees of freedom                               509         509
##   P-value (Chi-square)                           0.000       0.000
##   Scaling correction factor                                  1.066
##        Satorra-Bentler correction                                 
##   Test statistic for each group:
##     negative                                  2616.744    2453.838
##     neutral                                   2213.477    2075.677
##     positive                                   930.853     872.902
## 
## Parameter Estimates:
## 
##   Standard errors                           Robust.sem
##   Information                                 Expected
##   Information saturated (h1) model          Structured
## 
## 
## Group 1 [negative]:
## 
## Latent Variables:
##                    Estimate  Std.Err  z-value  P(>|z|)   Std.lv  Std.all
##   phc =~                                                                
##     contct1           1.000                               1.135    0.768
##     cntct2r (.p2.)    0.829    0.021   38.631    0.000    0.942    0.362
##     contct3 (.p3.)    1.085    0.018   60.617    0.000    1.232    0.591
##     contct4 (.p4.)    0.959    0.015   62.076    0.000    1.089    0.798
##   phs =~                                                                
##     pspprt1           1.000                               1.747    0.696
##     pspprt2 (.p6.)    1.022    0.010  106.700    0.000    1.786    0.896
##     pspprt3 (.p7.)    1.009    0.012   82.068    0.000    1.763    0.586
##     pspprt4 (.p8.)    0.862    0.011   78.077    0.000    1.506    0.839
##     pspprt5 (.p9.)    0.873    0.012   73.433    0.000    1.525    0.730
##   phg =~                                                                
##     hygien1           1.000                               1.496    0.764
##     hygien2 (.11.)    0.996    0.007  147.647    0.000    1.491    0.781
##     hygien3 (.12.)    0.968    0.016   61.731    0.000    1.449    0.729
##     hygien4 (.13.)    1.159    0.021   53.929    0.000    1.734    0.569
##     hygien5 (.14.)    0.892    0.020   45.536    0.000    1.334    0.578
##   om1 =~                                                                
##     omind2            1.000                               1.180    0.837
##     omind3  (.18.)    1.080    0.012   88.708    0.000    1.274    0.898
##     omind4  (.19.)    1.065    0.013   80.697    0.000    1.257    0.810
##     omind1  (.20.)    0.450    0.020   22.240    0.000    0.531    0.203
##     omind5  (.21.)    0.764    0.019   40.701    0.000    0.902    0.365
##     omind6  (.22.)    0.753    0.019   38.668    0.000    0.888    0.351
## 
## Covariances:
##                    Estimate  Std.Err  z-value  P(>|z|)   Std.lv  Std.all
##  .hygiene1 ~~                                                           
##    .hygiene2          1.113    0.075   14.807    0.000    1.113    0.739
##  .psupport4 ~~                                                          
##    .psupport5         0.262    0.041    6.340    0.000    0.262    0.188
##  .omind1 ~~                                                             
##    .omind5            2.070    0.123   16.829    0.000    2.070    0.350
##  .omind5 ~~                                                             
##    .omind6            2.723    0.141   19.313    0.000    2.723    0.499
##  .omind1 ~~                                                             
##    .omind6            2.501    0.118   21.198    0.000    2.501    0.411
##   phc ~~                                                                
##     phs               1.179    0.050   23.520    0.000    0.595    0.595
##     phg               0.883    0.040   22.144    0.000    0.520    0.520
##     om1               0.705    0.034   20.802    0.000    0.526    0.526
##   phs ~~                                                                
##     phg               1.001    0.057   17.672    0.000    0.383    0.383
##     om1               0.698    0.039   17.810    0.000    0.339    0.339
##   phg ~~                                                                
##     om1               0.510    0.034   15.130    0.000    0.289    0.289
## 
## Intercepts:
##                    Estimate  Std.Err  z-value  P(>|z|)   Std.lv  Std.all
##    .contact1          8.910    0.022  402.869    0.000    8.910    6.025
##    .contact2r         8.116    0.040  202.165    0.000    8.116    3.116
##    .contact3          8.288    0.032  262.127    0.000    8.288    3.978
##    .contact4          9.010    0.020  441.722    0.000    9.010    6.600
##    .psupport1         7.850    0.038  206.680    0.000    7.850    3.127
##    .psupport2         8.584    0.030  290.384    0.000    8.584    4.308
##    .psupport3         6.795    0.046  148.778    0.000    6.795    2.261
##    .psupport4         8.758    0.027  318.892    0.000    8.758    4.883
##    .psupport5         8.529    0.032  270.376    0.000    8.529    4.080
##    .hygiene1          8.227    0.029  282.302    0.000    8.227    4.202
##    .hygiene2          8.353    0.028  296.356    0.000    8.353    4.376
##    .hygiene3          8.437    0.030  277.465    0.000    8.437    4.247
##    .hygiene4          6.485    0.046  139.971    0.000    6.485    2.129
##    .hygiene5          8.045    0.035  231.746    0.000    8.045    3.483
##    .omind2            8.680    0.022  398.678    0.000    8.680    6.158
##    .omind3            8.610    0.021  405.088    0.000    8.610    6.069
##    .omind4            8.513    0.023  373.650    0.000    8.513    5.485
##    .omind1            5.986    0.040  150.521    0.000    5.986    2.283
##    .omind5            8.426    0.038  224.212    0.000    8.426    3.407
##    .omind6            7.550    0.038  196.408    0.000    7.550    2.984
##     phc               0.000                               0.000    0.000
##     phs               0.000                               0.000    0.000
##     phg               0.000                               0.000    0.000
##     om1               0.000                               0.000    0.000
## 
## Variances:
##                    Estimate  Std.Err  z-value  P(>|z|)   Std.lv  Std.all
##    .contact1          0.898    0.054   16.533    0.000    0.898    0.411
##    .contact2r         5.897    0.191   30.826    0.000    5.897    0.869
##    .contact3          2.823    0.129   21.958    0.000    2.823    0.650
##    .contact4          0.677    0.038   17.612    0.000    0.677    0.364
##    .psupport1         3.252    0.133   24.429    0.000    3.252    0.516
##    .psupport2         0.783    0.048   16.403    0.000    0.783    0.197
##    .psupport3         5.927    0.158   37.565    0.000    5.927    0.656
##    .psupport4         0.951    0.043   21.989    0.000    0.951    0.295
##    .psupport5         2.044    0.087   23.562    0.000    2.044    0.468
##    .hygiene1          1.594    0.083   19.291    0.000    1.594    0.416
##    .hygiene2          1.422    0.078   18.219    0.000    1.422    0.390
##    .hygiene3          1.847    0.087   21.264    0.000    1.847    0.468
##    .hygiene4          6.270    0.169   37.142    0.000    6.270    0.676
##    .hygiene5          3.554    0.146   24.366    0.000    3.554    0.666
##    .omind2            0.593    0.033   17.918    0.000    0.593    0.299
##    .omind3            0.389    0.026   15.113    0.000    0.389    0.193
##    .omind4            0.828    0.047   17.777    0.000    0.828    0.344
##    .omind1            6.593    0.131   50.497    0.000    6.593    0.959
##    .omind5            5.304    0.202   26.231    0.000    5.304    0.867
##    .omind6            5.611    0.159   35.213    0.000    5.611    0.877
##     phc               1.289    0.052   24.906    0.000    1.000    1.000
##     phs               3.050    0.118   25.912    0.000    1.000    1.000
##     phg               2.238    0.089   25.093    0.000    1.000    1.000
##     om1               1.393    0.048   28.887    0.000    1.000    1.000
## 
## 
## Group 2 [neutral]:
## 
## Latent Variables:
##                    Estimate  Std.Err  z-value  P(>|z|)   Std.lv  Std.all
##   phc =~                                                                
##     contct1           1.000                               1.364    0.737
##     cntct2r (.p2.)    0.829    0.021   38.631    0.000    1.131    0.401
##     contct3 (.p3.)    1.085    0.018   60.617    0.000    1.480    0.642
##     contct4 (.p4.)    0.959    0.015   62.076    0.000    1.309    0.660
##   phs =~                                                                
##     pspprt1           1.000                               2.046    0.804
##     pspprt2 (.p6.)    1.022    0.010  106.700    0.000    2.092    0.875
##     pspprt3 (.p7.)    1.009    0.012   82.068    0.000    2.065    0.647
##     pspprt4 (.p8.)    0.862    0.011   78.077    0.000    1.764    0.806
##     pspprt5 (.p9.)    0.873    0.012   73.433    0.000    1.787    0.726
##   phg =~                                                                
##     hygien1           1.000                               1.406    0.676
##     hygien2 (.11.)    0.996    0.007  147.647    0.000    1.401    0.695
##     hygien3 (.12.)    0.968    0.016   61.731    0.000    1.362    0.765
##     hygien4 (.13.)    1.159    0.021   53.929    0.000    1.630    0.515
##     hygien5 (.14.)    0.892    0.020   45.536    0.000    1.255    0.524
##   om1 =~                                                                
##     omind2            1.000                               1.232    0.642
##     omind3  (.18.)    1.080    0.012   88.708    0.000    1.330    0.871
##     omind4  (.19.)    1.065    0.013   80.697    0.000    1.312    0.782
##     omind1  (.20.)    0.450    0.020   22.240    0.000    0.555    0.201
##     omind5  (.21.)    0.764    0.019   40.701    0.000    0.942    0.369
##     omind6  (.22.)    0.753    0.019   38.668    0.000    0.927    0.353
## 
## Covariances:
##                    Estimate  Std.Err  z-value  P(>|z|)   Std.lv  Std.all
##  .hygiene1 ~~                                                           
##    .hygiene2          1.642    0.082   19.971    0.000    1.642    0.740
##  .psupport4 ~~                                                          
##    .psupport5         0.477    0.049    9.755    0.000    0.477    0.218
##  .omind1 ~~                                                             
##    .omind5            1.762    0.104   16.922    0.000    1.762    0.274
##  .omind5 ~~                                                             
##    .omind6            1.834    0.104   17.641    0.000    1.834    0.315
##  .omind1 ~~                                                             
##    .omind6            2.630    0.104   25.226    0.000    2.630    0.396
##   phc ~~                                                                
##     phs               1.397    0.059   23.600    0.000    0.501    0.501
##     phg               1.154    0.046   24.979    0.000    0.601    0.601
##     om1               0.603    0.030   20.342    0.000    0.359    0.359
##   phs ~~                                                                
##     phg               1.160    0.056   20.571    0.000    0.403    0.403
##     om1               0.616    0.038   16.290    0.000    0.244    0.244
##   phg ~~                                                                
##     om1               0.567    0.032   17.913    0.000    0.327    0.327
## 
## Intercepts:
##                    Estimate  Std.Err  z-value  P(>|z|)   Std.lv  Std.all
##    .contact1          8.541    0.023  365.072    0.000    8.541    4.613
##    .contact2r         7.741    0.035  221.455    0.000    7.741    2.744
##    .contact3          8.012    0.029  275.958    0.000    8.012    3.474
##    .contact4          8.366    0.025  333.764    0.000    8.366    4.221
##    .psupport1         7.682    0.032  237.857    0.000    7.682    3.019
##    .psupport2         8.063    0.030  266.316    0.000    8.063    3.374
##    .psupport3         6.267    0.040  156.003    0.000    6.267    1.965
##    .psupport4         8.484    0.027  314.295    0.000    8.484    3.877
##    .psupport5         8.213    0.031  266.041    0.000    8.213    3.337
##    .hygiene1          8.060    0.026  306.295    0.000    8.060    3.876
##    .hygiene2          8.190    0.026  319.242    0.000    8.190    4.063
##    .hygiene3          8.865    0.022  396.654    0.000    8.865    4.980
##    .hygiene4          5.899    0.040  148.965    0.000    5.899    1.863
##    .hygiene5          8.063    0.030  268.566    0.000    8.063    3.366
##    .omind2            8.743    0.023  376.978    0.000    8.743    4.555
##    .omind3            8.652    0.019  447.324    0.000    8.652    5.666
##    .omind4            8.516    0.022  394.797    0.000    8.516    5.077
##    .omind1            6.363    0.035  183.608    0.000    6.363    2.303
##    .omind5            8.260    0.032  259.996    0.000    8.260    3.236
##    .omind6            7.178    0.033  219.387    0.000    7.178    2.734
##     phc               0.000                               0.000    0.000
##     phs               0.000                               0.000    0.000
##     phg               0.000                               0.000    0.000
##     om1               0.000                               0.000    0.000
## 
## Variances:
##                    Estimate  Std.Err  z-value  P(>|z|)   Std.lv  Std.all
##    .contact1          1.568    0.069   22.656    0.000    1.568    0.457
##    .contact2r         6.680    0.175   38.120    0.000    6.680    0.839
##    .contact3          3.128    0.119   26.237    0.000    3.128    0.588
##    .contact4          2.217    0.090   24.626    0.000    2.217    0.564
##    .psupport1         2.290    0.084   27.253    0.000    2.290    0.354
##    .psupport2         1.337    0.057   23.447    0.000    1.337    0.234
##    .psupport3         5.913    0.131   45.086    0.000    5.913    0.581
##    .psupport4         1.678    0.059   28.545    0.000    1.678    0.350
##    .psupport5         2.866    0.094   30.524    0.000    2.866    0.473
##    .hygiene1          2.345    0.091   25.845    0.000    2.345    0.542
##    .hygiene2          2.100    0.087   24.186    0.000    2.100    0.517
##    .hygiene3          1.314    0.052   25.245    0.000    1.314    0.415
##    .hygiene4          7.370    0.140   52.783    0.000    7.370    0.735
##    .hygiene5          4.165    0.128   32.606    0.000    4.165    0.726
##    .omind2            2.165    0.139   15.619    0.000    2.165    0.588
##    .omind3            0.562    0.034   16.394    0.000    0.562    0.241
##    .omind4            1.091    0.055   19.726    0.000    1.091    0.388
##    .omind1            7.329    0.122   59.948    0.000    7.329    0.960
##    .omind5            5.629    0.172   32.640    0.000    5.629    0.864
##    .omind6            6.033    0.129   46.824    0.000    6.033    0.875
##     phc               1.860    0.064   29.232    0.000    1.000    1.000
##     phs               4.187    0.120   34.785    0.000    1.000    1.000
##     phg               1.978    0.076   26.023    0.000    1.000    1.000
##     om1               1.518    0.047   32.507    0.000    1.000    1.000
## 
## 
## Group 3 [positive]:
## 
## Latent Variables:
##                    Estimate  Std.Err  z-value  P(>|z|)   Std.lv  Std.all
##   phc =~                                                                
##     contct1           1.000                               1.555    0.771
##     cntct2r (.p2.)    0.829    0.021   38.631    0.000    1.290    0.450
##     contct3 (.p3.)    1.085    0.018   60.617    0.000    1.688    0.774
##     contct4 (.p4.)    0.959    0.015   62.076    0.000    1.492    0.852
##   phs =~                                                                
##     pspprt1           1.000                               2.190    0.876
##     pspprt2 (.p6.)    1.022    0.010  106.700    0.000    2.239    0.919
##     pspprt3 (.p7.)    1.009    0.012   82.068    0.000    2.210    0.611
##     pspprt4 (.p8.)    0.862    0.011   78.077    0.000    1.887    0.828
##     pspprt5 (.p9.)    0.873    0.012   73.433    0.000    1.912    0.797
##   phg =~                                                                
##     hygien1           1.000                               1.580    0.764
##     hygien2 (.11.)    0.996    0.007  147.647    0.000    1.575    0.779
##     hygien3 (.12.)    0.968    0.016   61.731    0.000    1.530    0.818
##     hygien4 (.13.)    1.159    0.021   53.929    0.000    1.832    0.604
##     hygien5 (.14.)    0.892    0.020   45.536    0.000    1.410    0.494
##   om1 =~                                                                
##     omind2            1.000                               1.307    0.842
##     omind3  (.18.)    1.080    0.012   88.708    0.000    1.411    0.915
##     omind4  (.19.)    1.065    0.013   80.697    0.000    1.392    0.860
##     omind1  (.20.)    0.450    0.020   22.240    0.000    0.589    0.223
##     omind5  (.21.)    0.764    0.019   40.701    0.000    0.999    0.405
##     omind6  (.22.)    0.753    0.019   38.668    0.000    0.984    0.389
## 
## Covariances:
##                    Estimate  Std.Err  z-value  P(>|z|)   Std.lv  Std.all
##  .hygiene1 ~~                                                           
##    .hygiene2          1.371    0.122   11.215    0.000    1.371    0.811
##  .psupport4 ~~                                                          
##    .psupport5         0.610    0.084    7.268    0.000    0.610    0.329
##  .omind1 ~~                                                             
##    .omind5            1.510    0.159    9.494    0.000    1.510    0.259
##  .omind5 ~~                                                             
##    .omind6            2.159    0.186   11.623    0.000    2.159    0.410
##  .omind1 ~~                                                             
##    .omind6            2.353    0.171   13.780    0.000    2.353    0.392
##   phc ~~                                                                
##     phs               2.133    0.152   14.052    0.000    0.626    0.626
##     phg               1.610    0.122   13.226    0.000    0.655    0.655
##     om1               0.959    0.066   14.562    0.000    0.472    0.472
##   phs ~~                                                                
##     phg               1.799    0.144   12.508    0.000    0.520    0.520
##     om1               0.786    0.080    9.774    0.000    0.275    0.275
##   phg ~~                                                                
##     om1               0.638    0.065    9.828    0.000    0.309    0.309
## 
## Intercepts:
##                    Estimate  Std.Err  z-value  P(>|z|)   Std.lv  Std.all
##    .contact1          8.379    0.048  173.912    0.000    8.379    4.156
##    .contact2r         7.680    0.068  112.739    0.000    7.680    2.676
##    .contact3          8.179    0.051  159.718    0.000    8.179    3.751
##    .contact4          8.617    0.042  205.576    0.000    8.617    4.922
##    .psupport1         7.916    0.058  136.600    0.000    7.916    3.167
##    .psupport2         8.027    0.059  136.697    0.000    8.027    3.296
##    .psupport3         5.551    0.084   66.137    0.000    5.551    1.535
##    .psupport4         8.334    0.055  150.879    0.000    8.334    3.654
##    .psupport5         8.171    0.057  142.320    0.000    8.171    3.407
##    .hygiene1          7.962    0.050  159.541    0.000    7.962    3.850
##    .hygiene2          8.067    0.049  164.854    0.000    8.067    3.991
##    .hygiene3          8.678    0.043  199.534    0.000    8.678    4.640
##    .hygiene4          6.467    0.072   89.985    0.000    6.467    2.133
##    .hygiene5          7.169    0.069  103.495    0.000    7.169    2.514
##    .omind2            8.511    0.037  229.195    0.000    8.511    5.485
##    .omind3            8.432    0.037  230.957    0.000    8.432    5.470
##    .omind4            8.450    0.038  220.690    0.000    8.450    5.219
##    .omind1            5.576    0.063   89.051    0.000    5.576    2.108
##    .omind5            8.060    0.060  134.408    0.000    8.060    3.264
##    .omind6            7.359    0.061  120.445    0.000    7.359    2.910
##     phc               0.000                               0.000    0.000
##     phs               0.000                               0.000    0.000
##     phg               0.000                               0.000    0.000
##     om1               0.000                               0.000    0.000
## 
## Variances:
##                    Estimate  Std.Err  z-value  P(>|z|)   Std.lv  Std.all
##    .contact1          1.647    0.119   13.806    0.000    1.647    0.405
##    .contact2r         6.570    0.305   21.573    0.000    6.570    0.798
##    .contact3          1.906    0.143   13.323    0.000    1.906    0.401
##    .contact4          0.839    0.057   14.721    0.000    0.839    0.274
##    .psupport1         1.452    0.114   12.790    0.000    1.452    0.232
##    .psupport2         0.920    0.074   12.436    0.000    0.920    0.155
##    .psupport3         8.195    0.276   29.671    0.000    8.195    0.627
##    .psupport4         1.640    0.110   14.972    0.000    1.640    0.315
##    .psupport5         2.095    0.132   15.860    0.000    2.095    0.364
##    .hygiene1          1.778    0.125   14.279    0.000    1.778    0.416
##    .hygiene2          1.606    0.128   12.564    0.000    1.606    0.393
##    .hygiene3          1.157    0.078   14.904    0.000    1.157    0.331
##    .hygiene4          5.838    0.252   23.124    0.000    5.838    0.635
##    .hygiene5          6.148    0.281   21.909    0.000    6.148    0.756
##    .omind2            0.699    0.054   12.983    0.000    0.699    0.290
##    .omind3            0.385    0.038   10.094    0.000    0.385    0.162
##    .omind4            0.683    0.057   12.084    0.000    0.683    0.260
##    .omind1            6.649    0.203   32.748    0.000    6.649    0.950
##    .omind5            5.099    0.295   17.287    0.000    5.099    0.836
##    .omind6            5.426    0.238   22.834    0.000    5.426    0.849
##     phc               2.419    0.148   16.323    0.000    1.000    1.000
##     phs               4.794    0.235   20.392    0.000    1.000    1.000
##     phg               2.497    0.144   17.373    0.000    1.000    1.000
##     om1               1.709    0.072   23.818    0.000    1.000    1.000
```

```
phymodcs <- cfa(phc, estimator = "MLM", data = pls4, group = "cpol", group.equal = c("loadings", "intercepts"))
summary(phymodcs, standardized = T)
```

```
## lavaan 0.6-8 ended normally after 225 iterations
## 
##   Estimator                                         ML
##   Optimization method                           NLMINB
##   Number of model parameters                       221
##   Number of equality constraints                    72
##                                                       
##   Number of observations per group:                   
##     negative                                      4408
##     neutral                                       6315
##     positive                                      1767
##                                                       
## Model Test User Model:
##                                               Standard      Robust
##   Test Statistic                              7296.345    6985.173
##   Degrees of freedom                               541         541
##   P-value (Chi-square)                           0.000       0.000
##   Scaling correction factor                                  1.045
##        Satorra-Bentler correction                                 
##   Test statistic for each group:
##     negative                                  3153.808    3019.305
##     neutral                                   2674.552    2560.489
##     positive                                  1467.985    1405.379
## 
## Parameter Estimates:
## 
##   Standard errors                           Robust.sem
##   Information                                 Expected
##   Information saturated (h1) model          Structured
## 
## 
## Group 1 [negative]:
## 
## Latent Variables:
##                    Estimate  Std.Err  z-value  P(>|z|)   Std.lv  Std.all
##   phc =~                                                                
##     contct1           1.000                               1.125    0.763
##     cntct2r (.p2.)    0.836    0.021   39.695    0.000    0.940    0.361
##     contct3 (.p3.)    1.075    0.018   61.373    0.000    1.209    0.581
##     contct4 (.p4.)    0.979    0.015   63.982    0.000    1.102    0.802
##   phs =~                                                                
##     pspprt1           1.000                               1.729    0.688
##     pspprt2 (.p6.)    1.035    0.010  106.672    0.000    1.790    0.896
##     pspprt3 (.p7.)    1.025    0.012   82.376    0.000    1.772    0.588
##     pspprt4 (.p8.)    0.869    0.011   78.376    0.000    1.502    0.839
##     pspprt5 (.p9.)    0.881    0.012   73.715    0.000    1.523    0.729
##   phg =~                                                                
##     hygien1           1.000                               1.495    0.764
##     hygien2 (.11.)    0.997    0.007  147.515    0.000    1.490    0.781
##     hygien3 (.12.)    0.959    0.016   61.458    0.000    1.433    0.712
##     hygien4 (.13.)    1.147    0.021   53.819    0.000    1.715    0.562
##     hygien5 (.14.)    0.896    0.020   45.582    0.000    1.339    0.580
##   om1 =~                                                                
##     omind2            1.000                               1.181    0.838
##     omind3  (.18.)    1.080    0.012   88.381    0.000    1.275    0.899
##     omind4  (.19.)    1.062    0.013   80.489    0.000    1.254    0.809
##     omind1  (.20.)    0.460    0.020   22.732    0.000    0.543    0.207
##     omind5  (.21.)    0.765    0.019   40.718    0.000    0.904    0.365
##     omind6  (.22.)    0.748    0.019   38.508    0.000    0.883    0.348
## 
## Covariances:
##                    Estimate  Std.Err  z-value  P(>|z|)   Std.lv  Std.all
##  .hygiene1 ~~                                                           
##    .hygiene2          1.115    0.075   14.829    0.000    1.115    0.739
##  .psupport4 ~~                                                          
##    .psupport5         0.261    0.041    6.334    0.000    0.261    0.188
##  .omind1 ~~                                                             
##    .omind5            2.051    0.123   16.662    0.000    2.051    0.346
##  .omind5 ~~                                                             
##    .omind6            2.754    0.141   19.535    0.000    2.754    0.502
##  .omind1 ~~                                                             
##    .omind6            2.475    0.118   20.970    0.000    2.475    0.405
##   phc ~~                                                                
##     phs               1.157    0.049   23.484    0.000    0.595    0.595
##     phg               0.876    0.040   22.144    0.000    0.521    0.521
##     om1               0.701    0.034   20.758    0.000    0.527    0.527
##   phs ~~                                                                
##     phg               0.993    0.056   17.634    0.000    0.384    0.384
##     om1               0.692    0.039   17.783    0.000    0.339    0.339
##   phg ~~                                                                
##     om1               0.512    0.034   15.123    0.000    0.290    0.290
## 
## Intercepts:
##                    Estimate  Std.Err  z-value  P(>|z|)   Std.lv  Std.all
##    .contct1 (.56.)    8.929    0.020  436.667    0.000    8.929    6.056
##    .cntct2r (.57.)    8.102    0.028  285.342    0.000    8.102    3.111
##    .contct3 (.58.)    8.444    0.025  332.666    0.000    8.444    4.059
##    .contct4 (.59.)    8.957    0.019  461.768    0.000    8.957    6.524
##    .pspprt1 (.60.)    8.083    0.031  262.047    0.000    8.083    3.216
##    .pspprt2 (.61.)    8.528    0.029  295.606    0.000    8.528    4.269
##    .pspprt3 (.62.)    6.654    0.036  182.550    0.000    6.654    2.207
##    .pspprt4 (.63.)    8.783    0.025  346.946    0.000    8.783    4.905
##    .pspprt5 (.64.)    8.552    0.027  315.151    0.000    8.552    4.094
##    .hygien1 (.65.)    8.128    0.027  302.165    0.000    8.128    4.153
##    .hygien2 (.66.)    8.252    0.026  312.567    0.000    8.252    4.322
##    .hygien3 (.67.)    8.729    0.026  329.668    0.000    8.729    4.338
##    .hygien4 (.68.)    6.224    0.037  169.497    0.000    6.224    2.040
##    .hygien5 (.69.)    7.978    0.028  282.833    0.000    7.978    3.454
##    .omind2  (.70.)    8.684    0.020  424.511    0.000    8.684    6.158
##    .omind3  (.71.)    8.614    0.021  416.199    0.000    8.614    6.069
##    .omind4  (.72.)    8.522    0.021  403.514    0.000    8.522    5.498
##    .omind1  (.73.)    6.107    0.025  241.421    0.000    6.107    2.325
##    .omind5  (.74.)    8.283    0.025  327.477    0.000    8.283    3.342
##    .omind6  (.75.)    7.344    0.026  284.864    0.000    7.344    2.895
##     phc               0.000                               0.000    0.000
##     phs               0.000                               0.000    0.000
##     phg               0.000                               0.000    0.000
##     om1               0.000                               0.000    0.000
## 
## Variances:
##                    Estimate  Std.Err  z-value  P(>|z|)   Std.lv  Std.all
##    .contact1          0.908    0.055   16.515    0.000    0.908    0.418
##    .contact2r         5.898    0.192   30.709    0.000    5.898    0.870
##    .contact3          2.867    0.129   22.228    0.000    2.867    0.662
##    .contact4          0.672    0.039   17.295    0.000    0.672    0.356
##    .psupport1         3.325    0.133   24.952    0.000    3.325    0.527
##    .psupport2         0.786    0.048   16.345    0.000    0.786    0.197
##    .psupport3         5.951    0.159   37.506    0.000    5.951    0.655
##    .psupport4         0.950    0.043   21.912    0.000    0.950    0.296
##    .psupport5         2.044    0.087   23.479    0.000    2.044    0.469
##    .hygiene1          1.596    0.083   19.319    0.000    1.596    0.417
##    .hygiene2          1.424    0.078   18.237    0.000    1.424    0.391
##    .hygiene3          1.995    0.087   22.925    0.000    1.995    0.493
##    .hygiene4          6.363    0.168   37.793    0.000    6.363    0.684
##    .hygiene5          3.543    0.146   24.292    0.000    3.543    0.664
##    .omind2            0.593    0.033   17.893    0.000    0.593    0.298
##    .omind3            0.388    0.026   15.050    0.000    0.388    0.193
##    .omind4            0.829    0.047   17.816    0.000    0.829    0.345
##    .omind1            6.604    0.131   50.483    0.000    6.604    0.957
##    .omind5            5.324    0.202   26.329    0.000    5.324    0.867
##    .omind6            5.656    0.159   35.524    0.000    5.656    0.879
##     phc               1.265    0.051   24.926    0.000    1.000    1.000
##     phs               2.990    0.116   25.678    0.000    1.000    1.000
##     phg               2.235    0.089   25.012    0.000    1.000    1.000
##     om1               1.396    0.048   28.914    0.000    1.000    1.000
## 
## 
## Group 2 [neutral]:
## 
## Latent Variables:
##                    Estimate  Std.Err  z-value  P(>|z|)   Std.lv  Std.all
##   phc =~                                                                
##     contct1           1.000                               1.357    0.733
##     cntct2r (.p2.)    0.836    0.021   39.695    0.000    1.134    0.402
##     contct3 (.p3.)    1.075    0.018   61.373    0.000    1.458    0.635
##     contct4 (.p4.)    0.979    0.015   63.982    0.000    1.329    0.665
##   phs =~                                                                
##     pspprt1           1.000                               2.029    0.801
##     pspprt2 (.p6.)    1.035    0.010  106.672    0.000    2.100    0.876
##     pspprt3 (.p7.)    1.025    0.012   82.376    0.000    2.079    0.650
##     pspprt4 (.p8.)    0.869    0.011   78.376    0.000    1.762    0.805
##     pspprt5 (.p9.)    0.881    0.012   73.715    0.000    1.786    0.726
##   phg =~                                                                
##     hygien1           1.000                               1.411    0.677
##     hygien2 (.11.)    0.997    0.007  147.515    0.000    1.406    0.696
##     hygien3 (.12.)    0.959    0.016   61.458    0.000    1.352    0.759
##     hygien4 (.13.)    1.147    0.021   53.819    0.000    1.618    0.509
##     hygien5 (.14.)    0.896    0.020   45.582    0.000    1.264    0.527
##   om1 =~                                                                
##     omind2            1.000                               1.233    0.642
##     omind3  (.18.)    1.080    0.012   88.381    0.000    1.332    0.872
##     omind4  (.19.)    1.062    0.013   80.489    0.000    1.309    0.781
##     omind1  (.20.)    0.460    0.020   22.732    0.000    0.567    0.204
##     omind5  (.21.)    0.765    0.019   40.718    0.000    0.944    0.369
##     omind6  (.22.)    0.748    0.019   38.508    0.000    0.922    0.351
## 
## Covariances:
##                    Estimate  Std.Err  z-value  P(>|z|)   Std.lv  Std.all
##  .hygiene1 ~~                                                           
##    .hygiene2          1.644    0.083   19.915    0.000    1.644    0.740
##  .psupport4 ~~                                                          
##    .psupport5         0.481    0.049    9.828    0.000    0.481    0.219
##  .omind1 ~~                                                             
##    .omind5            1.751    0.104   16.797    0.000    1.751    0.271
##  .omind5 ~~                                                             
##    .omind6            1.842    0.104   17.718    0.000    1.842    0.315
##  .omind1 ~~                                                             
##    .omind6            2.584    0.104   24.765    0.000    2.584    0.386
##   phc ~~                                                                
##     phs               1.378    0.059   23.519    0.000    0.501    0.501
##     phg               1.156    0.046   25.005    0.000    0.604    0.604
##     om1               0.602    0.030   20.354    0.000    0.359    0.359
##   phs ~~                                                                
##     phg               1.155    0.056   20.562    0.000    0.403    0.403
##     om1               0.611    0.037   16.294    0.000    0.244    0.244
##   phg ~~                                                                
##     om1               0.571    0.032   17.923    0.000    0.328    0.328
## 
## Intercepts:
##                    Estimate  Std.Err  z-value  P(>|z|)   Std.lv  Std.all
##    .contct1 (.56.)    8.929    0.020  436.667    0.000    8.929    4.825
##    .cntct2r (.57.)    8.102    0.028  285.342    0.000    8.102    2.871
##    .contct3 (.58.)    8.444    0.025  332.666    0.000    8.444    3.676
##    .contct4 (.59.)    8.957    0.019  461.768    0.000    8.957    4.483
##    .pspprt1 (.60.)    8.083    0.031  262.047    0.000    8.083    3.191
##    .pspprt2 (.61.)    8.528    0.029  295.606    0.000    8.528    3.559
##    .pspprt3 (.62.)    6.654    0.036  182.550    0.000    6.654    2.081
##    .pspprt4 (.63.)    8.783    0.025  346.946    0.000    8.783    4.014
##    .pspprt5 (.64.)    8.552    0.027  315.151    0.000    8.552    3.474
##    .hygien1 (.65.)    8.128    0.027  302.165    0.000    8.128    3.903
##    .hygien2 (.66.)    8.252    0.026  312.567    0.000    8.252    4.086
##    .hygien3 (.67.)    8.729    0.026  329.668    0.000    8.729    4.897
##    .hygien4 (.68.)    6.224    0.037  169.497    0.000    6.224    1.956
##    .hygien5 (.69.)    7.978    0.028  282.833    0.000    7.978    3.325
##    .omind2  (.70.)    8.684    0.020  424.511    0.000    8.684    4.522
##    .omind3  (.71.)    8.614    0.021  416.199    0.000    8.614    5.639
##    .omind4  (.72.)    8.522    0.021  403.514    0.000    8.522    5.084
##    .omind1  (.73.)    6.107    0.025  241.421    0.000    6.107    2.199
##    .omind5  (.74.)    8.283    0.025  327.477    0.000    8.283    3.244
##    .omind6  (.75.)    7.344    0.026  284.864    0.000    7.344    2.792
##     phc              -0.455    0.028  -16.246    0.000   -0.335   -0.335
##     phs              -0.405    0.039  -10.462    0.000   -0.200   -0.200
##     phg               0.020    0.033    0.618    0.537    0.014    0.014
##     om1               0.021    0.025    0.841    0.400    0.017    0.017
## 
## Variances:
##                    Estimate  Std.Err  z-value  P(>|z|)   Std.lv  Std.all
##    .contact1          1.584    0.068   23.146    0.000    1.584    0.462
##    .contact2r         6.678    0.174   38.352    0.000    6.678    0.838
##    .contact3          3.150    0.118   26.769    0.000    3.150    0.597
##    .contact4          2.227    0.089   24.898    0.000    2.227    0.558
##    .psupport1         2.303    0.084   27.571    0.000    2.303    0.359
##    .psupport2         1.331    0.057   23.401    0.000    1.331    0.232
##    .psupport3         5.908    0.131   45.060    0.000    5.908    0.578
##    .psupport4         1.682    0.059   28.683    0.000    1.682    0.351
##    .psupport5         2.868    0.094   30.643    0.000    2.868    0.473
##    .hygiene1          2.348    0.091   25.774    0.000    2.348    0.541
##    .hygiene2          2.101    0.087   24.101    0.000    2.101    0.515
##    .hygiene3          1.348    0.052   25.833    0.000    1.348    0.424
##    .hygiene4          7.510    0.139   53.843    0.000    7.510    0.741
##    .hygiene5          4.159    0.128   32.477    0.000    4.159    0.723
##    .omind2            2.166    0.139   15.615    0.000    2.166    0.587
##    .omind3            0.561    0.034   16.281    0.000    0.561    0.240
##    .omind4            1.095    0.055   19.766    0.000    1.095    0.390
##    .omind1            7.388    0.123   60.251    0.000    7.388    0.958
##    .omind5            5.631    0.173   32.622    0.000    5.631    0.863
##    .omind6            6.068    0.129   47.125    0.000    6.068    0.877
##     phc               1.841    0.063   29.195    0.000    1.000    1.000
##     phs               4.115    0.119   34.562    0.000    1.000    1.000
##     phg               1.990    0.077   26.012    0.000    1.000    1.000
##     om1               1.521    0.047   32.469    0.000    1.000    1.000
## 
## 
## Group 3 [positive]:
## 
## Latent Variables:
##                    Estimate  Std.Err  z-value  P(>|z|)   Std.lv  Std.all
##   phc =~                                                                
##     contct1           1.000                               1.544    0.764
##     cntct2r (.p2.)    0.836    0.021   39.695    0.000    1.290    0.449
##     contct3 (.p3.)    1.075    0.018   61.373    0.000    1.659    0.765
##     contct4 (.p4.)    0.979    0.015   63.982    0.000    1.512    0.858
##   phs =~                                                                
##     pspprt1           1.000                               2.173    0.866
##     pspprt2 (.p6.)    1.035    0.010  106.672    0.000    2.249    0.921
##     pspprt3 (.p7.)    1.025    0.012   82.376    0.000    2.226    0.603
##     pspprt4 (.p8.)    0.869    0.011   78.376    0.000    1.887    0.828
##     pspprt5 (.p9.)    0.881    0.012   73.715    0.000    1.913    0.799
##   phg =~                                                                
##     hygien1           1.000                               1.587    0.766
##     hygien2 (.11.)    0.997    0.007  147.515    0.000    1.581    0.781
##     hygien3 (.12.)    0.959    0.016   61.458    0.000    1.521    0.816
##     hygien4 (.13.)    1.147    0.021   53.819    0.000    1.820    0.596
##     hygien5 (.14.)    0.896    0.020   45.582    0.000    1.421    0.482
##   om1 =~                                                                
##     omind2            1.000                               1.308    0.843
##     omind3  (.18.)    1.080    0.012   88.381    0.000    1.412    0.916
##     omind4  (.19.)    1.062    0.013   80.489    0.000    1.389    0.857
##     omind1  (.20.)    0.460    0.020   22.732    0.000    0.601    0.224
##     omind5  (.21.)    0.765    0.019   40.718    0.000    1.001    0.405
##     omind6  (.22.)    0.748    0.019   38.508    0.000    0.978    0.387
## 
## Covariances:
##                    Estimate  Std.Err  z-value  P(>|z|)   Std.lv  Std.all
##  .hygiene1 ~~                                                           
##    .hygiene2          1.365    0.122   11.151    0.000    1.365    0.811
##  .psupport4 ~~                                                          
##    .psupport5         0.598    0.084    7.156    0.000    0.598    0.324
##  .omind1 ~~                                                             
##    .omind5            1.561    0.159    9.809    0.000    1.561    0.263
##  .omind5 ~~                                                             
##    .omind6            2.146    0.186   11.562    0.000    2.146    0.407
##  .omind1 ~~                                                             
##    .omind6            2.294    0.171   13.425    0.000    2.294    0.375
##   phc ~~                                                                
##     phs               2.100    0.150   14.004    0.000    0.626    0.626
##     phg               1.610    0.122   13.244    0.000    0.658    0.658
##     om1               0.955    0.066   14.567    0.000    0.473    0.473
##   phs ~~                                                                
##     phg               1.795    0.144   12.488    0.000    0.521    0.521
##     om1               0.782    0.080    9.781    0.000    0.275    0.275
##   phg ~~                                                                
##     om1               0.643    0.065    9.848    0.000    0.310    0.310
## 
## Intercepts:
##                    Estimate  Std.Err  z-value  P(>|z|)   Std.lv  Std.all
##    .contct1 (.56.)    8.929    0.020  436.667    0.000    8.929    4.417
##    .cntct2r (.57.)    8.102    0.028  285.342    0.000    8.102    2.821
##    .contct3 (.58.)    8.444    0.025  332.666    0.000    8.444    3.894
##    .contct4 (.59.)    8.957    0.019  461.768    0.000    8.957    5.086
##    .pspprt1 (.60.)    8.083    0.031  262.047    0.000    8.083    3.222
##    .pspprt2 (.61.)    8.528    0.029  295.606    0.000    8.528    3.491
##    .pspprt3 (.62.)    6.654    0.036  182.550    0.000    6.654    1.803
##    .pspprt4 (.63.)    8.783    0.025  346.946    0.000    8.783    3.852
##    .pspprt5 (.64.)    8.552    0.027  315.151    0.000    8.552    3.570
##    .hygien1 (.65.)    8.128    0.027  302.165    0.000    8.128    3.925
##    .hygien2 (.66.)    8.252    0.026  312.567    0.000    8.252    4.074
##    .hygien3 (.67.)    8.729    0.026  329.668    0.000    8.729    4.681
##    .hygien4 (.68.)    6.224    0.037  169.497    0.000    6.224    2.037
##    .hygien5 (.69.)    7.978    0.028  282.833    0.000    7.978    2.705
##    .omind2  (.70.)    8.684    0.020  424.511    0.000    8.684    5.594
##    .omind3  (.71.)    8.614    0.021  416.199    0.000    8.614    5.585
##    .omind4  (.72.)    8.522    0.021  403.514    0.000    8.522    5.261
##    .omind1  (.73.)    6.107    0.025  241.421    0.000    6.107    2.271
##    .omind5  (.74.)    8.283    0.025  327.477    0.000    8.283    3.350
##    .omind6  (.75.)    7.344    0.026  284.864    0.000    7.344    2.903
##     phc              -0.378    0.044   -8.558    0.000   -0.245   -0.245
##     phs              -0.433    0.060   -7.155    0.000   -0.199   -0.199
##     phg              -0.124    0.049   -2.534    0.011   -0.078   -0.078
##     om1              -0.144    0.038   -3.835    0.000   -0.110   -0.110
## 
## Variances:
##                    Estimate  Std.Err  z-value  P(>|z|)   Std.lv  Std.all
##    .contact1          1.703    0.119   14.291    0.000    1.703    0.417
##    .contact2r         6.585    0.304   21.640    0.000    6.585    0.798
##    .contact3          1.951    0.142   13.738    0.000    1.951    0.415
##    .contact4          0.817    0.057   14.390    0.000    0.817    0.263
##    .psupport1         1.575    0.113   13.935    0.000    1.575    0.250
##    .psupport2         0.907    0.074   12.243    0.000    0.907    0.152
##    .psupport3         8.659    0.277   31.307    0.000    8.659    0.636
##    .psupport4         1.638    0.109   14.997    0.000    1.638    0.315
##    .psupport5         2.079    0.132   15.792    0.000    2.079    0.362
##    .hygiene1          1.772    0.125   14.205    0.000    1.772    0.413
##    .hygiene2          1.601    0.128   12.508    0.000    1.601    0.390
##    .hygiene3          1.164    0.077   15.031    0.000    1.164    0.335
##    .hygiene4          6.028    0.252   23.940    0.000    6.028    0.645
##    .hygiene5          6.678    0.281   23.768    0.000    6.678    0.768
##    .omind2            0.699    0.054   12.997    0.000    0.699    0.290
##    .omind3            0.384    0.038   10.093    0.000    0.384    0.162
##    .omind4            0.695    0.056   12.338    0.000    0.695    0.265
##    .omind1            6.867    0.203   33.752    0.000    6.867    0.950
##    .omind5            5.112    0.295   17.334    0.000    5.112    0.836
##    .omind6            5.443    0.237   22.942    0.000    5.443    0.850
##     phc               2.383    0.146   16.299    0.000    1.000    1.000
##     phs               4.720    0.233   20.285    0.000    1.000    1.000
##     phg               2.517    0.145   17.361    0.000    1.000    1.000
##     om1               1.711    0.072   23.790    0.000    1.000    1.000
```

```
print(compareFit(phymodcc, phymodcw, phymodcs, nested = T))
```

```
## ################### Nested Model Comparison #########################
## Scaled Chi-Squared Difference Test (method = "satorra.bentler.2001")
## 
## lavaan NOTE:
##     The "Chisq" column contains standard test statistics, not the
##     robust test that should be reported per model. A robust difference
##     test is a function of two standard (not robust) statistics.
##  
##           Df    AIC    BIC  Chisq Chisq diff Df diff Pr(>Chisq)    
## phymodcc 477 989995 991578 5475.4                                  
## phymodcw 509 990217 991562 5761.1     253.69      32  < 2.2e-16 ***
## phymodcs 541 991688 992795 7296.3    2202.23      32  < 2.2e-16 ***
## ---
## Signif. codes:  0 '***' 0.001 '**' 0.01 '*' 0.05 '.' 0.1 ' ' 1
## 
## ####################### Model Fit Indices ###########################
##          chisq.scaled df.scaled pvalue.scaled cfi.robust tli.robust         aic
## phymodcc    5153.892†       477          .000      .957†      .948  989994.891†
## phymodcw    5402.417        509          .000      .954       .949† 990216.601 
## phymodcs    6985.173        541          .000      .941       .938  991687.873 
##                  bic rmsea.robust  srmr
## phymodcc 991578.052         .050  .044†
## phymodcw 991561.917†        .050† .047 
## phymodcs 992795.343         .055  .050 
## 
## ################## Differences in Fit Indices #######################
##                     df.scaled cfi.robust tli.robust      aic      bic
## phymodcw - phymodcc        32     -0.002      0.001  221.711  -16.135
## phymodcs - phymodcw        32     -0.013     -0.011 1471.272 1233.426
##                     rmsea.robust  srmr
## phymodcw - phymodcc        0.000 0.003
## phymodcs - phymodcw        0.005 0.003
```

```
miobj <- modindices(phymodcs, free.remove = F)
x <- as.data.frame(miobj %>% group_by(op, lhs) %>% summarize(mi_tot = sum(mi)))
```

```
## `summarise()` has grouped output by 'op'. You can override using the `.groups` argument.
```

```
y <- as.data.frame(subset(x, op == "~1"))
top_n(y, 5)
```

```
## Selecting by mi_tot
```

```
##   op       lhs   mi_tot
## 1 ~1  hygiene3 337.7670
## 2 ~1  hygiene4 210.1153
## 3 ~1  hygiene5 149.0005
## 4 ~1    omind1 220.5877
## 5 ~1 psupport1 180.6288
```

```
phymodcs2 <- cfa(phc, estimator = "MLM", data = pls4, group = "cpol", group.equal = c("loadings", "intercepts"), group.partial = c("psupport1 ~ 1"))
summary(phymodcs2, standardized = T)
```

```
## lavaan 0.6-8 ended normally after 229 iterations
## 
##   Estimator                                         ML
##   Optimization method                           NLMINB
##   Number of model parameters                       221
##   Number of equality constraints                    70
##                                                       
##   Number of observations per group:                   
##     negative                                      4408
##     neutral                                       6315
##     positive                                      1767
##                                                       
## Model Test User Model:
##                                               Standard      Robust
##   Test Statistic                              7104.324    6779.524
##   Degrees of freedom                               539         539
##   P-value (Chi-square)                           0.000       0.000
##   Scaling correction factor                                  1.048
##        Satorra-Bentler correction                                 
##   Test statistic for each group:
##     negative                                  3069.312    2928.987
##     neutral                                   2671.671    2549.526
##     positive                                  1363.342    1301.011
## 
## Parameter Estimates:
## 
##   Standard errors                           Robust.sem
##   Information                                 Expected
##   Information saturated (h1) model          Structured
## 
## 
## Group 1 [negative]:
## 
## Latent Variables:
##                    Estimate  Std.Err  z-value  P(>|z|)   Std.lv  Std.all
##   phc =~                                                                
##     contct1           1.000                               1.125    0.763
##     cntct2r (.p2.)    0.836    0.021   39.694    0.000    0.940    0.361
##     contct3 (.p3.)    1.075    0.018   61.375    0.000    1.209    0.581
##     contct4 (.p4.)    0.979    0.015   63.982    0.000    1.101    0.802
##   phs =~                                                                
##     pspprt1           1.000                               1.747    0.696
##     pspprt2 (.p6.)    1.024    0.010  106.966    0.000    1.788    0.897
##     pspprt3 (.p7.)    1.016    0.012   82.923    0.000    1.776    0.589
##     pspprt4 (.p8.)    0.858    0.011   78.130    0.000    1.499    0.837
##     pspprt5 (.p9.)    0.869    0.012   73.523    0.000    1.519    0.727
##   phg =~                                                                
##     hygien1           1.000                               1.495    0.764
##     hygien2 (.11.)    0.997    0.007  147.513    0.000    1.490    0.781
##     hygien3 (.12.)    0.959    0.016   61.461    0.000    1.433    0.712
##     hygien4 (.13.)    1.147    0.021   53.821    0.000    1.715    0.562
##     hygien5 (.14.)    0.896    0.020   45.584    0.000    1.340    0.580
##   om1 =~                                                                
##     omind2            1.000                               1.181    0.838
##     omind3  (.18.)    1.080    0.012   88.381    0.000    1.275    0.899
##     omind4  (.19.)    1.062    0.013   80.489    0.000    1.254    0.809
##     omind1  (.20.)    0.460    0.020   22.733    0.000    0.543    0.207
##     omind5  (.21.)    0.765    0.019   40.718    0.000    0.904    0.365
##     omind6  (.22.)    0.748    0.019   38.508    0.000    0.883    0.348
## 
## Covariances:
##                    Estimate  Std.Err  z-value  P(>|z|)   Std.lv  Std.all
##  .hygiene1 ~~                                                           
##    .hygiene2          1.115    0.075   14.832    0.000    1.115    0.739
##  .psupport4 ~~                                                          
##    .psupport5         0.271    0.041    6.567    0.000    0.271    0.193
##  .omind1 ~~                                                             
##    .omind5            2.051    0.123   16.662    0.000    2.051    0.346
##  .omind5 ~~                                                             
##    .omind6            2.754    0.141   19.535    0.000    2.754    0.502
##  .omind1 ~~                                                             
##    .omind6            2.475    0.118   20.970    0.000    2.475    0.405
##   phc ~~                                                                
##     phs               1.168    0.050   23.549    0.000    0.594    0.594
##     phg               0.876    0.040   22.144    0.000    0.521    0.521
##     om1               0.701    0.034   20.757    0.000    0.527    0.527
##   phs ~~                                                                
##     phg               1.004    0.057   17.665    0.000    0.384    0.384
##     om1               0.698    0.039   17.796    0.000    0.338    0.338
##   phg ~~                                                                
##     om1               0.512    0.034   15.122    0.000    0.290    0.290
## 
## Intercepts:
##                    Estimate  Std.Err  z-value  P(>|z|)   Std.lv  Std.all
##    .contct1 (.56.)    8.929    0.020  436.664    0.000    8.929    6.056
##    .cntct2r (.57.)    8.102    0.028  285.345    0.000    8.102    3.111
##    .contct3 (.58.)    8.444    0.025  332.660    0.000    8.444    4.059
##    .contct4 (.59.)    8.957    0.019  461.773    0.000    8.957    6.524
##    .pspprt1           7.850    0.038  206.680    0.000    7.850    3.127
##    .pspprt2 (.61.)    8.559    0.029  295.670    0.000    8.559    4.291
##    .pspprt3 (.62.)    6.683    0.036  183.154    0.000    6.683    2.217
##    .pspprt4 (.63.)    8.805    0.025  346.818    0.000    8.805    4.917
##    .pspprt5 (.64.)    8.577    0.027  315.424    0.000    8.577    4.107
##    .hygien1 (.65.)    8.128    0.027  302.167    0.000    8.128    4.153
##    .hygien2 (.66.)    8.252    0.026  312.571    0.000    8.252    4.322
##    .hygien3 (.67.)    8.729    0.026  329.666    0.000    8.729    4.338
##    .hygien4 (.68.)    6.224    0.037  169.494    0.000    6.224    2.040
##    .hygien5 (.69.)    7.978    0.028  282.830    0.000    7.978    3.454
##    .omind2  (.70.)    8.684    0.020  424.511    0.000    8.684    6.158
##    .omind3  (.71.)    8.614    0.021  416.199    0.000    8.614    6.069
##    .omind4  (.72.)    8.522    0.021  403.514    0.000    8.522    5.498
##    .omind1  (.73.)    6.107    0.025  241.421    0.000    6.107    2.325
##    .omind5  (.74.)    8.283    0.025  327.477    0.000    8.283    3.342
##    .omind6  (.75.)    7.344    0.026  284.863    0.000    7.344    2.895
##     phc               0.000                               0.000    0.000
##     phs               0.000                               0.000    0.000
##     phg               0.000                               0.000    0.000
##     om1               0.000                               0.000    0.000
## 
## Variances:
##                    Estimate  Std.Err  z-value  P(>|z|)   Std.lv  Std.all
##    .contact1          0.908    0.055   16.515    0.000    0.908    0.418
##    .contact2r         5.898    0.192   30.709    0.000    5.898    0.870
##    .contact3          2.867    0.129   22.226    0.000    2.867    0.662
##    .contact4          0.672    0.039   17.297    0.000    0.672    0.356
##    .psupport1         3.248    0.133   24.409    0.000    3.248    0.515
##    .psupport2         0.780    0.048   16.246    0.000    0.780    0.196
##    .psupport3         5.935    0.159   37.399    0.000    5.935    0.653
##    .psupport4         0.960    0.043   22.109    0.000    0.960    0.299
##    .psupport5         2.054    0.087   23.578    0.000    2.054    0.471
##    .hygiene1          1.596    0.083   19.321    0.000    1.596    0.417
##    .hygiene2          1.425    0.078   18.240    0.000    1.425    0.391
##    .hygiene3          1.995    0.087   22.925    0.000    1.995    0.493
##    .hygiene4          6.363    0.168   37.791    0.000    6.363    0.684
##    .hygiene5          3.543    0.146   24.291    0.000    3.543    0.664
##    .omind2            0.593    0.033   17.893    0.000    0.593    0.298
##    .omind3            0.388    0.026   15.050    0.000    0.388    0.193
##    .omind4            0.829    0.047   17.816    0.000    0.829    0.345
##    .omind1            6.604    0.131   50.483    0.000    6.604    0.957
##    .omind5            5.324    0.202   26.329    0.000    5.324    0.867
##    .omind6            5.656    0.159   35.524    0.000    5.656    0.879
##     phc               1.265    0.051   24.927    0.000    1.000    1.000
##     phs               3.053    0.118   25.914    0.000    1.000    1.000
##     phg               2.235    0.089   25.012    0.000    1.000    1.000
##     om1               1.396    0.048   28.914    0.000    1.000    1.000
## 
## 
## Group 2 [neutral]:
## 
## Latent Variables:
##                    Estimate  Std.Err  z-value  P(>|z|)   Std.lv  Std.all
##   phc =~                                                                
##     contct1           1.000                               1.357    0.733
##     cntct2r (.p2.)    0.836    0.021   39.694    0.000    1.134    0.402
##     contct3 (.p3.)    1.075    0.018   61.375    0.000    1.458    0.635
##     contct4 (.p4.)    0.979    0.015   63.982    0.000    1.329    0.665
##   phs =~                                                                
##     pspprt1           1.000                               2.047    0.804
##     pspprt2 (.p6.)    1.024    0.010  106.966    0.000    2.095    0.876
##     pspprt3 (.p7.)    1.016    0.012   82.923    0.000    2.080    0.650
##     pspprt4 (.p8.)    0.858    0.011   78.130    0.000    1.756    0.804
##     pspprt5 (.p9.)    0.869    0.012   73.523    0.000    1.779    0.724
##   phg =~                                                                
##     hygien1           1.000                               1.411    0.677
##     hygien2 (.11.)    0.997    0.007  147.513    0.000    1.406    0.696
##     hygien3 (.12.)    0.959    0.016   61.461    0.000    1.352    0.759
##     hygien4 (.13.)    1.147    0.021   53.821    0.000    1.618    0.509
##     hygien5 (.14.)    0.896    0.020   45.584    0.000    1.264    0.527
##   om1 =~                                                                
##     omind2            1.000                               1.233    0.642
##     omind3  (.18.)    1.080    0.012   88.381    0.000    1.332    0.872
##     omind4  (.19.)    1.062    0.013   80.489    0.000    1.309    0.781
##     omind1  (.20.)    0.460    0.020   22.733    0.000    0.567    0.204
##     omind5  (.21.)    0.765    0.019   40.718    0.000    0.944    0.369
##     omind6  (.22.)    0.748    0.019   38.508    0.000    0.922    0.351
## 
## Covariances:
##                    Estimate  Std.Err  z-value  P(>|z|)   Std.lv  Std.all
##  .hygiene1 ~~                                                           
##    .hygiene2          1.645    0.083   19.917    0.000    1.645    0.740
##  .psupport4 ~~                                                          
##    .psupport5         0.485    0.049    9.928    0.000    0.485    0.220
##  .omind1 ~~                                                             
##    .omind5            1.751    0.104   16.797    0.000    1.751    0.271
##  .omind5 ~~                                                             
##    .omind6            1.842    0.104   17.717    0.000    1.842    0.315
##  .omind1 ~~                                                             
##    .omind6            2.584    0.104   24.765    0.000    2.584    0.386
##   phc ~~                                                                
##     phs               1.390    0.059   23.541    0.000    0.501    0.501
##     phg               1.156    0.046   25.006    0.000    0.604    0.604
##     om1               0.602    0.030   20.354    0.000    0.359    0.359
##   phs ~~                                                                
##     phg               1.165    0.057   20.579    0.000    0.403    0.403
##     om1               0.616    0.038   16.276    0.000    0.244    0.244
##   phg ~~                                                                
##     om1               0.571    0.032   17.923    0.000    0.328    0.328
## 
## Intercepts:
##                    Estimate  Std.Err  z-value  P(>|z|)   Std.lv  Std.all
##    .contct1 (.56.)    8.929    0.020  436.664    0.000    8.929    4.825
##    .cntct2r (.57.)    8.102    0.028  285.345    0.000    8.102    2.871
##    .contct3 (.58.)    8.444    0.025  332.660    0.000    8.444    3.676
##    .contct4 (.59.)    8.957    0.019  461.773    0.000    8.957    4.483
##    .pspprt1           8.122    0.035  230.178    0.000    8.122    3.191
##    .pspprt2 (.61.)    8.559    0.029  295.670    0.000    8.559    3.577
##    .pspprt3 (.62.)    6.683    0.036  183.154    0.000    6.683    2.089
##    .pspprt4 (.63.)    8.805    0.025  346.818    0.000    8.805    4.031
##    .pspprt5 (.64.)    8.577    0.027  315.424    0.000    8.577    3.491
##    .hygien1 (.65.)    8.128    0.027  302.167    0.000    8.128    3.903
##    .hygien2 (.66.)    8.252    0.026  312.571    0.000    8.252    4.086
##    .hygien3 (.67.)    8.729    0.026  329.666    0.000    8.729    4.897
##    .hygien4 (.68.)    6.224    0.037  169.494    0.000    6.224    1.956
##    .hygien5 (.69.)    7.978    0.028  282.830    0.000    7.978    3.325
##    .omind2  (.70.)    8.684    0.020  424.511    0.000    8.684    4.522
##    .omind3  (.71.)    8.614    0.021  416.199    0.000    8.614    5.639
##    .omind4  (.72.)    8.522    0.021  403.514    0.000    8.522    5.084
##    .omind1  (.73.)    6.107    0.025  241.421    0.000    6.107    2.199
##    .omind5  (.74.)    8.283    0.025  327.477    0.000    8.283    3.244
##    .omind6  (.75.)    7.344    0.026  284.863    0.000    7.344    2.792
##     phc              -0.455    0.028  -16.245    0.000   -0.335   -0.335
##     phs              -0.440    0.039  -11.149    0.000   -0.215   -0.215
##     phg               0.020    0.033    0.618    0.537    0.014    0.014
##     om1               0.021    0.025    0.841    0.400    0.017    0.017
## 
## Variances:
##                    Estimate  Std.Err  z-value  P(>|z|)   Std.lv  Std.all
##    .contact1          1.583    0.068   23.145    0.000    1.583    0.462
##    .contact2r         6.679    0.174   38.352    0.000    6.679    0.839
##    .contact3          3.150    0.118   26.768    0.000    3.150    0.597
##    .contact4          2.227    0.089   24.900    0.000    2.227    0.558
##    .psupport1         2.288    0.084   27.236    0.000    2.288    0.353
##    .psupport2         1.337    0.057   23.531    0.000    1.337    0.233
##    .psupport3         5.905    0.131   45.036    0.000    5.905    0.577
##    .psupport4         1.687    0.059   28.818    0.000    1.687    0.354
##    .psupport5         2.872    0.093   30.726    0.000    2.872    0.476
##    .hygiene1          2.348    0.091   25.775    0.000    2.348    0.541
##    .hygiene2          2.101    0.087   24.102    0.000    2.101    0.515
##    .hygiene3          1.348    0.052   25.834    0.000    1.348    0.424
##    .hygiene4          7.510    0.139   53.842    0.000    7.510    0.741
##    .hygiene5          4.159    0.128   32.478    0.000    4.159    0.722
##    .omind2            2.166    0.139   15.615    0.000    2.166    0.587
##    .omind3            0.561    0.034   16.280    0.000    0.561    0.240
##    .omind4            1.095    0.055   19.766    0.000    1.095    0.390
##    .omind1            7.388    0.123   60.250    0.000    7.388    0.958
##    .omind5            5.631    0.173   32.622    0.000    5.631    0.863
##    .omind6            6.068    0.129   47.124    0.000    6.068    0.877
##     phc               1.841    0.063   29.196    0.000    1.000    1.000
##     phs               4.190    0.120   34.799    0.000    1.000    1.000
##     phg               1.990    0.077   26.012    0.000    1.000    1.000
##     om1               1.521    0.047   32.469    0.000    1.000    1.000
## 
## 
## Group 3 [positive]:
## 
## Latent Variables:
##                    Estimate  Std.Err  z-value  P(>|z|)   Std.lv  Std.all
##   phc =~                                                                
##     contct1           1.000                               1.544    0.764
##     cntct2r (.p2.)    0.836    0.021   39.694    0.000    1.290    0.449
##     contct3 (.p3.)    1.075    0.018   61.375    0.000    1.659    0.765
##     contct4 (.p4.)    0.979    0.015   63.982    0.000    1.512    0.858
##   phs =~                                                                
##     pspprt1           1.000                               2.190    0.876
##     pspprt2 (.p6.)    1.024    0.010  106.966    0.000    2.242    0.920
##     pspprt3 (.p7.)    1.016    0.012   82.923    0.000    2.226    0.606
##     pspprt4 (.p8.)    0.858    0.011   78.130    0.000    1.879    0.826
##     pspprt5 (.p9.)    0.869    0.012   73.523    0.000    1.904    0.795
##   phg =~                                                                
##     hygien1           1.000                               1.587    0.766
##     hygien2 (.11.)    0.997    0.007  147.513    0.000    1.581    0.781
##     hygien3 (.12.)    0.959    0.016   61.461    0.000    1.521    0.816
##     hygien4 (.13.)    1.147    0.021   53.821    0.000    1.820    0.596
##     hygien5 (.14.)    0.896    0.020   45.584    0.000    1.421    0.482
##   om1 =~                                                                
##     omind2            1.000                               1.308    0.843
##     omind3  (.18.)    1.080    0.012   88.381    0.000    1.412    0.916
##     omind4  (.19.)    1.062    0.013   80.489    0.000    1.389    0.857
##     omind1  (.20.)    0.460    0.020   22.733    0.000    0.601    0.224
##     omind5  (.21.)    0.765    0.019   40.718    0.000    1.001    0.405
##     omind6  (.22.)    0.748    0.019   38.508    0.000    0.978    0.387
## 
## Covariances:
##                    Estimate  Std.Err  z-value  P(>|z|)   Std.lv  Std.all
##  .hygiene1 ~~                                                           
##    .hygiene2          1.365    0.122   11.151    0.000    1.365    0.811
##  .psupport4 ~~                                                          
##    .psupport5         0.617    0.084    7.353    0.000    0.617    0.331
##  .omind1 ~~                                                             
##    .omind5            1.561    0.159    9.808    0.000    1.561    0.263
##  .omind5 ~~                                                             
##    .omind6            2.146    0.186   11.562    0.000    2.146    0.407
##  .omind1 ~~                                                             
##    .omind6            2.294    0.171   13.424    0.000    2.294    0.375
##   phc ~~                                                                
##     phs               2.114    0.151   14.019    0.000    0.625    0.625
##     phg               1.611    0.122   13.245    0.000    0.658    0.658
##     om1               0.955    0.066   14.568    0.000    0.473    0.473
##   phs ~~                                                                
##     phg               1.809    0.145   12.511    0.000    0.520    0.520
##     om1               0.788    0.081    9.781    0.000    0.275    0.275
##   phg ~~                                                                
##     om1               0.643    0.065    9.849    0.000    0.310    0.310
## 
## Intercepts:
##                    Estimate  Std.Err  z-value  P(>|z|)   Std.lv  Std.all
##    .contct1 (.56.)    8.929    0.020  436.664    0.000    8.929    4.417
##    .cntct2r (.57.)    8.102    0.028  285.345    0.000    8.102    2.821
##    .contct3 (.58.)    8.444    0.025  332.660    0.000    8.444    3.894
##    .contct4 (.59.)    8.957    0.019  461.773    0.000    8.957    5.086
##    .pspprt1           8.472    0.044  194.442    0.000    8.472    3.390
##    .pspprt2 (.61.)    8.559    0.029  295.670    0.000    8.559    3.511
##    .pspprt3 (.62.)    6.683    0.036  183.154    0.000    6.683    1.819
##    .pspprt4 (.63.)    8.805    0.025  346.818    0.000    8.805    3.870
##    .pspprt5 (.64.)    8.577    0.027  315.424    0.000    8.577    3.582
##    .hygien1 (.65.)    8.128    0.027  302.167    0.000    8.128    3.925
##    .hygien2 (.66.)    8.252    0.026  312.571    0.000    8.252    4.074
##    .hygien3 (.67.)    8.729    0.026  329.666    0.000    8.729    4.681
##    .hygien4 (.68.)    6.224    0.037  169.494    0.000    6.224    2.036
##    .hygien5 (.69.)    7.978    0.028  282.830    0.000    7.978    2.705
##    .omind2  (.70.)    8.684    0.020  424.511    0.000    8.684    5.594
##    .omind3  (.71.)    8.614    0.021  416.199    0.000    8.614    5.585
##    .omind4  (.72.)    8.522    0.021  403.514    0.000    8.522    5.261
##    .omind1  (.73.)    6.107    0.025  241.421    0.000    6.107    2.272
##    .omind5  (.74.)    8.283    0.025  327.477    0.000    8.283    3.350
##    .omind6  (.75.)    7.344    0.026  284.863    0.000    7.344    2.903
##     phc              -0.378    0.044   -8.558    0.000   -0.245   -0.245
##     phs              -0.556    0.062   -8.951    0.000   -0.254   -0.254
##     phg              -0.124    0.049   -2.534    0.011   -0.078   -0.078
##     om1              -0.144    0.038   -3.835    0.000   -0.110   -0.110
## 
## Variances:
##                    Estimate  Std.Err  z-value  P(>|z|)   Std.lv  Std.all
##    .contact1          1.703    0.119   14.292    0.000    1.703    0.417
##    .contact2r         6.585    0.304   21.642    0.000    6.585    0.798
##    .contact3          1.950    0.142   13.735    0.000    1.950    0.415
##    .contact4          0.817    0.057   14.386    0.000    0.817    0.263
##    .psupport1         1.448    0.114   12.746    0.000    1.448    0.232
##    .psupport2         0.917    0.074   12.400    0.000    0.917    0.154
##    .psupport3         8.547    0.277   30.858    0.000    8.547    0.633
##    .psupport4         1.645    0.109   15.022    0.000    1.645    0.318
##    .psupport5         2.109    0.132   15.984    0.000    2.109    0.368
##    .hygiene1          1.772    0.125   14.206    0.000    1.772    0.413
##    .hygiene2          1.601    0.128   12.508    0.000    1.601    0.390
##    .hygiene3          1.164    0.077   15.030    0.000    1.164    0.335
##    .hygiene4          6.028    0.252   23.941    0.000    6.028    0.645
##    .hygiene5          6.678    0.281   23.767    0.000    6.678    0.768
##    .omind2            0.699    0.054   12.996    0.000    0.699    0.290
##    .omind3            0.384    0.038   10.093    0.000    0.384    0.162
##    .omind4            0.695    0.056   12.338    0.000    0.695    0.265
##    .omind1            6.867    0.203   33.751    0.000    6.867    0.950
##    .omind5            5.112    0.295   17.334    0.000    5.112    0.836
##    .omind6            5.443    0.237   22.942    0.000    5.443    0.850
##     phc               2.383    0.146   16.301    0.000    1.000    1.000
##     phs               4.798    0.235   20.391    0.000    1.000    1.000
##     phg               2.517    0.145   17.361    0.000    1.000    1.000
##     om1               1.711    0.072   23.790    0.000    1.000    1.000
```

```
print(compareFit(phymodcc, phymodcw, phymodcs2, nested = T))
```

```
## ################### Nested Model Comparison #########################
## Scaled Chi-Squared Difference Test (method = "satorra.bentler.2001")
## 
## lavaan NOTE:
##     The "Chisq" column contains standard test statistics, not the
##     robust test that should be reported per model. A robust difference
##     test is a function of two standard (not robust) statistics.
##  
##            Df    AIC    BIC  Chisq Chisq diff Df diff Pr(>Chisq)    
## phymodcc  477 989995 991578 5475.4                                  
## phymodcw  509 990217 991562 5761.1     253.69      32  < 2.2e-16 ***
## phymodcs2 539 991500 992622 7104.3    1829.10      30  < 2.2e-16 ***
## ---
## Signif. codes:  0 '***' 0.001 '**' 0.01 '*' 0.05 '.' 0.1 ' ' 1
## 
## ####################### Model Fit Indices ###########################
##           chisq.scaled df.scaled pvalue.scaled cfi.robust tli.robust
## phymodcc     5153.892†       477          .000      .957†      .948 
## phymodcw     5402.417        509          .000      .954       .949†
## phymodcs2    6779.524        539          .000      .943       .940 
##                   aic         bic rmsea.robust  srmr
## phymodcc  989994.891† 991578.052         .050  .044†
## phymodcw  990216.601  991561.917†        .050† .047 
## phymodcs2 991499.852  992622.188         .054  .050 
## 
## ################## Differences in Fit Indices #######################
##                      df.scaled cfi.robust tli.robust      aic      bic
## phymodcw - phymodcc         32     -0.002      0.001  221.711  -16.135
## phymodcs2 - phymodcw        30     -0.012     -0.009 1283.251 1060.270
##                      rmsea.robust  srmr
## phymodcw - phymodcc         0.000 0.003
## phymodcs2 - phymodcw        0.004 0.003
```

## SEM

```
phczx <- 'phc =~ contact1 + contact2r + contact3 + contact4
        phs =~ psupport1 + psupport2 + psupport3 + psupport4 + psupport5
        phg =~ hygiene1 + hygiene2 + hygiene3 + hygiene4 + hygiene5
        hygiene1 ~~ hygiene2
        psupport4 ~~ psupport5
        om1 =~ omind2 + omind3 + omind4 + omind1 + omind5 + omind6
        omind1 ~~ omind5
        omind5 ~~ omind6
        omind1 ~~ omind6
        phc ~ ccrt + om1 + age + sex1 + political_ideology
        phs ~ ccrt + om1 + age + sex1 + political_ideology
        phg ~ ccrt + om1 + age + sex1 + political_ideology
        ccrt ~~ om1
        ccrt ~~ age
        ccrt ~~ sex1
        ccrt ~~ political_ideology
        om1 ~~ age
        om1 ~~ sex1
        om1 ~~ political_ideology
        age ~~ sex1
        age ~~ political_ideology
        sex1 ~~ political_ideology'

phymodzx <- sem(phczx, estimator = "MLM", data = pls4)
```

```
## Warning in lav_data_full(data = data, group = group, cluster = cluster, : lavaan
## WARNING: some observed variances are (at least) a factor 1000 times larger than
## others; use varTable(fit) to investigate
```

```
fitmeasures(phymodzx, fit.measures = c("chisq.scaled", "df.scaled", "cfi.robust", "rmsea.robust", "srmr"))
```

```
## chisq.scaled    df.scaled   cfi.robust rmsea.robust         srmr 
##     6202.351      223.000        0.944        0.048        0.044
```

```
semPaths(phymodzx, what = "std", edge.label.cex = 1.2, label.cex = 1.2)
```

```
summary(phymodzx, standardized = T, rsq = T)
```

```
## lavaan 0.6-8 ended normally after 149 iterations
## 
##   Estimator                                         ML
##   Optimization method                           NLMINB
##   Number of model parameters                        77
##                                                       
##   Number of observations                         12490
##                                                       
## Model Test User Model:
##                                               Standard      Robust
##   Test Statistic                              6573.822    6202.351
##   Degrees of freedom                               223         223
##   P-value (Chi-square)                           0.000       0.000
##   Scaling correction factor                                  1.060
##        Satorra-Bentler correction                                 
## 
## Parameter Estimates:
## 
##   Standard errors                           Robust.sem
##   Information                                 Expected
##   Information saturated (h1) model          Structured
## 
## Latent Variables:
##                    Estimate  Std.Err  z-value  P(>|z|)   Std.lv  Std.all
##   phc =~                                                                
##     contact1          1.000                               1.330    0.752
##     contact2r         0.842    0.021   39.501    0.000    1.120    0.406
##     contact3          1.080    0.019   58.353    0.000    1.437    0.648
##     contact4          0.976    0.016   59.679    0.000    1.297    0.728
##   phs =~                                                                
##     psupport1         1.000                               1.977    0.780
##     psupport2         1.024    0.010  105.330    0.000    2.023    0.886
##     psupport3         1.025    0.012   83.030    0.000    2.027    0.631
##     psupport4         0.854    0.011   76.599    0.000    1.689    0.816
##     psupport5         0.873    0.012   74.307    0.000    1.726    0.740
##   phg =~                                                                
##     hygiene1          1.000                               1.475    0.723
##     hygiene2          0.997    0.007  144.324    0.000    1.471    0.741
##     hygiene3          0.941    0.015   60.842    0.000    1.389    0.738
##     hygiene4          1.180    0.022   54.012    0.000    1.741    0.558
##     hygiene5          0.902    0.020   46.176    0.000    1.330    0.541
##   om1 =~                                                                
##     omind2            1.000                               1.175    0.701
##     omind3            1.128    0.015   77.519    0.000    1.326    0.886
##     omind4            1.119    0.015   72.468    0.000    1.315    0.806
##     omind1            0.487    0.021   22.914    0.000    0.572    0.211
##     omind5            0.803    0.020   39.921    0.000    0.943    0.375
##     omind6            0.785    0.021   37.623    0.000    0.922    0.357
## 
## Regressions:
##                    Estimate  Std.Err  z-value  P(>|z|)   Std.lv  Std.all
##   phc ~                                                                 
##     ccrt             -0.023    0.012   -1.898    0.058   -0.018   -0.019
##     om1               0.461    0.014   31.848    0.000    0.408    0.408
##     age               0.010    0.001   13.122    0.000    0.007    0.125
##     sex1              0.369    0.027   13.876    0.000    0.277    0.139
##     political_dlgy    0.001    0.006    0.086    0.932    0.000    0.001
##   phs ~                                                                 
##     ccrt             -0.119    0.018   -6.761    0.000   -0.060   -0.064
##     om1               0.467    0.018   25.601    0.000    0.277    0.277
##     age              -0.003    0.001   -3.111    0.002   -0.002   -0.027
##     sex1              0.231    0.037    6.241    0.000    0.117    0.058
##     political_dlgy   -0.005    0.009   -0.601    0.548   -0.003   -0.006
##   phg ~                                                                 
##     ccrt             -0.234    0.014  -16.251    0.000   -0.159   -0.170
##     om1               0.396    0.015   26.144    0.000    0.315    0.315
##     age               0.001    0.001    0.947    0.344    0.001    0.009
##     sex1              0.311    0.029   10.606    0.000    0.211    0.105
##     political_dlgy    0.030    0.007    4.600    0.000    0.020    0.045
## 
## Covariances:
##                    Estimate  Std.Err  z-value  P(>|z|)   Std.lv  Std.all
##  .hygiene1 ~~                                                           
##    .hygiene2          1.406    0.054   25.929    0.000    1.406    0.747
##  .psupport4 ~~                                                          
##    .psupport5         0.426    0.032   13.269    0.000    0.426    0.227
##  .omind1 ~~                                                             
##    .omind5            1.819    0.073   25.082    0.000    1.819    0.294
##  .omind5 ~~                                                             
##    .omind6            2.201    0.077   28.588    0.000    2.201    0.391
##  .omind1 ~~                                                             
##    .omind6            2.494    0.072   34.561    0.000    2.494    0.390
##   om1 ~~                                                                
##     ccrt              0.116    0.012    9.946    0.000    0.098    0.092
##   ccrt ~~                                                               
##     age              -0.554    0.163   -3.400    0.001   -0.554   -0.030
##     sex1             -0.087    0.005  -18.453    0.000   -0.087   -0.163
##     political_dlgy   -0.256    0.022  -11.919    0.000   -0.256   -0.108
##   om1 ~~                                                                
##     age               0.267    0.187    1.423    0.155    0.227    0.013
##     sex1              0.048    0.006    8.579    0.000    0.041    0.082
##     political_dlgy   -0.135    0.024   -5.669    0.000   -0.115   -0.052
##   age ~~                                                                
##     sex1             -0.981    0.076  -12.921    0.000   -0.981   -0.115
##     political_dlgy   -0.531    0.345   -1.537    0.124   -0.531   -0.014
##   sex1 ~~                                                               
##     political_dlgy   -0.089    0.010   -9.021    0.000   -0.089   -0.081
##  .phc ~~                                                                
##    .phs               1.144    0.042   27.274    0.000    0.511    0.511
##    .phg               0.834    0.032   25.797    0.000    0.515    0.515
##  .phs ~~                                                                
##    .phg               0.917    0.040   22.926    0.000    0.355    0.355
## 
## Variances:
##                    Estimate  Std.Err  z-value  P(>|z|)   Std.lv  Std.all
##    .contact1          1.360    0.044   31.150    0.000    1.360    0.435
##    .contact2r         6.349    0.122   52.114    0.000    6.349    0.835
##    .contact3          2.853    0.081   35.421    0.000    2.853    0.580
##    .contact4          1.496    0.051   29.170    0.000    1.496    0.470
##    .psupport1         2.515    0.068   36.837    0.000    2.515    0.392
##    .psupport2         1.118    0.037   30.253    0.000    1.118    0.215
##    .psupport3         6.217    0.099   62.699    0.000    6.217    0.602
##    .psupport4         1.434    0.038   37.857    0.000    1.434    0.334
##    .psupport5         2.461    0.061   40.564    0.000    2.461    0.452
##    .hygiene1          1.987    0.060   33.330    0.000    1.987    0.477
##    .hygiene2          1.782    0.057   31.271    0.000    1.782    0.452
##    .hygiene3          1.616    0.045   35.883    0.000    1.616    0.456
##    .hygiene4          6.691    0.105   63.693    0.000    6.691    0.688
##    .hygiene5          4.278    0.097   44.126    0.000    4.278    0.707
##    .omind2            1.425    0.073   19.644    0.000    1.425    0.508
##    .omind3            0.479    0.022   22.232    0.000    0.479    0.214
##    .omind4            0.931    0.035   26.692    0.000    0.931    0.350
##    .omind1            7.027    0.083   84.326    0.000    7.027    0.956
##    .omind5            5.443    0.121   44.971    0.000    5.443    0.859
##    .omind6            5.829    0.094   62.194    0.000    5.829    0.873
##     ccrt              1.141    0.011  105.655    0.000    1.141    1.000
##     age             292.109    2.495  117.084    0.000  292.109    1.000
##     sex1              0.250    0.000 3524.469    0.000    0.250    1.000
##     political_dlgy    4.933    0.063   78.834    0.000    4.933    1.000
##    .phc               1.402    0.047   29.842    0.000    0.793    0.793
##    .phs               3.572    0.087   41.046    0.000    0.914    0.914
##    .phg               1.867    0.060   31.358    0.000    0.858    0.858
##     om1               1.380    0.038   36.075    0.000    1.000    1.000
## 
## R-Square:
##                    Estimate
##     contact1          0.565
##     contact2r         0.165
##     contact3          0.420
##     contact4          0.530
##     psupport1         0.608
##     psupport2         0.785
##     psupport3         0.398
##     psupport4         0.666
##     psupport5         0.548
##     hygiene1          0.523
##     hygiene2          0.548
##     hygiene3          0.544
##     hygiene4          0.312
##     hygiene5          0.293
##     omind2            0.492
##     omind3            0.786
##     omind4            0.650
##     omind1            0.044
##     omind5            0.141
##     omind6            0.127
##     phc               0.207
##     phs               0.086
##     phg               0.142
```

```
lavInspect(phymodzx, "cor.all")
```

```
##                    cntct1 cntct2 cntct3 cntct4 psppr1 psppr2 psppr3 psppr4
## contact1            1.000                                                 
## contact2r           0.305  1.000                                          
## contact3            0.487  0.263  1.000                                   
## contact4            0.547  0.296  0.472  1.000                            
## psupport1           0.327  0.177  0.282  0.317  1.000                     
## psupport2           0.372  0.201  0.321  0.360  0.691  1.000              
## psupport3           0.265  0.143  0.228  0.256  0.492  0.559  1.000       
## psupport4           0.342  0.185  0.295  0.331  0.636  0.723  0.515  1.000
## psupport5           0.311  0.168  0.268  0.301  0.577  0.656  0.467  0.692
## hygiene1            0.312  0.169  0.269  0.302  0.236  0.268  0.191  0.247
## hygiene2            0.320  0.173  0.276  0.310  0.242  0.275  0.195  0.253
## hygiene3            0.319  0.172  0.275  0.309  0.241  0.274  0.195  0.252
## hygiene4            0.241  0.130  0.208  0.234  0.182  0.207  0.147  0.191
## hygiene5            0.234  0.126  0.201  0.226  0.177  0.201  0.143  0.185
## omind2              0.221  0.119  0.190  0.214  0.151  0.172  0.122  0.158
## omind3              0.279  0.151  0.241  0.270  0.191  0.217  0.154  0.200
## omind4              0.254  0.137  0.219  0.246  0.174  0.197  0.140  0.182
## omind1              0.066  0.036  0.057  0.064  0.045  0.052  0.037  0.048
## omind5              0.118  0.064  0.102  0.114  0.081  0.092  0.065  0.084
## omind6              0.112  0.061  0.097  0.109  0.077  0.087  0.062  0.080
## ccrt               -0.006 -0.003 -0.005 -0.006 -0.037 -0.042 -0.030 -0.038
## age                 0.086  0.047  0.074  0.084 -0.022 -0.025 -0.018 -0.023
## sex1                0.121  0.065  0.104  0.117  0.074  0.085  0.060  0.078
## political_ideology -0.023 -0.013 -0.020 -0.023 -0.014 -0.016 -0.011 -0.014
## phc                 0.752  0.406  0.648  0.728  0.435  0.495  0.352  0.455
## phs                 0.420  0.227  0.362  0.406  0.780  0.886  0.631  0.816
## phg                 0.432  0.233  0.372  0.418  0.326  0.371  0.264  0.341
## om1                 0.315  0.170  0.271  0.305  0.215  0.245  0.174  0.225
##                    psppr5 hygin1 hygin2 hygin3 hygin4 hygin5 omind2 omind3
## contact1                                                                  
## contact2r                                                                 
## contact3                                                                  
## contact4                                                                  
## psupport1                                                                 
## psupport2                                                                 
## psupport3                                                                 
## psupport4                                                                 
## psupport5           1.000                                                 
## hygiene1            0.224  1.000                                          
## hygiene2            0.229  0.882  1.000                                   
## hygiene3            0.228  0.533  0.546  1.000                            
## hygiene4            0.173  0.404  0.413  0.412  1.000                     
## hygiene5            0.168  0.391  0.401  0.399  0.302  1.000              
## omind2              0.143  0.155  0.159  0.158  0.120  0.116  1.000       
## omind3              0.181  0.196  0.201  0.200  0.151  0.147  0.622  1.000
## omind4              0.165  0.178  0.183  0.182  0.138  0.133  0.565  0.715
## omind1              0.043  0.047  0.048  0.048  0.036  0.035  0.148  0.187
## omind5              0.077  0.083  0.085  0.085  0.064  0.062  0.263  0.332
## omind6              0.073  0.079  0.081  0.081  0.061  0.059  0.250  0.316
## ccrt               -0.035 -0.118 -0.121 -0.120 -0.091 -0.088  0.065  0.082
## age                -0.021  0.004  0.004  0.004  0.003  0.003  0.009  0.012
## sex1                0.071  0.112  0.114  0.114  0.086  0.083  0.058  0.073
## political_ideology -0.013  0.028  0.029  0.029  0.022  0.021 -0.036 -0.046
## phc                 0.413  0.416  0.426  0.424  0.321  0.311  0.294  0.371
## phs                 0.740  0.303  0.310  0.309  0.234  0.226  0.194  0.245
## phg                 0.310  0.723  0.741  0.738  0.558  0.541  0.215  0.271
## om1                 0.204  0.221  0.227  0.226  0.171  0.166  0.701  0.886
##                    omind4 omind1 omind5 omind6 ccrt   age    sex1   pltcl_
## contact1                                                                  
## contact2r                                                                 
## contact3                                                                  
## contact4                                                                  
## psupport1                                                                 
## psupport2                                                                 
## psupport3                                                                 
## psupport4                                                                 
## psupport5                                                                 
## hygiene1                                                                  
## hygiene2                                                                  
## hygiene3                                                                  
## hygiene4                                                                  
## hygiene5                                                                  
## omind2                                                                    
## omind3                                                                    
## omind4              1.000                                                 
## omind1              0.170  1.000                                          
## omind5              0.302  0.346  1.000                                   
## omind6              0.288  0.431  0.472  1.000                            
## ccrt                0.074  0.019  0.035  0.033  1.000                     
## age                 0.011  0.003  0.005  0.005 -0.030  1.000              
## sex1                0.066  0.017  0.031  0.029 -0.163 -0.115  1.000       
## political_ideology -0.042 -0.011 -0.019 -0.018 -0.108 -0.014 -0.081  1.000
## phc                 0.338  0.088  0.157  0.149 -0.008  0.115  0.161 -0.031
## phs                 0.223  0.058  0.104  0.098 -0.047 -0.028  0.095 -0.018
## phg                 0.247  0.065  0.115  0.109 -0.163  0.006  0.154  0.039
## om1                 0.806  0.211  0.375  0.357  0.092  0.013  0.082 -0.052
##                    phc    phs    phg    om1   
## contact1                                      
## contact2r                                     
## contact3                                      
## contact4                                      
## psupport1                                     
## psupport2                                     
## psupport3                                     
## psupport4                                     
## psupport5                                     
## hygiene1                                      
## hygiene2                                      
## hygiene3                                      
## hygiene4                                      
## hygiene5                                      
## omind2                                        
## omind3                                        
## omind4                                        
## omind1                                        
## omind5                                        
## omind6                                        
## ccrt                                          
## age                                           
## sex1                                          
## political_ideology                            
## phc                 1.000                     
## phs                 0.558  1.000              
## phg                 0.575  0.419  1.000       
## om1                 0.419  0.276  0.306  1.000
```

```
#invariance
phczx <- 'phc =~ contact1 + contact2r + contact3 + contact4
        phs =~ psupport1 + psupport2 + psupport3 + psupport4 + psupport5
        phg =~ hygiene1 + hygiene2 + hygiene3 + hygiene4 + hygiene5
        hygiene1 ~~ hygiene2
        psupport4 ~~ psupport5
        om1 =~ omind2 + omind3 + omind4 + omind1 + omind5 + omind6
        omind1 ~~ omind5
        omind5 ~~ omind6
        omind1 ~~ omind6
        phc ~ ccrt + om1 + age + sex1 + political_ideology
        phs ~ ccrt + om1 + age + sex1 + political_ideology
        phg ~ ccrt + om1 + age + sex1 + political_ideology'

phymodcczx <- sem(phczx, estimator = "MLM", data = pls4, group = "cpol")
summary(phymodcczx, standardized = T)
```

```
## lavaan 0.6-8 ended normally after 277 iterations
## 
##   Estimator                                         ML
##   Optimization method                           NLMINB
##   Number of model parameters                       249
##                                                       
##   Number of observations per group:                   
##     negative                                      4408
##     neutral                                       6315
##     positive                                      1767
##                                                       
## Model Test User Model:
##                                               Standard      Robust
##   Test Statistic                              8477.971    8118.914
##   Degrees of freedom                               681         681
##   P-value (Chi-square)                           0.000       0.000
##   Scaling correction factor                                  1.044
##        Satorra-Bentler correction                                 
##   Test statistic for each group:
##     negative                                  3648.195    3493.688
##     neutral                                   3521.076    3371.952
##     positive                                  1308.700    1253.274
## 
## Parameter Estimates:
## 
##   Standard errors                           Robust.sem
##   Information                                 Expected
##   Information saturated (h1) model          Structured
## 
## 
## Group 1 [negative]:
## 
## Latent Variables:
##                    Estimate  Std.Err  z-value  P(>|z|)   Std.lv  Std.all
##   phc =~                                                                
##     contact1          1.000                               1.094    0.750
##     contact2r         1.053    0.043   24.402    0.000    1.152    0.433
##     contact3          1.140    0.033   34.269    0.000    1.247    0.596
##     contact4          0.977    0.025   38.622    0.000    1.069    0.795
##   phs =~                                                                
##     psupport1         1.000                               1.758    0.699
##     psupport2         0.983    0.019   52.454    0.000    1.729    0.884
##     psupport3         1.031    0.022   47.689    0.000    1.812    0.599
##     psupport4         0.878    0.021   41.992    0.000    1.544    0.850
##     psupport5         0.873    0.023   38.273    0.000    1.535    0.735
##   phg =~                                                                
##     hygiene1          1.000                               1.436    0.739
##     hygiene2          0.973    0.011   87.325    0.000    1.397    0.743
##     hygiene3          1.059    0.027   39.096    0.000    1.521    0.750
##     hygiene4          1.314    0.038   34.181    0.000    1.886    0.612
##     hygiene5          0.937    0.032   29.456    0.000    1.345    0.582
##   om1 =~                                                                
##     omind2            1.000                               1.237    0.856
##     omind3            1.019    0.017   60.524    0.000    1.260    0.893
##     omind4            0.972    0.019   52.288    0.000    1.202    0.795
##     omind1            0.509    0.033   15.223    0.000    0.630    0.239
##     omind5            0.783    0.030   25.962    0.000    0.968    0.388
##     omind6            0.774    0.031   24.718    0.000    0.958    0.375
## 
## Regressions:
##                    Estimate  Std.Err  z-value  P(>|z|)   Std.lv  Std.all
##   phc ~                                                                 
##     ccrt             -0.035    0.016   -2.180    0.029   -0.032   -0.034
##     om1               0.451    0.021   21.871    0.000    0.510    0.510
##     age               0.010    0.001   10.474    0.000    0.009    0.159
##     sex1              0.277    0.035    7.974    0.000    0.253    0.126
##     political_dlgy   -0.028    0.007   -3.883    0.000   -0.025   -0.058
##   phs ~                                                                 
##     ccrt             -0.073    0.026   -2.868    0.004   -0.042   -0.045
##     om1               0.463    0.026   17.807    0.000    0.326    0.326
##     age               0.004    0.001    2.469    0.014    0.002    0.036
##     sex1              0.258    0.054    4.776    0.000    0.147    0.073
##     political_dlgy   -0.089    0.012   -7.238    0.000   -0.051   -0.116
##   phg ~                                                                 
##     ccrt             -0.326    0.023  -13.896    0.000   -0.227   -0.244
##     om1               0.357    0.022   16.239    0.000    0.307    0.307
##     age               0.001    0.001    0.540    0.589    0.000    0.009
##     sex1              0.313    0.046    6.748    0.000    0.218    0.109
##     political_dlgy    0.035    0.010    3.447    0.001    0.024    0.055
## 
## Covariances:
##                    Estimate  Std.Err  z-value  P(>|z|)   Std.lv  Std.all
##  .hygiene1 ~~                                                           
##    .hygiene2          1.250    0.075   16.656    0.000    1.250    0.761
##  .psupport4 ~~                                                          
##    .psupport5         0.229    0.044    5.270    0.000    0.229    0.169
##  .omind1 ~~                                                             
##    .omind5            2.053    0.124   16.584    0.000    2.053    0.348
##  .omind5 ~~                                                             
##    .omind6            2.708    0.142   19.128    0.000    2.708    0.498
##  .omind1 ~~                                                             
##    .omind6            2.485    0.119   20.872    0.000    2.485    0.410
##  .phc ~~                                                                
##    .phs               0.775    0.043   17.858    0.000    0.516    0.516
##    .phg               0.542    0.034   16.115    0.000    0.455    0.455
##  .phs ~~                                                                
##    .phg               0.687    0.052   13.254    0.000    0.322    0.322
## 
## Intercepts:
##                    Estimate  Std.Err  z-value  P(>|z|)   Std.lv  Std.all
##    .contact1          8.194    0.098   83.722    0.000    8.194    5.615
##    .contact2r         7.363    0.108   68.271    0.000    7.363    2.768
##    .contact3          7.472    0.111   67.165    0.000    7.472    3.574
##    .contact4          8.311    0.097   85.255    0.000    8.311    6.179
##    .psupport1         7.822    0.149   52.645    0.000    7.822    3.109
##    .psupport2         8.557    0.145   58.870    0.000    8.557    4.375
##    .psupport3         6.767    0.155   43.747    0.000    6.767    2.235
##    .psupport4         8.734    0.131   66.919    0.000    8.734    4.805
##    .psupport5         8.505    0.130   65.484    0.000    8.505    4.071
##    .hygiene1          7.858    0.129   60.901    0.000    7.858    4.046
##    .hygiene2          7.994    0.126   63.345    0.000    7.994    4.255
##    .hygiene3          8.046    0.137   58.861    0.000    8.046    3.970
##    .hygiene4          6.000    0.171   35.180    0.000    6.000    1.946
##    .hygiene5          7.699    0.121   63.522    0.000    7.699    3.333
##    .omind2            8.680    0.021  403.986    0.000    8.680    6.005
##    .omind3            8.610    0.021  411.433    0.000    8.610    6.101
##    .omind4            8.513    0.023  376.614    0.000    8.513    5.628
##    .omind1            5.986    0.039  154.848    0.000    5.986    2.267
##    .omind5            8.426    0.036  235.596    0.000    8.426    3.377
##    .omind6            7.550    0.037  203.764    0.000    7.550    2.958
##    .phc               0.000                               0.000    0.000
##    .phs               0.000                               0.000    0.000
##    .phg               0.000                               0.000    0.000
##     om1               0.000                               0.000    0.000
## 
## Variances:
##                    Estimate  Std.Err  z-value  P(>|z|)   Std.lv  Std.all
##    .contact1          0.933    0.054   17.243    0.000    0.933    0.438
##    .contact2r         5.749    0.198   29.064    0.000    5.749    0.813
##    .contact3          2.818    0.132   21.388    0.000    2.818    0.645
##    .contact4          0.666    0.038   17.623    0.000    0.666    0.368
##    .psupport1         3.239    0.135   23.995    0.000    3.239    0.512
##    .psupport2         0.835    0.049   17.115    0.000    0.835    0.218
##    .psupport3         5.883    0.163   36.182    0.000    5.883    0.642
##    .psupport4         0.919    0.045   20.403    0.000    0.919    0.278
##    .psupport5         2.010    0.088   22.814    0.000    2.010    0.460
##    .hygiene1          1.710    0.083   20.683    0.000    1.710    0.453
##    .hygiene2          1.579    0.078   20.262    0.000    1.579    0.447
##    .hygiene3          1.795    0.088   20.513    0.000    1.795    0.437
##    .hygiene4          5.953    0.178   33.459    0.000    5.953    0.626
##    .hygiene5          3.528    0.148   23.865    0.000    3.528    0.661
##    .omind2            0.560    0.033   16.956    0.000    0.560    0.268
##    .omind3            0.405    0.027   14.864    0.000    0.405    0.203
##    .omind4            0.843    0.047   17.917    0.000    0.843    0.368
##    .omind1            6.575    0.134   49.209    0.000    6.575    0.943
##    .omind5            5.288    0.203   25.995    0.000    5.288    0.849
##    .omind6            5.596    0.163   34.432    0.000    5.596    0.859
##    .phc               0.837    0.045   18.526    0.000    0.699    0.699
##    .phs               2.693    0.126   21.379    0.000    0.871    0.871
##    .phg               1.694    0.089   18.985    0.000    0.822    0.822
##     om1               1.529    0.057   26.665    0.000    1.000    1.000
## 
## 
## Group 2 [neutral]:
## 
## Latent Variables:
##                    Estimate  Std.Err  z-value  P(>|z|)   Std.lv  Std.all
##   phc =~                                                                
##     contact1          1.000                               1.356    0.730
##     contact2r         0.746    0.030   25.003    0.000    1.011    0.364
##     contact3          1.093    0.028   38.528    0.000    1.482    0.643
##     contact4          0.989    0.027   37.222    0.000    1.341    0.674
##   phs =~                                                                
##     psupport1         1.000                               2.083    0.812
##     psupport2         1.016    0.013   77.923    0.000    2.116    0.880
##     psupport3         0.999    0.016   62.016    0.000    2.081    0.652
##     psupport4         0.811    0.015   54.239    0.000    1.690    0.788
##     psupport5         0.846    0.015   54.770    0.000    1.763    0.719
##   phg =~                                                                
##     hygiene1          1.000                               1.434    0.686
##     hygiene2          1.012    0.011   90.796    0.000    1.451    0.711
##     hygiene3          0.937    0.023   41.439    0.000    1.344    0.756
##     hygiene4          1.122    0.032   35.122    0.000    1.610    0.512
##     hygiene5          0.860    0.029   29.352    0.000    1.233    0.517
##   om1 =~                                                                
##     omind2            1.000                               1.089    0.591
##     omind3            1.234    0.028   44.775    0.000    1.344    0.875
##     omind4            1.264    0.029   42.866    0.000    1.377    0.803
##     omind1            0.460    0.033   14.104    0.000    0.501    0.182
##     omind5            0.783    0.032   24.802    0.000    0.853    0.338
##     omind6            0.777    0.033   23.648    0.000    0.847    0.326
## 
## Regressions:
##                    Estimate  Std.Err  z-value  P(>|z|)   Std.lv  Std.all
##   phc ~                                                                 
##     ccrt             -0.001    0.018   -0.037    0.971   -0.000   -0.001
##     om1               0.442    0.022   19.929    0.000    0.355    0.355
##     age               0.007    0.001    5.718    0.000    0.005    0.080
##     sex1              0.366    0.039    9.403    0.000    0.270    0.135
##     political_dlgy    0.005    0.009    0.596    0.551    0.004    0.009
##   phs ~                                                                 
##     ccrt             -0.127    0.026   -4.887    0.000   -0.061   -0.065
##     om1               0.460    0.029   16.009    0.000    0.241    0.241
##     age              -0.013    0.002   -7.931    0.000   -0.006   -0.100
##     sex1              0.108    0.055    1.974    0.048    0.052    0.026
##     political_dlgy    0.016    0.013    1.249    0.212    0.008    0.017
##   phg ~                                                                 
##     ccrt             -0.151    0.019   -7.786    0.000   -0.105   -0.112
##     om1               0.432    0.024   18.365    0.000    0.328    0.328
##     age              -0.000    0.001   -0.045    0.964   -0.000   -0.001
##     sex1              0.292    0.040    7.252    0.000    0.203    0.102
##     political_dlgy    0.017    0.009    1.892    0.058    0.012    0.026
## 
## Covariances:
##                    Estimate  Std.Err  z-value  P(>|z|)   Std.lv  Std.all
##  .hygiene1 ~~                                                           
##    .hygiene2          1.606    0.086   18.684    0.000    1.606    0.736
##  .psupport4 ~~                                                          
##    .psupport5         0.532    0.050   10.678    0.000    0.532    0.236
##  .omind1 ~~                                                             
##    .omind5            1.771    0.104   17.068    0.000    1.771    0.275
##  .omind5 ~~                                                             
##    .omind6            1.847    0.103   17.856    0.000    1.847    0.316
##  .omind1 ~~                                                             
##    .omind6            2.637    0.104   25.374    0.000    2.637    0.396
##  .phc ~~                                                                
##    .phs               1.167    0.061   19.133    0.000    0.466    0.466
##    .phg               0.916    0.047   19.550    0.000    0.549    0.549
##  .phs ~~                                                                
##    .phg               0.923    0.056   16.553    0.000    0.346    0.346
## 
## Intercepts:
##                    Estimate  Std.Err  z-value  P(>|z|)   Std.lv  Std.all
##    .contact1          7.686    0.108   71.105    0.000    7.686    4.137
##    .contact2r         7.103    0.087   81.260    0.000    7.103    2.558
##    .contact3          7.077    0.118   59.818    0.000    7.077    3.069
##    .contact4          7.521    0.111   67.748    0.000    7.521    3.778
##    .psupport1         8.108    0.142   56.975    0.000    8.108    3.160
##    .psupport2         8.496    0.144   58.873    0.000    8.496    3.532
##    .psupport3         6.693    0.146   45.990    0.000    6.693    2.097
##    .psupport4         8.829    0.116   75.948    0.000    8.829    4.117
##    .psupport5         8.574    0.122   70.521    0.000    8.574    3.496
##    .hygiene1          7.695    0.108   71.495    0.000    7.695    3.679
##    .hygiene2          7.822    0.108   72.137    0.000    7.822    3.835
##    .hygiene3          8.523    0.101   84.129    0.000    8.523    4.798
##    .hygiene4          5.489    0.123   44.675    0.000    5.489    1.744
##    .hygiene5          7.750    0.095   81.856    0.000    7.750    3.248
##    .omind2            8.743    0.023  377.762    0.000    8.743    4.744
##    .omind3            8.652    0.019  449.907    0.000    8.652    5.629
##    .omind4            8.516    0.021  396.715    0.000    8.516    4.968
##    .omind1            6.363    0.034  187.781    0.000    6.363    2.311
##    .omind5            8.260    0.031  266.408    0.000    8.260    3.272
##    .omind6            7.178    0.032  222.708    0.000    7.178    2.761
##    .phc               0.000                               0.000    0.000
##    .phs               0.000                               0.000    0.000
##    .phg               0.000                               0.000    0.000
##     om1               0.000                               0.000    0.000
## 
## Variances:
##                    Estimate  Std.Err  z-value  P(>|z|)   Std.lv  Std.all
##    .contact1          1.613    0.071   22.805    0.000    1.613    0.467
##    .contact2r         6.691    0.174   38.447    0.000    6.691    0.867
##    .contact3          3.120    0.123   25.275    0.000    3.120    0.587
##    .contact4          2.165    0.092   23.509    0.000    2.165    0.546
##    .psupport1         2.246    0.086   26.110    0.000    2.246    0.341
##    .psupport2         1.309    0.059   22.026    0.000    1.309    0.226
##    .psupport3         5.856    0.134   43.729    0.000    5.856    0.575
##    .psupport4         1.742    0.060   29.115    0.000    1.742    0.379
##    .psupport5         2.908    0.095   30.477    0.000    2.908    0.483
##    .hygiene1          2.317    0.095   24.371    0.000    2.317    0.530
##    .hygiene2          2.054    0.091   22.580    0.000    2.054    0.494
##    .hygiene3          1.351    0.053   25.652    0.000    1.351    0.428
##    .hygiene4          7.312    0.144   50.851    0.000    7.312    0.738
##    .hygiene5          4.173    0.130   32.058    0.000    4.173    0.733
##    .omind2            2.210    0.139   15.944    0.000    2.210    0.651
##    .omind3            0.556    0.037   14.941    0.000    0.556    0.235
##    .omind4            1.043    0.058   17.957    0.000    1.043    0.355
##    .omind1            7.334    0.122   60.232    0.000    7.334    0.967
##    .omind5            5.647    0.172   32.875    0.000    5.647    0.886
##    .omind6            6.042    0.128   47.104    0.000    6.042    0.894
##    .phc               1.564    0.072   21.709    0.000    0.851    0.851
##    .phs               4.016    0.128   31.416    0.000    0.926    0.926
##    .phg               1.778    0.084   21.141    0.000    0.864    0.864
##     om1               1.187    0.056   21.038    0.000    1.000    1.000
## 
## 
## Group 3 [positive]:
## 
## Latent Variables:
##                    Estimate  Std.Err  z-value  P(>|z|)   Std.lv  Std.all
##   phc =~                                                                
##     contact1          1.000                               1.548    0.770
##     contact2r         0.816    0.049   16.788    0.000    1.264    0.442
##     contact3          1.050    0.036   29.562    0.000    1.626    0.761
##     contact4          0.961    0.032   30.013    0.000    1.488    0.852
##   phs =~                                                                
##     psupport1         1.000                               2.101    0.865
##     psupport2         1.083    0.021   51.368    0.000    2.275    0.924
##     psupport3         0.980    0.033   29.465    0.000    2.058    0.584
##     psupport4         0.921    0.026   35.427    0.000    1.936    0.836
##     psupport5         0.918    0.029   31.906    0.000    1.929    0.801
##   phg =~                                                                
##     hygiene1          1.000                               1.642    0.785
##     hygiene2          1.001    0.012   84.757    0.000    1.644    0.801
##     hygiene3          0.870    0.032   27.407    0.000    1.429    0.784
##     hygiene4          1.121    0.042   26.439    0.000    1.841    0.610
##     hygiene5          0.960    0.042   23.007    0.000    1.576    0.542
##   om1 =~                                                                
##     omind2            1.000                               1.321    0.846
##     omind3            1.060    0.024   44.517    0.000    1.400    0.912
##     omind4            1.045    0.026   40.407    0.000    1.381    0.858
##     omind1            0.398    0.048    8.344    0.000    0.526    0.200
##     omind5            0.865    0.046   18.852    0.000    1.143    0.453
##     omind6            0.832    0.046   17.901    0.000    1.099    0.428
## 
## Regressions:
##                    Estimate  Std.Err  z-value  P(>|z|)   Std.lv  Std.all
##   phc ~                                                                 
##     ccrt             -0.047    0.038   -1.242    0.214   -0.030   -0.031
##     om1               0.525    0.035   15.090    0.000    0.448    0.448
##     age               0.009    0.002    4.027    0.000    0.006    0.095
##     sex1              0.549    0.076    7.250    0.000    0.354    0.177
##     political_dlgy    0.035    0.017    2.003    0.045    0.022    0.051
##   phs ~                                                                 
##     ccrt             -0.149    0.052   -2.872    0.004   -0.071   -0.073
##     om1               0.420    0.041   10.148    0.000    0.264    0.264
##     age               0.004    0.003    1.461    0.144    0.002    0.034
##     sex1              0.554    0.101    5.509    0.000    0.264    0.132
##     political_dlgy    0.104    0.023    4.410    0.000    0.049    0.112
##   phg ~                                                                 
##     ccrt             -0.208    0.042   -4.972    0.000   -0.126   -0.131
##     om1               0.354    0.036    9.882    0.000    0.285    0.285
##     age               0.006    0.003    2.233    0.026    0.003    0.056
##     sex1              0.520    0.083    6.266    0.000    0.317    0.158
##     political_dlgy    0.050    0.019    2.606    0.009    0.030    0.069
## 
## Covariances:
##                    Estimate  Std.Err  z-value  P(>|z|)   Std.lv  Std.all
##  .hygiene1 ~~                                                           
##    .hygiene2          1.273    0.126   10.106    0.000    1.273    0.800
##  .psupport4 ~~                                                          
##    .psupport5         0.594    0.089    6.657    0.000    0.594    0.323
##  .omind1 ~~                                                             
##    .omind5            1.514    0.160    9.480    0.000    1.514    0.261
##  .omind5 ~~                                                             
##    .omind6            2.116    0.190   11.148    0.000    2.116    0.406
##  .omind1 ~~                                                             
##    .omind6            2.357    0.172   13.730    0.000    2.357    0.394
##  .phc ~~                                                                
##    .phs               1.534    0.149   10.321    0.000    0.573    0.573
##    .phg               1.212    0.123    9.830    0.000    0.589    0.589
##  .phs ~~                                                                
##    .phg               1.365    0.145    9.426    0.000    0.451    0.451
## 
## Intercepts:
##                    Estimate  Std.Err  z-value  P(>|z|)   Std.lv  Std.all
##    .contact1          6.997    0.222   31.511    0.000    6.997    3.481
##    .contact2r         6.552    0.194   33.722    0.000    6.552    2.294
##    .contact3          6.727    0.236   28.537    0.000    6.727    3.148
##    .contact4          7.289    0.212   34.308    0.000    7.289    4.175
##    .psupport1         6.481    0.279   23.218    0.000    6.481    2.666
##    .psupport2         6.473    0.303   21.341    0.000    6.473    2.629
##    .psupport3         4.145    0.276   15.037    0.000    4.145    1.176
##    .psupport4         7.012    0.262   26.731    0.000    7.012    3.026
##    .psupport5         6.854    0.262   26.188    0.000    6.854    2.845
##    .hygiene1          6.850    0.224   30.644    0.000    6.850    3.274
##    .hygiene2          6.954    0.225   30.904    0.000    6.954    3.390
##    .hygiene3          7.711    0.205   37.557    0.000    7.711    4.228
##    .hygiene4          5.221    0.255   20.476    0.000    5.221    1.731
##    .hygiene5          6.102    0.218   27.952    0.000    6.102    2.098
##    .omind2            8.511    0.036  233.207    0.000    8.511    5.452
##    .omind3            8.432    0.036  235.474    0.000    8.432    5.494
##    .omind4            8.450    0.038  224.899    0.000    8.450    5.250
##    .omind1            5.576    0.062   89.957    0.000    5.576    2.118
##    .omind5            8.060    0.059  137.252    0.000    8.060    3.198
##    .omind6            7.359    0.060  121.899    0.000    7.359    2.865
##    .phc               0.000                               0.000    0.000
##    .phs               0.000                               0.000    0.000
##    .phg               0.000                               0.000    0.000
##     om1               0.000                               0.000    0.000
## 
## Variances:
##                    Estimate  Std.Err  z-value  P(>|z|)   Std.lv  Std.all
##    .contact1          1.644    0.121   13.631    0.000    1.644    0.407
##    .contact2r         6.563    0.312   21.025    0.000    6.563    0.804
##    .contact3          1.921    0.144   13.312    0.000    1.921    0.421
##    .contact4          0.835    0.059   14.121    0.000    0.835    0.274
##    .psupport1         1.492    0.117   12.757    0.000    1.492    0.253
##    .psupport2         0.886    0.076   11.703    0.000    0.886    0.146
##    .psupport3         8.187    0.274   29.862    0.000    8.187    0.659
##    .psupport4         1.621    0.115   14.086    0.000    1.621    0.302
##    .psupport5         2.082    0.139   14.944    0.000    2.082    0.359
##    .hygiene1          1.681    0.129   13.065    0.000    1.681    0.384
##    .hygiene2          1.507    0.132   11.438    0.000    1.507    0.358
##    .hygiene3          1.283    0.082   15.658    0.000    1.283    0.386
##    .hygiene4          5.710    0.263   21.715    0.000    5.710    0.628
##    .hygiene5          5.975    0.297   20.141    0.000    5.975    0.706
##    .omind2            0.691    0.056   12.369    0.000    0.691    0.284
##    .omind3            0.395    0.041    9.699    0.000    0.395    0.168
##    .omind4            0.684    0.059   11.695    0.000    0.684    0.264
##    .omind1            6.650    0.202   32.859    0.000    6.650    0.960
##    .omind5            5.048    0.304   16.606    0.000    5.048    0.794
##    .omind6            5.390    0.247   21.789    0.000    5.390    0.817
##    .phc               1.819    0.160   11.379    0.000    0.758    0.758
##    .phs               3.939    0.244   16.137    0.000    0.892    0.892
##    .phg               2.330    0.169   13.761    0.000    0.864    0.864
##     om1               1.745    0.089   19.590    0.000    1.000    1.000
```

```
phymodcwzx <- sem(phczx, estimator = "MLM", data = pls4, group = "cpol", group.equal = "loadings")
summary(phymodcwzx, standardized = T)
```

```
## lavaan 0.6-8 ended normally after 274 iterations
## 
##   Estimator                                         ML
##   Optimization method                           NLMINB
##   Number of model parameters                       249
##   Number of equality constraints                    32
##                                                       
##   Number of observations per group:                   
##     negative                                      4408
##     neutral                                       6315
##     positive                                      1767
##                                                       
## Model Test User Model:
##                                               Standard      Robust
##   Test Statistic                              8772.756    8370.471
##   Degrees of freedom                               713         713
##   P-value (Chi-square)                           0.000       0.000
##   Scaling correction factor                                  1.048
##        Satorra-Bentler correction                                 
##   Test statistic for each group:
##     negative                                  3767.574    3594.807
##     neutral                                   3643.914    3476.817
##     positive                                  1361.268    1298.846
## 
## Parameter Estimates:
## 
##   Standard errors                           Robust.sem
##   Information                                 Expected
##   Information saturated (h1) model          Structured
## 
## 
## Group 1 [negative]:
## 
## Latent Variables:
##                    Estimate  Std.Err  z-value  P(>|z|)   Std.lv  Std.all
##   phc =~                                                                
##     contct1           1.000                               1.111    0.755
##     cntct2r (.p2.)    0.851    0.022   38.529    0.000    0.946    0.364
##     contct3 (.p3.)    1.097    0.018   59.450    0.000    1.220    0.587
##     contct4 (.p4.)    0.977    0.016   61.188    0.000    1.086    0.802
##   phs =~                                                                
##     pspprt1           1.000                               1.742    0.695
##     pspprt2 (.p6.)    1.021    0.010  106.470    0.000    1.778    0.895
##     pspprt3 (.p7.)    1.011    0.012   82.040    0.000    1.762    0.586
##     pspprt4 (.p8.)    0.859    0.011   77.673    0.000    1.496    0.837
##     pspprt5 (.p9.)    0.871    0.012   73.100    0.000    1.517    0.728
##   phg =~                                                                
##     hygien1           1.000                               1.499    0.761
##     hygien2 (.11.)    0.994    0.007  148.892    0.000    1.490    0.775
##     hygien3 (.12.)    0.966    0.015   62.768    0.000    1.449    0.727
##     hygien4 (.13.)    1.192    0.022   55.157    0.000    1.786    0.587
##     hygien5 (.14.)    0.902    0.019   46.326    0.000    1.353    0.585
##   om1 =~                                                                
##     omind2            1.000                               1.180    0.837
##     omind3  (.18.)    1.079    0.012   88.708    0.000    1.274    0.898
##     omind4  (.19.)    1.066    0.013   80.664    0.000    1.258    0.811
##     omind1  (.20.)    0.451    0.020   22.266    0.000    0.532    0.203
##     omind5  (.21.)    0.764    0.019   40.687    0.000    0.902    0.364
##     omind6  (.22.)    0.753    0.019   38.654    0.000    0.888    0.351
## 
## Regressions:
##                    Estimate  Std.Err  z-value  P(>|z|)   Std.lv  Std.all
##   phc ~                                                                 
##     ccrt             -0.037    0.016   -2.307    0.021   -0.033   -0.036
##     om1               0.480    0.021   22.959    0.000    0.510    0.510
##     age               0.010    0.001   10.486    0.000    0.009    0.158
##     sex1              0.279    0.035    7.972    0.000    0.251    0.126
##     political_dlgy   -0.027    0.007   -3.697    0.000   -0.024   -0.055
##   phs ~                                                                 
##     ccrt             -0.071    0.025   -2.802    0.005   -0.041   -0.044
##     om1               0.479    0.027   17.924    0.000    0.325    0.325
##     age               0.004    0.001    2.504    0.012    0.002    0.037
##     sex1              0.256    0.054    4.768    0.000    0.147    0.073
##     political_dlgy   -0.089    0.012   -7.267    0.000   -0.051   -0.117
##   phg ~                                                                 
##     ccrt             -0.336    0.024  -13.945    0.000   -0.224   -0.241
##     om1               0.394    0.024   16.695    0.000    0.310    0.310
##     age               0.001    0.001    0.855    0.392    0.001    0.013
##     sex1              0.320    0.048    6.617    0.000    0.214    0.107
##     political_dlgy    0.035    0.010    3.358    0.001    0.023    0.054
## 
## Covariances:
##                    Estimate  Std.Err  z-value  P(>|z|)   Std.lv  Std.all
##  .hygiene1 ~~                                                           
##    .hygiene2          1.160    0.074   15.776    0.000    1.160    0.747
##  .psupport4 ~~                                                          
##    .psupport5         0.264    0.041    6.402    0.000    0.264    0.189
##  .omind1 ~~                                                             
##    .omind5            2.074    0.122   16.972    0.000    2.074    0.350
##  .omind5 ~~                                                             
##    .omind6            2.729    0.140   19.479    0.000    2.729    0.500
##  .omind1 ~~                                                             
##    .omind6            2.505    0.117   21.317    0.000    2.505    0.412
##  .phc ~~                                                                
##    .phs               0.776    0.040   19.242    0.000    0.513    0.513
##    .phg               0.576    0.033   17.251    0.000    0.455    0.455
##  .phs ~~                                                                
##    .phg               0.708    0.052   13.707    0.000    0.320    0.320
## 
## Intercepts:
##                    Estimate  Std.Err  z-value  P(>|z|)   Std.lv  Std.all
##    .contact1          8.182    0.099   82.977    0.000    8.182    5.559
##    .contact2r         7.496    0.091   82.317    0.000    7.496    2.883
##    .contact3          7.489    0.109   68.646    0.000    7.489    3.605
##    .contact4          8.299    0.098   84.972    0.000    8.299    6.129
##    .psupport1         7.819    0.147   53.180    0.000    7.819    3.121
##    .psupport2         8.553    0.149   57.398    0.000    8.553    4.306
##    .psupport3         6.764    0.151   44.942    0.000    6.764    2.252
##    .psupport4         8.732    0.126   69.096    0.000    8.732    4.886
##    .psupport5         8.503    0.128   66.291    0.000    8.503    4.079
##    .hygiene1          7.833    0.134   58.436    0.000    7.833    3.978
##    .hygiene2          7.962    0.134   59.561    0.000    7.962    4.139
##    .hygiene3          8.056    0.130   61.827    0.000    8.056    4.043
##    .hygiene4          6.015    0.162   37.051    0.000    6.015    1.976
##    .hygiene5          7.689    0.122   63.148    0.000    7.689    3.323
##    .omind2            8.680    0.021  403.986    0.000    8.680    6.158
##    .omind3            8.610    0.021  411.433    0.000    8.610    6.070
##    .omind4            8.513    0.023  376.614    0.000    8.513    5.486
##    .omind1            5.986    0.039  154.848    0.000    5.986    2.282
##    .omind5            8.426    0.036  235.596    0.000    8.426    3.405
##    .omind6            7.550    0.037  203.764    0.000    7.550    2.983
##    .phc               0.000                               0.000    0.000
##    .phs               0.000                               0.000    0.000
##    .phg               0.000                               0.000    0.000
##     om1               0.000                               0.000    0.000
## 
## Variances:
##                    Estimate  Std.Err  z-value  P(>|z|)   Std.lv  Std.all
##    .contact1          0.931    0.055   16.987    0.000    0.931    0.430
##    .contact2r         5.867    0.190   30.898    0.000    5.867    0.868
##    .contact3          2.827    0.128   22.035    0.000    2.827    0.655
##    .contact4          0.654    0.038   17.377    0.000    0.654    0.357
##    .psupport1         3.241    0.133   24.445    0.000    3.241    0.517
##    .psupport2         0.784    0.048   16.475    0.000    0.784    0.199
##    .psupport3         5.923    0.158   37.536    0.000    5.923    0.656
##    .psupport4         0.955    0.043   22.095    0.000    0.955    0.299
##    .psupport5         2.043    0.087   23.589    0.000    2.043    0.470
##    .hygiene1          1.630    0.081   20.146    0.000    1.630    0.420
##    .hygiene2          1.480    0.077   19.309    0.000    1.480    0.400
##    .hygiene3          1.873    0.085   21.943    0.000    1.873    0.472
##    .hygiene4          6.079    0.167   36.485    0.000    6.079    0.656
##    .hygiene5          3.525    0.145   24.332    0.000    3.525    0.658
##    .omind2            0.594    0.033   17.894    0.000    0.594    0.299
##    .omind3            0.390    0.026   15.084    0.000    0.390    0.194
##    .omind4            0.825    0.047   17.737    0.000    0.825    0.343
##    .omind1            6.594    0.130   50.638    0.000    6.594    0.959
##    .omind5            5.311    0.201   26.389    0.000    5.311    0.867
##    .omind6            5.617    0.159   35.383    0.000    5.617    0.877
##    .phc               0.865    0.039   21.960    0.000    0.700    0.700
##    .phs               2.645    0.114   23.238    0.000    0.872    0.872
##    .phg               1.848    0.083   22.260    0.000    0.822    0.822
##     om1               1.393    0.048   28.880    0.000    1.000    1.000
## 
## 
## Group 2 [neutral]:
## 
## Latent Variables:
##                    Estimate  Std.Err  z-value  P(>|z|)   Std.lv  Std.all
##   phc =~                                                                
##     contct1           1.000                               1.347    0.727
##     cntct2r (.p2.)    0.851    0.022   38.529    0.000    1.146    0.407
##     contct3 (.p3.)    1.097    0.018   59.450    0.000    1.478    0.641
##     contct4 (.p4.)    0.977    0.016   61.188    0.000    1.316    0.665
##   phs =~                                                                
##     pspprt1           1.000                               2.048    0.805
##     pspprt2 (.p6.)    1.021    0.010  106.470    0.000    2.091    0.875
##     pspprt3 (.p7.)    1.011    0.012   82.040    0.000    2.071    0.650
##     pspprt4 (.p8.)    0.859    0.011   77.673    0.000    1.759    0.803
##     pspprt5 (.p9.)    0.871    0.012   73.100    0.000    1.784    0.724
##   phg =~                                                                
##     hygien1           1.000                               1.400    0.674
##     hygien2 (.11.)    0.994    0.007  148.892    0.000    1.391    0.691
##     hygien3 (.12.)    0.966    0.015   62.768    0.000    1.353    0.760
##     hygien4 (.13.)    1.192    0.022   55.157    0.000    1.668    0.526
##     hygien5 (.14.)    0.902    0.019   46.326    0.000    1.263    0.527
##   om1 =~                                                                
##     omind2            1.000                               1.232    0.642
##     omind3  (.18.)    1.079    0.012   88.708    0.000    1.330    0.870
##     omind4  (.19.)    1.066    0.013   80.664    0.000    1.313    0.783
##     omind1  (.20.)    0.451    0.020   22.266    0.000    0.556    0.201
##     omind5  (.21.)    0.764    0.019   40.687    0.000    0.941    0.369
##     omind6  (.22.)    0.753    0.019   38.654    0.000    0.927    0.353
## 
## Regressions:
##                    Estimate  Std.Err  z-value  P(>|z|)   Std.lv  Std.all
##   phc ~                                                                 
##     ccrt              0.001    0.018    0.080    0.936    0.001    0.001
##     om1               0.389    0.018   21.392    0.000    0.356    0.356
##     age               0.007    0.001    5.822    0.000    0.005    0.081
##     sex1              0.365    0.039    9.453    0.000    0.271    0.135
##     political_dlgy    0.005    0.009    0.581    0.561    0.004    0.008
##   phs ~                                                                 
##     ccrt             -0.125    0.026   -4.877    0.000   -0.061   -0.065
##     om1               0.402    0.024   16.658    0.000    0.242    0.242
##     age              -0.012    0.002   -7.806    0.000   -0.006   -0.098
##     sex1              0.109    0.054    2.032    0.042    0.053    0.027
##     political_dlgy    0.016    0.013    1.276    0.202    0.008    0.017
##   phg ~                                                                 
##     ccrt             -0.148    0.019   -7.874    0.000   -0.106   -0.113
##     om1               0.373    0.019   19.520    0.000    0.329    0.329
##     age              -0.000    0.001   -0.280    0.779   -0.000   -0.004
##     sex1              0.290    0.039    7.385    0.000    0.207    0.104
##     political_dlgy    0.017    0.009    1.940    0.052    0.012    0.026
## 
## Covariances:
##                    Estimate  Std.Err  z-value  P(>|z|)   Std.lv  Std.all
##  .hygiene1 ~~                                                           
##    .hygiene2          1.658    0.082   20.334    0.000    1.658    0.742
##  .psupport4 ~~                                                          
##    .psupport5         0.498    0.049   10.120    0.000    0.498    0.225
##  .omind1 ~~                                                             
##    .omind5            1.758    0.104   16.911    0.000    1.758    0.274
##  .omind5 ~~                                                             
##    .omind6            1.830    0.104   17.650    0.000    1.830    0.314
##  .omind1 ~~                                                             
##    .omind6            2.626    0.104   25.226    0.000    2.626    0.395
##  .phc ~~                                                                
##    .phs               1.144    0.057   20.027    0.000    0.468    0.468
##    .phg               0.887    0.043   20.828    0.000    0.549    0.549
##  .phs ~~                                                                
##    .phg               0.890    0.053   16.817    0.000    0.347    0.347
## 
## Intercepts:
##                    Estimate  Std.Err  z-value  P(>|z|)   Std.lv  Std.all
##    .contact1          7.683    0.107   71.758    0.000    7.683    4.149
##    .contact2r         7.011    0.095   73.432    0.000    7.011    2.487
##    .contact3          7.070    0.118   60.024    0.000    7.070    3.067
##    .contact4          7.528    0.107   70.067    0.000    7.528    3.804
##    .psupport1         8.085    0.140   57.766    0.000    8.085    3.179
##    .psupport2         8.474    0.143   59.404    0.000    8.474    3.547
##    .psupport3         6.674    0.145   46.174    0.000    6.674    2.094
##    .psupport4         8.829    0.121   72.853    0.000    8.829    4.032
##    .psupport5         8.564    0.123   69.502    0.000    8.564    3.477
##    .hygiene1          7.706    0.105   73.192    0.000    7.706    3.708
##    .hygiene2          7.839    0.104   75.094    0.000    7.839    3.894
##    .hygiene3          8.523    0.102   83.770    0.000    8.523    4.786
##    .hygiene4          5.477    0.127   42.990    0.000    5.477    1.728
##    .hygiene5          7.744    0.097   80.212    0.000    7.744    3.230
##    .omind2            8.743    0.023  377.762    0.000    8.743    4.556
##    .omind3            8.652    0.019  449.907    0.000    8.652    5.664
##    .omind4            8.516    0.021  396.715    0.000    8.516    5.078
##    .omind1            6.363    0.034  187.781    0.000    6.363    2.303
##    .omind5            8.260    0.031  266.408    0.000    8.260    3.237
##    .omind6            7.178    0.032  222.708    0.000    7.178    2.735
##    .phc               0.000                               0.000    0.000
##    .phs               0.000                               0.000    0.000
##    .phg               0.000                               0.000    0.000
##     om1               0.000                               0.000    0.000
## 
## Variances:
##                    Estimate  Std.Err  z-value  P(>|z|)   Std.lv  Std.all
##    .contact1          1.615    0.069   23.330    0.000    1.615    0.471
##    .contact2r         6.634    0.175   37.977    0.000    6.634    0.835
##    .contact3          3.130    0.119   26.346    0.000    3.130    0.589
##    .contact4          2.185    0.089   24.513    0.000    2.185    0.558
##    .psupport1         2.273    0.084   27.176    0.000    2.273    0.351
##    .psupport2         1.336    0.057   23.487    0.000    1.336    0.234
##    .psupport3         5.870    0.131   44.872    0.000    5.870    0.578
##    .psupport4         1.701    0.059   28.786    0.000    1.701    0.355
##    .psupport5         2.882    0.094   30.648    0.000    2.882    0.475
##    .hygiene1          2.358    0.090   26.188    0.000    2.358    0.546
##    .hygiene2          2.117    0.086   24.621    0.000    2.117    0.522
##    .hygiene3          1.341    0.052   25.924    0.000    1.341    0.423
##    .hygiene4          7.267    0.139   52.119    0.000    7.267    0.723
##    .hygiene5          4.151    0.127   32.564    0.000    4.151    0.722
##    .omind2            2.165    0.139   15.610    0.000    2.165    0.588
##    .omind3            0.565    0.034   16.439    0.000    0.565    0.242
##    .omind4            1.089    0.055   19.701    0.000    1.089    0.387
##    .omind1            7.326    0.122   59.981    0.000    7.326    0.960
##    .omind5            5.626    0.172   32.656    0.000    5.626    0.864
##    .omind6            6.029    0.129   46.885    0.000    6.029    0.875
##    .phc               1.542    0.060   25.654    0.000    0.850    0.850
##    .phs               3.884    0.122   31.951    0.000    0.926    0.926
##    .phg               1.692    0.071   23.697    0.000    0.863    0.863
##     om1               1.518    0.047   32.496    0.000    1.000    1.000
## 
## 
## Group 3 [positive]:
## 
## Latent Variables:
##                    Estimate  Std.Err  z-value  P(>|z|)   Std.lv  Std.all
##   phc =~                                                                
##     contct1           1.000                               1.517    0.762
##     cntct2r (.p2.)    0.851    0.022   38.529    0.000    1.291    0.450
##     contct3 (.p3.)    1.097    0.018   59.450    0.000    1.665    0.770
##     contct4 (.p4.)    0.977    0.016   61.188    0.000    1.482    0.851
##   phs =~                                                                
##     pspprt1           1.000                               2.186    0.876
##     pspprt2 (.p6.)    1.021    0.010  106.470    0.000    2.231    0.918
##     pspprt3 (.p7.)    1.011    0.012   82.040    0.000    2.211    0.612
##     pspprt4 (.p8.)    0.859    0.011   77.673    0.000    1.877    0.826
##     pspprt5 (.p9.)    0.871    0.012   73.100    0.000    1.904    0.796
##   phg =~                                                                
##     hygien1           1.000                               1.568    0.761
##     hygien2 (.11.)    0.994    0.007  148.892    0.000    1.558    0.775
##     hygien3 (.12.)    0.966    0.015   62.768    0.000    1.515    0.812
##     hygien4 (.13.)    1.192    0.022   55.157    0.000    1.868    0.615
##     hygien5 (.14.)    0.902    0.019   46.326    0.000    1.415    0.497
##   om1 =~                                                                
##     omind2            1.000                               1.307    0.842
##     omind3  (.18.)    1.079    0.012   88.708    0.000    1.411    0.915
##     omind4  (.19.)    1.066    0.013   80.664    0.000    1.393    0.860
##     omind1  (.20.)    0.451    0.020   22.266    0.000    0.590    0.223
##     omind5  (.21.)    0.764    0.019   40.687    0.000    0.999    0.405
##     omind6  (.22.)    0.753    0.019   38.654    0.000    0.984    0.389
## 
## Regressions:
##                    Estimate  Std.Err  z-value  P(>|z|)   Std.lv  Std.all
##   phc ~                                                                 
##     ccrt             -0.045    0.037   -1.230    0.219   -0.030   -0.031
##     om1               0.519    0.033   15.739    0.000    0.447    0.447
##     age               0.009    0.002    4.048    0.000    0.006    0.096
##     sex1              0.539    0.073    7.353    0.000    0.355    0.178
##     political_dlgy    0.034    0.017    1.978    0.048    0.022    0.050
##   phs ~                                                                 
##     ccrt             -0.158    0.054   -2.942    0.003   -0.072   -0.075
##     om1               0.441    0.043   10.219    0.000    0.264    0.264
##     age               0.004    0.003    1.409    0.159    0.002    0.032
##     sex1              0.576    0.104    5.518    0.000    0.264    0.132
##     political_dlgy    0.108    0.024    4.429    0.000    0.050    0.113
##   phg ~                                                                 
##     ccrt             -0.189    0.039   -4.787    0.000   -0.121   -0.125
##     om1               0.349    0.035   10.099    0.000    0.291    0.291
##     age               0.005    0.002    2.283    0.022    0.003    0.057
##     sex1              0.507    0.080    6.366    0.000    0.323    0.162
##     political_dlgy    0.046    0.018    2.521    0.012    0.029    0.067
## 
## Covariances:
##                    Estimate  Std.Err  z-value  P(>|z|)   Std.lv  Std.all
##  .hygiene1 ~~                                                           
##    .hygiene2          1.376    0.121   11.356    0.000    1.376    0.812
##  .psupport4 ~~                                                          
##    .psupport5         0.613    0.084    7.302    0.000    0.613    0.330
##  .omind1 ~~                                                             
##    .omind5            1.508    0.159    9.477    0.000    1.508    0.259
##  .omind5 ~~                                                             
##    .omind6            2.158    0.186   11.613    0.000    2.158    0.410
##  .omind1 ~~                                                             
##    .omind6            2.352    0.171   13.768    0.000    2.352    0.392
##  .phc ~~                                                                
##    .phs               1.562    0.144   10.858    0.000    0.572    0.572
##    .phg               1.137    0.110   10.345    0.000    0.591    0.591
##  .phs ~~                                                                
##    .phg               1.361    0.140    9.699    0.000    0.453    0.453
## 
## Intercepts:
##                    Estimate  Std.Err  z-value  P(>|z|)   Std.lv  Std.all
##    .contact1          7.022    0.216   32.476    0.000    7.022    3.527
##    .contact2r         6.525    0.192   34.024    0.000    6.525    2.275
##    .contact3          6.690    0.238   28.134    0.000    6.690    3.096
##    .contact4          7.291    0.210   34.707    0.000    7.291    4.186
##    .psupport1         6.431    0.290   22.207    0.000    6.431    2.576
##    .psupport2         6.512    0.297   21.904    0.000    6.512    2.680
##    .psupport3         4.050    0.296   13.693    0.000    4.050    1.121
##    .psupport4         7.059    0.252   28.047    0.000    7.059    3.105
##    .psupport5         6.878    0.255   26.929    0.000    6.878    2.875
##    .hygiene1          6.879    0.215   31.943    0.000    6.879    3.341
##    .hygiene2          6.990    0.215   32.568    0.000    6.990    3.477
##    .hygiene3          7.631    0.211   36.095    0.000    7.631    4.089
##    .hygiene4          5.176    0.259   19.968    0.000    5.176    1.703
##    .hygiene5          6.192    0.199   31.040    0.000    6.192    2.175
##    .omind2            8.511    0.036  233.207    0.000    8.511    5.485
##    .omind3            8.432    0.036  235.474    0.000    8.432    5.471
##    .omind4            8.450    0.038  224.899    0.000    8.450    5.216
##    .omind1            5.576    0.062   89.957    0.000    5.576    2.108
##    .omind5            8.060    0.059  137.251    0.000    8.060    3.265
##    .omind6            7.359    0.060  121.899    0.000    7.359    2.910
##    .phc               0.000                               0.000    0.000
##    .phs               0.000                               0.000    0.000
##    .phg               0.000                               0.000    0.000
##     om1               0.000                               0.000    0.000
## 
## Variances:
##                    Estimate  Std.Err  z-value  P(>|z|)   Std.lv  Std.all
##    .contact1          1.662    0.119   13.976    0.000    1.662    0.419
##    .contact2r         6.559    0.306   21.445    0.000    6.559    0.797
##    .contact3          1.898    0.142   13.372    0.000    1.898    0.406
##    .contact4          0.836    0.057   14.592    0.000    0.836    0.276
##    .psupport1         1.452    0.114   12.751    0.000    1.452    0.233
##    .psupport2         0.924    0.074   12.549    0.000    0.924    0.157
##    .psupport3         8.159    0.276   29.583    0.000    8.159    0.625
##    .psupport4         1.646    0.109   15.062    0.000    1.646    0.318
##    .psupport5         2.097    0.132   15.895    0.000    2.097    0.366
##    .hygiene1          1.782    0.123   14.447    0.000    1.782    0.420
##    .hygiene2          1.613    0.127   12.743    0.000    1.613    0.399
##    .hygiene3          1.189    0.078   15.250    0.000    1.189    0.341
##    .hygiene4          5.745    0.253   22.746    0.000    5.745    0.622
##    .hygiene5          6.102    0.280   21.806    0.000    6.102    0.753
##    .omind2            0.699    0.054   12.979    0.000    0.699    0.290
##    .omind3            0.386    0.038   10.106    0.000    0.386    0.162
##    .omind4            0.683    0.057   12.031    0.000    0.683    0.260
##    .omind1            6.648    0.203   32.730    0.000    6.648    0.950
##    .omind5            5.097    0.295   17.285    0.000    5.097    0.836
##    .omind6            5.426    0.238   22.834    0.000    5.426    0.849
##    .phc               1.747    0.136   12.856    0.000    0.759    0.759
##    .phs               4.263    0.237   18.019    0.000    0.892    0.892
##    .phg               2.118    0.137   15.434    0.000    0.862    0.862
##     om1               1.708    0.072   23.812    0.000    1.000    1.000
```

```
phymodcs5xyx <- sem(phczx, estimator = "MLM", data = pls4, group = "cpol", group.equal = c("loadings", "intercepts"), group.partial = c("psupport1 ~ 1"))
summary(phymodcs5xyx, standardized = T)
```

```
## lavaan 0.6-8 ended normally after 332 iterations
## 
##   Estimator                                         ML
##   Optimization method                           NLMINB
##   Number of model parameters                       257
##   Number of equality constraints                    70
##                                                       
##   Number of observations per group:                   
##     negative                                      4408
##     neutral                                       6315
##     positive                                      1767
##                                                       
## Model Test User Model:
##                                                Standard      Robust
##   Test Statistic                              10112.587    9794.829
##   Degrees of freedom                                743         743
##   P-value (Chi-square)                            0.000       0.000
##   Scaling correction factor                                   1.032
##        Satorra-Bentler correction                                  
##   Test statistic for each group:
##     negative                                  4216.173    4083.693
##     neutral                                   4099.859    3971.033
##     positive                                  1796.555    1740.104
## 
## Parameter Estimates:
## 
##   Standard errors                           Robust.sem
##   Information                                 Expected
##   Information saturated (h1) model          Structured
## 
## 
## Group 1 [negative]:
## 
## Latent Variables:
##                    Estimate  Std.Err  z-value  P(>|z|)   Std.lv  Std.all
##   phc =~                                                                
##     contct1           1.000                               1.101    0.750
##     cntct2r (.p2.)    0.857    0.022   39.573    0.000    0.944    0.363
##     contct3 (.p3.)    1.086    0.018   60.221    0.000    1.196    0.577
##     contct4 (.p4.)    0.997    0.016   63.082    0.000    1.098    0.806
##   phs =~                                                                
##     pspprt1           1.000                               1.743    0.696
##     pspprt2 (.p6.)    1.022    0.010  106.740    0.000    1.781    0.896
##     pspprt3 (.p7.)    1.018    0.012   82.832    0.000    1.774    0.589
##     pspprt4 (.p8.)    0.855    0.011   77.737    0.000    1.490    0.835
##     pspprt5 (.p9.)    0.867    0.012   73.198    0.000    1.511    0.726
##   phg =~                                                                
##     hygien1           1.000                               1.497    0.760
##     hygien2 (.11.)    0.994    0.007  148.723    0.000    1.489    0.773
##     hygien3 (.12.)    0.957    0.015   62.506    0.000    1.434    0.710
##     hygien4 (.13.)    1.181    0.021   55.109    0.000    1.768    0.580
##     hygien5 (.14.)    0.907    0.020   46.389    0.000    1.358    0.587
##   om1 =~                                                                
##     omind2            1.000                               1.181    0.838
##     omind3  (.18.)    1.079    0.012   88.380    0.000    1.275    0.898
##     omind4  (.19.)    1.062    0.013   80.461    0.000    1.255    0.810
##     omind1  (.20.)    0.461    0.020   22.762    0.000    0.544    0.207
##     omind5  (.21.)    0.765    0.019   40.710    0.000    0.903    0.364
##     omind6  (.22.)    0.748    0.019   38.498    0.000    0.883    0.348
## 
## Regressions:
##                    Estimate  Std.Err  z-value  P(>|z|)   Std.lv  Std.all
##   phc ~                                                                 
##     ccrt             -0.037    0.016   -2.304    0.021   -0.033   -0.036
##     om1               0.476    0.021   22.941    0.000    0.511    0.511
##     age               0.010    0.001   10.538    0.000    0.009    0.160
##     sex1              0.276    0.035    7.959    0.000    0.251    0.125
##     political_dlgy   -0.027    0.007   -3.706    0.000   -0.024   -0.055
##   phs ~                                                                 
##     ccrt             -0.071    0.025   -2.814    0.005   -0.041   -0.044
##     om1               0.478    0.027   17.908    0.000    0.324    0.324
##     age               0.004    0.001    2.485    0.013    0.002    0.036
##     sex1              0.256    0.054    4.765    0.000    0.147    0.073
##     political_dlgy   -0.089    0.012   -7.268    0.000   -0.051   -0.117
##   phg ~                                                                 
##     ccrt             -0.338    0.024  -13.989    0.000   -0.226   -0.243
##     om1               0.394    0.024   16.689    0.000    0.311    0.311
##     age               0.001    0.001    0.869    0.385    0.001    0.014
##     sex1              0.320    0.049    6.599    0.000    0.214    0.107
##     political_dlgy    0.035    0.010    3.378    0.001    0.024    0.054
## 
## Covariances:
##                    Estimate  Std.Err  z-value  P(>|z|)   Std.lv  Std.all
##  .hygiene1 ~~                                                           
##    .hygiene2          1.167    0.073   15.882    0.000    1.167    0.748
##  .psupport4 ~~                                                          
##    .psupport5         0.273    0.041    6.626    0.000    0.273    0.194
##  .omind1 ~~                                                             
##    .omind5            2.054    0.122   16.802    0.000    2.054    0.346
##  .omind5 ~~                                                             
##    .omind6            2.760    0.140   19.703    0.000    2.760    0.502
##  .omind1 ~~                                                             
##    .omind6            2.479    0.118   21.088    0.000    2.479    0.405
##  .phc ~~                                                                
##    .phs               0.768    0.040   19.301    0.000    0.513    0.513
##    .phg               0.570    0.033   17.255    0.000    0.457    0.457
##  .phs ~~                                                                
##    .phg               0.710    0.052   13.703    0.000    0.322    0.322
## 
## Intercepts:
##                    Estimate  Std.Err  z-value  P(>|z|)   Std.lv  Std.all
##    .contct1 (.78.)    8.204    0.098   84.125    0.000    8.204    5.591
##    .cntct2r (.79.)    7.487    0.086   86.813    0.000    7.487    2.879
##    .contct3 (.80.)    7.660    0.106   72.127    0.000    7.660    3.693
##    .contct4 (.81.)    8.238    0.098   84.177    0.000    8.238    6.052
##    .pspprt1           7.821    0.147   53.165    0.000    7.821    3.122
##    .pspprt2 (.83.)    8.530    0.149   57.195    0.000    8.530    4.291
##    .pspprt3 (.84.)    6.655    0.150   44.378    0.000    6.655    2.209
##    .pspprt4 (.85.)    8.780    0.125   70.154    0.000    8.780    4.921
##    .pspprt5 (.86.)    8.552    0.127   67.346    0.000    8.552    4.107
##    .hygien1 (.87.)    7.734    0.134   57.767    0.000    7.734    3.926
##    .hygien2 (.88.)    7.859    0.133   58.957    0.000    7.859    4.083
##    .hygien3 (.89.)    8.353    0.128   65.007    0.000    8.353    4.139
##    .hygien4 (.90.)    5.762    0.159   36.226    0.000    5.762    1.890
##    .hygien5 (.91.)    7.622    0.122   62.666    0.000    7.622    3.293
##    .omind2  (.92.)    8.684    0.020  430.930    0.000    8.684    6.158
##    .omind3  (.93.)    8.614    0.020  423.374    0.000    8.614    6.069
##    .omind4  (.94.)    8.522    0.021  408.739    0.000    8.522    5.499
##    .omind1  (.95.)    6.107    0.025  247.531    0.000    6.107    2.325
##    .omind5  (.96.)    8.283    0.024  339.106    0.000    8.283    3.340
##    .omind6  (.97.)    7.344    0.025  291.995    0.000    7.344    2.893
##    .phc               0.000                               0.000    0.000
##    .phs               0.000                               0.000    0.000
##    .phg               0.000                               0.000    0.000
##     om1               0.000                               0.000    0.000
## 
## Variances:
##                    Estimate  Std.Err  z-value  P(>|z|)   Std.lv  Std.all
##    .contact1          0.941    0.055   16.961    0.000    0.941    0.437
##    .contact2r         5.869    0.191   30.790    0.000    5.869    0.868
##    .contact3          2.872    0.129   22.313    0.000    2.872    0.667
##    .contact4          0.648    0.038   17.061    0.000    0.648    0.350
##    .psupport1         3.237    0.133   24.425    0.000    3.237    0.516
##    .psupport2         0.781    0.048   16.323    0.000    0.781    0.198
##    .psupport3         5.930    0.159   37.372    0.000    5.930    0.653
##    .psupport4         0.965    0.043   22.212    0.000    0.965    0.303
##    .psupport5         2.053    0.087   23.603    0.000    2.053    0.474
##    .hygiene1          1.637    0.081   20.247    0.000    1.637    0.422
##    .hygiene2          1.488    0.077   19.413    0.000    1.488    0.402
##    .hygiene3          2.018    0.085   23.603    0.000    2.018    0.495
##    .hygiene4          6.165    0.166   37.111    0.000    6.165    0.663
##    .hygiene5          3.513    0.145   24.255    0.000    3.513    0.656
##    .omind2            0.593    0.033   17.870    0.000    0.593    0.298
##    .omind3            0.389    0.026   15.020    0.000    0.389    0.193
##    .omind4            0.826    0.046   17.776    0.000    0.826    0.344
##    .omind1            6.605    0.130   50.622    0.000    6.605    0.957
##    .omind5            5.332    0.201   26.487    0.000    5.332    0.867
##    .omind6            5.662    0.159   35.695    0.000    5.662    0.879
##    .phc               0.847    0.038   22.075    0.000    0.699    0.699
##    .phs               2.648    0.114   23.245    0.000    0.872    0.872
##    .phg               1.841    0.083   22.145    0.000    0.821    0.821
##     om1               1.396    0.048   28.907    0.000    1.000    1.000
## 
## 
## Group 2 [neutral]:
## 
## Latent Variables:
##                    Estimate  Std.Err  z-value  P(>|z|)   Std.lv  Std.all
##   phc =~                                                                
##     contct1           1.000                               1.340    0.724
##     cntct2r (.p2.)    0.857    0.022   39.573    0.000    1.148    0.407
##     contct3 (.p3.)    1.086    0.018   60.221    0.000    1.455    0.634
##     contct4 (.p4.)    0.997    0.016   63.082    0.000    1.336    0.670
##   phs =~                                                                
##     pspprt1           1.000                               2.049    0.806
##     pspprt2 (.p6.)    1.022    0.010  106.740    0.000    2.094    0.875
##     pspprt3 (.p7.)    1.018    0.012   82.832    0.000    2.086    0.653
##     pspprt4 (.p8.)    0.855    0.011   77.737    0.000    1.751    0.801
##     pspprt5 (.p9.)    0.867    0.012   73.198    0.000    1.776    0.723
##   phg =~                                                                
##     hygien1           1.000                               1.404    0.674
##     hygien2 (.11.)    0.994    0.007  148.723    0.000    1.395    0.692
##     hygien3 (.12.)    0.957    0.015   62.506    0.000    1.344    0.754
##     hygien4 (.13.)    1.181    0.021   55.109    0.000    1.657    0.520
##     hygien5 (.14.)    0.907    0.020   46.389    0.000    1.273    0.530
##   om1 =~                                                                
##     omind2            1.000                               1.233    0.642
##     omind3  (.18.)    1.079    0.012   88.380    0.000    1.331    0.871
##     omind4  (.19.)    1.062    0.013   80.461    0.000    1.310    0.782
##     omind1  (.20.)    0.461    0.020   22.762    0.000    0.568    0.205
##     omind5  (.21.)    0.765    0.019   40.710    0.000    0.943    0.369
##     omind6  (.22.)    0.748    0.019   38.498    0.000    0.922    0.351
## 
## Regressions:
##                    Estimate  Std.Err  z-value  P(>|z|)   Std.lv  Std.all
##   phc ~                                                                 
##     ccrt              0.002    0.018    0.108    0.914    0.001    0.002
##     om1               0.388    0.018   21.406    0.000    0.357    0.357
##     age               0.007    0.001    5.887    0.000    0.005    0.082
##     sex1              0.363    0.038    9.456    0.000    0.271    0.136
##     political_dlgy    0.005    0.009    0.585    0.558    0.004    0.008
##   phs ~                                                                 
##     ccrt             -0.125    0.026   -4.888    0.000   -0.061   -0.065
##     om1               0.401    0.024   16.643    0.000    0.241    0.241
##     age              -0.012    0.002   -7.841    0.000   -0.006   -0.099
##     sex1              0.109    0.054    2.021    0.043    0.053    0.026
##     political_dlgy    0.016    0.013    1.287    0.198    0.008    0.017
##   phg ~                                                                 
##     ccrt             -0.149    0.019   -7.887    0.000   -0.106   -0.113
##     om1               0.375    0.019   19.534    0.000    0.329    0.329
##     age              -0.000    0.001   -0.239    0.811   -0.000   -0.003
##     sex1              0.290    0.039    7.361    0.000    0.207    0.103
##     political_dlgy    0.017    0.009    1.936    0.053    0.012    0.026
## 
## Covariances:
##                    Estimate  Std.Err  z-value  P(>|z|)   Std.lv  Std.all
##  .hygiene1 ~~                                                           
##    .hygiene2          1.660    0.082   20.297    0.000    1.660    0.742
##  .psupport4 ~~                                                          
##    .psupport5         0.505    0.049   10.291    0.000    0.505    0.227
##  .omind1 ~~                                                             
##    .omind5            1.747    0.104   16.788    0.000    1.747    0.271
##  .omind5 ~~                                                             
##    .omind6            1.839    0.104   17.728    0.000    1.839    0.315
##  .omind1 ~~                                                             
##    .omind6            2.581    0.104   24.765    0.000    2.581    0.386
##  .phc ~~                                                                
##    .phs               1.138    0.057   19.989    0.000    0.468    0.468
##    .phg               0.888    0.043   20.850    0.000    0.552    0.552
##  .phs ~~                                                                
##    .phg               0.894    0.053   16.819    0.000    0.348    0.348
## 
## Intercepts:
##                    Estimate  Std.Err  z-value  P(>|z|)   Std.lv  Std.all
##    .contct1 (.78.)    8.204    0.098   84.125    0.000    8.204    4.433
##    .cntct2r (.79.)    7.487    0.086   86.813    0.000    7.487    2.655
##    .contct3 (.80.)    7.660    0.106   72.127    0.000    7.660    3.336
##    .contct4 (.81.)    8.238    0.098   84.177    0.000    8.238    4.131
##    .pspprt1           8.094    0.148   54.834    0.000    8.094    3.182
##    .pspprt2 (.83.)    8.530    0.149   57.195    0.000    8.530    3.566
##    .pspprt3 (.84.)    6.655    0.150   44.378    0.000    6.655    2.082
##    .pspprt4 (.85.)    8.780    0.125   70.154    0.000    8.780    4.016
##    .pspprt5 (.86.)    8.552    0.127   67.346    0.000    8.552    3.479
##    .hygien1 (.87.)    7.734    0.134   57.767    0.000    7.734    3.716
##    .hygien2 (.88.)    7.859    0.133   58.957    0.000    7.859    3.898
##    .hygien3 (.89.)    8.353    0.128   65.007    0.000    8.353    4.684
##    .hygien4 (.90.)    5.762    0.159   36.226    0.000    5.762    1.808
##    .hygien5 (.91.)    7.622    0.122   62.666    0.000    7.622    3.175
##    .omind2  (.92.)    8.684    0.020  430.930    0.000    8.684    4.523
##    .omind3  (.93.)    8.614    0.020  423.374    0.000    8.614    5.638
##    .omind4  (.94.)    8.522    0.021  408.739    0.000    8.522    5.084
##    .omind1  (.95.)    6.107    0.025  247.531    0.000    6.107    2.200
##    .omind5  (.96.)    8.283    0.024  339.106    0.000    8.283    3.244
##    .omind6  (.97.)    7.344    0.025  291.995    0.000    7.344    2.793
##    .phc              -0.597    0.143   -4.161    0.000   -0.445   -0.445
##    .phs              -0.015    0.201   -0.077    0.939   -0.008   -0.008
##    .phg               0.048    0.169    0.282    0.778    0.034    0.034
##     om1               0.021    0.025    0.851    0.395    0.017    0.017
## 
## Variances:
##                    Estimate  Std.Err  z-value  P(>|z|)   Std.lv  Std.all
##    .contact1          1.630    0.068   23.814    0.000    1.630    0.476
##    .contact2r         6.632    0.174   38.218    0.000    6.632    0.834
##    .contact3          3.153    0.117   26.894    0.000    3.153    0.598
##    .contact4          2.194    0.089   24.777    0.000    2.194    0.552
##    .psupport1         2.271    0.084   27.160    0.000    2.271    0.351
##    .psupport2         1.337    0.057   23.579    0.000    1.337    0.234
##    .psupport3         5.862    0.131   44.821    0.000    5.862    0.574
##    .psupport4         1.711    0.059   29.057    0.000    1.711    0.358
##    .psupport5         2.888    0.094   30.849    0.000    2.888    0.478
##    .hygiene1          2.362    0.090   26.136    0.000    2.362    0.545
##    .hygiene2          2.119    0.086   24.559    0.000    2.119    0.521
##    .hygiene3          1.374    0.052   26.504    0.000    1.374    0.432
##    .hygiene4          7.407    0.139   53.183    0.000    7.407    0.729
##    .hygiene5          4.145    0.128   32.443    0.000    4.145    0.719
##    .omind2            2.166    0.139   15.607    0.000    2.166    0.588
##    .omind3            0.564    0.035   16.326    0.000    0.564    0.241
##    .omind4            1.093    0.055   19.742    0.000    1.093    0.389
##    .omind1            7.385    0.123   60.286    0.000    7.385    0.958
##    .omind5            5.628    0.172   32.638    0.000    5.628    0.864
##    .omind6            6.065    0.129   47.186    0.000    6.065    0.877
##    .phc               1.525    0.060   25.604    0.000    0.849    0.849
##    .phs               3.887    0.122   31.965    0.000    0.926    0.926
##    .phg               1.699    0.072   23.664    0.000    0.863    0.863
##     om1               1.521    0.047   32.459    0.000    1.000    1.000
## 
## 
## Group 3 [positive]:
## 
## Latent Variables:
##                    Estimate  Std.Err  z-value  P(>|z|)   Std.lv  Std.all
##   phc =~                                                                
##     contct1           1.000                               1.506    0.754
##     cntct2r (.p2.)    0.857    0.022   39.573    0.000    1.291    0.450
##     contct3 (.p3.)    1.086    0.018   60.221    0.000    1.636    0.761
##     contct4 (.p4.)    0.997    0.016   63.082    0.000    1.501    0.857
##   phs =~                                                                
##     pspprt1           1.000                               2.187    0.876
##     pspprt2 (.p6.)    1.022    0.010  106.740    0.000    2.234    0.919
##     pspprt3 (.p7.)    1.018    0.012   82.832    0.000    2.227    0.607
##     pspprt4 (.p8.)    0.855    0.011   77.737    0.000    1.869    0.824
##     pspprt5 (.p9.)    0.867    0.012   73.198    0.000    1.896    0.794
##   phg =~                                                                
##     hygien1           1.000                               1.573    0.763
##     hygien2 (.11.)    0.994    0.007  148.723    0.000    1.564    0.777
##     hygien3 (.12.)    0.957    0.015   62.506    0.000    1.506    0.810
##     hygien4 (.13.)    1.181    0.021   55.109    0.000    1.858    0.606
##     hygien5 (.14.)    0.907    0.020   46.389    0.000    1.427    0.485
##   om1 =~                                                                
##     omind2            1.000                               1.308    0.843
##     omind3  (.18.)    1.079    0.012   88.380    0.000    1.412    0.916
##     omind4  (.19.)    1.062    0.013   80.461    0.000    1.390    0.857
##     omind1  (.20.)    0.461    0.020   22.762    0.000    0.603    0.224
##     omind5  (.21.)    0.765    0.019   40.710    0.000    1.000    0.405
##     omind6  (.22.)    0.748    0.019   38.498    0.000    0.978    0.387
## 
## Regressions:
##                    Estimate  Std.Err  z-value  P(>|z|)   Std.lv  Std.all
##   phc ~                                                                 
##     ccrt             -0.046    0.037   -1.256    0.209   -0.031   -0.032
##     om1               0.516    0.033   15.760    0.000    0.448    0.448
##     age               0.009    0.002    4.059    0.000    0.006    0.096
##     sex1              0.532    0.073    7.304    0.000    0.353    0.176
##     political_dlgy    0.033    0.017    1.974    0.048    0.022    0.050
##   phs ~                                                                 
##     ccrt             -0.158    0.054   -2.939    0.003   -0.072   -0.075
##     om1               0.441    0.043   10.227    0.000    0.264    0.264
##     age               0.004    0.003    1.407    0.159    0.002    0.032
##     sex1              0.575    0.104    5.508    0.000    0.263    0.132
##     political_dlgy    0.108    0.024    4.411    0.000    0.049    0.112
##   phg ~                                                                 
##     ccrt             -0.188    0.040   -4.739    0.000   -0.120   -0.124
##     om1               0.352    0.035   10.128    0.000    0.292    0.292
##     age               0.006    0.002    2.293    0.022    0.004    0.057
##     sex1              0.507    0.080    6.335    0.000    0.322    0.161
##     political_dlgy    0.046    0.018    2.510    0.012    0.029    0.066
## 
## Covariances:
##                    Estimate  Std.Err  z-value  P(>|z|)   Std.lv  Std.all
##  .hygiene1 ~~                                                           
##    .hygiene2          1.371    0.121   11.297    0.000    1.371    0.811
##  .psupport4 ~~                                                          
##    .psupport5         0.620    0.084    7.387    0.000    0.620    0.332
##  .omind1 ~~                                                             
##    .omind5            1.559    0.159    9.791    0.000    1.559    0.263
##  .omind5 ~~                                                             
##    .omind6            2.146    0.186   11.551    0.000    2.146    0.407
##  .omind1 ~~                                                             
##    .omind6            2.293    0.171   13.413    0.000    2.293    0.375
##  .phc ~~                                                                
##    .phs               1.547    0.143   10.821    0.000    0.571    0.571
##    .phg               1.137    0.110   10.354    0.000    0.594    0.594
##  .phs ~~                                                                
##    .phg               1.368    0.141    9.696    0.000    0.453    0.453
## 
## Intercepts:
##                    Estimate  Std.Err  z-value  P(>|z|)   Std.lv  Std.all
##    .contct1 (.78.)    8.204    0.098   84.125    0.000    8.204    4.109
##    .cntct2r (.79.)    7.487    0.086   86.813    0.000    7.487    2.608
##    .contct3 (.80.)    7.660    0.106   72.127    0.000    7.660    3.565
##    .contct4 (.81.)    8.238    0.098   84.177    0.000    8.238    4.703
##    .pspprt1           8.445    0.150   56.361    0.000    8.445    3.383
##    .pspprt2 (.83.)    8.530    0.149   57.195    0.000    8.530    3.507
##    .pspprt3 (.84.)    6.655    0.150   44.378    0.000    6.655    1.813
##    .pspprt4 (.85.)    8.780    0.125   70.154    0.000    8.780    3.871
##    .pspprt5 (.86.)    8.552    0.127   67.346    0.000    8.552    3.580
##    .hygien1 (.87.)    7.734    0.134   57.767    0.000    7.734    3.751
##    .hygien2 (.88.)    7.859    0.133   58.957    0.000    7.859    3.903
##    .hygien3 (.89.)    8.353    0.128   65.007    0.000    8.353    4.489
##    .hygien4 (.90.)    5.762    0.159   36.226    0.000    5.762    1.880
##    .hygien5 (.91.)    7.622    0.122   62.666    0.000    7.622    2.589
##    .omind2  (.92.)    8.684    0.020  430.930    0.000    8.684    5.594
##    .omind3  (.93.)    8.614    0.020  423.374    0.000    8.614    5.587
##    .omind4  (.94.)    8.522    0.021  408.739    0.000    8.522    5.258
##    .omind1  (.95.)    6.107    0.025  247.531    0.000    6.107    2.271
##    .omind5  (.96.)    8.283    0.024  339.106    0.000    8.283    3.351
##    .omind6  (.97.)    7.344    0.025  291.995    0.000    7.344    2.903
##    .phc              -0.918    0.232   -3.963    0.000   -0.610   -0.610
##    .phs              -1.947    0.323   -6.029    0.000   -0.890   -0.890
##    .phg              -0.765    0.253   -3.027    0.002   -0.486   -0.486
##     om1              -0.144    0.037   -3.915    0.000   -0.110   -0.110
## 
## Variances:
##                    Estimate  Std.Err  z-value  P(>|z|)   Std.lv  Std.all
##    .contact1          1.718    0.119   14.464    0.000    1.718    0.431
##    .contact2r         6.574    0.306   21.519    0.000    6.574    0.798
##    .contact3          1.942    0.141   13.785    0.000    1.942    0.420
##    .contact4          0.815    0.057   14.258    0.000    0.815    0.265
##    .psupport1         1.448    0.114   12.706    0.000    1.448    0.232
##    .psupport2         0.922    0.074   12.515    0.000    0.922    0.156
##    .psupport3         8.509    0.277   30.767    0.000    8.509    0.632
##    .psupport4         1.650    0.109   15.111    0.000    1.650    0.321
##    .psupport5         2.111    0.132   16.019    0.000    2.111    0.370
##    .hygiene1          1.776    0.124   14.377    0.000    1.776    0.418
##    .hygiene2          1.609    0.127   12.692    0.000    1.609    0.397
##    .hygiene3          1.193    0.078   15.343    0.000    1.193    0.345
##    .hygiene4          5.939    0.252   23.568    0.000    5.939    0.632
##    .hygiene5          6.633    0.280   23.674    0.000    6.633    0.765
##    .omind2            0.699    0.054   12.992    0.000    0.699    0.290
##    .omind3            0.385    0.038   10.106    0.000    0.385    0.162
##    .omind4            0.696    0.057   12.285    0.000    0.696    0.265
##    .omind1            6.866    0.204   33.734    0.000    6.866    0.950
##    .omind5            5.110    0.295   17.332    0.000    5.110    0.836
##    .omind6            5.444    0.237   22.943    0.000    5.444    0.851
##    .phc               1.720    0.134   12.814    0.000    0.758    0.758
##    .phs               4.266    0.237   18.017    0.000    0.892    0.892
##    .phg               2.132    0.139   15.390    0.000    0.861    0.861
##     om1               1.711    0.072   23.784    0.000    1.000    1.000
```

```
phymodcsrx <- sem(phczx, estimator = "MLM", data = pls4, group = "cpol", group.equal = c("loadings", "intercepts", "regressions"), group.partial = c("psupport1 ~ 1"))
summary(phymodcsrx, standardized = T)
```

```
## lavaan 0.6-8 ended normally after 233 iterations
## 
##   Estimator                                         ML
##   Optimization method                           NLMINB
##   Number of model parameters                       257
##   Number of equality constraints                   100
##                                                       
##   Number of observations per group:                   
##     negative                                      4408
##     neutral                                       6315
##     positive                                      1767
##                                                       
## Model Test User Model:
##                                                Standard      Robust
##   Test Statistic                              10382.985   10050.015
##   Degrees of freedom                                773         773
##   P-value (Chi-square)                            0.000       0.000
##   Scaling correction factor                                   1.033
##        Satorra-Bentler correction                                  
##   Test statistic for each group:
##     negative                                  4333.727    4194.750
##     neutral                                   4185.686    4051.456
##     positive                                  1863.572    1803.809
## 
## Parameter Estimates:
## 
##   Standard errors                           Robust.sem
##   Information                                 Expected
##   Information saturated (h1) model          Structured
## 
## 
## Group 1 [negative]:
## 
## Latent Variables:
##                    Estimate  Std.Err  z-value  P(>|z|)   Std.lv  Std.all
##   phc =~                                                                
##     contct1           1.000                               1.086    0.746
##     cntct2r (.p2.)    0.857    0.022   39.776    0.000    0.931    0.359
##     contct3 (.p3.)    1.088    0.018   60.231    0.000    1.182    0.572
##     contct4 (.p4.)    0.999    0.016   62.585    0.000    1.086    0.803
##   phs =~                                                                
##     pspprt1           1.000                               1.736    0.695
##     pspprt2 (.p6.)    1.022    0.010  107.258    0.000    1.774    0.894
##     pspprt3 (.p7.)    1.017    0.012   83.272    0.000    1.766    0.588
##     pspprt4 (.p8.)    0.856    0.011   78.077    0.000    1.485    0.833
##     pspprt5 (.p9.)    0.868    0.012   73.561    0.000    1.506    0.725
##   phg =~                                                                
##     hygien1           1.000                               1.473    0.757
##     hygien2 (.11.)    0.995    0.007  148.097    0.000    1.466    0.772
##     hygien3 (.12.)    0.952    0.015   62.285    0.000    1.402    0.703
##     hygien4 (.13.)    1.175    0.021   54.791    0.000    1.731    0.570
##     hygien5 (.14.)    0.904    0.020   46.181    0.000    1.331    0.579
##   om1 =~                                                                
##     omind2            1.000                               1.183    0.838
##     omind3  (.18.)    1.079    0.012   88.325    0.000    1.277    0.899
##     omind4  (.19.)    1.063    0.013   80.335    0.000    1.258    0.811
##     omind1  (.20.)    0.462    0.020   22.785    0.000    0.547    0.208
##     omind5  (.21.)    0.766    0.019   40.647    0.000    0.907    0.365
##     omind6  (.22.)    0.750    0.019   38.516    0.000    0.887    0.349
## 
## Regressions:
##                    Estimate  Std.Err  z-value  P(>|z|)   Std.lv  Std.all
##   phc ~                                                                 
##     ccrt    (.26.)   -0.021    0.011   -1.877    0.060   -0.020   -0.021
##     om1     (.27.)    0.451    0.014   33.364    0.000    0.491    0.491
##     age     (.28.)    0.009    0.001   12.470    0.000    0.008    0.138
##     sex1    (.29.)    0.333    0.024   13.711    0.000    0.307    0.153
##     pltcl_d (.30.)   -0.008    0.005   -1.432    0.152   -0.007   -0.016
##   phs ~                                                                 
##     ccrt    (.31.)   -0.102    0.017   -5.950    0.000   -0.059   -0.063
##     om1     (.32.)    0.453    0.017   26.688    0.000    0.309    0.309
##     age     (.33.)   -0.003    0.001   -3.268    0.001   -0.002   -0.033
##     sex1    (.34.)    0.219    0.036    6.155    0.000    0.126    0.063
##     pltcl_d (.35.)   -0.021    0.008   -2.536    0.011   -0.012   -0.028
##   phg ~                                                                 
##     ccrt    (.36.)   -0.218    0.014  -15.605    0.000   -0.148   -0.160
##     om1     (.37.)    0.383    0.014   26.953    0.000    0.307    0.307
##     age     (.38.)    0.001    0.001    1.443    0.149    0.001    0.014
##     sex1    (.39.)    0.314    0.028   11.028    0.000    0.213    0.106
##     pltcl_d (.40.)    0.024    0.006    3.745    0.000    0.016    0.037
## 
## Covariances:
##                    Estimate  Std.Err  z-value  P(>|z|)   Std.lv  Std.all
##  .hygiene1 ~~                                                           
##    .hygiene2          1.145    0.074   15.489    0.000    1.145    0.744
##  .psupport4 ~~                                                          
##    .psupport5         0.276    0.041    6.665    0.000    0.276    0.196
##  .omind1 ~~                                                             
##    .omind5            2.053    0.122   16.795    0.000    2.053    0.346
##  .omind5 ~~                                                             
##    .omind6            2.759    0.140   19.701    0.000    2.759    0.502
##  .omind1 ~~                                                             
##    .omind6            2.477    0.118   21.078    0.000    2.477    0.405
##  .phc ~~                                                                
##    .phs               0.780    0.040   19.737    0.000    0.515    0.515
##    .phg               0.573    0.033   17.392    0.000    0.454    0.454
##  .phs ~~                                                                
##    .phg               0.705    0.052   13.504    0.000    0.314    0.314
## 
## Intercepts:
##                    Estimate  Std.Err  z-value  P(>|z|)   Std.lv  Std.all
##    .contct1 (.78.)    8.073    0.070  114.655    0.000    8.073    5.544
##    .cntct2r (.79.)    7.374    0.064  115.055    0.000    7.374    2.841
##    .contct3 (.80.)    7.516    0.077   97.445    0.000    7.516    3.639
##    .contct4 (.81.)    8.106    0.071  113.506    0.000    8.106    5.995
##    .pspprt1           7.883    0.102   77.656    0.000    7.883    3.156
##    .pspprt2 (.83.)    8.593    0.101   85.026    0.000    8.593    4.333
##    .pspprt3 (.84.)    6.718    0.103   65.221    0.000    6.718    2.237
##    .pspprt4 (.85.)    8.833    0.085  103.687    0.000    8.833    4.957
##    .pspprt5 (.86.)    8.606    0.087   99.384    0.000    8.606    4.139
##    .hygien1 (.87.)    7.687    0.081   94.678    0.000    7.687    3.948
##    .hygien2 (.88.)    7.813    0.081   96.631    0.000    7.813    4.111
##    .hygien3 (.89.)    8.309    0.078  106.115    0.000    8.309    4.164
##    .hygien4 (.90.)    5.707    0.097   58.944    0.000    5.707    1.878
##    .hygien5 (.91.)    7.580    0.074  101.917    0.000    7.580    3.296
##    .omind2  (.92.)    8.684    0.020  430.999    0.000    8.684    6.153
##    .omind3  (.93.)    8.614    0.020  423.422    0.000    8.614    6.063
##    .omind4  (.94.)    8.522    0.021  408.613    0.000    8.522    5.490
##    .omind1  (.95.)    6.107    0.025  247.476    0.000    6.107    2.324
##    .omind5  (.96.)    8.283    0.024  338.993    0.000    8.283    3.339
##    .omind6  (.97.)    7.344    0.025  291.874    0.000    7.344    2.892
##    .phc               0.000                               0.000    0.000
##    .phs               0.000                               0.000    0.000
##    .phg               0.000                               0.000    0.000
##     om1               0.000                               0.000    0.000
## 
## Variances:
##                    Estimate  Std.Err  z-value  P(>|z|)   Std.lv  Std.all
##    .contact1          0.940    0.055   16.991    0.000    0.940    0.443
##    .contact2r         5.873    0.191   30.815    0.000    5.873    0.871
##    .contact3          2.868    0.128   22.324    0.000    2.868    0.672
##    .contact4          0.649    0.038   17.054    0.000    0.649    0.355
##    .psupport1         3.225    0.132   24.365    0.000    3.225    0.517
##    .psupport2         0.787    0.048   16.320    0.000    0.787    0.200
##    .psupport3         5.903    0.158   37.340    0.000    5.903    0.654
##    .psupport4         0.970    0.044   22.238    0.000    0.970    0.306
##    .psupport5         2.053    0.087   23.605    0.000    2.053    0.475
##    .hygiene1          1.620    0.081   19.925    0.000    1.620    0.427
##    .hygiene2          1.461    0.077   18.967    0.000    1.461    0.405
##    .hygiene3          2.015    0.086   23.442    0.000    2.015    0.506
##    .hygiene4          6.241    0.167   37.320    0.000    6.241    0.676
##    .hygiene5          3.517    0.145   24.251    0.000    3.517    0.665
##    .omind2            0.593    0.033   17.858    0.000    0.593    0.297
##    .omind3            0.387    0.026   14.955    0.000    0.387    0.192
##    .omind4            0.826    0.047   17.748    0.000    0.826    0.343
##    .omind1            6.604    0.131   50.602    0.000    6.604    0.957
##    .omind5            5.331    0.201   26.481    0.000    5.331    0.866
##    .omind6            5.661    0.159   35.676    0.000    5.661    0.878
##    .phc               0.852    0.038   22.284    0.000    0.722    0.722
##    .phs               2.690    0.114   23.515    0.000    0.893    0.893
##    .phg               1.871    0.084   22.244    0.000    0.862    0.862
##     om1               1.400    0.048   29.085    0.000    1.000    1.000
## 
## 
## Group 2 [neutral]:
## 
## Latent Variables:
##                    Estimate  Std.Err  z-value  P(>|z|)   Std.lv  Std.all
##   phc =~                                                                
##     contct1           1.000                               1.367    0.730
##     cntct2r (.p2.)    0.857    0.022   39.776    0.000    1.172    0.414
##     contct3 (.p3.)    1.088    0.018   60.231    0.000    1.488    0.642
##     contct4 (.p4.)    0.999    0.016   62.585    0.000    1.366    0.678
##   phs =~                                                                
##     pspprt1           1.000                               2.062    0.807
##     pspprt2 (.p6.)    1.022    0.010  107.258    0.000    2.107    0.877
##     pspprt3 (.p7.)    1.017    0.012   83.272    0.000    2.098    0.654
##     pspprt4 (.p8.)    0.856    0.011   78.077    0.000    1.764    0.805
##     pspprt5 (.p9.)    0.868    0.012   73.561    0.000    1.789    0.726
##   phg =~                                                                
##     hygien1           1.000                               1.426    0.681
##     hygien2 (.11.)    0.995    0.007  148.097    0.000    1.419    0.699
##     hygien3 (.12.)    0.952    0.015   62.285    0.000    1.357    0.755
##     hygien4 (.13.)    1.175    0.021   54.791    0.000    1.675    0.525
##     hygien5 (.14.)    0.904    0.020   46.181    0.000    1.288    0.535
##   om1 =~                                                                
##     omind2            1.000                               1.228    0.641
##     omind3  (.18.)    1.079    0.012   88.325    0.000    1.326    0.869
##     omind4  (.19.)    1.063    0.013   80.335    0.000    1.306    0.781
##     omind1  (.20.)    0.462    0.020   22.785    0.000    0.567    0.204
##     omind5  (.21.)    0.766    0.019   40.647    0.000    0.941    0.369
##     omind6  (.22.)    0.750    0.019   38.516    0.000    0.921    0.350
## 
## Regressions:
##                    Estimate  Std.Err  z-value  P(>|z|)   Std.lv  Std.all
##   phc ~                                                                 
##     ccrt    (.26.)   -0.021    0.011   -1.877    0.060   -0.016   -0.017
##     om1     (.27.)    0.451    0.014   33.364    0.000    0.405    0.405
##     age     (.28.)    0.009    0.001   12.470    0.000    0.006    0.104
##     sex1    (.29.)    0.333    0.024   13.711    0.000    0.244    0.122
##     pltcl_d (.30.)   -0.008    0.005   -1.432    0.152   -0.006   -0.012
##   phs ~                                                                 
##     ccrt    (.31.)   -0.102    0.017   -5.950    0.000   -0.049   -0.053
##     om1     (.32.)    0.453    0.017   26.688    0.000    0.270    0.270
##     age     (.33.)   -0.003    0.001   -3.268    0.001   -0.002   -0.026
##     sex1    (.34.)    0.219    0.036    6.155    0.000    0.106    0.053
##     pltcl_d (.35.)   -0.021    0.008   -2.536    0.011   -0.010   -0.022
##   phg ~                                                                 
##     ccrt    (.36.)   -0.218    0.014  -15.605    0.000   -0.153   -0.164
##     om1     (.37.)    0.383    0.014   26.953    0.000    0.330    0.330
##     age     (.38.)    0.001    0.001    1.443    0.149    0.001    0.014
##     sex1    (.39.)    0.314    0.028   11.028    0.000    0.220    0.110
##     pltcl_d (.40.)    0.024    0.006    3.745    0.000    0.017    0.036
## 
## Covariances:
##                    Estimate  Std.Err  z-value  P(>|z|)   Std.lv  Std.all
##  .hygiene1 ~~                                                           
##    .hygiene2          1.652    0.082   20.262    0.000    1.652    0.741
##  .psupport4 ~~                                                          
##    .psupport5         0.491    0.049   10.059    0.000    0.491    0.222
##  .omind1 ~~                                                             
##    .omind5            1.746    0.104   16.774    0.000    1.746    0.271
##  .omind5 ~~                                                             
##    .omind6            1.836    0.104   17.714    0.000    1.836    0.314
##  .omind1 ~~                                                             
##    .omind6            2.579    0.104   24.754    0.000    2.579    0.385
##  .phc ~~                                                                
##    .phs               1.135    0.057   19.788    0.000    0.466    0.466
##    .phg               0.886    0.043   20.753    0.000    0.549    0.549
##  .phs ~~                                                                
##    .phg               0.893    0.053   16.785    0.000    0.345    0.345
## 
## Intercepts:
##                    Estimate  Std.Err  z-value  P(>|z|)   Std.lv  Std.all
##    .contct1 (.78.)    8.073    0.070  114.655    0.000    8.073    4.312
##    .cntct2r (.79.)    7.374    0.064  115.055    0.000    7.374    2.606
##    .contct3 (.80.)    7.516    0.077   97.445    0.000    7.516    3.245
##    .contct4 (.81.)    8.106    0.071  113.506    0.000    8.106    4.025
##    .pspprt1           8.156    0.101   80.865    0.000    8.156    3.191
##    .pspprt2 (.83.)    8.593    0.101   85.026    0.000    8.593    3.575
##    .pspprt3 (.84.)    6.718    0.103   65.221    0.000    6.718    2.094
##    .pspprt4 (.85.)    8.833    0.085  103.687    0.000    8.833    4.028
##    .pspprt5 (.86.)    8.606    0.087   99.384    0.000    8.606    3.491
##    .hygien1 (.87.)    7.687    0.081   94.678    0.000    7.687    3.670
##    .hygien2 (.88.)    7.813    0.081   96.631    0.000    7.813    3.848
##    .hygien3 (.89.)    8.309    0.078  106.115    0.000    8.309    4.623
##    .hygien4 (.90.)    5.707    0.097   58.944    0.000    5.707    1.789
##    .hygien5 (.91.)    7.580    0.074  101.917    0.000    7.580    3.145
##    .omind2  (.92.)    8.684    0.020  430.999    0.000    8.684    4.531
##    .omind3  (.93.)    8.614    0.020  423.422    0.000    8.614    5.645
##    .omind4  (.94.)    8.522    0.021  408.613    0.000    8.522    5.094
##    .omind1  (.95.)    6.107    0.025  247.476    0.000    6.107    2.200
##    .omind5  (.96.)    8.283    0.024  338.993    0.000    8.283    3.246
##    .omind6  (.97.)    7.344    0.025  291.874    0.000    7.344    2.794
##    .phc              -0.412    0.026  -15.939    0.000   -0.301   -0.301
##    .phs              -0.456    0.038  -11.895    0.000   -0.221   -0.221
##    .phg               0.037    0.031    1.173    0.241    0.026    0.026
##     om1               0.021    0.025    0.846    0.397    0.017    0.017
## 
## Variances:
##                    Estimate  Std.Err  z-value  P(>|z|)   Std.lv  Std.all
##    .contact1          1.635    0.069   23.808    0.000    1.635    0.467
##    .contact2r         6.633    0.174   38.204    0.000    6.633    0.828
##    .contact3          3.151    0.118   26.816    0.000    3.151    0.587
##    .contact4          2.189    0.089   24.645    0.000    2.189    0.540
##    .psupport1         2.280    0.084   27.195    0.000    2.280    0.349
##    .psupport2         1.337    0.057   23.559    0.000    1.337    0.231
##    .psupport3         5.892    0.131   44.923    0.000    5.892    0.572
##    .psupport4         1.696    0.059   28.921    0.000    1.696    0.353
##    .psupport5         2.877    0.093   30.769    0.000    2.877    0.473
##    .hygiene1          2.354    0.090   26.118    0.000    2.354    0.537
##    .hygiene2          2.110    0.086   24.543    0.000    2.110    0.512
##    .hygiene3          1.389    0.052   26.810    0.000    1.389    0.430
##    .hygiene4          7.368    0.139   53.112    0.000    7.368    0.724
##    .hygiene5          4.149    0.128   32.474    0.000    4.149    0.714
##    .omind2            2.166    0.139   15.617    0.000    2.166    0.590
##    .omind3            0.571    0.035   16.544    0.000    0.571    0.245
##    .omind4            1.092    0.055   19.762    0.000    1.092    0.390
##    .omind1            7.384    0.122   60.286    0.000    7.384    0.958
##    .omind5            5.626    0.172   32.638    0.000    5.626    0.864
##    .omind6            6.062    0.128   47.189    0.000    6.062    0.877
##    .phc               1.515    0.060   25.436    0.000    0.811    0.811
##    .phs               3.909    0.122   31.932    0.000    0.919    0.919
##    .phg               1.715    0.072   23.695    0.000    0.844    0.844
##     om1               1.508    0.046   32.618    0.000    1.000    1.000
## 
## 
## Group 3 [positive]:
## 
## Latent Variables:
##                    Estimate  Std.Err  z-value  P(>|z|)   Std.lv  Std.all
##   phc =~                                                                
##     contct1           1.000                               1.464    0.745
##     cntct2r (.p2.)    0.857    0.022   39.776    0.000    1.255    0.440
##     contct3 (.p3.)    1.088    0.018   60.231    0.000    1.593    0.753
##     contct4 (.p4.)    0.999    0.016   62.585    0.000    1.463    0.851
##   phs =~                                                                
##     pspprt1           1.000                               2.186    0.876
##     pspprt2 (.p6.)    1.022    0.010  107.258    0.000    2.233    0.919
##     pspprt3 (.p7.)    1.017    0.012   83.272    0.000    2.223    0.605
##     pspprt4 (.p8.)    0.856    0.011   78.077    0.000    1.870    0.824
##     pspprt5 (.p9.)    0.868    0.012   73.561    0.000    1.897    0.794
##   phg =~                                                                
##     hygien1           1.000                               1.581    0.765
##     hygien2 (.11.)    0.995    0.007  148.097    0.000    1.574    0.780
##     hygien3 (.12.)    0.952    0.015   62.285    0.000    1.505    0.808
##     hygien4 (.13.)    1.175    0.021   54.791    0.000    1.858    0.606
##     hygien5 (.14.)    0.904    0.020   46.181    0.000    1.429    0.485
##   om1 =~                                                                
##     omind2            1.000                               1.310    0.843
##     omind3  (.18.)    1.079    0.012   88.325    0.000    1.414    0.916
##     omind4  (.19.)    1.063    0.013   80.335    0.000    1.393    0.858
##     omind1  (.20.)    0.462    0.020   22.785    0.000    0.605    0.225
##     omind5  (.21.)    0.766    0.019   40.647    0.000    1.004    0.406
##     omind6  (.22.)    0.750    0.019   38.516    0.000    0.982    0.388
## 
## Regressions:
##                    Estimate  Std.Err  z-value  P(>|z|)   Std.lv  Std.all
##   phc ~                                                                 
##     ccrt    (.26.)   -0.021    0.011   -1.877    0.060   -0.015   -0.015
##     om1     (.27.)    0.451    0.014   33.364    0.000    0.403    0.403
##     age     (.28.)    0.009    0.001   12.470    0.000    0.006    0.096
##     sex1    (.29.)    0.333    0.024   13.711    0.000    0.228    0.114
##     pltcl_d (.30.)   -0.008    0.005   -1.432    0.152   -0.005   -0.012
##   phs ~                                                                 
##     ccrt    (.31.)   -0.102    0.017   -5.950    0.000   -0.047   -0.048
##     om1     (.32.)    0.453    0.017   26.688    0.000    0.272    0.272
##     age     (.33.)   -0.003    0.001   -3.268    0.001   -0.002   -0.025
##     sex1    (.34.)    0.219    0.036    6.155    0.000    0.100    0.050
##     pltcl_d (.35.)   -0.021    0.008   -2.536    0.011   -0.010   -0.022
##   phg ~                                                                 
##     ccrt    (.36.)   -0.218    0.014  -15.605    0.000   -0.138   -0.143
##     om1     (.37.)    0.383    0.014   26.953    0.000    0.317    0.317
##     age     (.38.)    0.001    0.001    1.443    0.149    0.001    0.012
##     sex1    (.39.)    0.314    0.028   11.028    0.000    0.199    0.099
##     pltcl_d (.40.)    0.024    0.006    3.745    0.000    0.015    0.034
## 
## Covariances:
##                    Estimate  Std.Err  z-value  P(>|z|)   Std.lv  Std.all
##  .hygiene1 ~~                                                           
##    .hygiene2          1.361    0.122   11.169    0.000    1.361    0.810
##  .psupport4 ~~                                                          
##    .psupport5         0.619    0.084    7.369    0.000    0.619    0.332
##  .omind1 ~~                                                             
##    .omind5            1.560    0.159    9.795    0.000    1.560    0.263
##  .omind5 ~~                                                             
##    .omind6            2.148    0.186   11.552    0.000    2.148    0.407
##  .omind1 ~~                                                             
##    .omind6            2.294    0.171   13.413    0.000    2.294    0.375
##  .phc ~~                                                                
##    .phs               1.593    0.143   11.137    0.000    0.575    0.575
##    .phg               1.151    0.111   10.409    0.000    0.592    0.592
##  .phs ~~                                                                
##    .phg               1.402    0.141    9.966    0.000    0.455    0.455
## 
## Intercepts:
##                    Estimate  Std.Err  z-value  P(>|z|)   Std.lv  Std.all
##    .contct1 (.78.)    8.073    0.070  114.655    0.000    8.073    4.106
##    .cntct2r (.79.)    7.374    0.064  115.055    0.000    7.374    2.584
##    .contct3 (.80.)    7.516    0.077   97.445    0.000    7.516    3.552
##    .contct4 (.81.)    8.106    0.071  113.506    0.000    8.106    4.714
##    .pspprt1           8.506    0.104   81.465    0.000    8.506    3.411
##    .pspprt2 (.83.)    8.593    0.101   85.026    0.000    8.593    3.535
##    .pspprt3 (.84.)    6.718    0.103   65.221    0.000    6.718    1.829
##    .pspprt4 (.85.)    8.833    0.085  103.687    0.000    8.833    3.895
##    .pspprt5 (.86.)    8.606    0.087   99.384    0.000    8.606    3.602
##    .hygien1 (.87.)    7.687    0.081   94.678    0.000    7.687    3.722
##    .hygien2 (.88.)    7.813    0.081   96.631    0.000    7.813    3.871
##    .hygien3 (.89.)    8.309    0.078  106.115    0.000    8.309    4.463
##    .hygien4 (.90.)    5.707    0.097   58.944    0.000    5.707    1.862
##    .hygien5 (.91.)    7.580    0.074  101.917    0.000    7.580    2.575
##    .omind2  (.92.)    8.684    0.020  430.999    0.000    8.684    5.589
##    .omind3  (.93.)    8.614    0.020  423.422    0.000    8.614    5.582
##    .omind4  (.94.)    8.522    0.021  408.613    0.000    8.522    5.249
##    .omind1  (.95.)    6.107    0.025  247.476    0.000    6.107    2.271
##    .omind5  (.96.)    8.283    0.024  338.993    0.000    8.283    3.348
##    .omind6  (.97.)    7.344    0.025  291.874    0.000    7.344    2.901
##    .phc              -0.296    0.039   -7.623    0.000   -0.202   -0.202
##    .phs              -0.501    0.059   -8.471    0.000   -0.229   -0.229
##    .phg              -0.080    0.046   -1.734    0.083   -0.051   -0.051
##     om1              -0.144    0.037   -3.917    0.000   -0.110   -0.110
## 
## Variances:
##                    Estimate  Std.Err  z-value  P(>|z|)   Std.lv  Std.all
##    .contact1          1.722    0.119   14.490    0.000    1.722    0.445
##    .contact2r         6.567    0.305   21.553    0.000    6.567    0.807
##    .contact3          1.940    0.141   13.789    0.000    1.940    0.433
##    .contact4          0.815    0.058   14.164    0.000    0.815    0.276
##    .psupport1         1.442    0.114   12.695    0.000    1.442    0.232
##    .psupport2         0.921    0.074   12.373    0.000    0.921    0.156
##    .psupport3         8.553    0.278   30.814    0.000    8.553    0.634
##    .psupport4         1.647    0.110   15.028    0.000    1.647    0.320
##    .psupport5         2.111    0.132   15.974    0.000    2.111    0.370
##    .hygiene1          1.766    0.124   14.246    0.000    1.766    0.414
##    .hygiene2          1.598    0.127   12.559    0.000    1.598    0.392
##    .hygiene3          1.201    0.078   15.416    0.000    1.201    0.347
##    .hygiene4          5.943    0.253   23.508    0.000    5.943    0.633
##    .hygiene5          6.624    0.280   23.622    0.000    6.624    0.764
##    .omind2            0.699    0.054   12.993    0.000    0.699    0.289
##    .omind3            0.382    0.038    9.993    0.000    0.382    0.160
##    .omind4            0.696    0.057   12.267    0.000    0.696    0.264
##    .omind1            6.867    0.204   33.716    0.000    6.867    0.949
##    .omind5            5.113    0.295   17.328    0.000    5.113    0.835
##    .omind6            5.446    0.238   22.918    0.000    5.446    0.850
##    .phc               1.750    0.133   13.118    0.000    0.816    0.816
##    .phs               4.392    0.238   18.435    0.000    0.919    0.919
##    .phg               2.158    0.140   15.423    0.000    0.863    0.863
##     om1               1.716    0.072   23.834    0.000    1.000    1.000
```

```
print(compareFit(phymodcczx, phymodcwzx, phymodcs5xyx, phymodcsrx, nested = T))
```

```
## ################### Nested Model Comparison #########################
## Scaled Chi-Squared Difference Test (method = "satorra.bentler.2001")
## 
## lavaan NOTE:
##     The "Chisq" column contains standard test statistics, not the
##     robust test that should be reported per model. A robust difference
##     test is a function of two standard (not robust) statistics.
##  
##               Df    AIC    BIC   Chisq Chisq diff Df diff Pr(>Chisq)    
## phymodcczx   681 989026 990877  8478.0                                  
## phymodcwzx   713 989257 990870  8772.8     260.95      32  < 2.2e-16 ***
## phymodcs5xyx 743 990537 991926 10112.6    2026.25      30  < 2.2e-16 ***
## phymodcsrx   773 990747 991914 10383.0     257.47      30  < 2.2e-16 ***
## ---
## Signif. codes:  0 '***' 0.001 '**' 0.01 '*' 0.05 '.' 0.1 ' ' 1
## 
## ####################### Model Fit Indices ###########################
##              chisq.scaled df.scaled pvalue.scaled cfi.robust tli.robust
## phymodcczx      8118.914†       681          .000      .934†      .922 
## phymodcwzx      8370.471        713          .000      .932       .923†
## phymodcs5xyx    9794.829        743          .000      .921       .914 
## phymodcsrx     10050.015        773          .000      .919       .915 
##                      aic         bic rmsea.robust  srmr
## phymodcczx   989025.944† 990876.682         .052  .050†
## phymodcwzx   989256.729  990869.622†        .052† .052 
## phymodcs5xyx 990536.560  991926.472         .055  .054 
## phymodcsrx   990746.958  991913.889         .055  .056 
## 
## ################## Differences in Fit Indices #######################
##                           df.scaled cfi.robust tli.robust      aic      bic
## phymodcwzx - phymodcczx          32     -0.002      0.001  230.786   -7.060
## phymodcs5xyx - phymodcwzx        30     -0.011     -0.009 1279.831 1056.850
## phymodcsrx - phymodcs5xyx        30     -0.002      0.001  210.398  -12.583
##                           rmsea.robust  srmr
## phymodcwzx - phymodcczx          0.000 0.002
## phymodcs5xyx - phymodcwzx        0.003 0.002
## phymodcsrx - phymodcs5xyx        0.000 0.003
```

```
listarezx <- lavInspect(phymodcs5xyx, "cor.all")
minirezx <- list()
for(i in seq_along(listarezx)){
  nam <- names(listarezx)
  matrix <- listarezx[[i]]
  minirezx[[i]] <- round(matrix[21:28, 21:28], 2)
  names(minirezx)[i] <- nam[i]
}
minirezx #strong invariance
```

```
## $negative
##                     ccrt   age  sex1 political_ideology   phc   phs   phg  om1
## ccrt                1.00 -0.03 -0.15              -0.11 -0.05 -0.04 -0.27 0.00
## age                -0.03  1.00 -0.19               0.04  0.13  0.02  0.00 0.00
## sex1               -0.15 -0.19  1.00              -0.12  0.11  0.09  0.13 0.00
## political_ideology -0.11  0.04 -0.12               1.00 -0.06 -0.12  0.07 0.00
## phc                -0.05  0.13  0.11              -0.06  1.00  0.59  0.53 0.51
## phs                -0.04  0.02  0.09              -0.12  0.59  1.00  0.39 0.32
## phg                -0.27  0.00  0.13               0.07  0.53  0.39  1.00 0.31
## om1                 0.00  0.00  0.00               0.00  0.51  0.32  0.31 1.00
## 
## $neutral
##                     ccrt   age  sex1 political_ideology   phc   phs   phg  om1
## ccrt                1.00 -0.02 -0.18              -0.12 -0.03 -0.07 -0.13 0.00
## age                -0.02  1.00 -0.07              -0.05  0.07 -0.10 -0.01 0.00
## sex1               -0.18 -0.07  1.00              -0.06  0.13  0.04  0.12 0.00
## political_ideology -0.12 -0.05 -0.06               1.00  0.00  0.03  0.03 0.00
## phc                -0.03  0.07  0.13               0.00  1.00  0.50  0.61 0.36
## phs                -0.07 -0.10  0.04               0.03  0.50  1.00  0.40 0.24
## phg                -0.13 -0.01  0.12               0.03  0.61  0.40  1.00 0.33
## om1                 0.00  0.00  0.00               0.00  0.36  0.24  0.33 1.00
## 
## $positive
##                     ccrt   age  sex1 political_ideology   phc   phs   phg  om1
## ccrt                1.00 -0.03 -0.15              -0.06 -0.06 -0.10 -0.15 0.00
## age                -0.03  1.00 -0.11              -0.06  0.07  0.01  0.04 0.00
## sex1               -0.15 -0.11  1.00              -0.04  0.17  0.14  0.17 0.00
## political_ideology -0.06 -0.06 -0.04               1.00  0.04  0.11  0.06 0.00
## phc                -0.06  0.07  0.17               0.04  1.00  0.62  0.65 0.45
## phs                -0.10  0.01  0.14               0.11  0.62  1.00  0.52 0.26
## phg                -0.15  0.04  0.17               0.06  0.65  0.52  1.00 0.29
## om1                 0.00  0.00  0.00               0.00  0.45  0.26  0.29 1.00
```

## Mediation

```
narc <- 'consp =~ ctheory1 + ctheory2 + ctheory3 + ctheory4'

nm <- sem(narc, estimator = "MLM", data = pls4) 
fitmeasures(nm, fit.measures = c("chisq.scaled", "df.scaled", "cfi.robust", "rmsea.robust", "srmr"))
```

```
## chisq.scaled    df.scaled   cfi.robust rmsea.robust         srmr 
##      430.612        2.000        0.978        0.191        0.023
```

```
reliability(nm)
```

```
##            consp
## alpha  0.9254145
## omega  0.9257502
## omega2 0.9257502
## omega3 0.9254256
## avevar 0.7573903
```

```
narc <- 'consp =~ ctheory1 + ctheory2 + ctheory3 + ctheory4
         ctheory1 ~~ ctheory2'

nm <- sem(narc, estimator = "MLM", data = pls4) 
fitmeasures(nm, fit.measures = c("chisq.scaled", "df.scaled", "cfi.robust", "rmsea.robust", "srmr"))
```

```
## chisq.scaled    df.scaled   cfi.robust rmsea.robust         srmr 
##       77.040        1.000        0.997        0.102        0.007
```

```
reliability(nm)
```

```
##            consp
## alpha  0.9254145
## omega  0.9060737
## omega2 0.9060737
## omega3 0.9067764
## avevar 0.7432496
```

```
semPaths(nm, what = "std", edge.label.cex = 1.2, label.cex = 1.2)
```

```
phczx <-'phc =~ contact1 + contact2r + contact3 + contact4
        phs =~ psupport1 + psupport2 + psupport3 + psupport4 + psupport5
        phg =~ hygiene1 + hygiene2 + hygiene3 + hygiene4 + hygiene5
        hygiene1 ~~ hygiene2
        psupport4 ~~ psupport5
        om1 =~ omind2 + omind3 + omind4 + omind1 + omind5 + omind6
        omind1 ~~ omind5
        omind5 ~~ omind6
        omind1 ~~ omind6
        consp =~ ctheory1 + ctheory2 + ctheory3 + ctheory4
        ctheory1 ~~ ctheory2
        phc ~ e1*ccrt + c1*om1 + age + sex1 + political_ideology + b1*consp
        phs ~ e2*ccrt + c2*om1 + age + sex1 + political_ideology + b2*consp
        phg ~ e3*ccrt + c3*om1 + age + sex1 + political_ideology + b3*consp
        consp ~ a*om1 + d*ccrt
        om_indirectphc := a*b1
        om_indirectphs := a*b2
        om_indirectphg := a*b3
        om_completephc := a*b1 + c1
        om_completephs := a*b2 + c2
        om_completephg := a*b3 + c3
        crt_indirectphc := d*b1
        crt_indirectphs := d*b2
        crt_indirectphg := d*b3
        crt_completephc := d*b1 + e1
        crt_completephs := d*b2 + e2
        crt_completephg := d*b3 + e3
        ccrt ~~ om1
        ccrt ~~ age
        ccrt ~~ sex1
        ccrt ~~ political_ideology
        om1 ~~ age
        om1 ~~ sex1
        om1 ~~ political_ideology
        age ~~ consp
        age ~~ sex1
        age ~~ political_ideology
        sex1 ~~ consp
        sex1 ~~ political_ideology
        political_ideology ~~ consp'

phymodzx <- sem(phczx, estimator = "MLM", data = pls4)
```

```
## Warning in lav_data_full(data = data, group = group, cluster = cluster, : lavaan
## WARNING: some observed variances are (at least) a factor 1000 times larger than
## others; use varTable(fit) to investigate
```

```
fitmeasures(phymodzx, fit.measures = c("chisq.scaled", "df.scaled", "cfi.robust", "rmsea.robust", "srmr"))
```

```
## chisq.scaled    df.scaled   cfi.robust rmsea.robust         srmr 
##     9022.597      312.000        0.943        0.048        0.060
```

```
summary(phymodzx, standardized = T, rsq = T)
```

```
## lavaan 0.6-8 ended normally after 187 iterations
## 
##   Estimator                                         ML
##   Optimization method                           NLMINB
##   Number of model parameters                        94
##                                                       
##   Number of observations                         12490
##                                                       
## Model Test User Model:
##                                               Standard      Robust
##   Test Statistic                              9430.948    9022.597
##   Degrees of freedom                               312         312
##   P-value (Chi-square)                           0.000       0.000
##   Scaling correction factor                                  1.045
##        Satorra-Bentler correction                                 
## 
## Parameter Estimates:
## 
##   Standard errors                           Robust.sem
##   Information                                 Expected
##   Information saturated (h1) model          Structured
## 
## Latent Variables:
##                    Estimate  Std.Err  z-value  P(>|z|)   Std.lv  Std.all
##   phc =~                                                                
##     contact1          1.000                               1.329    0.751
##     contact2r         0.855    0.021   39.954    0.000    1.135    0.412
##     contact3          1.077    0.018   58.405    0.000    1.431    0.645
##     contact4          0.977    0.016   60.020    0.000    1.298    0.728
##   phs =~                                                                
##     psupport1         1.000                               1.974    0.779
##     psupport2         1.025    0.010  105.311    0.000    2.024    0.886
##     psupport3         1.025    0.012   82.981    0.000    2.024    0.630
##     psupport4         0.857    0.011   76.782    0.000    1.692    0.817
##     psupport5         0.875    0.012   74.352    0.000    1.727    0.740
##   phg =~                                                                
##     hygiene1          1.000                               1.473    0.722
##     hygiene2          0.997    0.007  144.223    0.000    1.468    0.739
##     hygiene3          0.943    0.016   60.771    0.000    1.388    0.738
##     hygiene4          1.189    0.022   54.081    0.000    1.750    0.561
##     hygiene5          0.902    0.020   46.082    0.000    1.328    0.540
##   om1 =~                                                                
##     omind2            1.000                               1.175    0.701
##     omind3            1.126    0.015   77.369    0.000    1.323    0.885
##     omind4            1.120    0.015   72.415    0.000    1.316    0.807
##     omind1            0.495    0.021   23.263    0.000    0.582    0.215
##     omind5            0.812    0.020   40.279    0.000    0.953    0.379
##     omind6            0.792    0.021   37.886    0.000    0.930    0.360
##   consp =~                                                              
##     ctheory1          1.000                               2.429    0.736
##     ctheory2          1.116    0.009  120.525    0.000    2.712    0.884
##     ctheory3          1.104    0.011  101.723    0.000    2.681    0.906
##     ctheory4          1.163    0.011  108.077    0.000    2.825    0.935
## 
## Regressions:
##                    Estimate  Std.Err  z-value  P(>|z|)   Std.lv  Std.all
##   phc ~                                                                 
##     ccrt      (e1)   -0.071    0.013   -5.638    0.000   -0.053   -0.057
##     om1       (c1)    0.435    0.014   30.336    0.000    0.385    0.385
##     age               0.008    0.001   11.426    0.000    0.006    0.109
##     sex1              0.370    0.026   14.061    0.000    0.278    0.139
##     pltcl_dlg         0.015    0.006    2.463    0.014    0.011    0.024
##     consp     (b1)   -0.088    0.006  -13.531    0.000   -0.160   -0.160
##   phs ~                                                                 
##     ccrt      (e2)   -0.189    0.018  -10.486    0.000   -0.096   -0.102
##     om1       (c2)    0.427    0.018   23.350    0.000    0.254    0.254
##     age              -0.005    0.001   -4.937    0.000   -0.003   -0.044
##     sex1              0.233    0.037    6.348    0.000    0.118    0.059
##     pltcl_dlg         0.016    0.009    1.821    0.069    0.008    0.018
##     consp     (b2)   -0.130    0.010  -13.523    0.000   -0.159   -0.159
##   phg ~                                                                 
##     ccrt      (e3)   -0.240    0.015  -16.301    0.000   -0.163   -0.174
##     om1       (c3)    0.392    0.015   25.471    0.000    0.313    0.313
##     age               0.001    0.001    0.711    0.477    0.000    0.007
##     sex1              0.311    0.029   10.625    0.000    0.211    0.106
##     pltcl_dlg         0.032    0.007    4.835    0.000    0.022    0.048
##     consp     (b3)   -0.011    0.007   -1.580    0.114   -0.018   -0.018
##   consp ~                                                               
##     om1        (a)   -0.318    0.020  -16.076    0.000   -0.154   -0.154
##     ccrt       (d)   -0.572    0.019  -29.593    0.000   -0.235   -0.251
## 
## Covariances:
##                    Estimate  Std.Err  z-value  P(>|z|)   Std.lv  Std.all
##  .hygiene1 ~~                                                           
##    .hygiene2          1.415    0.054   26.126    0.000    1.415    0.749
##  .psupport4 ~~                                                          
##    .psupport5         0.419    0.032   13.118    0.000    0.419    0.224
##  .omind1 ~~                                                             
##    .omind5            1.804    0.072   24.925    0.000    1.804    0.292
##  .omind5 ~~                                                             
##    .omind6            2.184    0.077   28.446    0.000    2.184    0.389
##  .omind1 ~~                                                             
##    .omind6            2.480    0.072   34.415    0.000    2.480    0.388
##  .ctheory1 ~~                                                           
##    .ctheory2          0.944    0.057   16.655    0.000    0.944    0.294
##   om1 ~~                                                                
##     ccrt              0.116    0.012    9.975    0.000    0.099    0.092
##   ccrt ~~                                                               
##     age              -0.554    0.163   -3.400    0.001   -0.554   -0.030
##     sex1             -0.087    0.005  -18.453    0.000   -0.087   -0.163
##     political_dlgy   -0.256    0.022  -11.919    0.000   -0.256   -0.108
##   om1 ~~                                                                
##     age               0.267    0.188    1.422    0.155    0.227    0.013
##     sex1              0.048    0.006    8.578    0.000    0.041    0.082
##     political_dlgy   -0.136    0.024   -5.703    0.000   -0.116   -0.052
##  .consp ~~                                                              
##     age              -4.419    0.356  -12.397    0.000   -1.911   -0.112
##   age ~~                                                                
##     sex1             -0.981    0.076  -12.921    0.000   -0.981   -0.115
##     political_dlgy   -0.531    0.345   -1.537    0.124   -0.531   -0.014
##  .consp ~~                                                              
##     sex1              0.001    0.011    0.125    0.901    0.001    0.001
##   sex1 ~~                                                               
##     political_dlgy   -0.089    0.010   -9.021    0.000   -0.089   -0.081
##  .consp ~~                                                              
##     political_dlgy    0.793    0.052   15.141    0.000    0.343    0.154
##  .phc ~~                                                                
##    .phs               1.083    0.040   27.360    0.000    0.498    0.498
##    .phg               0.826    0.032   26.179    0.000    0.520    0.520
##  .phs ~~                                                                
##    .phg               0.907    0.039   23.355    0.000    0.357    0.357
## 
## Variances:
##                    Estimate  Std.Err  z-value  P(>|z|)   Std.lv  Std.all
##    .contact1          1.364    0.044   31.176    0.000    1.364    0.436
##    .contact2r         6.314    0.122   51.946    0.000    6.314    0.830
##    .contact3          2.872    0.081   35.637    0.000    2.872    0.584
##    .contact4          1.493    0.051   29.236    0.000    1.493    0.470
##    .psupport1         2.525    0.068   36.881    0.000    2.525    0.393
##    .psupport2         1.118    0.037   30.230    0.000    1.118    0.214
##    .psupport3         6.229    0.099   62.887    0.000    6.229    0.603
##    .psupport4         1.424    0.038   37.703    0.000    1.424    0.332
##    .psupport5         2.458    0.061   40.461    0.000    2.458    0.452
##    .hygiene1          1.995    0.060   33.530    0.000    1.995    0.479
##    .hygiene2          1.791    0.057   31.494    0.000    1.791    0.454
##    .hygiene3          1.616    0.045   36.094    0.000    1.616    0.456
##    .hygiene4          6.657    0.105   63.461    0.000    6.657    0.685
##    .hygiene5          4.284    0.097   44.168    0.000    4.284    0.708
##    .omind2            1.425    0.073   19.598    0.000    1.425    0.508
##    .omind3            0.486    0.022   22.443    0.000    0.486    0.217
##    .omind4            0.929    0.035   26.643    0.000    0.929    0.349
##    .omind1            7.015    0.083   84.134    0.000    7.015    0.954
##    .omind5            5.424    0.121   44.870    0.000    5.424    0.856
##    .omind6            5.814    0.094   62.042    0.000    5.814    0.870
##    .ctheory1          4.994    0.093   53.984    0.000    4.994    0.458
##    .ctheory2          2.058    0.064   32.078    0.000    2.058    0.219
##    .ctheory3          1.576    0.053   30.003    0.000    1.576    0.180
##    .ctheory4          1.156    0.052   22.316    0.000    1.156    0.127
##     ccrt              1.141    0.011  105.655    0.000    1.141    1.000
##     age             292.108    2.495  117.084    0.000  292.108    1.000
##     sex1              0.250    0.000 3524.471    0.000    0.250    1.000
##     political_dlgy    4.933    0.063   78.834    0.000    4.933    1.000
##    .phc               1.359    0.045   30.097    0.000    0.769    0.769
##    .phs               3.475    0.084   41.400    0.000    0.892    0.892
##    .phg               1.859    0.059   31.319    0.000    0.857    0.857
##     om1               1.380    0.038   36.042    0.000    1.000    1.000
##    .consp             5.347    0.101   53.081    0.000    0.906    0.906
## 
## R-Square:
##                    Estimate
##     contact1          0.564
##     contact2r         0.170
##     contact3          0.416
##     contact4          0.530
##     psupport1         0.607
##     psupport2         0.786
##     psupport3         0.397
##     psupport4         0.668
##     psupport5         0.548
##     hygiene1          0.521
##     hygiene2          0.546
##     hygiene3          0.544
##     hygiene4          0.315
##     hygiene5          0.292
##     omind2            0.492
##     omind3            0.783
##     omind4            0.651
##     omind1            0.046
##     omind5            0.144
##     omind6            0.130
##     ctheory1          0.542
##     ctheory2          0.781
##     ctheory3          0.820
##     ctheory4          0.873
##     phc               0.231
##     phs               0.108
##     phg               0.143
##     consp             0.094
## 
## Defined Parameters:
##                    Estimate  Std.Err  z-value  P(>|z|)   Std.lv  Std.all
##     om_indirectphc    0.028    0.003   10.806    0.000    0.025    0.025
##     om_indirectphs    0.041    0.004   10.713    0.000    0.025    0.025
##     om_indirectphg    0.003    0.002    1.588    0.112    0.003    0.003
##     om_completephc    0.463    0.014   31.965    0.000    0.409    0.409
##     om_completephs    0.469    0.018   25.709    0.000    0.279    0.279
##     om_completephg    0.395    0.015   26.133    0.000    0.315    0.315
##     crt_indirctphc    0.050    0.004   12.562    0.000    0.038    0.040
##     crt_indirctphs    0.074    0.006   12.670    0.000    0.038    0.040
##     crt_indirctphg    0.006    0.004    1.580    0.114    0.004    0.004
##     crt_completphc   -0.021    0.012   -1.678    0.093   -0.016   -0.017
##     crt_completphs   -0.115    0.018   -6.541    0.000   -0.058   -0.062
##     crt_completphg   -0.234    0.014  -16.260    0.000   -0.159   -0.170
```

```
lavInspect(phymodzx, "cor.all")
```

```
##                    cntct1 cntct2 cntct3 cntct4 psppr1 psppr2 psppr3 psppr4
## contact1            1.000                                                 
## contact2r           0.309  1.000                                          
## contact3            0.485  0.266  1.000                                   
## contact4            0.547  0.300  0.470  1.000                            
## psupport1           0.327  0.179  0.281  0.317  1.000                     
## psupport2           0.372  0.204  0.319  0.360  0.690  1.000              
## psupport3           0.264  0.145  0.227  0.256  0.491  0.558  1.000       
## psupport4           0.343  0.188  0.294  0.332  0.637  0.724  0.515  1.000
## psupport5           0.311  0.170  0.267  0.301  0.577  0.656  0.466  0.692
## hygiene1            0.312  0.171  0.268  0.302  0.235  0.268  0.190  0.247
## hygiene2            0.319  0.175  0.274  0.309  0.241  0.274  0.195  0.253
## hygiene3            0.318  0.175  0.273  0.309  0.240  0.274  0.194  0.252
## hygiene4            0.242  0.133  0.208  0.235  0.183  0.208  0.148  0.192
## hygiene5            0.233  0.128  0.200  0.226  0.176  0.200  0.142  0.185
## omind2              0.221  0.121  0.190  0.214  0.151  0.172  0.122  0.158
## omind3              0.279  0.153  0.239  0.270  0.191  0.217  0.154  0.200
## omind4              0.254  0.139  0.218  0.246  0.174  0.198  0.140  0.182
## omind1              0.068  0.037  0.058  0.066  0.046  0.053  0.037  0.048
## omind5              0.119  0.065  0.103  0.116  0.082  0.093  0.066  0.086
## omind6              0.113  0.062  0.097  0.110  0.078  0.088  0.063  0.081
## ctheory1           -0.119 -0.065 -0.102 -0.116 -0.096 -0.110 -0.078 -0.101
## ctheory2           -0.143 -0.078 -0.123 -0.139 -0.116 -0.132 -0.093 -0.121
## ctheory3           -0.147 -0.080 -0.126 -0.142 -0.118 -0.135 -0.096 -0.124
## ctheory4           -0.151 -0.083 -0.130 -0.147 -0.122 -0.139 -0.099 -0.128
## ccrt               -0.006 -0.003 -0.005 -0.005 -0.036 -0.042 -0.029 -0.038
## age                 0.087  0.048  0.074  0.084 -0.022 -0.025 -0.018 -0.023
## sex1                0.121  0.066  0.104  0.117  0.074  0.085  0.060  0.078
## political_ideology -0.024 -0.013 -0.020 -0.023 -0.014 -0.016 -0.011 -0.014
## phc                 0.751  0.412  0.645  0.728  0.435  0.495  0.352  0.456
## phs                 0.419  0.230  0.360  0.407  0.779  0.886  0.630  0.817
## phg                 0.432  0.237  0.371  0.419  0.326  0.371  0.264  0.342
## om1                 0.315  0.173  0.271  0.306  0.215  0.245  0.174  0.226
## consp              -0.162 -0.089 -0.139 -0.157 -0.131 -0.149 -0.106 -0.137
##                    psppr5 hygin1 hygin2 hygin3 hygin4 hygin5 omind2 omind3
## contact1                                                                  
## contact2r                                                                 
## contact3                                                                  
## contact4                                                                  
## psupport1                                                                 
## psupport2                                                                 
## psupport3                                                                 
## psupport4                                                                 
## psupport5           1.000                                                 
## hygiene1            0.224  1.000                                          
## hygiene2            0.229  0.882  1.000                                   
## hygiene3            0.229  0.532  0.545  1.000                            
## hygiene4            0.174  0.405  0.415  0.414  1.000                     
## hygiene5            0.167  0.390  0.399  0.398  0.303  1.000              
## omind2              0.144  0.155  0.159  0.158  0.120  0.116  1.000       
## omind3              0.181  0.195  0.200  0.200  0.152  0.146  0.621  1.000
## omind4              0.165  0.178  0.182  0.182  0.139  0.133  0.566  0.714
## omind1              0.044  0.047  0.048  0.048  0.037  0.035  0.150  0.190
## omind5              0.078  0.084  0.086  0.085  0.065  0.063  0.266  0.335
## omind6              0.074  0.079  0.081  0.081  0.062  0.059  0.252  0.318
## ctheory1           -0.091 -0.008 -0.009 -0.009 -0.006 -0.006 -0.091 -0.115
## ctheory2           -0.110 -0.010 -0.010 -0.010 -0.008 -0.007 -0.110 -0.139
## ctheory3           -0.113 -0.010 -0.011 -0.010 -0.008 -0.008 -0.113 -0.142
## ctheory4           -0.116 -0.011 -0.011 -0.011 -0.008 -0.008 -0.116 -0.146
## ccrt               -0.035 -0.118 -0.121 -0.121 -0.092 -0.088  0.065  0.082
## age                -0.021  0.004  0.004  0.004  0.003  0.003  0.009  0.012
## sex1                0.071  0.112  0.114  0.114  0.087  0.083  0.058  0.073
## political_ideology -0.013  0.028  0.029  0.029  0.022  0.021 -0.037 -0.046
## phc                 0.413  0.415  0.425  0.424  0.323  0.310  0.294  0.371
## phs                 0.740  0.302  0.309  0.309  0.235  0.226  0.194  0.245
## phg                 0.310  0.722  0.739  0.738  0.561  0.540  0.215  0.271
## om1                 0.205  0.221  0.226  0.226  0.172  0.165  0.701  0.885
## consp              -0.124 -0.011 -0.012 -0.012 -0.009 -0.008 -0.124 -0.157
##                    omind4 omind1 omind5 omind6 cthry1 cthry2 cthry3 cthry4
## contact1                                                                  
## contact2r                                                                 
## contact3                                                                  
## contact4                                                                  
## psupport1                                                                 
## psupport2                                                                 
## psupport3                                                                 
## psupport4                                                                 
## psupport5                                                                 
## hygiene1                                                                  
## hygiene2                                                                  
## hygiene3                                                                  
## hygiene4                                                                  
## hygiene5                                                                  
## omind2                                                                    
## omind3                                                                    
## omind4              1.000                                                 
## omind1              0.173  1.000                                          
## omind5              0.306  0.346  1.000                                   
## omind6              0.290  0.431  0.472  1.000                            
## ctheory1           -0.105 -0.028 -0.049 -0.047  1.000                     
## ctheory2           -0.126 -0.034 -0.059 -0.056  0.744  1.000              
## ctheory3           -0.129 -0.034 -0.061 -0.058  0.667  0.800  1.000       
## ctheory4           -0.134 -0.036 -0.063 -0.060  0.688  0.826  0.846  1.000
## ccrt                0.075  0.020  0.035  0.033 -0.195 -0.235 -0.241 -0.248
## age                 0.011  0.003  0.005  0.005 -0.074 -0.089 -0.091 -0.094
## sex1                0.066  0.018  0.031  0.030  0.022  0.026  0.027  0.027
## political_ideology -0.042 -0.011 -0.020 -0.019  0.134  0.161  0.165  0.170
## phc                 0.338  0.090  0.159  0.151 -0.159 -0.191 -0.195 -0.202
## phs                 0.223  0.059  0.105  0.099 -0.124 -0.148 -0.152 -0.157
## phg                 0.247  0.066  0.116  0.110 -0.012 -0.014 -0.014 -0.015
## om1                 0.807  0.215  0.379  0.360 -0.130 -0.157 -0.160 -0.166
## consp              -0.143 -0.038 -0.067 -0.064  0.736  0.884  0.906  0.935
##                    ccrt   age    sex1   pltcl_ phc    phs    phg    om1   
## contact1                                                                  
## contact2r                                                                 
## contact3                                                                  
## contact4                                                                  
## psupport1                                                                 
## psupport2                                                                 
## psupport3                                                                 
## psupport4                                                                 
## psupport5                                                                 
## hygiene1                                                                  
## hygiene2                                                                  
## hygiene3                                                                  
## hygiene4                                                                  
## hygiene5                                                                  
## omind2                                                                    
## omind3                                                                    
## omind4                                                                    
## omind1                                                                    
## omind5                                                                    
## omind6                                                                    
## ctheory1                                                                  
## ctheory2                                                                  
## ctheory3                                                                  
## ctheory4                                                                  
## ccrt                1.000                                                 
## age                -0.030  1.000                                          
## sex1               -0.163 -0.115  1.000                                   
## political_ideology -0.108 -0.014 -0.081  1.000                            
## phc                -0.007  0.115  0.161 -0.031  1.000                     
## phs                -0.047 -0.028  0.095 -0.018  0.558  1.000              
## phg                -0.163  0.005  0.155  0.039  0.575  0.419  1.000       
## om1                 0.092  0.013  0.082 -0.052  0.420  0.276  0.306  1.000
## consp              -0.266 -0.101  0.029  0.182 -0.216 -0.168 -0.016 -0.177
##                    consp 
## contact1                 
## contact2r                
## contact3                 
## contact4                 
## psupport1                
## psupport2                
## psupport3                
## psupport4                
## psupport5                
## hygiene1                 
## hygiene2                 
## hygiene3                 
## hygiene4                 
## hygiene5                 
## omind2                   
## omind3                   
## omind4                   
## omind1                   
## omind5                   
## omind6                   
## ctheory1                 
## ctheory2                 
## ctheory3                 
## ctheory4                 
## ccrt                     
## age                      
## sex1                     
## political_ideology       
## phc                      
## phs                      
## phg                      
## om1                      
## consp               1.000
```

```
##testing significance of mediations across contexts

phczxbez <-'phc =~ contact1 + contact2r + contact3 + contact4
        phs =~ psupport1 + psupport2 + psupport3 + psupport4 + psupport5
        phg =~ hygiene1 + hygiene2 + hygiene3 + hygiene4 + hygiene5
        hygiene1 ~~ hygiene2
        psupport4 ~~ psupport5
        om1 =~ omind2 + omind3 + omind4 + omind1 + omind5 + omind6
        omind1 ~~ omind5
        omind5 ~~ omind6
        omind1 ~~ omind6
        consp =~ ctheory1 + ctheory2 + ctheory3 + ctheory4
        ctheory1 ~~ ctheory2
        phc ~ ccrt + om1 + age + sex1 + political_ideology + consp
        phs ~ ccrt + om1 + age + sex1 + political_ideology + consp
        phg ~ ccrt + om1 + age + sex1 + political_ideology + consp
        consp ~ om1 + ccrt'

phymodcczxy <- sem(phczxbez, estimator = "MLM", data = pls4, group = "cpol")
phymodcwzxy <- sem(phczxbez, estimator = "MLM", data = pls4, group = "cpol", group.equal = "loadings")
phymodcszxy <- sem(phczxbez, estimator = "MLM", data = pls4, group = "cpol", group.equal = c("loadings", "intercepts"), group.partial = c("psupport1 ~ 1"))
phymodcrzxy <- sem(phczxbez, estimator = "MLM", data = pls4, group = "cpol", group.equal = c("loadings", "intercepts", "regressions"), group.partial = c("psupport1 ~ 1"))
print(compareFit(phymodcczxy, phymodcwzxy, phymodcszxy, phymodcrzxy, nested = T))
```

```
## ################### Nested Model Comparison #########################
## Scaled Chi-Squared Difference Test (method = "satorra.bentler.2001")
## 
## lavaan NOTE:
##     The "Chisq" column contains standard test statistics, not the
##     robust test that should be reported per model. A robust difference
##     test is a function of two standard (not robust) statistics.
##  
##               Df     AIC     BIC Chisq Chisq diff Df diff Pr(>Chisq)    
## phymodcczxy  957 1198406 1200658 12471                                  
## phymodcwzxy  995 1198735 1200704 12875     358.83      38  < 2.2e-16 ***
## phymodcszxy 1031 1200114 1201816 14327    2241.16      36  < 2.2e-16 ***
## phymodcrzxy 1071 1200356 1201761 14649     307.31      40  < 2.2e-16 ***
## ---
## Signif. codes:  0 '***' 0.001 '**' 0.01 '*' 0.05 '.' 0.1 ' ' 1
## 
## ####################### Model Fit Indices ###########################
##             chisq.scaled df.scaled pvalue.scaled cfi.robust tli.robust
## phymodcczxy   12108.522†       957          .000      .930†      .919 
## phymodcwzxy   12456.315        995          .000      .928       .919†
## phymodcszxy   14043.411       1031          .000      .919       .913 
## phymodcrzxy   14344.304       1071          .000      .918       .914 
##                      aic          bic rmsea.robust  srmr
## phymodcczxy 1198406.197† 1200658.301†        .054  .068†
## phymodcwzxy 1198734.649  1200704.310         .053† .069 
## phymodcszxy 1200113.797  1201815.881         .056  .070 
## phymodcrzxy 1200356.111  1201760.888         .055  .073 
## 
## ################## Differences in Fit Indices #######################
##                           df.scaled cfi.robust tli.robust      aic      bic
## phymodcwzxy - phymodcczxy        38     -0.002      0.001  328.451   46.009
## phymodcszxy - phymodcwzxy        36     -0.009     -0.007 1379.148 1111.572
## phymodcrzxy - phymodcszxy        40     -0.002      0.001  242.314  -54.993
##                           rmsea.robust  srmr
## phymodcwzxy - phymodcczxy        0.000 0.002
## phymodcszxy - phymodcwzxy        0.002 0.001
## phymodcrzxy - phymodcszxy        0.000 0.003
```

Since there is no variation in regression slopes, there should be no variation in mediations.

## Data extraction - interactions per group

```
phczxbez <- 'phc =~ contact1 + contact2r + contact3 + contact4 #just the measurement model for data extraction
        phs =~ psupport1 + psupport2 + psupport3 + psupport4 + psupport5
        phg =~ hygiene1 + hygiene2 + hygiene3 + hygiene4 + hygiene5
        hygiene1 ~~ hygiene2
        psupport4 ~~ psupport5
        om1 =~ omind2 + omind3 + omind4 + omind1 + omind5 + omind6
        omind1 ~~ omind5
        omind5 ~~ omind6
        omind1 ~~ omind6
        consp =~ ctheory1 + ctheory2 + ctheory3 + ctheory4
        ctheory1 ~~ ctheory2'

phymodcczxy <- sem(phczxbez, estimator = "MLM", data = pls4, group = "cpol")
phymodcwzxy <- sem(phczxbez, estimator = "MLM", data = pls4, group = "cpol", group.equal = "loadings")
phymodcszxy <- sem(phczxbez, estimator = "MLM", data = pls4, group = "cpol", group.equal = c("loadings", "intercepts"), group.partial = c("psupport1 ~ 1"))
print(compareFit(phymodcczxy, phymodcwzxy, phymodcszxy, nested = T))
```

```
## ################### Nested Model Comparison #########################
## Scaled Chi-Squared Difference Test (method = "satorra.bentler.2001")
## 
## lavaan NOTE:
##     The "Chisq" column contains standard test statistics, not the
##     robust test that should be reported per model. A robust difference
##     test is a function of two standard (not robust) statistics.
##  
##              Df     AIC     BIC   Chisq Chisq diff Df diff Pr(>Chisq)    
## phymodcczxy 708 1200105 1202067  9028.6                                  
## phymodcwzxy 746 1200429 1202109  9428.7     355.99      38  < 2.2e-16 ***
## phymodcszxy 782 1201812 1203225 10883.9    2037.61      36  < 2.2e-16 ***
## ---
## Signif. codes:  0 '***' 0.001 '**' 0.01 '*' 0.05 '.' 0.1 ' ' 1
## 
## ####################### Model Fit Indices ###########################
##             chisq.scaled df.scaled pvalue.scaled cfi.robust tli.robust
## phymodcczxy    8671.811†       708          .000      .948†      .939 
## phymodcwzxy    9019.575        746          .000      .946       .940†
## phymodcszxy   10565.761        782          .000      .937       .933 
##                      aic          bic rmsea.robust  srmr
## phymodcczxy 1200104.995† 1202067.223†        .053  .066†
## phymodcwzxy 1200429.109  1202108.896         .053† .068 
## phymodcszxy 1201812.345  1203224.555         .056  .070 
## 
## ################## Differences in Fit Indices #######################
##                           df.scaled cfi.robust tli.robust      aic      bic
## phymodcwzxy - phymodcczxy        38     -0.002      0.001  324.114   41.672
## phymodcszxy - phymodcwzxy        36     -0.009     -0.007 1383.236 1115.659
##                           rmsea.robust  srmr
## phymodcwzxy - phymodcczxy        0.000 0.002
## phymodcszxy - phymodcwzxy        0.003 0.002
```

```
finalcovidxyx <- estimate_lavaan_ten_berge_per_group(phymodcszxy)
fcxx <- lapply(finalcovidxyx, as.data.frame)
dfsx <- list()
for(i in 1:length(fcxx)){
  nam <- names(fcxx)
  df <- fcxx[[i]]
  df$country <- nam[i]
  dfsx[[i]] <- df
}

dffsx <- bind_rows(dfsx)
names(dffsx) <- c("phc", "phs", "phg", "om1", "consp")
pls5 <- pls4[order(pls4$cpol), ]
dffsx$polid <- pls5$political_ideology
dffsx$crt <- pls5$ccrt
dffsx$age <- pls5$age
dffsx$sex <- pls5$sex1
dffsx$cpol <- pls5$cpol

dffsx$crtpolid <- scale(dffsx$crt)*scale(dffsx$polid)
dffsx$om1polid <- scale(dffsx$om1)*scale(dffsx$polid)

dfffsxy <- split.data.frame(dffsx, dffsx$cpol)
```

### Testing the stability of interactions

```
intmod <- 'phc ~ age + sex + polid + om1 + crt + om1polid
           phg ~ age + sex + polid + om1 + crt + om1polid
           phs ~ age + sex + polid + om1 + crt + om1polid'
intsem <- sem(intmod, estimator = "MLM", data = dffsx)
summary(intsem, standardized = T, rsq = T)
```

```
## lavaan 0.6-8 ended normally after 43 iterations
## 
##   Estimator                                         ML
##   Optimization method                           NLMINB
##   Number of model parameters                        24
##                                                       
##   Number of observations                         12490
##                                                       
## Model Test User Model:
##                                               Standard      Robust
##   Test Statistic                                 0.000       0.000
##   Degrees of freedom                                 0           0
## 
## Parameter Estimates:
## 
##   Standard errors                           Robust.sem
##   Information                                 Expected
##   Information saturated (h1) model          Structured
## 
## Regressions:
##                    Estimate  Std.Err  z-value  P(>|z|)   Std.lv  Std.all
##   phc ~                                                                 
##     age               0.004    0.000    8.817    0.000    0.004    0.071
##     sex               0.205    0.017   12.336    0.000    0.205    0.103
##     polid            -0.002    0.004   -0.398    0.691   -0.002   -0.003
##     om1               0.432    0.009   50.472    0.000    0.432    0.432
##     crt              -0.039    0.008   -4.978    0.000   -0.039   -0.042
##     om1polid         -0.020    0.009   -2.322    0.020   -0.020   -0.020
##   phg ~                                                                 
##     age              -0.001    0.000   -1.315    0.188   -0.001   -0.011
##     sex               0.194    0.017   11.343    0.000    0.194    0.097
##     polid             0.017    0.004    4.450    0.000    0.017    0.038
##     om1               0.318    0.009   36.466    0.000    0.318    0.318
##     crt              -0.144    0.008  -17.477    0.000   -0.144   -0.154
##     om1polid         -0.019    0.009   -2.138    0.033   -0.019   -0.019
##   phs ~                                                                 
##     age              -0.003    0.000   -6.457    0.000   -0.003   -0.054
##     sex               0.083    0.018    4.733    0.000    0.083    0.041
##     polid            -0.004    0.004   -0.947    0.344   -0.004   -0.009
##     om1               0.282    0.009   31.829    0.000    0.282    0.282
##     crt              -0.070    0.008   -8.426    0.000   -0.070   -0.075
##     om1polid         -0.020    0.009   -2.085    0.037   -0.020   -0.020
## 
## Covariances:
##                    Estimate  Std.Err  z-value  P(>|z|)   Std.lv  Std.all
##  .phc ~~                                                                
##    .phg               0.428    0.009   45.764    0.000    0.428    0.518
##    .phs               0.423    0.010   43.319    0.000    0.423    0.498
##  .phg ~~                                                                
##    .phs               0.307    0.010   32.231    0.000    0.307    0.347
## 
## Variances:
##                    Estimate  Std.Err  z-value  P(>|z|)   Std.lv  Std.all
##    .phc               0.793    0.012   64.715    0.000    0.793    0.793
##    .phg               0.862    0.013   66.370    0.000    0.862    0.862
##    .phs               0.908    0.015   61.874    0.000    0.908    0.909
## 
## R-Square:
##                    Estimate
##     phc               0.207
##     phg               0.138
##     phs               0.091
```

```
intsems <- sem(intmod, estimator = "MLM", data = dffsx, group = "cpol", group.equal = c("loadings", "intercepts"))
summary(intsems, standardized = T, rsq = T)
```

```
## lavaan 0.6-8 ended normally after 144 iterations
## 
##   Estimator                                         ML
##   Optimization method                           NLMINB
##   Number of model parameters                        81
##   Number of equality constraints                     6
##                                                       
##   Number of observations per group:                   
##     negative                                      4408
##     neutral                                       6315
##     positive                                      1767
##                                                       
## Model Test User Model:
##                                               Standard      Robust
##   Test Statistic                                36.392      35.293
##   Degrees of freedom                                 6           6
##   P-value (Chi-square)                           0.000       0.000
##   Scaling correction factor                                  1.031
##        Satorra-Bentler correction                                 
##   Test statistic for each group:
##     negative                                     0.961       0.932
##     neutral                                      5.840       5.663
##     positive                                    29.592      28.698
## 
## Parameter Estimates:
## 
##   Standard errors                           Robust.sem
##   Information                                 Expected
##   Information saturated (h1) model          Structured
## 
## 
## Group 1 [negative]:
## 
## Regressions:
##                    Estimate  Std.Err  z-value  P(>|z|)   Std.lv  Std.all
##   phc ~                                                                 
##     age               0.006    0.001    9.349    0.000    0.006    0.104
##     sex               0.200    0.022    9.136    0.000    0.200    0.100
##     polid            -0.017    0.005   -3.251    0.001   -0.017   -0.040
##     om1               0.516    0.014   36.136    0.000    0.516    0.515
##     crt              -0.060    0.012   -5.126    0.000   -0.060   -0.065
##     om1polid         -0.019    0.013   -1.416    0.157   -0.019   -0.019
##   phg ~                                                                 
##     age              -0.000    0.001   -0.316    0.752   -0.000   -0.004
##     sex               0.197    0.024    8.350    0.000    0.197    0.098
##     polid             0.021    0.006    3.620    0.000    0.021    0.047
##     om1               0.302    0.015   20.418    0.000    0.302    0.302
##     crt              -0.201    0.014  -14.834    0.000   -0.201   -0.216
##     om1polid         -0.020    0.014   -1.390    0.165   -0.020   -0.020
##   phs ~                                                                 
##     age              -0.000    0.001   -0.095    0.924   -0.000   -0.001
##     sex               0.125    0.024    5.239    0.000    0.125    0.062
##     polid            -0.040    0.006   -6.558    0.000   -0.040   -0.092
##     om1               0.327    0.015   22.062    0.000    0.327    0.327
##     crt              -0.081    0.013   -6.248    0.000   -0.081   -0.087
##     om1polid         -0.035    0.014   -2.437    0.015   -0.035   -0.035
## 
## Covariances:
##                    Estimate  Std.Err  z-value  P(>|z|)   Std.lv  Std.all
##  .phc ~~                                                                
##    .phg               0.345    0.013   25.922    0.000    0.345    0.447
##    .phs               0.399    0.014   27.573    0.000    0.399    0.515
##  .phg ~~                                                                
##    .phs               0.263    0.015   17.093    0.000    0.263    0.307
## 
## Intercepts:
##                    Estimate  Std.Err  z-value  P(>|z|)   Std.lv  Std.all
##    .phc     (.46.)   -0.443    0.046   -9.672    0.000   -0.443   -0.443
##    .phg     (.47.)   -0.203    0.046   -4.383    0.000   -0.203   -0.203
##    .phs     (.48.)    0.095    0.046    2.051    0.040    0.095    0.095
## 
## Variances:
##                    Estimate  Std.Err  z-value  P(>|z|)   Std.lv  Std.all
##    .phc               0.697    0.017   40.096    0.000    0.697    0.696
##    .phg               0.850    0.022   38.588    0.000    0.850    0.850
##    .phs               0.862    0.026   32.787    0.000    0.862    0.863
## 
## R-Square:
##                    Estimate
##     phc               0.304
##     phg               0.150
##     phs               0.137
## 
## 
## Group 2 [neutral]:
## 
## Regressions:
##                    Estimate  Std.Err  z-value  P(>|z|)   Std.lv  Std.all
##   phc ~                                                                 
##     age               0.003    0.001    4.941    0.000    0.003    0.053
##     sex               0.202    0.021    9.698    0.000    0.202    0.101
##     polid             0.004    0.005    0.829    0.407    0.004    0.009
##     om1               0.361    0.012   29.983    0.000    0.361    0.361
##     crt              -0.020    0.011   -1.827    0.068   -0.020   -0.021
##     om1polid         -0.024    0.013   -1.868    0.062   -0.024   -0.023
##   phg ~                                                                 
##     age              -0.001    0.001   -1.448    0.148   -0.001   -0.015
##     sex               0.194    0.021    9.176    0.000    0.194    0.097
##     polid             0.012    0.005    2.297    0.022    0.012    0.025
##     om1               0.332    0.012   27.533    0.000    0.332    0.332
##     crt              -0.106    0.011   -9.590    0.000   -0.106   -0.114
##     om1polid         -0.027    0.013   -2.127    0.033   -0.027   -0.026
##   phs ~                                                                 
##     age              -0.005    0.001   -7.380    0.000   -0.005   -0.083
##     sex               0.062    0.022    2.876    0.004    0.062    0.031
##     polid             0.014    0.005    2.647    0.008    0.014    0.031
##     om1               0.242    0.013   19.312    0.000    0.242    0.242
##     crt              -0.051    0.011   -4.497    0.000   -0.051   -0.055
##     om1polid         -0.015    0.014   -1.091    0.275   -0.015   -0.015
## 
## Covariances:
##                    Estimate  Std.Err  z-value  P(>|z|)   Std.lv  Std.all
##  .phc ~~                                                                
##    .phg               0.473    0.014   34.615    0.000    0.473    0.550
##    .phs               0.413    0.014   29.196    0.000    0.413    0.463
##  .phg ~~                                                                
##    .phs               0.311    0.013   23.486    0.000    0.311    0.347
## 
## Intercepts:
##                    Estimate  Std.Err  z-value  P(>|z|)   Std.lv  Std.all
##    .phc     (.46.)   -0.443    0.046   -9.672    0.000   -0.443   -0.443
##    .phg     (.47.)   -0.203    0.046   -4.383    0.000   -0.203   -0.203
##    .phs     (.48.)    0.095    0.046    2.051    0.040    0.095    0.095
## 
## Variances:
##                    Estimate  Std.Err  z-value  P(>|z|)   Std.lv  Std.all
##    .phc               0.857    0.018   47.787    0.000    0.857    0.856
##    .phg               0.865    0.018   47.575    0.000    0.865    0.865
##    .phs               0.926    0.020   46.831    0.000    0.926    0.927
## 
## R-Square:
##                    Estimate
##     phc               0.144
##     phg               0.135
##     phs               0.073
## 
## 
## Group 3 [positive]:
## 
## Regressions:
##                    Estimate  Std.Err  z-value  P(>|z|)   Std.lv  Std.all
##   phc ~                                                                 
##     age               0.002    0.001    1.791    0.073    0.002    0.030
##     sex               0.197    0.034    5.728    0.000    0.197    0.099
##     polid             0.024    0.009    2.797    0.005    0.024    0.056
##     om1               0.462    0.022   20.763    0.000    0.462    0.464
##     crt              -0.060    0.020   -3.038    0.002   -0.060   -0.063
##     om1polid         -0.043    0.022   -1.933    0.053   -0.043   -0.045
##   phg ~                                                                 
##     age              -0.001    0.001   -0.763    0.446   -0.001   -0.014
##     sex               0.190    0.037    5.159    0.000    0.190    0.095
##     polid             0.019    0.009    2.056    0.040    0.019    0.043
##     om1               0.303    0.024   12.649    0.000    0.303    0.303
##     crt              -0.142    0.021   -6.887    0.000   -0.142   -0.148
##     om1polid          0.017    0.024    0.710    0.478    0.017    0.017
##   phs ~                                                                 
##     age              -0.005    0.001   -4.301    0.000   -0.005   -0.078
##     sex               0.055    0.036    1.520    0.128    0.055    0.027
##     polid             0.032    0.009    3.502    0.000    0.032    0.072
##     om1               0.283    0.024   11.906    0.000    0.283    0.282
##     crt              -0.107    0.021   -5.037    0.000   -0.107   -0.110
##     om1polid         -0.022    0.024   -0.931    0.352   -0.022   -0.023
## 
## Covariances:
##                    Estimate  Std.Err  z-value  P(>|z|)   Std.lv  Std.all
##  .phc ~~                                                                
##    .phg               0.480    0.029   16.410    0.000    0.480    0.599
##    .phs               0.471    0.029   16.326    0.000    0.471    0.574
##  .phg ~~                                                                
##    .phs               0.405    0.028   14.590    0.000    0.405    0.461
## 
## Intercepts:
##                    Estimate  Std.Err  z-value  P(>|z|)   Std.lv  Std.all
##    .phc     (.46.)   -0.443    0.046   -9.672    0.000   -0.443   -0.445
##    .phg     (.47.)   -0.203    0.046   -4.383    0.000   -0.203   -0.204
##    .phs     (.48.)    0.095    0.046    2.051    0.040    0.095    0.095
## 
## Variances:
##                    Estimate  Std.Err  z-value  P(>|z|)   Std.lv  Std.all
##    .phc               0.747    0.038   19.789    0.000    0.747    0.754
##    .phg               0.860    0.033   26.210    0.000    0.860    0.865
##    .phs               0.901    0.036   25.102    0.000    0.901    0.898
## 
## R-Square:
##                    Estimate
##     phc               0.246
##     phg               0.135
##     phs               0.102
```

```
intmod <- 'phc ~ age + sex + polid + om1 + crt + a*om1polid
           phg ~ age + sex + polid + om1 + crt + b*om1polid
           phs ~ age + sex + polid + om1 + crt + c*om1polid'
intsemr <- sem(intmod, estimator = "MLM", data = dffsx, group = "cpol", group.equal = c("loadings", "intercepts"))
print(compareFit(intsems, intsemr, nested = T))
```

```
## ################### Nested Model Comparison #########################
## Scaled Chi-Squared Difference Test (method = "satorra.bentler.2001")
## 
## lavaan NOTE:
##     The "Chisq" column contains standard test statistics, not the
##     robust test that should be reported per model. A robust difference
##     test is a function of two standard (not robust) statistics.
##  
##         Df   AIC   BIC  Chisq Chisq diff Df diff Pr(>Chisq)
## intsems  6 92355 92913 36.392                              
## intsemr 12 92355 92868 47.991      10.48       6     0.1058
## 
## ####################### Model Fit Indices ###########################
##         chisq.scaled df.scaled pvalue.scaled cfi.robust tli.robust        aic
## intsems      35.293†         6          .000     0.998†      .978  92355.085 
## intsemr      44.896         12          .000     0.998       .987† 92354.684†
##                bic rmsea.robust  srmr
## intsems 92912.537         .035  .005†
## intsemr 92867.539†        .027† .006 
## 
## ################## Differences in Fit Indices #######################
##                   df.scaled cfi.robust tli.robust    aic     bic rmsea.robust
## intsemr - intsems         6          0      0.009 -0.401 -44.998       -0.008
##                    srmr
## intsemr - intsems 0.001
```

```
intmod <- 'phc ~ age + sex + polid + om1 + crt + crtpolid
           phg ~ age + sex + polid + om1 + crt + crtpolid
           phs ~ age + sex + polid + om1 + crt + crtpolid'

intsem <- sem(intmod, estimator = "MLM", data = dffsx)
summary(intsem, standardized = T, rsq = T)
```

```
## lavaan 0.6-8 ended normally after 44 iterations
## 
##   Estimator                                         ML
##   Optimization method                           NLMINB
##   Number of model parameters                        24
##                                                       
##   Number of observations                         12490
##                                                       
## Model Test User Model:
##                                               Standard      Robust
##   Test Statistic                                 0.000       0.000
##   Degrees of freedom                                 0           0
## 
## Parameter Estimates:
## 
##   Standard errors                           Robust.sem
##   Information                                 Expected
##   Information saturated (h1) model          Structured
## 
## Regressions:
##                    Estimate  Std.Err  z-value  P(>|z|)   Std.lv  Std.all
##   phc ~                                                                 
##     age               0.004    0.000    8.854    0.000    0.004    0.071
##     sex               0.205    0.017   12.305    0.000    0.205    0.102
##     polid            -0.003    0.004   -0.667    0.504   -0.003   -0.006
##     om1               0.431    0.009   50.381    0.000    0.431    0.431
##     crt              -0.040    0.008   -5.111    0.000   -0.040   -0.043
##     crtpolid         -0.018    0.008   -2.163    0.031   -0.018   -0.018
##   phg ~                                                                 
##     age              -0.001    0.000   -1.190    0.234   -0.001   -0.010
##     sex               0.194    0.017   11.341    0.000    0.194    0.097
##     polid             0.016    0.004    4.201    0.000    0.016    0.036
##     om1               0.317    0.009   36.464    0.000    0.317    0.317
##     crt              -0.146    0.008  -17.682    0.000   -0.146   -0.156
##     crtpolid         -0.030    0.009   -3.400    0.001   -0.030   -0.030
##   phs ~                                                                 
##     age              -0.003    0.000   -6.394    0.000   -0.003   -0.054
##     sex               0.082    0.018    4.712    0.000    0.082    0.041
##     polid            -0.005    0.004   -1.187    0.235   -0.005   -0.011
##     om1               0.281    0.009   31.757    0.000    0.281    0.281
##     crt              -0.071    0.008   -8.521    0.000   -0.071   -0.076
##     crtpolid         -0.019    0.009   -2.048    0.041   -0.019   -0.019
## 
## Covariances:
##                    Estimate  Std.Err  z-value  P(>|z|)   Std.lv  Std.all
##  .phc ~~                                                                
##    .phg               0.428    0.009   45.821    0.000    0.428    0.518
##    .phs               0.423    0.010   43.415    0.000    0.423    0.498
##  .phg ~~                                                                
##    .phs               0.307    0.010   32.251    0.000    0.307    0.346
## 
## Variances:
##                    Estimate  Std.Err  z-value  P(>|z|)   Std.lv  Std.all
##    .phc               0.793    0.012   64.796    0.000    0.793    0.793
##    .phg               0.862    0.013   66.433    0.000    0.862    0.862
##    .phs               0.908    0.015   61.891    0.000    0.908    0.909
## 
## R-Square:
##                    Estimate
##     phc               0.207
##     phg               0.138
##     phs               0.091
```

```
intsems <- sem(intmod, estimator = "MLM", data = dffsx, group = "cpol", group.equal = c("loadings", "intercepts"))
summary(intsems, standardized = T, rsq = T)
```

```
## lavaan 0.6-8 ended normally after 143 iterations
## 
##   Estimator                                         ML
##   Optimization method                           NLMINB
##   Number of model parameters                        81
##   Number of equality constraints                     6
##                                                       
##   Number of observations per group:                   
##     negative                                      4408
##     neutral                                       6315
##     positive                                      1767
##                                                       
## Model Test User Model:
##                                               Standard      Robust
##   Test Statistic                                35.665      34.601
##   Degrees of freedom                                 6           6
##   P-value (Chi-square)                           0.000       0.000
##   Scaling correction factor                                  1.031
##        Satorra-Bentler correction                                 
##   Test statistic for each group:
##     negative                                     0.853       0.827
##     neutral                                      5.606       5.439
##     positive                                    29.206      28.335
## 
## Parameter Estimates:
## 
##   Standard errors                           Robust.sem
##   Information                                 Expected
##   Information saturated (h1) model          Structured
## 
## 
## Group 1 [negative]:
## 
## Regressions:
##                    Estimate  Std.Err  z-value  P(>|z|)   Std.lv  Std.all
##   phc ~                                                                 
##     age               0.006    0.001    9.292    0.000    0.006    0.104
##     sex               0.197    0.022    9.043    0.000    0.197    0.098
##     polid            -0.018    0.005   -3.453    0.001   -0.018   -0.042
##     om1               0.514    0.014   36.225    0.000    0.514    0.514
##     crt              -0.062    0.012   -5.250    0.000   -0.062   -0.067
##     crtpolid         -0.022    0.012   -1.768    0.077   -0.022   -0.023
##   phg ~                                                                 
##     age              -0.000    0.001   -0.244    0.807   -0.000   -0.003
##     sex               0.197    0.024    8.360    0.000    0.197    0.099
##     polid             0.020    0.006    3.506    0.000    0.020    0.046
##     om1               0.301    0.015   20.390    0.000    0.301    0.301
##     crt              -0.202    0.014  -14.952    0.000   -0.202   -0.217
##     crtpolid         -0.028    0.015   -1.939    0.053   -0.028   -0.030
##   phs ~                                                                 
##     age              -0.000    0.001   -0.075    0.940   -0.000   -0.001
##     sex               0.123    0.024    5.198    0.000    0.123    0.062
##     polid            -0.041    0.006   -6.738    0.000   -0.041   -0.095
##     om1               0.324    0.015   21.826    0.000    0.324    0.324
##     crt              -0.083    0.013   -6.337    0.000   -0.083   -0.089
##     crtpolid         -0.033    0.015   -2.222    0.026   -0.033   -0.035
## 
## Covariances:
##                    Estimate  Std.Err  z-value  P(>|z|)   Std.lv  Std.all
##  .phc ~~                                                                
##    .phg               0.344    0.013   25.911    0.000    0.344    0.447
##    .phs               0.399    0.014   27.642    0.000    0.399    0.515
##  .phg ~~                                                                
##    .phs               0.262    0.015   17.053    0.000    0.262    0.306
## 
## Intercepts:
##                    Estimate  Std.Err  z-value  P(>|z|)   Std.lv  Std.all
##    .phc     (.46.)   -0.431    0.045   -9.499    0.000   -0.431   -0.431
##    .phg     (.47.)   -0.201    0.046   -4.367    0.000   -0.201   -0.201
##    .phs     (.48.)    0.106    0.046    2.304    0.021    0.106    0.106
## 
## Variances:
##                    Estimate  Std.Err  z-value  P(>|z|)   Std.lv  Std.all
##    .phc               0.697    0.017   40.111    0.000    0.697    0.696
##    .phg               0.850    0.022   38.641    0.000    0.850    0.850
##    .phs               0.862    0.026   32.736    0.000    0.862    0.863
## 
## R-Square:
##                    Estimate
##     phc               0.304
##     phg               0.150
##     phs               0.137
## 
## 
## Group 2 [neutral]:
## 
## Regressions:
##                    Estimate  Std.Err  z-value  P(>|z|)   Std.lv  Std.all
##   phc ~                                                                 
##     age               0.003    0.001    4.884    0.000    0.003    0.053
##     sex               0.200    0.021    9.642    0.000    0.200    0.100
##     polid             0.003    0.005    0.589    0.556    0.003    0.007
##     om1               0.360    0.012   29.924    0.000    0.360    0.360
##     crt              -0.020    0.011   -1.849    0.064   -0.020   -0.022
##     crtpolid         -0.001    0.012   -0.109    0.914   -0.001   -0.001
##   phg ~                                                                 
##     age              -0.001    0.001   -1.357    0.175   -0.001   -0.015
##     sex               0.194    0.021    9.199    0.000    0.194    0.097
##     polid             0.011    0.005    2.145    0.032    0.011    0.023
##     om1               0.331    0.012   27.529    0.000    0.331    0.331
##     crt              -0.108    0.011   -9.762    0.000   -0.108   -0.116
##     crtpolid         -0.029    0.012   -2.384    0.017   -0.029   -0.029
##   phs ~                                                                 
##     age              -0.005    0.001   -7.435    0.000   -0.005   -0.084
##     sex               0.061    0.022    2.791    0.005    0.061    0.030
##     polid             0.013    0.005    2.491    0.013    0.013    0.029
##     om1               0.242    0.013   19.316    0.000    0.242    0.242
##     crt              -0.051    0.011   -4.501    0.000   -0.051   -0.055
##     crtpolid          0.002    0.013    0.121    0.904    0.002    0.002
## 
## Covariances:
##                    Estimate  Std.Err  z-value  P(>|z|)   Std.lv  Std.all
##  .phc ~~                                                                
##    .phg               0.474    0.014   34.655    0.000    0.474    0.550
##    .phs               0.413    0.014   29.217    0.000    0.413    0.463
##  .phg ~~                                                                
##    .phs               0.311    0.013   23.530    0.000    0.311    0.348
## 
## Intercepts:
##                    Estimate  Std.Err  z-value  P(>|z|)   Std.lv  Std.all
##    .phc     (.46.)   -0.431    0.045   -9.499    0.000   -0.431   -0.431
##    .phg     (.47.)   -0.201    0.046   -4.367    0.000   -0.201   -0.201
##    .phs     (.48.)    0.106    0.046    2.304    0.021    0.106    0.106
## 
## Variances:
##                    Estimate  Std.Err  z-value  P(>|z|)   Std.lv  Std.all
##    .phc               0.857    0.018   47.749    0.000    0.857    0.857
##    .phg               0.865    0.018   47.591    0.000    0.865    0.864
##    .phs               0.926    0.020   46.833    0.000    0.926    0.927
## 
## R-Square:
##                    Estimate
##     phc               0.143
##     phg               0.136
##     phs               0.073
## 
## 
## Group 3 [positive]:
## 
## Regressions:
##                    Estimate  Std.Err  z-value  P(>|z|)   Std.lv  Std.all
##   phc ~                                                                 
##     age               0.002    0.001    2.063    0.039    0.002    0.035
##     sex               0.201    0.034    5.848    0.000    0.201    0.101
##     polid             0.017    0.009    1.941    0.052    0.017    0.040
##     om1               0.458    0.022   20.457    0.000    0.458    0.460
##     crt              -0.062    0.020   -3.112    0.002   -0.062   -0.064
##     crtpolid         -0.067    0.026   -2.532    0.011   -0.067   -0.066
##   phg ~                                                                 
##     age              -0.001    0.001   -0.655    0.513   -0.001   -0.012
##     sex               0.189    0.037    5.120    0.000    0.189    0.095
##     polid             0.018    0.010    1.857    0.063    0.018    0.041
##     om1               0.303    0.024   12.698    0.000    0.303    0.304
##     crt              -0.144    0.021   -6.943    0.000   -0.144   -0.149
##     crtpolid         -0.030    0.026   -1.160    0.246   -0.030   -0.030
##   phs ~                                                                 
##     age              -0.005    0.001   -4.085    0.000   -0.005   -0.075
##     sex               0.056    0.036    1.554    0.120    0.056    0.028
##     polid             0.027    0.010    2.854    0.004    0.027    0.062
##     om1               0.281    0.024   11.905    0.000    0.281    0.280
##     crt              -0.108    0.021   -5.105    0.000   -0.108   -0.112
##     crtpolid         -0.047    0.027   -1.736    0.083   -0.047   -0.046
## 
## Covariances:
##                    Estimate  Std.Err  z-value  P(>|z|)   Std.lv  Std.all
##  .phc ~~                                                                
##    .phg               0.478    0.029   16.537    0.000    0.478    0.597
##    .phs               0.470    0.028   16.646    0.000    0.470    0.573
##  .phg ~~                                                                
##    .phs               0.404    0.028   14.678    0.000    0.404    0.459
## 
## Intercepts:
##                    Estimate  Std.Err  z-value  P(>|z|)   Std.lv  Std.all
##    .phc     (.46.)   -0.431    0.045   -9.499    0.000   -0.431   -0.433
##    .phg     (.47.)   -0.201    0.046   -4.367    0.000   -0.201   -0.202
##    .phs     (.48.)    0.106    0.046    2.304    0.021    0.106    0.106
## 
## Variances:
##                    Estimate  Std.Err  z-value  P(>|z|)   Std.lv  Std.all
##    .phc               0.745    0.037   20.237    0.000    0.745    0.752
##    .phg               0.860    0.033   26.298    0.000    0.860    0.865
##    .phs               0.900    0.036   25.306    0.000    0.900    0.897
## 
## R-Square:
##                    Estimate
##     phc               0.248
##     phg               0.135
##     phs               0.103
```

```
intmod <- 'phc ~ age + sex + polid + om1 + crt + a*crtpolid
           phg ~ age + sex + polid + om1 + crt + b*crtpolid
           phs ~ age + sex + polid + om1 + crt + c*crtpolid'
intsemr <- sem(intmod, estimator = "MLM", data = dffsx, group = "cpol", group.equal = c("loadings", "intercepts"))
print(compareFit(intsems, intsemr, nested = T))
```

```
## ################### Nested Model Comparison #########################
## Scaled Chi-Squared Difference Test (method = "satorra.bentler.2001")
## 
## lavaan NOTE:
##     The "Chisq" column contains standard test statistics, not the
##     robust test that should be reported per model. A robust difference
##     test is a function of two standard (not robust) statistics.
##  
##         Df   AIC   BIC  Chisq Chisq diff Df diff Pr(>Chisq)  
## intsems  6 92352 92909 35.665                                
## intsemr 12 92353 92866 49.356     11.681       6    0.06947 .
## ---
## Signif. codes:  0 '***' 0.001 '**' 0.01 '*' 0.05 '.' 0.1 ' ' 1
## 
## ####################### Model Fit Indices ###########################
##         chisq.scaled df.scaled pvalue.scaled cfi.robust tli.robust        aic
## intsems      34.601†         6          .000     0.998†      .978  92351.735†
## intsemr      44.812         12          .000     0.997       .987† 92353.425 
##                bic rmsea.robust  srmr
## intsems 92909.186         .034  .005†
## intsemr 92866.281†        .027† .006 
## 
## ################## Differences in Fit Indices #######################
##                   df.scaled cfi.robust tli.robust   aic     bic rmsea.robust
## intsemr - intsems         6          0      0.008 1.691 -42.905       -0.007
##                    srmr
## intsemr - intsems 0.001
```

## Data extraction - interactions per country

```
phczxbez <- 'phc =~ contact1 + contact2r + contact3 + contact4 #just the measurement model for data extraction
        phs =~ psupport1 + psupport2 + psupport3 + psupport4 + psupport5
        phg =~ hygiene1 + hygiene2 + hygiene3 + hygiene4 + hygiene5
        hygiene1 ~~ hygiene2
        psupport4 ~~ psupport5
        om1 =~ omind2 + omind3 + omind4 + omind1 + omind5 + omind6
        omind1 ~~ omind5
        omind5 ~~ omind6
        omind1 ~~ omind6
        consp =~ ctheory1 + ctheory2 + ctheory3 + ctheory4
        ctheory1 ~~ ctheory2'

phymodcczxy <- sem(phczxbez, estimator = "MLM", data = pls4, group = "country")
phymodcwzxy <- sem(phczxbez, estimator = "MLM", data = pls4, group = "country", group.equal = "loadings")
phymodcszxy <- sem(phczxbez, estimator = "MLM", data = pls4, group = "country", group.equal = c("loadings", "intercepts"), group.partial = c("psupport1 ~ 1", "psupport3 ~ 1", "hygiene4 ~ 1", "hygiene5 ~ 1", "contact2r ~ 1", "psupport2 ~ 1", "ctheory1 ~ 1", "contact4~1", "omind2~1", "omind1 ~ 1"))
print(compareFit(phymodcczxy, phymodcwzxy, phymodcszxy, nested = T))
```

```
## ################### Nested Model Comparison #########################
## Scaled Chi-Squared Difference Test (method = "satorra.bentler.2001")
## 
## lavaan NOTE:
##     The "Chisq" column contains standard test statistics, not the
[truncated: 57,869 more chars]
