# Supplementary material for: Analytic Thinking and Political Orientation in the Corona Crisis
Source: Front Psychol. 2021 Jul 22;12:631800. doi: 10.3389/fpsyg.2021.631800 (PMC8341110; doi:10.3389/fpsyg.2021.631800)
Supplement: Supplementary file 1 [file Data_Sheet_1.zip › Supplementary Materials/Data cleaning, CFAs, descriptives, and correlations.docx]

**Supplementary Materials**

# **Data Cleaning Procedure**

The data cleaning strategy applied in this study consisted of multiple steps carried out in R (R Core Team, 2021). The initial sample consisted of 51,717 citizens aged 18 to 100 from 67 countries/regions. Firstly, participants who failed attention checks^[[1]](#footnote-1)^ were excluded (*n* = 3899), with the exceptions of Ghana and Romania data sets which did not include attention checks and were forwarded in the following step without any exclusions. In the next step, participants who did not answer more than 25% of questions were excluded (*n* = 548), followed by participants who responded carelessly, which was operationalized as using the same response more than 10 times in a row on questions unrelated to country-level data, attention checks, maintaining hygiene, avoiding contact and policy support (*n* = 1,583). Finally, we excluded participants who solved the entire questionnaire in less than 9 minutes due to the questionable effort invested in the task, as described in our preregistration. We did not exclude any participant from national data files where this information was not shown (Argentina, Bulgaria, Bolivia, one sample from Brazil, Chile, Costa Rica, Cuba, Dominican Republic, Ecuador, Ghana, Guatemala, Honduras, both samples from India, one sample from Italy and Mexico, Nicaragua, Panama, Peru, Porto Rico, Paraguay, one sample from Romania, Russia, El Salvador, Uruguay, Venezuela and South African Republic). This led to a sample size of 43,793 participants. Computational requirements of planned analyses motivated us to group samples from Bolivia, Chile, Ecuador, Peru, Paraguay, Uruguay, and Venezuela into LATAM (LATin AMerican countries) and samples from Costa Rica, Cuba, Dominican Republic, Guatemala, Honduras, Nicaragua, Panama, Puerto Rico, and El Salvador into CENAM (CENtral AMerican countries) due to the inadequately low number of participants. In the next step, the data were grouped by country and imputed using predictive mean matching with ten multiple imputations and ten iterations. Next, we tested the variability per country: variables where the ratio of occurrence between dominant response and the next most frequently chosen response was higher than 6:1 were marked as variables with low variability. To avoid biases due to low variance, which implies that only a small portion of the possible specter of the construct has been measured, we omitted all countries shad low variability from further analyses. This left us with participants from United Arab Emirates, Australia, Belgium, Canada, Switzerland, Germany, Greece, Iraq, Israel, Japan, South Korea, Nigeria, New Zealand, Pakistan, Poland, Singapore, Slovakia, and the USA in the sample (*n* = 15,565). Then, outliers were excluded from each national subsample using the robust Mahalanobis procedure (Leys et al., 2018). In brief, this procedure represents a stricter version of Mahalanobis procedure (Mahalanobis, 1936), based on the minimum covariance determinant approach (see Rousseeuw 1984, 1985) which selects the subset of data that is most homogeneous with respect to studied characteristics – in other words, which creates a matrix with a minimum determinant. This step resulted in 12,674 participants. In the following step, we excluded one country (United Arab Emirates (ARE), *n* = 153) due to having an insufficient number of participants to establish stable correlation patterns (below 200-250, see Schönbrodt and Perugini, 2013). The remaining national data sets contained more than 250 participants, which should have been sufficient to establish relatively stable patterns of correlations. From these participants, we also excluded those who selected “other” as their gender due to extremely rare occurrences (*n* = 31), resulting in a final sample of 12490 participants. The main analytical approach focused on establishing strong invariance using multi-group structural equation modeling, followed by extraction of specific factors from models using Ten Berge method (see Ten Berge, 1977) to preserve relationships and manual computation of interactions.

**References**

Leys, C., Klein, O., Dominicy, Y., and Ley, C. (2018). Detecting multivariate outliers: Use a robust variant of the Mahalanobis distance. *J. Exp. Soc. Psychol.* 74, 150-156. [doi: 10.1016/j.jesp.2017.09.011](https://doi.org/10.1016/j.jesp.2017.09.011)

Mahalanobis, P. C. (1936). *On the generalized distance in statistics.* National Institute of Science of India.

Rousseeuw, P. J. (1984). Least median of squares regression. *J. Am. Stat. Assoc.* Journal of the American Statistical Association. 79, 871–880. doi: 10.1080/01621459.1984.10477105

Rousseeuw, P. J. (1985). “Multivariate estimation with high breakdown point,” in *Mathematical Statistics and Applications, Vol. B*, eds W. Grossmann, G. Pflug, I. Vincze, and W. Wertz (Netherlands: Reidel), 283–297

Schönbrodt, F. D., and Perugini, M. (2013). At what sample size do correlations stabilize?. *J. Res. Pers.* 47. 609-612. doi: 10.1016/j.jrp.2013.05.009

Ten Berge, J. M. F. (1977). Orthogonal Procrustes rotation for two or more matrices. *Psychometrika*. 42, 267-276. doi: 10.1007/BF02294053

# **Measurement Models (Confirmatory Factor Analysis (CFA)) of the Research Instruments**

## **Measurement Model of the Outcome Variables**

Measurement model (Figure S1) of the examined preventive behaviors (avoiding physical contact and stricter physical hygiene) and COVID-19 policy support achieved adequate fit: robust CFI = 0.975, robust RMSEA = 0.048, SRMR = 0.030, indicating construct validity of the three related yet distinct constructs. All three measures exhibited acceptable reliability: ꞷ_contact_ = 0.69, ꞷ_hygiene_ = 0.74, ꞷ_support_ = 0.86. The results indicate the possibility of a general factor of attitudes and behaviors related to COVID-19.


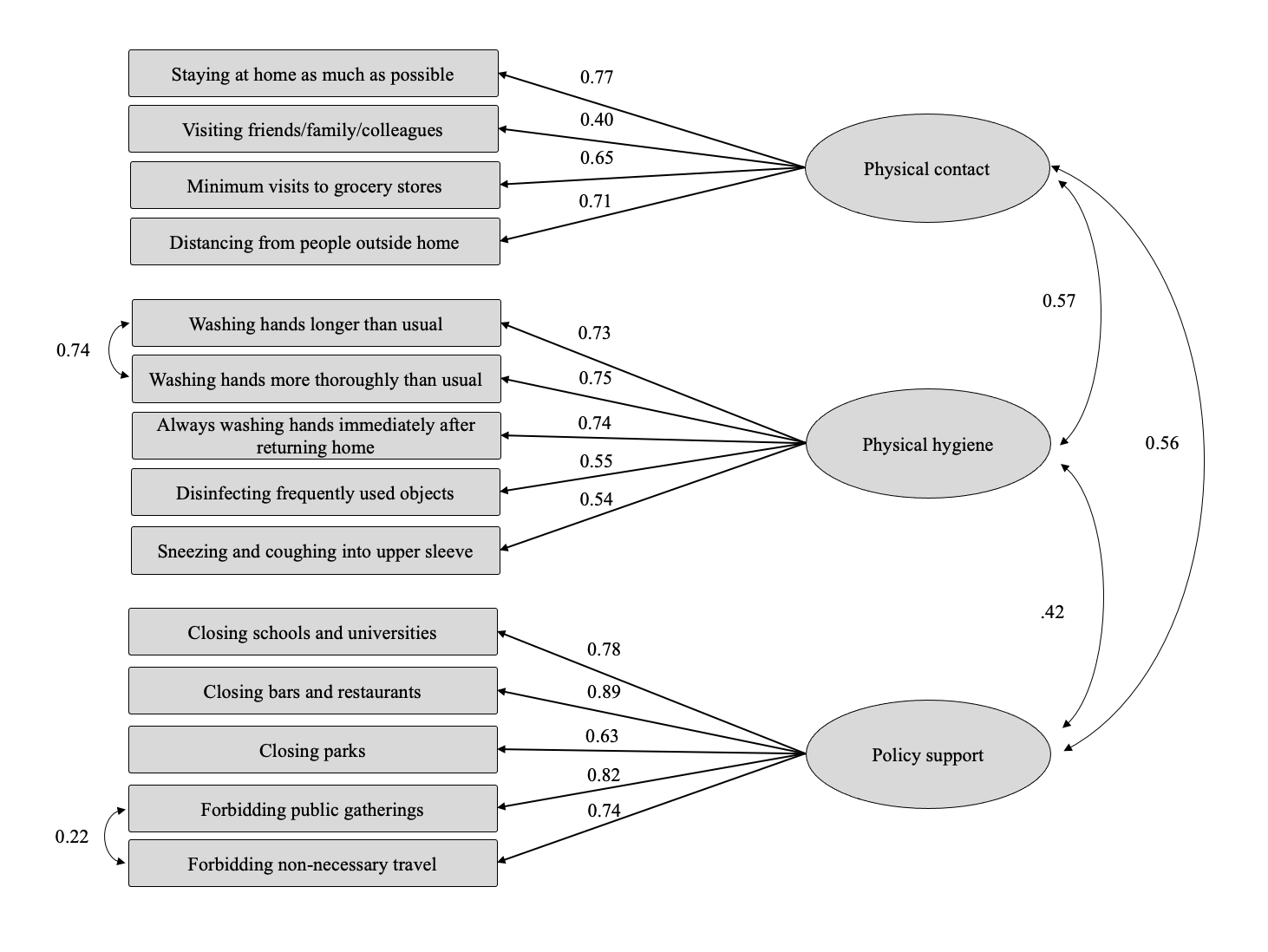


**FIGURE S1 |** Measurement model of the three outcome variables (*N* = 12,490). Values shown are standardized factor loadings. Latent variables are denoted as ellipse nodes and observed variables are denoted as rectangle nodes. Two-headed arrows between ellipses represent correlations between the constructs, and between rectangles denote correlation between unique variances of manifest variables. Error terms are omitted for clarity. All the parameters are significant at *p* < 0.001.

## **Measurement Model of Open-mindedness**

In this study, a unitary latent factor of open-mindedness was extracted (Figure S2), allowing for the correlations of unique item variances (items 1, 5, and 6), which achieved adequate fit: robust CFI = 0.998, robust RMSEA = 0.028, SRMR = 0.009, indicating construct validity. The measure of open-mindedness exhibited somewhat lower level of reliability: ꞷ = 0.53, comparable to the results of Erceg et al. (2020b; ꞷ = 0.65; also Thoma et al. (2021) reported that the Cronbach α of their measure of open-mindedness was 0.73 comparable to our α coefficient of 0.74) The results indicate the possibility of the existence of two correlated latent dimensions or a method factor based on the correlations of unique item variances of the negatively worded items. It should be mentioned that there are different versions of the scales intended to measure the tendency of open-minded thinking (comprising different dimensions of the construct (e.g., Alfano et al., 2017; Haran et al., 2013) up to 41 items (Stanovich and West, 2007) and both theoreticaly and empirically, openminded-thinking has shown to encapsulate different, albeit related dimensions deemed important in thinking and reasoning (see Deniz et al., 2008; Svedholm-Häkkinen and Lindeman, 2018; Sá et al., 1999; Stanovich and West, 1997). sFurther instigation of the latent structure of open-mindedness and its psychometric properties would be valuable for a clearer conceptualization and operationalization of the concept.


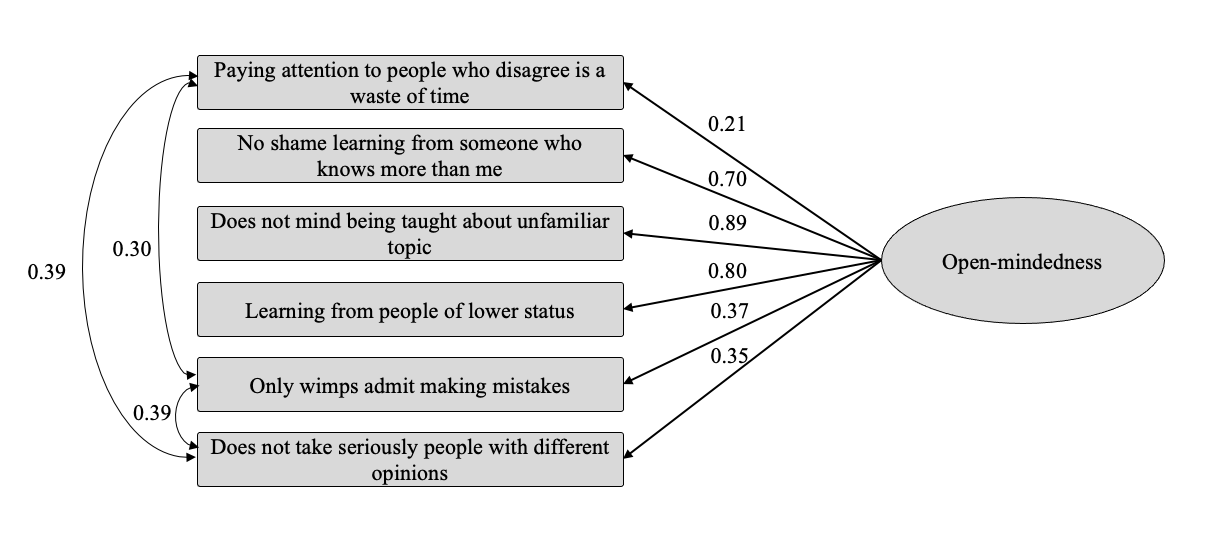


**FIGURE S2 |** Measurement model of open-mindedness (*N* = 12,490). Values shown are standardized factor loadings. Latent variables are denoted as ellipse nodes and observed variables are denoted as rectangle nodes. Two-headed arrows between ellipses represent correlations between the constructs. Error terms are omitted for clarity. All the parameters are significant at *p* < .001.

**References**

Deniz, H., Donnelly, L. A. and Yilmaz, I. (2008). Exploring the factors related to acceptance of evolutionary theory among Turkish preservice biology teachers: Toward a more informative conceptual ecology for biological evolution. *J. Res. Sci. Teach*. 45, 420–443. doi: 10.1002/tea.20223

Erceg, N., Ružojčić, M., and Galić, Z. (2020b) Misbehaving in the Corona crisis: The role of anxiety and unfounded beliefs. *Curr. Psychol.* doi: 10.1007/s12144-020-01040-4

Sá, W. C., West, R. F. i Stanovich, K. E. (1999). The domain specificity and generality of belief bias: Searching for a generalizable critical thinking skill. *J. Educ. Psychol*. *91*, 497-510. [doi: 10.1037/0022-0663.91.3.497](https://psycnet.apa.org/doi/10.1037/0022-0663.91.3.497)

Stanovich, K. E. and West, R. F. (1997). Reasoning independently of prior belief and individual differences in actively open-minded thinking. *J. Educ. Psychol.* 89, 342–357. dio: 10.1037/0022-0663.89.2.342

Thoma V, Weiss-Cohen L, Filkuková P and Ayton P (2021) Cognitive Predictors of Precautionary Behavior During the COVID-19 Pandemic. *Front. Psychol.* 12:589800. doi: 10.3389/fpsyg.2021.589800

## **Measurement Model of COVID-19 Conspiracy Beliefs**

Measurement model (Figure S3) of the examined COVID-19 conspiracy beliefs allowing for the correlation of unique item (items 1 and 2) variances achieved acceptable fit: χ2(1) = 77.040, CFI = 0.997, RMSEA = 0.102, SRMR = 0.007, indicating construct validity. A single factor extracted from these items exhibited a high level of reliability: ꞷ = 0.91.


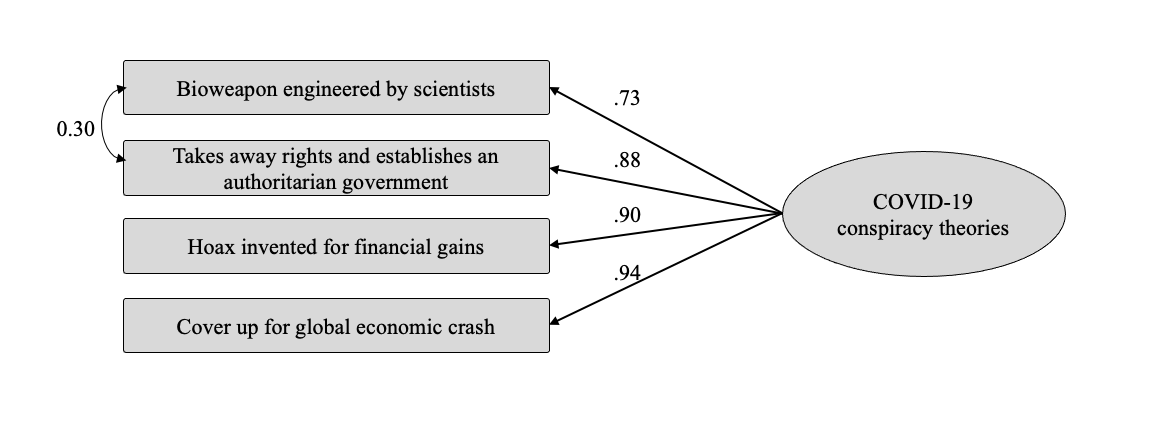


**FIGURE S3 |** Measurement models of COVID-19 conspiracy beliefs (*N* = 12,490). Values shown are standardized factor loadings. Latent variables are denoted as ellipse nodes and observed variables are denoted as rectangle nodes. Two-headed arrows between ellipses represent correlations between the constructs, and between rectangles denote correlation between unique variances of manifest variables. Error terms are omitted for clarity. All the parameters are significant at *p* < 0.001.

# **Sample Demographic Data and Descriptive Statistics and Intercorrelations of all the variables used in the study**

**TABLE S1 |** Basic demographic data across 17 countries of the final study sample (*N* = 12,490).

| Country | *N* (before cleaning) | *N* (after cleaning) | Mean age (*SD*) | % men | Employment status (in %) | | | | | | Sample representative (yes/no) |
| --- | --- | --- | --- | --- | --- | --- | --- | --- | --- | --- | --- |
|  |  |  |  |  | full time employed | part-time employed | unemployed | student | retired | other |  |
| Australia | 1819 | 1480 | 50.01 (17.09) | 47 | 33 | 19 | 10 | 5 | 27 | 7 | yes |
| Belgium | 1140 | 950 | 46.22 (18.91) | 57 | 27 | 4 | 3 | 26 | 26 | 13 | no |
| Canada | 941 | 740 | 44.05 (17.55) | 36 | 34 | 9 | 18 | 9 | 20 | 9 | yes |
| Switzerland | 969 | 801 | 49.33 (16.72) | 48 | 36 | 19 | 6 | 6 | 22 | 11 | yes |
| Germany | 1416 | 1117 | 50.07 (16.15) | 51 | 37 | 12 | 5 | 8 | 30 | 7 | yes |
| Greece | 614 | 479 | 30.05 (11.72) | 62 | 34 | 10 | 14 | 35 | 4 | 2 | no |
| Iraq | 414 | 320 | 32.40 (13.78) | 53 | 25 | 17 | 14 | 31 | 4 | 10 | no |
| Israel | 1174 | 994 | 41.27 (15.05) | 48 | 39 | 14 | 14 | 6 | 9 | 18 | yes |
| Japan | 891 | 747 | 50.63 (14.41) | 48 | 43 | 14 | 19 | 3 | 13 | 7 | yes |
| South Korea | 308 | 253 | 46.16 (12.87) | 53 | 53 | 12 | 6 | 5 | 10 | 14 | yes |
| Nigeria | 509 | 403 | 32.63 (11.37) | 51 | 36 | 17 | 19 | 20 | 1 | 7 | yes |
| New Zealand | 448 | 333 | 49.52 (16.96) | 54 | 39 | 15 | 8 | 3 | 21 | 14 | yes |
| Pakistan | 451 | 367 | 26.97 (8.41) | 44 | 29 | 6 | 10 | 50 | 1 | 5 | no |
| Poland | 1677 | 1394 | 47.61 (16.99) | 50 | 37 | 7 | 11 | 7 | 28 | 10 | yes |
| Singapore | 454 | 373 | 43.43 (13.59) | 49 | 66 | 6 | 9 | 5 | 6 | 7 | yes |
| Slovakia | 1010 | 834 | 44.31 (15.91) | 48 | 48 | 5 | 6 | 8 | 24 | 8 | yes |
| USA | 1147 | 905 | 48.29 (16.39) | 47 | 45 | 10 | 12 | 2 | 24 | 7 | yes |

**TABLE S2 |** Descriptive statistics and intercorrelations of all the variables used in the study presented by each country.

| **Australia** | |  |  |  |  |  |  |  |  |  |  | |  | |  |  |  |  |  |  |  |  |  |
| --- | --- | --- | --- | --- | --- | --- | --- | --- | --- | --- | --- | --- | --- | --- | --- | --- | --- | --- | --- | --- | --- | --- | --- |
|  |  | **Descriptives** | | | | | | | | | |  | | **Bivariate correlations** | | | | | | | | | |
|  |  | *n* | *M* | *SD* | *Median* | *Min* | *Max* | *Skew* | *Kurtosis* | *SE* |  | | (1) | | (2) | (3) | (4) | (5) | (6) | (7) | (8) | (9) |  |
| (1) | Physical contact | 1480 | 0 | 1 | 0.09 | -3.43 | 1.97 | -0.28 | -0.43 | 0.03 |  | | 1 | |  |  |  |  |  |  |  |  |  |
| (2) | Physical hygiene | 1480 | 0 | 1 | 0.06 | -4.13 | 2.03 | -0.38 | -0.16 | 0.03 |  | | 0.54 | | 1 |  |  |  |  |  |  |  |  |
| (3) | Policy support | 1480 | 0 | 1 | 0.11 | -3.89 | 1.69 | -0.68 | 0.30 | 0.03 |  | | 0.63 | | 0.42 | 1 |  |  |  |  |  |  |  |
| (4) | CRT | 1480 | 0.87 | 1.00 | 1 | 0 | 3 | 0.84 | -0.49 | 0.03 |  | | 0.01 | | -0.1 | 0.03 | 1 |  |  |  |  |  |  |
| (5) | Open-mindedness | 1480 | 0 | 1 | 0.04 | -3.54 | 2.27 | -0.16 | -0.41 | 0.03 |  | | 0.64 | | 0.4 | 0.44 | 0.05 | 1 |  |  |  |  |  |
| (6) | Conspiracy beliefs | 1480 | 0 | 1 | -0.16 | -3.21 | 4.38 | 0.71 | 0.83 | 0.03 |  | | -0.34 | | -0.05 | -0.24 | -0.12 | -0.34 | 1 |  |  |  |  |
| (7) | Political orientation | 1480 | 5.31 | 2.01 | 5 | 0 | 10 | -0.01 | 0.40 | 0.05 |  | | -0.13 | | -0.02 | -0.14 | -0.09 | -0.17 | 0.17 | 1 |  |  |  |
| (8) | Age | 1480 | 50.01 | 17.09 | 51 | 18 | 89 | -0.08 | -1.13 | 0.44 |  | | 0.12 | | -0.02 | -0.01 | 0.04 | 0.06 | -0.08 | 0.05 | 1 |  |  |
| (9) | Sex | 1480 | 1.53 | 0.50 | 2 | 1 | 2 | -0.14 | -1.98 | 0.01 |  | | 0.13 | | 0.12 | 0.1 | -0.16 | 0.1 | 0.01 | -0.13 | -0.18 | 1 |  |

| **Belgium** | |  |  |  |  |  |  |  |  |  |  |  |  |  |  |  |  |  |  |  |
| --- | --- | --- | --- | --- | --- | --- | --- | --- | --- | --- | --- | --- | --- | --- | --- | --- | --- | --- | --- | --- |
|  |  | **Descriptives** | | | | | | | | |  | **Bivariate correlations** | | | | | | | | |
|  |  | *n* | *M* | *SD* | *Median* | *Min* | *Max* | *Skew* | *Kurtosis* | *SE* |  | (1) | (2) | (3) | (4) | (5) | (6) | (7) | (8) | (9) |
| (1) | Physical contact | 950 | 0 | 1 | 0.15 | -3.85 | 1.86 | -0.68 | 0.19 | 0.03 |  | 1 |  |  |  |  |  |  |  |  |
| (2) | Physical hygiene | 950 | 0 | 1 | 0.05 | -3.74 | 2.22 | -0.47 | 0.19 | 0.03 |  | 0.53 | 1 |  |  |  |  |  |  |  |
| (3) | Policy support | 950 | 0 | 1 | 0.16 | -3.80 | 1.72 | -0.82 | 0.61 | 0.03 |  | 0.41 | 0.28 | 1 |  |  |  |  |  |  |
| (4) | CRT | 950 | 1.68 | 1.12 | 2 | 0 | 3 | -0.22 | -1.32 | 0.04 |  | -0.03 | -0.15 | -0.04 | 1 |  |  |  |  |  |
| (5) | Open-mindedness | 950 | 0 | 1 | 0.10 | -3.91 | 1.77 | -0.61 | 0.10 | 0.03 |  | 0.37 | 0.2 | 0.12 | 0.11 | 1 |  |  |  |  |
| (6) | Conspiracy beliefs | 950 | 0 | 1 | -0.28 | -1.86 | 4.56 | 1.11 | 1.05 | 0.03 |  | -0.17 | -0.02 | -0.23 | -0.2 | -0.18 | 1 |  |  |  |
| (7) | Political orientation | 950 | 5.01 | 2.67 | 5 | 0 | 10 | -0.12 | -1.00 | 0.09 |  | -0.07 | 0 | -0.12 | -0.06 | -0.15 | 0.16 | 1 |  |  |
| (8) | Age | 950 | 46.22 | 18.91 | 48 | 18 | 87 | -0.02 | -1.42 | 0.61 |  | -0.01 | 0.01 | -0.11 | -0.17 | -0.14 | 0.1 | 0.11 | 1 |  |
| (9) | Sex | 950 | 1.43 | 0.49 | 1 | 1 | 2 | 0.29 | -1.92 | 0.02 |  | 0.14 | 0.16 | 0.14 | -0.12 | 0.12 | -0.03 | -0.18 | -0.39 | 1 |

| **Canada** | |  |  |  |  |  |  |  |  |  |  |  |  |  |  |  |  |  |  |  |
| --- | --- | --- | --- | --- | --- | --- | --- | --- | --- | --- | --- | --- | --- | --- | --- | --- | --- | --- | --- | --- |
|  |  | **Descriptives** | | | | | | | | |  | **Bivariate correlations** | | | | | | | | |
|  |  | *n* | *M* | *SD* | *Median* | *Min* | *Max* | *Skew* | *Kurtosis* | *SE* |  | (1) | (2) | (3) | (4) | (5) | (6) | (7) | (8) | (9) |
| (1) | Physical contact | 740 | 0 | 1 | 0.22 | -3.40 | 1.54 | -0.94 | 0.49 | 0.04 |  | 1 |  |  |  |  |  |  |  |  |
| (2) | Physical hygiene | 740 | 0 | 1 | 0.17 | -5.20 | 1.80 | -0.95 | 1.21 | 0.04 |  | 0.6 | 1 |  |  |  |  |  |  |  |
| (3) | Policy support | 740 | 0 | 1 | 0.25 | -4.41 | 1.64 | -1.19 | 1.32 | 0.04 |  | 0.6 | 0.43 | 1 |  |  |  |  |  |  |
| (4) | CRT | 740 | 0.83 | 1.01 | 0 | 0 | 3 | 0.96 | -0.28 | 0.04 |  | -0.04 | -0.12 | -0.02 | 1 |  |  |  |  |  |
| (5) | Open-mindedness | 740 | 0 | 1 | 0.06 | -4.63 | 1.89 | -0.73 | 0.61 | 0.04 |  | 0.37 | 0.35 | 0.25 | 0.04 | 1 |  |  |  |  |
| (6) | Conspiracy beliefs | 740 | 0 | 1 | -0.35 | -1.73 | 3.43 | 1.07 | 0.40 | 0.04 |  | -0.16 | -0.03 | -0.29 | -0.13 | -0.05 | 1 |  |  |  |
| (7) | Political orientation | 740 | 4.72 | 1.80 | 5 | 0 | 10 | -0.08 | 0.51 | 0.07 |  | -0.1 | -0.04 | -0.12 | -0.13 | -0.05 | 0.22 | 1 |  |  |
| (8) | Age | 740 | 44.05 | 17.55 | 42 | 18 | 99 | 0.26 | -1.02 | 0.65 |  | 0.07 | -0.07 | 0.07 | -0.05 | -0.04 | -0.08 | 0.13 | 1 |  |
| (9) | Sex | 740 | 1.64 | 0.48 | 2 | 1 | 2 | -0.57 | -1.67 | 0.02 |  | 0.1 | 0.15 | 0.06 | -0.17 | 0.09 | 0.03 | -0.08 | -0.22 | 1 |

| **Switzerland** | |  |  |  |  |  |  |  |  |  |  |  |  |  |  |  |  |  |  |  |  |
| --- | --- | --- | --- | --- | --- | --- | --- | --- | --- | --- | --- | --- | --- | --- | --- | --- | --- | --- | --- | --- | --- |
|  |  | **Descriptives** | | | | | | | | |  | **Bivariate correlations** | | | | | | | | | |
|  |  | *n* | *M* | *SD* | *Median* | *Min* | *Max* | *Skew* | *Kurtosis* | *SE* |  | (1) | (2) | (3) | (4) | (5) | (6) | (7) | (8) | (9) |  |
| (1) | Physical contact | 801 | 0 | 1 | 0.14 | -4.10 | 1.87 | -0.78 | 0.67 | 0.04 |  | 1 |  |  |  |  |  |  |  |  |  |
| (2) | Physical hygiene | 801 | 0 | 1 | 0.06 | -4.14 | 2.06 | -0.68 | 0.84 | 0.04 |  | 0.57 | 1 |  |  |  |  |  |  |  |  |
| (3) | Policy support | 801 | 0 | 1 | 0.18 | -3.68 | 1.91 | -0.96 | 0.92 | 0.04 |  | 0.62 | 0.48 | 1 |  |  |  |  |  |  |  |
| (4) | CRT | 801 | 1.12 | 1.06 | 1 | 0 | 3 | 0.52 | -0.99 | 0.04 |  | -0.02 | -0.11 | -0.06 | 1 |  |  |  |  |  |  |
| (5) | Open-mindedness | 801 | 0 | 1 | 0.15 | -3.56 | 1.89 | -0.72 | 0.30 | 0.04 |  | 0.37 | 0.27 | 0.24 | 0.09 | 1 |  |  |  |  |  |
| (6) | Conspiracy beliefs | 801 | 0 | 1 | -0.34 | -1.59 | 3.66 | 0.93 | 0.06 | 0.04 |  | -0.23 | -0.11 | -0.27 | -0.17 | -0.2 | 1 |  |  |  |  |
| (7) | Political orientation | 801 | 5.04 | 2.11 | 5 | 0 | 10 | 0.03 | 0.20 | 0.07 |  | -0.09 | -0.04 | -0.09 | -0.13 | -0.14 | 0.16 | 1 |  |  |  |
| (8) | Age | 801 | 49.33 | 16.72 | 51 | 18 | 100 | -0.04 | -0.87 | 0.59 |  | 0.14 | 0.09 | 0.01 | -0.15 | -0.03 | -0.01 | 0.03 | 1 |  |  |
| (9) | Sex | 801 | 1.52 | 0.50 | 2 | 1 | 2 | -0.07 | -2.00 | 0.02 |  | 0.11 | 0.08 | 0.04 | -0.17 | 0.12 | -0.02 | -0.07 | -0.13 | 1 |  |

| **Germany** | |  |  |  |  |  |  |  |  |  |  |  |  |  |  |  |  |  |  |  |
| --- | --- | --- | --- | --- | --- | --- | --- | --- | --- | --- | --- | --- | --- | --- | --- | --- | --- | --- | --- | --- |
|  |  | **Descriptives** | | | | | | | | |  | **Bivariate correlations** | | | | | | | | |
|  |  | *n* | *M* | *SD* | *Median* | *Min* | *Max* | *Skew* | *Kurtosis* | *SE* |  | (1) | (2) | (3) | (4) | (5) | (6) | (7) | (8) | (9) |
| (1) | Physical contact | 1117 | 0 | 1 | 0.14 | -3.95 | 2.00 | -0.61 | 0.16 | 0.03 |  | 1 |  |  |  |  |  |  |  |  |
| (2) | Physical hygiene | 1117 | 0 | 1 | 0.03 | -3.76 | 2.07 | -0.39 | 0.22 | 0.03 |  | 0.6 | 1 |  |  |  |  |  |  |  |
| (3) | Policy support | 1117 | 0 | 1 | 0.11 | -3.22 | 1.80 | -0.63 | 0.09 | 0.03 |  | 0.6 | 0.41 | 1 |  |  |  |  |  |  |
| (4) | CRT | 1117 | 1.25 | 1.07 | 1 | 0 | 3 | 0.36 | -1.13 | 0.03 |  | -0.11 | -0.18 | -0.09 | 1 |  |  |  |  |  |
| (5) | Open-mindedness | 1117 | 0 | 1 | 0.14 | -4.77 | 2.08 | -0.79 | 0.90 | 0.03 |  | 0.3 | 0.22 | 0.14 | 0.07 | 1 |  |  |  |  |
| (6) | Conspiracy beliefs | 1117 | 0 | 1 | -0.33 | -1.50 | 3.97 | 1.49 | 1.90 | 0.03 |  | -0.21 | -0.1 | -0.36 | -0.1 | -0.32 | 1 |  |  |  |
| (7) | Political orientation | 1117 | 4.69 | 1.73 | 5 | 0 | 10 | -0.02 | 0.60 | 0.05 |  | -0.04 | 0.02 | -0.05 | -0.07 | -0.11 | 0.17 | 1 |  |  |
| (8) | Age | 1117 | 50.07 | 16.15 | 52 | 18 | 83 | -0.31 | -1.09 | 0.48 |  | 0.13 | 0.07 | 0.04 | -0.17 | 0.04 | 0.08 | 0.04 | 1 |  |
| (9) | Sex | 1117 | 1.49 | 0.50 | 1 | 1 | 2 | 0.04 | -2.00 | 0.01 |  | 0.14 | 0.21 | 0.05 | -0.23 | 0.01 | 0.1 | 0.06 | -0.02 | 1 |

| **Greece** | |  |  |  |  |  |  |  |  |  |  |  |  |  |  |  |  |  |  |  |
| --- | --- | --- | --- | --- | --- | --- | --- | --- | --- | --- | --- | --- | --- | --- | --- | --- | --- | --- | --- | --- |
|  |  | **Descriptives** | | | | | | | | |  | **Bivariate correlations** | | | | | | | | |
|  |  | *n* | *M* | *SD* | *Median* | *Min* | *Max* | *Skew* | *Kurtosis* | *SE* |  | (1) | (2) | (3) | (4) | (5) | (6) | (7) | (8) | (9) |
| (1) | Physical contact | 479 | 0 | 1 | 0.19 | -4.22 | 1.81 | -0.91 | phc | 0.05 |  | 1 |  |  |  |  |  |  |  |  |
| (2) | Physical hygiene | 479 | 0 | 1 | 0.21 | -3.17 | 2.04 | -0.73 | phg | 0.05 |  | 0.43 | 1 |  |  |  |  |  |  |  |
| (3) | Policy support | 479 | 0 | 1 | 0.17 | -3.66 | 1.69 | -0.83 | phs | 0.05 |  | 0.47 | 0.56 | 1 |  |  |  |  |  |  |
| (4) | CRT | 479 | 1.82 | 1.18 | 2 | 0 | 3 | -0.43 | crt | 0.05 |  | 0.02 | -0.13 | -0.05 | 1 |  |  |  |  |  |
| (5) | Open-mindedness | 479 | 0 | 1 | 0.12 | -3.11 | 2.11 | -0.62 | om1 | 0.05 |  | 0.23 | 0.35 | 0.18 | 0.02 | 1 |  |  |  |  |
| (6) | Conspiracy beliefs | 479 | 0 | 1 | -0.26 | -1.73 | 3.83 | 1.02 | consp | 0.05 |  | -0.19 | -0.11 | -0.22 | -0.21 | -0.07 | 1 |  |  |  |
| (7) | Political orientation | 479 | 4.30 | 1.95 | 4 | 0 | 10 | 0.00 | polid | 0.09 |  | 0.01 | 0 | 0.08 | -0.23 | -0.06 | 0.21 | 1 |  |  |
| (8) | Age | 479 | 30.05 | 11.72 | 26 | 18 | 80 | 1.92 | age | 0.54 |  | 0.07 | 0.16 | 0.06 | -0.11 | -0.01 | 0 | 0.06 | 1 |  |
| (9) | Sex | 479 | 1.38 | 0.49 | 1 | 1 | 2 | 0.49 | sex | 0.02 |  | 0.03 | 0.05 | -0.01 | -0.12 | -0.01 | 0.13 | -0.07 | 0 | 1 |

| **Iraq** | |  |  |  |  |  |  |  |  |  |  |  |  |  |  |  |  |  |  |  |
| --- | --- | --- | --- | --- | --- | --- | --- | --- | --- | --- | --- | --- | --- | --- | --- | --- | --- | --- | --- | --- |
|  |  | **Descriptives** | | | | | | | | |  | **Bivariate correlations** | | | | | | | | |
|  |  | *n* | *M* | *SD* | *Median* | *Min* | *Max* | *Skew* | *Kurtosis* | *SE* |  | (1) | (2) | (3) | (4) | (5) | (6) | (7) | (8) | (9) |
| (1) | Physical contact | 320 | 0 | 1 | 0.09 | -3.48 | 2.18 | -0.39 | 0.19 | 0.06 |  | 1 |  |  |  |  |  |  |  |  |
| (2) | Physical hygiene | 320 | 0 | 1 | 0.08 | -2.79 | 2.00 | -0.40 | -0.17 | 0.06 |  | 0.7 | 1 |  |  |  |  |  |  |  |
| (3) | Policy support | 320 | 0 | 1 | 0.20 | -4.80 | 1.67 | -1.63 | 3.82 | 0.06 |  | 0.51 | 0.38 | 1 |  |  |  |  |  |  |
| (4) | CRT | 320 | 0.36 | 0.70 | 0 | 0 | 3 | 2.00 | 3.48 | 0.04 |  | 0.09 | -0.01 | 0.11 | 1 |  |  |  |  |  |
| (5) | Open-mindedness | 320 | 0 | 1 | 0.16 | -3.81 | 2.05 | -0.76 | 0.51 | 0.06 |  | 0.19 | 0.1 | 0.26 | 0.16 | 1 |  |  |  |  |
| (6) | Conspiracy beliefs | 320 | 0 | 1 | 0.10 | -1.99 | 2.42 | 0.09 | -0.77 | 0.06 |  | -0.32 | -0.16 | -0.16 | -0.12 | 0.02 | 1 |  |  |  |
| (7) | Political orientation | 320 | 5.26 | 2.74 | 5 | 0 | 10 | -0.10 | -0.38 | 0.15 |  | -0.08 | 0 | -0.07 | 0.02 | -0.02 | 0.03 | 1 |  |  |
| (8) | Age | 320 | 32.40 | 13.78 | 28 | 18 | 100 | 1.83 | 5.44 | 0.77 |  | 0.1 | 0.07 | 0.01 | 0.16 | 0.01 | -0.12 | -0.02 | 1 |  |
| (9) | Sex | 320 | 1.47 | 0.50 | 1 | 1 | 2 | 0.14 | -1.99 | 0.03 |  | 0.09 | 0.11 | 0.06 | -0.17 | 0.01 | -0.02 | -0.11 | -0.29 | 1 |

| **Israel** | |  |  |  |  |  |  |  |  |  |  |  |  |  |  |  |  |  |  |  |
| --- | --- | --- | --- | --- | --- | --- | --- | --- | --- | --- | --- | --- | --- | --- | --- | --- | --- | --- | --- | --- |
|  |  | **Descriptives** | | | | | | | | |  | **Bivariate correlations** | | | | | | | | |
|  |  | *n* | *M* | *SD* | *Median* | *Min* | *Max* | *Skew* | *Kurtosis* | *SE* |  | (1) | (2) | (3) | (4) | (5) | (6) | (7) | (8) | (9) |
| (1) | Physical contact | 994 | 0 | 1 | 0.14 | -3.62 | 2.00 | -0.67 | 0.20 | 0.03 |  | 1 |  |  |  |  |  |  |  |  |
| (2) | Physical hygiene | 994 | 0 | 1 | 0.10 | -4.28 | 1.94 | -0.57 | 0.26 | 0.03 |  | 0.59 | 1 |  |  |  |  |  |  |  |
| (3) | Policy support | 994 | 0 | 1 | 0.17 | -3.34 | 1.89 | -0.69 | -0.07 | 0.03 |  | 0.43 | 0.29 | 1 |  |  |  |  |  |  |
| (4) | CRT | 994 | 1.24 | 1.13 | 1 | 0 | 3 | 0.31 | -1.33 | 0.04 |  | 0 | -0.14 | -0.05 | 1 |  |  |  |  |  |
| (5) | Open-mindedness | 994 | 0 | 1 | 0.18 | -4.07 | 1.68 | -0.70 | 0.10 | 0.03 |  | 0.28 | 0.19 | 0.13 | 0.11 | 1 |  |  |  |  |
| (6) | Conspiracy beliefs | 994 | 0 | 1 | -0.18 | -2.32 | 3.46 | 0.71 | 0.20 | 0.03 |  | -0.13 | 0.06 | -0.04 | -0.2 | -0.13 | 1 |  |  |  |
| (7) | Political orientation | 994 | 5.65 | 2.81 | 6 | 0 | 10 | -0.24 | -0.91 | 0.09 |  | -0.02 | -0.03 | 0.09 | -0.11 | -0.11 | 0.08 | 1 |  |  |
| (8) | Age | 994 | 41.27 | 15.05 | 40 | 18 | 74 | 0.31 | -1.06 | 0.48 |  | -0.01 | -0.02 | -0.17 | -0.01 | 0 | -0.06 | -0.16 | 1 |  |
| (9) | Sex | 994 | 1.52 | 0.50 | 2 | 1 | 2 | -0.08 | -2.00 | 0.02 |  | 0.12 | 0.12 | 0.07 | -0.18 | 0.18 | 0.09 | -0.15 | -0.03 | 1 |

| **Japan** | |  |  |  |  |  |  |  |  |  |  |  |  |  |  |  |  |  |  |  |
| --- | --- | --- | --- | --- | --- | --- | --- | --- | --- | --- | --- | --- | --- | --- | --- | --- | --- | --- | --- | --- |
|  |  | **Descriptives** | | | | | | | | |  | **Bivariate correlations** | | | | | | | | |
|  |  | *n* | *M* | *SD* | *Median* | *Min* | *Max* | *Skew* | *Kurtosis* | *SE* |  | (1) | (2) | (3) | (4) | (5) | (6) | (7) | (8) | (9) |
| (1) | Physical contact | 747 | 0 | 1 | -0.01 | -3.07 | 2.41 | -0.06 | -0.47 | 0.04 |  | 1 |  |  |  |  |  |  |  |  |
| (2) | Physical hygiene | 747 | 0 | 1 | -0.04 | -3.40 | 2.36 | 0.03 | -0.01 | 0.04 |  | 0.64 | 1 |  |  |  |  |  |  |  |
| (3) | Policy support | 747 | 0 | 1 | 0.04 | -3.46 | 2.28 | -0.21 | -0.29 | 0.04 |  | 0.56 | 0.48 | 1 |  |  |  |  |  |  |
| (4) | CRT | 747 | 1.02 | 0.89 | 1 | 0 | 3 | 0.42 | -0.76 | 0.03 |  | -0.14 | -0.14 | -0.09 | 1 |  |  |  |  |  |
| (5) | Open-mindedness | 747 | 0 | 1 | -0.08 | -3.01 | 2.82 | 0.18 | -0.33 | 0.04 |  | 0.37 | 0.32 | 0.29 | 0.05 | 1 |  |  |  |  |
| (6) | Conspiracy beliefs | 747 | 0 | 1 | -0.01 | -2.61 | 3.29 | 0.18 | -0.29 | 0.04 |  | -0.1 | 0 | -0.11 | -0.13 | -0.34 | 1 |  |  |  |
| (7) | Political orientation | 747 | 5.14 | 1.20 | 5 | 0 | 10 | 0.04 | 3.20 | 0.04 |  | -0.01 | 0.05 | 0.02 | 0.05 | 0.04 | -0.01 | 1 |  |  |
| (8) | Age | 747 | 50.43 | 14.61 | 52 | 18 | 74 | -0.36 | -0.93 | 0.53 |  | 0.04 | -0.02 | 0.1 | 0.14 | 0.08 | -0.04 | 0.1 | 1 |  |
| (9) | Sex | 747 | 1.52 | 0.50 | 2 | 1 | 2 | -0.08 | -2.00 | 0.02 |  | 0.17 | 0.2 | 0.16 | -0.2 | 0.09 | 0.13 | -0.13 | -0.19 | 1 |

| **South Korea** | |  |  |  |  |  |  |  |  |  |  |  |  |  |  |  |  |  |  |  |
| --- | --- | --- | --- | --- | --- | --- | --- | --- | --- | --- | --- | --- | --- | --- | --- | --- | --- | --- | --- | --- |
|  |  | **Descriptives** | | | | | | | | |  | **Bivariate correlations** | | | | | | | | |
|  |  | *n* | *M* | *SD* | *Median* | *Min* | *Max* | *Skew* | *Kurtosis* | *SE* |  | (1) | (2) | (3) | (4) | (5) | (6) | (7) | (8) | (9) |
| (1) | Physical contact | 253 | 0 | 1 | -0.06 | -2.07 | 2.21 | 0.07 | -0.83 | 0.06 |  | 1 |  |  |  |  |  |  |  |  |
| (2) | Physical hygiene | 253 | 0 | 1 | 0.01 | -2.31 | 2.21 | -0.04 | -0.77 | 0.06 |  | 0.69 | 1 |  |  |  |  |  |  |  |
| (3) | Policy support | 253 | 0 | 1 | 0.02 | -3.23 | 2.03 | -0.28 | -0.42 | 0.06 |  | 0.65 | 0.63 | 1 |  |  |  |  |  |  |
| (4) | CRT | 253 | 0.92 | 1.02 | 1 | 0 | 3 | 0.79 | -0.61 | 0.06 |  | -0.07 | -0.09 | -0.07 | 1 |  |  |  |  |  |
| (5) | Open-mindedness | 253 | 0 | 1 | 0.02 | -2.78 | 2.52 | -0.01 | -0.32 | 0.06 |  | 0.45 | 0.55 | 0.36 | 0.06 | 1 |  |  |  |  |
| (6) | Conspiracy beliefs | 253 | 0 | 1 | -0.09 | -2.25 | 3.71 | 0.44 | 0.14 | 0.06 |  | -0.19 | -0.19 | -0.12 | -0.1 | -0.27 | 1 |  |  |  |
| (7) | Political orientation | 253 | 5.03 | 2.16 | 5 | 0 | 10 | -0.08 | 0.02 | 0.14 |  | -0.08 | -0.06 | -0.04 | 0.04 | -0.08 | 0.05 | 1 |  |  |
| (8) | Age | 253 | 46.16 | 12.87 | 47 | 18 | 72 | -0.33 | -0.71 | 0.81 |  | -0.03 | -0.04 | -0.04 | 0.01 | 0.01 | -0.17 | 0.17 | 1 |  |
| (9) | Sex | 253 | 1.47 | 0.50 | 1 | 1 | 2 | 0.12 | -1.99 | 0.03 |  | 0.12 | 0.22 | 0.08 | -0.07 | -0.03 | 0 | -0.22 | -0.26 | 1 |

| **Nigeria** | |  |  |  |  |  |  |  |  |  |  | |  | |  |  |  |  |  |  |  |  |  |
| --- | --- | --- | --- | --- | --- | --- | --- | --- | --- | --- | --- | --- | --- | --- | --- | --- | --- | --- | --- | --- | --- | --- | --- |
|  |  | **Descriptives** | | | | | | | | | |  | | **Bivariate correlations** | | | | | | | | | |
|  |  | *n* | *M* | *SD* | *Median* | *Min* | *Max* | *Skew* | *Kurtosis* | *SE* |  | | (1) | | (2) | (3) | (4) | (5) | (6) | (7) | (8) | (9) |  |
| (1) | Physical contact | 403 | 0 | 1 | 0.12 | -3.08 | 2.03 | -0.53 | -0.24 | 0.05 |  | | 1 | |  |  |  |  |  |  |  |  |  |
| (2) | Physical hygiene | 403 | 0 | 1 | 0.14 | -3.86 | 1.68 | -0.70 | 0.36 | 0.05 |  | | 0.58 | | 1 |  |  |  |  |  |  |  |  |
| (3) | Policy support | 403 | 0 | 1 | 0.28 | -4.42 | 1.47 | -1.61 | 2.75 | 0.05 |  | | 0.34 | | 0.35 | 1 |  |  |  |  |  |  |  |
| (4) | CRT | 403 | 0.54 | 0.80 | 0 | 0 | 3 | 1.47 | 1.48 | 0.04 |  | | -0.01 | | -0.12 | -0.07 | 1 |  |  |  |  |  |  |
| (5) | Open-mindedness | 403 | 0 | 1 | 0.13 | -3.60 | 1.71 | -0.58 | 0.11 | 0.05 |  | | 0.41 | | 0.38 | 0.29 | 0.01 | 1 |  |  |  |  |  |
| (6) | Conspiracy beliefs | 403 | 0 | 1 | 0.08 | -2.40 | 2.70 | 0.03 | -0.50 | 0.05 |  | | -0.15 | | -0.01 | -0.09 | -0.12 | -0.08 | 1 |  |  |  |  |
| (7) | Political orientation | 403 | 6.16 | 2.16 | 6 | 0 | 10 | -0.16 | 0.07 | 0.11 |  | | 0.01 | | 0.12 | 0.06 | -0.13 | 0.06 | 0.04 | 1 |  |  |  |
| (8) | Age | 403 | 32.63 | 11.37 | 30 | 18 | 100 | 1.29 | 2.58 | 0.57 |  | | 0.04 | | 0.02 | 0.08 | 0.05 | -0.02 | 0.02 | 0.16 | 1 |  |  |
| (9) | Sex | 403 | 1.49 | 0.50 | 1 | 1 | 2 | 0.05 | -2.00 | 0.02 |  | | 0.06 | | 0.01 | -0.05 | -0.07 | 0.06 | -0.06 | -0.12 | -0.31 | 1 |  |

| **New Zealand** | |  |  |  |  |  |  |  |  |  |  |  |  |  |  |  |  |  |  |  |  |
| --- | --- | --- | --- | --- | --- | --- | --- | --- | --- | --- | --- | --- | --- | --- | --- | --- | --- | --- | --- | --- | --- |
|  |  | **Descriptives** | | | | | | | | |  | **Bivariate correlations** | | | | | | | | | |
|  |  | *n* | *M* | *SD* | *Median* | *Min* | *Max* | *Skew* | *Kurtosis* | *SE* |  | (1) | (2) | (3) | (4) | (5) | (6) | (7) | (8) | (9) |  |
| (1) | Physical contact | 333 | 0 | 1 | 0.22 | -3.22 | 2.08 | -0.73 | 0.06 | 0.05 |  | 1 |  |  |  |  |  |  |  |  |  |
| (2) | Physical hygiene | 333 | 0 | 1 | 0.14 | -3.36 | 1.60 | -0.58 | 0.14 | 0.05 |  | 0.38 | 1 |  |  |  |  |  |  |  |  |
| (3) | Policy support | 333 | 0 | 1 | 0.22 | -3.38 | 1.49 | -0.87 | 0.34 | 0.05 |  | 0.74 | 0.26 | 1 |  |  |  |  |  |  |  |
| (4) | CRT | 333 | 0.95 | 1.03 | 1 | 0 | 3 | 0.71 | -0.74 | 0.06 |  | 0.04 | -0.13 | 0 | 1 |  |  |  |  |  |  |
| (5) | Open-mindedness | 333 | 0 | 1 | 0.08 | -2.90 | 1.90 | -0.37 | -0.46 | 0.05 |  | 0.58 | 0.26 | 0.52 | 0.08 | 1 |  |  |  |  |  |
| (6) | Conspiracy beliefs | 333 | 0 | 1 | -0.23 | -2.08 | 3.42 | 0.90 | 0.62 | 0.05 |  | -0.13 | 0.09 | -0.18 | -0.14 | -0.12 | 1 |  |  |  |  |
| (7) | Political orientation | 333 | 5.04 | 1.99 | 5 | 0 | 10 | -0.09 | 0.54 | 0.11 |  | -0.08 | -0.04 | -0.14 | 0 | -0.15 | 0.16 | 1 |  |  |  |
| (8) | Age | 333 | 49.52 | 16.96 | 48 | 18 | 89 | 0.07 | -1.00 | 0.93 |  | 0.03 | -0.18 | 0.02 | 0.07 | 0.02 | -0.16 | -0.04 | 1 |  |  |
| (9) | Sex | 333 | 1.46 | 0.50 | 1 | 1 | 2 | 0.15 | -1.98 | 0.03 |  | -0.18 | -0.12 | -0.13 | 0.07 | -0.16 | -0.01 | 0.1 | 0.07 | 1 |  |

| **Pakistan** | |  |  |  |  |  |  |  |  |  |  |  |  |  |  |  |  |  |  |  |
| --- | --- | --- | --- | --- | --- | --- | --- | --- | --- | --- | --- | --- | --- | --- | --- | --- | --- | --- | --- | --- |
|  |  | **Descriptives** | | | | | | | | |  | **Bivariate correlations** | | | | | | | | |
|  |  | *n* | *M* | *SD* | *Median* | *Min* | *Max* | *Skew* | *Kurtosis* | *SE* |  | (1) | (2) | (3) | (4) | (5) | (6) | (7) | (8) | (9) |
| (1) | Physical contact | 367 | 0 | 1 | 0.22 | -4.07 | 1.66 | -0.79 | phc | 0.05 |  | 1 |  |  |  |  |  |  |  |  |
| (2) | Physical hygiene | 367 | 0 | 1 | 0.13 | -3.37 | 1.99 | -0.68 | phg | 0.05 |  | 0.46 | 1 |  |  |  |  |  |  |  |
| (3) | Policy support | 367 | 0 | 1 | 0.28 | -4.15 | 1.50 | -1.36 | phs | 0.05 |  | 0.26 | 0.26 | 1 |  |  |  |  |  |  |
| (4) | CRT | 367 | 0.94 | 1.01 | 1 | 0 | 3 | 0.76 | crt | 0.05 |  | 0.14 | -0.06 | 0.07 | 1 |  |  |  |  |  |
| (5) | Open-mindedness | 367 | 0 | 1 | 0.13 | -3.84 | 2.10 | -0.71 | om1 | 0.05 |  | 0.31 | 0.2 | 0.44 | 0.16 | 1 |  |  |  |  |
| (6) | Conspiracy beliefs | 367 | 0 | 1 | 0.05 | -2.21 | 2.71 | 0.15 | consp | 0.05 |  | -0.05 | -0.01 | -0.05 | -0.21 | -0.05 | 1 |  |  |  |
| (7) | Political orientation | 367 | 5.41 | 2.18 | 5 | 0 | 10 | -0.27 | polid | 0.11 |  | -0.03 | 0.01 | -0.08 | -0.09 | -0.11 | 0.11 | 1 |  |  |
| (8) | Age | 367 | 26.97 | 8.41 | 24 | 18 | 100 | 3.15 | age | 0.44 |  | -0.12 | 0.01 | -0.09 | -0.04 | -0.02 | 0 | 0.03 | 1 |  |
| (9) | Sex | 367 | 1.56 | 0.50 | 2 | 1 | 2 | -0.23 | sex | 0.03 |  | 0.17 | 0.08 | 0.08 | -0.17 | 0.04 | 0.04 | -0.06 | -0.2 | 1 |

| **Poland** | |  |  |  |  |  |  |  |  |  |  |  |  |  |  |  |  |  |  |  |  |
| --- | --- | --- | --- | --- | --- | --- | --- | --- | --- | --- | --- | --- | --- | --- | --- | --- | --- | --- | --- | --- | --- |
|  |  | **Descriptives** | | | | | | | | |  | **Bivariate correlations** | | | | | | | | | |
|  |  | *n* | *M* | *SD* | *Median* | *Min* | *Max* | *Skew* | *Kurtosis* | *SE* |  | (1) | (2) | (3) | (4) | (5) | (6) | (7) | (8) | (9) |  |
| (1) | Physical contact | 1394 | 0 | 1 | 0.11 | -4.15 | 2.14 | -0.68 | 0.33 | 0.03 |  | 1 |  |  |  |  |  |  |  |  |  |
| (2) | Physical hygiene | 1394 | 0 | 1 | 0.10 | -3.58 | 2.02 | -0.56 | -0.09 | 0.03 |  | 0.68 | 1 |  |  |  |  |  |  |  |  |
| (3) | Policy support | 1394 | 0 | 1 | 0.09 | -3.72 | 2.50 | -0.57 | 0.06 | 0.03 |  | 0.63 | 0.53 | 1 |  |  |  |  |  |  |  |
| (4) | CRT | 1394 | 0.81 | 0.97 | 1 | 0 | 3 | 0.98 | -0.16 | 0.03 |  | -0.05 | -0.12 | -0.1 | 1 |  |  |  |  |  |  |
| (5) | Open-mindedness | 1394 | 0 | 1 | 0.11 | -3.30 | 2.19 | -0.41 | -0.34 | 0.03 |  | 0.49 | 0.3 | 0.28 | 0.08 | 1 |  |  |  |  |  |
| (6) | Conspiracy beliefs | 1394 | 0 | 1 | 0.05 | -2.33 | 2.68 | 0.01 | -0.58 | 0.03 |  | -0.17 | -0.06 | -0.19 | -0.2 | -0.15 | 1 |  |  |  |  |
| (7) | Political orientation | 1394 | 5.07 | 2.43 | 5 | 0 | 10 | 0.09 | -0.25 | 0.07 |  | 0.06 | 0.06 | 0.11 | -0.05 | 0 | 0.09 | 1 |  |  |  |
| (8) | Age | 1394 | 47.61 | 16.99 | 47 | 18 | 100 | -0.02 | -0.99 | 0.45 |  | 0.1 | 0.03 | 0 | 0.03 | 0.08 | -0.1 | -0.07 | 1 |  |  |
| (9) | Sex | 1394 | 1.50 | 0.50 | 2 | 1 | 2 | -0.02 | -2.00 | 0.01 |  | 0.21 | 0.22 | 0.17 | -0.16 | 0.16 | 0.11 | -0.04 | -0.13 | 1 |  |

| **Singapore** | |  |  |  |  |  |  |  |  |  |  |  |  |  |  |  |  |  |  |  |
| --- | --- | --- | --- | --- | --- | --- | --- | --- | --- | --- | --- | --- | --- | --- | --- | --- | --- | --- | --- | --- |
|  |  | **Descriptives** | | | | | | | | |  | **Bivariate correlations** | | | | | | | | |
|  |  | *n* | *M* | *SD* | *Median* | *Min* | *Max* | *Skew* | *Kurtosis* | *SE* |  | (1) | (2) | (3) | (4) | (5) | (6) | (7) | (8) | (9) |
| (1) | Physical contact | 373 | 0 | 1 | 0.08 | -2.70 | 2.01 | -0.31 | -0.60 | 0.05 |  | 1 |  |  |  |  |  |  |  |  |
| (2) | Physical hygiene | 373 | 0 | 1 | -0.03 | -2.79 | 2.24 | -0.10 | -0.38 | 0.05 |  | 0.56 | 1 |  |  |  |  |  |  |  |
| (3) | Policy support | 373 | 0 | 1 | 0.10 | -4.46 | 1.95 | -0.68 | 0.76 | 0.05 |  | 0.53 | 0.49 | 1 |  |  |  |  |  |  |
| (4) | CRT | 373 | 1.30 | 1.18 | 1 | 0 | 3 | 0.29 | -1.41 | 0.06 |  | -0.12 | -0.17 | -0.12 | 1 |  |  |  |  |  |
| (5) | Open-mindedness | 373 | 0 | 1 | 0.02 | -3.23 | 2.48 | -0.09 | -0.08 | 0.05 |  | 0.44 | 0.35 | 0.33 | 0.02 | 1 |  |  |  |  |
| (6) | Conspiracy beliefs | 373 | 0 | 1 | -0.08 | -2.52 | 2.73 | 0.21 | -0.59 | 0.05 |  | -0.23 | 0.02 | -0.1 | -0.17 | -0.27 | 1 |  |  |  |
| (7) | Political orientation | 373 | 5.53 | 1.55 | 5 | 0 | 10 | -0.20 | 1.50 | 0.08 |  | 0.05 | 0.14 | 0.12 | -0.23 | -0.07 | 0.09 | 1 |  |  |
| (8) | Age | 373 | 43.43 | 13.59 | 46 | 18 | 73 | -0.01 | -0.88 | 0.7 |  | 0.05 | 0.05 | 0.09 | -0.15 | -0.04 | -0.04 | 0.09 | 1 |  |
| (9) | Sex | 373 | 1.51 | 0.50 | 2 | 1 | 2 | -0.05 | -2.00 | 0.03 |  | 0.15 | 0.16 | 0.12 | -0.13 | 0.08 | 0.05 | -0.04 | -0.03 | 1 |

| **Slovakia** | |  |  |  |  |  |  |  |  |  |  |  |  |  |  |  |  |  |  |  |
| --- | --- | --- | --- | --- | --- | --- | --- | --- | --- | --- | --- | --- | --- | --- | --- | --- | --- | --- | --- | --- |
|  |  | **Descriptives** | | | | | | | | |  | **Bivariate correlations** | | | | | | | | |
|  |  | *n* | *M* | *SD* | *Median* | *Min* | *Max* | *Skew* | *Kurtosis* | *SE* |  | (1) | (2) | (3) | (4) | (5) | (6) | (7) | (8) | (9) |
| (1) | Physical contact | 834 | 0 | 1 | 0.20 | -3.69 | 2.06 | -0.81 | 0.29 | 0.03 |  | 1 |  |  |  |  |  |  |  |  |
| (2) | Physical hygiene | 834 | 0 | 1 | 0.10 | -4.66 | 1.84 | -0.62 | 0.23 | 0.03 |  | 0.55 | 1 |  |  |  |  |  |  |  |
| (3) | Policy support | 834 | 0 | 1 | 0.11 | -3.55 | 2.17 | -0.74 | 0.52 | 0.03 |  | 0.51 | 0.36 | 1 |  |  |  |  |  |  |
| (4) | CRT | 834 | 0.71 | 0.93 | 0 | 0 | 3 | 1.19 | 0.41 | 0.03 |  | -0.06 | -0.09 | -0.03 | 1 |  |  |  |  |  |
| (5) | Open-mindedness | 834 | 0 | 1 | 0.13 | -3.72 | 2.36 | -0.50 | -0.08 | 0.03 |  | 0.39 | 0.37 | 0.18 | 0.08 | 1 |  |  |  |  |
| (6) | Conspiracy beliefs | 834 | 0 | 1 | 0.01 | -2.34 | 2.60 | 0.19 | -0.74 | 0.03 |  | -0.07 | -0.01 | -0.31 | -0.07 | -0.09 | 1 |  |  |  |
| (7) | Political orientation | 834 | 5.25 | 1.93 | 5 | 0 | 10 | -0.03 | 0.89 | 0.07 |  | 0.03 | 0.01 | 0.06 | -0.01 | -0.04 | -0.12 | 1 |  |  |
| (8) | Age | 834 | 44.31 | 15.91 | 43 | 18 | 88 | 0.13 | -1.12 | 0.55 |  | 0.07 | -0.01 | -0.04 | 0.02 | -0.02 | 0.14 | -0.21 | 1 |  |
| (9) | Sex | 834 | 1.52 | 0.50 | 2 | 1 | 2 | -0.08 | -2.00 | 0.02 |  | 0.13 | 0.17 | 0.03 | -0.21 | 0.05 | 0.09 | -0.02 | -0.01 | 1 |

| **USA** | |  |  |  |  |  |  |  |  |  |  |  |  |  |  |  |  |  |  |  |  |
| --- | --- | --- | --- | --- | --- | --- | --- | --- | --- | --- | --- | --- | --- | --- | --- | --- | --- | --- | --- | --- | --- |
|  |  | **Descriptives** | | | | | | | | |  | **Bivariate correlations** | | | | | | | | | |
|  |  | *n* | *M* | *SD* | *Median* | *Min* | *Max* | *Skew* | *Kurtosis* | *SE* |  | (1) | (2) | (3) | (4) | (5) | (6) | (7) | (8) | (9) |  |
| (1) | Physical contact | 905 | 0 | 1 | 0.02 | -3.22 | 2.39 | -0.34 | -0.48 | 0.03 |  | 1 |  |  |  |  |  |  |  |  |  |
| (2) | Physical hygiene | 905 | 0 | 1 | 0.13 | -4.16 | 1.82 | -0.80 | 0.79 | 0.03 |  | 0.59 | 1 |  |  |  |  |  |  |  |  |
| (3) | Policy support | 905 | 0 | 1 | 0.16 | -4.24 | 1.73 | -1.20 | 1.87 | 0.03 |  | 0.61 | 0.43 | 1 |  |  |  |  |  |  |  |
| (4) | CRT | 905 | 0.59 | 0.89 | 0 | 0 | 3 | 1.41 | 0.98 | 0.03 |  | -0.01 | -0.18 | -0.01 | 1 |  |  |  |  |  |  |
| (5) | Open-mindedness | 905 | 0 | 1 | 0.09 | -3.46 | 2.22 | -0.35 | -0.46 | 0.03 |  | 0.54 | 0.38 | 0.4 | 0.07 | 1 |  |  |  |  |  |
| (6) | Conspiracy beliefs | 905 | 0 | 1 | -0.17 | -2.94 | 3.67 | 0.66 | 0.50 | 0.03 |  | -0.3 | -0.03 | -0.24 | -0.16 | -0.26 | 1 |  |  |  |  |
| (7) | Political orientation | 905 | 5.81 | 2.63 | 5 | 0 | 10 | -0.25 | -0.55 | 0.09 |  | -0.13 | 0.05 | -0.2 | -0.15 | -0.15 | 0.26 | 1 |  |  |  |
| (8) | Age | 905 | 48.29 | 16.39 | 48 | 18 | 100 | 0.11 | -0.95 | 0.54 |  | 0.16 | 0.01 | 0.1 | 0.12 | 0.12 | -0.16 | -0.12 | 1 |  |  |
| (9) | Sex | 905 | 1.53 | 0.50 | 2 | 1 | 2 | -0.11 | -1.99 | 0.02 |  | 0.17 | 0.17 | 0.11 | -0.1 | 0.17 | -0.05 | -0.13 | -0.02 | 1 |  |
| ^a^Sex coded as males = 1 and females = 2 | | | | | | | | | | | | | | | | | | | | | |
| Intercorrelations among latent factors scores on physical contact, physical hygiene, policy support open-mindedness and conspiracy beliefs with manifest variables: CRT (sum of correct responses), political orientation, age and sex. | | | | | | | | | | | | | | | | | | | | | |

1. Two questions included in the survey as attention checks (one in the middle of the survey and the other at the end of the survey) were used to identify and exclude participants who did not put sufficient effort into responding.

   1. One of the following versions was applied depending on the application employed by the country team: Please confirm that you have read this question by moving the all the way to the left, providing the value 0 as your answer. / Please confirm that you have read this question by selecting the value 0 as your answer.
   2. Help us get rid of bots: Please write the number 213 into the comment box.

   [↑](#footnote-ref-1)
