# Supplementary material for: Analytic Thinking and Political Orientation in the Corona Crisis
Source: Front Psychol. 2021 Jul 22;12:631800. doi: 10.3389/fpsyg.2021.631800 (PMC8341110; doi:10.3389/fpsyg.2021.631800)
Supplement: Supplementary file 1 [file Data_Sheet_1.zip › Supplementary Materials/Data preparations and cleaning.html]

Data preparations - Analytic Thinking and Political Orientation in the Corona Crisis


# Data preparations - Analytic Thinking and Political Orientation in the Corona Crisis

#### Deadpool

#### 27 04 2021

## Brief introduction

This html file presents initial database preparations, before the factor extraction phase. The outputs of these analyses were used in factor extraction, which will be presented in a separate file due to size of analyses.

## Activating packages

```
lapply(c("psych", "haven", "dplyr", "mice", "careless"), library, character.only = T)
```

## Dealing with bots

```
dat <- read_sav("ICSMP_cleaned_data.sav")
dim(dat)
```

```
## [1] 51717    96
```

```
# Dealing with bots
table(dat$att_check_nobots, exclude = NULL)
```

```
## 
##     0     1  <NA> 
##   652 46928  4137
```

```
table(dat[is.na(dat$att_check_nobots), "country"])
```

```
## 
##  AE  AT  AU  BD  BE  BG BR1 BR2  CO  FI  GB  GH  GR  IE  IL  IN IN2  IQ IT1  JP 
## 126 207   3 197   2  15  11 433  36  13   1 390   3  41   1  63  45 586   5  74 
##  KR  LV  MA  MK  MX  NG  NP  PH  PK  PL  RO  RS  SE  SG  SN  TR  US  ZA 
##  56   2  42  25  59  83 214  19  84   8 500 331   2  43 100   2   4 311
```

```
table(dat$country)
```

```
## 
##    AE    AR    AT    AU    BD    BE    BG    BO   BR1   BR2   BR3   CAe   CAf 
##   313   721  1605  2161   596  1159   666    29   961  1301     6   792   171 
##    CH    CL    CN    CO   CO2    CR    CU    DE    DK    DO    EC    ES    FI 
##  1056    97  1030   731   546    25    43  1587   566    36   148  1090   698 
##    FR    GB    GH    GR    GT    HN    HR    HU    IE    IL    IN   IN2    IQ 
##  1119   550   390   640    48    24   515   506   785  1253   312   429  1142 
##   IT1   IT2    JP    KR    LV    MA mixed    MK    MX   MX2    NG    NI    NL 
##   998   284  1239   555  1008   812   313   726   804   507   608    16  1297 
##    NO    NP    NZ    PA    PE    PH    PK    PL    PR    PY    RO   RO2    RS 
##   532   563   510    18    91   524   565  1817     2    16   500   505  1070 
##    RU    SE    SG    SK    SN    SV    TR    TW    UA    US    UY    VE    ZA 
##   558  1568   564  1265   552    28  1455   833   577  1506    49    96   939
```

```
dat2 <- subset(dat, att_check_nobots == 1 | country %in% c("GH", "RO"))
dim(dat2)
```

```
## [1] 47818    96
```

## Dealing with excess missing values

```
# excluding participants with more than 25 % of missing inputs
dim(dat2)
```

```
## [1] 47818    96
```

```
dat3 <- dat2[-c(which(rowSums(is.na(dat2)) > .25*ncol(dat))), ]
dim(dat3)
```

```
## [1] 47270    96
```

## Dealing with careless participants

```
dat4 <- dat3[-which(longstring(dat3[, names(dat3)[-grep("psuppo|hygie|contac|duration|att_check|sample_c|revision_c|country|ISO3", names(dat3))]]) > 10), ]
dim(dat4)
```

```
## [1] 45687    96
```

## Dealing with too fast and too slow participants

```
describe(dat4$duration)
```

```
##    vars     n    mean       sd median trimmed    mad min    max  range skew
## X1    1 39806 4896.04 26636.59   1380 1505.86 687.93  55 604881 604826   11
##    kurtosis     se
## X1   143.03 133.51
```

```
table(dat4$country)
```

```
## 
##    AE    AR    AT    AU    BD    BE    BG    BO   BR1   BR2   BR3   CAe   CAf 
##   185   715  1370  1985   370  1142   631    29   866   853     6   779   162 
##    CH    CL    CN    CO   CO2    CR    CU    DE    DK    DO    EC    ES    FI 
##  1023    97  1030   665   540    25    42  1472   495    36   148  1081   675 
##    FR    GB    GH    GR    GT    HN    HR    HU    IE    IL    IN   IN2    IQ 
##  1035   543   193   633    48    23   494   493   727  1234   237   370   526 
##   IT1   IT2    JP    KR    LV    MA mixed    MK    MX   MX2    NG    NI    NL 
##   937   280  1059   455   927   610   185   682   730   507   509    16  1289 
##    NO    NP    NZ    PA    PE    PH    PK    PL    PR    PY    RO   RO2    RS 
##   507   340   492    18    90   482   456  1700     2    15   487   480   717 
##    RU    SE    SG    SK    SN    SV    TR    TW    UA    US    UY    VE    ZA 
##   498  1553   494  1040   301    28  1383   786   564  1343    49    96   602
```

```
table(dat4[is.na(dat4$duration), "country"])
```

```
## 
##  AR  BG  BO BR3  CL CO2  CR  CU  DE  DO  EC  GH  GT  HN  IN IN2 IT2 MX2  NI  PA 
## 715 631  29   6  97 540  25  42  53  36 148 193  48  23 237 370 280 507  16  18 
##  PE  PR  PY  RO  RU  SV  UY  VE  ZA 
##  90   2  15 487 498  28  49  96 602
```

```
dat5 <- subset(dat4, duration > 540 | country %in% c("AR", "BG", "BO", "BR3", "CL", "CO2", "CR", "CU", "DO", "EC", "GH", "GT", "HN", "IN", "IN2", "IT2", "MX2", "NI", "PA", "PE", "PR", "PY", "RO", "RU", "SV", "UY", "VE", "ZA"))
```

## Grouping countries to ensure sufficient N

```
as.data.frame(table(dat5$ISO3))[which(as.data.frame(table(dat5$ISO3))$Freq < 150), ]
```

```
##    Var1 Freq
## 8   BOL   29
## 12  CHL   97
## 15  CRI   25
## 16  CUB   42
## 19  DOM   36
## 20  ECU  148
## 27  GTM   48
## 28  HND   23
## 44  NIC   16
## 50  PAN   18
## 51  PER   90
## 54  PRI    2
## 55  PRY   15
## 60  SLV   28
## 67  URY   49
## 69  VEN   96
```

```
dat5$ISO3 <- ifelse(dat5$ISO3 %in% c("BOL", "CHL", "ECU", "PER", "PRY", "URY", "VEN"), "LATAM", ifelse(dat5$ISO3 %in% c("CRI", "CUB", "DOM", "GTM", "HND", "NIC", "PAN", "PRI", "SLV"), "CENAM", dat5$ISO3))
as.data.frame(table(dat5$ISO3))
```

```
##     Var1 Freq
## 1    ARE  183
## 2    ARG  715
## 3    AUS 1819
## 4    AUT 1368
## 5    BEL 1140
## 6    BGD  323
## 7    BGR  631
## 8    BRA 1666
## 9    CAN  941
## 10 CENAM  238
## 11   CHE  969
## 12   CHN 1030
## 13   COL 1200
## 14   DEU 1416
## 15   DNK  453
## 16   ESP 1081
## 17   FIN  662
## 18   FRA  800
## 19   GBR  543
## 20   GHA  193
## 21   GRC  614
## 22   HRV  493
## 23   HUN  479
## 24   IND  607
## 25   IRL  712
## 26   IRQ  414
## 27   ISR 1174
## 28   ITA 1137
## 29   JPN  891
## 30   KOR  308
## 31 LATAM  524
## 32   LVA  921
## 33   MAR  561
## 34   MEX 1237
## 35 mixed  183
## 36   MKD  678
## 37   NGA  509
## 38   NLD 1289
## 39   NOR  505
## 40   NPL  338
## 41   NZL  448
## 42   PAK  451
## 43   PHL  482
## 44   POL 1677
## 45   ROU  932
## 46   RUS  498
## 47   SEN  301
## 48   SGP  454
## 49   SRB  712
## 50   SVK 1010
## 51   SWE 1546
## 52   TUR 1256
## 53   TWN  786
## 54   UKR  546
## 55   USA 1147
## 56   ZAF  602
```

## Imputations (group-wise)

```
dat7 <- dat5[, sapply(dat5, is.numeric)]
dat7 <- as.data.frame(apply(dat7, 2, as.numeric))
dat7 <- cbind(dat7, dat5$ISO3)
names(dat7)[90] <- "country"
dat7s <- split.data.frame(dat7, dat7$country)

outloops <- list() #creating an empty list that will be filled by the following for loop
for(i in 1:length(dat7s)){
  m1 <- dat7s[[i]]
  set.seed(i)
  m2 <- mice(m1, m = 10, maxit = 10, method = "pmm")
  outloops[[i]] <- complete(m2, 10)
}
```

```
## 
##  iter imp variable
##   1   1  age  children  cnarc1  cnarc2  cnarc3  ctheory1  ctheory2  ctheory3  ctheory4  CRT1  CRT2  CRT3  ladder  mor_circle  moralid1  moralid10  moralid2  moralid3  moralid4  moralid5  moralid6  moralid7  moralid8  moralid9  mcoop3  mcoop4  mcoop5  mcoop6  mcoop7  narc1  narc4  narc5  omind1  omind2  omind3  omind4  omind5  omind6  contact1  psupport1  psupport3  political_ideology  happy  riskperc1  riskperc2  optim2
##   1   2  age  children  cnarc1  cnarc2  cnarc3  ctheory1  ctheory2  ctheory3  ctheory4  CRT1  CRT2  CRT3  ladder  mor_circle  moralid1  moralid10  moralid2  moralid3  moralid4  moralid5  moralid6  moralid7  moralid8  moralid9  mcoop3  mcoop4  mcoop5  mcoop6  mcoop7  narc1  narc4  narc5  omind1  omind2  omind3  omind4  omind5  omind6  contact1  psupport1  psupport3  political_ideology  happy  riskperc1  riskperc2  optim2
##   1   3  age  children  cnarc1  cnarc2  cnarc3  ctheory1  ctheory2  ctheory3  ctheory4  CRT1  CRT2  CRT3  ladder  mor_circle  moralid1  moralid10  moralid2  moralid3  moralid4  moralid5  moralid6  moralid7  moralid8  moralid9  mcoop3  mcoop4  mcoop5  mcoop6  mcoop7  narc1  narc4  narc5  omind1  omind2  omind3  omind4  omind5  omind6  contact1  psupport1  psupport3  political_ideology  happy  riskperc1  riskperc2  optim2
##   1   4  age  children  cnarc1  cnarc2  cnarc3  ctheory1  ctheory2  ctheory3  ctheory4  CRT1  CRT2  CRT3  ladder  mor_circle  moralid1  moralid10  moralid2  moralid3  moralid4  moralid5  moralid6  moralid7  moralid8  moralid9  mcoop3  mcoop4  mcoop5  mcoop6  mcoop7  narc1  narc4  narc5  omind1  omind2  omind3  omind4  omind5  omind6  contact1  psupport1  psupport3  political_ideology  happy  riskperc1  riskperc2  optim2
##   1   5  age  children  cnarc1  cnarc2  cnarc3  ctheory1  ctheory2  ctheory3  ctheory4  CRT1  CRT2  CRT3  ladder  mor_circle  moralid1  moralid10  moralid2  moralid3  moralid4  moralid5  moralid6  moralid7  moralid8  moralid9  mcoop3  mcoop4  mcoop5  mcoop6  mcoop7  narc1  narc4  narc5  omind1  omind2  omind3  omind4  omind5  omind6  contact1  psupport1  psupport3  political_ideology  happy  riskperc1  riskperc2  optim2
##   1   6  age  children  cnarc1  cnarc2  cnarc3  ctheory1  ctheory2  ctheory3  ctheory4  CRT1  CRT2  CRT3  ladder  mor_circle  moralid1  moralid10  moralid2  moralid3  moralid4  moralid5  moralid6  moralid7  moralid8  moralid9  mcoop3  mcoop4  mcoop5  mcoop6  mcoop7  narc1  narc4  narc5  omind1  omind2  omind3  omind4  omind5  omind6  contact1  psupport1  psupport3  political_ideology  happy  riskperc1  riskperc2  optim2
##   1   7  age  children  cnarc1  cnarc2  cnarc3  ctheory1  ctheory2  ctheory3  ctheory4  CRT1  CRT2  CRT3  ladder  mor_circle  moralid1  moralid10  moralid2  moralid3  moralid4  moralid5  moralid6  moralid7  moralid8  moralid9  mcoop3  mcoop4  mcoop5  mcoop6  mcoop7  narc1  narc4  narc5  omind1  omind2  omind3  omind4  omind5  omind6  contact1  psupport1  psupport3  political_ideology  happy  riskperc1  riskperc2  optim2
##   1   8  age  children  cnarc1  cnarc2  cnarc3  ctheory1  ctheory2  ctheory3  ctheory4  CRT1  CRT2  CRT3  ladder  mor_circle  moralid1  moralid10  moralid2  moralid3  moralid4  moralid5  moralid6  moralid7  moralid8  moralid9  mcoop3  mcoop4  mcoop5  mcoop6  mcoop7  narc1  narc4  narc5  omind1  omind2  omind3  omind4  omind5  omind6  contact1  psupport1  psupport3  political_ideology  happy  riskperc1  riskperc2  optim2
##   1   9  age  children  cnarc1  cnarc2  cnarc3  ctheory1  ctheory2  ctheory3  ctheory4  CRT1  CRT2  CRT3  ladder  mor_circle  moralid1  moralid10  moralid2  moralid3  moralid4  moralid5  moralid6  moralid7  moralid8  moralid9  mcoop3  mcoop4  mcoop5  mcoop6  mcoop7  narc1  narc4  narc5  omind1  omind2  omind3  omind4  omind5  omind6  contact1  psupport1  psupport3  political_ideology  happy  riskperc1  riskperc2  optim2
##   1   10  age  children  cnarc1  cnarc2  cnarc3  ctheory1  ctheory2  ctheory3  ctheory4  CRT1  CRT2  CRT3  ladder  mor_circle  moralid1  moralid10  moralid2  moralid3  moralid4  moralid5  moralid6  moralid7  moralid8  moralid9  mcoop3  mcoop4  mcoop5  mcoop6  mcoop7  narc1  narc4  narc5  omind1  omind2  omind3  omind4  omind5  omind6  contact1  psupport1  psupport3  political_ideology  happy  riskperc1  riskperc2  optim2
##   2   1  age  children  cnarc1  cnarc2  cnarc3  ctheory1  ctheory2  ctheory3  ctheory4  CRT1  CRT2  CRT3  ladder  mor_circle  moralid1  moralid10  moralid2  moralid3  moralid4  moralid5  moralid6  moralid7  moralid8  moralid9  mcoop3  mcoop4  mcoop5  mcoop6  mcoop7  narc1  narc4  narc5  omind1  omind2  omind3  omind4  omind5  omind6  contact1  psupport1  psupport3  political_ideology  happy  riskperc1  riskperc2  optim2
##   2   2  age  children  cnarc1  cnarc2  cnarc3  ctheory1  ctheory2  ctheory3  ctheory4  CRT1  CRT2  CRT3  ladder  mor_circle  moralid1  moralid10  moralid2  moralid3  moralid4  moralid5  moralid6  moralid7  moralid8  moralid9  mcoop3  mcoop4  mcoop5  mcoop6  mcoop7  narc1  narc4  narc5  omind1  omind2  omind3  omind4  omind5  omind6  contact1  psupport1  psupport3  political_ideology  happy  riskperc1  riskperc2  optim2
##   2   3  age  children  cnarc1  cnarc2  cnarc3  ctheory1  ctheory2  ctheory3  ctheory4  CRT1  CRT2  CRT3  ladder  mor_circle  moralid1  moralid10  moralid2  moralid3  moralid4  moralid5  moralid6  moralid7  moralid8  moralid9  mcoop3  mcoop4  mcoop5  mcoop6  mcoop7  narc1  narc4  narc5  omind1  omind2  omind3  omind4  omind5  omind6  contact1  psupport1  psupport3  political_ideology  happy  riskperc1  riskperc2  optim2
##   2   4  age  children  cnarc1  cnarc2  cnarc3  ctheory1  ctheory2  ctheory3  ctheory4  CRT1  CRT2  CRT3  ladder  mor_circle  moralid1  moralid10  moralid2  moralid3  moralid4  moralid5  moralid6  moralid7  moralid8  moralid9  mcoop3  mcoop4  mcoop5  mcoop6  mcoop7  narc1  narc4  narc5  omind1  omind2  omind3  omind4  omind5  omind6  contact1  psupport1  psupport3  political_ideology  happy  riskperc1  riskperc2  optim2
##   2   5  age  children  cnarc1  cnarc2  cnarc3  ctheory1  ctheory2  ctheory3  ctheory4  CRT1  CRT2  CRT3  ladder  mor_circle  moralid1  moralid10  moralid2  moralid3  moralid4  moralid5  moralid6  moralid7  moralid8  moralid9  mcoop3  mcoop4  mcoop5  mcoop6  mcoop7  narc1  narc4  narc5  omind1  omind2  omind3  omind4  omind5  omind6  contact1  psupport1  psupport3  political_ideology  happy  riskperc1  riskperc2  optim2
##   2   6  age  children  cnarc1  cnarc2  cnarc3  ctheory1  ctheory2  ctheory3  ctheory4  CRT1  CRT2  CRT3  ladder  mor_circle  moralid1  moralid10  moralid2  moralid3  moralid4  moralid5  moralid6  moralid7  moralid8  moralid9  mcoop3  mcoop4  mcoop5  mcoop6  mcoop7  narc1  narc4  narc5  omind1  omind2  omind3  omind4  omind5  omind6  contact1  psupport1  psupport3  political_ideology  happy  riskperc1  riskperc2  optim2
##   2   7  age  children  cnarc1  cnarc2  cnarc3  ctheory1  ctheory2  ctheory3  ctheory4  CRT1  CRT2  CRT3  ladder  mor_circle  moralid1  moralid10  moralid2  moralid3  moralid4  moralid5  moralid6  moralid7  moralid8  moralid9  mcoop3  mcoop4  mcoop5  mcoop6  mcoop7  narc1  narc4  narc5  omind1  omind2  omind3  omind4  omind5  omind6  contact1  psupport1  psupport3  political_ideology  happy  riskperc1  riskperc2  optim2
##   2   8  age  children  cnarc1  cnarc2  cnarc3  ctheory1  ctheory2  ctheory3  ctheory4  CRT1  CRT2  CRT3  ladder  mor_circle  moralid1  moralid10  moralid2  moralid3  moralid4  moralid5  moralid6  moralid7  moralid8  moralid9  mcoop3  mcoop4  mcoop5  mcoop6  mcoop7  narc1  narc4  narc5  omind1  omind2  omind3  omind4  omind5  omind6  contact1  psupport1  psupport3  political_ideology  happy  riskperc1  riskperc2  optim2
##   2   9  age  children  cnarc1  cnarc2  cnarc3  ctheory1  ctheory2  ctheory3  ctheory4  CRT1  CRT2  CRT3  ladder  mor_circle  moralid1  moralid10  moralid2  moralid3  moralid4  moralid5  moralid6  moralid7  moralid8  moralid9  mcoop3  mcoop4  mcoop5  mcoop6  mcoop7  narc1  narc4  narc5  omind1  omind2  omind3  omind4  omind5  omind6  contact1  psupport1  psupport3  political_ideology  happy  riskperc1  riskperc2  optim2
##   2   10  age  children  cnarc1  cnarc2  cnarc3  ctheory1  ctheory2  ctheory3  ctheory4  CRT1  CRT2  CRT3  ladder  mor_circle  moralid1  moralid10  moralid2  moralid3  moralid4  moralid5  moralid6  moralid7  moralid8  moralid9  mcoop3  mcoop4  mcoop5  mcoop6  mcoop7  narc1  narc4  narc5  omind1  omind2  omind3  omind4  omind5  omind6  contact1  psupport1  psupport3  political_ideology  happy  riskperc1  riskperc2  optim2
##   3   1  age  children  cnarc1  cnarc2  cnarc3  ctheory1  ctheory2  ctheory3  ctheory4  CRT1  CRT2  CRT3  ladder  mor_circle  moralid1  moralid10  moralid2  moralid3  moralid4  moralid5  moralid6  moralid7  moralid8  moralid9  mcoop3  mcoop4  mcoop5  mcoop6  mcoop7  narc1  narc4  narc5  omind1  omind2  omind3  omind4  omind5  omind6  contact1  psupport1  psupport3  political_ideology  happy  riskperc1  riskperc2  optim2
##   3   2  age  children  cnarc1  cnarc2  cnarc3  ctheory1  ctheory2  ctheory3  ctheory4  CRT1  CRT2  CRT3  ladder  mor_circle  moralid1  moralid10  moralid2  moralid3  moralid4  moralid5  moralid6  moralid7  moralid8  moralid9  mcoop3  mcoop4  mcoop5  mcoop6  mcoop7  narc1  narc4  narc5  omind1  omind2  omind3  omind4  omind5  omind6  contact1  psupport1  psupport3  political_ideology  happy  riskperc1  riskperc2  optim2
##   3   3  age  children  cnarc1  cnarc2  cnarc3  ctheory1  ctheory2  ctheory3  ctheory4  CRT1  CRT2  CRT3  ladder  mor_circle  moralid1  moralid10  moralid2  moralid3  moralid4  moralid5  moralid6  moralid7  moralid8  moralid9  mcoop3  mcoop4  mcoop5  mcoop6  mcoop7  narc1  narc4  narc5  omind1  omind2  omind3  omind4  omind5  omind6  contact1  psupport1  psupport3  political_ideology  happy  riskperc1  riskperc2  optim2
##   3   4  age  children  cnarc1  cnarc2  cnarc3  ctheory1  ctheory2  ctheory3  ctheory4  CRT1  CRT2  CRT3  ladder  mor_circle  moralid1  moralid10  moralid2  moralid3  moralid4  moralid5  moralid6  moralid7  moralid8  moralid9  mcoop3  mcoop4  mcoop5  mcoop6  mcoop7  narc1  narc4  narc5  omind1  omind2  omind3  omind4  omind5  omind6  contact1  psupport1  psupport3  political_ideology  happy  riskperc1  riskperc2  optim2
##   3   5  age  children  cnarc1  cnarc2  cnarc3  ctheory1  ctheory2  ctheory3  ctheory4  CRT1  CRT2  CRT3  ladder  mor_circle  moralid1  moralid10  moralid2  moralid3  moralid4  moralid5  moralid6  moralid7  moralid8  moralid9  mcoop3  mcoop4  mcoop5  mcoop6  mcoop7  narc1  narc4  narc5  omind1  omind2  omind3  omind4  omind5  omind6  contact1  psupport1  psupport3  political_ideology  happy  riskperc1  riskperc2  optim2
##   3   6  age  children  cnarc1  cnarc2  cnarc3  ctheory1  ctheory2  ctheory3  ctheory4  CRT1  CRT2  CRT3  ladder  mor_circle  moralid1  moralid10  moralid2  moralid3  moralid4  moralid5  moralid6  moralid7  moralid8  moralid9  mcoop3  mcoop4  mcoop5  mcoop6  mcoop7  narc1  narc4  narc5  omind1  omind2  omind3  omind4  omind5  omind6  contact1  psupport1  psupport3  political_ideology  happy  riskperc1  riskperc2  optim2
##   3   7  age  children  cnarc1  cnarc2  cnarc3  ctheory1  ctheory2  ctheory3  ctheory4  CRT1  CRT2  CRT3  ladder  mor_circle  moralid1  moralid10  moralid2  moralid3  moralid4  moralid5  moralid6  moralid7  moralid8  moralid9  mcoop3  mcoop4  mcoop5  mcoop6  mcoop7  narc1  narc4  narc5  omind1  omind2  omind3  omind4  omind5  omind6  contact1  psupport1  psupport3  political_ideology  happy  riskperc1  riskperc2  optim2
##   3   8  age  children  cnarc1  cnarc2  cnarc3  ctheory1  ctheory2  ctheory3  ctheory4  CRT1  CRT2  CRT3  ladder  mor_circle  moralid1  moralid10  moralid2  moralid3  moralid4  moralid5  moralid6  moralid7  moralid8  moralid9  mcoop3  mcoop4  mcoop5  mcoop6  mcoop7  narc1  narc4  narc5  omind1  omind2  omind3  omind4  omind5  omind6  contact1  psupport1  psupport3  political_ideology  happy  riskperc1  riskperc2  optim2
##   3   9  age  children  cnarc1  cnarc2  cnarc3  ctheory1  ctheory2  ctheory3  ctheory4  CRT1  CRT2  CRT3  ladder  mor_circle  moralid1  moralid10  moralid2  moralid3  moralid4  moralid5  moralid6  moralid7  moralid8  moralid9  mcoop3  mcoop4  mcoop5  mcoop6  mcoop7  narc1  narc4  narc5  omind1  omind2  omind3  omind4  omind5  omind6  contact1  psupport1  psupport3  political_ideology  happy  riskperc1  riskperc2  optim2
##   3   10  age  children  cnarc1  cnarc2  cnarc3  ctheory1  ctheory2  ctheory3  ctheory4  CRT1  CRT2  CRT3  ladder  mor_circle  moralid1  moralid10  moralid2  moralid3  moralid4  moralid5  moralid6  moralid7  moralid8  moralid9  mcoop3  mcoop4  mcoop5  mcoop6  mcoop7  narc1  narc4  narc5  omind1  omind2  omind3  omind4  omind5  omind6  contact1  psupport1  psupport3  political_ideology  happy  riskperc1  riskperc2  optim2
##   4   1  age  children  cnarc1  cnarc2  cnarc3  ctheory1  ctheory2  ctheory3  ctheory4  CRT1  CRT2  CRT3  ladder  mor_circle  moralid1  moralid10  moralid2  moralid3  moralid4  moralid5  moralid6  moralid7  moralid8  moralid9  mcoop3  mcoop4  mcoop5  mcoop6  mcoop7  narc1  narc4  narc5  omind1  omind2  omind3  omind4  omind5  omind6  contact1  psupport1  psupport3  political_ideology  happy  riskperc1  riskperc2  optim2
##   4   2  age  children  cnarc1  cnarc2  cnarc3  ctheory1  ctheory2  ctheory3  ctheory4  CRT1  CRT2  CRT3  ladder  mor_circle  moralid1  moralid10  moralid2  moralid3  moralid4  moralid5  moralid6  moralid7  moralid8  moralid9  mcoop3  mcoop4  mcoop5  mcoop6  mcoop7  narc1  narc4  narc5  omind1  omind2  omind3  omind4  omind5  omind6  contact1  psupport1  psupport3  political_ideology  happy  riskperc1  riskperc2  optim2
##   4   3  age  children  cnarc1  cnarc2  cnarc3  ctheory1  ctheory2  ctheory3  ctheory4  CRT1  CRT2  CRT3  ladder  mor_circle  moralid1  moralid10  moralid2  moralid3  moralid4  moralid5  moralid6  moralid7  moralid8  moralid9  mcoop3  mcoop4  mcoop5  mcoop6  mcoop7  narc1  narc4  narc5  omind1  omind2  omind3  omind4  omind5  omind6  contact1  psupport1  psupport3  political_ideology  happy  riskperc1  riskperc2  optim2
##   4   4  age  children  cnarc1  cnarc2  cnarc3  ctheory1  ctheory2  ctheory3  ctheory4  CRT1  CRT2  CRT3  ladder  mor_circle  moralid1  moralid10  moralid2  moralid3  moralid4  moralid5  moralid6  moralid7  moralid8  moralid9  mcoop3  mcoop4  mcoop5  mcoop6  mcoop7  narc1  narc4  narc5  omind1  omind2  omind3  omind4  omind5  omind6  contact1  psupport1  psupport3  political_ideology  happy  riskperc1  riskperc2  optim2
##   4   5  age  children  cnarc1  cnarc2  cnarc3  ctheory1  ctheory2  ctheory3  ctheory4  CRT1  CRT2  CRT3  ladder  mor_circle  moralid1  moralid10  moralid2  moralid3  moralid4  moralid5  moralid6  moralid7  moralid8  moralid9  mcoop3  mcoop4  mcoop5  mcoop6  mcoop7  narc1  narc4  narc5  omind1  omind2  omind3  omind4  omind5  omind6  contact1  psupport1  psupport3  political_ideology  happy  riskperc1  riskperc2  optim2
##   4   6  age  children  cnarc1  cnarc2  cnarc3  ctheory1  ctheory2  ctheory3  ctheory4  CRT1  CRT2  CRT3  ladder  mor_circle  moralid1  moralid10  moralid2  moralid3  moralid4  moralid5  moralid6  moralid7  moralid8  moralid9  mcoop3  mcoop4  mcoop5  mcoop6  mcoop7  narc1  narc4  narc5  omind1  omind2  omind3  omind4  omind5  omind6  contact1  psupport1  psupport3  political_ideology  happy  riskperc1  riskperc2  optim2
##   4   7  age  children  cnarc1  cnarc2  cnarc3  ctheory1  ctheory2  ctheory3  ctheory4  CRT1  CRT2  CRT3  ladder  mor_circle  moralid1  moralid10  moralid2  moralid3  moralid4  moralid5  moralid6  moralid7  moralid8  moralid9  mcoop3  mcoop4  mcoop5  mcoop6  mcoop7  narc1  narc4  narc5  omind1  omind2  omind3  omind4  omind5  omind6  contact1  psupport1  psupport3  political_ideology  happy  riskperc1  riskperc2  optim2
##   4   8  age  children  cnarc1  cnarc2  cnarc3  ctheory1  ctheory2  ctheory3  ctheory4  CRT1  CRT2  CRT3  ladder  mor_circle  moralid1  moralid10  moralid2  moralid3  moralid4  moralid5  moralid6  moralid7  moralid8  moralid9  mcoop3  mcoop4  mcoop5  mcoop6  mcoop7  narc1  narc4  narc5  omind1  omind2  omind3  omind4  omind5  omind6  contact1  psupport1  psupport3  political_ideology  happy  riskperc1  riskperc2  optim2
##   4   9  age  children  cnarc1  cnarc2  cnarc3  ctheory1  ctheory2  ctheory3  ctheory4  CRT1  CRT2  CRT3  ladder  mor_circle  moralid1  moralid10  moralid2  moralid3  moralid4  moralid5  moralid6  moralid7  moralid8  moralid9  mcoop3  mcoop4  mcoop5  mcoop6  mcoop7  narc1  narc4  narc5  omind1  omind2  omind3  omind4  omind5  omind6  contact1  psupport1  psupport3  political_ideology  happy  riskperc1  riskperc2  optim2
##   4   10  age  children  cnarc1  cnarc2  cnarc3  ctheory1  ctheory2  ctheory3  ctheory4  CRT1  CRT2  CRT3  ladder  mor_circle  moralid1  moralid10  moralid2  moralid3  moralid4  moralid5  moralid6  moralid7  moralid8  moralid9  mcoop3  mcoop4  mcoop5  mcoop6  mcoop7  narc1  narc4  narc5  omind1  omind2  omind3  omind4  omind5  omind6  contact1  psupport1  psupport3  political_ideology  happy  riskperc1  riskperc2  optim2
##   5   1  age  children  cnarc1  cnarc2  cnarc3  ctheory1  ctheory2  ctheory3  ctheory4  CRT1  CRT2  CRT3  ladder  mor_circle  moralid1  moralid10  moralid2  moralid3  moralid4  moralid5  moralid6  moralid7  moralid8  moralid9  mcoop3  mcoop4  mcoop5  mcoop6  mcoop7  narc1  narc4  narc5  omind1  omind2  omind3  omind4  omind5  omind6  contact1  psupport1  psupport3  political_ideology  happy  riskperc1  riskperc2  optim2
##   5   2  age  children  cnarc1  cnarc2  cnarc3  ctheory1  ctheory2  ctheory3  ctheory4  CRT1  CRT2  CRT3  ladder  mor_circle  moralid1  moralid10  moralid2  moralid3  moralid4  moralid5  moralid6  moralid7  moralid8  moralid9  mcoop3  mcoop4  mcoop5  mcoop6  mcoop7  narc1  narc4  narc5  omind1  omind2  omind3  omind4  omind5  omind6  contact1  psupport1  psupport3  political_ideology  happy  riskperc1  riskperc2  optim2
##   5   3  age  children  cnarc1  cnarc2  cnarc3  ctheory1  ctheory2  ctheory3  ctheory4  CRT1  CRT2  CRT3  ladder  mor_circle  moralid1  moralid10  moralid2  moralid3  moralid4  moralid5  moralid6  moralid7  moralid8  moralid9  mcoop3  mcoop4  mcoop5  mcoop6  mcoop7  narc1  narc4  narc5  omind1  omind2  omind3  omind4  omind5  omind6  contact1  psupport1  psupport3  political_ideology  happy  riskperc1  riskperc2  optim2
##   5   4  age  children  cnarc1  cnarc2  cnarc3  ctheory1  ctheory2  ctheory3  ctheory4  CRT1  CRT2  CRT3  ladder  mor_circle  moralid1  moralid10  moralid2  moralid3  moralid4  moralid5  moralid6  moralid7  moralid8  moralid9  mcoop3  mcoop4  mcoop5  mcoop6  mcoop7  narc1  narc4  narc5  omind1  omind2  omind3  omind4  omind5  omind6  contact1  psupport1  psupport3  political_ideology  happy  riskperc1  riskperc2  optim2
##   5   5  age  children  cnarc1  cnarc2  cnarc3  ctheory1  ctheory2  ctheory3  ctheory4  CRT1  CRT2  CRT3  ladder  mor_circle  moralid1  moralid10  moralid2  moralid3  moralid4  moralid5  moralid6  moralid7  moralid8  moralid9  mcoop3  mcoop4  mcoop5  mcoop6  mcoop7  narc1  narc4  narc5  omind1  omind2  omind3  omind4  omind5  omind6  contact1  psupport1  psupport3  political_ideology  happy  riskperc1  riskperc2  optim2
##   5   6  age  children  cnarc1  cnarc2  cnarc3  ctheory1  ctheory2  ctheory3  ctheory4  CRT1  CRT2  CRT3  ladder  mor_circle  moralid1  moralid10  moralid2  moralid3  moralid4  moralid5  moralid6  moralid7  moralid8  moralid9  mcoop3  mcoop4  mcoop5  mcoop6  mcoop7  narc1  narc4  narc5  omind1  omind2  omind3  omind4  omind5  omind6  contact1  psupport1  psupport3  political_ideology  happy  riskperc1  riskperc2  optim2
##   5   7  age  children  cnarc1  cnarc2  cnarc3  ctheory1  ctheory2  ctheory3  ctheory4  CRT1  CRT2  CRT3  ladder  mor_circle  moralid1  moralid10  moralid2  moralid3  moralid4  moralid5  moralid6  moralid7  moralid8  moralid9  mcoop3  mcoop4  mcoop5  mcoop6  mcoop7  narc1  narc4  narc5  omind1  omind2  omind3  omind4  omind5  omind6  contact1  psupport1  psupport3  political_ideology  happy  riskperc1  riskperc2  optim2
##   5   8  age  children  cnarc1  cnarc2  cnarc3  ctheory1  ctheory2  ctheory3  ctheory4  CRT1  CRT2  CRT3  ladder  mor_circle  moralid1  moralid10  moralid2  moralid3  moralid4  moralid5  moralid6  moralid7  moralid8  moralid9  mcoop3  mcoop4  mcoop5  mcoop6  mcoop7  narc1  narc4  narc5  omind1  omind2  omind3  omind4  omind5  omind6  contact1  psupport1  psupport3  political_ideology  happy  riskperc1  riskperc2  optim2
##   5   9  age  children  cnarc1  cnarc2  cnarc3  ctheory1  ctheory2  ctheory3  ctheory4  CRT1  CRT2  CRT3  ladder  mor_circle  moralid1  moralid10  moralid2  moralid3  moralid4  moralid5  moralid6  moralid7  moralid8  moralid9  mcoop3  mcoop4  mcoop5  mcoop6  mcoop7  narc1  narc4  narc5  omind1  omind2  omind3  omind4  omind5  omind6  contact1  psupport1  psupport3  political_ideology  happy  riskperc1  riskperc2  optim2
##   5   10  age  children  cnarc1  cnarc2  cnarc3  ctheory1  ctheory2  ctheory3  ctheory4  CRT1  CRT2  CRT3  ladder  mor_circle  moralid1  moralid10  moralid2  moralid3  moralid4  moralid5  moralid6  moralid7  moralid8  moralid9  mcoop3  mcoop4  mcoop5  mcoop6  mcoop7  narc1  narc4  narc5  omind1  omind2  omind3  omind4  omind5  omind6  contact1  psupport1  psupport3  political_ideology  happy  riskperc1  riskperc2  optim2
##   6   1  age  children  cnarc1  cnarc2  cnarc3  ctheory1  ctheory2  ctheory3  ctheory4  CRT1  CRT2  CRT3  ladder  mor_circle  moralid1  moralid10  moralid2  moralid3  moralid4  moralid5  moralid6  moralid7  moralid8  moralid9  mcoop3  mcoop4  mcoop5  mcoop6  mcoop7  narc1  narc4  narc5  omind1  omind2  omind3  omind4  omind5  omind6  contact1  psupport1  psupport3  political_ideology  happy  riskperc1  riskperc2  optim2
##   6   2  age  children  cnarc1  cnarc2  cnarc3  ctheory1  ctheory2  ctheory3  ctheory4  CRT1  CRT2  CRT3  ladder  mor_circle  moralid1  moralid10  moralid2  moralid3  moralid4  moralid5  moralid6  moralid7  moralid8  moralid9  mcoop3  mcoop4  mcoop5  mcoop6  mcoop7  narc1  narc4  narc5  omind1  omind2  omind3  omind4  omind5  omind6  contact1  psupport1  psupport3  political_ideology  happy  riskperc1  riskperc2  optim2
##   6   3  age  children  cnarc1  cnarc2  cnarc3  ctheory1  ctheory2  ctheory3  ctheory4  CRT1  CRT2  CRT3  ladder  mor_circle  moralid1  moralid10  moralid2  moralid3  moralid4  moralid5  moralid6  moralid7  moralid8  moralid9  mcoop3  mcoop4  mcoop5  mcoop6  mcoop7  narc1  narc4  narc5  omind1  omind2  omind3  omind4  omind5  omind6  contact1  psupport1  psupport3  political_ideology  happy  riskperc1  riskperc2  optim2
##   6   4  age  children  cnarc1  cnarc2  cnarc3  ctheory1  ctheory2  ctheory3  ctheory4  CRT1  CRT2  CRT3  ladder  mor_circle  moralid1  moralid10  moralid2  moralid3  moralid4  moralid5  moralid6  moralid7  moralid8  moralid9  mcoop3  mcoop4  mcoop5  mcoop6  mcoop7  narc1  narc4  narc5  omind1  omind2  omind3  omind4  omind5  omind6  contact1  psupport1  psupport3  political_ideology  happy  riskperc1  riskperc2  optim2
##   6   5  age  children  cnarc1  cnarc2  cnarc3  ctheory1  ctheory2  ctheory3  ctheory4  CRT1  CRT2  CRT3  ladder  mor_circle  moralid1  moralid10  moralid2  moralid3  moralid4  moralid5  moralid6  moralid7  moralid8  moralid9  mcoop3  mcoop4  mcoop5  mcoop6  mcoop7  narc1  narc4  narc5  omind1  omind2  omind3  omind4  omind5  omind6  contact1  psupport1  psupport3  political_ideology  happy  riskperc1  riskperc2  optim2
##   6   6  age  children  cnarc1  cnarc2  cnarc3  ctheory1  ctheory2  ctheory3  ctheory4  CRT1  CRT2  CRT3  ladder  mor_circle  moralid1  moralid10  moralid2  moralid3  moralid4  moralid5  moralid6  moralid7  moralid8  moralid9  mcoop3  mcoop4  mcoop5  mcoop6  mcoop7  narc1  narc4  narc5  omind1  omind2  omind3  omind4  omind5  omind6  contact1  psupport1  psupport3  political_ideology  happy  riskperc1  riskperc2  optim2
##   6   7  age  children  cnarc1  cnarc2  cnarc3  ctheory1  ctheory2  ctheory3  ctheory4  CRT1  CRT2  CRT3  ladder  mor_circle  moralid1  moralid10  moralid2  moralid3  moralid4  moralid5  moralid6  moralid7  moralid8  moralid9  mcoop3  mcoop4  mcoop5  mcoop6  mcoop7  narc1  narc4  narc5  omind1  omind2  omind3  omind4  omind5  omind6  contact1  psupport1  psupport3  political_ideology  happy  riskperc1  riskperc2  optim2
##   6   8  age  children  cnarc1  cnarc2  cnarc3  ctheory1  ctheory2  ctheory3  ctheory4  CRT1  CRT2  CRT3  ladder  mor_circle  moralid1  moralid10  moralid2  moralid3  moralid4  moralid5  moralid6  moralid7  moralid8  moralid9  mcoop3  mcoop4  mcoop5  mcoop6  mcoop7  narc1  narc4  narc5  omind1  omind2  omind3  omind4  omind5  omind6  contact1  psupport1  psupport3  political_ideology  happy  riskperc1  riskperc2  optim2
##   6   9  age  children  cnarc1  cnarc2  cnarc3  ctheory1  ctheory2  ctheory3  ctheory4  CRT1  CRT2  CRT3  ladder  mor_circle  moralid1  moralid10  moralid2  moralid3  moralid4  moralid5  moralid6  moralid7  moralid8  moralid9  mcoop3  mcoop4  mcoop5  mcoop6  mcoop7  narc1  narc4  narc5  omind1  omind2  omind3  omind4  omind5  omind6  contact1  psupport1  psupport3  political_ideology  happy  riskperc1  riskperc2  optim2
##   6   10  age  children  cnarc1  cnarc2  cnarc3  ctheory1  ctheory2  ctheory3  ctheory4  CRT1  CRT2  CRT3  ladder  mor_circle  moralid1  moralid10  moralid2  moralid3  moralid4  moralid5  moralid6  moralid7  moralid8  moralid9  mcoop3  mcoop4  mcoop5  mcoop6  mcoop7  narc1  narc4  narc5  omind1  omind2  omind3  omind4  omind5  omind6  contact1  psupport1  psupport3  political_ideology  happy  riskperc1  riskperc2  optim2
##   7   1  age  children  cnarc1  cnarc2  cnarc3  ctheory1  ctheory2  ctheory3  ctheory4  CRT1  CRT2  CRT3  ladder  mor_circle  moralid1  moralid10  moralid2  moralid3  moralid4  moralid5  moralid6  moralid7  moralid8  moralid9  mcoop3  mcoop4  mcoop5  mcoop6  mcoop7  narc1  narc4  narc5  omind1  omind2  omind3  omind4  omind5  omind6  contact1  psupport1  psupport3  political_ideology  happy  riskperc1  riskperc2  optim2
##   7   2  age  children  cnarc1  cnarc2  cnarc3  ctheory1  ctheory2  ctheory3  ctheory4  CRT1  CRT2  CRT3  ladder  mor_circle  moralid1  moralid10  moralid2  moralid3  moralid4  moralid5  moralid6  moralid7  moralid8  moralid9  mcoop3  mcoop4  mcoop5  mcoop6  mcoop7  narc1  narc4  narc5  omind1  omind2  omind3  omind4  omind5  omind6  contact1  psupport1  psupport3  political_ideology  happy  riskperc1  riskperc2  optim2
##   7   3  age  children  cnarc1  cnarc2  cnarc3  ctheory1  ctheory2  ctheory3  ctheory4  CRT1  CRT2  CRT3  ladder  mor_circle  moralid1  moralid10  moralid2  moralid3  moralid4  moralid5  moralid6  moralid7  moralid8  moralid9  mcoop3  mcoop4  mcoop5  mcoop6  mcoop7  narc1  narc4  narc5  omind1  omind2  omind3  omind4  omind5  omind6  contact1  psupport1  psupport3  political_ideology  happy  riskperc1  riskperc2  optim2
##   7   4  age  children  cnarc1  cnarc2  cnarc3  ctheory1  ctheory2  ctheory3  ctheory4  CRT1  CRT2  CRT3  ladder  mor_circle  moralid1  moralid10  moralid2  moralid3  moralid4  moralid5  moralid6  moralid7  moralid8  moralid9  mcoop3  mcoop4  mcoop5  mcoop6  mcoop7  narc1  narc4  narc5  omind1  omind2  omind3  omind4  omind5  omind6  contact1  psupport1  psupport3  political_ideology  happy  riskperc1  riskperc2  optim2
##   7   5  age  children  cnarc1  cnarc2  cnarc3  ctheory1  ctheory2  ctheory3  ctheory4  CRT1  CRT2  CRT3  ladder  mor_circle  moralid1  moralid10  moralid2  moralid3  moralid4  moralid5  moralid6  moralid7  moralid8  moralid9  mcoop3  mcoop4  mcoop5  mcoop6  mcoop7  narc1  narc4  narc5  omind1  omind2  omind3  omind4  omind5  omind6  contact1  psupport1  psupport3  political_ideology  happy  riskperc1  riskperc2  optim2
##   7   6  age  children  cnarc1  cnarc2  cnarc3  ctheory1  ctheory2  ctheory3  ctheory4  CRT1  CRT2  CRT3  ladder  mor_circle  moralid1  moralid10  moralid2  moralid3  moralid4  moralid5  moralid6  moralid7  moralid8  moralid9  mcoop3  mcoop4  mcoop5  mcoop6  mcoop7  narc1  narc4  narc5  omind1  omind2  omind3  omind4  omind5  omind6  contact1  psupport1  psupport3  political_ideology  happy  riskperc1  riskperc2  optim2
##   7   7  age  children  cnarc1  cnarc2  cnarc3  ctheory1  ctheory2  ctheory3  ctheory4  CRT1  CRT2  CRT3  ladder  mor_circle  moralid1  moralid10  moralid2  moralid3  moralid4  moralid5  moralid6  moralid7  moralid8  moralid9  mcoop3  mcoop4  mcoop5  mcoop6  mcoop7  narc1  narc4  narc5  omind1  omind2  omind3  omind4  omind5  omind6  contact1  psupport1  psupport3  political_ideology  happy  riskperc1  riskperc2  optim2
##   7   8  age  children  cnarc1  cnarc2  cnarc3  ctheory1  ctheory2  ctheory3  ctheory4  CRT1  CRT2  CRT3  ladder  mor_circle  moralid1  moralid10  moralid2  moralid3  moralid4  moralid5  moralid6  moralid7  moralid8  moralid9  mcoop3  mcoop4  mcoop5  mcoop6  mcoop7  narc1  narc4  narc5  omind1  omind2  omind3  omind4  omind5  omind6  contact1  psupport1  psupport3  political_ideology  happy  riskperc1  riskperc2  optim2
##   7   9  age  children  cnarc1  cnarc2  cnarc3  ctheory1  ctheory2  ctheory3  ctheory4  CRT1  CRT2  CRT3  ladder  mor_circle  moralid1  moralid10  moralid2  moralid3  moralid4  moralid5  moralid6  moralid7  moralid8  moralid9  mcoop3  mcoop4  mcoop5  mcoop6  mcoop7  narc1  narc4  narc5  omind1  omind2  omind3  omind4  omind5  omind6  contact1  psupport1  psupport3  political_ideology  happy  riskperc1  riskperc2  optim2
##   7   10  age  children  cnarc1  cnarc2  cnarc3  ctheory1  ctheory2  ctheory3  ctheory4  CRT1  CRT2  CRT3  ladder  mor_circle  moralid1  moralid10  moralid2  moralid3  moralid4  moralid5  moralid6  moralid7  moralid8  moralid9  mcoop3  mcoop4  mcoop5  mcoop6  mcoop7  narc1  narc4  narc5  omind1  omind2  omind3  omind4  omind5  omind6  contact1  psupport1  psupport3  political_ideology  happy  riskperc1  riskperc2  optim2
##   8   1  age  children  cnarc1  cnarc2  cnarc3  ctheory1  ctheory2  ctheory3  ctheory4  CRT1  CRT2  CRT3  ladder  mor_circle  moralid1  moralid10  moralid2  moralid3  moralid4  moralid5  moralid6  moralid7  moralid8  moralid9  mcoop3  mcoop4  mcoop5  mcoop6  mcoop7  narc1  narc4  narc5  omind1  omind2  omind3  omind4  omind5  omind6  contact1  psupport1  psupport3  political_ideology  happy  riskperc1  riskperc2  optim2
##   8   2  age  children  cnarc1  cnarc2  cnarc3  ctheory1  ctheory2  ctheory3  ctheory4  CRT1  CRT2  CRT3  ladder  mor_circle  moralid1  moralid10  moralid2  moralid3  moralid4  moralid5  moralid6  moralid7  moralid8  moralid9  mcoop3  mcoop4  mcoop5  mcoop6  mcoop7  narc1  narc4  narc5  omind1  omind2  omind3  omind4  omind5  omind6  contact1  psupport1  psupport3  political_ideology  happy  riskperc1  riskperc2  optim2
##   8   3  age  children  cnarc1  cnarc2  cnarc3  ctheory1  ctheory2  ctheory3  ctheory4  CRT1  CRT2  CRT3  ladder  mor_circle  moralid1  moralid10  moralid2  moralid3  moralid4  moralid5  moralid6  moralid7  moralid8  moralid9  mcoop3  mcoop4  mcoop5  mcoop6  mcoop7  narc1  narc4  narc5  omind1  omind2  omind3  omind4  omind5  omind6  contact1  psupport1  psupport3  political_ideology  happy  riskperc1  riskperc2  optim2
##   8   4  age  children  cnarc1  cnarc2  cnarc3  ctheory1  ctheory2  ctheory3  ctheory4  CRT1  CRT2  CRT3  ladder  mor_circle  moralid1  moralid10  moralid2  moralid3  moralid4  moralid5  moralid6  moralid7  moralid8  moralid9  mcoop3  mcoop4  mcoop5  mcoop6  mcoop7  narc1  narc4  narc5  omind1  omind2  omind3  omind4  omind5  omind6  contact1  psupport1  psupport3  political_ideology  happy  riskperc1  riskperc2  optim2
##   8   5  age  children  cnarc1  cnarc2  cnarc3  ctheory1  ctheory2  ctheory3  ctheory4  CRT1  CRT2  CRT3  ladder  mor_circle  moralid1  moralid10  moralid2  moralid3  moralid4  moralid5  moralid6  moralid7  moralid8  moralid9  mcoop3  mcoop4  mcoop5  mcoop6  mcoop7  narc1  narc4  narc5  omind1  omind2  omind3  omind4  omind5  omind6  contact1  psupport1  psupport3  political_ideology  happy  riskperc1  riskperc2  optim2
##   8   6  age  children  cnarc1  cnarc2  cnarc3  ctheory1  ctheory2  ctheory3  ctheory4  CRT1  CRT2  CRT3  ladder  mor_circle  moralid1  moralid10  moralid2  moralid3  moralid4  moralid5  moralid6  moralid7  moralid8  moralid9  mcoop3  mcoop4  mcoop5  mcoop6  mcoop7  narc1  narc4  narc5  omind1  omind2  omind3  omind4  omind5  omind6  contact1  psupport1  psupport3  political_ideology  happy  riskperc1  riskperc2  optim2
##   8   7  age  children  cnarc1  cnarc2  cnarc3  ctheory1  ctheory2  ctheory3  ctheory4  CRT1  CRT2  CRT3  ladder  mor_circle  moralid1  moralid10  moralid2  moralid3  moralid4  moralid5  moralid6  moralid7  moralid8  moralid9  mcoop3  mcoop4  mcoop5  mcoop6  mcoop7  narc1  narc4  narc5  omind1  omind2  omind3  omind4  omind5  omind6  contact1  psupport1  psupport3  political_ideology  happy  riskperc1  riskperc2  optim2
##   8   8  age  children  cnarc1  cnarc2  cnarc3  ctheory1  ctheory2  ctheory3  ctheory4  CRT1  CRT2  CRT3  ladder  mor_circle  moralid1  moralid10  moralid2  moralid3  moralid4  moralid5  moralid6  moralid7  moralid8  moralid9  mcoop3  mcoop4  mcoop5  mcoop6  mcoop7  narc1  narc4  narc5  omind1  omind2  omind3  omind4  omind5  omind6  contact1  psupport1  psupport3  political_ideology  happy  riskperc1  riskperc2  optim2
##   8   9  age  children  cnarc1  cnarc2  cnarc3  ctheory1  ctheory2  ctheory3  ctheory4  CRT1  CRT2  CRT3  ladder  mor_circle  moralid1  moralid10  moralid2  moralid3  moralid4  moralid5  moralid6  moralid7  moralid8  moralid9  mcoop3  mcoop4  mcoop5  mcoop6  mcoop7  narc1  narc4  narc5  omind1  omind2  omind3  omind4  omind5  omind6  contact1  psupport1  psupport3  political_ideology  happy  riskperc1  riskperc2  optim2
##   8   10  age  children  cnarc1  cnarc2  cnarc3  ctheory1  ctheory2  ctheory3  ctheory4  CRT1  CRT2  CRT3  ladder  mor_circle  moralid1  moralid10  moralid2  moralid3  moralid4  moralid5  moralid6  moralid7  moralid8  moralid9  mcoop3  mcoop4  mcoop5  mcoop6  mcoop7  narc1  narc4  narc5  omind1  omind2  omind3  omind4  omind5  omind6  contact1  psupport1  psupport3  political_ideology  happy  riskperc1  riskperc2  optim2
##   9   1  age  children  cnarc1  cnarc2  cnarc3  ctheory1  ctheory2  ctheory3  ctheory4  CRT1  CRT2  CRT3  ladder  mor_circle  moralid1  moralid10  moralid2  moralid3  moralid4  moralid5  moralid6  moralid7  moralid8  moralid9  mcoop3  mcoop4  mcoop5  mcoop6  mcoop7  narc1  narc4  narc5  omind1  omind2  omind3  omind4  omind5  omind6  contact1  psupport1  psupport3  political_ideology  happy  riskperc1  riskperc2  optim2
##   9   2  age  children  cnarc1  cnarc2  cnarc3  ctheory1  ctheory2  ctheory3  ctheory4  CRT1  CRT2  CRT3  ladder  mor_circle  moralid1  moralid10  moralid2  moralid3  moralid4  moralid5  moralid6  moralid7  moralid8  moralid9  mcoop3  mcoop4  mcoop5  mcoop6  mcoop7  narc1  narc4  narc5  omind1  omind2  omind3  omind4  omind5  omind6  contact1  psupport1  psupport3  political_ideology  happy  riskperc1  riskperc2  optim2
##   9   3  age  children  cnarc1  cnarc2  cnarc3  ctheory1  ctheory2  ctheory3  ctheory4  CRT1  CRT2  CRT3  ladder  mor_circle  moralid1  moralid10  moralid2  moralid3  moralid4  moralid5  moralid6  moralid7  moralid8  moralid9  mcoop3  mcoop4  mcoop5  mcoop6  mcoop7  narc1  narc4  narc5  omind1  omind2  omind3  omind4  omind5  omind6  contact1  psupport1  psupport3  political_ideology  happy  riskperc1  riskperc2  optim2
##   9   4  age  children  cnarc1  cnarc2  cnarc3  ctheory1  ctheory2  ctheory3  ctheory4  CRT1  CRT2  CRT3  ladder  mor_circle  moralid1  moralid10  moralid2  moralid3  moralid4  moralid5  moralid6  moralid7  moralid8  moralid9  mcoop3  mcoop4  mcoop5  mcoop6  mcoop7  narc1  narc4  narc5  omind1  omind2  omind3  omind4  omind5  omind6  contact1  psupport1  psupport3  political_ideology  happy  riskperc1  riskperc2  optim2
##   9   5  age  children  cnarc1  cnarc2  cnarc3  ctheory1  ctheory2  ctheory3  ctheory4  CRT1  CRT2  CRT3  ladder  mor_circle  moralid1  moralid10  moralid2  moralid3  moralid4  moralid5  moralid6  moralid7  moralid8  moralid9  mcoop3  mcoop4  mcoop5  mcoop6  mcoop7  narc1  narc4  narc5  omind1  omind2  omind3  omind4  omind5  omind6  contact1  psupport1  psupport3  political_ideology  happy  riskperc1  riskperc2  optim2
##   9   6  age  children  cnarc1  cnarc2  cnarc3  ctheory1  ctheory2  ctheory3  ctheory4  CRT1  CRT2  CRT3  ladder  mor_circle  moralid1  moralid10  moralid2  moralid3  moralid4  moralid5  moralid6  moralid7  moralid8  moralid9  mcoop3  mcoop4  mcoop5  mcoop6  mcoop7  narc1  narc4  narc5  omind1  omind2  omind3  omind4  omind5  omind6  contact1  psupport1  psupport3  political_ideology  happy  riskperc1  riskperc2  optim2
##   9   7  age  children  cnarc1  cnarc2  cnarc3  ctheory1  ctheory2  ctheory3  ctheory4  CRT1  CRT2  CRT3  ladder  mor_circle  moralid1  moralid10  moralid2  moralid3  moralid4  moralid5  moralid6  moralid7  moralid8  moralid9  mcoop3  mcoop4  mcoop5  mcoop6  mcoop7  narc1  narc4  narc5  omind1  omind2  omind3  omind4  omind5  omind6  contact1  psupport1  psupport3  political_ideology  happy  riskperc1  riskperc2  optim2
##   9   8  age  children  cnarc1  cnarc2  cnarc3  ctheory1  ctheory2  ctheory3  ctheory4  CRT1  CRT2  CRT3  ladder  mor_circle  moralid1  moralid10  moralid2  moralid3  moralid4  moralid5  moralid6  moralid7  moralid8  moralid9  mcoop3  mcoop4  mcoop5  mcoop6  mcoop7  narc1  narc4  narc5  omind1  omind2  omind3  omind4  omind5  omind6  contact1  psupport1  psupport3  political_ideology  happy  riskperc1  riskperc2  optim2
##   9   9  age  children  cnarc1  cnarc2  cnarc3  ctheory1  ctheory2  ctheory3  ctheory4  CRT1  CRT2  CRT3  ladder  mor_circle  moralid1  moralid10  moralid2  moralid3  moralid4  moralid5  moralid6  moralid7  moralid8  moralid9  mcoop3  mcoop4  mcoop5  mcoop6  mcoop7  narc1  narc4  narc5  omind1  omind2  omind3  omind4  omind5  omind6  contact1  psupport1  psupport3  political_ideology  happy  riskperc1  riskperc2  optim2
##   9   10  age  children  cnarc1  cnarc2  cnarc3  ctheory1  ctheory2  ctheory3  ctheory4  CRT1  CRT2  CRT3  ladder  mor_circle  moralid1  moralid10  moralid2  moralid3  moralid4  moralid5  moralid6  moralid7  moralid8  moralid9  mcoop3  mcoop4  mcoop5  mcoop6  mcoop7  narc1  narc4  narc5  omind1  omind2  omind3  omind4  omind5  omind6  contact1  psupport1  psupport3  political_ideology  happy  riskperc1  riskperc2  optim2
##   10   1  age  children  cnarc1  cnarc2  cnarc3  ctheory1  ctheory2  ctheory3  ctheory4  CRT1  CRT2  CRT3  ladder  mor_circle  moralid1  moralid10  moralid2  moralid3  moralid4  moralid5  moralid6  moralid7  moralid8  moralid9  mcoop3  mcoop4  mcoop5  mcoop6  mcoop7  narc1  narc4  narc5  omind1  omind2  omind3  omind4  omind5  omind6  contact1  psupport1  psupport3  political_ideology  happy  riskperc1  riskperc2  optim2
##   10   2  age  children  cnarc1  cnarc2  cnarc3  ctheory1  ctheory2  ctheory3  ctheory4  CRT1  CRT2  CRT3  ladder  mor_circle  moralid1  moralid10  moralid2  moralid3  moralid4  moralid5  moralid6  moralid7  moralid8  moralid9  mcoop3  mcoop4  mcoop5  mcoop6  mcoop7  narc1  narc4  narc5  omind1  omind2  omind3  omind4  omind5  omind6  contact1  psupport1  psupport3  political_ideology  happy  riskperc1  riskperc2  optim2
##   10   3  age  children  cnarc1  cnarc2  cnarc3  ctheory1  ctheory2  ctheory3  ctheory4  CRT1  CRT2  CRT3  ladder  mor_circle  moralid1  moralid10  moralid2  moralid3  moralid4  moralid5  moralid6  moralid7  moralid8  moralid9  mcoop3  mcoop4  mcoop5  mcoop6  mcoop7  narc1  narc4  narc5  omind1  omind2  omind3  omind4  omind5  omind6  contact1  psupport1  psupport3  political_ideology  happy  riskperc1  riskperc2  optim2
##   10   4  age  children  cnarc1  cnarc2  cnarc3  ctheory1  ctheory2  ctheory3  ctheory4  CRT1  CRT2  CRT3  ladder  mor_circle  moralid1  moralid10  moralid2  moralid3  moralid4  moralid5  moralid6  moralid7  moralid8  moralid9  mcoop3  mcoop4  mcoop5  mcoop6  mcoop7  narc1  narc4  narc5  omind1  omind2  omind3  omind4  omind5  omind6  contact1  psupport1  psupport3  political_ideology  happy  riskperc1  riskperc2  optim2
##   10   5  age  children  cnarc1  cnarc2  cnarc3  ctheory1  ctheory2  ctheory3  ctheory4  CRT1  CRT2  CRT3  ladder  mor_circle  moralid1  moralid10  moralid2  moralid3  moralid4  moralid5  moralid6  moralid7  moralid8  moralid9  mcoop3  mcoop4  mcoop5  mcoop6  mcoop7  narc1  narc4  narc5  omind1  omind2  omind3  omind4  omind5  omind6  contact1  psupport1  psupport3  political_ideology  happy  riskperc1  riskperc2  optim2
##   10   6  age  children  cnarc1  cnarc2  cnarc3  ctheory1  ctheory2  ctheory3  ctheory4  CRT1  CRT2  CRT3  ladder  mor_circle  moralid1  moralid10  moralid2  moralid3  moralid4  moralid5  moralid6  moralid7  moralid8  moralid9  mcoop3  mcoop4  mcoop5  mcoop6  mcoop7  narc1  narc4  narc5  omind1  omind2  omind3  omind4  omind5  omind6  contact1  psupport1  psupport3  political_ideology  happy  riskperc1  riskperc2  optim2
##   10   7  age  children  cnarc1  cnarc2  cnarc3  ctheory1  ctheory2  ctheory3  ctheory4  CRT1  CRT2  CRT3  ladder  mor_circle  moralid1  moralid10  moralid2  moralid3  moralid4  moralid5  moralid6  moralid7  moralid8  moralid9  mcoop3  mcoop4  mcoop5  mcoop6  mcoop7  narc1  narc4  narc5  omind1  omind2  omind3  omind4  omind5  omind6  contact1  psupport1  psupport3  political_ideology  happy  riskperc1  riskperc2  optim2
##   10   8  age  children  cnarc1  cnarc2  cnarc3  ctheory1  ctheory2  ctheory3  ctheory4  CRT1  CRT2  CRT3  ladder  mor_circle  moralid1  moralid10  moralid2  moralid3  moralid4  moralid5  moralid6  moralid7  moralid8  moralid9  mcoop3  mcoop4  mcoop5  mcoop6  mcoop7  narc1  narc4  narc5  omind1  omind2  omind3  omind4  omind5  omind6  contact1  psupport1  psupport3  political_ideology  happy  riskperc1  riskperc2  optim2
##   10   9  age  children  cnarc1  cnarc2  cnarc3  ctheory1  ctheory2  ctheory3  ctheory4  CRT1  CRT2  CRT3  ladder  mor_circle  moralid1  moralid10  moralid2  moralid3  moralid4  moralid5  moralid6  moralid7  moralid8  moralid9  mcoop3  mcoop4  mcoop5  mcoop6  mcoop7  narc1  narc4  narc5  omind1  omind2  omind3  omind4  omind5  omind6  contact1  psupport1  psupport3  political_ideology  happy  riskperc1  riskperc2  optim2
##   10   10  age  children  cnarc1  cnarc2  cnarc3  ctheory1  ctheory2  ctheory3  ctheory4  CRT1  CRT2  CRT3  ladder  mor_circle  moralid1  moralid10  moralid2  moralid3  moralid4  moralid5  moralid6  moralid7  moralid8  moralid9  mcoop3  mcoop4  mcoop5  mcoop6  mcoop7  narc1  narc4  narc5  omind1  omind2  omind3  omind4  omind5  omind6  contact1  psupport1  psupport3  political_ideology  happy  riskperc1  riskperc2  optim2
```

```
## Warning: Number of logged events: 4603
```

```
## 
##  iter imp variable
##   1   1  generosity1  generosity2  generosity3
##   1   2  generosity1  generosity2  generosity3
##   1   3  generosity1  generosity2  generosity3
##   1   4  generosity1  generosity2  generosity3
##   1   5  generosity1  generosity2  generosity3
##   1   6  generosity1  generosity2  generosity3
##   1   7  generosity1  generosity2  generosity3
##   1   8  generosity1  generosity2  generosity3
##   1   9  generosity1  generosity2  generosity3
##   1   10  generosity1  generosity2  generosity3
##   2   1  generosity1  generosity2  generosity3
##   2   2  generosity1  generosity2  generosity3
##   2   3  generosity1  generosity2  generosity3
##   2   4  generosity1  generosity2  generosity3
##   2   5  generosity1  generosity2  generosity3
##   2   6  generosity1  generosity2  generosity3
##   2   7  generosity1  generosity2  generosity3
##   2   8  generosity1  generosity2  generosity3
##   2   9  generosity1  generosity2  generosity3
##   2   10  generosity1  generosity2  generosity3
##   3   1  generosity1  generosity2  generosity3
##   3   2  generosity1  generosity2  generosity3
##   3   3  generosity1  generosity2  generosity3
##   3   4  generosity1  generosity2  generosity3
##   3   5  generosity1  generosity2  generosity3
##   3   6  generosity1  generosity2  generosity3
##   3   7  generosity1  generosity2  generosity3
##   3   8  generosity1  generosity2  generosity3
##   3   9  generosity1  generosity2  generosity3
##   3   10  generosity1  generosity2  generosity3
##   4   1  generosity1  generosity2  generosity3
##   4   2  generosity1  generosity2  generosity3
##   4   3  generosity1  generosity2  generosity3
##   4   4  generosity1  generosity2  generosity3
##   4   5  generosity1  generosity2  generosity3
##   4   6  generosity1  generosity2  generosity3
##   4   7  generosity1  generosity2  generosity3
##   4   8  generosity1  generosity2  generosity3
##   4   9  generosity1  generosity2  generosity3
##   4   10  generosity1  generosity2  generosity3
##   5   1  generosity1  generosity2  generosity3
##   5   2  generosity1  generosity2  generosity3
##   5   3  generosity1  generosity2  generosity3
##   5   4  generosity1  generosity2  generosity3
##   5   5  generosity1  generosity2  generosity3
##   5   6  generosity1  generosity2  generosity3
##   5   7  generosity1  generosity2  generosity3
##   5   8  generosity1  generosity2  generosity3
##   5   9  generosity1  generosity2  generosity3
##   5   10  generosity1  generosity2  generosity3
##   6   1  generosity1  generosity2  generosity3
##   6   2  generosity1  generosity2  generosity3
##   6   3  generosity1  generosity2  generosity3
##   6   4  generosity1  generosity2  generosity3
##   6   5  generosity1  generosity2  generosity3
##   6   6  generosity1  generosity2  generosity3
##   6   7  generosity1  generosity2  generosity3
##   6   8  generosity1  generosity2  generosity3
##   6   9  generosity1  generosity2  generosity3
##   6   10  generosity1  generosity2  generosity3
##   7   1  generosity1  generosity2  generosity3
##   7   2  generosity1  generosity2  generosity3
##   7   3  generosity1  generosity2  generosity3
##   7   4  generosity1  generosity2  generosity3
##   7   5  generosity1  generosity2  generosity3
##   7   6  generosity1  generosity2  generosity3
##   7   7  generosity1  generosity2  generosity3
##   7   8  generosity1  generosity2  generosity3
##   7   9  generosity1  generosity2  generosity3
##   7   10  generosity1  generosity2  generosity3
##   8   1  generosity1  generosity2  generosity3
##   8   2  generosity1  generosity2  generosity3
##   8   3  generosity1  generosity2  generosity3
##   8   4  generosity1  generosity2  generosity3
##   8   5  generosity1  generosity2  generosity3
##   8   6  generosity1  generosity2  generosity3
##   8   7  generosity1  generosity2  generosity3
##   8   8  generosity1  generosity2  generosity3
##   8   9  generosity1  generosity2  generosity3
##   8   10  generosity1  generosity2  generosity3
##   9   1  generosity1  generosity2  generosity3
##   9   2  generosity1  generosity2  generosity3
##   9   3  generosity1  generosity2  generosity3
##   9   4  generosity1  generosity2  generosity3
##   9   5  generosity1  generosity2  generosity3
##   9   6  generosity1  generosity2  generosity3
##   9   7  generosity1  generosity2  generosity3
##   9   8  generosity1  generosity2  generosity3
##   9   9  generosity1  generosity2  generosity3
##   9   10  generosity1  generosity2  generosity3
##   10   1  generosity1  generosity2  generosity3
##   10   2  generosity1  generosity2  generosity3
##   10   3  generosity1  generosity2  generosity3
##   10   4  generosity1  generosity2  generosity3
##   10   5  generosity1  generosity2  generosity3
##   10   6  generosity1  generosity2  generosity3
##   10   7  generosity1  generosity2  generosity3
##   10   8  generosity1  generosity2  generosity3
##   10   9  generosity1  generosity2  generosity3
##   10   10  generosity1  generosity2  generosity3
```

```
## Warning: Number of logged events: 4
```

```
## 
##  iter imp variable
##   1   1  age  cnarc1  cnarc2  cnarc3  ctheory1  ctheory3  ctheory4  CRT1  CRT2  CRT3  health_cond  know_tested_positive  moralid10  moralid4  moralid5  moralid6  moralid9  mcoop2  mcoop3  mcoop4  mcoop5  mcoop6  mcoop7  narc2  narc4  narc5  narc6  nidentity1  nidentity2  omind1  contact2  contact3  contact4  hygiene2  hygiene4  hygiene5  psupport1  psupport5  political_ideology  happy  slf_ladder  riskperc2  self_esteem  sbelong1  sbelong2  sbelong3  optim1  slfcont1  slfcont2  slfcont3  slfcont4
##   1   2  age  cnarc1  cnarc2  cnarc3  ctheory1  ctheory3  ctheory4  CRT1  CRT2  CRT3  health_cond  know_tested_positive  moralid10  moralid4  moralid5  moralid6  moralid9  mcoop2  mcoop3  mcoop4  mcoop5  mcoop6  mcoop7  narc2  narc4  narc5  narc6  nidentity1  nidentity2  omind1  contact2  contact3  contact4  hygiene2  hygiene4  hygiene5  psupport1  psupport5  political_ideology  happy  slf_ladder  riskperc2  self_esteem  sbelong1  sbelong2  sbelong3  optim1  slfcont1  slfcont2  slfcont3  slfcont4
##   1   3  age  cnarc1  cnarc2  cnarc3  ctheory1  ctheory3  ctheory4  CRT1  CRT2  CRT3  health_cond  know_tested_positive  moralid10  moralid4  moralid5  moralid6  moralid9  mcoop2  mcoop3  mcoop4  mcoop5  mcoop6  mcoop7  narc2  narc4  narc5  narc6  nidentity1  nidentity2  omind1  contact2  contact3  contact4  hygiene2  hygiene4  hygiene5  psupport1  psupport5  political_ideology  happy  slf_ladder  riskperc2  self_esteem  sbelong1  sbelong2  sbelong3  optim1  slfcont1  slfcont2  slfcont3  slfcont4
##   1   4  age  cnarc1  cnarc2  cnarc3  ctheory1  ctheory3  ctheory4  CRT1  CRT2  CRT3  health_cond  know_tested_positive  moralid10  moralid4  moralid5  moralid6  moralid9  mcoop2  mcoop3  mcoop4  mcoop5  mcoop6  mcoop7  narc2  narc4  narc5  narc6  nidentity1  nidentity2  omind1  contact2  contact3  contact4  hygiene2  hygiene4  hygiene5  psupport1  psupport5  political_ideology  happy  slf_ladder  riskperc2  self_esteem  sbelong1  sbelong2  sbelong3  optim1  slfcont1  slfcont2  slfcont3  slfcont4
##   1   5  age  cnarc1  cnarc2  cnarc3  ctheory1  ctheory3  ctheory4  CRT1  CRT2  CRT3  health_cond  know_tested_positive  moralid10  moralid4  moralid5  moralid6  moralid9  mcoop2  mcoop3  mcoop4  mcoop5  mcoop6  mcoop7  narc2  narc4  narc5  narc6  nidentity1  nidentity2  omind1  contact2  contact3  contact4  hygiene2  hygiene4  hygiene5  psupport1  psupport5  political_ideology  happy  slf_ladder  riskperc2  self_esteem  sbelong1  sbelong2  sbelong3  optim1  slfcont1  slfcont2  slfcont3  slfcont4
##   1   6  age  cnarc1  cnarc2  cnarc3  ctheory1  ctheory3  ctheory4  CRT1  CRT2  CRT3  health_cond  know_tested_positive  moralid10  moralid4  moralid5  moralid6  moralid9  mcoop2  mcoop3  mcoop4  mcoop5  mcoop6  mcoop7  narc2  narc4  narc5  narc6  nidentity1  nidentity2  omind1  contact2  contact3  contact4  hygiene2  hygiene4  hygiene5  psupport1  psupport5  political_ideology  happy  slf_ladder  riskperc2  self_esteem  sbelong1  sbelong2  sbelong3  optim1  slfcont1  slfcont2  slfcont3  slfcont4
##   1   7  age  cnarc1  cnarc2  cnarc3  ctheory1  ctheory3  ctheory4  CRT1  CRT2  CRT3  health_cond  know_tested_positive  moralid10  moralid4  moralid5  moralid6  moralid9  mcoop2  mcoop3  mcoop4  mcoop5  mcoop6  mcoop7  narc2  narc4  narc5  narc6  nidentity1  nidentity2  omind1  contact2  contact3  contact4  hygiene2  hygiene4  hygiene5  psupport1  psupport5  political_ideology  happy  slf_ladder  riskperc2  self_esteem  sbelong1  sbelong2  sbelong3  optim1  slfcont1  slfcont2  slfcont3  slfcont4
##   1   8  age  cnarc1  cnarc2  cnarc3  ctheory1  ctheory3  ctheory4  CRT1  CRT2  CRT3  health_cond  know_tested_positive  moralid10  moralid4  moralid5  moralid6  moralid9  mcoop2  mcoop3  mcoop4  mcoop5  mcoop6  mcoop7  narc2  narc4  narc5  narc6  nidentity1  nidentity2  omind1  contact2  contact3  contact4  hygiene2  hygiene4  hygiene5  psupport1  psupport5  political_ideology  happy  slf_ladder  riskperc2  self_esteem  sbelong1  sbelong2  sbelong3  optim1  slfcont1  slfcont2  slfcont3  slfcont4
##   1   9  age  cnarc1  cnarc2  cnarc3  ctheory1  ctheory3  ctheory4  CRT1  CRT2  CRT3  health_cond  know_tested_positive  moralid10  moralid4  moralid5  moralid6  moralid9  mcoop2  mcoop3  mcoop4  mcoop5  mcoop6  mcoop7  narc2  narc4  narc5  narc6  nidentity1  nidentity2  omind1  contact2  contact3  contact4  hygiene2  hygiene4  hygiene5  psupport1  psupport5  political_ideology  happy  slf_ladder  riskperc2  self_esteem  sbelong1  sbelong2  sbelong3  optim1  slfcont1  slfcont2  slfcont3  slfcont4
##   1   10  age  cnarc1  cnarc2  cnarc3  ctheory1  ctheory3  ctheory4  CRT1  CRT2  CRT3  health_cond  know_tested_positive  moralid10  moralid4  moralid5  moralid6  moralid9  mcoop2  mcoop3  mcoop4  mcoop5  mcoop6  mcoop7  narc2  narc4  narc5  narc6  nidentity1  nidentity2  omind1  contact2  contact3  contact4  hygiene2  hygiene4  hygiene5  psupport1  psupport5  political_ideology  happy  slf_ladder  riskperc2  self_esteem  sbelong1  sbelong2  sbelong3  optim1  slfcont1  slfcont2  slfcont3  slfcont4
##   2   1  age  cnarc1  cnarc2  cnarc3  ctheory1  ctheory3  ctheory4  CRT1  CRT2  CRT3  health_cond  know_tested_positive  moralid10  moralid4  moralid5  moralid6  moralid9  mcoop2  mcoop3  mcoop4  mcoop5  mcoop6  mcoop7  narc2  narc4  narc5  narc6  nidentity1  nidentity2  omind1  contact2  contact3  contact4  hygiene2  hygiene4  hygiene5  psupport1  psupport5  political_ideology  happy  slf_ladder  riskperc2  self_esteem  sbelong1  sbelong2  sbelong3  optim1  slfcont1  slfcont2  slfcont3  slfcont4
##   2   2  age  cnarc1  cnarc2  cnarc3  ctheory1  ctheory3  ctheory4  CRT1  CRT2  CRT3  health_cond  know_tested_positive  moralid10  moralid4  moralid5  moralid6  moralid9  mcoop2  mcoop3  mcoop4  mcoop5  mcoop6  mcoop7  narc2  narc4  narc5  narc6  nidentity1  nidentity2  omind1  contact2  contact3  contact4  hygiene2  hygiene4  hygiene5  psupport1  psupport5  political_ideology  happy  slf_ladder  riskperc2  self_esteem  sbelong1  sbelong2  sbelong3  optim1  slfcont1  slfcont2  slfcont3  slfcont4
##   2   3  age  cnarc1  cnarc2  cnarc3  ctheory1  ctheory3  ctheory4  CRT1  CRT2  CRT3  health_cond  know_tested_positive  moralid10  moralid4  moralid5  moralid6  moralid9  mcoop2  mcoop3  mcoop4  mcoop5  mcoop6  mcoop7  narc2  narc4  narc5  narc6  nidentity1  nidentity2  omind1  contact2  contact3  contact4  hygiene2  hygiene4  hygiene5  psupport1  psupport5  political_ideology  happy  slf_ladder  riskperc2  self_esteem  sbelong1  sbelong2  sbelong3  optim1  slfcont1  slfcont2  slfcont3  slfcont4
##   2   4  age  cnarc1  cnarc2  cnarc3  ctheory1  ctheory3  ctheory4  CRT1  CRT2  CRT3  health_cond  know_tested_positive  moralid10  moralid4  moralid5  moralid6  moralid9  mcoop2  mcoop3  mcoop4  mcoop5  mcoop6  mcoop7  narc2  narc4  narc5  narc6  nidentity1  nidentity2  omind1  contact2  contact3  contact4  hygiene2  hygiene4  hygiene5  psupport1  psupport5  political_ideology  happy  slf_ladder  riskperc2  self_esteem  sbelong1  sbelong2  sbelong3  optim1  slfcont1  slfcont2  slfcont3  slfcont4
##   2   5  age  cnarc1  cnarc2  cnarc3  ctheory1  ctheory3  ctheory4  CRT1  CRT2  CRT3  health_cond  know_tested_positive  moralid10  moralid4  moralid5  moralid6  moralid9  mcoop2  mcoop3  mcoop4  mcoop5  mcoop6  mcoop7  narc2  narc4  narc5  narc6  nidentity1  nidentity2  omind1  contact2  contact3  contact4  hygiene2  hygiene4  hygiene5  psupport1  psupport5  political_ideology  happy  slf_ladder  riskperc2  self_esteem  sbelong1  sbelong2  sbelong3  optim1  slfcont1  slfcont2  slfcont3  slfcont4
##   2   6  age  cnarc1  cnarc2  cnarc3  ctheory1  ctheory3  ctheory4  CRT1  CRT2  CRT3  health_cond  know_tested_positive  moralid10  moralid4  moralid5  moralid6  moralid9  mcoop2  mcoop3  mcoop4  mcoop5  mcoop6  mcoop7  narc2  narc4  narc5  narc6  nidentity1  nidentity2  omind1  contact2  contact3  contact4  hygiene2  hygiene4  hygiene5  psupport1  psupport5  political_ideology  happy  slf_ladder  riskperc2  self_esteem  sbelong1  sbelong2  sbelong3  optim1  slfcont1  slfcont2  slfcont3  slfcont4
##   2   7  age  cnarc1  cnarc2  cnarc3  ctheory1  ctheory3  ctheory4  CRT1  CRT2  CRT3  health_cond  know_tested_positive  moralid10  moralid4  moralid5  moralid6  moralid9  mcoop2  mcoop3  mcoop4  mcoop5  mcoop6  mcoop7  narc2  narc4  narc5  narc6  nidentity1  nidentity2  omind1  contact2  contact3  contact4  hygiene2  hygiene4  hygiene5  psupport1  psupport5  political_ideology  happy  slf_ladder  riskperc2  self_esteem  sbelong1  sbelong2  sbelong3  optim1  slfcont1  slfcont2  slfcont3  slfcont4
##   2   8  age  cnarc1  cnarc2  cnarc3  ctheory1  ctheory3  ctheory4  CRT1  CRT2  CRT3  health_cond  know_tested_positive  moralid10  moralid4  moralid5  moralid6  moralid9  mcoop2  mcoop3  mcoop4  mcoop5  mcoop6  mcoop7  narc2  narc4  narc5  narc6  nidentity1  nidentity2  omind1  contact2  contact3  contact4  hygiene2  hygiene4  hygiene5  psupport1  psupport5  political_ideology  happy  slf_ladder  riskperc2  self_esteem  sbelong1  sbelong2  sbelong3  optim1  slfcont1  slfcont2  slfcont3  slfcont4
##   2   9  age  cnarc1  cnarc2  cnarc3  ctheory1  ctheory3  ctheory4  CRT1  CRT2  CRT3  health_cond  know_tested_positive  moralid10  moralid4  moralid5  moralid6  moralid9  mcoop2  mcoop3  mcoop4  mcoop5  mcoop6  mcoop7  narc2  narc4  narc5  narc6  nidentity1  nidentity2  omind1  contact2  contact3  contact4  hygiene2  hygiene4  hygiene5  psupport1  psupport5  political_ideology  happy  slf_ladder  riskperc2  self_esteem  sbelong1  sbelong2  sbelong3  optim1  slfcont1  slfcont2  slfcont3  slfcont4
##   2   10  age  cnarc1  cnarc2  cnarc3  ctheory1  ctheory3  ctheory4  CRT1  CRT2  CRT3  health_cond  know_tested_positive  moralid10  moralid4  moralid5  moralid6  moralid9  mcoop2  mcoop3  mcoop4  mcoop5  mcoop6  mcoop7  narc2  narc4  narc5  narc6  nidentity1  nidentity2  omind1  contact2  contact3  contact4  hygiene2  hygiene4  hygiene5  psupport1  psupport5  political_ideology  happy  slf_ladder  riskperc2  self_esteem  sbelong1  sbelong2  sbelong3  optim1  slfcont1  slfcont2  slfcont3  slfcont4
##   3   1  age  cnarc1  cnarc2  cnarc3  ctheory1  ctheory3  ctheory4  CRT1  CRT2  CRT3  health_cond  know_tested_positive  moralid10  moralid4  moralid5  moralid6  moralid9  mcoop2  mcoop3  mcoop4  mcoop5  mcoop6  mcoop7  narc2  narc4  narc5  narc6  nidentity1  nidentity2  omind1  contact2  contact3  contact4  hygiene2  hygiene4  hygiene5  psupport1  psupport5  political_ideology  happy  slf_ladder  riskperc2  self_esteem  sbelong1  sbelong2  sbelong3  optim1  slfcont1  slfcont2  slfcont3  slfcont4
##   3   2  age  cnarc1  cnarc2  cnarc3  ctheory1  ctheory3  ctheory4  CRT1  CRT2  CRT3  health_cond  know_tested_positive  moralid10  moralid4  moralid5  moralid6  moralid9  mcoop2  mcoop3  mcoop4  mcoop5  mcoop6  mcoop7  narc2  narc4  narc5  narc6  nidentity1  nidentity2  omind1  contact2  contact3  contact4  hygiene2  hygiene4  hygiene5  psupport1  psupport5  political_ideology  happy  slf_ladder  riskperc2  self_esteem  sbelong1  sbelong2  sbelong3  optim1  slfcont1  slfcont2  slfcont3  slfcont4
##   3   3  age  cnarc1  cnarc2  cnarc3  ctheory1  ctheory3  ctheory4  CRT1  CRT2  CRT3  health_cond  know_tested_positive  moralid10  moralid4  moralid5  moralid6  moralid9  mcoop2  mcoop3  mcoop4  mcoop5  mcoop6  mcoop7  narc2  narc4  narc5  narc6  nidentity1  nidentity2  omind1  contact2  contact3  contact4  hygiene2  hygiene4  hygiene5  psupport1  psupport5  political_ideology  happy  slf_ladder  riskperc2  self_esteem  sbelong1  sbelong2  sbelong3  optim1  slfcont1  slfcont2  slfcont3  slfcont4
##   3   4  age  cnarc1  cnarc2  cnarc3  ctheory1  ctheory3  ctheory4  CRT1  CRT2  CRT3  health_cond  know_tested_positive  moralid10  moralid4  moralid5  moralid6  moralid9  mcoop2  mcoop3  mcoop4  mcoop5  mcoop6  mcoop7  narc2  narc4  narc5  narc6  nidentity1  nidentity2  omind1  contact2  contact3  contact4  hygiene2  hygiene4  hygiene5  psupport1  psupport5  political_ideology  happy  slf_ladder  riskperc2  self_esteem  sbelong1  sbelong2  sbelong3  optim1  slfcont1  slfcont2  slfcont3  slfcont4
##   3   5  age  cnarc1  cnarc2  cnarc3  ctheory1  ctheory3  ctheory4  CRT1  CRT2  CRT3  health_cond  know_tested_positive  moralid10  moralid4  moralid5  moralid6  moralid9  mcoop2  mcoop3  mcoop4  mcoop5  mcoop6  mcoop7  narc2  narc4  narc5  narc6  nidentity1  nidentity2  omind1  contact2  contact3  contact4  hygiene2  hygiene4  hygiene5  psupport1  psupport5  political_ideology  happy  slf_ladder  riskperc2  self_esteem  sbelong1  sbelong2  sbelong3  optim1  slfcont1  slfcont2  slfcont3  slfcont4
##   3   6  age  cnarc1  cnarc2  cnarc3  ctheory1  ctheory3  ctheory4  CRT1  CRT2  CRT3  health_cond  know_tested_positive  moralid10  moralid4  moralid5  moralid6  moralid9  mcoop2  mcoop3  mcoop4  mcoop5  mcoop6  mcoop7  narc2  narc4  narc5  narc6  nidentity1  nidentity2  omind1  contact2  contact3  contact4  hygiene2  hygiene4  hygiene5  psupport1  psupport5  political_ideology  happy  slf_ladder  riskperc2  self_esteem  sbelong1  sbelong2  sbelong3  optim1  slfcont1  slfcont2  slfcont3  slfcont4
##   3   7  age  cnarc1  cnarc2  cnarc3  ctheory1  ctheory3  ctheory4  CRT1  CRT2  CRT3  health_cond  know_tested_positive  moralid10  moralid4  moralid5  moralid6  moralid9  mcoop2  mcoop3  mcoop4  mcoop5  mcoop6  mcoop7  narc2  narc4  narc5  narc6  nidentity1  nidentity2  omind1  contact2  contact3  contact4  hygiene2  hygiene4  hygiene5  psupport1  psupport5  political_ideology  happy  slf_ladder  riskperc2  self_esteem  sbelong1  sbelong2  sbelong3  optim1  slfcont1  slfcont2  slfcont3  slfcont4
##   3   8  age  cnarc1  cnarc2  cnarc3  ctheory1  ctheory3  ctheory4  CRT1  CRT2  CRT3  health_cond  know_tested_positive  moralid10  moralid4  moralid5  moralid6  moralid9  mcoop2  mcoop3  mcoop4  mcoop5  mcoop6  mcoop7  narc2  narc4  narc5  narc6  nidentity1  nidentity2  omind1  contact2  contact3  contact4  hygiene2  hygiene4  hygiene5  psupport1  psupport5  political_ideology  happy  slf_ladder  riskperc2  self_esteem  sbelong1  sbelong2  sbelong3  optim1  slfcont1  slfcont2  slfcont3  slfcont4
##   3   9  age  cnarc1  cnarc2  cnarc3  ctheory1  ctheory3  ctheory4  CRT1  CRT2  CRT3  health_cond  know_tested_positive  moralid10  moralid4  moralid5  moralid6  moralid9  mcoop2  mcoop3  mcoop4  mcoop5  mcoop6  mcoop7  narc2  narc4  narc5  narc6  nidentity1  nidentity2  omind1  contact2  contact3  contact4  hygiene2  hygiene4  hygiene5  psupport1  psupport5  political_ideology  happy  slf_ladder  riskperc2  self_esteem  sbelong1  sbelong2  sbelong3  optim1  slfcont1  slfcont2  slfcont3  slfcont4
##   3   10  age  cnarc1  cnarc2  cnarc3  ctheory1  ctheory3  ctheory4  CRT1  CRT2  CRT3  health_cond  know_tested_positive  moralid10  moralid4  moralid5  moralid6  moralid9  mcoop2  mcoop3  mcoop4  mcoop5  mcoop6  mcoop7  narc2  narc4  narc5  narc6  nidentity1  nidentity2  omind1  contact2  contact3  contact4  hygiene2  hygiene4  hygiene5  psupport1  psupport5  political_ideology  happy  slf_ladder  riskperc2  self_esteem  sbelong1  sbelong2  sbelong3  optim1  slfcont1  slfcont2  slfcont3  slfcont4
##   4   1  age  cnarc1  cnarc2  cnarc3  ctheory1  ctheory3  ctheory4  CRT1  CRT2  CRT3  health_cond  know_tested_positive  moralid10  moralid4  moralid5  moralid6  moralid9  mcoop2  mcoop3  mcoop4  mcoop5  mcoop6  mcoop7  narc2  narc4  narc5  narc6  nidentity1  nidentity2  omind1  contact2  contact3  contact4  hygiene2  hygiene4  hygiene5  psupport1  psupport5  political_ideology  happy  slf_ladder  riskperc2  self_esteem  sbelong1  sbelong2  sbelong3  optim1  slfcont1  slfcont2  slfcont3  slfcont4
##   4   2  age  cnarc1  cnarc2  cnarc3  ctheory1  ctheory3  ctheory4  CRT1  CRT2  CRT3  health_cond  know_tested_positive  moralid10  moralid4  moralid5  moralid6  moralid9  mcoop2  mcoop3  mcoop4  mcoop5  mcoop6  mcoop7  narc2  narc4  narc5  narc6  nidentity1  nidentity2  omind1  contact2  contact3  contact4  hygiene2  hygiene4  hygiene5  psupport1  psupport5  political_ideology  happy  slf_ladder  riskperc2  self_esteem  sbelong1  sbelong2  sbelong3  optim1  slfcont1  slfcont2  slfcont3  slfcont4
##   4   3  age  cnarc1  cnarc2  cnarc3  ctheory1  ctheory3  ctheory4  CRT1  CRT2  CRT3  health_cond  know_tested_positive  moralid10  moralid4  moralid5  moralid6  moralid9  mcoop2  mcoop3  mcoop4  mcoop5  mcoop6  mcoop7  narc2  narc4  narc5  narc6  nidentity1  nidentity2  omind1  contact2  contact3  contact4  hygiene2  hygiene4  hygiene5  psupport1  psupport5  political_ideology  happy  slf_ladder  riskperc2  self_esteem  sbelong1  sbelong2  sbelong3  optim1  slfcont1  slfcont2  slfcont3  slfcont4
##   4   4  age  cnarc1  cnarc2  cnarc3  ctheory1  ctheory3  ctheory4  CRT1  CRT2  CRT3  health_cond  know_tested_positive  moralid10  moralid4  moralid5  moralid6  moralid9  mcoop2  mcoop3  mcoop4  mcoop5  mcoop6  mcoop7  narc2  narc4  narc5  narc6  nidentity1  nidentity2  omind1  contact2  contact3  contact4  hygiene2  hygiene4  hygiene5  psupport1  psupport5  political_ideology  happy  slf_ladder  riskperc2  self_esteem  sbelong1  sbelong2  sbelong3  optim1  slfcont1  slfcont2  slfcont3  slfcont4
##   4   5  age  cnarc1  cnarc2  cnarc3  ctheory1  ctheory3  ctheory4  CRT1  CRT2  CRT3  health_cond  know_tested_positive  moralid10  moralid4  moralid5  moralid6  moralid9  mcoop2  mcoop3  mcoop4  mcoop5  mcoop6  mcoop7  narc2  narc4  narc5  narc6  nidentity1  nidentity2  omind1  contact2  contact3  contact4  hygiene2  hygiene4  hygiene5  psupport1  psupport5  political_ideology  happy  slf_ladder  riskperc2  self_esteem  sbelong1  sbelong2  sbelong3  optim1  slfcont1  slfcont2  slfcont3  slfcont4
##   4   6  age  cnarc1  cnarc2  cnarc3  ctheory1  ctheory3  ctheory4  CRT1  CRT2  CRT3  health_cond  know_tested_positive  moralid10  moralid4  moralid5  moralid6  moralid9  mcoop2  mcoop3  mcoop4  mcoop5  mcoop6  mcoop7  narc2  narc4  narc5  narc6  nidentity1  nidentity2  omind1  contact2  contact3  contact4  hygiene2  hygiene4  hygiene5  psupport1  psupport5  political_ideology  happy  slf_ladder  riskperc2  self_esteem  sbelong1  sbelong2  sbelong3  optim1  slfcont1  slfcont2  slfcont3  slfcont4
##   4   7  age  cnarc1  cnarc2  cnarc3  ctheory1  ctheory3  ctheory4  CRT1  CRT2  CRT3  health_cond  know_tested_positive  moralid10  moralid4  moralid5  moralid6  moralid9  mcoop2  mcoop3  mcoop4  mcoop5  mcoop6  mcoop7  narc2  narc4  narc5  narc6  nidentity1  nidentity2  omind1  contact2  contact3  contact4  hygiene2  hygiene4  hygiene5  psupport1  psupport5  political_ideology  happy  slf_ladder  riskperc2  self_esteem  sbelong1  sbelong2  sbelong3  optim1  slfcont1  slfcont2  slfcont3  slfcont4
##   4   8  age  cnarc1  cnarc2  cnarc3  ctheory1  ctheory3  ctheory4  CRT1  CRT2  CRT3  health_cond  know_tested_positive  moralid10  moralid4  moralid5  moralid6  moralid9  mcoop2  mcoop3  mcoop4  mcoop5  mcoop6  mcoop7  narc2  narc4  narc5  narc6  nidentity1  nidentity2  omind1  contact2  contact3  contact4  hygiene2  hygiene4  hygiene5  psupport1  psupport5  political_ideology  happy  slf_ladder  riskperc2  self_esteem  sbelong1  sbelong2  sbelong3  optim1  slfcont1  slfcont2  slfcont3  slfcont4
##   4   9  age  cnarc1  cnarc2  cnarc3  ctheory1  ctheory3  ctheory4  CRT1  CRT2  CRT3  health_cond  know_tested_positive  moralid10  moralid4  moralid5  moralid6  moralid9  mcoop2  mcoop3  mcoop4  mcoop5  mcoop6  mcoop7  narc2  narc4  narc5  narc6  nidentity1  nidentity2  omind1  contact2  contact3  contact4  hygiene2  hygiene4  hygiene5  psupport1  psupport5  political_ideology  happy  slf_ladder  riskperc2  self_esteem  sbelong1  sbelong2  sbelong3  optim1  slfcont1  slfcont2  slfcont3  slfcont4
##   4   10  age  cnarc1  cnarc2  cnarc3  ctheory1  ctheory3  ctheory4  CRT1  CRT2  CRT3  health_cond  know_tested_positive  moralid10  moralid4  moralid5  moralid6  moralid9  mcoop2  mcoop3  mcoop4  mcoop5  mcoop6  mcoop7  narc2  narc4  narc5  narc6  nidentity1  nidentity2  omind1  contact2  contact3  contact4  hygiene2  hygiene4  hygiene5  psupport1  psupport5  political_ideology  happy  slf_ladder  riskperc2  self_esteem  sbelong1  sbelong2  sbelong3  optim1  slfcont1  slfcont2  slfcont3  slfcont4
##   5   1  age  cnarc1  cnarc2  cnarc3  ctheory1  ctheory3  ctheory4  CRT1  CRT2  CRT3  health_cond  know_tested_positive  moralid10  moralid4  moralid5  moralid6  moralid9  mcoop2  mcoop3  mcoop4  mcoop5  mcoop6  mcoop7  narc2  narc4  narc5  narc6  nidentity1  nidentity2  omind1  contact2  contact3  contact4  hygiene2  hygiene4  hygiene5  psupport1  psupport5  political_ideology  happy  slf_ladder  riskperc2  self_esteem  sbelong1  sbelong2  sbelong3  optim1  slfcont1  slfcont2  slfcont3  slfcont4
##   5   2  age  cnarc1  cnarc2  cnarc3  ctheory1  ctheory3  ctheory4  CRT1  CRT2  CRT3  health_cond  know_tested_positive  moralid10  moralid4  moralid5  moralid6  moralid9  mcoop2  mcoop3  mcoop4  mcoop5  mcoop6  mcoop7  narc2  narc4  narc5  narc6  nidentity1  nidentity2  omind1  contact2  contact3  contact4  hygiene2  hygiene4  hygiene5  psupport1  psupport5  political_ideology  happy  slf_ladder  riskperc2  self_esteem  sbelong1  sbelong2  sbelong3  optim1  slfcont1  slfcont2  slfcont3  slfcont4
##   5   3  age  cnarc1  cnarc2  cnarc3  ctheory1  ctheory3  ctheory4  CRT1  CRT2  CRT3  health_cond  know_tested_positive  moralid10  moralid4  moralid5  moralid6  moralid9  mcoop2  mcoop3  mcoop4  mcoop5  mcoop6  mcoop7  narc2  narc4  narc5  narc6  nidentity1  nidentity2  omind1  contact2  contact3  contact4  hygiene2  hygiene4  hygiene5  psupport1  psupport5  political_ideology  happy  slf_ladder  riskperc2  self_esteem  sbelong1  sbelong2  sbelong3  optim1  slfcont1  slfcont2  slfcont3  slfcont4
##   5   4  age  cnarc1  cnarc2  cnarc3  ctheory1  ctheory3  ctheory4  CRT1  CRT2  CRT3  health_cond  know_tested_positive  moralid10  moralid4  moralid5  moralid6  moralid9  mcoop2  mcoop3  mcoop4  mcoop5  mcoop6  mcoop7  narc2  narc4  narc5  narc6  nidentity1  nidentity2  omind1  contact2  contact3  contact4  hygiene2  hygiene4  hygiene5  psupport1  psupport5  political_ideology  happy  slf_ladder  riskperc2  self_esteem  sbelong1  sbelong2  sbelong3  optim1  slfcont1  slfcont2  slfcont3  slfcont4
##   5   5  age  cnarc1  cnarc2  cnarc3  ctheory1  ctheory3  ctheory4  CRT1  CRT2  CRT3  health_cond  know_tested_positive  moralid10  moralid4  moralid5  moralid6  moralid9  mcoop2  mcoop3  mcoop4  mcoop5  mcoop6  mcoop7  narc2  narc4  narc5  narc6  nidentity1  nidentity2  omind1  contact2  contact3  contact4  hygiene2  hygiene4  hygiene5  psupport1  psupport5  political_ideology  happy  slf_ladder  riskperc2  self_esteem  sbelong1  sbelong2  sbelong3  optim1  slfcont1  slfcont2  slfcont3  slfcont4
##   5   6  age  cnarc1  cnarc2  cnarc3  ctheory1  ctheory3  ctheory4  CRT1  CRT2  CRT3  health_cond  know_tested_positive  moralid10  moralid4  moralid5  moralid6  moralid9  mcoop2  mcoop3  mcoop4  mcoop5  mcoop6  mcoop7  narc2  narc4  narc5  narc6  nidentity1  nidentity2  omind1  contact2  contact3  contact4  hygiene2  hygiene4  hygiene5  psupport1  psupport5  political_ideology  happy  slf_ladder  riskperc2  self_esteem  sbelong1  sbelong2  sbelong3  optim1  slfcont1  slfcont2  slfcont3  slfcont4
##   5   7  age  cnarc1  cnarc2  cnarc3  ctheory1  ctheory3  ctheory4  CRT1  CRT2  CRT3  health_cond  know_tested_positive  moralid10  moralid4  moralid5  moralid6  moralid9  mcoop2  mcoop3  mcoop4  mcoop5  mcoop6  mcoop7  narc2  narc4  narc5  narc6  nidentity1  nidentity2  omind1  contact2  contact3  contact4  hygiene2  hygiene4  hygiene5  psupport1  psupport5  political_ideology  happy  slf_ladder  riskperc2  self_esteem  sbelong1  sbelong2  sbelong3  optim1  slfcont1  slfcont2  slfcont3  slfcont4
##   5   8  age  cnarc1  cnarc2  cnarc3  ctheory1  ctheory3  ctheory4  CRT1  CRT2  CRT3  health_cond  know_tested_positive  moralid10  moralid4  moralid5  moralid6  moralid9  mcoop2  mcoop3  mcoop4  mcoop5  mcoop6  mcoop7  narc2  narc4  narc5  narc6  nidentity1  nidentity2  omind1  contact2  contact3  contact4  hygiene2  hygiene4  hygiene5  psupport1  psupport5  political_ideology  happy  slf_ladder  riskperc2  self_esteem  sbelong1  sbelong2  sbelong3  optim1  slfcont1  slfcont2  slfcont3  slfcont4
##   5   9  age  cnarc1  cnarc2  cnarc3  ctheory1  ctheory3  ctheory4  CRT1  CRT2  CRT3  health_cond  know_tested_positive  moralid10  moralid4  moralid5  moralid6  moralid9  mcoop2  mcoop3  mcoop4  mcoop5  mcoop6  mcoop7  narc2  narc4  narc5  narc6  nidentity1  nidentity2  omind1  contact2  contact3  contact4  hygiene2  hygiene4  hygiene5  psupport1  psupport5  political_ideology  happy  slf_ladder  riskperc2  self_esteem  sbelong1  sbelong2  sbelong3  optim1  slfcont1  slfcont2  slfcont3  slfcont4
##   5   10  age  cnarc1  cnarc2  cnarc3  ctheory1  ctheory3  ctheory4  CRT1  CRT2  CRT3  health_cond  know_tested_positive  moralid10  moralid4  moralid5  moralid6  moralid9  mcoop2  mcoop3  mcoop4  mcoop5  mcoop6  mcoop7  narc2  narc4  narc5  narc6  nidentity1  nidentity2  omind1  contact2  contact3  contact4  hygiene2  hygiene4  hygiene5  psupport1  psupport5  political_ideology  happy  slf_ladder  riskperc2  self_esteem  sbelong1  sbelong2  sbelong3  optim1  slfcont1  slfcont2  slfcont3  slfcont4
##   6   1  age  cnarc1  cnarc2  cnarc3  ctheory1  ctheory3  ctheory4  CRT1  CRT2  CRT3  health_cond  know_tested_positive  moralid10  moralid4  moralid5  moralid6  moralid9  mcoop2  mcoop3  mcoop4  mcoop5  mcoop6  mcoop7  narc2  narc4  narc5  narc6  nidentity1  nidentity2  omind1  contact2  contact3  contact4  hygiene2  hygiene4  hygiene5  psupport1  psupport5  political_ideology  happy  slf_ladder  riskperc2  self_esteem  sbelong1  sbelong2  sbelong3  optim1  slfcont1  slfcont2  slfcont3  slfcont4
##   6   2  age  cnarc1  cnarc2  cnarc3  ctheory1  ctheory3  ctheory4  CRT1  CRT2  CRT3  health_cond  know_tested_positive  moralid10  moralid4  moralid5  moralid6  moralid9  mcoop2  mcoop3  mcoop4  mcoop5  mcoop6  mcoop7  narc2  narc4  narc5  narc6  nidentity1  nidentity2  omind1  contact2  contact3  contact4  hygiene2  hygiene4  hygiene5  psupport1  psupport5  political_ideology  happy  slf_ladder  riskperc2  self_esteem  sbelong1  sbelong2  sbelong3  optim1  slfcont1  slfcont2  slfcont3  slfcont4
##   6   3  age  cnarc1  cnarc2  cnarc3  ctheory1  ctheory3  ctheory4  CRT1  CRT2  CRT3  health_cond  know_tested_positive  moralid10  moralid4  moralid5  moralid6  moralid9  mcoop2  mcoop3  mcoop4  mcoop5  mcoop6  mcoop7  narc2  narc4  narc5  narc6  nidentity1  nidentity2  omind1  contact2  contact3  contact4  hygiene2  hygiene4  hygiene5  psupport1  psupport5  political_ideology  happy  slf_ladder  riskperc2  self_esteem  sbelong1  sbelong2  sbelong3  optim1  slfcont1  slfcont2  slfcont3  slfcont4
##   6   4  age  cnarc1  cnarc2  cnarc3  ctheory1  ctheory3  ctheory4  CRT1  CRT2  CRT3  health_cond  know_tested_positive  moralid10  moralid4  moralid5  moralid6  moralid9  mcoop2  mcoop3  mcoop4  mcoop5  mcoop6  mcoop7  narc2  narc4  narc5  narc6  nidentity1  nidentity2  omind1  contact2  contact3  contact4  hygiene2  hygiene4  hygiene5  psupport1  psupport5  political_ideology  happy  slf_ladder  riskperc2  self_esteem  sbelong1  sbelong2  sbelong3  optim1  slfcont1  slfcont2  slfcont3  slfcont4
##   6   5  age  cnarc1  cnarc2  cnarc3  ctheory1  ctheory3  ctheory4  CRT1  CRT2  CRT3  health_cond  know_tested_positive  moralid10  moralid4  moralid5  moralid6  moralid9  mcoop2  mcoop3  mcoop4  mcoop5  mcoop6  mcoop7  narc2  narc4  narc5  narc6  nidentity1  nidentity2  omind1  contact2  contact3  contact4  hygiene2  hygiene4  hygiene5  psupport1  psupport5  political_ideology  happy  slf_ladder  riskperc2  self_esteem  sbelong1  sbelong2  sbelong3  optim1  slfcont1  slfcont2  slfcont3  slfcont4
##   6   6  age  cnarc1  cnarc2  cnarc3  ctheory1  ctheory3  ctheory4  CRT1  CRT2  CRT3  health_cond  know_tested_positive  moralid10  moralid4  moralid5  moralid6  moralid9  mcoop2  mcoop3  mcoop4  mcoop5  mcoop6  mcoop7  narc2  narc4  narc5  narc6  nidentity1  nidentity2  omind1  contact2  contact3  contact4  hygiene2  hygiene4  hygiene5  psupport1  psupport5  political_ideology  happy  slf_ladder  riskperc2  self_esteem  sbelong1  sbelong2  sbelong3  optim1  slfcont1  slfcont2  slfcont3  slfcont4
##   6   7  age  cnarc1  cnarc2  cnarc3  ctheory1  ctheory3  ctheory4  CRT1  CRT2  CRT3  health_cond  know_tested_positive  moralid10  moralid4  moralid5  moralid6  moralid9  mcoop2  mcoop3  mcoop4  mcoop5  mcoop6  mcoop7  narc2  narc4  narc5  narc6  nidentity1  nidentity2  omind1  contact2  contact3  contact4  hygiene2  hygiene4  hygiene5  psupport1  psupport5  political_ideology  happy  slf_ladder  riskperc2  self_esteem  sbelong1  sbelong2  sbelong3  optim1  slfcont1  slfcont2  slfcont3  slfcont4
##   6   8  age  cnarc1  cnarc2  cnarc3  ctheory1  ctheory3  ctheory4  CRT1  CRT2  CRT3  health_cond  know_tested_positive  moralid10  moralid4  moralid5  moralid6  moralid9  mcoop2  mcoop3  mcoop4  mcoop5  mcoop6  mcoop7  narc2  narc4  narc5  narc6  nidentity1  nidentity2  omind1  contact2  contact3  contact4  hygiene2  hygiene4  hygiene5  psupport1  psupport5  political_ideology  happy  slf_ladder  riskperc2  self_esteem  sbelong1  sbelong2  sbelong3  optim1  slfcont1  slfcont2  slfcont3  slfcont4
##   6   9  age  cnarc1  cnarc2  cnarc3  ctheory1  ctheory3  ctheory4  CRT1  CRT2  CRT3  health_cond  know_tested_positive  moralid10  moralid4  moralid5  moralid6  moralid9  mcoop2  mcoop3  mcoop4  mcoop5  mcoop6  mcoop7  narc2  narc4  narc5  narc6  nidentity1  nidentity2  omind1  contact2  contact3  contact4  hygiene2  hygiene4  hygiene5  psupport1  psupport5  political_ideology  happy  slf_ladder  riskperc2  self_esteem  sbelong1  sbelong2  sbelong3  optim1  slfcont1  slfcont2  slfcont3  slfcont4
##   6   10  age  cnarc1  cnarc2  cnarc3  ctheory1  ctheory3  ctheory4  CRT1  CRT2  CRT3  health_cond  know_tested_positive  moralid10  moralid4  moralid5  moralid6  moralid9  mcoop2  mcoop3  mcoop4  mcoop5  mcoop6  mcoop7  narc2  narc4  narc5  narc6  nidentity1  nidentity2  omind1  contact2  contact3  contact4  hygiene2  hygiene4  hygiene5  psupport1  psupport5  political_ideology  happy  slf_ladder  riskperc2  self_esteem  sbelong1  sbelong2  sbelong3  optim1  slfcont1  slfcont2  slfcont3  slfcont4
##   7   1  age  cnarc1  cnarc2  cnarc3  ctheory1  ctheory3  ctheory4  CRT1  CRT2  CRT3  health_cond  know_tested_positive  moralid10  moralid4  moralid5  moralid6  moralid9  mcoop2  mcoop3  mcoop4  mcoop5  mcoop6  mcoop7  narc2  narc4  narc5  narc6  nidentity1  nidentity2  omind1  contact2  contact3  contact4  hygiene2  hygiene4  hygiene5  psupport1  psupport5  political_ideology  happy  slf_ladder  riskperc2  self_esteem  sbelong1  sbelong2  sbelong3  optim1  slfcont1  slfcont2  slfcont3  slfcont4
##   7   2  age  cnarc1  cnarc2  cnarc3  ctheory1  ctheory3  ctheory4  CRT1  CRT2  CRT3  health_cond  know_tested_positive  moralid10  moralid4  moralid5  moralid6  moralid9  mcoop2  mcoop3  mcoop4  mcoop5  mcoop6  mcoop7  narc2  narc4  narc5  narc6  nidentity1  nidentity2  omind1  contact2  contact3  contact4  hygiene2  hygiene4  hygiene5  psupport1  psupport5  political_ideology  happy  slf_ladder  riskperc2  self_esteem  sbelong1  sbelong2  sbelong3  optim1  slfcont1  slfcont2  slfcont3  slfcont4
##   7   3  age  cnarc1  cnarc2  cnarc3  ctheory1  ctheory3  ctheory4  CRT1  CRT2  CRT3  health_cond  know_tested_positive  moralid10  moralid4  moralid5  moralid6  moralid9  mcoop2  mcoop3  mcoop4  mcoop5  mcoop6  mcoop7  narc2  narc4  narc5  narc6  nidentity1  nidentity2  omind1  contact2  contact3  contact4  hygiene2  hygiene4  hygiene5  psupport1  psupport5  political_ideology  happy  slf_ladder  riskperc2  self_esteem  sbelong1  sbelong2  sbelong3  optim1  slfcont1  slfcont2  slfcont3  slfcont4
##   7   4  age  cnarc1  cnarc2  cnarc3  ctheory1  ctheory3  ctheory4  CRT1  CRT2  CRT3  health_cond  know_tested_positive  moralid10  moralid4  moralid5  moralid6  moralid9  mcoop2  mcoop3  mcoop4  mcoop5  mcoop6  mcoop7  narc2  narc4  narc5  narc6  nidentity1  nidentity2  omind1  contact2  contact3  contact4  hygiene2  hygiene4  hygiene5  psupport1  psupport5  political_ideology  happy  slf_ladder  riskperc2  self_esteem  sbelong1  sbelong2  sbelong3  optim1  slfcont1  slfcont2  slfcont3  slfcont4
##   7   5  age  cnarc1  cnarc2  cnarc3  ctheory1  ctheory3  ctheory4  CRT1  CRT2  CRT3  health_cond  know_tested_positive  moralid10  moralid4  moralid5  moralid6  moralid9  mcoop2  mcoop3  mcoop4  mcoop5  mcoop6  mcoop7  narc2  narc4  narc5  narc6  nidentity1  nidentity2  omind1  contact2  contact3  contact4  hygiene2  hygiene4  hygiene5  psupport1  psupport5  political_ideology  happy  slf_ladder  riskperc2  self_esteem  sbelong1  sbelong2  sbelong3  optim1  slfcont1  slfcont2  slfcont3  slfcont4
##   7   6  age  cnarc1  cnarc2  cnarc3  ctheory1  ctheory3  ctheory4  CRT1  CRT2  CRT3  health_cond  know_tested_positive  moralid10  moralid4  moralid5  moralid6  moralid9  mcoop2  mcoop3  mcoop4  mcoop5  mcoop6  mcoop7  narc2  narc4  narc5  narc6  nidentity1  nidentity2  omind1  contact2  contact3  contact4  hygiene2  hygiene4  hygiene5  psupport1  psupport5  political_ideology  happy  slf_ladder  riskperc2  self_esteem  sbelong1  sbelong2  sbelong3  optim1  slfcont1  slfcont2  slfcont3  slfcont4
##   7   7  age  cnarc1  cnarc2  cnarc3  ctheory1  ctheory3  ctheory4  CRT1  CRT2  CRT3  health_cond  know_tested_positive  moralid10  moralid4  moralid5  moralid6  moralid9  mcoop2  mcoop3  mcoop4  mcoop5  mcoop6  mcoop7  narc2  narc4  narc5  narc6  nidentity1  nidentity2  omind1  contact2  contact3  contact4  hygiene2  hygiene4  hygiene5  psupport1  psupport5  political_ideology  happy  slf_ladder  riskperc2  self_esteem  sbelong1  sbelong2  sbelong3  optim1  slfcont1  slfcont2  slfcont3  slfcont4
##   7   8  age  cnarc1  cnarc2  cnarc3  ctheory1  ctheory3  ctheory4  CRT1  CRT2  CRT3  health_cond  know_tested_positive  moralid10  moralid4  moralid5  moralid6  moralid9  mcoop2  mcoop3  mcoop4  mcoop5  mcoop6  mcoop7  narc2  narc4  narc5  narc6  nidentity1  nidentity2  omind1  contact2  contact3  contact4  hygiene2  hygiene4  hygiene5  psupport1  psupport5  political_ideology  happy  slf_ladder  riskperc2  self_esteem  sbelong1  sbelong2  sbelong3  optim1  slfcont1  slfcont2  slfcont3  slfcont4
##   7   9  age  cnarc1  cnarc2  cnarc3  ctheory1  ctheory3  ctheory4  CRT1  CRT2  CRT3  health_cond  know_tested_positive  moralid10  moralid4  moralid5  moralid6  moralid9  mcoop2  mcoop3  mcoop4  mcoop5  mcoop6  mcoop7  narc2  narc4  narc5  narc6  nidentity1  nidentity2  omind1  contact2  contact3  contact4  hygiene2  hygiene4  hygiene5  psupport1  psupport5  political_ideology  happy  slf_ladder  riskperc2  self_esteem  sbelong1  sbelong2  sbelong3  optim1  slfcont1  slfcont2  slfcont3  slfcont4
##   7   10  age  cnarc1  cnarc2  cnarc3  ctheory1  ctheory3  ctheory4  CRT1  CRT2  CRT3  health_cond  know_tested_positive  moralid10  moralid4  moralid5  moralid6  moralid9  mcoop2  mcoop3  mcoop4  mcoop5  mcoop6  mcoop7  narc2  narc4  narc5  narc6  nidentity1  nidentity2  omind1  contact2  contact3  contact4  hygiene2  hygiene4  hygiene5  psupport1  psupport5  political_ideology  happy  slf_ladder  riskperc2  self_esteem  sbelong1  sbelong2  sbelong3  optim1  slfcont1  slfcont2  slfcont3  slfcont4
##   8   1  age  cnarc1  cnarc2  cnarc3  ctheory1  ctheory3  ctheory4  CRT1  CRT2  CRT3  health_cond  know_tested_positive  moralid10  moralid4  moralid5  moralid6  moralid9  mcoop2  mcoop3  mcoop4  mcoop5  mcoop6  mcoop7  narc2  narc4  narc5  narc6  nidentity1  nidentity2  omind1  contact2  contact3  contact4  hygiene2  hygiene4  hygiene5  psupport1  psupport5  political_ideology  happy  slf_ladder  riskperc2  self_esteem  sbelong1  sbelong2  sbelong3  optim1  slfcont1  slfcont2  slfcont3  slfcont4
##   8   2  age  cnarc1  cnarc2  cnarc3  ctheory1  ctheory3  ctheory4  CRT1  CRT2  CRT3  health_cond  know_tested_positive  moralid10  moralid4  moralid5  moralid6  moralid9  mcoop2  mcoop3  mcoop4  mcoop5  mcoop6  mcoop7  narc2  narc4  narc5  narc6  nidentity1  nidentity2  omind1  contact2  contact3  contact4  hygiene2  hygiene4  hygiene5  psupport1  psupport5  political_ideology  happy  slf_ladder  riskperc2  self_esteem  sbelong1  sbelong2  sbelong3  optim1  slfcont1  slfcont2  slfcont3  slfcont4
##   8   3  age  cnarc1  cnarc2  cnarc3  ctheory1  ctheory3  ctheory4  CRT1  CRT2  CRT3  health_cond  know_tested_positive  moralid10  moralid4  moralid5  moralid6  moralid9  mcoop2  mcoop3  mcoop4  mcoop5  mcoop6  mcoop7  narc2  narc4  narc5  narc6  nidentity1  nidentity2  omind1  contact2  contact3  contact4  hygiene2  hygiene4  hygiene5  psupport1  psupport5  political_ideology  happy  slf_ladder  riskperc2  self_esteem  sbelong1  sbelong2  sbelong3  optim1  slfcont1  slfcont2  slfcont3  slfcont4
##   8   4  age  cnarc1  cnarc2  cnarc3  ctheory1  ctheory3  ctheory4  CRT1  CRT2  CRT3  health_cond  know_tested_positive  moralid10  moralid4  moralid5  moralid6  moralid9  mcoop2  mcoop3  mcoop4  mcoop5  mcoop6  mcoop7  narc2  narc4  narc5  narc6  nidentity1  nidentity2  omind1  contact2  contact3  contact4  hygiene2  hygiene4  hygiene5  psupport1  psupport5  political_ideology  happy  slf_ladder  riskperc2  self_esteem  sbelong1  sbelong2  sbelong3  optim1  slfcont1  slfcont2  slfcont3  slfcont4
##   8   5  age  cnarc1  cnarc2  cnarc3  ctheory1  ctheory3  ctheory4  CRT1  CRT2  CRT3  health_cond  know_tested_positive  moralid10  moralid4  moralid5  moralid6  moralid9  mcoop2  mcoop3  mcoop4  mcoop5  mcoop6  mcoop7  narc2  narc4  narc5  narc6  nidentity1  nidentity2  omind1  contact2  contact3  contact4  hygiene2  hygiene4  hygiene5  psupport1  psupport5  political_ideology  happy  slf_ladder  riskperc2  self_esteem  sbelong1  sbelong2  sbelong3  optim1  slfcont1  slfcont2  slfcont3  slfcont4
##   8   6  age  cnarc1  cnarc2  cnarc3  ctheory1  ctheory3  ctheory4  CRT1  CRT2  CRT3  health_cond  know_tested_positive  moralid10  moralid4  moralid5  moralid6  moralid9  mcoop2  mcoop3  mcoop4  mcoop5  mcoop6  mcoop7  narc2  narc4  narc5  narc6  nidentity1  nidentity2  omind1  contact2  contact3  contact4  hygiene2  hygiene4  hygiene5  psupport1  psupport5  political_ideology  happy  slf_ladder  riskperc2  self_esteem  sbelong1  sbelong2  sbelong3  optim1  slfcont1  slfcont2  slfcont3  slfcont4
##   8   7  age  cnarc1  cnarc2  cnarc3  ctheory1  ctheory3  ctheory4  CRT1  CRT2  CRT3  health_cond  know_tested_positive  moralid10  moralid4  moralid5  moralid6  moralid9  mcoop2  mcoop3  mcoop4  mcoop5  mcoop6  mcoop7  narc2  narc4  narc5  narc6  nidentity1  nidentity2  omind1  contact2  contact3  contact4  hygiene2  hygiene4  hygiene5  psupport1  psupport5  political_ideology  happy  slf_ladder  riskperc2  self_esteem  sbelong1  sbelong2  sbelong3  optim1  slfcont1  slfcont2  slfcont3  slfcont4
##   8   8  age  cnarc1  cnarc2  cnarc3  ctheory1  ctheory3  ctheory4  CRT1  CRT2  CRT3  health_cond  know_tested_positive  moralid10  moralid4  moralid5  moralid6  moralid9  mcoop2  mcoop3  mcoop4  mcoop5  mcoop6  mcoop7  narc2  narc4  narc5  narc6  nidentity1  nidentity2  omind1  contact2  contact3  contact4  hygiene2  hygiene4  hygiene5  psupport1  psupport5  political_ideology  happy  slf_ladder  riskperc2  self_esteem  sbelong1  sbelong2  sbelong3  optim1  slfcont1  slfcont2  slfcont3  slfcont4
##   8   9  age  cnarc1  cnarc2  cnarc3  ctheory1  ctheory3  ctheory4  CRT1  CRT2  CRT3  health_cond  know_tested_positive  moralid10  moralid4  moralid5  moralid6  moralid9  mcoop2  mcoop3  mcoop4  mcoop5  mcoop6  mcoop7  narc2  narc4  narc5  narc6  nidentity1  nidentity2  omind1  contact2  contact3  contact4  hygiene2  hygiene4  hygiene5  psupport1  psupport5  political_ideology  happy  slf_ladder  riskperc2  self_esteem  sbelong1  sbelong2  sbelong3  optim1  slfcont1  slfcont2  slfcont3  slfcont4
##   8   10  age  cnarc1  cnarc2  cnarc3  ctheory1  ctheory3  ctheory4  CRT1  CRT2  CRT3  health_cond  know_tested_positive  moralid10  moralid4  moralid5  moralid6  moralid9  mcoop2  mcoop3  mcoop4  mcoop5  mcoop6  mcoop7  narc2  narc4  narc5  narc6  nidentity1  nidentity2  omind1  contact2  contact3  contact4  hygiene2  hygiene4  hygiene5  psupport1  psupport5  political_ideology  happy  slf_ladder  riskperc2  self_esteem  sbelong1  sbelong2  sbelong3  optim1  slfcont1  slfcont2  slfcont3  slfcont4
##   9   1  age  cnarc1  cnarc2  cnarc3  ctheory1  ctheory3  ctheory4  CRT1  CRT2  CRT3  health_cond  know_tested_positive  moralid10  moralid4  moralid5  moralid6  moralid9  mcoop2  mcoop3  mcoop4  mcoop5  mcoop6  mcoop7  narc2  narc4  narc5  narc6  nidentity1  nidentity2  omind1  contact2  contact3  contact4  hygiene2  hygiene4  hygiene5  psupport1  psupport5  political_ideology  happy  slf_ladder  riskperc2  self_esteem  sbelong1  sbelong2  sbelong3  optim1  slfcont1  slfcont2  slfcont3  slfcont4
##   9   2  age  cnarc1  cnarc2  cnarc3  ctheory1  ctheory3  ctheory4  CRT1  CRT2  CRT3  health_cond  know_tested_positive  moralid10  moralid4  moralid5  moralid6  moralid9  mcoop2  mcoop3  mcoop4  mcoop5  mcoop6  mcoop7  narc2  narc4  narc5  narc6  nidentity1  nidentity2  omind1  contact2  contact3  contact4  hygiene2  hygiene4  hygiene5  psupport1  psupport5  political_ideology  happy  slf_ladder  riskperc2  self_esteem  sbelong1  sbelong2  sbelong3  optim1  slfcont1  slfcont2  slfcont3  slfcont4
##   9   3  age  cnarc1  cnarc2  cnarc3  ctheory1  ctheory3  ctheory4  CRT1  CRT2  CRT3  health_cond  know_tested_positive  moralid10  moralid4  moralid5  moralid6  moralid9  mcoop2  mcoop3  mcoop4  mcoop5  mcoop6  mcoop7  narc2  narc4  narc5  narc6  nidentity1  nidentity2  omind1  contact2  contact3  contact4  hygiene2  hygiene4  hygiene5  psupport1  psupport5  political_ideology  happy  slf_ladder  riskperc2  self_esteem  sbelong1  sbelong2  sbelong3  optim1  slfcont1  slfcont2  slfcont3  slfcont4
##   9   4  age  cnarc1  cnarc2  cnarc3  ctheory1  ctheory3  ctheory4  CRT1  CRT2  CRT3  health_cond  know_tested_positive  moralid10  moralid4  moralid5  moralid6  moralid9  mcoop2  mcoop3  mcoop4  mcoop5  mcoop6  mcoop7  narc2  narc4  narc5  narc6  nidentity1  nidentity2  omind1  contact2  contact3  contact4  hygiene2  hygiene4  hygiene5  psupport1  psupport5  political_ideology  happy  slf_ladder  riskperc2  self_esteem  sbelong1  sbelong2  sbelong3  optim1  slfcont1  slfcont2  slfcont3  slfcont4
##   9   5  age  cnarc1  cnarc2  cnarc3  ctheory1  ctheory3  ctheory4  CRT1  CRT2  CRT3  health_cond  know_tested_positive  moralid10  moralid4  moralid5  moralid6  moralid9  mcoop2  mcoop3  mcoop4  mcoop5  mcoop6  mcoop7  narc2  narc4  narc5  narc6  nidentity1  nidentity2  omind1  contact2  contact3  contact4  hygiene2  hygiene4  hygiene5  psupport1  psupport5  political_ideology  happy  slf_ladder  riskperc2  self_esteem  sbelong1  sbelong2  sbelong3  optim1  slfcont1  slfcont2  slfcont3  slfcont4
##   9   6  age  cnarc1  cnarc2  cnarc3  ctheory1  ctheory3  ctheory4  CRT1  CRT2  CRT3  health_cond  know_tested_positive  moralid10  moralid4  moralid5  moralid6  moralid9  mcoop2  mcoop3  mcoop4  mcoop5  mcoop6  mcoop7  narc2  narc4  narc5  narc6  nidentity1  nidentity2  omind1  contact2  contact3  contact4  hygiene2  hygiene4  hygiene5  psupport1  psupport5  political_ideology  happy  slf_ladder  riskperc2  self_esteem  sbelong1  sbelong2  sbelong3  optim1  slfcont1  slfcont2  slfcont3  slfcont4
##   9   7  age  cnarc1  cnarc2  cnarc3  ctheory1  ctheory3  ctheory4  CRT1  CRT2  CRT3  health_cond  know_tested_positive  moralid10  moralid4  moralid5  moralid6  moralid9  mcoop2  mcoop3  mcoop4  mcoop5  mcoop6  mcoop7  narc2  narc4  narc5  narc6  nidentity1  nidentity2  omind1  contact2  contact3  contact4  hygiene2  hygiene4  hygiene5  psupport1  psupport5  political_ideology  happy  slf_ladder  riskperc2  self_esteem  sbelong1  sbelong2  sbelong3  optim1  slfcont1  slfcont2  slfcont3  slfcont4
##   9   8  age  cnarc1  cnarc2  cnarc3  ctheory1  ctheory3  ctheory4  CRT1  CRT2  CRT3  health_cond  know_tested_positive  moralid10  moralid4  moralid5  moralid6  moralid9  mcoop2  mcoop3  mcoop4  mcoop5  mcoop6  mcoop7  narc2  narc4  narc5  narc6  nidentity1  nidentity2  omind1  contact2  contact3  contact4  hygiene2  hygiene4  hygiene5  psupport1  psupport5  political_ideology  happy  slf_ladder  riskperc2  self_esteem  sbelong1  sbelong2  sbelong3  optim1  slfcont1  slfcont2  slfcont3  slfcont4
##   9   9  age  cnarc1  cnarc2  cnarc3  ctheory1  ctheory3  ctheory4  CRT1  CRT2  CRT3  health_cond  know_tested_positive  moralid10  moralid4  moralid5  moralid6  moralid9  mcoop2  mcoop3  mcoop4  mcoop5  mcoop6  mcoop7  narc2  narc4  narc5  narc6  nidentity1  nidentity2  omind1  contact2  contact3  contact4  hygiene2  hygiene4  hygiene5  psupport1  psupport5  political_ideology  happy  slf_ladder  riskperc2  self_esteem  sbelong1  sbelong2  sbelong3  optim1  slfcont1  slfcont2  slfcont3  slfcont4
##   9   10  age  cnarc1  cnarc2  cnarc3  ctheory1  ctheory3  ctheory4  CRT1  CRT2  CRT3  health_cond  know_tested_positive  moralid10  moralid4  moralid5  moralid6  moralid9  mcoop2  mcoop3  mcoop4  mcoop5  mcoop6  mcoop7  narc2  narc4  narc5  narc6  nidentity1  nidentity2  omind1  contact2  contact3  contact4  hygiene2  hygiene4  hygiene5  psupport1  psupport5  political_ideology  happy  slf_ladder  riskperc2  self_esteem  sbelong1  sbelong2  sbelong3  optim1  slfcont1  slfcont2  slfcont3  slfcont4
##   10   1  age  cnarc1  cnarc2  cnarc3  ctheory1  ctheory3  ctheory4  CRT1  CRT2  CRT3  health_cond  know_tested_positive  moralid10  moralid4  moralid5  moralid6  moralid9  mcoop2  mcoop3  mcoop4  mcoop5  mcoop6  mcoop7  narc2  narc4  narc5  narc6  nidentity1  nidentity2  omind1  contact2  contact3  contact4  hygiene2  hygiene4  hygiene5  psupport1  psupport5  political_ideology  happy  slf_ladder  riskperc2  self_esteem  sbelong1  sbelong2  sbelong3  optim1  slfcont1  slfcont2  slfcont3  slfcont4
##   10   2  age  cnarc1  cnarc2  cnarc3  ctheory1  ctheory3  ctheory4  CRT1  CRT2  CRT3  health_cond  know_tested_positive  moralid10  moralid4  moralid5  moralid6  moralid9  mcoop2  mcoop3  mcoop4  mcoop5  mcoop6  mcoop7  narc2  narc4  narc5  narc6  nidentity1  nidentity2  omind1  contact2  contact3  contact4  hygiene2  hygiene4  hygiene5  psupport1  psupport5  political_ideology  happy  slf_ladder  riskperc2  self_esteem  sbelong1  sbelong2  sbelong3  optim1  slfcont1  slfcont2  slfcont3  slfcont4
##   10   3  age  cnarc1  cnarc2  cnarc3  ctheory1  ctheory3  ctheory4  CRT1  CRT2  CRT3  health_cond  know_tested_positive  moralid10  moralid4  moralid5  moralid6  moralid9  mcoop2  mcoop3  mcoop4  mcoop5  mcoop6  mcoop7  narc2  narc4  narc5  narc6  nidentity1  nidentity2  omind1  contact2  contact3  contact4  hygiene2  hygiene4  hygiene5  psupport1  psupport5  political_ideology  happy  slf_ladder  riskperc2  self_esteem  sbelong1  sbelong2  sbelong3  optim1  slfcont1  slfcont2  slfcont3  slfcont4
##   10   4  age  cnarc1  cnarc2  cnarc3  ctheory1  ctheory3  ctheory4  CRT1  CRT2  CRT3  health_cond  know_tested_positive  moralid10  moralid4  moralid5  moralid6  moralid9  mcoop2  mcoop3  mcoop4  mcoop5  mcoop6  mcoop7  narc2  narc4  narc5  narc6  nidentity1  nidentity2  omind1  contact2  contact3  contact4  hygiene2  hygiene4  hygiene5  psupport1  psupport5  political_ideology  happy  slf_ladder  riskperc2  self_esteem  sbelong1  sbelong2  sbelong3  optim1  slfcont1  slfcont2  slfcont3  slfcont4
##   10   5  age  cnarc1  cnarc2  cnarc3  ctheory1  ctheory3  ctheory4  CRT1  CRT2  CRT3  health_cond  know_tested_positive  moralid10  moralid4  moralid5  moralid6  moralid9  mcoop2  mcoop3  mcoop4  mcoop5  mcoop6  mcoop7  narc2  narc4  narc5  narc6  nidentity1  nidentity2  omind1  contact2  contact3  contact4  hygiene2  hygiene4  hygiene5  psupport1  psupport5  political_ideology  happy  slf_ladder  riskperc2  self_esteem  sbelong1  sbelong2  sbelong3  optim1  slfcont1  slfcont2  slfcont3  slfcont4
##   10   6  age  cnarc1  cnarc2  cnarc3  ctheory1  ctheory3  ctheory4  CRT1  CRT2  CRT3  health_cond  know_tested_positive  moralid10  moralid4  moralid5  moralid6  moralid9  mcoop2  mcoop3  mcoop4  mcoop5  mcoop6  mcoop7  narc2  narc4  narc5  narc6  nidentity1  nidentity2  omind1  contact2  contact3  contact4  hygiene2  hygiene4  hygiene5  psupport1  psupport5  political_ideology  happy  slf_ladder  riskperc2  self_esteem  sbelong1  sbelong2  sbelong3  optim1  slfcont1  slfcont2  slfcont3  slfcont4
##   10   7  age  cnarc1  cnarc2  cnarc3  ctheory1  ctheory3  ctheory4  CRT1  CRT2  CRT3  health_cond  know_tested_positive  moralid10  moralid4  moralid5  moralid6  moralid9  mcoop2  mcoop3  mcoop4  mcoop5  mcoop6  mcoop7  narc2  narc4  narc5  narc6  nidentity1  nidentity2  omind1  contact2  contact3  contact4  hygiene2  hygiene4  hygiene5  psupport1  psupport5  political_ideology  happy  slf_ladder  riskperc2  self_esteem  sbelong1  sbelong2  sbelong3  optim1  slfcont1  slfcont2  slfcont3  slfcont4
##   10   8  age  cnarc1  cnarc2  cnarc3  ctheory1  ctheory3  ctheory4  CRT1  CRT2  CRT3  health_cond  know_tested_positive  moralid10  moralid4  moralid5  moralid6  moralid9  mcoop2  mcoop3  mcoop4  mcoop5  mcoop6  mcoop7  narc2  narc4  narc5  narc6  nidentity1  nidentity2  omind1  contact2  contact3  contact4  hygiene2  hygiene4  hygiene5  psupport1  psupport5  political_ideology  happy  slf_ladder  riskperc2  self_esteem  sbelong1  sbelong2  sbelong3  optim1  slfcont1  slfcont2  slfcont3  slfcont4
##   10   9  age  cnarc1  cnarc2  cnarc3  ctheory1  ctheory3  ctheory4  CRT1  CRT2  CRT3  health_cond  know_tested_positive  moralid10  moralid4  moralid5  moralid6  moralid9  mcoop2  mcoop3  mcoop4  mcoop5  mcoop6  mcoop7  narc2  narc4  narc5  narc6  nidentity1  nidentity2  omind1  contact2  contact3  contact4  hygiene2  hygiene4  hygiene5  psupport1  psupport5  political_ideology  happy  slf_ladder  riskperc2  self_esteem  sbelong1  sbelong2  sbelong3  optim1  slfcont1  slfcont2  slfcont3  slfcont4
##   10   10  age  cnarc1  cnarc2  cnarc3  ctheory1  ctheory3  ctheory4  CRT1  CRT2  CRT3  health_cond  know_tested_positive  moralid10  moralid4  moralid5  moralid6  moralid9  mcoop2  mcoop3  mcoop4  mcoop5  mcoop6  mcoop7  narc2  narc4  narc5  narc6  nidentity1  nidentity2  omind1  contact2  contact3  contact4  hygiene2  hygiene4  hygiene5  psupport1  psupport5  political_ideology  happy  slf_ladder  riskperc2  self_esteem  sbelong1  sbelong2  sbelong3  optim1  slfcont1  slfcont2  slfcont3  slfcont4
```

```
## Warning: Number of logged events: 5103
```

```
## 
##  iter imp variable
##   1   1  age  children  cnarc1  cnarc2  cnarc3  CRT1  CRT2  CRT3  employ_status1  ladder  marital1  mor_circle  moralid1  moralid10  moralid2  moralid3  moralid4  moralid5  moralid6  moralid7  moralid8  moralid9  mcoop1  mcoop2  mcoop3  mcoop4  mcoop5  mcoop6  mcoop7  narc1  narc2  narc3  narc4  narc5  narc6  nidentity1  nidentity2  omind1  omind2  omind3  omind4  omind5  omind6  hygiene1  hygiene2  hygiene3  hygiene4  hygiene5  psupport2  political_ideology  happy  slf_ladder  riskperc1  riskperc2  self_esteem  sex1  sbelong1  sbelong2  sbelong3  sbelong4  tested_positive  optim1  optim2  slfcont1  slfcont3  slfcont4
##   1   2  age  children  cnarc1  cnarc2  cnarc3  CRT1  CRT2  CRT3  employ_status1  ladder  marital1  mor_circle  moralid1  moralid10  moralid2  moralid3  moralid4  moralid5  moralid6  moralid7  moralid8  moralid9  mcoop1  mcoop2  mcoop3  mcoop4  mcoop5  mcoop6  mcoop7  narc1  narc2  narc3  narc4  narc5  narc6  nidentity1  nidentity2  omind1  omind2  omind3  omind4  omind5  omind6  hygiene1  hygiene2  hygiene3  hygiene4  hygiene5  psupport2  political_ideology  happy  slf_ladder  riskperc1  riskperc2  self_esteem  sex1  sbelong1  sbelong2  sbelong3  sbelong4  tested_positive  optim1  optim2  slfcont1  slfcont3  slfcont4
##   1   3  age  children  cnarc1  cnarc2  cnarc3  CRT1  CRT2  CRT3  employ_status1  ladder  marital1  mor_circle  moralid1  moralid10  moralid2  moralid3  moralid4  moralid5  moralid6  moralid7  moralid8  moralid9  mcoop1  mcoop2  mcoop3  mcoop4  mcoop5  mcoop6  mcoop7  narc1  narc2  narc3  narc4  narc5  narc6  nidentity1  nidentity2  omind1  omind2  omind3  omind4  omind5  omind6  hygiene1  hygiene2  hygiene3  hygiene4  hygiene5  psupport2  political_ideology  happy  slf_ladder  riskperc1  riskperc2  self_esteem  sex1  sbelong1  sbelong2  sbelong3  sbelong4  tested_positive  optim1  optim2  slfcont1  slfcont3  slfcont4
##   1   4  age  children  cnarc1  cnarc2  cnarc3  CRT1  CRT2  CRT3  employ_status1  ladder  marital1  mor_circle  moralid1  moralid10  moralid2  moralid3  moralid4  moralid5  moralid6  moralid7  moralid8  moralid9  mcoop1  mcoop2  mcoop3  mcoop4  mcoop5  mcoop6  mcoop7  narc1  narc2  narc3  narc4  narc5  narc6  nidentity1  nidentity2  omind1  omind2  omind3  omind4  omind5  omind6  hygiene1  hygiene2  hygiene3  hygiene4  hygiene5  psupport2  political_ideology  happy  slf_ladder  riskperc1  riskperc2  self_esteem  sex1  sbelong1  sbelong2  sbelong3  sbelong4  tested_positive  optim1  optim2  slfcont1  slfcont3  slfcont4
##   1   5  age  children  cnarc1  cnarc2  cnarc3  CRT1  CRT2  CRT3  employ_status1  ladder  marital1  mor_circle  moralid1  moralid10  moralid2  moralid3  moralid4  moralid5  moralid6  moralid7  moralid8  moralid9  mcoop1  mcoop2  mcoop3  mcoop4  mcoop5  mcoop6  mcoop7  narc1  narc2  narc3  narc4  narc5  narc6  nidentity1  nidentity2  omind1  omind2  omind3  omind4  omind5  omind6  hygiene1  hygiene2  hygiene3  hygiene4  hygiene5  psupport2  political_ideology  happy  slf_ladder  riskperc1  riskperc2  self_esteem  sex1  sbelong1  sbelong2  sbelong3  sbelong4  tested_positive  optim1  optim2  slfcont1  slfcont3  slfcont4
##   1   6  age  children  cnarc1  cnarc2  cnarc3  CRT1  CRT2  CRT3  employ_status1  ladder  marital1  mor_circle  moralid1  moralid10  moralid2  moralid3  moralid4  moralid5  moralid6  moralid7  moralid8  moralid9  mcoop1  mcoop2  mcoop3  mcoop4  mcoop5  mcoop6  mcoop7  narc1  narc2  narc3  narc4  narc5  narc6  nidentity1  nidentity2  omind1  omind2  omind3  omind4  omind5  omind6  hygiene1  hygiene2  hygiene3  hygiene4  hygiene5  psupport2  political_ideology  happy  slf_ladder  riskperc1  riskperc2  self_esteem  sex1  sbelong1  sbelong2  sbelong3  sbelong4  tested_positive  optim1  optim2  slfcont1  slfcont3  slfcont4
##   1   7  age  children  cnarc1  cnarc2  cnarc3  CRT1  CRT2  CRT3  employ_status1  ladder  marital1  mor_circle  moralid1  moralid10  moralid2  moralid3  moralid4  moralid5  moralid6  moralid7  moralid8  moralid9  mcoop1  mcoop2  mcoop3  mcoop4  mcoop5  mcoop6  mcoop7  narc1  narc2  narc3  narc4  narc5  narc6  nidentity1  nidentity2  omind1  omind2  omind3  omind4  omind5  omind6  hygiene1  hygiene2  hygiene3  hygiene4  hygiene5  psupport2  political_ideology  happy  slf_ladder  riskperc1  riskperc2  self_esteem  sex1  sbelong1  sbelong2  sbelong3  sbelong4  tested_positive  optim1  optim2  slfcont1  slfcont3  slfcont4
##   1   8  age  children  cnarc1  cnarc2  cnarc3  CRT1  CRT2  CRT3  employ_status1  ladder  marital1  mor_circle  moralid1  moralid10  moralid2  moralid3  moralid4  moralid5  moralid6  moralid7  moralid8  moralid9  mcoop1  mcoop2  mcoop3  mcoop4  mcoop5  mcoop6  mcoop7  narc1  narc2  narc3  narc4  narc5  narc6  nidentity1  nidentity2  omind1  omind2  omind3  omind4  omind5  omind6  hygiene1  hygiene2  hygiene3  hygiene4  hygiene5  psupport2  political_ideology  happy  slf_ladder  riskperc1  riskperc2  self_esteem  sex1  sbelong1  sbelong2  sbelong3  sbelong4  tested_positive  optim1  optim2  slfcont1  slfcont3  slfcont4
##   1   9  age  children  cnarc1  cnarc2  cnarc3  CRT1  CRT2  CRT3  employ_status1  ladder  marital1  mor_circle  moralid1  moralid10  moralid2  moralid3  moralid4  moralid5  moralid6  moralid7  moralid8  moralid9  mcoop1  mcoop2  mcoop3  mcoop4  mcoop5  mcoop6  mcoop7  narc1  narc2  narc3  narc4  narc5  narc6  nidentity1  nidentity2  omind1  omind2  omind3  omind4  omind5  omind6  hygiene1  hygiene2  hygiene3  hygiene4  hygiene5  psupport2  political_ideology  happy  slf_ladder  riskperc1  riskperc2  self_esteem  sex1  sbelong1  sbelong2  sbelong3  sbelong4  tested_positive  optim1  optim2  slfcont1  slfcont3  slfcont4
##   1   10  age  children  cnarc1  cnarc2  cnarc3  CRT1  CRT2  CRT3  employ_status1  ladder  marital1  mor_circle  moralid1  moralid10  moralid2  moralid3  moralid4  moralid5  moralid6  moralid7  moralid8  moralid9  mcoop1  mcoop2  mcoop3  mcoop4  mcoop5  mcoop6  mcoop7  narc1  narc2  narc3  narc4  narc5  narc6  nidentity1  nidentity2  omind1  omind2  omind3  omind4  omind5  omind6  hygiene1  hygiene2  hygiene3  hygiene4  hygiene5  psupport2  political_ideology  happy  slf_ladder  riskperc1  riskperc2  self_esteem  sex1  sbelong1  sbelong2  sbelong3  sbelong4  tested_positive  optim1  optim2  slfcont1  slfcont3  slfcont4
##   2   1  age  children  cnarc1  cnarc2  cnarc3  CRT1  CRT2  CRT3  employ_status1  ladder  marital1  mor_circle  moralid1  moralid10  moralid2  moralid3  moralid4  moralid5  moralid6  moralid7  moralid8  moralid9  mcoop1  mcoop2  mcoop3  mcoop4  mcoop5  mcoop6  mcoop7  narc1  narc2  narc3  narc4  narc5  narc6  nidentity1  nidentity2  omind1  omind2  omind3  omind4  omind5  omind6  hygiene1  hygiene2  hygiene3  hygiene4  hygiene5  psupport2  political_ideology  happy  slf_ladder  riskperc1  riskperc2  self_esteem  sex1  sbelong1  sbelong2  sbelong3  sbelong4  tested_positive  optim1  optim2  slfcont1  slfcont3  slfcont4
##   2   2  age  children  cnarc1  cnarc2  cnarc3  CRT1  CRT2  CRT3  employ_status1  ladder  marital1  mor_circle  moralid1  moralid10  moralid2  moralid3  moralid4  moralid5  moralid6  moralid7  moralid8  moralid9  mcoop1  mcoop2  mcoop3  mcoop4  mcoop5  mcoop6  mcoop7  narc1  narc2  narc3  narc4  narc5  narc6  nidentity1  nidentity2  omind1  omind2  omind3  omind4  omind5  omind6  hygiene1  hygiene2  hygiene3  hygiene4  hygiene5  psupport2  political_ideology  happy  slf_ladder  riskperc1  riskperc2  self_esteem  sex1  sbelong1  sbelong2  sbelong3  sbelong4  tested_positive  optim1  optim2  slfcont1  slfcont3  slfcont4
##   2   3  age  children  cnarc1  cnarc2  cnarc3  CRT1  CRT2  CRT3  employ_status1  ladder  marital1  mor_circle  moralid1  moralid10  moralid2  moralid3  moralid4  moralid5  moralid6  moralid7  moralid8  moralid9  mcoop1  mcoop2  mcoop3  mcoop4  mcoop5  mcoop6  mcoop7  narc1  narc2  narc3  narc4  narc5  narc6  nidentity1  nidentity2  omind1  omind2  omind3  omind4  omind5  omind6  hygiene1  hygiene2  hygiene3  hygiene4  hygiene5  psupport2  political_ideology  happy  slf_ladder  riskperc1  riskperc2  self_esteem  sex1  sbelong1  sbelong2  sbelong3  sbelong4  tested_positive  optim1  optim2  slfcont1  slfcont3  slfcont4
##   2   4  age  children  cnarc1  cnarc2  cnarc3  CRT1  CRT2  CRT3  employ_status1  ladder  marital1  mor_circle  moralid1  moralid10  moralid2  moralid3  moralid4  moralid5  moralid6  moralid7  moralid8  moralid9  mcoop1  mcoop2  mcoop3  mcoop4  mcoop5  mcoop6  mcoop7  narc1  narc2  narc3  narc4  narc5  narc6  nidentity1  nidentity2  omind1  omind2  omind3  omind4  omind5  omind6  hygiene1  hygiene2  hygiene3  hygiene4  hygiene5  psupport2  political_ideology  happy  slf_ladder  riskperc1  riskperc2  self_esteem  sex1  sbelong1  sbelong2  sbelong3  sbelong4  tested_positive  optim1  optim2  slfcont1  slfcont3  slfcont4
##   2   5  age  children  cnarc1  cnarc2  cnarc3  CRT1  CRT2  CRT3  employ_status1  ladder  marital1  mor_circle  moralid1  moralid10  moralid2  moralid3  moralid4  moralid5  moralid6  moralid7  moralid8  moralid9  mcoop1  mcoop2  mcoop3  mcoop4  mcoop5  mcoop6  mcoop7  narc1  narc2  narc3  narc4  narc5  narc6  nidentity1  nidentity2  omind1  omind2  omind3  omind4  omind5  omind6  hygiene1  hygiene2  hygiene3  hygiene4  hygiene5  psupport2  political_ideology  happy  slf_ladder  riskperc1  riskperc2  self_esteem  sex1  sbelong1  sbelong2  sbelong3  sbelong4  tested_positive  optim1  optim2  slfcont1  slfcont3  slfcont4
##   2   6  age  children  cnarc1  cnarc2  cnarc3  CRT1  CRT2  CRT3  employ_status1  ladder  marital1  mor_circle  moralid1  moralid10  moralid2  moralid3  moralid4  moralid5  moralid6  moralid7  moralid8  moralid9  mcoop1  mcoop2  mcoop3  mcoop4  mcoop5  mcoop6  mcoop7  narc1  narc2  narc3  narc4  narc5  narc6  nidentity1  nidentity2  omind1  omind2  omind3  omind4  omind5  omind6  hygiene1  hygiene2  hygiene3  hygiene4  hygiene5  psupport2  political_ideology  happy  slf_ladder  riskperc1  riskperc2  self_esteem  sex1  sbelong1  sbelong2  sbelong3  sbelong4  tested_positive  optim1  optim2  slfcont1  slfcont3  slfcont4
##   2   7  age  children  cnarc1  cnarc2  cnarc3  CRT1  CRT2  CRT3  employ_status1  ladder  marital1  mor_circle  moralid1  moralid10  moralid2  moralid3  moralid4  moralid5  moralid6  moralid7  moralid8  moralid9  mcoop1  mcoop2  mcoop3  mcoop4  mcoop5  mcoop6  mcoop7  narc1  narc2  narc3  narc4  narc5  narc6  nidentity1  nidentity2  omind1  omind2  omind3  omind4  omind5  omind6  hygiene1  hygiene2  hygiene3  hygiene4  hygiene5  psupport2  political_ideology  happy  slf_ladder  riskperc1  riskperc2  self_esteem  sex1  sbelong1  sbelong2  sbelong3  sbelong4  tested_positive  optim1  optim2  slfcont1  slfcont3  slfcont4
##   2   8  age  children  cnarc1  cnarc2  cnarc3  CRT1  CRT2  CRT3  employ_status1  ladder  marital1  mor_circle  moralid1  moralid10  moralid2  moralid3  moralid4  moralid5  moralid6  moralid7  moralid8  moralid9  mcoop1  mcoop2  mcoop3  mcoop4  mcoop5  mcoop6  mcoop7  narc1  narc2  narc3  narc4  narc5  narc6  nidentity1  nidentity2  omind1  omind2  omind3  omind4  omind5  omind6  hygiene1  hygiene2  hygiene3  hygiene4  hygiene5  psupport2  political_ideology  happy  slf_ladder  riskperc1  riskperc2  self_esteem  sex1  sbelong1  sbelong2  sbelong3  sbelong4  tested_positive  optim1  optim2  slfcont1  slfcont3  slfcont4
##   2   9  age  children  cnarc1  cnarc2  cnarc3  CRT1  CRT2  CRT3  employ_status1  ladder  marital1  mor_circle  moralid1  moralid10  moralid2  moralid3  moralid4  moralid5  moralid6  moralid7  moralid8  moralid9  mcoop1  mcoop2  mcoop3  mcoop4  mcoop5  mcoop6  mcoop7  narc1  narc2  narc3  narc4  narc5  narc6  nidentity1  nidentity2  omind1  omind2  omind3  omind4  omind5  omind6  hygiene1  hygiene2  hygiene3  hygiene4  hygiene5  psupport2  political_ideology  happy  slf_ladder  riskperc1  riskperc2  self_esteem  sex1  sbelong1  sbelong2  sbelong3  sbelong4  tested_positive  optim1  optim2  slfcont1  slfcont3  slfcont4
##   2   10  age  children  cnarc1  cnarc2  cnarc3  CRT1  CRT2  CRT3  employ_status1  ladder  marital1  mor_circle  moralid1  moralid10  moralid2  moralid3  moralid4  moralid5  moralid6  moralid7  moralid8  moralid9  mcoop1  mcoop2  mcoop3  mcoop4  mcoop5  mcoop6  mcoop7  narc1  narc2  narc3  narc4  narc5  narc6  nidentity1  nidentity2  omind1  omind2  omind3  omind4  omind5  omind6  hygiene1  hygiene2  hygiene3  hygiene4  hygiene5  psupport2  political_ideology  happy  slf_ladder  riskperc1  riskperc2  self_esteem  sex1  sbelong1  sbelong2  sbelong3  sbelong4  tested_positive  optim1  optim2  slfcont1  slfcont3  slfcont4
##   3   1  age  children  cnarc1  cnarc2  cnarc3  CRT1  CRT2  CRT3  employ_status1  ladder  marital1  mor_circle  moralid1  moralid10  moralid2  moralid3  moralid4  moralid5  moralid6  moralid7  moralid8  moralid9  mcoop1  mcoop2  mcoop3  mcoop4  mcoop5  mcoop6  mcoop7  narc1  narc2  narc3  narc4  narc5  narc6  nidentity1  nidentity2  omind1  omind2  omind3  omind4  omind5  omind6  hygiene1  hygiene2  hygiene3  hygiene4  hygiene5  psupport2  political_ideology  happy  slf_ladder  riskperc1  riskperc2  self_esteem  sex1  sbelong1  sbelong2  sbelong3  sbelong4  tested_positive  optim1  optim2  slfcont1  slfcont3  slfcont4
##   3   2  age  children  cnarc1  cnarc2  cnarc3  CRT1  CRT2  CRT3  employ_status1  ladder  marital1  mor_circle  moralid1  moralid10  moralid2  moralid3  moralid4  moralid5  moralid6  moralid7  moralid8  moralid9  mcoop1  mcoop2  mcoop3  mcoop4  mcoop5  mcoop6  mcoop7  narc1  narc2  narc3  narc4  narc5  narc6  nidentity1  nidentity2  omind1  omind2  omind3  omind4  omind5  omind6  hygiene1  hygiene2  hygiene3  hygiene4  hygiene5  psupport2  political_ideology  happy  slf_ladder  riskperc1  riskperc2  self_esteem  sex1  sbelong1  sbelong2  sbelong3  sbelong4  tested_positive  optim1  optim2  slfcont1  slfcont3  slfcont4
##   3   3  age  children  cnarc1  cnarc2  cnarc3  CRT1  CRT2  CRT3  employ_status1  ladder  marital1  mor_circle  moralid1  moralid10  moralid2  moralid3  moralid4  moralid5  moralid6  moralid7  moralid8  moralid9  mcoop1  mcoop2  mcoop3  mcoop4  mcoop5  mcoop6  mcoop7  narc1  narc2  narc3  narc4  narc5  narc6  nidentity1  nidentity2  omind1  omind2  omind3  omind4  omind5  omind6  hygiene1  hygiene2  hygiene3  hygiene4  hygiene5  psupport2  political_ideology  happy  slf_ladder  riskperc1  riskperc2  self_esteem  sex1  sbelong1  sbelong2  sbelong3  sbelong4  tested_positive  optim1  optim2  slfcont1  slfcont3  slfcont4
##   3   4  age  children  cnarc1  cnarc2  cnarc3  CRT1  CRT2  CRT3  employ_status1  ladder  marital1  mor_circle  moralid1  moralid10  moralid2  moralid3  moralid4  moralid5  moralid6  moralid7  moralid8  moralid9  mcoop1  mcoop2  mcoop3  mcoop4  mcoop5  mcoop6  mcoop7  narc1  narc2  narc3  narc4  narc5  narc6  nidentity1  nidentity2  omind1  omind2  omind3  omind4  omind5  omind6  hygiene1  hygiene2  hygiene3  hygiene4  hygiene5  psupport2  political_ideology  happy  slf_ladder  riskperc1  riskperc2  self_esteem  sex1  sbelong1  sbelong2  sbelong3  sbelong4  tested_positive  optim1  optim2  slfcont1  slfcont3  slfcont4
##   3   5  age  children  cnarc1  cnarc2  cnarc3  CRT1  CRT2  CRT3  employ_status1  ladder  marital1  mor_circle  moralid1  moralid10  moralid2  moralid3  moralid4  moralid5  moralid6  moralid7  moralid8  moralid9  mcoop1  mcoop2  mcoop3  mcoop4  mcoop5  mcoop6  mcoop7  narc1  narc2  narc3  narc4  narc5  narc6  nidentity1  nidentity2  omind1  omind2  omind3  omind4  omind5  omind6  hygiene1  hygiene2  hygiene3  hygiene4  hygiene5  psupport2  political_ideology  happy  slf_ladder  riskperc1  riskperc2  self_esteem  sex1  sbelong1  sbelong2  sbelong3  sbelong4  tested_positive  optim1  optim2  slfcont1  slfcont3  slfcont4
##   3   6  age  children  cnarc1  cnarc2  cnarc3  CRT1  CRT2  CRT3  employ_status1  ladder  marital1  mor_circle  moralid1  moralid10  moralid2  moralid3  moralid4  moralid5  moralid6  moralid7  moralid8  moralid9  mcoop1  mcoop2  mcoop3  mcoop4  mcoop5  mcoop6  mcoop7  narc1  narc2  narc3  narc4  narc5  narc6  nidentity1  nidentity2  omind1  omind2  omind3  omind4  omind5  omind6  hygiene1  hygiene2  hygiene3  hygiene4  hygiene5  psupport2  political_ideology  happy  slf_ladder  riskperc1  riskperc2  self_esteem  sex1  sbelong1  sbelong2  sbelong3  sbelong4  tested_positive  optim1  optim2  slfcont1  slfcont3  slfcont4
##   3   7  age  children  cnarc1  cnarc2  cnarc3  CRT1  CRT2  CRT3  employ_status1  ladder  marital1  mor_circle  moralid1  moralid10  moralid2  moralid3  moralid4  moralid5  moralid6  moralid7  moralid8  moralid9  mcoop1  mcoop2  mcoop3  mcoop4  mcoop5  mcoop6  mcoop7  narc1  narc2  narc3  narc4  narc5  narc6  nidentity1  nidentity2  omind1  omind2  omind3  omind4  omind5  omind6  hygiene1  hygiene2  hygiene3  hygiene4  hygiene5  psupport2  political_ideology  happy  slf_ladder  riskperc1  riskperc2  self_esteem  sex1  sbelong1  sbelong2  sbelong3  sbelong4  tested_positive  optim1  optim2  slfcont1  slfcont3  slfcont4
##   3   8  age  children  cnarc1  cnarc2  cnarc3  CRT1  CRT2  CRT3  employ_status1  ladder  marital1  mor_circle  moralid1  moralid10  moralid2  moralid3  moralid4  moralid5  moralid6  moralid7  moralid8  moralid9  mcoop1  mcoop2  mcoop3  mcoop4  mcoop5  mcoop6  mcoop7  narc1  narc2  narc3  narc4  narc5  narc6  nidentity1  nidentity2  omind1  omind2  omind3  omind4  omind5  omind6  hygiene1  hygiene2  hygiene3  hygiene4  hygiene5  psupport2  political_ideology  happy  slf_ladder  riskperc1  riskperc2  self_esteem  sex1  sbelong1  sbelong2  sbelong3  sbelong4  tested_positive  optim1  optim2  slfcont1  slfcont3  slfcont4
##   3   9  age  children  cnarc1  cnarc2  cnarc3  CRT1  CRT2  CRT3  employ_status1  ladder  marital1  mor_circle  moralid1  moralid10  moralid2  moralid3  moralid4  moralid5  moralid6  moralid7  moralid8  moralid9  mcoop1  mcoop2  mcoop3  mcoop4  mcoop5  mcoop6  mcoop7  narc1  narc2  narc3  narc4  narc5  narc6  nidentity1  nidentity2  omind1  omind2  omind3  omind4  omind5  omind6  hygiene1  hygiene2  hygiene3  hygiene4  hygiene5  psupport2  political_ideology  happy  slf_ladder  riskperc1  riskperc2  self_esteem  sex1  sbelong1  sbelong2  sbelong3  sbelong4  tested_positive  optim1  optim2  slfcont1  slfcont3  slfcont4
##   3   10  age  children  cnarc1  cnarc2  cnarc3  CRT1  CRT2  CRT3  employ_status1  ladder  marital1  mor_circle  moralid1  moralid10  moralid2  moralid3  moralid4  moralid5  moralid6  moralid7  moralid8  moralid9  mcoop1  mcoop2  mcoop3  mcoop4  mcoop5  mcoop6  mcoop7  narc1  narc2  narc3  narc4  narc5  narc6  nidentity1  nidentity2  omind1  omind2  omind3  omind4  omind5  omind6  hygiene1  hygiene2  hygiene3  hygiene4  hygiene5  psupport2  political_ideology  happy  slf_ladder  riskperc1  riskperc2  self_esteem  sex1  sbelong1  sbelong2  sbelong3  sbelong4  tested_positive  optim1  optim2  slfcont1  slfcont3  slfcont4
##   4   1  age  children  cnarc1  cnarc2  cnarc3  CRT1  CRT2  CRT3  employ_status1  ladder  marital1  mor_circle  moralid1  moralid10  moralid2  moralid3  moralid4  moralid5  moralid6  moralid7  moralid8  moralid9  mcoop1  mcoop2  mcoop3  mcoop4  mcoop5  mcoop6  mcoop7  narc1  narc2  narc3  narc4  narc5  narc6  nidentity1  nidentity2  omind1  omind2  omind3  omind4  omind5  omind6  hygiene1  hygiene2  hygiene3  hygiene4  hygiene5  psupport2  political_ideology  happy  slf_ladder  riskperc1  riskperc2  self_esteem  sex1  sbelong1  sbelong2  sbelong3  sbelong4  tested_positive  optim1  optim2  slfcont1  slfcont3  slfcont4
##   4   2  age  children  cnarc1  cnarc2  cnarc3  CRT1  CRT2  CRT3  employ_status1  ladder  marital1  mor_circle  moralid1  moralid10  moralid2  moralid3  moralid4  moralid5  moralid6  moralid7  moralid8  moralid9  mcoop1  mcoop2  mcoop3  mcoop4  mcoop5  mcoop6  mcoop7  narc1  narc2  narc3  narc4  narc5  narc6  nidentity1  nidentity2  omind1  omind2  omind3  omind4  omind5  omind6  hygiene1  hygiene2  hygiene3  hygiene4  hygiene5  psupport2  political_ideology  happy  slf_ladder  riskperc1  riskperc2  self_esteem  sex1  sbelong1  sbelong2  sbelong3  sbelong4  tested_positive  optim1  optim2  slfcont1  slfcont3  slfcont4
##   4   3  age  children  cnarc1  cnarc2  cnarc3  CRT1  CRT2  CRT3  employ_status1  ladder  marital1  mor_circle  moralid1  moralid10  moralid2  moralid3  moralid4  moralid5  moralid6  moralid7  moralid8  moralid9  mcoop1  mcoop2  mcoop3  mcoop4  mcoop5  mcoop6  mcoop7  narc1  narc2  narc3  narc4  narc5  narc6  nidentity1  nidentity2  omind1  omind2  omind3  omind4  omind5  omind6  hygiene1  hygiene2  hygiene3  hygiene4  hygiene5  psupport2  political_ideology  happy  slf_ladder  riskperc1  riskperc2  self_esteem  sex1  sbelong1  sbelong2  sbelong3  sbelong4  tested_positive  optim1  optim2  slfcont1  slfcont3  slfcont4
##   4   4  age  children  cnarc1  cnarc2  cnarc3  CRT1  CRT2  CRT3  employ_status1  ladder  marital1  mor_circle  moralid1  moralid10  moralid2  moralid3  moralid4  moralid5  moralid6  moralid7  moralid8  moralid9  mcoop1  mcoop2  mcoop3  mcoop4  mcoop5  mcoop6  mcoop7  narc1  narc2  narc3  narc4  narc5  narc6  nidentity1  nidentity2  omind1  omind2  omind3  omind4  omind5  omind6  hygiene1  hygiene2  hygiene3  hygiene4  hygiene5  psupport2  political_ideology  happy  slf_ladder  riskperc1  riskperc2  self_esteem  sex1  sbelong1  sbelong2  sbelong3  sbelong4  tested_positive  optim1  optim2  slfcont1  slfcont3  slfcont4
##   4   5  age  children  cnarc1  cnarc2  cnarc3  CRT1  CRT2  CRT3  employ_status1  ladder  marital1  mor_circle  moralid1  moralid10  moralid2  moralid3  moralid4  moralid5  moralid6  moralid7  moralid8  moralid9  mcoop1  mcoop2  mcoop3  mcoop4  mcoop5  mcoop6  mcoop7  narc1  narc2  narc3  narc4  narc5  narc6  nidentity1  nidentity2  omind1  omind2  omind3  omind4  omind5  omind6  hygiene1  hygiene2  hygiene3  hygiene4  hygiene5  psupport2  political_ideology  happy  slf_ladder  riskperc1  riskperc2  self_esteem  sex1  sbelong1  sbelong2  sbelong3  sbelong4  tested_positive  optim1  optim2  slfcont1  slfcont3  slfcont4
##   4   6  age  children  cnarc1  cnarc2  cnarc3  CRT1  CRT2  CRT3  employ_status1  ladder  marital1  mor_circle  moralid1  moralid10  moralid2  moralid3  moralid4  moralid5  moralid6  moralid7  moralid8  moralid9  mcoop1  mcoop2  mcoop3  mcoop4  mcoop5  mcoop6  mcoop7  narc1  narc2  narc3  narc4  narc5  narc6  nidentity1  nidentity2  omind1  omind2  omind3  omind4  omind5  omind6  hygiene1  hygiene2  hygiene3  hygiene4  hygiene5  psupport2  political_ideology  happy  slf_ladder  riskperc1  riskperc2  self_esteem  sex1  sbelong1  sbelong2  sbelong3  sbelong4  tested_positive  optim1  optim2  slfcont1  slfcont3  slfcont4
##   4   7  age  children  cnarc1  cnarc2  cnarc3  CRT1  CRT2  CRT3  employ_status1  ladder  marital1  mor_circle  moralid1  moralid10  moralid2  moralid3  moralid4  moralid5  moralid6  moralid7  moralid8  moralid9  mcoop1  mcoop2  mcoop3  mcoop4  mcoop5  mcoop6  mcoop7  narc1  narc2  narc3  narc4  narc5  narc6  nidentity1  nidentity2  omind1  omind2  omind3  omind4  omind5  omind6  hygiene1  hygiene2  hygiene3  hygiene4  hygiene5  psupport2  political_ideology  happy  slf_ladder  riskperc1  riskperc2  self_esteem  sex1  sbelong1  sbelong2  sbelong3  sbelong4  tested_positive  optim1  optim2  slfcont1  slfcont3  slfcont4
##   4   8  age  children  cnarc1  cnarc2  cnarc3  CRT1  CRT2  CRT3  employ_status1  ladder  marital1  mor_circle  moralid1  moralid10  moralid2  moralid3  moralid4  moralid5  moralid6  moralid7  moralid8  moralid9  mcoop1  mcoop2  mcoop3  mcoop4  mcoop5  mcoop6  mcoop7  narc1  narc2  narc3  narc4  narc5  narc6  nidentity1  nidentity2  omind1  omind2  omind3  omind4  omind5  omind6  hygiene1  hygiene2  hygiene3  hygiene4  hygiene5  psupport2  political_ideology  happy  slf_ladder  riskperc1  riskperc2  self_esteem  sex1  sbelong1  sbelong2  sbelong3  sbelong4  tested_positive  optim1  optim2  slfcont1  slfcont3  slfcont4
##   4   9  age  children  cnarc1  cnarc2  cnarc3  CRT1  CRT2  CRT3  employ_status1  ladder  marital1  mor_circle  moralid1  moralid10  moralid2  moralid3  moralid4  moralid5  moralid6  moralid7  moralid8  moralid9  mcoop1  mcoop2  mcoop3  mcoop4  mcoop5  mcoop6  mcoop7  narc1  narc2  narc3  narc4  narc5  narc6  nidentity1  nidentity2  omind1  omind2  omind3  omind4  omind5  omind6  hygiene1  hygiene2  hygiene3  hygiene4  hygiene5  psupport2  political_ideology  happy  slf_ladder  riskperc1  riskperc2  self_esteem  sex1  sbelong1  sbelong2  sbelong3  sbelong4  tested_positive  optim1  optim2  slfcont1  slfcont3  slfcont4
##   4   10  age  children  cnarc1  cnarc2  cnarc3  CRT1  CRT2  CRT3  employ_status1  ladder  marital1  mor_circle  moralid1  moralid10  moralid2  moralid3  moralid4  moralid5  moralid6  moralid7  moralid8  moralid9  mcoop1  mcoop2  mcoop3  mcoop4  mcoop5  mcoop6  mcoop7  narc1  narc2  narc3  narc4  narc5  narc6  nidentity1  nidentity2  omind1  omind2  omind3  omind4  omind5  omind6  hygiene1  hygiene2  hygiene3  hygiene4  hygiene5  psupport2  political_ideology  happy  slf_ladder  riskperc1  riskperc2  self_esteem  sex1  sbelong1  sbelong2  sbelong3  sbelong4  tested_positive  optim1  optim2  slfcont1  slfcont3  slfcont4
##   5   1  age  children  cnarc1  cnarc2  cnarc3  CRT1  CRT2  CRT3  employ_status1  ladder  marital1  mor_circle  moralid1  moralid10  moralid2  moralid3  moralid4  moralid5  moralid6  moralid7  moralid8  moralid9  mcoop1  mcoop2  mcoop3  mcoop4  mcoop5  mcoop6  mcoop7  narc1  narc2  narc3  narc4  narc5  narc6  nidentity1  nidentity2  omind1  omind2  omind3  omind4  omind5  omind6  hygiene1  hygiene2  hygiene3  hygiene4  hygiene5  psupport2  political_ideology  happy  slf_ladder  riskperc1  riskperc2  self_esteem  sex1  sbelong1  sbelong2  sbelong3  sbelong4  tested_positive  optim1  optim2  slfcont1  slfcont3  slfcont4
##   5   2  age  children  cnarc1  cnarc2  cnarc3  CRT1  CRT2  CRT3  employ_status1  ladder  marital1  mor_circle  moralid1  moralid10  moralid2  moralid3  moralid4  moralid5  moralid6  moralid7  moralid8  moralid9  mcoop1  mcoop2  mcoop3  mcoop4  mcoop5  mcoop6  mcoop7  narc1  narc2  narc3  narc4  narc5  narc6  nidentity1  nidentity2  omind1  omind2  omind3  omind4  omind5  omind6  hygiene1  hygiene2  hygiene3  hygiene4  hygiene5  psupport2  political_ideology  happy  slf_ladder  riskperc1  riskperc2  self_esteem  sex1  sbelong1  sbelong2  sbelong3  sbelong4  tested_positive  optim1  optim2  slfcont1  slfcont3  slfcont4
##   5   3  age  children  cnarc1  cnarc2  cnarc3  CRT1  CRT2  CRT3  employ_status1  ladder  marital1  mor_circle  moralid1  moralid10  moralid2  moralid3  moralid4  moralid5  moralid6  moralid7  moralid8  moralid9  mcoop1  mcoop2  mcoop3  mcoop4  mcoop5  mcoop6  mcoop7  narc1  narc2  narc3  narc4  narc5  narc6  nidentity1  nidentity2  omind1  omind2  omind3  omind4  omind5  omind6  hygiene1  hygiene2  hygiene3  hygiene4  hygiene5  psupport2  political_ideology  happy  slf_ladder  riskperc1  riskperc2  self_esteem  sex1  sbelong1  sbelong2  sbelong3  sbelong4  tested_positive  optim1  optim2  slfcont1  slfcont3  slfcont4
##   5   4  age  children  cnarc1  cnarc2  cnarc3  CRT1  CRT2  CRT3  employ_status1  ladder  marital1  mor_circle  moralid1  moralid10  moralid2  moralid3  moralid4  moralid5  moralid6  moralid7  moralid8  moralid9  mcoop1  mcoop2  mcoop3  mcoop4  mcoop5  mcoop6  mcoop7  narc1  narc2  narc3  narc4  narc5  narc6  nidentity1  nidentity2  omind1  omind2  omind3  omind4  omind5  omind6  hygiene1  hygiene2  hygiene3  hygiene4  hygiene5  psupport2  political_ideology  happy  slf_ladder  riskperc1  riskperc2  self_esteem  sex1  sbelong1  sbelong2  sbelong3  sbelong4  tested_positive  optim1  optim2  slfcont1  slfcont3  slfcont4
##   5   5  age  children  cnarc1  cnarc2  cnarc3  CRT1  CRT2  CRT3  employ_status1  ladder  marital1  mor_circle  moralid1  moralid10  moralid2  moralid3  moralid4  moralid5  moralid6  moralid7  moralid8  moralid9  mcoop1  mcoop2  mcoop3  mcoop4  mcoop5  mcoop6  mcoop7  narc1  narc2  narc3  narc4  narc5  narc6  nidentity1  nidentity2  omind1  omind2  omind3  omind4  omind5  omind6  hygiene1  hygiene2  hygiene3  hygiene4  hygiene5  psupport2  political_ideology  happy  slf_ladder  riskperc1  riskperc2  self_esteem  sex1  sbelong1  sbelong2  sbelong3  sbelong4  tested_positive  optim1  optim2  slfcont1  slfcont3  slfcont4
##   5   6  age  children  cnarc1  cnarc2  cnarc3  CRT1  CRT2  CRT3  employ_status1  ladder  marital1  mor_circle  moralid1  moralid10  moralid2  moralid3  moralid4  moralid5  moralid6  moralid7  moralid8  moralid9  mcoop1  mcoop2  mcoop3  mcoop4  mcoop5  mcoop6  mcoop7  narc1  narc2  narc3  narc4  narc5  narc6  nidentity1  nidentity2  omind1  omind2  omind3  omind4  omind5  omind6  hygiene1  hygiene2  hygiene3  hygiene4  hygiene5  psupport2  political_ideology  happy  slf_ladder  riskperc1  riskperc2  self_esteem  sex1  sbelong1  sbelong2  sbelong3  sbelong4  tested_positive  optim1  optim2  slfcont1  slfcont3  slfcont4
##   5   7  age  children  cnarc1  cnarc2  cnarc3  CRT1  CRT2  CRT3  employ_status1  ladder  marital1  mor_circle  moralid1  moralid10  moralid2  moralid3  moralid4  moralid5  moralid6  moralid7  moralid8  moralid9  mcoop1  mcoop2  mcoop3  mcoop4  mcoop5  mcoop6  mcoop7  narc1  narc2  narc3  narc4  narc5  narc6  nidentity1  nidentity2  omind1  omind2  omind3  omind4  omind5  omind6  hygiene1  hygiene2  hygiene3  hygiene4  hygiene5  psupport2  political_ideology  happy  slf_ladder  riskperc1  riskperc2  self_esteem  sex1  sbelong1  sbelong2  sbelong3  sbelong4  tested_positive  optim1  optim2  slfcont1  slfcont3  slfcont4
##   5   8  age  children  cnarc1  cnarc2  cnarc3  CRT1  CRT2  CRT3  employ_status1  ladder  marital1  mor_circle  moralid1  moralid10  moralid2  moralid3  moralid4  moralid5  moralid6  moralid7  moralid8  moralid9  mcoop1  mcoop2  mcoop3  mcoop4  mcoop5  mcoop6  mcoop7  narc1  narc2  narc3  narc4  narc5  narc6  nidentity1  nidentity2  omind1  omind2  omind3  omind4  omind5  omind6  hygiene1  hygiene2  hygiene3  hygiene4  hygiene5  psupport2  political_ideology  happy  slf_ladder  riskperc1  riskperc2  self_esteem  sex1  sbelong1  sbelong2  sbelong3  sbelong4  tested_positive  optim1  optim2  slfcont1  slfcont3  slfcont4
##   5   9  age  children  cnarc1  cnarc2  cnarc3  CRT1  CRT2  CRT3  employ_status1  ladder  marital1  mor_circle  moralid1  moralid10  moralid2  moralid3  moralid4  moralid5  moralid6  moralid7  moralid8  moralid9  mcoop1  mcoop2  mcoop3  mcoop4  mcoop5  mcoop6  mcoop7  narc1  narc2  narc3  narc4  narc5  narc6  nidentity1  nidentity2  omind1  omind2  omind3  omind4  omind5  omind6  hygiene1  hygiene2  hygiene3  hygiene4  hygiene5  psupport2  political_ideology  happy  slf_ladder  riskperc1  riskperc2  self_esteem  sex1  sbelong1  sbelong2  sbelong3  sbelong4  tested_positive  optim1  optim2  slfcont1  slfcont3  slfcont4
##   5   10  age  children  cnarc1  cnarc2  cnarc3  CRT1  CRT2  CRT3  employ_status1  ladder  marital1  mor_circle  moralid1  moralid10  moralid2  moralid3  moralid4  moralid5  moralid6  moralid7  moralid8  moralid9  mcoop1  mcoop2  mcoop3  mcoop4  mcoop5  mcoop6  mcoop7  narc1  narc2  narc3  narc4  narc5  narc6  nidentity1  nidentity2  omind1  omind2  omind3  omind4  omind5  omind6  hygiene1  hygiene2  hygiene3  hygiene4  hygiene5  psupport2  political_ideology  happy  slf_ladder  riskperc1  riskperc2  self_esteem  sex1  sbelong1  sbelong2  sbelong3  sbelong4  tested_positive  optim1  optim2  slfcont1  slfcont3  slfcont4
##   6   1  age  children  cnarc1  cnarc2  cnarc3  CRT1  CRT2  CRT3  employ_status1  ladder  marital1  mor_circle  moralid1  moralid10  moralid2  moralid3  moralid4  moralid5  moralid6  moralid7  moralid8  moralid9  mcoop1  mcoop2  mcoop3  mcoop4  mcoop5  mcoop6  mcoop7  narc1  narc2  narc3  narc4  narc5  narc6  nidentity1  nidentity2  omind1  omind2  omind3  omind4  omind5  omind6  hygiene1  hygiene2  hygiene3  hygiene4  hygiene5  psupport2  political_ideology  happy  slf_ladder  riskperc1  riskperc2  self_esteem  sex1  sbelong1  sbelong2  sbelong3  sbelong4  tested_positive  optim1  optim2  slfcont1  slfcont3  slfcont4
##   6   2  age  children  cnarc1  cnarc2  cnarc3  CRT1  CRT2  CRT3  employ_status1  ladder  marital1  mor_circle  moralid1  moralid10  moralid2  moralid3  moralid4  moralid5  moralid6  moralid7  moralid8  moralid9  mcoop1  mcoop2  mcoop3  mcoop4  mcoop5  mcoop6  mcoop7  narc1  narc2  narc3  narc4  narc5  narc6  nidentity1  nidentity2  omind1  omind2  omind3  omind4  omind5  omind6  hygiene1  hygiene2  hygiene3  hygiene4  hygiene5  psupport2  political_ideology  happy  slf_ladder  riskperc1  riskperc2  self_esteem  sex1  sbelong1  sbelong2  sbelong3  sbelong4  tested_positive  optim1  optim2  slfcont1  slfcont3  slfcont4
##   6   3  age  children  cnarc1  cnarc2  cnarc3  CRT1  CRT2  CRT3  employ_status1  ladder  marital1  mor_circle  moralid1  moralid10  moralid2  moralid3  moralid4  moralid5  moralid6  moralid7  moralid8  moralid9  mcoop1  mcoop2  mcoop3  mcoop4  mcoop5  mcoop6  mcoop7  narc1  narc2  narc3  narc4  narc5  narc6  nidentity1  nidentity2  omind1  omind2  omind3  omind4  omind5  omind6  hygiene1  hygiene2  hygiene3  hygiene4  hygiene5  psupport2  political_ideology  happy  slf_ladder  riskperc1  riskperc2  self_esteem  sex1  sbelong1  sbelong2  sbelong3  sbelong4  tested_positive  optim1  optim2  slfcont1  slfcont3  slfcont4
##   6   4  age  children  cnarc1  cnarc2  cnarc3  CRT1  CRT2  CRT3  employ_status1  ladder  marital1  mor_circle  moralid1  moralid10  moralid2  moralid3  moralid4  moralid5  moralid6  moralid7  moralid8  moralid9  mcoop1  mcoop2  mcoop3  mcoop4  mcoop5  mcoop6  mcoop7  narc1  narc2  narc3  narc4  narc5  narc6  nidentity1  nidentity2  omind1  omind2  omind3  omind4  omind5  omind6  hygiene1  hygiene2  hygiene3  hygiene4  hygiene5  psupport2  political_ideology  happy  slf_ladder  riskperc1  riskperc2  self_esteem  sex1  sbelong1  sbelong2  sbelong3  sbelong4  tested_positive  optim1  optim2  slfcont1  slfcont3  slfcont4
##   6   5  age  children  cnarc1  cnarc2  cnarc3  CRT1  CRT2  CRT3  employ_status1  ladder  marital1  mor_circle  moralid1  moralid10  moralid2  moralid3  moralid4  moralid5  moralid6  moralid7  moralid8  moralid9  mcoop1  mcoop2  mcoop3  mcoop4  mcoop5  mcoop6  mcoop7  narc1  narc2  narc3  narc4  narc5  narc6  nidentity1  nidentity2  omind1  omind2  omind3  omind4  omind5  omind6  hygiene1  hygiene2  hygiene3  hygiene4  hygiene5  psupport2  political_ideology  happy  slf_ladder  riskperc1  riskperc2  self_esteem  sex1  sbelong1  sbelong2  sbelong3  sbelong4  tested_positive  optim1  optim2  slfcont1  slfcont3  slfcont4
##   6   6  age  children  cnarc1  cnarc2  cnarc3  CRT1  CRT2  CRT3  employ_status1  ladder  marital1  mor_circle  moralid1  moralid10  moralid2  moralid3  moralid4  moralid5  moralid6  moralid7  moralid8  moralid9  mcoop1  mcoop2  mcoop3  mcoop4  mcoop5  mcoop6  mcoop7  narc1  narc2  narc3  narc4  narc5  narc6  nidentity1  nidentity2  omind1  omind2  omind3  omind4  omind5  omind6  hygiene1  hygiene2  hygiene3  hygiene4  hygiene5  psupport2  political_ideology  happy  slf_ladder  riskperc1  riskperc2  self_esteem  sex1  sbelong1  sbelong2  sbelong3  sbelong4  tested_positive  optim1  optim2  slfcont1  slfcont3  slfcont4
##   6   7  age  children  cnarc1  cnarc2  cnarc3  CRT1  CRT2  CRT3  employ_status1  ladder  marital1  mor_circle  moralid1  moralid10  moralid2  moralid3  moralid4  moralid5  moralid6  moralid7  moralid8  moralid9  mcoop1  mcoop2  mcoop3  mcoop4  mcoop5  mcoop6  mcoop7  narc1  narc2  narc3  narc4  narc5  narc6  nidentity1  nidentity2  omind1  omind2  omind3  omind4  omind5  omind6  hygiene1  hygiene2  hygiene3  hygiene4  hygiene5  psupport2  political_ideology  happy  slf_ladder  riskperc1  riskperc2  self_esteem  sex1  sbelong1  sbelong2  sbelong3  sbelong4  tested_positive  optim1  optim2  slfcont1  slfcont3  slfcont4
##   6   8  age  children  cnarc1  cnarc2  cnarc3  CRT1  CRT2  CRT3  employ_status1  ladder  marital1  mor_circle  moralid1  moralid10  moralid2  moralid3  moralid4  moralid5  moralid6  moralid7  moralid8  moralid9  mcoop1  mcoop2  mcoop3  mcoop4  mcoop5  mcoop6  mcoop7  narc1  narc2  narc3  narc4  narc5  narc6  nidentity1  nidentity2  omind1  omind2  omind3  omind4  omind5  omind6  hygiene1  hygiene2  hygiene3  hygiene4  hygiene5  psupport2  political_ideology  happy  slf_ladder  riskperc1  riskperc2  self_esteem  sex1  sbelong1  sbelong2  sbelong3  sbelong4  tested_positive  optim1  optim2  slfcont1  slfcont3  slfcont4
##   6   9  age  children  cnarc1  cnarc2  cnarc3  CRT1  CRT2  CRT3  employ_status1  ladder  marital1  mor_circle  moralid1  moralid10  moralid2  moralid3  moralid4  moralid5  moralid6  moralid7  moralid8  moralid9  mcoop1  mcoop2  mcoop3  mcoop4  mcoop5  mcoop6  mcoop7  narc1  narc2  narc3  narc4  narc5  narc6  nidentity1  nidentity2  omind1  omind2  omind3  omind4  omind5  omind6  hygiene1  hygiene2  hygiene3  hygiene4  hygiene5  psupport2  political_ideology  happy  slf_ladder  riskperc1  riskperc2  self_esteem  sex1  sbelong1  sbelong2  sbelong3  sbelong4  tested_positive  optim1  optim2  slfcont1  slfcont3  slfcont4
##   6   10  age  children  cnarc1  cnarc2  cnarc3  CRT1  CRT2  CRT3  employ_status1  ladder  marital1  mor_circle  moralid1  moralid10  moralid2  moralid3  moralid4  moralid5  moralid6  moralid7  moralid8  moralid9  mcoop1  mcoop2  mcoop3  mcoop4  mcoop5  mcoop6  mcoop7  narc1  narc2  narc3  narc4  narc5  narc6  nidentity1  nidentity2  omind1  omind2  omind3  omind4  omind5  omind6  hygiene1  hygiene2  hygiene3  hygiene4  hygiene5  psupport2  political_ideology  happy  slf_ladder  riskperc1  riskperc2  self_esteem  sex1  sbelong1  sbelong2  sbelong3  sbelong4  tested_positive  optim1  optim2  slfcont1  slfcont3  slfcont4
##   7   1  age  children  cnarc1  cnarc2  cnarc3  CRT1  CRT2  CRT3  employ_status1  ladder  marital1  mor_circle  moralid1  moralid10  moralid2  moralid3  moralid4  moralid5  moralid6  moralid7  moralid8  moralid9  mcoop1  mcoop2  mcoop3  mcoop4  mcoop5  mcoop6  mcoop7  narc1  narc2  narc3  narc4  narc5  narc6  nidentity1  nidentity2  omind1  omind2  omind3  omind4  omind5  omind6  hygiene1  hygiene2  hygiene3  hygiene4  hygiene5  psupport2  political_ideology  happy  slf_ladder  riskperc1  riskperc2  self_esteem  sex1  sbelong1  sbelong2  sbelong3  sbelong4  tested_positive  optim1  optim2  slfcont1  slfcont3  slfcont4
##   7   2  age  children  cnarc1  cnarc2  cnarc3  CRT1  CRT2  CRT3  employ_status1  ladder  marital1  mor_circle  moralid1  moralid10  moralid2  moralid3  moralid4  moralid5  moralid6  moralid7  moralid8  moralid9  mcoop1  mcoop2  mcoop3  mcoop4  mcoop5  mcoop6  mcoop7  narc1  narc2  narc3  narc4  narc5  narc6  nidentity1  nidentity2  omind1  omind2  omind3  omind4  omind5  omind6  hygiene1  hygiene2  hygiene3  hygiene4  hygiene5  psupport2  political_ideology  happy  slf_ladder  riskperc1  riskperc2  self_esteem  sex1  sbelong1  sbelong2  sbelong3  sbelong4  tested_positive  optim1  optim2  slfcont1  slfcont3  slfcont4
##   7   3  age  children  cnarc1  cnarc2  cnarc3  CRT1  CRT2  CRT3  employ_status1  ladder  marital1  mor_circle  moralid1  moralid10  moralid2  moralid3  moralid4  moralid5  moralid6  moralid7  moralid8  moralid9  mcoop1  mcoop2  mcoop3  mcoop4  mcoop5  mcoop6  mcoop7  narc1  narc2  narc3  narc4  narc5  narc6  nidentity1  nidentity2  omind1  omind2  omind3  omind4  omind5  omind6  hygiene1  hygiene2  hygiene3  hygiene4  hygiene5  psupport2  political_ideology  happy  slf_ladder  riskperc1  riskperc2  self_esteem  sex1  sbelong1  sbelong2  sbelong3  sbelong4  tested_positive  optim1  optim2  slfcont1  slfcont3  slfcont4
##   7   4  age  children  cnarc1  cnarc2  cnarc3  CRT1  CRT2  CRT3  employ_status1  ladder  marital1  mor_circle  moralid1  moralid10  moralid2  moralid3  moralid4  moralid5  moralid6  moralid7  moralid8  moralid9  mcoop1  mcoop2  mcoop3  mcoop4  mcoop5  mcoop6  mcoop7  narc1  narc2  narc3  narc4  narc5  narc6  nidentity1  nidentity2  omind1  omind2  omind3  omind4  omind5  omind6  hygiene1  hygiene2  hygiene3  hygiene4  hygiene5  psupport2  political_ideology  happy  slf_ladder  riskperc1  riskperc2  self_esteem  sex1  sbelong1  sbelong2  sbelong3  sbelong4  tested_positive  optim1  optim2  slfcont1  slfcont3  slfcont4
##   7   5  age  children  cnarc1  cnarc2  cnarc3  CRT1  CRT2  CRT3  employ_status1  ladder  marital1  mor_circle  moralid1  moralid10  moralid2  moralid3  moralid4  moralid5  moralid6  moralid7  moralid8  moralid9  mcoop1  mcoop2  mcoop3  mcoop4  mcoop5  mcoop6  mcoop7  narc1  narc2  narc3  narc4  narc5  narc6  nidentity1  nidentity2  omind1  omind2  omind3  omind4  omind5  omind6  hygiene1  hygiene2  hygiene3  hygiene4  hygiene5  psupport2  political_ideology  happy  slf_ladder  riskperc1  riskperc2  self_esteem  sex1  sbelong1  sbelong2  sbelong3  sbelong4  tested_positive  optim1  optim2  slfcont1  slfcont3  slfcont4
##   7   6  age  children  cnarc1  cnarc2  cnarc3  CRT1  CRT2  CRT3  employ_status1  ladder  marital1  mor_circle  moralid1  moralid10  moralid2  moralid3  moralid4  moralid5  moralid6  moralid7  moralid8  moralid9  mcoop1  mcoop2  mcoop3  mcoop4  mcoop5  mcoop6  mcoop7  narc1  narc2  narc3  narc4  narc5  narc6  nidentity1  nidentity2  omind1  omind2  omind3  omind4  omind5  omind6  hygiene1  hygiene2  hygiene3  hygiene4  hygiene5  psupport2  political_ideology  happy  slf_ladder  riskperc1  riskperc2  self_esteem  sex1  sbelong1  sbelong2  sbelong3  sbelong4  tested_positive  optim1  optim2  slfcont1  slfcont3  slfcont4
##   7   7  age  children  cnarc1  cnarc2  cnarc3  CRT1  CRT2  CRT3  employ_status1  ladder  marital1  mor_circle  moralid1  moralid10  moralid2  moralid3  moralid4  moralid5  moralid6  moralid7  moralid8  moralid9  mcoop1  mcoop2  mcoop3  mcoop4  mcoop5  mcoop6  mcoop7  narc1  narc2  narc3  narc4  narc5  narc6  nidentity1  nidentity2  omind1  omind2  omind3  omind4  omind5  omind6  hygiene1  hygiene2  hygiene3  hygiene4  hygiene5  psupport2  political_ideology  happy  slf_ladder  riskperc1  riskperc2  self_esteem  sex1  sbelong1  sbelong2  sbelong3  sbelong4  tested_positive  optim1  optim2  slfcont1  slfcont3  slfcont4
##   7   8  age  children  cnarc1  cnarc2  cnarc3  CRT1  CRT2  CRT3  employ_status1  ladder  marital1  mor_circle  moralid1  moralid10  moralid2  moralid3  moralid4  moralid5  moralid6  moralid7  moralid8  moralid9  mcoop1  mcoop2  mcoop3  mcoop4  mcoop5  mcoop6  mcoop7  narc1  narc2  narc3  narc4  narc5  narc6  nidentity1  nidentity2  omind1  omind2  omind3  omind4  omind5  omind6  hygiene1  hygiene2  hygiene3  hygiene4  hygiene5  psupport2  political_ideology  happy  slf_ladder  riskperc1  riskperc2  self_esteem  sex1  sbelong1  sbelong2  sbelong3  sbelong4  tested_positive  optim1  optim2  slfcont1  slfcont3  slfcont4
##   7   9  age  children  cnarc1  cnarc2  cnarc3  CRT1  CRT2  CRT3  employ_status1  ladder  marital1  mor_circle  moralid1  moralid10  moralid2  moralid3  moralid4  moralid5  moralid6  moralid7  moralid8  moralid9  mcoop1  mcoop2  mcoop3  mcoop4  mcoop5  mcoop6  mcoop7  narc1  narc2  narc3  narc4  narc5  narc6  nidentity1  nidentity2  omind1  omind2  omind3  omind4  omind5  omind6  hygiene1  hygiene2  hygiene3  hygiene4  hygiene5  psupport2  political_ideology  happy  slf_ladder  riskperc1  riskperc2  self_esteem  sex1  sbelong1  sbelong2  sbelong3  sbelong4  tested_positive  optim1  optim2  slfcont1  slfcont3  slfcont4
##   7   10  age  children  cnarc1  cnarc2  cnarc3  CRT1  CRT2  CRT3  employ_status1  ladder  marital1  mor_circle  moralid1  moralid10  moralid2  moralid3  moralid4  moralid5  moralid6  moralid7  moralid8  moralid9  mcoop1  mcoop2  mcoop3  mcoop4  mcoop5  mcoop6  mcoop7  narc1  narc2  narc3  narc4  narc5  narc6  nidentity1  nidentity2  omind1  omind2  omind3  omind4  omind5  omind6  hygiene1  hygiene2  hygiene3  hygiene4  hygiene5  psupport2  political_ideology  happy  slf_ladder  riskperc1  riskperc2  self_esteem  sex1  sbelong1  sbelong2  sbelong3  sbelong4  tested_positive  optim1  optim2  slfcont1  slfcont3  slfcont4
##   8   1  age  children  cnarc1  cnarc2  cnarc3  CRT1  CRT2  CRT3  employ_status1  ladder  marital1  mor_circle  moralid1  moralid10  moralid2  moralid3  moralid4  moralid5  moralid6  moralid7  moralid8  moralid9  mcoop1  mcoop2  mcoop3  mcoop4  mcoop5  mcoop6  mcoop7  narc1  narc2  narc3  narc4  narc5  narc6  nidentity1  nidentity2  omind1  omind2  omind3  omind4  omind5  omind6  hygiene1  hygiene2  hygiene3  hygiene4  hygiene5  psupport2  political_ideology  happy  slf_ladder  riskperc1  riskperc2  self_esteem  sex1  sbelong1  sbelong2  sbelong3  sbelong4  tested_positive  optim1  optim2  slfcont1  slfcont3  slfcont4
##   8   2  age  children  cnarc1  cnarc2  cnarc3  CRT1  CRT2  CRT3  employ_status1  ladder  marital1  mor_circle  moralid1  moralid10  moralid2  moralid3  moralid4  moralid5  moralid6  moralid7  moralid8  moralid9  mcoop1  mcoop2  mcoop3  mcoop4  mcoop5  mcoop6  mcoop7  narc1  narc2  narc3  narc4  narc5  narc6  nidentity1  nidentity2  omind1  omind2  omind3  omind4  omind5  omind6  hygiene1  hygiene2  hygiene3  hygiene4  hygiene5  psupport2  political_ideology  happy  slf_ladder  riskperc1  riskperc2  self_esteem  sex1  sbelong1  sbelong2  sbelong3  sbelong4  tested_positive  optim1  optim2  slfcont1  slfcont3  slfcont4
##   8   3  age  children  cnarc1  cnarc2  cnarc3  CRT1  CRT2  CRT3  employ_status1  ladder  marital1  mor_circle  moralid1  moralid10  moralid2  moralid3  moralid4  moralid5  moralid6  moralid7  moralid8  moralid9  mcoop1  mcoop2  mcoop3  mcoop4  mcoop5  mcoop6  mcoop7  narc1  narc2  narc3  narc4  narc5  narc6  nidentity1  nidentity2  omind1  omind2  omind3  omind4  omind5  omind6  hygiene1  hygiene2  hygiene3  hygiene4  hygiene5  psupport2  political_ideology  happy  slf_ladder  riskperc1  riskperc2  self_esteem  sex1  sbelong1  sbelong2  sbelong3  sbelong4  tested_positive  optim1  optim2  slfcont1  slfcont3  slfcont4
##   8   4  age  children  cnarc1  cnarc2  cnarc3  CRT1  CRT2  CRT3  employ_status1  ladder  marital1  mor_circle  moralid1  moralid10  moralid2  moralid3  moralid4  moralid5  moralid6  moralid7  moralid8  moralid9  mcoop1  mcoop2  mcoop3  mcoop4  mcoop5  mcoop6  mcoop7  narc1  narc2  narc3  narc4  narc5  narc6  nidentity1  nidentity2  omind1  omind2  omind3  omind4  omind5  omind6  hygiene1  hygiene2  hygiene3  hygiene4  hygiene5  psupport2  political_ideology  happy  slf_ladder  riskperc1  riskperc2  self_esteem  sex1  sbelong1  sbelong2  sbelong3  sbelong4  tested_positive  optim1  optim2  slfcont1  slfcont3  slfcont4
##   8   5  age  children  cnarc1  cnarc2  cnarc3  CRT1  CRT2  CRT3  employ_status1  ladder  marital1  mor_circle  moralid1  moralid10  moralid2  moralid3  moralid4  moralid5  moralid6  moralid7  moralid8  moralid9  mcoop1  mcoop2  mcoop3  mcoop4  mcoop5  mcoop6  mcoop7  narc1  narc2  narc3  narc4  narc5  narc6  nidentity1  nidentity2  omind1  omind2  omind3  omind4  omind5  omind6  hygiene1  hygiene2  hygiene3  hygiene4  hygiene5  psupport2  political_ideology  happy  slf_ladder  riskperc1  riskperc2  self_esteem  sex1  sbelong1  sbelong2  sbelong3  sbelong4  tested_positive  optim1  optim2  slfcont1  slfcont3  slfcont4
##   8   6  age  children  cnarc1  cnarc2  cnarc3  CRT1  CRT2  CRT3  employ_status1  ladder  marital1  mor_circle  moralid1  moralid10  moralid2  moralid3  moralid4  moralid5  moralid6  moralid7  moralid8  moralid9  mcoop1  mcoop2  mcoop3  mcoop4  mcoop5  mcoop6  mcoop7  narc1  narc2  narc3  narc4  narc5  narc6  nidentity1  nidentity2  omind1  omind2  omind3  omind4  omind5  omind6  hygiene1  hygiene2  hygiene3  hygiene4  hygiene5  psupport2  political_ideology  happy  slf_ladder  riskperc1  riskperc2  self_esteem  sex1  sbelong1  sbelong2  sbelong3  sbelong4  tested_positive  optim1  optim2  slfcont1  slfcont3  slfcont4
##   8   7  age  children  cnarc1  cnarc2  cnarc3  CRT1  CRT2  CRT3  employ_status1  ladder  marital1  mor_circle  moralid1  moralid10  moralid2  moralid3  moralid4  moralid5  moralid6  moralid7  moralid8  moralid9  mcoop1  mcoop2  mcoop3  mcoop4  mcoop5  mcoop6  mcoop7  narc1  narc2  narc3  narc4  narc5  narc6  nidentity1  nidentity2  omind1  omind2  omind3  omind4  omind5  omind6  hygiene1  hygiene2  hygiene3  hygiene4  hygiene5  psupport2  political_ideology  happy  slf_ladder  riskperc1  riskperc2  self_esteem  sex1  sbelong1  sbelong2  sbelong3  sbelong4  tested_positive  optim1  optim2  slfcont1  slfcont3  slfcont4
##   8   8  age  children  cnarc1  cnarc2  cnarc3  CRT1  CRT2  CRT3  employ_status1  ladder  marital1  mor_circle  moralid1  moralid10  moralid2  moralid3  moralid4  moralid5  moralid6  moralid7  moralid8  moralid9  mcoop1  mcoop2  mcoop3  mcoop4  mcoop5  mcoop6  mcoop7  narc1  narc2  narc3  narc4  narc5  narc6  nidentity1  nidentity2  omind1  omind2  omind3  omind4  omind5  omind6  hygiene1  hygiene2  hygiene3  hygiene4  hygiene5  psupport2  political_ideology  happy  slf_ladder  riskperc1  riskperc2  self_esteem  sex1  sbelong1  sbelong2  sbelong3  sbelong4  tested_positive  optim1  optim2  slfcont1  slfcont3  slfcont4
##   8   9  age  children  cnarc1  cnarc2  cnarc3  CRT1  CRT2  CRT3  employ_status1  ladder  marital1  mor_circle  moralid1  moralid10  moralid2  moralid3  moralid4  moralid5  moralid6  moralid7  moralid8  moralid9  mcoop1  mcoop2  mcoop3  mcoop4  mcoop5  mcoop6  mcoop7  narc1  narc2  narc3  narc4  narc5  narc6  nidentity1  nidentity2  omind1  omind2  omind3  omind4  omind5  omind6  hygiene1  hygiene2  hygiene3  hygiene4  hygiene5  psupport2  political_ideology  happy  slf_ladder  riskperc1  riskperc2  self_esteem  sex1  sbelong1  sbelong2  sbelong3  sbelong4  tested_positive  optim1  optim2  slfcont1  slfcont3  slfcont4
##   8   10  age  children  cnarc1  cnarc2  cnarc3  CRT1  CRT2  CRT3  employ_status1  ladder  marital1  mor_circle  moralid1  moralid10  moralid2  moralid3  moralid4  moralid5  moralid6  moralid7  moralid8  moralid9  mcoop1  mcoop2  mcoop3  mcoop4  mcoop5  mcoop6  mcoop7  narc1  narc2  narc3  narc4  narc5  narc6  nidentity1  nidentity2  omind1  omind2  omind3  omind4  omind5  omind6  hygiene1  hygiene2  hygiene3  hygiene4  hygiene5  psupport2  political_ideology  happy  slf_ladder  riskperc1  riskperc2  self_esteem  sex1  sbelong1  sbelong2  sbelong3  sbelong4  tested_positive  optim1  optim2  slfcont1  slfcont3  slfcont4
##   9   1  age  children  cnarc1  cnarc2  cnarc3  CRT1  CRT2  CRT3  employ_status1  ladder  marital1  mor_circle  moralid1  moralid10  moralid2  moralid3  moralid4  moralid5  moralid6  moralid7  moralid8  moralid9  mcoop1  mcoop2  mcoop3  mcoop4  mcoop5  mcoop6  mcoop7  narc1  narc2  narc3  narc4  narc5  narc6  nidentity1  nidentity2  omind1  omind2  omind3  omind4  omind5  omind6  hygiene1  hygiene2  hygiene3  hygiene4  hygiene5  psupport2  political_ideology  happy  slf_ladder  riskperc1  riskperc2  self_esteem  sex1  sbelong1  sbelong2  sbelong3  sbelong4  tested_positive  optim1  optim2  slfcont1  slfcont3  slfcont4
##   9   2  age  children  cnarc1  cnarc2  cnarc3  CRT1  CRT2  CRT3  employ_status1  ladder  marital1  mor_circle  moralid1  moralid10  moralid2  moralid3  moralid4  moralid5  moralid6  moralid7  moralid8  moralid9  mcoop1  mcoop2  mcoop3  mcoop4  mcoop5  mcoop6  mcoop7  narc1  narc2  narc3  narc4  narc5  narc6  nidentity1  nidentity2  omind1  omind2  omind3  omind4  omind5  omind6  hygiene1  hygiene2  hygiene3  hygiene4  hygiene5  psupport2  political_ideology  happy  slf_ladder  riskperc1  riskperc2  self_esteem  sex1  sbelong1  sbelong2  sbelong3  sbelong4  tested_positive  optim1  optim2  slfcont1  slfcont3  slfcont4
##   9   3  age  children  cnarc1  cnarc2  cnarc3  CRT1  CRT2  CRT3  employ_status1  ladder  marital1  mor_circle  moralid1  moralid10  moralid2  moralid3  moralid4  moralid5  moralid6  moralid7  moralid8  moralid9  mcoop1  mcoop2  mcoop3  mcoop4  mcoop5  mcoop6  mcoop7  narc1  narc2  narc3  narc4  narc5  narc6  nidentity1  nidentity2  omind1  omind2  omind3  omind4  omind5  omind6  hygiene1  hygiene2  hygiene3  hygiene4  hygiene5  psupport2  political_ideology  happy  slf_ladder  riskperc1  riskperc2  self_esteem  sex1  sbelong1  sbelong2  sbelong3  sbelong4  tested_positive  optim1  optim2  slfcont1  slfcont3  slfcont4
##   9   4  age  children  cnarc1  cnarc2  cnarc3  CRT1  CRT2  CRT3  employ_status1  ladder  marital1  mor_circle  moralid1  moralid10  moralid2  moralid3  moralid4  moralid5  moralid6  moralid7  moralid8  moralid9  mcoop1  mcoop2  mcoop3  mcoop4  mcoop5  mcoop6  mcoop7  narc1  narc2  narc3  narc4  narc5  narc6  nidentity1  nidentity2  omind1  omind2  omind3  omind4  omind5  omind6  hygiene1  hygiene2  hygiene3  hygiene4  hygiene5  psupport2  political_ideology  happy  slf_ladder  riskperc1  riskperc2  self_esteem  sex1  sbelong1  sbelong2  sbelong3  sbelong4  tested_positive  optim1  optim2  slfcont1  slfcont3  slfcont4
##   9   5  age  children  cnarc1  cnarc2  cnarc3  CRT1  CRT2  CRT3  employ_status1  ladder  marital1  mor_circle  moralid1  moralid10  moralid2  moralid3  moralid4  moralid5  moralid6  moralid7  moralid8  moralid9  mcoop1  mcoop2  mcoop3  mcoop4  mcoop5  mcoop6  mcoop7  narc1  narc2  narc3  narc4  narc5  narc6  nidentity1  nidentity2  omind1  omind2  omind3  omind4  omind5  omind6  hygiene1  hygiene2  hygiene3  hygiene4  hygiene5  psupport2  political_ideology  happy  slf_ladder  riskperc1  riskperc2  self_esteem  sex1  sbelong1  sbelong2  sbelong3  sbelong4  tested_positive  optim1  optim2  slfcont1  slfcont3  slfcont4
##   9   6  age  children  cnarc1  cnarc2  cnarc3  CRT1  CRT2  CRT3  employ_status1  ladder  marital1  mor_circle  moralid1  moralid10  moralid2  moralid3  moralid4  moralid5  moralid6  moralid7  moralid8  moralid9  mcoop1  mcoop2  mcoop3  mcoop4  mcoop5  mcoop6  mcoop7  narc1  narc2  narc3  narc4  narc5  narc6  nidentity1  nidentity2  omind1  omind2  omind3  omind4  omind5  omind6  hygiene1  hygiene2  hygiene3  hygiene4  hygiene5  psupport2  political_ideology  happy  slf_ladder  riskperc1  riskperc2  self_esteem  sex1  sbelong1  sbelong2  sbelong3  sbelong4  tested_positive  optim1  optim2  slfcont1  slfcont3  slfcont4
##   9   7  age  children  cnarc1  cnarc2  cnarc3  CRT1  CRT2  CRT3  employ_status1  ladder  marital1  mor_circle  moralid1  moralid10  moralid2  moralid3  moralid4  moralid5  moralid6  moralid7  moralid8  moralid9  mcoop1  mcoop2  mcoop3  mcoop4  mcoop5  mcoop6  mcoop7  narc1  narc2  narc3  narc4  narc5  narc6  nidentity1  nidentity2  omind1  omind2  omind3  omind4  omind5  omind6  hygiene1  hygiene2  hygiene3  hygiene4  hygiene5  psupport2  political_ideology  happy  slf_ladder  riskperc1  riskperc2  self_esteem  sex1  sbelong1  sbelong2  sbelong3  sbelong4  tested_positive  optim1  optim2  slfcont1  slfcont3  slfcont4
##   9   8  age  children  cnarc1  cnarc2  cnarc3  CRT1  CRT2  CRT3  employ_status1  ladder  marital1  mor_circle  moralid1  moralid10  moralid2  moralid3  moralid4  moralid5  moralid6  moralid7  moralid8  moralid9  mcoop1  mcoop2  mcoop3  mcoop4  mcoop5  mcoop6  mcoop7  narc1  narc2  narc3  narc4  narc5  narc6  nidentity1  nidentity2  omind1  omind2  omind3  omind4  omind5  omind6  hygiene1  hygiene2  hygiene3  hygiene4  hygiene5  psupport2  political_ideology  happy  slf_ladder  riskperc1  riskperc2  self_esteem  sex1  sbelong1  sbelong2  sbelong3  sbelong4  tested_positive  optim1  optim2  slfcont1  slfcont3  slfcont4
##   9   9  age  children  cnarc1  cnarc2  cnarc3  CRT1  CRT2  CRT3  employ_status1  ladder  marital1  mor_circle  moralid1  moralid10  moralid2  moralid3  moralid4  moralid5  moralid6  moralid7  moralid8  moralid9  mcoop1  mcoop2  mcoop3  mcoop4  mcoop5  mcoop6  mcoop7  narc1  narc2  narc3  narc4  narc5  narc6  nidentity1  nidentity2  omind1  omind2  omind3  omind4  omind5  omind6  hygiene1  hygiene2  hygiene3  hygiene4  hygiene5  psupport2  political_ideology  happy  slf_ladder  riskperc1  riskperc2  self_esteem  sex1  sbelong1  sbelong2  sbelong3  sbelong4  tested_positive  optim1  optim2  slfcont1  slfcont3  slfcont4
##   9   10  age  children  cnarc1  cnarc2  cnarc3  CRT1  CRT2  CRT3  employ_status1  ladder  marital1  mor_circle  moralid1  moralid10  moralid2  moralid3  moralid4  moralid5  moralid6  moralid7  moralid8  moralid9  mcoop1  mcoop2  mcoop3  mcoop4  mcoop5  mcoop6  mcoop7  narc1  narc2  narc3  narc4  narc5  narc6  nidentity1  nidentity2  omind1  omind2  omind3  omind4  omind5  omind6  hygiene1  hygiene2  hygiene3  hygiene4  hygiene5  psupport2  political_ideology  happy  slf_ladder  riskperc1  riskperc2  self_esteem  sex1  sbelong1  sbelong2  sbelong3  sbelong4  tested_positive  optim1  optim2  slfcont1  slfcont3  slfcont4
##   10   1  age  children  cnarc1  cnarc2  cnarc3  CRT1  CRT2  CRT3  employ_status1  ladder  marital1  mor_circle  moralid1  moralid10  moralid2  moralid3  moralid4  moralid5  moralid6  moralid7  moralid8  moralid9  mcoop1  mcoop2  mcoop3  mcoop4  mcoop5  mcoop6  mcoop7  narc1  narc2  narc3  narc4  narc5  narc6  nidentity1  nidentity2  omind1  omind2  omind3  omind4  omind5  omind6  hygiene1  hygiene2  hygiene3  hygiene4  hygiene5  psupport2  political_ideology  happy  slf_ladder  riskperc1  riskperc2  self_esteem  sex1  sbelong1  sbelong2  sbelong3  sbelong4  tested_positive  optim1  optim2  slfcont1  slfcont3  slfcont4
##   10   2  age  children  cnarc1  cnarc2  cnarc3  CRT1  CRT2  CRT3  employ_status1  ladder  marital1  mor_circle  moralid1  moralid10  moralid2  moralid3  moralid4  moralid5  moralid6  moralid7  moralid8  moralid9  mcoop1  mcoop2  mcoop3  mcoop4  mcoop5  mcoop6  mcoop7  narc1  narc2  narc3  narc4  narc5  narc6  nidentity1  nidentity2  omind1  omind2  omind3  omind4  omind5  omind6  hygiene1  hygiene2  hygiene3  hygiene4  hygiene5  psupport2  political_ideology  happy  slf_ladder  riskperc1  riskperc2  self_esteem  sex1  sbelong1  sbelong2  sbelong3  sbelong4  tested_positive  optim1  optim2  slfcont1  slfcont3  slfcont4
##   10   3  age  children  cnarc1  cnarc2  cnarc3  CRT1  CRT2  CRT3  employ_status1  ladder  marital1  mor_circle  moralid1  moralid10  moralid2  moralid3  moralid4  moralid5  moralid6  moralid7  moralid8  moralid9  mcoop1  mcoop2  mcoop3  mcoop4  mcoop5  mcoop6  mcoop7  narc1  narc2  narc3  narc4  narc5  narc6  nidentity1  nidentity2  omind1  omind2  omind3  omind4  omind5  omind6  hygiene1  hygiene2  hygiene3  hygiene4  hygiene5  psupport2  political_ideology  happy  slf_ladder  riskperc1  riskperc2  self_esteem  sex1  sbelong1  sbelong2  sbelong3  sbelong4  tested_positive  optim1  optim2  slfcont1  slfcont3  slfcont4
##   10   4  age  children  cnarc1  cnarc2  cnarc3  CRT1  CRT2  CRT3  employ_status1  ladder  marital1  mor_circle  moralid1  moralid10  moralid2  moralid3  moralid4  moralid5  moralid6  moralid7  moralid8  moralid9  mcoop1  mcoop2  mcoop3  mcoop4  mcoop5  mcoop6  mcoop7  narc1  narc2  narc3  narc4  narc5  narc6  nidentity1  nidentity2  omind1  omind2  omind3  omind4  omind5  omind6  hygiene1  hygiene2  hygiene3  hygiene4  hygiene5  psupport2  political_ideology  happy  slf_ladder  riskperc1  riskperc2  self_esteem  sex1  sbelong1  sbelong2  sbelong3  sbelong4  tested_positive  optim1  optim2  slfcont1  slfcont3  slfcont4
##   10   5  age  children  cnarc1  cnarc2  cnarc3  CRT1  CRT2  CRT3  employ_status1  ladder  marital1  mor_circle  moralid1  moralid10  moralid2  moralid3  moralid4  moralid5  moralid6  moralid7  moralid8  moralid9  mcoop1  mcoop2  mcoop3  mcoop4  mcoop5  mcoop6  mcoop7  narc1  narc2  narc3  narc4  narc5  narc6  nidentity1  nidentity2  omind1  omind2  omind3  omind4  omind5  omind6  hygiene1  hygiene2  hygiene3  hygiene4  hygiene5  psupport2  political_ideology  happy  slf_ladder  riskperc1  riskperc2  self_esteem  sex1  sbelong1  sbelong2  sbelong3  sbelong4  tested_positive  optim1  optim2  slfcont1  slfcont3  slfcont4
##   10   6  age  children  cnarc1  cnarc2  cnarc3  CRT1  CRT2  CRT3  employ_status1  ladder  marital1  mor_circle  moralid1  moralid10  moralid2  moralid3  moralid4  moralid5  moralid6  moralid7  moralid8  moralid9  mcoop1  mcoop2  mcoop3  mcoop4  mcoop5  mcoop6  mcoop7  narc1  narc2  narc3  narc4  narc5  narc6  nidentity1  nidentity2  omind1  omind2  omind3  omind4  omind5  omind6  hygiene1  hygiene2  hygiene3  hygiene4  hygiene5  psupport2  political_ideology  happy  slf_ladder  riskperc1  riskperc2  self_esteem  sex1  sbelong1  sbelong2  sbelong3  sbelong4  tested_positive  optim1  optim2  slfcont1  slfcont3  slfcont4
##   10   7  age  children  cnarc1  cnarc2  cnarc3  CRT1  CRT2  CRT3  employ_status1  ladder  marital1  mor_circle  moralid1  moralid10  moralid2  moralid3  moralid4  moralid5  moralid6  moralid7  moralid8  moralid9  mcoop1  mcoop2  mcoop3  mcoop4  mcoop5  mcoop6  mcoop7  narc1  narc2  narc3  narc4  narc5  narc6  nidentity1  nidentity2  omind1  omind2  omind3  omind4  omind5  omind6  hygiene1  hygiene2  hygiene3  hygiene4  hygiene5  psupport2  political_ideology  happy  slf_ladder  riskperc1  riskperc2  self_esteem  sex1  sbelong1  sbelong2  sbelong3  sbelong4  tested_positive  optim1  optim2  slfcont1  slfcont3  slfcont4
##   10   8  age  children  cnarc1  cnarc2  cnarc3  CRT1  CRT2  CRT3  employ_status1  ladder  marital1  mor_circle  moralid1  moralid10  moralid2  moralid3  moralid4  moralid5  moralid6  moralid7  moralid8  moralid9  mcoop1  mcoop2  mcoop3  mcoop4  mcoop5  mcoop6  mcoop7  narc1  narc2  narc3  narc4  narc5  narc6  nidentity1  nidentity2  omind1  omind2  omind3  omind4  omind5  omind6  hygiene1  hygiene2  hygiene3  hygiene4  hygiene5  psupport2  political_ideology  happy  slf_ladder  riskperc1  riskperc2  self_esteem  sex1  sbelong1  sbelong2  sbelong3  sbelong4  tested_positive  optim1  optim2  slfcont1  slfcont3  slfcont4
##   10   9  age  children  cnarc1  cnarc2  cnarc3  CRT1  CRT2  CRT3  employ_status1  ladder  marital1  mor_circle  moralid1  moralid10  moralid2  moralid3  moralid4  moralid5  moralid6  moralid7  moralid8  moralid9  mcoop1  mcoop2  mcoop3  mcoop4  mcoop5  mcoop6  mcoop7  narc1  narc2  narc3  narc4  narc5  narc6  nidentity1  nidentity2  omind1  omind2  omind3  omind4  omind5  omind6  hygiene1  hygiene2  hygiene3  hygiene4  hygiene5  psupport2  political_ideology  happy  slf_ladder  riskperc1  riskperc2  self_esteem  sex1  sbelong1  sbelong2  sbelong3  sbelong4  tested_positive  optim1  optim2  slfcont1  slfcont3  slfcont4
##   10   10  age  children  cnarc1  cnarc2  cnarc3  CRT1  CRT2  CRT3  employ_status1  ladder  marital1  mor_circle  moralid1  moralid10  moralid2  moralid3  moralid4  moralid5  moralid6  moralid7  moralid8  moralid9  mcoop1  mcoop2  mcoop3  mcoop4  mcoop5  mcoop6  mcoop7  narc1  narc2  narc3  narc4  narc5  narc6  nidentity1  nidentity2  omind1  omind2  omind3  omind4  omind5  omind6  hygiene1  hygiene2  hygiene3  hygiene4  hygiene5  psupport2  political_ideology  happy  slf_ladder  riskperc1  riskperc2  self_esteem  sex1  sbelong1  sbelong2  sbelong3  sbelong4  tested_positive  optim1  optim2  slfcont1  slfcont3  slfcont4
```

```
## Warning: Number of logged events: 6603
```

```
## 
##  iter imp variable
##   1   1  age  children  cnarc1  cnarc2  cnarc3  CRT1  CRT2  CRT3  health_cond  know_tested_positive  marital1  mor_circle  moralid1  moralid10  moralid2  moralid3  moralid4  moralid5  moralid6  moralid7  moralid8  moralid9  mcoop1  mcoop2  mcoop3  mcoop4  mcoop5  mcoop6  mcoop7  narc1  narc2  narc3  narc4  narc5  narc6  nidentity1  nidentity2  omind1  omind4  contact1  contact2  contact3  hygiene1  hygiene2  hygiene3  hygiene4  hygiene5  psupport1  psupport2  psupport3  psupport4  psupport5  political_ideology  happy  riskperc1  riskperc2  self_esteem  sbelong1  sbelong2  sbelong3  tested_positive  optim1  optim2  slfcont1  slfcont2  slfcont3
##   1   2  age  children  cnarc1  cnarc2  cnarc3  CRT1  CRT2  CRT3  health_cond  know_tested_positive  marital1  mor_circle  moralid1  moralid10  moralid2  moralid3  moralid4  moralid5  moralid6  moralid7  moralid8  moralid9  mcoop1  mcoop2  mcoop3  mcoop4  mcoop5  mcoop6  mcoop7  narc1  narc2  narc3  narc4  narc5  narc6  nidentity1  nidentity2  omind1  omind4  contact1  contact2  contact3  hygiene1  hygiene2  hygiene3  hygiene4  hygiene5  psupport1  psupport2  psupport3  psupport4  psupport5  political_ideology  happy  riskperc1  riskperc2  self_esteem  sbelong1  sbelong2  sbelong3  tested_positive  optim1  optim2  slfcont1  slfcont2  slfcont3
##   1   3  age  children  cnarc1  cnarc2  cnarc3  CRT1  CRT2  CRT3  health_cond  know_tested_positive  marital1  mor_circle  moralid1  moralid10  moralid2  moralid3  moralid4  moralid5  moralid6  moralid7  moralid8  moralid9  mcoop1  mcoop2  mcoop3  mcoop4  mcoop5  mcoop6  mcoop7  narc1  narc2  narc3  narc4  narc5  narc6  nidentity1  nidentity2  omind1  omind4  contact1  contact2  contact3  hygiene1  hygiene2  hygiene3  hygiene4  hygiene5  psupport1  psupport2  psupport3  psupport4  psupport5  political_ideology  happy  riskperc1  riskperc2  self_esteem  sbelong1  sbelong2  sbelong3  tested_positive  optim1  optim2  slfcont1  slfcont2  slfcont3
##   1   4  age  children  cnarc1  cnarc2  cnarc3  CRT1  CRT2  CRT3  health_cond  know_tested_positive  marital1  mor_circle  moralid1  moralid10  moralid2  moralid3  moralid4  moralid5  moralid6  moralid7  moralid8  moralid9  mcoop1  mcoop2  mcoop3  mcoop4  mcoop5  mcoop6  mcoop7  narc1  narc2  narc3  narc4  narc5  narc6  nidentity1  nidentity2  omind1  omind4  contact1  contact2  contact3  hygiene1  hygiene2  hygiene3  hygiene4  hygiene5  psupport1  psupport2  psupport3  psupport4  psupport5  political_ideology  happy  riskperc1  riskperc2  self_esteem  sbelong1  sbelong2  sbelong3  tested_positive  optim1  optim2  slfcont1  slfcont2  slfcont3
##   1   5  age  children  cnarc1  cnarc2  cnarc3  CRT1  CRT2  CRT3  health_cond  know_tested_positive  marital1  mor_circle  moralid1  moralid10  moralid2  moralid3  moralid4  moralid5  moralid6  moralid7  moralid8  moralid9  mcoop1  mcoop2  mcoop3  mcoop4  mcoop5  mcoop6  mcoop7  narc1  narc2  narc3  narc4  narc5  narc6  nidentity1  nidentity2  omind1  omind4  contact1  contact2  contact3  hygiene1  hygiene2  hygiene3  hygiene4  hygiene5  psupport1  psupport2  psupport3  psupport4  psupport5  political_ideology  happy  riskperc1  riskperc2  self_esteem  sbelong1  sbelong2  sbelong3  tested_positive  optim1  optim2  slfcont1  slfcont2  slfcont3
##   1   6  age  children  cnarc1  cnarc2  cnarc3  CRT1  CRT2  CRT3  health_cond  know_tested_positive  marital1  mor_circle  moralid1  moralid10  moralid2  moralid3  moralid4  moralid5  moralid6  moralid7  moralid8  moralid9  mcoop1  mcoop2  mcoop3  mcoop4  mcoop5  mcoop6  mcoop7  narc1  narc2  narc3  narc4  narc5  narc6  nidentity1  nidentity2  omind1  omind4  contact1  contact2  contact3  hygiene1  hygiene2  hygiene3  hygiene4  hygiene5  psupport1  psupport2  psupport3  psupport4  psupport5  political_ideology  happy  riskperc1  riskperc2  self_esteem  sbelong1  sbelong2  sbelong3  tested_positive  optim1  optim2  slfcont1  slfcont2  slfcont3
##   1   7  age  children  cnarc1  cnarc2  cnarc3  CRT1  CRT2  CRT3  health_cond  know_tested_positive  marital1  mor_circle  moralid1  moralid10  moralid2  moralid3  moralid4  moralid5  moralid6  moralid7  moralid8  moralid9  mcoop1  mcoop2  mcoop3  mcoop4  mcoop5  mcoop6  mcoop7  narc1  narc2  narc3  narc4  narc5  narc6  nidentity1  nidentity2  omind1  omind4  contact1  contact2  contact3  hygiene1  hygiene2  hygiene3  hygiene4  hygiene5  psupport1  psupport2  psupport3  psupport4  psupport5  political_ideology  happy  riskperc1  riskperc2  self_esteem  sbelong1  sbelong2  sbelong3  tested_positive  optim1  optim2  slfcont1  slfcont2  slfcont3
##   1   8  age  children  cnarc1  cnarc2  cnarc3  CRT1  CRT2  CRT3  health_cond  know_tested_positive  marital1  mor_circle  moralid1  moralid10  moralid2  moralid3  moralid4  moralid5  moralid6  moralid7  moralid8  moralid9  mcoop1  mcoop2  mcoop3  mcoop4  mcoop5  mcoop6  mcoop7  narc1  narc2  narc3  narc4  narc5  narc6  nidentity1  nidentity2  omind1  omind4  contact1  contact2  contact3  hygiene1  hygiene2  hygiene3  hygiene4  hygiene5  psupport1  psupport2  psupport3  psupport4  psupport5  political_ideology  happy  riskperc1  riskperc2  self_esteem  sbelong1  sbelong2  sbelong3  tested_positive  optim1  optim2  slfcont1  slfcont2  slfcont3
##   1   9  age  children  cnarc1  cnarc2  cnarc3  CRT1  CRT2  CRT3  health_cond  know_tested_positive  marital1  mor_circle  moralid1  moralid10  moralid2  moralid3  moralid4  moralid5  moralid6  moralid7  moralid8  moralid9  mcoop1  mcoop2  mcoop3  mcoop4  mcoop5  mcoop6  mcoop7  narc1  narc2  narc3  narc4  narc5  narc6  nidentity1  nidentity2  omind1  omind4  contact1  contact2  contact3  hygiene1  hygiene2  hygiene3  hygiene4  hygiene5  psupport1  psupport2  psupport3  psupport4  psupport5  political_ideology  happy  riskperc1  riskperc2  self_esteem  sbelong1  sbelong2  sbelong3  tested_positive  optim1  optim2  slfcont1  slfcont2  slfcont3
##   1   10  age  children  cnarc1  cnarc2  cnarc3  CRT1  CRT2  CRT3  health_cond  know_tested_positive  marital1  mor_circle  moralid1  moralid10  moralid2  moralid3  moralid4  moralid5  moralid6  moralid7  moralid8  moralid9  mcoop1  mcoop2  mcoop3  mcoop4  mcoop5  mcoop6  mcoop7  narc1  narc2  narc3  narc4  narc5  narc6  nidentity1  nidentity2  omind1  omind4  contact1  contact2  contact3  hygiene1  hygiene2  hygiene3  hygiene4  hygiene5  psupport1  psupport2  psupport3  psupport4  psupport5  political_ideology  happy  riskperc1  riskperc2  self_esteem  sbelong1  sbelong2  sbelong3  tested_positive  optim1  optim2  slfcont1  slfcont2  slfcont3
##   2   1  age  children  cnarc1  cnarc2  cnarc3  CRT1  CRT2  CRT3  health_cond  know_tested_positive  marital1  mor_circle  moralid1  moralid10  moralid2  moralid3  moralid4  moralid5  moralid6  moralid7  moralid8  moralid9  mcoop1  mcoop2  mcoop3  mcoop4  mcoop5  mcoop6  mcoop7  narc1  narc2  narc3  narc4  narc5  narc6  nidentity1  nidentity2  omind1  omind4  contact1  contact2  contact3  hygiene1  hygiene2  hygiene3  hygiene4  hygiene5  psupport1  psupport2  psupport3  psupport4  psupport5  political_ideology  happy  riskperc1  riskperc2  self_esteem  sbelong1  sbelong2  sbelong3  tested_positive  optim1  optim2  slfcont1  slfcont2  slfcont3
##   2   2  age  children  cnarc1  cnarc2  cnarc3  CRT1  CRT2  CRT3  health_cond  know_tested_positive  marital1  mor_circle  moralid1  moralid10  moralid2  moralid3  moralid4  moralid5  moralid6  moralid7  moralid8  moralid9  mcoop1  mcoop2  mcoop3  mcoop4  mcoop5  mcoop6  mcoop7  narc1  narc2  narc3  narc4  narc5  narc6  nidentity1  nidentity2  omind1  omind4  contact1  contact2  contact3  hygiene1  hygiene2  hygiene3  hygiene4  hygiene5  psupport1  psupport2  psupport3  psupport4  psupport5  political_ideology  happy  riskperc1  riskperc2  self_esteem  sbelong1  sbelong2  sbelong3  tested_positive  optim1  optim2  slfcont1  slfcont2  slfcont3
##   2   3  age  children  cnarc1  cnarc2  cnarc3  CRT1  CRT2  CRT3  health_cond  know_tested_positive  marital1  mor_circle  moralid1  moralid10  moralid2  moralid3  moralid4  moralid5  moralid6  moralid7  moralid8  moralid9  mcoop1  mcoop2  mcoop3  mcoop4  mcoop5  mcoop6  mcoop7  narc1  narc2  narc3  narc4  narc5  narc6  nidentity1  nidentity2  omind1  omind4  contact1  contact2  contact3  hygiene1  hygiene2  hygiene3  hygiene4  hygiene5  psupport1  psupport2  psupport3  psupport4  psupport5  political_ideology  happy  riskperc1  riskperc2  self_esteem  sbelong1  sbelong2  sbelong3  tested_positive  optim1  optim2  slfcont1  slfcont2  slfcont3
##   2   4  age  children  cnarc1  cnarc2  cnarc3  CRT1  CRT2  CRT3  health_cond  know_tested_positive  marital1  mor_circle  moralid1  moralid10  moralid2  moralid3  moralid4  moralid5  moralid6  moralid7  moralid8  moralid9  mcoop1  mcoop2  mcoop3  mcoop4  mcoop5  mcoop6  mcoop7  narc1  narc2  narc3  narc4  narc5  narc6  nidentity1  nidentity2  omind1  omind4  contact1  contact2  contact3  hygiene1  hygiene2  hygiene3  hygiene4  hygiene5  psupport1  psupport2  psupport3  psupport4  psupport5  political_ideology  happy  riskperc1  riskperc2  self_esteem  sbelong1  sbelong2  sbelong3  tested_positive  optim1  optim2  slfcont1  slfcont2  slfcont3
##   2   5  age  children  cnarc1  cnarc2  cnarc3  CRT1  CRT2  CRT3  health_cond  know_tested_positive  marital1  mor_circle  moralid1  moralid10  moralid2  moralid3  moralid4  moralid5  moralid6  moralid7  moralid8  moralid9  mcoop1  mcoop2  mcoop3  mcoop4  mcoop5  mcoop6  mcoop7  narc1  narc2  narc3  narc4  narc5  narc6  nidentity1  nidentity2  omind1  omind4  contact1  contact2  contact3  hygiene1  hygiene2  hygiene3  hygiene4  hygiene5  psupport1  psupport2  psupport3  psupport4  psupport5  political_ideology  happy  riskperc1  riskperc2  self_esteem  sbelong1  sbelong2  sbelong3  tested_positive  optim1  optim2  slfcont1  slfcont2  slfcont3
##   2   6  age  children  cnarc1  cnarc2  cnarc3  CRT1  CRT2  CRT3  health_cond  know_tested_positive  marital1  mor_circle  moralid1  moralid10  moralid2  moralid3  moralid4  moralid5  moralid6  moralid7  moralid8  moralid9  mcoop1  mcoop2  mcoop3  mcoop4  mcoop5  mcoop6  mcoop7  narc1  narc2  narc3  narc4  narc5  narc6  nidentity1  nidentity2  omind1  omind4  contact1  contact2  contact3  hygiene1  hygiene2  hygiene3  hygiene4  hygiene5  psupport1  psupport2  psupport3  psupport4  psupport5  political_ideology  happy  riskperc1  riskperc2  self_esteem  sbelong1  sbelong2  sbelong3  tested_positive  optim1  optim2  slfcont1  slfcont2  slfcont3
##   2   7  age  children  cnarc1  cnarc2  cnarc3  CRT1  CRT2  CRT3  health_cond  know_tested_positive  marital1  mor_circle  moralid1  moralid10  moralid2  moralid3  moralid4  moralid5  moralid6  moralid7  moralid8  moralid9  mcoop1  mcoop2  mcoop3  mcoop4  mcoop5  mcoop6  mcoop7  narc1  narc2  narc3  narc4  narc5  narc6  nidentity1  nidentity2  omind1  omind4  contact1  contact2  contact3  hygiene1  hygiene2  hygiene3  hygiene4  hygiene5  psupport1  psupport2  psupport3  psupport4  psupport5  political_ideology  happy  riskperc1  riskperc2  self_esteem  sbelong1  sbelong2  sbelong3  tested_positive  optim1  optim2  slfcont1  slfcont2  slfcont3
##   2   8  age  children  cnarc1  cnarc2  cnarc3  CRT1  CRT2  CRT3  health_cond  know_tested_positive  marital1  mor_circle  moralid1  moralid10  moralid2  moralid3  moralid4  moralid5  moralid6  moralid7  moralid8  moralid9  mcoop1  mcoop2  mcoop3  mcoop4  mcoop5  mcoop6  mcoop7  narc1  narc2  narc3  narc4  narc5  narc6  nidentity1  nidentity2  omind1  omind4  contact1  contact2  contact3  hygiene1  hygiene2  hygiene3  hygiene4  hygiene5  psupport1  psupport2  psupport3  psupport4  psupport5  political_ideology  happy  riskperc1  riskperc2  self_esteem  sbelong1  sbelong2  sbelong3  tested_positive  optim1  optim2  slfcont1  slfcont2  slfcont3
##   2   9  age  children  cnarc1  cnarc2  cnarc3  CRT1  CRT2  CRT3  health_cond  know_tested_positive  marital1  mor_circle  moralid1  moralid10  moralid2  moralid3  moralid4  moralid5  moralid6  moralid7  moralid8  moralid9  mcoop1  mcoop2  mcoop3  mcoop4  mcoop5  mcoop6  mcoop7  narc1  narc2  narc3  narc4  narc5  narc6  nidentity1  nidentity2  omind1  omind4  contact1  contact2  contact3  hygiene1  hygiene2  hygiene3  hygiene4  hygiene5  psupport1  psupport2  psupport3  psupport4  psupport5  political_ideology  happy  riskperc1  riskperc2  self_esteem  sbelong1  sbelong2  sbelong3  tested_positive  optim1  optim2  slfcont1  slfcont2  slfcont3
##   2   10  age  children  cnarc1  cnarc2  cnarc3  CRT1  CRT2  CRT3  health_cond  know_tested_positive  marital1  mor_circle  moralid1  moralid10  moralid2  moralid3  moralid4  moralid5  moralid6  moralid7  moralid8  moralid9  mcoop1  mcoop2  mcoop3  mcoop4  mcoop5  mcoop6  mcoop7  narc1  narc2  narc3  narc4  narc5  narc6  nidentity1  nidentity2  omind1  omind4  contact1  contact2  contact3  hygiene1  hygiene2  hygiene3  hygiene4  hygiene5  psupport1  psupport2  psupport3  psupport4  psupport5  political_ideology  happy  riskperc1  riskperc2  self_esteem  sbelong1  sbelong2  sbelong3  tested_positive  optim1  optim2  slfcont1  slfcont2  slfcont3
##   3   1  age  children  cnarc1  cnarc2  cnarc3  CRT1  CRT2  CRT3  health_cond  know_tested_positive  marital1  mor_circle  moralid1  moralid10  moralid2  moralid3  moralid4  moralid5  moralid6  moralid7  moralid8  moralid9  mcoop1  mcoop2  mcoop3  mcoop4  mcoop5  mcoop6  mcoop7  narc1  narc2  narc3  narc4  narc5  narc6  nidentity1  nidentity2  omind1  omind4  contact1  contact2  contact3  hygiene1  hygiene2  hygiene3  hygiene4  hygiene5  psupport1  psupport2  psupport3  psupport4  psupport5  political_ideology  happy  riskperc1  riskperc2  self_esteem  sbelong1  sbelong2  sbelong3  tested_positive  optim1  optim2  slfcont1  slfcont2  slfcont3
##   3   2  age  children  cnarc1  cnarc2  cnarc3  CRT1  CRT2  CRT3  health_cond  know_tested_positive  marital1  mor_circle  moralid1  moralid10  moralid2  moralid3  moralid4  moralid5  moralid6  moralid7  moralid8  moralid9  mcoop1  mcoop2  mcoop3  mcoop4  mcoop5  mcoop6  mcoop7  narc1  narc2  narc3  narc4  narc5  narc6  nidentity1  nidentity2  omind1  omind4  contact1  contact2  contact3  hygiene1  hygiene2  hygiene3  hygiene4  hygiene5  psupport1  psupport2  psupport3  psupport4  psupport5  political_ideology  happy  riskperc1  riskperc2  self_esteem  sbelong1  sbelong2  sbelong3  tested_positive  optim1  optim2  slfcont1  slfcont2  slfcont3
##   3   3  age  children  cnarc1  cnarc2  cnarc3  CRT1  CRT2  CRT3  health_cond  know_tested_positive  marital1  mor_circle  moralid1  moralid10  moralid2  moralid3  moralid4  moralid5  moralid6  moralid7  moralid8  moralid9  mcoop1  mcoop2  mcoop3  mcoop4  mcoop5  mcoop6  mcoop7  narc1  narc2  narc3  narc4  narc5  narc6  nidentity1  nidentity2  omind1  omind4  contact1  contact2  contact3  hygiene1  hygiene2  hygiene3  hygiene4  hygiene5  psupport1  psupport2  psupport3  psupport4  psupport5  political_ideology  happy  riskperc1  riskperc2  self_esteem  sbelong1  sbelong2  sbelong3  tested_positive  optim1  optim2  slfcont1  slfcont2  slfcont3
##   3   4  age  children  cnarc1  cnarc2  cnarc3  CRT1  CRT2  CRT3  health_cond  know_tested_positive  marital1  mor_circle  moralid1  moralid10  moralid2  moralid3  moralid4  moralid5  moralid6  moralid7  moralid8  moralid9  mcoop1  mcoop2  mcoop3  mcoop4  mcoop5  mcoop6  mcoop7  narc1  narc2  narc3  narc4  narc5  narc6  nidentity1  nidentity2  omind1  omind4  contact1  contact2  contact3  hygiene1  hygiene2  hygiene3  hygiene4  hygiene5  psupport1  psupport2  psupport3  psupport4  psupport5  political_ideology  happy  riskperc1  riskperc2  self_esteem  sbelong1  sbelong2  sbelong3  tested_positive  optim1  optim2  slfcont1  slfcont2  slfcont3
##   3   5  age  children  cnarc1  cnarc2  cnarc3  CRT1  CRT2  CRT3  health_cond  know_tested_positive  marital1  mor_circle  moralid1  moralid10  moralid2  moralid3  moralid4  moralid5  moralid6  moralid7  moralid8  moralid9  mcoop1  mcoop2  mcoop3  mcoop4  mcoop5  mcoop6  mcoop7  narc1  narc2  narc3  narc4  narc5  narc6  nidentity1  nidentity2  omind1  omind4  contact1  contact2  contact3  hygiene1  hygiene2  hygiene3  hygiene4  hygiene5  psupport1  psupport2  psupport3  psupport4  psupport5  political_ideology  happy  riskperc1  riskperc2  self_esteem  sbelong1  sbelong2  sbelong3  tested_positive  optim1  optim2  slfcont1  slfcont2  slfcont3
##   3   6  age  children  cnarc1  cnarc2  cnarc3  CRT1  CRT2  CRT3  health_cond  know_tested_positive  marital1  mor_circle  moralid1  moralid10  moralid2  moralid3  moralid4  moralid5  moralid6  moralid7  moralid8  moralid9  mcoop1  mcoop2  mcoop3  mcoop4  mcoop5  mcoop6  mcoop7  narc1  narc2  narc3  narc4  narc5  narc6  nidentity1  nidentity2  omind1  omind4  contact1  contact2  contact3  hygiene1  hygiene2  hygiene3  hygiene4  hygiene5  psupport1  psupport2  psupport3  psupport4  psupport5  political_ideology  happy  riskperc1  riskperc2  self_esteem  sbelong1  sbelong2  sbelong3  tested_positive  optim1  optim2  slfcont1  slfcont2  slfcont3
##   3   7  age  children  cnarc1  cnarc2  cnarc3  CRT1  CRT2  CRT3  health_cond  know_tested_positive  marital1  mor_circle  moralid1  moralid10  moralid2  moralid3  moralid4  moralid5  moralid6  moralid7  moralid8  moralid9  mcoop1  mcoop2  mcoop3  mcoop4  mcoop5  mcoop6  mcoop7  narc1  narc2  narc3  narc4  narc5  narc6  nidentity1  nidentity2  omind1  omind4  contact1  contact2  contact3  hygiene1  hygiene2  hygiene3  hygiene4  hygiene5  psupport1  psupport2  psupport3  psupport4  psupport5  political_ideology  happy  riskperc1  riskperc2  self_esteem  sbelong1  sbelong2  sbelong3  tested_positive  optim1  optim2  slfcont1  slfcont2  slfcont3
##   3   8  age  children  cnarc1  cnarc2  cnarc3  CRT1  CRT2  CRT3  health_cond  know_tested_positive  marital1  mor_circle  moralid1  moralid10  moralid2  moralid3  moralid4  moralid5  moralid6  moralid7  moralid8  moralid9  mcoop1  mcoop2  mcoop3  mcoop4  mcoop5  mcoop6  mcoop7  narc1  narc2  narc3  narc4  narc5  narc6  nidentity1  nidentity2  omind1  omind4  contact1  contact2  contact3  hygiene1  hygiene2  hygiene3  hygiene4  hygiene5  psupport1  psupport2  psupport3  psupport4  psupport5  political_ideology  happy  riskperc1  riskperc2  self_esteem  sbelong1  sbelong2  sbelong3  tested_positive  optim1  optim2  slfcont1  slfcont2  slfcont3
##   3   9  age  children  cnarc1  cnarc2  cnarc3  CRT1  CRT2  CRT3  health_cond  know_tested_positive  marital1  mor_circle  moralid1  moralid10  moralid2  moralid3  moralid4  moralid5  moralid6  moralid7  moralid8  moralid9  mcoop1  mcoop2  mcoop3  mcoop4  mcoop5  mcoop6  mcoop7  narc1  narc2  narc3  narc4  narc5  narc6  nidentity1  nidentity2  omind1  omind4  contact1  contact2  contact3  hygiene1  hygiene2  hygiene3  hygiene4  hygiene5  psupport1  psupport2  psupport3  psupport4  psupport5  political_ideology  happy  riskperc1  riskperc2  self_esteem  sbelong1  sbelong2  sbelong3  tested_positive  optim1  optim2  slfcont1  slfcont2  slfcont3
##   3   10  age  children  cnarc1  cnarc2  cnarc3  CRT1  CRT2  CRT3  health_cond  know_tested_positive  marital1  mor_circle  moralid1  moralid10  moralid2  moralid3  moralid4  moralid5  moralid6  moralid7  moralid8  moralid9  mcoop1  mcoop2  mcoop3  mcoop4  mcoop5  mcoop6  mcoop7  narc1  narc2  narc3  narc4  narc5  narc6  nidentity1  nidentity2  omind1  omind4  contact1  contact2  contact3  hygiene1  hygiene2  hygiene3  hygiene4  hygiene5  psupport1  psupport2  psupport3  psupport4  psupport5  political_ideology  happy  riskperc1  riskperc2  self_esteem  sbelong1  sbelong2  sbelong3  tested_positive  optim1  optim2  slfcont1  slfcont2  slfcont3
##   4   1  age  children  cnarc1  cnarc2  cnarc3  CRT1  CRT2  CRT3  health_cond  know_tested_positive  marital1  mor_circle  moralid1  moralid10  moralid2  moralid3  moralid4  moralid5  moralid6  moralid7  moralid8  moralid9  mcoop1  mcoop2  mcoop3  mcoop4  mcoop5  mcoop6  mcoop7  narc1  narc2  narc3  narc4  narc5  narc6  nidentity1  nidentity2  omind1  omind4  contact1  contact2  contact3  hygiene1  hygiene2  hygiene3  hygiene4  hygiene5  psupport1  psupport2  psupport3  psupport4  psupport5  political_ideology  happy  riskperc1  riskperc2  self_esteem  sbelong1  sbelong2  sbelong3  tested_positive  optim1  optim2  slfcont1  slfcont2  slfcont3
##   4   2  age  children  cnarc1  cnarc2  cnarc3  CRT1  CRT2  CRT3  health_cond  know_tested_positive  marital1  mor_circle  moralid1  moralid10  moralid2  moralid3  moralid4  moralid5  moralid6  moralid7  moralid8  moralid9  mcoop1  mcoop2  mcoop3  mcoop4  mcoop5  mcoop6  mcoop7  narc1  narc2  narc3  narc4  narc5  narc6  nidentity1  nidentity2  omind1  omind4  contact1  contact2  contact3  hygiene1  hygiene2  hygiene3  hygiene4  hygiene5  psupport1  psupport2  psupport3  psupport4  psupport5  political_ideology  happy  riskperc1  riskperc2  self_esteem  sbelong1  sbelong2  sbelong3  tested_positive  optim1  optim2  slfcont1  slfcont2  slfcont3
##   4   3  age  children  cnarc1  cnarc2  cnarc3  CRT1  CRT2  CRT3  health_cond  know_tested_positive  marital1  mor_circle  moralid1  moralid10  moralid2  moralid3  moralid4  moralid5  moralid6  moralid7  moralid8  moralid9  mcoop1  mcoop2  mcoop3  mcoop4  mcoop5  mcoop6  mcoop7  narc1  narc2  narc3  narc4  narc5  narc6  nidentity1  nidentity2  omind1  omind4  contact1  contact2  contact3  hygiene1  hygiene2  hygiene3  hygiene4  hygiene5  psupport1  psupport2  psupport3  psupport4  psupport5  political_ideology  happy  riskperc1  riskperc2  self_esteem  sbelong1  sbelong2  sbelong3  tested_positive  optim1  optim2  slfcont1  slfcont2  slfcont3
##   4   4  age  children  cnarc1  cnarc2  cnarc3  CRT1  CRT2  CRT3  health_cond  know_tested_positive  marital1  mor_circle  moralid1  moralid10  moralid2  moralid3  moralid4  moralid5  moralid6  moralid7  moralid8  moralid9  mcoop1  mcoop2  mcoop3  mcoop4  mcoop5  mcoop6  mcoop7  narc1  narc2  narc3  narc4  narc5  narc6  nidentity1  nidentity2  omind1  omind4  contact1  contact2  contact3  hygiene1  hygiene2  hygiene3  hygiene4  hygiene5  psupport1  psupport2  psupport3  psupport4  psupport5  political_ideology  happy  riskperc1  riskperc2  self_esteem  sbelong1  sbelong2  sbelong3  tested_positive  optim1  optim2  slfcont1  slfcont2  slfcont3
##   4   5  age  children  cnarc1  cnarc2  cnarc3  CRT1  CRT2  CRT3  health_cond  know_tested_positive  marital1  mor_circle  moralid1  moralid10  moralid2  moralid3  moralid4  moralid5  moralid6  moralid7  moralid8  moralid9  mcoop1  mcoop2  mcoop3  mcoop4  mcoop5  mcoop6  mcoop7  narc1  narc2  narc3  narc4  narc5  narc6  nidentity1  nidentity2  omind1  omind4  contact1  contact2  contact3  hygiene1  hygiene2  hygiene3  hygiene4  hygiene5  psupport1  psupport2  psupport3  psupport4  psupport5  political_ideology  happy  riskperc1  riskperc2  self_esteem  sbelong1  sbelong2  sbelong3  tested_positive  optim1  optim2  slfcont1  slfcont2  slfcont3
##   4   6  age  children  cnarc1  cnarc2  cnarc3  CRT1  CRT2  CRT3  health_cond  know_tested_positive  marital1  mor_circle  moralid1  moralid10  moralid2  moralid3  moralid4  moralid5  moralid6  moralid7  moralid8  moralid9  mcoop1  mcoop2  mcoop3  mcoop4  mcoop5  mcoop6  mcoop7  narc1  narc2  narc3  narc4  narc5  narc6  nidentity1  nidentity2  omind1  omind4  contact1  contact2  contact3  hygiene1  hygiene2  hygiene3  hygiene4  hygiene5  psupport1  psupport2  psupport3  psupport4  psupport5  political_ideology  happy  riskperc1  riskperc2  self_esteem  sbelong1  sbelong2  sbelong3  tested_positive  optim1  optim2  slfcont1  slfcont2  slfcont3
##   4   7  age  children  cnarc1  cnarc2  cnarc3  CRT1  CRT2  CRT3  health_cond  know_tested_positive  marital1  mor_circle  moralid1  moralid10  moralid2  moralid3  moralid4  moralid5  moralid6  moralid7  moralid8  moralid9  mcoop1  mcoop2  mcoop3  mcoop4  mcoop5  mcoop6  mcoop7  narc1  narc2  narc3  narc4  narc5  narc6  nidentity1  nidentity2  omind1  omind4  contact1  contact2  contact3  hygiene1  hygiene2  hygiene3  hygiene4  hygiene5  psupport1  psupport2  psupport3  psupport4  psupport5  political_ideology  happy  riskperc1  riskperc2  self_esteem  sbelong1  sbelong2  sbelong3  tested_positive  optim1  optim2  slfcont1  slfcont2  slfcont3
##   4   8  age  children  cnarc1  cnarc2  cnarc3  CRT1  CRT2  CRT3  health_cond  know_tested_positive  marital1  mor_circle  moralid1  moralid10  moralid2  moralid3  moralid4  moralid5  moralid6  moralid7  moralid8  moralid9  mcoop1  mcoop2  mcoop3  mcoop4  mcoop5  mcoop6  mcoop7  narc1  narc2  narc3  narc4  narc5  narc6  nidentity1  nidentity2  omind1  omind4  contact1  contact2  contact3  hygiene1  hygiene2  hygiene3  hygiene4  hygiene5  psupport1  psupport2  psupport3  psupport4  psupport5  political_ideology  happy  riskperc1  riskperc2  self_esteem  sbelong1  sbelong2  sbelong3  tested_positive  optim1  optim2  slfcont1  slfcont2  slfcont3
##   4   9  age  children  cnarc1  cnarc2  cnarc3  CRT1  CRT2  CRT3  health_cond  know_tested_positive  marital1  mor_circle  moralid1  moralid10  moralid2  moralid3  moralid4  moralid5  moralid6  moralid7  moralid8  moralid9  mcoop1  mcoop2  mcoop3  mcoop4  mcoop5  mcoop6  mcoop7  narc1  narc2  narc3  narc4  narc5  narc6  nidentity1  nidentity2  omind1  omind4  contact1  contact2  contact3  hygiene1  hygiene2  hygiene3  hygiene4  hygiene5  psupport1  psupport2  psupport3  psupport4  psupport5  political_ideology  happy  riskperc1  riskperc2  self_esteem  sbelong1  sbelong2  sbelong3  tested_positive  optim1  optim2  slfcont1  slfcont2  slfcont3
##   4   10  age  children  cnarc1  cnarc2  cnarc3  CRT1  CRT2  CRT3  health_cond  know_tested_positive  marital1  mor_circle  moralid1  moralid10  moralid2  moralid3  moralid4  moralid5  moralid6  moralid7  moralid8  moralid9  mcoop1  mcoop2  mcoop3  mcoop4  mcoop5  mcoop6  mcoop7  narc1  narc2  narc3  narc4  narc5  narc6  nidentity1  nidentity2  omind1  omind4  contact1  contact2  contact3  hygiene1  hygiene2  hygiene3  hygiene4  hygiene5  psupport1  psupport2  psupport3  psupport4  psupport5  political_ideology  happy  riskperc1  riskperc2  self_esteem  sbelong1  sbelong2  sbelong3  tested_positive  optim1  optim2  slfcont1  slfcont2  slfcont3
##   5   1  age  children  cnarc1  cnarc2  cnarc3  CRT1  CRT2  CRT3  health_cond  know_tested_positive  marital1  mor_circle  moralid1  moralid10  moralid2  moralid3  moralid4  moralid5  moralid6  moralid7  moralid8  moralid9  mcoop1  mcoop2  mcoop3  mcoop4  mcoop5  mcoop6  mcoop7  narc1  narc2  narc3  narc4  narc5  narc6  nidentity1  nidentity2  omind1  omind4  contact1  contact2  contact3  hygiene1  hygiene2  hygiene3  hygiene4  hygiene5  psupport1  psupport2  psupport3  psupport4  psupport5  political_ideology  happy  riskperc1  riskperc2  self_esteem  sbelong1  sbelong2  sbelong3  tested_positive  optim1  optim2  slfcont1  slfcont2  slfcont3
##   5   2  age  children  cnarc1  cnarc2  cnarc3  CRT1  CRT2  CRT3  health_cond  know_tested_positive  marital1  mor_circle  moralid1  moralid10  moralid2  moralid3  moralid4  moralid5  moralid6  moralid7  moralid8  moralid9  mcoop1  mcoop2  mcoop3  mcoop4  mcoop5  mcoop6  mcoop7  narc1  narc2  narc3  narc4  narc5  narc6  nidentity1  nidentity2  omind1  omind4  contact1  contact2  contact3  hygiene1  hygiene2  hygiene3  hygiene4  hygiene5  psupport1  psupport2  psupport3  psupport4  psupport5  political_ideology  happy  riskperc1  riskperc2  self_esteem  sbelong1  sbelong2  sbelong3  tested_positive  optim1  optim2  slfcont1  slfcont2  slfcont3
##   5   3  age  children  cnarc1  cnarc2  cnarc3  CRT1  CRT2  CRT3  health_cond  know_tested_positive  marital1  mor_circle  moralid1  moralid10  moralid2  moralid3  moralid4  moralid5  moralid6  moralid7  moralid8  moralid9  mcoop1  mcoop2  mcoop3  mcoop4  mcoop5  mcoop6  mcoop7  narc1  narc2  narc3  narc4  narc5  narc6  nidentity1  nidentity2  omind1  omind4  contact1  contact2  contact3  hygiene1  hygiene2  hygiene3  hygiene4  hygiene5  psupport1  psupport2  psupport3  psupport4  psupport5  political_ideology  happy  riskperc1  riskperc2  self_esteem  sbelong1  sbelong2  sbelong3  tested_positive  optim1  optim2  slfcont1  slfcont2  slfcont3
##   5   4  age  children  cnarc1  cnarc2  cnarc3  CRT1  CRT2  CRT3  health_cond  know_tested_positive  marital1  mor_circle  moralid1  moralid10  moralid2  moralid3  moralid4  moralid5  moralid6  moralid7  moralid8  moralid9  mcoop1  mcoop2  mcoop3  mcoop4  mcoop5  mcoop6  mcoop7  narc1  narc2  narc3  narc4  narc5  narc6  nidentity1  nidentity2  omind1  omind4  contact1  contact2  contact3  hygiene1  hygiene2  hygiene3  hygiene4  hygiene5  psupport1  psupport2  psupport3  psupport4  psupport5  political_ideology  happy  riskperc1  riskperc2  self_esteem  sbelong1  sbelong2  sbelong3  tested_positive  optim1  optim2  slfcont1  slfcont2  slfcont3
##   5   5  age  children  cnarc1  cnarc2  cnarc3  CRT1  CRT2  CRT3  health_cond  know_tested_positive  marital1  mor_circle  moralid1  moralid10  moralid2  moralid3  moralid4  moralid5  moralid6  moralid7  moralid8  moralid9  mcoop1  mcoop2  mcoop3  mcoop4  mcoop5  mcoop6  mcoop7  narc1  narc2  narc3  narc4  narc5  narc6  nidentity1  nidentity2  omind1  omind4  contact1  contact2  contact3  hygiene1  hygiene2  hygiene3  hygiene4  hygiene5  psupport1  psupport2  psupport3  psupport4  psupport5  political_ideology  happy  riskperc1  riskperc2  self_esteem  sbelong1  sbelong2  sbelong3  tested_positive  optim1  optim2  slfcont1  slfcont2  slfcont3
##   5   6  age  children  cnarc1  cnarc2  cnarc3  CRT1  CRT2  CRT3  health_cond  know_tested_positive  marital1  mor_circle  moralid1  moralid10  moralid2  moralid3  moralid4  moralid5  moralid6  moralid7  moralid8  moralid9  mcoop1  mcoop2  mcoop3  mcoop4  mcoop5  mcoop6  mcoop7  narc1  narc2  narc3  narc4  narc5  narc6  nidentity1  nidentity2  omind1  omind4  contact1  contact2  contact3  hygiene1  hygiene2  hygiene3  hygiene4  hygiene5  psupport1  psupport2  psupport3  psupport4  psupport5  political_ideology  happy  riskperc1  riskperc2  self_esteem  sbelong1  sbelong2  sbelong3  tested_positive  optim1  optim2  slfcont1  slfcont2  slfcont3
##   5   7  age  children  cnarc1  cnarc2  cnarc3  CRT1  CRT2  CRT3  health_cond  know_tested_positive  marital1  mor_circle  moralid1  moralid10  moralid2  moralid3  moralid4  moralid5  moralid6  moralid7  moralid8  moralid9  mcoop1  mcoop2  mcoop3  mcoop4  mcoop5  mcoop6  mcoop7  narc1  narc2  narc3  narc4  narc5  narc6  nidentity1  nidentity2  omind1  omind4  contact1  contact2  contact3  hygiene1  hygiene2  hygiene3  hygiene4  hygiene5  psupport1  psupport2  psupport3  psupport4  psupport5  political_ideology  happy  riskperc1  riskperc2  self_esteem  sbelong1  sbelong2  sbelong3  tested_positive  optim1  optim2  slfcont1  slfcont2  slfcont3
##   5   8  age  children  cnarc1  cnarc2  cnarc3  CRT1  CRT2  CRT3  health_cond  know_tested_positive  marital1  mor_circle  moralid1  moralid10  moralid2  moralid3  moralid4  moralid5  moralid6  moralid7  moralid8  moralid9  mcoop1  mcoop2  mcoop3  mcoop4  mcoop5  mcoop6  mcoop7  narc1  narc2  narc3  narc4  narc5  narc6  nidentity1  nidentity2  omind1  omind4  contact1  contact2  contact3  hygiene1  hygiene2  hygiene3  hygiene4  hygiene5  psupport1  psupport2  psupport3  psupport4  psupport5  political_ideology  happy  riskperc1  riskperc2  self_esteem  sbelong1  sbelong2  sbelong3  tested_positive  optim1  optim2  slfcont1  slfcont2  slfcont3
##   5   9  age  children  cnarc1  cnarc2  cnarc3  CRT1  CRT2  CRT3  health_cond  know_tested_positive  marital1  mor_circle  moralid1  moralid10  moralid2  moralid3  moralid4  moralid5  moralid6  moralid7  moralid8  moralid9  mcoop1  mcoop2  mcoop3  mcoop4  mcoop5  mcoop6  mcoop7  narc1  narc2  narc3  narc4  narc5  narc6  nidentity1  nidentity2  omind1  omind4  contact1  contact2  contact3  hygiene1  hygiene2  hygiene3  hygiene4  hygiene5  psupport1  psupport2  psupport3  psupport4  psupport5  political_ideology  happy  riskperc1  riskperc2  self_esteem  sbelong1  sbelong2  sbelong3  tested_positive  optim1  optim2  slfcont1  slfcont2  slfcont3
##   5   10  age  children  cnarc1  cnarc2  cnarc3  CRT1  CRT2  CRT3  health_cond  know_tested_positive  marital1  mor_circle  moralid1  moralid10  moralid2  moralid3  moralid4  moralid5  moralid6  moralid7  moralid8  moralid9  mcoop1  mcoop2  mcoop3  mcoop4  mcoop5  mcoop6  mcoop7  narc1  narc2  narc3  narc4  narc5  narc6  nidentity1  nidentity2  omind1  omind4  contact1  contact2  contact3  hygiene1  hygiene2  hygiene3  hygiene4  hygiene5  psupport1  psupport2  psupport3  psupport4  psupport5  political_ideology  happy  riskperc1  riskperc2  self_esteem  sbelong1  sbelong2  sbelong3  tested_positive  optim1  optim2  slfcont1  slfcont2  slfcont3
##   6   1  age  children  cnarc1  cnarc2  cnarc3  CRT1  CRT2  CRT3  health_cond  know_tested_positive  marital1  mor_circle  moralid1  moralid10  moralid2  moralid3  moralid4  moralid5  moralid6  moralid7  moralid8  moralid9  mcoop1  mcoop2  mcoop3  mcoop4  mcoop5  mcoop6  mcoop7  narc1  narc2  narc3  narc4  narc5  narc6  nidentity1  nidentity2  omind1  omind4  contact1  contact2  contact3  hygiene1  hygiene2  hygiene3  hygiene4  hygiene5  psupport1  psupport2  psupport3  psupport4  psupport5  political_ideology  happy  riskperc1  riskperc2  self_esteem  sbelong1  sbelong2  sbelong3  tested_positive  optim1  optim2  slfcont1  slfcont2  slfcont3
##   6   2  age  children  cnarc1  cnarc2  cnarc3  CRT1  CRT2  CRT3  health_cond  know_tested_positive  marital1  mor_circle  moralid1  moralid10  moralid2  moralid3  moralid4  moralid5  moralid6  moralid7  moralid8  moralid9  mcoop1  mcoop2  mcoop3  mcoop4  mcoop5  mcoop6  mcoop7  narc1  narc2  narc3  narc4  narc5  narc6  nidentity1  nidentity2  omind1  omind4  contact1  contact2  contact3  hygiene1  hygiene2  hygiene3  hygiene4  hygiene5  psupport1  psupport2  psupport3  psupport4  psupport5  political_ideology  happy  riskperc1  riskperc2  self_esteem  sbelong1  sbelong2  sbelong3  tested_positive  optim1  optim2  slfcont1  slfcont2  slfcont3
##   6   3  age  children  cnarc1  cnarc2  cnarc3  CRT1  CRT2  CRT3  health_cond  know_tested_positive  marital1  mor_circle  moralid1  moralid10  moralid2  moralid3  moralid4  moralid5  moralid6  moralid7  moralid8  moralid9  mcoop1  mcoop2  mcoop3  mcoop4  mcoop5  mcoop6  mcoop7  narc1  narc2  narc3  narc4  narc5  narc6  nidentity1  nidentity2  omind1  omind4  contact1  contact2  contact3  hygiene1  hygiene2  hygiene3  hygiene4  hygiene5  psupport1  psupport2  psupport3  psupport4  psupport5  political_ideology  happy  riskperc1  riskperc2  self_esteem  sbelong1  sbelong2  sbelong3  tested_positive  optim1  optim2  slfcont1  slfcont2  slfcont3
##   6   4  age  children  cnarc1  cnarc2  cnarc3  CRT1  CRT2  CRT3  health_cond  know_tested_positive  marital1  mor_circle  moralid1  moralid10  moralid2  moralid3  moralid4  moralid5  moralid6  moralid7  moralid8  moralid9  mcoop1  mcoop2  mcoop3  mcoop4  mcoop5  mcoop6  mcoop7  narc1  narc2  narc3  narc4  narc5  narc6  nidentity1  nidentity2  omind1  omind4  contact1  contact2  contact3  hygiene1  hygiene2  hygiene3  hygiene4  hygiene5  psupport1  psupport2  psupport3  psupport4  psupport5  political_ideology  happy  riskperc1  riskperc2  self_esteem  sbelong1  sbelong2  sbelong3  tested_positive  optim1  optim2  slfcont1  slfcont2  slfcont3
##   6   5  age  children  cnarc1  cnarc2  cnarc3  CRT1  CRT2  CRT3  health_cond  know_tested_positive  marital1  mor_circle  moralid1  moralid10  moralid2  moralid3  moralid4  moralid5  moralid6  moralid7  moralid8  moralid9  mcoop1  mcoop2  mcoop3  mcoop4  mcoop5  mcoop6  mcoop7  narc1  narc2  narc3  narc4  narc5  narc6  nidentity1  nidentity2  omind1  omind4  contact1  contact2  contact3  hygiene1  hygiene2  hygiene3  hygiene4  hygiene5  psupport1  psupport2  psupport3  psupport4  psupport5  political_ideology  happy  riskperc1  riskperc2  self_esteem  sbelong1  sbelong2  sbelong3  tested_positive  optim1  optim2  slfcont1  slfcont2  slfcont3
##   6   6  age  children  cnarc1  cnarc2  cnarc3  CRT1  CRT2  CRT3  health_cond  know_tested_positive  marital1  mor_circle  moralid1  moralid10  moralid2  moralid3  moralid4  moralid5  moralid6  moralid7  moralid8  moralid9  mcoop1  mcoop2  mcoop3  mcoop4  mcoop5  mcoop6  mcoop7  narc1  narc2  narc3  narc4  narc5  narc6  nidentity1  nidentity2  omind1  omind4  contact1  contact2  contact3  hygiene1  hygiene2  hygiene3  hygiene4  hygiene5  psupport1  psupport2  psupport3  psupport4  psupport5  political_ideology  happy  riskperc1  riskperc2  self_esteem  sbelong1  sbelong2  sbelong3  tested_positive  optim1  optim2  slfcont1  slfcont2  slfcont3
##   6   7  age  children  cnarc1  cnarc2  cnarc3  CRT1  CRT2  CRT3  health_cond  know_tested_positive  marital1  mor_circle  moralid1  moralid10  moralid2  moralid3  moralid4  moralid5  moralid6  moralid7  moralid8  moralid9  mcoop1  mcoop2  mcoop3  mcoop4  mcoop5  mcoop6  mcoop7  narc1  narc2  narc3  narc4  narc5  narc6  nidentity1  nidentity2  omind1  omind4  contact1  contact2  contact3  hygiene1  hygiene2  hygiene3  hygiene4  hygiene5  psupport1  psupport2  psupport3  psupport4  psupport5  political_ideology  happy  riskperc1  riskperc2  self_esteem  sbelong1  sbelong2  sbelong3  tested_positive  optim1  optim2  slfcont1  slfcont2  slfcont3
##   6   8  age  children  cnarc1  cnarc2  cnarc3  CRT1  CRT2  CRT3  health_cond  know_tested_positive  marital1  mor_circle  moralid1  moralid10  moralid2  moralid3  moralid4  moralid5  moralid6  moralid7  moralid8  moralid9  mcoop1  mcoop2  mcoop3  mcoop4  mcoop5  mcoop6  mcoop7  narc1  narc2  narc3  narc4  narc5  narc6  nidentity1  nidentity2  omind1  omind4  contact1  contact2  contact3  hygiene1  hygiene2  hygiene3  hygiene4  hygiene5  psupport1  psupport2  psupport3  psupport4  psupport5  political_ideology  happy  riskperc1  riskperc2  self_esteem  sbelong1  sbelong2  sbelong3  tested_positive  optim1  optim2  slfcont1  slfcont2  slfcont3
##   6   9  age  children  cnarc1  cnarc2  cnarc3  CRT1  CRT2  CRT3  health_cond  know_tested_positive  marital1  mor_circle  moralid1  moralid10  moralid2  moralid3  moralid4  moralid5  moralid6  moralid7  moralid8  moralid9  mcoop1  mcoop2  mcoop3  mcoop4  mcoop5  mcoop6  mcoop7  narc1  narc2  narc3  narc4  narc5  narc6  nidentity1  nidentity2  omind1  omind4  contact1  contact2  contact3  hygiene1  hygiene2  hygiene3  hygiene4  hygiene5  psupport1  psupport2  psupport3  psupport4  psupport5  political_ideology  happy  riskperc1  riskperc2  self_esteem  sbelong1  sbelong2  sbelong3  tested_positive  optim1  optim2  slfcont1  slfcont2  slfcont3
##   6   10  age  children  cnarc1  cnarc2  cnarc3  CRT1  CRT2  CRT3  health_cond  know_tested_positive  marital1  mor_circle  moralid1  moralid10  moralid2  moralid3  moralid4  moralid5  moralid6  moralid7  moralid8  moralid9  mcoop1  mcoop2  mcoop3  mcoop4  mcoop5  mcoop6  mcoop7  narc1  narc2  narc3  narc4  narc5  narc6  nidentity1  nidentity2  omind1  omind4  contact1  contact2  contact3  hygiene1  hygiene2  hygiene3  hygiene4  hygiene5  psupport1  psupport2  psupport3  psupport4  psupport5  political_ideology  happy  riskperc1  riskperc2  self_esteem  sbelong1  sbelong2  sbelong3  tested_positive  optim1  optim2  slfcont1  slfcont2  slfcont3
##   7   1  age  children  cnarc1  cnarc2  cnarc3  CRT1  CRT2  CRT3  health_cond  know_tested_positive  marital1  mor_circle  moralid1  moralid10  moralid2  moralid3  moralid4  moralid5  moralid6  moralid7  moralid8  moralid9  mcoop1  mcoop2  mcoop3  mcoop4  mcoop5  mcoop6  mcoop7  narc1  narc2  narc3  narc4  narc5  narc6  nidentity1  nidentity2  omind1  omind4  contact1  contact2  contact3  hygiene1  hygiene2  hygiene3  hygiene4  hygiene5  psupport1  psupport2  psupport3  psupport4  psupport5  political_ideology  happy  riskperc1  riskperc2  self_esteem  sbelong1  sbelong2  sbelong3  tested_positive  optim1  optim2  slfcont1  slfcont2  slfcont3
##   7   2  age  children  cnarc1  cnarc2  cnarc3  CRT1  CRT2  CRT3  health_cond  know_tested_positive  marital1  mor_circle  moralid1  moralid10  moralid2  moralid3  moralid4  moralid5  moralid6  moralid7  moralid8  moralid9  mcoop1  mcoop2  mcoop3  mcoop4  mcoop5  mcoop6  mcoop7  narc1  narc2  narc3  narc4  narc5  narc6  nidentity1  nidentity2  omind1  omind4  contact1  contact2  contact3  hygiene1  hygiene2  hygiene3  hygiene4  hygiene5  psupport1  psupport2  psupport3  psupport4  psupport5  political_ideology  happy  riskperc1  riskperc2  self_esteem  sbelong1  sbelong2  sbelong3  tested_positive  optim1  optim2  slfcont1  slfcont2  slfcont3
##   7   3  age  children  cnarc1  cnarc2  cnarc3  CRT1  CRT2  CRT3  health_cond  know_tested_positive  marital1  mor_circle  moralid1  moralid10  moralid2  moralid3  moralid4  moralid5  moralid6  moralid7  moralid8  moralid9  mcoop1  mcoop2  mcoop3  mcoop4  mcoop5  mcoop6  mcoop7  narc1  narc2  narc3  narc4  narc5  narc6  nidentity1  nidentity2  omind1  omind4  contact1  contact2  contact3  hygiene1  hygiene2  hygiene3  hygiene4  hygiene5  psupport1  psupport2  psupport3  psupport4  psupport5  political_ideology  happy  riskperc1  riskperc2  self_esteem  sbelong1  sbelong2  sbelong3  tested_positive  optim1  optim2  slfcont1  slfcont2  slfcont3
##   7   4  age  children  cnarc1  cnarc2  cnarc3  CRT1  CRT2  CRT3  health_cond  know_tested_positive  marital1  mor_circle  moralid1  moralid10  moralid2  moralid3  moralid4  moralid5  moralid6  moralid7  moralid8  moralid9  mcoop1  mcoop2  mcoop3  mcoop4  mcoop5  mcoop6  mcoop7  narc1  narc2  narc3  narc4  narc5  narc6  nidentity1  nidentity2  omind1  omind4  contact1  contact2  contact3  hygiene1  hygiene2  hygiene3  hygiene4  hygiene5  psupport1  psupport2  psupport3  psupport4  psupport5  political_ideology  happy  riskperc1  riskperc2  self_esteem  sbelong1  sbelong2  sbelong3  tested_positive  optim1  optim2  slfcont1  slfcont2  slfcont3
##   7   5  age  children  cnarc1  cnarc2  cnarc3  CRT1  CRT2  CRT3  health_cond  know_tested_positive  marital1  mor_circle  moralid1  moralid10  moralid2  moralid3  moralid4  moralid5  moralid6  moralid7  moralid8  moralid9  mcoop1  mcoop2  mcoop3  mcoop4  mcoop5  mcoop6  mcoop7  narc1  narc2  narc3  narc4  narc5  narc6  nidentity1  nidentity2  omind1  omind4  contact1  contact2  contact3  hygiene1  hygiene2  hygiene3  hygiene4  hygiene5  psupport1  psupport2  psupport3  psupport4  psupport5  political_ideology  happy  riskperc1  riskperc2  self_esteem  sbelong1  sbelong2  sbelong3  tested_positive  optim1  optim2  slfcont1  slfcont2  slfcont3
##   7   6  age  children  cnarc1  cnarc2  cnarc3  CRT1  CRT2  CRT3  health_cond  know_tested_positive  marital1  mor_circle  moralid1  moralid10  moralid2  moralid3  moralid4  moralid5  moralid6  moralid7  moralid8  moralid9  mcoop1  mcoop2  mcoop3  mcoop4  mcoop5  mcoop6  mcoop7  narc1  narc2  narc3  narc4  narc5  narc6  nidentity1  nidentity2  omind1  omind4  contact1  contact2  contact3  hygiene1  hygiene2  hygiene3  hygiene4  hygiene5  psupport1  psupport2  psupport3  psupport4  psupport5  political_ideology  happy  riskperc1  riskperc2  self_esteem  sbelong1  sbelong2  sbelong3  tested_positive  optim1  optim2  slfcont1  slfcont2  slfcont3
##   7   7  age  children  cnarc1  cnarc2  cnarc3  CRT1  CRT2  CRT3  health_cond  know_tested_positive  marital1  mor_circle  moralid1  moralid10  moralid2  moralid3  moralid4  moralid5  moralid6  moralid7  moralid8  moralid9  mcoop1  mcoop2  mcoop3  mcoop4  mcoop5  mcoop6  mcoop7  narc1  narc2  narc3  narc4  narc5  narc6  nidentity1  nidentity2  omind1  omind4  contact1  contact2  contact3  hygiene1  hygiene2  hygiene3  hygiene4  hygiene5  psupport1  psupport2  psupport3  psupport4  psupport5  political_ideology  happy  riskperc1  riskperc2  self_esteem  sbelong1  sbelong2  sbelong3  tested_positive  optim1  optim2  slfcont1  slfcont2  slfcont3
##   7   8  age  children  cnarc1  cnarc2  cnarc3  CRT1  CRT2  CRT3  health_cond  know_tested_positive  marital1  mor_circle  moralid1  moralid10  moralid2  moralid3  moralid4  moralid5  moralid6  moralid7  moralid8  moralid9  mcoop1  mcoop2  mcoop3  mcoop4  mcoop5  mcoop6  mcoop7  narc1  narc2  narc3  narc4  narc5  narc6  nidentity1  nidentity2  omind1  omind4  contact1  contact2  contact3  hygiene1  hygiene2  hygiene3  hygiene4  hygiene5  psupport1  psupport2  psupport3  psupport4  psupport5  political_ideology  happy  riskperc1  riskperc2  self_esteem  sbelong1  sbelong2  sbelong3  tested_positive  optim1  optim2  slfcont1  slfcont2  slfcont3
##   7   9  age  children  cnarc1  cnarc2  cnarc3  CRT1  CRT2  CRT3  health_cond  know_tested_positive  marital1  mor_circle  moralid1  moralid10  moralid2  moralid3  moralid4  moralid5  moralid6  moralid7  moralid8  moralid9  mcoop1  mcoop2  mcoop3  mcoop4  mcoop5  mcoop6  mcoop7  narc1  narc2  narc3  narc4  narc5  narc6  nidentity1  nidentity2  omind1  omind4  contact1  contact2  contact3  hygiene1  hygiene2  hygiene3  hygiene4  hygiene5  psupport1  psupport2  psupport3  psupport4  psupport5  political_ideology  happy  riskperc1  riskperc2  self_esteem  sbelong1  sbelong2  sbelong3  tested_positive  optim1  optim2  slfcont1  slfcont2  slfcont3
##   7   10  age  children  cnarc1  cnarc2  cnarc3  CRT1  CRT2  CRT3  health_cond  know_tested_positive  marital1  mor_circle  moralid1  moralid10  moralid2  moralid3  moralid4  moralid5  moralid6  moralid7  moralid8  moralid9  mcoop1  mcoop2  mcoop3  mcoop4  mcoop5  mcoop6  mcoop7  narc1  narc2  narc3  narc4  narc5  narc6  nidentity1  nidentity2  omind1  omind4  contact1  contact2  contact3  hygiene1  hygiene2  hygiene3  hygiene4  hygiene5  psupport1  psupport2  psupport3  psupport4  psupport5  political_ideology  happy  riskperc1  riskperc2  self_esteem  sbelong1  sbelong2  sbelong3  tested_positive  optim1  optim2  slfcont1  slfcont2  slfcont3
##   8   1  age  children  cnarc1  cnarc2  cnarc3  CRT1  CRT2  CRT3  health_cond  know_tested_positive  marital1  mor_circle  moralid1  moralid10  moralid2  moralid3  moralid4  moralid5  moralid6  moralid7  moralid8  moralid9  mcoop1  mcoop2  mcoop3  mcoop4  mcoop5  mcoop6  mcoop7  narc1  narc2  narc3  narc4  narc5  narc6  nidentity1  nidentity2  omind1  omind4  contact1  contact2  contact3  hygiene1  hygiene2  hygiene3  hygiene4  hygiene5  psupport1  psupport2  psupport3  psupport4  psupport5  political_ideology  happy  riskperc1  riskperc2  self_esteem  sbelong1  sbelong2  sbelong3  tested_positive  optim1  optim2  slfcont1  slfcont2  slfcont3
##   8   2  age  children  cnarc1  cnarc2  cnarc3  CRT1  CRT2  CRT3  health_cond  know_tested_positive  marital1  mor_circle  moralid1  moralid10  moralid2  moralid3  moralid4  moralid5  moralid6  moralid7  moralid8  moralid9  mcoop1  mcoop2  mcoop3  mcoop4  mcoop5  mcoop6  mcoop7  narc1  narc2  narc3  narc4  narc5  narc6  nidentity1  nidentity2  omind1  omind4  contact1  contact2  contact3  hygiene1  hygiene2  hygiene3  hygiene4  hygiene5  psupport1  psupport2  psupport3  psupport4  psupport5  political_ideology  happy  riskperc1  riskperc2  self_esteem  sbelong1  sbelong2  sbelong3  tested_positive  optim1  optim2  slfcont1  slfcont2  slfcont3
##   8   3  age  children  cnarc1  cnarc2  cnarc3  CRT1  CRT2  CRT3  health_cond  know_tested_positive  marital1  mor_circle  moralid1  moralid10  moralid2  moralid3  moralid4  moralid5  moralid6  moralid7  moralid8  moralid9  mcoop1  mcoop2  mcoop3  mcoop4  mcoop5  mcoop6  mcoop7  narc1  narc2  narc3  narc4  narc5  narc6  nidentity1  nidentity2  omind1  omind4  contact1  contact2  contact3  hygiene1  hygiene2  hygiene3  hygiene4  hygiene5  psupport1  psupport2  psupport3  psupport4  psupport5  political_ideology  happy  riskperc1  riskperc2  self_esteem  sbelong1  sbelong2  sbelong3  tested_positive  optim1  optim2  slfcont1  slfcont2  slfcont3
##   8   4  age  children  cnarc1  cnarc2  cnarc3  CRT1  CRT2  CRT3  health_cond  know_tested_positive  marital1  mor_circle  moralid1  moralid10  moralid2  moralid3  moralid4  moralid5  moralid6  moralid7  moralid8  moralid9  mcoop1  mcoop2  mcoop3  mcoop4  mcoop5  mcoop6  mcoop7  narc1  narc2  narc3  narc4  narc5  narc6  nidentity1  nidentity2  omind1  omind4  contact1  contact2  contact3  hygiene1  hygiene2  hygiene3  hygiene4  hygiene5  psupport1  psupport2  psupport3  psupport4  psupport5  political_ideology  happy  riskperc1  riskperc2  self_esteem  sbelong1  sbelong2  sbelong3  tested_positive  optim1  optim2  slfcont1  slfcont2  slfcont3
##   8   5  age  children  cnarc1  cnarc2  cnarc3  CRT1  CRT2  CRT3  health_cond  know_tested_positive  marital1  mor_circle  moralid1  moralid10  moralid2  moralid3  moralid4  moralid5  moralid6  moralid7  moralid8  moralid9  mcoop1  mcoop2  mcoop3  mcoop4  mcoop5  mcoop6  mcoop7  narc1  narc2  narc3  narc4  narc5  narc6  nidentity1  nidentity2  omind1  omind4  contact1  contact2  contact3  hygiene1  hygiene2  hygiene3  hygiene4  hygiene5  psupport1  psupport2  psupport3  psupport4  psupport5  political_ideology  happy  riskperc1  riskperc2  self_esteem  sbelong1  sbelong2  sbelong3  tested_positive  optim1  optim2  slfcont1  slfcont2  slfcont3
##   8   6  age  children  cnarc1  cnarc2  cnarc3  CRT1  CRT2  CRT3  health_cond  know_tested_positive  marital1  mor_circle  moralid1  moralid10  moralid2  moralid3  moralid4  moralid5  moralid6  moralid7  moralid8  moralid9  mcoop1  mcoop2  mcoop3  mcoop4  mcoop5  mcoop6  mcoop7  narc1  narc2  narc3  narc4  narc5  narc6  nidentity1  nidentity2  omind1  omind4  contact1  contact2  contact3  hygiene1  hygiene2  hygiene3  hygiene4  hygiene5  psupport1  psupport2  psupport3  psupport4  psupport5  political_ideology  happy  riskperc1  riskperc2  self_esteem  sbelong1  sbelong2  sbelong3  tested_positive  optim1  optim2  slfcont1  slfcont2  slfcont3
##   8   7  age  children  cnarc1  cnarc2  cnarc3  CRT1  CRT2  CRT3  health_cond  know_tested_positive  marital1  mor_circle  moralid1  moralid10  moralid2  moralid3  moralid4  moralid5  moralid6  moralid7  moralid8  moralid9  mcoop1  mcoop2  mcoop3  mcoop4  mcoop5  mcoop6  mcoop7  narc1  narc2  narc3  narc4  narc5  narc6  nidentity1  nidentity2  omind1  omind4  contact1  contact2  contact3  hygiene1  hygiene2  hygiene3  hygiene4  hygiene5  psupport1  psupport2  psupport3  psupport4  psupport5  political_ideology  happy  riskperc1  riskperc2  self_esteem  sbelong1  sbelong2  sbelong3  tested_positive  optim1  optim2  slfcont1  slfcont2  slfcont3
##   8   8  age  children  cnarc1  cnarc2  cnarc3  CRT1  CRT2  CRT3  health_cond  know_tested_positive  marital1  mor_circle  moralid1  moralid10  moralid2  moralid3  moralid4  moralid5  moralid6  moralid7  moralid8  moralid9  mcoop1  mcoop2  mcoop3  mcoop4  mcoop5  mcoop6  mcoop7  narc1  narc2  narc3  narc4  narc5  narc6  nidentity1  nidentity2  omind1  omind4  contact1  contact2  contact3  hygiene1  hygiene2  hygiene3  hygiene4  hygiene5  psupport1  psupport2  psupport3  psupport4  psupport5  political_ideology  happy  riskperc1  riskperc2  self_esteem  sbelong1  sbelong2  sbelong3  tested_positive  optim1  optim2  slfcont1  slfcont2  slfcont3
##   8   9  age  children  cnarc1  cnarc2  cnarc3  CRT1  CRT2  CRT3  health_cond  know_tested_positive  marital1  mor_circle  moralid1  moralid10  moralid2  moralid3  moralid4  moralid5  moralid6  moralid7  moralid8  moralid9  mcoop1  mcoop2  mcoop3  mcoop4  mcoop5  mcoop6  mcoop7  narc1  narc2  narc3  narc4  narc5  narc6  nidentity1  nidentity2  omind1  omind4  contact1  contact2  contact3  hygiene1  hygiene2  hygiene3  hygiene4  hygiene5  psupport1  psupport2  psupport3  psupport4  psupport5  political_ideology  happy  riskperc1  riskperc2  self_esteem  sbelong1  sbelong2  sbelong3  tested_positive  optim1  optim2  slfcont1  slfcont2  slfcont3
##   8   10  age  children  cnarc1  cnarc2  cnarc3  CRT1  CRT2  CRT3  health_cond  know_tested_positive  marital1  mor_circle  moralid1  moralid10  moralid2  moralid3  moralid4  moralid5  moralid6  moralid7  moralid8  moralid9  mcoop1  mcoop2  mcoop3  mcoop4  mcoop5  mcoop6  mcoop7  narc1  narc2  narc3  narc4  narc5  narc6  nidentity1  nidentity2  omind1  omind4  contact1  contact2  contact3  hygiene1  hygiene2  hygiene3  hygiene4  hygiene5  psupport1  psupport2  psupport3  psupport4  psupport5  political_ideology  happy  riskperc1  riskperc2  self_esteem  sbelong1  sbelong2  sbelong3  tested_positive  optim1  optim2  slfcont1  slfcont2  slfcont3
##   9   1  age  children  cnarc1  cnarc2  cnarc3  CRT1  CRT2  CRT3  health_cond  know_tested_positive  marital1  mor_circle  moralid1  moralid10  moralid2  moralid3  moralid4  moralid5  moralid6  moralid7  moralid8  moralid9  mcoop1  mcoop2  mcoop3  mcoop4  mcoop5  mcoop6  mcoop7  narc1  narc2  narc3  narc4  narc5  narc6  nidentity1  nidentity2  omind1  omind4  contact1  contact2  contact3  hygiene1  hygiene2  hygiene3  hygiene4  hygiene5  psupport1  psupport2  psupport3  psupport4  psupport5  political_ideology  happy  riskperc1  riskperc2  self_esteem  sbelong1  sbelong2  sbelong3  tested_positive  optim1  optim2  slfcont1  slfcont2  slfcont3
##   9   2  age  children  cnarc1  cnarc2  cnarc3  CRT1  CRT2  CRT3  health_cond  know_tested_positive  marital1  mor_circle  moralid1  moralid10  moralid2  moralid3  moralid4  moralid5  moralid6  moralid7  moralid8  moralid9  mcoop1  mcoop2  mcoop3  mcoop4  mcoop5  mcoop6  mcoop7  narc1  narc2  narc3  narc4  narc5  narc6  nidentity1  nidentity2  omind1  omind4  contact1  contact2  contact3  hygiene1  hygiene2  hygiene3  hygiene4  hygiene5  psupport1  psupport2  psupport3  psupport4  psupport5  political_ideology  happy  riskperc1  riskperc2  self_esteem  sbelong1  sbelong2  sbelong3  tested_positive  optim1  optim2  slfcont1  slfcont2  slfcont3
##   9   3  age  children  cnarc1  cnarc2  cnarc3  CRT1  CRT2  CRT3  health_cond  know_tested_positive  marital1  mor_circle  moralid1  moralid10  moralid2  moralid3  moralid4  moralid5  moralid6  moralid7  moralid8  moralid9  mcoop1  mcoop2  mcoop3  mcoop4  mcoop5  mcoop6  mcoop7  narc1  narc2  narc3  narc4  narc5  narc6  nidentity1  nidentity2  omind1  omind4  contact1  contact2  contact3  hygiene1  hygiene2  hygiene3  hygiene4  hygiene5  psupport1  psupport2  psupport3  psupport4  psupport5  political_ideology  happy  riskperc1  riskperc2  self_esteem  sbelong1  sbelong2  sbelong3  tested_positive  optim1  optim2  slfcont1  slfcont2  slfcont3
##   9   4  age  children  cnarc1  cnarc2  cnarc3  CRT1  CRT2  CRT3  health_cond  know_tested_positive  marital1  mor_circle  moralid1  moralid10  moralid2  moralid3  moralid4  moralid5  moralid6  moralid7  moralid8  moralid9  mcoop1  mcoop2  mcoop3  mcoop4  mcoop5  mcoop6  mcoop7  narc1  narc2  narc3  narc4  narc5  narc6  nidentity1  nidentity2  omind1  omind4  contact1  contact2  contact3  hygiene1  hygiene2  hygiene3  hygiene4  hygiene5  psupport1  psupport2  psupport3  psupport4  psupport5  political_ideology  happy  riskperc1  riskperc2  self_esteem  sbelong1  sbelong2  sbelong3  tested_positive  optim1  optim2  slfcont1  slfcont2  slfcont3
##   9   5  age  children  cnarc1  cnarc2  cnarc3  CRT1  CRT2  CRT3  health_cond  know_tested_positive  marital1  mor_circle  moralid1  moralid10  moralid2  moralid3  moralid4  moralid5  moralid6  moralid7  moralid8  moralid9  mcoop1  mcoop2  mcoop3  mcoop4  mcoop5  mcoop6  mcoop7  narc1  narc2  narc3  narc4  narc5  narc6  nidentity1  nidentity2  omind1  omind4  contact1  contact2  contact3  hygiene1  hygiene2  hygiene3  hygiene4  hygiene5  psupport1  psupport2  psupport3  psupport4  psupport5  political_ideology  happy  riskperc1  riskperc2  self_esteem  sbelong1  sbelong2  sbelong3  tested_positive  optim1  optim2  slfcont1  slfcont2  slfcont3
##   9   6  age  children  cnarc1  cnarc2  cnarc3  CRT1  CRT2  CRT3  health_cond  know_tested_positive  marital1  mor_circle  moralid1  moralid10  moralid2  moralid3  moralid4  moralid5  moralid6  moralid7  moralid8  moralid9  mcoop1  mcoop2  mcoop3  mcoop4  mcoop5  mcoop6  mcoop7  narc1  narc2  narc3  narc4  narc5  narc6  nidentity1  nidentity2  omind1  omind4  contact1  contact2  contact3  hygiene1  hygiene2  hygiene3  hygiene4  hygiene5  psupport1  psupport2  psupport3  psupport4  psupport5  political_ideology  happy  riskperc1  riskperc2  self_esteem  sbelong1  sbelong2  sbelong3  tested_positive  optim1  optim2  slfcont1  slfcont2  slfcont3
##   9   7  age  children  cnarc1  cnarc2  cnarc3  CRT1  CRT2  CRT3  health_cond  know_tested_positive  marital1  mor_circle  moralid1  moralid10  moralid2  moralid3  moralid4  moralid5  moralid6  moralid7  moralid8  moralid9  mcoop1  mcoop2  mcoop3  mcoop4  mcoop5  mcoop6  mcoop7  narc1  narc2  narc3  narc4  narc5  narc6  nidentity1  nidentity2  omind1  omind4  contact1  contact2  contact3  hygiene1  hygiene2  hygiene3  hygiene4  hygiene5  psupport1  psupport2  psupport3  psupport4  psupport5  political_ideology  happy  riskperc1  riskperc2  self_esteem  sbelong1  sbelong2  sbelong3  tested_positive  optim1  optim2  slfcont1  slfcont2  slfcont3
##   9   8  age  children  cnarc1  cnarc2  cnarc3  CRT1  CRT2  CRT3  health_cond  know_tested_positive  marital1  mor_circle  moralid1  moralid10  moralid2  moralid3  moralid4  moralid5  moralid6  moralid7  moralid8  moralid9  mcoop1  mcoop2  mcoop3  mcoop4  mcoop5  mcoop6  mcoop7  narc1  narc2  narc3  narc4  narc5  narc6  nidentity1  nidentity2  omind1  omind4  contact1  contact2  contact3  hygiene1  hygiene2  hygiene3  hygiene4  hygiene5  psupport1  psupport2  psupport3  psupport4  psupport5  political_ideology  happy  riskperc1  riskperc2  self_esteem  sbelong1  sbelong2  sbelong3  tested_positive  optim1  optim2  slfcont1  slfcont2  slfcont3
##   9   9  age  children  cnarc1  cnarc2  cnarc3  CRT1  CRT2  CRT3  health_cond  know_tested_positive  marital1  mor_circle  moralid1  moralid10  moralid2  moralid3  moralid4  moralid5  moralid6  moralid7  moralid8  moralid9  mcoop1  mcoop2  mcoop3  mcoop4  mcoop5  mcoop6  mcoop7  narc1  narc2  narc3  narc4  narc5  narc6  nidentity1  nidentity2  omind1  omind4  contact1  contact2  contact3  hygiene1  hygiene2  hygiene3  hygiene4  hygiene5  psupport1  psupport2  psupport3  psupport4  psupport5  political_ideology  happy  riskperc1  riskperc2  self_esteem  sbelong1  sbelong2  sbelong3  tested_positive  optim1  optim2  slfcont1  slfcont2  slfcont3
##   9   10  age  children  cnarc1  cnarc2  cnarc3  CRT1  CRT2  CRT3  health_cond  know_tested_positive  marital1  mor_circle  moralid1  moralid10  moralid2  moralid3  moralid4  moralid5  moralid6  moralid7  moralid8  moralid9  mcoop1  mcoop2  mcoop3  mcoop4  mcoop5  mcoop6  mcoop7  narc1  narc2  narc3  narc4  narc5  narc6  nidentity1  nidentity2  omind1  omind4  contact1  contact2  contact3  hygiene1  hygiene2  hygiene3  hygiene4  hygiene5  psupport1  psupport2  psupport3  psupport4  psupport5  political_ideology  happy  riskperc1  riskperc2  self_esteem  sbelong1  sbelong2  sbelong3  tested_positive  optim1  optim2  slfcont1  slfcont2  slfcont3
##   10   1  age  children  cnarc1  cnarc2  cnarc3  CRT1  CRT2  CRT3  health_cond  know_tested_positive  marital1  mor_circle  moralid1  moralid10  moralid2  moralid3  moralid4  moralid5  moralid6  moralid7  moralid8  moralid9  mcoop1  mcoop2  mcoop3  mcoop4  mcoop5  mcoop6  mcoop7  narc1  narc2  narc3  narc4  narc5  narc6  nidentity1  nidentity2  omind1  omind4  contact1  contact2  contact3  hygiene1  hygiene2  hygiene3  hygiene4  hygiene5  psupport1  psupport2  psupport3  psupport4  psupport5  political_ideology  happy  riskperc1  riskperc2  self_esteem  sbelong1  sbelong2  sbelong3  tested_positive  optim1  optim2  slfcont1  slfcont2  slfcont3
##   10   2  age  children  cnarc1  cnarc2  cnarc3  CRT1  CRT2  CRT3  health_cond  know_tested_positive  marital1  mor_circle  moralid1  moralid10  moralid2  moralid3  moralid4  moralid5  moralid6  moralid7  moralid8  moralid9  mcoop1  mcoop2  mcoop3  mcoop4  mcoop5  mcoop6  mcoop7  narc1  narc2  narc3  narc4  narc5  narc6  nidentity1  nidentity2  omind1  omind4  contact1  contact2  contact3  hygiene1  hygiene2  hygiene3  hygiene4  hygiene5  psupport1  psupport2  psupport3  psupport4  psupport5  political_ideology  happy  riskperc1  riskperc2  self_esteem  sbelong1  sbelong2  sbelong3  tested_positive  optim1  optim2  slfcont1  slfcont2  slfcont3
##   10   3  age  children  cnarc1  cnarc2  cnarc3  CRT1  CRT2  CRT3  health_cond  know_tested_positive  marital1  mor_circle  moralid1  moralid10  moralid2  moralid3  moralid4  moralid5  moralid6  moralid7  moralid8  moralid9  mcoop1  mcoop2  mcoop3  mcoop4  mcoop5  mcoop6  mcoop7  narc1  narc2  narc3  narc4  narc5  narc6  nidentity1  nidentity2  omind1  omind4  contact1  contact2  contact3  hygiene1  hygiene2  hygiene3  hygiene4  hygiene5  psupport1  psupport2  psupport3  psupport4  psupport5  political_ideology  happy  riskperc1  riskperc2  self_esteem  sbelong1  sbelong2  sbelong3  tested_positive  optim1  optim2  slfcont1  slfcont2  slfcont3
##   10   4  age  children  cnarc1  cnarc2  cnarc3  CRT1  CRT2  CRT3  health_cond  know_tested_positive  marital1  mor_circle  moralid1  moralid10  moralid2  moralid3  moralid4  moralid5  moralid6  moralid7  moralid8  moralid9  mcoop1  mcoop2  mcoop3  mcoop4  mcoop5  mcoop6  mcoop7  narc1  narc2  narc3  narc4  narc5  narc6  nidentity1  nidentity2  omind1  omind4  contact1  contact2  contact3  hygiene1  hygiene2  hygiene3  hygiene4  hygiene5  psupport1  psupport2  psupport3  psupport4  psupport5  political_ideology  happy  riskperc1  riskperc2  self_esteem  sbelong1  sbelong2  sbelong3  tested_positive  optim1  optim2  slfcont1  slfcont2  slfcont3
##   10   5  age  children  cnarc1  cnarc2  cnarc3  CRT1  CRT2  CRT3  health_cond  know_tested_positive  marital1  mor_circle  moralid1  moralid10  moralid2  moralid3  moralid4  moralid5  moralid6  moralid7  moralid8  moralid9  mcoop1  mcoop2  mcoop3  mcoop4  mcoop5  mcoop6  mcoop7  narc1  narc2  narc3  narc4  narc5  narc6  nidentity1  nidentity2  omind1  omind4  contact1  contact2  contact3  hygiene1  hygiene2  hygiene3  hygiene4  hygiene5  psupport1  psupport2  psupport3  psupport4  psupport5  political_ideology  happy  riskperc1  riskperc2  self_esteem  sbelong1  sbelong2  sbelong3  tested_positive  optim1  optim2  slfcont1  slfcont2  slfcont3
##   10   6  age  children  cnarc1  cnarc2  cnarc3  CRT1  CRT2  CRT3  health_cond  know_tested_positive  marital1  mor_circle  moralid1  moralid10  moralid2  moralid3  moralid4  moralid5  moralid6  moralid7  moralid8  moralid9  mcoop1  mcoop2  mcoop3  mcoop4  mcoop5  mcoop6  mcoop7  narc1  narc2  narc3  narc4  narc5  narc6  nidentity1  nidentity2  omind1  omind4  contact1  contact2  contact3  hygiene1  hygiene2  hygiene3  hygiene4  hygiene5  psupport1  psupport2  psupport3  psupport4  psupport5  political_ideology  happy  riskperc1  riskperc2  self_esteem  sbelong1  sbelong2  sbelong3  tested_positive  optim1  optim2  slfcont1  slfcont2  slfcont3
##   10   7  age  children  cnarc1  cnarc2  cnarc3  CRT1  CRT2  CRT3  health_cond  know_tested_positive  marital1  mor_circle  moralid1  moralid10  moralid2  moralid3  moralid4  moralid5  moralid6  moralid7  moralid8  moralid9  mcoop1  mcoop2  mcoop3  mcoop4  mcoop5  mcoop6  mcoop7  narc1  narc2  narc3  narc4  narc5  narc6  nidentity1  nidentity2  omind1  omind4  contact1  contact2  contact3  hygiene1  hygiene2  hygiene3  hygiene4  hygiene5  psupport1  psupport2  psupport3  psupport4  psupport5  political_ideology  happy  riskperc1  riskperc2  self_esteem  sbelong1  sbelong2  sbelong3  tested_positive  optim1  optim2  slfcont1  slfcont2  slfcont3
##   10   8  age  children  cnarc1  cnarc2  cnarc3  CRT1  CRT2  CRT3  health_cond  know_tested_positive  marital1  mor_circle  moralid1  moralid10  moralid2  moralid3  moralid4  moralid5  moralid6  moralid7  moralid8  moralid9  mcoop1  mcoop2  mcoop3  mcoop4  mcoop5  mcoop6  mcoop7  narc1  narc2  narc3  narc4  narc5  narc6  nidentity1  nidentity2  omind1  omind4  contact1  contact2  contact3  hygiene1  hygiene2  hygiene3  hygiene4  hygiene5  psupport1  psupport2  psupport3  psupport4  psupport5  political_ideology  happy  riskperc1  riskperc2  self_esteem  sbelong1  sbelong2  sbelong3  tested_positive  optim1  optim2  slfcont1  slfcont2  slfcont3
##   10   9  age  children  cnarc1  cnarc2  cnarc3  CRT1  CRT2  CRT3  health_cond  know_tested_positive  marital1  mor_circle  moralid1  moralid10  moralid2  moralid3  moralid4  moralid5  moralid6  moralid7  moralid8  moralid9  mcoop1  mcoop2  mcoop3  mcoop4  mcoop5  mcoop6  mcoop7  narc1  narc2  narc3  narc4  narc5  narc6  nidentity1  nidentity2  omind1  omind4  contact1  contact2  contact3  hygiene1  hygiene2  hygiene3  hygiene4  hygiene5  psupport1  psupport2  psupport3  psupport4  psupport5  political_ideology  happy  riskperc1  riskperc2  self_esteem  sbelong1  sbelong2  sbelong3  tested_positive  optim1  optim2  slfcont1  slfcont2  slfcont3
##   10   10  age  children  cnarc1  cnarc2  cnarc3  CRT1  CRT2  CRT3  health_cond  know_tested_positive  marital1  mor_circle  moralid1  moralid10  moralid2  moralid3  moralid4  moralid5  moralid6  moralid7  moralid8  moralid9  mcoop1  mcoop2  mcoop3  mcoop4  mcoop5  mcoop6  mcoop7  narc1  narc2  narc3  narc4  narc5  narc6  nidentity1  nidentity2  omind1  omind4  contact1  contact2  contact3  hygiene1  hygiene2  hygiene3  hygiene4  hygiene5  psupport1  psupport2  psupport3  psupport4  psupport5  political_ideology  happy  riskperc1  riskperc2  self_esteem  sbelong1  sbelong2  sbelong3  tested_positive  optim1  optim2  slfcont1  slfcont2  slfcont3
```

```
## Warning: Number of logged events: 6603
```

```
## 
##  iter imp variable
##   1   1  age  children  cnarc1  cnarc2  cnarc3  CRT1  CRT2  CRT3  employ_status1  health_cond  know_tested_positive  ladder  marital1  mor_circle  moralid10  moralid2  moralid3  moralid4  moralid6  moralid8  moralid9  mcoop1  mcoop2  mcoop3  mcoop4  mcoop5  mcoop6  narc2  narc3  narc5  nidentity1  nidentity2  omind5  omind6  contact2  contact3  contact4  hygiene4  hygiene5  psupport2  political_ideology  happy  slf_ladder  riskperc1  riskperc2  self_esteem  sex1  tested_positive  optim1  optim2  slfcont4  urban
##   1   2  age  children  cnarc1  cnarc2  cnarc3  CRT1  CRT2  CRT3  employ_status1  health_cond  know_tested_positive  ladder  marital1  mor_circle  moralid10  moralid2  moralid3  moralid4  moralid6  moralid8  moralid9  mcoop1  mcoop2  mcoop3  mcoop4  mcoop5  mcoop6  narc2  narc3  narc5  nidentity1  nidentity2  omind5  omind6  contact2  contact3  contact4  hygiene4  hygiene5  psupport2  political_ideology  happy  slf_ladder  riskperc1  riskperc2  self_esteem  sex1  tested_positive  optim1  optim2  slfcont4  urban
##   1   3  age  children  cnarc1  cnarc2  cnarc3  CRT1  CRT2  CRT3  employ_status1  health_cond  know_tested_positive  ladder  marital1  mor_circle  moralid10  moralid2  moralid3  moralid4  moralid6  moralid8  moralid9  mcoop1  mcoop2  mcoop3  mcoop4  mcoop5  mcoop6  narc2  narc3  narc5  nidentity1  nidentity2  omind5  omind6  contact2  contact3  contact4  hygiene4  hygiene5  psupport2  political_ideology  happy  slf_ladder  riskperc1  riskperc2  self_esteem  sex1  tested_positive  optim1  optim2  slfcont4  urban
##   1   4  age  children  cnarc1  cnarc2  cnarc3  CRT1  CRT2  CRT3  employ_status1  health_cond  know_tested_positive  ladder  marital1  mor_circle  moralid10  moralid2  moralid3  moralid4  moralid6  moralid8  moralid9  mcoop1  mcoop2  mcoop3  mcoop4  mcoop5  mcoop6  narc2  narc3  narc5  nidentity1  nidentity2  omind5  omind6  contact2  contact3  contact4  hygiene4  hygiene5  psupport2  political_ideology  happy  slf_ladder  riskperc1  riskperc2  self_esteem  sex1  tested_positive  optim1  optim2  slfcont4  urban
##   1   5  age  children  cnarc1  cnarc2  cnarc3  CRT1  CRT2  CRT3  employ_status1  health_cond  know_tested_positive  ladder  marital1  mor_circle  moralid10  moralid2  moralid3  moralid4  moralid6  moralid8  moralid9  mcoop1  mcoop2  mcoop3  mcoop4  mcoop5  mcoop6  narc2  narc3  narc5  nidentity1  nidentity2  omind5  omind6  contact2  contact3  contact4  hygiene4  hygiene5  psupport2  political_ideology  happy  slf_ladder  riskperc1  riskperc2  self_esteem  sex1  tested_positive  optim1  optim2  slfcont4  urban
##   1   6  age  children  cnarc1  cnarc2  cnarc3  CRT1  CRT2  CRT3  employ_status1  health_cond  know_tested_positive  ladder  marital1  mor_circle  moralid10  moralid2  moralid3  moralid4  moralid6  moralid8  moralid9  mcoop1  mcoop2  mcoop3  mcoop4  mcoop5  mcoop6  narc2  narc3  narc5  nidentity1  nidentity2  omind5  omind6  contact2  contact3  contact4  hygiene4  hygiene5  psupport2  political_ideology  happy  slf_ladder  riskperc1  riskperc2  self_esteem  sex1  tested_positive  optim1  optim2  slfcont4  urban
##   1   7  age  children  cnarc1  cnarc2  cnarc3  CRT1  CRT2  CRT3  employ_status1  health_cond  know_tested_positive  ladder  marital1  mor_circle  moralid10  moralid2  moralid3  moralid4  moralid6  moralid8  moralid9  mcoop1  mcoop2  mcoop3  mcoop4  mcoop5  mcoop6  narc2  narc3  narc5  nidentity1  nidentity2  omind5  omind6  contact2  contact3  contact4  hygiene4  hygiene5  psupport2  political_ideology  happy  slf_ladder  riskperc1  riskperc2  self_esteem  sex1  tested_positive  optim1  optim2  slfcont4  urban
##   1   8  age  children  cnarc1  cnarc2  cnarc3  CRT1  CRT2  CRT3  employ_status1  health_cond  know_tested_positive  ladder  marital1  mor_circle  moralid10  moralid2  moralid3  moralid4  moralid6  moralid8  moralid9  mcoop1  mcoop2  mcoop3  mcoop4  mcoop5  mcoop6  narc2  narc3  narc5  nidentity1  nidentity2  omind5  omind6  contact2  contact3  contact4  hygiene4  hygiene5  psupport2  political_ideology  happy  slf_ladder  riskperc1  riskperc2  self_esteem  sex1  tested_positive  optim1  optim2  slfcont4  urban
##   1   9  age  children  cnarc1  cnarc2  cnarc3  CRT1  CRT2  CRT3  employ_status1  health_cond  know_tested_positive  ladder  marital1  mor_circle  moralid10  moralid2  moralid3  moralid4  moralid6  moralid8  moralid9  mcoop1  mcoop2  mcoop3  mcoop4  mcoop5  mcoop6  narc2  narc3  narc5  nidentity1  nidentity2  omind5  omind6  contact2  contact3  contact4  hygiene4  hygiene5  psupport2  political_ideology  happy  slf_ladder  riskperc1  riskperc2  self_esteem  sex1  tested_positive  optim1  optim2  slfcont4  urban
##   1   10  age  children  cnarc1  cnarc2  cnarc3  CRT1  CRT2  CRT3  employ_status1  health_cond  know_tested_positive  ladder  marital1  mor_circle  moralid10  moralid2  moralid3  moralid4  moralid6  moralid8  moralid9  mcoop1  mcoop2  mcoop3  mcoop4  mcoop5  mcoop6  narc2  narc3  narc5  nidentity1  nidentity2  omind5  omind6  contact2  contact3  contact4  hygiene4  hygiene5  psupport2  political_ideology  happy  slf_ladder  riskperc1  riskperc2  self_esteem  sex1  tested_positive  optim1  optim2  slfcont4  urban
##   2   1  age  children  cnarc1  cnarc2  cnarc3  CRT1  CRT2  CRT3  employ_status1  health_cond  know_tested_positive  ladder  marital1  mor_circle  moralid10  moralid2  moralid3  moralid4  moralid6  moralid8  moralid9  mcoop1  mcoop2  mcoop3  mcoop4  mcoop5  mcoop6  narc2  narc3  narc5  nidentity1  nidentity2  omind5  omind6  contact2  contact3  contact4  hygiene4  hygiene5  psupport2  political_ideology  happy  slf_ladder  riskperc1  riskperc2  self_esteem  sex1  tested_positive  optim1  optim2  slfcont4  urban
##   2   2  age  children  cnarc1  cnarc2  cnarc3  CRT1  CRT2  CRT3  employ_status1  health_cond  know_tested_positive  ladder  marital1  mor_circle  moralid10  moralid2  moralid3  moralid4  moralid6  moralid8  moralid9  mcoop1  mcoop2  mcoop3  mcoop4  mcoop5  mcoop6  narc2  narc3  narc5  nidentity1  nidentity2  omind5  omind6  contact2  contact3  contact4  hygiene4  hygiene5  psupport2  political_ideology  happy  slf_ladder  riskperc1  riskperc2  self_esteem  sex1  tested_positive  optim1  optim2  slfcont4  urban
##   2   3  age  children  cnarc1  cnarc2  cnarc3  CRT1  CRT2  CRT3  employ_status1  health_cond  know_tested_positive  ladder  marital1  mor_circle  moralid10  moralid2  moralid3  moralid4  moralid6  moralid8  moralid9  mcoop1  mcoop2  mcoop3  mcoop4  mcoop5  mcoop6  narc2  narc3  narc5  nidentity1  nidentity2  omind5  omind6  contact2  contact3  contact4  hygiene4  hygiene5  psupport2  political_ideology  happy  slf_ladder  riskperc1  riskperc2  self_esteem  sex1  tested_positive  optim1  optim2  slfcont4  urban
##   2   4  age  children  cnarc1  cnarc2  cnarc3  CRT1  CRT2  CRT3  employ_status1  health_cond  know_tested_positive  ladder  marital1  mor_circle  moralid10  moralid2  moralid3  moralid4  moralid6  moralid8  moralid9  mcoop1  mcoop2  mcoop3  mcoop4  mcoop5  mcoop6  narc2  narc3  narc5  nidentity1  nidentity2  omind5  omind6  contact2  contact3  contact4  hygiene4  hygiene5  psupport2  political_ideology  happy  slf_ladder  riskperc1  riskperc2  self_esteem  sex1  tested_positive  optim1  optim2  slfcont4  urban
##   2   5  age  children  cnarc1  cnarc2  cnarc3  CRT1  CRT2  CRT3  employ_status1  health_cond  know_tested_positive  ladder  marital1  mor_circle  moralid10  moralid2  moralid3  moralid4  moralid6  moralid8  moralid9  mcoop1  mcoop2  mcoop3  mcoop4  mcoop5  mcoop6  narc2  narc3  narc5  nidentity1  nidentity2  omind5  omind6  contact2  contact3  contact4  hygiene4  hygiene5  psupport2  political_ideology  happy  slf_ladder  riskperc1  riskperc2  self_esteem  sex1  tested_positive  optim1  optim2  slfcont4  urban
##   2   6  age  children  cnarc1  cnarc2  cnarc3  CRT1  CRT2  CRT3  employ_status1  health_cond  know_tested_positive  ladder  marital1  mor_circle  moralid10  moralid2  moralid3  moralid4  moralid6  moralid8  moralid9  mcoop1  mcoop2  mcoop3  mcoop4  mcoop5  mcoop6  narc2  narc3  narc5  nidentity1  nidentity2  omind5  omind6  contact2  contact3  contact4  hygiene4  hygiene5  psupport2  political_ideology  happy  slf_ladder  riskperc1  riskperc2  self_esteem  sex1  tested_positive  optim1  optim2  slfcont4  urban
##   2   7  age  children  cnarc1  cnarc2  cnarc3  CRT1  CRT2  CRT3  employ_status1  health_cond  know_tested_positive  ladder  marital1  mor_circle  moralid10  moralid2  moralid3  moralid4  moralid6  moralid8  moralid9  mcoop1  mcoop2  mcoop3  mcoop4  mcoop5  mcoop6  narc2  narc3  narc5  nidentity1  nidentity2  omind5  omind6  contact2  contact3  contact4  hygiene4  hygiene5  psupport2  political_ideology  happy  slf_ladder  riskperc1  riskperc2  self_esteem  sex1  tested_positive  optim1  optim2  slfcont4  urban
##   2   8  age  children  cnarc1  cnarc2  cnarc3  CRT1  CRT2  CRT3  employ_status1  health_cond  know_tested_positive  ladder  marital1  mor_circle  moralid10  moralid2  moralid3  moralid4  moralid6  moralid8  moralid9  mcoop1  mcoop2  mcoop3  mcoop4  mcoop5  mcoop6  narc2  narc3  narc5  nidentity1  nidentity2  omind5  omind6  contact2  contact3  contact4  hygiene4  hygiene5  psupport2  political_ideology  happy  slf_ladder  riskperc1  riskperc2  self_esteem  sex1  tested_positive  optim1  optim2  slfcont4  urban
##   2   9  age  children  cnarc1  cnarc2  cnarc3  CRT1  CRT2  CRT3  employ_status1  health_cond  know_tested_positive  ladder  marital1  mor_circle  moralid10  moralid2  moralid3  moralid4  moralid6  moralid8  moralid9  mcoop1  mcoop2  mcoop3  mcoop4  mcoop5  mcoop6  narc2  narc3  narc5  nidentity1  nidentity2  omind5  omind6  contact2  contact3  contact4  hygiene4  hygiene5  psupport2  political_ideology  happy  slf_ladder  riskperc1  riskperc2  self_esteem  sex1  tested_positive  optim1  optim2  slfcont4  urban
##   2   10  age  children  cnarc1  cnarc2  cnarc3  CRT1  CRT2  CRT3  employ_status1  health_cond  know_tested_positive  ladder  marital1  mor_circle  moralid10  moralid2  moralid3  moralid4  moralid6  moralid8  moralid9  mcoop1  mcoop2  mcoop3  mcoop4  mcoop5  mcoop6  narc2  narc3  narc5  nidentity1  nidentity2  omind5  omind6  contact2  contact3  contact4  hygiene4  hygiene5  psupport2  political_ideology  happy  slf_ladder  riskperc1  riskperc2  self_esteem  sex1  tested_positive  optim1  optim2  slfcont4  urban
##   3   1  age  children  cnarc1  cnarc2  cnarc3  CRT1  CRT2  CRT3  employ_status1  health_cond  know_tested_positive  ladder  marital1  mor_circle  moralid10  moralid2  moralid3  moralid4  moralid6  moralid8  moralid9  mcoop1  mcoop2  mcoop3  mcoop4  mcoop5  mcoop6  narc2  narc3  narc5  nidentity1  nidentity2  omind5  omind6  contact2  contact3  contact4  hygiene4  hygiene5  psupport2  political_ideology  happy  slf_ladder  riskperc1  riskperc2  self_esteem  sex1  tested_positive  optim1  optim2  slfcont4  urban
##   3   2  age  children  cnarc1  cnarc2  cnarc3  CRT1  CRT2  CRT3  employ_status1  health_cond  know_tested_positive  ladder  marital1  mor_circle  moralid10  moralid2  moralid3  moralid4  moralid6  moralid8  moralid9  mcoop1  mcoop2  mcoop3  mcoop4  mcoop5  mcoop6  narc2  narc3  narc5  nidentity1  nidentity2  omind5  omind6  contact2  contact3  contact4  hygiene4  hygiene5  psupport2  political_ideology  happy  slf_ladder  riskperc1  riskperc2  self_esteem  sex1  tested_positive  optim1  optim2  slfcont4  urban
##   3   3  age  children  cnarc1  cnarc2  cnarc3  CRT1  CRT2  CRT3  employ_status1  health_cond  know_tested_positive  ladder  marital1  mor_circle  moralid10  moralid2  moralid3  moralid4  moralid6  moralid8  moralid9  mcoop1  mcoop2  mcoop3  mcoop4  mcoop5  mcoop6  narc2  narc3  narc5  nidentity1  nidentity2  omind5  omind6  contact2  contact3  contact4  hygiene4  hygiene5  psupport2  political_ideology  happy  slf_ladder  riskperc1  riskperc2  self_esteem  sex1  tested_positive  optim1  optim2  slfcont4  urban
##   3   4  age  children  cnarc1  cnarc2  cnarc3  CRT1  CRT2  CRT3  employ_status1  health_cond  know_tested_positive  ladder  marital1  mor_circle  moralid10  moralid2  moralid3  moralid4  moralid6  moralid8  moralid9  mcoop1  mcoop2  mcoop3  mcoop4  mcoop5  mcoop6  narc2  narc3  narc5  nidentity1  nidentity2  omind5  omind6  contact2  contact3  contact4  hygiene4  hygiene5  psupport2  political_ideology  happy  slf_ladder  riskperc1  riskperc2  self_esteem  sex1  tested_positive  optim1  optim2  slfcont4  urban
##   3   5  age  children  cnarc1  cnarc2  cnarc3  CRT1  CRT2  CRT3  employ_status1  health_cond  know_tested_positive  ladder  marital1  mor_circle  moralid10  moralid2  moralid3  moralid4  moralid6  moralid8  moralid9  mcoop1  mcoop2  mcoop3  mcoop4  mcoop5  mcoop6  narc2  narc3  narc5  nidentity1  nidentity2  omind5  omind6  contact2  contact3  contact4  hygiene4  hygiene5  psupport2  political_ideology  happy  slf_ladder  riskperc1  riskperc2  self_esteem  sex1  tested_positive  optim1  optim2  slfcont4  urban
##   3   6  age  children  cnarc1  cnarc2  cnarc3  CRT1  CRT2  CRT3  employ_status1  health_cond  know_tested_positive  ladder  marital1  mor_circle  moralid10  moralid2  moralid3  moralid4  moralid6  moralid8  moralid9  mcoop1  mcoop2  mcoop3  mcoop4  mcoop5  mcoop6  narc2  narc3  narc5  nidentity1  nidentity2  omind5  omind6  contact2  contact3  contact4  hygiene4  hygiene5  psupport2  political_ideology  happy  slf_ladder  riskperc1  riskperc2  self_esteem  sex1  tested_positive  optim1  optim2  slfcont4  urban
##   3   7  age  children  cnarc1  cnarc2  cnarc3  CRT1  CRT2  CRT3  employ_status1  health_cond  know_tested_positive  ladder  marital1  mor_circle  moralid10  moralid2  moralid3  moralid4  moralid6  moralid8  moralid9  mcoop1  mcoop2  mcoop3  mcoop4  mcoop5  mcoop6  narc2  narc3  narc5  nidentity1  nidentity2  omind5  omind6  contact2  contact3  contact4  hygiene4  hygiene5  psupport2  political_ideology  happy  slf_ladder  riskperc1  riskperc2  self_esteem  sex1  tested_positive  optim1  optim2  slfcont4  urban
##   3   8  age  children  cnarc1  cnarc2  cnarc3  CRT1  CRT2  CRT3  employ_status1  health_cond  know_tested_positive  ladder  marital1  mor_circle  moralid10  moralid2  moralid3  moralid4  moralid6  moralid8  moralid9  mcoop1  mcoop2  mcoop3  mcoop4  mcoop5  mcoop6  narc2  narc3  narc5  nidentity1  nidentity2  omind5  omind6  contact2  contact3  contact4  hygiene4  hygiene5  psupport2  political_ideology  happy  slf_ladder  riskperc1  riskperc2  self_esteem  sex1  tested_positive  optim1  optim2  slfcont4  urban
##   3   9  age  children  cnarc1  cnarc2  cnarc3  CRT1  CRT2  CRT3  employ_status1  health_cond  know_tested_positive  ladder  marital1  mor_circle  moralid10  moralid2  moralid3  moralid4  moralid6  moralid8  moralid9  mcoop1  mcoop2  mcoop3  mcoop4  mcoop5  mcoop6  narc2  narc3  narc5  nidentity1  nidentity2  omind5  omind6  contact2  contact3  contact4  hygiene4  hygiene5  psupport2  political_ideology  happy  slf_ladder  riskperc1  riskperc2  self_esteem  sex1  tested_positive  optim1  optim2  slfcont4  urban
##   3   10  age  children  cnarc1  cnarc2  cnarc3  CRT1  CRT2  CRT3  employ_status1  health_cond  know_tested_positive  ladder  marital1  mor_circle  moralid10  moralid2  moralid3  moralid4  moralid6  moralid8  moralid9  mcoop1  mcoop2  mcoop3  mcoop4  mcoop5  mcoop6  narc2  narc3  narc5  nidentity1  nidentity2  omind5  omind6  contact2  contact3  contact4  hygiene4  hygiene5  psupport2  political_ideology  happy  slf_ladder  riskperc1  riskperc2  self_esteem  sex1  tested_positive  optim1  optim2  slfcont4  urban
##   4   1  age  children  cnarc1  cnarc2  cnarc3  CRT1  CRT2  CRT3  employ_status1  health_cond  know_tested_positive  ladder  marital1  mor_circle  moralid10  moralid2  moralid3  moralid4  moralid6  moralid8  moralid9  mcoop1  mcoop2  mcoop3  mcoop4  mcoop5  mcoop6  narc2  narc3  narc5  nidentity1  nidentity2  omind5  omind6  contact2  contact3  contact4  hygiene4  hygiene5  psupport2  political_ideology  happy  slf_ladder  riskperc1  riskperc2  self_esteem  sex1  tested_positive  optim1  optim2  slfcont4  urban
##   4   2  age  children  cnarc1  cnarc2  cnarc3  CRT1  CRT2  CRT3  employ_status1  health_cond  know_tested_positive  ladder  marital1  mor_circle  moralid10  moralid2  moralid3  moralid4  moralid6  moralid8  moralid9  mcoop1  mcoop2  mcoop3  mcoop4  mcoop5  mcoop6  narc2  narc3  narc5  nidentity1  nidentity2  omind5  omind6  contact2  contact3  contact4  hygiene4  hygiene5  psupport2  political_ideology  happy  slf_ladder  riskperc1  riskperc2  self_esteem  sex1  tested_positive  optim1  optim2  slfcont4  urban
##   4   3  age  children  cnarc1  cnarc2  cnarc3  CRT1  CRT2  CRT3  employ_status1  health_cond  know_tested_positive  ladder  marital1  mor_circle  moralid10  moralid2  moralid3  moralid4  moralid6  moralid8  moralid9  mcoop1  mcoop2  mcoop3  mcoop4  mcoop5  mcoop6  narc2  narc3  narc5  nidentity1  nidentity2  omind5  omind6  contact2  contact3  contact4  hygiene4  hygiene5  psupport2  political_ideology  happy  slf_ladder  riskperc1  riskperc2  self_esteem  sex1  tested_positive  optim1  optim2  slfcont4  urban
##   4   4  age  children  cnarc1  cnarc2  cnarc3  CRT1  CRT2  CRT3  employ_status1  health_cond  know_tested_positive  ladder  marital1  mor_circle  moralid10  moralid2  moralid3  moralid4  moralid6  moralid8  moralid9  mcoop1  mcoop2  mcoop3  mcoop4  mcoop5  mcoop6  narc2  narc3  narc5  nidentity1  nidentity2  omind5  omind6  contact2  contact3  contact4  hygiene4  hygiene5  psupport2  political_ideology  happy  slf_ladder  riskperc1  riskperc2  self_esteem  sex1  tested_positive  optim1  optim2  slfcont4  urban
##   4   5  age  children  cnarc1  cnarc2  cnarc3  CRT1  CRT2  CRT3  employ_status1  health_cond  know_tested_positive  ladder  marital1  mor_circle  moralid10  moralid2  moralid3  moralid4  moralid6  moralid8  moralid9  mcoop1  mcoop2  mcoop3  mcoop4  mcoop5  mcoop6  narc2  narc3  narc5  nidentity1  nidentity2  omind5  omind6  contact2  contact3  contact4  hygiene4  hygiene5  psupport2  political_ideology  happy  slf_ladder  riskperc1  riskperc2  self_esteem  sex1  tested_positive  optim1  optim2  slfcont4  urban
##   4   6  age  children  cnarc1  cnarc2  cnarc3  CRT1  CRT2  CRT3  employ_status1  health_cond  know_tested_positive  ladder  marital1  mor_circle  moralid10  moralid2  moralid3  moralid4  moralid6  moralid8  moralid9  mcoop1  mcoop2  mcoop3  mcoop4  mcoop5  mcoop6  narc2  narc3  narc5  nidentity1  nidentity2  omind5  omind6  contact2  contact3  contact4  hygiene4  hygiene5  psupport2  political_ideology  happy  slf_ladder  riskperc1  riskperc2  self_esteem  sex1  tested_positive  optim1  optim2  slfcont4  urban
##   4   7  age  children  cnarc1  cnarc2  cnarc3  CRT1  CRT2  CRT3  employ_status1  health_cond  know_tested_positive  ladder  marital1  mor_circle  moralid10  moralid2  moralid3  moralid4  moralid6  moralid8  moralid9  mcoop1  mcoop2  mcoop3  mcoop4  mcoop5  mcoop6  narc2  narc3  narc5  nidentity1  nidentity2  omind5  omind6  contact2  contact3  contact4  hygiene4  hygiene5  psupport2  political_ideology  happy  slf_ladder  riskperc1  riskperc2  self_esteem  sex1  tested_positive  optim1  optim2  slfcont4  urban
##   4   8  age  children  cnarc1  cnarc2  cnarc3  CRT1  CRT2  CRT3  employ_status1  health_cond  know_tested_positive  ladder  marital1  mor_circle  moralid10  moralid2  moralid3  moralid4  moralid6  moralid8  moralid9  mcoop1  mcoop2  mcoop3  mcoop4  mcoop5  mcoop6  narc2  narc3  narc5  nidentity1  nidentity2  omind5  omind6  contact2  contact3  contact4  hygiene4  hygiene5  psupport2  political_ideology  happy  slf_ladder  riskperc1  riskperc2  self_esteem  sex1  tested_positive  optim1  optim2  slfcont4  urban
##   4   9  age  children  cnarc1  cnarc2  cnarc3  CRT1  CRT2  CRT3  employ_status1  health_cond  know_tested_positive  ladder  marital1  mor_circle  moralid10  moralid2  moralid3  moralid4  moralid6  moralid8  moralid9  mcoop1  mcoop2  mcoop3  mcoop4  mcoop5  mcoop6  narc2  narc3  narc5  nidentity1  nidentity2  omind5  omind6  contact2  contact3  contact4  hygiene4  hygiene5  psupport2  political_ideology  happy  slf_ladder  riskperc1  riskperc2  self_esteem  sex1  tested_positive  optim1  optim2  slfcont4  urban
##   4   10  age  children  cnarc1  cnarc2  cnarc3  CRT1  CRT2  CRT3  employ_status1  health_cond  know_tested_positive  ladder  marital1  mor_circle  moralid10  moralid2  moralid3  moralid4  moralid6  moralid8  moralid9  mcoop1  mcoop2  mcoop3  mcoop4  mcoop5  mcoop6  narc2  narc3  narc5  nidentity1  nidentity2  omind5  omind6  contact2  contact3  contact4  hygiene4  hygiene5  psupport2  political_ideology  happy  slf_ladder  riskperc1  riskperc2  self_esteem  sex1  tested_positive  optim1  optim2  slfcont4  urban
##   5   1  age  children  cnarc1  cnarc2  cnarc3  CRT1  CRT2  CRT3  employ_status1  health_cond  know_tested_positive  ladder  marital1  mor_circle  moralid10  moralid2  moralid3  moralid4  moralid6  moralid8  moralid9  mcoop1  mcoop2  mcoop3  mcoop4  mcoop5  mcoop6  narc2  narc3  narc5  nidentity1  nidentity2  omind5  omind6  contact2  contact3  contact4  hygiene4  hygiene5  psupport2  political_ideology  happy  slf_ladder  riskperc1  riskperc2  self_esteem  sex1  tested_positive  optim1  optim2  slfcont4  urban
##   5   2  age  children  cnarc1  cnarc2  cnarc3  CRT1  CRT2  CRT3  employ_status1  health_cond  know_tested_positive  ladder  marital1  mor_circle  moralid10  moralid2  moralid3  moralid4  moralid6  moralid8  moralid9  mcoop1  mcoop2  mcoop3  mcoop4  mcoop5  mcoop6  narc2  narc3  narc5  nidentity1  nidentity2  omind5  omind6  contact2  contact3  contact4  hygiene4  hygiene5  psupport2  political_ideology  happy  slf_ladder  riskperc1  riskperc2  self_esteem  sex1  tested_positive  optim1  optim2  slfcont4  urban
##   5   3  age  children  cnarc1  cnarc2  cnarc3  CRT1  CRT2  CRT3  employ_status1  health_cond  know_tested_positive  ladder  marital1  mor_circle  moralid10  moralid2  moralid3  moralid4  moralid6  moralid8  moralid9  mcoop1  mcoop2  mcoop3  mcoop4  mcoop5  mcoop6  narc2  narc3  narc5  nidentity1  nidentity2  omind5  omind6  contact2  contact3  contact4  hygiene4  hygiene5  psupport2  political_ideology  happy  slf_ladder  riskperc1  riskperc2  self_esteem  sex1  tested_positive  optim1  optim2  slfcont4  urban
##   5   4  age  children  cnarc1  cnarc2  cnarc3  CRT1  CRT2  CRT3  employ_status1  health_cond  know_tested_positive  ladder  marital1  mor_circle  moralid10  moralid2  moralid3  moralid4  moralid6  moralid8  moralid9  mcoop1  mcoop2  mcoop3  mcoop4  mcoop5  mcoop6  narc2  narc3  narc5  nidentity1  nidentity2  omind5  omind6  contact2  contact3  contact4  hygiene4  hygiene5  psupport2  political_ideology  happy  slf_ladder  riskperc1  riskperc2  self_esteem  sex1  tested_positive  optim1  optim2  slfcont4  urban
##   5   5  age  children  cnarc1  cnarc2  cnarc3  CRT1  CRT2  CRT3  employ_status1  health_cond  know_tested_positive  ladder  marital1  mor_circle  moralid10  moralid2  moralid3  moralid4  moralid6  moralid8  moralid9  mcoop1  mcoop2  mcoop3  mcoop4  mcoop5  mcoop6  narc2  narc3  narc5  nidentity1  nidentity2  omind5  omind6  contact2  contact3  contact4  hygiene4  hygiene5  psupport2  political_ideology  happy  slf_ladder  riskperc1  riskperc2  self_esteem  sex1  tested_positive  optim1  optim2  slfcont4  urban
##   5   6  age  children  cnarc1  cnarc2  cnarc3  CRT1  CRT2  CRT3  employ_status1  health_cond  know_tested_positive  ladder  marital1  mor_circle  moralid10  moralid2  moralid3  moralid4  moralid6  moralid8  moralid9  mcoop1  mcoop2  mcoop3  mcoop4  mcoop5  mcoop6  narc2  narc3  narc5  nidentity1  nidentity2  omind5  omind6  contact2  contact3  contact4  hygiene4  hygiene5  psupport2  political_ideology  happy  slf_ladder  riskperc1  riskperc2  self_esteem  sex1  tested_positive  optim1  optim2  slfcont4  urban
##   5   7  age  children  cnarc1  cnarc2  cnarc3  CRT1  CRT2  CRT3  employ_status1  health_cond  know_tested_positive  ladder  marital1  mor_circle  moralid10  moralid2  moralid3  moralid4  moralid6  moralid8  moralid9  mcoop1  mcoop2  mcoop3  mcoop4  mcoop5  mcoop6  narc2  narc3  narc5  nidentity1  nidentity2  omind5  omind6  contact2  contact3  contact4  hygiene4  hygiene5  psupport2  political_ideology  happy  slf_ladder  riskperc1  riskperc2  self_esteem  sex1  tested_positive  optim1  optim2  slfcont4  urban
##   5   8  age  children  cnarc1  cnarc2  cnarc3  CRT1  CRT2  CRT3  employ_status1  health_cond  know_tested_positive  ladder  marital1  mor_circle  moralid10  moralid2  moralid3  moralid4  moralid6  moralid8  moralid9  mcoop1  mcoop2  mcoop3  mcoop4  mcoop5  mcoop6  narc2  narc3  narc5  nidentity1  nidentity2  omind5  omind6  contact2  contact3  contact4  hygiene4  hygiene5  psupport2  political_ideology  happy  slf_ladder  riskperc1  riskperc2  self_esteem  sex1  tested_positive  optim1  optim2  slfcont4  urban
##   5   9  age  children  cnarc1  cnarc2  cnarc3  CRT1  CRT2  CRT3  employ_status1  health_cond  know_tested_positive  ladder  marital1  mor_circle  moralid10  moralid2  moralid3  moralid4  moralid6  moralid8  moralid9  mcoop1  mcoop2  mcoop3  mcoop4  mcoop5  mcoop6  narc2  narc3  narc5  nidentity1  nidentity2  omind5  omind6  contact2  contact3  contact4  hygiene4  hygiene5  psupport2  political_ideology  happy  slf_ladder  riskperc1  riskperc2  self_esteem  sex1  tested_positive  optim1  optim2  slfcont4  urban
##   5   10  age  children  cnarc1  cnarc2  cnarc3  CRT1  CRT2  CRT3  employ_status1  health_cond  know_tested_positive  ladder  marital1  mor_circle  moralid10  moralid2  moralid3  moralid4  moralid6  moralid8  moralid9  mcoop1  mcoop2  mcoop3  mcoop4  mcoop5  mcoop6  narc2  narc3  narc5  nidentity1  nidentity2  omind5  omind6  contact2  contact3  contact4  hygiene4  hygiene5  psupport2  political_ideology  happy  slf_ladder  riskperc1  riskperc2  self_esteem  sex1  tested_positive  optim1  optim2  slfcont4  urban
##   6   1  age  children  cnarc1  cnarc2  cnarc3  CRT1  CRT2  CRT3  employ_status1  health_cond  know_tested_positive  ladder  marital1  mor_circle  moralid10  moralid2  moralid3  moralid4  moralid6  moralid8  moralid9  mcoop1  mcoop2  mcoop3  mcoop4  mcoop5  mcoop6  narc2  narc3  narc5  nidentity1  nidentity2  omind5  omind6  contact2  contact3  contact4  hygiene4  hygiene5  psupport2  political_ideology  happy  slf_ladder  riskperc1  riskperc2  self_esteem  sex1  tested_positive  optim1  optim2  slfcont4  urban
##   6   2  age  children  cnarc1  cnarc2  cnarc3  CRT1  CRT2  CRT3  employ_status1  health_cond  know_tested_positive  ladder  marital1  mor_circle  moralid10  moralid2  moralid3  moralid4  moralid6  moralid8  moralid9  mcoop1  mcoop2  mcoop3  mcoop4  mcoop5  mcoop6  narc2  narc3  narc5  nidentity1  nidentity2  omind5  omind6  contact2  contact3  contact4  hygiene4  hygiene5  psupport2  political_ideology  happy  slf_ladder  riskperc1  riskperc2  self_esteem  sex1  tested_positive  optim1  optim2  slfcont4  urban
##   6   3  age  children  cnarc1  cnarc2  cnarc3  CRT1  CRT2  CRT3  employ_status1  health_cond  know_tested_positive  ladder  marital1  mor_circle  moralid10  moralid2  moralid3  moralid4  moralid6  moralid8  moralid9  mcoop1  mcoop2  mcoop3  mcoop4  mcoop5  mcoop6  narc2  narc3  narc5  nidentity1  nidentity2  omind5  omind6  contact2  contact3  contact4  hygiene4  hygiene5  psupport2  political_ideology  happy  slf_ladder  riskperc1  riskperc2  self_esteem  sex1  tested_positive  optim1  optim2  slfcont4  urban
##   6   4  age  children  cnarc1  cnarc2  cnarc3  CRT1  CRT2  CRT3  employ_status1  health_cond  know_tested_positive  ladder  marital1  mor_circle  moralid10  moralid2  moralid3  moralid4  moralid6  moralid8  moralid9  mcoop1  mcoop2  mcoop3  mcoop4  mcoop5  mcoop6  narc2  narc3  narc5  nidentity1  nidentity2  omind5  omind6  contact2  contact3  contact4  hygiene4  hygiene5  psupport2  political_ideology  happy  slf_ladder  riskperc1  riskperc2  self_esteem  sex1  tested_positive  optim1  optim2  slfcont4  urban
##   6   5  age  children  cnarc1  cnarc2  cnarc3  CRT1  CRT2  CRT3  employ_status1  health_cond  know_tested_positive  ladder  marital1  mor_circle  moralid10  moralid2  moralid3  moralid4  moralid6  moralid8  moralid9  mcoop1  mcoop2  mcoop3  mcoop4  mcoop5  mcoop6  narc2  narc3  narc5  nidentity1  nidentity2  omind5  omind6  contact2  contact3  contact4  hygiene4  hygiene5  psupport2  political_ideology  happy  slf_ladder  riskperc1  riskperc2  self_esteem  sex1  tested_positive  optim1  optim2  slfcont4  urban
##   6   6  age  children  cnarc1  cnarc2  cnarc3  CRT1  CRT2  CRT3  employ_status1  health_cond  know_tested_positive  ladder  marital1  mor_circle  moralid10  moralid2  moralid3  moralid4  moralid6  moralid8  moralid9  mcoop1  mcoop2  mcoop3  mcoop4  mcoop5  mcoop6  narc2  narc3  narc5  nidentity1  nidentity2  omind5  omind6  contact2  contact3  contact4  hygiene4  hygiene5  psupport2  political_ideology  happy  slf_ladder  riskperc1  riskperc2  self_esteem  sex1  tested_positive  optim1  optim2  slfcont4  urban
##   6   7  age  children  cnarc1  cnarc2  cnarc3  CRT1  CRT2  CRT3  employ_status1  health_cond  know_tested_positive  ladder  marital1  mor_circle  moralid10  moralid2  moralid3  moralid4  moralid6  moralid8  moralid9  mcoop1  mcoop2  mcoop3  mcoop4  mcoop5  mcoop6  narc2  narc3  narc5  nidentity1  nidentity2  omind5  omind6  contact2  contact3  contact4  hygiene4  hygiene5  psupport2  political_ideology  happy  slf_ladder  riskperc1  riskperc2  self_esteem  sex1  tested_positive  optim1  optim2  slfcont4  urban
##   6   8  age  children  cnarc1  cnarc2  cnarc3  CRT1  CRT2  CRT3  employ_status1  health_cond  know_tested_positive  ladder  marital1  mor_circle  moralid10  moralid2  moralid3  moralid4  moralid6  moralid8  moralid9  mcoop1  mcoop2  mcoop3  mcoop4  mcoop5  mcoop6  narc2  narc3  narc5  nidentity1  nidentity2  omind5  omind6  contact2  contact3  contact4  hygiene4  hygiene5  psupport2  political_ideology  happy  slf_ladder  riskperc1  riskperc2  self_esteem  sex1  tested_positive  optim1  optim2  slfcont4  urban
##   6   9  age  children  cnarc1  cnarc2  cnarc3  CRT1  CRT2  CRT3  employ_status1  health_cond  know_tested_positive  ladder  marital1  mor_circle  moralid10  moralid2  moralid3  moralid4  moralid6  moralid8  moralid9  mcoop1  mcoop2  mcoop3  mcoop4  mcoop5  mcoop6  narc2  narc3  narc5  nidentity1  nidentity2  omind5  omind6  contact2  contact3  contact4  hygiene4  hygiene5  psupport2  political_ideology  happy  slf_ladder  riskperc1  riskperc2  self_esteem  sex1  tested_positive  optim1  optim2  slfcont4  urban
##   6   10  age  children  cnarc1  cnarc2  cnarc3  CRT1  CRT2  CRT3  employ_status1  health_cond  know_tested_positive  ladder  marital1  mor_circle  moralid10  moralid2  moralid3  moralid4  moralid6  moralid8  moralid9  mcoop1  mcoop2  mcoop3  mcoop4  mcoop5  mcoop6  narc2  narc3  narc5  nidentity1  nidentity2  omind5  omind6  contact2  contact3  contact4  hygiene4  hygiene5  psupport2  political_ideology  happy  slf_ladder  riskperc1  riskperc2  self_esteem  sex1  tested_positive  optim1  optim2  slfcont4  urban
##   7   1  age  children  cnarc1  cnarc2  cnarc3  CRT1  CRT2  CRT3  employ_status1  health_cond  know_tested_positive  ladder  marital1  mor_circle  moralid10  moralid2  moralid3  moralid4  moralid6  moralid8  moralid9  mcoop1  mcoop2  mcoop3  mcoop4  mcoop5  mcoop6  narc2  narc3  narc5  nidentity1  nidentity2  omind5  omind6  contact2  contact3  contact4  hygiene4  hygiene5  psupport2  political_ideology  happy  slf_ladder  riskperc1  riskperc2  self_esteem  sex1  tested_positive  optim1  optim2  slfcont4  urban
##   7   2  age  children  cnarc1  cnarc2  cnarc3  CRT1  CRT2  CRT3  employ_status1  health_cond  know_tested_positive  ladder  marital1  mor_circle  moralid10  moralid2  moralid3  moralid4  moralid6  moralid8  moralid9  mcoop1  mcoop2  mcoop3  mcoop4  mcoop5  mcoop6  narc2  narc3  narc5  nidentity1  nidentity2  omind5  omind6  contact2  contact3  contact4  hygiene4  hygiene5  psupport2  political_ideology  happy  slf_ladder  riskperc1  riskperc2  self_esteem  sex1  tested_positive  optim1  optim2  slfcont4  urban
##   7   3  age  children  cnarc1  cnarc2  cnarc3  CRT1  CRT2  CRT3  employ_status1  health_cond  know_tested_positive  ladder  marital1  mor_circle  moralid10  moralid2  moralid3  moralid4  moralid6  moralid8  moralid9  mcoop1  mcoop2  mcoop3  mcoop4  mcoop5  mcoop6  narc2  narc3  narc5  nidentity1  nidentity2  omind5  omind6  contact2  contact3  contact4  hygiene4  hygiene5  psupport2  political_ideology  happy  slf_ladder  riskperc1  riskperc2  self_esteem  sex1  tested_positive  optim1  optim2  slfcont4  urban
##   7   4  age  children  cnarc1  cnarc2  cnarc3  CRT1  CRT2  CRT3  employ_status1  health_cond  know_tested_positive  ladder  marital1  mor_circle  moralid10  moralid2  moralid3  moralid4  moralid6  moralid8  moralid9  mcoop1  mcoop2  mcoop3  mcoop4  mcoop5  mcoop6  narc2  narc3  narc5  nidentity1  nidentity2  omind5  omind6  contact2  contact3  contact4  hygiene4  hygiene5  psupport2  political_ideology  happy  slf_ladder  riskperc1  riskperc2  self_esteem  sex1  tested_positive  optim1  optim2  slfcont4  urban
##   7   5  age  children  cnarc1  cnarc2  cnarc3  CRT1  CRT2  CRT3  employ_status1  health_cond  know_tested_positive  ladder  marital1  mor_circle  moralid10  moralid2  moralid3  moralid4  moralid6  moralid8  moralid9  mcoop1  mcoop2  mcoop3  mcoop4  mcoop5  mcoop6  narc2  narc3  narc5  nidentity1  nidentity2  omind5  omind6  contact2  contact3  contact4  hygiene4  hygiene5  psupport2  political_ideology  happy  slf_ladder  riskperc1  riskperc2  self_esteem  sex1  tested_positive  optim1  optim2  slfcont4  urban
##   7   6  age  children  cnarc1  cnarc2  cnarc3  CRT1  CRT2  CRT3  employ_status1  health_cond  know_tested_positive  ladder  marital1  mor_circle  moralid10  moralid2  moralid3  moralid4  moralid6  moralid8  moralid9  mcoop1  mcoop2  mcoop3  mcoop4  mcoop5  mcoop6  narc2  narc3  narc5  nidentity1  nidentity2  omind5  omind6  contact2  contact3  contact4  hygiene4  hygiene5  psupport2  political_ideology  happy  slf_ladder  riskperc1  riskperc2  self_esteem  sex1  tested_positive  optim1  optim2  slfcont4  urban
##   7   7  age  children  cnarc1  cnarc2  cnarc3  CRT1  CRT2  CRT3  employ_status1  health_cond  know_tested_positive  ladder  marital1  mor_circle  moralid10  moralid2  moralid3  moralid4  moralid6  moralid8  moralid9  mcoop1  mcoop2  mcoop3  mcoop4  mcoop5  mcoop6  narc2  narc3  narc5  nidentity1  nidentity2  omind5  omind6  contact2  contact3  contact4  hygiene4  hygiene5  psupport2  political_ideology  happy  slf_ladder  riskperc1  riskperc2  self_esteem  sex1  tested_positive  optim1  optim2  slfcont4  urban
##   7   8  age  children  cnarc1  cnarc2  cnarc3  CRT1  CRT2  CRT3  employ_status1  health_cond  know_tested_positive  ladder  marital1  mor_circle  moralid10  moralid2  moralid3  moralid4  moralid6  moralid8  moralid9  mcoop1  mcoop2  mcoop3  mcoop4  mcoop5  mcoop6  narc2  narc3  narc5  nidentity1  nidentity2  omind5  omind6  contact2  contact3  contact4  hygiene4  hygiene5  psupport2  political_ideology  happy  slf_ladder  riskperc1  riskperc2  self_esteem  sex1  tested_positive  optim1  optim2  slfcont4  urban
##   7   9  age  children  cnarc1  cnarc2  cnarc3  CRT1  CRT2  CRT3  employ_status1  health_cond  know_tested_positive  ladder  marital1  mor_circle  moralid10  moralid2  moralid3  moralid4  moralid6  moralid8  moralid9  mcoop1  mcoop2  mcoop3  mcoop4  mcoop5  mcoop6  narc2  narc3  narc5  nidentity1  nidentity2  omind5  omind6  contact2  contact3  contact4  hygiene4  hygiene5  psupport2  political_ideology  happy  slf_ladder  riskperc1  riskperc2  self_esteem  sex1  tested_positive  optim1  optim2  slfcont4  urban
##   7   10  age  children  cnarc1  cnarc2  cnarc3  CRT1  CRT2  CRT3  employ_status1  health_cond  know_tested_positive  ladder  marital1  mor_circle  moralid10  moralid2  moralid3  moralid4  moralid6  moralid8  moralid9  mcoop1  mcoop2  mcoop3  mcoop4  mcoop5  mcoop6  narc2  narc3  narc5  nidentity1  nidentity2  omind5  omind6  contact2  contact3  contact4  hygiene4  hygiene5  psupport2  political_ideology  happy  slf_ladder  riskperc1  riskperc2  self_esteem  sex1  tested_positive  optim1  optim2  slfcont4  urban
##   8   1  age  children  cnarc1  cnarc2  cnarc3  CRT1  CRT2  CRT3  employ_status1  health_cond  know_tested_positive  ladder  marital1  mor_circle  moralid10  moralid2  moralid3  moralid4  moralid6  moralid8  moralid9  mcoop1  mcoop2  mcoop3  mcoop4  mcoop5  mcoop6  narc2  narc3  narc5  nidentity1  nidentity2  omind5  omind6  contact2  contact3  contact4  hygiene4  hygiene5  psupport2  political_ideology  happy  slf_ladder  riskperc1  riskperc2  self_esteem  sex1  tested_positive  optim1  optim2  slfcont4  urban
##   8   2  age  children  cnarc1  cnarc2  cnarc3  CRT1  CRT2  CRT3  employ_status1  health_cond  know_tested_positive  ladder  marital1  mor_circle  moralid10  moralid2  moralid3  moralid4  moralid6  moralid8  moralid9  mcoop1  mcoop2  mcoop3  mcoop4  mcoop5  mcoop6  narc2  narc3  narc5  nidentity1  nidentity2  omind5  omind6  contact2  contact3  contact4  hygiene4  hygiene5  psupport2  political_ideology  happy  slf_ladder  riskperc1  riskperc2  self_esteem  sex1  tested_positive  optim1  optim2  slfcont4  urban
##   8   3  age  children  cnarc1  cnarc2  cnarc3  CRT1  CRT2  CRT3  employ_status1  health_cond  know_tested_positive  ladder  marital1  mor_circle  moralid10  moralid2  moralid3  moralid4  moralid6  moralid8  moralid9  mcoop1  mcoop2  mcoop3  mcoop4  mcoop5  mcoop6  narc2  narc3  narc5  nidentity1  nidentity2  omind5  omind6  contact2  contact3  contact4  hygiene4  hygiene5  psupport2  political_ideology  happy  slf_ladder  riskperc1  riskperc2  self_esteem  sex1  tested_positive  optim1  optim2  slfcont4  urban
##   8   4  age  children  cnarc1  cnarc2  cnarc3  CRT1  CRT2  CRT3  employ_status1  health_cond  know_tested_positive  ladder  marital1  mor_circle  moralid10  moralid2  moralid3  moralid4  moralid6  moralid8  moralid9  mcoop1  mcoop2  mcoop3  mcoop4  mcoop5  mcoop6  narc2  narc3  narc5  nidentity1  nidentity2  omind5  omind6  contact2  contact3  contact4  hygiene4  hygiene5  psupport2  political_ideology  happy  slf_ladder  riskperc1  riskperc2  self_esteem  sex1  tested_positive  optim1  optim2  slfcont4  urban
##   8   5  age  children  cnarc1  cnarc2  cnarc3  CRT1  CRT2  CRT3  employ_status1  health_cond  know_tested_positive  ladder  marital1  mor_circle  moralid10  moralid2  moralid3  moralid4  moralid6  moralid8  moralid9  mcoop1  mcoop2  mcoop3  mcoop4  mcoop5  mcoop6  narc2  narc3  narc5  nidentity1  nidentity2  omind5  omind6  contact2  contact3  contact4  hygiene4  hygiene5  psupport2  political_ideology  happy  slf_ladder  riskperc1  riskperc2  self_esteem  sex1  tested_positive  optim1  optim2  slfcont4  urban
##   8   6  age  children  cnarc1  cnarc2  cnarc3  CRT1  CRT2  CRT3  employ_status1  health_cond  know_tested_positive  ladder  marital1  mor_circle  moralid10  moralid2  moralid3  moralid4  moralid6  moralid8  moralid9  mcoop1  mcoop2  mcoop3  mcoop4  mcoop5  mcoop6  narc2  narc3  narc5  nidentity1  nidentity2  omind5  omind6  contact2  contact3  contact4  hygiene4  hygiene5  psupport2  political_ideology  happy  slf_ladder  riskperc1  riskperc2  self_esteem  sex1  tested_positive  optim1  optim2  slfcont4  urban
##   8   7  age  children  cnarc1  cnarc2  cnarc3  CRT1  CRT2  CRT3  employ_status1  health_cond  know_tested_positive  ladder  marital1  mor_circle  moralid10  moralid2  moralid3  moralid4  moralid6  moralid8  moralid9  mcoop1  mcoop2  mcoop3  mcoop4  mcoop5  mcoop6  narc2  narc3  narc5  nidentity1  nidentity2  omind5  omind6  contact2  contact3  contact4  hygiene4  hygiene5  psupport2  political_ideology  happy  slf_ladder  riskperc1  riskperc2  self_esteem  sex1  tested_positive  optim1  optim2  slfcont4  urban
##   8   8  age  children  cnarc1  cnarc2  cnarc3  CRT1  CRT2  CRT3  employ_status1  health_cond  know_tested_positive  ladder  marital1  mor_circle  moralid10  moralid2  moralid3  moralid4  moralid6  moralid8  moralid9  mcoop1  mcoop2  mcoop3  mcoop4  mcoop5  mcoop6  narc2  narc3  narc5  nidentity1  nidentity2  omind5  omind6  contact2  contact3  contact4  hygiene4  hygiene5  psupport2  political_ideology  happy  slf_ladder  riskperc1  riskperc2  self_esteem  sex1  tested_positive  optim1  optim2  slfcont4  urban
##   8   9  age  children  cnarc1  cnarc2  cnarc3  CRT1  CRT2  CRT3  employ_status1  health_cond  know_tested_positive  ladder  marital1  mor_circle  moralid10  moralid2  moralid3  moralid4  moralid6  moralid8  moralid9  mcoop1  mcoop2  mcoop3  mcoop4  mcoop5  mcoop6  narc2  narc3  narc5  nidentity1  nidentity2  omind5  omind6  contact2  contact3  contact4  hygiene4  hygiene5  psupport2  political_ideology  happy  slf_ladder  riskperc1  riskperc2  self_esteem  sex1  tested_positive  optim1  optim2  slfcont4  urban
##   8   10  age  children  cnarc1  cnarc2  cnarc3  CRT1  CRT2  CRT3  employ_status1  health_cond  know_tested_positive  ladder  marital1  mor_circle  moralid10  moralid2  moralid3  moralid4  moralid6  moralid8  moralid9  mcoop1  mcoop2  mcoop3  mcoop4  mcoop5  mcoop6  narc2  narc3  narc5  nidentity1  nidentity2  omind5  omind6  contact2  contact3  contact4  hygiene4  hygiene5  psupport2  political_ideology  happy  slf_ladder  riskperc1  riskperc2  self_esteem  sex1  tested_positive  optim1  optim2  slfcont4  urban
##   9   1  age  children  cnarc1  cnarc2  cnarc3  CRT1  CRT2  CRT3  employ_status1  health_cond  know_tested_positive  ladder  marital1  mor_circle  moralid10  moralid2  moralid3  moralid4  moralid6  moralid8  moralid9  mcoop1  mcoop2  mcoop3  mcoop4  mcoop5  mcoop6  narc2  narc3  narc5  nidentity1  nidentity2  omind5  omind6  contact2  contact3  contact4  hygiene4  hygiene5  psupport2  political_ideology  happy  slf_ladder  riskperc1  riskperc2  self_esteem  sex1  tested_positive  optim1  optim2  slfcont4  urban
##   9   2  age  children  cnarc1  cnarc2  cnarc3  CRT1  CRT2  CRT3  employ_status1  health_cond  know_tested_positive  ladder  marital1  mor_circle  moralid10  moralid2  moralid3  moralid4  moralid6  moralid8  moralid9  mcoop1  mcoop2  mcoop3  mcoop4  mcoop5  mcoop6  narc2  narc3  narc5  nidentity1  nidentity2  omind5  omind6  contact2  contact3  contact4  hygiene4  hygiene5  psupport2  political_ideology  happy  slf_ladder  riskperc1  riskperc2  self_esteem  sex1  tested_positive  optim1  optim2  slfcont4  urban
##   9   3  age  children  cnarc1  cnarc2  cnarc3  CRT1  CRT2  CRT3  employ_status1  health_cond  know_tested_positive  ladder  marital1  mor_circle  moralid10  moralid2  moralid3  moralid4  moralid6  moralid8  moralid9  mcoop1  mcoop2  mcoop3  mcoop4  mcoop5  mcoop6  narc2  narc3  narc5  nidentity1  nidentity2  omind5  omind6  contact2  contact3  contact4  hygiene4  hygiene5  psupport2  political_ideology  happy  slf_ladder  riskperc1  riskperc2  self_esteem  sex1  tested_positive  optim1  optim2  slfcont4  urban
##   9   4  age  children  cnarc1  cnarc2  cnarc3  CRT1  CRT2  CRT3  employ_status1  health_cond  know_tested_positive  ladder  marital1  mor_circle  moralid10  moralid2  moralid3  moralid4  moralid6  moralid8  moralid9  mcoop1  mcoop2  mcoop3  mcoop4  mcoop5  mcoop6  narc2  narc3  narc5  nidentity1  nidentity2  omind5  omind6  contact2  contact3  contact4  hygiene4  hygiene5  psupport2  political_ideology  happy  slf_ladder  riskperc1  riskperc2  self_esteem  sex1  tested_positive  optim1  optim2  slfcont4  urban
##   9   5  age  children  cnarc1  cnarc2  cnarc3  CRT1  CRT2  CRT3  employ_status1  health_cond  know_tested_positive  ladder  marital1  mor_circle  moralid10  moralid2  moralid3  moralid4  moralid6  moralid8  moralid9  mcoop1  mcoop2  mcoop3  mcoop4  mcoop5  mcoop6  narc2  narc3  narc5  nidentity1  nidentity2  omind5  omind6  contact2  contact3  contact4  hygiene4  hygiene5  psupport2  political_ideology  happy  slf_ladder  riskperc1  riskperc2  self_esteem  sex1  tested_positive  optim1  optim2  slfcont4  urban
##   9   6  age  children  cnarc1  cnarc2  cnarc3  CRT1  CRT2  CRT3  employ_status1  health_cond  know_tested_positive  ladder  marital1  mor_circle  moralid10  moralid2  moralid3  moralid4  moralid6  moralid8  moralid9  mcoop1  mcoop2  mcoop3  mcoop4  mcoop5  mcoop6  narc2  narc3  narc5  nidentity1  nidentity2  omind5  omind6  contact2  contact3  contact4  hygiene4  hygiene5  psupport2  political_ideology  happy  slf_ladder  riskperc1  riskperc2  self_esteem  sex1  tested_positive  optim1  optim2  slfcont4  urban
##   9   7  age  children  cnarc1  cnarc2  cnarc3  CRT1  CRT2  CRT3  employ_status1  health_cond  know_tested_positive  ladder  marital1  mor_circle  moralid10  moralid2  moralid3  moralid4  moralid6  moralid8  moralid9  mcoop1  mcoop2  mcoop3  mcoop4  mcoop5  mcoop6  narc2  narc3  narc5  nidentity1  nidentity2  omind5  omind6  contact2  contact3  contact4  hygiene4  hygiene5  psupport2  political_ideology  happy  slf_ladder  riskperc1  riskperc2  self_esteem  sex1  tested_positive  optim1  optim2  slfcont4  urban
##   9   8  age  children  cnarc1  cnarc2  cnarc3  CRT1  CRT2  CRT3  employ_status1  health_cond  know_tested_positive  ladder  marital1  mor_circle  moralid10  moralid2  moralid3  moralid4  moralid6  moralid8  moralid9  mcoop1  mcoop2  mcoop3  mcoop4  mcoop5  mcoop6  narc2  narc3  narc5  nidentity1  nidentity2  omind5  omind6  contact2  contact3  contact4  hygiene4  hygiene5  psupport2  political_ideology  happy  slf_ladder  riskperc1  riskperc2  self_esteem  sex1  tested_positive  optim1  optim2  slfcont4  urban
##   9   9  age  children  cnarc1  cnarc2  cnarc3  CRT1  CRT2  CRT3  employ_status1  health_cond  know_tested_positive  ladder  marital1  mor_circle  moralid10  moralid2  moralid3  moralid4  moralid6  moralid8  moralid9  mcoop1  mcoop2  mcoop3  mcoop4  mcoop5  mcoop6  narc2  narc3  narc5  nidentity1  nidentity2  omind5  omind6  contact2  contact3  contact4  hygiene4  hygiene5  psupport2  political_ideology  happy  slf_ladder  riskperc1  riskperc2  self_esteem  sex1  tested_positive  optim1  optim2  slfcont4  urban
##   9   10  age  children  cnarc1  cnarc2  cnarc3  CRT1  CRT2  CRT3  employ_status1  health_cond  know_tested_positive  ladder  marital1  mor_circle  moralid10  moralid2  moralid3  moralid4  moralid6  moralid8  moralid9  mcoop1  mcoop2  mcoop3  mcoop4  mcoop5  mcoop6  narc2  narc3  narc5  nidentity1  nidentity2  omind5  omind6  contact2  contact3  contact4  hygiene4  hygiene5  psupport2  political_ideology  happy  slf_ladder  riskperc1  riskperc2  self_esteem  sex1  tested_positive  optim1  optim2  slfcont4  urban
##   10   1  age  children  cnarc1  cnarc2  cnarc3  CRT1  CRT2  CRT3  employ_status1  health_cond  know_tested_positive  ladder  marital1  mor_circle  moralid10  moralid2  moralid3  moralid4  moralid6  moralid8  moralid9  mcoop1  mcoop2  mcoop3  mcoop4  mcoop5  mcoop6  narc2  narc3  narc5  nidentity1  nidentity2  omind5  omind6  contact2  contact3  contact4  hygiene4  hygiene5  psupport2  political_ideology  happy  slf_ladder  riskperc1  riskperc2  self_esteem  sex1  tested_positive  optim1  optim2  slfcont4  urban
##   10   2  age  children  cnarc1  cnarc2  cnarc3  CRT1  CRT2  CRT3  employ_status1  health_cond  know_tested_positive  ladder  marital1  mor_circle  moralid10  moralid2  moralid3  moralid4  moralid6  moralid8  moralid9  mcoop1  mcoop2  mcoop3  mcoop4  mcoop5  mcoop6  narc2  narc3  narc5  nidentity1  nidentity2  omind5  omind6  contact2  contact3  contact4  hygiene4  hygiene5  psupport2  political_ideology  happy  slf_ladder  riskperc1  riskperc2  self_esteem  sex1  tested_positive  optim1  optim2  slfcont4  urban
##   10   3  age  children  cnarc1  cnarc2  cnarc3  CRT1  CRT2  CRT3  employ_status1  health_cond  know_tested_positive  ladder  marital1  mor_circle  moralid10  moralid2  moralid3  moralid4  moralid6  moralid8  moralid9  mcoop1  mcoop2  mcoop3  mcoop4  mcoop5  mcoop6  narc2  narc3  narc5  nidentity1  nidentity2  omind5  omind6  contact2  contact3  contact4  hygiene4  hygiene5  psupport2  political_ideology  happy  slf_ladder  riskperc1  riskperc2  self_esteem  sex1  tested_positive  optim1  optim2  slfcont4  urban
##   10   4  age  children  cnarc1  cnarc2  cnarc3  CRT1  CRT2  CRT3  employ_status1  health_cond  know_tested_positive  ladder  marital1  mor_circle  moralid10  moralid2  moralid3  moralid4  moralid6  moralid8  moralid9  mcoop1  mcoop2  mcoop3  mcoop4  mcoop5  mcoop6  narc2  narc3  narc5  nidentity1  nidentity2  omind5  omind6  contact2  contact3  contact4  hygiene4  hygiene5  psupport2  political_ideology  happy  slf_ladder  riskperc1  riskperc2  self_esteem  sex1  tested_positive  optim1  optim2  slfcont4  urban
##   10   5  age  children  cnarc1  cnarc2  cnarc3  CRT1  CRT2  CRT3  employ_status1  health_cond  know_tested_positive  ladder  marital1  mor_circle  moralid10  moralid2  moralid3  moralid4  moralid6  moralid8  moralid9  mcoop1  mcoop2  mcoop3  mcoop4  mcoop5  mcoop6  narc2  narc3  narc5  nidentity1  nidentity2  omind5  omind6  contact2  contact3  contact4  hygiene4  hygiene5  psupport2  political_ideology  happy  slf_ladder  riskperc1  riskperc2  self_esteem  sex1  tested_positive  optim1  optim2  slfcont4  urban
##   10   6  age  children  cnarc1  cnarc2  cnarc3  CRT1  CRT2  CRT3  employ_status1  health_cond  know_tested_positive  ladder  marital1  mor_circle  moralid10  moralid2  moralid3  moralid4  moralid6  moralid8  moralid9  mcoop1  mcoop2  mcoop3  mcoop4  mcoop5  mcoop6  narc2  narc3  narc5  nidentity1  nidentity2  omind5  omind6  contact2  contact3  contact4  hygiene4  hygiene5  psupport2  political_ideology  happy  slf_ladder  riskperc1  riskperc2  self_esteem  sex1  tested_positive  optim1  optim2  slfcont4  urban
##   10   7  age  children  cnarc1  cnarc2  cnarc3  CRT1  CRT2  CRT3  employ_status1  health_cond  know_tested_positive  ladder  marital1  mor_circle  moralid10  moralid2  moralid3  moralid4  moralid6  moralid8  moralid9  mcoop1  mcoop2  mcoop3  mcoop4  mcoop5  mcoop6  narc2  narc3  narc5  nidentity1  nidentity2  omind5  omind6  contact2  contact3  contact4  hygiene4  hygiene5  psupport2  political_ideology  happy  slf_ladder  riskperc1  riskperc2  self_esteem  sex1  tested_positive  optim1  optim2  slfcont4  urban
##   10   8  age  children  cnarc1  cnarc2  cnarc3  CRT1  CRT2  CRT3  employ_status1  health_cond  know_tested_positive  ladder  marital1  mor_circle  moralid10  moralid2  moralid3  moralid4  moralid6  moralid8  moralid9  mcoop1  mcoop2  mcoop3  mcoop4  mcoop5  mcoop6  narc2  narc3  narc5  nidentity1  nidentity2  omind5  omind6  contact2  contact3  contact4  hygiene4  hygiene5  psupport2  political_ideology  happy  slf_ladder  riskperc1  riskperc2  self_esteem  sex1  tested_positive  optim1  optim2  slfcont4  urban
##   10   9  age  children  cnarc1  cnarc2  cnarc3  CRT1  CRT2  CRT3  employ_status1  health_cond  know_tested_positive  ladder  marital1  mor_circle  moralid10  moralid2  moralid3  moralid4  moralid6  moralid8  moralid9  mcoop1  mcoop2  mcoop3  mcoop4  mcoop5  mcoop6  narc2  narc3  narc5  nidentity1  nidentity2  omind5  omind6  contact2  contact3  contact4  hygiene4  hygiene5  psupport2  political_ideology  happy  slf_ladder  riskperc1  riskperc2  self_esteem  sex1  tested_positive  optim1  optim2  slfcont4  urban
##   10   10  age  children  cnarc1  cnarc2  cnarc3  CRT1  CRT2  CRT3  employ_status1  health_cond  know_tested_positive  ladder  marital1  mor_circle  moralid10  moralid2  moralid3  moralid4  moralid6  moralid8  moralid9  mcoop1  mcoop2  mcoop3  mcoop4  mcoop5  mcoop6  narc2  narc3  narc5  nidentity1  nidentity2  omind5  omind6  contact2  contact3  contact4  hygiene4  hygiene5  psupport2  political_ideology  happy  slf_ladder  riskperc1  riskperc2  self_esteem  sex1  tested_positive  optim1  optim2  slfcont4  urban
```

```
## Warning: Number of logged events: 5203
```

```
## 
##  iter imp variable
##   1   1  age  children  cnarc2  cnarc3  ctheory1  ctheory3  ctheory4  CRT1  CRT2  CRT3  employ_status1  generosity1  generosity2  generosity3  health_cond  know_tested_positive  ladder  marital1  mor_circle  moralid1  moralid10  moralid2  moralid3  moralid4  moralid5  moralid6  moralid7  moralid8  moralid9  mcoop4  mcoop5  narc1  narc2  narc3  narc4  narc5  narc6  nidentity1  nidentity2  omind4  omind5  omind6  contact1  contact3  contact4  hygiene1  hygiene2  hygiene4  hygiene5  psupport1  psupport2  psupport3  psupport5  political_ideology  happy  slf_ladder  riskperc1  riskperc2  self_esteem  sex1  sbelong1  sbelong2  tested_positive  slfcont1  slfcont2
##   1   2  age  children  cnarc2  cnarc3  ctheory1  ctheory3  ctheory4  CRT1  CRT2  CRT3  employ_status1  generosity1  generosity2  generosity3  health_cond  know_tested_positive  ladder  marital1  mor_circle  moralid1  moralid10  moralid2  moralid3  moralid4  moralid5  moralid6  moralid7  moralid8  moralid9  mcoop4  mcoop5  narc1  narc2  narc3  narc4  narc5  narc6  nidentity1  nidentity2  omind4  omind5  omind6  contact1  contact3  contact4  hygiene1  hygiene2  hygiene4  hygiene5  psupport1  psupport2  psupport3  psupport5  political_ideology  happy  slf_ladder  riskperc1  riskperc2  self_esteem  sex1  sbelong1  sbelong2  tested_positive  slfcont1  slfcont2
##   1   3  age  children  cnarc2  cnarc3  ctheory1  ctheory3  ctheory4  CRT1  CRT2  CRT3  employ_status1  generosity1  generosity2  generosity3  health_cond  know_tested_positive  ladder  marital1  mor_circle  moralid1  moralid10  moralid2  moralid3  moralid4  moralid5  moralid6  moralid7  moralid8  moralid9  mcoop4  mcoop5  narc1  narc2  narc3  narc4  narc5  narc6  nidentity1  nidentity2  omind4  omind5  omind6  contact1  contact3  contact4  hygiene1  hygiene2  hygiene4  hygiene5  psupport1  psupport2  psupport3  psupport5  political_ideology  happy  slf_ladder  riskperc1  riskperc2  self_esteem  sex1  sbelong1  sbelong2  tested_positive  slfcont1  slfcont2
##   1   4  age  children  cnarc2  cnarc3  ctheory1  ctheory3  ctheory4  CRT1  CRT2  CRT3  employ_status1  generosity1  generosity2  generosity3  health_cond  know_tested_positive  ladder  marital1  mor_circle  moralid1  moralid10  moralid2  moralid3  moralid4  moralid5  moralid6  moralid7  moralid8  moralid9  mcoop4  mcoop5  narc1  narc2  narc3  narc4  narc5  narc6  nidentity1  nidentity2  omind4  omind5  omind6  contact1  contact3  contact4  hygiene1  hygiene2  hygiene4  hygiene5  psupport1  psupport2  psupport3  psupport5  political_ideology  happy  slf_ladder  riskperc1  riskperc2  self_esteem  sex1  sbelong1  sbelong2  tested_positive  slfcont1  slfcont2
##   1   5  age  children  cnarc2  cnarc3  ctheory1  ctheory3  ctheory4  CRT1  CRT2  CRT3  employ_status1  generosity1  generosity2  generosity3  health_cond  know_tested_positive  ladder  marital1  mor_circle  moralid1  moralid10  moralid2  moralid3  moralid4  moralid5  moralid6  moralid7  moralid8  moralid9  mcoop4  mcoop5  narc1  narc2  narc3  narc4  narc5  narc6  nidentity1  nidentity2  omind4  omind5  omind6  contact1  contact3  contact4  hygiene1  hygiene2  hygiene4  hygiene5  psupport1  psupport2  psupport3  psupport5  political_ideology  happy  slf_ladder  riskperc1  riskperc2  self_esteem  sex1  sbelong1  sbelong2  tested_positive  slfcont1  slfcont2
##   1   6  age  children  cnarc2  cnarc3  ctheory1  ctheory3  ctheory4  CRT1  CRT2  CRT3  employ_status1  generosity1  generosity2  generosity3  health_cond  know_tested_positive  ladder  marital1  mor_circle  moralid1  moralid10  moralid2  moralid3  moralid4  moralid5  moralid6  moralid7  moralid8  moralid9  mcoop4  mcoop5  narc1  narc2  narc3  narc4  narc5  narc6  nidentity1  nidentity2  omind4  omind5  omind6  contact1  contact3  contact4  hygiene1  hygiene2  hygiene4  hygiene5  psupport1  psupport2  psupport3  psupport5  political_ideology  happy  slf_ladder  riskperc1  riskperc2  self_esteem  sex1  sbelong1  sbelong2  tested_positive  slfcont1  slfcont2
##   1   7  age  children  cnarc2  cnarc3  ctheory1  ctheory3  ctheory4  CRT1  CRT2  CRT3  employ_status1  generosity1  generosity2  generosity3  health_cond  know_tested_positive  ladder  marital1  mor_circle  moralid1  moralid10  moralid2  moralid3  moralid4  moralid5  moralid6  moralid7  moralid8  moralid9  mcoop4  mcoop5  narc1  narc2  narc3  narc4  narc5  narc6  nidentity1  nidentity2  omind4  omind5  omind6  contact1  contact3  contact4  hygiene1  hygiene2  hygiene4  hygiene5  psupport1  psupport2  psupport3  psupport5  political_ideology  happy  slf_ladder  riskperc1  riskperc2  self_esteem  sex1  sbelong1  sbelong2  tested_positive  slfcont1  slfcont2
##   1   8  age  children  cnarc2  cnarc3  ctheory1  ctheory3  ctheory4  CRT1  CRT2  CRT3  employ_status1  generosity1  generosity2  generosity3  health_cond  know_tested_positive  ladder  marital1  mor_circle  moralid1  moralid10  moralid2  moralid3  moralid4  moralid5  moralid6  moralid7  moralid8  moralid9  mcoop4  mcoop5  narc1  narc2  narc3  narc4  narc5  narc6  nidentity1  nidentity2  omind4  omind5  omind6  contact1  contact3  contact4  hygiene1  hygiene2  hygiene4  hygiene5  psupport1  psupport2  psupport3  psupport5  political_ideology  happy  slf_ladder  riskperc1  riskperc2  self_esteem  sex1  sbelong1  sbelong2  tested_positive  slfcont1  slfcont2
##   1   9  age  children  cnarc2  cnarc3  ctheory1  ctheory3  ctheory4  CRT1  CRT2  CRT3  employ_status1  generosity1  generosity2  generosity3  health_cond  know_tested_positive  ladder  marital1  mor_circle  moralid1  moralid10  moralid2  moralid3  moralid4  moralid5  moralid6  moralid7  moralid8  moralid9  mcoop4  mcoop5  narc1  narc2  narc3  narc4  narc5  narc6  nidentity1  nidentity2  omind4  omind5  omind6  contact1  contact3  contact4  hygiene1  hygiene2  hygiene4  hygiene5  psupport1  psupport2  psupport3  psupport5  political_ideology  happy  slf_ladder  riskperc1  riskperc2  self_esteem  sex1  sbelong1  sbelong2  tested_positive  slfcont1  slfcont2
##   1   10  age  children  cnarc2  cnarc3  ctheory1  ctheory3  ctheory4  CRT1  CRT2  CRT3  employ_status1  generosity1  generosity2  generosity3  health_cond  know_tested_positive  ladder  marital1  mor_circle  moralid1  moralid10  moralid2  moralid3  moralid4  moralid5  moralid6  moralid7  moralid8  moralid9  mcoop4  mcoop5  narc1  narc2  narc3  narc4  narc5  narc6  nidentity1  nidentity2  omind4  omind5  omind6  contact1  contact3  contact4  hygiene1  hygiene2  hygiene4  hygiene5  psupport1  psupport2  psupport3  psupport5  political_ideology  happy  slf_ladder  riskperc1  riskperc2  self_esteem  sex1  sbelong1  sbelong2  tested_positive  slfcont1  slfcont2
##   2   1  age  children  cnarc2  cnarc3  ctheory1  ctheory3  ctheory4  CRT1  CRT2  CRT3  employ_status1  generosity1  generosity2  generosity3  health_cond  know_tested_positive  ladder  marital1  mor_circle  moralid1  moralid10  moralid2  moralid3  moralid4  moralid5  moralid6  moralid7  moralid8  moralid9  mcoop4  mcoop5  narc1  narc2  narc3  narc4  narc5  narc6  nidentity1  nidentity2  omind4  omind5  omind6  contact1  contact3  contact4  hygiene1  hygiene2  hygiene4  hygiene5  psupport1  psupport2  psupport3  psupport5  political_ideology  happy  slf_ladder  riskperc1  riskperc2  self_esteem  sex1  sbelong1  sbelong2  tested_positive  slfcont1  slfcont2
##   2   2  age  children  cnarc2  cnarc3  ctheory1  ctheory3  ctheory4  CRT1  CRT2  CRT3  employ_status1  generosity1  generosity2  generosity3  health_cond  know_tested_positive  ladder  marital1  mor_circle  moralid1  moralid10  moralid2  moralid3  moralid4  moralid5  moralid6  moralid7  moralid8  moralid9  mcoop4  mcoop5  narc1  narc2  narc3  narc4  narc5  narc6  nidentity1  nidentity2  omind4  omind5  omind6  contact1  contact3  contact4  hygiene1  hygiene2  hygiene4  hygiene5  psupport1  psupport2  psupport3  psupport5  political_ideology  happy  slf_ladder  riskperc1  riskperc2  self_esteem  sex1  sbelong1  sbelong2  tested_positive  slfcont1  slfcont2
##   2   3  age  children  cnarc2  cnarc3  ctheory1  ctheory3  ctheory4  CRT1  CRT2  CRT3  employ_status1  generosity1  generosity2  generosity3  health_cond  know_tested_positive  ladder  marital1  mor_circle  moralid1  moralid10  moralid2  moralid3  moralid4  moralid5  moralid6  moralid7  moralid8  moralid9  mcoop4  mcoop5  narc1  narc2  narc3  narc4  narc5  narc6  nidentity1  nidentity2  omind4  omind5  omind6  contact1  contact3  contact4  hygiene1  hygiene2  hygiene4  hygiene5  psupport1  psupport2  psupport3  psupport5  political_ideology  happy  slf_ladder  riskperc1  riskperc2  self_esteem  sex1  sbelong1  sbelong2  tested_positive  slfcont1  slfcont2
##   2   4  age  children  cnarc2  cnarc3  ctheory1  ctheory3  ctheory4  CRT1  CRT2  CRT3  employ_status1  generosity1  generosity2  generosity3  health_cond  know_tested_positive  ladder  marital1  mor_circle  moralid1  moralid10  moralid2  moralid3  moralid4  moralid5  moralid6  moralid7  moralid8  moralid9  mcoop4  mcoop5  narc1  narc2  narc3  narc4  narc5  narc6  nidentity1  nidentity2  omind4  omind5  omind6  contact1  contact3  contact4  hygiene1  hygiene2  hygiene4  hygiene5  psupport1  psupport2  psupport3  psupport5  political_ideology  happy  slf_ladder  riskperc1  riskperc2  self_esteem  sex1  sbelong1  sbelong2  tested_positive  slfcont1  slfcont2
##   2   5  age  children  cnarc2  cnarc3  ctheory1  ctheory3  ctheory4  CRT1  CRT2  CRT3  employ_status1  generosity1  generosity2  generosity3  health_cond  know_tested_positive  ladder  marital1  mor_circle  moralid1  moralid10  moralid2  moralid3  moralid4  moralid5  moralid6  moralid7  moralid8  moralid9  mcoop4  mcoop5  narc1  narc2  narc3  narc4  narc5  narc6  nidentity1  nidentity2  omind4  omind5  omind6  contact1  contact3  contact4  hygiene1  hygiene2  hygiene4  hygiene5  psupport1  psupport2  psupport3  psupport5  political_ideology  happy  slf_ladder  riskperc1  riskperc2  self_esteem  sex1  sbelong1  sbelong2  tested_positive  slfcont1  slfcont2
##   2   6  age  children  cnarc2  cnarc3  ctheory1  ctheory3  ctheory4  CRT1  CRT2  CRT3  employ_status1  generosity1  generosity2  generosity3  health_cond  know_tested_positive  ladder  marital1  mor_circle  moralid1  moralid10  moralid2  moralid3  moralid4  moralid5  moralid6  moralid7  moralid8  moralid9  mcoop4  mcoop5  narc1  narc2  narc3  narc4  narc5  narc6  nidentity1  nidentity2  omind4  omind5  omind6  contact1  contact3  contact4  hygiene1  hygiene2  hygiene4  hygiene5  psupport1  psupport2  psupport3  psupport5  political_ideology  happy  slf_ladder  riskperc1  riskperc2  self_esteem  sex1  sbelong1  sbelong2  tested_positive  slfcont1  slfcont2
##   2   7  age  children  cnarc2  cnarc3  ctheory1  ctheory3  ctheory4  CRT1  CRT2  CRT3  employ_status1  generosity1  generosity2  generosity3  health_cond  know_tested_positive  ladder  marital1  mor_circle  moralid1  moralid10  moralid2  moralid3  moralid4  moralid5  moralid6  moralid7  moralid8  moralid9  mcoop4  mcoop5  narc1  narc2  narc3  narc4  narc5  narc6  nidentity1  nidentity2  omind4  omind5  omind6  contact1  contact3  contact4  hygiene1  hygiene2  hygiene4  hygiene5  psupport1  psupport2  psupport3  psupport5  political_ideology  happy  slf_ladder  riskperc1  riskperc2  self_esteem  sex1  sbelong1  sbelong2  tested_positive  slfcont1  slfcont2
##   2   8  age  children  cnarc2  cnarc3  ctheory1  ctheory3  ctheory4  CRT1  CRT2  CRT3  employ_status1  generosity1  generosity2  generosity3  health_cond  know_tested_positive  ladder  marital1  mor_circle  moralid1  moralid10  moralid2  moralid3  moralid4  moralid5  moralid6  moralid7  moralid8  moralid9  mcoop4  mcoop5  narc1  narc2  narc3  narc4  narc5  narc6  nidentity1  nidentity2  omind4  omind5  omind6  contact1  contact3  contact4  hygiene1  hygiene2  hygiene4  hygiene5  psupport1  psupport2  psupport3  psupport5  political_ideology  happy  slf_ladder  riskperc1  riskperc2  self_esteem  sex1  sbelong1  sbelong2  tested_positive  slfcont1  slfcont2
##   2   9  age  children  cnarc2  cnarc3  ctheory1  ctheory3  ctheory4  CRT1  CRT2  CRT3  employ_status1  generosity1  generosity2  generosity3  health_cond  know_tested_positive  ladder  marital1  mor_circle  moralid1  moralid10  moralid2  moralid3  moralid4  moralid5  moralid6  moralid7  moralid8  moralid9  mcoop4  mcoop5  narc1  narc2  narc3  narc4  narc5  narc6  nidentity1  nidentity2  omind4  omind5  omind6  contact1  contact3  contact4  hygiene1  hygiene2  hygiene4  hygiene5  psupport1  psupport2  psupport3  psupport5  political_ideology  happy  slf_ladder  riskperc1  riskperc2  self_esteem  sex1  sbelong1  sbelong2  tested_positive  slfcont1  slfcont2
##   2   10  age  children  cnarc2  cnarc3  ctheory1  ctheory3  ctheory4  CRT1  CRT2  CRT3  employ_status1  generosity1  generosity2  generosity3  health_cond  know_tested_positive  ladder  marital1  mor_circle  moralid1  moralid10  moralid2  moralid3  moralid4  moralid5  moralid6  moralid7  moralid8  moralid9  mcoop4  mcoop5  narc1  narc2  narc3  narc4  narc5  narc6  nidentity1  nidentity2  omind4  omind5  omind6  contact1  contact3  contact4  hygiene1  hygiene2  hygiene4  hygiene5  psupport1  psupport2  psupport3  psupport5  political_ideology  happy  slf_ladder  riskperc1  riskperc2  self_esteem  sex1  sbelong1  sbelong2  tested_positive  slfcont1  slfcont2
##   3   1  age  children  cnarc2  cnarc3  ctheory1  ctheory3  ctheory4  CRT1  CRT2  CRT3  employ_status1  generosity1  generosity2  generosity3  health_cond  know_tested_positive  ladder  marital1  mor_circle  moralid1  moralid10  moralid2  moralid3  moralid4  moralid5  moralid6  moralid7  moralid8  moralid9  mcoop4  mcoop5  narc1  narc2  narc3  narc4  narc5  narc6  nidentity1  nidentity2  omind4  omind5  omind6  contact1  contact3  contact4  hygiene1  hygiene2  hygiene4  hygiene5  psupport1  psupport2  psupport3  psupport5  political_ideology  happy  slf_ladder  riskperc1  riskperc2  self_esteem  sex1  sbelong1  sbelong2  tested_positive  slfcont1  slfcont2
##   3   2  age  children  cnarc2  cnarc3  ctheory1  ctheory3  ctheory4  CRT1  CRT2  CRT3  employ_status1  generosity1  generosity2  generosity3  health_cond  know_tested_positive  ladder  marital1  mor_circle  moralid1  moralid10  moralid2  moralid3  moralid4  moralid5  moralid6  moralid7  moralid8  moralid9  mcoop4  mcoop5  narc1  narc2  narc3  narc4  narc5  narc6  nidentity1  nidentity2  omind4  omind5  omind6  contact1  contact3  contact4  hygiene1  hygiene2  hygiene4  hygiene5  psupport1  psupport2  psupport3  psupport5  political_ideology  happy  slf_ladder  riskperc1  riskperc2  self_esteem  sex1  sbelong1  sbelong2  tested_positive  slfcont1  slfcont2
##   3   3  age  children  cnarc2  cnarc3  ctheory1  ctheory3  ctheory4  CRT1  CRT2  CRT3  employ_status1  generosity1  generosity2  generosity3  health_cond  know_tested_positive  ladder  marital1  mor_circle  moralid1  moralid10  moralid2  moralid3  moralid4  moralid5  moralid6  moralid7  moralid8  moralid9  mcoop4  mcoop5  narc1  narc2  narc3  narc4  narc5  narc6  nidentity1  nidentity2  omind4  omind5  omind6  contact1  contact3  contact4  hygiene1  hygiene2  hygiene4  hygiene5  psupport1  psupport2  psupport3  psupport5  political_ideology  happy  slf_ladder  riskperc1  riskperc2  self_esteem  sex1  sbelong1  sbelong2  tested_positive  slfcont1  slfcont2
##   3   4  age  children  cnarc2  cnarc3  ctheory1  ctheory3  ctheory4  CRT1  CRT2  CRT3  employ_status1  generosity1  generosity2  generosity3  health_cond  know_tested_positive  ladder  marital1  mor_circle  moralid1  moralid10  moralid2  moralid3  moralid4  moralid5  moralid6  moralid7  moralid8  moralid9  mcoop4  mcoop5  narc1  narc2  narc3  narc4  narc5  narc6  nidentity1  nidentity2  omind4  omind5  omind6  contact1  contact3  contact4  hygiene1  hygiene2  hygiene4  hygiene5  psupport1  psupport2  psupport3  psupport5  political_ideology  happy  slf_ladder  riskperc1  riskperc2  self_esteem  sex1  sbelong1  sbelong2  tested_positive  slfcont1  slfcont2
##   3   5  age  children  cnarc2  cnarc3  ctheory1  ctheory3  ctheory4  CRT1  CRT2  CRT3  employ_status1  generosity1  generosity2  generosity3  health_cond  know_tested_positive  ladder  marital1  mor_circle  moralid1  moralid10  moralid2  moralid3  moralid4  moralid5  moralid6  moralid7  moralid8  moralid9  mcoop4  mcoop5  narc1  narc2  narc3  narc4  narc5  narc6  nidentity1  nidentity2  omind4  omind5  omind6  contact1  contact3  contact4  hygiene1  hygiene2  hygiene4  hygiene5  psupport1  psupport2  psupport3  psupport5  political_ideology  happy  slf_ladder  riskperc1  riskperc2  self_esteem  sex1  sbelong1  sbelong2  tested_positive  slfcont1  slfcont2
##   3   6  age  children  cnarc2  cnarc3  ctheory1  ctheory3  ctheory4  CRT1  CRT2  CRT3  employ_status1  generosity1  generosity2  generosity3  health_cond  know_tested_positive  ladder  marital1  mor_circle  moralid1  moralid10  moralid2  moralid3  moralid4  moralid5  moralid6  moralid7  moralid8  moralid9  mcoop4  mcoop5  narc1  narc2  narc3  narc4  narc5  narc6  nidentity1  nidentity2  omind4  omind5  omind6  contact1  contact3  contact4  hygiene1  hygiene2  hygiene4  hygiene5  psupport1  psupport2  psupport3  psupport5  political_ideology  happy  slf_ladder  riskperc1  riskperc2  self_esteem  sex1  sbelong1  sbelong2  tested_positive  slfcont1  slfcont2
##   3   7  age  children  cnarc2  cnarc3  ctheory1  ctheory3  ctheory4  CRT1  CRT2  CRT3  employ_status1  generosity1  generosity2  generosity3  health_cond  know_tested_positive  ladder  marital1  mor_circle  moralid1  moralid10  moralid2  moralid3  moralid4  moralid5  moralid6  moralid7  moralid8  moralid9  mcoop4  mcoop5  narc1  narc2  narc3  narc4  narc5  narc6  nidentity1  nidentity2  omind4  omind5  omind6  contact1  contact3  contact4  hygiene1  hygiene2  hygiene4  hygiene5  psupport1  psupport2  psupport3  psupport5  political_ideology  happy  slf_ladder  riskperc1  riskperc2  self_esteem  sex1  sbelong1  sbelong2  tested_positive  slfcont1  slfcont2
##   3   8  age  children  cnarc2  cnarc3  ctheory1  ctheory3  ctheory4  CRT1  CRT2  CRT3  employ_status1  generosity1  generosity2  generosity3  health_cond  know_tested_positive  ladder  marital1  mor_circle  moralid1  moralid10  moralid2  moralid3  moralid4  moralid5  moralid6  moralid7  moralid8  moralid9  mcoop4  mcoop5  narc1  narc2  narc3  narc4  narc5  narc6  nidentity1  nidentity2  omind4  omind5  omind6  contact1  contact3  contact4  hygiene1  hygiene2  hygiene4  hygiene5  psupport1  psupport2  psupport3  psupport5  political_ideology  happy  slf_ladder  riskperc1  riskperc2  self_esteem  sex1  sbelong1  sbelong2  tested_positive  slfcont1  slfcont2
##   3   9  age  children  cnarc2  cnarc3  ctheory1  ctheory3  ctheory4  CRT1  CRT2  CRT3  employ_status1  generosity1  generosity2  generosity3  health_cond  know_tested_positive  ladder  marital1  mor_circle  moralid1  moralid10  moralid2  moralid3  moralid4  moralid5  moralid6  moralid7  moralid8  moralid9  mcoop4  mcoop5  narc1  narc2  narc3  narc4  narc5  narc6  nidentity1  nidentity2  omind4  omind5  omind6  contact1  contact3  contact4  hygiene1  hygiene2  hygiene4  hygiene5  psupport1  psupport2  psupport3  psupport5  political_ideology  happy  slf_ladder  riskperc1  riskperc2  self_esteem  sex1  sbelong1  sbelong2  tested_positive  slfcont1  slfcont2
##   3   10  age  children  cnarc2  cnarc3  ctheory1  ctheory3  ctheory4  CRT1  CRT2  CRT3  employ_status1  generosity1  generosity2  generosity3  health_cond  know_tested_positive  ladder  marital1  mor_circle  moralid1  moralid10  moralid2  moralid3  moralid4  moralid5  moralid6  moralid7  moralid8  moralid9  mcoop4  mcoop5  narc1  narc2  narc3  narc4  narc5  narc6  nidentity1  nidentity2  omind4  omind5  omind6  contact1  contact3  contact4  hygiene1  hygiene2  hygiene4  hygiene5  psupport1  psupport2  psupport3  psupport5  political_ideology  happy  slf_ladder  riskperc1  riskperc2  self_esteem  sex1  sbelong1  sbelong2  tested_positive  slfcont1  slfcont2
##   4   1  age  children  cnarc2  cnarc3  ctheory1  ctheory3  ctheory4  CRT1  CRT2  CRT3  employ_status1  generosity1  generosity2  generosity3  health_cond  know_tested_positive  ladder  marital1  mor_circle  moralid1  moralid10  moralid2  moralid3  moralid4  moralid5  moralid6  moralid7  moralid8  moralid9  mcoop4  mcoop5  narc1  narc2  narc3  narc4  narc5  narc6  nidentity1  nidentity2  omind4  omind5  omind6  contact1  contact3  contact4  hygiene1  hygiene2  hygiene4  hygiene5  psupport1  psupport2  psupport3  psupport5  political_ideology  happy  slf_ladder  riskperc1  riskperc2  self_esteem  sex1  sbelong1  sbelong2  tested_positive  slfcont1  slfcont2
##   4   2  age  children  cnarc2  cnarc3  ctheory1  ctheory3  ctheory4  CRT1  CRT2  CRT3  employ_status1  generosity1  generosity2  generosity3  health_cond  know_tested_positive  ladder  marital1  mor_circle  moralid1  moralid10  moralid2  moralid3  moralid4  moralid5  moralid6  moralid7  moralid8  moralid9  mcoop4  mcoop5  narc1  narc2  narc3  narc4  narc5  narc6  nidentity1  nidentity2  omind4  omind5  omind6  contact1  contact3  contact4  hygiene1  hygiene2  hygiene4  hygiene5  psupport1  psupport2  psupport3  psupport5  political_ideology  happy  slf_ladder  riskperc1  riskperc2  self_esteem  sex1  sbelong1  sbelong2  tested_positive  slfcont1  slfcont2
##   4   3  age  children  cnarc2  cnarc3  ctheory1  ctheory3  ctheory4  CRT1  CRT2  CRT3  employ_status1  generosity1  generosity2  generosity3  health_cond  know_tested_positive  ladder  marital1  mor_circle  moralid1  moralid10  moralid2  moralid3  moralid4  moralid5  moralid6  moralid7  moralid8  moralid9  mcoop4  mcoop5  narc1  narc2  narc3  narc4  narc5  narc6  nidentity1  nidentity2  omind4  omind5  omind6  contact1  contact3  contact4  hygiene1  hygiene2  hygiene4  hygiene5  psupport1  psupport2  psupport3  psupport5  political_ideology  happy  slf_ladder  riskperc1  riskperc2  self_esteem  sex1  sbelong1  sbelong2  tested_positive  slfcont1  slfcont2
##   4   4  age  children  cnarc2  cnarc3  ctheory1  ctheory3  ctheory4  CRT1  CRT2  CRT3  employ_status1  generosity1  generosity2  generosity3  health_cond  know_tested_positive  ladder  marital1  mor_circle  moralid1  moralid10  moralid2  moralid3  moralid4  moralid5  moralid6  moralid7  moralid8  moralid9  mcoop4  mcoop5  narc1  narc2  narc3  narc4  narc5  narc6  nidentity1  nidentity2  omind4  omind5  omind6  contact1  contact3  contact4  hygiene1  hygiene2  hygiene4  hygiene5  psupport1  psupport2  psupport3  psupport5  political_ideology  happy  slf_ladder  riskperc1  riskperc2  self_esteem  sex1  sbelong1  sbelong2  tested_positive  slfcont1  slfcont2
##   4   5  age  children  cnarc2  cnarc3  ctheory1  ctheory3  ctheory4  CRT1  CRT2  CRT3  employ_status1  generosity1  generosity2  generosity3  health_cond  know_tested_positive  ladder  marital1  mor_circle  moralid1  moralid10  moralid2  moralid3  moralid4  moralid5  moralid6  moralid7  moralid8  moralid9  mcoop4  mcoop5  narc1  narc2  narc3  narc4  narc5  narc6  nidentity1  nidentity2  omind4  omind5  omind6  contact1  contact3  contact4  hygiene1  hygiene2  hygiene4  hygiene5  psupport1  psupport2  psupport3  psupport5  political_ideology  happy  slf_ladder  riskperc1  riskperc2  self_esteem  sex1  sbelong1  sbelong2  tested_positive  slfcont1  slfcont2
##   4   6  age  children  cnarc2  cnarc3  ctheory1  ctheory3  ctheory4  CRT1  CRT2  CRT3  employ_status1  generosity1  generosity2  generosity3  health_cond  know_tested_positive  ladder  marital1  mor_circle  moralid1  moralid10  moralid2  moralid3  moralid4  moralid5  moralid6  moralid7  moralid8  moralid9  mcoop4  mcoop5  narc1  narc2  narc3  narc4  narc5  narc6  nidentity1  nidentity2  omind4  omind5  omind6  contact1  contact3  contact4  hygiene1  hygiene2  hygiene4  hygiene5  psupport1  psupport2  psupport3  psupport5  political_ideology  happy  slf_ladder  riskperc1  riskperc2  self_esteem  sex1  sbelong1  sbelong2  tested_positive  slfcont1  slfcont2
##   4   7  age  children  cnarc2  cnarc3  ctheory1  ctheory3  ctheory4  CRT1  CRT2  CRT3  employ_status1  generosity1  generosity2  generosity3  health_cond  know_tested_positive  ladder  marital1  mor_circle  moralid1  moralid10  moralid2  moralid3  moralid4  moralid5  moralid6  moralid7  moralid8  moralid9  mcoop4  mcoop5  narc1  narc2  narc3  narc4  narc5  narc6  nidentity1  nidentity2  omind4  omind5  omind6  contact1  contact3  contact4  hygiene1  hygiene2  hygiene4  hygiene5  psupport1  psupport2  psupport3  psupport5  political_ideology  happy  slf_ladder  riskperc1  riskperc2  self_esteem  sex1  sbelong1  sbelong2  tested_positive  slfcont1  slfcont2
##   4   8  age  children  cnarc2  cnarc3  ctheory1  ctheory3  ctheory4  CRT1  CRT2  CRT3  employ_status1  generosity1  generosity2  generosity3  health_cond  know_tested_positive  ladder  marital1  mor_circle  moralid1  moralid10  moralid2  moralid3  moralid4  moralid5  moralid6  moralid7  moralid8  moralid9  mcoop4  mcoop5  narc1  narc2  narc3  narc4  narc5  narc6  nidentity1  nidentity2  omind4  omind5  omind6  contact1  contact3  contact4  hygiene1  hygiene2  hygiene4  hygiene5  psupport1  psupport2  psupport3  psupport5  political_ideology  happy  slf_ladder  riskperc1  riskperc2  self_esteem  sex1  sbelong1  sbelong2  tested_positive  slfcont1  slfcont2
##   4   9  age  children  cnarc2  cnarc3  ctheory1  ctheory3  ctheory4  CRT1  CRT2  CRT3  employ_status1  generosity1  generosity2  generosity3  health_cond  know_tested_positive  ladder  marital1  mor_circle  moralid1  moralid10  moralid2  moralid3  moralid4  moralid5  moralid6  moralid7  moralid8  moralid9  mcoop4  mcoop5  narc1  narc2  narc3  narc4  narc5  narc6  nidentity1  nidentity2  omind4  omind5  omind6  contact1  contact3  contact4  hygiene1  hygiene2  hygiene4  hygiene5  psupport1  psupport2  psupport3  psupport5  political_ideology  happy  slf_ladder  riskperc1  riskperc2  self_esteem  sex1  sbelong1  sbelong2  tested_positive  slfcont1  slfcont2
##   4   10  age  children  cnarc2  cnarc3  ctheory1  ctheory3  ctheory4  CRT1  CRT2  CRT3  employ_status1  generosity1  generosity2  generosity3  health_cond  know_tested_positive  ladder  marital1  mor_circle  moralid1  moralid10  moralid2  moralid3  moralid4  moralid5  moralid6  moralid7  moralid8  moralid9  mcoop4  mcoop5  narc1  narc2  narc3  narc4  narc5  narc6  nidentity1  nidentity2  omind4  omind5  omind6  contact1  contact3  contact4  hygiene1  hygiene2  hygiene4  hygiene5  psupport1  psupport2  psupport3  psupport5  political_ideology  happy  slf_ladder  riskperc1  riskperc2  self_esteem  sex1  sbelong1  sbelong2  tested_positive  slfcont1  slfcont2
##   5   1  age  children  cnarc2  cnarc3  ctheory1  ctheory3  ctheory4  CRT1  CRT2  CRT3  employ_status1  generosity1  generosity2  generosity3  health_cond  know_tested_positive  ladder  marital1  mor_circle  moralid1  moralid10  moralid2  moralid3  moralid4  moralid5  moralid6  moralid7  moralid8  moralid9  mcoop4  mcoop5  narc1  narc2  narc3  narc4  narc5  narc6  nidentity1  nidentity2  omind4  omind5  omind6  contact1  contact3  contact4  hygiene1  hygiene2  hygiene4  hygiene5  psupport1  psupport2  psupport3  psupport5  political_ideology  happy  slf_ladder  riskperc1  riskperc2  self_esteem  sex1  sbelong1  sbelong2  tested_positive  slfcont1  slfcont2
##   5   2  age  children  cnarc2  cnarc3  ctheory1  ctheory3  ctheory4  CRT1  CRT2  CRT3  employ_status1  generosity1  generosity2  generosity3  health_cond  know_tested_positive  ladder  marital1  mor_circle  moralid1  moralid10  moralid2  moralid3  moralid4  moralid5  moralid6  moralid7  moralid8  moralid9  mcoop4  mcoop5  narc1  narc2  narc3  narc4  narc5  narc6  nidentity1  nidentity2  omind4  omind5  omind6  contact1  contact3  contact4  hygiene1  hygiene2  hygiene4  hygiene5  psupport1  psupport2  psupport3  psupport5  political_ideology  happy  slf_ladder  riskperc1  riskperc2  self_esteem  sex1  sbelong1  sbelong2  tested_positive  slfcont1  slfcont2
##   5   3  age  children  cnarc2  cnarc3  ctheory1  ctheory3  ctheory4  CRT1  CRT2  CRT3  employ_status1  generosity1  generosity2  generosity3  health_cond  know_tested_positive  ladder  marital1  mor_circle  moralid1  moralid10  moralid2  moralid3  moralid4  moralid5  moralid6  moralid7  moralid8  moralid9  mcoop4  mcoop5  narc1  narc2  narc3  narc4  narc5  narc6  nidentity1  nidentity2  omind4  omind5  omind6  contact1  contact3  contact4  hygiene1  hygiene2  hygiene4  hygiene5  psupport1  psupport2  psupport3  psupport5  political_ideology  happy  slf_ladder  riskperc1  riskperc2  self_esteem  sex1  sbelong1  sbelong2  tested_positive  slfcont1  slfcont2
##   5   4  age  children  cnarc2  cnarc3  ctheory1  ctheory3  ctheory4  CRT1  CRT2  CRT3  employ_status1  generosity1  generosity2  generosity3  health_cond  know_tested_positive  ladder  marital1  mor_circle  moralid1  moralid10  moralid2  moralid3  moralid4  moralid5  moralid6  moralid7  moralid8  moralid9  mcoop4  mcoop5  narc1  narc2  narc3  narc4  narc5  narc6  nidentity1  nidentity2  omind4  omind5  omind6  contact1  contact3  contact4  hygiene1  hygiene2  hygiene4  hygiene5  psupport1  psupport2  psupport3  psupport5  political_ideology  happy  slf_ladder  riskperc1  riskperc2  self_esteem  sex1  sbelong1  sbelong2  tested_positive  slfcont1  slfcont2
##   5   5  age  children  cnarc2  cnarc3  ctheory1  ctheory3  ctheory4  CRT1  CRT2  CRT3  employ_status1  generosity1  generosity2  generosity3  health_cond  know_tested_positive  ladder  marital1  mor_circle  moralid1  moralid10  moralid2  moralid3  moralid4  moralid5  moralid6  moralid7  moralid8  moralid9  mcoop4  mcoop5  narc1  narc2  narc3  narc4  narc5  narc6  nidentity1  nidentity2  omind4  omind5  omind6  contact1  contact3  contact4  hygiene1  hygiene2  hygiene4  hygiene5  psupport1  psupport2  psupport3  psupport5  political_ideology  happy  slf_ladder  riskperc1  riskperc2  self_esteem  sex1  sbelong1  sbelong2  tested_positive  slfcont1  slfcont2
##   5   6  age  children  cnarc2  cnarc3  ctheory1  ctheory3  ctheory4  CRT1  CRT2  CRT3  employ_status1  generosity1  generosity2  generosity3  health_cond  know_tested_positive  ladder  marital1  mor_circle  moralid1  moralid10  moralid2  moralid3  moralid4  moralid5  moralid6  moralid7  moralid8  moralid9  mcoop4  mcoop5  narc1  narc2  narc3  narc4  narc5  narc6  nidentity1  nidentity2  omind4  omind5  omind6  contact1  contact3  contact4  hygiene1  hygiene2  hygiene4  hygiene5  psupport1  psupport2  psupport3  psupport5  political_ideology  happy  slf_ladder  riskperc1  riskperc2  self_esteem  sex1  sbelong1  sbelong2  tested_positive  slfcont1  slfcont2
##   5   7  age  children  cnarc2  cnarc3  ctheory1  ctheory3  ctheory4  CRT1  CRT2  CRT3  employ_status1  generosity1  generosity2  generosity3  health_cond  know_tested_positive  ladder  marital1  mor_circle  moralid1  moralid10  moralid2  moralid3  moralid4  moralid5  moralid6  moralid7  moralid8  moralid9  mcoop4  mcoop5  narc1  narc2  narc3  narc4  narc5  narc6  nidentity1  nidentity2  omind4  omind5  omind6  contact1  contact3  contact4  hygiene1  hygiene2  hygiene4  hygiene5  psupport1  psupport2  psupport3  psupport5  political_ideology  happy  slf_ladder  riskperc1  riskperc2  self_esteem  sex1  sbelong1  sbelong2  tested_positive  slfcont1  slfcont2
##   5   8  age  children  cnarc2  cnarc3  ctheory1  ctheory3  ctheory4  CRT1  CRT2  CRT3  employ_status1  generosity1  generosity2  generosity3  health_cond  know_tested_positive  ladder  marital1  mor_circle  moralid1  moralid10  moralid2  moralid3  moralid4  moralid5  moralid6  moralid7  moralid8  moralid9  mcoop4  mcoop5  narc1  narc2  narc3  narc4  narc5  narc6  nidentity1  nidentity2  omind4  omind5  omind6  contact1  contact3  contact4  hygiene1  hygiene2  hygiene4  hygiene5  psupport1  psupport2  psupport3  psupport5  political_ideology  happy  slf_ladder  riskperc1  riskperc2  self_esteem  sex1  sbelong1  sbelong2  tested_positive  slfcont1  slfcont2
##   5   9  age  children  cnarc2  cnarc3  ctheory1  ctheory3  ctheory4  CRT1  CRT2  CRT3  employ_status1  generosity1  generosity2  generosity3  health_cond  know_tested_positive  ladder  marital1  mor_circle  moralid1  moralid10  moralid2  moralid3  moralid4  moralid5  moralid6  moralid7  moralid8  moralid9  mcoop4  mcoop5  narc1  narc2  narc3  narc4  narc5  narc6  nidentity1  nidentity2  omind4  omind5  omind6  contact1  contact3  contact4  hygiene1  hygiene2  hygiene4  hygiene5  psupport1  psupport2  psupport3  psupport5  political_ideology  happy  slf_ladder  riskperc1  riskperc2  self_esteem  sex1  sbelong1  sbelong2  tested_positive  slfcont1  slfcont2
##   5   10  age  children  cnarc2  cnarc3  ctheory1  ctheory3  ctheory4  CRT1  CRT2  CRT3  employ_status1  generosity1  generosity2  generosity3  health_cond  know_tested_positive  ladder  marital1  mor_circle  moralid1  moralid10  moralid2  moralid3  moralid4  moralid5  moralid6  moralid7  moralid8  moralid9  mcoop4  mcoop5  narc1  narc2  narc3  narc4  narc5  narc6  nidentity1  nidentity2  omind4  omind5  omind6  contact1  contact3  contact4  hygiene1  hygiene2  hygiene4  hygiene5  psupport1  psupport2  psupport3  psupport5  political_ideology  happy  slf_ladder  riskperc1  riskperc2  self_esteem  sex1  sbelong1  sbelong2  tested_positive  slfcont1  slfcont2
##   6   1  age  children  cnarc2  cnarc3  ctheory1  ctheory3  ctheory4  CRT1  CRT2  CRT3  employ_status1  generosity1  generosity2  generosity3  health_cond  know_tested_positive  ladder  marital1  mor_circle  moralid1  moralid10  moralid2  moralid3  moralid4  moralid5  moralid6  moralid7  moralid8  moralid9  mcoop4  mcoop5  narc1  narc2  narc3  narc4  narc5  narc6  nidentity1  nidentity2  omind4  omind5  omind6  contact1  contact3  contact4  hygiene1  hygiene2  hygiene4  hygiene5  psupport1  psupport2  psupport3  psupport5  political_ideology  happy  slf_ladder  riskperc1  riskperc2  self_esteem  sex1  sbelong1  sbelong2  tested_positive  slfcont1  slfcont2
##   6   2  age  children  cnarc2  cnarc3  ctheory1  ctheory3  ctheory4  CRT1  CRT2  CRT3  employ_status1  generosity1  generosity2  generosity3  health_cond  know_tested_positive  ladder  marital1  mor_circle  moralid1  moralid10  moralid2  moralid3  moralid4  moralid5  moralid6  moralid7  moralid8  moralid9  mcoop4  mcoop5  narc1  narc2  narc3  narc4  narc5  narc6  nidentity1  nidentity2  omind4  omind5  omind6  contact1  contact3  contact4  hygiene1  hygiene2  hygiene4  hygiene5  psupport1  psupport2  psupport3  psupport5  political_ideology  happy  slf_ladder  riskperc1  riskperc2  self_esteem  sex1  sbelong1  sbelong2  tested_positive  slfcont1  slfcont2
##   6   3  age  children  cnarc2  cnarc3  ctheory1  ctheory3  ctheory4  CRT1  CRT2  CRT3  employ_status1  generosity1  generosity2  generosity3  health_cond  know_tested_positive  ladder  marital1  mor_circle  moralid1  moralid10  moralid2  moralid3  moralid4  moralid5  moralid6  moralid7  moralid8  moralid9  mcoop4  mcoop5  narc1  narc2  narc3  narc4  narc5  narc6  nidentity1  nidentity2  omind4  omind5  omind6  contact1  contact3  contact4  hygiene1  hygiene2  hygiene4  hygiene5  psupport1  psupport2  psupport3  psupport5  political_ideology  happy  slf_ladder  riskperc1  riskperc2  self_esteem  sex1  sbelong1  sbelong2  tested_positive  slfcont1  slfcont2
##   6   4  age  children  cnarc2  cnarc3  ctheory1  ctheory3  ctheory4  CRT1  CRT2  CRT3  employ_status1  generosity1  generosity2  generosity3  health_cond  know_tested_positive  ladder  marital1  mor_circle  moralid1  moralid10  moralid2  moralid3  moralid4  moralid5  moralid6  moralid7  moralid8  moralid9  mcoop4  mcoop5  narc1  narc2  narc3  narc4  narc5  narc6  nidentity1  nidentity2  omind4  omind5  omind6  contact1  contact3  contact4  hygiene1  hygiene2  hygiene4  hygiene5  psupport1  psupport2  psupport3  psupport5  political_ideology  happy  slf_ladder  riskperc1  riskperc2  self_esteem  sex1  sbelong1  sbelong2  tested_positive  slfcont1  slfcont2
##   6   5  age  children  cnarc2  cnarc3  ctheory1  ctheory3  ctheory4  CRT1  CRT2  CRT3  employ_status1  generosity1  generosity2  generosity3  health_cond  know_tested_positive  ladder  marital1  mor_circle  moralid1  moralid10  moralid2  moralid3  moralid4  moralid5  moralid6  moralid7  moralid8  moralid9  mcoop4  mcoop5  narc1  narc2  narc3  narc4  narc5  narc6  nidentity1  nidentity2  omind4  omind5  omind6  contact1  contact3  contact4  hygiene1  hygiene2  hygiene4  hygiene5  psupport1  psupport2  psupport3  psupport5  political_ideology  happy  slf_ladder  riskperc1  riskperc2  self_esteem  sex1  sbelong1  sbelong2  tested_positive  slfcont1  slfcont2
##   6   6  age  children  cnarc2  cnarc3  ctheory1  ctheory3  ctheory4  CRT1  CRT2  CRT3  employ_status1  generosity1  generosity2  generosity3  health_cond  know_tested_positive  ladder  marital1  mor_circle  moralid1  moralid10  moralid2  moralid3  moralid4  moralid5  moralid6  moralid7  moralid8  moralid9  mcoop4  mcoop5  narc1  narc2  narc3  narc4  narc5  narc6  nidentity1  nidentity2  omind4  omind5  omind6  contact1  contact3  contact4  hygiene1  hygiene2  hygiene4  hygiene5  psupport1  psupport2  psupport3  psupport5  political_ideology  happy  slf_ladder  riskperc1  riskperc2  self_esteem  sex1  sbelong1  sbelong2  tested_positive  slfcont1  slfcont2
##   6   7  age  children  cnarc2  cnarc3  ctheory1  ctheory3  ctheory4  CRT1  CRT2  CRT3  employ_status1  generosity1  generosity2  generosity3  health_cond  know_tested_positive  ladder  marital1  mor_circle  moralid1  moralid10  moralid2  moralid3  moralid4  moralid5  moralid6  moralid7  moralid8  moralid9  mcoop4  mcoop5  narc1  narc2  narc3  narc4  narc5  narc6  nidentity1  nidentity2  omind4  omind5  omind6  contact1  contact3  contact4  hygiene1  hygiene2  hygiene4  hygiene5  psupport1  psupport2  psupport3  psupport5  political_ideology  happy  slf_ladder  riskperc1  riskperc2  self_esteem  sex1  sbelong1  sbelong2  tested_positive  slfcont1  slfcont2
##   6   8  age  children  cnarc2  cnarc3  ctheory1  ctheory3  ctheory4  CRT1  CRT2  CRT3  employ_status1  generosity1  generosity2  generosity3  health_cond  know_tested_positive  ladder  marital1  mor_circle  moralid1  moralid10  moralid2  moralid3  moralid4  moralid5  moralid6  moralid7  moralid8  moralid9  mcoop4  mcoop5  narc1  narc2  narc3  narc4  narc5  narc6  nidentity1  nidentity2  omind4  omind5  omind6  contact1  contact3  contact4  hygiene1  hygiene2  hygiene4  hygiene5  psupport1  psupport2  psupport3  psupport5  political_ideology  happy  slf_ladder  riskperc1  riskperc2  self_esteem  sex1  sbelong1  sbelong2  tested_positive  slfcont1  slfcont2
##   6   9  age  children  cnarc2  cnarc3  ctheory1  ctheory3  ctheory4  CRT1  CRT2  CRT3  employ_status1  generosity1  generosity2  generosity3  health_cond  know_tested_positive  ladder  marital1  mor_circle  moralid1  moralid10  moralid2  moralid3  moralid4  moralid5  moralid6  moralid7  moralid8  moralid9  mcoop4  mcoop5  narc1  narc2  narc3  narc4  narc5  narc6  nidentity1  nidentity2  omind4  omind5  omind6  contact1  contact3  contact4  hygiene1  hygiene2  hygiene4  hygiene5  psupport1  psupport2  psupport3  psupport5  political_ideology  happy  slf_ladder  riskperc1  riskperc2  self_esteem  sex1  sbelong1  sbelong2  tested_positive  slfcont1  slfcont2
##   6   10  age  children  cnarc2  cnarc3  ctheory1  ctheory3  ctheory4  CRT1  CRT2  CRT3  employ_status1  generosity1  generosity2  generosity3  health_cond  know_tested_positive  ladder  marital1  mor_circle  moralid1  moralid10  moralid2  moralid3  moralid4  moralid5  moralid6  moralid7  moralid8  moralid9  mcoop4  mcoop5  narc1  narc2  narc3  narc4  narc5  narc6  nidentity1  nidentity2  omind4  omind5  omind6  contact1  contact3  contact4  hygiene1  hygiene2  hygiene4  hygiene5  psupport1  psupport2  psupport3  psupport5  political_ideology  happy  slf_ladder  riskperc1  riskperc2  self_esteem  sex1  sbelong1  sbelong2  tested_positive  slfcont1  slfcont2
##   7   1  age  children  cnarc2  cnarc3  ctheory1  ctheory3  ctheory4  CRT1  CRT2  CRT3  employ_status1  generosity1  generosity2  generosity3  health_cond  know_tested_positive  ladder  marital1  mor_circle  moralid1  moralid10  moralid2  moralid3  moralid4  moralid5  moralid6  moralid7  moralid8  moralid9  mcoop4  mcoop5  narc1  narc2  narc3  narc4  narc5  narc6  nidentity1  nidentity2  omind4  omind5  omind6  contact1  contact3  contact4  hygiene1  hygiene2  hygiene4  hygiene5  psupport1  psupport2  psupport3  psupport5  political_ideology  happy  slf_ladder  riskperc1  riskperc2  self_esteem  sex1  sbelong1  sbelong2  tested_positive  slfcont1  slfcont2
##   7   2  age  children  cnarc2  cnarc3  ctheory1  ctheory3  ctheory4  CRT1  CRT2  CRT3  employ_status1  generosity1  generosity2  generosity3  health_cond  know_tested_positive  ladder  marital1  mor_circle  moralid1  moralid10  moralid2  moralid3  moralid4  moralid5  moralid6  moralid7  moralid8  moralid9  mcoop4  mcoop5  narc1  narc2  narc3  narc4  narc5  narc6  nidentity1  nidentity2  omind4  omind5  omind6  contact1  contact3  contact4  hygiene1  hygiene2  hygiene4  hygiene5  psupport1  psupport2  psupport3  psupport5  political_ideology  happy  slf_ladder  riskperc1  riskperc2  self_esteem  sex1  sbelong1  sbelong2  tested_positive  slfcont1  slfcont2
##   7   3  age  children  cnarc2  cnarc3  ctheory1  ctheory3  ctheory4  CRT1  CRT2  CRT3  employ_status1  generosity1  generosity2  generosity3  health_cond  know_tested_positive  ladder  marital1  mor_circle  moralid1  moralid10  moralid2  moralid3  moralid4  moralid5  moralid6  moralid7  moralid8  moralid9  mcoop4  mcoop5  narc1  narc2  narc3  narc4  narc5  narc6  nidentity1  nidentity2  omind4  omind5  omind6  contact1  contact3  contact4  hygiene1  hygiene2  hygiene4  hygiene5  psupport1  psupport2  psupport3  psupport5  political_ideology  happy  slf_ladder  riskperc1  riskperc2  self_esteem  sex1  sbelong1  sbelong2  tested_positive  slfcont1  slfcont2
##   7   4  age  children  cnarc2  cnarc3  ctheory1  ctheory3  ctheory4  CRT1  CRT2  CRT3  employ_status1  generosity1  generosity2  generosity3  health_cond  know_tested_positive  ladder  marital1  mor_circle  moralid1  moralid10  moralid2  moralid3  moralid4  moralid5  moralid6  moralid7  moralid8  moralid9  mcoop4  mcoop5  narc1  narc2  narc3  narc4  narc5  narc6  nidentity1  nidentity2  omind4  omind5  omind6  contact1  contact3  contact4  hygiene1  hygiene2  hygiene4  hygiene5  psupport1  psupport2  psupport3  psupport5  political_ideology  happy  slf_ladder  riskperc1  riskperc2  self_esteem  sex1  sbelong1  sbelong2  tested_positive  slfcont1  slfcont2
##   7   5  age  children  cnarc2  cnarc3  ctheory1  ctheory3  ctheory4  CRT1  CRT2  CRT3  employ_status1  generosity1  generosity2  generosity3  health_cond  know_tested_positive  ladder  marital1  mor_circle  moralid1  moralid10  moralid2  moralid3  moralid4  moralid5  moralid6  moralid7  moralid8  moralid9  mcoop4  mcoop5  narc1  narc2  narc3  narc4  narc5  narc6  nidentity1  nidentity2  omind4  omind5  omind6  contact1  contact3  contact4  hygiene1  hygiene2  hygiene4  hygiene5  psupport1  psupport2  psupport3  psupport5  political_ideology  happy  slf_ladder  riskperc1  riskperc2  self_esteem  sex1  sbelong1  sbelong2  tested_positive  slfcont1  slfcont2
##   7   6  age  children  cnarc2  cnarc3  ctheory1  ctheory3  ctheory4  CRT1  CRT2  CRT3  employ_status1  generosity1  generosity2  generosity3  health_cond  know_tested_positive  ladder  marital1  mor_circle  moralid1  moralid10  moralid2  moralid3  moralid4  moralid5  moralid6  moralid7  moralid8  moralid9  mcoop4  mcoop5  narc1  narc2  narc3  narc4  narc5  narc6  nidentity1  nidentity2  omind4  omind5  omind6  contact1  contact3  contact4  hygiene1  hygiene2  hygiene4  hygiene5  psupport1  psupport2  psupport3  psupport5  political_ideology  happy  slf_ladder  riskperc1  riskperc2  self_esteem  sex1  sbelong1  sbelong2  tested_positive  slfcont1  slfcont2
##   7   7  age  children  cnarc2  cnarc3  ctheory1  ctheory3  ctheory4  CRT1  CRT2  CRT3  employ_status1  generosity1  generosity2  generosity3  health_cond  know_tested_positive  ladder  marital1  mor_circle  moralid1  moralid10  moralid2  moralid3  moralid4  moralid5  moralid6  moralid7  moralid8  moralid9  mcoop4  mcoop5  narc1  narc2  narc3  narc4  narc5  narc6  nidentity1  nidentity2  omind4  omind5  omind6  contact1  contact3  contact4  hygiene1  hygiene2  hygiene4  hygiene5  psupport1  psupport2  psupport3  psupport5  political_ideology  happy  slf_ladder  riskperc1  riskperc2  self_esteem  sex1  sbelong1  sbelong2  tested_positive  slfcont1  slfcont2
##   7   8  age  children  cnarc2  cnarc3  ctheory1  ctheory3  ctheory4  CRT1  CRT2  CRT3  employ_status1  generosity1  generosity2  generosity3  health_cond  know_tested_positive  ladder  marital1  mor_circle  moralid1  moralid10  moralid2  moralid3  moralid4  moralid5  moralid6  moralid7  moralid8  moralid9  mcoop4  mcoop5  narc1  narc2  narc3  narc4  narc5  narc6  nidentity1  nidentity2  omind4  omind5  omind6  contact1  contact3  contact4  hygiene1  hygiene2  hygiene4  hygiene5  psupport1  psupport2  psupport3  psupport5  political_ideology  happy  slf_ladder  riskperc1  riskperc2  self_esteem  sex1  sbelong1  sbelong2  tested_positive  slfcont1  slfcont2
##   7   9  age  children  cnarc2  cnarc3  ctheory1  ctheory3  ctheory4  CRT1  CRT2  CRT3  employ_status1  generosity1  generosity2  generosity3  health_cond  know_tested_positive  ladder  marital1  mor_circle  moralid1  moralid10  moralid2  moralid3  moralid4  moralid5  moralid6  moralid7  moralid8  moralid9  mcoop4  mcoop5  narc1  narc2  narc3  narc4  narc5  narc6  nidentity1  nidentity2  omind4  omind5  omind6  contact1  contact3  contact4  hygiene1  hygiene2  hygiene4  hygiene5  psupport1  psupport2  psupport3  psupport5  political_ideology  happy  slf_ladder  riskperc1  riskperc2  self_esteem  sex1  sbelong1  sbelong2  tested_positive  slfcont1  slfcont2
##   7   10  age  children  cnarc2  cnarc3  ctheory1  ctheory3  ctheory4  CRT1  CRT2  CRT3  employ_status1  generosity1  generosity2  generosity3  health_cond  know_tested_positive  ladder  marital1  mor_circle  moralid1  moralid10  moralid2  moralid3  moralid4  moralid5  moralid6  moralid7  moralid8  moralid9  mcoop4  mcoop5  narc1  narc2  narc3  narc4  narc5  narc6  nidentity1  nidentity2  omind4  omind5  omind6  contact1  contact3  contact4  hygiene1  hygiene2  hygiene4  hygiene5  psupport1  psupport2  psupport3  psupport5  political_ideology  happy  slf_ladder  riskperc1  riskperc2  self_esteem  sex1  sbelong1  sbelong2  tested_positive  slfcont1  slfcont2
##   8   1  age  children  cnarc2  cnarc3  ctheory1  ctheory3  ctheory4  CRT1  CRT2  CRT3  employ_status1  generosity1  generosity2  generosity3  health_cond  know_tested_positive  ladder  marital1  mor_circle  moralid1  moralid10  moralid2  moralid3  moralid4  moralid5  moralid6  moralid7  moralid8  moralid9  mcoop4  mcoop5  narc1  narc2  narc3  narc4  narc5  narc6  nidentity1  nidentity2  omind4  omind5  omind6  contact1  contact3  contact4  hygiene1  hygiene2  hygiene4  hygiene5  psupport1  psupport2  psupport3  psupport5  political_ideology  happy  slf_ladder  riskperc1  riskperc2  self_esteem  sex1  sbelong1  sbelong2  tested_positive  slfcont1  slfcont2
##   8   2  age  children  cnarc2  cnarc3  ctheory1  ctheory3  ctheory4  CRT1  CRT2  CRT3  employ_status1  generosity1  generosity2  generosity3  health_cond  know_tested_positive  ladder  marital1  mor_circle  moralid1  moralid10  moralid2  moralid3  moralid4  moralid5  moralid6  moralid7  moralid8  moralid9  mcoop4  mcoop5  narc1  narc2  narc3  narc4  narc5  narc6  nidentity1  nidentity2  omind4  omind5  omind6  contact1  contact3  contact4  hygiene1  hygiene2  hygiene4  hygiene5  psupport1  psupport2  psupport3  psupport5  political_ideology  happy  slf_ladder  riskperc1  riskperc2  self_esteem  sex1  sbelong1  sbelong2  tested_positive  slfcont1  slfcont2
##   8   3  age  children  cnarc2  cnarc3  ctheory1  ctheory3  ctheory4  CRT1  CRT2  CRT3  employ_status1  generosity1  generosity2  generosity3  health_cond  know_tested_positive  ladder  marital1  mor_circle  moralid1  moralid10  moralid2  moralid3  moralid4  moralid5  moralid6  moralid7  moralid8  moralid9  mcoop4  mcoop5  narc1  narc2  narc3  narc4  narc5  narc6  nidentity1  nidentity2  omind4  omind5  omind6  contact1  contact3  contact4  hygiene1  hygiene2  hygiene4  hygiene5  psupport1  psupport2  psupport3  psupport5  political_ideology  happy  slf_ladder  riskperc1  riskperc2  self_esteem  sex1  sbelong1  sbelong2  tested_positive  slfcont1  slfcont2
##   8   4  age  children  cnarc2  cnarc3  ctheory1  ctheory3  ctheory4  CRT1  CRT2  CRT3  employ_status1  generosity1  generosity2  generosity3  health_cond  know_tested_positive  ladder  marital1  mor_circle  moralid1  moralid10  moralid2  moralid3  moralid4  moralid5  moralid6  moralid7  moralid8  moralid9  mcoop4  mcoop5  narc1  narc2  narc3  narc4  narc5  narc6  nidentity1  nidentity2  omind4  omind5  omind6  contact1  contact3  contact4  hygiene1  hygiene2  hygiene4  hygiene5  psupport1  psupport2  psupport3  psupport5  political_ideology  happy  slf_ladder  riskperc1  riskperc2  self_esteem  sex1  sbelong1  sbelong2  tested_positive  slfcont1  slfcont2
##   8   5  age  children  cnarc2  cnarc3  ctheory1  ctheory3  ctheory4  CRT1  CRT2  CRT3  employ_status1  generosity1  generosity2  generosity3  health_cond  know_tested_positive  ladder  marital1  mor_circle  moralid1  moralid10  moralid2  moralid3  moralid4  moralid5  moralid6  moralid7  moralid8  moralid9  mcoop4  mcoop5  narc1  narc2  narc3  narc4  narc5  narc6  nidentity1  nidentity2  omind4  omind5  omind6  contact1  contact3  contact4  hygiene1  hygiene2  hygiene4  hygiene5  psupport1  psupport2  psupport3  psupport5  political_ideology  happy  slf_ladder  riskperc1  riskperc2  self_esteem  sex1  sbelong1  sbelong2  tested_positive  slfcont1  slfcont2
##   8   6  age  children  cnarc2  cnarc3  ctheory1  ctheory3  ctheory4  CRT1  CRT2  CRT3  employ_status1  generosity1  generosity2  generosity3  health_cond  know_tested_positive  ladder  marital1  mor_circle  moralid1  moralid10  moralid2  moralid3  moralid4  moralid5  moralid6  moralid7  moralid8  moralid9  mcoop4  mcoop5  narc1  narc2  narc3  narc4  narc5  narc6  nidentity1  nidentity2  omind4  omind5  omind6  contact1  contact3  contact4  hygiene1  hygiene2  hygiene4  hygiene5  psupport1  psupport2  psupport3  psupport5  political_ideology  happy  slf_ladder  riskperc1  riskperc2  self_esteem  sex1  sbelong1  sbelong2  tested_positive  slfcont1  slfcont2
##   8   7  age  children  cnarc2  cnarc3  ctheory1  ctheory3  ctheory4  CRT1  CRT2  CRT3  employ_status1  generosity1  generosity2  generosity3  health_cond  know_tested_positive  ladder  marital1  mor_circle  moralid1  moralid10  moralid2  moralid3  moralid4  moralid5  moralid6  moralid7  moralid8  moralid9  mcoop4  mcoop5  narc1  narc2  narc3  narc4  narc5  narc6  nidentity1  nidentity2  omind4  omind5  omind6  contact1  contact3  contact4  hygiene1  hygiene2  hygiene4  hygiene5  psupport1  psupport2  psupport3  psupport5  political_ideology  happy  slf_ladder  riskperc1  riskperc2  self_esteem  sex1  sbelong1  sbelong2  tested_positive  slfcont1  slfcont2
##   8   8  age  children  cnarc2  cnarc3  ctheory1  ctheory3  ctheory4  CRT1  CRT2  CRT3  employ_status1  generosity1  generosity2  generosity3  health_cond  know_tested_positive  ladder  marital1  mor_circle  moralid1  moralid10  moralid2  moralid3  moralid4  moralid5  moralid6  moralid7  moralid8  moralid9  mcoop4  mcoop5  narc1  narc2  narc3  narc4  narc5  narc6  nidentity1  nidentity2  omind4  omind5  omind6  contact1  contact3  contact4  hygiene1  hygiene2  hygiene4  hygiene5  psupport1  psupport2  psupport3  psupport5  political_ideology  happy  slf_ladder  riskperc1  riskperc2  self_esteem  sex1  sbelong1  sbelong2  tested_positive  slfcont1  slfcont2
##   8   9  age  children  cnarc2  cnarc3  ctheory1  ctheory3  ctheory4  CRT1  CRT2  CRT3  employ_status1  generosity1  generosity2  generosity3  health_cond  know_tested_positive  ladder  marital1  mor_circle  moralid1  moralid10  moralid2  moralid3  moralid4  moralid5  moralid6  moralid7  moralid8  moralid9  mcoop4  mcoop5  narc1  narc2  narc3  narc4  narc5  narc6  nidentity1  nidentity2  omind4  omind5  omind6  contact1  contact3  contact4  hygiene1  hygiene2  hygiene4  hygiene5  psupport1  psupport2  psupport3  psupport5  political_ideology  happy  slf_ladder  riskperc1  riskperc2  self_esteem  sex1  sbelong1  sbelong2  tested_positive  slfcont1  slfcont2
##   8   10  age  children  cnarc2  cnarc3  ctheory1  ctheory3  ctheory4  CRT1  CRT2  CRT3  employ_status1  generosity1  generosity2  generosity3  health_cond  know_tested_positive  ladder  marital1  mor_circle  moralid1  moralid10  moralid2  moralid3  moralid4  moralid5  moralid6  moralid7  moralid8  moralid9  mcoop4  mcoop5  narc1  narc2  narc3  narc4  narc5  narc6  nidentity1  nidentity2  omind4  omind5  omind6  contact1  contact3  contact4  hygiene1  hygiene2  hygiene4  hygiene5  psupport1  psupport2  psupport3  psupport5  political_ideology  happy  slf_ladder  riskperc1  riskperc2  self_esteem  sex1  sbelong1  sbelong2  tested_positive  slfcont1  slfcont2
##   9   1  age  children  cnarc2  cnarc3  ctheory1  ctheory3  ctheory4  CRT1  CRT2  CRT3  employ_status1  generosity1  generosity2  generosity3  health_cond  know_tested_positive  ladder  marital1  mor_circle  moralid1  moralid10  moralid2  moralid3  moralid4  moralid5  moralid6  moralid7  moralid8  moralid9  mcoop4  mcoop5  narc1  narc2  narc3  narc4  narc5  narc6  nidentity1  nidentity2  omind4  omind5  omind6  contact1  contact3  contact4  hygiene1  hygiene2  hygiene4  hygiene5  psupport1  psupport2  psupport3  psupport5  political_ideology  happy  slf_ladder  riskperc1  riskperc2  self_esteem  sex1  sbelong1  sbelong2  tested_positive  slfcont1  slfcont2
##   9   2  age  children  cnarc2  cnarc3  ctheory1  ctheory3  ctheory4  CRT1  CRT2  CRT3  employ_status1  generosity1  generosity2  generosity3  health_cond  know_tested_positive  ladder  marital1  mor_circle  moralid1  moralid10  moralid2  moralid3  moralid4  moralid5  moralid6  moralid7  moralid8  moralid9  mcoop4  mcoop5  narc1  narc2  narc3  narc4  narc5  narc6  nidentity1  nidentity2  omind4  omind5  omind6  contact1  contact3  contact4  hygiene1  hygiene2  hygiene4  hygiene5  psupport1  psupport2  psupport3  psupport5  political_ideology  happy  slf_ladder  riskperc1  riskperc2  self_esteem  sex1  sbelong1  sbelong2  tested_positive  slfcont1  slfcont2
##   9   3  age  children  cnarc2  cnarc3  ctheory1  ctheory3  ctheory4  CRT1  CRT2  CRT3  employ_status1  generosity1  generosity2  generosity3  health_cond  know_tested_positive  ladder  marital1  mor_circle  moralid1  moralid10  moralid2  moralid3  moralid4  moralid5  moralid6  moralid7  moralid8  moralid9  mcoop4  mcoop5  narc1  narc2  narc3  narc4  narc5  narc6  nidentity1  nidentity2  omind4  omind5  omind6  contact1  contact3  contact4  hygiene1  hygiene2  hygiene4  hygiene5  psupport1  psupport2  psupport3  psupport5  political_ideology  happy  slf_ladder  riskperc1  riskperc2  self_esteem  sex1  sbelong1  sbelong2  tested_positive  slfcont1  slfcont2
##   9   4  age  children  cnarc2  cnarc3  ctheory1  ctheory3  ctheory4  CRT1  CRT2  CRT3  employ_status1  generosity1  generosity2  generosity3  health_cond  know_tested_positive  ladder  marital1  mor_circle  moralid1  moralid10  moralid2  moralid3  moralid4  moralid5  moralid6  moralid7  moralid8  moralid9  mcoop4  mcoop5  narc1  narc2  narc3  narc4  narc5  narc6  nidentity1  nidentity2  omind4  omind5  omind6  contact1  contact3  contact4  hygiene1  hygiene2  hygiene4  hygiene5  psupport1  psupport2  psupport3  psupport5  political_ideology  happy  slf_ladder  riskperc1  riskperc2  self_esteem  sex1  sbelong1  sbelong2  tested_positive  slfcont1  slfcont2
##   9   5  age  children  cnarc2  cnarc3  ctheory1  ctheory3  ctheory4  CRT1  CRT2  CRT3  employ_status1  generosity1  generosity2  generosity3  health_cond  know_tested_positive  ladder  marital1  mor_circle  moralid1  moralid10  moralid2  moralid3  moralid4  moralid5  moralid6  moralid7  moralid8  moralid9  mcoop4  mcoop5  narc1  narc2  narc3  narc4  narc5  narc6  nidentity1  nidentity2  omind4  omind5  omind6  contact1  contact3  contact4  hygiene1  hygiene2  hygiene4  hygiene5  psupport1  psupport2  psupport3  psupport5  political_ideology  happy  slf_ladder  riskperc1  riskperc2  self_esteem  sex1  sbelong1  sbelong2  tested_positive  slfcont1  slfcont2
##   9   6  age  children  cnarc2  cnarc3  ctheory1  ctheory3  ctheory4  CRT1  CRT2  CRT3  employ_status1  generosity1  generosity2  generosity3  health_cond  know_tested_positive  ladder  marital1  mor_circle  moralid1  moralid10  moralid2  moralid3  moralid4  moralid5  moralid6  moralid7  moralid8  moralid9  mcoop4  mcoop5  narc1  narc2  narc3  narc4  narc5  narc6  nidentity1  nidentity2  omind4  omind5  omind6  contact1  contact3  contact4  hygiene1  hygiene2  hygiene4  hygiene5  psupport1  psupport2  psupport3  psupport5  political_ideology  happy  slf_ladder  riskperc1  riskperc2  self_esteem  sex1  sbelong1  sbelong2  tested_positive  slfcont1  slfcont2
##   9   7  age  children  cnarc2  cnarc3  ctheory1  ctheory3  ctheory4  CRT1  CRT2  CRT3  employ_status1  generosity1  generosity2  generosity3  health_cond  know_tested_positive  ladder  marital1  mor_circle  moralid1  moralid10  moralid2  moralid3  moralid4  moralid5  moralid6  moralid7  moralid8  moralid9  mcoop4  mcoop5  narc1  narc2  narc3  narc4  narc5  narc6  nidentity1  nidentity2  omind4  omind5  omind6  contact1  contact3  contact4  hygiene1  hygiene2  hygiene4  hygiene5  psupport1  psupport2  psupport3  psupport5  political_ideology  happy  slf_ladder  riskperc1  riskperc2  self_esteem  sex1  sbelong1  sbelong2  tested_positive  slfcont1  slfcont2
##   9   8  age  children  cnarc2  cnarc3  ctheory1  ctheory3  ctheory4  CRT1  CRT2  CRT3  employ_status1  generosity1  generosity2  generosity3  health_cond  know_tested_positive  ladder  marital1  mor_circle  moralid1  moralid10  moralid2  moralid3  moralid4  moralid5  moralid6  moralid7  moralid8  moralid9  mcoop4  mcoop5  narc1  narc2  narc3  narc4  narc5  narc6  nidentity1  nidentity2  omind4  omind5  omind6  contact1  contact3  contact4  hygiene1  hygiene2  hygiene4  hygiene5  psupport1  psupport2  psupport3  psupport5  political_ideology  happy  slf_ladder  riskperc1  riskperc2  self_esteem  sex1  sbelong1  sbelong2  tested_positive  slfcont1  slfcont2
##   9   9  age  children  cnarc2  cnarc3  ctheory1  ctheory3  ctheory4  CRT1  CRT2  CRT3  employ_status1  generosity1  generosity2  generosity3  health_cond  know_tested_positive  ladder  marital1  mor_circle  moralid1  moralid10  moralid2  moralid3  moralid4  moralid5  moralid6  moralid7  moralid8  moralid9  mcoop4  mcoop5  narc1  narc2  narc3  narc4  narc5  narc6  nidentity1  nidentity2  omind4  omind5  omind6  contact1  contact3  contact4  hygiene1  hygiene2  hygiene4  hygiene5  psupport1  psupport2  psupport3  psupport5  political_ideology  happy  slf_ladder  riskperc1  riskperc2  self_esteem  sex1  sbelong1  sbelong2  tested_positive  slfcont1  slfcont2
##   9   10  age  children  cnarc2  cnarc3  ctheory1  ctheory3  ctheory4  CRT1  CRT2  CRT3  employ_status1  generosity1  generosity2  generosity3  health_cond  know_tested_positive  ladder  marital1  mor_circle  moralid1  moralid10  moralid2  moralid3  moralid4  moralid5  moralid6  moralid7  moralid8  moralid9  mcoop4  mcoop5  narc1  narc2  narc3  narc4  narc5  narc6  nidentity1  nidentity2  omind4  omind5  omind6  contact1  contact3  contact4  hygiene1  hygiene2  hygiene4  hygiene5  psupport1  psupport2  psupport3  psupport5  political_ideology  happy  slf_ladder  riskperc1  riskperc2  self_esteem  sex1  sbelong1  sbelong2  tested_positive  slfcont1  slfcont2
##   10   1  age  children  cnarc2  cnarc3  ctheory1  ctheory3  ctheory4  CRT1  CRT2  CRT3  employ_status1  generosity1  generosity2  generosity3  health_cond  know_tested_positive  ladder  marital1  mor_circle  moralid1  moralid10  moralid2  moralid3  moralid4  moralid5  moralid6  moralid7  moralid8  moralid9  mcoop4  mcoop5  narc1  narc2  narc3  narc4  narc5  narc6  nidentity1  nidentity2  omind4  omind5  omind6  contact1  contact3  contact4  hygiene1  hygiene2  hygiene4  hygiene5  psupport1  psupport2  psupport3  psupport5  political_ideology  happy  slf_ladder  riskperc1  riskperc2  self_esteem  sex1  sbelong1  sbelong2  tested_positive  slfcont1  slfcont2
##   10   2  age  children  cnarc2  cnarc3  ctheory1  ctheory3  ctheory4  CRT1  CRT2  CRT3  employ_status1  generosity1  generosity2  generosity3  health_cond  know_tested_positive  ladder  marital1  mor_circle  moralid1  moralid10  moralid2  moralid3  moralid4  moralid5  moralid6  moralid7  moralid8  moralid9  mcoop4  mcoop5  narc1  narc2  narc3  narc4  narc5  narc6  nidentity1  nidentity2  omind4  omind5  omind6  contact1  contact3  contact4  hygiene1  hygiene2  hygiene4  hygiene5  psupport1  psupport2  psupport3  psupport5  political_ideology  happy  slf_ladder  riskperc1  riskperc2  self_esteem  sex1  sbelong1  sbelong2  tested_positive  slfcont1  slfcont2
##   10   3  age  children  cnarc2  cnarc3  ctheory1  ctheory3  ctheory4  CRT1  CRT2  CRT3  employ_status1  generosity1  generosity2  generosity3  health_cond  know_tested_positive  ladder  marital1  mor_circle  moralid1  moralid10  moralid2  moralid3  moralid4  moralid5  moralid6  moralid7  moralid8  moralid9  mcoop4  mcoop5  narc1  narc2  narc3  narc4  narc5  narc6  nidentity1  nidentity2  omind4  omind5  omind6  contact1  contact3  contact4  hygiene1  hygiene2  hygiene4  hygiene5  psupport1  psupport2  psupport3  psupport5  political_ideology  happy  slf_ladder  riskperc1  riskperc2  self_esteem  sex1  sbelong1  sbelong2  tested_positive  slfcont1  slfcont2
##   10   4  age  children  cnarc2  cnarc3  ctheory1  ctheory3  ctheory4  CRT1  CRT2  CRT3  employ_status1  generosity1  generosity2  generosity3  health_cond  know_tested_positive  ladder  marital1  mor_circle  moralid1  moralid10  moralid2  moralid3  moralid4  moralid5  moralid6  moralid7  moralid8  moralid9  mcoop4  mcoop5  narc1  narc2  narc3  narc4  narc5  narc6  nidentity1  nidentity2  omind4  omind5  omind6  contact1  contact3  contact4  hygiene1  hygiene2  hygiene4  hygiene5  psupport1  psupport2  psupport3  psupport5  political_ideology  happy  slf_ladder  riskperc1  riskperc2  self_esteem  sex1  sbelong1  sbelong2  tested_positive  slfcont1  slfcont2
##   10   5  age  children  cnarc2  cnarc3  ctheory1  ctheory3  ctheory4  CRT1  CRT2  CRT3  employ_status1  generosity1  generosity2  generosity3  health_cond  know_tested_positive  ladder  marital1  mor_circle  moralid1  moralid10  moralid2  moralid3  moralid4  moralid5  moralid6  moralid7  moralid8  moralid9  mcoop4  mcoop5  narc1  narc2  narc3  narc4  narc5  narc6  nidentity1  nidentity2  omind4  omind5  omind6  contact1  contact3  contact4  hygiene1  hygiene2  hygiene4  hygiene5  psupport1  psupport2  psupport3  psupport5  political_ideology  happy  slf_ladder  riskperc1  riskperc2  self_esteem  sex1  sbelong1  sbelong2  tested_positive  slfcont1  slfcont2
##   10   6  age  children  cnarc2  cnarc3  ctheory1  ctheory3  ctheory4  CRT1  CRT2  CRT3  employ_status1  generosity1  generosity2  generosity3  health_cond  know_tested_positive  ladder  marital1  mor_circle  moralid1  moralid10  moralid2  moralid3  moralid4  moralid5  moralid6  moralid7  moralid8  moralid9  mcoop4  mcoop5  narc1  narc2  narc3  narc4  narc5  narc6  nidentity1  nidentity2  omind4  omind5  omind6  contact1  contact3  contact4  hygiene1  hygiene2  hygiene4  hygiene5  psupport1  psupport2  psupport3  psupport5  political_ideology  happy  slf_ladder  riskperc1  riskperc2  self_esteem  sex1  sbelong1  sbelong2  tested_positive  slfcont1  slfcont2
##   10   7  age  children  cnarc2  cnarc3  ctheory1  ctheory3  ctheory4  CRT1  CRT2  CRT3  employ_status1  generosity1  generosity2  generosity3  health_cond  know_tested_positive  ladder  marital1  mor_circle  moralid1  moralid10  moralid2  moralid3  moralid4  moralid5  moralid6  moralid7  moralid8  moralid9  mcoop4  mcoop5  narc1  narc2  narc3  narc4  narc5  narc6  nidentity1  nidentity2  omind4  omind5  omind6  contact1  contact3  contact4  hygiene1  hygiene2  hygiene4  hygiene5  psupport1  psupport2  psupport3  psupport5  political_ideology  happy  slf_ladder  riskperc1  riskperc2  self_esteem  sex1  sbelong1  sbelong2  tested_positive  slfcont1  slfcont2
##   10   8  age  children  cnarc2  cnarc3  ctheory1  ctheory3  ctheory4  CRT1  CRT2  CRT3  employ_status1  generosity1  generosity2  generosity3  health_cond  know_tested_positive  ladder  marital1  mor_circle  moralid1  moralid10  moralid2  moralid3  moralid4  moralid5  moralid6  moralid7  moralid8  moralid9  mcoop4  mcoop5  narc1  narc2  narc3  narc4  narc5  narc6  nidentity1  nidentity2  omind4  omind5  omind6  contact1  contact3  contact4  hygiene1  hygiene2  hygiene4  hygiene5  psupport1  psupport2  psupport3  psupport5  political_ideology  happy  slf_ladder  riskperc1  riskperc2  self_esteem  sex1  sbelong1  sbelong2  tested_positive  slfcont1  slfcont2
##   10   9  age  children  cnarc2  cnarc3  ctheory1  ctheory3  ctheory4  CRT1  CRT2  CRT3  employ_status1  generosity1  generosity2  generosity3  health_cond  know_tested_positive  ladder  marital1  mor_circle  moralid1  moralid10  moralid2  moralid3  moralid4  moralid5  moralid6  moralid7  moralid8  moralid9  mcoop4  mcoop5  narc1  narc2  narc3  narc4  narc5  narc6  nidentity1  nidentity2  omind4  omind5  omind6  contact1  contact3  contact4  hygiene1  hygiene2  hygiene4  hygiene5  psupport1  psupport2  psupport3  psupport5  political_ideology  happy  slf_ladder  riskperc1  riskperc2  self_esteem  sex1  sbelong1  sbelong2  tested_positive  slfcont1  slfcont2
##   10   10  age  children  cnarc2  cnarc3  ctheory1  ctheory3  ctheory4  CRT1  CRT2  CRT3  employ_status1  generosity1  generosity2  generosity3  health_cond  know_tested_positive  ladder  marital1  mor_circle  moralid1  moralid10  moralid2  moralid3  moralid4  moralid5  moralid6  moralid7  moralid8  moralid9  mcoop4  mcoop5  narc1  narc2  narc3  narc4  narc5  narc6  nidentity1  nidentity2  omind4  omind5  omind6  contact1  contact3  contact4  hygiene1  hygiene2  hygiene4  hygiene5  psupport1  psupport2  psupport3  psupport5  political_ideology  happy  slf_ladder  riskperc1  riskperc2  self_esteem  sex1  sbelong1  sbelong2  tested_positive  slfcont1  slfcont2
```

```
## Warning: Number of logged events: 6395
```

```
## 
##  iter imp variable
##   1   1  age  children  cnarc1  cnarc2  cnarc3  ctheory1  ctheory2  ctheory3  ctheory4  CRT1  CRT2  CRT3  duration  generosity1  generosity2  generosity3  know_tested_positive  ladder  marital1  mor_circle  moralid1  moralid10  moralid2  moralid3  moralid4  moralid5  moralid6  moralid7  moralid8  moralid9  mcoop1  mcoop2  mcoop3  mcoop4  mcoop5  mcoop6  mcoop7  narc1  narc2  narc3  narc4  narc5  narc6  nidentity1  nidentity2  omind1  omind4  omind5  omind6  contact3  hygiene1  psupport3  political_ideology  happy  riskperc1  riskperc2  self_esteem  sbelong1  sbelong2  sbelong3  sbelong4  optim1  optim2  slfcont1  slfcont2  slfcont3  slfcont4  urban
##   1   2  age  children  cnarc1  cnarc2  cnarc3  ctheory1  ctheory2  ctheory3  ctheory4  CRT1  CRT2  CRT3  duration  generosity1  generosity2  generosity3  know_tested_positive  ladder  marital1  mor_circle  moralid1  moralid10  moralid2  moralid3  moralid4  moralid5  moralid6  moralid7  moralid8  moralid9  mcoop1  mcoop2  mcoop3  mcoop4  mcoop5  mcoop6  mcoop7  narc1  narc2  narc3  narc4  narc5  narc6  nidentity1  nidentity2  omind1  omind4  omind5  omind6  contact3  hygiene1  psupport3  political_ideology  happy  riskperc1  riskperc2  self_esteem  sbelong1  sbelong2  sbelong3  sbelong4  optim1  optim2  slfcont1  slfcont2  slfcont3  slfcont4  urban
##   1   3  age  children  cnarc1  cnarc2  cnarc3  ctheory1  ctheory2  ctheory3  ctheory4  CRT1  CRT2  CRT3  duration  generosity1  generosity2  generosity3  know_tested_positive  ladder  marital1  mor_circle  moralid1  moralid10  moralid2  moralid3  moralid4  moralid5  moralid6  moralid7  moralid8  moralid9  mcoop1  mcoop2  mcoop3  mcoop4  mcoop5  mcoop6  mcoop7  narc1  narc2  narc3  narc4  narc5  narc6  nidentity1  nidentity2  omind1  omind4  omind5  omind6  contact3  hygiene1  psupport3  political_ideology  happy  riskperc1  riskperc2  self_esteem  sbelong1  sbelong2  sbelong3  sbelong4  optim1  optim2  slfcont1  slfcont2  slfcont3  slfcont4  urban
##   1   4  age  children  cnarc1  cnarc2  cnarc3  ctheory1  ctheory2  ctheory3  ctheory4  CRT1  CRT2  CRT3  duration  generosity1  generosity2  generosity3  know_tested_positive  ladder  marital1  mor_circle  moralid1  moralid10  moralid2  moralid3  moralid4  moralid5  moralid6  moralid7  moralid8  moralid9  mcoop1  mcoop2  mcoop3  mcoop4  mcoop5  mcoop6  mcoop7  narc1  narc2  narc3  narc4  narc5  narc6  nidentity1  nidentity2  omind1  omind4  omind5  omind6  contact3  hygiene1  psupport3  political_ideology  happy  riskperc1  riskperc2  self_esteem  sbelong1  sbelong2  sbelong3  sbelong4  optim1  optim2  slfcont1  slfcont2  slfcont3  slfcont4  urban
##   1   5  age  children  cnarc1  cnarc2  cnarc3  ctheory1  ctheory2  ctheory3  ctheory4  CRT1  CRT2  CRT3  duration  generosity1  generosity2  generosity3  know_tested_positive  ladder  marital1  mor_circle  moralid1  moralid10  moralid2  moralid3  moralid4  moralid5  moralid6  moralid7  moralid8  moralid9  mcoop1  mcoop2  mcoop3  mcoop4  mcoop5  mcoop6  mcoop7  narc1  narc2  narc3  narc4  narc5  narc6  nidentity1  nidentity2  omind1  omind4  omind5  omind6  contact3  hygiene1  psupport3  political_ideology  happy  riskperc1  riskperc2  self_esteem  sbelong1  sbelong2  sbelong3  sbelong4  optim1  optim2  slfcont1  slfcont2  slfcont3  slfcont4  urban
##   1   6  age  children  cnarc1  cnarc2  cnarc3  ctheory1  ctheory2  ctheory3  ctheory4  CRT1  CRT2  CRT3  duration  generosity1  generosity2  generosity3  know_tested_positive  ladder  marital1  mor_circle  moralid1  moralid10  moralid2  moralid3  moralid4  moralid5  moralid6  moralid7  moralid8  moralid9  mcoop1  mcoop2  mcoop3  mcoop4  mcoop5  mcoop6  mcoop7  narc1  narc2  narc3  narc4  narc5  narc6  nidentity1  nidentity2  omind1  omind4  omind5  omind6  contact3  hygiene1  psupport3  political_ideology  happy  riskperc1  riskperc2  self_esteem  sbelong1  sbelong2  sbelong3  sbelong4  optim1  optim2  slfcont1  slfcont2  slfcont3  slfcont4  urban
##   1   7  age  children  cnarc1  cnarc2  cnarc3  ctheory1  ctheory2  ctheory3  ctheory4  CRT1  CRT2  CRT3  duration  generosity1  generosity2  generosity3  know_tested_positive  ladder  marital1  mor_circle  moralid1  moralid10  moralid2  moralid3  moralid4  moralid5  moralid6  moralid7  moralid8  moralid9  mcoop1  mcoop2  mcoop3  mcoop4  mcoop5  mcoop6  mcoop7  narc1  narc2  narc3  narc4  narc5  narc6  nidentity1  nidentity2  omind1  omind4  omind5  omind6  contact3  hygiene1  psupport3  political_ideology  happy  riskperc1  riskperc2  self_esteem  sbelong1  sbelong2  sbelong3  sbelong4  optim1  optim2  slfcont1  slfcont2  slfcont3  slfcont4  urban
##   1   8  age  children  cnarc1  cnarc2  cnarc3  ctheory1  ctheory2  ctheory3  ctheory4  CRT1  CRT2  CRT3  duration  generosity1  generosity2  generosity3  know_tested_positive  ladder  marital1  mor_circle  moralid1  moralid10  moralid2  moralid3  moralid4  moralid5  moralid6  moralid7  moralid8  moralid9  mcoop1  mcoop2  mcoop3  mcoop4  mcoop5  mcoop6  mcoop7  narc1  narc2  narc3  narc4  narc5  narc6  nidentity1  nidentity2  omind1  omind4  omind5  omind6  contact3  hygiene1  psupport3  political_ideology  happy  riskperc1  riskperc2  self_esteem  sbelong1  sbelong2  sbelong3  sbelong4  optim1  optim2  slfcont1  slfcont2  slfcont3  slfcont4  urban
##   1   9  age  children  cnarc1  cnarc2  cnarc3  ctheory1  ctheory2  ctheory3  ctheory4  CRT1  CRT2  CRT3  duration  generosity1  generosity2  generosity3  know_tested_positive  ladder  marital1  mor_circle  moralid1  moralid10  moralid2  moralid3  moralid4  moralid5  moralid6  moralid7  moralid8  moralid9  mcoop1  mcoop2  mcoop3  mcoop4  mcoop5  mcoop6  mcoop7  narc1  narc2  narc3  narc4  narc5  narc6  nidentity1  nidentity2  omind1  omind4  omind5  omind6  contact3  hygiene1  psupport3  political_ideology  happy  riskperc1  riskperc2  self_esteem  sbelong1  sbelong2  sbelong3  sbelong4  optim1  optim2  slfcont1  slfcont2  slfcont3  slfcont4  urban
##   1   10  age  children  cnarc1  cnarc2  cnarc3  ctheory1  ctheory2  ctheory3  ctheory4  CRT1  CRT2  CRT3  duration  generosity1  generosity2  generosity3  know_tested_positive  ladder  marital1  mor_circle  moralid1  moralid10  moralid2  moralid3  moralid4  moralid5  moralid6  moralid7  moralid8  moralid9  mcoop1  mcoop2  mcoop3  mcoop4  mcoop5  mcoop6  mcoop7  narc1  narc2  narc3  narc4  narc5  narc6  nidentity1  nidentity2  omind1  omind4  omind5  omind6  contact3  hygiene1  psupport3  political_ideology  happy  riskperc1  riskperc2  self_esteem  sbelong1  sbelong2  sbelong3  sbelong4  optim1  optim2  slfcont1  slfcont2  slfcont3  slfcont4  urban
##   2   1  age  children  cnarc1  cnarc2  cnarc3  ctheory1  ctheory2  ctheory3  ctheory4  CRT1  CRT2  CRT3  duration  generosity1  generosity2  generosity3  know_tested_positive  ladder  marital1  mor_circle  moralid1  moralid10  moralid2  moralid3  moralid4  moralid5  moralid6  moralid7  moralid8  moralid9  mcoop1  mcoop2  mcoop3  mcoop4  mcoop5  mcoop6  mcoop7  narc1  narc2  narc3  narc4  narc5  narc6  nidentity1  nidentity2  omind1  omind4  omind5  omind6  contact3  hygiene1  psupport3  political_ideology  happy  riskperc1  riskperc2  self_esteem  sbelong1  sbelong2  sbelong3  sbelong4  optim1  optim2  slfcont1  slfcont2  slfcont3  slfcont4  urban
##   2   2  age  children  cnarc1  cnarc2  cnarc3  ctheory1  ctheory2  ctheory3  ctheory4  CRT1  CRT2  CRT3  duration  generosity1  generosity2  generosity3  know_tested_positive  ladder  marital1  mor_circle  moralid1  moralid10  moralid2  moralid3  moralid4  moralid5  moralid6  moralid7  moralid8  moralid9  mcoop1  mcoop2  mcoop3  mcoop4  mcoop5  mcoop6  mcoop7  narc1  narc2  narc3  narc4  narc5  narc6  nidentity1  nidentity2  omind1  omind4  omind5  omind6  contact3  hygiene1  psupport3  political_ideology  happy  riskperc1  riskperc2  self_esteem  sbelong1  sbelong2  sbelong3  sbelong4  optim1  optim2  slfcont1  slfcont2  slfcont3  slfcont4  urban
##   2   3  age  children  cnarc1  cnarc2  cnarc3  ctheory1  ctheory2  ctheory3  ctheory4  CRT1  CRT2  CRT3  duration  generosity1  generosity2  generosity3  know_tested_positive  ladder  marital1  mor_circle  moralid1  moralid10  moralid2  moralid3  moralid4  moralid5  moralid6  moralid7  moralid8  moralid9  mcoop1  mcoop2  mcoop3  mcoop4  mcoop5  mcoop6  mcoop7  narc1  narc2  narc3  narc4  narc5  narc6  nidentity1  nidentity2  omind1  omind4  omind5  omind6  contact3  hygiene1  psupport3  political_ideology  happy  riskperc1  riskperc2  self_esteem  sbelong1  sbelong2  sbelong3  sbelong4  optim1  optim2  slfcont1  slfcont2  slfcont3  slfcont4  urban
##   2   4  age  children  cnarc1  cnarc2  cnarc3  ctheory1  ctheory2  ctheory3  ctheory4  CRT1  CRT2  CRT3  duration  generosity1  generosity2  generosity3  know_tested_positive  ladder  marital1  mor_circle  moralid1  moralid10  moralid2  moralid3  moralid4  moralid5  moralid6  moralid7  moralid8  moralid9  mcoop1  mcoop2  mcoop3  mcoop4  mcoop5  mcoop6  mcoop7  narc1  narc2  narc3  narc4  narc5  narc6  nidentity1  nidentity2  omind1  omind4  omind5  omind6  contact3  hygiene1  psupport3  political_ideology  happy  riskperc1  riskperc2  self_esteem  sbelong1  sbelong2  sbelong3  sbelong4  optim1  optim2  slfcont1  slfcont2  slfcont3  slfcont4  urban
##   2   5  age  children  cnarc1  cnarc2  cnarc3  ctheory1  ctheory2  ctheory3  ctheory4  CRT1  CRT2  CRT3  duration  generosity1  generosity2  generosity3  know_tested_positive  ladder  marital1  mor_circle  moralid1  moralid10  moralid2  moralid3  moralid4  moralid5  moralid6  moralid7  moralid8  moralid9  mcoop1  mcoop2  mcoop3  mcoop4  mcoop5  mcoop6  mcoop7  narc1  narc2  narc3  narc4  narc5  narc6  nidentity1  nidentity2  omind1  omind4  omind5  omind6  contact3  hygiene1  psupport3  political_ideology  happy  riskperc1  riskperc2  self_esteem  sbelong1  sbelong2  sbelong3  sbelong4  optim1  optim2  slfcont1  slfcont2  slfcont3  slfcont4  urban
##   2   6  age  children  cnarc1  cnarc2  cnarc3  ctheory1  ctheory2  ctheory3  ctheory4  CRT1  CRT2  CRT3  duration  generosity1  generosity2  generosity3  know_tested_positive  ladder  marital1  mor_circle  moralid1  moralid10  moralid2  moralid3  moralid4  moralid5  moralid6  moralid7  moralid8  moralid9  mcoop1  mcoop2  mcoop3  mcoop4  mcoop5  mcoop6  mcoop7  narc1  narc2  narc3  narc4  narc5  narc6  nidentity1  nidentity2  omind1  omind4  omind5  omind6  contact3  hygiene1  psupport3  political_ideology  happy  riskperc1  riskperc2  self_esteem  sbelong1  sbelong2  sbelong3  sbelong4  optim1  optim2  slfcont1  slfcont2  slfcont3  slfcont4  urban
##   2   7  age  children  cnarc1  cnarc2  cnarc3  ctheory1  ctheory2  ctheory3  ctheory4  CRT1  CRT2  CRT3  duration  generosity1  generosity2  generosity3  know_tested_positive  ladder  marital1  mor_circle  moralid1  moralid10  moralid2  moralid3  moralid4  moralid5  moralid6  moralid7  moralid8  moralid9  mcoop1  mcoop2  mcoop3  mcoop4  mcoop5  mcoop6  mcoop7  narc1  narc2  narc3  narc4  narc5  narc6  nidentity1  nidentity2  omind1  omind4  omind5  omind6  contact3  hygiene1  psupport3  political_ideology  happy  riskperc1  riskperc2  self_esteem  sbelong1  sbelong2  sbelong3  sbelong4  optim1  optim2  slfcont1  slfcont2  slfcont3  slfcont4  urban
##   2   8  age  children  cnarc1  cnarc2  cnarc3  ctheory1  ctheory2  ctheory3  ctheory4  CRT1  CRT2  CRT3  duration  generosity1  generosity2  generosity3  know_tested_positive  ladder  marital1  mor_circle  moralid1  moralid10  moralid2  moralid3  moralid4  moralid5  moralid6  moralid7  moralid8  moralid9  mcoop1  mcoop2  mcoop3  mcoop4  mcoop5  mcoop6  mcoop7  narc1  narc2  narc3  narc4  narc5  narc6  nidentity1  nidentity2  omind1  omind4  omind5  omind6  contact3  hygiene1  psupport3  political_ideology  happy  riskperc1  riskperc2  self_esteem  sbelong1  sbelong2  sbelong3  sbelong4  optim1  optim2  slfcont1  slfcont2  slfcont3  slfcont4  urban
##   2   9  age  children  cnarc1  cnarc2  cnarc3  ctheory1  ctheory2  ctheory3  ctheory4  CRT1  CRT2  CRT3  duration  generosity1  generosity2  generosity3  know_tested_positive  ladder  marital1  mor_circle  moralid1  moralid10  moralid2  moralid3  moralid4  moralid5  moralid6  moralid7  moralid8  moralid9  mcoop1  mcoop2  mcoop3  mcoop4  mcoop5  mcoop6  mcoop7  narc1  narc2  narc3  narc4  narc5  narc6  nidentity1  nidentity2  omind1  omind4  omind5  omind6  contact3  hygiene1  psupport3  political_ideology  happy  riskperc1  riskperc2  self_esteem  sbelong1  sbelong2  sbelong3  sbelong4  optim1  optim2  slfcont1  slfcont2  slfcont3  slfcont4  urban
##   2   10  age  children  cnarc1  cnarc2  cnarc3  ctheory1  ctheory2  ctheory3  ctheory4  CRT1  CRT2  CRT3  duration  generosity1  generosity2  generosity3  know_tested_positive  ladder  marital1  mor_circle  moralid1  moralid10  moralid2  moralid3  moralid4  moralid5  moralid6  moralid7  moralid8  moralid9  mcoop1  mcoop2  mcoop3  mcoop4  mcoop5  mcoop6  mcoop7  narc1  narc2  narc3  narc4  narc5  narc6  nidentity1  nidentity2  omind1  omind4  omind5  omind6  contact3  hygiene1  psupport3  political_ideology  happy  riskperc1  riskperc2  self_esteem  sbelong1  sbelong2  sbelong3  sbelong4  optim1  optim2  slfcont1  slfcont2  slfcont3  slfcont4  urban
##   3   1  age  children  cnarc1  cnarc2  cnarc3  ctheory1  ctheory2  ctheory3  ctheory4  CRT1  CRT2  CRT3  duration  generosity1  generosity2  generosity3  know_tested_positive  ladder  marital1  mor_circle  moralid1  moralid10  moralid2  moralid3  moralid4  moralid5  moralid6  moralid7  moralid8  moralid9  mcoop1  mcoop2  mcoop3  mcoop4  mcoop5  mcoop6  mcoop7  narc1  narc2  narc3  narc4  narc5  narc6  nidentity1  nidentity2  omind1  omind4  omind5  omind6  contact3  hygiene1  psupport3  political_ideology  happy  riskperc1  riskperc2  self_esteem  sbelong1  sbelong2  sbelong3  sbelong4  optim1  optim2  slfcont1  slfcont2  slfcont3  slfcont4  urban
##   3   2  age  children  cnarc1  cnarc2  cnarc3  ctheory1  ctheory2  ctheory3  ctheory4  CRT1  CRT2  CRT3  duration  generosity1  generosity2  generosity3  know_tested_positive  ladder  marital1  mor_circle  moralid1  moralid10  moralid2  moralid3  moralid4  moralid5  moralid6  moralid7  moralid8  moralid9  mcoop1  mcoop2  mcoop3  mcoop4  mcoop5  mcoop6  mcoop7  narc1  narc2  narc3  narc4  narc5  narc6  nidentity1  nidentity2  omind1  omind4  omind5  omind6  contact3  hygiene1  psupport3  political_ideology  happy  riskperc1  riskperc2  self_esteem  sbelong1  sbelong2  sbelong3  sbelong4  optim1  optim2  slfcont1  slfcont2  slfcont3  slfcont4  urban
##   3   3  age  children  cnarc1  cnarc2  cnarc3  ctheory1  ctheory2  ctheory3  ctheory4  CRT1  CRT2  CRT3  duration  generosity1  generosity2  generosity3  know_tested_positive  ladder  marital1  mor_circle  moralid1  moralid10  moralid2  moralid3  moralid4  moralid5  moralid6  moralid7  moralid8  moralid9  mcoop1  mcoop2  mcoop3  mcoop4  mcoop5  mcoop6  mcoop7  narc1  narc2  narc3  narc4  narc5  narc6  nidentity1  nidentity2  omind1  omind4  omind5  omind6  contact3  hygiene1  psupport3  political_ideology  happy  riskperc1  riskperc2  self_esteem  sbelong1  sbelong2  sbelong3  sbelong4  optim1  optim2  slfcont1  slfcont2  slfcont3  slfcont4  urban
##   3   4  age  children  cnarc1  cnarc2  cnarc3  ctheory1  ctheory2  ctheory3  ctheory4  CRT1  CRT2  CRT3  duration  generosity1  generosity2  generosity3  know_tested_positive  ladder  marital1  mor_circle  moralid1  moralid10  moralid2  moralid3  moralid4  moralid5  moralid6  moralid7  moralid8  moralid9  mcoop1  mcoop2  mcoop3  mcoop4  mcoop5  mcoop6  mcoop7  narc1  narc2  narc3  narc4  narc5  narc6  nidentity1  nidentity2  omind1  omind4  omind5  omind6  contact3  hygiene1  psupport3  political_ideology  happy  riskperc1  riskperc2  self_esteem  sbelong1  sbelong2  sbelong3  sbelong4  optim1  optim2  slfcont1  slfcont2  slfcont3  slfcont4  urban
##   3   5  age  children  cnarc1  cnarc2  cnarc3  ctheory1  ctheory2  ctheory3  ctheory4  CRT1  CRT2  CRT3  duration  generosity1  generosity2  generosity3  know_tested_positive  ladder  marital1  mor_circle  moralid1  moralid10  moralid2  moralid3  moralid4  moralid5  moralid6  moralid7  moralid8  moralid9  mcoop1  mcoop2  mcoop3  mcoop4  mcoop5  mcoop6  mcoop7  narc1  narc2  narc3  narc4  narc5  narc6  nidentity1  nidentity2  omind1  omind4  omind5  omind6  contact3  hygiene1  psupport3  political_ideology  happy  riskperc1  riskperc2  self_esteem  sbelong1  sbelong2  sbelong3  sbelong4  optim1  optim2  slfcont1  slfcont2  slfcont3  slfcont4  urban
##   3   6  age  children  cnarc1  cnarc2  cnarc3  ctheory1  ctheory2  ctheory3  ctheory4  CRT1  CRT2  CRT3  duration  generosity1  generosity2  generosity3  know_tested_positive  ladder  marital1  mor_circle  moralid1  moralid10  moralid2  moralid3  moralid4  moralid5  moralid6  moralid7  moralid8  moralid9  mcoop1  mcoop2  mcoop3  mcoop4  mcoop5  mcoop6  mcoop7  narc1  narc2  narc3  narc4  narc5  narc6  nidentity1  nidentity2  omind1  omind4  omind5  omind6  contact3  hygiene1  psupport3  political_ideology  happy  riskperc1  riskperc2  self_esteem  sbelong1  sbelong2  sbelong3  sbelong4  optim1  optim2  slfcont1  slfcont2  slfcont3  slfcont4  urban
##   3   7  age  children  cnarc1  cnarc2  cnarc3  ctheory1  ctheory2  ctheory3  ctheory4  CRT1  CRT2  CRT3  duration  generosity1  generosity2  generosity3  know_tested_positive  ladder  marital1  mor_circle  moralid1  moralid10  moralid2  moralid3  moralid4  moralid5  moralid6  moralid7  moralid8  moralid9  mcoop1  mcoop2  mcoop3  mcoop4  mcoop5  mcoop6  mcoop7  narc1  narc2  narc3  narc4  narc5  narc6  nidentity1  nidentity2  omind1  omind4  omind5  omind6  contact3  hygiene1  psupport3  political_ideology  happy  riskperc1  riskperc2  self_esteem  sbelong1  sbelong2  sbelong3  sbelong4  optim1  optim2  slfcont1  slfcont2  slfcont3  slfcont4  urban
##   3   8  age  children  cnarc1  cnarc2  cnarc3  ctheory1  ctheory2  ctheory3  ctheory4  CRT1  CRT2  CRT3  duration  generosity1  generosity2  generosity3  know_tested_positive  ladder  marital1  mor_circle  moralid1  moralid10  moralid2  moralid3  moralid4  moralid5  moralid6  moralid7  moralid8  moralid9  mcoop1  mcoop2  mcoop3  mcoop4  mcoop5  mcoop6  mcoop7  narc1  narc2  narc3  narc4  narc5  narc6  nidentity1  nidentity2  omind1  omind4  omind5  omind6  contact3  hygiene1  psupport3  political_ideology  happy  riskperc1  riskperc2  self_esteem  sbelong1  sbelong2  sbelong3  sbelong4  optim1  optim2  slfcont1  slfcont2  slfcont3  slfcont4  urban
##   3   9  age  children  cnarc1  cnarc2  cnarc3  ctheory1  ctheory2  ctheory3  ctheory4  CRT1  CRT2  CRT3  duration  generosity1  generosity2  generosity3  know_tested_positive  ladder  marital1  mor_circle  moralid1  moralid10  moralid2  moralid3  moralid4  moralid5  moralid6  moralid7  moralid8  moralid9  mcoop1  mcoop2  mcoop3  mcoop4  mcoop5  mcoop6  mcoop7  narc1  narc2  narc3  narc4  narc5  narc6  nidentity1  nidentity2  omind1  omind4  omind5  omind6  contact3  hygiene1  psupport3  political_ideology  happy  riskperc1  riskperc2  self_esteem  sbelong1  sbelong2  sbelong3  sbelong4  optim1  optim2  slfcont1  slfcont2  slfcont3  slfcont4  urban
##   3   10  age  children  cnarc1  cnarc2  cnarc3  ctheory1  ctheory2  ctheory3  ctheory4  CRT1  CRT2  CRT3  duration  generosity1  generosity2  generosity3  know_tested_positive  ladder  marital1  mor_circle  moralid1  moralid10  moralid2  moralid3  moralid4  moralid5  moralid6  moralid7  moralid8  moralid9  mcoop1  mcoop2  mcoop3  mcoop4  mcoop5  mcoop6  mcoop7  narc1  narc2  narc3  narc4  narc5  narc6  nidentity1  nidentity2  omind1  omind4  omind5  omind6  contact3  hygiene1  psupport3  political_ideology  happy  riskperc1  riskperc2  self_esteem  sbelong1  sbelong2  sbelong3  sbelong4  optim1  optim2  slfcont1  slfcont2  slfcont3  slfcont4  urban
##   4   1  age  children  cnarc1  cnarc2  cnarc3  ctheory1  ctheory2  ctheory3  ctheory4  CRT1  CRT2  CRT3  duration  generosity1  generosity2  generosity3  know_tested_positive  ladder  marital1  mor_circle  moralid1  moralid10  moralid2  moralid3  moralid4  moralid5  moralid6  moralid7  moralid8  moralid9  mcoop1  mcoop2  mcoop3  mcoop4  mcoop5  mcoop6  mcoop7  narc1  narc2  narc3  narc4  narc5  narc6  nidentity1  nidentity2  omind1  omind4  omind5  omind6  contact3  hygiene1  psupport3  political_ideology  happy  riskperc1  riskperc2  self_esteem  sbelong1  sbelong2  sbelong3  sbelong4  optim1  optim2  slfcont1  slfcont2  slfcont3  slfcont4  urban
##   4   2  age  children  cnarc1  cnarc2  cnarc3  ctheory1  ctheory2  ctheory3  ctheory4  CRT1  CRT2  CRT3  duration  generosity1  generosity2  generosity3  know_tested_positive  ladder  marital1  mor_circle  moralid1  moralid10  moralid2  moralid3  moralid4  moralid5  moralid6  moralid7  moralid8  moralid9  mcoop1  mcoop2  mcoop3  mcoop4  mcoop5  mcoop6  mcoop7  narc1  narc2  narc3  narc4  narc5  narc6  nidentity1  nidentity2  omind1  omind4  omind5  omind6  contact3  hygiene1  psupport3  political_ideology  happy  riskperc1  riskperc2  self_esteem  sbelong1  sbelong2  sbelong3  sbelong4  optim1  optim2  slfcont1  slfcont2  slfcont3  slfcont4  urban
##   4   3  age  children  cnarc1  cnarc2  cnarc3  ctheory1  ctheory2  ctheory3  ctheory4  CRT1  CRT2  CRT3  duration  generosity1  generosity2  generosity3  know_tested_positive  ladder  marital1  mor_circle  moralid1  moralid10  moralid2  moralid3  moralid4  moralid5  moralid6  moralid7  moralid8  moralid9  mcoop1  mcoop2  mcoop3  mcoop4  mcoop5  mcoop6  mcoop7  narc1  narc2  narc3  narc4  narc5  narc6  nidentity1  nidentity2  omind1  omind4  omind5  omind6  contact3  hygiene1  psupport3  political_ideology  happy  riskperc1  riskperc2  self_esteem  sbelong1  sbelong2  sbelong3  sbelong4  optim1  optim2  slfcont1  slfcont2  slfcont3  slfcont4  urban
##   4   4  age  children  cnarc1  cnarc2  cnarc3  ctheory1  ctheory2  ctheory3  ctheory4  CRT1  CRT2  CRT3  duration  generosity1  generosity2  generosity3  know_tested_positive  ladder  marital1  mor_circle  moralid1  moralid10  moralid2  moralid3  moralid4  moralid5  moralid6  moralid7  moralid8  moralid9  mcoop1  mcoop2  mcoop3  mcoop4  mcoop5  mcoop6  mcoop7  narc1  narc2  narc3  narc4  narc5  narc6  nidentity1  nidentity2  omind1  omind4  omind5  omind6  contact3  hygiene1  psupport3  political_ideology  happy  riskperc1  riskperc2  self_esteem  sbelong1  sbelong2  sbelong3  sbelong4  optim1  optim2  slfcont1  slfcont2  slfcont3  slfcont4  urban
##   4   5  age  children  cnarc1  cnarc2  cnarc3  ctheory1  ctheory2  ctheory3  ctheory4  CRT1  CRT2  CRT3  duration  generosity1  generosity2  generosity3  know_tested_positive  ladder  marital1  mor_circle  moralid1  moralid10  moralid2  moralid3  moralid4  moralid5  moralid6  moralid7  moralid8  moralid9  mcoop1  mcoop2  mcoop3  mcoop4  mcoop5  mcoop6  mcoop7  narc1  narc2  narc3  narc4  narc5  narc6  nidentity1  nidentity2  omind1  omind4  omind5  omind6  contact3  hygiene1  psupport3  political_ideology  happy  riskperc1  riskperc2  self_esteem  sbelong1  sbelong2  sbelong3  sbelong4  optim1  optim2  slfcont1  slfcont2  slfcont3  slfcont4  urban
##   4   6  age  children  cnarc1  cnarc2  cnarc3  ctheory1  ctheory2  ctheory3  ctheory4  CRT1  CRT2  CRT3  duration  generosity1  generosity2  generosity3  know_tested_positive  ladder  marital1  mor_circle  moralid1  moralid10  moralid2  moralid3  moralid4  moralid5  moralid6  moralid7  moralid8  moralid9  mcoop1  mcoop2  mcoop3  mcoop4  mcoop5  mcoop6  mcoop7  narc1  narc2  narc3  narc4  narc5  narc6  nidentity1  nidentity2  omind1  omind4  omind5  omind6  contact3  hygiene1  psupport3  political_ideology  happy  riskperc1  riskperc2  self_esteem  sbelong1  sbelong2  sbelong3  sbelong4  optim1  optim2  slfcont1  slfcont2  slfcont3  slfcont4  urban
##   4   7  age  children  cnarc1  cnarc2  cnarc3  ctheory1  ctheory2  ctheory3  ctheory4  CRT1  CRT2  CRT3  duration  generosity1  generosity2  generosity3  know_tested_positive  ladder  marital1  mor_circle  moralid1  moralid10  moralid2  moralid3  moralid4  moralid5  moralid6  moralid7  moralid8  moralid9  mcoop1  mcoop2  mcoop3  mcoop4  mcoop5  mcoop6  mcoop7  narc1  narc2  narc3  narc4  narc5  narc6  nidentity1  nidentity2  omind1  omind4  omind5  omind6  contact3  hygiene1  psupport3  political_ideology  happy  riskperc1  riskperc2  self_esteem  sbelong1  sbelong2  sbelong3  sbelong4  optim1  optim2  slfcont1  slfcont2  slfcont3  slfcont4  urban
##   4   8  age  children  cnarc1  cnarc2  cnarc3  ctheory1  ctheory2  ctheory3  ctheory4  CRT1  CRT2  CRT3  duration  generosity1  generosity2  generosity3  know_tested_positive  ladder  marital1  mor_circle  moralid1  moralid10  moralid2  moralid3  moralid4  moralid5  moralid6  moralid7  moralid8  moralid9  mcoop1  mcoop2  mcoop3  mcoop4  mcoop5  mcoop6  mcoop7  narc1  narc2  narc3  narc4  narc5  narc6  nidentity1  nidentity2  omind1  omind4  omind5  omind6  contact3  hygiene1  psupport3  political_ideology  happy  riskperc1  riskperc2  self_esteem  sbelong1  sbelong2  sbelong3  sbelong4  optim1  optim2  slfcont1  slfcont2  slfcont3  slfcont4  urban
##   4   9  age  children  cnarc1  cnarc2  cnarc3  ctheory1  ctheory2  ctheory3  ctheory4  CRT1  CRT2  CRT3  duration  generosity1  generosity2  generosity3  know_tested_positive  ladder  marital1  mor_circle  moralid1  moralid10  moralid2  moralid3  moralid4  moralid5  moralid6  moralid7  moralid8  moralid9  mcoop1  mcoop2  mcoop3  mcoop4  mcoop5  mcoop6  mcoop7  narc1  narc2  narc3  narc4  narc5  narc6  nidentity1  nidentity2  omind1  omind4  omind5  omind6  contact3  hygiene1  psupport3  political_ideology  happy  riskperc1  riskperc2  self_esteem  sbelong1  sbelong2  sbelong3  sbelong4  optim1  optim2  slfcont1  slfcont2  slfcont3  slfcont4  urban
##   4   10  age  children  cnarc1  cnarc2  cnarc3  ctheory1  ctheory2  ctheory3  ctheory4  CRT1  CRT2  CRT3  duration  generosity1  generosity2  generosity3  know_tested_positive  ladder  marital1  mor_circle  moralid1  moralid10  moralid2  moralid3  moralid4  moralid5  moralid6  moralid7  moralid8  moralid9  mcoop1  mcoop2  mcoop3  mcoop4  mcoop5  mcoop6  mcoop7  narc1  narc2  narc3  narc4  narc5  narc6  nidentity1  nidentity2  omind1  omind4  omind5  omind6  contact3  hygiene1  psupport3  political_ideology  happy  riskperc1  riskperc2  self_esteem  sbelong1  sbelong2  sbelong3  sbelong4  optim1  optim2  slfcont1  slfcont2  slfcont3  slfcont4  urban
##   5   1  age  children  cnarc1  cnarc2  cnarc3  ctheory1  ctheory2  ctheory3  ctheory4  CRT1  CRT2  CRT3  duration  generosity1  generosity2  generosity3  know_tested_positive  ladder  marital1  mor_circle  moralid1  moralid10  moralid2  moralid3  moralid4  moralid5  moralid6  moralid7  moralid8  moralid9  mcoop1  mcoop2  mcoop3  mcoop4  mcoop5  mcoop6  mcoop7  narc1  narc2  narc3  narc4  narc5  narc6  nidentity1  nidentity2  omind1  omind4  omind5  omind6  contact3  hygiene1  psupport3  political_ideology  happy  riskperc1  riskperc2  self_esteem  sbelong1  sbelong2  sbelong3  sbelong4  optim1  optim2  slfcont1  slfcont2  slfcont3  slfcont4  urban
##   5   2  age  children  cnarc1  cnarc2  cnarc3  ctheory1  ctheory2  ctheory3  ctheory4  CRT1  CRT2  CRT3  duration  generosity1  generosity2  generosity3  know_tested_positive  ladder  marital1  mor_circle  moralid1  moralid10  moralid2  moralid3  moralid4  moralid5  moralid6  moralid7  moralid8  moralid9  mcoop1  mcoop2  mcoop3  mcoop4  mcoop5  mcoop6  mcoop7  narc1  narc2  narc3  narc4  narc5  narc6  nidentity1  nidentity2  omind1  omind4  omind5  omind6  contact3  hygiene1  psupport3  political_ideology  happy  riskperc1  riskperc2  self_esteem  sbelong1  sbelong2  sbelong3  sbelong4  optim1  optim2  slfcont1  slfcont2  slfcont3  slfcont4  urban
##   5   3  age  children  cnarc1  cnarc2  cnarc3  ctheory1  ctheory2  ctheory3  ctheory4  CRT1  CRT2  CRT3  duration  generosity1  generosity2  generosity3  know_tested_positive  ladder  marital1  mor_circle  moralid1  moralid10  moralid2  moralid3  moralid4  moralid5  moralid6  moralid7  moralid8  moralid9  mcoop1  mcoop2  mcoop3  mcoop4  mcoop5  mcoop6  mcoop7  narc1  narc2  narc3  narc4  narc5  narc6  nidentity1  nidentity2  omind1  omind4  omind5  omind6  contact3  hygiene1  psupport3  political_ideology  happy  riskperc1  riskperc2  self_esteem  sbelong1  sbelong2  sbelong3  sbelong4  optim1  optim2  slfcont1  slfcont2  slfcont3  slfcont4  urban
##   5   4  age  children  cnarc1  cnarc2  cnarc3  ctheory1  ctheory2  ctheory3  ctheory4  CRT1  CRT2  CRT3  duration  generosity1  generosity2  generosity3  know_tested_positive  ladder  marital1  mor_circle  moralid1  moralid10  moralid2  moralid3  moralid4  moralid5  moralid6  moralid7  moralid8  moralid9  mcoop1  mcoop2  mcoop3  mcoop4  mcoop5  mcoop6  mcoop7  narc1  narc2  narc3  narc4  narc5  narc6  nidentity1  nidentity2  omind1  omind4  omind5  omind6  contact3  hygiene1  psupport3  political_ideology  happy  riskperc1  riskperc2  self_esteem  sbelong1  sbelong2  sbelong3  sbelong4  optim1  optim2  slfcont1  slfcont2  slfcont3  slfcont4  urban
##   5   5  age  children  cnarc1  cnarc2  cnarc3  ctheory1  ctheory2  ctheory3  ctheory4  CRT1  CRT2  CRT3  duration  generosity1  generosity2  generosity3  know_tested_positive  ladder  marital1  mor_circle  moralid1  moralid10  moralid2  moralid3  moralid4  moralid5  moralid6  moralid7  moralid8  moralid9  mcoop1  mcoop2  mcoop3  mcoop4  mcoop5  mcoop6  mcoop7  narc1  narc2  narc3  narc4  narc5  narc6  nidentity1  nidentity2  omind1  omind4  omind5  omind6  contact3  hygiene1  psupport3  political_ideology  happy  riskperc1  riskperc2  self_esteem  sbelong1  sbelong2  sbelong3  sbelong4  optim1  optim2  slfcont1  slfcont2  slfcont3  slfcont4  urban
##   5   6  age  children  cnarc1  cnarc2  cnarc3  ctheory1  ctheory2  ctheory3  ctheory4  CRT1  CRT2  CRT3  duration  generosity1  generosity2  generosity3  know_tested_positive  ladder  marital1  mor_circle  moralid1  moralid10  moralid2  moralid3  moralid4  moralid5  moralid6  moralid7  moralid8  moralid9  mcoop1  mcoop2  mcoop3  mcoop4  mcoop5  mcoop6  mcoop7  narc1  narc2  narc3  narc4  narc5  narc6  nidentity1  nidentity2  omind1  omind4  omind5  omind6  contact3  hygiene1  psupport3  political_ideology  happy  riskperc1  riskperc2  self_esteem  sbelong1  sbelong2  sbelong3  sbelong4  optim1  optim2  slfcont1  slfcont2  slfcont3  slfcont4  urban
##   5   7  age  children  cnarc1  cnarc2  cnarc3  ctheory1  ctheory2  ctheory3  ctheory4  CRT1  CRT2  CRT3  duration  generosity1  generosity2  generosity3  know_tested_positive  ladder  marital1  mor_circle  moralid1  moralid10  moralid2  moralid3  moralid4  moralid5  moralid6  moralid7  moralid8  moralid9  mcoop1  mcoop2  mcoop3  mcoop4  mcoop5  mcoop6  mcoop7  narc1  narc2  narc3  narc4  narc5  narc6  nidentity1  nidentity2  omind1  omind4  omind5  omind6  contact3  hygiene1  psupport3  political_ideology  happy  riskperc1  riskperc2  self_esteem  sbelong1  sbelong2  sbelong3  sbelong4  optim1  optim2  slfcont1  slfcont2  slfcont3  slfcont4  urban
##   5   8  age  children  cnarc1  cnarc2  cnarc3  ctheory1  ctheory2  ctheory3  ctheory4  CRT1  CRT2  CRT3  duration  generosity1  generosity2  generosity3  know_tested_positive  ladder  marital1  mor_circle  moralid1  moralid10  moralid2  moralid3  moralid4  moralid5  moralid6  moralid7  moralid8  moralid9  mcoop1  mcoop2  mcoop3  mcoop4  mcoop5  mcoop6  mcoop7  narc1  narc2  narc3  narc4  narc5  narc6  nidentity1  nidentity2  omind1  omind4  omind5  omind6  contact3  hygiene1  psupport3  political_ideology  happy  riskperc1  riskperc2  self_esteem  sbelong1  sbelong2  sbelong3  sbelong4  optim1  optim2  slfcont1  slfcont2  slfcont3  slfcont4  urban
##   5   9  age  children  cnarc1  cnarc2  cnarc3  ctheory1  ctheory2  ctheory3  ctheory4  CRT1  CRT2  CRT3  duration  generosity1  generosity2  generosity3  know_tested_positive  ladder  marital1  mor_circle  moralid1  moralid10  moralid2  moralid3  moralid4  moralid5  moralid6  moralid7  moralid8  moralid9  mcoop1  mcoop2  mcoop3  mcoop4  mcoop5  mcoop6  mcoop7  narc1  narc2  narc3  narc4  narc5  narc6  nidentity1  nidentity2  omind1  omind4  omind5  omind6  contact3  hygiene1  psupport3  political_ideology  happy  riskperc1  riskperc2  self_esteem  sbelong1  sbelong2  sbelong3  sbelong4  optim1  optim2  slfcont1  slfcont2  slfcont3  slfcont4  urban
##   5   10  age  children  cnarc1  cnarc2  cnarc3  ctheory1  ctheory2  ctheory3  ctheory4  CRT1  CRT2  CRT3  duration  generosity1  generosity2  generosity3  know_tested_positive  ladder  marital1  mor_circle  moralid1  moralid10  moralid2  moralid3  moralid4  moralid5  moralid6  moralid7  moralid8  moralid9  mcoop1  mcoop2  mcoop3  mcoop4  mcoop5  mcoop6  mcoop7  narc1  narc2  narc3  narc4  narc5  narc6  nidentity1  nidentity2  omind1  omind4  omind5  omind6  contact3  hygiene1  psupport3  political_ideology  happy  riskperc1  riskperc2  self_esteem  sbelong1  sbelong2  sbelong3  sbelong4  optim1  optim2  slfcont1  slfcont2  slfcont3  slfcont4  urban
##   6   1  age  children  cnarc1  cnarc2  cnarc3  ctheory1  ctheory2  ctheory3  ctheory4  CRT1  CRT2  CRT3  duration  generosity1  generosity2  generosity3  know_tested_positive  ladder  marital1  mor_circle  moralid1  moralid10  moralid2  moralid3  moralid4  moralid5  moralid6  moralid7  moralid8  moralid9  mcoop1  mcoop2  mcoop3  mcoop4  mcoop5  mcoop6  mcoop7  narc1  narc2  narc3  narc4  narc5  narc6  nidentity1  nidentity2  omind1  omind4  omind5  omind6  contact3  hygiene1  psupport3  political_ideology  happy  riskperc1  riskperc2  self_esteem  sbelong1  sbelong2  sbelong3  sbelong4  optim1  optim2  slfcont1  slfcont2  slfcont3  slfcont4  urban
##   6   2  age  children  cnarc1  cnarc2  cnarc3  ctheory1  ctheory2  ctheory3  ctheory4  CRT1  CRT2  CRT3  duration  generosity1  generosity2  generosity3  know_tested_positive  ladder  marital1  mor_circle  moralid1  moralid10  moralid2  moralid3  moralid4  moralid5  moralid6  moralid7  moralid8  moralid9  mcoop1  mcoop2  mcoop3  mcoop4  mcoop5  mcoop6  mcoop7  narc1  narc2  narc3  narc4  narc5  narc6  nidentity1  nidentity2  omind1  omind4  omind5  omind6  contact3  hygiene1  psupport3  political_ideology  happy  riskperc1  riskperc2  self_esteem  sbelong1  sbelong2  sbelong3  sbelong4  optim1  optim2  slfcont1  slfcont2  slfcont3  slfcont4  urban
##   6   3  age  children  cnarc1  cnarc2  cnarc3  ctheory1  ctheory2  ctheory3  ctheory4  CRT1  CRT2  CRT3  duration  generosity1  generosity2  generosity3  know_tested_positive  ladder  marital1  mor_circle  moralid1  moralid10  moralid2  moralid3  moralid4  moralid5  moralid6  moralid7  moralid8  moralid9  mcoop1  mcoop2  mcoop3  mcoop4  mcoop5  mcoop6  mcoop7  narc1  narc2  narc3  narc4  narc5  narc6  nidentity1  nidentity2  omind1  omind4  omind5  omind6  contact3  hygiene1  psupport3  political_ideology  happy  riskperc1  riskperc2  self_esteem  sbelong1  sbelong2  sbelong3  sbelong4  optim1  optim2  slfcont1  slfcont2  slfcont3  slfcont4  urban
##   6   4  age  children  cnarc1  cnarc2  cnarc3  ctheory1  ctheory2  ctheory3  ctheory4  CRT1  CRT2  CRT3  duration  generosity1  generosity2  generosity3  know_tested_positive  ladder  marital1  mor_circle  moralid1  moralid10  moralid2  moralid3  moralid4  moralid5  moralid6  moralid7  moralid8  moralid9  mcoop1  mcoop2  mcoop3  mcoop4  mcoop5  mcoop6  mcoop7  narc1  narc2  narc3  narc4  narc5  narc6  nidentity1  nidentity2  omind1  omind4  omind5  omind6  contact3  hygiene1  psupport3  political_ideology  happy  riskperc1  riskperc2  self_esteem  sbelong1  sbelong2  sbelong3  sbelong4  optim1  optim2  slfcont1  slfcont2  slfcont3  slfcont4  urban
##   6   5  age  children  cnarc1  cnarc2  cnarc3  ctheory1  ctheory2  ctheory3  ctheory4  CRT1  CRT2  CRT3  duration  generosity1  generosity2  generosity3  know_tested_positive  ladder  marital1  mor_circle  moralid1  moralid10  moralid2  moralid3  moralid4  moralid5  moralid6  moralid7  moralid8  moralid9  mcoop1  mcoop2  mcoop3  mcoop4  mcoop5  mcoop6  mcoop7  narc1  narc2  narc3  narc4  narc5  narc6  nidentity1  nidentity2  omind1  omind4  omind5  omind6  contact3  hygiene1  psupport3  political_ideology  happy  riskperc1  riskperc2  self_esteem  sbelong1  sbelong2  sbelong3  sbelong4  optim1  optim2  slfcont1  slfcont2  slfcont3  slfcont4  urban
##   6   6  age  children  cnarc1  cnarc2  cnarc3  ctheory1  ctheory2  ctheory3  ctheory4  CRT1  CRT2  CRT3  duration  generosity1  generosity2  generosity3  know_tested_positive  ladder  marital1  mor_circle  moralid1  moralid10  moralid2  moralid3  moralid4  moralid5  moralid6  moralid7  moralid8  moralid9  mcoop1  mcoop2  mcoop3  mcoop4  mcoop5  mcoop6  mcoop7  narc1  narc2  narc3  narc4  narc5  narc6  nidentity1  nidentity2  omind1  omind4  omind5  omind6  contact3  hygiene1  psupport3  political_ideology  happy  riskperc1  riskperc2  self_esteem  sbelong1  sbelong2  sbelong3  sbelong4  optim1  optim2  slfcont1  slfcont2  slfcont3  slfcont4  urban
##   6   7  age  children  cnarc1  cnarc2  cnarc3  ctheory1  ctheory2  ctheory3  ctheory4  CRT1  CRT2  CRT3  duration  generosity1  generosity2  generosity3  know_tested_positive  ladder  marital1  mor_circle  moralid1  moralid10  moralid2  moralid3  moralid4  moralid5  moralid6  moralid7  moralid8  moralid9  mcoop1  mcoop2  mcoop3  mcoop4  mcoop5  mcoop6  mcoop7  narc1  narc2  narc3  narc4  narc5  narc6  nidentity1  nidentity2  omind1  omind4  omind5  omind6  contact3  hygiene1  psupport3  political_ideology  happy  riskperc1  riskperc2  self_esteem  sbelong1  sbelong2  sbelong3  sbelong4  optim1  optim2  slfcont1  slfcont2  slfcont3  slfcont4  urban
##   6   8  age  children  cnarc1  cnarc2  cnarc3  ctheory1  ctheory2  ctheory3  ctheory4  CRT1  CRT2  CRT3  duration  generosity1  generosity2  generosity3  know_tested_positive  ladder  marital1  mor_circle  moralid1  moralid10  moralid2  moralid3  moralid4  moralid5  moralid6  moralid7  moralid8  moralid9  mcoop1  mcoop2  mcoop3  mcoop4  mcoop5  mcoop6  mcoop7  narc1  narc2  narc3  narc4  narc5  narc6  nidentity1  nidentity2  omind1  omind4  omind5  omind6  contact3  hygiene1  psupport3  political_ideology  happy  riskperc1  riskperc2  self_esteem  sbelong1  sbelong2  sbelong3  sbelong4  optim1  optim2  slfcont1  slfcont2  slfcont3  slfcont4  urban
##   6   9  age  children  cnarc1  cnarc2  cnarc3  ctheory1  ctheory2  ctheory3  ctheory4  CRT1  CRT2  CRT3  duration  generosity1  generosity2  generosity3  know_tested_positive  ladder  marital1  mor_circle  moralid1  moralid10  moralid2  moralid3  moralid4  moralid5  moralid6  moralid7  moralid8  moralid9  mcoop1  mcoop2  mcoop3  mcoop4  mcoop5  mcoop6  mcoop7  narc1  narc2  narc3  narc4  narc5  narc6  nidentity1  nidentity2  omind1  omind4  omind5  omind6  contact3  hygiene1  psupport3  political_ideology  happy  riskperc1  riskperc2  self_esteem  sbelong1  sbelong2  sbelong3  sbelong4  optim1  optim2  slfcont1  slfcont2  slfcont3  slfcont4  urban
##   6   10  age  children  cnarc1  cnarc2  cnarc3  ctheory1  ctheory2  ctheory3  ctheory4  CRT1  CRT2  CRT3  duration  generosity1  generosity2  generosity3  know_tested_positive  ladder  marital1  mor_circle  moralid1  moralid10  moralid2  moralid3  moralid4  moralid5  moralid6  moralid7  moralid8  moralid9  mcoop1  mcoop2  mcoop3  mcoop4  mcoop5  mcoop6  mcoop7  narc1  narc2  narc3  narc4  narc5  narc6  nidentity1  nidentity2  omind1  omind4  omind5  omind6  contact3  hygiene1  psupport3  political_ideology  happy  riskperc1  riskperc2  self_esteem  sbelong1  sbelong2  sbelong3  sbelong4  optim1  optim2  slfcont1  slfcont2  slfcont3  slfcont4  urban
##   7   1  age  children  cnarc1  cnarc2  cnarc3  ctheory1  ctheory2  ctheory3  ctheory4  CRT1  CRT2  CRT3  duration  generosity1  generosity2  generosity3  know_tested_positive  ladder  marital1  mor_circle  moralid1  moralid10  moralid2  moralid3  moralid4  moralid5  moralid6  moralid7  moralid8  moralid9  mcoop1  mcoop2  mcoop3  mcoop4  mcoop5  mcoop6  mcoop7  narc1  narc2  narc3  narc4  narc5  narc6  nidentity1  nidentity2  omind1  omind4  omind5  omind6  contact3  hygiene1  psupport3  political_ideology  happy  riskperc1  riskperc2  self_esteem  sbelong1  sbelong2  sbelong3  sbelong4  optim1  optim2  slfcont1  slfcont2  slfcont3  slfcont4  urban
##   7   2  age  children  cnarc1  cnarc2  cnarc3  ctheory1  ctheory2  ctheory3  ctheory4  CRT1  CRT2  CRT3  duration  generosity1  generosity2  generosity3  know_tested_positive  ladder  marital1  mor_circle  moralid1  moralid10  moralid2  moralid3  moralid4  moralid5  moralid6  moralid7  moralid8  moralid9  mcoop1  mcoop2  mcoop3  mcoop4  mcoop5  mcoop6  mcoop7  narc1  narc2  narc3  narc4  narc5  narc6  nidentity1  nidentity2  omind1  omind4  omind5  omind6  contact3  hygiene1  psupport3  political_ideology  happy  riskperc1  riskperc2  self_esteem  sbelong1  sbelong2  sbelong3  sbelong4  optim1  optim2  slfcont1  slfcont2  slfcont3  slfcont4  urban
##   7   3  age  children  cnarc1  cnarc2  cnarc3  ctheory1  ctheory2  ctheory3  ctheory4  CRT1  CRT2  CRT3  duration  generosity1  generosity2  generosity3  know_tested_positive  ladder  marital1  mor_circle  moralid1  moralid10  moralid2  moralid3  moralid4  moralid5  moralid6  moralid7  moralid8  moralid9  mcoop1  mcoop2  mcoop3  mcoop4  mcoop5  mcoop6  mcoop7  narc1  narc2  narc3  narc4  narc5  narc6  nidentity1  nidentity2  omind1  omind4  omind5  omind6  contact3  hygiene1  psupport3  political_ideology  happy  riskperc1  riskperc2  self_esteem  sbelong1  sbelong2  sbelong3  sbelong4  optim1  optim2  slfcont1  slfcont2  slfcont3  slfcont4  urban
##   7   4  age  children  cnarc1  cnarc2  cnarc3  ctheory1  ctheory2  ctheory3  ctheory4  CRT1  CRT2  CRT3  duration  generosity1  generosity2  generosity3  know_tested_positive  ladder  marital1  mor_circle  moralid1  moralid10  moralid2  moralid3  moralid4  moralid5  moralid6  moralid7  moralid8  moralid9  mcoop1  mcoop2  mcoop3  mcoop4  mcoop5  mcoop6  mcoop7  narc1  narc2  narc3  narc4  narc5  narc6  nidentity1  nidentity2  omind1  omind4  omind5  omind6  contact3  hygiene1  psupport3  political_ideology  happy  riskperc1  riskperc2  self_esteem  sbelong1  sbelong2  sbelong3  sbelong4  optim1  optim2  slfcont1  slfcont2  slfcont3  slfcont4  urban
##   7   5  age  children  cnarc1  cnarc2  cnarc3  ctheory1  ctheory2  ctheory3  ctheory4  CRT1  CRT2  CRT3  duration  generosity1  generosity2  generosity3  know_tested_positive  ladder  marital1  mor_circle  moralid1  moralid10  moralid2  moralid3  moralid4  moralid5  moralid6  moralid7  moralid8  moralid9  mcoop1  mcoop2  mcoop3  mcoop4  mcoop5  mcoop6  mcoop7  narc1  narc2  narc3  narc4  narc5  narc6  nidentity1  nidentity2  omind1  omind4  omind5  omind6  contact3  hygiene1  psupport3  political_ideology  happy  riskperc1  riskperc2  self_esteem  sbelong1  sbelong2  sbelong3  sbelong4  optim1  optim2  slfcont1  slfcont2  slfcont3  slfcont4  urban
##   7   6  age  children  cnarc1  cnarc2  cnarc3  ctheory1  ctheory2  ctheory3  ctheory4  CRT1  CRT2  CRT3  duration  generosity1  generosity2  generosity3  know_tested_positive  ladder  marital1  mor_circle  moralid1  moralid10  moralid2  moralid3  moralid4  moralid5  moralid6  moralid7  moralid8  moralid9  mcoop1  mcoop2  mcoop3  mcoop4  mcoop5  mcoop6  mcoop7  narc1  narc2  narc3  narc4  narc5  narc6  nidentity1  nidentity2  omind1  omind4  omind5  omind6  contact3  hygiene1  psupport3  political_ideology  happy  riskperc1  riskperc2  self_esteem  sbelong1  sbelong2  sbelong3  sbelong4  optim1  optim2  slfcont1  slfcont2  slfcont3  slfcont4  urban
##   7   7  age  children  cnarc1  cnarc2  cnarc3  ctheory1  ctheory2  ctheory3  ctheory4  CRT1  CRT2  CRT3  duration  generosity1  generosity2  generosity3  know_tested_positive  ladder  marital1  mor_circle  moralid1  moralid10  moralid2  moralid3  moralid4  moralid5  moralid6  moralid7  moralid8  moralid9  mcoop1  mcoop2  mcoop3  mcoop4  mcoop5  mcoop6  mcoop7  narc1  narc2  narc3  narc4  narc5  narc6  nidentity1  nidentity2  omind1  omind4  omind5  omind6  contact3  hygiene1  psupport3  political_ideology  happy  riskperc1  riskperc2  self_esteem  sbelong1  sbelong2  sbelong3  sbelong4  optim1  optim2  slfcont1  slfcont2  slfcont3  slfcont4  urban
##   7   8  age  children  cnarc1  cnarc2  cnarc3  ctheory1  ctheory2  ctheory3  ctheory4  CRT1  CRT2  CRT3  duration  generosity1  generosity2  generosity3  know_tested_positive  ladder  marital1  mor_circle  moralid1  moralid10  moralid2  moralid3  moralid4  moralid5  moralid6  moralid7  moralid8  moralid9  mcoop1  mcoop2  mcoop3  mcoop4  mcoop5  mcoop6  mcoop7  narc1  narc2  narc3  narc4  narc5  narc6  nidentity1  nidentity2  omind1  omind4  omind5  omind6  contact3  hygiene1  psupport3  political_ideology  happy  riskperc1  riskperc2  self_esteem  sbelong1  sbelong2  sbelong3  sbelong4  optim1  optim2  slfcont1  slfcont2  slfcont3  slfcont4  urban
##   7   9  age  children  cnarc1  cnarc2  cnarc3  ctheory1  ctheory2  ctheory3  ctheory4  CRT1  CRT2  CRT3  duration  generosity1  generosity2  generosity3  know_tested_positive  ladder  marital1  mor_circle  moralid1  moralid10  moralid2  moralid3  moralid4  moralid5  moralid6  moralid7  moralid8  moralid9  mcoop1  mcoop2  mcoop3  mcoop4  mcoop5  mcoop6  mcoop7  narc1  narc2  narc3  narc4  narc5  narc6  nidentity1  nidentity2  omind1  omind4  omind5  omind6  contact3  hygiene1  psupport3  political_ideology  happy  riskperc1  riskperc2  self_esteem  sbelong1  sbelong2  sbelong3  sbelong4  optim1  optim2  slfcont1  slfcont2  slfcont3  slfcont4  urban
##   7   10  age  children  cnarc1  cnarc2  cnarc3  ctheory1  ctheory2  ctheory3  ctheory4  CRT1  CRT2  CRT3  duration  generosity1  generosity2  generosity3  know_tested_positive  ladder  marital1  mor_circle  moralid1  moralid10  moralid2  moralid3  moralid4  moralid5  moralid6  moralid7  moralid8  moralid9  mcoop1  mcoop2  mcoop3  mcoop4  mcoop5  mcoop6  mcoop7  narc1  narc2  narc3  narc4  narc5  narc6  nidentity1  nidentity2  omind1  omind4  omind5  omind6  contact3  hygiene1  psupport3  political_ideology  happy  riskperc1  riskperc2  self_esteem  sbelong1  sbelong2  sbelong3  sbelong4  optim1  optim2  slfcont1  slfcont2  slfcont3  slfcont4  urban
##   8   1  age  children  cnarc1  cnarc2  cnarc3  ctheory1  ctheory2  ctheory3  ctheory4  CRT1  CRT2  CRT3  duration  generosity1  generosity2  generosity3  know_tested_positive  ladder  marital1  mor_circle  moralid1  moralid10  moralid2  moralid3  moralid4  moralid5  moralid6  moralid7  moralid8  moralid9  mcoop1  mcoop2  mcoop3  mcoop4  mcoop5  mcoop6  mcoop7  narc1  narc2  narc3  narc4  narc5  narc6  nidentity1  nidentity2  omind1  omind4  omind5  omind6  contact3  hygiene1  psupport3  political_ideology  happy  riskperc1  riskperc2  self_esteem  sbelong1  sbelong2  sbelong3  sbelong4  optim1  optim2  slfcont1  slfcont2  slfcont3  slfcont4  urban
##   8   2  age  children  cnarc1  cnarc2  cnarc3  ctheory1  ctheory2  ctheory3  ctheory4  CRT1  CRT2  CRT3  duration  generosity1  generosity2  generosity3  know_tested_positive  ladder  marital1  mor_circle  moralid1  moralid10  moralid2  moralid3  moralid4  moralid5  moralid6  moralid7  moralid8  moralid9  mcoop1  mcoop2  mcoop3  mcoop4  mcoop5  mcoop6  mcoop7  narc1  narc2  narc3  narc4  narc5  narc6  nidentity1  nidentity2  omind1  omind4  omind5  omind6  contact3  hygiene1  psupport3  political_ideology  happy  riskperc1  riskperc2  self_esteem  sbelong1  sbelong2  sbelong3  sbelong4  optim1  optim2  slfcont1  slfcont2  slfcont3  slfcont4  urban
##   8   3  age  children  cnarc1  cnarc2  cnarc3  ctheory1  ctheory2  ctheory3  ctheory4  CRT1  CRT2  CRT3  duration  generosity1  generosity2  generosity3  know_tested_positive  ladder  marital1  mor_circle  moralid1  moralid10  moralid2  moralid3  moralid4  moralid5  moralid6  moralid7  moralid8  moralid9  mcoop1  mcoop2  mcoop3  mcoop4  mcoop5  mcoop6  mcoop7  narc1  narc2  narc3  narc4  narc5  narc6  nidentity1  nidentity2  omind1  omind4  omind5  omind6  contact3  hygiene1  psupport3  political_ideology  happy  riskperc1  riskperc2  self_esteem  sbelong1  sbelong2  sbelong3  sbelong4  optim1  optim2  slfcont1  slfcont2  slfcont3  slfcont4  urban
##   8   4  age  children  cnarc1  cnarc2  cnarc3  ctheory1  ctheory2  ctheory3  ctheory4  CRT1  CRT2  CRT3  duration  generosity1  generosity2  generosity3  know_tested_positive  ladder  marital1  mor_circle  moralid1  moralid10  moralid2  moralid3  moralid4  moralid5  moralid6  moralid7  moralid8  moralid9  mcoop1  mcoop2  mcoop3  mcoop4  mcoop5  mcoop6  mcoop7  narc1  narc2  narc3  narc4  narc5  narc6  nidentity1  nidentity2  omind1  omind4  omind5  omind6  contact3  hygiene1  psupport3  political_ideology  happy  riskperc1  riskperc2  self_esteem  sbelong1  sbelong2  sbelong3  sbelong4  optim1  optim2  slfcont1  slfcont2  slfcont3  slfcont4  urban
##   8   5  age  children  cnarc1  cnarc2  cnarc3  ctheory1  ctheory2  ctheory3  ctheory4  CRT1  CRT2  CRT3  duration  generosity1  generosity2  generosity3  know_tested_positive  ladder  marital1  mor_circle  moralid1  moralid10  moralid2  moralid3  moralid4  moralid5  moralid6  moralid7  moralid8  moralid9  mcoop1  mcoop2  mcoop3  mcoop4  mcoop5  mcoop6  mcoop7  narc1  narc2  narc3  narc4  narc5  narc6  nidentity1  nidentity2  omind1  omind4  omind5  omind6  contact3  hygiene1  psupport3  political_ideology  happy  riskperc1  riskperc2  self_esteem  sbelong1  sbelong2  sbelong3  sbelong4  optim1  optim2  slfcont1  slfcont2  slfcont3  slfcont4  urban
##   8   6  age  children  cnarc1  cnarc2  cnarc3  ctheory1  ctheory2  ctheory3  ctheory4  CRT1  CRT2  CRT3  duration  generosity1  generosity2  generosity3  know_tested_positive  ladder  marital1  mor_circle  moralid1  moralid10  moralid2  moralid3  moralid4  moralid5  moralid6  moralid7  moralid8  moralid9  mcoop1  mcoop2  mcoop3  mcoop4  mcoop5  mcoop6  mcoop7  narc1  narc2  narc3  narc4  narc5  narc6  nidentity1  nidentity2  omind1  omind4  omind5  omind6  contact3  hygiene1  psupport3  political_ideology  happy  riskperc1  riskperc2  self_esteem  sbelong1  sbelong2  sbelong3  sbelong4  optim1  optim2  slfcont1  slfcont2  slfcont3  slfcont4  urban
##   8   7  age  children  cnarc1  cnarc2  cnarc3  ctheory1  ctheory2  ctheory3  ctheory4  CRT1  CRT2  CRT3  duration  generosity1  generosity2  generosity3  know_tested_positive  ladder  marital1  mor_circle  moralid1  moralid10  moralid2  moralid3  moralid4  moralid5  moralid6  moralid7  moralid8  moralid9  mcoop1  mcoop2  mcoop3  mcoop4  mcoop5  mcoop6  mcoop7  narc1  narc2  narc3  narc4  narc5  narc6  nidentity1  nidentity2  omind1  omind4  omind5  omind6  contact3  hygiene1  psupport3  political_ideology  happy  riskperc1  riskperc2  self_esteem  sbelong1  sbelong2  sbelong3  sbelong4  optim1  optim2  slfcont1  slfcont2  slfcont3  slfcont4  urban
##   8   8  age  children  cnarc1  cnarc2  cnarc3  ctheory1  ctheory2  ctheory3  ctheory4  CRT1  CRT2  CRT3  duration  generosity1  generosity2  generosity3  know_tested_positive  ladder  marital1  mor_circle  moralid1  moralid10  moralid2  moralid3  moralid4  moralid5  moralid6  moralid7  moralid8  moralid9  mcoop1  mcoop2  mcoop3  mcoop4  mcoop5  mcoop6  mcoop7  narc1  narc2  narc3  narc4  narc5  narc6  nidentity1  nidentity2  omind1  omind4  omind5  omind6  contact3  hygiene1  psupport3  political_ideology  happy  riskperc1  riskperc2  self_esteem  sbelong1  sbelong2  sbelong3  sbelong4  optim1  optim2  slfcont1  slfcont2  slfcont3  slfcont4  urban
##   8   9  age  children  cnarc1  cnarc2  cnarc3  ctheory1  ctheory2  ctheory3  ctheory4  CRT1  CRT2  CRT3  duration  generosity1  generosity2  generosity3  know_tested_positive  ladder  marital1  mor_circle  moralid1  moralid10  moralid2  moralid3  moralid4  moralid5  moralid6  moralid7  moralid8  moralid9  mcoop1  mcoop2  mcoop3  mcoop4  mcoop5  mcoop6  mcoop7  narc1  narc2  narc3  narc4  narc5  narc6  nidentity1  nidentity2  omind1  omind4  omind5  omind6  contact3  hygiene1  psupport3  political_ideology  happy  riskperc1  riskperc2  self_esteem  sbelong1  sbelong2  sbelong3  sbelong4  optim1  optim2  slfcont1  slfcont2  slfcont3  slfcont4  urban
##   8   10  age  children  cnarc1  cnarc2  cnarc3  ctheory1  ctheory2  ctheory3  ctheory4  CRT1  CRT2  CRT3  duration  generosity1  generosity2  generosity3  know_tested_positive  ladder  marital1  mor_circle  moralid1  moralid10  moralid2  moralid3  moralid4  moralid5  moralid6  moralid7  moralid8  moralid9  mcoop1  mcoop2  mcoop3  mcoop4  mcoop5  mcoop6  mcoop7  narc1  narc2  narc3  narc4  narc5  narc6  nidentity1  nidentity2  omind1  omind4  omind5  omind6  contact3  hygiene1  psupport3  political_ideology  happy  riskperc1  riskperc2  self_esteem  sbelong1  sbelong2  sbelong3  sbelong4  optim1  optim2  slfcont1  slfcont2  slfcont3  slfcont4  urban
##   9   1  age  children  cnarc1  cnarc2  cnarc3  ctheory1  ctheory2  ctheory3  ctheory4  CRT1  CRT2  CRT3  duration  generosity1  generosity2  generosity3  know_tested_positive  ladder  marital1  mor_circle  moralid1  moralid10  moralid2  moralid3  moralid4  moralid5  moralid6  moralid7  moralid8  moralid9  mcoop1  mcoop2  mcoop3  mcoop4  mcoop5  mcoop6  mcoop7  narc1  narc2  narc3  narc4  narc5  narc6  nidentity1  nidentity2  omind1  omind4  omind5  omind6  contact3  hygiene1  psupport3  political_ideology  happy  riskperc1  riskperc2  self_esteem  sbelong1  sbelong2  sbelong3  sbelong4  optim1  optim2  slfcont1  slfcont2  slfcont3  slfcont4  urban
##   9   2  age  children  cnarc1  cnarc2  cnarc3  ctheory1  ctheory2  ctheory3  ctheory4  CRT1  CRT2  CRT3  duration  generosity1  generosity2  generosity3  know_tested_positive  ladder  marital1  mor_circle  moralid1  moralid10  moralid2  moralid3  moralid4  moralid5  moralid6  moralid7  moralid8  moralid9  mcoop1  mcoop2  mcoop3  mcoop4  mcoop5  mcoop6  mcoop7  narc1  narc2  narc3  narc4  narc5  narc6  nidentity1  nidentity2  omind1  omind4  omind5  omind6  contact3  hygiene1  psupport3  political_ideology  happy  riskperc1  riskperc2  self_esteem  sbelong1  sbelong2  sbelong3  sbelong4  optim1  optim2  slfcont1  slfcont2  slfcont3  slfcont4  urban
##   9   3  age  children  cnarc1  cnarc2  cnarc3  ctheory1  ctheory2  ctheory3  ctheory4  CRT1  CRT2  CRT3  duration  generosity1  generosity2  generosity3  know_tested_positive  ladder  marital1  mor_circle  moralid1  moralid10  moralid2  moralid3  moralid4  moralid5  moralid6  moralid7  moralid8  moralid9  mcoop1  mcoop2  mcoop3  mcoop4  mcoop5  mcoop6  mcoop7  narc1  narc2  narc3  narc4  narc5  narc6  nidentity1  nidentity2  omind1  omind4  omind5  omind6  contact3  hygiene1  psupport3  political_ideology  happy  riskperc1  riskperc2  self_esteem  sbelong1  sbelong2  sbelong3  sbelong4  optim1  optim2  slfcont1  slfcont2  slfcont3  slfcont4  urban
##   9   4  age  children  cnarc1  cnarc2  cnarc3  ctheory1  ctheory2  ctheory3  ctheory4  CRT1  CRT2  CRT3  duration  generosity1  generosity2  generosity3  know_tested_positive  ladder  marital1  mor_circle  moralid1  moralid10  moralid2  moralid3  moralid4  moralid5  moralid6  moralid7  moralid8  moralid9  mcoop1  mcoop2  mcoop3  mcoop4  mcoop5  mcoop6  mcoop7  narc1  narc2  narc3  narc4  narc5  narc6  nidentity1  nidentity2  omind1  omind4  omind5  omind6  contact3  hygiene1  psupport3  political_ideology  happy  riskperc1  riskperc2  self_esteem  sbelong1  sbelong2  sbelong3  sbelong4  optim1  optim2  slfcont1  slfcont2  slfcont3  slfcont4  urban
##   9   5  age  children  cnarc1  cnarc2  cnarc3  ctheory1  ctheory2  ctheory3  ctheory4  CRT1  CRT2  CRT3  duration  generosity1  generosity2  generosity3  know_tested_positive  ladder  marital1  mor_circle  moralid1  moralid10  moralid2  moralid3  moralid4  moralid5  moralid6  moralid7  moralid8  moralid9  mcoop1  mcoop2  mcoop3  mcoop4  mcoop5  mcoop6  mcoop7  narc1  narc2  narc3  narc4  narc5  narc6  nidentity1  nidentity2  omind1  omind4  omind5  omind6  contact3  hygiene1  psupport3  political_ideology  happy  riskperc1  riskperc2  self_esteem  sbelong1  sbelong2  sbelong3  sbelong4  optim1  optim2  slfcont1  slfcont2  slfcont3  slfcont4  urban
##   9   6  age  children  cnarc1  cnarc2  cnarc3  ctheory1  ctheory2  ctheory3  ctheory4  CRT1  CRT2  CRT3  duration  generosity1  generosity2  generosity3  know_tested_positive  ladder  marital1  mor_circle  moralid1  moralid10  moralid2  moralid3  moralid4  moralid5  moralid6  moralid7  moralid8  moralid9  mcoop1  mcoop2  mcoop3  mcoop4  mcoop5  mcoop6  mcoop7  narc1  narc2  narc3  narc4  narc5  narc6  nidentity1  nidentity2  omind1  omind4  omind5  omind6  contact3  hygiene1  psupport3  political_ideology  happy  riskperc1  riskperc2  self_esteem  sbelong1  sbelong2  sbelong3  sbelong4  optim1  optim2  slfcont1  slfcont2  slfcont3  slfcont4  urban
##   9   7  age  children  cnarc1  cnarc2  cnarc3  ctheory1  ctheory2  ctheory3  ctheory4  CRT1  CRT2  CRT3  duration  generosity1  generosity2  generosity3  know_tested_positive  ladder  marital1  mor_circle  moralid1  moralid10  moralid2  moralid3  moralid4  moralid5  moralid6  moralid7  moralid8  moralid9  mcoop1  mcoop2  mcoop3  mcoop4  mcoop5  mcoop6  mcoop7  narc1  narc2  narc3  narc4  narc5  narc6  nidentity1  nidentity2  omind1  omind4  omind5  omind6  contact3  hygiene1  psupport3  political_ideology  happy  riskperc1  riskperc2  self_esteem  sbelong1  sbelong2  sbelong3  sbelong4  optim1  optim2  slfcont1  slfcont2  slfcont3  slfcont4  urban
##   9   8  age  children  cnarc1  cnarc2  cnarc3  ctheory1  ctheory2  ctheory3  ctheory4  CRT1  CRT2  CRT3  duration  generosity1  generosity2  generosity3  know_tested_positive  ladder  marital1  mor_circle  moralid1  moralid10  moralid2  moralid3  moralid4  moralid5  moralid6  moralid7  moralid8  moralid9  mcoop1  mcoop2  mcoop3  mcoop4  mcoop5  mcoop6  mcoop7  narc1  narc2  narc3  narc4  narc5  narc6  nidentity1  nidentity2  omind1  omind4  omind5  omind6  contact3  hygiene1  psupport3  political_ideology  happy  riskperc1  riskperc2  self_esteem  sbelong1  sbelong2  sbelong3  sbelong4  optim1  optim2  slfcont1  slfcont2  slfcont3  slfcont4  urban
##   9   9  age  children  cnarc1  cnarc2  cnarc3  ctheory1  ctheory2  ctheory3  ctheory4  CRT1  CRT2  CRT3  duration  generosity1  generosity2  generosity3  know_tested_positive  ladder  marital1  mor_circle  moralid1  moralid10  moralid2  moralid3  moralid4  moralid5  moralid6  moralid7  moralid8  moralid9  mcoop1  mcoop2  mcoop3  mcoop4  mcoop5  mcoop6  mcoop7  narc1  narc2  narc3  narc4  narc5  narc6  nidentity1  nidentity2  omind1  omind4  omind5  omind6  contact3  hygiene1  psupport3  political_ideology  happy  riskperc1  riskperc2  self_esteem  sbelong1  sbelong2  sbelong3  sbelong4  optim1  optim2  slfcont1  slfcont2  slfcont3  slfcont4  urban
##   9   10  age  children  cnarc1  cnarc2  cnarc3  ctheory1  ctheory2  ctheory3  ctheory4  CRT1  CRT2  CRT3  duration  generosity1  generosity2  generosity3  know_tested_positive  ladder  marital1  mor_circle  moralid1  moralid10  moralid2  moralid3  moralid4  moralid5  moralid6  moralid7  moralid8  moralid9  mcoop1  mcoop2  mcoop3  mcoop4  mcoop5  mcoop6  mcoop7  narc1  narc2  narc3  narc4  narc5  narc6  nidentity1  nidentity2  omind1  omind4  omind5  omind6  contact3  hygiene1  psupport3  political_ideology  happy  riskperc1  riskperc2  self_esteem  sbelong1  sbelong2  sbelong3  sbelong4  optim1  optim2  slfcont1  slfcont2  slfcont3  slfcont4  urban
##   10   1  age  children  cnarc1  cnarc2  cnarc3  ctheory1  ctheory2  ctheory3  ctheory4  CRT1  CRT2  CRT3  duration  generosity1  generosity2  generosity3  know_tested_positive  ladder  marital1  mor_circle  moralid1  moralid10  moralid2  moralid3  moralid4  moralid5  moralid6  moralid7  moralid8  moralid9  mcoop1  mcoop2  mcoop3  mcoop4  mcoop5  mcoop6  mcoop7  narc1  narc2  narc3  narc4  narc5  narc6  nidentity1  nidentity2  omind1  omind4  omind5  omind6  contact3  hygiene1  psupport3  political_ideology  happy  riskperc1  riskperc2  self_esteem  sbelong1  sbelong2  sbelong3  sbelong4  optim1  optim2  slfcont1  slfcont2  slfcont3  slfcont4  urban
##   10   2  age  children  cnarc1  cnarc2  cnarc3  ctheory1  ctheory2  ctheory3  ctheory4  CRT1  CRT2  CRT3  duration  generosity1  generosity2  generosity3  know_tested_positive  ladder  marital1  mor_circle  moralid1  moralid10  moralid2  moralid3  moralid4  moralid5  moralid6  moralid7  moralid8  moralid9  mcoop1  mcoop2  mcoop3  mcoop4  mcoop5  mcoop6  mcoop7  narc1  narc2  narc3  narc4  narc5  narc6  nidentity1  nidentity2  omind1  omind4  omind5  omind6  contact3  hygiene1  psupport3  political_ideology  happy  riskperc1  riskperc2  self_esteem  sbelong1  sbelong2  sbelong3  sbelong4  optim1  optim2  slfcont1  slfcont2  slfcont3  slfcont4  urban
##   10   3  age  children  cnarc1  cnarc2  cnarc3  ctheory1  ctheory2  ctheory3  ctheory4  CRT1  CRT2  CRT3  duration  generosity1  generosity2  generosity3  know_tested_positive  ladder  marital1  mor_circle  moralid1  moralid10  moralid2  moralid3  moralid4  moralid5  moralid6  moralid7  moralid8  moralid9  mcoop1  mcoop2  mcoop3  mcoop4  mcoop5  mcoop6  mcoop7  narc1  narc2  narc3  narc4  narc5  narc6  nidentity1  nidentity2  omind1  omind4  omind5  omind6  contact3  hygiene1  psupport3  political_ideology  happy  riskperc1  riskperc2  self_esteem  sbelong1  sbelong2  sbelong3  sbelong4  optim1  optim2  slfcont1  slfcont2  slfcont3  slfcont4  urban
##   10   4  age  children  cnarc1  cnarc2  cnarc3  ctheory1  ctheory2  ctheory3  ctheory4  CRT1  CRT2  CRT3  duration  generosity1  generosity2  generosity3  know_tested_positive  ladder  marital1  mor_circle  moralid1  moralid10  moralid2  moralid3  moralid4  moralid5  moralid6  moralid7  moralid8  moralid9  mcoop1  mcoop2  mcoop3  mcoop4  mcoop5  mcoop6  mcoop7  narc1  narc2  narc3  narc4  narc5  narc6  nidentity1  nidentity2  omind1  omind4  omind5  omind6  contact3  hygiene1  psupport3  political_ideology  happy  riskperc1  riskperc2  self_esteem  sbelong1  sbelong2  sbelong3  sbelong4  optim1  optim2  slfcont1  slfcont2  slfcont3  slfcont4  urban
##   10   5  age  children  cnarc1  cnarc2  cnarc3  ctheory1  ctheory2  ctheory3  ctheory4  CRT1  CRT2  CRT3  duration  generosity1  generosity2  generosity3  know_tested_positive  ladder  marital1  mor_circle  moralid1  moralid10  moralid2  moralid3  moralid4  moralid5  moralid6  moralid7  moralid8  moralid9  mcoop1  mcoop2  mcoop3  mcoop4  mcoop5  mcoop6  mcoop7  narc1  narc2  narc3  narc4  narc5  narc6  nidentity1  nidentity2  omind1  omind4  omind5  omind6  contact3  hygiene1  psupport3  political_ideology  happy  riskperc1  riskperc2  self_esteem  sbelong1  sbelong2  sbelong3  sbelong4  optim1  optim2  slfcont1  slfcont2  slfcont3  slfcont4  urban
##   10   6  age  children  cnarc1  cnarc2  cnarc3  ctheory1  ctheory2  ctheory3  ctheory4  CRT1  CRT2  CRT3  duration  generosity1  generosity2  generosity3  know_tested_positive  ladder  marital1  mor_circle  moralid1  moralid10  moralid2  moralid3  moralid4  moralid5  moralid6  moralid7  moralid8  moralid9  mcoop1  mcoop2  mcoop3  mcoop4  mcoop5  mcoop6  mcoop7  narc1  narc2  narc3  narc4  narc5  narc6  nidentity1  nidentity2  omind1  omind4  omind5  omind6  contact3  hygiene1  psupport3  political_ideology  happy  riskperc1  riskperc2  self_esteem  sbelong1  sbelong2  sbelong3  sbelong4  optim1  optim2  slfcont1  slfcont2  slfcont3  slfcont4  urban
##   10   7  age  children  cnarc1  cnarc2  cnarc3  ctheory1  ctheory2  ctheory3  ctheory4  CRT1  CRT2  CRT3  duration  generosity1  generosity2  generosity3  know_tested_positive  ladder  marital1  mor_circle  moralid1  moralid10  moralid2  moralid3  moralid4  moralid5  moralid6  moralid7  moralid8  moralid9  mcoop1  mcoop2  mcoop3  mcoop4  mcoop5  mcoop6  mcoop7  narc1  narc2  narc3  narc4  narc5  narc6  nidentity1  nidentity2  omind1  omind4  omind5  omind6  contact3  hygiene1  psupport3  political_ideology  happy  riskperc1  riskperc2  self_esteem  sbelong1  sbelong2  sbelong3  sbelong4  optim1  optim2  slfcont1  slfcont2  slfcont3  slfcont4  urban
##   10   8  age  children  cnarc1  cnarc2  cnarc3  ctheory1  ctheory2  ctheory3  ctheory4  CRT1  CRT2  CRT3  duration  generosity1  generosity2  generosity3  know_tested_positive  ladder  marital1  mor_circle  moralid1  moralid10  moralid2  moralid3  moralid4  moralid5  moralid6  moralid7  moralid8  moralid9  mcoop1  mcoop2  mcoop3  mcoop4  mcoop5  mcoop6  mcoop7  narc1  narc2  narc3  narc4  narc5  narc6  nidentity1  nidentity2  omind1  omind4  omind5  omind6  contact3  hygiene1  psupport3  political_ideology  happy  riskperc1  riskperc2  self_esteem  sbelong1  sbelong2  sbelong3  sbelong4  optim1  optim2  slfcont1  slfcont2  slfcont3  slfcont4  urban
##   10   9  age  children  cnarc1  cnarc2  cnarc3  ctheory1  ctheory2  ctheory3  ctheory4  CRT1  CRT2  CRT3  duration  generosity1  generosity2  generosity3  know_tested_positive  ladder  marital1  mor_circle  moralid1  moralid10  moralid2  moralid3  moralid4  moralid5  moralid6  moralid7  moralid8  moralid9  mcoop1  mcoop2  mcoop3  mcoop4  mcoop5  mcoop6  mcoop7  narc1  narc2  narc3  narc4  narc5  narc6  nidentity1  nidentity2  omind1  omind4  omind5  omind6  contact3  hygiene1  psupport3  political_ideology  happy  riskperc1  riskperc2  self_esteem  sbelong1  sbelong2  sbelong3  sbelong4  optim1  optim2  slfcont1  slfcont2  slfcont3  slfcont4  urban
##   10   10  age  children  cnarc1  cnarc2  cnarc3  ctheory1  ctheory2  ctheory3  ctheory4  CRT1  CRT2  CRT3  duration  generosity1  generosity2  generosity3  know_tested_positive  ladder  marital1  mor_circle  moralid1  moralid10  moralid2  moralid3  moralid4  moralid5  moralid6  moralid7  moralid8  moralid9  mcoop1  mcoop2  mcoop3  mcoop4  mcoop5  mcoop6  mcoop7  narc1  narc2  narc3  narc4  narc5  narc6  nidentity1  nidentity2  omind1  omind4  omind5  omind6  contact3  hygiene1  psupport3  political_ideology  happy  riskperc1  riskperc2  self_esteem  sbelong1  sbelong2  sbelong3  sbelong4  optim1  optim2  slfcont1  slfcont2  slfcont3  slfcont4  urban
```

```
## Warning: Number of logged events: 6478
```

```
## 
##  iter imp variable
##   1   1  cnarc1  cnarc2  cnarc3  ctheory1  ctheory2  ctheory3  ctheory4  CRT1  CRT2  CRT3  generosity1  generosity2  generosity3  health_cond  know_tested_positive  moralid10  moralid3  moralid4  moralid5  moralid6  moralid7  moralid8  moralid9  mcoop1  mcoop2  mcoop3  mcoop4  mcoop5  mcoop6  mcoop7  narc2  narc4  narc5  narc6  nidentity1  omind2  omind3  omind4  hygiene4  hygiene5  psupport4  political_ideology  happy  slf_ladder  riskperc1  riskperc2  self_esteem  sbelong2  sbelong3  tested_positive  slfcont1  slfcont2
##   1   2  cnarc1  cnarc2  cnarc3  ctheory1  ctheory2  ctheory3  ctheory4  CRT1  CRT2  CRT3  generosity1  generosity2  generosity3  health_cond  know_tested_positive  moralid10  moralid3  moralid4  moralid5  moralid6  moralid7  moralid8  moralid9  mcoop1  mcoop2  mcoop3  mcoop4  mcoop5  mcoop6  mcoop7  narc2  narc4  narc5  narc6  nidentity1  omind2  omind3  omind4  hygiene4  hygiene5  psupport4  political_ideology  happy  slf_ladder  riskperc1  riskperc2  self_esteem  sbelong2  sbelong3  tested_positive  slfcont1  slfcont2
##   1   3  cnarc1  cnarc2  cnarc3  ctheory1  ctheory2  ctheory3  ctheory4  CRT1  CRT2  CRT3  generosity1  generosity2  generosity3  health_cond  know_tested_positive  moralid10  moralid3  moralid4  moralid5  moralid6  moralid7  moralid8  moralid9  mcoop1  mcoop2  mcoop3  mcoop4  mcoop5  mcoop6  mcoop7  narc2  narc4  narc5  narc6  nidentity1  omind2  omind3  omind4  hygiene4  hygiene5  psupport4  political_ideology  happy  slf_ladder  riskperc1  riskperc2  self_esteem  sbelong2  sbelong3  tested_positive  slfcont1  slfcont2
##   1   4  cnarc1  cnarc2  cnarc3  ctheory1  ctheory2  ctheory3  ctheory4  CRT1  CRT2  CRT3  generosity1  generosity2  generosity3  health_cond  know_tested_positive  moralid10  moralid3  moralid4  moralid5  moralid6  moralid7  moralid8  moralid9  mcoop1  mcoop2  mcoop3  mcoop4  mcoop5  mcoop6  mcoop7  narc2  narc4  narc5  narc6  nidentity1  omind2  omind3  omind4  hygiene4  hygiene5  psupport4  political_ideology  happy  slf_ladder  riskperc1  riskperc2  self_esteem  sbelong2  sbelong3  tested_positive  slfcont1  slfcont2
##   1   5  cnarc1  cnarc2  cnarc3  ctheory1  ctheory2  ctheory3  ctheory4  CRT1  CRT2  CRT3  generosity1  generosity2  generosity3  health_cond  know_tested_positive  moralid10  moralid3  moralid4  moralid5  moralid6  moralid7  moralid8  moralid9  mcoop1  mcoop2  mcoop3  mcoop4  mcoop5  mcoop6  mcoop7  narc2  narc4  narc5  narc6  nidentity1  omind2  omind3  omind4  hygiene4  hygiene5  psupport4  political_ideology  happy  slf_ladder  riskperc1  riskperc2  self_esteem  sbelong2  sbelong3  tested_positive  slfcont1  slfcont2
##   1   6  cnarc1  cnarc2  cnarc3  ctheory1  ctheory2  ctheory3  ctheory4  CRT1  CRT2  CRT3  generosity1  generosity2  generosity3  health_cond  know_tested_positive  moralid10  moralid3  moralid4  moralid5  moralid6  moralid7  moralid8  moralid9  mcoop1  mcoop2  mcoop3  mcoop4  mcoop5  mcoop6  mcoop7  narc2  narc4  narc5  narc6  nidentity1  omind2  omind3  omind4  hygiene4  hygiene5  psupport4  political_ideology  happy  slf_ladder  riskperc1  riskperc2  self_esteem  sbelong2  sbelong3  tested_positive  slfcont1  slfcont2
##   1   7  cnarc1  cnarc2  cnarc3  ctheory1  ctheory2  ctheory3  ctheory4  CRT1  CRT2  CRT3  generosity1  generosity2  generosity3  health_cond  know_tested_positive  moralid10  moralid3  moralid4  moralid5  moralid6  moralid7  moralid8  moralid9  mcoop1  mcoop2  mcoop3  mcoop4  mcoop5  mcoop6  mcoop7  narc2  narc4  narc5  narc6  nidentity1  omind2  omind3  omind4  hygiene4  hygiene5  psupport4  political_ideology  happy  slf_ladder  riskperc1  riskperc2  self_esteem  sbelong2  sbelong3  tested_positive  slfcont1  slfcont2
##   1   8  cnarc1  cnarc2  cnarc3  ctheory1  ctheory2  ctheory3  ctheory4  CRT1  CRT2  CRT3  generosity1  generosity2  generosity3  health_cond  know_tested_positive  moralid10  moralid3  moralid4  moralid5  moralid6  moralid7  moralid8  moralid9  mcoop1  mcoop2  mcoop3  mcoop4  mcoop5  mcoop6  mcoop7  narc2  narc4  narc5  narc6  nidentity1  omind2  omind3  omind4  hygiene4  hygiene5  psupport4  political_ideology  happy  slf_ladder  riskperc1  riskperc2  self_esteem  sbelong2  sbelong3  tested_positive  slfcont1  slfcont2
##   1   9  cnarc1  cnarc2  cnarc3  ctheory1  ctheory2  ctheory3  ctheory4  CRT1  CRT2  CRT3  generosity1  generosity2  generosity3  health_cond  know_tested_positive  moralid10  moralid3  moralid4  moralid5  moralid6  moralid7  moralid8  moralid9  mcoop1  mcoop2  mcoop3  mcoop4  mcoop5  mcoop6  mcoop7  narc2  narc4  narc5  narc6  nidentity1  omind2  omind3  omind4  hygiene4  hygiene5  psupport4  political_ideology  happy  slf_ladder  riskperc1  riskperc2  self_esteem  sbelong2  sbelong3  tested_positive  slfcont1  slfcont2
##   1   10  cnarc1  cnarc2  cnarc3  ctheory1  ctheory2  ctheory3  ctheory4  CRT1  CRT2  CRT3  generosity1  generosity2  generosity3  health_cond  know_tested_positive  moralid10  moralid3  moralid4  moralid5  moralid6  moralid7  moralid8  moralid9  mcoop1  mcoop2  mcoop3  mcoop4  mcoop5  mcoop6  mcoop7  narc2  narc4  narc5  narc6  nidentity1  omind2  omind3  omind4  hygiene4  hygiene5  psupport4  political_ideology  happy  slf_ladder  riskperc1  riskperc2  self_esteem  sbelong2  sbelong3  tested_positive  slfcont1  slfcont2
##   2   1  cnarc1  cnarc2  cnarc3  ctheory1  ctheory2  ctheory3  ctheory4  CRT1  CRT2  CRT3  generosity1  generosity2  generosity3  health_cond  know_tested_positive  moralid10  moralid3  moralid4  moralid5  moralid6  moralid7  moralid8  moralid9  mcoop1  mcoop2  mcoop3  mcoop4  mcoop5  mcoop6  mcoop7  narc2  narc4  narc5  narc6  nidentity1  omind2  omind3  omind4  hygiene4  hygiene5  psupport4  political_ideology  happy  slf_ladder  riskperc1  riskperc2  self_esteem  sbelong2  sbelong3  tested_positive  slfcont1  slfcont2
##   2   2  cnarc1  cnarc2  cnarc3  ctheory1  ctheory2  ctheory3  ctheory4  CRT1  CRT2  CRT3  generosity1  generosity2  generosity3  health_cond  know_tested_positive  moralid10  moralid3  moralid4  moralid5  moralid6  moralid7  moralid8  moralid9  mcoop1  mcoop2  mcoop3  mcoop4  mcoop5  mcoop6  mcoop7  narc2  narc4  narc5  narc6  nidentity1  omind2  omind3  omind4  hygiene4  hygiene5  psupport4  political_ideology  happy  slf_ladder  riskperc1  riskperc2  self_esteem  sbelong2  sbelong3  tested_positive  slfcont1  slfcont2
##   2   3  cnarc1  cnarc2  cnarc3  ctheory1  ctheory2  ctheory3  ctheory4  CRT1  CRT2  CRT3  generosity1  generosity2  generosity3  health_cond  know_tested_positive  moralid10  moralid3  moralid4  moralid5  moralid6  moralid7  moralid8  moralid9  mcoop1  mcoop2  mcoop3  mcoop4  mcoop5  mcoop6  mcoop7  narc2  narc4  narc5  narc6  nidentity1  omind2  omind3  omind4  hygiene4  hygiene5  psupport4  political_ideology  happy  slf_ladder  riskperc1  riskperc2  self_esteem  sbelong2  sbelong3  tested_positive  slfcont1  slfcont2
##   2   4  cnarc1  cnarc2  cnarc3  ctheory1  ctheory2  ctheory3  ctheory4  CRT1  CRT2  CRT3  generosity1  generosity2  generosity3  health_cond  know_tested_positive  moralid10  moralid3  moralid4  moralid5  moralid6  moralid7  moralid8  moralid9  mcoop1  mcoop2  mcoop3  mcoop4  mcoop5  mcoop6  mcoop7  narc2  narc4  narc5  narc6  nidentity1  omind2  omind3  omind4  hygiene4  hygiene5  psupport4  political_ideology  happy  slf_ladder  riskperc1  riskperc2  self_esteem  sbelong2  sbelong3  tested_positive  slfcont1  slfcont2
##   2   5  cnarc1  cnarc2  cnarc3  ctheory1  ctheory2  ctheory3  ctheory4  CRT1  CRT2  CRT3  generosity1  generosity2  generosity3  health_cond  know_tested_positive  moralid10  moralid3  moralid4  moralid5  moralid6  moralid7  moralid8  moralid9  mcoop1  mcoop2  mcoop3  mcoop4  mcoop5  mcoop6  mcoop7  narc2  narc4  narc5  narc6  nidentity1  omind2  omind3  omind4  hygiene4  hygiene5  psupport4  political_ideology  happy  slf_ladder  riskperc1  riskperc2  self_esteem  sbelong2  sbelong3  tested_positive  slfcont1  slfcont2
##   2   6  cnarc1  cnarc2  cnarc3  ctheory1  ctheory2  ctheory3  ctheory4  CRT1  CRT2  CRT3  generosity1  generosity2  generosity3  health_cond  know_tested_positive  moralid10  moralid3  moralid4  moralid5  moralid6  moralid7  moralid8  moralid9  mcoop1  mcoop2  mcoop3  mcoop4  mcoop5  mcoop6  mcoop7  narc2  narc4  narc5  narc6  nidentity1  omind2  omind3  omind4  hygiene4  hygiene5  psupport4  political_ideology  happy  slf_ladder  riskperc1  riskperc2  self_esteem  sbelong2  sbelong3  tested_positive  slfcont1  slfcont2
##   2   7  cnarc1  cnarc2  cnarc3  ctheory1  ctheory2  ctheory3  ctheory4  CRT1  CRT2  CRT3  generosity1  generosity2  generosity3  health_cond  know_tested_positive  moralid10  moralid3  moralid4  moralid5  moralid6  moralid7  moralid8  moralid9  mcoop1  mcoop2  mcoop3  mcoop4  mcoop5  mcoop6  mcoop7  narc2  narc4  narc5  narc6  nidentity1  omind2  omind3  omind4  hygiene4  hygiene5  psupport4  political_ideology  happy  slf_ladder  riskperc1  riskperc2  self_esteem  sbelong2  sbelong3  tested_positive  slfcont1  slfcont2
##   2   8  cnarc1  cnarc2  cnarc3  ctheory1  ctheory2  ctheory3  ctheory4  CRT1  CRT2  CRT3  generosity1  generosity2  generosity3  health_cond  know_tested_positive  moralid10  moralid3  moralid4  moralid5  moralid6  moralid7  moralid8  moralid9  mcoop1  mcoop2  mcoop3  mcoop4  mcoop5  mcoop6  mcoop7  narc2  narc4  narc5  narc6  nidentity1  omind2  omind3  omind4  hygiene4  hygiene5  psupport4  political_ideology  happy  slf_ladder  riskperc1  riskperc2  self_esteem  sbelong2  sbelong3  tested_positive  slfcont1  slfcont2
##   2   9  cnarc1  cnarc2  cnarc3  ctheory1  ctheory2  ctheory3  ctheory4  CRT1  CRT2  CRT3  generosity1  generosity2  generosity3  health_cond  know_tested_positive  moralid10  moralid3  moralid4  moralid5  moralid6  moralid7  moralid8  moralid9  mcoop1  mcoop2  mcoop3  mcoop4  mcoop5  mcoop6  mcoop7  narc2  narc4  narc5  narc6  nidentity1  omind2  omind3  omind4  hygiene4  hygiene5  psupport4  political_ideology  happy  slf_ladder  riskperc1  riskperc2  self_esteem  sbelong2  sbelong3  tested_positive  slfcont1  slfcont2
##   2   10  cnarc1  cnarc2  cnarc3  ctheory1  ctheory2  ctheory3  ctheory4  CRT1  CRT2  CRT3  generosity1  generosity2  generosity3  health_cond  know_tested_positive  moralid10  moralid3  moralid4  moralid5  moralid6  moralid7  moralid8  moralid9  mcoop1  mcoop2  mcoop3  mcoop4  mcoop5  mcoop6  mcoop7  narc2  narc4  narc5  narc6  nidentity1  omind2  omind3  omind4  hygiene4  hygiene5  psupport4  political_ideology  happy  slf_ladder  riskperc1  riskperc2  self_esteem  sbelong2  sbelong3  tested_positive  slfcont1  slfcont2
##   3   1  cnarc1  cnarc2  cnarc3  ctheory1  ctheory2  ctheory3  ctheory4  CRT1  CRT2  CRT3  generosity1  generosity2  generosity3  health_cond  know_tested_positive  moralid10  moralid3  moralid4  moralid5  moralid6  moralid7  moralid8  moralid9  mcoop1  mcoop2  mcoop3  mcoop4  mcoop5  mcoop6  mcoop7  narc2  narc4  narc5  narc6  nidentity1  omind2  omind3  omind4  hygiene4  hygiene5  psupport4  political_ideology  happy  slf_ladder  riskperc1  riskperc2  self_esteem  sbelong2  sbelong3  tested_positive  slfcont1  slfcont2
##   3   2  cnarc1  cnarc2  cnarc3  ctheory1  ctheory2  ctheory3  ctheory4  CRT1  CRT2  CRT3  generosity1  generosity2  generosity3  health_cond  know_tested_positive  moralid10  moralid3  moralid4  moralid5  moralid6  moralid7  moralid8  moralid9  mcoop1  mcoop2  mcoop3  mcoop4  mcoop5  mcoop6  mcoop7  narc2  narc4  narc5  narc6  nidentity1  omind2  omind3  omind4  hygiene4  hygiene5  psupport4  political_ideology  happy  slf_ladder  riskperc1  riskperc2  self_esteem  sbelong2  sbelong3  tested_positive  slfcont1  slfcont2
##   3   3  cnarc1  cnarc2  cnarc3  ctheory1  ctheory2  ctheory3  ctheory4  CRT1  CRT2  CRT3  generosity1  generosity2  generosity3  health_cond  know_tested_positive  moralid10  moralid3  moralid4  moralid5  moralid6  moralid7  moralid8  moralid9  mcoop1  mcoop2  mcoop3  mcoop4  mcoop5  mcoop6  mcoop7  narc2  narc4  narc5  narc6  nidentity1  omind2  omind3  omind4  hygiene4  hygiene5  psupport4  political_ideology  happy  slf_ladder  riskperc1  riskperc2  self_esteem  sbelong2  sbelong3  tested_positive  slfcont1  slfcont2
##   3   4  cnarc1  cnarc2  cnarc3  ctheory1  ctheory2  ctheory3  ctheory4  CRT1  CRT2  CRT3  generosity1  generosity2  generosity3  health_cond  know_tested_positive  moralid10  moralid3  moralid4  moralid5  moralid6  moralid7  moralid8  moralid9  mcoop1  mcoop2  mcoop3  mcoop4  mcoop5  mcoop6  mcoop7  narc2  narc4  narc5  narc6  nidentity1  omind2  omind3  omind4  hygiene4  hygiene5  psupport4  political_ideology  happy  slf_ladder  riskperc1  riskperc2  self_esteem  sbelong2  sbelong3  tested_positive  slfcont1  slfcont2
##   3   5  cnarc1  cnarc2  cnarc3  ctheory1  ctheory2  ctheory3  ctheory4  CRT1  CRT2  CRT3  generosity1  generosity2  generosity3  health_cond  know_tested_positive  moralid10  moralid3  moralid4  moralid5  moralid6  moralid7  moralid8  moralid9  mcoop1  mcoop2  mcoop3  mcoop4  mcoop5  mcoop6  mcoop7  narc2  narc4  narc5  narc6  nidentity1  omind2  omind3  omind4  hygiene4  hygiene5  psupport4  political_ideology  happy  slf_ladder  riskperc1  riskperc2  self_esteem  sbelong2  sbelong3  tested_positive  slfcont1  slfcont2
##   3   6  cnarc1  cnarc2  cnarc3  ctheory1  ctheory2  ctheory3  ctheory4  CRT1  CRT2  CRT3  generosity1  generosity2  generosity3  health_cond  know_tested_positive  moralid10  moralid3  moralid4  moralid5  moralid6  moralid7  moralid8  moralid9  mcoop1  mcoop2  mcoop3  mcoop4  mcoop5  mcoop6  mcoop7  narc2  narc4  narc5  narc6  nidentity1  omind2  omind3  omind4  hygiene4  hygiene5  psupport4  political_ideology  happy  slf_ladder  riskperc1  riskperc2  self_esteem  sbelong2  sbelong3  tested_positive  slfcont1  slfcont2
##   3   7  cnarc1  cnarc2  cnarc3  ctheory1  ctheory2  ctheory3  ctheory4  CRT1  CRT2  CRT3  generosity1  generosity2  generosity3  health_cond  know_tested_positive  moralid10  moralid3  moralid4  moralid5  moralid6  moralid7  moralid8  moralid9  mcoop1  mcoop2  mcoop3  mcoop4  mcoop5  mcoop6  mcoop7  narc2  narc4  narc5  narc6  nidentity1  omind2  omind3  omind4  hygiene4  hygiene5  psupport4  political_ideology  happy  slf_ladder  riskperc1  riskperc2  self_esteem  sbelong2  sbelong3  tested_positive  slfcont1  slfcont2
##   3   8  cnarc1  cnarc2  cnarc3  ctheory1  ctheory2  ctheory3  ctheory4  CRT1  CRT2  CRT3  generosity1  generosity2  generosity3  health_cond  know_tested_positive  moralid10  moralid3  moralid4  moralid5  moralid6  moralid7  moralid8  moralid9  mcoop1  mcoop2  mcoop3  mcoop4  mcoop5  mcoop6  mcoop7  narc2  narc4  narc5  narc6  nidentity1  omind2  omind3  omind4  hygiene4  hygiene5  psupport4  political_ideology  happy  slf_ladder  riskperc1  riskperc2  self_esteem  sbelong2  sbelong3  tested_positive  slfcont1  slfcont2
##   3   9  cnarc1  cnarc2  cnarc3  ctheory1  ctheory2  ctheory3  ctheory4  CRT1  CRT2  CRT3  generosity1  generosity2  generosity3  health_cond  know_tested_positive  moralid10  moralid3  moralid4  moralid5  moralid6  moralid7  moralid8  moralid9  mcoop1  mcoop2  mcoop3  mcoop4  mcoop5  mcoop6  mcoop7  narc2  narc4  narc5  narc6  nidentity1  omind2  omind3  omind4  hygiene4  hygiene5  psupport4  political_ideology  happy  slf_ladder  riskperc1  riskperc2  self_esteem  sbelong2  sbelong3  tested_positive  slfcont1  slfcont2
##   3   10  cnarc1  cnarc2  cnarc3  ctheory1  ctheory2  ctheory3  ctheory4  CRT1  CRT2  CRT3  generosity1  generosity2  generosity3  health_cond  know_tested_positive  moralid10  moralid3  moralid4  moralid5  moralid6  moralid7  moralid8  moralid9  mcoop1  mcoop2  mcoop3  mcoop4  mcoop5  mcoop6  mcoop7  narc2  narc4  narc5  narc6  nidentity1  omind2  omind3  omind4  hygiene4  hygiene5  psupport4  political_ideology  happy  slf_ladder  riskperc1  riskperc2  self_esteem  sbelong2  sbelong3  tested_positive  slfcont1  slfcont2
##   4   1  cnarc1  cnarc2  cnarc3  ctheory1  ctheory2  ctheory3  ctheory4  CRT1  CRT2  CRT3  generosity1  generosity2  generosity3  health_cond  know_tested_positive  moralid10  moralid3  moralid4  moralid5  moralid6  moralid7  moralid8  moralid9  mcoop1  mcoop2  mcoop3  mcoop4  mcoop5  mcoop6  mcoop7  narc2  narc4  narc5  narc6  nidentity1  omind2  omind3  omind4  hygiene4  hygiene5  psupport4  political_ideology  happy  slf_ladder  riskperc1  riskperc2  self_esteem  sbelong2  sbelong3  tested_positive  slfcont1  slfcont2
##   4   2  cnarc1  cnarc2  cnarc3  ctheory1  ctheory2  ctheory3  ctheory4  CRT1  CRT2  CRT3  generosity1  generosity2  generosity3  health_cond  know_tested_positive  moralid10  moralid3  moralid4  moralid5  moralid6  moralid7  moralid8  moralid9  mcoop1  mcoop2  mcoop3  mcoop4  mcoop5  mcoop6  mcoop7  narc2  narc4  narc5  narc6  nidentity1  omind2  omind3  omind4  hygiene4  hygiene5  psupport4  political_ideology  happy  slf_ladder  riskperc1  riskperc2  self_esteem  sbelong2  sbelong3  tested_positive  slfcont1  slfcont2
##   4   3  cnarc1  cnarc2  cnarc3  ctheory1  ctheory2  ctheory3  ctheory4  CRT1  CRT2  CRT3  generosity1  generosity2  generosity3  health_cond  know_tested_positive  moralid10  moralid3  moralid4  moralid5  moralid6  moralid7  moralid8  moralid9  mcoop1  mcoop2  mcoop3  mcoop4  mcoop5  mcoop6  mcoop7  narc2  narc4  narc5  narc6  nidentity1  omind2  omind3  omind4  hygiene4  hygiene5  psupport4  political_ideology  happy  slf_ladder  riskperc1  riskperc2  self_esteem  sbelong2  sbelong3  tested_positive  slfcont1  slfcont2
##   4   4  cnarc1  cnarc2  cnarc3  ctheory1  ctheory2  ctheory3  ctheory4  CRT1  CRT2  CRT3  generosity1  generosity2  generosity3  health_cond  know_tested_positive  moralid10  moralid3  moralid4  moralid5  moralid6  moralid7  moralid8  moralid9  mcoop1  mcoop2  mcoop3  mcoop4  mcoop5  mcoop6  mcoop7  narc2  narc4  narc5  narc6  nidentity1  omind2  omind3  omind4  hygiene4  hygiene5  psupport4  political_ideology  happy  slf_ladder  riskperc1  riskperc2  self_esteem  sbelong2  sbelong3  tested_positive  slfcont1  slfcont2
##   4   5  cnarc1  cnarc2  cnarc3  ctheory1  ctheory2  ctheory3  ctheory4  CRT1  CRT2  CRT3  generosity1  generosity2  generosity3  health_cond  know_tested_positive  moralid10  moralid3  moralid4  moralid5  moralid6  moralid7  moralid8  moralid9  mcoop1  mcoop2  mcoop3  mcoop4  mcoop5  mcoop6  mcoop7  narc2  narc4  narc5  narc6  nidentity1  omind2  omind3  omind4  hygiene4  hygiene5  psupport4  political_ideology  happy  slf_ladder  riskperc1  riskperc2  self_esteem  sbelong2  sbelong3  tested_positive  slfcont1  slfcont2
##   4   6  cnarc1  cnarc2  cnarc3  ctheory1  ctheory2  ctheory3  ctheory4  CRT1  CRT2  CRT3  generosity1  generosity2  generosity3  health_cond  know_tested_positive  moralid10  moralid3  moralid4  moralid5  moralid6  moralid7  moralid8  moralid9  mcoop1  mcoop2  mcoop3  mcoop4  mcoop5  mcoop6  mcoop7  narc2  narc4  narc5  narc6  nidentity1  omind2  omind3  omind4  hygiene4  hygiene5  psupport4  political_ideology  happy  slf_ladder  riskperc1  riskperc2  self_esteem  sbelong2  sbelong3  tested_positive  slfcont1  slfcont2
##   4   7  cnarc1  cnarc2  cnarc3  ctheory1  ctheory2  ctheory3  ctheory4  CRT1  CRT2  CRT3  generosity1  generosity2  generosity3  health_cond  know_tested_positive  moralid10  moralid3  moralid4  moralid5  moralid6  moralid7  moralid8  moralid9  mcoop1  mcoop2  mcoop3  mcoop4  mcoop5  mcoop6  mcoop7  narc2  narc4  narc5  narc6  nidentity1  omind2  omind3  omind4  hygiene4  hygiene5  psupport4  political_ideology  happy  slf_ladder  riskperc1  riskperc2  self_esteem  sbelong2  sbelong3  tested_positive  slfcont1  slfcont2
##   4   8  cnarc1  cnarc2  cnarc3  ctheory1  ctheory2  ctheory3  ctheory4  CRT1  CRT2  CRT3  generosity1  generosity2  generosity3  health_cond  know_tested_positive  moralid10  moralid3  moralid4  moralid5  moralid6  moralid7  moralid8  moralid9  mcoop1  mcoop2  mcoop3  mcoop4  mcoop5  mcoop6  mcoop7  narc2  narc4  narc5  narc6  nidentity1  omind2  omind3  omind4  hygiene4  hygiene5  psupport4  political_ideology  happy  slf_ladder  riskperc1  riskperc2  self_esteem  sbelong2  sbelong3  tested_positive  slfcont1  slfcont2
##   4   9  cnarc1  cnarc2  cnarc3  ctheory1  ctheory2  ctheory3  ctheory4  CRT1  CRT2  CRT3  generosity1  generosity2  generosity3  health_cond  know_tested_positive  moralid10  moralid3  moralid4  moralid5  moralid6  moralid7  moralid8  moralid9  mcoop1  mcoop2  mcoop3  mcoop4  mcoop5  mcoop6  mcoop7  narc2  narc4  narc5  narc6  nidentity1  omind2  omind3  omind4  hygiene4  hygiene5  psupport4  political_ideology  happy  slf_ladder  riskperc1  riskperc2  self_esteem  sbelong2  sbelong3  tested_positive  slfcont1  slfcont2
##   4   10  cnarc1  cnarc2  cnarc3  ctheory1  ctheory2  ctheory3  ctheory4  CRT1  CRT2  CRT3  generosity1  generosity2  generosity3  health_cond  know_tested_positive  moralid10  moralid3  moralid4  moralid5  moralid6  moralid7  moralid8  moralid9  mcoop1  mcoop2  mcoop3  mcoop4  mcoop5  mcoop6  mcoop7  narc2  narc4  narc5  narc6  nidentity1  omind2  omind3  omind4  hygiene4  hygiene5  psupport4  political_ideology  happy  slf_ladder  riskperc1  riskperc2  self_esteem  sbelong2  sbelong3  tested_positive  slfcont1  slfcont2
##   5   1  cnarc1  cnarc2  cnarc3  ctheory1  ctheory2  ctheory3  ctheory4  CRT1  CRT2  CRT3  generosity1  generosity2  generosity3  health_cond  know_tested_positive  moralid10  moralid3  moralid4  moralid5  moralid6  moralid7  moralid8  moralid9  mcoop1  mcoop2  mcoop3  mcoop4  mcoop5  mcoop6  mcoop7  narc2  narc4  narc5  narc6  nidentity1  omind2  omind3  omind4  hygiene4  hygiene5  psupport4  political_ideology  happy  slf_ladder  riskperc1  riskperc2  self_esteem  sbelong2  sbelong3  tested_positive  slfcont1  slfcont2
##   5   2  cnarc1  cnarc2  cnarc3  ctheory1  ctheory2  ctheory3  ctheory4  CRT1  CRT2  CRT3  generosity1  generosity2  generosity3  health_cond  know_tested_positive  moralid10  moralid3  moralid4  moralid5  moralid6  moralid7  moralid8  moralid9  mcoop1  mcoop2  mcoop3  mcoop4  mcoop5  mcoop6  mcoop7  narc2  narc4  narc5  narc6  nidentity1  omind2  omind3  omind4  hygiene4  hygiene5  psupport4  political_ideology  happy  slf_ladder  riskperc1  riskperc2  self_esteem  sbelong2  sbelong3  tested_positive  slfcont1  slfcont2
##   5   3  cnarc1  cnarc2  cnarc3  ctheory1  ctheory2  ctheory3  ctheory4  CRT1  CRT2  CRT3  generosity1  generosity2  generosity3  health_cond  know_tested_positive  moralid10  moralid3  moralid4  moralid5  moralid6  moralid7  moralid8  moralid9  mcoop1  mcoop2  mcoop3  mcoop4  mcoop5  mcoop6  mcoop7  narc2  narc4  narc5  narc6  nidentity1  omind2  omind3  omind4  hygiene4  hygiene5  psupport4  political_ideology  happy  slf_ladder  riskperc1  riskperc2  self_esteem  sbelong2  sbelong3  tested_positive  slfcont1  slfcont2
##   5   4  cnarc1  cnarc2  cnarc3  ctheory1  ctheory2  ctheory3  ctheory4  CRT1  CRT2  CRT3  generosity1  generosity2  generosity3  health_cond  know_tested_positive  moralid10  moralid3  moralid4  moralid5  moralid6  moralid7  moralid8  moralid9  mcoop1  mcoop2  mcoop3  mcoop4  mcoop5  mcoop6  mcoop7  narc2  narc4  narc5  narc6  nidentity1  omind2  omind3  omind4  hygiene4  hygiene5  psupport4  political_ideology  happy  slf_ladder  riskperc1  riskperc2  self_esteem  sbelong2  sbelong3  tested_positive  slfcont1  slfcont2
##   5   5  cnarc1  cnarc2  cnarc3  ctheory1  ctheory2  ctheory3  ctheory4  CRT1  CRT2  CRT3  generosity1  generosity2  generosity3  health_cond  know_tested_positive  moralid10  moralid3  moralid4  moralid5  moralid6  moralid7  moralid8  moralid9  mcoop1  mcoop2  mcoop3  mcoop4  mcoop5  mcoop6  mcoop7  narc2  narc4  narc5  narc6  nidentity1  omind2  omind3  omind4  hygiene4  hygiene5  psupport4  political_ideology  happy  slf_ladder  riskperc1  riskperc2  self_esteem  sbelong2  sbelong3  tested_positive  slfcont1  slfcont2
##   5   6  cnarc1  cnarc2  cnarc3  ctheory1  ctheory2  ctheory3  ctheory4  CRT1  CRT2  CRT3  generosity1  generosity2  generosity3  health_cond  know_tested_positive  moralid10  moralid3  moralid4  moralid5  moralid6  moralid7  moralid8  moralid9  mcoop1  mcoop2  mcoop3  mcoop4  mcoop5  mcoop6  mcoop7  narc2  narc4  narc5  narc6  nidentity1  omind2  omind3  omind4  hygiene4  hygiene5  psupport4  political_ideology  happy  slf_ladder  riskperc1  riskperc2  self_esteem  sbelong2  sbelong3  tested_positive  slfcont1  slfcont2
##   5   7  cnarc1  cnarc2  cnarc3  ctheory1  ctheory2  ctheory3  ctheory4  CRT1  CRT2  CRT3  generosity1  generosity2  generosity3  health_cond  know_tested_positive  moralid10  moralid3  moralid4  moralid5  moralid6  moralid7  moralid8  moralid9  mcoop1  mcoop2  mcoop3  mcoop4  mcoop5  mcoop6  mcoop7  narc2  narc4  narc5  narc6  nidentity1  omind2  omind3  omind4  hygiene4  hygiene5  psupport4  political_ideology  happy  slf_ladder  riskperc1  riskperc2  self_esteem  sbelong2  sbelong3  tested_positive  slfcont1  slfcont2
##   5   8  cnarc1  cnarc2  cnarc3  ctheory1  ctheory2  ctheory3  ctheory4  CRT1  CRT2  CRT3  generosity1  generosity2  generosity3  health_cond  know_tested_positive  moralid10  moralid3  moralid4  moralid5  moralid6  moralid7  moralid8  moralid9  mcoop1  mcoop2  mcoop3  mcoop4  mcoop5  mcoop6  mcoop7  narc2  narc4  narc5  narc6  nidentity1  omind2  omind3  omind4  hygiene4  hygiene5  psupport4  political_ideology  happy  slf_ladder  riskperc1  riskperc2  self_esteem  sbelong2  sbelong3  tested_positive  slfcont1  slfcont2
##   5   9  cnarc1  cnarc2  cnarc3  ctheory1  ctheory2  ctheory3  ctheory4  CRT1  CRT2  CRT3  generosity1  generosity2  generosity3  health_cond  know_tested_positive  moralid10  moralid3  moralid4  moralid5  moralid6  moralid7  moralid8  moralid9  mcoop1  mcoop2  mcoop3  mcoop4  mcoop5  mcoop6  mcoop7  narc2  narc4  narc5  narc6  nidentity1  omind2  omind3  omind4  hygiene4  hygiene5  psupport4  political_ideology  happy  slf_ladder  riskperc1  riskperc2  self_esteem  sbelong2  sbelong3  tested_positive  slfcont1  slfcont2
##   5   10  cnarc1  cnarc2  cnarc3  ctheory1  ctheory2  ctheory3  ctheory4  CRT1  CRT2  CRT3  generosity1  generosity2  generosity3  health_cond  know_tested_positive  moralid10  moralid3  moralid4  moralid5  moralid6  moralid7  moralid8  moralid9  mcoop1  mcoop2  mcoop3  mcoop4  mcoop5  mcoop6  mcoop7  narc2  narc4  narc5  narc6  nidentity1  omind2  omind3  omind4  hygiene4  hygiene5  psupport4  political_ideology  happy  slf_ladder  riskperc1  riskperc2  self_esteem  sbelong2  sbelong3  tested_positive  slfcont1  slfcont2
##   6   1  cnarc1  cnarc2  cnarc3  ctheory1  ctheory2  ctheory3  ctheory4  CRT1  CRT2  CRT3  generosity1  generosity2  generosity3  health_cond  know_tested_positive  moralid10  moralid3  moralid4  moralid5  moralid6  moralid7  moralid8  moralid9  mcoop1  mcoop2  mcoop3  mcoop4  mcoop5  mcoop6  mcoop7  narc2  narc4  narc5  narc6  nidentity1  omind2  omind3  omind4  hygiene4  hygiene5  psupport4  political_ideology  happy  slf_ladder  riskperc1  riskperc2  self_esteem  sbelong2  sbelong3  tested_positive  slfcont1  slfcont2
##   6   2  cnarc1  cnarc2  cnarc3  ctheory1  ctheory2  ctheory3  ctheory4  CRT1  CRT2  CRT3  generosity1  generosity2  generosity3  health_cond  know_tested_positive  moralid10  moralid3  moralid4  moralid5  moralid6  moralid7  moralid8  moralid9  mcoop1  mcoop2  mcoop3  mcoop4  mcoop5  mcoop6  mcoop7  narc2  narc4  narc5  narc6  nidentity1  omind2  omind3  omind4  hygiene4  hygiene5  psupport4  political_ideology  happy  slf_ladder  riskperc1  riskperc2  self_esteem  sbelong2  sbelong3  tested_positive  slfcont1  slfcont2
##   6   3  cnarc1  cnarc2  cnarc3  ctheory1  ctheory2  ctheory3  ctheory4  CRT1  CRT2  CRT3  generosity1  generosity2  generosity3  health_cond  know_tested_positive  moralid10  moralid3  moralid4  moralid5  moralid6  moralid7  moralid8  moralid9  mcoop1  mcoop2  mcoop3  mcoop4  mcoop5  mcoop6  mcoop7  narc2  narc4  narc5  narc6  nidentity1  omind2  omind3  omind4  hygiene4  hygiene5  psupport4  political_ideology  happy  slf_ladder  riskperc1  riskperc2  self_esteem  sbelong2  sbelong3  tested_positive  slfcont1  slfcont2
##   6   4  cnarc1  cnarc2  cnarc3  ctheory1  ctheory2  ctheory3  ctheory4  CRT1  CRT2  CRT3  generosity1  generosity2  generosity3  health_cond  know_tested_positive  moralid10  moralid3  moralid4  moralid5  moralid6  moralid7  moralid8  moralid9  mcoop1  mcoop2  mcoop3  mcoop4  mcoop5  mcoop6  mcoop7  narc2  narc4  narc5  narc6  nidentity1  omind2  omind3  omind4  hygiene4  hygiene5  psupport4  political_ideology  happy  slf_ladder  riskperc1  riskperc2  self_esteem  sbelong2  sbelong3  tested_positive  slfcont1  slfcont2
##   6   5  cnarc1  cnarc2  cnarc3  ctheory1  ctheory2  ctheory3  ctheory4  CRT1  CRT2  CRT3  generosity1  generosity2  generosity3  health_cond  know_tested_positive  moralid10  moralid3  moralid4  moralid5  moralid6  moralid7  moralid8  moralid9  mcoop1  mcoop2  mcoop3  mcoop4  mcoop5  mcoop6  mcoop7  narc2  narc4  narc5  narc6  nidentity1  omind2  omind3  omind4  hygiene4  hygiene5  psupport4  political_ideology  happy  slf_ladder  riskperc1  riskperc2  self_esteem  sbelong2  sbelong3  tested_positive  slfcont1  slfcont2
##   6   6  cnarc1  cnarc2  cnarc3  ctheory1  ctheory2  ctheory3  ctheory4  CRT1  CRT2  CRT3  generosity1  generosity2  generosity3  health_cond  know_tested_positive  moralid10  moralid3  moralid4  moralid5  moralid6  moralid7  moralid8  moralid9  mcoop1  mcoop2  mcoop3  mcoop4  mcoop5  mcoop6  mcoop7  narc2  narc4  narc5  narc6  nidentity1  omind2  omind3  omind4  hygiene4  hygiene5  psupport4  political_ideology  happy  slf_ladder  riskperc1  riskperc2  self_esteem  sbelong2  sbelong3  tested_positive  slfcont1  slfcont2
##   6   7  cnarc1  cnarc2  cnarc3  ctheory1  ctheory2  ctheory3  ctheory4  CRT1  CRT2  CRT3  generosity1  generosity2  generosity3  health_cond  know_tested_positive  moralid10  moralid3  moralid4  moralid5  moralid6  moralid7  moralid8  moralid9  mcoop1  mcoop2  mcoop3  mcoop4  mcoop5  mcoop6  mcoop7  narc2  narc4  narc5  narc6  nidentity1  omind2  omind3  omind4  hygiene4  hygiene5  psupport4  political_ideology  happy  slf_ladder  riskperc1  riskperc2  self_esteem  sbelong2  sbelong3  tested_positive  slfcont1  slfcont2
##   6   8  cnarc1  cnarc2  cnarc3  ctheory1  ctheory2  ctheory3  ctheory4  CRT1  CRT2  CRT3  generosity1  generosity2  generosity3  health_cond  know_tested_positive  moralid10  moralid3  moralid4  moralid5  moralid6  moralid7  moralid8  moralid9  mcoop1  mcoop2  mcoop3  mcoop4  mcoop5  mcoop6  mcoop7  narc2  narc4  narc5  narc6  nidentity1  omind2  omind3  omind4  hygiene4  hygiene5  psupport4  political_ideology  happy  slf_ladder  riskperc1  riskperc2  self_esteem  sbelong2  sbelong3  tested_positive  slfcont1  slfcont2
##   6   9  cnarc1  cnarc2  cnarc3  ctheory1  ctheory2  ctheory3  ctheory4  CRT1  CRT2  CRT3  generosity1  generosity2  generosity3  health_cond  know_tested_positive  moralid10  moralid3  moralid4  moralid5  moralid6  moralid7  moralid8  moralid9  mcoop1  mcoop2  mcoop3  mcoop4  mcoop5  mcoop6  mcoop7  narc2  narc4  narc5  narc6  nidentity1  omind2  omind3  omind4  hygiene4  hygiene5  psupport4  political_ideology  happy  slf_ladder  riskperc1  riskperc2  self_esteem  sbelong2  sbelong3  tested_positive  slfcont1  slfcont2
##   6   10  cnarc1  cnarc2  cnarc3  ctheory1  ctheory2  ctheory3  ctheory4  CRT1  CRT2  CRT3  generosity1  generosity2  generosity3  health_cond  know_tested_positive  moralid10  moralid3  moralid4  moralid5  moralid6  moralid7  moralid8  moralid9  mcoop1  mcoop2  mcoop3  mcoop4  mcoop5  mcoop6  mcoop7  narc2  narc4  narc5  narc6  nidentity1  omind2  omind3  omind4  hygiene4  hygiene5  psupport4  political_ideology  happy  slf_ladder  riskperc1  riskperc2  self_esteem  sbelong2  sbelong3  tested_positive  slfcont1  slfcont2
##   7   1  cnarc1  cnarc2  cnarc3  ctheory1  ctheory2  ctheory3  ctheory4  CRT1  CRT2  CRT3  generosity1  generosity2  generosity3  health_cond  know_tested_positive  moralid10  moralid3  moralid4  moralid5  moralid6  moralid7  moralid8  moralid9  mcoop1  mcoop2  mcoop3  mcoop4  mcoop5  mcoop6  mcoop7  narc2  narc4  narc5  narc6  nidentity1  omind2  omind3  omind4  hygiene4  hygiene5  psupport4  political_ideology  happy  slf_ladder  riskperc1  riskperc2  self_esteem  sbelong2  sbelong3  tested_positive  slfcont1  slfcont2
##   7   2  cnarc1  cnarc2  cnarc3  ctheory1  ctheory2  ctheory3  ctheory4  CRT1  CRT2  CRT3  generosity1  generosity2  generosity3  health_cond  know_tested_positive  moralid10  moralid3  moralid4  moralid5  moralid6  moralid7  moralid8  moralid9  mcoop1  mcoop2  mcoop3  mcoop4  mcoop5  mcoop6  mcoop7  narc2  narc4  narc5  narc6  nidentity1  omind2  omind3  omind4  hygiene4  hygiene5  psupport4  political_ideology  happy  slf_ladder  riskperc1  riskperc2  self_esteem  sbelong2  sbelong3  tested_positive  slfcont1  slfcont2
##   7   3  cnarc1  cnarc2  cnarc3  ctheory1  ctheory2  ctheory3  ctheory4  CRT1  CRT2  CRT3  generosity1  generosity2  generosity3  health_cond  know_tested_positive  moralid10  moralid3  moralid4  moralid5  moralid6  moralid7  moralid8  moralid9  mcoop1  mcoop2  mcoop3  mcoop4  mcoop5  mcoop6  mcoop7  narc2  narc4  narc5  narc6  nidentity1  omind2  omind3  omind4  hygiene4  hygiene5  psupport4  political_ideology  happy  slf_ladder  riskperc1  riskperc2  self_esteem  sbelong2  sbelong3  tested_positive  slfcont1  slfcont2
##   7   4  cnarc1  cnarc2  cnarc3  ctheory1  ctheory2  ctheory3  ctheory4  CRT1  CRT2  CRT3  generosity1  generosity2  generosity3  health_cond  know_tested_positive  moralid10  moralid3  moralid4  moralid5  moralid6  moralid7  moralid8  moralid9  mcoop1  mcoop2  mcoop3  mcoop4  mcoop5  mcoop6  mcoop7  narc2  narc4  narc5  narc6  nidentity1  omind2  omind3  omind4  hygiene4  hygiene5  psupport4  political_ideology  happy  slf_ladder  riskperc1  riskperc2  self_esteem  sbelong2  sbelong3  tested_positive  slfcont1  slfcont2
##   7   5  cnarc1  cnarc2  cnarc3  ctheory1  ctheory2  ctheory3  ctheory4  CRT1  CRT2  CRT3  generosity1  generosity2  generosity3  health_cond  know_tested_positive  moralid10  moralid3  moralid4  moralid5  moralid6  moralid7  moralid8  moralid9  mcoop1  mcoop2  mcoop3  mcoop4  mcoop5  mcoop6  mcoop7  narc2  narc4  narc5  narc6  nidentity1  omind2  omind3  omind4  hygiene4  hygiene5  psupport4  political_ideology  happy  slf_ladder  riskperc1  riskperc2  self_esteem  sbelong2  sbelong3  tested_positive  slfcont1  slfcont2
##   7   6  cnarc1  cnarc2  cnarc3  ctheory1  ctheory2  ctheory3  ctheory4  CRT1  CRT2  CRT3  generosity1  generosity2  generosity3  health_cond  know_tested_positive  moralid10  moralid3  moralid4  moralid5  moralid6  moralid7  moralid8  moralid9  mcoop1  mcoop2  mcoop3  mcoop4  mcoop5  mcoop6  mcoop7  narc2  narc4  narc5  narc6  nidentity1  omind2  omind3  omind4  hygiene4  hygiene5  psupport4  political_ideology  happy  slf_ladder  riskperc1  riskperc2  self_esteem  sbelong2  sbelong3  tested_positive  slfcont1  slfcont2
##   7   7  cnarc1  cnarc2  cnarc3  ctheory1  ctheory2  ctheory3  ctheory4  CRT1  CRT2  CRT3  generosity1  generosity2  generosity3  health_cond  know_tested_positive  moralid10  moralid3  moralid4  moralid5  moralid6  moralid7  moralid8  moralid9  mcoop1  mcoop2  mcoop3  mcoop4  mcoop5  mcoop6  mcoop7  narc2  narc4  narc5  narc6  nidentity1  omind2  omind3  omind4  hygiene4  hygiene5  psupport4  political_ideology  happy  slf_ladder  riskperc1  riskperc2  self_esteem  sbelong2  sbelong3  tested_positive  slfcont1  slfcont2
##   7   8  cnarc1  cnarc2  cnarc3  ctheory1  ctheory2  ctheory3  ctheory4  CRT1  CRT2  CRT3  generosity1  generosity2  generosity3  health_cond  know_tested_positive  moralid10  moralid3  moralid4  moralid5  moralid6  moralid7  moralid8  moralid9  mcoop1  mcoop2  mcoop3  mcoop4  mcoop5  mcoop6  mcoop7  narc2  narc4  narc5  narc6  nidentity1  omind2  omind3  omind4  hygiene4  hygiene5  psupport4  political_ideology  happy  slf_ladder  riskperc1  riskperc2  self_esteem  sbelong2  sbelong3  tested_positive  slfcont1  slfcont2
##   7   9  cnarc1  cnarc2  cnarc3  ctheory1  ctheory2  ctheory3  ctheory4  CRT1  CRT2  CRT3  generosity1  generosity2  generosity3  health_cond  know_tested_positive  moralid10  moralid3  moralid4  moralid5  moralid6  moralid7  moralid8  moralid9  mcoop1  mcoop2  mcoop3  mcoop4  mcoop5  mcoop6  mcoop7  narc2  narc4  narc5  narc6  nidentity1  omind2  omind3  omind4  hygiene4  hygiene5  psupport4  political_ideology  happy  slf_ladder  riskperc1  riskperc2  self_esteem  sbelong2  sbelong3  tested_positive  slfcont1  slfcont2
##   7   10  cnarc1  cnarc2  cnarc3  ctheory1  ctheory2  ctheory3  ctheory4  CRT1  CRT2  CRT3  generosity1  generosity2  generosity3  health_cond  know_tested_positive  moralid10  moralid3  moralid4  moralid5  moralid6  moralid7  moralid8  moralid9  mcoop1  mcoop2  mcoop3  mcoop4  mcoop5  mcoop6  mcoop7  narc2  narc4  narc5  narc6  nidentity1  omind2  omind3  omind4  hygiene4  hygiene5  psupport4  political_ideology  happy  slf_ladder  riskperc1  riskperc2  self_esteem  sbelong2  sbelong3  tested_positive  slfcont1  slfcont2
##   8   1  cnarc1  cnarc2  cnarc3  ctheory1  ctheory2  ctheory3  ctheory4  CRT1  CRT2  CRT3  generosity1  generosity2  generosity3  health_cond  know_tested_positive  moralid10  moralid3  moralid4  moralid5  moralid6  moralid7  moralid8  moralid9  mcoop1  mcoop2  mcoop3  mcoop4  mcoop5  mcoop6  mcoop7  narc2  narc4  narc5  narc6  nidentity1  omind2  omind3  omind4  hygiene4  hygiene5  psupport4  political_ideology  happy  slf_ladder  riskperc1  riskperc2  self_esteem  sbelong2  sbelong3  tested_positive  slfcont1  slfcont2
##   8   2  cnarc1  cnarc2  cnarc3  ctheory1  ctheory2  ctheory3  ctheory4  CRT1  CRT2  CRT3  generosity1  generosity2  generosity3  health_cond  know_tested_positive  moralid10  moralid3  moralid4  moralid5  moralid6  moralid7  moralid8  moralid9  mcoop1  mcoop2  mcoop3  mcoop4  mcoop5  mcoop6  mcoop7  narc2  narc4  narc5  narc6  nidentity1  omind2  omind3  omind4  hygiene4  hygiene5  psupport4  political_ideology  happy  slf_ladder  riskperc1  riskperc2  self_esteem  sbelong2  sbelong3  tested_positive  slfcont1  slfcont2
##   8   3  cnarc1  cnarc2  cnarc3  ctheory1  ctheory2  ctheory3  ctheory4  CRT1  CRT2  CRT3  generosity1  generosity2  generosity3  health_cond  know_tested_positive  moralid10  moralid3  moralid4  moralid5  moralid6  moralid7  moralid8  moralid9  mcoop1  mcoop2  mcoop3  mcoop4  mcoop5  mcoop6  mcoop7  narc2  narc4  narc5  narc6  nidentity1  omind2  omind3  omind4  hygiene4  hygiene5  psupport4  political_ideology  happy  slf_ladder  riskperc1  riskperc2  self_esteem  sbelong2  sbelong3  tested_positive  slfcont1  slfcont2
##   8   4  cnarc1  cnarc2  cnarc3  ctheory1  ctheory2  ctheory3  ctheory4  CRT1  CRT2  CRT3  generosity1  generosity2  generosity3  health_cond  know_tested_positive  moralid10  moralid3  moralid4  moralid5  moralid6  moralid7  moralid8  moralid9  mcoop1  mcoop2  mcoop3  mcoop4  mcoop5  mcoop6  mcoop7  narc2  narc4  narc5  narc6  nidentity1  omind2  omind3  omind4  hygiene4  hygiene5  psupport4  political_ideology  happy  slf_ladder  riskperc1  riskperc2  self_esteem  sbelong2  sbelong3  tested_positive  slfcont1  slfcont2
##   8   5  cnarc1  cnarc2  cnarc3  ctheory1  ctheory2  ctheory3  ctheory4  CRT1  CRT2  CRT3  generosity1  generosity2  generosity3  health_cond  know_tested_positive  moralid10  moralid3  moralid4  moralid5  moralid6  moralid7  moralid8  moralid9  mcoop1  mcoop2  mcoop3  mcoop4  mcoop5  mcoop6  mcoop7  narc2  narc4  narc5  narc6  nidentity1  omind2  omind3  omind4  hygiene4  hygiene5  psupport4  political_ideology  happy  slf_ladder  riskperc1  riskperc2  self_esteem  sbelong2  sbelong3  tested_positive  slfcont1  slfcont2
##   8   6  cnarc1  cnarc2  cnarc3  ctheory1  ctheory2  ctheory3  ctheory4  CRT1  CRT2  CRT3  generosity1  generosity2  generosity3  health_cond  know_tested_positive  moralid10  moralid3  moralid4  moralid5  moralid6  moralid7  moralid8  moralid9  mcoop1  mcoop2  mcoop3  mcoop4  mcoop5  mcoop6  mcoop7  narc2  narc4  narc5  narc6  nidentity1  omind2  omind3  omind4  hygiene4  hygiene5  psupport4  political_ideology  happy  slf_ladder  riskperc1  riskperc2  self_esteem  sbelong2  sbelong3  tested_positive  slfcont1  slfcont2
##   8   7  cnarc1  cnarc2  cnarc3  ctheory1  ctheory2  ctheory3  ctheory4  CRT1  CRT2  CRT3  generosity1  generosity2  generosity3  health_cond  know_tested_positive  moralid10  moralid3  moralid4  moralid5  moralid6  moralid7  moralid8  moralid9  mcoop1  mcoop2  mcoop3  mcoop4  mcoop5  mcoop6  mcoop7  narc2  narc4  narc5  narc6  nidentity1  omind2  omind3  omind4  hygiene4  hygiene5  psupport4  political_ideology  happy  slf_ladder  riskperc1  riskperc2  self_esteem  sbelong2  sbelong3  tested_positive  slfcont1  slfcont2
##   8   8  cnarc1  cnarc2  cnarc3  ctheory1  ctheory2  ctheory3  ctheory4  CRT1  CRT2  CRT3  generosity1  generosity2  generosity3  health_cond  know_tested_positive  moralid10  moralid3  moralid4  moralid5  moralid6  moralid7  moralid8  moralid9  mcoop1  mcoop2  mcoop3  mcoop4  mcoop5  mcoop6  mcoop7  narc2  narc4  narc5  narc6  nidentity1  omind2  omind3  omind4  hygiene4  hygiene5  psupport4  political_ideology  happy  slf_ladder  riskperc1  riskperc2  self_esteem  sbelong2  sbelong3  tested_positive  slfcont1  slfcont2
##   8   9  cnarc1  cnarc2  cnarc3  ctheory1  ctheory2  ctheory3  ctheory4  CRT1  CRT2  CRT3  generosity1  generosity2  generosity3  health_cond  know_tested_positive  moralid10  moralid3  moralid4  moralid5  moralid6  moralid7  moralid8  moralid9  mcoop1  mcoop2  mcoop3  mcoop4  mcoop5  mcoop6  mcoop7  narc2  narc4  narc5  narc6  nidentity1  omind2  omind3  omind4  hygiene4  hygiene5  psupport4  political_ideology  happy  slf_ladder  riskperc1  riskperc2  self_esteem  sbelong2  sbelong3  tested_positive  slfcont1  slfcont2
##   8   10  cnarc1  cnarc2  cnarc3  ctheory1  ctheory2  ctheory3  ctheory4  CRT1  CRT2  CRT3  generosity1  generosity2  generosity3  health_cond  know_tested_positive  moralid10  moralid3  moralid4  moralid5  moralid6  moralid7  moralid8  moralid9  mcoop1  mcoop2  mcoop3  mcoop4  mcoop5  mcoop6  mcoop7  narc2  narc4  narc5  narc6  nidentity1  omind2  omind3  omind4  hygiene4  hygiene5  psupport4  political_ideology  happy  slf_ladder  riskperc1  riskperc2  self_esteem  sbelong2  sbelong3  tested_positive  slfcont1  slfcont2
##   9   1  cnarc1  cnarc2  cnarc3  ctheory1  ctheory2  ctheory3  ctheory4  CRT1  CRT2  CRT3  generosity1  generosity2  generosity3  health_cond  know_tested_positive  moralid10  moralid3  moralid4  moralid5  moralid6  moralid7  moralid8  moralid9  mcoop1  mcoop2  mcoop3  mcoop4  mcoop5  mcoop6  mcoop7  narc2  narc4  narc5  narc6  nidentity1  omind2  omind3  omind4  hygiene4  hygiene5  psupport4  political_ideology  happy  slf_ladder  riskperc1  riskperc2  self_esteem  sbelong2  sbelong3  tested_positive  slfcont1  slfcont2
##   9   2  cnarc1  cnarc2  cnarc3  ctheory1  ctheory2  ctheory3  ctheory4  CRT1  CRT2  CRT3  generosity1  generosity2  generosity3  health_cond  know_tested_positive  moralid10  moralid3  moralid4  moralid5  moralid6  moralid7  moralid8  moralid9  mcoop1  mcoop2  mcoop3  mcoop4  mcoop5  mcoop6  mcoop7  narc2  narc4  narc5  narc6  nidentity1  omind2  omind3  omind4  hygiene4  hygiene5  psupport4  political_ideology  happy  slf_ladder  riskperc1  riskperc2  self_esteem  sbelong2  sbelong3  tested_positive  slfcont1  slfcont2
##   9   3  cnarc1  cnarc2  cnarc3  ctheory1  ctheory2  ctheory3  ctheory4  CRT1  CRT2  CRT3  generosity1  generosity2  generosity3  health_cond  know_tested_positive  moralid10  moralid3  moralid4  moralid5  moralid6  moralid7  moralid8  moralid9  mcoop1  mcoop2  mcoop3  mcoop4  mcoop5  mcoop6  mcoop7  narc2  narc4  narc5  narc6  nidentity1  omind2  omind3  omind4  hygiene4  hygiene5  psupport4  political_ideology  happy  slf_ladder  riskperc1  riskperc2  self_esteem  sbelong2  sbelong3  tested_positive  slfcont1  slfcont2
##   9   4  cnarc1  cnarc2  cnarc3  ctheory1  ctheory2  ctheory3  ctheory4  CRT1  CRT2  CRT3  generosity1  generosity2  generosity3  health_cond  know_tested_positive  moralid10  moralid3  moralid4  moralid5  moralid6  moralid7  moralid8  moralid9  mcoop1  mcoop2  mcoop3  mcoop4  mcoop5  mcoop6  mcoop7  narc2  narc4  narc5  narc6  nidentity1  omind2  omind3  omind4  hygiene4  hygiene5  psupport4  political_ideology  happy  slf_ladder  riskperc1  riskperc2  self_esteem  sbelong2  sbelong3  tested_positive  slfcont1  slfcont2
##   9   5  cnarc1  cnarc2  cnarc3  ctheory1  ctheory2  ctheory3  ctheory4  CRT1  CRT2  CRT3  generosity1  generosity2  generosity3  health_cond  know_tested_positive  moralid10  moralid3  moralid4  moralid5  moralid6  moralid7  moralid8  moralid9  mcoop1  mcoop2  mcoop3  mcoop4  mcoop5  mcoop6  mcoop7  narc2  narc4  narc5  narc6  nidentity1  omind2  omind3  omind4  hygiene4  hygiene5  psupport4  political_ideology  happy  slf_ladder  riskperc1  riskperc2  self_esteem  sbelong2  sbelong3  tested_positive  slfcont1  slfcont2
##   9   6  cnarc1  cnarc2  cnarc3  ctheory1  ctheory2  ctheory3  ctheory4  CRT1  CRT2  CRT3  generosity1  generosity2  generosity3  health_cond  know_tested_positive  moralid10  moralid3  moralid4  moralid5  moralid6  moralid7  moralid8  moralid9  mcoop1  mcoop2  mcoop3  mcoop4  mcoop5  mcoop6  mcoop7  narc2  narc4  narc5  narc6  nidentity1  omind2  omind3  omind4  hygiene4  hygiene5  psupport4  political_ideology  happy  slf_ladder  riskperc1  riskperc2  self_esteem  sbelong2  sbelong3  tested_positive  slfcont1  slfcont2
##   9   7  cnarc1  cnarc2  cnarc3  ctheory1  ctheory2  ctheory3  ctheory4  CRT1  CRT2  CRT3  generosity1  generosity2  generosity3  health_cond  know_tested_positive  moralid10  moralid3  moralid4  moralid5  moralid6  moralid7  moralid8  moralid9  mcoop1  mcoop2  mcoop3  mcoop4  mcoop5  mcoop6  mcoop7  narc2  narc4  narc5  narc6  nidentity1  omind2  omind3  omind4  hygiene4  hygiene5  psupport4  political_ideology  happy  slf_ladder  riskperc1  riskperc2  self_esteem  sbelong2  sbelong3  tested_positive  slfcont1  slfcont2
##   9   8  cnarc1  cnarc2  cnarc3  ctheory1  ctheory2  ctheory3  ctheory4  CRT1  CRT2  CRT3  generosity1  generosity2  generosity3  health_cond  know_tested_positive  moralid10  moralid3  moralid4  moralid5  moralid6  moralid7  moralid8  moralid9  mcoop1  mcoop2  mcoop3  mcoop4  mcoop5  mcoop6  mcoop7  narc2  narc4  narc5  narc6  nidentity1  omind2  omind3  omind4  hygiene4  hygiene5  psupport4  political_ideology  happy  slf_ladder  riskperc1  riskperc2  self_esteem  sbelong2  sbelong3  tested_positive  slfcont1  slfcont2
##   9   9  cnarc1  cnarc2  cnarc3  ctheory1  ctheory2  ctheory3  ctheory4  CRT1  CRT2  CRT3  generosity1  generosity2  generosity3  health_cond  know_tested_positive  moralid10  moralid3  moralid4  moralid5  moralid6  moralid7  moralid8  moralid9  mcoop1  mcoop2  mcoop3  mcoop4  mcoop5  mcoop6  mcoop7  narc2  narc4  narc5  narc6  nidentity1  omind2  omind3  omind4  hygiene4  hygiene5  psupport4  political_ideology  happy  slf_ladder  riskperc1  riskperc2  self_esteem  sbelong2  sbelong3  tested_positive  slfcont1  slfcont2
##   9   10  cnarc1  cnarc2  cnarc3  ctheory1  ctheory2  ctheory3  ctheory4  CRT1  CRT2  CRT3  generosity1  generosity2  generosity3  health_cond  know_tested_positive  moralid10  moralid3  moralid4  moralid5  moralid6  moralid7  moralid8  moralid9  mcoop1  mcoop2  mcoop3  mcoop4  mcoop5  mcoop6  mcoop7  narc2  narc4  narc5  narc6  nidentity1  omind2  omind3  omind4  hygiene4  hygiene5  psupport4  political_ideology  happy  slf_ladder  riskperc1  riskperc2  self_esteem  sbelong2  sbelong3  tested_positive  slfcont1  slfcont2
##   10   1  cnarc1  cnarc2  cnarc3  ctheory1  ctheory2  ctheory3  ctheory4  CRT1  CRT2  CRT3  generosity1  generosity2  generosity3  health_cond  know_tested_positive  moralid10  moralid3  moralid4  moralid5  moralid6  moralid7  moralid8  moralid9  mcoop1  mcoop2  mcoop3  mcoop4  mcoop5  mcoop6  mcoop7  narc2  narc4  narc5  narc6  nidentity1  omind2  omind3  omind4  hygiene4  hygiene5  psupport4  political_ideology  happy  slf_ladder  riskperc1  riskperc2  self_esteem  sbelong2  sbelong3  tested_positive  slfcont1  slfcont2
##   10   2  cnarc1  cnarc2  cnarc3  ctheory1  ctheory2  ctheory3  ctheory4  CRT1  CRT2  CRT3  generosity1  generosity2  generosity3  health_cond  know_tested_positive  moralid10  moralid3  moralid4  moralid5  moralid6  moralid7  moralid8  moralid9  mcoop1  mcoop2  mcoop3  mcoop4  mcoop5  mcoop6  mcoop7  narc2  narc4  narc5  narc6  nidentity1  omind2  omind3  omind4  hygiene4  hygiene5  psupport4  political_ideology  happy  slf_ladder  riskperc1  riskperc2  self_esteem  sbelong2  sbelong3  tested_positive  slfcont1  slfcont2
##   10   3  cnarc1  cnarc2  cnarc3  ctheory1  ctheory2  ctheory3  ctheory4  CRT1  CRT2  CRT3  generosity1  generosity2  generosity3  health_cond  know_tested_positive  moralid10  moralid3  moralid4  moralid5  moralid6  moralid7  moralid8  moralid9  mcoop1  mcoop2  mcoop3  mcoop4  mcoop5  mcoop6  mcoop7  narc2  narc4  narc5  narc6  nidentity1  omind2  omind3  omind4  hygiene4  hygiene5  psupport4  political_ideology  happy  slf_ladder  riskperc1  riskperc2  self_esteem  sbelong2  sbelong3  tested_positive  slfcont1  slfcont2
##   10   4  cnarc1  cnarc2  cnarc3  ctheory1  ctheory2  ctheory3  ctheory4  CRT1  CRT2  CRT3  generosity1  generosity2  generosity3  health_cond  know_tested_positive  moralid10  moralid3  moralid4  moralid5  moralid6  moralid7  moralid8  moralid9  mcoop1  mcoop2  mcoop3  mcoop4  mcoop5  mcoop6  mcoop7  narc2  narc4  narc5  narc6  nidentity1  omind2  omind3  omind4  hygiene4  hygiene5  psupport4  political_ideology  happy  slf_ladder  riskperc1  riskperc2  self_esteem  sbelong2  sbelong3  tested_positive  slfcont1  slfcont2
##   10   5  cnarc1  cnarc2  cnarc3  ctheory1  ctheory2  ctheory3  ctheory4  CRT1  CRT2  CRT3  generosity1  generosity2  generosity3  health_cond  know_tested_positive  moralid10  moralid3  moralid4  moralid5  moralid6  moralid7  moralid8  moralid9  mcoop1  mcoop2  mcoop3  mcoop4  mcoop5  mcoop6  mcoop7  narc2  narc4  narc5  narc6  nidentity1  omind2  omind3  omind4  hygiene4  hygiene5  psupport4  political_ideology  happy  slf_ladder  riskperc1  riskperc2  self_esteem  sbelong2  sbelong3  tested_positive  slfcont1  slfcont2
##   10   6  cnarc1  cnarc2  cnarc3  ctheory1  ctheory2  ctheory3  ctheory4  CRT1  CRT2  CRT3  generosity1  generosity2  generosity3  health_cond  know_tested_positive  moralid10  moralid3  moralid4  moralid5  moralid6  moralid7  moralid8  moralid9  mcoop1  mcoop2  mcoop3  mcoop4  mcoop5  mcoop6  mcoop7  narc2  narc4  narc5  narc6  nidentity1  omind2  omind3  omind4  hygiene4  hygiene5  psupport4  political_ideology  happy  slf_ladder  riskperc1  riskperc2  self_esteem  sbelong2  sbelong3  tested_positive  slfcont1  slfcont2
##   10   7  cnarc1  cnarc2  cnarc3  ctheory1  ctheory2  ctheory3  ctheory4  CRT1  CRT2  CRT3  generosity1  generosity2  generosity3  health_cond  know_tested_positive  moralid10  moralid3  moralid4  moralid5  moralid6  moralid7  moralid8  moralid9  mcoop1  mcoop2  mcoop3  mcoop4  mcoop5  mcoop6  mcoop7  narc2  narc4  narc5  narc6  nidentity1  omind2  omind3  omind4  hygiene4  hygiene5  psupport4  political_ideology  happy  slf_ladder  riskperc1  riskperc2  self_esteem  sbelong2  sbelong3  tested_positive  slfcont1  slfcont2
##   10   8  cnarc1  cnarc2  cnarc3  ctheory1  ctheory2  ctheory3  ctheory4  CRT1  CRT2  CRT3  generosity1  generosity2  generosity3  health_cond  know_tested_positive  moralid10  moralid3  moralid4  moralid5  moralid6  moralid7  moralid8  moralid9  mcoop1  mcoop2  mcoop3  mcoop4  mcoop5  mcoop6  mcoop7  narc2  narc4  narc5  narc6  nidentity1  omind2  omind3  omind4  hygiene4  hygiene5  psupport4  political_ideology  happy  slf_ladder  riskperc1  riskperc2  self_esteem  sbelong2  sbelong3  tested_positive  slfcont1  slfcont2
##   10   9  cnarc1  cnarc2  cnarc3  ctheory1  ctheory2  ctheory3  ctheory4  CRT1  CRT2  CRT3  generosity1  generosity2  generosity3  health_cond  know_tested_positive  moralid10  moralid3  moralid4  moralid5  moralid6  moralid7  moralid8  moralid9  mcoop1  mcoop2  mcoop3  mcoop4  mcoop5  mcoop6  mcoop7  narc2  narc4  narc5  narc6  nidentity1  omind2  omind3  omind4  hygiene4  hygiene5  psupport4  political_ideology  happy  slf_ladder  riskperc1  riskperc2  self_esteem  sbelong2  sbelong3  tested_positive  slfcont1  slfcont2
##   10   10  cnarc1  cnarc2  cnarc3  ctheory1  ctheory2  ctheory3  ctheory4  CRT1  CRT2  CRT3  generosity1  generosity2  generosity3  health_cond  know_tested_positive  moralid10  moralid3  moralid4  moralid5  moralid6  moralid7  moralid8  moralid9  mcoop1  mcoop2  mcoop3  mcoop4  mcoop5  mcoop6  mcoop7  narc2  narc4  narc5  narc6  nidentity1  omind2  omind3  omind4  hygiene4  hygiene5  psupport4  political_ideology  happy  slf_ladder  riskperc1  riskperc2  self_esteem  sbelong2  sbelong3  tested_positive  slfcont1  slfcont2
```

```
## Warning: Number of logged events: 4804
```

```
## 
##  iter imp variable
##   1   1  generosity1  generosity2  generosity3
##   1   2  generosity1  generosity2  generosity3
##   1   3  generosity1  generosity2  generosity3
##   1   4  generosity1  generosity2  generosity3
##   1   5  generosity1  generosity2  generosity3
##   1   6  generosity1  generosity2  generosity3
##   1   7  generosity1  generosity2  generosity3
##   1   8  generosity1  generosity2  generosity3
##   1   9  generosity1  generosity2  generosity3
##   1   10  generosity1  generosity2  generosity3
##   2   1  generosity1  generosity2  generosity3
##   2   2  generosity1  generosity2  generosity3
##   2   3  generosity1  generosity2  generosity3
##   2   4  generosity1  generosity2  generosity3
##   2   5  generosity1  generosity2  generosity3
##   2   6  generosity1  generosity2  generosity3
##   2   7  generosity1  generosity2  generosity3
##   2   8  generosity1  generosity2  generosity3
##   2   9  generosity1  generosity2  generosity3
##   2   10  generosity1  generosity2  generosity3
##   3   1  generosity1  generosity2  generosity3
##   3   2  generosity1  generosity2  generosity3
##   3   3  generosity1  generosity2  generosity3
##   3   4  generosity1  generosity2  generosity3
##   3   5  generosity1  generosity2  generosity3
##   3   6  generosity1  generosity2  generosity3
##   3   7  generosity1  generosity2  generosity3
##   3   8  generosity1  generosity2  generosity3
##   3   9  generosity1  generosity2  generosity3
##   3   10  generosity1  generosity2  generosity3
##   4   1  generosity1  generosity2  generosity3
##   4   2  generosity1  generosity2  generosity3
##   4   3  generosity1  generosity2  generosity3
##   4   4  generosity1  generosity2  generosity3
##   4   5  generosity1  generosity2  generosity3
##   4   6  generosity1  generosity2  generosity3
##   4   7  generosity1  generosity2  generosity3
##   4   8  generosity1  generosity2  generosity3
##   4   9  generosity1  generosity2  generosity3
##   4   10  generosity1  generosity2  generosity3
##   5   1  generosity1  generosity2  generosity3
##   5   2  generosity1  generosity2  generosity3
##   5   3  generosity1  generosity2  generosity3
##   5   4  generosity1  generosity2  generosity3
##   5   5  generosity1  generosity2  generosity3
##   5   6  generosity1  generosity2  generosity3
##   5   7  generosity1  generosity2  generosity3
##   5   8  generosity1  generosity2  generosity3
##   5   9  generosity1  generosity2  generosity3
##   5   10  generosity1  generosity2  generosity3
##   6   1  generosity1  generosity2  generosity3
##   6   2  generosity1  generosity2  generosity3
##   6   3  generosity1  generosity2  generosity3
##   6   4  generosity1  generosity2  generosity3
##   6   5  generosity1  generosity2  generosity3
##   6   6  generosity1  generosity2  generosity3
##   6   7  generosity1  generosity2  generosity3
##   6   8  generosity1  generosity2  generosity3
##   6   9  generosity1  generosity2  generosity3
##   6   10  generosity1  generosity2  generosity3
##   7   1  generosity1  generosity2  generosity3
##   7   2  generosity1  generosity2  generosity3
##   7   3  generosity1  generosity2  generosity3
##   7   4  generosity1  generosity2  generosity3
##   7   5  generosity1  generosity2  generosity3
##   7   6  generosity1  generosity2  generosity3
##   7   7  generosity1  generosity2  generosity3
##   7   8  generosity1  generosity2  generosity3
##   7   9  generosity1  generosity2  generosity3
##   7   10  generosity1  generosity2  generosity3
##   8   1  generosity1  generosity2  generosity3
##   8   2  generosity1  generosity2  generosity3
##   8   3  generosity1  generosity2  generosity3
##   8   4  generosity1  generosity2  generosity3
##   8   5  generosity1  generosity2  generosity3
##   8   6  generosity1  generosity2  generosity3
##   8   7  generosity1  generosity2  generosity3
##   8   8  generosity1  generosity2  generosity3
##   8   9  generosity1  generosity2  generosity3
##   8   10  generosity1  generosity2  generosity3
##   9   1  generosity1  generosity2  generosity3
##   9   2  generosity1  generosity2  generosity3
##   9   3  generosity1  generosity2  generosity3
##   9   4  generosity1  generosity2  generosity3
##   9   5  generosity1  generosity2  generosity3
##   9   6  generosity1  generosity2  generosity3
##   9   7  generosity1  generosity2  generosity3
##   9   8  generosity1  generosity2  generosity3
##   9   9  generosity1  generosity2  generosity3
##   9   10  generosity1  generosity2  generosity3
##   10   1  generosity1  generosity2  generosity3
##   10   2  generosity1  generosity2  generosity3
##   10   3  generosity1  generosity2  generosity3
##   10   4  generosity1  generosity2  generosity3
##   10   5  generosity1  generosity2  generosity3
##   10   6  generosity1  generosity2  generosity3
##   10   7  generosity1  generosity2  generosity3
##   10   8  generosity1  generosity2  generosity3
##   10   9  generosity1  generosity2  generosity3
##   10   10  generosity1  generosity2  generosity3
```

```
## Warning: Number of logged events: 4
```

```
## 
##  iter imp variable
##   1   1  children
##   1   2  children
##   1   3  children
##   1   4  children
##   1   5  children
##   1   6  children
##   1   7  children
##   1   8  children
##   1   9  children
##   1   10  children
##   2   1  children
##   2   2  children
##   2   3  children
##   2   4  children
##   2   5  children
##   2   6  children
##   2   7  children
##   2   8  children
##   2   9  children
##   2   10  children
##   3   1  children
##   3   2  children
##   3   3  children
##   3   4  children
##   3   5  children
##   3   6  children
##   3   7  children
##   3   8  children
##   3   9  children
##   3   10  children
##   4   1  children
##   4   2  children
##   4   3  children
##   4   4  children
##   4   5  children
##   4   6  children
##   4   7  children
##   4   8  children
##   4   9  children
##   4   10  children
##   5   1  children
##   5   2  children
##   5   3  children
##   5   4  children
##   5   5  children
##   5   6  children
##   5   7  children
##   5   8  children
##   5   9  children
##   5   10  children
##   6   1  children
##   6   2  children
##   6   3  children
##   6   4  children
##   6   5  children
##   6   6  children
##   6   7  children
##   6   8  children
##   6   9  children
##   6   10  children
##   7   1  children
##   7   2  children
##   7   3  children
##   7   4  children
##   7   5  children
##   7   6  children
##   7   7  children
##   7   8  children
##   7   9  children
##   7   10  children
##   8   1  children
##   8   2  children
##   8   3  children
##   8   4  children
##   8   5  children
##   8   6  children
##   8   7  children
##   8   8  children
##   8   9  children
##   8   10  children
##   9   1  children
##   9   2  children
##   9   3  children
##   9   4  children
##   9   5  children
##   9   6  children
##   9   7  children
##   9   8  children
##   9   9  children
##   9   10  children
##   10   1  children
##   10   2  children
##   10   3  children
##   10   4  children
##   10   5  children
##   10   6  children
##   10   7  children
##   10   8  children
##   10   9  children
##   10   10  children
```

```
## Warning: Number of logged events: 103
```

```
## 
##  iter imp variable
##   1   1
##   1   2
##   1   3
##   1   4
##   1   5
##   1   6
##   1   7
##   1   8
##   1   9
##   1   10
##   2   1
##   2   2
##   2   3
##   2   4
##   2   5
##   2   6
##   2   7
##   2   8
##   2   9
##   2   10
##   3   1
##   3   2
##   3   3
##   3   4
##   3   5
##   3   6
##   3   7
##   3   8
##   3   9
##   3   10
##   4   1
##   4   2
##   4   3
##   4   4
##   4   5
##   4   6
##   4   7
##   4   8
##   4   9
##   4   10
##   5   1
##   5   2
##   5   3
##   5   4
##   5   5
##   5   6
##   5   7
##   5   8
##   5   9
##   5   10
##   6   1
##   6   2
##   6   3
##   6   4
##   6   5
##   6   6
##   6   7
##   6   8
##   6   9
##   6   10
##   7   1
##   7   2
##   7   3
##   7   4
##   7   5
##   7   6
##   7   7
##   7   8
##   7   9
##   7   10
##   8   1
##   8   2
##   8   3
##   8   4
##   8   5
##   8   6
##   8   7
##   8   8
##   8   9
##   8   10
##   9   1
##   9   2
##   9   3
##   9   4
##   9   5
##   9   6
##   9   7
##   9   8
##   9   9
##   9   10
##   10   1
##   10   2
##   10   3
##   10   4
##   10   5
##   10   6
##   10   7
##   10   8
##   10   9
##   10   10
```

```
## Warning: Number of logged events: 4
```

```
## 
##  iter imp variable
##   1   1  age  children  cnarc1  cnarc2  cnarc3  ctheory1  ctheory2  ctheory3  ctheory4  CRT1  CRT2  CRT3  duration  employ_status1  generosity1  generosity2  generosity3  health_cond  know_tested_positive  ladder  marital1  mor_circle  moralid1  moralid10  moralid2  moralid3  moralid5  moralid6  moralid7  moralid8  moralid9  mcoop1  mcoop2  mcoop3  mcoop4  mcoop5  mcoop6  mcoop7  narc1  narc2  narc3  narc4  narc5  narc6  nidentity1  nidentity2  omind1  omind2  omind3  omind4  omind5  omind6  contact1  contact2  contact3  contact4  contact5  hygiene1  hygiene2  hygiene3  hygiene4  hygiene5  psupport1  psupport2  psupport3  psupport4  psupport5  political_ideology  happy  slf_ladder  riskperc1  riskperc2  self_esteem  sex1  sbelong1  sbelong2  sbelong3  sbelong4  tested_positive  optim1  optim2  slfcont1  slfcont3  slfcont4  urban
##   1   2  age  children  cnarc1  cnarc2  cnarc3  ctheory1  ctheory2  ctheory3  ctheory4  CRT1  CRT2  CRT3  duration  employ_status1  generosity1  generosity2  generosity3  health_cond  know_tested_positive  ladder  marital1  mor_circle  moralid1  moralid10  moralid2  moralid3  moralid5  moralid6  moralid7  moralid8  moralid9  mcoop1  mcoop2  mcoop3  mcoop4  mcoop5  mcoop6  mcoop7  narc1  narc2  narc3  narc4  narc5  narc6  nidentity1  nidentity2  omind1  omind2  omind3  omind4  omind5  omind6  contact1  contact2  contact3  contact4  contact5  hygiene1  hygiene2  hygiene3  hygiene4  hygiene5  psupport1  psupport2  psupport3  psupport4  psupport5  political_ideology  happy  slf_ladder  riskperc1  riskperc2  self_esteem  sex1  sbelong1  sbelong2  sbelong3  sbelong4  tested_positive  optim1  optim2  slfcont1  slfcont3  slfcont4  urban
##   1   3  age  children  cnarc1  cnarc2  cnarc3  ctheory1  ctheory2  ctheory3  ctheory4  CRT1  CRT2  CRT3  duration  employ_status1  generosity1  generosity2  generosity3  health_cond  know_tested_positive  ladder  marital1  mor_circle  moralid1  moralid10  moralid2  moralid3  moralid5  moralid6  moralid7  moralid8  moralid9  mcoop1  mcoop2  mcoop3  mcoop4  mcoop5  mcoop6  mcoop7  narc1  narc2  narc3  narc4  narc5  narc6  nidentity1  nidentity2  omind1  omind2  omind3  omind4  omind5  omind6  contact1  contact2  contact3  contact4  contact5  hygiene1  hygiene2  hygiene3  hygiene4  hygiene5  psupport1  psupport2  psupport3  psupport4  psupport5  political_ideology  happy  slf_ladder  riskperc1  riskperc2  self_esteem  sex1  sbelong1  sbelong2  sbelong3  sbelong4  tested_positive  optim1  optim2  slfcont1  slfcont3  slfcont4  urban
##   1   4  age  children  cnarc1  cnarc2  cnarc3  ctheory1  ctheory2  ctheory3  ctheory4  CRT1  CRT2  CRT3  duration  employ_status1  generosity1  generosity2  generosity3  health_cond  know_tested_positive  ladder  marital1  mor_circle  moralid1  moralid10  moralid2  moralid3  moralid5  moralid6  moralid7  moralid8  moralid9  mcoop1  mcoop2  mcoop3  mcoop4  mcoop5  mcoop6  mcoop7  narc1  narc2  narc3  narc4  narc5  narc6  nidentity1  nidentity2  omind1  omind2  omind3  omind4  omind5  omind6  contact1  contact2  contact3  contact4  contact5  hygiene1  hygiene2  hygiene3  hygiene4  hygiene5  psupport1  psupport2  psupport3  psupport4  psupport5  political_ideology  happy  slf_ladder  riskperc1  riskperc2  self_esteem  sex1  sbelong1  sbelong2  sbelong3  sbelong4  tested_positive  optim1  optim2  slfcont1  slfcont3  slfcont4  urban
##   1   5  age  children  cnarc1  cnarc2  cnarc3  ctheory1  ctheory2  ctheory3  ctheory4  CRT1  CRT2  CRT3  duration  employ_status1  generosity1  generosity2  generosity3  health_cond  know_tested_positive  ladder  marital1  mor_circle  moralid1  moralid10  moralid2  moralid3  moralid5  moralid6  moralid7  moralid8  moralid9  mcoop1  mcoop2  mcoop3  mcoop4  mcoop5  mcoop6  mcoop7  narc1  narc2  narc3  narc4  narc5  narc6  nidentity1  nidentity2  omind1  omind2  omind3  omind4  omind5  omind6  contact1  contact2  contact3  contact4  contact5  hygiene1  hygiene2  hygiene3  hygiene4  hygiene5  psupport1  psupport2  psupport3  psupport4  psupport5  political_ideology  happy  slf_ladder  riskperc1  riskperc2  self_esteem  sex1  sbelong1  sbelong2  sbelong3  sbelong4  tested_positive  optim1  optim2  slfcont1  slfcont3  slfcont4  urban
##   1   6  age  children  cnarc1  cnarc2  cnarc3  ctheory1  ctheory2  ctheory3  ctheory4  CRT1  CRT2  CRT3  duration  employ_status1  generosity1  generosity2  generosity3  health_cond  know_tested_positive  ladder  marital1  mor_circle  moralid1  moralid10  moralid2  moralid3  moralid5  moralid6  moralid7  moralid8  moralid9  mcoop1  mcoop2  mcoop3  mcoop4  mcoop5  mcoop6  mcoop7  narc1  narc2  narc3  narc4  narc5  narc6  nidentity1  nidentity2  omind1  omind2  omind3  omind4  omind5  omind6  contact1  contact2  contact3  contact4  contact5  hygiene1  hygiene2  hygiene3  hygiene4  hygiene5  psupport1  psupport2  psupport3  psupport4  psupport5  political_ideology  happy  slf_ladder  riskperc1  riskperc2  self_esteem  sex1  sbelong1  sbelong2  sbelong3  sbelong4  tested_positive  optim1  optim2  slfcont1  slfcont3  slfcont4  urban
##   1   7  age  children  cnarc1  cnarc2  cnarc3  ctheory1  ctheory2  ctheory3  ctheory4  CRT1  CRT2  CRT3  duration  employ_status1  generosity1  generosity2  generosity3  health_cond  know_tested_positive  ladder  marital1  mor_circle  moralid1  moralid10  moralid2  moralid3  moralid5  moralid6  moralid7  moralid8  moralid9  mcoop1  mcoop2  mcoop3  mcoop4  mcoop5  mcoop6  mcoop7  narc1  narc2  narc3  narc4  narc5  narc6  nidentity1  nidentity2  omind1  omind2  omind3  omind4  omind5  omind6  contact1  contact2  contact3  contact4  contact5  hygiene1  hygiene2  hygiene3  hygiene4  hygiene5  psupport1  psupport2  psupport3  psupport4  psupport5  political_ideology  happy  slf_ladder  riskperc1  riskperc2  self_esteem  sex1  sbelong1  sbelong2  sbelong3  sbelong4  tested_positive  optim1  optim2  slfcont1  slfcont3  slfcont4  urban
##   1   8  age  children  cnarc1  cnarc2  cnarc3  ctheory1  ctheory2  ctheory3  ctheory4  CRT1  CRT2  CRT3  duration  employ_status1  generosity1  generosity2  generosity3  health_cond  know_tested_positive  ladder  marital1  mor_circle  moralid1  moralid10  moralid2  moralid3  moralid5  moralid6  moralid7  moralid8  moralid9  mcoop1  mcoop2  mcoop3  mcoop4  mcoop5  mcoop6  mcoop7  narc1  narc2  narc3  narc4  narc5  narc6  nidentity1  nidentity2  omind1  omind2  omind3  omind4  omind5  omind6  contact1  contact2  contact3  contact4  contact5  hygiene1  hygiene2  hygiene3  hygiene4  hygiene5  psupport1  psupport2  psupport3  psupport4  psupport5  political_ideology  happy  slf_ladder  riskperc1  riskperc2  self_esteem  sex1  sbelong1  sbelong2  sbelong3  sbelong4  tested_positive  optim1  optim2  slfcont1  slfcont3  slfcont4  urban
##   1   9  age  children  cnarc1  cnarc2  cnarc3  ctheory1  ctheory2  ctheory3  ctheory4  CRT1  CRT2  CRT3  duration  employ_status1  generosity1  generosity2  generosity3  health_cond  know_tested_positive  ladder  marital1  mor_circle  moralid1  moralid10  moralid2  moralid3  moralid5  moralid6  moralid7  moralid8  moralid9  mcoop1  mcoop2  mcoop3  mcoop4  mcoop5  mcoop6  mcoop7  narc1  narc2  narc3  narc4  narc5  narc6  nidentity1  nidentity2  omind1  omind2  omind3  omind4  omind5  omind6  contact1  contact2  contact3  contact4  contact5  hygiene1  hygiene2  hygiene3  hygiene4  hygiene5  psupport1  psupport2  psupport3  psupport4  psupport5  political_ideology  happy  slf_ladder  riskperc1  riskperc2  self_esteem  sex1  sbelong1  sbelong2  sbelong3  sbelong4  tested_positive  optim1  optim2  slfcont1  slfcont3  slfcont4  urban
##   1   10  age  children  cnarc1  cnarc2  cnarc3  ctheory1  ctheory2  ctheory3  ctheory4  CRT1  CRT2  CRT3  duration  employ_status1  generosity1  generosity2  generosity3  health_cond  know_tested_positive  ladder  marital1  mor_circle  moralid1  moralid10  moralid2  moralid3  moralid5  moralid6  moralid7  moralid8  moralid9  mcoop1  mcoop2  mcoop3  mcoop4  mcoop5  mcoop6  mcoop7  narc1  narc2  narc3  narc4  narc5  narc6  nidentity1  nidentity2  omind1  omind2  omind3  omind4  omind5  omind6  contact1  contact2  contact3  contact4  contact5  hygiene1  hygiene2  hygiene3  hygiene4  hygiene5  psupport1  psupport2  psupport3  psupport4  psupport5  political_ideology  happy  slf_ladder  riskperc1  riskperc2  self_esteem  sex1  sbelong1  sbelong2  sbelong3  sbelong4  tested_positive  optim1  optim2  slfcont1  slfcont3  slfcont4  urban
##   2   1  age  children  cnarc1  cnarc2  cnarc3  ctheory1  ctheory2  ctheory3  ctheory4  CRT1  CRT2  CRT3  duration  employ_status1  generosity1  generosity2  generosity3  health_cond  know_tested_positive  ladder  marital1  mor_circle  moralid1  moralid10  moralid2  moralid3  moralid5  moralid6  moralid7  moralid8  moralid9  mcoop1  mcoop2  mcoop3  mcoop4  mcoop5  mcoop6  mcoop7  narc1  narc2  narc3  narc4  narc5  narc6  nidentity1  nidentity2  omind1  omind2  omind3  omind4  omind5  omind6  contact1  contact2  contact3  contact4  contact5  hygiene1  hygiene2  hygiene3  hygiene4  hygiene5  psupport1  psupport2  psupport3  psupport4  psupport5  political_ideology  happy  slf_ladder  riskperc1  riskperc2  self_esteem  sex1  sbelong1  sbelong2  sbelong3  sbelong4  tested_positive  optim1  optim2  slfcont1  slfcont3  slfcont4  urban
##   2   2  age  children  cnarc1  cnarc2  cnarc3  ctheory1  ctheory2  ctheory3  ctheory4  CRT1  CRT2  CRT3  duration  employ_status1  generosity1  generosity2  generosity3  health_cond  know_tested_positive  ladder  marital1  mor_circle  moralid1  moralid10  moralid2  moralid3  moralid5  moralid6  moralid7  moralid8  moralid9  mcoop1  mcoop2  mcoop3  mcoop4  mcoop5  mcoop6  mcoop7  narc1  narc2  narc3  narc4  narc5  narc6  nidentity1  nidentity2  omind1  omind2  omind3  omind4  omind5  omind6  contact1  contact2  contact3  contact4  contact5  hygiene1  hygiene2  hygiene3  hygiene4  hygiene5  psupport1  psupport2  psupport3  psupport4  psupport5  political_ideology  happy  slf_ladder  riskperc1  riskperc2  self_esteem  sex1  sbelong1  sbelong2  sbelong3  sbelong4  tested_positive  optim1  optim2  slfcont1  slfcont3  slfcont4  urban
##   2   3  age  children  cnarc1  cnarc2  cnarc3  ctheory1  ctheory2  ctheory3  ctheory4  CRT1  CRT2  CRT3  duration  employ_status1  generosity1  generosity2  generosity3  health_cond  know_tested_positive  ladder  marital1  mor_circle  moralid1  moralid10  moralid2  moralid3  moralid5  moralid6  moralid7  moralid8  moralid9  mcoop1  mcoop2  mcoop3  mcoop4  mcoop5  mcoop6  mcoop7  narc1  narc2  narc3  narc4  narc5  narc6  nidentity1  nidentity2  omind1  omind2  omind3  omind4  omind5  omind6  contact1  contact2  contact3  contact4  contact5  hygiene1  hygiene2  hygiene3  hygiene4  hygiene5  psupport1  psupport2  psupport3  psupport4  psupport5  political_ideology  happy  slf_ladder  riskperc1  riskperc2  self_esteem  sex1  sbelong1  sbelong2  sbelong3  sbelong4  tested_positive  optim1  optim2  slfcont1  slfcont3  slfcont4  urban
##   2   4  age  children  cnarc1  cnarc2  cnarc3  ctheory1  ctheory2  ctheory3  ctheory4  CRT1  CRT2  CRT3  duration  employ_status1  generosity1  generosity2  generosity3  health_cond  know_tested_positive  ladder  marital1  mor_circle  moralid1  moralid10  moralid2  moralid3  moralid5  moralid6  moralid7  moralid8  moralid9  mcoop1  mcoop2  mcoop3  mcoop4  mcoop5  mcoop6  mcoop7  narc1  narc2  narc3  narc4  narc5  narc6  nidentity1  nidentity2  omind1  omind2  omind3  omind4  omind5  omind6  contact1  contact2  contact3  contact4  contact5  hygiene1  hygiene2  hygiene3  hygiene4  hygiene5  psupport1  psupport2  psupport3  psupport4  psupport5  political_ideology  happy  slf_ladder  riskperc1  riskperc2  self_esteem  sex1  sbelong1  sbelong2  sbelong3  sbelong4  tested_positive  optim1  optim2  slfcont1  slfcont3  slfcont4  urban
##   2   5  age  children  cnarc1  cnarc2  cnarc3  ctheory1  ctheory2  ctheory3  ctheory4  CRT1  CRT2  CRT3  duration  employ_status1  generosity1  generosity2  generosity3  health_cond  know_tested_positive  ladder  marital1  mor_circle  moralid1  moralid10  moralid2  moralid3  moralid5  moralid6  moralid7  moralid8  moralid9  mcoop1  mcoop2  mcoop3  mcoop4  mcoop5  mcoop6  mcoop7  narc1  narc2  narc3  narc4  narc5  narc6  nidentity1  nidentity2  omind1  omind2  omind3  omind4  omind5  omind6  contact1  contact2  contact3  contact4  contact5  hygiene1  hygiene2  hygiene3  hygiene4  hygiene5  psupport1  psupport2  psupport3  psupport4  psupport5  political_ideology  happy  slf_ladder  riskperc1  riskperc2  self_esteem  sex1  sbelong1  sbelong2  sbelong3  sbelong4  tested_positive  optim1  optim2  slfcont1  slfcont3  slfcont4  urban
##   2   6  age  children  cnarc1  cnarc2  cnarc3  ctheory1  ctheory2  ctheory3  ctheory4  CRT1  CRT2  CRT3  duration  employ_status1  generosity1  generosity2  generosity3  health_cond  know_tested_positive  ladder  marital1  mor_circle  moralid1  moralid10  moralid2  moralid3  moralid5  moralid6  moralid7  moralid8  moralid9  mcoop1  mcoop2  mcoop3  mcoop4  mcoop5  mcoop6  mcoop7  narc1  narc2  narc3  narc4  narc5  narc6  nidentity1  nidentity2  omind1  omind2  omind3  omind4  omind5  omind6  contact1  contact2  contact3  contact4  contact5  hygiene1  hygiene2  hygiene3  hygiene4  hygiene5  psupport1  psupport2  psupport3  psupport4  psupport5  political_ideology  happy  slf_ladder  riskperc1  riskperc2  self_esteem  sex1  sbelong1  sbelong2  sbelong3  sbelong4  tested_positive  optim1  optim2  slfcont1  slfcont3  slfcont4  urban
##   2   7  age  children  cnarc1  cnarc2  cnarc3  ctheory1  ctheory2  ctheory3  ctheory4  CRT1  CRT2  CRT3  duration  employ_status1  generosity1  generosity2  generosity3  health_cond  know_tested_positive  ladder  marital1  mor_circle  moralid1  moralid10  moralid2  moralid3  moralid5  moralid6  moralid7  moralid8  moralid9  mcoop1  mcoop2  mcoop3  mcoop4  mcoop5  mcoop6  mcoop7  narc1  narc2  narc3  narc4  narc5  narc6  nidentity1  nidentity2  omind1  omind2  omind3  omind4  omind5  omind6  contact1  contact2  contact3  contact4  contact5  hygiene1  hygiene2  hygiene3  hygiene4  hygiene5  psupport1  psupport2  psupport3  psupport4  psupport5  political_ideology  happy  slf_ladder  riskperc1  riskperc2  self_esteem  sex1  sbelong1  sbelong2  sbelong3  sbelong4  tested_positive  optim1  optim2  slfcont1  slfcont3  slfcont4  urban
##   2   8  age  children  cnarc1  cnarc2  cnarc3  ctheory1  ctheory2  ctheory3  ctheory4  CRT1  CRT2  CRT3  duration  employ_status1  generosity1  generosity2  generosity3  health_cond  know_tested_positive  ladder  marital1  mor_circle  moralid1  moralid10  moralid2  moralid3  moralid5  moralid6  moralid7  moralid8  moralid9  mcoop1  mcoop2  mcoop3  mcoop4  mcoop5  mcoop6  mcoop7  narc1  narc2  narc3  narc4  narc5  narc6  nidentity1  nidentity2  omind1  omind2  omind3  omind4  omind5  omind6  contact1  contact2  contact3  contact4  contact5  hygiene1  hygiene2  hygiene3  hygiene4  hygiene5  psupport1  psupport2  psupport3  psupport4  psupport5  political_ideology  happy  slf_ladder  riskperc1  riskperc2  self_esteem  sex1  sbelong1  sbelong2  sbelong3  sbelong4  tested_positive  optim1  optim2  slfcont1  slfcont3  slfcont4  urban
##   2   9  age  children  cnarc1  cnarc2  cnarc3  ctheory1  ctheory2  ctheory3  ctheory4  CRT1  CRT2  CRT3  duration  employ_status1  generosity1  generosity2  generosity3  health_cond  know_tested_positive  ladder  marital1  mor_circle  moralid1  moralid10  moralid2  moralid3  moralid5  moralid6  moralid7  moralid8  moralid9  mcoop1  mcoop2  mcoop3  mcoop4  mcoop5  mcoop6  mcoop7  narc1  narc2  narc3  narc4  narc5  narc6  nidentity1  nidentity2  omind1  omind2  omind3  omind4  omind5  omind6  contact1  contact2  contact3  contact4  contact5  hygiene1  hygiene2  hygiene3  hygiene4  hygiene5  psupport1  psupport2  psupport3  psupport4  psupport5  political_ideology  happy  slf_ladder  riskperc1  riskperc2  self_esteem  sex1  sbelong1  sbelong2  sbelong3  sbelong4  tested_positive  optim1  optim2  slfcont1  slfcont3  slfcont4  urban
##   2   10  age  children  cnarc1  cnarc2  cnarc3  ctheory1  ctheory2  ctheory3  ctheory4  CRT1  CRT2  CRT3  duration  employ_status1  generosity1  generosity2  generosity3  health_cond  know_tested_positive  ladder  marital1  mor_circle  moralid1  moralid10  moralid2  moralid3  moralid5  moralid6  moralid7  moralid8  moralid9  mcoop1  mcoop2  mcoop3  mcoop4  mcoop5  mcoop6  mcoop7  narc1  narc2  narc3  narc4  narc5  narc6  nidentity1  nidentity2  omind1  omind2  omind3  omind4  omind5  omind6  contact1  contact2  contact3  contact4  contact5  hygiene1  hygiene2  hygiene3  hygiene4  hygiene5  psupport1  psupport2  psupport3  psupport4  psupport5  political_ideology  happy  slf_ladder  riskperc1  riskperc2  self_esteem  sex1  sbelong1  sbelong2  sbelong3  sbelong4  tested_positive  optim1  optim2  slfcont1  slfcont3  slfcont4  urban
##   3   1  age  children  cnarc1  cnarc2  cnarc3  ctheory1  ctheory2  ctheory3  ctheory4  CRT1  CRT2  CRT3  duration  employ_status1  generosity1  generosity2  generosity3  health_cond  know_tested_positive  ladder  marital1  mor_circle  moralid1  moralid10  moralid2  moralid3  moralid5  moralid6  moralid7  moralid8  moralid9  mcoop1  mcoop2  mcoop3  mcoop4  mcoop5  mcoop6  mcoop7  narc1  narc2  narc3  narc4  narc5  narc6  nidentity1  nidentity2  omind1  omind2  omind3  omind4  omind5  omind6  contact1  contact2  contact3  contact4  contact5  hygiene1  hygiene2  hygiene3  hygiene4  hygiene5  psupport1  psupport2  psupport3  psupport4  psupport5  political_ideology  happy  slf_ladder  riskperc1  riskperc2  self_esteem  sex1  sbelong1  sbelong2  sbelong3  sbelong4  tested_positive  optim1  optim2  slfcont1  slfcont3  slfcont4  urban
##   3   2  age  children  cnarc1  cnarc2  cnarc3  ctheory1  ctheory2  ctheory3  ctheory4  CRT1  CRT2  CRT3  duration  employ_status1  generosity1  generosity2  generosity3  health_cond  know_tested_positive  ladder  marital1  mor_circle  moralid1  moralid10  moralid2  moralid3  moralid5  moralid6  moralid7  moralid8  moralid9  mcoop1  mcoop2  mcoop3  mcoop4  mcoop5  mcoop6  mcoop7  narc1  narc2  narc3  narc4  narc5  narc6  nidentity1  nidentity2  omind1  omind2  omind3  omind4  omind5  omind6  contact1  contact2  contact3  contact4  contact5  hygiene1  hygiene2  hygiene3  hygiene4  hygiene5  psupport1  psupport2  psupport3  psupport4  psupport5  political_ideology  happy  slf_ladder  riskperc1  riskperc2  self_esteem  sex1  sbelong1  sbelong2  sbelong3  sbelong4  tested_positive  optim1  optim2  slfcont1  slfcont3  slfcont4  urban
##   3   3  age  children  cnarc1  cnarc2  cnarc3  ctheory1  ctheory2  ctheory3  ctheory4  CRT1  CRT2  CRT3  duration  employ_status1  generosity1  generosity2  generosity3  health_cond  know_tested_positive  ladder  marital1  mor_circle  moralid1  moralid10  moralid2  moralid3  moralid5  moralid6  moralid7  moralid8  moralid9  mcoop1  mcoop2  mcoop3  mcoop4  mcoop5  mcoop6  mcoop7  narc1  narc2  narc3  narc4  narc5  narc6  nidentity1  nidentity2  omind1  omind2  omind3  omind4  omind5  omind6  contact1  contact2  contact3  contact4  contact5  hygiene1  hygiene2  hygiene3  hygiene4  hygiene5  psupport1  psupport2  psupport3  psupport4  psupport5  political_ideology  happy  slf_ladder  riskperc1  riskperc2  self_esteem  sex1  sbelong1  sbelong2  sbelong3  sbelong4  tested_positive  optim1  optim2  slfcont1  slfcont3  slfcont4  urban
##   3   4  age  children  cnarc1  cnarc2  cnarc3  ctheory1  ctheory2  ctheory3  ctheory4  CRT1  CRT2  CRT3  duration  employ_status1  generosity1  generosity2  generosity3  health_cond  know_tested_positive  ladder  marital1  mor_circle  moralid1  moralid10  moralid2  moralid3  moralid5  moralid6  moralid7  moralid8  moralid9  mcoop1  mcoop2  mcoop3  mcoop4  mcoop5  mcoop6  mcoop7  narc1  narc2  narc3  narc4  narc5  narc6  nidentity1  nidentity2  omind1  omind2  omind3  omind4  omind5  omind6  contact1  contact2  contact3  contact4  contact5  hygiene1  hygiene2  hygiene3  hygiene4  hygiene5  psupport1  psupport2  psupport3  psupport4  psupport5  political_ideology  happy  slf_ladder  riskperc1  riskperc2  self_esteem  sex1  sbelong1  sbelong2  sbelong3  sbelong4  tested_positive  optim1  optim2  slfcont1  slfcont3  slfcont4  urban
##   3   5  age  children  cnarc1  cnarc2  cnarc3  ctheory1  ctheory2  ctheory3  ctheory4  CRT1  CRT2  CRT3  duration  employ_status1  generosity1  generosity2  generosity3  health_cond  know_tested_positive  ladder  marital1  mor_circle  moralid1  moralid10  moralid2  moralid3  moralid5  moralid6  moralid7  moralid8  moralid9  mcoop1  mcoop2  mcoop3  mcoop4  mcoop5  mcoop6  mcoop7  narc1  narc2  narc3  narc4  narc5  narc6  nidentity1  nidentity2  omind1  omind2  omind3  omind4  omind5  omind6  contact1  contact2  contact3  contact4  contact5  hygiene1  hygiene2  hygiene3  hygiene4  hygiene5  psupport1  psupport2  psupport3  psupport4  psupport5  political_ideology  happy  slf_ladder  riskperc1  riskperc2  self_esteem  sex1  sbelong1  sbelong2  sbelong3  sbelong4  tested_positive  optim1  optim2  slfcont1  slfcont3  slfcont4  urban
##   3   6  age  children  cnarc1  cnarc2  cnarc3  ctheory1  ctheory2  ctheory3  ctheory4  CRT1  CRT2  CRT3  duration  employ_status1  generosity1  generosity2  generosity3  health_cond  know_tested_positive  ladder  marital1  mor_circle  moralid1  moralid10  moralid2  moralid3  moralid5  moralid6  moralid7  moralid8  moralid9  mcoop1  mcoop2  mcoop3  mcoop4  mcoop5  mcoop6  mcoop7  narc1  narc2  narc3  narc4  narc5  narc6  nidentity1  nidentity2  omind1  omind2  omind3  omind4  omind5  omind6  contact1  contact2  contact3  contact4  contact5  hygiene1  hygiene2  hygiene3  hygiene4  hygiene5  psupport1  psupport2  psupport3  psupport4  psupport5  political_ideology  happy  slf_ladder  riskperc1  riskperc2  self_esteem  sex1  sbelong1  sbelong2  sbelong3  sbelong4  tested_positive  optim1  optim2  slfcont1  slfcont3  slfcont4  urban
##   3   7  age  children  cnarc1  cnarc2  cnarc3  ctheory1  ctheory2  ctheory3  ctheory4  CRT1  CRT2  CRT3  duration  employ_status1  generosity1  generosity2  generosity3  health_cond  know_tested_positive  ladder  marital1  mor_circle  moralid1  moralid10  moralid2  moralid3  moralid5  moralid6  moralid7  moralid8  moralid9  mcoop1  mcoop2  mcoop3  mcoop4  mcoop5  mcoop6  mcoop7  narc1  narc2  narc3  narc4  narc5  narc6  nidentity1  nidentity2  omind1  omind2  omind3  omind4  omind5  omind6  contact1  contact2  contact3  contact4  contact5  hygiene1  hygiene2  hygiene3  hygiene4  hygiene5  psupport1  psupport2  psupport3  psupport4  psupport5  political_ideology  happy  slf_ladder  riskperc1  riskperc2  self_esteem  sex1  sbelong1  sbelong2  sbelong3  sbelong4  tested_positive  optim1  optim2  slfcont1  slfcont3  slfcont4  urban
##   3   8  age  children  cnarc1  cnarc2  cnarc3  ctheory1  ctheory2  ctheory3  ctheory4  CRT1  CRT2  CRT3  duration  employ_status1  generosity1  generosity2  generosity3  health_cond  know_tested_positive  ladder  marital1  mor_circle  moralid1  moralid10  moralid2  moralid3  moralid5  moralid6  moralid7  moralid8  moralid9  mcoop1  mcoop2  mcoop3  mcoop4  mcoop5  mcoop6  mcoop7  narc1  narc2  narc3  narc4  narc5  narc6  nidentity1  nidentity2  omind1  omind2  omind3  omind4  omind5  omind6  contact1  contact2  contact3  contact4  contact5  hygiene1  hygiene2  hygiene3  hygiene4  hygiene5  psupport1  psupport2  psupport3  psupport4  psupport5  political_ideology  happy  slf_ladder  riskperc1  riskperc2  self_esteem  sex1  sbelong1  sbelong2  sbelong3  sbelong4  tested_positive  optim1  optim2  slfcont1  slfcont3  slfcont4  urban
##   3   9  age  children  cnarc1  cnarc2  cnarc3  ctheory1  ctheory2  ctheory3  ctheory4  CRT1  CRT2  CRT3  duration  employ_status1  generosity1  generosity2  generosity3  health_cond  know_tested_positive  ladder  marital1  mor_circle  moralid1  moralid10  moralid2  moralid3  moralid5  moralid6  moralid7  moralid8  moralid9  mcoop1  mcoop2  mcoop3  mcoop4  mcoop5  mcoop6  mcoop7  narc1  narc2  narc3  narc4  narc5  narc6  nidentity1  nidentity2  omind1  omind2  omind3  omind4  omind5  omind6  contact1  contact2  contact3  contact4  contact5  hygiene1  hygiene2  hygiene3  hygiene4  hygiene5  psupport1  psupport2  psupport3  psupport4  psupport5  political_ideology  happy  slf_ladder  riskperc1  riskperc2  self_esteem  sex1  sbelong1  sbelong2  sbelong3  sbelong4  tested_positive  optim1  optim2  slfcont1  slfcont3  slfcont4  urban
##   3   10  age  children  cnarc1  cnarc2  cnarc3  ctheory1  ctheory2  ctheory3  ctheory4  CRT1  CRT2  CRT3  duration  employ_status1  generosity1  generosity2  generosity3  health_cond  know_tested_positive  ladder  marital1  mor_circle  moralid1  moralid10  moralid2  moralid3  moralid5  moralid6  moralid7  moralid8  moralid9  mcoop1  mcoop2  mcoop3  mcoop4  mcoop5  mcoop6  mcoop7  narc1  narc2  narc3  narc4  narc5  narc6  nidentity1  nidentity2  omind1  omind2  omind3  omind4  omind5  omind6  contact1  contact2  contact3  contact4  contact5  hygiene1  hygiene2  hygiene3  hygiene4  hygiene5  psupport1  psupport2  psupport3  psupport4  psupport5  political_ideology  happy  slf_ladder  riskperc1  riskperc2  self_esteem  sex1  sbelong1  sbelong2  sbelong3  sbelong4  tested_positive  optim1  optim2  slfcont1  slfcont3  slfcont4  urban
##   4   1  age  children  cnarc1  cnarc2  cnarc3  ctheory1  ctheory2  ctheory3  ctheory4  CRT1  CRT2  CRT3  duration  employ_status1  generosity1  generosity2  generosity3  health_cond  know_tested_positive  ladder  marital1  mor_circle  moralid1  moralid10  moralid2  moralid3  moralid5  moralid6  moralid7  moralid8  moralid9  mcoop1  mcoop2  mcoop3  mcoop4  mcoop5  mcoop6  mcoop7  narc1  narc2  narc3  narc4  narc5  narc6  nidentity1  nidentity2  omind1  omind2  omind3  omind4  omind5  omind6  contact1  contact2  contact3  contact4  contact5  hygiene1  hygiene2  hygiene3  hygiene4  hygiene5  psupport1  psupport2  psupport3  psupport4  psupport5  political_ideology  happy  slf_ladder  riskperc1  riskperc2  self_esteem  sex1  sbelong1  sbelong2  sbelong3  sbelong4  tested_positive  optim1  optim2  slfcont1  slfcont3  slfcont4  urban
##   4   2  age  children  cnarc1  cnarc2  cnarc3  ctheory1  ctheory2  ctheory3  ctheory4  CRT1  CRT2  CRT3  duration  employ_status1  generosity1  generosity2  generosity3  health_cond  know_tested_positive  ladder  marital1  mor_circle  moralid1  moralid10  moralid2  moralid3  moralid5  moralid6  moralid7  moralid8  moralid9  mcoop1  mcoop2  mcoop3  mcoop4  mcoop5  mcoop6  mcoop7  narc1  narc2  narc3  narc4  narc5  narc6  nidentity1  nidentity2  omind1  omind2  omind3  omind4  omind5  omind6  contact1  contact2  contact3  contact4  contact5  hygiene1  hygiene2  hygiene3  hygiene4  hygiene5  psupport1  psupport2  psupport3  psupport4  psupport5  political_ideology  happy  slf_ladder  riskperc1  riskperc2  self_esteem  sex1  sbelong1  sbelong2  sbelong3  sbelong4  tested_positive  optim1  optim2  slfcont1  slfcont3  slfcont4  urban
##   4   3  age  children  cnarc1  cnarc2  cnarc3  ctheory1  ctheory2  ctheory3  ctheory4  CRT1  CRT2  CRT3  duration  employ_status1  generosity1  generosity2  generosity3  health_cond  know_tested_positive  ladder  marital1  mor_circle  moralid1  moralid10  moralid2  moralid3  moralid5  moralid6  moralid7  moralid8  moralid9  mcoop1  mcoop2  mcoop3  mcoop4  mcoop5  mcoop6  mcoop7  narc1  narc2  narc3  narc4  narc5  narc6  nidentity1  nidentity2  omind1  omind2  omind3  omind4  omind5  omind6  contact1  contact2  contact3  contact4  contact5  hygiene1  hygiene2  hygiene3  hygiene4  hygiene5  psupport1  psupport2  psupport3  psupport4  psupport5  political_ideology  happy  slf_ladder  riskperc1  riskperc2  self_esteem  sex1  sbelong1  sbelong2  sbelong3  sbelong4  tested_positive  optim1  optim2  slfcont1  slfcont3  slfcont4  urban
##   4   4  age  children  cnarc1  cnarc2  cnarc3  ctheory1  ctheory2  ctheory3  ctheory4  CRT1  CRT2  CRT3  duration  employ_status1  generosity1  generosity2  generosity3  health_cond  know_tested_positive  ladder  marital1  mor_circle  moralid1  moralid10  moralid2  moralid3  moralid5  moralid6  moralid7  moralid8  moralid9  mcoop1  mcoop2  mcoop3  mcoop4  mcoop5  mcoop6  mcoop7  narc1  narc2  narc3  narc4  narc5  narc6  nidentity1  nidentity2  omind1  omind2  omind3  omind4  omind5  omind6  contact1  contact2  contact3  contact4  contact5  hygiene1  hygiene2  hygiene3  hygiene4  hygiene5  psupport1  psupport2  psupport3  psupport4  psupport5  political_ideology  happy  slf_ladder  riskperc1  riskperc2  self_esteem  sex1  sbelong1  sbelong2  sbelong3  sbelong4  tested_positive  optim1  optim2  slfcont1  slfcont3  slfcont4  urban
##   4   5  age  children  cnarc1  cnarc2  cnarc3  ctheory1  ctheory2  ctheory3  ctheory4  CRT1  CRT2  CRT3  duration  employ_status1  generosity1  generosity2  generosity3  health_cond  know_tested_positive  ladder  marital1  mor_circle  moralid1  moralid10  moralid2  moralid3  moralid5  moralid6  moralid7  moralid8  moralid9  mcoop1  mcoop2  mcoop3  mcoop4  mcoop5  mcoop6  mcoop7  narc1  narc2  narc3  narc4  narc5  narc6  nidentity1  nidentity2  omind1  omind2  omind3  omind4  omind5  omind6  contact1  contact2  contact3  contact4  contact5  hygiene1  hygiene2  hygiene3  hygiene4  hygiene5  psupport1  psupport2  psupport3  psupport4  psupport5  political_ideology  happy  slf_ladder  riskperc1  riskperc2  self_esteem  sex1  sbelong1  sbelong2  sbelong3  sbelong4  tested_positive  optim1  optim2  slfcont1  slfcont3  slfcont4  urban
##   4   6  age  children  cnarc1  cnarc2  cnarc3  ctheory1  ctheory2  ctheory3  ctheory4  CRT1  CRT2  CRT3  duration  employ_status1  generosity1  generosity2  generosity3  health_cond  know_tested_positive  ladder  marital1  mor_circle  moralid1  moralid10  moralid2  moralid3  moralid5  moralid6  moralid7  moralid8  moralid9  mcoop1  mcoop2  mcoop3  mcoop4  mcoop5  mcoop6  mcoop7  narc1  narc2  narc3  narc4  narc5  narc6  nidentity1  nidentity2  omind1  omind2  omind3  omind4  omind5  omind6  contact1  contact2  contact3  contact4  contact5  hygiene1  hygiene2  hygiene3  hygiene4  hygiene5  psupport1  psupport2  psupport3  psupport4  psupport5  political_ideology  happy  slf_ladder  riskperc1  riskperc2  self_esteem  sex1  sbelong1  sbelong2  sbelong3  sbelong4  tested_positive  optim1  optim2  slfcont1  slfcont3  slfcont4  urban
##   4   7  age  children  cnarc1  cnarc2  cnarc3  ctheory1  ctheory2  ctheory3  ctheory4  CRT1  CRT2  CRT3  duration  employ_status1  generosity1  generosity2  generosity3  health_cond  know_tested_positive  ladder  marital1  mor_circle  moralid1  moralid10  moralid2  moralid3  moralid5  moralid6  moralid7  moralid8  moralid9  mcoop1  mcoop2  mcoop3  mcoop4  mcoop5  mcoop6  mcoop7  narc1  narc2  narc3  narc4  narc5  narc6  nidentity1  nidentity2  omind1  omind2  omind3  omind4  omind5  omind6  contact1  contact2  contact3  contact4  contact5  hygiene1  hygiene2  hygiene3  hygiene4  hygiene5  psupport1  psupport2  psupport3  psupport4  psupport5  political_ideology  happy  slf_ladder  riskperc1  riskperc2  self_esteem  sex1  sbelong1  sbelong2  sbelong3  sbelong4  tested_positive  optim1  optim2  slfcont1  slfcont3  slfcont4  urban
##   4   8  age  children  cnarc1  cnarc2  cnarc3  ctheory1  ctheory2  ctheory3  ctheory4  CRT1  CRT2  CRT3  duration  employ_status1  generosity1  generosity2  generosity3  health_cond  know_tested_positive  ladder  marital1  mor_circle  moralid1  moralid10  moralid2  moralid3  moralid5  moralid6  moralid7  moralid8  moralid9  mcoop1  mcoop2  mcoop3  mcoop4  mcoop5  mcoop6  mcoop7  narc1  narc2  narc3  narc4  narc5  narc6  nidentity1  nidentity2  omind1  omind2  omind3  omind4  omind5  omind6  contact1  contact2  contact3  contact4  contact5  hygiene1  hygiene2  hygiene3  hygiene4  hygiene5  psupport1  psupport2  psupport3  psupport4  psupport5  political_ideology  happy  slf_ladder  riskperc1  riskperc2  self_esteem  sex1  sbelong1  sbelong2  sbelong3  sbelong4  tested_positive  optim1  optim2  slfcont1  slfcont3  slfcont4  urban
##   4   9  age  children  cnarc1  cnarc2  cnarc3  ctheory1  ctheory2  ctheory3  ctheory4  CRT1  CRT2  CRT3  duration  employ_status1  generosity1  generosity2  generosity3  health_cond  know_tested_positive  ladder  marital1  mor_circle  moralid1  moralid10  moralid2  moralid3  moralid5  moralid6  moralid7  moralid8  moralid9  mcoop1  mcoop2  mcoop3  mcoop4  mcoop5  mcoop6  mcoop7  narc1  narc2  narc3  narc4  narc5  narc6  nidentity1  nidentity2  omind1  omind2  omind3  omind4  omind5  omind6  contact1  contact2  contact3  contact4  contact5  hygiene1  hygiene2  hygiene3  hygiene4  hygiene5  psupport1  psupport2  psupport3  psupport4  psupport5  political_ideology  happy  slf_ladder  riskperc1  riskperc2  self_esteem  sex1  sbelong1  sbelong2  sbelong3  sbelong4  tested_positive  optim1  optim2  slfcont1  slfcont3  slfcont4  urban
##   4   10  age  children  cnarc1  cnarc2  cnarc3  ctheory1  ctheory2  ctheory3  ctheory4  CRT1  CRT2  CRT3  duration  employ_status1  generosity1  generosity2  generosity3  health_cond  know_tested_positive  ladder  marital1  mor_circle  moralid1  moralid10  moralid2  moralid3  moralid5  moralid6  moralid7  moralid8  moralid9  mcoop1  mcoop2  mcoop3  mcoop4  mcoop5  mcoop6  mcoop7  narc1  narc2  narc3  narc4  narc5  narc6  nidentity1  nidentity2  omind1  omind2  omind3  omind4  omind5  omind6  contact1  contact2  contact3  contact4  contact5  hygiene1  hygiene2  hygiene3  hygiene4  hygiene5  psupport1  psupport2  psupport3  psupport4  psupport5  political_ideology  happy  slf_ladder  riskperc1  riskperc2  self_esteem  sex1  sbelong1  sbelong2  sbelong3  sbelong4  tested_positive  optim1  optim2  slfcont1  slfcont3  slfcont4  urban
##   5   1  age  children  cnarc1  cnarc2  cnarc3  ctheory1  ctheory2  ctheory3  ctheory4  CRT1  CRT2  CRT3  duration  employ_status1  generosity1  generosity2  generosity3  health_cond  know_tested_positive  ladder  marital1  mor_circle  moralid1  moralid10  moralid2  moralid3  moralid5  moralid6  moralid7  moralid8  moralid9  mcoop1  mcoop2  mcoop3  mcoop4  mcoop5  mcoop6  mcoop7  narc1  narc2  narc3  narc4  narc5  narc6  nidentity1  nidentity2  omind1  omind2  omind3  omind4  omind5  omind6  contact1  contact2  contact3  contact4  contact5  hygiene1  hygiene2  hygiene3  hygiene4  hygiene5  psupport1  psupport2  psupport3  psupport4  psupport5  political_ideology  happy  slf_ladder  riskperc1  riskperc2  self_esteem  sex1  sbelong1  sbelong2  sbelong3  sbelong4  tested_positive  optim1  optim2  slfcont1  slfcont3  slfcont4  urban
##   5   2  age  children  cnarc1  cnarc2  cnarc3  ctheory1  ctheory2  ctheory3  ctheory4  CRT1  CRT2  CRT3  duration  employ_status1  generosity1  generosity2  generosity3  health_cond  know_tested_positive  ladder  marital1  mor_circle  moralid1  moralid10  moralid2  moralid3  moralid5  moralid6  moralid7  moralid8  moralid9  mcoop1  mcoop2  mcoop3  mcoop4  mcoop5  mcoop6  mcoop7  narc1  narc2  narc3  narc4  narc5  narc6  nidentity1  nidentity2  omind1  omind2  omind3  omind4  omind5  omind6  contact1  contact2  contact3  contact4  contact5  hygiene1  hygiene2  hygiene3  hygiene4  hygiene5  psupport1  psupport2  psupport3  psupport4  psupport5  political_ideology  happy  slf_ladder  riskperc1  riskperc2  self_esteem  sex1  sbelong1  sbelong2  sbelong3  sbelong4  tested_positive  optim1  optim2  slfcont1  slfcont3  slfcont4  urban
##   5   3  age  children  cnarc1  cnarc2  cnarc3  ctheory1  ctheory2  ctheory3  ctheory4  CRT1  CRT2  CRT3  duration  employ_status1  generosity1  generosity2  generosity3  health_cond  know_tested_positive  ladder  marital1  mor_circle  moralid1  moralid10  moralid2  moralid3  moralid5  moralid6  moralid7  moralid8  moralid9  mcoop1  mcoop2  mcoop3  mcoop4  mcoop5  mcoop6  mcoop7  narc1  narc2  narc3  narc4  narc5  narc6  nidentity1  nidentity2  omind1  omind2  omind3  omind4  omind5  omind6  contact1  contact2  contact3  contact4  contact5  hygiene1  hygiene2  hygiene3  hygiene4  hygiene5  psupport1  psupport2  psupport3  psupport4  psupport5  political_ideology  happy  slf_ladder  riskperc1  riskperc2  self_esteem  sex1  sbelong1  sbelong2  sbelong3  sbelong4  tested_positive  optim1  optim2  slfcont1  slfcont3  slfcont4  urban
##   5   4  age  children  cnarc1  cnarc2  cnarc3  ctheory1  ctheory2  ctheory3  ctheory4  CRT1  CRT2  CRT3  duration  employ_status1  generosity1  generosity2  generosity3  health_cond  know_tested_positive  ladder  marital1  mor_circle  moralid1  moralid10  moralid2  moralid3  moralid5  moralid6  moralid7  moralid8  moralid9  mcoop1  mcoop2  mcoop3  mcoop4  mcoop5  mcoop6  mcoop7  narc1  narc2  narc3  narc4  narc5  narc6  nidentity1  nidentity2  omind1  omind2  omind3  omind4  omind5  omind6  contact1  contact2  contact3  contact4  contact5  hygiene1  hygiene2  hygiene3  hygiene4  hygiene5  psupport1  psupport2  psupport3  psupport4  psupport5  political_ideology  happy  slf_ladder  riskperc1  riskperc2  self_esteem  sex1  sbelong1  sbelong2  sbelong3  sbelong4  tested_positive  optim1  optim2  slfcont1  slfcont3  slfcont4  urban
##   5   5  age  children  cnarc1  cnarc2  cnarc3  ctheory1  ctheory2  ctheory3  ctheory4  CRT1  CRT2  CRT3  duration  employ_status1  generosity1  generosity2  generosity3  health_cond  know_tested_positive  ladder  marital1  mor_circle  moralid1  moralid10  moralid2  moralid3  moralid5  moralid6  moralid7  moralid8  moralid9  mcoop1  mcoop2  mcoop3  mcoop4  mcoop5  mcoop6  mcoop7  narc1  narc2  narc3  narc4  narc5  narc6  nidentity1  nidentity2  omind1  omind2  omind3  omind4  omind5  omind6  contact1  contact2  contact3  contact4  contact5  hygiene1  hygiene2  hygiene3  hygiene4  hygiene5  psupport1  psupport2  psupport3  psupport4  psupport5  political_ideology  happy  slf_ladder  riskperc1  riskperc2  self_esteem  sex1  sbelong1  sbelong2  sbelong3  sbelong4  tested_positive  optim1  optim2  slfcont1  slfcont3  slfcont4  urban
##   5   6  age  children  cnarc1  cnarc2  cnarc3  ctheory1  ctheory2  ctheory3  ctheory4  CRT1  CRT2  CRT3  duration  employ_status1  generosity1  generosity2  generosity3  health_cond  know_tested_positive  ladder  marital1  mor_circle  moralid1  moralid10  moralid2  moralid3  moralid5  moralid6  moralid7  moralid8  moralid9  mcoop1  mcoop2  mcoop3  mcoop4  mcoop5  mcoop6  mcoop7  narc1  narc2  narc3  narc4  narc5  narc6  nidentity1  nidentity2  omind1  omind2  omind3  omind4  omind5  omind6  contact1  contact2  contact3  contact4  contact5  hygiene1  hygiene2  hygiene3  hygiene4  hygiene5  psupport1  psupport2  psupport3  psupport4  psupport5  political_ideology  happy  slf_ladder  riskperc1  riskperc2  self_esteem  sex1  sbelong1  sbelong2  sbelong3  sbelong4  tested_positive  optim1  optim2  slfcont1  slfcont3  slfcont4  urban
##   5   7  age  children  cnarc1  cnarc2  cnarc3  ctheory1  ctheory2  ctheory3  ctheory4  CRT1  CRT2  CRT3  duration  employ_status1  generosity1  generosity2  generosity3  health_cond  know_tested_positive  ladder  marital1  mor_circle  moralid1  moralid10  moralid2  moralid3  moralid5  moralid6  moralid7  moralid8  moralid9  mcoop1  mcoop2  mcoop3  mcoop4  mcoop5  mcoop6  mcoop7  narc1  narc2  narc3  narc4  narc5  narc6  nidentity1  nidentity2  omind1  omind2  omind3  omind4  omind5  omind6  contact1  contact2  contact3  contact4  contact5  hygiene1  hygiene2  hygiene3  hygiene4  hygiene5  psupport1  psupport2  psupport3  psupport4  psupport5  political_ideology  happy  slf_ladder  riskperc1  riskperc2  self_esteem  sex1  sbelong1  sbelong2  sbelong3  sbelong4  tested_positive  optim1  optim2  slfcont1  slfcont3  slfcont4  urban
##   5   8  age  children  cnarc1  cnarc2  cnarc3  ctheory1  ctheory2  ctheory3  ctheory4  CRT1  CRT2  CRT3  duration  employ_status1  generosity1  generosity2  generosity3  health_cond  know_tested_positive  ladder  marital1  mor_circle  moralid1  moralid10  moralid2  moralid3  moralid5  moralid6  moralid7  moralid8  moralid9  mcoop1  mcoop2  mcoop3  mcoop4  mcoop5  mcoop6  mcoop7  narc1  narc2  narc3  narc4  narc5  narc6  nidentity1  nidentity2  omind1  omind2  omind3  omind4  omind5  omind6  contact1  contact2  contact3  contact4  contact5  hygiene1  hygiene2  hygiene3  hygiene4  hygiene5  psupport1  psupport2  psupport3  psupport4  psupport5  political_ideology  happy  slf_ladder  riskperc1  riskperc2  self_esteem  sex1  sbelong1  sbelong2  sbelong3  sbelong4  tested_positive  optim1  optim2  slfcont1  slfcont3  slfcont4  urban
##   5   9  age  children  cnarc1  cnarc2  cnarc3  ctheory1  ctheory2  ctheory3  ctheory4  CRT1  CRT2  CRT3  duration  employ_status1  generosity1  generosity2  generosity3  health_cond  know_tested_positive  ladder  marital1  mor_circle  moralid1  moralid10  moralid2  moralid3  moralid5  moralid6  moralid7  moralid8  moralid9  mcoop1  mcoop2  mcoop3  mcoop4  mcoop5  mcoop6  mcoop7  narc1  narc2  narc3  narc4  narc5  narc6  nidentity1  nidentity2  omind1  omind2  omind3  omind4  omind5  omind6  contact1  contact2  contact3  contact4  contact5  hygiene1  hygiene2  hygiene3  hygiene4  hygiene5  psupport1  psupport2  psupport3  psupport4  psupport5  political_ideology  happy  slf_ladder  riskperc1  riskperc2  self_esteem  sex1  sbelong1  sbelong2  sbelong3  sbelong4  tested_positive  optim1  optim2  slfcont1  slfcont3  slfcont4  urban
##   5   10  age  children  cnarc1  cnarc2  cnarc3  ctheory1  ctheory2  ctheory3  ctheory4  CRT1  CRT2  CRT3  duration  employ_status1  generosity1  generosity2  generosity3  health_cond  know_tested_positive  ladder  marital1  mor_circle  moralid1  moralid10  moralid2  moralid3  moralid5  moralid6  moralid7  moralid8  moralid9  mcoop1  mcoop2  mcoop3  mcoop4  mcoop5  mcoop6  mcoop7  narc1  narc2  narc3  narc4  narc5  narc6  nidentity1  nidentity2  omind1  omind2  omind3  omind4  omind5  omind6  contact1  contact2  contact3  contact4  contact5  hygiene1  hygiene2  hygiene3  hygiene4  hygiene5  psupport1  psupport2  psupport3  psupport4  psupport5  political_ideology  happy  slf_ladder  riskperc1  riskperc2  self_esteem  sex1  sbelong1  sbelong2  sbelong3  sbelong4  tested_positive  optim1  optim2  slfcont1  slfcont3  slfcont4  urban
##   6   1  age  children  cnarc1  cnarc2  cnarc3  ctheory1  ctheory2  ctheory3  ctheory4  CRT1  CRT2  CRT3  duration  employ_status1  generosity1  generosity2  generosity3  health_cond  know_tested_positive  ladder  marital1  mor_circle  moralid1  moralid10  moralid2  moralid3  moralid5  moralid6  moralid7  moralid8  moralid9  mcoop1  mcoop2  mcoop3  mcoop4  mcoop5  mcoop6  mcoop7  narc1  narc2  narc3  narc4  narc5  narc6  nidentity1  nidentity2  omind1  omind2  omind3  omind4  omind5  omind6  contact1  contact2  contact3  contact4  contact5  hygiene1  hygiene2  hygiene3  hygiene4  hygiene5  psupport1  psupport2  psupport3  psupport4  psupport5  political_ideology  happy  slf_ladder  riskperc1  riskperc2  self_esteem  sex1  sbelong1  sbelong2  sbelong3  sbelong4  tested_positive  optim1  optim2  slfcont1  slfcont3  slfcont4  urban
##   6   2  age  children  cnarc1  cnarc2  cnarc3  ctheory1  ctheory2  ctheory3  ctheory4  CRT1  CRT2  CRT3  duration  employ_status1  generosity1  generosity2  generosity3  health_cond  know_tested_positive  ladder  marital1  mor_circle  moralid1  moralid10  moralid2  moralid3  moralid5  moralid6  moralid7  moralid8  moralid9  mcoop1  mcoop2  mcoop3  mcoop4  mcoop5  mcoop6  mcoop7  narc1  narc2  narc3  narc4  narc5  narc6  nidentity1  nidentity2  omind1  omind2  omind3  omind4  omind5  omind6  contact1  contact2  contact3  contact4  contact5  hygiene1  hygiene2  hygiene3  hygiene4  hygiene5  psupport1  psupport2  psupport3  psupport4  psupport5  political_ideology  happy  slf_ladder  riskperc1  riskperc2  self_esteem  sex1  sbelong1  sbelong2  sbelong3  sbelong4  tested_positive  optim1  optim2  slfcont1  slfcont3  slfcont4  urban
##   6   3  age  children  cnarc1  cnarc2  cnarc3  ctheory1  ctheory2  ctheory3  ctheory4  CRT1  CRT2  CRT3  duration  employ_status1  generosity1  generosity2  generosity3  health_cond  know_tested_positive  ladder  marital1  mor_circle  moralid1  moralid10  moralid2  moralid3  moralid5  moralid6  moralid7  moralid8  moralid9  mcoop1  mcoop2  mcoop3  mcoop4  mcoop5  mcoop6  mcoop7  narc1  narc2  narc3  narc4  narc5  narc6  nidentity1  nidentity2  omind1  omind2  omind3  omind4  omind5  omind6  contact1  contact2  contact3  contact4  contact5  hygiene1  hygiene2  hygiene3  hygiene4  hygiene5  psupport1  psupport2  psupport3  psupport4  psupport5  political_ideology  happy  slf_ladder  riskperc1  riskperc2  self_esteem  sex1  sbelong1  sbelong2  sbelong3  sbelong4  tested_positive  optim1  optim2  slfcont1  slfcont3  slfcont4  urban
##   6   4  age  children  cnarc1  cnarc2  cnarc3  ctheory1  ctheory2  ctheory3  ctheory4  CRT1  CRT2  CRT3  duration  employ_status1  generosity1  generosity2  generosity3  health_cond  know_tested_positive  ladder  marital1  mor_circle  moralid1  moralid10  moralid2  moralid3  moralid5  moralid6  moralid7  moralid8  moralid9  mcoop1  mcoop2  mcoop3  mcoop4  mcoop5  mcoop6  mcoop7  narc1  narc2  narc3  narc4  narc5  narc6  nidentity1  nidentity2  omind1  omind2  omind3  omind4  omind5  omind6  contact1  contact2  contact3  contact4  contact5  hygiene1  hygiene2  hygiene3  hygiene4  hygiene5  psupport1  psupport2  psupport3  psupport4  psupport5  political_ideology  happy  slf_ladder  riskperc1  riskperc2  self_esteem  sex1  sbelong1  sbelong2  sbelong3  sbelong4  tested_positive  optim1  optim2  slfcont1  slfcont3  slfcont4  urban
##   6   5  age  children  cnarc1  cnarc2  cnarc3  ctheory1  ctheory2  ctheory3  ctheory4  CRT1  CRT2  CRT3  duration  employ_status1  generosity1  generosity2  generosity3  health_cond  know_tested_positive  ladder  marital1  mor_circle  moralid1  moralid10  moralid2  moralid3  moralid5  moralid6  moralid7  moralid8  moralid9  mcoop1  mcoop2  mcoop3  mcoop4  mcoop5  mcoop6  mcoop7  narc1  narc2  narc3  narc4  narc5  narc6  nidentity1  nidentity2  omind1  omind2  omind3  omind4  omind5  omind6  contact1  contact2  contact3  contact4  contact5  hygiene1  hygiene2  hygiene3  hygiene4  hygiene5  psupport1  psupport2  psupport3  psupport4  psupport5  political_ideology  happy  slf_ladder  riskperc1  riskperc2  self_esteem  sex1  sbelong1  sbelong2  sbelong3  sbelong4  tested_positive  optim1  optim2  slfcont1  slfcont3  slfcont4  urban
##   6   6  age  children  cnarc1  cnarc2  cnarc3  ctheory1  ctheory2  ctheory3  ctheory4  CRT1  CRT2  CRT3  duration  employ_status1  generosity1  generosity2  generosity3  health_cond  know_tested_positive  ladder  marital1  mor_circle  moralid1  moralid10  moralid2  moralid3  moralid5  moralid6  moralid7  moralid8  moralid9  mcoop1  mcoop2  mcoop3  mcoop4  mcoop5  mcoop6  mcoop7  narc1  narc2  narc3  narc4  narc5  narc6  nidentity1  nidentity2  omind1  omind2  omind3  omind4  omind5  omind6  contact1  contact2  contact3  contact4  contact5  hygiene1  hygiene2  hygiene3  hygiene4  hygiene5  psupport1  psupport2  psupport3  psupport4  psupport5  political_ideology  happy  slf_ladder  riskperc1  riskperc2  self_esteem  sex1  sbelong1  sbelong2  sbelong3  sbelong4  tested_positive  optim1  optim2  slfcont1  slfcont3  slfcont4  urban
##   6   7  age  children  cnarc1  cnarc2  cnarc3  ctheory1  ctheory2  ctheory3  ctheory4  CRT1  CRT2  CRT3  duration  employ_status1  generosity1  generosity2  generosity3  health_cond  know_tested_positive  ladder  marital1  mor_circle  moralid1  moralid10  moralid2  moralid3  moralid5  moralid6  moralid7  moralid8  moralid9  mcoop1  mcoop2  mcoop3  mcoop4  mcoop5  mcoop6  mcoop7  narc1  narc2  narc3  narc4  narc5  narc6  nidentity1  nidentity2  omind1  omind2  omind3  omind4  omind5  omind6  contact1  contact2  contact3  contact4  contact5  hygiene1  hygiene2  hygiene3  hygiene4  hygiene5  psupport1  psupport2  psupport3  psupport4  psupport5  political_ideology  happy  slf_ladder  riskperc1  riskperc2  self_esteem  sex1  sbelong1  sbelong2  sbelong3  sbelong4  tested_positive  optim1  optim2  slfcont1  slfcont3  slfcont4  urban
##   6   8  age  children  cnarc1  cnarc2  cnarc3  ctheory1  ctheory2  ctheory3  ctheory4  CRT1  CRT2  CRT3  duration  employ_status1  generosity1  generosity2  generosity3  health_cond  know_tested_positive  ladder  marital1  mor_circle  moralid1  moralid10  moralid2  moralid3  moralid5  moralid6  moralid7  moralid8  moralid9  mcoop1  mcoop2  mcoop3  mcoop4  mcoop5  mcoop6  mcoop7  narc1  narc2  narc3  narc4  narc5  narc6  nidentity1  nidentity2  omind1  omind2  omind3  omind4  omind5  omind6  contact1  contact2  contact3  contact4  contact5  hygiene1  hygiene2  hygiene3  hygiene4  hygiene5  psupport1  psupport2  psupport3  psupport4  psupport5  political_ideology  happy  slf_ladder  riskperc1  riskperc2  self_esteem  sex1  sbelong1  sbelong2  sbelong3  sbelong4  tested_positive  optim1  optim2  slfcont1  slfcont3  slfcont4  urban
##   6   9  age  children  cnarc1  cnarc2  cnarc3  ctheory1  ctheory2  ctheory3  ctheory4  CRT1  CRT2  CRT3  duration  employ_status1  generosity1  generosity2  generosity3  health_cond  know_tested_positive  ladder  marital1  mor_circle  moralid1  moralid10  moralid2  moralid3  moralid5  moralid6  moralid7  moralid8  moralid9  mcoop1  mcoop2  mcoop3  mcoop4  mcoop5  mcoop6  mcoop7  narc1  narc2  narc3  narc4  narc5  narc6  nidentity1  nidentity2  omind1  omind2  omind3  omind4  omind5  omind6  contact1  contact2  contact3  contact4  contact5  hygiene1  hygiene2  hygiene3  hygiene4  hygiene5  psupport1  psupport2  psupport3  psupport4  psupport5  political_ideology  happy  slf_ladder  riskperc1  riskperc2  self_esteem  sex1  sbelong1  sbelong2  sbelong3  sbelong4  tested_positive  optim1  optim2  slfcont1  slfcont3  slfcont4  urban
##   6   10  age  children  cnarc1  cnarc2  cnarc3  ctheory1  ctheory2  ctheory3  ctheory4  CRT1  CRT2  CRT3  duration  employ_status1  generosity1  generosity2  generosity3  health_cond  know_tested_positive  ladder  marital1  mor_circle  moralid1  moralid10  moralid2  moralid3  moralid5  moralid6  moralid7  moralid8  moralid9  mcoop1  mcoop2  mcoop3  mcoop4  mcoop5  mcoop6  mcoop7  narc1  narc2  narc3  narc4  narc5  narc6  nidentity1  nidentity2  omind1  omind2  omind3  omind4  omind5  omind6  contact1  contact2  contact3  contact4  contact5  hygiene1  hygiene2  hygiene3  hygiene4  hygiene5  psupport1  psupport2  psupport3  psupport4  psupport5  political_ideology  happy  slf_ladder  riskperc1  riskperc2  self_esteem  sex1  sbelong1  sbelong2  sbelong3  sbelong4  tested_positive  optim1  optim2  slfcont1  slfcont3  slfcont4  urban
##   7   1  age  children  cnarc1  cnarc2  cnarc3  ctheory1  ctheory2  ctheory3  ctheory4  CRT1  CRT2  CRT3  duration  employ_status1  generosity1  generosity2  generosity3  health_cond  know_tested_positive  ladder  marital1  mor_circle  moralid1  moralid10  moralid2  moralid3  moralid5  moralid6  moralid7  moralid8  moralid9  mcoop1  mcoop2  mcoop3  mcoop4  mcoop5  mcoop6  mcoop7  narc1  narc2  narc3  narc4  narc5  narc6  nidentity1  nidentity2  omind1  omind2  omind3  omind4  omind5  omind6  contact1  contact2  contact3  contact4  contact5  hygiene1  hygiene2  hygiene3  hygiene4  hygiene5  psupport1  psupport2  psupport3  psupport4  psupport5  political_ideology  happy  slf_ladder  riskperc1  riskperc2  self_esteem  sex1  sbelong1  sbelong2  sbelong3  sbelong4  tested_positive  optim1  optim2  slfcont1  slfcont3  slfcont4  urban
##   7   2  age  children  cnarc1  cnarc2  cnarc3  ctheory1  ctheory2  ctheory3  ctheory4  CRT1  CRT2  CRT3  duration  employ_status1  generosity1  generosity2  generosity3  health_cond  know_tested_positive  ladder  marital1  mor_circle  moralid1  moralid10  moralid2  moralid3  moralid5  moralid6  moralid7  moralid8  moralid9  mcoop1  mcoop2  mcoop3  mcoop4  mcoop5  mcoop6  mcoop7  narc1  narc2  narc3  narc4  narc5  narc6  nidentity1  nidentity2  omind1  omind2  omind3  omind4  omind5  omind6  contact1  contact2  contact3  contact4  contact5  hygiene1  hygiene2  hygiene3  hygiene4  hygiene5  psupport1  psupport2  psupport3  psupport4  psupport5  political_ideology  happy  slf_ladder  riskperc1  riskperc2  self_esteem  sex1  sbelong1  sbelong2  sbelong3  sbelong4  tested_positive  optim1  optim2  slfcont1  slfcont3  slfcont4  urban
##   7   3  age  children  cnarc1  cnarc2  cnarc3  ctheory1  ctheory2  ctheory3  ctheory4  CRT1  CRT2  CRT3  duration  employ_status1  generosity1  generosity2  generosity3  health_cond  know_tested_positive  ladder  marital1  mor_circle  moralid1  moralid10  moralid2  moralid3  moralid5  moralid6  moralid7  moralid8  moralid9  mcoop1  mcoop2  mcoop3  mcoop4  mcoop5  mcoop6  mcoop7  narc1  narc2  narc3  narc4  narc5  narc6  nidentity1  nidentity2  omind1  omind2  omind3  omind4  omind5  omind6  contact1  contact2  contact3  contact4  contact5  hygiene1  hygiene2  hygiene3  hygiene4  hygiene5  psupport1  psupport2  psupport3  psupport4  psupport5  political_ideology  happy  slf_ladder  riskperc1  riskperc2  self_esteem  sex1  sbelong1  sbelong2  sbelong3  sbelong4  tested_positive  optim1  optim2  slfcont1  slfcont3  slfcont4  urban
##   7   4  age  children  cnarc1  cnarc2  cnarc3  ctheory1  ctheory2  ctheory3  ctheory4  CRT1  CRT2  CRT3  duration  employ_status1  generosity1  generosity2  generosity3  health_cond  know_tested_positive  ladder  marital1  mor_circle  moralid1  moralid10  moralid2  moralid3  moralid5  moralid6  moralid7  moralid8  moralid9  mcoop1  mcoop2  mcoop3  mcoop4  mcoop5  mcoop6  mcoop7  narc1  narc2  narc3  narc4  narc5  narc6  nidentity1  nidentity2  omind1  omind2  omind3  omind4  omind5  omind6  contact1  contact2  contact3  contact4  contact5  hygiene1  hygiene2  hygiene3  hygiene4  hygiene5  psupport1  psupport2  psupport3  psupport4  psupport5  political_ideology  happy  slf_ladder  riskperc1  riskperc2  self_esteem  sex1  sbelong1  sbelong2  sbelong3  sbelong4  tested_positive  optim1  optim2  slfcont1  slfcont3  slfcont4  urban
##   7   5  age  children  cnarc1  cnarc2  cnarc3  ctheory1  ctheory2  ctheory3  ctheory4  CRT1  CRT2  CRT3  duration  employ_status1  generosity1  generosity2  generosity3  health_cond  know_tested_positive  ladder  marital1  mor_circle  moralid1  moralid10  moralid2  moralid3  moralid5  moralid6  moralid7  moralid8  moralid9  mcoop1  mcoop2  mcoop3  mcoop4  mcoop5  mcoop6  mcoop7  narc1  narc2  narc3  narc4  narc5  narc6  nidentity1  nidentity2  omind1  omind2  omind3  omind4  omind5  omind6  contact1  contact2  contact3  contact4  contact5  hygiene1  hygiene2  hygiene3  hygiene4  hygiene5  psupport1  psupport2  psupport3  psupport4  psupport5  political_ideology  happy  slf_ladder  riskperc1  riskperc2  self_esteem  sex1  sbelong1  sbelong2  sbelong3  sbelong4  tested_positive  optim1  optim2  slfcont1  slfcont3  slfcont4  urban
##   7   6  age  children  cnarc1  cnarc2  cnarc3  ctheory1  ctheory2  ctheory3  ctheory4  CRT1  CRT2  CRT3  duration  employ_status1  generosity1  generosity2  generosity3  health_cond  know_tested_positive  ladder  marital1  mor_circle  moralid1  moralid10  moralid2  moralid3  moralid5  moralid6  moralid7  moralid8  moralid9  mcoop1  mcoop2  mcoop3  mcoop4  mcoop5  mcoop6  mcoop7  narc1  narc2  narc3  narc4  narc5  narc6  nidentity1  nidentity2  omind1  omind2  omind3  omind4  omind5  omind6  contact1  contact2  contact3  contact4  contact5  hygiene1  hygiene2  hygiene3  hygiene4  hygiene5  psupport1  psupport2  psupport3  psupport4  psupport5  political_ideology  happy  slf_ladder  riskperc1  riskperc2  self_esteem  sex1  sbelong1  sbelong2  sbelong3  sbelong4  tested_positive  optim1  optim2  slfcont1  slfcont3  slfcont4  urban
##   7   7  age  children  cnarc1  cnarc2  cnarc3  ctheory1  ctheory2  ctheory3  ctheory4  CRT1  CRT2  CRT3  duration  employ_status1  generosity1  generosity2  generosity3  health_cond  know_tested_positive  ladder  marital1  mor_circle  moralid1  moralid10  moralid2  moralid3  moralid5  moralid6  moralid7  moralid8  moralid9  mcoop1  mcoop2  mcoop3  mcoop4  mcoop5  mcoop6  mcoop7  narc1  narc2  narc3  narc4  narc5  narc6  nidentity1  nidentity2  omind1  omind2  omind3  omind4  omind5  omind6  contact1  contact2  contact3  contact4  contact5  hygiene1  hygiene2  hygiene3  hygiene4  hygiene5  psupport1  psupport2  psupport3  psupport4  psupport5  political_ideology  happy  slf_ladder  riskperc1  riskperc2  self_esteem  sex1  sbelong1  sbelong2  sbelong3  sbelong4  tested_positive  optim1  optim2  slfcont1  slfcont3  slfcont4  urban
##   7   8  age  children  cnarc1  cnarc2  cnarc3  ctheory1  ctheory2  ctheory3  ctheory4  CRT1  CRT2  CRT3  duration  employ_status1  generosity1  generosity2  generosity3  health_cond  know_tested_positive  ladder  marital1  mor_circle  moralid1  moralid10  moralid2  moralid3  moralid5  moralid6  moralid7  moralid8  moralid9  mcoop1  mcoop2  mcoop3  mcoop4  mcoop5  mcoop6  mcoop7  narc1  narc2  narc3  narc4  narc5  narc6  nidentity1  nidentity2  omind1  omind2  omind3  omind4  omind5  omind6  contact1  contact2  contact3  contact4  contact5  hygiene1  hygiene2  hygiene3  hygiene4  hygiene5  psupport1  psupport2  psupport3  psupport4  psupport5  political_ideology  happy  slf_ladder  riskperc1  riskperc2  self_esteem  sex1  sbelong1  sbelong2  sbelong3  sbelong4  tested_positive  optim1  optim2  slfcont1  slfcont3  slfcont4  urban
##   7   9  age  children  cnarc1  cnarc2  cnarc3  ctheory1  ctheory2  ctheory3  ctheory4  CRT1  CRT2  CRT3  duration  employ_status1  generosity1  generosity2  generosity3  health_cond  know_tested_positive  ladder  marital1  mor_circle  moralid1  moralid10  moralid2  moralid3  moralid5  moralid6  moralid7  moralid8  moralid9  mcoop1  mcoop2  mcoop3  mcoop4  mcoop5  mcoop6  mcoop7  narc1  narc2  narc3  narc4  narc5  narc6  nidentity1  nidentity2  omind1  omind2  omind3  omind4  omind5  omind6  contact1  contact2  contact3  contact4  contact5  hygiene1  hygiene2  hygiene3  hygiene4  hygiene5  psupport1  psupport2  psupport3  psupport4  psupport5  political_ideology  happy  slf_ladder  riskperc1  riskperc2  self_esteem  sex1  sbelong1  sbelong2  sbelong3  sbelong4  tested_positive  optim1  optim2  slfcont1  slfcont3  slfcont4  urban
##   7   10  age  children  cnarc1  cnarc2  cnarc3  ctheory1  ctheory2  ctheory3  ctheory4  CRT1  CRT2  CRT3  duration  employ_status1  generosity1  generosity2  generosity3  health_cond  know_tested_positive  ladder  marital1  mor_circle  moralid1  moralid10  moralid2  moralid3  moralid5  moralid6  moralid7  moralid8  moralid9  mcoop1  mcoop2  mcoop3  mcoop4  mcoop5  mcoop6  mcoop7  narc1  narc2  narc3  narc4  narc5  narc6  nidentity1  nidentity2  omind1  omind2  omind3  omind4  omind5  omind6  contact1  contact2  contact3  contact4  contact5  hygiene1  hygiene2  hygiene3  hygiene4  hygiene5  psupport1  psupport2  psupport3  psupport4  psupport5  political_ideology  happy  slf_ladder  riskperc1  riskperc2  self_esteem  sex1  sbelong1  sbelong2  sbelong3  sbelong4  tested_positive  optim1  optim2  slfcont1  slfcont3  slfcont4  urban
##   8   1  age  children  cnarc1  cnarc2  cnarc3  ctheory1  ctheory2  ctheory3  ctheory4  CRT1  CRT2  CRT3  duration  employ_status1  generosity1  generosity2  generosity3  health_cond  know_tested_positive  ladder  marital1  mor_circle  moralid1  moralid10  moralid2  moralid3  moralid5  moralid6  moralid7  moralid8  moralid9  mcoop1  mcoop2  mcoop3  mcoop4  mcoop5  mcoop6  mcoop7  narc1  narc2  narc3  narc4  narc5  narc6  nidentity1  nidentity2  omind1  omind2  omind3  omind4  omind5  omind6  contact1  contact2  contact3  contact4  contact5  hygiene1  hygiene2  hygiene3  hygiene4  hygiene5  psupport1  psupport2  psupport3  psupport4  psupport5  political_ideology  happy  slf_ladder  riskperc1  riskperc2  self_esteem  sex1  sbelong1  sbelong2  sbelong3  sbelong4  tested_positive  optim1  optim2  slfcont1  slfcont3  slfcont4  urban
##   8   2  age  children  cnarc1  cnarc2  cnarc3  ctheory1  ctheory2  ctheory3  ctheory4  CRT1  CRT2  CRT3  duration  employ_status1  generosity1  generosity2  generosity3  health_cond  know_tested_positive  ladder  marital1  mor_circle  moralid1  moralid10  moralid2  moralid3  moralid5  moralid6  moralid7  moralid8  moralid9  mcoop1  mcoop2  mcoop3  mcoop4  mcoop5  mcoop6  mcoop7  narc1  narc2  narc3  narc4  narc5  narc6  nidentity1  nidentity2  omind1  omind2  omind3  omind4  omind5  omind6  contact1  contact2  contact3  contact4  contact5  hygiene1  hygiene2  hygiene3  hygiene4  hygiene5  psupport1  psupport2  psupport3  psupport4  psupport5  political_ideology  happy  slf_ladder  riskperc1  riskperc2  self_esteem  sex1  sbelong1  sbelong2  sbelong3  sbelong4  tested_positive  optim1  optim2  slfcont1  slfcont3  slfcont4  urban
##   8   3  age  children  cnarc1  cnarc2  cnarc3  ctheory1  ctheory2  ctheory3  ctheory4  CRT1  CRT2  CRT3  duration  employ_status1  generosity1  generosity2  generosity3  health_cond  know_tested_positive  ladder  marital1  mor_circle  moralid1  moralid10  moralid2  moralid3  moralid5  moralid6  moralid7  moralid8  moralid9  mcoop1  mcoop2  mcoop3  mcoop4  mcoop5  mcoop6  mcoop7  narc1  narc2  narc3  narc4  narc5  narc6  nidentity1  nidentity2  omind1  omind2  omind3  omind4  omind5  omind6  contact1  contact2  contact3  contact4  contact5  hygiene1  hygiene2  hygiene3  hygiene4  hygiene5  psupport1  psupport2  psupport3  psupport4  psupport5  political_ideology  happy  slf_ladder  riskperc1  riskperc2  self_esteem  sex1  sbelong1  sbelong2  sbelong3  sbelong4  tested_positive  optim1  optim2  slfcont1  slfcont3  slfcont4  urban
##   8   4  age  children  cnarc1  cnarc2  cnarc3  ctheory1  ctheory2  ctheory3  ctheory4  CRT1  CRT2  CRT3  duration  employ_status1  generosity1  generosity2  generosity3  health_cond  know_tested_positive  ladder  marital1  mor_circle  moralid1  moralid10  moralid2  moralid3  moralid5  moralid6  moralid7  moralid8  moralid9  mcoop1  mcoop2  mcoop3  mcoop4  mcoop5  mcoop6  mcoop7  narc1  narc2  narc3  narc4  narc5  narc6  nidentity1  nidentity2  omind1  omind2  omind3  omind4  omind5  omind6  contact1  contact2  contact3  contact4  contact5  hygiene1  hygiene2  hygiene3  hygiene4  hygiene5  psupport1  psupport2  psupport3  psupport4  psupport5  political_ideology  happy  slf_ladder  riskperc1  riskperc2  self_esteem  sex1  sbelong1  sbelong2  sbelong3  sbelong4  tested_positive  optim1  optim2  slfcont1  slfcont3  slfcont4  urban
##   8   5  age  children  cnarc1  cnarc2  cnarc3  ctheory1  ctheory2  ctheory3  ctheory4  CRT1  CRT2  CRT3  duration  employ_status1  generosity1  generosity2  generosity3  health_cond  know_tested_positive  ladder  marital1  mor_circle  moralid1  moralid10  moralid2  moralid3  moralid5  moralid6  moralid7  moralid8  moralid9  mcoop1  mcoop2  mcoop3  mcoop4  mcoop5  mcoop6  mcoop7  narc1  narc2  narc3  narc4  narc5  narc6  nidentity1  nidentity2  omind1  omind2  omind3  omind4  omind5  omind6  contact1  contact2  contact3  contact4  contact5  hygiene1  hygiene2  hygiene3  hygiene4  hygiene5  psupport1  psupport2  psupport3  psupport4  psupport5  political_ideology  happy  slf_ladder  riskperc1  riskperc2  self_esteem  sex1  sbelong1  sbelong2  sbelong3  sbelong4  tested_positive  optim1  optim2  slfcont1  slfcont3  slfcont4  urban
##   8   6  age  children  cnarc1  cnarc2  cnarc3  ctheory1  ctheory2  ctheory3  ctheory4  CRT1  CRT2  CRT3  duration  employ_status1  generosity1  generosity2  generosity3  health_cond  know_tested_positive  ladder  marital1  mor_circle  moralid1  moralid10  moralid2  moralid3  moralid5  moralid6  moralid7  moralid8  moralid9  mcoop1  mcoop2  mcoop3  mcoop4  mcoop5  mcoop6  mcoop7  narc1  narc2  narc3  narc4  narc5  narc6  nidentity1  nidentity2  omind1  omind2  omind3  omind4  omind5  omind6  contact1  contact2  contact3  contact4  contact5  hygiene1  hygiene2  hygiene3  hygiene4  hygiene5  psupport1  psupport2  psupport3  psupport4  psupport5  political_ideology  happy  slf_ladder  riskperc1  riskperc2  self_esteem  sex1  sbelong1  sbelong2  sbelong3  sbelong4  tested_positive  optim1  optim2  slfcont1  slfcont3  slfcont4  urban
##   8   7  age  children  cnarc1  cnarc2  cnarc3  ctheory1  ctheory2  ctheory3  ctheory4  CRT1  CRT2  CRT3  duration  employ_status1  generosity1  generosity2  generosity3  health_cond  know_tested_positive  ladder  marital1  mor_circle  moralid1  moralid10  moralid2  moralid3  moralid5  moralid6  moralid7  moralid8  moralid9  mcoop1  mcoop2  mcoop3  mcoop4  mcoop5  mcoop6  mcoop7  narc1  narc2  narc3  narc4  narc5  narc6  nidentity1  nidentity2  omind1  omind2  omind3  omind4  omind5  omind6  contact1  contact2  contact3  contact4  contact5  hygiene1  hygiene2  hygiene3  hygiene4  hygiene5  psupport1  psupport2  psupport3  psupport4  psupport5  political_ideology  happy  slf_ladder  riskperc1  riskperc2  self_esteem  sex1  sbelong1  sbelong2  sbelong3  sbelong4  tested_positive  optim1  optim2  slfcont1  slfcont3  slfcont4  urban
##   8   8  age  children  cnarc1  cnarc2  cnarc3  ctheory1  ctheory2  ctheory3  ctheory4  CRT1  CRT2  CRT3  duration  employ_status1  generosity1  generosity2  generosity3  health_cond  know_tested_positive  ladder  marital1  mor_circle  moralid1  moralid10  moralid2  moralid3  moralid5  moralid6  moralid7  moralid8  moralid9  mcoop1  mcoop2  mcoop3  mcoop4  mcoop5  mcoop6  mcoop7  narc1  narc2  narc3  narc4  narc5  narc6  nidentity1  nidentity2  omind1  omind2  omind3  omind4  omind5  omind6  contact1  contact2  contact3  contact4  contact5  hygiene1  hygiene2  hygiene3  hygiene4  hygiene5  psupport1  psupport2  psupport3  psupport4  psupport5  political_ideology  happy  slf_ladder  riskperc1  riskperc2  self_esteem  sex1  sbelong1  sbelong2  sbelong3  sbelong4  tested_positive  optim1  optim2  slfcont1  slfcont3  slfcont4  urban
##   8   9  age  children  cnarc1  cnarc2  cnarc3  ctheory1  ctheory2  ctheory3  ctheory4  CRT1  CRT2  CRT3  duration  employ_status1  generosity1  generosity2  generosity3  health_cond  know_tested_positive  ladder  marital1  mor_circle  moralid1  moralid10  moralid2  moralid3  moralid5  moralid6  moralid7  moralid8  moralid9  mcoop1  mcoop2  mcoop3  mcoop4  mcoop5  mcoop6  mcoop7  narc1  narc2  narc3  narc4  narc5  narc6  nidentity1  nidentity2  omind1  omind2  omind3  omind4  omind5  omind6  contact1  contact2  contact3  contact4  contact5  hygiene1  hygiene2  hygiene3  hygiene4  hygiene5  psupport1  psupport2  psupport3  psupport4  psupport5  political_ideology  happy  slf_ladder  riskperc1  riskperc2  self_esteem  sex1  sbelong1  sbelong2  sbelong3  sbelong4  tested_positive  optim1  optim2  slfcont1  slfcont3  slfcont4  urban
##   8   10  age  children  cnarc1  cnarc2  cnarc3  ctheory1  ctheory2  ctheory3  ctheory4  CRT1  CRT2  CRT3  duration  employ_status1  generosity1  generosity2  generosity3  health_cond  know_tested_positive  ladder  marital1  mor_circle  moralid1  moralid10  moralid2  moralid3  moralid5  moralid6  moralid7  moralid8  moralid9  mcoop1  mcoop2  mcoop3  mcoop4  mcoop5  mcoop6  mcoop7  narc1  narc2  narc3  narc4  narc5  narc6  nidentity1  nidentity2  omind1  omind2  omind3  omind4  omind5  omind6  contact1  contact2  contact3  contact4  contact5  hygiene1  hygiene2  hygiene3  hygiene4  hygiene5  psupport1  psupport2  psupport3  psupport4  psupport5  political_ideology  happy  slf_ladder  riskperc1  riskperc2  self_esteem  sex1  sbelong1  sbelong2  sbelong3  sbelong4  tested_positive  optim1  optim2  slfcont1  slfcont3  slfcont4  urban
##   9   1  age  children  cnarc1  cnarc2  cnarc3  ctheory1  ctheory2  ctheory3  ctheory4  CRT1  CRT2  CRT3  duration  employ_status1  generosity1  generosity2  generosity3  health_cond  know_tested_positive  ladder  marital1  mor_circle  moralid1  moralid10  moralid2  moralid3  moralid5  moralid6  moralid7  moralid8  moralid9  mcoop1  mcoop2  mcoop3  mcoop4  mcoop5  mcoop6  mcoop7  narc1  narc2  narc3  narc4  narc5  narc6  nidentity1  nidentity2  omind1  omind2  omind3  omind4  omind5  omind6  contact1  contact2  contact3  contact4  contact5  hygiene1  hygiene2  hygiene3  hygiene4  hygiene5  psupport1  psupport2  psupport3  psupport4  psupport5  political_ideology  happy  slf_ladder  riskperc1  riskperc2  self_esteem  sex1  sbelong1  sbelong2  sbelong3  sbelong4  tested_positive  optim1  optim2  slfcont1  slfcont3  slfcont4  urban
##   9   2  age  children  cnarc1  cnarc2  cnarc3  ctheory1  ctheory2  ctheory3  ctheory4  CRT1  CRT2  CRT3  duration  employ_status1  generosity1  generosity2  generosity3  health_cond  know_tested_positive  ladder  marital1  mor_circle  moralid1  moralid10  moralid2  moralid3  moralid5  moralid6  moralid7  moralid8  moralid9  mcoop1  mcoop2  mcoop3  mcoop4  mcoop5  mcoop6  mcoop7  narc1  narc2  narc3  narc4  narc5  narc6  nidentity1  nidentity2  omind1  omind2  omind3  omind4  omind5  omind6  contact1  contact2  contact3  contact4  contact5  hygiene1  hygiene2  hygiene3  hygiene4  hygiene5  psupport1  psupport2  psupport3  psupport4  psupport5  political_ideology  happy  slf_ladder  riskperc1  riskperc2  self_esteem  sex1  sbelong1  sbelong2  sbelong3  sbelong4  tested_positive  optim1  optim2  slfcont1  slfcont3  slfcont4  urban
##   9   3  age  children  cnarc1  cnarc2  cnarc3  ctheory1  ctheory2  ctheory3  ctheory4  CRT1  CRT2  CRT3  duration  employ_status1  generosity1  generosity2  generosity3  health_cond  know_tested_positive  ladder  marital1  mor_circle  moralid1  moralid10  moralid2  moralid3  moralid5  moralid6  moralid7  moralid8  moralid9  mcoop1  mcoop2  mcoop3  mcoop4  mcoop5  mcoop6  mcoop7  narc1  narc2  narc3  narc4  narc5  narc6  nidentity1  nidentity2  omind1  omind2  omind3  omind4  omind5  omind6  contact1  contact2  contact3  contact4  contact5  hygiene1  hygiene2  hygiene3  hygiene4  hygiene5  psupport1  psupport2  psupport3  psupport4  psupport5  political_ideology  happy  slf_ladder  riskperc1  riskperc2  self_esteem  sex1  sbelong1  sbelong2  sbelong3  sbelong4  tested_positive  optim1  optim2  slfcont1  slfcont3  slfcont4  urban
##   9   4  age  children  cnarc1  cnarc2  cnarc3  ctheory1  ctheory2  ctheory3  ctheory4  CRT1  CRT2  CRT3  duration  employ_status1  generosity1  generosity2  generosity3  health_cond  know_tested_positive  ladder  marital1  mor_circle  moralid1  moralid10  moralid2  moralid3  moralid5  moralid6  moralid7  moralid8  moralid9  mcoop1  mcoop2  mcoop3  mcoop4  mcoop5  mcoop6  mcoop7  narc1  narc2  narc3  narc4  narc5  narc6  nidentity1  nidentity2  omind1  omind2  omind3  omind4  omind5  omind6  contact1  contact2  contact3  contact4  contact5  hygiene1  hygiene2  hygiene3  hygiene4  hygiene5  psupport1  psupport2  psupport3  psupport4  psupport5  political_ideology  happy  slf_ladder  riskperc1  riskperc2  self_esteem  sex1  sbelong1  sbelong2  sbelong3  sbelong4  tested_positive  optim1  optim2  slfcont1  slfcont3  slfcont4  urban
##   9   5  age  children  cnarc1  cnarc2  cnarc3  ctheory1  ctheory2  ctheory3  ctheory4  CRT1  CRT2  CRT3  duration  employ_status1  generosity1  generosity2  generosity3  health_cond  know_tested_positive  ladder  marital1  mor_circle  moralid1  moralid10  moralid2  moralid3  moralid5  moralid6  moralid7  moralid8  moralid9  mcoop1  mcoop2  mcoop3  mcoop4  mcoop5  mcoop6  mcoop7  narc1  narc2  narc3  narc4  narc5  narc6  nidentity1  nidentity2  omind1  omind2  omind3  omind4  omind5  omind6  contact1  contact2  contact3  contact4  contact5  hygiene1  hygiene2  hygiene3  hygiene4  hygiene5  psupport1  psupport2  psupport3  psupport4  psupport5  political_ideology  happy  slf_ladder  riskperc1  riskperc2  self_esteem  sex1  sbelong1  sbelong2  sbelong3  sbelong4  tested_positive  optim1  optim2  slfcont1  slfcont3  slfcont4  urban
##   9   6  age  children  cnarc1  cnarc2  cnarc3  ctheory1  ctheory2  ctheory3  ctheory4  CRT1  CRT2  CRT3  duration  employ_status1  generosity1  generosity2  generosity3  health_cond  know_tested_positive  ladder  marital1  mor_circle  moralid1  moralid10  moralid2  moralid3  moralid5  moralid6  moralid7  moralid8  moralid9  mcoop1  mcoop2  mcoop3  mcoop4  mcoop5  mcoop6  mcoop7  narc1  narc2  narc3  narc4  narc5  narc6  nidentity1  nidentity2  omind1  omind2  omind3  omind4  omind5  omind6  contact1  contact2  contact3  contact4  contact5  hygiene1  hygiene2  hygiene3  hygiene4  hygiene5  psupport1  psupport2  psupport3  psupport4  psupport5  political_ideology  happy  slf_ladder  riskperc1  riskperc2  self_esteem  sex1  sbelong1  sbelong2  sbelong3  sbelong4  tested_positive  optim1  optim2  slfcont1  slfcont3  slfcont4  urban
##   9   7  age  children  cnarc1  cnarc2  cnarc3  ctheory1  ctheory2  ctheory3  ctheory4  CRT1  CRT2  CRT3  duration  employ_status1  generosity1  generosity2  generosity3  health_cond  know_tested_positive  ladder  marital1  mor_circle  moralid1  moralid10  moralid2  moralid3  moralid5  moralid6  moralid7  moralid8  moralid9  mcoop1  mcoop2  mcoop3  mcoop4  mcoop5  mcoop6  mcoop7  narc1  narc2  narc3  narc4  narc5  narc6  nidentity1  nidentity2  omind1  omind2  omind3  omind4  omind5  omind6  contact1  contact2  contact3  contact4  contact5  hygiene1  hygiene2  hygiene3  hygiene4  hygiene5  psupport1  psupport2  psupport3  psupport4  psupport5  political_ideology  happy  slf_ladder  riskperc1  riskperc2  self_esteem  sex1  sbelong1  sbelong2  sbelong3  sbelong4  tested_positive  optim1  optim2  slfcont1  slfcont3  slfcont4  urban
##   9   8  age  children  cnarc1  cnarc2  cnarc3  ctheory1  ctheory2  ctheory3  ctheory4  CRT1  CRT2  CRT3  duration  employ_status1  generosity1  generosity2  generosity3  health_cond  know_tested_positive  ladder  marital1  mor_circle  moralid1  moralid10  moralid2  moralid3  moralid5  moralid6  moralid7  moralid8  moralid9  mcoop1  mcoop2  mcoop3  mcoop4  mcoop5  mcoop6  mcoop7  narc1  narc2  narc3  narc4  narc5  narc6  nidentity1  nidentity2  omind1  omind2  omind3  omind4  omind5  omind6  contact1  contact2  contact3  contact4  contact5  hygiene1  hygiene2  hygiene3  hygiene4  hygiene5  psupport1  psupport2  psupport3  psupport4  psupport5  political_ideology  happy  slf_ladder  riskperc1  riskperc2  self_esteem  sex1  sbelong1  sbelong2  sbelong3  sbelong4  tested_positive  optim1  optim2  slfcont1  slfcont3  slfcont4  urban
##   9   9  age  children  cnarc1  cnarc2  cnarc3  ctheory1  ctheory2  ctheory3  ctheory4  CRT1  CRT2  CRT3  duration  employ_status1  generosity1  generosity2  generosity3  health_cond  know_tested_positive  ladder  marital1  mor_circle  moralid1  moralid10  moralid2  moralid3  moralid5  moralid6  moralid7  moralid8  moralid9  mcoop1  mcoop2  mcoop3  mcoop4  mcoop5  mcoop6  mcoop7  narc1  narc2  narc3  narc4  narc5  narc6  nidentity1  nidentity2  omind1  omind2  omind3  omind4  omind5  omind6  contact1  contact2  contact3  contact4  contact5  hygiene1  hygiene2  hygiene3  hygiene4  hygiene5  psupport1  psupport2  psupport3  psupport4  psupport5  political_ideology  happy  slf_ladder  riskperc1  riskperc2  self_esteem  sex1  sbelong1  sbelong2  sbelong3  sbelong4  tested_positive  optim1  optim2  slfcont1  slfcont3  slfcont4  urban
##   9   10  age  children  cnarc1  cnarc2  cnarc3  ctheory1  ctheory2  ctheory3  ctheory4  CRT1  CRT2  CRT3  duration  employ_status1  generosity1  generosity2  generosity3  health_cond  know_tested_positive  ladder  marital1  mor_circle  moralid1  moralid10  moralid2  moralid3  moralid5  moralid6  moralid7  moralid8  moralid9  mcoop1  mcoop2  mcoop3  mcoop4  mcoop5  mcoop6  mcoop7  narc1  narc2  narc3  narc4  narc5  narc6  nidentity1  nidentity2  omind1  omind2  omind3  omind4  omind5  omind6  contact1  contact2  contact3  contact4  contact5  hygiene1  hygiene2  hygiene3  hygiene4  hygiene5  psupport1  psupport2  psupport3  psupport4  psupport5  political_ideology  happy  slf_ladder  riskperc1  riskperc2  self_esteem  sex1  sbelong1  sbelong2  sbelong3  sbelong4  tested_positive  optim1  optim2  slfcont1  slfcont3  slfcont4  urban
##   10   1  age  children  cnarc1  cnarc2  cnarc3  ctheory1  ctheory2  ctheory3  ctheory4  CRT1  CRT2  CRT3  duration  employ_status1  generosity1  generosity2  generosity3  health_cond  know_tested_positive  ladder  marital1  mor_circle  moralid1  moralid10  moralid2  moralid3  moralid5  moralid6  moralid7  moralid8  moralid9  mcoop1  mcoop2  mcoop3  mcoop4  mcoop5  mcoop6  mcoop7  narc1  narc2  narc3  narc4  narc5  narc6  nidentity1  nidentity2  omind1  omind2  omind3  omind4  omind5  omind6  contact1  contact2  contact3  contact4  contact5  hygiene1  hygiene2  hygiene3  hygiene4  hygiene5  psupport1  psupport2  psupport3  psupport4  psupport5  political_ideology  happy  slf_ladder  riskperc1  riskperc2  self_esteem  sex1  sbelong1  sbelong2  sbelong3  sbelong4  tested_positive  optim1  optim2  slfcont1  slfcont3  slfcont4  urban
##   10   2  age  children  cnarc1  cnarc2  cnarc3  ctheory1  ctheory2  ctheory3  ctheory4  CRT1  CRT2  CRT3  duration  employ_status1  generosity1  generosity2  generosity3  health_cond  know_tested_positive  ladder  marital1  mor_circle  moralid1  moralid10  moralid2  moralid3  moralid5  moralid6  moralid7  moralid8  moralid9  mcoop1  mcoop2  mcoop3  mcoop4  mcoop5  mcoop6  mcoop7  narc1  narc2  narc3  narc4  narc5  narc6  nidentity1  nidentity2  omind1  omind2  omind3  omind4  omind5  omind6  contact1  contact2  contact3  contact4  contact5  hygiene1  hygiene2  hygiene3  hygiene4  hygiene5  psupport1  psupport2  psupport3  psupport4  psupport5  political_ideology  happy  slf_ladder  riskperc1  riskperc2  self_esteem  sex1  sbelong1  sbelong2  sbelong3  sbelong4  tested_positive  optim1  optim2  slfcont1  slfcont3  slfcont4  urban
##   10   3  age  children  cnarc1  cnarc2  cnarc3  ctheory1  ctheory2  ctheory3  ctheory4  CRT1  CRT2  CRT3  duration  employ_status1  generosity1  generosity2  generosity3  health_cond  know_tested_positive  ladder  marital1  mor_circle  moralid1  moralid10  moralid2  moralid3  moralid5  moralid6  moralid7  moralid8  moralid9  mcoop1  mcoop2  mcoop3  mcoop4  mcoop5  mcoop6  mcoop7  narc1  narc2  narc3  narc4  narc5  narc6  nidentity1  nidentity2  omind1  omind2  omind3  omind4  omind5  omind6  contact1  contact2  contact3  contact4  contact5  hygiene1  hygiene2  hygiene3  hygiene4  hygiene5  psupport1  psupport2  psupport3  psupport4  psupport5  political_ideology  happy  slf_ladder  riskperc1  riskperc2  self_esteem  sex1  sbelong1  sbelong2  sbelong3  sbelong4  tested_positive  optim1  optim2  slfcont1  slfcont3  slfcont4  urban
##   10   4  age  children  cnarc1  cnarc2  cnarc3  ctheory1  ctheory2  ctheory3  ctheory4  CRT1  CRT2  CRT3  duration  employ_status1  generosity1  generosity2  generosity3  health_cond  know_tested_positive  ladder  marital1  mor_circle  moralid1  moralid10  moralid2  moralid3  moralid5  moralid6  moralid7  moralid8  moralid9  mcoop1  mcoop2  mcoop3  mcoop4  mcoop5  mcoop6  mcoop7  narc1  narc2  narc3  narc4  narc5  narc6  nidentity1  nidentity2  omind1  omind2  omind3  omind4  omind5  omind6  contact1  contact2  contact3  contact4  contact5  hygiene1  hygiene2  hygiene3  hygiene4  hygiene5  psupport1  psupport2  psupport3  psupport4  psupport5  political_ideology  happy  slf_ladder  riskperc1  riskperc2  self_esteem  sex1  sbelong1  sbelong2  sbelong3  sbelong4  tested_positive  optim1  optim2  slfcont1  slfcont3  slfcont4  urban
##   10   5  age  children  cnarc1  cnarc2  cnarc3  ctheory1  ctheory2  ctheory3  ctheory4  CRT1  CRT2  CRT3  duration  employ_status1  generosity1  generosity2  generosity3  health_cond  know_tested_positive  ladder  marital1  mor_circle  moralid1  moralid10  moralid2  moralid3  moralid5  moralid6  moralid7  moralid8  moralid9  mcoop1  mcoop2  mcoop3  mcoop4  mcoop5  mcoop6  mcoop7  narc1  narc2  narc3  narc4  narc5  narc6  nidentity1  nidentity2  omind1  omind2  omind3  omind4  omind5  omind6  contact1  contact2  contact3  contact4  contact5  hygiene1  hygiene2  hygiene3  hygiene4  hygiene5  psupport1  psupport2  psupport3  psupport4  psupport5  political_ideology  happy  slf_ladder  riskperc1  riskperc2  self_esteem  sex1  sbelong1  sbelong2  sbelong3  sbelong4  tested_positive  optim1  optim2  slfcont1  slfcont3  slfcont4  urban
##   10   6  age  children  cnarc1  cnarc2  cnarc3  ctheory1  ctheory2  ctheory3  ctheory4  CRT1  CRT2  CRT3  duration  employ_status1  generosity1  generosity2  generosity3  health_cond  know_tested_positive  ladder  marital1  mor_circle  moralid1  moralid10  moralid2  moralid3  moralid5  moralid6  moralid7  moralid8  moralid9  mcoop1  mcoop2  mcoop3  mcoop4  mcoop5  mcoop6  mcoop7  narc1  narc2  narc3  narc4  narc5  narc6  nidentity1  nidentity2  omind1  omind2  omind3  omind4  omind5  omind6  contact1  contact2  contact3  contact4  contact5  hygiene1  hygiene2  hygiene3  hygiene4  hygiene5  psupport1  psupport2  psupport3  psupport4  psupport5  political_ideology  happy  slf_ladder  riskperc1  riskperc2  self_esteem  sex1  sbelong1  sbelong2  sbelong3  sbelong4  tested_positive  optim1  optim2  slfcont1  slfcont3  slfcont4  urban
##   10   7  age  children  cnarc1  cnarc2  cnarc3  ctheory1  ctheory2  ctheory3  ctheory4  CRT1  CRT2  CRT3  duration  employ_status1  generosity1  generosity2  generosity3  health_cond  know_tested_positive  ladder  marital1  mor_circle  moralid1  moralid10  moralid2  moralid3  moralid5  moralid6  moralid7  moralid8  moralid9  mcoop1  mcoop2  mcoop3  mcoop4  mcoop5  mcoop6  mcoop7  narc1  narc2  narc3  narc4  narc5  narc6  nidentity1  nidentity2  omind1  omind2  omind3  omind4  omind5  omind6  contact1  contact2  contact3  contact4  contact5  hygiene1  hygiene2  hygiene3  hygiene4  hygiene5  psupport1  psupport2  psupport3  psupport4  psupport5  political_ideology  happy  slf_ladder  riskperc1  riskperc2  self_esteem  sex1  sbelong1  sbelong2  sbelong3  sbelong4  tested_positive  optim1  optim2  slfcont1  slfcont3  slfcont4  urban
##   10   8  age  children  cnarc1  cnarc2  cnarc3  ctheory1  ctheory2  ctheory3  ctheory4  CRT1  CRT2  CRT3  duration  employ_status1  generosity1  generosity2  generosity3  health_cond  know_tested_positive  ladder  marital1  mor_circle  moralid1  moralid10  moralid2  moralid3  moralid5  moralid6  moralid7  moralid8  moralid9  mcoop1  mcoop2  mcoop3  mcoop4  mcoop5  mcoop6  mcoop7  narc1  narc2  narc3  narc4  narc5  narc6  nidentity1  nidentity2  omind1  omind2  omind3  omind4  omind5  omind6  contact1  contact2  contact3  contact4  contact5  hygiene1  hygiene2  hygiene3  hygiene4  hygiene5  psupport1  psupport2  psupport3  psupport4  psupport5  political_ideology  happy  slf_ladder  riskperc1  riskperc2  self_esteem  sex1  sbelong1  sbelong2  sbelong3  sbelong4  tested_positive  optim1  optim2  slfcont1  slfcont3  slfcont4  urban
##   10   9  age  children  cnarc1  cnarc2  cnarc3  ctheory1  ctheory2  ctheory3  ctheory4  CRT1  CRT2  CRT3  duration  employ_status1  generosity1  generosity2  generosity3  health_cond  know_tested_positive  ladder  marital1  mor_circle  moralid1  moralid10  moralid2  moralid3  moralid5  moralid6  moralid7  moralid8  moralid9  mcoop1  mcoop2  mcoop3  mcoop4  mcoop5  mcoop6  mcoop7  narc1  narc2  narc3  narc4  narc5  narc6  nidentity1  nidentity2  omind1  omind2  omind3  omind4  omind5  omind6  contact1  contact2  contact3  contact4  contact5  hygiene1  hygiene2  hygiene3  hygiene4  hygiene5  psupport1  psupport2  psupport3  psupport4  psupport5  political_ideology  happy  slf_ladder  riskperc1  riskperc2  self_esteem  sex1  sbelong1  sbelong2  sbelong3  sbelong4  tested_positive  optim1  optim2  slfcont1  slfcont3  slfcont4  urban
##   10   10  age  children  cnarc1  cnarc2  cnarc3  ctheory1  ctheory2  ctheory3  ctheory4  CRT1  CRT2  CRT3  duration  employ_status1  generosity1  generosity2  generosity3  health_cond  know_tested_positive  ladder  marital1  mor_circle  moralid1  moralid10  moralid2  moralid3  moralid5  moralid6  moralid7  moralid8  moralid9  mcoop1  mcoop2  mcoop3  mcoop4  mcoop5  mcoop6  mcoop7  narc1  narc2  narc3  narc4  narc5  narc6  nidentity1  nidentity2  omind1  omind2  omind3  omind4  omind5  omind6  contact1  contact2  contact3  contact4  contact5  hygiene1  hygiene2  hygiene3  hygiene4  hygiene5  psupport1  psupport2  psupport3  psupport4  psupport5  political_ideology  happy  slf_ladder  riskperc1  riskperc2  self_esteem  sex1  sbelong1  sbelong2  sbelong3  sbelong4  tested_positive  optim1  optim2  slfcont1  slfcont3  slfcont4  urban
```

```
## Warning: Number of logged events: 8073
```

```
## 
##  iter imp variable
##   1   1  children  cnarc1  cnarc2  cnarc3  ctheory1  ctheory2  ctheory3  ctheory4  CRT1  CRT2  CRT3  employ_status1  health_cond  marital1  mor_circle  moralid1  moralid10  moralid2  moralid3  moralid4  moralid5  moralid6  moralid7  moralid8  moralid9  mcoop1  mcoop2  mcoop3  mcoop4  mcoop5  mcoop6  mcoop7  nidentity1  omind1  omind2  omind3  omind4  omind5  omind6  political_ideology  riskperc1  riskperc2  sbelong1  sbelong2  sbelong3  sbelong4  tested_positive  optim1  optim2  slfcont1  slfcont2  slfcont3  slfcont4  urban
##   1   2  children  cnarc1  cnarc2  cnarc3  ctheory1  ctheory2  ctheory3  ctheory4  CRT1  CRT2  CRT3  employ_status1  health_cond  marital1  mor_circle  moralid1  moralid10  moralid2  moralid3  moralid4  moralid5  moralid6  moralid7  moralid8  moralid9  mcoop1  mcoop2  mcoop3  mcoop4  mcoop5  mcoop6  mcoop7  nidentity1  omind1  omind2  omind3  omind4  omind5  omind6  political_ideology  riskperc1  riskperc2  sbelong1  sbelong2  sbelong3  sbelong4  tested_positive  optim1  optim2  slfcont1  slfcont2  slfcont3  slfcont4  urban
##   1   3  children  cnarc1  cnarc2  cnarc3  ctheory1  ctheory2  ctheory3  ctheory4  CRT1  CRT2  CRT3  employ_status1  health_cond  marital1  mor_circle  moralid1  moralid10  moralid2  moralid3  moralid4  moralid5  moralid6  moralid7  moralid8  moralid9  mcoop1  mcoop2  mcoop3  mcoop4  mcoop5  mcoop6  mcoop7  nidentity1  omind1  omind2  omind3  omind4  omind5  omind6  political_ideology  riskperc1  riskperc2  sbelong1  sbelong2  sbelong3  sbelong4  tested_positive  optim1  optim2  slfcont1  slfcont2  slfcont3  slfcont4  urban
##   1   4  children  cnarc1  cnarc2  cnarc3  ctheory1  ctheory2  ctheory3  ctheory4  CRT1  CRT2  CRT3  employ_status1  health_cond  marital1  mor_circle  moralid1  moralid10  moralid2  moralid3  moralid4  moralid5  moralid6  moralid7  moralid8  moralid9  mcoop1  mcoop2  mcoop3  mcoop4  mcoop5  mcoop6  mcoop7  nidentity1  omind1  omind2  omind3  omind4  omind5  omind6  political_ideology  riskperc1  riskperc2  sbelong1  sbelong2  sbelong3  sbelong4  tested_positive  optim1  optim2  slfcont1  slfcont2  slfcont3  slfcont4  urban
##   1   5  children  cnarc1  cnarc2  cnarc3  ctheory1  ctheory2  ctheory3  ctheory4  CRT1  CRT2  CRT3  employ_status1  health_cond  marital1  mor_circle  moralid1  moralid10  moralid2  moralid3  moralid4  moralid5  moralid6  moralid7  moralid8  moralid9  mcoop1  mcoop2  mcoop3  mcoop4  mcoop5  mcoop6  mcoop7  nidentity1  omind1  omind2  omind3  omind4  omind5  omind6  political_ideology  riskperc1  riskperc2  sbelong1  sbelong2  sbelong3  sbelong4  tested_positive  optim1  optim2  slfcont1  slfcont2  slfcont3  slfcont4  urban
##   1   6  children  cnarc1  cnarc2  cnarc3  ctheory1  ctheory2  ctheory3  ctheory4  CRT1  CRT2  CRT3  employ_status1  health_cond  marital1  mor_circle  moralid1  moralid10  moralid2  moralid3  moralid4  moralid5  moralid6  moralid7  moralid8  moralid9  mcoop1  mcoop2  mcoop3  mcoop4  mcoop5  mcoop6  mcoop7  nidentity1  omind1  omind2  omind3  omind4  omind5  omind6  political_ideology  riskperc1  riskperc2  sbelong1  sbelong2  sbelong3  sbelong4  tested_positive  optim1  optim2  slfcont1  slfcont2  slfcont3  slfcont4  urban
##   1   7  children  cnarc1  cnarc2  cnarc3  ctheory1  ctheory2  ctheory3  ctheory4  CRT1  CRT2  CRT3  employ_status1  health_cond  marital1  mor_circle  moralid1  moralid10  moralid2  moralid3  moralid4  moralid5  moralid6  moralid7  moralid8  moralid9  mcoop1  mcoop2  mcoop3  mcoop4  mcoop5  mcoop6  mcoop7  nidentity1  omind1  omind2  omind3  omind4  omind5  omind6  political_ideology  riskperc1  riskperc2  sbelong1  sbelong2  sbelong3  sbelong4  tested_positive  optim1  optim2  slfcont1  slfcont2  slfcont3  slfcont4  urban
##   1   8  children  cnarc1  cnarc2  cnarc3  ctheory1  ctheory2  ctheory3  ctheory4  CRT1  CRT2  CRT3  employ_status1  health_cond  marital1  mor_circle  moralid1  moralid10  moralid2  moralid3  moralid4  moralid5  moralid6  moralid7  moralid8  moralid9  mcoop1  mcoop2  mcoop3  mcoop4  mcoop5  mcoop6  mcoop7  nidentity1  omind1  omind2  omind3  omind4  omind5  omind6  political_ideology  riskperc1  riskperc2  sbelong1  sbelong2  sbelong3  sbelong4  tested_positive  optim1  optim2  slfcont1  slfcont2  slfcont3  slfcont4  urban
##   1   9  children  cnarc1  cnarc2  cnarc3  ctheory1  ctheory2  ctheory3  ctheory4  CRT1  CRT2  CRT3  employ_status1  health_cond  marital1  mor_circle  moralid1  moralid10  moralid2  moralid3  moralid4  moralid5  moralid6  moralid7  moralid8  moralid9  mcoop1  mcoop2  mcoop3  mcoop4  mcoop5  mcoop6  mcoop7  nidentity1  omind1  omind2  omind3  omind4  omind5  omind6  political_ideology  riskperc1  riskperc2  sbelong1  sbelong2  sbelong3  sbelong4  tested_positive  optim1  optim2  slfcont1  slfcont2  slfcont3  slfcont4  urban
##   1   10  children  cnarc1  cnarc2  cnarc3  ctheory1  ctheory2  ctheory3  ctheory4  CRT1  CRT2  CRT3  employ_status1  health_cond  marital1  mor_circle  moralid1  moralid10  moralid2  moralid3  moralid4  moralid5  moralid6  moralid7  moralid8  moralid9  mcoop1  mcoop2  mcoop3  mcoop4  mcoop5  mcoop6  mcoop7  nidentity1  omind1  omind2  omind3  omind4  omind5  omind6  political_ideology  riskperc1  riskperc2  sbelong1  sbelong2  sbelong3  sbelong4  tested_positive  optim1  optim2  slfcont1  slfcont2  slfcont3  slfcont4  urban
##   2   1  children  cnarc1  cnarc2  cnarc3  ctheory1  ctheory2  ctheory3  ctheory4  CRT1  CRT2  CRT3  employ_status1  health_cond  marital1  mor_circle  moralid1  moralid10  moralid2  moralid3  moralid4  moralid5  moralid6  moralid7  moralid8  moralid9  mcoop1  mcoop2  mcoop3  mcoop4  mcoop5  mcoop6  mcoop7  nidentity1  omind1  omind2  omind3  omind4  omind5  omind6  political_ideology  riskperc1  riskperc2  sbelong1  sbelong2  sbelong3  sbelong4  tested_positive  optim1  optim2  slfcont1  slfcont2  slfcont3  slfcont4  urban
##   2   2  children  cnarc1  cnarc2  cnarc3  ctheory1  ctheory2  ctheory3  ctheory4  CRT1  CRT2  CRT3  employ_status1  health_cond  marital1  mor_circle  moralid1  moralid10  moralid2  moralid3  moralid4  moralid5  moralid6  moralid7  moralid8  moralid9  mcoop1  mcoop2  mcoop3  mcoop4  mcoop5  mcoop6  mcoop7  nidentity1  omind1  omind2  omind3  omind4  omind5  omind6  political_ideology  riskperc1  riskperc2  sbelong1  sbelong2  sbelong3  sbelong4  tested_positive  optim1  optim2  slfcont1  slfcont2  slfcont3  slfcont4  urban
##   2   3  children  cnarc1  cnarc2  cnarc3  ctheory1  ctheory2  ctheory3  ctheory4  CRT1  CRT2  CRT3  employ_status1  health_cond  marital1  mor_circle  moralid1  moralid10  moralid2  moralid3  moralid4  moralid5  moralid6  moralid7  moralid8  moralid9  mcoop1  mcoop2  mcoop3  mcoop4  mcoop5  mcoop6  mcoop7  nidentity1  omind1  omind2  omind3  omind4  omind5  omind6  political_ideology  riskperc1  riskperc2  sbelong1  sbelong2  sbelong3  sbelong4  tested_positive  optim1  optim2  slfcont1  slfcont2  slfcont3  slfcont4  urban
##   2   4  children  cnarc1  cnarc2  cnarc3  ctheory1  ctheory2  ctheory3  ctheory4  CRT1  CRT2  CRT3  employ_status1  health_cond  marital1  mor_circle  moralid1  moralid10  moralid2  moralid3  moralid4  moralid5  moralid6  moralid7  moralid8  moralid9  mcoop1  mcoop2  mcoop3  mcoop4  mcoop5  mcoop6  mcoop7  nidentity1  omind1  omind2  omind3  omind4  omind5  omind6  political_ideology  riskperc1  riskperc2  sbelong1  sbelong2  sbelong3  sbelong4  tested_positive  optim1  optim2  slfcont1  slfcont2  slfcont3  slfcont4  urban
##   2   5  children  cnarc1  cnarc2  cnarc3  ctheory1  ctheory2  ctheory3  ctheory4  CRT1  CRT2  CRT3  employ_status1  health_cond  marital1  mor_circle  moralid1  moralid10  moralid2  moralid3  moralid4  moralid5  moralid6  moralid7  moralid8  moralid9  mcoop1  mcoop2  mcoop3  mcoop4  mcoop5  mcoop6  mcoop7  nidentity1  omind1  omind2  omind3  omind4  omind5  omind6  political_ideology  riskperc1  riskperc2  sbelong1  sbelong2  sbelong3  sbelong4  tested_positive  optim1  optim2  slfcont1  slfcont2  slfcont3  slfcont4  urban
##   2   6  children  cnarc1  cnarc2  cnarc3  ctheory1  ctheory2  ctheory3  ctheory4  CRT1  CRT2  CRT3  employ_status1  health_cond  marital1  mor_circle  moralid1  moralid10  moralid2  moralid3  moralid4  moralid5  moralid6  moralid7  moralid8  moralid9  mcoop1  mcoop2  mcoop3  mcoop4  mcoop5  mcoop6  mcoop7  nidentity1  omind1  omind2  omind3  omind4  omind5  omind6  political_ideology  riskperc1  riskperc2  sbelong1  sbelong2  sbelong3  sbelong4  tested_positive  optim1  optim2  slfcont1  slfcont2  slfcont3  slfcont4  urban
##   2   7  children  cnarc1  cnarc2  cnarc3  ctheory1  ctheory2  ctheory3  ctheory4  CRT1  CRT2  CRT3  employ_status1  health_cond  marital1  mor_circle  moralid1  moralid10  moralid2  moralid3  moralid4  moralid5  moralid6  moralid7  moralid8  moralid9  mcoop1  mcoop2  mcoop3  mcoop4  mcoop5  mcoop6  mcoop7  nidentity1  omind1  omind2  omind3  omind4  omind5  omind6  political_ideology  riskperc1  riskperc2  sbelong1  sbelong2  sbelong3  sbelong4  tested_positive  optim1  optim2  slfcont1  slfcont2  slfcont3  slfcont4  urban
##   2   8  children  cnarc1  cnarc2  cnarc3  ctheory1  ctheory2  ctheory3  ctheory4  CRT1  CRT2  CRT3  employ_status1  health_cond  marital1  mor_circle  moralid1  moralid10  moralid2  moralid3  moralid4  moralid5  moralid6  moralid7  moralid8  moralid9  mcoop1  mcoop2  mcoop3  mcoop4  mcoop5  mcoop6  mcoop7  nidentity1  omind1  omind2  omind3  omind4  omind5  omind6  political_ideology  riskperc1  riskperc2  sbelong1  sbelong2  sbelong3  sbelong4  tested_positive  optim1  optim2  slfcont1  slfcont2  slfcont3  slfcont4  urban
##   2   9  children  cnarc1  cnarc2  cnarc3  ctheory1  ctheory2  ctheory3  ctheory4  CRT1  CRT2  CRT3  employ_status1  health_cond  marital1  mor_circle  moralid1  moralid10  moralid2  moralid3  moralid4  moralid5  moralid6  moralid7  moralid8  moralid9  mcoop1  mcoop2  mcoop3  mcoop4  mcoop5  mcoop6  mcoop7  nidentity1  omind1  omind2  omind3  omind4  omind5  omind6  political_ideology  riskperc1  riskperc2  sbelong1  sbelong2  sbelong3  sbelong4  tested_positive  optim1  optim2  slfcont1  slfcont2  slfcont3  slfcont4  urban
##   2   10  children  cnarc1  cnarc2  cnarc3  ctheory1  ctheory2  ctheory3  ctheory4  CRT1  CRT2  CRT3  employ_status1  health_cond  marital1  mor_circle  moralid1  moralid10  moralid2  moralid3  moralid4  moralid5  moralid6  moralid7  moralid8  moralid9  mcoop1  mcoop2  mcoop3  mcoop4  mcoop5  mcoop6  mcoop7  nidentity1  omind1  omind2  omind3  omind4  omind5  omind6  political_ideology  riskperc1  riskperc2  sbelong1  sbelong2  sbelong3  sbelong4  tested_positive  optim1  optim2  slfcont1  slfcont2  slfcont3  slfcont4  urban
##   3   1  children  cnarc1  cnarc2  cnarc3  ctheory1  ctheory2  ctheory3  ctheory4  CRT1  CRT2  CRT3  employ_status1  health_cond  marital1  mor_circle  moralid1  moralid10  moralid2  moralid3  moralid4  moralid5  moralid6  moralid7  moralid8  moralid9  mcoop1  mcoop2  mcoop3  mcoop4  mcoop5  mcoop6  mcoop7  nidentity1  omind1  omind2  omind3  omind4  omind5  omind6  political_ideology  riskperc1  riskperc2  sbelong1  sbelong2  sbelong3  sbelong4  tested_positive  optim1  optim2  slfcont1  slfcont2  slfcont3  slfcont4  urban
##   3   2  children  cnarc1  cnarc2  cnarc3  ctheory1  ctheory2  ctheory3  ctheory4  CRT1  CRT2  CRT3  employ_status1  health_cond  marital1  mor_circle  moralid1  moralid10  moralid2  moralid3  moralid4  moralid5  moralid6  moralid7  moralid8  moralid9  mcoop1  mcoop2  mcoop3  mcoop4  mcoop5  mcoop6  mcoop7  nidentity1  omind1  omind2  omind3  omind4  omind5  omind6  political_ideology  riskperc1  riskperc2  sbelong1  sbelong2  sbelong3  sbelong4  tested_positive  optim1  optim2  slfcont1  slfcont2  slfcont3  slfcont4  urban
##   3   3  children  cnarc1  cnarc2  cnarc3  ctheory1  ctheory2  ctheory3  ctheory4  CRT1  CRT2  CRT3  employ_status1  health_cond  marital1  mor_circle  moralid1  moralid10  moralid2  moralid3  moralid4  moralid5  moralid6  moralid7  moralid8  moralid9  mcoop1  mcoop2  mcoop3  mcoop4  mcoop5  mcoop6  mcoop7  nidentity1  omind1  omind2  omind3  omind4  omind5  omind6  political_ideology  riskperc1  riskperc2  sbelong1  sbelong2  sbelong3  sbelong4  tested_positive  optim1  optim2  slfcont1  slfcont2  slfcont3  slfcont4  urban
##   3   4  children  cnarc1  cnarc2  cnarc3  ctheory1  ctheory2  ctheory3  ctheory4  CRT1  CRT2  CRT3  employ_status1  health_cond  marital1  mor_circle  moralid1  moralid10  moralid2  moralid3  moralid4  moralid5  moralid6  moralid7  moralid8  moralid9  mcoop1  mcoop2  mcoop3  mcoop4  mcoop5  mcoop6  mcoop7  nidentity1  omind1  omind2  omind3  omind4  omind5  omind6  political_ideology  riskperc1  riskperc2  sbelong1  sbelong2  sbelong3  sbelong4  tested_positive  optim1  optim2  slfcont1  slfcont2  slfcont3  slfcont4  urban
##   3   5  children  cnarc1  cnarc2  cnarc3  ctheory1  ctheory2  ctheory3  ctheory4  CRT1  CRT2  CRT3  employ_status1  health_cond  marital1  mor_circle  moralid1  moralid10  moralid2  moralid3  moralid4  moralid5  moralid6  moralid7  moralid8  moralid9  mcoop1  mcoop2  mcoop3  mcoop4  mcoop5  mcoop6  mcoop7  nidentity1  omind1  omind2  omind3  omind4  omind5  omind6  political_ideology  riskperc1  riskperc2  sbelong1  sbelong2  sbelong3  sbelong4  tested_positive  optim1  optim2  slfcont1  slfcont2  slfcont3  slfcont4  urban
##   3   6  children  cnarc1  cnarc2  cnarc3  ctheory1  ctheory2  ctheory3  ctheory4  CRT1  CRT2  CRT3  employ_status1  health_cond  marital1  mor_circle  moralid1  moralid10  moralid2  moralid3  moralid4  moralid5  moralid6  moralid7  moralid8  moralid9  mcoop1  mcoop2  mcoop3  mcoop4  mcoop5  mcoop6  mcoop7  nidentity1  omind1  omind2  omind3  omind4  omind5  omind6  political_ideology  riskperc1  riskperc2  sbelong1  sbelong2  sbelong3  sbelong4  tested_positive  optim1  optim2  slfcont1  slfcont2  slfcont3  slfcont4  urban
##   3   7  children  cnarc1  cnarc2  cnarc3  ctheory1  ctheory2  ctheory3  ctheory4  CRT1  CRT2  CRT3  employ_status1  health_cond  marital1  mor_circle  moralid1  moralid10  moralid2  moralid3  moralid4  moralid5  moralid6  moralid7  moralid8  moralid9  mcoop1  mcoop2  mcoop3  mcoop4  mcoop5  mcoop6  mcoop7  nidentity1  omind1  omind2  omind3  omind4  omind5  omind6  political_ideology  riskperc1  riskperc2  sbelong1  sbelong2  sbelong3  sbelong4  tested_positive  optim1  optim2  slfcont1  slfcont2  slfcont3  slfcont4  urban
##   3   8  children  cnarc1  cnarc2  cnarc3  ctheory1  ctheory2  ctheory3  ctheory4  CRT1  CRT2  CRT3  employ_status1  health_cond  marital1  mor_circle  moralid1  moralid10  moralid2  moralid3  moralid4  moralid5  moralid6  moralid7  moralid8  moralid9  mcoop1  mcoop2  mcoop3  mcoop4  mcoop5  mcoop6  mcoop7  nidentity1  omind1  omind2  omind3  omind4  omind5  omind6  political_ideology  riskperc1  riskperc2  sbelong1  sbelong2  sbelong3  sbelong4  tested_positive  optim1  optim2  slfcont1  slfcont2  slfcont3  slfcont4  urban
##   3   9  children  cnarc1  cnarc2  cnarc3  ctheory1  ctheory2  ctheory3  ctheory4  CRT1  CRT2  CRT3  employ_status1  health_cond  marital1  mor_circle  moralid1  moralid10  moralid2  moralid3  moralid4  moralid5  moralid6  moralid7  moralid8  moralid9  mcoop1  mcoop2  mcoop3  mcoop4  mcoop5  mcoop6  mcoop7  nidentity1  omind1  omind2  omind3  omind4  omind5  omind6  political_ideology  riskperc1  riskperc2  sbelong1  sbelong2  sbelong3  sbelong4  tested_positive  optim1  optim2  slfcont1  slfcont2  slfcont3  slfcont4  urban
##   3   10  children  cnarc1  cnarc2  cnarc3  ctheory1  ctheory2  ctheory3  ctheory4  CRT1  CRT2  CRT3  employ_status1  health_cond  marital1  mor_circle  moralid1  moralid10  moralid2  moralid3  moralid4  moralid5  moralid6  moralid7  moralid8  moralid9  mcoop1  mcoop2  mcoop3  mcoop4  mcoop5  mcoop6  mcoop7  nidentity1  omind1  omind2  omind3  omind4  omind5  omind6  political_ideology  riskperc1  riskperc2  sbelong1  sbelong2  sbelong3  sbelong4  tested_positive  optim1  optim2  slfcont1  slfcont2  slfcont3  slfcont4  urban
##   4   1  children  cnarc1  cnarc2  cnarc3  ctheory1  ctheory2  ctheory3  ctheory4  CRT1  CRT2  CRT3  employ_status1  health_cond  marital1  mor_circle  moralid1  moralid10  moralid2  moralid3  moralid4  moralid5  moralid6  moralid7  moralid8  moralid9  mcoop1  mcoop2  mcoop3  mcoop4  mcoop5  mcoop6  mcoop7  nidentity1  omind1  omind2  omind3  omind4  omind5  omind6  political_ideology  riskperc1  riskperc2  sbelong1  sbelong2  sbelong3  sbelong4  tested_positive  optim1  optim2  slfcont1  slfcont2  slfcont3  slfcont4  urban
##   4   2  children  cnarc1  cnarc2  cnarc3  ctheory1  ctheory2  ctheory3  ctheory4  CRT1  CRT2  CRT3  employ_status1  health_cond  marital1  mor_circle  moralid1  moralid10  moralid2  moralid3  moralid4  moralid5  moralid6  moralid7  moralid8  moralid9  mcoop1  mcoop2  mcoop3  mcoop4  mcoop5  mcoop6  mcoop7  nidentity1  omind1  omind2  omind3  omind4  omind5  omind6  political_ideology  riskperc1  riskperc2  sbelong1  sbelong2  sbelong3  sbelong4  tested_positive  optim1  optim2  slfcont1  slfcont2  slfcont3  slfcont4  urban
##   4   3  children  cnarc1  cnarc2  cnarc3  ctheory1  ctheory2  ctheory3  ctheory4  CRT1  CRT2  CRT3  employ_status1  health_cond  marital1  mor_circle  moralid1  moralid10  moralid2  moralid3  moralid4  moralid5  moralid6  moralid7  moralid8  moralid9  mcoop1  mcoop2  mcoop3  mcoop4  mcoop5  mcoop6  mcoop7  nidentity1  omind1  omind2  omind3  omind4  omind5  omind6  political_ideology  riskperc1  riskperc2  sbelong1  sbelong2  sbelong3  sbelong4  tested_positive  optim1  optim2  slfcont1  slfcont2  slfcont3  slfcont4  urban
##   4   4  children  cnarc1  cnarc2  cnarc3  ctheory1  ctheory2  ctheory3  ctheory4  CRT1  CRT2  CRT3  employ_status1  health_cond  marital1  mor_circle  moralid1  moralid10  moralid2  moralid3  moralid4  moralid5  moralid6  moralid7  moralid8  moralid9  mcoop1  mcoop2  mcoop3  mcoop4  mcoop5  mcoop6  mcoop7  nidentity1  omind1  omind2  omind3  omind4  omind5  omind6  political_ideology  riskperc1  riskperc2  sbelong1  sbelong2  sbelong3  sbelong4  tested_positive  optim1  optim2  slfcont1  slfcont2  slfcont3  slfcont4  urban
##   4   5  children  cnarc1  cnarc2  cnarc3  ctheory1  ctheory2  ctheory3  ctheory4  CRT1  CRT2  CRT3  employ_status1  health_cond  marital1  mor_circle  moralid1  moralid10  moralid2  moralid3  moralid4  moralid5  moralid6  moralid7  moralid8  moralid9  mcoop1  mcoop2  mcoop3  mcoop4  mcoop5  mcoop6  mcoop7  nidentity1  omind1  omind2  omind3  omind4  omind5  omind6  political_ideology  riskperc1  riskperc2  sbelong1  sbelong2  sbelong3  sbelong4  tested_positive  optim1  optim2  slfcont1  slfcont2  slfcont3  slfcont4  urban
##   4   6  children  cnarc1  cnarc2  cnarc3  ctheory1  ctheory2  ctheory3  ctheory4  CRT1  CRT2  CRT3  employ_status1  health_cond  marital1  mor_circle  moralid1  moralid10  moralid2  moralid3  moralid4  moralid5  moralid6  moralid7  moralid8  moralid9  mcoop1  mcoop2  mcoop3  mcoop4  mcoop5  mcoop6  mcoop7  nidentity1  omind1  omind2  omind3  omind4  omind5  omind6  political_ideology  riskperc1  riskperc2  sbelong1  sbelong2  sbelong3  sbelong4  tested_positive  optim1  optim2  slfcont1  slfcont2  slfcont3  slfcont4  urban
##   4   7  children  cnarc1  cnarc2  cnarc3  ctheory1  ctheory2  ctheory3  ctheory4  CRT1  CRT2  CRT3  employ_status1  health_cond  marital1  mor_circle  moralid1  moralid10  moralid2  moralid3  moralid4  moralid5  moralid6  moralid7  moralid8  moralid9  mcoop1  mcoop2  mcoop3  mcoop4  mcoop5  mcoop6  mcoop7  nidentity1  omind1  omind2  omind3  omind4  omind5  omind6  political_ideology  riskperc1  riskperc2  sbelong1  sbelong2  sbelong3  sbelong4  tested_positive  optim1  optim2  slfcont1  slfcont2  slfcont3  slfcont4  urban
##   4   8  children  cnarc1  cnarc2  cnarc3  ctheory1  ctheory2  ctheory3  ctheory4  CRT1  CRT2  CRT3  employ_status1  health_cond  marital1  mor_circle  moralid1  moralid10  moralid2  moralid3  moralid4  moralid5  moralid6  moralid7  moralid8  moralid9  mcoop1  mcoop2  mcoop3  mcoop4  mcoop5  mcoop6  mcoop7  nidentity1  omind1  omind2  omind3  omind4  omind5  omind6  political_ideology  riskperc1  riskperc2  sbelong1  sbelong2  sbelong3  sbelong4  tested_positive  optim1  optim2  slfcont1  slfcont2  slfcont3  slfcont4  urban
##   4   9  children  cnarc1  cnarc2  cnarc3  ctheory1  ctheory2  ctheory3  ctheory4  CRT1  CRT2  CRT3  employ_status1  health_cond  marital1  mor_circle  moralid1  moralid10  moralid2  moralid3  moralid4  moralid5  moralid6  moralid7  moralid8  moralid9  mcoop1  mcoop2  mcoop3  mcoop4  mcoop5  mcoop6  mcoop7  nidentity1  omind1  omind2  omind3  omind4  omind5  omind6  political_ideology  riskperc1  riskperc2  sbelong1  sbelong2  sbelong3  sbelong4  tested_positive  optim1  optim2  slfcont1  slfcont2  slfcont3  slfcont4  urban
##   4   10  children  cnarc1  cnarc2  cnarc3  ctheory1  ctheory2  ctheory3  ctheory4  CRT1  CRT2  CRT3  employ_status1  health_cond  marital1  mor_circle  moralid1  moralid10  moralid2  moralid3  moralid4  moralid5  moralid6  moralid7  moralid8  moralid9  mcoop1  mcoop2  mcoop3  mcoop4  mcoop5  mcoop6  mcoop7  nidentity1  omind1  omind2  omind3  omind4  omind5  omind6  political_ideology  riskperc1  riskperc2  sbelong1  sbelong2  sbelong3  sbelong4  tested_positive  optim1  optim2  slfcont1  slfcont2  slfcont3  slfcont4  urban
##   5   1  children  cnarc1  cnarc2  cnarc3  ctheory1  ctheory2  ctheory3  ctheory4  CRT1  CRT2  CRT3  employ_status1  health_cond  marital1  mor_circle  moralid1  moralid10  moralid2  moralid3  moralid4  moralid5  moralid6  moralid7  moralid8  moralid9  mcoop1  mcoop2  mcoop3  mcoop4  mcoop5  mcoop6  mcoop7  nidentity1  omind1  omind2  omind3  omind4  omind5  omind6  political_ideology  riskperc1  riskperc2  sbelong1  sbelong2  sbelong3  sbelong4  tested_positive  optim1  optim2  slfcont1  slfcont2  slfcont3  slfcont4  urban
##   5   2  children  cnarc1  cnarc2  cnarc3  ctheory1  ctheory2  ctheory3  ctheory4  CRT1  CRT2  CRT3  employ_status1  health_cond  marital1  mor_circle  moralid1  moralid10  moralid2  moralid3  moralid4  moralid5  moralid6  moralid7  moralid8  moralid9  mcoop1  mcoop2  mcoop3  mcoop4  mcoop5  mcoop6  mcoop7  nidentity1  omind1  omind2  omind3  omind4  omind5  omind6  political_ideology  riskperc1  riskperc2  sbelong1  sbelong2  sbelong3  sbelong4  tested_positive  optim1  optim2  slfcont1  slfcont2  slfcont3  slfcont4  urban
##   5   3  children  cnarc1  cnarc2  cnarc3  ctheory1  ctheory2  ctheory3  ctheory4  CRT1  CRT2  CRT3  employ_status1  health_cond  marital1  mor_circle  moralid1  moralid10  moralid2  moralid3  moralid4  moralid5  moralid6  moralid7  moralid8  moralid9  mcoop1  mcoop2  mcoop3  mcoop4  mcoop5  mcoop6  mcoop7  nidentity1  omind1  omind2  omind3  omind4  omind5  omind6  political_ideology  riskperc1  riskperc2  sbelong1  sbelong2  sbelong3  sbelong4  tested_positive  optim1  optim2  slfcont1  slfcont2  slfcont3  slfcont4  urban
##   5   4  children  cnarc1  cnarc2  cnarc3  ctheory1  ctheory2  ctheory3  ctheory4  CRT1  CRT2  CRT3  employ_status1  health_cond  marital1  mor_circle  moralid1  moralid10  moralid2  moralid3  moralid4  moralid5  moralid6  moralid7  moralid8  moralid9  mcoop1  mcoop2  mcoop3  mcoop4  mcoop5  mcoop6  mcoop7  nidentity1  omind1  omind2  omind3  omind4  omind5  omind6  political_ideology  riskperc1  riskperc2  sbelong1  sbelong2  sbelong3  sbelong4  tested_positive  optim1  optim2  slfcont1  slfcont2  slfcont3  slfcont4  urban
##   5   5  children  cnarc1  cnarc2  cnarc3  ctheory1  ctheory2  ctheory3  ctheory4  CRT1  CRT2  CRT3  employ_status1  health_cond  marital1  mor_circle  moralid1  moralid10  moralid2  moralid3  moralid4  moralid5  moralid6  moralid7  moralid8  moralid9  mcoop1  mcoop2  mcoop3  mcoop4  mcoop5  mcoop6  mcoop7  nidentity1  omind1  omind2  omind3  omind4  omind5  omind6  political_ideology  riskperc1  riskperc2  sbelong1  sbelong2  sbelong3  sbelong4  tested_positive  optim1  optim2  slfcont1  slfcont2  slfcont3  slfcont4  urban
##   5   6  children  cnarc1  cnarc2  cnarc3  ctheory1  ctheory2  ctheory3  ctheory4  CRT1  CRT2  CRT3  employ_status1  health_cond  marital1  mor_circle  moralid1  moralid10  moralid2  moralid3  moralid4  moralid5  moralid6  moralid7  moralid8  moralid9  mcoop1  mcoop2  mcoop3  mcoop4  mcoop5  mcoop6  mcoop7  nidentity1  omind1  omind2  omind3  omind4  omind5  omind6  political_ideology  riskperc1  riskperc2  sbelong1  sbelong2  sbelong3  sbelong4  tested_positive  optim1  optim2  slfcont1  slfcont2  slfcont3  slfcont4  urban
##   5   7  children  cnarc1  cnarc2  cnarc3  ctheory1  ctheory2  ctheory3  ctheory4  CRT1  CRT2  CRT3  employ_status1  health_cond  marital1  mor_circle  moralid1  moralid10  moralid2  moralid3  moralid4  moralid5  moralid6  moralid7  moralid8  moralid9  mcoop1  mcoop2  mcoop3  mcoop4  mcoop5  mcoop6  mcoop7  nidentity1  omind1  omind2  omind3  omind4  omind5  omind6  political_ideology  riskperc1  riskperc2  sbelong1  sbelong2  sbelong3  sbelong4  tested_positive  optim1  optim2  slfcont1  slfcont2  slfcont3  slfcont4  urban
##   5   8  children  cnarc1  cnarc2  cnarc3  ctheory1  ctheory2  ctheory3  ctheory4  CRT1  CRT2  CRT3  employ_status1  health_cond  marital1  mor_circle  moralid1  moralid10  moralid2  moralid3  moralid4  moralid5  moralid6  moralid7  moralid8  moralid9  mcoop1  mcoop2  mcoop3  mcoop4  mcoop5  mcoop6  mcoop7  nidentity1  omind1  omind2  omind3  omind4  omind5  omind6  political_ideology  riskperc1  riskperc2  sbelong1  sbelong2  sbelong3  sbelong4  tested_positive  optim1  optim2  slfcont1  slfcont2  slfcont3  slfcont4  urban
##   5   9  children  cnarc1  cnarc2  cnarc3  ctheory1  ctheory2  ctheory3  ctheory4  CRT1  CRT2  CRT3  employ_status1  health_cond  marital1  mor_circle  moralid1  moralid10  moralid2  moralid3  moralid4  moralid5  moralid6  moralid7  moralid8  moralid9  mcoop1  mcoop2  mcoop3  mcoop4  mcoop5  mcoop6  mcoop7  nidentity1  omind1  omind2  omind3  omind4  omind5  omind6  political_ideology  riskperc1  riskperc2  sbelong1  sbelong2  sbelong3  sbelong4  tested_positive  optim1  optim2  slfcont1  slfcont2  slfcont3  slfcont4  urban
##   5   10  children  cnarc1  cnarc2  cnarc3  ctheory1  ctheory2  ctheory3  ctheory4  CRT1  CRT2  CRT3  employ_status1  health_cond  marital1  mor_circle  moralid1  moralid10  moralid2  moralid3  moralid4  moralid5  moralid6  moralid7  moralid8  moralid9  mcoop1  mcoop2  mcoop3  mcoop4  mcoop5  mcoop6  mcoop7  nidentity1  omind1  omind2  omind3  omind4  omind5  omind6  political_ideology  riskperc1  riskperc2  sbelong1  sbelong2  sbelong3  sbelong4  tested_positive  optim1  optim2  slfcont1  slfcont2  slfcont3  slfcont4  urban
##   6   1  children  cnarc1  cnarc2  cnarc3  ctheory1  ctheory2  ctheory3  ctheory4  CRT1  CRT2  CRT3  employ_status1  health_cond  marital1  mor_circle  moralid1  moralid10  moralid2  moralid3  moralid4  moralid5  moralid6  moralid7  moralid8  moralid9  mcoop1  mcoop2  mcoop3  mcoop4  mcoop5  mcoop6  mcoop7  nidentity1  omind1  omind2  omind3  omind4  omind5  omind6  political_ideology  riskperc1  riskperc2  sbelong1  sbelong2  sbelong3  sbelong4  tested_positive  optim1  optim2  slfcont1  slfcont2  slfcont3  slfcont4  urban
##   6   2  children  cnarc1  cnarc2  cnarc3  ctheory1  ctheory2  ctheory3  ctheory4  CRT1  CRT2  CRT3  employ_status1  health_cond  marital1  mor_circle  moralid1  moralid10  moralid2  moralid3  moralid4  moralid5  moralid6  moralid7  moralid8  moralid9  mcoop1  mcoop2  mcoop3  mcoop4  mcoop5  mcoop6  mcoop7  nidentity1  omind1  omind2  omind3  omind4  omind5  omind6  political_ideology  riskperc1  riskperc2  sbelong1  sbelong2  sbelong3  sbelong4  tested_positive  optim1  optim2  slfcont1  slfcont2  slfcont3  slfcont4  urban
##   6   3  children  cnarc1  cnarc2  cnarc3  ctheory1  ctheory2  ctheory3  ctheory4  CRT1  CRT2  CRT3  employ_status1  health_cond  marital1  mor_circle  moralid1  moralid10  moralid2  moralid3  moralid4  moralid5  moralid6  moralid7  moralid8  moralid9  mcoop1  mcoop2  mcoop3  mcoop4  mcoop5  mcoop6  mcoop7  nidentity1  omind1  omind2  omind3  omind4  omind5  omind6  political_ideology  riskperc1  riskperc2  sbelong1  sbelong2  sbelong3  sbelong4  tested_positive  optim1  optim2  slfcont1  slfcont2  slfcont3  slfcont4  urban
##   6   4  children  cnarc1  cnarc2  cnarc3  ctheory1  ctheory2  ctheory3  ctheory4  CRT1  CRT2  CRT3  employ_status1  health_cond  marital1  mor_circle  moralid1  moralid10  moralid2  moralid3  moralid4  moralid5  moralid6  moralid7  moralid8  moralid9  mcoop1  mcoop2  mcoop3  mcoop4  mcoop5  mcoop6  mcoop7  nidentity1  omind1  omind2  omind3  omind4  omind5  omind6  political_ideology  riskperc1  riskperc2  sbelong1  sbelong2  sbelong3  sbelong4  tested_positive  optim1  optim2  slfcont1  slfcont2  slfcont3  slfcont4  urban
##   6   5  children  cnarc1  cnarc2  cnarc3  ctheory1  ctheory2  ctheory3  ctheory4  CRT1  CRT2  CRT3  employ_status1  health_cond  marital1  mor_circle  moralid1  moralid10  moralid2  moralid3  moralid4  moralid5  moralid6  moralid7  moralid8  moralid9  mcoop1  mcoop2  mcoop3  mcoop4  mcoop5  mcoop6  mcoop7  nidentity1  omind1  omind2  omind3  omind4  omind5  omind6  political_ideology  riskperc1  riskperc2  sbelong1  sbelong2  sbelong3  sbelong4  tested_positive  optim1  optim2  slfcont1  slfcont2  slfcont3  slfcont4  urban
##   6   6  children  cnarc1  cnarc2  cnarc3  ctheory1  ctheory2  ctheory3  ctheory4  CRT1  CRT2  CRT3  employ_status1  health_cond  marital1  mor_circle  moralid1  moralid10  moralid2  moralid3  moralid4  moralid5  moralid6  moralid7  moralid8  moralid9  mcoop1  mcoop2  mcoop3  mcoop4  mcoop5  mcoop6  mcoop7  nidentity1  omind1  omind2  omind3  omind4  omind5  omind6  political_ideology  riskperc1  riskperc2  sbelong1  sbelong2  sbelong3  sbelong4  tested_positive  optim1  optim2  slfcont1  slfcont2  slfcont3  slfcont4  urban
##   6   7  children  cnarc1  cnarc2  cnarc3  ctheory1  ctheory2  ctheory3  ctheory4  CRT1  CRT2  CRT3  employ_status1  health_cond  marital1  mor_circle  moralid1  moralid10  moralid2  moralid3  moralid4  moralid5  moralid6  moralid7  moralid8  moralid9  mcoop1  mcoop2  mcoop3  mcoop4  mcoop5  mcoop6  mcoop7  nidentity1  omind1  omind2  omind3  omind4  omind5  omind6  political_ideology  riskperc1  riskperc2  sbelong1  sbelong2  sbelong3  sbelong4  tested_positive  optim1  optim2  slfcont1  slfcont2  slfcont3  slfcont4  urban
##   6   8  children  cnarc1  cnarc2  cnarc3  ctheory1  ctheory2  ctheory3  ctheory4  CRT1  CRT2  CRT3  employ_status1  health_cond  marital1  mor_circle  moralid1  moralid10  moralid2  moralid3  moralid4  moralid5  moralid6  moralid7  moralid8  moralid9  mcoop1  mcoop2  mcoop3  mcoop4  mcoop5  mcoop6  mcoop7  nidentity1  omind1  omind2  omind3  omind4  omind5  omind6  political_ideology  riskperc1  riskperc2  sbelong1  sbelong2  sbelong3  sbelong4  tested_positive  optim1  optim2  slfcont1  slfcont2  slfcont3  slfcont4  urban
##   6   9  children  cnarc1  cnarc2  cnarc3  ctheory1  ctheory2  ctheory3  ctheory4  CRT1  CRT2  CRT3  employ_status1  health_cond  marital1  mor_circle  moralid1  moralid10  moralid2  moralid3  moralid4  moralid5  moralid6  moralid7  moralid8  moralid9  mcoop1  mcoop2  mcoop3  mcoop4  mcoop5  mcoop6  mcoop7  nidentity1  omind1  omind2  omind3  omind4  omind5  omind6  political_ideology  riskperc1  riskperc2  sbelong1  sbelong2  sbelong3  sbelong4  tested_positive  optim1  optim2  slfcont1  slfcont2  slfcont3  slfcont4  urban
##   6   10  children  cnarc1  cnarc2  cnarc3  ctheory1  ctheory2  ctheory3  ctheory4  CRT1  CRT2  CRT3  employ_status1  health_cond  marital1  mor_circle  moralid1  moralid10  moralid2  moralid3  moralid4  moralid5  moralid6  moralid7  moralid8  moralid9  mcoop1  mcoop2  mcoop3  mcoop4  mcoop5  mcoop6  mcoop7  nidentity1  omind1  omind2  omind3  omind4  omind5  omind6  political_ideology  riskperc1  riskperc2  sbelong1  sbelong2  sbelong3  sbelong4  tested_positive  optim1  optim2  slfcont1  slfcont2  slfcont3  slfcont4  urban
##   7   1  children  cnarc1  cnarc2  cnarc3  ctheory1  ctheory2  ctheory3  ctheory4  CRT1  CRT2  CRT3  employ_status1  health_cond  marital1  mor_circle  moralid1  moralid10  moralid2  moralid3  moralid4  moralid5  moralid6  moralid7  moralid8  moralid9  mcoop1  mcoop2  mcoop3  mcoop4  mcoop5  mcoop6  mcoop7  nidentity1  omind1  omind2  omind3  omind4  omind5  omind6  political_ideology  riskperc1  riskperc2  sbelong1  sbelong2  sbelong3  sbelong4  tested_positive  optim1  optim2  slfcont1  slfcont2  slfcont3  slfcont4  urban
##   7   2  children  cnarc1  cnarc2  cnarc3  ctheory1  ctheory2  ctheory3  ctheory4  CRT1  CRT2  CRT3  employ_status1  health_cond  marital1  mor_circle  moralid1  moralid10  moralid2  moralid3  moralid4  moralid5  moralid6  moralid7  moralid8  moralid9  mcoop1  mcoop2  mcoop3  mcoop4  mcoop5  mcoop6  mcoop7  nidentity1  omind1  omind2  omind3  omind4  omind5  omind6  political_ideology  riskperc1  riskperc2  sbelong1  sbelong2  sbelong3  sbelong4  tested_positive  optim1  optim2  slfcont1  slfcont2  slfcont3  slfcont4  urban
##   7   3  children  cnarc1  cnarc2  cnarc3  ctheory1  ctheory2  ctheory3  ctheory4  CRT1  CRT2  CRT3  employ_status1  health_cond  marital1  mor_circle  moralid1  moralid10  moralid2  moralid3  moralid4  moralid5  moralid6  moralid7  moralid8  moralid9  mcoop1  mcoop2  mcoop3  mcoop4  mcoop5  mcoop6  mcoop7  nidentity1  omind1  omind2  omind3  omind4  omind5  omind6  political_ideology  riskperc1  riskperc2  sbelong1  sbelong2  sbelong3  sbelong4  tested_positive  optim1  optim2  slfcont1  slfcont2  slfcont3  slfcont4  urban
##   7   4  children  cnarc1  cnarc2  cnarc3  ctheory1  ctheory2  ctheory3  ctheory4  CRT1  CRT2  CRT3  employ_status1  health_cond  marital1  mor_circle  moralid1  moralid10  moralid2  moralid3  moralid4  moralid5  moralid6  moralid7  moralid8  moralid9  mcoop1  mcoop2  mcoop3  mcoop4  mcoop5  mcoop6  mcoop7  nidentity1  omind1  omind2  omind3  omind4  omind5  omind6  political_ideology  riskperc1  riskperc2  sbelong1  sbelong2  sbelong3  sbelong4  tested_positive  optim1  optim2  slfcont1  slfcont2  slfcont3  slfcont4  urban
##   7   5  children  cnarc1  cnarc2  cnarc3  ctheory1  ctheory2  ctheory3  ctheory4  CRT1  CRT2  CRT3  employ_status1  health_cond  marital1  mor_circle  moralid1  moralid10  moralid2  moralid3  moralid4  moralid5  moralid6  moralid7  moralid8  moralid9  mcoop1  mcoop2  mcoop3  mcoop4  mcoop5  mcoop6  mcoop7  nidentity1  omind1  omind2  omind3  omind4  omind5  omind6  political_ideology  riskperc1  riskperc2  sbelong1  sbelong2  sbelong3  sbelong4  tested_positive  optim1  optim2  slfcont1  slfcont2  slfcont3  slfcont4  urban
##   7   6  children  cnarc1  cnarc2  cnarc3  ctheory1  ctheory2  ctheory3  ctheory4  CRT1  CRT2  CRT3  employ_status1  health_cond  marital1  mor_circle  moralid1  moralid10  moralid2  moralid3  moralid4  moralid5  moralid6  moralid7  moralid8  moralid9  mcoop1  mcoop2  mcoop3  mcoop4  mcoop5  mcoop6  mcoop7  nidentity1  omind1  omind2  omind3  omind4  omind5  omind6  political_ideology  riskperc1  riskperc2  sbelong1  sbelong2  sbelong3  sbelong4  tested_positive  optim1  optim2  slfcont1  slfcont2  slfcont3  slfcont4  urban
##   7   7  children  cnarc1  cnarc2  cnarc3  ctheory1  ctheory2  ctheory3  ctheory4  CRT1  CRT2  CRT3  employ_status1  health_cond  marital1  mor_circle  moralid1  moralid10  moralid2  moralid3  moralid4  moralid5  moralid6  moralid7  moralid8  moralid9  mcoop1  mcoop2  mcoop3  mcoop4  mcoop5  mcoop6  mcoop7  nidentity1  omind1  omind2  omind3  omind4  omind5  omind6  political_ideology  riskperc1  riskperc2  sbelong1  sbelong2  sbelong3  sbelong4  tested_positive  optim1  optim2  slfcont1  slfcont2  slfcont3  slfcont4  urban
##   7   8  children  cnarc1  cnarc2  cnarc3  ctheory1  ctheory2  ctheory3  ctheory4  CRT1  CRT2  CRT3  employ_status1  health_cond  marital1  mor_circle  moralid1  moralid10  moralid2  moralid3  moralid4  moralid5  moralid6  moralid7  moralid8  moralid9  mcoop1  mcoop2  mcoop3  mcoop4  mcoop5  mcoop6  mcoop7  nidentity1  omind1  omind2  omind3  omind4  omind5  omind6  political_ideology  riskperc1  riskperc2  sbelong1  sbelong2  sbelong3  sbelong4  tested_positive  optim1  optim2  slfcont1  slfcont2  slfcont3  slfcont4  urban
##   7   9  children  cnarc1  cnarc2  cnarc3  ctheory1  ctheory2  ctheory3  ctheory4  CRT1  CRT2  CRT3  employ_status1  health_cond  marital1  mor_circle  moralid1  moralid10  moralid2  moralid3  moralid4  moralid5  moralid6  moralid7  moralid8  moralid9  mcoop1  mcoop2  mcoop3  mcoop4  mcoop5  mcoop6  mcoop7  nidentity1  omind1  omind2  omind3  omind4  omind5  omind6  political_ideology  riskperc1  riskperc2  sbelong1  sbelong2  sbelong3  sbelong4  tested_positive  optim1  optim2  slfcont1  slfcont2  slfcont3  slfcont4  urban
##   7   10  children  cnarc1  cnarc2  cnarc3  ctheory1  ctheory2  ctheory3  ctheory4  CRT1  CRT2  CRT3  employ_status1  health_cond  marital1  mor_circle  moralid1  moralid10  moralid2  moralid3  moralid4  moralid5  moralid6  moralid7  moralid8  moralid9  mcoop1  mcoop2  mcoop3  mcoop4  mcoop5  mcoop6  mcoop7  nidentity1  omind1  omind2  omind3  omind4  omind5  omind6  political_ideology  riskperc1  riskperc2  sbelong1  sbelong2  sbelong3  sbelong4  tested_positive  optim1  optim2  slfcont1  slfcont2  slfcont3  slfcont4  urban
##   8   1  children  cnarc1  cnarc2  cnarc3  ctheory1  ctheory2  ctheory3  ctheory4  CRT1  CRT2  CRT3  employ_status1  health_cond  marital1  mor_circle  moralid1  moralid10  moralid2  moralid3  moralid4  moralid5  moralid6  moralid7  moralid8  moralid9  mcoop1  mcoop2  mcoop3  mcoop4  mcoop5  mcoop6  mcoop7  nidentity1  omind1  omind2  omind3  omind4  omind5  omind6  political_ideology  riskperc1  riskperc2  sbelong1  sbelong2  sbelong3  sbelong4  tested_positive  optim1  optim2  slfcont1  slfcont2  slfcont3  slfcont4  urban
##   8   2  children  cnarc1  cnarc2  cnarc3  ctheory1  ctheory2  ctheory3  ctheory4  CRT1  CRT2  CRT3  employ_status1  health_cond  marital1  mor_circle  moralid1  moralid10  moralid2  moralid3  moralid4  moralid5  moralid6  moralid7  moralid8  moralid9  mcoop1  mcoop2  mcoop3  mcoop4  mcoop5  mcoop6  mcoop7  nidentity1  omind1  omind2  omind3  omind4  omind5  omind6  political_ideology  riskperc1  riskperc2  sbelong1  sbelong2  sbelong3  sbelong4  tested_positive  optim1  optim2  slfcont1  slfcont2  slfcont3  slfcont4  urban
##   8   3  children  cnarc1  cnarc2  cnarc3  ctheory1  ctheory2  ctheory3  ctheory4  CRT1  CRT2  CRT3  employ_status1  health_cond  marital1  mor_circle  moralid1  moralid10  moralid2  moralid3  moralid4  moralid5  moralid6  moralid7  moralid8  moralid9  mcoop1  mcoop2  mcoop3  mcoop4  mcoop5  mcoop6  mcoop7  nidentity1  omind1  omind2  omind3  omind4  omind5  omind6  political_ideology  riskperc1  riskperc2  sbelong1  sbelong2  sbelong3  sbelong4  tested_positive  optim1  optim2  slfcont1  slfcont2  slfcont3  slfcont4  urban
##   8   4  children  cnarc1  cnarc2  cnarc3  ctheory1  ctheory2  ctheory3  ctheory4  CRT1  CRT2  CRT3  employ_status1  health_cond  marital1  mor_circle  moralid1  moralid10  moralid2  moralid3  moralid4  moralid5  moralid6  moralid7  moralid8  moralid9  mcoop1  mcoop2  mcoop3  mcoop4  mcoop5  mcoop6  mcoop7  nidentity1  omind1  omind2  omind3  omind4  omind5  omind6  political_ideology  riskperc1  riskperc2  sbelong1  sbelong2  sbelong3  sbelong4  tested_positive  optim1  optim2  slfcont1  slfcont2  slfcont3  slfcont4  urban
##   8   5  children  cnarc1  cnarc2  cnarc3  ctheory1  ctheory2  ctheory3  ctheory4  CRT1  CRT2  CRT3  employ_status1  health_cond  marital1  mor_circle  moralid1  moralid10  moralid2  moralid3  moralid4  moralid5  moralid6  moralid7  moralid8  moralid9  mcoop1  mcoop2  mcoop3  mcoop4  mcoop5  mcoop6  mcoop7  nidentity1  omind1  omind2  omind3  omind4  omind5  omind6  political_ideology  riskperc1  riskperc2  sbelong1  sbelong2  sbelong3  sbelong4  tested_positive  optim1  optim2  slfcont1  slfcont2  slfcont3  slfcont4  urban
##   8   6  children  cnarc1  cnarc2  cnarc3  ctheory1  ctheory2  ctheory3  ctheory4  CRT1  CRT2  CRT3  employ_status1  health_cond  marital1  mor_circle  moralid1  moralid10  moralid2  moralid3  moralid4  moralid5  moralid6  moralid7  moralid8  moralid9  mcoop1  mcoop2  mcoop3  mcoop4  mcoop5  mcoop6  mcoop7  nidentity1  omind1  omind2  omind3  omind4  omind5  omind6  political_ideology  riskperc1  riskperc2  sbelong1  sbelong2  sbelong3  sbelong4  tested_positive  optim1  optim2  slfcont1  slfcont2  slfcont3  slfcont4  urban
##   8   7  children  cnarc1  cnarc2  cnarc3  ctheory1  ctheory2  ctheory3  ctheory4  CRT1  CRT2  CRT3  employ_status1  health_cond  marital1  mor_circle  moralid1  moralid10  moralid2  moralid3  moralid4  moralid5  moralid6  moralid7  moralid8  moralid9  mcoop1  mcoop2  mcoop3  mcoop4  mcoop5  mcoop6  mcoop7  nidentity1  omind1  omind2  omind3  omind4  omind5  omind6  political_ideology  riskperc1  riskperc2  sbelong1  sbelong2  sbelong3  sbelong4  tested_positive  optim1  optim2  slfcont1  slfcont2  slfcont3  slfcont4  urban
##   8   8  children  cnarc1  cnarc2  cnarc3  ctheory1  ctheory2  ctheory3  ctheory4  CRT1  CRT2  CRT3  employ_status1  health_cond  marital1  mor_circle  moralid1  moralid10  moralid2  moralid3  moralid4  moralid5  moralid6  moralid7  moralid8  moralid9  mcoop1  mcoop2  mcoop3  mcoop4  mcoop5  mcoop6  mcoop7  nidentity1  omind1  omind2  omind3  omind4  omind5  omind6  political_ideology  riskperc1  riskperc2  sbelong1  sbelong2  sbelong3  sbelong4  tested_positive  optim1  optim2  slfcont1  slfcont2  slfcont3  slfcont4  urban
##   8   9  children  cnarc1  cnarc2  cnarc3  ctheory1  ctheory2  ctheory3  ctheory4  CRT1  CRT2  CRT3  employ_status1  health_cond  marital1  mor_circle  moralid1  moralid10  moralid2  moralid3  moralid4  moralid5  moralid6  moralid7  moralid8  moralid9  mcoop1  mcoop2  mcoop3  mcoop4  mcoop5  mcoop6  mcoop7  nidentity1  omind1  omind2  omind3  omind4  omind5  omind6  political_ideology  riskperc1  riskperc2  sbelong1  sbelong2  sbelong3  sbelong4  tested_positive  optim1  optim2  slfcont1  slfcont2  slfcont3  slfcont4  urban
##   8   10  children  cnarc1  cnarc2  cnarc3  ctheory1  ctheory2  ctheory3  ctheory4  CRT1  CRT2  CRT3  employ_status1  health_cond  marital1  mor_circle  moralid1  moralid10  moralid2  moralid3  moralid4  moralid5  moralid6  moralid7  moralid8  moralid9  mcoop1  mcoop2  mcoop3  mcoop4  mcoop5  mcoop6  mcoop7  nidentity1  omind1  omind2  omind3  omind4  omind5  omind6  political_ideology  riskperc1  riskperc2  sbelong1  sbelong2  sbelong3  sbelong4  tested_positive  optim1  optim2  slfcont1  slfcont2  slfcont3  slfcont4  urban
##   9   1  children  cnarc1  cnarc2  cnarc3  ctheory1  ctheory2  ctheory3  ctheory4  CRT1  CRT2  CRT3  employ_status1  health_cond  marital1  mor_circle  moralid1  moralid10  moralid2  moralid3  moralid4  moralid5  moralid6  moralid7  moralid8  moralid9  mcoop1  mcoop2  mcoop3  mcoop4  mcoop5  mcoop6  mcoop7  nidentity1  omind1  omind2  omind3  omind4  omind5  omind6  political_ideology  riskperc1  riskperc2  sbelong1  sbelong2  sbelong3  sbelong4  tested_positive  optim1  optim2  slfcont1  slfcont2  slfcont3  slfcont4  urban
##   9   2  children  cnarc1  cnarc2  cnarc3  ctheory1  ctheory2  ctheory3  ctheory4  CRT1  CRT2  CRT3  employ_status1  health_cond  marital1  mor_circle  moralid1  moralid10  moralid2  moralid3  moralid4  moralid5  moralid6  moralid7  moralid8  moralid9  mcoop1  mcoop2  mcoop3  mcoop4  mcoop5  mcoop6  mcoop7  nidentity1  omind1  omind2  omind3  omind4  omind5  omind6  political_ideology  riskperc1  riskperc2  sbelong1  sbelong2  sbelong3  sbelong4  tested_positive  optim1  optim2  slfcont1  slfcont2  slfcont3  slfcont4  urban
##   9   3  children  cnarc1  cnarc2  cnarc3  ctheory1  ctheory2  ctheory3  ctheory4  CRT1  CRT2  CRT3  employ_status1  health_cond  marital1  mor_circle  moralid1  moralid10  moralid2  moralid3  moralid4  moralid5  moralid6  moralid7  moralid8  moralid9  mcoop1  mcoop2  mcoop3  mcoop4  mcoop5  mcoop6  mcoop7  nidentity1  omind1  omind2  omind3  omind4  omind5  omind6  political_ideology  riskperc1  riskperc2  sbelong1  sbelong2  sbelong3  sbelong4  tested_positive  optim1  optim2  slfcont1  slfcont2  slfcont3  slfcont4  urban
##   9   4  children  cnarc1  cnarc2  cnarc3  ctheory1  ctheory2  ctheory3  ctheory4  CRT1  CRT2  CRT3  employ_status1  health_cond  marital1  mor_circle  moralid1  moralid10  moralid2  moralid3  moralid4  moralid5  moralid6  moralid7  moralid8  moralid9  mcoop1  mcoop2  mcoop3  mcoop4  mcoop5  mcoop6  mcoop7  nidentity1  omind1  omind2  omind3  omind4  omind5  omind6  political_ideology  riskperc1  riskperc2  sbelong1  sbelong2  sbelong3  sbelong4  tested_positive  optim1  optim2  slfcont1  slfcont2  slfcont3  slfcont4  urban
##   9   5  children  cnarc1  cnarc2  cnarc3  ctheory1  ctheory2  ctheory3  ctheory4  CRT1  CRT2  CRT3  employ_status1  health_cond  marital1  mor_circle  moralid1  moralid10  moralid2  moralid3  moralid4  moralid5  moralid6  moralid7  moralid8  moralid9  mcoop1  mcoop2  mcoop3  mcoop4  mcoop5  mcoop6  mcoop7  nidentity1  omind1  omind2  omind3  omind4  omind5  omind6  political_ideology  riskperc1  riskperc2  sbelong1  sbelong2  sbelong3  sbelong4  tested_positive  optim1  optim2  slfcont1  slfcont2  slfcont3  slfcont4  urban
##   9   6  children  cnarc1  cnarc2  cnarc3  ctheory1  ctheory2  ctheory3  ctheory4  CRT1  CRT2  CRT3  employ_status1  health_cond  marital1  mor_circle  moralid1  moralid10  moralid2  moralid3  moralid4  moralid5  moralid6  moralid7  moralid8  moralid9  mcoop1  mcoop2  mcoop3  mcoop4  mcoop5  mcoop6  mcoop7  nidentity1  omind1  omind2  omind3  omind4  omind5  omind6  political_ideology  riskperc1  riskperc2  sbelong1  sbelong2  sbelong3  sbelong4  tested_positive  optim1  optim2  slfcont1  slfcont2  slfcont3  slfcont4  urban
##   9   7  children  cnarc1  cnarc2  cnarc3  ctheory1  ctheory2  ctheory3  ctheory4  CRT1  CRT2  CRT3  employ_status1  health_cond  marital1  mor_circle  moralid1  moralid10  moralid2  moralid3  moralid4  moralid5  moralid6  moralid7  moralid8  moralid9  mcoop1  mcoop2  mcoop3  mcoop4  mcoop5  mcoop6  mcoop7  nidentity1  omind1  omind2  omind3  omind4  omind5  omind6  political_ideology  riskperc1  riskperc2  sbelong1  sbelong2  sbelong3  sbelong4  tested_positive  optim1  optim2  slfcont1  slfcont2  slfcont3  slfcont4  urban
##   9   8  children  cnarc1  cnarc2  cnarc3  ctheory1  ctheory2  ctheory3  ctheory4  CRT1  CRT2  CRT3  employ_status1  health_cond  marital1  mor_circle  moralid1  moralid10  moralid2  moralid3  moralid4  moralid5  moralid6  moralid7  moralid8  moralid9  mcoop1  mcoop2  mcoop3  mcoop4  mcoop5  mcoop6  mcoop7  nidentity1  omind1  omind2  omind3  omind4  omind5  omind6  political_ideology  riskperc1  riskperc2  sbelong1  sbelong2  sbelong3  sbelong4  tested_positive  optim1  optim2  slfcont1  slfcont2  slfcont3  slfcont4  urban
##   9   9  children  cnarc1  cnarc2  cnarc3  ctheory1  ctheory2  ctheory3  ctheory4  CRT1  CRT2  CRT3  employ_status1  health_cond  marital1  mor_circle  moralid1  moralid10  moralid2  moralid3  moralid4  moralid5  moralid6  moralid7  moralid8  moralid9  mcoop1  mcoop2  mcoop3  mcoop4  mcoop5  mcoop6  mcoop7  nidentity1  omind1  omind2  omind3  omind4  omind5  omind6  political_ideology  riskperc1  riskperc2  sbelong1  sbelong2  sbelong3  sbelong4  tested_positive  optim1  optim2  slfcont1  slfcont2  slfcont3  slfcont4  urban
##   9   10  children  cnarc1  cnarc2  cnarc3  ctheory1  ctheory2  ctheory3  ctheory4  CRT1  CRT2  CRT3  employ_status1  health_cond  marital1  mor_circle  moralid1  moralid10  moralid2  moralid3  moralid4  moralid5  moralid6  moralid7  moralid8  moralid9  mcoop1  mcoop2  mcoop3  mcoop4  mcoop5  mcoop6  mcoop7  nidentity1  omind1  omind2  omind3  omind4  omind5  omind6  political_ideology  riskperc1  riskperc2  sbelong1  sbelong2  sbelong3  sbelong4  tested_positive  optim1  optim2  slfcont1  slfcont2  slfcont3  slfcont4  urban
##   10   1  children  cnarc1  cnarc2  cnarc3  ctheory1  ctheory2  ctheory3  ctheory4  CRT1  CRT2  CRT3  employ_status1  health_cond  marital1  mor_circle  moralid1  moralid10  moralid2  moralid3  moralid4  moralid5  moralid6  moralid7  moralid8  moralid9  mcoop1  mcoop2  mcoop3  mcoop4  mcoop5  mcoop6  mcoop7  nidentity1  omind1  omind2  omind3  omind4  omind5  omind6  political_ideology  riskperc1  riskperc2  sbelong1  sbelong2  sbelong3  sbelong4  tested_positive  optim1  optim2  slfcont1  slfcont2  slfcont3  slfcont4  urban
##   10   2  children  cnarc1  cnarc2  cnarc3  ctheory1  ctheory2  ctheory3  ctheory4  CRT1  CRT2  CRT3  employ_status1  health_cond  marital1  mor_circle  moralid1  moralid10  moralid2  moralid3  moralid4  moralid5  moralid6  moralid7  moralid8  moralid9  mcoop1  mcoop2  mcoop3  mcoop4  mcoop5  mcoop6  mcoop7  nidentity1  omind1  omind2  omind3  omind4  omind5  omind6  political_ideology  riskperc1  riskperc2  sbelong1  sbelong2  sbelong3  sbelong4  tested_positive  optim1  optim2  slfcont1  slfcont2  slfcont3  slfcont4  urban
##   10   3  children  cnarc1  cnarc2  cnarc3  ctheory1  ctheory2  ctheory3  ctheory4  CRT1  CRT2  CRT3  employ_status1  health_cond  marital1  mor_circle  moralid1  moralid10  moralid2  moralid3  moralid4  moralid5  moralid6  moralid7  moralid8  moralid9  mcoop1  mcoop2  mcoop3  mcoop4  mcoop5  mcoop6  mcoop7  nidentity1  omind1  omind2  omind3  omind4  omind5  omind6  political_ideology  riskperc1  riskperc2  sbelong1  sbelong2  sbelong3  sbelong4  tested_positive  optim1  optim2  slfcont1  slfcont2  slfcont3  slfcont4  urban
##   10   4  children  cnarc1  cnarc2  cnarc3  ctheory1  ctheory2  ctheory3  ctheory4  CRT1  CRT2  CRT3  employ_status1  health_cond  marital1  mor_circle  moralid1  moralid10  moralid2  moralid3  moralid4  moralid5  moralid6  moralid7  moralid8  moralid9  mcoop1  mcoop2  mcoop3  mcoop4  mcoop5  mcoop6  mcoop7  nidentity1  omind1  omind2  omind3  omind4  omind5  omind6  political_ideology  riskperc1  riskperc2  sbelong1  sbelong2  sbelong3  sbelong4  tested_positive  optim1  optim2  slfcont1  slfcont2  slfcont3  slfcont4  urban
##   10   5  children  cnarc1  cnarc2  cnarc3  ctheory1  ctheory2  ctheory3  ctheory4  CRT1  CRT2  CRT3  employ_status1  health_cond  marital1  mor_circle  moralid1  moralid10  moralid2  moralid3  moralid4  moralid5  moralid6  moralid7  moralid8  moralid9  mcoop1  mcoop2  mcoop3  mcoop4  mcoop5  mcoop6  mcoop7  nidentity1  omind1  omind2  omind3  omind4  omind5  omind6  political_ideology  riskperc1  riskperc2  sbelong1  sbelong2  sbelong3  sbelong4  tested_positive  optim1  optim2  slfcont1  slfcont2  slfcont3  slfcont4  urban
##   10   6  children  cnarc1  cnarc2  cnarc3  ctheory1  ctheory2  ctheory3  ctheory4  CRT1  CRT2  CRT3  employ_status1  health_cond  marital1  mor_circle  moralid1  moralid10  moralid2  moralid3  moralid4  moralid5  moralid6  moralid7  moralid8  moralid9  mcoop1  mcoop2  mcoop3  mcoop4  mcoop5  mcoop6  mcoop7  nidentity1  omind1  omind2  omind3  omind4  omind5  omind6  political_ideology  riskperc1  riskperc2  sbelong1  sbelong2  sbelong3  sbelong4  tested_positive  optim1  optim2  slfcont1  slfcont2  slfcont3  slfcont4  urban
##   10   7  children  cnarc1  cnarc2  cnarc3  ctheory1  ctheory2  ctheory3  ctheory4  CRT1  CRT2  CRT3  employ_status1  health_cond  marital1  mor_circle  moralid1  moralid10  moralid2  moralid3  moralid4  moralid5  moralid6  moralid7  moralid8  moralid9  mcoop1  mcoop2  mcoop3  mcoop4  mcoop5  mcoop6  mcoop7  nidentity1  omind1  omind2  omind3  omind4  omind5  omind6  political_ideology  riskperc1  riskperc2  sbelong1  sbelong2  sbelong3  sbelong4  tested_positive  optim1  optim2  slfcont1  slfcont2  slfcont3  slfcont4  urban
##   10   8  children  cnarc1  cnarc2  cnarc3  ctheory1  ctheory2  ctheory3  ctheory4  CRT1  CRT2  CRT3  employ_status1  health_cond  marital1  mor_circle  moralid1  moralid10  moralid2  moralid3  moralid4  moralid5  moralid6  moralid7  moralid8  moralid9  mcoop1  mcoop2  mcoop3  mcoop4  mcoop5  mcoop6  mcoop7  nidentity1  omind1  omind2  omind3  omind4  omind5  omind6  political_ideology  riskperc1  riskperc2  sbelong1  sbelong2  sbelong3  sbelong4  tested_positive  optim1  optim2  slfcont1  slfcont2  slfcont3  slfcont4  urban
##   10   9  children  cnarc1  cnarc2  cnarc3  ctheory1  ctheory2  ctheory3  ctheory4  CRT1  CRT2  CRT3  employ_status1  health_cond  marital1  mor_circle  moralid1  moralid10  moralid2  moralid3  moralid4  moralid5  moralid6  moralid7  moralid8  moralid9  mcoop1  mcoop2  mcoop3  mcoop4  mcoop5  mcoop6  mcoop7  nidentity1  omind1  omind2  omind3  omind4  omind5  omind6  political_ideology  riskperc1  riskperc2  sbelong1  sbelong2  sbelong3  sbelong4  tested_positive  optim1  optim2  slfcont1  slfcont2  slfcont3  slfcont4  urban
##   10   10  children  cnarc1  cnarc2  cnarc3  ctheory1  ctheory2  ctheory3  ctheory4  CRT1  CRT2  CRT3  employ_status1  health_cond  marital1  mor_circle  moralid1  moralid10  moralid2  moralid3  moralid4  moralid5  moralid6  moralid7  moralid8  moralid9  mcoop1  mcoop2  mcoop3  mcoop4  mcoop5  mcoop6  mcoop7  nidentity1  omind1  omind2  omind3  omind4  omind5  omind6  political_ideology  riskperc1  riskperc2  sbelong1  sbelong2  sbelong3  sbelong4  tested_positive  optim1  optim2  slfcont1  slfcont2  slfcont3  slfcont4  urban
```

```
## Warning: Number of logged events: 9
```

```
## 
##  iter imp variable
##   1   1  health_cond  know_tested_positive  tested_positive
##   1   2  health_cond  know_tested_positive  tested_positive
##   1   3  health_cond  know_tested_positive  tested_positive
##   1   4  health_cond  know_tested_positive  tested_positive
##   1   5  health_cond  know_tested_positive  tested_positive
##   1   6  health_cond  know_tested_positive  tested_positive
##   1   7  health_cond  know_tested_positive  tested_positive
##   1   8  health_cond  know_tested_positive  tested_positive
##   1   9  health_cond  know_tested_positive  tested_positive
##   1   10  health_cond  know_tested_positive  tested_positive
##   2   1  health_cond  know_tested_positive  tested_positive
##   2   2  health_cond  know_tested_positive  tested_positive
##   2   3  health_cond  know_tested_positive  tested_positive
##   2   4  health_cond  know_tested_positive  tested_positive
##   2   5  health_cond  know_tested_positive  tested_positive
##   2   6  health_cond  know_tested_positive  tested_positive
##   2   7  health_cond  know_tested_positive  tested_positive
##   2   8  health_cond  know_tested_positive  tested_positive
##   2   9  health_cond  know_tested_positive  tested_positive
##   2   10  health_cond  know_tested_positive  tested_positive
##   3   1  health_cond  know_tested_positive  tested_positive
##   3   2  health_cond  know_tested_positive  tested_positive
##   3   3  health_cond  know_tested_positive  tested_positive
##   3   4  health_cond  know_tested_positive  tested_positive
##   3   5  health_cond  know_tested_positive  tested_positive
##   3   6  health_cond  know_tested_positive  tested_positive
##   3   7  health_cond  know_tested_positive  tested_positive
##   3   8  health_cond  know_tested_positive  tested_positive
##   3   9  health_cond  know_tested_positive  tested_positive
##   3   10  health_cond  know_tested_positive  tested_positive
##   4   1  health_cond  know_tested_positive  tested_positive
##   4   2  health_cond  know_tested_positive  tested_positive
##   4   3  health_cond  know_tested_positive  tested_positive
##   4   4  health_cond  know_tested_positive  tested_positive
##   4   5  health_cond  know_tested_positive  tested_positive
##   4   6  health_cond  know_tested_positive  tested_positive
##   4   7  health_cond  know_tested_positive  tested_positive
##   4   8  health_cond  know_tested_positive  tested_positive
##   4   9  health_cond  know_tested_positive  tested_positive
##   4   10  health_cond  know_tested_positive  tested_positive
##   5   1  health_cond  know_tested_positive  tested_positive
##   5   2  health_cond  know_tested_positive  tested_positive
##   5   3  health_cond  know_tested_positive  tested_positive
##   5   4  health_cond  know_tested_positive  tested_positive
##   5   5  health_cond  know_tested_positive  tested_positive
##   5   6  health_cond  know_tested_positive  tested_positive
##   5   7  health_cond  know_tested_positive  tested_positive
##   5   8  health_cond  know_tested_positive  tested_positive
##   5   9  health_cond  know_tested_positive  tested_positive
##   5   10  health_cond  know_tested_positive  tested_positive
##   6   1  health_cond  know_tested_positive  tested_positive
##   6   2  health_cond  know_tested_positive  tested_positive
##   6   3  health_cond  know_tested_positive  tested_positive
##   6   4  health_cond  know_tested_positive  tested_positive
##   6   5  health_cond  know_tested_positive  tested_positive
##   6   6  health_cond  know_tested_positive  tested_positive
##   6   7  health_cond  know_tested_positive  tested_positive
##   6   8  health_cond  know_tested_positive  tested_positive
##   6   9  health_cond  know_tested_positive  tested_positive
##   6   10  health_cond  know_tested_positive  tested_positive
##   7   1  health_cond  know_tested_positive  tested_positive
##   7   2  health_cond  know_tested_positive  tested_positive
##   7   3  health_cond  know_tested_positive  tested_positive
##   7   4  health_cond  know_tested_positive  tested_positive
##   7   5  health_cond  know_tested_positive  tested_positive
##   7   6  health_cond  know_tested_positive  tested_positive
##   7   7  health_cond  know_tested_positive  tested_positive
##   7   8  health_cond  know_tested_positive  tested_positive
##   7   9  health_cond  know_tested_positive  tested_positive
##   7   10  health_cond  know_tested_positive  tested_positive
##   8   1  health_cond  know_tested_positive  tested_positive
##   8   2  health_cond  know_tested_positive  tested_positive
##   8   3  health_cond  know_tested_positive  tested_positive
##   8   4  health_cond  know_tested_positive  tested_positive
##   8   5  health_cond  know_tested_positive  tested_positive
##   8   6  health_cond  know_tested_positive  tested_positive
##   8   7  health_cond  know_tested_positive  tested_positive
##   8   8  health_cond  know_tested_positive  tested_positive
##   8   9  health_cond  know_tested_positive  tested_positive
##   8   10  health_cond  know_tested_positive  tested_positive
##   9   1  health_cond  know_tested_positive  tested_positive
##   9   2  health_cond  know_tested_positive  tested_positive
##   9   3  health_cond  know_tested_positive  tested_positive
##   9   4  health_cond  know_tested_positive  tested_positive
##   9   5  health_cond  know_tested_positive  tested_positive
##   9   6  health_cond  know_tested_positive  tested_positive
##   9   7  health_cond  know_tested_positive  tested_positive
##   9   8  health_cond  know_tested_positive  tested_positive
##   9   9  health_cond  know_tested_positive  tested_positive
##   9   10  health_cond  know_tested_positive  tested_positive
##   10   1  health_cond  know_tested_positive  tested_positive
##   10   2  health_cond  know_tested_positive  tested_positive
##   10   3  health_cond  know_tested_positive  tested_positive
##   10   4  health_cond  know_tested_positive  tested_positive
##   10   5  health_cond  know_tested_positive  tested_positive
##   10   6  health_cond  know_tested_positive  tested_positive
##   10   7  health_cond  know_tested_positive  tested_positive
##   10   8  health_cond  know_tested_positive  tested_positive
##   10   9  health_cond  know_tested_positive  tested_positive
##   10   10  health_cond  know_tested_positive  tested_positive
```

```
## Warning: Number of logged events: 303
```

```
## 
##  iter imp variable
##   1   1  children  cnarc1  cnarc2  cnarc3  CRT1  CRT2  CRT3  ladder  marital1  mor_circle  moralid1  moralid10  moralid2  moralid3  moralid4  moralid5  moralid6  moralid7  moralid8  moralid9  mcoop1  mcoop2  mcoop3  mcoop4  mcoop5  mcoop6  mcoop7  narc1  narc2  narc3  narc4  narc5  narc6  nidentity1  nidentity2  omind1  omind5  omind6  contact2  contact3  contact4  contact5  hygiene1  hygiene2  hygiene3  hygiene4  hygiene5  psupport1  psupport2  psupport3  psupport4  psupport5  happy  slf_ladder  riskperc2  self_esteem  sbelong1  sbelong2  sbelong3  sbelong4  tested_positive  optim1  optim2  slfcont1  slfcont2  slfcont3  slfcont4
##   1   2  children  cnarc1  cnarc2  cnarc3  CRT1  CRT2  CRT3  ladder  marital1  mor_circle  moralid1  moralid10  moralid2  moralid3  moralid4  moralid5  moralid6  moralid7  moralid8  moralid9  mcoop1  mcoop2  mcoop3  mcoop4  mcoop5  mcoop6  mcoop7  narc1  narc2  narc3  narc4  narc5  narc6  nidentity1  nidentity2  omind1  omind5  omind6  contact2  contact3  contact4  contact5  hygiene1  hygiene2  hygiene3  hygiene4  hygiene5  psupport1  psupport2  psupport3  psupport4  psupport5  happy  slf_ladder  riskperc2  self_esteem  sbelong1  sbelong2  sbelong3  sbelong4  tested_positive  optim1  optim2  slfcont1  slfcont2  slfcont3  slfcont4
##   1   3  children  cnarc1  cnarc2  cnarc3  CRT1  CRT2  CRT3  ladder  marital1  mor_circle  moralid1  moralid10  moralid2  moralid3  moralid4  moralid5  moralid6  moralid7  moralid8  moralid9  mcoop1  mcoop2  mcoop3  mcoop4  mcoop5  mcoop6  mcoop7  narc1  narc2  narc3  narc4  narc5  narc6  nidentity1  nidentity2  omind1  omind5  omind6  contact2  contact3  contact4  contact5  hygiene1  hygiene2  hygiene3  hygiene4  hygiene5  psupport1  psupport2  psupport3  psupport4  psupport5  happy  slf_ladder  riskperc2  self_esteem  sbelong1  sbelong2  sbelong3  sbelong4  tested_positive  optim1  optim2  slfcont1  slfcont2  slfcont3  slfcont4
##   1   4  children  cnarc1  cnarc2  cnarc3  CRT1  CRT2  CRT3  ladder  marital1  mor_circle  moralid1  moralid10  moralid2  moralid3  moralid4  moralid5  moralid6  moralid7  moralid8  moralid9  mcoop1  mcoop2  mcoop3  mcoop4  mcoop5  mcoop6  mcoop7  narc1  narc2  narc3  narc4  narc5  narc6  nidentity1  nidentity2  omind1  omind5  omind6  contact2  contact3  contact4  contact5  hygiene1  hygiene2  hygiene3  hygiene4  hygiene5  psupport1  psupport2  psupport3  psupport4  psupport5  happy  slf_ladder  riskperc2  self_esteem  sbelong1  sbelong2  sbelong3  sbelong4  tested_positive  optim1  optim2  slfcont1  slfcont2  slfcont3  slfcont4
##   1   5  children  cnarc1  cnarc2  cnarc3  CRT1  CRT2  CRT3  ladder  marital1  mor_circle  moralid1  moralid10  moralid2  moralid3  moralid4  moralid5  moralid6  moralid7  moralid8  moralid9  mcoop1  mcoop2  mcoop3  mcoop4  mcoop5  mcoop6  mcoop7  narc1  narc2  narc3  narc4  narc5  narc6  nidentity1  nidentity2  omind1  omind5  omind6  contact2  contact3  contact4  contact5  hygiene1  hygiene2  hygiene3  hygiene4  hygiene5  psupport1  psupport2  psupport3  psupport4  psupport5  happy  slf_ladder  riskperc2  self_esteem  sbelong1  sbelong2  sbelong3  sbelong4  tested_positive  optim1  optim2  slfcont1  slfcont2  slfcont3  slfcont4
##   1   6  children  cnarc1  cnarc2  cnarc3  CRT1  CRT2  CRT3  ladder  marital1  mor_circle  moralid1  moralid10  moralid2  moralid3  moralid4  moralid5  moralid6  moralid7  moralid8  moralid9  mcoop1  mcoop2  mcoop3  mcoop4  mcoop5  mcoop6  mcoop7  narc1  narc2  narc3  narc4  narc5  narc6  nidentity1  nidentity2  omind1  omind5  omind6  contact2  contact3  contact4  contact5  hygiene1  hygiene2  hygiene3  hygiene4  hygiene5  psupport1  psupport2  psupport3  psupport4  psupport5  happy  slf_ladder  riskperc2  self_esteem  sbelong1  sbelong2  sbelong3  sbelong4  tested_positive  optim1  optim2  slfcont1  slfcont2  slfcont3  slfcont4
##   1   7  children  cnarc1  cnarc2  cnarc3  CRT1  CRT2  CRT3  ladder  marital1  mor_circle  moralid1  moralid10  moralid2  moralid3  moralid4  moralid5  moralid6  moralid7  moralid8  moralid9  mcoop1  mcoop2  mcoop3  mcoop4  mcoop5  mcoop6  mcoop7  narc1  narc2  narc3  narc4  narc5  narc6  nidentity1  nidentity2  omind1  omind5  omind6  contact2  contact3  contact4  contact5  hygiene1  hygiene2  hygiene3  hygiene4  hygiene5  psupport1  psupport2  psupport3  psupport4  psupport5  happy  slf_ladder  riskperc2  self_esteem  sbelong1  sbelong2  sbelong3  sbelong4  tested_positive  optim1  optim2  slfcont1  slfcont2  slfcont3  slfcont4
##   1   8  children  cnarc1  cnarc2  cnarc3  CRT1  CRT2  CRT3  ladder  marital1  mor_circle  moralid1  moralid10  moralid2  moralid3  moralid4  moralid5  moralid6  moralid7  moralid8  moralid9  mcoop1  mcoop2  mcoop3  mcoop4  mcoop5  mcoop6  mcoop7  narc1  narc2  narc3  narc4  narc5  narc6  nidentity1  nidentity2  omind1  omind5  omind6  contact2  contact3  contact4  contact5  hygiene1  hygiene2  hygiene3  hygiene4  hygiene5  psupport1  psupport2  psupport3  psupport4  psupport5  happy  slf_ladder  riskperc2  self_esteem  sbelong1  sbelong2  sbelong3  sbelong4  tested_positive  optim1  optim2  slfcont1  slfcont2  slfcont3  slfcont4
##   1   9  children  cnarc1  cnarc2  cnarc3  CRT1  CRT2  CRT3  ladder  marital1  mor_circle  moralid1  moralid10  moralid2  moralid3  moralid4  moralid5  moralid6  moralid7  moralid8  moralid9  mcoop1  mcoop2  mcoop3  mcoop4  mcoop5  mcoop6  mcoop7  narc1  narc2  narc3  narc4  narc5  narc6  nidentity1  nidentity2  omind1  omind5  omind6  contact2  contact3  contact4  contact5  hygiene1  hygiene2  hygiene3  hygiene4  hygiene5  psupport1  psupport2  psupport3  psupport4  psupport5  happy  slf_ladder  riskperc2  self_esteem  sbelong1  sbelong2  sbelong3  sbelong4  tested_positive  optim1  optim2  slfcont1  slfcont2  slfcont3  slfcont4
##   1   10  children  cnarc1  cnarc2  cnarc3  CRT1  CRT2  CRT3  ladder  marital1  mor_circle  moralid1  moralid10  moralid2  moralid3  moralid4  moralid5  moralid6  moralid7  moralid8  moralid9  mcoop1  mcoop2  mcoop3  mcoop4  mcoop5  mcoop6  mcoop7  narc1  narc2  narc3  narc4  narc5  narc6  nidentity1  nidentity2  omind1  omind5  omind6  contact2  contact3  contact4  contact5  hygiene1  hygiene2  hygiene3  hygiene4  hygiene5  psupport1  psupport2  psupport3  psupport4  psupport5  happy  slf_ladder  riskperc2  self_esteem  sbelong1  sbelong2  sbelong3  sbelong4  tested_positive  optim1  optim2  slfcont1  slfcont2  slfcont3  slfcont4
##   2   1  children  cnarc1  cnarc2  cnarc3  CRT1  CRT2  CRT3  ladder  marital1  mor_circle  moralid1  moralid10  moralid2  moralid3  moralid4  moralid5  moralid6  moralid7  moralid8  moralid9  mcoop1  mcoop2  mcoop3  mcoop4  mcoop5  mcoop6  mcoop7  narc1  narc2  narc3  narc4  narc5  narc6  nidentity1  nidentity2  omind1  omind5  omind6  contact2  contact3  contact4  contact5  hygiene1  hygiene2  hygiene3  hygiene4  hygiene5  psupport1  psupport2  psupport3  psupport4  psupport5  happy  slf_ladder  riskperc2  self_esteem  sbelong1  sbelong2  sbelong3  sbelong4  tested_positive  optim1  optim2  slfcont1  slfcont2  slfcont3  slfcont4
##   2   2  children  cnarc1  cnarc2  cnarc3  CRT1  CRT2  CRT3  ladder  marital1  mor_circle  moralid1  moralid10  moralid2  moralid3  moralid4  moralid5  moralid6  moralid7  moralid8  moralid9  mcoop1  mcoop2  mcoop3  mcoop4  mcoop5  mcoop6  mcoop7  narc1  narc2  narc3  narc4  narc5  narc6  nidentity1  nidentity2  omind1  omind5  omind6  contact2  contact3  contact4  contact5  hygiene1  hygiene2  hygiene3  hygiene4  hygiene5  psupport1  psupport2  psupport3  psupport4  psupport5  happy  slf_ladder  riskperc2  self_esteem  sbelong1  sbelong2  sbelong3  sbelong4  tested_positive  optim1  optim2  slfcont1  slfcont2  slfcont3  slfcont4
##   2   3  children  cnarc1  cnarc2  cnarc3  CRT1  CRT2  CRT3  ladder  marital1  mor_circle  moralid1  moralid10  moralid2  moralid3  moralid4  moralid5  moralid6  moralid7  moralid8  moralid9  mcoop1  mcoop2  mcoop3  mcoop4  mcoop5  mcoop6  mcoop7  narc1  narc2  narc3  narc4  narc5  narc6  nidentity1  nidentity2  omind1  omind5  omind6  contact2  contact3  contact4  contact5  hygiene1  hygiene2  hygiene3  hygiene4  hygiene5  psupport1  psupport2  psupport3  psupport4  psupport5  happy  slf_ladder  riskperc2  self_esteem  sbelong1  sbelong2  sbelong3  sbelong4  tested_positive  optim1  optim2  slfcont1  slfcont2  slfcont3  slfcont4
##   2   4  children  cnarc1  cnarc2  cnarc3  CRT1  CRT2  CRT3  ladder  marital1  mor_circle  moralid1  moralid10  moralid2  moralid3  moralid4  moralid5  moralid6  moralid7  moralid8  moralid9  mcoop1  mcoop2  mcoop3  mcoop4  mcoop5  mcoop6  mcoop7  narc1  narc2  narc3  narc4  narc5  narc6  nidentity1  nidentity2  omind1  omind5  omind6  contact2  contact3  contact4  contact5  hygiene1  hygiene2  hygiene3  hygiene4  hygiene5  psupport1  psupport2  psupport3  psupport4  psupport5  happy  slf_ladder  riskperc2  self_esteem  sbelong1  sbelong2  sbelong3  sbelong4  tested_positive  optim1  optim2  slfcont1  slfcont2  slfcont3  slfcont4
##   2   5  children  cnarc1  cnarc2  cnarc3  CRT1  CRT2  CRT3  ladder  marital1  mor_circle  moralid1  moralid10  moralid2  moralid3  moralid4  moralid5  moralid6  moralid7  moralid8  moralid9  mcoop1  mcoop2  mcoop3  mcoop4  mcoop5  mcoop6  mcoop7  narc1  narc2  narc3  narc4  narc5  narc6  nidentity1  nidentity2  omind1  omind5  omind6  contact2  contact3  contact4  contact5  hygiene1  hygiene2  hygiene3  hygiene4  hygiene5  psupport1  psupport2  psupport3  psupport4  psupport5  happy  slf_ladder  riskperc2  self_esteem  sbelong1  sbelong2  sbelong3  sbelong4  tested_positive  optim1  optim2  slfcont1  slfcont2  slfcont3  slfcont4
##   2   6  children  cnarc1  cnarc2  cnarc3  CRT1  CRT2  CRT3  ladder  marital1  mor_circle  moralid1  moralid10  moralid2  moralid3  moralid4  moralid5  moralid6  moralid7  moralid8  moralid9  mcoop1  mcoop2  mcoop3  mcoop4  mcoop5  mcoop6  mcoop7  narc1  narc2  narc3  narc4  narc5  narc6  nidentity1  nidentity2  omind1  omind5  omind6  contact2  contact3  contact4  contact5  hygiene1  hygiene2  hygiene3  hygiene4  hygiene5  psupport1  psupport2  psupport3  psupport4  psupport5  happy  slf_ladder  riskperc2  self_esteem  sbelong1  sbelong2  sbelong3  sbelong4  tested_positive  optim1  optim2  slfcont1  slfcont2  slfcont3  slfcont4
##   2   7  children  cnarc1  cnarc2  cnarc3  CRT1  CRT2  CRT3  ladder  marital1  mor_circle  moralid1  moralid10  moralid2  moralid3  moralid4  moralid5  moralid6  moralid7  moralid8  moralid9  mcoop1  mcoop2  mcoop3  mcoop4  mcoop5  mcoop6  mcoop7  narc1  narc2  narc3  narc4  narc5  narc6  nidentity1  nidentity2  omind1  omind5  omind6  contact2  contact3  contact4  contact5  hygiene1  hygiene2  hygiene3  hygiene4  hygiene5  psupport1  psupport2  psupport3  psupport4  psupport5  happy  slf_ladder  riskperc2  self_esteem  sbelong1  sbelong2  sbelong3  sbelong4  tested_positive  optim1  optim2  slfcont1  slfcont2  slfcont3  slfcont4
##   2   8  children  cnarc1  cnarc2  cnarc3  CRT1  CRT2  CRT3  ladder  marital1  mor_circle  moralid1  moralid10  moralid2  moralid3  moralid4  moralid5  moralid6  moralid7  moralid8  moralid9  mcoop1  mcoop2  mcoop3  mcoop4  mcoop5  mcoop6  mcoop7  narc1  narc2  narc3  narc4  narc5  narc6  nidentity1  nidentity2  omind1  omind5  omind6  contact2  contact3  contact4  contact5  hygiene1  hygiene2  hygiene3  hygiene4  hygiene5  psupport1  psupport2  psupport3  psupport4  psupport5  happy  slf_ladder  riskperc2  self_esteem  sbelong1  sbelong2  sbelong3  sbelong4  tested_positive  optim1  optim2  slfcont1  slfcont2  slfcont3  slfcont4
##   2   9  children  cnarc1  cnarc2  cnarc3  CRT1  CRT2  CRT3  ladder  marital1  mor_circle  moralid1  moralid10  moralid2  moralid3  moralid4  moralid5  moralid6  moralid7  moralid8  moralid9  mcoop1  mcoop2  mcoop3  mcoop4  mcoop5  mcoop6  mcoop7  narc1  narc2  narc3  narc4  narc5  narc6  nidentity1  nidentity2  omind1  omind5  omind6  contact2  contact3  contact4  contact5  hygiene1  hygiene2  hygiene3  hygiene4  hygiene5  psupport1  psupport2  psupport3  psupport4  psupport5  happy  slf_ladder  riskperc2  self_esteem  sbelong1  sbelong2  sbelong3  sbelong4  tested_positive  optim1  optim2  slfcont1  slfcont2  slfcont3  slfcont4
##   2   10  children  cnarc1  cnarc2  cnarc3  CRT1  CRT2  CRT3  ladder  marital1  mor_circle  moralid1  moralid10  moralid2  moralid3  moralid4  moralid5  moralid6  moralid7  moralid8  moralid9  mcoop1  mcoop2  mcoop3  mcoop4  mcoop5  mcoop6  mcoop7  narc1  narc2  narc3  narc4  narc5  narc6  nidentity1  nidentity2  omind1  omind5  omind6  contact2  contact3  contact4  contact5  hygiene1  hygiene2  hygiene3  hygiene4  hygiene5  psupport1  psupport2  psupport3  psupport4  psupport5  happy  slf_ladder  riskperc2  self_esteem  sbelong1  sbelong2  sbelong3  sbelong4  tested_positive  optim1  optim2  slfcont1  slfcont2  slfcont3  slfcont4
##   3   1  children  cnarc1  cnarc2  cnarc3  CRT1  CRT2  CRT3  ladder  marital1  mor_circle  moralid1  moralid10  moralid2  moralid3  moralid4  moralid5  moralid6  moralid7  moralid8  moralid9  mcoop1  mcoop2  mcoop3  mcoop4  mcoop5  mcoop6  mcoop7  narc1  narc2  narc3  narc4  narc5  narc6  nidentity1  nidentity2  omind1  omind5  omind6  contact2  contact3  contact4  contact5  hygiene1  hygiene2  hygiene3  hygiene4  hygiene5  psupport1  psupport2  psupport3  psupport4  psupport5  happy  slf_ladder  riskperc2  self_esteem  sbelong1  sbelong2  sbelong3  sbelong4  tested_positive  optim1  optim2  slfcont1  slfcont2  slfcont3  slfcont4
##   3   2  children  cnarc1  cnarc2  cnarc3  CRT1  CRT2  CRT3  ladder  marital1  mor_circle  moralid1  moralid10  moralid2  moralid3  moralid4  moralid5  moralid6  moralid7  moralid8  moralid9  mcoop1  mcoop2  mcoop3  mcoop4  mcoop5  mcoop6  mcoop7  narc1  narc2  narc3  narc4  narc5  narc6  nidentity1  nidentity2  omind1  omind5  omind6  contact2  contact3  contact4  contact5  hygiene1  hygiene2  hygiene3  hygiene4  hygiene5  psupport1  psupport2  psupport3  psupport4  psupport5  happy  slf_ladder  riskperc2  self_esteem  sbelong1  sbelong2  sbelong3  sbelong4  tested_positive  optim1  optim2  slfcont1  slfcont2  slfcont3  slfcont4
##   3   3  children  cnarc1  cnarc2  cnarc3  CRT1  CRT2  CRT3  ladder  marital1  mor_circle  moralid1  moralid10  moralid2  moralid3  moralid4  moralid5  moralid6  moralid7  moralid8  moralid9  mcoop1  mcoop2  mcoop3  mcoop4  mcoop5  mcoop6  mcoop7  narc1  narc2  narc3  narc4  narc5  narc6  nidentity1  nidentity2  omind1  omind5  omind6  contact2  contact3  contact4  contact5  hygiene1  hygiene2  hygiene3  hygiene4  hygiene5  psupport1  psupport2  psupport3  psupport4  psupport5  happy  slf_ladder  riskperc2  self_esteem  sbelong1  sbelong2  sbelong3  sbelong4  tested_positive  optim1  optim2  slfcont1  slfcont2  slfcont3  slfcont4
##   3   4  children  cnarc1  cnarc2  cnarc3  CRT1  CRT2  CRT3  ladder  marital1  mor_circle  moralid1  moralid10  moralid2  moralid3  moralid4  moralid5  moralid6  moralid7  moralid8  moralid9  mcoop1  mcoop2  mcoop3  mcoop4  mcoop5  mcoop6  mcoop7  narc1  narc2  narc3  narc4  narc5  narc6  nidentity1  nidentity2  omind1  omind5  omind6  contact2  contact3  contact4  contact5  hygiene1  hygiene2  hygiene3  hygiene4  hygiene5  psupport1  psupport2  psupport3  psupport4  psupport5  happy  slf_ladder  riskperc2  self_esteem  sbelong1  sbelong2  sbelong3  sbelong4  tested_positive  optim1  optim2  slfcont1  slfcont2  slfcont3  slfcont4
##   3   5  children  cnarc1  cnarc2  cnarc3  CRT1  CRT2  CRT3  ladder  marital1  mor_circle  moralid1  moralid10  moralid2  moralid3  moralid4  moralid5  moralid6  moralid7  moralid8  moralid9  mcoop1  mcoop2  mcoop3  mcoop4  mcoop5  mcoop6  mcoop7  narc1  narc2  narc3  narc4  narc5  narc6  nidentity1  nidentity2  omind1  omind5  omind6  contact2  contact3  contact4  contact5  hygiene1  hygiene2  hygiene3  hygiene4  hygiene5  psupport1  psupport2  psupport3  psupport4  psupport5  happy  slf_ladder  riskperc2  self_esteem  sbelong1  sbelong2  sbelong3  sbelong4  tested_positive  optim1  optim2  slfcont1  slfcont2  slfcont3  slfcont4
##   3   6  children  cnarc1  cnarc2  cnarc3  CRT1  CRT2  CRT3  ladder  marital1  mor_circle  moralid1  moralid10  moralid2  moralid3  moralid4  moralid5  moralid6  moralid7  moralid8  moralid9  mcoop1  mcoop2  mcoop3  mcoop4  mcoop5  mcoop6  mcoop7  narc1  narc2  narc3  narc4  narc5  narc6  nidentity1  nidentity2  omind1  omind5  omind6  contact2  contact3  contact4  contact5  hygiene1  hygiene2  hygiene3  hygiene4  hygiene5  psupport1  psupport2  psupport3  psupport4  psupport5  happy  slf_ladder  riskperc2  self_esteem  sbelong1  sbelong2  sbelong3  sbelong4  tested_positive  optim1  optim2  slfcont1  slfcont2  slfcont3  slfcont4
##   3   7  children  cnarc1  cnarc2  cnarc3  CRT1  CRT2  CRT3  ladder  marital1  mor_circle  moralid1  moralid10  moralid2  moralid3  moralid4  moralid5  moralid6  moralid7  moralid8  moralid9  mcoop1  mcoop2  mcoop3  mcoop4  mcoop5  mcoop6  mcoop7  narc1  narc2  narc3  narc4  narc5  narc6  nidentity1  nidentity2  omind1  omind5  omind6  contact2  contact3  contact4  contact5  hygiene1  hygiene2  hygiene3  hygiene4  hygiene5  psupport1  psupport2  psupport3  psupport4  psupport5  happy  slf_ladder  riskperc2  self_esteem  sbelong1  sbelong2  sbelong3  sbelong4  tested_positive  optim1  optim2  slfcont1  slfcont2  slfcont3  slfcont4
##   3   8  children  cnarc1  cnarc2  cnarc3  CRT1  CRT2  CRT3  ladder  marital1  mor_circle  moralid1  moralid10  moralid2  moralid3  moralid4  moralid5  moralid6  moralid7  moralid8  moralid9  mcoop1  mcoop2  mcoop3  mcoop4  mcoop5  mcoop6  mcoop7  narc1  narc2  narc3  narc4  narc5  narc6  nidentity1  nidentity2  omind1  omind5  omind6  contact2  contact3  contact4  contact5  hygiene1  hygiene2  hygiene3  hygiene4  hygiene5  psupport1  psupport2  psupport3  psupport4  psupport5  happy  slf_ladder  riskperc2  self_esteem  sbelong1  sbelong2  sbelong3  sbelong4  tested_positive  optim1  optim2  slfcont1  slfcont2  slfcont3  slfcont4
##   3   9  children  cnarc1  cnarc2  cnarc3  CRT1  CRT2  CRT3  ladder  marital1  mor_circle  moralid1  moralid10  moralid2  moralid3  moralid4  moralid5  moralid6  moralid7  moralid8  moralid9  mcoop1  mcoop2  mcoop3  mcoop4  mcoop5  mcoop6  mcoop7  narc1  narc2  narc3  narc4  narc5  narc6  nidentity1  nidentity2  omind1  omind5  omind6  contact2  contact3  contact4  contact5  hygiene1  hygiene2  hygiene3  hygiene4  hygiene5  psupport1  psupport2  psupport3  psupport4  psupport5  happy  slf_ladder  riskperc2  self_esteem  sbelong1  sbelong2  sbelong3  sbelong4  tested_positive  optim1  optim2  slfcont1  slfcont2  slfcont3  slfcont4
##   3   10  children  cnarc1  cnarc2  cnarc3  CRT1  CRT2  CRT3  ladder  marital1  mor_circle  moralid1  moralid10  moralid2  moralid3  moralid4  moralid5  moralid6  moralid7  moralid8  moralid9  mcoop1  mcoop2  mcoop3  mcoop4  mcoop5  mcoop6  mcoop7  narc1  narc2  narc3  narc4  narc5  narc6  nidentity1  nidentity2  omind1  omind5  omind6  contact2  contact3  contact4  contact5  hygiene1  hygiene2  hygiene3  hygiene4  hygiene5  psupport1  psupport2  psupport3  psupport4  psupport5  happy  slf_ladder  riskperc2  self_esteem  sbelong1  sbelong2  sbelong3  sbelong4  tested_positive  optim1  optim2  slfcont1  slfcont2  slfcont3  slfcont4
##   4   1  children  cnarc1  cnarc2  cnarc3  CRT1  CRT2  CRT3  ladder  marital1  mor_circle  moralid1  moralid10  moralid2  moralid3  moralid4  moralid5  moralid6  moralid7  moralid8  moralid9  mcoop1  mcoop2  mcoop3  mcoop4  mcoop5  mcoop6  mcoop7  narc1  narc2  narc3  narc4  narc5  narc6  nidentity1  nidentity2  omind1  omind5  omind6  contact2  contact3  contact4  contact5  hygiene1  hygiene2  hygiene3  hygiene4  hygiene5  psupport1  psupport2  psupport3  psupport4  psupport5  happy  slf_ladder  riskperc2  self_esteem  sbelong1  sbelong2  sbelong3  sbelong4  tested_positive  optim1  optim2  slfcont1  slfcont2  slfcont3  slfcont4
##   4   2  children  cnarc1  cnarc2  cnarc3  CRT1  CRT2  CRT3  ladder  marital1  mor_circle  moralid1  moralid10  moralid2  moralid3  moralid4  moralid5  moralid6  moralid7  moralid8  moralid9  mcoop1  mcoop2  mcoop3  mcoop4  mcoop5  mcoop6  mcoop7  narc1  narc2  narc3  narc4  narc5  narc6  nidentity1  nidentity2  omind1  omind5  omind6  contact2  contact3  contact4  contact5  hygiene1  hygiene2  hygiene3  hygiene4  hygiene5  psupport1  psupport2  psupport3  psupport4  psupport5  happy  slf_ladder  riskperc2  self_esteem  sbelong1  sbelong2  sbelong3  sbelong4  tested_positive  optim1  optim2  slfcont1  slfcont2  slfcont3  slfcont4
##   4   3  children  cnarc1  cnarc2  cnarc3  CRT1  CRT2  CRT3  ladder  marital1  mor_circle  moralid1  moralid10  moralid2  moralid3  moralid4  moralid5  moralid6  moralid7  moralid8  moralid9  mcoop1  mcoop2  mcoop3  mcoop4  mcoop5  mcoop6  mcoop7  narc1  narc2  narc3  narc4  narc5  narc6  nidentity1  nidentity2  omind1  omind5  omind6  contact2  contact3  contact4  contact5  hygiene1  hygiene2  hygiene3  hygiene4  hygiene5  psupport1  psupport2  psupport3  psupport4  psupport5  happy  slf_ladder  riskperc2  self_esteem  sbelong1  sbelong2  sbelong3  sbelong4  tested_positive  optim1  optim2  slfcont1  slfcont2  slfcont3  slfcont4
##   4   4  children  cnarc1  cnarc2  cnarc3  CRT1  CRT2  CRT3  ladder  marital1  mor_circle  moralid1  moralid10  moralid2  moralid3  moralid4  moralid5  moralid6  moralid7  moralid8  moralid9  mcoop1  mcoop2  mcoop3  mcoop4  mcoop5  mcoop6  mcoop7  narc1  narc2  narc3  narc4  narc5  narc6  nidentity1  nidentity2  omind1  omind5  omind6  contact2  contact3  contact4  contact5  hygiene1  hygiene2  hygiene3  hygiene4  hygiene5  psupport1  psupport2  psupport3  psupport4  psupport5  happy  slf_ladder  riskperc2  self_esteem  sbelong1  sbelong2  sbelong3  sbelong4  tested_positive  optim1  optim2  slfcont1  slfcont2  slfcont3  slfcont4
##   4   5  children  cnarc1  cnarc2  cnarc3  CRT1  CRT2  CRT3  ladder  marital1  mor_circle  moralid1  moralid10  moralid2  moralid3  moralid4  moralid5  moralid6  moralid7  moralid8  moralid9  mcoop1  mcoop2  mcoop3  mcoop4  mcoop5  mcoop6  mcoop7  narc1  narc2  narc3  narc4  narc5  narc6  nidentity1  nidentity2  omind1  omind5  omind6  contact2  contact3  contact4  contact5  hygiene1  hygiene2  hygiene3  hygiene4  hygiene5  psupport1  psupport2  psupport3  psupport4  psupport5  happy  slf_ladder  riskperc2  self_esteem  sbelong1  sbelong2  sbelong3  sbelong4  tested_positive  optim1  optim2  slfcont1  slfcont2  slfcont3  slfcont4
##   4   6  children  cnarc1  cnarc2  cnarc3  CRT1  CRT2  CRT3  ladder  marital1  mor_circle  moralid1  moralid10  moralid2  moralid3  moralid4  moralid5  moralid6  moralid7  moralid8  moralid9  mcoop1  mcoop2  mcoop3  mcoop4  mcoop5  mcoop6  mcoop7  narc1  narc2  narc3  narc4  narc5  narc6  nidentity1  nidentity2  omind1  omind5  omind6  contact2  contact3  contact4  contact5  hygiene1  hygiene2  hygiene3  hygiene4  hygiene5  psupport1  psupport2  psupport3  psupport4  psupport5  happy  slf_ladder  riskperc2  self_esteem  sbelong1  sbelong2  sbelong3  sbelong4  tested_positive  optim1  optim2  slfcont1  slfcont2  slfcont3  slfcont4
##   4   7  children  cnarc1  cnarc2  cnarc3  CRT1  CRT2  CRT3  ladder  marital1  mor_circle  moralid1  moralid10  moralid2  moralid3  moralid4  moralid5  moralid6  moralid7  moralid8  moralid9  mcoop1  mcoop2  mcoop3  mcoop4  mcoop5  mcoop6  mcoop7  narc1  narc2  narc3  narc4  narc5  narc6  nidentity1  nidentity2  omind1  omind5  omind6  contact2  contact3  contact4  contact5  hygiene1  hygiene2  hygiene3  hygiene4  hygiene5  psupport1  psupport2  psupport3  psupport4  psupport5  happy  slf_ladder  riskperc2  self_esteem  sbelong1  sbelong2  sbelong3  sbelong4  tested_positive  optim1  optim2  slfcont1  slfcont2  slfcont3  slfcont4
##   4   8  children  cnarc1  cnarc2  cnarc3  CRT1  CRT2  CRT3  ladder  marital1  mor_circle  moralid1  moralid10  moralid2  moralid3  moralid4  moralid5  moralid6  moralid7  moralid8  moralid9  mcoop1  mcoop2  mcoop3  mcoop4  mcoop5  mcoop6  mcoop7  narc1  narc2  narc3  narc4  narc5  narc6  nidentity1  nidentity2  omind1  omind5  omind6  contact2  contact3  contact4  contact5  hygiene1  hygiene2  hygiene3  hygiene4  hygiene5  psupport1  psupport2  psupport3  psupport4  psupport5  happy  slf_ladder  riskperc2  self_esteem  sbelong1  sbelong2  sbelong3  sbelong4  tested_positive  optim1  optim2  slfcont1  slfcont2  slfcont3  slfcont4
##   4   9  children  cnarc1  cnarc2  cnarc3  CRT1  CRT2  CRT3  ladder  marital1  mor_circle  moralid1  moralid10  moralid2  moralid3  moralid4  moralid5  moralid6  moralid7  moralid8  moralid9  mcoop1  mcoop2  mcoop3  mcoop4  mcoop5  mcoop6  mcoop7  narc1  narc2  narc3  narc4  narc5  narc6  nidentity1  nidentity2  omind1  omind5  omind6  contact2  contact3  contact4  contact5  hygiene1  hygiene2  hygiene3  hygiene4  hygiene5  psupport1  psupport2  psupport3  psupport4  psupport5  happy  slf_ladder  riskperc2  self_esteem  sbelong1  sbelong2  sbelong3  sbelong4  tested_positive  optim1  optim2  slfcont1  slfcont2  slfcont3  slfcont4
##   4   10  children  cnarc1  cnarc2  cnarc3  CRT1  CRT2  CRT3  ladder  marital1  mor_circle  moralid1  moralid10  moralid2  moralid3  moralid4  moralid5  moralid6  moralid7  moralid8  moralid9  mcoop1  mcoop2  mcoop3  mcoop4  mcoop5  mcoop6  mcoop7  narc1  narc2  narc3  narc4  narc5  narc6  nidentity1  nidentity2  omind1  omind5  omind6  contact2  contact3  contact4  contact5  hygiene1  hygiene2  hygiene3  hygiene4  hygiene5  psupport1  psupport2  psupport3  psupport4  psupport5  happy  slf_ladder  riskperc2  self_esteem  sbelong1  sbelong2  sbelong3  sbelong4  tested_positive  optim1  optim2  slfcont1  slfcont2  slfcont3  slfcont4
##   5   1  children  cnarc1  cnarc2  cnarc3  CRT1  CRT2  CRT3  ladder  marital1  mor_circle  moralid1  moralid10  moralid2  moralid3  moralid4  moralid5  moralid6  moralid7  moralid8  moralid9  mcoop1  mcoop2  mcoop3  mcoop4  mcoop5  mcoop6  mcoop7  narc1  narc2  narc3  narc4  narc5  narc6  nidentity1  nidentity2  omind1  omind5  omind6  contact2  contact3  contact4  contact5  hygiene1  hygiene2  hygiene3  hygiene4  hygiene5  psupport1  psupport2  psupport3  psupport4  psupport5  happy  slf_ladder  riskperc2  self_esteem  sbelong1  sbelong2  sbelong3  sbelong4  tested_positive  optim1  optim2  slfcont1  slfcont2  slfcont3  slfcont4
##   5   2  children  cnarc1  cnarc2  cnarc3  CRT1  CRT2  CRT3  ladder  marital1  mor_circle  moralid1  moralid10  moralid2  moralid3  moralid4  moralid5  moralid6  moralid7  moralid8  moralid9  mcoop1  mcoop2  mcoop3  mcoop4  mcoop5  mcoop6  mcoop7  narc1  narc2  narc3  narc4  narc5  narc6  nidentity1  nidentity2  omind1  omind5  omind6  contact2  contact3  contact4  contact5  hygiene1  hygiene2  hygiene3  hygiene4  hygiene5  psupport1  psupport2  psupport3  psupport4  psupport5  happy  slf_ladder  riskperc2  self_esteem  sbelong1  sbelong2  sbelong3  sbelong4  tested_positive  optim1  optim2  slfcont1  slfcont2  slfcont3  slfcont4
##   5   3  children  cnarc1  cnarc2  cnarc3  CRT1  CRT2  CRT3  ladder  marital1  mor_circle  moralid1  moralid10  moralid2  moralid3  moralid4  moralid5  moralid6  moralid7  moralid8  moralid9  mcoop1  mcoop2  mcoop3  mcoop4  mcoop5  mcoop6  mcoop7  narc1  narc2  narc3  narc4  narc5  narc6  nidentity1  nidentity2  omind1  omind5  omind6  contact2  contact3  contact4  contact5  hygiene1  hygiene2  hygiene3  hygiene4  hygiene5  psupport1  psupport2  psupport3  psupport4  psupport5  happy  slf_ladder  riskperc2  self_esteem  sbelong1  sbelong2  sbelong3  sbelong4  tested_positive  optim1  optim2  slfcont1  slfcont2  slfcont3  slfcont4
##   5   4  children  cnarc1  cnarc2  cnarc3  CRT1  CRT2  CRT3  ladder  marital1  mor_circle  moralid1  moralid10  moralid2  moralid3  moralid4  moralid5  moralid6  moralid7  moralid8  moralid9  mcoop1  mcoop2  mcoop3  mcoop4  mcoop5  mcoop6  mcoop7  narc1  narc2  narc3  narc4  narc5  narc6  nidentity1  nidentity2  omind1  omind5  omind6  contact2  contact3  contact4  contact5  hygiene1  hygiene2  hygiene3  hygiene4  hygiene5  psupport1  psupport2  psupport3  psupport4  psupport5  happy  slf_ladder  riskperc2  self_esteem  sbelong1  sbelong2  sbelong3  sbelong4  tested_positive  optim1  optim2  slfcont1  slfcont2  slfcont3  slfcont4
##   5   5  children  cnarc1  cnarc2  cnarc3  CRT1  CRT2  CRT3  ladder  marital1  mor_circle  moralid1  moralid10  moralid2  moralid3  moralid4  moralid5  moralid6  moralid7  moralid8  moralid9  mcoop1  mcoop2  mcoop3  mcoop4  mcoop5  mcoop6  mcoop7  narc1  narc2  narc3  narc4  narc5  narc6  nidentity1  nidentity2  omind1  omind5  omind6  contact2  contact3  contact4  contact5  hygiene1  hygiene2  hygiene3  hygiene4  hygiene5  psupport1  psupport2  psupport3  psupport4  psupport5  happy  slf_ladder  riskperc2  self_esteem  sbelong1  sbelong2  sbelong3  sbelong4  tested_positive  optim1  optim2  slfcont1  slfcont2  slfcont3  slfcont4
##   5   6  children  cnarc1  cnarc2  cnarc3  CRT1  CRT2  CRT3  ladder  marital1  mor_circle  moralid1  moralid10  moralid2  moralid3  moralid4  moralid5  moralid6  moralid7  moralid8  moralid9  mcoop1  mcoop2  mcoop3  mcoop4  mcoop5  mcoop6  mcoop7  narc1  narc2  narc3  narc4  narc5  narc6  nidentity1  nidentity2  omind1  omind5  omind6  contact2  contact3  contact4  contact5  hygiene1  hygiene2  hygiene3  hygiene4  hygiene5  psupport1  psupport2  psupport3  psupport4  psupport5  happy  slf_ladder  riskperc2  self_esteem  sbelong1  sbelong2  sbelong3  sbelong4  tested_positive  optim1  optim2  slfcont1  slfcont2  slfcont3  slfcont4
##   5   7  children  cnarc1  cnarc2  cnarc3  CRT1  CRT2  CRT3  ladder  marital1  mor_circle  moralid1  moralid10  moralid2  moralid3  moralid4  moralid5  moralid6  moralid7  moralid8  moralid9  mcoop1  mcoop2  mcoop3  mcoop4  mcoop5  mcoop6  mcoop7  narc1  narc2  narc3  narc4  narc5  narc6  nidentity1  nidentity2  omind1  omind5  omind6  contact2  contact3  contact4  contact5  hygiene1  hygiene2  hygiene3  hygiene4  hygiene5  psupport1  psupport2  psupport3  psupport4  psupport5  happy  slf_ladder  riskperc2  self_esteem  sbelong1  sbelong2  sbelong3  sbelong4  tested_positive  optim1  optim2  slfcont1  slfcont2  slfcont3  slfcont4
##   5   8  children  cnarc1  cnarc2  cnarc3  CRT1  CRT2  CRT3  ladder  marital1  mor_circle  moralid1  moralid10  moralid2  moralid3  moralid4  moralid5  moralid6  moralid7  moralid8  moralid9  mcoop1  mcoop2  mcoop3  mcoop4  mcoop5  mcoop6  mcoop7  narc1  narc2  narc3  narc4  narc5  narc6  nidentity1  nidentity2  omind1  omind5  omind6  contact2  contact3  contact4  contact5  hygiene1  hygiene2  hygiene3  hygiene4  hygiene5  psupport1  psupport2  psupport3  psupport4  psupport5  happy  slf_ladder  riskperc2  self_esteem  sbelong1  sbelong2  sbelong3  sbelong4  tested_positive  optim1  optim2  slfcont1  slfcont2  slfcont3  slfcont4
##   5   9  children  cnarc1  cnarc2  cnarc3  CRT1  CRT2  CRT3  ladder  marital1  mor_circle  moralid1  moralid10  moralid2  moralid3  moralid4  moralid5  moralid6  moralid7  moralid8  moralid9  mcoop1  mcoop2  mcoop3  mcoop4  mcoop5  mcoop6  mcoop7  narc1  narc2  narc3  narc4  narc5  narc6  nidentity1  nidentity2  omind1  omind5  omind6  contact2  contact3  contact4  contact5  hygiene1  hygiene2  hygiene3  hygiene4  hygiene5  psupport1  psupport2  psupport3  psupport4  psupport5  happy  slf_ladder  riskperc2  self_esteem  sbelong1  sbelong2  sbelong3  sbelong4  tested_positive  optim1  optim2  slfcont1  slfcont2  slfcont3  slfcont4
##   5   10  children  cnarc1  cnarc2  cnarc3  CRT1  CRT2  CRT3  ladder  marital1  mor_circle  moralid1  moralid10  moralid2  moralid3  moralid4  moralid5  moralid6  moralid7  moralid8  moralid9  mcoop1  mcoop2  mcoop3  mcoop4  mcoop5  mcoop6  mcoop7  narc1  narc2  narc3  narc4  narc5  narc6  nidentity1  nidentity2  omind1  omind5  omind6  contact2  contact3  contact4  contact5  hygiene1  hygiene2  hygiene3  hygiene4  hygiene5  psupport1  psupport2  psupport3  psupport4  psupport5  happy  slf_ladder  riskperc2  self_esteem  sbelong1  sbelong2  sbelong3  sbelong4  tested_positive  optim1  optim2  slfcont1  slfcont2  slfcont3  slfcont4
##   6   1  children  cnarc1  cnarc2  cnarc3  CRT1  CRT2  CRT3  ladder  marital1  mor_circle  moralid1  moralid10  moralid2  moralid3  moralid4  moralid5  moralid6  moralid7  moralid8  moralid9  mcoop1  mcoop2  mcoop3  mcoop4  mcoop5  mcoop6  mcoop7  narc1  narc2  narc3  narc4  narc5  narc6  nidentity1  nidentity2  omind1  omind5  omind6  contact2  contact3  contact4  contact5  hygiene1  hygiene2  hygiene3  hygiene4  hygiene5  psupport1  psupport2  psupport3  psupport4  psupport5  happy  slf_ladder  riskperc2  self_esteem  sbelong1  sbelong2  sbelong3  sbelong4  tested_positive  optim1  optim2  slfcont1  slfcont2  slfcont3  slfcont4
##   6   2  children  cnarc1  cnarc2  cnarc3  CRT1  CRT2  CRT3  ladder  marital1  mor_circle  moralid1  moralid10  moralid2  moralid3  moralid4  moralid5  moralid6  moralid7  moralid8  moralid9  mcoop1  mcoop2  mcoop3  mcoop4  mcoop5  mcoop6  mcoop7  narc1  narc2  narc3  narc4  narc5  narc6  nidentity1  nidentity2  omind1  omind5  omind6  contact2  contact3  contact4  contact5  hygiene1  hygiene2  hygiene3  hygiene4  hygiene5  psupport1  psupport2  psupport3  psupport4  psupport5  happy  slf_ladder  riskperc2  self_esteem  sbelong1  sbelong2  sbelong3  sbelong4  tested_positive  optim1  optim2  slfcont1  slfcont2  slfcont3  slfcont4
##   6   3  children  cnarc1  cnarc2  cnarc3  CRT1  CRT2  CRT3  ladder  marital1  mor_circle  moralid1  moralid10  moralid2  moralid3  moralid4  moralid5  moralid6  moralid7  moralid8  moralid9  mcoop1  mcoop2  mcoop3  mcoop4  mcoop5  mcoop6  mcoop7  narc1  narc2  narc3  narc4  narc5  narc6  nidentity1  nidentity2  omind1  omind5  omind6  contact2  contact3  contact4  contact5  hygiene1  hygiene2  hygiene3  hygiene4  hygiene5  psupport1  psupport2  psupport3  psupport4  psupport5  happy  slf_ladder  riskperc2  self_esteem  sbelong1  sbelong2  sbelong3  sbelong4  tested_positive  optim1  optim2  slfcont1  slfcont2  slfcont3  slfcont4
##   6   4  children  cnarc1  cnarc2  cnarc3  CRT1  CRT2  CRT3  ladder  marital1  mor_circle  moralid1  moralid10  moralid2  moralid3  moralid4  moralid5  moralid6  moralid7  moralid8  moralid9  mcoop1  mcoop2  mcoop3  mcoop4  mcoop5  mcoop6  mcoop7  narc1  narc2  narc3  narc4  narc5  narc6  nidentity1  nidentity2  omind1  omind5  omind6  contact2  contact3  contact4  contact5  hygiene1  hygiene2  hygiene3  hygiene4  hygiene5  psupport1  psupport2  psupport3  psupport4  psupport5  happy  slf_ladder  riskperc2  self_esteem  sbelong1  sbelong2  sbelong3  sbelong4  tested_positive  optim1  optim2  slfcont1  slfcont2  slfcont3  slfcont4
##   6   5  children  cnarc1  cnarc2  cnarc3  CRT1  CRT2  CRT3  ladder  marital1  mor_circle  moralid1  moralid10  moralid2  moralid3  moralid4  moralid5  moralid6  moralid7  moralid8  moralid9  mcoop1  mcoop2  mcoop3  mcoop4  mcoop5  mcoop6  mcoop7  narc1  narc2  narc3  narc4  narc5  narc6  nidentity1  nidentity2  omind1  omind5  omind6  contact2  contact3  contact4  contact5  hygiene1  hygiene2  hygiene3  hygiene4  hygiene5  psupport1  psupport2  psupport3  psupport4  psupport5  happy  slf_ladder  riskperc2  self_esteem  sbelong1  sbelong2  sbelong3  sbelong4  tested_positive  optim1  optim2  slfcont1  slfcont2  slfcont3  slfcont4
##   6   6  children  cnarc1  cnarc2  cnarc3  CRT1  CRT2  CRT3  ladder  marital1  mor_circle  moralid1  moralid10  moralid2  moralid3  moralid4  moralid5  moralid6  moralid7  moralid8  moralid9  mcoop1  mcoop2  mcoop3  mcoop4  mcoop5  mcoop6  mcoop7  narc1  narc2  narc3  narc4  narc5  narc6  nidentity1  nidentity2  omind1  omind5  omind6  contact2  contact3  contact4  contact5  hygiene1  hygiene2  hygiene3  hygiene4  hygiene5  psupport1  psupport2  psupport3  psupport4  psupport5  happy  slf_ladder  riskperc2  self_esteem  sbelong1  sbelong2  sbelong3  sbelong4  tested_positive  optim1  optim2  slfcont1  slfcont2  slfcont3  slfcont4
##   6   7  children  cnarc1  cnarc2  cnarc3  CRT1  CRT2  CRT3  ladder  marital1  mor_circle  moralid1  moralid10  moralid2  moralid3  moralid4  moralid5  moralid6  moralid7  moralid8  moralid9  mcoop1  mcoop2  mcoop3  mcoop4  mcoop5  mcoop6  mcoop7  narc1  narc2  narc3  narc4  narc5  narc6  nidentity1  nidentity2  omind1  omind5  omind6  contact2  contact3  contact4  contact5  hygiene1  hygiene2  hygiene3  hygiene4  hygiene5  psupport1  psupport2  psupport3  psupport4  psupport5  happy  slf_ladder  riskperc2  self_esteem  sbelong1  sbelong2  sbelong3  sbelong4  tested_positive  optim1  optim2  slfcont1  slfcont2  slfcont3  slfcont4
##   6   8  children  cnarc1  cnarc2  cnarc3  CRT1  CRT2  CRT3  ladder  marital1  mor_circle  moralid1  moralid10  moralid2  moralid3  moralid4  moralid5  moralid6  moralid7  moralid8  moralid9  mcoop1  mcoop2  mcoop3  mcoop4  mcoop5  mcoop6  mcoop7  narc1  narc2  narc3  narc4  narc5  narc6  nidentity1  nidentity2  omind1  omind5  omind6  contact2  contact3  contact4  contact5  hygiene1  hygiene2  hygiene3  hygiene4  hygiene5  psupport1  psupport2  psupport3  psupport4  psupport5  happy  slf_ladder  riskperc2  self_esteem  sbelong1  sbelong2  sbelong3  sbelong4  tested_positive  optim1  optim2  slfcont1  slfcont2  slfcont3  slfcont4
##   6   9  children  cnarc1  cnarc2  cnarc3  CRT1  CRT2  CRT3  ladder  marital1  mor_circle  moralid1  moralid10  moralid2  moralid3  moralid4  moralid5  moralid6  moralid7  moralid8  moralid9  mcoop1  mcoop2  mcoop3  mcoop4  mcoop5  mcoop6  mcoop7  narc1  narc2  narc3  narc4  narc5  narc6  nidentity1  nidentity2  omind1  omind5  omind6  contact2  contact3  contact4  contact5  hygiene1  hygiene2  hygiene3  hygiene4  hygiene5  psupport1  psupport2  psupport3  psupport4  psupport5  happy  slf_ladder  riskperc2  self_esteem  sbelong1  sbelong2  sbelong3  sbelong4  tested_positive  optim1  optim2  slfcont1  slfcont2  slfcont3  slfcont4
##   6   10  children  cnarc1  cnarc2  cnarc3  CRT1  CRT2  CRT3  ladder  marital1  mor_circle  moralid1  moralid10  moralid2  moralid3  moralid4  moralid5  moralid6  moralid7  moralid8  moralid9  mcoop1  mcoop2  mcoop3  mcoop4  mcoop5  mcoop6  mcoop7  narc1  narc2  narc3  narc4  narc5  narc6  nidentity1  nidentity2  omind1  omind5  omind6  contact2  contact3  contact4  contact5  hygiene1  hygiene2  hygiene3  hygiene4  hygiene5  psupport1  psupport2  psupport3  psupport4  psupport5  happy  slf_ladder  riskperc2  self_esteem  sbelong1  sbelong2  sbelong3  sbelong4  tested_positive  optim1  optim2  slfcont1  slfcont2  slfcont3  slfcont4
##   7   1  children  cnarc1  cnarc2  cnarc3  CRT1  CRT2  CRT3  ladder  marital1  mor_circle  moralid1  moralid10  moralid2  moralid3  moralid4  moralid5  moralid6  moralid7  moralid8  moralid9  mcoop1  mcoop2  mcoop3  mcoop4  mcoop5  mcoop6  mcoop7  narc1  narc2  narc3  narc4  narc5  narc6  nidentity1  nidentity2  omind1  omind5  omind6  contact2  contact3  contact4  contact5  hygiene1  hygiene2  hygiene3  hygiene4  hygiene5  psupport1  psupport2  psupport3  psupport4  psupport5  happy  slf_ladder  riskperc2  self_esteem  sbelong1  sbelong2  sbelong3  sbelong4  tested_positive  optim1  optim2  slfcont1  slfcont2  slfcont3  slfcont4
##   7   2  children  cnarc1  cnarc2  cnarc3  CRT1  CRT2  CRT3  ladder  marital1  mor_circle  moralid1  moralid10  moralid2  moralid3  moralid4  moralid5  moralid6  moralid7  moralid8  moralid9  mcoop1  mcoop2  mcoop3  mcoop4  mcoop5  mcoop6  mcoop7  narc1  narc2  narc3  narc4  narc5  narc6  nidentity1  nidentity2  omind1  omind5  omind6  contact2  contact3  contact4  contact5  hygiene1  hygiene2  hygiene3  hygiene4  hygiene5  psupport1  psupport2  psupport3  psupport4  psupport5  happy  slf_ladder  riskperc2  self_esteem  sbelong1  sbelong2  sbelong3  sbelong4  tested_positive  optim1  optim2  slfcont1  slfcont2  slfcont3  slfcont4
##   7   3  children  cnarc1  cnarc2  cnarc3  CRT1  CRT2  CRT3  ladder  marital1  mor_circle  moralid1  moralid10  moralid2  moralid3  moralid4  moralid5  moralid6  moralid7  moralid8  moralid9  mcoop1  mcoop2  mcoop3  mcoop4  mcoop5  mcoop6  mcoop7  narc1  narc2  narc3  narc4  narc5  narc6  nidentity1  nidentity2  omind1  omind5  omind6  contact2  contact3  contact4  contact5  hygiene1  hygiene2  hygiene3  hygiene4  hygiene5  psupport1  psupport2  psupport3  psupport4  psupport5  happy  slf_ladder  riskperc2  self_esteem  sbelong1  sbelong2  sbelong3  sbelong4  tested_positive  optim1  optim2  slfcont1  slfcont2  slfcont3  slfcont4
##   7   4  children  cnarc1  cnarc2  cnarc3  CRT1  CRT2  CRT3  ladder  marital1  mor_circle  moralid1  moralid10  moralid2  moralid3  moralid4  moralid5  moralid6  moralid7  moralid8  moralid9  mcoop1  mcoop2  mcoop3  mcoop4  mcoop5  mcoop6  mcoop7  narc1  narc2  narc3  narc4  narc5  narc6  nidentity1  nidentity2  omind1  omind5  omind6  contact2  contact3  contact4  contact5  hygiene1  hygiene2  hygiene3  hygiene4  hygiene5  psupport1  psupport2  psupport3  psupport4  psupport5  happy  slf_ladder  riskperc2  self_esteem  sbelong1  sbelong2  sbelong3  sbelong4  tested_positive  optim1  optim2  slfcont1  slfcont2  slfcont3  slfcont4
##   7   5  children  cnarc1  cnarc2  cnarc3  CRT1  CRT2  CRT3  ladder  marital1  mor_circle  moralid1  moralid10  moralid2  moralid3  moralid4  moralid5  moralid6  moralid7  moralid8  moralid9  mcoop1  mcoop2  mcoop3  mcoop4  mcoop5  mcoop6  mcoop7  narc1  narc2  narc3  narc4  narc5  narc6  nidentity1  nidentity2  omind1  omind5  omind6  contact2  contact3  contact4  contact5  hygiene1  hygiene2  hygiene3  hygiene4  hygiene5  psupport1  psupport2  psupport3  psupport4  psupport5  happy  slf_ladder  riskperc2  self_esteem  sbelong1  sbelong2  sbelong3  sbelong4  tested_positive  optim1  optim2  slfcont1  slfcont2  slfcont3  slfcont4
##   7   6  children  cnarc1  cnarc2  cnarc3  CRT1  CRT2  CRT3  ladder  marital1  mor_circle  moralid1  moralid10  moralid2  moralid3  moralid4  moralid5  moralid6  moralid7  moralid8  moralid9  mcoop1  mcoop2  mcoop3  mcoop4  mcoop5  mcoop6  mcoop7  narc1  narc2  narc3  narc4  narc5  narc6  nidentity1  nidentity2  omind1  omind5  omind6  contact2  contact3  contact4  contact5  hygiene1  hygiene2  hygiene3  hygiene4  hygiene5  psupport1  psupport2  psupport3  psupport4  psupport5  happy  slf_ladder  riskperc2  self_esteem  sbelong1  sbelong2  sbelong3  sbelong4  tested_positive  optim1  optim2  slfcont1  slfcont2  slfcont3  slfcont4
##   7   7  children  cnarc1  cnarc2  cnarc3  CRT1  CRT2  CRT3  ladder  marital1  mor_circle  moralid1  moralid10  moralid2  moralid3  moralid4  moralid5  moralid6  moralid7  moralid8  moralid9  mcoop1  mcoop2  mcoop3  mcoop4  mcoop5  mcoop6  mcoop7  narc1  narc2  narc3  narc4  narc5  narc6  nidentity1  nidentity2  omind1  omind5  omind6  contact2  contact3  contact4  contact5  hygiene1  hygiene2  hygiene3  hygiene4  hygiene5  psupport1  psupport2  psupport3  psupport4  psupport5  happy  slf_ladder  riskperc2  self_esteem  sbelong1  sbelong2  sbelong3  sbelong4  tested_positive  optim1  optim2  slfcont1  slfcont2  slfcont3  slfcont4
##   7   8  children  cnarc1  cnarc2  cnarc3  CRT1  CRT2  CRT3  ladder  marital1  mor_circle  moralid1  moralid10  moralid2  moralid3  moralid4  moralid5  moralid6  moralid7  moralid8  moralid9  mcoop1  mcoop2  mcoop3  mcoop4  mcoop5  mcoop6  mcoop7  narc1  narc2  narc3  narc4  narc5  narc6  nidentity1  nidentity2  omind1  omind5  omind6  contact2  contact3  contact4  contact5  hygiene1  hygiene2  hygiene3  hygiene4  hygiene5  psupport1  psupport2  psupport3  psupport4  psupport5  happy  slf_ladder  riskperc2  self_esteem  sbelong1  sbelong2  sbelong3  sbelong4  tested_positive  optim1  optim2  slfcont1  slfcont2  slfcont3  slfcont4
##   7   9  children  cnarc1  cnarc2  cnarc3  CRT1  CRT2  CRT3  ladder  marital1  mor_circle  moralid1  moralid10  moralid2  moralid3  moralid4  moralid5  moralid6  moralid7  moralid8  moralid9  mcoop1  mcoop2  mcoop3  mcoop4  mcoop5  mcoop6  mcoop7  narc1  narc2  narc3  narc4  narc5  narc6  nidentity1  nidentity2  omind1  omind5  omind6  contact2  contact3  contact4  contact5  hygiene1  hygiene2  hygiene3  hygiene4  hygiene5  psupport1  psupport2  psupport3  psupport4  psupport5  happy  slf_ladder  riskperc2  self_esteem  sbelong1  sbelong2  sbelong3  sbelong4  tested_positive  optim1  optim2  slfcont1  slfcont2  slfcont3  slfcont4
##   7   10  children  cnarc1  cnarc2  cnarc3  CRT1  CRT2  CRT3  ladder  marital1  mor_circle  moralid1  moralid10  moralid2  moralid3  moralid4  moralid5  moralid6  moralid7  moralid8  moralid9  mcoop1  mcoop2  mcoop3  mcoop4  mcoop5  mcoop6  mcoop7  narc1  narc2  narc3  narc4  narc5  narc6  nidentity1  nidentity2  omind1  omind5  omind6  contact2  contact3  contact4  contact5  hygiene1  hygiene2  hygiene3  hygiene4  hygiene5  psupport1  psupport2  psupport3  psupport4  psupport5  happy  slf_ladder  riskperc2  self_esteem  sbelong1  sbelong2  sbelong3  sbelong4  tested_positive  optim1  optim2  slfcont1  slfcont2  slfcont3  slfcont4
##   8   1  children  cnarc1  cnarc2  cnarc3  CRT1  CRT2  CRT3  ladder  marital1  mor_circle  moralid1  moralid10  moralid2  moralid3  moralid4  moralid5  moralid6  moralid7  moralid8  moralid9  mcoop1  mcoop2  mcoop3  mcoop4  mcoop5  mcoop6  mcoop7  narc1  narc2  narc3  narc4  narc5  narc6  nidentity1  nidentity2  omind1  omind5  omind6  contact2  contact3  contact4  contact5  hygiene1  hygiene2  hygiene3  hygiene4  hygiene5  psupport1  psupport2  psupport3  psupport4  psupport5  happy  slf_ladder  riskperc2  self_esteem  sbelong1  sbelong2  sbelong3  sbelong4  tested_positive  optim1  optim2  slfcont1  slfcont2  slfcont3  slfcont4
##   8   2  children  cnarc1  cnarc2  cnarc3  CRT1  CRT2  CRT3  ladder  marital1  mor_circle  moralid1  moralid10  moralid2  moralid3  moralid4  moralid5  moralid6  moralid7  moralid8  moralid9  mcoop1  mcoop2  mcoop3  mcoop4  mcoop5  mcoop6  mcoop7  narc1  narc2  narc3  narc4  narc5  narc6  nidentity1  nidentity2  omind1  omind5  omind6  contact2  contact3  contact4  contact5  hygiene1  hygiene2  hygiene3  hygiene4  hygiene5  psupport1  psupport2  psupport3  psupport4  psupport5  happy  slf_ladder  riskperc2  self_esteem  sbelong1  sbelong2  sbelong3  sbelong4  tested_positive  optim1  optim2  slfcont1  slfcont2  slfcont3  slfcont4
##   8   3  children  cnarc1  cnarc2  cnarc3  CRT1  CRT2  CRT3  ladder  marital1  mor_circle  moralid1  moralid10  moralid2  moralid3  moralid4  moralid5  moralid6  moralid7  moralid8  moralid9  mcoop1  mcoop2  mcoop3  mcoop4  mcoop5  mcoop6  mcoop7  narc1  narc2  narc3  narc4  narc5  narc6  nidentity1  nidentity2  omind1  omind5  omind6  contact2  contact3  contact4  contact5  hygiene1  hygiene2  hygiene3  hygiene4  hygiene5  psupport1  psupport2  psupport3  psupport4  psupport5  happy  slf_ladder  riskperc2  self_esteem  sbelong1  sbelong2  sbelong3  sbelong4  tested_positive  optim1  optim2  slfcont1  slfcont2  slfcont3  slfcont4
##   8   4  children  cnarc1  cnarc2  cnarc3  CRT1  CRT2  CRT3  ladder  marital1  mor_circle  moralid1  moralid10  moralid2  moralid3  moralid4  moralid5  moralid6  moralid7  moralid8  moralid9  mcoop1  mcoop2  mcoop3  mcoop4  mcoop5  mcoop6  mcoop7  narc1  narc2  narc3  narc4  narc5  narc6  nidentity1  nidentity2  omind1  omind5  omind6  contact2  contact3  contact4  contact5  hygiene1  hygiene2  hygiene3  hygiene4  hygiene5  psupport1  psupport2  psupport3  psupport4  psupport5  happy  slf_ladder  riskperc2  self_esteem  sbelong1  sbelong2  sbelong3  sbelong4  tested_positive  optim1  optim2  slfcont1  slfcont2  slfcont3  slfcont4
##   8   5  children  cnarc1  cnarc2  cnarc3  CRT1  CRT2  CRT3  ladder  marital1  mor_circle  moralid1  moralid10  moralid2  moralid3  moralid4  moralid5  moralid6  moralid7  moralid8  moralid9  mcoop1  mcoop2  mcoop3  mcoop4  mcoop5  mcoop6  mcoop7  narc1  narc2  narc3  narc4  narc5  narc6  nidentity1  nidentity2  omind1  omind5  omind6  contact2  contact3  contact4  contact5  hygiene1  hygiene2  hygiene3  hygiene4  hygiene5  psupport1  psupport2  psupport3  psupport4  psupport5  happy  slf_ladder  riskperc2  self_esteem  sbelong1  sbelong2  sbelong3  sbelong4  tested_positive  optim1  optim2  slfcont1  slfcont2  slfcont3  slfcont4
##   8   6  children  cnarc1  cnarc2  cnarc3  CRT1  CRT2  CRT3  ladder  marital1  mor_circle  moralid1  moralid10  moralid2  moralid3  moralid4  moralid5  moralid6  moralid7  moralid8  moralid9  mcoop1  mcoop2  mcoop3  mcoop4  mcoop5  mcoop6  mcoop7  narc1  narc2  narc3  narc4  narc5  narc6  nidentity1  nidentity2  omind1  omind5  omind6  contact2  contact3  contact4  contact5  hygiene1  hygiene2  hygiene3  hygiene4  hygiene5  psupport1  psupport2  psupport3  psupport4  psupport5  happy  slf_ladder  riskperc2  self_esteem  sbelong1  sbelong2  sbelong3  sbelong4  tested_positive  optim1  optim2  slfcont1  slfcont2  slfcont3  slfcont4
##   8   7  children  cnarc1  cnarc2  cnarc3  CRT1  CRT2  CRT3  ladder  marital1  mor_circle  moralid1  moralid10  moralid2  moralid3  moralid4  moralid5  moralid6  moralid7  moralid8  moralid9  mcoop1  mcoop2  mcoop3  mcoop4  mcoop5  mcoop6  mcoop7  narc1  narc2  narc3  narc4  narc5  narc6  nidentity1  nidentity2  omind1  omind5  omind6  contact2  contact3  contact4  contact5  hygiene1  hygiene2  hygiene3  hygiene4  hygiene5  psupport1  psupport2  psupport3  psupport4  psupport5  happy  slf_ladder  riskperc2  self_esteem  sbelong1  sbelong2  sbelong3  sbelong4  tested_positive  optim1  optim2  slfcont1  slfcont2  slfcont3  slfcont4
##   8   8  children  cnarc1  cnarc2  cnarc3  CRT1  CRT2  CRT3  ladder  marital1  mor_circle  moralid1  moralid10  moralid2  moralid3  moralid4  moralid5  moralid6  moralid7  moralid8  moralid9  mcoop1  mcoop2  mcoop3  mcoop4  mcoop5  mcoop6  mcoop7  narc1  narc2  narc3  narc4  narc5  narc6  nidentity1  nidentity2  omind1  omind5  omind6  contact2  contact3  contact4  contact5  hygiene1  hygiene2  hygiene3  hygiene4  hygiene5  psupport1  psupport2  psupport3  psupport4  psupport5  happy  slf_ladder  riskperc2  self_esteem  sbelong1  sbelong2  sbelong3  sbelong4  tested_positive  optim1  optim2  slfcont1  slfcont2  slfcont3  slfcont4
##   8   9  children  cnarc1  cnarc2  cnarc3  CRT1  CRT2  CRT3  ladder  marital1  mor_circle  moralid1  moralid10  moralid2  moralid3  moralid4  moralid5  moralid6  moralid7  moralid8  moralid9  mcoop1  mcoop2  mcoop3  mcoop4  mcoop5  mcoop6  mcoop7  narc1  narc2  narc3  narc4  narc5  narc6  nidentity1  nidentity2  omind1  omind5  omind6  contact2  contact3  contact4  contact5  hygiene1  hygiene2  hygiene3  hygiene4  hygiene5  psupport1  psupport2  psupport3  psupport4  psupport5  happy  slf_ladder  riskperc2  self_esteem  sbelong1  sbelong2  sbelong3  sbelong4  tested_positive  optim1  optim2  slfcont1  slfcont2  slfcont3  slfcont4
##   8   10  children  cnarc1  cnarc2  cnarc3  CRT1  CRT2  CRT3  ladder  marital1  mor_circle  moralid1  moralid10  moralid2  moralid3  moralid4  moralid5  moralid6  moralid7  moralid8  moralid9  mcoop1  mcoop2  mcoop3  mcoop4  mcoop5  mcoop6  mcoop7  narc1  narc2  narc3  narc4  narc5  narc6  nidentity1  nidentity2  omind1  omind5  omind6  contact2  contact3  contact4  contact5  hygiene1  hygiene2  hygiene3  hygiene4  hygiene5  psupport1  psupport2  psupport3  psupport4  psupport5  happy  slf_ladder  riskperc2  self_esteem  sbelong1  sbelong2  sbelong3  sbelong4  tested_positive  optim1  optim2  slfcont1  slfcont2  slfcont3  slfcont4
##   9   1  children  cnarc1  cnarc2  cnarc3  CRT1  CRT2  CRT3  ladder  marital1  mor_circle  moralid1  moralid10  moralid2  moralid3  moralid4  moralid5  moralid6  moralid7  moralid8  moralid9  mcoop1  mcoop2  mcoop3  mcoop4  mcoop5  mcoop6  mcoop7  narc1  narc2  narc3  narc4  narc5  narc6  nidentity1  nidentity2  omind1  omind5  omind6  contact2  contact3  contact4  contact5  hygiene1  hygiene2  hygiene3  hygiene4  hygiene5  psupport1  psupport2  psupport3  psupport4  psupport5  happy  slf_ladder  riskperc2  self_esteem  sbelong1  sbelong2  sbelong3  sbelong4  tested_positive  optim1  optim2  slfcont1  slfcont2  slfcont3  slfcont4
##   9   2  children  cnarc1  cnarc2  cnarc3  CRT1  CRT2  CRT3  ladder  marital1  mor_circle  moralid1  moralid10  moralid2  moralid3  moralid4  moralid5  moralid6  moralid7  moralid8  moralid9  mcoop1  mcoop2  mcoop3  mcoop4  mcoop5  mcoop6  mcoop7  narc1  narc2  narc3  narc4  narc5  narc6  nidentity1  nidentity2  omind1  omind5  omind6  contact2  contact3  contact4  contact5  hygiene1  hygiene2  hygiene3  hygiene4  hygiene5  psupport1  psupport2  psupport3  psupport4  psupport5  happy  slf_ladder  riskperc2  self_esteem  sbelong1  sbelong2  sbelong3  sbelong4  tested_positive  optim1  optim2  slfcont1  slfcont2  slfcont3  slfcont4
##   9   3  children  cnarc1  cnarc2  cnarc3  CRT1  CRT2  CRT3  ladder  marital1  mor_circle  moralid1  moralid10  moralid2  moralid3  moralid4  moralid5  moralid6  moralid7  moralid8  moralid9  mcoop1  mcoop2  mcoop3  mcoop4  mcoop5  mcoop6  mcoop7  narc1  narc2  narc3  narc4  narc5  narc6  nidentity1  nidentity2  omind1  omind5  omind6  contact2  contact3  contact4  contact5  hygiene1  hygiene2  hygiene3  hygiene4  hygiene5  psupport1  psupport2  psupport3  psupport4  psupport5  happy  slf_ladder  riskperc2  self_esteem  sbelong1  sbelong2  sbelong3  sbelong4  tested_positive  optim1  optim2  slfcont1  slfcont2  slfcont3  slfcont4
##   9   4  children  cnarc1  cnarc2  cnarc3  CRT1  CRT2  CRT3  ladder  marital1  mor_circle  moralid1  moralid10  moralid2  moralid3  moralid4  moralid5  moralid6  moralid7  moralid8  moralid9  mcoop1  mcoop2  mcoop3  mcoop4  mcoop5  mcoop6  mcoop7  narc1  narc2  narc3  narc4  narc5  narc6  nidentity1  nidentity2  omind1  omind5  omind6  contact2  contact3  contact4  contact5  hygiene1  hygiene2  hygiene3  hygiene4  hygiene5  psupport1  psupport2  psupport3  psupport4  psupport5  happy  slf_ladder  riskperc2  self_esteem  sbelong1  sbelong2  sbelong3  sbelong4  tested_positive  optim1  optim2  slfcont1  slfcont2  slfcont3  slfcont4
##   9   5  children  cnarc1  cnarc2  cnarc3  CRT1  CRT2  CRT3  ladder  marital1  mor_circle  moralid1  moralid10  moralid2  moralid3  moralid4  moralid5  moralid6  moralid7  moralid8  moralid9  mcoop1  mcoop2  mcoop3  mcoop4  mcoop5  mcoop6  mcoop7  narc1  narc2  narc3  narc4  narc5  narc6  nidentity1  nidentity2  omind1  omind5  omind6  contact2  contact3  contact4  contact5  hygiene1  hygiene2  hygiene3  hygiene4  hygiene5  psupport1  psupport2  psupport3  psupport4  psupport5  happy  slf_ladder  riskperc2  self_esteem  sbelong1  sbelong2  sbelong3  sbelong4  tested_positive  optim1  optim2  slfcont1  slfcont2  slfcont3  slfcont4
##   9   6  children  cnarc1  cnarc2  cnarc3  CRT1  CRT2  CRT3  ladder  marital1  mor_circle  moralid1  moralid10  moralid2  moralid3  moralid4  moralid5  moralid6  moralid7  moralid8  moralid9  mcoop1  mcoop2  mcoop3  mcoop4  mcoop5  mcoop6  mcoop7  narc1  narc2  narc3  narc4  narc5  narc6  nidentity1  nidentity2  omind1  omind5  omind6  contact2  contact3  contact4  contact5  hygiene1  hygiene2  hygiene3  hygiene4  hygiene5  psupport1  psupport2  psupport3  psupport4  psupport5  happy  slf_ladder  riskperc2  self_esteem  sbelong1  sbelong2  sbelong3  sbelong4  tested_positive  optim1  optim2  slfcont1  slfcont2  slfcont3  slfcont4
##   9   7  children  cnarc1  cnarc2  cnarc3  CRT1  CRT2  CRT3  ladder  marital1  mor_circle  moralid1  moralid10  moralid2  moralid3  moralid4  moralid5  moralid6  moralid7  moralid8  moralid9  mcoop1  mcoop2  mcoop3  mcoop4  mcoop5  mcoop6  mcoop7  narc1  narc2  narc3  narc4  narc5  narc6  nidentity1  nidentity2  omind1  omind5  omind6  contact2  contact3  contact4  contact5  hygiene1  hygiene2  hygiene3  hygiene4  hygiene5  psupport1  psupport2  psupport3  psupport4  psupport5  happy  slf_ladder  riskperc2  self_esteem  sbelong1  sbelong2  sbelong3  sbelong4  tested_positive  optim1  optim2  slfcont1  slfcont2  slfcont3  slfcont4
##   9   8  children  cnarc1  cnarc2  cnarc3  CRT1  CRT2  CRT3  ladder  marital1  mor_circle  moralid1  moralid10  moralid2  moralid3  moralid4  moralid5  moralid6  moralid7  moralid8  moralid9  mcoop1  mcoop2  mcoop3  mcoop4  mcoop5  mcoop6  mcoop7  narc1  narc2  narc3  narc4  narc5  narc6  nidentity1  nidentity2  omind1  omind5  omind6  contact2  contact3  contact4  contact5  hygiene1  hygiene2  hygiene3  hygiene4  hygiene5  psupport1  psupport2  psupport3  psupport4  psupport5  happy  slf_ladder  riskperc2  self_esteem  sbelong1  sbelong2  sbelong3  sbelong4  tested_positive  optim1  optim2  slfcont1  slfcont2  slfcont3  slfcont4
##   9   9  children  cnarc1  cnarc2  cnarc3  CRT1  CRT2  CRT3  ladder  marital1  mor_circle  moralid1  moralid10  moralid2  moralid3  moralid4  moralid5  moralid6  moralid7  moralid8  moralid9  mcoop1  mcoop2  mcoop3  mcoop4  mcoop5  mcoop6  mcoop7  narc1  narc2  narc3  narc4  narc5  narc6  nidentity1  nidentity2  omind1  omind5  omind6  contact2  contact3  contact4  contact5  hygiene1  hygiene2  hygiene3  hygiene4  hygiene5  psupport1  psupport2  psupport3  psupport4  psupport5  happy  slf_ladder  riskperc2  self_esteem  sbelong1  sbelong2  sbelong3  sbelong4  tested_positive  optim1  optim2  slfcont1  slfcont2  slfcont3  slfcont4
##   9   10  children  cnarc1  cnarc2  cnarc3  CRT1  CRT2  CRT3  ladder  marital1  mor_circle  moralid1  moralid10  moralid2  moralid3  moralid4  moralid5  moralid6  moralid7  moralid8  moralid9  mcoop1  mcoop2  mcoop3  mcoop4  mcoop5  mcoop6  mcoop7  narc1  narc2  narc3  narc4  narc5  narc6  nidentity1  nidentity2  omind1  omind5  omind6  contact2  contact3  contact4  contact5  hygiene1  hygiene2  hygiene3  hygiene4  hygiene5  psupport1  psupport2  psupport3  psupport4  psupport5  happy  slf_ladder  riskperc2  self_esteem  sbelong1  sbelong2  sbelong3  sbelong4  tested_positive  optim1  optim2  slfcont1  slfcont2  slfcont3  slfcont4
##   10   1  children  cnarc1  cnarc2  cnarc3  CRT1  CRT2  CRT3  ladder  marital1  mor_circle  moralid1  moralid10  moralid2  moralid3  moralid4  moralid5  moralid6  moralid7  moralid8  moralid9  mcoop1  mcoop2  mcoop3  mcoop4  mcoop5  mcoop6  mcoop7  narc1  narc2  narc3  narc4  narc5  narc6  nidentity1  nidentity2  omind1  omind5  omind6  contact2  contact3  contact4  contact5  hygiene1  hygiene2  hygiene3  hygiene4  hygiene5  psupport1  psupport2  psupport3  psupport4  psupport5  happy  slf_ladder  riskperc2  self_esteem  sbelong1  sbelong2  sbelong3  sbelong4  tested_positive  optim1  optim2  slfcont1  slfcont2  slfcont3  slfcont4
##   10   2  children  cnarc1  cnarc2  cnarc3  CRT1  CRT2  CRT3  ladder  marital1  mor_circle  moralid1  moralid10  moralid2  moralid3  moralid4  moralid5  moralid6  moralid7  moralid8  moralid9  mcoop1  mcoop2  mcoop3  mcoop4  mcoop5  mcoop6  mcoop7  narc1  narc2  narc3  narc4  narc5  narc6  nidentity1  nidentity2  omind1  omind5  omind6  contact2  contact3  contact4  contact5  hygiene1  hygiene2  hygiene3  hygiene4  hygiene5  psupport1  psupport2  psupport3  psupport4  psupport5  happy  slf_ladder  riskperc2  self_esteem  sbelong1  sbelong2  sbelong3  sbelong4  tested_positive  optim1  optim2  slfcont1  slfcont2  slfcont3  slfcont4
##   10   3  children  cnarc1  cnarc2  cnarc3  CRT1  CRT2  CRT3  ladder  marital1  mor_circle  moralid1  moralid10  moralid2  moralid3  moralid4  moralid5  moralid6  moralid7  moralid8  moralid9  mcoop1  mcoop2  mcoop3  mcoop4  mcoop5  mcoop6  mcoop7  narc1  narc2  narc3  narc4  narc5  narc6  nidentity1  nidentity2  omind1  omind5  omind6  contact2  contact3  contact4  contact5  hygiene1  hygiene2  hygiene3  hygiene4  hygiene5  psupport1  psupport2  psupport3  psupport4  psupport5  happy  slf_ladder  riskperc2  self_esteem  sbelong1  sbelong2  sbelong3  sbelong4  tested_positive  optim1  optim2  slfcont1  slfcont2  slfcont3  slfcont4
##   10   4  children  cnarc1  cnarc2  cnarc3  CRT1  CRT2  CRT3  ladder  marital1  mor_circle  moralid1  moralid10  moralid2  moralid3  moralid4  moralid5  moralid6  moralid7  moralid8  moralid9  mcoop1  mcoop2  mcoop3  mcoop4  mcoop5  mcoop6  mcoop7  narc1  narc2  narc3  narc4  narc5  narc6  nidentity1  nidentity2  omind1  omind5  omind6  contact2  contact3  contact4  contact5  hygiene1  hygiene2  hygiene3  hygiene4  hygiene5  psupport1  psupport2  psupport3  psupport4  psupport5  happy  slf_ladder  riskperc2  self_esteem  sbelong1  sbelong2  sbelong3  sbelong4  tested_positive  optim1  optim2  slfcont1  slfcont2  slfcont3  slfcont4
##   10   5  children  cnarc1  cnarc2  cnarc3  CRT1  CRT2  CRT3  ladder  marital1  mor_circle  moralid1  moralid10  moralid2  moralid3  moralid4  moralid5  moralid6  moralid7  moralid8  moralid9  mcoop1  mcoop2  mcoop3  mcoop4  mcoop5  mcoop6  mcoop7  narc1  narc2  narc3  narc4  narc5  narc6  nidentity1  nidentity2  omind1  omind5  omind6  contact2  contact3  contact4  contact5  hygiene1  hygiene2  hygiene3  hygiene4  hygiene5  psupport1  psupport2  psupport3  psupport4  psupport5  happy  slf_ladder  riskperc2  self_esteem  sbelong1  sbelong2  sbelong3  sbelong4  tested_positive  optim1  optim2  slfcont1  slfcont2  slfcont3  slfcont4
##   10   6  children  cnarc1  cnarc2  cnarc3  CRT1  CRT2  CRT3  ladder  marital1  mor_circle  moralid1  moralid10  moralid2  moralid3  moralid4  moralid5  moralid6  moralid7  moralid8  moralid9  mcoop1  mcoop2  mcoop3  mcoop4  mcoop5  mcoop6  mcoop7  narc1  narc2  narc3  narc4  narc5  narc6  nidentity1  nidentity2  omind1  omind5  omind6  contact2  contact3  contact4  contact5  hygiene1  hygiene2  hygiene3  hygiene4  hygiene5  psupport1  psupport2  psupport3  psupport4  psupport5  happy  slf_ladder  riskperc2  self_esteem  sbelong1  sbelong2  sbelong3  sbelong4  tested_positive  optim1  optim2  slfcont1  slfcont2  slfcont3  slfcont4
##   10   7  children  cnarc1  cnarc2  cnarc3  CRT1  CRT2  CRT3  ladder  marital1  mor_circle  moralid1  moralid10  moralid2  moralid3  moralid4  moralid5  moralid6  moralid7  moralid8  moralid9  mcoop1  mcoop2  mcoop3  mcoop4  mcoop5  mcoop6  mcoop7  narc1  narc2  narc3  narc4  narc5  narc6  nidentity1  nidentity2  omind1  omind5  omind6  contact2  contact3  contact4  contact5  hygiene1  hygiene2  hygiene3  hygiene4  hygiene5  psupport1  psupport2  psupport3  psupport4  psupport5  happy  slf_ladder  riskperc2  self_esteem  sbelong1  sbelong2  sbelong3  sbelong4  tested_positive  optim1  optim2  slfcont1  slfcont2  slfcont3  slfcont4
##   10   8  children  cnarc1  cnarc2  cnarc3  CRT1  CRT2  CRT3  ladder  marital1  mor_circle  moralid1  moralid10  moralid2  moralid3  moralid4  moralid5  moralid6  moralid7  moralid8  moralid9  mcoop1  mcoop2  mcoop3  mcoop4  mcoop5  mcoop6  mcoop7  narc1  narc2  narc3  narc4  narc5  narc6  nidentity1  nidentity2  omind1  omind5  omind6  contact2  contact3  contact4  contact5  hygiene1  hygiene2  hygiene3  hygiene4  hygiene5  psupport1  psupport2  psupport3  psupport4  psupport5  happy  slf_ladder  riskperc2  self_esteem  sbelong1  sbelong2  sbelong3  sbelong4  tested_positive  optim1  optim2  slfcont1  slfcont2  slfcont3  slfcont4
##   10   9  children  cnarc1  cnarc2  cnarc3  CRT1  CRT2  CRT3  ladder  marital1  mor_circle  moralid1  moralid10  moralid2  moralid3  moralid4  moralid5  moralid6  moralid7  moralid8  moralid9  mcoop1  mcoop2  mcoop3  mcoop4  mcoop5  mcoop6  mcoop7  narc1  narc2  narc3  narc4  narc5  narc6  nidentity1  nidentity2  omind1  omind5  omind6  contact2  contact3  contact4  contact5  hygiene1  hygiene2  hygiene3  hygiene4  hygiene5  psupport1  psupport2  psupport3  psupport4  psupport5  happy  slf_ladder  riskperc2  self_esteem  sbelong1  sbelong2  sbelong3  sbelong4  tested_positive  optim1  optim2  slfcont1  slfcont2  slfcont3  slfcont4
##   10   10  children  cnarc1  cnarc2  cnarc3  CRT1  CRT2  CRT3  ladder  marital1  mor_circle  moralid1  moralid10  moralid2  moralid3  moralid4  moralid5  moralid6  moralid7  moralid8  moralid9  mcoop1  mcoop2  mcoop3  mcoop4  mcoop5  mcoop6  mcoop7  narc1  narc2  narc3  narc4  narc5  narc6  nidentity1  nidentity2  omind1  omind5  omind6  contact2  contact3  contact4  contact5  hygiene1  hygiene2  hygiene3  hygiene4  hygiene5  psupport1  psupport2  psupport3  psupport4  psupport5  happy  slf_ladder  riskperc2  self_esteem  sbelong1  sbelong2  sbelong3  sbelong4  tested_positive  optim1  optim2  slfcont1  slfcont2  slfcont3  slfcont4
```

```
## Warning: Number of logged events: 6703
```

```
## 
##  iter imp variable
##   1   1
##   1   2
##   1   3
##   1   4
##   1   5
##   1   6
##   1   7
##   1   8
##   1   9
##   1   10
##   2   1
##   2   2
##   2   3
##   2   4
##   2   5
##   2   6
##   2   7
##   2   8
##   2   9
##   2   10
##   3   1
##   3   2
##   3   3
##   3   4
##   3   5
##   3   6
##   3   7
##   3   8
##   3   9
##   3   10
##   4   1
##   4   2
##   4   3
##   4   4
##   4   5
##   4   6
##   4   7
##   4   8
##   4   9
##   4   10
##   5   1
##   5   2
##   5   3
##   5   4
##   5   5
##   5   6
##   5   7
##   5   8
##   5   9
##   5   10
##   6   1
##   6   2
##   6   3
##   6   4
##   6   5
##   6   6
##   6   7
##   6   8
##   6   9
##   6   10
##   7   1
##   7   2
##   7   3
##   7   4
##   7   5
##   7   6
##   7   7
##   7   8
##   7   9
##   7   10
##   8   1
##   8   2
##   8   3
##   8   4
##   8   5
##   8   6
##   8   7
##   8   8
##   8   9
##   8   10
##   9   1
##   9   2
##   9   3
##   9   4
##   9   5
##   9   6
##   9   7
##   9   8
##   9   9
##   9   10
##   10   1
##   10   2
##   10   3
##   10   4
##   10   5
##   10   6
##   10   7
##   10   8
##   10   9
##   10   10
```

```
## Warning: Number of logged events: 3
```

```
## 
##  iter imp variable
##   1   1  cnarc1  cnarc2  cnarc3  ctheory1  ctheory2  ctheory3  ctheory4  CRT1  CRT2  CRT3  health_cond  marital1  mor_circle  moralid1  moralid10  moralid2  moralid3  moralid4  moralid5  moralid6  moralid7  moralid8  moralid9  mcoop1  mcoop2  mcoop3  mcoop4  mcoop5  mcoop6  mcoop7  narc1  narc2  narc3  narc4  narc5  narc6  nidentity1  nidentity2  omind1  omind2  omind3  omind4  omind5  omind6  contact1  contact2  contact3  contact4  contact5  hygiene1  hygiene2  hygiene3  hygiene4  hygiene5  psupport1  psupport2  psupport3  psupport4  psupport5  political_ideology  happy  slf_ladder  riskperc2  self_esteem  sbelong1  sbelong2  sbelong3  sbelong4  optim1  optim2  slfcont1  slfcont2  slfcont3  slfcont4
##   1   2  cnarc1  cnarc2  cnarc3  ctheory1  ctheory2  ctheory3  ctheory4  CRT1  CRT2  CRT3  health_cond  marital1  mor_circle  moralid1  moralid10  moralid2  moralid3  moralid4  moralid5  moralid6  moralid7  moralid8  moralid9  mcoop1  mcoop2  mcoop3  mcoop4  mcoop5  mcoop6  mcoop7  narc1  narc2  narc3  narc4  narc5  narc6  nidentity1  nidentity2  omind1  omind2  omind3  omind4  omind5  omind6  contact1  contact2  contact3  contact4  contact5  hygiene1  hygiene2  hygiene3  hygiene4  hygiene5  psupport1  psupport2  psupport3  psupport4  psupport5  political_ideology  happy  slf_ladder  riskperc2  self_esteem  sbelong1  sbelong2  sbelong3  sbelong4  optim1  optim2  slfcont1  slfcont2  slfcont3  slfcont4
##   1   3  cnarc1  cnarc2  cnarc3  ctheory1  ctheory2  ctheory3  ctheory4  CRT1  CRT2  CRT3  health_cond  marital1  mor_circle  moralid1  moralid10  moralid2  moralid3  moralid4  moralid5  moralid6  moralid7  moralid8  moralid9  mcoop1  mcoop2  mcoop3  mcoop4  mcoop5  mcoop6  mcoop7  narc1  narc2  narc3  narc4  narc5  narc6  nidentity1  nidentity2  omind1  omind2  omind3  omind4  omind5  omind6  contact1  contact2  contact3  contact4  contact5  hygiene1  hygiene2  hygiene3  hygiene4  hygiene5  psupport1  psupport2  psupport3  psupport4  psupport5  political_ideology  happy  slf_ladder  riskperc2  self_esteem  sbelong1  sbelong2  sbelong3  sbelong4  optim1  optim2  slfcont1  slfcont2  slfcont3  slfcont4
##   1   4  cnarc1  cnarc2  cnarc3  ctheory1  ctheory2  ctheory3  ctheory4  CRT1  CRT2  CRT3  health_cond  marital1  mor_circle  moralid1  moralid10  moralid2  moralid3  moralid4  moralid5  moralid6  moralid7  moralid8  moralid9  mcoop1  mcoop2  mcoop3  mcoop4  mcoop5  mcoop6  mcoop7  narc1  narc2  narc3  narc4  narc5  narc6  nidentity1  nidentity2  omind1  omind2  omind3  omind4  omind5  omind6  contact1  contact2  contact3  contact4  contact5  hygiene1  hygiene2  hygiene3  hygiene4  hygiene5  psupport1  psupport2  psupport3  psupport4  psupport5  political_ideology  happy  slf_ladder  riskperc2  self_esteem  sbelong1  sbelong2  sbelong3  sbelong4  optim1  optim2  slfcont1  slfcont2  slfcont3  slfcont4
##   1   5  cnarc1  cnarc2  cnarc3  ctheory1  ctheory2  ctheory3  ctheory4  CRT1  CRT2  CRT3  health_cond  marital1  mor_circle  moralid1  moralid10  moralid2  moralid3  moralid4  moralid5  moralid6  moralid7  moralid8  moralid9  mcoop1  mcoop2  mcoop3  mcoop4  mcoop5  mcoop6  mcoop7  narc1  narc2  narc3  narc4  narc5  narc6  nidentity1  nidentity2  omind1  omind2  omind3  omind4  omind5  omind6  contact1  contact2  contact3  contact4  contact5  hygiene1  hygiene2  hygiene3  hygiene4  hygiene5  psupport1  psupport2  psupport3  psupport4  psupport5  political_ideology  happy  slf_ladder  riskperc2  self_esteem  sbelong1  sbelong2  sbelong3  sbelong4  optim1  optim2  slfcont1  slfcont2  slfcont3  slfcont4
##   1   6  cnarc1  cnarc2  cnarc3  ctheory1  ctheory2  ctheory3  ctheory4  CRT1  CRT2  CRT3  health_cond  marital1  mor_circle  moralid1  moralid10  moralid2  moralid3  moralid4  moralid5  moralid6  moralid7  moralid8  moralid9  mcoop1  mcoop2  mcoop3  mcoop4  mcoop5  mcoop6  mcoop7  narc1  narc2  narc3  narc4  narc5  narc6  nidentity1  nidentity2  omind1  omind2  omind3  omind4  omind5  omind6  contact1  contact2  contact3  contact4  contact5  hygiene1  hygiene2  hygiene3  hygiene4  hygiene5  psupport1  psupport2  psupport3  psupport4  psupport5  political_ideology  happy  slf_ladder  riskperc2  self_esteem  sbelong1  sbelong2  sbelong3  sbelong4  optim1  optim2  slfcont1  slfcont2  slfcont3  slfcont4
##   1   7  cnarc1  cnarc2  cnarc3  ctheory1  ctheory2  ctheory3  ctheory4  CRT1  CRT2  CRT3  health_cond  marital1  mor_circle  moralid1  moralid10  moralid2  moralid3  moralid4  moralid5  moralid6  moralid7  moralid8  moralid9  mcoop1  mcoop2  mcoop3  mcoop4  mcoop5  mcoop6  mcoop7  narc1  narc2  narc3  narc4  narc5  narc6  nidentity1  nidentity2  omind1  omind2  omind3  omind4  omind5  omind6  contact1  contact2  contact3  contact4  contact5  hygiene1  hygiene2  hygiene3  hygiene4  hygiene5  psupport1  psupport2  psupport3  psupport4  psupport5  political_ideology  happy  slf_ladder  riskperc2  self_esteem  sbelong1  sbelong2  sbelong3  sbelong4  optim1  optim2  slfcont1  slfcont2  slfcont3  slfcont4
##   1   8  cnarc1  cnarc2  cnarc3  ctheory1  ctheory2  ctheory3  ctheory4  CRT1  CRT2  CRT3  health_cond  marital1  mor_circle  moralid1  moralid10  moralid2  moralid3  moralid4  moralid5  moralid6  moralid7  moralid8  moralid9  mcoop1  mcoop2  mcoop3  mcoop4  mcoop5  mcoop6  mcoop7  narc1  narc2  narc3  narc4  narc5  narc6  nidentity1  nidentity2  omind1  omind2  omind3  omind4  omind5  omind6  contact1  contact2  contact3  contact4  contact5  hygiene1  hygiene2  hygiene3  hygiene4  hygiene5  psupport1  psupport2  psupport3  psupport4  psupport5  political_ideology  happy  slf_ladder  riskperc2  self_esteem  sbelong1  sbelong2  sbelong3  sbelong4  optim1  optim2  slfcont1  slfcont2  slfcont3  slfcont4
##   1   9  cnarc1  cnarc2  cnarc3  ctheory1  ctheory2  ctheory3  ctheory4  CRT1  CRT2  CRT3  health_cond  marital1  mor_circle  moralid1  moralid10  moralid2  moralid3  moralid4  moralid5  moralid6  moralid7  moralid8  moralid9  mcoop1  mcoop2  mcoop3  mcoop4  mcoop5  mcoop6  mcoop7  narc1  narc2  narc3  narc4  narc5  narc6  nidentity1  nidentity2  omind1  omind2  omind3  omind4  omind5  omind6  contact1  contact2  contact3  contact4  contact5  hygiene1  hygiene2  hygiene3  hygiene4  hygiene5  psupport1  psupport2  psupport3  psupport4  psupport5  political_ideology  happy  slf_ladder  riskperc2  self_esteem  sbelong1  sbelong2  sbelong3  sbelong4  optim1  optim2  slfcont1  slfcont2  slfcont3  slfcont4
##   1   10  cnarc1  cnarc2  cnarc3  ctheory1  ctheory2  ctheory3  ctheory4  CRT1  CRT2  CRT3  health_cond  marital1  mor_circle  moralid1  moralid10  moralid2  moralid3  moralid4  moralid5  moralid6  moralid7  moralid8  moralid9  mcoop1  mcoop2  mcoop3  mcoop4  mcoop5  mcoop6  mcoop7  narc1  narc2  narc3  narc4  narc5  narc6  nidentity1  nidentity2  omind1  omind2  omind3  omind4  omind5  omind6  contact1  contact2  contact3  contact4  contact5  hygiene1  hygiene2  hygiene3  hygiene4  hygiene5  psupport1  psupport2  psupport3  psupport4  psupport5  political_ideology  happy  slf_ladder  riskperc2  self_esteem  sbelong1  sbelong2  sbelong3  sbelong4  optim1  optim2  slfcont1  slfcont2  slfcont3  slfcont4
##   2   1  cnarc1  cnarc2  cnarc3  ctheory1  ctheory2  ctheory3  ctheory4  CRT1  CRT2  CRT3  health_cond  marital1  mor_circle  moralid1  moralid10  moralid2  moralid3  moralid4  moralid5  moralid6  moralid7  moralid8  moralid9  mcoop1  mcoop2  mcoop3  mcoop4  mcoop5  mcoop6  mcoop7  narc1  narc2  narc3  narc4  narc5  narc6  nidentity1  nidentity2  omind1  omind2  omind3  omind4  omind5  omind6  contact1  contact2  contact3  contact4  contact5  hygiene1  hygiene2  hygiene3  hygiene4  hygiene5  psupport1  psupport2  psupport3  psupport4  psupport5  political_ideology  happy  slf_ladder  riskperc2  self_esteem  sbelong1  sbelong2  sbelong3  sbelong4  optim1  optim2  slfcont1  slfcont2  slfcont3  slfcont4
##   2   2  cnarc1  cnarc2  cnarc3  ctheory1  ctheory2  ctheory3  ctheory4  CRT1  CRT2  CRT3  health_cond  marital1  mor_circle  moralid1  moralid10  moralid2  moralid3  moralid4  moralid5  moralid6  moralid7  moralid8  moralid9  mcoop1  mcoop2  mcoop3  mcoop4  mcoop5  mcoop6  mcoop7  narc1  narc2  narc3  narc4  narc5  narc6  nidentity1  nidentity2  omind1  omind2  omind3  omind4  omind5  omind6  contact1  contact2  contact3  contact4  contact5  hygiene1  hygiene2  hygiene3  hygiene4  hygiene5  psupport1  psupport2  psupport3  psupport4  psupport5  political_ideology  happy  slf_ladder  riskperc2  self_esteem  sbelong1  sbelong2  sbelong3  sbelong4  optim1  optim2  slfcont1  slfcont2  slfcont3  slfcont4
##   2   3  cnarc1  cnarc2  cnarc3  ctheory1  ctheory2  ctheory3  ctheory4  CRT1  CRT2  CRT3  health_cond  marital1  mor_circle  moralid1  moralid10  moralid2  moralid3  moralid4  moralid5  moralid6  moralid7  moralid8  moralid9  mcoop1  mcoop2  mcoop3  mcoop4  mcoop5  mcoop6  mcoop7  narc1  narc2  narc3  narc4  narc5  narc6  nidentity1  nidentity2  omind1  omind2  omind3  omind4  omind5  omind6  contact1  contact2  contact3  contact4  contact5  hygiene1  hygiene2  hygiene3  hygiene4  hygiene5  psupport1  psupport2  psupport3  psupport4  psupport5  political_ideology  happy  slf_ladder  riskperc2  self_esteem  sbelong1  sbelong2  sbelong3  sbelong4  optim1  optim2  slfcont1  slfcont2  slfcont3  slfcont4
##   2   4  cnarc1  cnarc2  cnarc3  ctheory1  ctheory2  ctheory3  ctheory4  CRT1  CRT2  CRT3  health_cond  marital1  mor_circle  moralid1  moralid10  moralid2  moralid3  moralid4  moralid5  moralid6  moralid7  moralid8  moralid9  mcoop1  mcoop2  mcoop3  mcoop4  mcoop5  mcoop6  mcoop7  narc1  narc2  narc3  narc4  narc5  narc6  nidentity1  nidentity2  omind1  omind2  omind3  omind4  omind5  omind6  contact1  contact2  contact3  contact4  contact5  hygiene1  hygiene2  hygiene3  hygiene4  hygiene5  psupport1  psupport2  psupport3  psupport4  psupport5  political_ideology  happy  slf_ladder  riskperc2  self_esteem  sbelong1  sbelong2  sbelong3  sbelong4  optim1  optim2  slfcont1  slfcont2  slfcont3  slfcont4
##   2   5  cnarc1  cnarc2  cnarc3  ctheory1  ctheory2  ctheory3  ctheory4  CRT1  CRT2  CRT3  health_cond  marital1  mor_circle  moralid1  moralid10  moralid2  moralid3  moralid4  moralid5  moralid6  moralid7  moralid8  moralid9  mcoop1  mcoop2  mcoop3  mcoop4  mcoop5  mcoop6  mcoop7  narc1  narc2  narc3  narc4  narc5  narc6  nidentity1  nidentity2  omind1  omind2  omind3  omind4  omind5  omind6  contact1  contact2  contact3  contact4  contact5  hygiene1  hygiene2  hygiene3  hygiene4  hygiene5  psupport1  psupport2  psupport3  psupport4  psupport5  political_ideology  happy  slf_ladder  riskperc2  self_esteem  sbelong1  sbelong2  sbelong3  sbelong4  optim1  optim2  slfcont1  slfcont2  slfcont3  slfcont4
##   2   6  cnarc1  cnarc2  cnarc3  ctheory1  ctheory2  ctheory3  ctheory4  CRT1  CRT2  CRT3  health_cond  marital1  mor_circle  moralid1  moralid10  moralid2  moralid3  moralid4  moralid5  moralid6  moralid7  moralid8  moralid9  mcoop1  mcoop2  mcoop3  mcoop4  mcoop5  mcoop6  mcoop7  narc1  narc2  narc3  narc4  narc5  narc6  nidentity1  nidentity2  omind1  omind2  omind3  omind4  omind5  omind6  contact1  contact2  contact3  contact4  contact5  hygiene1  hygiene2  hygiene3  hygiene4  hygiene5  psupport1  psupport2  psupport3  psupport4  psupport5  political_ideology  happy  slf_ladder  riskperc2  self_esteem  sbelong1  sbelong2  sbelong3  sbelong4  optim1  optim2  slfcont1  slfcont2  slfcont3  slfcont4
##   2   7  cnarc1  cnarc2  cnarc3  ctheory1  ctheory2  ctheory3  ctheory4  CRT1  CRT2  CRT3  health_cond  marital1  mor_circle  moralid1  moralid10  moralid2  moralid3  moralid4  moralid5  moralid6  moralid7  moralid8  moralid9  mcoop1  mcoop2  mcoop3  mcoop4  mcoop5  mcoop6  mcoop7  narc1  narc2  narc3  narc4  narc5  narc6  nidentity1  nidentity2  omind1  omind2  omind3  omind4  omind5  omind6  contact1  contact2  contact3  contact4  contact5  hygiene1  hygiene2  hygiene3  hygiene4  hygiene5  psupport1  psupport2  psupport3  psupport4  psupport5  political_ideology  happy  slf_ladder  riskperc2  self_esteem  sbelong1  sbelong2  sbelong3  sbelong4  optim1  optim2  slfcont1  slfcont2  slfcont3  slfcont4
##   2   8  cnarc1  cnarc2  cnarc3  ctheory1  ctheory2  ctheory3  ctheory4  CRT1  CRT2  CRT3  health_cond  marital1  mor_circle  moralid1  moralid10  moralid2  moralid3  moralid4  moralid5  moralid6  moralid7  moralid8  moralid9  mcoop1  mcoop2  mcoop3  mcoop4  mcoop5  mcoop6  mcoop7  narc1  narc2  narc3  narc4  narc5  narc6  nidentity1  nidentity2  omind1  omind2  omind3  omind4  omind5  omind6  contact1  contact2  contact3  contact4  contact5  hygiene1  hygiene2  hygiene3  hygiene4  hygiene5  psupport1  psupport2  psupport3  psupport4  psupport5  political_ideology  happy  slf_ladder  riskperc2  self_esteem  sbelong1  sbelong2  sbelong3  sbelong4  optim1  optim2  slfcont1  slfcont2  slfcont3  slfcont4
##   2   9  cnarc1  cnarc2  cnarc3  ctheory1  ctheory2  ctheory3  ctheory4  CRT1  CRT2  CRT3  health_cond  marital1  mor_circle  moralid1  moralid10  moralid2  moralid3  moralid4  moralid5  moralid6  moralid7  moralid8  moralid9  mcoop1  mcoop2  mcoop3  mcoop4  mcoop5  mcoop6  mcoop7  narc1  narc2  narc3  narc4  narc5  narc6  nidentity1  nidentity2  omind1  omind2  omind3  omind4  omind5  omind6  contact1  contact2  contact3  contact4  contact5  hygiene1  hygiene2  hygiene3  hygiene4  hygiene5  psupport1  psupport2  psupport3  psupport4  psupport5  political_ideology  happy  slf_ladder  riskperc2  self_esteem  sbelong1  sbelong2  sbelong3  sbelong4  optim1  optim2  slfcont1  slfcont2  slfcont3  slfcont4
##   2   10  cnarc1  cnarc2  cnarc3  ctheory1  ctheory2  ctheory3  ctheory4  CRT1  CRT2  CRT3  health_cond  marital1  mor_circle  moralid1  moralid10  moralid2  moralid3  moralid4  moralid5  moralid6  moralid7  moralid8  moralid9  mcoop1  mcoop2  mcoop3  mcoop4  mcoop5  mcoop6  mcoop7  narc1  narc2  narc3  narc4  narc5  narc6  nidentity1  nidentity2  omind1  omind2  omind3  omind4  omind5  omind6  contact1  contact2  contact3  contact4  contact5  hygiene1  hygiene2  hygiene3  hygiene4  hygiene5  psupport1  psupport2  psupport3  psupport4  psupport5  political_ideology  happy  slf_ladder  riskperc2  self_esteem  sbelong1  sbelong2  sbelong3  sbelong4  optim1  optim2  slfcont1  slfcont2  slfcont3  slfcont4
##   3   1  cnarc1  cnarc2  cnarc3  ctheory1  ctheory2  ctheory3  ctheory4  CRT1  CRT2  CRT3  health_cond  marital1  mor_circle  moralid1  moralid10  moralid2  moralid3  moralid4  moralid5  moralid6  moralid7  moralid8  moralid9  mcoop1  mcoop2  mcoop3  mcoop4  mcoop5  mcoop6  mcoop7  narc1  narc2  narc3  narc4  narc5  narc6  nidentity1  nidentity2  omind1  omind2  omind3  omind4  omind5  omind6  contact1  contact2  contact3  contact4  contact5  hygiene1  hygiene2  hygiene3  hygiene4  hygiene5  psupport1  psupport2  psupport3  psupport4  psupport5  political_ideology  happy  slf_ladder  riskperc2  self_esteem  sbelong1  sbelong2  sbelong3  sbelong4  optim1  optim2  slfcont1  slfcont2  slfcont3  slfcont4
##   3   2  cnarc1  cnarc2  cnarc3  ctheory1  ctheory2  ctheory3  ctheory4  CRT1  CRT2  CRT3  health_cond  marital1  mor_circle  moralid1  moralid10  moralid2  moralid3  moralid4  moralid5  moralid6  moralid7  moralid8  moralid9  mcoop1  mcoop2  mcoop3  mcoop4  mcoop5  mcoop6  mcoop7  narc1  narc2  narc3  narc4  narc5  narc6  nidentity1  nidentity2  omind1  omind2  omind3  omind4  omind5  omind6  contact1  contact2  contact3  contact4  contact5  hygiene1  hygiene2  hygiene3  hygiene4  hygiene5  psupport1  psupport2  psupport3  psupport4  psupport5  political_ideology  happy  slf_ladder  riskperc2  self_esteem  sbelong1  sbelong2  sbelong3  sbelong4  optim1  optim2  slfcont1  slfcont2  slfcont3  slfcont4
##   3   3  cnarc1  cnarc2  cnarc3  ctheory1  ctheory2  ctheory3  ctheory4  CRT1  CRT2  CRT3  health_cond  marital1  mor_circle  moralid1  moralid10  moralid2  moralid3  moralid4  moralid5  moralid6  moralid7  moralid8  moralid9  mcoop1  mcoop2  mcoop3  mcoop4  mcoop5  mcoop6  mcoop7  narc1  narc2  narc3  narc4  narc5  narc6  nidentity1  nidentity2  omind1  omind2  omind3  omind4  omind5  omind6  contact1  contact2  contact3  contact4  contact5  hygiene1  hygiene2  hygiene3  hygiene4  hygiene5  psupport1  psupport2  psupport3  psupport4  psupport5  political_ideology  happy  slf_ladder  riskperc2  self_esteem  sbelong1  sbelong2  sbelong3  sbelong4  optim1  optim2  slfcont1  slfcont2  slfcont3  slfcont4
##   3   4  cnarc1  cnarc2  cnarc3  ctheory1  ctheory2  ctheory3  ctheory4  CRT1  CRT2  CRT3  health_cond  marital1  mor_circle  moralid1  moralid10  moralid2  moralid3  moralid4  moralid5  moralid6  moralid7  moralid8  moralid9  mcoop1  mcoop2  mcoop3  mcoop4  mcoop5  mcoop6  mcoop7  narc1  narc2  narc3  narc4  narc5  narc6  nidentity1  nidentity2  omind1  omind2  omind3  omind4  omind5  omind6  contact1  contact2  contact3  contact4  contact5  hygiene1  hygiene2  hygiene3  hygiene4  hygiene5  psupport1  psupport2  psupport3  psupport4  psupport5  political_ideology  happy  slf_ladder  riskperc2  self_esteem  sbelong1  sbelong2  sbelong3  sbelong4  optim1  optim2  slfcont1  slfcont2  slfcont3  slfcont4
##   3   5  cnarc1  cnarc2  cnarc3  ctheory1  ctheory2  ctheory3  ctheory4  CRT1  CRT2  CRT3  health_cond  marital1  mor_circle  moralid1  moralid10  moralid2  moralid3  moralid4  moralid5  moralid6  moralid7  moralid8  moralid9  mcoop1  mcoop2  mcoop3  mcoop4  mcoop5  mcoop6  mcoop7  narc1  narc2  narc3  narc4  narc5  narc6  nidentity1  nidentity2  omind1  omind2  omind3  omind4  omind5  omind6  contact1  contact2  contact3  contact4  contact5  hygiene1  hygiene2  hygiene3  hygiene4  hygiene5  psupport1  psupport2  psupport3  psupport4  psupport5  political_ideology  happy  slf_ladder  riskperc2  self_esteem  sbelong1  sbelong2  sbelong3  sbelong4  optim1  optim2  slfcont1  slfcont2  slfcont3  slfcont4
##   3   6  cnarc1  cnarc2  cnarc3  ctheory1  ctheory2  ctheory3  ctheory4  CRT1  CRT2  CRT3  health_cond  marital1  mor_circle  moralid1  moralid10  moralid2  moralid3  moralid4  moralid5  moralid6  moralid7  moralid8  moralid9  mcoop1  mcoop2  mcoop3  mcoop4  mcoop5  mcoop6  mcoop7  narc1  narc2  narc3  narc4  narc5  narc6  nidentity1  nidentity2  omind1  omind2  omind3  omind4  omind5  omind6  contact1  contact2  contact3  contact4  contact5  hygiene1  hygiene2  hygiene3  hygiene4  hygiene5  psupport1  psupport2  psupport3  psupport4  psupport5  political_ideology  happy  slf_ladder  riskperc2  self_esteem  sbelong1  sbelong2  sbelong3  sbelong4  optim1  optim2  slfcont1  slfcont2  slfcont3  slfcont4
##   3   7  cnarc1  cnarc2  cnarc3  ctheory1  ctheory2  ctheory3  ctheory4  CRT1  CRT2  CRT3  health_cond  marital1  mor_circle  moralid1  moralid10  moralid2  moralid3  moralid4  moralid5  moralid6  moralid7  moralid8  moralid9  mcoop1  mcoop2  mcoop3  mcoop4  mcoop5  mcoop6  mcoop7  narc1  narc2  narc3  narc4  narc5  narc6  nidentity1  nidentity2  omind1  omind2  omind3  omind4  omind5  omind6  contact1  contact2  contact3  contact4  contact5  hygiene1  hygiene2  hygiene3  hygiene4  hygiene5  psupport1  psupport2  psupport3  psupport4  psupport5  political_ideology  happy  slf_ladder  riskperc2  self_esteem  sbelong1  sbelong2  sbelong3  sbelong4  optim1  optim2  slfcont1  slfcont2  slfcont3  slfcont4
##   3   8  cnarc1  cnarc2  cnarc3  ctheory1  ctheory2  ctheory3  ctheory4  CRT1  CRT2  CRT3  health_cond  marital1  mor_circle  moralid1  moralid10  moralid2  moralid3  moralid4  moralid5  moralid6  moralid7  moralid8  moralid9  mcoop1  mcoop2  mcoop3  mcoop4  mcoop5  mcoop6  mcoop7  narc1  narc2  narc3  narc4  narc5  narc6  nidentity1  nidentity2  omind1  omind2  omind3  omind4  omind5  omind6  contact1  contact2  contact3  contact4  contact5  hygiene1  hygiene2  hygiene3  hygiene4  hygiene5  psupport1  psupport2  psupport3  psupport4  psupport5  political_ideology  happy  slf_ladder  riskperc2  self_esteem  sbelong1  sbelong2  sbelong3  sbelong4  optim1  optim2  slfcont1  slfcont2  slfcont3  slfcont4
##   3   9  cnarc1  cnarc2  cnarc3  ctheory1  ctheory2  ctheory3  ctheory4  CRT1  CRT2  CRT3  health_cond  marital1  mor_circle  moralid1  moralid10  moralid2  moralid3  moralid4  moralid5  moralid6  moralid7  moralid8  moralid9  mcoop1  mcoop2  mcoop3  mcoop4  mcoop5  mcoop6  mcoop7  narc1  narc2  narc3  narc4  narc5  narc6  nidentity1  nidentity2  omind1  omind2  omind3  omind4  omind5  omind6  contact1  contact2  contact3  contact4  contact5  hygiene1  hygiene2  hygiene3  hygiene4  hygiene5  psupport1  psupport2  psupport3  psupport4  psupport5  political_ideology  happy  slf_ladder  riskperc2  self_esteem  sbelong1  sbelong2  sbelong3  sbelong4  optim1  optim2  slfcont1  slfcont2  slfcont3  slfcont4
##   3   10  cnarc1  cnarc2  cnarc3  ctheory1  ctheory2  ctheory3  ctheory4  CRT1  CRT2  CRT3  health_cond  marital1  mor_circle  moralid1  moralid10  moralid2  moralid3  moralid4  moralid5  moralid6  moralid7  moralid8  moralid9  mcoop1  mcoop2  mcoop3  mcoop4  mcoop5  mcoop6  mcoop7  narc1  narc2  narc3  narc4  narc5  narc6  nidentity1  nidentity2  omind1  omind2  omind3  omind4  omind5  omind6  contact1  contact2  contact3  contact4  contact5  hygiene1  hygiene2  hygiene3  hygiene4  hygiene5  psupport1  psupport2  psupport3  psupport4  psupport5  political_ideology  happy  slf_ladder  riskperc2  self_esteem  sbelong1  sbelong2  sbelong3  sbelong4  optim1  optim2  slfcont1  slfcont2  slfcont3  slfcont4
##   4   1  cnarc1  cnarc2  cnarc3  ctheory1  ctheory2  ctheory3  ctheory4  CRT1  CRT2  CRT3  health_cond  marital1  mor_circle  moralid1  moralid10  moralid2  moralid3  moralid4  moralid5  moralid6  moralid7  moralid8  moralid9  mcoop1  mcoop2  mcoop3  mcoop4  mcoop5  mcoop6  mcoop7  narc1  narc2  narc3  narc4  narc5  narc6  nidentity1  nidentity2  omind1  omind2  omind3  omind4  omind5  omind6  contact1  contact2  contact3  contact4  contact5  hygiene1  hygiene2  hygiene3  hygiene4  hygiene5  psupport1  psupport2  psupport3  psupport4  psupport5  political_ideology  happy  slf_ladder  riskperc2  self_esteem  sbelong1  sbelong2  sbelong3  sbelong4  optim1  optim2  slfcont1  slfcont2  slfcont3  slfcont4
##   4   2  cnarc1  cnarc2  cnarc3  ctheory1  ctheory2  ctheory3  ctheory4  CRT1  CRT2  CRT3  health_cond  marital1  mor_circle  moralid1  moralid10  moralid2  moralid3  moralid4  moralid5  moralid6  moralid7  moralid8  moralid9  mcoop1  mcoop2  mcoop3  mcoop4  mcoop5  mcoop6  mcoop7  narc1  narc2  narc3  narc4  narc5  narc6  nidentity1  nidentity2  omind1  omind2  omind3  omind4  omind5  omind6  contact1  contact2  contact3  contact4  contact5  hygiene1  hygiene2  hygiene3  hygiene4  hygiene5  psupport1  psupport2  psupport3  psupport4  psupport5  political_ideology  happy  slf_ladder  riskperc2  self_esteem  sbelong1  sbelong2  sbelong3  sbelong4  optim1  optim2  slfcont1  slfcont2  slfcont3  slfcont4
##   4   3  cnarc1  cnarc2  cnarc3  ctheory1  ctheory2  ctheory3  ctheory4  CRT1  CRT2  CRT3  health_cond  marital1  mor_circle  moralid1  moralid10  moralid2  moralid3  moralid4  moralid5  moralid6  moralid7  moralid8  moralid9  mcoop1  mcoop2  mcoop3  mcoop4  mcoop5  mcoop6  mcoop7  narc1  narc2  narc3  narc4  narc5  narc6  nidentity1  nidentity2  omind1  omind2  omind3  omind4  omind5  omind6  contact1  contact2  contact3  contact4  contact5  hygiene1  hygiene2  hygiene3  hygiene4  hygiene5  psupport1  psupport2  psupport3  psupport4  psupport5  political_ideology  happy  slf_ladder  riskperc2  self_esteem  sbelong1  sbelong2  sbelong3  sbelong4  optim1  optim2  slfcont1  slfcont2  slfcont3  slfcont4
##   4   4  cnarc1  cnarc2  cnarc3  ctheory1  ctheory2  ctheory3  ctheory4  CRT1  CRT2  CRT3  health_cond  marital1  mor_circle  moralid1  moralid10  moralid2  moralid3  moralid4  moralid5  moralid6  moralid7  moralid8  moralid9  mcoop1  mcoop2  mcoop3  mcoop4  mcoop5  mcoop6  mcoop7  narc1  narc2  narc3  narc4  narc5  narc6  nidentity1  nidentity2  omind1  omind2  omind3  omind4  omind5  omind6  contact1  contact2  contact3  contact4  contact5  hygiene1  hygiene2  hygiene3  hygiene4  hygiene5  psupport1  psupport2  psupport3  psupport4  psupport5  political_ideology  happy  slf_ladder  riskperc2  self_esteem  sbelong1  sbelong2  sbelong3  sbelong4  optim1  optim2  slfcont1  slfcont2  slfcont3  slfcont4
##   4   5  cnarc1  cnarc2  cnarc3  ctheory1  ctheory2  ctheory3  ctheory4  CRT1  CRT2  CRT3  health_cond  marital1  mor_circle  moralid1  moralid10  moralid2  moralid3  moralid4  moralid5  moralid6  moralid7  moralid8  moralid9  mcoop1  mcoop2  mcoop3  mcoop4  mcoop5  mcoop6  mcoop7  narc1  narc2  narc3  narc4  narc5  narc6  nidentity1  nidentity2  omind1  omind2  omind3  omind4  omind5  omind6  contact1  contact2  contact3  contact4  contact5  hygiene1  hygiene2  hygiene3  hygiene4  hygiene5  psupport1  psupport2  psupport3  psupport4  psupport5  political_ideology  happy  slf_ladder  riskperc2  self_esteem  sbelong1  sbelong2  sbelong3  sbelong4  optim1  optim2  slfcont1  slfcont2  slfcont3  slfcont4
##   4   6  cnarc1  cnarc2  cnarc3  ctheory1  ctheory2  ctheory3  ctheory4  CRT1  CRT2  CRT3  health_cond  marital1  mor_circle  moralid1  moralid10  moralid2  moralid3  moralid4  moralid5  moralid6  moralid7  moralid8  moralid9  mcoop1  mcoop2  mcoop3  mcoop4  mcoop5  mcoop6  mcoop7  narc1  narc2  narc3  narc4  narc5  narc6  nidentity1  nidentity2  omind1  omind2  omind3  omind4  omind5  omind6  contact1  contact2  contact3  contact4  contact5  hygiene1  hygiene2  hygiene3  hygiene4  hygiene5  psupport1  psupport2  psupport3  psupport4  psupport5  political_ideology  happy  slf_ladder  riskperc2  self_esteem  sbelong1  sbelong2  sbelong3  sbelong4  optim1  optim2  slfcont1  slfcont2  slfcont3  slfcont4
##   4   7  cnarc1  cnarc2  cnarc3  ctheory1  ctheory2  ctheory3  ctheory4  CRT1  CRT2  CRT3  health_cond  marital1  mor_circle  moralid1  moralid10  moralid2  moralid3  moralid4  moralid5  moralid6  moralid7  moralid8  moralid9  mcoop1  mcoop2  mcoop3  mcoop4  mcoop5  mcoop6  mcoop7  narc1  narc2  narc3  narc4  narc5  narc6  nidentity1  nidentity2  omind1  omind2  omind3  omind4  omind5  omind6  contact1  contact2  contact3  contact4  contact5  hygiene1  hygiene2  hygiene3  hygiene4  hygiene5  psupport1  psupport2  psupport3  psupport4  psupport5  political_ideology  happy  slf_ladder  riskperc2  self_esteem  sbelong1  sbelong2  sbelong3  sbelong4  optim1  optim2  slfcont1  slfcont2  slfcont3  slfcont4
##   4   8  cnarc1  cnarc2  cnarc3  ctheory1  ctheory2  ctheory3  ctheory4  CRT1  CRT2  CRT3  health_cond  marital1  mor_circle  moralid1  moralid10  moralid2  moralid3  moralid4  moralid5  moralid6  moralid7  moralid8  moralid9  mcoop1  mcoop2  mcoop3  mcoop4  mcoop5  mcoop6  mcoop7  narc1  narc2  narc3  narc4  narc5  narc6  nidentity1  nidentity2  omind1  omind2  omind3  omind4  omind5  omind6  contact1  contact2  contact3  contact4  contact5  hygiene1  hygiene2  hygiene3  hygiene4  hygiene5  psupport1  psupport2  psupport3  psupport4  psupport5  political_ideology  happy  slf_ladder  riskperc2  self_esteem  sbelong1  sbelong2  sbelong3  sbelong4  optim1  optim2  slfcont1  slfcont2  slfcont3  slfcont4
##   4   9  cnarc1  cnarc2  cnarc3  ctheory1  ctheory2  ctheory3  ctheory4  CRT1  CRT2  CRT3  health_cond  marital1  mor_circle  moralid1  moralid10  moralid2  moralid3  moralid4  moralid5  moralid6  moralid7  moralid8  moralid9  mcoop1  mcoop2  mcoop3  mcoop4  mcoop5  mcoop6  mcoop7  narc1  narc2  narc3  narc4  narc5  narc6  nidentity1  nidentity2  omind1  omind2  omind3  omind4  omind5  omind6  contact1  contact2  contact3  contact4  contact5  hygiene1  hygiene2  hygiene3  hygiene4  hygiene5  psupport1  psupport2  psupport3  psupport4  psupport5  political_ideology  happy  slf_ladder  riskperc2  self_esteem  sbelong1  sbelong2  sbelong3  sbelong4  optim1  optim2  slfcont1  slfcont2  slfcont3  slfcont4
##   4   10  cnarc1  cnarc2  cnarc3  ctheory1  ctheory2  ctheory3  ctheory4  CRT1  CRT2  CRT3  health_cond  marital1  mor_circle  moralid1  moralid10  moralid2  moralid3  moralid4  moralid5  moralid6  moralid7  moralid8  moralid9  mcoop1  mcoop2  mcoop3  mcoop4  mcoop5  mcoop6  mcoop7  narc1  narc2  narc3  narc4  narc5  narc6  nidentity1  nidentity2  omind1  omind2  omind3  omind4  omind5  omind6  contact1  contact2  contact3  contact4  contact5  hygiene1  hygiene2  hygiene3  hygiene4  hygiene5  psupport1  psupport2  psupport3  psupport4  psupport5  political_ideology  happy  slf_ladder  riskperc2  self_esteem  sbelong1  sbelong2  sbelong3  sbelong4  optim1  optim2  slfcont1  slfcont2  slfcont3  slfcont4
##   5   1  cnarc1  cnarc2  cnarc3  ctheory1  ctheory2  ctheory3  ctheory4  CRT1  CRT2  CRT3  health_cond  marital1  mor_circle  moralid1  moralid10  moralid2  moralid3  moralid4  moralid5  moralid6  moralid7  moralid8  moralid9  mcoop1  mcoop2  mcoop3  mcoop4  mcoop5  mcoop6  mcoop7  narc1  narc2  narc3  narc4  narc5  narc6  nidentity1  nidentity2  omind1  omind2  omind3  omind4  omind5  omind6  contact1  contact2  contact3  contact4  contact5  hygiene1  hygiene2  hygiene3  hygiene4  hygiene5  psupport1  psupport2  psupport3  psupport4  psupport5  political_ideology  happy  slf_ladder  riskperc2  self_esteem  sbelong1  sbelong2  sbelong3  sbelong4  optim1  optim2  slfcont1  slfcont2  slfcont3  slfcont4
##   5   2  cnarc1  cnarc2  cnarc3  ctheory1  ctheory2  ctheory3  ctheory4  CRT1  CRT2  CRT3  health_cond  marital1  mor_circle  moralid1  moralid10  moralid2  moralid3  moralid4  moralid5  moralid6  moralid7  moralid8  moralid9  mcoop1  mcoop2  mcoop3  mcoop4  mcoop5  mcoop6  mcoop7  narc1  narc2  narc3  narc4  narc5  narc6  nidentity1  nidentity2  omind1  omind2  omind3  omind4  omind5  omind6  contact1  contact2  contact3  contact4  contact5  hygiene1  hygiene2  hygiene3  hygiene4  hygiene5  psupport1  psupport2  psupport3  psupport4  psupport5  political_ideology  happy  slf_ladder  riskperc2  self_esteem  sbelong1  sbelong2  sbelong3  sbelong4  optim1  optim2  slfcont1  slfcont2  slfcont3  slfcont4
##   5   3  cnarc1  cnarc2  cnarc3  ctheory1  ctheory2  ctheory3  ctheory4  CRT1  CRT2  CRT3  health_cond  marital1  mor_circle  moralid1  moralid10  moralid2  moralid3  moralid4  moralid5  moralid6  moralid7  moralid8  moralid9  mcoop1  mcoop2  mcoop3  mcoop4  mcoop5  mcoop6  mcoop7  narc1  narc2  narc3  narc4  narc5  narc6  nidentity1  nidentity2  omind1  omind2  omind3  omind4  omind5  omind6  contact1  contact2  contact3  contact4  contact5  hygiene1  hygiene2  hygiene3  hygiene4  hygiene5  psupport1  psupport2  psupport3  psupport4  psupport5  political_ideology  happy  slf_ladder  riskperc2  self_esteem  sbelong1  sbelong2  sbelong3  sbelong4  optim1  optim2  slfcont1  slfcont2  slfcont3  slfcont4
##   5   4  cnarc1  cnarc2  cnarc3  ctheory1  ctheory2  ctheory3  ctheory4  CRT1  CRT2  CRT3  health_cond  marital1  mor_circle  moralid1  moralid10  moralid2  moralid3  moralid4  moralid5  moralid6  moralid7  moralid8  moralid9  mcoop1  mcoop2  mcoop3  mcoop4  mcoop5  mcoop6  mcoop7  narc1  narc2  narc3  narc4  narc5  narc6  nidentity1  nidentity2  omind1  omind2  omind3  omind4  omind5  omind6  contact1  contact2  contact3  contact4  contact5  hygiene1  hygiene2  hygiene3  hygiene4  hygiene5  psupport1  psupport2  psupport3  psupport4  psupport5  political_ideology  happy  slf_ladder  riskperc2  self_esteem  sbelong1  sbelong2  sbelong3  sbelong4  optim1  optim2  slfcont1  slfcont2  slfcont3  slfcont4
##   5   5  cnarc1  cnarc2  cnarc3  ctheory1  ctheory2  ctheory3  ctheory4  CRT1  CRT2  CRT3  health_cond  marital1  mor_circle  moralid1  moralid10  moralid2  moralid3  moralid4  moralid5  moralid6  moralid7  moralid8  moralid9  mcoop1  mcoop2  mcoop3  mcoop4  mcoop5  mcoop6  mcoop7  narc1  narc2  narc3  narc4  narc5  narc6  nidentity1  nidentity2  omind1  omind2  omind3  omind4  omind5  omind6  contact1  contact2  contact3  contact4  contact5  hygiene1  hygiene2  hygiene3  hygiene4  hygiene5  psupport1  psupport2  psupport3  psupport4  psupport5  political_ideology  happy  slf_ladder  riskperc2  self_esteem  sbelong1  sbelong2  sbelong3  sbelong4  optim1  optim2  slfcont1  slfcont2  slfcont3  slfcont4
##   5   6  cnarc1  cnarc2  cnarc3  ctheory1  ctheory2  ctheory3  ctheory4  CRT1  CRT2  CRT3  health_cond  marital1  mor_circle  moralid1  moralid10  moralid2  moralid3  moralid4  moralid5  moralid6  moralid7  moralid8  moralid9  mcoop1  mcoop2  mcoop3  mcoop4  mcoop5  mcoop6  mcoop7  narc1  narc2  narc3  narc4  narc5  narc6  nidentity1  nidentity2  omind1  omind2  omind3  omind4  omind5  omind6  contact1  contact2  contact3  contact4  contact5  hygiene1  hygiene2  hygiene3  hygiene4  hygiene5  psupport1  psupport2  psupport3  psupport4  psupport5  political_ideology  happy  slf_ladder  riskperc2  self_esteem  sbelong1  sbelong2  sbelong3  sbelong4  optim1  optim2  slfcont1  slfcont2  slfcont3  slfcont4
##   5   7  cnarc1  cnarc2  cnarc3  ctheory1  ctheory2  ctheory3  ctheory4  CRT1  CRT2  CRT3  health_cond  marital1  mor_circle  moralid1  moralid10  moralid2  moralid3  moralid4  moralid5  moralid6  moralid7  moralid8  moralid9  mcoop1  mcoop2  mcoop3  mcoop4  mcoop5  mcoop6  mcoop7  narc1  narc2  narc3  narc4  narc5  narc6  nidentity1  nidentity2  omind1  omind2  omind3  omind4  omind5  omind6  contact1  contact2  contact3  contact4  contact5  hygiene1  hygiene2  hygiene3  hygiene4  hygiene5  psupport1  psupport2  psupport3  psupport4  psupport5  political_ideology  happy  slf_ladder  riskperc2  self_esteem  sbelong1  sbelong2  sbelong3  sbelong4  optim1  optim2  slfcont1  slfcont2  slfcont3  slfcont4
##   5   8  cnarc1  cnarc2  cnarc3  ctheory1  ctheory2  ctheory3  ctheory4  CRT1  CRT2  CRT3  health_cond  marital1  mor_circle  moralid1  moralid10  moralid2  moralid3  moralid4  moralid5  moralid6  moralid7  moralid8  moralid9  mcoop1  mcoop2  mcoop3  mcoop4  mcoop5  mcoop6  mcoop7  narc1  narc2  narc3  narc4  narc5  narc6  nidentity1  nidentity2  omind1  omind2  omind3  omind4  omind5  omind6  contact1  contact2  contact3  contact4  contact5  hygiene1  hygiene2  hygiene3  hygiene4  hygiene5  psupport1  psupport2  psupport3  psupport4  psupport5  political_ideology  happy  slf_ladder  riskperc2  self_esteem  sbelong1  sbelong2  sbelong3  sbelong4  optim1  optim2  slfcont1  slfcont2  slfcont3  slfcont4
##   5   9  cnarc1  cnarc2  cnarc3  ctheory1  ctheory2  ctheory3  ctheory4  CRT1  CRT2  CRT3  health_cond  marital1  mor_circle  moralid1  moralid10  moralid2  moralid3  moralid4  moralid5  moralid6  moralid7  moralid8  moralid9  mcoop1  mcoop2  mcoop3  mcoop4  mcoop5  mcoop6  mcoop7  narc1  narc2  narc3  narc4  narc5  narc6  nidentity1  nidentity2  omind1  omind2  omind3  omind4  omind5  omind6  contact1  contact2  contact3  contact4  contact5  hygiene1  hygiene2  hygiene3  hygiene4  hygiene5  psupport1  psupport2  psupport3  psupport4  psupport5  political_ideology  happy  slf_ladder  riskperc2  self_esteem  sbelong1  sbelong2  sbelong3  sbelong4  optim1  optim2  slfcont1  slfcont2  slfcont3  slfcont4
##   5   10  cnarc1  cnarc2  cnarc3  ctheory1  ctheory2  ctheory3  ctheory4  CRT1  CRT2  CRT3  health_cond  marital1  mor_circle  moralid1  moralid10  moralid2  moralid3  moralid4  moralid5  moralid6  moralid7  moralid8  moralid9  mcoop1  mcoop2  mcoop3  mcoop4  mcoop5  mcoop6  mcoop7  narc1  narc2  narc3  narc4  narc5  narc6  nidentity1  nidentity2  omind1  omind2  omind3  omind4  omind5  omind6  contact1  contact2  contact3  contact4  contact5  hygiene1  hygiene2  hygiene3  hygiene4  hygiene5  psupport1  psupport2  psupport3  psupport4  psupport5  political_ideology  happy  slf_ladder  riskperc2  self_esteem  sbelong1  sbelong2  sbelong3  sbelong4  optim1  optim2  slfcont1  slfcont2  slfcont3  slfcont4
##   6   1  cnarc1  cnarc2  cnarc3  ctheory1  ctheory2  ctheory3  ctheory4  CRT1  CRT2  CRT3  health_cond  marital1  mor_circle  moralid1  moralid10  moralid2  moralid3  moralid4  moralid5  moralid6  moralid7  moralid8  moralid9  mcoop1  mcoop2  mcoop3  mcoop4  mcoop5  mcoop6  mcoop7  narc1  narc2  narc3  narc4  narc5  narc6  nidentity1  nidentity2  omind1  omind2  omind3  omind4  omind5  omind6  contact1  contact2  contact3  contact4  contact5  hygiene1  hygiene2  hygiene3  hygiene4  hygiene5  psupport1  psupport2  psupport3  psupport4  psupport5  political_ideology  happy  slf_ladder  riskperc2  self_esteem  sbelong1  sbelong2  sbelong3  sbelong4  optim1  optim2  slfcont1  slfcont2  slfcont3  slfcont4
##   6   2  cnarc1  cnarc2  cnarc3  ctheory1  ctheory2  ctheory3  ctheory4  CRT1  CRT2  CRT3  health_cond  marital1  mor_circle  moralid1  moralid10  moralid2  moralid3  moralid4  moralid5  moralid6  moralid7  moralid8  moralid9  mcoop1  mcoop2  mcoop3  mcoop4  mcoop5  mcoop6  mcoop7  narc1  narc2  narc3  narc4  narc5  narc6  nidentity1  nidentity2  omind1  omind2  omind3  omind4  omind5  omind6  contact1  contact2  contact3  contact4  contact5  hygiene1  hygiene2  hygiene3  hygiene4  hygiene5  psupport1  psupport2  psupport3  psupport4  psupport5  political_ideology  happy  slf_ladder  riskperc2  self_esteem  sbelong1  sbelong2  sbelong3  sbelong4  optim1  optim2  slfcont1  slfcont2  slfcont3  slfcont4
##   6   3  cnarc1  cnarc2  cnarc3  ctheory1  ctheory2  ctheory3  ctheory4  CRT1  CRT2  CRT3  health_cond  marital1  mor_circle  moralid1  moralid10  moralid2  moralid3  moralid4  moralid5  moralid6  moralid7  moralid8  moralid9  mcoop1  mcoop2  mcoop3  mcoop4  mcoop5  mcoop6  mcoop7  narc1  narc2  narc3  narc4  narc5  narc6  nidentity1  nidentity2  omind1  omind2  omind3  omind4  omind5  omind6  contact1  contact2  contact3  contact4  contact5  hygiene1  hygiene2  hygiene3  hygiene4  hygiene5  psupport1  psupport2  psupport3  psupport4  psupport5  political_ideology  happy  slf_ladder  riskperc2  self_esteem  sbelong1  sbelong2  sbelong3  sbelong4  optim1  optim2  slfcont1  slfcont2  slfcont3  slfcont4
##   6   4  cnarc1  cnarc2  cnarc3  ctheory1  ctheory2  ctheory3  ctheory4  CRT1  CRT2  CRT3  health_cond  marital1  mor_circle  moralid1  moralid10  moralid2  moralid3  moralid4  moralid5  moralid6  moralid7  moralid8  moralid9  mcoop1  mcoop2  mcoop3  mcoop4  mcoop5  mcoop6  mcoop7  narc1  narc2  narc3  narc4  narc5  narc6  nidentity1  nidentity2  omind1  omind2  omind3  omind4  omind5  omind6  contact1  contact2  contact3  contact4  contact5  hygiene1  hygiene2  hygiene3  hygiene4  hygiene5  psupport1  psupport2  psupport3  psupport4  psupport5  political_ideology  happy  slf_ladder  riskperc2  self_esteem  sbelong1  sbelong2  sbelong3  sbelong4  optim1  optim2  slfcont1  slfcont2  slfcont3  slfcont4
##   6   5  cnarc1  cnarc2  cnarc3  ctheory1  ctheory2  ctheory3  ctheory4  CRT1  CRT2  CRT3  health_cond  marital1  mor_circle  moralid1  moralid10  moralid2  moralid3  moralid4  moralid5  moralid6  moralid7  moralid8  moralid9  mcoop1  mcoop2  mcoop3  mcoop4  mcoop5  mcoop6  mcoop7  narc1  narc2  narc3  narc4  narc5  narc6  nidentity1  nidentity2  omind1  omind2  omind3  omind4  omind5  omind6  contact1  contact2  contact3  contact4  contact5  hygiene1  hygiene2  hygiene3  hygiene4  hygiene5  psupport1  psupport2  psupport3  psupport4  psupport5  political_ideology  happy  slf_ladder  riskperc2  self_esteem  sbelong1  sbelong2  sbelong3  sbelong4  optim1  optim2  slfcont1  slfcont2  slfcont3  slfcont4
##   6   6  cnarc1  cnarc2  cnarc3  ctheory1  ctheory2  ctheory3  ctheory4  CRT1  CRT2  CRT3  health_cond  marital1  mor_circle  moralid1  moralid10  moralid2  moralid3  moralid4  moralid5  moralid6  moralid7  moralid8  moralid9  mcoop1  mcoop2  mcoop3  mcoop4  mcoop5  mcoop6  mcoop7  narc1  narc2  narc3  narc4  narc5  narc6  nidentity1  nidentity2  omind1  omind2  omind3  omind4  omind5  omind6  contact1  contact2  contact3  contact4  contact5  hygiene1  hygiene2  hygiene3  hygiene4  hygiene5  psupport1  psupport2  psupport3  psupport4  psupport5  political_ideology  happy  slf_ladder  riskperc2  self_esteem  sbelong1  sbelong2  sbelong3  sbelong4  optim1  optim2  slfcont1  slfcont2  slfcont3  slfcont4
##   6   7  cnarc1  cnarc2  cnarc3  ctheory1  ctheory2  ctheory3  ctheory4  CRT1  CRT2  CRT3  health_cond  marital1  mor_circle  moralid1  moralid10  moralid2  moralid3  moralid4  moralid5  moralid6  moralid7  moralid8  moralid9  mcoop1  mcoop2  mcoop3  mcoop4  mcoop5  mcoop6  mcoop7  narc1  narc2  narc3  narc4  narc5  narc6  nidentity1  nidentity2  omind1  omind2  omind3  omind4  omind5  omind6  contact1  contact2  contact3  contact4  contact5  hygiene1  hygiene2  hygiene3  hygiene4  hygiene5  psupport1  psupport2  psupport3  psupport4  psupport5  political_ideology  happy  slf_ladder  riskperc2  self_esteem  sbelong1  sbelong2  sbelong3  sbelong4  optim1  optim2  slfcont1  slfcont2  slfcont3  slfcont4
##   6   8  cnarc1  cnarc2  cnarc3  ctheory1  ctheory2  ctheory3  ctheory4  CRT1  CRT2  CRT3  health_cond  marital1  mor_circle  moralid1  moralid10  moralid2  moralid3  moralid4  moralid5  moralid6  moralid7  moralid8  moralid9  mcoop1  mcoop2  mcoop3  mcoop4  mcoop5  mcoop6  mcoop7  narc1  narc2  narc3  narc4  narc5  narc6  nidentity1  nidentity2  omind1  omind2  omind3  omind4  omind5  omind6  contact1  contact2  contact3  contact4  contact5  hygiene1  hygiene2  hygiene3  hygiene4  hygiene5  psupport1  psupport2  psupport3  psupport4  psupport5  political_ideology  happy  slf_ladder  riskperc2  self_esteem  sbelong1  sbelong2  sbelong3  sbelong4  optim1  optim2  slfcont1  slfcont2  slfcont3  slfcont4
##   6   9  cnarc1  cnarc2  cnarc3  ctheory1  ctheory2  ctheory3  ctheory4  CRT1  CRT2  CRT3  health_cond  marital1  mor_circle  moralid1  moralid10  moralid2  moralid3  moralid4  moralid5  moralid6  moralid7  moralid8  moralid9  mcoop1  mcoop2  mcoop3  mcoop4  mcoop5  mcoop6  mcoop7  narc1  narc2  narc3  narc4  narc5  narc6  nidentity1  nidentity2  omind1  omind2  omind3  omind4  omind5  omind6  contact1  contact2  contact3  contact4  contact5  hygiene1  hygiene2  hygiene3  hygiene4  hygiene5  psupport1  psupport2  psupport3  psupport4  psupport5  political_ideology  happy  slf_ladder  riskperc2  self_esteem  sbelong1  sbelong2  sbelong3  sbelong4  optim1  optim2  slfcont1  slfcont2  slfcont3  slfcont4
##   6   10  cnarc1  cnarc2  cnarc3  ctheory1  ctheory2  ctheory3  ctheory4  CRT1  CRT2  CRT3  health_cond  marital1  mor_circle  moralid1  moralid10  moralid2  moralid3  moralid4  moralid5  moralid6  moralid7  moralid8  moralid9  mcoop1  mcoop2  mcoop3  mcoop4  mcoop5  mcoop6  mcoop7  narc1  narc2  narc3  narc4  narc5  narc6  nidentity1  nidentity2  omind1  omind2  omind3  omind4  omind5  omind6  contact1  contact2  contact3  contact4  contact5  hygiene1  hygiene2  hygiene3  hygiene4  hygiene5  psupport1  psupport2  psupport3  psupport4  psupport5  political_ideology  happy  slf_ladder  riskperc2  self_esteem  sbelong1  sbelong2  sbelong3  sbelong4  optim1  optim2  slfcont1  slfcont2  slfcont3  slfcont4
##   7   1  cnarc1  cnarc2  cnarc3  ctheory1  ctheory2  ctheory3  ctheory4  CRT1  CRT2  CRT3  health_cond  marital1  mor_circle  moralid1  moralid10  moralid2  moralid3  moralid4  moralid5  moralid6  moralid7  moralid8  moralid9  mcoop1  mcoop2  mcoop3  mcoop4  mcoop5  mcoop6  mcoop7  narc1  narc2  narc3  narc4  narc5  narc6  nidentity1  nidentity2  omind1  omind2  omind3  omind4  omind5  omind6  contact1  contact2  contact3  contact4  contact5  hygiene1  hygiene2  hygiene3  hygiene4  hygiene5  psupport1  psupport2  psupport3  psupport4  psupport5  political_ideology  happy  slf_ladder  riskperc2  self_esteem  sbelong1  sbelong2  sbelong3  sbelong4  optim1  optim2  slfcont1  slfcont2  slfcont3  slfcont4
##   7   2  cnarc1  cnarc2  cnarc3  ctheory1  ctheory2  ctheory3  ctheory4  CRT1  CRT2  CRT3  health_cond  marital1  mor_circle  moralid1  moralid10  moralid2  moralid3  moralid4  moralid5  moralid6  moralid7  moralid8  moralid9  mcoop1  mcoop2  mcoop3  mcoop4  mcoop5  mcoop6  mcoop7  narc1  narc2  narc3  narc4  narc5  narc6  nidentity1  nidentity2  omind1  omind2  omind3  omind4  omind5  omind6  contact1  contact2  contact3  contact4  contact5  hygiene1  hygiene2  hygiene3  hygiene4  hygiene5  psupport1  psupport2  psupport3  psupport4  psupport5  political_ideology  happy  slf_ladder  riskperc2  self_esteem  sbelong1  sbelong2  sbelong3  sbelong4  optim1  optim2  slfcont1  slfcont2  slfcont3  slfcont4
##   7   3  cnarc1  cnarc2  cnarc3  ctheory1  ctheory2  ctheory3  ctheory4  CRT1  CRT2  CRT3  health_cond  marital1  mor_circle  moralid1  moralid10  moralid2  moralid3  moralid4  moralid5  moralid6  moralid7  moralid8  moralid9  mcoop1  mcoop2  mcoop3  mcoop4  mcoop5  mcoop6  mcoop7  narc1  narc2  narc3  narc4  narc5  narc6  nidentity1  nidentity2  omind1  omind2  omind3  omind4  omind5  omind6  contact1  contact2  contact3  contact4  contact5  hygiene1  hygiene2  hygiene3  hygiene4  hygiene5  psupport1  psupport2  psupport3  psupport4  psupport5  political_ideology  happy  slf_ladder  riskperc2  self_esteem  sbelong1  sbelong2  sbelong3  sbelong4  optim1  optim2  slfcont1  slfcont2  slfcont3  slfcont4
##   7   4  cnarc1  cnarc2  cnarc3  ctheory1  ctheory2  ctheory3  ctheory4  CRT1  CRT2  CRT3  health_cond  marital1  mor_circle  moralid1  moralid10  moralid2  moralid3  moralid4  moralid5  moralid6  moralid7  moralid8  moralid9  mcoop1  mcoop2  mcoop3  mcoop4  mcoop5  mcoop6  mcoop7  narc1  narc2  narc3  narc4  narc5  narc6  nidentity1  nidentity2  omind1  omind2  omind3  omind4  omind5  omind6  contact1  contact2  contact3  contact4  contact5  hygiene1  hygiene2  hygiene3  hygiene4  hygiene5  psupport1  psupport2  psupport3  psupport4  psupport5  political_ideology  happy  slf_ladder  riskperc2  self_esteem  sbelong1  sbelong2  sbelong3  sbelong4  optim1  optim2  slfcont1  slfcont2  slfcont3  slfcont4
##   7   5  cnarc1  cnarc2  cnarc3  ctheory1  ctheory2  ctheory3  ctheory4  CRT1  CRT2  CRT3  health_cond  marital1  mor_circle  moralid1  moralid10  moralid2  moralid3  moralid4  moralid5  moralid6  moralid7  moralid8  moralid9  mcoop1  mcoop2  mcoop3  mcoop4  mcoop5  mcoop6  mcoop7  narc1  narc2  narc3  narc4  narc5  narc6  nidentity1  nidentity2  omind1  omind2  omind3  omind4  omind5  omind6  contact1  contact2  contact3  contact4  contact5  hygiene1  hygiene2  hygiene3  hygiene4  hygiene5  psupport1  psupport2  psupport3  psupport4  psupport5  political_ideology  happy  slf_ladder  riskperc2  self_esteem  sbelong1  sbelong2  sbelong3  sbelong4  optim1  optim2  slfcont1  slfcont2  slfcont3  slfcont4
##   7   6  cnarc1  cnarc2  cnarc3  ctheory1  ctheory2  ctheory3  ctheory4  CRT1  CRT2  CRT3  health_cond  marital1  mor_circle  moralid1  moralid10  moralid2  moralid3  moralid4  moralid5  moralid6  moralid7  moralid8  moralid9  mcoop1  mcoop2  mcoop3  mcoop4  mcoop5  mcoop6  mcoop7  narc1  narc2  narc3  narc4  narc5  narc6  nidentity1  nidentity2  omind1  omind2  omind3  omind4  omind5  omind6  contact1  contact2  contact3  contact4  contact5  hygiene1  hygiene2  hygiene3  hygiene4  hygiene5  psupport1  psupport2  psupport3  psupport4  psupport5  political_ideology  happy  slf_ladder  riskperc2  self_esteem  sbelong1  sbelong2  sbelong3  sbelong4  optim1  optim2  slfcont1  slfcont2  slfcont3  slfcont4
##   7   7  cnarc1  cnarc2  cnarc3  ctheory1  ctheory2  ctheory3  ctheory4  CRT1  CRT2  CRT3  health_cond  marital1  mor_circle  moralid1  moralid10  moralid2  moralid3  moralid4  moralid5  moralid6  moralid7  moralid8  moralid9  mcoop1  mcoop2  mcoop3  mcoop4  mcoop5  mcoop6  mcoop7  narc1  narc2  narc3  narc4  narc5  narc6  nidentity1  nidentity2  omind1  omind2  omind3  omind4  omind5  omind6  contact1  contact2  contact3  contact4  contact5  hygiene1  hygiene2  hygiene3  hygiene4  hygiene5  psupport1  psupport2  psupport3  psupport4  psupport5  political_ideology  happy  slf_ladder  riskperc2  self_esteem  sbelong1  sbelong2  sbelong3  sbelong4  optim1  optim2  slfcont1  slfcont2  slfcont3  slfcont4
##   7   8  cnarc1  cnarc2  cnarc3  ctheory1  ctheory2  ctheory3  ctheory4  CRT1  CRT2  CRT3  health_cond  marital1  mor_circle  moralid1  moralid10  moralid2  moralid3  moralid4  moralid5  moralid6  moralid7  moralid8  moralid9  mcoop1  mcoop2  mcoop3  mcoop4  mcoop5  mcoop6  mcoop7  narc1  narc2  narc3  narc4  narc5  narc6  nidentity1  nidentity2  omind1  omind2  omind3  omind4  omind5  omind6  contact1  contact2  contact3  contact4  contact5  hygiene1  hygiene2  hygiene3  hygiene4  hygiene5  psupport1  psupport2  psupport3  psupport4  psupport5  political_ideology  happy  slf_ladder  riskperc2  self_esteem  sbelong1  sbelong2  sbelong3  sbelong4  optim1  optim2  slfcont1  slfcont2  slfcont3  slfcont4
##   7   9  cnarc1  cnarc2  cnarc3  ctheory1  ctheory2  ctheory3  ctheory4  CRT1  CRT2  CRT3  health_cond  marital1  mor_circle  moralid1  moralid10  moralid2  moralid3  moralid4  moralid5  moralid6  moralid7  moralid8  moralid9  mcoop1  mcoop2  mcoop3  mcoop4  mcoop5  mcoop6  mcoop7  narc1  narc2  narc3  narc4  narc5  narc6  nidentity1  nidentity2  omind1  omind2  omind3  omind4  omind5  omind6  contact1  contact2  contact3  contact4  contact5  hygiene1  hygiene2  hygiene3  hygiene4  hygiene5  psupport1  psupport2  psupport3  psupport4  psupport5  political_ideology  happy  slf_ladder  riskperc2  self_esteem  sbelong1  sbelong2  sbelong3  sbelong4  optim1  optim2  slfcont1  slfcont2  slfcont3  slfcont4
##   7   10  cnarc1  cnarc2  cnarc3  ctheory1  ctheory2  ctheory3  ctheory4  CRT1  CRT2  CRT3  health_cond  marital1  mor_circle  moralid1  moralid10  moralid2  moralid3  moralid4  moralid5  moralid6  moralid7  moralid8  moralid9  mcoop1  mcoop2  mcoop3  mcoop4  mcoop5  mcoop6  mcoop7  narc1  narc2  narc3  narc4  narc5  narc6  nidentity1  nidentity2  omind1  omind2  omind3  omind4  omind5  omind6  contact1  contact2  contact3  contact4  contact5  hygiene1  hygiene2  hygiene3  hygiene4  hygiene5  psupport1  psupport2  psupport3  psupport4  psupport5  political_ideology  happy  slf_ladder  riskperc2  self_esteem  sbelong1  sbelong2  sbelong3  sbelong4  optim1  optim2  slfcont1  slfcont2  slfcont3  slfcont4
##   8   1  cnarc1  cnarc2  cnarc3  ctheory1  ctheory2  ctheory3  ctheory4  CRT1  CRT2  CRT3  health_cond  marital1  mor_circle  moralid1  moralid10  moralid2  moralid3  moralid4  moralid5  moralid6  moralid7  moralid8  moralid9  mcoop1  mcoop2  mcoop3  mcoop4  mcoop5  mcoop6  mcoop7  narc1  narc2  narc3  narc4  narc5  narc6  nidentity1  nidentity2  omind1  omind2  omind3  omind4  omind5  omind6  contact1  contact2  contact3  contact4  contact5  hygiene1  hygiene2  hygiene3  hygiene4  hygiene5  psupport1  psupport2  psupport3  psupport4  psupport5  political_ideology  happy  slf_ladder  riskperc2  self_esteem  sbelong1  sbelong2  sbelong3  sbelong4  optim1  optim2  slfcont1  slfcont2  slfcont3  slfcont4
##   8   2  cnarc1  cnarc2  cnarc3  ctheory1  ctheory2  ctheory3  ctheory4  CRT1  CRT2  CRT3  health_cond  marital1  mor_circle  moralid1  moralid10  moralid2  moralid3  moralid4  moralid5  moralid6  moralid7  moralid8  moralid9  mcoop1  mcoop2  mcoop3  mcoop4  mcoop5  mcoop6  mcoop7  narc1  narc2  narc3  narc4  narc5  narc6  nidentity1  nidentity2  omind1  omind2  omind3  omind4  omind5  omind6  contact1  contact2  contact3  contact4  contact5  hygiene1  hygiene2  hygiene3  hygiene4  hygiene5  psupport1  psupport2  psupport3  psupport4  psupport5  political_ideology  happy  slf_ladder  riskperc2  self_esteem  sbelong1  sbelong2  sbelong3  sbelong4  optim1  optim2  slfcont1  slfcont2  slfcont3  slfcont4
##   8   3  cnarc1  cnarc2  cnarc3  ctheory1  ctheory2  ctheory3  ctheory4  CRT1  CRT2  CRT3  health_cond  marital1  mor_circle  moralid1  moralid10  moralid2  moralid3  moralid4  moralid5  moralid6  moralid7  moralid8  moralid9  mcoop1  mcoop2  mcoop3  mcoop4  mcoop5  mcoop6  mcoop7  narc1  narc2  narc3  narc4  narc5  narc6  nidentity1  nidentity2  omind1  omind2  omind3  omind4  omind5  omind6  contact1  contact2  contact3  contact4  contact5  hygiene1  hygiene2  hygiene3  hygiene4  hygiene5  psupport1  psupport2  psupport3  psupport4  psupport5  political_ideology  happy  slf_ladder  riskperc2  self_esteem  sbelong1  sbelong2  sbelong3  sbelong4  optim1  optim2  slfcont1  slfcont2  slfcont3  slfcont4
##   8   4  cnarc1  cnarc2  cnarc3  ctheory1  ctheory2  ctheory3  ctheory4  CRT1  CRT2  CRT3  health_cond  marital1  mor_circle  moralid1  moralid10  moralid2  moralid3  moralid4  moralid5  moralid6  moralid7  moralid8  moralid9  mcoop1  mcoop2  mcoop3  mcoop4  mcoop5  mcoop6  mcoop7  narc1  narc2  narc3  narc4  narc5  narc6  nidentity1  nidentity2  omind1  omind2  omind3  omind4  omind5  omind6  contact1  contact2  contact3  contact4  contact5  hygiene1  hygiene2  hygiene3  hygiene4  hygiene5  psupport1  psupport2  psupport3  psupport4  psupport5  political_ideology  happy  slf_ladder  riskperc2  self_esteem  sbelong1  sbelong2  sbelong3  sbelong4  optim1  optim2  slfcont1  slfcont2  slfcont3  slfcont4
##   8   5  cnarc1  cnarc2  cnarc3  ctheory1  ctheory2  ctheory3  ctheory4  CRT1  CRT2  CRT3  health_cond  marital1  mor_circle  moralid1  moralid10  moralid2  moralid3  moralid4  moralid5  moralid6  moralid7  moralid8  moralid9  mcoop1  mcoop2  mcoop3  mcoop4  mcoop5  mcoop6  mcoop7  narc1  narc2  narc3  narc4  narc5  narc6  nidentity1  nidentity2  omind1  omind2  omind3  omind4  omind5  omind6  contact1  contact2  contact3  contact4  contact5  hygiene1  hygiene2  hygiene3  hygiene4  hygiene5  psupport1  psupport2  psupport3  psupport4  psupport5  political_ideology  happy  slf_ladder  riskperc2  self_esteem  sbelong1  sbelong2  sbelong3  sbelong4  optim1  optim2  slfcont1  slfcont2  slfcont3  slfcont4
##   8   6  cnarc1  cnarc2  cnarc3  ctheory1  ctheory2  ctheory3  ctheory4  CRT1  CRT2  CRT3  health_cond  marital1  mor_circle  moralid1  moralid10  moralid2  moralid3  moralid4  moralid5  moralid6  moralid7  moralid8  moralid9  mcoop1  mcoop2  mcoop3  mcoop4  mcoop5  mcoop6  mcoop7  narc1  narc2  narc3  narc4  narc5  narc6  nidentity1  nidentity2  omind1  omind2  omind3  omind4  omind5  omind6  contact1  contact2  contact3  contact4  contact5  hygiene1  hygiene2  hygiene3  hygiene4  hygiene5  psupport1  psupport2  psupport3  psupport4  psupport5  political_ideology  happy  slf_ladder  riskperc2  self_esteem  sbelong1  sbelong2  sbelong3  sbelong4  optim1  optim2  slfcont1  slfcont2  slfcont3  slfcont4
##   8   7  cnarc1  cnarc2  cnarc3  ctheory1  ctheory2  ctheory3  ctheory4  CRT1  CRT2  CRT3  health_cond  marital1  mor_circle  moralid1  moralid10  moralid2  moralid3  moralid4  moralid5  moralid6  moralid7  moralid8  moralid9  mcoop1  mcoop2  mcoop3  mcoop4  mcoop5  mcoop6  mcoop7  narc1  narc2  narc3  narc4  narc5  narc6  nidentity1  nidentity2  omind1  omind2  omind3  omind4  omind5  omind6  contact1  contact2  contact3  contact4  contact5  hygiene1  hygiene2  hygiene3  hygiene4  hygiene5  psupport1  psupport2  psupport3  psupport4  psupport5  political_ideology  happy  slf_ladder  riskperc2  self_esteem  sbelong1  sbelong2  sbelong3  sbelong4  optim1  optim2  slfcont1  slfcont2  slfcont3  slfcont4
##   8   8  cnarc1  cnarc2  cnarc3  ctheory1  ctheory2  ctheory3  ctheory4  CRT1  CRT2  CRT3  health_cond  marital1  mor_circle  moralid1  moralid10  moralid2  moralid3  moralid4  moralid5  moralid6  moralid7  moralid8  moralid9  mcoop1  mcoop2  mcoop3  mcoop4  mcoop5  mcoop6  mcoop7  narc1  narc2  narc3  narc4  narc5  narc6  nidentity1  nidentity2  omind1  omind2  omind3  omind4  omind5  omind6  contact1  contact2  contact3  contact4  contact5  hygiene1  hygiene2  hygiene3  hygiene4  hygiene5  psupport1  psupport2  psupport3  psupport4  psupport5  political_ideology  happy  slf_ladder  riskperc2  self_esteem  sbelong1  sbelong2  sbelong3  sbelong4  optim1  optim2  slfcont1  slfcont2  slfcont3  slfcont4
##   8   9  cnarc1  cnarc2  cnarc3  ctheory1  ctheory2  ctheory3  ctheory4  CRT1  CRT2  CRT3  health_cond  marital1  mor_circle  moralid1  moralid10  moralid2  moralid3  moralid4  moralid5  moralid6  moralid7  moralid8  moralid9  mcoop1  mcoop2  mcoop3  mcoop4  mcoop5  mcoop6  mcoop7  narc1  narc2  narc3  narc4  narc5  narc6  nidentity1  nidentity2  omind1  omind2  omind3  omind4  omind5  omind6  contact1  contact2  contact3  contact4  contact5  hygiene1  hygiene2  hygiene3  hygiene4  hygiene5  psupport1  psupport2  psupport3  psupport4  psupport5  political_ideology  happy  slf_ladder  riskperc2  self_esteem  sbelong1  sbelong2  sbelong3  sbelong4  optim1  optim2  slfcont1  slfcont2  slfcont3  slfcont4
##   8   10  cnarc1  cnarc2  cnarc3  ctheory1  ctheory2  ctheory3  ctheory4  CRT1  CRT2  CRT3  health_cond  marital1  mor_circle  moralid1  moralid10  moralid2  moralid3  moralid4  moralid5  moralid6  moralid7  moralid8  moralid9  mcoop1  mcoop2  mcoop3  mcoop4  mcoop5  mcoop6  mcoop7  narc1  narc2  narc3  narc4  narc5  narc6  nidentity1  nidentity2  omind1  omind2  omind3  omind4  omind5  omind6  contact1  contact2  contact3  contact4  contact5  hygiene1  hygiene2  hygiene3  hygiene4  hygiene5  psupport1  psupport2  psupport3  psupport4  psupport5  political_ideology  happy  slf_ladder  riskperc2  self_esteem  sbelong1  sbelong2  sbelong3  sbelong4  optim1  optim2  slfcont1  slfcont2  slfcont3  slfcont4
##   9   1  cnarc1  cnarc2  cnarc3  ctheory1  ctheory2  ctheory3  ctheory4  CRT1  CRT2  CRT3  health_cond  marital1  mor_circle  moralid1  moralid10  moralid2  moralid3  moralid4  moralid5  moralid6  moralid7  moralid8  moralid9  mcoop1  mcoop2  mcoop3  mcoop4  mcoop5  mcoop6  mcoop7  narc1  narc2  narc3  narc4  narc5  narc6  nidentity1  nidentity2  omind1  omind2  omind3  omind4  omind5  omind6  contact1  contact2  contact3  contact4  contact5  hygiene1  hygiene2  hygiene3  hygiene4  hygiene5  psupport1  psupport2  psupport3  psupport4  psupport5  political_ideology  happy  slf_ladder  riskperc2  self_esteem  sbelong1  sbelong2  sbelong3  sbelong4  optim1  optim2  slfcont1  slfcont2  slfcont3  slfcont4
##   9   2  cnarc1  cnarc2  cnarc3  ctheory1  ctheory2  ctheory3  ctheory4  CRT1  CRT2  CRT3  health_cond  marital1  mor_circle  moralid1  moralid10  moralid2  moralid3  moralid4  moralid5  moralid6  moralid7  moralid8  moralid9  mcoop1  mcoop2  mcoop3  mcoop4  mcoop5  mcoop6  mcoop7  narc1  narc2  narc3  narc4  narc5  narc6  nidentity1  nidentity2  omind1  omind2  omind3  omind4  omind5  omind6  contact1  contact2  contact3  contact4  contact5  hygiene1  hygiene2  hygiene3  hygiene4  hygiene5  psupport1  psupport2  psupport3  psupport4  psupport5  political_ideology  happy  slf_ladder  riskperc2  self_esteem  sbelong1  sbelong2  sbelong3  sbelong4  optim1  optim2  slfcont1  slfcont2  slfcont3  slfcont4
##   9   3  cnarc1  cnarc2  cnarc3  ctheory1  ctheory2  ctheory3  ctheory4  CRT1  CRT2  CRT3  health_cond  marital1  mor_circle  moralid1  moralid10  moralid2  moralid3  moralid4  moralid5  moralid6  moralid7  moralid8  moralid9  mcoop1  mcoop2  mcoop3  mcoop4  mcoop5  mcoop6  mcoop7  narc1  narc2  narc3  narc4  narc5  narc6  nidentity1  nidentity2  omind1  omind2  omind3  omind4  omind5  omind6  contact1  contact2  contact3  contact4  contact5  hygiene1  hygiene2  hygiene3  hygiene4  hygiene5  psupport1  psupport2  psupport3  psupport4  psupport5  political_ideology  happy  slf_ladder  riskperc2  self_esteem  sbelong1  sbelong2  sbelong3  sbelong4  optim1  optim2  slfcont1  slfcont2  slfcont3  slfcont4
##   9   4  cnarc1  cnarc2  cnarc3  ctheory1  ctheory2  ctheory3  ctheory4  CRT1  CRT2  CRT3  health_cond  marital1  mor_circle  moralid1  moralid10  moralid2  moralid3  moralid4  moralid5  moralid6  moralid7  moralid8  moralid9  mcoop1  mcoop2  mcoop3  mcoop4  mcoop5  mcoop6  mcoop7  narc1  narc2  narc3  narc4  narc5  narc6  nidentity1  nidentity2  omind1  omind2  omind3  omind4  omind5  omind6  contact1  contact2  contact3  contact4  contact5  hygiene1  hygiene2  hygiene3  hygiene4  hygiene5  psupport1  psupport2  psupport3  psupport4  psupport5  political_ideology  happy  slf_ladder  riskperc2  self_esteem  sbelong1  sbelong2  sbelong3  sbelong4  optim1  optim2  slfcont1  slfcont2  slfcont3  slfcont4
##   9   5  cnarc1  cnarc2  cnarc3  ctheory1  ctheory2  ctheory3  ctheory4  CRT1  CRT2  CRT3  health_cond  marital1  mor_circle  moralid1  moralid10  moralid2  moralid3  moralid4  moralid5  moralid6  moralid7  moralid8  moralid9  mcoop1  mcoop2  mcoop3  mcoop4  mcoop5  mcoop6  mcoop7  narc1  narc2  narc3  narc4  narc5  narc6  nidentity1  nidentity2  omind1  omind2  omind3  omind4  omind5  omind6  contact1  contact2  contact3  contact4  contact5  hygiene1  hygiene2  hygiene3  hygiene4  hygiene5  psupport1  psupport2  psupport3  psupport4  psupport5  political_ideology  happy  slf_ladder  riskperc2  self_esteem  sbelong1  sbelong2  sbelong3  sbelong4  optim1  optim2  slfcont1  slfcont2  slfcont3  slfcont4
##   9   6  cnarc1  cnarc2  cnarc3  ctheory1  ctheory2  ctheory3  ctheory4  CRT1  CRT2  CRT3  health_cond  marital1  mor_circle  moralid1  moralid10  moralid2  moralid3  moralid4  moralid5  moralid6  moralid7  moralid8  moralid9  mcoop1  mcoop2  mcoop3  mcoop4  mcoop5  mcoop6  mcoop7  narc1  narc2  narc3  narc4  narc5  narc6  nidentity1  nidentity2  omind1  omind2  omind3  omind4  omind5  omind6  contact1  contact2  contact3  contact4  contact5  hygiene1  hygiene2  hygiene3  hygiene4  hygiene5  psupport1  psupport2  psupport3  psupport4  psupport5  political_ideology  happy  slf_ladder  riskperc2  self_esteem  sbelong1  sbelong2  sbelong3  sbelong4  optim1  optim2  slfcont1  slfcont2  slfcont3  slfcont4
##   9   7  cnarc1  cnarc2  cnarc3  ctheory1  ctheory2  ctheory3  ctheory4  CRT1  CRT2  CRT3  health_cond  marital1  mor_circle  moralid1  moralid10  moralid2  moralid3  moralid4  moralid5  moralid6  moralid7  moralid8  moralid9  mcoop1  mcoop2  mcoop3  mcoop4  mcoop5  mcoop6  mcoop7  narc1  narc2  narc3  narc4  narc5  narc6  nidentity1  nidentity2  omind1  omind2  omind3  omind4  omind5  omind6  contact1  contact2  contact3  contact4  contact5  hygiene1  hygiene2  hygiene3  hygiene4  hygiene5  psupport1  psupport2  psupport3  psupport4  psupport5  political_ideology  happy  slf_ladder  riskperc2  self_esteem  sbelong1  sbelong2  sbelong3  sbelong4  optim1  optim2  slfcont1  slfcont2  slfcont3  slfcont4
##   9   8  cnarc1  cnarc2  cnarc3  ctheory1  ctheory2  ctheory3  ctheory4  CRT1  CRT2  CRT3  health_cond  marital1  mor_circle  moralid1  moralid10  moralid2  moralid3  moralid4  moralid5  moralid6  moralid7  moralid8  moralid9  mcoop1  mcoop2  mcoop3  mcoop4  mcoop5  mcoop6  mcoop7  narc1  narc2  narc3  narc4  narc5  narc6  nidentity1  nidentity2  omind1  omind2  omind3  omind4  omind5  omind6  contact1  contact2  contact3  contact4  contact5  hygiene1  hygiene2  hygiene3  hygiene4  hygiene5  psupport1  psupport2  psupport3  psupport4  psupport5  political_ideology  happy  slf_ladder  riskperc2  self_esteem  sbelong1  sbelong2  sbelong3  sbelong4  optim1  optim2  slfcont1  slfcont2  slfcont3  slfcont4
##   9   9  cnarc1  cnarc2  cnarc3  ctheory1  ctheory2  ctheory3  ctheory4  CRT1  CRT2  CRT3  health_cond  marital1  mor_circle  moralid1  moralid10  moralid2  moralid3  moralid4  moralid5  moralid6  moralid7  moralid8  moralid9  mcoop1  mcoop2  mcoop3  mcoop4  mcoop5  mcoop6  mcoop7  narc1  narc2  narc3  narc4  narc5  narc6  nidentity1  nidentity2  omind1  omind2  omind3  omind4  omind5  omind6  contact1  contact2  contact3  contact4  contact5  hygiene1  hygiene2  hygiene3  hygiene4  hygiene5  psupport1  psupport2  psupport3  psupport4  psupport5  political_ideology  happy  slf_ladder  riskperc2  self_esteem  sbelong1  sbelong2  sbelong3  sbelong4  optim1  optim2  slfcont1  slfcont2  slfcont3  slfcont4
##   9   10  cnarc1  cnarc2  cnarc3  ctheory1  ctheory2  ctheory3  ctheory4  CRT1  CRT2  CRT3  health_cond  marital1  mor_circle  moralid1  moralid10  moralid2  moralid3  moralid4  moralid5  moralid6  moralid7  moralid8  moralid9  mcoop1  mcoop2  mcoop3  mcoop4  mcoop5  mcoop6  mcoop7  narc1  narc2  narc3  narc4  narc5  narc6  nidentity1  nidentity2  omind1  omind2  omind3  omind4  omind5  omind6  contact1  contact2  contact3  contact4  contact5  hygiene1  hygiene2  hygiene3  hygiene4  hygiene5  psupport1  psupport2  psupport3  psupport4  psupport5  political_ideology  happy  slf_ladder  riskperc2  self_esteem  sbelong1  sbelong2  sbelong3  sbelong4  optim1  optim2  slfcont1  slfcont2  slfcont3  slfcont4
##   10   1  cnarc1  cnarc2  cnarc3  ctheory1  ctheory2  ctheory3  ctheory4  CRT1  CRT2  CRT3  health_cond  marital1  mor_circle  moralid1  moralid10  moralid2  moralid3  moralid4  moralid5  moralid6  moralid7  moralid8  moralid9  mcoop1  mcoop2  mcoop3  mcoop4  mcoop5  mcoop6  mcoop7  narc1  narc2  narc3  narc4  narc5  narc6  nidentity1  nidentity2  omind1  omind2  omind3  omind4  omind5  omind6  contact1  contact2  contact3  contact4  contact5  hygiene1  hygiene2  hygiene3  hygiene4  hygiene5  psupport1  psupport2  psupport3  psupport4  psupport5  political_ideology  happy  slf_ladder  riskperc2  self_esteem  sbelong1  sbelong2  sbelong3  sbelong4  optim1  optim2  slfcont1  slfcont2  slfcont3  slfcont4
##   10   2  cnarc1  cnarc2  cnarc3  ctheory1  ctheory2  ctheory3  ctheory4  CRT1  CRT2  CRT3  health_cond  marital1  mor_circle  moralid1  moralid10  moralid2  moralid3  moralid4  moralid5  moralid6  moralid7  moralid8  moralid9  mcoop1  mcoop2  mcoop3  mcoop4  mcoop5  mcoop6  mcoop7  narc1  narc2  narc3  narc4  narc5  narc6  nidentity1  nidentity2  omind1  omind2  omind3  omind4  omind5  omind6  contact1  contact2  contact3  contact4  contact5  hygiene1  hygiene2  hygiene3  hygiene4  hygiene5  psupport1  psupport2  psupport3  psupport4  psupport5  political_ideology  happy  slf_ladder  riskperc2  self_esteem  sbelong1  sbelong2  sbelong3  sbelong4  optim1  optim2  slfcont1  slfcont2  slfcont3  slfcont4
##   10   3  cnarc1  cnarc2  cnarc3  ctheory1  ctheory2  ctheory3  ctheory4  CRT1  CRT2  CRT3  health_cond  marital1  mor_circle  moralid1  moralid10  moralid2  moralid3  moralid4  moralid5  moralid6  moralid7  moralid8  moralid9  mcoop1  mcoop2  mcoop3  mcoop4  mcoop5  mcoop6  mcoop7  narc1  narc2  narc3  narc4  narc5  narc6  nidentity1  nidentity2  omind1  omind2  omind3  omind4  omind5  omind6  contact1  contact2  contact3  contact4  contact5  hygiene1  hygiene2  hygiene3  hygiene4  hygiene5  psupport1  psupport2  psupport3  psupport4  psupport5  political_ideology  happy  slf_ladder  riskperc2  self_esteem  sbelong1  sbelong2  sbelong3  sbelong4  optim1  optim2  slfcont1  slfcont2  slfcont3  slfcont4
##   10   4  cnarc1  cnarc2  cnarc3  ctheory1  ctheory2  ctheory3  ctheory4  CRT1  CRT2  CRT3  health_cond  marital1  mor_circle  moralid1  moralid10  moralid2  moralid3  moralid4  moralid5  moralid6  moralid7  moralid8  moralid9  mcoop1  mcoop2  mcoop3  mcoop4  mcoop5  mcoop6  mcoop7  narc1  narc2  narc3  narc4  narc5  narc6  nidentity1  nidentity2  omind1  omind2  omind3  omind4  omind5  omind6  contact1  contact2  contact3  contact4  contact5  hygiene1  hygiene2  hygiene3  hygiene4  hygiene5  psupport1  psupport2  psupport3  psupport4  psupport5  political_ideology  happy  slf_ladder  riskperc2  self_esteem  sbelong1  sbelong2  sbelong3  sbelong4  optim1  optim2  slfcont1  slfcont2  slfcont3  slfcont4
##   10   5  cnarc1  cnarc2  cnarc3  ctheory1  ctheory2  ctheory3  ctheory4  CRT1  CRT2  CRT3  health_cond  marital1  mor_circle  moralid1  moralid10  moralid2  moralid3  moralid4  moralid5  moralid6  moralid7  moralid8  moralid9  mcoop1  mcoop2  mcoop3  mcoop4  mcoop5  mcoop6  mcoop7  narc1  narc2  narc3  narc4  narc5  narc6  nidentity1  nidentity2  omind1  omind2  omind3  omind4  omind5  omind6  contact1  contact2  contact3  contact4  contact5  hygiene1  hygiene2  hygiene3  hygiene4  hygiene5  psupport1  psupport2  psupport3  psupport4  psupport5  political_ideology  happy  slf_ladder  riskperc2  self_esteem  sbelong1  sbelong2  sbelong3  sbelong4  optim1  optim2  slfcont1  slfcont2  slfcont3  slfcont4
##   10   6  cnarc1  cnarc2  cnarc3  ctheory1  ctheory2  ctheory3  ctheory4  CRT1  CRT2  CRT3  health_cond  marital1  mor_circle  moralid1  moralid10  moralid2  moralid3  moralid4  moralid5  moralid6  moralid7  moralid8  moralid9  mcoop1  mcoop2  mcoop3  mcoop4  mcoop5  mcoop6  mcoop7  narc1  narc2  narc3  narc4  narc5  narc6  nidentity1  nidentity2  omind1  omind2  omind3  omind4  omind5  omind6  contact1  contact2  contact3  contact4  contact5  hygiene1  hygiene2  hygiene3  hygiene4  hygiene5  psupport1  psupport2  psupport3  psupport4  psupport5  political_ideology  happy  slf_ladder  riskperc2  self_esteem  sbelong1  sbelong2  sbelong3  sbelong4  optim1  optim2  slfcont1  slfcont2  slfcont3  slfcont4
##   10   7  cnarc1  cnarc2  cnarc3  ctheory1  ctheory2  ctheory3  ctheory4  CRT1  CRT2  CRT3  health_cond  marital1  mor_circle  moralid1  moralid10  moralid2  moralid3  moralid4  moralid5  moralid6  moralid7  moralid8  moralid9  mcoop1  mcoop2  mcoop3  mcoop4  mcoop5  mcoop6  mcoop7  narc1  narc2  narc3  narc4  narc5  narc6  nidentity1  nidentity2  omind1  omind2  omind3  omind4  omind5  omind6  contact1  contact2  contact3  contact4  contact5  hygiene1  hygiene2  hygiene3  hygiene4  hygiene5  psupport1  psupport2  psupport3  psupport4  psupport5  political_ideology  happy  slf_ladder  riskperc2  self_esteem  sbelong1  sbelong2  sbelong3  sbelong4  optim1  optim2  slfcont1  slfcont2  slfcont3  slfcont4
##   10   8  cnarc1  cnarc2  cnarc3  ctheory1  ctheory2  ctheory3  ctheory4  CRT1  CRT2  CRT3  health_cond  marital1  mor_circle  moralid1  moralid10  moralid2  moralid3  moralid4  moralid5  moralid6  moralid7  moralid8  moralid9  mcoop1  mcoop2  mcoop3  mcoop4  mcoop5  mcoop6  mcoop7  narc1  narc2  narc3  narc4  narc5  narc6  nidentity1  nidentity2  omind1  omind2  omind3  omind4  omind5  omind6  contact1  contact2  contact3  contact4  contact5  hygiene1  hygiene2  hygiene3  hygiene4  hygiene5  psupport1  psupport2  psupport3  psupport4  psupport5  political_ideology  happy  slf_ladder  riskperc2  self_esteem  sbelong1  sbelong2  sbelong3  sbelong4  optim1  optim2  slfcont1  slfcont2  slfcont3  slfcont4
##   10   9  cnarc1  cnarc2  cnarc3  ctheory1  ctheory2  ctheory3  ctheory4  CRT1  CRT2  CRT3  health_cond  marital1  mor_circle  moralid1  moralid10  moralid2  moralid3  moralid4  moralid5  moralid6  moralid7  moralid8  moralid9  mcoop1  mcoop2  mcoop3  mcoop4  mcoop5  mcoop6  mcoop7  narc1  narc2  narc3  narc4  narc5  narc6  nidentity1  nidentity2  omind1  omind2  omind3  omind4  omind5  omind6  contact1  contact2  contact3  contact4  contact5  hygiene1  hygiene2  hygiene3  hygiene4  hygiene5  psupport1  psupport2  psupport3  psupport4  psupport5  political_ideology  happy  slf_ladder  riskperc2  self_esteem  sbelong1  sbelong2  sbelong3  sbelong4  optim1  optim2  slfcont1  slfcont2  slfcont3  slfcont4
##   10   10  cnarc1  cnarc2  cnarc3  ctheory1  ctheory2  ctheory3  ctheory4  CRT1  CRT2  CRT3  health_cond  marital1  mor_circle  moralid1  moralid10  moralid2  moralid3  moralid4  moralid5  moralid6  moralid7  moralid8  moralid9  mcoop1  mcoop2  mcoop3  mcoop4  mcoop5  mcoop6  mcoop7  narc1  narc2  narc3  narc4  narc5  narc6  nidentity1  nidentity2  omind1  omind2  omind3  omind4  omind5  omind6  contact1  contact2  contact3  contact4  contact5  hygiene1  hygiene2  hygiene3  hygiene4  hygiene5  psupport1  psupport2  psupport3  psupport4  psupport5  political_ideology  happy  slf_ladder  riskperc2  self_esteem  sbelong1  sbelong2  sbelong3  sbelong4  optim1  optim2  slfcont1  slfcont2  slfcont3  slfcont4
```

```
## Warning: Number of logged events: 7403
```

```
## 
##  iter imp variable
##   1   1  cnarc2  CRT2  CRT3  marital1  moralid10  moralid5  moralid9  mcoop5  mcoop6  narc2  narc3  narc4  narc5  political_ideology
##   1   2  cnarc2  CRT2  CRT3  marital1  moralid10  moralid5  moralid9  mcoop5  mcoop6  narc2  narc3  narc4  narc5  political_ideology
##   1   3  cnarc2  CRT2  CRT3  marital1  moralid10  moralid5  moralid9  mcoop5  mcoop6  narc2  narc3  narc4  narc5  political_ideology
##   1   4  cnarc2  CRT2  CRT3  marital1  moralid10  moralid5  moralid9  mcoop5  mcoop6  narc2  narc3  narc4  narc5  political_ideology
##   1   5  cnarc2  CRT2  CRT3  marital1  moralid10  moralid5  moralid9  mcoop5  mcoop6  narc2  narc3  narc4  narc5  political_ideology
##   1   6  cnarc2  CRT2  CRT3  marital1  moralid10  moralid5  moralid9  mcoop5  mcoop6  narc2  narc3  narc4  narc5  political_ideology
##   1   7  cnarc2  CRT2  CRT3  marital1  moralid10  moralid5  moralid9  mcoop5  mcoop6  narc2  narc3  narc4  narc5  political_ideology
##   1   8  cnarc2  CRT2  CRT3  marital1  moralid10  moralid5  moralid9  mcoop5  mcoop6  narc2  narc3  narc4  narc5  political_ideology
##   1   9  cnarc2  CRT2  CRT3  marital1  moralid10  moralid5  moralid9  mcoop5  mcoop6  narc2  narc3  narc4  narc5  political_ideology
##   1   10  cnarc2  CRT2  CRT3  marital1  moralid10  moralid5  moralid9  mcoop5  mcoop6  narc2  narc3  narc4  narc5  political_ideology
##   2   1  cnarc2  CRT2  CRT3  marital1  moralid10  moralid5  moralid9  mcoop5  mcoop6  narc2  narc3  narc4  narc5  political_ideology
##   2   2  cnarc2  CRT2  CRT3  marital1  moralid10  moralid5  moralid9  mcoop5  mcoop6  narc2  narc3  narc4  narc5  political_ideology
##   2   3  cnarc2  CRT2  CRT3  marital1  moralid10  moralid5  moralid9  mcoop5  mcoop6  narc2  narc3  narc4  narc5  political_ideology
##   2   4  cnarc2  CRT2  CRT3  marital1  moralid10  moralid5  moralid9  mcoop5  mcoop6  narc2  narc3  narc4  narc5  political_ideology
##   2   5  cnarc2  CRT2  CRT3  marital1  moralid10  moralid5  moralid9  mcoop5  mcoop6  narc2  narc3  narc4  narc5  political_ideology
##   2   6  cnarc2  CRT2  CRT3  marital1  moralid10  moralid5  moralid9  mcoop5  mcoop6  narc2  narc3  narc4  narc5  political_ideology
##   2   7  cnarc2  CRT2  CRT3  marital1  moralid10  moralid5  moralid9  mcoop5  mcoop6  narc2  narc3  narc4  narc5  political_ideology
##   2   8  cnarc2  CRT2  CRT3  marital1  moralid10  moralid5  moralid9  mcoop5  mcoop6  narc2  narc3  narc4  narc5  political_ideology
##   2   9  cnarc2  CRT2  CRT3  marital1  moralid10  moralid5  moralid9  mcoop5  mcoop6  narc2  narc3  narc4  narc5  political_ideology
##   2   10  cnarc2  CRT2  CRT3  marital1  moralid10  moralid5  moralid9  mcoop5  mcoop6  narc2  narc3  narc4  narc5  political_ideology
##   3   1  cnarc2  CRT2  CRT3  marital1  moralid10  moralid5  moralid9  mcoop5  mcoop6  narc2  narc3  narc4  narc5  political_ideology
##   3   2  cnarc2  CRT2  CRT3  marital1  moralid10  moralid5  moralid9  mcoop5  mcoop6  narc2  narc3  narc4  narc5  political_ideology
##   3   3  cnarc2  CRT2  CRT3  marital1  moralid10  moralid5  moralid9  mcoop5  mcoop6  narc2  narc3  narc4  narc5  political_ideology
##   3   4  cnarc2  CRT2  CRT3  marital1  moralid10  moralid5  moralid9  mcoop5  mcoop6  narc2  narc3  narc4  narc5  political_ideology
##   3   5  cnarc2  CRT2  CRT3  marital1  moralid10  moralid5  moralid9  mcoop5  mcoop6  narc2  narc3  narc4  narc5  political_ideology
##   3   6  cnarc2  CRT2  CRT3  marital1  moralid10  moralid5  moralid9  mcoop5  mcoop6  narc2  narc3  narc4  narc5  political_ideology
##   3   7  cnarc2  CRT2  CRT3  marital1  moralid10  moralid5  moralid9  mcoop5  mcoop6  narc2  narc3  narc4  narc5  political_ideology
##   3   8  cnarc2  CRT2  CRT3  marital1  moralid10  moralid5  moralid9  mcoop5  mcoop6  narc2  narc3  narc4  narc5  political_ideology
##   3   9  cnarc2  CRT2  CRT3  marital1  moralid10  moralid5  moralid9  mcoop5  mcoop6  narc2  narc3  narc4  narc5  political_ideology
##   3   10  cnarc2  CRT2  CRT3  marital1  moralid10  moralid5  moralid9  mcoop5  mcoop6  narc2  narc3  narc4  narc5  political_ideology
##   4   1  cnarc2  CRT2  CRT3  marital1  moralid10  moralid5  moralid9  mcoop5  mcoop6  narc2  narc3  narc4  narc5  political_ideology
##   4   2  cnarc2  CRT2  CRT3  marital1  moralid10  moralid5  moralid9  mcoop5  mcoop6  narc2  narc3  narc4  narc5  political_ideology
##   4   3  cnarc2  CRT2  CRT3  marital1  moralid10  moralid5  moralid9  mcoop5  mcoop6  narc2  narc3  narc4  narc5  political_ideology
##   4   4  cnarc2  CRT2  CRT3  marital1  moralid10  moralid5  moralid9  mcoop5  mcoop6  narc2  narc3  narc4  narc5  political_ideology
##   4   5  cnarc2  CRT2  CRT3  marital1  moralid10  moralid5  moralid9  mcoop5  mcoop6  narc2  narc3  narc4  narc5  political_ideology
##   4   6  cnarc2  CRT2  CRT3  marital1  moralid10  moralid5  moralid9  mcoop5  mcoop6  narc2  narc3  narc4  narc5  political_ideology
##   4   7  cnarc2  CRT2  CRT3  marital1  moralid10  moralid5  moralid9  mcoop5  mcoop6  narc2  narc3  narc4  narc5  political_ideology
##   4   8  cnarc2  CRT2  CRT3  marital1  moralid10  moralid5  moralid9  mcoop5  mcoop6  narc2  narc3  narc4  narc5  political_ideology
##   4   9  cnarc2  CRT2  CRT3  marital1  moralid10  moralid5  moralid9  mcoop5  mcoop6  narc2  narc3  narc4  narc5  political_ideology
##   4   10  cnarc2  CRT2  CRT3  marital1  moralid10  moralid5  moralid9  mcoop5  mcoop6  narc2  narc3  narc4  narc5  political_ideology
##   5   1  cnarc2  CRT2  CRT3  marital1  moralid10  moralid5  moralid9  mcoop5  mcoop6  narc2  narc3  narc4  narc5  political_ideology
##   5   2  cnarc2  CRT2  CRT3  marital1  moralid10  moralid5  moralid9  mcoop5  mcoop6  narc2  narc3  narc4  narc5  political_ideology
##   5   3  cnarc2  CRT2  CRT3  marital1  moralid10  moralid5  moralid9  mcoop5  mcoop6  narc2  narc3  narc4  narc5  political_ideology
##   5   4  cnarc2  CRT2  CRT3  marital1  moralid10  moralid5  moralid9  mcoop5  mcoop6  narc2  narc3  narc4  narc5  political_ideology
##   5   5  cnarc2  CRT2  CRT3  marital1  moralid10  moralid5  moralid9  mcoop5  mcoop6  narc2  narc3  narc4  narc5  political_ideology
##   5   6  cnarc2  CRT2  CRT3  marital1  moralid10  moralid5  moralid9  mcoop5  mcoop6  narc2  narc3  narc4  narc5  political_ideology
##   5   7  cnarc2  CRT2  CRT3  marital1  moralid10  moralid5  moralid9  mcoop5  mcoop6  narc2  narc3  narc4  narc5  political_ideology
##   5   8  cnarc2  CRT2  CRT3  marital1  moralid10  moralid5  moralid9  mcoop5  mcoop6  narc2  narc3  narc4  narc5  political_ideology
##   5   9  cnarc2  CRT2  CRT3  marital1  moralid10  moralid5  moralid9  mcoop5  mcoop6  narc2  narc3  narc4  narc5  political_ideology
##   5   10  cnarc2  CRT2  CRT3  marital1  moralid10  moralid5  moralid9  mcoop5  mcoop6  narc2  narc3  narc4  narc5  political_ideology
##   6   1  cnarc2  CRT2  CRT3  marital1  moralid10  moralid5  moralid9  mcoop5  mcoop6  narc2  narc3  narc4  narc5  political_ideology
##   6   2  cnarc2  CRT2  CRT3  marital1  moralid10  moralid5  moralid9  mcoop5  mcoop6  narc2  narc3  narc4  narc5  political_ideology
##   6   3  cnarc2  CRT2  CRT3  marital1  moralid10  moralid5  moralid9  mcoop5  mcoop6  narc2  narc3  narc4  narc5  political_ideology
##   6   4  cnarc2  CRT2  CRT3  marital1  moralid10  moralid5  moralid9  mcoop5  mcoop6  narc2  narc3  narc4  narc5  political_ideology
##   6   5  cnarc2  CRT2  CRT3  marital1  moralid10  moralid5  moralid9  mcoop5  mcoop6  narc2  narc3  narc4  narc5  political_ideology
##   6   6  cnarc2  CRT2  CRT3  marital1  moralid10  moralid5  moralid9  mcoop5  mcoop6  narc2  narc3  narc4  narc5  political_ideology
##   6   7  cnarc2  CRT2  CRT3  marital1  moralid10  moralid5  moralid9  mcoop5  mcoop6  narc2  narc3  narc4  narc5  political_ideology
##   6   8  cnarc2  CRT2  CRT3  marital1  moralid10  moralid5  moralid9  mcoop5  mcoop6  narc2  narc3  narc4  narc5  political_ideology
##   6   9  cnarc2  CRT2  CRT3  marital1  moralid10  moralid5  moralid9  mcoop5  mcoop6  narc2  narc3  narc4  narc5  political_ideology
##   6   10  cnarc2  CRT2  CRT3  marital1  moralid10  moralid5  moralid9  mcoop5  mcoop6  narc2  narc3  narc4  narc5  political_ideology
##   7   1  cnarc2  CRT2  CRT3  marital1  moralid10  moralid5  moralid9  mcoop5  mcoop6  narc2  narc3  narc4  narc5  political_ideology
##   7   2  cnarc2  CRT2  CRT3  marital1  moralid10  moralid5  moralid9  mcoop5  mcoop6  narc2  narc3  narc4  narc5  political_ideology
##   7   3  cnarc2  CRT2  CRT3  marital1  moralid10  moralid5  moralid9  mcoop5  mcoop6  narc2  narc3  narc4  narc5  political_ideology
##   7   4  cnarc2  CRT2  CRT3  marital1  moralid10  moralid5  moralid9  mcoop5  mcoop6  narc2  narc3  narc4  narc5  political_ideology
##   7   5  cnarc2  CRT2  CRT3  marital1  moralid10  moralid5  moralid9  mcoop5  mcoop6  narc2  narc3  narc4  narc5  political_ideology
##   7   6  cnarc2  CRT2  CRT3  marital1  moralid10  moralid5  moralid9  mcoop5  mcoop6  narc2  narc3  narc4  narc5  political_ideology
##   7   7  cnarc2  CRT2  CRT3  marital1  moralid10  moralid5  moralid9  mcoop5  mcoop6  narc2  narc3  narc4  narc5  political_ideology
##   7   8  cnarc2  CRT2  CRT3  marital1  moralid10  moralid5  moralid9  mcoop5  mcoop6  narc2  narc3  narc4  narc5  political_ideology
##   7   9  cnarc2  CRT2  CRT3  marital1  moralid10  moralid5  moralid9  mcoop5  mcoop6  narc2  narc3  narc4  narc5  political_ideology
##   7   10  cnarc2  CRT2  CRT3  marital1  moralid10  moralid5  moralid9  mcoop5  mcoop6  narc2  narc3  narc4  narc5  political_ideology
##   8   1  cnarc2  CRT2  CRT3  marital1  moralid10  moralid5  moralid9  mcoop5  mcoop6  narc2  narc3  narc4  narc5  political_ideology
##   8   2  cnarc2  CRT2  CRT3  marital1  moralid10  moralid5  moralid9  mcoop5  mcoop6  narc2  narc3  narc4  narc5  political_ideology
##   8   3  cnarc2  CRT2  CRT3  marital1  moralid10  moralid5  moralid9  mcoop5  mcoop6  narc2  narc3  narc4  narc5  political_ideology
##   8   4  cnarc2  CRT2  CRT3  marital1  moralid10  moralid5  moralid9  mcoop5  mcoop6  narc2  narc3  narc4  narc5  political_ideology
##   8   5  cnarc2  CRT2  CRT3  marital1  moralid10  moralid5  moralid9  mcoop5  mcoop6  narc2  narc3  narc4  narc5  political_ideology
##   8   6  cnarc2  CRT2  CRT3  marital1  moralid10  moralid5  moralid9  mcoop5  mcoop6  narc2  narc3  narc4  narc5  political_ideology
##   8   7  cnarc2  CRT2  CRT3  marital1  moralid10  moralid5  moralid9  mcoop5  mcoop6  narc2  narc3  narc4  narc5  political_ideology
##   8   8  cnarc2  CRT2  CRT3  marital1  moralid10  moralid5  moralid9  mcoop5  mcoop6  narc2  narc3  narc4  narc5  political_ideology
##   8   9  cnarc2  CRT2  CRT3  marital1  moralid10  moralid5  moralid9  mcoop5  mcoop6  narc2  narc3  narc4  narc5  political_ideology
##   8   10  cnarc2  CRT2  CRT3  marital1  moralid10  moralid5  moralid9  mcoop5  mcoop6  narc2  narc3  narc4  narc5  political_ideology
##   9   1  cnarc2  CRT2  CRT3  marital1  moralid10  moralid5  moralid9  mcoop5  mcoop6  narc2  narc3  narc4  narc5  political_ideology
##   9   2  cnarc2  CRT2  CRT3  marital1  moralid10  moralid5  moralid9  mcoop5  mcoop6  narc2  narc3  narc4  narc5  political_ideology
##   9   3  cnarc2  CRT2  CRT3  marital1  moralid10  moralid5  moralid9  mcoop5  mcoop6  narc2  narc3  narc4  narc5  political_ideology
##   9   4  cnarc2  CRT2  CRT3  marital1  moralid10  moralid5  moralid9  mcoop5  mcoop6  narc2  narc3  narc4  narc5  political_ideology
##   9   5  cnarc2  CRT2  CRT3  marital1  moralid10  moralid5  moralid9  mcoop5  mcoop6  narc2  narc3  narc4  narc5  political_ideology
##   9   6  cnarc2  CRT2  CRT3  marital1  moralid10  moralid5  moralid9  mcoop5  mcoop6  narc2  narc3  narc4  narc5  political_ideology
##   9   7  cnarc2  CRT2  CRT3  marital1  moralid10  moralid5  moralid9  mcoop5  mcoop6  narc2  narc3  narc4  narc5  political_ideology
##   9   8  cnarc2  CRT2  CRT3  marital1  moralid10  moralid5  moralid9  mcoop5  mcoop6  narc2  narc3  narc4  narc5  political_ideology
##   9   9  cnarc2  CRT2  CRT3  marital1  moralid10  moralid5  moralid9  mcoop5  mcoop6  narc2  narc3  narc4  narc5  political_ideology
##   9   10  cnarc2  CRT2  CRT3  marital1  moralid10  moralid5  moralid9  mcoop5  mcoop6  narc2  narc3  narc4  narc5  political_ideology
##   10   1  cnarc2  CRT2  CRT3  marital1  moralid10  moralid5  moralid9  mcoop5  mcoop6  narc2  narc3  narc4  narc5  political_ideology
##   10   2  cnarc2  CRT2  CRT3  marital1  moralid10  moralid5  moralid9  mcoop5  mcoop6  narc2  narc3  narc4  narc5  political_ideology
##   10   3  cnarc2  CRT2  CRT3  marital1  moralid10  moralid5  moralid9  mcoop5  mcoop6  narc2  narc3  narc4  narc5  political_ideology
##   10   4  cnarc2  CRT2  CRT3  marital1  moralid10  moralid5  moralid9  mcoop5  mcoop6  narc2  narc3  narc4  narc5  political_ideology
##   10   5  cnarc2  CRT2  CRT3  marital1  moralid10  moralid5  moralid9  mcoop5  mcoop6  narc2  narc3  narc4  narc5  political_ideology
##   10   6  cnarc2  CRT2  CRT3  marital1  moralid10  moralid5  moralid9  mcoop5  mcoop6  narc2  narc3  narc4  narc5  political_ideology
##   10   7  cnarc2  CRT2  CRT3  marital1  moralid10  moralid5  moralid9  mcoop5  mcoop6  narc2  narc3  narc4  narc5  political_ideology
##   10   8  cnarc2  CRT2  CRT3  marital1  moralid10  moralid5  moralid9  mcoop5  mcoop6  narc2  narc3  narc4  narc5  political_ideology
##   10   9  cnarc2  CRT2  CRT3  marital1  moralid10  moralid5  moralid9  mcoop5  mcoop6  narc2  narc3  narc4  narc5  political_ideology
##   10   10  cnarc2  CRT2  CRT3  marital1  moralid10  moralid5  moralid9  mcoop5  mcoop6  narc2  narc3  narc4  narc5  political_ideology
```

```
## Warning: Number of logged events: 1403
```

```
## 
##  iter imp variable
##   1   1  age  cnarc2  cnarc3  ctheory1  ctheory2  ctheory3  ctheory4  CRT1  CRT2  CRT3  employ_status1  generosity1  generosity2  generosity3  health_cond  marital1  mor_circle  moralid1  moralid10  moralid2  moralid3  moralid4  moralid5  moralid6  moralid7  moralid8  moralid9  mcoop3  mcoop5  narc1  narc2  narc3  narc4  narc5  narc6  nidentity1  omind1  omind2  omind5  omind6  contact2  contact3  contact4  hygiene1  hygiene2  hygiene4  hygiene5  psupport1  psupport2  psupport3  psupport4  psupport5  political_ideology  happy  slf_ladder  riskperc1  riskperc2  self_esteem  sex1  sbelong1  sbelong2  sbelong3  sbelong4  slfcont1  slfcont2  slfcont3  slfcont4  urban
##   1   2  age  cnarc2  cnarc3  ctheory1  ctheory2  ctheory3  ctheory4  CRT1  CRT2  CRT3  employ_status1  generosity1  generosity2  generosity3  health_cond  marital1  mor_circle  moralid1  moralid10  moralid2  moralid3  moralid4  moralid5  moralid6  moralid7  moralid8  moralid9  mcoop3  mcoop5  narc1  narc2  narc3  narc4  narc5  narc6  nidentity1  omind1  omind2  omind5  omind6  contact2  contact3  contact4  hygiene1  hygiene2  hygiene4  hygiene5  psupport1  psupport2  psupport3  psupport4  psupport5  political_ideology  happy  slf_ladder  riskperc1  riskperc2  self_esteem  sex1  sbelong1  sbelong2  sbelong3  sbelong4  slfcont1  slfcont2  slfcont3  slfcont4  urban
##   1   3  age  cnarc2  cnarc3  ctheory1  ctheory2  ctheory3  ctheory4  CRT1  CRT2  CRT3  employ_status1  generosity1  generosity2  generosity3  health_cond  marital1  mor_circle  moralid1  moralid10  moralid2  moralid3  moralid4  moralid5  moralid6  moralid7  moralid8  moralid9  mcoop3  mcoop5  narc1  narc2  narc3  narc4  narc5  narc6  nidentity1  omind1  omind2  omind5  omind6  contact2  contact3  contact4  hygiene1  hygiene2  hygiene4  hygiene5  psupport1  psupport2  psupport3  psupport4  psupport5  political_ideology  happy  slf_ladder  riskperc1  riskperc2  self_esteem  sex1  sbelong1  sbelong2  sbelong3  sbelong4  slfcont1  slfcont2  slfcont3  slfcont4  urban
##   1   4  age  cnarc2  cnarc3  ctheory1  ctheory2  ctheory3  ctheory4  CRT1  CRT2  CRT3  employ_status1  generosity1  generosity2  generosity3  health_cond  marital1  mor_circle  moralid1  moralid10  moralid2  moralid3  moralid4  moralid5  moralid6  moralid7  moralid8  moralid9  mcoop3  mcoop5  narc1  narc2  narc3  narc4  narc5  narc6  nidentity1  omind1  omind2  omind5  omind6  contact2  contact3  contact4  hygiene1  hygiene2  hygiene4  hygiene5  psupport1  psupport2  psupport3  psupport4  psupport5  political_ideology  happy  slf_ladder  riskperc1  riskperc2  self_esteem  sex1  sbelong1  sbelong2  sbelong3  sbelong4  slfcont1  slfcont2  slfcont3  slfcont4  urban
##   1   5  age  cnarc2  cnarc3  ctheory1  ctheory2  ctheory3  ctheory4  CRT1  CRT2  CRT3  employ_status1  generosity1  generosity2  generosity3  health_cond  marital1  mor_circle  moralid1  moralid10  moralid2  moralid3  moralid4  moralid5  moralid6  moralid7  moralid8  moralid9  mcoop3  mcoop5  narc1  narc2  narc3  narc4  narc5  narc6  nidentity1  omind1  omind2  omind5  omind6  contact2  contact3  contact4  hygiene1  hygiene2  hygiene4  hygiene5  psupport1  psupport2  psupport3  psupport4  psupport5  political_ideology  happy  slf_ladder  riskperc1  riskperc2  self_esteem  sex1  sbelong1  sbelong2  sbelong3  sbelong4  slfcont1  slfcont2  slfcont3  slfcont4  urban
##   1   6  age  cnarc2  cnarc3  ctheory1  ctheory2  ctheory3  ctheory4  CRT1  CRT2  CRT3  employ_status1  generosity1  generosity2  generosity3  health_cond  marital1  mor_circle  moralid1  moralid10  moralid2  moralid3  moralid4  moralid5  moralid6  moralid7  moralid8  moralid9  mcoop3  mcoop5  narc1  narc2  narc3  narc4  narc5  narc6  nidentity1  omind1  omind2  omind5  omind6  contact2  contact3  contact4  hygiene1  hygiene2  hygiene4  hygiene5  psupport1  psupport2  psupport3  psupport4  psupport5  political_ideology  happy  slf_ladder  riskperc1  riskperc2  self_esteem  sex1  sbelong1  sbelong2  sbelong3  sbelong4  slfcont1  slfcont2  slfcont3  slfcont4  urban
##   1   7  age  cnarc2  cnarc3  ctheory1  ctheory2  ctheory3  ctheory4  CRT1  CRT2  CRT3  employ_status1  generosity1  generosity2  generosity3  health_cond  marital1  mor_circle  moralid1  moralid10  moralid2  moralid3  moralid4  moralid5  moralid6  moralid7  moralid8  moralid9  mcoop3  mcoop5  narc1  narc2  narc3  narc4  narc5  narc6  nidentity1  omind1  omind2  omind5  omind6  contact2  contact3  contact4  hygiene1  hygiene2  hygiene4  hygiene5  psupport1  psupport2  psupport3  psupport4  psupport5  political_ideology  happy  slf_ladder  riskperc1  riskperc2  self_esteem  sex1  sbelong1  sbelong2  sbelong3  sbelong4  slfcont1  slfcont2  slfcont3  slfcont4  urban
##   1   8  age  cnarc2  cnarc3  ctheory1  ctheory2  ctheory3  ctheory4  CRT1  CRT2  CRT3  employ_status1  generosity1  generosity2  generosity3  health_cond  marital1  mor_circle  moralid1  moralid10  moralid2  moralid3  moralid4  moralid5  moralid6  moralid7  moralid8  moralid9  mcoop3  mcoop5  narc1  narc2  narc3  narc4  narc5  narc6  nidentity1  omind1  omind2  omind5  omind6  contact2  contact3  contact4  hygiene1  hygiene2  hygiene4  hygiene5  psupport1  psupport2  psupport3  psupport4  psupport5  political_ideology  happy  slf_ladder  riskperc1  riskperc2  self_esteem  sex1  sbelong1  sbelong2  sbelong3  sbelong4  slfcont1  slfcont2  slfcont3  slfcont4  urban
##   1   9  age  cnarc2  cnarc3  ctheory1  ctheory2  ctheory3  ctheory4  CRT1  CRT2  CRT3  employ_status1  generosity1  generosity2  generosity3  health_cond  marital1  mor_circle  moralid1  moralid10  moralid2  moralid3  moralid4  moralid5  moralid6  moralid7  moralid8  moralid9  mcoop3  mcoop5  narc1  narc2  narc3  narc4  narc5  narc6  nidentity1  omind1  omind2  omind5  omind6  contact2  contact3  contact4  hygiene1  hygiene2  hygiene4  hygiene5  psupport1  psupport2  psupport3  psupport4  psupport5  political_ideology  happy  slf_ladder  riskperc1  riskperc2  self_esteem  sex1  sbelong1  sbelong2  sbelong3  sbelong4  slfcont1  slfcont2  slfcont3  slfcont4  urban
##   1   10  age  cnarc2  cnarc3  ctheory1  ctheory2  ctheory3  ctheory4  CRT1  CRT2  CRT3  employ_status1  generosity1  generosity2  generosity3  health_cond  marital1  mor_circle  moralid1  moralid10  moralid2  moralid3  moralid4  moralid5  moralid6  moralid7  moralid8  moralid9  mcoop3  mcoop5  narc1  narc2  narc3  narc4  narc5  narc6  nidentity1  omind1  omind2  omind5  omind6  contact2  contact3  contact4  hygiene1  hygiene2  hygiene4  hygiene5  psupport1  psupport2  psupport3  psupport4  psupport5  political_ideology  happy  slf_ladder  riskperc1  riskperc2  self_esteem  sex1  sbelong1  sbelong2  sbelong3  sbelong4  slfcont1  slfcont2  slfcont3  slfcont4  urban
##   2   1  age  cnarc2  cnarc3  ctheory1  ctheory2  ctheory3  ctheory4  CRT1  CRT2  CRT3  employ_status1  generosity1  generosity2  generosity3  health_cond  marital1  mor_circle  moralid1  moralid10  moralid2  moralid3  moralid4  moralid5  moralid6  moralid7  moralid8  moralid9  mcoop3  mcoop5  narc1  narc2  narc3  narc4  narc5  narc6  nidentity1  omind1  omind2  omind5  omind6  contact2  contact3  contact4  hygiene1  hygiene2  hygiene4  hygiene5  psupport1  psupport2  psupport3  psupport4  psupport5  political_ideology  happy  slf_ladder  riskperc1  riskperc2  self_esteem  sex1  sbelong1  sbelong2  sbelong3  sbelong4  slfcont1  slfcont2  slfcont3  slfcont4  urban
##   2   2  age  cnarc2  cnarc3  ctheory1  ctheory2  ctheory3  ctheory4  CRT1  CRT2  CRT3  employ_status1  generosity1  generosity2  generosity3  health_cond  marital1  mor_circle  moralid1  moralid10  moralid2  moralid3  moralid4  moralid5  moralid6  moralid7  moralid8  moralid9  mcoop3  mcoop5  narc1  narc2  narc3  narc4  narc5  narc6  nidentity1  omind1  omind2  omind5  omind6  contact2  contact3  contact4  hygiene1  hygiene2  hygiene4  hygiene5  psupport1  psupport2  psupport3  psupport4  psupport5  political_ideology  happy  slf_ladder  riskperc1  riskperc2  self_esteem  sex1  sbelong1  sbelong2  sbelong3  sbelong4  slfcont1  slfcont2  slfcont3  slfcont4  urban
##   2   3  age  cnarc2  cnarc3  ctheory1  ctheory2  ctheory3  ctheory4  CRT1  CRT2  CRT3  employ_status1  generosity1  generosity2  generosity3  health_cond  marital1  mor_circle  moralid1  moralid10  moralid2  moralid3  moralid4  moralid5  moralid6  moralid7  moralid8  moralid9  mcoop3  mcoop5  narc1  narc2  narc3  narc4  narc5  narc6  nidentity1  omind1  omind2  omind5  omind6  contact2  contact3  contact4  hygiene1  hygiene2  hygiene4  hygiene5  psupport1  psupport2  psupport3  psupport4  psupport5  political_ideology  happy  slf_ladder  riskperc1  riskperc2  self_esteem  sex1  sbelong1  sbelong2  sbelong3  sbelong4  slfcont1  slfcont2  slfcont3  slfcont4  urban
##   2   4  age  cnarc2  cnarc3  ctheory1  ctheory2  ctheory3  ctheory4  CRT1  CRT2  CRT3  employ_status1  generosity1  generosity2  generosity3  health_cond  marital1  mor_circle  moralid1  moralid10  moralid2  moralid3  moralid4  moralid5  moralid6  moralid7  moralid8  moralid9  mcoop3  mcoop5  narc1  narc2  narc3  narc4  narc5  narc6  nidentity1  omind1  omind2  omind5  omind6  contact2  contact3  contact4  hygiene1  hygiene2  hygiene4  hygiene5  psupport1  psupport2  psupport3  psupport4  psupport5  political_ideology  happy  slf_ladder  riskperc1  riskperc2  self_esteem  sex1  sbelong1  sbelong2  sbelong3  sbelong4  slfcont1  slfcont2  slfcont3  slfcont4  urban
##   2   5  age  cnarc2  cnarc3  ctheory1  ctheory2  ctheory3  ctheory4  CRT1  CRT2  CRT3  employ_status1  generosity1  generosity2  generosity3  health_cond  marital1  mor_circle  moralid1  moralid10  moralid2  moralid3  moralid4  moralid5  moralid6  moralid7  moralid8  moralid9  mcoop3  mcoop5  narc1  narc2  narc3  narc4  narc5  narc6  nidentity1  omind1  omind2  omind5  omind6  contact2  contact3  contact4  hygiene1  hygiene2  hygiene4  hygiene5  psupport1  psupport2  psupport3  psupport4  psupport5  political_ideology  happy  slf_ladder  riskperc1  riskperc2  self_esteem  sex1  sbelong1  sbelong2  sbelong3  sbelong4  slfcont1  slfcont2  slfcont3  slfcont4  urban
##   2   6  age  cnarc2  cnarc3  ctheory1  ctheory2  ctheory3  ctheory4  CRT1  CRT2  CRT3  employ_status1  generosity1  generosity2  generosity3  health_cond  marital1  mor_circle  moralid1  moralid10  moralid2  moralid3  moralid4  moralid5  moralid6  moralid7  moralid8  moralid9  mcoop3  mcoop5  narc1  narc2  narc3  narc4  narc5  narc6  nidentity1  omind1  omind2  omind5  omind6  contact2  contact3  contact4  hygiene1  hygiene2  hygiene4  hygiene5  psupport1  psupport2  psupport3  psupport4  psupport5  political_ideology  happy  slf_ladder  riskperc1  riskperc2  self_esteem  sex1  sbelong1  sbelong2  sbelong3  sbelong4  slfcont1  slfcont2  slfcont3  slfcont4  urban
##   2   7  age  cnarc2  cnarc3  ctheory1  ctheory2  ctheory3  ctheory4  CRT1  CRT2  CRT3  employ_status1  generosity1  generosity2  generosity3  health_cond  marital1  mor_circle  moralid1  moralid10  moralid2  moralid3  moralid4  moralid5  moralid6  moralid7  moralid8  moralid9  mcoop3  mcoop5  narc1  narc2  narc3  narc4  narc5  narc6  nidentity1  omind1  omind2  omind5  omind6  contact2  contact3  contact4  hygiene1  hygiene2  hygiene4  hygiene5  psupport1  psupport2  psupport3  psupport4  psupport5  political_ideology  happy  slf_ladder  riskperc1  riskperc2  self_esteem  sex1  sbelong1  sbelong2  sbelong3  sbelong4  slfcont1  slfcont2  slfcont3  slfcont4  urban
##   2   8  age  cnarc2  cnarc3  ctheory1  ctheory2  ctheory3  ctheory4  CRT1  CRT2  CRT3  employ_status1  generosity1  generosity2  generosity3  health_cond  marital1  mor_circle  moralid1  moralid10  moralid2  moralid3  moralid4  moralid5  moralid6  moralid7  moralid8  moralid9  mcoop3  mcoop5  narc1  narc2  narc3  narc4  narc5  narc6  nidentity1  omind1  omind2  omind5  omind6  contact2  contact3  contact4  hygiene1  hygiene2  hygiene4  hygiene5  psupport1  psupport2  psupport3  psupport4  psupport5  political_ideology  happy  slf_ladder  riskperc1  riskperc2  self_esteem  sex1  sbelong1  sbelong2  sbelong3  sbelong4  slfcont1  slfcont2  slfcont3  slfcont4  urban
##   2   9  age  cnarc2  cnarc3  ctheory1  ctheory2  ctheory3  ctheory4  CRT1  CRT2  CRT3  employ_status1  generosity1  generosity2  generosity3  health_cond  marital1  mor_circle  moralid1  moralid10  moralid2  moralid3  moralid4  moralid5  moralid6  moralid7  moralid8  moralid9  mcoop3  mcoop5  narc1  narc2  narc3  narc4  narc5  narc6  nidentity1  omind1  omind2  omind5  omind6  contact2  contact3  contact4  hygiene1  hygiene2  hygiene4  hygiene5  psupport1  psupport2  psupport3  psupport4  psupport5  political_ideology  happy  slf_ladder  riskperc1  riskperc2  self_esteem  sex1  sbelong1  sbelong2  sbelong3  sbelong4  slfcont1  slfcont2  slfcont3  slfcont4  urban
##   2   10  age  cnarc2  cnarc3  ctheory1  ctheory2  ctheory3  ctheory4  CRT1  CRT2  CRT3  employ_status1  generosity1  generosity2  generosity3  health_cond  marital1  mor_circle  moralid1  moralid10  moralid2  moralid3  moralid4  moralid5  moralid6  moralid7  moralid8  moralid9  mcoop3  mcoop5  narc1  narc2  narc3  narc4  narc5  narc6  nidentity1  omind1  omind2  omind5  omind6  contact2  contact3  contact4  hygiene1  hygiene2  hygiene4  hygiene5  psupport1  psupport2  psupport3  psupport4  psupport5  political_ideology  happy  slf_ladder  riskperc1  riskperc2  self_esteem  sex1  sbelong1  sbelong2  sbelong3  sbelong4  slfcont1  slfcont2  slfcont3  slfcont4  urban
##   3   1  age  cnarc2  cnarc3  ctheory1  ctheory2  ctheory3  ctheory4  CRT1  CRT2  CRT3  employ_status1  generosity1  generosity2  generosity3  health_cond  marital1  mor_circle  moralid1  moralid10  moralid2  moralid3  moralid4  moralid5  moralid6  moralid7  moralid8  moralid9  mcoop3  mcoop5  narc1  narc2  narc3  narc4  narc5  narc6  nidentity1  omind1  omind2  omind5  omind6  contact2  contact3  contact4  hygiene1  hygiene2  hygiene4  hygiene5  psupport1  psupport2  psupport3  psupport4  psupport5  political_ideology  happy  slf_ladder  riskperc1  riskperc2  self_esteem  sex1  sbelong1  sbelong2  sbelong3  sbelong4  slfcont1  slfcont2  slfcont3  slfcont4  urban
##   3   2  age  cnarc2  cnarc3  ctheory1  ctheory2  ctheory3  ctheory4  CRT1  CRT2  CRT3  employ_status1  generosity1  generosity2  generosity3  health_cond  marital1  mor_circle  moralid1  moralid10  moralid2  moralid3  moralid4  moralid5  moralid6  moralid7  moralid8  moralid9  mcoop3  mcoop5  narc1  narc2  narc3  narc4  narc5  narc6  nidentity1  omind1  omind2  omind5  omind6  contact2  contact3  contact4  hygiene1  hygiene2  hygiene4  hygiene5  psupport1  psupport2  psupport3  psupport4  psupport5  political_ideology  happy  slf_ladder  riskperc1  riskperc2  self_esteem  sex1  sbelong1  sbelong2  sbelong3  sbelong4  slfcont1  slfcont2  slfcont3  slfcont4  urban
##   3   3  age  cnarc2  cnarc3  ctheory1  ctheory2  ctheory3  ctheory4  CRT1  CRT2  CRT3  employ_status1  generosity1  generosity2  generosity3  health_cond  marital1  mor_circle  moralid1  moralid10  moralid2  moralid3  moralid4  moralid5  moralid6  moralid7  moralid8  moralid9  mcoop3  mcoop5  narc1  narc2  narc3  narc4  narc5  narc6  nidentity1  omind1  omind2  omind5  omind6  contact2  contact3  contact4  hygiene1  hygiene2  hygiene4  hygiene5  psupport1  psupport2  psupport3  psupport4  psupport5  political_ideology  happy  slf_ladder  riskperc1  riskperc2  self_esteem  sex1  sbelong1  sbelong2  sbelong3  sbelong4  slfcont1  slfcont2  slfcont3  slfcont4  urban
##   3   4  age  cnarc2  cnarc3  ctheory1  ctheory2  ctheory3  ctheory4  CRT1  CRT2  CRT3  employ_status1  generosity1  generosity2  generosity3  health_cond  marital1  mor_circle  moralid1  moralid10  moralid2  moralid3  moralid4  moralid5  moralid6  moralid7  moralid8  moralid9  mcoop3  mcoop5  narc1  narc2  narc3  narc4  narc5  narc6  nidentity1  omind1  omind2  omind5  omind6  contact2  contact3  contact4  hygiene1  hygiene2  hygiene4  hygiene5  psupport1  psupport2  psupport3  psupport4  psupport5  political_ideology  happy  slf_ladder  riskperc1  riskperc2  self_esteem  sex1  sbelong1  sbelong2  sbelong3  sbelong4  slfcont1  slfcont2  slfcont3  slfcont4  urban
##   3   5  age  cnarc2  cnarc3  ctheory1  ctheory2  ctheory3  ctheory4  CRT1  CRT2  CRT3  employ_status1  generosity1  generosity2  generosity3  health_cond  marital1  mor_circle  moralid1  moralid10  moralid2  moralid3  moralid4  moralid5  moralid6  moralid7  moralid8  moralid9  mcoop3  mcoop5  narc1  narc2  narc3  narc4  narc5  narc6  nidentity1  omind1  omind2  omind5  omind6  contact2  contact3  contact4  hygiene1  hygiene2  hygiene4  hygiene5  psupport1  psupport2  psupport3  psupport4  psupport5  political_ideology  happy  slf_ladder  riskperc1  riskperc2  self_esteem  sex1  sbelong1  sbelong2  sbelong3  sbelong4  slfcont1  slfcont2  slfcont3  slfcont4  urban
##   3   6  age  cnarc2  cnarc3  ctheory1  ctheory2  ctheory3  ctheory4  CRT1  CRT2  CRT3  employ_status1  generosity1  generosity2  generosity3  health_cond  marital1  mor_circle  moralid1  moralid10  moralid2  moralid3  moralid4  moralid5  moralid6  moralid7  moralid8  moralid9  mcoop3  mcoop5  narc1  narc2  narc3  narc4  narc5  narc6  nidentity1  omind1  omind2  omind5  omind6  contact2  contact3  contact4  hygiene1  hygiene2  hygiene4  hygiene5  psupport1  psupport2  psupport3  psupport4  psupport5  political_ideology  happy  slf_ladder  riskperc1  riskperc2  self_esteem  sex1  sbelong1  sbelong2  sbelong3  sbelong4  slfcont1  slfcont2  slfcont3  slfcont4  urban
##   3   7  age  cnarc2  cnarc3  ctheory1  ctheory2  ctheory3  ctheory4  CRT1  CRT2  CRT3  employ_status1  generosity1  generosity2  generosity3  health_cond  marital1  mor_circle  moralid1  moralid10  moralid2  moralid3  moralid4  moralid5  moralid6  moralid7  moralid8  moralid9  mcoop3  mcoop5  narc1  narc2  narc3  narc4  narc5  narc6  nidentity1  omind1  omind2  omind5  omind6  contact2  contact3  contact4  hygiene1  hygiene2  hygiene4  hygiene5  psupport1  psupport2  psupport3  psupport4  psupport5  political_ideology  happy  slf_ladder  riskperc1  riskperc2  self_esteem  sex1  sbelong1  sbelong2  sbelong3  sbelong4  slfcont1  slfcont2  slfcont3  slfcont4  urban
##   3   8  age  cnarc2  cnarc3  ctheory1  ctheory2  ctheory3  ctheory4  CRT1  CRT2  CRT3  employ_status1  generosity1  generosity2  generosity3  health_cond  marital1  mor_circle  moralid1  moralid10  moralid2  moralid3  moralid4  moralid5  moralid6  moralid7  moralid8  moralid9  mcoop3  mcoop5  narc1  narc2  narc3  narc4  narc5  narc6  nidentity1  omind1  omind2  omind5  omind6  contact2  contact3  contact4  hygiene1  hygiene2  hygiene4  hygiene5  psupport1  psupport2  psupport3  psupport4  psupport5  political_ideology  happy  slf_ladder  riskperc1  riskperc2  self_esteem  sex1  sbelong1  sbelong2  sbelong3  sbelong4  slfcont1  slfcont2  slfcont3  slfcont4  urban
##   3   9  age  cnarc2  cnarc3  ctheory1  ctheory2  ctheory3  ctheory4  CRT1  CRT2  CRT3  employ_status1  generosity1  generosity2  generosity3  health_cond  marital1  mor_circle  moralid1  moralid10  moralid2  moralid3  moralid4  moralid5  moralid6  moralid7  moralid8  moralid9  mcoop3  mcoop5  narc1  narc2  narc3  narc4  narc5  narc6  nidentity1  omind1  omind2  omind5  omind6  contact2  contact3  contact4  hygiene1  hygiene2  hygiene4  hygiene5  psupport1  psupport2  psupport3  psupport4  psupport5  political_ideology  happy  slf_ladder  riskperc1  riskperc2  self_esteem  sex1  sbelong1  sbelong2  sbelong3  sbelong4  slfcont1  slfcont2  slfcont3  slfcont4  urban
##   3   10  age  cnarc2  cnarc3  ctheory1  ctheory2  ctheory3  ctheory4  CRT1  CRT2  CRT3  employ_status1  generosity1  generosity2  generosity3  health_cond  marital1  mor_circle  moralid1  moralid10  moralid2  moralid3  moralid4  moralid5  moralid6  moralid7  moralid8  moralid9  mcoop3  mcoop5  narc1  narc2  narc3  narc4  narc5  narc6  nidentity1  omind1  omind2  omind5  omind6  contact2  contact3  contact4  hygiene1  hygiene2  hygiene4  hygiene5  psupport1  psupport2  psupport3  psupport4  psupport5  political_ideology  happy  slf_ladder  riskperc1  riskperc2  self_esteem  sex1  sbelong1  sbelong2  sbelong3  sbelong4  slfcont1  slfcont2  slfcont3  slfcont4  urban
##   4   1  age  cnarc2  cnarc3  ctheory1  ctheory2  ctheory3  ctheory4  CRT1  CRT2  CRT3  employ_status1  generosity1  generosity2  generosity3  health_cond  marital1  mor_circle  moralid1  moralid10  moralid2  moralid3  moralid4  moralid5  moralid6  moralid7  moralid8  moralid9  mcoop3  mcoop5  narc1  narc2  narc3  narc4  narc5  narc6  nidentity1  omind1  omind2  omind5  omind6  contact2  contact3  contact4  hygiene1  hygiene2  hygiene4  hygiene5  psupport1  psupport2  psupport3  psupport4  psupport5  political_ideology  happy  slf_ladder  riskperc1  riskperc2  self_esteem  sex1  sbelong1  sbelong2  sbelong3  sbelong4  slfcont1  slfcont2  slfcont3  slfcont4  urban
##   4   2  age  cnarc2  cnarc3  ctheory1  ctheory2  ctheory3  ctheory4  CRT1  CRT2  CRT3  employ_status1  generosity1  generosity2  generosity3  health_cond  marital1  mor_circle  moralid1  moralid10  moralid2  moralid3  moralid4  moralid5  moralid6  moralid7  moralid8  moralid9  mcoop3  mcoop5  narc1  narc2  narc3  narc4  narc5  narc6  nidentity1  omind1  omind2  omind5  omind6  contact2  contact3  contact4  hygiene1  hygiene2  hygiene4  hygiene5  psupport1  psupport2  psupport3  psupport4  psupport5  political_ideology  happy  slf_ladder  riskperc1  riskperc2  self_esteem  sex1  sbelong1  sbelong2  sbelong3  sbelong4  slfcont1  slfcont2  slfcont3  slfcont4  urban
##   4   3  age  cnarc2  cnarc3  ctheory1  ctheory2  ctheory3  ctheory4  CRT1  CRT2  CRT3  employ_status1  generosity1  generosity2  generosity3  health_cond  marital1  mor_circle  moralid1  moralid10  moralid2  moralid3  moralid4  moralid5  moralid6  moralid7  moralid8  moralid9  mcoop3  mcoop5  narc1  narc2  narc3  narc4  narc5  narc6  nidentity1  omind1  omind2  omind5  omind6  contact2  contact3  contact4  hygiene1  hygiene2  hygiene4  hygiene5  psupport1  psupport2  psupport3  psupport4  psupport5  political_ideology  happy  slf_ladder  riskperc1  riskperc2  self_esteem  sex1  sbelong1  sbelong2  sbelong3  sbelong4  slfcont1  slfcont2  slfcont3  slfcont4  urban
##   4   4  age  cnarc2  cnarc3  ctheory1  ctheory2  ctheory3  ctheory4  CRT1  CRT2  CRT3  employ_status1  generosity1  generosity2  generosity3  health_cond  marital1  mor_circle  moralid1  moralid10  moralid2  moralid3  moralid4  moralid5  moralid6  moralid7  moralid8  moralid9  mcoop3  mcoop5  narc1  narc2  narc3  narc4  narc5  narc6  nidentity1  omind1  omind2  omind5  omind6  contact2  contact3  contact4  hygiene1  hygiene2  hygiene4  hygiene5  psupport1  psupport2  psupport3  psupport4  psupport5  political_ideology  happy  slf_ladder  riskperc1  riskperc2  self_esteem  sex1  sbelong1  sbelong2  sbelong3  sbelong4  slfcont1  slfcont2  slfcont3  slfcont4  urban
##   4   5  age  cnarc2  cnarc3  ctheory1  ctheory2  ctheory3  ctheory4  CRT1  CRT2  CRT3  employ_status1  generosity1  generosity2  generosity3  health_cond  marital1  mor_circle  moralid1  moralid10  moralid2  moralid3  moralid4  moralid5  moralid6  moralid7  moralid8  moralid9  mcoop3  mcoop5  narc1  narc2  narc3  narc4  narc5  narc6  nidentity1  omind1  omind2  omind5  omind6  contact2  contact3  contact4  hygiene1  hygiene2  hygiene4  hygiene5  psupport1  psupport2  psupport3  psupport4  psupport5  political_ideology  happy  slf_ladder  riskperc1  riskperc2  self_esteem  sex1  sbelong1  sbelong2  sbelong3  sbelong4  slfcont1  slfcont2  slfcont3  slfcont4  urban
##   4   6  age  cnarc2  cnarc3  ctheory1  ctheory2  ctheory3  ctheory4  CRT1  CRT2  CRT3  employ_status1  generosity1  generosity2  generosity3  health_cond  marital1  mor_circle  moralid1  moralid10  moralid2  moralid3  moralid4  moralid5  moralid6  moralid7  moralid8  moralid9  mcoop3  mcoop5  narc1  narc2  narc3  narc4  narc5  narc6  nidentity1  omind1  omind2  omind5  omind6  contact2  contact3  contact4  hygiene1  hygiene2  hygiene4  hygiene5  psupport1  psupport2  psupport3  psupport4  psupport5  political_ideology  happy  slf_ladder  riskperc1  riskperc2  self_esteem  sex1  sbelong1  sbelong2  sbelong3  sbelong4  slfcont1  slfcont2  slfcont3  slfcont4  urban
##   4   7  age  cnarc2  cnarc3  ctheory1  ctheory2  ctheory3  ctheory4  CRT1  CRT2  CRT3  employ_status1  generosity1  generosity2  generosity3  health_cond  marital1  mor_circle  moralid1  moralid10  moralid2  moralid3  moralid4  moralid5  moralid6  moralid7  moralid8  moralid9  mcoop3  mcoop5  narc1  narc2  narc3  narc4  narc5  narc6  nidentity1  omind1  omind2  omind5  omind6  contact2  contact3  contact4  hygiene1  hygiene2  hygiene4  hygiene5  psupport1  psupport2  psupport3  psupport4  psupport5  political_ideology  happy  slf_ladder  riskperc1  riskperc2  self_esteem  sex1  sbelong1  sbelong2  sbelong3  sbelong4  slfcont1  slfcont2  slfcont3  slfcont4  urban
##   4   8  age  cnarc2  cnarc3  ctheory1  ctheory2  ctheory3  ctheory4  CRT1  CRT2  CRT3  employ_status1  generosity1  generosity2  generosity3  health_cond  marital1  mor_circle  moralid1  moralid10  moralid2  moralid3  moralid4  moralid5  moralid6  moralid7  moralid8  moralid9  mcoop3  mcoop5  narc1  narc2  narc3  narc4  narc5  narc6  nidentity1  omind1  omind2  omind5  omind6  contact2  contact3  contact4  hygiene1  hygiene2  hygiene4  hygiene5  psupport1  psupport2  psupport3  psupport4  psupport5  political_ideology  happy  slf_ladder  riskperc1  riskperc2  self_esteem  sex1  sbelong1  sbelong2  sbelong3  sbelong4  slfcont1  slfcont2  slfcont3  slfcont4  urban
##   4   9  age  cnarc2  cnarc3  ctheory1  ctheory2  ctheory3  ctheory4  CRT1  CRT2  CRT3  employ_status1  generosity1  generosity2  generosity3  health_cond  marital1  mor_circle  moralid1  moralid10  moralid2  moralid3  moralid4  moralid5  moralid6  moralid7  moralid8  moralid9  mcoop3  mcoop5  narc1  narc2  narc3  narc4  narc5  narc6  nidentity1  omind1  omind2  omind5  omind6  contact2  contact3  contact4  hygiene1  hygiene2  hygiene4  hygiene5  psupport1  psupport2  psupport3  psupport4  psupport5  political_ideology  happy  slf_ladder  riskperc1  riskperc2  self_esteem  sex1  sbelong1  sbelong2  sbelong3  sbelong4  slfcont1  slfcont2  slfcont3  slfcont4  urban
##   4   10  age  cnarc2  cnarc3  ctheory1  ctheory2  ctheory3  ctheory4  CRT1  CRT2  CRT3  employ_status1  generosity1  generosity2  generosity3  health_cond  marital1  mor_circle  moralid1  moralid10  moralid2  moralid3  moralid4  moralid5  moralid6  moralid7  moralid8  moralid9  mcoop3  mcoop5  narc1  narc2  narc3  narc4  narc5  narc6  nidentity1  omind1  omind2  omind5  omind6  contact2  contact3  contact4  hygiene1  hygiene2  hygiene4  hygiene5  psupport1  psupport2  psupport3  psupport4  psupport5  political_ideology  happy  slf_ladder  riskperc1  riskperc2  self_esteem  sex1  sbelong1  sbelong2  sbelong3  sbelong4  slfcont1  slfcont2  slfcont3  slfcont4  urban
##   5   1  age  cnarc2  cnarc3  ctheory1  ctheory2  ctheory3  ctheory4  CRT1  CRT2  CRT3  employ_status1  generosity1  generosity2  generosity3  health_cond  marital1  mor_circle  moralid1  moralid10  moralid2  moralid3  moralid4  moralid5  moralid6  moralid7  moralid8  moralid9  mcoop3  mcoop5  narc1  narc2  narc3  narc4  narc5  narc6  nidentity1  omind1  omind2  omind5  omind6  contact2  contact3  contact4  hygiene1  hygiene2  hygiene4  hygiene5  psupport1  psupport2  psupport3  psupport4  psupport5  political_ideology  happy  slf_ladder  riskperc1  riskperc2  self_esteem  sex1  sbelong1  sbelong2  sbelong3  sbelong4  slfcont1  slfcont2  slfcont3  slfcont4  urban
##   5   2  age  cnarc2  cnarc3  ctheory1  ctheory2  ctheory3  ctheory4  CRT1  CRT2  CRT3  employ_status1  generosity1  generosity2  generosity3  health_cond  marital1  mor_circle  moralid1  moralid10  moralid2  moralid3  moralid4  moralid5  moralid6  moralid7  moralid8  moralid9  mcoop3  mcoop5  narc1  narc2  narc3  narc4  narc5  narc6  nidentity1  omind1  omind2  omind5  omind6  contact2  contact3  contact4  hygiene1  hygiene2  hygiene4  hygiene5  psupport1  psupport2  psupport3  psupport4  psupport5  political_ideology  happy  slf_ladder  riskperc1  riskperc2  self_esteem  sex1  sbelong1  sbelong2  sbelong3  sbelong4  slfcont1  slfcont2  slfcont3  slfcont4  urban
##   5   3  age  cnarc2  cnarc3  ctheory1  ctheory2  ctheory3  ctheory4  CRT1  CRT2  CRT3  employ_status1  generosity1  generosity2  generosity3  health_cond  marital1  mor_circle  moralid1  moralid10  moralid2  moralid3  moralid4  moralid5  moralid6  moralid7  moralid8  moralid9  mcoop3  mcoop5  narc1  narc2  narc3  narc4  narc5  narc6  nidentity1  omind1  omind2  omind5  omind6  contact2  contact3  contact4  hygiene1  hygiene2  hygiene4  hygiene5  psupport1  psupport2  psupport3  psupport4  psupport5  political_ideology  happy  slf_ladder  riskperc1  riskperc2  self_esteem  sex1  sbelong1  sbelong2  sbelong3  sbelong4  slfcont1  slfcont2  slfcont3  slfcont4  urban
##   5   4  age  cnarc2  cnarc3  ctheory1  ctheory2  ctheory3  ctheory4  CRT1  CRT2  CRT3  employ_status1  generosity1  generosity2  generosity3  health_cond  marital1  mor_circle  moralid1  moralid10  moralid2  moralid3  moralid4  moralid5  moralid6  moralid7  moralid8  moralid9  mcoop3  mcoop5  narc1  narc2  narc3  narc4  narc5  narc6  nidentity1  omind1  omind2  omind5  omind6  contact2  contact3  contact4  hygiene1  hygiene2  hygiene4  hygiene5  psupport1  psupport2  psupport3  psupport4  psupport5  political_ideology  happy  slf_ladder  riskperc1  riskperc2  self_esteem  sex1  sbelong1  sbelong2  sbelong3  sbelong4  slfcont1  slfcont2  slfcont3  slfcont4  urban
##   5   5  age  cnarc2  cnarc3  ctheory1  ctheory2  ctheory3  ctheory4  CRT1  CRT2  CRT3  employ_status1  generosity1  generosity2  generosity3  health_cond  marital1  mor_circle  moralid1  moralid10  moralid2  moralid3  moralid4  moralid5  moralid6  moralid7  moralid8  moralid9  mcoop3  mcoop5  narc1  narc2  narc3  narc4  narc5  narc6  nidentity1  omind1  omind2  omind5  omind6  contact2  contact3  contact4  hygiene1  hygiene2  hygiene4  hygiene5  psupport1  psupport2  psupport3  psupport4  psupport5  political_ideology  happy  slf_ladder  riskperc1  riskperc2  self_esteem  sex1  sbelong1  sbelong2  sbelong3  sbelong4  slfcont1  slfcont2  slfcont3  slfcont4  urban
##   5   6  age  cnarc2  cnarc3  ctheory1  ctheory2  ctheory3  ctheory4  CRT1  CRT2  CRT3  employ_status1  generosity1  generosity2  generosity3  health_cond  marital1  mor_circle  moralid1  moralid10  moralid2  moralid3  moralid4  moralid5  moralid6  moralid7  moralid8  moralid9  mcoop3  mcoop5  narc1  narc2  narc3  narc4  narc5  narc6  nidentity1  omind1  omind2  omind5  omind6  contact2  contact3  contact4  hygiene1  hygiene2  hygiene4  hygiene5  psupport1  psupport2  psupport3  psupport4  psupport5  political_ideology  happy  slf_ladder  riskperc1  riskperc2  self_esteem  sex1  sbelong1  sbelong2  sbelong3  sbelong4  slfcont1  slfcont2  slfcont3  slfcont4  urban
##   5   7  age  cnarc2  cnarc3  ctheory1  ctheory2  ctheory3  ctheory4  CRT1  CRT2  CRT3  employ_status1  generosity1  generosity2  generosity3  health_cond  marital1  mor_circle  moralid1  moralid10  moralid2  moralid3  moralid4  moralid5  moralid6  moralid7  moralid8  moralid9  mcoop3  mcoop5  narc1  narc2  narc3  narc4  narc5  narc6  nidentity1  omind1  omind2  omind5  omind6  contact2  contact3  contact4  hygiene1  hygiene2  hygiene4  hygiene5  psupport1  psupport2  psupport3  psupport4  psupport5  political_ideology  happy  slf_ladder  riskperc1  riskperc2  self_esteem  sex1  sbelong1  sbelong2  sbelong3  sbelong4  slfcont1  slfcont2  slfcont3  slfcont4  urban
##   5   8  age  cnarc2  cnarc3  ctheory1  ctheory2  ctheory3  ctheory4  CRT1  CRT2  CRT3  employ_status1  generosity1  generosity2  generosity3  health_cond  marital1  mor_circle  moralid1  moralid10  moralid2  moralid3  moralid4  moralid5  moralid6  moralid7  moralid8  moralid9  mcoop3  mcoop5  narc1  narc2  narc3  narc4  narc5  narc6  nidentity1  omind1  omind2  omind5  omind6  contact2  contact3  contact4  hygiene1  hygiene2  hygiene4  hygiene5  psupport1  psupport2  psupport3  psupport4  psupport5  political_ideology  happy  slf_ladder  riskperc1  riskperc2  self_esteem  sex1  sbelong1  sbelong2  sbelong3  sbelong4  slfcont1  slfcont2  slfcont3  slfcont4  urban
##   5   9  age  cnarc2  cnarc3  ctheory1  ctheory2  ctheory3  ctheory4  CRT1  CRT2  CRT3  employ_status1  generosity1  generosity2  generosity3  health_cond  marital1  mor_circle  moralid1  moralid10  moralid2  moralid3  moralid4  moralid5  moralid6  moralid7  moralid8  moralid9  mcoop3  mcoop5  narc1  narc2  narc3  narc4  narc5  narc6  nidentity1  omind1  omind2  omind5  omind6  contact2  contact3  contact4  hygiene1  hygiene2  hygiene4  hygiene5  psupport1  psupport2  psupport3  psupport4  psupport5  political_ideology  happy  slf_ladder  riskperc1  riskperc2  self_esteem  sex1  sbelong1  sbelong2  sbelong3  sbelong4  slfcont1  slfcont2  slfcont3  slfcont4  urban
##   5   10  age  cnarc2  cnarc3  ctheory1  ctheory2  ctheory3  ctheory4  CRT1  CRT2  CRT3  employ_status1  generosity1  generosity2  generosity3  health_cond  marital1  mor_circle  moralid1  moralid10  moralid2  moralid3  moralid4  moralid5  moralid6  moralid7  moralid8  moralid9  mcoop3  mcoop5  narc1  narc2  narc3  narc4  narc5  narc6  nidentity1  omind1  omind2  omind5  omind6  contact2  contact3  contact4  hygiene1  hygiene2  hygiene4  hygiene5  psupport1  psupport2  psupport3  psupport4  psupport5  political_ideology  happy  slf_ladder  riskperc1  riskperc2  self_esteem  sex1  sbelong1  sbelong2  sbelong3  sbelong4  slfcont1  slfcont2  slfcont3  slfcont4  urban
##   6   1  age  cnarc2  cnarc3  ctheory1  ctheory2  ctheory3  ctheory4  CRT1  CRT2  CRT3  employ_status1  generosity1  generosity2  generosity3  health_cond  marital1  mor_circle  moralid1  moralid10  moralid2  moralid3  moralid4  moralid5  moralid6  moralid7  moralid8  moralid9  mcoop3  mcoop5  narc1  narc2  narc3  narc4  narc5  narc6  nidentity1  omind1  omind2  omind5  omind6  contact2  contact3  contact4  hygiene1  hygiene2  hygiene4  hygiene5  psupport1  psupport2  psupport3  psupport4  psupport5  political_ideology  happy  slf_ladder  riskperc1  riskperc2  self_esteem  sex1  sbelong1  sbelong2  sbelong3  sbelong4  slfcont1  slfcont2  slfcont3  slfcont4  urban
##   6   2  age  cnarc2  cnarc3  ctheory1  ctheory2  ctheory3  ctheory4  CRT1  CRT2  CRT3  employ_status1  generosity1  generosity2  generosity3  health_cond  marital1  mor_circle  moralid1  moralid10  moralid2  moralid3  moralid4  moralid5  moralid6  moralid7  moralid8  moralid9  mcoop3  mcoop5  narc1  narc2  narc3  narc4  narc5  narc6  nidentity1  omind1  omind2  omind5  omind6  contact2  contact3  contact4  hygiene1  hygiene2  hygiene4  hygiene5  psupport1  psupport2  psupport3  psupport4  psupport5  political_ideology  happy  slf_ladder  riskperc1  riskperc2  self_esteem  sex1  sbelong1  sbelong2  sbelong3  sbelong4  slfcont1  slfcont2  slfcont3  slfcont4  urban
##   6   3  age  cnarc2  cnarc3  ctheory1  ctheory2  ctheory3  ctheory4  CRT1  CRT2  CRT3  employ_status1  generosity1  generosity2  generosity3  health_cond  marital1  mor_circle  moralid1  moralid10  moralid2  moralid3  moralid4  moralid5  moralid6  moralid7  moralid8  moralid9  mcoop3  mcoop5  narc1  narc2  narc3  narc4  narc5  narc6  nidentity1  omind1  omind2  omind5  omind6  contact2  contact3  contact4  hygiene1  hygiene2  hygiene4  hygiene5  psupport1  psupport2  psupport3  psupport4  psupport5  political_ideology  happy  slf_ladder  riskperc1  riskperc2  self_esteem  sex1  sbelong1  sbelong2  sbelong3  sbelong4  slfcont1  slfcont2  slfcont3  slfcont4  urban
##   6   4  age  cnarc2  cnarc3  ctheory1  ctheory2  ctheory3  ctheory4  CRT1  CRT2  CRT3  employ_status1  generosity1  generosity2  generosity3  health_cond  marital1  mor_circle  moralid1  moralid10  moralid2  moralid3  moralid4  moralid5  moralid6  moralid7  moralid8  moralid9  mcoop3  mcoop5  narc1  narc2  narc3  narc4  narc5  narc6  nidentity1  omind1  omind2  omind5  omind6  contact2  contact3  contact4  hygiene1  hygiene2  hygiene4  hygiene5  psupport1  psupport2  psupport3  psupport4  psupport5  political_ideology  happy  slf_ladder  riskperc1  riskperc2  self_esteem  sex1  sbelong1  sbelong2  sbelong3  sbelong4  slfcont1  slfcont2  slfcont3  slfcont4  urban
##   6   5  age  cnarc2  cnarc3  ctheory1  ctheory2  ctheory3  ctheory4  CRT1  CRT2  CRT3  employ_status1  generosity1  generosity2  generosity3  health_cond  marital1  mor_circle  moralid1  moralid10  moralid2  moralid3  moralid4  moralid5  moralid6  moralid7  moralid8  moralid9  mcoop3  mcoop5  narc1  narc2  narc3  narc4  narc5  narc6  nidentity1  omind1  omind2  omind5  omind6  contact2  contact3  contact4  hygiene1  hygiene2  hygiene4  hygiene5  psupport1  psupport2  psupport3  psupport4  psupport5  political_ideology  happy  slf_ladder  riskperc1  riskperc2  self_esteem  sex1  sbelong1  sbelong2  sbelong3  sbelong4  slfcont1  slfcont2  slfcont3  slfcont4  urban
##   6   6  age  cnarc2  cnarc3  ctheory1  ctheory2  ctheory3  ctheory4  CRT1  CRT2  CRT3  employ_status1  generosity1  generosity2  generosity3  health_cond  marital1  mor_circle  moralid1  moralid10  moralid2  moralid3  moralid4  moralid5  moralid6  moralid7  moralid8  moralid9  mcoop3  mcoop5  narc1  narc2  narc3  narc4  narc5  narc6  nidentity1  omind1  omind2  omind5  omind6  contact2  contact3  contact4  hygiene1  hygiene2  hygiene4  hygiene5  psupport1  psupport2  psupport3  psupport4  psupport5  political_ideology  happy  slf_ladder  riskperc1  riskperc2  self_esteem  sex1  sbelong1  sbelong2  sbelong3  sbelong4  slfcont1  slfcont2  slfcont3  slfcont4  urban
##   6   7  age  cnarc2  cnarc3  ctheory1  ctheory2  ctheory3  ctheory4  CRT1  CRT2  CRT3  employ_status1  generosity1  generosity2  generosity3  health_cond  marital1  mor_circle  moralid1  moralid10  moralid2  moralid3  moralid4  moralid5  moralid6  moralid7  moralid8  moralid9  mcoop3  mcoop5  narc1  narc2  narc3  narc4  narc5  narc6  nidentity1  omind1  omind2  omind5  omind6  contact2  contact3  contact4  hygiene1  hygiene2  hygiene4  hygiene5  psupport1  psupport2  psupport3  psupport4  psupport5  political_ideology  happy  slf_ladder  riskperc1  riskperc2  self_esteem  sex1  sbelong1  sbelong2  sbelong3  sbelong4  slfcont1  slfcont2  slfcont3  slfcont4  urban
##   6   8  age  cnarc2  cnarc3  ctheory1  ctheory2  ctheory3  ctheory4  CRT1  CRT2  CRT3  employ_status1  generosity1  generosity2  generosity3  health_cond  marital1  mor_circle  moralid1  moralid10  moralid2  moralid3  moralid4  moralid5  moralid6  moralid7  moralid8  moralid9  mcoop3  mcoop5  narc1  narc2  narc3  narc4  narc5  narc6  nidentity1  omind1  omind2  omind5  omind6  contact2  contact3  contact4  hygiene1  hygiene2  hygiene4  hygiene5  psupport1  psupport2  psupport3  psupport4  psupport5  political_ideology  happy  slf_ladder  riskperc1  riskperc2  self_esteem  sex1  sbelong1  sbelong2  sbelong3  sbelong4  slfcont1  slfcont2  slfcont3  slfcont4  urban
##   6   9  age  cnarc2  cnarc3  ctheory1  ctheory2  ctheory3  ctheory4  CRT1  CRT2  CRT3  employ_status1  generosity1  generosity2  generosity3  health_cond  marital1  mor_circle  moralid1  moralid10  moralid2  moralid3  moralid4  moralid5  moralid6  moralid7  moralid8  moralid9  mcoop3  mcoop5  narc1  narc2  narc3  narc4  narc5  narc6  nidentity1  omind1  omind2  omind5  omind6  contact2  contact3  contact4  hygiene1  hygiene2  hygiene4  hygiene5  psupport1  psupport2  psupport3  psupport4  psupport5  political_ideology  happy  slf_ladder  riskperc1  riskperc2  self_esteem  sex1  sbelong1  sbelong2  sbelong3  sbelong4  slfcont1  slfcont2  slfcont3  slfcont4  urban
##   6   10  age  cnarc2  cnarc3  ctheory1  ctheory2  ctheory3  ctheory4  CRT1  CRT2  CRT3  employ_status1  generosity1  generosity2  generosity3  health_cond  marital1  mor_circle  moralid1  moralid10  moralid2  moralid3  moralid4  moralid5  moralid6  moralid7  moralid8  moralid9  mcoop3  mcoop5  narc1  narc2  narc3  narc4  narc5  narc6  nidentity1  omind1  omind2  omind5  omind6  contact2  contact3  contact4  hygiene1  hygiene2  hygiene4  hygiene5  psupport1  psupport2  psupport3  psupport4  psupport5  political_ideology  happy  slf_ladder  riskperc1  riskperc2  self_esteem  sex1  sbelong1  sbelong2  sbelong3  sbelong4  slfcont1  slfcont2  slfcont3  slfcont4  urban
##   7   1  age  cnarc2  cnarc3  ctheory1  ctheory2  ctheory3  ctheory4  CRT1  CRT2  CRT3  employ_status1  generosity1  generosity2  generosity3  health_cond  marital1  mor_circle  moralid1  moralid10  moralid2  moralid3  moralid4  moralid5  moralid6  moralid7  moralid8  moralid9  mcoop3  mcoop5  narc1  narc2  narc3  narc4  narc5  narc6  nidentity1  omind1  omind2  omind5  omind6  contact2  contact3  contact4  hygiene1  hygiene2  hygiene4  hygiene5  psupport1  psupport2  psupport3  psupport4  psupport5  political_ideology  happy  slf_ladder  riskperc1  riskperc2  self_esteem  sex1  sbelong1  sbelong2  sbelong3  sbelong4  slfcont1  slfcont2  slfcont3  slfcont4  urban
##   7   2  age  cnarc2  cnarc3  ctheory1  ctheory2  ctheory3  ctheory4  CRT1  CRT2  CRT3  employ_status1  generosity1  generosity2  generosity3  health_cond  marital1  mor_circle  moralid1  moralid10  moralid2  moralid3  moralid4  moralid5  moralid6  moralid7  moralid8  moralid9  mcoop3  mcoop5  narc1  narc2  narc3  narc4  narc5  narc6  nidentity1  omind1  omind2  omind5  omind6  contact2  contact3  contact4  hygiene1  hygiene2  hygiene4  hygiene5  psupport1  psupport2  psupport3  psupport4  psupport5  political_ideology  happy  slf_ladder  riskperc1  riskperc2  self_esteem  sex1  sbelong1  sbelong2  sbelong3  sbelong4  slfcont1  slfcont2  slfcont3  slfcont4  urban
##   7   3  age  cnarc2  cnarc3  ctheory1  ctheory2  ctheory3  ctheory4  CRT1  CRT2  CRT3  employ_status1  generosity1  generosity2  generosity3  health_cond  marital1  mor_circle  moralid1  moralid10  moralid2  moralid3  moralid4  moralid5  moralid6  moralid7  moralid8  moralid9  mcoop3  mcoop5  narc1  narc2  narc3  narc4  narc5  narc6  nidentity1  omind1  omind2  omind5  omind6  contact2  contact3  contact4  hygiene1  hygiene2  hygiene4  hygiene5  psupport1  psupport2  psupport3  psupport4  psupport5  political_ideology  happy  slf_ladder  riskperc1  riskperc2  self_esteem  sex1  sbelong1  sbelong2  sbelong3  sbelong4  slfcont1  slfcont2  slfcont3  slfcont4  urban
##   7   4  age  cnarc2  cnarc3  ctheory1  ctheory2  ctheory3  ctheory4  CRT1  CRT2  CRT3  employ_status1  generosity1  generosity2  generosity3  health_cond  marital1  mor_circle  moralid1  moralid10  moralid2  moralid3  moralid4  moralid5  moralid6  moralid7  moralid8  moralid9  mcoop3  mcoop5  narc1  narc2  narc3  narc4  narc5  narc6  nidentity1  omind1  omind2  omind5  omind6  contact2  contact3  contact4  hygiene1  hygiene2  hygiene4  hygiene5  psupport1  psupport2  psupport3  psupport4  psupport5  political_ideology  happy  slf_ladder  riskperc1  riskperc2  self_esteem  sex1  sbelong1  sbelong2  sbelong3  sbelong4  slfcont1  slfcont2  slfcont3  slfcont4  urban
##   7   5  age  cnarc2  cnarc3  ctheory1  ctheory2  ctheory3  ctheory4  CRT1  CRT2  CRT3  employ_status1  generosity1  generosity2  generosity3  health_cond  marital1  mor_circle  moralid1  moralid10  moralid2  moralid3  moralid4  moralid5  moralid6  moralid7  moralid8  moralid9  mcoop3  mcoop5  narc1  narc2  narc3  narc4  narc5  narc6  nidentity1  omind1  omind2  omind5  omind6  contact2  contact3  contact4  hygiene1  hygiene2  hygiene4  hygiene5  psupport1  psupport2  psupport3  psupport4  psupport5  political_ideology  happy  slf_ladder  riskperc1  riskperc2  self_esteem  sex1  sbelong1  sbelong2  sbelong3  sbelong4  slfcont1  slfcont2  slfcont3  slfcont4  urban
##   7   6  age  cnarc2  cnarc3  ctheory1  ctheory2  ctheory3  ctheory4  CRT1  CRT2  CRT3  employ_status1  generosity1  generosity2  generosity3  health_cond  marital1  mor_circle  moralid1  moralid10  moralid2  moralid3  moralid4  moralid5  moralid6  moralid7  moralid8  moralid9  mcoop3  mcoop5  narc1  narc2  narc3  narc4  narc5  narc6  nidentity1  omind1  omind2  omind5  omind6  contact2  contact3  contact4  hygiene1  hygiene2  hygiene4  hygiene5  psupport1  psupport2  psupport3  psupport4  psupport5  political_ideology  happy  slf_ladder  riskperc1  riskperc2  self_esteem  sex1  sbelong1  sbelong2  sbelong3  sbelong4  slfcont1  slfcont2  slfcont3  slfcont4  urban
##   7   7  age  cnarc2  cnarc3  ctheory1  ctheory2  ctheory3  ctheory4  CRT1  CRT2  CRT3  employ_status1  generosity1  generosity2  generosity3  health_cond  marital1  mor_circle  moralid1  moralid10  moralid2  moralid3  moralid4  moralid5  moralid6  moralid7  moralid8  moralid9  mcoop3  mcoop5  narc1  narc2  narc3  narc4  narc5  narc6  nidentity1  omind1  omind2  omind5  omind6  contact2  contact3  contact4  hygiene1  hygiene2  hygiene4  hygiene5  psupport1  psupport2  psupport3  psupport4  psupport5  political_ideology  happy  slf_ladder  riskperc1  riskperc2  self_esteem  sex1  sbelong1  sbelong2  sbelong3  sbelong4  slfcont1  slfcont2  slfcont3  slfcont4  urban
##   7   8  age  cnarc2  cnarc3  ctheory1  ctheory2  ctheory3  ctheory4  CRT1  CRT2  CRT3  employ_status1  generosity1  generosity2  generosity3  health_cond  marital1  mor_circle  moralid1  moralid10  moralid2  moralid3  moralid4  moralid5  moralid6  moralid7  moralid8  moralid9  mcoop3  mcoop5  narc1  narc2  narc3  narc4  narc5  narc6  nidentity1  omind1  omind2  omind5  omind6  contact2  contact3  contact4  hygiene1  hygiene2  hygiene4  hygiene5  psupport1  psupport2  psupport3  psupport4  psupport5  political_ideology  happy  slf_ladder  riskperc1  riskperc2  self_esteem  sex1  sbelong1  sbelong2  sbelong3  sbelong4  slfcont1  slfcont2  slfcont3  slfcont4  urban
##   7   9  age  cnarc2  cnarc3  ctheory1  ctheory2  ctheory3  ctheory4  CRT1  CRT2  CRT3  employ_status1  generosity1  generosity2  generosity3  health_cond  marital1  mor_circle  moralid1  moralid10  moralid2  moralid3  moralid4  moralid5  moralid6  moralid7  moralid8  moralid9  mcoop3  mcoop5  narc1  narc2  narc3  narc4  narc5  narc6  nidentity1  omind1  omind2  omind5  omind6  contact2  contact3  contact4  hygiene1  hygiene2  hygiene4  hygiene5  psupport1  psupport2  psupport3  psupport4  psupport5  political_ideology  happy  slf_ladder  riskperc1  riskperc2  self_esteem  sex1  sbelong1  sbelong2  sbelong3  sbelong4  slfcont1  slfcont2  slfcont3  slfcont4  urban
##   7   10  age  cnarc2  cnarc3  ctheory1  ctheory2  ctheory3  ctheory4  CRT1  CRT2  CRT3  employ_status1  generosity1  generosity2  generosity3  health_cond  marital1  mor_circle  moralid1  moralid10  moralid2  moralid3  moralid4  moralid5  moralid6  moralid7  moralid8  moralid9  mcoop3  mcoop5  narc1  narc2  narc3  narc4  narc5  narc6  nidentity1  omind1  omind2  omind5  omind6  contact2  contact3  contact4  hygiene1  hygiene2  hygiene4  hygiene5  psupport1  psupport2  psupport3  psupport4  psupport5  political_ideology  happy  slf_ladder  riskperc1  riskperc2  self_esteem  sex1  sbelong1  sbelong2  sbelong3  sbelong4  slfcont1  slfcont2  slfcont3  slfcont4  urban
##   8   1  age  cnarc2  cnarc3  ctheory1  ctheory2  ctheory3  ctheory4  CRT1  CRT2  CRT3  employ_status1  generosity1  generosity2  generosity3  health_cond  marital1  mor_circle  moralid1  moralid10  moralid2  moralid3  moralid4  moralid5  moralid6  moralid7  moralid8  moralid9  mcoop3  mcoop5  narc1  narc2  narc3  narc4  narc5  narc6  nidentity1  omind1  omind2  omind5  omind6  contact2  contact3  contact4  hygiene1  hygiene2  hygiene4  hygiene5  psupport1  psupport2  psupport3  psupport4  psupport5  political_ideology  happy  slf_ladder  riskperc1  riskperc2  self_esteem  sex1  sbelong1  sbelong2  sbelong3  sbelong4  slfcont1  slfcont2  slfcont3  slfcont4  urban
##   8   2  age  cnarc2  cnarc3  ctheory1  ctheory2  ctheory3  ctheory4  CRT1  CRT2  CRT3  employ_status1  generosity1  generosity2  generosity3  health_cond  marital1  mor_circle  moralid1  moralid10  moralid2  moralid3  moralid4  moralid5  moralid6  moralid7  moralid8  moralid9  mcoop3  mcoop5  narc1  narc2  narc3  narc4  narc5  narc6  nidentity1  omind1  omind2  omind5  omind6  contact2  contact3  contact4  hygiene1  hygiene2  hygiene4  hygiene5  psupport1  psupport2  psupport3  psupport4  psupport5  political_ideology  happy  slf_ladder  riskperc1  riskperc2  self_esteem  sex1  sbelong1  sbelong2  sbelong3  sbelong4  slfcont1  slfcont2  slfcont3  slfcont4  urban
##   8   3  age  cnarc2  cnarc3  ctheory1  ctheory2  ctheory3  ctheory4  CRT1  CRT2  CRT3  employ_status1  generosity1  generosity2  generosity3  health_cond  marital1  mor_circle  moralid1  moralid10  moralid2  moralid3  moralid4  moralid5  moralid6  moralid7  moralid8  moralid9  mcoop3  mcoop5  narc1  narc2  narc3  narc4  narc5  narc6  nidentity1  omind1  omind2  omind5  omind6  contact2  contact3  contact4  hygiene1  hygiene2  hygiene4  hygiene5  psupport1  psupport2  psupport3  psupport4  psupport5  political_ideology  happy  slf_ladder  riskperc1  riskperc2  self_esteem  sex1  sbelong1  sbelong2  sbelong3  sbelong4  slfcont1  slfcont2  slfcont3  slfcont4  urban
##   8   4  age  cnarc2  cnarc3  ctheory1  ctheory2  ctheory3  ctheory4  CRT1  CRT2  CRT3  employ_status1  generosity1  generosity2  generosity3  health_cond  marital1  mor_circle  moralid1  moralid10  moralid2  moralid3  moralid4  moralid5  moralid6  moralid7  moralid8  moralid9  mcoop3  mcoop5  narc1  narc2  narc3  narc4  narc5  narc6  nidentity1  omind1  omind2  omind5  omind6  contact2  contact3  contact4  hygiene1  hygiene2  hygiene4  hygiene5  psupport1  psupport2  psupport3  psupport4  psupport5  political_ideology  happy  slf_ladder  riskperc1  riskperc2  self_esteem  sex1  sbelong1  sbelong2  sbelong3  sbelong4  slfcont1  slfcont2  slfcont3  slfcont4  urban
##   8   5  age  cnarc2  cnarc3  ctheory1  ctheory2  ctheory3  ctheory4  CRT1  CRT2  CRT3  employ_status1  generosity1  generosity2  generosity3  health_cond  marital1  mor_circle  moralid1  moralid10  moralid2  moralid3  moralid4  moralid5  moralid6  moralid7  moralid8  moralid9  mcoop3  mcoop5  narc1  narc2  narc3  narc4  narc5  narc6  nidentity1  omind1  omind2  omind5  omind6  contact2  contact3  contact4  hygiene1  hygiene2  hygiene4  hygiene5  psupport1  psupport2  psupport3  psupport4  psupport5  political_ideology  happy  slf_ladder  riskperc1  riskperc2  self_esteem  sex1  sbelong1  sbelong2  sbelong3  sbelong4  slfcont1  slfcont2  slfcont3  slfcont4  urban
##   8   6  age  cnarc2  cnarc3  ctheory1  ctheory2  ctheory3  ctheory4  CRT1  CRT2  CRT3  employ_status1  generosity1  generosity2  generosity3  health_cond  marital1  mor_circle  moralid1  moralid10  moralid2  moralid3  moralid4  moralid5  moralid6  moralid7  moralid8  moralid9  mcoop3  mcoop5  narc1  narc2  narc3  narc4  narc5  narc6  nidentity1  omind1  omind2  omind5  omind6  contact2  contact3  contact4  hygiene1  hygiene2  hygiene4  hygiene5  psupport1  psupport2  psupport3  psupport4  psupport5  political_ideology  happy  slf_ladder  riskperc1  riskperc2  self_esteem  sex1  sbelong1  sbelong2  sbelong3  sbelong4  slfcont1  slfcont2  slfcont3  slfcont4  urban
##   8   7  age  cnarc2  cnarc3  ctheory1  ctheory2  ctheory3  ctheory4  CRT1  CRT2  CRT3  employ_status1  generosity1  generosity2  generosity3  health_cond  marital1  mor_circle  moralid1  moralid10  moralid2  moralid3  moralid4  moralid5  moralid6  moralid7  moralid8  moralid9  mcoop3  mcoop5  narc1  narc2  narc3  narc4  narc5  narc6  nidentity1  omind1  omind2  omind5  omind6  contact2  contact3  contact4  hygiene1  hygiene2  hygiene4  hygiene5  psupport1  psupport2  psupport3  psupport4  psupport5  political_ideology  happy  slf_ladder  riskperc1  riskperc2  self_esteem  sex1  sbelong1  sbelong2  sbelong3  sbelong4  slfcont1  slfcont2  slfcont3  slfcont4  urban
##   8   8  age  cnarc2  cnarc3  ctheory1  ctheory2  ctheory3  ctheory4  CRT1  CRT2  CRT3  employ_status1  generosity1  generosity2  generosity3  health_cond  marital1  mor_circle  moralid1  moralid10  moralid2  moralid3  moralid4  moralid5  moralid6  moralid7  moralid8  moralid9  mcoop3  mcoop5  narc1  narc2  narc3  narc4  narc5  narc6  nidentity1  omind1  omind2  omind5  omind6  contact2  contact3  contact4  hygiene1  hygiene2  hygiene4  hygiene5  psupport1  psupport2  psupport3  psupport4  psupport5  political_ideology  happy  slf_ladder  riskperc1  riskperc2  self_esteem  sex1  sbelong1  sbelong2  sbelong3  sbelong4  slfcont1  slfcont2  slfcont3  slfcont4  urban
##   8   9  age  cnarc2  cnarc3  ctheory1  ctheory2  ctheory3  ctheory4  CRT1  CRT2  CRT3  employ_status1  generosity1  generosity2  generosity3  health_cond  marital1  mor_circle  moralid1  moralid10  moralid2  moralid3  moralid4  moralid5  moralid6  moralid7  moralid8  moralid9  mcoop3  mcoop5  narc1  narc2  narc3  narc4  narc5  narc6  nidentity1  omind1  omind2  omind5  omind6  contact2  contact3  contact4  hygiene1  hygiene2  hygiene4  hygiene5  psupport1  psupport2  psupport3  psupport4  psupport5  political_ideology  happy  slf_ladder  riskperc1  riskperc2  self_esteem  sex1  sbelong1  sbelong2  sbelong3  sbelong4  slfcont1  slfcont2  slfcont3  slfcont4  urban
##   8   10  age  cnarc2  cnarc3  ctheory1  ctheory2  ctheory3  ctheory4  CRT1  CRT2  CRT3  employ_status1  generosity1  generosity2  generosity3  health_cond  marital1  mor_circle  moralid1  moralid10  moralid2  moralid3  moralid4  moralid5  moralid6  moralid7  moralid8  moralid9  mcoop3  mcoop5  narc1  narc2  narc3  narc4  narc5  narc6  nidentity1  omind1  omind2  omind5  omind6  contact2  contact3  contact4  hygiene1  hygiene2  hygiene4  hygiene5  psupport1  psupport2  psupport3  psupport4  psupport5  political_ideology  happy  slf_ladder  riskperc1  riskperc2  self_esteem  sex1  sbelong1  sbelong2  sbelong3  sbelong4  slfcont1  slfcont2  slfcont3  slfcont4  urban
##   9   1  age  cnarc2  cnarc3  ctheory1  ctheory2  ctheory3  ctheory4  CRT1  CRT2  CRT3  employ_status1  generosity1  generosity2  generosity3  health_cond  marital1  mor_circle  moralid1  moralid10  moralid2  moralid3  moralid4  moralid5  moralid6  moralid7  moralid8  moralid9  mcoop3  mcoop5  narc1  narc2  narc3  narc4  narc5  narc6  nidentity1  omind1  omind2  omind5  omind6  contact2  contact3  contact4  hygiene1  hygiene2  hygiene4  hygiene5  psupport1  psupport2  psupport3  psupport4  psupport5  political_ideology  happy  slf_ladder  riskperc1  riskperc2  self_esteem  sex1  sbelong1  sbelong2  sbelong3  sbelong4  slfcont1  slfcont2  slfcont3  slfcont4  urban
##   9   2  age  cnarc2  cnarc3  ctheory1  ctheory2  ctheory3  ctheory4  CRT1  CRT2  CRT3  employ_status1  generosity1  generosity2  generosity3  health_cond  marital1  mor_circle  moralid1  moralid10  moralid2  moralid3  moralid4  moralid5  moralid6  moralid7  moralid8  moralid9  mcoop3  mcoop5  narc1  narc2  narc3  narc4  narc5  narc6  nidentity1  omind1  omind2  omind5  omind6  contact2  contact3  contact4  hygiene1  hygiene2  hygiene4  hygiene5  psupport1  psupport2  psupport3  psupport4  psupport5  political_ideology  happy  slf_ladder  riskperc1  riskperc2  self_esteem  sex1  sbelong1  sbelong2  sbelong3  sbelong4  slfcont1  slfcont2  slfcont3  slfcont4  urban
##   9   3  age  cnarc2  cnarc3  ctheory1  ctheory2  ctheory3  ctheory4  CRT1  CRT2  CRT3  employ_status1  generosity1  generosity2  generosity3  health_cond  marital1  mor_circle  moralid1  moralid10  moralid2  moralid3  moralid4  moralid5  moralid6  moralid7  moralid8  moralid9  mcoop3  mcoop5  narc1  narc2  narc3  narc4  narc5  narc6  nidentity1  omind1  omind2  omind5  omind6  contact2  contact3  contact4  hygiene1  hygiene2  hygiene4  hygiene5  psupport1  psupport2  psupport3  psupport4  psupport5  political_ideology  happy  slf_ladder  riskperc1  riskperc2  self_esteem  sex1  sbelong1  sbelong2  sbelong3  sbelong4  slfcont1  slfcont2  slfcont3  slfcont4  urban
##   9   4  age  cnarc2  cnarc3  ctheory1  ctheory2  ctheory3  ctheory4  CRT1  CRT2  CRT3  employ_status1  generosity1  generosity2  generosity3  health_cond  marital1  mor_circle  moralid1  moralid10  moralid2  moralid3  moralid4  moralid5  moralid6  moralid7  moralid8  moralid9  mcoop3  mcoop5  narc1  narc2  narc3  narc4  narc5  narc6  nidentity1  omind1  omind2  omind5  omind6  contact2  contact3  contact4  hygiene1  hygiene2  hygiene4  hygiene5  psupport1  psupport2  psupport3  psupport4  psupport5  political_ideology  happy  slf_ladder  riskperc1  riskperc2  self_esteem  sex1  sbelong1  sbelong2  sbelong3  sbelong4  slfcont1  slfcont2  slfcont3  slfcont4  urban
##   9   5  age  cnarc2  cnarc3  ctheory1  ctheory2  ctheory3  ctheory4  CRT1  CRT2  CRT3  employ_status1  generosity1  generosity2  generosity3  health_cond  marital1  mor_circle  moralid1  moralid10  moralid2  moralid3  moralid4  moralid5  moralid6  moralid7  moralid8  moralid9  mcoop3  mcoop5  narc1  narc2  narc3  narc4  narc5  narc6  nidentity1  omind1  omind2  omind5  omind6  contact2  contact3  contact4  hygiene1  hygiene2  hygiene4  hygiene5  psupport1  psupport2  psupport3  psupport4  psupport5  political_ideology  happy  slf_ladder  riskperc1  riskperc2  self_esteem  sex1  sbelong1  sbelong2  sbelong3  sbelong4  slfcont1  slfcont2  slfcont3  slfcont4  urban
##   9   6  age  cnarc2  cnarc3  ctheory1  ctheory2  ctheory3  ctheory4  CRT1  CRT2  CRT3  employ_status1  generosity1  generosity2  generosity3  health_cond  marital1  mor_circle  moralid1  moralid10  moralid2  moralid3  moralid4  moralid5  moralid6  moralid7  moralid8  moralid9  mcoop3  mcoop5  narc1  narc2  narc3  narc4  narc5  narc6  nidentity1  omind1  omind2  omind5  omind6  contact2  contact3  contact4  hygiene1  hygiene2  hygiene4  hygiene5  psupport1  psupport2  psupport3  psupport4  psupport5  political_ideology  happy  slf_ladder  riskperc1  riskperc2  self_esteem  sex1  sbelong1  sbelong2  sbelong3  sbelong4  slfcont1  slfcont2  slfcont3  slfcont4  urban
##   9   7  age  cnarc2  cnarc3  ctheory1  ctheory2  ctheory3  ctheory4  CRT1  CRT2  CRT3  employ_status1  generosity1  generosity2  generosity3  health_cond  marital1  mor_circle  moralid1  moralid10  moralid2  moralid3  moralid4  moralid5  moralid6  moralid7  moralid8  moralid9  mcoop3  mcoop5  narc1  narc2  narc3  narc4  narc5  narc6  nidentity1  omind1  omind2  omind5  omind6  contact2  contact3  contact4  hygiene1  hygiene2  hygiene4  hygiene5  psupport1  psupport2  psupport3  psupport4  psupport5  political_ideology  happy  slf_ladder  riskperc1  riskperc2  self_esteem  sex1  sbelong1  sbelong2  sbelong3  sbelong4  slfcont1  slfcont2  slfcont3  slfcont4  urban
##   9   8  age  cnarc2  cnarc3  ctheory1  ctheory2  ctheory3  ctheory4  CRT1  CRT2  CRT3  employ_status1  generosity1  generosity2  generosity3  health_cond  marital1  mor_circle  moralid1  moralid10  moralid2  moralid3  moralid4  moralid5  moralid6  moralid7  moralid8  moralid9  mcoop3  mcoop5  narc1  narc2  narc3  narc4  narc5  narc6  nidentity1  omind1  omind2  omind5  omind6  contact2  contact3  contact4  hygiene1  hygiene2  hygiene4  hygiene5  psupport1  psupport2  psupport3  psupport4  psupport5  political_ideology  happy  slf_ladder  riskperc1  riskperc2  self_esteem  sex1  sbelong1  sbelong2  sbelong3  sbelong4  slfcont1  slfcont2  slfcont3  slfcont4  urban
##   9   9  age  cnarc2  cnarc3  ctheory1  ctheory2  ctheory3  ctheory4  CRT1  CRT2  CRT3  employ_status1  generosity1  generosity2  generosity3  health_cond  marital1  mor_circle  moralid1  moralid10  moralid2  moralid3  moralid4  moralid5  moralid6  moralid7  moralid8  moralid9  mcoop3  mcoop5  narc1  narc2  narc3  narc4  narc5  narc6  nidentity1  omind1  omind2  omind5  omind6  contact2  contact3  contact4  hygiene1  hygiene2  hygiene4  hygiene5  psupport1  psupport2  psupport3  psupport4  psupport5  political_ideology  happy  slf_ladder  riskperc1  riskperc2  self_esteem  sex1  sbelong1  sbelong2  sbelong3  sbelong4  slfcont1  slfcont2  slfcont3  slfcont4  urban
##   9   10  age  cnarc2  cnarc3  ctheory1  ctheory2  ctheory3  ctheory4  CRT1  CRT2  CRT3  employ_status1  generosity1  generosity2  generosity3  health_cond  marital1  mor_circle  moralid1  moralid10  moralid2  moralid3  moralid4  moralid5  moralid6  moralid7  moralid8  moralid9  mcoop3  mcoop5  narc1  narc2  narc3  narc4  narc5  narc6  nidentity1  omind1  omind2  omind5  omind6  contact2  contact3  contact4  hygiene1  hygiene2  hygiene4  hygiene5  psupport1  psupport2  psupport3  psupport4  psupport5  political_ideology  happy  slf_ladder  riskperc1  riskperc2  self_esteem  sex1  sbelong1  sbelong2  sbelong3  sbelong4  slfcont1  slfcont2  slfcont3  slfcont4  urban
##   10   1  age  cnarc2  cnarc3  ctheory1  ctheory2  ctheory3  ctheory4  CRT1  CRT2  CRT3  employ_status1  generosity1  generosity2  generosity3  health_cond  marital1  mor_circle  moralid1  moralid10  moralid2  moralid3  moralid4  moralid5  moralid6  moralid7  moralid8  moralid9  mcoop3  mcoop5  narc1  narc2  narc3  narc4  narc5  narc6  nidentity1  omind1  omind2  omind5  omind6  contact2  contact3  contact4  hygiene1  hygiene2  hygiene4  hygiene5  psupport1  psupport2  psupport3  psupport4  psupport5  political_ideology  happy  slf_ladder  riskperc1  riskperc2  self_esteem  sex1  sbelong1  sbelong2  sbelong3  sbelong4  slfcont1  slfcont2  slfcont3  slfcont4  urban
##   10   2  age  cnarc2  cnarc3  ctheory1  ctheory2  ctheory3  ctheory4  CRT1  CRT2  CRT3  employ_status1  generosity1  generosity2  generosity3  health_cond  marital1  mor_circle  moralid1  moralid10  moralid2  moralid3  moralid4  moralid5  moralid6  moralid7  moralid8  moralid9  mcoop3  mcoop5  narc1  narc2  narc3  narc4  narc5  narc6  nidentity1  omind1  omind2  omind5  omind6  contact2  contact3  contact4  hygiene1  hygiene2  hygiene4  hygiene5  psupport1  psupport2  psupport3  psupport4  psupport5  political_ideology  happy  slf_ladder  riskperc1  riskperc2  self_esteem  sex1  sbelong1  sbelong2  sbelong3  sbelong4  slfcont1  slfcont2  slfcont3  slfcont4  urban
##   10   3  age  cnarc2  cnarc3  ctheory1  ctheory2  ctheory3  ctheory4  CRT1  CRT2  CRT3  employ_status1  generosity1  generosity2  generosity3  health_cond  marital1  mor_circle  moralid1  moralid10  moralid2  moralid3  moralid4  moralid5  moralid6  moralid7  moralid8  moralid9  mcoop3  mcoop5  narc1  narc2  narc3  narc4  narc5  narc6  nidentity1  omind1  omind2  omind5  omind6  contact2  contact3  contact4  hygiene1  hygiene2  hygiene4  hygiene5  psupport1  psupport2  psupport3  psupport4  psupport5  political_ideology  happy  slf_ladder  riskperc1  riskperc2  self_esteem  sex1  sbelong1  sbelong2  sbelong3  sbelong4  slfcont1  slfcont2  slfcont3  slfcont4  urban
##   10   4  age  cnarc2  cnarc3  ctheory1  ctheory2  ctheory3  ctheory4  CRT1  CRT2  CRT3  employ_status1  generosity1  generosity2  generosity3  health_cond  marital1  mor_circle  moralid1  moralid10  moralid2  moralid3  moralid4  moralid5  moralid6  moralid7  moralid8  moralid9  mcoop3  mcoop5  narc1  narc2  narc3  narc4  narc5  narc6  nidentity1  omind1  omind2  omind5  omind6  contact2  contact3  contact4  hygiene1  hygiene2  hygiene4  hygiene5  psupport1  psupport2  psupport3  psupport4  psupport5  political_ideology  happy  slf_ladder  riskperc1  riskperc2  self_esteem  sex1  sbelong1  sbelong2  sbelong3  sbelong4  slfcont1  slfcont2  slfcont3  slfcont4  urban
##   10   5  age  cnarc2  cnarc3  ctheory1  ctheory2  ctheory3  ctheory4  CRT1  CRT2  CRT3  employ_status1  generosity1  generosity2  generosity3  health_cond  marital1  mor_circle  moralid1  moralid10  moralid2  moralid3  moralid4  moralid5  moralid6  moralid7  moralid8  moralid9  mcoop3  mcoop5  narc1  narc2  narc3  narc4  narc5  narc6  nidentity1  omind1  omind2  omind5  omind6  contact2  contact3  contact4  hygiene1  hygiene2  hygiene4  hygiene5  psupport1  psupport2  psupport3  psupport4  psupport5  political_ideology  happy  slf_ladder  riskperc1  riskperc2  self_esteem  sex1  sbelong1  sbelong2  sbelong3  sbelong4  slfcont1  slfcont2  slfcont3  slfcont4  urban
##   10   6  age  cnarc2  cnarc3  ctheory1  ctheory2  ctheory3  ctheory4  CRT1  CRT2  CRT3  employ_status1  generosity1  generosity2  generosity3  health_cond  marital1  mor_circle  moralid1  moralid10  moralid2  moralid3  moralid4  moralid5  moralid6  moralid7  moralid8  moralid9  mcoop3  mcoop5  narc1  narc2  narc3  narc4  narc5  narc6  nidentity1  omind1  omind2  omind5  omind6  contact2  contact3  contact4  hygiene1  hygiene2  hygiene4  hygiene5  psupport1  psupport2  psupport3  psupport4  psupport5  political_ideology  happy  slf_ladder  riskperc1  riskperc2  self_esteem  sex1  sbelong1  sbelong2  sbelong3  sbelong4  slfcont1  slfcont2  slfcont3  slfcont4  urban
##   10   7  age  cnarc2  cnarc3  ctheory1  ctheory2  ctheory3  ctheory4  CRT1  CRT2  CRT3  employ_status1  generosity1  generosity2  generosity3  health_cond  marital1  mor_circle  moralid1  moralid10  moralid2  moralid3  moralid4  moralid5  moralid6  moralid7  moralid8  moralid9  mcoop3  mcoop5  narc1  narc2  narc3  narc4  narc5  narc6  nidentity1  omind1  omind2  omind5  omind6  contact2  contact3  contact4  hygiene1  hygiene2  hygiene4  hygiene5  psupport1  psupport2  psupport3  psupport4  psupport5  political_ideology  happy  slf_ladder  riskperc1  riskperc2  self_esteem  sex1  sbelong1  sbelong2  sbelong3  sbelong4  slfcont1  slfcont2  slfcont3  slfcont4  urban
##   10   8  age  cnarc2  cnarc3  ctheory1  ctheory2  ctheory3  ctheory4  CRT1  CRT2  CRT3  employ_status1  generosity1  generosity2  generosity3  health_cond  marital1  mor_circle  moralid1  moralid10  moralid2  moralid3  moralid4  moralid5  moralid6  moralid7  moralid8  moralid9  mcoop3  mcoop5  narc1  narc2  narc3  narc4  narc5  narc6  nidentity1  omind1  omind2  omind5  omind6  contact2  contact3  contact4  hygiene1  hygiene2  hygiene4  hygiene5  psupport1  psupport2  psupport3  psupport4  psupport5  political_ideology  happy  slf_ladder  riskperc1  riskperc2  self_esteem  sex1  sbelong1  sbelong2  sbelong3  sbelong4  slfcont1  slfcont2  slfcont3  slfcont4  urban
##   10   9  age  cnarc2  cnarc3  ctheory1  ctheory2  ctheory3  ctheory4  CRT1  CRT2  CRT3  employ_status1  generosity1  generosity2  generosity3  health_cond  marital1  mor_circle  moralid1  moralid10  moralid2  moralid3  moralid4  moralid5  moralid6  moralid7  moralid8  moralid9  mcoop3  mcoop5  narc1  narc2  narc3  narc4  narc5  narc6  nidentity1  omind1  omind2  omind5  omind6  contact2  contact3  contact4  hygiene1  hygiene2  hygiene4  hygiene5  psupport1  psupport2  psupport3  psupport4  psupport5  political_ideology  happy  slf_ladder  riskperc1  riskperc2  self_esteem  sex1  sbelong1  sbelong2  sbelong3  sbelong4  slfcont1  slfcont2  slfcont3  slfcont4  urban
##   10   10  age  cnarc2  cnarc3  ctheory1  ctheory2  ctheory3  ctheory4  CRT1  CRT2  CRT3  employ_status1  generosity1  generosity2  generosity3  health_cond  marital1  mor_circle  moralid1  moralid10  moralid2  moralid3  moralid4  moralid5  moralid6  moralid7  moralid8  moralid9  mcoop3  mcoop5  narc1  narc2  narc3  narc4  narc5  narc6  nidentity1  omind1  omind2  omind5  omind6  contact2  contact3  contact4  hygiene1  hygiene2  hygiene4  hygiene5  psupport1  psupport2  psupport3  psupport4  psupport5  political_ideology  happy  slf_ladder  riskperc1  riskperc2  self_esteem  sex1  sbelong1  sbelong2  sbelong3  sbelong4  slfcont1  slfcont2  slfcont3  slfcont4  urban
```

```
## Warning: Number of logged events: 6424
```

```
## 
##  iter imp variable
##   1   1  cnarc1  cnarc3  CRT1  CRT2  CRT3  moralid5  moralid6  moralid8  narc4  contact1  contact2  contact3  contact5  hygiene1  hygiene2  hygiene3  hygiene4  hygiene5  psupport1  psupport3  psupport4  psupport5  political_ideology  happy  riskperc1  riskperc2  sbelong1  sbelong2  optim1  optim2  slfcont3
##   1   2  cnarc1  cnarc3  CRT1  CRT2  CRT3  moralid5  moralid6  moralid8  narc4  contact1  contact2  contact3  contact5  hygiene1  hygiene2  hygiene3  hygiene4  hygiene5  psupport1  psupport3  psupport4  psupport5  political_ideology  happy  riskperc1  riskperc2  sbelong1  sbelong2  optim1  optim2  slfcont3
##   1   3  cnarc1  cnarc3  CRT1  CRT2  CRT3  moralid5  moralid6  moralid8  narc4  contact1  contact2  contact3  contact5  hygiene1  hygiene2  hygiene3  hygiene4  hygiene5  psupport1  psupport3  psupport4  psupport5  political_ideology  happy  riskperc1  riskperc2  sbelong1  sbelong2  optim1  optim2  slfcont3
##   1   4  cnarc1  cnarc3  CRT1  CRT2  CRT3  moralid5  moralid6  moralid8  narc4  contact1  contact2  contact3  contact5  hygiene1  hygiene2  hygiene3  hygiene4  hygiene5  psupport1  psupport3  psupport4  psupport5  political_ideology  happy  riskperc1  riskperc2  sbelong1  sbelong2  optim1  optim2  slfcont3
##   1   5  cnarc1  cnarc3  CRT1  CRT2  CRT3  moralid5  moralid6  moralid8  narc4  contact1  contact2  contact3  contact5  hygiene1  hygiene2  hygiene3  hygiene4  hygiene5  psupport1  psupport3  psupport4  psupport5  political_ideology  happy  riskperc1  riskperc2  sbelong1  sbelong2  optim1  optim2  slfcont3
##   1   6  cnarc1  cnarc3  CRT1  CRT2  CRT3  moralid5  moralid6  moralid8  narc4  contact1  contact2  contact3  contact5  hygiene1  hygiene2  hygiene3  hygiene4  hygiene5  psupport1  psupport3  psupport4  psupport5  political_ideology  happy  riskperc1  riskperc2  sbelong1  sbelong2  optim1  optim2  slfcont3
##   1   7  cnarc1  cnarc3  CRT1  CRT2  CRT3  moralid5  moralid6  moralid8  narc4  contact1  contact2  contact3  contact5  hygiene1  hygiene2  hygiene3  hygiene4  hygiene5  psupport1  psupport3  psupport4  psupport5  political_ideology  happy  riskperc1  riskperc2  sbelong1  sbelong2  optim1  optim2  slfcont3
##   1   8  cnarc1  cnarc3  CRT1  CRT2  CRT3  moralid5  moralid6  moralid8  narc4  contact1  contact2  contact3  contact5  hygiene1  hygiene2  hygiene3  hygiene4  hygiene5  psupport1  psupport3  psupport4  psupport5  political_ideology  happy  riskperc1  riskperc2  sbelong1  sbelong2  optim1  optim2  slfcont3
##   1   9  cnarc1  cnarc3  CRT1  CRT2  CRT3  moralid5  moralid6  moralid8  narc4  contact1  contact2  contact3  contact5  hygiene1  hygiene2  hygiene3  hygiene4  hygiene5  psupport1  psupport3  psupport4  psupport5  political_ideology  happy  riskperc1  riskperc2  sbelong1  sbelong2  optim1  optim2  slfcont3
##   1   10  cnarc1  cnarc3  CRT1  CRT2  CRT3  moralid5  moralid6  moralid8  narc4  contact1  contact2  contact3  contact5  hygiene1  hygiene2  hygiene3  hygiene4  hygiene5  psupport1  psupport3  psupport4  psupport5  political_ideology  happy  riskperc1  riskperc2  sbelong1  sbelong2  optim1  optim2  slfcont3
##   2   1  cnarc1  cnarc3  CRT1  CRT2  CRT3  moralid5  moralid6  moralid8  narc4  contact1  contact2  contact3  contact5  hygiene1  hygiene2  hygiene3  hygiene4  hygiene5  psupport1  psupport3  psupport4  psupport5  political_ideology  happy  riskperc1  riskperc2  sbelong1  sbelong2  optim1  optim2  slfcont3
##   2   2  cnarc1  cnarc3  CRT1  CRT2  CRT3  moralid5  moralid6  moralid8  narc4  contact1  contact2  contact3  contact5  hygiene1  hygiene2  hygiene3  hygiene4  hygiene5  psupport1  psupport3  psupport4  psupport5  political_ideology  happy  riskperc1  riskperc2  sbelong1  sbelong2  optim1  optim2  slfcont3
##   2   3  cnarc1  cnarc3  CRT1  CRT2  CRT3  moralid5  moralid6  moralid8  narc4  contact1  contact2  contact3  contact5  hygiene1  hygiene2  hygiene3  hygiene4  hygiene5  psupport1  psupport3  psupport4  psupport5  political_ideology  happy  riskperc1  riskperc2  sbelong1  sbelong2  optim1  optim2  slfcont3
##   2   4  cnarc1  cnarc3  CRT1  CRT2  CRT3  moralid5  moralid6  moralid8  narc4  contact1  contact2  contact3  contact5  hygiene1  hygiene2  hygiene3  hygiene4  hygiene5  psupport1  psupport3  psupport4  psupport5  political_ideology  happy  riskperc1  riskperc2  sbelong1  sbelong2  optim1  optim2  slfcont3
##   2   5  cnarc1  cnarc3  CRT1  CRT2  CRT3  moralid5  moralid6  moralid8  narc4  contact1  contact2  contact3  contact5  hygiene1  hygiene2  hygiene3  hygiene4  hygiene5  psupport1  psupport3  psupport4  psupport5  political_ideology  happy  riskperc1  riskperc2  sbelong1  sbelong2  optim1  optim2  slfcont3
##   2   6  cnarc1  cnarc3  CRT1  CRT2  CRT3  moralid5  moralid6  moralid8  narc4  contact1  contact2  contact3  contact5  hygiene1  hygiene2  hygiene3  hygiene4  hygiene5  psupport1  psupport3  psupport4  psupport5  political_ideology  happy  riskperc1  riskperc2  sbelong1  sbelong2  optim1  optim2  slfcont3
##   2   7  cnarc1  cnarc3  CRT1  CRT2  CRT3  moralid5  moralid6  moralid8  narc4  contact1  contact2  contact3  contact5  hygiene1  hygiene2  hygiene3  hygiene4  hygiene5  psupport1  psupport3  psupport4  psupport5  political_ideology  happy  riskperc1  riskperc2  sbelong1  sbelong2  optim1  optim2  slfcont3
##   2   8  cnarc1  cnarc3  CRT1  CRT2  CRT3  moralid5  moralid6  moralid8  narc4  contact1  contact2  contact3  contact5  hygiene1  hygiene2  hygiene3  hygiene4  hygiene5  psupport1  psupport3  psupport4  psupport5  political_ideology  happy  riskperc1  riskperc2  sbelong1  sbelong2  optim1  optim2  slfcont3
##   2   9  cnarc1  cnarc3  CRT1  CRT2  CRT3  moralid5  moralid6  moralid8  narc4  contact1  contact2  contact3  contact5  hygiene1  hygiene2  hygiene3  hygiene4  hygiene5  psupport1  psupport3  psupport4  psupport5  political_ideology  happy  riskperc1  riskperc2  sbelong1  sbelong2  optim1  optim2  slfcont3
##   2   10  cnarc1  cnarc3  CRT1  CRT2  CRT3  moralid5  moralid6  moralid8  narc4  contact1  contact2  contact3  contact5  hygiene1  hygiene2  hygiene3  hygiene4  hygiene5  psupport1  psupport3  psupport4  psupport5  political_ideology  happy  riskperc1  riskperc2  sbelong1  sbelong2  optim1  optim2  slfcont3
##   3   1  cnarc1  cnarc3  CRT1  CRT2  CRT3  moralid5  moralid6  moralid8  narc4  contact1  contact2  contact3  contact5  hygiene1  hygiene2  hygiene3  hygiene4  hygiene5  psupport1  psupport3  psupport4  psupport5  political_ideology  happy  riskperc1  riskperc2  sbelong1  sbelong2  optim1  optim2  slfcont3
##   3   2  cnarc1  cnarc3  CRT1  CRT2  CRT3  moralid5  moralid6  moralid8  narc4  contact1  contact2  contact3  contact5  hygiene1  hygiene2  hygiene3  hygiene4  hygiene5  psupport1  psupport3  psupport4  psupport5  political_ideology  happy  riskperc1  riskperc2  sbelong1  sbelong2  optim1  optim2  slfcont3
##   3   3  cnarc1  cnarc3  CRT1  CRT2  CRT3  moralid5  moralid6  moralid8  narc4  contact1  contact2  contact3  contact5  hygiene1  hygiene2  hygiene3  hygiene4  hygiene5  psupport1  psupport3  psupport4  psupport5  political_ideology  happy  riskperc1  riskperc2  sbelong1  sbelong2  optim1  optim2  slfcont3
##   3   4  cnarc1  cnarc3  CRT1  CRT2  CRT3  moralid5  moralid6  moralid8  narc4  contact1  contact2  contact3  contact5  hygiene1  hygiene2  hygiene3  hygiene4  hygiene5  psupport1  psupport3  psupport4  psupport5  political_ideology  happy  riskperc1  riskperc2  sbelong1  sbelong2  optim1  optim2  slfcont3
##   3   5  cnarc1  cnarc3  CRT1  CRT2  CRT3  moralid5  moralid6  moralid8  narc4  contact1  contact2  contact3  contact5  hygiene1  hygiene2  hygiene3  hygiene4  hygiene5  psupport1  psupport3  psupport4  psupport5  political_ideology  happy  riskperc1  riskperc2  sbelong1  sbelong2  optim1  optim2  slfcont3
##   3   6  cnarc1  cnarc3  CRT1  CRT2  CRT3  moralid5  moralid6  moralid8  narc4  contact1  contact2  contact3  contact5  hygiene1  hygiene2  hygiene3  hygiene4  hygiene5  psupport1  psupport3  psupport4  psupport5  political_ideology  happy  riskperc1  riskperc2  sbelong1  sbelong2  optim1  optim2  slfcont3
##   3   7  cnarc1  cnarc3  CRT1  CRT2  CRT3  moralid5  moralid6  moralid8  narc4  contact1  contact2  contact3  contact5  hygiene1  hygiene2  hygiene3  hygiene4  hygiene5  psupport1  psupport3  psupport4  psupport5  political_ideology  happy  riskperc1  riskperc2  sbelong1  sbelong2  optim1  optim2  slfcont3
##   3   8  cnarc1  cnarc3  CRT1  CRT2  CRT3  moralid5  moralid6  moralid8  narc4  contact1  contact2  contact3  contact5  hygiene1  hygiene2  hygiene3  hygiene4  hygiene5  psupport1  psupport3  psupport4  psupport5  political_ideology  happy  riskperc1  riskperc2  sbelong1  sbelong2  optim1  optim2  slfcont3
##   3   9  cnarc1  cnarc3  CRT1  CRT2  CRT3  moralid5  moralid6  moralid8  narc4  contact1  contact2  contact3  contact5  hygiene1  hygiene2  hygiene3  hygiene4  hygiene5  psupport1  psupport3  psupport4  psupport5  political_ideology  happy  riskperc1  riskperc2  sbelong1  sbelong2  optim1  optim2  slfcont3
##   3   10  cnarc1  cnarc3  CRT1  CRT2  CRT3  moralid5  moralid6  moralid8  narc4  contact1  contact2  contact3  contact5  hygiene1  hygiene2  hygiene3  hygiene4  hygiene5  psupport1  psupport3  psupport4  psupport5  political_ideology  happy  riskperc1  riskperc2  sbelong1  sbelong2  optim1  optim2  slfcont3
##   4   1  cnarc1  cnarc3  CRT1  CRT2  CRT3  moralid5  moralid6  moralid8  narc4  contact1  contact2  contact3  contact5  hygiene1  hygiene2  hygiene3  hygiene4  hygiene5  psupport1  psupport3  psupport4  psupport5  political_ideology  happy  riskperc1  riskperc2  sbelong1  sbelong2  optim1  optim2  slfcont3
##   4   2  cnarc1  cnarc3  CRT1  CRT2  CRT3  moralid5  moralid6  moralid8  narc4  contact1  contact2  contact3  contact5  hygiene1  hygiene2  hygiene3  hygiene4  hygiene5  psupport1  psupport3  psupport4  psupport5  political_ideology  happy  riskperc1  riskperc2  sbelong1  sbelong2  optim1  optim2  slfcont3
##   4   3  cnarc1  cnarc3  CRT1  CRT2  CRT3  moralid5  moralid6  moralid8  narc4  contact1  contact2  contact3  contact5  hygiene1  hygiene2  hygiene3  hygiene4  hygiene5  psupport1  psupport3  psupport4  psupport5  political_ideology  happy  riskperc1  riskperc2  sbelong1  sbelong2  optim1  optim2  slfcont3
##   4   4  cnarc1  cnarc3  CRT1  CRT2  CRT3  moralid5  moralid6  moralid8  narc4  contact1  contact2  contact3  contact5  hygiene1  hygiene2  hygiene3  hygiene4  hygiene5  psupport1  psupport3  psupport4  psupport5  political_ideology  happy  riskperc1  riskperc2  sbelong1  sbelong2  optim1  optim2  slfcont3
##   4   5  cnarc1  cnarc3  CRT1  CRT2  CRT3  moralid5  moralid6  moralid8  narc4  contact1  contact2  contact3  contact5  hygiene1  hygiene2  hygiene3  hygiene4  hygiene5  psupport1  psupport3  psupport4  psupport5  political_ideology  happy  riskperc1  riskperc2  sbelong1  sbelong2  optim1  optim2  slfcont3
##   4   6  cnarc1  cnarc3  CRT1  CRT2  CRT3  moralid5  moralid6  moralid8  narc4  contact1  contact2  contact3  contact5  hygiene1  hygiene2  hygiene3  hygiene4  hygiene5  psupport1  psupport3  psupport4  psupport5  political_ideology  happy  riskperc1  riskperc2  sbelong1  sbelong2  optim1  optim2  slfcont3
##   4   7  cnarc1  cnarc3  CRT1  CRT2  CRT3  moralid5  moralid6  moralid8  narc4  contact1  contact2  contact3  contact5  hygiene1  hygiene2  hygiene3  hygiene4  hygiene5  psupport1  psupport3  psupport4  psupport5  political_ideology  happy  riskperc1  riskperc2  sbelong1  sbelong2  optim1  optim2  slfcont3
##   4   8  cnarc1  cnarc3  CRT1  CRT2  CRT3  moralid5  moralid6  moralid8  narc4  contact1  contact2  contact3  contact5  hygiene1  hygiene2  hygiene3  hygiene4  hygiene5  psupport1  psupport3  psupport4  psupport5  political_ideology  happy  riskperc1  riskperc2  sbelong1  sbelong2  optim1  optim2  slfcont3
##   4   9  cnarc1  cnarc3  CRT1  CRT2  CRT3  moralid5  moralid6  moralid8  narc4  contact1  contact2  contact3  contact5  hygiene1  hygiene2  hygiene3  hygiene4  hygiene5  psupport1  psupport3  psupport4  psupport5  political_ideology  happy  riskperc1  riskperc2  sbelong1  sbelong2  optim1  optim2  slfcont3
##   4   10  cnarc1  cnarc3  CRT1  CRT2  CRT3  moralid5  moralid6  moralid8  narc4  contact1  contact2  contact3  contact5  hygiene1  hygiene2  hygiene3  hygiene4  hygiene5  psupport1  psupport3  psupport4  psupport5  political_ideology  happy  riskperc1  riskperc2  sbelong1  sbelong2  optim1  optim2  slfcont3
##   5   1  cnarc1  cnarc3  CRT1  CRT2  CRT3  moralid5  moralid6  moralid8  narc4  contact1  contact2  contact3  contact5  hygiene1  hygiene2  hygiene3  hygiene4  hygiene5  psupport1  psupport3  psupport4  psupport5  political_ideology  happy  riskperc1  riskperc2  sbelong1  sbelong2  optim1  optim2  slfcont3
##   5   2  cnarc1  cnarc3  CRT1  CRT2  CRT3  moralid5  moralid6  moralid8  narc4  contact1  contact2  contact3  contact5  hygiene1  hygiene2  hygiene3  hygiene4  hygiene5  psupport1  psupport3  psupport4  psupport5  political_ideology  happy  riskperc1  riskperc2  sbelong1  sbelong2  optim1  optim2  slfcont3
##   5   3  cnarc1  cnarc3  CRT1  CRT2  CRT3  moralid5  moralid6  moralid8  narc4  contact1  contact2  contact3  contact5  hygiene1  hygiene2  hygiene3  hygiene4  hygiene5  psupport1  psupport3  psupport4  psupport5  political_ideology  happy  riskperc1  riskperc2  sbelong1  sbelong2  optim1  optim2  slfcont3
##   5   4  cnarc1  cnarc3  CRT1  CRT2  CRT3  moralid5  moralid6  moralid8  narc4  contact1  contact2  contact3  contact5  hygiene1  hygiene2  hygiene3  hygiene4  hygiene5  psupport1  psupport3  psupport4  psupport5  political_ideology  happy  riskperc1  riskperc2  sbelong1  sbelong2  optim1  optim2  slfcont3
##   5   5  cnarc1  cnarc3  CRT1  CRT2  CRT3  moralid5  moralid6  moralid8  narc4  contact1  contact2  contact3  contact5  hygiene1  hygiene2  hygiene3  hygiene4  hygiene5  psupport1  psupport3  psupport4  psupport5  political_ideology  happy  riskperc1  riskperc2  sbelong1  sbelong2  optim1  optim2  slfcont3
##   5   6  cnarc1  cnarc3  CRT1  CRT2  CRT3  moralid5  moralid6  moralid8  narc4  contact1  contact2  contact3  contact5  hygiene1  hygiene2  hygiene3  hygiene4  hygiene5  psupport1  psupport3  psupport4  psupport5  political_ideology  happy  riskperc1  riskperc2  sbelong1  sbelong2  optim1  optim2  slfcont3
##   5   7  cnarc1  cnarc3  CRT1  CRT2  CRT3  moralid5  moralid6  moralid8  narc4  contact1  contact2  contact3  contact5  hygiene1  hygiene2  hygiene3  hygiene4  hygiene5  psupport1  psupport3  psupport4  psupport5  political_ideology  happy  riskperc1  riskperc2  sbelong1  sbelong2  optim1  optim2  slfcont3
##   5   8  cnarc1  cnarc3  CRT1  CRT2  CRT3  moralid5  moralid6  moralid8  narc4  contact1  contact2  contact3  contact5  hygiene1  hygiene2  hygiene3  hygiene4  hygiene5  psupport1  psupport3  psupport4  psupport5  political_ideology  happy  riskperc1  riskperc2  sbelong1  sbelong2  optim1  optim2  slfcont3
##   5   9  cnarc1  cnarc3  CRT1  CRT2  CRT3  moralid5  moralid6  moralid8  narc4  contact1  contact2  contact3  contact5  hygiene1  hygiene2  hygiene3  hygiene4  hygiene5  psupport1  psupport3  psupport4  psupport5  political_ideology  happy  riskperc1  riskperc2  sbelong1  sbelong2  optim1  optim2  slfcont3
##   5   10  cnarc1  cnarc3  CRT1  CRT2  CRT3  moralid5  moralid6  moralid8  narc4  contact1  contact2  contact3  contact5  hygiene1  hygiene2  hygiene3  hygiene4  hygiene5  psupport1  psupport3  psupport4  psupport5  political_ideology  happy  riskperc1  riskperc2  sbelong1  sbelong2  optim1  optim2  slfcont3
##   6   1  cnarc1  cnarc3  CRT1  CRT2  CRT3  moralid5  moralid6  moralid8  narc4  contact1  contact2  contact3  contact5  hygiene1  hygiene2  hygiene3  hygiene4  hygiene5  psupport1  psupport3  psupport4  psupport5  political_ideology  happy  riskperc1  riskperc2  sbelong1  sbelong2  optim1  optim2  slfcont3
##   6   2  cnarc1  cnarc3  CRT1  CRT2  CRT3  moralid5  moralid6  moralid8  narc4  contact1  contact2  contact3  contact5  hygiene1  hygiene2  hygiene3  hygiene4  hygiene5  psupport1  psupport3  psupport4  psupport5  political_ideology  happy  riskperc1  riskperc2  sbelong1  sbelong2  optim1  optim2  slfcont3
##   6   3  cnarc1  cnarc3  CRT1  CRT2  CRT3  moralid5  moralid6  moralid8  narc4  contact1  contact2  contact3  contact5  hygiene1  hygiene2  hygiene3  hygiene4  hygiene5  psupport1  psupport3  psupport4  psupport5  political_ideology  happy  riskperc1  riskperc2  sbelong1  sbelong2  optim1  optim2  slfcont3
##   6   4  cnarc1  cnarc3  CRT1  CRT2  CRT3  moralid5  moralid6  moralid8  narc4  contact1  contact2  contact3  contact5  hygiene1  hygiene2  hygiene3  hygiene4  hygiene5  psupport1  psupport3  psupport4  psupport5  political_ideology  happy  riskperc1  riskperc2  sbelong1  sbelong2  optim1  optim2  slfcont3
##   6   5  cnarc1  cnarc3  CRT1  CRT2  CRT3  moralid5  moralid6  moralid8  narc4  contact1  contact2  contact3  contact5  hygiene1  hygiene2  hygiene3  hygiene4  hygiene5  psupport1  psupport3  psupport4  psupport5  political_ideology  happy  riskperc1  riskperc2  sbelong1  sbelong2  optim1  optim2  slfcont3
##   6   6  cnarc1  cnarc3  CRT1  CRT2  CRT3  moralid5  moralid6  moralid8  narc4  contact1  contact2  contact3  contact5  hygiene1  hygiene2  hygiene3  hygiene4  hygiene5  psupport1  psupport3  psupport4  psupport5  political_ideology  happy  riskperc1  riskperc2  sbelong1  sbelong2  optim1  optim2  slfcont3
##   6   7  cnarc1  cnarc3  CRT1  CRT2  CRT3  moralid5  moralid6  moralid8  narc4  contact1  contact2  contact3  contact5  hygiene1  hygiene2  hygiene3  hygiene4  hygiene5  psupport1  psupport3  psupport4  psupport5  political_ideology  happy  riskperc1  riskperc2  sbelong1  sbelong2  optim1  optim2  slfcont3
##   6   8  cnarc1  cnarc3  CRT1  CRT2  CRT3  moralid5  moralid6  moralid8  narc4  contact1  contact2  contact3  contact5  hygiene1  hygiene2  hygiene3  hygiene4  hygiene5  psupport1  psupport3  psupport4  psupport5  political_ideology  happy  riskperc1  riskperc2  sbelong1  sbelong2  optim1  optim2  slfcont3
##   6   9  cnarc1  cnarc3  CRT1  CRT2  CRT3  moralid5  moralid6  moralid8  narc4  contact1  contact2  contact3  contact5  hygiene1  hygiene2  hygiene3  hygiene4  hygiene5  psupport1  psupport3  psupport4  psupport5  political_ideology  happy  riskperc1  riskperc2  sbelong1  sbelong2  optim1  optim2  slfcont3
##   6   10  cnarc1  cnarc3  CRT1  CRT2  CRT3  moralid5  moralid6  moralid8  narc4  contact1  contact2  contact3  contact5  hygiene1  hygiene2  hygiene3  hygiene4  hygiene5  psupport1  psupport3  psupport4  psupport5  political_ideology  happy  riskperc1  riskperc2  sbelong1  sbelong2  optim1  optim2  slfcont3
##   7   1  cnarc1  cnarc3  CRT1  CRT2  CRT3  moralid5  moralid6  moralid8  narc4  contact1  contact2  contact3  contact5  hygiene1  hygiene2  hygiene3  hygiene4  hygiene5  psupport1  psupport3  psupport4  psupport5  political_ideology  happy  riskperc1  riskperc2  sbelong1  sbelong2  optim1  optim2  slfcont3
##   7   2  cnarc1  cnarc3  CRT1  CRT2  CRT3  moralid5  moralid6  moralid8  narc4  contact1  contact2  contact3  contact5  hygiene1  hygiene2  hygiene3  hygiene4  hygiene5  psupport1  psupport3  psupport4  psupport5  political_ideology  happy  riskperc1  riskperc2  sbelong1  sbelong2  optim1  optim2  slfcont3
##   7   3  cnarc1  cnarc3  CRT1  CRT2  CRT3  moralid5  moralid6  moralid8  narc4  contact1  contact2  contact3  contact5  hygiene1  hygiene2  hygiene3  hygiene4  hygiene5  psupport1  psupport3  psupport4  psupport5  political_ideology  happy  riskperc1  riskperc2  sbelong1  sbelong2  optim1  optim2  slfcont3
##   7   4  cnarc1  cnarc3  CRT1  CRT2  CRT3  moralid5  moralid6  moralid8  narc4  contact1  contact2  contact3  contact5  hygiene1  hygiene2  hygiene3  hygiene4  hygiene5  psupport1  psupport3  psupport4  psupport5  political_ideology  happy  riskperc1  riskperc2  sbelong1  sbelong2  optim1  optim2  slfcont3
##   7   5  cnarc1  cnarc3  CRT1  CRT2  CRT3  moralid5  moralid6  moralid8  narc4  contact1  contact2  contact3  contact5  hygiene1  hygiene2  hygiene3  hygiene4  hygiene5  psupport1  psupport3  psupport4  psupport5  political_ideology  happy  riskperc1  riskperc2  sbelong1  sbelong2  optim1  optim2  slfcont3
##   7   6  cnarc1  cnarc3  CRT1  CRT2  CRT3  moralid5  moralid6  moralid8  narc4  contact1  contact2  contact3  contact5  hygiene1  hygiene2  hygiene3  hygiene4  hygiene5  psupport1  psupport3  psupport4  psupport5  political_ideology  happy  riskperc1  riskperc2  sbelong1  sbelong2  optim1  optim2  slfcont3
##   7   7  cnarc1  cnarc3  CRT1  CRT2  CRT3  moralid5  moralid6  moralid8  narc4  contact1  contact2  contact3  contact5  hygiene1  hygiene2  hygiene3  hygiene4  hygiene5  psupport1  psupport3  psupport4  psupport5  political_ideology  happy  riskperc1  riskperc2  sbelong1  sbelong2  optim1  optim2  slfcont3
##   7   8  cnarc1  cnarc3  CRT1  CRT2  CRT3  moralid5  moralid6  moralid8  narc4  contact1  contact2  contact3  contact5  hygiene1  hygiene2  hygiene3  hygiene4  hygiene5  psupport1  psupport3  psupport4  psupport5  political_ideology  happy  riskperc1  riskperc2  sbelong1  sbelong2  optim1  optim2  slfcont3
##   7   9  cnarc1  cnarc3  CRT1  CRT2  CRT3  moralid5  moralid6  moralid8  narc4  contact1  contact2  contact3  contact5  hygiene1  hygiene2  hygiene3  hygiene4  hygiene5  psupport1  psupport3  psupport4  psupport5  political_ideology  happy  riskperc1  riskperc2  sbelong1  sbelong2  optim1  optim2  slfcont3
##   7   10  cnarc1  cnarc3  CRT1  CRT2  CRT3  moralid5  moralid6  moralid8  narc4  contact1  contact2  contact3  contact5  hygiene1  hygiene2  hygiene3  hygiene4  hygiene5  psupport1  psupport3  psupport4  psupport5  political_ideology  happy  riskperc1  riskperc2  sbelong1  sbelong2  optim1  optim2  slfcont3
##   8   1  cnarc1  cnarc3  CRT1  CRT2  CRT3  moralid5  moralid6  moralid8  narc4  contact1  contact2  contact3  contact5  hygiene1  hygiene2  hygiene3  hygiene4  hygiene5  psupport1  psupport3  psupport4  psupport5  political_ideology  happy  riskperc1  riskperc2  sbelong1  sbelong2  optim1  optim2  slfcont3
##   8   2  cnarc1  cnarc3  CRT1  CRT2  CRT3  moralid5  moralid6  moralid8  narc4  contact1  contact2  contact3  contact5  hygiene1  hygiene2  hygiene3  hygiene4  hygiene5  psupport1  psupport3  psupport4  psupport5  political_ideology  happy  riskperc1  riskperc2  sbelong1  sbelong2  optim1  optim2  slfcont3
##   8   3  cnarc1  cnarc3  CRT1  CRT2  CRT3  moralid5  moralid6  moralid8  narc4  contact1  contact2  contact3  contact5  hygiene1  hygiene2  hygiene3  hygiene4  hygiene5  psupport1  psupport3  psupport4  psupport5  political_ideology  happy  riskperc1  riskperc2  sbelong1  sbelong2  optim1  optim2  slfcont3
##   8   4  cnarc1  cnarc3  CRT1  CRT2  CRT3  moralid5  moralid6  moralid8  narc4  contact1  contact2  contact3  contact5  hygiene1  hygiene2  hygiene3  hygiene4  hygiene5  psupport1  psupport3  psupport4  psupport5  political_ideology  happy  riskperc1  riskperc2  sbelong1  sbelong2  optim1  optim2  slfcont3
##   8   5  cnarc1  cnarc3  CRT1  CRT2  CRT3  moralid5  moralid6  moralid8  narc4  contact1  contact2  contact3  contact5  hygiene1  hygiene2  hygiene3  hygiene4  hygiene5  psupport1  psupport3  psupport4  psupport5  political_ideology  happy  riskperc1  riskperc2  sbelong1  sbelong2  optim1  optim2  slfcont3
##   8   6  cnarc1  cnarc3  CRT1  CRT2  CRT3  moralid5  moralid6  moralid8  narc4  contact1  contact2  contact3  contact5  hygiene1  hygiene2  hygiene3  hygiene4  hygiene5  psupport1  psupport3  psupport4  psupport5  political_ideology  happy  riskperc1  riskperc2  sbelong1  sbelong2  optim1  optim2  slfcont3
##   8   7  cnarc1  cnarc3  CRT1  CRT2  CRT3  moralid5  moralid6  moralid8  narc4  contact1  contact2  contact3  contact5  hygiene1  hygiene2  hygiene3  hygiene4  hygiene5  psupport1  psupport3  psupport4  psupport5  political_ideology  happy  riskperc1  riskperc2  sbelong1  sbelong2  optim1  optim2  slfcont3
##   8   8  cnarc1  cnarc3  CRT1  CRT2  CRT3  moralid5  moralid6  moralid8  narc4  contact1  contact2  contact3  contact5  hygiene1  hygiene2  hygiene3  hygiene4  hygiene5  psupport1  psupport3  psupport4  psupport5  political_ideology  happy  riskperc1  riskperc2  sbelong1  sbelong2  optim1  optim2  slfcont3
##   8   9  cnarc1  cnarc3  CRT1  CRT2  CRT3  moralid5  moralid6  moralid8  narc4  contact1  contact2  contact3  contact5  hygiene1  hygiene2  hygiene3  hygiene4  hygiene5  psupport1  psupport3  psupport4  psupport5  political_ideology  happy  riskperc1  riskperc2  sbelong1  sbelong2  optim1  optim2  slfcont3
##   8   10  cnarc1  cnarc3  CRT1  CRT2  CRT3  moralid5  moralid6  moralid8  narc4  contact1  contact2  contact3  contact5  hygiene1  hygiene2  hygiene3  hygiene4  hygiene5  psupport1  psupport3  psupport4  psupport5  political_ideology  happy  riskperc1  riskperc2  sbelong1  sbelong2  optim1  optim2  slfcont3
##   9   1  cnarc1  cnarc3  CRT1  CRT2  CRT3  moralid5  moralid6  moralid8  narc4  contact1  contact2  contact3  contact5  hygiene1  hygiene2  hygiene3  hygiene4  hygiene5  psupport1  psupport3  psupport4  psupport5  political_ideology  happy  riskperc1  riskperc2  sbelong1  sbelong2  optim1  optim2  slfcont3
##   9   2  cnarc1  cnarc3  CRT1  CRT2  CRT3  moralid5  moralid6  moralid8  narc4  contact1  contact2  contact3  contact5  hygiene1  hygiene2  hygiene3  hygiene4  hygiene5  psupport1  psupport3  psupport4  psupport5  political_ideology  happy  riskperc1  riskperc2  sbelong1  sbelong2  optim1  optim2  slfcont3
##   9   3  cnarc1  cnarc3  CRT1  CRT2  CRT3  moralid5  moralid6  moralid8  narc4  contact1  contact2  contact3  contact5  hygiene1  hygiene2  hygiene3  hygiene4  hygiene5  psupport1  psupport3  psupport4  psupport5  political_ideology  happy  riskperc1  riskperc2  sbelong1  sbelong2  optim1  optim2  slfcont3
##   9   4  cnarc1  cnarc3  CRT1  CRT2  CRT3  moralid5  moralid6  moralid8  narc4  contact1  contact2  contact3  contact5  hygiene1  hygiene2  hygiene3  hygiene4  hygiene5  psupport1  psupport3  psupport4  psupport5  political_ideology  happy  riskperc1  riskperc2  sbelong1  sbelong2  optim1  optim2  slfcont3
##   9   5  cnarc1  cnarc3  CRT1  CRT2  CRT3  moralid5  moralid6  moralid8  narc4  contact1  contact2  contact3  contact5  hygiene1  hygiene2  hygiene3  hygiene4  hygiene5  psupport1  psupport3  psupport4  psupport5  political_ideology  happy  riskperc1  riskperc2  sbelong1  sbelong2  optim1  optim2  slfcont3
##   9   6  cnarc1  cnarc3  CRT1  CRT2  CRT3  moralid5  moralid6  moralid8  narc4  contact1  contact2  contact3  contact5  hygiene1  hygiene2  hygiene3  hygiene4  hygiene5  psupport1  psupport3  psupport4  psupport5  political_ideology  happy  riskperc1  riskperc2  sbelong1  sbelong2  optim1  optim2  slfcont3
##   9   7  cnarc1  cnarc3  CRT1  CRT2  CRT3  moralid5  moralid6  moralid8  narc4  contact1  contact2  contact3  contact5  hygiene1  hygiene2  hygiene3  hygiene4  hygiene5  psupport1  psupport3  psupport4  psupport5  political_ideology  happy  riskperc1  riskperc2  sbelong1  sbelong2  optim1  optim2  slfcont3
##   9   8  cnarc1  cnarc3  CRT1  CRT2  CRT3  moralid5  moralid6  moralid8  narc4  contact1  contact2  contact3  contact5  hygiene1  hygiene2  hygiene3  hygiene4  hygiene5  psupport1  psupport3  psupport4  psupport5  political_ideology  happy  riskperc1  riskperc2  sbelong1  sbelong2  optim1  optim2  slfcont3
##   9   9  cnarc1  cnarc3  CRT1  CRT2  CRT3  moralid5  moralid6  moralid8  narc4  contact1  contact2  contact3  contact5  hygiene1  hygiene2  hygiene3  hygiene4  hygiene5  psupport1  psupport3  psupport4  psupport5  political_ideology  happy  riskperc1  riskperc2  sbelong1  sbelong2  optim1  optim2  slfcont3
##   9   10  cnarc1  cnarc3  CRT1  CRT2  CRT3  moralid5  moralid6  moralid8  narc4  contact1  contact2  contact3  contact5  hygiene1  hygiene2  hygiene3  hygiene4  hygiene5  psupport1  psupport3  psupport4  psupport5  political_ideology  happy  riskperc1  riskperc2  sbelong1  sbelong2  optim1  optim2  slfcont3
##   10   1  cnarc1  cnarc3  CRT1  CRT2  CRT3  moralid5  moralid6  moralid8  narc4  contact1  contact2  contact3  contact5  hygiene1  hygiene2  hygiene3  hygiene4  hygiene5  psupport1  psupport3  psupport4  psupport5  political_ideology  happy  riskperc1  riskperc2  sbelong1  sbelong2  optim1  optim2  slfcont3
##   10   2  cnarc1  cnarc3  CRT1  CRT2  CRT3  moralid5  moralid6  moralid8  narc4  contact1  contact2  contact3  contact5  hygiene1  hygiene2  hygiene3  hygiene4  hygiene5  psupport1  psupport3  psupport4  psupport5  political_ideology  happy  riskperc1  riskperc2  sbelong1  sbelong2  optim1  optim2  slfcont3
##   10   3  cnarc1  cnarc3  CRT1  CRT2  CRT3  moralid5  moralid6  moralid8  narc4  contact1  contact2  contact3  contact5  hygiene1  hygiene2  hygiene3  hygiene4  hygiene5  psupport1  psupport3  psupport4  psupport5  political_ideology  happy  riskperc1  riskperc2  sbelong1  sbelong2  optim1  optim2  slfcont3
##   10   4  cnarc1  cnarc3  CRT1  CRT2  CRT3  moralid5  moralid6  moralid8  narc4  contact1  contact2  contact3  contact5  hygiene1  hygiene2  hygiene3  hygiene4  hygiene5  psupport1  psupport3  psupport4  psupport5  political_ideology  happy  riskperc1  riskperc2  sbelong1  sbelong2  optim1  optim2  slfcont3
##   10   5  cnarc1  cnarc3  CRT1  CRT2  CRT3  moralid5  moralid6  moralid8  narc4  contact1  contact2  contact3  contact5  hygiene1  hygiene2  hygiene3  hygiene4  hygiene5  psupport1  psupport3  psupport4  psupport5  political_ideology  happy  riskperc1  riskperc2  sbelong1  sbelong2  optim1  optim2  slfcont3
##   10   6  cnarc1  cnarc3  CRT1  CRT2  CRT3  moralid5  moralid6  moralid8  narc4  contact1  contact2  contact3  contact5  hygiene1  hygiene2  hygiene3  hygiene4  hygiene5  psupport1  psupport3  psupport4  psupport5  political_ideology  happy  riskperc1  riskperc2  sbelong1  sbelong2  optim1  optim2  slfcont3
##   10   7  cnarc1  cnarc3  CRT1  CRT2  CRT3  moralid5  moralid6  moralid8  narc4  contact1  contact2  contact3  contact5  hygiene1  hygiene2  hygiene3  hygiene4  hygiene5  psupport1  psupport3  psupport4  psupport5  political_ideology  happy  riskperc1  riskperc2  sbelong1  sbelong2  optim1  optim2  slfcont3
##   10   8  cnarc1  cnarc3  CRT1  CRT2  CRT3  moralid5  moralid6  moralid8  narc4  contact1  contact2  contact3  contact5  hygiene1  hygiene2  hygiene3  hygiene4  hygiene5  psupport1  psupport3  psupport4  psupport5  political_ideology  happy  riskperc1  riskperc2  sbelong1  sbelong2  optim1  optim2  slfcont3
##   10   9  cnarc1  cnarc3  CRT1  CRT2  CRT3  moralid5  moralid6  moralid8  narc4  contact1  contact2  contact3  contact5  hygiene1  hygiene2  hygiene3  hygiene4  hygiene5  psupport1  psupport3  psupport4  psupport5  political_ideology  happy  riskperc1  riskperc2  sbelong1  sbelong2  optim1  optim2  slfcont3
##   10   10  cnarc1  cnarc3  CRT1  CRT2  CRT3  moralid5  moralid6  moralid8  narc4  contact1  contact2  contact3  contact5  hygiene1  hygiene2  hygiene3  hygiene4  hygiene5  psupport1  psupport3  psupport4  psupport5  political_ideology  happy  riskperc1  riskperc2  sbelong1  sbelong2  optim1  optim2  slfcont3
```

```
## Warning: Number of logged events: 3103
```

```
## 
##  iter imp variable
##   1   1  age  children  cnarc2  CRT1  CRT2  CRT3  generosity1  generosity2  generosity3  health_cond  know_tested_positive  marital1  mor_circle  moralid1  moralid10  moralid2  moralid3  moralid4  moralid5  moralid6  moralid7  moralid8  moralid9  mcoop1  mcoop2  mcoop3  mcoop4  mcoop5  mcoop6  mcoop7  narc1  narc2  narc3  narc4  narc5  nidentity1  omind2  omind4  omind6  contact2  contact4  contact5  hygiene1  hygiene3  hygiene4  hygiene5  psupport4  psupport5  political_ideology  riskperc2  sex1  sbelong2  sbelong4  optim2  slfcont1  slfcont3  urban
##   1   2  age  children  cnarc2  CRT1  CRT2  CRT3  generosity1  generosity2  generosity3  health_cond  know_tested_positive  marital1  mor_circle  moralid1  moralid10  moralid2  moralid3  moralid4  moralid5  moralid6  moralid7  moralid8  moralid9  mcoop1  mcoop2  mcoop3  mcoop4  mcoop5  mcoop6  mcoop7  narc1  narc2  narc3  narc4  narc5  nidentity1  omind2  omind4  omind6  contact2  contact4  contact5  hygiene1  hygiene3  hygiene4  hygiene5  psupport4  psupport5  political_ideology  riskperc2  sex1  sbelong2  sbelong4  optim2  slfcont1  slfcont3  urban
##   1   3  age  children  cnarc2  CRT1  CRT2  CRT3  generosity1  generosity2  generosity3  health_cond  know_tested_positive  marital1  mor_circle  moralid1  moralid10  moralid2  moralid3  moralid4  moralid5  moralid6  moralid7  moralid8  moralid9  mcoop1  mcoop2  mcoop3  mcoop4  mcoop5  mcoop6  mcoop7  narc1  narc2  narc3  narc4  narc5  nidentity1  omind2  omind4  omind6  contact2  contact4  contact5  hygiene1  hygiene3  hygiene4  hygiene5  psupport4  psupport5  political_ideology  riskperc2  sex1  sbelong2  sbelong4  optim2  slfcont1  slfcont3  urban
##   1   4  age  children  cnarc2  CRT1  CRT2  CRT3  generosity1  generosity2  generosity3  health_cond  know_tested_positive  marital1  mor_circle  moralid1  moralid10  moralid2  moralid3  moralid4  moralid5  moralid6  moralid7  moralid8  moralid9  mcoop1  mcoop2  mcoop3  mcoop4  mcoop5  mcoop6  mcoop7  narc1  narc2  narc3  narc4  narc5  nidentity1  omind2  omind4  omind6  contact2  contact4  contact5  hygiene1  hygiene3  hygiene4  hygiene5  psupport4  psupport5  political_ideology  riskperc2  sex1  sbelong2  sbelong4  optim2  slfcont1  slfcont3  urban
##   1   5  age  children  cnarc2  CRT1  CRT2  CRT3  generosity1  generosity2  generosity3  health_cond  know_tested_positive  marital1  mor_circle  moralid1  moralid10  moralid2  moralid3  moralid4  moralid5  moralid6  moralid7  moralid8  moralid9  mcoop1  mcoop2  mcoop3  mcoop4  mcoop5  mcoop6  mcoop7  narc1  narc2  narc3  narc4  narc5  nidentity1  omind2  omind4  omind6  contact2  contact4  contact5  hygiene1  hygiene3  hygiene4  hygiene5  psupport4  psupport5  political_ideology  riskperc2  sex1  sbelong2  sbelong4  optim2  slfcont1  slfcont3  urban
##   1   6  age  children  cnarc2  CRT1  CRT2  CRT3  generosity1  generosity2  generosity3  health_cond  know_tested_positive  marital1  mor_circle  moralid1  moralid10  moralid2  moralid3  moralid4  moralid5  moralid6  moralid7  moralid8  moralid9  mcoop1  mcoop2  mcoop3  mcoop4  mcoop5  mcoop6  mcoop7  narc1  narc2  narc3  narc4  narc5  nidentity1  omind2  omind4  omind6  contact2  contact4  contact5  hygiene1  hygiene3  hygiene4  hygiene5  psupport4  psupport5  political_ideology  riskperc2  sex1  sbelong2  sbelong4  optim2  slfcont1  slfcont3  urban
##   1   7  age  children  cnarc2  CRT1  CRT2  CRT3  generosity1  generosity2  generosity3  health_cond  know_tested_positive  marital1  mor_circle  moralid1  moralid10  moralid2  moralid3  moralid4  moralid5  moralid6  moralid7  moralid8  moralid9  mcoop1  mcoop2  mcoop3  mcoop4  mcoop5  mcoop6  mcoop7  narc1  narc2  narc3  narc4  narc5  nidentity1  omind2  omind4  omind6  contact2  contact4  contact5  hygiene1  hygiene3  hygiene4  hygiene5  psupport4  psupport5  political_ideology  riskperc2  sex1  sbelong2  sbelong4  optim2  slfcont1  slfcont3  urban
##   1   8  age  children  cnarc2  CRT1  CRT2  CRT3  generosity1  generosity2  generosity3  health_cond  know_tested_positive  marital1  mor_circle  moralid1  moralid10  moralid2  moralid3  moralid4  moralid5  moralid6  moralid7  moralid8  moralid9  mcoop1  mcoop2  mcoop3  mcoop4  mcoop5  mcoop6  mcoop7  narc1  narc2  narc3  narc4  narc5  nidentity1  omind2  omind4  omind6  contact2  contact4  contact5  hygiene1  hygiene3  hygiene4  hygiene5  psupport4  psupport5  political_ideology  riskperc2  sex1  sbelong2  sbelong4  optim2  slfcont1  slfcont3  urban
##   1   9  age  children  cnarc2  CRT1  CRT2  CRT3  generosity1  generosity2  generosity3  health_cond  know_tested_positive  marital1  mor_circle  moralid1  moralid10  moralid2  moralid3  moralid4  moralid5  moralid6  moralid7  moralid8  moralid9  mcoop1  mcoop2  mcoop3  mcoop4  mcoop5  mcoop6  mcoop7  narc1  narc2  narc3  narc4  narc5  nidentity1  omind2  omind4  omind6  contact2  contact4  contact5  hygiene1  hygiene3  hygiene4  hygiene5  psupport4  psupport5  political_ideology  riskperc2  sex1  sbelong2  sbelong4  optim2  slfcont1  slfcont3  urban
##   1   10  age  children  cnarc2  CRT1  CRT2  CRT3  generosity1  generosity2  generosity3  health_cond  know_tested_positive  marital1  mor_circle  moralid1  moralid10  moralid2  moralid3  moralid4  moralid5  moralid6  moralid7  moralid8  moralid9  mcoop1  mcoop2  mcoop3  mcoop4  mcoop5  mcoop6  mcoop7  narc1  narc2  narc3  narc4  narc5  nidentity1  omind2  omind4  omind6  contact2  contact4  contact5  hygiene1  hygiene3  hygiene4  hygiene5  psupport4  psupport5  political_ideology  riskperc2  sex1  sbelong2  sbelong4  optim2  slfcont1  slfcont3  urban
##   2   1  age  children  cnarc2  CRT1  CRT2  CRT3  generosity1  generosity2  generosity3  health_cond  know_tested_positive  marital1  mor_circle  moralid1  moralid10  moralid2  moralid3  moralid4  moralid5  moralid6  moralid7  moralid8  moralid9  mcoop1  mcoop2  mcoop3  mcoop4  mcoop5  mcoop6  mcoop7  narc1  narc2  narc3  narc4  narc5  nidentity1  omind2  omind4  omind6  contact2  contact4  contact5  hygiene1  hygiene3  hygiene4  hygiene5  psupport4  psupport5  political_ideology  riskperc2  sex1  sbelong2  sbelong4  optim2  slfcont1  slfcont3  urban
##   2   2  age  children  cnarc2  CRT1  CRT2  CRT3  generosity1  generosity2  generosity3  health_cond  know_tested_positive  marital1  mor_circle  moralid1  moralid10  moralid2  moralid3  moralid4  moralid5  moralid6  moralid7  moralid8  moralid9  mcoop1  mcoop2  mcoop3  mcoop4  mcoop5  mcoop6  mcoop7  narc1  narc2  narc3  narc4  narc5  nidentity1  omind2  omind4  omind6  contact2  contact4  contact5  hygiene1  hygiene3  hygiene4  hygiene5  psupport4  psupport5  political_ideology  riskperc2  sex1  sbelong2  sbelong4  optim2  slfcont1  slfcont3  urban
##   2   3  age  children  cnarc2  CRT1  CRT2  CRT3  generosity1  generosity2  generosity3  health_cond  know_tested_positive  marital1  mor_circle  moralid1  moralid10  moralid2  moralid3  moralid4  moralid5  moralid6  moralid7  moralid8  moralid9  mcoop1  mcoop2  mcoop3  mcoop4  mcoop5  mcoop6  mcoop7  narc1  narc2  narc3  narc4  narc5  nidentity1  omind2  omind4  omind6  contact2  contact4  contact5  hygiene1  hygiene3  hygiene4  hygiene5  psupport4  psupport5  political_ideology  riskperc2  sex1  sbelong2  sbelong4  optim2  slfcont1  slfcont3  urban
##   2   4  age  children  cnarc2  CRT1  CRT2  CRT3  generosity1  generosity2  generosity3  health_cond  know_tested_positive  marital1  mor_circle  moralid1  moralid10  moralid2  moralid3  moralid4  moralid5  moralid6  moralid7  moralid8  moralid9  mcoop1  mcoop2  mcoop3  mcoop4  mcoop5  mcoop6  mcoop7  narc1  narc2  narc3  narc4  narc5  nidentity1  omind2  omind4  omind6  contact2  contact4  contact5  hygiene1  hygiene3  hygiene4  hygiene5  psupport4  psupport5  political_ideology  riskperc2  sex1  sbelong2  sbelong4  optim2  slfcont1  slfcont3  urban
##   2   5  age  children  cnarc2  CRT1  CRT2  CRT3  generosity1  generosity2  generosity3  health_cond  know_tested_positive  marital1  mor_circle  moralid1  moralid10  moralid2  moralid3  moralid4  moralid5  moralid6  moralid7  moralid8  moralid9  mcoop1  mcoop2  mcoop3  mcoop4  mcoop5  mcoop6  mcoop7  narc1  narc2  narc3  narc4  narc5  nidentity1  omind2  omind4  omind6  contact2  contact4  contact5  hygiene1  hygiene3  hygiene4  hygiene5  psupport4  psupport5  political_ideology  riskperc2  sex1  sbelong2  sbelong4  optim2  slfcont1  slfcont3  urban
##   2   6  age  children  cnarc2  CRT1  CRT2  CRT3  generosity1  generosity2  generosity3  health_cond  know_tested_positive  marital1  mor_circle  moralid1  moralid10  moralid2  moralid3  moralid4  moralid5  moralid6  moralid7  moralid8  moralid9  mcoop1  mcoop2  mcoop3  mcoop4  mcoop5  mcoop6  mcoop7  narc1  narc2  narc3  narc4  narc5  nidentity1  omind2  omind4  omind6  contact2  contact4  contact5  hygiene1  hygiene3  hygiene4  hygiene5  psupport4  psupport5  political_ideology  riskperc2  sex1  sbelong2  sbelong4  optim2  slfcont1  slfcont3  urban
##   2   7  age  children  cnarc2  CRT1  CRT2  CRT3  generosity1  generosity2  generosity3  health_cond  know_tested_positive  marital1  mor_circle  moralid1  moralid10  moralid2  moralid3  moralid4  moralid5  moralid6  moralid7  moralid8  moralid9  mcoop1  mcoop2  mcoop3  mcoop4  mcoop5  mcoop6  mcoop7  narc1  narc2  narc3  narc4  narc5  nidentity1  omind2  omind4  omind6  contact2  contact4  contact5  hygiene1  hygiene3  hygiene4  hygiene5  psupport4  psupport5  political_ideology  riskperc2  sex1  sbelong2  sbelong4  optim2  slfcont1  slfcont3  urban
##   2   8  age  children  cnarc2  CRT1  CRT2  CRT3  generosity1  generosity2  generosity3  health_cond  know_tested_positive  marital1  mor_circle  moralid1  moralid10  moralid2  moralid3  moralid4  moralid5  moralid6  moralid7  moralid8  moralid9  mcoop1  mcoop2  mcoop3  mcoop4  mcoop5  mcoop6  mcoop7  narc1  narc2  narc3  narc4  narc5  nidentity1  omind2  omind4  omind6  contact2  contact4  contact5  hygiene1  hygiene3  hygiene4  hygiene5  psupport4  psupport5  political_ideology  riskperc2  sex1  sbelong2  sbelong4  optim2  slfcont1  slfcont3  urban
##   2   9  age  children  cnarc2  CRT1  CRT2  CRT3  generosity1  generosity2  generosity3  health_cond  know_tested_positive  marital1  mor_circle  moralid1  moralid10  moralid2  moralid3  moralid4  moralid5  moralid6  moralid7  moralid8  moralid9  mcoop1  mcoop2  mcoop3  mcoop4  mcoop5  mcoop6  mcoop7  narc1  narc2  narc3  narc4  narc5  nidentity1  omind2  omind4  omind6  contact2  contact4  contact5  hygiene1  hygiene3  hygiene4  hygiene5  psupport4  psupport5  political_ideology  riskperc2  sex1  sbelong2  sbelong4  optim2  slfcont1  slfcont3  urban
##   2   10  age  children  cnarc2  CRT1  CRT2  CRT3  generosity1  generosity2  generosity3  health_cond  know_tested_positive  marital1  mor_circle  moralid1  moralid10  moralid2  moralid3  moralid4  moralid5  moralid6  moralid7  moralid8  moralid9  mcoop1  mcoop2  mcoop3  mcoop4  mcoop5  mcoop6  mcoop7  narc1  narc2  narc3  narc4  narc5  nidentity1  omind2  omind4  omind6  contact2  contact4  contact5  hygiene1  hygiene3  hygiene4  hygiene5  psupport4  psupport5  political_ideology  riskperc2  sex1  sbelong2  sbelong4  optim2  slfcont1  slfcont3  urban
##   3   1  age  children  cnarc2  CRT1  CRT2  CRT3  generosity1  generosity2  generosity3  health_cond  know_tested_positive  marital1  mor_circle  moralid1  moralid10  moralid2  moralid3  moralid4  moralid5  moralid6  moralid7  moralid8  moralid9  mcoop1  mcoop2  mcoop3  mcoop4  mcoop5  mcoop6  mcoop7  narc1  narc2  narc3  narc4  narc5  nidentity1  omind2  omind4  omind6  contact2  contact4  contact5  hygiene1  hygiene3  hygiene4  hygiene5  psupport4  psupport5  political_ideology  riskperc2  sex1  sbelong2  sbelong4  optim2  slfcont1  slfcont3  urban
##   3   2  age  children  cnarc2  CRT1  CRT2  CRT3  generosity1  generosity2  generosity3  health_cond  know_tested_positive  marital1  mor_circle  moralid1  moralid10  moralid2  moralid3  moralid4  moralid5  moralid6  moralid7  moralid8  moralid9  mcoop1  mcoop2  mcoop3  mcoop4  mcoop5  mcoop6  mcoop7  narc1  narc2  narc3  narc4  narc5  nidentity1  omind2  omind4  omind6  contact2  contact4  contact5  hygiene1  hygiene3  hygiene4  hygiene5  psupport4  psupport5  political_ideology  riskperc2  sex1  sbelong2  sbelong4  optim2  slfcont1  slfcont3  urban
##   3   3  age  children  cnarc2  CRT1  CRT2  CRT3  generosity1  generosity2  generosity3  health_cond  know_tested_positive  marital1  mor_circle  moralid1  moralid10  moralid2  moralid3  moralid4  moralid5  moralid6  moralid7  moralid8  moralid9  mcoop1  mcoop2  mcoop3  mcoop4  mcoop5  mcoop6  mcoop7  narc1  narc2  narc3  narc4  narc5  nidentity1  omind2  omind4  omind6  contact2  contact4  contact5  hygiene1  hygiene3  hygiene4  hygiene5  psupport4  psupport5  political_ideology  riskperc2  sex1  sbelong2  sbelong4  optim2  slfcont1  slfcont3  urban
##   3   4  age  children  cnarc2  CRT1  CRT2  CRT3  generosity1  generosity2  generosity3  health_cond  know_tested_positive  marital1  mor_circle  moralid1  moralid10  moralid2  moralid3  moralid4  moralid5  moralid6  moralid7  moralid8  moralid9  mcoop1  mcoop2  mcoop3  mcoop4  mcoop5  mcoop6  mcoop7  narc1  narc2  narc3  narc4  narc5  nidentity1  omind2  omind4  omind6  contact2  contact4  contact5  hygiene1  hygiene3  hygiene4  hygiene5  psupport4  psupport5  political_ideology  riskperc2  sex1  sbelong2  sbelong4  optim2  slfcont1  slfcont3  urban
##   3   5  age  children  cnarc2  CRT1  CRT2  CRT3  generosity1  generosity2  generosity3  health_cond  know_tested_positive  marital1  mor_circle  moralid1  moralid10  moralid2  moralid3  moralid4  moralid5  moralid6  moralid7  moralid8  moralid9  mcoop1  mcoop2  mcoop3  mcoop4  mcoop5  mcoop6  mcoop7  narc1  narc2  narc3  narc4  narc5  nidentity1  omind2  omind4  omind6  contact2  contact4  contact5  hygiene1  hygiene3  hygiene4  hygiene5  psupport4  psupport5  political_ideology  riskperc2  sex1  sbelong2  sbelong4  optim2  slfcont1  slfcont3  urban
##   3   6  age  children  cnarc2  CRT1  CRT2  CRT3  generosity1  generosity2  generosity3  health_cond  know_tested_positive  marital1  mor_circle  moralid1  moralid10  moralid2  moralid3  moralid4  moralid5  moralid6  moralid7  moralid8  moralid9  mcoop1  mcoop2  mcoop3  mcoop4  mcoop5  mcoop6  mcoop7  narc1  narc2  narc3  narc4  narc5  nidentity1  omind2  omind4  omind6  contact2  contact4  contact5  hygiene1  hygiene3  hygiene4  hygiene5  psupport4  psupport5  political_ideology  riskperc2  sex1  sbelong2  sbelong4  optim2  slfcont1  slfcont3  urban
##   3   7  age  children  cnarc2  CRT1  CRT2  CRT3  generosity1  generosity2  generosity3  health_cond  know_tested_positive  marital1  mor_circle  moralid1  moralid10  moralid2  moralid3  moralid4  moralid5  moralid6  moralid7  moralid8  moralid9  mcoop1  mcoop2  mcoop3  mcoop4  mcoop5  mcoop6  mcoop7  narc1  narc2  narc3  narc4  narc5  nidentity1  omind2  omind4  omind6  contact2  contact4  contact5  hygiene1  hygiene3  hygiene4  hygiene5  psupport4  psupport5  political_ideology  riskperc2  sex1  sbelong2  sbelong4  optim2  slfcont1  slfcont3  urban
##   3   8  age  children  cnarc2  CRT1  CRT2  CRT3  generosity1  generosity2  generosity3  health_cond  know_tested_positive  marital1  mor_circle  moralid1  moralid10  moralid2  moralid3  moralid4  moralid5  moralid6  moralid7  moralid8  moralid9  mcoop1  mcoop2  mcoop3  mcoop4  mcoop5  mcoop6  mcoop7  narc1  narc2  narc3  narc4  narc5  nidentity1  omind2  omind4  omind6  contact2  contact4  contact5  hygiene1  hygiene3  hygiene4  hygiene5  psupport4  psupport5  political_ideology  riskperc2  sex1  sbelong2  sbelong4  optim2  slfcont1  slfcont3  urban
##   3   9  age  children  cnarc2  CRT1  CRT2  CRT3  generosity1  generosity2  generosity3  health_cond  know_tested_positive  marital1  mor_circle  moralid1  moralid10  moralid2  moralid3  moralid4  moralid5  moralid6  moralid7  moralid8  moralid9  mcoop1  mcoop2  mcoop3  mcoop4  mcoop5  mcoop6  mcoop7  narc1  narc2  narc3  narc4  narc5  nidentity1  omind2  omind4  omind6  contact2  contact4  contact5  hygiene1  hygiene3  hygiene4  hygiene5  psupport4  psupport5  political_ideology  riskperc2  sex1  sbelong2  sbelong4  optim2  slfcont1  slfcont3  urban
##   3   10  age  children  cnarc2  CRT1  CRT2  CRT3  generosity1  generosity2  generosity3  health_cond  know_tested_positive  marital1  mor_circle  moralid1  moralid10  moralid2  moralid3  moralid4  moralid5  moralid6  moralid7  moralid8  moralid9  mcoop1  mcoop2  mcoop3  mcoop4  mcoop5  mcoop6  mcoop7  narc1  narc2  narc3  narc4  narc5  nidentity1  omind2  omind4  omind6  contact2  contact4  contact5  hygiene1  hygiene3  hygiene4  hygiene5  psupport4  psupport5  political_ideology  riskperc2  sex1  sbelong2  sbelong4  optim2  slfcont1  slfcont3  urban
##   4   1  age  children  cnarc2  CRT1  CRT2  CRT3  generosity1  generosity2  generosity3  health_cond  know_tested_positive  marital1  mor_circle  moralid1  moralid10  moralid2  moralid3  moralid4  moralid5  moralid6  moralid7  moralid8  moralid9  mcoop1  mcoop2  mcoop3  mcoop4  mcoop5  mcoop6  mcoop7  narc1  narc2  narc3  narc4  narc5  nidentity1  omind2  omind4  omind6  contact2  contact4  contact5  hygiene1  hygiene3  hygiene4  hygiene5  psupport4  psupport5  political_ideology  riskperc2  sex1  sbelong2  sbelong4  optim2  slfcont1  slfcont3  urban
##   4   2  age  children  cnarc2  CRT1  CRT2  CRT3  generosity1  generosity2  generosity3  health_cond  know_tested_positive  marital1  mor_circle  moralid1  moralid10  moralid2  moralid3  moralid4  moralid5  moralid6  moralid7  moralid8  moralid9  mcoop1  mcoop2  mcoop3  mcoop4  mcoop5  mcoop6  mcoop7  narc1  narc2  narc3  narc4  narc5  nidentity1  omind2  omind4  omind6  contact2  contact4  contact5  hygiene1  hygiene3  hygiene4  hygiene5  psupport4  psupport5  political_ideology  riskperc2  sex1  sbelong2  sbelong4  optim2  slfcont1  slfcont3  urban
##   4   3  age  children  cnarc2  CRT1  CRT2  CRT3  generosity1  generosity2  generosity3  health_cond  know_tested_positive  marital1  mor_circle  moralid1  moralid10  moralid2  moralid3  moralid4  moralid5  moralid6  moralid7  moralid8  moralid9  mcoop1  mcoop2  mcoop3  mcoop4  mcoop5  mcoop6  mcoop7  narc1  narc2  narc3  narc4  narc5  nidentity1  omind2  omind4  omind6  contact2  contact4  contact5  hygiene1  hygiene3  hygiene4  hygiene5  psupport4  psupport5  political_ideology  riskperc2  sex1  sbelong2  sbelong4  optim2  slfcont1  slfcont3  urban
##   4   4  age  children  cnarc2  CRT1  CRT2  CRT3  generosity1  generosity2  generosity3  health_cond  know_tested_positive  marital1  mor_circle  moralid1  moralid10  moralid2  moralid3  moralid4  moralid5  moralid6  moralid7  moralid8  moralid9  mcoop1  mcoop2  mcoop3  mcoop4  mcoop5  mcoop6  mcoop7  narc1  narc2  narc3  narc4  narc5  nidentity1  omind2  omind4  omind6  contact2  contact4  contact5  hygiene1  hygiene3  hygiene4  hygiene5  psupport4  psupport5  political_ideology  riskperc2  sex1  sbelong2  sbelong4  optim2  slfcont1  slfcont3  urban
##   4   5  age  children  cnarc2  CRT1  CRT2  CRT3  generosity1  generosity2  generosity3  health_cond  know_tested_positive  marital1  mor_circle  moralid1  moralid10  moralid2  moralid3  moralid4  moralid5  moralid6  moralid7  moralid8  moralid9  mcoop1  mcoop2  mcoop3  mcoop4  mcoop5  mcoop6  mcoop7  narc1  narc2  narc3  narc4  narc5  nidentity1  omind2  omind4  omind6  contact2  contact4  contact5  hygiene1  hygiene3  hygiene4  hygiene5  psupport4  psupport5  political_ideology  riskperc2  sex1  sbelong2  sbelong4  optim2  slfcont1  slfcont3  urban
##   4   6  age  children  cnarc2  CRT1  CRT2  CRT3  generosity1  generosity2  generosity3  health_cond  know_tested_positive  marital1  mor_circle  moralid1  moralid10  moralid2  moralid3  moralid4  moralid5  moralid6  moralid7  moralid8  moralid9  mcoop1  mcoop2  mcoop3  mcoop4  mcoop5  mcoop6  mcoop7  narc1  narc2  narc3  narc4  narc5  nidentity1  omind2  omind4  omind6  contact2  contact4  contact5  hygiene1  hygiene3  hygiene4  hygiene5  psupport4  psupport5  political_ideology  riskperc2  sex1  sbelong2  sbelong4  optim2  slfcont1  slfcont3  urban
##   4   7  age  children  cnarc2  CRT1  CRT2  CRT3  generosity1  generosity2  generosity3  health_cond  know_tested_positive  marital1  mor_circle  moralid1  moralid10  moralid2  moralid3  moralid4  moralid5  moralid6  moralid7  moralid8  moralid9  mcoop1  mcoop2  mcoop3  mcoop4  mcoop5  mcoop6  mcoop7  narc1  narc2  narc3  narc4  narc5  nidentity1  omind2  omind4  omind6  contact2  contact4  contact5  hygiene1  hygiene3  hygiene4  hygiene5  psupport4  psupport5  political_ideology  riskperc2  sex1  sbelong2  sbelong4  optim2  slfcont1  slfcont3  urban
##   4   8  age  children  cnarc2  CRT1  CRT2  CRT3  generosity1  generosity2  generosity3  health_cond  know_tested_positive  marital1  mor_circle  moralid1  moralid10  moralid2  moralid3  moralid4  moralid5  moralid6  moralid7  moralid8  moralid9  mcoop1  mcoop2  mcoop3  mcoop4  mcoop5  mcoop6  mcoop7  narc1  narc2  narc3  narc4  narc5  nidentity1  omind2  omind4  omind6  contact2  contact4  contact5  hygiene1  hygiene3  hygiene4  hygiene5  psupport4  psupport5  political_ideology  riskperc2  sex1  sbelong2  sbelong4  optim2  slfcont1  slfcont3  urban
##   4   9  age  children  cnarc2  CRT1  CRT2  CRT3  generosity1  generosity2  generosity3  health_cond  know_tested_positive  marital1  mor_circle  moralid1  moralid10  moralid2  moralid3  moralid4  moralid5  moralid6  moralid7  moralid8  moralid9  mcoop1  mcoop2  mcoop3  mcoop4  mcoop5  mcoop6  mcoop7  narc1  narc2  narc3  narc4  narc5  nidentity1  omind2  omind4  omind6  contact2  contact4  contact5  hygiene1  hygiene3  hygiene4  hygiene5  psupport4  psupport5  political_ideology  riskperc2  sex1  sbelong2  sbelong4  optim2  slfcont1  slfcont3  urban
##   4   10  age  children  cnarc2  CRT1  CRT2  CRT3  generosity1  generosity2  generosity3  health_cond  know_tested_positive  marital1  mor_circle  moralid1  moralid10  moralid2  moralid3  moralid4  moralid5  moralid6  moralid7  moralid8  moralid9  mcoop1  mcoop2  mcoop3  mcoop4  mcoop5  mcoop6  mcoop7  narc1  narc2  narc3  narc4  narc5  nidentity1  omind2  omind4  omind6  contact2  contact4  contact5  hygiene1  hygiene3  hygiene4  hygiene5  psupport4  psupport5  political_ideology  riskperc2  sex1  sbelong2  sbelong4  optim2  slfcont1  slfcont3  urban
##   5   1  age  children  cnarc2  CRT1  CRT2  CRT3  generosity1  generosity2  generosity3  health_cond  know_tested_positive  marital1  mor_circle  moralid1  moralid10  moralid2  moralid3  moralid4  moralid5  moralid6  moralid7  moralid8  moralid9  mcoop1  mcoop2  mcoop3  mcoop4  mcoop5  mcoop6  mcoop7  narc1  narc2  narc3  narc4  narc5  nidentity1  omind2  omind4  omind6  contact2  contact4  contact5  hygiene1  hygiene3  hygiene4  hygiene5  psupport4  psupport5  political_ideology  riskperc2  sex1  sbelong2  sbelong4  optim2  slfcont1  slfcont3  urban
##   5   2  age  children  cnarc2  CRT1  CRT2  CRT3  generosity1  generosity2  generosity3  health_cond  know_tested_positive  marital1  mor_circle  moralid1  moralid10  moralid2  moralid3  moralid4  moralid5  moralid6  moralid7  moralid8  moralid9  mcoop1  mcoop2  mcoop3  mcoop4  mcoop5  mcoop6  mcoop7  narc1  narc2  narc3  narc4  narc5  nidentity1  omind2  omind4  omind6  contact2  contact4  contact5  hygiene1  hygiene3  hygiene4  hygiene5  psupport4  psupport5  political_ideology  riskperc2  sex1  sbelong2  sbelong4  optim2  slfcont1  slfcont3  urban
##   5   3  age  children  cnarc2  CRT1  CRT2  CRT3  generosity1  generosity2  generosity3  health_cond  know_tested_positive  marital1  mor_circle  moralid1  moralid10  moralid2  moralid3  moralid4  moralid5  moralid6  moralid7  moralid8  moralid9  mcoop1  mcoop2  mcoop3  mcoop4  mcoop5  mcoop6  mcoop7  narc1  narc2  narc3  narc4  narc5  nidentity1  omind2  omind4  omind6  contact2  contact4  contact5  hygiene1  hygiene3  hygiene4  hygiene5  psupport4  psupport5  political_ideology  riskperc2  sex1  sbelong2  sbelong4  optim2  slfcont1  slfcont3  urban
##   5   4  age  children  cnarc2  CRT1  CRT2  CRT3  generosity1  generosity2  generosity3  health_cond  know_tested_positive  marital1  mor_circle  moralid1  moralid10  moralid2  moralid3  moralid4  moralid5  moralid6  moralid7  moralid8  moralid9  mcoop1  mcoop2  mcoop3  mcoop4  mcoop5  mcoop6  mcoop7  narc1  narc2  narc3  narc4  narc5  nidentity1  omind2  omind4  omind6  contact2  contact4  contact5  hygiene1  hygiene3  hygiene4  hygiene5  psupport4  psupport5  political_ideology  riskperc2  sex1  sbelong2  sbelong4  optim2  slfcont1  slfcont3  urban
##   5   5  age  children  cnarc2  CRT1  CRT2  CRT3  generosity1  generosity2  generosity3  health_cond  know_tested_positive  marital1  mor_circle  moralid1  moralid10  moralid2  moralid3  moralid4  moralid5  moralid6  moralid7  moralid8  moralid9  mcoop1  mcoop2  mcoop3  mcoop4  mcoop5  mcoop6  mcoop7  narc1  narc2  narc3  narc4  narc5  nidentity1  omind2  omind4  omind6  contact2  contact4  contact5  hygiene1  hygiene3  hygiene4  hygiene5  psupport4  psupport5  political_ideology  riskperc2  sex1  sbelong2  sbelong4  optim2  slfcont1  slfcont3  urban
##   5   6  age  children  cnarc2  CRT1  CRT2  CRT3  generosity1  generosity2  generosity3  health_cond  know_tested_positive  marital1  mor_circle  moralid1  moralid10  moralid2  moralid3  moralid4  moralid5  moralid6  moralid7  moralid8  moralid9  mcoop1  mcoop2  mcoop3  mcoop4  mcoop5  mcoop6  mcoop7  narc1  narc2  narc3  narc4  narc5  nidentity1  omind2  omind4  omind6  contact2  contact4  contact5  hygiene1  hygiene3  hygiene4  hygiene5  psupport4  psupport5  political_ideology  riskperc2  sex1  sbelong2  sbelong4  optim2  slfcont1  slfcont3  urban
##   5   7  age  children  cnarc2  CRT1  CRT2  CRT3  generosity1  generosity2  generosity3  health_cond  know_tested_positive  marital1  mor_circle  moralid1  moralid10  moralid2  moralid3  moralid4  moralid5  moralid6  moralid7  moralid8  moralid9  mcoop1  mcoop2  mcoop3  mcoop4  mcoop5  mcoop6  mcoop7  narc1  narc2  narc3  narc4  narc5  nidentity1  omind2  omind4  omind6  contact2  contact4  contact5  hygiene1  hygiene3  hygiene4  hygiene5  psupport4  psupport5  political_ideology  riskperc2  sex1  sbelong2  sbelong4  optim2  slfcont1  slfcont3  urban
##   5   8  age  children  cnarc2  CRT1  CRT2  CRT3  generosity1  generosity2  generosity3  health_cond  know_tested_positive  marital1  mor_circle  moralid1  moralid10  moralid2  moralid3  moralid4  moralid5  moralid6  moralid7  moralid8  moralid9  mcoop1  mcoop2  mcoop3  mcoop4  mcoop5  mcoop6  mcoop7  narc1  narc2  narc3  narc4  narc5  nidentity1  omind2  omind4  omind6  contact2  contact4  contact5  hygiene1  hygiene3  hygiene4  hygiene5  psupport4  psupport5  political_ideology  riskperc2  sex1  sbelong2  sbelong4  optim2  slfcont1  slfcont3  urban
##   5   9  age  children  cnarc2  CRT1  CRT2  CRT3  generosity1  generosity2  generosity3  health_cond  know_tested_positive  marital1  mor_circle  moralid1  moralid10  moralid2  moralid3  moralid4  moralid5  moralid6  moralid7  moralid8  moralid9  mcoop1  mcoop2  mcoop3  mcoop4  mcoop5  mcoop6  mcoop7  narc1  narc2  narc3  narc4  narc5  nidentity1  omind2  omind4  omind6  contact2  contact4  contact5  hygiene1  hygiene3  hygiene4  hygiene5  psupport4  psupport5  political_ideology  riskperc2  sex1  sbelong2  sbelong4  optim2  slfcont1  slfcont3  urban
##   5   10  age  children  cnarc2  CRT1  CRT2  CRT3  generosity1  generosity2  generosity3  health_cond  know_tested_positive  marital1  mor_circle  moralid1  moralid10  moralid2  moralid3  moralid4  moralid5  moralid6  moralid7  moralid8  moralid9  mcoop1  mcoop2  mcoop3  mcoop4  mcoop5  mcoop6  mcoop7  narc1  narc2  narc3  narc4  narc5  nidentity1  omind2  omind4  omind6  contact2  contact4  contact5  hygiene1  hygiene3  hygiene4  hygiene5  psupport4  psupport5  political_ideology  riskperc2  sex1  sbelong2  sbelong4  optim2  slfcont1  slfcont3  urban
##   6   1  age  children  cnarc2  CRT1  CRT2  CRT3  generosity1  generosity2  generosity3  health_cond  know_tested_positive  marital1  mor_circle  moralid1  moralid10  moralid2  moralid3  moralid4  moralid5  moralid6  moralid7  moralid8  moralid9  mcoop1  mcoop2  mcoop3  mcoop4  mcoop5  mcoop6  mcoop7  narc1  narc2  narc3  narc4  narc5  nidentity1  omind2  omind4  omind6  contact2  contact4  contact5  hygiene1  hygiene3  hygiene4  hygiene5  psupport4  psupport5  political_ideology  riskperc2  sex1  sbelong2  sbelong4  optim2  slfcont1  slfcont3  urban
##   6   2  age  children  cnarc2  CRT1  CRT2  CRT3  generosity1  generosity2  generosity3  health_cond  know_tested_positive  marital1  mor_circle  moralid1  moralid10  moralid2  moralid3  moralid4  moralid5  moralid6  moralid7  moralid8  moralid9  mcoop1  mcoop2  mcoop3  mcoop4  mcoop5  mcoop6  mcoop7  narc1  narc2  narc3  narc4  narc5  nidentity1  omind2  omind4  omind6  contact2  contact4  contact5  hygiene1  hygiene3  hygiene4  hygiene5  psupport4  psupport5  political_ideology  riskperc2  sex1  sbelong2  sbelong4  optim2  slfcont1  slfcont3  urban
##   6   3  age  children  cnarc2  CRT1  CRT2  CRT3  generosity1  generosity2  generosity3  health_cond  know_tested_positive  marital1  mor_circle  moralid1  moralid10  moralid2  moralid3  moralid4  moralid5  moralid6  moralid7  moralid8  moralid9  mcoop1  mcoop2  mcoop3  mcoop4  mcoop5  mcoop6  mcoop7  narc1  narc2  narc3  narc4  narc5  nidentity1  omind2  omind4  omind6  contact2  contact4  contact5  hygiene1  hygiene3  hygiene4  hygiene5  psupport4  psupport5  political_ideology  riskperc2  sex1  sbelong2  sbelong4  optim2  slfcont1  slfcont3  urban
##   6   4  age  children  cnarc2  CRT1  CRT2  CRT3  generosity1  generosity2  generosity3  health_cond  know_tested_positive  marital1  mor_circle  moralid1  moralid10  moralid2  moralid3  moralid4  moralid5  moralid6  moralid7  moralid8  moralid9  mcoop1  mcoop2  mcoop3  mcoop4  mcoop5  mcoop6  mcoop7  narc1  narc2  narc3  narc4  narc5  nidentity1  omind2  omind4  omind6  contact2  contact4  contact5  hygiene1  hygiene3  hygiene4  hygiene5  psupport4  psupport5  political_ideology  riskperc2  sex1  sbelong2  sbelong4  optim2  slfcont1  slfcont3  urban
##   6   5  age  children  cnarc2  CRT1  CRT2  CRT3  generosity1  generosity2  generosity3  health_cond  know_tested_positive  marital1  mor_circle  moralid1  moralid10  moralid2  moralid3  moralid4  moralid5  moralid6  moralid7  moralid8  moralid9  mcoop1  mcoop2  mcoop3  mcoop4  mcoop5  mcoop6  mcoop7  narc1  narc2  narc3  narc4  narc5  nidentity1  omind2  omind4  omind6  contact2  contact4  contact5  hygiene1  hygiene3  hygiene4  hygiene5  psupport4  psupport5  political_ideology  riskperc2  sex1  sbelong2  sbelong4  optim2  slfcont1  slfcont3  urban
##   6   6  age  children  cnarc2  CRT1  CRT2  CRT3  generosity1  generosity2  generosity3  health_cond  know_tested_positive  marital1  mor_circle  moralid1  moralid10  moralid2  moralid3  moralid4  moralid5  moralid6  moralid7  moralid8  moralid9  mcoop1  mcoop2  mcoop3  mcoop4  mcoop5  mcoop6  mcoop7  narc1  narc2  narc3  narc4  narc5  nidentity1  omind2  omind4  omind6  contact2  contact4  contact5  hygiene1  hygiene3  hygiene4  hygiene5  psupport4  psupport5  political_ideology  riskperc2  sex1  sbelong2  sbelong4  optim2  slfcont1  slfcont3  urban
##   6   7  age  children  cnarc2  CRT1  CRT2  CRT3  generosity1  generosity2  generosity3  health_cond  know_tested_positive  marital1  mor_circle  moralid1  moralid10  moralid2  moralid3  moralid4  moralid5  moralid6  moralid7  moralid8  moralid9  mcoop1  mcoop2  mcoop3  mcoop4  mcoop5  mcoop6  mcoop7  narc1  narc2  narc3  narc4  narc5  nidentity1  omind2  omind4  omind6  contact2  contact4  contact5  hygiene1  hygiene3  hygiene4  hygiene5  psupport4  psupport5  political_ideology  riskperc2  sex1  sbelong2  sbelong4  optim2  slfcont1  slfcont3  urban
##   6   8  age  children  cnarc2  CRT1  CRT2  CRT3  generosity1  generosity2  generosity3  health_cond  know_tested_positive  marital1  mor_circle  moralid1  moralid10  moralid2  moralid3  moralid4  moralid5  moralid6  moralid7  moralid8  moralid9  mcoop1  mcoop2  mcoop3  mcoop4  mcoop5  mcoop6  mcoop7  narc1  narc2  narc3  narc4  narc5  nidentity1  omind2  omind4  omind6  contact2  contact4  contact5  hygiene1  hygiene3  hygiene4  hygiene5  psupport4  psupport5  political_ideology  riskperc2  sex1  sbelong2  sbelong4  optim2  slfcont1  slfcont3  urban
##   6   9  age  children  cnarc2  CRT1  CRT2  CRT3  generosity1  generosity2  generosity3  health_cond  know_tested_positive  marital1  mor_circle  moralid1  moralid10  moralid2  moralid3  moralid4  moralid5  moralid6  moralid7  moralid8  moralid9  mcoop1  mcoop2  mcoop3  mcoop4  mcoop5  mcoop6  mcoop7  narc1  narc2  narc3  narc4  narc5  nidentity1  omind2  omind4  omind6  contact2  contact4  contact5  hygiene1  hygiene3  hygiene4  hygiene5  psupport4  psupport5  political_ideology  riskperc2  sex1  sbelong2  sbelong4  optim2  slfcont1  slfcont3  urban
##   6   10  age  children  cnarc2  CRT1  CRT2  CRT3  generosity1  generosity2  generosity3  health_cond  know_tested_positive  marital1  mor_circle  moralid1  moralid10  moralid2  moralid3  moralid4  moralid5  moralid6  moralid7  moralid8  moralid9  mcoop1  mcoop2  mcoop3  mcoop4  mcoop5  mcoop6  mcoop7  narc1  narc2  narc3  narc4  narc5  nidentity1  omind2  omind4  omind6  contact2  contact4  contact5  hygiene1  hygiene3  hygiene4  hygiene5  psupport4  psupport5  political_ideology  riskperc2  sex1  sbelong2  sbelong4  optim2  slfcont1  slfcont3  urban
##   7   1  age  children  cnarc2  CRT1  CRT2  CRT3  generosity1  generosity2  generosity3  health_cond  know_tested_positive  marital1  mor_circle  moralid1  moralid10  moralid2  moralid3  moralid4  moralid5  moralid6  moralid7  moralid8  moralid9  mcoop1  mcoop2  mcoop3  mcoop4  mcoop5  mcoop6  mcoop7  narc1  narc2  narc3  narc4  narc5  nidentity1  omind2  omind4  omind6  contact2  contact4  contact5  hygiene1  hygiene3  hygiene4  hygiene5  psupport4  psupport5  political_ideology  riskperc2  sex1  sbelong2  sbelong4  optim2  slfcont1  slfcont3  urban
##   7   2  age  children  cnarc2  CRT1  CRT2  CRT3  generosity1  generosity2  generosity3  health_cond  know_tested_positive  marital1  mor_circle  moralid1  moralid10  moralid2  moralid3  moralid4  moralid5  moralid6  moralid7  moralid8  moralid9  mcoop1  mcoop2  mcoop3  mcoop4  mcoop5  mcoop6  mcoop7  narc1  narc2  narc3  narc4  narc5  nidentity1  omind2  omind4  omind6  contact2  contact4  contact5  hygiene1  hygiene3  hygiene4  hygiene5  psupport4  psupport5  political_ideology  riskperc2  sex1  sbelong2  sbelong4  optim2  slfcont1  slfcont3  urban
##   7   3  age  children  cnarc2  CRT1  CRT2  CRT3  generosity1  generosity2  generosity3  health_cond  know_tested_positive  marital1  mor_circle  moralid1  moralid10  moralid2  moralid3  moralid4  moralid5  moralid6  moralid7  moralid8  moralid9  mcoop1  mcoop2  mcoop3  mcoop4  mcoop5  mcoop6  mcoop7  narc1  narc2  narc3  narc4  narc5  nidentity1  omind2  omind4  omind6  contact2  contact4  contact5  hygiene1  hygiene3  hygiene4  hygiene5  psupport4  psupport5  political_ideology  riskperc2  sex1  sbelong2  sbelong4  optim2  slfcont1  slfcont3  urban
##   7   4  age  children  cnarc2  CRT1  CRT2  CRT3  generosity1  generosity2  generosity3  health_cond  know_tested_positive  marital1  mor_circle  moralid1  moralid10  moralid2  moralid3  moralid4  moralid5  moralid6  moralid7  moralid8  moralid9  mcoop1  mcoop2  mcoop3  mcoop4  mcoop5  mcoop6  mcoop7  narc1  narc2  narc3  narc4  narc5  nidentity1  omind2  omind4  omind6  contact2  contact4  contact5  hygiene1  hygiene3  hygiene4  hygiene5  psupport4  psupport5  political_ideology  riskperc2  sex1  sbelong2  sbelong4  optim2  slfcont1  slfcont3  urban
##   7   5  age  children  cnarc2  CRT1  CRT2  CRT3  generosity1  generosity2  generosity3  health_cond  know_tested_positive  marital1  mor_circle  moralid1  moralid10  moralid2  moralid3  moralid4  moralid5  moralid6  moralid7  moralid8  moralid9  mcoop1  mcoop2  mcoop3  mcoop4  mcoop5  mcoop6  mcoop7  narc1  narc2  narc3  narc4  narc5  nidentity1  omind2  omind4  omind6  contact2  contact4  contact5  hygiene1  hygiene3  hygiene4  hygiene5  psupport4  psupport5  political_ideology  riskperc2  sex1  sbelong2  sbelong4  optim2  slfcont1  slfcont3  urban
##   7   6  age  children  cnarc2  CRT1  CRT2  CRT3  generosity1  generosity2  generosity3  health_cond  know_tested_positive  marital1  mor_circle  moralid1  moralid10  moralid2  moralid3  moralid4  moralid5  moralid6  moralid7  moralid8  moralid9  mcoop1  mcoop2  mcoop3  mcoop4  mcoop5  mcoop6  mcoop7  narc1  narc2  narc3  narc4  narc5  nidentity1  omind2  omind4  omind6  contact2  contact4  contact5  hygiene1  hygiene3  hygiene4  hygiene5  psupport4  psupport5  political_ideology  riskperc2  sex1  sbelong2  sbelong4  optim2  slfcont1  slfcont3  urban
##   7   7  age  children  cnarc2  CRT1  CRT2  CRT3  generosity1  generosity2  generosity3  health_cond  know_tested_positive  marital1  mor_circle  moralid1  moralid10  moralid2  moralid3  moralid4  moralid5  moralid6  moralid7  moralid8  moralid9  mcoop1  mcoop2  mcoop3  mcoop4  mcoop5  mcoop6  mcoop7  narc1  narc2  narc3  narc4  narc5  nidentity1  omind2  omind4  omind6  contact2  contact4  contact5  hygiene1  hygiene3  hygiene4  hygiene5  psupport4  psupport5  political_ideology  riskperc2  sex1  sbelong2  sbelong4  optim2  slfcont1  slfcont3  urban
##   7   8  age  children  cnarc2  CRT1  CRT2  CRT3  generosity1  generosity2  generosity3  health_cond  know_tested_positive  marital1  mor_circle  moralid1  moralid10  moralid2  moralid3  moralid4  moralid5  moralid6  moralid7  moralid8  moralid9  mcoop1  mcoop2  mcoop3  mcoop4  mcoop5  mcoop6  mcoop7  narc1  narc2  narc3  narc4  narc5  nidentity1  omind2  omind4  omind6  contact2  contact4  contact5  hygiene1  hygiene3  hygiene4  hygiene5  psupport4  psupport5  political_ideology  riskperc2  sex1  sbelong2  sbelong4  optim2  slfcont1  slfcont3  urban
##   7   9  age  children  cnarc2  CRT1  CRT2  CRT3  generosity1  generosity2  generosity3  health_cond  know_tested_positive  marital1  mor_circle  moralid1  moralid10  moralid2  moralid3  moralid4  moralid5  moralid6  moralid7  moralid8  moralid9  mcoop1  mcoop2  mcoop3  mcoop4  mcoop5  mcoop6  mcoop7  narc1  narc2  narc3  narc4  narc5  nidentity1  omind2  omind4  omind6  contact2  contact4  contact5  hygiene1  hygiene3  hygiene4  hygiene5  psupport4  psupport5  political_ideology  riskperc2  sex1  sbelong2  sbelong4  optim2  slfcont1  slfcont3  urban
##   7   10  age  children  cnarc2  CRT1  CRT2  CRT3  generosity1  generosity2  generosity3  health_cond  know_tested_positive  marital1  mor_circle  moralid1  moralid10  moralid2  moralid3  moralid4  moralid5  moralid6  moralid7  moralid8  moralid9  mcoop1  mcoop2  mcoop3  mcoop4  mcoop5  mcoop6  mcoop7  narc1  narc2  narc3  narc4  narc5  nidentity1  omind2  omind4  omind6  contact2  contact4  contact5  hygiene1  hygiene3  hygiene4  hygiene5  psupport4  psupport5  political_ideology  riskperc2  sex1  sbelong2  sbelong4  optim2  slfcont1  slfcont3  urban
##   8   1  age  children  cnarc2  CRT1  CRT2  CRT3  generosity1  generosity2  generosity3  health_cond  know_tested_positive  marital1  mor_circle  moralid1  moralid10  moralid2  moralid3  moralid4  moralid5  moralid6  moralid7  moralid8  moralid9  mcoop1  mcoop2  mcoop3  mcoop4  mcoop5  mcoop6  mcoop7  narc1  narc2  narc3  narc4  narc5  nidentity1  omind2  omind4  omind6  contact2  contact4  contact5  hygiene1  hygiene3  hygiene4  hygiene5  psupport4  psupport5  political_ideology  riskperc2  sex1  sbelong2  sbelong4  optim2  slfcont1  slfcont3  urban
##   8   2  age  children  cnarc2  CRT1  CRT2  CRT3  generosity1  generosity2  generosity3  health_cond  know_tested_positive  marital1  mor_circle  moralid1  moralid10  moralid2  moralid3  moralid4  moralid5  moralid6  moralid7  moralid8  moralid9  mcoop1  mcoop2  mcoop3  mcoop4  mcoop5  mcoop6  mcoop7  narc1  narc2  narc3  narc4  narc5  nidentity1  omind2  omind4  omind6  contact2  contact4  contact5  hygiene1  hygiene3  hygiene4  hygiene5  psupport4  psupport5  political_ideology  riskperc2  sex1  sbelong2  sbelong4  optim2  slfcont1  slfcont3  urban
##   8   3  age  children  cnarc2  CRT1  CRT2  CRT3  generosity1  generosity2  generosity3  health_cond  know_tested_positive  marital1  mor_circle  moralid1  moralid10  moralid2  moralid3  moralid4  moralid5  moralid6  moralid7  moralid8  moralid9  mcoop1  mcoop2  mcoop3  mcoop4  mcoop5  mcoop6  mcoop7  narc1  narc2  narc3  narc4  narc5  nidentity1  omind2  omind4  omind6  contact2  contact4  contact5  hygiene1  hygiene3  hygiene4  hygiene5  psupport4  psupport5  political_ideology  riskperc2  sex1  sbelong2  sbelong4  optim2  slfcont1  slfcont3  urban
##   8   4  age  children  cnarc2  CRT1  CRT2  CRT3  generosity1  generosity2  generosity3  health_cond  know_tested_positive  marital1  mor_circle  moralid1  moralid10  moralid2  moralid3  moralid4  moralid5  moralid6  moralid7  moralid8  moralid9  mcoop1  mcoop2  mcoop3  mcoop4  mcoop5  mcoop6  mcoop7  narc1  narc2  narc3  narc4  narc5  nidentity1  omind2  omind4  omind6  contact2  contact4  contact5  hygiene1  hygiene3  hygiene4  hygiene5  psupport4  psupport5  political_ideology  riskperc2  sex1  sbelong2  sbelong4  optim2  slfcont1  slfcont3  urban
##   8   5  age  children  cnarc2  CRT1  CRT2  CRT3  generosity1  generosity2  generosity3  health_cond  know_tested_positive  marital1  mor_circle  moralid1  moralid10  moralid2  moralid3  moralid4  moralid5  moralid6  moralid7  moralid8  moralid9  mcoop1  mcoop2  mcoop3  mcoop4  mcoop5  mcoop6  mcoop7  narc1  narc2  narc3  narc4  narc5  nidentity1  omind2  omind4  omind6  contact2  contact4  contact5  hygiene1  hygiene3  hygiene4  hygiene5  psupport4  psupport5  political_ideology  riskperc2  sex1  sbelong2  sbelong4  optim2  slfcont1  slfcont3  urban
##   8   6  age  children  cnarc2  CRT1  CRT2  CRT3  generosity1  generosity2  generosity3  health_cond  know_tested_positive  marital1  mor_circle  moralid1  moralid10  moralid2  moralid3  moralid4  moralid5  moralid6  moralid7  moralid8  moralid9  mcoop1  mcoop2  mcoop3  mcoop4  mcoop5  mcoop6  mcoop7  narc1  narc2  narc3  narc4  narc5  nidentity1  omind2  omind4  omind6  contact2  contact4  contact5  hygiene1  hygiene3  hygiene4  hygiene5  psupport4  psupport5  political_ideology  riskperc2  sex1  sbelong2  sbelong4  optim2  slfcont1  slfcont3  urban
##   8   7  age  children  cnarc2  CRT1  CRT2  CRT3  generosity1  generosity2  generosity3  health_cond  know_tested_positive  marital1  mor_circle  moralid1  moralid10  moralid2  moralid3  moralid4  moralid5  moralid6  moralid7  moralid8  moralid9  mcoop1  mcoop2  mcoop3  mcoop4  mcoop5  mcoop6  mcoop7  narc1  narc2  narc3  narc4  narc5  nidentity1  omind2  omind4  omind6  contact2  contact4  contact5  hygiene1  hygiene3  hygiene4  hygiene5  psupport4  psupport5  political_ideology  riskperc2  sex1  sbelong2  sbelong4  optim2  slfcont1  slfcont3  urban
##   8   8  age  children  cnarc2  CRT1  CRT2  CRT3  generosity1  generosity2  generosity3  health_cond  know_tested_positive  marital1  mor_circle  moralid1  moralid10  moralid2  moralid3  moralid4  moralid5  moralid6  moralid7  moralid8  moralid9  mcoop1  mcoop2  mcoop3  mcoop4  mcoop5  mcoop6  mcoop7  narc1  narc2  narc3  narc4  narc5  nidentity1  omind2  omind4  omind6  contact2  contact4  contact5  hygiene1  hygiene3  hygiene4  hygiene5  psupport4  psupport5  political_ideology  riskperc2  sex1  sbelong2  sbelong4  optim2  slfcont1  slfcont3  urban
##   8   9  age  children  cnarc2  CRT1  CRT2  CRT3  generosity1  generosity2  generosity3  health_cond  know_tested_positive  marital1  mor_circle  moralid1  moralid10  moralid2  moralid3  moralid4  moralid5  moralid6  moralid7  moralid8  moralid9  mcoop1  mcoop2  mcoop3  mcoop4  mcoop5  mcoop6  mcoop7  narc1  narc2  narc3  narc4  narc5  nidentity1  omind2  omind4  omind6  contact2  contact4  contact5  hygiene1  hygiene3  hygiene4  hygiene5  psupport4  psupport5  political_ideology  riskperc2  sex1  sbelong2  sbelong4  optim2  slfcont1  slfcont3  urban
##   8   10  age  children  cnarc2  CRT1  CRT2  CRT3  generosity1  generosity2  generosity3  health_cond  know_tested_positive  marital1  mor_circle  moralid1  moralid10  moralid2  moralid3  moralid4  moralid5  moralid6  moralid7  moralid8  moralid9  mcoop1  mcoop2  mcoop3  mcoop4  mcoop5  mcoop6  mcoop7  narc1  narc2  narc3  narc4  narc5  nidentity1  omind2  omind4  omind6  contact2  contact4  contact5  hygiene1  hygiene3  hygiene4  hygiene5  psupport4  psupport5  political_ideology  riskperc2  sex1  sbelong2  sbelong4  optim2  slfcont1  slfcont3  urban
##   9   1  age  children  cnarc2  CRT1  CRT2  CRT3  generosity1  generosity2  generosity3  health_cond  know_tested_positive  marital1  mor_circle  moralid1  moralid10  moralid2  moralid3  moralid4  moralid5  moralid6  moralid7  moralid8  moralid9  mcoop1  mcoop2  mcoop3  mcoop4  mcoop5  mcoop6  mcoop7  narc1  narc2  narc3  narc4  narc5  nidentity1  omind2  omind4  omind6  contact2  contact4  contact5  hygiene1  hygiene3  hygiene4  hygiene5  psupport4  psupport5  political_ideology  riskperc2  sex1  sbelong2  sbelong4  optim2  slfcont1  slfcont3  urban
##   9   2  age  children  cnarc2  CRT1  CRT2  CRT3  generosity1  generosity2  generosity3  health_cond  know_tested_positive  marital1  mor_circle  moralid1  moralid10  moralid2  moralid3  moralid4  moralid5  moralid6  moralid7  moralid8  moralid9  mcoop1  mcoop2  mcoop3  mcoop4  mcoop5  mcoop6  mcoop7  narc1  narc2  narc3  narc4  narc5  nidentity1  omind2  omind4  omind6  contact2  contact4  contact5  hygiene1  hygiene3  hygiene4  hygiene5  psupport4  psupport5  political_ideology  riskperc2  sex1  sbelong2  sbelong4  optim2  slfcont1  slfcont3  urban
##   9   3  age  children  cnarc2  CRT1  CRT2  CRT3  generosity1  generosity2  generosity3  health_cond  know_tested_positive  marital1  mor_circle  moralid1  moralid10  moralid2  moralid3  moralid4  moralid5  moralid6  moralid7  moralid8  moralid9  mcoop1  mcoop2  mcoop3  mcoop4  mcoop5  mcoop6  mcoop7  narc1  narc2  narc3  narc4  narc5  nidentity1  omind2  omind4  omind6  contact2  contact4  contact5  hygiene1  hygiene3  hygiene4  hygiene5  psupport4  psupport5  political_ideology  riskperc2  sex1  sbelong2  sbelong4  optim2  slfcont1  slfcont3  urban
##   9   4  age  children  cnarc2  CRT1  CRT2  CRT3  generosity1  generosity2  generosity3  health_cond  know_tested_positive  marital1  mor_circle  moralid1  moralid10  moralid2  moralid3  moralid4  moralid5  moralid6  moralid7  moralid8  moralid9  mcoop1  mcoop2  mcoop3  mcoop4  mcoop5  mcoop6  mcoop7  narc1  narc2  narc3  narc4  narc5  nidentity1  omind2  omind4  omind6  contact2  contact4  contact5  hygiene1  hygiene3  hygiene4  hygiene5  psupport4  psupport5  political_ideology  riskperc2  sex1  sbelong2  sbelong4  optim2  slfcont1  slfcont3  urban
##   9   5  age  children  cnarc2  CRT1  CRT2  CRT3  generosity1  generosity2  generosity3  health_cond  know_tested_positive  marital1  mor_circle  moralid1  moralid10  moralid2  moralid3  moralid4  moralid5  moralid6  moralid7  moralid8  moralid9  mcoop1  mcoop2  mcoop3  mcoop4  mcoop5  mcoop6  mcoop7  narc1  narc2  narc3  narc4  narc5  nidentity1  omind2  omind4  omind6  contact2  contact4  contact5  hygiene1  hygiene3  hygiene4  hygiene5  psupport4  psupport5  political_ideology  riskperc2  sex1  sbelong2  sbelong4  optim2  slfcont1  slfcont3  urban
##   9   6  age  children  cnarc2  CRT1  CRT2  CRT3  generosity1  generosity2  generosity3  health_cond  know_tested_positive  marital1  mor_circle  moralid1  moralid10  moralid2  moralid3  moralid4  moralid5  moralid6  moralid7  moralid8  moralid9  mcoop1  mcoop2  mcoop3  mcoop4  mcoop5  mcoop6  mcoop7  narc1  narc2  narc3  narc4  narc5  nidentity1  omind2  omind4  omind6  contact2  contact4  contact5  hygiene1  hygiene3  hygiene4  hygiene5  psupport4  psupport5  political_ideology  riskperc2  sex1  sbelong2  sbelong4  optim2  slfcont1  slfcont3  urban
##   9   7  age  children  cnarc2  CRT1  CRT2  CRT3  generosity1  generosity2  generosity3  health_cond  know_tested_positive  marital1  mor_circle  moralid1  moralid10  moralid2  moralid3  moralid4  moralid5  moralid6  moralid7  moralid8  moralid9  mcoop1  mcoop2  mcoop3  mcoop4  mcoop5  mcoop6  mcoop7  narc1  narc2  narc3  narc4  narc5  nidentity1  omind2  omind4  omind6  contact2  contact4  contact5  hygiene1  hygiene3  hygiene4  hygiene5  psupport4  psupport5  political_ideology  riskperc2  sex1  sbelong2  sbelong4  optim2  slfcont1  slfcont3  urban
##   9   8  age  children  cnarc2  CRT1  CRT2  CRT3  generosity1  generosity2  generosity3  health_cond  know_tested_positive  marital1  mor_circle  moralid1  moralid10  moralid2  moralid3  moralid4  moralid5  moralid6  moralid7  moralid8  moralid9  mcoop1  mcoop2  mcoop3  mcoop4  mcoop5  mcoop6  mcoop7  narc1  narc2  narc3  narc4  narc5  nidentity1  omind2  omind4  omind6  contact2  contact4  contact5  hygiene1  hygiene3  hygiene4  hygiene5  psupport4  psupport5  political_ideology  riskperc2  sex1  sbelong2  sbelong4  optim2  slfcont1  slfcont3  urban
##   9   9  age  children  cnarc2  CRT1  CRT2  CRT3  generosity1  generosity2  generosity3  health_cond  know_tested_positive  marital1  mor_circle  moralid1  moralid10  moralid2  moralid3  moralid4  moralid5  moralid6  moralid7  moralid8  moralid9  mcoop1  mcoop2  mcoop3  mcoop4  mcoop5  mcoop6  mcoop7  narc1  narc2  narc3  narc4  narc5  nidentity1  omind2  omind4  omind6  contact2  contact4  contact5  hygiene1  hygiene3  hygiene4  hygiene5  psupport4  psupport5  political_ideology  riskperc2  sex1  sbelong2  sbelong4  optim2  slfcont1  slfcont3  urban
##   9   10  age  children  cnarc2  CRT1  CRT2  CRT3  generosity1  generosity2  generosity3  health_cond  know_tested_positive  marital1  mor_circle  moralid1  moralid10  moralid2  moralid3  moralid4  moralid5  moralid6  moralid7  moralid8  moralid9  mcoop1  mcoop2  mcoop3  mcoop4  mcoop5  mcoop6  mcoop7  narc1  narc2  narc3  narc4  narc5  nidentity1  omind2  omind4  omind6  contact2  contact4  contact5  hygiene1  hygiene3  hygiene4  hygiene5  psupport4  psupport5  political_ideology  riskperc2  sex1  sbelong2  sbelong4  optim2  slfcont1  slfcont3  urban
##   10   1  age  children  cnarc2  CRT1  CRT2  CRT3  generosity1  generosity2  generosity3  health_cond  know_tested_positive  marital1  mor_circle  moralid1  moralid10  moralid2  moralid3  moralid4  moralid5  moralid6  moralid7  moralid8  moralid9  mcoop1  mcoop2  mcoop3  mcoop4  mcoop5  mcoop6  mcoop7  narc1  narc2  narc3  narc4  narc5  nidentity1  omind2  omind4  omind6  contact2  contact4  contact5  hygiene1  hygiene3  hygiene4  hygiene5  psupport4  psupport5  political_ideology  riskperc2  sex1  sbelong2  sbelong4  optim2  slfcont1  slfcont3  urban
##   10   2  age  children  cnarc2  CRT1  CRT2  CRT3  generosity1  generosity2  generosity3  health_cond  know_tested_positive  marital1  mor_circle  moralid1  moralid10  moralid2  moralid3  moralid4  moralid5  moralid6  moralid7  moralid8  moralid9  mcoop1  mcoop2  mcoop3  mcoop4  mcoop5  mcoop6  mcoop7  narc1  narc2  narc3  narc4  narc5  nidentity1  omind2  omind4  omind6  contact2  contact4  contact5  hygiene1  hygiene3  hygiene4  hygiene5  psupport4  psupport5  political_ideology  riskperc2  sex1  sbelong2  sbelong4  optim2  slfcont1  slfcont3  urban
##   10   3  age  children  cnarc2  CRT1  CRT2  CRT3  generosity1  generosity2  generosity3  health_cond  know_tested_positive  marital1  mor_circle  moralid1  moralid10  moralid2  moralid3  moralid4  moralid5  moralid6  moralid7  moralid8  moralid9  mcoop1  mcoop2  mcoop3  mcoop4  mcoop5  mcoop6  mcoop7  narc1  narc2  narc3  narc4  narc5  nidentity1  omind2  omind4  omind6  contact2  contact4  contact5  hygiene1  hygiene3  hygiene4  hygiene5  psupport4  psupport5  political_ideology  riskperc2  sex1  sbelong2  sbelong4  optim2  slfcont1  slfcont3  urban
##   10   4  age  children  cnarc2  CRT1  CRT2  CRT3  generosity1  generosity2  generosity3  health_cond  know_tested_positive  marital1  mor_circle  moralid1  moralid10  moralid2  moralid3  moralid4  moralid5  moralid6  moralid7  moralid8  moralid9  mcoop1  mcoop2  mcoop3  mcoop4  mcoop5  mcoop6  mcoop7  narc1  narc2  narc3  narc4  narc5  nidentity1  omind2  omind4  omind6  contact2  contact4  contact5  hygiene1  hygiene3  hygiene4  hygiene5  psupport4  psupport5  political_ideology  riskperc2  sex1  sbelong2  sbelong4  optim2  slfcont1  slfcont3  urban
##   10   5  age  children  cnarc2  CRT1  CRT2  CRT3  generosity1  generosity2  generosity3  health_cond  know_tested_positive  marital1  mor_circle  moralid1  moralid10  moralid2  moralid3  moralid4  moralid5  moralid6  moralid7  moralid8  moralid9  mcoop1  mcoop2  mcoop3  mcoop4  mcoop5  mcoop6  mcoop7  narc1  narc2  narc3  narc4  narc5  nidentity1  omind2  omind4  omind6  contact2  contact4  contact5  hygiene1  hygiene3  hygiene4  hygiene5  psupport4  psupport5  political_ideology  riskperc2  sex1  sbelong2  sbelong4  optim2  slfcont1  slfcont3  urban
##   10   6  age  children  cnarc2  CRT1  CRT2  CRT3  generosity1  generosity2  generosity3  health_cond  know_tested_positive  marital1  mor_circle  moralid1  moralid10  moralid2  moralid3  moralid4  moralid5  moralid6  moralid7  moralid8  moralid9  mcoop1  mcoop2  mcoop3  mcoop4  mcoop5  mcoop6  mcoop7  narc1  narc2  narc3  narc4  narc5  nidentity1  omind2  omind4  omind6  contact2  contact4  contact5  hygiene1  hygiene3  hygiene4  hygiene5  psupport4  psupport5  political_ideology  riskperc2  sex1  sbelong2  sbelong4  optim2  slfcont1  slfcont3  urban
##   10   7  age  children  cnarc2  CRT1  CRT2  CRT3  generosity1  generosity2  generosity3  health_cond  know_tested_positive  marital1  mor_circle  moralid1  moralid10  moralid2  moralid3  moralid4  moralid5  moralid6  moralid7  moralid8  moralid9  mcoop1  mcoop2  mcoop3  mcoop4  mcoop5  mcoop6  mcoop7  narc1  narc2  narc3  narc4  narc5  nidentity1  omind2  omind4  omind6  contact2  contact4  contact5  hygiene1  hygiene3  hygiene4  hygiene5  psupport4  psupport5  political_ideology  riskperc2  sex1  sbelong2  sbelong4  optim2  slfcont1  slfcont3  urban
##   10   8  age  children  cnarc2  CRT1  CRT2  CRT3  generosity1  generosity2  generosity3  health_cond  know_tested_positive  marital1  mor_circle  moralid1  moralid10  moralid2  moralid3  moralid4  moralid5  moralid6  moralid7  moralid8  moralid9  mcoop1  mcoop2  mcoop3  mcoop4  mcoop5  mcoop6  mcoop7  narc1  narc2  narc3  narc4  narc5  nidentity1  omind2  omind4  omind6  contact2  contact4  contact5  hygiene1  hygiene3  hygiene4  hygiene5  psupport4  psupport5  political_ideology  riskperc2  sex1  sbelong2  sbelong4  optim2  slfcont1  slfcont3  urban
##   10   9  age  children  cnarc2  CRT1  CRT2  CRT3  generosity1  generosity2  generosity3  health_cond  know_tested_positive  marital1  mor_circle  moralid1  moralid10  moralid2  moralid3  moralid4  moralid5  moralid6  moralid7  moralid8  moralid9  mcoop1  mcoop2  mcoop3  mcoop4  mcoop5  mcoop6  mcoop7  narc1  narc2  narc3  narc4  narc5  nidentity1  omind2  omind4  omind6  contact2  contact4  contact5  hygiene1  hygiene3  hygiene4  hygiene5  psupport4  psupport5  political_ideology  riskperc2  sex1  sbelong2  sbelong4  optim2  slfcont1  slfcont3  urban
##   10   10  age  children  cnarc2  CRT1  CRT2  CRT3  generosity1  generosity2  generosity3  health_cond  know_tested_positive  marital1  mor_circle  moralid1  moralid10  moralid2  moralid3  moralid4  moralid5  moralid6  moralid7  moralid8  moralid9  mcoop1  mcoop2  mcoop3  mcoop4  mcoop5  mcoop6  mcoop7  narc1  narc2  narc3  narc4  narc5  nidentity1  omind2  omind4  omind6  contact2  contact4  contact5  hygiene1  hygiene3  hygiene4  hygiene5  psupport4  psupport5  political_ideology  riskperc2  sex1  sbelong2  sbelong4  optim2  slfcont1  slfcont3  urban
```

```
## Warning: Number of logged events: 5343
```

```
## 
##  iter imp variable
##   1   1  CRT1  CRT2  CRT3  mor_circle  moralid1  moralid10  moralid2  moralid3  moralid4  moralid5  moralid6  moralid7  moralid8  moralid9  political_ideology
##   1   2  CRT1  CRT2  CRT3  mor_circle  moralid1  moralid10  moralid2  moralid3  moralid4  moralid5  moralid6  moralid7  moralid8  moralid9  political_ideology
##   1   3  CRT1  CRT2  CRT3  mor_circle  moralid1  moralid10  moralid2  moralid3  moralid4  moralid5  moralid6  moralid7  moralid8  moralid9  political_ideology
##   1   4  CRT1  CRT2  CRT3  mor_circle  moralid1  moralid10  moralid2  moralid3  moralid4  moralid5  moralid6  moralid7  moralid8  moralid9  political_ideology
##   1   5  CRT1  CRT2  CRT3  mor_circle  moralid1  moralid10  moralid2  moralid3  moralid4  moralid5  moralid6  moralid7  moralid8  moralid9  political_ideology
##   1   6  CRT1  CRT2  CRT3  mor_circle  moralid1  moralid10  moralid2  moralid3  moralid4  moralid5  moralid6  moralid7  moralid8  moralid9  political_ideology
##   1   7  CRT1  CRT2  CRT3  mor_circle  moralid1  moralid10  moralid2  moralid3  moralid4  moralid5  moralid6  moralid7  moralid8  moralid9  political_ideology
##   1   8  CRT1  CRT2  CRT3  mor_circle  moralid1  moralid10  moralid2  moralid3  moralid4  moralid5  moralid6  moralid7  moralid8  moralid9  political_ideology
##   1   9  CRT1  CRT2  CRT3  mor_circle  moralid1  moralid10  moralid2  moralid3  moralid4  moralid5  moralid6  moralid7  moralid8  moralid9  political_ideology
##   1   10  CRT1  CRT2  CRT3  mor_circle  moralid1  moralid10  moralid2  moralid3  moralid4  moralid5  moralid6  moralid7  moralid8  moralid9  political_ideology
##   2   1  CRT1  CRT2  CRT3  mor_circle  moralid1  moralid10  moralid2  moralid3  moralid4  moralid5  moralid6  moralid7  moralid8  moralid9  political_ideology
##   2   2  CRT1  CRT2  CRT3  mor_circle  moralid1  moralid10  moralid2  moralid3  moralid4  moralid5  moralid6  moralid7  moralid8  moralid9  political_ideology
##   2   3  CRT1  CRT2  CRT3  mor_circle  moralid1  moralid10  moralid2  moralid3  moralid4  moralid5  moralid6  moralid7  moralid8  moralid9  political_ideology
##   2   4  CRT1  CRT2  CRT3  mor_circle  moralid1  moralid10  moralid2  moralid3  moralid4  moralid5  moralid6  moralid7  moralid8  moralid9  political_ideology
##   2   5  CRT1  CRT2  CRT3  mor_circle  moralid1  moralid10  moralid2  moralid3  moralid4  moralid5  moralid6  moralid7  moralid8  moralid9  political_ideology
##   2   6  CRT1  CRT2  CRT3  mor_circle  moralid1  moralid10  moralid2  moralid3  moralid4  moralid5  moralid6  moralid7  moralid8  moralid9  political_ideology
##   2   7  CRT1  CRT2  CRT3  mor_circle  moralid1  moralid10  moralid2  moralid3  moralid4  moralid5  moralid6  moralid7  moralid8  moralid9  political_ideology
##   2   8  CRT1  CRT2  CRT3  mor_circle  moralid1  moralid10  moralid2  moralid3  moralid4  moralid5  moralid6  moralid7  moralid8  moralid9  political_ideology
##   2   9  CRT1  CRT2  CRT3  mor_circle  moralid1  moralid10  moralid2  moralid3  moralid4  moralid5  moralid6  moralid7  moralid8  moralid9  political_ideology
##   2   10  CRT1  CRT2  CRT3  mor_circle  moralid1  moralid10  moralid2  moralid3  moralid4  moralid5  moralid6  moralid7  moralid8  moralid9  political_ideology
##   3   1  CRT1  CRT2  CRT3  mor_circle  moralid1  moralid10  moralid2  moralid3  moralid4  moralid5  moralid6  moralid7  moralid8  moralid9  political_ideology
##   3   2  CRT1  CRT2  CRT3  mor_circle  moralid1  moralid10  moralid2  moralid3  moralid4  moralid5  moralid6  moralid7  moralid8  moralid9  political_ideology
##   3   3  CRT1  CRT2  CRT3  mor_circle  moralid1  moralid10  moralid2  moralid3  moralid4  moralid5  moralid6  moralid7  moralid8  moralid9  political_ideology
##   3   4  CRT1  CRT2  CRT3  mor_circle  moralid1  moralid10  moralid2  moralid3  moralid4  moralid5  moralid6  moralid7  moralid8  moralid9  political_ideology
##   3   5  CRT1  CRT2  CRT3  mor_circle  moralid1  moralid10  moralid2  moralid3  moralid4  moralid5  moralid6  moralid7  moralid8  moralid9  political_ideology
##   3   6  CRT1  CRT2  CRT3  mor_circle  moralid1  moralid10  moralid2  moralid3  moralid4  moralid5  moralid6  moralid7  moralid8  moralid9  political_ideology
##   3   7  CRT1  CRT2  CRT3  mor_circle  moralid1  moralid10  moralid2  moralid3  moralid4  moralid5  moralid6  moralid7  moralid8  moralid9  political_ideology
##   3   8  CRT1  CRT2  CRT3  mor_circle  moralid1  moralid10  moralid2  moralid3  moralid4  moralid5  moralid6  moralid7  moralid8  moralid9  political_ideology
##   3   9  CRT1  CRT2  CRT3  mor_circle  moralid1  moralid10  moralid2  moralid3  moralid4  moralid5  moralid6  moralid7  moralid8  moralid9  political_ideology
##   3   10  CRT1  CRT2  CRT3  mor_circle  moralid1  moralid10  moralid2  moralid3  moralid4  moralid5  moralid6  moralid7  moralid8  moralid9  political_ideology
##   4   1  CRT1  CRT2  CRT3  mor_circle  moralid1  moralid10  moralid2  moralid3  moralid4  moralid5  moralid6  moralid7  moralid8  moralid9  political_ideology
##   4   2  CRT1  CRT2  CRT3  mor_circle  moralid1  moralid10  moralid2  moralid3  moralid4  moralid5  moralid6  moralid7  moralid8  moralid9  political_ideology
##   4   3  CRT1  CRT2  CRT3  mor_circle  moralid1  moralid10  moralid2  moralid3  moralid4  moralid5  moralid6  moralid7  moralid8  moralid9  political_ideology
##   4   4  CRT1  CRT2  CRT3  mor_circle  moralid1  moralid10  moralid2  moralid3  moralid4  moralid5  moralid6  moralid7  moralid8  moralid9  political_ideology
##   4   5  CRT1  CRT2  CRT3  mor_circle  moralid1  moralid10  moralid2  moralid3  moralid4  moralid5  moralid6  moralid7  moralid8  moralid9  political_ideology
##   4   6  CRT1  CRT2  CRT3  mor_circle  moralid1  moralid10  moralid2  moralid3  moralid4  moralid5  moralid6  moralid7  moralid8  moralid9  political_ideology
##   4   7  CRT1  CRT2  CRT3  mor_circle  moralid1  moralid10  moralid2  moralid3  moralid4  moralid5  moralid6  moralid7  moralid8  moralid9  political_ideology
##   4   8  CRT1  CRT2  CRT3  mor_circle  moralid1  moralid10  moralid2  moralid3  moralid4  moralid5  moralid6  moralid7  moralid8  moralid9  political_ideology
##   4   9  CRT1  CRT2  CRT3  mor_circle  moralid1  moralid10  moralid2  moralid3  moralid4  moralid5  moralid6  moralid7  moralid8  moralid9  political_ideology
##   4   10  CRT1  CRT2  CRT3  mor_circle  moralid1  moralid10  moralid2  moralid3  moralid4  moralid5  moralid6  moralid7  moralid8  moralid9  political_ideology
##   5   1  CRT1  CRT2  CRT3  mor_circle  moralid1  moralid10  moralid2  moralid3  moralid4  moralid5  moralid6  moralid7  moralid8  moralid9  political_ideology
##   5   2  CRT1  CRT2  CRT3  mor_circle  moralid1  moralid10  moralid2  moralid3  moralid4  moralid5  moralid6  moralid7  moralid8  moralid9  political_ideology
##   5   3  CRT1  CRT2  CRT3  mor_circle  moralid1  moralid10  moralid2  moralid3  moralid4  moralid5  moralid6  moralid7  moralid8  moralid9  political_ideology
##   5   4  CRT1  CRT2  CRT3  mor_circle  moralid1  moralid10  moralid2  moralid3  moralid4  moralid5  moralid6  moralid7  moralid8  moralid9  political_ideology
##   5   5  CRT1  CRT2  CRT3  mor_circle  moralid1  moralid10  moralid2  moralid3  moralid4  moralid5  moralid6  moralid7  moralid8  moralid9  political_ideology
##   5   6  CRT1  CRT2  CRT3  mor_circle  moralid1  moralid10  moralid2  moralid3  moralid4  moralid5  moralid6  moralid7  moralid8  moralid9  political_ideology
##   5   7  CRT1  CRT2  CRT3  mor_circle  moralid1  moralid10  moralid2  moralid3  moralid4  moralid5  moralid6  moralid7  moralid8  moralid9  political_ideology
##   5   8  CRT1  CRT2  CRT3  mor_circle  moralid1  moralid10  moralid2  moralid3  moralid4  moralid5  moralid6  moralid7  moralid8  moralid9  political_ideology
##   5   9  CRT1  CRT2  CRT3  mor_circle  moralid1  moralid10  moralid2  moralid3  moralid4  moralid5  moralid6  moralid7  moralid8  moralid9  political_ideology
##   5   10  CRT1  CRT2  CRT3  mor_circle  moralid1  moralid10  moralid2  moralid3  moralid4  moralid5  moralid6  moralid7  moralid8  moralid9  political_ideology
##   6   1  CRT1  CRT2  CRT3  mor_circle  moralid1  moralid10  moralid2  moralid3  moralid4  moralid5  moralid6  moralid7  moralid8  moralid9  political_ideology
##   6   2  CRT1  CRT2  CRT3  mor_circle  moralid1  moralid10  moralid2  moralid3  moralid4  moralid5  moralid6  moralid7  moralid8  moralid9  political_ideology
##   6   3  CRT1  CRT2  CRT3  mor_circle  moralid1  moralid10  moralid2  moralid3  moralid4  moralid5  moralid6  moralid7  moralid8  moralid9  political_ideology
##   6   4  CRT1  CRT2  CRT3  mor_circle  moralid1  moralid10  moralid2  moralid3  moralid4  moralid5  moralid6  moralid7  moralid8  moralid9  political_ideology
##   6   5  CRT1  CRT2  CRT3  mor_circle  moralid1  moralid10  moralid2  moralid3  moralid4  moralid5  moralid6  moralid7  moralid8  moralid9  political_ideology
##   6   6  CRT1  CRT2  CRT3  mor_circle  moralid1  moralid10  moralid2  moralid3  moralid4  moralid5  moralid6  moralid7  moralid8  moralid9  political_ideology
##   6   7  CRT1  CRT2  CRT3  mor_circle  moralid1  moralid10  moralid2  moralid3  moralid4  moralid5  moralid6  moralid7  moralid8  moralid9  political_ideology
##   6   8  CRT1  CRT2  CRT3  mor_circle  moralid1  moralid10  moralid2  moralid3  moralid4  moralid5  moralid6  moralid7  moralid8  moralid9  political_ideology
##   6   9  CRT1  CRT2  CRT3  mor_circle  moralid1  moralid10  moralid2  moralid3  moralid4  moralid5  moralid6  moralid7  moralid8  moralid9  political_ideology
##   6   10  CRT1  CRT2  CRT3  mor_circle  moralid1  moralid10  moralid2  moralid3  moralid4  moralid5  moralid6  moralid7  moralid8  moralid9  political_ideology
##   7   1  CRT1  CRT2  CRT3  mor_circle  moralid1  moralid10  moralid2  moralid3  moralid4  moralid5  moralid6  moralid7  moralid8  moralid9  political_ideology
##   7   2  CRT1  CRT2  CRT3  mor_circle  moralid1  moralid10  moralid2  moralid3  moralid4  moralid5  moralid6  moralid7  moralid8  moralid9  political_ideology
##   7   3  CRT1  CRT2  CRT3  mor_circle  moralid1  moralid10  moralid2  moralid3  moralid4  moralid5  moralid6  moralid7  moralid8  moralid9  political_ideology
##   7   4  CRT1  CRT2  CRT3  mor_circle  moralid1  moralid10  moralid2  moralid3  moralid4  moralid5  moralid6  moralid7  moralid8  moralid9  political_ideology
##   7   5  CRT1  CRT2  CRT3  mor_circle  moralid1  moralid10  moralid2  moralid3  moralid4  moralid5  moralid6  moralid7  moralid8  moralid9  political_ideology
##   7   6  CRT1  CRT2  CRT3  mor_circle  moralid1  moralid10  moralid2  moralid3  moralid4  moralid5  moralid6  moralid7  moralid8  moralid9  political_ideology
##   7   7  CRT1  CRT2  CRT3  mor_circle  moralid1  moralid10  moralid2  moralid3  moralid4  moralid5  moralid6  moralid7  moralid8  moralid9  political_ideology
##   7   8  CRT1  CRT2  CRT3  mor_circle  moralid1  moralid10  moralid2  moralid3  moralid4  moralid5  moralid6  moralid7  moralid8  moralid9  political_ideology
##   7   9  CRT1  CRT2  CRT3  mor_circle  moralid1  moralid10  moralid2  moralid3  moralid4  moralid5  moralid6  moralid7  moralid8  moralid9  political_ideology
##   7   10  CRT1  CRT2  CRT3  mor_circle  moralid1  moralid10  moralid2  moralid3  moralid4  moralid5  moralid6  moralid7  moralid8  moralid9  political_ideology
##   8   1  CRT1  CRT2  CRT3  mor_circle  moralid1  moralid10  moralid2  moralid3  moralid4  moralid5  moralid6  moralid7  moralid8  moralid9  political_ideology
##   8   2  CRT1  CRT2  CRT3  mor_circle  moralid1  moralid10  moralid2  moralid3  moralid4  moralid5  moralid6  moralid7  moralid8  moralid9  political_ideology
##   8   3  CRT1  CRT2  CRT3  mor_circle  moralid1  moralid10  moralid2  moralid3  moralid4  moralid5  moralid6  moralid7  moralid8  moralid9  political_ideology
##   8   4  CRT1  CRT2  CRT3  mor_circle  moralid1  moralid10  moralid2  moralid3  moralid4  moralid5  moralid6  moralid7  moralid8  moralid9  political_ideology
##   8   5  CRT1  CRT2  CRT3  mor_circle  moralid1  moralid10  moralid2  moralid3  moralid4  moralid5  moralid6  moralid7  moralid8  moralid9  political_ideology
##   8   6  CRT1  CRT2  CRT3  mor_circle  moralid1  moralid10  moralid2  moralid3  moralid4  moralid5  moralid6  moralid7  moralid8  moralid9  political_ideology
##   8   7  CRT1  CRT2  CRT3  mor_circle  moralid1  moralid10  moralid2  moralid3  moralid4  moralid5  moralid6  moralid7  moralid8  moralid9  political_ideology
##   8   8  CRT1  CRT2  CRT3  mor_circle  moralid1  moralid10  moralid2  moralid3  moralid4  moralid5  moralid6  moralid7  moralid8  moralid9  political_ideology
##   8   9  CRT1  CRT2  CRT3  mor_circle  moralid1  moralid10  moralid2  moralid3  moralid4  moralid5  moralid6  moralid7  moralid8  moralid9  political_ideology
##   8   10  CRT1  CRT2  CRT3  mor_circle  moralid1  moralid10  moralid2  moralid3  moralid4  moralid5  moralid6  moralid7  moralid8  moralid9  political_ideology
##   9   1  CRT1  CRT2  CRT3  mor_circle  moralid1  moralid10  moralid2  moralid3  moralid4  moralid5  moralid6  moralid7  moralid8  moralid9  political_ideology
##   9   2  CRT1  CRT2  CRT3  mor_circle  moralid1  moralid10  moralid2  moralid3  moralid4  moralid5  moralid6  moralid7  moralid8  moralid9  political_ideology
##   9   3  CRT1  CRT2  CRT3  mor_circle  moralid1  moralid10  moralid2  moralid3  moralid4  moralid5  moralid6  moralid7  moralid8  moralid9  political_ideology
##   9   4  CRT1  CRT2  CRT3  mor_circle  moralid1  moralid10  moralid2  moralid3  moralid4  moralid5  moralid6  moralid7  moralid8  moralid9  political_ideology
##   9   5  CRT1  CRT2  CRT3  mor_circle  moralid1  moralid10  moralid2  moralid3  moralid4  moralid5  moralid6  moralid7  moralid8  moralid9  political_ideology
##   9   6  CRT1  CRT2  CRT3  mor_circle  moralid1  moralid10  moralid2  moralid3  moralid4  moralid5  moralid6  moralid7  moralid8  moralid9  political_ideology
##   9   7  CRT1  CRT2  CRT3  mor_circle  moralid1  moralid10  moralid2  moralid3  moralid4  moralid5  moralid6  moralid7  moralid8  moralid9  political_ideology
##   9   8  CRT1  CRT2  CRT3  mor_circle  moralid1  moralid10  moralid2  moralid3  moralid4  moralid5  moralid6  moralid7  moralid8  moralid9  political_ideology
##   9   9  CRT1  CRT2  CRT3  mor_circle  moralid1  moralid10  moralid2  moralid3  moralid4  moralid5  moralid6  moralid7  moralid8  moralid9  political_ideology
##   9   10  CRT1  CRT2  CRT3  mor_circle  moralid1  moralid10  moralid2  moralid3  moralid4  moralid5  moralid6  moralid7  moralid8  moralid9  political_ideology
##   10   1  CRT1  CRT2  CRT3  mor_circle  moralid1  moralid10  moralid2  moralid3  moralid4  moralid5  moralid6  moralid7  moralid8  moralid9  political_ideology
##   10   2  CRT1  CRT2  CRT3  mor_circle  moralid1  moralid10  moralid2  moralid3  moralid4  moralid5  moralid6  moralid7  moralid8  moralid9  political_ideology
##   10   3  CRT1  CRT2  CRT3  mor_circle  moralid1  moralid10  moralid2  moralid3  moralid4  moralid5  moralid6  moralid7  moralid8  moralid9  political_ideology
##   10   4  CRT1  CRT2  CRT3  mor_circle  moralid1  moralid10  moralid2  moralid3  moralid4  moralid5  moralid6  moralid7  moralid8  moralid9  political_ideology
##   10   5  CRT1  CRT2  CRT3  mor_circle  moralid1  moralid10  moralid2  moralid3  moralid4  moralid5  moralid6  moralid7  moralid8  moralid9  political_ideology
##   10   6  CRT1  CRT2  CRT3  mor_circle  moralid1  moralid10  moralid2  moralid3  moralid4  moralid5  moralid6  moralid7  moralid8  moralid9  political_ideology
##   10   7  CRT1  CRT2  CRT3  mor_circle  moralid1  moralid10  moralid2  moralid3  moralid4  moralid5  moralid6  moralid7  moralid8  moralid9  political_ideology
##   10   8  CRT1  CRT2  CRT3  mor_circle  moralid1  moralid10  moralid2  moralid3  moralid4  moralid5  moralid6  moralid7  moralid8  moralid9  political_ideology
##   10   9  CRT1  CRT2  CRT3  mor_circle  moralid1  moralid10  moralid2  moralid3  moralid4  moralid5  moralid6  moralid7  moralid8  moralid9  political_ideology
##   10   10  CRT1  CRT2  CRT3  mor_circle  moralid1  moralid10  moralid2  moralid3  moralid4  moralid5  moralid6  moralid7  moralid8  moralid9  political_ideology
```

```
## Warning: Number of logged events: 1503
```

```
## 
##  iter imp variable
##   1   1  age  children  cnarc1  cnarc2  cnarc3  CRT1  CRT2  CRT3  employ_status1  health_cond  know_tested_positive  ladder  marital1  mor_circle  moralid1  moralid10  moralid2  moralid3  moralid4  moralid5  moralid6  moralid7  moralid8  moralid9  mcoop1  mcoop2  mcoop3  mcoop4  mcoop5  mcoop6  mcoop7  narc1  narc2  narc3  narc4  narc5  narc6  nidentity1  nidentity2  omind1  omind2  omind3  omind4  omind5  omind6  contact1  contact2  contact3  contact4  contact5  hygiene1  hygiene2  hygiene3  hygiene4  hygiene5  psupport1  psupport2  psupport3  psupport4  psupport5  political_ideology  happy  slf_ladder  riskperc1  riskperc2  self_esteem  sbelong3  tested_positive  optim1  optim2  slfcont1  slfcont2  slfcont3  slfcont4  urban
##   1   2  age  children  cnarc1  cnarc2  cnarc3  CRT1  CRT2  CRT3  employ_status1  health_cond  know_tested_positive  ladder  marital1  mor_circle  moralid1  moralid10  moralid2  moralid3  moralid4  moralid5  moralid6  moralid7  moralid8  moralid9  mcoop1  mcoop2  mcoop3  mcoop4  mcoop5  mcoop6  mcoop7  narc1  narc2  narc3  narc4  narc5  narc6  nidentity1  nidentity2  omind1  omind2  omind3  omind4  omind5  omind6  contact1  contact2  contact3  contact4  contact5  hygiene1  hygiene2  hygiene3  hygiene4  hygiene5  psupport1  psupport2  psupport3  psupport4  psupport5  political_ideology  happy  slf_ladder  riskperc1  riskperc2  self_esteem  sbelong3  tested_positive  optim1  optim2  slfcont1  slfcont2  slfcont3  slfcont4  urban
##   1   3  age  children  cnarc1  cnarc2  cnarc3  CRT1  CRT2  CRT3  employ_status1  health_cond  know_tested_positive  ladder  marital1  mor_circle  moralid1  moralid10  moralid2  moralid3  moralid4  moralid5  moralid6  moralid7  moralid8  moralid9  mcoop1  mcoop2  mcoop3  mcoop4  mcoop5  mcoop6  mcoop7  narc1  narc2  narc3  narc4  narc5  narc6  nidentity1  nidentity2  omind1  omind2  omind3  omind4  omind5  omind6  contact1  contact2  contact3  contact4  contact5  hygiene1  hygiene2  hygiene3  hygiene4  hygiene5  psupport1  psupport2  psupport3  psupport4  psupport5  political_ideology  happy  slf_ladder  riskperc1  riskperc2  self_esteem  sbelong3  tested_positive  optim1  optim2  slfcont1  slfcont2  slfcont3  slfcont4  urban
##   1   4  age  children  cnarc1  cnarc2  cnarc3  CRT1  CRT2  CRT3  employ_status1  health_cond  know_tested_positive  ladder  marital1  mor_circle  moralid1  moralid10  moralid2  moralid3  moralid4  moralid5  moralid6  moralid7  moralid8  moralid9  mcoop1  mcoop2  mcoop3  mcoop4  mcoop5  mcoop6  mcoop7  narc1  narc2  narc3  narc4  narc5  narc6  nidentity1  nidentity2  omind1  omind2  omind3  omind4  omind5  omind6  contact1  contact2  contact3  contact4  contact5  hygiene1  hygiene2  hygiene3  hygiene4  hygiene5  psupport1  psupport2  psupport3  psupport4  psupport5  political_ideology  happy  slf_ladder  riskperc1  riskperc2  self_esteem  sbelong3  tested_positive  optim1  optim2  slfcont1  slfcont2  slfcont3  slfcont4  urban
##   1   5  age  children  cnarc1  cnarc2  cnarc3  CRT1  CRT2  CRT3  employ_status1  health_cond  know_tested_positive  ladder  marital1  mor_circle  moralid1  moralid10  moralid2  moralid3  moralid4  moralid5  moralid6  moralid7  moralid8  moralid9  mcoop1  mcoop2  mcoop3  mcoop4  mcoop5  mcoop6  mcoop7  narc1  narc2  narc3  narc4  narc5  narc6  nidentity1  nidentity2  omind1  omind2  omind3  omind4  omind5  omind6  contact1  contact2  contact3  contact4  contact5  hygiene1  hygiene2  hygiene3  hygiene4  hygiene5  psupport1  psupport2  psupport3  psupport4  psupport5  political_ideology  happy  slf_ladder  riskperc1  riskperc2  self_esteem  sbelong3  tested_positive  optim1  optim2  slfcont1  slfcont2  slfcont3  slfcont4  urban
##   1   6  age  children  cnarc1  cnarc2  cnarc3  CRT1  CRT2  CRT3  employ_status1  health_cond  know_tested_positive  ladder  marital1  mor_circle  moralid1  moralid10  moralid2  moralid3  moralid4  moralid5  moralid6  moralid7  moralid8  moralid9  mcoop1  mcoop2  mcoop3  mcoop4  mcoop5  mcoop6  mcoop7  narc1  narc2  narc3  narc4  narc5  narc6  nidentity1  nidentity2  omind1  omind2  omind3  omind4  omind5  omind6  contact1  contact2  contact3  contact4  contact5  hygiene1  hygiene2  hygiene3  hygiene4  hygiene5  psupport1  psupport2  psupport3  psupport4  psupport5  political_ideology  happy  slf_ladder  riskperc1  riskperc2  self_esteem  sbelong3  tested_positive  optim1  optim2  slfcont1  slfcont2  slfcont3  slfcont4  urban
##   1   7  age  children  cnarc1  cnarc2  cnarc3  CRT1  CRT2  CRT3  employ_status1  health_cond  know_tested_positive  ladder  marital1  mor_circle  moralid1  moralid10  moralid2  moralid3  moralid4  moralid5  moralid6  moralid7  moralid8  moralid9  mcoop1  mcoop2  mcoop3  mcoop4  mcoop5  mcoop6  mcoop7  narc1  narc2  narc3  narc4  narc5  narc6  nidentity1  nidentity2  omind1  omind2  omind3  omind4  omind5  omind6  contact1  contact2  contact3  contact4  contact5  hygiene1  hygiene2  hygiene3  hygiene4  hygiene5  psupport1  psupport2  psupport3  psupport4  psupport5  political_ideology  happy  slf_ladder  riskperc1  riskperc2  self_esteem  sbelong3  tested_positive  optim1  optim2  slfcont1  slfcont2  slfcont3  slfcont4  urban
##   1   8  age  children  cnarc1  cnarc2  cnarc3  CRT1  CRT2  CRT3  employ_status1  health_cond  know_tested_positive  ladder  marital1  mor_circle  moralid1  moralid10  moralid2  moralid3  moralid4  moralid5  moralid6  moralid7  moralid8  moralid9  mcoop1  mcoop2  mcoop3  mcoop4  mcoop5  mcoop6  mcoop7  narc1  narc2  narc3  narc4  narc5  narc6  nidentity1  nidentity2  omind1  omind2  omind3  omind4  omind5  omind6  contact1  contact2  contact3  contact4  contact5  hygiene1  hygiene2  hygiene3  hygiene4  hygiene5  psupport1  psupport2  psupport3  psupport4  psupport5  political_ideology  happy  slf_ladder  riskperc1  riskperc2  self_esteem  sbelong3  tested_positive  optim1  optim2  slfcont1  slfcont2  slfcont3  slfcont4  urban
##   1   9  age  children  cnarc1  cnarc2  cnarc3  CRT1  CRT2  CRT3  employ_status1  health_cond  know_tested_positive  ladder  marital1  mor_circle  moralid1  moralid10  moralid2  moralid3  moralid4  moralid5  moralid6  moralid7  moralid8  moralid9  mcoop1  mcoop2  mcoop3  mcoop4  mcoop5  mcoop6  mcoop7  narc1  narc2  narc3  narc4  narc5  narc6  nidentity1  nidentity2  omind1  omind2  omind3  omind4  omind5  omind6  contact1  contact2  contact3  contact4  contact5  hygiene1  hygiene2  hygiene3  hygiene4  hygiene5  psupport1  psupport2  psupport3  psupport4  psupport5  political_ideology  happy  slf_ladder  riskperc1  riskperc2  self_esteem  sbelong3  tested_positive  optim1  optim2  slfcont1  slfcont2  slfcont3  slfcont4  urban
##   1   10  age  children  cnarc1  cnarc2  cnarc3  CRT1  CRT2  CRT3  employ_status1  health_cond  know_tested_positive  ladder  marital1  mor_circle  moralid1  moralid10  moralid2  moralid3  moralid4  moralid5  moralid6  moralid7  moralid8  moralid9  mcoop1  mcoop2  mcoop3  mcoop4  mcoop5  mcoop6  mcoop7  narc1  narc2  narc3  narc4  narc5  narc6  nidentity1  nidentity2  omind1  omind2  omind3  omind4  omind5  omind6  contact1  contact2  contact3  contact4  contact5  hygiene1  hygiene2  hygiene3  hygiene4  hygiene5  psupport1  psupport2  psupport3  psupport4  psupport5  political_ideology  happy  slf_ladder  riskperc1  riskperc2  self_esteem  sbelong3  tested_positive  optim1  optim2  slfcont1  slfcont2  slfcont3  slfcont4  urban
##   2   1  age  children  cnarc1  cnarc2  cnarc3  CRT1  CRT2  CRT3  employ_status1  health_cond  know_tested_positive  ladder  marital1  mor_circle  moralid1  moralid10  moralid2  moralid3  moralid4  moralid5  moralid6  moralid7  moralid8  moralid9  mcoop1  mcoop2  mcoop3  mcoop4  mcoop5  mcoop6  mcoop7  narc1  narc2  narc3  narc4  narc5  narc6  nidentity1  nidentity2  omind1  omind2  omind3  omind4  omind5  omind6  contact1  contact2  contact3  contact4  contact5  hygiene1  hygiene2  hygiene3  hygiene4  hygiene5  psupport1  psupport2  psupport3  psupport4  psupport5  political_ideology  happy  slf_ladder  riskperc1  riskperc2  self_esteem  sbelong3  tested_positive  optim1  optim2  slfcont1  slfcont2  slfcont3  slfcont4  urban
##   2   2  age  children  cnarc1  cnarc2  cnarc3  CRT1  CRT2  CRT3  employ_status1  health_cond  know_tested_positive  ladder  marital1  mor_circle  moralid1  moralid10  moralid2  moralid3  moralid4  moralid5  moralid6  moralid7  moralid8  moralid9  mcoop1  mcoop2  mcoop3  mcoop4  mcoop5  mcoop6  mcoop7  narc1  narc2  narc3  narc4  narc5  narc6  nidentity1  nidentity2  omind1  omind2  omind3  omind4  omind5  omind6  contact1  contact2  contact3  contact4  contact5  hygiene1  hygiene2  hygiene3  hygiene4  hygiene5  psupport1  psupport2  psupport3  psupport4  psupport5  political_ideology  happy  slf_ladder  riskperc1  riskperc2  self_esteem  sbelong3  tested_positive  optim1  optim2  slfcont1  slfcont2  slfcont3  slfcont4  urban
##   2   3  age  children  cnarc1  cnarc2  cnarc3  CRT1  CRT2  CRT3  employ_status1  health_cond  know_tested_positive  ladder  marital1  mor_circle  moralid1  moralid10  moralid2  moralid3  moralid4  moralid5  moralid6  moralid7  moralid8  moralid9  mcoop1  mcoop2  mcoop3  mcoop4  mcoop5  mcoop6  mcoop7  narc1  narc2  narc3  narc4  narc5  narc6  nidentity1  nidentity2  omind1  omind2  omind3  omind4  omind5  omind6  contact1  contact2  contact3  contact4  contact5  hygiene1  hygiene2  hygiene3  hygiene4  hygiene5  psupport1  psupport2  psupport3  psupport4  psupport5  political_ideology  happy  slf_ladder  riskperc1  riskperc2  self_esteem  sbelong3  tested_positive  optim1  optim2  slfcont1  slfcont2  slfcont3  slfcont4  urban
##   2   4  age  children  cnarc1  cnarc2  cnarc3  CRT1  CRT2  CRT3  employ_status1  health_cond  know_tested_positive  ladder  marital1  mor_circle  moralid1  moralid10  moralid2  moralid3  moralid4  moralid5  moralid6  moralid7  moralid8  moralid9  mcoop1  mcoop2  mcoop3  mcoop4  mcoop5  mcoop6  mcoop7  narc1  narc2  narc3  narc4  narc5  narc6  nidentity1  nidentity2  omind1  omind2  omind3  omind4  omind5  omind6  contact1  contact2  contact3  contact4  contact5  hygiene1  hygiene2  hygiene3  hygiene4  hygiene5  psupport1  psupport2  psupport3  psupport4  psupport5  political_ideology  happy  slf_ladder  riskperc1  riskperc2  self_esteem  sbelong3  tested_positive  optim1  optim2  slfcont1  slfcont2  slfcont3  slfcont4  urban
##   2   5  age  children  cnarc1  cnarc2  cnarc3  CRT1  CRT2  CRT3  employ_status1  health_cond  know_tested_positive  ladder  marital1  mor_circle  moralid1  moralid10  moralid2  moralid3  moralid4  moralid5  moralid6  moralid7  moralid8  moralid9  mcoop1  mcoop2  mcoop3  mcoop4  mcoop5  mcoop6  mcoop7  narc1  narc2  narc3  narc4  narc5  narc6  nidentity1  nidentity2  omind1  omind2  omind3  omind4  omind5  omind6  contact1  contact2  contact3  contact4  contact5  hygiene1  hygiene2  hygiene3  hygiene4  hygiene5  psupport1  psupport2  psupport3  psupport4  psupport5  political_ideology  happy  slf_ladder  riskperc1  riskperc2  self_esteem  sbelong3  tested_positive  optim1  optim2  slfcont1  slfcont2  slfcont3  slfcont4  urban
##   2   6  age  children  cnarc1  cnarc2  cnarc3  CRT1  CRT2  CRT3  employ_status1  health_cond  know_tested_positive  ladder  marital1  mor_circle  moralid1  moralid10  moralid2  moralid3  moralid4  moralid5  moralid6  moralid7  moralid8  moralid9  mcoop1  mcoop2  mcoop3  mcoop4  mcoop5  mcoop6  mcoop7  narc1  narc2  narc3  narc4  narc5  narc6  nidentity1  nidentity2  omind1  omind2  omind3  omind4  omind5  omind6  contact1  contact2  contact3  contact4  contact5  hygiene1  hygiene2  hygiene3  hygiene4  hygiene5  psupport1  psupport2  psupport3  psupport4  psupport5  political_ideology  happy  slf_ladder  riskperc1  riskperc2  self_esteem  sbelong3  tested_positive  optim1  optim2  slfcont1  slfcont2  slfcont3  slfcont4  urban
##   2   7  age  children  cnarc1  cnarc2  cnarc3  CRT1  CRT2  CRT3  employ_status1  health_cond  know_tested_positive  ladder  marital1  mor_circle  moralid1  moralid10  moralid2  moralid3  moralid4  moralid5  moralid6  moralid7  moralid8  moralid9  mcoop1  mcoop2  mcoop3  mcoop4  mcoop5  mcoop6  mcoop7  narc1  narc2  narc3  narc4  narc5  narc6  nidentity1  nidentity2  omind1  omind2  omind3  omind4  omind5  omind6  contact1  contact2  contact3  contact4  contact5  hygiene1  hygiene2  hygiene3  hygiene4  hygiene5  psupport1  psupport2  psupport3  psupport4  psupport5  political_ideology  happy  slf_ladder  riskperc1  riskperc2  self_esteem  sbelong3  tested_positive  optim1  optim2  slfcont1  slfcont2  slfcont3  slfcont4  urban
##   2   8  age  children  cnarc1  cnarc2  cnarc3  CRT1  CRT2  CRT3  employ_status1  health_cond  know_tested_positive  ladder  marital1  mor_circle  moralid1  moralid10  moralid2  moralid3  moralid4  moralid5  moralid6  moralid7  moralid8  moralid9  mcoop1  mcoop2  mcoop3  mcoop4  mcoop5  mcoop6  mcoop7  narc1  narc2  narc3  narc4  narc5  narc6  nidentity1  nidentity2  omind1  omind2  omind3  omind4  omind5  omind6  contact1  contact2  contact3  contact4  contact5  hygiene1  hygiene2  hygiene3  hygiene4  hygiene5  psupport1  psupport2  psupport3  psupport4  psupport5  political_ideology  happy  slf_ladder  riskperc1  riskperc2  self_esteem  sbelong3  tested_positive  optim1  optim2  slfcont1  slfcont2  slfcont3  slfcont4  urban
##   2   9  age  children  cnarc1  cnarc2  cnarc3  CRT1  CRT2  CRT3  employ_status1  health_cond  know_tested_positive  ladder  marital1  mor_circle  moralid1  moralid10  moralid2  moralid3  moralid4  moralid5  moralid6  moralid7  moralid8  moralid9  mcoop1  mcoop2  mcoop3  mcoop4  mcoop5  mcoop6  mcoop7  narc1  narc2  narc3  narc4  narc5  narc6  nidentity1  nidentity2  omind1  omind2  omind3  omind4  omind5  omind6  contact1  contact2  contact3  contact4  contact5  hygiene1  hygiene2  hygiene3  hygiene4  hygiene5  psupport1  psupport2  psupport3  psupport4  psupport5  political_ideology  happy  slf_ladder  riskperc1  riskperc2  self_esteem  sbelong3  tested_positive  optim1  optim2  slfcont1  slfcont2  slfcont3  slfcont4  urban
##   2   10  age  children  cnarc1  cnarc2  cnarc3  CRT1  CRT2  CRT3  employ_status1  health_cond  know_tested_positive  ladder  marital1  mor_circle  moralid1  moralid10  moralid2  moralid3  moralid4  moralid5  moralid6  moralid7  moralid8  moralid9  mcoop1  mcoop2  mcoop3  mcoop4  mcoop5  mcoop6  mcoop7  narc1  narc2  narc3  narc4  narc5  narc6  nidentity1  nidentity2  omind1  omind2  omind3  omind4  omind5  omind6  contact1  contact2  contact3  contact4  contact5  hygiene1  hygiene2  hygiene3  hygiene4  hygiene5  psupport1  psupport2  psupport3  psupport4  psupport5  political_ideology  happy  slf_ladder  riskperc1  riskperc2  self_esteem  sbelong3  tested_positive  optim1  optim2  slfcont1  slfcont2  slfcont3  slfcont4  urban
##   3   1  age  children  cnarc1  cnarc2  cnarc3  CRT1  CRT2  CRT3  employ_status1  health_cond  know_tested_positive  ladder  marital1  mor_circle  moralid1  moralid10  moralid2  moralid3  moralid4  moralid5  moralid6  moralid7  moralid8  moralid9  mcoop1  mcoop2  mcoop3  mcoop4  mcoop5  mcoop6  mcoop7  narc1  narc2  narc3  narc4  narc5  narc6  nidentity1  nidentity2  omind1  omind2  omind3  omind4  omind5  omind6  contact1  contact2  contact3  contact4  contact5  hygiene1  hygiene2  hygiene3  hygiene4  hygiene5  psupport1  psupport2  psupport3  psupport4  psupport5  political_ideology  happy  slf_ladder  riskperc1  riskperc2  self_esteem  sbelong3  tested_positive  optim1  optim2  slfcont1  slfcont2  slfcont3  slfcont4  urban
##   3   2  age  children  cnarc1  cnarc2  cnarc3  CRT1  CRT2  CRT3  employ_status1  health_cond  know_tested_positive  ladder  marital1  mor_circle  moralid1  moralid10  moralid2  moralid3  moralid4  moralid5  moralid6  moralid7  moralid8  moralid9  mcoop1  mcoop2  mcoop3  mcoop4  mcoop5  mcoop6  mcoop7  narc1  narc2  narc3  narc4  narc5  narc6  nidentity1  nidentity2  omind1  omind2  omind3  omind4  omind5  omind6  contact1  contact2  contact3  contact4  contact5  hygiene1  hygiene2  hygiene3  hygiene4  hygiene5  psupport1  psupport2  psupport3  psupport4  psupport5  political_ideology  happy  slf_ladder  riskperc1  riskperc2  self_esteem  sbelong3  tested_positive  optim1  optim2  slfcont1  slfcont2  slfcont3  slfcont4  urban
##   3   3  age  children  cnarc1  cnarc2  cnarc3  CRT1  CRT2  CRT3  employ_status1  health_cond  know_tested_positive  ladder  marital1  mor_circle  moralid1  moralid10  moralid2  moralid3  moralid4  moralid5  moralid6  moralid7  moralid8  moralid9  mcoop1  mcoop2  mcoop3  mcoop4  mcoop5  mcoop6  mcoop7  narc1  narc2  narc3  narc4  narc5  narc6  nidentity1  nidentity2  omind1  omind2  omind3  omind4  omind5  omind6  contact1  contact2  contact3  contact4  contact5  hygiene1  hygiene2  hygiene3  hygiene4  hygiene5  psupport1  psupport2  psupport3  psupport4  psupport5  political_ideology  happy  slf_ladder  riskperc1  riskperc2  self_esteem  sbelong3  tested_positive  optim1  optim2  slfcont1  slfcont2  slfcont3  slfcont4  urban
##   3   4  age  children  cnarc1  cnarc2  cnarc3  CRT1  CRT2  CRT3  employ_status1  health_cond  know_tested_positive  ladder  marital1  mor_circle  moralid1  moralid10  moralid2  moralid3  moralid4  moralid5  moralid6  moralid7  moralid8  moralid9  mcoop1  mcoop2  mcoop3  mcoop4  mcoop5  mcoop6  mcoop7  narc1  narc2  narc3  narc4  narc5  narc6  nidentity1  nidentity2  omind1  omind2  omind3  omind4  omind5  omind6  contact1  contact2  contact3  contact4  contact5  hygiene1  hygiene2  hygiene3  hygiene4  hygiene5  psupport1  psupport2  psupport3  psupport4  psupport5  political_ideology  happy  slf_ladder  riskperc1  riskperc2  self_esteem  sbelong3  tested_positive  optim1  optim2  slfcont1  slfcont2  slfcont3  slfcont4  urban
##   3   5  age  children  cnarc1  cnarc2  cnarc3  CRT1  CRT2  CRT3  employ_status1  health_cond  know_tested_positive  ladder  marital1  mor_circle  moralid1  moralid10  moralid2  moralid3  moralid4  moralid5  moralid6  moralid7  moralid8  moralid9  mcoop1  mcoop2  mcoop3  mcoop4  mcoop5  mcoop6  mcoop7  narc1  narc2  narc3  narc4  narc5  narc6  nidentity1  nidentity2  omind1  omind2  omind3  omind4  omind5  omind6  contact1  contact2  contact3  contact4  contact5  hygiene1  hygiene2  hygiene3  hygiene4  hygiene5  psupport1  psupport2  psupport3  psupport4  psupport5  political_ideology  happy  slf_ladder  riskperc1  riskperc2  self_esteem  sbelong3  tested_positive  optim1  optim2  slfcont1  slfcont2  slfcont3  slfcont4  urban
##   3   6  age  children  cnarc1  cnarc2  cnarc3  CRT1  CRT2  CRT3  employ_status1  health_cond  know_tested_positive  ladder  marital1  mor_circle  moralid1  moralid10  moralid2  moralid3  moralid4  moralid5  moralid6  moralid7  moralid8  moralid9  mcoop1  mcoop2  mcoop3  mcoop4  mcoop5  mcoop6  mcoop7  narc1  narc2  narc3  narc4  narc5  narc6  nidentity1  nidentity2  omind1  omind2  omind3  omind4  omind5  omind6  contact1  contact2  contact3  contact4  contact5  hygiene1  hygiene2  hygiene3  hygiene4  hygiene5  psupport1  psupport2  psupport3  psupport4  psupport5  political_ideology  happy  slf_ladder  riskperc1  riskperc2  self_esteem  sbelong3  tested_positive  optim1  optim2  slfcont1  slfcont2  slfcont3  slfcont4  urban
##   3   7  age  children  cnarc1  cnarc2  cnarc3  CRT1  CRT2  CRT3  employ_status1  health_cond  know_tested_positive  ladder  marital1  mor_circle  moralid1  moralid10  moralid2  moralid3  moralid4  moralid5  moralid6  moralid7  moralid8  moralid9  mcoop1  mcoop2  mcoop3  mcoop4  mcoop5  mcoop6  mcoop7  narc1  narc2  narc3  narc4  narc5  narc6  nidentity1  nidentity2  omind1  omind2  omind3  omind4  omind5  omind6  contact1  contact2  contact3  contact4  contact5  hygiene1  hygiene2  hygiene3  hygiene4  hygiene5  psupport1  psupport2  psupport3  psupport4  psupport5  political_ideology  happy  slf_ladder  riskperc1  riskperc2  self_esteem  sbelong3  tested_positive  optim1  optim2  slfcont1  slfcont2  slfcont3  slfcont4  urban
##   3   8  age  children  cnarc1  cnarc2  cnarc3  CRT1  CRT2  CRT3  employ_status1  health_cond  know_tested_positive  ladder  marital1  mor_circle  moralid1  moralid10  moralid2  moralid3  moralid4  moralid5  moralid6  moralid7  moralid8  moralid9  mcoop1  mcoop2  mcoop3  mcoop4  mcoop5  mcoop6  mcoop7  narc1  narc2  narc3  narc4  narc5  narc6  nidentity1  nidentity2  omind1  omind2  omind3  omind4  omind5  omind6  contact1  contact2  contact3  contact4  contact5  hygiene1  hygiene2  hygiene3  hygiene4  hygiene5  psupport1  psupport2  psupport3  psupport4  psupport5  political_ideology  happy  slf_ladder  riskperc1  riskperc2  self_esteem  sbelong3  tested_positive  optim1  optim2  slfcont1  slfcont2  slfcont3  slfcont4  urban
##   3   9  age  children  cnarc1  cnarc2  cnarc3  CRT1  CRT2  CRT3  employ_status1  health_cond  know_tested_positive  ladder  marital1  mor_circle  moralid1  moralid10  moralid2  moralid3  moralid4  moralid5  moralid6  moralid7  moralid8  moralid9  mcoop1  mcoop2  mcoop3  mcoop4  mcoop5  mcoop6  mcoop7  narc1  narc2  narc3  narc4  narc5  narc6  nidentity1  nidentity2  omind1  omind2  omind3  omind4  omind5  omind6  contact1  contact2  contact3  contact4  contact5  hygiene1  hygiene2  hygiene3  hygiene4  hygiene5  psupport1  psupport2  psupport3  psupport4  psupport5  political_ideology  happy  slf_ladder  riskperc1  riskperc2  self_esteem  sbelong3  tested_positive  optim1  optim2  slfcont1  slfcont2  slfcont3  slfcont4  urban
##   3   10  age  children  cnarc1  cnarc2  cnarc3  CRT1  CRT2  CRT3  employ_status1  health_cond  know_tested_positive  ladder  marital1  mor_circle  moralid1  moralid10  moralid2  moralid3  moralid4  moralid5  moralid6  moralid7  moralid8  moralid9  mcoop1  mcoop2  mcoop3  mcoop4  mcoop5  mcoop6  mcoop7  narc1  narc2  narc3  narc4  narc5  narc6  nidentity1  nidentity2  omind1  omind2  omind3  omind4  omind5  omind6  contact1  contact2  contact3  contact4  contact5  hygiene1  hygiene2  hygiene3  hygiene4  hygiene5  psupport1  psupport2  psupport3  psupport4  psupport5  political_ideology  happy  slf_ladder  riskperc1  riskperc2  self_esteem  sbelong3  tested_positive  optim1  optim2  slfcont1  slfcont2  slfcont3  slfcont4  urban
##   4   1  age  children  cnarc1  cnarc2  cnarc3  CRT1  CRT2  CRT3  employ_status1  health_cond  know_tested_positive  ladder  marital1  mor_circle  moralid1  moralid10  moralid2  moralid3  moralid4  moralid5  moralid6  moralid7  moralid8  moralid9  mcoop1  mcoop2  mcoop3  mcoop4  mcoop5  mcoop6  mcoop7  narc1  narc2  narc3  narc4  narc5  narc6  nidentity1  nidentity2  omind1  omind2  omind3  omind4  omind5  omind6  contact1  contact2  contact3  contact4  contact5  hygiene1  hygiene2  hygiene3  hygiene4  hygiene5  psupport1  psupport2  psupport3  psupport4  psupport5  political_ideology  happy  slf_ladder  riskperc1  riskperc2  self_esteem  sbelong3  tested_positive  optim1  optim2  slfcont1  slfcont2  slfcont3  slfcont4  urban
##   4   2  age  children  cnarc1  cnarc2  cnarc3  CRT1  CRT2  CRT3  employ_status1  health_cond  know_tested_positive  ladder  marital1  mor_circle  moralid1  moralid10  moralid2  moralid3  moralid4  moralid5  moralid6  moralid7  moralid8  moralid9  mcoop1  mcoop2  mcoop3  mcoop4  mcoop5  mcoop6  mcoop7  narc1  narc2  narc3  narc4  narc5  narc6  nidentity1  nidentity2  omind1  omind2  omind3  omind4  omind5  omind6  contact1  contact2  contact3  contact4  contact5  hygiene1  hygiene2  hygiene3  hygiene4  hygiene5  psupport1  psupport2  psupport3  psupport4  psupport5  political_ideology  happy  slf_ladder  riskperc1  riskperc2  self_esteem  sbelong3  tested_positive  optim1  optim2  slfcont1  slfcont2  slfcont3  slfcont4  urban
##   4   3  age  children  cnarc1  cnarc2  cnarc3  CRT1  CRT2  CRT3  employ_status1  health_cond  know_tested_positive  ladder  marital1  mor_circle  moralid1  moralid10  moralid2  moralid3  moralid4  moralid5  moralid6  moralid7  moralid8  moralid9  mcoop1  mcoop2  mcoop3  mcoop4  mcoop5  mcoop6  mcoop7  narc1  narc2  narc3  narc4  narc5  narc6  nidentity1  nidentity2  omind1  omind2  omind3  omind4  omind5  omind6  contact1  contact2  contact3  contact4  contact5  hygiene1  hygiene2  hygiene3  hygiene4  hygiene5  psupport1  psupport2  psupport3  psupport4  psupport5  political_ideology  happy  slf_ladder  riskperc1  riskperc2  self_esteem  sbelong3  tested_positive  optim1  optim2  slfcont1  slfcont2  slfcont3  slfcont4  urban
##   4   4  age  children  cnarc1  cnarc2  cnarc3  CRT1  CRT2  CRT3  employ_status1  health_cond  know_tested_positive  ladder  marital1  mor_circle  moralid1  moralid10  moralid2  moralid3  moralid4  moralid5  moralid6  moralid7  moralid8  moralid9  mcoop1  mcoop2  mcoop3  mcoop4  mcoop5  mcoop6  mcoop7  narc1  narc2  narc3  narc4  narc5  narc6  nidentity1  nidentity2  omind1  omind2  omind3  omind4  omind5  omind6  contact1  contact2  contact3  contact4  contact5  hygiene1  hygiene2  hygiene3  hygiene4  hygiene5  psupport1  psupport2  psupport3  psupport4  psupport5  political_ideology  happy  slf_ladder  riskperc1  riskperc2  self_esteem  sbelong3  tested_positive  optim1  optim2  slfcont1  slfcont2  slfcont3  slfcont4  urban
##   4   5  age  children  cnarc1  cnarc2  cnarc3  CRT1  CRT2  CRT3  employ_status1  health_cond  know_tested_positive  ladder  marital1  mor_circle  moralid1  moralid10  moralid2  moralid3  moralid4  moralid5  moralid6  moralid7  moralid8  moralid9  mcoop1  mcoop2  mcoop3  mcoop4  mcoop5  mcoop6  mcoop7  narc1  narc2  narc3  narc4  narc5  narc6  nidentity1  nidentity2  omind1  omind2  omind3  omind4  omind5  omind6  contact1  contact2  contact3  contact4  contact5  hygiene1  hygiene2  hygiene3  hygiene4  hygiene5  psupport1  psupport2  psupport3  psupport4  psupport5  political_ideology  happy  slf_ladder  riskperc1  riskperc2  self_esteem  sbelong3  tested_positive  optim1  optim2  slfcont1  slfcont2  slfcont3  slfcont4  urban
##   4   6  age  children  cnarc1  cnarc2  cnarc3  CRT1  CRT2  CRT3  employ_status1  health_cond  know_tested_positive  ladder  marital1  mor_circle  moralid1  moralid10  moralid2  moralid3  moralid4  moralid5  moralid6  moralid7  moralid8  moralid9  mcoop1  mcoop2  mcoop3  mcoop4  mcoop5  mcoop6  mcoop7  narc1  narc2  narc3  narc4  narc5  narc6  nidentity1  nidentity2  omind1  omind2  omind3  omind4  omind5  omind6  contact1  contact2  contact3  contact4  contact5  hygiene1  hygiene2  hygiene3  hygiene4  hygiene5  psupport1  psupport2  psupport3  psupport4  psupport5  political_ideology  happy  slf_ladder  riskperc1  riskperc2  self_esteem  sbelong3  tested_positive  optim1  optim2  slfcont1  slfcont2  slfcont3  slfcont4  urban
##   4   7  age  children  cnarc1  cnarc2  cnarc3  CRT1  CRT2  CRT3  employ_status1  health_cond  know_tested_positive  ladder  marital1  mor_circle  moralid1  moralid10  moralid2  moralid3  moralid4  moralid5  moralid6  moralid7  moralid8  moralid9  mcoop1  mcoop2  mcoop3  mcoop4  mcoop5  mcoop6  mcoop7  narc1  narc2  narc3  narc4  narc5  narc6  nidentity1  nidentity2  omind1  omind2  omind3  omind4  omind5  omind6  contact1  contact2  contact3  contact4  contact5  hygiene1  hygiene2  hygiene3  hygiene4  hygiene5  psupport1  psupport2  psupport3  psupport4  psupport5  political_ideology  happy  slf_ladder  riskperc1  riskperc2  self_esteem  sbelong3  tested_positive  optim1  optim2  slfcont1  slfcont2  slfcont3  slfcont4  urban
##   4   8  age  children  cnarc1  cnarc2  cnarc3  CRT1  CRT2  CRT3  employ_status1  health_cond  know_tested_positive  ladder  marital1  mor_circle  moralid1  moralid10  moralid2  moralid3  moralid4  moralid5  moralid6  moralid7  moralid8  moralid9  mcoop1  mcoop2  mcoop3  mcoop4  mcoop5  mcoop6  mcoop7  narc1  narc2  narc3  narc4  narc5  narc6  nidentity1  nidentity2  omind1  omind2  omind3  omind4  omind5  omind6  contact1  contact2  contact3  contact4  contact5  hygiene1  hygiene2  hygiene3  hygiene4  hygiene5  psupport1  psupport2  psupport3  psupport4  psupport5  political_ideology  happy  slf_ladder  riskperc1  riskperc2  self_esteem  sbelong3  tested_positive  optim1  optim2  slfcont1  slfcont2  slfcont3  slfcont4  urban
##   4   9  age  children  cnarc1  cnarc2  cnarc3  CRT1  CRT2  CRT3  employ_status1  health_cond  know_tested_positive  ladder  marital1  mor_circle  moralid1  moralid10  moralid2  moralid3  moralid4  moralid5  moralid6  moralid7  moralid8  moralid9  mcoop1  mcoop2  mcoop3  mcoop4  mcoop5  mcoop6  mcoop7  narc1  narc2  narc3  narc4  narc5  narc6  nidentity1  nidentity2  omind1  omind2  omind3  omind4  omind5  omind6  contact1  contact2  contact3  contact4  contact5  hygiene1  hygiene2  hygiene3  hygiene4  hygiene5  psupport1  psupport2  psupport3  psupport4  psupport5  political_ideology  happy  slf_ladder  riskperc1  riskperc2  self_esteem  sbelong3  tested_positive  optim1  optim2  slfcont1  slfcont2  slfcont3  slfcont4  urban
##   4   10  age  children  cnarc1  cnarc2  cnarc3  CRT1  CRT2  CRT3  employ_status1  health_cond  know_tested_positive  ladder  marital1  mor_circle  moralid1  moralid10  moralid2  moralid3  moralid4  moralid5  moralid6  moralid7  moralid8  moralid9  mcoop1  mcoop2  mcoop3  mcoop4  mcoop5  mcoop6  mcoop7  narc1  narc2  narc3  narc4  narc5  narc6  nidentity1  nidentity2  omind1  omind2  omind3  omind4  omind5  omind6  contact1  contact2  contact3  contact4  contact5  hygiene1  hygiene2  hygiene3  hygiene4  hygiene5  psupport1  psupport2  psupport3  psupport4  psupport5  political_ideology  happy  slf_ladder  riskperc1  riskperc2  self_esteem  sbelong3  tested_positive  optim1  optim2  slfcont1  slfcont2  slfcont3  slfcont4  urban
##   5   1  age  children  cnarc1  cnarc2  cnarc3  CRT1  CRT2  CRT3  employ_status1  health_cond  know_tested_positive  ladder  marital1  mor_circle  moralid1  moralid10  moralid2  moralid3  moralid4  moralid5  moralid6  moralid7  moralid8  moralid9  mcoop1  mcoop2  mcoop3  mcoop4  mcoop5  mcoop6  mcoop7  narc1  narc2  narc3  narc4  narc5  narc6  nidentity1  nidentity2  omind1  omind2  omind3  omind4  omind5  omind6  contact1  contact2  contact3  contact4  contact5  hygiene1  hygiene2  hygiene3  hygiene4  hygiene5  psupport1  psupport2  psupport3  psupport4  psupport5  political_ideology  happy  slf_ladder  riskperc1  riskperc2  self_esteem  sbelong3  tested_positive  optim1  optim2  slfcont1  slfcont2  slfcont3  slfcont4  urban
##   5   2  age  children  cnarc1  cnarc2  cnarc3  CRT1  CRT2  CRT3  employ_status1  health_cond  know_tested_positive  ladder  marital1  mor_circle  moralid1  moralid10  moralid2  moralid3  moralid4  moralid5  moralid6  moralid7  moralid8  moralid9  mcoop1  mcoop2  mcoop3  mcoop4  mcoop5  mcoop6  mcoop7  narc1  narc2  narc3  narc4  narc5  narc6  nidentity1  nidentity2  omind1  omind2  omind3  omind4  omind5  omind6  contact1  contact2  contact3  contact4  contact5  hygiene1  hygiene2  hygiene3  hygiene4  hygiene5  psupport1  psupport2  psupport3  psupport4  psupport5  political_ideology  happy  slf_ladder  riskperc1  riskperc2  self_esteem  sbelong3  tested_positive  optim1  optim2  slfcont1  slfcont2  slfcont3  slfcont4  urban
##   5   3  age  children  cnarc1  cnarc2  cnarc3  CRT1  CRT2  CRT3  employ_status1  health_cond  know_tested_positive  ladder  marital1  mor_circle  moralid1  moralid10  moralid2  moralid3  moralid4  moralid5  moralid6  moralid7  moralid8  moralid9  mcoop1  mcoop2  mcoop3  mcoop4  mcoop5  mcoop6  mcoop7  narc1  narc2  narc3  narc4  narc5  narc6  nidentity1  nidentity2  omind1  omind2  omind3  omind4  omind5  omind6  contact1  contact2  contact3  contact4  contact5  hygiene1  hygiene2  hygiene3  hygiene4  hygiene5  psupport1  psupport2  psupport3  psupport4  psupport5  political_ideology  happy  slf_ladder  riskperc1  riskperc2  self_esteem  sbelong3  tested_positive  optim1  optim2  slfcont1  slfcont2  slfcont3  slfcont4  urban
##   5   4  age  children  cnarc1  cnarc2  cnarc3  CRT1  CRT2  CRT3  employ_status1  health_cond  know_tested_positive  ladder  marital1  mor_circle  moralid1  moralid10  moralid2  moralid3  moralid4  moralid5  moralid6  moralid7  moralid8  moralid9  mcoop1  mcoop2  mcoop3  mcoop4  mcoop5  mcoop6  mcoop7  narc1  narc2  narc3  narc4  narc5  narc6  nidentity1  nidentity2  omind1  omind2  omind3  omind4  omind5  omind6  contact1  contact2  contact3  contact4  contact5  hygiene1  hygiene2  hygiene3  hygiene4  hygiene5  psupport1  psupport2  psupport3  psupport4  psupport5  political_ideology  happy  slf_ladder  riskperc1  riskperc2  self_esteem  sbelong3  tested_positive  optim1  optim2  slfcont1  slfcont2  slfcont3  slfcont4  urban
##   5   5  age  children  cnarc1  cnarc2  cnarc3  CRT1  CRT2  CRT3  employ_status1  health_cond  know_tested_positive  ladder  marital1  mor_circle  moralid1  moralid10  moralid2  moralid3  moralid4  moralid5  moralid6  moralid7  moralid8  moralid9  mcoop1  mcoop2  mcoop3  mcoop4  mcoop5  mcoop6  mcoop7  narc1  narc2  narc3  narc4  narc5  narc6  nidentity1  nidentity2  omind1  omind2  omind3  omind4  omind5  omind6  contact1  contact2  contact3  contact4  contact5  hygiene1  hygiene2  hygiene3  hygiene4  hygiene5  psupport1  psupport2  psupport3  psupport4  psupport5  political_ideology  happy  slf_ladder  riskperc1  riskperc2  self_esteem  sbelong3  tested_positive  optim1  optim2  slfcont1  slfcont2  slfcont3  slfcont4  urban
##   5   6  age  children  cnarc1  cnarc2  cnarc3  CRT1  CRT2  CRT3  employ_status1  health_cond  know_tested_positive  ladder  marital1  mor_circle  moralid1  moralid10  moralid2  moralid3  moralid4  moralid5  moralid6  moralid7  moralid8  moralid9  mcoop1  mcoop2  mcoop3  mcoop4  mcoop5  mcoop6  mcoop7  narc1  narc2  narc3  narc4  narc5  narc6  nidentity1  nidentity2  omind1  omind2  omind3  omind4  omind5  omind6  contact1  contact2  contact3  contact4  contact5  hygiene1  hygiene2  hygiene3  hygiene4  hygiene5  psupport1  psupport2  psupport3  psupport4  psupport5  political_ideology  happy  slf_ladder  riskperc1  riskperc2  self_esteem  sbelong3  tested_positive  optim1  optim2  slfcont1  slfcont2  slfcont3  slfcont4  urban
##   5   7  age  children  cnarc1  cnarc2  cnarc3  CRT1  CRT2  CRT3  employ_status1  health_cond  know_tested_positive  ladder  marital1  mor_circle  moralid1  moralid10  moralid2  moralid3  moralid4  moralid5  moralid6  moralid7  moralid8  moralid9  mcoop1  mcoop2  mcoop3  mcoop4  mcoop5  mcoop6  mcoop7  narc1  narc2  narc3  narc4  narc5  narc6  nidentity1  nidentity2  omind1  omind2  omind3  omind4  omind5  omind6  contact1  contact2  contact3  contact4  contact5  hygiene1  hygiene2  hygiene3  hygiene4  hygiene5  psupport1  psupport2  psupport3  psupport4  psupport5  political_ideology  happy  slf_ladder  riskperc1  riskperc2  self_esteem  sbelong3  tested_positive  optim1  optim2  slfcont1  slfcont2  slfcont3  slfcont4  urban
##   5   8  age  children  cnarc1  cnarc2  cnarc3  CRT1  CRT2  CRT3  employ_status1  health_cond  know_tested_positive  ladder  marital1  mor_circle  moralid1  moralid10  moralid2  moralid3  moralid4  moralid5  moralid6  moralid7  moralid8  moralid9  mcoop1  mcoop2  mcoop3  mcoop4  mcoop5  mcoop6  mcoop7  narc1  narc2  narc3  narc4  narc5  narc6  nidentity1  nidentity2  omind1  omind2  omind3  omind4  omind5  omind6  contact1  contact2  contact3  contact4  contact5  hygiene1  hygiene2  hygiene3  hygiene4  hygiene5  psupport1  psupport2  psupport3  psupport4  psupport5  political_ideology  happy  slf_ladder  riskperc1  riskperc2  self_esteem  sbelong3  tested_positive  optim1  optim2  slfcont1  slfcont2  slfcont3  slfcont4  urban
##   5   9  age  children  cnarc1  cnarc2  cnarc3  CRT1  CRT2  CRT3  employ_status1  health_cond  know_tested_positive  ladder  marital1  mor_circle  moralid1  moralid10  moralid2  moralid3  moralid4  moralid5  moralid6  moralid7  moralid8  moralid9  mcoop1  mcoop2  mcoop3  mcoop4  mcoop5  mcoop6  mcoop7  narc1  narc2  narc3  narc4  narc5  narc6  nidentity1  nidentity2  omind1  omind2  omind3  omind4  omind5  omind6  contact1  contact2  contact3  contact4  contact5  hygiene1  hygiene2  hygiene3  hygiene4  hygiene5  psupport1  psupport2  psupport3  psupport4  psupport5  political_ideology  happy  slf_ladder  riskperc1  riskperc2  self_esteem  sbelong3  tested_positive  optim1  optim2  slfcont1  slfcont2  slfcont3  slfcont4  urban
##   5   10  age  children  cnarc1  cnarc2  cnarc3  CRT1  CRT2  CRT3  employ_status1  health_cond  know_tested_positive  ladder  marital1  mor_circle  moralid1  moralid10  moralid2  moralid3  moralid4  moralid5  moralid6  moralid7  moralid8  moralid9  mcoop1  mcoop2  mcoop3  mcoop4  mcoop5  mcoop6  mcoop7  narc1  narc2  narc3  narc4  narc5  narc6  nidentity1  nidentity2  omind1  omind2  omind3  omind4  omind5  omind6  contact1  contact2  contact3  contact4  contact5  hygiene1  hygiene2  hygiene3  hygiene4  hygiene5  psupport1  psupport2  psupport3  psupport4  psupport5  political_ideology  happy  slf_ladder  riskperc1  riskperc2  self_esteem  sbelong3  tested_positive  optim1  optim2  slfcont1  slfcont2  slfcont3  slfcont4  urban
##   6   1  age  children  cnarc1  cnarc2  cnarc3  CRT1  CRT2  CRT3  employ_status1  health_cond  know_tested_positive  ladder  marital1  mor_circle  moralid1  moralid10  moralid2  moralid3  moralid4  moralid5  moralid6  moralid7  moralid8  moralid9  mcoop1  mcoop2  mcoop3  mcoop4  mcoop5  mcoop6  mcoop7  narc1  narc2  narc3  narc4  narc5  narc6  nidentity1  nidentity2  omind1  omind2  omind3  omind4  omind5  omind6  contact1  contact2  contact3  contact4  contact5  hygiene1  hygiene2  hygiene3  hygiene4  hygiene5  psupport1  psupport2  psupport3  psupport4  psupport5  political_ideology  happy  slf_ladder  riskperc1  riskperc2  self_esteem  sbelong3  tested_positive  optim1  optim2  slfcont1  slfcont2  slfcont3  slfcont4  urban
##   6   2  age  children  cnarc1  cnarc2  cnarc3  CRT1  CRT2  CRT3  employ_status1  health_cond  know_tested_positive  ladder  marital1  mor_circle  moralid1  moralid10  moralid2  moralid3  moralid4  moralid5  moralid6  moralid7  moralid8  moralid9  mcoop1  mcoop2  mcoop3  mcoop4  mcoop5  mcoop6  mcoop7  narc1  narc2  narc3  narc4  narc5  narc6  nidentity1  nidentity2  omind1  omind2  omind3  omind4  omind5  omind6  contact1  contact2  contact3  contact4  contact5  hygiene1  hygiene2  hygiene3  hygiene4  hygiene5  psupport1  psupport2  psupport3  psupport4  psupport5  political_ideology  happy  slf_ladder  riskperc1  riskperc2  self_esteem  sbelong3  tested_positive  optim1  optim2  slfcont1  slfcont2  slfcont3  slfcont4  urban
##   6   3  age  children  cnarc1  cnarc2  cnarc3  CRT1  CRT2  CRT3  employ_status1  health_cond  know_tested_positive  ladder  marital1  mor_circle  moralid1  moralid10  moralid2  moralid3  moralid4  moralid5  moralid6  moralid7  moralid8  moralid9  mcoop1  mcoop2  mcoop3  mcoop4  mcoop5  mcoop6  mcoop7  narc1  narc2  narc3  narc4  narc5  narc6  nidentity1  nidentity2  omind1  omind2  omind3  omind4  omind5  omind6  contact1  contact2  contact3  contact4  contact5  hygiene1  hygiene2  hygiene3  hygiene4  hygiene5  psupport1  psupport2  psupport3  psupport4  psupport5  political_ideology  happy  slf_ladder  riskperc1  riskperc2  self_esteem  sbelong3  tested_positive  optim1  optim2  slfcont1  slfcont2  slfcont3  slfcont4  urban
##   6   4  age  children  cnarc1  cnarc2  cnarc3  CRT1  CRT2  CRT3  employ_status1  health_cond  know_tested_positive  ladder  marital1  mor_circle  moralid1  moralid10  moralid2  moralid3  moralid4  moralid5  moralid6  moralid7  moralid8  moralid9  mcoop1  mcoop2  mcoop3  mcoop4  mcoop5  mcoop6  mcoop7  narc1  narc2  narc3  narc4  narc5  narc6  nidentity1  nidentity2  omind1  omind2  omind3  omind4  omind5  omind6  contact1  contact2  contact3  contact4  contact5  hygiene1  hygiene2  hygiene3  hygiene4  hygiene5  psupport1  psupport2  psupport3  psupport4  psupport5  political_ideology  happy  slf_ladder  riskperc1  riskperc2  self_esteem  sbelong3  tested_positive  optim1  optim2  slfcont1  slfcont2  slfcont3  slfcont4  urban
##   6   5  age  children  cnarc1  cnarc2  cnarc3  CRT1  CRT2  CRT3  employ_status1  health_cond  know_tested_positive  ladder  marital1  mor_circle  moralid1  moralid10  moralid2  moralid3  moralid4  moralid5  moralid6  moralid7  moralid8  moralid9  mcoop1  mcoop2  mcoop3  mcoop4  mcoop5  mcoop6  mcoop7  narc1  narc2  narc3  narc4  narc5  narc6  nidentity1  nidentity2  omind1  omind2  omind3  omind4  omind5  omind6  contact1  contact2  contact3  contact4  contact5  hygiene1  hygiene2  hygiene3  hygiene4  hygiene5  psupport1  psupport2  psupport3  psupport4  psupport5  political_ideology  happy  slf_ladder  riskperc1  riskperc2  self_esteem  sbelong3  tested_positive  optim1  optim2  slfcont1  slfcont2  slfcont3  slfcont4  urban
##   6   6  age  children  cnarc1  cnarc2  cnarc3  CRT1  CRT2  CRT3  employ_status1  health_cond  know_tested_positive  ladder  marital1  mor_circle  moralid1  moralid10  moralid2  moralid3  moralid4  moralid5  moralid6  moralid7  moralid8  moralid9  mcoop1  mcoop2  mcoop3  mcoop4  mcoop5  mcoop6  mcoop7  narc1  narc2  narc3  narc4  narc5  narc6  nidentity1  nidentity2  omind1  omind2  omind3  omind4  omind5  omind6  contact1  contact2  contact3  contact4  contact5  hygiene1  hygiene2  hygiene3  hygiene4  hygiene5  psupport1  psupport2  psupport3  psupport4  psupport5  political_ideology  happy  slf_ladder  riskperc1  riskperc2  self_esteem  sbelong3  tested_positive  optim1  optim2  slfcont1  slfcont2  slfcont3  slfcont4  urban
##   6   7  age  children  cnarc1  cnarc2  cnarc3  CRT1  CRT2  CRT3  employ_status1  health_cond  know_tested_positive  ladder  marital1  mor_circle  moralid1  moralid10  moralid2  moralid3  moralid4  moralid5  moralid6  moralid7  moralid8  moralid9  mcoop1  mcoop2  mcoop3  mcoop4  mcoop5  mcoop6  mcoop7  narc1  narc2  narc3  narc4  narc5  narc6  nidentity1  nidentity2  omind1  omind2  omind3  omind4  omind5  omind6  contact1  contact2  contact3  contact4  contact5  hygiene1  hygiene2  hygiene3  hygiene4  hygiene5  psupport1  psupport2  psupport3  psupport4  psupport5  political_ideology  happy  slf_ladder  riskperc1  riskperc2  self_esteem  sbelong3  tested_positive  optim1  optim2  slfcont1  slfcont2  slfcont3  slfcont4  urban
##   6   8  age  children  cnarc1  cnarc2  cnarc3  CRT1  CRT2  CRT3  employ_status1  health_cond  know_tested_positive  ladder  marital1  mor_circle  moralid1  moralid10  moralid2  moralid3  moralid4  moralid5  moralid6  moralid7  moralid8  moralid9  mcoop1  mcoop2  mcoop3  mcoop4  mcoop5  mcoop6  mcoop7  narc1  narc2  narc3  narc4  narc5  narc6  nidentity1  nidentity2  omind1  omind2  omind3  omind4  omind5  omind6  contact1  contact2  contact3  contact4  contact5  hygiene1  hygiene2  hygiene3  hygiene4  hygiene5  psupport1  psupport2  psupport3  psupport4  psupport5  political_ideology  happy  slf_ladder  riskperc1  riskperc2  self_esteem  sbelong3  tested_positive  optim1  optim2  slfcont1  slfcont2  slfcont3  slfcont4  urban
##   6   9  age  children  cnarc1  cnarc2  cnarc3  CRT1  CRT2  CRT3  employ_status1  health_cond  know_tested_positive  ladder  marital1  mor_circle  moralid1  moralid10  moralid2  moralid3  moralid4  moralid5  moralid6  moralid7  moralid8  moralid9  mcoop1  mcoop2  mcoop3  mcoop4  mcoop5  mcoop6  mcoop7  narc1  narc2  narc3  narc4  narc5  narc6  nidentity1  nidentity2  omind1  omind2  omind3  omind4  omind5  omind6  contact1  contact2  contact3  contact4  contact5  hygiene1  hygiene2  hygiene3  hygiene4  hygiene5  psupport1  psupport2  psupport3  psupport4  psupport5  political_ideology  happy  slf_ladder  riskperc1  riskperc2  self_esteem  sbelong3  tested_positive  optim1  optim2  slfcont1  slfcont2  slfcont3  slfcont4  urban
##   6   10  age  children  cnarc1  cnarc2  cnarc3  CRT1  CRT2  CRT3  employ_status1  health_cond  know_tested_positive  ladder  marital1  mor_circle  moralid1  moralid10  moralid2  moralid3  moralid4  moralid5  moralid6  moralid7  moralid8  moralid9  mcoop1  mcoop2  mcoop3  mcoop4  mcoop5  mcoop6  mcoop7  narc1  narc2  narc3  narc4  narc5  narc6  nidentity1  nidentity2  omind1  omind2  omind3  omind4  omind5  omind6  contact1  contact2  contact3  contact4  contact5  hygiene1  hygiene2  hygiene3  hygiene4  hygiene5  psupport1  psupport2  psupport3  psupport4  psupport5  political_ideology  happy  slf_ladder  riskperc1  riskperc2  self_esteem  sbelong3  tested_positive  optim1  optim2  slfcont1  slfcont2  slfcont3  slfcont4  urban
##   7   1  age  children  cnarc1  cnarc2  cnarc3  CRT1  CRT2  CRT3  employ_status1  health_cond  know_tested_positive  ladder  marital1  mor_circle  moralid1  moralid10  moralid2  moralid3  moralid4  moralid5  moralid6  moralid7  moralid8  moralid9  mcoop1  mcoop2  mcoop3  mcoop4  mcoop5  mcoop6  mcoop7  narc1  narc2  narc3  narc4  narc5  narc6  nidentity1  nidentity2  omind1  omind2  omind3  omind4  omind5  omind6  contact1  contact2  contact3  contact4  contact5  hygiene1  hygiene2  hygiene3  hygiene4  hygiene5  psupport1  psupport2  psupport3  psupport4  psupport5  political_ideology  happy  slf_ladder  riskperc1  riskperc2  self_esteem  sbelong3  tested_positive  optim1  optim2  slfcont1  slfcont2  slfcont3  slfcont4  urban
##   7   2  age  children  cnarc1  cnarc2  cnarc3  CRT1  CRT2  CRT3  employ_status1  health_cond  know_tested_positive  ladder  marital1  mor_circle  moralid1  moralid10  moralid2  moralid3  moralid4  moralid5  moralid6  moralid7  moralid8  moralid9  mcoop1  mcoop2  mcoop3  mcoop4  mcoop5  mcoop6  mcoop7  narc1  narc2  narc3  narc4  narc5  narc6  nidentity1  nidentity2  omind1  omind2  omind3  omind4  omind5  omind6  contact1  contact2  contact3  contact4  contact5  hygiene1  hygiene2  hygiene3  hygiene4  hygiene5  psupport1  psupport2  psupport3  psupport4  psupport5  political_ideology  happy  slf_ladder  riskperc1  riskperc2  self_esteem  sbelong3  tested_positive  optim1  optim2  slfcont1  slfcont2  slfcont3  slfcont4  urban
##   7   3  age  children  cnarc1  cnarc2  cnarc3  CRT1  CRT2  CRT3  employ_status1  health_cond  know_tested_positive  ladder  marital1  mor_circle  moralid1  moralid10  moralid2  moralid3  moralid4  moralid5  moralid6  moralid7  moralid8  moralid9  mcoop1  mcoop2  mcoop3  mcoop4  mcoop5  mcoop6  mcoop7  narc1  narc2  narc3  narc4  narc5  narc6  nidentity1  nidentity2  omind1  omind2  omind3  omind4  omind5  omind6  contact1  contact2  contact3  contact4  contact5  hygiene1  hygiene2  hygiene3  hygiene4  hygiene5  psupport1  psupport2  psupport3  psupport4  psupport5  political_ideology  happy  slf_ladder  riskperc1  riskperc2  self_esteem  sbelong3  tested_positive  optim1  optim2  slfcont1  slfcont2  slfcont3  slfcont4  urban
##   7   4  age  children  cnarc1  cnarc2  cnarc3  CRT1  CRT2  CRT3  employ_status1  health_cond  know_tested_positive  ladder  marital1  mor_circle  moralid1  moralid10  moralid2  moralid3  moralid4  moralid5  moralid6  moralid7  moralid8  moralid9  mcoop1  mcoop2  mcoop3  mcoop4  mcoop5  mcoop6  mcoop7  narc1  narc2  narc3  narc4  narc5  narc6  nidentity1  nidentity2  omind1  omind2  omind3  omind4  omind5  omind6  contact1  contact2  contact3  contact4  contact5  hygiene1  hygiene2  hygiene3  hygiene4  hygiene5  psupport1  psupport2  psupport3  psupport4  psupport5  political_ideology  happy  slf_ladder  riskperc1  riskperc2  self_esteem  sbelong3  tested_positive  optim1  optim2  slfcont1  slfcont2  slfcont3  slfcont4  urban
##   7   5  age  children  cnarc1  cnarc2  cnarc3  CRT1  CRT2  CRT3  employ_status1  health_cond  know_tested_positive  ladder  marital1  mor_circle  moralid1  moralid10  moralid2  moralid3  moralid4  moralid5  moralid6  moralid7  moralid8  moralid9  mcoop1  mcoop2  mcoop3  mcoop4  mcoop5  mcoop6  mcoop7  narc1  narc2  narc3  narc4  narc5  narc6  nidentity1  nidentity2  omind1  omind2  omind3  omind4  omind5  omind6  contact1  contact2  contact3  contact4  contact5  hygiene1  hygiene2  hygiene3  hygiene4  hygiene5  psupport1  psupport2  psupport3  psupport4  psupport5  political_ideology  happy  slf_ladder  riskperc1  riskperc2  self_esteem  sbelong3  tested_positive  optim1  optim2  slfcont1  slfcont2  slfcont3  slfcont4  urban
##   7   6  age  children  cnarc1  cnarc2  cnarc3  CRT1  CRT2  CRT3  employ_status1  health_cond  know_tested_positive  ladder  marital1  mor_circle  moralid1  moralid10  moralid2  moralid3  moralid4  moralid5  moralid6  moralid7  moralid8  moralid9  mcoop1  mcoop2  mcoop3  mcoop4  mcoop5  mcoop6  mcoop7  narc1  narc2  narc3  narc4  narc5  narc6  nidentity1  nidentity2  omind1  omind2  omind3  omind4  omind5  omind6  contact1  contact2  contact3  contact4  contact5  hygiene1  hygiene2  hygiene3  hygiene4  hygiene5  psupport1  psupport2  psupport3  psupport4  psupport5  political_ideology  happy  slf_ladder  riskperc1  riskperc2  self_esteem  sbelong3  tested_positive  optim1  optim2  slfcont1  slfcont2  slfcont3  slfcont4  urban
##   7   7  age  children  cnarc1  cnarc2  cnarc3  CRT1  CRT2  CRT3  employ_status1  health_cond  know_tested_positive  ladder  marital1  mor_circle  moralid1  moralid10  moralid2  moralid3  moralid4  moralid5  moralid6  moralid7  moralid8  moralid9  mcoop1  mcoop2  mcoop3  mcoop4  mcoop5  mcoop6  mcoop7  narc1  narc2  narc3  narc4  narc5  narc6  nidentity1  nidentity2  omind1  omind2  omind3  omind4  omind5  omind6  contact1  contact2  contact3  contact4  contact5  hygiene1  hygiene2  hygiene3  hygiene4  hygiene5  psupport1  psupport2  psupport3  psupport4  psupport5  political_ideology  happy  slf_ladder  riskperc1  riskperc2  self_esteem  sbelong3  tested_positive  optim1  optim2  slfcont1  slfcont2  slfcont3  slfcont4  urban
##   7   8  age  children  cnarc1  cnarc2  cnarc3  CRT1  CRT2  CRT3  employ_status1  health_cond  know_tested_positive  ladder  marital1  mor_circle  moralid1  moralid10  moralid2  moralid3  moralid4  moralid5  moralid6  moralid7  moralid8  moralid9  mcoop1  mcoop2  mcoop3  mcoop4  mcoop5  mcoop6  mcoop7  narc1  narc2  narc3  narc4  narc5  narc6  nidentity1  nidentity2  omind1  omind2  omind3  omind4  omind5  omind6  contact1  contact2  contact3  contact4  contact5  hygiene1  hygiene2  hygiene3  hygiene4  hygiene5  psupport1  psupport2  psupport3  psupport4  psupport5  political_ideology  happy  slf_ladder  riskperc1  riskperc2  self_esteem  sbelong3  tested_positive  optim1  optim2  slfcont1  slfcont2  slfcont3  slfcont4  urban
##   7   9  age  children  cnarc1  cnarc2  cnarc3  CRT1  CRT2  CRT3  employ_status1  health_cond  know_tested_positive  ladder  marital1  mor_circle  moralid1  moralid10  moralid2  moralid3  moralid4  moralid5  moralid6  moralid7  moralid8  moralid9  mcoop1  mcoop2  mcoop3  mcoop4  mcoop5  mcoop6  mcoop7  narc1  narc2  narc3  narc4  narc5  narc6  nidentity1  nidentity2  omind1  omind2  omind3  omind4  omind5  omind6  contact1  contact2  contact3  contact4  contact5  hygiene1  hygiene2  hygiene3  hygiene4  hygiene5  psupport1  psupport2  psupport3  psupport4  psupport5  political_ideology  happy  slf_ladder  riskperc1  riskperc2  self_esteem  sbelong3  tested_positive  optim1  optim2  slfcont1  slfcont2  slfcont3  slfcont4  urban
##   7   10  age  children  cnarc1  cnarc2  cnarc3  CRT1  CRT2  CRT3  employ_status1  health_cond  know_tested_positive  ladder  marital1  mor_circle  moralid1  moralid10  moralid2  moralid3  moralid4  moralid5  moralid6  moralid7  moralid8  moralid9  mcoop1  mcoop2  mcoop3  mcoop4  mcoop5  mcoop6  mcoop7  narc1  narc2  narc3  narc4  narc5  narc6  nidentity1  nidentity2  omind1  omind2  omind3  omind4  omind5  omind6  contact1  contact2  contact3  contact4  contact5  hygiene1  hygiene2  hygiene3  hygiene4  hygiene5  psupport1  psupport2  psupport3  psupport4  psupport5  political_ideology  happy  slf_ladder  riskperc1  riskperc2  self_esteem  sbelong3  tested_positive  optim1  optim2  slfcont1  slfcont2  slfcont3  slfcont4  urban
##   8   1  age  children  cnarc1  cnarc2  cnarc3  CRT1  CRT2  CRT3  employ_status1  health_cond  know_tested_positive  ladder  marital1  mor_circle  moralid1  moralid10  moralid2  moralid3  moralid4  moralid5  moralid6  moralid7  moralid8  moralid9  mcoop1  mcoop2  mcoop3  mcoop4  mcoop5  mcoop6  mcoop7  narc1  narc2  narc3  narc4  narc5  narc6  nidentity1  nidentity2  omind1  omind2  omind3  omind4  omind5  omind6  contact1  contact2  contact3  contact4  contact5  hygiene1  hygiene2  hygiene3  hygiene4  hygiene5  psupport1  psupport2  psupport3  psupport4  psupport5  political_ideology  happy  slf_ladder  riskperc1  riskperc2  self_esteem  sbelong3  tested_positive  optim1  optim2  slfcont1  slfcont2  slfcont3  slfcont4  urban
##   8   2  age  children  cnarc1  cnarc2  cnarc3  CRT1  CRT2  CRT3  employ_status1  health_cond  know_tested_positive  ladder  marital1  mor_circle  moralid1  moralid10  moralid2  moralid3  moralid4  moralid5  moralid6  moralid7  moralid8  moralid9  mcoop1  mcoop2  mcoop3  mcoop4  mcoop5  mcoop6  mcoop7  narc1  narc2  narc3  narc4  narc5  narc6  nidentity1  nidentity2  omind1  omind2  omind3  omind4  omind5  omind6  contact1  contact2  contact3  contact4  contact5  hygiene1  hygiene2  hygiene3  hygiene4  hygiene5  psupport1  psupport2  psupport3  psupport4  psupport5  political_ideology  happy  slf_ladder  riskperc1  riskperc2  self_esteem  sbelong3  tested_positive  optim1  optim2  slfcont1  slfcont2  slfcont3  slfcont4  urban
##   8   3  age  children  cnarc1  cnarc2  cnarc3  CRT1  CRT2  CRT3  employ_status1  health_cond  know_tested_positive  ladder  marital1  mor_circle  moralid1  moralid10  moralid2  moralid3  moralid4  moralid5  moralid6  moralid7  moralid8  moralid9  mcoop1  mcoop2  mcoop3  mcoop4  mcoop5  mcoop6  mcoop7  narc1  narc2  narc3  narc4  narc5  narc6  nidentity1  nidentity2  omind1  omind2  omind3  omind4  omind5  omind6  contact1  contact2  contact3  contact4  contact5  hygiene1  hygiene2  hygiene3  hygiene4  hygiene5  psupport1  psupport2  psupport3  psupport4  psupport5  political_ideology  happy  slf_ladder  riskperc1  riskperc2  self_esteem  sbelong3  tested_positive  optim1  optim2  slfcont1  slfcont2  slfcont3  slfcont4  urban
##   8   4  age  children  cnarc1  cnarc2  cnarc3  CRT1  CRT2  CRT3  employ_status1  health_cond  know_tested_positive  ladder  marital1  mor_circle  moralid1  moralid10  moralid2  moralid3  moralid4  moralid5  moralid6  moralid7  moralid8  moralid9  mcoop1  mcoop2  mcoop3  mcoop4  mcoop5  mcoop6  mcoop7  narc1  narc2  narc3  narc4  narc5  narc6  nidentity1  nidentity2  omind1  omind2  omind3  omind4  omind5  omind6  contact1  contact2  contact3  contact4  contact5  hygiene1  hygiene2  hygiene3  hygiene4  hygiene5  psupport1  psupport2  psupport3  psupport4  psupport5  political_ideology  happy  slf_ladder  riskperc1  riskperc2  self_esteem  sbelong3  tested_positive  optim1  optim2  slfcont1  slfcont2  slfcont3  slfcont4  urban
##   8   5  age  children  cnarc1  cnarc2  cnarc3  CRT1  CRT2  CRT3  employ_status1  health_cond  know_tested_positive  ladder  marital1  mor_circle  moralid1  moralid10  moralid2  moralid3  moralid4  moralid5  moralid6  moralid7  moralid8  moralid9  mcoop1  mcoop2  mcoop3  mcoop4  mcoop5  mcoop6  mcoop7  narc1  narc2  narc3  narc4  narc5  narc6  nidentity1  nidentity2  omind1  omind2  omind3  omind4  omind5  omind6  contact1  contact2  contact3  contact4  contact5  hygiene1  hygiene2  hygiene3  hygiene4  hygiene5  psupport1  psupport2  psupport3  psupport4  psupport5  political_ideology  happy  slf_ladder  riskperc1  riskperc2  self_esteem  sbelong3  tested_positive  optim1  optim2  slfcont1  slfcont2  slfcont3  slfcont4  urban
##   8   6  age  children  cnarc1  cnarc2  cnarc3  CRT1  CRT2  CRT3  employ_status1  health_cond  know_tested_positive  ladder  marital1  mor_circle  moralid1  moralid10  moralid2  moralid3  moralid4  moralid5  moralid6  moralid7  moralid8  moralid9  mcoop1  mcoop2  mcoop3  mcoop4  mcoop5  mcoop6  mcoop7  narc1  narc2  narc3  narc4  narc5  narc6  nidentity1  nidentity2  omind1  omind2  omind3  omind4  omind5  omind6  contact1  contact2  contact3  contact4  contact5  hygiene1  hygiene2  hygiene3  hygiene4  hygiene5  psupport1  psupport2  psupport3  psupport4  psupport5  political_ideology  happy  slf_ladder  riskperc1  riskperc2  self_esteem  sbelong3  tested_positive  optim1  optim2  slfcont1  slfcont2  slfcont3  slfcont4  urban
##   8   7  age  children  cnarc1  cnarc2  cnarc3  CRT1  CRT2  CRT3  employ_status1  health_cond  know_tested_positive  ladder  marital1  mor_circle  moralid1  moralid10  moralid2  moralid3  moralid4  moralid5  moralid6  moralid7  moralid8  moralid9  mcoop1  mcoop2  mcoop3  mcoop4  mcoop5  mcoop6  mcoop7  narc1  narc2  narc3  narc4  narc5  narc6  nidentity1  nidentity2  omind1  omind2  omind3  omind4  omind5  omind6  contact1  contact2  contact3  contact4  contact5  hygiene1  hygiene2  hygiene3  hygiene4  hygiene5  psupport1  psupport2  psupport3  psupport4  psupport5  political_ideology  happy  slf_ladder  riskperc1  riskperc2  self_esteem  sbelong3  tested_positive  optim1  optim2  slfcont1  slfcont2  slfcont3  slfcont4  urban
##   8   8  age  children  cnarc1  cnarc2  cnarc3  CRT1  CRT2  CRT3  employ_status1  health_cond  know_tested_positive  ladder  marital1  mor_circle  moralid1  moralid10  moralid2  moralid3  moralid4  moralid5  moralid6  moralid7  moralid8  moralid9  mcoop1  mcoop2  mcoop3  mcoop4  mcoop5  mcoop6  mcoop7  narc1  narc2  narc3  narc4  narc5  narc6  nidentity1  nidentity2  omind1  omind2  omind3  omind4  omind5  omind6  contact1  contact2  contact3  contact4  contact5  hygiene1  hygiene2  hygiene3  hygiene4  hygiene5  psupport1  psupport2  psupport3  psupport4  psupport5  political_ideology  happy  slf_ladder  riskperc1  riskperc2  self_esteem  sbelong3  tested_positive  optim1  optim2  slfcont1  slfcont2  slfcont3  slfcont4  urban
##   8   9  age  children  cnarc1  cnarc2  cnarc3  CRT1  CRT2  CRT3  employ_status1  health_cond  know_tested_positive  ladder  marital1  mor_circle  moralid1  moralid10  moralid2  moralid3  moralid4  moralid5  moralid6  moralid7  moralid8  moralid9  mcoop1  mcoop2  mcoop3  mcoop4  mcoop5  mcoop6  mcoop7  narc1  narc2  narc3  narc4  narc5  narc6  nidentity1  nidentity2  omind1  omind2  omind3  omind4  omind5  omind6  contact1  contact2  contact3  contact4  contact5  hygiene1  hygiene2  hygiene3  hygiene4  hygiene5  psupport1  psupport2  psupport3  psupport4  psupport5  political_ideology  happy  slf_ladder  riskperc1  riskperc2  self_esteem  sbelong3  tested_positive  optim1  optim2  slfcont1  slfcont2  slfcont3  slfcont4  urban
##   8   10  age  children  cnarc1  cnarc2  cnarc3  CRT1  CRT2  CRT3  employ_status1  health_cond  know_tested_positive  ladder  marital1  mor_circle  moralid1  moralid10  moralid2  moralid3  moralid4  moralid5  moralid6  moralid7  moralid8  moralid9  mcoop1  mcoop2  mcoop3  mcoop4  mcoop5  mcoop6  mcoop7  narc1  narc2  narc3  narc4  narc5  narc6  nidentity1  nidentity2  omind1  omind2  omind3  omind4  omind5  omind6  contact1  contact2  contact3  contact4  contact5  hygiene1  hygiene2  hygiene3  hygiene4  hygiene5  psupport1  psupport2  psupport3  psupport4  psupport5  political_ideology  happy  slf_ladder  riskperc1  riskperc2  self_esteem  sbelong3  tested_positive  optim1  optim2  slfcont1  slfcont2  slfcont3  slfcont4  urban
##   9   1  age  children  cnarc1  cnarc2  cnarc3  CRT1  CRT2  CRT3  employ_status1  health_cond  know_tested_positive  ladder  marital1  mor_circle  moralid1  moralid10  moralid2  moralid3  moralid4  moralid5  moralid6  moralid7  moralid8  moralid9  mcoop1  mcoop2  mcoop3  mcoop4  mcoop5  mcoop6  mcoop7  narc1  narc2  narc3  narc4  narc5  narc6  nidentity1  nidentity2  omind1  omind2  omind3  omind4  omind5  omind6  contact1  contact2  contact3  contact4  contact5  hygiene1  hygiene2  hygiene3  hygiene4  hygiene5  psupport1  psupport2  psupport3  psupport4  psupport5  political_ideology  happy  slf_ladder  riskperc1  riskperc2  self_esteem  sbelong3  tested_positive  optim1  optim2  slfcont1  slfcont2  slfcont3  slfcont4  urban
##   9   2  age  children  cnarc1  cnarc2  cnarc3  CRT1  CRT2  CRT3  employ_status1  health_cond  know_tested_positive  ladder  marital1  mor_circle  moralid1  moralid10  moralid2  moralid3  moralid4  moralid5  moralid6  moralid7  moralid8  moralid9  mcoop1  mcoop2  mcoop3  mcoop4  mcoop5  mcoop6  mcoop7  narc1  narc2  narc3  narc4  narc5  narc6  nidentity1  nidentity2  omind1  omind2  omind3  omind4  omind5  omind6  contact1  contact2  contact3  contact4  contact5  hygiene1  hygiene2  hygiene3  hygiene4  hygiene5  psupport1  psupport2  psupport3  psupport4  psupport5  political_ideology  happy  slf_ladder  riskperc1  riskperc2  self_esteem  sbelong3  tested_positive  optim1  optim2  slfcont1  slfcont2  slfcont3  slfcont4  urban
##   9   3  age  children  cnarc1  cnarc2  cnarc3  CRT1  CRT2  CRT3  employ_status1  health_cond  know_tested_positive  ladder  marital1  mor_circle  moralid1  moralid10  moralid2  moralid3  moralid4  moralid5  moralid6  moralid7  moralid8  moralid9  mcoop1  mcoop2  mcoop3  mcoop4  mcoop5  mcoop6  mcoop7  narc1  narc2  narc3  narc4  narc5  narc6  nidentity1  nidentity2  omind1  omind2  omind3  omind4  omind5  omind6  contact1  contact2  contact3  contact4  contact5  hygiene1  hygiene2  hygiene3  hygiene4  hygiene5  psupport1  psupport2  psupport3  psupport4  psupport5  political_ideology  happy  slf_ladder  riskperc1  riskperc2  self_esteem  sbelong3  tested_positive  optim1  optim2  slfcont1  slfcont2  slfcont3  slfcont4  urban
##   9   4  age  children  cnarc1  cnarc2  cnarc3  CRT1  CRT2  CRT3  employ_status1  health_cond  know_tested_positive  ladder  marital1  mor_circle  moralid1  moralid10  moralid2  moralid3  moralid4  moralid5  moralid6  moralid7  moralid8  moralid9  mcoop1  mcoop2  mcoop3  mcoop4  mcoop5  mcoop6  mcoop7  narc1  narc2  narc3  narc4  narc5  narc6  nidentity1  nidentity2  omind1  omind2  omind3  omind4  omind5  omind6  contact1  contact2  contact3  contact4  contact5  hygiene1  hygiene2  hygiene3  hygiene4  hygiene5  psupport1  psupport2  psupport3  psupport4  psupport5  political_ideology  happy  slf_ladder  riskperc1  riskperc2  self_esteem  sbelong3  tested_positive  optim1  optim2  slfcont1  slfcont2  slfcont3  slfcont4  urban
##   9   5  age  children  cnarc1  cnarc2  cnarc3  CRT1  CRT2  CRT3  employ_status1  health_cond  know_tested_positive  ladder  marital1  mor_circle  moralid1  moralid10  moralid2  moralid3  moralid4  moralid5  moralid6  moralid7  moralid8  moralid9  mcoop1  mcoop2  mcoop3  mcoop4  mcoop5  mcoop6  mcoop7  narc1  narc2  narc3  narc4  narc5  narc6  nidentity1  nidentity2  omind1  omind2  omind3  omind4  omind5  omind6  contact1  contact2  contact3  contact4  contact5  hygiene1  hygiene2  hygiene3  hygiene4  hygiene5  psupport1  psupport2  psupport3  psupport4  psupport5  political_ideology  happy  slf_ladder  riskperc1  riskperc2  self_esteem  sbelong3  tested_positive  optim1  optim2  slfcont1  slfcont2  slfcont3  slfcont4  urban
##   9   6  age  children  cnarc1  cnarc2  cnarc3  CRT1  CRT2  CRT3  employ_status1  health_cond  know_tested_positive  ladder  marital1  mor_circle  moralid1  moralid10  moralid2  moralid3  moralid4  moralid5  moralid6  moralid7  moralid8  moralid9  mcoop1  mcoop2  mcoop3  mcoop4  mcoop5  mcoop6  mcoop7  narc1  narc2  narc3  narc4  narc5  narc6  nidentity1  nidentity2  omind1  omind2  omind3  omind4  omind5  omind6  contact1  contact2  contact3  contact4  contact5  hygiene1  hygiene2  hygiene3  hygiene4  hygiene5  psupport1  psupport2  psupport3  psupport4  psupport5  political_ideology  happy  slf_ladder  riskperc1  riskperc2  self_esteem  sbelong3  tested_positive  optim1  optim2  slfcont1  slfcont2  slfcont3  slfcont4  urban
##   9   7  age  children  cnarc1  cnarc2  cnarc3  CRT1  CRT2  CRT3  employ_status1  health_cond  know_tested_positive  ladder  marital1  mor_circle  moralid1  moralid10  moralid2  moralid3  moralid4  moralid5  moralid6  moralid7  moralid8  moralid9  mcoop1  mcoop2  mcoop3  mcoop4  mcoop5  mcoop6  mcoop7  narc1  narc2  narc3  narc4  narc5  narc6  nidentity1  nidentity2  omind1  omind2  omind3  omind4  omind5  omind6  contact1  contact2  contact3  contact4  contact5  hygiene1  hygiene2  hygiene3  hygiene4  hygiene5  psupport1  psupport2  psupport3  psupport4  psupport5  political_ideology  happy  slf_ladder  riskperc1  riskperc2  self_esteem  sbelong3  tested_positive  optim1  optim2  slfcont1  slfcont2  slfcont3  slfcont4  urban
##   9   8  age  children  cnarc1  cnarc2  cnarc3  CRT1  CRT2  CRT3  employ_status1  health_cond  know_tested_positive  ladder  marital1  mor_circle  moralid1  moralid10  moralid2  moralid3  moralid4  moralid5  moralid6  moralid7  moralid8  moralid9  mcoop1  mcoop2  mcoop3  mcoop4  mcoop5  mcoop6  mcoop7  narc1  narc2  narc3  narc4  narc5  narc6  nidentity1  nidentity2  omind1  omind2  omind3  omind4  omind5  omind6  contact1  contact2  contact3  contact4  contact5  hygiene1  hygiene2  hygiene3  hygiene4  hygiene5  psupport1  psupport2  psupport3  psupport4  psupport5  political_ideology  happy  slf_ladder  riskperc1  riskperc2  self_esteem  sbelong3  tested_positive  optim1  optim2  slfcont1  slfcont2  slfcont3  slfcont4  urban
##   9   9  age  children  cnarc1  cnarc2  cnarc3  CRT1  CRT2  CRT3  employ_status1  health_cond  know_tested_positive  ladder  marital1  mor_circle  moralid1  moralid10  moralid2  moralid3  moralid4  moralid5  moralid6  moralid7  moralid8  moralid9  mcoop1  mcoop2  mcoop3  mcoop4  mcoop5  mcoop6  mcoop7  narc1  narc2  narc3  narc4  narc5  narc6  nidentity1  nidentity2  omind1  omind2  omind3  omind4  omind5  omind6  contact1  contact2  contact3  contact4  contact5  hygiene1  hygiene2  hygiene3  hygiene4  hygiene5  psupport1  psupport2  psupport3  psupport4  psupport5  political_ideology  happy  slf_ladder  riskperc1  riskperc2  self_esteem  sbelong3  tested_positive  optim1  optim2  slfcont1  slfcont2  slfcont3  slfcont4  urban
##   9   10  age  children  cnarc1  cnarc2  cnarc3  CRT1  CRT2  CRT3  employ_status1  health_cond  know_tested_positive  ladder  marital1  mor_circle  moralid1  moralid10  moralid2  moralid3  moralid4  moralid5  moralid6  moralid7  moralid8  moralid9  mcoop1  mcoop2  mcoop3  mcoop4  mcoop5  mcoop6  mcoop7  narc1  narc2  narc3  narc4  narc5  narc6  nidentity1  nidentity2  omind1  omind2  omind3  omind4  omind5  omind6  contact1  contact2  contact3  contact4  contact5  hygiene1  hygiene2  hygiene3  hygiene4  hygiene5  psupport1  psupport2  psupport3  psupport4  psupport5  political_ideology  happy  slf_ladder  riskperc1  riskperc2  self_esteem  sbelong3  tested_positive  optim1  optim2  slfcont1  slfcont2  slfcont3  slfcont4  urban
##   10   1  age  children  cnarc1  cnarc2  cnarc3  CRT1  CRT2  CRT3  employ_status1  health_cond  know_tested_positive  ladder  marital1  mor_circle  moralid1  moralid10  moralid2  moralid3  moralid4  moralid5  moralid6  moralid7  moralid8  moralid9  mcoop1  mcoop2  mcoop3  mcoop4  mcoop5  mcoop6  mcoop7  narc1  narc2  narc3  narc4  narc5  narc6  nidentity1  nidentity2  omind1  omind2  omind3  omind4  omind5  omind6  contact1  contact2  contact3  contact4  contact5  hygiene1  hygiene2  hygiene3  hygiene4  hygiene5  psupport1  psupport2  psupport3  psupport4  psupport5  political_ideology  happy  slf_ladder  riskperc1  riskperc2  self_esteem  sbelong3  tested_positive  optim1  optim2  slfcont1  slfcont2  slfcont3  slfcont4  urban
##   10   2  age  children  cnarc1  cnarc2  cnarc3  CRT1  CRT2  CRT3  employ_status1  health_cond  know_tested_positive  ladder  marital1  mor_circle  moralid1  moralid10  moralid2  moralid3  moralid4  moralid5  moralid6  moralid7  moralid8  moralid9  mcoop1  mcoop2  mcoop3  mcoop4  mcoop5  mcoop6  mcoop7  narc1  narc2  narc3  narc4  narc5  narc6  nidentity1  nidentity2  omind1  omind2  omind3  omind4  omind5  omind6  contact1  contact2  contact3  contact4  contact5  hygiene1  hygiene2  hygiene3  hygiene4  hygiene5  psupport1  psupport2  psupport3  psupport4  psupport5  political_ideology  happy  slf_ladder  riskperc1  riskperc2  self_esteem  sbelong3  tested_positive  optim1  optim2  slfcont1  slfcont2  slfcont3  slfcont4  urban
##   10   3  age  children  cnarc1  cnarc2  cnarc3  CRT1  CRT2  CRT3  employ_status1  health_cond  know_tested_positive  ladder  marital1  mor_circle  moralid1  moralid10  moralid2  moralid3  moralid4  moralid5  moralid6  moralid7  moralid8  moralid9  mcoop1  mcoop2  mcoop3  mcoop4  mcoop5  mcoop6  mcoop7  narc1  narc2  narc3  narc4  narc5  narc6  nidentity1  nidentity2  omind1  omind2  omind3  omind4  omind5  omind6  contact1  contact2  contact3  contact4  contact5  hygiene1  hygiene2  hygiene3  hygiene4  hygiene5  psupport1  psupport2  psupport3  psupport4  psupport5  political_ideology  happy  slf_ladder  riskperc1  riskperc2  self_esteem  sbelong3  tested_positive  optim1  optim2  slfcont1  slfcont2  slfcont3  slfcont4  urban
##   10   4  age  children  cnarc1  cnarc2  cnarc3  CRT1  CRT2  CRT3  employ_status1  health_cond  know_tested_positive  ladder  marital1  mor_circle  moralid1  moralid10  moralid2  moralid3  moralid4  moralid5  moralid6  moralid7  moralid8  moralid9  mcoop1  mcoop2  mcoop3  mcoop4  mcoop5  mcoop6  mcoop7  narc1  narc2  narc3  narc4  narc5  narc6  nidentity1  nidentity2  omind1  omind2  omind3  omind4  omind5  omind6  contact1  contact2  contact3  contact4  contact5  hygiene1  hygiene2  hygiene3  hygiene4  hygiene5  psupport1  psupport2  psupport3  psupport4  psupport5  political_ideology  happy  slf_ladder  riskperc1  riskperc2  self_esteem  sbelong3  tested_positive  optim1  optim2  slfcont1  slfcont2  slfcont3  slfcont4  urban
##   10   5  age  children  cnarc1  cnarc2  cnarc3  CRT1  CRT2  CRT3  employ_status1  health_cond  know_tested_positive  ladder  marital1  mor_circle  moralid1  moralid10  moralid2  moralid3  moralid4  moralid5  moralid6  moralid7  moralid8  moralid9  mcoop1  mcoop2  mcoop3  mcoop4  mcoop5  mcoop6  mcoop7  narc1  narc2  narc3  narc4  narc5  narc6  nidentity1  nidentity2  omind1  omind2  omind3  omind4  omind5  omind6  contact1  contact2  contact3  contact4  contact5  hygiene1  hygiene2  hygiene3  hygiene4  hygiene5  psupport1  psupport2  psupport3  psupport4  psupport5  political_ideology  happy  slf_ladder  riskperc1  riskperc2  self_esteem  sbelong3  tested_positive  optim1  optim2  slfcont1  slfcont2  slfcont3  slfcont4  urban
##   10   6  age  children  cnarc1  cnarc2  cnarc3  CRT1  CRT2  CRT3  employ_status1  health_cond  know_tested_positive  ladder  marital1  mor_circle  moralid1  moralid10  moralid2  moralid3  moralid4  moralid5  moralid6  moralid7  moralid8  moralid9  mcoop1  mcoop2  mcoop3  mcoop4  mcoop5  mcoop6  mcoop7  narc1  narc2  narc3  narc4  narc5  narc6  nidentity1  nidentity2  omind1  omind2  omind3  omind4  omind5  omind6  contact1  contact2  contact3  contact4  contact5  hygiene1  hygiene2  hygiene3  hygiene4  hygiene5  psupport1  psupport2  psupport3  psupport4  psupport5  political_ideology  happy  slf_ladder  riskperc1  riskperc2  self_esteem  sbelong3  tested_positive  optim1  optim2  slfcont1  slfcont2  slfcont3  slfcont4  urban
##   10   7  age  children  cnarc1  cnarc2  cnarc3  CRT1  CRT2  CRT3  employ_status1  health_cond  know_tested_positive  ladder  marital1  mor_circle  moralid1  moralid10  moralid2  moralid3  moralid4  moralid5  moralid6  moralid7  moralid8  moralid9  mcoop1  mcoop2  mcoop3  mcoop4  mcoop5  mcoop6  mcoop7  narc1  narc2  narc3  narc4  narc5  narc6  nidentity1  nidentity2  omind1  omind2  omind3  omind4  omind5  omind6  contact1  contact2  contact3  contact4  contact5  hygiene1  hygiene2  hygiene3  hygiene4  hygiene5  psupport1  psupport2  psupport3  psupport4  psupport5  political_ideology  happy  slf_ladder  riskperc1  riskperc2  self_esteem  sbelong3  tested_positive  optim1  optim2  slfcont1  slfcont2  slfcont3  slfcont4  urban
##   10   8  age  children  cnarc1  cnarc2  cnarc3  CRT1  CRT2  CRT3  employ_status1  health_cond  know_tested_positive  ladder  marital1  mor_circle  moralid1  moralid10  moralid2  moralid3  moralid4  moralid5  moralid6  moralid7  moralid8  moralid9  mcoop1  mcoop2  mcoop3  mcoop4  mcoop5  mcoop6  mcoop7  narc1  narc2  narc3  narc4  narc5  narc6  nidentity1  nidentity2  omind1  omind2  omind3  omind4  omind5  omind6  contact1  contact2  contact3  contact4  contact5  hygiene1  hygiene2  hygiene3  hygiene4  hygiene5  psupport1  psupport2  psupport3  psupport4  psupport5  political_ideology  happy  slf_ladder  riskperc1  riskperc2  self_esteem  sbelong3  tested_positive  optim1  optim2  slfcont1  slfcont2  slfcont3  slfcont4  urban
##   10   9  age  children  cnarc1  cnarc2  cnarc3  CRT1  CRT2  CRT3  employ_status1  health_cond  know_tested_positive  ladder  marital1  mor_circle  moralid1  moralid10  moralid2  moralid3  moralid4  moralid5  moralid6  moralid7  moralid8  moralid9  mcoop1  mcoop2  mcoop3  mcoop4  mcoop5  mcoop6  mcoop7  narc1  narc2  narc3  narc4  narc5  narc6  nidentity1  nidentity2  omind1  omind2  omind3  omind4  omind5  omind6  contact1  contact2  contact3  contact4  contact5  hygiene1  hygiene2  hygiene3  hygiene4  hygiene5  psupport1  psupport2  psupport3  psupport4  psupport5  political_ideology  happy  slf_ladder  riskperc1  riskperc2  self_esteem  sbelong3  tested_positive  optim1  optim2  slfcont1  slfcont2  slfcont3  slfcont4  urban
##   10   10  age  children  cnarc1  cnarc2  cnarc3  CRT1  CRT2  CRT3  employ_status1  health_cond  know_tested_positive  ladder  marital1  mor_circle  moralid1  moralid10  moralid2  moralid3  moralid4  moralid5  moralid6  moralid7  moralid8  moralid9  mcoop1  mcoop2  mcoop3  mcoop4  mcoop5  mcoop6  mcoop7  narc1  narc2  narc3  narc4  narc5  narc6  nidentity1  nidentity2  omind1  omind2  omind3  omind4  omind5  omind6  contact1  contact2  contact3  contact4  contact5  hygiene1  hygiene2  hygiene3  hygiene4  hygiene5  psupport1  psupport2  psupport3  psupport4  psupport5  political_ideology  happy  slf_ladder  riskperc1  riskperc2  self_esteem  sbelong3  tested_positive  optim1  optim2  slfcont1  slfcont2  slfcont3  slfcont4  urban
```

```
## Warning: Number of logged events: 7504
```

```
## 
##  iter imp variable
##   1   1  age  children  cnarc1  cnarc2  cnarc3  ctheory2  ctheory3  ctheory4  CRT1  CRT2  CRT3  employ_status1  health_cond  ladder  marital1  mor_circle  moralid1  moralid10  moralid3  moralid4  moralid5  moralid6  moralid7  moralid8  moralid9  mcoop1  mcoop2  mcoop3  mcoop4  mcoop5  mcoop6  mcoop7  narc1  narc2  narc3  narc4  narc5  narc6  nidentity2  omind1  omind3  omind4  omind5  contact3  hygiene3  hygiene5  political_ideology  happy  slf_ladder  riskperc1  riskperc2  self_esteem  sbelong1  sbelong2  sbelong3  sbelong4  optim1  optim2  slfcont1  slfcont3  slfcont4
##   1   2  age  children  cnarc1  cnarc2  cnarc3  ctheory2  ctheory3  ctheory4  CRT1  CRT2  CRT3  employ_status1  health_cond  ladder  marital1  mor_circle  moralid1  moralid10  moralid3  moralid4  moralid5  moralid6  moralid7  moralid8  moralid9  mcoop1  mcoop2  mcoop3  mcoop4  mcoop5  mcoop6  mcoop7  narc1  narc2  narc3  narc4  narc5  narc6  nidentity2  omind1  omind3  omind4  omind5  contact3  hygiene3  hygiene5  political_ideology  happy  slf_ladder  riskperc1  riskperc2  self_esteem  sbelong1  sbelong2  sbelong3  sbelong4  optim1  optim2  slfcont1  slfcont3  slfcont4
##   1   3  age  children  cnarc1  cnarc2  cnarc3  ctheory2  ctheory3  ctheory4  CRT1  CRT2  CRT3  employ_status1  health_cond  ladder  marital1  mor_circle  moralid1  moralid10  moralid3  moralid4  moralid5  moralid6  moralid7  moralid8  moralid9  mcoop1  mcoop2  mcoop3  mcoop4  mcoop5  mcoop6  mcoop7  narc1  narc2  narc3  narc4  narc5  narc6  nidentity2  omind1  omind3  omind4  omind5  contact3  hygiene3  hygiene5  political_ideology  happy  slf_ladder  riskperc1  riskperc2  self_esteem  sbelong1  sbelong2  sbelong3  sbelong4  optim1  optim2  slfcont1  slfcont3  slfcont4
##   1   4  age  children  cnarc1  cnarc2  cnarc3  ctheory2  ctheory3  ctheory4  CRT1  CRT2  CRT3  employ_status1  health_cond  ladder  marital1  mor_circle  moralid1  moralid10  moralid3  moralid4  moralid5  moralid6  moralid7  moralid8  moralid9  mcoop1  mcoop2  mcoop3  mcoop4  mcoop5  mcoop6  mcoop7  narc1  narc2  narc3  narc4  narc5  narc6  nidentity2  omind1  omind3  omind4  omind5  contact3  hygiene3  hygiene5  political_ideology  happy  slf_ladder  riskperc1  riskperc2  self_esteem  sbelong1  sbelong2  sbelong3  sbelong4  optim1  optim2  slfcont1  slfcont3  slfcont4
##   1   5  age  children  cnarc1  cnarc2  cnarc3  ctheory2  ctheory3  ctheory4  CRT1  CRT2  CRT3  employ_status1  health_cond  ladder  marital1  mor_circle  moralid1  moralid10  moralid3  moralid4  moralid5  moralid6  moralid7  moralid8  moralid9  mcoop1  mcoop2  mcoop3  mcoop4  mcoop5  mcoop6  mcoop7  narc1  narc2  narc3  narc4  narc5  narc6  nidentity2  omind1  omind3  omind4  omind5  contact3  hygiene3  hygiene5  political_ideology  happy  slf_ladder  riskperc1  riskperc2  self_esteem  sbelong1  sbelong2  sbelong3  sbelong4  optim1  optim2  slfcont1  slfcont3  slfcont4
##   1   6  age  children  cnarc1  cnarc2  cnarc3  ctheory2  ctheory3  ctheory4  CRT1  CRT2  CRT3  employ_status1  health_cond  ladder  marital1  mor_circle  moralid1  moralid10  moralid3  moralid4  moralid5  moralid6  moralid7  moralid8  moralid9  mcoop1  mcoop2  mcoop3  mcoop4  mcoop5  mcoop6  mcoop7  narc1  narc2  narc3  narc4  narc5  narc6  nidentity2  omind1  omind3  omind4  omind5  contact3  hygiene3  hygiene5  political_ideology  happy  slf_ladder  riskperc1  riskperc2  self_esteem  sbelong1  sbelong2  sbelong3  sbelong4  optim1  optim2  slfcont1  slfcont3  slfcont4
##   1   7  age  children  cnarc1  cnarc2  cnarc3  ctheory2  ctheory3  ctheory4  CRT1  CRT2  CRT3  employ_status1  health_cond  ladder  marital1  mor_circle  moralid1  moralid10  moralid3  moralid4  moralid5  moralid6  moralid7  moralid8  moralid9  mcoop1  mcoop2  mcoop3  mcoop4  mcoop5  mcoop6  mcoop7  narc1  narc2  narc3  narc4  narc5  narc6  nidentity2  omind1  omind3  omind4  omind5  contact3  hygiene3  hygiene5  political_ideology  happy  slf_ladder  riskperc1  riskperc2  self_esteem  sbelong1  sbelong2  sbelong3  sbelong4  optim1  optim2  slfcont1  slfcont3  slfcont4
##   1   8  age  children  cnarc1  cnarc2  cnarc3  ctheory2  ctheory3  ctheory4  CRT1  CRT2  CRT3  employ_status1  health_cond  ladder  marital1  mor_circle  moralid1  moralid10  moralid3  moralid4  moralid5  moralid6  moralid7  moralid8  moralid9  mcoop1  mcoop2  mcoop3  mcoop4  mcoop5  mcoop6  mcoop7  narc1  narc2  narc3  narc4  narc5  narc6  nidentity2  omind1  omind3  omind4  omind5  contact3  hygiene3  hygiene5  political_ideology  happy  slf_ladder  riskperc1  riskperc2  self_esteem  sbelong1  sbelong2  sbelong3  sbelong4  optim1  optim2  slfcont1  slfcont3  slfcont4
##   1   9  age  children  cnarc1  cnarc2  cnarc3  ctheory2  ctheory3  ctheory4  CRT1  CRT2  CRT3  employ_status1  health_cond  ladder  marital1  mor_circle  moralid1  moralid10  moralid3  moralid4  moralid5  moralid6  moralid7  moralid8  moralid9  mcoop1  mcoop2  mcoop3  mcoop4  mcoop5  mcoop6  mcoop7  narc1  narc2  narc3  narc4  narc5  narc6  nidentity2  omind1  omind3  omind4  omind5  contact3  hygiene3  hygiene5  political_ideology  happy  slf_ladder  riskperc1  riskperc2  self_esteem  sbelong1  sbelong2  sbelong3  sbelong4  optim1  optim2  slfcont1  slfcont3  slfcont4
##   1   10  age  children  cnarc1  cnarc2  cnarc3  ctheory2  ctheory3  ctheory4  CRT1  CRT2  CRT3  employ_status1  health_cond  ladder  marital1  mor_circle  moralid1  moralid10  moralid3  moralid4  moralid5  moralid6  moralid7  moralid8  moralid9  mcoop1  mcoop2  mcoop3  mcoop4  mcoop5  mcoop6  mcoop7  narc1  narc2  narc3  narc4  narc5  narc6  nidentity2  omind1  omind3  omind4  omind5  contact3  hygiene3  hygiene5  political_ideology  happy  slf_ladder  riskperc1  riskperc2  self_esteem  sbelong1  sbelong2  sbelong3  sbelong4  optim1  optim2  slfcont1  slfcont3  slfcont4
##   2   1  age  children  cnarc1  cnarc2  cnarc3  ctheory2  ctheory3  ctheory4  CRT1  CRT2  CRT3  employ_status1  health_cond  ladder  marital1  mor_circle  moralid1  moralid10  moralid3  moralid4  moralid5  moralid6  moralid7  moralid8  moralid9  mcoop1  mcoop2  mcoop3  mcoop4  mcoop5  mcoop6  mcoop7  narc1  narc2  narc3  narc4  narc5  narc6  nidentity2  omind1  omind3  omind4  omind5  contact3  hygiene3  hygiene5  political_ideology  happy  slf_ladder  riskperc1  riskperc2  self_esteem  sbelong1  sbelong2  sbelong3  sbelong4  optim1  optim2  slfcont1  slfcont3  slfcont4
##   2   2  age  children  cnarc1  cnarc2  cnarc3  ctheory2  ctheory3  ctheory4  CRT1  CRT2  CRT3  employ_status1  health_cond  ladder  marital1  mor_circle  moralid1  moralid10  moralid3  moralid4  moralid5  moralid6  moralid7  moralid8  moralid9  mcoop1  mcoop2  mcoop3  mcoop4  mcoop5  mcoop6  mcoop7  narc1  narc2  narc3  narc4  narc5  narc6  nidentity2  omind1  omind3  omind4  omind5  contact3  hygiene3  hygiene5  political_ideology  happy  slf_ladder  riskperc1  riskperc2  self_esteem  sbelong1  sbelong2  sbelong3  sbelong4  optim1  optim2  slfcont1  slfcont3  slfcont4
##   2   3  age  children  cnarc1  cnarc2  cnarc3  ctheory2  ctheory3  ctheory4  CRT1  CRT2  CRT3  employ_status1  health_cond  ladder  marital1  mor_circle  moralid1  moralid10  moralid3  moralid4  moralid5  moralid6  moralid7  moralid8  moralid9  mcoop1  mcoop2  mcoop3  mcoop4  mcoop5  mcoop6  mcoop7  narc1  narc2  narc3  narc4  narc5  narc6  nidentity2  omind1  omind3  omind4  omind5  contact3  hygiene3  hygiene5  political_ideology  happy  slf_ladder  riskperc1  riskperc2  self_esteem  sbelong1  sbelong2  sbelong3  sbelong4  optim1  optim2  slfcont1  slfcont3  slfcont4
##   2   4  age  children  cnarc1  cnarc2  cnarc3  ctheory2  ctheory3  ctheory4  CRT1  CRT2  CRT3  employ_status1  health_cond  ladder  marital1  mor_circle  moralid1  moralid10  moralid3  moralid4  moralid5  moralid6  moralid7  moralid8  moralid9  mcoop1  mcoop2  mcoop3  mcoop4  mcoop5  mcoop6  mcoop7  narc1  narc2  narc3  narc4  narc5  narc6  nidentity2  omind1  omind3  omind4  omind5  contact3  hygiene3  hygiene5  political_ideology  happy  slf_ladder  riskperc1  riskperc2  self_esteem  sbelong1  sbelong2  sbelong3  sbelong4  optim1  optim2  slfcont1  slfcont3  slfcont4
##   2   5  age  children  cnarc1  cnarc2  cnarc3  ctheory2  ctheory3  ctheory4  CRT1  CRT2  CRT3  employ_status1  health_cond  ladder  marital1  mor_circle  moralid1  moralid10  moralid3  moralid4  moralid5  moralid6  moralid7  moralid8  moralid9  mcoop1  mcoop2  mcoop3  mcoop4  mcoop5  mcoop6  mcoop7  narc1  narc2  narc3  narc4  narc5  narc6  nidentity2  omind1  omind3  omind4  omind5  contact3  hygiene3  hygiene5  political_ideology  happy  slf_ladder  riskperc1  riskperc2  self_esteem  sbelong1  sbelong2  sbelong3  sbelong4  optim1  optim2  slfcont1  slfcont3  slfcont4
##   2   6  age  children  cnarc1  cnarc2  cnarc3  ctheory2  ctheory3  ctheory4  CRT1  CRT2  CRT3  employ_status1  health_cond  ladder  marital1  mor_circle  moralid1  moralid10  moralid3  moralid4  moralid5  moralid6  moralid7  moralid8  moralid9  mcoop1  mcoop2  mcoop3  mcoop4  mcoop5  mcoop6  mcoop7  narc1  narc2  narc3  narc4  narc5  narc6  nidentity2  omind1  omind3  omind4  omind5  contact3  hygiene3  hygiene5  political_ideology  happy  slf_ladder  riskperc1  riskperc2  self_esteem  sbelong1  sbelong2  sbelong3  sbelong4  optim1  optim2  slfcont1  slfcont3  slfcont4
##   2   7  age  children  cnarc1  cnarc2  cnarc3  ctheory2  ctheory3  ctheory4  CRT1  CRT2  CRT3  employ_status1  health_cond  ladder  marital1  mor_circle  moralid1  moralid10  moralid3  moralid4  moralid5  moralid6  moralid7  moralid8  moralid9  mcoop1  mcoop2  mcoop3  mcoop4  mcoop5  mcoop6  mcoop7  narc1  narc2  narc3  narc4  narc5  narc6  nidentity2  omind1  omind3  omind4  omind5  contact3  hygiene3  hygiene5  political_ideology  happy  slf_ladder  riskperc1  riskperc2  self_esteem  sbelong1  sbelong2  sbelong3  sbelong4  optim1  optim2  slfcont1  slfcont3  slfcont4
##   2   8  age  children  cnarc1  cnarc2  cnarc3  ctheory2  ctheory3  ctheory4  CRT1  CRT2  CRT3  employ_status1  health_cond  ladder  marital1  mor_circle  moralid1  moralid10  moralid3  moralid4  moralid5  moralid6  moralid7  moralid8  moralid9  mcoop1  mcoop2  mcoop3  mcoop4  mcoop5  mcoop6  mcoop7  narc1  narc2  narc3  narc4  narc5  narc6  nidentity2  omind1  omind3  omind4  omind5  contact3  hygiene3  hygiene5  political_ideology  happy  slf_ladder  riskperc1  riskperc2  self_esteem  sbelong1  sbelong2  sbelong3  sbelong4  optim1  optim2  slfcont1  slfcont3  slfcont4
##   2   9  age  children  cnarc1  cnarc2  cnarc3  ctheory2  ctheory3  ctheory4  CRT1  CRT2  CRT3  employ_status1  health_cond  ladder  marital1  mor_circle  moralid1  moralid10  moralid3  moralid4  moralid5  moralid6  moralid7  moralid8  moralid9  mcoop1  mcoop2  mcoop3  mcoop4  mcoop5  mcoop6  mcoop7  narc1  narc2  narc3  narc4  narc5  narc6  nidentity2  omind1  omind3  omind4  omind5  contact3  hygiene3  hygiene5  political_ideology  happy  slf_ladder  riskperc1  riskperc2  self_esteem  sbelong1  sbelong2  sbelong3  sbelong4  optim1  optim2  slfcont1  slfcont3  slfcont4
##   2   10  age  children  cnarc1  cnarc2  cnarc3  ctheory2  ctheory3  ctheory4  CRT1  CRT2  CRT3  employ_status1  health_cond  ladder  marital1  mor_circle  moralid1  moralid10  moralid3  moralid4  moralid5  moralid6  moralid7  moralid8  moralid9  mcoop1  mcoop2  mcoop3  mcoop4  mcoop5  mcoop6  mcoop7  narc1  narc2  narc3  narc4  narc5  narc6  nidentity2  omind1  omind3  omind4  omind5  contact3  hygiene3  hygiene5  political_ideology  happy  slf_ladder  riskperc1  riskperc2  self_esteem  sbelong1  sbelong2  sbelong3  sbelong4  optim1  optim2  slfcont1  slfcont3  slfcont4
##   3   1  age  children  cnarc1  cnarc2  cnarc3  ctheory2  ctheory3  ctheory4  CRT1  CRT2  CRT3  employ_status1  health_cond  ladder  marital1  mor_circle  moralid1  moralid10  moralid3  moralid4  moralid5  moralid6  moralid7  moralid8  moralid9  mcoop1  mcoop2  mcoop3  mcoop4  mcoop5  mcoop6  mcoop7  narc1  narc2  narc3  narc4  narc5  narc6  nidentity2  omind1  omind3  omind4  omind5  contact3  hygiene3  hygiene5  political_ideology  happy  slf_ladder  riskperc1  riskperc2  self_esteem  sbelong1  sbelong2  sbelong3  sbelong4  optim1  optim2  slfcont1  slfcont3  slfcont4
##   3   2  age  children  cnarc1  cnarc2  cnarc3  ctheory2  ctheory3  ctheory4  CRT1  CRT2  CRT3  employ_status1  health_cond  ladder  marital1  mor_circle  moralid1  moralid10  moralid3  moralid4  moralid5  moralid6  moralid7  moralid8  moralid9  mcoop1  mcoop2  mcoop3  mcoop4  mcoop5  mcoop6  mcoop7  narc1  narc2  narc3  narc4  narc5  narc6  nidentity2  omind1  omind3  omind4  omind5  contact3  hygiene3  hygiene5  political_ideology  happy  slf_ladder  riskperc1  riskperc2  self_esteem  sbelong1  sbelong2  sbelong3  sbelong4  optim1  optim2  slfcont1  slfcont3  slfcont4
##   3   3  age  children  cnarc1  cnarc2  cnarc3  ctheory2  ctheory3  ctheory4  CRT1  CRT2  CRT3  employ_status1  health_cond  ladder  marital1  mor_circle  moralid1  moralid10  moralid3  moralid4  moralid5  moralid6  moralid7  moralid8  moralid9  mcoop1  mcoop2  mcoop3  mcoop4  mcoop5  mcoop6  mcoop7  narc1  narc2  narc3  narc4  narc5  narc6  nidentity2  omind1  omind3  omind4  omind5  contact3  hygiene3  hygiene5  political_ideology  happy  slf_ladder  riskperc1  riskperc2  self_esteem  sbelong1  sbelong2  sbelong3  sbelong4  optim1  optim2  slfcont1  slfcont3  slfcont4
##   3   4  age  children  cnarc1  cnarc2  cnarc3  ctheory2  ctheory3  ctheory4  CRT1  CRT2  CRT3  employ_status1  health_cond  ladder  marital1  mor_circle  moralid1  moralid10  moralid3  moralid4  moralid5  moralid6  moralid7  moralid8  moralid9  mcoop1  mcoop2  mcoop3  mcoop4  mcoop5  mcoop6  mcoop7  narc1  narc2  narc3  narc4  narc5  narc6  nidentity2  omind1  omind3  omind4  omind5  contact3  hygiene3  hygiene5  political_ideology  happy  slf_ladder  riskperc1  riskperc2  self_esteem  sbelong1  sbelong2  sbelong3  sbelong4  optim1  optim2  slfcont1  slfcont3  slfcont4
##   3   5  age  children  cnarc1  cnarc2  cnarc3  ctheory2  ctheory3  ctheory4  CRT1  CRT2  CRT3  employ_status1  health_cond  ladder  marital1  mor_circle  moralid1  moralid10  moralid3  moralid4  moralid5  moralid6  moralid7  moralid8  moralid9  mcoop1  mcoop2  mcoop3  mcoop4  mcoop5  mcoop6  mcoop7  narc1  narc2  narc3  narc4  narc5  narc6  nidentity2  omind1  omind3  omind4  omind5  contact3  hygiene3  hygiene5  political_ideology  happy  slf_ladder  riskperc1  riskperc2  self_esteem  sbelong1  sbelong2  sbelong3  sbelong4  optim1  optim2  slfcont1  slfcont3  slfcont4
##   3   6  age  children  cnarc1  cnarc2  cnarc3  ctheory2  ctheory3  ctheory4  CRT1  CRT2  CRT3  employ_status1  health_cond  ladder  marital1  mor_circle  moralid1  moralid10  moralid3  moralid4  moralid5  moralid6  moralid7  moralid8  moralid9  mcoop1  mcoop2  mcoop3  mcoop4  mcoop5  mcoop6  mcoop7  narc1  narc2  narc3  narc4  narc5  narc6  nidentity2  omind1  omind3  omind4  omind5  contact3  hygiene3  hygiene5  political_ideology  happy  slf_ladder  riskperc1  riskperc2  self_esteem  sbelong1  sbelong2  sbelong3  sbelong4  optim1  optim2  slfcont1  slfcont3  slfcont4
##   3   7  age  children  cnarc1  cnarc2  cnarc3  ctheory2  ctheory3  ctheory4  CRT1  CRT2  CRT3  employ_status1  health_cond  ladder  marital1  mor_circle  moralid1  moralid10  moralid3  moralid4  moralid5  moralid6  moralid7  moralid8  moralid9  mcoop1  mcoop2  mcoop3  mcoop4  mcoop5  mcoop6  mcoop7  narc1  narc2  narc3  narc4  narc5  narc6  nidentity2  omind1  omind3  omind4  omind5  contact3  hygiene3  hygiene5  political_ideology  happy  slf_ladder  riskperc1  riskperc2  self_esteem  sbelong1  sbelong2  sbelong3  sbelong4  optim1  optim2  slfcont1  slfcont3  slfcont4
##   3   8  age  children  cnarc1  cnarc2  cnarc3  ctheory2  ctheory3  ctheory4  CRT1  CRT2  CRT3  employ_status1  health_cond  ladder  marital1  mor_circle  moralid1  moralid10  moralid3  moralid4  moralid5  moralid6  moralid7  moralid8  moralid9  mcoop1  mcoop2  mcoop3  mcoop4  mcoop5  mcoop6  mcoop7  narc1  narc2  narc3  narc4  narc5  narc6  nidentity2  omind1  omind3  omind4  omind5  contact3  hygiene3  hygiene5  political_ideology  happy  slf_ladder  riskperc1  riskperc2  self_esteem  sbelong1  sbelong2  sbelong3  sbelong4  optim1  optim2  slfcont1  slfcont3  slfcont4
##   3   9  age  children  cnarc1  cnarc2  cnarc3  ctheory2  ctheory3  ctheory4  CRT1  CRT2  CRT3  employ_status1  health_cond  ladder  marital1  mor_circle  moralid1  moralid10  moralid3  moralid4  moralid5  moralid6  moralid7  moralid8  moralid9  mcoop1  mcoop2  mcoop3  mcoop4  mcoop5  mcoop6  mcoop7  narc1  narc2  narc3  narc4  narc5  narc6  nidentity2  omind1  omind3  omind4  omind5  contact3  hygiene3  hygiene5  political_ideology  happy  slf_ladder  riskperc1  riskperc2  self_esteem  sbelong1  sbelong2  sbelong3  sbelong4  optim1  optim2  slfcont1  slfcont3  slfcont4
##   3   10  age  children  cnarc1  cnarc2  cnarc3  ctheory2  ctheory3  ctheory4  CRT1  CRT2  CRT3  employ_status1  health_cond  ladder  marital1  mor_circle  moralid1  moralid10  moralid3  moralid4  moralid5  moralid6  moralid7  moralid8  moralid9  mcoop1  mcoop2  mcoop3  mcoop4  mcoop5  mcoop6  mcoop7  narc1  narc2  narc3  narc4  narc5  narc6  nidentity2  omind1  omind3  omind4  omind5  contact3  hygiene3  hygiene5  political_ideology  happy  slf_ladder  riskperc1  riskperc2  self_esteem  sbelong1  sbelong2  sbelong3  sbelong4  optim1  optim2  slfcont1  slfcont3  slfcont4
##   4   1  age  children  cnarc1  cnarc2  cnarc3  ctheory2  ctheory3  ctheory4  CRT1  CRT2  CRT3  employ_status1  health_cond  ladder  marital1  mor_circle  moralid1  moralid10  moralid3  moralid4  moralid5  moralid6  moralid7  moralid8  moralid9  mcoop1  mcoop2  mcoop3  mcoop4  mcoop5  mcoop6  mcoop7  narc1  narc2  narc3  narc4  narc5  narc6  nidentity2  omind1  omind3  omind4  omind5  contact3  hygiene3  hygiene5  political_ideology  happy  slf_ladder  riskperc1  riskperc2  self_esteem  sbelong1  sbelong2  sbelong3  sbelong4  optim1  optim2  slfcont1  slfcont3  slfcont4
##   4   2  age  children  cnarc1  cnarc2  cnarc3  ctheory2  ctheory3  ctheory4  CRT1  CRT2  CRT3  employ_status1  health_cond  ladder  marital1  mor_circle  moralid1  moralid10  moralid3  moralid4  moralid5  moralid6  moralid7  moralid8  moralid9  mcoop1  mcoop2  mcoop3  mcoop4  mcoop5  mcoop6  mcoop7  narc1  narc2  narc3  narc4  narc5  narc6  nidentity2  omind1  omind3  omind4  omind5  contact3  hygiene3  hygiene5  political_ideology  happy  slf_ladder  riskperc1  riskperc2  self_esteem  sbelong1  sbelong2  sbelong3  sbelong4  optim1  optim2  slfcont1  slfcont3  slfcont4
##   4   3  age  children  cnarc1  cnarc2  cnarc3  ctheory2  ctheory3  ctheory4  CRT1  CRT2  CRT3  employ_status1  health_cond  ladder  marital1  mor_circle  moralid1  moralid10  moralid3  moralid4  moralid5  moralid6  moralid7  moralid8  moralid9  mcoop1  mcoop2  mcoop3  mcoop4  mcoop5  mcoop6  mcoop7  narc1  narc2  narc3  narc4  narc5  narc6  nidentity2  omind1  omind3  omind4  omind5  contact3  hygiene3  hygiene5  political_ideology  happy  slf_ladder  riskperc1  riskperc2  self_esteem  sbelong1  sbelong2  sbelong3  sbelong4  optim1  optim2  slfcont1  slfcont3  slfcont4
##   4   4  age  children  cnarc1  cnarc2  cnarc3  ctheory2  ctheory3  ctheory4  CRT1  CRT2  CRT3  employ_status1  health_cond  ladder  marital1  mor_circle  moralid1  moralid10  moralid3  moralid4  moralid5  moralid6  moralid7  moralid8  moralid9  mcoop1  mcoop2  mcoop3  mcoop4  mcoop5  mcoop6  mcoop7  narc1  narc2  narc3  narc4  narc5  narc6  nidentity2  omind1  omind3  omind4  omind5  contact3  hygiene3  hygiene5  political_ideology  happy  slf_ladder  riskperc1  riskperc2  self_esteem  sbelong1  sbelong2  sbelong3  sbelong4  optim1  optim2  slfcont1  slfcont3  slfcont4
##   4   5  age  children  cnarc1  cnarc2  cnarc3  ctheory2  ctheory3  ctheory4  CRT1  CRT2  CRT3  employ_status1  health_cond  ladder  marital1  mor_circle  moralid1  moralid10  moralid3  moralid4  moralid5  moralid6  moralid7  moralid8  moralid9  mcoop1  mcoop2  mcoop3  mcoop4  mcoop5  mcoop6  mcoop7  narc1  narc2  narc3  narc4  narc5  narc6  nidentity2  omind1  omind3  omind4  omind5  contact3  hygiene3  hygiene5  political_ideology  happy  slf_ladder  riskperc1  riskperc2  self_esteem  sbelong1  sbelong2  sbelong3  sbelong4  optim1  optim2  slfcont1  slfcont3  slfcont4
##   4   6  age  children  cnarc1  cnarc2  cnarc3  ctheory2  ctheory3  ctheory4  CRT1  CRT2  CRT3  employ_status1  health_cond  ladder  marital1  mor_circle  moralid1  moralid10  moralid3  moralid4  moralid5  moralid6  moralid7  moralid8  moralid9  mcoop1  mcoop2  mcoop3  mcoop4  mcoop5  mcoop6  mcoop7  narc1  narc2  narc3  narc4  narc5  narc6  nidentity2  omind1  omind3  omind4  omind5  contact3  hygiene3  hygiene5  political_ideology  happy  slf_ladder  riskperc1  riskperc2  self_esteem  sbelong1  sbelong2  sbelong3  sbelong4  optim1  optim2  slfcont1  slfcont3  slfcont4
##   4   7  age  children  cnarc1  cnarc2  cnarc3  ctheory2  ctheory3  ctheory4  CRT1  CRT2  CRT3  employ_status1  health_cond  ladder  marital1  mor_circle  moralid1  moralid10  moralid3  moralid4  moralid5  moralid6  moralid7  moralid8  moralid9  mcoop1  mcoop2  mcoop3  mcoop4  mcoop5  mcoop6  mcoop7  narc1  narc2  narc3  narc4  narc5  narc6  nidentity2  omind1  omind3  omind4  omind5  contact3  hygiene3  hygiene5  political_ideology  happy  slf_ladder  riskperc1  riskperc2  self_esteem  sbelong1  sbelong2  sbelong3  sbelong4  optim1  optim2  slfcont1  slfcont3  slfcont4
##   4   8  age  children  cnarc1  cnarc2  cnarc3  ctheory2  ctheory3  ctheory4  CRT1  CRT2  CRT3  employ_status1  health_cond  ladder  marital1  mor_circle  moralid1  moralid10  moralid3  moralid4  moralid5  moralid6  moralid7  moralid8  moralid9  mcoop1  mcoop2  mcoop3  mcoop4  mcoop5  mcoop6  mcoop7  narc1  narc2  narc3  narc4  narc5  narc6  nidentity2  omind1  omind3  omind4  omind5  contact3  hygiene3  hygiene5  political_ideology  happy  slf_ladder  riskperc1  riskperc2  self_esteem  sbelong1  sbelong2  sbelong3  sbelong4  optim1  optim2  slfcont1  slfcont3  slfcont4
##   4   9  age  children  cnarc1  cnarc2  cnarc3  ctheory2  ctheory3  ctheory4  CRT1  CRT2  CRT3  employ_status1  health_cond  ladder  marital1  mor_circle  moralid1  moralid10  moralid3  moralid4  moralid5  moralid6  moralid7  moralid8  moralid9  mcoop1  mcoop2  mcoop3  mcoop4  mcoop5  mcoop6  mcoop7  narc1  narc2  narc3  narc4  narc5  narc6  nidentity2  omind1  omind3  omind4  omind5  contact3  hygiene3  hygiene5  political_ideology  happy  slf_ladder  riskperc1  riskperc2  self_esteem  sbelong1  sbelong2  sbelong3  sbelong4  optim1  optim2  slfcont1  slfcont3  slfcont4
##   4   10  age  children  cnarc1  cnarc2  cnarc3  ctheory2  ctheory3  ctheory4  CRT1  CRT2  CRT3  employ_status1  health_cond  ladder  marital1  mor_circle  moralid1  moralid10  moralid3  moralid4  moralid5  moralid6  moralid7  moralid8  moralid9  mcoop1  mcoop2  mcoop3  mcoop4  mcoop5  mcoop6  mcoop7  narc1  narc2  narc3  narc4  narc5  narc6  nidentity2  omind1  omind3  omind4  omind5  contact3  hygiene3  hygiene5  political_ideology  happy  slf_ladder  riskperc1  riskperc2  self_esteem  sbelong1  sbelong2  sbelong3  sbelong4  optim1  optim2  slfcont1  slfcont3  slfcont4
##   5   1  age  children  cnarc1  cnarc2  cnarc3  ctheory2  ctheory3  ctheory4  CRT1  CRT2  CRT3  employ_status1  health_cond  ladder  marital1  mor_circle  moralid1  moralid10  moralid3  moralid4  moralid5  moralid6  moralid7  moralid8  moralid9  mcoop1  mcoop2  mcoop3  mcoop4  mcoop5  mcoop6  mcoop7  narc1  narc2  narc3  narc4  narc5  narc6  nidentity2  omind1  omind3  omind4  omind5  contact3  hygiene3  hygiene5  political_ideology  happy  slf_ladder  riskperc1  riskperc2  self_esteem  sbelong1  sbelong2  sbelong3  sbelong4  optim1  optim2  slfcont1  slfcont3  slfcont4
##   5   2  age  children  cnarc1  cnarc2  cnarc3  ctheory2  ctheory3  ctheory4  CRT1  CRT2  CRT3  employ_status1  health_cond  ladder  marital1  mor_circle  moralid1  moralid10  moralid3  moralid4  moralid5  moralid6  moralid7  moralid8  moralid9  mcoop1  mcoop2  mcoop3  mcoop4  mcoop5  mcoop6  mcoop7  narc1  narc2  narc3  narc4  narc5  narc6  nidentity2  omind1  omind3  omind4  omind5  contact3  hygiene3  hygiene5  political_ideology  happy  slf_ladder  riskperc1  riskperc2  self_esteem  sbelong1  sbelong2  sbelong3  sbelong4  optim1  optim2  slfcont1  slfcont3  slfcont4
##   5   3  age  children  cnarc1  cnarc2  cnarc3  ctheory2  ctheory3  ctheory4  CRT1  CRT2  CRT3  employ_status1  health_cond  ladder  marital1  mor_circle  moralid1  moralid10  moralid3  moralid4  moralid5  moralid6  moralid7  moralid8  moralid9  mcoop1  mcoop2  mcoop3  mcoop4  mcoop5  mcoop6  mcoop7  narc1  narc2  narc3  narc4  narc5  narc6  nidentity2  omind1  omind3  omind4  omind5  contact3  hygiene3  hygiene5  political_ideology  happy  slf_ladder  riskperc1  riskperc2  self_esteem  sbelong1  sbelong2  sbelong3  sbelong4  optim1  optim2  slfcont1  slfcont3  slfcont4
##   5   4  age  children  cnarc1  cnarc2  cnarc3  ctheory2  ctheory3  ctheory4  CRT1  CRT2  CRT3  employ_status1  health_cond  ladder  marital1  mor_circle  moralid1  moralid10  moralid3  moralid4  moralid5  moralid6  moralid7  moralid8  moralid9  mcoop1  mcoop2  mcoop3  mcoop4  mcoop5  mcoop6  mcoop7  narc1  narc2  narc3  narc4  narc5  narc6  nidentity2  omind1  omind3  omind4  omind5  contact3  hygiene3  hygiene5  political_ideology  happy  slf_ladder  riskperc1  riskperc2  self_esteem  sbelong1  sbelong2  sbelong3  sbelong4  optim1  optim2  slfcont1  slfcont3  slfcont4
##   5   5  age  children  cnarc1  cnarc2  cnarc3  ctheory2  ctheory3  ctheory4  CRT1  CRT2  CRT3  employ_status1  health_cond  ladder  marital1  mor_circle  moralid1  moralid10  moralid3  moralid4  moralid5  moralid6  moralid7  moralid8  moralid9  mcoop1  mcoop2  mcoop3  mcoop4  mcoop5  mcoop6  mcoop7  narc1  narc2  narc3  narc4  narc5  narc6  nidentity2  omind1  omind3  omind4  omind5  contact3  hygiene3  hygiene5  political_ideology  happy  slf_ladder  riskperc1  riskperc2  self_esteem  sbelong1  sbelong2  sbelong3  sbelong4  optim1  optim2  slfcont1  slfcont3  slfcont4
##   5   6  age  children  cnarc1  cnarc2  cnarc3  ctheory2  ctheory3  ctheory4  CRT1  CRT2  CRT3  employ_status1  health_cond  ladder  marital1  mor_circle  moralid1  moralid10  moralid3  moralid4  moralid5  moralid6  moralid7  moralid8  moralid9  mcoop1  mcoop2  mcoop3  mcoop4  mcoop5  mcoop6  mcoop7  narc1  narc2  narc3  narc4  narc5  narc6  nidentity2  omind1  omind3  omind4  omind5  contact3  hygiene3  hygiene5  political_ideology  happy  slf_ladder  riskperc1  riskperc2  self_esteem  sbelong1  sbelong2  sbelong3  sbelong4  optim1  optim2  slfcont1  slfcont3  slfcont4
##   5   7  age  children  cnarc1  cnarc2  cnarc3  ctheory2  ctheory3  ctheory4  CRT1  CRT2  CRT3  employ_status1  health_cond  ladder  marital1  mor_circle  moralid1  moralid10  moralid3  moralid4  moralid5  moralid6  moralid7  moralid8  moralid9  mcoop1  mcoop2  mcoop3  mcoop4  mcoop5  mcoop6  mcoop7  narc1  narc2  narc3  narc4  narc5  narc6  nidentity2  omind1  omind3  omind4  omind5  contact3  hygiene3  hygiene5  political_ideology  happy  slf_ladder  riskperc1  riskperc2  self_esteem  sbelong1  sbelong2  sbelong3  sbelong4  optim1  optim2  slfcont1  slfcont3  slfcont4
##   5   8  age  children  cnarc1  cnarc2  cnarc3  ctheory2  ctheory3  ctheory4  CRT1  CRT2  CRT3  employ_status1  health_cond  ladder  marital1  mor_circle  moralid1  moralid10  moralid3  moralid4  moralid5  moralid6  moralid7  moralid8  moralid9  mcoop1  mcoop2  mcoop3  mcoop4  mcoop5  mcoop6  mcoop7  narc1  narc2  narc3  narc4  narc5  narc6  nidentity2  omind1  omind3  omind4  omind5  contact3  hygiene3  hygiene5  political_ideology  happy  slf_ladder  riskperc1  riskperc2  self_esteem  sbelong1  sbelong2  sbelong3  sbelong4  optim1  optim2  slfcont1  slfcont3  slfcont4
##   5   9  age  children  cnarc1  cnarc2  cnarc3  ctheory2  ctheory3  ctheory4  CRT1  CRT2  CRT3  employ_status1  health_cond  ladder  marital1  mor_circle  moralid1  moralid10  moralid3  moralid4  moralid5  moralid6  moralid7  moralid8  moralid9  mcoop1  mcoop2  mcoop3  mcoop4  mcoop5  mcoop6  mcoop7  narc1  narc2  narc3  narc4  narc5  narc6  nidentity2  omind1  omind3  omind4  omind5  contact3  hygiene3  hygiene5  political_ideology  happy  slf_ladder  riskperc1  riskperc2  self_esteem  sbelong1  sbelong2  sbelong3  sbelong4  optim1  optim2  slfcont1  slfcont3  slfcont4
##   5   10  age  children  cnarc1  cnarc2  cnarc3  ctheory2  ctheory3  ctheory4  CRT1  CRT2  CRT3  employ_status1  health_cond  ladder  marital1  mor_circle  moralid1  moralid10  moralid3  moralid4  moralid5  moralid6  moralid7  moralid8  moralid9  mcoop1  mcoop2  mcoop3  mcoop4  mcoop5  mcoop6  mcoop7  narc1  narc2  narc3  narc4  narc5  narc6  nidentity2  omind1  omind3  omind4  omind5  contact3  hygiene3  hygiene5  political_ideology  happy  slf_ladder  riskperc1  riskperc2  self_esteem  sbelong1  sbelong2  sbelong3  sbelong4  optim1  optim2  slfcont1  slfcont3  slfcont4
##   6   1  age  children  cnarc1  cnarc2  cnarc3  ctheory2  ctheory3  ctheory4  CRT1  CRT2  CRT3  employ_status1  health_cond  ladder  marital1  mor_circle  moralid1  moralid10  moralid3  moralid4  moralid5  moralid6  moralid7  moralid8  moralid9  mcoop1  mcoop2  mcoop3  mcoop4  mcoop5  mcoop6  mcoop7  narc1  narc2  narc3  narc4  narc5  narc6  nidentity2  omind1  omind3  omind4  omind5  contact3  hygiene3  hygiene5  political_ideology  happy  slf_ladder  riskperc1  riskperc2  self_esteem  sbelong1  sbelong2  sbelong3  sbelong4  optim1  optim2  slfcont1  slfcont3  slfcont4
##   6   2  age  children  cnarc1  cnarc2  cnarc3  ctheory2  ctheory3  ctheory4  CRT1  CRT2  CRT3  employ_status1  health_cond  ladder  marital1  mor_circle  moralid1  moralid10  moralid3  moralid4  moralid5  moralid6  moralid7  moralid8  moralid9  mcoop1  mcoop2  mcoop3  mcoop4  mcoop5  mcoop6  mcoop7  narc1  narc2  narc3  narc4  narc5  narc6  nidentity2  omind1  omind3  omind4  omind5  contact3  hygiene3  hygiene5  political_ideology  happy  slf_ladder  riskperc1  riskperc2  self_esteem  sbelong1  sbelong2  sbelong3  sbelong4  optim1  optim2  slfcont1  slfcont3  slfcont4
##   6   3  age  children  cnarc1  cnarc2  cnarc3  ctheory2  ctheory3  ctheory4  CRT1  CRT2  CRT3  employ_status1  health_cond  ladder  marital1  mor_circle  moralid1  moralid10  moralid3  moralid4  moralid5  moralid6  moralid7  moralid8  moralid9  mcoop1  mcoop2  mcoop3  mcoop4  mcoop5  mcoop6  mcoop7  narc1  narc2  narc3  narc4  narc5  narc6  nidentity2  omind1  omind3  omind4  omind5  contact3  hygiene3  hygiene5  political_ideology  happy  slf_ladder  riskperc1  riskperc2  self_esteem  sbelong1  sbelong2  sbelong3  sbelong4  optim1  optim2  slfcont1  slfcont3  slfcont4
##   6   4  age  children  cnarc1  cnarc2  cnarc3  ctheory2  ctheory3  ctheory4  CRT1  CRT2  CRT3  employ_status1  health_cond  ladder  marital1  mor_circle  moralid1  moralid10  moralid3  moralid4  moralid5  moralid6  moralid7  moralid8  moralid9  mcoop1  mcoop2  mcoop3  mcoop4  mcoop5  mcoop6  mcoop7  narc1  narc2  narc3  narc4  narc5  narc6  nidentity2  omind1  omind3  omind4  omind5  contact3  hygiene3  hygiene5  political_ideology  happy  slf_ladder  riskperc1  riskperc2  self_esteem  sbelong1  sbelong2  sbelong3  sbelong4  optim1  optim2  slfcont1  slfcont3  slfcont4
##   6   5  age  children  cnarc1  cnarc2  cnarc3  ctheory2  ctheory3  ctheory4  CRT1  CRT2  CRT3  employ_status1  health_cond  ladder  marital1  mor_circle  moralid1  moralid10  moralid3  moralid4  moralid5  moralid6  moralid7  moralid8  moralid9  mcoop1  mcoop2  mcoop3  mcoop4  mcoop5  mcoop6  mcoop7  narc1  narc2  narc3  narc4  narc5  narc6  nidentity2  omind1  omind3  omind4  omind5  contact3  hygiene3  hygiene5  political_ideology  happy  slf_ladder  riskperc1  riskperc2  self_esteem  sbelong1  sbelong2  sbelong3  sbelong4  optim1  optim2  slfcont1  slfcont3  slfcont4
##   6   6  age  children  cnarc1  cnarc2  cnarc3  ctheory2  ctheory3  ctheory4  CRT1  CRT2  CRT3  employ_status1  health_cond  ladder  marital1  mor_circle  moralid1  moralid10  moralid3  moralid4  moralid5  moralid6  moralid7  moralid8  moralid9  mcoop1  mcoop2  mcoop3  mcoop4  mcoop5  mcoop6  mcoop7  narc1  narc2  narc3  narc4  narc5  narc6  nidentity2  omind1  omind3  omind4  omind5  contact3  hygiene3  hygiene5  political_ideology  happy  slf_ladder  riskperc1  riskperc2  self_esteem  sbelong1  sbelong2  sbelong3  sbelong4  optim1  optim2  slfcont1  slfcont3  slfcont4
##   6   7  age  children  cnarc1  cnarc2  cnarc3  ctheory2  ctheory3  ctheory4  CRT1  CRT2  CRT3  employ_status1  health_cond  ladder  marital1  mor_circle  moralid1  moralid10  moralid3  moralid4  moralid5  moralid6  moralid7  moralid8  moralid9  mcoop1  mcoop2  mcoop3  mcoop4  mcoop5  mcoop6  mcoop7  narc1  narc2  narc3  narc4  narc5  narc6  nidentity2  omind1  omind3  omind4  omind5  contact3  hygiene3  hygiene5  political_ideology  happy  slf_ladder  riskperc1  riskperc2  self_esteem  sbelong1  sbelong2  sbelong3  sbelong4  optim1  optim2  slfcont1  slfcont3  slfcont4
##   6   8  age  children  cnarc1  cnarc2  cnarc3  ctheory2  ctheory3  ctheory4  CRT1  CRT2  CRT3  employ_status1  health_cond  ladder  marital1  mor_circle  moralid1  moralid10  moralid3  moralid4  moralid5  moralid6  moralid7  moralid8  moralid9  mcoop1  mcoop2  mcoop3  mcoop4  mcoop5  mcoop6  mcoop7  narc1  narc2  narc3  narc4  narc5  narc6  nidentity2  omind1  omind3  omind4  omind5  contact3  hygiene3  hygiene5  political_ideology  happy  slf_ladder  riskperc1  riskperc2  self_esteem  sbelong1  sbelong2  sbelong3  sbelong4  optim1  optim2  slfcont1  slfcont3  slfcont4
##   6   9  age  children  cnarc1  cnarc2  cnarc3  ctheory2  ctheory3  ctheory4  CRT1  CRT2  CRT3  employ_status1  health_cond  ladder  marital1  mor_circle  moralid1  moralid10  moralid3  moralid4  moralid5  moralid6  moralid7  moralid8  moralid9  mcoop1  mcoop2  mcoop3  mcoop4  mcoop5  mcoop6  mcoop7  narc1  narc2  narc3  narc4  narc5  narc6  nidentity2  omind1  omind3  omind4  omind5  contact3  hygiene3  hygiene5  political_ideology  happy  slf_ladder  riskperc1  riskperc2  self_esteem  sbelong1  sbelong2  sbelong3  sbelong4  optim1  optim2  slfcont1  slfcont3  slfcont4
##   6   10  age  children  cnarc1  cnarc2  cnarc3  ctheory2  ctheory3  ctheory4  CRT1  CRT2  CRT3  employ_status1  health_cond  ladder  marital1  mor_circle  moralid1  moralid10  moralid3  moralid4  moralid5  moralid6  moralid7  moralid8  moralid9  mcoop1  mcoop2  mcoop3  mcoop4  mcoop5  mcoop6  mcoop7  narc1  narc2  narc3  narc4  narc5  narc6  nidentity2  omind1  omind3  omind4  omind5  contact3  hygiene3  hygiene5  political_ideology  happy  slf_ladder  riskperc1  riskperc2  self_esteem  sbelong1  sbelong2  sbelong3  sbelong4  optim1  optim2  slfcont1  slfcont3  slfcont4
##   7   1  age  children  cnarc1  cnarc2  cnarc3  ctheory2  ctheory3  ctheory4  CRT1  CRT2  CRT3  employ_status1  health_cond  ladder  marital1  mor_circle  moralid1  moralid10  moralid3  moralid4  moralid5  moralid6  moralid7  moralid8  moralid9  mcoop1  mcoop2  mcoop3  mcoop4  mcoop5  mcoop6  mcoop7  narc1  narc2  narc3  narc4  narc5  narc6  nidentity2  omind1  omind3  omind4  omind5  contact3  hygiene3  hygiene5  political_ideology  happy  slf_ladder  riskperc1  riskperc2  self_esteem  sbelong1  sbelong2  sbelong3  sbelong4  optim1  optim2  slfcont1  slfcont3  slfcont4
##   7   2  age  children  cnarc1  cnarc2  cnarc3  ctheory2  ctheory3  ctheory4  CRT1  CRT2  CRT3  employ_status1  health_cond  ladder  marital1  mor_circle  moralid1  moralid10  moralid3  moralid4  moralid5  moralid6  moralid7  moralid8  moralid9  mcoop1  mcoop2  mcoop3  mcoop4  mcoop5  mcoop6  mcoop7  narc1  narc2  narc3  narc4  narc5  narc6  nidentity2  omind1  omind3  omind4  omind5  contact3  hygiene3  hygiene5  political_ideology  happy  slf_ladder  riskperc1  riskperc2  self_esteem  sbelong1  sbelong2  sbelong3  sbelong4  optim1  optim2  slfcont1  slfcont3  slfcont4
##   7   3  age  children  cnarc1  cnarc2  cnarc3  ctheory2  ctheory3  ctheory4  CRT1  CRT2  CRT3  employ_status1  health_cond  ladder  marital1  mor_circle  moralid1  moralid10  moralid3  moralid4  moralid5  moralid6  moralid7  moralid8  moralid9  mcoop1  mcoop2  mcoop3  mcoop4  mcoop5  mcoop6  mcoop7  narc1  narc2  narc3  narc4  narc5  narc6  nidentity2  omind1  omind3  omind4  omind5  contact3  hygiene3  hygiene5  political_ideology  happy  slf_ladder  riskperc1  riskperc2  self_esteem  sbelong1  sbelong2  sbelong3  sbelong4  optim1  optim2  slfcont1  slfcont3  slfcont4
##   7   4  age  children  cnarc1  cnarc2  cnarc3  ctheory2  ctheory3  ctheory4  CRT1  CRT2  CRT3  employ_status1  health_cond  ladder  marital1  mor_circle  moralid1  moralid10  moralid3  moralid4  moralid5  moralid6  moralid7  moralid8  moralid9  mcoop1  mcoop2  mcoop3  mcoop4  mcoop5  mcoop6  mcoop7  narc1  narc2  narc3  narc4  narc5  narc6  nidentity2  omind1  omind3  omind4  omind5  contact3  hygiene3  hygiene5  political_ideology  happy  slf_ladder  riskperc1  riskperc2  self_esteem  sbelong1  sbelong2  sbelong3  sbelong4  optim1  optim2  slfcont1  slfcont3  slfcont4
##   7   5  age  children  cnarc1  cnarc2  cnarc3  ctheory2  ctheory3  ctheory4  CRT1  CRT2  CRT3  employ_status1  health_cond  ladder  marital1  mor_circle  moralid1  moralid10  moralid3  moralid4  moralid5  moralid6  moralid7  moralid8  moralid9  mcoop1  mcoop2  mcoop3  mcoop4  mcoop5  mcoop6  mcoop7  narc1  narc2  narc3  narc4  narc5  narc6  nidentity2  omind1  omind3  omind4  omind5  contact3  hygiene3  hygiene5  political_ideology  happy  slf_ladder  riskperc1  riskperc2  self_esteem  sbelong1  sbelong2  sbelong3  sbelong4  optim1  optim2  slfcont1  slfcont3  slfcont4
##   7   6  age  children  cnarc1  cnarc2  cnarc3  ctheory2  ctheory3  ctheory4  CRT1  CRT2  CRT3  employ_status1  health_cond  ladder  marital1  mor_circle  moralid1  moralid10  moralid3  moralid4  moralid5  moralid6  moralid7  moralid8  moralid9  mcoop1  mcoop2  mcoop3  mcoop4  mcoop5  mcoop6  mcoop7  narc1  narc2  narc3  narc4  narc5  narc6  nidentity2  omind1  omind3  omind4  omind5  contact3  hygiene3  hygiene5  political_ideology  happy  slf_ladder  riskperc1  riskperc2  self_esteem  sbelong1  sbelong2  sbelong3  sbelong4  optim1  optim2  slfcont1  slfcont3  slfcont4
##   7   7  age  children  cnarc1  cnarc2  cnarc3  ctheory2  ctheory3  ctheory4  CRT1  CRT2  CRT3  employ_status1  health_cond  ladder  marital1  mor_circle  moralid1  moralid10  moralid3  moralid4  moralid5  moralid6  moralid7  moralid8  moralid9  mcoop1  mcoop2  mcoop3  mcoop4  mcoop5  mcoop6  mcoop7  narc1  narc2  narc3  narc4  narc5  narc6  nidentity2  omind1  omind3  omind4  omind5  contact3  hygiene3  hygiene5  political_ideology  happy  slf_ladder  riskperc1  riskperc2  self_esteem  sbelong1  sbelong2  sbelong3  sbelong4  optim1  optim2  slfcont1  slfcont3  slfcont4
##   7   8  age  children  cnarc1  cnarc2  cnarc3  ctheory2  ctheory3  ctheory4  CRT1  CRT2  CRT3  employ_status1  health_cond  ladder  marital1  mor_circle  moralid1  moralid10  moralid3  moralid4  moralid5  moralid6  moralid7  moralid8  moralid9  mcoop1  mcoop2  mcoop3  mcoop4  mcoop5  mcoop6  mcoop7  narc1  narc2  narc3  narc4  narc5  narc6  nidentity2  omind1  omind3  omind4  omind5  contact3  hygiene3  hygiene5  political_ideology  happy  slf_ladder  riskperc1  riskperc2  self_esteem  sbelong1  sbelong2  sbelong3  sbelong4  optim1  optim2  slfcont1  slfcont3  slfcont4
##   7   9  age  children  cnarc1  cnarc2  cnarc3  ctheory2  ctheory3  ctheory4  CRT1  CRT2  CRT3  employ_status1  health_cond  ladder  marital1  mor_circle  moralid1  moralid10  moralid3  moralid4  moralid5  moralid6  moralid7  moralid8  moralid9  mcoop1  mcoop2  mcoop3  mcoop4  mcoop5  mcoop6  mcoop7  narc1  narc2  narc3  narc4  narc5  narc6  nidentity2  omind1  omind3  omind4  omind5  contact3  hygiene3  hygiene5  political_ideology  happy  slf_ladder  riskperc1  riskperc2  self_esteem  sbelong1  sbelong2  sbelong3  sbelong4  optim1  optim2  slfcont1  slfcont3  slfcont4
##   7   10  age  children  cnarc1  cnarc2  cnarc3  ctheory2  ctheory3  ctheory4  CRT1  CRT2  CRT3  employ_status1  health_cond  ladder  marital1  mor_circle  moralid1  moralid10  moralid3  moralid4  moralid5  moralid6  moralid7  moralid8  moralid9  mcoop1  mcoop2  mcoop3  mcoop4  mcoop5  mcoop6  mcoop7  narc1  narc2  narc3  narc4  narc5  narc6  nidentity2  omind1  omind3  omind4  omind5  contact3  hygiene3  hygiene5  political_ideology  happy  slf_ladder  riskperc1  riskperc2  self_esteem  sbelong1  sbelong2  sbelong3  sbelong4  optim1  optim2  slfcont1  slfcont3  slfcont4
##   8   1  age  children  cnarc1  cnarc2  cnarc3  ctheory2  ctheory3  ctheory4  CRT1  CRT2  CRT3  employ_status1  health_cond  ladder  marital1  mor_circle  moralid1  moralid10  moralid3  moralid4  moralid5  moralid6  moralid7  moralid8  moralid9  mcoop1  mcoop2  mcoop3  mcoop4  mcoop5  mcoop6  mcoop7  narc1  narc2  narc3  narc4  narc5  narc6  nidentity2  omind1  omind3  omind4  omind5  contact3  hygiene3  hygiene5  political_ideology  happy  slf_ladder  riskperc1  riskperc2  self_esteem  sbelong1  sbelong2  sbelong3  sbelong4  optim1  optim2  slfcont1  slfcont3  slfcont4
##   8   2  age  children  cnarc1  cnarc2  cnarc3  ctheory2  ctheory3  ctheory4  CRT1  CRT2  CRT3  employ_status1  health_cond  ladder  marital1  mor_circle  moralid1  moralid10  moralid3  moralid4  moralid5  moralid6  moralid7  moralid8  moralid9  mcoop1  mcoop2  mcoop3  mcoop4  mcoop5  mcoop6  mcoop7  narc1  narc2  narc3  narc4  narc5  narc6  nidentity2  omind1  omind3  omind4  omind5  contact3  hygiene3  hygiene5  political_ideology  happy  slf_ladder  riskperc1  riskperc2  self_esteem  sbelong1  sbelong2  sbelong3  sbelong4  optim1  optim2  slfcont1  slfcont3  slfcont4
##   8   3  age  children  cnarc1  cnarc2  cnarc3  ctheory2  ctheory3  ctheory4  CRT1  CRT2  CRT3  employ_status1  health_cond  ladder  marital1  mor_circle  moralid1  moralid10  moralid3  moralid4  moralid5  moralid6  moralid7  moralid8  moralid9  mcoop1  mcoop2  mcoop3  mcoop4  mcoop5  mcoop6  mcoop7  narc1  narc2  narc3  narc4  narc5  narc6  nidentity2  omind1  omind3  omind4  omind5  contact3  hygiene3  hygiene5  political_ideology  happy  slf_ladder  riskperc1  riskperc2  self_esteem  sbelong1  sbelong2  sbelong3  sbelong4  optim1  optim2  slfcont1  slfcont3  slfcont4
##   8   4  age  children  cnarc1  cnarc2  cnarc3  ctheory2  ctheory3  ctheory4  CRT1  CRT2  CRT3  employ_status1  health_cond  ladder  marital1  mor_circle  moralid1  moralid10  moralid3  moralid4  moralid5  moralid6  moralid7  moralid8  moralid9  mcoop1  mcoop2  mcoop3  mcoop4  mcoop5  mcoop6  mcoop7  narc1  narc2  narc3  narc4  narc5  narc6  nidentity2  omind1  omind3  omind4  omind5  contact3  hygiene3  hygiene5  political_ideology  happy  slf_ladder  riskperc1  riskperc2  self_esteem  sbelong1  sbelong2  sbelong3  sbelong4  optim1  optim2  slfcont1  slfcont3  slfcont4
##   8   5  age  children  cnarc1  cnarc2  cnarc3  ctheory2  ctheory3  ctheory4  CRT1  CRT2  CRT3  employ_status1  health_cond  ladder  marital1  mor_circle  moralid1  moralid10  moralid3  moralid4  moralid5  moralid6  moralid7  moralid8  moralid9  mcoop1  mcoop2  mcoop3  mcoop4  mcoop5  mcoop6  mcoop7  narc1  narc2  narc3  narc4  narc5  narc6  nidentity2  omind1  omind3  omind4  omind5  contact3  hygiene3  hygiene5  political_ideology  happy  slf_ladder  riskperc1  riskperc2  self_esteem  sbelong1  sbelong2  sbelong3  sbelong4  optim1  optim2  slfcont1  slfcont3  slfcont4
##   8   6  age  children  cnarc1  cnarc2  cnarc3  ctheory2  ctheory3  ctheory4  CRT1  CRT2  CRT3  employ_status1  health_cond  ladder  marital1  mor_circle  moralid1  moralid10  moralid3  moralid4  moralid5  moralid6  moralid7  moralid8  moralid9  mcoop1  mcoop2  mcoop3  mcoop4  mcoop5  mcoop6  mcoop7  narc1  narc2  narc3  narc4  narc5  narc6  nidentity2  omind1  omind3  omind4  omind5  contact3  hygiene3  hygiene5  political_ideology  happy  slf_ladder  riskperc1  riskperc2  self_esteem  sbelong1  sbelong2  sbelong3  sbelong4  optim1  optim2  slfcont1  slfcont3  slfcont4
##   8   7  age  children  cnarc1  cnarc2  cnarc3  ctheory2  ctheory3  ctheory4  CRT1  CRT2  CRT3  employ_status1  health_cond  ladder  marital1  mor_circle  moralid1  moralid10  moralid3  moralid4  moralid5  moralid6  moralid7  moralid8  moralid9  mcoop1  mcoop2  mcoop3  mcoop4  mcoop5  mcoop6  mcoop7  narc1  narc2  narc3  narc4  narc5  narc6  nidentity2  omind1  omind3  omind4  omind5  contact3  hygiene3  hygiene5  political_ideology  happy  slf_ladder  riskperc1  riskperc2  self_esteem  sbelong1  sbelong2  sbelong3  sbelong4  optim1  optim2  slfcont1  slfcont3  slfcont4
##   8   8  age  children  cnarc1  cnarc2  cnarc3  ctheory2  ctheory3  ctheory4  CRT1  CRT2  CRT3  employ_status1  health_cond  ladder  marital1  mor_circle  moralid1  moralid10  moralid3  moralid4  moralid5  moralid6  moralid7  moralid8  moralid9  mcoop1  mcoop2  mcoop3  mcoop4  mcoop5  mcoop6  mcoop7  narc1  narc2  narc3  narc4  narc5  narc6  nidentity2  omind1  omind3  omind4  omind5  contact3  hygiene3  hygiene5  political_ideology  happy  slf_ladder  riskperc1  riskperc2  self_esteem  sbelong1  sbelong2  sbelong3  sbelong4  optim1  optim2  slfcont1  slfcont3  slfcont4
##   8   9  age  children  cnarc1  cnarc2  cnarc3  ctheory2  ctheory3  ctheory4  CRT1  CRT2  CRT3  employ_status1  health_cond  ladder  marital1  mor_circle  moralid1  moralid10  moralid3  moralid4  moralid5  moralid6  moralid7  moralid8  moralid9  mcoop1  mcoop2  mcoop3  mcoop4  mcoop5  mcoop6  mcoop7  narc1  narc2  narc3  narc4  narc5  narc6  nidentity2  omind1  omind3  omind4  omind5  contact3  hygiene3  hygiene5  political_ideology  happy  slf_ladder  riskperc1  riskperc2  self_esteem  sbelong1  sbelong2  sbelong3  sbelong4  optim1  optim2  slfcont1  slfcont3  slfcont4
##   8   10  age  children  cnarc1  cnarc2  cnarc3  ctheory2  ctheory3  ctheory4  CRT1  CRT2  CRT3  employ_status1  health_cond  ladder  marital1  mor_circle  moralid1  moralid10  moralid3  moralid4  moralid5  moralid6  moralid7  moralid8  moralid9  mcoop1  mcoop2  mcoop3  mcoop4  mcoop5  mcoop6  mcoop7  narc1  narc2  narc3  narc4  narc5  narc6  nidentity2  omind1  omind3  omind4  omind5  contact3  hygiene3  hygiene5  political_ideology  happy  slf_ladder  riskperc1  riskperc2  self_esteem  sbelong1  sbelong2  sbelong3  sbelong4  optim1  optim2  slfcont1  slfcont3  slfcont4
##   9   1  age  children  cnarc1  cnarc2  cnarc3  ctheory2  ctheory3  ctheory4  CRT1  CRT2  CRT3  employ_status1  health_cond  ladder  marital1  mor_circle  moralid1  moralid10  moralid3  moralid4  moralid5  moralid6  moralid7  moralid8  moralid9  mcoop1  mcoop2  mcoop3  mcoop4  mcoop5  mcoop6  mcoop7  narc1  narc2  narc3  narc4  narc5  narc6  nidentity2  omind1  omind3  omind4  omind5  contact3  hygiene3  hygiene5  political_ideology  happy  slf_ladder  riskperc1  riskperc2  self_esteem  sbelong1  sbelong2  sbelong3  sbelong4  optim1  optim2  slfcont1  slfcont3  slfcont4
##   9   2  age  children  cnarc1  cnarc2  cnarc3  ctheory2  ctheory3  ctheory4  CRT1  CRT2  CRT3  employ_status1  health_cond  ladder  marital1  mor_circle  moralid1  moralid10  moralid3  moralid4  moralid5  moralid6  moralid7  moralid8  moralid9  mcoop1  mcoop2  mcoop3  mcoop4  mcoop5  mcoop6  mcoop7  narc1  narc2  narc3  narc4  narc5  narc6  nidentity2  omind1  omind3  omind4  omind5  contact3  hygiene3  hygiene5  political_ideology  happy  slf_ladder  riskperc1  riskperc2  self_esteem  sbelong1  sbelong2  sbelong3  sbelong4  optim1  optim2  slfcont1  slfcont3  slfcont4
##   9   3  age  children  cnarc1  cnarc2  cnarc3  ctheory2  ctheory3  ctheory4  CRT1  CRT2  CRT3  employ_status1  health_cond  ladder  marital1  mor_circle  moralid1  moralid10  moralid3  moralid4  moralid5  moralid6  moralid7  moralid8  moralid9  mcoop1  mcoop2  mcoop3  mcoop4  mcoop5  mcoop6  mcoop7  narc1  narc2  narc3  narc4  narc5  narc6  nidentity2  omind1  omind3  omind4  omind5  contact3  hygiene3  hygiene5  political_ideology  happy  slf_ladder  riskperc1  riskperc2  self_esteem  sbelong1  sbelong2  sbelong3  sbelong4  optim1  optim2  slfcont1  slfcont3  slfcont4
##   9   4  age  children  cnarc1  cnarc2  cnarc3  ctheory2  ctheory3  ctheory4  CRT1  CRT2  CRT3  employ_status1  health_cond  ladder  marital1  mor_circle  moralid1  moralid10  moralid3  moralid4  moralid5  moralid6  moralid7  moralid8  moralid9  mcoop1  mcoop2  mcoop3  mcoop4  mcoop5  mcoop6  mcoop7  narc1  narc2  narc3  narc4  narc5  narc6  nidentity2  omind1  omind3  omind4  omind5  contact3  hygiene3  hygiene5  political_ideology  happy  slf_ladder  riskperc1  riskperc2  self_esteem  sbelong1  sbelong2  sbelong3  sbelong4  optim1  optim2  slfcont1  slfcont3  slfcont4
##   9   5  age  children  cnarc1  cnarc2  cnarc3  ctheory2  ctheory3  ctheory4  CRT1  CRT2  CRT3  employ_status1  health_cond  ladder  marital1  mor_circle  moralid1  moralid10  moralid3  moralid4  moralid5  moralid6  moralid7  moralid8  moralid9  mcoop1  mcoop2  mcoop3  mcoop4  mcoop5  mcoop6  mcoop7  narc1  narc2  narc3  narc4  narc5  narc6  nidentity2  omind1  omind3  omind4  omind5  contact3  hygiene3  hygiene5  political_ideology  happy  slf_ladder  riskperc1  riskperc2  self_esteem  sbelong1  sbelong2  sbelong3  sbelong4  optim1  optim2  slfcont1  slfcont3  slfcont4
##   9   6  age  children  cnarc1  cnarc2  cnarc3  ctheory2  ctheory3  ctheory4  CRT1  CRT2  CRT3  employ_status1  health_cond  ladder  marital1  mor_circle  moralid1  moralid10  moralid3  moralid4  moralid5  moralid6  moralid7  moralid8  moralid9  mcoop1  mcoop2  mcoop3  mcoop4  mcoop5  mcoop6  mcoop7  narc1  narc2  narc3  narc4  narc5  narc6  nidentity2  omind1  omind3  omind4  omind5  contact3  hygiene3  hygiene5  political_ideology  happy  slf_ladder  riskperc1  riskperc2  self_esteem  sbelong1  sbelong2  sbelong3  sbelong4  optim1  optim2  slfcont1  slfcont3  slfcont4
##   9   7  age  children  cnarc1  cnarc2  cnarc3  ctheory2  ctheory3  ctheory4  CRT1  CRT2  CRT3  employ_status1  health_cond  ladder  marital1  mor_circle  moralid1  moralid10  moralid3  moralid4  moralid5  moralid6  moralid7  moralid8  moralid9  mcoop1  mcoop2  mcoop3  mcoop4  mcoop5  mcoop6  mcoop7  narc1  narc2  narc3  narc4  narc5  narc6  nidentity2  omind1  omind3  omind4  omind5  contact3  hygiene3  hygiene5  political_ideology  happy  slf_ladder  riskperc1  riskperc2  self_esteem  sbelong1  sbelong2  sbelong3  sbelong4  optim1  optim2  slfcont1  slfcont3  slfcont4
##   9   8  age  children  cnarc1  cnarc2  cnarc3  ctheory2  ctheory3  ctheory4  CRT1  CRT2  CRT3  employ_status1  health_cond  ladder  marital1  mor_circle  moralid1  moralid10  moralid3  moralid4  moralid5  moralid6  moralid7  moralid8  moralid9  mcoop1  mcoop2  mcoop3  mcoop4  mcoop5  mcoop6  mcoop7  narc1  narc2  narc3  narc4  narc5  narc6  nidentity2  omind1  omind3  omind4  omind5  contact3  hygiene3  hygiene5  political_ideology  happy  slf_ladder  riskperc1  riskperc2  self_esteem  sbelong1  sbelong2  sbelong3  sbelong4  optim1  optim2  slfcont1  slfcont3  slfcont4
##   9   9  age  children  cnarc1  cnarc2  cnarc3  ctheory2  ctheory3  ctheory4  CRT1  CRT2  CRT3  employ_status1  health_cond  ladder  marital1  mor_circle  moralid1  moralid10  moralid3  moralid4  moralid5  moralid6  moralid7  moralid8  moralid9  mcoop1  mcoop2  mcoop3  mcoop4  mcoop5  mcoop6  mcoop7  narc1  narc2  narc3  narc4  narc5  narc6  nidentity2  omind1  omind3  omind4  omind5  contact3  hygiene3  hygiene5  political_ideology  happy  slf_ladder  riskperc1  riskperc2  self_esteem  sbelong1  sbelong2  sbelong3  sbelong4  optim1  optim2  slfcont1  slfcont3  slfcont4
##   9   10  age  children  cnarc1  cnarc2  cnarc3  ctheory2  ctheory3  ctheory4  CRT1  CRT2  CRT3  employ_status1  health_cond  ladder  marital1  mor_circle  moralid1  moralid10  moralid3  moralid4  moralid5  moralid6  moralid7  moralid8  moralid9  mcoop1  mcoop2  mcoop3  mcoop4  mcoop5  mcoop6  mcoop7  narc1  narc2  narc3  narc4  narc5  narc6  nidentity2  omind1  omind3  omind4  omind5  contact3  hygiene3  hygiene5  political_ideology  happy  slf_ladder  riskperc1  riskperc2  self_esteem  sbelong1  sbelong2  sbelong3  sbelong4  optim1  optim2  slfcont1  slfcont3  slfcont4
##   10   1  age  children  cnarc1  cnarc2  cnarc3  ctheory2  ctheory3  ctheory4  CRT1  CRT2  CRT3  employ_status1  health_cond  ladder  marital1  mor_circle  moralid1  moralid10  moralid3  moralid4  moralid5  moralid6  moralid7  moralid8  moralid9  mcoop1  mcoop2  mcoop3  mcoop4  mcoop5  mcoop6  mcoop7  narc1  narc2  narc3  narc4  narc5  narc6  nidentity2  omind1  omind3  omind4  omind5  contact3  hygiene3  hygiene5  political_ideology  happy  slf_ladder  riskperc1  riskperc2  self_esteem  sbelong1  sbelong2  sbelong3  sbelong4  optim1  optim2  slfcont1  slfcont3  slfcont4
##   10   2  age  children  cnarc1  cnarc2  cnarc3  ctheory2  ctheory3  ctheory4  CRT1  CRT2  CRT3  employ_status1  health_cond  ladder  marital1  mor_circle  moralid1  moralid10  moralid3  moralid4  moralid5  moralid6  moralid7  moralid8  moralid9  mcoop1  mcoop2  mcoop3  mcoop4  mcoop5  mcoop6  mcoop7  narc1  narc2  narc3  narc4  narc5  narc6  nidentity2  omind1  omind3  omind4  omind5  contact3  hygiene3  hygiene5  political_ideology  happy  slf_ladder  riskperc1  riskperc2  self_esteem  sbelong1  sbelong2  sbelong3  sbelong4  optim1  optim2  slfcont1  slfcont3  slfcont4
##   10   3  age  children  cnarc1  cnarc2  cnarc3  ctheory2  ctheory3  ctheory4  CRT1  CRT2  CRT3  employ_status1  health_cond  ladder  marital1  mor_circle  moralid1  moralid10  moralid3  moralid4  moralid5  moralid6  moralid7  moralid8  moralid9  mcoop1  mcoop2  mcoop3  mcoop4  mcoop5  mcoop6  mcoop7  narc1  narc2  narc3  narc4  narc5  narc6  nidentity2  omind1  omind3  omind4  omind5  contact3  hygiene3  hygiene5  political_ideology  happy  slf_ladder  riskperc1  riskperc2  self_esteem  sbelong1  sbelong2  sbelong3  sbelong4  optim1  optim2  slfcont1  slfcont3  slfcont4
##   10   4  age  children  cnarc1  cnarc2  cnarc3  ctheory2  ctheory3  ctheory4  CRT1  CRT2  CRT3  employ_status1  health_cond  ladder  marital1  mor_circle  moralid1  moralid10  moralid3  moralid4  moralid5  moralid6  moralid7  moralid8  moralid9  mcoop1  mcoop2  mcoop3  mcoop4  mcoop5  mcoop6  mcoop7  narc1  narc2  narc3  narc4  narc5  narc6  nidentity2  omind1  omind3  omind4  omind5  contact3  hygiene3  hygiene5  political_ideology  happy  slf_ladder  riskperc1  riskperc2  self_esteem  sbelong1  sbelong2  sbelong3  sbelong4  optim1  optim2  slfcont1  slfcont3  slfcont4
##   10   5  age  children  cnarc1  cnarc2  cnarc3  ctheory2  ctheory3  ctheory4  CRT1  CRT2  CRT3  employ_status1  health_cond  ladder  marital1  mor_circle  moralid1  moralid10  moralid3  moralid4  moralid5  moralid6  moralid7  moralid8  moralid9  mcoop1  mcoop2  mcoop3  mcoop4  mcoop5  mcoop6  mcoop7  narc1  narc2  narc3  narc4  narc5  narc6  nidentity2  omind1  omind3  omind4  omind5  contact3  hygiene3  hygiene5  political_ideology  happy  slf_ladder  riskperc1  riskperc2  self_esteem  sbelong1  sbelong2  sbelong3  sbelong4  optim1  optim2  slfcont1  slfcont3  slfcont4
##   10   6  age  children  cnarc1  cnarc2  cnarc3  ctheory2  ctheory3  ctheory4  CRT1  CRT2  CRT3  employ_status1  health_cond  ladder  marital1  mor_circle  moralid1  moralid10  moralid3  moralid4  moralid5  moralid6  moralid7  moralid8  moralid9  mcoop1  mcoop2  mcoop3  mcoop4  mcoop5  mcoop6  mcoop7  narc1  narc2  narc3  narc4  narc5  narc6  nidentity2  omind1  omind3  omind4  omind5  contact3  hygiene3  hygiene5  political_ideology  happy  slf_ladder  riskperc1  riskperc2  self_esteem  sbelong1  sbelong2  sbelong3  sbelong4  optim1  optim2  slfcont1  slfcont3  slfcont4
##   10   7  age  children  cnarc1  cnarc2  cnarc3  ctheory2  ctheory3  ctheory4  CRT1  CRT2  CRT3  employ_status1  health_cond  ladder  marital1  mor_circle  moralid1  moralid10  moralid3  moralid4  moralid5  moralid6  moralid7  moralid8  moralid9  mcoop1  mcoop2  mcoop3  mcoop4  mcoop5  mcoop6  mcoop7  narc1  narc2  narc3  narc4  narc5  narc6  nidentity2  omind1  omind3  omind4  omind5  contact3  hygiene3  hygiene5  political_ideology  happy  slf_ladder  riskperc1  riskperc2  self_esteem  sbelong1  sbelong2  sbelong3  sbelong4  optim1  optim2  slfcont1  slfcont3  slfcont4
##   10   8  age  children  cnarc1  cnarc2  cnarc3  ctheory2  ctheory3  ctheory4  CRT1  CRT2  CRT3  employ_status1  health_cond  ladder  marital1  mor_circle  moralid1  moralid10  moralid3  moralid4  moralid5  moralid6  moralid7  moralid8  moralid9  mcoop1  mcoop2  mcoop3  mcoop4  mcoop5  mcoop6  mcoop7  narc1  narc2  narc3  narc4  narc5  narc6  nidentity2  omind1  omind3  omind4  omind5  contact3  hygiene3  hygiene5  political_ideology  happy  slf_ladder  riskperc1  riskperc2  self_esteem  sbelong1  sbelong2  sbelong3  sbelong4  optim1  optim2  slfcont1  slfcont3  slfcont4
##   10   9  age  children  cnarc1  cnarc2  cnarc3  ctheory2  ctheory3  ctheory4  CRT1  CRT2  CRT3  employ_status1  health_cond  ladder  marital1  mor_circle  moralid1  moralid10  moralid3  moralid4  moralid5  moralid6  moralid7  moralid8  moralid9  mcoop1  mcoop2  mcoop3  mcoop4  mcoop5  mcoop6  mcoop7  narc1  narc2  narc3  narc4  narc5  narc6  nidentity2  omind1  omind3  omind4  omind5  contact3  hygiene3  hygiene5  political_ideology  happy  slf_ladder  riskperc1  riskperc2  self_esteem  sbelong1  sbelong2  sbelong3  sbelong4  optim1  optim2  slfcont1  slfcont3  slfcont4
##   10   10  age  children  cnarc1  cnarc2  cnarc3  ctheory2  ctheory3  ctheory4  CRT1  CRT2  CRT3  employ_status1  health_cond  ladder  marital1  mor_circle  moralid1  moralid10  moralid3  moralid4  moralid5  moralid6  moralid7  moralid8  moralid9  mcoop1  mcoop2  mcoop3  mcoop4  mcoop5  mcoop6  mcoop7  narc1  narc2  narc3  narc4  narc5  narc6  nidentity2  omind1  omind3  omind4  omind5  contact3  hygiene3  hygiene5  political_ideology  happy  slf_ladder  riskperc1  riskperc2  self_esteem  sbelong1  sbelong2  sbelong3  sbelong4  optim1  optim2  slfcont1  slfcont3  slfcont4
```

```
## Warning: Number of logged events: 6103
```

```
## 
##  iter imp variable
##   1   1  age  children  cnarc1  cnarc2  cnarc3  ctheory1  ctheory2  ctheory3  ctheory4  CRT1  CRT2  CRT3  employ_status1  health_cond  know_tested_positive  ladder  marital1  mor_circle  moralid1  moralid10  moralid2  moralid3  moralid4  moralid5  moralid6  moralid7  moralid8  moralid9  mcoop1  mcoop2  mcoop3  mcoop4  mcoop5  mcoop6  mcoop7  narc1  narc2  narc3  narc4  narc5  narc6  nidentity1  nidentity2  omind1  omind2  omind3  omind4  omind5  omind6  contact1  contact2  contact3  contact4  contact5  hygiene1  hygiene2  hygiene3  hygiene4  hygiene5  psupport1  psupport2  psupport3  psupport4  psupport5  political_ideology  happy  slf_ladder  riskperc1  riskperc2  self_esteem  sex1  sbelong1  sbelong2  sbelong3  sbelong4  tested_positive  optim1  optim2  slfcont1  slfcont3  slfcont4  urban
##   1   2  age  children  cnarc1  cnarc2  cnarc3  ctheory1  ctheory2  ctheory3  ctheory4  CRT1  CRT2  CRT3  employ_status1  health_cond  know_tested_positive  ladder  marital1  mor_circle  moralid1  moralid10  moralid2  moralid3  moralid4  moralid5  moralid6  moralid7  moralid8  moralid9  mcoop1  mcoop2  mcoop3  mcoop4  mcoop5  mcoop6  mcoop7  narc1  narc2  narc3  narc4  narc5  narc6  nidentity1  nidentity2  omind1  omind2  omind3  omind4  omind5  omind6  contact1  contact2  contact3  contact4  contact5  hygiene1  hygiene2  hygiene3  hygiene4  hygiene5  psupport1  psupport2  psupport3  psupport4  psupport5  political_ideology  happy  slf_ladder  riskperc1  riskperc2  self_esteem  sex1  sbelong1  sbelong2  sbelong3  sbelong4  tested_positive  optim1  optim2  slfcont1  slfcont3  slfcont4  urban
##   1   3  age  children  cnarc1  cnarc2  cnarc3  ctheory1  ctheory2  ctheory3  ctheory4  CRT1  CRT2  CRT3  employ_status1  health_cond  know_tested_positive  ladder  marital1  mor_circle  moralid1  moralid10  moralid2  moralid3  moralid4  moralid5  moralid6  moralid7  moralid8  moralid9  mcoop1  mcoop2  mcoop3  mcoop4  mcoop5  mcoop6  mcoop7  narc1  narc2  narc3  narc4  narc5  narc6  nidentity1  nidentity2  omind1  omind2  omind3  omind4  omind5  omind6  contact1  contact2  contact3  contact4  contact5  hygiene1  hygiene2  hygiene3  hygiene4  hygiene5  psupport1  psupport2  psupport3  psupport4  psupport5  political_ideology  happy  slf_ladder  riskperc1  riskperc2  self_esteem  sex1  sbelong1  sbelong2  sbelong3  sbelong4  tested_positive  optim1  optim2  slfcont1  slfcont3  slfcont4  urban
##   1   4  age  children  cnarc1  cnarc2  cnarc3  ctheory1  ctheory2  ctheory3  ctheory4  CRT1  CRT2  CRT3  employ_status1  health_cond  know_tested_positive  ladder  marital1  mor_circle  moralid1  moralid10  moralid2  moralid3  moralid4  moralid5  moralid6  moralid7  moralid8  moralid9  mcoop1  mcoop2  mcoop3  mcoop4  mcoop5  mcoop6  mcoop7  narc1  narc2  narc3  narc4  narc5  narc6  nidentity1  nidentity2  omind1  omind2  omind3  omind4  omind5  omind6  contact1  contact2  contact3  contact4  contact5  hygiene1  hygiene2  hygiene3  hygiene4  hygiene5  psupport1  psupport2  psupport3  psupport4  psupport5  political_ideology  happy  slf_ladder  riskperc1  riskperc2  self_esteem  sex1  sbelong1  sbelong2  sbelong3  sbelong4  tested_positive  optim1  optim2  slfcont1  slfcont3  slfcont4  urban
##   1   5  age  children  cnarc1  cnarc2  cnarc3  ctheory1  ctheory2  ctheory3  ctheory4  CRT1  CRT2  CRT3  employ_status1  health_cond  know_tested_positive  ladder  marital1  mor_circle  moralid1  moralid10  moralid2  moralid3  moralid4  moralid5  moralid6  moralid7  moralid8  moralid9  mcoop1  mcoop2  mcoop3  mcoop4  mcoop5  mcoop6  mcoop7  narc1  narc2  narc3  narc4  narc5  narc6  nidentity1  nidentity2  omind1  omind2  omind3  omind4  omind5  omind6  contact1  contact2  contact3  contact4  contact5  hygiene1  hygiene2  hygiene3  hygiene4  hygiene5  psupport1  psupport2  psupport3  psupport4  psupport5  political_ideology  happy  slf_ladder  riskperc1  riskperc2  self_esteem  sex1  sbelong1  sbelong2  sbelong3  sbelong4  tested_positive  optim1  optim2  slfcont1  slfcont3  slfcont4  urban
##   1   6  age  children  cnarc1  cnarc2  cnarc3  ctheory1  ctheory2  ctheory3  ctheory4  CRT1  CRT2  CRT3  employ_status1  health_cond  know_tested_positive  ladder  marital1  mor_circle  moralid1  moralid10  moralid2  moralid3  moralid4  moralid5  moralid6  moralid7  moralid8  moralid9  mcoop1  mcoop2  mcoop3  mcoop4  mcoop5  mcoop6  mcoop7  narc1  narc2  narc3  narc4  narc5  narc6  nidentity1  nidentity2  omind1  omind2  omind3  omind4  omind5  omind6  contact1  contact2  contact3  contact4  contact5  hygiene1  hygiene2  hygiene3  hygiene4  hygiene5  psupport1  psupport2  psupport3  psupport4  psupport5  political_ideology  happy  slf_ladder  riskperc1  riskperc2  self_esteem  sex1  sbelong1  sbelong2  sbelong3  sbelong4  tested_positive  optim1  optim2  slfcont1  slfcont3  slfcont4  urban
##   1   7  age  children  cnarc1  cnarc2  cnarc3  ctheory1  ctheory2  ctheory3  ctheory4  CRT1  CRT2  CRT3  employ_status1  health_cond  know_tested_positive  ladder  marital1  mor_circle  moralid1  moralid10  moralid2  moralid3  moralid4  moralid5  moralid6  moralid7  moralid8  moralid9  mcoop1  mcoop2  mcoop3  mcoop4  mcoop5  mcoop6  mcoop7  narc1  narc2  narc3  narc4  narc5  narc6  nidentity1  nidentity2  omind1  omind2  omind3  omind4  omind5  omind6  contact1  contact2  contact3  contact4  contact5  hygiene1  hygiene2  hygiene3  hygiene4  hygiene5  psupport1  psupport2  psupport3  psupport4  psupport5  political_ideology  happy  slf_ladder  riskperc1  riskperc2  self_esteem  sex1  sbelong1  sbelong2  sbelong3  sbelong4  tested_positive  optim1  optim2  slfcont1  slfcont3  slfcont4  urban
##   1   8  age  children  cnarc1  cnarc2  cnarc3  ctheory1  ctheory2  ctheory3  ctheory4  CRT1  CRT2  CRT3  employ_status1  health_cond  know_tested_positive  ladder  marital1  mor_circle  moralid1  moralid10  moralid2  moralid3  moralid4  moralid5  moralid6  moralid7  moralid8  moralid9  mcoop1  mcoop2  mcoop3  mcoop4  mcoop5  mcoop6  mcoop7  narc1  narc2  narc3  narc4  narc5  narc6  nidentity1  nidentity2  omind1  omind2  omind3  omind4  omind5  omind6  contact1  contact2  contact3  contact4  contact5  hygiene1  hygiene2  hygiene3  hygiene4  hygiene5  psupport1  psupport2  psupport3  psupport4  psupport5  political_ideology  happy  slf_ladder  riskperc1  riskperc2  self_esteem  sex1  sbelong1  sbelong2  sbelong3  sbelong4  tested_positive  optim1  optim2  slfcont1  slfcont3  slfcont4  urban
##   1   9  age  children  cnarc1  cnarc2  cnarc3  ctheory1  ctheory2  ctheory3  ctheory4  CRT1  CRT2  CRT3  employ_status1  health_cond  know_tested_positive  ladder  marital1  mor_circle  moralid1  moralid10  moralid2  moralid3  moralid4  moralid5  moralid6  moralid7  moralid8  moralid9  mcoop1  mcoop2  mcoop3  mcoop4  mcoop5  mcoop6  mcoop7  narc1  narc2  narc3  narc4  narc5  narc6  nidentity1  nidentity2  omind1  omind2  omind3  omind4  omind5  omind6  contact1  contact2  contact3  contact4  contact5  hygiene1  hygiene2  hygiene3  hygiene4  hygiene5  psupport1  psupport2  psupport3  psupport4  psupport5  political_ideology  happy  slf_ladder  riskperc1  riskperc2  self_esteem  sex1  sbelong1  sbelong2  sbelong3  sbelong4  tested_positive  optim1  optim2  slfcont1  slfcont3  slfcont4  urban
##   1   10  age  children  cnarc1  cnarc2  cnarc3  ctheory1  ctheory2  ctheory3  ctheory4  CRT1  CRT2  CRT3  employ_status1  health_cond  know_tested_positive  ladder  marital1  mor_circle  moralid1  moralid10  moralid2  moralid3  moralid4  moralid5  moralid6  moralid7  moralid8  moralid9  mcoop1  mcoop2  mcoop3  mcoop4  mcoop5  mcoop6  mcoop7  narc1  narc2  narc3  narc4  narc5  narc6  nidentity1  nidentity2  omind1  omind2  omind3  omind4  omind5  omind6  contact1  contact2  contact3  contact4  contact5  hygiene1  hygiene2  hygiene3  hygiene4  hygiene5  psupport1  psupport2  psupport3  psupport4  psupport5  political_ideology  happy  slf_ladder  riskperc1  riskperc2  self_esteem  sex1  sbelong1  sbelong2  sbelong3  sbelong4  tested_positive  optim1  optim2  slfcont1  slfcont3  slfcont4  urban
##   2   1  age  children  cnarc1  cnarc2  cnarc3  ctheory1  ctheory2  ctheory3  ctheory4  CRT1  CRT2  CRT3  employ_status1  health_cond  know_tested_positive  ladder  marital1  mor_circle  moralid1  moralid10  moralid2  moralid3  moralid4  moralid5  moralid6  moralid7  moralid8  moralid9  mcoop1  mcoop2  mcoop3  mcoop4  mcoop5  mcoop6  mcoop7  narc1  narc2  narc3  narc4  narc5  narc6  nidentity1  nidentity2  omind1  omind2  omind3  omind4  omind5  omind6  contact1  contact2  contact3  contact4  contact5  hygiene1  hygiene2  hygiene3  hygiene4  hygiene5  psupport1  psupport2  psupport3  psupport4  psupport5  political_ideology  happy  slf_ladder  riskperc1  riskperc2  self_esteem  sex1  sbelong1  sbelong2  sbelong3  sbelong4  tested_positive  optim1  optim2  slfcont1  slfcont3  slfcont4  urban
##   2   2  age  children  cnarc1  cnarc2  cnarc3  ctheory1  ctheory2  ctheory3  ctheory4  CRT1  CRT2  CRT3  employ_status1  health_cond  know_tested_positive  ladder  marital1  mor_circle  moralid1  moralid10  moralid2  moralid3  moralid4  moralid5  moralid6  moralid7  moralid8  moralid9  mcoop1  mcoop2  mcoop3  mcoop4  mcoop5  mcoop6  mcoop7  narc1  narc2  narc3  narc4  narc5  narc6  nidentity1  nidentity2  omind1  omind2  omind3  omind4  omind5  omind6  contact1  contact2  contact3  contact4  contact5  hygiene1  hygiene2  hygiene3  hygiene4  hygiene5  psupport1  psupport2  psupport3  psupport4  psupport5  political_ideology  happy  slf_ladder  riskperc1  riskperc2  self_esteem  sex1  sbelong1  sbelong2  sbelong3  sbelong4  tested_positive  optim1  optim2  slfcont1  slfcont3  slfcont4  urban
##   2   3  age  children  cnarc1  cnarc2  cnarc3  ctheory1  ctheory2  ctheory3  ctheory4  CRT1  CRT2  CRT3  employ_status1  health_cond  know_tested_positive  ladder  marital1  mor_circle  moralid1  moralid10  moralid2  moralid3  moralid4  moralid5  moralid6  moralid7  moralid8  moralid9  mcoop1  mcoop2  mcoop3  mcoop4  mcoop5  mcoop6  mcoop7  narc1  narc2  narc3  narc4  narc5  narc6  nidentity1  nidentity2  omind1  omind2  omind3  omind4  omind5  omind6  contact1  contact2  contact3  contact4  contact5  hygiene1  hygiene2  hygiene3  hygiene4  hygiene5  psupport1  psupport2  psupport3  psupport4  psupport5  political_ideology  happy  slf_ladder  riskperc1  riskperc2  self_esteem  sex1  sbelong1  sbelong2  sbelong3  sbelong4  tested_positive  optim1  optim2  slfcont1  slfcont3  slfcont4  urban
##   2   4  age  children  cnarc1  cnarc2  cnarc3  ctheory1  ctheory2  ctheory3  ctheory4  CRT1  CRT2  CRT3  employ_status1  health_cond  know_tested_positive  ladder  marital1  mor_circle  moralid1  moralid10  moralid2  moralid3  moralid4  moralid5  moralid6  moralid7  moralid8  moralid9  mcoop1  mcoop2  mcoop3  mcoop4  mcoop5  mcoop6  mcoop7  narc1  narc2  narc3  narc4  narc5  narc6  nidentity1  nidentity2  omind1  omind2  omind3  omind4  omind5  omind6  contact1  contact2  contact3  contact4  contact5  hygiene1  hygiene2  hygiene3  hygiene4  hygiene5  psupport1  psupport2  psupport3  psupport4  psupport5  political_ideology  happy  slf_ladder  riskperc1  riskperc2  self_esteem  sex1  sbelong1  sbelong2  sbelong3  sbelong4  tested_positive  optim1  optim2  slfcont1  slfcont3  slfcont4  urban
##   2   5  age  children  cnarc1  cnarc2  cnarc3  ctheory1  ctheory2  ctheory3  ctheory4  CRT1  CRT2  CRT3  employ_status1  health_cond  know_tested_positive  ladder  marital1  mor_circle  moralid1  moralid10  moralid2  moralid3  moralid4  moralid5  moralid6  moralid7  moralid8  moralid9  mcoop1  mcoop2  mcoop3  mcoop4  mcoop5  mcoop6  mcoop7  narc1  narc2  narc3  narc4  narc5  narc6  nidentity1  nidentity2  omind1  omind2  omind3  omind4  omind5  omind6  contact1  contact2  contact3  contact4  contact5  hygiene1  hygiene2  hygiene3  hygiene4  hygiene5  psupport1  psupport2  psupport3  psupport4  psupport5  political_ideology  happy  slf_ladder  riskperc1  riskperc2  self_esteem  sex1  sbelong1  sbelong2  sbelong3  sbelong4  tested_positive  optim1  optim2  slfcont1  slfcont3  slfcont4  urban
##   2   6  age  children  cnarc1  cnarc2  cnarc3  ctheory1  ctheory2  ctheory3  ctheory4  CRT1  CRT2  CRT3  employ_status1  health_cond  know_tested_positive  ladder  marital1  mor_circle  moralid1  moralid10  moralid2  moralid3  moralid4  moralid5  moralid6  moralid7  moralid8  moralid9  mcoop1  mcoop2  mcoop3  mcoop4  mcoop5  mcoop6  mcoop7  narc1  narc2  narc3  narc4  narc5  narc6  nidentity1  nidentity2  omind1  omind2  omind3  omind4  omind5  omind6  contact1  contact2  contact3  contact4  contact5  hygiene1  hygiene2  hygiene3  hygiene4  hygiene5  psupport1  psupport2  psupport3  psupport4  psupport5  political_ideology  happy  slf_ladder  riskperc1  riskperc2  self_esteem  sex1  sbelong1  sbelong2  sbelong3  sbelong4  tested_positive  optim1  optim2  slfcont1  slfcont3  slfcont4  urban
##   2   7  age  children  cnarc1  cnarc2  cnarc3  ctheory1  ctheory2  ctheory3  ctheory4  CRT1  CRT2  CRT3  employ_status1  health_cond  know_tested_positive  ladder  marital1  mor_circle  moralid1  moralid10  moralid2  moralid3  moralid4  moralid5  moralid6  moralid7  moralid8  moralid9  mcoop1  mcoop2  mcoop3  mcoop4  mcoop5  mcoop6  mcoop7  narc1  narc2  narc3  narc4  narc5  narc6  nidentity1  nidentity2  omind1  omind2  omind3  omind4  omind5  omind6  contact1  contact2  contact3  contact4  contact5  hygiene1  hygiene2  hygiene3  hygiene4  hygiene5  psupport1  psupport2  psupport3  psupport4  psupport5  political_ideology  happy  slf_ladder  riskperc1  riskperc2  self_esteem  sex1  sbelong1  sbelong2  sbelong3  sbelong4  tested_positive  optim1  optim2  slfcont1  slfcont3  slfcont4  urban
##   2   8  age  children  cnarc1  cnarc2  cnarc3  ctheory1  ctheory2  ctheory3  ctheory4  CRT1  CRT2  CRT3  employ_status1  health_cond  know_tested_positive  ladder  marital1  mor_circle  moralid1  moralid10  moralid2  moralid3  moralid4  moralid5  moralid6  moralid7  moralid8  moralid9  mcoop1  mcoop2  mcoop3  mcoop4  mcoop5  mcoop6  mcoop7  narc1  narc2  narc3  narc4  narc5  narc6  nidentity1  nidentity2  omind1  omind2  omind3  omind4  omind5  omind6  contact1  contact2  contact3  contact4  contact5  hygiene1  hygiene2  hygiene3  hygiene4  hygiene5  psupport1  psupport2  psupport3  psupport4  psupport5  political_ideology  happy  slf_ladder  riskperc1  riskperc2  self_esteem  sex1  sbelong1  sbelong2  sbelong3  sbelong4  tested_positive  optim1  optim2  slfcont1  slfcont3  slfcont4  urban
##   2   9  age  children  cnarc1  cnarc2  cnarc3  ctheory1  ctheory2  ctheory3  ctheory4  CRT1  CRT2  CRT3  employ_status1  health_cond  know_tested_positive  ladder  marital1  mor_circle  moralid1  moralid10  moralid2  moralid3  moralid4  moralid5  moralid6  moralid7  moralid8  moralid9  mcoop1  mcoop2  mcoop3  mcoop4  mcoop5  mcoop6  mcoop7  narc1  narc2  narc3  narc4  narc5  narc6  nidentity1  nidentity2  omind1  omind2  omind3  omind4  omind5  omind6  contact1  contact2  contact3  contact4  contact5  hygiene1  hygiene2  hygiene3  hygiene4  hygiene5  psupport1  psupport2  psupport3  psupport4  psupport5  political_ideology  happy  slf_ladder  riskperc1  riskperc2  self_esteem  sex1  sbelong1  sbelong2  sbelong3  sbelong4  tested_positive  optim1  optim2  slfcont1  slfcont3  slfcont4  urban
##   2   10  age  children  cnarc1  cnarc2  cnarc3  ctheory1  ctheory2  ctheory3  ctheory4  CRT1  CRT2  CRT3  employ_status1  health_cond  know_tested_positive  ladder  marital1  mor_circle  moralid1  moralid10  moralid2  moralid3  moralid4  moralid5  moralid6  moralid7  moralid8  moralid9  mcoop1  mcoop2  mcoop3  mcoop4  mcoop5  mcoop6  mcoop7  narc1  narc2  narc3  narc4  narc5  narc6  nidentity1  nidentity2  omind1  omind2  omind3  omind4  omind5  omind6  contact1  contact2  contact3  contact4  contact5  hygiene1  hygiene2  hygiene3  hygiene4  hygiene5  psupport1  psupport2  psupport3  psupport4  psupport5  political_ideology  happy  slf_ladder  riskperc1  riskperc2  self_esteem  sex1  sbelong1  sbelong2  sbelong3  sbelong4  tested_positive  optim1  optim2  slfcont1  slfcont3  slfcont4  urban
##   3   1  age  children  cnarc1  cnarc2  cnarc3  ctheory1  ctheory2  ctheory3  ctheory4  CRT1  CRT2  CRT3  employ_status1  health_cond  know_tested_positive  ladder  marital1  mor_circle  moralid1  moralid10  moralid2  moralid3  moralid4  moralid5  moralid6  moralid7  moralid8  moralid9  mcoop1  mcoop2  mcoop3  mcoop4  mcoop5  mcoop6  mcoop7  narc1  narc2  narc3  narc4  narc5  narc6  nidentity1  nidentity2  omind1  omind2  omind3  omind4  omind5  omind6  contact1  contact2  contact3  contact4  contact5  hygiene1  hygiene2  hygiene3  hygiene4  hygiene5  psupport1  psupport2  psupport3  psupport4  psupport5  political_ideology  happy  slf_ladder  riskperc1  riskperc2  self_esteem  sex1  sbelong1  sbelong2  sbelong3  sbelong4  tested_positive  optim1  optim2  slfcont1  slfcont3  slfcont4  urban
##   3   2  age  children  cnarc1  cnarc2  cnarc3  ctheory1  ctheory2  ctheory3  ctheory4  CRT1  CRT2  CRT3  employ_status1  health_cond  know_tested_positive  ladder  marital1  mor_circle  moralid1  moralid10  moralid2  moralid3  moralid4  moralid5  moralid6  moralid7  moralid8  moralid9  mcoop1  mcoop2  mcoop3  mcoop4  mcoop5  mcoop6  mcoop7  narc1  narc2  narc3  narc4  narc5  narc6  nidentity1  nidentity2  omind1  omind2  omind3  omind4  omind5  omind6  contact1  contact2  contact3  contact4  contact5  hygiene1  hygiene2  hygiene3  hygiene4  hygiene5  psupport1  psupport2  psupport3  psupport4  psupport5  political_ideology  happy  slf_ladder  riskperc1  riskperc2  self_esteem  sex1  sbelong1  sbelong2  sbelong3  sbelong4  tested_positive  optim1  optim2  slfcont1  slfcont3  slfcont4  urban
##   3   3  age  children  cnarc1  cnarc2  cnarc3  ctheory1  ctheory2  ctheory3  ctheory4  CRT1  CRT2  CRT3  employ_status1  health_cond  know_tested_positive  ladder  marital1  mor_circle  moralid1  moralid10  moralid2  moralid3  moralid4  moralid5  moralid6  moralid7  moralid8  moralid9  mcoop1  mcoop2  mcoop3  mcoop4  mcoop5  mcoop6  mcoop7  narc1  narc2  narc3  narc4  narc5  narc6  nidentity1  nidentity2  omind1  omind2  omind3  omind4  omind5  omind6  contact1  contact2  contact3  contact4  contact5  hygiene1  hygiene2  hygiene3  hygiene4  hygiene5  psupport1  psupport2  psupport3  psupport4  psupport5  political_ideology  happy  slf_ladder  riskperc1  riskperc2  self_esteem  sex1  sbelong1  sbelong2  sbelong3  sbelong4  tested_positive  optim1  optim2  slfcont1  slfcont3  slfcont4  urban
##   3   4  age  children  cnarc1  cnarc2  cnarc3  ctheory1  ctheory2  ctheory3  ctheory4  CRT1  CRT2  CRT3  employ_status1  health_cond  know_tested_positive  ladder  marital1  mor_circle  moralid1  moralid10  moralid2  moralid3  moralid4  moralid5  moralid6  moralid7  moralid8  moralid9  mcoop1  mcoop2  mcoop3  mcoop4  mcoop5  mcoop6  mcoop7  narc1  narc2  narc3  narc4  narc5  narc6  nidentity1  nidentity2  omind1  omind2  omind3  omind4  omind5  omind6  contact1  contact2  contact3  contact4  contact5  hygiene1  hygiene2  hygiene3  hygiene4  hygiene5  psupport1  psupport2  psupport3  psupport4  psupport5  political_ideology  happy  slf_ladder  riskperc1  riskperc2  self_esteem  sex1  sbelong1  sbelong2  sbelong3  sbelong4  tested_positive  optim1  optim2  slfcont1  slfcont3  slfcont4  urban
##   3   5  age  children  cnarc1  cnarc2  cnarc3  ctheory1  ctheory2  ctheory3  ctheory4  CRT1  CRT2  CRT3  employ_status1  health_cond  know_tested_positive  ladder  marital1  mor_circle  moralid1  moralid10  moralid2  moralid3  moralid4  moralid5  moralid6  moralid7  moralid8  moralid9  mcoop1  mcoop2  mcoop3  mcoop4  mcoop5  mcoop6  mcoop7  narc1  narc2  narc3  narc4  narc5  narc6  nidentity1  nidentity2  omind1  omind2  omind3  omind4  omind5  omind6  contact1  contact2  contact3  contact4  contact5  hygiene1  hygiene2  hygiene3  hygiene4  hygiene5  psupport1  psupport2  psupport3  psupport4  psupport5  political_ideology  happy  slf_ladder  riskperc1  riskperc2  self_esteem  sex1  sbelong1  sbelong2  sbelong3  sbelong4  tested_positive  optim1  optim2  slfcont1  slfcont3  slfcont4  urban
##   3   6  age  children  cnarc1  cnarc2  cnarc3  ctheory1  ctheory2  ctheory3  ctheory4  CRT1  CRT2  CRT3  employ_status1  health_cond  know_tested_positive  ladder  marital1  mor_circle  moralid1  moralid10  moralid2  moralid3  moralid4  moralid5  moralid6  moralid7  moralid8  moralid9  mcoop1  mcoop2  mcoop3  mcoop4  mcoop5  mcoop6  mcoop7  narc1  narc2  narc3  narc4  narc5  narc6  nidentity1  nidentity2  omind1  omind2  omind3  omind4  omind5  omind6  contact1  contact2  contact3  contact4  contact5  hygiene1  hygiene2  hygiene3  hygiene4  hygiene5  psupport1  psupport2  psupport3  psupport4  psupport5  political_ideology  happy  slf_ladder  riskperc1  riskperc2  self_esteem  sex1  sbelong1  sbelong2  sbelong3  sbelong4  tested_positive  optim1  optim2  slfcont1  slfcont3  slfcont4  urban
##   3   7  age  children  cnarc1  cnarc2  cnarc3  ctheory1  ctheory2  ctheory3  ctheory4  CRT1  CRT2  CRT3  employ_status1  health_cond  know_tested_positive  ladder  marital1  mor_circle  moralid1  moralid10  moralid2  moralid3  moralid4  moralid5  moralid6  moralid7  moralid8  moralid9  mcoop1  mcoop2  mcoop3  mcoop4  mcoop5  mcoop6  mcoop7  narc1  narc2  narc3  narc4  narc5  narc6  nidentity1  nidentity2  omind1  omind2  omind3  omind4  omind5  omind6  contact1  contact2  contact3  contact4  contact5  hygiene1  hygiene2  hygiene3  hygiene4  hygiene5  psupport1  psupport2  psupport3  psupport4  psupport5  political_ideology  happy  slf_ladder  riskperc1  riskperc2  self_esteem  sex1  sbelong1  sbelong2  sbelong3  sbelong4  tested_positive  optim1  optim2  slfcont1  slfcont3  slfcont4  urban
##   3   8  age  children  cnarc1  cnarc2  cnarc3  ctheory1  ctheory2  ctheory3  ctheory4  CRT1  CRT2  CRT3  employ_status1  health_cond  know_tested_positive  ladder  marital1  mor_circle  moralid1  moralid10  moralid2  moralid3  moralid4  moralid5  moralid6  moralid7  moralid8  moralid9  mcoop1  mcoop2  mcoop3  mcoop4  mcoop5  mcoop6  mcoop7  narc1  narc2  narc3  narc4  narc5  narc6  nidentity1  nidentity2  omind1  omind2  omind3  omind4  omind5  omind6  contact1  contact2  contact3  contact4  contact5  hygiene1  hygiene2  hygiene3  hygiene4  hygiene5  psupport1  psupport2  psupport3  psupport4  psupport5  political_ideology  happy  slf_ladder  riskperc1  riskperc2  self_esteem  sex1  sbelong1  sbelong2  sbelong3  sbelong4  tested_positive  optim1  optim2  slfcont1  slfcont3  slfcont4  urban
##   3   9  age  children  cnarc1  cnarc2  cnarc3  ctheory1  ctheory2  ctheory3  ctheory4  CRT1  CRT2  CRT3  employ_status1  health_cond  know_tested_positive  ladder  marital1  mor_circle  moralid1  moralid10  moralid2  moralid3  moralid4  moralid5  moralid6  moralid7  moralid8  moralid9  mcoop1  mcoop2  mcoop3  mcoop4  mcoop5  mcoop6  mcoop7  narc1  narc2  narc3  narc4  narc5  narc6  nidentity1  nidentity2  omind1  omind2  omind3  omind4  omind5  omind6  contact1  contact2  contact3  contact4  contact5  hygiene1  hygiene2  hygiene3  hygiene4  hygiene5  psupport1  psupport2  psupport3  psupport4  psupport5  political_ideology  happy  slf_ladder  riskperc1  riskperc2  self_esteem  sex1  sbelong1  sbelong2  sbelong3  sbelong4  tested_positive  optim1  optim2  slfcont1  slfcont3  slfcont4  urban
##   3   10  age  children  cnarc1  cnarc2  cnarc3  ctheory1  ctheory2  ctheory3  ctheory4  CRT1  CRT2  CRT3  employ_status1  health_cond  know_tested_positive  ladder  marital1  mor_circle  moralid1  moralid10  moralid2  moralid3  moralid4  moralid5  moralid6  moralid7  moralid8  moralid9  mcoop1  mcoop2  mcoop3  mcoop4  mcoop5  mcoop6  mcoop7  narc1  narc2  narc3  narc4  narc5  narc6  nidentity1  nidentity2  omind1  omind2  omind3  omind4  omind5  omind6  contact1  contact2  contact3  contact4  contact5  hygiene1  hygiene2  hygiene3  hygiene4  hygiene5  psupport1  psupport2  psupport3  psupport4  psupport5  political_ideology  happy  slf_ladder  riskperc1  riskperc2  self_esteem  sex1  sbelong1  sbelong2  sbelong3  sbelong4  tested_positive  optim1  optim2  slfcont1  slfcont3  slfcont4  urban
##   4   1  age  children  cnarc1  cnarc2  cnarc3  ctheory1  ctheory2  ctheory3  ctheory4  CRT1  CRT2  CRT3  employ_status1  health_cond  know_tested_positive  ladder  marital1  mor_circle  moralid1  moralid10  moralid2  moralid3  moralid4  moralid5  moralid6  moralid7  moralid8  moralid9  mcoop1  mcoop2  mcoop3  mcoop4  mcoop5  mcoop6  mcoop7  narc1  narc2  narc3  narc4  narc5  narc6  nidentity1  nidentity2  omind1  omind2  omind3  omind4  omind5  omind6  contact1  contact2  contact3  contact4  contact5  hygiene1  hygiene2  hygiene3  hygiene4  hygiene5  psupport1  psupport2  psupport3  psupport4  psupport5  political_ideology  happy  slf_ladder  riskperc1  riskperc2  self_esteem  sex1  sbelong1  sbelong2  sbelong3  sbelong4  tested_positive  optim1  optim2  slfcont1  slfcont3  slfcont4  urban
##   4   2  age  children  cnarc1  cnarc2  cnarc3  ctheory1  ctheory2  ctheory3  ctheory4  CRT1  CRT2  CRT3  employ_status1  health_cond  know_tested_positive  ladder  marital1  mor_circle  moralid1  moralid10  moralid2  moralid3  moralid4  moralid5  moralid6  moralid7  moralid8  moralid9  mcoop1  mcoop2  mcoop3  mcoop4  mcoop5  mcoop6  mcoop7  narc1  narc2  narc3  narc4  narc5  narc6  nidentity1  nidentity2  omind1  omind2  omind3  omind4  omind5  omind6  contact1  contact2  contact3  contact4  contact5  hygiene1  hygiene2  hygiene3  hygiene4  hygiene5  psupport1  psupport2  psupport3  psupport4  psupport5  political_ideology  happy  slf_ladder  riskperc1  riskperc2  self_esteem  sex1  sbelong1  sbelong2  sbelong3  sbelong4  tested_positive  optim1  optim2  slfcont1  slfcont3  slfcont4  urban
##   4   3  age  children  cnarc1  cnarc2  cnarc3  ctheory1  ctheory2  ctheory3  ctheory4  CRT1  CRT2  CRT3  employ_status1  health_cond  know_tested_positive  ladder  marital1  mor_circle  moralid1  moralid10  moralid2  moralid3  moralid4  moralid5  moralid6  moralid7  moralid8  moralid9  mcoop1  mcoop2  mcoop3  mcoop4  mcoop5  mcoop6  mcoop7  narc1  narc2  narc3  narc4  narc5  narc6  nidentity1  nidentity2  omind1  omind2  omind3  omind4  omind5  omind6  contact1  contact2  contact3  contact4  contact5  hygiene1  hygiene2  hygiene3  hygiene4  hygiene5  psupport1  psupport2  psupport3  psupport4  psupport5  political_ideology  happy  slf_ladder  riskperc1  riskperc2  self_esteem  sex1  sbelong1  sbelong2  sbelong3  sbelong4  tested_positive  optim1  optim2  slfcont1  slfcont3  slfcont4  urban
##   4   4  age  children  cnarc1  cnarc2  cnarc3  ctheory1  ctheory2  ctheory3  ctheory4  CRT1  CRT2  CRT3  employ_status1  health_cond  know_tested_positive  ladder  marital1  mor_circle  moralid1  moralid10  moralid2  moralid3  moralid4  moralid5  moralid6  moralid7  moralid8  moralid9  mcoop1  mcoop2  mcoop3  mcoop4  mcoop5  mcoop6  mcoop7  narc1  narc2  narc3  narc4  narc5  narc6  nidentity1  nidentity2  omind1  omind2  omind3  omind4  omind5  omind6  contact1  contact2  contact3  contact4  contact5  hygiene1  hygiene2  hygiene3  hygiene4  hygiene5  psupport1  psupport2  psupport3  psupport4  psupport5  political_ideology  happy  slf_ladder  riskperc1  riskperc2  self_esteem  sex1  sbelong1  sbelong2  sbelong3  sbelong4  tested_positive  optim1  optim2  slfcont1  slfcont3  slfcont4  urban
##   4   5  age  children  cnarc1  cnarc2  cnarc3  ctheory1  ctheory2  ctheory3  ctheory4  CRT1  CRT2  CRT3  employ_status1  health_cond  know_tested_positive  ladder  marital1  mor_circle  moralid1  moralid10  moralid2  moralid3  moralid4  moralid5  moralid6  moralid7  moralid8  moralid9  mcoop1  mcoop2  mcoop3  mcoop4  mcoop5  mcoop6  mcoop7  narc1  narc2  narc3  narc4  narc5  narc6  nidentity1  nidentity2  omind1  omind2  omind3  omind4  omind5  omind6  contact1  contact2  contact3  contact4  contact5  hygiene1  hygiene2  hygiene3  hygiene4  hygiene5  psupport1  psupport2  psupport3  psupport4  psupport5  political_ideology  happy  slf_ladder  riskperc1  riskperc2  self_esteem  sex1  sbelong1  sbelong2  sbelong3  sbelong4  tested_positive  optim1  optim2  slfcont1  slfcont3  slfcont4  urban
##   4   6  age  children  cnarc1  cnarc2  cnarc3  ctheory1  ctheory2  ctheory3  ctheory4  CRT1  CRT2  CRT3  employ_status1  health_cond  know_tested_positive  ladder  marital1  mor_circle  moralid1  moralid10  moralid2  moralid3  moralid4  moralid5  moralid6  moralid7  moralid8  moralid9  mcoop1  mcoop2  mcoop3  mcoop4  mcoop5  mcoop6  mcoop7  narc1  narc2  narc3  narc4  narc5  narc6  nidentity1  nidentity2  omind1  omind2  omind3  omind4  omind5  omind6  contact1  contact2  contact3  contact4  contact5  hygiene1  hygiene2  hygiene3  hygiene4  hygiene5  psupport1  psupport2  psupport3  psupport4  psupport5  political_ideology  happy  slf_ladder  riskperc1  riskperc2  self_esteem  sex1  sbelong1  sbelong2  sbelong3  sbelong4  tested_positive  optim1  optim2  slfcont1  slfcont3  slfcont4  urban
##   4   7  age  children  cnarc1  cnarc2  cnarc3  ctheory1  ctheory2  ctheory3  ctheory4  CRT1  CRT2  CRT3  employ_status1  health_cond  know_tested_positive  ladder  marital1  mor_circle  moralid1  moralid10  moralid2  moralid3  moralid4  moralid5  moralid6  moralid7  moralid8  moralid9  mcoop1  mcoop2  mcoop3  mcoop4  mcoop5  mcoop6  mcoop7  narc1  narc2  narc3  narc4  narc5  narc6  nidentity1  nidentity2  omind1  omind2  omind3  omind4  omind5  omind6  contact1  contact2  contact3  contact4  contact5  hygiene1  hygiene2  hygiene3  hygiene4  hygiene5  psupport1  psupport2  psupport3  psupport4  psupport5  political_ideology  happy  slf_ladder  riskperc1  riskperc2  self_esteem  sex1  sbelong1  sbelong2  sbelong3  sbelong4  tested_positive  optim1  optim2  slfcont1  slfcont3  slfcont4  urban
##   4   8  age  children  cnarc1  cnarc2  cnarc3  ctheory1  ctheory2  ctheory3  ctheory4  CRT1  CRT2  CRT3  employ_status1  health_cond  know_tested_positive  ladder  marital1  mor_circle  moralid1  moralid10  moralid2  moralid3  moralid4  moralid5  moralid6  moralid7  moralid8  moralid9  mcoop1  mcoop2  mcoop3  mcoop4  mcoop5  mcoop6  mcoop7  narc1  narc2  narc3  narc4  narc5  narc6  nidentity1  nidentity2  omind1  omind2  omind3  omind4  omind5  omind6  contact1  contact2  contact3  contact4  contact5  hygiene1  hygiene2  hygiene3  hygiene4  hygiene5  psupport1  psupport2  psupport3  psupport4  psupport5  political_ideology  happy  slf_ladder  riskperc1  riskperc2  self_esteem  sex1  sbelong1  sbelong2  sbelong3  sbelong4  tested_positive  optim1  optim2  slfcont1  slfcont3  slfcont4  urban
##   4   9  age  children  cnarc1  cnarc2  cnarc3  ctheory1  ctheory2  ctheory3  ctheory4  CRT1  CRT2  CRT3  employ_status1  health_cond  know_tested_positive  ladder  marital1  mor_circle  moralid1  moralid10  moralid2  moralid3  moralid4  moralid5  moralid6  moralid7  moralid8  moralid9  mcoop1  mcoop2  mcoop3  mcoop4  mcoop5  mcoop6  mcoop7  narc1  narc2  narc3  narc4  narc5  narc6  nidentity1  nidentity2  omind1  omind2  omind3  omind4  omind5  omind6  contact1  contact2  contact3  contact4  contact5  hygiene1  hygiene2  hygiene3  hygiene4  hygiene5  psupport1  psupport2  psupport3  psupport4  psupport5  political_ideology  happy  slf_ladder  riskperc1  riskperc2  self_esteem  sex1  sbelong1  sbelong2  sbelong3  sbelong4  tested_positive  optim1  optim2  slfcont1  slfcont3  slfcont4  urban
##   4   10  age  children  cnarc1  cnarc2  cnarc3  ctheory1  ctheory2  ctheory3  ctheory4  CRT1  CRT2  CRT3  employ_status1  health_cond  know_tested_positive  ladder  marital1  mor_circle  moralid1  moralid10  moralid2  moralid3  moralid4  moralid5  moralid6  moralid7  moralid8  moralid9  mcoop1  mcoop2  mcoop3  mcoop4  mcoop5  mcoop6  mcoop7  narc1  narc2  narc3  narc4  narc5  narc6  nidentity1  nidentity2  omind1  omind2  omind3  omind4  omind5  omind6  contact1  contact2  contact3  contact4  contact5  hygiene1  hygiene2  hygiene3  hygiene4  hygiene5  psupport1  psupport2  psupport3  psupport4  psupport5  political_ideology  happy  slf_ladder  riskperc1  riskperc2  self_esteem  sex1  sbelong1  sbelong2  sbelong3  sbelong4  tested_positive  optim1  optim2  slfcont1  slfcont3  slfcont4  urban
##   5   1  age  children  cnarc1  cnarc2  cnarc3  ctheory1  ctheory2  ctheory3  ctheory4  CRT1  CRT2  CRT3  employ_status1  health_cond  know_tested_positive  ladder  marital1  mor_circle  moralid1  moralid10  moralid2  moralid3  moralid4  moralid5  moralid6  moralid7  moralid8  moralid9  mcoop1  mcoop2  mcoop3  mcoop4  mcoop5  mcoop6  mcoop7  narc1  narc2  narc3  narc4  narc5  narc6  nidentity1  nidentity2  omind1  omind2  omind3  omind4  omind5  omind6  contact1  contact2  contact3  contact4  contact5  hygiene1  hygiene2  hygiene3  hygiene4  hygiene5  psupport1  psupport2  psupport3  psupport4  psupport5  political_ideology  happy  slf_ladder  riskperc1  riskperc2  self_esteem  sex1  sbelong1  sbelong2  sbelong3  sbelong4  tested_positive  optim1  optim2  slfcont1  slfcont3  slfcont4  urban
##   5   2  age  children  cnarc1  cnarc2  cnarc3  ctheory1  ctheory2  ctheory3  ctheory4  CRT1  CRT2  CRT3  employ_status1  health_cond  know_tested_positive  ladder  marital1  mor_circle  moralid1  moralid10  moralid2  moralid3  moralid4  moralid5  moralid6  moralid7  moralid8  moralid9  mcoop1  mcoop2  mcoop3  mcoop4  mcoop5  mcoop6  mcoop7  narc1  narc2  narc3  narc4  narc5  narc6  nidentity1  nidentity2  omind1  omind2  omind3  omind4  omind5  omind6  contact1  contact2  contact3  contact4  contact5  hygiene1  hygiene2  hygiene3  hygiene4  hygiene5  psupport1  psupport2  psupport3  psupport4  psupport5  political_ideology  happy  slf_ladder  riskperc1  riskperc2  self_esteem  sex1  sbelong1  sbelong2  sbelong3  sbelong4  tested_positive  optim1  optim2  slfcont1  slfcont3  slfcont4  urban
##   5   3  age  children  cnarc1  cnarc2  cnarc3  ctheory1  ctheory2  ctheory3  ctheory4  CRT1  CRT2  CRT3  employ_status1  health_cond  know_tested_positive  ladder  marital1  mor_circle  moralid1  moralid10  moralid2  moralid3  moralid4  moralid5  moralid6  moralid7  moralid8  moralid9  mcoop1  mcoop2  mcoop3  mcoop4  mcoop5  mcoop6  mcoop7  narc1  narc2  narc3  narc4  narc5  narc6  nidentity1  nidentity2  omind1  omind2  omind3  omind4  omind5  omind6  contact1  contact2  contact3  contact4  contact5  hygiene1  hygiene2  hygiene3  hygiene4  hygiene5  psupport1  psupport2  psupport3  psupport4  psupport5  political_ideology  happy  slf_ladder  riskperc1  riskperc2  self_esteem  sex1  sbelong1  sbelong2  sbelong3  sbelong4  tested_positive  optim1  optim2  slfcont1  slfcont3  slfcont4  urban
##   5   4  age  children  cnarc1  cnarc2  cnarc3  ctheory1  ctheory2  ctheory3  ctheory4  CRT1  CRT2  CRT3  employ_status1  health_cond  know_tested_positive  ladder  marital1  mor_circle  moralid1  moralid10  moralid2  moralid3  moralid4  moralid5  moralid6  moralid7  moralid8  moralid9  mcoop1  mcoop2  mcoop3  mcoop4  mcoop5  mcoop6  mcoop7  narc1  narc2  narc3  narc4  narc5  narc6  nidentity1  nidentity2  omind1  omind2  omind3  omind4  omind5  omind6  contact1  contact2  contact3  contact4  contact5  hygiene1  hygiene2  hygiene3  hygiene4  hygiene5  psupport1  psupport2  psupport3  psupport4  psupport5  political_ideology  happy  slf_ladder  riskperc1  riskperc2  self_esteem  sex1  sbelong1  sbelong2  sbelong3  sbelong4  tested_positive  optim1  optim2  slfcont1  slfcont3  slfcont4  urban
##   5   5  age  children  cnarc1  cnarc2  cnarc3  ctheory1  ctheory2  ctheory3  ctheory4  CRT1  CRT2  CRT3  employ_status1  health_cond  know_tested_positive  ladder  marital1  mor_circle  moralid1  moralid10  moralid2  moralid3  moralid4  moralid5  moralid6  moralid7  moralid8  moralid9  mcoop1  mcoop2  mcoop3  mcoop4  mcoop5  mcoop6  mcoop7  narc1  narc2  narc3  narc4  narc5  narc6  nidentity1  nidentity2  omind1  omind2  omind3  omind4  omind5  omind6  contact1  contact2  contact3  contact4  contact5  hygiene1  hygiene2  hygiene3  hygiene4  hygiene5  psupport1  psupport2  psupport3  psupport4  psupport5  political_ideology  happy  slf_ladder  riskperc1  riskperc2  self_esteem  sex1  sbelong1  sbelong2  sbelong3  sbelong4  tested_positive  optim1  optim2  slfcont1  slfcont3  slfcont4  urban
##   5   6  age  children  cnarc1  cnarc2  cnarc3  ctheory1  ctheory2  ctheory3  ctheory4  CRT1  CRT2  CRT3  employ_status1  health_cond  know_tested_positive  ladder  marital1  mor_circle  moralid1  moralid10  moralid2  moralid3  moralid4  moralid5  moralid6  moralid7  moralid8  moralid9  mcoop1  mcoop2  mcoop3  mcoop4  mcoop5  mcoop6  mcoop7  narc1  narc2  narc3  narc4  narc5  narc6  nidentity1  nidentity2  omind1  omind2  omind3  omind4  omind5  omind6  contact1  contact2  contact3  contact4  contact5  hygiene1  hygiene2  hygiene3  hygiene4  hygiene5  psupport1  psupport2  psupport3  psupport4  psupport5  political_ideology  happy  slf_ladder  riskperc1  riskperc2  self_esteem  sex1  sbelong1  sbelong2  sbelong3  sbelong4  tested_positive  optim1  optim2  slfcont1  slfcont3  slfcont4  urban
##   5   7  age  children  cnarc1  cnarc2  cnarc3  ctheory1  ctheory2  ctheory3  ctheory4  CRT1  CRT2  CRT3  employ_status1  health_cond  know_tested_positive  ladder  marital1  mor_circle  moralid1  moralid10  moralid2  moralid3  moralid4  moralid5  moralid6  moralid7  moralid8  moralid9  mcoop1  mcoop2  mcoop3  mcoop4  mcoop5  mcoop6  mcoop7  narc1  narc2  narc3  narc4  narc5  narc6  nidentity1  nidentity2  omind1  omind2  omind3  omind4  omind5  omind6  contact1  contact2  contact3  contact4  contact5  hygiene1  hygiene2  hygiene3  hygiene4  hygiene5  psupport1  psupport2  psupport3  psupport4  psupport5  political_ideology  happy  slf_ladder  riskperc1  riskperc2  self_esteem  sex1  sbelong1  sbelong2  sbelong3  sbelong4  tested_positive  optim1  optim2  slfcont1  slfcont3  slfcont4  urban
##   5   8  age  children  cnarc1  cnarc2  cnarc3  ctheory1  ctheory2  ctheory3  ctheory4  CRT1  CRT2  CRT3  employ_status1  health_cond  know_tested_positive  ladder  marital1  mor_circle  moralid1  moralid10  moralid2  moralid3  moralid4  moralid5  moralid6  moralid7  moralid8  moralid9  mcoop1  mcoop2  mcoop3  mcoop4  mcoop5  mcoop6  mcoop7  narc1  narc2  narc3  narc4  narc5  narc6  nidentity1  nidentity2  omind1  omind2  omind3  omind4  omind5  omind6  contact1  contact2  contact3  contact4  contact5  hygiene1  hygiene2  hygiene3  hygiene4  hygiene5  psupport1  psupport2  psupport3  psupport4  psupport5  political_ideology  happy  slf_ladder  riskperc1  riskperc2  self_esteem  sex1  sbelong1  sbelong2  sbelong3  sbelong4  tested_positive  optim1  optim2  slfcont1  slfcont3  slfcont4  urban
##   5   9  age  children  cnarc1  cnarc2  cnarc3  ctheory1  ctheory2  ctheory3  ctheory4  CRT1  CRT2  CRT3  employ_status1  health_cond  know_tested_positive  ladder  marital1  mor_circle  moralid1  moralid10  moralid2  moralid3  moralid4  moralid5  moralid6  moralid7  moralid8  moralid9  mcoop1  mcoop2  mcoop3  mcoop4  mcoop5  mcoop6  mcoop7  narc1  narc2  narc3  narc4  narc5  narc6  nidentity1  nidentity2  omind1  omind2  omind3  omind4  omind5  omind6  contact1  contact2  contact3  contact4  contact5  hygiene1  hygiene2  hygiene3  hygiene4  hygiene5  psupport1  psupport2  psupport3  psupport4  psupport5  political_ideology  happy  slf_ladder  riskperc1  riskperc2  self_esteem  sex1  sbelong1  sbelong2  sbelong3  sbelong4  tested_positive  optim1  optim2  slfcont1  slfcont3  slfcont4  urban
##   5   10  age  children  cnarc1  cnarc2  cnarc3  ctheory1  ctheory2  ctheory3  ctheory4  CRT1  CRT2  CRT3  employ_status1  health_cond  know_tested_positive  ladder  marital1  mor_circle  moralid1  moralid10  moralid2  moralid3  moralid4  moralid5  moralid6  moralid7  moralid8  moralid9  mcoop1  mcoop2  mcoop3  mcoop4  mcoop5  mcoop6  mcoop7  narc1  narc2  narc3  narc4  narc5  narc6  nidentity1  nidentity2  omind1  omind2  omind3  omind4  omind5  omind6  contact1  contact2  contact3  contact4  contact5  hygiene1  hygiene2  hygiene3  hygiene4  hygiene5  psupport1  psupport2  psupport3  psupport4  psupport5  political_ideology  happy  slf_ladder  riskperc1  riskperc2  self_esteem  sex1  sbelong1  sbelong2  sbelong3  sbelong4  tested_positive  optim1  optim2  slfcont1  slfcont3  slfcont4  urban
##   6   1  age  children  cnarc1  cnarc2  cnarc3  ctheory1  ctheory2  ctheory3  ctheory4  CRT1  CRT2  CRT3  employ_status1  health_cond  know_tested_positive  ladder  marital1  mor_circle  moralid1  moralid10  moralid2  moralid3  moralid4  moralid5  moralid6  moralid7  moralid8  moralid9  mcoop1  mcoop2  mcoop3  mcoop4  mcoop5  mcoop6  mcoop7  narc1  narc2  narc3  narc4  narc5  narc6  nidentity1  nidentity2  omind1  omind2  omind3  omind4  omind5  omind6  contact1  contact2  contact3  contact4  contact5  hygiene1  hygiene2  hygiene3  hygiene4  hygiene5  psupport1  psupport2  psupport3  psupport4  psupport5  political_ideology  happy  slf_ladder  riskperc1  riskperc2  self_esteem  sex1  sbelong1  sbelong2  sbelong3  sbelong4  tested_positive  optim1  optim2  slfcont1  slfcont3  slfcont4  urban
##   6   2  age  children  cnarc1  cnarc2  cnarc3  ctheory1  ctheory2  ctheory3  ctheory4  CRT1  CRT2  CRT3  employ_status1  health_cond  know_tested_positive  ladder  marital1  mor_circle  moralid1  moralid10  moralid2  moralid3  moralid4  moralid5  moralid6  moralid7  moralid8  moralid9  mcoop1  mcoop2  mcoop3  mcoop4  mcoop5  mcoop6  mcoop7  narc1  narc2  narc3  narc4  narc5  narc6  nidentity1  nidentity2  omind1  omind2  omind3  omind4  omind5  omind6  contact1  contact2  contact3  contact4  contact5  hygiene1  hygiene2  hygiene3  hygiene4  hygiene5  psupport1  psupport2  psupport3  psupport4  psupport5  political_ideology  happy  slf_ladder  riskperc1  riskperc2  self_esteem  sex1  sbelong1  sbelong2  sbelong3  sbelong4  tested_positive  optim1  optim2  slfcont1  slfcont3  slfcont4  urban
##   6   3  age  children  cnarc1  cnarc2  cnarc3  ctheory1  ctheory2  ctheory3  ctheory4  CRT1  CRT2  CRT3  employ_status1  health_cond  know_tested_positive  ladder  marital1  mor_circle  moralid1  moralid10  moralid2  moralid3  moralid4  moralid5  moralid6  moralid7  moralid8  moralid9  mcoop1  mcoop2  mcoop3  mcoop4  mcoop5  mcoop6  mcoop7  narc1  narc2  narc3  narc4  narc5  narc6  nidentity1  nidentity2  omind1  omind2  omind3  omind4  omind5  omind6  contact1  contact2  contact3  contact4  contact5  hygiene1  hygiene2  hygiene3  hygiene4  hygiene5  psupport1  psupport2  psupport3  psupport4  psupport5  political_ideology  happy  slf_ladder  riskperc1  riskperc2  self_esteem  sex1  sbelong1  sbelong2  sbelong3  sbelong4  tested_positive  optim1  optim2  slfcont1  slfcont3  slfcont4  urban
##   6   4  age  children  cnarc1  cnarc2  cnarc3  ctheory1  ctheory2  ctheory3  ctheory4  CRT1  CRT2  CRT3  employ_status1  health_cond  know_tested_positive  ladder  marital1  mor_circle  moralid1  moralid10  moralid2  moralid3  moralid4  moralid5  moralid6  moralid7  moralid8  moralid9  mcoop1  mcoop2  mcoop3  mcoop4  mcoop5  mcoop6  mcoop7  narc1  narc2  narc3  narc4  narc5  narc6  nidentity1  nidentity2  omind1  omind2  omind3  omind4  omind5  omind6  contact1  contact2  contact3  contact4  contact5  hygiene1  hygiene2  hygiene3  hygiene4  hygiene5  psupport1  psupport2  psupport3  psupport4  psupport5  political_ideology  happy  slf_ladder  riskperc1  riskperc2  self_esteem  sex1  sbelong1  sbelong2  sbelong3  sbelong4  tested_positive  optim1  optim2  slfcont1  slfcont3  slfcont4  urban
##   6   5  age  children  cnarc1  cnarc2  cnarc3  ctheory1  ctheory2  ctheory3  ctheory4  CRT1  CRT2  CRT3  employ_status1  health_cond  know_tested_positive  ladder  marital1  mor_circle  moralid1  moralid10  moralid2  moralid3  moralid4  moralid5  moralid6  moralid7  moralid8  moralid9  mcoop1  mcoop2  mcoop3  mcoop4  mcoop5  mcoop6  mcoop7  narc1  narc2  narc3  narc4  narc5  narc6  nidentity1  nidentity2  omind1  omind2  omind3  omind4  omind5  omind6  contact1  contact2  contact3  contact4  contact5  hygiene1  hygiene2  hygiene3  hygiene4  hygiene5  psupport1  psupport2  psupport3  psupport4  psupport5  political_ideology  happy  slf_ladder  riskperc1  riskperc2  self_esteem  sex1  sbelong1  sbelong2  sbelong3  sbelong4  tested_positive  optim1  optim2  slfcont1  slfcont3  slfcont4  urban
##   6   6  age  children  cnarc1  cnarc2  cnarc3  ctheory1  ctheory2  ctheory3  ctheory4  CRT1  CRT2  CRT3  employ_status1  health_cond  know_tested_positive  ladder  marital1  mor_circle  moralid1  moralid10  moralid2  moralid3  moralid4  moralid5  moralid6  moralid7  moralid8  moralid9  mcoop1  mcoop2  mcoop3  mcoop4  mcoop5  mcoop6  mcoop7  narc1  narc2  narc3  narc4  narc5  narc6  nidentity1  nidentity2  omind1  omind2  omind3  omind4  omind5  omind6  contact1  contact2  contact3  contact4  contact5  hygiene1  hygiene2  hygiene3  hygiene4  hygiene5  psupport1  psupport2  psupport3  psupport4  psupport5  political_ideology  happy  slf_ladder  riskperc1  riskperc2  self_esteem  sex1  sbelong1  sbelong2  sbelong3  sbelong4  tested_positive  optim1  optim2  slfcont1  slfcont3  slfcont4  urban
##   6   7  age  children  cnarc1  cnarc2  cnarc3  ctheory1  ctheory2  ctheory3  ctheory4  CRT1  CRT2  CRT3  employ_status1  health_cond  know_tested_positive  ladder  marital1  mor_circle  moralid1  moralid10  moralid2  moralid3  moralid4  moralid5  moralid6  moralid7  moralid8  moralid9  mcoop1  mcoop2  mcoop3  mcoop4  mcoop5  mcoop6  mcoop7  narc1  narc2  narc3  narc4  narc5  narc6  nidentity1  nidentity2  omind1  omind2  omind3  omind4  omind5  omind6  contact1  contact2  contact3  contact4  contact5  hygiene1  hygiene2  hygiene3  hygiene4  hygiene5  psupport1  psupport2  psupport3  psupport4  psupport5  political_ideology  happy  slf_ladder  riskperc1  riskperc2  self_esteem  sex1  sbelong1  sbelong2  sbelong3  sbelong4  tested_positive  optim1  optim2  slfcont1  slfcont3  slfcont4  urban
##   6   8  age  children  cnarc1  cnarc2  cnarc3  ctheory1  ctheory2  ctheory3  ctheory4  CRT1  CRT2  CRT3  employ_status1  health_cond  know_tested_positive  ladder  marital1  mor_circle  moralid1  moralid10  moralid2  moralid3  moralid4  moralid5  moralid6  moralid7  moralid8  moralid9  mcoop1  mcoop2  mcoop3  mcoop4  mcoop5  mcoop6  mcoop7  narc1  narc2  narc3  narc4  narc5  narc6  nidentity1  nidentity2  omind1  omind2  omind3  omind4  omind5  omind6  contact1  contact2  contact3  contact4  contact5  hygiene1  hygiene2  hygiene3  hygiene4  hygiene5  psupport1  psupport2  psupport3  psupport4  psupport5  political_ideology  happy  slf_ladder  riskperc1  riskperc2  self_esteem  sex1  sbelong1  sbelong2  sbelong3  sbelong4  tested_positive  optim1  optim2  slfcont1  slfcont3  slfcont4  urban
##   6   9  age  children  cnarc1  cnarc2  cnarc3  ctheory1  ctheory2  ctheory3  ctheory4  CRT1  CRT2  CRT3  employ_status1  health_cond  know_tested_positive  ladder  marital1  mor_circle  moralid1  moralid10  moralid2  moralid3  moralid4  moralid5  moralid6  moralid7  moralid8  moralid9  mcoop1  mcoop2  mcoop3  mcoop4  mcoop5  mcoop6  mcoop7  narc1  narc2  narc3  narc4  narc5  narc6  nidentity1  nidentity2  omind1  omind2  omind3  omind4  omind5  omind6  contact1  contact2  contact3  contact4  contact5  hygiene1  hygiene2  hygiene3  hygiene4  hygiene5  psupport1  psupport2  psupport3  psupport4  psupport5  political_ideology  happy  slf_ladder  riskperc1  riskperc2  self_esteem  sex1  sbelong1  sbelong2  sbelong3  sbelong4  tested_positive  optim1  optim2  slfcont1  slfcont3  slfcont4  urban
##   6   10  age  children  cnarc1  cnarc2  cnarc3  ctheory1  ctheory2  ctheory3  ctheory4  CRT1  CRT2  CRT3  employ_status1  health_cond  know_tested_positive  ladder  marital1  mor_circle  moralid1  moralid10  moralid2  moralid3  moralid4  moralid5  moralid6  moralid7  moralid8  moralid9  mcoop1  mcoop2  mcoop3  mcoop4  mcoop5  mcoop6  mcoop7  narc1  narc2  narc3  narc4  narc5  narc6  nidentity1  nidentity2  omind1  omind2  omind3  omind4  omind5  omind6  contact1  contact2  contact3  contact4  contact5  hygiene1  hygiene2  hygiene3  hygiene4  hygiene5  psupport1  psupport2  psupport3  psupport4  psupport5  political_ideology  happy  slf_ladder  riskperc1  riskperc2  self_esteem  sex1  sbelong1  sbelong2  sbelong3  sbelong4  tested_positive  optim1  optim2  slfcont1  slfcont3  slfcont4  urban
##   7   1  age  children  cnarc1  cnarc2  cnarc3  ctheory1  ctheory2  ctheory3  ctheory4  CRT1  CRT2  CRT3  employ_status1  health_cond  know_tested_positive  ladder  marital1  mor_circle  moralid1  moralid10  moralid2  moralid3  moralid4  moralid5  moralid6  moralid7  moralid8  moralid9  mcoop1  mcoop2  mcoop3  mcoop4  mcoop5  mcoop6  mcoop7  narc1  narc2  narc3  narc4  narc5  narc6  nidentity1  nidentity2  omind1  omind2  omind3  omind4  omind5  omind6  contact1  contact2  contact3  contact4  contact5  hygiene1  hygiene2  hygiene3  hygiene4  hygiene5  psupport1  psupport2  psupport3  psupport4  psupport5  political_ideology  happy  slf_ladder  riskperc1  riskperc2  self_esteem  sex1  sbelong1  sbelong2  sbelong3  sbelong4  tested_positive  optim1  optim2  slfcont1  slfcont3  slfcont4  urban
##   7   2  age  children  cnarc1  cnarc2  cnarc3  ctheory1  ctheory2  ctheory3  ctheory4  CRT1  CRT2  CRT3  employ_status1  health_cond  know_tested_positive  ladder  marital1  mor_circle  moralid1  moralid10  moralid2  moralid3  moralid4  moralid5  moralid6  moralid7  moralid8  moralid9  mcoop1  mcoop2  mcoop3  mcoop4  mcoop5  mcoop6  mcoop7  narc1  narc2  narc3  narc4  narc5  narc6  nidentity1  nidentity2  omind1  omind2  omind3  omind4  omind5  omind6  contact1  contact2  contact3  contact4  contact5  hygiene1  hygiene2  hygiene3  hygiene4  hygiene5  psupport1  psupport2  psupport3  psupport4  psupport5  political_ideology  happy  slf_ladder  riskperc1  riskperc2  self_esteem  sex1  sbelong1  sbelong2  sbelong3  sbelong4  tested_positive  optim1  optim2  slfcont1  slfcont3  slfcont4  urban
##   7   3  age  children  cnarc1  cnarc2  cnarc3  ctheory1  ctheory2  ctheory3  ctheory4  CRT1  CRT2  CRT3  employ_status1  health_cond  know_tested_positive  ladder  marital1  mor_circle  moralid1  moralid10  moralid2  moralid3  moralid4  moralid5  moralid6  moralid7  moralid8  moralid9  mcoop1  mcoop2  mcoop3  mcoop4  mcoop5  mcoop6  mcoop7  narc1  narc2  narc3  narc4  narc5  narc6  nidentity1  nidentity2  omind1  omind2  omind3  omind4  omind5  omind6  contact1  contact2  contact3  contact4  contact5  hygiene1  hygiene2  hygiene3  hygiene4  hygiene5  psupport1  psupport2  psupport3  psupport4  psupport5  political_ideology  happy  slf_ladder  riskperc1  riskperc2  self_esteem  sex1  sbelong1  sbelong2  sbelong3  sbelong4  tested_positive  optim1  optim2  slfcont1  slfcont3  slfcont4  urban
##   7   4  age  children  cnarc1  cnarc2  cnarc3  ctheory1  ctheory2  ctheory3  ctheory4  CRT1  CRT2  CRT3  employ_status1  health_cond  know_tested_positive  ladder  marital1  mor_circle  moralid1  moralid10  moralid2  moralid3  moralid4  moralid5  moralid6  moralid7  moralid8  moralid9  mcoop1  mcoop2  mcoop3  mcoop4  mcoop5  mcoop6  mcoop7  narc1  narc2  narc3  narc4  narc5  narc6  nidentity1  nidentity2  omind1  omind2  omind3  omind4  omind5  omind6  contact1  contact2  contact3  contact4  contact5  hygiene1  hygiene2  hygiene3  hygiene4  hygiene5  psupport1  psupport2  psupport3  psupport4  psupport5  political_ideology  happy  slf_ladder  riskperc1  riskperc2  self_esteem  sex1  sbelong1  sbelong2  sbelong3  sbelong4  tested_positive  optim1  optim2  slfcont1  slfcont3  slfcont4  urban
##   7   5  age  children  cnarc1  cnarc2  cnarc3  ctheory1  ctheory2  ctheory3  ctheory4  CRT1  CRT2  CRT3  employ_status1  health_cond  know_tested_positive  ladder  marital1  mor_circle  moralid1  moralid10  moralid2  moralid3  moralid4  moralid5  moralid6  moralid7  moralid8  moralid9  mcoop1  mcoop2  mcoop3  mcoop4  mcoop5  mcoop6  mcoop7  narc1  narc2  narc3  narc4  narc5  narc6  nidentity1  nidentity2  omind1  omind2  omind3  omind4  omind5  omind6  contact1  contact2  contact3  contact4  contact5  hygiene1  hygiene2  hygiene3  hygiene4  hygiene5  psupport1  psupport2  psupport3  psupport4  psupport5  political_ideology  happy  slf_ladder  riskperc1  riskperc2  self_esteem  sex1  sbelong1  sbelong2  sbelong3  sbelong4  tested_positive  optim1  optim2  slfcont1  slfcont3  slfcont4  urban
##   7   6  age  children  cnarc1  cnarc2  cnarc3  ctheory1  ctheory2  ctheory3  ctheory4  CRT1  CRT2  CRT3  employ_status1  health_cond  know_tested_positive  ladder  marital1  mor_circle  moralid1  moralid10  moralid2  moralid3  moralid4  moralid5  moralid6  moralid7  moralid8  moralid9  mcoop1  mcoop2  mcoop3  mcoop4  mcoop5  mcoop6  mcoop7  narc1  narc2  narc3  narc4  narc5  narc6  nidentity1  nidentity2  omind1  omind2  omind3  omind4  omind5  omind6  contact1  contact2  contact3  contact4  contact5  hygiene1  hygiene2  hygiene3  hygiene4  hygiene5  psupport1  psupport2  psupport3  psupport4  psupport5  political_ideology  happy  slf_ladder  riskperc1  riskperc2  self_esteem  sex1  sbelong1  sbelong2  sbelong3  sbelong4  tested_positive  optim1  optim2  slfcont1  slfcont3  slfcont4  urban
##   7   7  age  children  cnarc1  cnarc2  cnarc3  ctheory1  ctheory2  ctheory3  ctheory4  CRT1  CRT2  CRT3  employ_status1  health_cond  know_tested_positive  ladder  marital1  mor_circle  moralid1  moralid10  moralid2  moralid3  moralid4  moralid5  moralid6  moralid7  moralid8  moralid9  mcoop1  mcoop2  mcoop3  mcoop4  mcoop5  mcoop6  mcoop7  narc1  narc2  narc3  narc4  narc5  narc6  nidentity1  nidentity2  omind1  omind2  omind3  omind4  omind5  omind6  contact1  contact2  contact3  contact4  contact5  hygiene1  hygiene2  hygiene3  hygiene4  hygiene5  psupport1  psupport2  psupport3  psupport4  psupport5  political_ideology  happy  slf_ladder  riskperc1  riskperc2  self_esteem  sex1  sbelong1  sbelong2  sbelong3  sbelong4  tested_positive  optim1  optim2  slfcont1  slfcont3  slfcont4  urban
##   7   8  age  children  cnarc1  cnarc2  cnarc3  ctheory1  ctheory2  ctheory3  ctheory4  CRT1  CRT2  CRT3  employ_status1  health_cond  know_tested_positive  ladder  marital1  mor_circle  moralid1  moralid10  moralid2  moralid3  moralid4  moralid5  moralid6  moralid7  moralid8  moralid9  mcoop1  mcoop2  mcoop3  mcoop4  mcoop5  mcoop6  mcoop7  narc1  narc2  narc3  narc4  narc5  narc6  nidentity1  nidentity2  omind1  omind2  omind3  omind4  omind5  omind6  contact1  contact2  contact3  contact4  contact5  hygiene1  hygiene2  hygiene3  hygiene4  hygiene5  psupport1  psupport2  psupport3  psupport4  psupport5  political_ideology  happy  slf_ladder  riskperc1  riskperc2  self_esteem  sex1  sbelong1  sbelong2  sbelong3  sbelong4  tested_positive  optim1  optim2  slfcont1  slfcont3  slfcont4  urban
##   7   9  age  children  cnarc1  cnarc2  cnarc3  ctheory1  ctheory2  ctheory3  ctheory4  CRT1  CRT2  CRT3  employ_status1  health_cond  know_tested_positive  ladder  marital1  mor_circle  moralid1  moralid10  moralid2  moralid3  moralid4  moralid5  moralid6  moralid7  moralid8  moralid9  mcoop1  mcoop2  mcoop3  mcoop4  mcoop5  mcoop6  mcoop7  narc1  narc2  narc3  narc4  narc5  narc6  nidentity1  nidentity2  omind1  omind2  omind3  omind4  omind5  omind6  contact1  contact2  contact3  contact4  contact5  hygiene1  hygiene2  hygiene3  hygiene4  hygiene5  psupport1  psupport2  psupport3  psupport4  psupport5  political_ideology  happy  slf_ladder  riskperc1  riskperc2  self_esteem  sex1  sbelong1  sbelong2  sbelong3  sbelong4  tested_positive  optim1  optim2  slfcont1  slfcont3  slfcont4  urban
##   7   10  age  children  cnarc1  cnarc2  cnarc3  ctheory1  ctheory2  ctheory3  ctheory4  CRT1  CRT2  CRT3  employ_status1  health_cond  know_tested_positive  ladder  marital1  mor_circle  moralid1  moralid10  moralid2  moralid3  moralid4  moralid5  moralid6  moralid7  moralid8  moralid9  mcoop1  mcoop2  mcoop3  mcoop4  mcoop5  mcoop6  mcoop7  narc1  narc2  narc3  narc4  narc5  narc6  nidentity1  nidentity2  omind1  omind2  omind3  omind4  omind5  omind6  contact1  contact2  contact3  contact4  contact5  hygiene1  hygiene2  hygiene3  hygiene4  hygiene5  psupport1  psupport2  psupport3  psupport4  psupport5  political_ideology  happy  slf_ladder  riskperc1  riskperc2  self_esteem  sex1  sbelong1  sbelong2  sbelong3  sbelong4  tested_positive  optim1  optim2  slfcont1  slfcont3  slfcont4  urban
##   8   1  age  children  cnarc1  cnarc2  cnarc3  ctheory1  ctheory2  ctheory3  ctheory4  CRT1  CRT2  CRT3  employ_status1  health_cond  know_tested_positive  ladder  marital1  mor_circle  moralid1  moralid10  moralid2  moralid3  moralid4  moralid5  moralid6  moralid7  moralid8  moralid9  mcoop1  mcoop2  mcoop3  mcoop4  mcoop5  mcoop6  mcoop7  narc1  narc2  narc3  narc4  narc5  narc6  nidentity1  nidentity2  omind1  omind2  omind3  omind4  omind5  omind6  contact1  contact2  contact3  contact4  contact5  hygiene1  hygiene2  hygiene3  hygiene4  hygiene5  psupport1  psupport2  psupport3  psupport4  psupport5  political_ideology  happy  slf_ladder  riskperc1  riskperc2  self_esteem  sex1  sbelong1  sbelong2  sbelong3  sbelong4  tested_positive  optim1  optim2  slfcont1  slfcont3  slfcont4  urban
##   8   2  age  children  cnarc1  cnarc2  cnarc3  ctheory1  ctheory2  ctheory3  ctheory4  CRT1  CRT2  CRT3  employ_status1  health_cond  know_tested_positive  ladder  marital1  mor_circle  moralid1  moralid10  moralid2  moralid3  moralid4  moralid5  moralid6  moralid7  moralid8  moralid9  mcoop1  mcoop2  mcoop3  mcoop4  mcoop5  mcoop6  mcoop7  narc1  narc2  narc3  narc4  narc5  narc6  nidentity1  nidentity2  omind1  omind2  omind3  omind4  omind5  omind6  contact1  contact2  contact3  contact4  contact5  hygiene1  hygiene2  hygiene3  hygiene4  hygiene5  psupport1  psupport2  psupport3  psupport4  psupport5  political_ideology  happy  slf_ladder  riskperc1  riskperc2  self_esteem  sex1  sbelong1  sbelong2  sbelong3  sbelong4  tested_positive  optim1  optim2  slfcont1  slfcont3  slfcont4  urban
##   8   3  age  children  cnarc1  cnarc2  cnarc3  ctheory1  ctheory2  ctheory3  ctheory4  CRT1  CRT2  CRT3  employ_status1  health_cond  know_tested_positive  ladder  marital1  mor_circle  moralid1  moralid10  moralid2  moralid3  moralid4  moralid5  moralid6  moralid7  moralid8  moralid9  mcoop1  mcoop2  mcoop3  mcoop4  mcoop5  mcoop6  mcoop7  narc1  narc2  narc3  narc4  narc5  narc6  nidentity1  nidentity2  omind1  omind2  omind3  omind4  omind5  omind6  contact1  contact2  contact3  contact4  contact5  hygiene1  hygiene2  hygiene3  hygiene4  hygiene5  psupport1  psupport2  psupport3  psupport4  psupport5  political_ideology  happy  slf_ladder  riskperc1  riskperc2  self_esteem  sex1  sbelong1  sbelong2  sbelong3  sbelong4  tested_positive  optim1  optim2  slfcont1  slfcont3  slfcont4  urban
##   8   4  age  children  cnarc1  cnarc2  cnarc3  ctheory1  ctheory2  ctheory3  ctheory4  CRT1  CRT2  CRT3  employ_status1  health_cond  know_tested_positive  ladder  marital1  mor_circle  moralid1  moralid10  moralid2  moralid3  moralid4  moralid5  moralid6  moralid7  moralid8  moralid9  mcoop1  mcoop2  mcoop3  mcoop4  mcoop5  mcoop6  mcoop7  narc1  narc2  narc3  narc4  narc5  narc6  nidentity1  nidentity2  omind1  omind2  omind3  omind4  omind5  omind6  contact1  contact2  contact3  contact4  contact5  hygiene1  hygiene2  hygiene3  hygiene4  hygiene5  psupport1  psupport2  psupport3  psupport4  psupport5  political_ideology  happy  slf_ladder  riskperc1  riskperc2  self_esteem  sex1  sbelong1  sbelong2  sbelong3  sbelong4  tested_positive  optim1  optim2  slfcont1  slfcont3  slfcont4  urban
##   8   5  age  children  cnarc1  cnarc2  cnarc3  ctheory1  ctheory2  ctheory3  ctheory4  CRT1  CRT2  CRT3  employ_status1  health_cond  know_tested_positive  ladder  marital1  mor_circle  moralid1  moralid10  moralid2  moralid3  moralid4  moralid5  moralid6  moralid7  moralid8  moralid9  mcoop1  mcoop2  mcoop3  mcoop4  mcoop5  mcoop6  mcoop7  narc1  narc2  narc3  narc4  narc5  narc6  nidentity1  nidentity2  omind1  omind2  omind3  omind4  omind5  omind6  contact1  contact2  contact3  contact4  contact5  hygiene1  hygiene2  hygiene3  hygiene4  hygiene5  psupport1  psupport2  psupport3  psupport4  psupport5  political_ideology  happy  slf_ladder  riskperc1  riskperc2  self_esteem  sex1  sbelong1  sbelong2  sbelong3  sbelong4  tested_positive  optim1  optim2  slfcont1  slfcont3  slfcont4  urban
##   8   6  age  children  cnarc1  cnarc2  cnarc3  ctheory1  ctheory2  ctheory3  ctheory4  CRT1  CRT2  CRT3  employ_status1  health_cond  know_tested_positive  ladder  marital1  mor_circle  moralid1  moralid10  moralid2  moralid3  moralid4  moralid5  moralid6  moralid7  moralid8  moralid9  mcoop1  mcoop2  mcoop3  mcoop4  mcoop5  mcoop6  mcoop7  narc1  narc2  narc3  narc4  narc5  narc6  nidentity1  nidentity2  omind1  omind2  omind3  omind4  omind5  omind6  contact1  contact2  contact3  contact4  contact5  hygiene1  hygiene2  hygiene3  hygiene4  hygiene5  psupport1  psupport2  psupport3  psupport4  psupport5  political_ideology  happy  slf_ladder  riskperc1  riskperc2  self_esteem  sex1  sbelong1  sbelong2  sbelong3  sbelong4  tested_positive  optim1  optim2  slfcont1  slfcont3  slfcont4  urban
##   8   7  age  children  cnarc1  cnarc2  cnarc3  ctheory1  ctheory2  ctheory3  ctheory4  CRT1  CRT2  CRT3  employ_status1  health_cond  know_tested_positive  ladder  marital1  mor_circle  moralid1  moralid10  moralid2  moralid3  moralid4  moralid5  moralid6  moralid7  moralid8  moralid9  mcoop1  mcoop2  mcoop3  mcoop4  mcoop5  mcoop6  mcoop7  narc1  narc2  narc3  narc4  narc5  narc6  nidentity1  nidentity2  omind1  omind2  omind3  omind4  omind5  omind6  contact1  contact2  contact3  contact4  contact5  hygiene1  hygiene2  hygiene3  hygiene4  hygiene5  psupport1  psupport2  psupport3  psupport4  psupport5  political_ideology  happy  slf_ladder  riskperc1  riskperc2  self_esteem  sex1  sbelong1  sbelong2  sbelong3  sbelong4  tested_positive  optim1  optim2  slfcont1  slfcont3  slfcont4  urban
##   8   8  age  children  cnarc1  cnarc2  cnarc3  ctheory1  ctheory2  ctheory3  ctheory4  CRT1  CRT2  CRT3  employ_status1  health_cond  know_tested_positive  ladder  marital1  mor_circle  moralid1  moralid10  moralid2  moralid3  moralid4  moralid5  moralid6  moralid7  moralid8  moralid9  mcoop1  mcoop2  mcoop3  mcoop4  mcoop5  mcoop6  mcoop7  narc1  narc2  narc3  narc4  narc5  narc6  nidentity1  nidentity2  omind1  omind2  omind3  omind4  omind5  omind6  contact1  contact2  contact3  contact4  contact5  hygiene1  hygiene2  hygiene3  hygiene4  hygiene5  psupport1  psupport2  psupport3  psupport4  psupport5  political_ideology  happy  slf_ladder  riskperc1  riskperc2  self_esteem  sex1  sbelong1  sbelong2  sbelong3  sbelong4  tested_positive  optim1  optim2  slfcont1  slfcont3  slfcont4  urban
##   8   9  age  children  cnarc1  cnarc2  cnarc3  ctheory1  ctheory2  ctheory3  ctheory4  CRT1  CRT2  CRT3  employ_status1  health_cond  know_tested_positive  ladder  marital1  mor_circle  moralid1  moralid10  moralid2  moralid3  moralid4  moralid5  moralid6  moralid7  moralid8  moralid9  mcoop1  mcoop2  mcoop3  mcoop4  mcoop5  mcoop6  mcoop7  narc1  narc2  narc3  narc4  narc5  narc6  nidentity1  nidentity2  omind1  omind2  omind3  omind4  omind5  omind6  contact1  contact2  contact3  contact4  contact5  hygiene1  hygiene2  hygiene3  hygiene4  hygiene5  psupport1  psupport2  psupport3  psupport4  psupport5  political_ideology  happy  slf_ladder  riskperc1  riskperc2  self_esteem  sex1  sbelong1  sbelong2  sbelong3  sbelong4  tested_positive  optim1  optim2  slfcont1  slfcont3  slfcont4  urban
##   8   10  age  children  cnarc1  cnarc2  cnarc3  ctheory1  ctheory2  ctheory3  ctheory4  CRT1  CRT2  CRT3  employ_status1  health_cond  know_tested_positive  ladder  marital1  mor_circle  moralid1  moralid10  moralid2  moralid3  moralid4  moralid5  moralid6  moralid7  moralid8  moralid9  mcoop1  mcoop2  mcoop3  mcoop4  mcoop5  mcoop6  mcoop7  narc1  narc2  narc3  narc4  narc5  narc6  nidentity1  nidentity2  omind1  omind2  omind3  omind4  omind5  omind6  contact1  contact2  contact3  contact4  contact5  hygiene1  hygiene2  hygiene3  hygiene4  hygiene5  psupport1  psupport2  psupport3  psupport4  psupport5  political_ideology  happy  slf_ladder  riskperc1  riskperc2  self_esteem  sex1  sbelong1  sbelong2  sbelong3  sbelong4  tested_positive  optim1  optim2  slfcont1  slfcont3  slfcont4  urban
##   9   1  age  children  cnarc1  cnarc2  cnarc3  ctheory1  ctheory2  ctheory3  ctheory4  CRT1  CRT2  CRT3  employ_status1  health_cond  know_tested_positive  ladder  marital1  mor_circle  moralid1  moralid10  moralid2  moralid3  moralid4  moralid5  moralid6  moralid7  moralid8  moralid9  mcoop1  mcoop2  mcoop3  mcoop4  mcoop5  mcoop6  mcoop7  narc1  narc2  narc3  narc4  narc5  narc6  nidentity1  nidentity2  omind1  omind2  omind3  omind4  omind5  omind6  contact1  contact2  contact3  contact4  contact5  hygiene1  hygiene2  hygiene3  hygiene4  hygiene5  psupport1  psupport2  psupport3  psupport4  psupport5  political_ideology  happy  slf_ladder  riskperc1  riskperc2  self_esteem  sex1  sbelong1  sbelong2  sbelong3  sbelong4  tested_positive  optim1  optim2  slfcont1  slfcont3  slfcont4  urban
##   9   2  age  children  cnarc1  cnarc2  cnarc3  ctheory1  ctheory2  ctheory3  ctheory4  CRT1  CRT2  CRT3  employ_status1  health_cond  know_tested_positive  ladder  marital1  mor_circle  moralid1  moralid10  moralid2  moralid3  moralid4  moralid5  moralid6  moralid7  moralid8  moralid9  mcoop1  mcoop2  mcoop3  mcoop4  mcoop5  mcoop6  mcoop7  narc1  narc2  narc3  narc4  narc5  narc6  nidentity1  nidentity2  omind1  omind2  omind3  omind4  omind5  omind6  contact1  contact2  contact3  contact4  contact5  hygiene1  hygiene2  hygiene3  hygiene4  hygiene5  psupport1  psupport2  psupport3  psupport4  psupport5  political_ideology  happy  slf_ladder  riskperc1  riskperc2  self_esteem  sex1  sbelong1  sbelong2  sbelong3  sbelong4  tested_positive  optim1  optim2  slfcont1  slfcont3  slfcont4  urban
##   9   3  age  children  cnarc1  cnarc2  cnarc3  ctheory1  ctheory2  ctheory3  ctheory4  CRT1  CRT2  CRT3  employ_status1  health_cond  know_tested_positive  ladder  marital1  mor_circle  moralid1  moralid10  moralid2  moralid3  moralid4  moralid5  moralid6  moralid7  moralid8  moralid9  mcoop1  mcoop2  mcoop3  mcoop4  mcoop5  mcoop6  mcoop7  narc1  narc2  narc3  narc4  narc5  narc6  nidentity1  nidentity2  omind1  omind2  omind3  omind4  omind5  omind6  contact1  contact2  contact3  contact4  contact5  hygiene1  hygiene2  hygiene3  hygiene4  hygiene5  psupport1  psupport2  psupport3  psupport4  psupport5  political_ideology  happy  slf_ladder  riskperc1  riskperc2  self_esteem  sex1  sbelong1  sbelong2  sbelong3  sbelong4  tested_positive  optim1  optim2  slfcont1  slfcont3  slfcont4  urban
##   9   4  age  children  cnarc1  cnarc2  cnarc3  ctheory1  ctheory2  ctheory3  ctheory4  CRT1  CRT2  CRT3  employ_status1  health_cond  know_tested_positive  ladder  marital1  mor_circle  moralid1  moralid10  moralid2  moralid3  moralid4  moralid5  moralid6  moralid7  moralid8  moralid9  mcoop1  mcoop2  mcoop3  mcoop4  mcoop5  mcoop6  mcoop7  narc1  narc2  narc3  narc4  narc5  narc6  nidentity1  nidentity2  omind1  omind2  omind3  omind4  omind5  omind6  contact1  contact2  contact3  contact4  contact5  hygiene1  hygiene2  hygiene3  hygiene4  hygiene5  psupport1  psupport2  psupport3  psupport4  psupport5  political_ideology  happy  slf_ladder  riskperc1  riskperc2  self_esteem  sex1  sbelong1  sbelong2  sbelong3  sbelong4  tested_positive  optim1  optim2  slfcont1  slfcont3  slfcont4  urban
##   9   5  age  children  cnarc1  cnarc2  cnarc3  ctheory1  ctheory2  ctheory3  ctheory4  CRT1  CRT2  CRT3  employ_status1  health_cond  know_tested_positive  ladder  marital1  mor_circle  moralid1  moralid10  moralid2  moralid3  moralid4  moralid5  moralid6  moralid7  moralid8  moralid9  mcoop1  mcoop2  mcoop3  mcoop4  mcoop5  mcoop6  mcoop7  narc1  narc2  narc3  narc4  narc5  narc6  nidentity1  nidentity2  omind1  omind2  omind3  omind4  omind5  omind6  contact1  contact2  contact3  contact4  contact5  hygiene1  hygiene2  hygiene3  hygiene4  hygiene5  psupport1  psupport2  psupport3  psupport4  psupport5  political_ideology  happy  slf_ladder  riskperc1  riskperc2  self_esteem  sex1  sbelong1  sbelong2  sbelong3  sbelong4  tested_positive  optim1  optim2  slfcont1  slfcont3  slfcont4  urban
##   9   6  age  children  cnarc1  cnarc2  cnarc3  ctheory1  ctheory2  ctheory3  ctheory4  CRT1  CRT2  CRT3  employ_status1  health_cond  know_tested_positive  ladder  marital1  mor_circle  moralid1  moralid10  moralid2  moralid3  moralid4  moralid5  moralid6  moralid7  moralid8  moralid9  mcoop1  mcoop2  mcoop3  mcoop4  mcoop5  mcoop6  mcoop7  narc1  narc2  narc3  narc4  narc5  narc6  nidentity1  nidentity2  omind1  omind2  omind3  omind4  omind5  omind6  contact1  contact2  contact3  contact4  contact5  hygiene1  hygiene2  hygiene3  hygiene4  hygiene5  psupport1  psupport2  psupport3  psupport4  psupport5  political_ideology  happy  slf_ladder  riskperc1  riskperc2  self_esteem  sex1  sbelong1  sbelong2  sbelong3  sbelong4  tested_positive  optim1  optim2  slfcont1  slfcont3  slfcont4  urban
##   9   7  age  children  cnarc1  cnarc2  cnarc3  ctheory1  ctheory2  ctheory3  ctheory4  CRT1  CRT2  CRT3  employ_status1  health_cond  know_tested_positive  ladder  marital1  mor_circle  moralid1  moralid10  moralid2  moralid3  moralid4  moralid5  moralid6  moralid7  moralid8  moralid9  mcoop1  mcoop2  mcoop3  mcoop4  mcoop5  mcoop6  mcoop7  narc1  narc2  narc3  narc4  narc5  narc6  nidentity1  nidentity2  omind1  omind2  omind3  omind4  omind5  omind6  contact1  contact2  contact3  contact4  contact5  hygiene1  hygiene2  hygiene3  hygiene4  hygiene5  psupport1  psupport2  psupport3  psupport4  psupport5  political_ideology  happy  slf_ladder  riskperc1  riskperc2  self_esteem  sex1  sbelong1  sbelong2  sbelong3  sbelong4  tested_positive  optim1  optim2  slfcont1  slfcont3  slfcont4  urban
##   9   8  age  children  cnarc1  cnarc2  cnarc3  ctheory1  ctheory2  ctheory3  ctheory4  CRT1  CRT2  CRT3  employ_status1  health_cond  know_tested_positive  ladder  marital1  mor_circle  moralid1  moralid10  moralid2  moralid3  moralid4  moralid5  moralid6  moralid7  moralid8  moralid9  mcoop1  mcoop2  mcoop3  mcoop4  mcoop5  mcoop6  mcoop7  narc1  narc2  narc3  narc4  narc5  narc6  nidentity1  nidentity2  omind1  omind2  omind3  omind4  omind5  omind6  contact1  contact2  contact3  contact4  contact5  hygiene1  hygiene2  hygiene3  hygiene4  hygiene5  psupport1  psupport2  psupport3  psupport4  psupport5  political_ideology  happy  slf_ladder  riskperc1  riskperc2  self_esteem  sex1  sbelong1  sbelong2  sbelong3  sbelong4  tested_positive  optim1  optim2  slfcont1  slfcont3  slfcont4  urban
##   9   9  age  children  cnarc1  cnarc2  cnarc3  ctheory1  ctheory2  ctheory3  ctheory4  CRT1  CRT2  CRT3  employ_status1  health_cond  know_tested_positive  ladder  marital1  mor_circle  moralid1  moralid10  moralid2  moralid3  moralid4  moralid5  moralid6  moralid7  moralid8  moralid9  mcoop1  mcoop2  mcoop3  mcoop4  mcoop5  mcoop6  mcoop7  narc1  narc2  narc3  narc4  narc5  narc6  nidentity1  nidentity2  omind1  omind2  omind3  omind4  omind5  omind6  contact1  contact2  contact3  contact4  contact5  hygiene1  hygiene2  hygiene3  hygiene4  hygiene5  psupport1  psupport2  psupport3  psupport4  psupport5  political_ideology  happy  slf_ladder  riskperc1  riskperc2  self_esteem  sex1  sbelong1  sbelong2  sbelong3  sbelong4  tested_positive  optim1  optim2  slfcont1  slfcont3  slfcont4  urban
##   9   10  age  children  cnarc1  cnarc2  cnarc3  ctheory1  ctheory2  ctheory3  ctheory4  CRT1  CRT2  CRT3  employ_status1  health_cond  know_tested_positive  ladder  marital1  mor_circle  moralid1  moralid10  moralid2  moralid3  moralid4  moralid5  moralid6  moralid7  moralid8  moralid9  mcoop1  mcoop2  mcoop3  mcoop4  mcoop5  mcoop6  mcoop7  narc1  narc2  narc3  narc4  narc5  narc6  nidentity1  nidentity2  omind1  omind2  omind3  omind4  omind5  omind6  contact1  contact2  contact3  contact4  contact5  hygiene1  hygiene2  hygiene3  hygiene4  hygiene5  psupport1  psupport2  psupport3  psupport4  psupport5  political_ideology  happy  slf_ladder  riskperc1  riskperc2  self_esteem  sex1  sbelong1  sbelong2  sbelong3  sbelong4  tested_positive  optim1  optim2  slfcont1  slfcont3  slfcont4  urban
##   10   1  age  children  cnarc1  cnarc2  cnarc3  ctheory1  ctheory2  ctheory3  ctheory4  CRT1  CRT2  CRT3  employ_status1  health_cond  know_tested_positive  ladder  marital1  mor_circle  moralid1  moralid10  moralid2  moralid3  moralid4  moralid5  moralid6  moralid7  moralid8  moralid9  mcoop1  mcoop2  mcoop3  mcoop4  mcoop5  mcoop6  mcoop7  narc1  narc2  narc3  narc4  narc5  narc6  nidentity1  nidentity2  omind1  omind2  omind3  omind4  omind5  omind6  contact1  contact2  contact3  contact4  contact5  hygiene1  hygiene2  hygiene3  hygiene4  hygiene5  psupport1  psupport2  psupport3  psupport4  psupport5  political_ideology  happy  slf_ladder  riskperc1  riskperc2  self_esteem  sex1  sbelong1  sbelong2  sbelong3  sbelong4  tested_positive  optim1  optim2  slfcont1  slfcont3  slfcont4  urban
##   10   2  age  children  cnarc1  cnarc2  cnarc3  ctheory1  ctheory2  ctheory3  ctheory4  CRT1  CRT2  CRT3  employ_status1  health_cond  know_tested_positive  ladder  marital1  mor_circle  moralid1  moralid10  moralid2  moralid3  moralid4  moralid5  moralid6  moralid7  moralid8  moralid9  mcoop1  mcoop2  mcoop3  mcoop4  mcoop5  mcoop6  mcoop7  narc1  narc2  narc3  narc4  narc5  narc6  nidentity1  nidentity2  omind1  omind2  omind3  omind4  omind5  omind6  contact1  contact2  contact3  contact4  contact5  hygiene1  hygiene2  hygiene3  hygiene4  hygiene5  psupport1  psupport2  psupport3  psupport4  psupport5  political_ideology  happy  slf_ladder  riskperc1  riskperc2  self_esteem  sex1  sbelong1  sbelong2  sbelong3  sbelong4  tested_positive  optim1  optim2  slfcont1  slfcont3  slfcont4  urban
##   10   3  age  children  cnarc1  cnarc2  cnarc3  ctheory1  ctheory2  ctheory3  ctheory4  CRT1  CRT2  CRT3  employ_status1  health_cond  know_tested_positive  ladder  marital1  mor_circle  moralid1  moralid10  moralid2  moralid3  moralid4  moralid5  moralid6  moralid7  moralid8  moralid9  mcoop1  mcoop2  mcoop3  mcoop4  mcoop5  mcoop6  mcoop7  narc1  narc2  narc3  narc4  narc5  narc6  nidentity1  nidentity2  omind1  omind2  omind3  omind4  omind5  omind6  contact1  contact2  contact3  contact4  contact5  hygiene1  hygiene2  hygiene3  hygiene4  hygiene5  psupport1  psupport2  psupport3  psupport4  psupport5  political_ideology  happy  slf_ladder  riskperc1  riskperc2  self_esteem  sex1  sbelong1  sbelong2  sbelong3  sbelong4  tested_positive  optim1  optim2  slfcont1  slfcont3  slfcont4  urban
##   10   4  age  children  cnarc1  cnarc2  cnarc3  ctheory1  ctheory2  ctheory3  ctheory4  CRT1  CRT2  CRT3  employ_status1  health_cond  know_tested_positive  ladder  marital1  mor_circle  moralid1  moralid10  moralid2  moralid3  moralid4  moralid5  moralid6  moralid7  moralid8  moralid9  mcoop1  mcoop2  mcoop3  mcoop4  mcoop5  mcoop6  mcoop7  narc1  narc2  narc3  narc4  narc5  narc6  nidentity1  nidentity2  omind1  omind2  omind3  omind4  omind5  omind6  contact1  contact2  contact3  contact4  contact5  hygiene1  hygiene2  hygiene3  hygiene4  hygiene5  psupport1  psupport2  psupport3  psupport4  psupport5  political_ideology  happy  slf_ladder  riskperc1  riskperc2  self_esteem  sex1  sbelong1  sbelong2  sbelong3  sbelong4  tested_positive  optim1  optim2  slfcont1  slfcont3  slfcont4  urban
##   10   5  age  children  cnarc1  cnarc2  cnarc3  ctheory1  ctheory2  ctheory3  ctheory4  CRT1  CRT2  CRT3  employ_status1  health_cond  know_tested_positive  ladder  marital1  mor_circle  moralid1  moralid10  moralid2  moralid3  moralid4  moralid5  moralid6  moralid7  moralid8  moralid9  mcoop1  mcoop2  mcoop3  mcoop4  mcoop5  mcoop6  mcoop7  narc1  narc2  narc3  narc4  narc5  narc6  nidentity1  nidentity2  omind1  omind2  omind3  omind4  omind5  omind6  contact1  contact2  contact3  contact4  contact5  hygiene1  hygiene2  hygiene3  hygiene4  hygiene5  psupport1  psupport2  psupport3  psupport4  psupport5  political_ideology  happy  slf_ladder  riskperc1  riskperc2  self_esteem  sex1  sbelong1  sbelong2  sbelong3  sbelong4  tested_positive  optim1  optim2  slfcont1  slfcont3  slfcont4  urban
##   10   6  age  children  cnarc1  cnarc2  cnarc3  ctheory1  ctheory2  ctheory3  ctheory4  CRT1  CRT2  CRT3  employ_status1  health_cond  know_tested_positive  ladder  marital1  mor_circle  moralid1  moralid10  moralid2  moralid3  moralid4  moralid5  moralid6  moralid7  moralid8  moralid9  mcoop1  mcoop2  mcoop3  mcoop4  mcoop5  mcoop6  mcoop7  narc1  narc2  narc3  narc4  narc5  narc6  nidentity1  nidentity2  omind1  omind2  omind3  omind4  omind5  omind6  contact1  contact2  contact3  contact4  contact5  hygiene1  hygiene2  hygiene3  hygiene4  hygiene5  psupport1  psupport2  psupport3  psupport4  psupport5  political_ideology  happy  slf_ladder  riskperc1  riskperc2  self_esteem  sex1  sbelong1  sbelong2  sbelong3  sbelong4  tested_positive  optim1  optim2  slfcont1  slfcont3  slfcont4  urban
##   10   7  age  children  cnarc1  cnarc2  cnarc3  ctheory1  ctheory2  ctheory3  ctheory4  CRT1  CRT2  CRT3  employ_status1  health_cond  know_tested_positive  ladder  marital1  mor_circle  moralid1  moralid10  moralid2  moralid3  moralid4  moralid5  moralid6  moralid7  moralid8  moralid9  mcoop1  mcoop2  mcoop3  mcoop4  mcoop5  mcoop6  mcoop7  narc1  narc2  narc3  narc4  narc5  narc6  nidentity1  nidentity2  omind1  omind2  omind3  omind4  omind5  omind6  contact1  contact2  contact3  contact4  contact5  hygiene1  hygiene2  hygiene3  hygiene4  hygiene5  psupport1  psupport2  psupport3  psupport4  psupport5  political_ideology  happy  slf_ladder  riskperc1  riskperc2  self_esteem  sex1  sbelong1  sbelong2  sbelong3  sbelong4  tested_positive  optim1  optim2  slfcont1  slfcont3  slfcont4  urban
##   10   8  age  children  cnarc1  cnarc2  cnarc3  ctheory1  ctheory2  ctheory3  ctheory4  CRT1  CRT2  CRT3  employ_status1  health_cond  know_tested_positive  ladder  marital1  mor_circle  moralid1  moralid10  moralid2  moralid3  moralid4  moralid5  moralid6  moralid7  moralid8  moralid9  mcoop1  mcoop2  mcoop3  mcoop4  mcoop5  mcoop6  mcoop7  narc1  narc2  narc3  narc4  narc5  narc6  nidentity1  nidentity2  omind1  omind2  omind3  omind4  omind5  omind6  contact1  contact2  contact3  contact4  contact5  hygiene1  hygiene2  hygiene3  hygiene4  hygiene5  psupport1  psupport2  psupport3  psupport4  psupport5  political_ideology  happy  slf_ladder  riskperc1  riskperc2  self_esteem  sex1  sbelong1  sbelong2  sbelong3  sbelong4  tested_positive  optim1  optim2  slfcont1  slfcont3  slfcont4  urban
##   10   9  age  children  cnarc1  cnarc2  cnarc3  ctheory1  ctheory2  ctheory3  ctheory4  CRT1  CRT2  CRT3  employ_status1  health_cond  know_tested_positive  ladder  marital1  mor_circle  moralid1  moralid10  moralid2  moralid3  moralid4  moralid5  moralid6  moralid7  moralid8  moralid9  mcoop1  mcoop2  mcoop3  mcoop4  mcoop5  mcoop6  mcoop7  narc1  narc2  narc3  narc4  narc5  narc6  nidentity1  nidentity2  omind1  omind2  omind3  omind4  omind5  omind6  contact1  contact2  contact3  contact4  contact5  hygiene1  hygiene2  hygiene3  hygiene4  hygiene5  psupport1  psupport2  psupport3  psupport4  psupport5  political_ideology  happy  slf_ladder  riskperc1  riskperc2  self_esteem  sex1  sbelong1  sbelong2  sbelong3  sbelong4  tested_positive  optim1  optim2  slfcont1  slfcont3  slfcont4  urban
##   10   10  age  children  cnarc1  cnarc2  cnarc3  ctheory1  ctheory2  ctheory3  ctheory4  CRT1  CRT2  CRT3  employ_status1  health_cond  know_tested_positive  ladder  marital1  mor_circle  moralid1  moralid10  moralid2  moralid3  moralid4  moralid5  moralid6  moralid7  moralid8  moralid9  mcoop1  mcoop2  mcoop3  mcoop4  mcoop5  mcoop6  mcoop7  narc1  narc2  narc3  narc4  narc5  narc6  nidentity1  nidentity2  omind1  omind2  omind3  omind4  omind5  omind6  contact1  contact2  contact3  contact4  contact5  hygiene1  hygiene2  hygiene3  hygiene4  hygiene5  psupport1  psupport2  psupport3  psupport4  psupport5  political_ideology  happy  slf_ladder  riskperc1  riskperc2  self_esteem  sex1  sbelong1  sbelong2  sbelong3  sbelong4  tested_positive  optim1  optim2  slfcont1  slfcont3  slfcont4  urban
```

```
## Warning: Number of logged events: 8203
```

```
## 
##  iter imp variable
##   1   1  age  cnarc1  cnarc2  cnarc3  ctheory1  ctheory2  ctheory3  ctheory4  CRT1  CRT2  CRT3  health_cond  marital1  mor_circle  moralid10  moralid3  moralid5  moralid6  moralid7  moralid8  moralid9  mcoop1  mcoop2  mcoop3  mcoop4  mcoop5  mcoop6  mcoop7  narc1  narc2  narc3  narc4  narc5  nidentity1  nidentity2  omind1  omind6  contact1  contact2  contact3  contact4  hygiene1  hygiene2  hygiene3  hygiene4  hygiene5  political_ideology  happy  slf_ladder  riskperc1  riskperc2  sbelong1  sbelong3  sbelong4  optim1  optim2  slfcont1  slfcont2  slfcont3  slfcont4
##   1   2  age  cnarc1  cnarc2  cnarc3  ctheory1  ctheory2  ctheory3  ctheory4  CRT1  CRT2  CRT3  health_cond  marital1  mor_circle  moralid10  moralid3  moralid5  moralid6  moralid7  moralid8  moralid9  mcoop1  mcoop2  mcoop3  mcoop4  mcoop5  mcoop6  mcoop7  narc1  narc2  narc3  narc4  narc5  nidentity1  nidentity2  omind1  omind6  contact1  contact2  contact3  contact4  hygiene1  hygiene2  hygiene3  hygiene4  hygiene5  political_ideology  happy  slf_ladder  riskperc1  riskperc2  sbelong1  sbelong3  sbelong4  optim1  optim2  slfcont1  slfcont2  slfcont3  slfcont4
##   1   3  age  cnarc1  cnarc2  cnarc3  ctheory1  ctheory2  ctheory3  ctheory4  CRT1  CRT2  CRT3  health_cond  marital1  mor_circle  moralid10  moralid3  moralid5  moralid6  moralid7  moralid8  moralid9  mcoop1  mcoop2  mcoop3  mcoop4  mcoop5  mcoop6  mcoop7  narc1  narc2  narc3  narc4  narc5  nidentity1  nidentity2  omind1  omind6  contact1  contact2  contact3  contact4  hygiene1  hygiene2  hygiene3  hygiene4  hygiene5  political_ideology  happy  slf_ladder  riskperc1  riskperc2  sbelong1  sbelong3  sbelong4  optim1  optim2  slfcont1  slfcont2  slfcont3  slfcont4
##   1   4  age  cnarc1  cnarc2  cnarc3  ctheory1  ctheory2  ctheory3  ctheory4  CRT1  CRT2  CRT3  health_cond  marital1  mor_circle  moralid10  moralid3  moralid5  moralid6  moralid7  moralid8  moralid9  mcoop1  mcoop2  mcoop3  mcoop4  mcoop5  mcoop6  mcoop7  narc1  narc2  narc3  narc4  narc5  nidentity1  nidentity2  omind1  omind6  contact1  contact2  contact3  contact4  hygiene1  hygiene2  hygiene3  hygiene4  hygiene5  political_ideology  happy  slf_ladder  riskperc1  riskperc2  sbelong1  sbelong3  sbelong4  optim1  optim2  slfcont1  slfcont2  slfcont3  slfcont4
##   1   5  age  cnarc1  cnarc2  cnarc3  ctheory1  ctheory2  ctheory3  ctheory4  CRT1  CRT2  CRT3  health_cond  marital1  mor_circle  moralid10  moralid3  moralid5  moralid6  moralid7  moralid8  moralid9  mcoop1  mcoop2  mcoop3  mcoop4  mcoop5  mcoop6  mcoop7  narc1  narc2  narc3  narc4  narc5  nidentity1  nidentity2  omind1  omind6  contact1  contact2  contact3  contact4  hygiene1  hygiene2  hygiene3  hygiene4  hygiene5  political_ideology  happy  slf_ladder  riskperc1  riskperc2  sbelong1  sbelong3  sbelong4  optim1  optim2  slfcont1  slfcont2  slfcont3  slfcont4
##   1   6  age  cnarc1  cnarc2  cnarc3  ctheory1  ctheory2  ctheory3  ctheory4  CRT1  CRT2  CRT3  health_cond  marital1  mor_circle  moralid10  moralid3  moralid5  moralid6  moralid7  moralid8  moralid9  mcoop1  mcoop2  mcoop3  mcoop4  mcoop5  mcoop6  mcoop7  narc1  narc2  narc3  narc4  narc5  nidentity1  nidentity2  omind1  omind6  contact1  contact2  contact3  contact4  hygiene1  hygiene2  hygiene3  hygiene4  hygiene5  political_ideology  happy  slf_ladder  riskperc1  riskperc2  sbelong1  sbelong3  sbelong4  optim1  optim2  slfcont1  slfcont2  slfcont3  slfcont4
##   1   7  age  cnarc1  cnarc2  cnarc3  ctheory1  ctheory2  ctheory3  ctheory4  CRT1  CRT2  CRT3  health_cond  marital1  mor_circle  moralid10  moralid3  moralid5  moralid6  moralid7  moralid8  moralid9  mcoop1  mcoop2  mcoop3  mcoop4  mcoop5  mcoop6  mcoop7  narc1  narc2  narc3  narc4  narc5  nidentity1  nidentity2  omind1  omind6  contact1  contact2  contact3  contact4  hygiene1  hygiene2  hygiene3  hygiene4  hygiene5  political_ideology  happy  slf_ladder  riskperc1  riskperc2  sbelong1  sbelong3  sbelong4  optim1  optim2  slfcont1  slfcont2  slfcont3  slfcont4
##   1   8  age  cnarc1  cnarc2  cnarc3  ctheory1  ctheory2  ctheory3  ctheory4  CRT1  CRT2  CRT3  health_cond  marital1  mor_circle  moralid10  moralid3  moralid5  moralid6  moralid7  moralid8  moralid9  mcoop1  mcoop2  mcoop3  mcoop4  mcoop5  mcoop6  mcoop7  narc1  narc2  narc3  narc4  narc5  nidentity1  nidentity2  omind1  omind6  contact1  contact2  contact3  contact4  hygiene1  hygiene2  hygiene3  hygiene4  hygiene5  political_ideology  happy  slf_ladder  riskperc1  riskperc2  sbelong1  sbelong3  sbelong4  optim1  optim2  slfcont1  slfcont2  slfcont3  slfcont4
##   1   9  age  cnarc1  cnarc2  cnarc3  ctheory1  ctheory2  ctheory3  ctheory4  CRT1  CRT2  CRT3  health_cond  marital1  mor_circle  moralid10  moralid3  moralid5  moralid6  moralid7  moralid8  moralid9  mcoop1  mcoop2  mcoop3  mcoop4  mcoop5  mcoop6  mcoop7  narc1  narc2  narc3  narc4  narc5  nidentity1  nidentity2  omind1  omind6  contact1  contact2  contact3  contact4  hygiene1  hygiene2  hygiene3  hygiene4  hygiene5  political_ideology  happy  slf_ladder  riskperc1  riskperc2  sbelong1  sbelong3  sbelong4  optim1  optim2  slfcont1  slfcont2  slfcont3  slfcont4
##   1   10  age  cnarc1  cnarc2  cnarc3  ctheory1  ctheory2  ctheory3  ctheory4  CRT1  CRT2  CRT3  health_cond  marital1  mor_circle  moralid10  moralid3  moralid5  moralid6  moralid7  moralid8  moralid9  mcoop1  mcoop2  mcoop3  mcoop4  mcoop5  mcoop6  mcoop7  narc1  narc2  narc3  narc4  narc5  nidentity1  nidentity2  omind1  omind6  contact1  contact2  contact3  contact4  hygiene1  hygiene2  hygiene3  hygiene4  hygiene5  political_ideology  happy  slf_ladder  riskperc1  riskperc2  sbelong1  sbelong3  sbelong4  optim1  optim2  slfcont1  slfcont2  slfcont3  slfcont4
##   2   1  age  cnarc1  cnarc2  cnarc3  ctheory1  ctheory2  ctheory3  ctheory4  CRT1  CRT2  CRT3  health_cond  marital1  mor_circle  moralid10  moralid3  moralid5  moralid6  moralid7  moralid8  moralid9  mcoop1  mcoop2  mcoop3  mcoop4  mcoop5  mcoop6  mcoop7  narc1  narc2  narc3  narc4  narc5  nidentity1  nidentity2  omind1  omind6  contact1  contact2  contact3  contact4  hygiene1  hygiene2  hygiene3  hygiene4  hygiene5  political_ideology  happy  slf_ladder  riskperc1  riskperc2  sbelong1  sbelong3  sbelong4  optim1  optim2  slfcont1  slfcont2  slfcont3  slfcont4
##   2   2  age  cnarc1  cnarc2  cnarc3  ctheory1  ctheory2  ctheory3  ctheory4  CRT1  CRT2  CRT3  health_cond  marital1  mor_circle  moralid10  moralid3  moralid5  moralid6  moralid7  moralid8  moralid9  mcoop1  mcoop2  mcoop3  mcoop4  mcoop5  mcoop6  mcoop7  narc1  narc2  narc3  narc4  narc5  nidentity1  nidentity2  omind1  omind6  contact1  contact2  contact3  contact4  hygiene1  hygiene2  hygiene3  hygiene4  hygiene5  political_ideology  happy  slf_ladder  riskperc1  riskperc2  sbelong1  sbelong3  sbelong4  optim1  optim2  slfcont1  slfcont2  slfcont3  slfcont4
##   2   3  age  cnarc1  cnarc2  cnarc3  ctheory1  ctheory2  ctheory3  ctheory4  CRT1  CRT2  CRT3  health_cond  marital1  mor_circle  moralid10  moralid3  moralid5  moralid6  moralid7  moralid8  moralid9  mcoop1  mcoop2  mcoop3  mcoop4  mcoop5  mcoop6  mcoop7  narc1  narc2  narc3  narc4  narc5  nidentity1  nidentity2  omind1  omind6  contact1  contact2  contact3  contact4  hygiene1  hygiene2  hygiene3  hygiene4  hygiene5  political_ideology  happy  slf_ladder  riskperc1  riskperc2  sbelong1  sbelong3  sbelong4  optim1  optim2  slfcont1  slfcont2  slfcont3  slfcont4
##   2   4  age  cnarc1  cnarc2  cnarc3  ctheory1  ctheory2  ctheory3  ctheory4  CRT1  CRT2  CRT3  health_cond  marital1  mor_circle  moralid10  moralid3  moralid5  moralid6  moralid7  moralid8  moralid9  mcoop1  mcoop2  mcoop3  mcoop4  mcoop5  mcoop6  mcoop7  narc1  narc2  narc3  narc4  narc5  nidentity1  nidentity2  omind1  omind6  contact1  contact2  contact3  contact4  hygiene1  hygiene2  hygiene3  hygiene4  hygiene5  political_ideology  happy  slf_ladder  riskperc1  riskperc2  sbelong1  sbelong3  sbelong4  optim1  optim2  slfcont1  slfcont2  slfcont3  slfcont4
##   2   5  age  cnarc1  cnarc2  cnarc3  ctheory1  ctheory2  ctheory3  ctheory4  CRT1  CRT2  CRT3  health_cond  marital1  mor_circle  moralid10  moralid3  moralid5  moralid6  moralid7  moralid8  moralid9  mcoop1  mcoop2  mcoop3  mcoop4  mcoop5  mcoop6  mcoop7  narc1  narc2  narc3  narc4  narc5  nidentity1  nidentity2  omind1  omind6  contact1  contact2  contact3  contact4  hygiene1  hygiene2  hygiene3  hygiene4  hygiene5  political_ideology  happy  slf_ladder  riskperc1  riskperc2  sbelong1  sbelong3  sbelong4  optim1  optim2  slfcont1  slfcont2  slfcont3  slfcont4
##   2   6  age  cnarc1  cnarc2  cnarc3  ctheory1  ctheory2  ctheory3  ctheory4  CRT1  CRT2  CRT3  health_cond  marital1  mor_circle  moralid10  moralid3  moralid5  moralid6  moralid7  moralid8  moralid9  mcoop1  mcoop2  mcoop3  mcoop4  mcoop5  mcoop6  mcoop7  narc1  narc2  narc3  narc4  narc5  nidentity1  nidentity2  omind1  omind6  contact1  contact2  contact3  contact4  hygiene1  hygiene2  hygiene3  hygiene4  hygiene5  political_ideology  happy  slf_ladder  riskperc1  riskperc2  sbelong1  sbelong3  sbelong4  optim1  optim2  slfcont1  slfcont2  slfcont3  slfcont4
##   2   7  age  cnarc1  cnarc2  cnarc3  ctheory1  ctheory2  ctheory3  ctheory4  CRT1  CRT2  CRT3  health_cond  marital1  mor_circle  moralid10  moralid3  moralid5  moralid6  moralid7  moralid8  moralid9  mcoop1  mcoop2  mcoop3  mcoop4  mcoop5  mcoop6  mcoop7  narc1  narc2  narc3  narc4  narc5  nidentity1  nidentity2  omind1  omind6  contact1  contact2  contact3  contact4  hygiene1  hygiene2  hygiene3  hygiene4  hygiene5  political_ideology  happy  slf_ladder  riskperc1  riskperc2  sbelong1  sbelong3  sbelong4  optim1  optim2  slfcont1  slfcont2  slfcont3  slfcont4
##   2   8  age  cnarc1  cnarc2  cnarc3  ctheory1  ctheory2  ctheory3  ctheory4  CRT1  CRT2  CRT3  health_cond  marital1  mor_circle  moralid10  moralid3  moralid5  moralid6  moralid7  moralid8  moralid9  mcoop1  mcoop2  mcoop3  mcoop4  mcoop5  mcoop6  mcoop7  narc1  narc2  narc3  narc4  narc5  nidentity1  nidentity2  omind1  omind6  contact1  contact2  contact3  contact4  hygiene1  hygiene2  hygiene3  hygiene4  hygiene5  political_ideology  happy  slf_ladder  riskperc1  riskperc2  sbelong1  sbelong3  sbelong4  optim1  optim2  slfcont1  slfcont2  slfcont3  slfcont4
##   2   9  age  cnarc1  cnarc2  cnarc3  ctheory1  ctheory2  ctheory3  ctheory4  CRT1  CRT2  CRT3  health_cond  marital1  mor_circle  moralid10  moralid3  moralid5  moralid6  moralid7  moralid8  moralid9  mcoop1  mcoop2  mcoop3  mcoop4  mcoop5  mcoop6  mcoop7  narc1  narc2  narc3  narc4  narc5  nidentity1  nidentity2  omind1  omind6  contact1  contact2  contact3  contact4  hygiene1  hygiene2  hygiene3  hygiene4  hygiene5  political_ideology  happy  slf_ladder  riskperc1  riskperc2  sbelong1  sbelong3  sbelong4  optim1  optim2  slfcont1  slfcont2  slfcont3  slfcont4
##   2   10  age  cnarc1  cnarc2  cnarc3  ctheory1  ctheory2  ctheory3  ctheory4  CRT1  CRT2  CRT3  health_cond  marital1  mor_circle  moralid10  moralid3  moralid5  moralid6  moralid7  moralid8  moralid9  mcoop1  mcoop2  mcoop3  mcoop4  mcoop5  mcoop6  mcoop7  narc1  narc2  narc3  narc4  narc5  nidentity1  nidentity2  omind1  omind6  contact1  contact2  contact3  contact4  hygiene1  hygiene2  hygiene3  hygiene4  hygiene5  political_ideology  happy  slf_ladder  riskperc1  riskperc2  sbelong1  sbelong3  sbelong4  optim1  optim2  slfcont1  slfcont2  slfcont3  slfcont4
##   3   1  age  cnarc1  cnarc2  cnarc3  ctheory1  ctheory2  ctheory3  ctheory4  CRT1  CRT2  CRT3  health_cond  marital1  mor_circle  moralid10  moralid3  moralid5  moralid6  moralid7  moralid8  moralid9  mcoop1  mcoop2  mcoop3  mcoop4  mcoop5  mcoop6  mcoop7  narc1  narc2  narc3  narc4  narc5  nidentity1  nidentity2  omind1  omind6  contact1  contact2  contact3  contact4  hygiene1  hygiene2  hygiene3  hygiene4  hygiene5  political_ideology  happy  slf_ladder  riskperc1  riskperc2  sbelong1  sbelong3  sbelong4  optim1  optim2  slfcont1  slfcont2  slfcont3  slfcont4
##   3   2  age  cnarc1  cnarc2  cnarc3  ctheory1  ctheory2  ctheory3  ctheory4  CRT1  CRT2  CRT3  health_cond  marital1  mor_circle  moralid10  moralid3  moralid5  moralid6  moralid7  moralid8  moralid9  mcoop1  mcoop2  mcoop3  mcoop4  mcoop5  mcoop6  mcoop7  narc1  narc2  narc3  narc4  narc5  nidentity1  nidentity2  omind1  omind6  contact1  contact2  contact3  contact4  hygiene1  hygiene2  hygiene3  hygiene4  hygiene5  political_ideology  happy  slf_ladder  riskperc1  riskperc2  sbelong1  sbelong3  sbelong4  optim1  optim2  slfcont1  slfcont2  slfcont3  slfcont4
##   3   3  age  cnarc1  cnarc2  cnarc3  ctheory1  ctheory2  ctheory3  ctheory4  CRT1  CRT2  CRT3  health_cond  marital1  mor_circle  moralid10  moralid3  moralid5  moralid6  moralid7  moralid8  moralid9  mcoop1  mcoop2  mcoop3  mcoop4  mcoop5  mcoop6  mcoop7  narc1  narc2  narc3  narc4  narc5  nidentity1  nidentity2  omind1  omind6  contact1  contact2  contact3  contact4  hygiene1  hygiene2  hygiene3  hygiene4  hygiene5  political_ideology  happy  slf_ladder  riskperc1  riskperc2  sbelong1  sbelong3  sbelong4  optim1  optim2  slfcont1  slfcont2  slfcont3  slfcont4
##   3   4  age  cnarc1  cnarc2  cnarc3  ctheory1  ctheory2  ctheory3  ctheory4  CRT1  CRT2  CRT3  health_cond  marital1  mor_circle  moralid10  moralid3  moralid5  moralid6  moralid7  moralid8  moralid9  mcoop1  mcoop2  mcoop3  mcoop4  mcoop5  mcoop6  mcoop7  narc1  narc2  narc3  narc4  narc5  nidentity1  nidentity2  omind1  omind6  contact1  contact2  contact3  contact4  hygiene1  hygiene2  hygiene3  hygiene4  hygiene5  political_ideology  happy  slf_ladder  riskperc1  riskperc2  sbelong1  sbelong3  sbelong4  optim1  optim2  slfcont1  slfcont2  slfcont3  slfcont4
##   3   5  age  cnarc1  cnarc2  cnarc3  ctheory1  ctheory2  ctheory3  ctheory4  CRT1  CRT2  CRT3  health_cond  marital1  mor_circle  moralid10  moralid3  moralid5  moralid6  moralid7  moralid8  moralid9  mcoop1  mcoop2  mcoop3  mcoop4  mcoop5  mcoop6  mcoop7  narc1  narc2  narc3  narc4  narc5  nidentity1  nidentity2  omind1  omind6  contact1  contact2  contact3  contact4  hygiene1  hygiene2  hygiene3  hygiene4  hygiene5  political_ideology  happy  slf_ladder  riskperc1  riskperc2  sbelong1  sbelong3  sbelong4  optim1  optim2  slfcont1  slfcont2  slfcont3  slfcont4
##   3   6  age  cnarc1  cnarc2  cnarc3  ctheory1  ctheory2  ctheory3  ctheory4  CRT1  CRT2  CRT3  health_cond  marital1  mor_circle  moralid10  moralid3  moralid5  moralid6  moralid7  moralid8  moralid9  mcoop1  mcoop2  mcoop3  mcoop4  mcoop5  mcoop6  mcoop7  narc1  narc2  narc3  narc4  narc5  nidentity1  nidentity2  omind1  omind6  contact1  contact2  contact3  contact4  hygiene1  hygiene2  hygiene3  hygiene4  hygiene5  political_ideology  happy  slf_ladder  riskperc1  riskperc2  sbelong1  sbelong3  sbelong4  optim1  optim2  slfcont1  slfcont2  slfcont3  slfcont4
##   3   7  age  cnarc1  cnarc2  cnarc3  ctheory1  ctheory2  ctheory3  ctheory4  CRT1  CRT2  CRT3  health_cond  marital1  mor_circle  moralid10  moralid3  moralid5  moralid6  moralid7  moralid8  moralid9  mcoop1  mcoop2  mcoop3  mcoop4  mcoop5  mcoop6  mcoop7  narc1  narc2  narc3  narc4  narc5  nidentity1  nidentity2  omind1  omind6  contact1  contact2  contact3  contact4  hygiene1  hygiene2  hygiene3  hygiene4  hygiene5  political_ideology  happy  slf_ladder  riskperc1  riskperc2  sbelong1  sbelong3  sbelong4  optim1  optim2  slfcont1  slfcont2  slfcont3  slfcont4
##   3   8  age  cnarc1  cnarc2  cnarc3  ctheory1  ctheory2  ctheory3  ctheory4  CRT1  CRT2  CRT3  health_cond  marital1  mor_circle  moralid10  moralid3  moralid5  moralid6  moralid7  moralid8  moralid9  mcoop1  mcoop2  mcoop3  mcoop4  mcoop5  mcoop6  mcoop7  narc1  narc2  narc3  narc4  narc5  nidentity1  nidentity2  omind1  omind6  contact1  contact2  contact3  contact4  hygiene1  hygiene2  hygiene3  hygiene4  hygiene5  political_ideology  happy  slf_ladder  riskperc1  riskperc2  sbelong1  sbelong3  sbelong4  optim1  optim2  slfcont1  slfcont2  slfcont3  slfcont4
##   3   9  age  cnarc1  cnarc2  cnarc3  ctheory1  ctheory2  ctheory3  ctheory4  CRT1  CRT2  CRT3  health_cond  marital1  mor_circle  moralid10  moralid3  moralid5  moralid6  moralid7  moralid8  moralid9  mcoop1  mcoop2  mcoop3  mcoop4  mcoop5  mcoop6  mcoop7  narc1  narc2  narc3  narc4  narc5  nidentity1  nidentity2  omind1  omind6  contact1  contact2  contact3  contact4  hygiene1  hygiene2  hygiene3  hygiene4  hygiene5  political_ideology  happy  slf_ladder  riskperc1  riskperc2  sbelong1  sbelong3  sbelong4  optim1  optim2  slfcont1  slfcont2  slfcont3  slfcont4
##   3   10  age  cnarc1  cnarc2  cnarc3  ctheory1  ctheory2  ctheory3  ctheory4  CRT1  CRT2  CRT3  health_cond  marital1  mor_circle  moralid10  moralid3  moralid5  moralid6  moralid7  moralid8  moralid9  mcoop1  mcoop2  mcoop3  mcoop4  mcoop5  mcoop6  mcoop7  narc1  narc2  narc3  narc4  narc5  nidentity1  nidentity2  omind1  omind6  contact1  contact2  contact3  contact4  hygiene1  hygiene2  hygiene3  hygiene4  hygiene5  political_ideology  happy  slf_ladder  riskperc1  riskperc2  sbelong1  sbelong3  sbelong4  optim1  optim2  slfcont1  slfcont2  slfcont3  slfcont4
##   4   1  age  cnarc1  cnarc2  cnarc3  ctheory1  ctheory2  ctheory3  ctheory4  CRT1  CRT2  CRT3  health_cond  marital1  mor_circle  moralid10  moralid3  moralid5  moralid6  moralid7  moralid8  moralid9  mcoop1  mcoop2  mcoop3  mcoop4  mcoop5  mcoop6  mcoop7  narc1  narc2  narc3  narc4  narc5  nidentity1  nidentity2  omind1  omind6  contact1  contact2  contact3  contact4  hygiene1  hygiene2  hygiene3  hygiene4  hygiene5  political_ideology  happy  slf_ladder  riskperc1  riskperc2  sbelong1  sbelong3  sbelong4  optim1  optim2  slfcont1  slfcont2  slfcont3  slfcont4
##   4   2  age  cnarc1  cnarc2  cnarc3  ctheory1  ctheory2  ctheory3  ctheory4  CRT1  CRT2  CRT3  health_cond  marital1  mor_circle  moralid10  moralid3  moralid5  moralid6  moralid7  moralid8  moralid9  mcoop1  mcoop2  mcoop3  mcoop4  mcoop5  mcoop6  mcoop7  narc1  narc2  narc3  narc4  narc5  nidentity1  nidentity2  omind1  omind6  contact1  contact2  contact3  contact4  hygiene1  hygiene2  hygiene3  hygiene4  hygiene5  political_ideology  happy  slf_ladder  riskperc1  riskperc2  sbelong1  sbelong3  sbelong4  optim1  optim2  slfcont1  slfcont2  slfcont3  slfcont4
##   4   3  age  cnarc1  cnarc2  cnarc3  ctheory1  ctheory2  ctheory3  ctheory4  CRT1  CRT2  CRT3  health_cond  marital1  mor_circle  moralid10  moralid3  moralid5  moralid6  moralid7  moralid8  moralid9  mcoop1  mcoop2  mcoop3  mcoop4  mcoop5  mcoop6  mcoop7  narc1  narc2  narc3  narc4  narc5  nidentity1  nidentity2  omind1  omind6  contact1  contact2  contact3  contact4  hygiene1  hygiene2  hygiene3  hygiene4  hygiene5  political_ideology  happy  slf_ladder  riskperc1  riskperc2  sbelong1  sbelong3  sbelong4  optim1  optim2  slfcont1  slfcont2  slfcont3  slfcont4
##   4   4  age  cnarc1  cnarc2  cnarc3  ctheory1  ctheory2  ctheory3  ctheory4  CRT1  CRT2  CRT3  health_cond  marital1  mor_circle  moralid10  moralid3  moralid5  moralid6  moralid7  moralid8  moralid9  mcoop1  mcoop2  mcoop3  mcoop4  mcoop5  mcoop6  mcoop7  narc1  narc2  narc3  narc4  narc5  nidentity1  nidentity2  omind1  omind6  contact1  contact2  contact3  contact4  hygiene1  hygiene2  hygiene3  hygiene4  hygiene5  political_ideology  happy  slf_ladder  riskperc1  riskperc2  sbelong1  sbelong3  sbelong4  optim1  optim2  slfcont1  slfcont2  slfcont3  slfcont4
##   4   5  age  cnarc1  cnarc2  cnarc3  ctheory1  ctheory2  ctheory3  ctheory4  CRT1  CRT2  CRT3  health_cond  marital1  mor_circle  moralid10  moralid3  moralid5  moralid6  moralid7  moralid8  moralid9  mcoop1  mcoop2  mcoop3  mcoop4  mcoop5  mcoop6  mcoop7  narc1  narc2  narc3  narc4  narc5  nidentity1  nidentity2  omind1  omind6  contact1  contact2  contact3  contact4  hygiene1  hygiene2  hygiene3  hygiene4  hygiene5  political_ideology  happy  slf_ladder  riskperc1  riskperc2  sbelong1  sbelong3  sbelong4  optim1  optim2  slfcont1  slfcont2  slfcont3  slfcont4
##   4   6  age  cnarc1  cnarc2  cnarc3  ctheory1  ctheory2  ctheory3  ctheory4  CRT1  CRT2  CRT3  health_cond  marital1  mor_circle  moralid10  moralid3  moralid5  moralid6  moralid7  moralid8  moralid9  mcoop1  mcoop2  mcoop3  mcoop4  mcoop5  mcoop6  mcoop7  narc1  narc2  narc3  narc4  narc5  nidentity1  nidentity2  omind1  omind6  contact1  contact2  contact3  contact4  hygiene1  hygiene2  hygiene3  hygiene4  hygiene5  political_ideology  happy  slf_ladder  riskperc1  riskperc2  sbelong1  sbelong3  sbelong4  optim1  optim2  slfcont1  slfcont2  slfcont3  slfcont4
##   4   7  age  cnarc1  cnarc2  cnarc3  ctheory1  ctheory2  ctheory3  ctheory4  CRT1  CRT2  CRT3  health_cond  marital1  mor_circle  moralid10  moralid3  moralid5  moralid6  moralid7  moralid8  moralid9  mcoop1  mcoop2  mcoop3  mcoop4  mcoop5  mcoop6  mcoop7  narc1  narc2  narc3  narc4  narc5  nidentity1  nidentity2  omind1  omind6  contact1  contact2  contact3  contact4  hygiene1  hygiene2  hygiene3  hygiene4  hygiene5  political_ideology  happy  slf_ladder  riskperc1  riskperc2  sbelong1  sbelong3  sbelong4  optim1  optim2  slfcont1  slfcont2  slfcont3  slfcont4
##   4   8  age  cnarc1  cnarc2  cnarc3  ctheory1  ctheory2  ctheory3  ctheory4  CRT1  CRT2  CRT3  health_cond  marital1  mor_circle  moralid10  moralid3  moralid5  moralid6  moralid7  moralid8  moralid9  mcoop1  mcoop2  mcoop3  mcoop4  mcoop5  mcoop6  mcoop7  narc1  narc2  narc3  narc4  narc5  nidentity1  nidentity2  omind1  omind6  contact1  contact2  contact3  contact4  hygiene1  hygiene2  hygiene3  hygiene4  hygiene5  political_ideology  happy  slf_ladder  riskperc1  riskperc2  sbelong1  sbelong3  sbelong4  optim1  optim2  slfcont1  slfcont2  slfcont3  slfcont4
##   4   9  age  cnarc1  cnarc2  cnarc3  ctheory1  ctheory2  ctheory3  ctheory4  CRT1  CRT2  CRT3  health_cond  marital1  mor_circle  moralid10  moralid3  moralid5  moralid6  moralid7  moralid8  moralid9  mcoop1  mcoop2  mcoop3  mcoop4  mcoop5  mcoop6  mcoop7  narc1  narc2  narc3  narc4  narc5  nidentity1  nidentity2  omind1  omind6  contact1  contact2  contact3  contact4  hygiene1  hygiene2  hygiene3  hygiene4  hygiene5  political_ideology  happy  slf_ladder  riskperc1  riskperc2  sbelong1  sbelong3  sbelong4  optim1  optim2  slfcont1  slfcont2  slfcont3  slfcont4
##   4   10  age  cnarc1  cnarc2  cnarc3  ctheory1  ctheory2  ctheory3  ctheory4  CRT1  CRT2  CRT3  health_cond  marital1  mor_circle  moralid10  moralid3  moralid5  moralid6  moralid7  moralid8  moralid9  mcoop1  mcoop2  mcoop3  mcoop4  mcoop5  mcoop6  mcoop7  narc1  narc2  narc3  narc4  narc5  nidentity1  nidentity2  omind1  omind6  contact1  contact2  contact3  contact4  hygiene1  hygiene2  hygiene3  hygiene4  hygiene5  political_ideology  happy  slf_ladder  riskperc1  riskperc2  sbelong1  sbelong3  sbelong4  optim1  optim2  slfcont1  slfcont2  slfcont3  slfcont4
##   5   1  age  cnarc1  cnarc2  cnarc3  ctheory1  ctheory2  ctheory3  ctheory4  CRT1  CRT2  CRT3  health_cond  marital1  mor_circle  moralid10  moralid3  moralid5  moralid6  moralid7  moralid8  moralid9  mcoop1  mcoop2  mcoop3  mcoop4  mcoop5  mcoop6  mcoop7  narc1  narc2  narc3  narc4  narc5  nidentity1  nidentity2  omind1  omind6  contact1  contact2  contact3  contact4  hygiene1  hygiene2  hygiene3  hygiene4  hygiene5  political_ideology  happy  slf_ladder  riskperc1  riskperc2  sbelong1  sbelong3  sbelong4  optim1  optim2  slfcont1  slfcont2  slfcont3  slfcont4
##   5   2  age  cnarc1  cnarc2  cnarc3  ctheory1  ctheory2  ctheory3  ctheory4  CRT1  CRT2  CRT3  health_cond  marital1  mor_circle  moralid10  moralid3  moralid5  moralid6  moralid7  moralid8  moralid9  mcoop1  mcoop2  mcoop3  mcoop4  mcoop5  mcoop6  mcoop7  narc1  narc2  narc3  narc4  narc5  nidentity1  nidentity2  omind1  omind6  contact1  contact2  contact3  contact4  hygiene1  hygiene2  hygiene3  hygiene4  hygiene5  political_ideology  happy  slf_ladder  riskperc1  riskperc2  sbelong1  sbelong3  sbelong4  optim1  optim2  slfcont1  slfcont2  slfcont3  slfcont4
##   5   3  age  cnarc1  cnarc2  cnarc3  ctheory1  ctheory2  ctheory3  ctheory4  CRT1  CRT2  CRT3  health_cond  marital1  mor_circle  moralid10  moralid3  moralid5  moralid6  moralid7  moralid8  moralid9  mcoop1  mcoop2  mcoop3  mcoop4  mcoop5  mcoop6  mcoop7  narc1  narc2  narc3  narc4  narc5  nidentity1  nidentity2  omind1  omind6  contact1  contact2  contact3  contact4  hygiene1  hygiene2  hygiene3  hygiene4  hygiene5  political_ideology  happy  slf_ladder  riskperc1  riskperc2  sbelong1  sbelong3  sbelong4  optim1  optim2  slfcont1  slfcont2  slfcont3  slfcont4
##   5   4  age  cnarc1  cnarc2  cnarc3  ctheory1  ctheory2  ctheory3  ctheory4  CRT1  CRT2  CRT3  health_cond  marital1  mor_circle  moralid10  moralid3  moralid5  moralid6  moralid7  moralid8  moralid9  mcoop1  mcoop2  mcoop3  mcoop4  mcoop5  mcoop6  mcoop7  narc1  narc2  narc3  narc4  narc5  nidentity1  nidentity2  omind1  omind6  contact1  contact2  contact3  contact4  hygiene1  hygiene2  hygiene3  hygiene4  hygiene5  political_ideology  happy  slf_ladder  riskperc1  riskperc2  sbelong1  sbelong3  sbelong4  optim1  optim2  slfcont1  slfcont2  slfcont3  slfcont4
##   5   5  age  cnarc1  cnarc2  cnarc3  ctheory1  ctheory2  ctheory3  ctheory4  CRT1  CRT2  CRT3  health_cond  marital1  mor_circle  moralid10  moralid3  moralid5  moralid6  moralid7  moralid8  moralid9  mcoop1  mcoop2  mcoop3  mcoop4  mcoop5  mcoop6  mcoop7  narc1  narc2  narc3  narc4  narc5  nidentity1  nidentity2  omind1  omind6  contact1  contact2  contact3  contact4  hygiene1  hygiene2  hygiene3  hygiene4  hygiene5  political_ideology  happy  slf_ladder  riskperc1  riskperc2  sbelong1  sbelong3  sbelong4  optim1  optim2  slfcont1  slfcont2  slfcont3  slfcont4
##   5   6  age  cnarc1  cnarc2  cnarc3  ctheory1  ctheory2  ctheory3  ctheory4  CRT1  CRT2  CRT3  health_cond  marital1  mor_circle  moralid10  moralid3  moralid5  moralid6  moralid7  moralid8  moralid9  mcoop1  mcoop2  mcoop3  mcoop4  mcoop5  mcoop6  mcoop7  narc1  narc2  narc3  narc4  narc5  nidentity1  nidentity2  omind1  omind6  contact1  contact2  contact3  contact4  hygiene1  hygiene2  hygiene3  hygiene4  hygiene5  political_ideology  happy  slf_ladder  riskperc1  riskperc2  sbelong1  sbelong3  sbelong4  optim1  optim2  slfcont1  slfcont2  slfcont3  slfcont4
##   5   7  age  cnarc1  cnarc2  cnarc3  ctheory1  ctheory2  ctheory3  ctheory4  CRT1  CRT2  CRT3  health_cond  marital1  mor_circle  moralid10  moralid3  moralid5  moralid6  moralid7  moralid8  moralid9  mcoop1  mcoop2  mcoop3  mcoop4  mcoop5  mcoop6  mcoop7  narc1  narc2  narc3  narc4  narc5  nidentity1  nidentity2  omind1  omind6  contact1  contact2  contact3  contact4  hygiene1  hygiene2  hygiene3  hygiene4  hygiene5  political_ideology  happy  slf_ladder  riskperc1  riskperc2  sbelong1  sbelong3  sbelong4  optim1  optim2  slfcont1  slfcont2  slfcont3  slfcont4
##   5   8  age  cnarc1  cnarc2  cnarc3  ctheory1  ctheory2  ctheory3  ctheory4  CRT1  CRT2  CRT3  health_cond  marital1  mor_circle  moralid10  moralid3  moralid5  moralid6  moralid7  moralid8  moralid9  mcoop1  mcoop2  mcoop3  mcoop4  mcoop5  mcoop6  mcoop7  narc1  narc2  narc3  narc4  narc5  nidentity1  nidentity2  omind1  omind6  contact1  contact2  contact3  contact4  hygiene1  hygiene2  hygiene3  hygiene4  hygiene5  political_ideology  happy  slf_ladder  riskperc1  riskperc2  sbelong1  sbelong3  sbelong4  optim1  optim2  slfcont1  slfcont2  slfcont3  slfcont4
##   5   9  age  cnarc1  cnarc2  cnarc3  ctheory1  ctheory2  ctheory3  ctheory4  CRT1  CRT2  CRT3  health_cond  marital1  mor_circle  moralid10  moralid3  moralid5  moralid6  moralid7  moralid8  moralid9  mcoop1  mcoop2  mcoop3  mcoop4  mcoop5  mcoop6  mcoop7  narc1  narc2  narc3  narc4  narc5  nidentity1  nidentity2  omind1  omind6  contact1  contact2  contact3  contact4  hygiene1  hygiene2  hygiene3  hygiene4  hygiene5  political_ideology  happy  slf_ladder  riskperc1  riskperc2  sbelong1  sbelong3  sbelong4  optim1  optim2  slfcont1  slfcont2  slfcont3  slfcont4
##   5   10  age  cnarc1  cnarc2  cnarc3  ctheory1  ctheory2  ctheory3  ctheory4  CRT1  CRT2  CRT3  health_cond  marital1  mor_circle  moralid10  moralid3  moralid5  moralid6  moralid7  moralid8  moralid9  mcoop1  mcoop2  mcoop3  mcoop4  mcoop5  mcoop6  mcoop7  narc1  narc2  narc3  narc4  narc5  nidentity1  nidentity2  omind1  omind6  contact1  contact2  contact3  contact4  hygiene1  hygiene2  hygiene3  hygiene4  hygiene5  political_ideology  happy  slf_ladder  riskperc1  riskperc2  sbelong1  sbelong3  sbelong4  optim1  optim2  slfcont1  slfcont2  slfcont3  slfcont4
##   6   1  age  cnarc1  cnarc2  cnarc3  ctheory1  ctheory2  ctheory3  ctheory4  CRT1  CRT2  CRT3  health_cond  marital1  mor_circle  moralid10  moralid3  moralid5  moralid6  moralid7  moralid8  moralid9  mcoop1  mcoop2  mcoop3  mcoop4  mcoop5  mcoop6  mcoop7  narc1  narc2  narc3  narc4  narc5  nidentity1  nidentity2  omind1  omind6  contact1  contact2  contact3  contact4  hygiene1  hygiene2  hygiene3  hygiene4  hygiene5  political_ideology  happy  slf_ladder  riskperc1  riskperc2  sbelong1  sbelong3  sbelong4  optim1  optim2  slfcont1  slfcont2  slfcont3  slfcont4
##   6   2  age  cnarc1  cnarc2  cnarc3  ctheory1  ctheory2  ctheory3  ctheory4  CRT1  CRT2  CRT3  health_cond  marital1  mor_circle  moralid10  moralid3  moralid5  moralid6  moralid7  moralid8  moralid9  mcoop1  mcoop2  mcoop3  mcoop4  mcoop5  mcoop6  mcoop7  narc1  narc2  narc3  narc4  narc5  nidentity1  nidentity2  omind1  omind6  contact1  contact2  contact3  contact4  hygiene1  hygiene2  hygiene3  hygiene4  hygiene5  political_ideology  happy  slf_ladder  riskperc1  riskperc2  sbelong1  sbelong3  sbelong4  optim1  optim2  slfcont1  slfcont2  slfcont3  slfcont4
##   6   3  age  cnarc1  cnarc2  cnarc3  ctheory1  ctheory2  ctheory3  ctheory4  CRT1  CRT2  CRT3  health_cond  marital1  mor_circle  moralid10  moralid3  moralid5  moralid6  moralid7  moralid8  moralid9  mcoop1  mcoop2  mcoop3  mcoop4  mcoop5  mcoop6  mcoop7  narc1  narc2  narc3  narc4  narc5  nidentity1  nidentity2  omind1  omind6  contact1  contact2  contact3  contact4  hygiene1  hygiene2  hygiene3  hygiene4  hygiene5  political_ideology  happy  slf_ladder  riskperc1  riskperc2  sbelong1  sbelong3  sbelong4  optim1  optim2  slfcont1  slfcont2  slfcont3  slfcont4
##   6   4  age  cnarc1  cnarc2  cnarc3  ctheory1  ctheory2  ctheory3  ctheory4  CRT1  CRT2  CRT3  health_cond  marital1  mor_circle  moralid10  moralid3  moralid5  moralid6  moralid7  moralid8  moralid9  mcoop1  mcoop2  mcoop3  mcoop4  mcoop5  mcoop6  mcoop7  narc1  narc2  narc3  narc4  narc5  nidentity1  nidentity2  omind1  omind6  contact1  contact2  contact3  contact4  hygiene1  hygiene2  hygiene3  hygiene4  hygiene5  political_ideology  happy  slf_ladder  riskperc1  riskperc2  sbelong1  sbelong3  sbelong4  optim1  optim2  slfcont1  slfcont2  slfcont3  slfcont4
##   6   5  age  cnarc1  cnarc2  cnarc3  ctheory1  ctheory2  ctheory3  ctheory4  CRT1  CRT2  CRT3  health_cond  marital1  mor_circle  moralid10  moralid3  moralid5  moralid6  moralid7  moralid8  moralid9  mcoop1  mcoop2  mcoop3  mcoop4  mcoop5  mcoop6  mcoop7  narc1  narc2  narc3  narc4  narc5  nidentity1  nidentity2  omind1  omind6  contact1  contact2  contact3  contact4  hygiene1  hygiene2  hygiene3  hygiene4  hygiene5  political_ideology  happy  slf_ladder  riskperc1  riskperc2  sbelong1  sbelong3  sbelong4  optim1  optim2  slfcont1  slfcont2  slfcont3  slfcont4
##   6   6  age  cnarc1  cnarc2  cnarc3  ctheory1  ctheory2  ctheory3  ctheory4  CRT1  CRT2  CRT3  health_cond  marital1  mor_circle  moralid10  moralid3  moralid5  moralid6  moralid7  moralid8  moralid9  mcoop1  mcoop2  mcoop3  mcoop4  mcoop5  mcoop6  mcoop7  narc1  narc2  narc3  narc4  narc5  nidentity1  nidentity2  omind1  omind6  contact1  contact2  contact3  contact4  hygiene1  hygiene2  hygiene3  hygiene4  hygiene5  political_ideology  happy  slf_ladder  riskperc1  riskperc2  sbelong1  sbelong3  sbelong4  optim1  optim2  slfcont1  slfcont2  slfcont3  slfcont4
##   6   7  age  cnarc1  cnarc2  cnarc3  ctheory1  ctheory2  ctheory3  ctheory4  CRT1  CRT2  CRT3  health_cond  marital1  mor_circle  moralid10  moralid3  moralid5  moralid6  moralid7  moralid8  moralid9  mcoop1  mcoop2  mcoop3  mcoop4  mcoop5  mcoop6  mcoop7  narc1  narc2  narc3  narc4  narc5  nidentity1  nidentity2  omind1  omind6  contact1  contact2  contact3  contact4  hygiene1  hygiene2  hygiene3  hygiene4  hygiene5  political_ideology  happy  slf_ladder  riskperc1  riskperc2  sbelong1  sbelong3  sbelong4  optim1  optim2  slfcont1  slfcont2  slfcont3  slfcont4
##   6   8  age  cnarc1  cnarc2  cnarc3  ctheory1  ctheory2  ctheory3  ctheory4  CRT1  CRT2  CRT3  health_cond  marital1  mor_circle  moralid10  moralid3  moralid5  moralid6  moralid7  moralid8  moralid9  mcoop1  mcoop2  mcoop3  mcoop4  mcoop5  mcoop6  mcoop7  narc1  narc2  narc3  narc4  narc5  nidentity1  nidentity2  omind1  omind6  contact1  contact2  contact3  contact4  hygiene1  hygiene2  hygiene3  hygiene4  hygiene5  political_ideology  happy  slf_ladder  riskperc1  riskperc2  sbelong1  sbelong3  sbelong4  optim1  optim2  slfcont1  slfcont2  slfcont3  slfcont4
##   6   9  age  cnarc1  cnarc2  cnarc3  ctheory1  ctheory2  ctheory3  ctheory4  CRT1  CRT2  CRT3  health_cond  marital1  mor_circle  moralid10  moralid3  moralid5  moralid6  moralid7  moralid8  moralid9  mcoop1  mcoop2  mcoop3  mcoop4  mcoop5  mcoop6  mcoop7  narc1  narc2  narc3  narc4  narc5  nidentity1  nidentity2  omind1  omind6  contact1  contact2  contact3  contact4  hygiene1  hygiene2  hygiene3  hygiene4  hygiene5  political_ideology  happy  slf_ladder  riskperc1  riskperc2  sbelong1  sbelong3  sbelong4  optim1  optim2  slfcont1  slfcont2  slfcont3  slfcont4
##   6   10  age  cnarc1  cnarc2  cnarc3  ctheory1  ctheory2  ctheory3  ctheory4  CRT1  CRT2  CRT3  health_cond  marital1  mor_circle  moralid10  moralid3  moralid5  moralid6  moralid7  moralid8  moralid9  mcoop1  mcoop2  mcoop3  mcoop4  mcoop5  mcoop6  mcoop7  narc1  narc2  narc3  narc4  narc5  nidentity1  nidentity2  omind1  omind6  contact1  contact2  contact3  contact4  hygiene1  hygiene2  hygiene3  hygiene4  hygiene5  political_ideology  happy  slf_ladder  riskperc1  riskperc2  sbelong1  sbelong3  sbelong4  optim1  optim2  slfcont1  slfcont2  slfcont3  slfcont4
##   7   1  age  cnarc1  cnarc2  cnarc3  ctheory1  ctheory2  ctheory3  ctheory4  CRT1  CRT2  CRT3  health_cond  marital1  mor_circle  moralid10  moralid3  moralid5  moralid6  moralid7  moralid8  moralid9  mcoop1  mcoop2  mcoop3  mcoop4  mcoop5  mcoop6  mcoop7  narc1  narc2  narc3  narc4  narc5  nidentity1  nidentity2  omind1  omind6  contact1  contact2  contact3  contact4  hygiene1  hygiene2  hygiene3  hygiene4  hygiene5  political_ideology  happy  slf_ladder  riskperc1  riskperc2  sbelong1  sbelong3  sbelong4  optim1  optim2  slfcont1  slfcont2  slfcont3  slfcont4
##   7   2  age  cnarc1  cnarc2  cnarc3  ctheory1  ctheory2  ctheory3  ctheory4  CRT1  CRT2  CRT3  health_cond  marital1  mor_circle  moralid10  moralid3  moralid5  moralid6  moralid7  moralid8  moralid9  mcoop1  mcoop2  mcoop3  mcoop4  mcoop5  mcoop6  mcoop7  narc1  narc2  narc3  narc4  narc5  nidentity1  nidentity2  omind1  omind6  contact1  contact2  contact3  contact4  hygiene1  hygiene2  hygiene3  hygiene4  hygiene5  political_ideology  happy  slf_ladder  riskperc1  riskperc2  sbelong1  sbelong3  sbelong4  optim1  optim2  slfcont1  slfcont2  slfcont3  slfcont4
##   7   3  age  cnarc1  cnarc2  cnarc3  ctheory1  ctheory2  ctheory3  ctheory4  CRT1  CRT2  CRT3  health_cond  marital1  mor_circle  moralid10  moralid3  moralid5  moralid6  moralid7  moralid8  moralid9  mcoop1  mcoop2  mcoop3  mcoop4  mcoop5  mcoop6  mcoop7  narc1  narc2  narc3  narc4  narc5  nidentity1  nidentity2  omind1  omind6  contact1  contact2  contact3  contact4  hygiene1  hygiene2  hygiene3  hygiene4  hygiene5  political_ideology  happy  slf_ladder  riskperc1  riskperc2  sbelong1  sbelong3  sbelong4  optim1  optim2  slfcont1  slfcont2  slfcont3  slfcont4
##   7   4  age  cnarc1  cnarc2  cnarc3  ctheory1  ctheory2  ctheory3  ctheory4  CRT1  CRT2  CRT3  health_cond  marital1  mor_circle  moralid10  moralid3  moralid5  moralid6  moralid7  moralid8  moralid9  mcoop1  mcoop2  mcoop3  mcoop4  mcoop5  mcoop6  mcoop7  narc1  narc2  narc3  narc4  narc5  nidentity1  nidentity2  omind1  omind6  contact1  contact2  contact3  contact4  hygiene1  hygiene2  hygiene3  hygiene4  hygiene5  political_ideology  happy  slf_ladder  riskperc1  riskperc2  sbelong1  sbelong3  sbelong4  optim1  optim2  slfcont1  slfcont2  slfcont3  slfcont4
##   7   5  age  cnarc1  cnarc2  cnarc3  ctheory1  ctheory2  ctheory3  ctheory4  CRT1  CRT2  CRT3  health_cond  marital1  mor_circle  moralid10  moralid3  moralid5  moralid6  moralid7  moralid8  moralid9  mcoop1  mcoop2  mcoop3  mcoop4  mcoop5  mcoop6  mcoop7  narc1  narc2  narc3  narc4  narc5  nidentity1  nidentity2  omind1  omind6  contact1  contact2  contact3  contact4  hygiene1  hygiene2  hygiene3  hygiene4  hygiene5  political_ideology  happy  slf_ladder  riskperc1  riskperc2  sbelong1  sbelong3  sbelong4  optim1  optim2  slfcont1  slfcont2  slfcont3  slfcont4
##   7   6  age  cnarc1  cnarc2  cnarc3  ctheory1  ctheory2  ctheory3  ctheory4  CRT1  CRT2  CRT3  health_cond  marital1  mor_circle  moralid10  moralid3  moralid5  moralid6  moralid7  moralid8  moralid9  mcoop1  mcoop2  mcoop3  mcoop4  mcoop5  mcoop6  mcoop7  narc1  narc2  narc3  narc4  narc5  nidentity1  nidentity2  omind1  omind6  contact1  contact2  contact3  contact4  hygiene1  hygiene2  hygiene3  hygiene4  hygiene5  political_ideology  happy  slf_ladder  riskperc1  riskperc2  sbelong1  sbelong3  sbelong4  optim1  optim2  slfcont1  slfcont2  slfcont3  slfcont4
##   7   7  age  cnarc1  cnarc2  cnarc3  ctheory1  ctheory2  ctheory3  ctheory4  CRT1  CRT2  CRT3  health_cond  marital1  mor_circle  moralid10  moralid3  moralid5  moralid6  moralid7  moralid8  moralid9  mcoop1  mcoop2  mcoop3  mcoop4  mcoop5  mcoop6  mcoop7  narc1  narc2  narc3  narc4  narc5  nidentity1  nidentity2  omind1  omind6  contact1  contact2  contact3  contact4  hygiene1  hygiene2  hygiene3  hygiene4  hygiene5  political_ideology  happy  slf_ladder  riskperc1  riskperc2  sbelong1  sbelong3  sbelong4  optim1  optim2  slfcont1  slfcont2  slfcont3  slfcont4
##   7   8  age  cnarc1  cnarc2  cnarc3  ctheory1  ctheory2  ctheory3  ctheory4  CRT1  CRT2  CRT3  health_cond  marital1  mor_circle  moralid10  moralid3  moralid5  moralid6  moralid7  moralid8  moralid9  mcoop1  mcoop2  mcoop3  mcoop4  mcoop5  mcoop6  mcoop7  narc1  narc2  narc3  narc4  narc5  nidentity1  nidentity2  omind1  omind6  contact1  contact2  contact3  contact4  hygiene1  hygiene2  hygiene3  hygiene4  hygiene5  political_ideology  happy  slf_ladder  riskperc1  riskperc2  sbelong1  sbelong3  sbelong4  optim1  optim2  slfcont1  slfcont2  slfcont3  slfcont4
##   7   9  age  cnarc1  cnarc2  cnarc3  ctheory1  ctheory2  ctheory3  ctheory4  CRT1  CRT2  CRT3  health_cond  marital1  mor_circle  moralid10  moralid3  moralid5  moralid6  moralid7  moralid8  moralid9  mcoop1  mcoop2  mcoop3  mcoop4  mcoop5  mcoop6  mcoop7  narc1  narc2  narc3  narc4  narc5  nidentity1  nidentity2  omind1  omind6  contact1  contact2  contact3  contact4  hygiene1  hygiene2  hygiene3  hygiene4  hygiene5  political_ideology  happy  slf_ladder  riskperc1  riskperc2  sbelong1  sbelong3  sbelong4  optim1  optim2  slfcont1  slfcont2  slfcont3  slfcont4
##   7   10  age  cnarc1  cnarc2  cnarc3  ctheory1  ctheory2  ctheory3  ctheory4  CRT1  CRT2  CRT3  health_cond  marital1  mor_circle  moralid10  moralid3  moralid5  moralid6  moralid7  moralid8  moralid9  mcoop1  mcoop2  mcoop3  mcoop4  mcoop5  mcoop6  mcoop7  narc1  narc2  narc3  narc4  narc5  nidentity1  nidentity2  omind1  omind6  contact1  contact2  contact3  contact4  hygiene1  hygiene2  hygiene3  hygiene4  hygiene5  political_ideology  happy  slf_ladder  riskperc1  riskperc2  sbelong1  sbelong3  sbelong4  optim1  optim2  slfcont1  slfcont2  slfcont3  slfcont4
##   8   1  age  cnarc1  cnarc2  cnarc3  ctheory1  ctheory2  ctheory3  ctheory4  CRT1  CRT2  CRT3  health_cond  marital1  mor_circle  moralid10  moralid3  moralid5  moralid6  moralid7  moralid8  moralid9  mcoop1  mcoop2  mcoop3  mcoop4  mcoop5  mcoop6  mcoop7  narc1  narc2  narc3  narc4  narc5  nidentity1  nidentity2  omind1  omind6  contact1  contact2  contact3  contact4  hygiene1  hygiene2  hygiene3  hygiene4  hygiene5  political_ideology  happy  slf_ladder  riskperc1  riskperc2  sbelong1  sbelong3  sbelong4  optim1  optim2  slfcont1  slfcont2  slfcont3  slfcont4
##   8   2  age  cnarc1  cnarc2  cnarc3  ctheory1  ctheory2  ctheory3  ctheory4  CRT1  CRT2  CRT3  health_cond  marital1  mor_circle  moralid10  moralid3  moralid5  moralid6  moralid7  moralid8  moralid9  mcoop1  mcoop2  mcoop3  mcoop4  mcoop5  mcoop6  mcoop7  narc1  narc2  narc3  narc4  narc5  nidentity1  nidentity2  omind1  omind6  contact1  contact2  contact3  contact4  hygiene1  hygiene2  hygiene3  hygiene4  hygiene5  political_ideology  happy  slf_ladder  riskperc1  riskperc2  sbelong1  sbelong3  sbelong4  optim1  optim2  slfcont1  slfcont2  slfcont3  slfcont4
##   8   3  age  cnarc1  cnarc2  cnarc3  ctheory1  ctheory2  ctheory3  ctheory4  CRT1  CRT2  CRT3  health_cond  marital1  mor_circle  moralid10  moralid3  moralid5  moralid6  moralid7  moralid8  moralid9  mcoop1  mcoop2  mcoop3  mcoop4  mcoop5  mcoop6  mcoop7  narc1  narc2  narc3  narc4  narc5  nidentity1  nidentity2  omind1  omind6  contact1  contact2  contact3  contact4  hygiene1  hygiene2  hygiene3  hygiene4  hygiene5  political_ideology  happy  slf_ladder  riskperc1  riskperc2  sbelong1  sbelong3  sbelong4  optim1  optim2  slfcont1  slfcont2  slfcont3  slfcont4
##   8   4  age  cnarc1  cnarc2  cnarc3  ctheory1  ctheory2  ctheory3  ctheory4  CRT1  CRT2  CRT3  health_cond  marital1  mor_circle  moralid10  moralid3  moralid5  moralid6  moralid7  moralid8  moralid9  mcoop1  mcoop2  mcoop3  mcoop4  mcoop5  mcoop6  mcoop7  narc1  narc2  narc3  narc4  narc5  nidentity1  nidentity2  omind1  omind6  contact1  contact2  contact3  contact4  hygiene1  hygiene2  hygiene3  hygiene4  hygiene5  political_ideology  happy  slf_ladder  riskperc1  riskperc2  sbelong1  sbelong3  sbelong4  optim1  optim2  slfcont1  slfcont2  slfcont3  slfcont4
##   8   5  age  cnarc1  cnarc2  cnarc3  ctheory1  ctheory2  ctheory3  ctheory4  CRT1  CRT2  CRT3  health_cond  marital1  mor_circle  moralid10  moralid3  moralid5  moralid6  moralid7  moralid8  moralid9  mcoop1  mcoop2  mcoop3  mcoop4  mcoop5  mcoop6  mcoop7  narc1  narc2  narc3  narc4  narc5  nidentity1  nidentity2  omind1  omind6  contact1  contact2  contact3  contact4  hygiene1  hygiene2  hygiene3  hygiene4  hygiene5  political_ideology  happy  slf_ladder  riskperc1  riskperc2  sbelong1  sbelong3  sbelong4  optim1  optim2  slfcont1  slfcont2  slfcont3  slfcont4
##   8   6  age  cnarc1  cnarc2  cnarc3  ctheory1  ctheory2  ctheory3  ctheory4  CRT1  CRT2  CRT3  health_cond  marital1  mor_circle  moralid10  moralid3  moralid5  moralid6  moralid7  moralid8  moralid9  mcoop1  mcoop2  mcoop3  mcoop4  mcoop5  mcoop6  mcoop7  narc1  narc2  narc3  narc4  narc5  nidentity1  nidentity2  omind1  omind6  contact1  contact2  contact3  contact4  hygiene1  hygiene2  hygiene3  hygiene4  hygiene5  political_ideology  happy  slf_ladder  riskperc1  riskperc2  sbelong1  sbelong3  sbelong4  optim1  optim2  slfcont1  slfcont2  slfcont3  slfcont4
##   8   7  age  cnarc1  cnarc2  cnarc3  ctheory1  ctheory2  ctheory3  ctheory4  CRT1  CRT2  CRT3  health_cond  marital1  mor_circle  moralid10  moralid3  moralid5  moralid6  moralid7  moralid8  moralid9  mcoop1  mcoop2  mcoop3  mcoop4  mcoop5  mcoop6  mcoop7  narc1  narc2  narc3  narc4  narc5  nidentity1  nidentity2  omind1  omind6  contact1  contact2  contact3  contact4  hygiene1  hygiene2  hygiene3  hygiene4  hygiene5  political_ideology  happy  slf_ladder  riskperc1  riskperc2  sbelong1  sbelong3  sbelong4  optim1  optim2  slfcont1  slfcont2  slfcont3  slfcont4
##   8   8  age  cnarc1  cnarc2  cnarc3  ctheory1  ctheory2  ctheory3  ctheory4  CRT1  CRT2  CRT3  health_cond  marital1  mor_circle  moralid10  moralid3  moralid5  moralid6  moralid7  moralid8  moralid9  mcoop1  mcoop2  mcoop3  mcoop4  mcoop5  mcoop6  mcoop7  narc1  narc2  narc3  narc4  narc5  nidentity1  nidentity2  omind1  omind6  contact1  contact2  contact3  contact4  hygiene1  hygiene2  hygiene3  hygiene4  hygiene5  political_ideology  happy  slf_ladder  riskperc1  riskperc2  sbelong1  sbelong3  sbelong4  optim1  optim2  slfcont1  slfcont2  slfcont3  slfcont4
##   8   9  age  cnarc1  cnarc2  cnarc3  ctheory1  ctheory2  ctheory3  ctheory4  CRT1  CRT2  CRT3  health_cond  marital1  mor_circle  moralid10  moralid3  moralid5  moralid6  moralid7  moralid8  moralid9  mcoop1  mcoop2  mcoop3  mcoop4  mcoop5  mcoop6  mcoop7  narc1  narc2  narc3  narc4  narc5  nidentity1  nidentity2  omind1  omind6  contact1  contact2  contact3  contact4  hygiene1  hygiene2  hygiene3  hygiene4  hygiene5  political_ideology  happy  slf_ladder  riskperc1  riskperc2  sbelong1  sbelong3  sbelong4  optim1  optim2  slfcont1  slfcont2  slfcont3  slfcont4
##   8   10  age  cnarc1  cnarc2  cnarc3  ctheory1  ctheory2  ctheory3  ctheory4  CRT1  CRT2  CRT3  health_cond  marital1  mor_circle  moralid10  moralid3  moralid5  moralid6  moralid7  moralid8  moralid9  mcoop1  mcoop2  mcoop3  mcoop4  mcoop5  mcoop6  mcoop7  narc1  narc2  narc3  narc4  narc5  nidentity1  nidentity2  omind1  omind6  contact1  contact2  contact3  contact4  hygiene1  hygiene2  hygiene3  hygiene4  hygiene5  political_ideology  happy  slf_ladder  riskperc1  riskperc2  sbelong1  sbelong3  sbelong4  optim1  optim2  slfcont1  slfcont2  slfcont3  slfcont4
##   9   1  age  cnarc1  cnarc2  cnarc3  ctheory1  ctheory2  ctheory3  ctheory4  CRT1  CRT2  CRT3  health_cond  marital1  mor_circle  moralid10  moralid3  moralid5  moralid6  moralid7  moralid8  moralid9  mcoop1  mcoop2  mcoop3  mcoop4  mcoop5  mcoop6  mcoop7  narc1  narc2  narc3  narc4  narc5  nidentity1  nidentity2  omind1  omind6  contact1  contact2  contact3  contact4  hygiene1  hygiene2  hygiene3  hygiene4  hygiene5  political_ideology  happy  slf_ladder  riskperc1  riskperc2  sbelong1  sbelong3  sbelong4  optim1  optim2  slfcont1  slfcont2  slfcont3  slfcont4
##   9   2  age  cnarc1  cnarc2  cnarc3  ctheory1  ctheory2  ctheory3  ctheory4  CRT1  CRT2  CRT3  health_cond  marital1  mor_circle  moralid10  moralid3  moralid5  moralid6  moralid7  moralid8  moralid9  mcoop1  mcoop2  mcoop3  mcoop4  mcoop5  mcoop6  mcoop7  narc1  narc2  narc3  narc4  narc5  nidentity1  nidentity2  omind1  omind6  contact1  contact2  contact3  contact4  hygiene1  hygiene2  hygiene3  hygiene4  hygiene5  political_ideology  happy  slf_ladder  riskperc1  riskperc2  sbelong1  sbelong3  sbelong4  optim1  optim2  slfcont1  slfcont2  slfcont3  slfcont4
##   9   3  age  cnarc1  cnarc2  cnarc3  ctheory1  ctheory2  ctheory3  ctheory4  CRT1  CRT2  CRT3  health_cond  marital1  mor_circle  moralid10  moralid3  moralid5  moralid6  moralid7  moralid8  moralid9  mcoop1  mcoop2  mcoop3  mcoop4  mcoop5  mcoop6  mcoop7  narc1  narc2  narc3  narc4  narc5  nidentity1  nidentity2  omind1  omind6  contact1  contact2  contact3  contact4  hygiene1  hygiene2  hygiene3  hygiene4  hygiene5  political_ideology  happy  slf_ladder  riskperc1  riskperc2  sbelong1  sbelong3  sbelong4  optim1  optim2  slfcont1  slfcont2  slfcont3  slfcont4
##   9   4  age  cnarc1  cnarc2  cnarc3  ctheory1  ctheory2  ctheory3  ctheory4  CRT1  CRT2  CRT3  health_cond  marital1  mor_circle  moralid10  moralid3  moralid5  moralid6  moralid7  moralid8  moralid9  mcoop1  mcoop2  mcoop3  mcoop4  mcoop5  mcoop6  mcoop7  narc1  narc2  narc3  narc4  narc5  nidentity1  nidentity2  omind1  omind6  contact1  contact2  contact3  contact4  hygiene1  hygiene2  hygiene3  hygiene4  hygiene5  political_ideology  happy  slf_ladder  riskperc1  riskperc2  sbelong1  sbelong3  sbelong4  optim1  optim2  slfcont1  slfcont2  slfcont3  slfcont4
##   9   5  age  cnarc1  cnarc2  cnarc3  ctheory1  ctheory2  ctheory3  ctheory4  CRT1  CRT2  CRT3  health_cond  marital1  mor_circle  moralid10  moralid3  moralid5  moralid6  moralid7  moralid8  moralid9  mcoop1  mcoop2  mcoop3  mcoop4  mcoop5  mcoop6  mcoop7  narc1  narc2  narc3  narc4  narc5  nidentity1  nidentity2  omind1  omind6  contact1  contact2  contact3  contact4  hygiene1  hygiene2  hygiene3  hygiene4  hygiene5  political_ideology  happy  slf_ladder  riskperc1  riskperc2  sbelong1  sbelong3  sbelong4  optim1  optim2  slfcont1  slfcont2  slfcont3  slfcont4
##   9   6  age  cnarc1  cnarc2  cnarc3  ctheory1  ctheory2  ctheory3  ctheory4  CRT1  CRT2  CRT3  health_cond  marital1  mor_circle  moralid10  moralid3  moralid5  moralid6  moralid7  moralid8  moralid9  mcoop1  mcoop2  mcoop3  mcoop4  mcoop5  mcoop6  mcoop7  narc1  narc2  narc3  narc4  narc5  nidentity1  nidentity2  omind1  omind6  contact1  contact2  contact3  contact4  hygiene1  hygiene2  hygiene3  hygiene4  hygiene5  political_ideology  happy  slf_ladder  riskperc1  riskperc2  sbelong1  sbelong3  sbelong4  optim1  optim2  slfcont1  slfcont2  slfcont3  slfcont4
##   9   7  age  cnarc1  cnarc2  cnarc3  ctheory1  ctheory2  ctheory3  ctheory4  CRT1  CRT2  CRT3  health_cond  marital1  mor_circle  moralid10  moralid3  moralid5  moralid6  moralid7  moralid8  moralid9  mcoop1  mcoop2  mcoop3  mcoop4  mcoop5  mcoop6  mcoop7  narc1  narc2  narc3  narc4  narc5  nidentity1  nidentity2  omind1  omind6  contact1  contact2  contact3  contact4  hygiene1  hygiene2  hygiene3  hygiene4  hygiene5  political_ideology  happy  slf_ladder  riskperc1  riskperc2  sbelong1  sbelong3  sbelong4  optim1  optim2  slfcont1  slfcont2  slfcont3  slfcont4
##   9   8  age  cnarc1  cnarc2  cnarc3  ctheory1  ctheory2  ctheory3  ctheory4  CRT1  CRT2  CRT3  health_cond  marital1  mor_circle  moralid10  moralid3  moralid5  moralid6  moralid7  moralid8  moralid9  mcoop1  mcoop2  mcoop3  mcoop4  mcoop5  mcoop6  mcoop7  narc1  narc2  narc3  narc4  narc5  nidentity1  nidentity2  omind1  omind6  contact1  contact2  contact3  contact4  hygiene1  hygiene2  hygiene3  hygiene4  hygiene5  political_ideology  happy  slf_ladder  riskperc1  riskperc2  sbelong1  sbelong3  sbelong4  optim1  optim2  slfcont1  slfcont2  slfcont3  slfcont4
##   9   9  age  cnarc1  cnarc2  cnarc3  ctheory1  ctheory2  ctheory3  ctheory4  CRT1  CRT2  CRT3  health_cond  marital1  mor_circle  moralid10  moralid3  moralid5  moralid6  moralid7  moralid8  moralid9  mcoop1  mcoop2  mcoop3  mcoop4  mcoop5  mcoop6  mcoop7  narc1  narc2  narc3  narc4  narc5  nidentity1  nidentity2  omind1  omind6  contact1  contact2  contact3  contact4  hygiene1  hygiene2  hygiene3  hygiene4  hygiene5  political_ideology  happy  slf_ladder  riskperc1  riskperc2  sbelong1  sbelong3  sbelong4  optim1  optim2  slfcont1  slfcont2  slfcont3  slfcont4
##   9   10  age  cnarc1  cnarc2  cnarc3  ctheory1  ctheory2  ctheory3  ctheory4  CRT1  CRT2  CRT3  health_cond  marital1  mor_circle  moralid10  moralid3  moralid5  moralid6  moralid7  moralid8  moralid9  mcoop1  mcoop2  mcoop3  mcoop4  mcoop5  mcoop6  mcoop7  narc1  narc2  narc3  narc4  narc5  nidentity1  nidentity2  omind1  omind6  contact1  contact2  contact3  contact4  hygiene1  hygiene2  hygiene3  hygiene4  hygiene5  political_ideology  happy  slf_ladder  riskperc1  riskperc2  sbelong1  sbelong3  sbelong4  optim1  optim2  slfcont1  slfcont2  slfcont3  slfcont4
##   10   1  age  cnarc1  cnarc2  cnarc3  ctheory1  ctheory2  ctheory3  ctheory4  CRT1  CRT2  CRT3  health_cond  marital1  mor_circle  moralid10  moralid3  moralid5  moralid6  moralid7  moralid8  moralid9  mcoop1  mcoop2  mcoop3  mcoop4  mcoop5  mcoop6  mcoop7  narc1  narc2  narc3  narc4  narc5  nidentity1  nidentity2  omind1  omind6  contact1  contact2  contact3  contact4  hygiene1  hygiene2  hygiene3  hygiene4  hygiene5  political_ideology  happy  slf_ladder  riskperc1  riskperc2  sbelong1  sbelong3  sbelong4  optim1  optim2  slfcont1  slfcont2  slfcont3  slfcont4
##   10   2  age  cnarc1  cnarc2  cnarc3  ctheory1  ctheory2  ctheory3  ctheory4  CRT1  CRT2  CRT3  health_cond  marital1  mor_circle  moralid10  moralid3  moralid5  moralid6  moralid7  moralid8  moralid9  mcoop1  mcoop2  mcoop3  mcoop4  mcoop5  mcoop6  mcoop7  narc1  narc2  narc3  narc4  narc5  nidentity1  nidentity2  omind1  omind6  contact1  contact2  contact3  contact4  hygiene1  hygiene2  hygiene3  hygiene4  hygiene5  political_ideology  happy  slf_ladder  riskperc1  riskperc2  sbelong1  sbelong3  sbelong4  optim1  optim2  slfcont1  slfcont2  slfcont3  slfcont4
##   10   3  age  cnarc1  cnarc2  cnarc3  ctheory1  ctheory2  ctheory3  ctheory4  CRT1  CRT2  CRT3  health_cond  marital1  mor_circle  moralid10  moralid3  moralid5  moralid6  moralid7  moralid8  moralid9  mcoop1  mcoop2  mcoop3  mcoop4  mcoop5  mcoop6  mcoop7  narc1  narc2  narc3  narc4  narc5  nidentity1  nidentity2  omind1  omind6  contact1  contact2  contact3  contact4  hygiene1  hygiene2  hygiene3  hygiene4  hygiene5  political_ideology  happy  slf_ladder  riskperc1  riskperc2  sbelong1  sbelong3  sbelong4  optim1  optim2  slfcont1  slfcont2  slfcont3  slfcont4
##   10   4  age  cnarc1  cnarc2  cnarc3  ctheory1  ctheory2  ctheory3  ctheory4  CRT1  CRT2  CRT3  health_cond  marital1  mor_circle  moralid10  moralid3  moralid5  moralid6  moralid7  moralid8  moralid9  mcoop1  mcoop2  mcoop3  mcoop4  mcoop5  mcoop6  mcoop7  narc1  narc2  narc3  narc4  narc5  nidentity1  nidentity2  omind1  omind6  contact1  contact2  contact3  contact4  hygiene1  hygiene2  hygiene3  hygiene4  hygiene5  political_ideology  happy  slf_ladder  riskperc1  riskperc2  sbelong1  sbelong3  sbelong4  optim1  optim2  slfcont1  slfcont2  slfcont3  slfcont4
##   10   5  age  cnarc1  cnarc2  cnarc3  ctheory1  ctheory2  ctheory3  ctheory4  CRT1  CRT2  CRT3  health_cond  marital1  mor_circle  moralid10  moralid3  moralid5  moralid6  moralid7  moralid8  moralid9  mcoop1  mcoop2  mcoop3  mcoop4  mcoop5  mcoop6  mcoop7  narc1  narc2  narc3  narc4  narc5  nidentity1  nidentity2  omind1  omind6  contact1  contact2  contact3  contact4  hygiene1  hygiene2  hygiene3  hygiene4  hygiene5  political_ideology  happy  slf_ladder  riskperc1  riskperc2  sbelong1  sbelong3  sbelong4  optim1  optim2  slfcont1  slfcont2  slfcont3  slfcont4
##   10   6  age  cnarc1  cnarc2  cnarc3  ctheory1  ctheory2  ctheory3  ctheory4  CRT1  CRT2  CRT3  health_cond  marital1  mor_circle  moralid10  moralid3  moralid5  moralid6  moralid7  moralid8  moralid9  mcoop1  mcoop2  mcoop3  mcoop4  mcoop5  mcoop6  mcoop7  narc1  narc2  narc3  narc4  narc5  nidentity1  nidentity2  omind1  omind6  contact1  contact2  contact3  contact4  hygiene1  hygiene2  hygiene3  hygiene4  hygiene5  political_ideology  happy  slf_ladder  riskperc1  riskperc2  sbelong1  sbelong3  sbelong4  optim1  optim2  slfcont1  slfcont2  slfcont3  slfcont4
##   10   7  age  cnarc1  cnarc2  cnarc3  ctheory1  ctheory2  ctheory3  ctheory4  CRT1  CRT2  CRT3  health_cond  marital1  mor_circle  moralid10  moralid3  moralid5  moralid6  moralid7  moralid8  moralid9  mcoop1  mcoop2  mcoop3  mcoop4  mcoop5  mcoop6  mcoop7  narc1  narc2  narc3  narc4  narc5  nidentity1  nidentity2  omind1  omind6  contact1  contact2  contact3  contact4  hygiene1  hygiene2  hygiene3  hygiene4  hygiene5  political_ideology  happy  slf_ladder  riskperc1  riskperc2  sbelong1  sbelong3  sbelong4  optim1  optim2  slfcont1  slfcont2  slfcont3  slfcont4
##   10   8  age  cnarc1  cnarc2  cnarc3  ctheory1  ctheory2  ctheory3  ctheory4  CRT1  CRT2  CRT3  health_cond  marital1  mor_circle  moralid10  moralid3  moralid5  moralid6  moralid7  moralid8  moralid9  mcoop1  mcoop2  mcoop3  mcoop4  mcoop5  mcoop6  mcoop7  narc1  narc2  narc3  narc4  narc5  nidentity1  nidentity2  omind1  omind6  contact1  contact2  contact3  contact4  hygiene1  hygiene2  hygiene3  hygiene4  hygiene5  political_ideology  happy  slf_ladder  riskperc1  riskperc2  sbelong1  sbelong3  sbelong4  optim1  optim2  slfcont1  slfcont2  slfcont3  slfcont4
##   10   9  age  cnarc1  cnarc2  cnarc3  ctheory1  ctheory2  ctheory3  ctheory4  CRT1  CRT2  CRT3  health_cond  marital1  mor_circle  moralid10  moralid3  moralid5  moralid6  moralid7  moralid8  moralid9  mcoop1  mcoop2  mcoop3  mcoop4  mcoop5  mcoop6  mcoop7  narc1  narc2  narc3  narc4  narc5  nidentity1  nidentity2  omind1  omind6  contact1  contact2  contact3  contact4  hygiene1  hygiene2  hygiene3  hygiene4  hygiene5  political_ideology  happy  slf_ladder  riskperc1  riskperc2  sbelong1  sbelong3  sbelong4  optim1  optim2  slfcont1  slfcont2  slfcont3  slfcont4
##   10   10  age  cnarc1  cnarc2  cnarc3  ctheory1  ctheory2  ctheory3  ctheory4  CRT1  CRT2  CRT3  health_cond  marital1  mor_circle  moralid10  moralid3  moralid5  moralid6  moralid7  moralid8  moralid9  mcoop1  mcoop2  mcoop3  mcoop4  mcoop5  mcoop6  mcoop7  narc1  narc2  narc3  narc4  narc5  nidentity1  nidentity2  omind1  omind6  contact1  contact2  contact3  contact4  hygiene1  hygiene2  hygiene3  hygiene4  hygiene5  political_ideology  happy  slf_ladder  riskperc1  riskperc2  sbelong1  sbelong3  sbelong4  optim1  optim2  slfcont1  slfcont2  slfcont3  slfcont4
```

```
## Warning: Number of logged events: 6003
```

```
## 
##  iter imp variable
##   1   1  cnarc1  cnarc2  cnarc3  ctheory1  ctheory3  ctheory4  CRT1  CRT2  CRT3  duration  employ_status1  know_tested_positive  marital1  moralid10  moralid3  moralid4  moralid5  moralid6  moralid8  moralid9  mcoop2  mcoop3  mcoop4  mcoop5  mcoop6  narc4  narc5  nidentity1  nidentity2  omind5  contact1  contact2  contact3  contact4  contact5  hygiene4  hygiene5  psupport1  political_ideology  happy  slf_ladder  sex1  tested_positive  optim1  optim2  slfcont2  slfcont4
##   1   2  cnarc1  cnarc2  cnarc3  ctheory1  ctheory3  ctheory4  CRT1  CRT2  CRT3  duration  employ_status1  know_tested_positive  marital1  moralid10  moralid3  moralid4  moralid5  moralid6  moralid8  moralid9  mcoop2  mcoop3  mcoop4  mcoop5  mcoop6  narc4  narc5  nidentity1  nidentity2  omind5  contact1  contact2  contact3  contact4  contact5  hygiene4  hygiene5  psupport1  political_ideology  happy  slf_ladder  sex1  tested_positive  optim1  optim2  slfcont2  slfcont4
##   1   3  cnarc1  cnarc2  cnarc3  ctheory1  ctheory3  ctheory4  CRT1  CRT2  CRT3  duration  employ_status1  know_tested_positive  marital1  moralid10  moralid3  moralid4  moralid5  moralid6  moralid8  moralid9  mcoop2  mcoop3  mcoop4  mcoop5  mcoop6  narc4  narc5  nidentity1  nidentity2  omind5  contact1  contact2  contact3  contact4  contact5  hygiene4  hygiene5  psupport1  political_ideology  happy  slf_ladder  sex1  tested_positive  optim1  optim2  slfcont2  slfcont4
##   1   4  cnarc1  cnarc2  cnarc3  ctheory1  ctheory3  ctheory4  CRT1  CRT2  CRT3  duration  employ_status1  know_tested_positive  marital1  moralid10  moralid3  moralid4  moralid5  moralid6  moralid8  moralid9  mcoop2  mcoop3  mcoop4  mcoop5  mcoop6  narc4  narc5  nidentity1  nidentity2  omind5  contact1  contact2  contact3  contact4  contact5  hygiene4  hygiene5  psupport1  political_ideology  happy  slf_ladder  sex1  tested_positive  optim1  optim2  slfcont2  slfcont4
##   1   5  cnarc1  cnarc2  cnarc3  ctheory1  ctheory3  ctheory4  CRT1  CRT2  CRT3  duration  employ_status1  know_tested_positive  marital1  moralid10  moralid3  moralid4  moralid5  moralid6  moralid8  moralid9  mcoop2  mcoop3  mcoop4  mcoop5  mcoop6  narc4  narc5  nidentity1  nidentity2  omind5  contact1  contact2  contact3  contact4  contact5  hygiene4  hygiene5  psupport1  political_ideology  happy  slf_ladder  sex1  tested_positive  optim1  optim2  slfcont2  slfcont4
##   1   6  cnarc1  cnarc2  cnarc3  ctheory1  ctheory3  ctheory4  CRT1  CRT2  CRT3  duration  employ_status1  know_tested_positive  marital1  moralid10  moralid3  moralid4  moralid5  moralid6  moralid8  moralid9  mcoop2  mcoop3  mcoop4  mcoop5  mcoop6  narc4  narc5  nidentity1  nidentity2  omind5  contact1  contact2  contact3  contact4  contact5  hygiene4  hygiene5  psupport1  political_ideology  happy  slf_ladder  sex1  tested_positive  optim1  optim2  slfcont2  slfcont4
##   1   7  cnarc1  cnarc2  cnarc3  ctheory1  ctheory3  ctheory4  CRT1  CRT2  CRT3  duration  employ_status1  know_tested_positive  marital1  moralid10  moralid3  moralid4  moralid5  moralid6  moralid8  moralid9  mcoop2  mcoop3  mcoop4  mcoop5  mcoop6  narc4  narc5  nidentity1  nidentity2  omind5  contact1  contact2  contact3  contact4  contact5  hygiene4  hygiene5  psupport1  political_ideology  happy  slf_ladder  sex1  tested_positive  optim1  optim2  slfcont2  slfcont4
##   1   8  cnarc1  cnarc2  cnarc3  ctheory1  ctheory3  ctheory4  CRT1  CRT2  CRT3  duration  employ_status1  know_tested_positive  marital1  moralid10  moralid3  moralid4  moralid5  moralid6  moralid8  moralid9  mcoop2  mcoop3  mcoop4  mcoop5  mcoop6  narc4  narc5  nidentity1  nidentity2  omind5  contact1  contact2  contact3  contact4  contact5  hygiene4  hygiene5  psupport1  political_ideology  happy  slf_ladder  sex1  tested_positive  optim1  optim2  slfcont2  slfcont4
##   1   9  cnarc1  cnarc2  cnarc3  ctheory1  ctheory3  ctheory4  CRT1  CRT2  CRT3  duration  employ_status1  know_tested_positive  marital1  moralid10  moralid3  moralid4  moralid5  moralid6  moralid8  moralid9  mcoop2  mcoop3  mcoop4  mcoop5  mcoop6  narc4  narc5  nidentity1  nidentity2  omind5  contact1  contact2  contact3  contact4  contact5  hygiene4  hygiene5  psupport1  political_ideology  happy  slf_ladder  sex1  tested_positive  optim1  optim2  slfcont2  slfcont4
##   1   10  cnarc1  cnarc2  cnarc3  ctheory1  ctheory3  ctheory4  CRT1  CRT2  CRT3  duration  employ_status1  know_tested_positive  marital1  moralid10  moralid3  moralid4  moralid5  moralid6  moralid8  moralid9  mcoop2  mcoop3  mcoop4  mcoop5  mcoop6  narc4  narc5  nidentity1  nidentity2  omind5  contact1  contact2  contact3  contact4  contact5  hygiene4  hygiene5  psupport1  political_ideology  happy  slf_ladder  sex1  tested_positive  optim1  optim2  slfcont2  slfcont4
##   2   1  cnarc1  cnarc2  cnarc3  ctheory1  ctheory3  ctheory4  CRT1  CRT2  CRT3  duration  employ_status1  know_tested_positive  marital1  moralid10  moralid3  moralid4  moralid5  moralid6  moralid8  moralid9  mcoop2  mcoop3  mcoop4  mcoop5  mcoop6  narc4  narc5  nidentity1  nidentity2  omind5  contact1  contact2  contact3  contact4  contact5  hygiene4  hygiene5  psupport1  political_ideology  happy  slf_ladder  sex1  tested_positive  optim1  optim2  slfcont2  slfcont4
##   2   2  cnarc1  cnarc2  cnarc3  ctheory1  ctheory3  ctheory4  CRT1  CRT2  CRT3  duration  employ_status1  know_tested_positive  marital1  moralid10  moralid3  moralid4  moralid5  moralid6  moralid8  moralid9  mcoop2  mcoop3  mcoop4  mcoop5  mcoop6  narc4  narc5  nidentity1  nidentity2  omind5  contact1  contact2  contact3  contact4  contact5  hygiene4  hygiene5  psupport1  political_ideology  happy  slf_ladder  sex1  tested_positive  optim1  optim2  slfcont2  slfcont4
##   2   3  cnarc1  cnarc2  cnarc3  ctheory1  ctheory3  ctheory4  CRT1  CRT2  CRT3  duration  employ_status1  know_tested_positive  marital1  moralid10  moralid3  moralid4  moralid5  moralid6  moralid8  moralid9  mcoop2  mcoop3  mcoop4  mcoop5  mcoop6  narc4  narc5  nidentity1  nidentity2  omind5  contact1  contact2  contact3  contact4  contact5  hygiene4  hygiene5  psupport1  political_ideology  happy  slf_ladder  sex1  tested_positive  optim1  optim2  slfcont2  slfcont4
##   2   4  cnarc1  cnarc2  cnarc3  ctheory1  ctheory3  ctheory4  CRT1  CRT2  CRT3  duration  employ_status1  know_tested_positive  marital1  moralid10  moralid3  moralid4  moralid5  moralid6  moralid8  moralid9  mcoop2  mcoop3  mcoop4  mcoop5  mcoop6  narc4  narc5  nidentity1  nidentity2  omind5  contact1  contact2  contact3  contact4  contact5  hygiene4  hygiene5  psupport1  political_ideology  happy  slf_ladder  sex1  tested_positive  optim1  optim2  slfcont2  slfcont4
##   2   5  cnarc1  cnarc2  cnarc3  ctheory1  ctheory3  ctheory4  CRT1  CRT2  CRT3  duration  employ_status1  know_tested_positive  marital1  moralid10  moralid3  moralid4  moralid5  moralid6  moralid8  moralid9  mcoop2  mcoop3  mcoop4  mcoop5  mcoop6  narc4  narc5  nidentity1  nidentity2  omind5  contact1  contact2  contact3  contact4  contact5  hygiene4  hygiene5  psupport1  political_ideology  happy  slf_ladder  sex1  tested_positive  optim1  optim2  slfcont2  slfcont4
##   2   6  cnarc1  cnarc2  cnarc3  ctheory1  ctheory3  ctheory4  CRT1  CRT2  CRT3  duration  employ_status1  know_tested_positive  marital1  moralid10  moralid3  moralid4  moralid5  moralid6  moralid8  moralid9  mcoop2  mcoop3  mcoop4  mcoop5  mcoop6  narc4  narc5  nidentity1  nidentity2  omind5  contact1  contact2  contact3  contact4  contact5  hygiene4  hygiene5  psupport1  political_ideology  happy  slf_ladder  sex1  tested_positive  optim1  optim2  slfcont2  slfcont4
##   2   7  cnarc1  cnarc2  cnarc3  ctheory1  ctheory3  ctheory4  CRT1  CRT2  CRT3  duration  employ_status1  know_tested_positive  marital1  moralid10  moralid3  moralid4  moralid5  moralid6  moralid8  moralid9  mcoop2  mcoop3  mcoop4  mcoop5  mcoop6  narc4  narc5  nidentity1  nidentity2  omind5  contact1  contact2  contact3  contact4  contact5  hygiene4  hygiene5  psupport1  political_ideology  happy  slf_ladder  sex1  tested_positive  optim1  optim2  slfcont2  slfcont4
##   2   8  cnarc1  cnarc2  cnarc3  ctheory1  ctheory3  ctheory4  CRT1  CRT2  CRT3  duration  employ_status1  know_tested_positive  marital1  moralid10  moralid3  moralid4  moralid5  moralid6  moralid8  moralid9  mcoop2  mcoop3  mcoop4  mcoop5  mcoop6  narc4  narc5  nidentity1  nidentity2  omind5  contact1  contact2  contact3  contact4  contact5  hygiene4  hygiene5  psupport1  political_ideology  happy  slf_ladder  sex1  tested_positive  optim1  optim2  slfcont2  slfcont4
##   2   9  cnarc1  cnarc2  cnarc3  ctheory1  ctheory3  ctheory4  CRT1  CRT2  CRT3  duration  employ_status1  know_tested_positive  marital1  moralid10  moralid3  moralid4  moralid5  moralid6  moralid8  moralid9  mcoop2  mcoop3  mcoop4  mcoop5  mcoop6  narc4  narc5  nidentity1  nidentity2  omind5  contact1  contact2  contact3  contact4  contact5  hygiene4  hygiene5  psupport1  political_ideology  happy  slf_ladder  sex1  tested_positive  optim1  optim2  slfcont2  slfcont4
##   2   10  cnarc1  cnarc2  cnarc3  ctheory1  ctheory3  ctheory4  CRT1  CRT2  CRT3  duration  employ_status1  know_tested_positive  marital1  moralid10  moralid3  moralid4  moralid5  moralid6  moralid8  moralid9  mcoop2  mcoop3  mcoop4  mcoop5  mcoop6  narc4  narc5  nidentity1  nidentity2  omind5  contact1  contact2  contact3  contact4  contact5  hygiene4  hygiene5  psupport1  political_ideology  happy  slf_ladder  sex1  tested_positive  optim1  optim2  slfcont2  slfcont4
##   3   1  cnarc1  cnarc2  cnarc3  ctheory1  ctheory3  ctheory4  CRT1  CRT2  CRT3  duration  employ_status1  know_tested_positive  marital1  moralid10  moralid3  moralid4  moralid5  moralid6  moralid8  moralid9  mcoop2  mcoop3  mcoop4  mcoop5  mcoop6  narc4  narc5  nidentity1  nidentity2  omind5  contact1  contact2  contact3  contact4  contact5  hygiene4  hygiene5  psupport1  political_ideology  happy  slf_ladder  sex1  tested_positive  optim1  optim2  slfcont2  slfcont4
##   3   2  cnarc1  cnarc2  cnarc3  ctheory1  ctheory3  ctheory4  CRT1  CRT2  CRT3  duration  employ_status1  know_tested_positive  marital1  moralid10  moralid3  moralid4  moralid5  moralid6  moralid8  moralid9  mcoop2  mcoop3  mcoop4  mcoop5  mcoop6  narc4  narc5  nidentity1  nidentity2  omind5  contact1  contact2  contact3  contact4  contact5  hygiene4  hygiene5  psupport1  political_ideology  happy  slf_ladder  sex1  tested_positive  optim1  optim2  slfcont2  slfcont4
##   3   3  cnarc1  cnarc2  cnarc3  ctheory1  ctheory3  ctheory4  CRT1  CRT2  CRT3  duration  employ_status1  know_tested_positive  marital1  moralid10  moralid3  moralid4  moralid5  moralid6  moralid8  moralid9  mcoop2  mcoop3  mcoop4  mcoop5  mcoop6  narc4  narc5  nidentity1  nidentity2  omind5  contact1  contact2  contact3  contact4  contact5  hygiene4  hygiene5  psupport1  political_ideology  happy  slf_ladder  sex1  tested_positive  optim1  optim2  slfcont2  slfcont4
##   3   4  cnarc1  cnarc2  cnarc3  ctheory1  ctheory3  ctheory4  CRT1  CRT2  CRT3  duration  employ_status1  know_tested_positive  marital1  moralid10  moralid3  moralid4  moralid5  moralid6  moralid8  moralid9  mcoop2  mcoop3  mcoop4  mcoop5  mcoop6  narc4  narc5  nidentity1  nidentity2  omind5  contact1  contact2  contact3  contact4  contact5  hygiene4  hygiene5  psupport1  political_ideology  happy  slf_ladder  sex1  tested_positive  optim1  optim2  slfcont2  slfcont4
##   3   5  cnarc1  cnarc2  cnarc3  ctheory1  ctheory3  ctheory4  CRT1  CRT2  CRT3  duration  employ_status1  know_tested_positive  marital1  moralid10  moralid3  moralid4  moralid5  moralid6  moralid8  moralid9  mcoop2  mcoop3  mcoop4  mcoop5  mcoop6  narc4  narc5  nidentity1  nidentity2  omind5  contact1  contact2  contact3  contact4  contact5  hygiene4  hygiene5  psupport1  political_ideology  happy  slf_ladder  sex1  tested_positive  optim1  optim2  slfcont2  slfcont4
##   3   6  cnarc1  cnarc2  cnarc3  ctheory1  ctheory3  ctheory4  CRT1  CRT2  CRT3  duration  employ_status1  know_tested_positive  marital1  moralid10  moralid3  moralid4  moralid5  moralid6  moralid8  moralid9  mcoop2  mcoop3  mcoop4  mcoop5  mcoop6  narc4  narc5  nidentity1  nidentity2  omind5  contact1  contact2  contact3  contact4  contact5  hygiene4  hygiene5  psupport1  political_ideology  happy  slf_ladder  sex1  tested_positive  optim1  optim2  slfcont2  slfcont4
##   3   7  cnarc1  cnarc2  cnarc3  ctheory1  ctheory3  ctheory4  CRT1  CRT2  CRT3  duration  employ_status1  know_tested_positive  marital1  moralid10  moralid3  moralid4  moralid5  moralid6  moralid8  moralid9  mcoop2  mcoop3  mcoop4  mcoop5  mcoop6  narc4  narc5  nidentity1  nidentity2  omind5  contact1  contact2  contact3  contact4  contact5  hygiene4  hygiene5  psupport1  political_ideology  happy  slf_ladder  sex1  tested_positive  optim1  optim2  slfcont2  slfcont4
##   3   8  cnarc1  cnarc2  cnarc3  ctheory1  ctheory3  ctheory4  CRT1  CRT2  CRT3  duration  employ_status1  know_tested_positive  marital1  moralid10  moralid3  moralid4  moralid5  moralid6  moralid8  moralid9  mcoop2  mcoop3  mcoop4  mcoop5  mcoop6  narc4  narc5  nidentity1  nidentity2  omind5  contact1  contact2  contact3  contact4  contact5  hygiene4  hygiene5  psupport1  political_ideology  happy  slf_ladder  sex1  tested_positive  optim1  optim2  slfcont2  slfcont4
##   3   9  cnarc1  cnarc2  cnarc3  ctheory1  ctheory3  ctheory4  CRT1  CRT2  CRT3  duration  employ_status1  know_tested_positive  marital1  moralid10  moralid3  moralid4  moralid5  moralid6  moralid8  moralid9  mcoop2  mcoop3  mcoop4  mcoop5  mcoop6  narc4  narc5  nidentity1  nidentity2  omind5  contact1  contact2  contact3  contact4  contact5  hygiene4  hygiene5  psupport1  political_ideology  happy  slf_ladder  sex1  tested_positive  optim1  optim2  slfcont2  slfcont4
##   3   10  cnarc1  cnarc2  cnarc3  ctheory1  ctheory3  ctheory4  CRT1  CRT2  CRT3  duration  employ_status1  know_tested_positive  marital1  moralid10  moralid3  moralid4  moralid5  moralid6  moralid8  moralid9  mcoop2  mcoop3  mcoop4  mcoop5  mcoop6  narc4  narc5  nidentity1  nidentity2  omind5  contact1  contact2  contact3  contact4  contact5  hygiene4  hygiene5  psupport1  political_ideology  happy  slf_ladder  sex1  tested_positive  optim1  optim2  slfcont2  slfcont4
##   4   1  cnarc1  cnarc2  cnarc3  ctheory1  ctheory3  ctheory4  CRT1  CRT2  CRT3  duration  employ_status1  know_tested_positive  marital1  moralid10  moralid3  moralid4  moralid5  moralid6  moralid8  moralid9  mcoop2  mcoop3  mcoop4  mcoop5  mcoop6  narc4  narc5  nidentity1  nidentity2  omind5  contact1  contact2  contact3  contact4  contact5  hygiene4  hygiene5  psupport1  political_ideology  happy  slf_ladder  sex1  tested_positive  optim1  optim2  slfcont2  slfcont4
##   4   2  cnarc1  cnarc2  cnarc3  ctheory1  ctheory3  ctheory4  CRT1  CRT2  CRT3  duration  employ_status1  know_tested_positive  marital1  moralid10  moralid3  moralid4  moralid5  moralid6  moralid8  moralid9  mcoop2  mcoop3  mcoop4  mcoop5  mcoop6  narc4  narc5  nidentity1  nidentity2  omind5  contact1  contact2  contact3  contact4  contact5  hygiene4  hygiene5  psupport1  political_ideology  happy  slf_ladder  sex1  tested_positive  optim1  optim2  slfcont2  slfcont4
##   4   3  cnarc1  cnarc2  cnarc3  ctheory1  ctheory3  ctheory4  CRT1  CRT2  CRT3  duration  employ_status1  know_tested_positive  marital1  moralid10  moralid3  moralid4  moralid5  moralid6  moralid8  moralid9  mcoop2  mcoop3  mcoop4  mcoop5  mcoop6  narc4  narc5  nidentity1  nidentity2  omind5  contact1  contact2  contact3  contact4  contact5  hygiene4  hygiene5  psupport1  political_ideology  happy  slf_ladder  sex1  tested_positive  optim1  optim2  slfcont2  slfcont4
##   4   4  cnarc1  cnarc2  cnarc3  ctheory1  ctheory3  ctheory4  CRT1  CRT2  CRT3  duration  employ_status1  know_tested_positive  marital1  moralid10  moralid3  moralid4  moralid5  moralid6  moralid8  moralid9  mcoop2  mcoop3  mcoop4  mcoop5  mcoop6  narc4  narc5  nidentity1  nidentity2  omind5  contact1  contact2  contact3  contact4  contact5  hygiene4  hygiene5  psupport1  political_ideology  happy  slf_ladder  sex1  tested_positive  optim1  optim2  slfcont2  slfcont4
##   4   5  cnarc1  cnarc2  cnarc3  ctheory1  ctheory3  ctheory4  CRT1  CRT2  CRT3  duration  employ_status1  know_tested_positive  marital1  moralid10  moralid3  moralid4  moralid5  moralid6  moralid8  moralid9  mcoop2  mcoop3  mcoop4  mcoop5  mcoop6  narc4  narc5  nidentity1  nidentity2  omind5  contact1  contact2  contact3  contact4  contact5  hygiene4  hygiene5  psupport1  political_ideology  happy  slf_ladder  sex1  tested_positive  optim1  optim2  slfcont2  slfcont4
##   4   6  cnarc1  cnarc2  cnarc3  ctheory1  ctheory3  ctheory4  CRT1  CRT2  CRT3  duration  employ_status1  know_tested_positive  marital1  moralid10  moralid3  moralid4  moralid5  moralid6  moralid8  moralid9  mcoop2  mcoop3  mcoop4  mcoop5  mcoop6  narc4  narc5  nidentity1  nidentity2  omind5  contact1  contact2  contact3  contact4  contact5  hygiene4  hygiene5  psupport1  political_ideology  happy  slf_ladder  sex1  tested_positive  optim1  optim2  slfcont2  slfcont4
##   4   7  cnarc1  cnarc2  cnarc3  ctheory1  ctheory3  ctheory4  CRT1  CRT2  CRT3  duration  employ_status1  know_tested_positive  marital1  moralid10  moralid3  moralid4  moralid5  moralid6  moralid8  moralid9  mcoop2  mcoop3  mcoop4  mcoop5  mcoop6  narc4  narc5  nidentity1  nidentity2  omind5  contact1  contact2  contact3  contact4  contact5  hygiene4  hygiene5  psupport1  political_ideology  happy  slf_ladder  sex1  tested_positive  optim1  optim2  slfcont2  slfcont4
##   4   8  cnarc1  cnarc2  cnarc3  ctheory1  ctheory3  ctheory4  CRT1  CRT2  CRT3  duration  employ_status1  know_tested_positive  marital1  moralid10  moralid3  moralid4  moralid5  moralid6  moralid8  moralid9  mcoop2  mcoop3  mcoop4  mcoop5  mcoop6  narc4  narc5  nidentity1  nidentity2  omind5  contact1  contact2  contact3  contact4  contact5  hygiene4  hygiene5  psupport1  political_ideology  happy  slf_ladder  sex1  tested_positive  optim1  optim2  slfcont2  slfcont4
##   4   9  cnarc1  cnarc2  cnarc3  ctheory1  ctheory3  ctheory4  CRT1  CRT2  CRT3  duration  employ_status1  know_tested_positive  marital1  moralid10  moralid3  moralid4  moralid5  moralid6  moralid8  moralid9  mcoop2  mcoop3  mcoop4  mcoop5  mcoop6  narc4  narc5  nidentity1  nidentity2  omind5  contact1  contact2  contact3  contact4  contact5  hygiene4  hygiene5  psupport1  political_ideology  happy  slf_ladder  sex1  tested_positive  optim1  optim2  slfcont2  slfcont4
##   4   10  cnarc1  cnarc2  cnarc3  ctheory1  ctheory3  ctheory4  CRT1  CRT2  CRT3  duration  employ_status1  know_tested_positive  marital1  moralid10  moralid3  moralid4  moralid5  moralid6  moralid8  moralid9  mcoop2  mcoop3  mcoop4  mcoop5  mcoop6  narc4  narc5  nidentity1  nidentity2  omind5  contact1  contact2  contact3  contact4  contact5  hygiene4  hygiene5  psupport1  political_ideology  happy  slf_ladder  sex1  tested_positive  optim1  optim2  slfcont2  slfcont4
##   5   1  cnarc1  cnarc2  cnarc3  ctheory1  ctheory3  ctheory4  CRT1  CRT2  CRT3  duration  employ_status1  know_tested_positive  marital1  moralid10  moralid3  moralid4  moralid5  moralid6  moralid8  moralid9  mcoop2  mcoop3  mcoop4  mcoop5  mcoop6  narc4  narc5  nidentity1  nidentity2  omind5  contact1  contact2  contact3  contact4  contact5  hygiene4  hygiene5  psupport1  political_ideology  happy  slf_ladder  sex1  tested_positive  optim1  optim2  slfcont2  slfcont4
##   5   2  cnarc1  cnarc2  cnarc3  ctheory1  ctheory3  ctheory4  CRT1  CRT2  CRT3  duration  employ_status1  know_tested_positive  marital1  moralid10  moralid3  moralid4  moralid5  moralid6  moralid8  moralid9  mcoop2  mcoop3  mcoop4  mcoop5  mcoop6  narc4  narc5  nidentity1  nidentity2  omind5  contact1  contact2  contact3  contact4  contact5  hygiene4  hygiene5  psupport1  political_ideology  happy  slf_ladder  sex1  tested_positive  optim1  optim2  slfcont2  slfcont4
##   5   3  cnarc1  cnarc2  cnarc3  ctheory1  ctheory3  ctheory4  CRT1  CRT2  CRT3  duration  employ_status1  know_tested_positive  marital1  moralid10  moralid3  moralid4  moralid5  moralid6  moralid8  moralid9  mcoop2  mcoop3  mcoop4  mcoop5  mcoop6  narc4  narc5  nidentity1  nidentity2  omind5  contact1  contact2  contact3  contact4  contact5  hygiene4  hygiene5  psupport1  political_ideology  happy  slf_ladder  sex1  tested_positive  optim1  optim2  slfcont2  slfcont4
##   5   4  cnarc1  cnarc2  cnarc3  ctheory1  ctheory3  ctheory4  CRT1  CRT2  CRT3  duration  employ_status1  know_tested_positive  marital1  moralid10  moralid3  moralid4  moralid5  moralid6  moralid8  moralid9  mcoop2  mcoop3  mcoop4  mcoop5  mcoop6  narc4  narc5  nidentity1  nidentity2  omind5  contact1  contact2  contact3  contact4  contact5  hygiene4  hygiene5  psupport1  political_ideology  happy  slf_ladder  sex1  tested_positive  optim1  optim2  slfcont2  slfcont4
##   5   5  cnarc1  cnarc2  cnarc3  ctheory1  ctheory3  ctheory4  CRT1  CRT2  CRT3  duration  employ_status1  know_tested_positive  marital1  moralid10  moralid3  moralid4  moralid5  moralid6  moralid8  moralid9  mcoop2  mcoop3  mcoop4  mcoop5  mcoop6  narc4  narc5  nidentity1  nidentity2  omind5  contact1  contact2  contact3  contact4  contact5  hygiene4  hygiene5  psupport1  political_ideology  happy  slf_ladder  sex1  tested_positive  optim1  optim2  slfcont2  slfcont4
##   5   6  cnarc1  cnarc2  cnarc3  ctheory1  ctheory3  ctheory4  CRT1  CRT2  CRT3  duration  employ_status1  know_tested_positive  marital1  moralid10  moralid3  moralid4  moralid5  moralid6  moralid8  moralid9  mcoop2  mcoop3  mcoop4  mcoop5  mcoop6  narc4  narc5  nidentity1  nidentity2  omind5  contact1  contact2  contact3  contact4  contact5  hygiene4  hygiene5  psupport1  political_ideology  happy  slf_ladder  sex1  tested_positive  optim1  optim2  slfcont2  slfcont4
##   5   7  cnarc1  cnarc2  cnarc3  ctheory1  ctheory3  ctheory4  CRT1  CRT2  CRT3  duration  employ_status1  know_tested_positive  marital1  moralid10  moralid3  moralid4  moralid5  moralid6  moralid8  moralid9  mcoop2  mcoop3  mcoop4  mcoop5  mcoop6  narc4  narc5  nidentity1  nidentity2  omind5  contact1  contact2  contact3  contact4  contact5  hygiene4  hygiene5  psupport1  political_ideology  happy  slf_ladder  sex1  tested_positive  optim1  optim2  slfcont2  slfcont4
##   5   8  cnarc1  cnarc2  cnarc3  ctheory1  ctheory3  ctheory4  CRT1  CRT2  CRT3  duration  employ_status1  know_tested_positive  marital1  moralid10  moralid3  moralid4  moralid5  moralid6  moralid8  moralid9  mcoop2  mcoop3  mcoop4  mcoop5  mcoop6  narc4  narc5  nidentity1  nidentity2  omind5  contact1  contact2  contact3  contact4  contact5  hygiene4  hygiene5  psupport1  political_ideology  happy  slf_ladder  sex1  tested_positive  optim1  optim2  slfcont2  slfcont4
##   5   9  cnarc1  cnarc2  cnarc3  ctheory1  ctheory3  ctheory4  CRT1  CRT2  CRT3  duration  employ_status1  know_tested_positive  marital1  moralid10  moralid3  moralid4  moralid5  moralid6  moralid8  moralid9  mcoop2  mcoop3  mcoop4  mcoop5  mcoop6  narc4  narc5  nidentity1  nidentity2  omind5  contact1  contact2  contact3  contact4  contact5  hygiene4  hygiene5  psupport1  political_ideology  happy  slf_ladder  sex1  tested_positive  optim1  optim2  slfcont2  slfcont4
##   5   10  cnarc1  cnarc2  cnarc3  ctheory1  ctheory3  ctheory4  CRT1  CRT2  CRT3  duration  employ_status1  know_tested_positive  marital1  moralid10  moralid3  moralid4  moralid5  moralid6  moralid8  moralid9  mcoop2  mcoop3  mcoop4  mcoop5  mcoop6  narc4  narc5  nidentity1  nidentity2  omind5  contact1  contact2  contact3  contact4  contact5  hygiene4  hygiene5  psupport1  political_ideology  happy  slf_ladder  sex1  tested_positive  optim1  optim2  slfcont2  slfcont4
##   6   1  cnarc1  cnarc2  cnarc3  ctheory1  ctheory3  ctheory4  CRT1  CRT2  CRT3  duration  employ_status1  know_tested_positive  marital1  moralid10  moralid3  moralid4  moralid5  moralid6  moralid8  moralid9  mcoop2  mcoop3  mcoop4  mcoop5  mcoop6  narc4  narc5  nidentity1  nidentity2  omind5  contact1  contact2  contact3  contact4  contact5  hygiene4  hygiene5  psupport1  political_ideology  happy  slf_ladder  sex1  tested_positive  optim1  optim2  slfcont2  slfcont4
##   6   2  cnarc1  cnarc2  cnarc3  ctheory1  ctheory3  ctheory4  CRT1  CRT2  CRT3  duration  employ_status1  know_tested_positive  marital1  moralid10  moralid3  moralid4  moralid5  moralid6  moralid8  moralid9  mcoop2  mcoop3  mcoop4  mcoop5  mcoop6  narc4  narc5  nidentity1  nidentity2  omind5  contact1  contact2  contact3  contact4  contact5  hygiene4  hygiene5  psupport1  political_ideology  happy  slf_ladder  sex1  tested_positive  optim1  optim2  slfcont2  slfcont4
##   6   3  cnarc1  cnarc2  cnarc3  ctheory1  ctheory3  ctheory4  CRT1  CRT2  CRT3  duration  employ_status1  know_tested_positive  marital1  moralid10  moralid3  moralid4  moralid5  moralid6  moralid8  moralid9  mcoop2  mcoop3  mcoop4  mcoop5  mcoop6  narc4  narc5  nidentity1  nidentity2  omind5  contact1  contact2  contact3  contact4  contact5  hygiene4  hygiene5  psupport1  political_ideology  happy  slf_ladder  sex1  tested_positive  optim1  optim2  slfcont2  slfcont4
##   6   4  cnarc1  cnarc2  cnarc3  ctheory1  ctheory3  ctheory4  CRT1  CRT2  CRT3  duration  employ_status1  know_tested_positive  marital1  moralid10  moralid3  moralid4  moralid5  moralid6  moralid8  moralid9  mcoop2  mcoop3  mcoop4  mcoop5  mcoop6  narc4  narc5  nidentity1  nidentity2  omind5  contact1  contact2  contact3  contact4  contact5  hygiene4  hygiene5  psupport1  political_ideology  happy  slf_ladder  sex1  tested_positive  optim1  optim2  slfcont2  slfcont4
##   6   5  cnarc1  cnarc2  cnarc3  ctheory1  ctheory3  ctheory4  CRT1  CRT2  CRT3  duration  employ_status1  know_tested_positive  marital1  moralid10  moralid3  moralid4  moralid5  moralid6  moralid8  moralid9  mcoop2  mcoop3  mcoop4  mcoop5  mcoop6  narc4  narc5  nidentity1  nidentity2  omind5  contact1  contact2  contact3  contact4  contact5  hygiene4  hygiene5  psupport1  political_ideology  happy  slf_ladder  sex1  tested_positive  optim1  optim2  slfcont2  slfcont4
##   6   6  cnarc1  cnarc2  cnarc3  ctheory1  ctheory3  ctheory4  CRT1  CRT2  CRT3  duration  employ_status1  know_tested_positive  marital1  moralid10  moralid3  moralid4  moralid5  moralid6  moralid8  moralid9  mcoop2  mcoop3  mcoop4  mcoop5  mcoop6  narc4  narc5  nidentity1  nidentity2  omind5  contact1  contact2  contact3  contact4  contact5  hygiene4  hygiene5  psupport1  political_ideology  happy  slf_ladder  sex1  tested_positive  optim1  optim2  slfcont2  slfcont4
##   6   7  cnarc1  cnarc2  cnarc3  ctheory1  ctheory3  ctheory4  CRT1  CRT2  CRT3  duration  employ_status1  know_tested_positive  marital1  moralid10  moralid3  moralid4  moralid5  moralid6  moralid8  moralid9  mcoop2  mcoop3  mcoop4  mcoop5  mcoop6  narc4  narc5  nidentity1  nidentity2  omind5  contact1  contact2  contact3  contact4  contact5  hygiene4  hygiene5  psupport1  political_ideology  happy  slf_ladder  sex1  tested_positive  optim1  optim2  slfcont2  slfcont4
##   6   8  cnarc1  cnarc2  cnarc3  ctheory1  ctheory3  ctheory4  CRT1  CRT2  CRT3  duration  employ_status1  know_tested_positive  marital1  moralid10  moralid3  moralid4  moralid5  moralid6  moralid8  moralid9  mcoop2  mcoop3  mcoop4  mcoop5  mcoop6  narc4  narc5  nidentity1  nidentity2  omind5  contact1  contact2  contact3  contact4  contact5  hygiene4  hygiene5  psupport1  political_ideology  happy  slf_ladder  sex1  tested_positive  optim1  optim2  slfcont2  slfcont4
##   6   9  cnarc1  cnarc2  cnarc3  ctheory1  ctheory3  ctheory4  CRT1  CRT2  CRT3  duration  employ_status1  know_tested_positive  marital1  moralid10  moralid3  moralid4  moralid5  moralid6  moralid8  moralid9  mcoop2  mcoop3  mcoop4  mcoop5  mcoop6  narc4  narc5  nidentity1  nidentity2  omind5  contact1  contact2  contact3  contact4  contact5  hygiene4  hygiene5  psupport1  political_ideology  happy  slf_ladder  sex1  tested_positive  optim1  optim2  slfcont2  slfcont4
##   6   10  cnarc1  cnarc2  cnarc3  ctheory1  ctheory3  ctheory4  CRT1  CRT2  CRT3  duration  employ_status1  know_tested_positive  marital1  moralid10  moralid3  moralid4  moralid5  moralid6  moralid8  moralid9  mcoop2  mcoop3  mcoop4  mcoop5  mcoop6  narc4  narc5  nidentity1  nidentity2  omind5  contact1  contact2  contact3  contact4  contact5  hygiene4  hygiene5  psupport1  political_ideology  happy  slf_ladder  sex1  tested_positive  optim1  optim2  slfcont2  slfcont4
##   7   1  cnarc1  cnarc2  cnarc3  ctheory1  ctheory3  ctheory4  CRT1  CRT2  CRT3  duration  employ_status1  know_tested_positive  marital1  moralid10  moralid3  moralid4  moralid5  moralid6  moralid8  moralid9  mcoop2  mcoop3  mcoop4  mcoop5  mcoop6  narc4  narc5  nidentity1  nidentity2  omind5  contact1  contact2  contact3  contact4  contact5  hygiene4  hygiene5  psupport1  political_ideology  happy  slf_ladder  sex1  tested_positive  optim1  optim2  slfcont2  slfcont4
##   7   2  cnarc1  cnarc2  cnarc3  ctheory1  ctheory3  ctheory4  CRT1  CRT2  CRT3  duration  employ_status1  know_tested_positive  marital1  moralid10  moralid3  moralid4  moralid5  moralid6  moralid8  moralid9  mcoop2  mcoop3  mcoop4  mcoop5  mcoop6  narc4  narc5  nidentity1  nidentity2  omind5  contact1  contact2  contact3  contact4  contact5  hygiene4  hygiene5  psupport1  political_ideology  happy  slf_ladder  sex1  tested_positive  optim1  optim2  slfcont2  slfcont4
##   7   3  cnarc1  cnarc2  cnarc3  ctheory1  ctheory3  ctheory4  CRT1  CRT2  CRT3  duration  employ_status1  know_tested_positive  marital1  moralid10  moralid3  moralid4  moralid5  moralid6  moralid8  moralid9  mcoop2  mcoop3  mcoop4  mcoop5  mcoop6  narc4  narc5  nidentity1  nidentity2  omind5  contact1  contact2  contact3  contact4  contact5  hygiene4  hygiene5  psupport1  political_ideology  happy  slf_ladder  sex1  tested_positive  optim1  optim2  slfcont2  slfcont4
##   7   4  cnarc1  cnarc2  cnarc3  ctheory1  ctheory3  ctheory4  CRT1  CRT2  CRT3  duration  employ_status1  know_tested_positive  marital1  moralid10  moralid3  moralid4  moralid5  moralid6  moralid8  moralid9  mcoop2  mcoop3  mcoop4  mcoop5  mcoop6  narc4  narc5  nidentity1  nidentity2  omind5  contact1  contact2  contact3  contact4  contact5  hygiene4  hygiene5  psupport1  political_ideology  happy  slf_ladder  sex1  tested_positive  optim1  optim2  slfcont2  slfcont4
##   7   5  cnarc1  cnarc2  cnarc3  ctheory1  ctheory3  ctheory4  CRT1  CRT2  CRT3  duration  employ_status1  know_tested_positive  marital1  moralid10  moralid3  moralid4  moralid5  moralid6  moralid8  moralid9  mcoop2  mcoop3  mcoop4  mcoop5  mcoop6  narc4  narc5  nidentity1  nidentity2  omind5  contact1  contact2  contact3  contact4  contact5  hygiene4  hygiene5  psupport1  political_ideology  happy  slf_ladder  sex1  tested_positive  optim1  optim2  slfcont2  slfcont4
##   7   6  cnarc1  cnarc2  cnarc3  ctheory1  ctheory3  ctheory4  CRT1  CRT2  CRT3  duration  employ_status1  know_tested_positive  marital1  moralid10  moralid3  moralid4  moralid5  moralid6  moralid8  moralid9  mcoop2  mcoop3  mcoop4  mcoop5  mcoop6  narc4  narc5  nidentity1  nidentity2  omind5  contact1  contact2  contact3  contact4  contact5  hygiene4  hygiene5  psupport1  political_ideology  happy  slf_ladder  sex1  tested_positive  optim1  optim2  slfcont2  slfcont4
##   7   7  cnarc1  cnarc2  cnarc3  ctheory1  ctheory3  ctheory4  CRT1  CRT2  CRT3  duration  employ_status1  know_tested_positive  marital1  moralid10  moralid3  moralid4  moralid5  moralid6  moralid8  moralid9  mcoop2  mcoop3  mcoop4  mcoop5  mcoop6  narc4  narc5  nidentity1  nidentity2  omind5  contact1  contact2  contact3  contact4  contact5  hygiene4  hygiene5  psupport1  political_ideology  happy  slf_ladder  sex1  tested_positive  optim1  optim2  slfcont2  slfcont4
##   7   8  cnarc1  cnarc2  cnarc3  ctheory1  ctheory3  ctheory4  CRT1  CRT2  CRT3  duration  employ_status1  know_tested_positive  marital1  moralid10  moralid3  moralid4  moralid5  moralid6  moralid8  moralid9  mcoop2  mcoop3  mcoop4  mcoop5  mcoop6  narc4  narc5  nidentity1  nidentity2  omind5  contact1  contact2  contact3  contact4  contact5  hygiene4  hygiene5  psupport1  political_ideology  happy  slf_ladder  sex1  tested_positive  optim1  optim2  slfcont2  slfcont4
##   7   9  cnarc1  cnarc2  cnarc3  ctheory1  ctheory3  ctheory4  CRT1  CRT2  CRT3  duration  employ_status1  know_tested_positive  marital1  moralid10  moralid3  moralid4  moralid5  moralid6  moralid8  moralid9  mcoop2  mcoop3  mcoop4  mcoop5  mcoop6  narc4  narc5  nidentity1  nidentity2  omind5  contact1  contact2  contact3  contact4  contact5  hygiene4  hygiene5  psupport1  political_ideology  happy  slf_ladder  sex1  tested_positive  optim1  optim2  slfcont2  slfcont4
##   7   10  cnarc1  cnarc2  cnarc3  ctheory1  ctheory3  ctheory4  CRT1  CRT2  CRT3  duration  employ_status1  know_tested_positive  marital1  moralid10  moralid3  moralid4  moralid5  moralid6  moralid8  moralid9  mcoop2  mcoop3  mcoop4  mcoop5  mcoop6  narc4  narc5  nidentity1  nidentity2  omind5  contact1  contact2  contact3  contact4  contact5  hygiene4  hygiene5  psupport1  political_ideology  happy  slf_ladder  sex1  tested_positive  optim1  optim2  slfcont2  slfcont4
##   8   1  cnarc1  cnarc2  cnarc3  ctheory1  ctheory3  ctheory4  CRT1  CRT2  CRT3  duration  employ_status1  know_tested_positive  marital1  moralid10  moralid3  moralid4  moralid5  moralid6  moralid8  moralid9  mcoop2  mcoop3  mcoop4  mcoop5  mcoop6  narc4  narc5  nidentity1  nidentity2  omind5  contact1  contact2  contact3  contact4  contact5  hygiene4  hygiene5  psupport1  political_ideology  happy  slf_ladder  sex1  tested_positive  optim1  optim2  slfcont2  slfcont4
##   8   2  cnarc1  cnarc2  cnarc3  ctheory1  ctheory3  ctheory4  CRT1  CRT2  CRT3  duration  employ_status1  know_tested_positive  marital1  moralid10  moralid3  moralid4  moralid5  moralid6  moralid8  moralid9  mcoop2  mcoop3  mcoop4  mcoop5  mcoop6  narc4  narc5  nidentity1  nidentity2  omind5  contact1  contact2  contact3  contact4  contact5  hygiene4  hygiene5  psupport1  political_ideology  happy  slf_ladder  sex1  tested_positive  optim1  optim2  slfcont2  slfcont4
##   8   3  cnarc1  cnarc2  cnarc3  ctheory1  ctheory3  ctheory4  CRT1  CRT2  CRT3  duration  employ_status1  know_tested_positive  marital1  moralid10  moralid3  moralid4  moralid5  moralid6  moralid8  moralid9  mcoop2  mcoop3  mcoop4  mcoop5  mcoop6  narc4  narc5  nidentity1  nidentity2  omind5  contact1  contact2  contact3  contact4  contact5  hygiene4  hygiene5  psupport1  political_ideology  happy  slf_ladder  sex1  tested_positive  optim1  optim2  slfcont2  slfcont4
##   8   4  cnarc1  cnarc2  cnarc3  ctheory1  ctheory3  ctheory4  CRT1  CRT2  CRT3  duration  employ_status1  know_tested_positive  marital1  moralid10  moralid3  moralid4  moralid5  moralid6  moralid8  moralid9  mcoop2  mcoop3  mcoop4  mcoop5  mcoop6  narc4  narc5  nidentity1  nidentity2  omind5  contact1  contact2  contact3  contact4  contact5  hygiene4  hygiene5  psupport1  political_ideology  happy  slf_ladder  sex1  tested_positive  optim1  optim2  slfcont2  slfcont4
##   8   5  cnarc1  cnarc2  cnarc3  ctheory1  ctheory3  ctheory4  CRT1  CRT2  CRT3  duration  employ_status1  know_tested_positive  marital1  moralid10  moralid3  moralid4  moralid5  moralid6  moralid8  moralid9  mcoop2  mcoop3  mcoop4  mcoop5  mcoop6  narc4  narc5  nidentity1  nidentity2  omind5  contact1  contact2  contact3  contact4  contact5  hygiene4  hygiene5  psupport1  political_ideology  happy  slf_ladder  sex1  tested_positive  optim1  optim2  slfcont2  slfcont4
##   8   6  cnarc1  cnarc2  cnarc3  ctheory1  ctheory3  ctheory4  CRT1  CRT2  CRT3  duration  employ_status1  know_tested_positive  marital1  moralid10  moralid3  moralid4  moralid5  moralid6  moralid8  moralid9  mcoop2  mcoop3  mcoop4  mcoop5  mcoop6  narc4  narc5  nidentity1  nidentity2  omind5  contact1  contact2  contact3  contact4  contact5  hygiene4  hygiene5  psupport1  political_ideology  happy  slf_ladder  sex1  tested_positive  optim1  optim2  slfcont2  slfcont4
##   8   7  cnarc1  cnarc2  cnarc3  ctheory1  ctheory3  ctheory4  CRT1  CRT2  CRT3  duration  employ_status1  know_tested_positive  marital1  moralid10  moralid3  moralid4  moralid5  moralid6  moralid8  moralid9  mcoop2  mcoop3  mcoop4  mcoop5  mcoop6  narc4  narc5  nidentity1  nidentity2  omind5  contact1  contact2  contact3  contact4  contact5  hygiene4  hygiene5  psupport1  political_ideology  happy  slf_ladder  sex1  tested_positive  optim1  optim2  slfcont2  slfcont4
##   8   8  cnarc1  cnarc2  cnarc3  ctheory1  ctheory3  ctheory4  CRT1  CRT2  CRT3  duration  employ_status1  know_tested_positive  marital1  moralid10  moralid3  moralid4  moralid5  moralid6  moralid8  moralid9  mcoop2  mcoop3  mcoop4  mcoop5  mcoop6  narc4  narc5  nidentity1  nidentity2  omind5  contact1  contact2  contact3  contact4  contact5  hygiene4  hygiene5  psupport1  political_ideology  happy  slf_ladder  sex1  tested_positive  optim1  optim2  slfcont2  slfcont4
##   8   9  cnarc1  cnarc2  cnarc3  ctheory1  ctheory3  ctheory4  CRT1  CRT2  CRT3  duration  employ_status1  know_tested_positive  marital1  moralid10  moralid3  moralid4  moralid5  moralid6  moralid8  moralid9  mcoop2  mcoop3  mcoop4  mcoop5  mcoop6  narc4  narc5  nidentity1  nidentity2  omind5  contact1  contact2  contact3  contact4  contact5  hygiene4  hygiene5  psupport1  political_ideology  happy  slf_ladder  sex1  tested_positive  optim1  optim2  slfcont2  slfcont4
##   8   10  cnarc1  cnarc2  cnarc3  ctheory1  ctheory3  ctheory4  CRT1  CRT2  CRT3  duration  employ_status1  know_tested_positive  marital1  moralid10  moralid3  moralid4  moralid5  moralid6  moralid8  moralid9  mcoop2  mcoop3  mcoop4  mcoop5  mcoop6  narc4  narc5  nidentity1  nidentity2  omind5  contact1  contact2  contact3  contact4  contact5  hygiene4  hygiene5  psupport1  political_ideology  happy  slf_ladder  sex1  tested_positive  optim1  optim2  slfcont2  slfcont4
##   9   1  cnarc1  cnarc2  cnarc3  ctheory1  ctheory3  ctheory4  CRT1  CRT2  CRT3  duration  employ_status1  know_tested_positive  marital1  moralid10  moralid3  moralid4  moralid5  moralid6  moralid8  moralid9  mcoop2  mcoop3  mcoop4  mcoop5  mcoop6  narc4  narc5  nidentity1  nidentity2  omind5  contact1  contact2  contact3  contact4  contact5  hygiene4  hygiene5  psupport1  political_ideology  happy  slf_ladder  sex1  tested_positive  optim1  optim2  slfcont2  slfcont4
##   9   2  cnarc1  cnarc2  cnarc3  ctheory1  ctheory3  ctheory4  CRT1  CRT2  CRT3  duration  employ_status1  know_tested_positive  marital1  moralid10  moralid3  moralid4  moralid5  moralid6  moralid8  moralid9  mcoop2  mcoop3  mcoop4  mcoop5  mcoop6  narc4  narc5  nidentity1  nidentity2  omind5  contact1  contact2  contact3  contact4  contact5  hygiene4  hygiene5  psupport1  political_ideology  happy  slf_ladder  sex1  tested_positive  optim1  optim2  slfcont2  slfcont4
##   9   3  cnarc1  cnarc2  cnarc3  ctheory1  ctheory3  ctheory4  CRT1  CRT2  CRT3  duration  employ_status1  know_tested_positive  marital1  moralid10  moralid3  moralid4  moralid5  moralid6  moralid8  moralid9  mcoop2  mcoop3  mcoop4  mcoop5  mcoop6  narc4  narc5  nidentity1  nidentity2  omind5  contact1  contact2  contact3  contact4  contact5  hygiene4  hygiene5  psupport1  political_ideology  happy  slf_ladder  sex1  tested_positive  optim1  optim2  slfcont2  slfcont4
##   9   4  cnarc1  cnarc2  cnarc3  ctheory1  ctheory3  ctheory4  CRT1  CRT2  CRT3  duration  employ_status1  know_tested_positive  marital1  moralid10  moralid3  moralid4  moralid5  moralid6  moralid8  moralid9  mcoop2  mcoop3  mcoop4  mcoop5  mcoop6  narc4  narc5  nidentity1  nidentity2  omind5  contact1  contact2  contact3  contact4  contact5  hygiene4  hygiene5  psupport1  political_ideology  happy  slf_ladder  sex1  tested_positive  optim1  optim2  slfcont2  slfcont4
##   9   5  cnarc1  cnarc2  cnarc3  ctheory1  ctheory3  ctheory4  CRT1  CRT2  CRT3  duration  employ_status1  know_tested_positive  marital1  moralid10  moralid3  moralid4  moralid5  moralid6  moralid8  moralid9  mcoop2  mcoop3  mcoop4  mcoop5  mcoop6  narc4  narc5  nidentity1  nidentity2  omind5  contact1  contact2  contact3  contact4  contact5  hygiene4  hygiene5  psupport1  political_ideology  happy  slf_ladder  sex1  tested_positive  optim1  optim2  slfcont2  slfcont4
##   9   6  cnarc1  cnarc2  cnarc3  ctheory1  ctheory3  ctheory4  CRT1  CRT2  CRT3  duration  employ_status1  know_tested_positive  marital1  moralid10  moralid3  moralid4  moralid5  moralid6  moralid8  moralid9  mcoop2  mcoop3  mcoop4  mcoop5  mcoop6  narc4  narc5  nidentity1  nidentity2  omind5  contact1  contact2  contact3  contact4  contact5  hygiene4  hygiene5  psupport1  political_ideology  happy  slf_ladder  sex1  tested_positive  optim1  optim2  slfcont2  slfcont4
##   9   7  cnarc1  cnarc2  cnarc3  ctheory1  ctheory3  ctheory4  CRT1  CRT2  CRT3  duration  employ_status1  know_tested_positive  marital1  moralid10  moralid3  moralid4  moralid5  moralid6  moralid8  moralid9  mcoop2  mcoop3  mcoop4  mcoop5  mcoop6  narc4  narc5  nidentity1  nidentity2  omind5  contact1  contact2  contact3  contact4  contact5  hygiene4  hygiene5  psupport1  political_ideology  happy  slf_ladder  sex1  tested_positive  optim1  optim2  slfcont2  slfcont4
##   9   8  cnarc1  cnarc2  cnarc3  ctheory1  ctheory3  ctheory4  CRT1  CRT2  CRT3  duration  employ_status1  know_tested_positive  marital1  moralid10  moralid3  moralid4  moralid5  moralid6  moralid8  moralid9  mcoop2  mcoop3  mcoop4  mcoop5  mcoop6  narc4  narc5  nidentity1  nidentity2  omind5  contact1  contact2  contact3  contact4  contact5  hygiene4  hygiene5  psupport1  political_ideology  happy  slf_ladder  sex1  tested_positive  optim1  optim2  slfcont2  slfcont4
##   9   9  cnarc1  cnarc2  cnarc3  ctheory1  ctheory3  ctheory4  CRT1  CRT2  CRT3  duration  employ_status1  know_tested_positive  marital1  moralid10  moralid3  moralid4  moralid5  moralid6  moralid8  moralid9  mcoop2  mcoop3  mcoop4  mcoop5  mcoop6  narc4  narc5  nidentity1  nidentity2  omind5  contact1  contact2  contact3  contact4  contact5  hygiene4  hygiene5  psupport1  political_ideology  happy  slf_ladder  sex1  tested_positive  optim1  optim2  slfcont2  slfcont4
##   9   10  cnarc1  cnarc2  cnarc3  ctheory1  ctheory3  ctheory4  CRT1  CRT2  CRT3  duration  employ_status1  know_tested_positive  marital1  moralid10  moralid3  moralid4  moralid5  moralid6  moralid8  moralid9  mcoop2  mcoop3  mcoop4  mcoop5  mcoop6  narc4  narc5  nidentity1  nidentity2  omind5  contact1  contact2  contact3  contact4  contact5  hygiene4  hygiene5  psupport1  political_ideology  happy  slf_ladder  sex1  tested_positive  optim1  optim2  slfcont2  slfcont4
##   10   1  cnarc1  cnarc2  cnarc3  ctheory1  ctheory3  ctheory4  CRT1  CRT2  CRT3  duration  employ_status1  know_tested_positive  marital1  moralid10  moralid3  moralid4  moralid5  moralid6  moralid8  moralid9  mcoop2  mcoop3  mcoop4  mcoop5  mcoop6  narc4  narc5  nidentity1  nidentity2  omind5  contact1  contact2  contact3  contact4  contact5  hygiene4  hygiene5  psupport1  political_ideology  happy  slf_ladder  sex1  tested_positive  optim1  optim2  slfcont2  slfcont4
##   10   2  cnarc1  cnarc2  cnarc3  ctheory1  ctheory3  ctheory4  CRT1  CRT2  CRT3  duration  employ_status1  know_tested_positive  marital1  moralid10  moralid3  moralid4  moralid5  moralid6  moralid8  moralid9  mcoop2  mcoop3  mcoop4  mcoop5  mcoop6  narc4  narc5  nidentity1  nidentity2  omind5  contact1  contact2  contact3  contact4  contact5  hygiene4  hygiene5  psupport1  political_ideology  happy  slf_ladder  sex1  tested_positive  optim1  optim2  slfcont2  slfcont4
##   10   3  cnarc1  cnarc2  cnarc3  ctheory1  ctheory3  ctheory4  CRT1  CRT2  CRT3  duration  employ_status1  know_tested_positive  marital1  moralid10  moralid3  moralid4  moralid5  moralid6  moralid8  moralid9  mcoop2  mcoop3  mcoop4  mcoop5  mcoop6  narc4  narc5  nidentity1  nidentity2  omind5  contact1  contact2  contact3  contact4  contact5  hygiene4  hygiene5  psupport1  political_ideology  happy  slf_ladder  sex1  tested_positive  optim1  optim2  slfcont2  slfcont4
##   10   4  cnarc1  cnarc2  cnarc3  ctheory1  ctheory3  ctheory4  CRT1  CRT2  CRT3  duration  employ_status1  know_tested_positive  marital1  moralid10  moralid3  moralid4  moralid5  moralid6  moralid8  moralid9  mcoop2  mcoop3  mcoop4  mcoop5  mcoop6  narc4  narc5  nidentity1  nidentity2  omind5  contact1  contact2  contact3  contact4  contact5  hygiene4  hygiene5  psupport1  political_ideology  happy  slf_ladder  sex1  tested_positive  optim1  optim2  slfcont2  slfcont4
##   10   5  cnarc1  cnarc2  cnarc3  ctheory1  ctheory3  ctheory4  CRT1  CRT2  CRT3  duration  employ_status1  know_tested_positive  marital1  moralid10  moralid3  moralid4  moralid5  moralid6  moralid8  moralid9  mcoop2  mcoop3  mcoop4  mcoop5  mcoop6  narc4  narc5  nidentity1  nidentity2  omind5  contact1  contact2  contact3  contact4  contact5  hygiene4  hygiene5  psupport1  political_ideology  happy  slf_ladder  sex1  tested_positive  optim1  optim2  slfcont2  slfcont4
##   10   6  cnarc1  cnarc2  cnarc3  ctheory1  ctheory3  ctheory4  CRT1  CRT2  CRT3  duration  employ_status1  know_tested_positive  marital1  moralid10  moralid3  moralid4  moralid5  moralid6  moralid8  moralid9  mcoop2  mcoop3  mcoop4  mcoop5  mcoop6  narc4  narc5  nidentity1  nidentity2  omind5  contact1  contact2  contact3  contact4  contact5  hygiene4  hygiene5  psupport1  political_ideology  happy  slf_ladder  sex1  tested_positive  optim1  optim2  slfcont2  slfcont4
##   10   7  cnarc1  cnarc2  cnarc3  ctheory1  ctheory3  ctheory4  CRT1  CRT2  CRT3  duration  employ_status1  know_tested_positive  marital1  moralid10  moralid3  moralid4  moralid5  moralid6  moralid8  moralid9  mcoop2  mcoop3  mcoop4  mcoop5  mcoop6  narc4  narc5  nidentity1  nidentity2  omind5  contact1  contact2  contact3  contact4  contact5  hygiene4  hygiene5  psupport1  political_ideology  happy  slf_ladder  sex1  tested_positive  optim1  optim2  slfcont2  slfcont4
##   10   8  cnarc1  cnarc2  cnarc3  ctheory1  ctheory3  ctheory4  CRT1  CRT2  CRT3  duration  employ_status1  know_tested_positive  marital1  moralid10  moralid3  moralid4  moralid5  moralid6  moralid8  moralid9  mcoop2  mcoop3  mcoop4  mcoop5  mcoop6  narc4  narc5  nidentity1  nidentity2  omind5  contact1  contact2  contact3  contact4  contact5  hygiene4  hygiene5  psupport1  political_ideology  happy  slf_ladder  sex1  tested_positive  optim1  optim2  slfcont2  slfcont4
##   10   9  cnarc1  cnarc2  cnarc3  ctheory1  ctheory3  ctheory4  CRT1  CRT2  CRT3  duration  employ_status1  know_tested_positive  marital1  moralid10  moralid3  moralid4  moralid5  moralid6  moralid8  moralid9  mcoop2  mcoop3  mcoop4  mcoop5  mcoop6  narc4  narc5  nidentity1  nidentity2  omind5  contact1  contact2  contact3  contact4  contact5  hygiene4  hygiene5  psupport1  political_ideology  happy  slf_ladder  sex1  tested_positive  optim1  optim2  slfcont2  slfcont4
##   10   10  cnarc1  cnarc2  cnarc3  ctheory1  ctheory3  ctheory4  CRT1  CRT2  CRT3  duration  employ_status1  know_tested_positive  marital1  moralid10  moralid3  moralid4  moralid5  moralid6  moralid8  moralid9  mcoop2  mcoop3  mcoop4  mcoop5  mcoop6  narc4  narc5  nidentity1  nidentity2  omind5  contact1  contact2  contact3  contact4  contact5  hygiene4  hygiene5  psupport1  political_ideology  happy  slf_ladder  sex1  tested_positive  optim1  optim2  slfcont2  slfcont4
```

```
## Warning: Number of logged events: 102
```

```
## 
##  iter imp variable
##   1   1  age  children  cnarc2  cnarc3  ctheory1  ctheory2  ctheory3  ctheory4  CRT1  CRT2  CRT3  health_cond  ladder  mor_circle  moralid1  moralid10  moralid2  moralid3  moralid4  moralid5  moralid6  moralid7  moralid8  moralid9  mcoop2  mcoop3  mcoop4  mcoop5  mcoop6  mcoop7  narc2  narc3  narc4  narc5  narc6  nidentity1  nidentity2  omind1  omind3  omind4  omind5  omind6  contact1  contact2  contact3  contact4  contact5  hygiene4  psupport1  psupport2  psupport3  political_ideology  happy  slf_ladder  riskperc1  riskperc2  self_esteem  sbelong2  sbelong4  optim1  optim2  slfcont1  slfcont3  slfcont4
##   1   2  age  children  cnarc2  cnarc3  ctheory1  ctheory2  ctheory3  ctheory4  CRT1  CRT2  CRT3  health_cond  ladder  mor_circle  moralid1  moralid10  moralid2  moralid3  moralid4  moralid5  moralid6  moralid7  moralid8  moralid9  mcoop2  mcoop3  mcoop4  mcoop5  mcoop6  mcoop7  narc2  narc3  narc4  narc5  narc6  nidentity1  nidentity2  omind1  omind3  omind4  omind5  omind6  contact1  contact2  contact3  contact4  contact5  hygiene4  psupport1  psupport2  psupport3  political_ideology  happy  slf_ladder  riskperc1  riskperc2  self_esteem  sbelong2  sbelong4  optim1  optim2  slfcont1  slfcont3  slfcont4
##   1   3  age  children  cnarc2  cnarc3  ctheory1  ctheory2  ctheory3  ctheory4  CRT1  CRT2  CRT3  health_cond  ladder  mor_circle  moralid1  moralid10  moralid2  moralid3  moralid4  moralid5  moralid6  moralid7  moralid8  moralid9  mcoop2  mcoop3  mcoop4  mcoop5  mcoop6  mcoop7  narc2  narc3  narc4  narc5  narc6  nidentity1  nidentity2  omind1  omind3  omind4  omind5  omind6  contact1  contact2  contact3  contact4  contact5  hygiene4  psupport1  psupport2  psupport3  political_ideology  happy  slf_ladder  riskperc1  riskperc2  self_esteem  sbelong2  sbelong4  optim1  optim2  slfcont1  slfcont3  slfcont4
##   1   4  age  children  cnarc2  cnarc3  ctheory1  ctheory2  ctheory3  ctheory4  CRT1  CRT2  CRT3  health_cond  ladder  mor_circle  moralid1  moralid10  moralid2  moralid3  moralid4  moralid5  moralid6  moralid7  moralid8  moralid9  mcoop2  mcoop3  mcoop4  mcoop5  mcoop6  mcoop7  narc2  narc3  narc4  narc5  narc6  nidentity1  nidentity2  omind1  omind3  omind4  omind5  omind6  contact1  contact2  contact3  contact4  contact5  hygiene4  psupport1  psupport2  psupport3  political_ideology  happy  slf_ladder  riskperc1  riskperc2  self_esteem  sbelong2  sbelong4  optim1  optim2  slfcont1  slfcont3  slfcont4
##   1   5  age  children  cnarc2  cnarc3  ctheory1  ctheory2  ctheory3  ctheory4  CRT1  CRT2  CRT3  health_cond  ladder  mor_circle  moralid1  moralid10  moralid2  moralid3  moralid4  moralid5  moralid6  moralid7  moralid8  moralid9  mcoop2  mcoop3  mcoop4  mcoop5  mcoop6  mcoop7  narc2  narc3  narc4  narc5  narc6  nidentity1  nidentity2  omind1  omind3  omind4  omind5  omind6  contact1  contact2  contact3  contact4  contact5  hygiene4  psupport1  psupport2  psupport3  political_ideology  happy  slf_ladder  riskperc1  riskperc2  self_esteem  sbelong2  sbelong4  optim1  optim2  slfcont1  slfcont3  slfcont4
##   1   6  age  children  cnarc2  cnarc3  ctheory1  ctheory2  ctheory3  ctheory4  CRT1  CRT2  CRT3  health_cond  ladder  mor_circle  moralid1  moralid10  moralid2  moralid3  moralid4  moralid5  moralid6  moralid7  moralid8  moralid9  mcoop2  mcoop3  mcoop4  mcoop5  mcoop6  mcoop7  narc2  narc3  narc4  narc5  narc6  nidentity1  nidentity2  omind1  omind3  omind4  omind5  omind6  contact1  contact2  contact3  contact4  contact5  hygiene4  psupport1  psupport2  psupport3  political_ideology  happy  slf_ladder  riskperc1  riskperc2  self_esteem  sbelong2  sbelong4  optim1  optim2  slfcont1  slfcont3  slfcont4
##   1   7  age  children  cnarc2  cnarc3  ctheory1  ctheory2  ctheory3  ctheory4  CRT1  CRT2  CRT3  health_cond  ladder  mor_circle  moralid1  moralid10  moralid2  moralid3  moralid4  moralid5  moralid6  moralid7  moralid8  moralid9  mcoop2  mcoop3  mcoop4  mcoop5  mcoop6  mcoop7  narc2  narc3  narc4  narc5  narc6  nidentity1  nidentity2  omind1  omind3  omind4  omind5  omind6  contact1  contact2  contact3  contact4  contact5  hygiene4  psupport1  psupport2  psupport3  political_ideology  happy  slf_ladder  riskperc1  riskperc2  self_esteem  sbelong2  sbelong4  optim1  optim2  slfcont1  slfcont3  slfcont4
##   1   8  age  children  cnarc2  cnarc3  ctheory1  ctheory2  ctheory3  ctheory4  CRT1  CRT2  CRT3  health_cond  ladder  mor_circle  moralid1  moralid10  moralid2  moralid3  moralid4  moralid5  moralid6  moralid7  moralid8  moralid9  mcoop2  mcoop3  mcoop4  mcoop5  mcoop6  mcoop7  narc2  narc3  narc4  narc5  narc6  nidentity1  nidentity2  omind1  omind3  omind4  omind5  omind6  contact1  contact2  contact3  contact4  contact5  hygiene4  psupport1  psupport2  psupport3  political_ideology  happy  slf_ladder  riskperc1  riskperc2  self_esteem  sbelong2  sbelong4  optim1  optim2  slfcont1  slfcont3  slfcont4
##   1   9  age  children  cnarc2  cnarc3  ctheory1  ctheory2  ctheory3  ctheory4  CRT1  CRT2  CRT3  health_cond  ladder  mor_circle  moralid1  moralid10  moralid2  moralid3  moralid4  moralid5  moralid6  moralid7  moralid8  moralid9  mcoop2  mcoop3  mcoop4  mcoop5  mcoop6  mcoop7  narc2  narc3  narc4  narc5  narc6  nidentity1  nidentity2  omind1  omind3  omind4  omind5  omind6  contact1  contact2  contact3  contact4  contact5  hygiene4  psupport1  psupport2  psupport3  political_ideology  happy  slf_ladder  riskperc1  riskperc2  self_esteem  sbelong2  sbelong4  optim1  optim2  slfcont1  slfcont3  slfcont4
##   1   10  age  children  cnarc2  cnarc3  ctheory1  ctheory2  ctheory3  ctheory4  CRT1  CRT2  CRT3  health_cond  ladder  mor_circle  moralid1  moralid10  moralid2  moralid3  moralid4  moralid5  moralid6  moralid7  moralid8  moralid9  mcoop2  mcoop3  mcoop4  mcoop5  mcoop6  mcoop7  narc2  narc3  narc4  narc5  narc6  nidentity1  nidentity2  omind1  omind3  omind4  omind5  omind6  contact1  contact2  contact3  contact4  contact5  hygiene4  psupport1  psupport2  psupport3  political_ideology  happy  slf_ladder  riskperc1  riskperc2  self_esteem  sbelong2  sbelong4  optim1  optim2  slfcont1  slfcont3  slfcont4
##   2   1  age  children  cnarc2  cnarc3  ctheory1  ctheory2  ctheory3  ctheory4  CRT1  CRT2  CRT3  health_cond  ladder  mor_circle  moralid1  moralid10  moralid2  moralid3  moralid4  moralid5  moralid6  moralid7  moralid8  moralid9  mcoop2  mcoop3  mcoop4  mcoop5  mcoop6  mcoop7  narc2  narc3  narc4  narc5  narc6  nidentity1  nidentity2  omind1  omind3  omind4  omind5  omind6  contact1  contact2  contact3  contact4  contact5  hygiene4  psupport1  psupport2  psupport3  political_ideology  happy  slf_ladder  riskperc1  riskperc2  self_esteem  sbelong2  sbelong4  optim1  optim2  slfcont1  slfcont3  slfcont4
##   2   2  age  children  cnarc2  cnarc3  ctheory1  ctheory2  ctheory3  ctheory4  CRT1  CRT2  CRT3  health_cond  ladder  mor_circle  moralid1  moralid10  moralid2  moralid3  moralid4  moralid5  moralid6  moralid7  moralid8  moralid9  mcoop2  mcoop3  mcoop4  mcoop5  mcoop6  mcoop7  narc2  narc3  narc4  narc5  narc6  nidentity1  nidentity2  omind1  omind3  omind4  omind5  omind6  contact1  contact2  contact3  contact4  contact5  hygiene4  psupport1  psupport2  psupport3  political_ideology  happy  slf_ladder  riskperc1  riskperc2  self_esteem  sbelong2  sbelong4  optim1  optim2  slfcont1  slfcont3  slfcont4
##   2   3  age  children  cnarc2  cnarc3  ctheory1  ctheory2  ctheory3  ctheory4  CRT1  CRT2  CRT3  health_cond  ladder  mor_circle  moralid1  moralid10  moralid2  moralid3  moralid4  moralid5  moralid6  moralid7  moralid8  moralid9  mcoop2  mcoop3  mcoop4  mcoop5  mcoop6  mcoop7  narc2  narc3  narc4  narc5  narc6  nidentity1  nidentity2  omind1  omind3  omind4  omind5  omind6  contact1  contact2  contact3  contact4  contact5  hygiene4  psupport1  psupport2  psupport3  political_ideology  happy  slf_ladder  riskperc1  riskperc2  self_esteem  sbelong2  sbelong4  optim1  optim2  slfcont1  slfcont3  slfcont4
##   2   4  age  children  cnarc2  cnarc3  ctheory1  ctheory2  ctheory3  ctheory4  CRT1  CRT2  CRT3  health_cond  ladder  mor_circle  moralid1  moralid10  moralid2  moralid3  moralid4  moralid5  moralid6  moralid7  moralid8  moralid9  mcoop2  mcoop3  mcoop4  mcoop5  mcoop6  mcoop7  narc2  narc3  narc4  narc5  narc6  nidentity1  nidentity2  omind1  omind3  omind4  omind5  omind6  contact1  contact2  contact3  contact4  contact5  hygiene4  psupport1  psupport2  psupport3  political_ideology  happy  slf_ladder  riskperc1  riskperc2  self_esteem  sbelong2  sbelong4  optim1  optim2  slfcont1  slfcont3  slfcont4
##   2   5  age  children  cnarc2  cnarc3  ctheory1  ctheory2  ctheory3  ctheory4  CRT1  CRT2  CRT3  health_cond  ladder  mor_circle  moralid1  moralid10  moralid2  moralid3  moralid4  moralid5  moralid6  moralid7  moralid8  moralid9  mcoop2  mcoop3  mcoop4  mcoop5  mcoop6  mcoop7  narc2  narc3  narc4  narc5  narc6  nidentity1  nidentity2  omind1  omind3  omind4  omind5  omind6  contact1  contact2  contact3  contact4  contact5  hygiene4  psupport1  psupport2  psupport3  political_ideology  happy  slf_ladder  riskperc1  riskperc2  self_esteem  sbelong2  sbelong4  optim1  optim2  slfcont1  slfcont3  slfcont4
##   2   6  age  children  cnarc2  cnarc3  ctheory1  ctheory2  ctheory3  ctheory4  CRT1  CRT2  CRT3  health_cond  ladder  mor_circle  moralid1  moralid10  moralid2  moralid3  moralid4  moralid5  moralid6  moralid7  moralid8  moralid9  mcoop2  mcoop3  mcoop4  mcoop5  mcoop6  mcoop7  narc2  narc3  narc4  narc5  narc6  nidentity1  nidentity2  omind1  omind3  omind4  omind5  omind6  contact1  contact2  contact3  contact4  contact5  hygiene4  psupport1  psupport2  psupport3  political_ideology  happy  slf_ladder  riskperc1  riskperc2  self_esteem  sbelong2  sbelong4  optim1  optim2  slfcont1  slfcont3  slfcont4
##   2   7  age  children  cnarc2  cnarc3  ctheory1  ctheory2  ctheory3  ctheory4  CRT1  CRT2  CRT3  health_cond  ladder  mor_circle  moralid1  moralid10  moralid2  moralid3  moralid4  moralid5  moralid6  moralid7  moralid8  moralid9  mcoop2  mcoop3  mcoop4  mcoop5  mcoop6  mcoop7  narc2  narc3  narc4  narc5  narc6  nidentity1  nidentity2  omind1  omind3  omind4  omind5  omind6  contact1  contact2  contact3  contact4  contact5  hygiene4  psupport1  psupport2  psupport3  political_ideology  happy  slf_ladder  riskperc1  riskperc2  self_esteem  sbelong2  sbelong4  optim1  optim2  slfcont1  slfcont3  slfcont4
##   2   8  age  children  cnarc2  cnarc3  ctheory1  ctheory2  ctheory3  ctheory4  CRT1  CRT2  CRT3  health_cond  ladder  mor_circle  moralid1  moralid10  moralid2  moralid3  moralid4  moralid5  moralid6  moralid7  moralid8  moralid9  mcoop2  mcoop3  mcoop4  mcoop5  mcoop6  mcoop7  narc2  narc3  narc4  narc5  narc6  nidentity1  nidentity2  omind1  omind3  omind4  omind5  omind6  contact1  contact2  contact3  contact4  contact5  hygiene4  psupport1  psupport2  psupport3  political_ideology  happy  slf_ladder  riskperc1  riskperc2  self_esteem  sbelong2  sbelong4  optim1  optim2  slfcont1  slfcont3  slfcont4
##   2   9  age  children  cnarc2  cnarc3  ctheory1  ctheory2  ctheory3  ctheory4  CRT1  CRT2  CRT3  health_cond  ladder  mor_circle  moralid1  moralid10  moralid2  moralid3  moralid4  moralid5  moralid6  moralid7  moralid8  moralid9  mcoop2  mcoop3  mcoop4  mcoop5  mcoop6  mcoop7  narc2  narc3  narc4  narc5  narc6  nidentity1  nidentity2  omind1  omind3  omind4  omind5  omind6  contact1  contact2  contact3  contact4  contact5  hygiene4  psupport1  psupport2  psupport3  political_ideology  happy  slf_ladder  riskperc1  riskperc2  self_esteem  sbelong2  sbelong4  optim1  optim2  slfcont1  slfcont3  slfcont4
##   2   10  age  children  cnarc2  cnarc3  ctheory1  ctheory2  ctheory3  ctheory4  CRT1  CRT2  CRT3  health_cond  ladder  mor_circle  moralid1  moralid10  moralid2  moralid3  moralid4  moralid5  moralid6  moralid7  moralid8  moralid9  mcoop2  mcoop3  mcoop4  mcoop5  mcoop6  mcoop7  narc2  narc3  narc4  narc5  narc6  nidentity1  nidentity2  omind1  omind3  omind4  omind5  omind6  contact1  contact2  contact3  contact4  contact5  hygiene4  psupport1  psupport2  psupport3  political_ideology  happy  slf_ladder  riskperc1  riskperc2  self_esteem  sbelong2  sbelong4  optim1  optim2  slfcont1  slfcont3  slfcont4
##   3   1  age  children  cnarc2  cnarc3  ctheory1  ctheory2  ctheory3  ctheory4  CRT1  CRT2  CRT3  health_cond  ladder  mor_circle  moralid1  moralid10  moralid2  moralid3  moralid4  moralid5  moralid6  moralid7  moralid8  moralid9  mcoop2  mcoop3  mcoop4  mcoop5  mcoop6  mcoop7  narc2  narc3  narc4  narc5  narc6  nidentity1  nidentity2  omind1  omind3  omind4  omind5  omind6  contact1  contact2  contact3  contact4  contact5  hygiene4  psupport1  psupport2  psupport3  political_ideology  happy  slf_ladder  riskperc1  riskperc2  self_esteem  sbelong2  sbelong4  optim1  optim2  slfcont1  slfcont3  slfcont4
##   3   2  age  children  cnarc2  cnarc3  ctheory1  ctheory2  ctheory3  ctheory4  CRT1  CRT2  CRT3  health_cond  ladder  mor_circle  moralid1  moralid10  moralid2  moralid3  moralid4  moralid5  moralid6  moralid7  moralid8  moralid9  mcoop2  mcoop3  mcoop4  mcoop5  mcoop6  mcoop7  narc2  narc3  narc4  narc5  narc6  nidentity1  nidentity2  omind1  omind3  omind4  omind5  omind6  contact1  contact2  contact3  contact4  contact5  hygiene4  psupport1  psupport2  psupport3  political_ideology  happy  slf_ladder  riskperc1  riskperc2  self_esteem  sbelong2  sbelong4  optim1  optim2  slfcont1  slfcont3  slfcont4
##   3   3  age  children  cnarc2  cnarc3  ctheory1  ctheory2  ctheory3  ctheory4  CRT1  CRT2  CRT3  health_cond  ladder  mor_circle  moralid1  moralid10  moralid2  moralid3  moralid4  moralid5  moralid6  moralid7  moralid8  moralid9  mcoop2  mcoop3  mcoop4  mcoop5  mcoop6  mcoop7  narc2  narc3  narc4  narc5  narc6  nidentity1  nidentity2  omind1  omind3  omind4  omind5  omind6  contact1  contact2  contact3  contact4  contact5  hygiene4  psupport1  psupport2  psupport3  political_ideology  happy  slf_ladder  riskperc1  riskperc2  self_esteem  sbelong2  sbelong4  optim1  optim2  slfcont1  slfcont3  slfcont4
##   3   4  age  children  cnarc2  cnarc3  ctheory1  ctheory2  ctheory3  ctheory4  CRT1  CRT2  CRT3  health_cond  ladder  mor_circle  moralid1  moralid10  moralid2  moralid3  moralid4  moralid5  moralid6  moralid7  moralid8  moralid9  mcoop2  mcoop3  mcoop4  mcoop5  mcoop6  mcoop7  narc2  narc3  narc4  narc5  narc6  nidentity1  nidentity2  omind1  omind3  omind4  omind5  omind6  contact1  contact2  contact3  contact4  contact5  hygiene4  psupport1  psupport2  psupport3  political_ideology  happy  slf_ladder  riskperc1  riskperc2  self_esteem  sbelong2  sbelong4  optim1  optim2  slfcont1  slfcont3  slfcont4
##   3   5  age  children  cnarc2  cnarc3  ctheory1  ctheory2  ctheory3  ctheory4  CRT1  CRT2  CRT3  health_cond  ladder  mor_circle  moralid1  moralid10  moralid2  moralid3  moralid4  moralid5  moralid6  moralid7  moralid8  moralid9  mcoop2  mcoop3  mcoop4  mcoop5  mcoop6  mcoop7  narc2  narc3  narc4  narc5  narc6  nidentity1  nidentity2  omind1  omind3  omind4  omind5  omind6  contact1  contact2  contact3  contact4  contact5  hygiene4  psupport1  psupport2  psupport3  political_ideology  happy  slf_ladder  riskperc1  riskperc2  self_esteem  sbelong2  sbelong4  optim1  optim2  slfcont1  slfcont3  slfcont4
##   3   6  age  children  cnarc2  cnarc3  ctheory1  ctheory2  ctheory3  ctheory4  CRT1  CRT2  CRT3  health_cond  ladder  mor_circle  moralid1  moralid10  moralid2  moralid3  moralid4  moralid5  moralid6  moralid7  moralid8  moralid9  mcoop2  mcoop3  mcoop4  mcoop5  mcoop6  mcoop7  narc2  narc3  narc4  narc5  narc6  nidentity1  nidentity2  omind1  omind3  omind4  omind5  omind6  contact1  contact2  contact3  contact4  contact5  hygiene4  psupport1  psupport2  psupport3  political_ideology  happy  slf_ladder  riskperc1  riskperc2  self_esteem  sbelong2  sbelong4  optim1  optim2  slfcont1  slfcont3  slfcont4
##   3   7  age  children  cnarc2  cnarc3  ctheory1  ctheory2  ctheory3  ctheory4  CRT1  CRT2  CRT3  health_cond  ladder  mor_circle  moralid1  moralid10  moralid2  moralid3  moralid4  moralid5  moralid6  moralid7  moralid8  moralid9  mcoop2  mcoop3  mcoop4  mcoop5  mcoop6  mcoop7  narc2  narc3  narc4  narc5  narc6  nidentity1  nidentity2  omind1  omind3  omind4  omind5  omind6  contact1  contact2  contact3  contact4  contact5  hygiene4  psupport1  psupport2  psupport3  political_ideology  happy  slf_ladder  riskperc1  riskperc2  self_esteem  sbelong2  sbelong4  optim1  optim2  slfcont1  slfcont3  slfcont4
##   3   8  age  children  cnarc2  cnarc3  ctheory1  ctheory2  ctheory3  ctheory4  CRT1  CRT2  CRT3  health_cond  ladder  mor_circle  moralid1  moralid10  moralid2  moralid3  moralid4  moralid5  moralid6  moralid7  moralid8  moralid9  mcoop2  mcoop3  mcoop4  mcoop5  mcoop6  mcoop7  narc2  narc3  narc4  narc5  narc6  nidentity1  nidentity2  omind1  omind3  omind4  omind5  omind6  contact1  contact2  contact3  contact4  contact5  hygiene4  psupport1  psupport2  psupport3  political_ideology  happy  slf_ladder  riskperc1  riskperc2  self_esteem  sbelong2  sbelong4  optim1  optim2  slfcont1  slfcont3  slfcont4
##   3   9  age  children  cnarc2  cnarc3  ctheory1  ctheory2  ctheory3  ctheory4  CRT1  CRT2  CRT3  health_cond  ladder  mor_circle  moralid1  moralid10  moralid2  moralid3  moralid4  moralid5  moralid6  moralid7  moralid8  moralid9  mcoop2  mcoop3  mcoop4  mcoop5  mcoop6  mcoop7  narc2  narc3  narc4  narc5  narc6  nidentity1  nidentity2  omind1  omind3  omind4  omind5  omind6  contact1  contact2  contact3  contact4  contact5  hygiene4  psupport1  psupport2  psupport3  political_ideology  happy  slf_ladder  riskperc1  riskperc2  self_esteem  sbelong2  sbelong4  optim1  optim2  slfcont1  slfcont3  slfcont4
##   3   10  age  children  cnarc2  cnarc3  ctheory1  ctheory2  ctheory3  ctheory4  CRT1  CRT2  CRT3  health_cond  ladder  mor_circle  moralid1  moralid10  moralid2  moralid3  moralid4  moralid5  moralid6  moralid7  moralid8  moralid9  mcoop2  mcoop3  mcoop4  mcoop5  mcoop6  mcoop7  narc2  narc3  narc4  narc5  narc6  nidentity1  nidentity2  omind1  omind3  omind4  omind5  omind6  contact1  contact2  contact3  contact4  contact5  hygiene4  psupport1  psupport2  psupport3  political_ideology  happy  slf_ladder  riskperc1  riskperc2  self_esteem  sbelong2  sbelong4  optim1  optim2  slfcont1  slfcont3  slfcont4
##   4   1  age  children  cnarc2  cnarc3  ctheory1  ctheory2  ctheory3  ctheory4  CRT1  CRT2  CRT3  health_cond  ladder  mor_circle  moralid1  moralid10  moralid2  moralid3  moralid4  moralid5  moralid6  moralid7  moralid8  moralid9  mcoop2  mcoop3  mcoop4  mcoop5  mcoop6  mcoop7  narc2  narc3  narc4  narc5  narc6  nidentity1  nidentity2  omind1  omind3  omind4  omind5  omind6  contact1  contact2  contact3  contact4  contact5  hygiene4  psupport1  psupport2  psupport3  political_ideology  happy  slf_ladder  riskperc1  riskperc2  self_esteem  sbelong2  sbelong4  optim1  optim2  slfcont1  slfcont3  slfcont4
##   4   2  age  children  cnarc2  cnarc3  ctheory1  ctheory2  ctheory3  ctheory4  CRT1  CRT2  CRT3  health_cond  ladder  mor_circle  moralid1  moralid10  moralid2  moralid3  moralid4  moralid5  moralid6  moralid7  moralid8  moralid9  mcoop2  mcoop3  mcoop4  mcoop5  mcoop6  mcoop7  narc2  narc3  narc4  narc5  narc6  nidentity1  nidentity2  omind1  omind3  omind4  omind5  omind6  contact1  contact2  contact3  contact4  contact5  hygiene4  psupport1  psupport2  psupport3  political_ideology  happy  slf_ladder  riskperc1  riskperc2  self_esteem  sbelong2  sbelong4  optim1  optim2  slfcont1  slfcont3  slfcont4
##   4   3  age  children  cnarc2  cnarc3  ctheory1  ctheory2  ctheory3  ctheory4  CRT1  CRT2  CRT3  health_cond  ladder  mor_circle  moralid1  moralid10  moralid2  moralid3  moralid4  moralid5  moralid6  moralid7  moralid8  moralid9  mcoop2  mcoop3  mcoop4  mcoop5  mcoop6  mcoop7  narc2  narc3  narc4  narc5  narc6  nidentity1  nidentity2  omind1  omind3  omind4  omind5  omind6  contact1  contact2  contact3  contact4  contact5  hygiene4  psupport1  psupport2  psupport3  political_ideology  happy  slf_ladder  riskperc1  riskperc2  self_esteem  sbelong2  sbelong4  optim1  optim2  slfcont1  slfcont3  slfcont4
##   4   4  age  children  cnarc2  cnarc3  ctheory1  ctheory2  ctheory3  ctheory4  CRT1  CRT2  CRT3  health_cond  ladder  mor_circle  moralid1  moralid10  moralid2  moralid3  moralid4  moralid5  moralid6  moralid7  moralid8  moralid9  mcoop2  mcoop3  mcoop4  mcoop5  mcoop6  mcoop7  narc2  narc3  narc4  narc5  narc6  nidentity1  nidentity2  omind1  omind3  omind4  omind5  omind6  contact1  contact2  contact3  contact4  contact5  hygiene4  psupport1  psupport2  psupport3  political_ideology  happy  slf_ladder  riskperc1  riskperc2  self_esteem  sbelong2  sbelong4  optim1  optim2  slfcont1  slfcont3  slfcont4
##   4   5  age  children  cnarc2  cnarc3  ctheory1  ctheory2  ctheory3  ctheory4  CRT1  CRT2  CRT3  health_cond  ladder  mor_circle  moralid1  moralid10  moralid2  moralid3  moralid4  moralid5  moralid6  moralid7  moralid8  moralid9  mcoop2  mcoop3  mcoop4  mcoop5  mcoop6  mcoop7  narc2  narc3  narc4  narc5  narc6  nidentity1  nidentity2  omind1  omind3  omind4  omind5  omind6  contact1  contact2  contact3  contact4  contact5  hygiene4  psupport1  psupport2  psupport3  political_ideology  happy  slf_ladder  riskperc1  riskperc2  self_esteem  sbelong2  sbelong4  optim1  optim2  slfcont1  slfcont3  slfcont4
##   4   6  age  children  cnarc2  cnarc3  ctheory1  ctheory2  ctheory3  ctheory4  CRT1  CRT2  CRT3  health_cond  ladder  mor_circle  moralid1  moralid10  moralid2  moralid3  moralid4  moralid5  moralid6  moralid7  moralid8  moralid9  mcoop2  mcoop3  mcoop4  mcoop5  mcoop6  mcoop7  narc2  narc3  narc4  narc5  narc6  nidentity1  nidentity2  omind1  omind3  omind4  omind5  omind6  contact1  contact2  contact3  contact4  contact5  hygiene4  psupport1  psupport2  psupport3  political_ideology  happy  slf_ladder  riskperc1  riskperc2  self_esteem  sbelong2  sbelong4  optim1  optim2  slfcont1  slfcont3  slfcont4
##   4   7  age  children  cnarc2  cnarc3  ctheory1  ctheory2  ctheory3  ctheory4  CRT1  CRT2  CRT3  health_cond  ladder  mor_circle  moralid1  moralid10  moralid2  moralid3  moralid4  moralid5  moralid6  moralid7  moralid8  moralid9  mcoop2  mcoop3  mcoop4  mcoop5  mcoop6  mcoop7  narc2  narc3  narc4  narc5  narc6  nidentity1  nidentity2  omind1  omind3  omind4  omind5  omind6  contact1  contact2  contact3  contact4  contact5  hygiene4  psupport1  psupport2  psupport3  political_ideology  happy  slf_ladder  riskperc1  riskperc2  self_esteem  sbelong2  sbelong4  optim1  optim2  slfcont1  slfcont3  slfcont4
##   4   8  age  children  cnarc2  cnarc3  ctheory1  ctheory2  ctheory3  ctheory4  CRT1  CRT2  CRT3  health_cond  ladder  mor_circle  moralid1  moralid10  moralid2  moralid3  moralid4  moralid5  moralid6  moralid7  moralid8  moralid9  mcoop2  mcoop3  mcoop4  mcoop5  mcoop6  mcoop7  narc2  narc3  narc4  narc5  narc6  nidentity1  nidentity2  omind1  omind3  omind4  omind5  omind6  contact1  contact2  contact3  contact4  contact5  hygiene4  psupport1  psupport2  psupport3  political_ideology  happy  slf_ladder  riskperc1  riskperc2  self_esteem  sbelong2  sbelong4  optim1  optim2  slfcont1  slfcont3  slfcont4
##   4   9  age  children  cnarc2  cnarc3  ctheory1  ctheory2  ctheory3  ctheory4  CRT1  CRT2  CRT3  health_cond  ladder  mor_circle  moralid1  moralid10  moralid2  moralid3  moralid4  moralid5  moralid6  moralid7  moralid8  moralid9  mcoop2  mcoop3  mcoop4  mcoop5  mcoop6  mcoop7  narc2  narc3  narc4  narc5  narc6  nidentity1  nidentity2  omind1  omind3  omind4  omind5  omind6  contact1  contact2  contact3  contact4  contact5  hygiene4  psupport1  psupport2  psupport3  political_ideology  happy  slf_ladder  riskperc1  riskperc2  self_esteem  sbelong2  sbelong4  optim1  optim2  slfcont1  slfcont3  slfcont4
##   4   10  age  children  cnarc2  cnarc3  ctheory1  ctheory2  ctheory3  ctheory4  CRT1  CRT2  CRT3  health_cond  ladder  mor_circle  moralid1  moralid10  moralid2  moralid3  moralid4  moralid5  moralid6  moralid7  moralid8  moralid9  mcoop2  mcoop3  mcoop4  mcoop5  mcoop6  mcoop7  narc2  narc3  narc4  narc5  narc6  nidentity1  nidentity2  omind1  omind3  omind4  omind5  omind6  contact1  contact2  contact3  contact4  contact5  hygiene4  psupport1  psupport2  psupport3  political_ideology  happy  slf_ladder  riskperc1  riskperc2  self_esteem  sbelong2  sbelong4  optim1  optim2  slfcont1  slfcont3  slfcont4
##   5   1  age  children  cnarc2  cnarc3  ctheory1  ctheory2  ctheory3  ctheory4  CRT1  CRT2  CRT3  health_cond  ladder  mor_circle  moralid1  moralid10  moralid2  moralid3  moralid4  moralid5  moralid6  moralid7  moralid8  moralid9  mcoop2  mcoop3  mcoop4  mcoop5  mcoop6  mcoop7  narc2  narc3  narc4  narc5  narc6  nidentity1  nidentity2  omind1  omind3  omind4  omind5  omind6  contact1  contact2  contact3  contact4  contact5  hygiene4  psupport1  psupport2  psupport3  political_ideology  happy  slf_ladder  riskperc1  riskperc2  self_esteem  sbelong2  sbelong4  optim1  optim2  slfcont1  slfcont3  slfcont4
##   5   2  age  children  cnarc2  cnarc3  ctheory1  ctheory2  ctheory3  ctheory4  CRT1  CRT2  CRT3  health_cond  ladder  mor_circle  moralid1  moralid10  moralid2  moralid3  moralid4  moralid5  moralid6  moralid7  moralid8  moralid9  mcoop2  mcoop3  mcoop4  mcoop5  mcoop6  mcoop7  narc2  narc3  narc4  narc5  narc6  nidentity1  nidentity2  omind1  omind3  omind4  omind5  omind6  contact1  contact2  contact3  contact4  contact5  hygiene4  psupport1  psupport2  psupport3  political_ideology  happy  slf_ladder  riskperc1  riskperc2  self_esteem  sbelong2  sbelong4  optim1  optim2  slfcont1  slfcont3  slfcont4
##   5   3  age  children  cnarc2  cnarc3  ctheory1  ctheory2  ctheory3  ctheory4  CRT1  CRT2  CRT3  health_cond  ladder  mor_circle  moralid1  moralid10  moralid2  moralid3  moralid4  moralid5  moralid6  moralid7  moralid8  moralid9  mcoop2  mcoop3  mcoop4  mcoop5  mcoop6  mcoop7  narc2  narc3  narc4  narc5  narc6  nidentity1  nidentity2  omind1  omind3  omind4  omind5  omind6  contact1  contact2  contact3  contact4  contact5  hygiene4  psupport1  psupport2  psupport3  political_ideology  happy  slf_ladder  riskperc1  riskperc2  self_esteem  sbelong2  sbelong4  optim1  optim2  slfcont1  slfcont3  slfcont4
##   5   4  age  children  cnarc2  cnarc3  ctheory1  ctheory2  ctheory3  ctheory4  CRT1  CRT2  CRT3  health_cond  ladder  mor_circle  moralid1  moralid10  moralid2  moralid3  moralid4  moralid5  moralid6  moralid7  moralid8  moralid9  mcoop2  mcoop3  mcoop4  mcoop5  mcoop6  mcoop7  narc2  narc3  narc4  narc5  narc6  nidentity1  nidentity2  omind1  omind3  omind4  omind5  omind6  contact1  contact2  contact3  contact4  contact5  hygiene4  psupport1  psupport2  psupport3  political_ideology  happy  slf_ladder  riskperc1  riskperc2  self_esteem  sbelong2  sbelong4  optim1  optim2  slfcont1  slfcont3  slfcont4
##   5   5  age  children  cnarc2  cnarc3  ctheory1  ctheory2  ctheory3  ctheory4  CRT1  CRT2  CRT3  health_cond  ladder  mor_circle  moralid1  moralid10  moralid2  moralid3  moralid4  moralid5  moralid6  moralid7  moralid8  moralid9  mcoop2  mcoop3  mcoop4  mcoop5  mcoop6  mcoop7  narc2  narc3  narc4  narc5  narc6  nidentity1  nidentity2  omind1  omind3  omind4  omind5  omind6  contact1  contact2  contact3  contact4  contact5  hygiene4  psupport1  psupport2  psupport3  political_ideology  happy  slf_ladder  riskperc1  riskperc2  self_esteem  sbelong2  sbelong4  optim1  optim2  slfcont1  slfcont3  slfcont4
##   5   6  age  children  cnarc2  cnarc3  ctheory1  ctheory2  ctheory3  ctheory4  CRT1  CRT2  CRT3  health_cond  ladder  mor_circle  moralid1  moralid10  moralid2  moralid3  moralid4  moralid5  moralid6  moralid7  moralid8  moralid9  mcoop2  mcoop3  mcoop4  mcoop5  mcoop6  mcoop7  narc2  narc3  narc4  narc5  narc6  nidentity1  nidentity2  omind1  omind3  omind4  omind5  omind6  contact1  contact2  contact3  contact4  contact5  hygiene4  psupport1  psupport2  psupport3  political_ideology  happy  slf_ladder  riskperc1  riskperc2  self_esteem  sbelong2  sbelong4  optim1  optim2  slfcont1  slfcont3  slfcont4
##   5   7  age  children  cnarc2  cnarc3  ctheory1  ctheory2  ctheory3  ctheory4  CRT1  CRT2  CRT3  health_cond  ladder  mor_circle  moralid1  moralid10  moralid2  moralid3  moralid4  moralid5  moralid6  moralid7  moralid8  moralid9  mcoop2  mcoop3  mcoop4  mcoop5  mcoop6  mcoop7  narc2  narc3  narc4  narc5  narc6  nidentity1  nidentity2  omind1  omind3  omind4  omind5  omind6  contact1  contact2  contact3  contact4  contact5  hygiene4  psupport1  psupport2  psupport3  political_ideology  happy  slf_ladder  riskperc1  riskperc2  self_esteem  sbelong2  sbelong4  optim1  optim2  slfcont1  slfcont3  slfcont4
##   5   8  age  children  cnarc2  cnarc3  ctheory1  ctheory2  ctheory3  ctheory4  CRT1  CRT2  CRT3  health_cond  ladder  mor_circle  moralid1  moralid10  moralid2  moralid3  moralid4  moralid5  moralid6  moralid7  moralid8  moralid9  mcoop2  mcoop3  mcoop4  mcoop5  mcoop6  mcoop7  narc2  narc3  narc4  narc5  narc6  nidentity1  nidentity2  omind1  omind3  omind4  omind5  omind6  contact1  contact2  contact3  contact4  contact5  hygiene4  psupport1  psupport2  psupport3  political_ideology  happy  slf_ladder  riskperc1  riskperc2  self_esteem  sbelong2  sbelong4  optim1  optim2  slfcont1  slfcont3  slfcont4
##   5   9  age  children  cnarc2  cnarc3  ctheory1  ctheory2  ctheory3  ctheory4  CRT1  CRT2  CRT3  health_cond  ladder  mor_circle  moralid1  moralid10  moralid2  moralid3  moralid4  moralid5  moralid6  moralid7  moralid8  moralid9  mcoop2  mcoop3  mcoop4  mcoop5  mcoop6  mcoop7  narc2  narc3  narc4  narc5  narc6  nidentity1  nidentity2  omind1  omind3  omind4  omind5  omind6  contact1  contact2  contact3  contact4  contact5  hygiene4  psupport1  psupport2  psupport3  political_ideology  happy  slf_ladder  riskperc1  riskperc2  self_esteem  sbelong2  sbelong4  optim1  optim2  slfcont1  slfcont3  slfcont4
##   5   10  age  children  cnarc2  cnarc3  ctheory1  ctheory2  ctheory3  ctheory4  CRT1  CRT2  CRT3  health_cond  ladder  mor_circle  moralid1  moralid10  moralid2  moralid3  moralid4  moralid5  moralid6  moralid7  moralid8  moralid9  mcoop2  mcoop3  mcoop4  mcoop5  mcoop6  mcoop7  narc2  narc3  narc4  narc5  narc6  nidentity1  nidentity2  omind1  omind3  omind4  omind5  omind6  contact1  contact2  contact3  contact4  contact5  hygiene4  psupport1  psupport2  psupport3  political_ideology  happy  slf_ladder  riskperc1  riskperc2  self_esteem  sbelong2  sbelong4  optim1  optim2  slfcont1  slfcont3  slfcont4
##   6   1  age  children  cnarc2  cnarc3  ctheory1  ctheory2  ctheory3  ctheory4  CRT1  CRT2  CRT3  health_cond  ladder  mor_circle  moralid1  moralid10  moralid2  moralid3  moralid4  moralid5  moralid6  moralid7  moralid8  moralid9  mcoop2  mcoop3  mcoop4  mcoop5  mcoop6  mcoop7  narc2  narc3  narc4  narc5  narc6  nidentity1  nidentity2  omind1  omind3  omind4  omind5  omind6  contact1  contact2  contact3  contact4  contact5  hygiene4  psupport1  psupport2  psupport3  political_ideology  happy  slf_ladder  riskperc1  riskperc2  self_esteem  sbelong2  sbelong4  optim1  optim2  slfcont1  slfcont3  slfcont4
##   6   2  age  children  cnarc2  cnarc3  ctheory1  ctheory2  ctheory3  ctheory4  CRT1  CRT2  CRT3  health_cond  ladder  mor_circle  moralid1  moralid10  moralid2  moralid3  moralid4  moralid5  moralid6  moralid7  moralid8  moralid9  mcoop2  mcoop3  mcoop4  mcoop5  mcoop6  mcoop7  narc2  narc3  narc4  narc5  narc6  nidentity1  nidentity2  omind1  omind3  omind4  omind5  omind6  contact1  contact2  contact3  contact4  contact5  hygiene4  psupport1  psupport2  psupport3  political_ideology  happy  slf_ladder  riskperc1  riskperc2  self_esteem  sbelong2  sbelong4  optim1  optim2  slfcont1  slfcont3  slfcont4
##   6   3  age  children  cnarc2  cnarc3  ctheory1  ctheory2  ctheory3  ctheory4  CRT1  CRT2  CRT3  health_cond  ladder  mor_circle  moralid1  moralid10  moralid2  moralid3  moralid4  moralid5  moralid6  moralid7  moralid8  moralid9  mcoop2  mcoop3  mcoop4  mcoop5  mcoop6  mcoop7  narc2  narc3  narc4  narc5  narc6  nidentity1  nidentity2  omind1  omind3  omind4  omind5  omind6  contact1  contact2  contact3  contact4  contact5  hygiene4  psupport1  psupport2  psupport3  political_ideology  happy  slf_ladder  riskperc1  riskperc2  self_esteem  sbelong2  sbelong4  optim1  optim2  slfcont1  slfcont3  slfcont4
##   6   4  age  children  cnarc2  cnarc3  ctheory1  ctheory2  ctheory3  ctheory4  CRT1  CRT2  CRT3  health_cond  ladder  mor_circle  moralid1  moralid10  moralid2  moralid3  moralid4  moralid5  moralid6  moralid7  moralid8  moralid9  mcoop2  mcoop3  mcoop4  mcoop5  mcoop6  mcoop7  narc2  narc3  narc4  narc5  narc6  nidentity1  nidentity2  omind1  omind3  omind4  omind5  omind6  contact1  contact2  contact3  contact4  contact5  hygiene4  psupport1  psupport2  psupport3  political_ideology  happy  slf_ladder  riskperc1  riskperc2  self_esteem  sbelong2  sbelong4  optim1  optim2  slfcont1  slfcont3  slfcont4
##   6   5  age  children  cnarc2  cnarc3  ctheory1  ctheory2  ctheory3  ctheory4  CRT1  CRT2  CRT3  health_cond  ladder  mor_circle  moralid1  moralid10  moralid2  moralid3  moralid4  moralid5  moralid6  moralid7  moralid8  moralid9  mcoop2  mcoop3  mcoop4  mcoop5  mcoop6  mcoop7  narc2  narc3  narc4  narc5  narc6  nidentity1  nidentity2  omind1  omind3  omind4  omind5  omind6  contact1  contact2  contact3  contact4  contact5  hygiene4  psupport1  psupport2  psupport3  political_ideology  happy  slf_ladder  riskperc1  riskperc2  self_esteem  sbelong2  sbelong4  optim1  optim2  slfcont1  slfcont3  slfcont4
##   6   6  age  children  cnarc2  cnarc3  ctheory1  ctheory2  ctheory3  ctheory4  CRT1  CRT2  CRT3  health_cond  ladder  mor_circle  moralid1  moralid10  moralid2  moralid3  moralid4  moralid5  moralid6  moralid7  moralid8  moralid9  mcoop2  mcoop3  mcoop4  mcoop5  mcoop6  mcoop7  narc2  narc3  narc4  narc5  narc6  nidentity1  nidentity2  omind1  omind3  omind4  omind5  omind6  contact1  contact2  contact3  contact4  contact5  hygiene4  psupport1  psupport2  psupport3  political_ideology  happy  slf_ladder  riskperc1  riskperc2  self_esteem  sbelong2  sbelong4  optim1  optim2  slfcont1  slfcont3  slfcont4
##   6   7  age  children  cnarc2  cnarc3  ctheory1  ctheory2  ctheory3  ctheory4  CRT1  CRT2  CRT3  health_cond  ladder  mor_circle  moralid1  moralid10  moralid2  moralid3  moralid4  moralid5  moralid6  moralid7  moralid8  moralid9  mcoop2  mcoop3  mcoop4  mcoop5  mcoop6  mcoop7  narc2  narc3  narc4  narc5  narc6  nidentity1  nidentity2  omind1  omind3  omind4  omind5  omind6  contact1  contact2  contact3  contact4  contact5  hygiene4  psupport1  psupport2  psupport3  political_ideology  happy  slf_ladder  riskperc1  riskperc2  self_esteem  sbelong2  sbelong4  optim1  optim2  slfcont1  slfcont3  slfcont4
##   6   8  age  children  cnarc2  cnarc3  ctheory1  ctheory2  ctheory3  ctheory4  CRT1  CRT2  CRT3  health_cond  ladder  mor_circle  moralid1  moralid10  moralid2  moralid3  moralid4  moralid5  moralid6  moralid7  moralid8  moralid9  mcoop2  mcoop3  mcoop4  mcoop5  mcoop6  mcoop7  narc2  narc3  narc4  narc5  narc6  nidentity1  nidentity2  omind1  omind3  omind4  omind5  omind6  contact1  contact2  contact3  contact4  contact5  hygiene4  psupport1  psupport2  psupport3  political_ideology  happy  slf_ladder  riskperc1  riskperc2  self_esteem  sbelong2  sbelong4  optim1  optim2  slfcont1  slfcont3  slfcont4
##   6   9  age  children  cnarc2  cnarc3  ctheory1  ctheory2  ctheory3  ctheory4  CRT1  CRT2  CRT3  health_cond  ladder  mor_circle  moralid1  moralid10  moralid2  moralid3  moralid4  moralid5  moralid6  moralid7  moralid8  moralid9  mcoop2  mcoop3  mcoop4  mcoop5  mcoop6  mcoop7  narc2  narc3  narc4  narc5  narc6  nidentity1  nidentity2  omind1  omind3  omind4  omind5  omind6  contact1  contact2  contact3  contact4  contact5  hygiene4  psupport1  psupport2  psupport3  political_ideology  happy  slf_ladder  riskperc1  riskperc2  self_esteem  sbelong2  sbelong4  optim1  optim2  slfcont1  slfcont3  slfcont4
##   6   10  age  children  cnarc2  cnarc3  ctheory1  ctheory2  ctheory3  ctheory4  CRT1  CRT2  CRT3  health_cond  ladder  mor_circle  moralid1  moralid10  moralid2  moralid3  moralid4  moralid5  moralid6  moralid7  moralid8  moralid9  mcoop2  mcoop3  mcoop4  mcoop5  mcoop6  mcoop7  narc2  narc3  narc4  narc5  narc6  nidentity1  nidentity2  omind1  omind3  omind4  omind5  omind6  contact1  contact2  contact3  contact4  contact5  hygiene4  psupport1  psupport2  psupport3  political_ideology  happy  slf_ladder  riskperc1  riskperc2  self_esteem  sbelong2  sbelong4  optim1  optim2  slfcont1  slfcont3  slfcont4
##   7   1  age  children  cnarc2  cnarc3  ctheory1  ctheory2  ctheory3  ctheory4  CRT1  CRT2  CRT3  health_cond  ladder  mor_circle  moralid1  moralid10  moralid2  moralid3  moralid4  moralid5  moralid6  moralid7  moralid8  moralid9  mcoop2  mcoop3  mcoop4  mcoop5  mcoop6  mcoop7  narc2  narc3  narc4  narc5  narc6  nidentity1  nidentity2  omind1  omind3  omind4  omind5  omind6  contact1  contact2  contact3  contact4  contact5  hygiene4  psupport1  psupport2  psupport3  political_ideology  happy  slf_ladder  riskperc1  riskperc2  self_esteem  sbelong2  sbelong4  optim1  optim2  slfcont1  slfcont3  slfcont4
##   7   2  age  children  cnarc2  cnarc3  ctheory1  ctheory2  ctheory3  ctheory4  CRT1  CRT2  CRT3  health_cond  ladder  mor_circle  moralid1  moralid10  moralid2  moralid3  moralid4  moralid5  moralid6  moralid7  moralid8  moralid9  mcoop2  mcoop3  mcoop4  mcoop5  mcoop6  mcoop7  narc2  narc3  narc4  narc5  narc6  nidentity1  nidentity2  omind1  omind3  omind4  omind5  omind6  contact1  contact2  contact3  contact4  contact5  hygiene4  psupport1  psupport2  psupport3  political_ideology  happy  slf_ladder  riskperc1  riskperc2  self_esteem  sbelong2  sbelong4  optim1  optim2  slfcont1  slfcont3  slfcont4
##   7   3  age  children  cnarc2  cnarc3  ctheory1  ctheory2  ctheory3  ctheory4  CRT1  CRT2  CRT3  health_cond  ladder  mor_circle  moralid1  moralid10  moralid2  moralid3  moralid4  moralid5  moralid6  moralid7  moralid8  moralid9  mcoop2  mcoop3  mcoop4  mcoop5  mcoop6  mcoop7  narc2  narc3  narc4  narc5  narc6  nidentity1  nidentity2  omind1  omind3  omind4  omind5  omind6  contact1  contact2  contact3  contact4  contact5  hygiene4  psupport1  psupport2  psupport3  political_ideology  happy  slf_ladder  riskperc1  riskperc2  self_esteem  sbelong2  sbelong4  optim1  optim2  slfcont1  slfcont3  slfcont4
##   7   4  age  children  cnarc2  cnarc3  ctheory1  ctheory2  ctheory3  ctheory4  CRT1  CRT2  CRT3  health_cond  ladder  mor_circle  moralid1  moralid10  moralid2  moralid3  moralid4  moralid5  moralid6  moralid7  moralid8  moralid9  mcoop2  mcoop3  mcoop4  mcoop5  mcoop6  mcoop7  narc2  narc3  narc4  narc5  narc6  nidentity1  nidentity2  omind1  omind3  omind4  omind5  omind6  contact1  contact2  contact3  contact4  contact5  hygiene4  psupport1  psupport2  psupport3  political_ideology  happy  slf_ladder  riskperc1  riskperc2  self_esteem  sbelong2  sbelong4  optim1  optim2  slfcont1  slfcont3  slfcont4
##   7   5  age  children  cnarc2  cnarc3  ctheory1  ctheory2  ctheory3  ctheory4  CRT1  CRT2  CRT3  health_cond  ladder  mor_circle  moralid1  moralid10  moralid2  moralid3  moralid4  moralid5  moralid6  moralid7  moralid8  moralid9  mcoop2  mcoop3  mcoop4  mcoop5  mcoop6  mcoop7  narc2  narc3  narc4  narc5  narc6  nidentity1  nidentity2  omind1  omind3  omind4  omind5  omind6  contact1  contact2  contact3  contact4  contact5  hygiene4  psupport1  psupport2  psupport3  political_ideology  happy  slf_ladder  riskperc1  riskperc2  self_esteem  sbelong2  sbelong4  optim1  optim2  slfcont1  slfcont3  slfcont4
##   7   6  age  children  cnarc2  cnarc3  ctheory1  ctheory2  ctheory3  ctheory4  CRT1  CRT2  CRT3  health_cond  ladder  mor_circle  moralid1  moralid10  moralid2  moralid3  moralid4  moralid5  moralid6  moralid7  moralid8  moralid9  mcoop2  mcoop3  mcoop4  mcoop5  mcoop6  mcoop7  narc2  narc3  narc4  narc5  narc6  nidentity1  nidentity2  omind1  omind3  omind4  omind5  omind6  contact1  contact2  contact3  contact4  contact5  hygiene4  psupport1  psupport2  psupport3  political_ideology  happy  slf_ladder  riskperc1  riskperc2  self_esteem  sbelong2  sbelong4  optim1  optim2  slfcont1  slfcont3  slfcont4
##   7   7  age  children  cnarc2  cnarc3  ctheory1  ctheory2  ctheory3  ctheory4  CRT1  CRT2  CRT3  health_cond  ladder  mor_circle  moralid1  moralid10  moralid2  moralid3  moralid4  moralid5  moralid6  moralid7  moralid8  moralid9  mcoop2  mcoop3  mcoop4  mcoop5  mcoop6  mcoop7  narc2  narc3  narc4  narc5  narc6  nidentity1  nidentity2  omind1  omind3  omind4  omind5  omind6  contact1  contact2  contact3  contact4  contact5  hygiene4  psupport1  psupport2  psupport3  political_ideology  happy  slf_ladder  riskperc1  riskperc2  self_esteem  sbelong2  sbelong4  optim1  optim2  slfcont1  slfcont3  slfcont4
##   7   8  age  children  cnarc2  cnarc3  ctheory1  ctheory2  ctheory3  ctheory4  CRT1  CRT2  CRT3  health_cond  ladder  mor_circle  moralid1  moralid10  moralid2  moralid3  moralid4  moralid5  moralid6  moralid7  moralid8  moralid9  mcoop2  mcoop3  mcoop4  mcoop5  mcoop6  mcoop7  narc2  narc3  narc4  narc5  narc6  nidentity1  nidentity2  omind1  omind3  omind4  omind5  omind6  contact1  contact2  contact3  contact4  contact5  hygiene4  psupport1  psupport2  psupport3  political_ideology  happy  slf_ladder  riskperc1  riskperc2  self_esteem  sbelong2  sbelong4  optim1  optim2  slfcont1  slfcont3  slfcont4
##   7   9  age  children  cnarc2  cnarc3  ctheory1  ctheory2  ctheory3  ctheory4  CRT1  CRT2  CRT3  health_cond  ladder  mor_circle  moralid1  moralid10  moralid2  moralid3  moralid4  moralid5  moralid6  moralid7  moralid8  moralid9  mcoop2  mcoop3  mcoop4  mcoop5  mcoop6  mcoop7  narc2  narc3  narc4  narc5  narc6  nidentity1  nidentity2  omind1  omind3  omind4  omind5  omind6  contact1  contact2  contact3  contact4  contact5  hygiene4  psupport1  psupport2  psupport3  political_ideology  happy  slf_ladder  riskperc1  riskperc2  self_esteem  sbelong2  sbelong4  optim1  optim2  slfcont1  slfcont3  slfcont4
##   7   10  age  children  cnarc2  cnarc3  ctheory1  ctheory2  ctheory3  ctheory4  CRT1  CRT2  CRT3  health_cond  ladder  mor_circle  moralid1  moralid10  moralid2  moralid3  moralid4  moralid5  moralid6  moralid7  moralid8  moralid9  mcoop2  mcoop3  mcoop4  mcoop5  mcoop6  mcoop7  narc2  narc3  narc4  narc5  narc6  nidentity1  nidentity2  omind1  omind3  omind4  omind5  omind6  contact1  contact2  contact3  contact4  contact5  hygiene4  psupport1  psupport2  psupport3  political_ideology  happy  slf_ladder  riskperc1  riskperc2  self_esteem  sbelong2  sbelong4  optim1  optim2  slfcont1  slfcont3  slfcont4
##   8   1  age  children  cnarc2  cnarc3  ctheory1  ctheory2  ctheory3  ctheory4  CRT1  CRT2  CRT3  health_cond  ladder  mor_circle  moralid1  moralid10  moralid2  moralid3  moralid4  moralid5  moralid6  moralid7  moralid8  moralid9  mcoop2  mcoop3  mcoop4  mcoop5  mcoop6  mcoop7  narc2  narc3  narc4  narc5  narc6  nidentity1  nidentity2  omind1  omind3  omind4  omind5  omind6  contact1  contact2  contact3  contact4  contact5  hygiene4  psupport1  psupport2  psupport3  political_ideology  happy  slf_ladder  riskperc1  riskperc2  self_esteem  sbelong2  sbelong4  optim1  optim2  slfcont1  slfcont3  slfcont4
##   8   2  age  children  cnarc2  cnarc3  ctheory1  ctheory2  ctheory3  ctheory4  CRT1  CRT2  CRT3  health_cond  ladder  mor_circle  moralid1  moralid10  moralid2  moralid3  moralid4  moralid5  moralid6  moralid7  moralid8  moralid9  mcoop2  mcoop3  mcoop4  mcoop5  mcoop6  mcoop7  narc2  narc3  narc4  narc5  narc6  nidentity1  nidentity2  omind1  omind3  omind4  omind5  omind6  contact1  contact2  contact3  contact4  contact5  hygiene4  psupport1  psupport2  psupport3  political_ideology  happy  slf_ladder  riskperc1  riskperc2  self_esteem  sbelong2  sbelong4  optim1  optim2  slfcont1  slfcont3  slfcont4
##   8   3  age  children  cnarc2  cnarc3  ctheory1  ctheory2  ctheory3  ctheory4  CRT1  CRT2  CRT3  health_cond  ladder  mor_circle  moralid1  moralid10  moralid2  moralid3  moralid4  moralid5  moralid6  moralid7  moralid8  moralid9  mcoop2  mcoop3  mcoop4  mcoop5  mcoop6  mcoop7  narc2  narc3  narc4  narc5  narc6  nidentity1  nidentity2  omind1  omind3  omind4  omind5  omind6  contact1  contact2  contact3  contact4  contact5  hygiene4  psupport1  psupport2  psupport3  political_ideology  happy  slf_ladder  riskperc1  riskperc2  self_esteem  sbelong2  sbelong4  optim1  optim2  slfcont1  slfcont3  slfcont4
##   8   4  age  children  cnarc2  cnarc3  ctheory1  ctheory2  ctheory3  ctheory4  CRT1  CRT2  CRT3  health_cond  ladder  mor_circle  moralid1  moralid10  moralid2  moralid3  moralid4  moralid5  moralid6  moralid7  moralid8  moralid9  mcoop2  mcoop3  mcoop4  mcoop5  mcoop6  mcoop7  narc2  narc3  narc4  narc5  narc6  nidentity1  nidentity2  omind1  omind3  omind4  omind5  omind6  contact1  contact2  contact3  contact4  contact5  hygiene4  psupport1  psupport2  psupport3  political_ideology  happy  slf_ladder  riskperc1  riskperc2  self_esteem  sbelong2  sbelong4  optim1  optim2  slfcont1  slfcont3  slfcont4
##   8   5  age  children  cnarc2  cnarc3  ctheory1  ctheory2  ctheory3  ctheory4  CRT1  CRT2  CRT3  health_cond  ladder  mor_circle  moralid1  moralid10  moralid2  moralid3  moralid4  moralid5  moralid6  moralid7  moralid8  moralid9  mcoop2  mcoop3  mcoop4  mcoop5  mcoop6  mcoop7  narc2  narc3  narc4  narc5  narc6  nidentity1  nidentity2  omind1  omind3  omind4  omind5  omind6  contact1  contact2  contact3  contact4  contact5  hygiene4  psupport1  psupport2  psupport3  political_ideology  happy  slf_ladder  riskperc1  riskperc2  self_esteem  sbelong2  sbelong4  optim1  optim2  slfcont1  slfcont3  slfcont4
##   8   6  age  children  cnarc2  cnarc3  ctheory1  ctheory2  ctheory3  ctheory4  CRT1  CRT2  CRT3  health_cond  ladder  mor_circle  moralid1  moralid10  moralid2  moralid3  moralid4  moralid5  moralid6  moralid7  moralid8  moralid9  mcoop2  mcoop3  mcoop4  mcoop5  mcoop6  mcoop7  narc2  narc3  narc4  narc5  narc6  nidentity1  nidentity2  omind1  omind3  omind4  omind5  omind6  contact1  contact2  contact3  contact4  contact5  hygiene4  psupport1  psupport2  psupport3  political_ideology  happy  slf_ladder  riskperc1  riskperc2  self_esteem  sbelong2  sbelong4  optim1  optim2  slfcont1  slfcont3  slfcont4
##   8   7  age  children  cnarc2  cnarc3  ctheory1  ctheory2  ctheory3  ctheory4  CRT1  CRT2  CRT3  health_cond  ladder  mor_circle  moralid1  moralid10  moralid2  moralid3  moralid4  moralid5  moralid6  moralid7  moralid8  moralid9  mcoop2  mcoop3  mcoop4  mcoop5  mcoop6  mcoop7  narc2  narc3  narc4  narc5  narc6  nidentity1  nidentity2  omind1  omind3  omind4  omind5  omind6  contact1  contact2  contact3  contact4  contact5  hygiene4  psupport1  psupport2  psupport3  political_ideology  happy  slf_ladder  riskperc1  riskperc2  self_esteem  sbelong2  sbelong4  optim1  optim2  slfcont1  slfcont3  slfcont4
##   8   8  age  children  cnarc2  cnarc3  ctheory1  ctheory2  ctheory3  ctheory4  CRT1  CRT2  CRT3  health_cond  ladder  mor_circle  moralid1  moralid10  moralid2  moralid3  moralid4  moralid5  moralid6  moralid7  moralid8  moralid9  mcoop2  mcoop3  mcoop4  mcoop5  mcoop6  mcoop7  narc2  narc3  narc4  narc5  narc6  nidentity1  nidentity2  omind1  omind3  omind4  omind5  omind6  contact1  contact2  contact3  contact4  contact5  hygiene4  psupport1  psupport2  psupport3  political_ideology  happy  slf_ladder  riskperc1  riskperc2  self_esteem  sbelong2  sbelong4  optim1  optim2  slfcont1  slfcont3  slfcont4
##   8   9  age  children  cnarc2  cnarc3  ctheory1  ctheory2  ctheory3  ctheory4  CRT1  CRT2  CRT3  health_cond  ladder  mor_circle  moralid1  moralid10  moralid2  moralid3  moralid4  moralid5  moralid6  moralid7  moralid8  moralid9  mcoop2  mcoop3  mcoop4  mcoop5  mcoop6  mcoop7  narc2  narc3  narc4  narc5  narc6  nidentity1  nidentity2  omind1  omind3  omind4  omind5  omind6  contact1  contact2  contact3  contact4  contact5  hygiene4  psupport1  psupport2  psupport3  political_ideology  happy  slf_ladder  riskperc1  riskperc2  self_esteem  sbelong2  sbelong4  optim1  optim2  slfcont1  slfcont3  slfcont4
##   8   10  age  children  cnarc2  cnarc3  ctheory1  ctheory2  ctheory3  ctheory4  CRT1  CRT2  CRT3  health_cond  ladder  mor_circle  moralid1  moralid10  moralid2  moralid3  moralid4  moralid5  moralid6  moralid7  moralid8  moralid9  mcoop2  mcoop3  mcoop4  mcoop5  mcoop6  mcoop7  narc2  narc3  narc4  narc5  narc6  nidentity1  nidentity2  omind1  omind3  omind4  omind5  omind6  contact1  contact2  contact3  contact4  contact5  hygiene4  psupport1  psupport2  psupport3  political_ideology  happy  slf_ladder  riskperc1  riskperc2  self_esteem  sbelong2  sbelong4  optim1  optim2  slfcont1  slfcont3  slfcont4
##   9   1  age  children  cnarc2  cnarc3  ctheory1  ctheory2  ctheory3  ctheory4  CRT1  CRT2  CRT3  health_cond  ladder  mor_circle  moralid1  moralid10  moralid2  moralid3  moralid4  moralid5  moralid6  moralid7  moralid8  moralid9  mcoop2  mcoop3  mcoop4  mcoop5  mcoop6  mcoop7  narc2  narc3  narc4  narc5  narc6  nidentity1  nidentity2  omind1  omind3  omind4  omind5  omind6  contact1  contact2  contact3  contact4  contact5  hygiene4  psupport1  psupport2  psupport3  political_ideology  happy  slf_ladder  riskperc1  riskperc2  self_esteem  sbelong2  sbelong4  optim1  optim2  slfcont1  slfcont3  slfcont4
##   9   2  age  children  cnarc2  cnarc3  ctheory1  ctheory2  ctheory3  ctheory4  CRT1  CRT2  CRT3  health_cond  ladder  mor_circle  moralid1  moralid10  moralid2  moralid3  moralid4  moralid5  moralid6  moralid7  moralid8  moralid9  mcoop2  mcoop3  mcoop4  mcoop5  mcoop6  mcoop7  narc2  narc3  narc4  narc5  narc6  nidentity1  nidentity2  omind1  omind3  omind4  omind5  omind6  contact1  contact2  contact3  contact4  contact5  hygiene4  psupport1  psupport2  psupport3  political_ideology  happy  slf_ladder  riskperc1  riskperc2  self_esteem  sbelong2  sbelong4  optim1  optim2  slfcont1  slfcont3  slfcont4
##   9   3  age  children  cnarc2  cnarc3  ctheory1  ctheory2  ctheory3  ctheory4  CRT1  CRT2  CRT3  health_cond  ladder  mor_circle  moralid1  moralid10  moralid2  moralid3  moralid4  moralid5  moralid6  moralid7  moralid8  moralid9  mcoop2  mcoop3  mcoop4  mcoop5  mcoop6  mcoop7  narc2  narc3  narc4  narc5  narc6  nidentity1  nidentity2  omind1  omind3  omind4  omind5  omind6  contact1  contact2  contact3  contact4  contact5  hygiene4  psupport1  psupport2  psupport3  political_ideology  happy  slf_ladder  riskperc1  riskperc2  self_esteem  sbelong2  sbelong4  optim1  optim2  slfcont1  slfcont3  slfcont4
##   9   4  age  children  cnarc2  cnarc3  ctheory1  ctheory2  ctheory3  ctheory4  CRT1  CRT2  CRT3  health_cond  ladder  mor_circle  moralid1  moralid10  moralid2  moralid3  moralid4  moralid5  moralid6  moralid7  moralid8  moralid9  mcoop2  mcoop3  mcoop4  mcoop5  mcoop6  mcoop7  narc2  narc3  narc4  narc5  narc6  nidentity1  nidentity2  omind1  omind3  omind4  omind5  omind6  contact1  contact2  contact3  contact4  contact5  hygiene4  psupport1  psupport2  psupport3  political_ideology  happy  slf_ladder  riskperc1  riskperc2  self_esteem  sbelong2  sbelong4  optim1  optim2  slfcont1  slfcont3  slfcont4
##   9   5  age  children  cnarc2  cnarc3  ctheory1  ctheory2  ctheory3  ctheory4  CRT1  CRT2  CRT3  health_cond  ladder  mor_circle  moralid1  moralid10  moralid2  moralid3  moralid4  moralid5  moralid6  moralid7  moralid8  moralid9  mcoop2  mcoop3  mcoop4  mcoop5  mcoop6  mcoop7  narc2  narc3  narc4  narc5  narc6  nidentity1  nidentity2  omind1  omind3  omind4  omind5  omind6  contact1  contact2  contact3  contact4  contact5  hygiene4  psupport1  psupport2  psupport3  political_ideology  happy  slf_ladder  riskperc1  riskperc2  self_esteem  sbelong2  sbelong4  optim1  optim2  slfcont1  slfcont3  slfcont4
##   9   6  age  children  cnarc2  cnarc3  ctheory1  ctheory2  ctheory3  ctheory4  CRT1  CRT2  CRT3  health_cond  ladder  mor_circle  moralid1  moralid10  moralid2  moralid3  moralid4  moralid5  moralid6  moralid7  moralid8  moralid9  mcoop2  mcoop3  mcoop4  mcoop5  mcoop6  mcoop7  narc2  narc3  narc4  narc5  narc6  nidentity1  nidentity2  omind1  omind3  omind4  omind5  omind6  contact1  contact2  contact3  contact4  contact5  hygiene4  psupport1  psupport2  psupport3  political_ideology  happy  slf_ladder  riskperc1  riskperc2  self_esteem  sbelong2  sbelong4  optim1  optim2  slfcont1  slfcont3  slfcont4
##   9   7  age  children  cnarc2  cnarc3  ctheory1  ctheory2  ctheory3  ctheory4  CRT1  CRT2  CRT3  health_cond  ladder  mor_circle  moralid1  moralid10  moralid2  moralid3  moralid4  moralid5  moralid6  moralid7  moralid8  moralid9  mcoop2  mcoop3  mcoop4  mcoop5  mcoop6  mcoop7  narc2  narc3  narc4  narc5  narc6  nidentity1  nidentity2  omind1  omind3  omind4  omind5  omind6  contact1  contact2  contact3  contact4  contact5  hygiene4  psupport1  psupport2  psupport3  political_ideology  happy  slf_ladder  riskperc1  riskperc2  self_esteem  sbelong2  sbelong4  optim1  optim2  slfcont1  slfcont3  slfcont4
##   9   8  age  children  cnarc2  cnarc3  ctheory1  ctheory2  ctheory3  ctheory4  CRT1  CRT2  CRT3  health_cond  ladder  mor_circle  moralid1  moralid10  moralid2  moralid3  moralid4  moralid5  moralid6  moralid7  moralid8  moralid9  mcoop2  mcoop3  mcoop4  mcoop5  mcoop6  mcoop7  narc2  narc3  narc4  narc5  narc6  nidentity1  nidentity2  omind1  omind3  omind4  omind5  omind6  contact1  contact2  contact3  contact4  contact5  hygiene4  psupport1  psupport2  psupport3  political_ideology  happy  slf_ladder  riskperc1  riskperc2  self_esteem  sbelong2  sbelong4  optim1  optim2  slfcont1  slfcont3  slfcont4
##   9   9  age  children  cnarc2  cnarc3  ctheory1  ctheory2  ctheory3  ctheory4  CRT1  CRT2  CRT3  health_cond  ladder  mor_circle  moralid1  moralid10  moralid2  moralid3  moralid4  moralid5  moralid6  moralid7  moralid8  moralid9  mcoop2  mcoop3  mcoop4  mcoop5  mcoop6  mcoop7  narc2  narc3  narc4  narc5  narc6  nidentity1  nidentity2  omind1  omind3  omind4  omind5  omind6  contact1  contact2  contact3  contact4  contact5  hygiene4  psupport1  psupport2  psupport3  political_ideology  happy  slf_ladder  riskperc1  riskperc2  self_esteem  sbelong2  sbelong4  optim1  optim2  slfcont1  slfcont3  slfcont4
##   9   10  age  children  cnarc2  cnarc3  ctheory1  ctheory2  ctheory3  ctheory4  CRT1  CRT2  CRT3  health_cond  ladder  mor_circle  moralid1  moralid10  moralid2  moralid3  moralid4  moralid5  moralid6  moralid7  moralid8  moralid9  mcoop2  mcoop3  mcoop4  mcoop5  mcoop6  mcoop7  narc2  narc3  narc4  narc5  narc6  nidentity1  nidentity2  omind1  omind3  omind4  omind5  omind6  contact1  contact2  contact3  contact4  contact5  hygiene4  psupport1  psupport2  psupport3  political_ideology  happy  slf_ladder  riskperc1  riskperc2  self_esteem  sbelong2  sbelong4  optim1  optim2  slfcont1  slfcont3  slfcont4
##   10   1  age  children  cnarc2  cnarc3  ctheory1  ctheory2  ctheory3  ctheory4  CRT1  CRT2  CRT3  health_cond  ladder  mor_circle  moralid1  moralid10  moralid2  moralid3  moralid4  moralid5  moralid6  moralid7  moralid8  moralid9  mcoop2  mcoop3  mcoop4  mcoop5  mcoop6  mcoop7  narc2  narc3  narc4  narc5  narc6  nidentity1  nidentity2  omind1  omind3  omind4  omind5  omind6  contact1  contact2  contact3  contact4  contact5  hygiene4  psupport1  psupport2  psupport3  political_ideology  happy  slf_ladder  riskperc1  riskperc2  self_esteem  sbelong2  sbelong4  optim1  optim2  slfcont1  slfcont3  slfcont4
##   10   2  age  children  cnarc2  cnarc3  ctheory1  ctheory2  ctheory3  ctheory4  CRT1  CRT2  CRT3  health_cond  ladder  mor_circle  moralid1  moralid10  moralid2  moralid3  moralid4  moralid5  moralid6  moralid7  moralid8  moralid9  mcoop2  mcoop3  mcoop4  mcoop5  mcoop6  mcoop7  narc2  narc3  narc4  narc5  narc6  nidentity1  nidentity2  omind1  omind3  omind4  omind5  omind6  contact1  contact2  contact3  contact4  contact5  hygiene4  psupport1  psupport2  psupport3  political_ideology  happy  slf_ladder  riskperc1  riskperc2  self_esteem  sbelong2  sbelong4  optim1  optim2  slfcont1  slfcont3  slfcont4
##   10   3  age  children  cnarc2  cnarc3  ctheory1  ctheory2  ctheory3  ctheory4  CRT1  CRT2  CRT3  health_cond  ladder  mor_circle  moralid1  moralid10  moralid2  moralid3  moralid4  moralid5  moralid6  moralid7  moralid8  moralid9  mcoop2  mcoop3  mcoop4  mcoop5  mcoop6  mcoop7  narc2  narc3  narc4  narc5  narc6  nidentity1  nidentity2  omind1  omind3  omind4  omind5  omind6  contact1  contact2  contact3  contact4  contact5  hygiene4  psupport1  psupport2  psupport3  political_ideology  happy  slf_ladder  riskperc1  riskperc2  self_esteem  sbelong2  sbelong4  optim1  optim2  slfcont1  slfcont3  slfcont4
##   10   4  age  children  cnarc2  cnarc3  ctheory1  ctheory2  ctheory3  ctheory4  CRT1  CRT2  CRT3  health_cond  ladder  mor_circle  moralid1  moralid10  moralid2  moralid3  moralid4  moralid5  moralid6  moralid7  moralid8  moralid9  mcoop2  mcoop3  mcoop4  mcoop5  mcoop6  mcoop7  narc2  narc3  narc4  narc5  narc6  nidentity1  nidentity2  omind1  omind3  omind4  omind5  omind6  contact1  contact2  contact3  contact4  contact5  hygiene4  psupport1  psupport2  psupport3  political_ideology  happy  slf_ladder  riskperc1  riskperc2  self_esteem  sbelong2  sbelong4  optim1  optim2  slfcont1  slfcont3  slfcont4
##   10   5  age  children  cnarc2  cnarc3  ctheory1  ctheory2  ctheory3  ctheory4  CRT1  CRT2  CRT3  health_cond  ladder  mor_circle  moralid1  moralid10  moralid2  moralid3  moralid4  moralid5  moralid6  moralid7  moralid8  moralid9  mcoop2  mcoop3  mcoop4  mcoop5  mcoop6  mcoop7  narc2  narc3  narc4  narc5  narc6  nidentity1  nidentity2  omind1  omind3  omind4  omind5  omind6  contact1  contact2  contact3  contact4  contact5  hygiene4  psupport1  psupport2  psupport3  political_ideology  happy  slf_ladder  riskperc1  riskperc2  self_esteem  sbelong2  sbelong4  optim1  optim2  slfcont1  slfcont3  slfcont4
##   10   6  age  children  cnarc2  cnarc3  ctheory1  ctheory2  ctheory3  ctheory4  CRT1  CRT2  CRT3  health_cond  ladder  mor_circle  moralid1  moralid10  moralid2  moralid3  moralid4  moralid5  moralid6  moralid7  moralid8  moralid9  mcoop2  mcoop3  mcoop4  mcoop5  mcoop6  mcoop7  narc2  narc3  narc4  narc5  narc6  nidentity1  nidentity2  omind1  omind3  omind4  omind5  omind6  contact1  contact2  contact3  contact4  contact5  hygiene4  psupport1  psupport2  psupport3  political_ideology  happy  slf_ladder  riskperc1  riskperc2  self_esteem  sbelong2  sbelong4  optim1  optim2  slfcont1  slfcont3  slfcont4
##   10   7  age  children  cnarc2  cnarc3  ctheory1  ctheory2  ctheory3  ctheory4  CRT1  CRT2  CRT3  health_cond  ladder  mor_circle  moralid1  moralid10  moralid2  moralid3  moralid4  moralid5  moralid6  moralid7  moralid8  moralid9  mcoop2  mcoop3  mcoop4  mcoop5  mcoop6  mcoop7  narc2  narc3  narc4  narc5  narc6  nidentity1  nidentity2  omind1  omind3  omind4  omind5  omind6  contact1  contact2  contact3  contact4  contact5  hygiene4  psupport1  psupport2  psupport3  political_ideology  happy  slf_ladder  riskperc1  riskperc2  self_esteem  sbelong2  sbelong4  optim1  optim2  slfcont1  slfcont3  slfcont4
##   10   8  age  children  cnarc2  cnarc3  ctheory1  ctheory2  ctheory3  ctheory4  CRT1  CRT2  CRT3  health_cond  ladder  mor_circle  moralid1  moralid10  moralid2  moralid3  moralid4  moralid5  moralid6  moralid7  moralid8  moralid9  mcoop2  mcoop3  mcoop4  mcoop5  mcoop6  mcoop7  narc2  narc3  narc4  narc5  narc6  nidentity1  nidentity2  omind1  omind3  omind4  omind5  omind6  contact1  contact2  contact3  contact4  contact5  hygiene4  psupport1  psupport2  psupport3  political_ideology  happy  slf_ladder  riskperc1  riskperc2  self_esteem  sbelong2  sbelong4  optim1  optim2  slfcont1  slfcont3  slfcont4
##   10   9  age  children  cnarc2  cnarc3  ctheory1  ctheory2  ctheory3  ctheory4  CRT1  CRT2  CRT3  health_cond  ladder  mor_circle  moralid1  moralid10  moralid2  moralid3  moralid4  moralid5  moralid6  moralid7  moralid8  moralid9  mcoop2  mcoop3  mcoop4  mcoop5  mcoop6  mcoop7  narc2  narc3  narc4  narc5  narc6  nidentity1  nidentity2  omind1  omind3  omind4  omind5  omind6  contact1  contact2  contact3  contact4  contact5  hygiene4  psupport1  psupport2  psupport3  political_ideology  happy  slf_ladder  riskperc1  riskperc2  self_esteem  sbelong2  sbelong4  optim1  optim2  slfcont1  slfcont3  slfcont4
##   10   10  age  children  cnarc2  cnarc3  ctheory1  ctheory2  ctheory3  ctheory4  CRT1  CRT2  CRT3  health_cond  ladder  mor_circle  moralid1  moralid10  moralid2  moralid3  moralid4  moralid5  moralid6  moralid7  moralid8  moralid9  mcoop2  mcoop3  mcoop4  mcoop5  mcoop6  mcoop7  narc2  narc3  narc4  narc5  narc6  nidentity1  nidentity2  omind1  omind3  omind4  omind5  omind6  contact1  contact2  contact3  contact4  contact5  hygiene4  psupport1  psupport2  psupport3  political_ideology  happy  slf_ladder  riskperc1  riskperc2  self_esteem  sbelong2  sbelong4  optim1  optim2  slfcont1  slfcont3  slfcont4
```

```
## Warning: Number of logged events: 6403
```

```
## 
##  iter imp variable
##   1   1  children  CRT1  CRT2  CRT3  moralid2  moralid5  moralid6  moralid8  contact2  hygiene2  hygiene3  hygiene4  political_ideology  happy  optim2
##   1   2  children  CRT1  CRT2  CRT3  moralid2  moralid5  moralid6  moralid8  contact2  hygiene2  hygiene3  hygiene4  political_ideology  happy  optim2
##   1   3  children  CRT1  CRT2  CRT3  moralid2  moralid5  moralid6  moralid8  contact2  hygiene2  hygiene3  hygiene4  political_ideology  happy  optim2
##   1   4  children  CRT1  CRT2  CRT3  moralid2  moralid5  moralid6  moralid8  contact2  hygiene2  hygiene3  hygiene4  political_ideology  happy  optim2
##   1   5  children  CRT1  CRT2  CRT3  moralid2  moralid5  moralid6  moralid8  contact2  hygiene2  hygiene3  hygiene4  political_ideology  happy  optim2
##   1   6  children  CRT1  CRT2  CRT3  moralid2  moralid5  moralid6  moralid8  contact2  hygiene2  hygiene3  hygiene4  political_ideology  happy  optim2
##   1   7  children  CRT1  CRT2  CRT3  moralid2  moralid5  moralid6  moralid8  contact2  hygiene2  hygiene3  hygiene4  political_ideology  happy  optim2
##   1   8  children  CRT1  CRT2  CRT3  moralid2  moralid5  moralid6  moralid8  contact2  hygiene2  hygiene3  hygiene4  political_ideology  happy  optim2
##   1   9  children  CRT1  CRT2  CRT3  moralid2  moralid5  moralid6  moralid8  contact2  hygiene2  hygiene3  hygiene4  political_ideology  happy  optim2
##   1   10  children  CRT1  CRT2  CRT3  moralid2  moralid5  moralid6  moralid8  contact2  hygiene2  hygiene3  hygiene4  political_ideology  happy  optim2
##   2   1  children  CRT1  CRT2  CRT3  moralid2  moralid5  moralid6  moralid8  contact2  hygiene2  hygiene3  hygiene4  political_ideology  happy  optim2
##   2   2  children  CRT1  CRT2  CRT3  moralid2  moralid5  moralid6  moralid8  contact2  hygiene2  hygiene3  hygiene4  political_ideology  happy  optim2
##   2   3  children  CRT1  CRT2  CRT3  moralid2  moralid5  moralid6  moralid8  contact2  hygiene2  hygiene3  hygiene4  political_ideology  happy  optim2
##   2   4  children  CRT1  CRT2  CRT3  moralid2  moralid5  moralid6  moralid8  contact2  hygiene2  hygiene3  hygiene4  political_ideology  happy  optim2
##   2   5  children  CRT1  CRT2  CRT3  moralid2  moralid5  moralid6  moralid8  contact2  hygiene2  hygiene3  hygiene4  political_ideology  happy  optim2
##   2   6  children  CRT1  CRT2  CRT3  moralid2  moralid5  moralid6  moralid8  contact2  hygiene2  hygiene3  hygiene4  political_ideology  happy  optim2
##   2   7  children  CRT1  CRT2  CRT3  moralid2  moralid5  moralid6  moralid8  contact2  hygiene2  hygiene3  hygiene4  political_ideology  happy  optim2
##   2   8  children  CRT1  CRT2  CRT3  moralid2  moralid5  moralid6  moralid8  contact2  hygiene2  hygiene3  hygiene4  political_ideology  happy  optim2
##   2   9  children  CRT1  CRT2  CRT3  moralid2  moralid5  moralid6  moralid8  contact2  hygiene2  hygiene3  hygiene4  political_ideology  happy  optim2
##   2   10  children  CRT1  CRT2  CRT3  moralid2  moralid5  moralid6  moralid8  contact2  hygiene2  hygiene3  hygiene4  political_ideology  happy  optim2
##   3   1  children  CRT1  CRT2  CRT3  moralid2  moralid5  moralid6  moralid8  contact2  hygiene2  hygiene3  hygiene4  political_ideology  happy  optim2
##   3   2  children  CRT1  CRT2  CRT3  moralid2  moralid5  moralid6  moralid8  contact2  hygiene2  hygiene3  hygiene4  political_ideology  happy  optim2
##   3   3  children  CRT1  CRT2  CRT3  moralid2  moralid5  moralid6  moralid8  contact2  hygiene2  hygiene3  hygiene4  political_ideology  happy  optim2
##   3   4  children  CRT1  CRT2  CRT3  moralid2  moralid5  moralid6  moralid8  contact2  hygiene2  hygiene3  hygiene4  political_ideology  happy  optim2
##   3   5  children  CRT1  CRT2  CRT3  moralid2  moralid5  moralid6  moralid8  contact2  hygiene2  hygiene3  hygiene4  political_ideology  happy  optim2
##   3   6  children  CRT1  CRT2  CRT3  moralid2  moralid5  moralid6  moralid8  contact2  hygiene2  hygiene3  hygiene4  political_ideology  happy  optim2
##   3   7  children  CRT1  CRT2  CRT3  moralid2  moralid5  moralid6  moralid8  contact2  hygiene2  hygiene3  hygiene4  political_ideology  happy  optim2
##   3   8  children  CRT1  CRT2  CRT3  moralid2  moralid5  moralid6  moralid8  contact2  hygiene2  hygiene3  hygiene4  political_ideology  happy  optim2
##   3   9  children  CRT1  CRT2  CRT3  moralid2  moralid5  moralid6  moralid8  contact2  hygiene2  hygiene3  hygiene4  political_ideology  happy  optim2
##   3   10  children  CRT1  CRT2  CRT3  moralid2  moralid5  moralid6  moralid8  contact2  hygiene2  hygiene3  hygiene4  political_ideology  happy  optim2
##   4   1  children  CRT1  CRT2  CRT3  moralid2  moralid5  moralid6  moralid8  contact2  hygiene2  hygiene3  hygiene4  political_ideology  happy  optim2
##   4   2  children  CRT1  CRT2  CRT3  moralid2  moralid5  moralid6  moralid8  contact2  hygiene2  hygiene3  hygiene4  political_ideology  happy  optim2
##   4   3  children  CRT1  CRT2  CRT3  moralid2  moralid5  moralid6  moralid8  contact2  hygiene2  hygiene3  hygiene4  political_ideology  happy  optim2
##   4   4  children  CRT1  CRT2  CRT3  moralid2  moralid5  moralid6  moralid8  contact2  hygiene2  hygiene3  hygiene4  political_ideology  happy  optim2
##   4   5  children  CRT1  CRT2  CRT3  moralid2  moralid5  moralid6  moralid8  contact2  hygiene2  hygiene3  hygiene4  political_ideology  happy  optim2
##   4   6  children  CRT1  CRT2  CRT3  moralid2  moralid5  moralid6  moralid8  contact2  hygiene2  hygiene3  hygiene4  political_ideology  happy  optim2
##   4   7  children  CRT1  CRT2  CRT3  moralid2  moralid5  moralid6  moralid8  contact2  hygiene2  hygiene3  hygiene4  political_ideology  happy  optim2
##   4   8  children  CRT1  CRT2  CRT3  moralid2  moralid5  moralid6  moralid8  contact2  hygiene2  hygiene3  hygiene4  political_ideology  happy  optim2
##   4   9  children  CRT1  CRT2  CRT3  moralid2  moralid5  moralid6  moralid8  contact2  hygiene2  hygiene3  hygiene4  political_ideology  happy  optim2
##   4   10  children  CRT1  CRT2  CRT3  moralid2  moralid5  moralid6  moralid8  contact2  hygiene2  hygiene3  hygiene4  political_ideology  happy  optim2
##   5   1  children  CRT1  CRT2  CRT3  moralid2  moralid5  moralid6  moralid8  contact2  hygiene2  hygiene3  hygiene4  political_ideology  happy  optim2
##   5   2  children  CRT1  CRT2  CRT3  moralid2  moralid5  moralid6  moralid8  contact2  hygiene2  hygiene3  hygiene4  political_ideology  happy  optim2
##   5   3  children  CRT1  CRT2  CRT3  moralid2  moralid5  moralid6  moralid8  contact2  hygiene2  hygiene3  hygiene4  political_ideology  happy  optim2
##   5   4  children  CRT1  CRT2  CRT3  moralid2  moralid5  moralid6  moralid8  contact2  hygiene2  hygiene3  hygiene4  political_ideology  happy  optim2
##   5   5  children  CRT1  CRT2  CRT3  moralid2  moralid5  moralid6  moralid8  contact2  hygiene2  hygiene3  hygiene4  political_ideology  happy  optim2
##   5   6  children  CRT1  CRT2  CRT3  moralid2  moralid5  moralid6  moralid8  contact2  hygiene2  hygiene3  hygiene4  political_ideology  happy  optim2
##   5   7  children  CRT1  CRT2  CRT3  moralid2  moralid5  moralid6  moralid8  contact2  hygiene2  hygiene3  hygiene4  political_ideology  happy  optim2
##   5   8  children  CRT1  CRT2  CRT3  moralid2  moralid5  moralid6  moralid8  contact2  hygiene2  hygiene3  hygiene4  political_ideology  happy  optim2
##   5   9  children  CRT1  CRT2  CRT3  moralid2  moralid5  moralid6  moralid8  contact2  hygiene2  hygiene3  hygiene4  political_ideology  happy  optim2
##   5   10  children  CRT1  CRT2  CRT3  moralid2  moralid5  moralid6  moralid8  contact2  hygiene2  hygiene3  hygiene4  political_ideology  happy  optim2
##   6   1  children  CRT1  CRT2  CRT3  moralid2  moralid5  moralid6  moralid8  contact2  hygiene2  hygiene3  hygiene4  political_ideology  happy  optim2
##   6   2  children  CRT1  CRT2  CRT3  moralid2  moralid5  moralid6  moralid8  contact2  hygiene2  hygiene3  hygiene4  political_ideology  happy  optim2
##   6   3  children  CRT1  CRT2  CRT3  moralid2  moralid5  moralid6  moralid8  contact2  hygiene2  hygiene3  hygiene4  political_ideology  happy  optim2
##   6   4  children  CRT1  CRT2  CRT3  moralid2  moralid5  moralid6  moralid8  contact2  hygiene2  hygiene3  hygiene4  political_ideology  happy  optim2
##   6   5  children  CRT1  CRT2  CRT3  moralid2  moralid5  moralid6  moralid8  contact2  hygiene2  hygiene3  hygiene4  political_ideology  happy  optim2
##   6   6  children  CRT1  CRT2  CRT3  moralid2  moralid5  moralid6  moralid8  contact2  hygiene2  hygiene3  hygiene4  political_ideology  happy  optim2
##   6   7  children  CRT1  CRT2  CRT3  moralid2  moralid5  moralid6  moralid8  contact2  hygiene2  hygiene3  hygiene4  political_ideology  happy  optim2
##   6   8  children  CRT1  CRT2  CRT3  moralid2  moralid5  moralid6  moralid8  contact2  hygiene2  hygiene3  hygiene4  political_ideology  happy  optim2
##   6   9  children  CRT1  CRT2  CRT3  moralid2  moralid5  moralid6  moralid8  contact2  hygiene2  hygiene3  hygiene4  political_ideology  happy  optim2
##   6   10  children  CRT1  CRT2  CRT3  moralid2  moralid5  moralid6  moralid8  contact2  hygiene2  hygiene3  hygiene4  political_ideology  happy  optim2
##   7   1  children  CRT1  CRT2  CRT3  moralid2  moralid5  moralid6  moralid8  contact2  hygiene2  hygiene3  hygiene4  political_ideology  happy  optim2
##   7   2  children  CRT1  CRT2  CRT3  moralid2  moralid5  moralid6  moralid8  contact2  hygiene2  hygiene3  hygiene4  political_ideology  happy  optim2
##   7   3  children  CRT1  CRT2  CRT3  moralid2  moralid5  moralid6  moralid8  contact2  hygiene2  hygiene3  hygiene4  political_ideology  happy  optim2
##   7   4  children  CRT1  CRT2  CRT3  moralid2  moralid5  moralid6  moralid8  contact2  hygiene2  hygiene3  hygiene4  political_ideology  happy  optim2
##   7   5  children  CRT1  CRT2  CRT3  moralid2  moralid5  moralid6  moralid8  contact2  hygiene2  hygiene3  hygiene4  political_ideology  happy  optim2
##   7   6  children  CRT1  CRT2  CRT3  moralid2  moralid5  moralid6  moralid8  contact2  hygiene2  hygiene3  hygiene4  political_ideology  happy  optim2
##   7   7  children  CRT1  CRT2  CRT3  moralid2  moralid5  moralid6  moralid8  contact2  hygiene2  hygiene3  hygiene4  political_ideology  happy  optim2
##   7   8  children  CRT1  CRT2  CRT3  moralid2  moralid5  moralid6  moralid8  contact2  hygiene2  hygiene3  hygiene4  political_ideology  happy  optim2
##   7   9  children  CRT1  CRT2  CRT3  moralid2  moralid5  moralid6  moralid8  contact2  hygiene2  hygiene3  hygiene4  political_ideology  happy  optim2
##   7   10  children  CRT1  CRT2  CRT3  moralid2  moralid5  moralid6  moralid8  contact2  hygiene2  hygiene3  hygiene4  political_ideology  happy  optim2
##   8   1  children  CRT1  CRT2  CRT3  moralid2  moralid5  moralid6  moralid8  contact2  hygiene2  hygiene3  hygiene4  political_ideology  happy  optim2
##   8   2  children  CRT1  CRT2  CRT3  moralid2  moralid5  moralid6  moralid8  contact2  hygiene2  hygiene3  hygiene4  political_ideology  happy  optim2
##   8   3  children  CRT1  CRT2  CRT3  moralid2  moralid5  moralid6  moralid8  contact2  hygiene2  hygiene3  hygiene4  political_ideology  happy  optim2
##   8   4  children  CRT1  CRT2  CRT3  moralid2  moralid5  moralid6  moralid8  contact2  hygiene2  hygiene3  hygiene4  political_ideology  happy  optim2
##   8   5  children  CRT1  CRT2  CRT3  moralid2  moralid5  moralid6  moralid8  contact2  hygiene2  hygiene3  hygiene4  political_ideology  happy  optim2
##   8   6  children  CRT1  CRT2  CRT3  moralid2  moralid5  moralid6  moralid8  contact2  hygiene2  hygiene3  hygiene4  political_ideology  happy  optim2
##   8   7  children  CRT1  CRT2  CRT3  moralid2  moralid5  moralid6  moralid8  contact2  hygiene2  hygiene3  hygiene4  political_ideology  happy  optim2
##   8   8  children  CRT1  CRT2  CRT3  moralid2  moralid5  moralid6  moralid8  contact2  hygiene2  hygiene3  hygiene4  political_ideology  happy  optim2
##   8   9  children  CRT1  CRT2  CRT3  moralid2  moralid5  moralid6  moralid8  contact2  hygiene2  hygiene3  hygiene4  political_ideology  happy  optim2
##   8   10  children  CRT1  CRT2  CRT3  moralid2  moralid5  moralid6  moralid8  contact2  hygiene2  hygiene3  hygiene4  political_ideology  happy  optim2
##   9   1  children  CRT1  CRT2  CRT3  moralid2  moralid5  moralid6  moralid8  contact2  hygiene2  hygiene3  hygiene4  political_ideology  happy  optim2
##   9   2  children  CRT1  CRT2  CRT3  moralid2  moralid5  moralid6  moralid8  contact2  hygiene2  hygiene3  hygiene4  political_ideology  happy  optim2
##   9   3  children  CRT1  CRT2  CRT3  moralid2  moralid5  moralid6  moralid8  contact2  hygiene2  hygiene3  hygiene4  political_ideology  happy  optim2
##   9   4  children  CRT1  CRT2  CRT3  moralid2  moralid5  moralid6  moralid8  contact2  hygiene2  hygiene3  hygiene4  political_ideology  happy  optim2
##   9   5  children  CRT1  CRT2  CRT3  moralid2  moralid5  moralid6  moralid8  contact2  hygiene2  hygiene3  hygiene4  political_ideology  happy  optim2
##   9   6  children  CRT1  CRT2  CRT3  moralid2  moralid5  moralid6  moralid8  contact2  hygiene2  hygiene3  hygiene4  political_ideology  happy  optim2
##   9   7  children  CRT1  CRT2  CRT3  moralid2  moralid5  moralid6  moralid8  contact2  hygiene2  hygiene3  hygiene4  political_ideology  happy  optim2
##   9   8  children  CRT1  CRT2  CRT3  moralid2  moralid5  moralid6  moralid8  contact2  hygiene2  hygiene3  hygiene4  political_ideology  happy  optim2
##   9   9  children  CRT1  CRT2  CRT3  moralid2  moralid5  moralid6  moralid8  contact2  hygiene2  hygiene3  hygiene4  political_ideology  happy  optim2
##   9   10  children  CRT1  CRT2  CRT3  moralid2  moralid5  moralid6  moralid8  contact2  hygiene2  hygiene3  hygiene4  political_ideology  happy  optim2
##   10   1  children  CRT1  CRT2  CRT3  moralid2  moralid5  moralid6  moralid8  contact2  hygiene2  hygiene3  hygiene4  political_ideology  happy  optim2
##   10   2  children  CRT1  CRT2  CRT3  moralid2  moralid5  moralid6  moralid8  contact2  hygiene2  hygiene3  hygiene4  political_ideology  happy  optim2
##   10   3  children  CRT1  CRT2  CRT3  moralid2  moralid5  moralid6  moralid8  contact2  hygiene2  hygiene3  hygiene4  political_ideology  happy  optim2
##   10   4  children  CRT1  CRT2  CRT3  moralid2  moralid5  moralid6  moralid8  contact2  hygiene2  hygiene3  hygiene4  political_ideology  happy  optim2
##   10   5  children  CRT1  CRT2  CRT3  moralid2  moralid5  moralid6  moralid8  contact2  hygiene2  hygiene3  hygiene4  political_ideology  happy  optim2
##   10   6  children  CRT1  CRT2  CRT3  moralid2  moralid5  moralid6  moralid8  contact2  hygiene2  hygiene3  hygiene4  political_ideology  happy  optim2
##   10   7  children  CRT1  CRT2  CRT3  moralid2  moralid5  moralid6  moralid8  contact2  hygiene2  hygiene3  hygiene4  political_ideology  happy  optim2
##   10   8  children  CRT1  CRT2  CRT3  moralid2  moralid5  moralid6  moralid8  contact2  hygiene2  hygiene3  hygiene4  political_ideology  happy  optim2
##   10   9  children  CRT1  CRT2  CRT3  moralid2  moralid5  moralid6  moralid8  contact2  hygiene2  hygiene3  hygiene4  political_ideology  happy  optim2
##   10   10  children  CRT1  CRT2  CRT3  moralid2  moralid5  moralid6  moralid8  contact2  hygiene2  hygiene3  hygiene4  political_ideology  happy  optim2
```

```
## Warning: Number of logged events: 1503
```

```
## 
##  iter imp variable
##   1   1  CRT3  generosity1  generosity2  generosity3  riskperc2
##   1   2  CRT3  generosity1  generosity2  generosity3  riskperc2
##   1   3  CRT3  generosity1  generosity2  generosity3  riskperc2
##   1   4  CRT3  generosity1  generosity2  generosity3  riskperc2
##   1   5  CRT3  generosity1  generosity2  generosity3  riskperc2
##   1   6  CRT3  generosity1  generosity2  generosity3  riskperc2
##   1   7  CRT3  generosity1  generosity2  generosity3  riskperc2
##   1   8  CRT3  generosity1  generosity2  generosity3  riskperc2
##   1   9  CRT3  generosity1  generosity2  generosity3  riskperc2
##   1   10  CRT3  generosity1  generosity2  generosity3  riskperc2
##   2   1  CRT3  generosity1  generosity2  generosity3  riskperc2
##   2   2  CRT3  generosity1  generosity2  generosity3  riskperc2
##   2   3  CRT3  generosity1  generosity2  generosity3  riskperc2
##   2   4  CRT3  generosity1  generosity2  generosity3  riskperc2
##   2   5  CRT3  generosity1  generosity2  generosity3  riskperc2
##   2   6  CRT3  generosity1  generosity2  generosity3  riskperc2
##   2   7  CRT3  generosity1  generosity2  generosity3  riskperc2
##   2   8  CRT3  generosity1  generosity2  generosity3  riskperc2
##   2   9  CRT3  generosity1  generosity2  generosity3  riskperc2
##   2   10  CRT3  generosity1  generosity2  generosity3  riskperc2
##   3   1  CRT3  generosity1  generosity2  generosity3  riskperc2
##   3   2  CRT3  generosity1  generosity2  generosity3  riskperc2
##   3   3  CRT3  generosity1  generosity2  generosity3  riskperc2
##   3   4  CRT3  generosity1  generosity2  generosity3  riskperc2
##   3   5  CRT3  generosity1  generosity2  generosity3  riskperc2
##   3   6  CRT3  generosity1  generosity2  generosity3  riskperc2
##   3   7  CRT3  generosity1  generosity2  generosity3  riskperc2
##   3   8  CRT3  generosity1  generosity2  generosity3  riskperc2
##   3   9  CRT3  generosity1  generosity2  generosity3  riskperc2
##   3   10  CRT3  generosity1  generosity2  generosity3  riskperc2
##   4   1  CRT3  generosity1  generosity2  generosity3  riskperc2
##   4   2  CRT3  generosity1  generosity2  generosity3  riskperc2
##   4   3  CRT3  generosity1  generosity2  generosity3  riskperc2
##   4   4  CRT3  generosity1  generosity2  generosity3  riskperc2
##   4   5  CRT3  generosity1  generosity2  generosity3  riskperc2
##   4   6  CRT3  generosity1  generosity2  generosity3  riskperc2
##   4   7  CRT3  generosity1  generosity2  generosity3  riskperc2
##   4   8  CRT3  generosity1  generosity2  generosity3  riskperc2
##   4   9  CRT3  generosity1  generosity2  generosity3  riskperc2
##   4   10  CRT3  generosity1  generosity2  generosity3  riskperc2
##   5   1  CRT3  generosity1  generosity2  generosity3  riskperc2
##   5   2  CRT3  generosity1  generosity2  generosity3  riskperc2
##   5   3  CRT3  generosity1  generosity2  generosity3  riskperc2
##   5   4  CRT3  generosity1  generosity2  generosity3  riskperc2
##   5   5  CRT3  generosity1  generosity2  generosity3  riskperc2
##   5   6  CRT3  generosity1  generosity2  generosity3  riskperc2
##   5   7  CRT3  generosity1  generosity2  generosity3  riskperc2
##   5   8  CRT3  generosity1  generosity2  generosity3  riskperc2
##   5   9  CRT3  generosity1  generosity2  generosity3  riskperc2
##   5   10  CRT3  generosity1  generosity2  generosity3  riskperc2
##   6   1  CRT3  generosity1  generosity2  generosity3  riskperc2
##   6   2  CRT3  generosity1  generosity2  generosity3  riskperc2
##   6   3  CRT3  generosity1  generosity2  generosity3  riskperc2
##   6   4  CRT3  generosity1  generosity2  generosity3  riskperc2
##   6   5  CRT3  generosity1  generosity2  generosity3  riskperc2
##   6   6  CRT3  generosity1  generosity2  generosity3  riskperc2
##   6   7  CRT3  generosity1  generosity2  generosity3  riskperc2
##   6   8  CRT3  generosity1  generosity2  generosity3  riskperc2
##   6   9  CRT3  generosity1  generosity2  generosity3  riskperc2
##   6   10  CRT3  generosity1  generosity2  generosity3  riskperc2
##   7   1  CRT3  generosity1  generosity2  generosity3  riskperc2
##   7   2  CRT3  generosity1  generosity2  generosity3  riskperc2
##   7   3  CRT3  generosity1  generosity2  generosity3  riskperc2
##   7   4  CRT3  generosity1  generosity2  generosity3  riskperc2
##   7   5  CRT3  generosity1  generosity2  generosity3  riskperc2
##   7   6  CRT3  generosity1  generosity2  generosity3  riskperc2
##   7   7  CRT3  generosity1  generosity2  generosity3  riskperc2
##   7   8  CRT3  generosity1  generosity2  generosity3  riskperc2
##   7   9  CRT3  generosity1  generosity2  generosity3  riskperc2
##   7   10  CRT3  generosity1  generosity2  generosity3  riskperc2
##   8   1  CRT3  generosity1  generosity2  generosity3  riskperc2
##   8   2  CRT3  generosity1  generosity2  generosity3  riskperc2
##   8   3  CRT3  generosity1  generosity2  generosity3  riskperc2
##   8   4  CRT3  generosity1  generosity2  generosity3  riskperc2
##   8   5  CRT3  generosity1  generosity2  generosity3  riskperc2
##   8   6  CRT3  generosity1  generosity2  generosity3  riskperc2
##   8   7  CRT3  generosity1  generosity2  generosity3  riskperc2
##   8   8  CRT3  generosity1  generosity2  generosity3  riskperc2
##   8   9  CRT3  generosity1  generosity2  generosity3  riskperc2
##   8   10  CRT3  generosity1  generosity2  generosity3  riskperc2
##   9   1  CRT3  generosity1  generosity2  generosity3  riskperc2
##   9   2  CRT3  generosity1  generosity2  generosity3  riskperc2
##   9   3  CRT3  generosity1  generosity2  generosity3  riskperc2
##   9   4  CRT3  generosity1  generosity2  generosity3  riskperc2
##   9   5  CRT3  generosity1  generosity2  generosity3  riskperc2
##   9   6  CRT3  generosity1  generosity2  generosity3  riskperc2
##   9   7  CRT3  generosity1  generosity2  generosity3  riskperc2
##   9   8  CRT3  generosity1  generosity2  generosity3  riskperc2
##   9   9  CRT3  generosity1  generosity2  generosity3  riskperc2
##   9   10  CRT3  generosity1  generosity2  generosity3  riskperc2
##   10   1  CRT3  generosity1  generosity2  generosity3  riskperc2
##   10   2  CRT3  generosity1  generosity2  generosity3  riskperc2
##   10   3  CRT3  generosity1  generosity2  generosity3  riskperc2
##   10   4  CRT3  generosity1  generosity2  generosity3  riskperc2
##   10   5  CRT3  generosity1  generosity2  generosity3  riskperc2
##   10   6  CRT3  generosity1  generosity2  generosity3  riskperc2
##   10   7  CRT3  generosity1  generosity2  generosity3  riskperc2
##   10   8  CRT3  generosity1  generosity2  generosity3  riskperc2
##   10   9  CRT3  generosity1  generosity2  generosity3  riskperc2
##   10   10  CRT3  generosity1  generosity2  generosity3  riskperc2
```

```
## Warning: Number of logged events: 194
```

```
## 
##  iter imp variable
##   1   1  age  CRT1  CRT2  CRT3
##   1   2  age  CRT1  CRT2  CRT3
##   1   3  age  CRT1  CRT2  CRT3
##   1   4  age  CRT1  CRT2  CRT3
##   1   5  age  CRT1  CRT2  CRT3
##   1   6  age  CRT1  CRT2  CRT3
##   1   7  age  CRT1  CRT2  CRT3
##   1   8  age  CRT1  CRT2  CRT3
##   1   9  age  CRT1  CRT2  CRT3
##   1   10  age  CRT1  CRT2  CRT3
##   2   1  age  CRT1  CRT2  CRT3
##   2   2  age  CRT1  CRT2  CRT3
##   2   3  age  CRT1  CRT2  CRT3
##   2   4  age  CRT1  CRT2  CRT3
##   2   5  age  CRT1  CRT2  CRT3
##   2   6  age  CRT1  CRT2  CRT3
##   2   7  age  CRT1  CRT2  CRT3
##   2   8  age  CRT1  CRT2  CRT3
##   2   9  age  CRT1  CRT2  CRT3
##   2   10  age  CRT1  CRT2  CRT3
##   3   1  age  CRT1  CRT2  CRT3
##   3   2  age  CRT1  CRT2  CRT3
##   3   3  age  CRT1  CRT2  CRT3
##   3   4  age  CRT1  CRT2  CRT3
##   3   5  age  CRT1  CRT2  CRT3
##   3   6  age  CRT1  CRT2  CRT3
##   3   7  age  CRT1  CRT2  CRT3
##   3   8  age  CRT1  CRT2  CRT3
##   3   9  age  CRT1  CRT2  CRT3
##   3   10  age  CRT1  CRT2  CRT3
##   4   1  age  CRT1  CRT2  CRT3
##   4   2  age  CRT1  CRT2  CRT3
##   4   3  age  CRT1  CRT2  CRT3
##   4   4  age  CRT1  CRT2  CRT3
##   4   5  age  CRT1  CRT2  CRT3
##   4   6  age  CRT1  CRT2  CRT3
##   4   7  age  CRT1  CRT2  CRT3
##   4   8  age  CRT1  CRT2  CRT3
##   4   9  age  CRT1  CRT2  CRT3
##   4   10  age  CRT1  CRT2  CRT3
##   5   1  age  CRT1  CRT2  CRT3
##   5   2  age  CRT1  CRT2  CRT3
##   5   3  age  CRT1  CRT2  CRT3
##   5   4  age  CRT1  CRT2  CRT3
##   5   5  age  CRT1  CRT2  CRT3
##   5   6  age  CRT1  CRT2  CRT3
##   5   7  age  CRT1  CRT2  CRT3
##   5   8  age  CRT1  CRT2  CRT3
##   5   9  age  CRT1  CRT2  CRT3
##   5   10  age  CRT1  CRT2  CRT3
##   6   1  age  CRT1  CRT2  CRT3
##   6   2  age  CRT1  CRT2  CRT3
##   6   3  age  CRT1  CRT2  CRT3
##   6   4  age  CRT1  CRT2  CRT3
##   6   5  age  CRT1  CRT2  CRT3
##   6   6  age  CRT1  CRT2  CRT3
##   6   7  age  CRT1  CRT2  CRT3
##   6   8  age  CRT1  CRT2  CRT3
##   6   9  age  CRT1  CRT2  CRT3
##   6   10  age  CRT1  CRT2  CRT3
##   7   1  age  CRT1  CRT2  CRT3
##   7   2  age  CRT1  CRT2  CRT3
##   7   3  age  CRT1  CRT2  CRT3
##   7   4  age  CRT1  CRT2  CRT3
##   7   5  age  CRT1  CRT2  CRT3
##   7   6  age  CRT1  CRT2  CRT3
##   7   7  age  CRT1  CRT2  CRT3
##   7   8  age  CRT1  CRT2  CRT3
##   7   9  age  CRT1  CRT2  CRT3
##   7   10  age  CRT1  CRT2  CRT3
##   8   1  age  CRT1  CRT2  CRT3
##   8   2  age  CRT1  CRT2  CRT3
##   8   3  age  CRT1  CRT2  CRT3
##   8   4  age  CRT1  CRT2  CRT3
##   8   5  age  CRT1  CRT2  CRT3
##   8   6  age  CRT1  CRT2  CRT3
##   8   7  age  CRT1  CRT2  CRT3
##   8   8  age  CRT1  CRT2  CRT3
##   8   9  age  CRT1  CRT2  CRT3
##   8   10  age  CRT1  CRT2  CRT3
##   9   1  age  CRT1  CRT2  CRT3
##   9   2  age  CRT1  CRT2  CRT3
##   9   3  age  CRT1  CRT2  CRT3
##   9   4  age  CRT1  CRT2  CRT3
##   9   5  age  CRT1  CRT2  CRT3
##   9   6  age  CRT1  CRT2  CRT3
##   9   7  age  CRT1  CRT2  CRT3
##   9   8  age  CRT1  CRT2  CRT3
##   9   9  age  CRT1  CRT2  CRT3
##   9   10  age  CRT1  CRT2  CRT3
##   10   1  age  CRT1  CRT2  CRT3
##   10   2  age  CRT1  CRT2  CRT3
##   10   3  age  CRT1  CRT2  CRT3
##   10   4  age  CRT1  CRT2  CRT3
##   10   5  age  CRT1  CRT2  CRT3
##   10   6  age  CRT1  CRT2  CRT3
##   10   7  age  CRT1  CRT2  CRT3
##   10   8  age  CRT1  CRT2  CRT3
##   10   9  age  CRT1  CRT2  CRT3
##   10   10  age  CRT1  CRT2  CRT3
```

```
## Warning: Number of logged events: 403
```

```
## 
##  iter imp variable
##   1   1  children  cnarc1  cnarc2  cnarc3  ctheory1  ctheory2  ctheory3  ctheory4  CRT1  CRT2  CRT3  employ_status1  generosity1  generosity2  generosity3  health_cond  marital1  mor_circle  moralid1  moralid10  moralid2  moralid3  moralid4  moralid5  moralid6  moralid7  moralid8  moralid9  mcoop1  mcoop2  mcoop3  mcoop4  mcoop5  mcoop6  mcoop7  narc1  narc2  narc3  narc4  narc5  narc6  nidentity1  nidentity2  omind1  omind2  omind3  omind4  omind5  omind6  contact1  contact2  contact3  contact4  contact5  hygiene1  hygiene2  hygiene3  hygiene4  hygiene5  psupport1  psupport2  psupport3  psupport4  psupport5  political_ideology  happy  slf_ladder  riskperc1  riskperc2  self_esteem  sex1  sbelong1  sbelong2  sbelong3  sbelong4  optim1  optim2  slfcont1  slfcont2  slfcont3  slfcont4  urban
##   1   2  children  cnarc1  cnarc2  cnarc3  ctheory1  ctheory2  ctheory3  ctheory4  CRT1  CRT2  CRT3  employ_status1  generosity1  generosity2  generosity3  health_cond  marital1  mor_circle  moralid1  moralid10  moralid2  moralid3  moralid4  moralid5  moralid6  moralid7  moralid8  moralid9  mcoop1  mcoop2  mcoop3  mcoop4  mcoop5  mcoop6  mcoop7  narc1  narc2  narc3  narc4  narc5  narc6  nidentity1  nidentity2  omind1  omind2  omind3  omind4  omind5  omind6  contact1  contact2  contact3  contact4  contact5  hygiene1  hygiene2  hygiene3  hygiene4  hygiene5  psupport1  psupport2  psupport3  psupport4  psupport5  political_ideology  happy  slf_ladder  riskperc1  riskperc2  self_esteem  sex1  sbelong1  sbelong2  sbelong3  sbelong4  optim1  optim2  slfcont1  slfcont2  slfcont3  slfcont4  urban
##   1   3  children  cnarc1  cnarc2  cnarc3  ctheory1  ctheory2  ctheory3  ctheory4  CRT1  CRT2  CRT3  employ_status1  generosity1  generosity2  generosity3  health_cond  marital1  mor_circle  moralid1  moralid10  moralid2  moralid3  moralid4  moralid5  moralid6  moralid7  moralid8  moralid9  mcoop1  mcoop2  mcoop3  mcoop4  mcoop5  mcoop6  mcoop7  narc1  narc2  narc3  narc4  narc5  narc6  nidentity1  nidentity2  omind1  omind2  omind3  omind4  omind5  omind6  contact1  contact2  contact3  contact4  contact5  hygiene1  hygiene2  hygiene3  hygiene4  hygiene5  psupport1  psupport2  psupport3  psupport4  psupport5  political_ideology  happy  slf_ladder  riskperc1  riskperc2  self_esteem  sex1  sbelong1  sbelong2  sbelong3  sbelong4  optim1  optim2  slfcont1  slfcont2  slfcont3  slfcont4  urban
##   1   4  children  cnarc1  cnarc2  cnarc3  ctheory1  ctheory2  ctheory3  ctheory4  CRT1  CRT2  CRT3  employ_status1  generosity1  generosity2  generosity3  health_cond  marital1  mor_circle  moralid1  moralid10  moralid2  moralid3  moralid4  moralid5  moralid6  moralid7  moralid8  moralid9  mcoop1  mcoop2  mcoop3  mcoop4  mcoop5  mcoop6  mcoop7  narc1  narc2  narc3  narc4  narc5  narc6  nidentity1  nidentity2  omind1  omind2  omind3  omind4  omind5  omind6  contact1  contact2  contact3  contact4  contact5  hygiene1  hygiene2  hygiene3  hygiene4  hygiene5  psupport1  psupport2  psupport3  psupport4  psupport5  political_ideology  happy  slf_ladder  riskperc1  riskperc2  self_esteem  sex1  sbelong1  sbelong2  sbelong3  sbelong4  optim1  optim2  slfcont1  slfcont2  slfcont3  slfcont4  urban
##   1   5  children  cnarc1  cnarc2  cnarc3  ctheory1  ctheory2  ctheory3  ctheory4  CRT1  CRT2  CRT3  employ_status1  generosity1  generosity2  generosity3  health_cond  marital1  mor_circle  moralid1  moralid10  moralid2  moralid3  moralid4  moralid5  moralid6  moralid7  moralid8  moralid9  mcoop1  mcoop2  mcoop3  mcoop4  mcoop5  mcoop6  mcoop7  narc1  narc2  narc3  narc4  narc5  narc6  nidentity1  nidentity2  omind1  omind2  omind3  omind4  omind5  omind6  contact1  contact2  contact3  contact4  contact5  hygiene1  hygiene2  hygiene3  hygiene4  hygiene5  psupport1  psupport2  psupport3  psupport4  psupport5  political_ideology  happy  slf_ladder  riskperc1  riskperc2  self_esteem  sex1  sbelong1  sbelong2  sbelong3  sbelong4  optim1  optim2  slfcont1  slfcont2  slfcont3  slfcont4  urban
##   1   6  children  cnarc1  cnarc2  cnarc3  ctheory1  ctheory2  ctheory3  ctheory4  CRT1  CRT2  CRT3  employ_status1  generosity1  generosity2  generosity3  health_cond  marital1  mor_circle  moralid1  moralid10  moralid2  moralid3  moralid4  moralid5  moralid6  moralid7  moralid8  moralid9  mcoop1  mcoop2  mcoop3  mcoop4  mcoop5  mcoop6  mcoop7  narc1  narc2  narc3  narc4  narc5  narc6  nidentity1  nidentity2  omind1  omind2  omind3  omind4  omind5  omind6  contact1  contact2  contact3  contact4  contact5  hygiene1  hygiene2  hygiene3  hygiene4  hygiene5  psupport1  psupport2  psupport3  psupport4  psupport5  political_ideology  happy  slf_ladder  riskperc1  riskperc2  self_esteem  sex1  sbelong1  sbelong2  sbelong3  sbelong4  optim1  optim2  slfcont1  slfcont2  slfcont3  slfcont4  urban
##   1   7  children  cnarc1  cnarc2  cnarc3  ctheory1  ctheory2  ctheory3  ctheory4  CRT1  CRT2  CRT3  employ_status1  generosity1  generosity2  generosity3  health_cond  marital1  mor_circle  moralid1  moralid10  moralid2  moralid3  moralid4  moralid5  moralid6  moralid7  moralid8  moralid9  mcoop1  mcoop2  mcoop3  mcoop4  mcoop5  mcoop6  mcoop7  narc1  narc2  narc3  narc4  narc5  narc6  nidentity1  nidentity2  omind1  omind2  omind3  omind4  omind5  omind6  contact1  contact2  contact3  contact4  contact5  hygiene1  hygiene2  hygiene3  hygiene4  hygiene5  psupport1  psupport2  psupport3  psupport4  psupport5  political_ideology  happy  slf_ladder  riskperc1  riskperc2  self_esteem  sex1  sbelong1  sbelong2  sbelong3  sbelong4  optim1  optim2  slfcont1  slfcont2  slfcont3  slfcont4  urban
##   1   8  children  cnarc1  cnarc2  cnarc3  ctheory1  ctheory2  ctheory3  ctheory4  CRT1  CRT2  CRT3  employ_status1  generosity1  generosity2  generosity3  health_cond  marital1  mor_circle  moralid1  moralid10  moralid2  moralid3  moralid4  moralid5  moralid6  moralid7  moralid8  moralid9  mcoop1  mcoop2  mcoop3  mcoop4  mcoop5  mcoop6  mcoop7  narc1  narc2  narc3  narc4  narc5  narc6  nidentity1  nidentity2  omind1  omind2  omind3  omind4  omind5  omind6  contact1  contact2  contact3  contact4  contact5  hygiene1  hygiene2  hygiene3  hygiene4  hygiene5  psupport1  psupport2  psupport3  psupport4  psupport5  political_ideology  happy  slf_ladder  riskperc1  riskperc2  self_esteem  sex1  sbelong1  sbelong2  sbelong3  sbelong4  optim1  optim2  slfcont1  slfcont2  slfcont3  slfcont4  urban
##   1   9  children  cnarc1  cnarc2  cnarc3  ctheory1  ctheory2  ctheory3  ctheory4  CRT1  CRT2  CRT3  employ_status1  generosity1  generosity2  generosity3  health_cond  marital1  mor_circle  moralid1  moralid10  moralid2  moralid3  moralid4  moralid5  moralid6  moralid7  moralid8  moralid9  mcoop1  mcoop2  mcoop3  mcoop4  mcoop5  mcoop6  mcoop7  narc1  narc2  narc3  narc4  narc5  narc6  nidentity1  nidentity2  omind1  omind2  omind3  omind4  omind5  omind6  contact1  contact2  contact3  contact4  contact5  hygiene1  hygiene2  hygiene3  hygiene4  hygiene5  psupport1  psupport2  psupport3  psupport4  psupport5  political_ideology  happy  slf_ladder  riskperc1  riskperc2  self_esteem  sex1  sbelong1  sbelong2  sbelong3  sbelong4  optim1  optim2  slfcont1  slfcont2  slfcont3  slfcont4  urban
##   1   10  children  cnarc1  cnarc2  cnarc3  ctheory1  ctheory2  ctheory3  ctheory4  CRT1  CRT2  CRT3  employ_status1  generosity1  generosity2  generosity3  health_cond  marital1  mor_circle  moralid1  moralid10  moralid2  moralid3  moralid4  moralid5  moralid6  moralid7  moralid8  moralid9  mcoop1  mcoop2  mcoop3  mcoop4  mcoop5  mcoop6  mcoop7  narc1  narc2  narc3  narc4  narc5  narc6  nidentity1  nidentity2  omind1  omind2  omind3  omind4  omind5  omind6  contact1  contact2  contact3  contact4  contact5  hygiene1  hygiene2  hygiene3  hygiene4  hygiene5  psupport1  psupport2  psupport3  psupport4  psupport5  political_ideology  happy  slf_ladder  riskperc1  riskperc2  self_esteem  sex1  sbelong1  sbelong2  sbelong3  sbelong4  optim1  optim2  slfcont1  slfcont2  slfcont3  slfcont4  urban
##   2   1  children  cnarc1  cnarc2  cnarc3  ctheory1  ctheory2  ctheory3  ctheory4  CRT1  CRT2  CRT3  employ_status1  generosity1  generosity2  generosity3  health_cond  marital1  mor_circle  moralid1  moralid10  moralid2  moralid3  moralid4  moralid5  moralid6  moralid7  moralid8  moralid9  mcoop1  mcoop2  mcoop3  mcoop4  mcoop5  mcoop6  mcoop7  narc1  narc2  narc3  narc4  narc5  narc6  nidentity1  nidentity2  omind1  omind2  omind3  omind4  omind5  omind6  contact1  contact2  contact3  contact4  contact5  hygiene1  hygiene2  hygiene3  hygiene4  hygiene5  psupport1  psupport2  psupport3  psupport4  psupport5  political_ideology  happy  slf_ladder  riskperc1  riskperc2  self_esteem  sex1  sbelong1  sbelong2  sbelong3  sbelong4  optim1  optim2  slfcont1  slfcont2  slfcont3  slfcont4  urban
##   2   2  children  cnarc1  cnarc2  cnarc3  ctheory1  ctheory2  ctheory3  ctheory4  CRT1  CRT2  CRT3  employ_status1  generosity1  generosity2  generosity3  health_cond  marital1  mor_circle  moralid1  moralid10  moralid2  moralid3  moralid4  moralid5  moralid6  moralid7  moralid8  moralid9  mcoop1  mcoop2  mcoop3  mcoop4  mcoop5  mcoop6  mcoop7  narc1  narc2  narc3  narc4  narc5  narc6  nidentity1  nidentity2  omind1  omind2  omind3  omind4  omind5  omind6  contact1  contact2  contact3  contact4  contact5  hygiene1  hygiene2  hygiene3  hygiene4  hygiene5  psupport1  psupport2  psupport3  psupport4  psupport5  political_ideology  happy  slf_ladder  riskperc1  riskperc2  self_esteem  sex1  sbelong1  sbelong2  sbelong3  sbelong4  optim1  optim2  slfcont1  slfcont2  slfcont3  slfcont4  urban
##   2   3  children  cnarc1  cnarc2  cnarc3  ctheory1  ctheory2  ctheory3  ctheory4  CRT1  CRT2  CRT3  employ_status1  generosity1  generosity2  generosity3  health_cond  marital1  mor_circle  moralid1  moralid10  moralid2  moralid3  moralid4  moralid5  moralid6  moralid7  moralid8  moralid9  mcoop1  mcoop2  mcoop3  mcoop4  mcoop5  mcoop6  mcoop7  narc1  narc2  narc3  narc4  narc5  narc6  nidentity1  nidentity2  omind1  omind2  omind3  omind4  omind5  omind6  contact1  contact2  contact3  contact4  contact5  hygiene1  hygiene2  hygiene3  hygiene4  hygiene5  psupport1  psupport2  psupport3  psupport4  psupport5  political_ideology  happy  slf_ladder  riskperc1  riskperc2  self_esteem  sex1  sbelong1  sbelong2  sbelong3  sbelong4  optim1  optim2  slfcont1  slfcont2  slfcont3  slfcont4  urban
##   2   4  children  cnarc1  cnarc2  cnarc3  ctheory1  ctheory2  ctheory3  ctheory4  CRT1  CRT2  CRT3  employ_status1  generosity1  generosity2  generosity3  health_cond  marital1  mor_circle  moralid1  moralid10  moralid2  moralid3  moralid4  moralid5  moralid6  moralid7  moralid8  moralid9  mcoop1  mcoop2  mcoop3  mcoop4  mcoop5  mcoop6  mcoop7  narc1  narc2  narc3  narc4  narc5  narc6  nidentity1  nidentity2  omind1  omind2  omind3  omind4  omind5  omind6  contact1  contact2  contact3  contact4  contact5  hygiene1  hygiene2  hygiene3  hygiene4  hygiene5  psupport1  psupport2  psupport3  psupport4  psupport5  political_ideology  happy  slf_ladder  riskperc1  riskperc2  self_esteem  sex1  sbelong1  sbelong2  sbelong3  sbelong4  optim1  optim2  slfcont1  slfcont2  slfcont3  slfcont4  urban
##   2   5  children  cnarc1  cnarc2  cnarc3  ctheory1  ctheory2  ctheory3  ctheory4  CRT1  CRT2  CRT3  employ_status1  generosity1  generosity2  generosity3  health_cond  marital1  mor_circle  moralid1  moralid10  moralid2  moralid3  moralid4  moralid5  moralid6  moralid7  moralid8  moralid9  mcoop1  mcoop2  mcoop3  mcoop4  mcoop5  mcoop6  mcoop7  narc1  narc2  narc3  narc4  narc5  narc6  nidentity1  nidentity2  omind1  omind2  omind3  omind4  omind5  omind6  contact1  contact2  contact3  contact4  contact5  hygiene1  hygiene2  hygiene3  hygiene4  hygiene5  psupport1  psupport2  psupport3  psupport4  psupport5  political_ideology  happy  slf_ladder  riskperc1  riskperc2  self_esteem  sex1  sbelong1  sbelong2  sbelong3  sbelong4  optim1  optim2  slfcont1  slfcont2  slfcont3  slfcont4  urban
##   2   6  children  cnarc1  cnarc2  cnarc3  ctheory1  ctheory2  ctheory3  ctheory4  CRT1  CRT2  CRT3  employ_status1  generosity1  generosity2  generosity3  health_cond  marital1  mor_circle  moralid1  moralid10  moralid2  moralid3  moralid4  moralid5  moralid6  moralid7  moralid8  moralid9  mcoop1  mcoop2  mcoop3  mcoop4  mcoop5  mcoop6  mcoop7  narc1  narc2  narc3  narc4  narc5  narc6  nidentity1  nidentity2  omind1  omind2  omind3  omind4  omind5  omind6  contact1  contact2  contact3  contact4  contact5  hygiene1  hygiene2  hygiene3  hygiene4  hygiene5  psupport1  psupport2  psupport3  psupport4  psupport5  political_ideology  happy  slf_ladder  riskperc1  riskperc2  self_esteem  sex1  sbelong1  sbelong2  sbelong3  sbelong4  optim1  optim2  slfcont1  slfcont2  slfcont3  slfcont4  urban
##   2   7  children  cnarc1  cnarc2  cnarc3  ctheory1  ctheory2  ctheory3  ctheory4  CRT1  CRT2  CRT3  employ_status1  generosity1  generosity2  generosity3  health_cond  marital1  mor_circle  moralid1  moralid10  moralid2  moralid3  moralid4  moralid5  moralid6  moralid7  moralid8  moralid9  mcoop1  mcoop2  mcoop3  mcoop4  mcoop5  mcoop6  mcoop7  narc1  narc2  narc3  narc4  narc5  narc6  nidentity1  nidentity2  omind1  omind2  omind3  omind4  omind5  omind6  contact1  contact2  contact3  contact4  contact5  hygiene1  hygiene2  hygiene3  hygiene4  hygiene5  psupport1  psupport2  psupport3  psupport4  psupport5  political_ideology  happy  slf_ladder  riskperc1  riskperc2  self_esteem  sex1  sbelong1  sbelong2  sbelong3  sbelong4  optim1  optim2  slfcont1  slfcont2  slfcont3  slfcont4  urban
##   2   8  children  cnarc1  cnarc2  cnarc3  ctheory1  ctheory2  ctheory3  ctheory4  CRT1  CRT2  CRT3  employ_status1  generosity1  generosity2  generosity3  health_cond  marital1  mor_circle  moralid1  moralid10  moralid2  moralid3  moralid4  moralid5  moralid6  moralid7  moralid8  moralid9  mcoop1  mcoop2  mcoop3  mcoop4  mcoop5  mcoop6  mcoop7  narc1  narc2  narc3  narc4  narc5  narc6  nidentity1  nidentity2  omind1  omind2  omind3  omind4  omind5  omind6  contact1  contact2  contact3  contact4  contact5  hygiene1  hygiene2  hygiene3  hygiene4  hygiene5  psupport1  psupport2  psupport3  psupport4  psupport5  political_ideology  happy  slf_ladder  riskperc1  riskperc2  self_esteem  sex1  sbelong1  sbelong2  sbelong3  sbelong4  optim1  optim2  slfcont1  slfcont2  slfcont3  slfcont4  urban
##   2   9  children  cnarc1  cnarc2  cnarc3  ctheory1  ctheory2  ctheory3  ctheory4  CRT1  CRT2  CRT3  employ_status1  generosity1  generosity2  generosity3  health_cond  marital1  mor_circle  moralid1  moralid10  moralid2  moralid3  moralid4  moralid5  moralid6  moralid7  moralid8  moralid9  mcoop1  mcoop2  mcoop3  mcoop4  mcoop5  mcoop6  mcoop7  narc1  narc2  narc3  narc4  narc5  narc6  nidentity1  nidentity2  omind1  omind2  omind3  omind4  omind5  omind6  contact1  contact2  contact3  contact4  contact5  hygiene1  hygiene2  hygiene3  hygiene4  hygiene5  psupport1  psupport2  psupport3  psupport4  psupport5  political_ideology  happy  slf_ladder  riskperc1  riskperc2  self_esteem  sex1  sbelong1  sbelong2  sbelong3  sbelong4  optim1  optim2  slfcont1  slfcont2  slfcont3  slfcont4  urban
##   2   10  children  cnarc1  cnarc2  cnarc3  ctheory1  ctheory2  ctheory3  ctheory4  CRT1  CRT2  CRT3  employ_status1  generosity1  generosity2  generosity3  health_cond  marital1  mor_circle  moralid1  moralid10  moralid2  moralid3  moralid4  moralid5  moralid6  moralid7  moralid8  moralid9  mcoop1  mcoop2  mcoop3  mcoop4  mcoop5  mcoop6  mcoop7  narc1  narc2  narc3  narc4  narc5  narc6  nidentity1  nidentity2  omind1  omind2  omind3  omind4  omind5  omind6  contact1  contact2  contact3  contact4  contact5  hygiene1  hygiene2  hygiene3  hygiene4  hygiene5  psupport1  psupport2  psupport3  psupport4  psupport5  political_ideology  happy  slf_ladder  riskperc1  riskperc2  self_esteem  sex1  sbelong1  sbelong2  sbelong3  sbelong4  optim1  optim2  slfcont1  slfcont2  slfcont3  slfcont4  urban
##   3   1  children  cnarc1  cnarc2  cnarc3  ctheory1  ctheory2  ctheory3  ctheory4  CRT1  CRT2  CRT3  employ_status1  generosity1  generosity2  generosity3  health_cond  marital1  mor_circle  moralid1  moralid10  moralid2  moralid3  moralid4  moralid5  moralid6  moralid7  moralid8  moralid9  mcoop1  mcoop2  mcoop3  mcoop4  mcoop5  mcoop6  mcoop7  narc1  narc2  narc3  narc4  narc5  narc6  nidentity1  nidentity2  omind1  omind2  omind3  omind4  omind5  omind6  contact1  contact2  contact3  contact4  contact5  hygiene1  hygiene2  hygiene3  hygiene4  hygiene5  psupport1  psupport2  psupport3  psupport4  psupport5  political_ideology  happy  slf_ladder  riskperc1  riskperc2  self_esteem  sex1  sbelong1  sbelong2  sbelong3  sbelong4  optim1  optim2  slfcont1  slfcont2  slfcont3  slfcont4  urban
##   3   2  children  cnarc1  cnarc2  cnarc3  ctheory1  ctheory2  ctheory3  ctheory4  CRT1  CRT2  CRT3  employ_status1  generosity1  generosity2  generosity3  health_cond  marital1  mor_circle  moralid1  moralid10  moralid2  moralid3  moralid4  moralid5  moralid6  moralid7  moralid8  moralid9  mcoop1  mcoop2  mcoop3  mcoop4  mcoop5  mcoop6  mcoop7  narc1  narc2  narc3  narc4  narc5  narc6  nidentity1  nidentity2  omind1  omind2  omind3  omind4  omind5  omind6  contact1  contact2  contact3  contact4  contact5  hygiene1  hygiene2  hygiene3  hygiene4  hygiene5  psupport1  psupport2  psupport3  psupport4  psupport5  political_ideology  happy  slf_ladder  riskperc1  riskperc2  self_esteem  sex1  sbelong1  sbelong2  sbelong3  sbelong4  optim1  optim2  slfcont1  slfcont2  slfcont3  slfcont4  urban
##   3   3  children  cnarc1  cnarc2  cnarc3  ctheory1  ctheory2  ctheory3  ctheory4  CRT1  CRT2  CRT3  employ_status1  generosity1  generosity2  generosity3  health_cond  marital1  mor_circle  moralid1  moralid10  moralid2  moralid3  moralid4  moralid5  moralid6  moralid7  moralid8  moralid9  mcoop1  mcoop2  mcoop3  mcoop4  mcoop5  mcoop6  mcoop7  narc1  narc2  narc3  narc4  narc5  narc6  nidentity1  nidentity2  omind1  omind2  omind3  omind4  omind5  omind6  contact1  contact2  contact3  contact4  contact5  hygiene1  hygiene2  hygiene3  hygiene4  hygiene5  psupport1  psupport2  psupport3  psupport4  psupport5  political_ideology  happy  slf_ladder  riskperc1  riskperc2  self_esteem  sex1  sbelong1  sbelong2  sbelong3  sbelong4  optim1  optim2  slfcont1  slfcont2  slfcont3  slfcont4  urban
##   3   4  children  cnarc1  cnarc2  cnarc3  ctheory1  ctheory2  ctheory3  ctheory4  CRT1  CRT2  CRT3  employ_status1  generosity1  generosity2  generosity3  health_cond  marital1  mor_circle  moralid1  moralid10  moralid2  moralid3  moralid4  moralid5  moralid6  moralid7  moralid8  moralid9  mcoop1  mcoop2  mcoop3  mcoop4  mcoop5  mcoop6  mcoop7  narc1  narc2  narc3  narc4  narc5  narc6  nidentity1  nidentity2  omind1  omind2  omind3  omind4  omind5  omind6  contact1  contact2  contact3  contact4  contact5  hygiene1  hygiene2  hygiene3  hygiene4  hygiene5  psupport1  psupport2  psupport3  psupport4  psupport5  political_ideology  happy  slf_ladder  riskperc1  riskperc2  self_esteem  sex1  sbelong1  sbelong2  sbelong3  sbelong4  optim1  optim2  slfcont1  slfcont2  slfcont3  slfcont4  urban
##   3   5  children  cnarc1  cnarc2  cnarc3  ctheory1  ctheory2  ctheory3  ctheory4  CRT1  CRT2  CRT3  employ_status1  generosity1  generosity2  generosity3  health_cond  marital1  mor_circle  moralid1  moralid10  moralid2  moralid3  moralid4  moralid5  moralid6  moralid7  moralid8  moralid9  mcoop1  mcoop2  mcoop3  mcoop4  mcoop5  mcoop6  mcoop7  narc1  narc2  narc3  narc4  narc5  narc6  nidentity1  nidentity2  omind1  omind2  omind3  omind4  omind5  omind6  contact1  contact2  contact3  contact4  contact5  hygiene1  hygiene2  hygiene3  hygiene4  hygiene5  psupport1  psupport2  psupport3  psupport4  psupport5  political_ideology  happy  slf_ladder  riskperc1  riskperc2  self_esteem  sex1  sbelong1  sbelong2  sbelong3  sbelong4  optim1  optim2  slfcont1  slfcont2  slfcont3  slfcont4  urban
##   3   6  children  cnarc1  cnarc2  cnarc3  ctheory1  ctheory2  ctheory3  ctheory4  CRT1  CRT2  CRT3  employ_status1  generosity1  generosity2  generosity3  health_cond  marital1  mor_circle  moralid1  moralid10  moralid2  moralid3  moralid4  moralid5  moralid6  moralid7  moralid8  moralid9  mcoop1  mcoop2  mcoop3  mcoop4  mcoop5  mcoop6  mcoop7  narc1  narc2  narc3  narc4  narc5  narc6  nidentity1  nidentity2  omind1  omind2  omind3  omind4  omind5  omind6  contact1  contact2  contact3  contact4  contact5  hygiene1  hygiene2  hygiene3  hygiene4  hygiene5  psupport1  psupport2  psupport3  psupport4  psupport5  political_ideology  happy  slf_ladder  riskperc1  riskperc2  self_esteem  sex1  sbelong1  sbelong2  sbelong3  sbelong4  optim1  optim2  slfcont1  slfcont2  slfcont3  slfcont4  urban
##   3   7  children  cnarc1  cnarc2  cnarc3  ctheory1  ctheory2  ctheory3  ctheory4  CRT1  CRT2  CRT3  employ_status1  generosity1  generosity2  generosity3  health_cond  marital1  mor_circle  moralid1  moralid10  moralid2  moralid3  moralid4  moralid5  moralid6  moralid7  moralid8  moralid9  mcoop1  mcoop2  mcoop3  mcoop4  mcoop5  mcoop6  mcoop7  narc1  narc2  narc3  narc4  narc5  narc6  nidentity1  nidentity2  omind1  omind2  omind3  omind4  omind5  omind6  contact1  contact2  contact3  contact4  contact5  hygiene1  hygiene2  hygiene3  hygiene4  hygiene5  psupport1  psupport2  psupport3  psupport4  psupport5  political_ideology  happy  slf_ladder  riskperc1  riskperc2  self_esteem  sex1  sbelong1  sbelong2  sbelong3  sbelong4  optim1  optim2  slfcont1  slfcont2  slfcont3  slfcont4  urban
##   3   8  children  cnarc1  cnarc2  cnarc3  ctheory1  ctheory2  ctheory3  ctheory4  CRT1  CRT2  CRT3  employ_status1  generosity1  generosity2  generosity3  health_cond  marital1  mor_circle  moralid1  moralid10  moralid2  moralid3  moralid4  moralid5  moralid6  moralid7  moralid8  moralid9  mcoop1  mcoop2  mcoop3  mcoop4  mcoop5  mcoop6  mcoop7  narc1  narc2  narc3  narc4  narc5  narc6  nidentity1  nidentity2  omind1  omind2  omind3  omind4  omind5  omind6  contact1  contact2  contact3  contact4  contact5  hygiene1  hygiene2  hygiene3  hygiene4  hygiene5  psupport1  psupport2  psupport3  psupport4  psupport5  political_ideology  happy  slf_ladder  riskperc1  riskperc2  self_esteem  sex1  sbelong1  sbelong2  sbelong3  sbelong4  optim1  optim2  slfcont1  slfcont2  slfcont3  slfcont4  urban
##   3   9  children  cnarc1  cnarc2  cnarc3  ctheory1  ctheory2  ctheory3  ctheory4  CRT1  CRT2  CRT3  employ_status1  generosity1  generosity2  generosity3  health_cond  marital1  mor_circle  moralid1  moralid10  moralid2  moralid3  moralid4  moralid5  moralid6  moralid7  moralid8  moralid9  mcoop1  mcoop2  mcoop3  mcoop4  mcoop5  mcoop6  mcoop7  narc1  narc2  narc3  narc4  narc5  narc6  nidentity1  nidentity2  omind1  omind2  omind3  omind4  omind5  omind6  contact1  contact2  contact3  contact4  contact5  hygiene1  hygiene2  hygiene3  hygiene4  hygiene5  psupport1  psupport2  psupport3  psupport4  psupport5  political_ideology  happy  slf_ladder  riskperc1  riskperc2  self_esteem  sex1  sbelong1  sbelong2  sbelong3  sbelong4  optim1  optim2  slfcont1  slfcont2  slfcont3  slfcont4  urban
##   3   10  children  cnarc1  cnarc2  cnarc3  ctheory1  ctheory2  ctheory3  ctheory4  CRT1  CRT2  CRT3  employ_status1  generosity1  generosity2  generosity3  health_cond  marital1  mor_circle  moralid1  moralid10  moralid2  moralid3  moralid4  moralid5  moralid6  moralid7  moralid8  moralid9  mcoop1  mcoop2  mcoop3  mcoop4  mcoop5  mcoop6  mcoop7  narc1  narc2  narc3  narc4  narc5  narc6  nidentity1  nidentity2  omind1  omind2  omind3  omind4  omind5  omind6  contact1  contact2  contact3  contact4  contact5  hygiene1  hygiene2  hygiene3  hygiene4  hygiene5  psupport1  psupport2  psupport3  psupport4  psupport5  political_ideology  happy  slf_ladder  riskperc1  riskperc2  self_esteem  sex1  sbelong1  sbelong2  sbelong3  sbelong4  optim1  optim2  slfcont1  slfcont2  slfcont3  slfcont4  urban
##   4   1  children  cnarc1  cnarc2  cnarc3  ctheory1  ctheory2  ctheory3  ctheory4  CRT1  CRT2  CRT3  employ_status1  generosity1  generosity2  generosity3  health_cond  marital1  mor_circle  moralid1  moralid10  moralid2  moralid3  moralid4  moralid5  moralid6  moralid7  moralid8  moralid9  mcoop1  mcoop2  mcoop3  mcoop4  mcoop5  mcoop6  mcoop7  narc1  narc2  narc3  narc4  narc5  narc6  nidentity1  nidentity2  omind1  omind2  omind3  omind4  omind5  omind6  contact1  contact2  contact3  contact4  contact5  hygiene1  hygiene2  hygiene3  hygiene4  hygiene5  psupport1  psupport2  psupport3  psupport4  psupport5  political_ideology  happy  slf_ladder  riskperc1  riskperc2  self_esteem  sex1  sbelong1  sbelong2  sbelong3  sbelong4  optim1  optim2  slfcont1  slfcont2  slfcont3  slfcont4  urban
##   4   2  children  cnarc1  cnarc2  cnarc3  ctheory1  ctheory2  ctheory3  ctheory4  CRT1  CRT2  CRT3  employ_status1  generosity1  generosity2  generosity3  health_cond  marital1  mor_circle  moralid1  moralid10  moralid2  moralid3  moralid4  moralid5  moralid6  moralid7  moralid8  moralid9  mcoop1  mcoop2  mcoop3  mcoop4  mcoop5  mcoop6  mcoop7  narc1  narc2  narc3  narc4  narc5  narc6  nidentity1  nidentity2  omind1  omind2  omind3  omind4  omind5  omind6  contact1  contact2  contact3  contact4  contact5  hygiene1  hygiene2  hygiene3  hygiene4  hygiene5  psupport1  psupport2  psupport3  psupport4  psupport5  political_ideology  happy  slf_ladder  riskperc1  riskperc2  self_esteem  sex1  sbelong1  sbelong2  sbelong3  sbelong4  optim1  optim2  slfcont1  slfcont2  slfcont3  slfcont4  urban
##   4   3  children  cnarc1  cnarc2  cnarc3  ctheory1  ctheory2  ctheory3  ctheory4  CRT1  CRT2  CRT3  employ_status1  generosity1  generosity2  generosity3  health_cond  marital1  mor_circle  moralid1  moralid10  moralid2  moralid3  moralid4  moralid5  moralid6  moralid7  moralid8  moralid9  mcoop1  mcoop2  mcoop3  mcoop4  mcoop5  mcoop6  mcoop7  narc1  narc2  narc3  narc4  narc5  narc6  nidentity1  nidentity2  omind1  omind2  omind3  omind4  omind5  omind6  contact1  contact2  contact3  contact4  contact5  hygiene1  hygiene2  hygiene3  hygiene4  hygiene5  psupport1  psupport2  psupport3  psupport4  psupport5  political_ideology  happy  slf_ladder  riskperc1  riskperc2  self_esteem  sex1  sbelong1  sbelong2  sbelong3  sbelong4  optim1  optim2  slfcont1  slfcont2  slfcont3  slfcont4  urban
##   4   4  children  cnarc1  cnarc2  cnarc3  ctheory1  ctheory2  ctheory3  ctheory4  CRT1  CRT2  CRT3  employ_status1  generosity1  generosity2  generosity3  health_cond  marital1  mor_circle  moralid1  moralid10  moralid2  moralid3  moralid4  moralid5  moralid6  moralid7  moralid8  moralid9  mcoop1  mcoop2  mcoop3  mcoop4  mcoop5  mcoop6  mcoop7  narc1  narc2  narc3  narc4  narc5  narc6  nidentity1  nidentity2  omind1  omind2  omind3  omind4  omind5  omind6  contact1  contact2  contact3  contact4  contact5  hygiene1  hygiene2  hygiene3  hygiene4  hygiene5  psupport1  psupport2  psupport3  psupport4  psupport5  political_ideology  happy  slf_ladder  riskperc1  riskperc2  self_esteem  sex1  sbelong1  sbelong2  sbelong3  sbelong4  optim1  optim2  slfcont1  slfcont2  slfcont3  slfcont4  urban
##   4   5  children  cnarc1  cnarc2  cnarc3  ctheory1  ctheory2  ctheory3  ctheory4  CRT1  CRT2  CRT3  employ_status1  generosity1  generosity2  generosity3  health_cond  marital1  mor_circle  moralid1  moralid10  moralid2  moralid3  moralid4  moralid5  moralid6  moralid7  moralid8  moralid9  mcoop1  mcoop2  mcoop3  mcoop4  mcoop5  mcoop6  mcoop7  narc1  narc2  narc3  narc4  narc5  narc6  nidentity1  nidentity2  omind1  omind2  omind3  omind4  omind5  omind6  contact1  contact2  contact3  contact4  contact5  hygiene1  hygiene2  hygiene3  hygiene4  hygiene5  psupport1  psupport2  psupport3  psupport4  psupport5  political_ideology  happy  slf_ladder  riskperc1  riskperc2  self_esteem  sex1  sbelong1  sbelong2  sbelong3  sbelong4  optim1  optim2  slfcont1  slfcont2  slfcont3  slfcont4  urban
##   4   6  children  cnarc1  cnarc2  cnarc3  ctheory1  ctheory2  ctheory3  ctheory4  CRT1  CRT2  CRT3  employ_status1  generosity1  generosity2  generosity3  health_cond  marital1  mor_circle  moralid1  moralid10  moralid2  moralid3  moralid4  moralid5  moralid6  moralid7  moralid8  moralid9  mcoop1  mcoop2  mcoop3  mcoop4  mcoop5  mcoop6  mcoop7  narc1  narc2  narc3  narc4  narc5  narc6  nidentity1  nidentity2  omind1  omind2  omind3  omind4  omind5  omind6  contact1  contact2  contact3  contact4  contact5  hygiene1  hygiene2  hygiene3  hygiene4  hygiene5  psupport1  psupport2  psupport3  psupport4  psupport5  political_ideology  happy  slf_ladder  riskperc1  riskperc2  self_esteem  sex1  sbelong1  sbelong2  sbelong3  sbelong4  optim1  optim2  slfcont1  slfcont2  slfcont3  slfcont4  urban
##   4   7  children  cnarc1  cnarc2  cnarc3  ctheory1  ctheory2  ctheory3  ctheory4  CRT1  CRT2  CRT3  employ_status1  generosity1  generosity2  generosity3  health_cond  marital1  mor_circle  moralid1  moralid10  moralid2  moralid3  moralid4  moralid5  moralid6  moralid7  moralid8  moralid9  mcoop1  mcoop2  mcoop3  mcoop4  mcoop5  mcoop6  mcoop7  narc1  narc2  narc3  narc4  narc5  narc6  nidentity1  nidentity2  omind1  omind2  omind3  omind4  omind5  omind6  contact1  contact2  contact3  contact4  contact5  hygiene1  hygiene2  hygiene3  hygiene4  hygiene5  psupport1  psupport2  psupport3  psupport4  psupport5  political_ideology  happy  slf_ladder  riskperc1  riskperc2  self_esteem  sex1  sbelong1  sbelong2  sbelong3  sbelong4  optim1  optim2  slfcont1  slfcont2  slfcont3  slfcont4  urban
##   4   8  children  cnarc1  cnarc2  cnarc3  ctheory1  ctheory2  ctheory3  ctheory4  CRT1  CRT2  CRT3  employ_status1  generosity1  generosity2  generosity3  health_cond  marital1  mor_circle  moralid1  moralid10  moralid2  moralid3  moralid4  moralid5  moralid6  moralid7  moralid8  moralid9  mcoop1  mcoop2  mcoop3  mcoop4  mcoop5  mcoop6  mcoop7  narc1  narc2  narc3  narc4  narc5  narc6  nidentity1  nidentity2  omind1  omind2  omind3  omind4  omind5  omind6  contact1  contact2  contact3  contact4  contact5  hygiene1  hygiene2  hygiene3  hygiene4  hygiene5  psupport1  psupport2  psupport3  psupport4  psupport5  political_ideology  happy  slf_ladder  riskperc1  riskperc2  self_esteem  sex1  sbelong1  sbelong2  sbelong3  sbelong4  optim1  optim2  slfcont1  slfcont2  slfcont3  slfcont4  urban
##   4   9  children  cnarc1  cnarc2  cnarc3  ctheory1  ctheory2  ctheory3  ctheory4  CRT1  CRT2  CRT3  employ_status1  generosity1  generosity2  generosity3  health_cond  marital1  mor_circle  moralid1  moralid10  moralid2  moralid3  moralid4  moralid5  moralid6  moralid7  moralid8  moralid9  mcoop1  mcoop2  mcoop3  mcoop4  mcoop5  mcoop6  mcoop7  narc1  narc2  narc3  narc4  narc5  narc6  nidentity1  nidentity2  omind1  omind2  omind3  omind4  omind5  omind6  contact1  contact2  contact3  contact4  contact5  hygiene1  hygiene2  hygiene3  hygiene4  hygiene5  psupport1  psupport2  psupport3  psupport4  psupport5  political_ideology  happy  slf_ladder  riskperc1  riskperc2  self_esteem  sex1  sbelong1  sbelong2  sbelong3  sbelong4  optim1  optim2  slfcont1  slfcont2  slfcont3  slfcont4  urban
##   4   10  children  cnarc1  cnarc2  cnarc3  ctheory1  ctheory2  ctheory3  ctheory4  CRT1  CRT2  CRT3  employ_status1  generosity1  generosity2  generosity3  health_cond  marital1  mor_circle  moralid1  moralid10  moralid2  moralid3  moralid4  moralid5  moralid6  moralid7  moralid8  moralid9  mcoop1  mcoop2  mcoop3  mcoop4  mcoop5  mcoop6  mcoop7  narc1  narc2  narc3  narc4  narc5  narc6  nidentity1  nidentity2  omind1  omind2  omind3  omind4  omind5  omind6  contact1  contact2  contact3  contact4  contact5  hygiene1  hygiene2  hygiene3  hygiene4  hygiene5  psupport1  psupport2  psupport3  psupport4  psupport5  political_ideology  happy  slf_ladder  riskperc1  riskperc2  self_esteem  sex1  sbelong1  sbelong2  sbelong3  sbelong4  optim1  optim2  slfcont1  slfcont2  slfcont3  slfcont4  urban
##   5   1  children  cnarc1  cnarc2  cnarc3  ctheory1  ctheory2  ctheory3  ctheory4  CRT1  CRT2  CRT3  employ_status1  generosity1  generosity2  generosity3  health_cond  marital1  mor_circle  moralid1  moralid10  moralid2  moralid3  moralid4  moralid5  moralid6  moralid7  moralid8  moralid9  mcoop1  mcoop2  mcoop3  mcoop4  mcoop5  mcoop6  mcoop7  narc1  narc2  narc3  narc4  narc5  narc6  nidentity1  nidentity2  omind1  omind2  omind3  omind4  omind5  omind6  contact1  contact2  contact3  contact4  contact5  hygiene1  hygiene2  hygiene3  hygiene4  hygiene5  psupport1  psupport2  psupport3  psupport4  psupport5  political_ideology  happy  slf_ladder  riskperc1  riskperc2  self_esteem  sex1  sbelong1  sbelong2  sbelong3  sbelong4  optim1  optim2  slfcont1  slfcont2  slfcont3  slfcont4  urban
##   5   2  children  cnarc1  cnarc2  cnarc3  ctheory1  ctheory2  ctheory3  ctheory4  CRT1  CRT2  CRT3  employ_status1  generosity1  generosity2  generosity3  health_cond  marital1  mor_circle  moralid1  moralid10  moralid2  moralid3  moralid4  moralid5  moralid6  moralid7  moralid8  moralid9  mcoop1  mcoop2  mcoop3  mcoop4  mcoop5  mcoop6  mcoop7  narc1  narc2  narc3  narc4  narc5  narc6  nidentity1  nidentity2  omind1  omind2  omind3  omind4  omind5  omind6  contact1  contact2  contact3  contact4  contact5  hygiene1  hygiene2  hygiene3  hygiene4  hygiene5  psupport1  psupport2  psupport3  psupport4  psupport5  political_ideology  happy  slf_ladder  riskperc1  riskperc2  self_esteem  sex1  sbelong1  sbelong2  sbelong3  sbelong4  optim1  optim2  slfcont1  slfcont2  slfcont3  slfcont4  urban
##   5   3  children  cnarc1  cnarc2  cnarc3  ctheory1  ctheory2  ctheory3  ctheory4  CRT1  CRT2  CRT3  employ_status1  generosity1  generosity2  generosity3  health_cond  marital1  mor_circle  moralid1  moralid10  moralid2  moralid3  moralid4  moralid5  moralid6  moralid7  moralid8  moralid9  mcoop1  mcoop2  mcoop3  mcoop4  mcoop5  mcoop6  mcoop7  narc1  narc2  narc3  narc4  narc5  narc6  nidentity1  nidentity2  omind1  omind2  omind3  omind4  omind5  omind6  contact1  contact2  contact3  contact4  contact5  hygiene1  hygiene2  hygiene3  hygiene4  hygiene5  psupport1  psupport2  psupport3  psupport4  psupport5  political_ideology  happy  slf_ladder  riskperc1  riskperc2  self_esteem  sex1  sbelong1  sbelong2  sbelong3  sbelong4  optim1  optim2  slfcont1  slfcont2  slfcont3  slfcont4  urban
##   5   4  children  cnarc1  cnarc2  cnarc3  ctheory1  ctheory2  ctheory3  ctheory4  CRT1  CRT2  CRT3  employ_status1  generosity1  generosity2  generosity3  health_cond  marital1  mor_circle  moralid1  moralid10  moralid2  moralid3  moralid4  moralid5  moralid6  moralid7  moralid8  moralid9  mcoop1  mcoop2  mcoop3  mcoop4  mcoop5  mcoop6  mcoop7  narc1  narc2  narc3  narc4  narc5  narc6  nidentity1  nidentity2  omind1  omind2  omind3  omind4  omind5  omind6  contact1  contact2  contact3  contact4  contact5  hygiene1  hygiene2  hygiene3  hygiene4  hygiene5  psupport1  psupport2  psupport3  psupport4  psupport5  political_ideology  happy  slf_ladder  riskperc1  riskperc2  self_esteem  sex1  sbelong1  sbelong2  sbelong3  sbelong4  optim1  optim2  slfcont1  slfcont2  slfcont3  slfcont4  urban
##   5   5  children  cnarc1  cnarc2  cnarc3  ctheory1  ctheory2  ctheory3  ctheory4  CRT1  CRT2  CRT3  employ_status1  generosity1  generosity2  generosity3  health_cond  marital1  mor_circle  moralid1  moralid10  moralid2  moralid3  moralid4  moralid5  moralid6  moralid7  moralid8  moralid9  mcoop1  mcoop2  mcoop3  mcoop4  mcoop5  mcoop6  mcoop7  narc1  narc2  narc3  narc4  narc5  narc6  nidentity1  nidentity2  omind1  omind2  omind3  omind4  omind5  omind6  contact1  contact2  contact3  contact4  contact5  hygiene1  hygiene2  hygiene3  hygiene4  hygiene5  psupport1  psupport2  psupport3  psupport4  psupport5  political_ideology  happy  slf_ladder  riskperc1  riskperc2  self_esteem  sex1  sbelong1  sbelong2  sbelong3  sbelong4  optim1  optim2  slfcont1  slfcont2  slfcont3  slfcont4  urban
##   5   6  children  cnarc1  cnarc2  cnarc3  ctheory1  ctheory2  ctheory3  ctheory4  CRT1  CRT2  CRT3  employ_status1  generosity1  generosity2  generosity3  health_cond  marital1  mor_circle  moralid1  moralid10  moralid2  moralid3  moralid4  moralid5  moralid6  moralid7  moralid8  moralid9  mcoop1  mcoop2  mcoop3  mcoop4  mcoop5  mcoop6  mcoop7  narc1  narc2  narc3  narc4  narc5  narc6  nidentity1  nidentity2  omind1  omind2  omind3  omind4  omind5  omind6  contact1  contact2  contact3  contact4  contact5  hygiene1  hygiene2  hygiene3  hygiene4  hygiene5  psupport1  psupport2  psupport3  psupport4  psupport5  political_ideology  happy  slf_ladder  riskperc1  riskperc2  self_esteem  sex1  sbelong1  sbelong2  sbelong3  sbelong4  optim1  optim2  slfcont1  slfcont2  slfcont3  slfcont4  urban
##   5   7  children  cnarc1  cnarc2  cnarc3  ctheory1  ctheory2  ctheory3  ctheory4  CRT1  CRT2  CRT3  employ_status1  generosity1  generosity2  generosity3  health_cond  marital1  mor_circle  moralid1  moralid10  moralid2  moralid3  moralid4  moralid5  moralid6  moralid7  moralid8  moralid9  mcoop1  mcoop2  mcoop3  mcoop4  mcoop5  mcoop6  mcoop7  narc1  narc2  narc3  narc4  narc5  narc6  nidentity1  nidentity2  omind1  omind2  omind3  omind4  omind5  omind6  contact1  contact2  contact3  contact4  contact5  hygiene1  hygiene2  hygiene3  hygiene4  hygiene5  psupport1  psupport2  psupport3  psupport4  psupport5  political_ideology  happy  slf_ladder  riskperc1  riskperc2  self_esteem  sex1  sbelong1  sbelong2  sbelong3  sbelong4  optim1  optim2  slfcont1  slfcont2  slfcont3  slfcont4  urban
##   5   8  children  cnarc1  cnarc2  cnarc3  ctheory1  ctheory2  ctheory3  ctheory4  CRT1  CRT2  CRT3  employ_status1  generosity1  generosity2  generosity3  health_cond  marital1  mor_circle  moralid1  moralid10  moralid2  moralid3  moralid4  moralid5  moralid6  moralid7  moralid8  moralid9  mcoop1  mcoop2  mcoop3  mcoop4  mcoop5  mcoop6  mcoop7  narc1  narc2  narc3  narc4  narc5  narc6  nidentity1  nidentity2  omind1  omind2  omind3  omind4  omind5  omind6  contact1  contact2  contact3  contact4  contact5  hygiene1  hygiene2  hygiene3  hygiene4  hygiene5  psupport1  psupport2  psupport3  psupport4  psupport5  political_ideology  happy  slf_ladder  riskperc1  riskperc2  self_esteem  sex1  sbelong1  sbelong2  sbelong3  sbelong4  optim1  optim2  slfcont1  slfcont2  slfcont3  slfcont4  urban
##   5   9  children  cnarc1  cnarc2  cnarc3  ctheory1  ctheory2  ctheory3  ctheory4  CRT1  CRT2  CRT3  employ_status1  generosity1  generosity2  generosity3  health_cond  marital1  mor_circle  moralid1  moralid10  moralid2  moralid3  moralid4  moralid5  moralid6  moralid7  moralid8  moralid9  mcoop1  mcoop2  mcoop3  mcoop4  mcoop5  mcoop6  mcoop7  narc1  narc2  narc3  narc4  narc5  narc6  nidentity1  nidentity2  omind1  omind2  omind3  omind4  omind5  omind6  contact1  contact2  contact3  contact4  contact5  hygiene1  hygiene2  hygiene3  hygiene4  hygiene5  psupport1  psupport2  psupport3  psupport4  psupport5  political_ideology  happy  slf_ladder  riskperc1  riskperc2  self_esteem  sex1  sbelong1  sbelong2  sbelong3  sbelong4  optim1  optim2  slfcont1  slfcont2  slfcont3  slfcont4  urban
##   5   10  children  cnarc1  cnarc2  cnarc3  ctheory1  ctheory2  ctheory3  ctheory4  CRT1  CRT2  CRT3  employ_status1  generosity1  generosity2  generosity3  health_cond  marital1  mor_circle  moralid1  moralid10  moralid2  moralid3  moralid4  moralid5  moralid6  moralid7  moralid8  moralid9  mcoop1  mcoop2  mcoop3  mcoop4  mcoop5  mcoop6  mcoop7  narc1  narc2  narc3  narc4  narc5  narc6  nidentity1  nidentity2  omind1  omind2  omind3  omind4  omind5  omind6  contact1  contact2  contact3  contact4  contact5  hygiene1  hygiene2  hygiene3  hygiene4  hygiene5  psupport1  psupport2  psupport3  psupport4  psupport5  political_ideology  happy  slf_ladder  riskperc1  riskperc2  self_esteem  sex1  sbelong1  sbelong2  sbelong3  sbelong4  optim1  optim2  slfcont1  slfcont2  slfcont3  slfcont4  urban
##   6   1  children  cnarc1  cnarc2  cnarc3  ctheory1  ctheory2  ctheory3  ctheory4  CRT1  CRT2  CRT3  employ_status1  generosity1  generosity2  generosity3  health_cond  marital1  mor_circle  moralid1  moralid10  moralid2  moralid3  moralid4  moralid5  moralid6  moralid7  moralid8  moralid9  mcoop1  mcoop2  mcoop3  mcoop4  mcoop5  mcoop6  mcoop7  narc1  narc2  narc3  narc4  narc5  narc6  nidentity1  nidentity2  omind1  omind2  omind3  omind4  omind5  omind6  contact1  contact2  contact3  contact4  contact5  hygiene1  hygiene2  hygiene3  hygiene4  hygiene5  psupport1  psupport2  psupport3  psupport4  psupport5  political_ideology  happy  slf_ladder  riskperc1  riskperc2  self_esteem  sex1  sbelong1  sbelong2  sbelong3  sbelong4  optim1  optim2  slfcont1  slfcont2  slfcont3  slfcont4  urban
##   6   2  children  cnarc1  cnarc2  cnarc3  ctheory1  ctheory2  ctheory3  ctheory4  CRT1  CRT2  CRT3  employ_status1  generosity1  generosity2  generosity3  health_cond  marital1  mor_circle  moralid1  moralid10  moralid2  moralid3  moralid4  moralid5  moralid6  moralid7  moralid8  moralid9  mcoop1  mcoop2  mcoop3  mcoop4  mcoop5  mcoop6  mcoop7  narc1  narc2  narc3  narc4  narc5  narc6  nidentity1  nidentity2  omind1  omind2  omind3  omind4  omind5  omind6  contact1  contact2  contact3  contact4  contact5  hygiene1  hygiene2  hygiene3  hygiene4  hygiene5  psupport1  psupport2  psupport3  psupport4  psupport5  political_ideology  happy  slf_ladder  riskperc1  riskperc2  self_esteem  sex1  sbelong1  sbelong2  sbelong3  sbelong4  optim1  optim2  slfcont1  slfcont2  slfcont3  slfcont4  urban
##   6   3  children  cnarc1  cnarc2  cnarc3  ctheory1  ctheory2  ctheory3  ctheory4  CRT1  CRT2  CRT3  employ_status1  generosity1  generosity2  generosity3  health_cond  marital1  mor_circle  moralid1  moralid10  moralid2  moralid3  moralid4  moralid5  moralid6  moralid7  moralid8  moralid9  mcoop1  mcoop2  mcoop3  mcoop4  mcoop5  mcoop6  mcoop7  narc1  narc2  narc3  narc4  narc5  narc6  nidentity1  nidentity2  omind1  omind2  omind3  omind4  omind5  omind6  contact1  contact2  contact3  contact4  contact5  hygiene1  hygiene2  hygiene3  hygiene4  hygiene5  psupport1  psupport2  psupport3  psupport4  psupport5  political_ideology  happy  slf_ladder  riskperc1  riskperc2  self_esteem  sex1  sbelong1  sbelong2  sbelong3  sbelong4  optim1  optim2  slfcont1  slfcont2  slfcont3  slfcont4  urban
##   6   4  children  cnarc1  cnarc2  cnarc3  ctheory1  ctheory2  ctheory3  ctheory4  CRT1  CRT2  CRT3  employ_status1  generosity1  generosity2  generosity3  health_cond  marital1  mor_circle  moralid1  moralid10  moralid2  moralid3  moralid4  moralid5  moralid6  moralid7  moralid8  moralid9  mcoop1  mcoop2  mcoop3  mcoop4  mcoop5  mcoop6  mcoop7  narc1  narc2  narc3  narc4  narc5  narc6  nidentity1  nidentity2  omind1  omind2  omind3  omind4  omind5  omind6  contact1  contact2  contact3  contact4  contact5  hygiene1  hygiene2  hygiene3  hygiene4  hygiene5  psupport1  psupport2  psupport3  psupport4  psupport5  political_ideology  happy  slf_ladder  riskperc1  riskperc2  self_esteem  sex1  sbelong1  sbelong2  sbelong3  sbelong4  optim1  optim2  slfcont1  slfcont2  slfcont3  slfcont4  urban
##   6   5  children  cnarc1  cnarc2  cnarc3  ctheory1  ctheory2  ctheory3  ctheory4  CRT1  CRT2  CRT3  employ_status1  generosity1  generosity2  generosity3  health_cond  marital1  mor_circle  moralid1  moralid10  moralid2  moralid3  moralid4  moralid5  moralid6  moralid7  moralid8  moralid9  mcoop1  mcoop2  mcoop3  mcoop4  mcoop5  mcoop6  mcoop7  narc1  narc2  narc3  narc4  narc5  narc6  nidentity1  nidentity2  omind1  omind2  omind3  omind4  omind5  omind6  contact1  contact2  contact3  contact4  contact5  hygiene1  hygiene2  hygiene3  hygiene4  hygiene5  psupport1  psupport2  psupport3  psupport4  psupport5  political_ideology  happy  slf_ladder  riskperc1  riskperc2  self_esteem  sex1  sbelong1  sbelong2  sbelong3  sbelong4  optim1  optim2  slfcont1  slfcont2  slfcont3  slfcont4  urban
##   6   6  children  cnarc1  cnarc2  cnarc3  ctheory1  ctheory2  ctheory3  ctheory4  CRT1  CRT2  CRT3  employ_status1  generosity1  generosity2  generosity3  health_cond  marital1  mor_circle  moralid1  moralid10  moralid2  moralid3  moralid4  moralid5  moralid6  moralid7  moralid8  moralid9  mcoop1  mcoop2  mcoop3  mcoop4  mcoop5  mcoop6  mcoop7  narc1  narc2  narc3  narc4  narc5  narc6  nidentity1  nidentity2  omind1  omind2  omind3  omind4  omind5  omind6  contact1  contact2  contact3  contact4  contact5  hygiene1  hygiene2  hygiene3  hygiene4  hygiene5  psupport1  psupport2  psupport3  psupport4  psupport5  political_ideology  happy  slf_ladder  riskperc1  riskperc2  self_esteem  sex1  sbelong1  sbelong2  sbelong3  sbelong4  optim1  optim2  slfcont1  slfcont2  slfcont3  slfcont4  urban
##   6   7  children  cnarc1  cnarc2  cnarc3  ctheory1  ctheory2  ctheory3  ctheory4  CRT1  CRT2  CRT3  employ_status1  generosity1  generosity2  generosity3  health_cond  marital1  mor_circle  moralid1  moralid10  moralid2  moralid3  moralid4  moralid5  moralid6  moralid7  moralid8  moralid9  mcoop1  mcoop2  mcoop3  mcoop4  mcoop5  mcoop6  mcoop7  narc1  narc2  narc3  narc4  narc5  narc6  nidentity1  nidentity2  omind1  omind2  omind3  omind4  omind5  omind6  contact1  contact2  contact3  contact4  contact5  hygiene1  hygiene2  hygiene3  hygiene4  hygiene5  psupport1  psupport2  psupport3  psupport4  psupport5  political_ideology  happy  slf_ladder  riskperc1  riskperc2  self_esteem  sex1  sbelong1  sbelong2  sbelong3  sbelong4  optim1  optim2  slfcont1  slfcont2  slfcont3  slfcont4  urban
##   6   8  children  cnarc1  cnarc2  cnarc3  ctheory1  ctheory2  ctheory3  ctheory4  CRT1  CRT2  CRT3  employ_status1  generosity1  generosity2  generosity3  health_cond  marital1  mor_circle  moralid1  moralid10  moralid2  moralid3  moralid4  moralid5  moralid6  moralid7  moralid8  moralid9  mcoop1  mcoop2  mcoop3  mcoop4  mcoop5  mcoop6  mcoop7  narc1  narc2  narc3  narc4  narc5  narc6  nidentity1  nidentity2  omind1  omind2  omind3  omind4  omind5  omind6  contact1  contact2  contact3  contact4  contact5  hygiene1  hygiene2  hygiene3  hygiene4  hygiene5  psupport1  psupport2  psupport3  psupport4  psupport5  political_ideology  happy  slf_ladder  riskperc1  riskperc2  self_esteem  sex1  sbelong1  sbelong2  sbelong3  sbelong4  optim1  optim2  slfcont1  slfcont2  slfcont3  slfcont4  urban
##   6   9  children  cnarc1  cnarc2  cnarc3  ctheory1  ctheory2  ctheory3  ctheory4  CRT1  CRT2  CRT3  employ_status1  generosity1  generosity2  generosity3  health_cond  marital1  mor_circle  moralid1  moralid10  moralid2  moralid3  moralid4  moralid5  moralid6  moralid7  moralid8  moralid9  mcoop1  mcoop2  mcoop3  mcoop4  mcoop5  mcoop6  mcoop7  narc1  narc2  narc3  narc4  narc5  narc6  nidentity1  nidentity2  omind1  omind2  omind3  omind4  omind5  omind6  contact1  contact2  contact3  contact4  contact5  hygiene1  hygiene2  hygiene3  hygiene4  hygiene5  psupport1  psupport2  psupport3  psupport4  psupport5  political_ideology  happy  slf_ladder  riskperc1  riskperc2  self_esteem  sex1  sbelong1  sbelong2  sbelong3  sbelong4  optim1  optim2  slfcont1  slfcont2  slfcont3  slfcont4  urban
##   6   10  children  cnarc1  cnarc2  cnarc3  ctheory1  ctheory2  ctheory3  ctheory4  CRT1  CRT2  CRT3  employ_status1  generosity1  generosity2  generosity3  health_cond  marital1  mor_circle  moralid1  moralid10  moralid2  moralid3  moralid4  moralid5  moralid6  moralid7  moralid8  moralid9  mcoop1  mcoop2  mcoop3  mcoop4  mcoop5  mcoop6  mcoop7  narc1  narc2  narc3  narc4  narc5  narc6  nidentity1  nidentity2  omind1  omind2  omind3  omind4  omind5  omind6  contact1  contact2  contact3  contact4  contact5  hygiene1  hygiene2  hygiene3  hygiene4  hygiene5  psupport1  psupport2  psupport3  psupport4  psupport5  political_ideology  happy  slf_ladder  riskperc1  riskperc2  self_esteem  sex1  sbelong1  sbelong2  sbelong3  sbelong4  optim1  optim2  slfcont1  slfcont2  slfcont3  slfcont4  urban
##   7   1  children  cnarc1  cnarc2  cnarc3  ctheory1  ctheory2  ctheory3  ctheory4  CRT1  CRT2  CRT3  employ_status1  generosity1  generosity2  generosity3  health_cond  marital1  mor_circle  moralid1  moralid10  moralid2  moralid3  moralid4  moralid5  moralid6  moralid7  moralid8  moralid9  mcoop1  mcoop2  mcoop3  mcoop4  mcoop5  mcoop6  mcoop7  narc1  narc2  narc3  narc4  narc5  narc6  nidentity1  nidentity2  omind1  omind2  omind3  omind4  omind5  omind6  contact1  contact2  contact3  contact4  contact5  hygiene1  hygiene2  hygiene3  hygiene4  hygiene5  psupport1  psupport2  psupport3  psupport4  psupport5  political_ideology  happy  slf_ladder  riskperc1  riskperc2  self_esteem  sex1  sbelong1  sbelong2  sbelong3  sbelong4  optim1  optim2  slfcont1  slfcont2  slfcont3  slfcont4  urban
##   7   2  children  cnarc1  cnarc2  cnarc3  ctheory1  ctheory2  ctheory3  ctheory4  CRT1  CRT2  CRT3  employ_status1  generosity1  generosity2  generosity3  health_cond  marital1  mor_circle  moralid1  moralid10  moralid2  moralid3  moralid4  moralid5  moralid6  moralid7  moralid8  moralid9  mcoop1  mcoop2  mcoop3  mcoop4  mcoop5  mcoop6  mcoop7  narc1  narc2  narc3  narc4  narc5  narc6  nidentity1  nidentity2  omind1  omind2  omind3  omind4  omind5  omind6  contact1  contact2  contact3  contact4  contact5  hygiene1  hygiene2  hygiene3  hygiene4  hygiene5  psupport1  psupport2  psupport3  psupport4  psupport5  political_ideology  happy  slf_ladder  riskperc1  riskperc2  self_esteem  sex1  sbelong1  sbelong2  sbelong3  sbelong4  optim1  optim2  slfcont1  slfcont2  slfcont3  slfcont4  urban
##   7   3  children  cnarc1  cnarc2  cnarc3  ctheory1  ctheory2  ctheory3  ctheory4  CRT1  CRT2  CRT3  employ_status1  generosity1  generosity2  generosity3  health_cond  marital1  mor_circle  moralid1  moralid10  moralid2  moralid3  moralid4  moralid5  moralid6  moralid7  moralid8  moralid9  mcoop1  mcoop2  mcoop3  mcoop4  mcoop5  mcoop6  mcoop7  narc1  narc2  narc3  narc4  narc5  narc6  nidentity1  nidentity2  omind1  omind2  omind3  omind4  omind5  omind6  contact1  contact2  contact3  contact4  contact5  hygiene1  hygiene2  hygiene3  hygiene4  hygiene5  psupport1  psupport2  psupport3  psupport4  psupport5  political_ideology  happy  slf_ladder  riskperc1  riskperc2  self_esteem  sex1  sbelong1  sbelong2  sbelong3  sbelong4  optim1  optim2  slfcont1  slfcont2  slfcont3  slfcont4  urban
##   7   4  children  cnarc1  cnarc2  cnarc3  ctheory1  ctheory2  ctheory3  ctheory4  CRT1  CRT2  CRT3  employ_status1  generosity1  generosity2  generosity3  health_cond  marital1  mor_circle  moralid1  moralid10  moralid2  moralid3  moralid4  moralid5  moralid6  moralid7  moralid8  moralid9  mcoop1  mcoop2  mcoop3  mcoop4  mcoop5  mcoop6  mcoop7  narc1  narc2  narc3  narc4  narc5  narc6  nidentity1  nidentity2  omind1  omind2  omind3  omind4  omind5  omind6  contact1  contact2  contact3  contact4  contact5  hygiene1  hygiene2  hygiene3  hygiene4  hygiene5  psupport1  psupport2  psupport3  psupport4  psupport5  political_ideology  happy  slf_ladder  riskperc1  riskperc2  self_esteem  sex1  sbelong1  sbelong2  sbelong3  sbelong4  optim1  optim2  slfcont1  slfcont2  slfcont3  slfcont4  urban
##   7   5  children  cnarc1  cnarc2  cnarc3  ctheory1  ctheory2  ctheory3  ctheory4  CRT1  CRT2  CRT3  employ_status1  generosity1  generosity2  generosity3  health_cond  marital1  mor_circle  moralid1  moralid10  moralid2  moralid3  moralid4  moralid5  moralid6  moralid7  moralid8  moralid9  mcoop1  mcoop2  mcoop3  mcoop4  mcoop5  mcoop6  mcoop7  narc1  narc2  narc3  narc4  narc5  narc6  nidentity1  nidentity2  omind1  omind2  omind3  omind4  omind5  omind6  contact1  contact2  contact3  contact4  contact5  hygiene1  hygiene2  hygiene3  hygiene4  hygiene5  psupport1  psupport2  psupport3  psupport4  psupport5  political_ideology  happy  slf_ladder  riskperc1  riskperc2  self_esteem  sex1  sbelong1  sbelong2  sbelong3  sbelong4  optim1  optim2  slfcont1  slfcont2  slfcont3  slfcont4  urban
##   7   6  children  cnarc1  cnarc2  cnarc3  ctheory1  ctheory2  ctheory3  ctheory4  CRT1  CRT2  CRT3  employ_status1  generosity1  generosity2  generosity3  health_cond  marital1  mor_circle  moralid1  moralid10  moralid2  moralid3  moralid4  moralid5  moralid6  moralid7  moralid8  moralid9  mcoop1  mcoop2  mcoop3  mcoop4  mcoop5  mcoop6  mcoop7  narc1  narc2  narc3  narc4  narc5  narc6  nidentity1  nidentity2  omind1  omind2  omind3  omind4  omind5  omind6  contact1  contact2  contact3  contact4  contact5  hygiene1  hygiene2  hygiene3  hygiene4  hygiene5  psupport1  psupport2  psupport3  psupport4  psupport5  political_ideology  happy  slf_ladder  riskperc1  riskperc2  self_esteem  sex1  sbelong1  sbelong2  sbelong3  sbelong4  optim1  optim2  slfcont1  slfcont2  slfcont3  slfcont4  urban
##   7   7  children  cnarc1  cnarc2  cnarc3  ctheory1  ctheory2  ctheory3  ctheory4  CRT1  CRT2  CRT3  employ_status1  generosity1  generosity2  generosity3  health_cond  marital1  mor_circle  moralid1  moralid10  moralid2  moralid3  moralid4  moralid5  moralid6  moralid7  moralid8  moralid9  mcoop1  mcoop2  mcoop3  mcoop4  mcoop5  mcoop6  mcoop7  narc1  narc2  narc3  narc4  narc5  narc6  nidentity1  nidentity2  omind1  omind2  omind3  omind4  omind5  omind6  contact1  contact2  contact3  contact4  contact5  hygiene1  hygiene2  hygiene3  hygiene4  hygiene5  psupport1  psupport2  psupport3  psupport4  psupport5  political_ideology  happy  slf_ladder  riskperc1  riskperc2  self_esteem  sex1  sbelong1  sbelong2  sbelong3  sbelong4  optim1  optim2  slfcont1  slfcont2  slfcont3  slfcont4  urban
##   7   8  children  cnarc1  cnarc2  cnarc3  ctheory1  ctheory2  ctheory3  ctheory4  CRT1  CRT2  CRT3  employ_status1  generosity1  generosity2  generosity3  health_cond  marital1  mor_circle  moralid1  moralid10  moralid2  moralid3  moralid4  moralid5  moralid6  moralid7  moralid8  moralid9  mcoop1  mcoop2  mcoop3  mcoop4  mcoop5  mcoop6  mcoop7  narc1  narc2  narc3  narc4  narc5  narc6  nidentity1  nidentity2  omind1  omind2  omind3  omind4  omind5  omind6  contact1  contact2  contact3  contact4  contact5  hygiene1  hygiene2  hygiene3  hygiene4  hygiene5  psupport1  psupport2  psupport3  psupport4  psupport5  political_ideology  happy  slf_ladder  riskperc1  riskperc2  self_esteem  sex1  sbelong1  sbelong2  sbelong3  sbelong4  optim1  optim2  slfcont1  slfcont2  slfcont3  slfcont4  urban
##   7   9  children  cnarc1  cnarc2  cnarc3  ctheory1  ctheory2  ctheory3  ctheory4  CRT1  CRT2  CRT3  employ_status1  generosity1  generosity2  generosity3  health_cond  marital1  mor_circle  moralid1  moralid10  moralid2  moralid3  moralid4  moralid5  moralid6  moralid7  moralid8  moralid9  mcoop1  mcoop2  mcoop3  mcoop4  mcoop5  mcoop6  mcoop7  narc1  narc2  narc3  narc4  narc5  narc6  nidentity1  nidentity2  omind1  omind2  omind3  omind4  omind5  omind6  contact1  contact2  contact3  contact4  contact5  hygiene1  hygiene2  hygiene3  hygiene4  hygiene5  psupport1  psupport2  psupport3  psupport4  psupport5  political_ideology  happy  slf_ladder  riskperc1  riskperc2  self_esteem  sex1  sbelong1  sbelong2  sbelong3  sbelong4  optim1  optim2  slfcont1  slfcont2  slfcont3  slfcont4  urban
##   7   10  children  cnarc1  cnarc2  cnarc3  ctheory1  ctheory2  ctheory3  ctheory4  CRT1  CRT2  CRT3  employ_status1  generosity1  generosity2  generosity3  health_cond  marital1  mor_circle  moralid1  moralid10  moralid2  moralid3  moralid4  moralid5  moralid6  moralid7  moralid8  moralid9  mcoop1  mcoop2  mcoop3  mcoop4  mcoop5  mcoop6  mcoop7  narc1  narc2  narc3  narc4  narc5  narc6  nidentity1  nidentity2  omind1  omind2  omind3  omind4  omind5  omind6  contact1  contact2  contact3  contact4  contact5  hygiene1  hygiene2  hygiene3  hygiene4  hygiene5  psupport1  psupport2  psupport3  psupport4  psupport5  political_ideology  happy  slf_ladder  riskperc1  riskperc2  self_esteem  sex1  sbelong1  sbelong2  sbelong3  sbelong4  optim1  optim2  slfcont1  slfcont2  slfcont3  slfcont4  urban
##   8   1  children  cnarc1  cnarc2  cnarc3  ctheory1  ctheory2  ctheory3  ctheory4  CRT1  CRT2  CRT3  employ_status1  generosity1  generosity2  generosity3  health_cond  marital1  mor_circle  moralid1  moralid10  moralid2  moralid3  moralid4  moralid5  moralid6  moralid7  moralid8  moralid9  mcoop1  mcoop2  mcoop3  mcoop4  mcoop5  mcoop6  mcoop7  narc1  narc2  narc3  narc4  narc5  narc6  nidentity1  nidentity2  omind1  omind2  omind3  omind4  omind5  omind6  contact1  contact2  contact3  contact4  contact5  hygiene1  hygiene2  hygiene3  hygiene4  hygiene5  psupport1  psupport2  psupport3  psupport4  psupport5  political_ideology  happy  slf_ladder  riskperc1  riskperc2  self_esteem  sex1  sbelong1  sbelong2  sbelong3  sbelong4  optim1  optim2  slfcont1  slfcont2  slfcont3  slfcont4  urban
##   8   2  children  cnarc1  cnarc2  cnarc3  ctheory1  ctheory2  ctheory3  ctheory4  CRT1  CRT2  CRT3  employ_status1  generosity1  generosity2  generosity3  health_cond  marital1  mor_circle  moralid1  moralid10  moralid2  moralid3  moralid4  moralid5  moralid6  moralid7  moralid8  moralid9  mcoop1  mcoop2  mcoop3  mcoop4  mcoop5  mcoop6  mcoop7  narc1  narc2  narc3  narc4  narc5  narc6  nidentity1  nidentity2  omind1  omind2  omind3  omind4  omind5  omind6  contact1  contact2  contact3  contact4  contact5  hygiene1  hygiene2  hygiene3  hygiene4  hygiene5  psupport1  psupport2  psupport3  psupport4  psupport5  political_ideology  happy  slf_ladder  riskperc1  riskperc2  self_esteem  sex1  sbelong1  sbelong2  sbelong3  sbelong4  optim1  optim2  slfcont1  slfcont2  slfcont3  slfcont4  urban
##   8   3  children  cnarc1  cnarc2  cnarc3  ctheory1  ctheory2  ctheory3  ctheory4  CRT1  CRT2  CRT3  employ_status1  generosity1  generosity2  generosity3  health_cond  marital1  mor_circle  moralid1  moralid10  moralid2  moralid3  moralid4  moralid5  moralid6  moralid7  moralid8  moralid9  mcoop1  mcoop2  mcoop3  mcoop4  mcoop5  mcoop6  mcoop7  narc1  narc2  narc3  narc4  narc5  narc6  nidentity1  nidentity2  omind1  omind2  omind3  omind4  omind5  omind6  contact1  contact2  contact3  contact4  contact5  hygiene1  hygiene2  hygiene3  hygiene4  hygiene5  psupport1  psupport2  psupport3  psupport4  psupport5  political_ideology  happy  slf_ladder  riskperc1  riskperc2  self_esteem  sex1  sbelong1  sbelong2  sbelong3  sbelong4  optim1  optim2  slfcont1  slfcont2  slfcont3  slfcont4  urban
##   8   4  children  cnarc1  cnarc2  cnarc3  ctheory1  ctheory2  ctheory3  ctheory4  CRT1  CRT2  CRT3  employ_status1  generosity1  generosity2  generosity3  health_cond  marital1  mor_circle  moralid1  moralid10  moralid2  moralid3  moralid4  moralid5  moralid6  moralid7  moralid8  moralid9  mcoop1  mcoop2  mcoop3  mcoop4  mcoop5  mcoop6  mcoop7  narc1  narc2  narc3  narc4  narc5  narc6  nidentity1  nidentity2  omind1  omind2  omind3  omind4  omind5  omind6  contact1  contact2  contact3  contact4  contact5  hygiene1  hygiene2  hygiene3  hygiene4  hygiene5  psupport1  psupport2  psupport3  psupport4  psupport5  political_ideology  happy  slf_ladder  riskperc1  riskperc2  self_esteem  sex1  sbelong1  sbelong2  sbelong3  sbelong4  optim1  optim2  slfcont1  slfcont2  slfcont3  slfcont4  urban
##   8   5  children  cnarc1  cnarc2  cnarc3  ctheory1  ctheory2  ctheory3  ctheory4  CRT1  CRT2  CRT3  employ_status1  generosity1  generosity2  generosity3  health_cond  marital1  mor_circle  moralid1  moralid10  moralid2  moralid3  moralid4  moralid5  moralid6  moralid7  moralid8  moralid9  mcoop1  mcoop2  mcoop3  mcoop4  mcoop5  mcoop6  mcoop7  narc1  narc2  narc3  narc4  narc5  narc6  nidentity1  nidentity2  omind1  omind2  omind3  omind4  omind5  omind6  contact1  contact2  contact3  contact4  contact5  hygiene1  hygiene2  hygiene3  hygiene4  hygiene5  psupport1  psupport2  psupport3  psupport4  psupport5  political_ideology  happy  slf_ladder  riskperc1  riskperc2  self_esteem  sex1  sbelong1  sbelong2  sbelong3  sbelong4  optim1  optim2  slfcont1  slfcont2  slfcont3  slfcont4  urban
##   8   6  children  cnarc1  cnarc2  cnarc3  ctheory1  ctheory2  ctheory3  ctheory4  CRT1  CRT2  CRT3  employ_status1  generosity1  generosity2  generosity3  health_cond  marital1  mor_circle  moralid1  moralid10  moralid2  moralid3  moralid4  moralid5  moralid6  moralid7  moralid8  moralid9  mcoop1  mcoop2  mcoop3  mcoop4  mcoop5  mcoop6  mcoop7  narc1  narc2  narc3  narc4  narc5  narc6  nidentity1  nidentity2  omind1  omind2  omind3  omind4  omind5  omind6  contact1  contact2  contact3  contact4  contact5  hygiene1  hygiene2  hygiene3  hygiene4  hygiene5  psupport1  psupport2  psupport3  psupport4  psupport5  political_ideology  happy  slf_ladder  riskperc1  riskperc2  self_esteem  sex1  sbelong1  sbelong2  sbelong3  sbelong4  optim1  optim2  slfcont1  slfcont2  slfcont3  slfcont4  urban
##   8   7  children  cnarc1  cnarc2  cnarc3  ctheory1  ctheory2  ctheory3  ctheory4  CRT1  CRT2  CRT3  employ_status1  generosity1  generosity2  generosity3  health_cond  marital1  mor_circle  moralid1  moralid10  moralid2  moralid3  moralid4  moralid5  moralid6  moralid7  moralid8  moralid9  mcoop1  mcoop2  mcoop3  mcoop4  mcoop5  mcoop6  mcoop7  narc1  narc2  narc3  narc4  narc5  narc6  nidentity1  nidentity2  omind1  omind2  omind3  omind4  omind5  omind6  contact1  contact2  contact3  contact4  contact5  hygiene1  hygiene2  hygiene3  hygiene4  hygiene5  psupport1  psupport2  psupport3  psupport4  psupport5  political_ideology  happy  slf_ladder  riskperc1  riskperc2  self_esteem  sex1  sbelong1  sbelong2  sbelong3  sbelong4  optim1  optim2  slfcont1  slfcont2  slfcont3  slfcont4  urban
##   8   8  children  cnarc1  cnarc2  cnarc3  ctheory1  ctheory2  ctheory3  ctheory4  CRT1  CRT2  CRT3  employ_status1  generosity1  generosity2  generosity3  health_cond  marital1  mor_circle  moralid1  moralid10  moralid2  moralid3  moralid4  moralid5  moralid6  moralid7  moralid8  moralid9  mcoop1  mcoop2  mcoop3  mcoop4  mcoop5  mcoop6  mcoop7  narc1  narc2  narc3  narc4  narc5  narc6  nidentity1  nidentity2  omind1  omind2  omind3  omind4  omind5  omind6  contact1  contact2  contact3  contact4  contact5  hygiene1  hygiene2  hygiene3  hygiene4  hygiene5  psupport1  psupport2  psupport3  psupport4  psupport5  political_ideology  happy  slf_ladder  riskperc1  riskperc2  self_esteem  sex1  sbelong1  sbelong2  sbelong3  sbelong4  optim1  optim2  slfcont1  slfcont2  slfcont3  slfcont4  urban
##   8   9  children  cnarc1  cnarc2  cnarc3  ctheory1  ctheory2  ctheory3  ctheory4  CRT1  CRT2  CRT3  employ_status1  generosity1  generosity2  generosity3  health_cond  marital1  mor_circle  moralid1  moralid10  moralid2  moralid3  moralid4  moralid5  moralid6  moralid7  moralid8  moralid9  mcoop1  mcoop2  mcoop3  mcoop4  mcoop5  mcoop6  mcoop7  narc1  narc2  narc3  narc4  narc5  narc6  nidentity1  nidentity2  omind1  omind2  omind3  omind4  omind5  omind6  contact1  contact2  contact3  contact4  contact5  hygiene1  hygiene2  hygiene3  hygiene4  hygiene5  psupport1  psupport2  psupport3  psupport4  psupport5  political_ideology  happy  slf_ladder  riskperc1  riskperc2  self_esteem  sex1  sbelong1  sbelong2  sbelong3  sbelong4  optim1  optim2  slfcont1  slfcont2  slfcont3  slfcont4  urban
##   8   10  children  cnarc1  cnarc2  cnarc3  ctheory1  ctheory2  ctheory3  ctheory4  CRT1  CRT2  CRT3  employ_status1  generosity1  generosity2  generosity3  health_cond  marital1  mor_circle  moralid1  moralid10  moralid2  moralid3  moralid4  moralid5  moralid6  moralid7  moralid8  moralid9  mcoop1  mcoop2  mcoop3  mcoop4  mcoop5  mcoop6  mcoop7  narc1  narc2  narc3  narc4  narc5  narc6  nidentity1  nidentity2  omind1  omind2  omind3  omind4  omind5  omind6  contact1  contact2  contact3  contact4  contact5  hygiene1  hygiene2  hygiene3  hygiene4  hygiene5  psupport1  psupport2  psupport3  psupport4  psupport5  political_ideology  happy  slf_ladder  riskperc1  riskperc2  self_esteem  sex1  sbelong1  sbelong2  sbelong3  sbelong4  optim1  optim2  slfcont1  slfcont2  slfcont3  slfcont4  urban
##   9   1  children  cnarc1  cnarc2  cnarc3  ctheory1  ctheory2  ctheory3  ctheory4  CRT1  CRT2  CRT3  employ_status1  generosity1  generosity2  generosity3  health_cond  marital1  mor_circle  moralid1  moralid10  moralid2  moralid3  moralid4  moralid5  moralid6  moralid7  moralid8  moralid9  mcoop1  mcoop2  mcoop3  mcoop4  mcoop5  mcoop6  mcoop7  narc1  narc2  narc3  narc4  narc5  narc6  nidentity1  nidentity2  omind1  omind2  omind3  omind4  omind5  omind6  contact1  contact2  contact3  contact4  contact5  hygiene1  hygiene2  hygiene3  hygiene4  hygiene5  psupport1  psupport2  psupport3  psupport4  psupport5  political_ideology  happy  slf_ladder  riskperc1  riskperc2  self_esteem  sex1  sbelong1  sbelong2  sbelong3  sbelong4  optim1  optim2  slfcont1  slfcont2  slfcont3  slfcont4  urban
##   9   2  children  cnarc1  cnarc2  cnarc3  ctheory1  ctheory2  ctheory3  ctheory4  CRT1  CRT2  CRT3  employ_status1  generosity1  generosity2  generosity3  health_cond  marital1  mor_circle  moralid1  moralid10  moralid2  moralid3  moralid4  moralid5  moralid6  moralid7  moralid8  moralid9  mcoop1  mcoop2  mcoop3  mcoop4  mcoop5  mcoop6  mcoop7  narc1  narc2  narc3  narc4  narc5  narc6  nidentity1  nidentity2  omind1  omind2  omind3  omind4  omind5  omind6  contact1  contact2  contact3  contact4  contact5  hygiene1  hygiene2  hygiene3  hygiene4  hygiene5  psupport1  psupport2  psupport3  psupport4  psupport5  political_ideology  happy  slf_ladder  riskperc1  riskperc2  self_esteem  sex1  sbelong1  sbelong2  sbelong3  sbelong4  optim1  optim2  slfcont1  slfcont2  slfcont3  slfcont4  urban
##   9   3  children  cnarc1  cnarc2  cnarc3  ctheory1  ctheory2  ctheory3  ctheory4  CRT1  CRT2  CRT3  employ_status1  generosity1  generosity2  generosity3  health_cond  marital1  mor_circle  moralid1  moralid10  moralid2  moralid3  moralid4  moralid5  moralid6  moralid7  moralid8  moralid9  mcoop1  mcoop2  mcoop3  mcoop4  mcoop5  mcoop6  mcoop7  narc1  narc2  narc3  narc4  narc5  narc6  nidentity1  nidentity2  omind1  omind2  omind3  omind4  omind5  omind6  contact1  contact2  contact3  contact4  contact5  hygiene1  hygiene2  hygiene3  hygiene4  hygiene5  psupport1  psupport2  psupport3  psupport4  psupport5  political_ideology  happy  slf_ladder  riskperc1  riskperc2  self_esteem  sex1  sbelong1  sbelong2  sbelong3  sbelong4  optim1  optim2  slfcont1  slfcont2  slfcont3  slfcont4  urban
##   9   4  children  cnarc1  cnarc2  cnarc3  ctheory1  ctheory2  ctheory3  ctheory4  CRT1  CRT2  CRT3  employ_status1  generosity1  generosity2  generosity3  health_cond  marital1  mor_circle  moralid1  moralid10  moralid2  moralid3  moralid4  moralid5  moralid6  moralid7  moralid8  moralid9  mcoop1  mcoop2  mcoop3  mcoop4  mcoop5  mcoop6  mcoop7  narc1  narc2  narc3  narc4  narc5  narc6  nidentity1  nidentity2  omind1  omind2  omind3  omind4  omind5  omind6  contact1  contact2  contact3  contact4  contact5  hygiene1  hygiene2  hygiene3  hygiene4  hygiene5  psupport1  psupport2  psupport3  psupport4  psupport5  political_ideology  happy  slf_ladder  riskperc1  riskperc2  self_esteem  sex1  sbelong1  sbelong2  sbelong3  sbelong4  optim1  optim2  slfcont1  slfcont2  slfcont3  slfcont4  urban
##   9   5  children  cnarc1  cnarc2  cnarc3  ctheory1  ctheory2  ctheory3  ctheory4  CRT1  CRT2  CRT3  employ_status1  generosity1  generosity2  generosity3  health_cond  marital1  mor_circle  moralid1  moralid10  moralid2  moralid3  moralid4  moralid5  moralid6  moralid7  moralid8  moralid9  mcoop1  mcoop2  mcoop3  mcoop4  mcoop5  mcoop6  mcoop7  narc1  narc2  narc3  narc4  narc5  narc6  nidentity1  nidentity2  omind1  omind2  omind3  omind4  omind5  omind6  contact1  contact2  contact3  contact4  contact5  hygiene1  hygiene2  hygiene3  hygiene4  hygiene5  psupport1  psupport2  psupport3  psupport4  psupport5  political_ideology  happy  slf_ladder  riskperc1  riskperc2  self_esteem  sex1  sbelong1  sbelong2  sbelong3  sbelong4  optim1  optim2  slfcont1  slfcont2  slfcont3  slfcont4  urban
##   9   6  children  cnarc1  cnarc2  cnarc3  ctheory1  ctheory2  ctheory3  ctheory4  CRT1  CRT2  CRT3  employ_status1  generosity1  generosity2  generosity3  health_cond  marital1  mor_circle  moralid1  moralid10  moralid2  moralid3  moralid4  moralid5  moralid6  moralid7  moralid8  moralid9  mcoop1  mcoop2  mcoop3  mcoop4  mcoop5  mcoop6  mcoop7  narc1  narc2  narc3  narc4  narc5  narc6  nidentity1  nidentity2  omind1  omind2  omind3  omind4  omind5  omind6  contact1  contact2  contact3  contact4  contact5  hygiene1  hygiene2  hygiene3  hygiene4  hygiene5  psupport1  psupport2  psupport3  psupport4  psupport5  political_ideology  happy  slf_ladder  riskperc1  riskperc2  self_esteem  sex1  sbelong1  sbelong2  sbelong3  sbelong4  optim1  optim2  slfcont1  slfcont2  slfcont3  slfcont4  urban
##   9   7  children  cnarc1  cnarc2  cnarc3  ctheory1  ctheory2  ctheory3  ctheory4  CRT1  CRT2  CRT3  employ_status1  generosity1  generosity2  generosity3  health_cond  marital1  mor_circle  moralid1  moralid10  moralid2  moralid3  moralid4  moralid5  moralid6  moralid7  moralid8  moralid9  mcoop1  mcoop2  mcoop3  mcoop4  mcoop5  mcoop6  mcoop7  narc1  narc2  narc3  narc4  narc5  narc6  nidentity1  nidentity2  omind1  omind2  omind3  omind4  omind5  omind6  contact1  contact2  contact3  contact4  contact5  hygiene1  hygiene2  hygiene3  hygiene4  hygiene5  psupport1  psupport2  psupport3  psupport4  psupport5  political_ideology  happy  slf_ladder  riskperc1  riskperc2  self_esteem  sex1  sbelong1  sbelong2  sbelong3  sbelong4  optim1  optim2  slfcont1  slfcont2  slfcont3  slfcont4  urban
##   9   8  children  cnarc1  cnarc2  cnarc3  ctheory1  ctheory2  ctheory3  ctheory4  CRT1  CRT2  CRT3  employ_status1  generosity1  generosity2  generosity3  health_cond  marital1  mor_circle  moralid1  moralid10  moralid2  moralid3  moralid4  moralid5  moralid6  moralid7  moralid8  moralid9  mcoop1  mcoop2  mcoop3  mcoop4  mcoop5  mcoop6  mcoop7  narc1  narc2  narc3  narc4  narc5  narc6  nidentity1  nidentity2  omind1  omind2  omind3  omind4  omind5  omind6  contact1  contact2  contact3  contact4  contact5  hygiene1  hygiene2  hygiene3  hygiene4  hygiene5  psupport1  psupport2  psupport3  psupport4  psupport5  political_ideology  happy  slf_ladder  riskperc1  riskperc2  self_esteem  sex1  sbelong1  sbelong2  sbelong3  sbelong4  optim1  optim2  slfcont1  slfcont2  slfcont3  slfcont4  urban
##   9   9  children  cnarc1  cnarc2  cnarc3  ctheory1  ctheory2  ctheory3  ctheory4  CRT1  CRT2  CRT3  employ_status1  generosity1  generosity2  generosity3  health_cond  marital1  mor_circle  moralid1  moralid10  moralid2  moralid3  moralid4  moralid5  moralid6  moralid7  moralid8  moralid9  mcoop1  mcoop2  mcoop3  mcoop4  mcoop5  mcoop6  mcoop7  narc1  narc2  narc3  narc4  narc5  narc6  nidentity1  nidentity2  omind1  omind2  omind3  omind4  omind5  omind6  contact1  contact2  contact3  contact4  contact5  hygiene1  hygiene2  hygiene3  hygiene4  hygiene5  psupport1  psupport2  psupport3  psupport4  psupport5  political_ideology  happy  slf_ladder  riskperc1  riskperc2  self_esteem  sex1  sbelong1  sbelong2  sbelong3  sbelong4  optim1  optim2  slfcont1  slfcont2  slfcont3  slfcont4  urban
##   9   10  children  cnarc1  cnarc2  cnarc3  ctheory1  ctheory2  ctheory3  ctheory4  CRT1  CRT2  CRT3  employ_status1  generosity1  generosity2  generosity3  health_cond  marital1  mor_circle  moralid1  moralid10  moralid2  moralid3  moralid4  moralid5  moralid6  moralid7  moralid8  moralid9  mcoop1  mcoop2  mcoop3  mcoop4  mcoop5  mcoop6  mcoop7  narc1  narc2  narc3  narc4  narc5  narc6  nidentity1  nidentity2  omind1  omind2  omind3  omind4  omind5  omind6  contact1  contact2  contact3  contact4  contact5  hygiene1  hygiene2  hygiene3  hygiene4  hygiene5  psupport1  psupport2  psupport3  psupport4  psupport5  political_ideology  happy  slf_ladder  riskperc1  riskperc2  self_esteem  sex1  sbelong1  sbelong2  sbelong3  sbelong4  optim1  optim2  slfcont1  slfcont2  slfcont3  slfcont4  urban
##   10   1  children  cnarc1  cnarc2  cnarc3  ctheory1  ctheory2  ctheory3  ctheory4  CRT1  CRT2  CRT3  employ_status1  generosity1  generosity2  generosity3  health_cond  marital1  mor_circle  moralid1  moralid10  moralid2  moralid3  moralid4  moralid5  moralid6  moralid7  moralid8  moralid9  mcoop1  mcoop2  mcoop3  mcoop4  mcoop5  mcoop6  mcoop7  narc1  narc2  narc3  narc4  narc5  narc6  nidentity1  nidentity2  omind1  omind2  omind3  omind4  omind5  omind6  contact1  contact2  contact3  contact4  contact5  hygiene1  hygiene2  hygiene3  hygiene4  hygiene5  psupport1  psupport2  psupport3  psupport4  psupport5  political_ideology  happy  slf_ladder  riskperc1  riskperc2  self_esteem  sex1  sbelong1  sbelong2  sbelong3  sbelong4  optim1  optim2  slfcont1  slfcont2  slfcont3  slfcont4  urban
##   10   2  children  cnarc1  cnarc2  cnarc3  ctheory1  ctheory2  ctheory3  ctheory4  CRT1  CRT2  CRT3  employ_status1  generosity1  generosity2  generosity3  health_cond  marital1  mor_circle  moralid1  moralid10  moralid2  moralid3  moralid4  moralid5  moralid6  moralid7  moralid8  moralid9  mcoop1  mcoop2  mcoop3  mcoop4  mcoop5  mcoop6  mcoop7  narc1  narc2  narc3  narc4  narc5  narc6  nidentity1  nidentity2  omind1  omind2  omind3  omind4  omind5  omind6  contact1  contact2  contact3  contact4  contact5  hygiene1  hygiene2  hygiene3  hygiene4  hygiene5  psupport1  psupport2  psupport3  psupport4  psupport5  political_ideology  happy  slf_ladder  riskperc1  riskperc2  self_esteem  sex1  sbelong1  sbelong2  sbelong3  sbelong4  optim1  optim2  slfcont1  slfcont2  slfcont3  slfcont4  urban
##   10   3  children  cnarc1  cnarc2  cnarc3  ctheory1  ctheory2  ctheory3  ctheory4  CRT1  CRT2  CRT3  employ_status1  generosity1  generosity2  generosity3  health_cond  marital1  mor_circle  moralid1  moralid10  moralid2  moralid3  moralid4  moralid5  moralid6  moralid7  moralid8  moralid9  mcoop1  mcoop2  mcoop3  mcoop4  mcoop5  mcoop6  mcoop7  narc1  narc2  narc3  narc4  narc5  narc6  nidentity1  nidentity2  omind1  omind2  omind3  omind4  omind5  omind6  contact1  contact2  contact3  contact4  contact5  hygiene1  hygiene2  hygiene3  hygiene4  hygiene5  psupport1  psupport2  psupport3  psupport4  psupport5  political_ideology  happy  slf_ladder  riskperc1  riskperc2  self_esteem  sex1  sbelong1  sbelong2  sbelong3  sbelong4  optim1  optim2  slfcont1  slfcont2  slfcont3  slfcont4  urban
##   10   4  children  cnarc1  cnarc2  cnarc3  ctheory1  ctheory2  ctheory3  ctheory4  CRT1  CRT2  CRT3  employ_status1  generosity1  generosity2  generosity3  health_cond  marital1  mor_circle  moralid1  moralid10  moralid2  moralid3  moralid4  moralid5  moralid6  moralid7  moralid8  moralid9  mcoop1  mcoop2  mcoop3  mcoop4  mcoop5  mcoop6  mcoop7  narc1  narc2  narc3  narc4  narc5  narc6  nidentity1  nidentity2  omind1  omind2  omind3  omind4  omind5  omind6  contact1  contact2  contact3  contact4  contact5  hygiene1  hygiene2  hygiene3  hygiene4  hygiene5  psupport1  psupport2  psupport3  psupport4  psupport5  political_ideology  happy  slf_ladder  riskperc1  riskperc2  self_esteem  sex1  sbelong1  sbelong2  sbelong3  sbelong4  optim1  optim2  slfcont1  slfcont2  slfcont3  slfcont4  urban
##   10   5  children  cnarc1  cnarc2  cnarc3  ctheory1  ctheory2  ctheory3  ctheory4  CRT1  CRT2  CRT3  employ_status1  generosity1  generosity2  generosity3  health_cond  marital1  mor_circle  moralid1  moralid10  moralid2  moralid3  moralid4  moralid5  moralid6  moralid7  moralid8  moralid9  mcoop1  mcoop2  mcoop3  mcoop4  mcoop5  mcoop6  mcoop7  narc1  narc2  narc3  narc4  narc5  narc6  nidentity1  nidentity2  omind1  omind2  omind3  omind4  omind5  omind6  contact1  contact2  contact3  contact4  contact5  hygiene1  hygiene2  hygiene3  hygiene4  hygiene5  psupport1  psupport2  psupport3  psupport4  psupport5  political_ideology  happy  slf_ladder  riskperc1  riskperc2  self_esteem  sex1  sbelong1  sbelong2  sbelong3  sbelong4  optim1  optim2  slfcont1  slfcont2  slfcont3  slfcont4  urban
##   10   6  children  cnarc1  cnarc2  cnarc3  ctheory1  ctheory2  ctheory3  ctheory4  CRT1  CRT2  CRT3  employ_status1  generosity1  generosity2  generosity3  health_cond  marital1  mor_circle  moralid1  moralid10  moralid2  moralid3  moralid4  moralid5  moralid6  moralid7  moralid8  moralid9  mcoop1  mcoop2  mcoop3  mcoop4  mcoop5  mcoop6  mcoop7  narc1  narc2  narc3  narc4  narc5  narc6  nidentity1  nidentity2  omind1  omind2  omind3  omind4  omind5  omind6  contact1  contact2  contact3  contact4  contact5  hygiene1  hygiene2  hygiene3  hygiene4  hygiene5  psupport1  psupport2  psupport3  psupport4  psupport5  political_ideology  happy  slf_ladder  riskperc1  riskperc2  self_esteem  sex1  sbelong1  sbelong2  sbelong3  sbelong4  optim1  optim2  slfcont1  slfcont2  slfcont3  slfcont4  urban
##   10   7  children  cnarc1  cnarc2  cnarc3  ctheory1  ctheory2  ctheory3  ctheory4  CRT1  CRT2  CRT3  employ_status1  generosity1  generosity2  generosity3  health_cond  marital1  mor_circle  moralid1  moralid10  moralid2  moralid3  moralid4  moralid5  moralid6  moralid7  moralid8  moralid9  mcoop1  mcoop2  mcoop3  mcoop4  mcoop5  mcoop6  mcoop7  narc1  narc2  narc3  narc4  narc5  narc6  nidentity1  nidentity2  omind1  omind2  omind3  omind4  omind5  omind6  contact1  contact2  contact3  contact4  contact5  hygiene1  hygiene2  hygiene3  hygiene4  hygiene5  psupport1  psupport2  psupport3  psupport4  psupport5  political_ideology  happy  slf_ladder  riskperc1  riskperc2  self_esteem  sex1  sbelong1  sbelong2  sbelong3  sbelong4  optim1  optim2  slfcont1  slfcont2  slfcont3  slfcont4  urban
##   10   8  children  cnarc1  cnarc2  cnarc3  ctheory1  ctheory2  ctheory3  ctheory4  CRT1  CRT2  CRT3  employ_status1  generosity1  generosity2  generosity3  health_cond  marital1  mor_circle  moralid1  moralid10  moralid2  moralid3  moralid4  moralid5  moralid6  moralid7  moralid8  moralid9  mcoop1  mcoop2  mcoop3  mcoop4  mcoop5  mcoop6  mcoop7  narc1  narc2  narc3  narc4  narc5  narc6  nidentity1  nidentity2  omind1  omind2  omind3  omind4  omind5  omind6  contact1  contact2  contact3  contact4  contact5  hygiene1  hygiene2  hygiene3  hygiene4  hygiene5  psupport1  psupport2  psupport3  psupport4  psupport5  political_ideology  happy  slf_ladder  riskperc1  riskperc2  self_esteem  sex1  sbelong1  sbelong2  sbelong3  sbelong4  optim1  optim2  slfcont1  slfcont2  slfcont3  slfcont4  urban
##   10   9  children  cnarc1  cnarc2  cnarc3  ctheory1  ctheory2  ctheory3  ctheory4  CRT1  CRT2  CRT3  employ_status1  generosity1  generosity2  generosity3  health_cond  marital1  mor_circle  moralid1  moralid10  moralid2  moralid3  moralid4  moralid5  moralid6  moralid7  moralid8  moralid9  mcoop1  mcoop2  mcoop3  mcoop4  mcoop5  mcoop6  mcoop7  narc1  narc2  narc3  narc4  narc5  narc6  nidentity1  nidentity2  omind1  omind2  omind3  omind4  omind5  omind6  contact1  contact2  contact3  contact4  contact5  hygiene1  hygiene2  hygiene3  hygiene4  hygiene5  psupport1  psupport2  psupport3  psupport4  psupport5  political_ideology  happy  slf_ladder  riskperc1  riskperc2  self_esteem  sex1  sbelong1  sbelong2  sbelong3  sbelong4  optim1  optim2  slfcont1  slfcont2  slfcont3  slfcont4  urban
##   10   10  children  cnarc1  cnarc2  cnarc3  ctheory1  ctheory2  ctheory3  ctheory4  CRT1  CRT2  CRT3  employ_status1  generosity1  generosity2  generosity3  health_cond  marital1  mor_circle  moralid1  moralid10  moralid2  moralid3  moralid4  moralid5  moralid6  moralid7  moralid8  moralid9  mcoop1  mcoop2  mcoop3  mcoop4  mcoop5  mcoop6  mcoop7  narc1  narc2  narc3  narc4  narc5  narc6  nidentity1  nidentity2  omind1  omind2  omind3  omind4  omind5  omind6  contact1  contact2  contact3  contact4  contact5  hygiene1  hygiene2  hygiene3  hygiene4  hygiene5  psupport1  psupport2  psupport3  psupport4  psupport5  political_ideology  happy  slf_ladder  riskperc1  riskperc2  self_esteem  sex1  sbelong1  sbelong2  sbelong3  sbelong4  optim1  optim2  slfcont1  slfcont2  slfcont3  slfcont4  urban
```

```
## Warning: Number of logged events: 7813
```

```
## 
##  iter imp variable
##   1   1  age  cnarc1  cnarc2  cnarc3  CRT1  CRT2  CRT3  duration  generosity1  generosity2  generosity3  marital1  mor_circle  moralid1  moralid10  moralid2  moralid3  moralid4  moralid5  moralid6  moralid7  moralid8  moralid9  mcoop1  mcoop2  mcoop3  mcoop4  mcoop5  mcoop6  mcoop7  narc1  narc3  narc4  narc5  omind5  omind6  contact1  contact2  contact3  contact4  contact5  hygiene1  hygiene2  hygiene3  hygiene4  hygiene5  psupport1  psupport2  psupport3  psupport4  psupport5  political_ideology  happy  riskperc1  riskperc2  self_esteem  sbelong1  sbelong2  sbelong3  slfcont3  urban
##   1   2  age  cnarc1  cnarc2  cnarc3  CRT1  CRT2  CRT3  duration  generosity1  generosity2  generosity3  marital1  mor_circle  moralid1  moralid10  moralid2  moralid3  moralid4  moralid5  moralid6  moralid7  moralid8  moralid9  mcoop1  mcoop2  mcoop3  mcoop4  mcoop5  mcoop6  mcoop7  narc1  narc3  narc4  narc5  omind5  omind6  contact1  contact2  contact3  contact4  contact5  hygiene1  hygiene2  hygiene3  hygiene4  hygiene5  psupport1  psupport2  psupport3  psupport4  psupport5  political_ideology  happy  riskperc1  riskperc2  self_esteem  sbelong1  sbelong2  sbelong3  slfcont3  urban
##   1   3  age  cnarc1  cnarc2  cnarc3  CRT1  CRT2  CRT3  duration  generosity1  generosity2  generosity3  marital1  mor_circle  moralid1  moralid10  moralid2  moralid3  moralid4  moralid5  moralid6  moralid7  moralid8  moralid9  mcoop1  mcoop2  mcoop3  mcoop4  mcoop5  mcoop6  mcoop7  narc1  narc3  narc4  narc5  omind5  omind6  contact1  contact2  contact3  contact4  contact5  hygiene1  hygiene2  hygiene3  hygiene4  hygiene5  psupport1  psupport2  psupport3  psupport4  psupport5  political_ideology  happy  riskperc1  riskperc2  self_esteem  sbelong1  sbelong2  sbelong3  slfcont3  urban
##   1   4  age  cnarc1  cnarc2  cnarc3  CRT1  CRT2  CRT3  duration  generosity1  generosity2  generosity3  marital1  mor_circle  moralid1  moralid10  moralid2  moralid3  moralid4  moralid5  moralid6  moralid7  moralid8  moralid9  mcoop1  mcoop2  mcoop3  mcoop4  mcoop5  mcoop6  mcoop7  narc1  narc3  narc4  narc5  omind5  omind6  contact1  contact2  contact3  contact4  contact5  hygiene1  hygiene2  hygiene3  hygiene4  hygiene5  psupport1  psupport2  psupport3  psupport4  psupport5  political_ideology  happy  riskperc1  riskperc2  self_esteem  sbelong1  sbelong2  sbelong3  slfcont3  urban
##   1   5  age  cnarc1  cnarc2  cnarc3  CRT1  CRT2  CRT3  duration  generosity1  generosity2  generosity3  marital1  mor_circle  moralid1  moralid10  moralid2  moralid3  moralid4  moralid5  moralid6  moralid7  moralid8  moralid9  mcoop1  mcoop2  mcoop3  mcoop4  mcoop5  mcoop6  mcoop7  narc1  narc3  narc4  narc5  omind5  omind6  contact1  contact2  contact3  contact4  contact5  hygiene1  hygiene2  hygiene3  hygiene4  hygiene5  psupport1  psupport2  psupport3  psupport4  psupport5  political_ideology  happy  riskperc1  riskperc2  self_esteem  sbelong1  sbelong2  sbelong3  slfcont3  urban
##   1   6  age  cnarc1  cnarc2  cnarc3  CRT1  CRT2  CRT3  duration  generosity1  generosity2  generosity3  marital1  mor_circle  moralid1  moralid10  moralid2  moralid3  moralid4  moralid5  moralid6  moralid7  moralid8  moralid9  mcoop1  mcoop2  mcoop3  mcoop4  mcoop5  mcoop6  mcoop7  narc1  narc3  narc4  narc5  omind5  omind6  contact1  contact2  contact3  contact4  contact5  hygiene1  hygiene2  hygiene3  hygiene4  hygiene5  psupport1  psupport2  psupport3  psupport4  psupport5  political_ideology  happy  riskperc1  riskperc2  self_esteem  sbelong1  sbelong2  sbelong3  slfcont3  urban
##   1   7  age  cnarc1  cnarc2  cnarc3  CRT1  CRT2  CRT3  duration  generosity1  generosity2  generosity3  marital1  mor_circle  moralid1  moralid10  moralid2  moralid3  moralid4  moralid5  moralid6  moralid7  moralid8  moralid9  mcoop1  mcoop2  mcoop3  mcoop4  mcoop5  mcoop6  mcoop7  narc1  narc3  narc4  narc5  omind5  omind6  contact1  contact2  contact3  contact4  contact5  hygiene1  hygiene2  hygiene3  hygiene4  hygiene5  psupport1  psupport2  psupport3  psupport4  psupport5  political_ideology  happy  riskperc1  riskperc2  self_esteem  sbelong1  sbelong2  sbelong3  slfcont3  urban
##   1   8  age  cnarc1  cnarc2  cnarc3  CRT1  CRT2  CRT3  duration  generosity1  generosity2  generosity3  marital1  mor_circle  moralid1  moralid10  moralid2  moralid3  moralid4  moralid5  moralid6  moralid7  moralid8  moralid9  mcoop1  mcoop2  mcoop3  mcoop4  mcoop5  mcoop6  mcoop7  narc1  narc3  narc4  narc5  omind5  omind6  contact1  contact2  contact3  contact4  contact5  hygiene1  hygiene2  hygiene3  hygiene4  hygiene5  psupport1  psupport2  psupport3  psupport4  psupport5  political_ideology  happy  riskperc1  riskperc2  self_esteem  sbelong1  sbelong2  sbelong3  slfcont3  urban
##   1   9  age  cnarc1  cnarc2  cnarc3  CRT1  CRT2  CRT3  duration  generosity1  generosity2  generosity3  marital1  mor_circle  moralid1  moralid10  moralid2  moralid3  moralid4  moralid5  moralid6  moralid7  moralid8  moralid9  mcoop1  mcoop2  mcoop3  mcoop4  mcoop5  mcoop6  mcoop7  narc1  narc3  narc4  narc5  omind5  omind6  contact1  contact2  contact3  contact4  contact5  hygiene1  hygiene2  hygiene3  hygiene4  hygiene5  psupport1  psupport2  psupport3  psupport4  psupport5  political_ideology  happy  riskperc1  riskperc2  self_esteem  sbelong1  sbelong2  sbelong3  slfcont3  urban
##   1   10  age  cnarc1  cnarc2  cnarc3  CRT1  CRT2  CRT3  duration  generosity1  generosity2  generosity3  marital1  mor_circle  moralid1  moralid10  moralid2  moralid3  moralid4  moralid5  moralid6  moralid7  moralid8  moralid9  mcoop1  mcoop2  mcoop3  mcoop4  mcoop5  mcoop6  mcoop7  narc1  narc3  narc4  narc5  omind5  omind6  contact1  contact2  contact3  contact4  contact5  hygiene1  hygiene2  hygiene3  hygiene4  hygiene5  psupport1  psupport2  psupport3  psupport4  psupport5  political_ideology  happy  riskperc1  riskperc2  self_esteem  sbelong1  sbelong2  sbelong3  slfcont3  urban
##   2   1  age  cnarc1  cnarc2  cnarc3  CRT1  CRT2  CRT3  duration  generosity1  generosity2  generosity3  marital1  mor_circle  moralid1  moralid10  moralid2  moralid3  moralid4  moralid5  moralid6  moralid7  moralid8  moralid9  mcoop1  mcoop2  mcoop3  mcoop4  mcoop5  mcoop6  mcoop7  narc1  narc3  narc4  narc5  omind5  omind6  contact1  contact2  contact3  contact4  contact5  hygiene1  hygiene2  hygiene3  hygiene4  hygiene5  psupport1  psupport2  psupport3  psupport4  psupport5  political_ideology  happy  riskperc1  riskperc2  self_esteem  sbelong1  sbelong2  sbelong3  slfcont3  urban
##   2   2  age  cnarc1  cnarc2  cnarc3  CRT1  CRT2  CRT3  duration  generosity1  generosity2  generosity3  marital1  mor_circle  moralid1  moralid10  moralid2  moralid3  moralid4  moralid5  moralid6  moralid7  moralid8  moralid9  mcoop1  mcoop2  mcoop3  mcoop4  mcoop5  mcoop6  mcoop7  narc1  narc3  narc4  narc5  omind5  omind6  contact1  contact2  contact3  contact4  contact5  hygiene1  hygiene2  hygiene3  hygiene4  hygiene5  psupport1  psupport2  psupport3  psupport4  psupport5  political_ideology  happy  riskperc1  riskperc2  self_esteem  sbelong1  sbelong2  sbelong3  slfcont3  urban
##   2   3  age  cnarc1  cnarc2  cnarc3  CRT1  CRT2  CRT3  duration  generosity1  generosity2  generosity3  marital1  mor_circle  moralid1  moralid10  moralid2  moralid3  moralid4  moralid5  moralid6  moralid7  moralid8  moralid9  mcoop1  mcoop2  mcoop3  mcoop4  mcoop5  mcoop6  mcoop7  narc1  narc3  narc4  narc5  omind5  omind6  contact1  contact2  contact3  contact4  contact5  hygiene1  hygiene2  hygiene3  hygiene4  hygiene5  psupport1  psupport2  psupport3  psupport4  psupport5  political_ideology  happy  riskperc1  riskperc2  self_esteem  sbelong1  sbelong2  sbelong3  slfcont3  urban
##   2   4  age  cnarc1  cnarc2  cnarc3  CRT1  CRT2  CRT3  duration  generosity1  generosity2  generosity3  marital1  mor_circle  moralid1  moralid10  moralid2  moralid3  moralid4  moralid5  moralid6  moralid7  moralid8  moralid9  mcoop1  mcoop2  mcoop3  mcoop4  mcoop5  mcoop6  mcoop7  narc1  narc3  narc4  narc5  omind5  omind6  contact1  contact2  contact3  contact4  contact5  hygiene1  hygiene2  hygiene3  hygiene4  hygiene5  psupport1  psupport2  psupport3  psupport4  psupport5  political_ideology  happy  riskperc1  riskperc2  self_esteem  sbelong1  sbelong2  sbelong3  slfcont3  urban
##   2   5  age  cnarc1  cnarc2  cnarc3  CRT1  CRT2  CRT3  duration  generosity1  generosity2  generosity3  marital1  mor_circle  moralid1  moralid10  moralid2  moralid3  moralid4  moralid5  moralid6  moralid7  moralid8  moralid9  mcoop1  mcoop2  mcoop3  mcoop4  mcoop5  mcoop6  mcoop7  narc1  narc3  narc4  narc5  omind5  omind6  contact1  contact2  contact3  contact4  contact5  hygiene1  hygiene2  hygiene3  hygiene4  hygiene5  psupport1  psupport2  psupport3  psupport4  psupport5  political_ideology  happy  riskperc1  riskperc2  self_esteem  sbelong1  sbelong2  sbelong3  slfcont3  urban
##   2   6  age  cnarc1  cnarc2  cnarc3  CRT1  CRT2  CRT3  duration  generosity1  generosity2  generosity3  marital1  mor_circle  moralid1  moralid10  moralid2  moralid3  moralid4  moralid5  moralid6  moralid7  moralid8  moralid9  mcoop1  mcoop2  mcoop3  mcoop4  mcoop5  mcoop6  mcoop7  narc1  narc3  narc4  narc5  omind5  omind6  contact1  contact2  contact3  contact4  contact5  hygiene1  hygiene2  hygiene3  hygiene4  hygiene5  psupport1  psupport2  psupport3  psupport4  psupport5  political_ideology  happy  riskperc1  riskperc2  self_esteem  sbelong1  sbelong2  sbelong3  slfcont3  urban
##   2   7  age  cnarc1  cnarc2  cnarc3  CRT1  CRT2  CRT3  duration  generosity1  generosity2  generosity3  marital1  mor_circle  moralid1  moralid10  moralid2  moralid3  moralid4  moralid5  moralid6  moralid7  moralid8  moralid9  mcoop1  mcoop2  mcoop3  mcoop4  mcoop5  mcoop6  mcoop7  narc1  narc3  narc4  narc5  omind5  omind6  contact1  contact2  contact3  contact4  contact5  hygiene1  hygiene2  hygiene3  hygiene4  hygiene5  psupport1  psupport2  psupport3  psupport4  psupport5  political_ideology  happy  riskperc1  riskperc2  self_esteem  sbelong1  sbelong2  sbelong3  slfcont3  urban
##   2   8  age  cnarc1  cnarc2  cnarc3  CRT1  CRT2  CRT3  duration  generosity1  generosity2  generosity3  marital1  mor_circle  moralid1  moralid10  moralid2  moralid3  moralid4  moralid5  moralid6  moralid7  moralid8  moralid9  mcoop1  mcoop2  mcoop3  mcoop4  mcoop5  mcoop6  mcoop7  narc1  narc3  narc4  narc5  omind5  omind6  contact1  contact2  contact3  contact4  contact5  hygiene1  hygiene2  hygiene3  hygiene4  hygiene5  psupport1  psupport2  psupport3  psupport4  psupport5  political_ideology  happy  riskperc1  riskperc2  self_esteem  sbelong1  sbelong2  sbelong3  slfcont3  urban
##   2   9  age  cnarc1  cnarc2  cnarc3  CRT1  CRT2  CRT3  duration  generosity1  generosity2  generosity3  marital1  mor_circle  moralid1  moralid10  moralid2  moralid3  moralid4  moralid5  moralid6  moralid7  moralid8  moralid9  mcoop1  mcoop2  mcoop3  mcoop4  mcoop5  mcoop6  mcoop7  narc1  narc3  narc4  narc5  omind5  omind6  contact1  contact2  contact3  contact4  contact5  hygiene1  hygiene2  hygiene3  hygiene4  hygiene5  psupport1  psupport2  psupport3  psupport4  psupport5  political_ideology  happy  riskperc1  riskperc2  self_esteem  sbelong1  sbelong2  sbelong3  slfcont3  urban
##   2   10  age  cnarc1  cnarc2  cnarc3  CRT1  CRT2  CRT3  duration  generosity1  generosity2  generosity3  marital1  mor_circle  moralid1  moralid10  moralid2  moralid3  moralid4  moralid5  moralid6  moralid7  moralid8  moralid9  mcoop1  mcoop2  mcoop3  mcoop4  mcoop5  mcoop6  mcoop7  narc1  narc3  narc4  narc5  omind5  omind6  contact1  contact2  contact3  contact4  contact5  hygiene1  hygiene2  hygiene3  hygiene4  hygiene5  psupport1  psupport2  psupport3  psupport4  psupport5  political_ideology  happy  riskperc1  riskperc2  self_esteem  sbelong1  sbelong2  sbelong3  slfcont3  urban
##   3   1  age  cnarc1  cnarc2  cnarc3  CRT1  CRT2  CRT3  duration  generosity1  generosity2  generosity3  marital1  mor_circle  moralid1  moralid10  moralid2  moralid3  moralid4  moralid5  moralid6  moralid7  moralid8  moralid9  mcoop1  mcoop2  mcoop3  mcoop4  mcoop5  mcoop6  mcoop7  narc1  narc3  narc4  narc5  omind5  omind6  contact1  contact2  contact3  contact4  contact5  hygiene1  hygiene2  hygiene3  hygiene4  hygiene5  psupport1  psupport2  psupport3  psupport4  psupport5  political_ideology  happy  riskperc1  riskperc2  self_esteem  sbelong1  sbelong2  sbelong3  slfcont3  urban
##   3   2  age  cnarc1  cnarc2  cnarc3  CRT1  CRT2  CRT3  duration  generosity1  generosity2  generosity3  marital1  mor_circle  moralid1  moralid10  moralid2  moralid3  moralid4  moralid5  moralid6  moralid7  moralid8  moralid9  mcoop1  mcoop2  mcoop3  mcoop4  mcoop5  mcoop6  mcoop7  narc1  narc3  narc4  narc5  omind5  omind6  contact1  contact2  contact3  contact4  contact5  hygiene1  hygiene2  hygiene3  hygiene4  hygiene5  psupport1  psupport2  psupport3  psupport4  psupport5  political_ideology  happy  riskperc1  riskperc2  self_esteem  sbelong1  sbelong2  sbelong3  slfcont3  urban
##   3   3  age  cnarc1  cnarc2  cnarc3  CRT1  CRT2  CRT3  duration  generosity1  generosity2  generosity3  marital1  mor_circle  moralid1  moralid10  moralid2  moralid3  moralid4  moralid5  moralid6  moralid7  moralid8  moralid9  mcoop1  mcoop2  mcoop3  mcoop4  mcoop5  mcoop6  mcoop7  narc1  narc3  narc4  narc5  omind5  omind6  contact1  contact2  contact3  contact4  contact5  hygiene1  hygiene2  hygiene3  hygiene4  hygiene5  psupport1  psupport2  psupport3  psupport4  psupport5  political_ideology  happy  riskperc1  riskperc2  self_esteem  sbelong1  sbelong2  sbelong3  slfcont3  urban
##   3   4  age  cnarc1  cnarc2  cnarc3  CRT1  CRT2  CRT3  duration  generosity1  generosity2  generosity3  marital1  mor_circle  moralid1  moralid10  moralid2  moralid3  moralid4  moralid5  moralid6  moralid7  moralid8  moralid9  mcoop1  mcoop2  mcoop3  mcoop4  mcoop5  mcoop6  mcoop7  narc1  narc3  narc4  narc5  omind5  omind6  contact1  contact2  contact3  contact4  contact5  hygiene1  hygiene2  hygiene3  hygiene4  hygiene5  psupport1  psupport2  psupport3  psupport4  psupport5  political_ideology  happy  riskperc1  riskperc2  self_esteem  sbelong1  sbelong2  sbelong3  slfcont3  urban
##   3   5  age  cnarc1  cnarc2  cnarc3  CRT1  CRT2  CRT3  duration  generosity1  generosity2  generosity3  marital1  mor_circle  moralid1  moralid10  moralid2  moralid3  moralid4  moralid5  moralid6  moralid7  moralid8  moralid9  mcoop1  mcoop2  mcoop3  mcoop4  mcoop5  mcoop6  mcoop7  narc1  narc3  narc4  narc5  omind5  omind6  contact1  contact2  contact3  contact4  contact5  hygiene1  hygiene2  hygiene3  hygiene4  hygiene5  psupport1  psupport2  psupport3  psupport4  psupport5  political_ideology  happy  riskperc1  riskperc2  self_esteem  sbelong1  sbelong2  sbelong3  slfcont3  urban
##   3   6  age  cnarc1  cnarc2  cnarc3  CRT1  CRT2  CRT3  duration  generosity1  generosity2  generosity3  marital1  mor_circle  moralid1  moralid10  moralid2  moralid3  moralid4  moralid5  moralid6  moralid7  moralid8  moralid9  mcoop1  mcoop2  mcoop3  mcoop4  mcoop5  mcoop6  mcoop7  narc1  narc3  narc4  narc5  omind5  omind6  contact1  contact2  contact3  contact4  contact5  hygiene1  hygiene2  hygiene3  hygiene4  hygiene5  psupport1  psupport2  psupport3  psupport4  psupport5  political_ideology  happy  riskperc1  riskperc2  self_esteem  sbelong1  sbelong2  sbelong3  slfcont3  urban
##   3   7  age  cnarc1  cnarc2  cnarc3  CRT1  CRT2  CRT3  duration  generosity1  generosity2  generosity3  marital1  mor_circle  moralid1  moralid10  moralid2  moralid3  moralid4  moralid5  moralid6  moralid7  moralid8  moralid9  mcoop1  mcoop2  mcoop3  mcoop4  mcoop5  mcoop6  mcoop7  narc1  narc3  narc4  narc5  omind5  omind6  contact1  contact2  contact3  contact4  contact5  hygiene1  hygiene2  hygiene3  hygiene4  hygiene5  psupport1  psupport2  psupport3  psupport4  psupport5  political_ideology  happy  riskperc1  riskperc2  self_esteem  sbelong1  sbelong2  sbelong3  slfcont3  urban
##   3   8  age  cnarc1  cnarc2  cnarc3  CRT1  CRT2  CRT3  duration  generosity1  generosity2  generosity3  marital1  mor_circle  moralid1  moralid10  moralid2  moralid3  moralid4  moralid5  moralid6  moralid7  moralid8  moralid9  mcoop1  mcoop2  mcoop3  mcoop4  mcoop5  mcoop6  mcoop7  narc1  narc3  narc4  narc5  omind5  omind6  contact1  contact2  contact3  contact4  contact5  hygiene1  hygiene2  hygiene3  hygiene4  hygiene5  psupport1  psupport2  psupport3  psupport4  psupport5  political_ideology  happy  riskperc1  riskperc2  self_esteem  sbelong1  sbelong2  sbelong3  slfcont3  urban
##   3   9  age  cnarc1  cnarc2  cnarc3  CRT1  CRT2  CRT3  duration  generosity1  generosity2  generosity3  marital1  mor_circle  moralid1  moralid10  moralid2  moralid3  moralid4  moralid5  moralid6  moralid7  moralid8  moralid9  mcoop1  mcoop2  mcoop3  mcoop4  mcoop5  mcoop6  mcoop7  narc1  narc3  narc4  narc5  omind5  omind6  contact1  contact2  contact3  contact4  contact5  hygiene1  hygiene2  hygiene3  hygiene4  hygiene5  psupport1  psupport2  psupport3  psupport4  psupport5  political_ideology  happy  riskperc1  riskperc2  self_esteem  sbelong1  sbelong2  sbelong3  slfcont3  urban
##   3   10  age  cnarc1  cnarc2  cnarc3  CRT1  CRT2  CRT3  duration  generosity1  generosity2  generosity3  marital1  mor_circle  moralid1  moralid10  moralid2  moralid3  moralid4  moralid5  moralid6  moralid7  moralid8  moralid9  mcoop1  mcoop2  mcoop3  mcoop4  mcoop5  mcoop6  mcoop7  narc1  narc3  narc4  narc5  omind5  omind6  contact1  contact2  contact3  contact4  contact5  hygiene1  hygiene2  hygiene3  hygiene4  hygiene5  psupport1  psupport2  psupport3  psupport4  psupport5  political_ideology  happy  riskperc1  riskperc2  self_esteem  sbelong1  sbelong2  sbelong3  slfcont3  urban
##   4   1  age  cnarc1  cnarc2  cnarc3  CRT1  CRT2  CRT3  duration  generosity1  generosity2  generosity3  marital1  mor_circle  moralid1  moralid10  moralid2  moralid3  moralid4  moralid5  moralid6  moralid7  moralid8  moralid9  mcoop1  mcoop2  mcoop3  mcoop4  mcoop5  mcoop6  mcoop7  narc1  narc3  narc4  narc5  omind5  omind6  contact1  contact2  contact3  contact4  contact5  hygiene1  hygiene2  hygiene3  hygiene4  hygiene5  psupport1  psupport2  psupport3  psupport4  psupport5  political_ideology  happy  riskperc1  riskperc2  self_esteem  sbelong1  sbelong2  sbelong3  slfcont3  urban
##   4   2  age  cnarc1  cnarc2  cnarc3  CRT1  CRT2  CRT3  duration  generosity1  generosity2  generosity3  marital1  mor_circle  moralid1  moralid10  moralid2  moralid3  moralid4  moralid5  moralid6  moralid7  moralid8  moralid9  mcoop1  mcoop2  mcoop3  mcoop4  mcoop5  mcoop6  mcoop7  narc1  narc3  narc4  narc5  omind5  omind6  contact1  contact2  contact3  contact4  contact5  hygiene1  hygiene2  hygiene3  hygiene4  hygiene5  psupport1  psupport2  psupport3  psupport4  psupport5  political_ideology  happy  riskperc1  riskperc2  self_esteem  sbelong1  sbelong2  sbelong3  slfcont3  urban
##   4   3  age  cnarc1  cnarc2  cnarc3  CRT1  CRT2  CRT3  duration  generosity1  generosity2  generosity3  marital1  mor_circle  moralid1  moralid10  moralid2  moralid3  moralid4  moralid5  moralid6  moralid7  moralid8  moralid9  mcoop1  mcoop2  mcoop3  mcoop4  mcoop5  mcoop6  mcoop7  narc1  narc3  narc4  narc5  omind5  omind6  contact1  contact2  contact3  contact4  contact5  hygiene1  hygiene2  hygiene3  hygiene4  hygiene5  psupport1  psupport2  psupport3  psupport4  psupport5  political_ideology  happy  riskperc1  riskperc2  self_esteem  sbelong1  sbelong2  sbelong3  slfcont3  urban
##   4   4  age  cnarc1  cnarc2  cnarc3  CRT1  CRT2  CRT3  duration  generosity1  generosity2  generosity3  marital1  mor_circle  moralid1  moralid10  moralid2  moralid3  moralid4  moralid5  moralid6  moralid7  moralid8  moralid9  mcoop1  mcoop2  mcoop3  mcoop4  mcoop5  mcoop6  mcoop7  narc1  narc3  narc4  narc5  omind5  omind6  contact1  contact2  contact3  contact4  contact5  hygiene1  hygiene2  hygiene3  hygiene4  hygiene5  psupport1  psupport2  psupport3  psupport4  psupport5  political_ideology  happy  riskperc1  riskperc2  self_esteem  sbelong1  sbelong2  sbelong3  slfcont3  urban
##   4   5  age  cnarc1  cnarc2  cnarc3  CRT1  CRT2  CRT3  duration  generosity1  generosity2  generosity3  marital1  mor_circle  moralid1  moralid10  moralid2  moralid3  moralid4  moralid5  moralid6  moralid7  moralid8  moralid9  mcoop1  mcoop2  mcoop3  mcoop4  mcoop5  mcoop6  mcoop7  narc1  narc3  narc4  narc5  omind5  omind6  contact1  contact2  contact3  contact4  contact5  hygiene1  hygiene2  hygiene3  hygiene4  hygiene5  psupport1  psupport2  psupport3  psupport4  psupport5  political_ideology  happy  riskperc1  riskperc2  self_esteem  sbelong1  sbelong2  sbelong3  slfcont3  urban
##   4   6  age  cnarc1  cnarc2  cnarc3  CRT1  CRT2  CRT3  duration  generosity1  generosity2  generosity3  marital1  mor_circle  moralid1  moralid10  moralid2  moralid3  moralid4  moralid5  moralid6  moralid7  moralid8  moralid9  mcoop1  mcoop2  mcoop3  mcoop4  mcoop5  mcoop6  mcoop7  narc1  narc3  narc4  narc5  omind5  omind6  contact1  contact2  contact3  contact4  contact5  hygiene1  hygiene2  hygiene3  hygiene4  hygiene5  psupport1  psupport2  psupport3  psupport4  psupport5  political_ideology  happy  riskperc1  riskperc2  self_esteem  sbelong1  sbelong2  sbelong3  slfcont3  urban
##   4   7  age  cnarc1  cnarc2  cnarc3  CRT1  CRT2  CRT3  duration  generosity1  generosity2  generosity3  marital1  mor_circle  moralid1  moralid10  moralid2  moralid3  moralid4  moralid5  moralid6  moralid7  moralid8  moralid9  mcoop1  mcoop2  mcoop3  mcoop4  mcoop5  mcoop6  mcoop7  narc1  narc3  narc4  narc5  omind5  omind6  contact1  contact2  contact3  contact4  contact5  hygiene1  hygiene2  hygiene3  hygiene4  hygiene5  psupport1  psupport2  psupport3  psupport4  psupport5  political_ideology  happy  riskperc1  riskperc2  self_esteem  sbelong1  sbelong2  sbelong3  slfcont3  urban
##   4   8  age  cnarc1  cnarc2  cnarc3  CRT1  CRT2  CRT3  duration  generosity1  generosity2  generosity3  marital1  mor_circle  moralid1  moralid10  moralid2  moralid3  moralid4  moralid5  moralid6  moralid7  moralid8  moralid9  mcoop1  mcoop2  mcoop3  mcoop4  mcoop5  mcoop6  mcoop7  narc1  narc3  narc4  narc5  omind5  omind6  contact1  contact2  contact3  contact4  contact5  hygiene1  hygiene2  hygiene3  hygiene4  hygiene5  psupport1  psupport2  psupport3  psupport4  psupport5  political_ideology  happy  riskperc1  riskperc2  self_esteem  sbelong1  sbelong2  sbelong3  slfcont3  urban
##   4   9  age  cnarc1  cnarc2  cnarc3  CRT1  CRT2  CRT3  duration  generosity1  generosity2  generosity3  marital1  mor_circle  moralid1  moralid10  moralid2  moralid3  moralid4  moralid5  moralid6  moralid7  moralid8  moralid9  mcoop1  mcoop2  mcoop3  mcoop4  mcoop5  mcoop6  mcoop7  narc1  narc3  narc4  narc5  omind5  omind6  contact1  contact2  contact3  contact4  contact5  hygiene1  hygiene2  hygiene3  hygiene4  hygiene5  psupport1  psupport2  psupport3  psupport4  psupport5  political_ideology  happy  riskperc1  riskperc2  self_esteem  sbelong1  sbelong2  sbelong3  slfcont3  urban
##   4   10  age  cnarc1  cnarc2  cnarc3  CRT1  CRT2  CRT3  duration  generosity1  generosity2  generosity3  marital1  mor_circle  moralid1  moralid10  moralid2  moralid3  moralid4  moralid5  moralid6  moralid7  moralid8  moralid9  mcoop1  mcoop2  mcoop3  mcoop4  mcoop5  mcoop6  mcoop7  narc1  narc3  narc4  narc5  omind5  omind6  contact1  contact2  contact3  contact4  contact5  hygiene1  hygiene2  hygiene3  hygiene4  hygiene5  psupport1  psupport2  psupport3  psupport4  psupport5  political_ideology  happy  riskperc1  riskperc2  self_esteem  sbelong1  sbelong2  sbelong3  slfcont3  urban
##   5   1  age  cnarc1  cnarc2  cnarc3  CRT1  CRT2  CRT3  duration  generosity1  generosity2  generosity3  marital1  mor_circle  moralid1  moralid10  moralid2  moralid3  moralid4  moralid5  moralid6  moralid7  moralid8  moralid9  mcoop1  mcoop2  mcoop3  mcoop4  mcoop5  mcoop6  mcoop7  narc1  narc3  narc4  narc5  omind5  omind6  contact1  contact2  contact3  contact4  contact5  hygiene1  hygiene2  hygiene3  hygiene4  hygiene5  psupport1  psupport2  psupport3  psupport4  psupport5  political_ideology  happy  riskperc1  riskperc2  self_esteem  sbelong1  sbelong2  sbelong3  slfcont3  urban
##   5   2  age  cnarc1  cnarc2  cnarc3  CRT1  CRT2  CRT3  duration  generosity1  generosity2  generosity3  marital1  mor_circle  moralid1  moralid10  moralid2  moralid3  moralid4  moralid5  moralid6  moralid7  moralid8  moralid9  mcoop1  mcoop2  mcoop3  mcoop4  mcoop5  mcoop6  mcoop7  narc1  narc3  narc4  narc5  omind5  omind6  contact1  contact2  contact3  contact4  contact5  hygiene1  hygiene2  hygiene3  hygiene4  hygiene5  psupport1  psupport2  psupport3  psupport4  psupport5  political_ideology  happy  riskperc1  riskperc2  self_esteem  sbelong1  sbelong2  sbelong3  slfcont3  urban
##   5   3  age  cnarc1  cnarc2  cnarc3  CRT1  CRT2  CRT3  duration  generosity1  generosity2  generosity3  marital1  mor_circle  moralid1  moralid10  moralid2  moralid3  moralid4  moralid5  moralid6  moralid7  moralid8  moralid9  mcoop1  mcoop2  mcoop3  mcoop4  mcoop5  mcoop6  mcoop7  narc1  narc3  narc4  narc5  omind5  omind6  contact1  contact2  contact3  contact4  contact5  hygiene1  hygiene2  hygiene3  hygiene4  hygiene5  psupport1  psupport2  psupport3  psupport4  psupport5  political_ideology  happy  riskperc1  riskperc2  self_esteem  sbelong1  sbelong2  sbelong3  slfcont3  urban
##   5   4  age  cnarc1  cnarc2  cnarc3  CRT1  CRT2  CRT3  duration  generosity1  generosity2  generosity3  marital1  mor_circle  moralid1  moralid10  moralid2  moralid3  moralid4  moralid5  moralid6  moralid7  moralid8  moralid9  mcoop1  mcoop2  mcoop3  mcoop4  mcoop5  mcoop6  mcoop7  narc1  narc3  narc4  narc5  omind5  omind6  contact1  contact2  contact3  contact4  contact5  hygiene1  hygiene2  hygiene3  hygiene4  hygiene5  psupport1  psupport2  psupport3  psupport4  psupport5  political_ideology  happy  riskperc1  riskperc2  self_esteem  sbelong1  sbelong2  sbelong3  slfcont3  urban
##   5   5  age  cnarc1  cnarc2  cnarc3  CRT1  CRT2  CRT3  duration  generosity1  generosity2  generosity3  marital1  mor_circle  moralid1  moralid10  moralid2  moralid3  moralid4  moralid5  moralid6  moralid7  moralid8  moralid9  mcoop1  mcoop2  mcoop3  mcoop4  mcoop5  mcoop6  mcoop7  narc1  narc3  narc4  narc5  omind5  omind6  contact1  contact2  contact3  contact4  contact5  hygiene1  hygiene2  hygiene3  hygiene4  hygiene5  psupport1  psupport2  psupport3  psupport4  psupport5  political_ideology  happy  riskperc1  riskperc2  self_esteem  sbelong1  sbelong2  sbelong3  slfcont3  urban
##   5   6  age  cnarc1  cnarc2  cnarc3  CRT1  CRT2  CRT3  duration  generosity1  generosity2  generosity3  marital1  mor_circle  moralid1  moralid10  moralid2  moralid3  moralid4  moralid5  moralid6  moralid7  moralid8  moralid9  mcoop1  mcoop2  mcoop3  mcoop4  mcoop5  mcoop6  mcoop7  narc1  narc3  narc4  narc5  omind5  omind6  contact1  contact2  contact3  contact4  contact5  hygiene1  hygiene2  hygiene3  hygiene4  hygiene5  psupport1  psupport2  psupport3  psupport4  psupport5  political_ideology  happy  riskperc1  riskperc2  self_esteem  sbelong1  sbelong2  sbelong3  slfcont3  urban
##   5   7  age  cnarc1  cnarc2  cnarc3  CRT1  CRT2  CRT3  duration  generosity1  generosity2  generosity3  marital1  mor_circle  moralid1  moralid10  moralid2  moralid3  moralid4  moralid5  moralid6  moralid7  moralid8  moralid9  mcoop1  mcoop2  mcoop3  mcoop4  mcoop5  mcoop6  mcoop7  narc1  narc3  narc4  narc5  omind5  omind6  contact1  contact2  contact3  contact4  contact5  hygiene1  hygiene2  hygiene3  hygiene4  hygiene5  psupport1  psupport2  psupport3  psupport4  psupport5  political_ideology  happy  riskperc1  riskperc2  self_esteem  sbelong1  sbelong2  sbelong3  slfcont3  urban
##   5   8  age  cnarc1  cnarc2  cnarc3  CRT1  CRT2  CRT3  duration  generosity1  generosity2  generosity3  marital1  mor_circle  moralid1  moralid10  moralid2  moralid3  moralid4  moralid5  moralid6  moralid7  moralid8  moralid9  mcoop1  mcoop2  mcoop3  mcoop4  mcoop5  mcoop6  mcoop7  narc1  narc3  narc4  narc5  omind5  omind6  contact1  contact2  contact3  contact4  contact5  hygiene1  hygiene2  hygiene3  hygiene4  hygiene5  psupport1  psupport2  psupport3  psupport4  psupport5  political_ideology  happy  riskperc1  riskperc2  self_esteem  sbelong1  sbelong2  sbelong3  slfcont3  urban
##   5   9  age  cnarc1  cnarc2  cnarc3  CRT1  CRT2  CRT3  duration  generosity1  generosity2  generosity3  marital1  mor_circle  moralid1  moralid10  moralid2  moralid3  moralid4  moralid5  moralid6  moralid7  moralid8  moralid9  mcoop1  mcoop2  mcoop3  mcoop4  mcoop5  mcoop6  mcoop7  narc1  narc3  narc4  narc5  omind5  omind6  contact1  contact2  contact3  contact4  contact5  hygiene1  hygiene2  hygiene3  hygiene4  hygiene5  psupport1  psupport2  psupport3  psupport4  psupport5  political_ideology  happy  riskperc1  riskperc2  self_esteem  sbelong1  sbelong2  sbelong3  slfcont3  urban
##   5   10  age  cnarc1  cnarc2  cnarc3  CRT1  CRT2  CRT3  duration  generosity1  generosity2  generosity3  marital1  mor_circle  moralid1  moralid10  moralid2  moralid3  moralid4  moralid5  moralid6  moralid7  moralid8  moralid9  mcoop1  mcoop2  mcoop3  mcoop4  mcoop5  mcoop6  mcoop7  narc1  narc3  narc4  narc5  omind5  omind6  contact1  contact2  contact3  contact4  contact5  hygiene1  hygiene2  hygiene3  hygiene4  hygiene5  psupport1  psupport2  psupport3  psupport4  psupport5  political_ideology  happy  riskperc1  riskperc2  self_esteem  sbelong1  sbelong2  sbelong3  slfcont3  urban
##   6   1  age  cnarc1  cnarc2  cnarc3  CRT1  CRT2  CRT3  duration  generosity1  generosity2  generosity3  marital1  mor_circle  moralid1  moralid10  moralid2  moralid3  moralid4  moralid5  moralid6  moralid7  moralid8  moralid9  mcoop1  mcoop2  mcoop3  mcoop4  mcoop5  mcoop6  mcoop7  narc1  narc3  narc4  narc5  omind5  omind6  contact1  contact2  contact3  contact4  contact5  hygiene1  hygiene2  hygiene3  hygiene4  hygiene5  psupport1  psupport2  psupport3  psupport4  psupport5  political_ideology  happy  riskperc1  riskperc2  self_esteem  sbelong1  sbelong2  sbelong3  slfcont3  urban
##   6   2  age  cnarc1  cnarc2  cnarc3  CRT1  CRT2  CRT3  duration  generosity1  generosity2  generosity3  marital1  mor_circle  moralid1  moralid10  moralid2  moralid3  moralid4  moralid5  moralid6  moralid7  moralid8  moralid9  mcoop1  mcoop2  mcoop3  mcoop4  mcoop5  mcoop6  mcoop7  narc1  narc3  narc4  narc5  omind5  omind6  contact1  contact2  contact3  contact4  contact5  hygiene1  hygiene2  hygiene3  hygiene4  hygiene5  psupport1  psupport2  psupport3  psupport4  psupport5  political_ideology  happy  riskperc1  riskperc2  self_esteem  sbelong1  sbelong2  sbelong3  slfcont3  urban
##   6   3  age  cnarc1  cnarc2  cnarc3  CRT1  CRT2  CRT3  duration  generosity1  generosity2  generosity3  marital1  mor_circle  moralid1  moralid10  moralid2  moralid3  moralid4  moralid5  moralid6  moralid7  moralid8  moralid9  mcoop1  mcoop2  mcoop3  mcoop4  mcoop5  mcoop6  mcoop7  narc1  narc3  narc4  narc5  omind5  omind6  contact1  contact2  contact3  contact4  contact5  hygiene1  hygiene2  hygiene3  hygiene4  hygiene5  psupport1  psupport2  psupport3  psupport4  psupport5  political_ideology  happy  riskperc1  riskperc2  self_esteem  sbelong1  sbelong2  sbelong3  slfcont3  urban
##   6   4  age  cnarc1  cnarc2  cnarc3  CRT1  CRT2  CRT3  duration  generosity1  generosity2  generosity3  marital1  mor_circle  moralid1  moralid10  moralid2  moralid3  moralid4  moralid5  moralid6  moralid7  moralid8  moralid9  mcoop1  mcoop2  mcoop3  mcoop4  mcoop5  mcoop6  mcoop7  narc1  narc3  narc4  narc5  omind5  omind6  contact1  contact2  contact3  contact4  contact5  hygiene1  hygiene2  hygiene3  hygiene4  hygiene5  psupport1  psupport2  psupport3  psupport4  psupport5  political_ideology  happy  riskperc1  riskperc2  self_esteem  sbelong1  sbelong2  sbelong3  slfcont3  urban
##   6   5  age  cnarc1  cnarc2  cnarc3  CRT1  CRT2  CRT3  duration  generosity1  generosity2  generosity3  marital1  mor_circle  moralid1  moralid10  moralid2  moralid3  moralid4  moralid5  moralid6  moralid7  moralid8  moralid9  mcoop1  mcoop2  mcoop3  mcoop4  mcoop5  mcoop6  mcoop7  narc1  narc3  narc4  narc5  omind5  omind6  contact1  contact2  contact3  contact4  contact5  hygiene1  hygiene2  hygiene3  hygiene4  hygiene5  psupport1  psupport2  psupport3  psupport4  psupport5  political_ideology  happy  riskperc1  riskperc2  self_esteem  sbelong1  sbelong2  sbelong3  slfcont3  urban
##   6   6  age  cnarc1  cnarc2  cnarc3  CRT1  CRT2  CRT3  duration  generosity1  generosity2  generosity3  marital1  mor_circle  moralid1  moralid10  moralid2  moralid3  moralid4  moralid5  moralid6  moralid7  moralid8  moralid9  mcoop1  mcoop2  mcoop3  mcoop4  mcoop5  mcoop6  mcoop7  narc1  narc3  narc4  narc5  omind5  omind6  contact1  contact2  contact3  contact4  contact5  hygiene1  hygiene2  hygiene3  hygiene4  hygiene5  psupport1  psupport2  psupport3  psupport4  psupport5  political_ideology  happy  riskperc1  riskperc2  self_esteem  sbelong1  sbelong2  sbelong3  slfcont3  urban
##   6   7  age  cnarc1  cnarc2  cnarc3  CRT1  CRT2  CRT3  duration  generosity1  generosity2  generosity3  marital1  mor_circle  moralid1  moralid10  moralid2  moralid3  moralid4  moralid5  moralid6  moralid7  moralid8  moralid9  mcoop1  mcoop2  mcoop3  mcoop4  mcoop5  mcoop6  mcoop7  narc1  narc3  narc4  narc5  omind5  omind6  contact1  contact2  contact3  contact4  contact5  hygiene1  hygiene2  hygiene3  hygiene4  hygiene5  psupport1  psupport2  psupport3  psupport4  psupport5  political_ideology  happy  riskperc1  riskperc2  self_esteem  sbelong1  sbelong2  sbelong3  slfcont3  urban
##   6   8  age  cnarc1  cnarc2  cnarc3  CRT1  CRT2  CRT3  duration  generosity1  generosity2  generosity3  marital1  mor_circle  moralid1  moralid10  moralid2  moralid3  moralid4  moralid5  moralid6  moralid7  moralid8  moralid9  mcoop1  mcoop2  mcoop3  mcoop4  mcoop5  mcoop6  mcoop7  narc1  narc3  narc4  narc5  omind5  omind6  contact1  contact2  contact3  contact4  contact5  hygiene1  hygiene2  hygiene3  hygiene4  hygiene5  psupport1  psupport2  psupport3  psupport4  psupport5  political_ideology  happy  riskperc1  riskperc2  self_esteem  sbelong1  sbelong2  sbelong3  slfcont3  urban
##   6   9  age  cnarc1  cnarc2  cnarc3  CRT1  CRT2  CRT3  duration  generosity1  generosity2  generosity3  marital1  mor_circle  moralid1  moralid10  moralid2  moralid3  moralid4  moralid5  moralid6  moralid7  moralid8  moralid9  mcoop1  mcoop2  mcoop3  mcoop4  mcoop5  mcoop6  mcoop7  narc1  narc3  narc4  narc5  omind5  omind6  contact1  contact2  contact3  contact4  contact5  hygiene1  hygiene2  hygiene3  hygiene4  hygiene5  psupport1  psupport2  psupport3  psupport4  psupport5  political_ideology  happy  riskperc1  riskperc2  self_esteem  sbelong1  sbelong2  sbelong3  slfcont3  urban
##   6   10  age  cnarc1  cnarc2  cnarc3  CRT1  CRT2  CRT3  duration  generosity1  generosity2  generosity3  marital1  mor_circle  moralid1  moralid10  moralid2  moralid3  moralid4  moralid5  moralid6  moralid7  moralid8  moralid9  mcoop1  mcoop2  mcoop3  mcoop4  mcoop5  mcoop6  mcoop7  narc1  narc3  narc4  narc5  omind5  omind6  contact1  contact2  contact3  contact4  contact5  hygiene1  hygiene2  hygiene3  hygiene4  hygiene5  psupport1  psupport2  psupport3  psupport4  psupport5  political_ideology  happy  riskperc1  riskperc2  self_esteem  sbelong1  sbelong2  sbelong3  slfcont3  urban
##   7   1  age  cnarc1  cnarc2  cnarc3  CRT1  CRT2  CRT3  duration  generosity1  generosity2  generosity3  marital1  mor_circle  moralid1  moralid10  moralid2  moralid3  moralid4  moralid5  moralid6  moralid7  moralid8  moralid9  mcoop1  mcoop2  mcoop3  mcoop4  mcoop5  mcoop6  mcoop7  narc1  narc3  narc4  narc5  omind5  omind6  contact1  contact2  contact3  contact4  contact5  hygiene1  hygiene2  hygiene3  hygiene4  hygiene5  psupport1  psupport2  psupport3  psupport4  psupport5  political_ideology  happy  riskperc1  riskperc2  self_esteem  sbelong1  sbelong2  sbelong3  slfcont3  urban
##   7   2  age  cnarc1  cnarc2  cnarc3  CRT1  CRT2  CRT3  duration  generosity1  generosity2  generosity3  marital1  mor_circle  moralid1  moralid10  moralid2  moralid3  moralid4  moralid5  moralid6  moralid7  moralid8  moralid9  mcoop1  mcoop2  mcoop3  mcoop4  mcoop5  mcoop6  mcoop7  narc1  narc3  narc4  narc5  omind5  omind6  contact1  contact2  contact3  contact4  contact5  hygiene1  hygiene2  hygiene3  hygiene4  hygiene5  psupport1  psupport2  psupport3  psupport4  psupport5  political_ideology  happy  riskperc1  riskperc2  self_esteem  sbelong1  sbelong2  sbelong3  slfcont3  urban
##   7   3  age  cnarc1  cnarc2  cnarc3  CRT1  CRT2  CRT3  duration  generosity1  generosity2  generosity3  marital1  mor_circle  moralid1  moralid10  moralid2  moralid3  moralid4  moralid5  moralid6  moralid7  moralid8  moralid9  mcoop1  mcoop2  mcoop3  mcoop4  mcoop5  mcoop6  mcoop7  narc1  narc3  narc4  narc5  omind5  omind6  contact1  contact2  contact3  contact4  contact5  hygiene1  hygiene2  hygiene3  hygiene4  hygiene5  psupport1  psupport2  psupport3  psupport4  psupport5  political_ideology  happy  riskperc1  riskperc2  self_esteem  sbelong1  sbelong2  sbelong3  slfcont3  urban
##   7   4  age  cnarc1  cnarc2  cnarc3  CRT1  CRT2  CRT3  duration  generosity1  generosity2  generosity3  marital1  mor_circle  moralid1  moralid10  moralid2  moralid3  moralid4  moralid5  moralid6  moralid7  moralid8  moralid9  mcoop1  mcoop2  mcoop3  mcoop4  mcoop5  mcoop6  mcoop7  narc1  narc3  narc4  narc5  omind5  omind6  contact1  contact2  contact3  contact4  contact5  hygiene1  hygiene2  hygiene3  hygiene4  hygiene5  psupport1  psupport2  psupport3  psupport4  psupport5  political_ideology  happy  riskperc1  riskperc2  self_esteem  sbelong1  sbelong2  sbelong3  slfcont3  urban
##   7   5  age  cnarc1  cnarc2  cnarc3  CRT1  CRT2  CRT3  duration  generosity1  generosity2  generosity3  marital1  mor_circle  moralid1  moralid10  moralid2  moralid3  moralid4  moralid5  moralid6  moralid7  moralid8  moralid9  mcoop1  mcoop2  mcoop3  mcoop4  mcoop5  mcoop6  mcoop7  narc1  narc3  narc4  narc5  omind5  omind6  contact1  contact2  contact3  contact4  contact5  hygiene1  hygiene2  hygiene3  hygiene4  hygiene5  psupport1  psupport2  psupport3  psupport4  psupport5  political_ideology  happy  riskperc1  riskperc2  self_esteem  sbelong1  sbelong2  sbelong3  slfcont3  urban
##   7   6  age  cnarc1  cnarc2  cnarc3  CRT1  CRT2  CRT3  duration  generosity1  generosity2  generosity3  marital1  mor_circle  moralid1  moralid10  moralid2  moralid3  moralid4  moralid5  moralid6  moralid7  moralid8  moralid9  mcoop1  mcoop2  mcoop3  mcoop4  mcoop5  mcoop6  mcoop7  narc1  narc3  narc4  narc5  omind5  omind6  contact1  contact2  contact3  contact4  contact5  hygiene1  hygiene2  hygiene3  hygiene4  hygiene5  psupport1  psupport2  psupport3  psupport4  psupport5  political_ideology  happy  riskperc1  riskperc2  self_esteem  sbelong1  sbelong2  sbelong3  slfcont3  urban
##   7   7  age  cnarc1  cnarc2  cnarc3  CRT1  CRT2  CRT3  duration  generosity1  generosity2  generosity3  marital1  mor_circle  moralid1  moralid10  moralid2  moralid3  moralid4  moralid5  moralid6  moralid7  moralid8  moralid9  mcoop1  mcoop2  mcoop3  mcoop4  mcoop5  mcoop6  mcoop7  narc1  narc3  narc4  narc5  omind5  omind6  contact1  contact2  contact3  contact4  contact5  hygiene1  hygiene2  hygiene3  hygiene4  hygiene5  psupport1  psupport2  psupport3  psupport4  psupport5  political_ideology  happy  riskperc1  riskperc2  self_esteem  sbelong1  sbelong2  sbelong3  slfcont3  urban
##   7   8  age  cnarc1  cnarc2  cnarc3  CRT1  CRT2  CRT3  duration  generosity1  generosity2  generosity3  marital1  mor_circle  moralid1  moralid10  moralid2  moralid3  moralid4  moralid5  moralid6  moralid7  moralid8  moralid9  mcoop1  mcoop2  mcoop3  mcoop4  mcoop5  mcoop6  mcoop7  narc1  narc3  narc4  narc5  omind5  omind6  contact1  contact2  contact3  contact4  contact5  hygiene1  hygiene2  hygiene3  hygiene4  hygiene5  psupport1  psupport2  psupport3  psupport4  psupport5  political_ideology  happy  riskperc1  riskperc2  self_esteem  sbelong1  sbelong2  sbelong3  slfcont3  urban
##   7   9  age  cnarc1  cnarc2  cnarc3  CRT1  CRT2  CRT3  duration  generosity1  generosity2  generosity3  marital1  mor_circle  moralid1  moralid10  moralid2  moralid3  moralid4  moralid5  moralid6  moralid7  moralid8  moralid9  mcoop1  mcoop2  mcoop3  mcoop4  mcoop5  mcoop6  mcoop7  narc1  narc3  narc4  narc5  omind5  omind6  contact1  contact2  contact3  contact4  contact5  hygiene1  hygiene2  hygiene3  hygiene4  hygiene5  psupport1  psupport2  psupport3  psupport4  psupport5  political_ideology  happy  riskperc1  riskperc2  self_esteem  sbelong1  sbelong2  sbelong3  slfcont3  urban
##   7   10  age  cnarc1  cnarc2  cnarc3  CRT1  CRT2  CRT3  duration  generosity1  generosity2  generosity3  marital1  mor_circle  moralid1  moralid10  moralid2  moralid3  moralid4  moralid5  moralid6  moralid7  moralid8  moralid9  mcoop1  mcoop2  mcoop3  mcoop4  mcoop5  mcoop6  mcoop7  narc1  narc3  narc4  narc5  omind5  omind6  contact1  contact2  contact3  contact4  contact5  hygiene1  hygiene2  hygiene3  hygiene4  hygiene5  psupport1  psupport2  psupport3  psupport4  psupport5  political_ideology  happy  riskperc1  riskperc2  self_esteem  sbelong1  sbelong2  sbelong3  slfcont3  urban
##   8   1  age  cnarc1  cnarc2  cnarc3  CRT1  CRT2  CRT3  duration  generosity1  generosity2  generosity3  marital1  mor_circle  moralid1  moralid10  moralid2  moralid3  moralid4  moralid5  moralid6  moralid7  moralid8  moralid9  mcoop1  mcoop2  mcoop3  mcoop4  mcoop5  mcoop6  mcoop7  narc1  narc3  narc4  narc5  omind5  omind6  contact1  contact2  contact3  contact4  contact5  hygiene1  hygiene2  hygiene3  hygiene4  hygiene5  psupport1  psupport2  psupport3  psupport4  psupport5  political_ideology  happy  riskperc1  riskperc2  self_esteem  sbelong1  sbelong2  sbelong3  slfcont3  urban
##   8   2  age  cnarc1  cnarc2  cnarc3  CRT1  CRT2  CRT3  duration  generosity1  generosity2  generosity3  marital1  mor_circle  moralid1  moralid10  moralid2  moralid3  moralid4  moralid5  moralid6  moralid7  moralid8  moralid9  mcoop1  mcoop2  mcoop3  mcoop4  mcoop5  mcoop6  mcoop7  narc1  narc3  narc4  narc5  omind5  omind6  contact1  contact2  contact3  contact4  contact5  hygiene1  hygiene2  hygiene3  hygiene4  hygiene5  psupport1  psupport2  psupport3  psupport4  psupport5  political_ideology  happy  riskperc1  riskperc2  self_esteem  sbelong1  sbelong2  sbelong3  slfcont3  urban
##   8   3  age  cnarc1  cnarc2  cnarc3  CRT1  CRT2  CRT3  duration  generosity1  generosity2  generosity3  marital1  mor_circle  moralid1  moralid10  moralid2  moralid3  moralid4  moralid5  moralid6  moralid7  moralid8  moralid9  mcoop1  mcoop2  mcoop3  mcoop4  mcoop5  mcoop6  mcoop7  narc1  narc3  narc4  narc5  omind5  omind6  contact1  contact2  contact3  contact4  contact5  hygiene1  hygiene2  hygiene3  hygiene4  hygiene5  psupport1  psupport2  psupport3  psupport4  psupport5  political_ideology  happy  riskperc1  riskperc2  self_esteem  sbelong1  sbelong2  sbelong3  slfcont3  urban
##   8   4  age  cnarc1  cnarc2  cnarc3  CRT1  CRT2  CRT3  duration  generosity1  generosity2  generosity3  marital1  mor_circle  moralid1  moralid10  moralid2  moralid3  moralid4  moralid5  moralid6  moralid7  moralid8  moralid9  mcoop1  mcoop2  mcoop3  mcoop4  mcoop5  mcoop6  mcoop7  narc1  narc3  narc4  narc5  omind5  omind6  contact1  contact2  contact3  contact4  contact5  hygiene1  hygiene2  hygiene3  hygiene4  hygiene5  psupport1  psupport2  psupport3  psupport4  psupport5  political_ideology  happy  riskperc1  riskperc2  self_esteem  sbelong1  sbelong2  sbelong3  slfcont3  urban
##   8   5  age  cnarc1  cnarc2  cnarc3  CRT1  CRT2  CRT3  duration  generosity1  generosity2  generosity3  marital1  mor_circle  moralid1  moralid10  moralid2  moralid3  moralid4  moralid5  moralid6  moralid7  moralid8  moralid9  mcoop1  mcoop2  mcoop3  mcoop4  mcoop5  mcoop6  mcoop7  narc1  narc3  narc4  narc5  omind5  omind6  contact1  contact2  contact3  contact4  contact5  hygiene1  hygiene2  hygiene3  hygiene4  hygiene5  psupport1  psupport2  psupport3  psupport4  psupport5  political_ideology  happy  riskperc1  riskperc2  self_esteem  sbelong1  sbelong2  sbelong3  slfcont3  urban
##   8   6  age  cnarc1  cnarc2  cnarc3  CRT1  CRT2  CRT3  duration  generosity1  generosity2  generosity3  marital1  mor_circle  moralid1  moralid10  moralid2  moralid3  moralid4  moralid5  moralid6  moralid7  moralid8  moralid9  mcoop1  mcoop2  mcoop3  mcoop4  mcoop5  mcoop6  mcoop7  narc1  narc3  narc4  narc5  omind5  omind6  contact1  contact2  contact3  contact4  contact5  hygiene1  hygiene2  hygiene3  hygiene4  hygiene5  psupport1  psupport2  psupport3  psupport4  psupport5  political_ideology  happy  riskperc1  riskperc2  self_esteem  sbelong1  sbelong2  sbelong3  slfcont3  urban
##   8   7  age  cnarc1  cnarc2  cnarc3  CRT1  CRT2  CRT3  duration  generosity1  generosity2  generosity3  marital1  mor_circle  moralid1  moralid10  moralid2  moralid3  moralid4  moralid5  moralid6  moralid7  moralid8  moralid9  mcoop1  mcoop2  mcoop3  mcoop4  mcoop5  mcoop6  mcoop7  narc1  narc3  narc4  narc5  omind5  omind6  contact1  contact2  contact3  contact4  contact5  hygiene1  hygiene2  hygiene3  hygiene4  hygiene5  psupport1  psupport2  psupport3  psupport4  psupport5  political_ideology  happy  riskperc1  riskperc2  self_esteem  sbelong1  sbelong2  sbelong3  slfcont3  urban
##   8   8  age  cnarc1  cnarc2  cnarc3  CRT1  CRT2  CRT3  duration  generosity1  generosity2  generosity3  marital1  mor_circle  moralid1  moralid10  moralid2  moralid3  moralid4  moralid5  moralid6  moralid7  moralid8  moralid9  mcoop1  mcoop2  mcoop3  mcoop4  mcoop5  mcoop6  mcoop7  narc1  narc3  narc4  narc5  omind5  omind6  contact1  contact2  contact3  contact4  contact5  hygiene1  hygiene2  hygiene3  hygiene4  hygiene5  psupport1  psupport2  psupport3  psupport4  psupport5  political_ideology  happy  riskperc1  riskperc2  self_esteem  sbelong1  sbelong2  sbelong3  slfcont3  urban
##   8   9  age  cnarc1  cnarc2  cnarc3  CRT1  CRT2  CRT3  duration  generosity1  generosity2  generosity3  marital1  mor_circle  moralid1  moralid10  moralid2  moralid3  moralid4  moralid5  moralid6  moralid7  moralid8  moralid9  mcoop1  mcoop2  mcoop3  mcoop4  mcoop5  mcoop6  mcoop7  narc1  narc3  narc4  narc5  omind5  omind6  contact1  contact2  contact3  contact4  contact5  hygiene1  hygiene2  hygiene3  hygiene4  hygiene5  psupport1  psupport2  psupport3  psupport4  psupport5  political_ideology  happy  riskperc1  riskperc2  self_esteem  sbelong1  sbelong2  sbelong3  slfcont3  urban
##   8   10  age  cnarc1  cnarc2  cnarc3  CRT1  CRT2  CRT3  duration  generosity1  generosity2  generosity3  marital1  mor_circle  moralid1  moralid10  moralid2  moralid3  moralid4  moralid5  moralid6  moralid7  moralid8  moralid9  mcoop1  mcoop2  mcoop3  mcoop4  mcoop5  mcoop6  mcoop7  narc1  narc3  narc4  narc5  omind5  omind6  contact1  contact2  contact3  contact4  contact5  hygiene1  hygiene2  hygiene3  hygiene4  hygiene5  psupport1  psupport2  psupport3  psupport4  psupport5  political_ideology  happy  riskperc1  riskperc2  self_esteem  sbelong1  sbelong2  sbelong3  slfcont3  urban
##   9   1  age  cnarc1  cnarc2  cnarc3  CRT1  CRT2  CRT3  duration  generosity1  generosity2  generosity3  marital1  mor_circle  moralid1  moralid10  moralid2  moralid3  moralid4  moralid5  moralid6  moralid7  moralid8  moralid9  mcoop1  mcoop2  mcoop3  mcoop4  mcoop5  mcoop6  mcoop7  narc1  narc3  narc4  narc5  omind5  omind6  contact1  contact2  contact3  contact4  contact5  hygiene1  hygiene2  hygiene3  hygiene4  hygiene5  psupport1  psupport2  psupport3  psupport4  psupport5  political_ideology  happy  riskperc1  riskperc2  self_esteem  sbelong1  sbelong2  sbelong3  slfcont3  urban
##   9   2  age  cnarc1  cnarc2  cnarc3  CRT1  CRT2  CRT3  duration  generosity1  generosity2  generosity3  marital1  mor_circle  moralid1  moralid10  moralid2  moralid3  moralid4  moralid5  moralid6  moralid7  moralid8  moralid9  mcoop1  mcoop2  mcoop3  mcoop4  mcoop5  mcoop6  mcoop7  narc1  narc3  narc4  narc5  omind5  omind6  contact1  contact2  contact3  contact4  contact5  hygiene1  hygiene2  hygiene3  hygiene4  hygiene5  psupport1  psupport2  psupport3  psupport4  psupport5  political_ideology  happy  riskperc1  riskperc2  self_esteem  sbelong1  sbelong2  sbelong3  slfcont3  urban
##   9   3  age  cnarc1  cnarc2  cnarc3  CRT1  CRT2  CRT3  duration  generosity1  generosity2  generosity3  marital1  mor_circle  moralid1  moralid10  moralid2  moralid3  moralid4  moralid5  moralid6  moralid7  moralid8  moralid9  mcoop1  mcoop2  mcoop3  mcoop4  mcoop5  mcoop6  mcoop7  narc1  narc3  narc4  narc5  omind5  omind6  contact1  contact2  contact3  contact4  contact5  hygiene1  hygiene2  hygiene3  hygiene4  hygiene5  psupport1  psupport2  psupport3  psupport4  psupport5  political_ideology  happy  riskperc1  riskperc2  self_esteem  sbelong1  sbelong2  sbelong3  slfcont3  urban
##   9   4  age  cnarc1  cnarc2  cnarc3  CRT1  CRT2  CRT3  duration  generosity1  generosity2  generosity3  marital1  mor_circle  moralid1  moralid10  moralid2  moralid3  moralid4  moralid5  moralid6  moralid7  moralid8  moralid9  mcoop1  mcoop2  mcoop3  mcoop4  mcoop5  mcoop6  mcoop7  narc1  narc3  narc4  narc5  omind5  omind6  contact1  contact2  contact3  contact4  contact5  hygiene1  hygiene2  hygiene3  hygiene4  hygiene5  psupport1  psupport2  psupport3  psupport4  psupport5  political_ideology  happy  riskperc1  riskperc2  self_esteem  sbelong1  sbelong2  sbelong3  slfcont3  urban
##   9   5  age  cnarc1  cnarc2  cnarc3  CRT1  CRT2  CRT3  duration  generosity1  generosity2  generosity3  marital1  mor_circle  moralid1  moralid10  moralid2  moralid3  moralid4  moralid5  moralid6  moralid7  moralid8  moralid9  mcoop1  mcoop2  mcoop3  mcoop4  mcoop5  mcoop6  mcoop7  narc1  narc3  narc4  narc5  omind5  omind6  contact1  contact2  contact3  contact4  contact5  hygiene1  hygiene2  hygiene3  hygiene4  hygiene5  psupport1  psupport2  psupport3  psupport4  psupport5  political_ideology  happy  riskperc1  riskperc2  self_esteem  sbelong1  sbelong2  sbelong3  slfcont3  urban
##   9   6  age  cnarc1  cnarc2  cnarc3  CRT1  CRT2  CRT3  duration  generosity1  generosity2  generosity3  marital1  mor_circle  moralid1  moralid10  moralid2  moralid3  moralid4  moralid5  moralid6  moralid7  moralid8  moralid9  mcoop1  mcoop2  mcoop3  mcoop4  mcoop5  mcoop6  mcoop7  narc1  narc3  narc4  narc5  omind5  omind6  contact1  contact2  contact3  contact4  contact5  hygiene1  hygiene2  hygiene3  hygiene4  hygiene5  psupport1  psupport2  psupport3  psupport4  psupport5  political_ideology  happy  riskperc1  riskperc2  self_esteem  sbelong1  sbelong2  sbelong3  slfcont3  urban
##   9   7  age  cnarc1  cnarc2  cnarc3  CRT1  CRT2  CRT3  duration  generosity1  generosity2  generosity3  marital1  mor_circle  moralid1  moralid10  moralid2  moralid3  moralid4  moralid5  moralid6  moralid7  moralid8  moralid9  mcoop1  mcoop2  mcoop3  mcoop4  mcoop5  mcoop6  mcoop7  narc1  narc3  narc4  narc5  omind5  omind6  contact1  contact2  contact3  contact4  contact5  hygiene1  hygiene2  hygiene3  hygiene4  hygiene5  psupport1  psupport2  psupport3  psupport4  psupport5  political_ideology  happy  riskperc1  riskperc2  self_esteem  sbelong1  sbelong2  sbelong3  slfcont3  urban
##   9   8  age  cnarc1  cnarc2  cnarc3  CRT1  CRT2  CRT3  duration  generosity1  generosity2  generosity3  marital1  mor_circle  moralid1  moralid10  moralid2  moralid3  moralid4  moralid5  moralid6  moralid7  moralid8  moralid9  mcoop1  mcoop2  mcoop3  mcoop4  mcoop5  mcoop6  mcoop7  narc1  narc3  narc4  narc5  omind5  omind6  contact1  contact2  contact3  contact4  contact5  hygiene1  hygiene2  hygiene3  hygiene4  hygiene5  psupport1  psupport2  psupport3  psupport4  psupport5  political_ideology  happy  riskperc1  riskperc2  self_esteem  sbelong1  sbelong2  sbelong3  slfcont3  urban
##   9   9  age  cnarc1  cnarc2  cnarc3  CRT1  CRT2  CRT3  duration  generosity1  generosity2  generosity3  marital1  mor_circle  moralid1  moralid10  moralid2  moralid3  moralid4  moralid5  moralid6  moralid7  moralid8  moralid9  mcoop1  mcoop2  mcoop3  mcoop4  mcoop5  mcoop6  mcoop7  narc1  narc3  narc4  narc5  omind5  omind6  contact1  contact2  contact3  contact4  contact5  hygiene1  hygiene2  hygiene3  hygiene4  hygiene5  psupport1  psupport2  psupport3  psupport4  psupport5  political_ideology  happy  riskperc1  riskperc2  self_esteem  sbelong1  sbelong2  sbelong3  slfcont3  urban
##   9   10  age  cnarc1  cnarc2  cnarc3  CRT1  CRT2  CRT3  duration  generosity1  generosity2  generosity3  marital1  mor_circle  moralid1  moralid10  moralid2  moralid3  moralid4  moralid5  moralid6  moralid7  moralid8  moralid9  mcoop1  mcoop2  mcoop3  mcoop4  mcoop5  mcoop6  mcoop7  narc1  narc3  narc4  narc5  omind5  omind6  contact1  contact2  contact3  contact4  contact5  hygiene1  hygiene2  hygiene3  hygiene4  hygiene5  psupport1  psupport2  psupport3  psupport4  psupport5  political_ideology  happy  riskperc1  riskperc2  self_esteem  sbelong1  sbelong2  sbelong3  slfcont3  urban
##   10   1  age  cnarc1  cnarc2  cnarc3  CRT1  CRT2  CRT3  duration  generosity1  generosity2  generosity3  marital1  mor_circle  moralid1  moralid10  moralid2  moralid3  moralid4  moralid5  moralid6  moralid7  moralid8  moralid9  mcoop1  mcoop2  mcoop3  mcoop4  mcoop5  mcoop6  mcoop7  narc1  narc3  narc4  narc5  omind5  omind6  contact1  contact2  contact3  contact4  contact5  hygiene1  hygiene2  hygiene3  hygiene4  hygiene5  psupport1  psupport2  psupport3  psupport4  psupport5  political_ideology  happy  riskperc1  riskperc2  self_esteem  sbelong1  sbelong2  sbelong3  slfcont3  urban
##   10   2  age  cnarc1  cnarc2  cnarc3  CRT1  CRT2  CRT3  duration  generosity1  generosity2  generosity3  marital1  mor_circle  moralid1  moralid10  moralid2  moralid3  moralid4  moralid5  moralid6  moralid7  moralid8  moralid9  mcoop1  mcoop2  mcoop3  mcoop4  mcoop5  mcoop6  mcoop7  narc1  narc3  narc4  narc5  omind5  omind6  contact1  contact2  contact3  contact4  contact5  hygiene1  hygiene2  hygiene3  hygiene4  hygiene5  psupport1  psupport2  psupport3  psupport4  psupport5  political_ideology  happy  riskperc1  riskperc2  self_esteem  sbelong1  sbelong2  sbelong3  slfcont3  urban
##   10   3  age  cnarc1  cnarc2  cnarc3  CRT1  CRT2  CRT3  duration  generosity1  generosity2  generosity3  marital1  mor_circle  moralid1  moralid10  moralid2  moralid3  moralid4  moralid5  moralid6  moralid7  moralid8  moralid9  mcoop1  mcoop2  mcoop3  mcoop4  mcoop5  mcoop6  mcoop7  narc1  narc3  narc4  narc5  omind5  omind6  contact1  contact2  contact3  contact4  contact5  hygiene1  hygiene2  hygiene3  hygiene4  hygiene5  psupport1  psupport2  psupport3  psupport4  psupport5  political_ideology  happy  riskperc1  riskperc2  self_esteem  sbelong1  sbelong2  sbelong3  slfcont3  urban
##   10   4  age  cnarc1  cnarc2  cnarc3  CRT1  CRT2  CRT3  duration  generosity1  generosity2  generosity3  marital1  mor_circle  moralid1  moralid10  moralid2  moralid3  moralid4  moralid5  moralid6  moralid7  moralid8  moralid9  mcoop1  mcoop2  mcoop3  mcoop4  mcoop5  mcoop6  mcoop7  narc1  narc3  narc4  narc5  omind5  omind6  contact1  contact2  contact3  contact4  contact5  hygiene1  hygiene2  hygiene3  hygiene4  hygiene5  psupport1  psupport2  psupport3  psupport4  psupport5  political_ideology  happy  riskperc1  riskperc2  self_esteem  sbelong1  sbelong2  sbelong3  slfcont3  urban
##   10   5  age  cnarc1  cnarc2  cnarc3  CRT1  CRT2  CRT3  duration  generosity1  generosity2  generosity3  marital1  mor_circle  moralid1  moralid10  moralid2  moralid3  moralid4  moralid5  moralid6  moralid7  moralid8  moralid9  mcoop1  mcoop2  mcoop3  mcoop4  mcoop5  mcoop6  mcoop7  narc1  narc3  narc4  narc5  omind5  omind6  contact1  contact2  contact3  contact4  contact5  hygiene1  hygiene2  hygiene3  hygiene4  hygiene5  psupport1  psupport2  psupport3  psupport4  psupport5  political_ideology  happy  riskperc1  riskperc2  self_esteem  sbelong1  sbelong2  sbelong3  slfcont3  urban
##   10   6  age  cnarc1  cnarc2  cnarc3  CRT1  CRT2  CRT3  duration  generosity1  generosity2  generosity3  marital1  mor_circle  moralid1  moralid10  moralid2  moralid3  moralid4  moralid5  moralid6  moralid7  moralid8  moralid9  mcoop1  mcoop2  mcoop3  mcoop4  mcoop5  mcoop6  mcoop7  narc1  narc3  narc4  narc5  omind5  omind6  contact1  contact2  contact3  contact4  contact5  hygiene1  hygiene2  hygiene3  hygiene4  hygiene5  psupport1  psupport2  psupport3  psupport4  psupport5  political_ideology  happy  riskperc1  riskperc2  self_esteem  sbelong1  sbelong2  sbelong3  slfcont3  urban
##   10   7  age  cnarc1  cnarc2  cnarc3  CRT1  CRT2  CRT3  duration  generosity1  generosity2  generosity3  marital1  mor_circle  moralid1  moralid10  moralid2  moralid3  moralid4  moralid5  moralid6  moralid7  moralid8  moralid9  mcoop1  mcoop2  mcoop3  mcoop4  mcoop5  mcoop6  mcoop7  narc1  narc3  narc4  narc5  omind5  omind6  contact1  contact2  contact3  contact4  contact5  hygiene1  hygiene2  hygiene3  hygiene4  hygiene5  psupport1  psupport2  psupport3  psupport4  psupport5  political_ideology  happy  riskperc1  riskperc2  self_esteem  sbelong1  sbelong2  sbelong3  slfcont3  urban
##   10   8  age  cnarc1  cnarc2  cnarc3  CRT1  CRT2  CRT3  duration  generosity1  generosity2  generosity3  marital1  mor_circle  moralid1  moralid10  moralid2  moralid3  moralid4  moralid5  moralid6  moralid7  moralid8  moralid9  mcoop1  mcoop2  mcoop3  mcoop4  mcoop5  mcoop6  mcoop7  narc1  narc3  narc4  narc5  omind5  omind6  contact1  contact2  contact3  contact4  contact5  hygiene1  hygiene2  hygiene3  hygiene4  hygiene5  psupport1  psupport2  psupport3  psupport4  psupport5  political_ideology  happy  riskperc1  riskperc2  self_esteem  sbelong1  sbelong2  sbelong3  slfcont3  urban
##   10   9  age  cnarc1  cnarc2  cnarc3  CRT1  CRT2  CRT3  duration  generosity1  generosity2  generosity3  marital1  mor_circle  moralid1  moralid10  moralid2  moralid3  moralid4  moralid5  moralid6  moralid7  moralid8  moralid9  mcoop1  mcoop2  mcoop3  mcoop4  mcoop5  mcoop6  mcoop7  narc1  narc3  narc4  narc5  omind5  omind6  contact1  contact2  contact3  contact4  contact5  hygiene1  hygiene2  hygiene3  hygiene4  hygiene5  psupport1  psupport2  psupport3  psupport4  psupport5  political_ideology  happy  riskperc1  riskperc2  self_esteem  sbelong1  sbelong2  sbelong3  slfcont3  urban
##   10   10  age  cnarc1  cnarc2  cnarc3  CRT1  CRT2  CRT3  duration  generosity1  generosity2  generosity3  marital1  mor_circle  moralid1  moralid10  moralid2  moralid3  moralid4  moralid5  moralid6  moralid7  moralid8  moralid9  mcoop1  mcoop2  mcoop3  mcoop4  mcoop5  mcoop6  mcoop7  narc1  narc3  narc4  narc5  omind5  omind6  contact1  contact2  contact3  contact4  contact5  hygiene1  hygiene2  hygiene3  hygiene4  hygiene5  psupport1  psupport2  psupport3  psupport4  psupport5  political_ideology  happy  riskperc1  riskperc2  self_esteem  sbelong1  sbelong2  sbelong3  slfcont3  urban
```

```
## Warning: Number of logged events: 5733
```

```
## 
##  iter imp variable
##   1   1  age  children  cnarc1  cnarc2  cnarc3  ctheory1  ctheory2  ctheory3  ctheory4  CRT1  CRT2  CRT3  ladder  mor_circle  moralid1  moralid10  moralid2  moralid3  moralid4  moralid5  moralid6  moralid7  moralid8  moralid9  mcoop3  mcoop4  mcoop5  mcoop6  mcoop7  narc1  narc4  narc5  omind1  omind2  omind3  omind4  omind5  omind6  contact1  psupport1  psupport3  political_ideology  happy  riskperc1  riskperc2  optim2
##   1   2  age  children  cnarc1  cnarc2  cnarc3  ctheory1  ctheory2  ctheory3  ctheory4  CRT1  CRT2  CRT3  ladder  mor_circle  moralid1  moralid10  moralid2  moralid3  moralid4  moralid5  moralid6  moralid7  moralid8  moralid9  mcoop3  mcoop4  mcoop5  mcoop6  mcoop7  narc1  narc4  narc5  omind1  omind2  omind3  omind4  omind5  omind6  contact1  psupport1  psupport3  political_ideology  happy  riskperc1  riskperc2  optim2
##   1   3  age  children  cnarc1  cnarc2  cnarc3  ctheory1  ctheory2  ctheory3  ctheory4  CRT1  CRT2  CRT3  ladder  mor_circle  moralid1  moralid10  moralid2  moralid3  moralid4  moralid5  moralid6  moralid7  moralid8  moralid9  mcoop3  mcoop4  mcoop5  mcoop6  mcoop7  narc1  narc4  narc5  omind1  omind2  omind3  omind4  omind5  omind6  contact1  psupport1  psupport3  political_ideology  happy  riskperc1  riskperc2  optim2
##   1   4  age  children  cnarc1  cnarc2  cnarc3  ctheory1  ctheory2  ctheory3  ctheory4  CRT1  CRT2  CRT3  ladder  mor_circle  moralid1  moralid10  moralid2  moralid3  moralid4  moralid5  moralid6  moralid7  moralid8  moralid9  mcoop3  mcoop4  mcoop5  mcoop6  mcoop7  narc1  narc4  narc5  omind1  omind2  omind3  omind4  omind5  omind6  contact1  psupport1  psupport3  political_ideology  happy  riskperc1  riskperc2  optim2
##   1   5  age  children  cnarc1  cnarc2  cnarc3  ctheory1  ctheory2  ctheory3  ctheory4  CRT1  CRT2  CRT3  ladder  mor_circle  moralid1  moralid10  moralid2  moralid3  moralid4  moralid5  moralid6  moralid7  moralid8  moralid9  mcoop3  mcoop4  mcoop5  mcoop6  mcoop7  narc1  narc4  narc5  omind1  omind2  omind3  omind4  omind5  omind6  contact1  psupport1  psupport3  political_ideology  happy  riskperc1  riskperc2  optim2
##   1   6  age  children  cnarc1  cnarc2  cnarc3  ctheory1  ctheory2  ctheory3  ctheory4  CRT1  CRT2  CRT3  ladder  mor_circle  moralid1  moralid10  moralid2  moralid3  moralid4  moralid5  moralid6  moralid7  moralid8  moralid9  mcoop3  mcoop4  mcoop5  mcoop6  mcoop7  narc1  narc4  narc5  omind1  omind2  omind3  omind4  omind5  omind6  contact1  psupport1  psupport3  political_ideology  happy  riskperc1  riskperc2  optim2
##   1   7  age  children  cnarc1  cnarc2  cnarc3  ctheory1  ctheory2  ctheory3  ctheory4  CRT1  CRT2  CRT3  ladder  mor_circle  moralid1  moralid10  moralid2  moralid3  moralid4  moralid5  moralid6  moralid7  moralid8  moralid9  mcoop3  mcoop4  mcoop5  mcoop6  mcoop7  narc1  narc4  narc5  omind1  omind2  omind3  omind4  omind5  omind6  contact1  psupport1  psupport3  political_ideology  happy  riskperc1  riskperc2  optim2
##   1   8  age  children  cnarc1  cnarc2  cnarc3  ctheory1  ctheory2  ctheory3  ctheory4  CRT1  CRT2  CRT3  ladder  mor_circle  moralid1  moralid10  moralid2  moralid3  moralid4  moralid5  moralid6  moralid7  moralid8  moralid9  mcoop3  mcoop4  mcoop5  mcoop6  mcoop7  narc1  narc4  narc5  omind1  omind2  omind3  omind4  omind5  omind6  contact1  psupport1  psupport3  political_ideology  happy  riskperc1  riskperc2  optim2
##   1   9  age  children  cnarc1  cnarc2  cnarc3  ctheory1  ctheory2  ctheory3  ctheory4  CRT1  CRT2  CRT3  ladder  mor_circle  moralid1  moralid10  moralid2  moralid3  moralid4  moralid5  moralid6  moralid7  moralid8  moralid9  mcoop3  mcoop4  mcoop5  mcoop6  mcoop7  narc1  narc4  narc5  omind1  omind2  omind3  omind4  omind5  omind6  contact1  psupport1  psupport3  political_ideology  happy  riskperc1  riskperc2  optim2
##   1   10  age  children  cnarc1  cnarc2  cnarc3  ctheory1  ctheory2  ctheory3  ctheory4  CRT1  CRT2  CRT3  ladder  mor_circle  moralid1  moralid10  moralid2  moralid3  moralid4  moralid5  moralid6  moralid7  moralid8  moralid9  mcoop3  mcoop4  mcoop5  mcoop6  mcoop7  narc1  narc4  narc5  omind1  omind2  omind3  omind4  omind5  omind6  contact1  psupport1  psupport3  political_ideology  happy  riskperc1  riskperc2  optim2
##   2   1  age  children  cnarc1  cnarc2  cnarc3  ctheory1  ctheory2  ctheory3  ctheory4  CRT1  CRT2  CRT3  ladder  mor_circle  moralid1  moralid10  moralid2  moralid3  moralid4  moralid5  moralid6  moralid7  moralid8  moralid9  mcoop3  mcoop4  mcoop5  mcoop6  mcoop7  narc1  narc4  narc5  omind1  omind2  omind3  omind4  omind5  omind6  contact1  psupport1  psupport3  political_ideology  happy  riskperc1  riskperc2  optim2
##   2   2  age  children  cnarc1  cnarc2  cnarc3  ctheory1  ctheory2  ctheory3  ctheory4  CRT1  CRT2  CRT3  ladder  mor_circle  moralid1  moralid10  moralid2  moralid3  moralid4  moralid5  moralid6  moralid7  moralid8  moralid9  mcoop3  mcoop4  mcoop5  mcoop6  mcoop7  narc1  narc4  narc5  omind1  omind2  omind3  omind4  omind5  omind6  contact1  psupport1  psupport3  political_ideology  happy  riskperc1  riskperc2  optim2
##   2   3  age  children  cnarc1  cnarc2  cnarc3  ctheory1  ctheory2  ctheory3  ctheory4  CRT1  CRT2  CRT3  ladder  mor_circle  moralid1  moralid10  moralid2  moralid3  moralid4  moralid5  moralid6  moralid7  moralid8  moralid9  mcoop3  mcoop4  mcoop5  mcoop6  mcoop7  narc1  narc4  narc5  omind1  omind2  omind3  omind4  omind5  omind6  contact1  psupport1  psupport3  political_ideology  happy  riskperc1  riskperc2  optim2
##   2   4  age  children  cnarc1  cnarc2  cnarc3  ctheory1  ctheory2  ctheory3  ctheory4  CRT1  CRT2  CRT3  ladder  mor_circle  moralid1  moralid10  moralid2  moralid3  moralid4  moralid5  moralid6  moralid7  moralid8  moralid9  mcoop3  mcoop4  mcoop5  mcoop6  mcoop7  narc1  narc4  narc5  omind1  omind2  omind3  omind4  omind5  omind6  contact1  psupport1  psupport3  political_ideology  happy  riskperc1  riskperc2  optim2
##   2   5  age  children  cnarc1  cnarc2  cnarc3  ctheory1  ctheory2  ctheory3  ctheory4  CRT1  CRT2  CRT3  ladder  mor_circle  moralid1  moralid10  moralid2  moralid3  moralid4  moralid5  moralid6  moralid7  moralid8  moralid9  mcoop3  mcoop4  mcoop5  mcoop6  mcoop7  narc1  narc4  narc5  omind1  omind2  omind3  omind4  omind5  omind6  contact1  psupport1  psupport3  political_ideology  happy  riskperc1  riskperc2  optim2
##   2   6  age  children  cnarc1  cnarc2  cnarc3  ctheory1  ctheory2  ctheory3  ctheory4  CRT1  CRT2  CRT3  ladder  mor_circle  moralid1  moralid10  moralid2  moralid3  moralid4  moralid5  moralid6  moralid7  moralid8  moralid9  mcoop3  mcoop4  mcoop5  mcoop6  mcoop7  narc1  narc4  narc5  omind1  omind2  omind3  omind4  omind5  omind6  contact1  psupport1  psupport3  political_ideology  happy  riskperc1  riskperc2  optim2
##   2   7  age  children  cnarc1  cnarc2  cnarc3  ctheory1  ctheory2  ctheory3  ctheory4  CRT1  CRT2  CRT3  ladder  mor_circle  moralid1  moralid10  moralid2  moralid3  moralid4  moralid5  moralid6  moralid7  moralid8  moralid9  mcoop3  mcoop4  mcoop5  mcoop6  mcoop7  narc1  narc4  narc5  omind1  omind2  omind3  omind4  omind5  omind6  contact1  psupport1  psupport3  political_ideology  happy  riskperc1  riskperc2  optim2
##   2   8  age  children  cnarc1  cnarc2  cnarc3  ctheory1  ctheory2  ctheory3  ctheory4  CRT1  CRT2  CRT3  ladder  mor_circle  moralid1  moralid10  moralid2  moralid3  moralid4  moralid5  moralid6  moralid7  moralid8  moralid9  mcoop3  mcoop4  mcoop5  mcoop6  mcoop7  narc1  narc4  narc5  omind1  omind2  omind3  omind4  omind5  omind6  contact1  psupport1  psupport3  political_ideology  happy  riskperc1  riskperc2  optim2
##   2   9  age  children  cnarc1  cnarc2  cnarc3  ctheory1  ctheory2  ctheory3  ctheory4  CRT1  CRT2  CRT3  ladder  mor_circle  moralid1  moralid10  moralid2  moralid3  moralid4  moralid5  moralid6  moralid7  moralid8  moralid9  mcoop3  mcoop4  mcoop5  mcoop6  mcoop7  narc1  narc4  narc5  omind1  omind2  omind3  omind4  omind5  omind6  contact1  psupport1  psupport3  political_ideology  happy  riskperc1  riskperc2  optim2
##   2   10  age  children  cnarc1  cnarc2  cnarc3  ctheory1  ctheory2  ctheory3  ctheory4  CRT1  CRT2  CRT3  ladder  mor_circle  moralid1  moralid10  moralid2  moralid3  moralid4  moralid5  moralid6  moralid7  moralid8  moralid9  mcoop3  mcoop4  mcoop5  mcoop6  mcoop7  narc1  narc4  narc5  omind1  omind2  omind3  omind4  omind5  omind6  contact1  psupport1  psupport3  political_ideology  happy  riskperc1  riskperc2  optim2
##   3   1  age  children  cnarc1  cnarc2  cnarc3  ctheory1  ctheory2  ctheory3  ctheory4  CRT1  CRT2  CRT3  ladder  mor_circle  moralid1  moralid10  moralid2  moralid3  moralid4  moralid5  moralid6  moralid7  moralid8  moralid9  mcoop3  mcoop4  mcoop5  mcoop6  mcoop7  narc1  narc4  narc5  omind1  omind2  omind3  omind4  omind5  omind6  contact1  psupport1  psupport3  political_ideology  happy  riskperc1  riskperc2  optim2
##   3   2  age  children  cnarc1  cnarc2  cnarc3  ctheory1  ctheory2  ctheory3  ctheory4  CRT1  CRT2  CRT3  ladder  mor_circle  moralid1  moralid10  moralid2  moralid3  moralid4  moralid5  moralid6  moralid7  moralid8  moralid9  mcoop3  mcoop4  mcoop5  mcoop6  mcoop7  narc1  narc4  narc5  omind1  omind2  omind3  omind4  omind5  omind6  contact1  psupport1  psupport3  political_ideology  happy  riskperc1  riskperc2  optim2
##   3   3  age  children  cnarc1  cnarc2  cnarc3  ctheory1  ctheory2  ctheory3  ctheory4  CRT1  CRT2  CRT3  ladder  mor_circle  moralid1  moralid10  moralid2  moralid3  moralid4  moralid5  moralid6  moralid7  moralid8  moralid9  mcoop3  mcoop4  mcoop5  mcoop6  mcoop7  narc1  narc4  narc5  omind1  omind2  omind3  omind4  omind5  omind6  contact1  psupport1  psupport3  political_ideology  happy  riskperc1  riskperc2  optim2
##   3   4  age  children  cnarc1  cnarc2  cnarc3  ctheory1  ctheory2  ctheory3  ctheory4  CRT1  CRT2  CRT3  ladder  mor_circle  moralid1  moralid10  moralid2  moralid3  moralid4  moralid5  moralid6  moralid7  moralid8  moralid9  mcoop3  mcoop4  mcoop5  mcoop6  mcoop7  narc1  narc4  narc5  omind1  omind2  omind3  omind4  omind5  omind6  contact1  psupport1  psupport3  political_ideology  happy  riskperc1  riskperc2  optim2
##   3   5  age  children  cnarc1  cnarc2  cnarc3  ctheory1  ctheory2  ctheory3  ctheory4  CRT1  CRT2  CRT3  ladder  mor_circle  moralid1  moralid10  moralid2  moralid3  moralid4  moralid5  moralid6  moralid7  moralid8  moralid9  mcoop3  mcoop4  mcoop5  mcoop6  mcoop7  narc1  narc4  narc5  omind1  omind2  omind3  omind4  omind5  omind6  contact1  psupport1  psupport3  political_ideology  happy  riskperc1  riskperc2  optim2
##   3   6  age  children  cnarc1  cnarc2  cnarc3  ctheory1  ctheory2  ctheory3  ctheory4  CRT1  CRT2  CRT3  ladder  mor_circle  moralid1  moralid10  moralid2  moralid3  moralid4  moralid5  moralid6  moralid7  moralid8  moralid9  mcoop3  mcoop4  mcoop5  mcoop6  mcoop7  narc1  narc4  narc5  omind1  omind2  omind3  omind4  omind5  omind6  contact1  psupport1  psupport3  political_ideology  happy  riskperc1  riskperc2  optim2
##   3   7  age  children  cnarc1  cnarc2  cnarc3  ctheory1  ctheory2  ctheory3  ctheory4  CRT1  CRT2  CRT3  ladder  mor_circle  moralid1  moralid10  moralid2  moralid3  moralid4  moralid5  moralid6  moralid7  moralid8  moralid9  mcoop3  mcoop4  mcoop5  mcoop6  mcoop7  narc1  narc4  narc5  omind1  omind2  omind3  omind4  omind5  omind6  contact1  psupport1  psupport3  political_ideology  happy  riskperc1  riskperc2  optim2
##   3   8  age  children  cnarc1  cnarc2  cnarc3  ctheory1  ctheory2  ctheory3  ctheory4  CRT1  CRT2  CRT3  ladder  mor_circle  moralid1  moralid10  moralid2  moralid3  moralid4  moralid5  moralid6  moralid7  moralid8  moralid9  mcoop3  mcoop4  mcoop5  mcoop6  mcoop7  narc1  narc4  narc5  omind1  omind2  omind3  omind4  omind5  omind6  contact1  psupport1  psupport3  political_ideology  happy  riskperc1  riskperc2  optim2
##   3   9  age  children  cnarc1  cnarc2  cnarc3  ctheory1  ctheory2  ctheory3  ctheory4  CRT1  CRT2  CRT3  ladder  mor_circle  moralid1  moralid10  moralid2  moralid3  moralid4  moralid5  moralid6  moralid7  moralid8  moralid9  mcoop3  mcoop4  mcoop5  mcoop6  mcoop7  narc1  narc4  narc5  omind1  omind2  omind3  omind4  omind5  omind6  contact1  psupport1  psupport3  political_ideology  happy  riskperc1  riskperc2  optim2
##   3   10  age  children  cnarc1  cnarc2  cnarc3  ctheory1  ctheory2  ctheory3  ctheory4  CRT1  CRT2  CRT3  ladder  mor_circle  moralid1  moralid10  moralid2  moralid3  moralid4  moralid5  moralid6  moralid7  moralid8  moralid9  mcoop3  mcoop4  mcoop5  mcoop6  mcoop7  narc1  narc4  narc5  omind1  omind2  omind3  omind4  omind5  omind6  contact1  psupport1  psupport3  political_ideology  happy  riskperc1  riskperc2  optim2
##   4   1  age  children  cnarc1  cnarc2  cnarc3  ctheory1  ctheory2  ctheory3  ctheory4  CRT1  CRT2  CRT3  ladder  mor_circle  moralid1  moralid10  moralid2  moralid3  moralid4  moralid5  moralid6  moralid7  moralid8  moralid9  mcoop3  mcoop4  mcoop5  mcoop6  mcoop7  narc1  narc4  narc5  omind1  omind2  omind3  omind4  omind5  omind6  contact1  psupport1  psupport3  political_ideology  happy  riskperc1  riskperc2  optim2
##   4   2  age  children  cnarc1  cnarc2  cnarc3  ctheory1  ctheory2  ctheory3  ctheory4  CRT1  CRT2  CRT3  ladder  mor_circle  moralid1  moralid10  moralid2  moralid3  moralid4  moralid5  moralid6  moralid7  moralid8  moralid9  mcoop3  mcoop4  mcoop5  mcoop6  mcoop7  narc1  narc4  narc5  omind1  omind2  omind3  omind4  omind5  omind6  contact1  psupport1  psupport3  political_ideology  happy  riskperc1  riskperc2  optim2
##   4   3  age  children  cnarc1  cnarc2  cnarc3  ctheory1  ctheory2  ctheory3  ctheory4  CRT1  CRT2  CRT3  ladder  mor_circle  moralid1  moralid10  moralid2  moralid3  moralid4  moralid5  moralid6  moralid7  moralid8  moralid9  mcoop3  mcoop4  mcoop5  mcoop6  mcoop7  narc1  narc4  narc5  omind1  omind2  omind3  omind4  omind5  omind6  contact1  psupport1  psupport3  political_ideology  happy  riskperc1  riskperc2  optim2
##   4   4  age  children  cnarc1  cnarc2  cnarc3  ctheory1  ctheory2  ctheory3  ctheory4  CRT1  CRT2  CRT3  ladder  mor_circle  moralid1  moralid10  moralid2  moralid3  moralid4  moralid5  moralid6  moralid7  moralid8  moralid9  mcoop3  mcoop4  mcoop5  mcoop6  mcoop7  narc1  narc4  narc5  omind1  omind2  omind3  omind4  omind5  omind6  contact1  psupport1  psupport3  political_ideology  happy  riskperc1  riskperc2  optim2
##   4   5  age  children  cnarc1  cnarc2  cnarc3  ctheory1  ctheory2  ctheory3  ctheory4  CRT1  CRT2  CRT3  ladder  mor_circle  moralid1  moralid10  moralid2  moralid3  moralid4  moralid5  moralid6  moralid7  moralid8  moralid9  mcoop3  mcoop4  mcoop5  mcoop6  mcoop7  narc1  narc4  narc5  omind1  omind2  omind3  omind4  omind5  omind6  contact1  psupport1  psupport3  political_ideology  happy  riskperc1  riskperc2  optim2
##   4   6  age  children  cnarc1  cnarc2  cnarc3  ctheory1  ctheory2  ctheory3  ctheory4  CRT1  CRT2  CRT3  ladder  mor_circle  moralid1  moralid10  moralid2  moralid3  moralid4  moralid5  moralid6  moralid7  moralid8  moralid9  mcoop3  mcoop4  mcoop5  mcoop6  mcoop7  narc1  narc4  narc5  omind1  omind2  omind3  omind4  omind5  omind6  contact1  psupport1  psupport3  political_ideology  happy  riskperc1  riskperc2  optim2
##   4   7  age  children  cnarc1  cnarc2  cnarc3  ctheory1  ctheory2  ctheory3  ctheory4  CRT1  CRT2  CRT3  ladder  mor_circle  moralid1  moralid10  moralid2  moralid3  moralid4  moralid5  moralid6  moralid7  moralid8  moralid9  mcoop3  mcoop4  mcoop5  mcoop6  mcoop7  narc1  narc4  narc5  omind1  omind2  omind3  omind4  omind5  omind6  contact1  psupport1  psupport3  political_ideology  happy  riskperc1  riskperc2  optim2
##   4   8  age  children  cnarc1  cnarc2  cnarc3  ctheory1  ctheory2  ctheory3  ctheory4  CRT1  CRT2  CRT3  ladder  mor_circle  moralid1  moralid10  moralid2  moralid3  moralid4  moralid5  moralid6  moralid7  moralid8  moralid9  mcoop3  mcoop4  mcoop5  mcoop6  mcoop7  narc1  narc4  narc5  omind1  omind2  omind3  omind4  omind5  omind6  contact1  psupport1  psupport3  political_ideology  happy  riskperc1  riskperc2  optim2
##   4   9  age  children  cnarc1  cnarc2  cnarc3  ctheory1  ctheory2  ctheory3  ctheory4  CRT1  CRT2  CRT3  ladder  mor_circle  moralid1  moralid10  moralid2  moralid3  moralid4  moralid5  moralid6  moralid7  moralid8  moralid9  mcoop3  mcoop4  mcoop5  mcoop6  mcoop7  narc1  narc4  narc5  omind1  omind2  omind3  omind4  omind5  omind6  contact1  psupport1  psupport3  political_ideology  happy  riskperc1  riskperc2  optim2
##   4   10  age  children  cnarc1  cnarc2  cnarc3  ctheory1  ctheory2  ctheory3  ctheory4  CRT1  CRT2  CRT3  ladder  mor_circle  moralid1  moralid10  moralid2  moralid3  moralid4  moralid5  moralid6  moralid7  moralid8  moralid9  mcoop3  mcoop4  mcoop5  mcoop6  mcoop7  narc1  narc4  narc5  omind1  omind2  omind3  omind4  omind5  omind6  contact1  psupport1  psupport3  political_ideology  happy  riskperc1  riskperc2  optim2
##   5   1  age  children  cnarc1  cnarc2  cnarc3  ctheory1  ctheory2  ctheory3  ctheory4  CRT1  CRT2  CRT3  ladder  mor_circle  moralid1  moralid10  moralid2  moralid3  moralid4  moralid5  moralid6  moralid7  moralid8  moralid9  mcoop3  mcoop4  mcoop5  mcoop6  mcoop7  narc1  narc4  narc5  omind1  omind2  omind3  omind4  omind5  omind6  contact1  psupport1  psupport3  political_ideology  happy  riskperc1  riskperc2  optim2
##   5   2  age  children  cnarc1  cnarc2  cnarc3  ctheory1  ctheory2  ctheory3  ctheory4  CRT1  CRT2  CRT3  ladder  mor_circle  moralid1  moralid10  moralid2  moralid3  moralid4  moralid5  moralid6  moralid7  moralid8  moralid9  mcoop3  mcoop4  mcoop5  mcoop6  mcoop7  narc1  narc4  narc5  omind1  omind2  omind3  omind4  omind5  omind6  contact1  psupport1  psupport3  political_ideology  happy  riskperc1  riskperc2  optim2
##   5   3  age  children  cnarc1  cnarc2  cnarc3  ctheory1  ctheory2  ctheory3  ctheory4  CRT1  CRT2  CRT3  ladder  mor_circle  moralid1  moralid10  moralid2  moralid3  moralid4  moralid5  moralid6  moralid7  moralid8  moralid9  mcoop3  mcoop4  mcoop5  mcoop6  mcoop7  narc1  narc4  narc5  omind1  omind2  omind3  omind4  omind5  omind6  contact1  psupport1  psupport3  political_ideology  happy  riskperc1  riskperc2  optim2
##   5   4  age  children  cnarc1  cnarc2  cnarc3  ctheory1  ctheory2  ctheory3  ctheory4  CRT1  CRT2  CRT3  ladder  mor_circle  moralid1  moralid10  moralid2  moralid3  moralid4  moralid5  moralid6  moralid7  moralid8  moralid9  mcoop3  mcoop4  mcoop5  mcoop6  mcoop7  narc1  narc4  narc5  omind1  omind2  omind3  omind4  omind5  omind6  contact1  psupport1  psupport3  political_ideology  happy  riskperc1  riskperc2  optim2
##   5   5  age  children  cnarc1  cnarc2  cnarc3  ctheory1  ctheory2  ctheory3  ctheory4  CRT1  CRT2  CRT3  ladder  mor_circle  moralid1  moralid10  moralid2  moralid3  moralid4  moralid5  moralid6  moralid7  moralid8  moralid9  mcoop3  mcoop4  mcoop5  mcoop6  mcoop7  narc1  narc4  narc5  omind1  omind2  omind3  omind4  omind5  omind6  contact1  psupport1  psupport3  political_ideology  happy  riskperc1  riskperc2  optim2
##   5   6  age  children  cnarc1  cnarc2  cnarc3  ctheory1  ctheory2  ctheory3  ctheory4  CRT1  CRT2  CRT3  ladder  mor_circle  moralid1  moralid10  moralid2  moralid3  moralid4  moralid5  moralid6  moralid7  moralid8  moralid9  mcoop3  mcoop4  mcoop5  mcoop6  mcoop7  narc1  narc4  narc5  omind1  omind2  omind3  omind4  omind5  omind6  contact1  psupport1  psupport3  political_ideology  happy  riskperc1  riskperc2  optim2
##   5   7  age  children  cnarc1  cnarc2  cnarc3  ctheory1  ctheory2  ctheory3  ctheory4  CRT1  CRT2  CRT3  ladder  mor_circle  moralid1  moralid10  moralid2  moralid3  moralid4  moralid5  moralid6  moralid7  moralid8  moralid9  mcoop3  mcoop4  mcoop5  mcoop6  mcoop7  narc1  narc4  narc5  omind1  omind2  omind3  omind4  omind5  omind6  contact1  psupport1  psupport3  political_ideology  happy  riskperc1  riskperc2  optim2
##   5   8  age  children  cnarc1  cnarc2  cnarc3  ctheory1  ctheory2  ctheory3  ctheory4  CRT1  CRT2  CRT3  ladder  mor_circle  moralid1  moralid10  moralid2  moralid3  moralid4  moralid5  moralid6  moralid7  moralid8  moralid9  mcoop3  mcoop4  mcoop5  mcoop6  mcoop7  narc1  narc4  narc5  omind1  omind2  omind3  omind4  omind5  omind6  contact1  psupport1  psupport3  political_ideology  happy  riskperc1  riskperc2  optim2
##   5   9  age  children  cnarc1  cnarc2  cnarc3  ctheory1  ctheory2  ctheory3  ctheory4  CRT1  CRT2  CRT3  ladder  mor_circle  moralid1  moralid10  moralid2  moralid3  moralid4  moralid5  moralid6  moralid7  moralid8  moralid9  mcoop3  mcoop4  mcoop5  mcoop6  mcoop7  narc1  narc4  narc5  omind1  omind2  omind3  omind4  omind5  omind6  contact1  psupport1  psupport3  political_ideology  happy  riskperc1  riskperc2  optim2
##   5   10  age  children  cnarc1  cnarc2  cnarc3  ctheory1  ctheory2  ctheory3  ctheory4  CRT1  CRT2  CRT3  ladder  mor_circle  moralid1  moralid10  moralid2  moralid3  moralid4  moralid5  moralid6  moralid7  moralid8  moralid9  mcoop3  mcoop4  mcoop5  mcoop6  mcoop7  narc1  narc4  narc5  omind1  omind2  omind3  omind4  omind5  omind6  contact1  psupport1  psupport3  political_ideology  happy  riskperc1  riskperc2  optim2
##   6   1  age  children  cnarc1  cnarc2  cnarc3  ctheory1  ctheory2  ctheory3  ctheory4  CRT1  CRT2  CRT3  ladder  mor_circle  moralid1  moralid10  moralid2  moralid3  moralid4  moralid5  moralid6  moralid7  moralid8  moralid9  mcoop3  mcoop4  mcoop5  mcoop6  mcoop7  narc1  narc4  narc5  omind1  omind2  omind3  omind4  omind5  omind6  contact1  psupport1  psupport3  political_ideology  happy  riskperc1  riskperc2  optim2
##   6   2  age  children  cnarc1  cnarc2  cnarc3  ctheory1  ctheory2  ctheory3  ctheory4  CRT1  CRT2  CRT3  ladder  mor_circle  moralid1  moralid10  moralid2  moralid3  moralid4  moralid5  moralid6  moralid7  moralid8  moralid9  mcoop3  mcoop4  mcoop5  mcoop6  mcoop7  narc1  narc4  narc5  omind1  omind2  omind3  omind4  omind5  omind6  contact1  psupport1  psupport3  political_ideology  happy  riskperc1  riskperc2  optim2
##   6   3  age  children  cnarc1  cnarc2  cnarc3  ctheory1  ctheory2  ctheory3  ctheory4  CRT1  CRT2  CRT3  ladder  mor_circle  moralid1  moralid10  moralid2  moralid3  moralid4  moralid5  moralid6  moralid7  moralid8  moralid9  mcoop3  mcoop4  mcoop5  mcoop6  mcoop7  narc1  narc4  narc5  omind1  omind2  omind3  omind4  omind5  omind6  contact1  psupport1  psupport3  political_ideology  happy  riskperc1  riskperc2  optim2
##   6   4  age  children  cnarc1  cnarc2  cnarc3  ctheory1  ctheory2  ctheory3  ctheory4  CRT1  CRT2  CRT3  ladder  mor_circle  moralid1  moralid10  moralid2  moralid3  moralid4  moralid5  moralid6  moralid7  moralid8  moralid9  mcoop3  mcoop4  mcoop5  mcoop6  mcoop7  narc1  narc4  narc5  omind1  omind2  omind3  omind4  omind5  omind6  contact1  psupport1  psupport3  political_ideology  happy  riskperc1  riskperc2  optim2
##   6   5  age  children  cnarc1  cnarc2  cnarc3  ctheory1  ctheory2  ctheory3  ctheory4  CRT1  CRT2  CRT3  ladder  mor_circle  moralid1  moralid10  moralid2  moralid3  moralid4  moralid5  moralid6  moralid7  moralid8  moralid9  mcoop3  mcoop4  mcoop5  mcoop6  mcoop7  narc1  narc4  narc5  omind1  omind2  omind3  omind4  omind5  omind6  contact1  psupport1  psupport3  political_ideology  happy  riskperc1  riskperc2  optim2
##   6   6  age  children  cnarc1  cnarc2  cnarc3  ctheory1  ctheory2  ctheory3  ctheory4  CRT1  CRT2  CRT3  ladder  mor_circle  moralid1  moralid10  moralid2  moralid3  moralid4  moralid5  moralid6  moralid7  moralid8  moralid9  mcoop3  mcoop4  mcoop5  mcoop6  mcoop7  narc1  narc4  narc5  omind1  omind2  omind3  omind4  omind5  omind6  contact1  psupport1  psupport3  political_ideology  happy  riskperc1  riskperc2  optim2
##   6   7  age  children  cnarc1  cnarc2  cnarc3  ctheory1  ctheory2  ctheory3  ctheory4  CRT1  CRT2  CRT3  ladder  mor_circle  moralid1  moralid10  moralid2  moralid3  moralid4  moralid5  moralid6  moralid7  moralid8  moralid9  mcoop3  mcoop4  mcoop5  mcoop6  mcoop7  narc1  narc4  narc5  omind1  omind2  omind3  omind4  omind5  omind6  contact1  psupport1  psupport3  political_ideology  happy  riskperc1  riskperc2  optim2
##   6   8  age  children  cnarc1  cnarc2  cnarc3  ctheory1  ctheory2  ctheory3  ctheory4  CRT1  CRT2  CRT3  ladder  mor_circle  moralid1  moralid10  moralid2  moralid3  moralid4  moralid5  moralid6  moralid7  moralid8  moralid9  mcoop3  mcoop4  mcoop5  mcoop6  mcoop7  narc1  narc4  narc5  omind1  omind2  omind3  omind4  omind5  omind6  contact1  psupport1  psupport3  political_ideology  happy  riskperc1  riskperc2  optim2
##   6   9  age  children  cnarc1  cnarc2  cnarc3  ctheory1  ctheory2  ctheory3  ctheory4  CRT1  CRT2  CRT3  ladder  mor_circle  moralid1  moralid10  moralid2  moralid3  moralid4  moralid5  moralid6  moralid7  moralid8  moralid9  mcoop3  mcoop4  mcoop5  mcoop6  mcoop7  narc1  narc4  narc5  omind1  omind2  omind3  omind4  omind5  omind6  contact1  psupport1  psupport3  political_ideology  happy  riskperc1  riskperc2  optim2
##   6   10  age  children  cnarc1  cnarc2  cnarc3  ctheory1  ctheory2  ctheory3  ctheory4  CRT1  CRT2  CRT3  ladder  mor_circle  moralid1  moralid10  moralid2  moralid3  moralid4  moralid5  moralid6  moralid7  moralid8  moralid9  mcoop3  mcoop4  mcoop5  mcoop6  mcoop7  narc1  narc4  narc5  omind1  omind2  omind3  omind4  omind5  omind6  contact1  psupport1  psupport3  political_ideology  happy  riskperc1  riskperc2  optim2
##   7   1  age  children  cnarc1  cnarc2  cnarc3  ctheory1  ctheory2  ctheory3  ctheory4  CRT1  CRT2  CRT3  ladder  mor_circle  moralid1  moralid10  moralid2  moralid3  moralid4  moralid5  moralid6  moralid7  moralid8  moralid9  mcoop3  mcoop4  mcoop5  mcoop6  mcoop7  narc1  narc4  narc5  omind1  omind2  omind3  omind4  omind5  omind6  contact1  psupport1  psupport3  political_ideology  happy  riskperc1  riskperc2  optim2
##   7   2  age  children  cnarc1  cnarc2  cnarc3  ctheory1  ctheory2  ctheory3  ctheory4  CRT1  CRT2  CRT3  ladder  mor_circle  moralid1  moralid10  moralid2  moralid3  moralid4  moralid5  moralid6  moralid7  moralid8  moralid9  mcoop3  mcoop4  mcoop5  mcoop6  mcoop7  narc1  narc4  narc5  omind1  omind2  omind3  omind4  omind5  omind6  contact1  psupport1  psupport3  political_ideology  happy  riskperc1  riskperc2  optim2
##   7   3  age  children  cnarc1  cnarc2  cnarc3  ctheory1  ctheory2  ctheory3  ctheory4  CRT1  CRT2  CRT3  ladder  mor_circle  moralid1  moralid10  moralid2  moralid3  moralid4  moralid5  moralid6  moralid7  moralid8  moralid9  mcoop3  mcoop4  mcoop5  mcoop6  mcoop7  narc1  narc4  narc5  omind1  omind2  omind3  omind4  omind5  omind6  contact1  psupport1  psupport3  political_ideology  happy  riskperc1  riskperc2  optim2
##   7   4  age  children  cnarc1  cnarc2  cnarc3  ctheory1  ctheory2  ctheory3  ctheory4  CRT1  CRT2  CRT3  ladder  mor_circle  moralid1  moralid10  moralid2  moralid3  moralid4  moralid5  moralid6  moralid7  moralid8  moralid9  mcoop3  mcoop4  mcoop5  mcoop6  mcoop7  narc1  narc4  narc5  omind1  omind2  omind3  omind4  omind5  omind6  contact1  psupport1  psupport3  political_ideology  happy  riskperc1  riskperc2  optim2
##   7   5  age  children  cnarc1  cnarc2  cnarc3  ctheory1  ctheory2  ctheory3  ctheory4  CRT1  CRT2  CRT3  ladder  mor_circle  moralid1  moralid10  moralid2  moralid3  moralid4  moralid5  moralid6  moralid7  moralid8  moralid9  mcoop3  mcoop4  mcoop5  mcoop6  mcoop7  narc1  narc4  narc5  omind1  omind2  omind3  omind4  omind5  omind6  contact1  psupport1  psupport3  political_ideology  happy  riskperc1  riskperc2  optim2
##   7   6  age  children  cnarc1  cnarc2  cnarc3  ctheory1  ctheory2  ctheory3  ctheory4  CRT1  CRT2  CRT3  ladder  mor_circle  moralid1  moralid10  moralid2  moralid3  moralid4  moralid5  moralid6  moralid7  moralid8  moralid9  mcoop3  mcoop4  mcoop5  mcoop6  mcoop7  narc1  narc4  narc5  omind1  omind2  omind3  omind4  omind5  omind6  contact1  psupport1  psupport3  political_ideology  happy  riskperc1  riskperc2  optim2
##   7   7  age  children  cnarc1  cnarc2  cnarc3  ctheory1  ctheory2  ctheory3  ctheory4  CRT1  CRT2  CRT3  ladder  mor_circle  moralid1  moralid10  moralid2  moralid3  moralid4  moralid5  moralid6  moralid7  moralid8  moralid9  mcoop3  mcoop4  mcoop5  mcoop6  mcoop7  narc1  narc4  narc5  omind1  omind2  omind3  omind4  omind5  omind6  contact1  psupport1  psupport3  political_ideology  happy  riskperc1  riskperc2  optim2
##   7   8  age  children  cnarc1  cnarc2  cnarc3  ctheory1  ctheory2  ctheory3  ctheory4  CRT1  CRT2  CRT3  ladder  mor_circle  moralid1  moralid10  moralid2  moralid3  moralid4  moralid5  moralid6  moralid7  moralid8  moralid9  mcoop3  mcoop4  mcoop5  mcoop6  mcoop7  narc1  narc4  narc5  omind1  omind2  omind3  omind4  omind5  omind6  contact1  psupport1  psupport3  political_ideology  happy  riskperc1  riskperc2  optim2
##   7   9  age  children  cnarc1  cnarc2  cnarc3  ctheory1  ctheory2  ctheory3  ctheory4  CRT1  CRT2  CRT3  ladder  mor_circle  moralid1  moralid10  moralid2  moralid3  moralid4  moralid5  moralid6  moralid7  moralid8  moralid9  mcoop3  mcoop4  mcoop5  mcoop6  mcoop7  narc1  narc4  narc5  omind1  omind2  omind3  omind4  omind5  omind6  contact1  psupport1  psupport3  political_ideology  happy  riskperc1  riskperc2  optim2
##   7   10  age  children  cnarc1  cnarc2  cnarc3  ctheory1  ctheory2  ctheory3  ctheory4  CRT1  CRT2  CRT3  ladder  mor_circle  moralid1  moralid10  moralid2  moralid3  moralid4  moralid5  moralid6  moralid7  moralid8  moralid9  mcoop3  mcoop4  mcoop5  mcoop6  mcoop7  narc1  narc4  narc5  omind1  omind2  omind3  omind4  omind5  omind6  contact1  psupport1  psupport3  political_ideology  happy  riskperc1  riskperc2  optim2
##   8   1  age  children  cnarc1  cnarc2  cnarc3  ctheory1  ctheory2  ctheory3  ctheory4  CRT1  CRT2  CRT3  ladder  mor_circle  moralid1  moralid10  moralid2  moralid3  moralid4  moralid5  moralid6  moralid7  moralid8  moralid9  mcoop3  mcoop4  mcoop5  mcoop6  mcoop7  narc1  narc4  narc5  omind1  omind2  omind3  omind4  omind5  omind6  contact1  psupport1  psupport3  political_ideology  happy  riskperc1  riskperc2  optim2
##   8   2  age  children  cnarc1  cnarc2  cnarc3  ctheory1  ctheory2  ctheory3  ctheory4  CRT1  CRT2  CRT3  ladder  mor_circle  moralid1  moralid10  moralid2  moralid3  moralid4  moralid5  moralid6  moralid7  moralid8  moralid9  mcoop3  mcoop4  mcoop5  mcoop6  mcoop7  narc1  narc4  narc5  omind1  omind2  omind3  omind4  omind5  omind6  contact1  psupport1  psupport3  political_ideology  happy  riskperc1  riskperc2  optim2
##   8   3  age  children  cnarc1  cnarc2  cnarc3  ctheory1  ctheory2  ctheory3  ctheory4  CRT1  CRT2  CRT3  ladder  mor_circle  moralid1  moralid10  moralid2  moralid3  moralid4  moralid5  moralid6  moralid7  moralid8  moralid9  mcoop3  mcoop4  mcoop5  mcoop6  mcoop7  narc1  narc4  narc5  omind1  omind2  omind3  omind4  omind5  omind6  contact1  psupport1  psupport3  political_ideology  happy  riskperc1  riskperc2  optim2
##   8   4  age  children  cnarc1  cnarc2  cnarc3  ctheory1  ctheory2  ctheory3  ctheory4  CRT1  CRT2  CRT3  ladder  mor_circle  moralid1  moralid10  moralid2  moralid3  moralid4  moralid5  moralid6  moralid7  moralid8  moralid9  mcoop3  mcoop4  mcoop5  mcoop6  mcoop7  narc1  narc4  narc5  omind1  omind2  omind3  omind4  omind5  omind6  contact1  psupport1  psupport3  political_ideology  happy  riskperc1  riskperc2  optim2
##   8   5  age  children  cnarc1  cnarc2  cnarc3  ctheory1  ctheory2  ctheory3  ctheory4  CRT1  CRT2  CRT3  ladder  mor_circle  moralid1  moralid10  moralid2  moralid3  moralid4  moralid5  moralid6  moralid7  moralid8  moralid9  mcoop3  mcoop4  mcoop5  mcoop6  mcoop7  narc1  narc4  narc5  omind1  omind2  omind3  omind4  omind5  omind6  contact1  psupport1  psupport3  political_ideology  happy  riskperc1  riskperc2  optim2
##   8   6  age  children  cnarc1  cnarc2  cnarc3  ctheory1  ctheory2  ctheory3  ctheory4  CRT1  CRT2  CRT3  ladder  mor_circle  moralid1  moralid10  moralid2  moralid3  moralid4  moralid5  moralid6  moralid7  moralid8  moralid9  mcoop3  mcoop4  mcoop5  mcoop6  mcoop7  narc1  narc4  narc5  omind1  omind2  omind3  omind4  omind5  omind6  contact1  psupport1  psupport3  political_ideology  happy  riskperc1  riskperc2  optim2
##   8   7  age  children  cnarc1  cnarc2  cnarc3  ctheory1  ctheory2  ctheory3  ctheory4  CRT1  CRT2  CRT3  ladder  mor_circle  moralid1  moralid10  moralid2  moralid3  moralid4  moralid5  moralid6  moralid7  moralid8  moralid9  mcoop3  mcoop4  mcoop5  mcoop6  mcoop7  narc1  narc4  narc5  omind1  omind2  omind3  omind4  omind5  omind6  contact1  psupport1  psupport3  political_ideology  happy  riskperc1  riskperc2  optim2
##   8   8  age  children  cnarc1  cnarc2  cnarc3  ctheory1  ctheory2  ctheory3  ctheory4  CRT1  CRT2  CRT3  ladder  mor_circle  moralid1  moralid10  moralid2  moralid3  moralid4  moralid5  moralid6  moralid7  moralid8  moralid9  mcoop3  mcoop4  mcoop5  mcoop6  mcoop7  narc1  narc4  narc5  omind1  omind2  omind3  omind4  omind5  omind6  contact1  psupport1  psupport3  political_ideology  happy  riskperc1  riskperc2  optim2
##   8   9  age  children  cnarc1  cnarc2  cnarc3  ctheory1  ctheory2  ctheory3  ctheory4  CRT1  CRT2  CRT3  ladder  mor_circle  moralid1  moralid10  moralid2  moralid3  moralid4  moralid5  moralid6  moralid7  moralid8  moralid9  mcoop3  mcoop4  mcoop5  mcoop6  mcoop7  narc1  narc4  narc5  omind1  omind2  omind3  omind4  omind5  omind6  contact1  psupport1  psupport3  political_ideology  happy  riskperc1  riskperc2  optim2
##   8   10  age  children  cnarc1  cnarc2  cnarc3  ctheory1  ctheory2  ctheory3  ctheory4  CRT1  CRT2  CRT3  ladder  mor_circle  moralid1  moralid10  moralid2  moralid3  moralid4  moralid5  moralid6  moralid7  moralid8  moralid9  mcoop3  mcoop4  mcoop5  mcoop6  mcoop7  narc1  narc4  narc5  omind1  omind2  omind3  omind4  omind5  omind6  contact1  psupport1  psupport3  political_ideology  happy  riskperc1  riskperc2  optim2
##   9   1  age  children  cnarc1  cnarc2  cnarc3  ctheory1  ctheory2  ctheory3  ctheory4  CRT1  CRT2  CRT3  ladder  mor_circle  moralid1  moralid10  moralid2  moralid3  moralid4  moralid5  moralid6  moralid7  moralid8  moralid9  mcoop3  mcoop4  mcoop5  mcoop6  mcoop7  narc1  narc4  narc5  omind1  omind2  omind3  omind4  omind5  omind6  contact1  psupport1  psupport3  political_ideology  happy  riskperc1  riskperc2  optim2
##   9   2  age  children  cnarc1  cnarc2  cnarc3  ctheory1  ctheory2  ctheory3  ctheory4  CRT1  CRT2  CRT3  ladder  mor_circle  moralid1  moralid10  moralid2  moralid3  moralid4  moralid5  moralid6  moralid7  moralid8  moralid9  mcoop3  mcoop4  mcoop5  mcoop6  mcoop7  narc1  narc4  narc5  omind1  omind2  omind3  omind4  omind5  omind6  contact1  psupport1  psupport3  political_ideology  happy  riskperc1  riskperc2  optim2
##   9   3  age  children  cnarc1  cnarc2  cnarc3  ctheory1  ctheory2  ctheory3  ctheory4  CRT1  CRT2  CRT3  ladder  mor_circle  moralid1  moralid10  moralid2  moralid3  moralid4  moralid5  moralid6  moralid7  moralid8  moralid9  mcoop3  mcoop4  mcoop5  mcoop6  mcoop7  narc1  narc4  narc5  omind1  omind2  omind3  omind4  omind5  omind6  contact1  psupport1  psupport3  political_ideology  happy  riskperc1  riskperc2  optim2
##   9   4  age  children  cnarc1  cnarc2  cnarc3  ctheory1  ctheory2  ctheory3  ctheory4  CRT1  CRT2  CRT3  ladder  mor_circle  moralid1  moralid10  moralid2  moralid3  moralid4  moralid5  moralid6  moralid7  moralid8  moralid9  mcoop3  mcoop4  mcoop5  mcoop6  mcoop7  narc1  narc4  narc5  omind1  omind2  omind3  omind4  omind5  omind6  contact1  psupport1  psupport3  political_ideology  happy  riskperc1  riskperc2  optim2
##   9   5  age  children  cnarc1  cnarc2  cnarc3  ctheory1  ctheory2  ctheory3  ctheory4  CRT1  CRT2  CRT3  ladder  mor_circle  moralid1  moralid10  moralid2  moralid3  moralid4  moralid5  moralid6  moralid7  moralid8  moralid9  mcoop3  mcoop4  mcoop5  mcoop6  mcoop7  narc1  narc4  narc5  omind1  omind2  omind3  omind4  omind5  omind6  contact1  psupport1  psupport3  political_ideology  happy  riskperc1  riskperc2  optim2
##   9   6  age  children  cnarc1  cnarc2  cnarc3  ctheory1  ctheory2  ctheory3  ctheory4  CRT1  CRT2  CRT3  ladder  mor_circle  moralid1  moralid10  moralid2  moralid3  moralid4  moralid5  moralid6  moralid7  moralid8  moralid9  mcoop3  mcoop4  mcoop5  mcoop6  mcoop7  narc1  narc4  narc5  omind1  omind2  omind3  omind4  omind5  omind6  contact1  psupport1  psupport3  political_ideology  happy  riskperc1  riskperc2  optim2
##   9   7  age  children  cnarc1  cnarc2  cnarc3  ctheory1  ctheory2  ctheory3  ctheory4  CRT1  CRT2  CRT3  ladder  mor_circle  moralid1  moralid10  moralid2  moralid3  moralid4  moralid5  moralid6  moralid7  moralid8  moralid9  mcoop3  mcoop4  mcoop5  mcoop6  mcoop7  narc1  narc4  narc5  omind1  omind2  omind3  omind4  omind5  omind6  contact1  psupport1  psupport3  political_ideology  happy  riskperc1  riskperc2  optim2
##   9   8  age  children  cnarc1  cnarc2  cnarc3  ctheory1  ctheory2  ctheory3  ctheory4  CRT1  CRT2  CRT3  ladder  mor_circle  moralid1  moralid10  moralid2  moralid3  moralid4  moralid5  moralid6  moralid7  moralid8  moralid9  mcoop3  mcoop4  mcoop5  mcoop6  mcoop7  narc1  narc4  narc5  omind1  omind2  omind3  omind4  omind5  omind6  contact1  psupport1  psupport3  political_ideology  happy  riskperc1  riskperc2  optim2
##   9   9  age  children  cnarc1  cnarc2  cnarc3  ctheory1  ctheory2  ctheory3  ctheory4  CRT1  CRT2  CRT3  ladder  mor_circle  moralid1  moralid10  moralid2  moralid3  moralid4  moralid5  moralid6  moralid7  moralid8  moralid9  mcoop3  mcoop4  mcoop5  mcoop6  mcoop7  narc1  narc4  narc5  omind1  omind2  omind3  omind4  omind5  omind6  contact1  psupport1  psupport3  political_ideology  happy  riskperc1  riskperc2  optim2
##   9   10  age  children  cnarc1  cnarc2  cnarc3  ctheory1  ctheory2  ctheory3  ctheory4  CRT1  CRT2  CRT3  ladder  mor_circle  moralid1  moralid10  moralid2  moralid3  moralid4  moralid5  moralid6  moralid7  moralid8  moralid9  mcoop3  mcoop4  mcoop5  mcoop6  mcoop7  narc1  narc4  narc5  omind1  omind2  omind3  omind4  omind5  omind6  contact1  psupport1  psupport3  political_ideology  happy  riskperc1  riskperc2  optim2
##   10   1  age  children  cnarc1  cnarc2  cnarc3  ctheory1  ctheory2  ctheory3  ctheory4  CRT1  CRT2  CRT3  ladder  mor_circle  moralid1  moralid10  moralid2  moralid3  moralid4  moralid5  moralid6  moralid7  moralid8  moralid9  mcoop3  mcoop4  mcoop5  mcoop6  mcoop7  narc1  narc4  narc5  omind1  omind2  omind3  omind4  omind5  omind6  contact1  psupport1  psupport3  political_ideology  happy  riskperc1  riskperc2  optim2
##   10   2  age  children  cnarc1  cnarc2  cnarc3  ctheory1  ctheory2  ctheory3  ctheory4  CRT1  CRT2  CRT3  ladder  mor_circle  moralid1  moralid10  moralid2  moralid3  moralid4  moralid5  moralid6  moralid7  moralid8  moralid9  mcoop3  mcoop4  mcoop5  mcoop6  mcoop7  narc1  narc4  narc5  omind1  omind2  omind3  omind4  omind5  omind6  contact1  psupport1  psupport3  political_ideology  happy  riskperc1  riskperc2  optim2
##   10   3  age  children  cnarc1  cnarc2  cnarc3  ctheory1  ctheory2  ctheory3  ctheory4  CRT1  CRT2  CRT3  ladder  mor_circle  moralid1  moralid10  moralid2  moralid3  moralid4  moralid5  moralid6  moralid7  moralid8  moralid9  mcoop3  mcoop4  mcoop5  mcoop6  mcoop7  narc1  narc4  narc5  omind1  omind2  omind3  omind4  omind5  omind6  contact1  psupport1  psupport3  political_ideology  happy  riskperc1  riskperc2  optim2
##   10   4  age  children  cnarc1  cnarc2  cnarc3  ctheory1  ctheory2  ctheory3  ctheory4  CRT1  CRT2  CRT3  ladder  mor_circle  moralid1  moralid10  moralid2  moralid3  moralid4  moralid5  moralid6  moralid7  moralid8  moralid9  mcoop3  mcoop4  mcoop5  mcoop6  mcoop7  narc1  narc4  narc5  omind1  omind2  omind3  omind4  omind5  omind6  contact1  psupport1  psupport3  political_ideology  happy  riskperc1  riskperc2  optim2
##   10   5  age  children  cnarc1  cnarc2  cnarc3  ctheory1  ctheory2  ctheory3  ctheory4  CRT1  CRT2  CRT3  ladder  mor_circle  moralid1  moralid10  moralid2  moralid3  moralid4  moralid5  moralid6  moralid7  moralid8  moralid9  mcoop3  mcoop4  mcoop5  mcoop6  mcoop7  narc1  narc4  narc5  omind1  omind2  omind3  omind4  omind5  omind6  contact1  psupport1  psupport3  political_ideology  happy  riskperc1  riskperc2  optim2
##   10   6  age  children  cnarc1  cnarc2  cnarc3  ctheory1  ctheory2  ctheory3  ctheory4  CRT1  CRT2  CRT3  ladder  mor_circle  moralid1  moralid10  moralid2  moralid3  moralid4  moralid5  moralid6  moralid7  moralid8  moralid9  mcoop3  mcoop4  mcoop5  mcoop6  mcoop7  narc1  narc4  narc5  omind1  omind2  omind3  omind4  omind5  omind6  contact1  psupport1  psupport3  political_ideology  happy  riskperc1  riskperc2  optim2
##   10   7  age  children  cnarc1  cnarc2  cnarc3  ctheory1  ctheory2  ctheory3  ctheory4  CRT1  CRT2  CRT3  ladder  mor_circle  moralid1  moralid10  moralid2  moralid3  moralid4  moralid5  moralid6  moralid7  moralid8  moralid9  mcoop3  mcoop4  mcoop5  mcoop6  mcoop7  narc1  narc4  narc5  omind1  omind2  omind3  omind4  omind5  omind6  contact1  psupport1  psupport3  political_ideology  happy  riskperc1  riskperc2  optim2
##   10   8  age  children  cnarc1  cnarc2  cnarc3  ctheory1  ctheory2  ctheory3  ctheory4  CRT1  CRT2  CRT3  ladder  mor_circle  moralid1  moralid10  moralid2  moralid3  moralid4  moralid5  moralid6  moralid7  moralid8  moralid9  mcoop3  mcoop4  mcoop5  mcoop6  mcoop7  narc1  narc4  narc5  omind1  omind2  omind3  omind4  omind5  omind6  contact1  psupport1  psupport3  political_ideology  happy  riskperc1  riskperc2  optim2
##   10   9  age  children  cnarc1  cnarc2  cnarc3  ctheory1  ctheory2  ctheory3  ctheory4  CRT1  CRT2  CRT3  ladder  mor_circle  moralid1  moralid10  moralid2  moralid3  moralid4  moralid5  moralid6  moralid7  moralid8  moralid9  mcoop3  mcoop4  mcoop5  mcoop6  mcoop7  narc1  narc4  narc5  omind1  omind2  omind3  omind4  omind5  omind6  contact1  psupport1  psupport3  political_ideology  happy  riskperc1  riskperc2  optim2
##   10   10  age  children  cnarc1  cnarc2  cnarc3  ctheory1  ctheory2  ctheory3  ctheory4  CRT1  CRT2  CRT3  ladder  mor_circle  moralid1  moralid10  moralid2  moralid3  moralid4  moralid5  moralid6  moralid7  moralid8  moralid9  mcoop3  mcoop4  mcoop5  mcoop6  mcoop7  narc1  narc4  narc5  omind1  omind2  omind3  omind4  omind5  omind6  contact1  psupport1  psupport3  political_ideology  happy  riskperc1  riskperc2  optim2
```

```
## Warning: Number of logged events: 4603
```

```
## 
##  iter imp variable
##   1   1  age  children  cnarc1  cnarc2  cnarc3  ctheory1  ctheory2  ctheory3  ctheory4  CRT1  CRT2  CRT3  employ_status1  health_cond  ladder  marital1  mor_circle  moralid1  moralid10  moralid2  moralid3  moralid4  moralid5  moralid6  moralid7  moralid8  moralid9  mcoop1  mcoop2  mcoop3  mcoop4  mcoop5  mcoop6  mcoop7  narc1  narc2  narc3  narc4  narc5  narc6  nidentity1  nidentity2  omind1  omind2  omind3  omind4  omind5  omind6  contact1  contact2  hygiene1  hygiene5  political_ideology  happy  slf_ladder  riskperc2  self_esteem  sex1  sbelong1  sbelong2  sbelong3  sbelong4  optim1  optim2  slfcont1  slfcont3  slfcont4
##   1   2  age  children  cnarc1  cnarc2  cnarc3  ctheory1  ctheory2  ctheory3  ctheory4  CRT1  CRT2  CRT3  employ_status1  health_cond  ladder  marital1  mor_circle  moralid1  moralid10  moralid2  moralid3  moralid4  moralid5  moralid6  moralid7  moralid8  moralid9  mcoop1  mcoop2  mcoop3  mcoop4  mcoop5  mcoop6  mcoop7  narc1  narc2  narc3  narc4  narc5  narc6  nidentity1  nidentity2  omind1  omind2  omind3  omind4  omind5  omind6  contact1  contact2  hygiene1  hygiene5  political_ideology  happy  slf_ladder  riskperc2  self_esteem  sex1  sbelong1  sbelong2  sbelong3  sbelong4  optim1  optim2  slfcont1  slfcont3  slfcont4
##   1   3  age  children  cnarc1  cnarc2  cnarc3  ctheory1  ctheory2  ctheory3  ctheory4  CRT1  CRT2  CRT3  employ_status1  health_cond  ladder  marital1  mor_circle  moralid1  moralid10  moralid2  moralid3  moralid4  moralid5  moralid6  moralid7  moralid8  moralid9  mcoop1  mcoop2  mcoop3  mcoop4  mcoop5  mcoop6  mcoop7  narc1  narc2  narc3  narc4  narc5  narc6  nidentity1  nidentity2  omind1  omind2  omind3  omind4  omind5  omind6  contact1  contact2  hygiene1  hygiene5  political_ideology  happy  slf_ladder  riskperc2  self_esteem  sex1  sbelong1  sbelong2  sbelong3  sbelong4  optim1  optim2  slfcont1  slfcont3  slfcont4
##   1   4  age  children  cnarc1  cnarc2  cnarc3  ctheory1  ctheory2  ctheory3  ctheory4  CRT1  CRT2  CRT3  employ_status1  health_cond  ladder  marital1  mor_circle  moralid1  moralid10  moralid2  moralid3  moralid4  moralid5  moralid6  moralid7  moralid8  moralid9  mcoop1  mcoop2  mcoop3  mcoop4  mcoop5  mcoop6  mcoop7  narc1  narc2  narc3  narc4  narc5  narc6  nidentity1  nidentity2  omind1  omind2  omind3  omind4  omind5  omind6  contact1  contact2  hygiene1  hygiene5  political_ideology  happy  slf_ladder  riskperc2  self_esteem  sex1  sbelong1  sbelong2  sbelong3  sbelong4  optim1  optim2  slfcont1  slfcont3  slfcont4
##   1   5  age  children  cnarc1  cnarc2  cnarc3  ctheory1  ctheory2  ctheory3  ctheory4  CRT1  CRT2  CRT3  employ_status1  health_cond  ladder  marital1  mor_circle  moralid1  moralid10  moralid2  moralid3  moralid4  moralid5  moralid6  moralid7  moralid8  moralid9  mcoop1  mcoop2  mcoop3  mcoop4  mcoop5  mcoop6  mcoop7  narc1  narc2  narc3  narc4  narc5  narc6  nidentity1  nidentity2  omind1  omind2  omind3  omind4  omind5  omind6  contact1  contact2  hygiene1  hygiene5  political_ideology  happy  slf_ladder  riskperc2  self_esteem  sex1  sbelong1  sbelong2  sbelong3  sbelong4  optim1  optim2  slfcont1  slfcont3  slfcont4
##   1   6  age  children  cnarc1  cnarc2  cnarc3  ctheory1  ctheory2  ctheory3  ctheory4  CRT1  CRT2  CRT3  employ_status1  health_cond  ladder  marital1  mor_circle  moralid1  moralid10  moralid2  moralid3  moralid4  moralid5  moralid6  moralid7  moralid8  moralid9  mcoop1  mcoop2  mcoop3  mcoop4  mcoop5  mcoop6  mcoop7  narc1  narc2  narc3  narc4  narc5  narc6  nidentity1  nidentity2  omind1  omind2  omind3  omind4  omind5  omind6  contact1  contact2  hygiene1  hygiene5  political_ideology  happy  slf_ladder  riskperc2  self_esteem  sex1  sbelong1  sbelong2  sbelong3  sbelong4  optim1  optim2  slfcont1  slfcont3  slfcont4
##   1   7  age  children  cnarc1  cnarc2  cnarc3  ctheory1  ctheory2  ctheory3  ctheory4  CRT1  CRT2  CRT3  employ_status1  health_cond  ladder  marital1  mor_circle  moralid1  moralid10  moralid2  moralid3  moralid4  moralid5  moralid6  moralid7  moralid8  moralid9  mcoop1  mcoop2  mcoop3  mcoop4  mcoop5  mcoop6  mcoop7  narc1  narc2  narc3  narc4  narc5  narc6  nidentity1  nidentity2  omind1  omind2  omind3  omind4  omind5  omind6  contact1  contact2  hygiene1  hygiene5  political_ideology  happy  slf_ladder  riskperc2  self_esteem  sex1  sbelong1  sbelong2  sbelong3  sbelong4  optim1  optim2  slfcont1  slfcont3  slfcont4
##   1   8  age  children  cnarc1  cnarc2  cnarc3  ctheory1  ctheory2  ctheory3  ctheory4  CRT1  CRT2  CRT3  employ_status1  health_cond  ladder  marital1  mor_circle  moralid1  moralid10  moralid2  moralid3  moralid4  moralid5  moralid6  moralid7  moralid8  moralid9  mcoop1  mcoop2  mcoop3  mcoop4  mcoop5  mcoop6  mcoop7  narc1  narc2  narc3  narc4  narc5  narc6  nidentity1  nidentity2  omind1  omind2  omind3  omind4  omind5  omind6  contact1  contact2  hygiene1  hygiene5  political_ideology  happy  slf_ladder  riskperc2  self_esteem  sex1  sbelong1  sbelong2  sbelong3  sbelong4  optim1  optim2  slfcont1  slfcont3  slfcont4
##   1   9  age  children  cnarc1  cnarc2  cnarc3  ctheory1  ctheory2  ctheory3  ctheory4  CRT1  CRT2  CRT3  employ_status1  health_cond  ladder  marital1  mor_circle  moralid1  moralid10  moralid2  moralid3  moralid4  moralid5  moralid6  moralid7  moralid8  moralid9  mcoop1  mcoop2  mcoop3  mcoop4  mcoop5  mcoop6  mcoop7  narc1  narc2  narc3  narc4  narc5  narc6  nidentity1  nidentity2  omind1  omind2  omind3  omind4  omind5  omind6  contact1  contact2  hygiene1  hygiene5  political_ideology  happy  slf_ladder  riskperc2  self_esteem  sex1  sbelong1  sbelong2  sbelong3  sbelong4  optim1  optim2  slfcont1  slfcont3  slfcont4
##   1   10  age  children  cnarc1  cnarc2  cnarc3  ctheory1  ctheory2  ctheory3  ctheory4  CRT1  CRT2  CRT3  employ_status1  health_cond  ladder  marital1  mor_circle  moralid1  moralid10  moralid2  moralid3  moralid4  moralid5  moralid6  moralid7  moralid8  moralid9  mcoop1  mcoop2  mcoop3  mcoop4  mcoop5  mcoop6  mcoop7  narc1  narc2  narc3  narc4  narc5  narc6  nidentity1  nidentity2  omind1  omind2  omind3  omind4  omind5  omind6  contact1  contact2  hygiene1  hygiene5  political_ideology  happy  slf_ladder  riskperc2  self_esteem  sex1  sbelong1  sbelong2  sbelong3  sbelong4  optim1  optim2  slfcont1  slfcont3  slfcont4
##   2   1  age  children  cnarc1  cnarc2  cnarc3  ctheory1  ctheory2  ctheory3  ctheory4  CRT1  CRT2  CRT3  employ_status1  health_cond  ladder  marital1  mor_circle  moralid1  moralid10  moralid2  moralid3  moralid4  moralid5  moralid6  moralid7  moralid8  moralid9  mcoop1  mcoop2  mcoop3  mcoop4  mcoop5  mcoop6  mcoop7  narc1  narc2  narc3  narc4  narc5  narc6  nidentity1  nidentity2  omind1  omind2  omind3  omind4  omind5  omind6  contact1  contact2  hygiene1  hygiene5  political_ideology  happy  slf_ladder  riskperc2  self_esteem  sex1  sbelong1  sbelong2  sbelong3  sbelong4  optim1  optim2  slfcont1  slfcont3  slfcont4
##   2   2  age  children  cnarc1  cnarc2  cnarc3  ctheory1  ctheory2  ctheory3  ctheory4  CRT1  CRT2  CRT3  employ_status1  health_cond  ladder  marital1  mor_circle  moralid1  moralid10  moralid2  moralid3  moralid4  moralid5  moralid6  moralid7  moralid8  moralid9  mcoop1  mcoop2  mcoop3  mcoop4  mcoop5  mcoop6  mcoop7  narc1  narc2  narc3  narc4  narc5  narc6  nidentity1  nidentity2  omind1  omind2  omind3  omind4  omind5  omind6  contact1  contact2  hygiene1  hygiene5  political_ideology  happy  slf_ladder  riskperc2  self_esteem  sex1  sbelong1  sbelong2  sbelong3  sbelong4  optim1  optim2  slfcont1  slfcont3  slfcont4
##   2   3  age  children  cnarc1  cnarc2  cnarc3  ctheory1  ctheory2  ctheory3  ctheory4  CRT1  CRT2  CRT3  employ_status1  health_cond  ladder  marital1  mor_circle  moralid1  moralid10  moralid2  moralid3  moralid4  moralid5  moralid6  moralid7  moralid8  moralid9  mcoop1  mcoop2  mcoop3  mcoop4  mcoop5  mcoop6  mcoop7  narc1  narc2  narc3  narc4  narc5  narc6  nidentity1  nidentity2  omind1  omind2  omind3  omind4  omind5  omind6  contact1  contact2  hygiene1  hygiene5  political_ideology  happy  slf_ladder  riskperc2  self_esteem  sex1  sbelong1  sbelong2  sbelong3  sbelong4  optim1  optim2  slfcont1  slfcont3  slfcont4
##   2   4  age  children  cnarc1  cnarc2  cnarc3  ctheory1  ctheory2  ctheory3  ctheory4  CRT1  CRT2  CRT3  employ_status1  health_cond  ladder  marital1  mor_circle  moralid1  moralid10  moralid2  moralid3  moralid4  moralid5  moralid6  moralid7  moralid8  moralid9  mcoop1  mcoop2  mcoop3  mcoop4  mcoop5  mcoop6  mcoop7  narc1  narc2  narc3  narc4  narc5  narc6  nidentity1  nidentity2  omind1  omind2  omind3  omind4  omind5  omind6  contact1  contact2  hygiene1  hygiene5  political_ideology  happy  slf_ladder  riskperc2  self_esteem  sex1  sbelong1  sbelong2  sbelong3  sbelong4  optim1  optim2  slfcont1  slfcont3  slfcont4
##   2   5  age  children  cnarc1  cnarc2  cnarc3  ctheory1  ctheory2  ctheory3  ctheory4  CRT1  CRT2  CRT3  employ_status1  health_cond  ladder  marital1  mor_circle  moralid1  moralid10  moralid2  moralid3  moralid4  moralid5  moralid6  moralid7  moralid8  moralid9  mcoop1  mcoop2  mcoop3  mcoop4  mcoop5  mcoop6  mcoop7  narc1  narc2  narc3  narc4  narc5  narc6  nidentity1  nidentity2  omind1  omind2  omind3  omind4  omind5  omind6  contact1  contact2  hygiene1  hygiene5  political_ideology  happy  slf_ladder  riskperc2  self_esteem  sex1  sbelong1  sbelong2  sbelong3  sbelong4  optim1  optim2  slfcont1  slfcont3  slfcont4
##   2   6  age  children  cnarc1  cnarc2  cnarc3  ctheory1  ctheory2  ctheory3  ctheory4  CRT1  CRT2  CRT3  employ_status1  health_cond  ladder  marital1  mor_circle  moralid1  moralid10  moralid2  moralid3  moralid4  moralid5  moralid6  moralid7  moralid8  moralid9  mcoop1  mcoop2  mcoop3  mcoop4  mcoop5  mcoop6  mcoop7  narc1  narc2  narc3  narc4  narc5  narc6  nidentity1  nidentity2  omind1  omind2  omind3  omind4  omind5  omind6  contact1  contact2  hygiene1  hygiene5  political_ideology  happy  slf_ladder  riskperc2  self_esteem  sex1  sbelong1  sbelong2  sbelong3  sbelong4  optim1  optim2  slfcont1  slfcont3  slfcont4
##   2   7  age  children  cnarc1  cnarc2  cnarc3  ctheory1  ctheory2  ctheory3  ctheory4  CRT1  CRT2  CRT3  employ_status1  health_cond  ladder  marital1  mor_circle  moralid1  moralid10  moralid2  moralid3  moralid4  moralid5  moralid6  moralid7  moralid8  moralid9  mcoop1  mcoop2  mcoop3  mcoop4  mcoop5  mcoop6  mcoop7  narc1  narc2  narc3  narc4  narc5  narc6  nidentity1  nidentity2  omind1  omind2  omind3  omind4  omind5  omind6  contact1  contact2  hygiene1  hygiene5  political_ideology  happy  slf_ladder  riskperc2  self_esteem  sex1  sbelong1  sbelong2  sbelong3  sbelong4  optim1  optim2  slfcont1  slfcont3  slfcont4
##   2   8  age  children  cnarc1  cnarc2  cnarc3  ctheory1  ctheory2  ctheory3  ctheory4  CRT1  CRT2  CRT3  employ_status1  health_cond  ladder  marital1  mor_circle  moralid1  moralid10  moralid2  moralid3  moralid4  moralid5  moralid6  moralid7  moralid8  moralid9  mcoop1  mcoop2  mcoop3  mcoop4  mcoop5  mcoop6  mcoop7  narc1  narc2  narc3  narc4  narc5  narc6  nidentity1  nidentity2  omind1  omind2  omind3  omind4  omind5  omind6  contact1  contact2  hygiene1  hygiene5  political_ideology  happy  slf_ladder  riskperc2  self_esteem  sex1  sbelong1  sbelong2  sbelong3  sbelong4  optim1  optim2  slfcont1  slfcont3  slfcont4
##   2   9  age  children  cnarc1  cnarc2  cnarc3  ctheory1  ctheory2  ctheory3  ctheory4  CRT1  CRT2  CRT3  employ_status1  health_cond  ladder  marital1  mor_circle  moralid1  moralid10  moralid2  moralid3  moralid4  moralid5  moralid6  moralid7  moralid8  moralid9  mcoop1  mcoop2  mcoop3  mcoop4  mcoop5  mcoop6  mcoop7  narc1  narc2  narc3  narc4  narc5  narc6  nidentity1  nidentity2  omind1  omind2  omind3  omind4  omind5  omind6  contact1  contact2  hygiene1  hygiene5  political_ideology  happy  slf_ladder  riskperc2  self_esteem  sex1  sbelong1  sbelong2  sbelong3  sbelong4  optim1  optim2  slfcont1  slfcont3  slfcont4
##   2   10  age  children  cnarc1  cnarc2  cnarc3  ctheory1  ctheory2  ctheory3  ctheory4  CRT1  CRT2  CRT3  employ_status1  health_cond  ladder  marital1  mor_circle  moralid1  moralid10  moralid2  moralid3  moralid4  moralid5  moralid6  moralid7  moralid8  moralid9  mcoop1  mcoop2  mcoop3  mcoop4  mcoop5  mcoop6  mcoop7  narc1  narc2  narc3  narc4  narc5  narc6  nidentity1  nidentity2  omind1  omind2  omind3  omind4  omind5  omind6  contact1  contact2  hygiene1  hygiene5  political_ideology  happy  slf_ladder  riskperc2  self_esteem  sex1  sbelong1  sbelong2  sbelong3  sbelong4  optim1  optim2  slfcont1  slfcont3  slfcont4
##   3   1  age  children  cnarc1  cnarc2  cnarc3  ctheory1  ctheory2  ctheory3  ctheory4  CRT1  CRT2  CRT3  employ_status1  health_cond  ladder  marital1  mor_circle  moralid1  moralid10  moralid2  moralid3  moralid4  moralid5  moralid6  moralid7  moralid8  moralid9  mcoop1  mcoop2  mcoop3  mcoop4  mcoop5  mcoop6  mcoop7  narc1  narc2  narc3  narc4  narc5  narc6  nidentity1  nidentity2  omind1  omind2  omind3  omind4  omind5  omind6  contact1  contact2  hygiene1  hygiene5  political_ideology  happy  slf_ladder  riskperc2  self_esteem  sex1  sbelong1  sbelong2  sbelong3  sbelong4  optim1  optim2  slfcont1  slfcont3  slfcont4
##   3   2  age  children  cnarc1  cnarc2  cnarc3  ctheory1  ctheory2  ctheory3  ctheory4  CRT1  CRT2  CRT3  employ_status1  health_cond  ladder  marital1  mor_circle  moralid1  moralid10  moralid2  moralid3  moralid4  moralid5  moralid6  moralid7  moralid8  moralid9  mcoop1  mcoop2  mcoop3  mcoop4  mcoop5  mcoop6  mcoop7  narc1  narc2  narc3  narc4  narc5  narc6  nidentity1  nidentity2  omind1  omind2  omind3  omind4  omind5  omind6  contact1  contact2  hygiene1  hygiene5  political_ideology  happy  slf_ladder  riskperc2  self_esteem  sex1  sbelong1  sbelong2  sbelong3  sbelong4  optim1  optim2  slfcont1  slfcont3  slfcont4
##   3   3  age  children  cnarc1  cnarc2  cnarc3  ctheory1  ctheory2  ctheory3  ctheory4  CRT1  CRT2  CRT3  employ_status1  health_cond  ladder  marital1  mor_circle  moralid1  moralid10  moralid2  moralid3  moralid4  moralid5  moralid6  moralid7  moralid8  moralid9  mcoop1  mcoop2  mcoop3  mcoop4  mcoop5  mcoop6  mcoop7  narc1  narc2  narc3  narc4  narc5  narc6  nidentity1  nidentity2  omind1  omind2  omind3  omind4  omind5  omind6  contact1  contact2  hygiene1  hygiene5  political_ideology  happy  slf_ladder  riskperc2  self_esteem  sex1  sbelong1  sbelong2  sbelong3  sbelong4  optim1  optim2  slfcont1  slfcont3  slfcont4
##   3   4  age  children  cnarc1  cnarc2  cnarc3  ctheory1  ctheory2  ctheory3  ctheory4  CRT1  CRT2  CRT3  employ_status1  health_cond  ladder  marital1  mor_circle  moralid1  moralid10  moralid2  moralid3  moralid4  moralid5  moralid6  moralid7  moralid8  moralid9  mcoop1  mcoop2  mcoop3  mcoop4  mcoop5  mcoop6  mcoop7  narc1  narc2  narc3  narc4  narc5  narc6  nidentity1  nidentity2  omind1  omind2  omind3  omind4  omind5  omind6  contact1  contact2  hygiene1  hygiene5  political_ideology  happy  slf_ladder  riskperc2  self_esteem  sex1  sbelong1  sbelong2  sbelong3  sbelong4  optim1  optim2  slfcont1  slfcont3  slfcont4
##   3   5  age  children  cnarc1  cnarc2  cnarc3  ctheory1  ctheory2  ctheory3  ctheory4  CRT1  CRT2  CRT3  employ_status1  health_cond  ladder  marital1  mor_circle  moralid1  moralid10  moralid2  moralid3  moralid4  moralid5  moralid6  moralid7  moralid8  moralid9  mcoop1  mcoop2  mcoop3  mcoop4  mcoop5  mcoop6  mcoop7  narc1  narc2  narc3  narc4  narc5  narc6  nidentity1  nidentity2  omind1  omind2  omind3  omind4  omind5  omind6  contact1  contact2  hygiene1  hygiene5  political_ideology  happy  slf_ladder  riskperc2  self_esteem  sex1  sbelong1  sbelong2  sbelong3  sbelong4  optim1  optim2  slfcont1  slfcont3  slfcont4
##   3   6  age  children  cnarc1  cnarc2  cnarc3  ctheory1  ctheory2  ctheory3  ctheory4  CRT1  CRT2  CRT3  employ_status1  health_cond  ladder  marital1  mor_circle  moralid1  moralid10  moralid2  moralid3  moralid4  moralid5  moralid6  moralid7  moralid8  moralid9  mcoop1  mcoop2  mcoop3  mcoop4  mcoop5  mcoop6  mcoop7  narc1  narc2  narc3  narc4  narc5  narc6  nidentity1  nidentity2  omind1  omind2  omind3  omind4  omind5  omind6  contact1  contact2  hygiene1  hygiene5  political_ideology  happy  slf_ladder  riskperc2  self_esteem  sex1  sbelong1  sbelong2  sbelong3  sbelong4  optim1  optim2  slfcont1  slfcont3  slfcont4
##   3   7  age  children  cnarc1  cnarc2  cnarc3  ctheory1  ctheory2  ctheory3  ctheory4  CRT1  CRT2  CRT3  employ_status1  health_cond  ladder  marital1  mor_circle  moralid1  moralid10  moralid2  moralid3  moralid4  moralid5  moralid6  moralid7  moralid8  moralid9  mcoop1  mcoop2  mcoop3  mcoop4  mcoop5  mcoop6  mcoop7  narc1  narc2  narc3  narc4  narc5  narc6  nidentity1  nidentity2  omind1  omind2  omind3  omind4  omind5  omind6  contact1  contact2  hygiene1  hygiene5  political_ideology  happy  slf_ladder  riskperc2  self_esteem  sex1  sbelong1  sbelong2  sbelong3  sbelong4  optim1  optim2  slfcont1  slfcont3  slfcont4
##   3   8  age  children  cnarc1  cnarc2  cnarc3  ctheory1  ctheory2  ctheory3  ctheory4  CRT1  CRT2  CRT3  employ_status1  health_cond  ladder  marital1  mor_circle  moralid1  moralid10  moralid2  moralid3  moralid4  moralid5  moralid6  moralid7  moralid8  moralid9  mcoop1  mcoop2  mcoop3  mcoop4  mcoop5  mcoop6  mcoop7  narc1  narc2  narc3  narc4  narc5  narc6  nidentity1  nidentity2  omind1  omind2  omind3  omind4  omind5  omind6  contact1  contact2  hygiene1  hygiene5  political_ideology  happy  slf_ladder  riskperc2  self_esteem  sex1  sbelong1  sbelong2  sbelong3  sbelong4  optim1  optim2  slfcont1  slfcont3  slfcont4
##   3   9  age  children  cnarc1  cnarc2  cnarc3  ctheory1  ctheory2  ctheory3  ctheory4  CRT1  CRT2  CRT3  employ_status1  health_cond  ladder  marital1  mor_circle  moralid1  moralid10  moralid2  moralid3  moralid4  moralid5  moralid6  moralid7  moralid8  moralid9  mcoop1  mcoop2  mcoop3  mcoop4  mcoop5  mcoop6  mcoop7  narc1  narc2  narc3  narc4  narc5  narc6  nidentity1  nidentity2  omind1  omind2  omind3  omind4  omind5  omind6  contact1  contact2  hygiene1  hygiene5  political_ideology  happy  slf_ladder  riskperc2  self_esteem  sex1  sbelong1  sbelong2  sbelong3  sbelong4  optim1  optim2  slfcont1  slfcont3  slfcont4
##   3   10  age  children  cnarc1  cnarc2  cnarc3  ctheory1  ctheory2  ctheory3  ctheory4  CRT1  CRT2  CRT3  employ_status1  health_cond  ladder  marital1  mor_circle  moralid1  moralid10  moralid2  moralid3  moralid4  moralid5  moralid6  moralid7  moralid8  moralid9  mcoop1  mcoop2  mcoop3  mcoop4  mcoop5  mcoop6  mcoop7  narc1  narc2  narc3  narc4  narc5  narc6  nidentity1  nidentity2  omind1  omind2  omind3  omind4  omind5  omind6  contact1  contact2  hygiene1  hygiene5  political_ideology  happy  slf_ladder  riskperc2  self_esteem  sex1  sbelong1  sbelong2  sbelong3  sbelong4  optim1  optim2  slfcont1  slfcont3  slfcont4
##   4   1  age  children  cnarc1  cnarc2  cnarc3  ctheory1  ctheory2  ctheory3  ctheory4  CRT1  CRT2  CRT3  employ_status1  health_cond  ladder  marital1  mor_circle  moralid1  moralid10  moralid2  moralid3  moralid4  moralid5  moralid6  moralid7  moralid8  moralid9  mcoop1  mcoop2  mcoop3  mcoop4  mcoop5  mcoop6  mcoop7  narc1  narc2  narc3  narc4  narc5  narc6  nidentity1  nidentity2  omind1  omind2  omind3  omind4  omind5  omind6  contact1  contact2  hygiene1  hygiene5  political_ideology  happy  slf_ladder  riskperc2  self_esteem  sex1  sbelong1  sbelong2  sbelong3  sbelong4  optim1  optim2  slfcont1  slfcont3  slfcont4
##   4   2  age  children  cnarc1  cnarc2  cnarc3  ctheory1  ctheory2  ctheory3  ctheory4  CRT1  CRT2  CRT3  employ_status1  health_cond  ladder  marital1  mor_circle  moralid1  moralid10  moralid2  moralid3  moralid4  moralid5  moralid6  moralid7  moralid8  moralid9  mcoop1  mcoop2  mcoop3  mcoop4  mcoop5  mcoop6  mcoop7  narc1  narc2  narc3  narc4  narc5  narc6  nidentity1  nidentity2  omind1  omind2  omind3  omind4  omind5  omind6  contact1  contact2  hygiene1  hygiene5  political_ideology  happy  slf_ladder  riskperc2  self_esteem  sex1  sbelong1  sbelong2  sbelong3  sbelong4  optim1  optim2  slfcont1  slfcont3  slfcont4
##   4   3  age  children  cnarc1  cnarc2  cnarc3  ctheory1  ctheory2  ctheory3  ctheory4  CRT1  CRT2  CRT3  employ_status1  health_cond  ladder  marital1  mor_circle  moralid1  moralid10  moralid2  moralid3  moralid4  moralid5  moralid6  moralid7  moralid8  moralid9  mcoop1  mcoop2  mcoop3  mcoop4  mcoop5  mcoop6  mcoop7  narc1  narc2  narc3  narc4  narc5  narc6  nidentity1  nidentity2  omind1  omind2  omind3  omind4  omind5  omind6  contact1  contact2  hygiene1  hygiene5  political_ideology  happy  slf_ladder  riskperc2  self_esteem  sex1  sbelong1  sbelong2  sbelong3  sbelong4  optim1  optim2  slfcont1  slfcont3  slfcont4
##   4   4  age  children  cnarc1  cnarc2  cnarc3  ctheory1  ctheory2  ctheory3  ctheory4  CRT1  CRT2  CRT3  employ_status1  health_cond  ladder  marital1  mor_circle  moralid1  moralid10  moralid2  moralid3  moralid4  moralid5  moralid6  moralid7  moralid8  moralid9  mcoop1  mcoop2  mcoop3  mcoop4  mcoop5  mcoop6  mcoop7  narc1  narc2  narc3  narc4  narc5  narc6  nidentity1  nidentity2  omind1  omind2  omind3  omind4  omind5  omind6  contact1  contact2  hygiene1  hygiene5  political_ideology  happy  slf_ladder  riskperc2  self_esteem  sex1  sbelong1  sbelong2  sbelong3  sbelong4  optim1  optim2  slfcont1  slfcont3  slfcont4
##   4   5  age  children  cnarc1  cnarc2  cnarc3  ctheory1  ctheory2  ctheory3  ctheory4  CRT1  CRT2  CRT3  employ_status1  health_cond  ladder  marital1  mor_circle  moralid1  moralid10  moralid2  moralid3  moralid4  moralid5  moralid6  moralid7  moralid8  moralid9  mcoop1  mcoop2  mcoop3  mcoop4  mcoop5  mcoop6  mcoop7  narc1  narc2  narc3  narc4  narc5  narc6  nidentity1  nidentity2  omind1  omind2  omind3  omind4  omind5  omind6  contact1  contact2  hygiene1  hygiene5  political_ideology  happy  slf_ladder  riskperc2  self_esteem  sex1  sbelong1  sbelong2  sbelong3  sbelong4  optim1  optim2  slfcont1  slfcont3  slfcont4
##   4   6  age  children  cnarc1  cnarc2  cnarc3  ctheory1  ctheory2  ctheory3  ctheory4  CRT1  CRT2  CRT3  employ_status1  health_cond  ladder  marital1  mor_circle  moralid1  moralid10  moralid2  moralid3  moralid4  moralid5  moralid6  moralid7  moralid8  moralid9  mcoop1  mcoop2  mcoop3  mcoop4  mcoop5  mcoop6  mcoop7  narc1  narc2  narc3  narc4  narc5  narc6  nidentity1  nidentity2  omind1  omind2  omind3  omind4  omind5  omind6  contact1  contact2  hygiene1  hygiene5  political_ideology  happy  slf_ladder  riskperc2  self_esteem  sex1  sbelong1  sbelong2  sbelong3  sbelong4  optim1  optim2  slfcont1  slfcont3  slfcont4
##   4   7  age  children  cnarc1  cnarc2  cnarc3  ctheory1  ctheory2  ctheory3  ctheory4  CRT1  CRT2  CRT3  employ_status1  health_cond  ladder  marital1  mor_circle  moralid1  moralid10  moralid2  moralid3  moralid4  moralid5  moralid6  moralid7  moralid8  moralid9  mcoop1  mcoop2  mcoop3  mcoop4  mcoop5  mcoop6  mcoop7  narc1  narc2  narc3  narc4  narc5  narc6  nidentity1  nidentity2  omind1  omind2  omind3  omind4  omind5  omind6  contact1  contact2  hygiene1  hygiene5  political_ideology  happy  slf_ladder  riskperc2  self_esteem  sex1  sbelong1  sbelong2  sbelong3  sbelong4  optim1  optim2  slfcont1  slfcont3  slfcont4
##   4   8  age  children  cnarc1  cnarc2  cnarc3  ctheory1  ctheory2  ctheory3  ctheory4  CRT1  CRT2  CRT3  employ_status1  health_cond  ladder  marital1  mor_circle  moralid1  moralid10  moralid2  moralid3  moralid4  moralid5  moralid6  moralid7  moralid8  moralid9  mcoop1  mcoop2  mcoop3  mcoop4  mcoop5  mcoop6  mcoop7  narc1  narc2  narc3  narc4  narc5  narc6  nidentity1  nidentity2  omind1  omind2  omind3  omind4  omind5  omind6  contact1  contact2  hygiene1  hygiene5  political_ideology  happy  slf_ladder  riskperc2  self_esteem  sex1  sbelong1  sbelong2  sbelong3  sbelong4  optim1  optim2  slfcont1  slfcont3  slfcont4
##   4   9  age  children  cnarc1  cnarc2  cnarc3  ctheory1  ctheory2  ctheory3  ctheory4  CRT1  CRT2  CRT3  employ_status1  health_cond  ladder  marital1  mor_circle  moralid1  moralid10  moralid2  moralid3  moralid4  moralid5  moralid6  moralid7  moralid8  moralid9  mcoop1  mcoop2  mcoop3  mcoop4  mcoop5  mcoop6  mcoop7  narc1  narc2  narc3  narc4  narc5  narc6  nidentity1  nidentity2  omind1  omind2  omind3  omind4  omind5  omind6  contact1  contact2  hygiene1  hygiene5  political_ideology  happy  slf_ladder  riskperc2  self_esteem  sex1  sbelong1  sbelong2  sbelong3  sbelong4  optim1  optim2  slfcont1  slfcont3  slfcont4
##   4   10  age  children  cnarc1  cnarc2  cnarc3  ctheory1  ctheory2  ctheory3  ctheory4  CRT1  CRT2  CRT3  employ_status1  health_cond  ladder  marital1  mor_circle  moralid1  moralid10  moralid2  moralid3  moralid4  moralid5  moralid6  moralid7  moralid8  moralid9  mcoop1  mcoop2  mcoop3  mcoop4  mcoop5  mcoop6  mcoop7  narc1  narc2  narc3  narc4  narc5  narc6  nidentity1  nidentity2  omind1  omind2  omind3  omind4  omind5  omind6  contact1  contact2  hygiene1  hygiene5  political_ideology  happy  slf_ladder  riskperc2  self_esteem  sex1  sbelong1  sbelong2  sbelong3  sbelong4  optim1  optim2  slfcont1  slfcont3  slfcont4
##   5   1  age  children  cnarc1  cnarc2  cnarc3  ctheory1  ctheory2  ctheory3  ctheory4  CRT1  CRT2  CRT3  employ_status1  health_cond  ladder  marital1  mor_circle  moralid1  moralid10  moralid2  moralid3  moralid4  moralid5  moralid6  moralid7  moralid8  moralid9  mcoop1  mcoop2  mcoop3  mcoop4  mcoop5  mcoop6  mcoop7  narc1  narc2  narc3  narc4  narc5  narc6  nidentity1  nidentity2  omind1  omind2  omind3  omind4  omind5  omind6  contact1  contact2  hygiene1  hygiene5  political_ideology  happy  slf_ladder  riskperc2  self_esteem  sex1  sbelong1  sbelong2  sbelong3  sbelong4  optim1  optim2  slfcont1  slfcont3  slfcont4
##   5   2  age  children  cnarc1  cnarc2  cnarc3  ctheory1  ctheory2  ctheory3  ctheory4  CRT1  CRT2  CRT3  employ_status1  health_cond  ladder  marital1  mor_circle  moralid1  moralid10  moralid2  moralid3  moralid4  moralid5  moralid6  moralid7  moralid8  moralid9  mcoop1  mcoop2  mcoop3  mcoop4  mcoop5  mcoop6  mcoop7  narc1  narc2  narc3  narc4  narc5  narc6  nidentity1  nidentity2  omind1  omind2  omind3  omind4  omind5  omind6  contact1  contact2  hygiene1  hygiene5  political_ideology  happy  slf_ladder  riskperc2  self_esteem  sex1  sbelong1  sbelong2  sbelong3  sbelong4  optim1  optim2  slfcont1  slfcont3  slfcont4
##   5   3  age  children  cnarc1  cnarc2  cnarc3  ctheory1  ctheory2  ctheory3  ctheory4  CRT1  CRT2  CRT3  employ_status1  health_cond  ladder  marital1  mor_circle  moralid1  moralid10  moralid2  moralid3  moralid4  moralid5  moralid6  moralid7  moralid8  moralid9  mcoop1  mcoop2  mcoop3  mcoop4  mcoop5  mcoop6  mcoop7  narc1  narc2  narc3  narc4  narc5  narc6  nidentity1  nidentity2  omind1  omind2  omind3  omind4  omind5  omind6  contact1  contact2  hygiene1  hygiene5  political_ideology  happy  slf_ladder  riskperc2  self_esteem  sex1  sbelong1  sbelong2  sbelong3  sbelong4  optim1  optim2  slfcont1  slfcont3  slfcont4
##   5   4  age  children  cnarc1  cnarc2  cnarc3  ctheory1  ctheory2  ctheory3  ctheory4  CRT1  CRT2  CRT3  employ_status1  health_cond  ladder  marital1  mor_circle  moralid1  moralid10  moralid2  moralid3  moralid4  moralid5  moralid6  moralid7  moralid8  moralid9  mcoop1  mcoop2  mcoop3  mcoop4  mcoop5  mcoop6  mcoop7  narc1  narc2  narc3  narc4  narc5  narc6  nidentity1  nidentity2  omind1  omind2  omind3  omind4  omind5  omind6  contact1  contact2  hygiene1  hygiene5  political_ideology  happy  slf_ladder  riskperc2  self_esteem  sex1  sbelong1  sbelong2  sbelong3  sbelong4  optim1  optim2  slfcont1  slfcont3  slfcont4
##   5   5  age  children  cnarc1  cnarc2  cnarc3  ctheory1  ctheory2  ctheory3  ctheory4  CRT1  CRT2  CRT3  employ_status1  health_cond  ladder  marital1  mor_circle  moralid1  moralid10  moralid2  moralid3  moralid4  moralid5  moralid6  moralid7  moralid8  moralid9  mcoop1  mcoop2  mcoop3  mcoop4  mcoop5  mcoop6  mcoop7  narc1  narc2  narc3  narc4  narc5  narc6  nidentity1  nidentity2  omind1  omind2  omind3  omind4  omind5  omind6  contact1  contact2  hygiene1  hygiene5  political_ideology  happy  slf_ladder  riskperc2  self_esteem  sex1  sbelong1  sbelong2  sbelong3  sbelong4  optim1  optim2  slfcont1  slfcont3  slfcont4
##   5   6  age  children  cnarc1  cnarc2  cnarc3  ctheory1  ctheory2  ctheory3  ctheory4  CRT1  CRT2  CRT3  employ_status1  health_cond  ladder  marital1  mor_circle  moralid1  moralid10  moralid2  moralid3  moralid4  moralid5  moralid6  moralid7  moralid8  moralid9  mcoop1  mcoop2  mcoop3  mcoop4  mcoop5  mcoop6  mcoop7  narc1  narc2  narc3  narc4  narc5  narc6  nidentity1  nidentity2  omind1  omind2  omind3  omind4  omind5  omind6  contact1  contact2  hygiene1  hygiene5  political_ideology  happy  slf_ladder  riskperc2  self_esteem  sex1  sbelong1  sbelong2  sbelong3  sbelong4  optim1  optim2  slfcont1  slfcont3  slfcont4
##   5   7  age  children  cnarc1  cnarc2  cnarc3  ctheory1  ctheory2  ctheory3  ctheory4  CRT1  CRT2  CRT3  employ_status1  health_cond  ladder  marital1  mor_circle  moralid1  moralid10  moralid2  moralid3  moralid4  moralid5  moralid6  moralid7  moralid8  moralid9  mcoop1  mcoop2  mcoop3  mcoop4  mcoop5  mcoop6  mcoop7  narc1  narc2  narc3  narc4  narc5  narc6  nidentity1  nidentity2  omind1  omind2  omind3  omind4  omind5  omind6  contact1  contact2  hygiene1  hygiene5  political_ideology  happy  slf_ladder  riskperc2  self_esteem  sex1  sbelong1  sbelong2  sbelong3  sbelong4  optim1  optim2  slfcont1  slfcont3  slfcont4
##   5   8  age  children  cnarc1  cnarc2  cnarc3  ctheory1  ctheory2  ctheory3  ctheory4  CRT1  CRT2  CRT3  employ_status1  health_cond  ladder  marital1  mor_circle  moralid1  moralid10  moralid2  moralid3  moralid4  moralid5  moralid6  moralid7  moralid8  moralid9  mcoop1  mcoop2  mcoop3  mcoop4  mcoop5  mcoop6  mcoop7  narc1  narc2  narc3  narc4  narc5  narc6  nidentity1  nidentity2  omind1  omind2  omind3  omind4  omind5  omind6  contact1  contact2  hygiene1  hygiene5  political_ideology  happy  slf_ladder  riskperc2  self_esteem  sex1  sbelong1  sbelong2  sbelong3  sbelong4  optim1  optim2  slfcont1  slfcont3  slfcont4
##   5   9  age  children  cnarc1  cnarc2  cnarc3  ctheory1  ctheory2  ctheory3  ctheory4  CRT1  CRT2  CRT3  employ_status1  health_cond  ladder  marital1  mor_circle  moralid1  moralid10  moralid2  moralid3  moralid4  moralid5  moralid6  moralid7  moralid8  moralid9  mcoop1  mcoop2  mcoop3  mcoop4  mcoop5  mcoop6  mcoop7  narc1  narc2  narc3  narc4  narc5  narc6  nidentity1  nidentity2  omind1  omind2  omind3  omind4  omind5  omind6  contact1  contact2  hygiene1  hygiene5  political_ideology  happy  slf_ladder  riskperc2  self_esteem  sex1  sbelong1  sbelong2  sbelong3  sbelong4  optim1  optim2  slfcont1  slfcont3  slfcont4
##   5   10  age  children  cnarc1  cnarc2  cnarc3  ctheory1  ctheory2  ctheory3  ctheory4  CRT1  CRT2  CRT3  employ_status1  health_cond  ladder  marital1  mor_circle  moralid1  moralid10  moralid2  moralid3  moralid4  moralid5  moralid6  moralid7  moralid8  moralid9  mcoop1  mcoop2  mcoop3  mcoop4  mcoop5  mcoop6  mcoop7  narc1  narc2  narc3  narc4  narc5  narc6  nidentity1  nidentity2  omind1  omind2  omind3  omind4  omind5  omind6  contact1  contact2  hygiene1  hygiene5  political_ideology  happy  slf_ladder  riskperc2  self_esteem  sex1  sbelong1  sbelong2  sbelong3  sbelong4  optim1  optim2  slfcont1  slfcont3  slfcont4
##   6   1  age  children  cnarc1  cnarc2  cnarc3  ctheory1  ctheory2  ctheory3  ctheory4  CRT1  CRT2  CRT3  employ_status1  health_cond  ladder  marital1  mor_circle  moralid1  moralid10  moralid2  moralid3  moralid4  moralid5  moralid6  moralid7  moralid8  moralid9  mcoop1  mcoop2  mcoop3  mcoop4  mcoop5  mcoop6  mcoop7  narc1  narc2  narc3  narc4  narc5  narc6  nidentity1  nidentity2  omind1  omind2  omind3  omind4  omind5  omind6  contact1  contact2  hygiene1  hygiene5  political_ideology  happy  slf_ladder  riskperc2  self_esteem  sex1  sbelong1  sbelong2  sbelong3  sbelong4  optim1  optim2  slfcont1  slfcont3  slfcont4
##   6   2  age  children  cnarc1  cnarc2  cnarc3  ctheory1  ctheory2  ctheory3  ctheory4  CRT1  CRT2  CRT3  employ_status1  health_cond  ladder  marital1  mor_circle  moralid1  moralid10  moralid2  moralid3  moralid4  moralid5  moralid6  moralid7  moralid8  moralid9  mcoop1  mcoop2  mcoop3  mcoop4  mcoop5  mcoop6  mcoop7  narc1  narc2  narc3  narc4  narc5  narc6  nidentity1  nidentity2  omind1  omind2  omind3  omind4  omind5  omind6  contact1  contact2  hygiene1  hygiene5  political_ideology  happy  slf_ladder  riskperc2  self_esteem  sex1  sbelong1  sbelong2  sbelong3  sbelong4  optim1  optim2  slfcont1  slfcont3  slfcont4
##   6   3  age  children  cnarc1  cnarc2  cnarc3  ctheory1  ctheory2  ctheory3  ctheory4  CRT1  CRT2  CRT3  employ_status1  health_cond  ladder  marital1  mor_circle  moralid1  moralid10  moralid2  moralid3  moralid4  moralid5  moralid6  moralid7  moralid8  moralid9  mcoop1  mcoop2  mcoop3  mcoop4  mcoop5  mcoop6  mcoop7  narc1  narc2  narc3  narc4  narc5  narc6  nidentity1  nidentity2  omind1  omind2  omind3  omind4  omind5  omind6  contact1  contact2  hygiene1  hygiene5  political_ideology  happy  slf_ladder  riskperc2  self_esteem  sex1  sbelong1  sbelong2  sbelong3  sbelong4  optim1  optim2  slfcont1  slfcont3  slfcont4
##   6   4  age  children  cnarc1  cnarc2  cnarc3  ctheory1  ctheory2  ctheory3  ctheory4  CRT1  CRT2  CRT3  employ_status1  health_cond  ladder  marital1  mor_circle  moralid1  moralid10  moralid2  moralid3  moralid4  moralid5  moralid6  moralid7  moralid8  moralid9  mcoop1  mcoop2  mcoop3  mcoop4  mcoop5  mcoop6  mcoop7  narc1  narc2  narc3  narc4  narc5  narc6  nidentity1  nidentity2  omind1  omind2  omind3  omind4  omind5  omind6  contact1  contact2  hygiene1  hygiene5  political_ideology  happy  slf_ladder  riskperc2  self_esteem  sex1  sbelong1  sbelong2  sbelong3  sbelong4  optim1  optim2  slfcont1  slfcont3  slfcont4
##   6   5  age  children  cnarc1  cnarc2  cnarc3  ctheory1  ctheory2  ctheory3  ctheory4  CRT1  CRT2  CRT3  employ_status1  health_cond  ladder  marital1  mor_circle  moralid1  moralid10  moralid2  moralid3  moralid4  moralid5  moralid6  moralid7  moralid8  moralid9  mcoop1  mcoop2  mcoop3  mcoop4  mcoop5  mcoop6  mcoop7  narc1  narc2  narc3  narc4  narc5  narc6  nidentity1  nidentity2  omind1  omind2  omind3  omind4  omind5  omind6  contact1  contact2  hygiene1  hygiene5  political_ideology  happy  slf_ladder  riskperc2  self_esteem  sex1  sbelong1  sbelong2  sbelong3  sbelong4  optim1  optim2  slfcont1  slfcont3  slfcont4
##   6   6  age  children  cnarc1  cnarc2  cnarc3  ctheory1  ctheory2  ctheory3  ctheory4  CRT1  CRT2  CRT3  employ_status1  health_cond  ladder  marital1  mor_circle  moralid1  moralid10  moralid2  moralid3  moralid4  moralid5  moralid6  moralid7  moralid8  moralid9  mcoop1  mcoop2  mcoop3  mcoop4  mcoop5  mcoop6  mcoop7  narc1  narc2  narc3  narc4  narc5  narc6  nidentity1  nidentity2  omind1  omind2  omind3  omind4  omind5  omind6  contact1  contact2  hygiene1  hygiene5  political_ideology  happy  slf_ladder  riskperc2  self_esteem  sex1  sbelong1  sbelong2  sbelong3  sbelong4  optim1  optim2  slfcont1  slfcont3  slfcont4
##   6   7  age  children  cnarc1  cnarc2  cnarc3  ctheory1  ctheory2  ctheory3  ctheory4  CRT1  CRT2  CRT3  employ_status1  health_cond  ladder  marital1  mor_circle  moralid1  moralid10  moralid2  moralid3  moralid4  moralid5  moralid6  moralid7  moralid8  moralid9  mcoop1  mcoop2  mcoop3  mcoop4  mcoop5  mcoop6  mcoop7  narc1  narc2  narc3  narc4  narc5  narc6  nidentity1  nidentity2  omind1  omind2  omind3  omind4  omind5  omind6  contact1  contact2  hygiene1  hygiene5  political_ideology  happy  slf_ladder  riskperc2  self_esteem  sex1  sbelong1  sbelong2  sbelong3  sbelong4  optim1  optim2  slfcont1  slfcont3  slfcont4
##   6   8  age  children  cnarc1  cnarc2  cnarc3  ctheory1  ctheory2  ctheory3  ctheory4  CRT1  CRT2  CRT3  employ_status1  health_cond  ladder  marital1  mor_circle  moralid1  moralid10  moralid2  moralid3  moralid4  moralid5  moralid6  moralid7  moralid8  moralid9  mcoop1  mcoop2  mcoop3  mcoop4  mcoop5  mcoop6  mcoop7  narc1  narc2  narc3  narc4  narc5  narc6  nidentity1  nidentity2  omind1  omind2  omind3  omind4  omind5  omind6  contact1  contact2  hygiene1  hygiene5  political_ideology  happy  slf_ladder  riskperc2  self_esteem  sex1  sbelong1  sbelong2  sbelong3  sbelong4  optim1  optim2  slfcont1  slfcont3  slfcont4
##   6   9  age  children  cnarc1  cnarc2  cnarc3  ctheory1  ctheory2  ctheory3  ctheory4  CRT1  CRT2  CRT3  employ_status1  health_cond  ladder  marital1  mor_circle  moralid1  moralid10  moralid2  moralid3  moralid4  moralid5  moralid6  moralid7  moralid8  moralid9  mcoop1  mcoop2  mcoop3  mcoop4  mcoop5  mcoop6  mcoop7  narc1  narc2  narc3  narc4  narc5  narc6  nidentity1  nidentity2  omind1  omind2  omind3  omind4  omind5  omind6  contact1  contact2  hygiene1  hygiene5  political_ideology  happy  slf_ladder  riskperc2  self_esteem  sex1  sbelong1  sbelong2  sbelong3  sbelong4  optim1  optim2  slfcont1  slfcont3  slfcont4
##   6   10  age  children  cnarc1  cnarc2  cnarc3  ctheory1  ctheory2  ctheory3  ctheory4  CRT1  CRT2  CRT3  employ_status1  health_cond  ladder  marital1  mor_circle  moralid1  moralid10  moralid2  moralid3  moralid4  moralid5  moralid6  moralid7  moralid8  moralid9  mcoop1  mcoop2  mcoop3  mcoop4  mcoop5  mcoop6  mcoop7  narc1  narc2  narc3  narc4  narc5  narc6  nidentity1  nidentity2  omind1  omind2  omind3  omind4  omind5  omind6  contact1  contact2  hygiene1  hygiene5  political_ideology  happy  slf_ladder  riskperc2  self_esteem  sex1  sbelong1  sbelong2  sbelong3  sbelong4  optim1  optim2  slfcont1  slfcont3  slfcont4
##   7   1  age  children  cnarc1  cnarc2  cnarc3  ctheory1  ctheory2  ctheory3  ctheory4  CRT1  CRT2  CRT3  employ_status1  health_cond  ladder  marital1  mor_circle  moralid1  moralid10  moralid2  moralid3  moralid4  moralid5  moralid6  moralid7  moralid8  moralid9  mcoop1  mcoop2  mcoop3  mcoop4  mcoop5  mcoop6  mcoop7  narc1  narc2  narc3  narc4  narc5  narc6  nidentity1  nidentity2  omind1  omind2  omind3  omind4  omind5  omind6  contact1  contact2  hygiene1  hygiene5  political_ideology  happy  slf_ladder  riskperc2  self_esteem  sex1  sbelong1  sbelong2  sbelong3  sbelong4  optim1  optim2  slfcont1  slfcont3  slfcont4
##   7   2  age  children  cnarc1  cnarc2  cnarc3  ctheory1  ctheory2  ctheory3  ctheory4  CRT1  CRT2  CRT3  employ_status1  health_cond  ladder  marital1  mor_circle  moralid1  moralid10  moralid2  moralid3  moralid4  moralid5  moralid6  moralid7  moralid8  moralid9  mcoop1  mcoop2  mcoop3  mcoop4  mcoop5  mcoop6  mcoop7  narc1  narc2  narc3  narc4  narc5  narc6  nidentity1  nidentity2  omind1  omind2  omind3  omind4  omind5  omind6  contact1  contact2  hygiene1  hygiene5  political_ideology  happy  slf_ladder  riskperc2  self_esteem  sex1  sbelong1  sbelong2  sbelong3  sbelong4  optim1  optim2  slfcont1  slfcont3  slfcont4
##   7   3  age  children  cnarc1  cnarc2  cnarc3  ctheory1  ctheory2  ctheory3  ctheory4  CRT1  CRT2  CRT3  employ_status1  health_cond  ladder  marital1  mor_circle  moralid1  moralid10  moralid2  moralid3  moralid4  moralid5  moralid6  moralid7  moralid8  moralid9  mcoop1  mcoop2  mcoop3  mcoop4  mcoop5  mcoop6  mcoop7  narc1  narc2  narc3  narc4  narc5  narc6  nidentity1  nidentity2  omind1  omind2  omind3  omind4  omind5  omind6  contact1  contact2  hygiene1  hygiene5  political_ideology  happy  slf_ladder  riskperc2  self_esteem  sex1  sbelong1  sbelong2  sbelong3  sbelong4  optim1  optim2  slfcont1  slfcont3  slfcont4
##   7   4  age  children  cnarc1  cnarc2  cnarc3  ctheory1  ctheory2  ctheory3  ctheory4  CRT1  CRT2  CRT3  employ_status1  health_cond  ladder  marital1  mor_circle  moralid1  moralid10  moralid2  moralid3  moralid4  moralid5  moralid6  moralid7  moralid8  moralid9  mcoop1  mcoop2  mcoop3  mcoop4  mcoop5  mcoop6  mcoop7  narc1  narc2  narc3  narc4  narc5  narc6  nidentity1  nidentity2  omind1  omind2  omind3  omind4  omind5  omind6  contact1  contact2  hygiene1  hygiene5  political_ideology  happy  slf_ladder  riskperc2  self_esteem  sex1  sbelong1  sbelong2  sbelong3  sbelong4  optim1  optim2  slfcont1  slfcont3  slfcont4
##   7   5  age  children  cnarc1  cnarc2  cnarc3  ctheory1  ctheory2  ctheory3  ctheory4  CRT1  CRT2  CRT3  employ_status1  health_cond  ladder  marital1  mor_circle  moralid1  moralid10  moralid2  moralid3  moralid4  moralid5  moralid6  moralid7  moralid8  moralid9  mcoop1  mcoop2  mcoop3  mcoop4  mcoop5  mcoop6  mcoop7  narc1  narc2  narc3  narc4  narc5  narc6  nidentity1  nidentity2  omind1  omind2  omind3  omind4  omind5  omind6  contact1  contact2  hygiene1  hygiene5  political_ideology  happy  slf_ladder  riskperc2  self_esteem  sex1  sbelong1  sbelong2  sbelong3  sbelong4  optim1  optim2  slfcont1  slfcont3  slfcont4
##   7   6  age  children  cnarc1  cnarc2  cnarc3  ctheory1  ctheory2  ctheory3  ctheory4  CRT1  CRT2  CRT3  employ_status1  health_cond  ladder  marital1  mor_circle  moralid1  moralid10  moralid2  moralid3  moralid4  moralid5  moralid6  moralid7  moralid8  moralid9  mcoop1  mcoop2  mcoop3  mcoop4  mcoop5  mcoop6  mcoop7  narc1  narc2  narc3  narc4  narc5  narc6  nidentity1  nidentity2  omind1  omind2  omind3  omind4  omind5  omind6  contact1  contact2  hygiene1  hygiene5  political_ideology  happy  slf_ladder  riskperc2  self_esteem  sex1  sbelong1  sbelong2  sbelong3  sbelong4  optim1  optim2  slfcont1  slfcont3  slfcont4
##   7   7  age  children  cnarc1  cnarc2  cnarc3  ctheory1  ctheory2  ctheory3  ctheory4  CRT1  CRT2  CRT3  employ_status1  health_cond  ladder  marital1  mor_circle  moralid1  moralid10  moralid2  moralid3  moralid4  moralid5  moralid6  moralid7  moralid8  moralid9  mcoop1  mcoop2  mcoop3  mcoop4  mcoop5  mcoop6  mcoop7  narc1  narc2  narc3  narc4  narc5  narc6  nidentity1  nidentity2  omind1  omind2  omind3  omind4  omind5  omind6  contact1  contact2  hygiene1  hygiene5  political_ideology  happy  slf_ladder  riskperc2  self_esteem  sex1  sbelong1  sbelong2  sbelong3  sbelong4  optim1  optim2  slfcont1  slfcont3  slfcont4
##   7   8  age  children  cnarc1  cnarc2  cnarc3  ctheory1  ctheory2  ctheory3  ctheory4  CRT1  CRT2  CRT3  employ_status1  health_cond  ladder  marital1  mor_circle  moralid1  moralid10  moralid2  moralid3  moralid4  moralid5  moralid6  moralid7  moralid8  moralid9  mcoop1  mcoop2  mcoop3  mcoop4  mcoop5  mcoop6  mcoop7  narc1  narc2  narc3  narc4  narc5  narc6  nidentity1  nidentity2  omind1  omind2  omind3  omind4  omind5  omind6  contact1  contact2  hygiene1  hygiene5  political_ideology  happy  slf_ladder  riskperc2  self_esteem  sex1  sbelong1  sbelong2  sbelong3  sbelong4  optim1  optim2  slfcont1  slfcont3  slfcont4
##   7   9  age  children  cnarc1  cnarc2  cnarc3  ctheory1  ctheory2  ctheory3  ctheory4  CRT1  CRT2  CRT3  employ_status1  health_cond  ladder  marital1  mor_circle  moralid1  moralid10  moralid2  moralid3  moralid4  moralid5  moralid6  moralid7  moralid8  moralid9  mcoop1  mcoop2  mcoop3  mcoop4  mcoop5  mcoop6  mcoop7  narc1  narc2  narc3  narc4  narc5  narc6  nidentity1  nidentity2  omind1  omind2  omind3  omind4  omind5  omind6  contact1  contact2  hygiene1  hygiene5  political_ideology  happy  slf_ladder  riskperc2  self_esteem  sex1  sbelong1  sbelong2  sbelong3  sbelong4  optim1  optim2  slfcont1  slfcont3  slfcont4
##   7   10  age  children  cnarc1  cnarc2  cnarc3  ctheory1  ctheory2  ctheory3  ctheory4  CRT1  CRT2  CRT3  employ_status1  health_cond  ladder  marital1  mor_circle  moralid1  moralid10  moralid2  moralid3  moralid4  moralid5  moralid6  moralid7  moralid8  moralid9  mcoop1  mcoop2  mcoop3  mcoop4  mcoop5  mcoop6  mcoop7  narc1  narc2  narc3  narc4  narc5  narc6  nidentity1  nidentity2  omind1  omind2  omind3  omind4  omind5  omind6  contact1  contact2  hygiene1  hygiene5  political_ideology  happy  slf_ladder  riskperc2  self_esteem  sex1  sbelong1  sbelong2  sbelong3  sbelong4  optim1  optim2  slfcont1  slfcont3  slfcont4
##   8   1  age  children  cnarc1  cnarc2  cnarc3  ctheory1  ctheory2  ctheory3  ctheory4  CRT1  CRT2  CRT3  employ_status1  health_cond  ladder  marital1  mor_circle  moralid1  moralid10  moralid2  moralid3  moralid4  moralid5  moralid6  moralid7  moralid8  moralid9  mcoop1  mcoop2  mcoop3  mcoop4  mcoop5  mcoop6  mcoop7  narc1  narc2  narc3  narc4  narc5  narc6  nidentity1  nidentity2  omind1  omind2  omind3  omind4  omind5  omind6  contact1  contact2  hygiene1  hygiene5  political_ideology  happy  slf_ladder  riskperc2  self_esteem  sex1  sbelong1  sbelong2  sbelong3  sbelong4  optim1  optim2  slfcont1  slfcont3  slfcont4
##   8   2  age  children  cnarc1  cnarc2  cnarc3  ctheory1  ctheory2  ctheory3  ctheory4  CRT1  CRT2  CRT3  employ_status1  health_cond  ladder  marital1  mor_circle  moralid1  moralid10  moralid2  moralid3  moralid4  moralid5  moralid6  moralid7  moralid8  moralid9  mcoop1  mcoop2  mcoop3  mcoop4  mcoop5  mcoop6  mcoop7  narc1  narc2  narc3  narc4  narc5  narc6  nidentity1  nidentity2  omind1  omind2  omind3  omind4  omind5  omind6  contact1  contact2  hygiene1  hygiene5  political_ideology  happy  slf_ladder  riskperc2  self_esteem  sex1  sbelong1  sbelong2  sbelong3  sbelong4  optim1  optim2  slfcont1  slfcont3  slfcont4
##   8   3  age  children  cnarc1  cnarc2  cnarc3  ctheory1  ctheory2  ctheory3  ctheory4  CRT1  CRT2  CRT3  employ_status1  health_cond  ladder  marital1  mor_circle  moralid1  moralid10  moralid2  moralid3  moralid4  moralid5  moralid6  moralid7  moralid8  moralid9  mcoop1  mcoop2  mcoop3  mcoop4  mcoop5  mcoop6  mcoop7  narc1  narc2  narc3  narc4  narc5  narc6  nidentity1  nidentity2  omind1  omind2  omind3  omind4  omind5  omind6  contact1  contact2  hygiene1  hygiene5  political_ideology  happy  slf_ladder  riskperc2  self_esteem  sex1  sbelong1  sbelong2  sbelong3  sbelong4  optim1  optim2  slfcont1  slfcont3  slfcont4
##   8   4  age  children  cnarc1  cnarc2  cnarc3  ctheory1  ctheory2  ctheory3  ctheory4  CRT1  CRT2  CRT3  employ_status1  health_cond  ladder  marital1  mor_circle  moralid1  moralid10  moralid2  moralid3  moralid4  moralid5  moralid6  moralid7  moralid8  moralid9  mcoop1  mcoop2  mcoop3  mcoop4  mcoop5  mcoop6  mcoop7  narc1  narc2  narc3  narc4  narc5  narc6  nidentity1  nidentity2  omind1  omind2  omind3  omind4  omind5  omind6  contact1  contact2  hygiene1  hygiene5  political_ideology  happy  slf_ladder  riskperc2  self_esteem  sex1  sbelong1  sbelong2  sbelong3  sbelong4  optim1  optim2  slfcont1  slfcont3  slfcont4
##   8   5  age  children  cnarc1  cnarc2  cnarc3  ctheory1  ctheory2  ctheory3  ctheory4  CRT1  CRT2  CRT3  employ_status1  health_cond  ladder  marital1  mor_circle  moralid1  moralid10  moralid2  moralid3  moralid4  moralid5  moralid6  moralid7  moralid8  moralid9  mcoop1  mcoop2  mcoop3  mcoop4  mcoop5  mcoop6  mcoop7  narc1  narc2  narc3  narc4  narc5  narc6  nidentity1  nidentity2  omind1  omind2  omind3  omind4  omind5  omind6  contact1  contact2  hygiene1  hygiene5  political_ideology  happy  slf_ladder  riskperc2  self_esteem  sex1  sbelong1  sbelong2  sbelong3  sbelong4  optim1  optim2  slfcont1  slfcont3  slfcont4
##   8   6  age  children  cnarc1  cnarc2  cnarc3  ctheory1  ctheory2  ctheory3  ctheory4  CRT1  CRT2  CRT3  employ_status1  health_cond  ladder  marital1  mor_circle  moralid1  moralid10  moralid2  moralid3  moralid4  moralid5  moralid6  moralid7  moralid8  moralid9  mcoop1  mcoop2  mcoop3  mcoop4  mcoop5  mcoop6  mcoop7  narc1  narc2  narc3  narc4  narc5  narc6  nidentity1  nidentity2  omind1  omind2  omind3  omind4  omind5  omind6  contact1  contact2  hygiene1  hygiene5  political_ideology  happy  slf_ladder  riskperc2  self_esteem  sex1  sbelong1  sbelong2  sbelong3  sbelong4  optim1  optim2  slfcont1  slfcont3  slfcont4
##   8   7  age  children  cnarc1  cnarc2  cnarc3  ctheory1  ctheory2  ctheory3  ctheory4  CRT1  CRT2  CRT3  employ_status1  health_cond  ladder  marital1  mor_circle  moralid1  moralid10  moralid2  moralid3  moralid4  moralid5  moralid6  moralid7  moralid8  moralid9  mcoop1  mcoop2  mcoop3  mcoop4  mcoop5  mcoop6  mcoop7  narc1  narc2  narc3  narc4  narc5  narc6  nidentity1  nidentity2  omind1  omind2  omind3  omind4  omind5  omind6  contact1  contact2  hygiene1  hygiene5  political_ideology  happy  slf_ladder  riskperc2  self_esteem  sex1  sbelong1  sbelong2  sbelong3  sbelong4  optim1  optim2  slfcont1  slfcont3  slfcont4
##   8   8  age  children  cnarc1  cnarc2  cnarc3  ctheory1  ctheory2  ctheory3  ctheory4  CRT1  CRT2  CRT3  employ_status1  health_cond  ladder  marital1  mor_circle  moralid1  moralid10  moralid2  moralid3  moralid4  moralid5  moralid6  moralid7  moralid8  moralid9  mcoop1  mcoop2  mcoop3  mcoop4  mcoop5  mcoop6  mcoop7  narc1  narc2  narc3  narc4  narc5  narc6  nidentity1  nidentity2  omind1  omind2  omind3  omind4  omind5  omind6  contact1  contact2  hygiene1  hygiene5  political_ideology  happy  slf_ladder  riskperc2  self_esteem  sex1  sbelong1  sbelong2  sbelong3  sbelong4  optim1  optim2  slfcont1  slfcont3  slfcont4
##   8   9  age  children  cnarc1  cnarc2  cnarc3  ctheory1  ctheory2  ctheory3  ctheory4  CRT1  CRT2  CRT3  employ_status1  health_cond  ladder  marital1  mor_circle  moralid1  moralid10  moralid2  moralid3  moralid4  moralid5  moralid6  moralid7  moralid8  moralid9  mcoop1  mcoop2  mcoop3  mcoop4  mcoop5  mcoop6  mcoop7  narc1  narc2  narc3  narc4  narc5  narc6  nidentity1  nidentity2  omind1  omind2  omind3  omind4  omind5  omind6  contact1  contact2  hygiene1  hygiene5  political_ideology  happy  slf_ladder  riskperc2  self_esteem  sex1  sbelong1  sbelong2  sbelong3  sbelong4  optim1  optim2  slfcont1  slfcont3  slfcont4
##   8   10  age  children  cnarc1  cnarc2  cnarc3  ctheory1  ctheory2  ctheory3  ctheory4  CRT1  CRT2  CRT3  employ_status1  health_cond  ladder  marital1  mor_circle  moralid1  moralid10  moralid2  moralid3  moralid4  moralid5  moralid6  moralid7  moralid8  moralid9  mcoop1  mcoop2  mcoop3  mcoop4  mcoop5  mcoop6  mcoop7  narc1  narc2  narc3  narc4  narc5  narc6  nidentity1  nidentity2  omind1  omind2  omind3  omind4  omind5  omind6  contact1  contact2  hygiene1  hygiene5  political_ideology  happy  slf_ladder  riskperc2  self_esteem  sex1  sbelong1  sbelong2  sbelong3  sbelong4  optim1  optim2  slfcont1  slfcont3  slfcont4
##   9   1  age  children  cnarc1  cnarc2  cnarc3  ctheory1  ctheory2  ctheory3  ctheory4  CRT1  CRT2  CRT3  employ_status1  health_cond  ladder  marital1  mor_circle  moralid1  moralid10  moralid2  moralid3  moralid4  moralid5  moralid6  moralid7  moralid8  moralid9  mcoop1  mcoop2  mcoop3  mcoop4  mcoop5  mcoop6  mcoop7  narc1  narc2  narc3  narc4  narc5  narc6  nidentity1  nidentity2  omind1  omind2  omind3  omind4  omind5  omind6  contact1  contact2  hygiene1  hygiene5  political_ideology  happy  slf_ladder  riskperc2  self_esteem  sex1  sbelong1  sbelong2  sbelong3  sbelong4  optim1  optim2  slfcont1  slfcont3  slfcont4
##   9   2  age  children  cnarc1  cnarc2  cnarc3  ctheory1  ctheory2  ctheory3  ctheory4  CRT1  CRT2  CRT3  employ_status1  health_cond  ladder  marital1  mor_circle  moralid1  moralid10  moralid2  moralid3  moralid4  moralid5  moralid6  moralid7  moralid8  moralid9  mcoop1  mcoop2  mcoop3  mcoop4  mcoop5  mcoop6  mcoop7  narc1  narc2  narc3  narc4  narc5  narc6  nidentity1  nidentity2  omind1  omind2  omind3  omind4  omind5  omind6  contact1  contact2  hygiene1  hygiene5  political_ideology  happy  slf_ladder  riskperc2  self_esteem  sex1  sbelong1  sbelong2  sbelong3  sbelong4  optim1  optim2  slfcont1  slfcont3  slfcont4
##   9   3  age  children  cnarc1  cnarc2  cnarc3  ctheory1  ctheory2  ctheory3  ctheory4  CRT1  CRT2  CRT3  employ_status1  health_cond  ladder  marital1  mor_circle  moralid1  moralid10  moralid2  moralid3  moralid4  moralid5  moralid6  moralid7  moralid8  moralid9  mcoop1  mcoop2  mcoop3  mcoop4  mcoop5  mcoop6  mcoop7  narc1  narc2  narc3  narc4  narc5  narc6  nidentity1  nidentity2  omind1  omind2  omind3  omind4  omind5  omind6  contact1  contact2  hygiene1  hygiene5  political_ideology  happy  slf_ladder  riskperc2  self_esteem  sex1  sbelong1  sbelong2  sbelong3  sbelong4  optim1  optim2  slfcont1  slfcont3  slfcont4
##   9   4  age  children  cnarc1  cnarc2  cnarc3  ctheory1  ctheory2  ctheory3  ctheory4  CRT1  CRT2  CRT3  employ_status1  health_cond  ladder  marital1  mor_circle  moralid1  moralid10  moralid2  moralid3  moralid4  moralid5  moralid6  moralid7  moralid8  moralid9  mcoop1  mcoop2  mcoop3  mcoop4  mcoop5  mcoop6  mcoop7  narc1  narc2  narc3  narc4  narc5  narc6  nidentity1  nidentity2  omind1  omind2  omind3  omind4  omind5  omind6  contact1  contact2  hygiene1  hygiene5  political_ideology  happy  slf_ladder  riskperc2  self_esteem  sex1  sbelong1  sbelong2  sbelong3  sbelong4  optim1  optim2  slfcont1  slfcont3  slfcont4
##   9   5  age  children  cnarc1  cnarc2  cnarc3  ctheory1  ctheory2  ctheory3  ctheory4  CRT1  CRT2  CRT3  employ_status1  health_cond  ladder  marital1  mor_circle  moralid1  moralid10  moralid2  moralid3  moralid4  moralid5  moralid6  moralid7  moralid8  moralid9  mcoop1  mcoop2  mcoop3  mcoop4  mcoop5  mcoop6  mcoop7  narc1  narc2  narc3  narc4  narc5  narc6  nidentity1  nidentity2  omind1  omind2  omind3  omind4  omind5  omind6  contact1  contact2  hygiene1  hygiene5  political_ideology  happy  slf_ladder  riskperc2  self_esteem  sex1  sbelong1  sbelong2  sbelong3  sbelong4  optim1  optim2  slfcont1  slfcont3  slfcont4
##   9   6  age  children  cnarc1  cnarc2  cnarc3  ctheory1  ctheory2  ctheory3  ctheory4  CRT1  CRT2  CRT3  employ_status1  health_cond  ladder  marital1  mor_circle  moralid1  moralid10  moralid2  moralid3  moralid4  moralid5  moralid6  moralid7  moralid8  moralid9  mcoop1  mcoop2  mcoop3  mcoop4  mcoop5  mcoop6  mcoop7  narc1  narc2  narc3  narc4  narc5  narc6  nidentity1  nidentity2  omind1  omind2  omind3  omind4  omind5  omind6  contact1  contact2  hygiene1  hygiene5  political_ideology  happy  slf_ladder  riskperc2  self_esteem  sex1  sbelong1  sbelong2  sbelong3  sbelong4  optim1  optim2  slfcont1  slfcont3  slfcont4
##   9   7  age  children  cnarc1  cnarc2  cnarc3  ctheory1  ctheory2  ctheory3  ctheory4  CRT1  CRT2  CRT3  employ_status1  health_cond  ladder  marital1  mor_circle  moralid1  moralid10  moralid2  moralid3  moralid4  moralid5  moralid6  moralid7  moralid8  moralid9  mcoop1  mcoop2  mcoop3  mcoop4  mcoop5  mcoop6  mcoop7  narc1  narc2  narc3  narc4  narc5  narc6  nidentity1  nidentity2  omind1  omind2  omind3  omind4  omind5  omind6  contact1  contact2  hygiene1  hygiene5  political_ideology  happy  slf_ladder  riskperc2  self_esteem  sex1  sbelong1  sbelong2  sbelong3  sbelong4  optim1  optim2  slfcont1  slfcont3  slfcont4
##   9   8  age  children  cnarc1  cnarc2  cnarc3  ctheory1  ctheory2  ctheory3  ctheory4  CRT1  CRT2  CRT3  employ_status1  health_cond  ladder  marital1  mor_circle  moralid1  moralid10  moralid2  moralid3  moralid4  moralid5  moralid6  moralid7  moralid8  moralid9  mcoop1  mcoop2  mcoop3  mcoop4  mcoop5  mcoop6  mcoop7  narc1  narc2  narc3  narc4  narc5  narc6  nidentity1  nidentity2  omind1  omind2  omind3  omind4  omind5  omind6  contact1  contact2  hygiene1  hygiene5  political_ideology  happy  slf_ladder  riskperc2  self_esteem  sex1  sbelong1  sbelong2  sbelong3  sbelong4  optim1  optim2  slfcont1  slfcont3  slfcont4
##   9   9  age  children  cnarc1  cnarc2  cnarc3  ctheory1  ctheory2  ctheory3  ctheory4  CRT1  CRT2  CRT3  employ_status1  health_cond  ladder  marital1  mor_circle  moralid1  moralid10  moralid2  moralid3  moralid4  moralid5  moralid6  moralid7  moralid8  moralid9  mcoop1  mcoop2  mcoop3  mcoop4  mcoop5  mcoop6  mcoop7  narc1  narc2  narc3  narc4  narc5  narc6  nidentity1  nidentity2  omind1  omind2  omind3  omind4  omind5  omind6  contact1  contact2  hygiene1  hygiene5  political_ideology  happy  slf_ladder  riskperc2  self_esteem  sex1  sbelong1  sbelong2  sbelong3  sbelong4  optim1  optim2  slfcont1  slfcont3  slfcont4
##   9   10  age  children  cnarc1  cnarc2  cnarc3  ctheory1  ctheory2  ctheory3  ctheory4  CRT1  CRT2  CRT3  employ_status1  health_cond  ladder  marital1  mor_circle  moralid1  moralid10  moralid2  moralid3  moralid4  moralid5  moralid6  moralid7  moralid8  moralid9  mcoop1  mcoop2  mcoop3  mcoop4  mcoop5  mcoop6  mcoop7  narc1  narc2  narc3  narc4  narc5  narc6  nidentity1  nidentity2  omind1  omind2  omind3  omind4  omind5  omind6  contact1  contact2  hygiene1  hygiene5  political_ideology  happy  slf_ladder  riskperc2  self_esteem  sex1  sbelong1  sbelong2  sbelong3  sbelong4  optim1  optim2  slfcont1  slfcont3  slfcont4
##   10   1  age  children  cnarc1  cnarc2  cnarc3  ctheory1  ctheory2  ctheory3  ctheory4  CRT1  CRT2  CRT3  employ_status1  health_cond  ladder  marital1  mor_circle  moralid1  moralid10  moralid2  moralid3  moralid4  moralid5  moralid6  moralid7  moralid8  moralid9  mcoop1  mcoop2  mcoop3  mcoop4  mcoop5  mcoop6  mcoop7  narc1  narc2  narc3  narc4  narc5  narc6  nidentity1  nidentity2  omind1  omind2  omind3  omind4  omind5  omind6  contact1  contact2  hygiene1  hygiene5  political_ideology  happy  slf_ladder  riskperc2  self_esteem  sex1  sbelong1  sbelong2  sbelong3  sbelong4  optim1  optim2  slfcont1  slfcont3  slfcont4
##   10   2  age  children  cnarc1  cnarc2  cnarc3  ctheory1  ctheory2  ctheory3  ctheory4  CRT1  CRT2  CRT3  employ_status1  health_cond  ladder  marital1  mor_circle  moralid1  moralid10  moralid2  moralid3  moralid4  moralid5  moralid6  moralid7  moralid8  moralid9  mcoop1  mcoop2  mcoop3  mcoop4  mcoop5  mcoop6  mcoop7  narc1  narc2  narc3  narc4  narc5  narc6  nidentity1  nidentity2  omind1  omind2  omind3  omind4  omind5  omind6  contact1  contact2  hygiene1  hygiene5  political_ideology  happy  slf_ladder  riskperc2  self_esteem  sex1  sbelong1  sbelong2  sbelong3  sbelong4  optim1  optim2  slfcont1  slfcont3  slfcont4
##   10   3  age  children  cnarc1  cnarc2  cnarc3  ctheory1  ctheory2  ctheory3  ctheory4  CRT1  CRT2  CRT3  employ_status1  health_cond  ladder  marital1  mor_circle  moralid1  moralid10  moralid2  moralid3  moralid4  moralid5  moralid6  moralid7  moralid8  moralid9  mcoop1  mcoop2  mcoop3  mcoop4  mcoop5  mcoop6  mcoop7  narc1  narc2  narc3  narc4  narc5  narc6  nidentity1  nidentity2  omind1  omind2  omind3  omind4  omind5  omind6  contact1  contact2  hygiene1  hygiene5  political_ideology  happy  slf_ladder  riskperc2  self_esteem  sex1  sbelong1  sbelong2  sbelong3  sbelong4  optim1  optim2  slfcont1  slfcont3  slfcont4
##   10   4  age  children  cnarc1  cnarc2  cnarc3  ctheory1  ctheory2  ctheory3  ctheory4  CRT1  CRT2  CRT3  employ_status1  health_cond  ladder  marital1  mor_circle  moralid1  moralid10  moralid2  moralid3  moralid4  moralid5  moralid6  moralid7  moralid8  moralid9  mcoop1  mcoop2  mcoop3  mcoop4  mcoop5  mcoop6  mcoop7  narc1  narc2  narc3  narc4  narc5  narc6  nidentity1  nidentity2  omind1  omind2  omind3  omind4  omind5  omind6  contact1  contact2  hygiene1  hygiene5  political_ideology  happy  slf_ladder  riskperc2  self_esteem  sex1  sbelong1  sbelong2  sbelong3  sbelong4  optim1  optim2  slfcont1  slfcont3  slfcont4
##   10   5  age  children  cnarc1  cnarc2  cnarc3  ctheory1  ctheory2  ctheory3  ctheory4  CRT1  CRT2  CRT3  employ_status1  health_cond  ladder  marital1  mor_circle  moralid1  moralid10  moralid2  moralid3  moralid4  moralid5  moralid6  moralid7  moralid8  moralid9  mcoop1  mcoop2  mcoop3  mcoop4  mcoop5  mcoop6  mcoop7  narc1  narc2  narc3  narc4  narc5  narc6  nidentity1  nidentity2  omind1  omind2  omind3  omind4  omind5  omind6  contact1  contact2  hygiene1  hygiene5  political_ideology  happy  slf_ladder  riskperc2  self_esteem  sex1  sbelong1  sbelong2  sbelong3  sbelong4  optim1  optim2  slfcont1  slfcont3  slfcont4
##   10   6  age  children  cnarc1  cnarc2  cnarc3  ctheory1  ctheory2  ctheory3  ctheory4  CRT1  CRT2  CRT3  employ_status1  health_cond  ladder  marital1  mor_circle  moralid1  moralid10  moralid2  moralid3  moralid4  moralid5  moralid6  moralid7  moralid8  moralid9  mcoop1  mcoop2  mcoop3  mcoop4  mcoop5  mcoop6  mcoop7  narc1  narc2  narc3  narc4  narc5  narc6  nidentity1  nidentity2  omind1  omind2  omind3  omind4  omind5  omind6  contact1  contact2  hygiene1  hygiene5  political_ideology  happy  slf_ladder  riskperc2  self_esteem  sex1  sbelong1  sbelong2  sbelong3  sbelong4  optim1  optim2  slfcont1  slfcont3  slfcont4
##   10   7  age  children  cnarc1  cnarc2  cnarc3  ctheory1  ctheory2  ctheory3  ctheory4  CRT1  CRT2  CRT3  employ_status1  health_cond  ladder  marital1  mor_circle  moralid1  moralid10  moralid2  moralid3  moralid4  moralid5  moralid6  moralid7  moralid8  moralid9  mcoop1  mcoop2  mcoop3  mcoop4  mcoop5  mcoop6  mcoop7  narc1  narc2  narc3  narc4  narc5  narc6  nidentity1  nidentity2  omind1  omind2  omind3  omind4  omind5  omind6  contact1  contact2  hygiene1  hygiene5  political_ideology  happy  slf_ladder  riskperc2  self_esteem  sex1  sbelong1  sbelong2  sbelong3  sbelong4  optim1  optim2  slfcont1  slfcont3  slfcont4
##   10   8  age  children  cnarc1  cnarc2  cnarc3  ctheory1  ctheory2  ctheory3  ctheory4  CRT1  CRT2  CRT3  employ_status1  health_cond  ladder  marital1  mor_circle  moralid1  moralid10  moralid2  moralid3  moralid4  moralid5  moralid6  moralid7  moralid8  moralid9  mcoop1  mcoop2  mcoop3  mcoop4  mcoop5  mcoop6  mcoop7  narc1  narc2  narc3  narc4  narc5  narc6  nidentity1  nidentity2  omind1  omind2  omind3  omind4  omind5  omind6  contact1  contact2  hygiene1  hygiene5  political_ideology  happy  slf_ladder  riskperc2  self_esteem  sex1  sbelong1  sbelong2  sbelong3  sbelong4  optim1  optim2  slfcont1  slfcont3  slfcont4
##   10   9  age  children  cnarc1  cnarc2  cnarc3  ctheory1  ctheory2  ctheory3  ctheory4  CRT1  CRT2  CRT3  employ_status1  health_cond  ladder  marital1  mor_circle  moralid1  moralid10  moralid2  moralid3  moralid4  moralid5  moralid6  moralid7  moralid8  moralid9  mcoop1  mcoop2  mcoop3  mcoop4  mcoop5  mcoop6  mcoop7  narc1  narc2  narc3  narc4  narc5  narc6  nidentity1  nidentity2  omind1  omind2  omind3  omind4  omind5  omind6  contact1  contact2  hygiene1  hygiene5  political_ideology  happy  slf_ladder  riskperc2  self_esteem  sex1  sbelong1  sbelong2  sbelong3  sbelong4  optim1  optim2  slfcont1  slfcont3  slfcont4
##   10   10  age  children  cnarc1  cnarc2  cnarc3  ctheory1  ctheory2  ctheory3  ctheory4  CRT1  CRT2  CRT3  employ_status1  health_cond  ladder  marital1  mor_circle  moralid1  moralid10  moralid2  moralid3  moralid4  moralid5  moralid6  moralid7  moralid8  moralid9  mcoop1  mcoop2  mcoop3  mcoop4  mcoop5  mcoop6  mcoop7  narc1  narc2  narc3  narc4  narc5  narc6  nidentity1  nidentity2  omind1  omind2  omind3  omind4  omind5  omind6  contact1  contact2  hygiene1  hygiene5  political_ideology  happy  slf_ladder  riskperc2  self_esteem  sex1  sbelong1  sbelong2  sbelong3  sbelong4  optim1  optim2  slfcont1  slfcont3  slfcont4
```

```
## Warning: Number of logged events: 6703
```

```
## 
##  iter imp variable
##   1   1  age  cnarc1  cnarc2  cnarc3  ctheory1  ctheory2  ctheory3  CRT1  CRT2  CRT3  moralid10  moralid2  moralid3  moralid4  moralid5  moralid6  moralid7  moralid8  moralid9  mcoop1  mcoop2  mcoop3  mcoop4  mcoop5  mcoop6  mcoop7  narc1  narc2  narc3  narc4  narc5  narc6  contact1  contact2  contact3  contact4  contact5  hygiene3  psupport3  political_ideology  happy  slf_ladder  riskperc2  self_esteem  sbelong1  sbelong2  slfcont1  slfcont2  slfcont3  slfcont4
##   1   2  age  cnarc1  cnarc2  cnarc3  ctheory1  ctheory2  ctheory3  CRT1  CRT2  CRT3  moralid10  moralid2  moralid3  moralid4  moralid5  moralid6  moralid7  moralid8  moralid9  mcoop1  mcoop2  mcoop3  mcoop4  mcoop5  mcoop6  mcoop7  narc1  narc2  narc3  narc4  narc5  narc6  contact1  contact2  contact3  contact4  contact5  hygiene3  psupport3  political_ideology  happy  slf_ladder  riskperc2  self_esteem  sbelong1  sbelong2  slfcont1  slfcont2  slfcont3  slfcont4
##   1   3  age  cnarc1  cnarc2  cnarc3  ctheory1  ctheory2  ctheory3  CRT1  CRT2  CRT3  moralid10  moralid2  moralid3  moralid4  moralid5  moralid6  moralid7  moralid8  moralid9  mcoop1  mcoop2  mcoop3  mcoop4  mcoop5  mcoop6  mcoop7  narc1  narc2  narc3  narc4  narc5  narc6  contact1  contact2  contact3  contact4  contact5  hygiene3  psupport3  political_ideology  happy  slf_ladder  riskperc2  self_esteem  sbelong1  sbelong2  slfcont1  slfcont2  slfcont3  slfcont4
##   1   4  age  cnarc1  cnarc2  cnarc3  ctheory1  ctheory2  ctheory3  CRT1  CRT2  CRT3  moralid10  moralid2  moralid3  moralid4  moralid5  moralid6  moralid7  moralid8  moralid9  mcoop1  mcoop2  mcoop3  mcoop4  mcoop5  mcoop6  mcoop7  narc1  narc2  narc3  narc4  narc5  narc6  contact1  contact2  contact3  contact4  contact5  hygiene3  psupport3  political_ideology  happy  slf_ladder  riskperc2  self_esteem  sbelong1  sbelong2  slfcont1  slfcont2  slfcont3  slfcont4
##   1   5  age  cnarc1  cnarc2  cnarc3  ctheory1  ctheory2  ctheory3  CRT1  CRT2  CRT3  moralid10  moralid2  moralid3  moralid4  moralid5  moralid6  moralid7  moralid8  moralid9  mcoop1  mcoop2  mcoop3  mcoop4  mcoop5  mcoop6  mcoop7  narc1  narc2  narc3  narc4  narc5  narc6  contact1  contact2  contact3  contact4  contact5  hygiene3  psupport3  political_ideology  happy  slf_ladder  riskperc2  self_esteem  sbelong1  sbelong2  slfcont1  slfcont2  slfcont3  slfcont4
##   1   6  age  cnarc1  cnarc2  cnarc3  ctheory1  ctheory2  ctheory3  CRT1  CRT2  CRT3  moralid10  moralid2  moralid3  moralid4  moralid5  moralid6  moralid7  moralid8  moralid9  mcoop1  mcoop2  mcoop3  mcoop4  mcoop5  mcoop6  mcoop7  narc1  narc2  narc3  narc4  narc5  narc6  contact1  contact2  contact3  contact4  contact5  hygiene3  psupport3  political_ideology  happy  slf_ladder  riskperc2  self_esteem  sbelong1  sbelong2  slfcont1  slfcont2  slfcont3  slfcont4
##   1   7  age  cnarc1  cnarc2  cnarc3  ctheory1  ctheory2  ctheory3  CRT1  CRT2  CRT3  moralid10  moralid2  moralid3  moralid4  moralid5  moralid6  moralid7  moralid8  moralid9  mcoop1  mcoop2  mcoop3  mcoop4  mcoop5  mcoop6  mcoop7  narc1  narc2  narc3  narc4  narc5  narc6  contact1  contact2  contact3  contact4  contact5  hygiene3  psupport3  political_ideology  happy  slf_ladder  riskperc2  self_esteem  sbelong1  sbelong2  slfcont1  slfcont2  slfcont3  slfcont4
##   1   8  age  cnarc1  cnarc2  cnarc3  ctheory1  ctheory2  ctheory3  CRT1  CRT2  CRT3  moralid10  moralid2  moralid3  moralid4  moralid5  moralid6  moralid7  moralid8  moralid9  mcoop1  mcoop2  mcoop3  mcoop4  mcoop5  mcoop6  mcoop7  narc1  narc2  narc3  narc4  narc5  narc6  contact1  contact2  contact3  contact4  contact5  hygiene3  psupport3  political_ideology  happy  slf_ladder  riskperc2  self_esteem  sbelong1  sbelong2  slfcont1  slfcont2  slfcont3  slfcont4
##   1   9  age  cnarc1  cnarc2  cnarc3  ctheory1  ctheory2  ctheory3  CRT1  CRT2  CRT3  moralid10  moralid2  moralid3  moralid4  moralid5  moralid6  moralid7  moralid8  moralid9  mcoop1  mcoop2  mcoop3  mcoop4  mcoop5  mcoop6  mcoop7  narc1  narc2  narc3  narc4  narc5  narc6  contact1  contact2  contact3  contact4  contact5  hygiene3  psupport3  political_ideology  happy  slf_ladder  riskperc2  self_esteem  sbelong1  sbelong2  slfcont1  slfcont2  slfcont3  slfcont4
##   1   10  age  cnarc1  cnarc2  cnarc3  ctheory1  ctheory2  ctheory3  CRT1  CRT2  CRT3  moralid10  moralid2  moralid3  moralid4  moralid5  moralid6  moralid7  moralid8  moralid9  mcoop1  mcoop2  mcoop3  mcoop4  mcoop5  mcoop6  mcoop7  narc1  narc2  narc3  narc4  narc5  narc6  contact1  contact2  contact3  contact4  contact5  hygiene3  psupport3  political_ideology  happy  slf_ladder  riskperc2  self_esteem  sbelong1  sbelong2  slfcont1  slfcont2  slfcont3  slfcont4
##   2   1  age  cnarc1  cnarc2  cnarc3  ctheory1  ctheory2  ctheory3  CRT1  CRT2  CRT3  moralid10  moralid2  moralid3  moralid4  moralid5  moralid6  moralid7  moralid8  moralid9  mcoop1  mcoop2  mcoop3  mcoop4  mcoop5  mcoop6  mcoop7  narc1  narc2  narc3  narc4  narc5  narc6  contact1  contact2  contact3  contact4  contact5  hygiene3  psupport3  political_ideology  happy  slf_ladder  riskperc2  self_esteem  sbelong1  sbelong2  slfcont1  slfcont2  slfcont3  slfcont4
##   2   2  age  cnarc1  cnarc2  cnarc3  ctheory1  ctheory2  ctheory3  CRT1  CRT2  CRT3  moralid10  moralid2  moralid3  moralid4  moralid5  moralid6  moralid7  moralid8  moralid9  mcoop1  mcoop2  mcoop3  mcoop4  mcoop5  mcoop6  mcoop7  narc1  narc2  narc3  narc4  narc5  narc6  contact1  contact2  contact3  contact4  contact5  hygiene3  psupport3  political_ideology  happy  slf_ladder  riskperc2  self_esteem  sbelong1  sbelong2  slfcont1  slfcont2  slfcont3  slfcont4
##   2   3  age  cnarc1  cnarc2  cnarc3  ctheory1  ctheory2  ctheory3  CRT1  CRT2  CRT3  moralid10  moralid2  moralid3  moralid4  moralid5  moralid6  moralid7  moralid8  moralid9  mcoop1  mcoop2  mcoop3  mcoop4  mcoop5  mcoop6  mcoop7  narc1  narc2  narc3  narc4  narc5  narc6  contact1  contact2  contact3  contact4  contact5  hygiene3  psupport3  political_ideology  happy  slf_ladder  riskperc2  self_esteem  sbelong1  sbelong2  slfcont1  slfcont2  slfcont3  slfcont4
##   2   4  age  cnarc1  cnarc2  cnarc3  ctheory1  ctheory2  ctheory3  CRT1  CRT2  CRT3  moralid10  moralid2  moralid3  moralid4  moralid5  moralid6  moralid7  moralid8  moralid9  mcoop1  mcoop2  mcoop3  mcoop4  mcoop5  mcoop6  mcoop7  narc1  narc2  narc3  narc4  narc5  narc6  contact1  contact2  contact3  contact4  contact5  hygiene3  psupport3  political_ideology  happy  slf_ladder  riskperc2  self_esteem  sbelong1  sbelong2  slfcont1  slfcont2  slfcont3  slfcont4
##   2   5  age  cnarc1  cnarc2  cnarc3  ctheory1  ctheory2  ctheory3  CRT1  CRT2  CRT3  moralid10  moralid2  moralid3  moralid4  moralid5  moralid6  moralid7  moralid8  moralid9  mcoop1  mcoop2  mcoop3  mcoop4  mcoop5  mcoop6  mcoop7  narc1  narc2  narc3  narc4  narc5  narc6  contact1  contact2  contact3  contact4  contact5  hygiene3  psupport3  political_ideology  happy  slf_ladder  riskperc2  self_esteem  sbelong1  sbelong2  slfcont1  slfcont2  slfcont3  slfcont4
##   2   6  age  cnarc1  cnarc2  cnarc3  ctheory1  ctheory2  ctheory3  CRT1  CRT2  CRT3  moralid10  moralid2  moralid3  moralid4  moralid5  moralid6  moralid7  moralid8  moralid9  mcoop1  mcoop2  mcoop3  mcoop4  mcoop5  mcoop6  mcoop7  narc1  narc2  narc3  narc4  narc5  narc6  contact1  contact2  contact3  contact4  contact5  hygiene3  psupport3  political_ideology  happy  slf_ladder  riskperc2  self_esteem  sbelong1  sbelong2  slfcont1  slfcont2  slfcont3  slfcont4
##   2   7  age  cnarc1  cnarc2  cnarc3  ctheory1  ctheory2  ctheory3  CRT1  CRT2  CRT3  moralid10  moralid2  moralid3  moralid4  moralid5  moralid6  moralid7  moralid8  moralid9  mcoop1  mcoop2  mcoop3  mcoop4  mcoop5  mcoop6  mcoop7  narc1  narc2  narc3  narc4  narc5  narc6  contact1  contact2  contact3  contact4  contact5  hygiene3  psupport3  political_ideology  happy  slf_ladder  riskperc2  self_esteem  sbelong1  sbelong2  slfcont1  slfcont2  slfcont3  slfcont4
##   2   8  age  cnarc1  cnarc2  cnarc3  ctheory1  ctheory2  ctheory3  CRT1  CRT2  CRT3  moralid10  moralid2  moralid3  moralid4  moralid5  moralid6  moralid7  moralid8  moralid9  mcoop1  mcoop2  mcoop3  mcoop4  mcoop5  mcoop6  mcoop7  narc1  narc2  narc3  narc4  narc5  narc6  contact1  contact2  contact3  contact4  contact5  hygiene3  psupport3  political_ideology  happy  slf_ladder  riskperc2  self_esteem  sbelong1  sbelong2  slfcont1  slfcont2  slfcont3  slfcont4
##   2   9  age  cnarc1  cnarc2  cnarc3  ctheory1  ctheory2  ctheory3  CRT1  CRT2  CRT3  moralid10  moralid2  moralid3  moralid4  moralid5  moralid6  moralid7  moralid8  moralid9  mcoop1  mcoop2  mcoop3  mcoop4  mcoop5  mcoop6  mcoop7  narc1  narc2  narc3  narc4  narc5  narc6  contact1  contact2  contact3  contact4  contact5  hygiene3  psupport3  political_ideology  happy  slf_ladder  riskperc2  self_esteem  sbelong1  sbelong2  slfcont1  slfcont2  slfcont3  slfcont4
##   2   10  age  cnarc1  cnarc2  cnarc3  ctheory1  ctheory2  ctheory3  CRT1  CRT2  CRT3  moralid10  moralid2  moralid3  moralid4  moralid5  moralid6  moralid7  moralid8  moralid9  mcoop1  mcoop2  mcoop3  mcoop4  mcoop5  mcoop6  mcoop7  narc1  narc2  narc3  narc4  narc5  narc6  contact1  contact2  contact3  contact4  contact5  hygiene3  psupport3  political_ideology  happy  slf_ladder  riskperc2  self_esteem  sbelong1  sbelong2  slfcont1  slfcont2  slfcont3  slfcont4
##   3   1  age  cnarc1  cnarc2  cnarc3  ctheory1  ctheory2  ctheory3  CRT1  CRT2  CRT3  moralid10  moralid2  moralid3  moralid4  moralid5  moralid6  moralid7  moralid8  moralid9  mcoop1  mcoop2  mcoop3  mcoop4  mcoop5  mcoop6  mcoop7  narc1  narc2  narc3  narc4  narc5  narc6  contact1  contact2  contact3  contact4  contact5  hygiene3  psupport3  political_ideology  happy  slf_ladder  riskperc2  self_esteem  sbelong1  sbelong2  slfcont1  slfcont2  slfcont3  slfcont4
##   3   2  age  cnarc1  cnarc2  cnarc3  ctheory1  ctheory2  ctheory3  CRT1  CRT2  CRT3  moralid10  moralid2  moralid3  moralid4  moralid5  moralid6  moralid7  moralid8  moralid9  mcoop1  mcoop2  mcoop3  mcoop4  mcoop5  mcoop6  mcoop7  narc1  narc2  narc3  narc4  narc5  narc6  contact1  contact2  contact3  contact4  contact5  hygiene3  psupport3  political_ideology  happy  slf_ladder  riskperc2  self_esteem  sbelong1  sbelong2  slfcont1  slfcont2  slfcont3  slfcont4
##   3   3  age  cnarc1  cnarc2  cnarc3  ctheory1  ctheory2  ctheory3  CRT1  CRT2  CRT3  moralid10  moralid2  moralid3  moralid4  moralid5  moralid6  moralid7  moralid8  moralid9  mcoop1  mcoop2  mcoop3  mcoop4  mcoop5  mcoop6  mcoop7  narc1  narc2  narc3  narc4  narc5  narc6  contact1  contact2  contact3  contact4  contact5  hygiene3  psupport3  political_ideology  happy  slf_ladder  riskperc2  self_esteem  sbelong1  sbelong2  slfcont1  slfcont2  slfcont3  slfcont4
##   3   4  age  cnarc1  cnarc2  cnarc3  ctheory1  ctheory2  ctheory3  CRT1  CRT2  CRT3  moralid10  moralid2  moralid3  moralid4  moralid5  moralid6  moralid7  moralid8  moralid9  mcoop1  mcoop2  mcoop3  mcoop4  mcoop5  mcoop6  mcoop7  narc1  narc2  narc3  narc4  narc5  narc6  contact1  contact2  contact3  contact4  contact5  hygiene3  psupport3  political_ideology  happy  slf_ladder  riskperc2  self_esteem  sbelong1  sbelong2  slfcont1  slfcont2  slfcont3  slfcont4
##   3   5  age  cnarc1  cnarc2  cnarc3  ctheory1  ctheory2  ctheory3  CRT1  CRT2  CRT3  moralid10  moralid2  moralid3  moralid4  moralid5  moralid6  moralid7  moralid8  moralid9  mcoop1  mcoop2  mcoop3  mcoop4  mcoop5  mcoop6  mcoop7  narc1  narc2  narc3  narc4  narc5  narc6  contact1  contact2  contact3  contact4  contact5  hygiene3  psupport3  political_ideology  happy  slf_ladder  riskperc2  self_esteem  sbelong1  sbelong2  slfcont1  slfcont2  slfcont3  slfcont4
##   3   6  age  cnarc1  cnarc2  cnarc3  ctheory1  ctheory2  ctheory3  CRT1  CRT2  CRT3  moralid10  moralid2  moralid3  moralid4  moralid5  moralid6  moralid7  moralid8  moralid9  mcoop1  mcoop2  mcoop3  mcoop4  mcoop5  mcoop6  mcoop7  narc1  narc2  narc3  narc4  narc5  narc6  contact1  contact2  contact3  contact4  contact5  hygiene3  psupport3  political_ideology  happy  slf_ladder  riskperc2  self_esteem  sbelong1  sbelong2  slfcont1  slfcont2  slfcont3  slfcont4
##   3   7  age  cnarc1  cnarc2  cnarc3  ctheory1  ctheory2  ctheory3  CRT1  CRT2  CRT3  moralid10  moralid2  moralid3  moralid4  moralid5  moralid6  moralid7  moralid8  moralid9  mcoop1  mcoop2  mcoop3  mcoop4  mcoop5  mcoop6  mcoop7  narc1  narc2  narc3  narc4  narc5  narc6  contact1  contact2  contact3  contact4  contact5  hygiene3  psupport3  political_ideology  happy  slf_ladder  riskperc2  self_esteem  sbelong1  sbelong2  slfcont1  slfcont2  slfcont3  slfcont4
##   3   8  age  cnarc1  cnarc2  cnarc3  ctheory1  ctheory2  ctheory3  CRT1  CRT2  CRT3  moralid10  moralid2  moralid3  moralid4  moralid5  moralid6  moralid7  moralid8  moralid9  mcoop1  mcoop2  mcoop3  mcoop4  mcoop5  mcoop6  mcoop7  narc1  narc2  narc3  narc4  narc5  narc6  contact1  contact2  contact3  contact4  contact5  hygiene3  psupport3  political_ideology  happy  slf_ladder  riskperc2  self_esteem  sbelong1  sbelong2  slfcont1  slfcont2  slfcont3  slfcont4
##   3   9  age  cnarc1  cnarc2  cnarc3  ctheory1  ctheory2  ctheory3  CRT1  CRT2  CRT3  moralid10  moralid2  moralid3  moralid4  moralid5  moralid6  moralid7  moralid8  moralid9  mcoop1  mcoop2  mcoop3  mcoop4  mcoop5  mcoop6  mcoop7  narc1  narc2  narc3  narc4  narc5  narc6  contact1  contact2  contact3  contact4  contact5  hygiene3  psupport3  political_ideology  happy  slf_ladder  riskperc2  self_esteem  sbelong1  sbelong2  slfcont1  slfcont2  slfcont3  slfcont4
##   3   10  age  cnarc1  cnarc2  cnarc3  ctheory1  ctheory2  ctheory3  CRT1  CRT2  CRT3  moralid10  moralid2  moralid3  moralid4  moralid5  moralid6  moralid7  moralid8  moralid9  mcoop1  mcoop2  mcoop3  mcoop4  mcoop5  mcoop6  mcoop7  narc1  narc2  narc3  narc4  narc5  narc6  contact1  contact2  contact3  contact4  contact5  hygiene3  psupport3  political_ideology  happy  slf_ladder  riskperc2  self_esteem  sbelong1  sbelong2  slfcont1  slfcont2  slfcont3  slfcont4
##   4   1  age  cnarc1  cnarc2  cnarc3  ctheory1  ctheory2  ctheory3  CRT1  CRT2  CRT3  moralid10  moralid2  moralid3  moralid4  moralid5  moralid6  moralid7  moralid8  moralid9  mcoop1  mcoop2  mcoop3  mcoop4  mcoop5  mcoop6  mcoop7  narc1  narc2  narc3  narc4  narc5  narc6  contact1  contact2  contact3  contact4  contact5  hygiene3  psupport3  political_ideology  happy  slf_ladder  riskperc2  self_esteem  sbelong1  sbelong2  slfcont1  slfcont2  slfcont3  slfcont4
##   4   2  age  cnarc1  cnarc2  cnarc3  ctheory1  ctheory2  ctheory3  CRT1  CRT2  CRT3  moralid10  moralid2  moralid3  moralid4  moralid5  moralid6  moralid7  moralid8  moralid9  mcoop1  mcoop2  mcoop3  mcoop4  mcoop5  mcoop6  mcoop7  narc1  narc2  narc3  narc4  narc5  narc6  contact1  contact2  contact3  contact4  contact5  hygiene3  psupport3  political_ideology  happy  slf_ladder  riskperc2  self_esteem  sbelong1  sbelong2  slfcont1  slfcont2  slfcont3  slfcont4
##   4   3  age  cnarc1  cnarc2  cnarc3  ctheory1  ctheory2  ctheory3  CRT1  CRT2  CRT3  moralid10  moralid2  moralid3  moralid4  moralid5  moralid6  moralid7  moralid8  moralid9  mcoop1  mcoop2  mcoop3  mcoop4  mcoop5  mcoop6  mcoop7  narc1  narc2  narc3  narc4  narc5  narc6  contact1  contact2  contact3  contact4  contact5  hygiene3  psupport3  political_ideology  happy  slf_ladder  riskperc2  self_esteem  sbelong1  sbelong2  slfcont1  slfcont2  slfcont3  slfcont4
##   4   4  age  cnarc1  cnarc2  cnarc3  ctheory1  ctheory2  ctheory3  CRT1  CRT2  CRT3  moralid10  moralid2  moralid3  moralid4  moralid5  moralid6  moralid7  moralid8  moralid9  mcoop1  mcoop2  mcoop3  mcoop4  mcoop5  mcoop6  mcoop7  narc1  narc2  narc3  narc4  narc5  narc6  contact1  contact2  contact3  contact4  contact5  hygiene3  psupport3  political_ideology  happy  slf_ladder  riskperc2  self_esteem  sbelong1  sbelong2  slfcont1  slfcont2  slfcont3  slfcont4
##   4   5  age  cnarc1  cnarc2  cnarc3  ctheory1  ctheory2  ctheory3  CRT1  CRT2  CRT3  moralid10  moralid2  moralid3  moralid4  moralid5  moralid6  moralid7  moralid8  moralid9  mcoop1  mcoop2  mcoop3  mcoop4  mcoop5  mcoop6  mcoop7  narc1  narc2  narc3  narc4  narc5  narc6  contact1  contact2  contact3  contact4  contact5  hygiene3  psupport3  political_ideology  happy  slf_ladder  riskperc2  self_esteem  sbelong1  sbelong2  slfcont1  slfcont2  slfcont3  slfcont4
##   4   6  age  cnarc1  cnarc2  cnarc3  ctheory1  ctheory2  ctheory3  CRT1  CRT2  CRT3  moralid10  moralid2  moralid3  moralid4  moralid5  moralid6  moralid7  moralid8  moralid9  mcoop1  mcoop2  mcoop3  mcoop4  mcoop5  mcoop6  mcoop7  narc1  narc2  narc3  narc4  narc5  narc6  contact1  contact2  contact3  contact4  contact5  hygiene3  psupport3  political_ideology  happy  slf_ladder  riskperc2  self_esteem  sbelong1  sbelong2  slfcont1  slfcont2  slfcont3  slfcont4
##   4   7  age  cnarc1  cnarc2  cnarc3  ctheory1  ctheory2  ctheory3  CRT1  CRT2  CRT3  moralid10  moralid2  moralid3  moralid4  moralid5  moralid6  moralid7  moralid8  moralid9  mcoop1  mcoop2  mcoop3  mcoop4  mcoop5  mcoop6  mcoop7  narc1  narc2  narc3  narc4  narc5  narc6  contact1  contact2  contact3  contact4  contact5  hygiene3  psupport3  political_ideology  happy  slf_ladder  riskperc2  self_esteem  sbelong1  sbelong2  slfcont1  slfcont2  slfcont3  slfcont4
##   4   8  age  cnarc1  cnarc2  cnarc3  ctheory1  ctheory2  ctheory3  CRT1  CRT2  CRT3  moralid10  moralid2  moralid3  moralid4  moralid5  moralid6  moralid7  moralid8  moralid9  mcoop1  mcoop2  mcoop3  mcoop4  mcoop5  mcoop6  mcoop7  narc1  narc2  narc3  narc4  narc5  narc6  contact1  contact2  contact3  contact4  contact5  hygiene3  psupport3  political_ideology  happy  slf_ladder  riskperc2  self_esteem  sbelong1  sbelong2  slfcont1  slfcont2  slfcont3  slfcont4
##   4   9  age  cnarc1  cnarc2  cnarc3  ctheory1  ctheory2  ctheory3  CRT1  CRT2  CRT3  moralid10  moralid2  moralid3  moralid4  moralid5  moralid6  moralid7  moralid8  moralid9  mcoop1  mcoop2  mcoop3  mcoop4  mcoop5  mcoop6  mcoop7  narc1  narc2  narc3  narc4  narc5  narc6  contact1  contact2  contact3  contact4  contact5  hygiene3  psupport3  political_ideology  happy  slf_ladder  riskperc2  self_esteem  sbelong1  sbelong2  slfcont1  slfcont2  slfcont3  slfcont4
##   4   10  age  cnarc1  cnarc2  cnarc3  ctheory1  ctheory2  ctheory3  CRT1  CRT2  CRT3  moralid10  moralid2  moralid3  moralid4  moralid5  moralid6  moralid7  moralid8  moralid9  mcoop1  mcoop2  mcoop3  mcoop4  mcoop5  mcoop6  mcoop7  narc1  narc2  narc3  narc4  narc5  narc6  contact1  contact2  contact3  contact4  contact5  hygiene3  psupport3  political_ideology  happy  slf_ladder  riskperc2  self_esteem  sbelong1  sbelong2  slfcont1  slfcont2  slfcont3  slfcont4
##   5   1  age  cnarc1  cnarc2  cnarc3  ctheory1  ctheory2  ctheory3  CRT1  CRT2  CRT3  moralid10  moralid2  moralid3  moralid4  moralid5  moralid6  moralid7  moralid8  moralid9  mcoop1  mcoop2  mcoop3  mcoop4  mcoop5  mcoop6  mcoop7  narc1  narc2  narc3  narc4  narc5  narc6  contact1  contact2  contact3  contact4  contact5  hygiene3  psupport3  political_ideology  happy  slf_ladder  riskperc2  self_esteem  sbelong1  sbelong2  slfcont1  slfcont2  slfcont3  slfcont4
##   5   2  age  cnarc1  cnarc2  cnarc3  ctheory1  ctheory2  ctheory3  CRT1  CRT2  CRT3  moralid10  moralid2  moralid3  moralid4  moralid5  moralid6  moralid7  moralid8  moralid9  mcoop1  mcoop2  mcoop3  mcoop4  mcoop5  mcoop6  mcoop7  narc1  narc2  narc3  narc4  narc5  narc6  contact1  contact2  contact3  contact4  contact5  hygiene3  psupport3  political_ideology  happy  slf_ladder  riskperc2  self_esteem  sbelong1  sbelong2  slfcont1  slfcont2  slfcont3  slfcont4
##   5   3  age  cnarc1  cnarc2  cnarc3  ctheory1  ctheory2  ctheory3  CRT1  CRT2  CRT3  moralid10  moralid2  moralid3  moralid4  moralid5  moralid6  moralid7  moralid8  moralid9  mcoop1  mcoop2  mcoop3  mcoop4  mcoop5  mcoop6  mcoop7  narc1  narc2  narc3  narc4  narc5  narc6  contact1  contact2  contact3  contact4  contact5  hygiene3  psupport3  political_ideology  happy  slf_ladder  riskperc2  self_esteem  sbelong1  sbelong2  slfcont1  slfcont2  slfcont3  slfcont4
##   5   4  age  cnarc1  cnarc2  cnarc3  ctheory1  ctheory2  ctheory3  CRT1  CRT2  CRT3  moralid10  moralid2  moralid3  moralid4  moralid5  moralid6  moralid7  moralid8  moralid9  mcoop1  mcoop2  mcoop3  mcoop4  mcoop5  mcoop6  mcoop7  narc1  narc2  narc3  narc4  narc5  narc6  contact1  contact2  contact3  contact4  contact5  hygiene3  psupport3  political_ideology  happy  slf_ladder  riskperc2  self_esteem  sbelong1  sbelong2  slfcont1  slfcont2  slfcont3  slfcont4
##   5   5  age  cnarc1  cnarc2  cnarc3  ctheory1  ctheory2  ctheory3  CRT1  CRT2  CRT3  moralid10  moralid2  moralid3  moralid4  moralid5  moralid6  moralid7  moralid8  moralid9  mcoop1  mcoop2  mcoop3  mcoop4  mcoop5  mcoop6  mcoop7  narc1  narc2  narc3  narc4  narc5  narc6  contact1  contact2  contact3  contact4  contact5  hygiene3  psupport3  political_ideology  happy  slf_ladder  riskperc2  self_esteem  sbelong1  sbelong2  slfcont1  slfcont2  slfcont3  slfcont4
##   5   6  age  cnarc1  cnarc2  cnarc3  ctheory1  ctheory2  ctheory3  CRT1  CRT2  CRT3  moralid10  moralid2  moralid3  moralid4  moralid5  moralid6  moralid7  moralid8  moralid9  mcoop1  mcoop2  mcoop3  mcoop4  mcoop5  mcoop6  mcoop7  narc1  narc2  narc3  narc4  narc5  narc6  contact1  contact2  contact3  contact4  contact5  hygiene3  psupport3  political_ideology  happy  slf_ladder  riskperc2  self_esteem  sbelong1  sbelong2  slfcont1  slfcont2  slfcont3  slfcont4
##   5   7  age  cnarc1  cnarc2  cnarc3  ctheory1  ctheory2  ctheory3  CRT1  CRT2  CRT3  moralid10  moralid2  moralid3  moralid4  moralid5  moralid6  moralid7  moralid8  moralid9  mcoop1  mcoop2  mcoop3  mcoop4  mcoop5  mcoop6  mcoop7  narc1  narc2  narc3  narc4  narc5  narc6  contact1  contact2  contact3  contact4  contact5  hygiene3  psupport3  political_ideology  happy  slf_ladder  riskperc2  self_esteem  sbelong1  sbelong2  slfcont1  slfcont2  slfcont3  slfcont4
##   5   8  age  cnarc1  cnarc2  cnarc3  ctheory1  ctheory2  ctheory3  CRT1  CRT2  CRT3  moralid10  moralid2  moralid3  moralid4  moralid5  moralid6  moralid7  moralid8  moralid9  mcoop1  mcoop2  mcoop3  mcoop4  mcoop5  mcoop6  mcoop7  narc1  narc2  narc3  narc4  narc5  narc6  contact1  contact2  contact3  contact4  contact5  hygiene3  psupport3  political_ideology  happy  slf_ladder  riskperc2  self_esteem  sbelong1  sbelong2  slfcont1  slfcont2  slfcont3  slfcont4
##   5   9  age  cnarc1  cnarc2  cnarc3  ctheory1  ctheory2  ctheory3  CRT1  CRT2  CRT3  moralid10  moralid2  moralid3  moralid4  moralid5  moralid6  moralid7  moralid8  moralid9  mcoop1  mcoop2  mcoop3  mcoop4  mcoop5  mcoop6  mcoop7  narc1  narc2  narc3  narc4  narc5  narc6  contact1  contact2  contact3  contact4  contact5  hygiene3  psupport3  political_ideology  happy  slf_ladder  riskperc2  self_esteem  sbelong1  sbelong2  slfcont1  slfcont2  slfcont3  slfcont4
##   5   10  age  cnarc1  cnarc2  cnarc3  ctheory1  ctheory2  ctheory3  CRT1  CRT2  CRT3  moralid10  moralid2  moralid3  moralid4  moralid5  moralid6  moralid7  moralid8  moralid9  mcoop1  mcoop2  mcoop3  mcoop4  mcoop5  mcoop6  mcoop7  narc1  narc2  narc3  narc4  narc5  narc6  contact1  contact2  contact3  contact4  contact5  hygiene3  psupport3  political_ideology  happy  slf_ladder  riskperc2  self_esteem  sbelong1  sbelong2  slfcont1  slfcont2  slfcont3  slfcont4
##   6   1  age  cnarc1  cnarc2  cnarc3  ctheory1  ctheory2  ctheory3  CRT1  CRT2  CRT3  moralid10  moralid2  moralid3  moralid4  moralid5  moralid6  moralid7  moralid8  moralid9  mcoop1  mcoop2  mcoop3  mcoop4  mcoop5  mcoop6  mcoop7  narc1  narc2  narc3  narc4  narc5  narc6  contact1  contact2  contact3  contact4  contact5  hygiene3  psupport3  political_ideology  happy  slf_ladder  riskperc2  self_esteem  sbelong1  sbelong2  slfcont1  slfcont2  slfcont3  slfcont4
##   6   2  age  cnarc1  cnarc2  cnarc3  ctheory1  ctheory2  ctheory3  CRT1  CRT2  CRT3  moralid10  moralid2  moralid3  moralid4  moralid5  moralid6  moralid7  moralid8  moralid9  mcoop1  mcoop2  mcoop3  mcoop4  mcoop5  mcoop6  mcoop7  narc1  narc2  narc3  narc4  narc5  narc6  contact1  contact2  contact3  contact4  contact5  hygiene3  psupport3  political_ideology  happy  slf_ladder  riskperc2  self_esteem  sbelong1  sbelong2  slfcont1  slfcont2  slfcont3  slfcont4
##   6   3  age  cnarc1  cnarc2  cnarc3  ctheory1  ctheory2  ctheory3  CRT1  CRT2  CRT3  moralid10  moralid2  moralid3  moralid4  moralid5  moralid6  moralid7  moralid8  moralid9  mcoop1  mcoop2  mcoop3  mcoop4  mcoop5  mcoop6  mcoop7  narc1  narc2  narc3  narc4  narc5  narc6  contact1  contact2  contact3  contact4  contact5  hygiene3  psupport3  political_ideology  happy  slf_ladder  riskperc2  self_esteem  sbelong1  sbelong2  slfcont1  slfcont2  slfcont3  slfcont4
##   6   4  age  cnarc1  cnarc2  cnarc3  ctheory1  ctheory2  ctheory3  CRT1  CRT2  CRT3  moralid10  moralid2  moralid3  moralid4  moralid5  moralid6  moralid7  moralid8  moralid9  mcoop1  mcoop2  mcoop3  mcoop4  mcoop5  mcoop6  mcoop7  narc1  narc2  narc3  narc4  narc5  narc6  contact1  contact2  contact3  contact4  contact5  hygiene3  psupport3  political_ideology  happy  slf_ladder  riskperc2  self_esteem  sbelong1  sbelong2  slfcont1  slfcont2  slfcont3  slfcont4
##   6   5  age  cnarc1  cnarc2  cnarc3  ctheory1  ctheory2  ctheory3  CRT1  CRT2  CRT3  moralid10  moralid2  moralid3  moralid4  moralid5  moralid6  moralid7  moralid8  moralid9  mcoop1  mcoop2  mcoop3  mcoop4  mcoop5  mcoop6  mcoop7  narc1  narc2  narc3  narc4  narc5  narc6  contact1  contact2  contact3  contact4  contact5  hygiene3  psupport3  political_ideology  happy  slf_ladder  riskperc2  self_esteem  sbelong1  sbelong2  slfcont1  slfcont2  slfcont3  slfcont4
##   6   6  age  cnarc1  cnarc2  cnarc3  ctheory1  ctheory2  ctheory3  CRT1  CRT2  CRT3  moralid10  moralid2  moralid3  moralid4  moralid5  moralid6  moralid7  moralid8  moralid9  mcoop1  mcoop2  mcoop3  mcoop4  mcoop5  mcoop6  mcoop7  narc1  narc2  narc3  narc4  narc5  narc6  contact1  contact2  contact3  contact4  contact5  hygiene3  psupport3  political_ideology  happy  slf_ladder  riskperc2  self_esteem  sbelong1  sbelong2  slfcont1  slfcont2  slfcont3  slfcont4
##   6   7  age  cnarc1  cnarc2  cnarc3  ctheory1  ctheory2  ctheory3  CRT1  CRT2  CRT3  moralid10  moralid2  moralid3  moralid4  moralid5  moralid6  moralid7  moralid8  moralid9  mcoop1  mcoop2  mcoop3  mcoop4  mcoop5  mcoop6  mcoop7  narc1  narc2  narc3  narc4  narc5  narc6  contact1  contact2  contact3  contact4  contact5  hygiene3  psupport3  political_ideology  happy  slf_ladder  riskperc2  self_esteem  sbelong1  sbelong2  slfcont1  slfcont2  slfcont3  slfcont4
##   6   8  age  cnarc1  cnarc2  cnarc3  ctheory1  ctheory2  ctheory3  CRT1  CRT2  CRT3  moralid10  moralid2  moralid3  moralid4  moralid5  moralid6  moralid7  moralid8  moralid9  mcoop1  mcoop2  mcoop3  mcoop4  mcoop5  mcoop6  mcoop7  narc1  narc2  narc3  narc4  narc5  narc6  contact1  contact2  contact3  contact4  contact5  hygiene3  psupport3  political_ideology  happy  slf_ladder  riskperc2  self_esteem  sbelong1  sbelong2  slfcont1  slfcont2  slfcont3  slfcont4
##   6   9  age  cnarc1  cnarc2  cnarc3  ctheory1  ctheory2  ctheory3  CRT1  CRT2  CRT3  moralid10  moralid2  moralid3  moralid4  moralid5  moralid6  moralid7  moralid8  moralid9  mcoop1  mcoop2  mcoop3  mcoop4  mcoop5  mcoop6  mcoop7  narc1  narc2  narc3  narc4  narc5  narc6  contact1  contact2  contact3  contact4  contact5  hygiene3  psupport3  political_ideology  happy  slf_ladder  riskperc2  self_esteem  sbelong1  sbelong2  slfcont1  slfcont2  slfcont3  slfcont4
##   6   10  age  cnarc1  cnarc2  cnarc3  ctheory1  ctheory2  ctheory3  CRT1  CRT2  CRT3  moralid10  moralid2  moralid3  moralid4  moralid5  moralid6  moralid7  moralid8  moralid9  mcoop1  mcoop2  mcoop3  mcoop4  mcoop5  mcoop6  mcoop7  narc1  narc2  narc3  narc4  narc5  narc6  contact1  contact2  contact3  contact4  contact5  hygiene3  psupport3  political_ideology  happy  slf_ladder  riskperc2  self_esteem  sbelong1  sbelong2  slfcont1  slfcont2  slfcont3  slfcont4
##   7   1  age  cnarc1  cnarc2  cnarc3  ctheory1  ctheory2  ctheory3  CRT1  CRT2  CRT3  moralid10  moralid2  moralid3  moralid4  moralid5  moralid6  moralid7  moralid8  moralid9  mcoop1  mcoop2  mcoop3  mcoop4  mcoop5  mcoop6  mcoop7  narc1  narc2  narc3  narc4  narc5  narc6  contact1  contact2  contact3  contact4  contact5  hygiene3  psupport3  political_ideology  happy  slf_ladder  riskperc2  self_esteem  sbelong1  sbelong2  slfcont1  slfcont2  slfcont3  slfcont4
##   7   2  age  cnarc1  cnarc2  cnarc3  ctheory1  ctheory2  ctheory3  CRT1  CRT2  CRT3  moralid10  moralid2  moralid3  moralid4  moralid5  moralid6  moralid7  moralid8  moralid9  mcoop1  mcoop2  mcoop3  mcoop4  mcoop5  mcoop6  mcoop7  narc1  narc2  narc3  narc4  narc5  narc6  contact1  contact2  contact3  contact4  contact5  hygiene3  psupport3  political_ideology  happy  slf_ladder  riskperc2  self_esteem  sbelong1  sbelong2  slfcont1  slfcont2  slfcont3  slfcont4
##   7   3  age  cnarc1  cnarc2  cnarc3  ctheory1  ctheory2  ctheory3  CRT1  CRT2  CRT3  moralid10  moralid2  moralid3  moralid4  moralid5  moralid6  moralid7  moralid8  moralid9  mcoop1  mcoop2  mcoop3  mcoop4  mcoop5  mcoop6  mcoop7  narc1  narc2  narc3  narc4  narc5  narc6  contact1  contact2  contact3  contact4  contact5  hygiene3  psupport3  political_ideology  happy  slf_ladder  riskperc2  self_esteem  sbelong1  sbelong2  slfcont1  slfcont2  slfcont3  slfcont4
##   7   4  age  cnarc1  cnarc2  cnarc3  ctheory1  ctheory2  ctheory3  CRT1  CRT2  CRT3  moralid10  moralid2  moralid3  moralid4  moralid5  moralid6  moralid7  moralid8  moralid9  mcoop1  mcoop2  mcoop3  mcoop4  mcoop5  mcoop6  mcoop7  narc1  narc2  narc3  narc4  narc5  narc6  contact1  contact2  contact3  contact4  contact5  hygiene3  psupport3  political_ideology  happy  slf_ladder  riskperc2  self_esteem  sbelong1  sbelong2  slfcont1  slfcont2  slfcont3  slfcont4
##   7   5  age  cnarc1  cnarc2  cnarc3  ctheory1  ctheory2  ctheory3  CRT1  CRT2  CRT3  moralid10  moralid2  moralid3  moralid4  moralid5  moralid6  moralid7  moralid8  moralid9  mcoop1  mcoop2  mcoop3  mcoop4  mcoop5  mcoop6  mcoop7  narc1  narc2  narc3  narc4  narc5  narc6  contact1  contact2  contact3  contact4  contact5  hygiene3  psupport3  political_ideology  happy  slf_ladder  riskperc2  self_esteem  sbelong1  sbelong2  slfcont1  slfcont2  slfcont3  slfcont4
##   7   6  age  cnarc1  cnarc2  cnarc3  ctheory1  ctheory2  ctheory3  CRT1  CRT2  CRT3  moralid10  moralid2  moralid3  moralid4  moralid5  moralid6  moralid7  moralid8  moralid9  mcoop1  mcoop2  mcoop3  mcoop4  mcoop5  mcoop6  mcoop7  narc1  narc2  narc3  narc4  narc5  narc6  contact1  contact2  contact3  contact4  contact5  hygiene3  psupport3  political_ideology  happy  slf_ladder  riskperc2  self_esteem  sbelong1  sbelong2  slfcont1  slfcont2  slfcont3  slfcont4
##   7   7  age  cnarc1  cnarc2  cnarc3  ctheory1  ctheory2  ctheory3  CRT1  CRT2  CRT3  moralid10  moralid2  moralid3  moralid4  moralid5  moralid6  moralid7  moralid8  moralid9  mcoop1  mcoop2  mcoop3  mcoop4  mcoop5  mcoop6  mcoop7  narc1  narc2  narc3  narc4  narc5  narc6  contact1  contact2  contact3  contact4  contact5  hygiene3  psupport3  political_ideology  happy  slf_ladder  riskperc2  self_esteem  sbelong1  sbelong2  slfcont1  slfcont2  slfcont3  slfcont4
##   7   8  age  cnarc1  cnarc2  cnarc3  ctheory1  ctheory2  ctheory3  CRT1  CRT2  CRT3  moralid10  moralid2  moralid3  moralid4  moralid5  moralid6  moralid7  moralid8  moralid9  mcoop1  mcoop2  mcoop3  mcoop4  mcoop5  mcoop6  mcoop7  narc1  narc2  narc3  narc4  narc5  narc6  contact1  contact2  contact3  contact4  contact5  hygiene3  psupport3  political_ideology  happy  slf_ladder  riskperc2  self_esteem  sbelong1  sbelong2  slfcont1  slfcont2  slfcont3  slfcont4
##   7   9  age  cnarc1  cnarc2  cnarc3  ctheory1  ctheory2  ctheory3  CRT1  CRT2  CRT3  moralid10  moralid2  moralid3  moralid4  moralid5  moralid6  moralid7  moralid8  moralid9  mcoop1  mcoop2  mcoop3  mcoop4  mcoop5  mcoop6  mcoop7  narc1  narc2  narc3  narc4  narc5  narc6  contact1  contact2  contact3  contact4  contact5  hygiene3  psupport3  political_ideology  happy  slf_ladder  riskperc2  self_esteem  sbelong1  sbelong2  slfcont1  slfcont2  slfcont3  slfcont4
##   7   10  age  cnarc1  cnarc2  cnarc3  ctheory1  ctheory2  ctheory3  CRT1  CRT2  CRT3  moralid10  moralid2  moralid3  moralid4  moralid5  moralid6  moralid7  moralid8  moralid9  mcoop1  mcoop2  mcoop3  mcoop4  mcoop5  mcoop6  mcoop7  narc1  narc2  narc3  narc4  narc5  narc6  contact1  contact2  contact3  contact4  contact5  hygiene3  psupport3  political_ideology  happy  slf_ladder  riskperc2  self_esteem  sbelong1  sbelong2  slfcont1  slfcont2  slfcont3  slfcont4
##   8   1  age  cnarc1  cnarc2  cnarc3  ctheory1  ctheory2  ctheory3  CRT1  CRT2  CRT3  moralid10  moralid2  moralid3  moralid4  moralid5  moralid6  moralid7  moralid8  moralid9  mcoop1  mcoop2  mcoop3  mcoop4  mcoop5  mcoop6  mcoop7  narc1  narc2  narc3  narc4  narc5  narc6  contact1  contact2  contact3  contact4  contact5  hygiene3  psupport3  political_ideology  happy  slf_ladder  riskperc2  self_esteem  sbelong1  sbelong2  slfcont1  slfcont2  slfcont3  slfcont4
##   8   2  age  cnarc1  cnarc2  cnarc3  ctheory1  ctheory2  ctheory3  CRT1  CRT2  CRT3  moralid10  moralid2  moralid3  moralid4  moralid5  moralid6  moralid7  moralid8  moralid9  mcoop1  mcoop2  mcoop3  mcoop4  mcoop5  mcoop6  mcoop7  narc1  narc2  narc3  narc4  narc5  narc6  contact1  contact2  contact3  contact4  contact5  hygiene3  psupport3  political_ideology  happy  slf_ladder  riskperc2  self_esteem  sbelong1  sbelong2  slfcont1  slfcont2  slfcont3  slfcont4
##   8   3  age  cnarc1  cnarc2  cnarc3  ctheory1  ctheory2  ctheory3  CRT1  CRT2  CRT3  moralid10  moralid2  moralid3  moralid4  moralid5  moralid6  moralid7  moralid8  moralid9  mcoop1  mcoop2  mcoop3  mcoop4  mcoop5  mcoop6  mcoop7  narc1  narc2  narc3  narc4  narc5  narc6  contact1  contact2  contact3  contact4  contact5  hygiene3  psupport3  political_ideology  happy  slf_ladder  riskperc2  self_esteem  sbelong1  sbelong2  slfcont1  slfcont2  slfcont3  slfcont4
##   8   4  age  cnarc1  cnarc2  cnarc3  ctheory1  ctheory2  ctheory3  CRT1  CRT2  CRT3  moralid10  moralid2  moralid3  moralid4  moralid5  moralid6  moralid7  moralid8  moralid9  mcoop1  mcoop2  mcoop3  mcoop4  mcoop5  mcoop6  mcoop7  narc1  narc2  narc3  narc4  narc5  narc6  contact1  contact2  contact3  contact4  contact5  hygiene3  psupport3  political_ideology  happy  slf_ladder  riskperc2  self_esteem  sbelong1  sbelong2  slfcont1  slfcont2  slfcont3  slfcont4
##   8   5  age  cnarc1  cnarc2  cnarc3  ctheory1  ctheory2  ctheory3  CRT1  CRT2  CRT3  moralid10  moralid2  moralid3  moralid4  moralid5  moralid6  moralid7  moralid8  moralid9  mcoop1  mcoop2  mcoop3  mcoop4  mcoop5  mcoop6  mcoop7  narc1  narc2  narc3  narc4  narc5  narc6  contact1  contact2  contact3  contact4  contact5  hygiene3  psupport3  political_ideology  happy  slf_ladder  riskperc2  self_esteem  sbelong1  sbelong2  slfcont1  slfcont2  slfcont3  slfcont4
##   8   6  age  cnarc1  cnarc2  cnarc3  ctheory1  ctheory2  ctheory3  CRT1  CRT2  CRT3  moralid10  moralid2  moralid3  moralid4  moralid5  moralid6  moralid7  moralid8  moralid9  mcoop1  mcoop2  mcoop3  mcoop4  mcoop5  mcoop6  mcoop7  narc1  narc2  narc3  narc4  narc5  narc6  contact1  contact2  contact3  contact4  contact5  hygiene3  psupport3  political_ideology  happy  slf_ladder  riskperc2  self_esteem  sbelong1  sbelong2  slfcont1  slfcont2  slfcont3  slfcont4
##   8   7  age  cnarc1  cnarc2  cnarc3  ctheory1  ctheory2  ctheory3  CRT1  CRT2  CRT3  moralid10  moralid2  moralid3  moralid4  moralid5  moralid6  moralid7  moralid8  moralid9  mcoop1  mcoop2  mcoop3  mcoop4  mcoop5  mcoop6  mcoop7  narc1  narc2  narc3  narc4  narc5  narc6  contact1  contact2  contact3  contact4  contact5  hygiene3  psupport3  political_ideology  happy  slf_ladder  riskperc2  self_esteem  sbelong1  sbelong2  slfcont1  slfcont2  slfcont3  slfcont4
##   8   8  age  cnarc1  cnarc2  cnarc3  ctheory1  ctheory2  ctheory3  CRT1  CRT2  CRT3  moralid10  moralid2  moralid3  moralid4  moralid5  moralid6  moralid7  moralid8  moralid9  mcoop1  mcoop2  mcoop3  mcoop4  mcoop5  mcoop6  mcoop7  narc1  narc2  narc3  narc4  narc5  narc6  contact1  contact2  contact3  contact4  contact5  hygiene3  psupport3  political_ideology  happy  slf_ladder  riskperc2  self_esteem  sbelong1  sbelong2  slfcont1  slfcont2  slfcont3  slfcont4
##   8   9  age  cnarc1  cnarc2  cnarc3  ctheory1  ctheory2  ctheory3  CRT1  CRT2  CRT3  moralid10  moralid2  moralid3  moralid4  moralid5  moralid6  moralid7  moralid8  moralid9  mcoop1  mcoop2  mcoop3  mcoop4  mcoop5  mcoop6  mcoop7  narc1  narc2  narc3  narc4  narc5  narc6  contact1  contact2  contact3  contact4  contact5  hygiene3  psupport3  political_ideology  happy  slf_ladder  riskperc2  self_esteem  sbelong1  sbelong2  slfcont1  slfcont2  slfcont3  slfcont4
##   8   10  age  cnarc1  cnarc2  cnarc3  ctheory1  ctheory2  ctheory3  CRT1  CRT2  CRT3  moralid10  moralid2  moralid3  moralid4  moralid5  moralid6  moralid7  moralid8  moralid9  mcoop1  mcoop2  mcoop3  mcoop4  mcoop5  mcoop6  mcoop7  narc1  narc2  narc3  narc4  narc5  narc6  contact1  contact2  contact3  contact4  contact5  hygiene3  psupport3  political_ideology  happy  slf_ladder  riskperc2  self_esteem  sbelong1  sbelong2  slfcont1  slfcont2  slfcont3  slfcont4
##   9   1  age  cnarc1  cnarc2  cnarc3  ctheory1  ctheory2  ctheory3  CRT1  CRT2  CRT3  moralid10  moralid2  moralid3  moralid4  moralid5  moralid6  moralid7  moralid8  moralid9  mcoop1  mcoop2  mcoop3  mcoop4  mcoop5  mcoop6  mcoop7  narc1  narc2  narc3  narc4  narc5  narc6  contact1  contact2  contact3  contact4  contact5  hygiene3  psupport3  political_ideology  happy  slf_ladder  riskperc2  self_esteem  sbelong1  sbelong2  slfcont1  slfcont2  slfcont3  slfcont4
##   9   2  age  cnarc1  cnarc2  cnarc3  ctheory1  ctheory2  ctheory3  CRT1  CRT2  CRT3  moralid10  moralid2  moralid3  moralid4  moralid5  moralid6  moralid7  moralid8  moralid9  mcoop1  mcoop2  mcoop3  mcoop4  mcoop5  mcoop6  mcoop7  narc1  narc2  narc3  narc4  narc5  narc6  contact1  contact2  contact3  contact4  contact5  hygiene3  psupport3  political_ideology  happy  slf_ladder  riskperc2  self_esteem  sbelong1  sbelong2  slfcont1  slfcont2  slfcont3  slfcont4
##   9   3  age  cnarc1  cnarc2  cnarc3  ctheory1  ctheory2  ctheory3  CRT1  CRT2  CRT3  moralid10  moralid2  moralid3  moralid4  moralid5  moralid6  moralid7  moralid8  moralid9  mcoop1  mcoop2  mcoop3  mcoop4  mcoop5  mcoop6  mcoop7  narc1  narc2  narc3  narc4  narc5  narc6  contact1  contact2  contact3  contact4  contact5  hygiene3  psupport3  political_ideology  happy  slf_ladder  riskperc2  self_esteem  sbelong1  sbelong2  slfcont1  slfcont2  slfcont3  slfcont4
##   9   4  age  cnarc1  cnarc2  cnarc3  ctheory1  ctheory2  ctheory3  CRT1  CRT2  CRT3  moralid10  moralid2  moralid3  moralid4  moralid5  moralid6  moralid7  moralid8  moralid9  mcoop1  mcoop2  mcoop3  mcoop4  mcoop5  mcoop6  mcoop7  narc1  narc2  narc3  narc4  narc5  narc6  contact1  contact2  contact3  contact4  contact5  hygiene3  psupport3  political_ideology  happy  slf_ladder  riskperc2  self_esteem  sbelong1  sbelong2  slfcont1  slfcont2  slfcont3  slfcont4
##   9   5  age  cnarc1  cnarc2  cnarc3  ctheory1  ctheory2  ctheory3  CRT1  CRT2  CRT3  moralid10  moralid2  moralid3  moralid4  moralid5  moralid6  moralid7  moralid8  moralid9  mcoop1  mcoop2  mcoop3  mcoop4  mcoop5  mcoop6  mcoop7  narc1  narc2  narc3  narc4  narc5  narc6  contact1  contact2  contact3  contact4  contact5  hygiene3  psupport3  political_ideology  happy  slf_ladder  riskperc2  self_esteem  sbelong1  sbelong2  slfcont1  slfcont2  slfcont3  slfcont4
##   9   6  age  cnarc1  cnarc2  cnarc3  ctheory1  ctheory2  ctheory3  CRT1  CRT2  CRT3  moralid10  moralid2  moralid3  moralid4  moralid5  moralid6  moralid7  moralid8  moralid9  mcoop1  mcoop2  mcoop3  mcoop4  mcoop5  mcoop6  mcoop7  narc1  narc2  narc3  narc4  narc5  narc6  contact1  contact2  contact3  contact4  contact5  hygiene3  psupport3  political_ideology  happy  slf_ladder  riskperc2  self_esteem  sbelong1  sbelong2  slfcont1  slfcont2  slfcont3  slfcont4
##   9   7  age  cnarc1  cnarc2  cnarc3  ctheory1  ctheory2  ctheory3  CRT1  CRT2  CRT3  moralid10  moralid2  moralid3  moralid4  moralid5  moralid6  moralid7  moralid8  moralid9  mcoop1  mcoop2  mcoop3  mcoop4  mcoop5  mcoop6  mcoop7  narc1  narc2  narc3  narc4  narc5  narc6  contact1  contact2  contact3  contact4  contact5  hygiene3  psupport3  political_ideology  happy  slf_ladder  riskperc2  self_esteem  sbelong1  sbelong2  slfcont1  slfcont2  slfcont3  slfcont4
##   9   8  age  cnarc1  cnarc2  cnarc3  ctheory1  ctheory2  ctheory3  CRT1  CRT2  CRT3  moralid10  moralid2  moralid3  moralid4  moralid5  moralid6  moralid7  moralid8  moralid9  mcoop1  mcoop2  mcoop3  mcoop4  mcoop5  mcoop6  mcoop7  narc1  narc2  narc3  narc4  narc5  narc6  contact1  contact2  contact3  contact4  contact5  hygiene3  psupport3  political_ideology  happy  slf_ladder  riskperc2  self_esteem  sbelong1  sbelong2  slfcont1  slfcont2  slfcont3  slfcont4
##   9   9  age  cnarc1  cnarc2  cnarc3  ctheory1  ctheory2  ctheory3  CRT1  CRT2  CRT3  moralid10  moralid2  moralid3  moralid4  moralid5  moralid6  moralid7  moralid8  moralid9  mcoop1  mcoop2  mcoop3  mcoop4  mcoop5  mcoop6  mcoop7  narc1  narc2  narc3  narc4  narc5  narc6  contact1  contact2  contact3  contact4  contact5  hygiene3  psupport3  political_ideology  happy  slf_ladder  riskperc2  self_esteem  sbelong1  sbelong2  slfcont1  slfcont2  slfcont3  slfcont4
##   9   10  age  cnarc1  cnarc2  cnarc3  ctheory1  ctheory2  ctheory3  CRT1  CRT2  CRT3  moralid10  moralid2  moralid3  moralid4  moralid5  moralid6  moralid7  moralid8  moralid9  mcoop1  mcoop2  mcoop3  mcoop4  mcoop5  mcoop6  mcoop7  narc1  narc2  narc3  narc4  narc5  narc6  contact1  contact2  contact3  contact4  contact5  hygiene3  psupport3  political_ideology  happy  slf_ladder  riskperc2  self_esteem  sbelong1  sbelong2  slfcont1  slfcont2  slfcont3  slfcont4
##   10   1  age  cnarc1  cnarc2  cnarc3  ctheory1  ctheory2  ctheory3  CRT1  CRT2  CRT3  moralid10  moralid2  moralid3  moralid4  moralid5  moralid6  moralid7  moralid8  moralid9  mcoop1  mcoop2  mcoop3  mcoop4  mcoop5  mcoop6  mcoop7  narc1  narc2  narc3  narc4  narc5  narc6  contact1  contact2  contact3  contact4  contact5  hygiene3  psupport3  political_ideology  happy  slf_ladder  riskperc2  self_esteem  sbelong1  sbelong2  slfcont1  slfcont2  slfcont3  slfcont4
##   10   2  age  cnarc1  cnarc2  cnarc3  ctheory1  ctheory2  ctheory3  CRT1  CRT2  CRT3  moralid10  moralid2  moralid3  moralid4  moralid5  moralid6  moralid7  moralid8  moralid9  mcoop1  mcoop2  mcoop3  mcoop4  mcoop5  mcoop6  mcoop7  narc1  narc2  narc3  narc4  narc5  narc6  contact1  contact2  contact3  contact4  contact5  hygiene3  psupport3  political_ideology  happy  slf_ladder  riskperc2  self_esteem  sbelong1  sbelong2  slfcont1  slfcont2  slfcont3  slfcont4
##   10   3  age  cnarc1  cnarc2  cnarc3  ctheory1  ctheory2  ctheory3  CRT1  CRT2  CRT3  moralid10  moralid2  moralid3  moralid4  moralid5  moralid6  moralid7  moralid8  moralid9  mcoop1  mcoop2  mcoop3  mcoop4  mcoop5  mcoop6  mcoop7  narc1  narc2  narc3  narc4  narc5  narc6  contact1  contact2  contact3  contact4  contact5  hygiene3  psupport3  political_ideology  happy  slf_ladder  riskperc2  self_esteem  sbelong1  sbelong2  slfcont1  slfcont2  slfcont3  slfcont4
##   10   4  age  cnarc1  cnarc2  cnarc3  ctheory1  ctheory2  ctheory3  CRT1  CRT2  CRT3  moralid10  moralid2  moralid3  moralid4  moralid5  moralid6  moralid7  moralid8  moralid9  mcoop1  mcoop2  mcoop3  mcoop4  mcoop5  mcoop6  mcoop7  narc1  narc2  narc3  narc4  narc5  narc6  contact1  contact2  contact3  contact4  contact5  hygiene3  psupport3  political_ideology  happy  slf_ladder  riskperc2  self_esteem  sbelong1  sbelong2  slfcont1  slfcont2  slfcont3  slfcont4
##   10   5  age  cnarc1  cnarc2  cnarc3  ctheory1  ctheory2  ctheory3  CRT1  CRT2  CRT3  moralid10  moralid2  moralid3  moralid4  moralid5  moralid6  moralid7  moralid8  moralid9  mcoop1  mcoop2  mcoop3  mcoop4  mcoop5  mcoop6  mcoop7  narc1  narc2  narc3  narc4  narc5  narc6  contact1  contact2  contact3  contact4  contact5  hygiene3  psupport3  political_ideology  happy  slf_ladder  riskperc2  self_esteem  sbelong1  sbelong2  slfcont1  slfcont2  slfcont3  slfcont4
##   10   6  age  cnarc1  cnarc2  cnarc3  ctheory1  ctheory2  ctheory3  CRT1  CRT2  CRT3  moralid10  moralid2  moralid3  moralid4  moralid5  moralid6  moralid7  moralid8  moralid9  mcoop1  mcoop2  mcoop3  mcoop4  mcoop5  mcoop6  mcoop7  narc1  narc2  narc3  narc4  narc5  narc6  contact1  contact2  contact3  contact4  contact5  hygiene3  psupport3  political_ideology  happy  slf_ladder  riskperc2  self_esteem  sbelong1  sbelong2  slfcont1  slfcont2  slfcont3  slfcont4
##   10   7  age  cnarc1  cnarc2  cnarc3  ctheory1  ctheory2  ctheory3  CRT1  CRT2  CRT3  moralid10  moralid2  moralid3  moralid4  moralid5  moralid6  moralid7  moralid8  moralid9  mcoop1  mcoop2  mcoop3  mcoop4  mcoop5  mcoop6  mcoop7  narc1  narc2  narc3  narc4  narc5  narc6  contact1  contact2  contact3  contact4  contact5  hygiene3  psupport3  political_ideology  happy  slf_ladder  riskperc2  self_esteem  sbelong1  sbelong2  slfcont1  slfcont2  slfcont3  slfcont4
##   10   8  age  cnarc1  cnarc2  cnarc3  ctheory1  ctheory2  ctheory3  CRT1  CRT2  CRT3  moralid10  moralid2  moralid3  moralid4  moralid5  moralid6  moralid7  moralid8  moralid9  mcoop1  mcoop2  mcoop3  mcoop4  mcoop5  mcoop6  mcoop7  narc1  narc2  narc3  narc4  narc5  narc6  contact1  contact2  contact3  contact4  contact5  hygiene3  psupport3  political_ideology  happy  slf_ladder  riskperc2  self_esteem  sbelong1  sbelong2  slfcont1  slfcont2  slfcont3  slfcont4
##   10   9  age  cnarc1  cnarc2  cnarc3  ctheory1  ctheory2  ctheory3  CRT1  CRT2  CRT3  moralid10  moralid2  moralid3  moralid4  moralid5  moralid6  moralid7  moralid8  moralid9  mcoop1  mcoop2  mcoop3  mcoop4  mcoop5  mcoop6  mcoop7  narc1  narc2  narc3  narc4  narc5  narc6  contact1  contact2  contact3  contact4  contact5  hygiene3  psupport3  political_ideology  happy  slf_ladder  riskperc2  self_esteem  sbelong1  sbelong2  slfcont1  slfcont2  slfcont3  slfcont4
##   10   10  age  cnarc1  cnarc2  cnarc3  ctheory1  ctheory2  ctheory3  CRT1  CRT2  CRT3  moralid10  moralid2  moralid3  moralid4  moralid5  moralid6  moralid7  moralid8  moralid9  mcoop1  mcoop2  mcoop3  mcoop4  mcoop5  mcoop6  mcoop7  narc1  narc2  narc3  narc4  narc5  narc6  contact1  contact2  contact3  contact4  contact5  hygiene3  psupport3  political_ideology  happy  slf_ladder  riskperc2  self_esteem  sbelong1  sbelong2  slfcont1  slfcont2  slfcont3  slfcont4
```

```
## Warning: Number of logged events: 5003
```

```
## 
##  iter imp variable
##   1   1  cnarc1  cnarc2  cnarc3  CRT1  CRT2  CRT3  employ_status1  health_cond  ladder  marital1  mor_circle  moralid1  moralid10  moralid2  moralid3  moralid4  moralid5  moralid6  moralid7  moralid8  moralid9  mcoop1  mcoop2  mcoop3  mcoop4  mcoop5  mcoop6  mcoop7  narc1  narc2  narc3  narc4  narc5  narc6  nidentity1  nidentity2  omind1  omind2  omind3  omind4  omind5  omind6  contact1  contact2  contact3  contact4  contact5  hygiene2  hygiene4  psupport1  psupport2  psupport3  psupport4  psupport5  political_ideology  happy  riskperc1  riskperc2  self_esteem  sbelong1  sbelong2  sbelong3  sbelong4  tested_positive  optim1  optim2  slfcont1  slfcont2  slfcont3  slfcont4
##   1   2  cnarc1  cnarc2  cnarc3  CRT1  CRT2  CRT3  employ_status1  health_cond  ladder  marital1  mor_circle  moralid1  moralid10  moralid2  moralid3  moralid4  moralid5  moralid6  moralid7  moralid8  moralid9  mcoop1  mcoop2  mcoop3  mcoop4  mcoop5  mcoop6  mcoop7  narc1  narc2  narc3  narc4  narc5  narc6  nidentity1  nidentity2  omind1  omind2  omind3  omind4  omind5  omind6  contact1  contact2  contact3  contact4  contact5  hygiene2  hygiene4  psupport1  psupport2  psupport3  psupport4  psupport5  political_ideology  happy  riskperc1  riskperc2  self_esteem  sbelong1  sbelong2  sbelong3  sbelong4  tested_positive  optim1  optim2  slfcont1  slfcont2  slfcont3  slfcont4
##   1   3  cnarc1  cnarc2  cnarc3  CRT1  CRT2  CRT3  employ_status1  health_cond  ladder  marital1  mor_circle  moralid1  moralid10  moralid2  moralid3  moralid4  moralid5  moralid6  moralid7  moralid8  moralid9  mcoop1  mcoop2  mcoop3  mcoop4  mcoop5  mcoop6  mcoop7  narc1  narc2  narc3  narc4  narc5  narc6  nidentity1  nidentity2  omind1  omind2  omind3  omind4  omind5  omind6  contact1  contact2  contact3  contact4  contact5  hygiene2  hygiene4  psupport1  psupport2  psupport3  psupport4  psupport5  political_ideology  happy  riskperc1  riskperc2  self_esteem  sbelong1  sbelong2  sbelong3  sbelong4  tested_positive  optim1  optim2  slfcont1  slfcont2  slfcont3  slfcont4
##   1   4  cnarc1  cnarc2  cnarc3  CRT1  CRT2  CRT3  employ_status1  health_cond  ladder  marital1  mor_circle  moralid1  moralid10  moralid2  moralid3  moralid4  moralid5  moralid6  moralid7  moralid8  moralid9  mcoop1  mcoop2  mcoop3  mcoop4  mcoop5  mcoop6  mcoop7  narc1  narc2  narc3  narc4  narc5  narc6  nidentity1  nidentity2  omind1  omind2  omind3  omind4  omind5  omind6  contact1  contact2  contact3  contact4  contact5  hygiene2  hygiene4  psupport1  psupport2  psupport3  psupport4  psupport5  political_ideology  happy  riskperc1  riskperc2  self_esteem  sbelong1  sbelong2  sbelong3  sbelong4  tested_positive  optim1  optim2  slfcont1  slfcont2  slfcont3  slfcont4
##   1   5  cnarc1  cnarc2  cnarc3  CRT1  CRT2  CRT3  employ_status1  health_cond  ladder  marital1  mor_circle  moralid1  moralid10  moralid2  moralid3  moralid4  moralid5  moralid6  moralid7  moralid8  moralid9  mcoop1  mcoop2  mcoop3  mcoop4  mcoop5  mcoop6  mcoop7  narc1  narc2  narc3  narc4  narc5  narc6  nidentity1  nidentity2  omind1  omind2  omind3  omind4  omind5  omind6  contact1  contact2  contact3  contact4  contact5  hygiene2  hygiene4  psupport1  psupport2  psupport3  psupport4  psupport5  political_ideology  happy  riskperc1  riskperc2  self_esteem  sbelong1  sbelong2  sbelong3  sbelong4  tested_positive  optim1  optim2  slfcont1  slfcont2  slfcont3  slfcont4
##   1   6  cnarc1  cnarc2  cnarc3  CRT1  CRT2  CRT3  employ_status1  health_cond  ladder  marital1  mor_circle  moralid1  moralid10  moralid2  moralid3  moralid4  moralid5  moralid6  moralid7  moralid8  moralid9  mcoop1  mcoop2  mcoop3  mcoop4  mcoop5  mcoop6  mcoop7  narc1  narc2  narc3  narc4  narc5  narc6  nidentity1  nidentity2  omind1  omind2  omind3  omind4  omind5  omind6  contact1  contact2  contact3  contact4  contact5  hygiene2  hygiene4  psupport1  psupport2  psupport3  psupport4  psupport5  political_ideology  happy  riskperc1  riskperc2  self_esteem  sbelong1  sbelong2  sbelong3  sbelong4  tested_positive  optim1  optim2  slfcont1  slfcont2  slfcont3  slfcont4
##   1   7  cnarc1  cnarc2  cnarc3  CRT1  CRT2  CRT3  employ_status1  health_cond  ladder  marital1  mor_circle  moralid1  moralid10  moralid2  moralid3  moralid4  moralid5  moralid6  moralid7  moralid8  moralid9  mcoop1  mcoop2  mcoop3  mcoop4  mcoop5  mcoop6  mcoop7  narc1  narc2  narc3  narc4  narc5  narc6  nidentity1  nidentity2  omind1  omind2  omind3  omind4  omind5  omind6  contact1  contact2  contact3  contact4  contact5  hygiene2  hygiene4  psupport1  psupport2  psupport3  psupport4  psupport5  political_ideology  happy  riskperc1  riskperc2  self_esteem  sbelong1  sbelong2  sbelong3  sbelong4  tested_positive  optim1  optim2  slfcont1  slfcont2  slfcont3  slfcont4
##   1   8  cnarc1  cnarc2  cnarc3  CRT1  CRT2  CRT3  employ_status1  health_cond  ladder  marital1  mor_circle  moralid1  moralid10  moralid2  moralid3  moralid4  moralid5  moralid6  moralid7  moralid8  moralid9  mcoop1  mcoop2  mcoop3  mcoop4  mcoop5  mcoop6  mcoop7  narc1  narc2  narc3  narc4  narc5  narc6  nidentity1  nidentity2  omind1  omind2  omind3  omind4  omind5  omind6  contact1  contact2  contact3  contact4  contact5  hygiene2  hygiene4  psupport1  psupport2  psupport3  psupport4  psupport5  political_ideology  happy  riskperc1  riskperc2  self_esteem  sbelong1  sbelong2  sbelong3  sbelong4  tested_positive  optim1  optim2  slfcont1  slfcont2  slfcont3  slfcont4
##   1   9  cnarc1  cnarc2  cnarc3  CRT1  CRT2  CRT3  employ_status1  health_cond  ladder  marital1  mor_circle  moralid1  moralid10  moralid2  moralid3  moralid4  moralid5  moralid6  moralid7  moralid8  moralid9  mcoop1  mcoop2  mcoop3  mcoop4  mcoop5  mcoop6  mcoop7  narc1  narc2  narc3  narc4  narc5  narc6  nidentity1  nidentity2  omind1  omind2  omind3  omind4  omind5  omind6  contact1  contact2  contact3  contact4  contact5  hygiene2  hygiene4  psupport1  psupport2  psupport3  psupport4  psupport5  political_ideology  happy  riskperc1  riskperc2  self_esteem  sbelong1  sbelong2  sbelong3  sbelong4  tested_positive  optim1  optim2  slfcont1  slfcont2  slfcont3  slfcont4
##   1   10  cnarc1  cnarc2  cnarc3  CRT1  CRT2  CRT3  employ_status1  health_cond  ladder  marital1  mor_circle  moralid1  moralid10  moralid2  moralid3  moralid4  moralid5  moralid6  moralid7  moralid8  moralid9  mcoop1  mcoop2  mcoop3  mcoop4  mcoop5  mcoop6  mcoop7  narc1  narc2  narc3  narc4  narc5  narc6  nidentity1  nidentity2  omind1  omind2  omind3  omind4  omind5  omind6  contact1  contact2  contact3  contact4  contact5  hygiene2  hygiene4  psupport1  psupport2  psupport3  psupport4  psupport5  political_ideology  happy  riskperc1  riskperc2  self_esteem  sbelong1  sbelong2  sbelong3  sbelong4  tested_positive  optim1  optim2  slfcont1  slfcont2  slfcont3  slfcont4
##   2   1  cnarc1  cnarc2  cnarc3  CRT1  CRT2  CRT3  employ_status1  health_cond  ladder  marital1  mor_circle  moralid1  moralid10  moralid2  moralid3  moralid4  moralid5  moralid6  moralid7  moralid8  moralid9  mcoop1  mcoop2  mcoop3  mcoop4  mcoop5  mcoop6  mcoop7  narc1  narc2  narc3  narc4  narc5  narc6  nidentity1  nidentity2  omind1  omind2  omind3  omind4  omind5  omind6  contact1  contact2  contact3  contact4  contact5  hygiene2  hygiene4  psupport1  psupport2  psupport3  psupport4  psupport5  political_ideology  happy  riskperc1  riskperc2  self_esteem  sbelong1  sbelong2  sbelong3  sbelong4  tested_positive  optim1  optim2  slfcont1  slfcont2  slfcont3  slfcont4
##   2   2  cnarc1  cnarc2  cnarc3  CRT1  CRT2  CRT3  employ_status1  health_cond  ladder  marital1  mor_circle  moralid1  moralid10  moralid2  moralid3  moralid4  moralid5  moralid6  moralid7  moralid8  moralid9  mcoop1  mcoop2  mcoop3  mcoop4  mcoop5  mcoop6  mcoop7  narc1  narc2  narc3  narc4  narc5  narc6  nidentity1  nidentity2  omind1  omind2  omind3  omind4  omind5  omind6  contact1  contact2  contact3  contact4  contact5  hygiene2  hygiene4  psupport1  psupport2  psupport3  psupport4  psupport5  political_ideology  happy  riskperc1  riskperc2  self_esteem  sbelong1  sbelong2  sbelong3  sbelong4  tested_positive  optim1  optim2  slfcont1  slfcont2  slfcont3  slfcont4
##   2   3  cnarc1  cnarc2  cnarc3  CRT1  CRT2  CRT3  employ_status1  health_cond  ladder  marital1  mor_circle  moralid1  moralid10  moralid2  moralid3  moralid4  moralid5  moralid6  moralid7  moralid8  moralid9  mcoop1  mcoop2  mcoop3  mcoop4  mcoop5  mcoop6  mcoop7  narc1  narc2  narc3  narc4  narc5  narc6  nidentity1  nidentity2  omind1  omind2  omind3  omind4  omind5  omind6  contact1  contact2  contact3  contact4  contact5  hygiene2  hygiene4  psupport1  psupport2  psupport3  psupport4  psupport5  political_ideology  happy  riskperc1  riskperc2  self_esteem  sbelong1  sbelong2  sbelong3  sbelong4  tested_positive  optim1  optim2  slfcont1  slfcont2  slfcont3  slfcont4
##   2   4  cnarc1  cnarc2  cnarc3  CRT1  CRT2  CRT3  employ_status1  health_cond  ladder  marital1  mor_circle  moralid1  moralid10  moralid2  moralid3  moralid4  moralid5  moralid6  moralid7  moralid8  moralid9  mcoop1  mcoop2  mcoop3  mcoop4  mcoop5  mcoop6  mcoop7  narc1  narc2  narc3  narc4  narc5  narc6  nidentity1  nidentity2  omind1  omind2  omind3  omind4  omind5  omind6  contact1  contact2  contact3  contact4  contact5  hygiene2  hygiene4  psupport1  psupport2  psupport3  psupport4  psupport5  political_ideology  happy  riskperc1  riskperc2  self_esteem  sbelong1  sbelong2  sbelong3  sbelong4  tested_positive  optim1  optim2  slfcont1  slfcont2  slfcont3  slfcont4
##   2   5  cnarc1  cnarc2  cnarc3  CRT1  CRT2  CRT3  employ_status1  health_cond  ladder  marital1  mor_circle  moralid1  moralid10  moralid2  moralid3  moralid4  moralid5  moralid6  moralid7  moralid8  moralid9  mcoop1  mcoop2  mcoop3  mcoop4  mcoop5  mcoop6  mcoop7  narc1  narc2  narc3  narc4  narc5  narc6  nidentity1  nidentity2  omind1  omind2  omind3  omind4  omind5  omind6  contact1  contact2  contact3  contact4  contact5  hygiene2  hygiene4  psupport1  psupport2  psupport3  psupport4  psupport5  political_ideology  happy  riskperc1  riskperc2  self_esteem  sbelong1  sbelong2  sbelong3  sbelong4  tested_positive  optim1  optim2  slfcont1  slfcont2  slfcont3  slfcont4
##   2   6  cnarc1  cnarc2  cnarc3  CRT1  CRT2  CRT3  employ_status1  health_cond  ladder  marital1  mor_circle  moralid1  moralid10  moralid2  moralid3  moralid4  moralid5  moralid6  moralid7  moralid8  moralid9  mcoop1  mcoop2  mcoop3  mcoop4  mcoop5  mcoop6  mcoop7  narc1  narc2  narc3  narc4  narc5  narc6  nidentity1  nidentity2  omind1  omind2  omind3  omind4  omind5  omind6  contact1  contact2  contact3  contact4  contact5  hygiene2  hygiene4  psupport1  psupport2  psupport3  psupport4  psupport5  political_ideology  happy  riskperc1  riskperc2  self_esteem  sbelong1  sbelong2  sbelong3  sbelong4  tested_positive  optim1  optim2  slfcont1  slfcont2  slfcont3  slfcont4
##   2   7  cnarc1  cnarc2  cnarc3  CRT1  CRT2  CRT3  employ_status1  health_cond  ladder  marital1  mor_circle  moralid1  moralid10  moralid2  moralid3  moralid4  moralid5  moralid6  moralid7  moralid8  moralid9  mcoop1  mcoop2  mcoop3  mcoop4  mcoop5  mcoop6  mcoop7  narc1  narc2  narc3  narc4  narc5  narc6  nidentity1  nidentity2  omind1  omind2  omind3  omind4  omind5  omind6  contact1  contact2  contact3  contact4  contact5  hygiene2  hygiene4  psupport1  psupport2  psupport3  psupport4  psupport5  political_ideology  happy  riskperc1  riskperc2  self_esteem  sbelong1  sbelong2  sbelong3  sbelong4  tested_positive  optim1  optim2  slfcont1  slfcont2  slfcont3  slfcont4
##   2   8  cnarc1  cnarc2  cnarc3  CRT1  CRT2  CRT3  employ_status1  health_cond  ladder  marital1  mor_circle  moralid1  moralid10  moralid2  moralid3  moralid4  moralid5  moralid6  moralid7  moralid8  moralid9  mcoop1  mcoop2  mcoop3  mcoop4  mcoop5  mcoop6  mcoop7  narc1  narc2  narc3  narc4  narc5  narc6  nidentity1  nidentity2  omind1  omind2  omind3  omind4  omind5  omind6  contact1  contact2  contact3  contact4  contact5  hygiene2  hygiene4  psupport1  psupport2  psupport3  psupport4  psupport5  political_ideology  happy  riskperc1  riskperc2  self_esteem  sbelong1  sbelong2  sbelong3  sbelong4  tested_positive  optim1  optim2  slfcont1  slfcont2  slfcont3  slfcont4
##   2   9  cnarc1  cnarc2  cnarc3  CRT1  CRT2  CRT3  employ_status1  health_cond  ladder  marital1  mor_circle  moralid1  moralid10  moralid2  moralid3  moralid4  moralid5  moralid6  moralid7  moralid8  moralid9  mcoop1  mcoop2  mcoop3  mcoop4  mcoop5  mcoop6  mcoop7  narc1  narc2  narc3  narc4  narc5  narc6  nidentity1  nidentity2  omind1  omind2  omind3  omind4  omind5  omind6  contact1  contact2  contact3  contact4  contact5  hygiene2  hygiene4  psupport1  psupport2  psupport3  psupport4  psupport5  political_ideology  happy  riskperc1  riskperc2  self_esteem  sbelong1  sbelong2  sbelong3  sbelong4  tested_positive  optim1  optim2  slfcont1  slfcont2  slfcont3  slfcont4
##   2   10  cnarc1  cnarc2  cnarc3  CRT1  CRT2  CRT3  employ_status1  health_cond  ladder  marital1  mor_circle  moralid1  moralid10  moralid2  moralid3  moralid4  moralid5  moralid6  moralid7  moralid8  moralid9  mcoop1  mcoop2  mcoop3  mcoop4  mcoop5  mcoop6  mcoop7  narc1  narc2  narc3  narc4  narc5  narc6  nidentity1  nidentity2  omind1  omind2  omind3  omind4  omind5  omind6  contact1  contact2  contact3  contact4  contact5  hygiene2  hygiene4  psupport1  psupport2  psupport3  psupport4  psupport5  political_ideology  happy  riskperc1  riskperc2  self_esteem  sbelong1  sbelong2  sbelong3  sbelong4  tested_positive  optim1  optim2  slfcont1  slfcont2  slfcont3  slfcont4
##   3   1  cnarc1  cnarc2  cnarc3  CRT1  CRT2  CRT3  employ_status1  health_cond  ladder  marital1  mor_circle  moralid1  moralid10  moralid2  moralid3  moralid4  moralid5  moralid6  moralid7  moralid8  moralid9  mcoop1  mcoop2  mcoop3  mcoop4  mcoop5  mcoop6  mcoop7  narc1  narc2  narc3  narc4  narc5  narc6  nidentity1  nidentity2  omind1  omind2  omind3  omind4  omind5  omind6  contact1  contact2  contact3  contact4  contact5  hygiene2  hygiene4  psupport1  psupport2  psupport3  psupport4  psupport5  political_ideology  happy  riskperc1  riskperc2  self_esteem  sbelong1  sbelong2  sbelong3  sbelong4  tested_positive  optim1  optim2  slfcont1  slfcont2  slfcont3  slfcont4
##   3   2  cnarc1  cnarc2  cnarc3  CRT1  CRT2  CRT3  employ_status1  health_cond  ladder  marital1  mor_circle  moralid1  moralid10  moralid2  moralid3  moralid4  moralid5  moralid6  moralid7  moralid8  moralid9  mcoop1  mcoop2  mcoop3  mcoop4  mcoop5  mcoop6  mcoop7  narc1  narc2  narc3  narc4  narc5  narc6  nidentity1  nidentity2  omind1  omind2  omind3  omind4  omind5  omind6  contact1  contact2  contact3  contact4  contact5  hygiene2  hygiene4  psupport1  psupport2  psupport3  psupport4  psupport5  political_ideology  happy  riskperc1  riskperc2  self_esteem  sbelong1  sbelong2  sbelong3  sbelong4  tested_positive  optim1  optim2  slfcont1  slfcont2  slfcont3  slfcont4
##   3   3  cnarc1  cnarc2  cnarc3  CRT1  CRT2  CRT3  employ_status1  health_cond  ladder  marital1  mor_circle  moralid1  moralid10  moralid2  moralid3  moralid4  moralid5  moralid6  moralid7  moralid8  moralid9  mcoop1  mcoop2  mcoop3  mcoop4  mcoop5  mcoop6  mcoop7  narc1  narc2  narc3  narc4  narc5  narc6  nidentity1  nidentity2  omind1  omind2  omind3  omind4  omind5  omind6  contact1  contact2  contact3  contact4  contact5  hygiene2  hygiene4  psupport1  psupport2  psupport3  psupport4  psupport5  political_ideology  happy  riskperc1  riskperc2  self_esteem  sbelong1  sbelong2  sbelong3  sbelong4  tested_positive  optim1  optim2  slfcont1  slfcont2  slfcont3  slfcont4
##   3   4  cnarc1  cnarc2  cnarc3  CRT1  CRT2  CRT3  employ_status1  health_cond  ladder  marital1  mor_circle  moralid1  moralid10  moralid2  moralid3  moralid4  moralid5  moralid6  moralid7  moralid8  moralid9  mcoop1  mcoop2  mcoop3  mcoop4  mcoop5  mcoop6  mcoop7  narc1  narc2  narc3  narc4  narc5  narc6  nidentity1  nidentity2  omind1  omind2  omind3  omind4  omind5  omind6  contact1  contact2  contact3  contact4  contact5  hygiene2  hygiene4  psupport1  psupport2  psupport3  psupport4  psupport5  political_ideology  happy  riskperc1  riskperc2  self_esteem  sbelong1  sbelong2  sbelong3  sbelong4  tested_positive  optim1  optim2  slfcont1  slfcont2  slfcont3  slfcont4
##   3   5  cnarc1  cnarc2  cnarc3  CRT1  CRT2  CRT3  employ_status1  health_cond  ladder  marital1  mor_circle  moralid1  moralid10  moralid2  moralid3  moralid4  moralid5  moralid6  moralid7  moralid8  moralid9  mcoop1  mcoop2  mcoop3  mcoop4  mcoop5  mcoop6  mcoop7  narc1  narc2  narc3  narc4  narc5  narc6  nidentity1  nidentity2  omind1  omind2  omind3  omind4  omind5  omind6  contact1  contact2  contact3  contact4  contact5  hygiene2  hygiene4  psupport1  psupport2  psupport3  psupport4  psupport5  political_ideology  happy  riskperc1  riskperc2  self_esteem  sbelong1  sbelong2  sbelong3  sbelong4  tested_positive  optim1  optim2  slfcont1  slfcont2  slfcont3  slfcont4
##   3   6  cnarc1  cnarc2  cnarc3  CRT1  CRT2  CRT3  employ_status1  health_cond  ladder  marital1  mor_circle  moralid1  moralid10  moralid2  moralid3  moralid4  moralid5  moralid6  moralid7  moralid8  moralid9  mcoop1  mcoop2  mcoop3  mcoop4  mcoop5  mcoop6  mcoop7  narc1  narc2  narc3  narc4  narc5  narc6  nidentity1  nidentity2  omind1  omind2  omind3  omind4  omind5  omind6  contact1  contact2  contact3  contact4  contact5  hygiene2  hygiene4  psupport1  psupport2  psupport3  psupport4  psupport5  political_ideology  happy  riskperc1  riskperc2  self_esteem  sbelong1  sbelong2  sbelong3  sbelong4  tested_positive  optim1  optim2  slfcont1  slfcont2  slfcont3  slfcont4
##   3   7  cnarc1  cnarc2  cnarc3  CRT1  CRT2  CRT3  employ_status1  health_cond  ladder  marital1  mor_circle  moralid1  moralid10  moralid2  moralid3  moralid4  moralid5  moralid6  moralid7  moralid8  moralid9  mcoop1  mcoop2  mcoop3  mcoop4  mcoop5  mcoop6  mcoop7  narc1  narc2  narc3  narc4  narc5  narc6  nidentity1  nidentity2  omind1  omind2  omind3  omind4  omind5  omind6  contact1  contact2  contact3  contact4  contact5  hygiene2  hygiene4  psupport1  psupport2  psupport3  psupport4  psupport5  political_ideology  happy  riskperc1  riskperc2  self_esteem  sbelong1  sbelong2  sbelong3  sbelong4  tested_positive  optim1  optim2  slfcont1  slfcont2  slfcont3  slfcont4
##   3   8  cnarc1  cnarc2  cnarc3  CRT1  CRT2  CRT3  employ_status1  health_cond  ladder  marital1  mor_circle  moralid1  moralid10  moralid2  moralid3  moralid4  moralid5  moralid6  moralid7  moralid8  moralid9  mcoop1  mcoop2  mcoop3  mcoop4  mcoop5  mcoop6  mcoop7  narc1  narc2  narc3  narc4  narc5  narc6  nidentity1  nidentity2  omind1  omind2  omind3  omind4  omind5  omind6  contact1  contact2  contact3  contact4  contact5  hygiene2  hygiene4  psupport1  psupport2  psupport3  psupport4  psupport5  political_ideology  happy  riskperc1  riskperc2  self_esteem  sbelong1  sbelong2  sbelong3  sbelong4  tested_positive  optim1  optim2  slfcont1  slfcont2  slfcont3  slfcont4
##   3   9  cnarc1  cnarc2  cnarc3  CRT1  CRT2  CRT3  employ_status1  health_cond  ladder  marital1  mor_circle  moralid1  moralid10  moralid2  moralid3  moralid4  moralid5  moralid6  moralid7  moralid8  moralid9  mcoop1  mcoop2  mcoop3  mcoop4  mcoop5  mcoop6  mcoop7  narc1  narc2  narc3  narc4  narc5  narc6  nidentity1  nidentity2  omind1  omind2  omind3  omind4  omind5  omind6  contact1  contact2  contact3  contact4  contact5  hygiene2  hygiene4  psupport1  psupport2  psupport3  psupport4  psupport5  political_ideology  happy  riskperc1  riskperc2  self_esteem  sbelong1  sbelong2  sbelong3  sbelong4  tested_positive  optim1  optim2  slfcont1  slfcont2  slfcont3  slfcont4
##   3   10  cnarc1  cnarc2  cnarc3  CRT1  CRT2  CRT3  employ_status1  health_cond  ladder  marital1  mor_circle  moralid1  moralid10  moralid2  moralid3  moralid4  moralid5  moralid6  moralid7  moralid8  moralid9  mcoop1  mcoop2  mcoop3  mcoop4  mcoop5  mcoop6  mcoop7  narc1  narc2  narc3  narc4  narc5  narc6  nidentity1  nidentity2  omind1  omind2  omind3  omind4  omind5  omind6  contact1  contact2  contact3  contact4  contact5  hygiene2  hygiene4  psupport1  psupport2  psupport3  psupport4  psupport5  political_ideology  happy  riskperc1  riskperc2  self_esteem  sbelong1  sbelong2  sbelong3  sbelong4  tested_positive  optim1  optim2  slfcont1  slfcont2  slfcont3  slfcont4
##   4   1  cnarc1  cnarc2  cnarc3  CRT1  CRT2  CRT3  employ_status1  health_cond  ladder  marital1  mor_circle  moralid1  moralid10  moralid2  moralid3  moralid4  moralid5  moralid6  moralid7  moralid8  moralid9  mcoop1  mcoop2  mcoop3  mcoop4  mcoop5  mcoop6  mcoop7  narc1  narc2  narc3  narc4  narc5  narc6  nidentity1  nidentity2  omind1  omind2  omind3  omind4  omind5  omind6  contact1  contact2  contact3  contact4  contact5  hygiene2  hygiene4  psupport1  psupport2  psupport3  psupport4  psupport5  political_ideology  happy  riskperc1  riskperc2  self_esteem  sbelong1  sbelong2  sbelong3  sbelong4  tested_positive  optim1  optim2  slfcont1  slfcont2  slfcont3  slfcont4
##   4   2  cnarc1  cnarc2  cnarc3  CRT1  CRT2  CRT3  employ_status1  health_cond  ladder  marital1  mor_circle  moralid1  moralid10  moralid2  moralid3  moralid4  moralid5  moralid6  moralid7  moralid8  moralid9  mcoop1  mcoop2  mcoop3  mcoop4  mcoop5  mcoop6  mcoop7  narc1  narc2  narc3  narc4  narc5  narc6  nidentity1  nidentity2  omind1  omind2  omind3  omind4  omind5  omind6  contact1  contact2  contact3  contact4  contact5  hygiene2  hygiene4  psupport1  psupport2  psupport3  psupport4  psupport5  political_ideology  happy  riskperc1  riskperc2  self_esteem  sbelong1  sbelong2  sbelong3  sbelong4  tested_positive  optim1  optim2  slfcont1  slfcont2  slfcont3  slfcont4
##   4   3  cnarc1  cnarc2  cnarc3  CRT1  CRT2  CRT3  employ_status1  health_cond  ladder  marital1  mor_circle  moralid1  moralid10  moralid2  moralid3  moralid4  moralid5  moralid6  moralid7  moralid8  moralid9  mcoop1  mcoop2  mcoop3  mcoop4  mcoop5  mcoop6  mcoop7  narc1  narc2  narc3  narc4  narc5  narc6  nidentity1  nidentity2  omind1  omind2  omind3  omind4  omind5  omind6  contact1  contact2  contact3  contact4  contact5  hygiene2  hygiene4  psupport1  psupport2  psupport3  psupport4  psupport5  political_ideology  happy  riskperc1  riskperc2  self_esteem  sbelong1  sbelong2  sbelong3  sbelong4  tested_positive  optim1  optim2  slfcont1  slfcont2  slfcont3  slfcont4
##   4   4  cnarc1  cnarc2  cnarc3  CRT1  CRT2  CRT3  employ_status1  health_cond  ladder  marital1  mor_circle  moralid1  moralid10  moralid2  moralid3  moralid4  moralid5  moralid6  moralid7  moralid8  moralid9  mcoop1  mcoop2  mcoop3  mcoop4  mcoop5  mcoop6  mcoop7  narc1  narc2  narc3  narc4  narc5  narc6  nidentity1  nidentity2  omind1  omind2  omind3  omind4  omind5  omind6  contact1  contact2  contact3  contact4  contact5  hygiene2  hygiene4  psupport1  psupport2  psupport3  psupport4  psupport5  political_ideology  happy  riskperc1  riskperc2  self_esteem  sbelong1  sbelong2  sbelong3  sbelong4  tested_positive  optim1  optim2  slfcont1  slfcont2  slfcont3  slfcont4
##   4   5  cnarc1  cnarc2  cnarc3  CRT1  CRT2  CRT3  employ_status1  health_cond  ladder  marital1  mor_circle  moralid1  moralid10  moralid2  moralid3  moralid4  moralid5  moralid6  moralid7  moralid8  moralid9  mcoop1  mcoop2  mcoop3  mcoop4  mcoop5  mcoop6  mcoop7  narc1  narc2  narc3  narc4  narc5  narc6  nidentity1  nidentity2  omind1  omind2  omind3  omind4  omind5  omind6  contact1  contact2  contact3  contact4  contact5  hygiene2  hygiene4  psupport1  psupport2  psupport3  psupport4  psupport5  political_ideology  happy  riskperc1  riskperc2  self_esteem  sbelong1  sbelong2  sbelong3  sbelong4  tested_positive  optim1  optim2  slfcont1  slfcont2  slfcont3  slfcont4
##   4   6  cnarc1  cnarc2  cnarc3  CRT1  CRT2  CRT3  employ_status1  health_cond  ladder  marital1  mor_circle  moralid1  moralid10  moralid2  moralid3  moralid4  moralid5  moralid6  moralid7  moralid8  moralid9  mcoop1  mcoop2  mcoop3  mcoop4  mcoop5  mcoop6  mcoop7  narc1  narc2  narc3  narc4  narc5  narc6  nidentity1  nidentity2  omind1  omind2  omind3  omind4  omind5  omind6  contact1  contact2  contact3  contact4  contact5  hygiene2  hygiene4  psupport1  psupport2  psupport3  psupport4  psupport5  political_ideology  happy  riskperc1  riskperc2  self_esteem  sbelong1  sbelong2  sbelong3  sbelong4  tested_positive  optim1  optim2  slfcont1  slfcont2  slfcont3  slfcont4
##   4   7  cnarc1  cnarc2  cnarc3  CRT1  CRT2  CRT3  employ_status1  health_cond  ladder  marital1  mor_circle  moralid1  moralid10  moralid2  moralid3  moralid4  moralid5  moralid6  moralid7  moralid8  moralid9  mcoop1  mcoop2  mcoop3  mcoop4  mcoop5  mcoop6  mcoop7  narc1  narc2  narc3  narc4  narc5  narc6  nidentity1  nidentity2  omind1  omind2  omind3  omind4  omind5  omind6  contact1  contact2  contact3  contact4  contact5  hygiene2  hygiene4  psupport1  psupport2  psupport3  psupport4  psupport5  political_ideology  happy  riskperc1  riskperc2  self_esteem  sbelong1  sbelong2  sbelong3  sbelong4  tested_positive  optim1  optim2  slfcont1  slfcont2  slfcont3  slfcont4
##   4   8  cnarc1  cnarc2  cnarc3  CRT1  CRT2  CRT3  employ_status1  health_cond  ladder  marital1  mor_circle  moralid1  moralid10  moralid2  moralid3  moralid4  moralid5  moralid6  moralid7  moralid8  moralid9  mcoop1  mcoop2  mcoop3  mcoop4  mcoop5  mcoop6  mcoop7  narc1  narc2  narc3  narc4  narc5  narc6  nidentity1  nidentity2  omind1  omind2  omind3  omind4  omind5  omind6  contact1  contact2  contact3  contact4  contact5  hygiene2  hygiene4  psupport1  psupport2  psupport3  psupport4  psupport5  political_ideology  happy  riskperc1  riskperc2  self_esteem  sbelong1  sbelong2  sbelong3  sbelong4  tested_positive  optim1  optim2  slfcont1  slfcont2  slfcont3  slfcont4
##   4   9  cnarc1  cnarc2  cnarc3  CRT1  CRT2  CRT3  employ_status1  health_cond  ladder  marital1  mor_circle  moralid1  moralid10  moralid2  moralid3  moralid4  moralid5  moralid6  moralid7  moralid8  moralid9  mcoop1  mcoop2  mcoop3  mcoop4  mcoop5  mcoop6  mcoop7  narc1  narc2  narc3  narc4  narc5  narc6  nidentity1  nidentity2  omind1  omind2  omind3  omind4  omind5  omind6  contact1  contact2  contact3  contact4  contact5  hygiene2  hygiene4  psupport1  psupport2  psupport3  psupport4  psupport5  political_ideology  happy  riskperc1  riskperc2  self_esteem  sbelong1  sbelong2  sbelong3  sbelong4  tested_positive  optim1  optim2  slfcont1  slfcont2  slfcont3  slfcont4
##   4   10  cnarc1  cnarc2  cnarc3  CRT1  CRT2  CRT3  employ_status1  health_cond  ladder  marital1  mor_circle  moralid1  moralid10  moralid2  moralid3  moralid4  moralid5  moralid6  moralid7  moralid8  moralid9  mcoop1  mcoop2  mcoop3  mcoop4  mcoop5  mcoop6  mcoop7  narc1  narc2  narc3  narc4  narc5  narc6  nidentity1  nidentity2  omind1  omind2  omind3  omind4  omind5  omind6  contact1  contact2  contact3  contact4  contact5  hygiene2  hygiene4  psupport1  psupport2  psupport3  psupport4  psupport5  political_ideology  happy  riskperc1  riskperc2  self_esteem  sbelong1  sbelong2  sbelong3  sbelong4  tested_positive  optim1  optim2  slfcont1  slfcont2  slfcont3  slfcont4
##   5   1  cnarc1  cnarc2  cnarc3  CRT1  CRT2  CRT3  employ_status1  health_cond  ladder  marital1  mor_circle  moralid1  moralid10  moralid2  moralid3  moralid4  moralid5  moralid6  moralid7  moralid8  moralid9  mcoop1  mcoop2  mcoop3  mcoop4  mcoop5  mcoop6  mcoop7  narc1  narc2  narc3  narc4  narc5  narc6  nidentity1  nidentity2  omind1  omind2  omind3  omind4  omind5  omind6  contact1  contact2  contact3  contact4  contact5  hygiene2  hygiene4  psupport1  psupport2  psupport3  psupport4  psupport5  political_ideology  happy  riskperc1  riskperc2  self_esteem  sbelong1  sbelong2  sbelong3  sbelong4  tested_positive  optim1  optim2  slfcont1  slfcont2  slfcont3  slfcont4
##   5   2  cnarc1  cnarc2  cnarc3  CRT1  CRT2  CRT3  employ_status1  health_cond  ladder  marital1  mor_circle  moralid1  moralid10  moralid2  moralid3  moralid4  moralid5  moralid6  moralid7  moralid8  moralid9  mcoop1  mcoop2  mcoop3  mcoop4  mcoop5  mcoop6  mcoop7  narc1  narc2  narc3  narc4  narc5  narc6  nidentity1  nidentity2  omind1  omind2  omind3  omind4  omind5  omind6  contact1  contact2  contact3  contact4  contact5  hygiene2  hygiene4  psupport1  psupport2  psupport3  psupport4  psupport5  political_ideology  happy  riskperc1  riskperc2  self_esteem  sbelong1  sbelong2  sbelong3  sbelong4  tested_positive  optim1  optim2  slfcont1  slfcont2  slfcont3  slfcont4
##   5   3  cnarc1  cnarc2  cnarc3  CRT1  CRT2  CRT3  employ_status1  health_cond  ladder  marital1  mor_circle  moralid1  moralid10  moralid2  moralid3  moralid4  moralid5  moralid6  moralid7  moralid8  moralid9  mcoop1  mcoop2  mcoop3  mcoop4  mcoop5  mcoop6  mcoop7  narc1  narc2  narc3  narc4  narc5  narc6  nidentity1  nidentity2  omind1  omind2  omind3  omind4  omind5  omind6  contact1  contact2  contact3  contact4  contact5  hygiene2  hygiene4  psupport1  psupport2  psupport3  psupport4  psupport5  political_ideology  happy  riskperc1  riskperc2  self_esteem  sbelong1  sbelong2  sbelong3  sbelong4  tested_positive  optim1  optim2  slfcont1  slfcont2  slfcont3  slfcont4
##   5   4  cnarc1  cnarc2  cnarc3  CRT1  CRT2  CRT3  employ_status1  health_cond  ladder  marital1  mor_circle  moralid1  moralid10  moralid2  moralid3  moralid4  moralid5  moralid6  moralid7  moralid8  moralid9  mcoop1  mcoop2  mcoop3  mcoop4  mcoop5  mcoop6  mcoop7  narc1  narc2  narc3  narc4  narc5  narc6  nidentity1  nidentity2  omind1  omind2  omind3  omind4  omind5  omind6  contact1  contact2  contact3  contact4  contact5  hygiene2  hygiene4  psupport1  psupport2  psupport3  psupport4  psupport5  political_ideology  happy  riskperc1  riskperc2  self_esteem  sbelong1  sbelong2  sbelong3  sbelong4  tested_positive  optim1  optim2  slfcont1  slfcont2  slfcont3  slfcont4
##   5   5  cnarc1  cnarc2  cnarc3  CRT1  CRT2  CRT3  employ_status1  health_cond  ladder  marital1  mor_circle  moralid1  moralid10  moralid2  moralid3  moralid4  moralid5  moralid6  moralid7  moralid8  moralid9  mcoop1  mcoop2  mcoop3  mcoop4  mcoop5  mcoop6  mcoop7  narc1  narc2  narc3  narc4  narc5  narc6  nidentity1  nidentity2  omind1  omind2  omind3  omind4  omind5  omind6  contact1  contact2  contact3  contact4  contact5  hygiene2  hygiene4  psupport1  psupport2  psupport3  psupport4  psupport5  political_ideology  happy  riskperc1  riskperc2  self_esteem  sbelong1  sbelong2  sbelong3  sbelong4  tested_positive  optim1  optim2  slfcont1  slfcont2  slfcont3  slfcont4
##   5   6  cnarc1  cnarc2  cnarc3  CRT1  CRT2  CRT3  employ_status1  health_cond  ladder  marital1  mor_circle  moralid1  moralid10  moralid2  moralid3  moralid4  moralid5  moralid6  moralid7  moralid8  moralid9  mcoop1  mcoop2  mcoop3  mcoop4  mcoop5  mcoop6  mcoop7  narc1  narc2  narc3  narc4  narc5  narc6  nidentity1  nidentity2  omind1  omind2  omind3  omind4  omind5  omind6  contact1  contact2  contact3  contact4  contact5  hygiene2  hygiene4  psupport1  psupport2  psupport3  psupport4  psupport5  political_ideology  happy  riskperc1  riskperc2  self_esteem  sbelong1  sbelong2  sbelong3  sbelong4  tested_positive  optim1  optim2  slfcont1  slfcont2  slfcont3  slfcont4
##   5   7  cnarc1  cnarc2  cnarc3  CRT1  CRT2  CRT3  employ_status1  health_cond  ladder  marital1  mor_circle  moralid1  moralid10  moralid2  moralid3  moralid4  moralid5  moralid6  moralid7  moralid8  moralid9  mcoop1  mcoop2  mcoop3  mcoop4  mcoop5  mcoop6  mcoop7  narc1  narc2  narc3  narc4  narc5  narc6  nidentity1  nidentity2  omind1  omind2  omind3  omind4  omind5  omind6  contact1  contact2  contact3  contact4  contact5  hygiene2  hygiene4  psupport1  psupport2  psupport3  psupport4  psupport5  political_ideology  happy  riskperc1  riskperc2  self_esteem  sbelong1  sbelong2  sbelong3  sbelong4  tested_positive  optim1  optim2  slfcont1  slfcont2  slfcont3  slfcont4
##   5   8  cnarc1  cnarc2  cnarc3  CRT1  CRT2  CRT3  employ_status1  health_cond  ladder  marital1  mor_circle  moralid1  moralid10  moralid2  moralid3  moralid4  moralid5  moralid6  moralid7  moralid8  moralid9  mcoop1  mcoop2  mcoop3  mcoop4  mcoop5  mcoop6  mcoop7  narc1  narc2  narc3  narc4  narc5  narc6  nidentity1  nidentity2  omind1  omind2  omind3  omind4  omind5  omind6  contact1  contact2  contact3  contact4  contact5  hygiene2  hygiene4  psupport1  psupport2  psupport3  psupport4  psupport5  political_ideology  happy  riskperc1  riskperc2  self_esteem  sbelong1  sbelong2  sbelong3  sbelong4  tested_positive  optim1  optim2  slfcont1  slfcont2  slfcont3  slfcont4
##   5   9  cnarc1  cnarc2  cnarc3  CRT1  CRT2  CRT3  employ_status1  health_cond  ladder  marital1  mor_circle  moralid1  moralid10  moralid2  moralid3  moralid4  moralid5  moralid6  moralid7  moralid8  moralid9  mcoop1  mcoop2  mcoop3  mcoop4  mcoop5  mcoop6  mcoop7  narc1  narc2  narc3  narc4  narc5  narc6  nidentity1  nidentity2  omind1  omind2  omind3  omind4  omind5  omind6  contact1  contact2  contact3  contact4  contact5  hygiene2  hygiene4  psupport1  psupport2  psupport3  psupport4  psupport5  political_ideology  happy  riskperc1  riskperc2  self_esteem  sbelong1  sbelong2  sbelong3  sbelong4  tested_positive  optim1  optim2  slfcont1  slfcont2  slfcont3  slfcont4
##   5   10  cnarc1  cnarc2  cnarc3  CRT1  CRT2  CRT3  employ_status1  health_cond  ladder  marital1  mor_circle  moralid1  moralid10  moralid2  moralid3  moralid4  moralid5  moralid6  moralid7  moralid8  moralid9  mcoop1  mcoop2  mcoop3  mcoop4  mcoop5  mcoop6  mcoop7  narc1  narc2  narc3  narc4  narc5  narc6  nidentity1  nidentity2  omind1  omind2  omind3  omind4  omind5  omind6  contact1  contact2  contact3  contact4  contact5  hygiene2  hygiene4  psupport1  psupport2  psupport3  psupport4  psupport5  political_ideology  happy  riskperc1  riskperc2  self_esteem  sbelong1  sbelong2  sbelong3  sbelong4  tested_positive  optim1  optim2  slfcont1  slfcont2  slfcont3  slfcont4
##   6   1  cnarc1  cnarc2  cnarc3  CRT1  CRT2  CRT3  employ_status1  health_cond  ladder  marital1  mor_circle  moralid1  moralid10  moralid2  moralid3  moralid4  moralid5  moralid6  moralid7  moralid8  moralid9  mcoop1  mcoop2  mcoop3  mcoop4  mcoop5  mcoop6  mcoop7  narc1  narc2  narc3  narc4  narc5  narc6  nidentity1  nidentity2  omind1  omind2  omind3  omind4  omind5  omind6  contact1  contact2  contact3  contact4  contact5  hygiene2  hygiene4  psupport1  psupport2  psupport3  psupport4  psupport5  political_ideology  happy  riskperc1  riskperc2  self_esteem  sbelong1  sbelong2  sbelong3  sbelong4  tested_positive  optim1  optim2  slfcont1  slfcont2  slfcont3  slfcont4
##   6   2  cnarc1  cnarc2  cnarc3  CRT1  CRT2  CRT3  employ_status1  health_cond  ladder  marital1  mor_circle  moralid1  moralid10  moralid2  moralid3  moralid4  moralid5  moralid6  moralid7  moralid8  moralid9  mcoop1  mcoop2  mcoop3  mcoop4  mcoop5  mcoop6  mcoop7  narc1  narc2  narc3  narc4  narc5  narc6  nidentity1  nidentity2  omind1  omind2  omind3  omind4  omind5  omind6  contact1  contact2  contact3  contact4  contact5  hygiene2  hygiene4  psupport1  psupport2  psupport3  psupport4  psupport5  political_ideology  happy  riskperc1  riskperc2  self_esteem  sbelong1  sbelong2  sbelong3  sbelong4  tested_positive  optim1  optim2  slfcont1  slfcont2  slfcont3  slfcont4
##   6   3  cnarc1  cnarc2  cnarc3  CRT1  CRT2  CRT3  employ_status1  health_cond  ladder  marital1  mor_circle  moralid1  moralid10  moralid2  moralid3  moralid4  moralid5  moralid6  moralid7  moralid8  moralid9  mcoop1  mcoop2  mcoop3  mcoop4  mcoop5  mcoop6  mcoop7  narc1  narc2  narc3  narc4  narc5  narc6  nidentity1  nidentity2  omind1  omind2  omind3  omind4  omind5  omind6  contact1  contact2  contact3  contact4  contact5  hygiene2  hygiene4  psupport1  psupport2  psupport3  psupport4  psupport5  political_ideology  happy  riskperc1  riskperc2  self_esteem  sbelong1  sbelong2  sbelong3  sbelong4  tested_positive  optim1  optim2  slfcont1  slfcont2  slfcont3  slfcont4
##   6   4  cnarc1  cnarc2  cnarc3  CRT1  CRT2  CRT3  employ_status1  health_cond  ladder  marital1  mor_circle  moralid1  moralid10  moralid2  moralid3  moralid4  moralid5  moralid6  moralid7  moralid8  moralid9  mcoop1  mcoop2  mcoop3  mcoop4  mcoop5  mcoop6  mcoop7  narc1  narc2  narc3  narc4  narc5  narc6  nidentity1  nidentity2  omind1  omind2  omind3  omind4  omind5  omind6  contact1  contact2  contact3  contact4  contact5  hygiene2  hygiene4  psupport1  psupport2  psupport3  psupport4  psupport5  political_ideology  happy  riskperc1  riskperc2  self_esteem  sbelong1  sbelong2  sbelong3  sbelong4  tested_positive  optim1  optim2  slfcont1  slfcont2  slfcont3  slfcont4
##   6   5  cnarc1  cnarc2  cnarc3  CRT1  CRT2  CRT3  employ_status1  health_cond  ladder  marital1  mor_circle  moralid1  moralid10  moralid2  moralid3  moralid4  moralid5  moralid6  moralid7  moralid8  moralid9  mcoop1  mcoop2  mcoop3  mcoop4  mcoop5  mcoop6  mcoop7  narc1  narc2  narc3  narc4  narc5  narc6  nidentity1  nidentity2  omind1  omind2  omind3  omind4  omind5  omind6  contact1  contact2  contact3  contact4  contact5  hygiene2  hygiene4  psupport1  psupport2  psupport3  psupport4  psupport5  political_ideology  happy  riskperc1  riskperc2  self_esteem  sbelong1  sbelong2  sbelong3  sbelong4  tested_positive  optim1  optim2  slfcont1  slfcont2  slfcont3  slfcont4
##   6   6  cnarc1  cnarc2  cnarc3  CRT1  CRT2  CRT3  employ_status1  health_cond  ladder  marital1  mor_circle  moralid1  moralid10  moralid2  moralid3  moralid4  moralid5  moralid6  moralid7  moralid8  moralid9  mcoop1  mcoop2  mcoop3  mcoop4  mcoop5  mcoop6  mcoop7  narc1  narc2  narc3  narc4  narc5  narc6  nidentity1  nidentity2  omind1  omind2  omind3  omind4  omind5  omind6  contact1  contact2  contact3  contact4  contact5  hygiene2  hygiene4  psupport1  psupport2  psupport3  psupport4  psupport5  political_ideology  happy  riskperc1  riskperc2  self_esteem  sbelong1  sbelong2  sbelong3  sbelong4  tested_positive  optim1  optim2  slfcont1  slfcont2  slfcont3  slfcont4
##   6   7  cnarc1  cnarc2  cnarc3  CRT1  CRT2  CRT3  employ_status1  health_cond  ladder  marital1  mor_circle  moralid1  moralid10  moralid2  moralid3  moralid4  moralid5  moralid6  moralid7  moralid8  moralid9  mcoop1  mcoop2  mcoop3  mcoop4  mcoop5  mcoop6  mcoop7  narc1  narc2  narc3  narc4  narc5  narc6  nidentity1  nidentity2  omind1  omind2  omind3  omind4  omind5  omind6  contact1  contact2  contact3  contact4  contact5  hygiene2  hygiene4  psupport1  psupport2  psupport3  psupport4  psupport5  political_ideology  happy  riskperc1  riskperc2  self_esteem  sbelong1  sbelong2  sbelong3  sbelong4  tested_positive  optim1  optim2  slfcont1  slfcont2  slfcont3  slfcont4
##   6   8  cnarc1  cnarc2  cnarc3  CRT1  CRT2  CRT3  employ_status1  health_cond  ladder  marital1  mor_circle  moralid1  moralid10  moralid2  moralid3  moralid4  moralid5  moralid6  moralid7  moralid8  moralid9  mcoop1  mcoop2  mcoop3  mcoop4  mcoop5  mcoop6  mcoop7  narc1  narc2  narc3  narc4  narc5  narc6  nidentity1  nidentity2  omind1  omind2  omind3  omind4  omind5  omind6  contact1  contact2  contact3  contact4  contact5  hygiene2  hygiene4  psupport1  psupport2  psupport3  psupport4  psupport5  political_ideology  happy  riskperc1  riskperc2  self_esteem  sbelong1  sbelong2  sbelong3  sbelong4  tested_positive  optim1  optim2  slfcont1  slfcont2  slfcont3  slfcont4
##   6   9  cnarc1  cnarc2  cnarc3  CRT1  CRT2  CRT3  employ_status1  health_cond  ladder  marital1  mor_circle  moralid1  moralid10  moralid2  moralid3  moralid4  moralid5  moralid6  moralid7  moralid8  moralid9  mcoop1  mcoop2  mcoop3  mcoop4  mcoop5  mcoop6  mcoop7  narc1  narc2  narc3  narc4  narc5  narc6  nidentity1  nidentity2  omind1  omind2  omind3  omind4  omind5  omind6  contact1  contact2  contact3  contact4  contact5  hygiene2  hygiene4  psupport1  psupport2  psupport3  psupport4  psupport5  political_ideology  happy  riskperc1  riskperc2  self_esteem  sbelong1  sbelong2  sbelong3  sbelong4  tested_positive  optim1  optim2  slfcont1  slfcont2  slfcont3  slfcont4
##   6   10  cnarc1  cnarc2  cnarc3  CRT1  CRT2  CRT3  employ_status1  health_cond  ladder  marital1  mor_circle  moralid1  moralid10  moralid2  moralid3  moralid4  moralid5  moralid6  moralid7  moralid8  moralid9  mcoop1  mcoop2  mcoop3  mcoop4  mcoop5  mcoop6  mcoop7  narc1  narc2  narc3  narc4  narc5  narc6  nidentity1  nidentity2  omind1  omind2  omind3  omind4  omind5  omind6  contact1  contact2  contact3  contact4  contact5  hygiene2  hygiene4  psupport1  psupport2  psupport3  psupport4  psupport5  political_ideology  happy  riskperc1  riskperc2  self_esteem  sbelong1  sbelong2  sbelong3  sbelong4  tested_positive  optim1  optim2  slfcont1  slfcont2  slfcont3  slfcont4
##   7   1  cnarc1  cnarc2  cnarc3  CRT1  CRT2  CRT3  employ_status1  health_cond  ladder  marital1  mor_circle  moralid1  moralid10  moralid2  moralid3  moralid4  moralid5  moralid6  moralid7  moralid8  moralid9  mcoop1  mcoop2  mcoop3  mcoop4  mcoop5  mcoop6  mcoop7  narc1  narc2  narc3  narc4  narc5  narc6  nidentity1  nidentity2  omind1  omind2  omind3  omind4  omind5  omind6  contact1  contact2  contact3  contact4  contact5  hygiene2  hygiene4  psupport1  psupport2  psupport3  psupport4  psupport5  political_ideology  happy  riskperc1  riskperc2  self_esteem  sbelong1  sbelong2  sbelong3  sbelong4  tested_positive  optim1  optim2  slfcont1  slfcont2  slfcont3  slfcont4
##   7   2  cnarc1  cnarc2  cnarc3  CRT1  CRT2  CRT3  employ_status1  health_cond  ladder  marital1  mor_circle  moralid1  moralid10  moralid2  moralid3  moralid4  moralid5  moralid6  moralid7  moralid8  moralid9  mcoop1  mcoop2  mcoop3  mcoop4  mcoop5  mcoop6  mcoop7  narc1  narc2  narc3  narc4  narc5  narc6  nidentity1  nidentity2  omind1  omind2  omind3  omind4  omind5  omind6  contact1  contact2  contact3  contact4  contact5  hygiene2  hygiene4  psupport1  psupport2  psupport3  psupport4  psupport5  political_ideology  happy  riskperc1  riskperc2  self_esteem  sbelong1  sbelong2  sbelong3  sbelong4  tested_positive  optim1  optim2  slfcont1  slfcont2  slfcont3  slfcont4
##   7   3  cnarc1  cnarc2  cnarc3  CRT1  CRT2  CRT3  employ_status1  health_cond  ladder  marital1  mor_circle  moralid1  moralid10  moralid2  moralid3  moralid4  moralid5  moralid6  moralid7  moralid8  moralid9  mcoop1  mcoop2  mcoop3  mcoop4  mcoop5  mcoop6  mcoop7  narc1  narc2  narc3  narc4  narc5  narc6  nidentity1  nidentity2  omind1  omind2  omind3  omind4  omind5  omind6  contact1  contact2  contact3  contact4  contact5  hygiene2  hygiene4  psupport1  psupport2  psupport3  psupport4  psupport5  political_ideology  happy  riskperc1  riskperc2  self_esteem  sbelong1  sbelong2  sbelong3  sbelong4  tested_positive  optim1  optim2  slfcont1  slfcont2  slfcont3  slfcont4
##   7   4  cnarc1  cnarc2  cnarc3  CRT1  CRT2  CRT3  employ_status1  health_cond  ladder  marital1  mor_circle  moralid1  moralid10  moralid2  moralid3  moralid4  moralid5  moralid6  moralid7  moralid8  moralid9  mcoop1  mcoop2  mcoop3  mcoop4  mcoop5  mcoop6  mcoop7  narc1  narc2  narc3  narc4  narc5  narc6  nidentity1  nidentity2  omind1  omind2  omind3  omind4  omind5  omind6  contact1  contact2  contact3  contact4  contact5  hygiene2  hygiene4  psupport1  psupport2  psupport3  psupport4  psupport5  political_ideology  happy  riskperc1  riskperc2  self_esteem  sbelong1  sbelong2  sbelong3  sbelong4  tested_positive  optim1  optim2  slfcont1  slfcont2  slfcont3  slfcont4
##   7   5  cnarc1  cnarc2  cnarc3  CRT1  CRT2  CRT3  employ_status1  health_cond  ladder  marital1  mor_circle  moralid1  moralid10  moralid2  moralid3  moralid4  moralid5  moralid6  moralid7  moralid8  moralid9  mcoop1  mcoop2  mcoop3  mcoop4  mcoop5  mcoop6  mcoop7  narc1  narc2  narc3  narc4  narc5  narc6  nidentity1  nidentity2  omind1  omind2  omind3  omind4  omind5  omind6  contact1  contact2  contact3  contact4  contact5  hygiene2  hygiene4  psupport1  psupport2  psupport3  psupport4  psupport5  political_ideology  happy  riskperc1  riskperc2  self_esteem  sbelong1  sbelong2  sbelong3  sbelong4  tested_positive  optim1  optim2  slfcont1  slfcont2  slfcont3  slfcont4
##   7   6  cnarc1  cnarc2  cnarc3  CRT1  CRT2  CRT3  employ_status1  health_cond  ladder  marital1  mor_circle  moralid1  moralid10  moralid2  moralid3  moralid4  moralid5  moralid6  moralid7  moralid8  moralid9  mcoop1  mcoop2  mcoop3  mcoop4  mcoop5  mcoop6  mcoop7  narc1  narc2  narc3  narc4  narc5  narc6  nidentity1  nidentity2  omind1  omind2  omind3  omind4  omind5  omind6  contact1  contact2  contact3  contact4  contact5  hygiene2  hygiene4  psupport1  psupport2  psupport3  psupport4  psupport5  political_ideology  happy  riskperc1  riskperc2  self_esteem  sbelong1  sbelong2  sbelong3  sbelong4  tested_positive  optim1  optim2  slfcont1  slfcont2  slfcont3  slfcont4
##   7   7  cnarc1  cnarc2  cnarc3  CRT1  CRT2  CRT3  employ_status1  health_cond  ladder  marital1  mor_circle  moralid1  moralid10  moralid2  moralid3  moralid4  moralid5  moralid6  moralid7  moralid8  moralid9  mcoop1  mcoop2  mcoop3  mcoop4  mcoop5  mcoop6  mcoop7  narc1  narc2  narc3  narc4  narc5  narc6  nidentity1  nidentity2  omind1  omind2  omind3  omind4  omind5  omind6  contact1  contact2  contact3  contact4  contact5  hygiene2  hygiene4  psupport1  psupport2  psupport3  psupport4  psupport5  political_ideology  happy  riskperc1  riskperc2  self_esteem  sbelong1  sbelong2  sbelong3  sbelong4  tested_positive  optim1  optim2  slfcont1  slfcont2  slfcont3  slfcont4
##   7   8  cnarc1  cnarc2  cnarc3  CRT1  CRT2  CRT3  employ_status1  health_cond  ladder  marital1  mor_circle  moralid1  moralid10  moralid2  moralid3  moralid4  moralid5  moralid6  moralid7  moralid8  moralid9  mcoop1  mcoop2  mcoop3  mcoop4  mcoop5  mcoop6  mcoop7  narc1  narc2  narc3  narc4  narc5  narc6  nidentity1  nidentity2  omind1  omind2  omind3  omind4  omind5  omind6  contact1  contact2  contact3  contact4  contact5  hygiene2  hygiene4  psupport1  psupport2  psupport3  psupport4  psupport5  political_ideology  happy  riskperc1  riskperc2  self_esteem  sbelong1  sbelong2  sbelong3  sbelong4  tested_positive  optim1  optim2  slfcont1  slfcont2  slfcont3  slfcont4
##   7   9  cnarc1  cnarc2  cnarc3  CRT1  CRT2  CRT3  employ_status1  health_cond  ladder  marital1  mor_circle  moralid1  moralid10  moralid2  moralid3  moralid4  moralid5  moralid6  moralid7  moralid8  moralid9  mcoop1  mcoop2  mcoop3  mcoop4  mcoop5  mcoop6  mcoop7  narc1  narc2  narc3  narc4  narc5  narc6  nidentity1  nidentity2  omind1  omind2  omind3  omind4  omind5  omind6  contact1  contact2  contact3  contact4  contact5  hygiene2  hygiene4  psupport1  psupport2  psupport3  psupport4  psupport5  political_ideology  happy  riskperc1  riskperc2  self_esteem  sbelong1  sbelong2  sbelong3  sbelong4  tested_positive  optim1  optim2  slfcont1  slfcont2  slfcont3  slfcont4
##   7   10  cnarc1  cnarc2  cnarc3  CRT1  CRT2  CRT3  employ_status1  health_cond  ladder  marital1  mor_circle  moralid1  moralid10  moralid2  moralid3  moralid4  moralid5  moralid6  moralid7  moralid8  moralid9  mcoop1  mcoop2  mcoop3  mcoop4  mcoop5  mcoop6  mcoop7  narc1  narc2  narc3  narc4  narc5  narc6  nidentity1  nidentity2  omind1  omind2  omind3  omind4  omind5  omind6  contact1  contact2  contact3  contact4  contact5  hygiene2  hygiene4  psupport1  psupport2  psupport3  psupport4  psupport5  political_ideology  happy  riskperc1  riskperc2  self_esteem  sbelong1  sbelong2  sbelong3  sbelong4  tested_positive  optim1  optim2  slfcont1  slfcont2  slfcont3  slfcont4
##   8   1  cnarc1  cnarc2  cnarc3  CRT1  CRT2  CRT3  employ_status1  health_cond  ladder  marital1  mor_circle  moralid1  moralid10  moralid2  moralid3  moralid4  moralid5  moralid6  moralid7  moralid8  moralid9  mcoop1  mcoop2  mcoop3  mcoop4  mcoop5  mcoop6  mcoop7  narc1  narc2  narc3  narc4  narc5  narc6  nidentity1  nidentity2  omind1  omind2  omind3  omind4  omind5  omind6  contact1  contact2  contact3  contact4  contact5  hygiene2  hygiene4  psupport1  psupport2  psupport3  psupport4  psupport5  political_ideology  happy  riskperc1  riskperc2  self_esteem  sbelong1  sbelong2  sbelong3  sbelong4  tested_positive  optim1  optim2  slfcont1  slfcont2  slfcont3  slfcont4
##   8   2  cnarc1  cnarc2  cnarc3  CRT1  CRT2  CRT3  employ_status1  health_cond  ladder  marital1  mor_circle  moralid1  moralid10  moralid2  moralid3  moralid4  moralid5  moralid6  moralid7  moralid8  moralid9  mcoop1  mcoop2  mcoop3  mcoop4  mcoop5  mcoop6  mcoop7  narc1  narc2  narc3  narc4  narc5  narc6  nidentity1  nidentity2  omind1  omind2  omind3  omind4  omind5  omind6  contact1  contact2  contact3  contact4  contact5  hygiene2  hygiene4  psupport1  psupport2  psupport3  psupport4  psupport5  political_ideology  happy  riskperc1  riskperc2  self_esteem  sbelong1  sbelong2  sbelong3  sbelong4  tested_positive  optim1  optim2  slfcont1  slfcont2  slfcont3  slfcont4
##   8   3  cnarc1  cnarc2  cnarc3  CRT1  CRT2  CRT3  employ_status1  health_cond  ladder  marital1  mor_circle  moralid1  moralid10  moralid2  moralid3  moralid4  moralid5  moralid6  moralid7  moralid8  moralid9  mcoop1  mcoop2  mcoop3  mcoop4  mcoop5  mcoop6  mcoop7  narc1  narc2  narc3  narc4  narc5  narc6  nidentity1  nidentity2  omind1  omind2  omind3  omind4  omind5  omind6  contact1  contact2  contact3  contact4  contact5  hygiene2  hygiene4  psupport1  psupport2  psupport3  psupport4  psupport5  political_ideology  happy  riskperc1  riskperc2  self_esteem  sbelong1  sbelong2  sbelong3  sbelong4  tested_positive  optim1  optim2  slfcont1  slfcont2  slfcont3  slfcont4
##   8   4  cnarc1  cnarc2  cnarc3  CRT1  CRT2  CRT3  employ_status1  health_cond  ladder  marital1  mor_circle  moralid1  moralid10  moralid2  moralid3  moralid4  moralid5  moralid6  moralid7  moralid8  moralid9  mcoop1  mcoop2  mcoop3  mcoop4  mcoop5  mcoop6  mcoop7  narc1  narc2  narc3  narc4  narc5  narc6  nidentity1  nidentity2  omind1  omind2  omind3  omind4  omind5  omind6  contact1  contact2  contact3  contact4  contact5  hygiene2  hygiene4  psupport1  psupport2  psupport3  psupport4  psupport5  political_ideology  happy  riskperc1  riskperc2  self_esteem  sbelong1  sbelong2  sbelong3  sbelong4  tested_positive  optim1  optim2  slfcont1  slfcont2  slfcont3  slfcont4
##   8   5  cnarc1  cnarc2  cnarc3  CRT1  CRT2  CRT3  employ_status1  health_cond  ladder  marital1  mor_circle  moralid1  moralid10  moralid2  moralid3  moralid4  moralid5  moralid6  moralid7  moralid8  moralid9  mcoop1  mcoop2  mcoop3  mcoop4  mcoop5  mcoop6  mcoop7  narc1  narc2  narc3  narc4  narc5  narc6  nidentity1  nidentity2  omind1  omind2  omind3  omind4  omind5  omind6  contact1  contact2  contact3  contact4  contact5  hygiene2  hygiene4  psupport1  psupport2  psupport3  psupport4  psupport5  political_ideology  happy  riskperc1  riskperc2  self_esteem  sbelong1  sbelong2  sbelong3  sbelong4  tested_positive  optim1  optim2  slfcont1  slfcont2  slfcont3  slfcont4
##   8   6  cnarc1  cnarc2  cnarc3  CRT1  CRT2  CRT3  employ_status1  health_cond  ladder  marital1  mor_circle  moralid1  moralid10  moralid2  moralid3  moralid4  moralid5  moralid6  moralid7  moralid8  moralid9  mcoop1  mcoop2  mcoop3  mcoop4  mcoop5  mcoop6  mcoop7  narc1  narc2  narc3  narc4  narc5  narc6  nidentity1  nidentity2  omind1  omind2  omind3  omind4  omind5  omind6  contact1  contact2  contact3  contact4  contact5  hygiene2  hygiene4  psupport1  psupport2  psupport3  psupport4  psupport5  political_ideology  happy  riskperc1  riskperc2  self_esteem  sbelong1  sbelong2  sbelong3  sbelong4  tested_positive  optim1  optim2  slfcont1  slfcont2  slfcont3  slfcont4
##   8   7  cnarc1  cnarc2  cnarc3  CRT1  CRT2  CRT3  employ_status1  health_cond  ladder  marital1  mor_circle  moralid1  moralid10  moralid2  moralid3  moralid4  moralid5  moralid6  moralid7  moralid8  moralid9  mcoop1  mcoop2  mcoop3  mcoop4  mcoop5  mcoop6  mcoop7  narc1  narc2  narc3  narc4  narc5  narc6  nidentity1  nidentity2  omind1  omind2  omind3  omind4  omind5  omind6  contact1  contact2  contact3  contact4  contact5  hygiene2  hygiene4  psupport1  psupport2  psupport3  psupport4  psupport5  political_ideology  happy  riskperc1  riskperc2  self_esteem  sbelong1  sbelong2  sbelong3  sbelong4  tested_positive  optim1  optim2  slfcont1  slfcont2  slfcont3  slfcont4
##   8   8  cnarc1  cnarc2  cnarc3  CRT1  CRT2  CRT3  employ_status1  health_cond  ladder  marital1  mor_circle  moralid1  moralid10  moralid2  moralid3  moralid4  moralid5  moralid6  moralid7  moralid8  moralid9  mcoop1  mcoop2  mcoop3  mcoop4  mcoop5  mcoop6  mcoop7  narc1  narc2  narc3  narc4  narc5  narc6  nidentity1  nidentity2  omind1  omind2  omind3  omind4  omind5  omind6  contact1  contact2  contact3  contact4  contact5  hygiene2  hygiene4  psupport1  psupport2  psupport3  psupport4  psupport5  political_ideology  happy  riskperc1  riskperc2  self_esteem  sbelong1  sbelong2  sbelong3  sbelong4  tested_positive  optim1  optim2  slfcont1  slfcont2  slfcont3  slfcont4
##   8   9  cnarc1  cnarc2  cnarc3  CRT1  CRT2  CRT3  employ_status1  health_cond  ladder  marital1  mor_circle  moralid1  moralid10  moralid2  moralid3  moralid4  moralid5  moralid6  moralid7  moralid8  moralid9  mcoop1  mcoop2  mcoop3  mcoop4  mcoop5  mcoop6  mcoop7  narc1  narc2  narc3  narc4  narc5  narc6  nidentity1  nidentity2  omind1  omind2  omind3  omind4  omind5  omind6  contact1  contact2  contact3  contact4  contact5  hygiene2  hygiene4  psupport1  psupport2  psupport3  psupport4  psupport5  political_ideology  happy  riskperc1  riskperc2  self_esteem  sbelong1  sbelong2  sbelong3  sbelong4  tested_positive  optim1  optim2  slfcont1  slfcont2  slfcont3  slfcont4
##   8   10  cnarc1  cnarc2  cnarc3  CRT1  CRT2  CRT3  employ_status1  health_cond  ladder  marital1  mor_circle  moralid1  moralid10  moralid2  moralid3  moralid4  moralid5  moralid6  moralid7  moralid8  moralid9  mcoop1  mcoop2  mcoop3  mcoop4  mcoop5  mcoop6  mcoop7  narc1  narc2  narc3  narc4  narc5  narc6  nidentity1  nidentity2  omind1  omind2  omind3  omind4  omind5  omind6  contact1  contact2  contact3  contact4  contact5  hygiene2  hygiene4  psupport1  psupport2  psupport3  psupport4  psupport5  political_ideology  happy  riskperc1  riskperc2  self_esteem  sbelong1  sbelong2  sbelong3  sbelong4  tested_positive  optim1  optim2  slfcont1  slfcont2  slfcont3  slfcont4
##   9   1  cnarc1  cnarc2  cnarc3  CRT1  CRT2  CRT3  employ_status1  health_cond  ladder  marital1  mor_circle  moralid1  moralid10  moralid2  moralid3  moralid4  moralid5  moralid6  moralid7  moralid8  moralid9  mcoop1  mcoop2  mcoop3  mcoop4  mcoop5  mcoop6  mcoop7  narc1  narc2  narc3  narc4  narc5  narc6  nidentity1  nidentity2  omind1  omind2  omind3  omind4  omind5  omind6  contact1  contact2  contact3  contact4  contact5  hygiene2  hygiene4  psupport1  psupport2  psupport3  psupport4  psupport5  political_ideology  happy  riskperc1  riskperc2  self_esteem  sbelong1  sbelong2  sbelong3  sbelong4  tested_positive  optim1  optim2  slfcont1  slfcont2  slfcont3  slfcont4
##   9   2  cnarc1  cnarc2  cnarc3  CRT1  CRT2  CRT3  employ_status1  health_cond  ladder  marital1  mor_circle  moralid1  moralid10  moralid2  moralid3  moralid4  moralid5  moralid6  moralid7  moralid8  moralid9  mcoop1  mcoop2  mcoop3  mcoop4  mcoop5  mcoop6  mcoop7  narc1  narc2  narc3  narc4  narc5  narc6  nidentity1  nidentity2  omind1  omind2  omind3  omind4  omind5  omind6  contact1  contact2  contact3  contact4  contact5  hygiene2  hygiene4  psupport1  psupport2  psupport3  psupport4  psupport5  political_ideology  happy  riskperc1  riskperc2  self_esteem  sbelong1  sbelong2  sbelong3  sbelong4  tested_positive  optim1  optim2  slfcont1  slfcont2  slfcont3  slfcont4
##   9   3  cnarc1  cnarc2  cnarc3  CRT1  CRT2  CRT3  employ_status1  health_cond  ladder  marital1  mor_circle  moralid1  moralid10  moralid2  moralid3  moralid4  moralid5  moralid6  moralid7  moralid8  moralid9  mcoop1  mcoop2  mcoop3  mcoop4  mcoop5  mcoop6  mcoop7  narc1  narc2  narc3  narc4  narc5  narc6  nidentity1  nidentity2  omind1  omind2  omind3  omind4  omind5  omind6  contact1  contact2  contact3  contact4  contact5  hygiene2  hygiene4  psupport1  psupport2  psupport3  psupport4  psupport5  political_ideology  happy  riskperc1  riskperc2  self_esteem  sbelong1  sbelong2  sbelong3  sbelong4  tested_positive  optim1  optim2  slfcont1  slfcont2  slfcont3  slfcont4
##   9   4  cnarc1  cnarc2  cnarc3  CRT1  CRT2  CRT3  employ_status1  health_cond  ladder  marital1  mor_circle  moralid1  moralid10  moralid2  moralid3  moralid4  moralid5  moralid6  moralid7  moralid8  moralid9  mcoop1  mcoop2  mcoop3  mcoop4  mcoop5  mcoop6  mcoop7  narc1  narc2  narc3  narc4  narc5  narc6  nidentity1  nidentity2  omind1  omind2  omind3  omind4  omind5  omind6  contact1  contact2  contact3  contact4  contact5  hygiene2  hygiene4  psupport1  psupport2  psupport3  psupport4  psupport5  political_ideology  happy  riskperc1  riskperc2  self_esteem  sbelong1  sbelong2  sbelong3  sbelong4  tested_positive  optim1  optim2  slfcont1  slfcont2  slfcont3  slfcont4
##   9   5  cnarc1  cnarc2  cnarc3  CRT1  CRT2  CRT3  employ_status1  health_cond  ladder  marital1  mor_circle  moralid1  moralid10  moralid2  moralid3  moralid4  moralid5  moralid6  moralid7  moralid8  moralid9  mcoop1  mcoop2  mcoop3  mcoop4  mcoop5  mcoop6  mcoop7  narc1  narc2  narc3  narc4  narc5  narc6  nidentity1  nidentity2  omind1  omind2  omind3  omind4  omind5  omind6  contact1  contact2  contact3  contact4  contact5  hygiene2  hygiene4  psupport1  psupport2  psupport3  psupport4  psupport5  political_ideology  happy  riskperc1  riskperc2  self_esteem  sbelong1  sbelong2  sbelong3  sbelong4  tested_positive  optim1  optim2  slfcont1  slfcont2  slfcont3  slfcont4
##   9   6  cnarc1  cnarc2  cnarc3  CRT1  CRT2  CRT3  employ_status1  health_cond  ladder  marital1  mor_circle  moralid1  moralid10  moralid2  moralid3  moralid4  moralid5  moralid6  moralid7  moralid8  moralid9  mcoop1  mcoop2  mcoop3  mcoop4  mcoop5  mcoop6  mcoop7  narc1  narc2  narc3  narc4  narc5  narc6  nidentity1  nidentity2  omind1  omind2  omind3  omind4  omind5  omind6  contact1  contact2  contact3  contact4  contact5  hygiene2  hygiene4  psupport1  psupport2  psupport3  psupport4  psupport5  political_ideology  happy  riskperc1  riskperc2  self_esteem  sbelong1  sbelong2  sbelong3  sbelong4  tested_positive  optim1  optim2  slfcont1  slfcont2  slfcont3  slfcont4
##   9   7  cnarc1  cnarc2  cnarc3  CRT1  CRT2  CRT3  employ_status1  health_cond  ladder  marital1  mor_circle  moralid1  moralid10  moralid2  moralid3  moralid4  moralid5  moralid6  moralid7  moralid8  moralid9  mcoop1  mcoop2  mcoop3  mcoop4  mcoop5  mcoop6  mcoop7  narc1  narc2  narc3  narc4  narc5  narc6  nidentity1  nidentity2  omind1  omind2  omind3  omind4  omind5  omind6  contact1  contact2  contact3  contact4  contact5  hygiene2  hygiene4  psupport1  psupport2  psupport3  psupport4  psupport5  political_ideology  happy  riskperc1  riskperc2  self_esteem  sbelong1  sbelong2  sbelong3  sbelong4  tested_positive  optim1  optim2  slfcont1  slfcont2  slfcont3  slfcont4
##   9   8  cnarc1  cnarc2  cnarc3  CRT1  CRT2  CRT3  employ_status1  health_cond  ladder  marital1  mor_circle  moralid1  moralid10  moralid2  moralid3  moralid4  moralid5  moralid6  moralid7  moralid8  moralid9  mcoop1  mcoop2  mcoop3  mcoop4  mcoop5  mcoop6  mcoop7  narc1  narc2  narc3  narc4  narc5  narc6  nidentity1  nidentity2  omind1  omind2  omind3  omind4  omind5  omind6  contact1  contact2  contact3  contact4  contact5  hygiene2  hygiene4  psupport1  psupport2  psupport3  psupport4  psupport5  political_ideology  happy  riskperc1  riskperc2  self_esteem  sbelong1  sbelong2  sbelong3  sbelong4  tested_positive  optim1  optim2  slfcont1  slfcont2  slfcont3  slfcont4
##   9   9  cnarc1  cnarc2  cnarc3  CRT1  CRT2  CRT3  employ_status1  health_cond  ladder  marital1  mor_circle  moralid1  moralid10  moralid2  moralid3  moralid4  moralid5  moralid6  moralid7  moralid8  moralid9  mcoop1  mcoop2  mcoop3  mcoop4  mcoop5  mcoop6  mcoop7  narc1  narc2  narc3  narc4  narc5  narc6  nidentity1  nidentity2  omind1  omind2  omind3  omind4  omind5  omind6  contact1  contact2  contact3  contact4  contact5  hygiene2  hygiene4  psupport1  psupport2  psupport3  psupport4  psupport5  political_ideology  happy  riskperc1  riskperc2  self_esteem  sbelong1  sbelong2  sbelong3  sbelong4  tested_positive  optim1  optim2  slfcont1  slfcont2  slfcont3  slfcont4
##   9   10  cnarc1  cnarc2  cnarc3  CRT1  CRT2  CRT3  employ_status1  health_cond  ladder  marital1  mor_circle  moralid1  moralid10  moralid2  moralid3  moralid4  moralid5  moralid6  moralid7  moralid8  moralid9  mcoop1  mcoop2  mcoop3  mcoop4  mcoop5  mcoop6  mcoop7  narc1  narc2  narc3  narc4  narc5  narc6  nidentity1  nidentity2  omind1  omind2  omind3  omind4  omind5  omind6  contact1  contact2  contact3  contact4  contact5  hygiene2  hygiene4  psupport1  psupport2  psupport3  psupport4  psupport5  political_ideology  happy  riskperc1  riskperc2  self_esteem  sbelong1  sbelong2  sbelong3  sbelong4  tested_positive  optim1  optim2  slfcont1  slfcont2  slfcont3  slfcont4
##   10   1  cnarc1  cnarc2  cnarc3  CRT1  CRT2  CRT3  employ_status1  health_cond  ladder  marital1  mor_circle  moralid1  moralid10  moralid2  moralid3  moralid4  moralid5  moralid6  moralid7  moralid8  moralid9  mcoop1  mcoop2  mcoop3  mcoop4  mcoop5  mcoop6  mcoop7  narc1  narc2  narc3  narc4  narc5  narc6  nidentity1  nidentity2  omind1  omind2  omind3  omind4  omind5  omind6  contact1  contact2  contact3  contact4  contact5  hygiene2  hygiene4  psupport1  psupport2  psupport3  psupport4  psupport5  political_ideology  happy  riskperc1  riskperc2  self_esteem  sbelong1  sbelong2  sbelong3  sbelong4  tested_positive  optim1  optim2  slfcont1  slfcont2  slfcont3  slfcont4
##   10   2  cnarc1  cnarc2  cnarc3  CRT1  CRT2  CRT3  employ_status1  health_cond  ladder  marital1  mor_circle  moralid1  moralid10  moralid2  moralid3  moralid4  moralid5  moralid6  moralid7  moralid8  moralid9  mcoop1  mcoop2  mcoop3  mcoop4  mcoop5  mcoop6  mcoop7  narc1  narc2  narc3  narc4  narc5  narc6  nidentity1  nidentity2  omind1  omind2  omind3  omind4  omind5  omind6  contact1  contact2  contact3  contact4  contact5  hygiene2  hygiene4  psupport1  psupport2  psupport3  psupport4  psupport5  political_ideology  happy  riskperc1  riskperc2  self_esteem  sbelong1  sbelong2  sbelong3  sbelong4  tested_positive  optim1  optim2  slfcont1  slfcont2  slfcont3  slfcont4
##   10   3  cnarc1  cnarc2  cnarc3  CRT1  CRT2  CRT3  employ_status1  health_cond  ladder  marital1  mor_circle  moralid1  moralid10  moralid2  moralid3  moralid4  moralid5  moralid6  moralid7  moralid8  moralid9  mcoop1  mcoop2  mcoop3  mcoop4  mcoop5  mcoop6  mcoop7  narc1  narc2  narc3  narc4  narc5  narc6  nidentity1  nidentity2  omind1  omind2  omind3  omind4  omind5  omind6  contact1  contact2  contact3  contact4  contact5  hygiene2  hygiene4  psupport1  psupport2  psupport3  psupport4  psupport5  political_ideology  happy  riskperc1  riskperc2  self_esteem  sbelong1  sbelong2  sbelong3  sbelong4  tested_positive  optim1  optim2  slfcont1  slfcont2  slfcont3  slfcont4
##   10   4  cnarc1  cnarc2  cnarc3  CRT1  CRT2  CRT3  employ_status1  health_cond  ladder  marital1  mor_circle  moralid1  moralid10  moralid2  moralid3  moralid4  moralid5  moralid6  moralid7  moralid8  moralid9  mcoop1  mcoop2  mcoop3  mcoop4  mcoop5  mcoop6  mcoop7  narc1  narc2  narc3  narc4  narc5  narc6  nidentity1  nidentity2  omind1  omind2  omind3  omind4  omind5  omind6  contact1  contact2  contact3  contact4  contact5  hygiene2  hygiene4  psupport1  psupport2  psupport3  psupport4  psupport5  political_ideology  happy  riskperc1  riskperc2  self_esteem  sbelong1  sbelong2  sbelong3  sbelong4  tested_positive  optim1  optim2  slfcont1  slfcont2  slfcont3  slfcont4
##   10   5  cnarc1  cnarc2  cnarc3  CRT1  CRT2  CRT3  employ_status1  health_cond  ladder  marital1  mor_circle  moralid1  moralid10  moralid2  moralid3  moralid4  moralid5  moralid6  moralid7  moralid8  moralid9  mcoop1  mcoop2  mcoop3  mcoop4  mcoop5  mcoop6  mcoop7  narc1  narc2  narc3  narc4  narc5  narc6  nidentity1  nidentity2  omind1  omind2  omind3  omind4  omind5  omind6  contact1  contact2  contact3  contact4  contact5  hygiene2  hygiene4  psupport1  psupport2  psupport3  psupport4  psupport5  political_ideology  happy  riskperc1  riskperc2  self_esteem  sbelong1  sbelong2  sbelong3  sbelong4  tested_positive  optim1  optim2  slfcont1  slfcont2  slfcont3  slfcont4
##   10   6  cnarc1  cnarc2  cnarc3  CRT1  CRT2  CRT3  employ_status1  health_cond  ladder  marital1  mor_circle  moralid1  moralid10  moralid2  moralid3  moralid4  moralid5  moralid6  moralid7  moralid8  moralid9  mcoop1  mcoop2  mcoop3  mcoop4  mcoop5  mcoop6  mcoop7  narc1  narc2  narc3  narc4  narc5  narc6  nidentity1  nidentity2  omind1  omind2  omind3  omind4  omind5  omind6  contact1  contact2  contact3  contact4  contact5  hygiene2  hygiene4  psupport1  psupport2  psupport3  psupport4  psupport5  political_ideology  happy  riskperc1  riskperc2  self_esteem  sbelong1  sbelong2  sbelong3  sbelong4  tested_positive  optim1  optim2  slfcont1  slfcont2  slfcont3  slfcont4
##   10   7  cnarc1  cnarc2  cnarc3  CRT1  CRT2  CRT3  employ_status1  health_cond  ladder  marital1  mor_circle  moralid1  moralid10  moralid2  moralid3  moralid4  moralid5  moralid6  moralid7  moralid8  moralid9  mcoop1  mcoop2  mcoop3  mcoop4  mcoop5  mcoop6  mcoop7  narc1  narc2  narc3  narc4  narc5  narc6  nidentity1  nidentity2  omind1  omind2  omind3  omind4  omind5  omind6  contact1  contact2  contact3  contact4  contact5  hygiene2  hygiene4  psupport1  psupport2  psupport3  psupport4  psupport5  political_ideology  happy  riskperc1  riskperc2  self_esteem  sbelong1  sbelong2  sbelong3  sbelong4  tested_positive  optim1  optim2  slfcont1  slfcont2  slfcont3  slfcont4
##   10   8  cnarc1  cnarc2  cnarc3  CRT1  CRT2  CRT3  employ_status1  health_cond  ladder  marital1  mor_circle  moralid1  moralid10  moralid2  moralid3  moralid4  moralid5  moralid6  moralid7  moralid8  moralid9  mcoop1  mcoop2  mcoop3  mcoop4  mcoop5  mcoop6  mcoop7  narc1  narc2  narc3  narc4  narc5  narc6  nidentity1  nidentity2  omind1  omind2  omind3  omind4  omind5  omind6  contact1  contact2  contact3  contact4  contact5  hygiene2  hygiene4  psupport1  psupport2  psupport3  psupport4  psupport5  political_ideology  happy  riskperc1  riskperc2  self_esteem  sbelong1  sbelong2  sbelong3  sbelong4  tested_positive  optim1  optim2  slfcont1  slfcont2  slfcont3  slfcont4
##   10   9  cnarc1  cnarc2  cnarc3  CRT1  CRT2  CRT3  employ_status1  health_cond  ladder  marital1  mor_circle  moralid1  moralid10  moralid2  moralid3  moralid4  moralid5  moralid6  moralid7  moralid8  moralid9  mcoop1  mcoop2  mcoop3  mcoop4  mcoop5  mcoop6  mcoop7  narc1  narc2  narc3  narc4  narc5  narc6  nidentity1  nidentity2  omind1  omind2  omind3  omind4  omind5  omind6  contact1  contact2  contact3  contact4  contact5  hygiene2  hygiene4  psupport1  psupport2  psupport3  psupport4  psupport5  political_ideology  happy  riskperc1  riskperc2  self_esteem  sbelong1  sbelong2  sbelong3  sbelong4  tested_positive  optim1  optim2  slfcont1  slfcont2  slfcont3  slfcont4
##   10   10  cnarc1  cnarc2  cnarc3  CRT1  CRT2  CRT3  employ_status1  health_cond  ladder  marital1  mor_circle  moralid1  moralid10  moralid2  moralid3  moralid4  moralid5  moralid6  moralid7  moralid8  moralid9  mcoop1  mcoop2  mcoop3  mcoop4  mcoop5  mcoop6  mcoop7  narc1  narc2  narc3  narc4  narc5  narc6  nidentity1  nidentity2  omind1  omind2  omind3  omind4  omind5  omind6  contact1  contact2  contact3  contact4  contact5  hygiene2  hygiene4  psupport1  psupport2  psupport3  psupport4  psupport5  political_ideology  happy  riskperc1  riskperc2  self_esteem  sbelong1  sbelong2  sbelong3  sbelong4  tested_positive  optim1  optim2  slfcont1  slfcont2  slfcont3  slfcont4
```

```
## Warning: Number of logged events: 7003
```

```
## 
##  iter imp variable
##   1   1  cnarc1  cnarc2  cnarc3  CRT1  CRT2  CRT3  employ_status1  marital1  mor_circle  moralid10  moralid3  moralid5  moralid6  moralid7  moralid8  moralid9  mcoop1  mcoop2  mcoop3  mcoop4  mcoop5  mcoop6  mcoop7  narc1  narc2  narc3  narc4  narc5  narc6  nidentity1  nidentity2  omind1  omind2  omind6  contact3  hygiene4  psupport1  psupport2  psupport3  psupport4  psupport5  political_ideology  happy  slf_ladder  self_esteem  sbelong3  sbelong4  optim1  optim2
##   1   2  cnarc1  cnarc2  cnarc3  CRT1  CRT2  CRT3  employ_status1  marital1  mor_circle  moralid10  moralid3  moralid5  moralid6  moralid7  moralid8  moralid9  mcoop1  mcoop2  mcoop3  mcoop4  mcoop5  mcoop6  mcoop7  narc1  narc2  narc3  narc4  narc5  narc6  nidentity1  nidentity2  omind1  omind2  omind6  contact3  hygiene4  psupport1  psupport2  psupport3  psupport4  psupport5  political_ideology  happy  slf_ladder  self_esteem  sbelong3  sbelong4  optim1  optim2
##   1   3  cnarc1  cnarc2  cnarc3  CRT1  CRT2  CRT3  employ_status1  marital1  mor_circle  moralid10  moralid3  moralid5  moralid6  moralid7  moralid8  moralid9  mcoop1  mcoop2  mcoop3  mcoop4  mcoop5  mcoop6  mcoop7  narc1  narc2  narc3  narc4  narc5  narc6  nidentity1  nidentity2  omind1  omind2  omind6  contact3  hygiene4  psupport1  psupport2  psupport3  psupport4  psupport5  political_ideology  happy  slf_ladder  self_esteem  sbelong3  sbelong4  optim1  optim2
##   1   4  cnarc1  cnarc2  cnarc3  CRT1  CRT2  CRT3  employ_status1  marital1  mor_circle  moralid10  moralid3  moralid5  moralid6  moralid7  moralid8  moralid9  mcoop1  mcoop2  mcoop3  mcoop4  mcoop5  mcoop6  mcoop7  narc1  narc2  narc3  narc4  narc5  narc6  nidentity1  nidentity2  omind1  omind2  omind6  contact3  hygiene4  psupport1  psupport2  psupport3  psupport4  psupport5  political_ideology  happy  slf_ladder  self_esteem  sbelong3  sbelong4  optim1  optim2
##   1   5  cnarc1  cnarc2  cnarc3  CRT1  CRT2  CRT3  employ_status1  marital1  mor_circle  moralid10  moralid3  moralid5  moralid6  moralid7  moralid8  moralid9  mcoop1  mcoop2  mcoop3  mcoop4  mcoop5  mcoop6  mcoop7  narc1  narc2  narc3  narc4  narc5  narc6  nidentity1  nidentity2  omind1  omind2  omind6  contact3  hygiene4  psupport1  psupport2  psupport3  psupport4  psupport5  political_ideology  happy  slf_ladder  self_esteem  sbelong3  sbelong4  optim1  optim2
##   1   6  cnarc1  cnarc2  cnarc3  CRT1  CRT2  CRT3  employ_status1  marital1  mor_circle  moralid10  moralid3  moralid5  moralid6  moralid7  moralid8  moralid9  mcoop1  mcoop2  mcoop3  mcoop4  mcoop5  mcoop6  mcoop7  narc1  narc2  narc3  narc4  narc5  narc6  nidentity1  nidentity2  omind1  omind2  omind6  contact3  hygiene4  psupport1  psupport2  psupport3  psupport4  psupport5  political_ideology  happy  slf_ladder  self_esteem  sbelong3  sbelong4  optim1  optim2
##   1   7  cnarc1  cnarc2  cnarc3  CRT1  CRT2  CRT3  employ_status1  marital1  mor_circle  moralid10  moralid3  moralid5  moralid6  moralid7  moralid8  moralid9  mcoop1  mcoop2  mcoop3  mcoop4  mcoop5  mcoop6  mcoop7  narc1  narc2  narc3  narc4  narc5  narc6  nidentity1  nidentity2  omind1  omind2  omind6  contact3  hygiene4  psupport1  psupport2  psupport3  psupport4  psupport5  political_ideology  happy  slf_ladder  self_esteem  sbelong3  sbelong4  optim1  optim2
##   1   8  cnarc1  cnarc2  cnarc3  CRT1  CRT2  CRT3  employ_status1  marital1  mor_circle  moralid10  moralid3  moralid5  moralid6  moralid7  moralid8  moralid9  mcoop1  mcoop2  mcoop3  mcoop4  mcoop5  mcoop6  mcoop7  narc1  narc2  narc3  narc4  narc5  narc6  nidentity1  nidentity2  omind1  omind2  omind6  contact3  hygiene4  psupport1  psupport2  psupport3  psupport4  psupport5  political_ideology  happy  slf_ladder  self_esteem  sbelong3  sbelong4  optim1  optim2
##   1   9  cnarc1  cnarc2  cnarc3  CRT1  CRT2  CRT3  employ_status1  marital1  mor_circle  moralid10  moralid3  moralid5  moralid6  moralid7  moralid8  moralid9  mcoop1  mcoop2  mcoop3  mcoop4  mcoop5  mcoop6  mcoop7  narc1  narc2  narc3  narc4  narc5  narc6  nidentity1  nidentity2  omind1  omind2  omind6  contact3  hygiene4  psupport1  psupport2  psupport3  psupport4  psupport5  political_ideology  happy  slf_ladder  self_esteem  sbelong3  sbelong4  optim1  optim2
##   1   10  cnarc1  cnarc2  cnarc3  CRT1  CRT2  CRT3  employ_status1  marital1  mor_circle  moralid10  moralid3  moralid5  moralid6  moralid7  moralid8  moralid9  mcoop1  mcoop2  mcoop3  mcoop4  mcoop5  mcoop6  mcoop7  narc1  narc2  narc3  narc4  narc5  narc6  nidentity1  nidentity2  omind1  omind2  omind6  contact3  hygiene4  psupport1  psupport2  psupport3  psupport4  psupport5  political_ideology  happy  slf_ladder  self_esteem  sbelong3  sbelong4  optim1  optim2
##   2   1  cnarc1  cnarc2  cnarc3  CRT1  CRT2  CRT3  employ_status1  marital1  mor_circle  moralid10  moralid3  moralid5  moralid6  moralid7  moralid8  moralid9  mcoop1  mcoop2  mcoop3  mcoop4  mcoop5  mcoop6  mcoop7  narc1  narc2  narc3  narc4  narc5  narc6  nidentity1  nidentity2  omind1  omind2  omind6  contact3  hygiene4  psupport1  psupport2  psupport3  psupport4  psupport5  political_ideology  happy  slf_ladder  self_esteem  sbelong3  sbelong4  optim1  optim2
##   2   2  cnarc1  cnarc2  cnarc3  CRT1  CRT2  CRT3  employ_status1  marital1  mor_circle  moralid10  moralid3  moralid5  moralid6  moralid7  moralid8  moralid9  mcoop1  mcoop2  mcoop3  mcoop4  mcoop5  mcoop6  mcoop7  narc1  narc2  narc3  narc4  narc5  narc6  nidentity1  nidentity2  omind1  omind2  omind6  contact3  hygiene4  psupport1  psupport2  psupport3  psupport4  psupport5  political_ideology  happy  slf_ladder  self_esteem  sbelong3  sbelong4  optim1  optim2
##   2   3  cnarc1  cnarc2  cnarc3  CRT1  CRT2  CRT3  employ_status1  marital1  mor_circle  moralid10  moralid3  moralid5  moralid6  moralid7  moralid8  moralid9  mcoop1  mcoop2  mcoop3  mcoop4  mcoop5  mcoop6  mcoop7  narc1  narc2  narc3  narc4  narc5  narc6  nidentity1  nidentity2  omind1  omind2  omind6  contact3  hygiene4  psupport1  psupport2  psupport3  psupport4  psupport5  political_ideology  happy  slf_ladder  self_esteem  sbelong3  sbelong4  optim1  optim2
##   2   4  cnarc1  cnarc2  cnarc3  CRT1  CRT2  CRT3  employ_status1  marital1  mor_circle  moralid10  moralid3  moralid5  moralid6  moralid7  moralid8  moralid9  mcoop1  mcoop2  mcoop3  mcoop4  mcoop5  mcoop6  mcoop7  narc1  narc2  narc3  narc4  narc5  narc6  nidentity1  nidentity2  omind1  omind2  omind6  contact3  hygiene4  psupport1  psupport2  psupport3  psupport4  psupport5  political_ideology  happy  slf_ladder  self_esteem  sbelong3  sbelong4  optim1  optim2
##   2   5  cnarc1  cnarc2  cnarc3  CRT1  CRT2  CRT3  employ_status1  marital1  mor_circle  moralid10  moralid3  moralid5  moralid6  moralid7  moralid8  moralid9  mcoop1  mcoop2  mcoop3  mcoop4  mcoop5  mcoop6  mcoop7  narc1  narc2  narc3  narc4  narc5  narc6  nidentity1  nidentity2  omind1  omind2  omind6  contact3  hygiene4  psupport1  psupport2  psupport3  psupport4  psupport5  political_ideology  happy  slf_ladder  self_esteem  sbelong3  sbelong4  optim1  optim2
##   2   6  cnarc1  cnarc2  cnarc3  CRT1  CRT2  CRT3  employ_status1  marital1  mor_circle  moralid10  moralid3  moralid5  moralid6  moralid7  moralid8  moralid9  mcoop1  mcoop2  mcoop3  mcoop4  mcoop5  mcoop6  mcoop7  narc1  narc2  narc3  narc4  narc5  narc6  nidentity1  nidentity2  omind1  omind2  omind6  contact3  hygiene4  psupport1  psupport2  psupport3  psupport4  psupport5  political_ideology  happy  slf_ladder  self_esteem  sbelong3  sbelong4  optim1  optim2
##   2   7  cnarc1  cnarc2  cnarc3  CRT1  CRT2  CRT3  employ_status1  marital1  mor_circle  moralid10  moralid3  moralid5  moralid6  moralid7  moralid8  moralid9  mcoop1  mcoop2  mcoop3  mcoop4  mcoop5  mcoop6  mcoop7  narc1  narc2  narc3  narc4  narc5  narc6  nidentity1  nidentity2  omind1  omind2  omind6  contact3  hygiene4  psupport1  psupport2  psupport3  psupport4  psupport5  political_ideology  happy  slf_ladder  self_esteem  sbelong3  sbelong4  optim1  optim2
##   2   8  cnarc1  cnarc2  cnarc3  CRT1  CRT2  CRT3  employ_status1  marital1  mor_circle  moralid10  moralid3  moralid5  moralid6  moralid7  moralid8  moralid9  mcoop1  mcoop2  mcoop3  mcoop4  mcoop5  mcoop6  mcoop7  narc1  narc2  narc3  narc4  narc5  narc6  nidentity1  nidentity2  omind1  omind2  omind6  contact3  hygiene4  psupport1  psupport2  psupport3  psupport4  psupport5  political_ideology  happy  slf_ladder  self_esteem  sbelong3  sbelong4  optim1  optim2
##   2   9  cnarc1  cnarc2  cnarc3  CRT1  CRT2  CRT3  employ_status1  marital1  mor_circle  moralid10  moralid3  moralid5  moralid6  moralid7  moralid8  moralid9  mcoop1  mcoop2  mcoop3  mcoop4  mcoop5  mcoop6  mcoop7  narc1  narc2  narc3  narc4  narc5  narc6  nidentity1  nidentity2  omind1  omind2  omind6  contact3  hygiene4  psupport1  psupport2  psupport3  psupport4  psupport5  political_ideology  happy  slf_ladder  self_esteem  sbelong3  sbelong4  optim1  optim2
##   2   10  cnarc1  cnarc2  cnarc3  CRT1  CRT2  CRT3  employ_status1  marital1  mor_circle  moralid10  moralid3  moralid5  moralid6  moralid7  moralid8  moralid9  mcoop1  mcoop2  mcoop3  mcoop4  mcoop5  mcoop6  mcoop7  narc1  narc2  narc3  narc4  narc5  narc6  nidentity1  nidentity2  omind1  omind2  omind6  contact3  hygiene4  psupport1  psupport2  psupport3  psupport4  psupport5  political_ideology  happy  slf_ladder  self_esteem  sbelong3  sbelong4  optim1  optim2
##   3   1  cnarc1  cnarc2  cnarc3  CRT1  CRT2  CRT3  employ_status1  marital1  mor_circle  moralid10  moralid3  moralid5  moralid6  moralid7  moralid8  moralid9  mcoop1  mcoop2  mcoop3  mcoop4  mcoop5  mcoop6  mcoop7  narc1  narc2  narc3  narc4  narc5  narc6  nidentity1  nidentity2  omind1  omind2  omind6  contact3  hygiene4  psupport1  psupport2  psupport3  psupport4  psupport5  political_ideology  happy  slf_ladder  self_esteem  sbelong3  sbelong4  optim1  optim2
##   3   2  cnarc1  cnarc2  cnarc3  CRT1  CRT2  CRT3  employ_status1  marital1  mor_circle  moralid10  moralid3  moralid5  moralid6  moralid7  moralid8  moralid9  mcoop1  mcoop2  mcoop3  mcoop4  mcoop5  mcoop6  mcoop7  narc1  narc2  narc3  narc4  narc5  narc6  nidentity1  nidentity2  omind1  omind2  omind6  contact3  hygiene4  psupport1  psupport2  psupport3  psupport4  psupport5  political_ideology  happy  slf_ladder  self_esteem  sbelong3  sbelong4  optim1  optim2
##   3   3  cnarc1  cnarc2  cnarc3  CRT1  CRT2  CRT3  employ_status1  marital1  mor_circle  moralid10  moralid3  moralid5  moralid6  moralid7  moralid8  moralid9  mcoop1  mcoop2  mcoop3  mcoop4  mcoop5  mcoop6  mcoop7  narc1  narc2  narc3  narc4  narc5  narc6  nidentity1  nidentity2  omind1  omind2  omind6  contact3  hygiene4  psupport1  psupport2  psupport3  psupport4  psupport5  political_ideology  happy  slf_ladder  self_esteem  sbelong3  sbelong4  optim1  optim2
##   3   4  cnarc1  cnarc2  cnarc3  CRT1  CRT2  CRT3  employ_status1  marital1  mor_circle  moralid10  moralid3  moralid5  moralid6  moralid7  moralid8  moralid9  mcoop1  mcoop2  mcoop3  mcoop4  mcoop5  mcoop6  mcoop7  narc1  narc2  narc3  narc4  narc5  narc6  nidentity1  nidentity2  omind1  omind2  omind6  contact3  hygiene4  psupport1  psupport2  psupport3  psupport4  psupport5  political_ideology  happy  slf_ladder  self_esteem  sbelong3  sbelong4  optim1  optim2
##   3   5  cnarc1  cnarc2  cnarc3  CRT1  CRT2  CRT3  employ_status1  marital1  mor_circle  moralid10  moralid3  moralid5  moralid6  moralid7  moralid8  moralid9  mcoop1  mcoop2  mcoop3  mcoop4  mcoop5  mcoop6  mcoop7  narc1  narc2  narc3  narc4  narc5  narc6  nidentity1  nidentity2  omind1  omind2  omind6  contact3  hygiene4  psupport1  psupport2  psupport3  psupport4  psupport5  political_ideology  happy  slf_ladder  self_esteem  sbelong3  sbelong4  optim1  optim2
##   3   6  cnarc1  cnarc2  cnarc3  CRT1  CRT2  CRT3  employ_status1  marital1  mor_circle  moralid10  moralid3  moralid5  moralid6  moralid7  moralid8  moralid9  mcoop1  mcoop2  mcoop3  mcoop4  mcoop5  mcoop6  mcoop7  narc1  narc2  narc3  narc4  narc5  narc6  nidentity1  nidentity2  omind1  omind2  omind6  contact3  hygiene4  psupport1  psupport2  psupport3  psupport4  psupport5  political_ideology  happy  slf_ladder  self_esteem  sbelong3  sbelong4  optim1  optim2
##   3   7  cnarc1  cnarc2  cnarc3  CRT1  CRT2  CRT3  employ_status1  marital1  mor_circle  moralid10  moralid3  moralid5  moralid6  moralid7  moralid8  moralid9  mcoop1  mcoop2  mcoop3  mcoop4  mcoop5  mcoop6  mcoop7  narc1  narc2  narc3  narc4  narc5  narc6  nidentity1  nidentity2  omind1  omind2  omind6  contact3  hygiene4  psupport1  psupport2  psupport3  psupport4  psupport5  political_ideology  happy  slf_ladder  self_esteem  sbelong3  sbelong4  optim1  optim2
##   3   8  cnarc1  cnarc2  cnarc3  CRT1  CRT2  CRT3  employ_status1  marital1  mor_circle  moralid10  moralid3  moralid5  moralid6  moralid7  moralid8  moralid9  mcoop1  mcoop2  mcoop3  mcoop4  mcoop5  mcoop6  mcoop7  narc1  narc2  narc3  narc4  narc5  narc6  nidentity1  nidentity2  omind1  omind2  omind6  contact3  hygiene4  psupport1  psupport2  psupport3  psupport4  psupport5  political_ideology  happy  slf_ladder  self_esteem  sbelong3  sbelong4  optim1  optim2
##   3   9  cnarc1  cnarc2  cnarc3  CRT1  CRT2  CRT3  employ_status1  marital1  mor_circle  moralid10  moralid3  moralid5  moralid6  moralid7  moralid8  moralid9  mcoop1  mcoop2  mcoop3  mcoop4  mcoop5  mcoop6  mcoop7  narc1  narc2  narc3  narc4  narc5  narc6  nidentity1  nidentity2  omind1  omind2  omind6  contact3  hygiene4  psupport1  psupport2  psupport3  psupport4  psupport5  political_ideology  happy  slf_ladder  self_esteem  sbelong3  sbelong4  optim1  optim2
##   3   10  cnarc1  cnarc2  cnarc3  CRT1  CRT2  CRT3  employ_status1  marital1  mor_circle  moralid10  moralid3  moralid5  moralid6  moralid7  moralid8  moralid9  mcoop1  mcoop2  mcoop3  mcoop4  mcoop5  mcoop6  mcoop7  narc1  narc2  narc3  narc4  narc5  narc6  nidentity1  nidentity2  omind1  omind2  omind6  contact3  hygiene4  psupport1  psupport2  psupport3  psupport4  psupport5  political_ideology  happy  slf_ladder  self_esteem  sbelong3  sbelong4  optim1  optim2
##   4   1  cnarc1  cnarc2  cnarc3  CRT1  CRT2  CRT3  employ_status1  marital1  mor_circle  moralid10  moralid3  moralid5  moralid6  moralid7  moralid8  moralid9  mcoop1  mcoop2  mcoop3  mcoop4  mcoop5  mcoop6  mcoop7  narc1  narc2  narc3  narc4  narc5  narc6  nidentity1  nidentity2  omind1  omind2  omind6  contact3  hygiene4  psupport1  psupport2  psupport3  psupport4  psupport5  political_ideology  happy  slf_ladder  self_esteem  sbelong3  sbelong4  optim1  optim2
##   4   2  cnarc1  cnarc2  cnarc3  CRT1  CRT2  CRT3  employ_status1  marital1  mor_circle  moralid10  moralid3  moralid5  moralid6  moralid7  moralid8  moralid9  mcoop1  mcoop2  mcoop3  mcoop4  mcoop5  mcoop6  mcoop7  narc1  narc2  narc3  narc4  narc5  narc6  nidentity1  nidentity2  omind1  omind2  omind6  contact3  hygiene4  psupport1  psupport2  psupport3  psupport4  psupport5  political_ideology  happy  slf_ladder  self_esteem  sbelong3  sbelong4  optim1  optim2
##   4   3  cnarc1  cnarc2  cnarc3  CRT1  CRT2  CRT3  employ_status1  marital1  mor_circle  moralid10  moralid3  moralid5  moralid6  moralid7  moralid8  moralid9  mcoop1  mcoop2  mcoop3  mcoop4  mcoop5  mcoop6  mcoop7  narc1  narc2  narc3  narc4  narc5  narc6  nidentity1  nidentity2  omind1  omind2  omind6  contact3  hygiene4  psupport1  psupport2  psupport3  psupport4  psupport5  political_ideology  happy  slf_ladder  self_esteem  sbelong3  sbelong4  optim1  optim2
##   4   4  cnarc1  cnarc2  cnarc3  CRT1  CRT2  CRT3  employ_status1  marital1  mor_circle  moralid10  moralid3  moralid5  moralid6  moralid7  moralid8  moralid9  mcoop1  mcoop2  mcoop3  mcoop4  mcoop5  mcoop6  mcoop7  narc1  narc2  narc3  narc4  narc5  narc6  nidentity1  nidentity2  omind1  omind2  omind6  contact3  hygiene4  psupport1  psupport2  psupport3  psupport4  psupport5  political_ideology  happy  slf_ladder  self_esteem  sbelong3  sbelong4  optim1  optim2
##   4   5  cnarc1  cnarc2  cnarc3  CRT1  CRT2  CRT3  employ_status1  marital1  mor_circle  moralid10  moralid3  moralid5  moralid6  moralid7  moralid8  moralid9  mcoop1  mcoop2  mcoop3  mcoop4  mcoop5  mcoop6  mcoop7  narc1  narc2  narc3  narc4  narc5  narc6  nidentity1  nidentity2  omind1  omind2  omind6  contact3  hygiene4  psupport1  psupport2  psupport3  psupport4  psupport5  political_ideology  happy  slf_ladder  self_esteem  sbelong3  sbelong4  optim1  optim2
##   4   6  cnarc1  cnarc2  cnarc3  CRT1  CRT2  CRT3  employ_status1  marital1  mor_circle  moralid10  moralid3  moralid5  moralid6  moralid7  moralid8  moralid9  mcoop1  mcoop2  mcoop3  mcoop4  mcoop5  mcoop6  mcoop7  narc1  narc2  narc3  narc4  narc5  narc6  nidentity1  nidentity2  omind1  omind2  omind6  contact3  hygiene4  psupport1  psupport2  psupport3  psupport4  psupport5  political_ideology  happy  slf_ladder  self_esteem  sbelong3  sbelong4  optim1  optim2
##   4   7  cnarc1  cnarc2  cnarc3  CRT1  CRT2  CRT3  employ_status1  marital1  mor_circle  moralid10  moralid3  moralid5  moralid6  moralid7  moralid8  moralid9  mcoop1  mcoop2  mcoop3  mcoop4  mcoop5  mcoop6  mcoop7  narc1  narc2  narc3  narc4  narc5  narc6  nidentity1  nidentity2  omind1  omind2  omind6  contact3  hygiene4  psupport1  psupport2  psupport3  psupport4  psupport5  political_ideology  happy  slf_ladder  self_esteem  sbelong3  sbelong4  optim1  optim2
##   4   8  cnarc1  cnarc2  cnarc3  CRT1  CRT2  CRT3  employ_status1  marital1  mor_circle  moralid10  moralid3  moralid5  moralid6  moralid7  moralid8  moralid9  mcoop1  mcoop2  mcoop3  mcoop4  mcoop5  mcoop6  mcoop7  narc1  narc2  narc3  narc4  narc5  narc6  nidentity1  nidentity2  omind1  omind2  omind6  contact3  hygiene4  psupport1  psupport2  psupport3  psupport4  psupport5  political_ideology  happy  slf_ladder  self_esteem  sbelong3  sbelong4  optim1  optim2
##   4   9  cnarc1  cnarc2  cnarc3  CRT1  CRT2  CRT3  employ_status1  marital1  mor_circle  moralid10  moralid3  moralid5  moralid6  moralid7  moralid8  moralid9  mcoop1  mcoop2  mcoop3  mcoop4  mcoop5  mcoop6  mcoop7  narc1  narc2  narc3  narc4  narc5  narc6  nidentity1  nidentity2  omind1  omind2  omind6  contact3  hygiene4  psupport1  psupport2  psupport3  psupport4  psupport5  political_ideology  happy  slf_ladder  self_esteem  sbelong3  sbelong4  optim1  optim2
##   4   10  cnarc1  cnarc2  cnarc3  CRT1  CRT2  CRT3  employ_status1  marital1  mor_circle  moralid10  moralid3  moralid5  moralid6  moralid7  moralid8  moralid9  mcoop1  mcoop2  mcoop3  mcoop4  mcoop5  mcoop6  mcoop7  narc1  narc2  narc3  narc4  narc5  narc6  nidentity1  nidentity2  omind1  omind2  omind6  contact3  hygiene4  psupport1  psupport2  psupport3  psupport4  psupport5  political_ideology  happy  slf_ladder  self_esteem  sbelong3  sbelong4  optim1  optim2
##   5   1  cnarc1  cnarc2  cnarc3  CRT1  CRT2  CRT3  employ_status1  marital1  mor_circle  moralid10  moralid3  moralid5  moralid6  moralid7  moralid8  moralid9  mcoop1  mcoop2  mcoop3  mcoop4  mcoop5  mcoop6  mcoop7  narc1  narc2  narc3  narc4  narc5  narc6  nidentity1  nidentity2  omind1  omind2  omind6  contact3  hygiene4  psupport1  psupport2  psupport3  psupport4  psupport5  political_ideology  happy  slf_ladder  self_esteem  sbelong3  sbelong4  optim1  optim2
##   5   2  cnarc1  cnarc2  cnarc3  CRT1  CRT2  CRT3  employ_status1  marital1  mor_circle  moralid10  moralid3  moralid5  moralid6  moralid7  moralid8  moralid9  mcoop1  mcoop2  mcoop3  mcoop4  mcoop5  mcoop6  mcoop7  narc1  narc2  narc3  narc4  narc5  narc6  nidentity1  nidentity2  omind1  omind2  omind6  contact3  hygiene4  psupport1  psupport2  psupport3  psupport4  psupport5  political_ideology  happy  slf_ladder  self_esteem  sbelong3  sbelong4  optim1  optim2
##   5   3  cnarc1  cnarc2  cnarc3  CRT1  CRT2  CRT3  employ_status1  marital1  mor_circle  moralid10  moralid3  moralid5  moralid6  moralid7  moralid8  moralid9  mcoop1  mcoop2  mcoop3  mcoop4  mcoop5  mcoop6  mcoop7  narc1  narc2  narc3  narc4  narc5  narc6  nidentity1  nidentity2  omind1  omind2  omind6  contact3  hygiene4  psupport1  psupport2  psupport3  psupport4  psupport5  political_ideology  happy  slf_ladder  self_esteem  sbelong3  sbelong4  optim1  optim2
##   5   4  cnarc1  cnarc2  cnarc3  CRT1  CRT2  CRT3  employ_status1  marital1  mor_circle  moralid10  moralid3  moralid5  moralid6  moralid7  moralid8  moralid9  mcoop1  mcoop2  mcoop3  mcoop4  mcoop5  mcoop6  mcoop7  narc1  narc2  narc3  narc4  narc5  narc6  nidentity1  nidentity2  omind1  omind2  omind6  contact3  hygiene4  psupport1  psupport2  psupport3  psupport4  psupport5  political_ideology  happy  slf_ladder  self_esteem  sbelong3  sbelong4  optim1  optim2
##   5   5  cnarc1  cnarc2  cnarc3  CRT1  CRT2  CRT3  employ_status1  marital1  mor_circle  moralid10  moralid3  moralid5  moralid6  moralid7  moralid8  moralid9  mcoop1  mcoop2  mcoop3  mcoop4  mcoop5  mcoop6  mcoop7  narc1  narc2  narc3  narc4  narc5  narc6  nidentity1  nidentity2  omind1  omind2  omind6  contact3  hygiene4  psupport1  psupport2  psupport3  psupport4  psupport5  political_ideology  happy  slf_ladder  self_esteem  sbelong3  sbelong4  optim1  optim2
##   5   6  cnarc1  cnarc2  cnarc3  CRT1  CRT2  CRT3  employ_status1  marital1  mor_circle  moralid10  moralid3  moralid5  moralid6  moralid7  moralid8  moralid9  mcoop1  mcoop2  mcoop3  mcoop4  mcoop5  mcoop6  mcoop7  narc1  narc2  narc3  narc4  narc5  narc6  nidentity1  nidentity2  omind1  omind2  omind6  contact3  hygiene4  psupport1  psupport2  psupport3  psupport4  psupport5  political_ideology  happy  slf_ladder  self_esteem  sbelong3  sbelong4  optim1  optim2
##   5   7  cnarc1  cnarc2  cnarc3  CRT1  CRT2  CRT3  employ_status1  marital1  mor_circle  moralid10  moralid3  moralid5  moralid6  moralid7  moralid8  moralid9  mcoop1  mcoop2  mcoop3  mcoop4  mcoop5  mcoop6  mcoop7  narc1  narc2  narc3  narc4  narc5  narc6  nidentity1  nidentity2  omind1  omind2  omind6  contact3  hygiene4  psupport1  psupport2  psupport3  psupport4  psupport5  political_ideology  happy  slf_ladder  self_esteem  sbelong3  sbelong4  optim1  optim2
##   5   8  cnarc1  cnarc2  cnarc3  CRT1  CRT2  CRT3  employ_status1  marital1  mor_circle  moralid10  moralid3  moralid5  moralid6  moralid7  moralid8  moralid9  mcoop1  mcoop2  mcoop3  mcoop4  mcoop5  mcoop6  mcoop7  narc1  narc2  narc3  narc4  narc5  narc6  nidentity1  nidentity2  omind1  omind2  omind6  contact3  hygiene4  psupport1  psupport2  psupport3  psupport4  psupport5  political_ideology  happy  slf_ladder  self_esteem  sbelong3  sbelong4  optim1  optim2
##   5   9  cnarc1  cnarc2  cnarc3  CRT1  CRT2  CRT3  employ_status1  marital1  mor_circle  moralid10  moralid3  moralid5  moralid6  moralid7  moralid8  moralid9  mcoop1  mcoop2  mcoop3  mcoop4  mcoop5  mcoop6  mcoop7  narc1  narc2  narc3  narc4  narc5  narc6  nidentity1  nidentity2  omind1  omind2  omind6  contact3  hygiene4  psupport1  psupport2  psupport3  psupport4  psupport5  political_ideology  happy  slf_ladder  self_esteem  sbelong3  sbelong4  optim1  optim2
##   5   10  cnarc1  cnarc2  cnarc3  CRT1  CRT2  CRT3  employ_status1  marital1  mor_circle  moralid10  moralid3  moralid5  moralid6  moralid7  moralid8  moralid9  mcoop1  mcoop2  mcoop3  mcoop4  mcoop5  mcoop6  mcoop7  narc1  narc2  narc3  narc4  narc5  narc6  nidentity1  nidentity2  omind1  omind2  omind6  contact3  hygiene4  psupport1  psupport2  psupport3  psupport4  psupport5  political_ideology  happy  slf_ladder  self_esteem  sbelong3  sbelong4  optim1  optim2
##   6   1  cnarc1  cnarc2  cnarc3  CRT1  CRT2  CRT3  employ_status1  marital1  mor_circle  moralid10  moralid3  moralid5  moralid6  moralid7  moralid8  moralid9  mcoop1  mcoop2  mcoop3  mcoop4  mcoop5  mcoop6  mcoop7  narc1  narc2  narc3  narc4  narc5  narc6  nidentity1  nidentity2  omind1  omind2  omind6  contact3  hygiene4  psupport1  psupport2  psupport3  psupport4  psupport5  political_ideology  happy  slf_ladder  self_esteem  sbelong3  sbelong4  optim1  optim2
##   6   2  cnarc1  cnarc2  cnarc3  CRT1  CRT2  CRT3  employ_status1  marital1  mor_circle  moralid10  moralid3  moralid5  moralid6  moralid7  moralid8  moralid9  mcoop1  mcoop2  mcoop3  mcoop4  mcoop5  mcoop6  mcoop7  narc1  narc2  narc3  narc4  narc5  narc6  nidentity1  nidentity2  omind1  omind2  omind6  contact3  hygiene4  psupport1  psupport2  psupport3  psupport4  psupport5  political_ideology  happy  slf_ladder  self_esteem  sbelong3  sbelong4  optim1  optim2
##   6   3  cnarc1  cnarc2  cnarc3  CRT1  CRT2  CRT3  employ_status1  marital1  mor_circle  moralid10  moralid3  moralid5  moralid6  moralid7  moralid8  moralid9  mcoop1  mcoop2  mcoop3  mcoop4  mcoop5  mcoop6  mcoop7  narc1  narc2  narc3  narc4  narc5  narc6  nidentity1  nidentity2  omind1  omind2  omind6  contact3  hygiene4  psupport1  psupport2  psupport3  psupport4  psupport5  political_ideology  happy  slf_ladder  self_esteem  sbelong3  sbelong4  optim1  optim2
##   6   4  cnarc1  cnarc2  cnarc3  CRT1  CRT2  CRT3  employ_status1  marital1  mor_circle  moralid10  moralid3  moralid5  moralid6  moralid7  moralid8  moralid9  mcoop1  mcoop2  mcoop3  mcoop4  mcoop5  mcoop6  mcoop7  narc1  narc2  narc3  narc4  narc5  narc6  nidentity1  nidentity2  omind1  omind2  omind6  contact3  hygiene4  psupport1  psupport2  psupport3  psupport4  psupport5  political_ideology  happy  slf_ladder  self_esteem  sbelong3  sbelong4  optim1  optim2
##   6   5  cnarc1  cnarc2  cnarc3  CRT1  CRT2  CRT3  employ_status1  marital1  mor_circle  moralid10  moralid3  moralid5  moralid6  moralid7  moralid8  moralid9  mcoop1  mcoop2  mcoop3  mcoop4  mcoop5  mcoop6  mcoop7  narc1  narc2  narc3  narc4  narc5  narc6  nidentity1  nidentity2  omind1  omind2  omind6  contact3  hygiene4  psupport1  psupport2  psupport3  psupport4  psupport5  political_ideology  happy  slf_ladder  self_esteem  sbelong3  sbelong4  optim1  optim2
##   6   6  cnarc1  cnarc2  cnarc3  CRT1  CRT2  CRT3  employ_status1  marital1  mor_circle  moralid10  moralid3  moralid5  moralid6  moralid7  moralid8  moralid9  mcoop1  mcoop2  mcoop3  mcoop4  mcoop5  mcoop6  mcoop7  narc1  narc2  narc3  narc4  narc5  narc6  nidentity1  nidentity2  omind1  omind2  omind6  contact3  hygiene4  psupport1  psupport2  psupport3  psupport4  psupport5  political_ideology  happy  slf_ladder  self_esteem  sbelong3  sbelong4  optim1  optim2
##   6   7  cnarc1  cnarc2  cnarc3  CRT1  CRT2  CRT3  employ_status1  marital1  mor_circle  moralid10  moralid3  moralid5  moralid6  moralid7  moralid8  moralid9  mcoop1  mcoop2  mcoop3  mcoop4  mcoop5  mcoop6  mcoop7  narc1  narc2  narc3  narc4  narc5  narc6  nidentity1  nidentity2  omind1  omind2  omind6  contact3  hygiene4  psupport1  psupport2  psupport3  psupport4  psupport5  political_ideology  happy  slf_ladder  self_esteem  sbelong3  sbelong4  optim1  optim2
##   6   8  cnarc1  cnarc2  cnarc3  CRT1  CRT2  CRT3  employ_status1  marital1  mor_circle  moralid10  moralid3  moralid5  moralid6  moralid7  moralid8  moralid9  mcoop1  mcoop2  mcoop3  mcoop4  mcoop5  mcoop6  mcoop7  narc1  narc2  narc3  narc4  narc5  narc6  nidentity1  nidentity2  omind1  omind2  omind6  contact3  hygiene4  psupport1  psupport2  psupport3  psupport4  psupport5  political_ideology  happy  slf_ladder  self_esteem  sbelong3  sbelong4  optim1  optim2
##   6   9  cnarc1  cnarc2  cnarc3  CRT1  CRT2  CRT3  employ_status1  marital1  mor_circle  moralid10  moralid3  moralid5  moralid6  moralid7  moralid8  moralid9  mcoop1  mcoop2  mcoop3  mcoop4  mcoop5  mcoop6  mcoop7  narc1  narc2  narc3  narc4  narc5  narc6  nidentity1  nidentity2  omind1  omind2  omind6  contact3  hygiene4  psupport1  psupport2  psupport3  psupport4  psupport5  political_ideology  happy  slf_ladder  self_esteem  sbelong3  sbelong4  optim1  optim2
##   6   10  cnarc1  cnarc2  cnarc3  CRT1  CRT2  CRT3  employ_status1  marital1  mor_circle  moralid10  moralid3  moralid5  moralid6  moralid7  moralid8  moralid9  mcoop1  mcoop2  mcoop3  mcoop4  mcoop5  mcoop6  mcoop7  narc1  narc2  narc3  narc4  narc5  narc6  nidentity1  nidentity2  omind1  omind2  omind6  contact3  hygiene4  psupport1  psupport2  psupport3  psupport4  psupport5  political_ideology  happy  slf_ladder  self_esteem  sbelong3  sbelong4  optim1  optim2
##   7   1  cnarc1  cnarc2  cnarc3  CRT1  CRT2  CRT3  employ_status1  marital1  mor_circle  moralid10  moralid3  moralid5  moralid6  moralid7  moralid8  moralid9  mcoop1  mcoop2  mcoop3  mcoop4  mcoop5  mcoop6  mcoop7  narc1  narc2  narc3  narc4  narc5  narc6  nidentity1  nidentity2  omind1  omind2  omind6  contact3  hygiene4  psupport1  psupport2  psupport3  psupport4  psupport5  political_ideology  happy  slf_ladder  self_esteem  sbelong3  sbelong4  optim1  optim2
##   7   2  cnarc1  cnarc2  cnarc3  CRT1  CRT2  CRT3  employ_status1  marital1  mor_circle  moralid10  moralid3  moralid5  moralid6  moralid7  moralid8  moralid9  mcoop1  mcoop2  mcoop3  mcoop4  mcoop5  mcoop6  mcoop7  narc1  narc2  narc3  narc4  narc5  narc6  nidentity1  nidentity2  omind1  omind2  omind6  contact3  hygiene4  psupport1  psupport2  psupport3  psupport4  psupport5  political_ideology  happy  slf_ladder  self_esteem  sbelong3  sbelong4  optim1  optim2
##   7   3  cnarc1  cnarc2  cnarc3  CRT1  CRT2  CRT3  employ_status1  marital1  mor_circle  moralid10  moralid3  moralid5  moralid6  moralid7  moralid8  moralid9  mcoop1  mcoop2  mcoop3  mcoop4  mcoop5  mcoop6  mcoop7  narc1  narc2  narc3  narc4  narc5  narc6  nidentity1  nidentity2  omind1  omind2  omind6  contact3  hygiene4  psupport1  psupport2  psupport3  psupport4  psupport5  political_ideology  happy  slf_ladder  self_esteem  sbelong3  sbelong4  optim1  optim2
##   7   4  cnarc1  cnarc2  cnarc3  CRT1  CRT2  CRT3  employ_status1  marital1  mor_circle  moralid10  moralid3  moralid5  moralid6  moralid7  moralid8  moralid9  mcoop1  mcoop2  mcoop3  mcoop4  mcoop5  mcoop6  mcoop7  narc1  narc2  narc3  narc4  narc5  narc6  nidentity1  nidentity2  omind1  omind2  omind6  contact3  hygiene4  psupport1  psupport2  psupport3  psupport4  psupport5  political_ideology  happy  slf_ladder  self_esteem  sbelong3  sbelong4  optim1  optim2
##   7   5  cnarc1  cnarc2  cnarc3  CRT1  CRT2  CRT3  employ_status1  marital1  mor_circle  moralid10  moralid3  moralid5  moralid6  moralid7  moralid8  moralid9  mcoop1  mcoop2  mcoop3  mcoop4  mcoop5  mcoop6  mcoop7  narc1  narc2  narc3  narc4  narc5  narc6  nidentity1  nidentity2  omind1  omind2  omind6  contact3  hygiene4  psupport1  psupport2  psupport3  psupport4  psupport5  political_ideology  happy  slf_ladder  self_esteem  sbelong3  sbelong4  optim1  optim2
##   7   6  cnarc1  cnarc2  cnarc3  CRT1  CRT2  CRT3  employ_status1  marital1  mor_circle  moralid10  moralid3  moralid5  moralid6  moralid7  moralid8  moralid9  mcoop1  mcoop2  mcoop3  mcoop4  mcoop5  mcoop6  mcoop7  narc1  narc2  narc3  narc4  narc5  narc6  nidentity1  nidentity2  omind1  omind2  omind6  contact3  hygiene4  psupport1  psupport2  psupport3  psupport4  psupport5  political_ideology  happy  slf_ladder  self_esteem  sbelong3  sbelong4  optim1  optim2
##   7   7  cnarc1  cnarc2  cnarc3  CRT1  CRT2  CRT3  employ_status1  marital1  mor_circle  moralid10  moralid3  moralid5  moralid6  moralid7  moralid8  moralid9  mcoop1  mcoop2  mcoop3  mcoop4  mcoop5  mcoop6  mcoop7  narc1  narc2  narc3  narc4  narc5  narc6  nidentity1  nidentity2  omind1  omind2  omind6  contact3  hygiene4  psupport1  psupport2  psupport3  psupport4  psupport5  political_ideology  happy  slf_ladder  self_esteem  sbelong3  sbelong4  optim1  optim2
##   7   8  cnarc1  cnarc2  cnarc3  CRT1  CRT2  CRT3  employ_status1  marital1  mor_circle  moralid10  moralid3  moralid5  moralid6  moralid7  moralid8  moralid9  mcoop1  mcoop2  mcoop3  mcoop4  mcoop5  mcoop6  mcoop7  narc1  narc2  narc3  narc4  narc5  narc6  nidentity1  nidentity2  omind1  omind2  omind6  contact3  hygiene4  psupport1  psupport2  psupport3  psupport4  psupport5  political_ideology  happy  slf_ladder  self_esteem  sbelong3  sbelong4  optim1  optim2
##   7   9  cnarc1  cnarc2  cnarc3  CRT1  CRT2  CRT3  employ_status1  marital1  mor_circle  moralid10  moralid3  moralid5  moralid6  moralid7  moralid8  moralid9  mcoop1  mcoop2  mcoop3  mcoop4  mcoop5  mcoop6  mcoop7  narc1  narc2  narc3  narc4  narc5  narc6  nidentity1  nidentity2  omind1  omind2  omind6  contact3  hygiene4  psupport1  psupport2  psupport3  psupport4  psupport5  political_ideology  happy  slf_ladder  self_esteem  sbelong3  sbelong4  optim1  optim2
##   7   10  cnarc1  cnarc2  cnarc3  CRT1  CRT2  CRT3  employ_status1  marital1  mor_circle  moralid10  moralid3  moralid5  moralid6  moralid7  moralid8  moralid9  mcoop1  mcoop2  mcoop3  mcoop4  mcoop5  mcoop6  mcoop7  narc1  narc2  narc3  narc4  narc5  narc6  nidentity1  nidentity2  omind1  omind2  omind6  contact3  hygiene4  psupport1  psupport2  psupport3  psupport4  psupport5  political_ideology  happy  slf_ladder  self_esteem  sbelong3  sbelong4  optim1  optim2
##   8   1  cnarc1  cnarc2  cnarc3  CRT1  CRT2  CRT3  employ_status1  marital1  mor_circle  moralid10  moralid3  moralid5  moralid6  moralid7  moralid8  moralid9  mcoop1  mcoop2  mcoop3  mcoop4  mcoop5  mcoop6  mcoop7  narc1  narc2  narc3  narc4  narc5  narc6  nidentity1  nidentity2  omind1  omind2  omind6  contact3  hygiene4  psupport1  psupport2  psupport3  psupport4  psupport5  political_ideology  happy  slf_ladder  self_esteem  sbelong3  sbelong4  optim1  optim2
##   8   2  cnarc1  cnarc2  cnarc3  CRT1  CRT2  CRT3  employ_status1  marital1  mor_circle  moralid10  moralid3  moralid5  moralid6  moralid7  moralid8  moralid9  mcoop1  mcoop2  mcoop3  mcoop4  mcoop5  mcoop6  mcoop7  narc1  narc2  narc3  narc4  narc5  narc6  nidentity1  nidentity2  omind1  omind2  omind6  contact3  hygiene4  psupport1  psupport2  psupport3  psupport4  psupport5  political_ideology  happy  slf_ladder  self_esteem  sbelong3  sbelong4  optim1  optim2
##   8   3  cnarc1  cnarc2  cnarc3  CRT1  CRT2  CRT3  employ_status1  marital1  mor_circle  moralid10  moralid3  moralid5  moralid6  moralid7  moralid8  moralid9  mcoop1  mcoop2  mcoop3  mcoop4  mcoop5  mcoop6  mcoop7  narc1  narc2  narc3  narc4  narc5  narc6  nidentity1  nidentity2  omind1  omind2  omind6  contact3  hygiene4  psupport1  psupport2  psupport3  psupport4  psupport5  political_ideology  happy  slf_ladder  self_esteem  sbelong3  sbelong4  optim1  optim2
##   8   4  cnarc1  cnarc2  cnarc3  CRT1  CRT2  CRT3  employ_status1  marital1  mor_circle  moralid10  moralid3  moralid5  moralid6  moralid7  moralid8  moralid9  mcoop1  mcoop2  mcoop3  mcoop4  mcoop5  mcoop6  mcoop7  narc1  narc2  narc3  narc4  narc5  narc6  nidentity1  nidentity2  omind1  omind2  omind6  contact3  hygiene4  psupport1  psupport2  psupport3  psupport4  psupport5  political_ideology  happy  slf_ladder  self_esteem  sbelong3  sbelong4  optim1  optim2
##   8   5  cnarc1  cnarc2  cnarc3  CRT1  CRT2  CRT3  employ_status1  marital1  mor_circle  moralid10  moralid3  moralid5  moralid6  moralid7  moralid8  moralid9  mcoop1  mcoop2  mcoop3  mcoop4  mcoop5  mcoop6  mcoop7  narc1  narc2  narc3  narc4  narc5  narc6  nidentity1  nidentity2  omind1  omind2  omind6  contact3  hygiene4  psupport1  psupport2  psupport3  psupport4  psupport5  political_ideology  happy  slf_ladder  self_esteem  sbelong3  sbelong4  optim1  optim2
##   8   6  cnarc1  cnarc2  cnarc3  CRT1  CRT2  CRT3  employ_status1  marital1  mor_circle  moralid10  moralid3  moralid5  moralid6  moralid7  moralid8  moralid9  mcoop1  mcoop2  mcoop3  mcoop4  mcoop5  mcoop6  mcoop7  narc1  narc2  narc3  narc4  narc5  narc6  nidentity1  nidentity2  omind1  omind2  omind6  contact3  hygiene4  psupport1  psupport2  psupport3  psupport4  psupport5  political_ideology  happy  slf_ladder  self_esteem  sbelong3  sbelong4  optim1  optim2
##   8   7  cnarc1  cnarc2  cnarc3  CRT1  CRT2  CRT3  employ_status1  marital1  mor_circle  moralid10  moralid3  moralid5  moralid6  moralid7  moralid8  moralid9  mcoop1  mcoop2  mcoop3  mcoop4  mcoop5  mcoop6  mcoop7  narc1  narc2  narc3  narc4  narc5  narc6  nidentity1  nidentity2  omind1  omind2  omind6  contact3  hygiene4  psupport1  psupport2  psupport3  psupport4  psupport5  political_ideology  happy  slf_ladder  self_esteem  sbelong3  sbelong4  optim1  optim2
##   8   8  cnarc1  cnarc2  cnarc3  CRT1  CRT2  CRT3  employ_status1  marital1  mor_circle  moralid10  moralid3  moralid5  moralid6  moralid7  moralid8  moralid9  mcoop1  mcoop2  mcoop3  mcoop4  mcoop5  mcoop6  mcoop7  narc1  narc2  narc3  narc4  narc5  narc6  nidentity1  nidentity2  omind1  omind2  omind6  contact3  hygiene4  psupport1  psupport2  psupport3  psupport4  psupport5  political_ideology  happy  slf_ladder  self_esteem  sbelong3  sbelong4  optim1  optim2
##   8   9  cnarc1  cnarc2  cnarc3  CRT1  CRT2  CRT3  employ_status1  marital1  mor_circle  moralid10  moralid3  moralid5  moralid6  moralid7  moralid8  moralid9  mcoop1  mcoop2  mcoop3  mcoop4  mcoop5  mcoop6  mcoop7  narc1  narc2  narc3  narc4  narc5  narc6  nidentity1  nidentity2  omind1  omind2  omind6  contact3  hygiene4  psupport1  psupport2  psupport3  psupport4  psupport5  political_ideology  happy  slf_ladder  self_esteem  sbelong3  sbelong4  optim1  optim2
##   8   10  cnarc1  cnarc2  cnarc3  CRT1  CRT2  CRT3  employ_status1  marital1  mor_circle  moralid10  moralid3  moralid5  moralid6  moralid7  moralid8  moralid9  mcoop1  mcoop2  mcoop3  mcoop4  mcoop5  mcoop6  mcoop7  narc1  narc2  narc3  narc4  narc5  narc6  nidentity1  nidentity2  omind1  omind2  omind6  contact3  hygiene4  psupport1  psupport2  psupport3  psupport4  psupport5  political_ideology  happy  slf_ladder  self_esteem  sbelong3  sbelong4  optim1  optim2
##   9   1  cnarc1  cnarc2  cnarc3  CRT1  CRT2  CRT3  employ_status1  marital1  mor_circle  moralid10  moralid3  moralid5  moralid6  moralid7  moralid8  moralid9  mcoop1  mcoop2  mcoop3  mcoop4  mcoop5  mcoop6  mcoop7  narc1  narc2  narc3  narc4  narc5  narc6  nidentity1  nidentity2  omind1  omind2  omind6  contact3  hygiene4  psupport1  psupport2  psupport3  psupport4  psupport5  political_ideology  happy  slf_ladder  self_esteem  sbelong3  sbelong4  optim1  optim2
##   9   2  cnarc1  cnarc2  cnarc3  CRT1  CRT2  CRT3  employ_status1  marital1  mor_circle  moralid10  moralid3  moralid5  moralid6  moralid7  moralid8  moralid9  mcoop1  mcoop2  mcoop3  mcoop4  mcoop5  mcoop6  mcoop7  narc1  narc2  narc3  narc4  narc5  narc6  nidentity1  nidentity2  omind1  omind2  omind6  contact3  hygiene4  psupport1  psupport2  psupport3  psupport4  psupport5  political_ideology  happy  slf_ladder  self_esteem  sbelong3  sbelong4  optim1  optim2
##   9   3  cnarc1  cnarc2  cnarc3  CRT1  CRT2  CRT3  employ_status1  marital1  mor_circle  moralid10  moralid3  moralid5  moralid6  moralid7  moralid8  moralid9  mcoop1  mcoop2  mcoop3  mcoop4  mcoop5  mcoop6  mcoop7  narc1  narc2  narc3  narc4  narc5  narc6  nidentity1  nidentity2  omind1  omind2  omind6  contact3  hygiene4  psupport1  psupport2  psupport3  psupport4  psupport5  political_ideology  happy  slf_ladder  self_esteem  sbelong3  sbelong4  optim1  optim2
##   9   4  cnarc1  cnarc2  cnarc3  CRT1  CRT2  CRT3  employ_status1  marital1  mor_circle  moralid10  moralid3  moralid5  moralid6  moralid7  moralid8  moralid9  mcoop1  mcoop2  mcoop3  mcoop4  mcoop5  mcoop6  mcoop7  narc1  narc2  narc3  narc4  narc5  narc6  nidentity1  nidentity2  omind1  omind2  omind6  contact3  hygiene4  psupport1  psupport2  psupport3  psupport4  psupport5  political_ideology  happy  slf_ladder  self_esteem  sbelong3  sbelong4  optim1  optim2
##   9   5  cnarc1  cnarc2  cnarc3  CRT1  CRT2  CRT3  employ_status1  marital1  mor_circle  moralid10  moralid3  moralid5  moralid6  moralid7  moralid8  moralid9  mcoop1  mcoop2  mcoop3  mcoop4  mcoop5  mcoop6  mcoop7  narc1  narc2  narc3  narc4  narc5  narc6  nidentity1  nidentity2  omind1  omind2  omind6  contact3  hygiene4  psupport1  psupport2  psupport3  psupport4  psupport5  political_ideology  happy  slf_ladder  self_esteem  sbelong3  sbelong4  optim1  optim2
##   9   6  cnarc1  cnarc2  cnarc3  CRT1  CRT2  CRT3  employ_status1  marital1  mor_circle  moralid10  moralid3  moralid5  moralid6  moralid7  moralid8  moralid9  mcoop1  mcoop2  mcoop3  mcoop4  mcoop5  mcoop6  mcoop7  narc1  narc2  narc3  narc4  narc5  narc6  nidentity1  nidentity2  omind1  omind2  omind6  contact3  hygiene4  psupport1  psupport2  psupport3  psupport4  psupport5  political_ideology  happy  slf_ladder  self_esteem  sbelong3  sbelong4  optim1  optim2
##   9   7  cnarc1  cnarc2  cnarc3  CRT1  CRT2  CRT3  employ_status1  marital1  mor_circle  moralid10  moralid3  moralid5  moralid6  moralid7  moralid8  moralid9  mcoop1  mcoop2  mcoop3  mcoop4  mcoop5  mcoop6  mcoop7  narc1  narc2  narc3  narc4  narc5  narc6  nidentity1  nidentity2  omind1  omind2  omind6  contact3  hygiene4  psupport1  psupport2  psupport3  psupport4  psupport5  political_ideology  happy  slf_ladder  self_esteem  sbelong3  sbelong4  optim1  optim2
##   9   8  cnarc1  cnarc2  cnarc3  CRT1  CRT2  CRT3  employ_status1  marital1  mor_circle  moralid10  moralid3  moralid5  moralid6  moralid7  moralid8  moralid9  mcoop1  mcoop2  mcoop3  mcoop4  mcoop5  mcoop6  mcoop7  narc1  narc2  narc3  narc4  narc5  narc6  nidentity1  nidentity2  omind1  omind2  omind6  contact3  hygiene4  psupport1  psupport2  psupport3  psupport4  psupport5  political_ideology  happy  slf_ladder  self_esteem  sbelong3  sbelong4  optim1  optim2
##   9   9  cnarc1  cnarc2  cnarc3  CRT1  CRT2  CRT3  employ_status1  marital1  mor_circle  moralid10  moralid3  moralid5  moralid6  moralid7  moralid8  moralid9  mcoop1  mcoop2  mcoop3  mcoop4  mcoop5  mcoop6  mcoop7  narc1  narc2  narc3  narc4  narc5  narc6  nidentity1  nidentity2  omind1  omind2  omind6  contact3  hygiene4  psupport1  psupport2  psupport3  psupport4  psupport5  political_ideology  happy  slf_ladder  self_esteem  sbelong3  sbelong4  optim1  optim2
##   9   10  cnarc1  cnarc2  cnarc3  CRT1  CRT2  CRT3  employ_status1  marital1  mor_circle  moralid10  moralid3  moralid5  moralid6  moralid7  moralid8  moralid9  mcoop1  mcoop2  mcoop3  mcoop4  mcoop5  mcoop6  mcoop7  narc1  narc2  narc3  narc4  narc5  narc6  nidentity1  nidentity2  omind1  omind2  omind6  contact3  hygiene4  psupport1  psupport2  psupport3  psupport4  psupport5  political_ideology  happy  slf_ladder  self_esteem  sbelong3  sbelong4  optim1  optim2
##   10   1  cnarc1  cnarc2  cnarc3  CRT1  CRT2  CRT3  employ_status1  marital1  mor_circle  moralid10  moralid3  moralid5  moralid6  moralid7  moralid8  moralid9  mcoop1  mcoop2  mcoop3  mcoop4  mcoop5  mcoop6  mcoop7  narc1  narc2  narc3  narc4  narc5  narc6  nidentity1  nidentity2  omind1  omind2  omind6  contact3  hygiene4  psupport1  psupport2  psupport3  psupport4  psupport5  political_ideology  happy  slf_ladder  self_esteem  sbelong3  sbelong4  optim1  optim2
##   10   2  cnarc1  cnarc2  cnarc3  CRT1  CRT2  CRT3  employ_status1  marital1  mor_circle  moralid10  moralid3  moralid5  moralid6  moralid7  moralid8  moralid9  mcoop1  mcoop2  mcoop3  mcoop4  mcoop5  mcoop6  mcoop7  narc1  narc2  narc3  narc4  narc5  narc6  nidentity1  nidentity2  omind1  omind2  omind6  contact3  hygiene4  psupport1  psupport2  psupport3  psupport4  psupport5  political_ideology  happy  slf_ladder  self_esteem  sbelong3  sbelong4  optim1  optim2
##   10   3  cnarc1  cnarc2  cnarc3  CRT1  CRT2  CRT3  employ_status1  marital1  mor_circle  moralid10  moralid3  moralid5  moralid6  moralid7  moralid8  moralid9  mcoop1  mcoop2  mcoop3  mcoop4  mcoop5  mcoop6  mcoop7  narc1  narc2  narc3  narc4  narc5  narc6  nidentity1  nidentity2  omind1  omind2  omind6  contact3  hygiene4  psupport1  psupport2  psupport3  psupport4  psupport5  political_ideology  happy  slf_ladder  self_esteem  sbelong3  sbelong4  optim1  optim2
##   10   4  cnarc1  cnarc2  cnarc3  CRT1  CRT2  CRT3  employ_status1  marital1  mor_circle  moralid10  moralid3  moralid5  moralid6  moralid7  moralid8  moralid9  mcoop1  mcoop2  mcoop3  mcoop4  mcoop5  mcoop6  mcoop7  narc1  narc2  narc3  narc4  narc5  narc6  nidentity1  nidentity2  omind1  omind2  omind6  contact3  hygiene4  psupport1  psupport2  psupport3  psupport4  psupport5  political_ideology  happy  slf_ladder  self_esteem  sbelong3  sbelong4  optim1  optim2
##   10   5  cnarc1  cnarc2  cnarc3  CRT1  CRT2  CRT3  employ_status1  marital1  mor_circle  moralid10  moralid3  moralid5  moralid6  moralid7  moralid8  moralid9  mcoop1  mcoop2  mcoop3  mcoop4  mcoop5  mcoop6  mcoop7  narc1  narc2  narc3  narc4  narc5  narc6  nidentity1  nidentity2  omind1  omind2  omind6  contact3  hygiene4  psupport1  psupport2  psupport3  psupport4  psupport5  political_ideology  happy  slf_ladder  self_esteem  sbelong3  sbelong4  optim1  optim2
##   10   6  cnarc1  cnarc2  cnarc3  CRT1  CRT2  CRT3  employ_status1  marital1  mor_circle  moralid10  moralid3  moralid5  moralid6  moralid7  moralid8  moralid9  mcoop1  mcoop2  mcoop3  mcoop4  mcoop5  mcoop6  mcoop7  narc1  narc2  narc3  narc4  narc5  narc6  nidentity1  nidentity2  omind1  omind2  omind6  contact3  hygiene4  psupport1  psupport2  psupport3  psupport4  psupport5  political_ideology  happy  slf_ladder  self_esteem  sbelong3  sbelong4  optim1  optim2
##   10   7  cnarc1  cnarc2  cnarc3  CRT1  CRT2  CRT3  employ_status1  marital1  mor_circle  moralid10  moralid3  moralid5  moralid6  moralid7  moralid8  moralid9  mcoop1  mcoop2  mcoop3  mcoop4  mcoop5  mcoop6  mcoop7  narc1  narc2  narc3  narc4  narc5  narc6  nidentity1  nidentity2  omind1  omind2  omind6  contact3  hygiene4  psupport1  psupport2  psupport3  psupport4  psupport5  political_ideology  happy  slf_ladder  self_esteem  sbelong3  sbelong4  optim1  optim2
##   10   8  cnarc1  cnarc2  cnarc3  CRT1  CRT2  CRT3  employ_status1  marital1  mor_circle  moralid10  moralid3  moralid5  moralid6  moralid7  moralid8  moralid9  mcoop1  mcoop2  mcoop3  mcoop4  mcoop5  mcoop6  mcoop7  narc1  narc2  narc3  narc4  narc5  narc6  nidentity1  nidentity2  omind1  omind2  omind6  contact3  hygiene4  psupport1  psupport2  psupport3  psupport4  psupport5  political_ideology  happy  slf_ladder  self_esteem  sbelong3  sbelong4  optim1  optim2
##   10   9  cnarc1  cnarc2  cnarc3  CRT1  CRT2  CRT3  employ_status1  marital1  mor_circle  moralid10  moralid3  moralid5  moralid6  moralid7  moralid8  moralid9  mcoop1  mcoop2  mcoop3  mcoop4  mcoop5  mcoop6  mcoop7  narc1  narc2  narc3  narc4  narc5  narc6  nidentity1  nidentity2  omind1  omind2  omind6  contact3  hygiene4  psupport1  psupport2  psupport3  psupport4  psupport5  political_ideology  happy  slf_ladder  self_esteem  sbelong3  sbelong4  optim1  optim2
##   10   10  cnarc1  cnarc2  cnarc3  CRT1  CRT2  CRT3  employ_status1  marital1  mor_circle  moralid10  moralid3  moralid5  moralid6  moralid7  moralid8  moralid9  mcoop1  mcoop2  mcoop3  mcoop4  mcoop5  mcoop6  mcoop7  narc1  narc2  narc3  narc4  narc5  narc6  nidentity1  nidentity2  omind1  omind2  omind6  contact3  hygiene4  psupport1  psupport2  psupport3  psupport4  psupport5  political_ideology  happy  slf_ladder  self_esteem  sbelong3  sbelong4  optim1  optim2
```

```
## Warning: Number of logged events: 4903
```

```
## 
##  iter imp variable
##   1   1  age  children  cnarc1  cnarc2  cnarc3  CRT1  CRT2  CRT3  health_cond  know_tested_positive  ladder  marital1  mor_circle  moralid1  moralid2  moralid3  moralid4  moralid5  moralid6  moralid7  moralid8  moralid9  mcoop1  mcoop2  mcoop3  mcoop4  mcoop5  mcoop6  mcoop7  narc1  narc2  narc3  narc4  narc5  narc6  nidentity2  omind1  omind2  omind5  omind6  contact1  contact2  contact3  contact4  contact5  hygiene1  hygiene2  hygiene3  hygiene4  hygiene5  psupport1  psupport2  psupport3  psupport4  psupport5  political_ideology  happy  slf_ladder  riskperc1  riskperc2  sbelong2  sbelong3  sbelong4  tested_positive  optim2  slfcont1  slfcont3  slfcont4  urban
##   1   2  age  children  cnarc1  cnarc2  cnarc3  CRT1  CRT2  CRT3  health_cond  know_tested_positive  ladder  marital1  mor_circle  moralid1  moralid2  moralid3  moralid4  moralid5  moralid6  moralid7  moralid8  moralid9  mcoop1  mcoop2  mcoop3  mcoop4  mcoop5  mcoop6  mcoop7  narc1  narc2  narc3  narc4  narc5  narc6  nidentity2  omind1  omind2  omind5  omind6  contact1  contact2  contact3  contact4  contact5  hygiene1  hygiene2  hygiene3  hygiene4  hygiene5  psupport1  psupport2  psupport3  psupport4  psupport5  political_ideology  happy  slf_ladder  riskperc1  riskperc2  sbelong2  sbelong3  sbelong4  tested_positive  optim2  slfcont1  slfcont3  slfcont4  urban
##   1   3  age  children  cnarc1  cnarc2  cnarc3  CRT1  CRT2  CRT3  health_cond  know_tested_positive  ladder  marital1  mor_circle  moralid1  moralid2  moralid3  moralid4  moralid5  moralid6  moralid7  moralid8  moralid9  mcoop1  mcoop2  mcoop3  mcoop4  mcoop5  mcoop6  mcoop7  narc1  narc2  narc3  narc4  narc5  narc6  nidentity2  omind1  omind2  omind5  omind6  contact1  contact2  contact3  contact4  contact5  hygiene1  hygiene2  hygiene3  hygiene4  hygiene5  psupport1  psupport2  psupport3  psupport4  psupport5  political_ideology  happy  slf_ladder  riskperc1  riskperc2  sbelong2  sbelong3  sbelong4  tested_positive  optim2  slfcont1  slfcont3  slfcont4  urban
##   1   4  age  children  cnarc1  cnarc2  cnarc3  CRT1  CRT2  CRT3  health_cond  know_tested_positive  ladder  marital1  mor_circle  moralid1  moralid2  moralid3  moralid4  moralid5  moralid6  moralid7  moralid8  moralid9  mcoop1  mcoop2  mcoop3  mcoop4  mcoop5  mcoop6  mcoop7  narc1  narc2  narc3  narc4  narc5  narc6  nidentity2  omind1  omind2  omind5  omind6  contact1  contact2  contact3  contact4  contact5  hygiene1  hygiene2  hygiene3  hygiene4  hygiene5  psupport1  psupport2  psupport3  psupport4  psupport5  political_ideology  happy  slf_ladder  riskperc1  riskperc2  sbelong2  sbelong3  sbelong4  tested_positive  optim2  slfcont1  slfcont3  slfcont4  urban
##   1   5  age  children  cnarc1  cnarc2  cnarc3  CRT1  CRT2  CRT3  health_cond  know_tested_positive  ladder  marital1  mor_circle  moralid1  moralid2  moralid3  moralid4  moralid5  moralid6  moralid7  moralid8  moralid9  mcoop1  mcoop2  mcoop3  mcoop4  mcoop5  mcoop6  mcoop7  narc1  narc2  narc3  narc4  narc5  narc6  nidentity2  omind1  omind2  omind5  omind6  contact1  contact2  contact3  contact4  contact5  hygiene1  hygiene2  hygiene3  hygiene4  hygiene5  psupport1  psupport2  psupport3  psupport4  psupport5  political_ideology  happy  slf_ladder  riskperc1  riskperc2  sbelong2  sbelong3  sbelong4  tested_positive  optim2  slfcont1  slfcont3  slfcont4  urban
##   1   6  age  children  cnarc1  cnarc2  cnarc3  CRT1  CRT2  CRT3  health_cond  know_tested_positive  ladder  marital1  mor_circle  moralid1  moralid2  moralid3  moralid4  moralid5  moralid6  moralid7  moralid8  moralid9  mcoop1  mcoop2  mcoop3  mcoop4  mcoop5  mcoop6  mcoop7  narc1  narc2  narc3  narc4  narc5  narc6  nidentity2  omind1  omind2  omind5  omind6  contact1  contact2  contact3  contact4  contact5  hygiene1  hygiene2  hygiene3  hygiene4  hygiene5  psupport1  psupport2  psupport3  psupport4  psupport5  political_ideology  happy  slf_ladder  riskperc1  riskperc2  sbelong2  sbelong3  sbelong4  tested_positive  optim2  slfcont1  slfcont3  slfcont4  urban
##   1   7  age  children  cnarc1  cnarc2  cnarc3  CRT1  CRT2  CRT3  health_cond  know_tested_positive  ladder  marital1  mor_circle  moralid1  moralid2  moralid3  moralid4  moralid5  moralid6  moralid7  moralid8  moralid9  mcoop1  mcoop2  mcoop3  mcoop4  mcoop5  mcoop6  mcoop7  narc1  narc2  narc3  narc4  narc5  narc6  nidentity2  omind1  omind2  omind5  omind6  contact1  contact2  contact3  contact4  contact5  hygiene1  hygiene2  hygiene3  hygiene4  hygiene5  psupport1  psupport2  psupport3  psupport4  psupport5  political_ideology  happy  slf_ladder  riskperc1  riskperc2  sbelong2  sbelong3  sbelong4  tested_positive  optim2  slfcont1  slfcont3  slfcont4  urban
##   1   8  age  children  cnarc1  cnarc2  cnarc3  CRT1  CRT2  CRT3  health_cond  know_tested_positive  ladder  marital1  mor_circle  moralid1  moralid2  moralid3  moralid4  moralid5  moralid6  moralid7  moralid8  moralid9  mcoop1  mcoop2  mcoop3  mcoop4  mcoop5  mcoop6  mcoop7  narc1  narc2  narc3  narc4  narc5  narc6  nidentity2  omind1  omind2  omind5  omind6  contact1  contact2  contact3  contact4  contact5  hygiene1  hygiene2  hygiene3  hygiene4  hygiene5  psupport1  psupport2  psupport3  psupport4  psupport5  political_ideology  happy  slf_ladder  riskperc1  riskperc2  sbelong2  sbelong3  sbelong4  tested_positive  optim2  slfcont1  slfcont3  slfcont4  urban
##   1   9  age  children  cnarc1  cnarc2  cnarc3  CRT1  CRT2  CRT3  health_cond  know_tested_positive  ladder  marital1  mor_circle  moralid1  moralid2  moralid3  moralid4  moralid5  moralid6  moralid7  moralid8  moralid9  mcoop1  mcoop2  mcoop3  mcoop4  mcoop5  mcoop6  mcoop7  narc1  narc2  narc3  narc4  narc5  narc6  nidentity2  omind1  omind2  omind5  omind6  contact1  contact2  contact3  contact4  contact5  hygiene1  hygiene2  hygiene3  hygiene4  hygiene5  psupport1  psupport2  psupport3  psupport4  psupport5  political_ideology  happy  slf_ladder  riskperc1  riskperc2  sbelong2  sbelong3  sbelong4  tested_positive  optim2  slfcont1  slfcont3  slfcont4  urban
##   1   10  age  children  cnarc1  cnarc2  cnarc3  CRT1  CRT2  CRT3  health_cond  know_tested_positive  ladder  marital1  mor_circle  moralid1  moralid2  moralid3  moralid4  moralid5  moralid6  moralid7  moralid8  moralid9  mcoop1  mcoop2  mcoop3  mcoop4  mcoop5  mcoop6  mcoop7  narc1  narc2  narc3  narc4  narc5  narc6  nidentity2  omind1  omind2  omind5  omind6  contact1  contact2  contact3  contact4  contact5  hygiene1  hygiene2  hygiene3  hygiene4  hygiene5  psupport1  psupport2  psupport3  psupport4  psupport5  political_ideology  happy  slf_ladder  riskperc1  riskperc2  sbelong2  sbelong3  sbelong4  tested_positive  optim2  slfcont1  slfcont3  slfcont4  urban
##   2   1  age  children  cnarc1  cnarc2  cnarc3  CRT1  CRT2  CRT3  health_cond  know_tested_positive  ladder  marital1  mor_circle  moralid1  moralid2  moralid3  moralid4  moralid5  moralid6  moralid7  moralid8  moralid9  mcoop1  mcoop2  mcoop3  mcoop4  mcoop5  mcoop6  mcoop7  narc1  narc2  narc3  narc4  narc5  narc6  nidentity2  omind1  omind2  omind5  omind6  contact1  contact2  contact3  contact4  contact5  hygiene1  hygiene2  hygiene3  hygiene4  hygiene5  psupport1  psupport2  psupport3  psupport4  psupport5  political_ideology  happy  slf_ladder  riskperc1  riskperc2  sbelong2  sbelong3  sbelong4  tested_positive  optim2  slfcont1  slfcont3  slfcont4  urban
##   2   2  age  children  cnarc1  cnarc2  cnarc3  CRT1  CRT2  CRT3  health_cond  know_tested_positive  ladder  marital1  mor_circle  moralid1  moralid2  moralid3  moralid4  moralid5  moralid6  moralid7  moralid8  moralid9  mcoop1  mcoop2  mcoop3  mcoop4  mcoop5  mcoop6  mcoop7  narc1  narc2  narc3  narc4  narc5  narc6  nidentity2  omind1  omind2  omind5  omind6  contact1  contact2  contact3  contact4  contact5  hygiene1  hygiene2  hygiene3  hygiene4  hygiene5  psupport1  psupport2  psupport3  psupport4  psupport5  political_ideology  happy  slf_ladder  riskperc1  riskperc2  sbelong2  sbelong3  sbelong4  tested_positive  optim2  slfcont1  slfcont3  slfcont4  urban
##   2   3  age  children  cnarc1  cnarc2  cnarc3  CRT1  CRT2  CRT3  health_cond  know_tested_positive  ladder  marital1  mor_circle  moralid1  moralid2  moralid3  moralid4  moralid5  moralid6  moralid7  moralid8  moralid9  mcoop1  mcoop2  mcoop3  mcoop4  mcoop5  mcoop6  mcoop7  narc1  narc2  narc3  narc4  narc5  narc6  nidentity2  omind1  omind2  omind5  omind6  contact1  contact2  contact3  contact4  contact5  hygiene1  hygiene2  hygiene3  hygiene4  hygiene5  psupport1  psupport2  psupport3  psupport4  psupport5  political_ideology  happy  slf_ladder  riskperc1  riskperc2  sbelong2  sbelong3  sbelong4  tested_positive  optim2  slfcont1  slfcont3  slfcont4  urban
##   2   4  age  children  cnarc1  cnarc2  cnarc3  CRT1  CRT2  CRT3  health_cond  know_tested_positive  ladder  marital1  mor_circle  moralid1  moralid2  moralid3  moralid4  moralid5  moralid6  moralid7  moralid8  moralid9  mcoop1  mcoop2  mcoop3  mcoop4  mcoop5  mcoop6  mcoop7  narc1  narc2  narc3  narc4  narc5  narc6  nidentity2  omind1  omind2  omind5  omind6  contact1  contact2  contact3  contact4  contact5  hygiene1  hygiene2  hygiene3  hygiene4  hygiene5  psupport1  psupport2  psupport3  psupport4  psupport5  political_ideology  happy  slf_ladder  riskperc1  riskperc2  sbelong2  sbelong3  sbelong4  tested_positive  optim2  slfcont1  slfcont3  slfcont4  urban
##   2   5  age  children  cnarc1  cnarc2  cnarc3  CRT1  CRT2  CRT3  health_cond  know_tested_positive  ladder  marital1  mor_circle  moralid1  moralid2  moralid3  moralid4  moralid5  moralid6  moralid7  moralid8  moralid9  mcoop1  mcoop2  mcoop3  mcoop4  mcoop5  mcoop6  mcoop7  narc1  narc2  narc3  narc4  narc5  narc6  nidentity2  omind1  omind2  omind5  omind6  contact1  contact2  contact3  contact4  contact5  hygiene1  hygiene2  hygiene3  hygiene4  hygiene5  psupport1  psupport2  psupport3  psupport4  psupport5  political_ideology  happy  slf_ladder  riskperc1  riskperc2  sbelong2  sbelong3  sbelong4  tested_positive  optim2  slfcont1  slfcont3  slfcont4  urban
##   2   6  age  children  cnarc1  cnarc2  cnarc3  CRT1  CRT2  CRT3  health_cond  know_tested_positive  ladder  marital1  mor_circle  moralid1  moralid2  moralid3  moralid4  moralid5  moralid6  moralid7  moralid8  moralid9  mcoop1  mcoop2  mcoop3  mcoop4  mcoop5  mcoop6  mcoop7  narc1  narc2  narc3  narc4  narc5  narc6  nidentity2  omind1  omind2  omind5  omind6  contact1  contact2  contact3  contact4  contact5  hygiene1  hygiene2  hygiene3  hygiene4  hygiene5  psupport1  psupport2  psupport3  psupport4  psupport5  political_ideology  happy  slf_ladder  riskperc1  riskperc2  sbelong2  sbelong3  sbelong4  tested_positive  optim2  slfcont1  slfcont3  slfcont4  urban
##   2   7  age  children  cnarc1  cnarc2  cnarc3  CRT1  CRT2  CRT3  health_cond  know_tested_positive  ladder  marital1  mor_circle  moralid1  moralid2  moralid3  moralid4  moralid5  moralid6  moralid7  moralid8  moralid9  mcoop1  mcoop2  mcoop3  mcoop4  mcoop5  mcoop6  mcoop7  narc1  narc2  narc3  narc4  narc5  narc6  nidentity2  omind1  omind2  omind5  omind6  contact1  contact2  contact3  contact4  contact5  hygiene1  hygiene2  hygiene3  hygiene4  hygiene5  psupport1  psupport2  psupport3  psupport4  psupport5  political_ideology  happy  slf_ladder  riskperc1  riskperc2  sbelong2  sbelong3  sbelong4  tested_positive  optim2  slfcont1  slfcont3  slfcont4  urban
##   2   8  age  children  cnarc1  cnarc2  cnarc3  CRT1  CRT2  CRT3  health_cond  know_tested_positive  ladder  marital1  mor_circle  moralid1  moralid2  moralid3  moralid4  moralid5  moralid6  moralid7  moralid8  moralid9  mcoop1  mcoop2  mcoop3  mcoop4  mcoop5  mcoop6  mcoop7  narc1  narc2  narc3  narc4  narc5  narc6  nidentity2  omind1  omind2  omind5  omind6  contact1  contact2  contact3  contact4  contact5  hygiene1  hygiene2  hygiene3  hygiene4  hygiene5  psupport1  psupport2  psupport3  psupport4  psupport5  political_ideology  happy  slf_ladder  riskperc1  riskperc2  sbelong2  sbelong3  sbelong4  tested_positive  optim2  slfcont1  slfcont3  slfcont4  urban
##   2   9  age  children  cnarc1  cnarc2  cnarc3  CRT1  CRT2  CRT3  health_cond  know_tested_positive  ladder  marital1  mor_circle  moralid1  moralid2  moralid3  moralid4  moralid5  moralid6  moralid7  moralid8  moralid9  mcoop1  mcoop2  mcoop3  mcoop4  mcoop5  mcoop6  mcoop7  narc1  narc2  narc3  narc4  narc5  narc6  nidentity2  omind1  omind2  omind5  omind6  contact1  contact2  contact3  contact4  contact5  hygiene1  hygiene2  hygiene3  hygiene4  hygiene5  psupport1  psupport2  psupport3  psupport4  psupport5  political_ideology  happy  slf_ladder  riskperc1  riskperc2  sbelong2  sbelong3  sbelong4  tested_positive  optim2  slfcont1  slfcont3  slfcont4  urban
##   2   10  age  children  cnarc1  cnarc2  cnarc3  CRT1  CRT2  CRT3  health_cond  know_tested_positive  ladder  marital1  mor_circle  moralid1  moralid2  moralid3  moralid4  moralid5  moralid6  moralid7  moralid8  moralid9  mcoop1  mcoop2  mcoop3  mcoop4  mcoop5  mcoop6  mcoop7  narc1  narc2  narc3  narc4  narc5  narc6  nidentity2  omind1  omind2  omind5  omind6  contact1  contact2  contact3  contact4  contact5  hygiene1  hygiene2  hygiene3  hygiene4  hygiene5  psupport1  psupport2  psupport3  psupport4  psupport5  political_ideology  happy  slf_ladder  riskperc1  riskperc2  sbelong2  sbelong3  sbelong4  tested_positive  optim2  slfcont1  slfcont3  slfcont4  urban
##   3   1  age  children  cnarc1  cnarc2  cnarc3  CRT1  CRT2  CRT3  health_cond  know_tested_positive  ladder  marital1  mor_circle  moralid1  moralid2  moralid3  moralid4  moralid5  moralid6  moralid7  moralid8  moralid9  mcoop1  mcoop2  mcoop3  mcoop4  mcoop5  mcoop6  mcoop7  narc1  narc2  narc3  narc4  narc5  narc6  nidentity2  omind1  omind2  omind5  omind6  contact1  contact2  contact3  contact4  contact5  hygiene1  hygiene2  hygiene3  hygiene4  hygiene5  psupport1  psupport2  psupport3  psupport4  psupport5  political_ideology  happy  slf_ladder  riskperc1  riskperc2  sbelong2  sbelong3  sbelong4  tested_positive  optim2  slfcont1  slfcont3  slfcont4  urban
##   3   2  age  children  cnarc1  cnarc2  cnarc3  CRT1  CRT2  CRT3  health_cond  know_tested_positive  ladder  marital1  mor_circle  moralid1  moralid2  moralid3  moralid4  moralid5  moralid6  moralid7  moralid8  moralid9  mcoop1  mcoop2  mcoop3  mcoop4  mcoop5  mcoop6  mcoop7  narc1  narc2  narc3  narc4  narc5  narc6  nidentity2  omind1  omind2  omind5  omind6  contact1  contact2  contact3  contact4  contact5  hygiene1  hygiene2  hygiene3  hygiene4  hygiene5  psupport1  psupport2  psupport3  psupport4  psupport5  political_ideology  happy  slf_ladder  riskperc1  riskperc2  sbelong2  sbelong3  sbelong4  tested_positive  optim2  slfcont1  slfcont3  slfcont4  urban
##   3   3  age  children  cnarc1  cnarc2  cnarc3  CRT1  CRT2  CRT3  health_cond  know_tested_positive  ladder  marital1  mor_circle  moralid1  moralid2  moralid3  moralid4  moralid5  moralid6  moralid7  moralid8  moralid9  mcoop1  mcoop2  mcoop3  mcoop4  mcoop5  mcoop6  mcoop7  narc1  narc2  narc3  narc4  narc5  narc6  nidentity2  omind1  omind2  omind5  omind6  contact1  contact2  contact3  contact4  contact5  hygiene1  hygiene2  hygiene3  hygiene4  hygiene5  psupport1  psupport2  psupport3  psupport4  psupport5  political_ideology  happy  slf_ladder  riskperc1  riskperc2  sbelong2  sbelong3  sbelong4  tested_positive  optim2  slfcont1  slfcont3  slfcont4  urban
##   3   4  age  children  cnarc1  cnarc2  cnarc3  CRT1  CRT2  CRT3  health_cond  know_tested_positive  ladder  marital1  mor_circle  moralid1  moralid2  moralid3  moralid4  moralid5  moralid6  moralid7  moralid8  moralid9  mcoop1  mcoop2  mcoop3  mcoop4  mcoop5  mcoop6  mcoop7  narc1  narc2  narc3  narc4  narc5  narc6  nidentity2  omind1  omind2  omind5  omind6  contact1  contact2  contact3  contact4  contact5  hygiene1  hygiene2  hygiene3  hygiene4  hygiene5  psupport1  psupport2  psupport3  psupport4  psupport5  political_ideology  happy  slf_ladder  riskperc1  riskperc2  sbelong2  sbelong3  sbelong4  tested_positive  optim2  slfcont1  slfcont3  slfcont4  urban
##   3   5  age  children  cnarc1  cnarc2  cnarc3  CRT1  CRT2  CRT3  health_cond  know_tested_positive  ladder  marital1  mor_circle  moralid1  moralid2  moralid3  moralid4  moralid5  moralid6  moralid7  moralid8  moralid9  mcoop1  mcoop2  mcoop3  mcoop4  mcoop5  mcoop6  mcoop7  narc1  narc2  narc3  narc4  narc5  narc6  nidentity2  omind1  omind2  omind5  omind6  contact1  contact2  contact3  contact4  contact5  hygiene1  hygiene2  hygiene3  hygiene4  hygiene5  psupport1  psupport2  psupport3  psupport4  psupport5  political_ideology  happy  slf_ladder  riskperc1  riskperc2  sbelong2  sbelong3  sbelong4  tested_positive  optim2  slfcont1  slfcont3  slfcont4  urban
##   3   6  age  children  cnarc1  cnarc2  cnarc3  CRT1  CRT2  CRT3  health_cond  know_tested_positive  ladder  marital1  mor_circle  moralid1  moralid2  moralid3  moralid4  moralid5  moralid6  moralid7  moralid8  moralid9  mcoop1  mcoop2  mcoop3  mcoop4  mcoop5  mcoop6  mcoop7  narc1  narc2  narc3  narc4  narc5  narc6  nidentity2  omind1  omind2  omind5  omind6  contact1  contact2  contact3  contact4  contact5  hygiene1  hygiene2  hygiene3  hygiene4  hygiene5  psupport1  psupport2  psupport3  psupport4  psupport5  political_ideology  happy  slf_ladder  riskperc1  riskperc2  sbelong2  sbelong3  sbelong4  tested_positive  optim2  slfcont1  slfcont3  slfcont4  urban
##   3   7  age  children  cnarc1  cnarc2  cnarc3  CRT1  CRT2  CRT3  health_cond  know_tested_positive  ladder  marital1  mor_circle  moralid1  moralid2  moralid3  moralid4  moralid5  moralid6  moralid7  moralid8  moralid9  mcoop1  mcoop2  mcoop3  mcoop4  mcoop5  mcoop6  mcoop7  narc1  narc2  narc3  narc4  narc5  narc6  nidentity2  omind1  omind2  omind5  omind6  contact1  contact2  contact3  contact4  contact5  hygiene1  hygiene2  hygiene3  hygiene4  hygiene5  psupport1  psupport2  psupport3  psupport4  psupport5  political_ideology  happy  slf_ladder  riskperc1  riskperc2  sbelong2  sbelong3  sbelong4  tested_positive  optim2  slfcont1  slfcont3  slfcont4  urban
##   3   8  age  children  cnarc1  cnarc2  cnarc3  CRT1  CRT2  CRT3  health_cond  know_tested_positive  ladder  marital1  mor_circle  moralid1  moralid2  moralid3  moralid4  moralid5  moralid6  moralid7  moralid8  moralid9  mcoop1  mcoop2  mcoop3  mcoop4  mcoop5  mcoop6  mcoop7  narc1  narc2  narc3  narc4  narc5  narc6  nidentity2  omind1  omind2  omind5  omind6  contact1  contact2  contact3  contact4  contact5  hygiene1  hygiene2  hygiene3  hygiene4  hygiene5  psupport1  psupport2  psupport3  psupport4  psupport5  political_ideology  happy  slf_ladder  riskperc1  riskperc2  sbelong2  sbelong3  sbelong4  tested_positive  optim2  slfcont1  slfcont3  slfcont4  urban
##   3   9  age  children  cnarc1  cnarc2  cnarc3  CRT1  CRT2  CRT3  health_cond  know_tested_positive  ladder  marital1  mor_circle  moralid1  moralid2  moralid3  moralid4  moralid5  moralid6  moralid7  moralid8  moralid9  mcoop1  mcoop2  mcoop3  mcoop4  mcoop5  mcoop6  mcoop7  narc1  narc2  narc3  narc4  narc5  narc6  nidentity2  omind1  omind2  omind5  omind6  contact1  contact2  contact3  contact4  contact5  hygiene1  hygiene2  hygiene3  hygiene4  hygiene5  psupport1  psupport2  psupport3  psupport4  psupport5  political_ideology  happy  slf_ladder  riskperc1  riskperc2  sbelong2  sbelong3  sbelong4  tested_positive  optim2  slfcont1  slfcont3  slfcont4  urban
##   3   10  age  children  cnarc1  cnarc2  cnarc3  CRT1  CRT2  CRT3  health_cond  know_tested_positive  ladder  marital1  mor_circle  moralid1  moralid2  moralid3  moralid4  moralid5  moralid6  moralid7  moralid8  moralid9  mcoop1  mcoop2  mcoop3  mcoop4  mcoop5  mcoop6  mcoop7  narc1  narc2  narc3  narc4  narc5  narc6  nidentity2  omind1  omind2  omind5  omind6  contact1  contact2  contact3  contact4  contact5  hygiene1  hygiene2  hygiene3  hygiene4  hygiene5  psupport1  psupport2  psupport3  psupport4  psupport5  political_ideology  happy  slf_ladder  riskperc1  riskperc2  sbelong2  sbelong3  sbelong4  tested_positive  optim2  slfcont1  slfcont3  slfcont4  urban
##   4   1  age  children  cnarc1  cnarc2  cnarc3  CRT1  CRT2  CRT3  health_cond  know_tested_positive  ladder  marital1  mor_circle  moralid1  moralid2  moralid3  moralid4  moralid5  moralid6  moralid7  moralid8  moralid9  mcoop1  mcoop2  mcoop3  mcoop4  mcoop5  mcoop6  mcoop7  narc1  narc2  narc3  narc4  narc5  narc6  nidentity2  omind1  omind2  omind5  omind6  contact1  contact2  contact3  contact4  contact5  hygiene1  hygiene2  hygiene3  hygiene4  hygiene5  psupport1  psupport2  psupport3  psupport4  psupport5  political_ideology  happy  slf_ladder  riskperc1  riskperc2  sbelong2  sbelong3  sbelong4  tested_positive  optim2  slfcont1  slfcont3  slfcont4  urban
##   4   2  age  children  cnarc1  cnarc2  cnarc3  CRT1  CRT2  CRT3  health_cond  know_tested_positive  ladder  marital1  mor_circle  moralid1  moralid2  moralid3  moralid4  moralid5  moralid6  moralid7  moralid8  moralid9  mcoop1  mcoop2  mcoop3  mcoop4  mcoop5  mcoop6  mcoop7  narc1  narc2  narc3  narc4  narc5  narc6  nidentity2  omind1  omind2  omind5  omind6  contact1  contact2  contact3  contact4  contact5  hygiene1  hygiene2  hygiene3  hygiene4  hygiene5  psupport1  psupport2  psupport3  psupport4  psupport5  political_ideology  happy  slf_ladder  riskperc1  riskperc2  sbelong2  sbelong3  sbelong4  tested_positive  optim2  slfcont1  slfcont3  slfcont4  urban
##   4   3  age  children  cnarc1  cnarc2  cnarc3  CRT1  CRT2  CRT3  health_cond  know_tested_positive  ladder  marital1  mor_circle  moralid1  moralid2  moralid3  moralid4  moralid5  moralid6  moralid7  moralid8  moralid9  mcoop1  mcoop2  mcoop3  mcoop4  mcoop5  mcoop6  mcoop7  narc1  narc2  narc3  narc4  narc5  narc6  nidentity2  omind1  omind2  omind5  omind6  contact1  contact2  contact3  contact4  contact5  hygiene1  hygiene2  hygiene3  hygiene4  hygiene5  psupport1  psupport2  psupport3  psupport4  psupport5  political_ideology  happy  slf_ladder  riskperc1  riskperc2  sbelong2  sbelong3  sbelong4  tested_positive  optim2  slfcont1  slfcont3  slfcont4  urban
##   4   4  age  children  cnarc1  cnarc2  cnarc3  CRT1  CRT2  CRT3  health_cond  know_tested_positive  ladder  marital1  mor_circle  moralid1  moralid2  moralid3  moralid4  moralid5  moralid6  moralid7  moralid8  moralid9  mcoop1  mcoop2  mcoop3  mcoop4  mcoop5  mcoop6  mcoop7  narc1  narc2  narc3  narc4  narc5  narc6  nidentity2  omind1  omind2  omind5  omind6  contact1  contact2  contact3  contact4  contact5  hygiene1  hygiene2  hygiene3  hygiene4  hygiene5  psupport1  psupport2  psupport3  psupport4  psupport5  political_ideology  happy  slf_ladder  riskperc1  riskperc2  sbelong2  sbelong3  sbelong4  tested_positive  optim2  slfcont1  slfcont3  slfcont4  urban
##   4   5  age  children  cnarc1  cnarc2  cnarc3  CRT1  CRT2  CRT3  health_cond  know_tested_positive  ladder  marital1  mor_circle  moralid1  moralid2  moralid3  moralid4  moralid5  moralid6  moralid7  moralid8  moralid9  mcoop1  mcoop2  mcoop3  mcoop4  mcoop5  mcoop6  mcoop7  narc1  narc2  narc3  narc4  narc5  narc6  nidentity2  omind1  omind2  omind5  omind6  contact1  contact2  contact3  contact4  contact5  hygiene1  hygiene2  hygiene3  hygiene4  hygiene5  psupport1  psupport2  psupport3  psupport4  psupport5  political_ideology  happy  slf_ladder  riskperc1  riskperc2  sbelong2  sbelong3  sbelong4  tested_positive  optim2  slfcont1  slfcont3  slfcont4  urban
##   4   6  age  children  cnarc1  cnarc2  cnarc3  CRT1  CRT2  CRT3  health_cond  know_tested_positive  ladder  marital1  mor_circle  moralid1  moralid2  moralid3  moralid4  moralid5  moralid6  moralid7  moralid8  moralid9  mcoop1  mcoop2  mcoop3  mcoop4  mcoop5  mcoop6  mcoop7  narc1  narc2  narc3  narc4  narc5  narc6  nidentity2  omind1  omind2  omind5  omind6  contact1  contact2  contact3  contact4  contact5  hygiene1  hygiene2  hygiene3  hygiene4  hygiene5  psupport1  psupport2  psupport3  psupport4  psupport5  political_ideology  happy  slf_ladder  riskperc1  riskperc2  sbelong2  sbelong3  sbelong4  tested_positive  optim2  slfcont1  slfcont3  slfcont4  urban
##   4   7  age  children  cnarc1  cnarc2  cnarc3  CRT1  CRT2  CRT3  health_cond  know_tested_positive  ladder  marital1  mor_circle  moralid1  moralid2  moralid3  moralid4  moralid5  moralid6  moralid7  moralid8  moralid9  mcoop1  mcoop2  mcoop3  mcoop4  mcoop5  mcoop6  mcoop7  narc1  narc2  narc3  narc4  narc5  narc6  nidentity2  omind1  omind2  omind5  omind6  contact1  contact2  contact3  contact4  contact5  hygiene1  hygiene2  hygiene3  hygiene4  hygiene5  psupport1  psupport2  psupport3  psupport4  psupport5  political_ideology  happy  slf_ladder  riskperc1  riskperc2  sbelong2  sbelong3  sbelong4  tested_positive  optim2  slfcont1  slfcont3  slfcont4  urban
##   4   8  age  children  cnarc1  cnarc2  cnarc3  CRT1  CRT2  CRT3  health_cond  know_tested_positive  ladder  marital1  mor_circle  moralid1  moralid2  moralid3  moralid4  moralid5  moralid6  moralid7  moralid8  moralid9  mcoop1  mcoop2  mcoop3  mcoop4  mcoop5  mcoop6  mcoop7  narc1  narc2  narc3  narc4  narc5  narc6  nidentity2  omind1  omind2  omind5  omind6  contact1  contact2  contact3  contact4  contact5  hygiene1  hygiene2  hygiene3  hygiene4  hygiene5  psupport1  psupport2  psupport3  psupport4  psupport5  political_ideology  happy  slf_ladder  riskperc1  riskperc2  sbelong2  sbelong3  sbelong4  tested_positive  optim2  slfcont1  slfcont3  slfcont4  urban
##   4   9  age  children  cnarc1  cnarc2  cnarc3  CRT1  CRT2  CRT3  health_cond  know_tested_positive  ladder  marital1  mor_circle  moralid1  moralid2  moralid3  moralid4  moralid5  moralid6  moralid7  moralid8  moralid9  mcoop1  mcoop2  mcoop3  mcoop4  mcoop5  mcoop6  mcoop7  narc1  narc2  narc3  narc4  narc5  narc6  nidentity2  omind1  omind2  omind5  omind6  contact1  contact2  contact3  contact4  contact5  hygiene1  hygiene2  hygiene3  hygiene4  hygiene5  psupport1  psupport2  psupport3  psupport4  psupport5  political_ideology  happy  slf_ladder  riskperc1  riskperc2  sbelong2  sbelong3  sbelong4  tested_positive  optim2  slfcont1  slfcont3  slfcont4  urban
##   4   10  age  children  cnarc1  cnarc2  cnarc3  CRT1  CRT2  CRT3  health_cond  know_tested_positive  ladder  marital1  mor_circle  moralid1  moralid2  moralid3  moralid4  moralid5  moralid6  moralid7  moralid8  moralid9  mcoop1  mcoop2  mcoop3  mcoop4  mcoop5  mcoop6  mcoop7  narc1  narc2  narc3  narc4  narc5  narc6  nidentity2  omind1  omind2  omind5  omind6  contact1  contact2  contact3  contact4  contact5  hygiene1  hygiene2  hygiene3  hygiene4  hygiene5  psupport1  psupport2  psupport3  psupport4  psupport5  political_ideology  happy  slf_ladder  riskperc1  riskperc2  sbelong2  sbelong3  sbelong4  tested_positive  optim2  slfcont1  slfcont3  slfcont4  urban
##   5   1  age  children  cnarc1  cnarc2  cnarc3  CRT1  CRT2  CRT3  health_cond  know_tested_positive  ladder  marital1  mor_circle  moralid1  moralid2  moralid3  moralid4  moralid5  moralid6  moralid7  moralid8  moralid9  mcoop1  mcoop2  mcoop3  mcoop4  mcoop5  mcoop6  mcoop7  narc1  narc2  narc3  narc4  narc5  narc6  nidentity2  omind1  omind2  omind5  omind6  contact1  contact2  contact3  contact4  contact5  hygiene1  hygiene2  hygiene3  hygiene4  hygiene5  psupport1  psupport2  psupport3  psupport4  psupport5  political_ideology  happy  slf_ladder  riskperc1  riskperc2  sbelong2  sbelong3  sbelong4  tested_positive  optim2  slfcont1  slfcont3  slfcont4  urban
##   5   2  age  children  cnarc1  cnarc2  cnarc3  CRT1  CRT2  CRT3  health_cond  know_tested_positive  ladder  marital1  mor_circle  moralid1  moralid2  moralid3  moralid4  moralid5  moralid6  moralid7  moralid8  moralid9  mcoop1  mcoop2  mcoop3  mcoop4  mcoop5  mcoop6  mcoop7  narc1  narc2  narc3  narc4  narc5  narc6  nidentity2  omind1  omind2  omind5  omind6  contact1  contact2  contact3  contact4  contact5  hygiene1  hygiene2  hygiene3  hygiene4  hygiene5  psupport1  psupport2  psupport3  psupport4  psupport5  political_ideology  happy  slf_ladder  riskperc1  riskperc2  sbelong2  sbelong3  sbelong4  tested_positive  optim2  slfcont1  slfcont3  slfcont4  urban
##   5   3  age  children  cnarc1  cnarc2  cnarc3  CRT1  CRT2  CRT3  health_cond  know_tested_positive  ladder  marital1  mor_circle  moralid1  moralid2  moralid3  moralid4  moralid5  moralid6  moralid7  moralid8  moralid9  mcoop1  mcoop2  mcoop3  mcoop4  mcoop5  mcoop6  mcoop7  narc1  narc2  narc3  narc4  narc5  narc6  nidentity2  omind1  omind2  omind5  omind6  contact1  contact2  contact3  contact4  contact5  hygiene1  hygiene2  hygiene3  hygiene4  hygiene5  psupport1  psupport2  psupport3  psupport4  psupport5  political_ideology  happy  slf_ladder  riskperc1  riskperc2  sbelong2  sbelong3  sbelong4  tested_positive  optim2  slfcont1  slfcont3  slfcont4  urban
##   5   4  age  children  cnarc1  cnarc2  cnarc3  CRT1  CRT2  CRT3  health_cond  know_tested_positive  ladder  marital1  mor_circle  moralid1  moralid2  moralid3  moralid4  moralid5  moralid6  moralid7  moralid8  moralid9  mcoop1  mcoop2  mcoop3  mcoop4  mcoop5  mcoop6  mcoop7  narc1  narc2  narc3  narc4  narc5  narc6  nidentity2  omind1  omind2  omind5  omind6  contact1  contact2  contact3  contact4  contact5  hygiene1  hygiene2  hygiene3  hygiene4  hygiene5  psupport1  psupport2  psupport3  psupport4  psupport5  political_ideology  happy  slf_ladder  riskperc1  riskperc2  sbelong2  sbelong3  sbelong4  tested_positive  optim2  slfcont1  slfcont3  slfcont4  urban
##   5   5  age  children  cnarc1  cnarc2  cnarc3  CRT1  CRT2  CRT3  health_cond  know_tested_positive  ladder  marital1  mor_circle  moralid1  moralid2  moralid3  moralid4  moralid5  moralid6  moralid7  moralid8  moralid9  mcoop1  mcoop2  mcoop3  mcoop4  mcoop5  mcoop6  mcoop7  narc1  narc2  narc3  narc4  narc5  narc6  nidentity2  omind1  omind2  omind5  omind6  contact1  contact2  contact3  contact4  contact5  hygiene1  hygiene2  hygiene3  hygiene4  hygiene5  psupport1  psupport2  psupport3  psupport4  psupport5  political_ideology  happy  slf_ladder  riskperc1  riskperc2  sbelong2  sbelong3  sbelong4  tested_positive  optim2  slfcont1  slfcont3  slfcont4  urban
##   5   6  age  children  cnarc1  cnarc2  cnarc3  CRT1  CRT2  CRT3  health_cond  know_tested_positive  ladder  marital1  mor_circle  moralid1  moralid2  moralid3  moralid4  moralid5  moralid6  moralid7  moralid8  moralid9  mcoop1  mcoop2  mcoop3  mcoop4  mcoop5  mcoop6  mcoop7  narc1  narc2  narc3  narc4  narc5  narc6  nidentity2  omind1  omind2  omind5  omind6  contact1  contact2  contact3  contact4  contact5  hygiene1  hygiene2  hygiene3  hygiene4  hygiene5  psupport1  psupport2  psupport3  psupport4  psupport5  political_ideology  happy  slf_ladder  riskperc1  riskperc2  sbelong2  sbelong3  sbelong4  tested_positive  optim2  slfcont1  slfcont3  slfcont4  urban
##   5   7  age  children  cnarc1  cnarc2  cnarc3  CRT1  CRT2  CRT3  health_cond  know_tested_positive  ladder  marital1  mor_circle  moralid1  moralid2  moralid3  moralid4  moralid5  moralid6  moralid7  moralid8  moralid9  mcoop1  mcoop2  mcoop3  mcoop4  mcoop5  mcoop6  mcoop7  narc1  narc2  narc3  narc4  narc5  narc6  nidentity2  omind1  omind2  omind5  omind6  contact1  contact2  contact3  contact4  contact5  hygiene1  hygiene2  hygiene3  hygiene4  hygiene5  psupport1  psupport2  psupport3  psupport4  psupport5  political_ideology  happy  slf_ladder  riskperc1  riskperc2  sbelong2  sbelong3  sbelong4  tested_positive  optim2  slfcont1  slfcont3  slfcont4  urban
##   5   8  age  children  cnarc1  cnarc2  cnarc3  CRT1  CRT2  CRT3  health_cond  know_tested_positive  ladder  marital1  mor_circle  moralid1  moralid2  moralid3  moralid4  moralid5  moralid6  moralid7  moralid8  moralid9  mcoop1  mcoop2  mcoop3  mcoop4  mcoop5  mcoop6  mcoop7  narc1  narc2  narc3  narc4  narc5  narc6  nidentity2  omind1  omind2  omind5  omind6  contact1  contact2  contact3  contact4  contact5  hygiene1  hygiene2  hygiene3  hygiene4  hygiene5  psupport1  psupport2  psupport3  psupport4  psupport5  political_ideology  happy  slf_ladder  riskperc1  riskperc2  sbelong2  sbelong3  sbelong4  tested_positive  optim2  slfcont1  slfcont3  slfcont4  urban
##   5   9  age  children  cnarc1  cnarc2  cnarc3  CRT1  CRT2  CRT3  health_cond  know_tested_positive  ladder  marital1  mor_circle  moralid1  moralid2  moralid3  moralid4  moralid5  moralid6  moralid7  moralid8  moralid9  mcoop1  mcoop2  mcoop3  mcoop4  mcoop5  mcoop6  mcoop7  narc1  narc2  narc3  narc4  narc5  narc6  nidentity2  omind1  omind2  omind5  omind6  contact1  contact2  contact3  contact4  contact5  hygiene1  hygiene2  hygiene3  hygiene4  hygiene5  psupport1  psupport2  psupport3  psupport4  psupport5  political_ideology  happy  slf_ladder  riskperc1  riskperc2  sbelong2  sbelong3  sbelong4  tested_positive  optim2  slfcont1  slfcont3  slfcont4  urban
##   5   10  age  children  cnarc1  cnarc2  cnarc3  CRT1  CRT2  CRT3  health_cond  know_tested_positive  ladder  marital1  mor_circle  moralid1  moralid2  moralid3  moralid4  moralid5  moralid6  moralid7  moralid8  moralid9  mcoop1  mcoop2  mcoop3  mcoop4  mcoop5  mcoop6  mcoop7  narc1  narc2  narc3  narc4  narc5  narc6  nidentity2  omind1  omind2  omind5  omind6  contact1  contact2  contact3  contact4  contact5  hygiene1  hygiene2  hygiene3  hygiene4  hygiene5  psupport1  psupport2  psupport3  psupport4  psupport5  political_ideology  happy  slf_ladder  riskperc1  riskperc2  sbelong2  sbelong3  sbelong4  tested_positive  optim2  slfcont1  slfcont3  slfcont4  urban
##   6   1  age  children  cnarc1  cnarc2  cnarc3  CRT1  CRT2  CRT3  health_cond  know_tested_positive  ladder  marital1  mor_circle  moralid1  moralid2  moralid3  moralid4  moralid5  moralid6  moralid7  moralid8  moralid9  mcoop1  mcoop2  mcoop3  mcoop4  mcoop5  mcoop6  mcoop7  narc1  narc2  narc3  narc4  narc5  narc6  nidentity2  omind1  omind2  omind5  omind6  contact1  contact2  contact3  contact4  contact5  hygiene1  hygiene2  hygiene3  hygiene4  hygiene5  psupport1  psupport2  psupport3  psupport4  psupport5  political_ideology  happy  slf_ladder  riskperc1  riskperc2  sbelong2  sbelong3  sbelong4  tested_positive  optim2  slfcont1  slfcont3  slfcont4  urban
##   6   2  age  children  cnarc1  cnarc2  cnarc3  CRT1  CRT2  CRT3  health_cond  know_tested_positive  ladder  marital1  mor_circle  moralid1  moralid2  moralid3  moralid4  moralid5  moralid6  moralid7  moralid8  moralid9  mcoop1  mcoop2  mcoop3  mcoop4  mcoop5  mcoop6  mcoop7  narc1  narc2  narc3  narc4  narc5  narc6  nidentity2  omind1  omind2  omind5  omind6  contact1  contact2  contact3  contact4  contact5  hygiene1  hygiene2  hygiene3  hygiene4  hygiene5  psupport1  psupport2  psupport3  psupport4  psupport5  political_ideology  happy  slf_ladder  riskperc1  riskperc2  sbelong2  sbelong3  sbelong4  tested_positive  optim2  slfcont1  slfcont3  slfcont4  urban
##   6   3  age  children  cnarc1  cnarc2  cnarc3  CRT1  CRT2  CRT3  health_cond  know_tested_positive  ladder  marital1  mor_circle  moralid1  moralid2  moralid3  moralid4  moralid5  moralid6  moralid7  moralid8  moralid9  mcoop1  mcoop2  mcoop3  mcoop4  mcoop5  mcoop6  mcoop7  narc1  narc2  narc3  narc4  narc5  narc6  nidentity2  omind1  omind2  omind5  omind6  contact1  contact2  contact3  contact4  contact5  hygiene1  hygiene2  hygiene3  hygiene4  hygiene5  psupport1  psupport2  psupport3  psupport4  psupport5  political_ideology  happy  slf_ladder  riskperc1  riskperc2  sbelong2  sbelong3  sbelong4  tested_positive  optim2  slfcont1  slfcont3  slfcont4  urban
##   6   4  age  children  cnarc1  cnarc2  cnarc3  CRT1  CRT2  CRT3  health_cond  know_tested_positive  ladder  marital1  mor_circle  moralid1  moralid2  moralid3  moralid4  moralid5  moralid6  moralid7  moralid8  moralid9  mcoop1  mcoop2  mcoop3  mcoop4  mcoop5  mcoop6  mcoop7  narc1  narc2  narc3  narc4  narc5  narc6  nidentity2  omind1  omind2  omind5  omind6  contact1  contact2  contact3  contact4  contact5  hygiene1  hygiene2  hygiene3  hygiene4  hygiene5  psupport1  psupport2  psupport3  psupport4  psupport5  political_ideology  happy  slf_ladder  riskperc1  riskperc2  sbelong2  sbelong3  sbelong4  tested_positive  optim2  slfcont1  slfcont3  slfcont4  urban
##   6   5  age  children  cnarc1  cnarc2  cnarc3  CRT1  CRT2  CRT3  health_cond  know_tested_positive  ladder  marital1  mor_circle  moralid1  moralid2  moralid3  moralid4  moralid5  moralid6  moralid7  moralid8  moralid9  mcoop1  mcoop2  mcoop3  mcoop4  mcoop5  mcoop6  mcoop7  narc1  narc2  narc3  narc4  narc5  narc6  nidentity2  omind1  omind2  omind5  omind6  contact1  contact2  contact3  contact4  contact5  hygiene1  hygiene2  hygiene3  hygiene4  hygiene5  psupport1  psupport2  psupport3  psupport4  psupport5  political_ideology  happy  slf_ladder  riskperc1  riskperc2  sbelong2  sbelong3  sbelong4  tested_positive  optim2  slfcont1  slfcont3  slfcont4  urban
##   6   6  age  children  cnarc1  cnarc2  cnarc3  CRT1  CRT2  CRT3  health_cond  know_tested_positive  ladder  marital1  mor_circle  moralid1  moralid2  moralid3  moralid4  moralid5  moralid6  moralid7  moralid8  moralid9  mcoop1  mcoop2  mcoop3  mcoop4  mcoop5  mcoop6  mcoop7  narc1  narc2  narc3  narc4  narc5  narc6  nidentity2  omind1  omind2  omind5  omind6  contact1  contact2  contact3  contact4  contact5  hygiene1  hygiene2  hygiene3  hygiene4  hygiene5  psupport1  psupport2  psupport3  psupport4  psupport5  political_ideology  happy  slf_ladder  riskperc1  riskperc2  sbelong2  sbelong3  sbelong4  tested_positive  optim2  slfcont1  slfcont3  slfcont4  urban
##   6   7  age  children  cnarc1  cnarc2  cnarc3  CRT1  CRT2  CRT3  health_cond  know_tested_positive  ladder  marital1  mor_circle  moralid1  moralid2  moralid3  moralid4  moralid5  moralid6  moralid7  moralid8  moralid9  mcoop1  mcoop2  mcoop3  mcoop4  mcoop5  mcoop6  mcoop7  narc1  narc2  narc3  narc4  narc5  narc6  nidentity2  omind1  omind2  omind5  omind6  contact1  contact2  contact3  contact4  contact5  hygiene1  hygiene2  hygiene3  hygiene4  hygiene5  psupport1  psupport2  psupport3  psupport4  psupport5  political_ideology  happy  slf_ladder  riskperc1  riskperc2  sbelong2  sbelong3  sbelong4  tested_positive  optim2  slfcont1  slfcont3  slfcont4  urban
##   6   8  age  children  cnarc1  cnarc2  cnarc3  CRT1  CRT2  CRT3  health_cond  know_tested_positive  ladder  marital1  mor_circle  moralid1  moralid2  moralid3  moralid4  moralid5  moralid6  moralid7  moralid8  moralid9  mcoop1  mcoop2  mcoop3  mcoop4  mcoop5  mcoop6  mcoop7  narc1  narc2  narc3  narc4  narc5  narc6  nidentity2  omind1  omind2  omind5  omind6  contact1  contact2  contact3  contact4  contact5  hygiene1  hygiene2  hygiene3  hygiene4  hygiene5  psupport1  psupport2  psupport3  psupport4  psupport5  political_ideology  happy  slf_ladder  riskperc1  riskperc2  sbelong2  sbelong3  sbelong4  tested_positive  optim2  slfcont1  slfcont3  slfcont4  urban
##   6   9  age  children  cnarc1  cnarc2  cnarc3  CRT1  CRT2  CRT3  health_cond  know_tested_positive  ladder  marital1  mor_circle  moralid1  moralid2  moralid3  moralid4  moralid5  moralid6  moralid7  moralid8  moralid9  mcoop1  mcoop2  mcoop3  mcoop4  mcoop5  mcoop6  mcoop7  narc1  narc2  narc3  narc4  narc5  narc6  nidentity2  omind1  omind2  omind5  omind6  contact1  contact2  contact3  contact4  contact5  hygiene1  hygiene2  hygiene3  hygiene4  hygiene5  psupport1  psupport2  psupport3  psupport4  psupport5  political_ideology  happy  slf_ladder  riskperc1  riskperc2  sbelong2  sbelong3  sbelong4  tested_positive  optim2  slfcont1  slfcont3  slfcont4  urban
##   6   10  age  children  cnarc1  cnarc2  cnarc3  CRT1  CRT2  CRT3  health_cond  know_tested_positive  ladder  marital1  mor_circle  moralid1  moralid2  moralid3  moralid4  moralid5  moralid6  moralid7  moralid8  moralid9  mcoop1  mcoop2  mcoop3  mcoop4  mcoop5  mcoop6  mcoop7  narc1  narc2  narc3  narc4  narc5  narc6  nidentity2  omind1  omind2  omind5  omind6  contact1  contact2  contact3  contact4  contact5  hygiene1  hygiene2  hygiene3  hygiene4  hygiene5  psupport1  psupport2  psupport3  psupport4  psupport5  political_ideology  happy  slf_ladder  riskperc1  riskperc2  sbelong2  sbelong3  sbelong4  tested_positive  optim2  slfcont1  slfcont3  slfcont4  urban
##   7   1  age  children  cnarc1  cnarc2  cnarc3  CRT1  CRT2  CRT3  health_cond  know_tested_positive  ladder  marital1  mor_circle  moralid1  moralid2  moralid3  moralid4  moralid5  moralid6  moralid7  moralid8  moralid9  mcoop1  mcoop2  mcoop3  mcoop4  mcoop5  mcoop6  mcoop7  narc1  narc2  narc3  narc4  narc5  narc6  nidentity2  omind1  omind2  omind5  omind6  contact1  contact2  contact3  contact4  contact5  hygiene1  hygiene2  hygiene3  hygiene4  hygiene5  psupport1  psupport2  psupport3  psupport4  psupport5  political_ideology  happy  slf_ladder  riskperc1  riskperc2  sbelong2  sbelong3  sbelong4  tested_positive  optim2  slfcont1  slfcont3  slfcont4  urban
##   7   2  age  children  cnarc1  cnarc2  cnarc3  CRT1  CRT2  CRT3  health_cond  know_tested_positive  ladder  marital1  mor_circle  moralid1  moralid2  moralid3  moralid4  moralid5  moralid6  moralid7  moralid8  moralid9  mcoop1  mcoop2  mcoop3  mcoop4  mcoop5  mcoop6  mcoop7  narc1  narc2  narc3  narc4  narc5  narc6  nidentity2  omind1  omind2  omind5  omind6  contact1  contact2  contact3  contact4  contact5  hygiene1  hygiene2  hygiene3  hygiene4  hygiene5  psupport1  psupport2  psupport3  psupport4  psupport5  political_ideology  happy  slf_ladder  riskperc1  riskperc2  sbelong2  sbelong3  sbelong4  tested_positive  optim2  slfcont1  slfcont3  slfcont4  urban
##   7   3  age  children  cnarc1  cnarc2  cnarc3  CRT1  CRT2  CRT3  health_cond  know_tested_positive  ladder  marital1  mor_circle  moralid1  moralid2  moralid3  moralid4  moralid5  moralid6  moralid7  moralid8  moralid9  mcoop1  mcoop2  mcoop3  mcoop4  mcoop5  mcoop6  mcoop7  narc1  narc2  narc3  narc4  narc5  narc6  nidentity2  omind1  omind2  omind5  omind6  contact1  contact2  contact3  contact4  contact5  hygiene1  hygiene2  hygiene3  hygiene4  hygiene5  psupport1  psupport2  psupport3  psupport4  psupport5  political_ideology  happy  slf_ladder  riskperc1  riskperc2  sbelong2  sbelong3  sbelong4  tested_positive  optim2  slfcont1  slfcont3  slfcont4  urban
##   7   4  age  children  cnarc1  cnarc2  cnarc3  CRT1  CRT2  CRT3  health_cond  know_tested_positive  ladder  marital1  mor_circle  moralid1  moralid2  moralid3  moralid4  moralid5  moralid6  moralid7  moralid8  moralid9  mcoop1  mcoop2  mcoop3  mcoop4  mcoop5  mcoop6  mcoop7  narc1  narc2  narc3  narc4  narc5  narc6  nidentity2  omind1  omind2  omind5  omind6  contact1  contact2  contact3  contact4  contact5  hygiene1  hygiene2  hygiene3  hygiene4  hygiene5  psupport1  psupport2  psupport3  psupport4  psupport5  political_ideology  happy  slf_ladder  riskperc1  riskperc2  sbelong2  sbelong3  sbelong4  tested_positive  optim2  slfcont1  slfcont3  slfcont4  urban
##   7   5  age  children  cnarc1  cnarc2  cnarc3  CRT1  CRT2  CRT3  health_cond  know_tested_positive  ladder  marital1  mor_circle  moralid1  moralid2  moralid3  moralid4  moralid5  moralid6  moralid7  moralid8  moralid9  mcoop1  mcoop2  mcoop3  mcoop4  mcoop5  mcoop6  mcoop7  narc1  narc2  narc3  narc4  narc5  narc6  nidentity2  omind1  omind2  omind5  omind6  contact1  contact2  contact3  contact4  contact5  hygiene1  hygiene2  hygiene3  hygiene4  hygiene5  psupport1  psupport2  psupport3  psupport4  psupport5  political_ideology  happy  slf_ladder  riskperc1  riskperc2  sbelong2  sbelong3  sbelong4  tested_positive  optim2  slfcont1  slfcont3  slfcont4  urban
##   7   6  age  children  cnarc1  cnarc2  cnarc3  CRT1  CRT2  CRT3  health_cond  know_tested_positive  ladder  marital1  mor_circle  moralid1  moralid2  moralid3  moralid4  moralid5  moralid6  moralid7  moralid8  moralid9  mcoop1  mcoop2  mcoop3  mcoop4  mcoop5  mcoop6  mcoop7  narc1  narc2  narc3  narc4  narc5  narc6  nidentity2  omind1  omind2  omind5  omind6  contact1  contact2  contact3  contact4  contact5  hygiene1  hygiene2  hygiene3  hygiene4  hygiene5  psupport1  psupport2  psupport3  psupport4  psupport5  political_ideology  happy  slf_ladder  riskperc1  riskperc2  sbelong2  sbelong3  sbelong4  tested_positive  optim2  slfcont1  slfcont3  slfcont4  urban
##   7   7  age  children  cnarc1  cnarc2  cnarc3  CRT1  CRT2  CRT3  health_cond  know_tested_positive  ladder  marital1  mor_circle  moralid1  moralid2  moralid3  moralid4  moralid5  moralid6  moralid7  moralid8  moralid9  mcoop1  mcoop2  mcoop3  mcoop4  mcoop5  mcoop6  mcoop7  narc1  narc2  narc3  narc4  narc5  narc6  nidentity2  omind1  omind2  omind5  omind6  contact1  contact2  contact3  contact4  contact5  hygiene1  hygiene2  hygiene3  hygiene4  hygiene5  psupport1  psupport2  psupport3  psupport4  psupport5  political_ideology  happy  slf_ladder  riskperc1  riskperc2  sbelong2  sbelong3  sbelong4  tested_positive  optim2  slfcont1  slfcont3  slfcont4  urban
##   7   8  age  children  cnarc1  cnarc2  cnarc3  CRT1  CRT2  CRT3  health_cond  know_tested_positive  ladder  marital1  mor_circle  moralid1  moralid2  moralid3  moralid4  moralid5  moralid6  moralid7  moralid8  moralid9  mcoop1  mcoop2  mcoop3  mcoop4  mcoop5  mcoop6  mcoop7  narc1  narc2  narc3  narc4  narc5  narc6  nidentity2  omind1  omind2  omind5  omind6  contact1  contact2  contact3  contact4  contact5  hygiene1  hygiene2  hygiene3  hygiene4  hygiene5  psupport1  psupport2  psupport3  psupport4  psupport5  political_ideology  happy  slf_ladder  riskperc1  riskperc2  sbelong2  sbelong3  sbelong4  tested_positive  optim2  slfcont1  slfcont3  slfcont4  urban
##   7   9  age  children  cnarc1  cnarc2  cnarc3  CRT1  CRT2  CRT3  health_cond  know_tested_positive  ladder  marital1  mor_circle  moralid1  moralid2  moralid3  moralid4  moralid5  moralid6  moralid7  moralid8  moralid9  mcoop1  mcoop2  mcoop3  mcoop4  mcoop5  mcoop6  mcoop7  narc1  narc2  narc3  narc4  narc5  narc6  nidentity2  omind1  omind2  omind5  omind6  contact1  contact2  contact3  contact4  contact5  hygiene1  hygiene2  hygiene3  hygiene4  hygiene5  psupport1  psupport2  psupport3  psupport4  psupport5  political_ideology  happy  slf_ladder  riskperc1  riskperc2  sbelong2  sbelong3  sbelong4  tested_positive  optim2  slfcont1  slfcont3  slfcont4  urban
##   7   10  age  children  cnarc1  cnarc2  cnarc3  CRT1  CRT2  CRT3  health_cond  know_tested_positive  ladder  marital1  mor_circle  moralid1  moralid2  moralid3  moralid4  moralid5  moralid6  moralid7  moralid8  moralid9  mcoop1  mcoop2  mcoop3  mcoop4  mcoop5  mcoop6  mcoop7  narc1  narc2  narc3  narc4  narc5  narc6  nidentity2  omind1  omind2  omind5  omind6  contact1  contact2  contact3  contact4  contact5  hygiene1  hygiene2  hygiene3  hygiene4  hygiene5  psupport1  psupport2  psupport3  psupport4  psupport5  political_ideology  happy  slf_ladder  riskperc1  riskperc2  sbelong2  sbelong3  sbelong4  tested_positive  optim2  slfcont1  slfcont3  slfcont4  urban
##   8   1  age  children  cnarc1  cnarc2  cnarc3  CRT1  CRT2  CRT3  health_cond  know_tested_positive  ladder  marital1  mor_circle  moralid1  moralid2  moralid3  moralid4  moralid5  moralid6  moralid7  moralid8  moralid9  mcoop1  mcoop2  mcoop3  mcoop4  mcoop5  mcoop6  mcoop7  narc1  narc2  narc3  narc4  narc5  narc6  nidentity2  omind1  omind2  omind5  omind6  contact1  contact2  contact3  contact4  contact5  hygiene1  hygiene2  hygiene3  hygiene4  hygiene5  psupport1  psupport2  psupport3  psupport4  psupport5  political_ideology  happy  slf_ladder  riskperc1  riskperc2  sbelong2  sbelong3  sbelong4  tested_positive  optim2  slfcont1  slfcont3  slfcont4  urban
##   8   2  age  children  cnarc1  cnarc2  cnarc3  CRT1  CRT2  CRT3  health_cond  know_tested_positive  ladder  marital1  mor_circle  moralid1  moralid2  moralid3  moralid4  moralid5  moralid6  moralid7  moralid8  moralid9  mcoop1  mcoop2  mcoop3  mcoop4  mcoop5  mcoop6  mcoop7  narc1  narc2  narc3  narc4  narc5  narc6  nidentity2  omind1  omind2  omind5  omind6  contact1  contact2  contact3  contact4  contact5  hygiene1  hygiene2  hygiene3  hygiene4  hygiene5  psupport1  psupport2  psupport3  psupport4  psupport5  political_ideology  happy  slf_ladder  riskperc1  riskperc2  sbelong2  sbelong3  sbelong4  tested_positive  optim2  slfcont1  slfcont3  slfcont4  urban
##   8   3  age  children  cnarc1  cnarc2  cnarc3  CRT1  CRT2  CRT3  health_cond  know_tested_positive  ladder  marital1  mor_circle  moralid1  moralid2  moralid3  moralid4  moralid5  moralid6  moralid7  moralid8  moralid9  mcoop1  mcoop2  mcoop3  mcoop4  mcoop5  mcoop6  mcoop7  narc1  narc2  narc3  narc4  narc5  narc6  nidentity2  omind1  omind2  omind5  omind6  contact1  contact2  contact3  contact4  contact5  hygiene1  hygiene2  hygiene3  hygiene4  hygiene5  psupport1  psupport2  psupport3  psupport4  psupport5  political_ideology  happy  slf_ladder  riskperc1  riskperc2  sbelong2  sbelong3  sbelong4  tested_positive  optim2  slfcont1  slfcont3  slfcont4  urban
##   8   4  age  children  cnarc1  cnarc2  cnarc3  CRT1  CRT2  CRT3  health_cond  know_tested_positive  ladder  marital1  mor_circle  moralid1  moralid2  moralid3  moralid4  moralid5  moralid6  moralid7  moralid8  moralid9  mcoop1  mcoop2  mcoop3  mcoop4  mcoop5  mcoop6  mcoop7  narc1  narc2  narc3  narc4  narc5  narc6  nidentity2  omind1  omind2  omind5  omind6  contact1  contact2  contact3  contact4  contact5  hygiene1  hygiene2  hygiene3  hygiene4  hygiene5  psupport1  psupport2  psupport3  psupport4  psupport5  political_ideology  happy  slf_ladder  riskperc1  riskperc2  sbelong2  sbelong3  sbelong4  tested_positive  optim2  slfcont1  slfcont3  slfcont4  urban
##   8   5  age  children  cnarc1  cnarc2  cnarc3  CRT1  CRT2  CRT3  health_cond  know_tested_positive  ladder  marital1  mor_circle  moralid1  moralid2  moralid3  moralid4  moralid5  moralid6  moralid7  moralid8  moralid9  mcoop1  mcoop2  mcoop3  mcoop4  mcoop5  mcoop6  mcoop7  narc1  narc2  narc3  narc4  narc5  narc6  nidentity2  omind1  omind2  omind5  omind6  contact1  contact2  contact3  contact4  contact5  hygiene1  hygiene2  hygiene3  hygiene4  hygiene5  psupport1  psupport2  psupport3  psupport4  psupport5  political_ideology  happy  slf_ladder  riskperc1  riskperc2  sbelong2  sbelong3  sbelong4  tested_positive  optim2  slfcont1  slfcont3  slfcont4  urban
##   8   6  age  children  cnarc1  cnarc2  cnarc3  CRT1  CRT2  CRT3  health_cond  know_tested_positive  ladder  marital1  mor_circle  moralid1  moralid2  moralid3  moralid4  moralid5  moralid6  moralid7  moralid8  moralid9  mcoop1  mcoop2  mcoop3  mcoop4  mcoop5  mcoop6  mcoop7  narc1  narc2  narc3  narc4  narc5  narc6  nidentity2  omind1  omind2  omind5  omind6  contact1  contact2  contact3  contact4  contact5  hygiene1  hygiene2  hygiene3  hygiene4  hygiene5  psupport1  psupport2  psupport3  psupport4  psupport5  political_ideology  happy  slf_ladder  riskperc1  riskperc2  sbelong2  sbelong3  sbelong4  tested_positive  optim2  slfcont1  slfcont3  slfcont4  urban
##   8   7  age  children  cnarc1  cnarc2  cnarc3  CRT1  CRT2  CRT3  health_cond  know_tested_positive  ladder  marital1  mor_circle  moralid1  moralid2  moralid3  moralid4  moralid5  moralid6  moralid7  moralid8  moralid9  mcoop1  mcoop2  mcoop3  mcoop4  mcoop5  mcoop6  mcoop7  narc1  narc2  narc3  narc4  narc5  narc6  nidentity2  omind1  omind2  omind5  omind6  contact1  contact2  contact3  contact4  contact5  hygiene1  hygiene2  hygiene3  hygiene4  hygiene5  psupport1  psupport2  psupport3  psupport4  psupport5  political_ideology  happy  slf_ladder  riskperc1  riskperc2  sbelong2  sbelong3  sbelong4  tested_positive  optim2  slfcont1  slfcont3  slfcont4  urban
##   8   8  age  children  cnarc1  cnarc2  cnarc3  CRT1  CRT2  CRT3  health_cond  know_tested_positive  ladder  marital1  mor_circle  moralid1  moralid2  moralid3  moralid4  moralid5  moralid6  moralid7  moralid8  moralid9  mcoop1  mcoop2  mcoop3  mcoop4  mcoop5  mcoop6  mcoop7  narc1  narc2  narc3  narc4  narc5  narc6  nidentity2  omind1  omind2  omind5  omind6  contact1  contact2  contact3  contact4  contact5  hygiene1  hygiene2  hygiene3  hygiene4  hygiene5  psupport1  psupport2  psupport3  psupport4  psupport5  political_ideology  happy  slf_ladder  riskperc1  riskperc2  sbelong2  sbelong3  sbelong4  tested_positive  optim2  slfcont1  slfcont3  slfcont4  urban
##   8   9  age  children  cnarc1  cnarc2  cnarc3  CRT1  CRT2  CRT3  health_cond  know_tested_positive  ladder  marital1  mor_circle  moralid1  moralid2  moralid3  moralid4  moralid5  moralid6  moralid7  moralid8  moralid9  mcoop1  mcoop2  mcoop3  mcoop4  mcoop5  mcoop6  mcoop7  narc1  narc2  narc3  narc4  narc5  narc6  nidentity2  omind1  omind2  omind5  omind6  contact1  contact2  contact3  contact4  contact5  hygiene1  hygiene2  hygiene3  hygiene4  hygiene5  psupport1  psupport2  psupport3  psupport4  psupport5  political_ideology  happy  slf_ladder  riskperc1  riskperc2  sbelong2  sbelong3  sbelong4  tested_positive  optim2  slfcont1  slfcont3  slfcont4  urban
##   8   10  age  children  cnarc1  cnarc2  cnarc3  CRT1  CRT2  CRT3  health_cond  know_tested_positive  ladder  marital1  mor_circle  moralid1  moralid2  moralid3  moralid4  moralid5  moralid6  moralid7  moralid8  moralid9  mcoop1  mcoop2  mcoop3  mcoop4  mcoop5  mcoop6  mcoop7  narc1  narc2  narc3  narc4  narc5  narc6  nidentity2  omind1  omind2  omind5  omind6  contact1  contact2  contact3  contact4  contact5  hygiene1  hygiene2  hygiene3  hygiene4  hygiene5  psupport1  psupport2  psupport3  psupport4  psupport5  political_ideology  happy  slf_ladder  riskperc1  riskperc2  sbelong2  sbelong3  sbelong4  tested_positive  optim2  slfcont1  slfcont3  slfcont4  urban
##   9   1  age  children  cnarc1  cnarc2  cnarc3  CRT1  CRT2  CRT3  health_cond  know_tested_positive  ladder  marital1  mor_circle  moralid1  moralid2  moralid3  moralid4  moralid5  moralid6  moralid7  moralid8  moralid9  mcoop1  mcoop2  mcoop3  mcoop4  mcoop5  mcoop6  mcoop7  narc1  narc2  narc3  narc4  narc5  narc6  nidentity2  omind1  omind2  omind5  omind6  contact1  contact2  contact3  contact4  contact5  hygiene1  hygiene2  hygiene3  hygiene4  hygiene5  psupport1  psupport2  psupport3  psupport4  psupport5  political_ideology  happy  slf_ladder  riskperc1  riskperc2  sbelong2  sbelong3  sbelong4  tested_positive  optim2  slfcont1  slfcont3  slfcont4  urban
##   9   2  age  children  cnarc1  cnarc2  cnarc3  CRT1  CRT2  CRT3  health_cond  know_tested_positive  ladder  marital1  mor_circle  moralid1  moralid2  moralid3  moralid4  moralid5  moralid6  moralid7  moralid8  moralid9  mcoop1  mcoop2  mcoop3  mcoop4  mcoop5  mcoop6  mcoop7  narc1  narc2  narc3  narc4  narc5  narc6  nidentity2  omind1  omind2  omind5  omind6  contact1  contact2  contact3  contact4  contact5  hygiene1  hygiene2  hygiene3  hygiene4  hygiene5  psupport1  psupport2  psupport3  psupport4  psupport5  political_ideology  happy  slf_ladder  riskperc1  riskperc2  sbelong2  sbelong3  sbelong4  tested_positive  optim2  slfcont1  slfcont3  slfcont4  urban
##   9   3  age  children  cnarc1  cnarc2  cnarc3  CRT1  CRT2  CRT3  health_cond  know_tested_positive  ladder  marital1  mor_circle  moralid1  moralid2  moralid3  moralid4  moralid5  moralid6  moralid7  moralid8  moralid9  mcoop1  mcoop2  mcoop3  mcoop4  mcoop5  mcoop6  mcoop7  narc1  narc2  narc3  narc4  narc5  narc6  nidentity2  omind1  omind2  omind5  omind6  contact1  contact2  contact3  contact4  contact5  hygiene1  hygiene2  hygiene3  hygiene4  hygiene5  psupport1  psupport2  psupport3  psupport4  psupport5  political_ideology  happy  slf_ladder  riskperc1  riskperc2  sbelong2  sbelong3  sbelong4  tested_positive  optim2  slfcont1  slfcont3  slfcont4  urban
##   9   4  age  children  cnarc1  cnarc2  cnarc3  CRT1  CRT2  CRT3  health_cond  know_tested_positive  ladder  marital1  mor_circle  moralid1  moralid2  moralid3  moralid4  moralid5  moralid6  moralid7  moralid8  moralid9  mcoop1  mcoop2  mcoop3  mcoop4  mcoop5  mcoop6  mcoop7  narc1  narc2  narc3  narc4  narc5  narc6  nidentity2  omind1  omind2  omind5  omind6  contact1  contact2  contact3  contact4  contact5  hygiene1  hygiene2  hygiene3  hygiene4  hygiene5  psupport1  psupport2  psupport3  psupport4  psupport5  political_ideology  happy  slf_ladder  riskperc1  riskperc2  sbelong2  sbelong3  sbelong4  tested_positive  optim2  slfcont1  slfcont3  slfcont4  urban
##   9   5  age  children  cnarc1  cnarc2  cnarc3  CRT1  CRT2  CRT3  health_cond  know_tested_positive  ladder  marital1  mor_circle  moralid1  moralid2  moralid3  moralid4  moralid5  moralid6  moralid7  moralid8  moralid9  mcoop1  mcoop2  mcoop3  mcoop4  mcoop5  mcoop6  mcoop7  narc1  narc2  narc3  narc4  narc5  narc6  nidentity2  omind1  omind2  omind5  omind6  contact1  contact2  contact3  contact4  contact5  hygiene1  hygiene2  hygiene3  hygiene4  hygiene5  psupport1  psupport2  psupport3  psupport4  psupport5  political_ideology  happy  slf_ladder  riskperc1  riskperc2  sbelong2  sbelong3  sbelong4  tested_positive  optim2  slfcont1  slfcont3  slfcont4  urban
##   9   6  age  children  cnarc1  cnarc2  cnarc3  CRT1  CRT2  CRT3  health_cond  know_tested_positive  ladder  marital1  mor_circle  moralid1  moralid2  moralid3  moralid4  moralid5  moralid6  moralid7  moralid8  moralid9  mcoop1  mcoop2  mcoop3  mcoop4  mcoop5  mcoop6  mcoop7  narc1  narc2  narc3  narc4  narc5  narc6  nidentity2  omind1  omind2  omind5  omind6  contact1  contact2  contact3  contact4  contact5  hygiene1  hygiene2  hygiene3  hygiene4  hygiene5  psupport1  psupport2  psupport3  psupport4  psupport5  political_ideology  happy  slf_ladder  riskperc1  riskperc2  sbelong2  sbelong3  sbelong4  tested_positive  optim2  slfcont1  slfcont3  slfcont4  urban
##   9   7  age  children  cnarc1  cnarc2  cnarc3  CRT1  CRT2  CRT3  health_cond  know_tested_positive  ladder  marital1  mor_circle  moralid1  moralid2  moralid3  moralid4  moralid5  moralid6  moralid7  moralid8  moralid9  mcoop1  mcoop2  mcoop3  mcoop4  mcoop5  mcoop6  mcoop7  narc1  narc2  narc3  narc4  narc5  narc6  nidentity2  omind1  omind2  omind5  omind6  contact1  contact2  contact3  contact4  contact5  hygiene1  hygiene2  hygiene3  hygiene4  hygiene5  psupport1  psupport2  psupport3  psupport4  psupport5  political_ideology  happy  slf_ladder  riskperc1  riskperc2  sbelong2  sbelong3  sbelong4  tested_positive  optim2  slfcont1  slfcont3  slfcont4  urban
##   9   8  age  children  cnarc1  cnarc2  cnarc3  CRT1  CRT2  CRT3  health_cond  know_tested_positive  ladder  marital1  mor_circle  moralid1  moralid2  moralid3  moralid4  moralid5  moralid6  moralid7  moralid8  moralid9  mcoop1  mcoop2  mcoop3  mcoop4  mcoop5  mcoop6  mcoop7  narc1  narc2  narc3  narc4  narc5  narc6  nidentity2  omind1  omind2  omind5  omind6  contact1  contact2  contact3  contact4  contact5  hygiene1  hygiene2  hygiene3  hygiene4  hygiene5  psupport1  psupport2  psupport3  psupport4  psupport5  political_ideology  happy  slf_ladder  riskperc1  riskperc2  sbelong2  sbelong3  sbelong4  tested_positive  optim2  slfcont1  slfcont3  slfcont4  urban
##   9   9  age  children  cnarc1  cnarc2  cnarc3  CRT1  CRT2  CRT3  health_cond  know_tested_positive  ladder  marital1  mor_circle  moralid1  moralid2  moralid3  moralid4  moralid5  moralid6  moralid7  moralid8  moralid9  mcoop1  mcoop2  mcoop3  mcoop4  mcoop5  mcoop6  mcoop7  narc1  narc2  narc3  narc4  narc5  narc6  nidentity2  omind1  omind2  omind5  omind6  contact1  contact2  contact3  contact4  contact5  hygiene1  hygiene2  hygiene3  hygiene4  hygiene5  psupport1  psupport2  psupport3  psupport4  psupport5  political_ideology  happy  slf_ladder  riskperc1  riskperc2  sbelong2  sbelong3  sbelong4  tested_positive  optim2  slfcont1  slfcont3  slfcont4  urban
##   9   10  age  children  cnarc1  cnarc2  cnarc3  CRT1  CRT2  CRT3  health_cond  know_tested_positive  ladder  marital1  mor_circle  moralid1  moralid2  moralid3  moralid4  moralid5  moralid6  moralid7  moralid8  moralid9  mcoop1  mcoop2  mcoop3  mcoop4  mcoop5  mcoop6  mcoop7  narc1  narc2  narc3  narc4  narc5  narc6  nidentity2  omind1  omind2  omind5  omind6  contact1  contact2  contact3  contact4  contact5  hygiene1  hygiene2  hygiene3  hygiene4  hygiene5  psupport1  psupport2  psupport3  psupport4  psupport5  political_ideology  happy  slf_ladder  riskperc1  riskperc2  sbelong2  sbelong3  sbelong4  tested_positive  optim2  slfcont1  slfcont3  slfcont4  urban
##   10   1  age  children  cnarc1  cnarc2  cnarc3  CRT1  CRT2  CRT3  health_cond  know_tested_positive  ladder  marital1  mor_circle  moralid1  moralid2  moralid3  moralid4  moralid5  moralid6  moralid7  moralid8  moralid9  mcoop1  mcoop2  mcoop3  mcoop4  mcoop5  mcoop6  mcoop7  narc1  narc2  narc3  narc4  narc5  narc6  nidentity2  omind1  omind2  omind5  omind6  contact1  contact2  contact3  contact4  contact5  hygiene1  hygiene2  hygiene3  hygiene4  hygiene5  psupport1  psupport2  psupport3  psupport4  psupport5  political_ideology  happy  slf_ladder  riskperc1  riskperc2  sbelong2  sbelong3  sbelong4  tested_positive  optim2  slfcont1  slfcont3  slfcont4  urban
##   10   2  age  children  cnarc1  cnarc2  cnarc3  CRT1  CRT2  CRT3  health_cond  know_tested_positive  ladder  marital1  mor_circle  moralid1  moralid2  moralid3  moralid4  moralid5  moralid6  moralid7  moralid8  moralid9  mcoop1  mcoop2  mcoop3  mcoop4  mcoop5  mcoop6  mcoop7  narc1  narc2  narc3  narc4  narc5  narc6  nidentity2  omind1  omind2  omind5  omind6  contact1  contact2  contact3  contact4  contact5  hygiene1  hygiene2  hygiene3  hygiene4  hygiene5  psupport1  psupport2  psupport3  psupport4  psupport5  political_ideology  happy  slf_ladder  riskperc1  riskperc2  sbelong2  sbelong3  sbelong4  tested_positive  optim2  slfcont1  slfcont3  slfcont4  urban
##   10   3  age  children  cnarc1  cnarc2  cnarc3  CRT1  CRT2  CRT3  health_cond  know_tested_positive  ladder  marital1  mor_circle  moralid1  moralid2  moralid3  moralid4  moralid5  moralid6  moralid7  moralid8  moralid9  mcoop1  mcoop2  mcoop3  mcoop4  mcoop5  mcoop6  mcoop7  narc1  narc2  narc3  narc4  narc5  narc6  nidentity2  omind1  omind2  omind5  omind6  contact1  contact2  contact3  contact4  contact5  hygiene1  hygiene2  hygiene3  hygiene4  hygiene5  psupport1  psupport2  psupport3  psupport4  psupport5  political_ideology  happy  slf_ladder  riskperc1  riskperc2  sbelong2  sbelong3  sbelong4  tested_positive  optim2  slfcont1  slfcont3  slfcont4  urban
##   10   4  age  children  cnarc1  cnarc2  cnarc3  CRT1  CRT2  CRT3  health_cond  know_tested_positive  ladder  marital1  mor_circle  moralid1  moralid2  moralid3  moralid4  moralid5  moralid6  moralid7  moralid8  moralid9  mcoop1  mcoop2  mcoop3  mcoop4  mcoop5  mcoop6  mcoop7  narc1  narc2  narc3  narc4  narc5  narc6  nidentity2  omind1  omind2  omind5  omind6  contact1  contact2  contact3  contact4  contact5  hygiene1  hygiene2  hygiene3  hygiene4  hygiene5  psupport1  psupport2  psupport3  psupport4  psupport5  political_ideology  happy  slf_ladder  riskperc1  riskperc2  sbelong2  sbelong3  sbelong4  tested_positive  optim2  slfcont1  slfcont3  slfcont4  urban
##   10   5  age  children  cnarc1  cnarc2  cnarc3  CRT1  CRT2  CRT3  health_cond  know_tested_positive  ladder  marital1  mor_circle  moralid1  moralid2  moralid3  moralid4  moralid5  moralid6  moralid7  moralid8  moralid9  mcoop1  mcoop2  mcoop3  mcoop4  mcoop5  mcoop6  mcoop7  narc1  narc2  narc3  narc4  narc5  narc6  nidentity2  omind1  omind2  omind5  omind6  contact1  contact2  contact3  contact4  contact5  hygiene1  hygiene2  hygiene3  hygiene4  hygiene5  psupport1  psupport2  psupport3  psupport4  psupport5  political_ideology  happy  slf_ladder  riskperc1  riskperc2  sbelong2  sbelong3  sbelong4  tested_positive  optim2  slfcont1  slfcont3  slfcont4  urban
##   10   6  age  children  cnarc1  cnarc2  cnarc3  CRT1  CRT2  CRT3  health_cond  know_tested_positive  ladder  marital1  mor_circle  moralid1  moralid2  moralid3  moralid4  moralid5  moralid6  moralid7  moralid8  moralid9  mcoop1  mcoop2  mcoop3  mcoop4  mcoop5  mcoop6  mcoop7  narc1  narc2  narc3  narc4  narc5  narc6  nidentity2  omind1  omind2  omind5  omind6  contact1  contact2  contact3  contact4  contact5  hygiene1  hygiene2  hygiene3  hygiene4  hygiene5  psupport1  psupport2  psupport3  psupport4  psupport5  political_ideology  happy  slf_ladder  riskperc1  riskperc2  sbelong2  sbelong3  sbelong4  tested_positive  optim2  slfcont1  slfcont3  slfcont4  urban
##   10   7  age  children  cnarc1  cnarc2  cnarc3  CRT1  CRT2  CRT3  health_cond  know_tested_positive  ladder  marital1  mor_circle  moralid1  moralid2  moralid3  moralid4  moralid5  moralid6  moralid7  moralid8  moralid9  mcoop1  mcoop2  mcoop3  mcoop4  mcoop5  mcoop6  mcoop7  narc1  narc2  narc3  narc4  narc5  narc6  nidentity2  omind1  omind2  omind5  omind6  contact1  contact2  contact3  contact4  contact5  hygiene1  hygiene2  hygiene3  hygiene4  hygiene5  psupport1  psupport2  psupport3  psupport4  psupport5  political_ideology  happy  slf_ladder  riskperc1  riskperc2  sbelong2  sbelong3  sbelong4  tested_positive  optim2  slfcont1  slfcont3  slfcont4  urban
##   10   8  age  children  cnarc1  cnarc2  cnarc3  CRT1  CRT2  CRT3  health_cond  know_tested_positive  ladder  marital1  mor_circle  moralid1  moralid2  moralid3  moralid4  moralid5  moralid6  moralid7  moralid8  moralid9  mcoop1  mcoop2  mcoop3  mcoop4  mcoop5  mcoop6  mcoop7  narc1  narc2  narc3  narc4  narc5  narc6  nidentity2  omind1  omind2  omind5  omind6  contact1  contact2  contact3  contact4  contact5  hygiene1  hygiene2  hygiene3  hygiene4  hygiene5  psupport1  psupport2  psupport3  psupport4  psupport5  political_ideology  happy  slf_ladder  riskperc1  riskperc2  sbelong2  sbelong3  sbelong4  tested_positive  optim2  slfcont1  slfcont3  slfcont4  urban
##   10   9  age  children  cnarc1  cnarc2  cnarc3  CRT1  CRT2  CRT3  health_cond  know_tested_positive  ladder  marital1  mor_circle  moralid1  moralid2  moralid3  moralid4  moralid5  moralid6  moralid7  moralid8  moralid9  mcoop1  mcoop2  mcoop3  mcoop4  mcoop5  mcoop6  mcoop7  narc1  narc2  narc3  narc4  narc5  narc6  nidentity2  omind1  omind2  omind5  omind6  contact1  contact2  contact3  contact4  contact5  hygiene1  hygiene2  hygiene3  hygiene4  hygiene5  psupport1  psupport2  psupport3  psupport4  psupport5  political_ideology  happy  slf_ladder  riskperc1  riskperc2  sbelong2  sbelong3  sbelong4  tested_positive  optim2  slfcont1  slfcont3  slfcont4  urban
##   10   10  age  children  cnarc1  cnarc2  cnarc3  CRT1  CRT2  CRT3  health_cond  know_tested_positive  ladder  marital1  mor_circle  moralid1  moralid2  moralid3  moralid4  moralid5  moralid6  moralid7  moralid8  moralid9  mcoop1  mcoop2  mcoop3  mcoop4  mcoop5  mcoop6  mcoop7  narc1  narc2  narc3  narc4  narc5  narc6  nidentity2  omind1  omind2  omind5  omind6  contact1  contact2  contact3  contact4  contact5  hygiene1  hygiene2  hygiene3  hygiene4  hygiene5  psupport1  psupport2  psupport3  psupport4  psupport5  political_ideology  happy  slf_ladder  riskperc1  riskperc2  sbelong2  sbelong3  sbelong4  tested_positive  optim2  slfcont1  slfcont3  slfcont4  urban
```

```
## Warning: Number of logged events: 6903
```

```
## 
##  iter imp variable
##   1   1  children  cnarc1  cnarc2  cnarc3  ctheory1  ctheory2  ctheory3  ctheory4  CRT1  CRT2  CRT3  ladder  mor_circle  moralid10  moralid3  moralid5  moralid6  moralid8  moralid9  mcoop2  mcoop5  mcoop6  mcoop7  narc1  narc2  narc3  narc4  narc5  narc6  omind5  omind6  contact2  hygiene4  hygiene5  psupport3  psupport5  political_ideology  self_esteem  sbelong1  sbelong2  sbelong3  slfcont3  slfcont4
##   1   2  children  cnarc1  cnarc2  cnarc3  ctheory1  ctheory2  ctheory3  ctheory4  CRT1  CRT2  CRT3  ladder  mor_circle  moralid10  moralid3  moralid5  moralid6  moralid8  moralid9  mcoop2  mcoop5  mcoop6  mcoop7  narc1  narc2  narc3  narc4  narc5  narc6  omind5  omind6  contact2  hygiene4  hygiene5  psupport3  psupport5  political_ideology  self_esteem  sbelong1  sbelong2  sbelong3  slfcont3  slfcont4
##   1   3  children  cnarc1  cnarc2  cnarc3  ctheory1  ctheory2  ctheory3  ctheory4  CRT1  CRT2  CRT3  ladder  mor_circle  moralid10  moralid3  moralid5  moralid6  moralid8  moralid9  mcoop2  mcoop5  mcoop6  mcoop7  narc1  narc2  narc3  narc4  narc5  narc6  omind5  omind6  contact2  hygiene4  hygiene5  psupport3  psupport5  political_ideology  self_esteem  sbelong1  sbelong2  sbelong3  slfcont3  slfcont4
##   1   4  children  cnarc1  cnarc2  cnarc3  ctheory1  ctheory2  ctheory3  ctheory4  CRT1  CRT2  CRT3  ladder  mor_circle  moralid10  moralid3  moralid5  moralid6  moralid8  moralid9  mcoop2  mcoop5  mcoop6  mcoop7  narc1  narc2  narc3  narc4  narc5  narc6  omind5  omind6  contact2  hygiene4  hygiene5  psupport3  psupport5  political_ideology  self_esteem  sbelong1  sbelong2  sbelong3  slfcont3  slfcont4
##   1   5  children  cnarc1  cnarc2  cnarc3  ctheory1  ctheory2  ctheory3  ctheory4  CRT1  CRT2  CRT3  ladder  mor_circle  moralid10  moralid3  moralid5  moralid6  moralid8  moralid9  mcoop2  mcoop5  mcoop6  mcoop7  narc1  narc2  narc3  narc4  narc5  narc6  omind5  omind6  contact2  hygiene4  hygiene5  psupport3  psupport5  political_ideology  self_esteem  sbelong1  sbelong2  sbelong3  slfcont3  slfcont4
##   1   6  children  cnarc1  cnarc2  cnarc3  ctheory1  ctheory2  ctheory3  ctheory4  CRT1  CRT2  CRT3  ladder  mor_circle  moralid10  moralid3  moralid5  moralid6  moralid8  moralid9  mcoop2  mcoop5  mcoop6  mcoop7  narc1  narc2  narc3  narc4  narc5  narc6  omind5  omind6  contact2  hygiene4  hygiene5  psupport3  psupport5  political_ideology  self_esteem  sbelong1  sbelong2  sbelong3  slfcont3  slfcont4
##   1   7  children  cnarc1  cnarc2  cnarc3  ctheory1  ctheory2  ctheory3  ctheory4  CRT1  CRT2  CRT3  ladder  mor_circle  moralid10  moralid3  moralid5  moralid6  moralid8  moralid9  mcoop2  mcoop5  mcoop6  mcoop7  narc1  narc2  narc3  narc4  narc5  narc6  omind5  omind6  contact2  hygiene4  hygiene5  psupport3  psupport5  political_ideology  self_esteem  sbelong1  sbelong2  sbelong3  slfcont3  slfcont4
##   1   8  children  cnarc1  cnarc2  cnarc3  ctheory1  ctheory2  ctheory3  ctheory4  CRT1  CRT2  CRT3  ladder  mor_circle  moralid10  moralid3  moralid5  moralid6  moralid8  moralid9  mcoop2  mcoop5  mcoop6  mcoop7  narc1  narc2  narc3  narc4  narc5  narc6  omind5  omind6  contact2  hygiene4  hygiene5  psupport3  psupport5  political_ideology  self_esteem  sbelong1  sbelong2  sbelong3  slfcont3  slfcont4
##   1   9  children  cnarc1  cnarc2  cnarc3  ctheory1  ctheory2  ctheory3  ctheory4  CRT1  CRT2  CRT3  ladder  mor_circle  moralid10  moralid3  moralid5  moralid6  moralid8  moralid9  mcoop2  mcoop5  mcoop6  mcoop7  narc1  narc2  narc3  narc4  narc5  narc6  omind5  omind6  contact2  hygiene4  hygiene5  psupport3  psupport5  political_ideology  self_esteem  sbelong1  sbelong2  sbelong3  slfcont3  slfcont4
##   1   10  children  cnarc1  cnarc2  cnarc3  ctheory1  ctheory2  ctheory3  ctheory4  CRT1  CRT2  CRT3  ladder  mor_circle  moralid10  moralid3  moralid5  moralid6  moralid8  moralid9  mcoop2  mcoop5  mcoop6  mcoop7  narc1  narc2  narc3  narc4  narc5  narc6  omind5  omind6  contact2  hygiene4  hygiene5  psupport3  psupport5  political_ideology  self_esteem  sbelong1  sbelong2  sbelong3  slfcont3  slfcont4
##   2   1  children  cnarc1  cnarc2  cnarc3  ctheory1  ctheory2  ctheory3  ctheory4  CRT1  CRT2  CRT3  ladder  mor_circle  moralid10  moralid3  moralid5  moralid6  moralid8  moralid9  mcoop2  mcoop5  mcoop6  mcoop7  narc1  narc2  narc3  narc4  narc5  narc6  omind5  omind6  contact2  hygiene4  hygiene5  psupport3  psupport5  political_ideology  self_esteem  sbelong1  sbelong2  sbelong3  slfcont3  slfcont4
##   2   2  children  cnarc1  cnarc2  cnarc3  ctheory1  ctheory2  ctheory3  ctheory4  CRT1  CRT2  CRT3  ladder  mor_circle  moralid10  moralid3  moralid5  moralid6  moralid8  moralid9  mcoop2  mcoop5  mcoop6  mcoop7  narc1  narc2  narc3  narc4  narc5  narc6  omind5  omind6  contact2  hygiene4  hygiene5  psupport3  psupport5  political_ideology  self_esteem  sbelong1  sbelong2  sbelong3  slfcont3  slfcont4
##   2   3  children  cnarc1  cnarc2  cnarc3  ctheory1  ctheory2  ctheory3  ctheory4  CRT1  CRT2  CRT3  ladder  mor_circle  moralid10  moralid3  moralid5  moralid6  moralid8  moralid9  mcoop2  mcoop5  mcoop6  mcoop7  narc1  narc2  narc3  narc4  narc5  narc6  omind5  omind6  contact2  hygiene4  hygiene5  psupport3  psupport5  political_ideology  self_esteem  sbelong1  sbelong2  sbelong3  slfcont3  slfcont4
##   2   4  children  cnarc1  cnarc2  cnarc3  ctheory1  ctheory2  ctheory3  ctheory4  CRT1  CRT2  CRT3  ladder  mor_circle  moralid10  moralid3  moralid5  moralid6  moralid8  moralid9  mcoop2  mcoop5  mcoop6  mcoop7  narc1  narc2  narc3  narc4  narc5  narc6  omind5  omind6  contact2  hygiene4  hygiene5  psupport3  psupport5  political_ideology  self_esteem  sbelong1  sbelong2  sbelong3  slfcont3  slfcont4
##   2   5  children  cnarc1  cnarc2  cnarc3  ctheory1  ctheory2  ctheory3  ctheory4  CRT1  CRT2  CRT3  ladder  mor_circle  moralid10  moralid3  moralid5  moralid6  moralid8  moralid9  mcoop2  mcoop5  mcoop6  mcoop7  narc1  narc2  narc3  narc4  narc5  narc6  omind5  omind6  contact2  hygiene4  hygiene5  psupport3  psupport5  political_ideology  self_esteem  sbelong1  sbelong2  sbelong3  slfcont3  slfcont4
##   2   6  children  cnarc1  cnarc2  cnarc3  ctheory1  ctheory2  ctheory3  ctheory4  CRT1  CRT2  CRT3  ladder  mor_circle  moralid10  moralid3  moralid5  moralid6  moralid8  moralid9  mcoop2  mcoop5  mcoop6  mcoop7  narc1  narc2  narc3  narc4  narc5  narc6  omind5  omind6  contact2  hygiene4  hygiene5  psupport3  psupport5  political_ideology  self_esteem  sbelong1  sbelong2  sbelong3  slfcont3  slfcont4
##   2   7  children  cnarc1  cnarc2  cnarc3  ctheory1  ctheory2  ctheory3  ctheory4  CRT1  CRT2  CRT3  ladder  mor_circle  moralid10  moralid3  moralid5  moralid6  moralid8  moralid9  mcoop2  mcoop5  mcoop6  mcoop7  narc1  narc2  narc3  narc4  narc5  narc6  omind5  omind6  contact2  hygiene4  hygiene5  psupport3  psupport5  political_ideology  self_esteem  sbelong1  sbelong2  sbelong3  slfcont3  slfcont4
##   2   8  children  cnarc1  cnarc2  cnarc3  ctheory1  ctheory2  ctheory3  ctheory4  CRT1  CRT2  CRT3  ladder  mor_circle  moralid10  moralid3  moralid5  moralid6  moralid8  moralid9  mcoop2  mcoop5  mcoop6  mcoop7  narc1  narc2  narc3  narc4  narc5  narc6  omind5  omind6  contact2  hygiene4  hygiene5  psupport3  psupport5  political_ideology  self_esteem  sbelong1  sbelong2  sbelong3  slfcont3  slfcont4
##   2   9  children  cnarc1  cnarc2  cnarc3  ctheory1  ctheory2  ctheory3  ctheory4  CRT1  CRT2  CRT3  ladder  mor_circle  moralid10  moralid3  moralid5  moralid6  moralid8  moralid9  mcoop2  mcoop5  mcoop6  mcoop7  narc1  narc2  narc3  narc4  narc5  narc6  omind5  omind6  contact2  hygiene4  hygiene5  psupport3  psupport5  political_ideology  self_esteem  sbelong1  sbelong2  sbelong3  slfcont3  slfcont4
##   2   10  children  cnarc1  cnarc2  cnarc3  ctheory1  ctheory2  ctheory3  ctheory4  CRT1  CRT2  CRT3  ladder  mor_circle  moralid10  moralid3  moralid5  moralid6  moralid8  moralid9  mcoop2  mcoop5  mcoop6  mcoop7  narc1  narc2  narc3  narc4  narc5  narc6  omind5  omind6  contact2  hygiene4  hygiene5  psupport3  psupport5  political_ideology  self_esteem  sbelong1  sbelong2  sbelong3  slfcont3  slfcont4
##   3   1  children  cnarc1  cnarc2  cnarc3  ctheory1  ctheory2  ctheory3  ctheory4  CRT1  CRT2  CRT3  ladder  mor_circle  moralid10  moralid3  moralid5  moralid6  moralid8  moralid9  mcoop2  mcoop5  mcoop6  mcoop7  narc1  narc2  narc3  narc4  narc5  narc6  omind5  omind6  contact2  hygiene4  hygiene5  psupport3  psupport5  political_ideology  self_esteem  sbelong1  sbelong2  sbelong3  slfcont3  slfcont4
##   3   2  children  cnarc1  cnarc2  cnarc3  ctheory1  ctheory2  ctheory3  ctheory4  CRT1  CRT2  CRT3  ladder  mor_circle  moralid10  moralid3  moralid5  moralid6  moralid8  moralid9  mcoop2  mcoop5  mcoop6  mcoop7  narc1  narc2  narc3  narc4  narc5  narc6  omind5  omind6  contact2  hygiene4  hygiene5  psupport3  psupport5  political_ideology  self_esteem  sbelong1  sbelong2  sbelong3  slfcont3  slfcont4
##   3   3  children  cnarc1  cnarc2  cnarc3  ctheory1  ctheory2  ctheory3  ctheory4  CRT1  CRT2  CRT3  ladder  mor_circle  moralid10  moralid3  moralid5  moralid6  moralid8  moralid9  mcoop2  mcoop5  mcoop6  mcoop7  narc1  narc2  narc3  narc4  narc5  narc6  omind5  omind6  contact2  hygiene4  hygiene5  psupport3  psupport5  political_ideology  self_esteem  sbelong1  sbelong2  sbelong3  slfcont3  slfcont4
##   3   4  children  cnarc1  cnarc2  cnarc3  ctheory1  ctheory2  ctheory3  ctheory4  CRT1  CRT2  CRT3  ladder  mor_circle  moralid10  moralid3  moralid5  moralid6  moralid8  moralid9  mcoop2  mcoop5  mcoop6  mcoop7  narc1  narc2  narc3  narc4  narc5  narc6  omind5  omind6  contact2  hygiene4  hygiene5  psupport3  psupport5  political_ideology  self_esteem  sbelong1  sbelong2  sbelong3  slfcont3  slfcont4
##   3   5  children  cnarc1  cnarc2  cnarc3  ctheory1  ctheory2  ctheory3  ctheory4  CRT1  CRT2  CRT3  ladder  mor_circle  moralid10  moralid3  moralid5  moralid6  moralid8  moralid9  mcoop2  mcoop5  mcoop6  mcoop7  narc1  narc2  narc3  narc4  narc5  narc6  omind5  omind6  contact2  hygiene4  hygiene5  psupport3  psupport5  political_ideology  self_esteem  sbelong1  sbelong2  sbelong3  slfcont3  slfcont4
##   3   6  children  cnarc1  cnarc2  cnarc3  ctheory1  ctheory2  ctheory3  ctheory4  CRT1  CRT2  CRT3  ladder  mor_circle  moralid10  moralid3  moralid5  moralid6  moralid8  moralid9  mcoop2  mcoop5  mcoop6  mcoop7  narc1  narc2  narc3  narc4  narc5  narc6  omind5  omind6  contact2  hygiene4  hygiene5  psupport3  psupport5  political_ideology  self_esteem  sbelong1  sbelong2  sbelong3  slfcont3  slfcont4
##   3   7  children  cnarc1  cnarc2  cnarc3  ctheory1  ctheory2  ctheory3  ctheory4  CRT1  CRT2  CRT3  ladder  mor_circle  moralid10  moralid3  moralid5  moralid6  moralid8  moralid9  mcoop2  mcoop5  mcoop6  mcoop7  narc1  narc2  narc3  narc4  narc5  narc6  omind5  omind6  contact2  hygiene4  hygiene5  psupport3  psupport5  political_ideology  self_esteem  sbelong1  sbelong2  sbelong3  slfcont3  slfcont4
##   3   8  children  cnarc1  cnarc2  cnarc3  ctheory1  ctheory2  ctheory3  ctheory4  CRT1  CRT2  CRT3  ladder  mor_circle  moralid10  moralid3  moralid5  moralid6  moralid8  moralid9  mcoop2  mcoop5  mcoop6  mcoop7  narc1  narc2  narc3  narc4  narc5  narc6  omind5  omind6  contact2  hygiene4  hygiene5  psupport3  psupport5  political_ideology  self_esteem  sbelong1  sbelong2  sbelong3  slfcont3  slfcont4
##   3   9  children  cnarc1  cnarc2  cnarc3  ctheory1  ctheory2  ctheory3  ctheory4  CRT1  CRT2  CRT3  ladder  mor_circle  moralid10  moralid3  moralid5  moralid6  moralid8  moralid9  mcoop2  mcoop5  mcoop6  mcoop7  narc1  narc2  narc3  narc4  narc5  narc6  omind5  omind6  contact2  hygiene4  hygiene5  psupport3  psupport5  political_ideology  self_esteem  sbelong1  sbelong2  sbelong3  slfcont3  slfcont4
##   3   10  children  cnarc1  cnarc2  cnarc3  ctheory1  ctheory2  ctheory3  ctheory4  CRT1  CRT2  CRT3  ladder  mor_circle  moralid10  moralid3  moralid5  moralid6  moralid8  moralid9  mcoop2  mcoop5  mcoop6  mcoop7  narc1  narc2  narc3  narc4  narc5  narc6  omind5  omind6  contact2  hygiene4  hygiene5  psupport3  psupport5  political_ideology  self_esteem  sbelong1  sbelong2  sbelong3  slfcont3  slfcont4
##   4   1  children  cnarc1  cnarc2  cnarc3  ctheory1  ctheory2  ctheory3  ctheory4  CRT1  CRT2  CRT3  ladder  mor_circle  moralid10  moralid3  moralid5  moralid6  moralid8  moralid9  mcoop2  mcoop5  mcoop6  mcoop7  narc1  narc2  narc3  narc4  narc5  narc6  omind5  omind6  contact2  hygiene4  hygiene5  psupport3  psupport5  political_ideology  self_esteem  sbelong1  sbelong2  sbelong3  slfcont3  slfcont4
##   4   2  children  cnarc1  cnarc2  cnarc3  ctheory1  ctheory2  ctheory3  ctheory4  CRT1  CRT2  CRT3  ladder  mor_circle  moralid10  moralid3  moralid5  moralid6  moralid8  moralid9  mcoop2  mcoop5  mcoop6  mcoop7  narc1  narc2  narc3  narc4  narc5  narc6  omind5  omind6  contact2  hygiene4  hygiene5  psupport3  psupport5  political_ideology  self_esteem  sbelong1  sbelong2  sbelong3  slfcont3  slfcont4
##   4   3  children  cnarc1  cnarc2  cnarc3  ctheory1  ctheory2  ctheory3  ctheory4  CRT1  CRT2  CRT3  ladder  mor_circle  moralid10  moralid3  moralid5  moralid6  moralid8  moralid9  mcoop2  mcoop5  mcoop6  mcoop7  narc1  narc2  narc3  narc4  narc5  narc6  omind5  omind6  contact2  hygiene4  hygiene5  psupport3  psupport5  political_ideology  self_esteem  sbelong1  sbelong2  sbelong3  slfcont3  slfcont4
##   4   4  children  cnarc1  cnarc2  cnarc3  ctheory1  ctheory2  ctheory3  ctheory4  CRT1  CRT2  CRT3  ladder  mor_circle  moralid10  moralid3  moralid5  moralid6  moralid8  moralid9  mcoop2  mcoop5  mcoop6  mcoop7  narc1  narc2  narc3  narc4  narc5  narc6  omind5  omind6  contact2  hygiene4  hygiene5  psupport3  psupport5  political_ideology  self_esteem  sbelong1  sbelong2  sbelong3  slfcont3  slfcont4
##   4   5  children  cnarc1  cnarc2  cnarc3  ctheory1  ctheory2  ctheory3  ctheory4  CRT1  CRT2  CRT3  ladder  mor_circle  moralid10  moralid3  moralid5  moralid6  moralid8  moralid9  mcoop2  mcoop5  mcoop6  mcoop7  narc1  narc2  narc3  narc4  narc5  narc6  omind5  omind6  contact2  hygiene4  hygiene5  psupport3  psupport5  political_ideology  self_esteem  sbelong1  sbelong2  sbelong3  slfcont3  slfcont4
##   4   6  children  cnarc1  cnarc2  cnarc3  ctheory1  ctheory2  ctheory3  ctheory4  CRT1  CRT2  CRT3  ladder  mor_circle  moralid10  moralid3  moralid5  moralid6  moralid8  moralid9  mcoop2  mcoop5  mcoop6  mcoop7  narc1  narc2  narc3  narc4  narc5  narc6  omind5  omind6  contact2  hygiene4  hygiene5  psupport3  psupport5  political_ideology  self_esteem  sbelong1  sbelong2  sbelong3  slfcont3  slfcont4
##   4   7  children  cnarc1  cnarc2  cnarc3  ctheory1  ctheory2  ctheory3  ctheory4  CRT1  CRT2  CRT3  ladder  mor_circle  moralid10  moralid3  moralid5  moralid6  moralid8  moralid9  mcoop2  mcoop5  mcoop6  mcoop7  narc1  narc2  narc3  narc4  narc5  narc6  omind5  omind6  contact2  hygiene4  hygiene5  psupport3  psupport5  political_ideology  self_esteem  sbelong1  sbelong2  sbelong3  slfcont3  slfcont4
##   4   8  children  cnarc1  cnarc2  cnarc3  ctheory1  ctheory2  ctheory3  ctheory4  CRT1  CRT2  CRT3  ladder  mor_circle  moralid10  moralid3  moralid5  moralid6  moralid8  moralid9  mcoop2  mcoop5  mcoop6  mcoop7  narc1  narc2  narc3  narc4  narc5  narc6  omind5  omind6  contact2  hygiene4  hygiene5  psupport3  psupport5  political_ideology  self_esteem  sbelong1  sbelong2  sbelong3  slfcont3  slfcont4
##   4   9  children  cnarc1  cnarc2  cnarc3  ctheory1  ctheory2  ctheory3  ctheory4  CRT1  CRT2  CRT3  ladder  mor_circle  moralid10  moralid3  moralid5  moralid6  moralid8  moralid9  mcoop2  mcoop5  mcoop6  mcoop7  narc1  narc2  narc3  narc4  narc5  narc6  omind5  omind6  contact2  hygiene4  hygiene5  psupport3  psupport5  political_ideology  self_esteem  sbelong1  sbelong2  sbelong3  slfcont3  slfcont4
##   4   10  children  cnarc1  cnarc2  cnarc3  ctheory1  ctheory2  ctheory3  ctheory4  CRT1  CRT2  CRT3  ladder  mor_circle  moralid10  moralid3  moralid5  moralid6  moralid8  moralid9  mcoop2  mcoop5  mcoop6  mcoop7  narc1  narc2  narc3  narc4  narc5  narc6  omind5  omind6  contact2  hygiene4  hygiene5  psupport3  psupport5  political_ideology  self_esteem  sbelong1  sbelong2  sbelong3  slfcont3  slfcont4
##   5   1  children  cnarc1  cnarc2  cnarc3  ctheory1  ctheory2  ctheory3  ctheory4  CRT1  CRT2  CRT3  ladder  mor_circle  moralid10  moralid3  moralid5  moralid6  moralid8  moralid9  mcoop2  mcoop5  mcoop6  mcoop7  narc1  narc2  narc3  narc4  narc5  narc6  omind5  omind6  contact2  hygiene4  hygiene5  psupport3  psupport5  political_ideology  self_esteem  sbelong1  sbelong2  sbelong3  slfcont3  slfcont4
##   5   2  children  cnarc1  cnarc2  cnarc3  ctheory1  ctheory2  ctheory3  ctheory4  CRT1  CRT2  CRT3  ladder  mor_circle  moralid10  moralid3  moralid5  moralid6  moralid8  moralid9  mcoop2  mcoop5  mcoop6  mcoop7  narc1  narc2  narc3  narc4  narc5  narc6  omind5  omind6  contact2  hygiene4  hygiene5  psupport3  psupport5  political_ideology  self_esteem  sbelong1  sbelong2  sbelong3  slfcont3  slfcont4
##   5   3  children  cnarc1  cnarc2  cnarc3  ctheory1  ctheory2  ctheory3  ctheory4  CRT1  CRT2  CRT3  ladder  mor_circle  moralid10  moralid3  moralid5  moralid6  moralid8  moralid9  mcoop2  mcoop5  mcoop6  mcoop7  narc1  narc2  narc3  narc4  narc5  narc6  omind5  omind6  contact2  hygiene4  hygiene5  psupport3  psupport5  political_ideology  self_esteem  sbelong1  sbelong2  sbelong3  slfcont3  slfcont4
##   5   4  children  cnarc1  cnarc2  cnarc3  ctheory1  ctheory2  ctheory3  ctheory4  CRT1  CRT2  CRT3  ladder  mor_circle  moralid10  moralid3  moralid5  moralid6  moralid8  moralid9  mcoop2  mcoop5  mcoop6  mcoop7  narc1  narc2  narc3  narc4  narc5  narc6  omind5  omind6  contact2  hygiene4  hygiene5  psupport3  psupport5  political_ideology  self_esteem  sbelong1  sbelong2  sbelong3  slfcont3  slfcont4
##   5   5  children  cnarc1  cnarc2  cnarc3  ctheory1  ctheory2  ctheory3  ctheory4  CRT1  CRT2  CRT3  ladder  mor_circle  moralid10  moralid3  moralid5  moralid6  moralid8  moralid9  mcoop2  mcoop5  mcoop6  mcoop7  narc1  narc2  narc3  narc4  narc5  narc6  omind5  omind6  contact2  hygiene4  hygiene5  psupport3  psupport5  political_ideology  self_esteem  sbelong1  sbelong2  sbelong3  slfcont3  slfcont4
##   5   6  children  cnarc1  cnarc2  cnarc3  ctheory1  ctheory2  ctheory3  ctheory4  CRT1  CRT2  CRT3  ladder  mor_circle  moralid10  moralid3  moralid5  moralid6  moralid8  moralid9  mcoop2  mcoop5  mcoop6  mcoop7  narc1  narc2  narc3  narc4  narc5  narc6  omind5  omind6  contact2  hygiene4  hygiene5  psupport3  psupport5  political_ideology  self_esteem  sbelong1  sbelong2  sbelong3  slfcont3  slfcont4
##   5   7  children  cnarc1  cnarc2  cnarc3  ctheory1  ctheory2  ctheory3  ctheory4  CRT1  CRT2  CRT3  ladder  mor_circle  moralid10  moralid3  moralid5  moralid6  moralid8  moralid9  mcoop2  mcoop5  mcoop6  mcoop7  narc1  narc2  narc3  narc4  narc5  narc6  omind5  omind6  contact2  hygiene4  hygiene5  psupport3  psupport5  political_ideology  self_esteem  sbelong1  sbelong2  sbelong3  slfcont3  slfcont4
##   5   8  children  cnarc1  cnarc2  cnarc3  ctheory1  ctheory2  ctheory3  ctheory4  CRT1  CRT2  CRT3  ladder  mor_circle  moralid10  moralid3  moralid5  moralid6  moralid8  moralid9  mcoop2  mcoop5  mcoop6  mcoop7  narc1  narc2  narc3  narc4  narc5  narc6  omind5  omind6  contact2  hygiene4  hygiene5  psupport3  psupport5  political_ideology  self_esteem  sbelong1  sbelong2  sbelong3  slfcont3  slfcont4
##   5   9  children  cnarc1  cnarc2  cnarc3  ctheory1  ctheory2  ctheory3  ctheory4  CRT1  CRT2  CRT3  ladder  mor_circle  moralid10  moralid3  moralid5  moralid6  moralid8  moralid9  mcoop2  mcoop5  mcoop6  mcoop7  narc1  narc2  narc3  narc4  narc5  narc6  omind5  omind6  contact2  hygiene4  hygiene5  psupport3  psupport5  political_ideology  self_esteem  sbelong1  sbelong2  sbelong3  slfcont3  slfcont4
##   5   10  children  cnarc1  cnarc2  cnarc3  ctheory1  ctheory2  ctheory3  ctheory4  CRT1  CRT2  CRT3  ladder  mor_circle  moralid10  moralid3  moralid5  moralid6  moralid8  moralid9  mcoop2  mcoop5  mcoop6  mcoop7  narc1  narc2  narc3  narc4  narc5  narc6  omind5  omind6  contact2  hygiene4  hygiene5  psupport3  psupport5  political_ideology  self_esteem  sbelong1  sbelong2  sbelong3  slfcont3  slfcont4
##   6   1  children  cnarc1  cnarc2  cnarc3  ctheory1  ctheory2  ctheory3  ctheory4  CRT1  CRT2  CRT3  ladder  mor_circle  moralid10  moralid3  moralid5  moralid6  moralid8  moralid9  mcoop2  mcoop5  mcoop6  mcoop7  narc1  narc2  narc3  narc4  narc5  narc6  omind5  omind6  contact2  hygiene4  hygiene5  psupport3  psupport5  political_ideology  self_esteem  sbelong1  sbelong2  sbelong3  slfcont3  slfcont4
##   6   2  children  cnarc1  cnarc2  cnarc3  ctheory1  ctheory2  ctheory3  ctheory4  CRT1  CRT2  CRT3  ladder  mor_circle  moralid10  moralid3  moralid5  moralid6  moralid8  moralid9  mcoop2  mcoop5  mcoop6  mcoop7  narc1  narc2  narc3  narc4  narc5  narc6  omind5  omind6  contact2  hygiene4  hygiene5  psupport3  psupport5  political_ideology  self_esteem  sbelong1  sbelong2  sbelong3  slfcont3  slfcont4
##   6   3  children  cnarc1  cnarc2  cnarc3  ctheory1  ctheory2  ctheory3  ctheory4  CRT1  CRT2  CRT3  ladder  mor_circle  moralid10  moralid3  moralid5  moralid6  moralid8  moralid9  mcoop2  mcoop5  mcoop6  mcoop7  narc1  narc2  narc3  narc4  narc5  narc6  omind5  omind6  contact2  hygiene4  hygiene5  psupport3  psupport5  political_ideology  self_esteem  sbelong1  sbelong2  sbelong3  slfcont3  slfcont4
##   6   4  children  cnarc1  cnarc2  cnarc3  ctheory1  ctheory2  ctheory3  ctheory4  CRT1  CRT2  CRT3  ladder  mor_circle  moralid10  moralid3  moralid5  moralid6  moralid8  moralid9  mcoop2  mcoop5  mcoop6  mcoop7  narc1  narc2  narc3  narc4  narc5  narc6  omind5  omind6  contact2  hygiene4  hygiene5  psupport3  psupport5  political_ideology  self_esteem  sbelong1  sbelong2  sbelong3  slfcont3  slfcont4
##   6   5  children  cnarc1  cnarc2  cnarc3  ctheory1  ctheory2  ctheory3  ctheory4  CRT1  CRT2  CRT3  ladder  mor_circle  moralid10  moralid3  moralid5  moralid6  moralid8  moralid9  mcoop2  mcoop5  mcoop6  mcoop7  narc1  narc2  narc3  narc4  narc5  narc6  omind5  omind6  contact2  hygiene4  hygiene5  psupport3  psupport5  political_ideology  self_esteem  sbelong1  sbelong2  sbelong3  slfcont3  slfcont4
##   6   6  children  cnarc1  cnarc2  cnarc3  ctheory1  ctheory2  ctheory3  ctheory4  CRT1  CRT2  CRT3  ladder  mor_circle  moralid10  moralid3  moralid5  moralid6  moralid8  moralid9  mcoop2  mcoop5  mcoop6  mcoop7  narc1  narc2  narc3  narc4  narc5  narc6  omind5  omind6  contact2  hygiene4  hygiene5  psupport3  psupport5  political_ideology  self_esteem  sbelong1  sbelong2  sbelong3  slfcont3  slfcont4
##   6   7  children  cnarc1  cnarc2  cnarc3  ctheory1  ctheory2  ctheory3  ctheory4  CRT1  CRT2  CRT3  ladder  mor_circle  moralid10  moralid3  moralid5  moralid6  moralid8  moralid9  mcoop2  mcoop5  mcoop6  mcoop7  narc1  narc2  narc3  narc4  narc5  narc6  omind5  omind6  contact2  hygiene4  hygiene5  psupport3  psupport5  political_ideology  self_esteem  sbelong1  sbelong2  sbelong3  slfcont3  slfcont4
##   6   8  children  cnarc1  cnarc2  cnarc3  ctheory1  ctheory2  ctheory3  ctheory4  CRT1  CRT2  CRT3  ladder  mor_circle  moralid10  moralid3  moralid5  moralid6  moralid8  moralid9  mcoop2  mcoop5  mcoop6  mcoop7  narc1  narc2  narc3  narc4  narc5  narc6  omind5  omind6  contact2  hygiene4  hygiene5  psupport3  psupport5  political_ideology  self_esteem  sbelong1  sbelong2  sbelong3  slfcont3  slfcont4
##   6   9  children  cnarc1  cnarc2  cnarc3  ctheory1  ctheory2  ctheory3  ctheory4  CRT1  CRT2  CRT3  ladder  mor_circle  moralid10  moralid3  moralid5  moralid6  moralid8  moralid9  mcoop2  mcoop5  mcoop6  mcoop7  narc1  narc2  narc3  narc4  narc5  narc6  omind5  omind6  contact2  hygiene4  hygiene5  psupport3  psupport5  political_ideology  self_esteem  sbelong1  sbelong2  sbelong3  slfcont3  slfcont4
##   6   10  children  cnarc1  cnarc2  cnarc3  ctheory1  ctheory2  ctheory3  ctheory4  CRT1  CRT2  CRT3  ladder  mor_circle  moralid10  moralid3  moralid5  moralid6  moralid8  moralid9  mcoop2  mcoop5  mcoop6  mcoop7  narc1  narc2  narc3  narc4  narc5  narc6  omind5  omind6  contact2  hygiene4  hygiene5  psupport3  psupport5  political_ideology  self_esteem  sbelong1  sbelong2  sbelong3  slfcont3  slfcont4
##   7   1  children  cnarc1  cnarc2  cnarc3  ctheory1  ctheory2  ctheory3  ctheory4  CRT1  CRT2  CRT3  ladder  mor_circle  moralid10  moralid3  moralid5  moralid6  moralid8  moralid9  mcoop2  mcoop5  mcoop6  mcoop7  narc1  narc2  narc3  narc4  narc5  narc6  omind5  omind6  contact2  hygiene4  hygiene5  psupport3  psupport5  political_ideology  self_esteem  sbelong1  sbelong2  sbelong3  slfcont3  slfcont4
##   7   2  children  cnarc1  cnarc2  cnarc3  ctheory1  ctheory2  ctheory3  ctheory4  CRT1  CRT2  CRT3  ladder  mor_circle  moralid10  moralid3  moralid5  moralid6  moralid8  moralid9  mcoop2  mcoop5  mcoop6  mcoop7  narc1  narc2  narc3  narc4  narc5  narc6  omind5  omind6  contact2  hygiene4  hygiene5  psupport3  psupport5  political_ideology  self_esteem  sbelong1  sbelong2  sbelong3  slfcont3  slfcont4
##   7   3  children  cnarc1  cnarc2  cnarc3  ctheory1  ctheory2  ctheory3  ctheory4  CRT1  CRT2  CRT3  ladder  mor_circle  moralid10  moralid3  moralid5  moralid6  moralid8  moralid9  mcoop2  mcoop5  mcoop6  mcoop7  narc1  narc2  narc3  narc4  narc5  narc6  omind5  omind6  contact2  hygiene4  hygiene5  psupport3  psupport5  political_ideology  self_esteem  sbelong1  sbelong2  sbelong3  slfcont3  slfcont4
##   7   4  children  cnarc1  cnarc2  cnarc3  ctheory1  ctheory2  ctheory3  ctheory4  CRT1  CRT2  CRT3  ladder  mor_circle  moralid10  moralid3  moralid5  moralid6  moralid8  moralid9  mcoop2  mcoop5  mcoop6  mcoop7  narc1  narc2  narc3  narc4  narc5  narc6  omind5  omind6  contact2  hygiene4  hygiene5  psupport3  psupport5  political_ideology  self_esteem  sbelong1  sbelong2  sbelong3  slfcont3  slfcont4
##   7   5  children  cnarc1  cnarc2  cnarc3  ctheory1  ctheory2  ctheory3  ctheory4  CRT1  CRT2  CRT3  ladder  mor_circle  moralid10  moralid3  moralid5  moralid6  moralid8  moralid9  mcoop2  mcoop5  mcoop6  mcoop7  narc1  narc2  narc3  narc4  narc5  narc6  omind5  omind6  contact2  hygiene4  hygiene5  psupport3  psupport5  political_ideology  self_esteem  sbelong1  sbelong2  sbelong3  slfcont3  slfcont4
##   7   6  children  cnarc1  cnarc2  cnarc3  ctheory1  ctheory2  ctheory3  ctheory4  CRT1  CRT2  CRT3  ladder  mor_circle  moralid10  moralid3  moralid5  moralid6  moralid8  moralid9  mcoop2  mcoop5  mcoop6  mcoop7  narc1  narc2  narc3  narc4  narc5  narc6  omind5  omind6  contact2  hygiene4  hygiene5  psupport3  psupport5  political_ideology  self_esteem  sbelong1  sbelong2  sbelong3  slfcont3  slfcont4
##   7   7  children  cnarc1  cnarc2  cnarc3  ctheory1  ctheory2  ctheory3  ctheory4  CRT1  CRT2  CRT3  ladder  mor_circle  moralid10  moralid3  moralid5  moralid6  moralid8  moralid9  mcoop2  mcoop5  mcoop6  mcoop7  narc1  narc2  narc3  narc4  narc5  narc6  omind5  omind6  contact2  hygiene4  hygiene5  psupport3  psupport5  political_ideology  self_esteem  sbelong1  sbelong2  sbelong3  slfcont3  slfcont4
##   7   8  children  cnarc1  cnarc2  cnarc3  ctheory1  ctheory2  ctheory3  ctheory4  CRT1  CRT2  CRT3  ladder  mor_circle  moralid10  moralid3  moralid5  moralid6  moralid8  moralid9  mcoop2  mcoop5  mcoop6  mcoop7  narc1  narc2  narc3  narc4  narc5  narc6  omind5  omind6  contact2  hygiene4  hygiene5  psupport3  psupport5  political_ideology  self_esteem  sbelong1  sbelong2  sbelong3  slfcont3  slfcont4
##   7   9  children  cnarc1  cnarc2  cnarc3  ctheory1  ctheory2  ctheory3  ctheory4  CRT1  CRT2  CRT3  ladder  mor_circle  moralid10  moralid3  moralid5  moralid6  moralid8  moralid9  mcoop2  mcoop5  mcoop6  mcoop7  narc1  narc2  narc3  narc4  narc5  narc6  omind5  omind6  contact2  hygiene4  hygiene5  psupport3  psupport5  political_ideology  self_esteem  sbelong1  sbelong2  sbelong3  slfcont3  slfcont4
##   7   10  children  cnarc1  cnarc2  cnarc3  ctheory1  ctheory2  ctheory3  ctheory4  CRT1  CRT2  CRT3  ladder  mor_circle  moralid10  moralid3  moralid5  moralid6  moralid8  moralid9  mcoop2  mcoop5  mcoop6  mcoop7  narc1  narc2  narc3  narc4  narc5  narc6  omind5  omind6  contact2  hygiene4  hygiene5  psupport3  psupport5  political_ideology  self_esteem  sbelong1  sbelong2  sbelong3  slfcont3  slfcont4
##   8   1  children  cnarc1  cnarc2  cnarc3  ctheory1  ctheory2  ctheory3  ctheory4  CRT1  CRT2  CRT3  ladder  mor_circle  moralid10  moralid3  moralid5  moralid6  moralid8  moralid9  mcoop2  mcoop5  mcoop6  mcoop7  narc1  narc2  narc3  narc4  narc5  narc6  omind5  omind6  contact2  hygiene4  hygiene5  psupport3  psupport5  political_ideology  self_esteem  sbelong1  sbelong2  sbelong3  slfcont3  slfcont4
##   8   2  children  cnarc1  cnarc2  cnarc3  ctheory1  ctheory2  ctheory3  ctheory4  CRT1  CRT2  CRT3  ladder  mor_circle  moralid10  moralid3  moralid5  moralid6  moralid8  moralid9  mcoop2  mcoop5  mcoop6  mcoop7  narc1  narc2  narc3  narc4  narc5  narc6  omind5  omind6  contact2  hygiene4  hygiene5  psupport3  psupport5  political_ideology  self_esteem  sbelong1  sbelong2  sbelong3  slfcont3  slfcont4
##   8   3  children  cnarc1  cnarc2  cnarc3  ctheory1  ctheory2  ctheory3  ctheory4  CRT1  CRT2  CRT3  ladder  mor_circle  moralid10  moralid3  moralid5  moralid6  moralid8  moralid9  mcoop2  mcoop5  mcoop6  mcoop7  narc1  narc2  narc3  narc4  narc5  narc6  omind5  omind6  contact2  hygiene4  hygiene5  psupport3  psupport5  political_ideology  self_esteem  sbelong1  sbelong2  sbelong3  slfcont3  slfcont4
##   8   4  children  cnarc1  cnarc2  cnarc3  ctheory1  ctheory2  ctheory3  ctheory4  CRT1  CRT2  CRT3  ladder  mor_circle  moralid10  moralid3  moralid5  moralid6  moralid8  moralid9  mcoop2  mcoop5  mcoop6  mcoop7  narc1  narc2  narc3  narc4  narc5  narc6  omind5  omind6  contact2  hygiene4  hygiene5  psupport3  psupport5  political_ideology  self_esteem  sbelong1  sbelong2  sbelong3  slfcont3  slfcont4
##   8   5  children  cnarc1  cnarc2  cnarc3  ctheory1  ctheory2  ctheory3  ctheory4  CRT1  CRT2  CRT3  ladder  mor_circle  moralid10  moralid3  moralid5  moralid6  moralid8  moralid9  mcoop2  mcoop5  mcoop6  mcoop7  narc1  narc2  narc3  narc4  narc5  narc6  omind5  omind6  contact2  hygiene4  hygiene5  psupport3  psupport5  political_ideology  self_esteem  sbelong1  sbelong2  sbelong3  slfcont3  slfcont4
##   8   6  children  cnarc1  cnarc2  cnarc3  ctheory1  ctheory2  ctheory3  ctheory4  CRT1  CRT2  CRT3  ladder  mor_circle  moralid10  moralid3  moralid5  moralid6  moralid8  moralid9  mcoop2  mcoop5  mcoop6  mcoop7  narc1  narc2  narc3  narc4  narc5  narc6  omind5  omind6  contact2  hygiene4  hygiene5  psupport3  psupport5  political_ideology  self_esteem  sbelong1  sbelong2  sbelong3  slfcont3  slfcont4
##   8   7  children  cnarc1  cnarc2  cnarc3  ctheory1  ctheory2  ctheory3  ctheory4  CRT1  CRT2  CRT3  ladder  mor_circle  moralid10  moralid3  moralid5  moralid6  moralid8  moralid9  mcoop2  mcoop5  mcoop6  mcoop7  narc1  narc2  narc3  narc4  narc5  narc6  omind5  omind6  contact2  hygiene4  hygiene5  psupport3  psupport5  political_ideology  self_esteem  sbelong1  sbelong2  sbelong3  slfcont3  slfcont4
##   8   8  children  cnarc1  cnarc2  cnarc3  ctheory1  ctheory2  ctheory3  ctheory4  CRT1  CRT2  CRT3  ladder  mor_circle  moralid10  moralid3  moralid5  moralid6  moralid8  moralid9  mcoop2  mcoop5  mcoop6  mcoop7  narc1  narc2  narc3  narc4  narc5  narc6  omind5  omind6  contact2  hygiene4  hygiene5  psupport3  psupport5  political_ideology  self_esteem  sbelong1  sbelong2  sbelong3  slfcont3  slfcont4
##   8   9  children  cnarc1  cnarc2  cnarc3  ctheory1  ctheory2  ctheory3  ctheory4  CRT1  CRT2  CRT3  ladder  mor_circle  moralid10  moralid3  moralid5  moralid6  moralid8  moralid9  mcoop2  mcoop5  mcoop6  mcoop7  narc1  narc2  narc3  narc4  narc5  narc6  omind5  omind6  contact2  hygiene4  hygiene5  psupport3  psupport5  political_ideology  self_esteem  sbelong1  sbelong2  sbelong3  slfcont3  slfcont4
##   8   10  children  cnarc1  cnarc2  cnarc3  ctheory1  ctheory2  ctheory3  ctheory4  CRT1  CRT2  CRT3  ladder  mor_circle  moralid10  moralid3  moralid5  moralid6  moralid8  moralid9  mcoop2  mcoop5  mcoop6  mcoop7  narc1  narc2  narc3  narc4  narc5  narc6  omind5  omind6  contact2  hygiene4  hygiene5  psupport3  psupport5  political_ideology  self_esteem  sbelong1  sbelong2  sbelong3  slfcont3  slfcont4
##   9   1  children  cnarc1  cnarc2  cnarc3  ctheory1  ctheory2  ctheory3  ctheory4  CRT1  CRT2  CRT3  ladder  mor_circle  moralid10  moralid3  moralid5  moralid6  moralid8  moralid9  mcoop2  mcoop5  mcoop6  mcoop7  narc1  narc2  narc3  narc4  narc5  narc6  omind5  omind6  contact2  hygiene4  hygiene5  psupport3  psupport5  political_ideology  self_esteem  sbelong1  sbelong2  sbelong3  slfcont3  slfcont4
##   9   2  children  cnarc1  cnarc2  cnarc3  ctheory1  ctheory2  ctheory3  ctheory4  CRT1  CRT2  CRT3  ladder  mor_circle  moralid10  moralid3  moralid5  moralid6  moralid8  moralid9  mcoop2  mcoop5  mcoop6  mcoop7  narc1  narc2  narc3  narc4  narc5  narc6  omind5  omind6  contact2  hygiene4  hygiene5  psupport3  psupport5  political_ideology  self_esteem  sbelong1  sbelong2  sbelong3  slfcont3  slfcont4
##   9   3  children  cnarc1  cnarc2  cnarc3  ctheory1  ctheory2  ctheory3  ctheory4  CRT1  CRT2  CRT3  ladder  mor_circle  moralid10  moralid3  moralid5  moralid6  moralid8  moralid9  mcoop2  mcoop5  mcoop6  mcoop7  narc1  narc2  narc3  narc4  narc5  narc6  omind5  omind6  contact2  hygiene4  hygiene5  psupport3  psupport5  political_ideology  self_esteem  sbelong1  sbelong2  sbelong3  slfcont3  slfcont4
##   9   4  children  cnarc1  cnarc2  cnarc3  ctheory1  ctheory2  ctheory3  ctheory4  CRT1  CRT2  CRT3  ladder  mor_circle  moralid10  moralid3  moralid5  moralid6  moralid8  moralid9  mcoop2  mcoop5  mcoop6  mcoop7  narc1  narc2  narc3  narc4  narc5  narc6  omind5  omind6  contact2  hygiene4  hygiene5  psupport3  psupport5  political_ideology  self_esteem  sbelong1  sbelong2  sbelong3  slfcont3  slfcont4
##   9   5  children  cnarc1  cnarc2  cnarc3  ctheory1  ctheory2  ctheory3  ctheory4  CRT1  CRT2  CRT3  ladder  mor_circle  moralid10  moralid3  moralid5  moralid6  moralid8  moralid9  mcoop2  mcoop5  mcoop6  mcoop7  narc1  narc2  narc3  narc4  narc5  narc6  omind5  omind6  contact2  hygiene4  hygiene5  psupport3  psupport5  political_ideology  self_esteem  sbelong1  sbelong2  sbelong3  slfcont3  slfcont4
##   9   6  children  cnarc1  cnarc2  cnarc3  ctheory1  ctheory2  ctheory3  ctheory4  CRT1  CRT2  CRT3  ladder  mor_circle  moralid10  moralid3  moralid5  moralid6  moralid8  moralid9  mcoop2  mcoop5  mcoop6  mcoop7  narc1  narc2  narc3  narc4  narc5  narc6  omind5  omind6  contact2  hygiene4  hygiene5  psupport3  psupport5  political_ideology  self_esteem  sbelong1  sbelong2  sbelong3  slfcont3  slfcont4
##   9   7  children  cnarc1  cnarc2  cnarc3  ctheory1  ctheory2  ctheory3  ctheory4  CRT1  CRT2  CRT3  ladder  mor_circle  moralid10  moralid3  moralid5  moralid6  moralid8  moralid9  mcoop2  mcoop5  mcoop6  mcoop7  narc1  narc2  narc3  narc4  narc5  narc6  omind5  omind6  contact2  hygiene4  hygiene5  psupport3  psupport5  political_ideology  self_esteem  sbelong1  sbelong2  sbelong3  slfcont3  slfcont4
##   9   8  children  cnarc1  cnarc2  cnarc3  ctheory1  ctheory2  ctheory3  ctheory4  CRT1  CRT2  CRT3  ladder  mor_circle  moralid10  moralid3  moralid5  moralid6  moralid8  moralid9  mcoop2  mcoop5  mcoop6  mcoop7  narc1  narc2  narc3  narc4  narc5  narc6  omind5  omind6  contact2  hygiene4  hygiene5  psupport3  psupport5  political_ideology  self_esteem  sbelong1  sbelong2  sbelong3  slfcont3  slfcont4
##   9   9  children  cnarc1  cnarc2  cnarc3  ctheory1  ctheory2  ctheory3  ctheory4  CRT1  CRT2  CRT3  ladder  mor_circle  moralid10  moralid3  moralid5  moralid6  moralid8  moralid9  mcoop2  mcoop5  mcoop6  mcoop7  narc1  narc2  narc3  narc4  narc5  narc6  omind5  omind6  contact2  hygiene4  hygiene5  psupport3  psupport5  political_ideology  self_esteem  sbelong1  sbelong2  sbelong3  slfcont3  slfcont4
##   9   10  children  cnarc1  cnarc2  cnarc3  ctheory1  ctheory2  ctheory3  ctheory4  CRT1  CRT2  CRT3  ladder  mor_circle  moralid10  moralid3  moralid5  moralid6  moralid8  moralid9  mcoop2  mcoop5  mcoop6  mcoop7  narc1  narc2  narc3  narc4  narc5  narc6  omind5  omind6  contact2  hygiene4  hygiene5  psupport3  psupport5  political_ideology  self_esteem  sbelong1  sbelong2  sbelong3  slfcont3  slfcont4
##   10   1  children  cnarc1  cnarc2  cnarc3  ctheory1  ctheory2  ctheory3  ctheory4  CRT1  CRT2  CRT3  ladder  mor_circle  moralid10  moralid3  moralid5  moralid6  moralid8  moralid9  mcoop2  mcoop5  mcoop6  mcoop7  narc1  narc2  narc3  narc4  narc5  narc6  omind5  omind6  contact2  hygiene4  hygiene5  psupport3  psupport5  political_ideology  self_esteem  sbelong1  sbelong2  sbelong3  slfcont3  slfcont4
##   10   2  children  cnarc1  cnarc2  cnarc3  ctheory1  ctheory2  ctheory3  ctheory4  CRT1  CRT2  CRT3  ladder  mor_circle  moralid10  moralid3  moralid5  moralid6  moralid8  moralid9  mcoop2  mcoop5  mcoop6  mcoop7  narc1  narc2  narc3  narc4  narc5  narc6  omind5  omind6  contact2  hygiene4  hygiene5  psupport3  psupport5  political_ideology  self_esteem  sbelong1  sbelong2  sbelong3  slfcont3  slfcont4
##   10   3  children  cnarc1  cnarc2  cnarc3  ctheory1  ctheory2  ctheory3  ctheory4  CRT1  CRT2  CRT3  ladder  mor_circle  moralid10  moralid3  moralid5  moralid6  moralid8  moralid9  mcoop2  mcoop5  mcoop6  mcoop7  narc1  narc2  narc3  narc4  narc5  narc6  omind5  omind6  contact2  hygiene4  hygiene5  psupport3  psupport5  political_ideology  self_esteem  sbelong1  sbelong2  sbelong3  slfcont3  slfcont4
##   10   4  children  cnarc1  cnarc2  cnarc3  ctheory1  ctheory2  ctheory3  ctheory4  CRT1  CRT2  CRT3  ladder  mor_circle  moralid10  moralid3  moralid5  moralid6  moralid8  moralid9  mcoop2  mcoop5  mcoop6  mcoop7  narc1  narc2  narc3  narc4  narc5  narc6  omind5  omind6  contact2  hygiene4  hygiene5  psupport3  psupport5  political_ideology  self_esteem  sbelong1  sbelong2  sbelong3  slfcont3  slfcont4
##   10   5  children  cnarc1  cnarc2  cnarc3  ctheory1  ctheory2  ctheory3  ctheory4  CRT1  CRT2  CRT3  ladder  mor_circle  moralid10  moralid3  moralid5  moralid6  moralid8  moralid9  mcoop2  mcoop5  mcoop6  mcoop7  narc1  narc2  narc3  narc4  narc5  narc6  omind5  omind6  contact2  hygiene4  hygiene5  psupport3  psupport5  political_ideology  self_esteem  sbelong1  sbelong2  sbelong3  slfcont3  slfcont4
##   10   6  children  cnarc1  cnarc2  cnarc3  ctheory1  ctheory2  ctheory3  ctheory4  CRT1  CRT2  CRT3  ladder  mor_circle  moralid10  moralid3  moralid5  moralid6  moralid8  moralid9  mcoop2  mcoop5  mcoop6  mcoop7  narc1  narc2  narc3  narc4  narc5  narc6  omind5  omind6  contact2  hygiene4  hygiene5  psupport3  psupport5  political_ideology  self_esteem  sbelong1  sbelong2  sbelong3  slfcont3  slfcont4
##   10   7  children  cnarc1  cnarc2  cnarc3  ctheory1  ctheory2  ctheory3  ctheory4  CRT1  CRT2  CRT3  ladder  mor_circle  moralid10  moralid3  moralid5  moralid6  moralid8  moralid9  mcoop2  mcoop5  mcoop6  mcoop7  narc1  narc2  narc3  narc4  narc5  narc6  omind5  omind6  contact2  hygiene4  hygiene5  psupport3  psupport5  political_ideology  self_esteem  sbelong1  sbelong2  sbelong3  slfcont3  slfcont4
##   10   8  children  cnarc1  cnarc2  cnarc3  ctheory1  ctheory2  ctheory3  ctheory4  CRT1  CRT2  CRT3  ladder  mor_circle  moralid10  moralid3  moralid5  moralid6  moralid8  moralid9  mcoop2  mcoop5  mcoop6  mcoop7  narc1  narc2  narc3  narc4  narc5  narc6  omind5  omind6  contact2  hygiene4  hygiene5  psupport3  psupport5  political_ideology  self_esteem  sbelong1  sbelong2  sbelong3  slfcont3  slfcont4
##   10   9  children  cnarc1  cnarc2  cnarc3  ctheory1  ctheory2  ctheory3  ctheory4  CRT1  CRT2  CRT3  ladder  mor_circle  moralid10  moralid3  moralid5  moralid6  moralid8  moralid9  mcoop2  mcoop5  mcoop6  mcoop7  narc1  narc2  narc3  narc4  narc5  narc6  omind5  omind6  contact2  hygiene4  hygiene5  psupport3  psupport5  political_ideology  self_esteem  sbelong1  sbelong2  sbelong3  slfcont3  slfcont4
##   10   10  children  cnarc1  cnarc2  cnarc3  ctheory1  ctheory2  ctheory3  ctheory4  CRT1  CRT2  CRT3  ladder  mor_circle  moralid10  moralid3  moralid5  moralid6  moralid8  moralid9  mcoop2  mcoop5  mcoop6  mcoop7  narc1  narc2  narc3  narc4  narc5  narc6  omind5  omind6  contact2  hygiene4  hygiene5  psupport3  psupport5  political_ideology  self_esteem  sbelong1  sbelong2  sbelong3  slfcont3  slfcont4
```

```
## Warning: Number of logged events: 4303
```

```
## 
##  iter imp variable
##   1   1  age  children  cnarc1  cnarc2  cnarc3  CRT1  CRT2  CRT3  ladder  marital1  mor_circle  moralid1  moralid10  moralid2  moralid3  moralid4  moralid5  moralid6  moralid7  moralid8  moralid9  mcoop1  mcoop2  mcoop3  mcoop4  mcoop5  mcoop6  mcoop7  narc1  narc4  narc5  narc6  nidentity1  nidentity2  omind1  omind2  omind3  omind4  omind5  omind6  contact1  contact2  contact3  contact4  contact5  hygiene1  hygiene2  hygiene3  hygiene4  hygiene5  psupport1  psupport2  psupport3  psupport4  psupport5  political_ideology  happy  slf_ladder  riskperc1  riskperc2  self_esteem  sbelong2  sbelong3  optim1  optim2  slfcont1
##   1   2  age  children  cnarc1  cnarc2  cnarc3  CRT1  CRT2  CRT3  ladder  marital1  mor_circle  moralid1  moralid10  moralid2  moralid3  moralid4  moralid5  moralid6  moralid7  moralid8  moralid9  mcoop1  mcoop2  mcoop3  mcoop4  mcoop5  mcoop6  mcoop7  narc1  narc4  narc5  narc6  nidentity1  nidentity2  omind1  omind2  omind3  omind4  omind5  omind6  contact1  contact2  contact3  contact4  contact5  hygiene1  hygiene2  hygiene3  hygiene4  hygiene5  psupport1  psupport2  psupport3  psupport4  psupport5  political_ideology  happy  slf_ladder  riskperc1  riskperc2  self_esteem  sbelong2  sbelong3  optim1  optim2  slfcont1
##   1   3  age  children  cnarc1  cnarc2  cnarc3  CRT1  CRT2  CRT3  ladder  marital1  mor_circle  moralid1  moralid10  moralid2  moralid3  moralid4  moralid5  moralid6  moralid7  moralid8  moralid9  mcoop1  mcoop2  mcoop3  mcoop4  mcoop5  mcoop6  mcoop7  narc1  narc4  narc5  narc6  nidentity1  nidentity2  omind1  omind2  omind3  omind4  omind5  omind6  contact1  contact2  contact3  contact4  contact5  hygiene1  hygiene2  hygiene3  hygiene4  hygiene5  psupport1  psupport2  psupport3  psupport4  psupport5  political_ideology  happy  slf_ladder  riskperc1  riskperc2  self_esteem  sbelong2  sbelong3  optim1  optim2  slfcont1
##   1   4  age  children  cnarc1  cnarc2  cnarc3  CRT1  CRT2  CRT3  ladder  marital1  mor_circle  moralid1  moralid10  moralid2  moralid3  moralid4  moralid5  moralid6  moralid7  moralid8  moralid9  mcoop1  mcoop2  mcoop3  mcoop4  mcoop5  mcoop6  mcoop7  narc1  narc4  narc5  narc6  nidentity1  nidentity2  omind1  omind2  omind3  omind4  omind5  omind6  contact1  contact2  contact3  contact4  contact5  hygiene1  hygiene2  hygiene3  hygiene4  hygiene5  psupport1  psupport2  psupport3  psupport4  psupport5  political_ideology  happy  slf_ladder  riskperc1  riskperc2  self_esteem  sbelong2  sbelong3  optim1  optim2  slfcont1
##   1   5  age  children  cnarc1  cnarc2  cnarc3  CRT1  CRT2  CRT3  ladder  marital1  mor_circle  moralid1  moralid10  moralid2  moralid3  moralid4  moralid5  moralid6  moralid7  moralid8  moralid9  mcoop1  mcoop2  mcoop3  mcoop4  mcoop5  mcoop6  mcoop7  narc1  narc4  narc5  narc6  nidentity1  nidentity2  omind1  omind2  omind3  omind4  omind5  omind6  contact1  contact2  contact3  contact4  contact5  hygiene1  hygiene2  hygiene3  hygiene4  hygiene5  psupport1  psupport2  psupport3  psupport4  psupport5  political_ideology  happy  slf_ladder  riskperc1  riskperc2  self_esteem  sbelong2  sbelong3  optim1  optim2  slfcont1
##   1   6  age  children  cnarc1  cnarc2  cnarc3  CRT1  CRT2  CRT3  ladder  marital1  mor_circle  moralid1  moralid10  moralid2  moralid3  moralid4  moralid5  moralid6  moralid7  moralid8  moralid9  mcoop1  mcoop2  mcoop3  mcoop4  mcoop5  mcoop6  mcoop7  narc1  narc4  narc5  narc6  nidentity1  nidentity2  omind1  omind2  omind3  omind4  omind5  omind6  contact1  contact2  contact3  contact4  contact5  hygiene1  hygiene2  hygiene3  hygiene4  hygiene5  psupport1  psupport2  psupport3  psupport4  psupport5  political_ideology  happy  slf_ladder  riskperc1  riskperc2  self_esteem  sbelong2  sbelong3  optim1  optim2  slfcont1
##   1   7  age  children  cnarc1  cnarc2  cnarc3  CRT1  CRT2  CRT3  ladder  marital1  mor_circle  moralid1  moralid10  moralid2  moralid3  moralid4  moralid5  moralid6  moralid7  moralid8  moralid9  mcoop1  mcoop2  mcoop3  mcoop4  mcoop5  mcoop6  mcoop7  narc1  narc4  narc5  narc6  nidentity1  nidentity2  omind1  omind2  omind3  omind4  omind5  omind6  contact1  contact2  contact3  contact4  contact5  hygiene1  hygiene2  hygiene3  hygiene4  hygiene5  psupport1  psupport2  psupport3  psupport4  psupport5  political_ideology  happy  slf_ladder  riskperc1  riskperc2  self_esteem  sbelong2  sbelong3  optim1  optim2  slfcont1
##   1   8  age  children  cnarc1  cnarc2  cnarc3  CRT1  CRT2  CRT3  ladder  marital1  mor_circle  moralid1  moralid10  moralid2  moralid3  moralid4  moralid5  moralid6  moralid7  moralid8  moralid9  mcoop1  mcoop2  mcoop3  mcoop4  mcoop5  mcoop6  mcoop7  narc1  narc4  narc5  narc6  nidentity1  nidentity2  omind1  omind2  omind3  omind4  omind5  omind6  contact1  contact2  contact3  contact4  contact5  hygiene1  hygiene2  hygiene3  hygiene4  hygiene5  psupport1  psupport2  psupport3  psupport4  psupport5  political_ideology  happy  slf_ladder  riskperc1  riskperc2  self_esteem  sbelong2  sbelong3  optim1  optim2  slfcont1
##   1   9  age  children  cnarc1  cnarc2  cnarc3  CRT1  CRT2  CRT3  ladder  marital1  mor_circle  moralid1  moralid10  moralid2  moralid3  moralid4  moralid5  moralid6  moralid7  moralid8  moralid9  mcoop1  mcoop2  mcoop3  mcoop4  mcoop5  mcoop6  mcoop7  narc1  narc4  narc5  narc6  nidentity1  nidentity2  omind1  omind2  omind3  omind4  omind5  omind6  contact1  contact2  contact3  contact4  contact5  hygiene1  hygiene2  hygiene3  hygiene4  hygiene5  psupport1  psupport2  psupport3  psupport4  psupport5  political_ideology  happy  slf_ladder  riskperc1  riskperc2  self_esteem  sbelong2  sbelong3  optim1  optim2  slfcont1
##   1   10  age  children  cnarc1  cnarc2  cnarc3  CRT1  CRT2  CRT3  ladder  marital1  mor_circle  moralid1  moralid10  moralid2  moralid3  moralid4  moralid5  moralid6  moralid7  moralid8  moralid9  mcoop1  mcoop2  mcoop3  mcoop4  mcoop5  mcoop6  mcoop7  narc1  narc4  narc5  narc6  nidentity1  nidentity2  omind1  omind2  omind3  omind4  omind5  omind6  contact1  contact2  contact3  contact4  contact5  hygiene1  hygiene2  hygiene3  hygiene4  hygiene5  psupport1  psupport2  psupport3  psupport4  psupport5  political_ideology  happy  slf_ladder  riskperc1  riskperc2  self_esteem  sbelong2  sbelong3  optim1  optim2  slfcont1
##   2   1  age  children  cnarc1  cnarc2  cnarc3  CRT1  CRT2  CRT3  ladder  marital1  mor_circle  moralid1  moralid10  moralid2  moralid3  moralid4  moralid5  moralid6  moralid7  moralid8  moralid9  mcoop1  mcoop2  mcoop3  mcoop4  mcoop5  mcoop6  mcoop7  narc1  narc4  narc5  narc6  nidentity1  nidentity2  omind1  omind2  omind3  omind4  omind5  omind6  contact1  contact2  contact3  contact4  contact5  hygiene1  hygiene2  hygiene3  hygiene4  hygiene5  psupport1  psupport2  psupport3  psupport4  psupport5  political_ideology  happy  slf_ladder  riskperc1  riskperc2  self_esteem  sbelong2  sbelong3  optim1  optim2  slfcont1
##   2   2  age  children  cnarc1  cnarc2  cnarc3  CRT1  CRT2  CRT3  ladder  marital1  mor_circle  moralid1  moralid10  moralid2  moralid3  moralid4  moralid5  moralid6  moralid7  moralid8  moralid9  mcoop1  mcoop2  mcoop3  mcoop4  mcoop5  mcoop6  mcoop7  narc1  narc4  narc5  narc6  nidentity1  nidentity2  omind1  omind2  omind3  omind4  omind5  omind6  contact1  contact2  contact3  contact4  contact5  hygiene1  hygiene2  hygiene3  hygiene4  hygiene5  psupport1  psupport2  psupport3  psupport4  psupport5  political_ideology  happy  slf_ladder  riskperc1  riskperc2  self_esteem  sbelong2  sbelong3  optim1  optim2  slfcont1
##   2   3  age  children  cnarc1  cnarc2  cnarc3  CRT1  CRT2  CRT3  ladder  marital1  mor_circle  moralid1  moralid10  moralid2  moralid3  moralid4  moralid5  moralid6  moralid7  moralid8  moralid9  mcoop1  mcoop2  mcoop3  mcoop4  mcoop5  mcoop6  mcoop7  narc1  narc4  narc5  narc6  nidentity1  nidentity2  omind1  omind2  omind3  omind4  omind5  omind6  contact1  contact2  contact3  contact4  contact5  hygiene1  hygiene2  hygiene3  hygiene4  hygiene5  psupport1  psupport2  psupport3  psupport4  psupport5  political_ideology  happy  slf_ladder  riskperc1  riskperc2  self_esteem  sbelong2  sbelong3  optim1  optim2  slfcont1
##   2   4  age  children  cnarc1  cnarc2  cnarc3  CRT1  CRT2  CRT3  ladder  marital1  mor_circle  moralid1  moralid10  moralid2  moralid3  moralid4  moralid5  moralid6  moralid7  moralid8  moralid9  mcoop1  mcoop2  mcoop3  mcoop4  mcoop5  mcoop6  mcoop7  narc1  narc4  narc5  narc6  nidentity1  nidentity2  omind1  omind2  omind3  omind4  omind5  omind6  contact1  contact2  contact3  contact4  contact5  hygiene1  hygiene2  hygiene3  hygiene4  hygiene5  psupport1  psupport2  psupport3  psupport4  psupport5  political_ideology  happy  slf_ladder  riskperc1  riskperc2  self_esteem  sbelong2  sbelong3  optim1  optim2  slfcont1
##   2   5  age  children  cnarc1  cnarc2  cnarc3  CRT1  CRT2  CRT3  ladder  marital1  mor_circle  moralid1  moralid10  moralid2  moralid3  moralid4  moralid5  moralid6  moralid7  moralid8  moralid9  mcoop1  mcoop2  mcoop3  mcoop4  mcoop5  mcoop6  mcoop7  narc1  narc4  narc5  narc6  nidentity1  nidentity2  omind1  omind2  omind3  omind4  omind5  omind6  contact1  contact2  contact3  contact4  contact5  hygiene1  hygiene2  hygiene3  hygiene4  hygiene5  psupport1  psupport2  psupport3  psupport4  psupport5  political_ideology  happy  slf_ladder  riskperc1  riskperc2  self_esteem  sbelong2  sbelong3  optim1  optim2  slfcont1
##   2   6  age  children  cnarc1  cnarc2  cnarc3  CRT1  CRT2  CRT3  ladder  marital1  mor_circle  moralid1  moralid10  moralid2  moralid3  moralid4  moralid5  moralid6  moralid7  moralid8  moralid9  mcoop1  mcoop2  mcoop3  mcoop4  mcoop5  mcoop6  mcoop7  narc1  narc4  narc5  narc6  nidentity1  nidentity2  omind1  omind2  omind3  omind4  omind5  omind6  contact1  contact2  contact3  contact4  contact5  hygiene1  hygiene2  hygiene3  hygiene4  hygiene5  psupport1  psupport2  psupport3  psupport4  psupport5  political_ideology  happy  slf_ladder  riskperc1  riskperc2  self_esteem  sbelong2  sbelong3  optim1  optim2  slfcont1
##   2   7  age  children  cnarc1  cnarc2  cnarc3  CRT1  CRT2  CRT3  ladder  marital1  mor_circle  moralid1  moralid10  moralid2  moralid3  moralid4  moralid5  moralid6  moralid7  moralid8  moralid9  mcoop1  mcoop2  mcoop3  mcoop4  mcoop5  mcoop6  mcoop7  narc1  narc4  narc5  narc6  nidentity1  nidentity2  omind1  omind2  omind3  omind4  omind5  omind6  contact1  contact2  contact3  contact4  contact5  hygiene1  hygiene2  hygiene3  hygiene4  hygiene5  psupport1  psupport2  psupport3  psupport4  psupport5  political_ideology  happy  slf_ladder  riskperc1  riskperc2  self_esteem  sbelong2  sbelong3  optim1  optim2  slfcont1
##   2   8  age  children  cnarc1  cnarc2  cnarc3  CRT1  CRT2  CRT3  ladder  marital1  mor_circle  moralid1  moralid10  moralid2  moralid3  moralid4  moralid5  moralid6  moralid7  moralid8  moralid9  mcoop1  mcoop2  mcoop3  mcoop4  mcoop5  mcoop6  mcoop7  narc1  narc4  narc5  narc6  nidentity1  nidentity2  omind1  omind2  omind3  omind4  omind5  omind6  contact1  contact2  contact3  contact4  contact5  hygiene1  hygiene2  hygiene3  hygiene4  hygiene5  psupport1  psupport2  psupport3  psupport4  psupport5  political_ideology  happy  slf_ladder  riskperc1  riskperc2  self_esteem  sbelong2  sbelong3  optim1  optim2  slfcont1
##   2   9  age  children  cnarc1  cnarc2  cnarc3  CRT1  CRT2  CRT3  ladder  marital1  mor_circle  moralid1  moralid10  moralid2  moralid3  moralid4  moralid5  moralid6  moralid7  moralid8  moralid9  mcoop1  mcoop2  mcoop3  mcoop4  mcoop5  mcoop6  mcoop7  narc1  narc4  narc5  narc6  nidentity1  nidentity2  omind1  omind2  omind3  omind4  omind5  omind6  contact1  contact2  contact3  contact4  contact5  hygiene1  hygiene2  hygiene3  hygiene4  hygiene5  psupport1  psupport2  psupport3  psupport4  psupport5  political_ideology  happy  slf_ladder  riskperc1  riskperc2  self_esteem  sbelong2  sbelong3  optim1  optim2  slfcont1
##   2   10  age  children  cnarc1  cnarc2  cnarc3  CRT1  CRT2  CRT3  ladder  marital1  mor_circle  moralid1  moralid10  moralid2  moralid3  moralid4  moralid5  moralid6  moralid7  moralid8  moralid9  mcoop1  mcoop2  mcoop3  mcoop4  mcoop5  mcoop6  mcoop7  narc1  narc4  narc5  narc6  nidentity1  nidentity2  omind1  omind2  omind3  omind4  omind5  omind6  contact1  contact2  contact3  contact4  contact5  hygiene1  hygiene2  hygiene3  hygiene4  hygiene5  psupport1  psupport2  psupport3  psupport4  psupport5  political_ideology  happy  slf_ladder  riskperc1  riskperc2  self_esteem  sbelong2  sbelong3  optim1  optim2  slfcont1
##   3   1  age  children  cnarc1  cnarc2  cnarc3  CRT1  CRT2  CRT3  ladder  marital1  mor_circle  moralid1  moralid10  moralid2  moralid3  moralid4  moralid5  moralid6  moralid7  moralid8  moralid9  mcoop1  mcoop2  mcoop3  mcoop4  mcoop5  mcoop6  mcoop7  narc1  narc4  narc5  narc6  nidentity1  nidentity2  omind1  omind2  omind3  omind4  omind5  omind6  contact1  contact2  contact3  contact4  contact5  hygiene1  hygiene2  hygiene3  hygiene4  hygiene5  psupport1  psupport2  psupport3  psupport4  psupport5  political_ideology  happy  slf_ladder  riskperc1  riskperc2  self_esteem  sbelong2  sbelong3  optim1  optim2  slfcont1
##   3   2  age  children  cnarc1  cnarc2  cnarc3  CRT1  CRT2  CRT3  ladder  marital1  mor_circle  moralid1  moralid10  moralid2  moralid3  moralid4  moralid5  moralid6  moralid7  moralid8  moralid9  mcoop1  mcoop2  mcoop3  mcoop4  mcoop5  mcoop6  mcoop7  narc1  narc4  narc5  narc6  nidentity1  nidentity2  omind1  omind2  omind3  omind4  omind5  omind6  contact1  contact2  contact3  contact4  contact5  hygiene1  hygiene2  hygiene3  hygiene4  hygiene5  psupport1  psupport2  psupport3  psupport4  psupport5  political_ideology  happy  slf_ladder  riskperc1  riskperc2  self_esteem  sbelong2  sbelong3  optim1  optim2  slfcont1
##   3   3  age  children  cnarc1  cnarc2  cnarc3  CRT1  CRT2  CRT3  ladder  marital1  mor_circle  moralid1  moralid10  moralid2  moralid3  moralid4  moralid5  moralid6  moralid7  moralid8  moralid9  mcoop1  mcoop2  mcoop3  mcoop4  mcoop5  mcoop6  mcoop7  narc1  narc4  narc5  narc6  nidentity1  nidentity2  omind1  omind2  omind3  omind4  omind5  omind6  contact1  contact2  contact3  contact4  contact5  hygiene1  hygiene2  hygiene3  hygiene4  hygiene5  psupport1  psupport2  psupport3  psupport4  psupport5  political_ideology  happy  slf_ladder  riskperc1  riskperc2  self_esteem  sbelong2  sbelong3  optim1  optim2  slfcont1
##   3   4  age  children  cnarc1  cnarc2  cnarc3  CRT1  CRT2  CRT3  ladder  marital1  mor_circle  moralid1  moralid10  moralid2  moralid3  moralid4  moralid5  moralid6  moralid7  moralid8  moralid9  mcoop1  mcoop2  mcoop3  mcoop4  mcoop5  mcoop6  mcoop7  narc1  narc4  narc5  narc6  nidentity1  nidentity2  omind1  omind2  omind3  omind4  omind5  omind6  contact1  contact2  contact3  contact4  contact5  hygiene1  hygiene2  hygiene3  hygiene4  hygiene5  psupport1  psupport2  psupport3  psupport4  psupport5  political_ideology  happy  slf_ladder  riskperc1  riskperc2  self_esteem  sbelong2  sbelong3  optim1  optim2  slfcont1
##   3   5  age  children  cnarc1  cnarc2  cnarc3  CRT1  CRT2  CRT3  ladder  marital1  mor_circle  moralid1  moralid10  moralid2  moralid3  moralid4  moralid5  moralid6  moralid7  moralid8  moralid9  mcoop1  mcoop2  mcoop3  mcoop4  mcoop5  mcoop6  mcoop7  narc1  narc4  narc5  narc6  nidentity1  nidentity2  omind1  omind2  omind3  omind4  omind5  omind6  contact1  contact2  contact3  contact4  contact5  hygiene1  hygiene2  hygiene3  hygiene4  hygiene5  psupport1  psupport2  psupport3  psupport4  psupport5  political_ideology  happy  slf_ladder  riskperc1  riskperc2  self_esteem  sbelong2  sbelong3  optim1  optim2  slfcont1
##   3   6  age  children  cnarc1  cnarc2  cnarc3  CRT1  CRT2  CRT3  ladder  marital1  mor_circle  moralid1  moralid10  moralid2  moralid3  moralid4  moralid5  moralid6  moralid7  moralid8  moralid9  mcoop1  mcoop2  mcoop3  mcoop4  mcoop5  mcoop6  mcoop7  narc1  narc4  narc5  narc6  nidentity1  nidentity2  omind1  omind2  omind3  omind4  omind5  omind6  contact1  contact2  contact3  contact4  contact5  hygiene1  hygiene2  hygiene3  hygiene4  hygiene5  psupport1  psupport2  psupport3  psupport4  psupport5  political_ideology  happy  slf_ladder  riskperc1  riskperc2  self_esteem  sbelong2  sbelong3  optim1  optim2  slfcont1
##   3   7  age  children  cnarc1  cnarc2  cnarc3  CRT1  CRT2  CRT3  ladder  marital1  mor_circle  moralid1  moralid10  moralid2  moralid3  moralid4  moralid5  moralid6  moralid7  moralid8  moralid9  mcoop1  mcoop2  mcoop3  mcoop4  mcoop5  mcoop6  mcoop7  narc1  narc4  narc5  narc6  nidentity1  nidentity2  omind1  omind2  omind3  omind4  omind5  omind6  contact1  contact2  contact3  contact4  contact5  hygiene1  hygiene2  hygiene3  hygiene4  hygiene5  psupport1  psupport2  psupport3  psupport4  psupport5  political_ideology  happy  slf_ladder  riskperc1  riskperc2  self_esteem  sbelong2  sbelong3  optim1  optim2  slfcont1
##   3   8  age  children  cnarc1  cnarc2  cnarc3  CRT1  CRT2  CRT3  ladder  marital1  mor_circle  moralid1  moralid10  moralid2  moralid3  moralid4  moralid5  moralid6  moralid7  moralid8  moralid9  mcoop1  mcoop2  mcoop3  mcoop4  mcoop5  mcoop6  mcoop7  narc1  narc4  narc5  narc6  nidentity1  nidentity2  omind1  omind2  omind3  omind4  omind5  omind6  contact1  contact2  contact3  contact4  contact5  hygiene1  hygiene2  hygiene3  hygiene4  hygiene5  psupport1  psupport2  psupport3  psupport4  psupport5  political_ideology  happy  slf_ladder  riskperc1  riskperc2  self_esteem  sbelong2  sbelong3  optim1  optim2  slfcont1
##   3   9  age  children  cnarc1  cnarc2  cnarc3  CRT1  CRT2  CRT3  ladder  marital1  mor_circle  moralid1  moralid10  moralid2  moralid3  moralid4  moralid5  moralid6  moralid7  moralid8  moralid9  mcoop1  mcoop2  mcoop3  mcoop4  mcoop5  mcoop6  mcoop7  narc1  narc4  narc5  narc6  nidentity1  nidentity2  omind1  omind2  omind3  omind4  omind5  omind6  contact1  contact2  contact3  contact4  contact5  hygiene1  hygiene2  hygiene3  hygiene4  hygiene5  psupport1  psupport2  psupport3  psupport4  psupport5  political_ideology  happy  slf_ladder  riskperc1  riskperc2  self_esteem  sbelong2  sbelong3  optim1  optim2  slfcont1
##   3   10  age  children  cnarc1  cnarc2  cnarc3  CRT1  CRT2  CRT3  ladder  marital1  mor_circle  moralid1  moralid10  moralid2  moralid3  moralid4  moralid5  moralid6  moralid7  moralid8  moralid9  mcoop1  mcoop2  mcoop3  mcoop4  mcoop5  mcoop6  mcoop7  narc1  narc4  narc5  narc6  nidentity1  nidentity2  omind1  omind2  omind3  omind4  omind5  omind6  contact1  contact2  contact3  contact4  contact5  hygiene1  hygiene2  hygiene3  hygiene4  hygiene5  psupport1  psupport2  psupport3  psupport4  psupport5  political_ideology  happy  slf_ladder  riskperc1  riskperc2  self_esteem  sbelong2  sbelong3  optim1  optim2  slfcont1
##   4   1  age  children  cnarc1  cnarc2  cnarc3  CRT1  CRT2  CRT3  ladder  marital1  mor_circle  moralid1  moralid10  moralid2  moralid3  moralid4  moralid5  moralid6  moralid7  moralid8  moralid9  mcoop1  mcoop2  mcoop3  mcoop4  mcoop5  mcoop6  mcoop7  narc1  narc4  narc5  narc6  nidentity1  nidentity2  omind1  omind2  omind3  omind4  omind5  omind6  contact1  contact2  contact3  contact4  contact5  hygiene1  hygiene2  hygiene3  hygiene4  hygiene5  psupport1  psupport2  psupport3  psupport4  psupport5  political_ideology  happy  slf_ladder  riskperc1  riskperc2  self_esteem  sbelong2  sbelong3  optim1  optim2  slfcont1
##   4   2  age  children  cnarc1  cnarc2  cnarc3  CRT1  CRT2  CRT3  ladder  marital1  mor_circle  moralid1  moralid10  moralid2  moralid3  moralid4  moralid5  moralid6  moralid7  moralid8  moralid9  mcoop1  mcoop2  mcoop3  mcoop4  mcoop5  mcoop6  mcoop7  narc1  narc4  narc5  narc6  nidentity1  nidentity2  omind1  omind2  omind3  omind4  omind5  omind6  contact1  contact2  contact3  contact4  contact5  hygiene1  hygiene2  hygiene3  hygiene4  hygiene5  psupport1  psupport2  psupport3  psupport4  psupport5  political_ideology  happy  slf_ladder  riskperc1  riskperc2  self_esteem  sbelong2  sbelong3  optim1  optim2  slfcont1
##   4   3  age  children  cnarc1  cnarc2  cnarc3  CRT1  CRT2  CRT3  ladder  marital1  mor_circle  moralid1  moralid10  moralid2  moralid3  moralid4  moralid5  moralid6  moralid7  moralid8  moralid9  mcoop1  mcoop2  mcoop3  mcoop4  mcoop5  mcoop6  mcoop7  narc1  narc4  narc5  narc6  nidentity1  nidentity2  omind1  omind2  omind3  omind4  omind5  omind6  contact1  contact2  contact3  contact4  contact5  hygiene1  hygiene2  hygiene3  hygiene4  hygiene5  psupport1  psupport2  psupport3  psupport4  psupport5  political_ideology  happy  slf_ladder  riskperc1  riskperc2  self_esteem  sbelong2  sbelong3  optim1  optim2  slfcont1
##   4   4  age  children  cnarc1  cnarc2  cnarc3  CRT1  CRT2  CRT3  ladder  marital1  mor_circle  moralid1  moralid10  moralid2  moralid3  moralid4  moralid5  moralid6  moralid7  moralid8  moralid9  mcoop1  mcoop2  mcoop3  mcoop4  mcoop5  mcoop6  mcoop7  narc1  narc4  narc5  narc6  nidentity1  nidentity2  omind1  omind2  omind3  omind4  omind5  omind6  contact1  contact2  contact3  contact4  contact5  hygiene1  hygiene2  hygiene3  hygiene4  hygiene5  psupport1  psupport2  psupport3  psupport4  psupport5  political_ideology  happy  slf_ladder  riskperc1  riskperc2  self_esteem  sbelong2  sbelong3  optim1  optim2  slfcont1
##   4   5  age  children  cnarc1  cnarc2  cnarc3  CRT1  CRT2  CRT3  ladder  marital1  mor_circle  moralid1  moralid10  moralid2  moralid3  moralid4  moralid5  moralid6  moralid7  moralid8  moralid9  mcoop1  mcoop2  mcoop3  mcoop4  mcoop5  mcoop6  mcoop7  narc1  narc4  narc5  narc6  nidentity1  nidentity2  omind1  omind2  omind3  omind4  omind5  omind6  contact1  contact2  contact3  contact4  contact5  hygiene1  hygiene2  hygiene3  hygiene4  hygiene5  psupport1  psupport2  psupport3  psupport4  psupport5  political_ideology  happy  slf_ladder  riskperc1  riskperc2  self_esteem  sbelong2  sbelong3  optim1  optim2  slfcont1
##   4   6  age  children  cnarc1  cnarc2  cnarc3  CRT1  CRT2  CRT3  ladder  marital1  mor_circle  moralid1  moralid10  moralid2  moralid3  moralid4  moralid5  moralid6  moralid7  moralid8  moralid9  mcoop1  mcoop2  mcoop3  mcoop4  mcoop5  mcoop6  mcoop7  narc1  narc4  narc5  narc6  nidentity1  nidentity2  omind1  omind2  omind3  omind4  omind5  omind6  contact1  contact2  contact3  contact4  contact5  hygiene1  hygiene2  hygiene3  hygiene4  hygiene5  psupport1  psupport2  psupport3  psupport4  psupport5  political_ideology  happy  slf_ladder  riskperc1  riskperc2  self_esteem  sbelong2  sbelong3  optim1  optim2  slfcont1
##   4   7  age  children  cnarc1  cnarc2  cnarc3  CRT1  CRT2  CRT3  ladder  marital1  mor_circle  moralid1  moralid10  moralid2  moralid3  moralid4  moralid5  moralid6  moralid7  moralid8  moralid9  mcoop1  mcoop2  mcoop3  mcoop4  mcoop5  mcoop6  mcoop7  narc1  narc4  narc5  narc6  nidentity1  nidentity2  omind1  omind2  omind3  omind4  omind5  omind6  contact1  contact2  contact3  contact4  contact5  hygiene1  hygiene2  hygiene3  hygiene4  hygiene5  psupport1  psupport2  psupport3  psupport4  psupport5  political_ideology  happy  slf_ladder  riskperc1  riskperc2  self_esteem  sbelong2  sbelong3  optim1  optim2  slfcont1
##   4   8  age  children  cnarc1  cnarc2  cnarc3  CRT1  CRT2  CRT3  ladder  marital1  mor_circle  moralid1  moralid10  moralid2  moralid3  moralid4  moralid5  moralid6  moralid7  moralid8  moralid9  mcoop1  mcoop2  mcoop3  mcoop4  mcoop5  mcoop6  mcoop7  narc1  narc4  narc5  narc6  nidentity1  nidentity2  omind1  omind2  omind3  omind4  omind5  omind6  contact1  contact2  contact3  contact4  contact5  hygiene1  hygiene2  hygiene3  hygiene4  hygiene5  psupport1  psupport2  psupport3  psupport4  psupport5  political_ideology  happy  slf_ladder  riskperc1  riskperc2  self_esteem  sbelong2  sbelong3  optim1  optim2  slfcont1
##   4   9  age  children  cnarc1  cnarc2  cnarc3  CRT1  CRT2  CRT3  ladder  marital1  mor_circle  moralid1  moralid10  moralid2  moralid3  moralid4  moralid5  moralid6  moralid7  moralid8  moralid9  mcoop1  mcoop2  mcoop3  mcoop4  mcoop5  mcoop6  mcoop7  narc1  narc4  narc5  narc6  nidentity1  nidentity2  omind1  omind2  omind3  omind4  omind5  omind6  contact1  contact2  contact3  contact4  contact5  hygiene1  hygiene2  hygiene3  hygiene4  hygiene5  psupport1  psupport2  psupport3  psupport4  psupport5  political_ideology  happy  slf_ladder  riskperc1  riskperc2  self_esteem  sbelong2  sbelong3  optim1  optim2  slfcont1
##   4   10  age  children  cnarc1  cnarc2  cnarc3  CRT1  CRT2  CRT3  ladder  marital1  mor_circle  moralid1  moralid10  moralid2  moralid3  moralid4  moralid5  moralid6  moralid7  moralid8  moralid9  mcoop1  mcoop2  mcoop3  mcoop4  mcoop5  mcoop6  mcoop7  narc1  narc4  narc5  narc6  nidentity1  nidentity2  omind1  omind2  omind3  omind4  omind5  omind6  contact1  contact2  contact3  contact4  contact5  hygiene1  hygiene2  hygiene3  hygiene4  hygiene5  psupport1  psupport2  psupport3  psupport4  psupport5  political_ideology  happy  slf_ladder  riskperc1  riskperc2  self_esteem  sbelong2  sbelong3  optim1  optim2  slfcont1
##   5   1  age  children  cnarc1  cnarc2  cnarc3  CRT1  CRT2  CRT3  ladder  marital1  mor_circle  moralid1  moralid10  moralid2  moralid3  moralid4  moralid5  moralid6  moralid7  moralid8  moralid9  mcoop1  mcoop2  mcoop3  mcoop4  mcoop5  mcoop6  mcoop7  narc1  narc4  narc5  narc6  nidentity1  nidentity2  omind1  omind2  omind3  omind4  omind5  omind6  contact1  contact2  contact3  contact4  contact5  hygiene1  hygiene2  hygiene3  hygiene4  hygiene5  psupport1  psupport2  psupport3  psupport4  psupport5  political_ideology  happy  slf_ladder  riskperc1  riskperc2  self_esteem  sbelong2  sbelong3  optim1  optim2  slfcont1
##   5   2  age  children  cnarc1  cnarc2  cnarc3  CRT1  CRT2  CRT3  ladder  marital1  mor_circle  moralid1  moralid10  moralid2  moralid3  moralid4  moralid5  moralid6  moralid7  moralid8  moralid9  mcoop1  mcoop2  mcoop3  mcoop4  mcoop5  mcoop6  mcoop7  narc1  narc4  narc5  narc6  nidentity1  nidentity2  omind1  omind2  omind3  omind4  omind5  omind6  contact1  contact2  contact3  contact4  contact5  hygiene1  hygiene2  hygiene3  hygiene4  hygiene5  psupport1  psupport2  psupport3  psupport4  psupport5  political_ideology  happy  slf_ladder  riskperc1  riskperc2  self_esteem  sbelong2  sbelong3  optim1  optim2  slfcont1
##   5   3  age  children  cnarc1  cnarc2  cnarc3  CRT1  CRT2  CRT3  ladder  marital1  mor_circle  moralid1  moralid10  moralid2  moralid3  moralid4  moralid5  moralid6  moralid7  moralid8  moralid9  mcoop1  mcoop2  mcoop3  mcoop4  mcoop5  mcoop6  mcoop7  narc1  narc4  narc5  narc6  nidentity1  nidentity2  omind1  omind2  omind3  omind4  omind5  omind6  contact1  contact2  contact3  contact4  contact5  hygiene1  hygiene2  hygiene3  hygiene4  hygiene5  psupport1  psupport2  psupport3  psupport4  psupport5  political_ideology  happy  slf_ladder  riskperc1  riskperc2  self_esteem  sbelong2  sbelong3  optim1  optim2  slfcont1
##   5   4  age  children  cnarc1  cnarc2  cnarc3  CRT1  CRT2  CRT3  ladder  marital1  mor_circle  moralid1  moralid10  moralid2  moralid3  moralid4  moralid5  moralid6  moralid7  moralid8  moralid9  mcoop1  mcoop2  mcoop3  mcoop4  mcoop5  mcoop6  mcoop7  narc1  narc4  narc5  narc6  nidentity1  nidentity2  omind1  omind2  omind3  omind4  omind5  omind6  contact1  contact2  contact3  contact4  contact5  hygiene1  hygiene2  hygiene3  hygiene4  hygiene5  psupport1  psupport2  psupport3  psupport4  psupport5  political_ideology  happy  slf_ladder  riskperc1  riskperc2  self_esteem  sbelong2  sbelong3  optim1  optim2  slfcont1
##   5   5  age  children  cnarc1  cnarc2  cnarc3  CRT1  CRT2  CRT3  ladder  marital1  mor_circle  moralid1  moralid10  moralid2  moralid3  moralid4  moralid5  moralid6  moralid7  moralid8  moralid9  mcoop1  mcoop2  mcoop3  mcoop4  mcoop5  mcoop6  mcoop7  narc1  narc4  narc5  narc6  nidentity1  nidentity2  omind1  omind2  omind3  omind4  omind5  omind6  contact1  contact2  contact3  contact4  contact5  hygiene1  hygiene2  hygiene3  hygiene4  hygiene5  psupport1  psupport2  psupport3  psupport4  psupport5  political_ideology  happy  slf_ladder  riskperc1  riskperc2  self_esteem  sbelong2  sbelong3  optim1  optim2  slfcont1
##   5   6  age  children  cnarc1  cnarc2  cnarc3  CRT1  CRT2  CRT3  ladder  marital1  mor_circle  moralid1  moralid10  moralid2  moralid3  moralid4  moralid5  moralid6  moralid7  moralid8  moralid9  mcoop1  mcoop2  mcoop3  mcoop4  mcoop5  mcoop6  mcoop7  narc1  narc4  narc5  narc6  nidentity1  nidentity2  omind1  omind2  omind3  omind4  omind5  omind6  contact1  contact2  contact3  contact4  contact5  hygiene1  hygiene2  hygiene3  hygiene4  hygiene5  psupport1  psupport2  psupport3  psupport4  psupport5  political_ideology  happy  slf_ladder  riskperc1  riskperc2  self_esteem  sbelong2  sbelong3  optim1  optim2  slfcont1
##   5   7  age  children  cnarc1  cnarc2  cnarc3  CRT1  CRT2  CRT3  ladder  marital1  mor_circle  moralid1  moralid10  moralid2  moralid3  moralid4  moralid5  moralid6  moralid7  moralid8  moralid9  mcoop1  mcoop2  mcoop3  mcoop4  mcoop5  mcoop6  mcoop7  narc1  narc4  narc5  narc6  nidentity1  nidentity2  omind1  omind2  omind3  omind4  omind5  omind6  contact1  contact2  contact3  contact4  contact5  hygiene1  hygiene2  hygiene3  hygiene4  hygiene5  psupport1  psupport2  psupport3  psupport4  psupport5  political_ideology  happy  slf_ladder  riskperc1  riskperc2  self_esteem  sbelong2  sbelong3  optim1  optim2  slfcont1
##   5   8  age  children  cnarc1  cnarc2  cnarc3  CRT1  CRT2  CRT3  ladder  marital1  mor_circle  moralid1  moralid10  moralid2  moralid3  moralid4  moralid5  moralid6  moralid7  moralid8  moralid9  mcoop1  mcoop2  mcoop3  mcoop4  mcoop5  mcoop6  mcoop7  narc1  narc4  narc5  narc6  nidentity1  nidentity2  omind1  omind2  omind3  omind4  omind5  omind6  contact1  contact2  contact3  contact4  contact5  hygiene1  hygiene2  hygiene3  hygiene4  hygiene5  psupport1  psupport2  psupport3  psupport4  psupport5  political_ideology  happy  slf_ladder  riskperc1  riskperc2  self_esteem  sbelong2  sbelong3  optim1  optim2  slfcont1
##   5   9  age  children  cnarc1  cnarc2  cnarc3  CRT1  CRT2  CRT3  ladder  marital1  mor_circle  moralid1  moralid10  moralid2  moralid3  moralid4  moralid5  moralid6  moralid7  moralid8  moralid9  mcoop1  mcoop2  mcoop3  mcoop4  mcoop5  mcoop6  mcoop7  narc1  narc4  narc5  narc6  nidentity1  nidentity2  omind1  omind2  omind3  omind4  omind5  omind6  contact1  contact2  contact3  contact4  contact5  hygiene1  hygiene2  hygiene3  hygiene4  hygiene5  psupport1  psupport2  psupport3  psupport4  psupport5  political_ideology  happy  slf_ladder  riskperc1  riskperc2  self_esteem  sbelong2  sbelong3  optim1  optim2  slfcont1
##   5   10  age  children  cnarc1  cnarc2  cnarc3  CRT1  CRT2  CRT3  ladder  marital1  mor_circle  moralid1  moralid10  moralid2  moralid3  moralid4  moralid5  moralid6  moralid7  moralid8  moralid9  mcoop1  mcoop2  mcoop3  mcoop4  mcoop5  mcoop6  mcoop7  narc1  narc4  narc5  narc6  nidentity1  nidentity2  omind1  omind2  omind3  omind4  omind5  omind6  contact1  contact2  contact3  contact4  contact5  hygiene1  hygiene2  hygiene3  hygiene4  hygiene5  psupport1  psupport2  psupport3  psupport4  psupport5  political_ideology  happy  slf_ladder  riskperc1  riskperc2  self_esteem  sbelong2  sbelong3  optim1  optim2  slfcont1
##   6   1  age  children  cnarc1  cnarc2  cnarc3  CRT1  CRT2  CRT3  ladder  marital1  mor_circle  moralid1  moralid10  moralid2  moralid3  moralid4  moralid5  moralid6  moralid7  moralid8  moralid9  mcoop1  mcoop2  mcoop3  mcoop4  mcoop5  mcoop6  mcoop7  narc1  narc4  narc5  narc6  nidentity1  nidentity2  omind1  omind2  omind3  omind4  omind5  omind6  contact1  contact2  contact3  contact4  contact5  hygiene1  hygiene2  hygiene3  hygiene4  hygiene5  psupport1  psupport2  psupport3  psupport4  psupport5  political_ideology  happy  slf_ladder  riskperc1  riskperc2  self_esteem  sbelong2  sbelong3  optim1  optim2  slfcont1
##   6   2  age  children  cnarc1  cnarc2  cnarc3  CRT1  CRT2  CRT3  ladder  marital1  mor_circle  moralid1  moralid10  moralid2  moralid3  moralid4  moralid5  moralid6  moralid7  moralid8  moralid9  mcoop1  mcoop2  mcoop3  mcoop4  mcoop5  mcoop6  mcoop7  narc1  narc4  narc5  narc6  nidentity1  nidentity2  omind1  omind2  omind3  omind4  omind5  omind6  contact1  contact2  contact3  contact4  contact5  hygiene1  hygiene2  hygiene3  hygiene4  hygiene5  psupport1  psupport2  psupport3  psupport4  psupport5  political_ideology  happy  slf_ladder  riskperc1  riskperc2  self_esteem  sbelong2  sbelong3  optim1  optim2  slfcont1
##   6   3  age  children  cnarc1  cnarc2  cnarc3  CRT1  CRT2  CRT3  ladder  marital1  mor_circle  moralid1  moralid10  moralid2  moralid3  moralid4  moralid5  moralid6  moralid7  moralid8  moralid9  mcoop1  mcoop2  mcoop3  mcoop4  mcoop5  mcoop6  mcoop7  narc1  narc4  narc5  narc6  nidentity1  nidentity2  omind1  omind2  omind3  omind4  omind5  omind6  contact1  contact2  contact3  contact4  contact5  hygiene1  hygiene2  hygiene3  hygiene4  hygiene5  psupport1  psupport2  psupport3  psupport4  psupport5  political_ideology  happy  slf_ladder  riskperc1  riskperc2  self_esteem  sbelong2  sbelong3  optim1  optim2  slfcont1
##   6   4  age  children  cnarc1  cnarc2  cnarc3  CRT1  CRT2  CRT3  ladder  marital1  mor_circle  moralid1  moralid10  moralid2  moralid3  moralid4  moralid5  moralid6  moralid7  moralid8  moralid9  mcoop1  mcoop2  mcoop3  mcoop4  mcoop5  mcoop6  mcoop7  narc1  narc4  narc5  narc6  nidentity1  nidentity2  omind1  omind2  omind3  omind4  omind5  omind6  contact1  contact2  contact3  contact4  contact5  hygiene1  hygiene2  hygiene3  hygiene4  hygiene5  psupport1  psupport2  psupport3  psupport4  psupport5  political_ideology  happy  slf_ladder  riskperc1  riskperc2  self_esteem  sbelong2  sbelong3  optim1  optim2  slfcont1
##   6   5  age  children  cnarc1  cnarc2  cnarc3  CRT1  CRT2  CRT3  ladder  marital1  mor_circle  moralid1  moralid10  moralid2  moralid3  moralid4  moralid5  moralid6  moralid7  moralid8  moralid9  mcoop1  mcoop2  mcoop3  mcoop4  mcoop5  mcoop6  mcoop7  narc1  narc4  narc5  narc6  nidentity1  nidentity2  omind1  omind2  omind3  omind4  omind5  omind6  contact1  contact2  contact3  contact4  contact5  hygiene1  hygiene2  hygiene3  hygiene4  hygiene5  psupport1  psupport2  psupport3  psupport4  psupport5  political_ideology  happy  slf_ladder  riskperc1  riskperc2  self_esteem  sbelong2  sbelong3  optim1  optim2  slfcont1
##   6   6  age  children  cnarc1  cnarc2  cnarc3  CRT1  CRT2  CRT3  ladder  marital1  mor_circle  moralid1  moralid10  moralid2  moralid3  moralid4  moralid5  moralid6  moralid7  moralid8  moralid9  mcoop1  mcoop2  mcoop3  mcoop4  mcoop5  mcoop6  mcoop7  narc1  narc4  narc5  narc6  nidentity1  nidentity2  omind1  omind2  omind3  omind4  omind5  omind6  contact1  contact2  contact3  contact4  contact5  hygiene1  hygiene2  hygiene3  hygiene4  hygiene5  psupport1  psupport2  psupport3  psupport4  psupport5  political_ideology  happy  slf_ladder  riskperc1  riskperc2  self_esteem  sbelong2  sbelong3  optim1  optim2  slfcont1
##   6   7  age  children  cnarc1  cnarc2  cnarc3  CRT1  CRT2  CRT3  ladder  marital1  mor_circle  moralid1  moralid10  moralid2  moralid3  moralid4  moralid5  moralid6  moralid7  moralid8  moralid9  mcoop1  mcoop2  mcoop3  mcoop4  mcoop5  mcoop6  mcoop7  narc1  narc4  narc5  narc6  nidentity1  nidentity2  omind1  omind2  omind3  omind4  omind5  omind6  contact1  contact2  contact3  contact4  contact5  hygiene1  hygiene2  hygiene3  hygiene4  hygiene5  psupport1  psupport2  psupport3  psupport4  psupport5  political_ideology  happy  slf_ladder  riskperc1  riskperc2  self_esteem  sbelong2  sbelong3  optim1  optim2  slfcont1
##   6   8  age  children  cnarc1  cnarc2  cnarc3  CRT1  CRT2  CRT3  ladder  marital1  mor_circle  moralid1  moralid10  moralid2  moralid3  moralid4  moralid5  moralid6  moralid7  moralid8  moralid9  mcoop1  mcoop2  mcoop3  mcoop4  mcoop5  mcoop6  mcoop7  narc1  narc4  narc5  narc6  nidentity1  nidentity2  omind1  omind2  omind3  omind4  omind5  omind6  contact1  contact2  contact3  contact4  contact5  hygiene1  hygiene2  hygiene3  hygiene4  hygiene5  psupport1  psupport2  psupport3  psupport4  psupport5  political_ideology  happy  slf_ladder  riskperc1  riskperc2  self_esteem  sbelong2  sbelong3  optim1  optim2  slfcont1
##   6   9  age  children  cnarc1  cnarc2  cnarc3  CRT1  CRT2  CRT3  ladder  marital1  mor_circle  moralid1  moralid10  moralid2  moralid3  moralid4  moralid5  moralid6  moralid7  moralid8  moralid9  mcoop1  mcoop2  mcoop3  mcoop4  mcoop5  mcoop6  mcoop7  narc1  narc4  narc5  narc6  nidentity1  nidentity2  omind1  omind2  omind3  omind4  omind5  omind6  contact1  contact2  contact3  contact4  contact5  hygiene1  hygiene2  hygiene3  hygiene4  hygiene5  psupport1  psupport2  psupport3  psupport4  psupport5  political_ideology  happy  slf_ladder  riskperc1  riskperc2  self_esteem  sbelong2  sbelong3  optim1  optim2  slfcont1
##   6   10  age  children  cnarc1  cnarc2  cnarc3  CRT1  CRT2  CRT3  ladder  marital1  mor_circle  moralid1  moralid10  moralid2  moralid3  moralid4  moralid5  moralid6  moralid7  moralid8  moralid9  mcoop1  mcoop2  mcoop3  mcoop4  mcoop5  mcoop6  mcoop7  narc1  narc4  narc5  narc6  nidentity1  nidentity2  omind1  omind2  omind3  omind4  omind5  omind6  contact1  contact2  contact3  contact4  contact5  hygiene1  hygiene2  hygiene3  hygiene4  hygiene5  psupport1  psupport2  psupport3  psupport4  psupport5  political_ideology  happy  slf_ladder  riskperc1  riskperc2  self_esteem  sbelong2  sbelong3  optim1  optim2  slfcont1
##   7   1  age  children  cnarc1  cnarc2  cnarc3  CRT1  CRT2  CRT3  ladder  marital1  mor_circle  moralid1  moralid10  moralid2  moralid3  moralid4  moralid5  moralid6  moralid7  moralid8  moralid9  mcoop1  mcoop2  mcoop3  mcoop4  mcoop5  mcoop6  mcoop7  narc1  narc4  narc5  narc6  nidentity1  nidentity2  omind1  omind2  omind3  omind4  omind5  omind6  contact1  contact2  contact3  contact4  contact5  hygiene1  hygiene2  hygiene3  hygiene4  hygiene5  psupport1  psupport2  psupport3  psupport4  psupport5  political_ideology  happy  slf_ladder  riskperc1  riskperc2  self_esteem  sbelong2  sbelong3  optim1  optim2  slfcont1
##   7   2  age  children  cnarc1  cnarc2  cnarc3  CRT1  CRT2  CRT3  ladder  marital1  mor_circle  moralid1  moralid10  moralid2  moralid3  moralid4  moralid5  moralid6  moralid7  moralid8  moralid9  mcoop1  mcoop2  mcoop3  mcoop4  mcoop5  mcoop6  mcoop7  narc1  narc4  narc5  narc6  nidentity1  nidentity2  omind1  omind2  omind3  omind4  omind5  omind6  contact1  contact2  contact3  contact4  contact5  hygiene1  hygiene2  hygiene3  hygiene4  hygiene5  psupport1  psupport2  psupport3  psupport4  psupport5  political_ideology  happy  slf_ladder  riskperc1  riskperc2  self_esteem  sbelong2  sbelong3  optim1  optim2  slfcont1
##   7   3  age  children  cnarc1  cnarc2  cnarc3  CRT1  CRT2  CRT3  ladder  marital1  mor_circle  moralid1  moralid10  moralid2  moralid3  moralid4  moralid5  moralid6  moralid7  moralid8  moralid9  mcoop1  mcoop2  mcoop3  mcoop4  mcoop5  mcoop6  mcoop7  narc1  narc4  narc5  narc6  nidentity1  nidentity2  omind1  omind2  omind3  omind4  omind5  omind6  contact1  contact2  contact3  contact4  contact5  hygiene1  hygiene2  hygiene3  hygiene4  hygiene5  psupport1  psupport2  psupport3  psupport4  psupport5  political_ideology  happy  slf_ladder  riskperc1  riskperc2  self_esteem  sbelong2  sbelong3  optim1  optim2  slfcont1
##   7   4  age  children  cnarc1  cnarc2  cnarc3  CRT1  CRT2  CRT3  ladder  marital1  mor_circle  moralid1  moralid10  moralid2  moralid3  moralid4  moralid5  moralid6  moralid7  moralid8  moralid9  mcoop1  mcoop2  mcoop3  mcoop4  mcoop5  mcoop6  mcoop7  narc1  narc4  narc5  narc6  nidentity1  nidentity2  omind1  omind2  omind3  omind4  omind5  omind6  contact1  contact2  contact3  contact4  contact5  hygiene1  hygiene2  hygiene3  hygiene4  hygiene5  psupport1  psupport2  psupport3  psupport4  psupport5  political_ideology  happy  slf_ladder  riskperc1  riskperc2  self_esteem  sbelong2  sbelong3  optim1  optim2  slfcont1
##   7   5  age  children  cnarc1  cnarc2  cnarc3  CRT1  CRT2  CRT3  ladder  marital1  mor_circle  moralid1  moralid10  moralid2  moralid3  moralid4  moralid5  moralid6  moralid7  moralid8  moralid9  mcoop1  mcoop2  mcoop3  mcoop4  mcoop5  mcoop6  mcoop7  narc1  narc4  narc5  narc6  nidentity1  nidentity2  omind1  omind2  omind3  omind4  omind5  omind6  contact1  contact2  contact3  contact4  contact5  hygiene1  hygiene2  hygiene3  hygiene4  hygiene5  psupport1  psupport2  psupport3  psupport4  psupport5  political_ideology  happy  slf_ladder  riskperc1  riskperc2  self_esteem  sbelong2  sbelong3  optim1  optim2  slfcont1
##   7   6  age  children  cnarc1  cnarc2  cnarc3  CRT1  CRT2  CRT3  ladder  marital1  mor_circle  moralid1  moralid10  moralid2  moralid3  moralid4  moralid5  moralid6  moralid7  moralid8  moralid9  mcoop1  mcoop2  mcoop3  mcoop4  mcoop5  mcoop6  mcoop7  narc1  narc4  narc5  narc6  nidentity1  nidentity2  omind1  omind2  omind3  omind4  omind5  omind6  contact1  contact2  contact3  contact4  contact5  hygiene1  hygiene2  hygiene3  hygiene4  hygiene5  psupport1  psupport2  psupport3  psupport4  psupport5  political_ideology  happy  slf_ladder  riskperc1  riskperc2  self_esteem  sbelong2  sbelong3  optim1  optim2  slfcont1
##   7   7  age  children  cnarc1  cnarc2  cnarc3  CRT1  CRT2  CRT3  ladder  marital1  mor_circle  moralid1  moralid10  moralid2  moralid3  moralid4  moralid5  moralid6  moralid7  moralid8  moralid9  mcoop1  mcoop2  mcoop3  mcoop4  mcoop5  mcoop6  mcoop7  narc1  narc4  narc5  narc6  nidentity1  nidentity2  omind1  omind2  omind3  omind4  omind5  omind6  contact1  contact2  contact3  contact4  contact5  hygiene1  hygiene2  hygiene3  hygiene4  hygiene5  psupport1  psupport2  psupport3  psupport4  psupport5  political_ideology  happy  slf_ladder  riskperc1  riskperc2  self_esteem  sbelong2  sbelong3  optim1  optim2  slfcont1
##   7   8  age  children  cnarc1  cnarc2  cnarc3  CRT1  CRT2  CRT3  ladder  marital1  mor_circle  moralid1  moralid10  moralid2  moralid3  moralid4  moralid5  moralid6  moralid7  moralid8  moralid9  mcoop1  mcoop2  mcoop3  mcoop4  mcoop5  mcoop6  mcoop7  narc1  narc4  narc5  narc6  nidentity1  nidentity2  omind1  omind2  omind3  omind4  omind5  omind6  contact1  contact2  contact3  contact4  contact5  hygiene1  hygiene2  hygiene3  hygiene4  hygiene5  psupport1  psupport2  psupport3  psupport4  psupport5  political_ideology  happy  slf_ladder  riskperc1  riskperc2  self_esteem  sbelong2  sbelong3  optim1  optim2  slfcont1
##   7   9  age  children  cnarc1  cnarc2  cnarc3  CRT1  CRT2  CRT3  ladder  marital1  mor_circle  moralid1  moralid10  moralid2  moralid3  moralid4  moralid5  moralid6  moralid7  moralid8  moralid9  mcoop1  mcoop2  mcoop3  mcoop4  mcoop5  mcoop6  mcoop7  narc1  narc4  narc5  narc6  nidentity1  nidentity2  omind1  omind2  omind3  omind4  omind5  omind6  contact1  contact2  contact3  contact4  contact5  hygiene1  hygiene2  hygiene3  hygiene4  hygiene5  psupport1  psupport2  psupport3  psupport4  psupport5  political_ideology  happy  slf_ladder  riskperc1  riskperc2  self_esteem  sbelong2  sbelong3  optim1  optim2  slfcont1
##   7   10  age  children  cnarc1  cnarc2  cnarc3  CRT1  CRT2  CRT3  ladder  marital1  mor_circle  moralid1  moralid10  moralid2  moralid3  moralid4  moralid5  moralid6  moralid7  moralid8  moralid9  mcoop1  mcoop2  mcoop3  mcoop4  mcoop5  mcoop6  mcoop7  narc1  narc4  narc5  narc6  nidentity1  nidentity2  omind1  omind2  omind3  omind4  omind5  omind6  contact1  contact2  contact3  contact4  contact5  hygiene1  hygiene2  hygiene3  hygiene4  hygiene5  psupport1  psupport2  psupport3  psupport4  psupport5  political_ideology  happy  slf_ladder  riskperc1  riskperc2  self_esteem  sbelong2  sbelong3  optim1  optim2  slfcont1
##   8   1  age  children  cnarc1  cnarc2  cnarc3  CRT1  CRT2  CRT3  ladder  marital1  mor_circle  moralid1  moralid10  moralid2  moralid3  moralid4  moralid5  moralid6  moralid7  moralid8  moralid9  mcoop1  mcoop2  mcoop3  mcoop4  mcoop5  mcoop6  mcoop7  narc1  narc4  narc5  narc6  nidentity1  nidentity2  omind1  omind2  omind3  omind4  omind5  omind6  contact1  contact2  contact3  contact4  contact5  hygiene1  hygiene2  hygiene3  hygiene4  hygiene5  psupport1  psupport2  psupport3  psupport4  psupport5  political_ideology  happy  slf_ladder  riskperc1  riskperc2  self_esteem  sbelong2  sbelong3  optim1  optim2  slfcont1
##   8   2  age  children  cnarc1  cnarc2  cnarc3  CRT1  CRT2  CRT3  ladder  marital1  mor_circle  moralid1  moralid10  moralid2  moralid3  moralid4  moralid5  moralid6  moralid7  moralid8  moralid9  mcoop1  mcoop2  mcoop3  mcoop4  mcoop5  mcoop6  mcoop7  narc1  narc4  narc5  narc6  nidentity1  nidentity2  omind1  omind2  omind3  omind4  omind5  omind6  contact1  contact2  contact3  contact4  contact5  hygiene1  hygiene2  hygiene3  hygiene4  hygiene5  psupport1  psupport2  psupport3  psupport4  psupport5  political_ideology  happy  slf_ladder  riskperc1  riskperc2  self_esteem  sbelong2  sbelong3  optim1  optim2  slfcont1
##   8   3  age  children  cnarc1  cnarc2  cnarc3  CRT1  CRT2  CRT3  ladder  marital1  mor_circle  moralid1  moralid10  moralid2  moralid3  moralid4  moralid5  moralid6  moralid7  moralid8  moralid9  mcoop1  mcoop2  mcoop3  mcoop4  mcoop5  mcoop6  mcoop7  narc1  narc4  narc5  narc6  nidentity1  nidentity2  omind1  omind2  omind3  omind4  omind5  omind6  contact1  contact2  contact3  contact4  contact5  hygiene1  hygiene2  hygiene3  hygiene4  hygiene5  psupport1  psupport2  psupport3  psupport4  psupport5  political_ideology  happy  slf_ladder  riskperc1  riskperc2  self_esteem  sbelong2  sbelong3  optim1  optim2  slfcont1
##   8   4  age  children  cnarc1  cnarc2  cnarc3  CRT1  CRT2  CRT3  ladder  marital1  mor_circle  moralid1  moralid10  moralid2  moralid3  moralid4  moralid5  moralid6  moralid7  moralid8  moralid9  mcoop1  mcoop2  mcoop3  mcoop4  mcoop5  mcoop6  mcoop7  narc1  narc4  narc5  narc6  nidentity1  nidentity2  omind1  omind2  omind3  omind4  omind5  omind6  contact1  contact2  contact3  contact4  contact5  hygiene1  hygiene2  hygiene3  hygiene4  hygiene5  psupport1  psupport2  psupport3  psupport4  psupport5  political_ideology  happy  slf_ladder  riskperc1  riskperc2  self_esteem  sbelong2  sbelong3  optim1  optim2  slfcont1
##   8   5  age  children  cnarc1  cnarc2  cnarc3  CRT1  CRT2  CRT3  ladder  marital1  mor_circle  moralid1  moralid10  moralid2  moralid3  moralid4  moralid5  moralid6  moralid7  moralid8  moralid9  mcoop1  mcoop2  mcoop3  mcoop4  mcoop5  mcoop6  mcoop7  narc1  narc4  narc5  narc6  nidentity1  nidentity2  omind1  omind2  omind3  omind4  omind5  omind6  contact1  contact2  contact3  contact4  contact5  hygiene1  hygiene2  hygiene3  hygiene4  hygiene5  psupport1  psupport2  psupport3  psupport4  psupport5  political_ideology  happy  slf_ladder  riskperc1  riskperc2  self_esteem  sbelong2  sbelong3  optim1  optim2  slfcont1
##   8   6  age  children  cnarc1  cnarc2  cnarc3  CRT1  CRT2  CRT3  ladder  marital1  mor_circle  moralid1  moralid10  moralid2  moralid3  moralid4  moralid5  moralid6  moralid7  moralid8  moralid9  mcoop1  mcoop2  mcoop3  mcoop4  mcoop5  mcoop6  mcoop7  narc1  narc4  narc5  narc6  nidentity1  nidentity2  omind1  omind2  omind3  omind4  omind5  omind6  contact1  contact2  contact3  contact4  contact5  hygiene1  hygiene2  hygiene3  hygiene4  hygiene5  psupport1  psupport2  psupport3  psupport4  psupport5  political_ideology  happy  slf_ladder  riskperc1  riskperc2  self_esteem  sbelong2  sbelong3  optim1  optim2  slfcont1
##   8   7  age  children  cnarc1  cnarc2  cnarc3  CRT1  CRT2  CRT3  ladder  marital1  mor_circle  moralid1  moralid10  moralid2  moralid3  moralid4  moralid5  moralid6  moralid7  moralid8  moralid9  mcoop1  mcoop2  mcoop3  mcoop4  mcoop5  mcoop6  mcoop7  narc1  narc4  narc5  narc6  nidentity1  nidentity2  omind1  omind2  omind3  omind4  omind5  omind6  contact1  contact2  contact3  contact4  contact5  hygiene1  hygiene2  hygiene3  hygiene4  hygiene5  psupport1  psupport2  psupport3  psupport4  psupport5  political_ideology  happy  slf_ladder  riskperc1  riskperc2  self_esteem  sbelong2  sbelong3  optim1  optim2  slfcont1
##   8   8  age  children  cnarc1  cnarc2  cnarc3  CRT1  CRT2  CRT3  ladder  marital1  mor_circle  moralid1  moralid10  moralid2  moralid3  moralid4  moralid5  moralid6  moralid7  moralid8  moralid9  mcoop1  mcoop2  mcoop3  mcoop4  mcoop5  mcoop6  mcoop7  narc1  narc4  narc5  narc6  nidentity1  nidentity2  omind1  omind2  omind3  omind4  omind5  omind6  contact1  contact2  contact3  contact4  contact5  hygiene1  hygiene2  hygiene3  hygiene4  hygiene5  psupport1  psupport2  psupport3  psupport4  psupport5  political_ideology  happy  slf_ladder  riskperc1  riskperc2  self_esteem  sbelong2  sbelong3  optim1  optim2  slfcont1
##   8   9  age  children  cnarc1  cnarc2  cnarc3  CRT1  CRT2  CRT3  ladder  marital1  mor_circle  moralid1  moralid10  moralid2  moralid3  moralid4  moralid5  moralid6  moralid7  moralid8  moralid9  mcoop1  mcoop2  mcoop3  mcoop4  mcoop5  mcoop6  mcoop7  narc1  narc4  narc5  narc6  nidentity1  nidentity2  omind1  omind2  omind3  omind4  omind5  omind6  contact1  contact2  contact3  contact4  contact5  hygiene1  hygiene2  hygiene3  hygiene4  hygiene5  psupport1  psupport2  psupport3  psupport4  psupport5  political_ideology  happy  slf_ladder  riskperc1  riskperc2  self_esteem  sbelong2  sbelong3  optim1  optim2  slfcont1
##   8   10  age  children  cnarc1  cnarc2  cnarc3  CRT1  CRT2  CRT3  ladder  marital1  mor_circle  moralid1  moralid10  moralid2  moralid3  moralid4  moralid5  moralid6  moralid7  moralid8  moralid9  mcoop1  mcoop2  mcoop3  mcoop4  mcoop5  mcoop6  mcoop7  narc1  narc4  narc5  narc6  nidentity1  nidentity2  omind1  omind2  omind3  omind4  omind5  omind6  contact1  contact2  contact3  contact4  contact5  hygiene1  hygiene2  hygiene3  hygiene4  hygiene5  psupport1  psupport2  psupport3  psupport4  psupport5  political_ideology  happy  slf_ladder  riskperc1  riskperc2  self_esteem  sbelong2  sbelong3  optim1  optim2  slfcont1
##   9   1  age  children  cnarc1  cnarc2  cnarc3  CRT1  CRT2  CRT3  ladder  marital1  mor_circle  moralid1  moralid10  moralid2  moralid3  moralid4  moralid5  moralid6  moralid7  moralid8  moralid9  mcoop1  mcoop2  mcoop3  mcoop4  mcoop5  mcoop6  mcoop7  narc1  narc4  narc5  narc6  nidentity1  nidentity2  omind1  omind2  omind3  omind4  omind5  omind6  contact1  contact2  contact3  contact4  contact5  hygiene1  hygiene2  hygiene3  hygiene4  hygiene5  psupport1  psupport2  psupport3  psupport4  psupport5  political_ideology  happy  slf_ladder  riskperc1  riskperc2  self_esteem  sbelong2  sbelong3  optim1  optim2  slfcont1
##   9   2  age  children  cnarc1  cnarc2  cnarc3  CRT1  CRT2  CRT3  ladder  marital1  mor_circle  moralid1  moralid10  moralid2  moralid3  moralid4  moralid5  moralid6  moralid7  moralid8  moralid9  mcoop1  mcoop2  mcoop3  mcoop4  mcoop5  mcoop6  mcoop7  narc1  narc4  narc5  narc6  nidentity1  nidentity2  omind1  omind2  omind3  omind4  omind5  omind6  contact1  contact2  contact3  contact4  contact5  hygiene1  hygiene2  hygiene3  hygiene4  hygiene5  psupport1  psupport2  psupport3  psupport4  psupport5  political_ideology  happy  slf_ladder  riskperc1  riskperc2  self_esteem  sbelong2  sbelong3  optim1  optim2  slfcont1
##   9   3  age  children  cnarc1  cnarc2  cnarc3  CRT1  CRT2  CRT3  ladder  marital1  mor_circle  moralid1  moralid10  moralid2  moralid3  moralid4  moralid5  moralid6  moralid7  moralid8  moralid9  mcoop1  mcoop2  mcoop3  mcoop4  mcoop5  mcoop6  mcoop7  narc1  narc4  narc5  narc6  nidentity1  nidentity2  omind1  omind2  omind3  omind4  omind5  omind6  contact1  contact2  contact3  contact4  contact5  hygiene1  hygiene2  hygiene3  hygiene4  hygiene5  psupport1  psupport2  psupport3  psupport4  psupport5  political_ideology  happy  slf_ladder  riskperc1  riskperc2  self_esteem  sbelong2  sbelong3  optim1  optim2  slfcont1
##   9   4  age  children  cnarc1  cnarc2  cnarc3  CRT1  CRT2  CRT3  ladder  marital1  mor_circle  moralid1  moralid10  moralid2  moralid3  moralid4  moralid5  moralid6  moralid7  moralid8  moralid9  mcoop1  mcoop2  mcoop3  mcoop4  mcoop5  mcoop6  mcoop7  narc1  narc4  narc5  narc6  nidentity1  nidentity2  omind1  omind2  omind3  omind4  omind5  omind6  contact1  contact2  contact3  contact4  contact5  hygiene1  hygiene2  hygiene3  hygiene4  hygiene5  psupport1  psupport2  psupport3  psupport4  psupport5  political_ideology  happy  slf_ladder  riskperc1  riskperc2  self_esteem  sbelong2  sbelong3  optim1  optim2  slfcont1
##   9   5  age  children  cnarc1  cnarc2  cnarc3  CRT1  CRT2  CRT3  ladder  marital1  mor_circle  moralid1  moralid10  moralid2  moralid3  moralid4  moralid5  moralid6  moralid7  moralid8  moralid9  mcoop1  mcoop2  mcoop3  mcoop4  mcoop5  mcoop6  mcoop7  narc1  narc4  narc5  narc6  nidentity1  nidentity2  omind1  omind2  omind3  omind4  omind5  omind6  contact1  contact2  contact3  contact4  contact5  hygiene1  hygiene2  hygiene3  hygiene4  hygiene5  psupport1  psupport2  psupport3  psupport4  psupport5  political_ideology  happy  slf_ladder  riskperc1  riskperc2  self_esteem  sbelong2  sbelong3  optim1  optim2  slfcont1
##   9   6  age  children  cnarc1  cnarc2  cnarc3  CRT1  CRT2  CRT3  ladder  marital1  mor_circle  moralid1  moralid10  moralid2  moralid3  moralid4  moralid5  moralid6  moralid7  moralid8  moralid9  mcoop1  mcoop2  mcoop3  mcoop4  mcoop5  mcoop6  mcoop7  narc1  narc4  narc5  narc6  nidentity1  nidentity2  omind1  omind2  omind3  omind4  omind5  omind6  contact1  contact2  contact3  contact4  contact5  hygiene1  hygiene2  hygiene3  hygiene4  hygiene5  psupport1  psupport2  psupport3  psupport4  psupport5  political_ideology  happy  slf_ladder  riskperc1  riskperc2  self_esteem  sbelong2  sbelong3  optim1  optim2  slfcont1
##   9   7  age  children  cnarc1  cnarc2  cnarc3  CRT1  CRT2  CRT3  ladder  marital1  mor_circle  moralid1  moralid10  moralid2  moralid3  moralid4  moralid5  moralid6  moralid7  moralid8  moralid9  mcoop1  mcoop2  mcoop3  mcoop4  mcoop5  mcoop6  mcoop7  narc1  narc4  narc5  narc6  nidentity1  nidentity2  omind1  omind2  omind3  omind4  omind5  omind6  contact1  contact2  contact3  contact4  contact5  hygiene1  hygiene2  hygiene3  hygiene4  hygiene5  psupport1  psupport2  psupport3  psupport4  psupport5  political_ideology  happy  slf_ladder  riskperc1  riskperc2  self_esteem  sbelong2  sbelong3  optim1  optim2  slfcont1
##   9   8  age  children  cnarc1  cnarc2  cnarc3  CRT1  CRT2  CRT3  ladder  marital1  mor_circle  moralid1  moralid10  moralid2  moralid3  moralid4  moralid5  moralid6  moralid7  moralid8  moralid9  mcoop1  mcoop2  mcoop3  mcoop4  mcoop5  mcoop6  mcoop7  narc1  narc4  narc5  narc6  nidentity1  nidentity2  omind1  omind2  omind3  omind4  omind5  omind6  contact1  contact2  contact3  contact4  contact5  hygiene1  hygiene2  hygiene3  hygiene4  hygiene5  psupport1  psupport2  psupport3  psupport4  psupport5  political_ideology  happy  slf_ladder  riskperc1  riskperc2  self_esteem  sbelong2  sbelong3  optim1  optim2  slfcont1
##   9   9  age  children  cnarc1  cnarc2  cnarc3  CRT1  CRT2  CRT3  ladder  marital1  mor_circle  moralid1  moralid10  moralid2  moralid3  moralid4  moralid5  moralid6  moralid7  moralid8  moralid9  mcoop1  mcoop2  mcoop3  mcoop4  mcoop5  mcoop6  mcoop7  narc1  narc4  narc5  narc6  nidentity1  nidentity2  omind1  omind2  omind3  omind4  omind5  omind6  contact1  contact2  contact3  contact4  contact5  hygiene1  hygiene2  hygiene3  hygiene4  hygiene5  psupport1  psupport2  psupport3  psupport4  psupport5  political_ideology  happy  slf_ladder  riskperc1  riskperc2  self_esteem  sbelong2  sbelong3  optim1  optim2  slfcont1
##   9   10  age  children  cnarc1  cnarc2  cnarc3  CRT1  CRT2  CRT3  ladder  marital1  mor_circle  moralid1  moralid10  moralid2  moralid3  moralid4  moralid5  moralid6  moralid7  moralid8  moralid9  mcoop1  mcoop2  mcoop3  mcoop4  mcoop5  mcoop6  mcoop7  narc1  narc4  narc5  narc6  nidentity1  nidentity2  omind1  omind2  omind3  omind4  omind5  omind6  contact1  contact2  contact3  contact4  contact5  hygiene1  hygiene2  hygiene3  hygiene4  hygiene5  psupport1  psupport2  psupport3  psupport4  psupport5  political_ideology  happy  slf_ladder  riskperc1  riskperc2  self_esteem  sbelong2  sbelong3  optim1  optim2  slfcont1
##   10   1  age  children  cnarc1  cnarc2  cnarc3  CRT1  CRT2  CRT3  ladder  marital1  mor_circle  moralid1  moralid10  moralid2  moralid3  moralid4  moralid5  moralid6  moralid7  moralid8  moralid9  mcoop1  mcoop2  mcoop3  mcoop4  mcoop5  mcoop6  mcoop7  narc1  narc4  narc5  narc6  nidentity1  nidentity2  omind1  omind2  omind3  omind4  omind5  omind6  contact1  contact2  contact3  contact4  contact5  hygiene1  hygiene2  hygiene3  hygiene4  hygiene5  psupport1  psupport2  psupport3  psupport4  psupport5  political_ideology  happy  slf_ladder  riskperc1  riskperc2  self_esteem  sbelong2  sbelong3  optim1  optim2  slfcont1
##   10   2  age  children  cnarc1  cnarc2  cnarc3  CRT1  CRT2  CRT3  ladder  marital1  mor_circle  moralid1  moralid10  moralid2  moralid3  moralid4  moralid5  moralid6  moralid7  moralid8  moralid9  mcoop1  mcoop2  mcoop3  mcoop4  mcoop5  mcoop6  mcoop7  narc1  narc4  narc5  narc6  nidentity1  nidentity2  omind1  omind2  omind3  omind4  omind5  omind6  contact1  contact2  contact3  contact4  contact5  hygiene1  hygiene2  hygiene3  hygiene4  hygiene5  psupport1  psupport2  psupport3  psupport4  psupport5  political_ideology  happy  slf_ladder  riskperc1  riskperc2  self_esteem  sbelong2  sbelong3  optim1  optim2  slfcont1
##   10   3  age  children  cnarc1  cnarc2  cnarc3  CRT1  CRT2  CRT3  ladder  marital1  mor_circle  moralid1  moralid10  moralid2  moralid3  moralid4  moralid5  moralid6  moralid7  moralid8  moralid9  mcoop1  mcoop2  mcoop3  mcoop4  mcoop5  mcoop6  mcoop7  narc1  narc4  narc5  narc6  nidentity1  nidentity2  omind1  omind2  omind3  omind4  omind5  omind6  contact1  contact2  contact3  contact4  contact5  hygiene1  hygiene2  hygiene3  hygiene4  hygiene5  psupport1  psupport2  psupport3  psupport4  psupport5  political_ideology  happy  slf_ladder  riskperc1  riskperc2  self_esteem  sbelong2  sbelong3  optim1  optim2  slfcont1
##   10   4  age  children  cnarc1  cnarc2  cnarc3  CRT1  CRT2  CRT3  ladder  marital1  mor_circle  moralid1  moralid10  moralid2  moralid3  moralid4  moralid5  moralid6  moralid7  moralid8  moralid9  mcoop1  mcoop2  mcoop3  mcoop4  mcoop5  mcoop6  mcoop7  narc1  narc4  narc5  narc6  nidentity1  nidentity2  omind1  omind2  omind3  omind4  omind5  omind6  contact1  contact2  contact3  contact4  contact5  hygiene1  hygiene2  hygiene3  hygiene4  hygiene5  psupport1  psupport2  psupport3  psupport4  psupport5  political_ideology  happy  slf_ladder  riskperc1  riskperc2  self_esteem  sbelong2  sbelong3  optim1  optim2  slfcont1
##   10   5  age  children  cnarc1  cnarc2  cnarc3  CRT1  CRT2  CRT3  ladder  marital1  mor_circle  moralid1  moralid10  moralid2  moralid3  moralid4  moralid5  moralid6  moralid7  moralid8  moralid9  mcoop1  mcoop2  mcoop3  mcoop4  mcoop5  mcoop6  mcoop7  narc1  narc4  narc5  narc6  nidentity1  nidentity2  omind1  omind2  omind3  omind4  omind5  omind6  contact1  contact2  contact3  contact4  contact5  hygiene1  hygiene2  hygiene3  hygiene4  hygiene5  psupport1  psupport2  psupport3  psupport4  psupport5  political_ideology  happy  slf_ladder  riskperc1  riskperc2  self_esteem  sbelong2  sbelong3  optim1  optim2  slfcont1
##   10   6  age  children  cnarc1  cnarc2  cnarc3  CRT1  CRT2  CRT3  ladder  marital1  mor_circle  moralid1  moralid10  moralid2  moralid3  moralid4  moralid5  moralid6  moralid7  moralid8  moralid9  mcoop1  mcoop2  mcoop3  mcoop4  mcoop5  mcoop6  mcoop7  narc1  narc4  narc5  narc6  nidentity1  nidentity2  omind1  omind2  omind3  omind4  omind5  omind6  contact1  contact2  contact3  contact4  contact5  hygiene1  hygiene2  hygiene3  hygiene4  hygiene5  psupport1  psupport2  psupport3  psupport4  psupport5  political_ideology  happy  slf_ladder  riskperc1  riskperc2  self_esteem  sbelong2  sbelong3  optim1  optim2  slfcont1
##   10   7  age  children  cnarc1  cnarc2  cnarc3  CRT1  CRT2  CRT3  ladder  marital1  mor_circle  moralid1  moralid10  moralid2  moralid3  moralid4  moralid5  moralid6  moralid7  moralid8  moralid9  mcoop1  mcoop2  mcoop3  mcoop4  mcoop5  mcoop6  mcoop7  narc1  narc4  narc5  narc6  nidentity1  nidentity2  omind1  omind2  omind3  omind4  omind5  omind6  contact1  contact2  contact3  contact4  contact5  hygiene1  hygiene2  hygiene3  hygiene4  hygiene5  psupport1  psupport2  psupport3  psupport4  psupport5  political_ideology  happy  slf_ladder  riskperc1  riskperc2  self_esteem  sbelong2  sbelong3  optim1  optim2  slfcont1
##   10   8  age  children  cnarc1  cnarc2  cnarc3  CRT1  CRT2  CRT3  ladder  marital1  mor_circle  moralid1  moralid10  moralid2  moralid3  moralid4  moralid5  moralid6  moralid7  moralid8  moralid9  mcoop1  mcoop2  mcoop3  mcoop4  mcoop5  mcoop6  mcoop7  narc1  narc4  narc5  narc6  nidentity1  nidentity2  omind1  omind2  omind3  omind4  omind5  omind6  contact1  contact2  contact3  contact4  contact5  hygiene1  hygiene2  hygiene3  hygiene4  hygiene5  psupport1  psupport2  psupport3  psupport4  psupport5  political_ideology  happy  slf_ladder  riskperc1  riskperc2  self_esteem  sbelong2  sbelong3  optim1  optim2  slfcont1
##   10   9  age  children  cnarc1  cnarc2  cnarc3  CRT1  CRT2  CRT3  ladder  marital1  mor_circle  moralid1  moralid10  moralid2  moralid3  moralid4  moralid5  moralid6  moralid7  moralid8  moralid9  mcoop1  mcoop2  mcoop3  mcoop4  mcoop5  mcoop6  mcoop7  narc1  narc4  narc5  narc6  nidentity1  nidentity2  omind1  omind2  omind3  omind4  omind5  omind6  contact1  contact2  contact3  contact4  contact5  hygiene1  hygiene2  hygiene3  hygiene4  hygiene5  psupport1  psupport2  psupport3  psupport4  psupport5  political_ideology  happy  slf_ladder  riskperc1  riskperc2  self_esteem  sbelong2  sbelong3  optim1  optim2  slfcont1
##   10   10  age  children  cnarc1  cnarc2  cnarc3  CRT1  CRT2  CRT3  ladder  marital1  mor_circle  moralid1  moralid10  moralid2  moralid3  moralid4  moralid5  moralid6  moralid7  moralid8  moralid9  mcoop1  mcoop2  mcoop3  mcoop4  mcoop5  mcoop6  mcoop7  narc1  narc4  narc5  narc6  nidentity1  nidentity2  omind1  omind2  omind3  omind4  omind5  omind6  contact1  contact2  contact3  contact4  contact5  hygiene1  hygiene2  hygiene3  hygiene4  hygiene5  psupport1  psupport2  psupport3  psupport4  psupport5  political_ideology  happy  slf_ladder  riskperc1  riskperc2  self_esteem  sbelong2  sbelong3  optim1  optim2  slfcont1
```

```
## Warning: Number of logged events: 6603
```

```
## 
##  iter imp variable
##   1   1  cnarc3  ctheory1  ctheory2  ctheory3  ctheory4  CRT1  CRT2  CRT3  health_cond  ladder  marital1  moralid10  moralid2  moralid3  moralid4  moralid5  moralid6  moralid7  moralid8  moralid9  mcoop1  mcoop2  mcoop3  mcoop5  mcoop6  mcoop7  narc1  narc2  narc3  narc4  narc5  narc6  omind2  omind3  omind4  omind5  omind6  contact2  contact3  hygiene5  political_ideology  riskperc2  self_esteem  sbelong2  optim1  optim2  slfcont1  slfcont3  slfcont4
##   1   2  cnarc3  ctheory1  ctheory2  ctheory3  ctheory4  CRT1  CRT2  CRT3  health_cond  ladder  marital1  moralid10  moralid2  moralid3  moralid4  moralid5  moralid6  moralid7  moralid8  moralid9  mcoop1  mcoop2  mcoop3  mcoop5  mcoop6  mcoop7  narc1  narc2  narc3  narc4  narc5  narc6  omind2  omind3  omind4  omind5  omind6  contact2  contact3  hygiene5  political_ideology  riskperc2  self_esteem  sbelong2  optim1  optim2  slfcont1  slfcont3  slfcont4
##   1   3  cnarc3  ctheory1  ctheory2  ctheory3  ctheory4  CRT1  CRT2  CRT3  health_cond  ladder  marital1  moralid10  moralid2  moralid3  moralid4  moralid5  moralid6  moralid7  moralid8  moralid9  mcoop1  mcoop2  mcoop3  mcoop5  mcoop6  mcoop7  narc1  narc2  narc3  narc4  narc5  narc6  omind2  omind3  omind4  omind5  omind6  contact2  contact3  hygiene5  political_ideology  riskperc2  self_esteem  sbelong2  optim1  optim2  slfcont1  slfcont3  slfcont4
##   1   4  cnarc3  ctheory1  ctheory2  ctheory3  ctheory4  CRT1  CRT2  CRT3  health_cond  ladder  marital1  moralid10  moralid2  moralid3  moralid4  moralid5  moralid6  moralid7  moralid8  moralid9  mcoop1  mcoop2  mcoop3  mcoop5  mcoop6  mcoop7  narc1  narc2  narc3  narc4  narc5  narc6  omind2  omind3  omind4  omind5  omind6  contact2  contact3  hygiene5  political_ideology  riskperc2  self_esteem  sbelong2  optim1  optim2  slfcont1  slfcont3  slfcont4
##   1   5  cnarc3  ctheory1  ctheory2  ctheory3  ctheory4  CRT1  CRT2  CRT3  health_cond  ladder  marital1  moralid10  moralid2  moralid3  moralid4  moralid5  moralid6  moralid7  moralid8  moralid9  mcoop1  mcoop2  mcoop3  mcoop5  mcoop6  mcoop7  narc1  narc2  narc3  narc4  narc5  narc6  omind2  omind3  omind4  omind5  omind6  contact2  contact3  hygiene5  political_ideology  riskperc2  self_esteem  sbelong2  optim1  optim2  slfcont1  slfcont3  slfcont4
##   1   6  cnarc3  ctheory1  ctheory2  ctheory3  ctheory4  CRT1  CRT2  CRT3  health_cond  ladder  marital1  moralid10  moralid2  moralid3  moralid4  moralid5  moralid6  moralid7  moralid8  moralid9  mcoop1  mcoop2  mcoop3  mcoop5  mcoop6  mcoop7  narc1  narc2  narc3  narc4  narc5  narc6  omind2  omind3  omind4  omind5  omind6  contact2  contact3  hygiene5  political_ideology  riskperc2  self_esteem  sbelong2  optim1  optim2  slfcont1  slfcont3  slfcont4
##   1   7  cnarc3  ctheory1  ctheory2  ctheory3  ctheory4  CRT1  CRT2  CRT3  health_cond  ladder  marital1  moralid10  moralid2  moralid3  moralid4  moralid5  moralid6  moralid7  moralid8  moralid9  mcoop1  mcoop2  mcoop3  mcoop5  mcoop6  mcoop7  narc1  narc2  narc3  narc4  narc5  narc6  omind2  omind3  omind4  omind5  omind6  contact2  contact3  hygiene5  political_ideology  riskperc2  self_esteem  sbelong2  optim1  optim2  slfcont1  slfcont3  slfcont4
##   1   8  cnarc3  ctheory1  ctheory2  ctheory3  ctheory4  CRT1  CRT2  CRT3  health_cond  ladder  marital1  moralid10  moralid2  moralid3  moralid4  moralid5  moralid6  moralid7  moralid8  moralid9  mcoop1  mcoop2  mcoop3  mcoop5  mcoop6  mcoop7  narc1  narc2  narc3  narc4  narc5  narc6  omind2  omind3  omind4  omind5  omind6  contact2  contact3  hygiene5  political_ideology  riskperc2  self_esteem  sbelong2  optim1  optim2  slfcont1  slfcont3  slfcont4
##   1   9  cnarc3  ctheory1  ctheory2  ctheory3  ctheory4  CRT1  CRT2  CRT3  health_cond  ladder  marital1  moralid10  moralid2  moralid3  moralid4  moralid5  moralid6  moralid7  moralid8  moralid9  mcoop1  mcoop2  mcoop3  mcoop5  mcoop6  mcoop7  narc1  narc2  narc3  narc4  narc5  narc6  omind2  omind3  omind4  omind5  omind6  contact2  contact3  hygiene5  political_ideology  riskperc2  self_esteem  sbelong2  optim1  optim2  slfcont1  slfcont3  slfcont4
##   1   10  cnarc3  ctheory1  ctheory2  ctheory3  ctheory4  CRT1  CRT2  CRT3  health_cond  ladder  marital1  moralid10  moralid2  moralid3  moralid4  moralid5  moralid6  moralid7  moralid8  moralid9  mcoop1  mcoop2  mcoop3  mcoop5  mcoop6  mcoop7  narc1  narc2  narc3  narc4  narc5  narc6  omind2  omind3  omind4  omind5  omind6  contact2  contact3  hygiene5  political_ideology  riskperc2  self_esteem  sbelong2  optim1  optim2  slfcont1  slfcont3  slfcont4
##   2   1  cnarc3  ctheory1  ctheory2  ctheory3  ctheory4  CRT1  CRT2  CRT3  health_cond  ladder  marital1  moralid10  moralid2  moralid3  moralid4  moralid5  moralid6  moralid7  moralid8  moralid9  mcoop1  mcoop2  mcoop3  mcoop5  mcoop6  mcoop7  narc1  narc2  narc3  narc4  narc5  narc6  omind2  omind3  omind4  omind5  omind6  contact2  contact3  hygiene5  political_ideology  riskperc2  self_esteem  sbelong2  optim1  optim2  slfcont1  slfcont3  slfcont4
##   2   2  cnarc3  ctheory1  ctheory2  ctheory3  ctheory4  CRT1  CRT2  CRT3  health_cond  ladder  marital1  moralid10  moralid2  moralid3  moralid4  moralid5  moralid6  moralid7  moralid8  moralid9  mcoop1  mcoop2  mcoop3  mcoop5  mcoop6  mcoop7  narc1  narc2  narc3  narc4  narc5  narc6  omind2  omind3  omind4  omind5  omind6  contact2  contact3  hygiene5  political_ideology  riskperc2  self_esteem  sbelong2  optim1  optim2  slfcont1  slfcont3  slfcont4
##   2   3  cnarc3  ctheory1  ctheory2  ctheory3  ctheory4  CRT1  CRT2  CRT3  health_cond  ladder  marital1  moralid10  moralid2  moralid3  moralid4  moralid5  moralid6  moralid7  moralid8  moralid9  mcoop1  mcoop2  mcoop3  mcoop5  mcoop6  mcoop7  narc1  narc2  narc3  narc4  narc5  narc6  omind2  omind3  omind4  omind5  omind6  contact2  contact3  hygiene5  political_ideology  riskperc2  self_esteem  sbelong2  optim1  optim2  slfcont1  slfcont3  slfcont4
##   2   4  cnarc3  ctheory1  ctheory2  ctheory3  ctheory4  CRT1  CRT2  CRT3  health_cond  ladder  marital1  moralid10  moralid2  moralid3  moralid4  moralid5  moralid6  moralid7  moralid8  moralid9  mcoop1  mcoop2  mcoop3  mcoop5  mcoop6  mcoop7  narc1  narc2  narc3  narc4  narc5  narc6  omind2  omind3  omind4  omind5  omind6  contact2  contact3  hygiene5  political_ideology  riskperc2  self_esteem  sbelong2  optim1  optim2  slfcont1  slfcont3  slfcont4
##   2   5  cnarc3  ctheory1  ctheory2  ctheory3  ctheory4  CRT1  CRT2  CRT3  health_cond  ladder  marital1  moralid10  moralid2  moralid3  moralid4  moralid5  moralid6  moralid7  moralid8  moralid9  mcoop1  mcoop2  mcoop3  mcoop5  mcoop6  mcoop7  narc1  narc2  narc3  narc4  narc5  narc6  omind2  omind3  omind4  omind5  omind6  contact2  contact3  hygiene5  political_ideology  riskperc2  self_esteem  sbelong2  optim1  optim2  slfcont1  slfcont3  slfcont4
##   2   6  cnarc3  ctheory1  ctheory2  ctheory3  ctheory4  CRT1  CRT2  CRT3  health_cond  ladder  marital1  moralid10  moralid2  moralid3  moralid4  moralid5  moralid6  moralid7  moralid8  moralid9  mcoop1  mcoop2  mcoop3  mcoop5  mcoop6  mcoop7  narc1  narc2  narc3  narc4  narc5  narc6  omind2  omind3  omind4  omind5  omind6  contact2  contact3  hygiene5  political_ideology  riskperc2  self_esteem  sbelong2  optim1  optim2  slfcont1  slfcont3  slfcont4
##   2   7  cnarc3  ctheory1  ctheory2  ctheory3  ctheory4  CRT1  CRT2  CRT3  health_cond  ladder  marital1  moralid10  moralid2  moralid3  moralid4  moralid5  moralid6  moralid7  moralid8  moralid9  mcoop1  mcoop2  mcoop3  mcoop5  mcoop6  mcoop7  narc1  narc2  narc3  narc4  narc5  narc6  omind2  omind3  omind4  omind5  omind6  contact2  contact3  hygiene5  political_ideology  riskperc2  self_esteem  sbelong2  optim1  optim2  slfcont1  slfcont3  slfcont4
##   2   8  cnarc3  ctheory1  ctheory2  ctheory3  ctheory4  CRT1  CRT2  CRT3  health_cond  ladder  marital1  moralid10  moralid2  moralid3  moralid4  moralid5  moralid6  moralid7  moralid8  moralid9  mcoop1  mcoop2  mcoop3  mcoop5  mcoop6  mcoop7  narc1  narc2  narc3  narc4  narc5  narc6  omind2  omind3  omind4  omind5  omind6  contact2  contact3  hygiene5  political_ideology  riskperc2  self_esteem  sbelong2  optim1  optim2  slfcont1  slfcont3  slfcont4
##   2   9  cnarc3  ctheory1  ctheory2  ctheory3  ctheory4  CRT1  CRT2  CRT3  health_cond  ladder  marital1  moralid10  moralid2  moralid3  moralid4  moralid5  moralid6  moralid7  moralid8  moralid9  mcoop1  mcoop2  mcoop3  mcoop5  mcoop6  mcoop7  narc1  narc2  narc3  narc4  narc5  narc6  omind2  omind3  omind4  omind5  omind6  contact2  contact3  hygiene5  political_ideology  riskperc2  self_esteem  sbelong2  optim1  optim2  slfcont1  slfcont3  slfcont4
##   2   10  cnarc3  ctheory1  ctheory2  ctheory3  ctheory4  CRT1  CRT2  CRT3  health_cond  ladder  marital1  moralid10  moralid2  moralid3  moralid4  moralid5  moralid6  moralid7  moralid8  moralid9  mcoop1  mcoop2  mcoop3  mcoop5  mcoop6  mcoop7  narc1  narc2  narc3  narc4  narc5  narc6  omind2  omind3  omind4  omind5  omind6  contact2  contact3  hygiene5  political_ideology  riskperc2  self_esteem  sbelong2  optim1  optim2  slfcont1  slfcont3  slfcont4
##   3   1  cnarc3  ctheory1  ctheory2  ctheory3  ctheory4  CRT1  CRT2  CRT3  health_cond  ladder  marital1  moralid10  moralid2  moralid3  moralid4  moralid5  moralid6  moralid7  moralid8  moralid9  mcoop1  mcoop2  mcoop3  mcoop5  mcoop6  mcoop7  narc1  narc2  narc3  narc4  narc5  narc6  omind2  omind3  omind4  omind5  omind6  contact2  contact3  hygiene5  political_ideology  riskperc2  self_esteem  sbelong2  optim1  optim2  slfcont1  slfcont3  slfcont4
##   3   2  cnarc3  ctheory1  ctheory2  ctheory3  ctheory4  CRT1  CRT2  CRT3  health_cond  ladder  marital1  moralid10  moralid2  moralid3  moralid4  moralid5  moralid6  moralid7  moralid8  moralid9  mcoop1  mcoop2  mcoop3  mcoop5  mcoop6  mcoop7  narc1  narc2  narc3  narc4  narc5  narc6  omind2  omind3  omind4  omind5  omind6  contact2  contact3  hygiene5  political_ideology  riskperc2  self_esteem  sbelong2  optim1  optim2  slfcont1  slfcont3  slfcont4
##   3   3  cnarc3  ctheory1  ctheory2  ctheory3  ctheory4  CRT1  CRT2  CRT3  health_cond  ladder  marital1  moralid10  moralid2  moralid3  moralid4  moralid5  moralid6  moralid7  moralid8  moralid9  mcoop1  mcoop2  mcoop3  mcoop5  mcoop6  mcoop7  narc1  narc2  narc3  narc4  narc5  narc6  omind2  omind3  omind4  omind5  omind6  contact2  contact3  hygiene5  political_ideology  riskperc2  self_esteem  sbelong2  optim1  optim2  slfcont1  slfcont3  slfcont4
##   3   4  cnarc3  ctheory1  ctheory2  ctheory3  ctheory4  CRT1  CRT2  CRT3  health_cond  ladder  marital1  moralid10  moralid2  moralid3  moralid4  moralid5  moralid6  moralid7  moralid8  moralid9  mcoop1  mcoop2  mcoop3  mcoop5  mcoop6  mcoop7  narc1  narc2  narc3  narc4  narc5  narc6  omind2  omind3  omind4  omind5  omind6  contact2  contact3  hygiene5  political_ideology  riskperc2  self_esteem  sbelong2  optim1  optim2  slfcont1  slfcont3  slfcont4
##   3   5  cnarc3  ctheory1  ctheory2  ctheory3  ctheory4  CRT1  CRT2  CRT3  health_cond  ladder  marital1  moralid10  moralid2  moralid3  moralid4  moralid5  moralid6  moralid7  moralid8  moralid9  mcoop1  mcoop2  mcoop3  mcoop5  mcoop6  mcoop7  narc1  narc2  narc3  narc4  narc5  narc6  omind2  omind3  omind4  omind5  omind6  contact2  contact3  hygiene5  political_ideology  riskperc2  self_esteem  sbelong2  optim1  optim2  slfcont1  slfcont3  slfcont4
##   3   6  cnarc3  ctheory1  ctheory2  ctheory3  ctheory4  CRT1  CRT2  CRT3  health_cond  ladder  marital1  moralid10  moralid2  moralid3  moralid4  moralid5  moralid6  moralid7  moralid8  moralid9  mcoop1  mcoop2  mcoop3  mcoop5  mcoop6  mcoop7  narc1  narc2  narc3  narc4  narc5  narc6  omind2  omind3  omind4  omind5  omind6  contact2  contact3  hygiene5  political_ideology  riskperc2  self_esteem  sbelong2  optim1  optim2  slfcont1  slfcont3  slfcont4
##   3   7  cnarc3  ctheory1  ctheory2  ctheory3  ctheory4  CRT1  CRT2  CRT3  health_cond  ladder  marital1  moralid10  moralid2  moralid3  moralid4  moralid5  moralid6  moralid7  moralid8  moralid9  mcoop1  mcoop2  mcoop3  mcoop5  mcoop6  mcoop7  narc1  narc2  narc3  narc4  narc5  narc6  omind2  omind3  omind4  omind5  omind6  contact2  contact3  hygiene5  political_ideology  riskperc2  self_esteem  sbelong2  optim1  optim2  slfcont1  slfcont3  slfcont4
##   3   8  cnarc3  ctheory1  ctheory2  ctheory3  ctheory4  CRT1  CRT2  CRT3  health_cond  ladder  marital1  moralid10  moralid2  moralid3  moralid4  moralid5  moralid6  moralid7  moralid8  moralid9  mcoop1  mcoop2  mcoop3  mcoop5  mcoop6  mcoop7  narc1  narc2  narc3  narc4  narc5  narc6  omind2  omind3  omind4  omind5  omind6  contact2  contact3  hygiene5  political_ideology  riskperc2  self_esteem  sbelong2  optim1  optim2  slfcont1  slfcont3  slfcont4
##   3   9  cnarc3  ctheory1  ctheory2  ctheory3  ctheory4  CRT1  CRT2  CRT3  health_cond  ladder  marital1  moralid10  moralid2  moralid3  moralid4  moralid5  moralid6  moralid7  moralid8  moralid9  mcoop1  mcoop2  mcoop3  mcoop5  mcoop6  mcoop7  narc1  narc2  narc3  narc4  narc5  narc6  omind2  omind3  omind4  omind5  omind6  contact2  contact3  hygiene5  political_ideology  riskperc2  self_esteem  sbelong2  optim1  optim2  slfcont1  slfcont3  slfcont4
##   3   10  cnarc3  ctheory1  ctheory2  ctheory3  ctheory4  CRT1  CRT2  CRT3  health_cond  ladder  marital1  moralid10  moralid2  moralid3  moralid4  moralid5  moralid6  moralid7  moralid8  moralid9  mcoop1  mcoop2  mcoop3  mcoop5  mcoop6  mcoop7  narc1  narc2  narc3  narc4  narc5  narc6  omind2  omind3  omind4  omind5  omind6  contact2  contact3  hygiene5  political_ideology  riskperc2  self_esteem  sbelong2  optim1  optim2  slfcont1  slfcont3  slfcont4
##   4   1  cnarc3  ctheory1  ctheory2  ctheory3  ctheory4  CRT1  CRT2  CRT3  health_cond  ladder  marital1  moralid10  moralid2  moralid3  moralid4  moralid5  moralid6  moralid7  moralid8  moralid9  mcoop1  mcoop2  mcoop3  mcoop5  mcoop6  mcoop7  narc1  narc2  narc3  narc4  narc5  narc6  omind2  omind3  omind4  omind5  omind6  contact2  contact3  hygiene5  political_ideology  riskperc2  self_esteem  sbelong2  optim1  optim2  slfcont1  slfcont3  slfcont4
##   4   2  cnarc3  ctheory1  ctheory2  ctheory3  ctheory4  CRT1  CRT2  CRT3  health_cond  ladder  marital1  moralid10  moralid2  moralid3  moralid4  moralid5  moralid6  moralid7  moralid8  moralid9  mcoop1  mcoop2  mcoop3  mcoop5  mcoop6  mcoop7  narc1  narc2  narc3  narc4  narc5  narc6  omind2  omind3  omind4  omind5  omind6  contact2  contact3  hygiene5  political_ideology  riskperc2  self_esteem  sbelong2  optim1  optim2  slfcont1  slfcont3  slfcont4
##   4   3  cnarc3  ctheory1  ctheory2  ctheory3  ctheory4  CRT1  CRT2  CRT3  health_cond  ladder  marital1  moralid10  moralid2  moralid3  moralid4  moralid5  moralid6  moralid7  moralid8  moralid9  mcoop1  mcoop2  mcoop3  mcoop5  mcoop6  mcoop7  narc1  narc2  narc3  narc4  narc5  narc6  omind2  omind3  omind4  omind5  omind6  contact2  contact3  hygiene5  political_ideology  riskperc2  self_esteem  sbelong2  optim1  optim2  slfcont1  slfcont3  slfcont4
##   4   4  cnarc3  ctheory1  ctheory2  ctheory3  ctheory4  CRT1  CRT2  CRT3  health_cond  ladder  marital1  moralid10  moralid2  moralid3  moralid4  moralid5  moralid6  moralid7  moralid8  moralid9  mcoop1  mcoop2  mcoop3  mcoop5  mcoop6  mcoop7  narc1  narc2  narc3  narc4  narc5  narc6  omind2  omind3  omind4  omind5  omind6  contact2  contact3  hygiene5  political_ideology  riskperc2  self_esteem  sbelong2  optim1  optim2  slfcont1  slfcont3  slfcont4
##   4   5  cnarc3  ctheory1  ctheory2  ctheory3  ctheory4  CRT1  CRT2  CRT3  health_cond  ladder  marital1  moralid10  moralid2  moralid3  moralid4  moralid5  moralid6  moralid7  moralid8  moralid9  mcoop1  mcoop2  mcoop3  mcoop5  mcoop6  mcoop7  narc1  narc2  narc3  narc4  narc5  narc6  omind2  omind3  omind4  omind5  omind6  contact2  contact3  hygiene5  political_ideology  riskperc2  self_esteem  sbelong2  optim1  optim2  slfcont1  slfcont3  slfcont4
##   4   6  cnarc3  ctheory1  ctheory2  ctheory3  ctheory4  CRT1  CRT2  CRT3  health_cond  ladder  marital1  moralid10  moralid2  moralid3  moralid4  moralid5  moralid6  moralid7  moralid8  moralid9  mcoop1  mcoop2  mcoop3  mcoop5  mcoop6  mcoop7  narc1  narc2  narc3  narc4  narc5  narc6  omind2  omind3  omind4  omind5  omind6  contact2  contact3  hygiene5  political_ideology  riskperc2  self_esteem  sbelong2  optim1  optim2  slfcont1  slfcont3  slfcont4
##   4   7  cnarc3  ctheory1  ctheory2  ctheory3  ctheory4  CRT1  CRT2  CRT3  health_cond  ladder  marital1  moralid10  moralid2  moralid3  moralid4  moralid5  moralid6  moralid7  moralid8  moralid9  mcoop1  mcoop2  mcoop3  mcoop5  mcoop6  mcoop7  narc1  narc2  narc3  narc4  narc5  narc6  omind2  omind3  omind4  omind5  omind6  contact2  contact3  hygiene5  political_ideology  riskperc2  self_esteem  sbelong2  optim1  optim2  slfcont1  slfcont3  slfcont4
##   4   8  cnarc3  ctheory1  ctheory2  ctheory3  ctheory4  CRT1  CRT2  CRT3  health_cond  ladder  marital1  moralid10  moralid2  moralid3  moralid4  moralid5  moralid6  moralid7  moralid8  moralid9  mcoop1  mcoop2  mcoop3  mcoop5  mcoop6  mcoop7  narc1  narc2  narc3  narc4  narc5  narc6  omind2  omind3  omind4  omind5  omind6  contact2  contact3  hygiene5  political_ideology  riskperc2  self_esteem  sbelong2  optim1  optim2  slfcont1  slfcont3  slfcont4
##   4   9  cnarc3  ctheory1  ctheory2  ctheory3  ctheory4  CRT1  CRT2  CRT3  health_cond  ladder  marital1  moralid10  moralid2  moralid3  moralid4  moralid5  moralid6  moralid7  moralid8  moralid9  mcoop1  mcoop2  mcoop3  mcoop5  mcoop6  mcoop7  narc1  narc2  narc3  narc4  narc5  narc6  omind2  omind3  omind4  omind5  omind6  contact2  contact3  hygiene5  political_ideology  riskperc2  self_esteem  sbelong2  optim1  optim2  slfcont1  slfcont3  slfcont4
##   4   10  cnarc3  ctheory1  ctheory2  ctheory3  ctheory4  CRT1  CRT2  CRT3  health_cond  ladder  marital1  moralid10  moralid2  moralid3  moralid4  moralid5  moralid6  moralid7  moralid8  moralid9  mcoop1  mcoop2  mcoop3  mcoop5  mcoop6  mcoop7  narc1  narc2  narc3  narc4  narc5  narc6  omind2  omind3  omind4  omind5  omind6  contact2  contact3  hygiene5  political_ideology  riskperc2  self_esteem  sbelong2  optim1  optim2  slfcont1  slfcont3  slfcont4
##   5   1  cnarc3  ctheory1  ctheory2  ctheory3  ctheory4  CRT1  CRT2  CRT3  health_cond  ladder  marital1  moralid10  moralid2  moralid3  moralid4  moralid5  moralid6  moralid7  moralid8  moralid9  mcoop1  mcoop2  mcoop3  mcoop5  mcoop6  mcoop7  narc1  narc2  narc3  narc4  narc5  narc6  omind2  omind3  omind4  omind5  omind6  contact2  contact3  hygiene5  political_ideology  riskperc2  self_esteem  sbelong2  optim1  optim2  slfcont1  slfcont3  slfcont4
##   5   2  cnarc3  ctheory1  ctheory2  ctheory3  ctheory4  CRT1  CRT2  CRT3  health_cond  ladder  marital1  moralid10  moralid2  moralid3  moralid4  moralid5  moralid6  moralid7  moralid8  moralid9  mcoop1  mcoop2  mcoop3  mcoop5  mcoop6  mcoop7  narc1  narc2  narc3  narc4  narc5  narc6  omind2  omind3  omind4  omind5  omind6  contact2  contact3  hygiene5  political_ideology  riskperc2  self_esteem  sbelong2  optim1  optim2  slfcont1  slfcont3  slfcont4
##   5   3  cnarc3  ctheory1  ctheory2  ctheory3  ctheory4  CRT1  CRT2  CRT3  health_cond  ladder  marital1  moralid10  moralid2  moralid3  moralid4  moralid5  moralid6  moralid7  moralid8  moralid9  mcoop1  mcoop2  mcoop3  mcoop5  mcoop6  mcoop7  narc1  narc2  narc3  narc4  narc5  narc6  omind2  omind3  omind4  omind5  omind6  contact2  contact3  hygiene5  political_ideology  riskperc2  self_esteem  sbelong2  optim1  optim2  slfcont1  slfcont3  slfcont4
##   5   4  cnarc3  ctheory1  ctheory2  ctheory3  ctheory4  CRT1  CRT2  CRT3  health_cond  ladder  marital1  moralid10  moralid2  moralid3  moralid4  moralid5  moralid6  moralid7  moralid8  moralid9  mcoop1  mcoop2  mcoop3  mcoop5  mcoop6  mcoop7  narc1  narc2  narc3  narc4  narc5  narc6  omind2  omind3  omind4  omind5  omind6  contact2  contact3  hygiene5  political_ideology  riskperc2  self_esteem  sbelong2  optim1  optim2  slfcont1  slfcont3  slfcont4
##   5   5  cnarc3  ctheory1  ctheory2  ctheory3  ctheory4  CRT1  CRT2  CRT3  health_cond  ladder  marital1  moralid10  moralid2  moralid3  moralid4  moralid5  moralid6  moralid7  moralid8  moralid9  mcoop1  mcoop2  mcoop3  mcoop5  mcoop6  mcoop7  narc1  narc2  narc3  narc4  narc5  narc6  omind2  omind3  omind4  omind5  omind6  contact2  contact3  hygiene5  political_ideology  riskperc2  self_esteem  sbelong2  optim1  optim2  slfcont1  slfcont3  slfcont4
##   5   6  cnarc3  ctheory1  ctheory2  ctheory3  ctheory4  CRT1  CRT2  CRT3  health_cond  ladder  marital1  moralid10  moralid2  moralid3  moralid4  moralid5  moralid6  moralid7  moralid8  moralid9  mcoop1  mcoop2  mcoop3  mcoop5  mcoop6  mcoop7  narc1  narc2  narc3  narc4  narc5  narc6  omind2  omind3  omind4  omind5  omind6  contact2  contact3  hygiene5  political_ideology  riskperc2  self_esteem  sbelong2  optim1  optim2  slfcont1  slfcont3  slfcont4
##   5   7  cnarc3  ctheory1  ctheory2  ctheory3  ctheory4  CRT1  CRT2  CRT3  health_cond  ladder  marital1  moralid10  moralid2  moralid3  moralid4  moralid5  moralid6  moralid7  moralid8  moralid9  mcoop1  mcoop2  mcoop3  mcoop5  mcoop6  mcoop7  narc1  narc2  narc3  narc4  narc5  narc6  omind2  omind3  omind4  omind5  omind6  contact2  contact3  hygiene5  political_ideology  riskperc2  self_esteem  sbelong2  optim1  optim2  slfcont1  slfcont3  slfcont4
##   5   8  cnarc3  ctheory1  ctheory2  ctheory3  ctheory4  CRT1  CRT2  CRT3  health_cond  ladder  marital1  moralid10  moralid2  moralid3  moralid4  moralid5  moralid6  moralid7  moralid8  moralid9  mcoop1  mcoop2  mcoop3  mcoop5  mcoop6  mcoop7  narc1  narc2  narc3  narc4  narc5  narc6  omind2  omind3  omind4  omind5  omind6  contact2  contact3  hygiene5  political_ideology  riskperc2  self_esteem  sbelong2  optim1  optim2  slfcont1  slfcont3  slfcont4
##   5   9  cnarc3  ctheory1  ctheory2  ctheory3  ctheory4  CRT1  CRT2  CRT3  health_cond  ladder  marital1  moralid10  moralid2  moralid3  moralid4  moralid5  moralid6  moralid7  moralid8  moralid9  mcoop1  mcoop2  mcoop3  mcoop5  mcoop6  mcoop7  narc1  narc2  narc3  narc4  narc5  narc6  omind2  omind3  omind4  omind5  omind6  contact2  contact3  hygiene5  political_ideology  riskperc2  self_esteem  sbelong2  optim1  optim2  slfcont1  slfcont3  slfcont4
##   5   10  cnarc3  ctheory1  ctheory2  ctheory3  ctheory4  CRT1  CRT2  CRT3  health_cond  ladder  marital1  moralid10  moralid2  moralid3  moralid4  moralid5  moralid6  moralid7  moralid8  moralid9  mcoop1  mcoop2  mcoop3  mcoop5  mcoop6  mcoop7  narc1  narc2  narc3  narc4  narc5  narc6  omind2  omind3  omind4  omind5  omind6  contact2  contact3  hygiene5  political_ideology  riskperc2  self_esteem  sbelong2  optim1  optim2  slfcont1  slfcont3  slfcont4
##   6   1  cnarc3  ctheory1  ctheory2  ctheory3  ctheory4  CRT1  CRT2  CRT3  health_cond  ladder  marital1  moralid10  moralid2  moralid3  moralid4  moralid5  moralid6  moralid7  moralid8  moralid9  mcoop1  mcoop2  mcoop3  mcoop5  mcoop6  mcoop7  narc1  narc2  narc3  narc4  narc5  narc6  omind2  omind3  omind4  omind5  omind6  contact2  contact3  hygiene5  political_ideology  riskperc2  self_esteem  sbelong2  optim1  optim2  slfcont1  slfcont3  slfcont4
##   6   2  cnarc3  ctheory1  ctheory2  ctheory3  ctheory4  CRT1  CRT2  CRT3  health_cond  ladder  marital1  moralid10  moralid2  moralid3  moralid4  moralid5  moralid6  moralid7  moralid8  moralid9  mcoop1  mcoop2  mcoop3  mcoop5  mcoop6  mcoop7  narc1  narc2  narc3  narc4  narc5  narc6  omind2  omind3  omind4  omind5  omind6  contact2  contact3  hygiene5  political_ideology  riskperc2  self_esteem  sbelong2  optim1  optim2  slfcont1  slfcont3  slfcont4
##   6   3  cnarc3  ctheory1  ctheory2  ctheory3  ctheory4  CRT1  CRT2  CRT3  health_cond  ladder  marital1  moralid10  moralid2  moralid3  moralid4  moralid5  moralid6  moralid7  moralid8  moralid9  mcoop1  mcoop2  mcoop3  mcoop5  mcoop6  mcoop7  narc1  narc2  narc3  narc4  narc5  narc6  omind2  omind3  omind4  omind5  omind6  contact2  contact3  hygiene5  political_ideology  riskperc2  self_esteem  sbelong2  optim1  optim2  slfcont1  slfcont3  slfcont4
##   6   4  cnarc3  ctheory1  ctheory2  ctheory3  ctheory4  CRT1  CRT2  CRT3  health_cond  ladder  marital1  moralid10  moralid2  moralid3  moralid4  moralid5  moralid6  moralid7  moralid8  moralid9  mcoop1  mcoop2  mcoop3  mcoop5  mcoop6  mcoop7  narc1  narc2  narc3  narc4  narc5  narc6  omind2  omind3  omind4  omind5  omind6  contact2  contact3  hygiene5  political_ideology  riskperc2  self_esteem  sbelong2  optim1  optim2  slfcont1  slfcont3  slfcont4
##   6   5  cnarc3  ctheory1  ctheory2  ctheory3  ctheory4  CRT1  CRT2  CRT3  health_cond  ladder  marital1  moralid10  moralid2  moralid3  moralid4  moralid5  moralid6  moralid7  moralid8  moralid9  mcoop1  mcoop2  mcoop3  mcoop5  mcoop6  mcoop7  narc1  narc2  narc3  narc4  narc5  narc6  omind2  omind3  omind4  omind5  omind6  contact2  contact3  hygiene5  political_ideology  riskperc2  self_esteem  sbelong2  optim1  optim2  slfcont1  slfcont3  slfcont4
##   6   6  cnarc3  ctheory1  ctheory2  ctheory3  ctheory4  CRT1  CRT2  CRT3  health_cond  ladder  marital1  moralid10  moralid2  moralid3  moralid4  moralid5  moralid6  moralid7  moralid8  moralid9  mcoop1  mcoop2  mcoop3  mcoop5  mcoop6  mcoop7  narc1  narc2  narc3  narc4  narc5  narc6  omind2  omind3  omind4  omind5  omind6  contact2  contact3  hygiene5  political_ideology  riskperc2  self_esteem  sbelong2  optim1  optim2  slfcont1  slfcont3  slfcont4
##   6   7  cnarc3  ctheory1  ctheory2  ctheory3  ctheory4  CRT1  CRT2  CRT3  health_cond  ladder  marital1  moralid10  moralid2  moralid3  moralid4  moralid5  moralid6  moralid7  moralid8  moralid9  mcoop1  mcoop2  mcoop3  mcoop5  mcoop6  mcoop7  narc1  narc2  narc3  narc4  narc5  narc6  omind2  omind3  omind4  omind5  omind6  contact2  contact3  hygiene5  political_ideology  riskperc2  self_esteem  sbelong2  optim1  optim2  slfcont1  slfcont3  slfcont4
##   6   8  cnarc3  ctheory1  ctheory2  ctheory3  ctheory4  CRT1  CRT2  CRT3  health_cond  ladder  marital1  moralid10  moralid2  moralid3  moralid4  moralid5  moralid6  moralid7  moralid8  moralid9  mcoop1  mcoop2  mcoop3  mcoop5  mcoop6  mcoop7  narc1  narc2  narc3  narc4  narc5  narc6  omind2  omind3  omind4  omind5  omind6  contact2  contact3  hygiene5  political_ideology  riskperc2  self_esteem  sbelong2  optim1  optim2  slfcont1  slfcont3  slfcont4
##   6   9  cnarc3  ctheory1  ctheory2  ctheory3  ctheory4  CRT1  CRT2  CRT3  health_cond  ladder  marital1  moralid10  moralid2  moralid3  moralid4  moralid5  moralid6  moralid7  moralid8  moralid9  mcoop1  mcoop2  mcoop3  mcoop5  mcoop6  mcoop7  narc1  narc2  narc3  narc4  narc5  narc6  omind2  omind3  omind4  omind5  omind6  contact2  contact3  hygiene5  political_ideology  riskperc2  self_esteem  sbelong2  optim1  optim2  slfcont1  slfcont3  slfcont4
##   6   10  cnarc3  ctheory1  ctheory2  ctheory3  ctheory4  CRT1  CRT2  CRT3  health_cond  ladder  marital1  moralid10  moralid2  moralid3  moralid4  moralid5  moralid6  moralid7  moralid8  moralid9  mcoop1  mcoop2  mcoop3  mcoop5  mcoop6  mcoop7  narc1  narc2  narc3  narc4  narc5  narc6  omind2  omind3  omind4  omind5  omind6  contact2  contact3  hygiene5  political_ideology  riskperc2  self_esteem  sbelong2  optim1  optim2  slfcont1  slfcont3  slfcont4
##   7   1  cnarc3  ctheory1  ctheory2  ctheory3  ctheory4  CRT1  CRT2  CRT3  health_cond  ladder  marital1  moralid10  moralid2  moralid3  moralid4  moralid5  moralid6  moralid7  moralid8  moralid9  mcoop1  mcoop2  mcoop3  mcoop5  mcoop6  mcoop7  narc1  narc2  narc3  narc4  narc5  narc6  omind2  omind3  omind4  omind5  omind6  contact2  contact3  hygiene5  political_ideology  riskperc2  self_esteem  sbelong2  optim1  optim2  slfcont1  slfcont3  slfcont4
##   7   2  cnarc3  ctheory1  ctheory2  ctheory3  ctheory4  CRT1  CRT2  CRT3  health_cond  ladder  marital1  moralid10  moralid2  moralid3  moralid4  moralid5  moralid6  moralid7  moralid8  moralid9  mcoop1  mcoop2  mcoop3  mcoop5  mcoop6  mcoop7  narc1  narc2  narc3  narc4  narc5  narc6  omind2  omind3  omind4  omind5  omind6  contact2  contact3  hygiene5  political_ideology  riskperc2  self_esteem  sbelong2  optim1  optim2  slfcont1  slfcont3  slfcont4
##   7   3  cnarc3  ctheory1  ctheory2  ctheory3  ctheory4  CRT1  CRT2  CRT3  health_cond  ladder  marital1  moralid10  moralid2  moralid3  moralid4  moralid5  moralid6  moralid7  moralid8  moralid9  mcoop1  mcoop2  mcoop3  mcoop5  mcoop6  mcoop7  narc1  narc2  narc3  narc4  narc5  narc6  omind2  omind3  omind4  omind5  omind6  contact2  contact3  hygiene5  political_ideology  riskperc2  self_esteem  sbelong2  optim1  optim2  slfcont1  slfcont3  slfcont4
##   7   4  cnarc3  ctheory1  ctheory2  ctheory3  ctheory4  CRT1  CRT2  CRT3  health_cond  ladder  marital1  moralid10  moralid2  moralid3  moralid4  moralid5  moralid6  moralid7  moralid8  moralid9  mcoop1  mcoop2  mcoop3  mcoop5  mcoop6  mcoop7  narc1  narc2  narc3  narc4  narc5  narc6  omind2  omind3  omind4  omind5  omind6  contact2  contact3  hygiene5  political_ideology  riskperc2  self_esteem  sbelong2  optim1  optim2  slfcont1  slfcont3  slfcont4
##   7   5  cnarc3  ctheory1  ctheory2  ctheory3  ctheory4  CRT1  CRT2  CRT3  health_cond  ladder  marital1  moralid10  moralid2  moralid3  moralid4  moralid5  moralid6  moralid7  moralid8  moralid9  mcoop1  mcoop2  mcoop3  mcoop5  mcoop6  mcoop7  narc1  narc2  narc3  narc4  narc5  narc6  omind2  omind3  omind4  omind5  omind6  contact2  contact3  hygiene5  political_ideology  riskperc2  self_esteem  sbelong2  optim1  optim2  slfcont1  slfcont3  slfcont4
##   7   6  cnarc3  ctheory1  ctheory2  ctheory3  ctheory4  CRT1  CRT2  CRT3  health_cond  ladder  marital1  moralid10  moralid2  moralid3  moralid4  moralid5  moralid6  moralid7  moralid8  moralid9  mcoop1  mcoop2  mcoop3  mcoop5  mcoop6  mcoop7  narc1  narc2  narc3  narc4  narc5  narc6  omind2  omind3  omind4  omind5  omind6  contact2  contact3  hygiene5  political_ideology  riskperc2  self_esteem  sbelong2  optim1  optim2  slfcont1  slfcont3  slfcont4
##   7   7  cnarc3  ctheory1  ctheory2  ctheory3  ctheory4  CRT1  CRT2  CRT3  health_cond  ladder  marital1  moralid10  moralid2  moralid3  moralid4  moralid5  moralid6  moralid7  moralid8  moralid9  mcoop1  mcoop2  mcoop3  mcoop5  mcoop6  mcoop7  narc1  narc2  narc3  narc4  narc5  narc6  omind2  omind3  omind4  omind5  omind6  contact2  contact3  hygiene5  political_ideology  riskperc2  self_esteem  sbelong2  optim1  optim2  slfcont1  slfcont3  slfcont4
##   7   8  cnarc3  ctheory1  ctheory2  ctheory3  ctheory4  CRT1  CRT2  CRT3  health_cond  ladder  marital1  moralid10  moralid2  moralid3  moralid4  moralid5  moralid6  moralid7  moralid8  moralid9  mcoop1  mcoop2  mcoop3  mcoop5  mcoop6  mcoop7  narc1  narc2  narc3  narc4  narc5  narc6  omind2  omind3  omind4  omind5  omind6  contact2  contact3  hygiene5  political_ideology  riskperc2  self_esteem  sbelong2  optim1  optim2  slfcont1  slfcont3  slfcont4
##   7   9  cnarc3  ctheory1  ctheory2  ctheory3  ctheory4  CRT1  CRT2  CRT3  health_cond  ladder  marital1  moralid10  moralid2  moralid3  moralid4  moralid5  moralid6  moralid7  moralid8  moralid9  mcoop1  mcoop2  mcoop3  mcoop5  mcoop6  mcoop7  narc1  narc2  narc3  narc4  narc5  narc6  omind2  omind3  omind4  omind5  omind6  contact2  contact3  hygiene5  political_ideology  riskperc2  self_esteem  sbelong2  optim1  optim2  slfcont1  slfcont3  slfcont4
##   7   10  cnarc3  ctheory1  ctheory2  ctheory3  ctheory4  CRT1  CRT2  CRT3  health_cond  ladder  marital1  moralid10  moralid2  moralid3  moralid4  moralid5  moralid6  moralid7  moralid8  moralid9  mcoop1  mcoop2  mcoop3  mcoop5  mcoop6  mcoop7  narc1  narc2  narc3  narc4  narc5  narc6  omind2  omind3  omind4  omind5  omind6  contact2  contact3  hygiene5  political_ideology  riskperc2  self_esteem  sbelong2  optim1  optim2  slfcont1  slfcont3  slfcont4
##   8   1  cnarc3  ctheory1  ctheory2  ctheory3  ctheory4  CRT1  CRT2  CRT3  health_cond  ladder  marital1  moralid10  moralid2  moralid3  moralid4  moralid5  moralid6  moralid7  moralid8  moralid9  mcoop1  mcoop2  mcoop3  mcoop5  mcoop6  mcoop7  narc1  narc2  narc3  narc4  narc5  narc6  omind2  omind3  omind4  omind5  omind6  contact2  contact3  hygiene5  political_ideology  riskperc2  self_esteem  sbelong2  optim1  optim2  slfcont1  slfcont3  slfcont4
##   8   2  cnarc3  ctheory1  ctheory2  ctheory3  ctheory4  CRT1  CRT2  CRT3  health_cond  ladder  marital1  moralid10  moralid2  moralid3  moralid4  moralid5  moralid6  moralid7  moralid8  moralid9  mcoop1  mcoop2  mcoop3  mcoop5  mcoop6  mcoop7  narc1  narc2  narc3  narc4  narc5  narc6  omind2  omind3  omind4  omind5  omind6  contact2  contact3  hygiene5  political_ideology  riskperc2  self_esteem  sbelong2  optim1  optim2  slfcont1  slfcont3  slfcont4
##   8   3  cnarc3  ctheory1  ctheory2  ctheory3  ctheory4  CRT1  CRT2  CRT3  health_cond  ladder  marital1  moralid10  moralid2  moralid3  moralid4  moralid5  moralid6  moralid7  moralid8  moralid9  mcoop1  mcoop2  mcoop3  mcoop5  mcoop6  mcoop7  narc1  narc2  narc3  narc4  narc5  narc6  omind2  omind3  omind4  omind5  omind6  contact2  contact3  hygiene5  political_ideology  riskperc2  self_esteem  sbelong2  optim1  optim2  slfcont1  slfcont3  slfcont4
##   8   4  cnarc3  ctheory1  ctheory2  ctheory3  ctheory4  CRT1  CRT2  CRT3  health_cond  ladder  marital1  moralid10  moralid2  moralid3  moralid4  moralid5  moralid6  moralid7  moralid8  moralid9  mcoop1  mcoop2  mcoop3  mcoop5  mcoop6  mcoop7  narc1  narc2  narc3  narc4  narc5  narc6  omind2  omind3  omind4  omind5  omind6  contact2  contact3  hygiene5  political_ideology  riskperc2  self_esteem  sbelong2  optim1  optim2  slfcont1  slfcont3  slfcont4
##   8   5  cnarc3  ctheory1  ctheory2  ctheory3  ctheory4  CRT1  CRT2  CRT3  health_cond  ladder  marital1  moralid10  moralid2  moralid3  moralid4  moralid5  moralid6  moralid7  moralid8  moralid9  mcoop1  mcoop2  mcoop3  mcoop5  mcoop6  mcoop7  narc1  narc2  narc3  narc4  narc5  narc6  omind2  omind3  omind4  omind5  omind6  contact2  contact3  hygiene5  political_ideology  riskperc2  self_esteem  sbelong2  optim1  optim2  slfcont1  slfcont3  slfcont4
##   8   6  cnarc3  ctheory1  ctheory2  ctheory3  ctheory4  CRT1  CRT2  CRT3  health_cond  ladder  marital1  moralid10  moralid2  moralid3  moralid4  moralid5  moralid6  moralid7  moralid8  moralid9  mcoop1  mcoop2  mcoop3  mcoop5  mcoop6  mcoop7  narc1  narc2  narc3  narc4  narc5  narc6  omind2  omind3  omind4  omind5  omind6  contact2  contact3  hygiene5  political_ideology  riskperc2  self_esteem  sbelong2  optim1  optim2  slfcont1  slfcont3  slfcont4
##   8   7  cnarc3  ctheory1  ctheory2  ctheory3  ctheory4  CRT1  CRT2  CRT3  health_cond  ladder  marital1  moralid10  moralid2  moralid3  moralid4  moralid5  moralid6  moralid7  moralid8  moralid9  mcoop1  mcoop2  mcoop3  mcoop5  mcoop6  mcoop7  narc1  narc2  narc3  narc4  narc5  narc6  omind2  omind3  omind4  omind5  omind6  contact2  contact3  hygiene5  political_ideology  riskperc2  self_esteem  sbelong2  optim1  optim2  slfcont1  slfcont3  slfcont4
##   8   8  cnarc3  ctheory1  ctheory2  ctheory3  ctheory4  CRT1  CRT2  CRT3  health_cond  ladder  marital1  moralid10  moralid2  moralid3  moralid4  moralid5  moralid6  moralid7  moralid8  moralid9  mcoop1  mcoop2  mcoop3  mcoop5  mcoop6  mcoop7  narc1  narc2  narc3  narc4  narc5  narc6  omind2  omind3  omind4  omind5  omind6  contact2  contact3  hygiene5  political_ideology  riskperc2  self_esteem  sbelong2  optim1  optim2  slfcont1  slfcont3  slfcont4
##   8   9  cnarc3  ctheory1  ctheory2  ctheory3  ctheory4  CRT1  CRT2  CRT3  health_cond  ladder  marital1  moralid10  moralid2  moralid3  moralid4  moralid5  moralid6  moralid7  moralid8  moralid9  mcoop1  mcoop2  mcoop3  mcoop5  mcoop6  mcoop7  narc1  narc2  narc3  narc4  narc5  narc6  omind2  omind3  omind4  omind5  omind6  contact2  contact3  hygiene5  political_ideology  riskperc2  self_esteem  sbelong2  optim1  optim2  slfcont1  slfcont3  slfcont4
##   8   10  cnarc3  ctheory1  ctheory2  ctheory3  ctheory4  CRT1  CRT2  CRT3  health_cond  ladder  marital1  moralid10  moralid2  moralid3  moralid4  moralid5  moralid6  moralid7  moralid8  moralid9  mcoop1  mcoop2  mcoop3  mcoop5  mcoop6  mcoop7  narc1  narc2  narc3  narc4  narc5  narc6  omind2  omind3  omind4  omind5  omind6  contact2  contact3  hygiene5  political_ideology  riskperc2  self_esteem  sbelong2  optim1  optim2  slfcont1  slfcont3  slfcont4
##   9   1  cnarc3  ctheory1  ctheory2  ctheory3  ctheory4  CRT1  CRT2  CRT3  health_cond  ladder  marital1  moralid10  moralid2  moralid3  moralid4  moralid5  moralid6  moralid7  moralid8  moralid9  mcoop1  mcoop2  mcoop3  mcoop5  mcoop6  mcoop7  narc1  narc2  narc3  narc4  narc5  narc6  omind2  omind3  omind4  omind5  omind6  contact2  contact3  hygiene5  political_ideology  riskperc2  self_esteem  sbelong2  optim1  optim2  slfcont1  slfcont3  slfcont4
##   9   2  cnarc3  ctheory1  ctheory2  ctheory3  ctheory4  CRT1  CRT2  CRT3  health_cond  ladder  marital1  moralid10  moralid2  moralid3  moralid4  moralid5  moralid6  moralid7  moralid8  moralid9  mcoop1  mcoop2  mcoop3  mcoop5  mcoop6  mcoop7  narc1  narc2  narc3  narc4  narc5  narc6  omind2  omind3  omind4  omind5  omind6  contact2  contact3  hygiene5  political_ideology  riskperc2  self_esteem  sbelong2  optim1  optim2  slfcont1  slfcont3  slfcont4
##   9   3  cnarc3  ctheory1  ctheory2  ctheory3  ctheory4  CRT1  CRT2  CRT3  health_cond  ladder  marital1  moralid10  moralid2  moralid3  moralid4  moralid5  moralid6  moralid7  moralid8  moralid9  mcoop1  mcoop2  mcoop3  mcoop5  mcoop6  mcoop7  narc1  narc2  narc3  narc4  narc5  narc6  omind2  omind3  omind4  omind5  omind6  contact2  contact3  hygiene5  political_ideology  riskperc2  self_esteem  sbelong2  optim1  optim2  slfcont1  slfcont3  slfcont4
##   9   4  cnarc3  ctheory1  ctheory2  ctheory3  ctheory4  CRT1  CRT2  CRT3  health_cond  ladder  marital1  moralid10  moralid2  moralid3  moralid4  moralid5  moralid6  moralid7  moralid8  moralid9  mcoop1  mcoop2  mcoop3  mcoop5  mcoop6  mcoop7  narc1  narc2  narc3  narc4  narc5  narc6  omind2  omind3  omind4  omind5  omind6  contact2  contact3  hygiene5  political_ideology  riskperc2  self_esteem  sbelong2  optim1  optim2  slfcont1  slfcont3  slfcont4
##   9   5  cnarc3  ctheory1  ctheory2  ctheory3  ctheory4  CRT1  CRT2  CRT3  health_cond  ladder  marital1  moralid10  moralid2  moralid3  moralid4  moralid5  moralid6  moralid7  moralid8  moralid9  mcoop1  mcoop2  mcoop3  mcoop5  mcoop6  mcoop7  narc1  narc2  narc3  narc4  narc5  narc6  omind2  omind3  omind4  omind5  omind6  contact2  contact3  hygiene5  political_ideology  riskperc2  self_esteem  sbelong2  optim1  optim2  slfcont1  slfcont3  slfcont4
##   9   6  cnarc3  ctheory1  ctheory2  ctheory3  ctheory4  CRT1  CRT2  CRT3  health_cond  ladder  marital1  moralid10  moralid2  moralid3  moralid4  moralid5  moralid6  moralid7  moralid8  moralid9  mcoop1  mcoop2  mcoop3  mcoop5  mcoop6  mcoop7  narc1  narc2  narc3  narc4  narc5  narc6  omind2  omind3  omind4  omind5  omind6  contact2  contact3  hygiene5  political_ideology  riskperc2  self_esteem  sbelong2  optim1  optim2  slfcont1  slfcont3  slfcont4
##   9   7  cnarc3  ctheory1  ctheory2  ctheory3  ctheory4  CRT1  CRT2  CRT3  health_cond  ladder  marital1  moralid10  moralid2  moralid3  moralid4  moralid5  moralid6  moralid7  moralid8  moralid9  mcoop1  mcoop2  mcoop3  mcoop5  mcoop6  mcoop7  narc1  narc2  narc3  narc4  narc5  narc6  omind2  omind3  omind4  omind5  omind6  contact2  contact3  hygiene5  political_ideology  riskperc2  self_esteem  sbelong2  optim1  optim2  slfcont1  slfcont3  slfcont4
##   9   8  cnarc3  ctheory1  ctheory2  ctheory3  ctheory4  CRT1  CRT2  CRT3  health_cond  ladder  marital1  moralid10  moralid2  moralid3  moralid4  moralid5  moralid6  moralid7  moralid8  moralid9  mcoop1  mcoop2  mcoop3  mcoop5  mcoop6  mcoop7  narc1  narc2  narc3  narc4  narc5  narc6  omind2  omind3  omind4  omind5  omind6  contact2  contact3  hygiene5  political_ideology  riskperc2  self_esteem  sbelong2  optim1  optim2  slfcont1  slfcont3  slfcont4
##   9   9  cnarc3  ctheory1  ctheory2  ctheory3  ctheory4  CRT1  CRT2  CRT3  health_cond  ladder  marital1  moralid10  moralid2  moralid3  moralid4  moralid5  moralid6  moralid7  moralid8  moralid9  mcoop1  mcoop2  mcoop3  mcoop5  mcoop6  mcoop7  narc1  narc2  narc3  narc4  narc5  narc6  omind2  omind3  omind4  omind5  omind6  contact2  contact3  hygiene5  political_ideology  riskperc2  self_esteem  sbelong2  optim1  optim2  slfcont1  slfcont3  slfcont4
##   9   10  cnarc3  ctheory1  ctheory2  ctheory3  ctheory4  CRT1  CRT2  CRT3  health_cond  ladder  marital1  moralid10  moralid2  moralid3  moralid4  moralid5  moralid6  moralid7  moralid8  moralid9  mcoop1  mcoop2  mcoop3  mcoop5  mcoop6  mcoop7  narc1  narc2  narc3  narc4  narc5  narc6  omind2  omind3  omind4  omind5  omind6  contact2  contact3  hygiene5  political_ideology  riskperc2  self_esteem  sbelong2  optim1  optim2  slfcont1  slfcont3  slfcont4
##   10   1  cnarc3  ctheory1  ctheory2  ctheory3  ctheory4  CRT1  CRT2  CRT3  health_cond  ladder  marital1  moralid10  moralid2  moralid3  moralid4  moralid5  moralid6  moralid7  moralid8  moralid9  mcoop1  mcoop2  mcoop3  mcoop5  mcoop6  mcoop7  narc1  narc2  narc3  narc4  narc5  narc6  omind2  omind3  omind4  omind5  omind6  contact2  contact3  hygiene5  political_ideology  riskperc2  self_esteem  sbelong2  optim1  optim2  slfcont1  slfcont3  slfcont4
##   10   2  cnarc3  ctheory1  ctheory2  ctheory3  ctheory4  CRT1  CRT2  CRT3  health_cond  ladder  marital1  moralid10  moralid2  moralid3  moralid4  moralid5  moralid6  moralid7  moralid8  moralid9  mcoop1  mcoop2  mcoop3  mcoop5  mcoop6  mcoop7  narc1  narc2  narc3  narc4  narc5  narc6  omind2  omind3  omind4  omind5  omind6  contact2  contact3  hygiene5  political_ideology  riskperc2  self_esteem  sbelong2  optim1  optim2  slfcont1  slfcont3  slfcont4
##   10   3  cnarc3  ctheory1  ctheory2  ctheory3  ctheory4  CRT1  CRT2  CRT3  health_cond  ladder  marital1  moralid10  moralid2  moralid3  moralid4  moralid5  moralid6  moralid7  moralid8  moralid9  mcoop1  mcoop2  mcoop3  mcoop5  mcoop6  mcoop7  narc1  narc2  narc3  narc4  narc5  narc6  omind2  omind3  omind4  omind5  omind6  contact2  contact3  hygiene5  political_ideology  riskperc2  self_esteem  sbelong2  optim1  optim2  slfcont1  slfcont3  slfcont4
##   10   4  cnarc3  ctheory1  ctheory2  ctheory3  ctheory4  CRT1  CRT2  CRT3  health_cond  ladder  marital1  moralid10  moralid2  moralid3  moralid4  moralid5  moralid6  moralid7  moralid8  moralid9  mcoop1  mcoop2  mcoop3  mcoop5  mcoop6  mcoop7  narc1  narc2  narc3  narc4  narc5  narc6  omind2  omind3  omind4  omind5  omind6  contact2  contact3  hygiene5  political_ideology  riskperc2  self_esteem  sbelong2  optim1  optim2  slfcont1  slfcont3  slfcont4
##   10   5  cnarc3  ctheory1  ctheory2  ctheory3  ctheory4  CRT1  CRT2  CRT3  health_cond  ladder  marital1  moralid10  moralid2  moralid3  moralid4  moralid5  moralid6  moralid7  moralid8  moralid9  mcoop1  mcoop2  mcoop3  mcoop5  mcoop6  mcoop7  narc1  narc2  narc3  narc4  narc5  narc6  omind2  omind3  omind4  omind5  omind6  contact2  contact3  hygiene5  political_ideology  riskperc2  self_esteem  sbelong2  optim1  optim2  slfcont1  slfcont3  slfcont4
##   10   6  cnarc3  ctheory1  ctheory2  ctheory3  ctheory4  CRT1  CRT2  CRT3  health_cond  ladder  marital1  moralid10  moralid2  moralid3  moralid4  moralid5  moralid6  moralid7  moralid8  moralid9  mcoop1  mcoop2  mcoop3  mcoop5  mcoop6  mcoop7  narc1  narc2  narc3  narc4  narc5  narc6  omind2  omind3  omind4  omind5  omind6  contact2  contact3  hygiene5  political_ideology  riskperc2  self_esteem  sbelong2  optim1  optim2  slfcont1  slfcont3  slfcont4
##   10   7  cnarc3  ctheory1  ctheory2  ctheory3  ctheory4  CRT1  CRT2  CRT3  health_cond  ladder  marital1  moralid10  moralid2  moralid3  moralid4  moralid5  moralid6  moralid7  moralid8  moralid9  mcoop1  mcoop2  mcoop3  mcoop5  mcoop6  mcoop7  narc1  narc2  narc3  narc4  narc5  narc6  omind2  omind3  omind4  omind5  omind6  contact2  contact3  hygiene5  political_ideology  riskperc2  self_esteem  sbelong2  optim1  optim2  slfcont1  slfcont3  slfcont4
##   10   8  cnarc3  ctheory1  ctheory2  ctheory3  ctheory4  CRT1  CRT2  CRT3  health_cond  ladder  marital1  moralid10  moralid2  moralid3  moralid4  moralid5  moralid6  moralid7  moralid8  moralid9  mcoop1  mcoop2  mcoop3  mcoop5  mcoop6  mcoop7  narc1  narc2  narc3  narc4  narc5  narc6  omind2  omind3  omind4  omind5  omind6  contact2  contact3  hygiene5  political_ideology  riskperc2  self_esteem  sbelong2  optim1  optim2  slfcont1  slfcont3  slfcont4
##   10   9  cnarc3  ctheory1  ctheory2  ctheory3  ctheory4  CRT1  CRT2  CRT3  health_cond  ladder  marital1  moralid10  moralid2  moralid3  moralid4  moralid5  moralid6  moralid7  moralid8  moralid9  mcoop1  mcoop2  mcoop3  mcoop5  mcoop6  mcoop7  narc1  narc2  narc3  narc4  narc5  narc6  omind2  omind3  omind4  omind5  omind6  contact2  contact3  hygiene5  political_ideology  riskperc2  self_esteem  sbelong2  optim1  optim2  slfcont1  slfcont3  slfcont4
##   10   10  cnarc3  ctheory1  ctheory2  ctheory3  ctheory4  CRT1  CRT2  CRT3  health_cond  ladder  marital1  moralid10  moralid2  moralid3  moralid4  moralid5  moralid6  moralid7  moralid8  moralid9  mcoop1  mcoop2  mcoop3  mcoop5  mcoop6  mcoop7  narc1  narc2  narc3  narc4  narc5  narc6  omind2  omind3  omind4  omind5  omind6  contact2  contact3  hygiene5  political_ideology  riskperc2  self_esteem  sbelong2  optim1  optim2  slfcont1  slfcont3  slfcont4
```

```
## Warning: Number of logged events: 4903
```

```
## 
##  iter imp variable
##   1   1  CRT1  CRT2  CRT3
##   1   2  CRT1  CRT2  CRT3
##   1   3  CRT1  CRT2  CRT3
##   1   4  CRT1  CRT2  CRT3
##   1   5  CRT1  CRT2  CRT3
##   1   6  CRT1  CRT2  CRT3
##   1   7  CRT1  CRT2  CRT3
##   1   8  CRT1  CRT2  CRT3
##   1   9  CRT1  CRT2  CRT3
##   1   10  CRT1  CRT2  CRT3
##   2   1  CRT1  CRT2  CRT3
##   2   2  CRT1  CRT2  CRT3
##   2   3  CRT1  CRT2  CRT3
##   2   4  CRT1  CRT2  CRT3
##   2   5  CRT1  CRT2  CRT3
##   2   6  CRT1  CRT2  CRT3
##   2   7  CRT1  CRT2  CRT3
##   2   8  CRT1  CRT2  CRT3
##   2   9  CRT1  CRT2  CRT3
##   2   10  CRT1  CRT2  CRT3
##   3   1  CRT1  CRT2  CRT3
##   3   2  CRT1  CRT2  CRT3
##   3   3  CRT1  CRT2  CRT3
##   3   4  CRT1  CRT2  CRT3
##   3   5  CRT1  CRT2  CRT3
##   3   6  CRT1  CRT2  CRT3
##   3   7  CRT1  CRT2  CRT3
##   3   8  CRT1  CRT2  CRT3
##   3   9  CRT1  CRT2  CRT3
##   3   10  CRT1  CRT2  CRT3
##   4   1  CRT1  CRT2  CRT3
##   4   2  CRT1  CRT2  CRT3
##   4   3  CRT1  CRT2  CRT3
##   4   4  CRT1  CRT2  CRT3
##   4   5  CRT1  CRT2  CRT3
##   4   6  CRT1  CRT2  CRT3
##   4   7  CRT1  CRT2  CRT3
##   4   8  CRT1  CRT2  CRT3
##   4   9  CRT1  CRT2  CRT3
##   4   10  CRT1  CRT2  CRT3
##   5   1  CRT1  CRT2  CRT3
##   5   2  CRT1  CRT2  CRT3
##   5   3  CRT1  CRT2  CRT3
##   5   4  CRT1  CRT2  CRT3
##   5   5  CRT1  CRT2  CRT3
##   5   6  CRT1  CRT2  CRT3
##   5   7  CRT1  CRT2  CRT3
##   5   8  CRT1  CRT2  CRT3
##   5   9  CRT1  CRT2  CRT3
##   5   10  CRT1  CRT2  CRT3
##   6   1  CRT1  CRT2  CRT3
##   6   2  CRT1  CRT2  CRT3
##   6   3  CRT1  CRT2  CRT3
##   6   4  CRT1  CRT2  CRT3
##   6   5  CRT1  CRT2  CRT3
##   6   6  CRT1  CRT2  CRT3
##   6   7  CRT1  CRT2  CRT3
##   6   8  CRT1  CRT2  CRT3
##   6   9  CRT1  CRT2  CRT3
##   6   10  CRT1  CRT2  CRT3
##   7   1  CRT1  CRT2  CRT3
##   7   2  CRT1  CRT2  CRT3
##   7   3  CRT1  CRT2  CRT3
##   7   4  CRT1  CRT2  CRT3
##   7   5  CRT1  CRT2  CRT3
##   7   6  CRT1  CRT2  CRT3
##   7   7  CRT1  CRT2  CRT3
##   7   8  CRT1  CRT2  CRT3
##   7   9  CRT1  CRT2  CRT3
##   7   10  CRT1  CRT2  CRT3
##   8   1  CRT1  CRT2  CRT3
##   8   2  CRT1  CRT2  CRT3
##   8   3  CRT1  CRT2  CRT3
##   8   4  CRT1  CRT2  CRT3
##   8   5  CRT1  CRT2  CRT3
##   8   6  CRT1  CRT2  CRT3
##   8   7  CRT1  CRT2  CRT3
##   8   8  CRT1  CRT2  CRT3
##   8   9  CRT1  CRT2  CRT3
##   8   10  CRT1  CRT2  CRT3
##   9   1  CRT1  CRT2  CRT3
##   9   2  CRT1  CRT2  CRT3
##   9   3  CRT1  CRT2  CRT3
##   9   4  CRT1  CRT2  CRT3
##   9   5  CRT1  CRT2  CRT3
##   9   6  CRT1  CRT2  CRT3
##   9   7  CRT1  CRT2  CRT3
##   9   8  CRT1  CRT2  CRT3
##   9   9  CRT1  CRT2  CRT3
##   9   10  CRT1  CRT2  CRT3
##   10   1  CRT1  CRT2  CRT3
##   10   2  CRT1  CRT2  CRT3
##   10   3  CRT1  CRT2  CRT3
##   10   4  CRT1  CRT2  CRT3
##   10   5  CRT1  CRT2  CRT3
##   10   6  CRT1  CRT2  CRT3
##   10   7  CRT1  CRT2  CRT3
##   10   8  CRT1  CRT2  CRT3
##   10   9  CRT1  CRT2  CRT3
##   10   10  CRT1  CRT2  CRT3
```

```
## Warning: Number of logged events: 303
```

```
## 
##  iter imp variable
##   1   1  age  children  cnarc1  cnarc2  CRT1  CRT2  CRT3  duration  health_cond  moralid1  moralid10  moralid2  moralid3  moralid4  moralid5  moralid6  moralid7  moralid8  moralid9  mcoop3  mcoop5  mcoop6  mcoop7  narc1  narc2  narc3  narc4  narc6  nidentity1  nidentity2  omind5  omind6  contact2  contact4  contact5  hygiene1  hygiene2  hygiene3  hygiene4  hygiene5  psupport1  psupport2  psupport3  psupport4  psupport5  political_ideology  happy  slf_ladder  riskperc1  riskperc2  self_esteem  sbelong1  sbelong2  optim1  optim2  slfcont4  urban
##   1   2  age  children  cnarc1  cnarc2  CRT1  CRT2  CRT3  duration  health_cond  moralid1  moralid10  moralid2  moralid3  moralid4  moralid5  moralid6  moralid7  moralid8  moralid9  mcoop3  mcoop5  mcoop6  mcoop7  narc1  narc2  narc3  narc4  narc6  nidentity1  nidentity2  omind5  omind6  contact2  contact4  contact5  hygiene1  hygiene2  hygiene3  hygiene4  hygiene5  psupport1  psupport2  psupport3  psupport4  psupport5  political_ideology  happy  slf_ladder  riskperc1  riskperc2  self_esteem  sbelong1  sbelong2  optim1  optim2  slfcont4  urban
##   1   3  age  children  cnarc1  cnarc2  CRT1  CRT2  CRT3  duration  health_cond  moralid1  moralid10  moralid2  moralid3  moralid4  moralid5  moralid6  moralid7  moralid8  moralid9  mcoop3  mcoop5  mcoop6  mcoop7  narc1  narc2  narc3  narc4  narc6  nidentity1  nidentity2  omind5  omind6  contact2  contact4  contact5  hygiene1  hygiene2  hygiene3  hygiene4  hygiene5  psupport1  psupport2  psupport3  psupport4  psupport5  political_ideology  happy  slf_ladder  riskperc1  riskperc2  self_esteem  sbelong1  sbelong2  optim1  optim2  slfcont4  urban
##   1   4  age  children  cnarc1  cnarc2  CRT1  CRT2  CRT3  duration  health_cond  moralid1  moralid10  moralid2  moralid3  moralid4  moralid5  moralid6  moralid7  moralid8  moralid9  mcoop3  mcoop5  mcoop6  mcoop7  narc1  narc2  narc3  narc4  narc6  nidentity1  nidentity2  omind5  omind6  contact2  contact4  contact5  hygiene1  hygiene2  hygiene3  hygiene4  hygiene5  psupport1  psupport2  psupport3  psupport4  psupport5  political_ideology  happy  slf_ladder  riskperc1  riskperc2  self_esteem  sbelong1  sbelong2  optim1  optim2  slfcont4  urban
##   1   5  age  children  cnarc1  cnarc2  CRT1  CRT2  CRT3  duration  health_cond  moralid1  moralid10  moralid2  moralid3  moralid4  moralid5  moralid6  moralid7  moralid8  moralid9  mcoop3  mcoop5  mcoop6  mcoop7  narc1  narc2  narc3  narc4  narc6  nidentity1  nidentity2  omind5  omind6  contact2  contact4  contact5  hygiene1  hygiene2  hygiene3  hygiene4  hygiene5  psupport1  psupport2  psupport3  psupport4  psupport5  political_ideology  happy  slf_ladder  riskperc1  riskperc2  self_esteem  sbelong1  sbelong2  optim1  optim2  slfcont4  urban
##   1   6  age  children  cnarc1  cnarc2  CRT1  CRT2  CRT3  duration  health_cond  moralid1  moralid10  moralid2  moralid3  moralid4  moralid5  moralid6  moralid7  moralid8  moralid9  mcoop3  mcoop5  mcoop6  mcoop7  narc1  narc2  narc3  narc4  narc6  nidentity1  nidentity2  omind5  omind6  contact2  contact4  contact5  hygiene1  hygiene2  hygiene3  hygiene4  hygiene5  psupport1  psupport2  psupport3  psupport4  psupport5  political_ideology  happy  slf_ladder  riskperc1  riskperc2  self_esteem  sbelong1  sbelong2  optim1  optim2  slfcont4  urban
##   1   7  age  children  cnarc1  cnarc2  CRT1  CRT2  CRT3  duration  health_cond  moralid1  moralid10  moralid2  moralid3  moralid4  moralid5  moralid6  moralid7  moralid8  moralid9  mcoop3  mcoop5  mcoop6  mcoop7  narc1  narc2  narc3  narc4  narc6  nidentity1  nidentity2  omind5  omind6  contact2  contact4  contact5  hygiene1  hygiene2  hygiene3  hygiene4  hygiene5  psupport1  psupport2  psupport3  psupport4  psupport5  political_ideology  happy  slf_ladder  riskperc1  riskperc2  self_esteem  sbelong1  sbelong2  optim1  optim2  slfcont4  urban
##   1   8  age  children  cnarc1  cnarc2  CRT1  CRT2  CRT3  duration  health_cond  moralid1  moralid10  moralid2  moralid3  moralid4  moralid5  moralid6  moralid7  moralid8  moralid9  mcoop3  mcoop5  mcoop6  mcoop7  narc1  narc2  narc3  narc4  narc6  nidentity1  nidentity2  omind5  omind6  contact2  contact4  contact5  hygiene1  hygiene2  hygiene3  hygiene4  hygiene5  psupport1  psupport2  psupport3  psupport4  psupport5  political_ideology  happy  slf_ladder  riskperc1  riskperc2  self_esteem  sbelong1  sbelong2  optim1  optim2  slfcont4  urban
##   1   9  age  children  cnarc1  cnarc2  CRT1  CRT2  CRT3  duration  health_cond  moralid1  moralid10  moralid2  moralid3  moralid4  moralid5  moralid6  moralid7  moralid8  moralid9  mcoop3  mcoop5  mcoop6  mcoop7  narc1  narc2  narc3  narc4  narc6  nidentity1  nidentity2  omind5  omind6  contact2  contact4  contact5  hygiene1  hygiene2  hygiene3  hygiene4  hygiene5  psupport1  psupport2  psupport3  psupport4  psupport5  political_ideology  happy  slf_ladder  riskperc1  riskperc2  self_esteem  sbelong1  sbelong2  optim1  optim2  slfcont4  urban
##   1   10  age  children  cnarc1  cnarc2  CRT1  CRT2  CRT3  duration  health_cond  moralid1  moralid10  moralid2  moralid3  moralid4  moralid5  moralid6  moralid7  moralid8  moralid9  mcoop3  mcoop5  mcoop6  mcoop7  narc1  narc2  narc3  narc4  narc6  nidentity1  nidentity2  omind5  omind6  contact2  contact4  contact5  hygiene1  hygiene2  hygiene3  hygiene4  hygiene5  psupport1  psupport2  psupport3  psupport4  psupport5  political_ideology  happy  slf_ladder  riskperc1  riskperc2  self_esteem  sbelong1  sbelong2  optim1  optim2  slfcont4  urban
##   2   1  age  children  cnarc1  cnarc2  CRT1  CRT2  CRT3  duration  health_cond  moralid1  moralid10  moralid2  moralid3  moralid4  moralid5  moralid6  moralid7  moralid8  moralid9  mcoop3  mcoop5  mcoop6  mcoop7  narc1  narc2  narc3  narc4  narc6  nidentity1  nidentity2  omind5  omind6  contact2  contact4  contact5  hygiene1  hygiene2  hygiene3  hygiene4  hygiene5  psupport1  psupport2  psupport3  psupport4  psupport5  political_ideology  happy  slf_ladder  riskperc1  riskperc2  self_esteem  sbelong1  sbelong2  optim1  optim2  slfcont4  urban
##   2   2  age  children  cnarc1  cnarc2  CRT1  CRT2  CRT3  duration  health_cond  moralid1  moralid10  moralid2  moralid3  moralid4  moralid5  moralid6  moralid7  moralid8  moralid9  mcoop3  mcoop5  mcoop6  mcoop7  narc1  narc2  narc3  narc4  narc6  nidentity1  nidentity2  omind5  omind6  contact2  contact4  contact5  hygiene1  hygiene2  hygiene3  hygiene4  hygiene5  psupport1  psupport2  psupport3  psupport4  psupport5  political_ideology  happy  slf_ladder  riskperc1  riskperc2  self_esteem  sbelong1  sbelong2  optim1  optim2  slfcont4  urban
##   2   3  age  children  cnarc1  cnarc2  CRT1  CRT2  CRT3  duration  health_cond  moralid1  moralid10  moralid2  moralid3  moralid4  moralid5  moralid6  moralid7  moralid8  moralid9  mcoop3  mcoop5  mcoop6  mcoop7  narc1  narc2  narc3  narc4  narc6  nidentity1  nidentity2  omind5  omind6  contact2  contact4  contact5  hygiene1  hygiene2  hygiene3  hygiene4  hygiene5  psupport1  psupport2  psupport3  psupport4  psupport5  political_ideology  happy  slf_ladder  riskperc1  riskperc2  self_esteem  sbelong1  sbelong2  optim1  optim2  slfcont4  urban
##   2   4  age  children  cnarc1  cnarc2  CRT1  CRT2  CRT3  duration  health_cond  moralid1  moralid10  moralid2  moralid3  moralid4  moralid5  moralid6  moralid7  moralid8  moralid9  mcoop3  mcoop5  mcoop6  mcoop7  narc1  narc2  narc3  narc4  narc6  nidentity1  nidentity2  omind5  omind6  contact2  contact4  contact5  hygiene1  hygiene2  hygiene3  hygiene4  hygiene5  psupport1  psupport2  psupport3  psupport4  psupport5  political_ideology  happy  slf_ladder  riskperc1  riskperc2  self_esteem  sbelong1  sbelong2  optim1  optim2  slfcont4  urban
##   2   5  age  children  cnarc1  cnarc2  CRT1  CRT2  CRT3  duration  health_cond  moralid1  moralid10  moralid2  moralid3  moralid4  moralid5  moralid6  moralid7  moralid8  moralid9  mcoop3  mcoop5  mcoop6  mcoop7  narc1  narc2  narc3  narc4  narc6  nidentity1  nidentity2  omind5  omind6  contact2  contact4  contact5  hygiene1  hygiene2  hygiene3  hygiene4  hygiene5  psupport1  psupport2  psupport3  psupport4  psupport5  political_ideology  happy  slf_ladder  riskperc1  riskperc2  self_esteem  sbelong1  sbelong2  optim1  optim2  slfcont4  urban
##   2   6  age  children  cnarc1  cnarc2  CRT1  CRT2  CRT3  duration  health_cond  moralid1  moralid10  moralid2  moralid3  moralid4  moralid5  moralid6  moralid7  moralid8  moralid9  mcoop3  mcoop5  mcoop6  mcoop7  narc1  narc2  narc3  narc4  narc6  nidentity1  nidentity2  omind5  omind6  contact2  contact4  contact5  hygiene1  hygiene2  hygiene3  hygiene4  hygiene5  psupport1  psupport2  psupport3  psupport4  psupport5  political_ideology  happy  slf_ladder  riskperc1  riskperc2  self_esteem  sbelong1  sbelong2  optim1  optim2  slfcont4  urban
##   2   7  age  children  cnarc1  cnarc2  CRT1  CRT2  CRT3  duration  health_cond  moralid1  moralid10  moralid2  moralid3  moralid4  moralid5  moralid6  moralid7  moralid8  moralid9  mcoop3  mcoop5  mcoop6  mcoop7  narc1  narc2  narc3  narc4  narc6  nidentity1  nidentity2  omind5  omind6  contact2  contact4  contact5  hygiene1  hygiene2  hygiene3  hygiene4  hygiene5  psupport1  psupport2  psupport3  psupport4  psupport5  political_ideology  happy  slf_ladder  riskperc1  riskperc2  self_esteem  sbelong1  sbelong2  optim1  optim2  slfcont4  urban
##   2   8  age  children  cnarc1  cnarc2  CRT1  CRT2  CRT3  duration  health_cond  moralid1  moralid10  moralid2  moralid3  moralid4  moralid5  moralid6  moralid7  moralid8  moralid9  mcoop3  mcoop5  mcoop6  mcoop7  narc1  narc2  narc3  narc4  narc6  nidentity1  nidentity2  omind5  omind6  contact2  contact4  contact5  hygiene1  hygiene2  hygiene3  hygiene4  hygiene5  psupport1  psupport2  psupport3  psupport4  psupport5  political_ideology  happy  slf_ladder  riskperc1  riskperc2  self_esteem  sbelong1  sbelong2  optim1  optim2  slfcont4  urban
##   2   9  age  children  cnarc1  cnarc2  CRT1  CRT2  CRT3  duration  health_cond  moralid1  moralid10  moralid2  moralid3  moralid4  moralid5  moralid6  moralid7  moralid8  moralid9  mcoop3  mcoop5  mcoop6  mcoop7  narc1  narc2  narc3  narc4  narc6  nidentity1  nidentity2  omind5  omind6  contact2  contact4  contact5  hygiene1  hygiene2  hygiene3  hygiene4  hygiene5  psupport1  psupport2  psupport3  psupport4  psupport5  political_ideology  happy  slf_ladder  riskperc1  riskperc2  self_esteem  sbelong1  sbelong2  optim1  optim2  slfcont4  urban
##   2   10  age  children  cnarc1  cnarc2  CRT1  CRT2  CRT3  duration  health_cond  moralid1  moralid10  moralid2  moralid3  moralid4  moralid5  moralid6  moralid7  moralid8  moralid9  mcoop3  mcoop5  mcoop6  mcoop7  narc1  narc2  narc3  narc4  narc6  nidentity1  nidentity2  omind5  omind6  contact2  contact4  contact5  hygiene1  hygiene2  hygiene3  hygiene4  hygiene5  psupport1  psupport2  psupport3  psupport4  psupport5  political_ideology  happy  slf_ladder  riskperc1  riskperc2  self_esteem  sbelong1  sbelong2  optim1  optim2  slfcont4  urban
##   3   1  age  children  cnarc1  cnarc2  CRT1  CRT2  CRT3  duration  health_cond  moralid1  moralid10  moralid2  moralid3  moralid4  moralid5  moralid6  moralid7  moralid8  moralid9  mcoop3  mcoop5  mcoop6  mcoop7  narc1  narc2  narc3  narc4  narc6  nidentity1  nidentity2  omind5  omind6  contact2  contact4  contact5  hygiene1  hygiene2  hygiene3  hygiene4  hygiene5  psupport1  psupport2  psupport3  psupport4  psupport5  political_ideology  happy  slf_ladder  riskperc1  riskperc2  self_esteem  sbelong1  sbelong2  optim1  optim2  slfcont4  urban
##   3   2  age  children  cnarc1  cnarc2  CRT1  CRT2  CRT3  duration  health_cond  moralid1  moralid10  moralid2  moralid3  moralid4  moralid5  moralid6  moralid7  moralid8  moralid9  mcoop3  mcoop5  mcoop6  mcoop7  narc1  narc2  narc3  narc4  narc6  nidentity1  nidentity2  omind5  omind6  contact2  contact4  contact5  hygiene1  hygiene2  hygiene3  hygiene4  hygiene5  psupport1  psupport2  psupport3  psupport4  psupport5  political_ideology  happy  slf_ladder  riskperc1  riskperc2  self_esteem  sbelong1  sbelong2  optim1  optim2  slfcont4  urban
##   3   3  age  children  cnarc1  cnarc2  CRT1  CRT2  CRT3  duration  health_cond  moralid1  moralid10  moralid2  moralid3  moralid4  moralid5  moralid6  moralid7  moralid8  moralid9  mcoop3  mcoop5  mcoop6  mcoop7  narc1  narc2  narc3  narc4  narc6  nidentity1  nidentity2  omind5  omind6  contact2  contact4  contact5  hygiene1  hygiene2  hygiene3  hygiene4  hygiene5  psupport1  psupport2  psupport3  psupport4  psupport5  political_ideology  happy  slf_ladder  riskperc1  riskperc2  self_esteem  sbelong1  sbelong2  optim1  optim2  slfcont4  urban
##   3   4  age  children  cnarc1  cnarc2  CRT1  CRT2  CRT3  duration  health_cond  moralid1  moralid10  moralid2  moralid3  moralid4  moralid5  moralid6  moralid7  moralid8  moralid9  mcoop3  mcoop5  mcoop6  mcoop7  narc1  narc2  narc3  narc4  narc6  nidentity1  nidentity2  omind5  omind6  contact2  contact4  contact5  hygiene1  hygiene2  hygiene3  hygiene4  hygiene5  psupport1  psupport2  psupport3  psupport4  psupport5  political_ideology  happy  slf_ladder  riskperc1  riskperc2  self_esteem  sbelong1  sbelong2  optim1  optim2  slfcont4  urban
##   3   5  age  children  cnarc1  cnarc2  CRT1  CRT2  CRT3  duration  health_cond  moralid1  moralid10  moralid2  moralid3  moralid4  moralid5  moralid6  moralid7  moralid8  moralid9  mcoop3  mcoop5  mcoop6  mcoop7  narc1  narc2  narc3  narc4  narc6  nidentity1  nidentity2  omind5  omind6  contact2  contact4  contact5  hygiene1  hygiene2  hygiene3  hygiene4  hygiene5  psupport1  psupport2  psupport3  psupport4  psupport5  political_ideology  happy  slf_ladder  riskperc1  riskperc2  self_esteem  sbelong1  sbelong2  optim1  optim2  slfcont4  urban
##   3   6  age  children  cnarc1  cnarc2  CRT1  CRT2  CRT3  duration  health_cond  moralid1  moralid10  moralid2  moralid3  moralid4  moralid5  moralid6  moralid7  moralid8  moralid9  mcoop3  mcoop5  mcoop6  mcoop7  narc1  narc2  narc3  narc4  narc6  nidentity1  nidentity2  omind5  omind6  contact2  contact4  contact5  hygiene1  hygiene2  hygiene3  hygiene4  hygiene5  psupport1  psupport2  psupport3  psupport4  psupport5  political_ideology  happy  slf_ladder  riskperc1  riskperc2  self_esteem  sbelong1  sbelong2  optim1  optim2  slfcont4  urban
##   3   7  age  children  cnarc1  cnarc2  CRT1  CRT2  CRT3  duration  health_cond  moralid1  moralid10  moralid2  moralid3  moralid4  moralid5  moralid6  moralid7  moralid8  moralid9  mcoop3  mcoop5  mcoop6  mcoop7  narc1  narc2  narc3  narc4  narc6  nidentity1  nidentity2  omind5  omind6  contact2  contact4  contact5  hygiene1  hygiene2  hygiene3  hygiene4  hygiene5  psupport1  psupport2  psupport3  psupport4  psupport5  political_ideology  happy  slf_ladder  riskperc1  riskperc2  self_esteem  sbelong1  sbelong2  optim1  optim2  slfcont4  urban
##   3   8  age  children  cnarc1  cnarc2  CRT1  CRT2  CRT3  duration  health_cond  moralid1  moralid10  moralid2  moralid3  moralid4  moralid5  moralid6  moralid7  moralid8  moralid9  mcoop3  mcoop5  mcoop6  mcoop7  narc1  narc2  narc3  narc4  narc6  nidentity1  nidentity2  omind5  omind6  contact2  contact4  contact5  hygiene1  hygiene2  hygiene3  hygiene4  hygiene5  psupport1  psupport2  psupport3  psupport4  psupport5  political_ideology  happy  slf_ladder  riskperc1  riskperc2  self_esteem  sbelong1  sbelong2  optim1  optim2  slfcont4  urban
##   3   9  age  children  cnarc1  cnarc2  CRT1  CRT2  CRT3  duration  health_cond  moralid1  moralid10  moralid2  moralid3  moralid4  moralid5  moralid6  moralid7  moralid8  moralid9  mcoop3  mcoop5  mcoop6  mcoop7  narc1  narc2  narc3  narc4  narc6  nidentity1  nidentity2  omind5  omind6  contact2  contact4  contact5  hygiene1  hygiene2  hygiene3  hygiene4  hygiene5  psupport1  psupport2  psupport3  psupport4  psupport5  political_ideology  happy  slf_ladder  riskperc1  riskperc2  self_esteem  sbelong1  sbelong2  optim1  optim2  slfcont4  urban
##   3   10  age  children  cnarc1  cnarc2  CRT1  CRT2  CRT3  duration  health_cond  moralid1  moralid10  moralid2  moralid3  moralid4  moralid5  moralid6  moralid7  moralid8  moralid9  mcoop3  mcoop5  mcoop6  mcoop7  narc1  narc2  narc3  narc4  narc6  nidentity1  nidentity2  omind5  omind6  contact2  contact4  contact5  hygiene1  hygiene2  hygiene3  hygiene4  hygiene5  psupport1  psupport2  psupport3  psupport4  psupport5  political_ideology  happy  slf_ladder  riskperc1  riskperc2  self_esteem  sbelong1  sbelong2  optim1  optim2  slfcont4  urban
##   4   1  age  children  cnarc1  cnarc2  CRT1  CRT2  CRT3  duration  health_cond  moralid1  moralid10  moralid2  moralid3  moralid4  moralid5  moralid6  moralid7  moralid8  moralid9  mcoop3  mcoop5  mcoop6  mcoop7  narc1  narc2  narc3  narc4  narc6  nidentity1  nidentity2  omind5  omind6  contact2  contact4  contact5  hygiene1  hygiene2  hygiene3  hygiene4  hygiene5  psupport1  psupport2  psupport3  psupport4  psupport5  political_ideology  happy  slf_ladder  riskperc1  riskperc2  self_esteem  sbelong1  sbelong2  optim1  optim2  slfcont4  urban
##   4   2  age  children  cnarc1  cnarc2  CRT1  CRT2  CRT3  duration  health_cond  moralid1  moralid10  moralid2  moralid3  moralid4  moralid5  moralid6  moralid7  moralid8  moralid9  mcoop3  mcoop5  mcoop6  mcoop7  narc1  narc2  narc3  narc4  narc6  nidentity1  nidentity2  omind5  omind6  contact2  contact4  contact5  hygiene1  hygiene2  hygiene3  hygiene4  hygiene5  psupport1  psupport2  psupport3  psupport4  psupport5  political_ideology  happy  slf_ladder  riskperc1  riskperc2  self_esteem  sbelong1  sbelong2  optim1  optim2  slfcont4  urban
##   4   3  age  children  cnarc1  cnarc2  CRT1  CRT2  CRT3  duration  health_cond  moralid1  moralid10  moralid2  moralid3  moralid4  moralid5  moralid6  moralid7  moralid8  moralid9  mcoop3  mcoop5  mcoop6  mcoop7  narc1  narc2  narc3  narc4  narc6  nidentity1  nidentity2  omind5  omind6  contact2  contact4  contact5  hygiene1  hygiene2  hygiene3  hygiene4  hygiene5  psupport1  psupport2  psupport3  psupport4  psupport5  political_ideology  happy  slf_ladder  riskperc1  riskperc2  self_esteem  sbelong1  sbelong2  optim1  optim2  slfcont4  urban
##   4   4  age  children  cnarc1  cnarc2  CRT1  CRT2  CRT3  duration  health_cond  moralid1  moralid10  moralid2  moralid3  moralid4  moralid5  moralid6  moralid7  moralid8  moralid9  mcoop3  mcoop5  mcoop6  mcoop7  narc1  narc2  narc3  narc4  narc6  nidentity1  nidentity2  omind5  omind6  contact2  contact4  contact5  hygiene1  hygiene2  hygiene3  hygiene4  hygiene5  psupport1  psupport2  psupport3  psupport4  psupport5  political_ideology  happy  slf_ladder  riskperc1  riskperc2  self_esteem  sbelong1  sbelong2  optim1  optim2  slfcont4  urban
##   4   5  age  children  cnarc1  cnarc2  CRT1  CRT2  CRT3  duration  health_cond  moralid1  moralid10  moralid2  moralid3  moralid4  moralid5  moralid6  moralid7  moralid8  moralid9  mcoop3  mcoop5  mcoop6  mcoop7  narc1  narc2  narc3  narc4  narc6  nidentity1  nidentity2  omind5  omind6  contact2  contact4  contact5  hygiene1  hygiene2  hygiene3  hygiene4  hygiene5  psupport1  psupport2  psupport3  psupport4  psupport5  political_ideology  happy  slf_ladder  riskperc1  riskperc2  self_esteem  sbelong1  sbelong2  optim1  optim2  slfcont4  urban
##   4   6  age  children  cnarc1  cnarc2  CRT1  CRT2  CRT3  duration  health_cond  moralid1  moralid10  moralid2  moralid3  moralid4  moralid5  moralid6  moralid7  moralid8  moralid9  mcoop3  mcoop5  mcoop6  mcoop7  narc1  narc2  narc3  narc4  narc6  nidentity1  nidentity2  omind5  omind6  contact2  contact4  contact5  hygiene1  hygiene2  hygiene3  hygiene4  hygiene5  psupport1  psupport2  psupport3  psupport4  psupport5  political_ideology  happy  slf_ladder  riskperc1  riskperc2  self_esteem  sbelong1  sbelong2  optim1  optim2  slfcont4  urban
##   4   7  age  children  cnarc1  cnarc2  CRT1  CRT2  CRT3  duration  health_cond  moralid1  moralid10  moralid2  moralid3  moralid4  moralid5  moralid6  moralid7  moralid8  moralid9  mcoop3  mcoop5  mcoop6  mcoop7  narc1  narc2  narc3  narc4  narc6  nidentity1  nidentity2  omind5  omind6  contact2  contact4  contact5  hygiene1  hygiene2  hygiene3  hygiene4  hygiene5  psupport1  psupport2  psupport3  psupport4  psupport5  political_ideology  happy  slf_ladder  riskperc1  riskperc2  self_esteem  sbelong1  sbelong2  optim1  optim2  slfcont4  urban
##   4   8  age  children  cnarc1  cnarc2  CRT1  CRT2  CRT3  duration  health_cond  moralid1  moralid10  moralid2  moralid3  moralid4  moralid5  moralid6  moralid7  moralid8  moralid9  mcoop3  mcoop5  mcoop6  mcoop7  narc1  narc2  narc3  narc4  narc6  nidentity1  nidentity2  omind5  omind6  contact2  contact4  contact5  hygiene1  hygiene2  hygiene3  hygiene4  hygiene5  psupport1  psupport2  psupport3  psupport4  psupport5  political_ideology  happy  slf_ladder  riskperc1  riskperc2  self_esteem  sbelong1  sbelong2  optim1  optim2  slfcont4  urban
##   4   9  age  children  cnarc1  cnarc2  CRT1  CRT2  CRT3  duration  health_cond  moralid1  moralid10  moralid2  moralid3  moralid4  moralid5  moralid6  moralid7  moralid8  moralid9  mcoop3  mcoop5  mcoop6  mcoop7  narc1  narc2  narc3  narc4  narc6  nidentity1  nidentity2  omind5  omind6  contact2  contact4  contact5  hygiene1  hygiene2  hygiene3  hygiene4  hygiene5  psupport1  psupport2  psupport3  psupport4  psupport5  political_ideology  happy  slf_ladder  riskperc1  riskperc2  self_esteem  sbelong1  sbelong2  optim1  optim2  slfcont4  urban
##   4   10  age  children  cnarc1  cnarc2  CRT1  CRT2  CRT3  duration  health_cond  moralid1  moralid10  moralid2  moralid3  moralid4  moralid5  moralid6  moralid7  moralid8  moralid9  mcoop3  mcoop5  mcoop6  mcoop7  narc1  narc2  narc3  narc4  narc6  nidentity1  nidentity2  omind5  omind6  contact2  contact4  contact5  hygiene1  hygiene2  hygiene3  hygiene4  hygiene5  psupport1  psupport2  psupport3  psupport4  psupport5  political_ideology  happy  slf_ladder  riskperc1  riskperc2  self_esteem  sbelong1  sbelong2  optim1  optim2  slfcont4  urban
##   5   1  age  children  cnarc1  cnarc2  CRT1  CRT2  CRT3  duration  health_cond  moralid1  moralid10  moralid2  moralid3  moralid4  moralid5  moralid6  moralid7  moralid8  moralid9  mcoop3  mcoop5  mcoop6  mcoop7  narc1  narc2  narc3  narc4  narc6  nidentity1  nidentity2  omind5  omind6  contact2  contact4  contact5  hygiene1  hygiene2  hygiene3  hygiene4  hygiene5  psupport1  psupport2  psupport3  psupport4  psupport5  political_ideology  happy  slf_ladder  riskperc1  riskperc2  self_esteem  sbelong1  sbelong2  optim1  optim2  slfcont4  urban
##   5   2  age  children  cnarc1  cnarc2  CRT1  CRT2  CRT3  duration  health_cond  moralid1  moralid10  moralid2  moralid3  moralid4  moralid5  moralid6  moralid7  moralid8  moralid9  mcoop3  mcoop5  mcoop6  mcoop7  narc1  narc2  narc3  narc4  narc6  nidentity1  nidentity2  omind5  omind6  contact2  contact4  contact5  hygiene1  hygiene2  hygiene3  hygiene4  hygiene5  psupport1  psupport2  psupport3  psupport4  psupport5  political_ideology  happy  slf_ladder  riskperc1  riskperc2  self_esteem  sbelong1  sbelong2  optim1  optim2  slfcont4  urban
##   5   3  age  children  cnarc1  cnarc2  CRT1  CRT2  CRT3  duration  health_cond  moralid1  moralid10  moralid2  moralid3  moralid4  moralid5  moralid6  moralid7  moralid8  moralid9  mcoop3  mcoop5  mcoop6  mcoop7  narc1  narc2  narc3  narc4  narc6  nidentity1  nidentity2  omind5  omind6  contact2  contact4  contact5  hygiene1  hygiene2  hygiene3  hygiene4  hygiene5  psupport1  psupport2  psupport3  psupport4  psupport5  political_ideology  happy  slf_ladder  riskperc1  riskperc2  self_esteem  sbelong1  sbelong2  optim1  optim2  slfcont4  urban
##   5   4  age  children  cnarc1  cnarc2  CRT1  CRT2  CRT3  duration  health_cond  moralid1  moralid10  moralid2  moralid3  moralid4  moralid5  moralid6  moralid7  moralid8  moralid9  mcoop3  mcoop5  mcoop6  mcoop7  narc1  narc2  narc3  narc4  narc6  nidentity1  nidentity2  omind5  omind6  contact2  contact4  contact5  hygiene1  hygiene2  hygiene3  hygiene4  hygiene5  psupport1  psupport2  psupport3  psupport4  psupport5  political_ideology  happy  slf_ladder  riskperc1  riskperc2  self_esteem  sbelong1  sbelong2  optim1  optim2  slfcont4  urban
##   5   5  age  children  cnarc1  cnarc2  CRT1  CRT2  CRT3  duration  health_cond  moralid1  moralid10  moralid2  moralid3  moralid4  moralid5  moralid6  moralid7  moralid8  moralid9  mcoop3  mcoop5  mcoop6  mcoop7  narc1  narc2  narc3  narc4  narc6  nidentity1  nidentity2  omind5  omind6  contact2  contact4  contact5  hygiene1  hygiene2  hygiene3  hygiene4  hygiene5  psupport1  psupport2  psupport3  psupport4  psupport5  political_ideology  happy  slf_ladder  riskperc1  riskperc2  self_esteem  sbelong1  sbelong2  optim1  optim2  slfcont4  urban
##   5   6  age  children  cnarc1  cnarc2  CRT1  CRT2  CRT3  duration  health_cond  moralid1  moralid10  moralid2  moralid3  moralid4  moralid5  moralid6  moralid7  moralid8  moralid9  mcoop3  mcoop5  mcoop6  mcoop7  narc1  narc2  narc3  narc4  narc6  nidentity1  nidentity2  omind5  omind6  contact2  contact4  contact5  hygiene1  hygiene2  hygiene3  hygiene4  hygiene5  psupport1  psupport2  psupport3  psupport4  psupport5  political_ideology  happy  slf_ladder  riskperc1  riskperc2  self_esteem  sbelong1  sbelong2  optim1  optim2  slfcont4  urban
##   5   7  age  children  cnarc1  cnarc2  CRT1  CRT2  CRT3  duration  health_cond  moralid1  moralid10  moralid2  moralid3  moralid4  moralid5  moralid6  moralid7  moralid8  moralid9  mcoop3  mcoop5  mcoop6  mcoop7  narc1  narc2  narc3  narc4  narc6  nidentity1  nidentity2  omind5  omind6  contact2  contact4  contact5  hygiene1  hygiene2  hygiene3  hygiene4  hygiene5  psupport1  psupport2  psupport3  psupport4  psupport5  political_ideology  happy  slf_ladder  riskperc1  riskperc2  self_esteem  sbelong1  sbelong2  optim1  optim2  slfcont4  urban
##   5   8  age  children  cnarc1  cnarc2  CRT1  CRT2  CRT3  duration  health_cond  moralid1  moralid10  moralid2  moralid3  moralid4  moralid5  moralid6  moralid7  moralid8  moralid9  mcoop3  mcoop5  mcoop6  mcoop7  narc1  narc2  narc3  narc4  narc6  nidentity1  nidentity2  omind5  omind6  contact2  contact4  contact5  hygiene1  hygiene2  hygiene3  hygiene4  hygiene5  psupport1  psupport2  psupport3  psupport4  psupport5  political_ideology  happy  slf_ladder  riskperc1  riskperc2  self_esteem  sbelong1  sbelong2  optim1  optim2  slfcont4  urban
##   5   9  age  children  cnarc1  cnarc2  CRT1  CRT2  CRT3  duration  health_cond  moralid1  moralid10  moralid2  moralid3  moralid4  moralid5  moralid6  moralid7  moralid8  moralid9  mcoop3  mcoop5  mcoop6  mcoop7  narc1  narc2  narc3  narc4  narc6  nidentity1  nidentity2  omind5  omind6  contact2  contact4  contact5  hygiene1  hygiene2  hygiene3  hygiene4  hygiene5  psupport1  psupport2  psupport3  psupport4  psupport5  political_ideology  happy  slf_ladder  riskperc1  riskperc2  self_esteem  sbelong1  sbelong2  optim1  optim2  slfcont4  urban
##   5   10  age  children  cnarc1  cnarc2  CRT1  CRT2  CRT3  duration  health_cond  moralid1  moralid10  moralid2  moralid3  moralid4  moralid5  moralid6  moralid7  moralid8  moralid9  mcoop3  mcoop5  mcoop6  mcoop7  narc1  narc2  narc3  narc4  narc6  nidentity1  nidentity2  omind5  omind6  contact2  contact4  contact5  hygiene1  hygiene2  hygiene3  hygiene4  hygiene5  psupport1  psupport2  psupport3  psupport4  psupport5  political_ideology  happy  slf_ladder  riskperc1  riskperc2  self_esteem  sbelong1  sbelong2  optim1  optim2  slfcont4  urban
##   6   1  age  children  cnarc1  cnarc2  CRT1  CRT2  CRT3  duration  health_cond  moralid1  moralid10  moralid2  moralid3  moralid4  moralid5  moralid6  moralid7  moralid8  moralid9  mcoop3  mcoop5  mcoop6  mcoop7  narc1  narc2  narc3  narc4  narc6  nidentity1  nidentity2  omind5  omind6  contact2  contact4  contact5  hygiene1  hygiene2  hygiene3  hygiene4  hygiene5  psupport1  psupport2  psupport3  psupport4  psupport5  political_ideology  happy  slf_ladder  riskperc1  riskperc2  self_esteem  sbelong1  sbelong2  optim1  optim2  slfcont4  urban
##   6   2  age  children  cnarc1  cnarc2  CRT1  CRT2  CRT3  duration  health_cond  moralid1  moralid10  moralid2  moralid3  moralid4  moralid5  moralid6  moralid7  moralid8  moralid9  mcoop3  mcoop5  mcoop6  mcoop7  narc1  narc2  narc3  narc4  narc6  nidentity1  nidentity2  omind5  omind6  contact2  contact4  contact5  hygiene1  hygiene2  hygiene3  hygiene4  hygiene5  psupport1  psupport2  psupport3  psupport4  psupport5  political_ideology  happy  slf_ladder  riskperc1  riskperc2  self_esteem  sbelong1  sbelong2  optim1  optim2  slfcont4  urban
##   6   3  age  children  cnarc1  cnarc2  CRT1  CRT2  CRT3  duration  health_cond  moralid1  moralid10  moralid2  moralid3  moralid4  moralid5  moralid6  moralid7  moralid8  moralid9  mcoop3  mcoop5  mcoop6  mcoop7  narc1  narc2  narc3  narc4  narc6  nidentity1  nidentity2  omind5  omind6  contact2  contact4  contact5  hygiene1  hygiene2  hygiene3  hygiene4  hygiene5  psupport1  psupport2  psupport3  psupport4  psupport5  political_ideology  happy  slf_ladder  riskperc1  riskperc2  self_esteem  sbelong1  sbelong2  optim1  optim2  slfcont4  urban
##   6   4  age  children  cnarc1  cnarc2  CRT1  CRT2  CRT3  duration  health_cond  moralid1  moralid10  moralid2  moralid3  moralid4  moralid5  moralid6  moralid7  moralid8  moralid9  mcoop3  mcoop5  mcoop6  mcoop7  narc1  narc2  narc3  narc4  narc6  nidentity1  nidentity2  omind5  omind6  contact2  contact4  contact5  hygiene1  hygiene2  hygiene3  hygiene4  hygiene5  psupport1  psupport2  psupport3  psupport4  psupport5  political_ideology  happy  slf_ladder  riskperc1  riskperc2  self_esteem  sbelong1  sbelong2  optim1  optim2  slfcont4  urban
##   6   5  age  children  cnarc1  cnarc2  CRT1  CRT2  CRT3  duration  health_cond  moralid1  moralid10  moralid2  moralid3  moralid4  moralid5  moralid6  moralid7  moralid8  moralid9  mcoop3  mcoop5  mcoop6  mcoop7  narc1  narc2  narc3  narc4  narc6  nidentity1  nidentity2  omind5  omind6  contact2  contact4  contact5  hygiene1  hygiene2  hygiene3  hygiene4  hygiene5  psupport1  psupport2  psupport3  psupport4  psupport5  political_ideology  happy  slf_ladder  riskperc1  riskperc2  self_esteem  sbelong1  sbelong2  optim1  optim2  slfcont4  urban
##   6   6  age  children  cnarc1  cnarc2  CRT1  CRT2  CRT3  duration  health_cond  moralid1  moralid10  moralid2  moralid3  moralid4  moralid5  moralid6  moralid7  moralid8  moralid9  mcoop3  mcoop5  mcoop6  mcoop7  narc1  narc2  narc3  narc4  narc6  nidentity1  nidentity2  omind5  omind6  contact2  contact4  contact5  hygiene1  hygiene2  hygiene3  hygiene4  hygiene5  psupport1  psupport2  psupport3  psupport4  psupport5  political_ideology  happy  slf_ladder  riskperc1  riskperc2  self_esteem  sbelong1  sbelong2  optim1  optim2  slfcont4  urban
##   6   7  age  children  cnarc1  cnarc2  CRT1  CRT2  CRT3  duration  health_cond  moralid1  moralid10  moralid2  moralid3  moralid4  moralid5  moralid6  moralid7  moralid8  moralid9  mcoop3  mcoop5  mcoop6  mcoop7  narc1  narc2  narc3  narc4  narc6  nidentity1  nidentity2  omind5  omind6  contact2  contact4  contact5  hygiene1  hygiene2  hygiene3  hygiene4  hygiene5  psupport1  psupport2  psupport3  psupport4  psupport5  political_ideology  happy  slf_ladder  riskperc1  riskperc2  self_esteem  sbelong1  sbelong2  optim1  optim2  slfcont4  urban
##   6   8  age  children  cnarc1  cnarc2  CRT1  CRT2  CRT3  duration  health_cond  moralid1  moralid10  moralid2  moralid3  moralid4  moralid5  moralid6  moralid7  moralid8  moralid9  mcoop3  mcoop5  mcoop6  mcoop7  narc1  narc2  narc3  narc4  narc6  nidentity1  nidentity2  omind5  omind6  contact2  contact4  contact5  hygiene1  hygiene2  hygiene3  hygiene4  hygiene5  psupport1  psupport2  psupport3  psupport4  psupport5  political_ideology  happy  slf_ladder  riskperc1  riskperc2  self_esteem  sbelong1  sbelong2  optim1  optim2  slfcont4  urban
##   6   9  age  children  cnarc1  cnarc2  CRT1  CRT2  CRT3  duration  health_cond  moralid1  moralid10  moralid2  moralid3  moralid4  moralid5  moralid6  moralid7  moralid8  moralid9  mcoop3  mcoop5  mcoop6  mcoop7  narc1  narc2  narc3  narc4  narc6  nidentity1  nidentity2  omind5  omind6  contact2  contact4  contact5  hygiene1  hygiene2  hygiene3  hygiene4  hygiene5  psupport1  psupport2  psupport3  psupport4  psupport5  political_ideology  happy  slf_ladder  riskperc1  riskperc2  self_esteem  sbelong1  sbelong2  optim1  optim2  slfcont4  urban
##   6   10  age  children  cnarc1  cnarc2  CRT1  CRT2  CRT3  duration  health_cond  moralid1  moralid10  moralid2  moralid3  moralid4  moralid5  moralid6  moralid7  moralid8  moralid9  mcoop3  mcoop5  mcoop6  mcoop7  narc1  narc2  narc3  narc4  narc6  nidentity1  nidentity2  omind5  omind6  contact2  contact4  contact5  hygiene1  hygiene2  hygiene3  hygiene4  hygiene5  psupport1  psupport2  psupport3  psupport4  psupport5  political_ideology  happy  slf_ladder  riskperc1  riskperc2  self_esteem  sbelong1  sbelong2  optim1  optim2  slfcont4  urban
##   7   1  age  children  cnarc1  cnarc2  CRT1  CRT2  CRT3  duration  health_cond  moralid1  moralid10  moralid2  moralid3  moralid4  moralid5  moralid6  moralid7  moralid8  moralid9  mcoop3  mcoop5  mcoop6  mcoop7  narc1  narc2  narc3  narc4  narc6  nidentity1  nidentity2  omind5  omind6  contact2  contact4  contact5  hygiene1  hygiene2  hygiene3  hygiene4  hygiene5  psupport1  psupport2  psupport3  psupport4  psupport5  political_ideology  happy  slf_ladder  riskperc1  riskperc2  self_esteem  sbelong1  sbelong2  optim1  optim2  slfcont4  urban
##   7   2  age  children  cnarc1  cnarc2  CRT1  CRT2  CRT3  duration  health_cond  moralid1  moralid10  moralid2  moralid3  moralid4  moralid5  moralid6  moralid7  moralid8  moralid9  mcoop3  mcoop5  mcoop6  mcoop7  narc1  narc2  narc3  narc4  narc6  nidentity1  nidentity2  omind5  omind6  contact2  contact4  contact5  hygiene1  hygiene2  hygiene3  hygiene4  hygiene5  psupport1  psupport2  psupport3  psupport4  psupport5  political_ideology  happy  slf_ladder  riskperc1  riskperc2  self_esteem  sbelong1  sbelong2  optim1  optim2  slfcont4  urban
##   7   3  age  children  cnarc1  cnarc2  CRT1  CRT2  CRT3  duration  health_cond  moralid1  moralid10  moralid2  moralid3  moralid4  moralid5  moralid6  moralid7  moralid8  moralid9  mcoop3  mcoop5  mcoop6  mcoop7  narc1  narc2  narc3  narc4  narc6  nidentity1  nidentity2  omind5  omind6  contact2  contact4  contact5  hygiene1  hygiene2  hygiene3  hygiene4  hygiene5  psupport1  psupport2  psupport3  psupport4  psupport5  political_ideology  happy  slf_ladder  riskperc1  riskperc2  self_esteem  sbelong1  sbelong2  optim1  optim2  slfcont4  urban
##   7   4  age  children  cnarc1  cnarc2  CRT1  CRT2  CRT3  duration  health_cond  moralid1  moralid10  moralid2  moralid3  moralid4  moralid5  moralid6  moralid7  moralid8  moralid9  mcoop3  mcoop5  mcoop6  mcoop7  narc1  narc2  narc3  narc4  narc6  nidentity1  nidentity2  omind5  omind6  contact2  contact4  contact5  hygiene1  hygiene2  hygiene3  hygiene4  hygiene5  psupport1  psupport2  psupport3  psupport4  psupport5  political_ideology  happy  slf_ladder  riskperc1  riskperc2  self_esteem  sbelong1  sbelong2  optim1  optim2  slfcont4  urban
##   7   5  age  children  cnarc1  cnarc2  CRT1  CRT2  CRT3  duration  health_cond  moralid1  moralid10  moralid2  moralid3  moralid4  moralid5  moralid6  moralid7  moralid8  moralid9  mcoop3  mcoop5  mcoop6  mcoop7  narc1  narc2  narc3  narc4  narc6  nidentity1  nidentity2  omind5  omind6  contact2  contact4  contact5  hygiene1  hygiene2  hygiene3  hygiene4  hygiene5  psupport1  psupport2  psupport3  psupport4  psupport5  political_ideology  happy  slf_ladder  riskperc1  riskperc2  self_esteem  sbelong1  sbelong2  optim1  optim2  slfcont4  urban
##   7   6  age  children  cnarc1  cnarc2  CRT1  CRT2  CRT3  duration  health_cond  moralid1  moralid10  moralid2  moralid3  moralid4  moralid5  moralid6  moralid7  moralid8  moralid9  mcoop3  mcoop5  mcoop6  mcoop7  narc1  narc2  narc3  narc4  narc6  nidentity1  nidentity2  omind5  omind6  contact2  contact4  contact5  hygiene1  hygiene2  hygiene3  hygiene4  hygiene5  psupport1  psupport2  psupport3  psupport4  psupport5  political_ideology  happy  slf_ladder  riskperc1  riskperc2  self_esteem  sbelong1  sbelong2  optim1  optim2  slfcont4  urban
##   7   7  age  children  cnarc1  cnarc2  CRT1  CRT2  CRT3  duration  health_cond  moralid1  moralid10  moralid2  moralid3  moralid4  moralid5  moralid6  moralid7  moralid8  moralid9  mcoop3  mcoop5  mcoop6  mcoop7  narc1  narc2  narc3  narc4  narc6  nidentity1  nidentity2  omind5  omind6  contact2  contact4  contact5  hygiene1  hygiene2  hygiene3  hygiene4  hygiene5  psupport1  psupport2  psupport3  psupport4  psupport5  political_ideology  happy  slf_ladder  riskperc1  riskperc2  self_esteem  sbelong1  sbelong2  optim1  optim2  slfcont4  urban
##   7   8  age  children  cnarc1  cnarc2  CRT1  CRT2  CRT3  duration  health_cond  moralid1  moralid10  moralid2  moralid3  moralid4  moralid5  moralid6  moralid7  moralid8  moralid9  mcoop3  mcoop5  mcoop6  mcoop7  narc1  narc2  narc3  narc4  narc6  nidentity1  nidentity2  omind5  omind6  contact2  contact4  contact5  hygiene1  hygiene2  hygiene3  hygiene4  hygiene5  psupport1  psupport2  psupport3  psupport4  psupport5  political_ideology  happy  slf_ladder  riskperc1  riskperc2  self_esteem  sbelong1  sbelong2  optim1  optim2  slfcont4  urban
##   7   9  age  children  cnarc1  cnarc2  CRT1  CRT2  CRT3  duration  health_cond  moralid1  moralid10  moralid2  moralid3  moralid4  moralid5  moralid6  moralid7  moralid8  moralid9  mcoop3  mcoop5  mcoop6  mcoop7  narc1  narc2  narc3  narc4  narc6  nidentity1  nidentity2  omind5  omind6  contact2  contact4  contact5  hygiene1  hygiene2  hygiene3  hygiene4  hygiene5  psupport1  psupport2  psupport3  psupport4  psupport5  political_ideology  happy  slf_ladder  riskperc1  riskperc2  self_esteem  sbelong1  sbelong2  optim1  optim2  slfcont4  urban
##   7   10  age  children  cnarc1  cnarc2  CRT1  CRT2  CRT3  duration  health_cond  moralid1  moralid10  moralid2  moralid3  moralid4  moralid5  moralid6  moralid7  moralid8  moralid9  mcoop3  mcoop5  mcoop6  mcoop7  narc1  narc2  narc3  narc4  narc6  nidentity1  nidentity2  omind5  omind6  contact2  contact4  contact5  hygiene1  hygiene2  hygiene3  hygiene4  hygiene5  psupport1  psupport2  psupport3  psupport4  psupport5  political_ideology  happy  slf_ladder  riskperc1  riskperc2  self_esteem  sbelong1  sbelong2  optim1  optim2  slfcont4  urban
##   8   1  age  children  cnarc1  cnarc2  CRT1  CRT2  CRT3  duration  health_cond  moralid1  moralid10  moralid2  moralid3  moralid4  moralid5  moralid6  moralid7  moralid8  moralid9  mcoop3  mcoop5  mcoop6  mcoop7  narc1  narc2  narc3  narc4  narc6  nidentity1  nidentity2  omind5  omind6  contact2  contact4  contact5  hygiene1  hygiene2  hygiene3  hygiene4  hygiene5  psupport1  psupport2  psupport3  psupport4  psupport5  political_ideology  happy  slf_ladder  riskperc1  riskperc2  self_esteem  sbelong1  sbelong2  optim1  optim2  slfcont4  urban
##   8   2  age  children  cnarc1  cnarc2  CRT1  CRT2  CRT3  duration  health_cond  moralid1  moralid10  moralid2  moralid3  moralid4  moralid5  moralid6  moralid7  moralid8  moralid9  mcoop3  mcoop5  mcoop6  mcoop7  narc1  narc2  narc3  narc4  narc6  nidentity1  nidentity2  omind5  omind6  contact2  contact4  contact5  hygiene1  hygiene2  hygiene3  hygiene4  hygiene5  psupport1  psupport2  psupport3  psupport4  psupport5  political_ideology  happy  slf_ladder  riskperc1  riskperc2  self_esteem  sbelong1  sbelong2  optim1  optim2  slfcont4  urban
##   8   3  age  children  cnarc1  cnarc2  CRT1  CRT2  CRT3  duration  health_cond  moralid1  moralid10  moralid2  moralid3  moralid4  moralid5  moralid6  moralid7  moralid8  moralid9  mcoop3  mcoop5  mcoop6  mcoop7  narc1  narc2  narc3  narc4  narc6  nidentity1  nidentity2  omind5  omind6  contact2  contact4  contact5  hygiene1  hygiene2  hygiene3  hygiene4  hygiene5  psupport1  psupport2  psupport3  psupport4  psupport5  political_ideology  happy  slf_ladder  riskperc1  riskperc2  self_esteem  sbelong1  sbelong2  optim1  optim2  slfcont4  urban
##   8   4  age  children  cnarc1  cnarc2  CRT1  CRT2  CRT3  duration  health_cond  moralid1  moralid10  moralid2  moralid3  moralid4  moralid5  moralid6  moralid7  moralid8  moralid9  mcoop3  mcoop5  mcoop6  mcoop7  narc1  narc2  narc3  narc4  narc6  nidentity1  nidentity2  omind5  omind6  contact2  contact4  contact5  hygiene1  hygiene2  hygiene3  hygiene4  hygiene5  psupport1  psupport2  psupport3  psupport4  psupport5  political_ideology  happy  slf_ladder  riskperc1  riskperc2  self_esteem  sbelong1  sbelong2  optim1  optim2  slfcont4  urban
##   8   5  age  children  cnarc1  cnarc2  CRT1  CRT2  CRT3  duration  health_cond  moralid1  moralid10  moralid2  moralid3  moralid4  moralid5  moralid6  moralid7  moralid8  moralid9  mcoop3  mcoop5  mcoop6  mcoop7  narc1  narc2  narc3  narc4  narc6  nidentity1  nidentity2  omind5  omind6  contact2  contact4  contact5  hygiene1  hygiene2  hygiene3  hygiene4  hygiene5  psupport1  psupport2  psupport3  psupport4  psupport5  political_ideology  happy  slf_ladder  riskperc1  riskperc2  self_esteem  sbelong1  sbelong2  optim1  optim2  slfcont4  urban
##   8   6  age  children  cnarc1  cnarc2  CRT1  CRT2  CRT3  duration  health_cond  moralid1  moralid10  moralid2  moralid3  moralid4  moralid5  moralid6  moralid7  moralid8  moralid9  mcoop3  mcoop5  mcoop6  mcoop7  narc1  narc2  narc3  narc4  narc6  nidentity1  nidentity2  omind5  omind6  contact2  contact4  contact5  hygiene1  hygiene2  hygiene3  hygiene4  hygiene5  psupport1  psupport2  psupport3  psupport4  psupport5  political_ideology  happy  slf_ladder  riskperc1  riskperc2  self_esteem  sbelong1  sbelong2  optim1  optim2  slfcont4  urban
##   8   7  age  children  cnarc1  cnarc2  CRT1  CRT2  CRT3  duration  health_cond  moralid1  moralid10  moralid2  moralid3  moralid4  moralid5  moralid6  moralid7  moralid8  moralid9  mcoop3  mcoop5  mcoop6  mcoop7  narc1  narc2  narc3  narc4  narc6  nidentity1  nidentity2  omind5  omind6  contact2  contact4  contact5  hygiene1  hygiene2  hygiene3  hygiene4  hygiene5  psupport1  psupport2  psupport3  psupport4  psupport5  political_ideology  happy  slf_ladder  riskperc1  riskperc2  self_esteem  sbelong1  sbelong2  optim1  optim2  slfcont4  urban
##   8   8  age  children  cnarc1  cnarc2  CRT1  CRT2  CRT3  duration  health_cond  moralid1  moralid10  moralid2  moralid3  moralid4  moralid5  moralid6  moralid7  moralid8  moralid9  mcoop3  mcoop5  mcoop6  mcoop7  narc1  narc2  narc3  narc4  narc6  nidentity1  nidentity2  omind5  omind6  contact2  contact4  contact5  hygiene1  hygiene2  hygiene3  hygiene4  hygiene5  psupport1  psupport2  psupport3  psupport4  psupport5  political_ideology  happy  slf_ladder  riskperc1  riskperc2  self_esteem  sbelong1  sbelong2  optim1  optim2  slfcont4  urban
##   8   9  age  children  cnarc1  cnarc2  CRT1  CRT2  CRT3  duration  health_cond  moralid1  moralid10  moralid2  moralid3  moralid4  moralid5  moralid6  moralid7  moralid8  moralid9  mcoop3  mcoop5  mcoop6  mcoop7  narc1  narc2  narc3  narc4  narc6  nidentity1  nidentity2  omind5  omind6  contact2  contact4  contact5  hygiene1  hygiene2  hygiene3  hygiene4  hygiene5  psupport1  psupport2  psupport3  psupport4  psupport5  political_ideology  happy  slf_ladder  riskperc1  riskperc2  self_esteem  sbelong1  sbelong2  optim1  optim2  slfcont4  urban
##   8   10  age  children  cnarc1  cnarc2  CRT1  CRT2  CRT3  duration  health_cond  moralid1  moralid10  moralid2  moralid3  moralid4  moralid5  moralid6  moralid7  moralid8  moralid9  mcoop3  mcoop5  mcoop6  mcoop7  narc1  narc2  narc3  narc4  narc6  nidentity1  nidentity2  omind5  omind6  contact2  contact4  contact5  hygiene1  hygiene2  hygiene3  hygiene4  hygiene5  psupport1  psupport2  psupport3  psupport4  psupport5  political_ideology  happy  slf_ladder  riskperc1  riskperc2  self_esteem  sbelong1  sbelong2  optim1  optim2  slfcont4  urban
##   9   1  age  children  cnarc1  cnarc2  CRT1  CRT2  CRT3  duration  health_cond  moralid1  moralid10  moralid2  moralid3  moralid4  moralid5  moralid6  moralid7  moralid8  moralid9  mcoop3  mcoop5  mcoop6  mcoop7  narc1  narc2  narc3  narc4  narc6  nidentity1  nidentity2  omind5  omind6  contact2  contact4  contact5  hygiene1  hygiene2  hygiene3  hygiene4  hygiene5  psupport1  psupport2  psupport3  psupport4  psupport5  political_ideology  happy  slf_ladder  riskperc1  riskperc2  self_esteem  sbelong1  sbelong2  optim1  optim2  slfcont4  urban
##   9   2  age  children  cnarc1  cnarc2  CRT1  CRT2  CRT3  duration  health_cond  moralid1  moralid10  moralid2  moralid3  moralid4  moralid5  moralid6  moralid7  moralid8  moralid9  mcoop3  mcoop5  mcoop6  mcoop7  narc1  narc2  narc3  narc4  narc6  nidentity1  nidentity2  omind5  omind6  contact2  contact4  contact5  hygiene1  hygiene2  hygiene3  hygiene4  hygiene5  psupport1  psupport2  psupport3  psupport4  psupport5  political_ideology  happy  slf_ladder  riskperc1  riskperc2  self_esteem  sbelong1  sbelong2  optim1  optim2  slfcont4  urban
##   9   3  age  children  cnarc1  cnarc2  CRT1  CRT2  CRT3  duration  health_cond  moralid1  moralid10  moralid2  moralid3  moralid4  moralid5  moralid6  moralid7  moralid8  moralid9  mcoop3  mcoop5  mcoop6  mcoop7  narc1  narc2  narc3  narc4  narc6  nidentity1  nidentity2  omind5  omind6  contact2  contact4  contact5  hygiene1  hygiene2  hygiene3  hygiene4  hygiene5  psupport1  psupport2  psupport3  psupport4  psupport5  political_ideology  happy  slf_ladder  riskperc1  riskperc2  self_esteem  sbelong1  sbelong2  optim1  optim2  slfcont4  urban
##   9   4  age  children  cnarc1  cnarc2  CRT1  CRT2  CRT3  duration  health_cond  moralid1  moralid10  moralid2  moralid3  moralid4  moralid5  moralid6  moralid7  moralid8  moralid9  mcoop3  mcoop5  mcoop6  mcoop7  narc1  narc2  narc3  narc4  narc6  nidentity1  nidentity2  omind5  omind6  contact2  contact4  contact5  hygiene1  hygiene2  hygiene3  hygiene4  hygiene5  psupport1  psupport2  psupport3  psupport4  psupport5  political_ideology  happy  slf_ladder  riskperc1  riskperc2  self_esteem  sbelong1  sbelong2  optim1  optim2  slfcont4  urban
##   9   5  age  children  cnarc1  cnarc2  CRT1  CRT2  CRT3  duration  health_cond  moralid1  moralid10  moralid2  moralid3  moralid4  moralid5  moralid6  moralid7  moralid8  moralid9  mcoop3  mcoop5  mcoop6  mcoop7  narc1  narc2  narc3  narc4  narc6  nidentity1  nidentity2  omind5  omind6  contact2  contact4  contact5  hygiene1  hygiene2  hygiene3  hygiene4  hygiene5  psupport1  psupport2  psupport3  psupport4  psupport5  political_ideology  happy  slf_ladder  riskperc1  riskperc2  self_esteem  sbelong1  sbelong2  optim1  optim2  slfcont4  urban
##   9   6  age  children  cnarc1  cnarc2  CRT1  CRT2  CRT3  duration  health_cond  moralid1  moralid10  moralid2  moralid3  moralid4  moralid5  moralid6  moralid7  moralid8  moralid9  mcoop3  mcoop5  mcoop6  mcoop7  narc1  narc2  narc3  narc4  narc6  nidentity1  nidentity2  omind5  omind6  contact2  contact4  contact5  hygiene1  hygiene2  hygiene3  hygiene4  hygiene5  psupport1  psupport2  psupport3  psupport4  psupport5  political_ideology  happy  slf_ladder  riskperc1  riskperc2  self_esteem  sbelong1  sbelong2  optim1  optim2  slfcont4  urban
##   9   7  age  children  cnarc1  cnarc2  CRT1  CRT2  CRT3  duration  health_cond  moralid1  moralid10  moralid2  moralid3  moralid4  moralid5  moralid6  moralid7  moralid8  moralid9  mcoop3  mcoop5  mcoop6  mcoop7  narc1  narc2  narc3  narc4  narc6  nidentity1  nidentity2  omind5  omind6  contact2  contact4  contact5  hygiene1  hygiene2  hygiene3  hygiene4  hygiene5  psupport1  psupport2  psupport3  psupport4  psupport5  political_ideology  happy  slf_ladder  riskperc1  riskperc2  self_esteem  sbelong1  sbelong2  optim1  optim2  slfcont4  urban
##   9   8  age  children  cnarc1  cnarc2  CRT1  CRT2  CRT3  duration  health_cond  moralid1  moralid10  moralid2  moralid3  moralid4  moralid5  moralid6  moralid7  moralid8  moralid9  mcoop3  mcoop5  mcoop6  mcoop7  narc1  narc2  narc3  narc4  narc6  nidentity1  nidentity2  omind5  omind6  contact2  contact4  contact5  hygiene1  hygiene2  hygiene3  hygiene4  hygiene5  psupport1  psupport2  psupport3  psupport4  psupport5  political_ideology  happy  slf_ladder  riskperc1  riskperc2  self_esteem  sbelong1  sbelong2  optim1  optim2  slfcont4  urban
##   9   9  age  children  cnarc1  cnarc2  CRT1  CRT2  CRT3  duration  health_cond  moralid1  moralid10  moralid2  moralid3  moralid4  moralid5  moralid6  moralid7  moralid8  moralid9  mcoop3  mcoop5  mcoop6  mcoop7  narc1  narc2  narc3  narc4  narc6  nidentity1  nidentity2  omind5  omind6  contact2  contact4  contact5  hygiene1  hygiene2  hygiene3  hygiene4  hygiene5  psupport1  psupport2  psupport3  psupport4  psupport5  political_ideology  happy  slf_ladder  riskperc1  riskperc2  self_esteem  sbelong1  sbelong2  optim1  optim2  slfcont4  urban
##   9   10  age  children  cnarc1  cnarc2  CRT1  CRT2  CRT3  duration  health_cond  moralid1  moralid10  moralid2  moralid3  moralid4  moralid5  moralid6  moralid7  moralid8  moralid9  mcoop3  mcoop5  mcoop6  mcoop7  narc1  narc2  narc3  narc4  narc6  nidentity1  nidentity2  omind5  omind6  contact2  contact4  contact5  hygiene1  hygiene2  hygiene3  hygiene4  hygiene5  psupport1  psupport2  psupport3  psupport4  psupport5  political_ideology  happy  slf_ladder  riskperc1  riskperc2  self_esteem  sbelong1  sbelong2  optim1  optim2  slfcont4  urban
##   10   1  age  children  cnarc1  cnarc2  CRT1  CRT2  CRT3  duration  health_cond  moralid1  moralid10  moralid2  moralid3  moralid4  moralid5  moralid6  moralid7  moralid8  moralid9  mcoop3  mcoop5  mcoop6  mcoop7  narc1  narc2  narc3  narc4  narc6  nidentity1  nidentity2  omind5  omind6  contact2  contact4  contact5  hygiene1  hygiene2  hygiene3  hygiene4  hygiene5  psupport1  psupport2  psupport3  psupport4  psupport5  political_ideology  happy  slf_ladder  riskperc1  riskperc2  self_esteem  sbelong1  sbelong2  optim1  optim2  slfcont4  urban
##   10   2  age  children  cnarc1  cnarc2  CRT1  CRT2  CRT3  duration  health_cond  moralid1  moralid10  moralid2  moralid3  moralid4  moralid5  moralid6  moralid7  moralid8  moralid9  mcoop3  mcoop5  mcoop6  mcoop7  narc1  narc2  narc3  narc4  narc6  nidentity1  nidentity2  omind5  omind6  contact2  contact4  contact5  hygiene1  hygiene2  hygiene3  hygiene4  hygiene5  psupport1  psupport2  psupport3  psupport4  psupport5  political_ideology  happy  slf_ladder  riskperc1  riskperc2  self_esteem  sbelong1  sbelong2  optim1  optim2  slfcont4  urban
##   10   3  age  children  cnarc1  cnarc2  CRT1  CRT2  CRT3  duration  health_cond  moralid1  moralid10  moralid2  moralid3  moralid4  moralid5  moralid6  moralid7  moralid8  moralid9  mcoop3  mcoop5  mcoop6  mcoop7  narc1  narc2  narc3  narc4  narc6  nidentity1  nidentity2  omind5  omind6  contact2  contact4  contact5  hygiene1  hygiene2  hygiene3  hygiene4  hygiene5  psupport1  psupport2  psupport3  psupport4  psupport5  political_ideology  happy  slf_ladder  riskperc1  riskperc2  self_esteem  sbelong1  sbelong2  optim1  optim2  slfcont4  urban
##   10   4  age  children  cnarc1  cnarc2  CRT1  CRT2  CRT3  duration  health_cond  moralid1  moralid10  moralid2  moralid3  moralid4  moralid5  moralid6  moralid7  moralid8  moralid9  mcoop3  mcoop5  mcoop6  mcoop7  narc1  narc2  narc3  narc4  narc6  nidentity1  nidentity2  omind5  omind6  contact2  contact4  contact5  hygiene1  hygiene2  hygiene3  hygiene4  hygiene5  psupport1  psupport2  psupport3  psupport4  psupport5  political_ideology  happy  slf_ladder  riskperc1  riskperc2  self_esteem  sbelong1  sbelong2  optim1  optim2  slfcont4  urban
##   10   5  age  children  cnarc1  cnarc2  CRT1  CRT2  CRT3  duration  health_cond  moralid1  moralid10  moralid2  moralid3  moralid4  moralid5  moralid6  moralid7  moralid8  moralid9  mcoop3  mcoop5  mcoop6  mcoop7  narc1  narc2  narc3  narc4  narc6  nidentity1  nidentity2  omind5  omind6  contact2  contact4  contact5  hygiene1  hygiene2  hygiene3  hygiene4  hygiene5  psupport1  psupport2  psupport3  psupport4  psupport5  political_ideology  happy  slf_ladder  riskperc1  riskperc2  self_esteem  sbelong1  sbelong2  optim1  optim2  slfcont4  urban
##   10   6  age  children  cnarc1  cnarc2  CRT1  CRT2  CRT3  duration  health_cond  moralid1  moralid10  moralid2  moralid3  moralid4  moralid5  moralid6  moralid7  moralid8  moralid9  mcoop3  mcoop5  mcoop6  mcoop7  narc1  narc2  narc3  narc4  narc6  nidentity1  nidentity2  omind5  omind6  contact2  contact4  contact5  hygiene1  hygiene2  hygiene3  hygiene4  hygiene5  psupport1  psupport2  psupport3  psupport4  psupport5  political_ideology  happy  slf_ladder  riskperc1  riskperc2  self_esteem  sbelong1  sbelong2  optim1  optim2  slfcont4  urban
##   10   7  age  children  cnarc1  cnarc2  CRT1  CRT2  CRT3  duration  health_cond  moralid1  moralid10  moralid2  moralid3  moralid4  moralid5  moralid6  moralid7  moralid8  moralid9  mcoop3  mcoop5  mcoop6  mcoop7  narc1  narc2  narc3  narc4  narc6  nidentity1  nidentity2  omind5  omind6  contact2  contact4  contact5  hygiene1  hygiene2  hygiene3  hygiene4  hygiene5  psupport1  psupport2  psupport3  psupport4  psupport5  political_ideology  happy  slf_ladder  riskperc1  riskperc2  self_esteem  sbelong1  sbelong2  optim1  optim2  slfcont4  urban
##   10   8  age  children  cnarc1  cnarc2  CRT1  CRT2  CRT3  duration  health_cond  moralid1  moralid10  moralid2  moralid3  moralid4  moralid5  moralid6  moralid7  moralid8  moralid9  mcoop3  mcoop5  mcoop6  mcoop7  narc1  narc2  narc3  narc4  narc6  nidentity1  nidentity2  omind5  omind6  contact2  contact4  contact5  hygiene1  hygiene2  hygiene3  hygiene4  hygiene5  psupport1  psupport2  psupport3  psupport4  psupport5  political_ideology  happy  slf_ladder  riskperc1  riskperc2  self_esteem  sbelong1  sbelong2  optim1  optim2  slfcont4  urban
##   10   9  age  children  cnarc1  cnarc2  CRT1  CRT2  CRT3  duration  health_cond  moralid1  moralid10  moralid2  moralid3  moralid4  moralid5  moralid6  moralid7  moralid8  moralid9  mcoop3  mcoop5  mcoop6  mcoop7  narc1  narc2  narc3  narc4  narc6  nidentity1  nidentity2  omind5  omind6  contact2  contact4  contact5  hygiene1  hygiene2  hygiene3  hygiene4  hygiene5  psupport1  psupport2  psupport3  psupport4  psupport5  political_ideology  happy  slf_ladder  riskperc1  riskperc2  self_esteem  sbelong1  sbelong2  optim1  optim2  slfcont4  urban
##   10   10  age  children  cnarc1  cnarc2  CRT1  CRT2  CRT3  duration  health_cond  moralid1  moralid10  moralid2  moralid3  moralid4  moralid5  moralid6  moralid7  moralid8  moralid9  mcoop3  mcoop5  mcoop6  mcoop7  narc1  narc2  narc3  narc4  narc6  nidentity1  nidentity2  omind5  omind6  contact2  contact4  contact5  hygiene1  hygiene2  hygiene3  hygiene4  hygiene5  psupport1  psupport2  psupport3  psupport4  psupport5  political_ideology  happy  slf_ladder  riskperc1  riskperc2  self_esteem  sbelong1  sbelong2  optim1  optim2  slfcont4  urban
```

```
## Warning: Number of logged events: 5702
```

```
## 
##  iter imp variable
##   1   1  riskperc2
##   1   2  riskperc2
##   1   3  riskperc2
##   1   4  riskperc2
##   1   5  riskperc2
##   1   6  riskperc2
##   1   7  riskperc2
##   1   8  riskperc2
##   1   9  riskperc2
##   1   10  riskperc2
##   2   1  riskperc2
##   2   2  riskperc2
##   2   3  riskperc2
##   2   4  riskperc2
##   2   5  riskperc2
##   2   6  riskperc2
##   2   7  riskperc2
##   2   8  riskperc2
##   2   9  riskperc2
##   2   10  riskperc2
##   3   1  riskperc2
##   3   2  riskperc2
##   3   3  riskperc2
##   3   4  riskperc2
##   3   5  riskperc2
##   3   6  riskperc2
##   3   7  riskperc2
##   3   8  riskperc2
##   3   9  riskperc2
##   3   10  riskperc2
##   4   1  riskperc2
##   4   2  riskperc2
##   4   3  riskperc2
##   4   4  riskperc2
##   4   5  riskperc2
##   4   6  riskperc2
##   4   7  riskperc2
##   4   8  riskperc2
##   4   9  riskperc2
##   4   10  riskperc2
##   5   1  riskperc2
##   5   2  riskperc2
##   5   3  riskperc2
##   5   4  riskperc2
##   5   5  riskperc2
##   5   6  riskperc2
##   5   7  riskperc2
##   5   8  riskperc2
##   5   9  riskperc2
##   5   10  riskperc2
##   6   1  riskperc2
##   6   2  riskperc2
##   6   3  riskperc2
##   6   4  riskperc2
##   6   5  riskperc2
##   6   6  riskperc2
##   6   7  riskperc2
##   6   8  riskperc2
##   6   9  riskperc2
##   6   10  riskperc2
##   7   1  riskperc2
##   7   2  riskperc2
##   7   3  riskperc2
##   7   4  riskperc2
##   7   5  riskperc2
##   7   6  riskperc2
##   7   7  riskperc2
##   7   8  riskperc2
##   7   9  riskperc2
##   7   10  riskperc2
##   8   1  riskperc2
##   8   2  riskperc2
##   8   3  riskperc2
##   8   4  riskperc2
##   8   5  riskperc2
##   8   6  riskperc2
##   8   7  riskperc2
##   8   8  riskperc2
##   8   9  riskperc2
##   8   10  riskperc2
##   9   1  riskperc2
##   9   2  riskperc2
##   9   3  riskperc2
##   9   4  riskperc2
##   9   5  riskperc2
##   9   6  riskperc2
##   9   7  riskperc2
##   9   8  riskperc2
##   9   9  riskperc2
##   9   10  riskperc2
##   10   1  riskperc2
##   10   2  riskperc2
##   10   3  riskperc2
##   10   4  riskperc2
##   10   5  riskperc2
##   10   6  riskperc2
##   10   7  riskperc2
##   10   8  riskperc2
##   10   9  riskperc2
##   10   10  riskperc2
```

```
## Warning: Number of logged events: 4
```

```
## 
##  iter imp variable
##   1   1  children  cnarc1  cnarc2  cnarc3  ctheory1  ctheory2  ctheory3  ctheory4  CRT1  CRT2  CRT3  employ_status1  generosity1  generosity2  generosity3  health_cond  mor_circle  moralid1  moralid10  moralid2  moralid3  moralid4  moralid5  moralid6  moralid7  moralid8  moralid9  mcoop1  mcoop2  mcoop3  mcoop4  mcoop5  mcoop6  mcoop7  narc1  narc2  narc3  narc4  narc5  narc6  nidentity1  nidentity2  omind1  omind2  omind3  omind4  omind5  omind6  contact1  contact2  contact3  contact4  contact5  hygiene1  hygiene2  hygiene3  hygiene4  hygiene5  psupport1  psupport2  psupport3  psupport4  psupport5  political_ideology  happy  slf_ladder  riskperc1  riskperc2  self_esteem  sbelong1  sbelong2  sbelong3  sbelong4  optim1  optim2  slfcont1  slfcont2  slfcont3  slfcont4
##   1   2  children  cnarc1  cnarc2  cnarc3  ctheory1  ctheory2  ctheory3  ctheory4  CRT1  CRT2  CRT3  employ_status1  generosity1  generosity2  generosity3  health_cond  mor_circle  moralid1  moralid10  moralid2  moralid3  moralid4  moralid5  moralid6  moralid7  moralid8  moralid9  mcoop1  mcoop2  mcoop3  mcoop4  mcoop5  mcoop6  mcoop7  narc1  narc2  narc3  narc4  narc5  narc6  nidentity1  nidentity2  omind1  omind2  omind3  omind4  omind5  omind6  contact1  contact2  contact3  contact4  contact5  hygiene1  hygiene2  hygiene3  hygiene4  hygiene5  psupport1  psupport2  psupport3  psupport4  psupport5  political_ideology  happy  slf_ladder  riskperc1  riskperc2  self_esteem  sbelong1  sbelong2  sbelong3  sbelong4  optim1  optim2  slfcont1  slfcont2  slfcont3  slfcont4
##   1   3  children  cnarc1  cnarc2  cnarc3  ctheory1  ctheory2  ctheory3  ctheory4  CRT1  CRT2  CRT3  employ_status1  generosity1  generosity2  generosity3  health_cond  mor_circle  moralid1  moralid10  moralid2  moralid3  moralid4  moralid5  moralid6  moralid7  moralid8  moralid9  mcoop1  mcoop2  mcoop3  mcoop4  mcoop5  mcoop6  mcoop7  narc1  narc2  narc3  narc4  narc5  narc6  nidentity1  nidentity2  omind1  omind2  omind3  omind4  omind5  omind6  contact1  contact2  contact3  contact4  contact5  hygiene1  hygiene2  hygiene3  hygiene4  hygiene5  psupport1  psupport2  psupport3  psupport4  psupport5  political_ideology  happy  slf_ladder  riskperc1  riskperc2  self_esteem  sbelong1  sbelong2  sbelong3  sbelong4  optim1  optim2  slfcont1  slfcont2  slfcont3  slfcont4
##   1   4  children  cnarc1  cnarc2  cnarc3  ctheory1  ctheory2  ctheory3  ctheory4  CRT1  CRT2  CRT3  employ_status1  generosity1  generosity2  generosity3  health_cond  mor_circle  moralid1  moralid10  moralid2  moralid3  moralid4  moralid5  moralid6  moralid7  moralid8  moralid9  mcoop1  mcoop2  mcoop3  mcoop4  mcoop5  mcoop6  mcoop7  narc1  narc2  narc3  narc4  narc5  narc6  nidentity1  nidentity2  omind1  omind2  omind3  omind4  omind5  omind6  contact1  contact2  contact3  contact4  contact5  hygiene1  hygiene2  hygiene3  hygiene4  hygiene5  psupport1  psupport2  psupport3  psupport4  psupport5  political_ideology  happy  slf_ladder  riskperc1  riskperc2  self_esteem  sbelong1  sbelong2  sbelong3  sbelong4  optim1  optim2  slfcont1  slfcont2  slfcont3  slfcont4
##   1   5  children  cnarc1  cnarc2  cnarc3  ctheory1  ctheory2  ctheory3  ctheory4  CRT1  CRT2  CRT3  employ_status1  generosity1  generosity2  generosity3  health_cond  mor_circle  moralid1  moralid10  moralid2  moralid3  moralid4  moralid5  moralid6  moralid7  moralid8  moralid9  mcoop1  mcoop2  mcoop3  mcoop4  mcoop5  mcoop6  mcoop7  narc1  narc2  narc3  narc4  narc5  narc6  nidentity1  nidentity2  omind1  omind2  omind3  omind4  omind5  omind6  contact1  contact2  contact3  contact4  contact5  hygiene1  hygiene2  hygiene3  hygiene4  hygiene5  psupport1  psupport2  psupport3  psupport4  psupport5  political_ideology  happy  slf_ladder  riskperc1  riskperc2  self_esteem  sbelong1  sbelong2  sbelong3  sbelong4  optim1  optim2  slfcont1  slfcont2  slfcont3  slfcont4
##   1   6  children  cnarc1  cnarc2  cnarc3  ctheory1  ctheory2  ctheory3  ctheory4  CRT1  CRT2  CRT3  employ_status1  generosity1  generosity2  generosity3  health_cond  mor_circle  moralid1  moralid10  moralid2  moralid3  moralid4  moralid5  moralid6  moralid7  moralid8  moralid9  mcoop1  mcoop2  mcoop3  mcoop4  mcoop5  mcoop6  mcoop7  narc1  narc2  narc3  narc4  narc5  narc6  nidentity1  nidentity2  omind1  omind2  omind3  omind4  omind5  omind6  contact1  contact2  contact3  contact4  contact5  hygiene1  hygiene2  hygiene3  hygiene4  hygiene5  psupport1  psupport2  psupport3  psupport4  psupport5  political_ideology  happy  slf_ladder  riskperc1  riskperc2  self_esteem  sbelong1  sbelong2  sbelong3  sbelong4  optim1  optim2  slfcont1  slfcont2  slfcont3  slfcont4
##   1   7  children  cnarc1  cnarc2  cnarc3  ctheory1  ctheory2  ctheory3  ctheory4  CRT1  CRT2  CRT3  employ_status1  generosity1  generosity2  generosity3  health_cond  mor_circle  moralid1  moralid10  moralid2  moralid3  moralid4  moralid5  moralid6  moralid7  moralid8  moralid9  mcoop1  mcoop2  mcoop3  mcoop4  mcoop5  mcoop6  mcoop7  narc1  narc2  narc3  narc4  narc5  narc6  nidentity1  nidentity2  omind1  omind2  omind3  omind4  omind5  omind6  contact1  contact2  contact3  contact4  contact5  hygiene1  hygiene2  hygiene3  hygiene4  hygiene5  psupport1  psupport2  psupport3  psupport4  psupport5  political_ideology  happy  slf_ladder  riskperc1  riskperc2  self_esteem  sbelong1  sbelong2  sbelong3  sbelong4  optim1  optim2  slfcont1  slfcont2  slfcont3  slfcont4
##   1   8  children  cnarc1  cnarc2  cnarc3  ctheory1  ctheory2  ctheory3  ctheory4  CRT1  CRT2  CRT3  employ_status1  generosity1  generosity2  generosity3  health_cond  mor_circle  moralid1  moralid10  moralid2  moralid3  moralid4  moralid5  moralid6  moralid7  moralid8  moralid9  mcoop1  mcoop2  mcoop3  mcoop4  mcoop5  mcoop6  mcoop7  narc1  narc2  narc3  narc4  narc5  narc6  nidentity1  nidentity2  omind1  omind2  omind3  omind4  omind5  omind6  contact1  contact2  contact3  contact4  contact5  hygiene1  hygiene2  hygiene3  hygiene4  hygiene5  psupport1  psupport2  psupport3  psupport4  psupport5  political_ideology  happy  slf_ladder  riskperc1  riskperc2  self_esteem  sbelong1  sbelong2  sbelong3  sbelong4  optim1  optim2  slfcont1  slfcont2  slfcont3  slfcont4
##   1   9  children  cnarc1  cnarc2  cnarc3  ctheory1  ctheory2  ctheory3  ctheory4  CRT1  CRT2  CRT3  employ_status1  generosity1  generosity2  generosity3  health_cond  mor_circle  moralid1  moralid10  moralid2  moralid3  moralid4  moralid5  moralid6  moralid7  moralid8  moralid9  mcoop1  mcoop2  mcoop3  mcoop4  mcoop5  mcoop6  mcoop7  narc1  narc2  narc3  narc4  narc5  narc6  nidentity1  nidentity2  omind1  omind2  omind3  omind4  omind5  omind6  contact1  contact2  contact3  contact4  contact5  hygiene1  hygiene2  hygiene3  hygiene4  hygiene5  psupport1  psupport2  psupport3  psupport4  psupport5  political_ideology  happy  slf_ladder  riskperc1  riskperc2  self_esteem  sbelong1  sbelong2  sbelong3  sbelong4  optim1  optim2  slfcont1  slfcont2  slfcont3  slfcont4
##   1   10  children  cnarc1  cnarc2  cnarc3  ctheory1  ctheory2  ctheory3  ctheory4  CRT1  CRT2  CRT3  employ_status1  generosity1  generosity2  generosity3  health_cond  mor_circle  moralid1  moralid10  moralid2  moralid3  moralid4  moralid5  moralid6  moralid7  moralid8  moralid9  mcoop1  mcoop2  mcoop3  mcoop4  mcoop5  mcoop6  mcoop7  narc1  narc2  narc3  narc4  narc5  narc6  nidentity1  nidentity2  omind1  omind2  omind3  omind4  omind5  omind6  contact1  contact2  contact3  contact4  contact5  hygiene1  hygiene2  hygiene3  hygiene4  hygiene5  psupport1  psupport2  psupport3  psupport4  psupport5  political_ideology  happy  slf_ladder  riskperc1  riskperc2  self_esteem  sbelong1  sbelong2  sbelong3  sbelong4  optim1  optim2  slfcont1  slfcont2  slfcont3  slfcont4
##   2   1  children  cnarc1  cnarc2  cnarc3  ctheory1  ctheory2  ctheory3  ctheory4  CRT1  CRT2  CRT3  employ_status1  generosity1  generosity2  generosity3  health_cond  mor_circle  moralid1  moralid10  moralid2  moralid3  moralid4  moralid5  moralid6  moralid7  moralid8  moralid9  mcoop1  mcoop2  mcoop3  mcoop4  mcoop5  mcoop6  mcoop7  narc1  narc2  narc3  narc4  narc5  narc6  nidentity1  nidentity2  omind1  omind2  omind3  omind4  omind5  omind6  contact1  contact2  contact3  contact4  contact5  hygiene1  hygiene2  hygiene3  hygiene4  hygiene5  psupport1  psupport2  psupport3  psupport4  psupport5  political_ideology  happy  slf_ladder  riskperc1  riskperc2  self_esteem  sbelong1  sbelong2  sbelong3  sbelong4  optim1  optim2  slfcont1  slfcont2  slfcont3  slfcont4
##   2   2  children  cnarc1  cnarc2  cnarc3  ctheory1  ctheory2  ctheory3  ctheory4  CRT1  CRT2  CRT3  employ_status1  generosity1  generosity2  generosity3  health_cond  mor_circle  moralid1  moralid10  moralid2  moralid3  moralid4  moralid5  moralid6  moralid7  moralid8  moralid9  mcoop1  mcoop2  mcoop3  mcoop4  mcoop5  mcoop6  mcoop7  narc1  narc2  narc3  narc4  narc5  narc6  nidentity1  nidentity2  omind1  omind2  omind3  omind4  omind5  omind6  contact1  contact2  contact3  contact4  contact5  hygiene1  hygiene2  hygiene3  hygiene4  hygiene5  psupport1  psupport2  psupport3  psupport4  psupport5  political_ideology  happy  slf_ladder  riskperc1  riskperc2  self_esteem  sbelong1  sbelong2  sbelong3  sbelong4  optim1  optim2  slfcont1  slfcont2  slfcont3  slfcont4
##   2   3  children  cnarc1  cnarc2  cnarc3  ctheory1  ctheory2  ctheory3  ctheory4  CRT1  CRT2  CRT3  employ_status1  generosity1  generosity2  generosity3  health_cond  mor_circle  moralid1  moralid10  moralid2  moralid3  moralid4  moralid5  moralid6  moralid7  moralid8  moralid9  mcoop1  mcoop2  mcoop3  mcoop4  mcoop5  mcoop6  mcoop7  narc1  narc2  narc3  narc4  narc5  narc6  nidentity1  nidentity2  omind1  omind2  omind3  omind4  omind5  omind6  contact1  contact2  contact3  contact4  contact5  hygiene1  hygiene2  hygiene3  hygiene4  hygiene5  psupport1  psupport2  psupport3  psupport4  psupport5  political_ideology  happy  slf_ladder  riskperc1  riskperc2  self_esteem  sbelong1  sbelong2  sbelong3  sbelong4  optim1  optim2  slfcont1  slfcont2  slfcont3  slfcont4
##   2   4  children  cnarc1  cnarc2  cnarc3  ctheory1  ctheory2  ctheory3  ctheory4  CRT1  CRT2  CRT3  employ_status1  generosity1  generosity2  generosity3  health_cond  mor_circle  moralid1  moralid10  moralid2  moralid3  moralid4  moralid5  moralid6  moralid7  moralid8  moralid9  mcoop1  mcoop2  mcoop3  mcoop4  mcoop5  mcoop6  mcoop7  narc1  narc2  narc3  narc4  narc5  narc6  nidentity1  nidentity2  omind1  omind2  omind3  omind4  omind5  omind6  contact1  contact2  contact3  contact4  contact5  hygiene1  hygiene2  hygiene3  hygiene4  hygiene5  psupport1  psupport2  psupport3  psupport4  psupport5  political_ideology  happy  slf_ladder  riskperc1  riskperc2  self_esteem  sbelong1  sbelong2  sbelong3  sbelong4  optim1  optim2  slfcont1  slfcont2  slfcont3  slfcont4
##   2   5  children  cnarc1  cnarc2  cnarc3  ctheory1  ctheory2  ctheory3  ctheory4  CRT1  CRT2  CRT3  employ_status1  generosity1  generosity2  generosity3  health_cond  mor_circle  moralid1  moralid10  moralid2  moralid3  moralid4  moralid5  moralid6  moralid7  moralid8  moralid9  mcoop1  mcoop2  mcoop3  mcoop4  mcoop5  mcoop6  mcoop7  narc1  narc2  narc3  narc4  narc5  narc6  nidentity1  nidentity2  omind1  omind2  omind3  omind4  omind5  omind6  contact1  contact2  contact3  contact4  contact5  hygiene1  hygiene2  hygiene3  hygiene4  hygiene5  psupport1  psupport2  psupport3  psupport4  psupport5  political_ideology  happy  slf_ladder  riskperc1  riskperc2  self_esteem  sbelong1  sbelong2  sbelong3  sbelong4  optim1  optim2  slfcont1  slfcont2  slfcont3  slfcont4
##   2   6  children  cnarc1  cnarc2  cnarc3  ctheory1  ctheory2  ctheory3  ctheory4  CRT1  CRT2  CRT3  employ_status1  generosity1  generosity2  generosity3  health_cond  mor_circle  moralid1  moralid10  moralid2  moralid3  moralid4  moralid5  moralid6  moralid7  moralid8  moralid9  mcoop1  mcoop2  mcoop3  mcoop4  mcoop5  mcoop6  mcoop7  narc1  narc2  narc3  narc4  narc5  narc6  nidentity1  nidentity2  omind1  omind2  omind3  omind4  omind5  omind6  contact1  contact2  contact3  contact4  contact5  hygiene1  hygiene2  hygiene3  hygiene4  hygiene5  psupport1  psupport2  psupport3  psupport4  psupport5  political_ideology  happy  slf_ladder  riskperc1  riskperc2  self_esteem  sbelong1  sbelong2  sbelong3  sbelong4  optim1  optim2  slfcont1  slfcont2  slfcont3  slfcont4
##   2   7  children  cnarc1  cnarc2  cnarc3  ctheory1  ctheory2  ctheory3  ctheory4  CRT1  CRT2  CRT3  employ_status1  generosity1  generosity2  generosity3  health_cond  mor_circle  moralid1  moralid10  moralid2  moralid3  moralid4  moralid5  moralid6  moralid7  moralid8  moralid9  mcoop1  mcoop2  mcoop3  mcoop4  mcoop5  mcoop6  mcoop7  narc1  narc2  narc3  narc4  narc5  narc6  nidentity1  nidentity2  omind1  omind2  omind3  omind4  omind5  omind6  contact1  contact2  contact3  contact4  contact5  hygiene1  hygiene2  hygiene3  hygiene4  hygiene5  psupport1  psupport2  psupport3  psupport4  psupport5  political_ideology  happy  slf_ladder  riskperc1  riskperc2  self_esteem  sbelong1  sbelong2  sbelong3  sbelong4  optim1  optim2  slfcont1  slfcont2  slfcont3  slfcont4
##   2   8  children  cnarc1  cnarc2  cnarc3  ctheory1  ctheory2  ctheory3  ctheory4  CRT1  CRT2  CRT3  employ_status1  generosity1  generosity2  generosity3  health_cond  mor_circle  moralid1  moralid10  moralid2  moralid3  moralid4  moralid5  moralid6  moralid7  moralid8  moralid9  mcoop1  mcoop2  mcoop3  mcoop4  mcoop5  mcoop6  mcoop7  narc1  narc2  narc3  narc4  narc5  narc6  nidentity1  nidentity2  omind1  omind2  omind3  omind4  omind5  omind6  contact1  contact2  contact3  contact4  contact5  hygiene1  hygiene2  hygiene3  hygiene4  hygiene5  psupport1  psupport2  psupport3  psupport4  psupport5  political_ideology  happy  slf_ladder  riskperc1  riskperc2  self_esteem  sbelong1  sbelong2  sbelong3  sbelong4  optim1  optim2  slfcont1  slfcont2  slfcont3  slfcont4
##   2   9  children  cnarc1  cnarc2  cnarc3  ctheory1  ctheory2  ctheory3  ctheory4  CRT1  CRT2  CRT3  employ_status1  generosity1  generosity2  generosity3  health_cond  mor_circle  moralid1  moralid10  moralid2  moralid3  moralid4  moralid5  moralid6  moralid7  moralid8  moralid9  mcoop1  mcoop2  mcoop3  mcoop4  mcoop5  mcoop6  mcoop7  narc1  narc2  narc3  narc4  narc5  narc6  nidentity1  nidentity2  omind1  omind2  omind3  omind4  omind5  omind6  contact1  contact2  contact3  contact4  contact5  hygiene1  hygiene2  hygiene3  hygiene4  hygiene5  psupport1  psupport2  psupport3  psupport4  psupport5  political_ideology  happy  slf_ladder  riskperc1  riskperc2  self_esteem  sbelong1  sbelong2  sbelong3  sbelong4  optim1  optim2  slfcont1  slfcont2  slfcont3  slfcont4
##   2   10  children  cnarc1  cnarc2  cnarc3  ctheory1  ctheory2  ctheory3  ctheory4  CRT1  CRT2  CRT3  employ_status1  generosity1  generosity2  generosity3  health_cond  mor_circle  moralid1  moralid10  moralid2  moralid3  moralid4  moralid5  moralid6  moralid7  moralid8  moralid9  mcoop1  mcoop2  mcoop3  mcoop4  mcoop5  mcoop6  mcoop7  narc1  narc2  narc3  narc4  narc5  narc6  nidentity1  nidentity2  omind1  omind2  omind3  omind4  omind5  omind6  contact1  contact2  contact3  contact4  contact5  hygiene1  hygiene2  hygiene3  hygiene4  hygiene5  psupport1  psupport2  psupport3  psupport4  psupport5  political_ideology  happy  slf_ladder  riskperc1  riskperc2  self_esteem  sbelong1  sbelong2  sbelong3  sbelong4  optim1  optim2  slfcont1  slfcont2  slfcont3  slfcont4
##   3   1  children  cnarc1  cnarc2  cnarc3  ctheory1  ctheory2  ctheory3  ctheory4  CRT1  CRT2  CRT3  employ_status1  generosity1  generosity2  generosity3  health_cond  mor_circle  moralid1  moralid10  moralid2  moralid3  moralid4  moralid5  moralid6  moralid7  moralid8  moralid9  mcoop1  mcoop2  mcoop3  mcoop4  mcoop5  mcoop6  mcoop7  narc1  narc2  narc3  narc4  narc5  narc6  nidentity1  nidentity2  omind1  omind2  omind3  omind4  omind5  omind6  contact1  contact2  contact3  contact4  contact5  hygiene1  hygiene2  hygiene3  hygiene4  hygiene5  psupport1  psupport2  psupport3  psupport4  psupport5  political_ideology  happy  slf_ladder  riskperc1  riskperc2  self_esteem  sbelong1  sbelong2  sbelong3  sbelong4  optim1  optim2  slfcont1  slfcont2  slfcont3  slfcont4
##   3   2  children  cnarc1  cnarc2  cnarc3  ctheory1  ctheory2  ctheory3  ctheory4  CRT1  CRT2  CRT3  employ_status1  generosity1  generosity2  generosity3  health_cond  mor_circle  moralid1  moralid10  moralid2  moralid3  moralid4  moralid5  moralid6  moralid7  moralid8  moralid9  mcoop1  mcoop2  mcoop3  mcoop4  mcoop5  mcoop6  mcoop7  narc1  narc2  narc3  narc4  narc5  narc6  nidentity1  nidentity2  omind1  omind2  omind3  omind4  omind5  omind6  contact1  contact2  contact3  contact4  contact5  hygiene1  hygiene2  hygiene3  hygiene4  hygiene5  psupport1  psupport2  psupport3  psupport4  psupport5  political_ideology  happy  slf_ladder  riskperc1  riskperc2  self_esteem  sbelong1  sbelong2  sbelong3  sbelong4  optim1  optim2  slfcont1  slfcont2  slfcont3  slfcont4
##   3   3  children  cnarc1  cnarc2  cnarc3  ctheory1  ctheory2  ctheory3  ctheory4  CRT1  CRT2  CRT3  employ_status1  generosity1  generosity2  generosity3  health_cond  mor_circle  moralid1  moralid10  moralid2  moralid3  moralid4  moralid5  moralid6  moralid7  moralid8  moralid9  mcoop1  mcoop2  mcoop3  mcoop4  mcoop5  mcoop6  mcoop7  narc1  narc2  narc3  narc4  narc5  narc6  nidentity1  nidentity2  omind1  omind2  omind3  omind4  omind5  omind6  contact1  contact2  contact3  contact4  contact5  hygiene1  hygiene2  hygiene3  hygiene4  hygiene5  psupport1  psupport2  psupport3  psupport4  psupport5  political_ideology  happy  slf_ladder  riskperc1  riskperc2  self_esteem  sbelong1  sbelong2  sbelong3  sbelong4  optim1  optim2  slfcont1  slfcont2  slfcont3  slfcont4
##   3   4  children  cnarc1  cnarc2  cnarc3  ctheory1  ctheory2  ctheory3  ctheory4  CRT1  CRT2  CRT3  employ_status1  generosity1  generosity2  generosity3  health_cond  mor_circle  moralid1  moralid10  moralid2  moralid3  moralid4  moralid5  moralid6  moralid7  moralid8  moralid9  mcoop1  mcoop2  mcoop3  mcoop4  mcoop5  mcoop6  mcoop7  narc1  narc2  narc3  narc4  narc5  narc6  nidentity1  nidentity2  omind1  omind2  omind3  omind4  omind5  omind6  contact1  contact2  contact3  contact4  contact5  hygiene1  hygiene2  hygiene3  hygiene4  hygiene5  psupport1  psupport2  psupport3  psupport4  psupport5  political_ideology  happy  slf_ladder  riskperc1  riskperc2  self_esteem  sbelong1  sbelong2  sbelong3  sbelong4  optim1  optim2  slfcont1  slfcont2  slfcont3  slfcont4
##   3   5  children  cnarc1  cnarc2  cnarc3  ctheory1  ctheory2  ctheory3  ctheory4  CRT1  CRT2  CRT3  employ_status1  generosity1  generosity2  generosity3  health_cond  mor_circle  moralid1  moralid10  moralid2  moralid3  moralid4  moralid5  moralid6  moralid7  moralid8  moralid9  mcoop1  mcoop2  mcoop3  mcoop4  mcoop5  mcoop6  mcoop7  narc1  narc2  narc3  narc4  narc5  narc6  nidentity1  nidentity2  omind1  omind2  omind3  omind4  omind5  omind6  contact1  contact2  contact3  contact4  contact5  hygiene1  hygiene2  hygiene3  hygiene4  hygiene5  psupport1  psupport2  psupport3  psupport4  psupport5  political_ideology  happy  slf_ladder  riskperc1  riskperc2  self_esteem  sbelong1  sbelong2  sbelong3  sbelong4  optim1  optim2  slfcont1  slfcont2  slfcont3  slfcont4
##   3   6  children  cnarc1  cnarc2  cnarc3  ctheory1  ctheory2  ctheory3  ctheory4  CRT1  CRT2  CRT3  employ_status1  generosity1  generosity2  generosity3  health_cond  mor_circle  moralid1  moralid10  moralid2  moralid3  moralid4  moralid5  moralid6  moralid7  moralid8  moralid9  mcoop1  mcoop2  mcoop3  mcoop4  mcoop5  mcoop6  mcoop7  narc1  narc2  narc3  narc4  narc5  narc6  nidentity1  nidentity2  omind1  omind2  omind3  omind4  omind5  omind6  contact1  contact2  contact3  contact4  contact5  hygiene1  hygiene2  hygiene3  hygiene4  hygiene5  psupport1  psupport2  psupport3  psupport4  psupport5  political_ideology  happy  slf_ladder  riskperc1  riskperc2  self_esteem  sbelong1  sbelong2  sbelong3  sbelong4  optim1  optim2  slfcont1  slfcont2  slfcont3  slfcont4
##   3   7  children  cnarc1  cnarc2  cnarc3  ctheory1  ctheory2  ctheory3  ctheory4  CRT1  CRT2  CRT3  employ_status1  generosity1  generosity2  generosity3  health_cond  mor_circle  moralid1  moralid10  moralid2  moralid3  moralid4  moralid5  moralid6  moralid7  moralid8  moralid9  mcoop1  mcoop2  mcoop3  mcoop4  mcoop5  mcoop6  mcoop7  narc1  narc2  narc3  narc4  narc5  narc6  nidentity1  nidentity2  omind1  omind2  omind3  omind4  omind5  omind6  contact1  contact2  contact3  contact4  contact5  hygiene1  hygiene2  hygiene3  hygiene4  hygiene5  psupport1  psupport2  psupport3  psupport4  psupport5  political_ideology  happy  slf_ladder  riskperc1  riskperc2  self_esteem  sbelong1  sbelong2  sbelong3  sbelong4  optim1  optim2  slfcont1  slfcont2  slfcont3  slfcont4
##   3   8  children  cnarc1  cnarc2  cnarc3  ctheory1  ctheory2  ctheory3  ctheory4  CRT1  CRT2  CRT3  employ_status1  generosity1  generosity2  generosity3  health_cond  mor_circle  moralid1  moralid10  moralid2  moralid3  moralid4  moralid5  moralid6  moralid7  moralid8  moralid9  mcoop1  mcoop2  mcoop3  mcoop4  mcoop5  mcoop6  mcoop7  narc1  narc2  narc3  narc4  narc5  narc6  nidentity1  nidentity2  omind1  omind2  omind3  omind4  omind5  omind6  contact1  contact2  contact3  contact4  contact5  hygiene1  hygiene2  hygiene3  hygiene4  hygiene5  psupport1  psupport2  psupport3  psupport4  psupport5  political_ideology  happy  slf_ladder  riskperc1  riskperc2  self_esteem  sbelong1  sbelong2  sbelong3  sbelong4  optim1  optim2  slfcont1  slfcont2  slfcont3  slfcont4
##   3   9  children  cnarc1  cnarc2  cnarc3  ctheory1  ctheory2  ctheory3  ctheory4  CRT1  CRT2  CRT3  employ_status1  generosity1  generosity2  generosity3  health_cond  mor_circle  moralid1  moralid10  moralid2  moralid3  moralid4  moralid5  moralid6  moralid7  moralid8  moralid9  mcoop1  mcoop2  mcoop3  mcoop4  mcoop5  mcoop6  mcoop7  narc1  narc2  narc3  narc4  narc5  narc6  nidentity1  nidentity2  omind1  omind2  omind3  omind4  omind5  omind6  contact1  contact2  contact3  contact4  contact5  hygiene1  hygiene2  hygiene3  hygiene4  hygiene5  psupport1  psupport2  psupport3  psupport4  psupport5  political_ideology  happy  slf_ladder  riskperc1  riskperc2  self_esteem  sbelong1  sbelong2  sbelong3  sbelong4  optim1  optim2  slfcont1  slfcont2  slfcont3  slfcont4
##   3   10  children  cnarc1  cnarc2  cnarc3  ctheory1  ctheory2  ctheory3  ctheory4  CRT1  CRT2  CRT3  employ_status1  generosity1  generosity2  generosity3  health_cond  mor_circle  moralid1  moralid10  moralid2  moralid3  moralid4  moralid5  moralid6  moralid7  moralid8  moralid9  mcoop1  mcoop2  mcoop3  mcoop4  mcoop5  mcoop6  mcoop7  narc1  narc2  narc3  narc4  narc5  narc6  nidentity1  nidentity2  omind1  omind2  omind3  omind4  omind5  omind6  contact1  contact2  contact3  contact4  contact5  hygiene1  hygiene2  hygiene3  hygiene4  hygiene5  psupport1  psupport2  psupport3  psupport4  psupport5  political_ideology  happy  slf_ladder  riskperc1  riskperc2  self_esteem  sbelong1  sbelong2  sbelong3  sbelong4  optim1  optim2  slfcont1  slfcont2  slfcont3  slfcont4
##   4   1  children  cnarc1  cnarc2  cnarc3  ctheory1  ctheory2  ctheory3  ctheory4  CRT1  CRT2  CRT3  employ_status1  generosity1  generosity2  generosity3  health_cond  mor_circle  moralid1  moralid10  moralid2  moralid3  moralid4  moralid5  moralid6  moralid7  moralid8  moralid9  mcoop1  mcoop2  mcoop3  mcoop4  mcoop5  mcoop6  mcoop7  narc1  narc2  narc3  narc4  narc5  narc6  nidentity1  nidentity2  omind1  omind2  omind3  omind4  omind5  omind6  contact1  contact2  contact3  contact4  contact5  hygiene1  hygiene2  hygiene3  hygiene4  hygiene5  psupport1  psupport2  psupport3  psupport4  psupport5  political_ideology  happy  slf_ladder  riskperc1  riskperc2  self_esteem  sbelong1  sbelong2  sbelong3  sbelong4  optim1  optim2  slfcont1  slfcont2  slfcont3  slfcont4
##   4   2  children  cnarc1  cnarc2  cnarc3  ctheory1  ctheory2  ctheory3  ctheory4  CRT1  CRT2  CRT3  employ_status1  generosity1  generosity2  generosity3  health_cond  mor_circle  moralid1  moralid10  moralid2  moralid3  moralid4  moralid5  moralid6  moralid7  moralid8  moralid9  mcoop1  mcoop2  mcoop3  mcoop4  mcoop5  mcoop6  mcoop7  narc1  narc2  narc3  narc4  narc5  narc6  nidentity1  nidentity2  omind1  omind2  omind3  omind4  omind5  omind6  contact1  contact2  contact3  contact4  contact5  hygiene1  hygiene2  hygiene3  hygiene4  hygiene5  psupport1  psupport2  psupport3  psupport4  psupport5  political_ideology  happy  slf_ladder  riskperc1  riskperc2  self_esteem  sbelong1  sbelong2  sbelong3  sbelong4  optim1  optim2  slfcont1  slfcont2  slfcont3  slfcont4
##   4   3  children  cnarc1  cnarc2  cnarc3  ctheory1  ctheory2  ctheory3  ctheory4  CRT1  CRT2  CRT3  employ_status1  generosity1  generosity2  generosity3  health_cond  mor_circle  moralid1  moralid10  moralid2  moralid3  moralid4  moralid5  moralid6  moralid7  moralid8  moralid9  mcoop1  mcoop2  mcoop3  mcoop4  mcoop5  mcoop6  mcoop7  narc1  narc2  narc3  narc4  narc5  narc6  nidentity1  nidentity2  omind1  omind2  omind3  omind4  omind5  omind6  contact1  contact2  contact3  contact4  contact5  hygiene1  hygiene2  hygiene3  hygiene4  hygiene5  psupport1  psupport2  psupport3  psupport4  psupport5  political_ideology  happy  slf_ladder  riskperc1  riskperc2  self_esteem  sbelong1  sbelong2  sbelong3  sbelong4  optim1  optim2  slfcont1  slfcont2  slfcont3  slfcont4
##   4   4  children  cnarc1  cnarc2  cnarc3  ctheory1  ctheory2  ctheory3  ctheory4  CRT1  CRT2  CRT3  employ_status1  generosity1  generosity2  generosity3  health_cond  mor_circle  moralid1  moralid10  moralid2  moralid3  moralid4  moralid5  moralid6  moralid7  moralid8  moralid9  mcoop1  mcoop2  mcoop3  mcoop4  mcoop5  mcoop6  mcoop7  narc1  narc2  narc3  narc4  narc5  narc6  nidentity1  nidentity2  omind1  omind2  omind3  omind4  omind5  omind6  contact1  contact2  contact3  contact4  contact5  hygiene1  hygiene2  hygiene3  hygiene4  hygiene5  psupport1  psupport2  psupport3  psupport4  psupport5  political_ideology  happy  slf_ladder  riskperc1  riskperc2  self_esteem  sbelong1  sbelong2  sbelong3  sbelong4  optim1  optim2  slfcont1  slfcont2  slfcont3  slfcont4
##   4   5  children  cnarc1  cnarc2  cnarc3  ctheory1  ctheory2  ctheory3  ctheory4  CRT1  CRT2  CRT3  employ_status1  generosity1  generosity2  generosity3  health_cond  mor_circle  moralid1  moralid10  moralid2  moralid3  moralid4  moralid5  moralid6  moralid7  moralid8  moralid9  mcoop1  mcoop2  mcoop3  mcoop4  mcoop5  mcoop6  mcoop7  narc1  narc2  narc3  narc4  narc5  narc6  nidentity1  nidentity2  omind1  omind2  omind3  omind4  omind5  omind6  contact1  contact2  contact3  contact4  contact5  hygiene1  hygiene2  hygiene3  hygiene4  hygiene5  psupport1  psupport2  psupport3  psupport4  psupport5  political_ideology  happy  slf_ladder  riskperc1  riskperc2  self_esteem  sbelong1  sbelong2  sbelong3  sbelong4  optim1  optim2  slfcont1  slfcont2  slfcont3  slfcont4
##   4   6  children  cnarc1  cnarc2  cnarc3  ctheory1  ctheory2  ctheory3  ctheory4  CRT1  CRT2  CRT3  employ_status1  generosity1  generosity2  generosity3  health_cond  mor_circle  moralid1  moralid10  moralid2  moralid3  moralid4  moralid5  moralid6  moralid7  moralid8  moralid9  mcoop1  mcoop2  mcoop3  mcoop4  mcoop5  mcoop6  mcoop7  narc1  narc2  narc3  narc4  narc5  narc6  nidentity1  nidentity2  omind1  omind2  omind3  omind4  omind5  omind6  contact1  contact2  contact3  contact4  contact5  hygiene1  hygiene2  hygiene3  hygiene4  hygiene5  psupport1  psupport2  psupport3  psupport4  psupport5  political_ideology  happy  slf_ladder  riskperc1  riskperc2  self_esteem  sbelong1  sbelong2  sbelong3  sbelong4  optim1  optim2  slfcont1  slfcont2  slfcont3  slfcont4
##   4   7  children  cnarc1  cnarc2  cnarc3  ctheory1  ctheory2  ctheory3  ctheory4  CRT1  CRT2  CRT3  employ_status1  generosity1  generosity2  generosity3  health_cond  mor_circle  moralid1  moralid10  moralid2  moralid3  moralid4  moralid5  moralid6  moralid7  moralid8  moralid9  mcoop1  mcoop2  mcoop3  mcoop4  mcoop5  mcoop6  mcoop7  narc1  narc2  narc3  narc4  narc5  narc6  nidentity1  nidentity2  omind1  omind2  omind3  omind4  omind5  omind6  contact1  contact2  contact3  contact4  contact5  hygiene1  hygiene2  hygiene3  hygiene4  hygiene5  psupport1  psupport2  psupport3  psupport4  psupport5  political_ideology  happy  slf_ladder  riskperc1  riskperc2  self_esteem  sbelong1  sbelong2  sbelong3  sbelong4  optim1  optim2  slfcont1  slfcont2  slfcont3  slfcont4
##   4   8  children  cnarc1  cnarc2  cnarc3  ctheory1  ctheory2  ctheory3  ctheory4  CRT1  CRT2  CRT3  employ_status1  generosity1  generosity2  generosity3  health_cond  mor_circle  moralid1  moralid10  moralid2  moralid3  moralid4  moralid5  moralid6  moralid7  moralid8  moralid9  mcoop1  mcoop2  mcoop3  mcoop4  mcoop5  mcoop6  mcoop7  narc1  narc2  narc3  narc4  narc5  narc6  nidentity1  nidentity2  omind1  omind2  omind3  omind4  omind5  omind6  contact1  contact2  contact3  contact4  contact5  hygiene1  hygiene2  hygiene3  hygiene4  hygiene5  psupport1  psupport2  psupport3  psupport4  psupport5  political_ideology  happy  slf_ladder  riskperc1  riskperc2  self_esteem  sbelong1  sbelong2  sbelong3  sbelong4  optim1  optim2  slfcont1  slfcont2  slfcont3  slfcont4
##   4   9  children  cnarc1  cnarc2  cnarc3  ctheory1  ctheory2  ctheory3  ctheory4  CRT1  CRT2  CRT3  employ_status1  generosity1  generosity2  generosity3  health_cond  mor_circle  moralid1  moralid10  moralid2  moralid3  moralid4  moralid5  moralid6  moralid7  moralid8  moralid9  mcoop1  mcoop2  mcoop3  mcoop4  mcoop5  mcoop6  mcoop7  narc1  narc2  narc3  narc4  narc5  narc6  nidentity1  nidentity2  omind1  omind2  omind3  omind4  omind5  omind6  contact1  contact2  contact3  contact4  contact5  hygiene1  hygiene2  hygiene3  hygiene4  hygiene5  psupport1  psupport2  psupport3  psupport4  psupport5  political_ideology  happy  slf_ladder  riskperc1  riskperc2  self_esteem  sbelong1  sbelong2  sbelong3  sbelong4  optim1  optim2  slfcont1  slfcont2  slfcont3  slfcont4
##   4   10  children  cnarc1  cnarc2  cnarc3  ctheory1  ctheory2  ctheory3  ctheory4  CRT1  CRT2  CRT3  employ_status1  generosity1  generosity2  generosity3  health_cond  mor_circle  moralid1  moralid10  moralid2  moralid3  moralid4  moralid5  moralid6  moralid7  moralid8  moralid9  mcoop1  mcoop2  mcoop3  mcoop4  mcoop5  mcoop6  mcoop7  narc1  narc2  narc3  narc4  narc5  narc6  nidentity1  nidentity2  omind1  omind2  omind3  omind4  omind5  omind6  contact1  contact2  contact3  contact4  contact5  hygiene1  hygiene2  hygiene3  hygiene4  hygiene5  psupport1  psupport2  psupport3  psupport4  psupport5  political_ideology  happy  slf_ladder  riskperc1  riskperc2  self_esteem  sbelong1  sbelong2  sbelong3  sbelong4  optim1  optim2  slfcont1  slfcont2  slfcont3  slfcont4
##   5   1  children  cnarc1  cnarc2  cnarc3  ctheory1  ctheory2  ctheory3  ctheory4  CRT1  CRT2  CRT3  employ_status1  generosity1  generosity2  generosity3  health_cond  mor_circle  moralid1  moralid10  moralid2  moralid3  moralid4  moralid5  moralid6  moralid7  moralid8  moralid9  mcoop1  mcoop2  mcoop3  mcoop4  mcoop5  mcoop6  mcoop7  narc1  narc2  narc3  narc4  narc5  narc6  nidentity1  nidentity2  omind1  omind2  omind3  omind4  omind5  omind6  contact1  contact2  contact3  contact4  contact5  hygiene1  hygiene2  hygiene3  hygiene4  hygiene5  psupport1  psupport2  psupport3  psupport4  psupport5  political_ideology  happy  slf_ladder  riskperc1  riskperc2  self_esteem  sbelong1  sbelong2  sbelong3  sbelong4  optim1  optim2  slfcont1  slfcont2  slfcont3  slfcont4
##   5   2  children  cnarc1  cnarc2  cnarc3  ctheory1  ctheory2  ctheory3  ctheory4  CRT1  CRT2  CRT3  employ_status1  generosity1  generosity2  generosity3  health_cond  mor_circle  moralid1  moralid10  moralid2  moralid3  moralid4  moralid5  moralid6  moralid7  moralid8  moralid9  mcoop1  mcoop2  mcoop3  mcoop4  mcoop5  mcoop6  mcoop7  narc1  narc2  narc3  narc4  narc5  narc6  nidentity1  nidentity2  omind1  omind2  omind3  omind4  omind5  omind6  contact1  contact2  contact3  contact4  contact5  hygiene1  hygiene2  hygiene3  hygiene4  hygiene5  psupport1  psupport2  psupport3  psupport4  psupport5  political_ideology  happy  slf_ladder  riskperc1  riskperc2  self_esteem  sbelong1  sbelong2  sbelong3  sbelong4  optim1  optim2  slfcont1  slfcont2  slfcont3  slfcont4
##   5   3  children  cnarc1  cnarc2  cnarc3  ctheory1  ctheory2  ctheory3  ctheory4  CRT1  CRT2  CRT3  employ_status1  generosity1  generosity2  generosity3  health_cond  mor_circle  moralid1  moralid10  moralid2  moralid3  moralid4  moralid5  moralid6  moralid7  moralid8  moralid9  mcoop1  mcoop2  mcoop3  mcoop4  mcoop5  mcoop6  mcoop7  narc1  narc2  narc3  narc4  narc5  narc6  nidentity1  nidentity2  omind1  omind2  omind3  omind4  omind5  omind6  contact1  contact2  contact3  contact4  contact5  hygiene1  hygiene2  hygiene3  hygiene4  hygiene5  psupport1  psupport2  psupport3  psupport4  psupport5  political_ideology  happy  slf_ladder  riskperc1  riskperc2  self_esteem  sbelong1  sbelong2  sbelong3  sbelong4  optim1  optim2  slfcont1  slfcont2  slfcont3  slfcont4
##   5   4  children  cnarc1  cnarc2  cnarc3  ctheory1  ctheory2  ctheory3  ctheory4  CRT1  CRT2  CRT3  employ_status1  generosity1  generosity2  generosity3  health_cond  mor_circle  moralid1  moralid10  moralid2  moralid3  moralid4  moralid5  moralid6  moralid7  moralid8  moralid9  mcoop1  mcoop2  mcoop3  mcoop4  mcoop5  mcoop6  mcoop7  narc1  narc2  narc3  narc4  narc5  narc6  nidentity1  nidentity2  omind1  omind2  omind3  omind4  omind5  omind6  contact1  contact2  contact3  contact4  contact5  hygiene1  hygiene2  hygiene3  hygiene4  hygiene5  psupport1  psupport2  psupport3  psupport4  psupport5  political_ideology  happy  slf_ladder  riskperc1  riskperc2  self_esteem  sbelong1  sbelong2  sbelong3  sbelong4  optim1  optim2  slfcont1  slfcont2  slfcont3  slfcont4
##   5   5  children  cnarc1  cnarc2  cnarc3  ctheory1  ctheory2  ctheory3  ctheory4  CRT1  CRT2  CRT3  employ_status1  generosity1  generosity2  generosity3  health_cond  mor_circle  moralid1  moralid10  moralid2  moralid3  moralid4  moralid5  moralid6  moralid7  moralid8  moralid9  mcoop1  mcoop2  mcoop3  mcoop4  mcoop5  mcoop6  mcoop7  narc1  narc2  narc3  narc4  narc5  narc6  nidentity1  nidentity2  omind1  omind2  omind3  omind4  omind5  omind6  contact1  contact2  contact3  contact4  contact5  hygiene1  hygiene2  hygiene3  hygiene4  hygiene5  psupport1  psupport2  psupport3  psupport4  psupport5  political_ideology  happy  slf_ladder  riskperc1  riskperc2  self_esteem  sbelong1  sbelong2  sbelong3  sbelong4  optim1  optim2  slfcont1  slfcont2  slfcont3  slfcont4
##   5   6  children  cnarc1  cnarc2  cnarc3  ctheory1  ctheory2  ctheory3  ctheory4  CRT1  CRT2  CRT3  employ_status1  generosity1  generosity2  generosity3  health_cond  mor_circle  moralid1  moralid10  moralid2  moralid3  moralid4  moralid5  moralid6  moralid7  moralid8  moralid9  mcoop1  mcoop2  mcoop3  mcoop4  mcoop5  mcoop6  mcoop7  narc1  narc2  narc3  narc4  narc5  narc6  nidentity1  nidentity2  omind1  omind2  omind3  omind4  omind5  omind6  contact1  contact2  contact3  contact4  contact5  hygiene1  hygiene2  hygiene3  hygiene4  hygiene5  psupport1  psupport2  psupport3  psupport4  psupport5  political_ideology  happy  slf_ladder  riskperc1  riskperc2  self_esteem  sbelong1  sbelong2  sbelong3  sbelong4  optim1  optim2  slfcont1  slfcont2  slfcont3  slfcont4
##   5   7  children  cnarc1  cnarc2  cnarc3  ctheory1  ctheory2  ctheory3  ctheory4  CRT1  CRT2  CRT3  employ_status1  generosity1  generosity2  generosity3  health_cond  mor_circle  moralid1  moralid10  moralid2  moralid3  moralid4  moralid5  moralid6  moralid7  moralid8  moralid9  mcoop1  mcoop2  mcoop3  mcoop4  mcoop5  mcoop6  mcoop7  narc1  narc2  narc3  narc4  narc5  narc6  nidentity1  nidentity2  omind1  omind2  omind3  omind4  omind5  omind6  contact1  contact2  contact3  contact4  contact5  hygiene1  hygiene2  hygiene3  hygiene4  hygiene5  psupport1  psupport2  psupport3  psupport4  psupport5  political_ideology  happy  slf_ladder  riskperc1  riskperc2  self_esteem  sbelong1  sbelong2  sbelong3  sbelong4  optim1  optim2  slfcont1  slfcont2  slfcont3  slfcont4
##   5   8  children  cnarc1  cnarc2  cnarc3  ctheory1  ctheory2  ctheory3  ctheory4  CRT1  CRT2  CRT3  employ_status1  generosity1  generosity2  generosity3  health_cond  mor_circle  moralid1  moralid10  moralid2  moralid3  moralid4  moralid5  moralid6  moralid7  moralid8  moralid9  mcoop1  mcoop2  mcoop3  mcoop4  mcoop5  mcoop6  mcoop7  narc1  narc2  narc3  narc4  narc5  narc6  nidentity1  nidentity2  omind1  omind2  omind3  omind4  omind5  omind6  contact1  contact2  contact3  contact4  contact5  hygiene1  hygiene2  hygiene3  hygiene4  hygiene5  psupport1  psupport2  psupport3  psupport4  psupport5  political_ideology  happy  slf_ladder  riskperc1  riskperc2  self_esteem  sbelong1  sbelong2  sbelong3  sbelong4  optim1  optim2  slfcont1  slfcont2  slfcont3  slfcont4
##   5   9  children  cnarc1  cnarc2  cnarc3  ctheory1  ctheory2  ctheory3  ctheory4  CRT1  CRT2  CRT3  employ_status1  generosity1  generosity2  generosity3  health_cond  mor_circle  moralid1  moralid10  moralid2  moralid3  moralid4  moralid5  moralid6  moralid7  moralid8  moralid9  mcoop1  mcoop2  mcoop3  mcoop4  mcoop5  mcoop6  mcoop7  narc1  narc2  narc3  narc4  narc5  narc6  nidentity1  nidentity2  omind1  omind2  omind3  omind4  omind5  omind6  contact1  contact2  contact3  contact4  contact5  hygiene1  hygiene2  hygiene3  hygiene4  hygiene5  psupport1  psupport2  psupport3  psupport4  psupport5  political_ideology  happy  slf_ladder  riskperc1  riskperc2  self_esteem  sbelong1  sbelong2  sbelong3  sbelong4  optim1  optim2  slfcont1  slfcont2  slfcont3  slfcont4
##   5   10  children  cnarc1  cnarc2  cnarc3  ctheory1  ctheory2  ctheory3  ctheory4  CRT1  CRT2  CRT3  employ_status1  generosity1  generosity2  generosity3  health_cond  mor_circle  moralid1  moralid10  moralid2  moralid3  moralid4  moralid5  moralid6  moralid7  moralid8  moralid9  mcoop1  mcoop2  mcoop3  mcoop4  mcoop5  mcoop6  mcoop7  narc1  narc2  narc3  narc4  narc5  narc6  nidentity1  nidentity2  omind1  omind2  omind3  omind4  omind5  omind6  contact1  contact2  contact3  contact4  contact5  hygiene1  hygiene2  hygiene3  hygiene4  hygiene5  psupport1  psupport2  psupport3  psupport4  psupport5  political_ideology  happy  slf_ladder  riskperc1  riskperc2  self_esteem  sbelong1  sbelong2  sbelong3  sbelong4  optim1  optim2  slfcont1  slfcont2  slfcont3  slfcont4
##   6   1  children  cnarc1  cnarc2  cnarc3  ctheory1  ctheory2  ctheory3  ctheory4  CRT1  CRT2  CRT3  employ_status1  generosity1  generosity2  generosity3  health_cond  mor_circle  moralid1  moralid10  moralid2  moralid3  moralid4  moralid5  moralid6  moralid7  moralid8  moralid9  mcoop1  mcoop2  mcoop3  mcoop4  mcoop5  mcoop6  mcoop7  narc1  narc2  narc3  narc4  narc5  narc6  nidentity1  nidentity2  omind1  omind2  omind3  omind4  omind5  omind6  contact1  contact2  contact3  contact4  contact5  hygiene1  hygiene2  hygiene3  hygiene4  hygiene5  psupport1  psupport2  psupport3  psupport4  psupport5  political_ideology  happy  slf_ladder  riskperc1  riskperc2  self_esteem  sbelong1  sbelong2  sbelong3  sbelong4  optim1  optim2  slfcont1  slfcont2  slfcont3  slfcont4
##   6   2  children  cnarc1  cnarc2  cnarc3  ctheory1  ctheory2  ctheory3  ctheory4  CRT1  CRT2  CRT3  employ_status1  generosity1  generosity2  generosity3  health_cond  mor_circle  moralid1  moralid10  moralid2  moralid3  moralid4  moralid5  moralid6  moralid7  moralid8  moralid9  mcoop1  mcoop2  mcoop3  mcoop4  mcoop5  mcoop6  mcoop7  narc1  narc2  narc3  narc4  narc5  narc6  nidentity1  nidentity2  omind1  omind2  omind3  omind4  omind5  omind6  contact1  contact2  contact3  contact4  contact5  hygiene1  hygiene2  hygiene3  hygiene4  hygiene5  psupport1  psupport2  psupport3  psupport4  psupport5  political_ideology  happy  slf_ladder  riskperc1  riskperc2  self_esteem  sbelong1  sbelong2  sbelong3  sbelong4  optim1  optim2  slfcont1  slfcont2  slfcont3  slfcont4
##   6   3  children  cnarc1  cnarc2  cnarc3  ctheory1  ctheory2  ctheory3  ctheory4  CRT1  CRT2  CRT3  employ_status1  generosity1  generosity2  generosity3  health_cond  mor_circle  moralid1  moralid10  moralid2  moralid3  moralid4  moralid5  moralid6  moralid7  moralid8  moralid9  mcoop1  mcoop2  mcoop3  mcoop4  mcoop5  mcoop6  mcoop7  narc1  narc2  narc3  narc4  narc5  narc6  nidentity1  nidentity2  omind1  omind2  omind3  omind4  omind5  omind6  contact1  contact2  contact3  contact4  contact5  hygiene1  hygiene2  hygiene3  hygiene4  hygiene5  psupport1  psupport2  psupport3  psupport4  psupport5  political_ideology  happy  slf_ladder  riskperc1  riskperc2  self_esteem  sbelong1  sbelong2  sbelong3  sbelong4  optim1  optim2  slfcont1  slfcont2  slfcont3  slfcont4
##   6   4  children  cnarc1  cnarc2  cnarc3  ctheory1  ctheory2  ctheory3  ctheory4  CRT1  CRT2  CRT3  employ_status1  generosity1  generosity2  generosity3  health_cond  mor_circle  moralid1  moralid10  moralid2  moralid3  moralid4  moralid5  moralid6  moralid7  moralid8  moralid9  mcoop1  mcoop2  mcoop3  mcoop4  mcoop5  mcoop6  mcoop7  narc1  narc2  narc3  narc4  narc5  narc6  nidentity1  nidentity2  omind1  omind2  omind3  omind4  omind5  omind6  contact1  contact2  contact3  contact4  contact5  hygiene1  hygiene2  hygiene3  hygiene4  hygiene5  psupport1  psupport2  psupport3  psupport4  psupport5  political_ideology  happy  slf_ladder  riskperc1  riskperc2  self_esteem  sbelong1  sbelong2  sbelong3  sbelong4  optim1  optim2  slfcont1  slfcont2  slfcont3  slfcont4
##   6   5  children  cnarc1  cnarc2  cnarc3  ctheory1  ctheory2  ctheory3  ctheory4  CRT1  CRT2  CRT3  employ_status1  generosity1  generosity2  generosity3  health_cond  mor_circle  moralid1  moralid10  moralid2  moralid3  moralid4  moralid5  moralid6  moralid7  moralid8  moralid9  mcoop1  mcoop2  mcoop3  mcoop4  mcoop5  mcoop6  mcoop7  narc1  narc2  narc3  narc4  narc5  narc6  nidentity1  nidentity2  omind1  omind2  omind3  omind4  omind5  omind6  contact1  contact2  contact3  contact4  contact5  hygiene1  hygiene2  hygiene3  hygiene4  hygiene5  psupport1  psupport2  psupport3  psupport4  psupport5  political_ideology  happy  slf_ladder  riskperc1  riskperc2  self_esteem  sbelong1  sbelong2  sbelong3  sbelong4  optim1  optim2  slfcont1  slfcont2  slfcont3  slfcont4
##   6   6  children  cnarc1  cnarc2  cnarc3  ctheory1  ctheory2  ctheory3  ctheory4  CRT1  CRT2  CRT3  employ_status1  generosity1  generosity2  generosity3  health_cond  mor_circle  moralid1  moralid10  moralid2  moralid3  moralid4  moralid5  moralid6  moralid7  moralid8  moralid9  mcoop1  mcoop2  mcoop3  mcoop4  mcoop5  mcoop6  mcoop7  narc1  narc2  narc3  narc4  narc5  narc6  nidentity1  nidentity2  omind1  omind2  omind3  omind4  omind5  omind6  contact1  contact2  contact3  contact4  contact5  hygiene1  hygiene2  hygiene3  hygiene4  hygiene5  psupport1  psupport2  psupport3  psupport4  psupport5  political_ideology  happy  slf_ladder  riskperc1  riskperc2  self_esteem  sbelong1  sbelong2  sbelong3  sbelong4  optim1  optim2  slfcont1  slfcont2  slfcont3  slfcont4
##   6   7  children  cnarc1  cnarc2  cnarc3  ctheory1  ctheory2  ctheory3  ctheory4  CRT1  CRT2  CRT3  employ_status1  generosity1  generosity2  generosity3  health_cond  mor_circle  moralid1  moralid10  moralid2  moralid3  moralid4  moralid5  moralid6  moralid7  moralid8  moralid9  mcoop1  mcoop2  mcoop3  mcoop4  mcoop5  mcoop6  mcoop7  narc1  narc2  narc3  narc4  narc5  narc6  nidentity1  nidentity2  omind1  omind2  omind3  omind4  omind5  omind6  contact1  contact2  contact3  contact4  contact5  hygiene1  hygiene2  hygiene3  hygiene4  hygiene5  psupport1  psupport2  psupport3  psupport4  psupport5  political_ideology  happy  slf_ladder  riskperc1  riskperc2  self_esteem  sbelong1  sbelong2  sbelong3  sbelong4  optim1  optim2  slfcont1  slfcont2  slfcont3  slfcont4
##   6   8  children  cnarc1  cnarc2  cnarc3  ctheory1  ctheory2  ctheory3  ctheory4  CRT1  CRT2  CRT3  employ_status1  generosity1  generosity2  generosity3  health_cond  mor_circle  moralid1  moralid10  moralid2  moralid3  moralid4  moralid5  moralid6  moralid7  moralid8  moralid9  mcoop1  mcoop2  mcoop3  mcoop4  mcoop5  mcoop6  mcoop7  narc1  narc2  narc3  narc4  narc5  narc6  nidentity1  nidentity2  omind1  omind2  omind3  omind4  omind5  omind6  contact1  contact2  contact3  contact4  contact5  hygiene1  hygiene2  hygiene3  hygiene4  hygiene5  psupport1  psupport2  psupport3  psupport4  psupport5  political_ideology  happy  slf_ladder  riskperc1  riskperc2  self_esteem  sbelong1  sbelong2  sbelong3  sbelong4  optim1  optim2  slfcont1  slfcont2  slfcont3  slfcont4
##   6   9  children  cnarc1  cnarc2  cnarc3  ctheory1  ctheory2  ctheory3  ctheory4  CRT1  CRT2  CRT3  employ_status1  generosity1  generosity2  generosity3  health_cond  mor_circle  moralid1  moralid10  moralid2  moralid3  moralid4  moralid5  moralid6  moralid7  moralid8  moralid9  mcoop1  mcoop2  mcoop3  mcoop4  mcoop5  mcoop6  mcoop7  narc1  narc2  narc3  narc4  narc5  narc6  nidentity1  nidentity2  omind1  omind2  omind3  omind4  omind5  omind6  contact1  contact2  contact3  contact4  contact5  hygiene1  hygiene2  hygiene3  hygiene4  hygiene5  psupport1  psupport2  psupport3  psupport4  psupport5  political_ideology  happy  slf_ladder  riskperc1  riskperc2  self_esteem  sbelong1  sbelong2  sbelong3  sbelong4  optim1  optim2  slfcont1  slfcont2  slfcont3  slfcont4
##   6   10  children  cnarc1  cnarc2  cnarc3  ctheory1  ctheory2  ctheory3  ctheory4  CRT1  CRT2  CRT3  employ_status1  generosity1  generosity2  generosity3  health_cond  mor_circle  moralid1  moralid10  moralid2  moralid3  moralid4  moralid5  moralid6  moralid7  moralid8  moralid9  mcoop1  mcoop2  mcoop3  mcoop4  mcoop5  mcoop6  mcoop7  narc1  narc2  narc3  narc4  narc5  narc6  nidentity1  nidentity2  omind1  omind2  omind3  omind4  omind5  omind6  contact1  contact2  contact3  contact4  contact5  hygiene1  hygiene2  hygiene3  hygiene4  hygiene5  psupport1  psupport2  psupport3  psupport4  psupport5  political_ideology  happy  slf_ladder  riskperc1  riskperc2  self_esteem  sbelong1  sbelong2  sbelong3  sbelong4  optim1  optim2  slfcont1  slfcont2  slfcont3  slfcont4
##   7   1  children  cnarc1  cnarc2  cnarc3  ctheory1  ctheory2  ctheory3  ctheory4  CRT1  CRT2  CRT3  employ_status1  generosity1  generosity2  generosity3  health_cond  mor_circle  moralid1  moralid10  moralid2  moralid3  moralid4  moralid5  moralid6  moralid7  moralid8  moralid9  mcoop1  mcoop2  mcoop3  mcoop4  mcoop5  mcoop6  mcoop7  narc1  narc2  narc3  narc4  narc5  narc6  nidentity1  nidentity2  omind1  omind2  omind3  omind4  omind5  omind6  contact1  contact2  contact3  contact4  contact5  hygiene1  hygiene2  hygiene3  hygiene4  hygiene5  psupport1  psupport2  psupport3  psupport4  psupport5  political_ideology  happy  slf_ladder  riskperc1  riskperc2  self_esteem  sbelong1  sbelong2  sbelong3  sbelong4  optim1  optim2  slfcont1  slfcont2  slfcont3  slfcont4
##   7   2  children  cnarc1  cnarc2  cnarc3  ctheory1  ctheory2  ctheory3  ctheory4  CRT1  CRT2  CRT3  employ_status1  generosity1  generosity2  generosity3  health_cond  mor_circle  moralid1  moralid10  moralid2  moralid3  moralid4  moralid5  moralid6  moralid7  moralid8  moralid9  mcoop1  mcoop2  mcoop3  mcoop4  mcoop5  mcoop6  mcoop7  narc1  narc2  narc3  narc4  narc5  narc6  nidentity1  nidentity2  omind1  omind2  omind3  omind4  omind5  omind6  contact1  contact2  contact3  contact4  contact5  hygiene1  hygiene2  hygiene3  hygiene4  hygiene5  psupport1  psupport2  psupport3  psupport4  psupport5  political_ideology  happy  slf_ladder  riskperc1  riskperc2  self_esteem  sbelong1  sbelong2  sbelong3  sbelong4  optim1  optim2  slfcont1  slfcont2  slfcont3  slfcont4
##   7   3  children  cnarc1  cnarc2  cnarc3  ctheory1  ctheory2  ctheory3  ctheory4  CRT1  CRT2  CRT3  employ_status1  generosity1  generosity2  generosity3  health_cond  mor_circle  moralid1  moralid10  moralid2  moralid3  moralid4  moralid5  moralid6  moralid7  moralid8  moralid9  mcoop1  mcoop2  mcoop3  mcoop4  mcoop5  mcoop6  mcoop7  narc1  narc2  narc3  narc4  narc5  narc6  nidentity1  nidentity2  omind1  omind2  omind3  omind4  omind5  omind6  contact1  contact2  contact3  contact4  contact5  hygiene1  hygiene2  hygiene3  hygiene4  hygiene5  psupport1  psupport2  psupport3  psupport4  psupport5  political_ideology  happy  slf_ladder  riskperc1  riskperc2  self_esteem  sbelong1  sbelong2  sbelong3  sbelong4  optim1  optim2  slfcont1  slfcont2  slfcont3  slfcont4
##   7   4  children  cnarc1  cnarc2  cnarc3  ctheory1  ctheory2  ctheory3  ctheory4  CRT1  CRT2  CRT3  employ_status1  generosity1  generosity2  generosity3  health_cond  mor_circle  moralid1  moralid10  moralid2  moralid3  moralid4  moralid5  moralid6  moralid7  moralid8  moralid9  mcoop1  mcoop2  mcoop3  mcoop4  mcoop5  mcoop6  mcoop7  narc1  narc2  narc3  narc4  narc5  narc6  nidentity1  nidentity2  omind1  omind2  omind3  omind4  omind5  omind6  contact1  contact2  contact3  contact4  contact5  hygiene1  hygiene2  hygiene3  hygiene4  hygiene5  psupport1  psupport2  psupport3  psupport4  psupport5  political_ideology  happy  slf_ladder  riskperc1  riskperc2  self_esteem  sbelong1  sbelong2  sbelong3  sbelong4  optim1  optim2  slfcont1  slfcont2  slfcont3  slfcont4
##   7   5  children  cnarc1  cnarc2  cnarc3  ctheory1  ctheory2  ctheory3  ctheory4  CRT1  CRT2  CRT3  employ_status1  generosity1  generosity2  generosity3  health_cond  mor_circle  moralid1  moralid10  moralid2  moralid3  moralid4  moralid5  moralid6  moralid7  moralid8  moralid9  mcoop1  mcoop2  mcoop3  mcoop4  mcoop5  mcoop6  mcoop7  narc1  narc2  narc3  narc4  narc5  narc6  nidentity1  nidentity2  omind1  omind2  omind3  omind4  omind5  omind6  contact1  contact2  contact3  contact4  contact5  hygiene1  hygiene2  hygiene3  hygiene4  hygiene5  psupport1  psupport2  psupport3  psupport4  psupport5  political_ideology  happy  slf_ladder  riskperc1  riskperc2  self_esteem  sbelong1  sbelong2  sbelong3  sbelong4  optim1  optim2  slfcont1  slfcont2  slfcont3  slfcont4
##   7   6  children  cnarc1  cnarc2  cnarc3  ctheory1  ctheory2  ctheory3  ctheory4  CRT1  CRT2  CRT3  employ_status1  generosity1  generosity2  generosity3  health_cond  mor_circle  moralid1  moralid10  moralid2  moralid3  moralid4  moralid5  moralid6  moralid7  moralid8  moralid9  mcoop1  mcoop2  mcoop3  mcoop4  mcoop5  mcoop6  mcoop7  narc1  narc2  narc3  narc4  narc5  narc6  nidentity1  nidentity2  omind1  omind2  omind3  omind4  omind5  omind6  contact1  contact2  contact3  contact4  contact5  hygiene1  hygiene2  hygiene3  hygiene4  hygiene5  psupport1  psupport2  psupport3  psupport4  psupport5  political_ideology  happy  slf_ladder  riskperc1  riskperc2  self_esteem  sbelong1  sbelong2  sbelong3  sbelong4  optim1  optim2  slfcont1  slfcont2  slfcont3  slfcont4
##   7   7  children  cnarc1  cnarc2  cnarc3  ctheory1  ctheory2  ctheory3  ctheory4  CRT1  CRT2  CRT3  employ_status1  generosity1  generosity2  generosity3  health_cond  mor_circle  moralid1  moralid10  moralid2  moralid3  moralid4  moralid5  moralid6  moralid7  moralid8  moralid9  mcoop1  mcoop2  mcoop3  mcoop4  mcoop5  mcoop6  mcoop7  narc1  narc2  narc3  narc4  narc5  narc6  nidentity1  nidentity2  omind1  omind2  omind3  omind4  omind5  omind6  contact1  contact2  contact3  contact4  contact5  hygiene1  hygiene2  hygiene3  hygiene4  hygiene5  psupport1  psupport2  psupport3  psupport4  psupport5  political_ideology  happy  slf_ladder  riskperc1  riskperc2  self_esteem  sbelong1  sbelong2  sbelong3  sbelong4  optim1  optim2  slfcont1  slfcont2  slfcont3  slfcont4
##   7   8  children  cnarc1  cnarc2  cnarc3  ctheory1  ctheory2  ctheory3  ctheory4  CRT1  CRT2  CRT3  employ_status1  generosity1  generosity2  generosity3  health_cond  mor_circle  moralid1  moralid10  moralid2  moralid3  moralid4  moralid5  moralid6  moralid7  moralid8  moralid9  mcoop1  mcoop2  mcoop3  mcoop4  mcoop5  mcoop6  mcoop7  narc1  narc2  narc3  narc4  narc5  narc6  nidentity1  nidentity2  omind1  omind2  omind3  omind4  omind5  omind6  contact1  contact2  contact3  contact4  contact5  hygiene1  hygiene2  hygiene3  hygiene4  hygiene5  psupport1  psupport2  psupport3  psupport4  psupport5  political_ideology  happy  slf_ladder  riskperc1  riskperc2  self_esteem  sbelong1  sbelong2  sbelong3  sbelong4  optim1  optim2  slfcont1  slfcont2  slfcont3  slfcont4
##   7   9  children  cnarc1  cnarc2  cnarc3  ctheory1  ctheory2  ctheory3  ctheory4  CRT1  CRT2  CRT3  employ_status1  generosity1  generosity2  generosity3  health_cond  mor_circle  moralid1  moralid10  moralid2  moralid3  moralid4  moralid5  moralid6  moralid7  moralid8  moralid9  mcoop1  mcoop2  mcoop3  mcoop4  mcoop5  mcoop6  mcoop7  narc1  narc2  narc3  narc4  narc5  narc6  nidentity1  nidentity2  omind1  omind2  omind3  omind4  omind5  omind6  contact1  contact2  contact3  contact4  contact5  hygiene1  hygiene2  hygiene3  hygiene4  hygiene5  psupport1  psupport2  psupport3  psupport4  psupport5  political_ideology  happy  slf_ladder  riskperc1  riskperc2  self_esteem  sbelong1  sbelong2  sbelong3  sbelong4  optim1  optim2  slfcont1  slfcont2  slfcont3  slfcont4
##   7   10  children  cnarc1  cnarc2  cnarc3  ctheory1  ctheory2  ctheory3  ctheory4  CRT1  CRT2  CRT3  employ_status1  generosity1  generosity2  generosity3  health_cond  mor_circle  moralid1  moralid10  moralid2  moralid3  moralid4  moralid5  moralid6  moralid7  moralid8  moralid9  mcoop1  mcoop2  mcoop3  mcoop4  mcoop5  mcoop6  mcoop7  narc1  narc2  narc3  narc4  narc5  narc6  nidentity1  nidentity2  omind1  omind2  omind3  omind4  omind5  omind6  contact1  contact2  contact3  contact4  contact5  hygiene1  hygiene2  hygiene3  hygiene4  hygiene5  psupport1  psupport2  psupport3  psupport4  psupport5  political_ideology  happy  slf_ladder  riskperc1  riskperc2  self_esteem  sbelong1  sbelong2  sbelong3  sbelong4  optim1  optim2  slfcont1  slfcont2  slfcont3  slfcont4
##   8   1  children  cnarc1  cnarc2  cnarc3  ctheory1  ctheory2  ctheory3  ctheory4  CRT1  CRT2  CRT3  employ_status1  generosity1  generosity2  generosity3  health_cond  mor_circle  moralid1  moralid10  moralid2  moralid3  moralid4  moralid5  moralid6  moralid7  moralid8  moralid9  mcoop1  mcoop2  mcoop3  mcoop4  mcoop5  mcoop6  mcoop7  narc1  narc2  narc3  narc4  narc5  narc6  nidentity1  nidentity2  omind1  omind2  omind3  omind4  omind5  omind6  contact1  contact2  contact3  contact4  contact5  hygiene1  hygiene2  hygiene3  hygiene4  hygiene5  psupport1  psupport2  psupport3  psupport4  psupport5  political_ideology  happy  slf_ladder  riskperc1  riskperc2  self_esteem  sbelong1  sbelong2  sbelong3  sbelong4  optim1  optim2  slfcont1  slfcont2  slfcont3  slfcont4
##   8   2  children  cnarc1  cnarc2  cnarc3  ctheory1  ctheory2  ctheory3  ctheory4  CRT1  CRT2  CRT3  employ_status1  generosity1  generosity2  generosity3  health_cond  mor_circle  moralid1  moralid10  moralid2  moralid3  moralid4  moralid5  moralid6  moralid7  moralid8  moralid9  mcoop1  mcoop2  mcoop3  mcoop4  mcoop5  mcoop6  mcoop7  narc1  narc2  narc3  narc4  narc5  narc6  nidentity1  nidentity2  omind1  omind2  omind3  omind4  omind5  omind6  contact1  contact2  contact3  contact4  contact5  hygiene1  hygiene2  hygiene3  hygiene4  hygiene5  psupport1  psupport2  psupport3  psupport4  psupport5  political_ideology  happy  slf_ladder  riskperc1  riskperc2  self_esteem  sbelong1  sbelong2  sbelong3  sbelong4  optim1  optim2  slfcont1  slfcont2  slfcont3  slfcont4
##   8   3  children  cnarc1  cnarc2  cnarc3  ctheory1  ctheory2  ctheory3  ctheory4  CRT1  CRT2  CRT3  employ_status1  generosity1  generosity2  generosity3  health_cond  mor_circle  moralid1  moralid10  moralid2  moralid3  moralid4  moralid5  moralid6  moralid7  moralid8  moralid9  mcoop1  mcoop2  mcoop3  mcoop4  mcoop5  mcoop6  mcoop7  narc1  narc2  narc3  narc4  narc5  narc6  nidentity1  nidentity2  omind1  omind2  omind3  omind4  omind5  omind6  contact1  contact2  contact3  contact4  contact5  hygiene1  hygiene2  hygiene3  hygiene4  hygiene5  psupport1  psupport2  psupport3  psupport4  psupport5  political_ideology  happy  slf_ladder  riskperc1  riskperc2  self_esteem  sbelong1  sbelong2  sbelong3  sbelong4  optim1  optim2  slfcont1  slfcont2  slfcont3  slfcont4
##   8   4  children  cnarc1  cnarc2  cnarc3  ctheory1  ctheory2  ctheory3  ctheory4  CRT1  CRT2  CRT3  employ_status1  generosity1  generosity2  generosity3  health_cond  mor_circle  moralid1  moralid10  moralid2  moralid3  moralid4  moralid5  moralid6  moralid7  moralid8  moralid9  mcoop1  mcoop2  mcoop3  mcoop4  mcoop5  mcoop6  mcoop7  narc1  narc2  narc3  narc4  narc5  narc6  nidentity1  nidentity2  omind1  omind2  omind3  omind4  omind5  omind6  contact1  contact2  contact3  contact4  contact5  hygiene1  hygiene2  hygiene3  hygiene4  hygiene5  psupport1  psupport2  psupport3  psupport4  psupport5  political_ideology  happy  slf_ladder  riskperc1  riskperc2  self_esteem  sbelong1  sbelong2  sbelong3  sbelong4  optim1  optim2  slfcont1  slfcont2  slfcont3  slfcont4
##   8   5  children  cnarc1  cnarc2  cnarc3  ctheory1  ctheory2  ctheory3  ctheory4  CRT1  CRT2  CRT3  employ_status1  generosity1  generosity2  generosity3  health_cond  mor_circle  moralid1  moralid10  moralid2  moralid3  moralid4  moralid5  moralid6  moralid7  moralid8  moralid9  mcoop1  mcoop2  mcoop3  mcoop4  mcoop5  mcoop6  mcoop7  narc1  narc2  narc3  narc4  narc5  narc6  nidentity1  nidentity2  omind1  omind2  omind3  omind4  omind5  omind6  contact1  contact2  contact3  contact4  contact5  hygiene1  hygiene2  hygiene3  hygiene4  hygiene5  psupport1  psupport2  psupport3  psupport4  psupport5  political_ideology  happy  slf_ladder  riskperc1  riskperc2  self_esteem  sbelong1  sbelong2  sbelong3  sbelong4  optim1  optim2  slfcont1  slfcont2  slfcont3  slfcont4
##   8   6  children  cnarc1  cnarc2  cnarc3  ctheory1  ctheory2  ctheory3  ctheory4  CRT1  CRT2  CRT3  employ_status1  generosity1  generosity2  generosity3  health_cond  mor_circle  moralid1  moralid10  moralid2  moralid3  moralid4  moralid5  moralid6  moralid7  moralid8  moralid9  mcoop1  mcoop2  mcoop3  mcoop4  mcoop5  mcoop6  mcoop7  narc1  narc2  narc3  narc4  narc5  narc6  nidentity1  nidentity2  omind1  omind2  omind3  omind4  omind5  omind6  contact1  contact2  contact3  contact4  contact5  hygiene1  hygiene2  hygiene3  hygiene4  hygiene5  psupport1  psupport2  psupport3  psupport4  psupport5  political_ideology  happy  slf_ladder  riskperc1  riskperc2  self_esteem  sbelong1  sbelong2  sbelong3  sbelong4  optim1  optim2  slfcont1  slfcont2  slfcont3  slfcont4
##   8   7  children  cnarc1  cnarc2  cnarc3  ctheory1  ctheory2  ctheory3  ctheory4  CRT1  CRT2  CRT3  employ_status1  generosity1  generosity2  generosity3  health_cond  mor_circle  moralid1  moralid10  moralid2  moralid3  moralid4  moralid5  moralid6  moralid7  moralid8  moralid9  mcoop1  mcoop2  mcoop3  mcoop4  mcoop5  mcoop6  mcoop7  narc1  narc2  narc3  narc4  narc5  narc6  nidentity1  nidentity2  omind1  omind2  omind3  omind4  omind5  omind6  contact1  contact2  contact3  contact4  contact5  hygiene1  hygiene2  hygiene3  hygiene4  hygiene5  psupport1  psupport2  psupport3  psupport4  psupport5  political_ideology  happy  slf_ladder  riskperc1  riskperc2  self_esteem  sbelong1  sbelong2  sbelong3  sbelong4  optim1  optim2  slfcont1  slfcont2  slfcont3  slfcont4
##   8   8  children  cnarc1  cnarc2  cnarc3  ctheory1  ctheory2  ctheory3  ctheory4  CRT1  CRT2  CRT3  employ_status1  generosity1  generosity2  generosity3  health_cond  mor_circle  moralid1  moralid10  moralid2  moralid3  moralid4  moralid5  moralid6  moralid7  moralid8  moralid9  mcoop1  mcoop2  mcoop3  mcoop4  mcoop5  mcoop6  mcoop7  narc1  narc2  narc3  narc4  narc5  narc6  nidentity1  nidentity2  omind1  omind2  omind3  omind4  omind5  omind6  contact1  contact2  contact3  contact4  contact5  hygiene1  hygiene2  hygiene3  hygiene4  hygiene5  psupport1  psupport2  psupport3  psupport4  psupport5  political_ideology  happy  slf_ladder  riskperc1  riskperc2  self_esteem  sbelong1  sbelong2  sbelong3  sbelong4  optim1  optim2  slfcont1  slfcont2  slfcont3  slfcont4
##   8   9  children  cnarc1  cnarc2  cnarc3  ctheory1  ctheory2  ctheory3  ctheory4  CRT1  CRT2  CRT3  employ_status1  generosity1  generosity2  generosity3  health_cond  mor_circle  moralid1  moralid10  moralid2  moralid3  moralid4  moralid5  moralid6  moralid7  moralid8  moralid9  mcoop1  mcoop2  mcoop3  mcoop4  mcoop5  mcoop6  mcoop7  narc1  narc2  narc3  narc4  narc5  narc6  nidentity1  nidentity2  omind1  omind2  omind3  omind4  omind5  omind6  contact1  contact2  contact3  contact4  contact5  hygiene1  hygiene2  hygiene3  hygiene4  hygiene5  psupport1  psupport2  psupport3  psupport4  psupport5  political_ideology  happy  slf_ladder  riskperc1  riskperc2  self_esteem  sbelong1  sbelong2  sbelong3  sbelong4  optim1  optim2  slfcont1  slfcont2  slfcont3  slfcont4
##   8   10  children  cnarc1  cnarc2  cnarc3  ctheory1  ctheory2  ctheory3  ctheory4  CRT1  CRT2  CRT3  employ_status1  generosity1  generosity2  generosity3  health_cond  mor_circle  moralid1  moralid10  moralid2  moralid3  moralid4  moralid5  moralid6  moralid7  moralid8  moralid9  mcoop1  mcoop2  mcoop3  mcoop4  mcoop5  mcoop6  mcoop7  narc1  narc2  narc3  narc4  narc5  narc6  nidentity1  nidentity2  omind1  omind2  omind3  omind4  omind5  omind6  contact1  contact2  contact3  contact4  contact5  hygiene1  hygiene2  hygiene3  hygiene4  hygiene5  psupport1  psupport2  psupport3  psupport4  psupport5  political_ideology  happy  slf_ladder  riskperc1  riskperc2  self_esteem  sbelong1  sbelong2  sbelong3  sbelong4  optim1  optim2  slfcont1  slfcont2  slfcont3  slfcont4
##   9   1  children  cnarc1  cnarc2  cnarc3  ctheory1  ctheory2  ctheory3  ctheory4  CRT1  CRT2  CRT3  employ_status1  generosity1  generosity2  generosity3  health_cond  mor_circle  moralid1  moralid10  moralid2  moralid3  moralid4  moralid5  moralid6  moralid7  moralid8  moralid9  mcoop1  mcoop2  mcoop3  mcoop4  mcoop5  mcoop6  mcoop7  narc1  narc2  narc3  narc4  narc5  narc6  nidentity1  nidentity2  omind1  omind2  omind3  omind4  omind5  omind6  contact1  contact2  contact3  contact4  contact5  hygiene1  hygiene2  hygiene3  hygiene4  hygiene5  psupport1  psupport2  psupport3  psupport4  psupport5  political_ideology  happy  slf_ladder  riskperc1  riskperc2  self_esteem  sbelong1  sbelong2  sbelong3  sbelong4  optim1  optim2  slfcont1  slfcont2  slfcont3  slfcont4
##   9   2  children  cnarc1  cnarc2  cnarc3  ctheory1  ctheory2  ctheory3  ctheory4  CRT1  CRT2  CRT3  employ_status1  generosity1  generosity2  generosity3  health_cond  mor_circle  moralid1  moralid10  moralid2  moralid3  moralid4  moralid5  moralid6  moralid7  moralid8  moralid9  mcoop1  mcoop2  mcoop3  mcoop4  mcoop5  mcoop6  mcoop7  narc1  narc2  narc3  narc4  narc5  narc6  nidentity1  nidentity2  omind1  omind2  omind3  omind4  omind5  omind6  contact1  contact2  contact3  contact4  contact5  hygiene1  hygiene2  hygiene3  hygiene4  hygiene5  psupport1  psupport2  psupport3  psupport4  psupport5  political_ideology  happy  slf_ladder  riskperc1  riskperc2  self_esteem  sbelong1  sbelong2  sbelong3  sbelong4  optim1  optim2  slfcont1  slfcont2  slfcont3  slfcont4
##   9   3  children  cnarc1  cnarc2  cnarc3  ctheory1  ctheory2  ctheory3  ctheory4  CRT1  CRT2  CRT3  employ_status1  generosity1  generosity2  generosity3  health_cond  mor_circle  moralid1  moralid10  moralid2  moralid3  moralid4  moralid5  moralid6  moralid7  moralid8  moralid9  mcoop1  mcoop2  mcoop3  mcoop4  mcoop5  mcoop6  mcoop7  narc1  narc2  narc3  narc4  narc5  narc6  nidentity1  nidentity2  omind1  omind2  omind3  omind4  omind5  omind6  contact1  contact2  contact3  contact4  contact5  hygiene1  hygiene2  hygiene3  hygiene4  hygiene5  psupport1  psupport2  psupport3  psupport4  psupport5  political_ideology  happy  slf_ladder  riskperc1  riskperc2  self_esteem  sbelong1  sbelong2  sbelong3  sbelong4  optim1  optim2  slfcont1  slfcont2  slfcont3  slfcont4
##   9   4  children  cnarc1  cnarc2  cnarc3  ctheory1  ctheory2  ctheory3  ctheory4  CRT1  CRT2  CRT3  employ_status1  generosity1  generosity2  generosity3  health_cond  mor_circle  moralid1  moralid10  moralid2  moralid3  moralid4  moralid5  moralid6  moralid7  moralid8  moralid9  mcoop1  mcoop2  mcoop3  mcoop4  mcoop5  mcoop6  mcoop7  narc1  narc2  narc3  narc4  narc5  narc6  nidentity1  nidentity2  omind1  omind2  omind3  omind4  omind5  omind6  contact1  contact2  contact3  contact4  contact5  hygiene1  hygiene2  hygiene3  hygiene4  hygiene5  psupport1  psupport2  psupport3  psupport4  psupport5  political_ideology  happy  slf_ladder  riskperc1  riskperc2  self_esteem  sbelong1  sbelong2  sbelong3  sbelong4  optim1  optim2  slfcont1  slfcont2  slfcont3  slfcont4
##   9   5  children  cnarc1  cnarc2  cnarc3  ctheory1  ctheory2  ctheory3  ctheory4  CRT1  CRT2  CRT3  employ_status1  generosity1  generosity2  generosity3  health_cond  mor_circle  moralid1  moralid10  moralid2  moralid3  moralid4  moralid5  moralid6  moralid7  moralid8  moralid9  mcoop1  mcoop2  mcoop3  mcoop4  mcoop5  mcoop6  mcoop7  narc1  narc2  narc3  narc4  narc5  narc6  nidentity1  nidentity2  omind1  omind2  omind3  omind4  omind5  omind6  contact1  contact2  contact3  contact4  contact5  hygiene1  hygiene2  hygiene3  hygiene4  hygiene5  psupport1  psupport2  psupport3  psupport4  psupport5  political_ideology  happy  slf_ladder  riskperc1  riskperc2  self_esteem  sbelong1  sbelong2  sbelong3  sbelong4  optim1  optim2  slfcont1  slfcont2  slfcont3  slfcont4
##   9   6  children  cnarc1  cnarc2  cnarc3  ctheory1  ctheory2  ctheory3  ctheory4  CRT1  CRT2  CRT3  employ_status1  generosity1  generosity2  generosity3  health_cond  mor_circle  moralid1  moralid10  moralid2  moralid3  moralid4  moralid5  moralid6  moralid7  moralid8  moralid9  mcoop1  mcoop2  mcoop3  mcoop4  mcoop5  mcoop6  mcoop7  narc1  narc2  narc3  narc4  narc5  narc6  nidentity1  nidentity2  omind1  omind2  omind3  omind4  omind5  omind6  contact1  contact2  contact3  contact4  contact5  hygiene1  hygiene2  hygiene3  hygiene4  hygiene5  psupport1  psupport2  psupport3  psupport4  psupport5  political_ideology  happy  slf_ladder  riskperc1  riskperc2  self_esteem  sbelong1  sbelong2  sbelong3  sbelong4  optim1  optim2  slfcont1  slfcont2  slfcont3  slfcont4
##   9   7  children  cnarc1  cnarc2  cnarc3  ctheory1  ctheory2  ctheory3  ctheory4  CRT1  CRT2  CRT3  employ_status1  generosity1  generosity2  generosity3  health_cond  mor_circle  moralid1  moralid10  moralid2  moralid3  moralid4  moralid5  moralid6  moralid7  moralid8  moralid9  mcoop1  mcoop2  mcoop3  mcoop4  mcoop5  mcoop6  mcoop7  narc1  narc2  narc3  narc4  narc5  narc6  nidentity1  nidentity2  omind1  omind2  omind3  omind4  omind5  omind6  contact1  contact2  contact3  contact4  contact5  hygiene1  hygiene2  hygiene3  hygiene4  hygiene5  psupport1  psupport2  psupport3  psupport4  psupport5  political_ideology  happy  slf_ladder  riskperc1  riskperc2  self_esteem  sbelong1  sbelong2  sbelong3  sbelong4  optim1  optim2  slfcont1  slfcont2  slfcont3  slfcont4
##   9   8  children  cnarc1  cnarc2  cnarc3  ctheory1  ctheory2  ctheory3  ctheory4  CRT1  CRT2  CRT3  employ_status1  generosity1  generosity2  generosity3  health_cond  mor_circle  moralid1  moralid10  moralid2  moralid3  moralid4  moralid5  moralid6  moralid7  moralid8  moralid9  mcoop1  mcoop2  mcoop3  mcoop4  mcoop5  mcoop6  mcoop7  narc1  narc2  narc3  narc4  narc5  narc6  nidentity1  nidentity2  omind1  omind2  omind3  omind4  omind5  omind6  contact1  contact2  contact3  contact4  contact5  hygiene1  hygiene2  hygiene3  hygiene4  hygiene5  psupport1  psupport2  psupport3  psupport4  psupport5  political_ideology  happy  slf_ladder  riskperc1  riskperc2  self_esteem  sbelong1  sbelong2  sbelong3  sbelong4  optim1  optim2  slfcont1  slfcont2  slfcont3  slfcont4
##   9   9  children  cnarc1  cnarc2  cnarc3  ctheory1  ctheory2  ctheory3  ctheory4  CRT1  CRT2  CRT3  employ_status1  generosity1  generosity2  generosity3  health_cond  mor_circle  moralid1  moralid10  moralid2  moralid3  moralid4  moralid5  moralid6  moralid7  moralid8  moralid9  mcoop1  mcoop2  mcoop3  mcoop4  mcoop5  mcoop6  mcoop7  narc1  narc2  narc3  narc4  narc5  narc6  nidentity1  nidentity2  omind1  omind2  omind3  omind4  omind5  omind6  contact1  contact2  contact3  contact4  contact5  hygiene1  hygiene2  hygiene3  hygiene4  hygiene5  psupport1  psupport2  psupport3  psupport4  psupport5  political_ideology  happy  slf_ladder  riskperc1  riskperc2  self_esteem  sbelong1  sbelong2  sbelong3  sbelong4  optim1  optim2  slfcont1  slfcont2  slfcont3  slfcont4
##   9   10  children  cnarc1  cnarc2  cnarc3  ctheory1  ctheory2  ctheory3  ctheory4  CRT1  CRT2  CRT3  employ_status1  generosity1  generosity2  generosity3  health_cond  mor_circle  moralid1  moralid10  moralid2  moralid3  moralid4  moralid5  moralid6  moralid7  moralid8  moralid9  mcoop1  mcoop2  mcoop3  mcoop4  mcoop5  mcoop6  mcoop7  narc1  narc2  narc3  narc4  narc5  narc6  nidentity1  nidentity2  omind1  omind2  omind3  omind4  omind5  omind6  contact1  contact2  contact3  contact4  contact5  hygiene1  hygiene2  hygiene3  hygiene4  hygiene5  psupport1  psupport2  psupport3  psupport4  psupport5  political_ideology  happy  slf_ladder  riskperc1  riskperc2  self_esteem  sbelong1  sbelong2  sbelong3  sbelong4  optim1  optim2  slfcont1  slfcont2  slfcont3  slfcont4
##   10   1  children  cnarc1  cnarc2  cnarc3  ctheory1  ctheory2  ctheory3  ctheory4  CRT1  CRT2  CRT3  employ_status1  generosity1  generosity2  generosity3  health_cond  mor_circle  moralid1  moralid10  moralid2  moralid3  moralid4  moralid5  moralid6  moralid7  moralid8  moralid9  mcoop1  mcoop2  mcoop3  mcoop4  mcoop5  mcoop6  mcoop7  narc1  narc2  narc3  narc4  narc5  narc6  nidentity1  nidentity2  omind1  omind2  omind3  omind4  omind5  omind6  contact1  contact2  contact3  contact4  contact5  hygiene1  hygiene2  hygiene3  hygiene4  hygiene5  psupport1  psupport2  psupport3  psupport4  psupport5  political_ideology  happy  slf_ladder  riskperc1  riskperc2  self_esteem  sbelong1  sbelong2  sbelong3  sbelong4  optim1  optim2  slfcont1  slfcont2  slfcont3  slfcont4
##   10   2  children  cnarc1  cnarc2  cnarc3  ctheory1  ctheory2  ctheory3  ctheory4  CRT1  CRT2  CRT3  employ_status1  generosity1  generosity2  generosity3  health_cond  mor_circle  moralid1  moralid10  moralid2  moralid3  moralid4  moralid5  moralid6  moralid7  moralid8  moralid9  mcoop1  mcoop2  mcoop3  mcoop4  mcoop5  mcoop6  mcoop7  narc1  narc2  narc3  narc4  narc5  narc6  nidentity1  nidentity2  omind1  omind2  omind3  omind4  omind5  omind6  contact1  contact2  contact3  contact4  contact5  hygiene1  hygiene2  hygiene3  hygiene4  hygiene5  psupport1  psupport2  psupport3  psupport4  psupport5  political_ideology  happy  slf_ladder  riskperc1  riskperc2  self_esteem  sbelong1  sbelong2  sbelong3  sbelong4  optim1  optim2  slfcont1  slfcont2  slfcont3  slfcont4
##   10   3  children  cnarc1  cnarc2  cnarc3  ctheory1  ctheory2  ctheory3  ctheory4  CRT1  CRT2  CRT3  employ_status1  generosity1  generosity2  generosity3  health_cond  mor_circle  moralid1  moralid10  moralid2  moralid3  moralid4  moralid5  moralid6  moralid7  moralid8  moralid9  mcoop1  mcoop2  mcoop3  mcoop4  mcoop5  mcoop6  mcoop7  narc1  narc2  narc3  narc4  narc5  narc6  nidentity1  nidentity2  omind1  omind2  omind3  omind4  omind5  omind6  contact1  contact2  contact3  contact4  contact5  hygiene1  hygiene2  hygiene3  hygiene4  hygiene5  psupport1  psupport2  psupport3  psupport4  psupport5  political_ideology  happy  slf_ladder  riskperc1  riskperc2  self_esteem  sbelong1  sbelong2  sbelong3  sbelong4  optim1  optim2  slfcont1  slfcont2  slfcont3  slfcont4
##   10   4  children  cnarc1  cnarc2  cnarc3  ctheory1  ctheory2  ctheory3  ctheory4  CRT1  CRT2  CRT3  employ_status1  generosity1  generosity2  generosity3  health_cond  mor_circle  moralid1  moralid10  moralid2  moralid3  moralid4  moralid5  moralid6  moralid7  moralid8  moralid9  mcoop1  mcoop2  mcoop3  mcoop4  mcoop5  mcoop6  mcoop7  narc1  narc2  narc3  narc4  narc5  narc6  nidentity1  nidentity2  omind1  omind2  omind3  omind4  omind5  omind6  contact1  contact2  contact3  contact4  contact5  hygiene1  hygiene2  hygiene3  hygiene4  hygiene5  psupport1  psupport2  psupport3  psupport4  psupport5  political_ideology  happy  slf_ladder  riskperc1  riskperc2  self_esteem  sbelong1  sbelong2  sbelong3  sbelong4  optim1  optim2  slfcont1  slfcont2  slfcont3  slfcont4
##   10   5  children  cnarc1  cnarc2  cnarc3  ctheory1  ctheory2  ctheory3  ctheory4  CRT1  CRT2  CRT3  employ_status1  generosity1  generosity2  generosity3  health_cond  mor_circle  moralid1  moralid10  moralid2  moralid3  moralid4  moralid5  moralid6  moralid7  moralid8  moralid9  mcoop1  mcoop2  mcoop3  mcoop4  mcoop5  mcoop6  mcoop7  narc1  narc2  narc3  narc4  narc5  narc6  nidentity1  nidentity2  omind1  omind2  omind3  omind4  omind5  omind6  contact1  contact2  contact3  contact4  contact5  hygiene1  hygiene2  hygiene3  hygiene4  hygiene5  psupport1  psupport2  psupport3  psupport4  psupport5  political_ideology  happy  slf_ladder  riskperc1  riskperc2  self_esteem  sbelong1  sbelong2  sbelong3  sbelong4  optim1  optim2  slfcont1  slfcont2  slfcont3  slfcont4
##   10   6  children  cnarc1  cnarc2  cnarc3  ctheory1  ctheory2  ctheory3  ctheory4  CRT1  CRT2  CRT3  employ_status1  generosity1  generosity2  generosity3  health_cond  mor_circle  moralid1  moralid10  moralid2  moralid3  moralid4  moralid5  moralid6  moralid7  moralid8  moralid9  mcoop1  mcoop2  mcoop3  mcoop4  mcoop5  mcoop6  mcoop7  narc1  narc2  narc3  narc4  narc5  narc6  nidentity1  nidentity2  omind1  omind2  omind3  omind4  omind5  omind6  contact1  contact2  contact3  contact4  contact5  hygiene1  hygiene2  hygiene3  hygiene4  hygiene5  psupport1  psupport2  psupport3  psupport4  psupport5  political_ideology  happy  slf_ladder  riskperc1  riskperc2  self_esteem  sbelong1  sbelong2  sbelong3  sbelong4  optim1  optim2  slfcont1  slfcont2  slfcont3  slfcont4
##   10   7  children  cnarc1  cnarc2  cnarc3  ctheory1  ctheory2  ctheory3  ctheory4  CRT1  CRT2  CRT3  employ_status1  generosity1  generosity2  generosity3  health_cond  mor_circle  moralid1  moralid10  moralid2  moralid3  moralid4  moralid5  moralid6  moralid7  moralid8  moralid9  mcoop1  mcoop2  mcoop3  mcoop4  mcoop5  mcoop6  mcoop7  narc1  narc2  narc3  narc4  narc5  narc6  nidentity1  nidentity2  omind1  omind2  omind3  omind4  omind5  omind6  contact1  contact2  contact3  contact4  contact5  hygiene1  hygiene2  hygiene3  hygiene4  hygiene5  psupport1  psupport2  psupport3  psupport4  psupport5  political_ideology  happy  slf_ladder  riskperc1  riskperc2  self_esteem  sbelong1  sbelong2  sbelong3  sbelong4  optim1  optim2  slfcont1  slfcont2  slfcont3  slfcont4
##   10   8  children  cnarc1  cnarc2  cnarc3  ctheory1  ctheory2  ctheory3  ctheory4  CRT1  CRT2  CRT3  employ_status1  generosity1  generosity2  generosity3  health_cond  mor_circle  moralid1  moralid10  moralid2  moralid3  moralid4  moralid5  moralid6  moralid7  moralid8  moralid9  mcoop1  mcoop2  mcoop3  mcoop4  mcoop5  mcoop6  mcoop7  narc1  narc2  narc3  narc4  narc5  narc6  nidentity1  nidentity2  omind1  omind2  omind3  omind4  omind5  omind6  contact1  contact2  contact3  contact4  contact5  hygiene1  hygiene2  hygiene3  hygiene4  hygiene5  psupport1  psupport2  psupport3  psupport4  psupport5  political_ideology  happy  slf_ladder  riskperc1  riskperc2  self_esteem  sbelong1  sbelong2  sbelong3  sbelong4  optim1  optim2  slfcont1  slfcont2  slfcont3  slfcont4
##   10   9  children  cnarc1  cnarc2  cnarc3  ctheory1  ctheory2  ctheory3  ctheory4  CRT1  CRT2  CRT3  employ_status1  generosity1  generosity2  generosity3  health_cond  mor_circle  moralid1  moralid10  moralid2  moralid3  moralid4  moralid5  moralid6  moralid7  moralid8  moralid9  mcoop1  mcoop2  mcoop3  mcoop4  mcoop5  mcoop6  mcoop7  narc1  narc2  narc3  narc4  narc5  narc6  nidentity1  nidentity2  omind1  omind2  omind3  omind4  omind5  omind6  contact1  contact2  contact3  contact4  contact5  hygiene1  hygiene2  hygiene3  hygiene4  hygiene5  psupport1  psupport2  psupport3  psupport4  psupport5  political_ideology  happy  slf_ladder  riskperc1  riskperc2  self_esteem  sbelong1  sbelong2  sbelong3  sbelong4  optim1  optim2  slfcont1  slfcont2  slfcont3  slfcont4
##   10   10  children  cnarc1  cnarc2  cnarc3  ctheory1  ctheory2  ctheory3  ctheory4  CRT1  CRT2  CRT3  employ_status1  generosity1  generosity2  generosity3  health_cond  mor_circle  moralid1  moralid10  moralid2  moralid3  moralid4  moralid5  moralid6  moralid7  moralid8  moralid9  mcoop1  mcoop2  mcoop3  mcoop4  mcoop5  mcoop6  mcoop7  narc1  narc2  narc3  narc4  narc5  narc6  nidentity1  nidentity2  omind1  omind2  omind3  omind4  omind5  omind6  contact1  contact2  contact3  contact4  contact5  hygiene1  hygiene2  hygiene3  hygiene4  hygiene5  psupport1  psupport2  psupport3  psupport4  psupport5  political_ideology  happy  slf_ladder  riskperc1  riskperc2  self_esteem  sbelong1  sbelong2  sbelong3  sbelong4  optim1  optim2  slfcont1  slfcont2  slfcont3  slfcont4
```

```
## Warning: Number of logged events: 7567
```

```
## 
##  iter imp variable
##   1   1  cnarc1  cnarc2  cnarc3  ctheory4  CRT1  CRT2  CRT3  health_cond  moralid10  moralid2  moralid5  moralid6  moralid8  mcoop1  mcoop2  mcoop3  mcoop4  mcoop5  mcoop7  narc3  narc4  narc5  narc6  omind1  omind2  omind3  hygiene5  psupport2  political_ideology  happy  slf_ladder  riskperc1  riskperc2  self_esteem
##   1   2  cnarc1  cnarc2  cnarc3  ctheory4  CRT1  CRT2  CRT3  health_cond  moralid10  moralid2  moralid5  moralid6  moralid8  mcoop1  mcoop2  mcoop3  mcoop4  mcoop5  mcoop7  narc3  narc4  narc5  narc6  omind1  omind2  omind3  hygiene5  psupport2  political_ideology  happy  slf_ladder  riskperc1  riskperc2  self_esteem
##   1   3  cnarc1  cnarc2  cnarc3  ctheory4  CRT1  CRT2  CRT3  health_cond  moralid10  moralid2  moralid5  moralid6  moralid8  mcoop1  mcoop2  mcoop3  mcoop4  mcoop5  mcoop7  narc3  narc4  narc5  narc6  omind1  omind2  omind3  hygiene5  psupport2  political_ideology  happy  slf_ladder  riskperc1  riskperc2  self_esteem
##   1   4  cnarc1  cnarc2  cnarc3  ctheory4  CRT1  CRT2  CRT3  health_cond  moralid10  moralid2  moralid5  moralid6  moralid8  mcoop1  mcoop2  mcoop3  mcoop4  mcoop5  mcoop7  narc3  narc4  narc5  narc6  omind1  omind2  omind3  hygiene5  psupport2  political_ideology  happy  slf_ladder  riskperc1  riskperc2  self_esteem
##   1   5  cnarc1  cnarc2  cnarc3  ctheory4  CRT1  CRT2  CRT3  health_cond  moralid10  moralid2  moralid5  moralid6  moralid8  mcoop1  mcoop2  mcoop3  mcoop4  mcoop5  mcoop7  narc3  narc4  narc5  narc6  omind1  omind2  omind3  hygiene5  psupport2  political_ideology  happy  slf_ladder  riskperc1  riskperc2  self_esteem
##   1   6  cnarc1  cnarc2  cnarc3  ctheory4  CRT1  CRT2  CRT3  health_cond  moralid10  moralid2  moralid5  moralid6  moralid8  mcoop1  mcoop2  mcoop3  mcoop4  mcoop5  mcoop7  narc3  narc4  narc5  narc6  omind1  omind2  omind3  hygiene5  psupport2  political_ideology  happy  slf_ladder  riskperc1  riskperc2  self_esteem
##   1   7  cnarc1  cnarc2  cnarc3  ctheory4  CRT1  CRT2  CRT3  health_cond  moralid10  moralid2  moralid5  moralid6  moralid8  mcoop1  mcoop2  mcoop3  mcoop4  mcoop5  mcoop7  narc3  narc4  narc5  narc6  omind1  omind2  omind3  hygiene5  psupport2  political_ideology  happy  slf_ladder  riskperc1  riskperc2  self_esteem
##   1   8  cnarc1  cnarc2  cnarc3  ctheory4  CRT1  CRT2  CRT3  health_cond  moralid10  moralid2  moralid5  moralid6  moralid8  mcoop1  mcoop2  mcoop3  mcoop4  mcoop5  mcoop7  narc3  narc4  narc5  narc6  omind1  omind2  omind3  hygiene5  psupport2  political_ideology  happy  slf_ladder  riskperc1  riskperc2  self_esteem
##   1   9  cnarc1  cnarc2  cnarc3  ctheory4  CRT1  CRT2  CRT3  health_cond  moralid10  moralid2  moralid5  moralid6  moralid8  mcoop1  mcoop2  mcoop3  mcoop4  mcoop5  mcoop7  narc3  narc4  narc5  narc6  omind1  omind2  omind3  hygiene5  psupport2  political_ideology  happy  slf_ladder  riskperc1  riskperc2  self_esteem
##   1   10  cnarc1  cnarc2  cnarc3  ctheory4  CRT1  CRT2  CRT3  health_cond  moralid10  moralid2  moralid5  moralid6  moralid8  mcoop1  mcoop2  mcoop3  mcoop4  mcoop5  mcoop7  narc3  narc4  narc5  narc6  omind1  omind2  omind3  hygiene5  psupport2  political_ideology  happy  slf_ladder  riskperc1  riskperc2  self_esteem
##   2   1  cnarc1  cnarc2  cnarc3  ctheory4  CRT1  CRT2  CRT3  health_cond  moralid10  moralid2  moralid5  moralid6  moralid8  mcoop1  mcoop2  mcoop3  mcoop4  mcoop5  mcoop7  narc3  narc4  narc5  narc6  omind1  omind2  omind3  hygiene5  psupport2  political_ideology  happy  slf_ladder  riskperc1  riskperc2  self_esteem
##   2   2  cnarc1  cnarc2  cnarc3  ctheory4  CRT1  CRT2  CRT3  health_cond  moralid10  moralid2  moralid5  moralid6  moralid8  mcoop1  mcoop2  mcoop3  mcoop4  mcoop5  mcoop7  narc3  narc4  narc5  narc6  omind1  omind2  omind3  hygiene5  psupport2  political_ideology  happy  slf_ladder  riskperc1  riskperc2  self_esteem
##   2   3  cnarc1  cnarc2  cnarc3  ctheory4  CRT1  CRT2  CRT3  health_cond  moralid10  moralid2  moralid5  moralid6  moralid8  mcoop1  mcoop2  mcoop3  mcoop4  mcoop5  mcoop7  narc3  narc4  narc5  narc6  omind1  omind2  omind3  hygiene5  psupport2  political_ideology  happy  slf_ladder  riskperc1  riskperc2  self_esteem
##   2   4  cnarc1  cnarc2  cnarc3  ctheory4  CRT1  CRT2  CRT3  health_cond  moralid10  moralid2  moralid5  moralid6  moralid8  mcoop1  mcoop2  mcoop3  mcoop4  mcoop5  mcoop7  narc3  narc4  narc5  narc6  omind1  omind2  omind3  hygiene5  psupport2  political_ideology  happy  slf_ladder  riskperc1  riskperc2  self_esteem
##   2   5  cnarc1  cnarc2  cnarc3  ctheory4  CRT1  CRT2  CRT3  health_cond  moralid10  moralid2  moralid5  moralid6  moralid8  mcoop1  mcoop2  mcoop3  mcoop4  mcoop5  mcoop7  narc3  narc4  narc5  narc6  omind1  omind2  omind3  hygiene5  psupport2  political_ideology  happy  slf_ladder  riskperc1  riskperc2  self_esteem
##   2   6  cnarc1  cnarc2  cnarc3  ctheory4  CRT1  CRT2  CRT3  health_cond  moralid10  moralid2  moralid5  moralid6  moralid8  mcoop1  mcoop2  mcoop3  mcoop4  mcoop5  mcoop7  narc3  narc4  narc5  narc6  omind1  omind2  omind3  hygiene5  psupport2  political_ideology  happy  slf_ladder  riskperc1  riskperc2  self_esteem
##   2   7  cnarc1  cnarc2  cnarc3  ctheory4  CRT1  CRT2  CRT3  health_cond  moralid10  moralid2  moralid5  moralid6  moralid8  mcoop1  mcoop2  mcoop3  mcoop4  mcoop5  mcoop7  narc3  narc4  narc5  narc6  omind1  omind2  omind3  hygiene5  psupport2  political_ideology  happy  slf_ladder  riskperc1  riskperc2  self_esteem
##   2   8  cnarc1  cnarc2  cnarc3  ctheory4  CRT1  CRT2  CRT3  health_cond  moralid10  moralid2  moralid5  moralid6  moralid8  mcoop1  mcoop2  mcoop3  mcoop4  mcoop5  mcoop7  narc3  narc4  narc5  narc6  omind1  omind2  omind3  hygiene5  psupport2  political_ideology  happy  slf_ladder  riskperc1  riskperc2  self_esteem
##   2   9  cnarc1  cnarc2  cnarc3  ctheory4  CRT1  CRT2  CRT3  health_cond  moralid10  moralid2  moralid5  moralid6  moralid8  mcoop1  mcoop2  mcoop3  mcoop4  mcoop5  mcoop7  narc3  narc4  narc5  narc6  omind1  omind2  omind3  hygiene5  psupport2  political_ideology  happy  slf_ladder  riskperc1  riskperc2  self_esteem
##   2   10  cnarc1  cnarc2  cnarc3  ctheory4  CRT1  CRT2  CRT3  health_cond  moralid10  moralid2  moralid5  moralid6  moralid8  mcoop1  mcoop2  mcoop3  mcoop4  mcoop5  mcoop7  narc3  narc4  narc5  narc6  omind1  omind2  omind3  hygiene5  psupport2  political_ideology  happy  slf_ladder  riskperc1  riskperc2  self_esteem
##   3   1  cnarc1  cnarc2  cnarc3  ctheory4  CRT1  CRT2  CRT3  health_cond  moralid10  moralid2  moralid5  moralid6  moralid8  mcoop1  mcoop2  mcoop3  mcoop4  mcoop5  mcoop7  narc3  narc4  narc5  narc6  omind1  omind2  omind3  hygiene5  psupport2  political_ideology  happy  slf_ladder  riskperc1  riskperc2  self_esteem
##   3   2  cnarc1  cnarc2  cnarc3  ctheory4  CRT1  CRT2  CRT3  health_cond  moralid10  moralid2  moralid5  moralid6  moralid8  mcoop1  mcoop2  mcoop3  mcoop4  mcoop5  mcoop7  narc3  narc4  narc5  narc6  omind1  omind2  omind3  hygiene5  psupport2  political_ideology  happy  slf_ladder  riskperc1  riskperc2  self_esteem
##   3   3  cnarc1  cnarc2  cnarc3  ctheory4  CRT1  CRT2  CRT3  health_cond  moralid10  moralid2  moralid5  moralid6  moralid8  mcoop1  mcoop2  mcoop3  mcoop4  mcoop5  mcoop7  narc3  narc4  narc5  narc6  omind1  omind2  omind3  hygiene5  psupport2  political_ideology  happy  slf_ladder  riskperc1  riskperc2  self_esteem
##   3   4  cnarc1  cnarc2  cnarc3  ctheory4  CRT1  CRT2  CRT3  health_cond  moralid10  moralid2  moralid5  moralid6  moralid8  mcoop1  mcoop2  mcoop3  mcoop4  mcoop5  mcoop7  narc3  narc4  narc5  narc6  omind1  omind2  omind3  hygiene5  psupport2  political_ideology  happy  slf_ladder  riskperc1  riskperc2  self_esteem
##   3   5  cnarc1  cnarc2  cnarc3  ctheory4  CRT1  CRT2  CRT3  health_cond  moralid10  moralid2  moralid5  moralid6  moralid8  mcoop1  mcoop2  mcoop3  mcoop4  mcoop5  mcoop7  narc3  narc4  narc5  narc6  omind1  omind2  omind3  hygiene5  psupport2  political_ideology  happy  slf_ladder  riskperc1  riskperc2  self_esteem
##   3   6  cnarc1  cnarc2  cnarc3  ctheory4  CRT1  CRT2  CRT3  health_cond  moralid10  moralid2  moralid5  moralid6  moralid8  mcoop1  mcoop2  mcoop3  mcoop4  mcoop5  mcoop7  narc3  narc4  narc5  narc6  omind1  omind2  omind3  hygiene5  psupport2  political_ideology  happy  slf_ladder  riskperc1  riskperc2  self_esteem
##   3   7  cnarc1  cnarc2  cnarc3  ctheory4  CRT1  CRT2  CRT3  health_cond  moralid10  moralid2  moralid5  moralid6  moralid8  mcoop1  mcoop2  mcoop3  mcoop4  mcoop5  mcoop7  narc3  narc4  narc5  narc6  omind1  omind2  omind3  hygiene5  psupport2  political_ideology  happy  slf_ladder  riskperc1  riskperc2  self_esteem
##   3   8  cnarc1  cnarc2  cnarc3  ctheory4  CRT1  CRT2  CRT3  health_cond  moralid10  moralid2  moralid5  moralid6  moralid8  mcoop1  mcoop2  mcoop3  mcoop4  mcoop5  mcoop7  narc3  narc4  narc5  narc6  omind1  omind2  omind3  hygiene5  psupport2  political_ideology  happy  slf_ladder  riskperc1  riskperc2  self_esteem
##   3   9  cnarc1  cnarc2  cnarc3  ctheory4  CRT1  CRT2  CRT3  health_cond  moralid10  moralid2  moralid5  moralid6  moralid8  mcoop1  mcoop2  mcoop3  mcoop4  mcoop5  mcoop7  narc3  narc4  narc5  narc6  omind1  omind2  omind3  hygiene5  psupport2  political_ideology  happy  slf_ladder  riskperc1  riskperc2  self_esteem
##   3   10  cnarc1  cnarc2  cnarc3  ctheory4  CRT1  CRT2  CRT3  health_cond  moralid10  moralid2  moralid5  moralid6  moralid8  mcoop1  mcoop2  mcoop3  mcoop4  mcoop5  mcoop7  narc3  narc4  narc5  narc6  omind1  omind2  omind3  hygiene5  psupport2  political_ideology  happy  slf_ladder  riskperc1  riskperc2  self_esteem
##   4   1  cnarc1  cnarc2  cnarc3  ctheory4  CRT1  CRT2  CRT3  health_cond  moralid10  moralid2  moralid5  moralid6  moralid8  mcoop1  mcoop2  mcoop3  mcoop4  mcoop5  mcoop7  narc3  narc4  narc5  narc6  omind1  omind2  omind3  hygiene5  psupport2  political_ideology  happy  slf_ladder  riskperc1  riskperc2  self_esteem
##   4   2  cnarc1  cnarc2  cnarc3  ctheory4  CRT1  CRT2  CRT3  health_cond  moralid10  moralid2  moralid5  moralid6  moralid8  mcoop1  mcoop2  mcoop3  mcoop4  mcoop5  mcoop7  narc3  narc4  narc5  narc6  omind1  omind2  omind3  hygiene5  psupport2  political_ideology  happy  slf_ladder  riskperc1  riskperc2  self_esteem
##   4   3  cnarc1  cnarc2  cnarc3  ctheory4  CRT1  CRT2  CRT3  health_cond  moralid10  moralid2  moralid5  moralid6  moralid8  mcoop1  mcoop2  mcoop3  mcoop4  mcoop5  mcoop7  narc3  narc4  narc5  narc6  omind1  omind2  omind3  hygiene5  psupport2  political_ideology  happy  slf_ladder  riskperc1  riskperc2  self_esteem
##   4   4  cnarc1  cnarc2  cnarc3  ctheory4  CRT1  CRT2  CRT3  health_cond  moralid10  moralid2  moralid5  moralid6  moralid8  mcoop1  mcoop2  mcoop3  mcoop4  mcoop5  mcoop7  narc3  narc4  narc5  narc6  omind1  omind2  omind3  hygiene5  psupport2  political_ideology  happy  slf_ladder  riskperc1  riskperc2  self_esteem
##   4   5  cnarc1  cnarc2  cnarc3  ctheory4  CRT1  CRT2  CRT3  health_cond  moralid10  moralid2  moralid5  moralid6  moralid8  mcoop1  mcoop2  mcoop3  mcoop4  mcoop5  mcoop7  narc3  narc4  narc5  narc6  omind1  omind2  omind3  hygiene5  psupport2  political_ideology  happy  slf_ladder  riskperc1  riskperc2  self_esteem
##   4   6  cnarc1  cnarc2  cnarc3  ctheory4  CRT1  CRT2  CRT3  health_cond  moralid10  moralid2  moralid5  moralid6  moralid8  mcoop1  mcoop2  mcoop3  mcoop4  mcoop5  mcoop7  narc3  narc4  narc5  narc6  omind1  omind2  omind3  hygiene5  psupport2  political_ideology  happy  slf_ladder  riskperc1  riskperc2  self_esteem
##   4   7  cnarc1  cnarc2  cnarc3  ctheory4  CRT1  CRT2  CRT3  health_cond  moralid10  moralid2  moralid5  moralid6  moralid8  mcoop1  mcoop2  mcoop3  mcoop4  mcoop5  mcoop7  narc3  narc4  narc5  narc6  omind1  omind2  omind3  hygiene5  psupport2  political_ideology  happy  slf_ladder  riskperc1  riskperc2  self_esteem
##   4   8  cnarc1  cnarc2  cnarc3  ctheory4  CRT1  CRT2  CRT3  health_cond  moralid10  moralid2  moralid5  moralid6  moralid8  mcoop1  mcoop2  mcoop3  mcoop4  mcoop5  mcoop7  narc3  narc4  narc5  narc6  omind1  omind2  omind3  hygiene5  psupport2  political_ideology  happy  slf_ladder  riskperc1  riskperc2  self_esteem
##   4   9  cnarc1  cnarc2  cnarc3  ctheory4  CRT1  CRT2  CRT3  health_cond  moralid10  moralid2  moralid5  moralid6  moralid8  mcoop1  mcoop2  mcoop3  mcoop4  mcoop5  mcoop7  narc3  narc4  narc5  narc6  omind1  omind2  omind3  hygiene5  psupport2  political_ideology  happy  slf_ladder  riskperc1  riskperc2  self_esteem
##   4   10  cnarc1  cnarc2  cnarc3  ctheory4  CRT1  CRT2  CRT3  health_cond  moralid10  moralid2  moralid5  moralid6  moralid8  mcoop1  mcoop2  mcoop3  mcoop4  mcoop5  mcoop7  narc3  narc4  narc5  narc6  omind1  omind2  omind3  hygiene5  psupport2  political_ideology  happy  slf_ladder  riskperc1  riskperc2  self_esteem
##   5   1  cnarc1  cnarc2  cnarc3  ctheory4  CRT1  CRT2  CRT3  health_cond  moralid10  moralid2  moralid5  moralid6  moralid8  mcoop1  mcoop2  mcoop3  mcoop4  mcoop5  mcoop7  narc3  narc4  narc5  narc6  omind1  omind2  omind3  hygiene5  psupport2  political_ideology  happy  slf_ladder  riskperc1  riskperc2  self_esteem
##   5   2  cnarc1  cnarc2  cnarc3  ctheory4  CRT1  CRT2  CRT3  health_cond  moralid10  moralid2  moralid5  moralid6  moralid8  mcoop1  mcoop2  mcoop3  mcoop4  mcoop5  mcoop7  narc3  narc4  narc5  narc6  omind1  omind2  omind3  hygiene5  psupport2  political_ideology  happy  slf_ladder  riskperc1  riskperc2  self_esteem
##   5   3  cnarc1  cnarc2  cnarc3  ctheory4  CRT1  CRT2  CRT3  health_cond  moralid10  moralid2  moralid5  moralid6  moralid8  mcoop1  mcoop2  mcoop3  mcoop4  mcoop5  mcoop7  narc3  narc4  narc5  narc6  omind1  omind2  omind3  hygiene5  psupport2  political_ideology  happy  slf_ladder  riskperc1  riskperc2  self_esteem
##   5   4  cnarc1  cnarc2  cnarc3  ctheory4  CRT1  CRT2  CRT3  health_cond  moralid10  moralid2  moralid5  moralid6  moralid8  mcoop1  mcoop2  mcoop3  mcoop4  mcoop5  mcoop7  narc3  narc4  narc5  narc6  omind1  omind2  omind3  hygiene5  psupport2  political_ideology  happy  slf_ladder  riskperc1  riskperc2  self_esteem
##   5   5  cnarc1  cnarc2  cnarc3  ctheory4  CRT1  CRT2  CRT3  health_cond  moralid10  moralid2  moralid5  moralid6  moralid8  mcoop1  mcoop2  mcoop3  mcoop4  mcoop5  mcoop7  narc3  narc4  narc5  narc6  omind1  omind2  omind3  hygiene5  psupport2  political_ideology  happy  slf_ladder  riskperc1  riskperc2  self_esteem
##   5   6  cnarc1  cnarc2  cnarc3  ctheory4  CRT1  CRT2  CRT3  health_cond  moralid10  moralid2  moralid5  moralid6  moralid8  mcoop1  mcoop2  mcoop3  mcoop4  mcoop5  mcoop7  narc3  narc4  narc5  narc6  omind1  omind2  omind3  hygiene5  psupport2  political_ideology  happy  slf_ladder  riskperc1  riskperc2  self_esteem
##   5   7  cnarc1  cnarc2  cnarc3  ctheory4  CRT1  CRT2  CRT3  health_cond  moralid10  moralid2  moralid5  moralid6  moralid8  mcoop1  mcoop2  mcoop3  mcoop4  mcoop5  mcoop7  narc3  narc4  narc5  narc6  omind1  omind2  omind3  hygiene5  psupport2  political_ideology  happy  slf_ladder  riskperc1  riskperc2  self_esteem
##   5   8  cnarc1  cnarc2  cnarc3  ctheory4  CRT1  CRT2  CRT3  health_cond  moralid10  moralid2  moralid5  moralid6  moralid8  mcoop1  mcoop2  mcoop3  mcoop4  mcoop5  mcoop7  narc3  narc4  narc5  narc6  omind1  omind2  omind3  hygiene5  psupport2  political_ideology  happy  slf_ladder  riskperc1  riskperc2  self_esteem
##   5   9  cnarc1  cnarc2  cnarc3  ctheory4  CRT1  CRT2  CRT3  health_cond  moralid10  moralid2  moralid5  moralid6  moralid8  mcoop1  mcoop2  mcoop3  mcoop4  mcoop5  mcoop7  narc3  narc4  narc5  narc6  omind1  omind2  omind3  hygiene5  psupport2  political_ideology  happy  slf_ladder  riskperc1  riskperc2  self_esteem
##   5   10  cnarc1  cnarc2  cnarc3  ctheory4  CRT1  CRT2  CRT3  health_cond  moralid10  moralid2  moralid5  moralid6  moralid8  mcoop1  mcoop2  mcoop3  mcoop4  mcoop5  mcoop7  narc3  narc4  narc5  narc6  omind1  omind2  omind3  hygiene5  psupport2  political_ideology  happy  slf_ladder  riskperc1  riskperc2  self_esteem
##   6   1  cnarc1  cnarc2  cnarc3  ctheory4  CRT1  CRT2  CRT3  health_cond  moralid10  moralid2  moralid5  moralid6  moralid8  mcoop1  mcoop2  mcoop3  mcoop4  mcoop5  mcoop7  narc3  narc4  narc5  narc6  omind1  omind2  omind3  hygiene5  psupport2  political_ideology  happy  slf_ladder  riskperc1  riskperc2  self_esteem
##   6   2  cnarc1  cnarc2  cnarc3  ctheory4  CRT1  CRT2  CRT3  health_cond  moralid10  moralid2  moralid5  moralid6  moralid8  mcoop1  mcoop2  mcoop3  mcoop4  mcoop5  mcoop7  narc3  narc4  narc5  narc6  omind1  omind2  omind3  hygiene5  psupport2  political_ideology  happy  slf_ladder  riskperc1  riskperc2  self_esteem
##   6   3  cnarc1  cnarc2  cnarc3  ctheory4  CRT1  CRT2  CRT3  health_cond  moralid10  moralid2  moralid5  moralid6  moralid8  mcoop1  mcoop2  mcoop3  mcoop4  mcoop5  mcoop7  narc3  narc4  narc5  narc6  omind1  omind2  omind3  hygiene5  psupport2  political_ideology  happy  slf_ladder  riskperc1  riskperc2  self_esteem
##   6   4  cnarc1  cnarc2  cnarc3  ctheory4  CRT1  CRT2  CRT3  health_cond  moralid10  moralid2  moralid5  moralid6  moralid8  mcoop1  mcoop2  mcoop3  mcoop4  mcoop5  mcoop7  narc3  narc4  narc5  narc6  omind1  omind2  omind3  hygiene5  psupport2  political_ideology  happy  slf_ladder  riskperc1  riskperc2  self_esteem
##   6   5  cnarc1  cnarc2  cnarc3  ctheory4  CRT1  CRT2  CRT3  health_cond  moralid10  moralid2  moralid5  moralid6  moralid8  mcoop1  mcoop2  mcoop3  mcoop4  mcoop5  mcoop7  narc3  narc4  narc5  narc6  omind1  omind2  omind3  hygiene5  psupport2  political_ideology  happy  slf_ladder  riskperc1  riskperc2  self_esteem
##   6   6  cnarc1  cnarc2  cnarc3  ctheory4  CRT1  CRT2  CRT3  health_cond  moralid10  moralid2  moralid5  moralid6  moralid8  mcoop1  mcoop2  mcoop3  mcoop4  mcoop5  mcoop7  narc3  narc4  narc5  narc6  omind1  omind2  omind3  hygiene5  psupport2  political_ideology  happy  slf_ladder  riskperc1  riskperc2  self_esteem
##   6   7  cnarc1  cnarc2  cnarc3  ctheory4  CRT1  CRT2  CRT3  health_cond  moralid10  moralid2  moralid5  moralid6  moralid8  mcoop1  mcoop2  mcoop3  mcoop4  mcoop5  mcoop7  narc3  narc4  narc5  narc6  omind1  omind2  omind3  hygiene5  psupport2  political_ideology  happy  slf_ladder  riskperc1  riskperc2  self_esteem
##   6   8  cnarc1  cnarc2  cnarc3  ctheory4  CRT1  CRT2  CRT3  health_cond  moralid10  moralid2  moralid5  moralid6  moralid8  mcoop1  mcoop2  mcoop3  mcoop4  mcoop5  mcoop7  narc3  narc4  narc5  narc6  omind1  omind2  omind3  hygiene5  psupport2  political_ideology  happy  slf_ladder  riskperc1  riskperc2  self_esteem
##   6   9  cnarc1  cnarc2  cnarc3  ctheory4  CRT1  CRT2  CRT3  health_cond  moralid10  moralid2  moralid5  moralid6  moralid8  mcoop1  mcoop2  mcoop3  mcoop4  mcoop5  mcoop7  narc3  narc4  narc5  narc6  omind1  omind2  omind3  hygiene5  psupport2  political_ideology  happy  slf_ladder  riskperc1  riskperc2  self_esteem
##   6   10  cnarc1  cnarc2  cnarc3  ctheory4  CRT1  CRT2  CRT3  health_cond  moralid10  moralid2  moralid5  moralid6  moralid8  mcoop1  mcoop2  mcoop3  mcoop4  mcoop5  mcoop7  narc3  narc4  narc5  narc6  omind1  omind2  omind3  hygiene5  psupport2  political_ideology  happy  slf_ladder  riskperc1  riskperc2  self_esteem
##   7   1  cnarc1  cnarc2  cnarc3  ctheory4  CRT1  CRT2  CRT3  health_cond  moralid10  moralid2  moralid5  moralid6  moralid8  mcoop1  mcoop2  mcoop3  mcoop4  mcoop5  mcoop7  narc3  narc4  narc5  narc6  omind1  omind2  omind3  hygiene5  psupport2  political_ideology  happy  slf_ladder  riskperc1  riskperc2  self_esteem
##   7   2  cnarc1  cnarc2  cnarc3  ctheory4  CRT1  CRT2  CRT3  health_cond  moralid10  moralid2  moralid5  moralid6  moralid8  mcoop1  mcoop2  mcoop3  mcoop4  mcoop5  mcoop7  narc3  narc4  narc5  narc6  omind1  omind2  omind3  hygiene5  psupport2  political_ideology  happy  slf_ladder  riskperc1  riskperc2  self_esteem
##   7   3  cnarc1  cnarc2  cnarc3  ctheory4  CRT1  CRT2  CRT3  health_cond  moralid10  moralid2  moralid5  moralid6  moralid8  mcoop1  mcoop2  mcoop3  mcoop4  mcoop5  mcoop7  narc3  narc4  narc5  narc6  omind1  omind2  omind3  hygiene5  psupport2  political_ideology  happy  slf_ladder  riskperc1  riskperc2  self_esteem
##   7   4  cnarc1  cnarc2  cnarc3  ctheory4  CRT1  CRT2  CRT3  health_cond  moralid10  moralid2  moralid5  moralid6  moralid8  mcoop1  mcoop2  mcoop3  mcoop4  mcoop5  mcoop7  narc3  narc4  narc5  narc6  omind1  omind2  omind3  hygiene5  psupport2  political_ideology  happy  slf_ladder  riskperc1  riskperc2  self_esteem
##   7   5  cnarc1  cnarc2  cnarc3  ctheory4  CRT1  CRT2  CRT3  health_cond  moralid10  moralid2  moralid5  moralid6  moralid8  mcoop1  mcoop2  mcoop3  mcoop4  mcoop5  mcoop7  narc3  narc4  narc5  narc6  omind1  omind2  omind3  hygiene5  psupport2  political_ideology  happy  slf_ladder  riskperc1  riskperc2  self_esteem
##   7   6  cnarc1  cnarc2  cnarc3  ctheory4  CRT1  CRT2  CRT3  health_cond  moralid10  moralid2  moralid5  moralid6  moralid8  mcoop1  mcoop2  mcoop3  mcoop4  mcoop5  mcoop7  narc3  narc4  narc5  narc6  omind1  omind2  omind3  hygiene5  psupport2  political_ideology  happy  slf_ladder  riskperc1  riskperc2  self_esteem
##   7   7  cnarc1  cnarc2  cnarc3  ctheory4  CRT1  CRT2  CRT3  health_cond  moralid10  moralid2  moralid5  moralid6  moralid8  mcoop1  mcoop2  mcoop3  mcoop4  mcoop5  mcoop7  narc3  narc4  narc5  narc6  omind1  omind2  omind3  hygiene5  psupport2  political_ideology  happy  slf_ladder  riskperc1  riskperc2  self_esteem
##   7   8  cnarc1  cnarc2  cnarc3  ctheory4  CRT1  CRT2  CRT3  health_cond  moralid10  moralid2  moralid5  moralid6  moralid8  mcoop1  mcoop2  mcoop3  mcoop4  mcoop5  mcoop7  narc3  narc4  narc5  narc6  omind1  omind2  omind3  hygiene5  psupport2  political_ideology  happy  slf_ladder  riskperc1  riskperc2  self_esteem
##   7   9  cnarc1  cnarc2  cnarc3  ctheory4  CRT1  CRT2  CRT3  health_cond  moralid10  moralid2  moralid5  moralid6  moralid8  mcoop1  mcoop2  mcoop3  mcoop4  mcoop5  mcoop7  narc3  narc4  narc5  narc6  omind1  omind2  omind3  hygiene5  psupport2  political_ideology  happy  slf_ladder  riskperc1  riskperc2  self_esteem
##   7   10  cnarc1  cnarc2  cnarc3  ctheory4  CRT1  CRT2  CRT3  health_cond  moralid10  moralid2  moralid5  moralid6  moralid8  mcoop1  mcoop2  mcoop3  mcoop4  mcoop5  mcoop7  narc3  narc4  narc5  narc6  omind1  omind2  omind3  hygiene5  psupport2  political_ideology  happy  slf_ladder  riskperc1  riskperc2  self_esteem
##   8   1  cnarc1  cnarc2  cnarc3  ctheory4  CRT1  CRT2  CRT3  health_cond  moralid10  moralid2  moralid5  moralid6  moralid8  mcoop1  mcoop2  mcoop3  mcoop4  mcoop5  mcoop7  narc3  narc4  narc5  narc6  omind1  omind2  omind3  hygiene5  psupport2  political_ideology  happy  slf_ladder  riskperc1  riskperc2  self_esteem
##   8   2  cnarc1  cnarc2  cnarc3  ctheory4  CRT1  CRT2  CRT3  health_cond  moralid10  moralid2  moralid5  moralid6  moralid8  mcoop1  mcoop2  mcoop3  mcoop4  mcoop5  mcoop7  narc3  narc4  narc5  narc6  omind1  omind2  omind3  hygiene5  psupport2  political_ideology  happy  slf_ladder  riskperc1  riskperc2  self_esteem
##   8   3  cnarc1  cnarc2  cnarc3  ctheory4  CRT1  CRT2  CRT3  health_cond  moralid10  moralid2  moralid5  moralid6  moralid8  mcoop1  mcoop2  mcoop3  mcoop4  mcoop5  mcoop7  narc3  narc4  narc5  narc6  omind1  omind2  omind3  hygiene5  psupport2  political_ideology  happy  slf_ladder  riskperc1  riskperc2  self_esteem
##   8   4  cnarc1  cnarc2  cnarc3  ctheory4  CRT1  CRT2  CRT3  health_cond  moralid10  moralid2  moralid5  moralid6  moralid8  mcoop1  mcoop2  mcoop3  mcoop4  mcoop5  mcoop7  narc3  narc4  narc5  narc6  omind1  omind2  omind3  hygiene5  psupport2  political_ideology  happy  slf_ladder  riskperc1  riskperc2  self_esteem
##   8   5  cnarc1  cnarc2  cnarc3  ctheory4  CRT1  CRT2  CRT3  health_cond  moralid10  moralid2  moralid5  moralid6  moralid8  mcoop1  mcoop2  mcoop3  mcoop4  mcoop5  mcoop7  narc3  narc4  narc5  narc6  omind1  omind2  omind3  hygiene5  psupport2  political_ideology  happy  slf_ladder  riskperc1  riskperc2  self_esteem
##   8   6  cnarc1  cnarc2  cnarc3  ctheory4  CRT1  CRT2  CRT3  health_cond  moralid10  moralid2  moralid5  moralid6  moralid8  mcoop1  mcoop2  mcoop3  mcoop4  mcoop5  mcoop7  narc3  narc4  narc5  narc6  omind1  omind2  omind3  hygiene5  psupport2  political_ideology  happy  slf_ladder  riskperc1  riskperc2  self_esteem
##   8   7  cnarc1  cnarc2  cnarc3  ctheory4  CRT1  CRT2  CRT3  health_cond  moralid10  moralid2  moralid5  moralid6  moralid8  mcoop1  mcoop2  mcoop3  mcoop4  mcoop5  mcoop7  narc3  narc4  narc5  narc6  omind1  omind2  omind3  hygiene5  psupport2  political_ideology  happy  slf_ladder  riskperc1  riskperc2  self_esteem
##   8   8  cnarc1  cnarc2  cnarc3  ctheory4  CRT1  CRT2  CRT3  health_cond  moralid10  moralid2  moralid5  moralid6  moralid8  mcoop1  mcoop2  mcoop3  mcoop4  mcoop5  mcoop7  narc3  narc4  narc5  narc6  omind1  omind2  omind3  hygiene5  psupport2  political_ideology  happy  slf_ladder  riskperc1  riskperc2  self_esteem
##   8   9  cnarc1  cnarc2  cnarc3  ctheory4  CRT1  CRT2  CRT3  health_cond  moralid10  moralid2  moralid5  moralid6  moralid8  mcoop1  mcoop2  mcoop3  mcoop4  mcoop5  mcoop7  narc3  narc4  narc5  narc6  omind1  omind2  omind3  hygiene5  psupport2  political_ideology  happy  slf_ladder  riskperc1  riskperc2  self_esteem
##   8   10  cnarc1  cnarc2  cnarc3  ctheory4  CRT1  CRT2  CRT3  health_cond  moralid10  moralid2  moralid5  moralid6  moralid8  mcoop1  mcoop2  mcoop3  mcoop4  mcoop5  mcoop7  narc3  narc4  narc5  narc6  omind1  omind2  omind3  hygiene5  psupport2  political_ideology  happy  slf_ladder  riskperc1  riskperc2  self_esteem
##   9   1  cnarc1  cnarc2  cnarc3  ctheory4  CRT1  CRT2  CRT3  health_cond  moralid10  moralid2  moralid5  moralid6  moralid8  mcoop1  mcoop2  mcoop3  mcoop4  mcoop5  mcoop7  narc3  narc4  narc5  narc6  omind1  omind2  omind3  hygiene5  psupport2  political_ideology  happy  slf_ladder  riskperc1  riskperc2  self_esteem
##   9   2  cnarc1  cnarc2  cnarc3  ctheory4  CRT1  CRT2  CRT3  health_cond  moralid10  moralid2  moralid5  moralid6  moralid8  mcoop1  mcoop2  mcoop3  mcoop4  mcoop5  mcoop7  narc3  narc4  narc5  narc6  omind1  omind2  omind3  hygiene5  psupport2  political_ideology  happy  slf_ladder  riskperc1  riskperc2  self_esteem
##   9   3  cnarc1  cnarc2  cnarc3  ctheory4  CRT1  CRT2  CRT3  health_cond  moralid10  moralid2  moralid5  moralid6  moralid8  mcoop1  mcoop2  mcoop3  mcoop4  mcoop5  mcoop7  narc3  narc4  narc5  narc6  omind1  omind2  omind3  hygiene5  psupport2  political_ideology  happy  slf_ladder  riskperc1  riskperc2  self_esteem
##   9   4  cnarc1  cnarc2  cnarc3  ctheory4  CRT1  CRT2  CRT3  health_cond  moralid10  moralid2  moralid5  moralid6  moralid8  mcoop1  mcoop2  mcoop3  mcoop4  mcoop5  mcoop7  narc3  narc4  narc5  narc6  omind1  omind2  omind3  hygiene5  psupport2  political_ideology  happy  slf_ladder  riskperc1  riskperc2  self_esteem
##   9   5  cnarc1  cnarc2  cnarc3  ctheory4  CRT1  CRT2  CRT3  health_cond  moralid10  moralid2  moralid5  moralid6  moralid8  mcoop1  mcoop2  mcoop3  mcoop4  mcoop5  mcoop7  narc3  narc4  narc5  narc6  omind1  omind2  omind3  hygiene5  psupport2  political_ideology  happy  slf_ladder  riskperc1  riskperc2  self_esteem
##   9   6  cnarc1  cnarc2  cnarc3  ctheory4  CRT1  CRT2  CRT3  health_cond  moralid10  moralid2  moralid5  moralid6  moralid8  mcoop1  mcoop2  mcoop3  mcoop4  mcoop5  mcoop7  narc3  narc4  narc5  narc6  omind1  omind2  omind3  hygiene5  psupport2  political_ideology  happy  slf_ladder  riskperc1  riskperc2  self_esteem
##   9   7  cnarc1  cnarc2  cnarc3  ctheory4  CRT1  CRT2  CRT3  health_cond  moralid10  moralid2  moralid5  moralid6  moralid8  mcoop1  mcoop2  mcoop3  mcoop4  mcoop5  mcoop7  narc3  narc4  narc5  narc6  omind1  omind2  omind3  hygiene5  psupport2  political_ideology  happy  slf_ladder  riskperc1  riskperc2  self_esteem
##   9   8  cnarc1  cnarc2  cnarc3  ctheory4  CRT1  CRT2  CRT3  health_cond  moralid10  moralid2  moralid5  moralid6  moralid8  mcoop1  mcoop2  mcoop3  mcoop4  mcoop5  mcoop7  narc3  narc4  narc5  narc6  omind1  omind2  omind3  hygiene5  psupport2  political_ideology  happy  slf_ladder  riskperc1  riskperc2  self_esteem
##   9   9  cnarc1  cnarc2  cnarc3  ctheory4  CRT1  CRT2  CRT3  health_cond  moralid10  moralid2  moralid5  moralid6  moralid8  mcoop1  mcoop2  mcoop3  mcoop4  mcoop5  mcoop7  narc3  narc4  narc5  narc6  omind1  omind2  omind3  hygiene5  psupport2  political_ideology  happy  slf_ladder  riskperc1  riskperc2  self_esteem
##   9   10  cnarc1  cnarc2  cnarc3  ctheory4  CRT1  CRT2  CRT3  health_cond  moralid10  moralid2  moralid5  moralid6  moralid8  mcoop1  mcoop2  mcoop3  mcoop4  mcoop5  mcoop7  narc3  narc4  narc5  narc6  omind1  omind2  omind3  hygiene5  psupport2  political_ideology  happy  slf_ladder  riskperc1  riskperc2  self_esteem
##   10   1  cnarc1  cnarc2  cnarc3  ctheory4  CRT1  CRT2  CRT3  health_cond  moralid10  moralid2  moralid5  moralid6  moralid8  mcoop1  mcoop2  mcoop3  mcoop4  mcoop5  mcoop7  narc3  narc4  narc5  narc6  omind1  omind2  omind3  hygiene5  psupport2  political_ideology  happy  slf_ladder  riskperc1  riskperc2  self_esteem
##   10   2  cnarc1  cnarc2  cnarc3  ctheory4  CRT1  CRT2  CRT3  health_cond  moralid10  moralid2  moralid5  moralid6  moralid8  mcoop1  mcoop2  mcoop3  mcoop4  mcoop5  mcoop7  narc3  narc4  narc5  narc6  omind1  omind2  omind3  hygiene5  psupport2  political_ideology  happy  slf_ladder  riskperc1  riskperc2  self_esteem
##   10   3  cnarc1  cnarc2  cnarc3  ctheory4  CRT1  CRT2  CRT3  health_cond  moralid10  moralid2  moralid5  moralid6  moralid8  mcoop1  mcoop2  mcoop3  mcoop4  mcoop5  mcoop7  narc3  narc4  narc5  narc6  omind1  omind2  omind3  hygiene5  psupport2  political_ideology  happy  slf_ladder  riskperc1  riskperc2  self_esteem
##   10   4  cnarc1  cnarc2  cnarc3  ctheory4  CRT1  CRT2  CRT3  health_cond  moralid10  moralid2  moralid5  moralid6  moralid8  mcoop1  mcoop2  mcoop3  mcoop4  mcoop5  mcoop7  narc3  narc4  narc5  narc6  omind1  omind2  omind3  hygiene5  psupport2  political_ideology  happy  slf_ladder  riskperc1  riskperc2  self_esteem
##   10   5  cnarc1  cnarc2  cnarc3  ctheory4  CRT1  CRT2  CRT3  health_cond  moralid10  moralid2  moralid5  moralid6  moralid8  mcoop1  mcoop2  mcoop3  mcoop4  mcoop5  mcoop7  narc3  narc4  narc5  narc6  omind1  omind2  omind3  hygiene5  psupport2  political_ideology  happy  slf_ladder  riskperc1  riskperc2  self_esteem
##   10   6  cnarc1  cnarc2  cnarc3  ctheory4  CRT1  CRT2  CRT3  health_cond  moralid10  moralid2  moralid5  moralid6  moralid8  mcoop1  mcoop2  mcoop3  mcoop4  mcoop5  mcoop7  narc3  narc4  narc5  narc6  omind1  omind2  omind3  hygiene5  psupport2  political_ideology  happy  slf_ladder  riskperc1  riskperc2  self_esteem
##   10   7  cnarc1  cnarc2  cnarc3  ctheory4  CRT1  CRT2  CRT3  health_cond  moralid10  moralid2  moralid5  moralid6  moralid8  mcoop1  mcoop2  mcoop3  mcoop4  mcoop5  mcoop7  narc3  narc4  narc5  narc6  omind1  omind2  omind3  hygiene5  psupport2  political_ideology  happy  slf_ladder  riskperc1  riskperc2  self_esteem
##   10   8  cnarc1  cnarc2  cnarc3  ctheory4  CRT1  CRT2  CRT3  health_cond  moralid10  moralid2  moralid5  moralid6  moralid8  mcoop1  mcoop2  mcoop3  mcoop4  mcoop5  mcoop7  narc3  narc4  narc5  narc6  omind1  omind2  omind3  hygiene5  psupport2  political_ideology  happy  slf_ladder  riskperc1  riskperc2  self_esteem
##   10   9  cnarc1  cnarc2  cnarc3  ctheory4  CRT1  CRT2  CRT3  health_cond  moralid10  moralid2  moralid5  moralid6  moralid8  mcoop1  mcoop2  mcoop3  mcoop4  mcoop5  mcoop7  narc3  narc4  narc5  narc6  omind1  omind2  omind3  hygiene5  psupport2  political_ideology  happy  slf_ladder  riskperc1  riskperc2  self_esteem
##   10   10  cnarc1  cnarc2  cnarc3  ctheory4  CRT1  CRT2  CRT3  health_cond  moralid10  moralid2  moralid5  moralid6  moralid8  mcoop1  mcoop2  mcoop3  mcoop4  mcoop5  mcoop7  narc3  narc4  narc5  narc6  omind1  omind2  omind3  hygiene5  psupport2  political_ideology  happy  slf_ladder  riskperc1  riskperc2  self_esteem
```

```
## Warning: Number of logged events: 3403
```

```
## 
##  iter imp variable
##   1   1  age  children  cnarc1  cnarc2  cnarc3  ctheory2  CRT1  CRT2  CRT3  employ_status1  generosity1  generosity2  generosity3  ladder  marital1  mor_circle  moralid1  moralid10  moralid2  moralid3  moralid4  moralid5  moralid6  moralid7  moralid8  moralid9  mcoop1  mcoop2  mcoop3  mcoop4  mcoop5  mcoop6  mcoop7  narc4  narc5  narc6  nidentity1  nidentity2  omind1  omind2  omind6  contact1  contact2  contact3  contact4  contact5  hygiene3  hygiene5  psupport1  psupport3  psupport4  psupport5  political_ideology  riskperc1  sex1  sbelong3  tested_positive  slfcont2  slfcont3  slfcont4  urban
##   1   2  age  children  cnarc1  cnarc2  cnarc3  ctheory2  CRT1  CRT2  CRT3  employ_status1  generosity1  generosity2  generosity3  ladder  marital1  mor_circle  moralid1  moralid10  moralid2  moralid3  moralid4  moralid5  moralid6  moralid7  moralid8  moralid9  mcoop1  mcoop2  mcoop3  mcoop4  mcoop5  mcoop6  mcoop7  narc4  narc5  narc6  nidentity1  nidentity2  omind1  omind2  omind6  contact1  contact2  contact3  contact4  contact5  hygiene3  hygiene5  psupport1  psupport3  psupport4  psupport5  political_ideology  riskperc1  sex1  sbelong3  tested_positive  slfcont2  slfcont3  slfcont4  urban
##   1   3  age  children  cnarc1  cnarc2  cnarc3  ctheory2  CRT1  CRT2  CRT3  employ_status1  generosity1  generosity2  generosity3  ladder  marital1  mor_circle  moralid1  moralid10  moralid2  moralid3  moralid4  moralid5  moralid6  moralid7  moralid8  moralid9  mcoop1  mcoop2  mcoop3  mcoop4  mcoop5  mcoop6  mcoop7  narc4  narc5  narc6  nidentity1  nidentity2  omind1  omind2  omind6  contact1  contact2  contact3  contact4  contact5  hygiene3  hygiene5  psupport1  psupport3  psupport4  psupport5  political_ideology  riskperc1  sex1  sbelong3  tested_positive  slfcont2  slfcont3  slfcont4  urban
##   1   4  age  children  cnarc1  cnarc2  cnarc3  ctheory2  CRT1  CRT2  CRT3  employ_status1  generosity1  generosity2  generosity3  ladder  marital1  mor_circle  moralid1  moralid10  moralid2  moralid3  moralid4  moralid5  moralid6  moralid7  moralid8  moralid9  mcoop1  mcoop2  mcoop3  mcoop4  mcoop5  mcoop6  mcoop7  narc4  narc5  narc6  nidentity1  nidentity2  omind1  omind2  omind6  contact1  contact2  contact3  contact4  contact5  hygiene3  hygiene5  psupport1  psupport3  psupport4  psupport5  political_ideology  riskperc1  sex1  sbelong3  tested_positive  slfcont2  slfcont3  slfcont4  urban
##   1   5  age  children  cnarc1  cnarc2  cnarc3  ctheory2  CRT1  CRT2  CRT3  employ_status1  generosity1  generosity2  generosity3  ladder  marital1  mor_circle  moralid1  moralid10  moralid2  moralid3  moralid4  moralid5  moralid6  moralid7  moralid8  moralid9  mcoop1  mcoop2  mcoop3  mcoop4  mcoop5  mcoop6  mcoop7  narc4  narc5  narc6  nidentity1  nidentity2  omind1  omind2  omind6  contact1  contact2  contact3  contact4  contact5  hygiene3  hygiene5  psupport1  psupport3  psupport4  psupport5  political_ideology  riskperc1  sex1  sbelong3  tested_positive  slfcont2  slfcont3  slfcont4  urban
##   1   6  age  children  cnarc1  cnarc2  cnarc3  ctheory2  CRT1  CRT2  CRT3  employ_status1  generosity1  generosity2  generosity3  ladder  marital1  mor_circle  moralid1  moralid10  moralid2  moralid3  moralid4  moralid5  moralid6  moralid7  moralid8  moralid9  mcoop1  mcoop2  mcoop3  mcoop4  mcoop5  mcoop6  mcoop7  narc4  narc5  narc6  nidentity1  nidentity2  omind1  omind2  omind6  contact1  contact2  contact3  contact4  contact5  hygiene3  hygiene5  psupport1  psupport3  psupport4  psupport5  political_ideology  riskperc1  sex1  sbelong3  tested_positive  slfcont2  slfcont3  slfcont4  urban
##   1   7  age  children  cnarc1  cnarc2  cnarc3  ctheory2  CRT1  CRT2  CRT3  employ_status1  generosity1  generosity2  generosity3  ladder  marital1  mor_circle  moralid1  moralid10  moralid2  moralid3  moralid4  moralid5  moralid6  moralid7  moralid8  moralid9  mcoop1  mcoop2  mcoop3  mcoop4  mcoop5  mcoop6  mcoop7  narc4  narc5  narc6  nidentity1  nidentity2  omind1  omind2  omind6  contact1  contact2  contact3  contact4  contact5  hygiene3  hygiene5  psupport1  psupport3  psupport4  psupport5  political_ideology  riskperc1  sex1  sbelong3  tested_positive  slfcont2  slfcont3  slfcont4  urban
##   1   8  age  children  cnarc1  cnarc2  cnarc3  ctheory2  CRT1  CRT2  CRT3  employ_status1  generosity1  generosity2  generosity3  ladder  marital1  mor_circle  moralid1  moralid10  moralid2  moralid3  moralid4  moralid5  moralid6  moralid7  moralid8  moralid9  mcoop1  mcoop2  mcoop3  mcoop4  mcoop5  mcoop6  mcoop7  narc4  narc5  narc6  nidentity1  nidentity2  omind1  omind2  omind6  contact1  contact2  contact3  contact4  contact5  hygiene3  hygiene5  psupport1  psupport3  psupport4  psupport5  political_ideology  riskperc1  sex1  sbelong3  tested_positive  slfcont2  slfcont3  slfcont4  urban
##   1   9  age  children  cnarc1  cnarc2  cnarc3  ctheory2  CRT1  CRT2  CRT3  employ_status1  generosity1  generosity2  generosity3  ladder  marital1  mor_circle  moralid1  moralid10  moralid2  moralid3  moralid4  moralid5  moralid6  moralid7  moralid8  moralid9  mcoop1  mcoop2  mcoop3  mcoop4  mcoop5  mcoop6  mcoop7  narc4  narc5  narc6  nidentity1  nidentity2  omind1  omind2  omind6  contact1  contact2  contact3  contact4  contact5  hygiene3  hygiene5  psupport1  psupport3  psupport4  psupport5  political_ideology  riskperc1  sex1  sbelong3  tested_positive  slfcont2  slfcont3  slfcont4  urban
##   1   10  age  children  cnarc1  cnarc2  cnarc3  ctheory2  CRT1  CRT2  CRT3  employ_status1  generosity1  generosity2  generosity3  ladder  marital1  mor_circle  moralid1  moralid10  moralid2  moralid3  moralid4  moralid5  moralid6  moralid7  moralid8  moralid9  mcoop1  mcoop2  mcoop3  mcoop4  mcoop5  mcoop6  mcoop7  narc4  narc5  narc6  nidentity1  nidentity2  omind1  omind2  omind6  contact1  contact2  contact3  contact4  contact5  hygiene3  hygiene5  psupport1  psupport3  psupport4  psupport5  political_ideology  riskperc1  sex1  sbelong3  tested_positive  slfcont2  slfcont3  slfcont4  urban
##   2   1  age  children  cnarc1  cnarc2  cnarc3  ctheory2  CRT1  CRT2  CRT3  employ_status1  generosity1  generosity2  generosity3  ladder  marital1  mor_circle  moralid1  moralid10  moralid2  moralid3  moralid4  moralid5  moralid6  moralid7  moralid8  moralid9  mcoop1  mcoop2  mcoop3  mcoop4  mcoop5  mcoop6  mcoop7  narc4  narc5  narc6  nidentity1  nidentity2  omind1  omind2  omind6  contact1  contact2  contact3  contact4  contact5  hygiene3  hygiene5  psupport1  psupport3  psupport4  psupport5  political_ideology  riskperc1  sex1  sbelong3  tested_positive  slfcont2  slfcont3  slfcont4  urban
##   2   2  age  children  cnarc1  cnarc2  cnarc3  ctheory2  CRT1  CRT2  CRT3  employ_status1  generosity1  generosity2  generosity3  ladder  marital1  mor_circle  moralid1  moralid10  moralid2  moralid3  moralid4  moralid5  moralid6  moralid7  moralid8  moralid9  mcoop1  mcoop2  mcoop3  mcoop4  mcoop5  mcoop6  mcoop7  narc4  narc5  narc6  nidentity1  nidentity2  omind1  omind2  omind6  contact1  contact2  contact3  contact4  contact5  hygiene3  hygiene5  psupport1  psupport3  psupport4  psupport5  political_ideology  riskperc1  sex1  sbelong3  tested_positive  slfcont2  slfcont3  slfcont4  urban
##   2   3  age  children  cnarc1  cnarc2  cnarc3  ctheory2  CRT1  CRT2  CRT3  employ_status1  generosity1  generosity2  generosity3  ladder  marital1  mor_circle  moralid1  moralid10  moralid2  moralid3  moralid4  moralid5  moralid6  moralid7  moralid8  moralid9  mcoop1  mcoop2  mcoop3  mcoop4  mcoop5  mcoop6  mcoop7  narc4  narc5  narc6  nidentity1  nidentity2  omind1  omind2  omind6  contact1  contact2  contact3  contact4  contact5  hygiene3  hygiene5  psupport1  psupport3  psupport4  psupport5  political_ideology  riskperc1  sex1  sbelong3  tested_positive  slfcont2  slfcont3  slfcont4  urban
##   2   4  age  children  cnarc1  cnarc2  cnarc3  ctheory2  CRT1  CRT2  CRT3  employ_status1  generosity1  generosity2  generosity3  ladder  marital1  mor_circle  moralid1  moralid10  moralid2  moralid3  moralid4  moralid5  moralid6  moralid7  moralid8  moralid9  mcoop1  mcoop2  mcoop3  mcoop4  mcoop5  mcoop6  mcoop7  narc4  narc5  narc6  nidentity1  nidentity2  omind1  omind2  omind6  contact1  contact2  contact3  contact4  contact5  hygiene3  hygiene5  psupport1  psupport3  psupport4  psupport5  political_ideology  riskperc1  sex1  sbelong3  tested_positive  slfcont2  slfcont3  slfcont4  urban
##   2   5  age  children  cnarc1  cnarc2  cnarc3  ctheory2  CRT1  CRT2  CRT3  employ_status1  generosity1  generosity2  generosity3  ladder  marital1  mor_circle  moralid1  moralid10  moralid2  moralid3  moralid4  moralid5  moralid6  moralid7  moralid8  moralid9  mcoop1  mcoop2  mcoop3  mcoop4  mcoop5  mcoop6  mcoop7  narc4  narc5  narc6  nidentity1  nidentity2  omind1  omind2  omind6  contact1  contact2  contact3  contact4  contact5  hygiene3  hygiene5  psupport1  psupport3  psupport4  psupport5  political_ideology  riskperc1  sex1  sbelong3  tested_positive  slfcont2  slfcont3  slfcont4  urban
##   2   6  age  children  cnarc1  cnarc2  cnarc3  ctheory2  CRT1  CRT2  CRT3  employ_status1  generosity1  generosity2  generosity3  ladder  marital1  mor_circle  moralid1  moralid10  moralid2  moralid3  moralid4  moralid5  moralid6  moralid7  moralid8  moralid9  mcoop1  mcoop2  mcoop3  mcoop4  mcoop5  mcoop6  mcoop7  narc4  narc5  narc6  nidentity1  nidentity2  omind1  omind2  omind6  contact1  contact2  contact3  contact4  contact5  hygiene3  hygiene5  psupport1  psupport3  psupport4  psupport5  political_ideology  riskperc1  sex1  sbelong3  tested_positive  slfcont2  slfcont3  slfcont4  urban
##   2   7  age  children  cnarc1  cnarc2  cnarc3  ctheory2  CRT1  CRT2  CRT3  employ_status1  generosity1  generosity2  generosity3  ladder  marital1  mor_circle  moralid1  moralid10  moralid2  moralid3  moralid4  moralid5  moralid6  moralid7  moralid8  moralid9  mcoop1  mcoop2  mcoop3  mcoop4  mcoop5  mcoop6  mcoop7  narc4  narc5  narc6  nidentity1  nidentity2  omind1  omind2  omind6  contact1  contact2  contact3  contact4  contact5  hygiene3  hygiene5  psupport1  psupport3  psupport4  psupport5  political_ideology  riskperc1  sex1  sbelong3  tested_positive  slfcont2  slfcont3  slfcont4  urban
##   2   8  age  children  cnarc1  cnarc2  cnarc3  ctheory2  CRT1  CRT2  CRT3  employ_status1  generosity1  generosity2  generosity3  ladder  marital1  mor_circle  moralid1  moralid10  moralid2  moralid3  moralid4  moralid5  moralid6  moralid7  moralid8  moralid9  mcoop1  mcoop2  mcoop3  mcoop4  mcoop5  mcoop6  mcoop7  narc4  narc5  narc6  nidentity1  nidentity2  omind1  omind2  omind6  contact1  contact2  contact3  contact4  contact5  hygiene3  hygiene5  psupport1  psupport3  psupport4  psupport5  political_ideology  riskperc1  sex1  sbelong3  tested_positive  slfcont2  slfcont3  slfcont4  urban
##   2   9  age  children  cnarc1  cnarc2  cnarc3  ctheory2  CRT1  CRT2  CRT3  employ_status1  generosity1  generosity2  generosity3  ladder  marital1  mor_circle  moralid1  moralid10  moralid2  moralid3  moralid4  moralid5  moralid6  moralid7  moralid8  moralid9  mcoop1  mcoop2  mcoop3  mcoop4  mcoop5  mcoop6  mcoop7  narc4  narc5  narc6  nidentity1  nidentity2  omind1  omind2  omind6  contact1  contact2  contact3  contact4  contact5  hygiene3  hygiene5  psupport1  psupport3  psupport4  psupport5  political_ideology  riskperc1  sex1  sbelong3  tested_positive  slfcont2  slfcont3  slfcont4  urban
##   2   10  age  children  cnarc1  cnarc2  cnarc3  ctheory2  CRT1  CRT2  CRT3  employ_status1  generosity1  generosity2  generosity3  ladder  marital1  mor_circle  moralid1  moralid10  moralid2  moralid3  moralid4  moralid5  moralid6  moralid7  moralid8  moralid9  mcoop1  mcoop2  mcoop3  mcoop4  mcoop5  mcoop6  mcoop7  narc4  narc5  narc6  nidentity1  nidentity2  omind1  omind2  omind6  contact1  contact2  contact3  contact4  contact5  hygiene3  hygiene5  psupport1  psupport3  psupport4  psupport5  political_ideology  riskperc1  sex1  sbelong3  tested_positive  slfcont2  slfcont3  slfcont4  urban
##   3   1  age  children  cnarc1  cnarc2  cnarc3  ctheory2  CRT1  CRT2  CRT3  employ_status1  generosity1  generosity2  generosity3  ladder  marital1  mor_circle  moralid1  moralid10  moralid2  moralid3  moralid4  moralid5  moralid6  moralid7  moralid8  moralid9  mcoop1  mcoop2  mcoop3  mcoop4  mcoop5  mcoop6  mcoop7  narc4  narc5  narc6  nidentity1  nidentity2  omind1  omind2  omind6  contact1  contact2  contact3  contact4  contact5  hygiene3  hygiene5  psupport1  psupport3  psupport4  psupport5  political_ideology  riskperc1  sex1  sbelong3  tested_positive  slfcont2  slfcont3  slfcont4  urban
##   3   2  age  children  cnarc1  cnarc2  cnarc3  ctheory2  CRT1  CRT2  CRT3  employ_status1  generosity1  generosity2  generosity3  ladder  marital1  mor_circle  moralid1  moralid10  moralid2  moralid3  moralid4  moralid5  moralid6  moralid7  moralid8  moralid9  mcoop1  mcoop2  mcoop3  mcoop4  mcoop5  mcoop6  mcoop7  narc4  narc5  narc6  nidentity1  nidentity2  omind1  omind2  omind6  contact1  contact2  contact3  contact4  contact5  hygiene3  hygiene5  psupport1  psupport3  psupport4  psupport5  political_ideology  riskperc1  sex1  sbelong3  tested_positive  slfcont2  slfcont3  slfcont4  urban
##   3   3  age  children  cnarc1  cnarc2  cnarc3  ctheory2  CRT1  CRT2  CRT3  employ_status1  generosity1  generosity2  generosity3  ladder  marital1  mor_circle  moralid1  moralid10  moralid2  moralid3  moralid4  moralid5  moralid6  moralid7  moralid8  moralid9  mcoop1  mcoop2  mcoop3  mcoop4  mcoop5  mcoop6  mcoop7  narc4  narc5  narc6  nidentity1  nidentity2  omind1  omind2  omind6  contact1  contact2  contact3  contact4  contact5  hygiene3  hygiene5  psupport1  psupport3  psupport4  psupport5  political_ideology  riskperc1  sex1  sbelong3  tested_positive  slfcont2  slfcont3  slfcont4  urban
##   3   4  age  children  cnarc1  cnarc2  cnarc3  ctheory2  CRT1  CRT2  CRT3  employ_status1  generosity1  generosity2  generosity3  ladder  marital1  mor_circle  moralid1  moralid10  moralid2  moralid3  moralid4  moralid5  moralid6  moralid7  moralid8  moralid9  mcoop1  mcoop2  mcoop3  mcoop4  mcoop5  mcoop6  mcoop7  narc4  narc5  narc6  nidentity1  nidentity2  omind1  omind2  omind6  contact1  contact2  contact3  contact4  contact5  hygiene3  hygiene5  psupport1  psupport3  psupport4  psupport5  political_ideology  riskperc1  sex1  sbelong3  tested_positive  slfcont2  slfcont3  slfcont4  urban
##   3   5  age  children  cnarc1  cnarc2  cnarc3  ctheory2  CRT1  CRT2  CRT3  employ_status1  generosity1  generosity2  generosity3  ladder  marital1  mor_circle  moralid1  moralid10  moralid2  moralid3  moralid4  moralid5  moralid6  moralid7  moralid8  moralid9  mcoop1  mcoop2  mcoop3  mcoop4  mcoop5  mcoop6  mcoop7  narc4  narc5  narc6  nidentity1  nidentity2  omind1  omind2  omind6  contact1  contact2  contact3  contact4  contact5  hygiene3  hygiene5  psupport1  psupport3  psupport4  psupport5  political_ideology  riskperc1  sex1  sbelong3  tested_positive  slfcont2  slfcont3  slfcont4  urban
##   3   6  age  children  cnarc1  cnarc2  cnarc3  ctheory2  CRT1  CRT2  CRT3  employ_status1  generosity1  generosity2  generosity3  ladder  marital1  mor_circle  moralid1  moralid10  moralid2  moralid3  moralid4  moralid5  moralid6  moralid7  moralid8  moralid9  mcoop1  mcoop2  mcoop3  mcoop4  mcoop5  mcoop6  mcoop7  narc4  narc5  narc6  nidentity1  nidentity2  omind1  omind2  omind6  contact1  contact2  contact3  contact4  contact5  hygiene3  hygiene5  psupport1  psupport3  psupport4  psupport5  political_ideology  riskperc1  sex1  sbelong3  tested_positive  slfcont2  slfcont3  slfcont4  urban
##   3   7  age  children  cnarc1  cnarc2  cnarc3  ctheory2  CRT1  CRT2  CRT3  employ_status1  generosity1  generosity2  generosity3  ladder  marital1  mor_circle  moralid1  moralid10  moralid2  moralid3  moralid4  moralid5  moralid6  moralid7  moralid8  moralid9  mcoop1  mcoop2  mcoop3  mcoop4  mcoop5  mcoop6  mcoop7  narc4  narc5  narc6  nidentity1  nidentity2  omind1  omind2  omind6  contact1  contact2  contact3  contact4  contact5  hygiene3  hygiene5  psupport1  psupport3  psupport4  psupport5  political_ideology  riskperc1  sex1  sbelong3  tested_positive  slfcont2  slfcont3  slfcont4  urban
##   3   8  age  children  cnarc1  cnarc2  cnarc3  ctheory2  CRT1  CRT2  CRT3  employ_status1  generosity1  generosity2  generosity3  ladder  marital1  mor_circle  moralid1  moralid10  moralid2  moralid3  moralid4  moralid5  moralid6  moralid7  moralid8  moralid9  mcoop1  mcoop2  mcoop3  mcoop4  mcoop5  mcoop6  mcoop7  narc4  narc5  narc6  nidentity1  nidentity2  omind1  omind2  omind6  contact1  contact2  contact3  contact4  contact5  hygiene3  hygiene5  psupport1  psupport3  psupport4  psupport5  political_ideology  riskperc1  sex1  sbelong3  tested_positive  slfcont2  slfcont3  slfcont4  urban
##   3   9  age  children  cnarc1  cnarc2  cnarc3  ctheory2  CRT1  CRT2  CRT3  employ_status1  generosity1  generosity2  generosity3  ladder  marital1  mor_circle  moralid1  moralid10  moralid2  moralid3  moralid4  moralid5  moralid6  moralid7  moralid8  moralid9  mcoop1  mcoop2  mcoop3  mcoop4  mcoop5  mcoop6  mcoop7  narc4  narc5  narc6  nidentity1  nidentity2  omind1  omind2  omind6  contact1  contact2  contact3  contact4  contact5  hygiene3  hygiene5  psupport1  psupport3  psupport4  psupport5  political_ideology  riskperc1  sex1  sbelong3  tested_positive  slfcont2  slfcont3  slfcont4  urban
##   3   10  age  children  cnarc1  cnarc2  cnarc3  ctheory2  CRT1  CRT2  CRT3  employ_status1  generosity1  generosity2  generosity3  ladder  marital1  mor_circle  moralid1  moralid10  moralid2  moralid3  moralid4  moralid5  moralid6  moralid7  moralid8  moralid9  mcoop1  mcoop2  mcoop3  mcoop4  mcoop5  mcoop6  mcoop7  narc4  narc5  narc6  nidentity1  nidentity2  omind1  omind2  omind6  contact1  contact2  contact3  contact4  contact5  hygiene3  hygiene5  psupport1  psupport3  psupport4  psupport5  political_ideology  riskperc1  sex1  sbelong3  tested_positive  slfcont2  slfcont3  slfcont4  urban
##   4   1  age  children  cnarc1  cnarc2  cnarc3  ctheory2  CRT1  CRT2  CRT3  employ_status1  generosity1  generosity2  generosity3  ladder  marital1  mor_circle  moralid1  moralid10  moralid2  moralid3  moralid4  moralid5  moralid6  moralid7  moralid8  moralid9  mcoop1  mcoop2  mcoop3  mcoop4  mcoop5  mcoop6  mcoop7  narc4  narc5  narc6  nidentity1  nidentity2  omind1  omind2  omind6  contact1  contact2  contact3  contact4  contact5  hygiene3  hygiene5  psupport1  psupport3  psupport4  psupport5  political_ideology  riskperc1  sex1  sbelong3  tested_positive  slfcont2  slfcont3  slfcont4  urban
##   4   2  age  children  cnarc1  cnarc2  cnarc3  ctheory2  CRT1  CRT2  CRT3  employ_status1  generosity1  generosity2  generosity3  ladder  marital1  mor_circle  moralid1  moralid10  moralid2  moralid3  moralid4  moralid5  moralid6  moralid7  moralid8  moralid9  mcoop1  mcoop2  mcoop3  mcoop4  mcoop5  mcoop6  mcoop7  narc4  narc5  narc6  nidentity1  nidentity2  omind1  omind2  omind6  contact1  contact2  contact3  contact4  contact5  hygiene3  hygiene5  psupport1  psupport3  psupport4  psupport5  political_ideology  riskperc1  sex1  sbelong3  tested_positive  slfcont2  slfcont3  slfcont4  urban
##   4   3  age  children  cnarc1  cnarc2  cnarc3  ctheory2  CRT1  CRT2  CRT3  employ_status1  generosity1  generosity2  generosity3  ladder  marital1  mor_circle  moralid1  moralid10  moralid2  moralid3  moralid4  moralid5  moralid6  moralid7  moralid8  moralid9  mcoop1  mcoop2  mcoop3  mcoop4  mcoop5  mcoop6  mcoop7  narc4  narc5  narc6  nidentity1  nidentity2  omind1  omind2  omind6  contact1  contact2  contact3  contact4  contact5  hygiene3  hygiene5  psupport1  psupport3  psupport4  psupport5  political_ideology  riskperc1  sex1  sbelong3  tested_positive  slfcont2  slfcont3  slfcont4  urban
##   4   4  age  children  cnarc1  cnarc2  cnarc3  ctheory2  CRT1  CRT2  CRT3  employ_status1  generosity1  generosity2  generosity3  ladder  marital1  mor_circle  moralid1  moralid10  moralid2  moralid3  moralid4  moralid5  moralid6  moralid7  moralid8  moralid9  mcoop1  mcoop2  mcoop3  mcoop4  mcoop5  mcoop6  mcoop7  narc4  narc5  narc6  nidentity1  nidentity2  omind1  omind2  omind6  contact1  contact2  contact3  contact4  contact5  hygiene3  hygiene5  psupport1  psupport3  psupport4  psupport5  political_ideology  riskperc1  sex1  sbelong3  tested_positive  slfcont2  slfcont3  slfcont4  urban
##   4   5  age  children  cnarc1  cnarc2  cnarc3  ctheory2  CRT1  CRT2  CRT3  employ_status1  generosity1  generosity2  generosity3  ladder  marital1  mor_circle  moralid1  moralid10  moralid2  moralid3  moralid4  moralid5  moralid6  moralid7  moralid8  moralid9  mcoop1  mcoop2  mcoop3  mcoop4  mcoop5  mcoop6  mcoop7  narc4  narc5  narc6  nidentity1  nidentity2  omind1  omind2  omind6  contact1  contact2  contact3  contact4  contact5  hygiene3  hygiene5  psupport1  psupport3  psupport4  psupport5  political_ideology  riskperc1  sex1  sbelong3  tested_positive  slfcont2  slfcont3  slfcont4  urban
##   4   6  age  children  cnarc1  cnarc2  cnarc3  ctheory2  CRT1  CRT2  CRT3  employ_status1  generosity1  generosity2  generosity3  ladder  marital1  mor_circle  moralid1  moralid10  moralid2  moralid3  moralid4  moralid5  moralid6  moralid7  moralid8  moralid9  mcoop1  mcoop2  mcoop3  mcoop4  mcoop5  mcoop6  mcoop7  narc4  narc5  narc6  nidentity1  nidentity2  omind1  omind2  omind6  contact1  contact2  contact3  contact4  contact5  hygiene3  hygiene5  psupport1  psupport3  psupport4  psupport5  political_ideology  riskperc1  sex1  sbelong3  tested_positive  slfcont2  slfcont3  slfcont4  urban
##   4   7  age  children  cnarc1  cnarc2  cnarc3  ctheory2  CRT1  CRT2  CRT3  employ_status1  generosity1  generosity2  generosity3  ladder  marital1  mor_circle  moralid1  moralid10  moralid2  moralid3  moralid4  moralid5  moralid6  moralid7  moralid8  moralid9  mcoop1  mcoop2  mcoop3  mcoop4  mcoop5  mcoop6  mcoop7  narc4  narc5  narc6  nidentity1  nidentity2  omind1  omind2  omind6  contact1  contact2  contact3  contact4  contact5  hygiene3  hygiene5  psupport1  psupport3  psupport4  psupport5  political_ideology  riskperc1  sex1  sbelong3  tested_positive  slfcont2  slfcont3  slfcont4  urban
##   4   8  age  children  cnarc1  cnarc2  cnarc3  ctheory2  CRT1  CRT2  CRT3  employ_status1  generosity1  generosity2  generosity3  ladder  marital1  mor_circle  moralid1  moralid10  moralid2  moralid3  moralid4  moralid5  moralid6  moralid7  moralid8  moralid9  mcoop1  mcoop2  mcoop3  mcoop4  mcoop5  mcoop6  mcoop7  narc4  narc5  narc6  nidentity1  nidentity2  omind1  omind2  omind6  contact1  contact2  contact3  contact4  contact5  hygiene3  hygiene5  psupport1  psupport3  psupport4  psupport5  political_ideology  riskperc1  sex1  sbelong3  tested_positive  slfcont2  slfcont3  slfcont4  urban
##   4   9  age  children  cnarc1  cnarc2  cnarc3  ctheory2  CRT1  CRT2  CRT3  employ_status1  generosity1  generosity2  generosity3  ladder  marital1  mor_circle  moralid1  moralid10  moralid2  moralid3  moralid4  moralid5  moralid6  moralid7  moralid8  moralid9  mcoop1  mcoop2  mcoop3  mcoop4  mcoop5  mcoop6  mcoop7  narc4  narc5  narc6  nidentity1  nidentity2  omind1  omind2  omind6  contact1  contact2  contact3  contact4  contact5  hygiene3  hygiene5  psupport1  psupport3  psupport4  psupport5  political_ideology  riskperc1  sex1  sbelong3  tested_positive  slfcont2  slfcont3  slfcont4  urban
##   4   10  age  children  cnarc1  cnarc2  cnarc3  ctheory2  CRT1  CRT2  CRT3  employ_status1  generosity1  generosity2  generosity3  ladder  marital1  mor_circle  moralid1  moralid10  moralid2  moralid3  moralid4  moralid5  moralid6  moralid7  moralid8  moralid9  mcoop1  mcoop2  mcoop3  mcoop4  mcoop5  mcoop6  mcoop7  narc4  narc5  narc6  nidentity1  nidentity2  omind1  omind2  omind6  contact1  contact2  contact3  contact4  contact5  hygiene3  hygiene5  psupport1  psupport3  psupport4  psupport5  political_ideology  riskperc1  sex1  sbelong3  tested_positive  slfcont2  slfcont3  slfcont4  urban
##   5   1  age  children  cnarc1  cnarc2  cnarc3  ctheory2  CRT1  CRT2  CRT3  employ_status1  generosity1  generosity2  generosity3  ladder  marital1  mor_circle  moralid1  moralid10  moralid2  moralid3  moralid4  moralid5  moralid6  moralid7  moralid8  moralid9  mcoop1  mcoop2  mcoop3  mcoop4  mcoop5  mcoop6  mcoop7  narc4  narc5  narc6  nidentity1  nidentity2  omind1  omind2  omind6  contact1  contact2  contact3  contact4  contact5  hygiene3  hygiene5  psupport1  psupport3  psupport4  psupport5  political_ideology  riskperc1  sex1  sbelong3  tested_positive  slfcont2  slfcont3  slfcont4  urban
##   5   2  age  children  cnarc1  cnarc2  cnarc3  ctheory2  CRT1  CRT2  CRT3  employ_status1  generosity1  generosity2  generosity3  ladder  marital1  mor_circle  moralid1  moralid10  moralid2  moralid3  moralid4  moralid5  moralid6  moralid7  moralid8  moralid9  mcoop1  mcoop2  mcoop3  mcoop4  mcoop5  mcoop6  mcoop7  narc4  narc5  narc6  nidentity1  nidentity2  omind1  omind2  omind6  contact1  contact2  contact3  contact4  contact5  hygiene3  hygiene5  psupport1  psupport3  psupport4  psupport5  political_ideology  riskperc1  sex1  sbelong3  tested_positive  slfcont2  slfcont3  slfcont4  urban
##   5   3  age  children  cnarc1  cnarc2  cnarc3  ctheory2  CRT1  CRT2  CRT3  employ_status1  generosity1  generosity2  generosity3  ladder  marital1  mor_circle  moralid1  moralid10  moralid2  moralid3  moralid4  moralid5  moralid6  moralid7  moralid8  moralid9  mcoop1  mcoop2  mcoop3  mcoop4  mcoop5  mcoop6  mcoop7  narc4  narc5  narc6  nidentity1  nidentity2  omind1  omind2  omind6  contact1  contact2  contact3  contact4  contact5  hygiene3  hygiene5  psupport1  psupport3  psupport4  psupport5  political_ideology  riskperc1  sex1  sbelong3  tested_positive  slfcont2  slfcont3  slfcont4  urban
##   5   4  age  children  cnarc1  cnarc2  cnarc3  ctheory2  CRT1  CRT2  CRT3  employ_status1  generosity1  generosity2  generosity3  ladder  marital1  mor_circle  moralid1  moralid10  moralid2  moralid3  moralid4  moralid5  moralid6  moralid7  moralid8  moralid9  mcoop1  mcoop2  mcoop3  mcoop4  mcoop5  mcoop6  mcoop7  narc4  narc5  narc6  nidentity1  nidentity2  omind1  omind2  omind6  contact1  contact2  contact3  contact4  contact5  hygiene3  hygiene5  psupport1  psupport3  psupport4  psupport5  political_ideology  riskperc1  sex1  sbelong3  tested_positive  slfcont2  slfcont3  slfcont4  urban
##   5   5  age  children  cnarc1  cnarc2  cnarc3  ctheory2  CRT1  CRT2  CRT3  employ_status1  generosity1  generosity2  generosity3  ladder  marital1  mor_circle  moralid1  moralid10  moralid2  moralid3  moralid4  moralid5  moralid6  moralid7  moralid8  moralid9  mcoop1  mcoop2  mcoop3  mcoop4  mcoop5  mcoop6  mcoop7  narc4  narc5  narc6  nidentity1  nidentity2  omind1  omind2  omind6  contact1  contact2  contact3  contact4  contact5  hygiene3  hygiene5  psupport1  psupport3  psupport4  psupport5  political_ideology  riskperc1  sex1  sbelong3  tested_positive  slfcont2  slfcont3  slfcont4  urban
##   5   6  age  children  cnarc1  cnarc2  cnarc3  ctheory2  CRT1  CRT2  CRT3  employ_status1  generosity1  generosity2  generosity3  ladder  marital1  mor_circle  moralid1  moralid10  moralid2  moralid3  moralid4  moralid5  moralid6  moralid7  moralid8  moralid9  mcoop1  mcoop2  mcoop3  mcoop4  mcoop5  mcoop6  mcoop7  narc4  narc5  narc6  nidentity1  nidentity2  omind1  omind2  omind6  contact1  contact2  contact3  contact4  contact5  hygiene3  hygiene5  psupport1  psupport3  psupport4  psupport5  political_ideology  riskperc1  sex1  sbelong3  tested_positive  slfcont2  slfcont3  slfcont4  urban
##   5   7  age  children  cnarc1  cnarc2  cnarc3  ctheory2  CRT1  CRT2  CRT3  employ_status1  generosity1  generosity2  generosity3  ladder  marital1  mor_circle  moralid1  moralid10  moralid2  moralid3  moralid4  moralid5  moralid6  moralid7  moralid8  moralid9  mcoop1  mcoop2  mcoop3  mcoop4  mcoop5  mcoop6  mcoop7  narc4  narc5  narc6  nidentity1  nidentity2  omind1  omind2  omind6  contact1  contact2  contact3  contact4  contact5  hygiene3  hygiene5  psupport1  psupport3  psupport4  psupport5  political_ideology  riskperc1  sex1  sbelong3  tested_positive  slfcont2  slfcont3  slfcont4  urban
##   5   8  age  children  cnarc1  cnarc2  cnarc3  ctheory2  CRT1  CRT2  CRT3  employ_status1  generosity1  generosity2  generosity3  ladder  marital1  mor_circle  moralid1  moralid10  moralid2  moralid3  moralid4  moralid5  moralid6  moralid7  moralid8  moralid9  mcoop1  mcoop2  mcoop3  mcoop4  mcoop5  mcoop6  mcoop7  narc4  narc5  narc6  nidentity1  nidentity2  omind1  omind2  omind6  contact1  contact2  contact3  contact4  contact5  hygiene3  hygiene5  psupport1  psupport3  psupport4  psupport5  political_ideology  riskperc1  sex1  sbelong3  tested_positive  slfcont2  slfcont3  slfcont4  urban
##   5   9  age  children  cnarc1  cnarc2  cnarc3  ctheory2  CRT1  CRT2  CRT3  employ_status1  generosity1  generosity2  generosity3  ladder  marital1  mor_circle  moralid1  moralid10  moralid2  moralid3  moralid4  moralid5  moralid6  moralid7  moralid8  moralid9  mcoop1  mcoop2  mcoop3  mcoop4  mcoop5  mcoop6  mcoop7  narc4  narc5  narc6  nidentity1  nidentity2  omind1  omind2  omind6  contact1  contact2  contact3  contact4  contact5  hygiene3  hygiene5  psupport1  psupport3  psupport4  psupport5  political_ideology  riskperc1  sex1  sbelong3  tested_positive  slfcont2  slfcont3  slfcont4  urban
##   5   10  age  children  cnarc1  cnarc2  cnarc3  ctheory2  CRT1  CRT2  CRT3  employ_status1  generosity1  generosity2  generosity3  ladder  marital1  mor_circle  moralid1  moralid10  moralid2  moralid3  moralid4  moralid5  moralid6  moralid7  moralid8  moralid9  mcoop1  mcoop2  mcoop3  mcoop4  mcoop5  mcoop6  mcoop7  narc4  narc5  narc6  nidentity1  nidentity2  omind1  omind2  omind6  contact1  contact2  contact3  contact4  contact5  hygiene3  hygiene5  psupport1  psupport3  psupport4  psupport5  political_ideology  riskperc1  sex1  sbelong3  tested_positive  slfcont2  slfcont3  slfcont4  urban
##   6   1  age  children  cnarc1  cnarc2  cnarc3  ctheory2  CRT1  CRT2  CRT3  employ_status1  generosity1  generosity2  generosity3  ladder  marital1  mor_circle  moralid1  moralid10  moralid2  moralid3  moralid4  moralid5  moralid6  moralid7  moralid8  moralid9  mcoop1  mcoop2  mcoop3  mcoop4  mcoop5  mcoop6  mcoop7  narc4  narc5  narc6  nidentity1  nidentity2  omind1  omind2  omind6  contact1  contact2  contact3  contact4  contact5  hygiene3  hygiene5  psupport1  psupport3  psupport4  psupport5  political_ideology  riskperc1  sex1  sbelong3  tested_positive  slfcont2  slfcont3  slfcont4  urban
##   6   2  age  children  cnarc1  cnarc2  cnarc3  ctheory2  CRT1  CRT2  CRT3  employ_status1  generosity1  generosity2  generosity3  ladder  marital1  mor_circle  moralid1  moralid10  moralid2  moralid3  moralid4  moralid5  moralid6  moralid7  moralid8  moralid9  mcoop1  mcoop2  mcoop3  mcoop4  mcoop5  mcoop6  mcoop7  narc4  narc5  narc6  nidentity1  nidentity2  omind1  omind2  omind6  contact1  contact2  contact3  contact4  contact5  hygiene3  hygiene5  psupport1  psupport3  psupport4  psupport5  political_ideology  riskperc1  sex1  sbelong3  tested_positive  slfcont2  slfcont3  slfcont4  urban
##   6   3  age  children  cnarc1  cnarc2  cnarc3  ctheory2  CRT1  CRT2  CRT3  employ_status1  generosity1  generosity2  generosity3  ladder  marital1  mor_circle  moralid1  moralid10  moralid2  moralid3  moralid4  moralid5  moralid6  moralid7  moralid8  moralid9  mcoop1  mcoop2  mcoop3  mcoop4  mcoop5  mcoop6  mcoop7  narc4  narc5  narc6  nidentity1  nidentity2  omind1  omind2  omind6  contact1  contact2  contact3  contact4  contact5  hygiene3  hygiene5  psupport1  psupport3  psupport4  psupport5  political_ideology  riskperc1  sex1  sbelong3  tested_positive  slfcont2  slfcont3  slfcont4  urban
##   6   4  age  children  cnarc1  cnarc2  cnarc3  ctheory2  CRT1  CRT2  CRT3  employ_status1  generosity1  generosity2  generosity3  ladder  marital1  mor_circle  moralid1  moralid10  moralid2  moralid3  moralid4  moralid5  moralid6  moralid7  moralid8  moralid9  mcoop1  mcoop2  mcoop3  mcoop4  mcoop5  mcoop6  mcoop7  narc4  narc5  narc6  nidentity1  nidentity2  omind1  omind2  omind6  contact1  contact2  contact3  contact4  contact5  hygiene3  hygiene5  psupport1  psupport3  psupport4  psupport5  political_ideology  riskperc1  sex1  sbelong3  tested_positive  slfcont2  slfcont3  slfcont4  urban
##   6   5  age  children  cnarc1  cnarc2  cnarc3  ctheory2  CRT1  CRT2  CRT3  employ_status1  generosity1  generosity2  generosity3  ladder  marital1  mor_circle  moralid1  moralid10  moralid2  moralid3  moralid4  moralid5  moralid6  moralid7  moralid8  moralid9  mcoop1  mcoop2  mcoop3  mcoop4  mcoop5  mcoop6  mcoop7  narc4  narc5  narc6  nidentity1  nidentity2  omind1  omind2  omind6  contact1  contact2  contact3  contact4  contact5  hygiene3  hygiene5  psupport1  psupport3  psupport4  psupport5  political_ideology  riskperc1  sex1  sbelong3  tested_positive  slfcont2  slfcont3  slfcont4  urban
##   6   6  age  children  cnarc1  cnarc2  cnarc3  ctheory2  CRT1  CRT2  CRT3  employ_status1  generosity1  generosity2  generosity3  ladder  marital1  mor_circle  moralid1  moralid10  moralid2  moralid3  moralid4  moralid5  moralid6  moralid7  moralid8  moralid9  mcoop1  mcoop2  mcoop3  mcoop4  mcoop5  mcoop6  mcoop7  narc4  narc5  narc6  nidentity1  nidentity2  omind1  omind2  omind6  contact1  contact2  contact3  contact4  contact5  hygiene3  hygiene5  psupport1  psupport3  psupport4  psupport5  political_ideology  riskperc1  sex1  sbelong3  tested_positive  slfcont2  slfcont3  slfcont4  urban
##   6   7  age  children  cnarc1  cnarc2  cnarc3  ctheory2  CRT1  CRT2  CRT3  employ_status1  generosity1  generosity2  generosity3  ladder  marital1  mor_circle  moralid1  moralid10  moralid2  moralid3  moralid4  moralid5  moralid6  moralid7  moralid8  moralid9  mcoop1  mcoop2  mcoop3  mcoop4  mcoop5  mcoop6  mcoop7  narc4  narc5  narc6  nidentity1  nidentity2  omind1  omind2  omind6  contact1  contact2  contact3  contact4  contact5  hygiene3  hygiene5  psupport1  psupport3  psupport4  psupport5  political_ideology  riskperc1  sex1  sbelong3  tested_positive  slfcont2  slfcont3  slfcont4  urban
##   6   8  age  children  cnarc1  cnarc2  cnarc3  ctheory2  CRT1  CRT2  CRT3  employ_status1  generosity1  generosity2  generosity3  ladder  marital1  mor_circle  moralid1  moralid10  moralid2  moralid3  moralid4  moralid5  moralid6  moralid7  moralid8  moralid9  mcoop1  mcoop2  mcoop3  mcoop4  mcoop5  mcoop6  mcoop7  narc4  narc5  narc6  nidentity1  nidentity2  omind1  omind2  omind6  contact1  contact2  contact3  contact4  contact5  hygiene3  hygiene5  psupport1  psupport3  psupport4  psupport5  political_ideology  riskperc1  sex1  sbelong3  tested_positive  slfcont2  slfcont3  slfcont4  urban
##   6   9  age  children  cnarc1  cnarc2  cnarc3  ctheory2  CRT1  CRT2  CRT3  employ_status1  generosity1  generosity2  generosity3  ladder  marital1  mor_circle  moralid1  moralid10  moralid2  moralid3  moralid4  moralid5  moralid6  moralid7  moralid8  moralid9  mcoop1  mcoop2  mcoop3  mcoop4  mcoop5  mcoop6  mcoop7  narc4  narc5  narc6  nidentity1  nidentity2  omind1  omind2  omind6  contact1  contact2  contact3  contact4  contact5  hygiene3  hygiene5  psupport1  psupport3  psupport4  psupport5  political_ideology  riskperc1  sex1  sbelong3  tested_positive  slfcont2  slfcont3  slfcont4  urban
##   6   10  age  children  cnarc1  cnarc2  cnarc3  ctheory2  CRT1  CRT2  CRT3  employ_status1  generosity1  generosity2  generosity3  ladder  marital1  mor_circle  moralid1  moralid10  moralid2  moralid3  moralid4  moralid5  moralid6  moralid7  moralid8  moralid9  mcoop1  mcoop2  mcoop3  mcoop4  mcoop5  mcoop6  mcoop7  narc4  narc5  narc6  nidentity1  nidentity2  omind1  omind2  omind6  contact1  contact2  contact3  contact4  contact5  hygiene3  hygiene5  psupport1  psupport3  psupport4  psupport5  political_ideology  riskperc1  sex1  sbelong3  tested_positive  slfcont2  slfcont3  slfcont4  urban
##   7   1  age  children  cnarc1  cnarc2  cnarc3  ctheory2  CRT1  CRT2  CRT3  employ_status1  generosity1  generosity2  generosity3  ladder  marital1  mor_circle  moralid1  moralid10  moralid2  moralid3  moralid4  moralid5  moralid6  moralid7  moralid8  moralid9  mcoop1  mcoop2  mcoop3  mcoop4  mcoop5  mcoop6  mcoop7  narc4  narc5  narc6  nidentity1  nidentity2  omind1  omind2  omind6  contact1  contact2  contact3  contact4  contact5  hygiene3  hygiene5  psupport1  psupport3  psupport4  psupport5  political_ideology  riskperc1  sex1  sbelong3  tested_positive  slfcont2  slfcont3  slfcont4  urban
##   7   2  age  children  cnarc1  cnarc2  cnarc3  ctheory2  CRT1  CRT2  CRT3  employ_status1  generosity1  generosity2  generosity3  ladder  marital1  mor_circle  moralid1  moralid10  moralid2  moralid3  moralid4  moralid5  moralid6  moralid7  moralid8  moralid9  mcoop1  mcoop2  mcoop3  mcoop4  mcoop5  mcoop6  mcoop7  narc4  narc5  narc6  nidentity1  nidentity2  omind1  omind2  omind6  contact1  contact2  contact3  contact4  contact5  hygiene3  hygiene5  psupport1  psupport3  psupport4  psupport5  political_ideology  riskperc1  sex1  sbelong3  tested_positive  slfcont2  slfcont3  slfcont4  urban
##   7   3  age  children  cnarc1  cnarc2  cnarc3  ctheory2  CRT1  CRT2  CRT3  employ_status1  generosity1  generosity2  generosity3  ladder  marital1  mor_circle  moralid1  moralid10  moralid2  moralid3  moralid4  moralid5  moralid6  moralid7  moralid8  moralid9  mcoop1  mcoop2  mcoop3  mcoop4  mcoop5  mcoop6  mcoop7  narc4  narc5  narc6  nidentity1  nidentity2  omind1  omind2  omind6  contact1  contact2  contact3  contact4  contact5  hygiene3  hygiene5  psupport1  psupport3  psupport4  psupport5  political_ideology  riskperc1  sex1  sbelong3  tested_positive  slfcont2  slfcont3  slfcont4  urban
##   7   4  age  children  cnarc1  cnarc2  cnarc3  ctheory2  CRT1  CRT2  CRT3  employ_status1  generosity1  generosity2  generosity3  ladder  marital1  mor_circle  moralid1  moralid10  moralid2  moralid3  moralid4  moralid5  moralid6  moralid7  moralid8  moralid9  mcoop1  mcoop2  mcoop3  mcoop4  mcoop5  mcoop6  mcoop7  narc4  narc5  narc6  nidentity1  nidentity2  omind1  omind2  omind6  contact1  contact2  contact3  contact4  contact5  hygiene3  hygiene5  psupport1  psupport3  psupport4  psupport5  political_ideology  riskperc1  sex1  sbelong3  tested_positive  slfcont2  slfcont3  slfcont4  urban
##   7   5  age  children  cnarc1  cnarc2  cnarc3  ctheory2  CRT1  CRT2  CRT3  employ_status1  generosity1  generosity2  generosity3  ladder  marital1  mor_circle  moralid1  moralid10  moralid2  moralid3  moralid4  moralid5  moralid6  moralid7  moralid8  moralid9  mcoop1  mcoop2  mcoop3  mcoop4  mcoop5  mcoop6  mcoop7  narc4  narc5  narc6  nidentity1  nidentity2  omind1  omind2  omind6  contact1  contact2  contact3  contact4  contact5  hygiene3  hygiene5  psupport1  psupport3  psupport4  psupport5  political_ideology  riskperc1  sex1  sbelong3  tested_positive  slfcont2  slfcont3  slfcont4  urban
##   7   6  age  children  cnarc1  cnarc2  cnarc3  ctheory2  CRT1  CRT2  CRT3  employ_status1  generosity1  generosity2  generosity3  ladder  marital1  mor_circle  moralid1  moralid10  moralid2  moralid3  moralid4  moralid5  moralid6  moralid7  moralid8  moralid9  mcoop1  mcoop2  mcoop3  mcoop4  mcoop5  mcoop6  mcoop7  narc4  narc5  narc6  nidentity1  nidentity2  omind1  omind2  omind6  contact1  contact2  contact3  contact4  contact5  hygiene3  hygiene5  psupport1  psupport3  psupport4  psupport5  political_ideology  riskperc1  sex1  sbelong3  tested_positive  slfcont2  slfcont3  slfcont4  urban
##   7   7  age  children  cnarc1  cnarc2  cnarc3  ctheory2  CRT1  CRT2  CRT3  employ_status1  generosity1  generosity2  generosity3  ladder  marital1  mor_circle  moralid1  moralid10  moralid2  moralid3  moralid4  moralid5  moralid6  moralid7  moralid8  moralid9  mcoop1  mcoop2  mcoop3  mcoop4  mcoop5  mcoop6  mcoop7  narc4  narc5  narc6  nidentity1  nidentity2  omind1  omind2  omind6  contact1  contact2  contact3  contact4  contact5  hygiene3  hygiene5  psupport1  psupport3  psupport4  psupport5  political_ideology  riskperc1  sex1  sbelong3  tested_positive  slfcont2  slfcont3  slfcont4  urban
##   7   8  age  children  cnarc1  cnarc2  cnarc3  ctheory2  CRT1  CRT2  CRT3  employ_status1  generosity1  generosity2  generosity3  ladder  marital1  mor_circle  moralid1  moralid10  moralid2  moralid3  moralid4  moralid5  moralid6  moralid7  moralid8  moralid9  mcoop1  mcoop2  mcoop3  mcoop4  mcoop5  mcoop6  mcoop7  narc4  narc5  narc6  nidentity1  nidentity2  omind1  omind2  omind6  contact1  contact2  contact3  contact4  contact5  hygiene3  hygiene5  psupport1  psupport3  psupport4  psupport5  political_ideology  riskperc1  sex1  sbelong3  tested_positive  slfcont2  slfcont3  slfcont4  urban
##   7   9  age  children  cnarc1  cnarc2  cnarc3  ctheory2  CRT1  CRT2  CRT3  employ_status1  generosity1  generosity2  generosity3  ladder  marital1  mor_circle  moralid1  moralid10  moralid2  moralid3  moralid4  moralid5  moralid6  moralid7  moralid8  moralid9  mcoop1  mcoop2  mcoop3  mcoop4  mcoop5  mcoop6  mcoop7  narc4  narc5  narc6  nidentity1  nidentity2  omind1  omind2  omind6  contact1  contact2  contact3  contact4  contact5  hygiene3  hygiene5  psupport1  psupport3  psupport4  psupport5  political_ideology  riskperc1  sex1  sbelong3  tested_positive  slfcont2  slfcont3  slfcont4  urban
##   7   10  age  children  cnarc1  cnarc2  cnarc3  ctheory2  CRT1  CRT2  CRT3  employ_status1  generosity1  generosity2  generosity3  ladder  marital1  mor_circle  moralid1  moralid10  moralid2  moralid3  moralid4  moralid5  moralid6  moralid7  moralid8  moralid9  mcoop1  mcoop2  mcoop3  mcoop4  mcoop5  mcoop6  mcoop7  narc4  narc5  narc6  nidentity1  nidentity2  omind1  omind2  omind6  contact1  contact2  contact3  contact4  contact5  hygiene3  hygiene5  psupport1  psupport3  psupport4  psupport5  political_ideology  riskperc1  sex1  sbelong3  tested_positive  slfcont2  slfcont3  slfcont4  urban
##   8   1  age  children  cnarc1  cnarc2  cnarc3  ctheory2  CRT1  CRT2  CRT3  employ_status1  generosity1  generosity2  generosity3  ladder  marital1  mor_circle  moralid1  moralid10  moralid2  moralid3  moralid4  moralid5  moralid6  moralid7  moralid8  moralid9  mcoop1  mcoop2  mcoop3  mcoop4  mcoop5  mcoop6  mcoop7  narc4  narc5  narc6  nidentity1  nidentity2  omind1  omind2  omind6  contact1  contact2  contact3  contact4  contact5  hygiene3  hygiene5  psupport1  psupport3  psupport4  psupport5  political_ideology  riskperc1  sex1  sbelong3  tested_positive  slfcont2  slfcont3  slfcont4  urban
##   8   2  age  children  cnarc1  cnarc2  cnarc3  ctheory2  CRT1  CRT2  CRT3  employ_status1  generosity1  generosity2  generosity3  ladder  marital1  mor_circle  moralid1  moralid10  moralid2  moralid3  moralid4  moralid5  moralid6  moralid7  moralid8  moralid9  mcoop1  mcoop2  mcoop3  mcoop4  mcoop5  mcoop6  mcoop7  narc4  narc5  narc6  nidentity1  nidentity2  omind1  omind2  omind6  contact1  contact2  contact3  contact4  contact5  hygiene3  hygiene5  psupport1  psupport3  psupport4  psupport5  political_ideology  riskperc1  sex1  sbelong3  tested_positive  slfcont2  slfcont3  slfcont4  urban
##   8   3  age  children  cnarc1  cnarc2  cnarc3  ctheory2  CRT1  CRT2  CRT3  employ_status1  generosity1  generosity2  generosity3  ladder  marital1  mor_circle  moralid1  moralid10  moralid2  moralid3  moralid4  moralid5  moralid6  moralid7  moralid8  moralid9  mcoop1  mcoop2  mcoop3  mcoop4  mcoop5  mcoop6  mcoop7  narc4  narc5  narc6  nidentity1  nidentity2  omind1  omind2  omind6  contact1  contact2  contact3  contact4  contact5  hygiene3  hygiene5  psupport1  psupport3  psupport4  psupport5  political_ideology  riskperc1  sex1  sbelong3  tested_positive  slfcont2  slfcont3  slfcont4  urban
##   8   4  age  children  cnarc1  cnarc2  cnarc3  ctheory2  CRT1  CRT2  CRT3  employ_status1  generosity1  generosity2  generosity3  ladder  marital1  mor_circle  moralid1  moralid10  moralid2  moralid3  moralid4  moralid5  moralid6  moralid7  moralid8  moralid9  mcoop1  mcoop2  mcoop3  mcoop4  mcoop5  mcoop6  mcoop7  narc4  narc5  narc6  nidentity1  nidentity2  omind1  omind2  omind6  contact1  contact2  contact3  contact4  contact5  hygiene3  hygiene5  psupport1  psupport3  psupport4  psupport5  political_ideology  riskperc1  sex1  sbelong3  tested_positive  slfcont2  slfcont3  slfcont4  urban
##   8   5  age  children  cnarc1  cnarc2  cnarc3  ctheory2  CRT1  CRT2  CRT3  employ_status1  generosity1  generosity2  generosity3  ladder  marital1  mor_circle  moralid1  moralid10  moralid2  moralid3  moralid4  moralid5  moralid6  moralid7  moralid8  moralid9  mcoop1  mcoop2  mcoop3  mcoop4  mcoop5  mcoop6  mcoop7  narc4  narc5  narc6  nidentity1  nidentity2  omind1  omind2  omind6  contact1  contact2  contact3  contact4  contact5  hygiene3  hygiene5  psupport1  psupport3  psupport4  psupport5  political_ideology  riskperc1  sex1  sbelong3  tested_positive  slfcont2  slfcont3  slfcont4  urban
##   8   6  age  children  cnarc1  cnarc2  cnarc3  ctheory2  CRT1  CRT2  CRT3  employ_status1  generosity1  generosity2  generosity3  ladder  marital1  mor_circle  moralid1  moralid10  moralid2  moralid3  moralid4  moralid5  moralid6  moralid7  moralid8  moralid9  mcoop1  mcoop2  mcoop3  mcoop4  mcoop5  mcoop6  mcoop7  narc4  narc5  narc6  nidentity1  nidentity2  omind1  omind2  omind6  contact1  contact2  contact3  contact4  contact5  hygiene3  hygiene5  psupport1  psupport3  psupport4  psupport5  political_ideology  riskperc1  sex1  sbelong3  tested_positive  slfcont2  slfcont3  slfcont4  urban
##   8   7  age  children  cnarc1  cnarc2  cnarc3  ctheory2  CRT1  CRT2  CRT3  employ_status1  generosity1  generosity2  generosity3  ladder  marital1  mor_circle  moralid1  moralid10  moralid2  moralid3  moralid4  moralid5  moralid6  moralid7  moralid8  moralid9  mcoop1  mcoop2  mcoop3  mcoop4  mcoop5  mcoop6  mcoop7  narc4  narc5  narc6  nidentity1  nidentity2  omind1  omind2  omind6  contact1  contact2  contact3  contact4  contact5  hygiene3  hygiene5  psupport1  psupport3  psupport4  psupport5  political_ideology  riskperc1  sex1  sbelong3  tested_positive  slfcont2  slfcont3  slfcont4  urban
##   8   8  age  children  cnarc1  cnarc2  cnarc3  ctheory2  CRT1  CRT2  CRT3  employ_status1  generosity1  generosity2  generosity3  ladder  marital1  mor_circle  moralid1  moralid10  moralid2  moralid3  moralid4  moralid5  moralid6  moralid7  moralid8  moralid9  mcoop1  mcoop2  mcoop3  mcoop4  mcoop5  mcoop6  mcoop7  narc4  narc5  narc6  nidentity1  nidentity2  omind1  omind2  omind6  contact1  contact2  contact3  contact4  contact5  hygiene3  hygiene5  psupport1  psupport3  psupport4  psupport5  political_ideology  riskperc1  sex1  sbelong3  tested_positive  slfcont2  slfcont3  slfcont4  urban
##   8   9  age  children  cnarc1  cnarc2  cnarc3  ctheory2  CRT1  CRT2  CRT3  employ_status1  generosity1  generosity2  generosity3  ladder  marital1  mor_circle  moralid1  moralid10  moralid2  moralid3  moralid4  moralid5  moralid6  moralid7  moralid8  moralid9  mcoop1  mcoop2  mcoop3  mcoop4  mcoop5  mcoop6  mcoop7  narc4  narc5  narc6  nidentity1  nidentity2  omind1  omind2  omind6  contact1  contact2  contact3  contact4  contact5  hygiene3  hygiene5  psupport1  psupport3  psupport4  psupport5  political_ideology  riskperc1  sex1  sbelong3  tested_positive  slfcont2  slfcont3  slfcont4  urban
##   8   10  age  children  cnarc1  cnarc2  cnarc3  ctheory2  CRT1  CRT2  CRT3  employ_status1  generosity1  generosity2  generosity3  ladder  marital1  mor_circle  moralid1  moralid10  moralid2  moralid3  moralid4  moralid5  moralid6  moralid7  moralid8  moralid9  mcoop1  mcoop2  mcoop3  mcoop4  mcoop5  mcoop6  mcoop7  narc4  narc5  narc6  nidentity1  nidentity2  omind1  omind2  omind6  contact1  contact2  contact3  contact4  contact5  hygiene3  hygiene5  psupport1  psupport3  psupport4  psupport5  political_ideology  riskperc1  sex1  sbelong3  tested_positive  slfcont2  slfcont3  slfcont4  urban
##   9   1  age  children  cnarc1  cnarc2  cnarc3  ctheory2  CRT1  CRT2  CRT3  employ_status1  generosity1  generosity2  generosity3  ladder  marital1  mor_circle  moralid1  moralid10  moralid2  moralid3  moralid4  moralid5  moralid6  moralid7  moralid8  moralid9  mcoop1  mcoop2  mcoop3  mcoop4  mcoop5  mcoop6  mcoop7  narc4  narc5  narc6  nidentity1  nidentity2  omind1  omind2  omind6  contact1  contact2  contact3  contact4  contact5  hygiene3  hygiene5  psupport1  psupport3  psupport4  psupport5  political_ideology  riskperc1  sex1  sbelong3  tested_positive  slfcont2  slfcont3  slfcont4  urban
##   9   2  age  children  cnarc1  cnarc2  cnarc3  ctheory2  CRT1  CRT2  CRT3  employ_status1  generosity1  generosity2  generosity3  ladder  marital1  mor_circle  moralid1  moralid10  moralid2  moralid3  moralid4  moralid5  moralid6  moralid7  moralid8  moralid9  mcoop1  mcoop2  mcoop3  mcoop4  mcoop5  mcoop6  mcoop7  narc4  narc5  narc6  nidentity1  nidentity2  omind1  omind2  omind6  contact1  contact2  contact3  contact4  contact5  hygiene3  hygiene5  psupport1  psupport3  psupport4  psupport5  political_ideology  riskperc1  sex1  sbelong3  tested_positive  slfcont2  slfcont3  slfcont4  urban
##   9   3  age  children  cnarc1  cnarc2  cnarc3  ctheory2  CRT1  CRT2  CRT3  employ_status1  generosity1  generosity2  generosity3  ladder  marital1  mor_circle  moralid1  moralid10  moralid2  moralid3  moralid4  moralid5  moralid6  moralid7  moralid8  moralid9  mcoop1  mcoop2  mcoop3  mcoop4  mcoop5  mcoop6  mcoop7  narc4  narc5  narc6  nidentity1  nidentity2  omind1  omind2  omind6  contact1  contact2  contact3  contact4  contact5  hygiene3  hygiene5  psupport1  psupport3  psupport4  psupport5  political_ideology  riskperc1  sex1  sbelong3  tested_positive  slfcont2  slfcont3  slfcont4  urban
##   9   4  age  children  cnarc1  cnarc2  cnarc3  ctheory2  CRT1  CRT2  CRT3  employ_status1  generosity1  generosity2  generosity3  ladder  marital1  mor_circle  moralid1  moralid10  moralid2  moralid3  moralid4  moralid5  moralid6  moralid7  moralid8  moralid9  mcoop1  mcoop2  mcoop3  mcoop4  mcoop5  mcoop6  mcoop7  narc4  narc5  narc6  nidentity1  nidentity2  omind1  omind2  omind6  contact1  contact2  contact3  contact4  contact5  hygiene3  hygiene5  psupport1  psupport3  psupport4  psupport5  political_ideology  riskperc1  sex1  sbelong3  tested_positive  slfcont2  slfcont3  slfcont4  urban
##   9   5  age  children  cnarc1  cnarc2  cnarc3  ctheory2  CRT1  CRT2  CRT3  employ_status1  generosity1  generosity2  generosity3  ladder  marital1  mor_circle  moralid1  moralid10  moralid2  moralid3  moralid4  moralid5  moralid6  moralid7  moralid8  moralid9  mcoop1  mcoop2  mcoop3  mcoop4  mcoop5  mcoop6  mcoop7  narc4  narc5  narc6  nidentity1  nidentity2  omind1  omind2  omind6  contact1  contact2  contact3  contact4  contact5  hygiene3  hygiene5  psupport1  psupport3  psupport4  psupport5  political_ideology  riskperc1  sex1  sbelong3  tested_positive  slfcont2  slfcont3  slfcont4  urban
##   9   6  age  children  cnarc1  cnarc2  cnarc3  ctheory2  CRT1  CRT2  CRT3  employ_status1  generosity1  generosity2  generosity3  ladder  marital1  mor_circle  moralid1  moralid10  moralid2  moralid3  moralid4  moralid5  moralid6  moralid7  moralid8  moralid9  mcoop1  mcoop2  mcoop3  mcoop4  mcoop5  mcoop6  mcoop7  narc4  narc5  narc6  nidentity1  nidentity2  omind1  omind2  omind6  contact1  contact2  contact3  contact4  contact5  hygiene3  hygiene5  psupport1  psupport3  psupport4  psupport5  political_ideology  riskperc1  sex1  sbelong3  tested_positive  slfcont2  slfcont3  slfcont4  urban
##   9   7  age  children  cnarc1  cnarc2  cnarc3  ctheory2  CRT1  CRT2  CRT3  employ_status1  generosity1  generosity2  generosity3  ladder  marital1  mor_circle  moralid1  moralid10  moralid2  moralid3  moralid4  moralid5  moralid6  moralid7  moralid8  moralid9  mcoop1  mcoop2  mcoop3  mcoop4  mcoop5  mcoop6  mcoop7  narc4  narc5  narc6  nidentity1  nidentity2  omind1  omind2  omind6  contact1  contact2  contact3  contact4  contact5  hygiene3  hygiene5  psupport1  psupport3  psupport4  psupport5  political_ideology  riskperc1  sex1  sbelong3  tested_positive  slfcont2  slfcont3  slfcont4  urban
##   9   8  age  children  cnarc1  cnarc2  cnarc3  ctheory2  CRT1  CRT2  CRT3  employ_status1  generosity1  generosity2  generosity3  ladder  marital1  mor_circle  moralid1  moralid10  moralid2  moralid3  moralid4  moralid5  moralid6  moralid7  moralid8  moralid9  mcoop1  mcoop2  mcoop3  mcoop4  mcoop5  mcoop6  mcoop7  narc4  narc5  narc6  nidentity1  nidentity2  omind1  omind2  omind6  contact1  contact2  contact3  contact4  contact5  hygiene3  hygiene5  psupport1  psupport3  psupport4  psupport5  political_ideology  riskperc1  sex1  sbelong3  tested_positive  slfcont2  slfcont3  slfcont4  urban
##   9   9  age  children  cnarc1  cnarc2  cnarc3  ctheory2  CRT1  CRT2  CRT3  employ_status1  generosity1  generosity2  generosity3  ladder  marital1  mor_circle  moralid1  moralid10  moralid2  moralid3  moralid4  moralid5  moralid6  moralid7  moralid8  moralid9  mcoop1  mcoop2  mcoop3  mcoop4  mcoop5  mcoop6  mcoop7  narc4  narc5  narc6  nidentity1  nidentity2  omind1  omind2  omind6  contact1  contact2  contact3  contact4  contact5  hygiene3  hygiene5  psupport1  psupport3  psupport4  psupport5  political_ideology  riskperc1  sex1  sbelong3  tested_positive  slfcont2  slfcont3  slfcont4  urban
##   9   10  age  children  cnarc1  cnarc2  cnarc3  ctheory2  CRT1  CRT2  CRT3  employ_status1  generosity1  generosity2  generosity3  ladder  marital1  mor_circle  moralid1  moralid10  moralid2  moralid3  moralid4  moralid5  moralid6  moralid7  moralid8  moralid9  mcoop1  mcoop2  mcoop3  mcoop4  mcoop5  mcoop6  mcoop7  narc4  narc5  narc6  nidentity1  nidentity2  omind1  omind2  omind6  contact1  contact2  contact3  contact4  contact5  hygiene3  hygiene5  psupport1  psupport3  psupport4  psupport5  political_ideology  riskperc1  sex1  sbelong3  tested_positive  slfcont2  slfcont3  slfcont4  urban
##   10   1  age  children  cnarc1  cnarc2  cnarc3  ctheory2  CRT1  CRT2  CRT3  employ_status1  generosity1  generosity2  generosity3  ladder  marital1  mor_circle  moralid1  moralid10  moralid2  moralid3  moralid4  moralid5  moralid6  moralid7  moralid8  moralid9  mcoop1  mcoop2  mcoop3  mcoop4  mcoop5  mcoop6  mcoop7  narc4  narc5  narc6  nidentity1  nidentity2  omind1  omind2  omind6  contact1  contact2  contact3  contact4  contact5  hygiene3  hygiene5  psupport1  psupport3  psupport4  psupport5  political_ideology  riskperc1  sex1  sbelong3  tested_positive  slfcont2  slfcont3  slfcont4  urban
##   10   2  age  children  cnarc1  cnarc2  cnarc3  ctheory2  CRT1  CRT2  CRT3  employ_status1  generosity1  generosity2  generosity3  ladder  marital1  mor_circle  moralid1  moralid10  moralid2  moralid3  moralid4  moralid5  moralid6  moralid7  moralid8  moralid9  mcoop1  mcoop2  mcoop3  mcoop4  mcoop5  mcoop6  mcoop7  narc4  narc5  narc6  nidentity1  nidentity2  omind1  omind2  omind6  contact1  contact2  contact3  contact4  contact5  hygiene3  hygiene5  psupport1  psupport3  psupport4  psupport5  political_ideology  riskperc1  sex1  sbelong3  tested_positive  slfcont2  slfcont3  slfcont4  urban
##   10   3  age  children  cnarc1  cnarc2  cnarc3  ctheory2  CRT1  CRT2  CRT3  employ_status1  generosity1  generosity2  generosity3  ladder  marital1  mor_circle  moralid1  moralid10  moralid2  moralid3  moralid4  moralid5  moralid6  moralid7  moralid8  moralid9  mcoop1  mcoop2  mcoop3  mcoop4  mcoop5  mcoop6  mcoop7  narc4  narc5  narc6  nidentity1  nidentity2  omind1  omind2  omind6  contact1  contact2  contact3  contact4  contact5  hygiene3  hygiene5  psupport1  psupport3  psupport4  psupport5  political_ideology  riskperc1  sex1  sbelong3  tested_positive  slfcont2  slfcont3  slfcont4  urban
##   10   4  age  children  cnarc1  cnarc2  cnarc3  ctheory2  CRT1  CRT2  CRT3  employ_status1  generosity1  generosity2  generosity3  ladder  marital1  mor_circle  moralid1  moralid10  moralid2  moralid3  moralid4  moralid5  moralid6  moralid7  moralid8  moralid9  mcoop1  mcoop2  mcoop3  mcoop4  mcoop5  mcoop6  mcoop7  narc4  narc5  narc6  nidentity1  nidentity2  omind1  omind2  omind6  contact1  contact2  contact3  contact4  contact5  hygiene3  hygiene5  psupport1  psupport3  psupport4  psupport5  political_ideology  riskperc1  sex1  sbelong3  tested_positive  slfcont2  slfcont3  slfcont4  urban
##   10   5  age  children  cnarc1  cnarc2  cnarc3  ctheory2  CRT1  CRT2  CRT3  employ_status1  generosity1  generosity2  generosity3  ladder  marital1  mor_circle  moralid1  moralid10  moralid2  moralid3  moralid4  moralid5  moralid6  moralid7  moralid8  moralid9  mcoop1  mcoop2  mcoop3  mcoop4  mcoop5  mcoop6  mcoop7  narc4  narc5  narc6  nidentity1  nidentity2  omind1  omind2  omind6  contact1  contact2  contact3  contact4  contact5  hygiene3  hygiene5  psupport1  psupport3  psupport4  psupport5  political_ideology  riskperc1  sex1  sbelong3  tested_positive  slfcont2  slfcont3  slfcont4  urban
##   10   6  age  children  cnarc1  cnarc2  cnarc3  ctheory2  CRT1  CRT2  CRT3  employ_status1  generosity1  generosity2  generosity3  ladder  marital1  mor_circle  moralid1  moralid10  moralid2  moralid3  moralid4  moralid5  moralid6  moralid7  moralid8  moralid9  mcoop1  mcoop2  mcoop3  mcoop4  mcoop5  mcoop6  mcoop7  narc4  narc5  narc6  nidentity1  nidentity2  omind1  omind2  omind6  contact1  contact2  contact3  contact4  contact5  hygiene3  hygiene5  psupport1  psupport3  psupport4  psupport5  political_ideology  riskperc1  sex1  sbelong3  tested_positive  slfcont2  slfcont3  slfcont4  urban
##   10   7  age  children  cnarc1  cnarc2  cnarc3  ctheory2  CRT1  CRT2  CRT3  employ_status1  generosity1  generosity2  generosity3  ladder  marital1  mor_circle  moralid1  moralid10  moralid2  moralid3  moralid4  moralid5  moralid6  moralid7  moralid8  moralid9  mcoop1  mcoop2  mcoop3  mcoop4  mcoop5  mcoop6  mcoop7  narc4  narc5  narc6  nidentity1  nidentity2  omind1  omind2  omind6  contact1  contact2  contact3  contact4  contact5  hygiene3  hygiene5  psupport1  psupport3  psupport4  psupport5  political_ideology  riskperc1  sex1  sbelong3  tested_positive  slfcont2  slfcont3  slfcont4  urban
##   10   8  age  children  cnarc1  cnarc2  cnarc3  ctheory2  CRT1  CRT2  CRT3  employ_status1  generosity1  generosity2  generosity3  ladder  marital1  mor_circle  moralid1  moralid10  moralid2  moralid3  moralid4  moralid5  moralid6  moralid7  moralid8  moralid9  mcoop1  mcoop2  mcoop3  mcoop4  mcoop5  mcoop6  mcoop7  narc4  narc5  narc6  nidentity1  nidentity2  omind1  omind2  omind6  contact1  contact2  contact3  contact4  contact5  hygiene3  hygiene5  psupport1  psupport3  psupport4  psupport5  political_ideology  riskperc1  sex1  sbelong3  tested_positive  slfcont2  slfcont3  slfcont4  urban
##   10   9  age  children  cnarc1  cnarc2  cnarc3  ctheory2  CRT1  CRT2  CRT3  employ_status1  generosity1  generosity2  generosity3  ladder  marital1  mor_circle  moralid1  moralid10  moralid2  moralid3  moralid4  moralid5  moralid6  moralid7  moralid8  moralid9  mcoop1  mcoop2  mcoop3  mcoop4  mcoop5  mcoop6  mcoop7  narc4  narc5  narc6  nidentity1  nidentity2  omind1  omind2  omind6  contact1  contact2  contact3  contact4  contact5  hygiene3  hygiene5  psupport1  psupport3  psupport4  psupport5  political_ideology  riskperc1  sex1  sbelong3  tested_positive  slfcont2  slfcont3  slfcont4  urban
##   10   10  age  children  cnarc1  cnarc2  cnarc3  ctheory2  CRT1  CRT2  CRT3  employ_status1  generosity1  generosity2  generosity3  ladder  marital1  mor_circle  moralid1  moralid10  moralid2  moralid3  moralid4  moralid5  moralid6  moralid7  moralid8  moralid9  mcoop1  mcoop2  mcoop3  mcoop4  mcoop5  mcoop6  mcoop7  narc4  narc5  narc6  nidentity1  nidentity2  omind1  omind2  omind6  contact1  contact2  contact3  contact4  contact5  hygiene3  hygiene5  psupport1  psupport3  psupport4  psupport5  political_ideology  riskperc1  sex1  sbelong3  tested_positive  slfcont2  slfcont3  slfcont4  urban
```

```
## Warning: Number of logged events: 5703
```

```
## 
##  iter imp variable
##   1   1  cnarc1  cnarc2  cnarc3  ctheory1  ctheory2  ctheory3  ctheory4  CRT1  CRT2  CRT3  health_cond  mor_circle  moralid1  moralid10  moralid2  moralid3  moralid4  moralid5  moralid6  moralid7  moralid8  moralid9  mcoop1  mcoop2  mcoop3  mcoop4  mcoop5  mcoop6  mcoop7  narc1  narc2  narc3  narc4  narc5  narc6  omind1  omind2  omind4  omind5  omind6  contact2  contact5  hygiene1  hygiene2  hygiene5  psupport2  psupport3  psupport5  political_ideology  happy  riskperc1  riskperc2  sbelong1  sbelong2  sbelong3  sbelong4  optim1  optim2  slfcont1  slfcont2  slfcont3  urban
##   1   2  cnarc1  cnarc2  cnarc3  ctheory1  ctheory2  ctheory3  ctheory4  CRT1  CRT2  CRT3  health_cond  mor_circle  moralid1  moralid10  moralid2  moralid3  moralid4  moralid5  moralid6  moralid7  moralid8  moralid9  mcoop1  mcoop2  mcoop3  mcoop4  mcoop5  mcoop6  mcoop7  narc1  narc2  narc3  narc4  narc5  narc6  omind1  omind2  omind4  omind5  omind6  contact2  contact5  hygiene1  hygiene2  hygiene5  psupport2  psupport3  psupport5  political_ideology  happy  riskperc1  riskperc2  sbelong1  sbelong2  sbelong3  sbelong4  optim1  optim2  slfcont1  slfcont2  slfcont3  urban
##   1   3  cnarc1  cnarc2  cnarc3  ctheory1  ctheory2  ctheory3  ctheory4  CRT1  CRT2  CRT3  health_cond  mor_circle  moralid1  moralid10  moralid2  moralid3  moralid4  moralid5  moralid6  moralid7  moralid8  moralid9  mcoop1  mcoop2  mcoop3  mcoop4  mcoop5  mcoop6  mcoop7  narc1  narc2  narc3  narc4  narc5  narc6  omind1  omind2  omind4  omind5  omind6  contact2  contact5  hygiene1  hygiene2  hygiene5  psupport2  psupport3  psupport5  political_ideology  happy  riskperc1  riskperc2  sbelong1  sbelong2  sbelong3  sbelong4  optim1  optim2  slfcont1  slfcont2  slfcont3  urban
##   1   4  cnarc1  cnarc2  cnarc3  ctheory1  ctheory2  ctheory3  ctheory4  CRT1  CRT2  CRT3  health_cond  mor_circle  moralid1  moralid10  moralid2  moralid3  moralid4  moralid5  moralid6  moralid7  moralid8  moralid9  mcoop1  mcoop2  mcoop3  mcoop4  mcoop5  mcoop6  mcoop7  narc1  narc2  narc3  narc4  narc5  narc6  omind1  omind2  omind4  omind5  omind6  contact2  contact5  hygiene1  hygiene2  hygiene5  psupport2  psupport3  psupport5  political_ideology  happy  riskperc1  riskperc2  sbelong1  sbelong2  sbelong3  sbelong4  optim1  optim2  slfcont1  slfcont2  slfcont3  urban
##   1   5  cnarc1  cnarc2  cnarc3  ctheory1  ctheory2  ctheory3  ctheory4  CRT1  CRT2  CRT3  health_cond  mor_circle  moralid1  moralid10  moralid2  moralid3  moralid4  moralid5  moralid6  moralid7  moralid8  moralid9  mcoop1  mcoop2  mcoop3  mcoop4  mcoop5  mcoop6  mcoop7  narc1  narc2  narc3  narc4  narc5  narc6  omind1  omind2  omind4  omind5  omind6  contact2  contact5  hygiene1  hygiene2  hygiene5  psupport2  psupport3  psupport5  political_ideology  happy  riskperc1  riskperc2  sbelong1  sbelong2  sbelong3  sbelong4  optim1  optim2  slfcont1  slfcont2  slfcont3  urban
##   1   6  cnarc1  cnarc2  cnarc3  ctheory1  ctheory2  ctheory3  ctheory4  CRT1  CRT2  CRT3  health_cond  mor_circle  moralid1  moralid10  moralid2  moralid3  moralid4  moralid5  moralid6  moralid7  moralid8  moralid9  mcoop1  mcoop2  mcoop3  mcoop4  mcoop5  mcoop6  mcoop7  narc1  narc2  narc3  narc4  narc5  narc6  omind1  omind2  omind4  omind5  omind6  contact2  contact5  hygiene1  hygiene2  hygiene5  psupport2  psupport3  psupport5  political_ideology  happy  riskperc1  riskperc2  sbelong1  sbelong2  sbelong3  sbelong4  optim1  optim2  slfcont1  slfcont2  slfcont3  urban
##   1   7  cnarc1  cnarc2  cnarc3  ctheory1  ctheory2  ctheory3  ctheory4  CRT1  CRT2  CRT3  health_cond  mor_circle  moralid1  moralid10  moralid2  moralid3  moralid4  moralid5  moralid6  moralid7  moralid8  moralid9  mcoop1  mcoop2  mcoop3  mcoop4  mcoop5  mcoop6  mcoop7  narc1  narc2  narc3  narc4  narc5  narc6  omind1  omind2  omind4  omind5  omind6  contact2  contact5  hygiene1  hygiene2  hygiene5  psupport2  psupport3  psupport5  political_ideology  happy  riskperc1  riskperc2  sbelong1  sbelong2  sbelong3  sbelong4  optim1  optim2  slfcont1  slfcont2  slfcont3  urban
##   1   8  cnarc1  cnarc2  cnarc3  ctheory1  ctheory2  ctheory3  ctheory4  CRT1  CRT2  CRT3  health_cond  mor_circle  moralid1  moralid10  moralid2  moralid3  moralid4  moralid5  moralid6  moralid7  moralid8  moralid9  mcoop1  mcoop2  mcoop3  mcoop4  mcoop5  mcoop6  mcoop7  narc1  narc2  narc3  narc4  narc5  narc6  omind1  omind2  omind4  omind5  omind6  contact2  contact5  hygiene1  hygiene2  hygiene5  psupport2  psupport3  psupport5  political_ideology  happy  riskperc1  riskperc2  sbelong1  sbelong2  sbelong3  sbelong4  optim1  optim2  slfcont1  slfcont2  slfcont3  urban
##   1   9  cnarc1  cnarc2  cnarc3  ctheory1  ctheory2  ctheory3  ctheory4  CRT1  CRT2  CRT3  health_cond  mor_circle  moralid1  moralid10  moralid2  moralid3  moralid4  moralid5  moralid6  moralid7  moralid8  moralid9  mcoop1  mcoop2  mcoop3  mcoop4  mcoop5  mcoop6  mcoop7  narc1  narc2  narc3  narc4  narc5  narc6  omind1  omind2  omind4  omind5  omind6  contact2  contact5  hygiene1  hygiene2  hygiene5  psupport2  psupport3  psupport5  political_ideology  happy  riskperc1  riskperc2  sbelong1  sbelong2  sbelong3  sbelong4  optim1  optim2  slfcont1  slfcont2  slfcont3  urban
##   1   10  cnarc1  cnarc2  cnarc3  ctheory1  ctheory2  ctheory3  ctheory4  CRT1  CRT2  CRT3  health_cond  mor_circle  moralid1  moralid10  moralid2  moralid3  moralid4  moralid5  moralid6  moralid7  moralid8  moralid9  mcoop1  mcoop2  mcoop3  mcoop4  mcoop5  mcoop6  mcoop7  narc1  narc2  narc3  narc4  narc5  narc6  omind1  omind2  omind4  omind5  omind6  contact2  contact5  hygiene1  hygiene2  hygiene5  psupport2  psupport3  psupport5  political_ideology  happy  riskperc1  riskperc2  sbelong1  sbelong2  sbelong3  sbelong4  optim1  optim2  slfcont1  slfcont2  slfcont3  urban
##   2   1  cnarc1  cnarc2  cnarc3  ctheory1  ctheory2  ctheory3  ctheory4  CRT1  CRT2  CRT3  health_cond  mor_circle  moralid1  moralid10  moralid2  moralid3  moralid4  moralid5  moralid6  moralid7  moralid8  moralid9  mcoop1  mcoop2  mcoop3  mcoop4  mcoop5  mcoop6  mcoop7  narc1  narc2  narc3  narc4  narc5  narc6  omind1  omind2  omind4  omind5  omind6  contact2  contact5  hygiene1  hygiene2  hygiene5  psupport2  psupport3  psupport5  political_ideology  happy  riskperc1  riskperc2  sbelong1  sbelong2  sbelong3  sbelong4  optim1  optim2  slfcont1  slfcont2  slfcont3  urban
##   2   2  cnarc1  cnarc2  cnarc3  ctheory1  ctheory2  ctheory3  ctheory4  CRT1  CRT2  CRT3  health_cond  mor_circle  moralid1  moralid10  moralid2  moralid3  moralid4  moralid5  moralid6  moralid7  moralid8  moralid9  mcoop1  mcoop2  mcoop3  mcoop4  mcoop5  mcoop6  mcoop7  narc1  narc2  narc3  narc4  narc5  narc6  omind1  omind2  omind4  omind5  omind6  contact2  contact5  hygiene1  hygiene2  hygiene5  psupport2  psupport3  psupport5  political_ideology  happy  riskperc1  riskperc2  sbelong1  sbelong2  sbelong3  sbelong4  optim1  optim2  slfcont1  slfcont2  slfcont3  urban
##   2   3  cnarc1  cnarc2  cnarc3  ctheory1  ctheory2  ctheory3  ctheory4  CRT1  CRT2  CRT3  health_cond  mor_circle  moralid1  moralid10  moralid2  moralid3  moralid4  moralid5  moralid6  moralid7  moralid8  moralid9  mcoop1  mcoop2  mcoop3  mcoop4  mcoop5  mcoop6  mcoop7  narc1  narc2  narc3  narc4  narc5  narc6  omind1  omind2  omind4  omind5  omind6  contact2  contact5  hygiene1  hygiene2  hygiene5  psupport2  psupport3  psupport5  political_ideology  happy  riskperc1  riskperc2  sbelong1  sbelong2  sbelong3  sbelong4  optim1  optim2  slfcont1  slfcont2  slfcont3  urban
##   2   4  cnarc1  cnarc2  cnarc3  ctheory1  ctheory2  ctheory3  ctheory4  CRT1  CRT2  CRT3  health_cond  mor_circle  moralid1  moralid10  moralid2  moralid3  moralid4  moralid5  moralid6  moralid7  moralid8  moralid9  mcoop1  mcoop2  mcoop3  mcoop4  mcoop5  mcoop6  mcoop7  narc1  narc2  narc3  narc4  narc5  narc6  omind1  omind2  omind4  omind5  omind6  contact2  contact5  hygiene1  hygiene2  hygiene5  psupport2  psupport3  psupport5  political_ideology  happy  riskperc1  riskperc2  sbelong1  sbelong2  sbelong3  sbelong4  optim1  optim2  slfcont1  slfcont2  slfcont3  urban
##   2   5  cnarc1  cnarc2  cnarc3  ctheory1  ctheory2  ctheory3  ctheory4  CRT1  CRT2  CRT3  health_cond  mor_circle  moralid1  moralid10  moralid2  moralid3  moralid4  moralid5  moralid6  moralid7  moralid8  moralid9  mcoop1  mcoop2  mcoop3  mcoop4  mcoop5  mcoop6  mcoop7  narc1  narc2  narc3  narc4  narc5  narc6  omind1  omind2  omind4  omind5  omind6  contact2  contact5  hygiene1  hygiene2  hygiene5  psupport2  psupport3  psupport5  political_ideology  happy  riskperc1  riskperc2  sbelong1  sbelong2  sbelong3  sbelong4  optim1  optim2  slfcont1  slfcont2  slfcont3  urban
##   2   6  cnarc1  cnarc2  cnarc3  ctheory1  ctheory2  ctheory3  ctheory4  CRT1  CRT2  CRT3  health_cond  mor_circle  moralid1  moralid10  moralid2  moralid3  moralid4  moralid5  moralid6  moralid7  moralid8  moralid9  mcoop1  mcoop2  mcoop3  mcoop4  mcoop5  mcoop6  mcoop7  narc1  narc2  narc3  narc4  narc5  narc6  omind1  omind2  omind4  omind5  omind6  contact2  contact5  hygiene1  hygiene2  hygiene5  psupport2  psupport3  psupport5  political_ideology  happy  riskperc1  riskperc2  sbelong1  sbelong2  sbelong3  sbelong4  optim1  optim2  slfcont1  slfcont2  slfcont3  urban
##   2   7  cnarc1  cnarc2  cnarc3  ctheory1  ctheory2  ctheory3  ctheory4  CRT1  CRT2  CRT3  health_cond  mor_circle  moralid1  moralid10  moralid2  moralid3  moralid4  moralid5  moralid6  moralid7  moralid8  moralid9  mcoop1  mcoop2  mcoop3  mcoop4  mcoop5  mcoop6  mcoop7  narc1  narc2  narc3  narc4  narc5  narc6  omind1  omind2  omind4  omind5  omind6  contact2  contact5  hygiene1  hygiene2  hygiene5  psupport2  psupport3  psupport5  political_ideology  happy  riskperc1  riskperc2  sbelong1  sbelong2  sbelong3  sbelong4  optim1  optim2  slfcont1  slfcont2  slfcont3  urban
##   2   8  cnarc1  cnarc2  cnarc3  ctheory1  ctheory2  ctheory3  ctheory4  CRT1  CRT2  CRT3  health_cond  mor_circle  moralid1  moralid10  moralid2  moralid3  moralid4  moralid5  moralid6  moralid7  moralid8  moralid9  mcoop1  mcoop2  mcoop3  mcoop4  mcoop5  mcoop6  mcoop7  narc1  narc2  narc3  narc4  narc5  narc6  omind1  omind2  omind4  omind5  omind6  contact2  contact5  hygiene1  hygiene2  hygiene5  psupport2  psupport3  psupport5  political_ideology  happy  riskperc1  riskperc2  sbelong1  sbelong2  sbelong3  sbelong4  optim1  optim2  slfcont1  slfcont2  slfcont3  urban
##   2   9  cnarc1  cnarc2  cnarc3  ctheory1  ctheory2  ctheory3  ctheory4  CRT1  CRT2  CRT3  health_cond  mor_circle  moralid1  moralid10  moralid2  moralid3  moralid4  moralid5  moralid6  moralid7  moralid8  moralid9  mcoop1  mcoop2  mcoop3  mcoop4  mcoop5  mcoop6  mcoop7  narc1  narc2  narc3  narc4  narc5  narc6  omind1  omind2  omind4  omind5  omind6  contact2  contact5  hygiene1  hygiene2  hygiene5  psupport2  psupport3  psupport5  political_ideology  happy  riskperc1  riskperc2  sbelong1  sbelong2  sbelong3  sbelong4  optim1  optim2  slfcont1  slfcont2  slfcont3  urban
##   2   10  cnarc1  cnarc2  cnarc3  ctheory1  ctheory2  ctheory3  ctheory4  CRT1  CRT2  CRT3  health_cond  mor_circle  moralid1  moralid10  moralid2  moralid3  moralid4  moralid5  moralid6  moralid7  moralid8  moralid9  mcoop1  mcoop2  mcoop3  mcoop4  mcoop5  mcoop6  mcoop7  narc1  narc2  narc3  narc4  narc5  narc6  omind1  omind2  omind4  omind5  omind6  contact2  contact5  hygiene1  hygiene2  hygiene5  psupport2  psupport3  psupport5  political_ideology  happy  riskperc1  riskperc2  sbelong1  sbelong2  sbelong3  sbelong4  optim1  optim2  slfcont1  slfcont2  slfcont3  urban
##   3   1  cnarc1  cnarc2  cnarc3  ctheory1  ctheory2  ctheory3  ctheory4  CRT1  CRT2  CRT3  health_cond  mor_circle  moralid1  moralid10  moralid2  moralid3  moralid4  moralid5  moralid6  moralid7  moralid8  moralid9  mcoop1  mcoop2  mcoop3  mcoop4  mcoop5  mcoop6  mcoop7  narc1  narc2  narc3  narc4  narc5  narc6  omind1  omind2  omind4  omind5  omind6  contact2  contact5  hygiene1  hygiene2  hygiene5  psupport2  psupport3  psupport5  political_ideology  happy  riskperc1  riskperc2  sbelong1  sbelong2  sbelong3  sbelong4  optim1  optim2  slfcont1  slfcont2  slfcont3  urban
##   3   2  cnarc1  cnarc2  cnarc3  ctheory1  ctheory2  ctheory3  ctheory4  CRT1  CRT2  CRT3  health_cond  mor_circle  moralid1  moralid10  moralid2  moralid3  moralid4  moralid5  moralid6  moralid7  moralid8  moralid9  mcoop1  mcoop2  mcoop3  mcoop4  mcoop5  mcoop6  mcoop7  narc1  narc2  narc3  narc4  narc5  narc6  omind1  omind2  omind4  omind5  omind6  contact2  contact5  hygiene1  hygiene2  hygiene5  psupport2  psupport3  psupport5  political_ideology  happy  riskperc1  riskperc2  sbelong1  sbelong2  sbelong3  sbelong4  optim1  optim2  slfcont1  slfcont2  slfcont3  urban
##   3   3  cnarc1  cnarc2  cnarc3  ctheory1  ctheory2  ctheory3  ctheory4  CRT1  CRT2  CRT3  health_cond  mor_circle  moralid1  moralid10  moralid2  moralid3  moralid4  moralid5  moralid6  moralid7  moralid8  moralid9  mcoop1  mcoop2  mcoop3  mcoop4  mcoop5  mcoop6  mcoop7  narc1  narc2  narc3  narc4  narc5  narc6  omind1  omind2  omind4  omind5  omind6  contact2  contact5  hygiene1  hygiene2  hygiene5  psupport2  psupport3  psupport5  political_ideology  happy  riskperc1  riskperc2  sbelong1  sbelong2  sbelong3  sbelong4  optim1  optim2  slfcont1  slfcont2  slfcont3  urban
##   3   4  cnarc1  cnarc2  cnarc3  ctheory1  ctheory2  ctheory3  ctheory4  CRT1  CRT2  CRT3  health_cond  mor_circle  moralid1  moralid10  moralid2  moralid3  moralid4  moralid5  moralid6  moralid7  moralid8  moralid9  mcoop1  mcoop2  mcoop3  mcoop4  mcoop5  mcoop6  mcoop7  narc1  narc2  narc3  narc4  narc5  narc6  omind1  omind2  omind4  omind5  omind6  contact2  contact5  hygiene1  hygiene2  hygiene5  psupport2  psupport3  psupport5  political_ideology  happy  riskperc1  riskperc2  sbelong1  sbelong2  sbelong3  sbelong4  optim1  optim2  slfcont1  slfcont2  slfcont3  urban
##   3   5  cnarc1  cnarc2  cnarc3  ctheory1  ctheory2  ctheory3  ctheory4  CRT1  CRT2  CRT3  health_cond  mor_circle  moralid1  moralid10  moralid2  moralid3  moralid4  moralid5  moralid6  moralid7  moralid8  moralid9  mcoop1  mcoop2  mcoop3  mcoop4  mcoop5  mcoop6  mcoop7  narc1  narc2  narc3  narc4  narc5  narc6  omind1  omind2  omind4  omind5  omind6  contact2  contact5  hygiene1  hygiene2  hygiene5  psupport2  psupport3  psupport5  political_ideology  happy  riskperc1  riskperc2  sbelong1  sbelong2  sbelong3  sbelong4  optim1  optim2  slfcont1  slfcont2  slfcont3  urban
##   3   6  cnarc1  cnarc2  cnarc3  ctheory1  ctheory2  ctheory3  ctheory4  CRT1  CRT2  CRT3  health_cond  mor_circle  moralid1  moralid10  moralid2  moralid3  moralid4  moralid5  moralid6  moralid7  moralid8  moralid9  mcoop1  mcoop2  mcoop3  mcoop4  mcoop5  mcoop6  mcoop7  narc1  narc2  narc3  narc4  narc5  narc6  omind1  omind2  omind4  omind5  omind6  contact2  contact5  hygiene1  hygiene2  hygiene5  psupport2  psupport3  psupport5  political_ideology  happy  riskperc1  riskperc2  sbelong1  sbelong2  sbelong3  sbelong4  optim1  optim2  slfcont1  slfcont2  slfcont3  urban
##   3   7  cnarc1  cnarc2  cnarc3  ctheory1  ctheory2  ctheory3  ctheory4  CRT1  CRT2  CRT3  health_cond  mor_circle  moralid1  moralid10  moralid2  moralid3  moralid4  moralid5  moralid6  moralid7  moralid8  moralid9  mcoop1  mcoop2  mcoop3  mcoop4  mcoop5  mcoop6  mcoop7  narc1  narc2  narc3  narc4  narc5  narc6  omind1  omind2  omind4  omind5  omind6  contact2  contact5  hygiene1  hygiene2  hygiene5  psupport2  psupport3  psupport5  political_ideology  happy  riskperc1  riskperc2  sbelong1  sbelong2  sbelong3  sbelong4  optim1  optim2  slfcont1  slfcont2  slfcont3  urban
##   3   8  cnarc1  cnarc2  cnarc3  ctheory1  ctheory2  ctheory3  ctheory4  CRT1  CRT2  CRT3  health_cond  mor_circle  moralid1  moralid10  moralid2  moralid3  moralid4  moralid5  moralid6  moralid7  moralid8  moralid9  mcoop1  mcoop2  mcoop3  mcoop4  mcoop5  mcoop6  mcoop7  narc1  narc2  narc3  narc4  narc5  narc6  omind1  omind2  omind4  omind5  omind6  contact2  contact5  hygiene1  hygiene2  hygiene5  psupport2  psupport3  psupport5  political_ideology  happy  riskperc1  riskperc2  sbelong1  sbelong2  sbelong3  sbelong4  optim1  optim2  slfcont1  slfcont2  slfcont3  urban
##   3   9  cnarc1  cnarc2  cnarc3  ctheory1  ctheory2  ctheory3  ctheory4  CRT1  CRT2  CRT3  health_cond  mor_circle  moralid1  moralid10  moralid2  moralid3  moralid4  moralid5  moralid6  moralid7  moralid8  moralid9  mcoop1  mcoop2  mcoop3  mcoop4  mcoop5  mcoop6  mcoop7  narc1  narc2  narc3  narc4  narc5  narc6  omind1  omind2  omind4  omind5  omind6  contact2  contact5  hygiene1  hygiene2  hygiene5  psupport2  psupport3  psupport5  political_ideology  happy  riskperc1  riskperc2  sbelong1  sbelong2  sbelong3  sbelong4  optim1  optim2  slfcont1  slfcont2  slfcont3  urban
##   3   10  cnarc1  cnarc2  cnarc3  ctheory1  ctheory2  ctheory3  ctheory4  CRT1  CRT2  CRT3  health_cond  mor_circle  moralid1  moralid10  moralid2  moralid3  moralid4  moralid5  moralid6  moralid7  moralid8  moralid9  mcoop1  mcoop2  mcoop3  mcoop4  mcoop5  mcoop6  mcoop7  narc1  narc2  narc3  narc4  narc5  narc6  omind1  omind2  omind4  omind5  omind6  contact2  contact5  hygiene1  hygiene2  hygiene5  psupport2  psupport3  psupport5  political_ideology  happy  riskperc1  riskperc2  sbelong1  sbelong2  sbelong3  sbelong4  optim1  optim2  slfcont1  slfcont2  slfcont3  urban
##   4   1  cnarc1  cnarc2  cnarc3  ctheory1  ctheory2  ctheory3  ctheory4  CRT1  CRT2  CRT3  health_cond  mor_circle  moralid1  moralid10  moralid2  moralid3  moralid4  moralid5  moralid6  moralid7  moralid8  moralid9  mcoop1  mcoop2  mcoop3  mcoop4  mcoop5  mcoop6  mcoop7  narc1  narc2  narc3  narc4  narc5  narc6  omind1  omind2  omind4  omind5  omind6  contact2  contact5  hygiene1  hygiene2  hygiene5  psupport2  psupport3  psupport5  political_ideology  happy  riskperc1  riskperc2  sbelong1  sbelong2  sbelong3  sbelong4  optim1  optim2  slfcont1  slfcont2  slfcont3  urban
##   4   2  cnarc1  cnarc2  cnarc3  ctheory1  ctheory2  ctheory3  ctheory4  CRT1  CRT2  CRT3  health_cond  mor_circle  moralid1  moralid10  moralid2  moralid3  moralid4  moralid5  moralid6  moralid7  moralid8  moralid9  mcoop1  mcoop2  mcoop3  mcoop4  mcoop5  mcoop6  mcoop7  narc1  narc2  narc3  narc4  narc5  narc6  omind1  omind2  omind4  omind5  omind6  contact2  contact5  hygiene1  hygiene2  hygiene5  psupport2  psupport3  psupport5  political_ideology  happy  riskperc1  riskperc2  sbelong1  sbelong2  sbelong3  sbelong4  optim1  optim2  slfcont1  slfcont2  slfcont3  urban
##   4   3  cnarc1  cnarc2  cnarc3  ctheory1  ctheory2  ctheory3  ctheory4  CRT1  CRT2  CRT3  health_cond  mor_circle  moralid1  moralid10  moralid2  moralid3  moralid4  moralid5  moralid6  moralid7  moralid8  moralid9  mcoop1  mcoop2  mcoop3  mcoop4  mcoop5  mcoop6  mcoop7  narc1  narc2  narc3  narc4  narc5  narc6  omind1  omind2  omind4  omind5  omind6  contact2  contact5  hygiene1  hygiene2  hygiene5  psupport2  psupport3  psupport5  political_ideology  happy  riskperc1  riskperc2  sbelong1  sbelong2  sbelong3  sbelong4  optim1  optim2  slfcont1  slfcont2  slfcont3  urban
##   4   4  cnarc1  cnarc2  cnarc3  ctheory1  ctheory2  ctheory3  ctheory4  CRT1  CRT2  CRT3  health_cond  mor_circle  moralid1  moralid10  moralid2  moralid3  moralid4  moralid5  moralid6  moralid7  moralid8  moralid9  mcoop1  mcoop2  mcoop3  mcoop4  mcoop5  mcoop6  mcoop7  narc1  narc2  narc3  narc4  narc5  narc6  omind1  omind2  omind4  omind5  omind6  contact2  contact5  hygiene1  hygiene2  hygiene5  psupport2  psupport3  psupport5  political_ideology  happy  riskperc1  riskperc2  sbelong1  sbelong2  sbelong3  sbelong4  optim1  optim2  slfcont1  slfcont2  slfcont3  urban
##   4   5  cnarc1  cnarc2  cnarc3  ctheory1  ctheory2  ctheory3  ctheory4  CRT1  CRT2  CRT3  health_cond  mor_circle  moralid1  moralid10  moralid2  moralid3  moralid4  moralid5  moralid6  moralid7  moralid8  moralid9  mcoop1  mcoop2  mcoop3  mcoop4  mcoop5  mcoop6  mcoop7  narc1  narc2  narc3  narc4  narc5  narc6  omind1  omind2  omind4  omind5  omind6  contact2  contact5  hygiene1  hygiene2  hygiene5  psupport2  psupport3  psupport5  political_ideology  happy  riskperc1  riskperc2  sbelong1  sbelong2  sbelong3  sbelong4  optim1  optim2  slfcont1  slfcont2  slfcont3  urban
##   4   6  cnarc1  cnarc2  cnarc3  ctheory1  ctheory2  ctheory3  ctheory4  CRT1  CRT2  CRT3  health_cond  mor_circle  moralid1  moralid10  moralid2  moralid3  moralid4  moralid5  moralid6  moralid7  moralid8  moralid9  mcoop1  mcoop2  mcoop3  mcoop4  mcoop5  mcoop6  mcoop7  narc1  narc2  narc3  narc4  narc5  narc6  omind1  omind2  omind4  omind5  omind6  contact2  contact5  hygiene1  hygiene2  hygiene5  psupport2  psupport3  psupport5  political_ideology  happy  riskperc1  riskperc2  sbelong1  sbelong2  sbelong3  sbelong4  optim1  optim2  slfcont1  slfcont2  slfcont3  urban
##   4   7  cnarc1  cnarc2  cnarc3  ctheory1  ctheory2  ctheory3  ctheory4  CRT1  CRT2  CRT3  health_cond  mor_circle  moralid1  moralid10  moralid2  moralid3  moralid4  moralid5  moralid6  moralid7  moralid8  moralid9  mcoop1  mcoop2  mcoop3  mcoop4  mcoop5  mcoop6  mcoop7  narc1  narc2  narc3  narc4  narc5  narc6  omind1  omind2  omind4  omind5  omind6  contact2  contact5  hygiene1  hygiene2  hygiene5  psupport2  psupport3  psupport5  political_ideology  happy  riskperc1  riskperc2  sbelong1  sbelong2  sbelong3  sbelong4  optim1  optim2  slfcont1  slfcont2  slfcont3  urban
##   4   8  cnarc1  cnarc2  cnarc3  ctheory1  ctheory2  ctheory3  ctheory4  CRT1  CRT2  CRT3  health_cond  mor_circle  moralid1  moralid10  moralid2  moralid3  moralid4  moralid5  moralid6  moralid7  moralid8  moralid9  mcoop1  mcoop2  mcoop3  mcoop4  mcoop5  mcoop6  mcoop7  narc1  narc2  narc3  narc4  narc5  narc6  omind1  omind2  omind4  omind5  omind6  contact2  contact5  hygiene1  hygiene2  hygiene5  psupport2  psupport3  psupport5  political_ideology  happy  riskperc1  riskperc2  sbelong1  sbelong2  sbelong3  sbelong4  optim1  optim2  slfcont1  slfcont2  slfcont3  urban
##   4   9  cnarc1  cnarc2  cnarc3  ctheory1  ctheory2  ctheory3  ctheory4  CRT1  CRT2  CRT3  health_cond  mor_circle  moralid1  moralid10  moralid2  moralid3  moralid4  moralid5  moralid6  moralid7  moralid8  moralid9  mcoop1  mcoop2  mcoop3  mcoop4  mcoop5  mcoop6  mcoop7  narc1  narc2  narc3  narc4  narc5  narc6  omind1  omind2  omind4  omind5  omind6  contact2  contact5  hygiene1  hygiene2  hygiene5  psupport2  psupport3  psupport5  political_ideology  happy  riskperc1  riskperc2  sbelong1  sbelong2  sbelong3  sbelong4  optim1  optim2  slfcont1  slfcont2  slfcont3  urban
##   4   10  cnarc1  cnarc2  cnarc3  ctheory1  ctheory2  ctheory3  ctheory4  CRT1  CRT2  CRT3  health_cond  mor_circle  moralid1  moralid10  moralid2  moralid3  moralid4  moralid5  moralid6  moralid7  moralid8  moralid9  mcoop1  mcoop2  mcoop3  mcoop4  mcoop5  mcoop6  mcoop7  narc1  narc2  narc3  narc4  narc5  narc6  omind1  omind2  omind4  omind5  omind6  contact2  contact5  hygiene1  hygiene2  hygiene5  psupport2  psupport3  psupport5  political_ideology  happy  riskperc1  riskperc2  sbelong1  sbelong2  sbelong3  sbelong4  optim1  optim2  slfcont1  slfcont2  slfcont3  urban
##   5   1  cnarc1  cnarc2  cnarc3  ctheory1  ctheory2  ctheory3  ctheory4  CRT1  CRT2  CRT3  health_cond  mor_circle  moralid1  moralid10  moralid2  moralid3  moralid4  moralid5  moralid6  moralid7  moralid8  moralid9  mcoop1  mcoop2  mcoop3  mcoop4  mcoop5  mcoop6  mcoop7  narc1  narc2  narc3  narc4  narc5  narc6  omind1  omind2  omind4  omind5  omind6  contact2  contact5  hygiene1  hygiene2  hygiene5  psupport2  psupport3  psupport5  political_ideology  happy  riskperc1  riskperc2  sbelong1  sbelong2  sbelong3  sbelong4  optim1  optim2  slfcont1  slfcont2  slfcont3  urban
##   5   2  cnarc1  cnarc2  cnarc3  ctheory1  ctheory2  ctheory3  ctheory4  CRT1  CRT2  CRT3  health_cond  mor_circle  moralid1  moralid10  moralid2  moralid3  moralid4  moralid5  moralid6  moralid7  moralid8  moralid9  mcoop1  mcoop2  mcoop3  mcoop4  mcoop5  mcoop6  mcoop7  narc1  narc2  narc3  narc4  narc5  narc6  omind1  omind2  omind4  omind5  omind6  contact2  contact5  hygiene1  hygiene2  hygiene5  psupport2  psupport3  psupport5  political_ideology  happy  riskperc1  riskperc2  sbelong1  sbelong2  sbelong3  sbelong4  optim1  optim2  slfcont1  slfcont2  slfcont3  urban
##   5   3  cnarc1  cnarc2  cnarc3  ctheory1  ctheory2  ctheory3  ctheory4  CRT1  CRT2  CRT3  health_cond  mor_circle  moralid1  moralid10  moralid2  moralid3  moralid4  moralid5  moralid6  moralid7  moralid8  moralid9  mcoop1  mcoop2  mcoop3  mcoop4  mcoop5  mcoop6  mcoop7  narc1  narc2  narc3  narc4  narc5  narc6  omind1  omind2  omind4  omind5  omind6  contact2  contact5  hygiene1  hygiene2  hygiene5  psupport2  psupport3  psupport5  political_ideology  happy  riskperc1  riskperc2  sbelong1  sbelong2  sbelong3  sbelong4  optim1  optim2  slfcont1  slfcont2  slfcont3  urban
##   5   4  cnarc1  cnarc2  cnarc3  ctheory1  ctheory2  ctheory3  ctheory4  CRT1  CRT2  CRT3  health_cond  mor_circle  moralid1  moralid10  moralid2  moralid3  moralid4  moralid5  moralid6  moralid7  moralid8  moralid9  mcoop1  mcoop2  mcoop3  mcoop4  mcoop5  mcoop6  mcoop7  narc1  narc2  narc3  narc4  narc5  narc6  omind1  omind2  omind4  omind5  omind6  contact2  contact5  hygiene1  hygiene2  hygiene5  psupport2  psupport3  psupport5  political_ideology  happy  riskperc1  riskperc2  sbelong1  sbelong2  sbelong3  sbelong4  optim1  optim2  slfcont1  slfcont2  slfcont3  urban
##   5   5  cnarc1  cnarc2  cnarc3  ctheory1  ctheory2  ctheory3  ctheory4  CRT1  CRT2  CRT3  health_cond  mor_circle  moralid1  moralid10  moralid2  moralid3  moralid4  moralid5  moralid6  moralid7  moralid8  moralid9  mcoop1  mcoop2  mcoop3  mcoop4  mcoop5  mcoop6  mcoop7  narc1  narc2  narc3  narc4  narc5  narc6  omind1  omind2  omind4  omind5  omind6  contact2  contact5  hygiene1  hygiene2  hygiene5  psupport2  psupport3  psupport5  political_ideology  happy  riskperc1  riskperc2  sbelong1  sbelong2  sbelong3  sbelong4  optim1  optim2  slfcont1  slfcont2  slfcont3  urban
##   5   6  cnarc1  cnarc2  cnarc3  ctheory1  ctheory2  ctheory3  ctheory4  CRT1  CRT2  CRT3  health_cond  mor_circle  moralid1  moralid10  moralid2  moralid3  moralid4  moralid5  moralid6  moralid7  moralid8  moralid9  mcoop1  mcoop2  mcoop3  mcoop4  mcoop5  mcoop6  mcoop7  narc1  narc2  narc3  narc4  narc5  narc6  omind1  omind2  omind4  omind5  omind6  contact2  contact5  hygiene1  hygiene2  hygiene5  psupport2  psupport3  psupport5  political_ideology  happy  riskperc1  riskperc2  sbelong1  sbelong2  sbelong3  sbelong4  optim1  optim2  slfcont1  slfcont2  slfcont3  urban
##   5   7  cnarc1  cnarc2  cnarc3  ctheory1  ctheory2  ctheory3  ctheory4  CRT1  CRT2  CRT3  health_cond  mor_circle  moralid1  moralid10  moralid2  moralid3  moralid4  moralid5  moralid6  moralid7  moralid8  moralid9  mcoop1  mcoop2  mcoop3  mcoop4  mcoop5  mcoop6  mcoop7  narc1  narc2  narc3  narc4  narc5  narc6  omind1  omind2  omind4  omind5  omind6  contact2  contact5  hygiene1  hygiene2  hygiene5  psupport2  psupport3  psupport5  political_ideology  happy  riskperc1  riskperc2  sbelong1  sbelong2  sbelong3  sbelong4  optim1  optim2  slfcont1  slfcont2  slfcont3  urban
##   5   8  cnarc1  cnarc2  cnarc3  ctheory1  ctheory2  ctheory3  ctheory4  CRT1  CRT2  CRT3  health_cond  mor_circle  moralid1  moralid10  moralid2  moralid3  moralid4  moralid5  moralid6  moralid7  moralid8  moralid9  mcoop1  mcoop2  mcoop3  mcoop4  mcoop5  mcoop6  mcoop7  narc1  narc2  narc3  narc4  narc5  narc6  omind1  omind2  omind4  omind5  omind6  contact2  contact5  hygiene1  hygiene2  hygiene5  psupport2  psupport3  psupport5  political_ideology  happy  riskperc1  riskperc2  sbelong1  sbelong2  sbelong3  sbelong4  optim1  optim2  slfcont1  slfcont2  slfcont3  urban
##   5   9  cnarc1  cnarc2  cnarc3  ctheory1  ctheory2  ctheory3  ctheory4  CRT1  CRT2  CRT3  health_cond  mor_circle  moralid1  moralid10  moralid2  moralid3  moralid4  moralid5  moralid6  moralid7  moralid8  moralid9  mcoop1  mcoop2  mcoop3  mcoop4  mcoop5  mcoop6  mcoop7  narc1  narc2  narc3  narc4  narc5  narc6  omind1  omind2  omind4  omind5  omind6  contact2  contact5  hygiene1  hygiene2  hygiene5  psupport2  psupport3  psupport5  political_ideology  happy  riskperc1  riskperc2  sbelong1  sbelong2  sbelong3  sbelong4  optim1  optim2  slfcont1  slfcont2  slfcont3  urban
##   5   10  cnarc1  cnarc2  cnarc3  ctheory1  ctheory2  ctheory3  ctheory4  CRT1  CRT2  CRT3  health_cond  mor_circle  moralid1  moralid10  moralid2  moralid3  moralid4  moralid5  moralid6  moralid7  moralid8  moralid9  mcoop1  mcoop2  mcoop3  mcoop4  mcoop5  mcoop6  mcoop7  narc1  narc2  narc3  narc4  narc5  narc6  omind1  omind2  omind4  omind5  omind6  contact2  contact5  hygiene1  hygiene2  hygiene5  psupport2  psupport3  psupport5  political_ideology  happy  riskperc1  riskperc2  sbelong1  sbelong2  sbelong3  sbelong4  optim1  optim2  slfcont1  slfcont2  slfcont3  urban
##   6   1  cnarc1  cnarc2  cnarc3  ctheory1  ctheory2  ctheory3  ctheory4  CRT1  CRT2  CRT3  health_cond  mor_circle  moralid1  moralid10  moralid2  moralid3  moralid4  moralid5  moralid6  moralid7  moralid8  moralid9  mcoop1  mcoop2  mcoop3  mcoop4  mcoop5  mcoop6  mcoop7  narc1  narc2  narc3  narc4  narc5  narc6  omind1  omind2  omind4  omind5  omind6  contact2  contact5  hygiene1  hygiene2  hygiene5  psupport2  psupport3  psupport5  political_ideology  happy  riskperc1  riskperc2  sbelong1  sbelong2  sbelong3  sbelong4  optim1  optim2  slfcont1  slfcont2  slfcont3  urban
##   6   2  cnarc1  cnarc2  cnarc3  ctheory1  ctheory2  ctheory3  ctheory4  CRT1  CRT2  CRT3  health_cond  mor_circle  moralid1  moralid10  moralid2  moralid3  moralid4  moralid5  moralid6  moralid7  moralid8  moralid9  mcoop1  mcoop2  mcoop3  mcoop4  mcoop5  mcoop6  mcoop7  narc1  narc2  narc3  narc4  narc5  narc6  omind1  omind2  omind4  omind5  omind6  contact2  contact5  hygiene1  hygiene2  hygiene5  psupport2  psupport3  psupport5  political_ideology  happy  riskperc1  riskperc2  sbelong1  sbelong2  sbelong3  sbelong4  optim1  optim2  slfcont1  slfcont2  slfcont3  urban
##   6   3  cnarc1  cnarc2  cnarc3  ctheory1  ctheory2  ctheory3  ctheory4  CRT1  CRT2  CRT3  health_cond  mor_circle  moralid1  moralid10  moralid2  moralid3  moralid4  moralid5  moralid6  moralid7  moralid8  moralid9  mcoop1  mcoop2  mcoop3  mcoop4  mcoop5  mcoop6  mcoop7  narc1  narc2  narc3  narc4  narc5  narc6  omind1  omind2  omind4  omind5  omind6  contact2  contact5  hygiene1  hygiene2  hygiene5  psupport2  psupport3  psupport5  political_ideology  happy  riskperc1  riskperc2  sbelong1  sbelong2  sbelong3  sbelong4  optim1  optim2  slfcont1  slfcont2  slfcont3  urban
##   6   4  cnarc1  cnarc2  cnarc3  ctheory1  ctheory2  ctheory3  ctheory4  CRT1  CRT2  CRT3  health_cond  mor_circle  moralid1  moralid10  moralid2  moralid3  moralid4  moralid5  moralid6  moralid7  moralid8  moralid9  mcoop1  mcoop2  mcoop3  mcoop4  mcoop5  mcoop6  mcoop7  narc1  narc2  narc3  narc4  narc5  narc6  omind1  omind2  omind4  omind5  omind6  contact2  contact5  hygiene1  hygiene2  hygiene5  psupport2  psupport3  psupport5  political_ideology  happy  riskperc1  riskperc2  sbelong1  sbelong2  sbelong3  sbelong4  optim1  optim2  slfcont1  slfcont2  slfcont3  urban
##   6   5  cnarc1  cnarc2  cnarc3  ctheory1  ctheory2  ctheory3  ctheory4  CRT1  CRT2  CRT3  health_cond  mor_circle  moralid1  moralid10  moralid2  moralid3  moralid4  moralid5  moralid6  moralid7  moralid8  moralid9  mcoop1  mcoop2  mcoop3  mcoop4  mcoop5  mcoop6  mcoop7  narc1  narc2  narc3  narc4  narc5  narc6  omind1  omind2  omind4  omind5  omind6  contact2  contact5  hygiene1  hygiene2  hygiene5  psupport2  psupport3  psupport5  political_ideology  happy  riskperc1  riskperc2  sbelong1  sbelong2  sbelong3  sbelong4  optim1  optim2  slfcont1  slfcont2  slfcont3  urban
##   6   6  cnarc1  cnarc2  cnarc3  ctheory1  ctheory2  ctheory3  ctheory4  CRT1  CRT2  CRT3  health_cond  mor_circle  moralid1  moralid10  moralid2  moralid3  moralid4  moralid5  moralid6  moralid7  moralid8  moralid9  mcoop1  mcoop2  mcoop3  mcoop4  mcoop5  mcoop6  mcoop7  narc1  narc2  narc3  narc4  narc5  narc6  omind1  omind2  omind4  omind5  omind6  contact2  contact5  hygiene1  hygiene2  hygiene5  psupport2  psupport3  psupport5  political_ideology  happy  riskperc1  riskperc2  sbelong1  sbelong2  sbelong3  sbelong4  optim1  optim2  slfcont1  slfcont2  slfcont3  urban
##   6   7  cnarc1  cnarc2  cnarc3  ctheory1  ctheory2  ctheory3  ctheory4  CRT1  CRT2  CRT3  health_cond  mor_circle  moralid1  moralid10  moralid2  moralid3  moralid4  moralid5  moralid6  moralid7  moralid8  moralid9  mcoop1  mcoop2  mcoop3  mcoop4  mcoop5  mcoop6  mcoop7  narc1  narc2  narc3  narc4  narc5  narc6  omind1  omind2  omind4  omind5  omind6  contact2  contact5  hygiene1  hygiene2  hygiene5  psupport2  psupport3  psupport5  political_ideology  happy  riskperc1  riskperc2  sbelong1  sbelong2  sbelong3  sbelong4  optim1  optim2  slfcont1  slfcont2  slfcont3  urban
##   6   8  cnarc1  cnarc2  cnarc3  ctheory1  ctheory2  ctheory3  ctheory4  CRT1  CRT2  CRT3  health_cond  mor_circle  moralid1  moralid10  moralid2  moralid3  moralid4  moralid5  moralid6  moralid7  moralid8  moralid9  mcoop1  mcoop2  mcoop3  mcoop4  mcoop5  mcoop6  mcoop7  narc1  narc2  narc3  narc4  narc5  narc6  omind1  omind2  omind4  omind5  omind6  contact2  contact5  hygiene1  hygiene2  hygiene5  psupport2  psupport3  psupport5  political_ideology  happy  riskperc1  riskperc2  sbelong1  sbelong2  sbelong3  sbelong4  optim1  optim2  slfcont1  slfcont2  slfcont3  urban
##   6   9  cnarc1  cnarc2  cnarc3  ctheory1  ctheory2  ctheory3  ctheory4  CRT1  CRT2  CRT3  health_cond  mor_circle  moralid1  moralid10  moralid2  moralid3  moralid4  moralid5  moralid6  moralid7  moralid8  moralid9  mcoop1  mcoop2  mcoop3  mcoop4  mcoop5  mcoop6  mcoop7  narc1  narc2  narc3  narc4  narc5  narc6  omind1  omind2  omind4  omind5  omind6  contact2  contact5  hygiene1  hygiene2  hygiene5  psupport2  psupport3  psupport5  political_ideology  happy  riskperc1  riskperc2  sbelong1  sbelong2  sbelong3  sbelong4  optim1  optim2  slfcont1  slfcont2  slfcont3  urban
##   6   10  cnarc1  cnarc2  cnarc3  ctheory1  ctheory2  ctheory3  ctheory4  CRT1  CRT2  CRT3  health_cond  mor_circle  moralid1  moralid10  moralid2  moralid3  moralid4  moralid5  moralid6  moralid7  moralid8  moralid9  mcoop1  mcoop2  mcoop3  mcoop4  mcoop5  mcoop6  mcoop7  narc1  narc2  narc3  narc4  narc5  narc6  omind1  omind2  omind4  omind5  omind6  contact2  contact5  hygiene1  hygiene2  hygiene5  psupport2  psupport3  psupport5  political_ideology  happy  riskperc1  riskperc2  sbelong1  sbelong2  sbelong3  sbelong4  optim1  optim2  slfcont1  slfcont2  slfcont3  urban
##   7   1  cnarc1  cnarc2  cnarc3  ctheory1  ctheory2  ctheory3  ctheory4  CRT1  CRT2  CRT3  health_cond  mor_circle  moralid1  moralid10  moralid2  moralid3  moralid4  moralid5  moralid6  moralid7  moralid8  moralid9  mcoop1  mcoop2  mcoop3  mcoop4  mcoop5  mcoop6  mcoop7  narc1  narc2  narc3  narc4  narc5  narc6  omind1  omind2  omind4  omind5  omind6  contact2  contact5  hygiene1  hygiene2  hygiene5  psupport2  psupport3  psupport5  political_ideology  happy  riskperc1  riskperc2  sbelong1  sbelong2  sbelong3  sbelong4  optim1  optim2  slfcont1  slfcont2  slfcont3  urban
##   7   2  cnarc1  cnarc2  cnarc3  ctheory1  ctheory2  ctheory3  ctheory4  CRT1  CRT2  CRT3  health_cond  mor_circle  moralid1  moralid10  moralid2  moralid3  moralid4  moralid5  moralid6  moralid7  moralid8  moralid9  mcoop1  mcoop2  mcoop3  mcoop4  mcoop5  mcoop6  mcoop7  narc1  narc2  narc3  narc4  narc5  narc6  omind1  omind2  omind4  omind5  omind6  contact2  contact5  hygiene1  hygiene2  hygiene5  psupport2  psupport3  psupport5  political_ideology  happy  riskperc1  riskperc2  sbelong1  sbelong2  sbelong3  sbelong4  optim1  optim2  slfcont1  slfcont2  slfcont3  urban
##   7   3  cnarc1  cnarc2  cnarc3  ctheory1  ctheory2  ctheory3  ctheory4  CRT1  CRT2  CRT3  health_cond  mor_circle  moralid1  moralid10  moralid2  moralid3  moralid4  moralid5  moralid6  moralid7  moralid8  moralid9  mcoop1  mcoop2  mcoop3  mcoop4  mcoop5  mcoop6  mcoop7  narc1  narc2  narc3  narc4  narc5  narc6  omind1  omind2  omind4  omind5  omind6  contact2  contact5  hygiene1  hygiene2  hygiene5  psupport2  psupport3  psupport5  political_ideology  happy  riskperc1  riskperc2  sbelong1  sbelong2  sbelong3  sbelong4  optim1  optim2  slfcont1  slfcont2  slfcont3  urban
##   7   4  cnarc1  cnarc2  cnarc3  ctheory1  ctheory2  ctheory3  ctheory4  CRT1  CRT2  CRT3  health_cond  mor_circle  moralid1  moralid10  moralid2  moralid3  moralid4  moralid5  moralid6  moralid7  moralid8  moralid9  mcoop1  mcoop2  mcoop3  mcoop4  mcoop5  mcoop6  mcoop7  narc1  narc2  narc3  narc4  narc5  narc6  omind1  omind2  omind4  omind5  omind6  contact2  contact5  hygiene1  hygiene2  hygiene5  psupport2  psupport3  psupport5  political_ideology  happy  riskperc1  riskperc2  sbelong1  sbelong2  sbelong3  sbelong4  optim1  optim2  slfcont1  slfcont2  slfcont3  urban
##   7   5  cnarc1  cnarc2  cnarc3  ctheory1  ctheory2  ctheory3  ctheory4  CRT1  CRT2  CRT3  health_cond  mor_circle  moralid1  moralid10  moralid2  moralid3  moralid4  moralid5  moralid6  moralid7  moralid8  moralid9  mcoop1  mcoop2  mcoop3  mcoop4  mcoop5  mcoop6  mcoop7  narc1  narc2  narc3  narc4  narc5  narc6  omind1  omind2  omind4  omind5  omind6  contact2  contact5  hygiene1  hygiene2  hygiene5  psupport2  psupport3  psupport5  political_ideology  happy  riskperc1  riskperc2  sbelong1  sbelong2  sbelong3  sbelong4  optim1  optim2  slfcont1  slfcont2  slfcont3  urban
##   7   6  cnarc1  cnarc2  cnarc3  ctheory1  ctheory2  ctheory3  ctheory4  CRT1  CRT2  CRT3  health_cond  mor_circle  moralid1  moralid10  moralid2  moralid3  moralid4  moralid5  moralid6  moralid7  moralid8  moralid9  mcoop1  mcoop2  mcoop3  mcoop4  mcoop5  mcoop6  mcoop7  narc1  narc2  narc3  narc4  narc5  narc6  omind1  omind2  omind4  omind5  omind6  contact2  contact5  hygiene1  hygiene2  hygiene5  psupport2  psupport3  psupport5  political_ideology  happy  riskperc1  riskperc2  sbelong1  sbelong2  sbelong3  sbelong4  optim1  optim2  slfcont1  slfcont2  slfcont3  urban
##   7   7  cnarc1  cnarc2  cnarc3  ctheory1  ctheory2  ctheory3  ctheory4  CRT1  CRT2  CRT3  health_cond  mor_circle  moralid1  moralid10  moralid2  moralid3  moralid4  moralid5  moralid6  moralid7  moralid8  moralid9  mcoop1  mcoop2  mcoop3  mcoop4  mcoop5  mcoop6  mcoop7  narc1  narc2  narc3  narc4  narc5  narc6  omind1  omind2  omind4  omind5  omind6  contact2  contact5  hygiene1  hygiene2  hygiene5  psupport2  psupport3  psupport5  political_ideology  happy  riskperc1  riskperc2  sbelong1  sbelong2  sbelong3  sbelong4  optim1  optim2  slfcont1  slfcont2  slfcont3  urban
##   7   8  cnarc1  cnarc2  cnarc3  ctheory1  ctheory2  ctheory3  ctheory4  CRT1  CRT2  CRT3  health_cond  mor_circle  moralid1  moralid10  moralid2  moralid3  moralid4  moralid5  moralid6  moralid7  moralid8  moralid9  mcoop1  mcoop2  mcoop3  mcoop4  mcoop5  mcoop6  mcoop7  narc1  narc2  narc3  narc4  narc5  narc6  omind1  omind2  omind4  omind5  omind6  contact2  contact5  hygiene1  hygiene2  hygiene5  psupport2  psupport3  psupport5  political_ideology  happy  riskperc1  riskperc2  sbelong1  sbelong2  sbelong3  sbelong4  optim1  optim2  slfcont1  slfcont2  slfcont3  urban
##   7   9  cnarc1  cnarc2  cnarc3  ctheory1  ctheory2  ctheory3  ctheory4  CRT1  CRT2  CRT3  health_cond  mor_circle  moralid1  moralid10  moralid2  moralid3  moralid4  moralid5  moralid6  moralid7  moralid8  moralid9  mcoop1  mcoop2  mcoop3  mcoop4  mcoop5  mcoop6  mcoop7  narc1  narc2  narc3  narc4  narc5  narc6  omind1  omind2  omind4  omind5  omind6  contact2  contact5  hygiene1  hygiene2  hygiene5  psupport2  psupport3  psupport5  political_ideology  happy  riskperc1  riskperc2  sbelong1  sbelong2  sbelong3  sbelong4  optim1  optim2  slfcont1  slfcont2  slfcont3  urban
##   7   10  cnarc1  cnarc2  cnarc3  ctheory1  ctheory2  ctheory3  ctheory4  CRT1  CRT2  CRT3  health_cond  mor_circle  moralid1  moralid10  moralid2  moralid3  moralid4  moralid5  moralid6  moralid7  moralid8  moralid9  mcoop1  mcoop2  mcoop3  mcoop4  mcoop5  mcoop6  mcoop7  narc1  narc2  narc3  narc4  narc5  narc6  omind1  omind2  omind4  omind5  omind6  contact2  contact5  hygiene1  hygiene2  hygiene5  psupport2  psupport3  psupport5  political_ideology  happy  riskperc1  riskperc2  sbelong1  sbelong2  sbelong3  sbelong4  optim1  optim2  slfcont1  slfcont2  slfcont3  urban
##   8   1  cnarc1  cnarc2  cnarc3  ctheory1  ctheory2  ctheory3  ctheory4  CRT1  CRT2  CRT3  health_cond  mor_circle  moralid1  moralid10  moralid2  moralid3  moralid4  moralid5  moralid6  moralid7  moralid8  moralid9  mcoop1  mcoop2  mcoop3  mcoop4  mcoop5  mcoop6  mcoop7  narc1  narc2  narc3  narc4  narc5  narc6  omind1  omind2  omind4  omind5  omind6  contact2  contact5  hygiene1  hygiene2  hygiene5  psupport2  psupport3  psupport5  political_ideology  happy  riskperc1  riskperc2  sbelong1  sbelong2  sbelong3  sbelong4  optim1  optim2  slfcont1  slfcont2  slfcont3  urban
##   8   2  cnarc1  cnarc2  cnarc3  ctheory1  ctheory2  ctheory3  ctheory4  CRT1  CRT2  CRT3  health_cond  mor_circle  moralid1  moralid10  moralid2  moralid3  moralid4  moralid5  moralid6  moralid7  moralid8  moralid9  mcoop1  mcoop2  mcoop3  mcoop4  mcoop5  mcoop6  mcoop7  narc1  narc2  narc3  narc4  narc5  narc6  omind1  omind2  omind4  omind5  omind6  contact2  contact5  hygiene1  hygiene2  hygiene5  psupport2  psupport3  psupport5  political_ideology  happy  riskperc1  riskperc2  sbelong1  sbelong2  sbelong3  sbelong4  optim1  optim2  slfcont1  slfcont2  slfcont3  urban
##   8   3  cnarc1  cnarc2  cnarc3  ctheory1  ctheory2  ctheory3  ctheory4  CRT1  CRT2  CRT3  health_cond  mor_circle  moralid1  moralid10  moralid2  moralid3  moralid4  moralid5  moralid6  moralid7  moralid8  moralid9  mcoop1  mcoop2  mcoop3  mcoop4  mcoop5  mcoop6  mcoop7  narc1  narc2  narc3  narc4  narc5  narc6  omind1  omind2  omind4  omind5  omind6  contact2  contact5  hygiene1  hygiene2  hygiene5  psupport2  psupport3  psupport5  political_ideology  happy  riskperc1  riskperc2  sbelong1  sbelong2  sbelong3  sbelong4  optim1  optim2  slfcont1  slfcont2  slfcont3  urban
##   8   4  cnarc1  cnarc2  cnarc3  ctheory1  ctheory2  ctheory3  ctheory4  CRT1  CRT2  CRT3  health_cond  mor_circle  moralid1  moralid10  moralid2  moralid3  moralid4  moralid5  moralid6  moralid7  moralid8  moralid9  mcoop1  mcoop2  mcoop3  mcoop4  mcoop5  mcoop6  mcoop7  narc1  narc2  narc3  narc4  narc5  narc6  omind1  omind2  omind4  omind5  omind6  contact2  contact5  hygiene1  hygiene2  hygiene5  psupport2  psupport3  psupport5  political_ideology  happy  riskperc1  riskperc2  sbelong1  sbelong2  sbelong3  sbelong4  optim1  optim2  slfcont1  slfcont2  slfcont3  urban
##   8   5  cnarc1  cnarc2  cnarc3  ctheory1  ctheory2  ctheory3  ctheory4  CRT1  CRT2  CRT3  health_cond  mor_circle  moralid1  moralid10  moralid2  moralid3  moralid4  moralid5  moralid6  moralid7  moralid8  moralid9  mcoop1  mcoop2  mcoop3  mcoop4  mcoop5  mcoop6  mcoop7  narc1  narc2  narc3  narc4  narc5  narc6  omind1  omind2  omind4  omind5  omind6  contact2  contact5  hygiene1  hygiene2  hygiene5  psupport2  psupport3  psupport5  political_ideology  happy  riskperc1  riskperc2  sbelong1  sbelong2  sbelong3  sbelong4  optim1  optim2  slfcont1  slfcont2  slfcont3  urban
##   8   6  cnarc1  cnarc2  cnarc3  ctheory1  ctheory2  ctheory3  ctheory4  CRT1  CRT2  CRT3  health_cond  mor_circle  moralid1  moralid10  moralid2  moralid3  moralid4  moralid5  moralid6  moralid7  moralid8  moralid9  mcoop1  mcoop2  mcoop3  mcoop4  mcoop5  mcoop6  mcoop7  narc1  narc2  narc3  narc4  narc5  narc6  omind1  omind2  omind4  omind5  omind6  contact2  contact5  hygiene1  hygiene2  hygiene5  psupport2  psupport3  psupport5  political_ideology  happy  riskperc1  riskperc2  sbelong1  sbelong2  sbelong3  sbelong4  optim1  optim2  slfcont1  slfcont2  slfcont3  urban
##   8   7  cnarc1  cnarc2  cnarc3  ctheory1  ctheory2  ctheory3  ctheory4  CRT1  CRT2  CRT3  health_cond  mor_circle  moralid1  moralid10  moralid2  moralid3  moralid4  moralid5  moralid6  moralid7  moralid8  moralid9  mcoop1  mcoop2  mcoop3  mcoop4  mcoop5  mcoop6  mcoop7  narc1  narc2  narc3  narc4  narc5  narc6  omind1  omind2  omind4  omind5  omind6  contact2  contact5  hygiene1  hygiene2  hygiene5  psupport2  psupport3  psupport5  political_ideology  happy  riskperc1  riskperc2  sbelong1  sbelong2  sbelong3  sbelong4  optim1  optim2  slfcont1  slfcont2  slfcont3  urban
##   8   8  cnarc1  cnarc2  cnarc3  ctheory1  ctheory2  ctheory3  ctheory4  CRT1  CRT2  CRT3  health_cond  mor_circle  moralid1  moralid10  moralid2  moralid3  moralid4  moralid5  moralid6  moralid7  moralid8  moralid9  mcoop1  mcoop2  mcoop3  mcoop4  mcoop5  mcoop6  mcoop7  narc1  narc2  narc3  narc4  narc5  narc6  omind1  omind2  omind4  omind5  omind6  contact2  contact5  hygiene1  hygiene2  hygiene5  psupport2  psupport3  psupport5  political_ideology  happy  riskperc1  riskperc2  sbelong1  sbelong2  sbelong3  sbelong4  optim1  optim2  slfcont1  slfcont2  slfcont3  urban
##   8   9  cnarc1  cnarc2  cnarc3  ctheory1  ctheory2  ctheory3  ctheory4  CRT1  CRT2  CRT3  health_cond  mor_circle  moralid1  moralid10  moralid2  moralid3  moralid4  moralid5  moralid6  moralid7  moralid8  moralid9  mcoop1  mcoop2  mcoop3  mcoop4  mcoop5  mcoop6  mcoop7  narc1  narc2  narc3  narc4  narc5  narc6  omind1  omind2  omind4  omind5  omind6  contact2  contact5  hygiene1  hygiene2  hygiene5  psupport2  psupport3  psupport5  political_ideology  happy  riskperc1  riskperc2  sbelong1  sbelong2  sbelong3  sbelong4  optim1  optim2  slfcont1  slfcont2  slfcont3  urban
##   8   10  cnarc1  cnarc2  cnarc3  ctheory1  ctheory2  ctheory3  ctheory4  CRT1  CRT2  CRT3  health_cond  mor_circle  moralid1  moralid10  moralid2  moralid3  moralid4  moralid5  moralid6  moralid7  moralid8  moralid9  mcoop1  mcoop2  mcoop3  mcoop4  mcoop5  mcoop6  mcoop7  narc1  narc2  narc3  narc4  narc5  narc6  omind1  omind2  omind4  omind5  omind6  contact2  contact5  hygiene1  hygiene2  hygiene5  psupport2  psupport3  psupport5  political_ideology  happy  riskperc1  riskperc2  sbelong1  sbelong2  sbelong3  sbelong4  optim1  optim2  slfcont1  slfcont2  slfcont3  urban
##   9   1  cnarc1  cnarc2  cnarc3  ctheory1  ctheory2  ctheory3  ctheory4  CRT1  CRT2  CRT3  health_cond  mor_circle  moralid1  moralid10  moralid2  moralid3  moralid4  moralid5  moralid6  moralid7  moralid8  moralid9  mcoop1  mcoop2  mcoop3  mcoop4  mcoop5  mcoop6  mcoop7  narc1  narc2  narc3  narc4  narc5  narc6  omind1  omind2  omind4  omind5  omind6  contact2  contact5  hygiene1  hygiene2  hygiene5  psupport2  psupport3  psupport5  political_ideology  happy  riskperc1  riskperc2  sbelong1  sbelong2  sbelong3  sbelong4  optim1  optim2  slfcont1  slfcont2  slfcont3  urban
##   9   2  cnarc1  cnarc2  cnarc3  ctheory1  ctheory2  ctheory3  ctheory4  CRT1  CRT2  CRT3  health_cond  mor_circle  moralid1  moralid10  moralid2  moralid3  moralid4  moralid5  moralid6  moralid7  moralid8  moralid9  mcoop1  mcoop2  mcoop3  mcoop4  mcoop5  mcoop6  mcoop7  narc1  narc2  narc3  narc4  narc5  narc6  omind1  omind2  omind4  omind5  omind6  contact2  contact5  hygiene1  hygiene2  hygiene5  psupport2  psupport3  psupport5  political_ideology  happy  riskperc1  riskperc2  sbelong1  sbelong2  sbelong3  sbelong4  optim1  optim2  slfcont1  slfcont2  slfcont3  urban
##   9   3  cnarc1  cnarc2  cnarc3  ctheory1  ctheory2  ctheory3  ctheory4  CRT1  CRT2  CRT3  health_cond  mor_circle  moralid1  moralid10  moralid2  moralid3  moralid4  moralid5  moralid6  moralid7  moralid8  moralid9  mcoop1  mcoop2  mcoop3  mcoop4  mcoop5  mcoop6  mcoop7  narc1  narc2  narc3  narc4  narc5  narc6  omind1  omind2  omind4  omind5  omind6  contact2  contact5  hygiene1  hygiene2  hygiene5  psupport2  psupport3  psupport5  political_ideology  happy  riskperc1  riskperc2  sbelong1  sbelong2  sbelong3  sbelong4  optim1  optim2  slfcont1  slfcont2  slfcont3  urban
##   9   4  cnarc1  cnarc2  cnarc3  ctheory1  ctheory2  ctheory3  ctheory4  CRT1  CRT2  CRT3  health_cond  mor_circle  moralid1  moralid10  moralid2  moralid3  moralid4  moralid5  moralid6  moralid7  moralid8  moralid9  mcoop1  mcoop2  mcoop3  mcoop4  mcoop5  mcoop6  mcoop7  narc1  narc2  narc3  narc4  narc5  narc6  omind1  omind2  omind4  omind5  omind6  contact2  contact5  hygiene1  hygiene2  hygiene5  psupport2  psupport3  psupport5  political_ideology  happy  riskperc1  riskperc2  sbelong1  sbelong2  sbelong3  sbelong4  optim1  optim2  slfcont1  slfcont2  slfcont3  urban
##   9   5  cnarc1  cnarc2  cnarc3  ctheory1  ctheory2  ctheory3  ctheory4  CRT1  CRT2  CRT3  health_cond  mor_circle  moralid1  moralid10  moralid2  moralid3  moralid4  moralid5  moralid6  moralid7  moralid8  moralid9  mcoop1  mcoop2  mcoop3  mcoop4  mcoop5  mcoop6  mcoop7  narc1  narc2  narc3  narc4  narc5  narc6  omind1  omind2  omind4  omind5  omind6  contact2  contact5  hygiene1  hygiene2  hygiene5  psupport2  psupport3  psupport5  political_ideology  happy  riskperc1  riskperc2  sbelong1  sbelong2  sbelong3  sbelong4  optim1  optim2  slfcont1  slfcont2  slfcont3  urban
##   9   6  cnarc1  cnarc2  cnarc3  ctheory1  ctheory2  ctheory3  ctheory4  CRT1  CRT2  CRT3  health_cond  mor_circle  moralid1  moralid10  moralid2  moralid3  moralid4  moralid5  moralid6  moralid7  moralid8  moralid9  mcoop1  mcoop2  mcoop3  mcoop4  mcoop5  mcoop6  mcoop7  narc1  narc2  narc3  narc4  narc5  narc6  omind1  omind2  omind4  omind5  omind6  contact2  contact5  hygiene1  hygiene2  hygiene5  psupport2  psupport3  psupport5  political_ideology  happy  riskperc1  riskperc2  sbelong1  sbelong2  sbelong3  sbelong4  optim1  optim2  slfcont1  slfcont2  slfcont3  urban
##   9   7  cnarc1  cnarc2  cnarc3  ctheory1  ctheory2  ctheory3  ctheory4  CRT1  CRT2  CRT3  health_cond  mor_circle  moralid1  moralid10  moralid2  moralid3  moralid4  moralid5  moralid6  moralid7  moralid8  moralid9  mcoop1  mcoop2  mcoop3  mcoop4  mcoop5  mcoop6  mcoop7  narc1  narc2  narc3  narc4  narc5  narc6  omind1  omind2  omind4  omind5  omind6  contact2  contact5  hygiene1  hygiene2  hygiene5  psupport2  psupport3  psupport5  political_ideology  happy  riskperc1  riskperc2  sbelong1  sbelong2  sbelong3  sbelong4  optim1  optim2  slfcont1  slfcont2  slfcont3  urban
##   9   8  cnarc1  cnarc2  cnarc3  ctheory1  ctheory2  ctheory3  ctheory4  CRT1  CRT2  CRT3  health_cond  mor_circle  moralid1  moralid10  moralid2  moralid3  moralid4  moralid5  moralid6  moralid7  moralid8  moralid9  mcoop1  mcoop2  mcoop3  mcoop4  mcoop5  mcoop6  mcoop7  narc1  narc2  narc3  narc4  narc5  narc6  omind1  omind2  omind4  omind5  omind6  contact2  contact5  hygiene1  hygiene2  hygiene5  psupport2  psupport3  psupport5  political_ideology  happy  riskperc1  riskperc2  sbelong1  sbelong2  sbelong3  sbelong4  optim1  optim2  slfcont1  slfcont2  slfcont3  urban
##   9   9  cnarc1  cnarc2  cnarc3  ctheory1  ctheory2  ctheory3  ctheory4  CRT1  CRT2  CRT3  health_cond  mor_circle  moralid1  moralid10  moralid2  moralid3  moralid4  moralid5  moralid6  moralid7  moralid8  moralid9  mcoop1  mcoop2  mcoop3  mcoop4  mcoop5  mcoop6  mcoop7  narc1  narc2  narc3  narc4  narc5  narc6  omind1  omind2  omind4  omind5  omind6  contact2  contact5  hygiene1  hygiene2  hygiene5  psupport2  psupport3  psupport5  political_ideology  happy  riskperc1  riskperc2  sbelong1  sbelong2  sbelong3  sbelong4  optim1  optim2  slfcont1  slfcont2  slfcont3  urban
##   9   10  cnarc1  cnarc2  cnarc3  ctheory1  ctheory2  ctheory3  ctheory4  CRT1  CRT2  CRT3  health_cond  mor_circle  moralid1  moralid10  moralid2  moralid3  moralid4  moralid5  moralid6  moralid7  moralid8  moralid9  mcoop1  mcoop2  mcoop3  mcoop4  mcoop5  mcoop6  mcoop7  narc1  narc2  narc3  narc4  narc5  narc6  omind1  omind2  omind4  omind5  omind6  contact2  contact5  hygiene1  hygiene2  hygiene5  psupport2  psupport3  psupport5  political_ideology  happy  riskperc1  riskperc2  sbelong1  sbelong2  sbelong3  sbelong4  optim1  optim2  slfcont1  slfcont2  slfcont3  urban
##   10   1  cnarc1  cnarc2  cnarc3  ctheory1  ctheory2  ctheory3  ctheory4  CRT1  CRT2  CRT3  health_cond  mor_circle  moralid1  moralid10  moralid2  moralid3  moralid4  moralid5  moralid6  moralid7  moralid8  moralid9  mcoop1  mcoop2  mcoop3  mcoop4  mcoop5  mcoop6  mcoop7  narc1  narc2  narc3  narc4  narc5  narc6  omind1  omind2  omind4  omind5  omind6  contact2  contact5  hygiene1  hygiene2  hygiene5  psupport2  psupport3  psupport5  political_ideology  happy  riskperc1  riskperc2  sbelong1  sbelong2  sbelong3  sbelong4  optim1  optim2  slfcont1  slfcont2  slfcont3  urban
##   10   2  cnarc1  cnarc2  cnarc3  ctheory1  ctheory2  ctheory3  ctheory4  CRT1  CRT2  CRT3  health_cond  mor_circle  moralid1  moralid10  moralid2  moralid3  moralid4  moralid5  moralid6  moralid7  moralid8  moralid9  mcoop1  mcoop2  mcoop3  mcoop4  mcoop5  mcoop6  mcoop7  narc1  narc2  narc3  narc4  narc5  narc6  omind1  omind2  omind4  omind5  omind6  contact2  contact5  hygiene1  hygiene2  hygiene5  psupport2  psupport3  psupport5  political_ideology  happy  riskperc1  riskperc2  sbelong1  sbelong2  sbelong3  sbelong4  optim1  optim2  slfcont1  slfcont2  slfcont3  urban
##   10   3  cnarc1  cnarc2  cnarc3  ctheory1  ctheory2  ctheory3  ctheory4  CRT1  CRT2  CRT3  health_cond  mor_circle  moralid1  moralid10  moralid2  moralid3  moralid4  moralid5  moralid6  moralid7  moralid8  moralid9  mcoop1  mcoop2  mcoop3  mcoop4  mcoop5  mcoop6  mcoop7  narc1  narc2  narc3  narc4  narc5  narc6  omind1  omind2  omind4  omind5  omind6  contact2  contact5  hygiene1  hygiene2  hygiene5  psupport2  psupport3  psupport5  political_ideology  happy  riskperc1  riskperc2  sbelong1  sbelong2  sbelong3  sbelong4  optim1  optim2  slfcont1  slfcont2  slfcont3  urban
##   10   4  cnarc1  cnarc2  cnarc3  ctheory1  ctheory2  ctheory3  ctheory4  CRT1  CRT2  CRT3  health_cond  mor_circle  moralid1  moralid10  moralid2  moralid3  moralid4  moralid5  moralid6  moralid7  moralid8  moralid9  mcoop1  mcoop2  mcoop3  mcoop4  mcoop5  mcoop6  mcoop7  narc1  narc2  narc3  narc4  narc5  narc6  omind1  omind2  omind4  omind5  omind6  contact2  contact5  hygiene1  hygiene2  hygiene5  psupport2  psupport3  psupport5  political_ideology  happy  riskperc1  riskperc2  sbelong1  sbelong2  sbelong3  sbelong4  optim1  optim2  slfcont1  slfcont2  slfcont3  urban
##   10   5  cnarc1  cnarc2  cnarc3  ctheory1  ctheory2  ctheory3  ctheory4  CRT1  CRT2  CRT3  health_cond  mor_circle  moralid1  moralid10  moralid2  moralid3  moralid4  moralid5  moralid6  moralid7  moralid8  moralid9  mcoop1  mcoop2  mcoop3  mcoop4  mcoop5  mcoop6  mcoop7  narc1  narc2  narc3  narc4  narc5  narc6  omind1  omind2  omind4  omind5  omind6  contact2  contact5  hygiene1  hygiene2  hygiene5  psupport2  psupport3  psupport5  political_ideology  happy  riskperc1  riskperc2  sbelong1  sbelong2  sbelong3  sbelong4  optim1  optim2  slfcont1  slfcont2  slfcont3  urban
##   10   6  cnarc1  cnarc2  cnarc3  ctheory1  ctheory2  ctheory3  ctheory4  CRT1  CRT2  CRT3  health_cond  mor_circle  moralid1  moralid10  moralid2  moralid3  moralid4  moralid5  moralid6  moralid7  moralid8  moralid9  mcoop1  mcoop2  mcoop3  mcoop4  mcoop5  mcoop6  mcoop7  narc1  narc2  narc3  narc4  narc5  narc6  omind1  omind2  omind4  omind5  omind6  contact2  contact5  hygiene1  hygiene2  hygiene5  psupport2  psupport3  psupport5  political_ideology  happy  riskperc1  riskperc2  sbelong1  sbelong2  sbelong3  sbelong4  optim1  optim2  slfcont1  slfcont2  slfcont3  urban
##   10   7  cnarc1  cnarc2  cnarc3  ctheory1  ctheory2  ctheory3  ctheory4  CRT1  CRT2  CRT3  health_cond  mor_circle  moralid1  moralid10  moralid2  moralid3  moralid4  moralid5  moralid6  moralid7  moralid8  moralid9  mcoop1  mcoop2  mcoop3  mcoop4  mcoop5  mcoop6  mcoop7  narc1  narc2  narc3  narc4  narc5  narc6  omind1  omind2  omind4  omind5  omind6  contact2  contact5  hygiene1  hygiene2  hygiene5  psupport2  psupport3  psupport5  political_ideology  happy  riskperc1  riskperc2  sbelong1  sbelong2  sbelong3  sbelong4  optim1  optim2  slfcont1  slfcont2  slfcont3  urban
##   10   8  cnarc1  cnarc2  cnarc3  ctheory1  ctheory2  ctheory3  ctheory4  CRT1  CRT2  CRT3  health_cond  mor_circle  moralid1  moralid10  moralid2  moralid3  moralid4  moralid5  moralid6  moralid7  moralid8  moralid9  mcoop1  mcoop2  mcoop3  mcoop4  mcoop5  mcoop6  mcoop7  narc1  narc2  narc3  narc4  narc5  narc6  omind1  omind2  omind4  omind5  omind6  contact2  contact5  hygiene1  hygiene2  hygiene5  psupport2  psupport3  psupport5  political_ideology  happy  riskperc1  riskperc2  sbelong1  sbelong2  sbelong3  sbelong4  optim1  optim2  slfcont1  slfcont2  slfcont3  urban
##   10   9  cnarc1  cnarc2  cnarc3  ctheory1  ctheory2  ctheory3  ctheory4  CRT1  CRT2  CRT3  health_cond  mor_circle  moralid1  moralid10  moralid2  moralid3  moralid4  moralid5  moralid6  moralid7  moralid8  moralid9  mcoop1  mcoop2  mcoop3  mcoop4  mcoop5  mcoop6  mcoop7  narc1  narc2  narc3  narc4  narc5  narc6  omind1  omind2  omind4  omind5  omind6  contact2  contact5  hygiene1  hygiene2  hygiene5  psupport2  psupport3  psupport5  political_ideology  happy  riskperc1  riskperc2  sbelong1  sbelong2  sbelong3  sbelong4  optim1  optim2  slfcont1  slfcont2  slfcont3  urban
##   10   10  cnarc1  cnarc2  cnarc3  ctheory1  ctheory2  ctheory3  ctheory4  CRT1  CRT2  CRT3  health_cond  mor_circle  moralid1  moralid10  moralid2  moralid3  moralid4  moralid5  moralid6  moralid7  moralid8  moralid9  mcoop1  mcoop2  mcoop3  mcoop4  mcoop5  mcoop6  mcoop7  narc1  narc2  narc3  narc4  narc5  narc6  omind1  omind2  omind4  omind5  omind6  contact2  contact5  hygiene1  hygiene2  hygiene5  psupport2  psupport3  psupport5  political_ideology  happy  riskperc1  riskperc2  sbelong1  sbelong2  sbelong3  sbelong4  optim1  optim2  slfcont1  slfcont2  slfcont3  urban
```

```
## Warning: Number of logged events: 6203
```

```
## 
##  iter imp variable
##   1   1  children  cnarc1  cnarc2  cnarc3  CRT1  CRT2  CRT3  health_cond  marital1  mor_circle  moralid10  moralid2  moralid3  moralid4  moralid5  moralid6  moralid7  moralid8  moralid9  mcoop1  mcoop2  mcoop3  mcoop4  mcoop5  mcoop6  mcoop7  narc1  narc2  narc3  narc4  narc5  narc6  nidentity1  nidentity2  omind1  omind4  omind6  contact1  contact2  contact3  contact4  contact5  hygiene1  hygiene2  hygiene3  hygiene4  hygiene5  psupport1  psupport2  psupport3  psupport4  psupport5  political_ideology  riskperc1  riskperc2  self_esteem  sbelong2  sbelong3  optim2  slfcont1  slfcont2  slfcont4  urban
##   1   2  children  cnarc1  cnarc2  cnarc3  CRT1  CRT2  CRT3  health_cond  marital1  mor_circle  moralid10  moralid2  moralid3  moralid4  moralid5  moralid6  moralid7  moralid8  moralid9  mcoop1  mcoop2  mcoop3  mcoop4  mcoop5  mcoop6  mcoop7  narc1  narc2  narc3  narc4  narc5  narc6  nidentity1  nidentity2  omind1  omind4  omind6  contact1  contact2  contact3  contact4  contact5  hygiene1  hygiene2  hygiene3  hygiene4  hygiene5  psupport1  psupport2  psupport3  psupport4  psupport5  political_ideology  riskperc1  riskperc2  self_esteem  sbelong2  sbelong3  optim2  slfcont1  slfcont2  slfcont4  urban
##   1   3  children  cnarc1  cnarc2  cnarc3  CRT1  CRT2  CRT3  health_cond  marital1  mor_circle  moralid10  moralid2  moralid3  moralid4  moralid5  moralid6  moralid7  moralid8  moralid9  mcoop1  mcoop2  mcoop3  mcoop4  mcoop5  mcoop6  mcoop7  narc1  narc2  narc3  narc4  narc5  narc6  nidentity1  nidentity2  omind1  omind4  omind6  contact1  contact2  contact3  contact4  contact5  hygiene1  hygiene2  hygiene3  hygiene4  hygiene5  psupport1  psupport2  psupport3  psupport4  psupport5  political_ideology  riskperc1  riskperc2  self_esteem  sbelong2  sbelong3  optim2  slfcont1  slfcont2  slfcont4  urban
##   1   4  children  cnarc1  cnarc2  cnarc3  CRT1  CRT2  CRT3  health_cond  marital1  mor_circle  moralid10  moralid2  moralid3  moralid4  moralid5  moralid6  moralid7  moralid8  moralid9  mcoop1  mcoop2  mcoop3  mcoop4  mcoop5  mcoop6  mcoop7  narc1  narc2  narc3  narc4  narc5  narc6  nidentity1  nidentity2  omind1  omind4  omind6  contact1  contact2  contact3  contact4  contact5  hygiene1  hygiene2  hygiene3  hygiene4  hygiene5  psupport1  psupport2  psupport3  psupport4  psupport5  political_ideology  riskperc1  riskperc2  self_esteem  sbelong2  sbelong3  optim2  slfcont1  slfcont2  slfcont4  urban
##   1   5  children  cnarc1  cnarc2  cnarc3  CRT1  CRT2  CRT3  health_cond  marital1  mor_circle  moralid10  moralid2  moralid3  moralid4  moralid5  moralid6  moralid7  moralid8  moralid9  mcoop1  mcoop2  mcoop3  mcoop4  mcoop5  mcoop6  mcoop7  narc1  narc2  narc3  narc4  narc5  narc6  nidentity1  nidentity2  omind1  omind4  omind6  contact1  contact2  contact3  contact4  contact5  hygiene1  hygiene2  hygiene3  hygiene4  hygiene5  psupport1  psupport2  psupport3  psupport4  psupport5  political_ideology  riskperc1  riskperc2  self_esteem  sbelong2  sbelong3  optim2  slfcont1  slfcont2  slfcont4  urban
##   1   6  children  cnarc1  cnarc2  cnarc3  CRT1  CRT2  CRT3  health_cond  marital1  mor_circle  moralid10  moralid2  moralid3  moralid4  moralid5  moralid6  moralid7  moralid8  moralid9  mcoop1  mcoop2  mcoop3  mcoop4  mcoop5  mcoop6  mcoop7  narc1  narc2  narc3  narc4  narc5  narc6  nidentity1  nidentity2  omind1  omind4  omind6  contact1  contact2  contact3  contact4  contact5  hygiene1  hygiene2  hygiene3  hygiene4  hygiene5  psupport1  psupport2  psupport3  psupport4  psupport5  political_ideology  riskperc1  riskperc2  self_esteem  sbelong2  sbelong3  optim2  slfcont1  slfcont2  slfcont4  urban
##   1   7  children  cnarc1  cnarc2  cnarc3  CRT1  CRT2  CRT3  health_cond  marital1  mor_circle  moralid10  moralid2  moralid3  moralid4  moralid5  moralid6  moralid7  moralid8  moralid9  mcoop1  mcoop2  mcoop3  mcoop4  mcoop5  mcoop6  mcoop7  narc1  narc2  narc3  narc4  narc5  narc6  nidentity1  nidentity2  omind1  omind4  omind6  contact1  contact2  contact3  contact4  contact5  hygiene1  hygiene2  hygiene3  hygiene4  hygiene5  psupport1  psupport2  psupport3  psupport4  psupport5  political_ideology  riskperc1  riskperc2  self_esteem  sbelong2  sbelong3  optim2  slfcont1  slfcont2  slfcont4  urban
##   1   8  children  cnarc1  cnarc2  cnarc3  CRT1  CRT2  CRT3  health_cond  marital1  mor_circle  moralid10  moralid2  moralid3  moralid4  moralid5  moralid6  moralid7  moralid8  moralid9  mcoop1  mcoop2  mcoop3  mcoop4  mcoop5  mcoop6  mcoop7  narc1  narc2  narc3  narc4  narc5  narc6  nidentity1  nidentity2  omind1  omind4  omind6  contact1  contact2  contact3  contact4  contact5  hygiene1  hygiene2  hygiene3  hygiene4  hygiene5  psupport1  psupport2  psupport3  psupport4  psupport5  political_ideology  riskperc1  riskperc2  self_esteem  sbelong2  sbelong3  optim2  slfcont1  slfcont2  slfcont4  urban
##   1   9  children  cnarc1  cnarc2  cnarc3  CRT1  CRT2  CRT3  health_cond  marital1  mor_circle  moralid10  moralid2  moralid3  moralid4  moralid5  moralid6  moralid7  moralid8  moralid9  mcoop1  mcoop2  mcoop3  mcoop4  mcoop5  mcoop6  mcoop7  narc1  narc2  narc3  narc4  narc5  narc6  nidentity1  nidentity2  omind1  omind4  omind6  contact1  contact2  contact3  contact4  contact5  hygiene1  hygiene2  hygiene3  hygiene4  hygiene5  psupport1  psupport2  psupport3  psupport4  psupport5  political_ideology  riskperc1  riskperc2  self_esteem  sbelong2  sbelong3  optim2  slfcont1  slfcont2  slfcont4  urban
##   1   10  children  cnarc1  cnarc2  cnarc3  CRT1  CRT2  CRT3  health_cond  marital1  mor_circle  moralid10  moralid2  moralid3  moralid4  moralid5  moralid6  moralid7  moralid8  moralid9  mcoop1  mcoop2  mcoop3  mcoop4  mcoop5  mcoop6  mcoop7  narc1  narc2  narc3  narc4  narc5  narc6  nidentity1  nidentity2  omind1  omind4  omind6  contact1  contact2  contact3  contact4  contact5  hygiene1  hygiene2  hygiene3  hygiene4  hygiene5  psupport1  psupport2  psupport3  psupport4  psupport5  political_ideology  riskperc1  riskperc2  self_esteem  sbelong2  sbelong3  optim2  slfcont1  slfcont2  slfcont4  urban
##   2   1  children  cnarc1  cnarc2  cnarc3  CRT1  CRT2  CRT3  health_cond  marital1  mor_circle  moralid10  moralid2  moralid3  moralid4  moralid5  moralid6  moralid7  moralid8  moralid9  mcoop1  mcoop2  mcoop3  mcoop4  mcoop5  mcoop6  mcoop7  narc1  narc2  narc3  narc4  narc5  narc6  nidentity1  nidentity2  omind1  omind4  omind6  contact1  contact2  contact3  contact4  contact5  hygiene1  hygiene2  hygiene3  hygiene4  hygiene5  psupport1  psupport2  psupport3  psupport4  psupport5  political_ideology  riskperc1  riskperc2  self_esteem  sbelong2  sbelong3  optim2  slfcont1  slfcont2  slfcont4  urban
##   2   2  children  cnarc1  cnarc2  cnarc3  CRT1  CRT2  CRT3  health_cond  marital1  mor_circle  moralid10  moralid2  moralid3  moralid4  moralid5  moralid6  moralid7  moralid8  moralid9  mcoop1  mcoop2  mcoop3  mcoop4  mcoop5  mcoop6  mcoop7  narc1  narc2  narc3  narc4  narc5  narc6  nidentity1  nidentity2  omind1  omind4  omind6  contact1  contact2  contact3  contact4  contact5  hygiene1  hygiene2  hygiene3  hygiene4  hygiene5  psupport1  psupport2  psupport3  psupport4  psupport5  political_ideology  riskperc1  riskperc2  self_esteem  sbelong2  sbelong3  optim2  slfcont1  slfcont2  slfcont4  urban
##   2   3  children  cnarc1  cnarc2  cnarc3  CRT1  CRT2  CRT3  health_cond  marital1  mor_circle  moralid10  moralid2  moralid3  moralid4  moralid5  moralid6  moralid7  moralid8  moralid9  mcoop1  mcoop2  mcoop3  mcoop4  mcoop5  mcoop6  mcoop7  narc1  narc2  narc3  narc4  narc5  narc6  nidentity1  nidentity2  omind1  omind4  omind6  contact1  contact2  contact3  contact4  contact5  hygiene1  hygiene2  hygiene3  hygiene4  hygiene5  psupport1  psupport2  psupport3  psupport4  psupport5  political_ideology  riskperc1  riskperc2  self_esteem  sbelong2  sbelong3  optim2  slfcont1  slfcont2  slfcont4  urban
##   2   4  children  cnarc1  cnarc2  cnarc3  CRT1  CRT2  CRT3  health_cond  marital1  mor_circle  moralid10  moralid2  moralid3  moralid4  moralid5  moralid6  moralid7  moralid8  moralid9  mcoop1  mcoop2  mcoop3  mcoop4  mcoop5  mcoop6  mcoop7  narc1  narc2  narc3  narc4  narc5  narc6  nidentity1  nidentity2  omind1  omind4  omind6  contact1  contact2  contact3  contact4  contact5  hygiene1  hygiene2  hygiene3  hygiene4  hygiene5  psupport1  psupport2  psupport3  psupport4  psupport5  political_ideology  riskperc1  riskperc2  self_esteem  sbelong2  sbelong3  optim2  slfcont1  slfcont2  slfcont4  urban
##   2   5  children  cnarc1  cnarc2  cnarc3  CRT1  CRT2  CRT3  health_cond  marital1  mor_circle  moralid10  moralid2  moralid3  moralid4  moralid5  moralid6  moralid7  moralid8  moralid9  mcoop1  mcoop2  mcoop3  mcoop4  mcoop5  mcoop6  mcoop7  narc1  narc2  narc3  narc4  narc5  narc6  nidentity1  nidentity2  omind1  omind4  omind6  contact1  contact2  contact3  contact4  contact5  hygiene1  hygiene2  hygiene3  hygiene4  hygiene5  psupport1  psupport2  psupport3  psupport4  psupport5  political_ideology  riskperc1  riskperc2  self_esteem  sbelong2  sbelong3  optim2  slfcont1  slfcont2  slfcont4  urban
##   2   6  children  cnarc1  cnarc2  cnarc3  CRT1  CRT2  CRT3  health_cond  marital1  mor_circle  moralid10  moralid2  moralid3  moralid4  moralid5  moralid6  moralid7  moralid8  moralid9  mcoop1  mcoop2  mcoop3  mcoop4  mcoop5  mcoop6  mcoop7  narc1  narc2  narc3  narc4  narc5  narc6  nidentity1  nidentity2  omind1  omind4  omind6  contact1  contact2  contact3  contact4  contact5  hygiene1  hygiene2  hygiene3  hygiene4  hygiene5  psupport1  psupport2  psupport3  psupport4  psupport5  political_ideology  riskperc1  riskperc2  self_esteem  sbelong2  sbelong3  optim2  slfcont1  slfcont2  slfcont4  urban
##   2   7  children  cnarc1  cnarc2  cnarc3  CRT1  CRT2  CRT3  health_cond  marital1  mor_circle  moralid10  moralid2  moralid3  moralid4  moralid5  moralid6  moralid7  moralid8  moralid9  mcoop1  mcoop2  mcoop3  mcoop4  mcoop5  mcoop6  mcoop7  narc1  narc2  narc3  narc4  narc5  narc6  nidentity1  nidentity2  omind1  omind4  omind6  contact1  contact2  contact3  contact4  contact5  hygiene1  hygiene2  hygiene3  hygiene4  hygiene5  psupport1  psupport2  psupport3  psupport4  psupport5  political_ideology  riskperc1  riskperc2  self_esteem  sbelong2  sbelong3  optim2  slfcont1  slfcont2  slfcont4  urban
##   2   8  children  cnarc1  cnarc2  cnarc3  CRT1  CRT2  CRT3  health_cond  marital1  mor_circle  moralid10  moralid2  moralid3  moralid4  moralid5  moralid6  moralid7  moralid8  moralid9  mcoop1  mcoop2  mcoop3  mcoop4  mcoop5  mcoop6  mcoop7  narc1  narc2  narc3  narc4  narc5  narc6  nidentity1  nidentity2  omind1  omind4  omind6  contact1  contact2  contact3  contact4  contact5  hygiene1  hygiene2  hygiene3  hygiene4  hygiene5  psupport1  psupport2  psupport3  psupport4  psupport5  political_ideology  riskperc1  riskperc2  self_esteem  sbelong2  sbelong3  optim2  slfcont1  slfcont2  slfcont4  urban
##   2   9  children  cnarc1  cnarc2  cnarc3  CRT1  CRT2  CRT3  health_cond  marital1  mor_circle  moralid10  moralid2  moralid3  moralid4  moralid5  moralid6  moralid7  moralid8  moralid9  mcoop1  mcoop2  mcoop3  mcoop4  mcoop5  mcoop6  mcoop7  narc1  narc2  narc3  narc4  narc5  narc6  nidentity1  nidentity2  omind1  omind4  omind6  contact1  contact2  contact3  contact4  contact5  hygiene1  hygiene2  hygiene3  hygiene4  hygiene5  psupport1  psupport2  psupport3  psupport4  psupport5  political_ideology  riskperc1  riskperc2  self_esteem  sbelong2  sbelong3  optim2  slfcont1  slfcont2  slfcont4  urban
##   2   10  children  cnarc1  cnarc2  cnarc3  CRT1  CRT2  CRT3  health_cond  marital1  mor_circle  moralid10  moralid2  moralid3  moralid4  moralid5  moralid6  moralid7  moralid8  moralid9  mcoop1  mcoop2  mcoop3  mcoop4  mcoop5  mcoop6  mcoop7  narc1  narc2  narc3  narc4  narc5  narc6  nidentity1  nidentity2  omind1  omind4  omind6  contact1  contact2  contact3  contact4  contact5  hygiene1  hygiene2  hygiene3  hygiene4  hygiene5  psupport1  psupport2  psupport3  psupport4  psupport5  political_ideology  riskperc1  riskperc2  self_esteem  sbelong2  sbelong3  optim2  slfcont1  slfcont2  slfcont4  urban
##   3   1  children  cnarc1  cnarc2  cnarc3  CRT1  CRT2  CRT3  health_cond  marital1  mor_circle  moralid10  moralid2  moralid3  moralid4  moralid5  moralid6  moralid7  moralid8  moralid9  mcoop1  mcoop2  mcoop3  mcoop4  mcoop5  mcoop6  mcoop7  narc1  narc2  narc3  narc4  narc5  narc6  nidentity1  nidentity2  omind1  omind4  omind6  contact1  contact2  contact3  contact4  contact5  hygiene1  hygiene2  hygiene3  hygiene4  hygiene5  psupport1  psupport2  psupport3  psupport4  psupport5  political_ideology  riskperc1  riskperc2  self_esteem  sbelong2  sbelong3  optim2  slfcont1  slfcont2  slfcont4  urban
##   3   2  children  cnarc1  cnarc2  cnarc3  CRT1  CRT2  CRT3  health_cond  marital1  mor_circle  moralid10  moralid2  moralid3  moralid4  moralid5  moralid6  moralid7  moralid8  moralid9  mcoop1  mcoop2  mcoop3  mcoop4  mcoop5  mcoop6  mcoop7  narc1  narc2  narc3  narc4  narc5  narc6  nidentity1  nidentity2  omind1  omind4  omind6  contact1  contact2  contact3  contact4  contact5  hygiene1  hygiene2  hygiene3  hygiene4  hygiene5  psupport1  psupport2  psupport3  psupport4  psupport5  political_ideology  riskperc1  riskperc2  self_esteem  sbelong2  sbelong3  optim2  slfcont1  slfcont2  slfcont4  urban
##   3   3  children  cnarc1  cnarc2  cnarc3  CRT1  CRT2  CRT3  health_cond  marital1  mor_circle  moralid10  moralid2  moralid3  moralid4  moralid5  moralid6  moralid7  moralid8  moralid9  mcoop1  mcoop2  mcoop3  mcoop4  mcoop5  mcoop6  mcoop7  narc1  narc2  narc3  narc4  narc5  narc6  nidentity1  nidentity2  omind1  omind4  omind6  contact1  contact2  contact3  contact4  contact5  hygiene1  hygiene2  hygiene3  hygiene4  hygiene5  psupport1  psupport2  psupport3  psupport4  psupport5  political_ideology  riskperc1  riskperc2  self_esteem  sbelong2  sbelong3  optim2  slfcont1  slfcont2  slfcont4  urban
##   3   4  children  cnarc1  cnarc2  cnarc3  CRT1  CRT2  CRT3  health_cond  marital1  mor_circle  moralid10  moralid2  moralid3  moralid4  moralid5  moralid6  moralid7  moralid8  moralid9  mcoop1  mcoop2  mcoop3  mcoop4  mcoop5  mcoop6  mcoop7  narc1  narc2  narc3  narc4  narc5  narc6  nidentity1  nidentity2  omind1  omind4  omind6  contact1  contact2  contact3  contact4  contact5  hygiene1  hygiene2  hygiene3  hygiene4  hygiene5  psupport1  psupport2  psupport3  psupport4  psupport5  political_ideology  riskperc1  riskperc2  self_esteem  sbelong2  sbelong3  optim2  slfcont1  slfcont2  slfcont4  urban
##   3   5  children  cnarc1  cnarc2  cnarc3  CRT1  CRT2  CRT3  health_cond  marital1  mor_circle  moralid10  moralid2  moralid3  moralid4  moralid5  moralid6  moralid7  moralid8  moralid9  mcoop1  mcoop2  mcoop3  mcoop4  mcoop5  mcoop6  mcoop7  narc1  narc2  narc3  narc4  narc5  narc6  nidentity1  nidentity2  omind1  omind4  omind6  contact1  contact2  contact3  contact4  contact5  hygiene1  hygiene2  hygiene3  hygiene4  hygiene5  psupport1  psupport2  psupport3  psupport4  psupport5  political_ideology  riskperc1  riskperc2  self_esteem  sbelong2  sbelong3  optim2  slfcont1  slfcont2  slfcont4  urban
##   3   6  children  cnarc1  cnarc2  cnarc3  CRT1  CRT2  CRT3  health_cond  marital1  mor_circle  moralid10  moralid2  moralid3  moralid4  moralid5  moralid6  moralid7  moralid8  moralid9  mcoop1  mcoop2  mcoop3  mcoop4  mcoop5  mcoop6  mcoop7  narc1  narc2  narc3  narc4  narc5  narc6  nidentity1  nidentity2  omind1  omind4  omind6  contact1  contact2  contact3  contact4  contact5  hygiene1  hygiene2  hygiene3  hygiene4  hygiene5  psupport1  psupport2  psupport3  psupport4  psupport5  political_ideology  riskperc1  riskperc2  self_esteem  sbelong2  sbelong3  optim2  slfcont1  slfcont2  slfcont4  urban
##   3   7  children  cnarc1  cnarc2  cnarc3  CRT1  CRT2  CRT3  health_cond  marital1  mor_circle  moralid10  moralid2  moralid3  moralid4  moralid5  moralid6  moralid7  moralid8  moralid9  mcoop1  mcoop2  mcoop3  mcoop4  mcoop5  mcoop6  mcoop7  narc1  narc2  narc3  narc4  narc5  narc6  nidentity1  nidentity2  omind1  omind4  omind6  contact1  contact2  contact3  contact4  contact5  hygiene1  hygiene2  hygiene3  hygiene4  hygiene5  psupport1  psupport2  psupport3  psupport4  psupport5  political_ideology  riskperc1  riskperc2  self_esteem  sbelong2  sbelong3  optim2  slfcont1  slfcont2  slfcont4  urban
##   3   8  children  cnarc1  cnarc2  cnarc3  CRT1  CRT2  CRT3  health_cond  marital1  mor_circle  moralid10  moralid2  moralid3  moralid4  moralid5  moralid6  moralid7  moralid8  moralid9  mcoop1  mcoop2  mcoop3  mcoop4  mcoop5  mcoop6  mcoop7  narc1  narc2  narc3  narc4  narc5  narc6  nidentity1  nidentity2  omind1  omind4  omind6  contact1  contact2  contact3  contact4  contact5  hygiene1  hygiene2  hygiene3  hygiene4  hygiene5  psupport1  psupport2  psupport3  psupport4  psupport5  political_ideology  riskperc1  riskperc2  self_esteem  sbelong2  sbelong3  optim2  slfcont1  slfcont2  slfcont4  urban
##   3   9  children  cnarc1  cnarc2  cnarc3  CRT1  CRT2  CRT3  health_cond  marital1  mor_circle  moralid10  moralid2  moralid3  moralid4  moralid5  moralid6  moralid7  moralid8  moralid9  mcoop1  mcoop2  mcoop3  mcoop4  mcoop5  mcoop6  mcoop7  narc1  narc2  narc3  narc4  narc5  narc6  nidentity1  nidentity2  omind1  omind4  omind6  contact1  contact2  contact3  contact4  contact5  hygiene1  hygiene2  hygiene3  hygiene4  hygiene5  psupport1  psupport2  psupport3  psupport4  psupport5  political_ideology  riskperc1  riskperc2  self_esteem  sbelong2  sbelong3  optim2  slfcont1  slfcont2  slfcont4  urban
##   3   10  children  cnarc1  cnarc2  cnarc3  CRT1  CRT2  CRT3  health_cond  marital1  mor_circle  moralid10  moralid2  moralid3  moralid4  moralid5  moralid6  moralid7  moralid8  moralid9  mcoop1  mcoop2  mcoop3  mcoop4  mcoop5  mcoop6  mcoop7  narc1  narc2  narc3  narc4  narc5  narc6  nidentity1  nidentity2  omind1  omind4  omind6  contact1  contact2  contact3  contact4  contact5  hygiene1  hygiene2  hygiene3  hygiene4  hygiene5  psupport1  psupport2  psupport3  psupport4  psupport5  political_ideology  riskperc1  riskperc2  self_esteem  sbelong2  sbelong3  optim2  slfcont1  slfcont2  slfcont4  urban
##   4   1  children  cnarc1  cnarc2  cnarc3  CRT1  CRT2  CRT3  health_cond  marital1  mor_circle  moralid10  moralid2  moralid3  moralid4  moralid5  moralid6  moralid7  moralid8  moralid9  mcoop1  mcoop2  mcoop3  mcoop4  mcoop5  mcoop6  mcoop7  narc1  narc2  narc3  narc4  narc5  narc6  nidentity1  nidentity2  omind1  omind4  omind6  contact1  contact2  contact3  contact4  contact5  hygiene1  hygiene2  hygiene3  hygiene4  hygiene5  psupport1  psupport2  psupport3  psupport4  psupport5  political_ideology  riskperc1  riskperc2  self_esteem  sbelong2  sbelong3  optim2  slfcont1  slfcont2  slfcont4  urban
##   4   2  children  cnarc1  cnarc2  cnarc3  CRT1  CRT2  CRT3  health_cond  marital1  mor_circle  moralid10  moralid2  moralid3  moralid4  moralid5  moralid6  moralid7  moralid8  moralid9  mcoop1  mcoop2  mcoop3  mcoop4  mcoop5  mcoop6  mcoop7  narc1  narc2  narc3  narc4  narc5  narc6  nidentity1  nidentity2  omind1  omind4  omind6  contact1  contact2  contact3  contact4  contact5  hygiene1  hygiene2  hygiene3  hygiene4  hygiene5  psupport1  psupport2  psupport3  psupport4  psupport5  political_ideology  riskperc1  riskperc2  self_esteem  sbelong2  sbelong3  optim2  slfcont1  slfcont2  slfcont4  urban
##   4   3  children  cnarc1  cnarc2  cnarc3  CRT1  CRT2  CRT3  health_cond  marital1  mor_circle  moralid10  moralid2  moralid3  moralid4  moralid5  moralid6  moralid7  moralid8  moralid9  mcoop1  mcoop2  mcoop3  mcoop4  mcoop5  mcoop6  mcoop7  narc1  narc2  narc3  narc4  narc5  narc6  nidentity1  nidentity2  omind1  omind4  omind6  contact1  contact2  contact3  contact4  contact5  hygiene1  hygiene2  hygiene3  hygiene4  hygiene5  psupport1  psupport2  psupport3  psupport4  psupport5  political_ideology  riskperc1  riskperc2  self_esteem  sbelong2  sbelong3  optim2  slfcont1  slfcont2  slfcont4  urban
##   4   4  children  cnarc1  cnarc2  cnarc3  CRT1  CRT2  CRT3  health_cond  marital1  mor_circle  moralid10  moralid2  moralid3  moralid4  moralid5  moralid6  moralid7  moralid8  moralid9  mcoop1  mcoop2  mcoop3  mcoop4  mcoop5  mcoop6  mcoop7  narc1  narc2  narc3  narc4  narc5  narc6  nidentity1  nidentity2  omind1  omind4  omind6  contact1  contact2  contact3  contact4  contact5  hygiene1  hygiene2  hygiene3  hygiene4  hygiene5  psupport1  psupport2  psupport3  psupport4  psupport5  political_ideology  riskperc1  riskperc2  self_esteem  sbelong2  sbelong3  optim2  slfcont1  slfcont2  slfcont4  urban
##   4   5  children  cnarc1  cnarc2  cnarc3  CRT1  CRT2  CRT3  health_cond  marital1  mor_circle  moralid10  moralid2  moralid3  moralid4  moralid5  moralid6  moralid7  moralid8  moralid9  mcoop1  mcoop2  mcoop3  mcoop4  mcoop5  mcoop6  mcoop7  narc1  narc2  narc3  narc4  narc5  narc6  nidentity1  nidentity2  omind1  omind4  omind6  contact1  contact2  contact3  contact4  contact5  hygiene1  hygiene2  hygiene3  hygiene4  hygiene5  psupport1  psupport2  psupport3  psupport4  psupport5  political_ideology  riskperc1  riskperc2  self_esteem  sbelong2  sbelong3  optim2  slfcont1  slfcont2  slfcont4  urban
##   4   6  children  cnarc1  cnarc2  cnarc3  CRT1  CRT2  CRT3  health_cond  marital1  mor_circle  moralid10  moralid2  moralid3  moralid4  moralid5  moralid6  moralid7  moralid8  moralid9  mcoop1  mcoop2  mcoop3  mcoop4  mcoop5  mcoop6  mcoop7  narc1  narc2  narc3  narc4  narc5  narc6  nidentity1  nidentity2  omind1  omind4  omind6  contact1  contact2  contact3  contact4  contact5  hygiene1  hygiene2  hygiene3  hygiene4  hygiene5  psupport1  psupport2  psupport3  psupport4  psupport5  political_ideology  riskperc1  riskperc2  self_esteem  sbelong2  sbelong3  optim2  slfcont1  slfcont2  slfcont4  urban
##   4   7  children  cnarc1  cnarc2  cnarc3  CRT1  CRT2  CRT3  health_cond  marital1  mor_circle  moralid10  moralid2  moralid3  moralid4  moralid5  moralid6  moralid7  moralid8  moralid9  mcoop1  mcoop2  mcoop3  mcoop4  mcoop5  mcoop6  mcoop7  narc1  narc2  narc3  narc4  narc5  narc6  nidentity1  nidentity2  omind1  omind4  omind6  contact1  contact2  contact3  contact4  contact5  hygiene1  hygiene2  hygiene3  hygiene4  hygiene5  psupport1  psupport2  psupport3  psupport4  psupport5  political_ideology  riskperc1  riskperc2  self_esteem  sbelong2  sbelong3  optim2  slfcont1  slfcont2  slfcont4  urban
##   4   8  children  cnarc1  cnarc2  cnarc3  CRT1  CRT2  CRT3  health_cond  marital1  mor_circle  moralid10  moralid2  moralid3  moralid4  moralid5  moralid6  moralid7  moralid8  moralid9  mcoop1  mcoop2  mcoop3  mcoop4  mcoop5  mcoop6  mcoop7  narc1  narc2  narc3  narc4  narc5  narc6  nidentity1  nidentity2  omind1  omind4  omind6  contact1  contact2  contact3  contact4  contact5  hygiene1  hygiene2  hygiene3  hygiene4  hygiene5  psupport1  psupport2  psupport3  psupport4  psupport5  political_ideology  riskperc1  riskperc2  self_esteem  sbelong2  sbelong3  optim2  slfcont1  slfcont2  slfcont4  urban
##   4   9  children  cnarc1  cnarc2  cnarc3  CRT1  CRT2  CRT3  health_cond  marital1  mor_circle  moralid10  moralid2  moralid3  moralid4  moralid5  moralid6  moralid7  moralid8  moralid9  mcoop1  mcoop2  mcoop3  mcoop4  mcoop5  mcoop6  mcoop7  narc1  narc2  narc3  narc4  narc5  narc6  nidentity1  nidentity2  omind1  omind4  omind6  contact1  contact2  contact3  contact4  contact5  hygiene1  hygiene2  hygiene3  hygiene4  hygiene5  psupport1  psupport2  psupport3  psupport4  psupport5  political_ideology  riskperc1  riskperc2  self_esteem  sbelong2  sbelong3  optim2  slfcont1  slfcont2  slfcont4  urban
##   4   10  children  cnarc1  cnarc2  cnarc3  CRT1  CRT2  CRT3  health_cond  marital1  mor_circle  moralid10  moralid2  moralid3  moralid4  moralid5  moralid6  moralid7  moralid8  moralid9  mcoop1  mcoop2  mcoop3  mcoop4  mcoop5  mcoop6  mcoop7  narc1  narc2  narc3  narc4  narc5  narc6  nidentity1  nidentity2  omind1  omind4  omind6  contact1  contact2  contact3  contact4  contact5  hygiene1  hygiene2  hygiene3  hygiene4  hygiene5  psupport1  psupport2  psupport3  psupport4  psupport5  political_ideology  riskperc1  riskperc2  self_esteem  sbelong2  sbelong3  optim2  slfcont1  slfcont2  slfcont4  urban
##   5   1  children  cnarc1  cnarc2  cnarc3  CRT1  CRT2  CRT3  health_cond  marital1  mor_circle  moralid10  moralid2  moralid3  moralid4  moralid5  moralid6  moralid7  moralid8  moralid9  mcoop1  mcoop2  mcoop3  mcoop4  mcoop5  mcoop6  mcoop7  narc1  narc2  narc3  narc4  narc5  narc6  nidentity1  nidentity2  omind1  omind4  omind6  contact1  contact2  contact3  contact4  contact5  hygiene1  hygiene2  hygiene3  hygiene4  hygiene5  psupport1  psupport2  psupport3  psupport4  psupport5  political_ideology  riskperc1  riskperc2  self_esteem  sbelong2  sbelong3  optim2  slfcont1  slfcont2  slfcont4  urban
##   5   2  children  cnarc1  cnarc2  cnarc3  CRT1  CRT2  CRT3  health_cond  marital1  mor_circle  moralid10  moralid2  moralid3  moralid4  moralid5  moralid6  moralid7  moralid8  moralid9  mcoop1  mcoop2  mcoop3  mcoop4  mcoop5  mcoop6  mcoop7  narc1  narc2  narc3  narc4  narc5  narc6  nidentity1  nidentity2  omind1  omind4  omind6  contact1  contact2  contact3  contact4  contact5  hygiene1  hygiene2  hygiene3  hygiene4  hygiene5  psupport1  psupport2  psupport3  psupport4  psupport5  political_ideology  riskperc1  riskperc2  self_esteem  sbelong2  sbelong3  optim2  slfcont1  slfcont2  slfcont4  urban
##   5   3  children  cnarc1  cnarc2  cnarc3  CRT1  CRT2  CRT3  health_cond  marital1  mor_circle  moralid10  moralid2  moralid3  moralid4  moralid5  moralid6  moralid7  moralid8  moralid9  mcoop1  mcoop2  mcoop3  mcoop4  mcoop5  mcoop6  mcoop7  narc1  narc2  narc3  narc4  narc5  narc6  nidentity1  nidentity2  omind1  omind4  omind6  contact1  contact2  contact3  contact4  contact5  hygiene1  hygiene2  hygiene3  hygiene4  hygiene5  psupport1  psupport2  psupport3  psupport4  psupport5  political_ideology  riskperc1  riskperc2  self_esteem  sbelong2  sbelong3  optim2  slfcont1  slfcont2  slfcont4  urban
##   5   4  children  cnarc1  cnarc2  cnarc3  CRT1  CRT2  CRT3  health_cond  marital1  mor_circle  moralid10  moralid2  moralid3  moralid4  moralid5  moralid6  moralid7  moralid8  moralid9  mcoop1  mcoop2  mcoop3  mcoop4  mcoop5  mcoop6  mcoop7  narc1  narc2  narc3  narc4  narc5  narc6  nidentity1  nidentity2  omind1  omind4  omind6  contact1  contact2  contact3  contact4  contact5  hygiene1  hygiene2  hygiene3  hygiene4  hygiene5  psupport1  psupport2  psupport3  psupport4  psupport5  political_ideology  riskperc1  riskperc2  self_esteem  sbelong2  sbelong3  optim2  slfcont1  slfcont2  slfcont4  urban
##   5   5  children  cnarc1  cnarc2  cnarc3  CRT1  CRT2  CRT3  health_cond  marital1  mor_circle  moralid10  moralid2  moralid3  moralid4  moralid5  moralid6  moralid7  moralid8  moralid9  mcoop1  mcoop2  mcoop3  mcoop4  mcoop5  mcoop6  mcoop7  narc1  narc2  narc3  narc4  narc5  narc6  nidentity1  nidentity2  omind1  omind4  omind6  contact1  contact2  contact3  contact4  contact5  hygiene1  hygiene2  hygiene3  hygiene4  hygiene5  psupport1  psupport2  psupport3  psupport4  psupport5  political_ideology  riskperc1  riskperc2  self_esteem  sbelong2  sbelong3  optim2  slfcont1  slfcont2  slfcont4  urban
##   5   6  children  cnarc1  cnarc2  cnarc3  CRT1  CRT2  CRT3  health_cond  marital1  mor_circle  moralid10  moralid2  moralid3  moralid4  moralid5  moralid6  moralid7  moralid8  moralid9  mcoop1  mcoop2  mcoop3  mcoop4  mcoop5  mcoop6  mcoop7  narc1  narc2  narc3  narc4  narc5  narc6  nidentity1  nidentity2  omind1  omind4  omind6  contact1  contact2  contact3  contact4  contact5  hygiene1  hygiene2  hygiene3  hygiene4  hygiene5  psupport1  psupport2  psupport3  psupport4  psupport5  political_ideology  riskperc1  riskperc2  self_esteem  sbelong2  sbelong3  optim2  slfcont1  slfcont2  slfcont4  urban
##   5   7  children  cnarc1  cnarc2  cnarc3  CRT1  CRT2  CRT3  health_cond  marital1  mor_circle  moralid10  moralid2  moralid3  moralid4  moralid5  moralid6  moralid7  moralid8  moralid9  mcoop1  mcoop2  mcoop3  mcoop4  mcoop5  mcoop6  mcoop7  narc1  narc2  narc3  narc4  narc5  narc6  nidentity1  nidentity2  omind1  omind4  omind6  contact1  contact2  contact3  contact4  contact5  hygiene1  hygiene2  hygiene3  hygiene4  hygiene5  psupport1  psupport2  psupport3  psupport4  psupport5  political_ideology  riskperc1  riskperc2  self_esteem  sbelong2  sbelong3  optim2  slfcont1  slfcont2  slfcont4  urban
##   5   8  children  cnarc1  cnarc2  cnarc3  CRT1  CRT2  CRT3  health_cond  marital1  mor_circle  moralid10  moralid2  moralid3  moralid4  moralid5  moralid6  moralid7  moralid8  moralid9  mcoop1  mcoop2  mcoop3  mcoop4  mcoop5  mcoop6  mcoop7  narc1  narc2  narc3  narc4  narc5  narc6  nidentity1  nidentity2  omind1  omind4  omind6  contact1  contact2  contact3  contact4  contact5  hygiene1  hygiene2  hygiene3  hygiene4  hygiene5  psupport1  psupport2  psupport3  psupport4  psupport5  political_ideology  riskperc1  riskperc2  self_esteem  sbelong2  sbelong3  optim2  slfcont1  slfcont2  slfcont4  urban
##   5   9  children  cnarc1  cnarc2  cnarc3  CRT1  CRT2  CRT3  health_cond  marital1  mor_circle  moralid10  moralid2  moralid3  moralid4  moralid5  moralid6  moralid7  moralid8  moralid9  mcoop1  mcoop2  mcoop3  mcoop4  mcoop5  mcoop6  mcoop7  narc1  narc2  narc3  narc4  narc5  narc6  nidentity1  nidentity2  omind1  omind4  omind6  contact1  contact2  contact3  contact4  contact5  hygiene1  hygiene2  hygiene3  hygiene4  hygiene5  psupport1  psupport2  psupport3  psupport4  psupport5  political_ideology  riskperc1  riskperc2  self_esteem  sbelong2  sbelong3  optim2  slfcont1  slfcont2  slfcont4  urban
##   5   10  children  cnarc1  cnarc2  cnarc3  CRT1  CRT2  CRT3  health_cond  marital1  mor_circle  moralid10  moralid2  moralid3  moralid4  moralid5  moralid6  moralid7  moralid8  moralid9  mcoop1  mcoop2  mcoop3  mcoop4  mcoop5  mcoop6  mcoop7  narc1  narc2  narc3  narc4  narc5  narc6  nidentity1  nidentity2  omind1  omind4  omind6  contact1  contact2  contact3  contact4  contact5  hygiene1  hygiene2  hygiene3  hygiene4  hygiene5  psupport1  psupport2  psupport3  psupport4  psupport5  political_ideology  riskperc1  riskperc2  self_esteem  sbelong2  sbelong3  optim2  slfcont1  slfcont2  slfcont4  urban
##   6   1  children  cnarc1  cnarc2  cnarc3  CRT1  CRT2  CRT3  health_cond  marital1  mor_circle  moralid10  moralid2  moralid3  moralid4  moralid5  moralid6  moralid7  moralid8  moralid9  mcoop1  mcoop2  mcoop3  mcoop4  mcoop5  mcoop6  mcoop7  narc1  narc2  narc3  narc4  narc5  narc6  nidentity1  nidentity2  omind1  omind4  omind6  contact1  contact2  contact3  contact4  contact5  hygiene1  hygiene2  hygiene3  hygiene4  hygiene5  psupport1  psupport2  psupport3  psupport4  psupport5  political_ideology  riskperc1  riskperc2  self_esteem  sbelong2  sbelong3  optim2  slfcont1  slfcont2  slfcont4  urban
##   6   2  children  cnarc1  cnarc2  cnarc3  CRT1  CRT2  CRT3  health_cond  marital1  mor_circle  moralid10  moralid2  moralid3  moralid4  moralid5  moralid6  moralid7  moralid8  moralid9  mcoop1  mcoop2  mcoop3  mcoop4  mcoop5  mcoop6  mcoop7  narc1  narc2  narc3  narc4  narc5  narc6  nidentity1  nidentity2  omind1  omind4  omind6  contact1  contact2  contact3  contact4  contact5  hygiene1  hygiene2  hygiene3  hygiene4  hygiene5  psupport1  psupport2  psupport3  psupport4  psupport5  political_ideology  riskperc1  riskperc2  self_esteem  sbelong2  sbelong3  optim2  slfcont1  slfcont2  slfcont4  urban
##   6   3  children  cnarc1  cnarc2  cnarc3  CRT1  CRT2  CRT3  health_cond  marital1  mor_circle  moralid10  moralid2  moralid3  moralid4  moralid5  moralid6  moralid7  moralid8  moralid9  mcoop1  mcoop2  mcoop3  mcoop4  mcoop5  mcoop6  mcoop7  narc1  narc2  narc3  narc4  narc5  narc6  nidentity1  nidentity2  omind1  omind4  omind6  contact1  contact2  contact3  contact4  contact5  hygiene1  hygiene2  hygiene3  hygiene4  hygiene5  psupport1  psupport2  psupport3  psupport4  psupport5  political_ideology  riskperc1  riskperc2  self_esteem  sbelong2  sbelong3  optim2  slfcont1  slfcont2  slfcont4  urban
##   6   4  children  cnarc1  cnarc2  cnarc3  CRT1  CRT2  CRT3  health_cond  marital1  mor_circle  moralid10  moralid2  moralid3  moralid4  moralid5  moralid6  moralid7  moralid8  moralid9  mcoop1  mcoop2  mcoop3  mcoop4  mcoop5  mcoop6  mcoop7  narc1  narc2  narc3  narc4  narc5  narc6  nidentity1  nidentity2  omind1  omind4  omind6  contact1  contact2  contact3  contact4  contact5  hygiene1  hygiene2  hygiene3  hygiene4  hygiene5  psupport1  psupport2  psupport3  psupport4  psupport5  political_ideology  riskperc1  riskperc2  self_esteem  sbelong2  sbelong3  optim2  slfcont1  slfcont2  slfcont4  urban
##   6   5  children  cnarc1  cnarc2  cnarc3  CRT1  CRT2  CRT3  health_cond  marital1  mor_circle  moralid10  moralid2  moralid3  moralid4  moralid5  moralid6  moralid7  moralid8  moralid9  mcoop1  mcoop2  mcoop3  mcoop4  mcoop5  mcoop6  mcoop7  narc1  narc2  narc3  narc4  narc5  narc6  nidentity1  nidentity2  omind1  omind4  omind6  contact1  contact2  contact3  contact4  contact5  hygiene1  hygiene2  hygiene3  hygiene4  hygiene5  psupport1  psupport2  psupport3  psupport4  psupport5  political_ideology  riskperc1  riskperc2  self_esteem  sbelong2  sbelong3  optim2  slfcont1  slfcont2  slfcont4  urban
##   6   6  children  cnarc1  cnarc2  cnarc3  CRT1  CRT2  CRT3  health_cond  marital1  mor_circle  moralid10  moralid2  moralid3  moralid4  moralid5  moralid6  moralid7  moralid8  moralid9  mcoop1  mcoop2  mcoop3  mcoop4  mcoop5  mcoop6  mcoop7  narc1  narc2  narc3  narc4  narc5  narc6  nidentity1  nidentity2  omind1  omind4  omind6  contact1  contact2  contact3  contact4  contact5  hygiene1  hygiene2  hygiene3  hygiene4  hygiene5  psupport1  psupport2  psupport3  psupport4  psupport5  political_ideology  riskperc1  riskperc2  self_esteem  sbelong2  sbelong3  optim2  slfcont1  slfcont2  slfcont4  urban
##   6   7  children  cnarc1  cnarc2  cnarc3  CRT1  CRT2  CRT3  health_cond  marital1  mor_circle  moralid10  moralid2  moralid3  moralid4  moralid5  moralid6  moralid7  moralid8  moralid9  mcoop1  mcoop2  mcoop3  mcoop4  mcoop5  mcoop6  mcoop7  narc1  narc2  narc3  narc4  narc5  narc6  nidentity1  nidentity2  omind1  omind4  omind6  contact1  contact2  contact3  contact4  contact5  hygiene1  hygiene2  hygiene3  hygiene4  hygiene5  psupport1  psupport2  psupport3  psupport4  psupport5  political_ideology  riskperc1  riskperc2  self_esteem  sbelong2  sbelong3  optim2  slfcont1  slfcont2  slfcont4  urban
##   6   8  children  cnarc1  cnarc2  cnarc3  CRT1  CRT2  CRT3  health_cond  marital1  mor_circle  moralid10  moralid2  moralid3  moralid4  moralid5  moralid6  moralid7  moralid8  moralid9  mcoop1  mcoop2  mcoop3  mcoop4  mcoop5  mcoop6  mcoop7  narc1  narc2  narc3  narc4  narc5  narc6  nidentity1  nidentity2  omind1  omind4  omind6  contact1  contact2  contact3  contact4  contact5  hygiene1  hygiene2  hygiene3  hygiene4  hygiene5  psupport1  psupport2  psupport3  psupport4  psupport5  political_ideology  riskperc1  riskperc2  self_esteem  sbelong2  sbelong3  optim2  slfcont1  slfcont2  slfcont4  urban
##   6   9  children  cnarc1  cnarc2  cnarc3  CRT1  CRT2  CRT3  health_cond  marital1  mor_circle  moralid10  moralid2  moralid3  moralid4  moralid5  moralid6  moralid7  moralid8  moralid9  mcoop1  mcoop2  mcoop3  mcoop4  mcoop5  mcoop6  mcoop7  narc1  narc2  narc3  narc4  narc5  narc6  nidentity1  nidentity2  omind1  omind4  omind6  contact1  contact2  contact3  contact4  contact5  hygiene1  hygiene2  hygiene3  hygiene4  hygiene5  psupport1  psupport2  psupport3  psupport4  psupport5  political_ideology  riskperc1  riskperc2  self_esteem  sbelong2  sbelong3  optim2  slfcont1  slfcont2  slfcont4  urban
##   6   10  children  cnarc1  cnarc2  cnarc3  CRT1  CRT2  CRT3  health_cond  marital1  mor_circle  moralid10  moralid2  moralid3  moralid4  moralid5  moralid6  moralid7  moralid8  moralid9  mcoop1  mcoop2  mcoop3  mcoop4  mcoop5  mcoop6  mcoop7  narc1  narc2  narc3  narc4  narc5  narc6  nidentity1  nidentity2  omind1  omind4  omind6  contact1  contact2  contact3  contact4  contact5  hygiene1  hygiene2  hygiene3  hygiene4  hygiene5  psupport1  psupport2  psupport3  psupport4  psupport5  political_ideology  riskperc1  riskperc2  self_esteem  sbelong2  sbelong3  optim2  slfcont1  slfcont2  slfcont4  urban
##   7   1  children  cnarc1  cnarc2  cnarc3  CRT1  CRT2  CRT3  health_cond  marital1  mor_circle  moralid10  moralid2  moralid3  moralid4  moralid5  moralid6  moralid7  moralid8  moralid9  mcoop1  mcoop2  mcoop3  mcoop4  mcoop5  mcoop6  mcoop7  narc1  narc2  narc3  narc4  narc5  narc6  nidentity1  nidentity2  omind1  omind4  omind6  contact1  contact2  contact3  contact4  contact5  hygiene1  hygiene2  hygiene3  hygiene4  hygiene5  psupport1  psupport2  psupport3  psupport4  psupport5  political_ideology  riskperc1  riskperc2  self_esteem  sbelong2  sbelong3  optim2  slfcont1  slfcont2  slfcont4  urban
##   7   2  children  cnarc1  cnarc2  cnarc3  CRT1  CRT2  CRT3  health_cond  marital1  mor_circle  moralid10  moralid2  moralid3  moralid4  moralid5  moralid6  moralid7  moralid8  moralid9  mcoop1  mcoop2  mcoop3  mcoop4  mcoop5  mcoop6  mcoop7  narc1  narc2  narc3  narc4  narc5  narc6  nidentity1  nidentity2  omind1  omind4  omind6  contact1  contact2  contact3  contact4  contact5  hygiene1  hygiene2  hygiene3  hygiene4  hygiene5  psupport1  psupport2  psupport3  psupport4  psupport5  political_ideology  riskperc1  riskperc2  self_esteem  sbelong2  sbelong3  optim2  slfcont1  slfcont2  slfcont4  urban
##   7   3  children  cnarc1  cnarc2  cnarc3  CRT1  CRT2  CRT3  health_cond  marital1  mor_circle  moralid10  moralid2  moralid3  moralid4  moralid5  moralid6  moralid7  moralid8  moralid9  mcoop1  mcoop2  mcoop3  mcoop4  mcoop5  mcoop6  mcoop7  narc1  narc2  narc3  narc4  narc5  narc6  nidentity1  nidentity2  omind1  omind4  omind6  contact1  contact2  contact3  contact4  contact5  hygiene1  hygiene2  hygiene3  hygiene4  hygiene5  psupport1  psupport2  psupport3  psupport4  psupport5  political_ideology  riskperc1  riskperc2  self_esteem  sbelong2  sbelong3  optim2  slfcont1  slfcont2  slfcont4  urban
##   7   4  children  cnarc1  cnarc2  cnarc3  CRT1  CRT2  CRT3  health_cond  marital1  mor_circle  moralid10  moralid2  moralid3  moralid4  moralid5  moralid6  moralid7  moralid8  moralid9  mcoop1  mcoop2  mcoop3  mcoop4  mcoop5  mcoop6  mcoop7  narc1  narc2  narc3  narc4  narc5  narc6  nidentity1  nidentity2  omind1  omind4  omind6  contact1  contact2  contact3  contact4  contact5  hygiene1  hygiene2  hygiene3  hygiene4  hygiene5  psupport1  psupport2  psupport3  psupport4  psupport5  political_ideology  riskperc1  riskperc2  self_esteem  sbelong2  sbelong3  optim2  slfcont1  slfcont2  slfcont4  urban
##   7   5  children  cnarc1  cnarc2  cnarc3  CRT1  CRT2  CRT3  health_cond  marital1  mor_circle  moralid10  moralid2  moralid3  moralid4  moralid5  moralid6  moralid7  moralid8  moralid9  mcoop1  mcoop2  mcoop3  mcoop4  mcoop5  mcoop6  mcoop7  narc1  narc2  narc3  narc4  narc5  narc6  nidentity1  nidentity2  omind1  omind4  omind6  contact1  contact2  contact3  contact4  contact5  hygiene1  hygiene2  hygiene3  hygiene4  hygiene5  psupport1  psupport2  psupport3  psupport4  psupport5  political_ideology  riskperc1  riskperc2  self_esteem  sbelong2  sbelong3  optim2  slfcont1  slfcont2  slfcont4  urban
##   7   6  children  cnarc1  cnarc2  cnarc3  CRT1  CRT2  CRT3  health_cond  marital1  mor_circle  moralid10  moralid2  moralid3  moralid4  moralid5  moralid6  moralid7  moralid8  moralid9  mcoop1  mcoop2  mcoop3  mcoop4  mcoop5  mcoop6  mcoop7  narc1  narc2  narc3  narc4  narc5  narc6  nidentity1  nidentity2  omind1  omind4  omind6  contact1  contact2  contact3  contact4  contact5  hygiene1  hygiene2  hygiene3  hygiene4  hygiene5  psupport1  psupport2  psupport3  psupport4  psupport5  political_ideology  riskperc1  riskperc2  self_esteem  sbelong2  sbelong3  optim2  slfcont1  slfcont2  slfcont4  urban
##   7   7  children  cnarc1  cnarc2  cnarc3  CRT1  CRT2  CRT3  health_cond  marital1  mor_circle  moralid10  moralid2  moralid3  moralid4  moralid5  moralid6  moralid7  moralid8  moralid9  mcoop1  mcoop2  mcoop3  mcoop4  mcoop5  mcoop6  mcoop7  narc1  narc2  narc3  narc4  narc5  narc6  nidentity1  nidentity2  omind1  omind4  omind6  contact1  contact2  contact3  contact4  contact5  hygiene1  hygiene2  hygiene3  hygiene4  hygiene5  psupport1  psupport2  psupport3  psupport4  psupport5  political_ideology  riskperc1  riskperc2  self_esteem  sbelong2  sbelong3  optim2  slfcont1  slfcont2  slfcont4  urban
##   7   8  children  cnarc1  cnarc2  cnarc3  CRT1  CRT2  CRT3  health_cond  marital1  mor_circle  moralid10  moralid2  moralid3  moralid4  moralid5  moralid6  moralid7  moralid8  moralid9  mcoop1  mcoop2  mcoop3  mcoop4  mcoop5  mcoop6  mcoop7  narc1  narc2  narc3  narc4  narc5  narc6  nidentity1  nidentity2  omind1  omind4  omind6  contact1  contact2  contact3  contact4  contact5  hygiene1  hygiene2  hygiene3  hygiene4  hygiene5  psupport1  psupport2  psupport3  psupport4  psupport5  political_ideology  riskperc1  riskperc2  self_esteem  sbelong2  sbelong3  optim2  slfcont1  slfcont2  slfcont4  urban
##   7   9  children  cnarc1  cnarc2  cnarc3  CRT1  CRT2  CRT3  health_cond  marital1  mor_circle  moralid10  moralid2  moralid3  moralid4  moralid5  moralid6  moralid7  moralid8  moralid9  mcoop1  mcoop2  mcoop3  mcoop4  mcoop5  mcoop6  mcoop7  narc1  narc2  narc3  narc4  narc5  narc6  nidentity1  nidentity2  omind1  omind4  omind6  contact1  contact2  contact3  contact4  contact5  hygiene1  hygiene2  hygiene3  hygiene4  hygiene5  psupport1  psupport2  psupport3  psupport4  psupport5  political_ideology  riskperc1  riskperc2  self_esteem  sbelong2  sbelong3  optim2  slfcont1  slfcont2  slfcont4  urban
##   7   10  children  cnarc1  cnarc2  cnarc3  CRT1  CRT2  CRT3  health_cond  marital1  mor_circle  moralid10  moralid2  moralid3  moralid4  moralid5  moralid6  moralid7  moralid8  moralid9  mcoop1  mcoop2  mcoop3  mcoop4  mcoop5  mcoop6  mcoop7  narc1  narc2  narc3  narc4  narc5  narc6  nidentity1  nidentity2  omind1  omind4  omind6  contact1  contact2  contact3  contact4  contact5  hygiene1  hygiene2  hygiene3  hygiene4  hygiene5  psupport1  psupport2  psupport3  psupport4  psupport5  political_ideology  riskperc1  riskperc2  self_esteem  sbelong2  sbelong3  optim2  slfcont1  slfcont2  slfcont4  urban
##   8   1  children  cnarc1  cnarc2  cnarc3  CRT1  CRT2  CRT3  health_cond  marital1  mor_circle  moralid10  moralid2  moralid3  moralid4  moralid5  moralid6  moralid7  moralid8  moralid9  mcoop1  mcoop2  mcoop3  mcoop4  mcoop5  mcoop6  mcoop7  narc1  narc2  narc3  narc4  narc5  narc6  nidentity1  nidentity2  omind1  omind4  omind6  contact1  contact2  contact3  contact4  contact5  hygiene1  hygiene2  hygiene3  hygiene4  hygiene5  psupport1  psupport2  psupport3  psupport4  psupport5  political_ideology  riskperc1  riskperc2  self_esteem  sbelong2  sbelong3  optim2  slfcont1  slfcont2  slfcont4  urban
##   8   2  children  cnarc1  cnarc2  cnarc3  CRT1  CRT2  CRT3  health_cond  marital1  mor_circle  moralid10  moralid2  moralid3  moralid4  moralid5  moralid6  moralid7  moralid8  moralid9  mcoop1  mcoop2  mcoop3  mcoop4  mcoop5  mcoop6  mcoop7  narc1  narc2  narc3  narc4  narc5  narc6  nidentity1  nidentity2  omind1  omind4  omind6  contact1  contact2  contact3  contact4  contact5  hygiene1  hygiene2  hygiene3  hygiene4  hygiene5  psupport1  psupport2  psupport3  psupport4  psupport5  political_ideology  riskperc1  riskperc2  self_esteem  sbelong2  sbelong3  optim2  slfcont1  slfcont2  slfcont4  urban
##   8   3  children  cnarc1  cnarc2  cnarc3  CRT1  CRT2  CRT3  health_cond  marital1  mor_circle  moralid10  moralid2  moralid3  moralid4  moralid5  moralid6  moralid7  moralid8  moralid9  mcoop1  mcoop2  mcoop3  mcoop4  mcoop5  mcoop6  mcoop7  narc1  narc2  narc3  narc4  narc5  narc6  nidentity1  nidentity2  omind1  omind4  omind6  contact1  contact2  contact3  contact4  contact5  hygiene1  hygiene2  hygiene3  hygiene4  hygiene5  psupport1  psupport2  psupport3  psupport4  psupport5  political_ideology  riskperc1  riskperc2  self_esteem  sbelong2  sbelong3  optim2  slfcont1  slfcont2  slfcont4  urban
##   8   4  children  cnarc1  cnarc2  cnarc3  CRT1  CRT2  CRT3  health_cond  marital1  mor_circle  moralid10  moralid2  moralid3  moralid4  moralid5  moralid6  moralid7  moralid8  moralid9  mcoop1  mcoop2  mcoop3  mcoop4  mcoop5  mcoop6  mcoop7  narc1  narc2  narc3  narc4  narc5  narc6  nidentity1  nidentity2  omind1  omind4  omind6  contact1  contact2  contact3  contact4  contact5  hygiene1  hygiene2  hygiene3  hygiene4  hygiene5  psupport1  psupport2  psupport3  psupport4  psupport5  political_ideology  riskperc1  riskperc2  self_esteem  sbelong2  sbelong3  optim2  slfcont1  slfcont2  slfcont4  urban
##   8   5  children  cnarc1  cnarc2  cnarc3  CRT1  CRT2  CRT3  health_cond  marital1  mor_circle  moralid10  moralid2  moralid3  moralid4  moralid5  moralid6  moralid7  moralid8  moralid9  mcoop1  mcoop2  mcoop3  mcoop4  mcoop5  mcoop6  mcoop7  narc1  narc2  narc3  narc4  narc5  narc6  nidentity1  nidentity2  omind1  omind4  omind6  contact1  contact2  contact3  contact4  contact5  hygiene1  hygiene2  hygiene3  hygiene4  hygiene5  psupport1  psupport2  psupport3  psupport4  psupport5  political_ideology  riskperc1  riskperc2  self_esteem  sbelong2  sbelong3  optim2  slfcont1  slfcont2  slfcont4  urban
##   8   6  children  cnarc1  cnarc2  cnarc3  CRT1  CRT2  CRT3  health_cond  marital1  mor_circle  moralid10  moralid2  moralid3  moralid4  moralid5  moralid6  moralid7  moralid8  moralid9  mcoop1  mcoop2  mcoop3  mcoop4  mcoop5  mcoop6  mcoop7  narc1  narc2  narc3  narc4  narc5  narc6  nidentity1  nidentity2  omind1  omind4  omind6  contact1  contact2  contact3  contact4  contact5  hygiene1  hygiene2  hygiene3  hygiene4  hygiene5  psupport1  psupport2  psupport3  psupport4  psupport5  political_ideology  riskperc1  riskperc2  self_esteem  sbelong2  sbelong3  optim2  slfcont1  slfcont2  slfcont4  urban
##   8   7  children  cnarc1  cnarc2  cnarc3  CRT1  CRT2  CRT3  health_cond  marital1  mor_circle  moralid10  moralid2  moralid3  moralid4  moralid5  moralid6  moralid7  moralid8  moralid9  mcoop1  mcoop2  mcoop3  mcoop4  mcoop5  mcoop6  mcoop7  narc1  narc2  narc3  narc4  narc5  narc6  nidentity1  nidentity2  omind1  omind4  omind6  contact1  contact2  contact3  contact4  contact5  hygiene1  hygiene2  hygiene3  hygiene4  hygiene5  psupport1  psupport2  psupport3  psupport4  psupport5  political_ideology  riskperc1  riskperc2  self_esteem  sbelong2  sbelong3  optim2  slfcont1  slfcont2  slfcont4  urban
##   8   8  children  cnarc1  cnarc2  cnarc3  CRT1  CRT2  CRT3  health_cond  marital1  mor_circle  moralid10  moralid2  moralid3  moralid4  moralid5  moralid6  moralid7  moralid8  moralid9  mcoop1  mcoop2  mcoop3  mcoop4  mcoop5  mcoop6  mcoop7  narc1  narc2  narc3  narc4  narc5  narc6  nidentity1  nidentity2  omind1  omind4  omind6  contact1  contact2  contact3  contact4  contact5  hygiene1  hygiene2  hygiene3  hygiene4  hygiene5  psupport1  psupport2  psupport3  psupport4  psupport5  political_ideology  riskperc1  riskperc2  self_esteem  sbelong2  sbelong3  optim2  slfcont1  slfcont2  slfcont4  urban
##   8   9  children  cnarc1  cnarc2  cnarc3  CRT1  CRT2  CRT3  health_cond  marital1  mor_circle  moralid10  moralid2  moralid3  moralid4  moralid5  moralid6  moralid7  moralid8  moralid9  mcoop1  mcoop2  mcoop3  mcoop4  mcoop5  mcoop6  mcoop7  narc1  narc2  narc3  narc4  narc5  narc6  nidentity1  nidentity2  omind1  omind4  omind6  contact1  contact2  contact3  contact4  contact5  hygiene1  hygiene2  hygiene3  hygiene4  hygiene5  psupport1  psupport2  psupport3  psupport4  psupport5  political_ideology  riskperc1  riskperc2  self_esteem  sbelong2  sbelong3  optim2  slfcont1  slfcont2  slfcont4  urban
##   8   10  children  cnarc1  cnarc2  cnarc3  CRT1  CRT2  CRT3  health_cond  marital1  mor_circle  moralid10  moralid2  moralid3  moralid4  moralid5  moralid6  moralid7  moralid8  moralid9  mcoop1  mcoop2  mcoop3  mcoop4  mcoop5  mcoop6  mcoop7  narc1  narc2  narc3  narc4  narc5  narc6  nidentity1  nidentity2  omind1  omind4  omind6  contact1  contact2  contact3  contact4  contact5  hygiene1  hygiene2  hygiene3  hygiene4  hygiene5  psupport1  psupport2  psupport3  psupport4  psupport5  political_ideology  riskperc1  riskperc2  self_esteem  sbelong2  sbelong3  optim2  slfcont1  slfcont2  slfcont4  urban
##   9   1  children  cnarc1  cnarc2  cnarc3  CRT1  CRT2  CRT3  health_cond  marital1  mor_circle  moralid10  moralid2  moralid3  moralid4  moralid5  moralid6  moralid7  moralid8  moralid9  mcoop1  mcoop2  mcoop3  mcoop4  mcoop5  mcoop6  mcoop7  narc1  narc2  narc3  narc4  narc5  narc6  nidentity1  nidentity2  omind1  omind4  omind6  contact1  contact2  contact3  contact4  contact5  hygiene1  hygiene2  hygiene3  hygiene4  hygiene5  psupport1  psupport2  psupport3  psupport4  psupport5  political_ideology  riskperc1  riskperc2  self_esteem  sbelong2  sbelong3  optim2  slfcont1  slfcont2  slfcont4  urban
##   9   2  children  cnarc1  cnarc2  cnarc3  CRT1  CRT2  CRT3  health_cond  marital1  mor_circle  moralid10  moralid2  moralid3  moralid4  moralid5  moralid6  moralid7  moralid8  moralid9  mcoop1  mcoop2  mcoop3  mcoop4  mcoop5  mcoop6  mcoop7  narc1  narc2  narc3  narc4  narc5  narc6  nidentity1  nidentity2  omind1  omind4  omind6  contact1  contact2  contact3  contact4  contact5  hygiene1  hygiene2  hygiene3  hygiene4  hygiene5  psupport1  psupport2  psupport3  psupport4  psupport5  political_ideology  riskperc1  riskperc2  self_esteem  sbelong2  sbelong3  optim2  slfcont1  slfcont2  slfcont4  urban
##   9   3  children  cnarc1  cnarc2  cnarc3  CRT1  CRT2  CRT3  health_cond  marital1  mor_circle  moralid10  moralid2  moralid3  moralid4  moralid5  moralid6  moralid7  moralid8  moralid9  mcoop1  mcoop2  mcoop3  mcoop4  mcoop5  mcoop6  mcoop7  narc1  narc2  narc3  narc4  narc5  narc6  nidentity1  nidentity2  omind1  omind4  omind6  contact1  contact2  contact3  contact4  contact5  hygiene1  hygiene2  hygiene3  hygiene4  hygiene5  psupport1  psupport2  psupport3  psupport4  psupport5  political_ideology  riskperc1  riskperc2  self_esteem  sbelong2  sbelong3  optim2  slfcont1  slfcont2  slfcont4  urban
##   9   4  children  cnarc1  cnarc2  cnarc3  CRT1  CRT2  CRT3  health_cond  marital1  mor_circle  moralid10  moralid2  moralid3  moralid4  moralid5  moralid6  moralid7  moralid8  moralid9  mcoop1  mcoop2  mcoop3  mcoop4  mcoop5  mcoop6  mcoop7  narc1  narc2  narc3  narc4  narc5  narc6  nidentity1  nidentity2  omind1  omind4  omind6  contact1  contact2  contact3  contact4  contact5  hygiene1  hygiene2  hygiene3  hygiene4  hygiene5  psupport1  psupport2  psupport3  psupport4  psupport5  political_ideology  riskperc1  riskperc2  self_esteem  sbelong2  sbelong3  optim2  slfcont1  slfcont2  slfcont4  urban
##   9   5  children  cnarc1  cnarc2  cnarc3  CRT1  CRT2  CRT3  health_cond  marital1  mor_circle  moralid10  moralid2  moralid3  moralid4  moralid5  moralid6  moralid7  moralid8  moralid9  mcoop1  mcoop2  mcoop3  mcoop4  mcoop5  mcoop6  mcoop7  narc1  narc2  narc3  narc4  narc5  narc6  nidentity1  nidentity2  omind1  omind4  omind6  contact1  contact2  contact3  contact4  contact5  hygiene1  hygiene2  hygiene3  hygiene4  hygiene5  psupport1  psupport2  psupport3  psupport4  psupport5  political_ideology  riskperc1  riskperc2  self_esteem  sbelong2  sbelong3  optim2  slfcont1  slfcont2  slfcont4  urban
##   9   6  children  cnarc1  cnarc2  cnarc3  CRT1  CRT2  CRT3  health_cond  marital1  mor_circle  moralid10  moralid2  moralid3  moralid4  moralid5  moralid6  moralid7  moralid8  moralid9  mcoop1  mcoop2  mcoop3  mcoop4  mcoop5  mcoop6  mcoop7  narc1  narc2  narc3  narc4  narc5  narc6  nidentity1  nidentity2  omind1  omind4  omind6  contact1  contact2  contact3  contact4  contact5  hygiene1  hygiene2  hygiene3  hygiene4  hygiene5  psupport1  psupport2  psupport3  psupport4  psupport5  political_ideology  riskperc1  riskperc2  self_esteem  sbelong2  sbelong3  optim2  slfcont1  slfcont2  slfcont4  urban
##   9   7  children  cnarc1  cnarc2  cnarc3  CRT1  CRT2  CRT3  health_cond  marital1  mor_circle  moralid10  moralid2  moralid3  moralid4  moralid5  moralid6  moralid7  moralid8  moralid9  mcoop1  mcoop2  mcoop3  mcoop4  mcoop5  mcoop6  mcoop7  narc1  narc2  narc3  narc4  narc5  narc6  nidentity1  nidentity2  omind1  omind4  omind6  contact1  contact2  contact3  contact4  contact5  hygiene1  hygiene2  hygiene3  hygiene4  hygiene5  psupport1  psupport2  psupport3  psupport4  psupport5  political_ideology  riskperc1  riskperc2  self_esteem  sbelong2  sbelong3  optim2  slfcont1  slfcont2  slfcont4  urban
##   9   8  children  cnarc1  cnarc2  cnarc3  CRT1  CRT2  CRT3  health_cond  marital1  mor_circle  moralid10  moralid2  moralid3  moralid4  moralid5  moralid6  moralid7  moralid8  moralid9  mcoop1  mcoop2  mcoop3  mcoop4  mcoop5  mcoop6  mcoop7  narc1  narc2  narc3  narc4  narc5  narc6  nidentity1  nidentity2  omind1  omind4  omind6  contact1  contact2  contact3  contact4  contact5  hygiene1  hygiene2  hygiene3  hygiene4  hygiene5  psupport1  psupport2  psupport3  psupport4  psupport5  political_ideology  riskperc1  riskperc2  self_esteem  sbelong2  sbelong3  optim2  slfcont1  slfcont2  slfcont4  urban
##   9   9  children  cnarc1  cnarc2  cnarc3  CRT1  CRT2  CRT3  health_cond  marital1  mor_circle  moralid10  moralid2  moralid3  moralid4  moralid5  moralid6  moralid7  moralid8  moralid9  mcoop1  mcoop2  mcoop3  mcoop4  mcoop5  mcoop6  mcoop7  narc1  narc2  narc3  narc4  narc5  narc6  nidentity1  nidentity2  omind1  omind4  omind6  contact1  contact2  contact3  contact4  contact5  hygiene1  hygiene2  hygiene3  hygiene4  hygiene5  psupport1  psupport2  psupport3  psupport4  psupport5  political_ideology  riskperc1  riskperc2  self_esteem  sbelong2  sbelong3  optim2  slfcont1  slfcont2  slfcont4  urban
##   9   10  children  cnarc1  cnarc2  cnarc3  CRT1  CRT2  CRT3  health_cond  marital1  mor_circle  moralid10  moralid2  moralid3  moralid4  moralid5  moralid6  moralid7  moralid8  moralid9  mcoop1  mcoop2  mcoop3  mcoop4  mcoop5  mcoop6  mcoop7  narc1  narc2  narc3  narc4  narc5  narc6  nidentity1  nidentity2  omind1  omind4  omind6  contact1  contact2  contact3  contact4  contact5  hygiene1  hygiene2  hygiene3  hygiene4  hygiene5  psupport1  psupport2  psupport3  psupport4  psupport5  political_ideology  riskperc1  riskperc2  self_esteem  sbelong2  sbelong3  optim2  slfcont1  slfcont2  slfcont4  urban
##   10   1  children  cnarc1  cnarc2  cnarc3  CRT1  CRT2  CRT3  health_cond  marital1  mor_circle  moralid10  moralid2  moralid3  moralid4  moralid5  moralid6  moralid7  moralid8  moralid9  mcoop1  mcoop2  mcoop3  mcoop4  mcoop5  mcoop6  mcoop7  narc1  narc2  narc3  narc4  narc5  narc6  nidentity1  nidentity2  omind1  omind4  omind6  contact1  contact2  contact3  contact4  contact5  hygiene1  hygiene2  hygiene3  hygiene4  hygiene5  psupport1  psupport2  psupport3  psupport4  psupport5  political_ideology  riskperc1  riskperc2  self_esteem  sbelong2  sbelong3  optim2  slfcont1  slfcont2  slfcont4  urban
##   10   2  children  cnarc1  cnarc2  cnarc3  CRT1  CRT2  CRT3  health_cond  marital1  mor_circle  moralid10  moralid2  moralid3  moralid4  moralid5  moralid6  moralid7  moralid8  moralid9  mcoop1  mcoop2  mcoop3  mcoop4  mcoop5  mcoop6  mcoop7  narc1  narc2  narc3  narc4  narc5  narc6  nidentity1  nidentity2  omind1  omind4  omind6  contact1  contact2  contact3  contact4  contact5  hygiene1  hygiene2  hygiene3  hygiene4  hygiene5  psupport1  psupport2  psupport3  psupport4  psupport5  political_ideology  riskperc1  riskperc2  self_esteem  sbelong2  sbelong3  optim2  slfcont1  slfcont2  slfcont4  urban
##   10   3  children  cnarc1  cnarc2  cnarc3  CRT1  CRT2  CRT3  health_cond  marital1  mor_circle  moralid10  moralid2  moralid3  moralid4  moralid5  moralid6  moralid7  moralid8  moralid9  mcoop1  mcoop2  mcoop3  mcoop4  mcoop5  mcoop6  mcoop7  narc1  narc2  narc3  narc4  narc5  narc6  nidentity1  nidentity2  omind1  omind4  omind6  contact1  contact2  contact3  contact4  contact5  hygiene1  hygiene2  hygiene3  hygiene4  hygiene5  psupport1  psupport2  psupport3  psupport4  psupport5  political_ideology  riskperc1  riskperc2  self_esteem  sbelong2  sbelong3  optim2  slfcont1  slfcont2  slfcont4  urban
##   10   4  children  cnarc1  cnarc2  cnarc3  CRT1  CRT2  CRT3  health_cond  marital1  mor_circle  moralid10  moralid2  moralid3  moralid4  moralid5  moralid6  moralid7  moralid8  moralid9  mcoop1  mcoop2  mcoop3  mcoop4  mcoop5  mcoop6  mcoop7  narc1  narc2  narc3  narc4  narc5  narc6  nidentity1  nidentity2  omind1  omind4  omind6  contact1  contact2  contact3  contact4  contact5  hygiene1  hygiene2  hygiene3  hygiene4  hygiene5  psupport1  psupport2  psupport3  psupport4  psupport5  political_ideology  riskperc1  riskperc2  self_esteem  sbelong2  sbelong3  optim2  slfcont1  slfcont2  slfcont4  urban
##   10   5  children  cnarc1  cnarc2  cnarc3  CRT1  CRT2  CRT3  health_cond  marital1  mor_circle  moralid10  moralid2  moralid3  moralid4  moralid5  moralid6  moralid7  moralid8  moralid9  mcoop1  mcoop2  mcoop3  mcoop4  mcoop5  mcoop6  mcoop7  narc1  narc2  narc3  narc4  narc5  narc6  nidentity1  nidentity2  omind1  omind4  omind6  contact1  contact2  contact3  contact4  contact5  hygiene1  hygiene2  hygiene3  hygiene4  hygiene5  psupport1  psupport2  psupport3  psupport4  psupport5  political_ideology  riskperc1  riskperc2  self_esteem  sbelong2  sbelong3  optim2  slfcont1  slfcont2  slfcont4  urban
##   10   6  children  cnarc1  cnarc2  cnarc3  CRT1  CRT2  CRT3  health_cond  marital1  mor_circle  moralid10  moralid2  moralid3  moralid4  moralid5  moralid6  moralid7  moralid8  moralid9  mcoop1  mcoop2  mcoop3  mcoop4  mcoop5  mcoop6  mcoop7  narc1  narc2  narc3  narc4  narc5  narc6  nidentity1  nidentity2  omind1  omind4  omind6  contact1  contact2  contact3  contact4  contact5  hygiene1  hygiene2  hygiene3  hygiene4  hygiene5  psupport1  psupport2  psupport3  psupport4  psupport5  political_ideology  riskperc1  riskperc2  self_esteem  sbelong2  sbelong3  optim2  slfcont1  slfcont2  slfcont4  urban
##   10   7  children  cnarc1  cnarc2  cnarc3  CRT1  CRT2  CRT3  health_cond  marital1  mor_circle  moralid10  moralid2  moralid3  moralid4  moralid5  moralid6  moralid7  moralid8  moralid9  mcoop1  mcoop2  mcoop3  mcoop4  mcoop5  mcoop6  mcoop7  narc1  narc2  narc3  narc4  narc5  narc6  nidentity1  nidentity2  omind1  omind4  omind6  contact1  contact2  contact3  contact4  contact5  hygiene1  hygiene2  hygiene3  hygiene4  hygiene5  psupport1  psupport2  psupport3  psupport4  psupport5  political_ideology  riskperc1  riskperc2  self_esteem  sbelong2  sbelong3  optim2  slfcont1  slfcont2  slfcont4  urban
##   10   8  children  cnarc1  cnarc2  cnarc3  CRT1  CRT2  CRT3  health_cond  marital1  mor_circle  moralid10  moralid2  moralid3  moralid4  moralid5  moralid6  moralid7  moralid8  moralid9  mcoop1  mcoop2  mcoop3  mcoop4  mcoop5  mcoop6  mcoop7  narc1  narc2  narc3  narc4  narc5  narc6  nidentity1  nidentity2  omind1  omind4  omind6  contact1  contact2  contact3  contact4  contact5  hygiene1  hygiene2  hygiene3  hygiene4  hygiene5  psupport1  psupport2  psupport3  psupport4  psupport5  political_ideology  riskperc1  riskperc2  self_esteem  sbelong2  sbelong3  optim2  slfcont1  slfcont2  slfcont4  urban
##   10   9  children  cnarc1  cnarc2  cnarc3  CRT1  CRT2  CRT3  health_cond  marital1  mor_circle  moralid10  moralid2  moralid3  moralid4  moralid5  moralid6  moralid7  moralid8  moralid9  mcoop1  mcoop2  mcoop3  mcoop4  mcoop5  mcoop6  mcoop7  narc1  narc2  narc3  narc4  narc5  narc6  nidentity1  nidentity2  omind1  omind4  omind6  contact1  contact2  contact3  contact4  contact5  hygiene1  hygiene2  hygiene3  hygiene4  hygiene5  psupport1  psupport2  psupport3  psupport4  psupport5  political_ideology  riskperc1  riskperc2  self_esteem  sbelong2  sbelong3  optim2  slfcont1  slfcont2  slfcont4  urban
##   10   10  children  cnarc1  cnarc2  cnarc3  CRT1  CRT2  CRT3  health_cond  marital1  mor_circle  moralid10  moralid2  moralid3  moralid4  moralid5  moralid6  moralid7  moralid8  moralid9  mcoop1  mcoop2  mcoop3  mcoop4  mcoop5  mcoop6  mcoop7  narc1  narc2  narc3  narc4  narc5  narc6  nidentity1  nidentity2  omind1  omind4  omind6  contact1  contact2  contact3  contact4  contact5  hygiene1  hygiene2  hygiene3  hygiene4  hygiene5  psupport1  psupport2  psupport3  psupport4  psupport5  political_ideology  riskperc1  riskperc2  self_esteem  sbelong2  sbelong3  optim2  slfcont1  slfcont2  slfcont4  urban
```

```
## Warning: Number of logged events: 6303
```

```
## 
##  iter imp variable
##   1   1  cnarc1  cnarc2  cnarc3  ctheory1  ctheory2  ctheory3  ctheory4  CRT1  CRT2  CRT3  generosity1  generosity2  generosity3  health_cond  know_tested_positive  ladder  marital1  moralid1  moralid10  moralid2  moralid3  moralid4  moralid5  moralid6  moralid7  moralid8  moralid9  mcoop1  mcoop2  mcoop3  mcoop4  mcoop5  mcoop6  mcoop7  narc1  narc2  narc3  narc4  narc5  narc6  nidentity1  nidentity2  omind1  omind2  omind3  omind4  omind5  contact4  contact5  hygiene1  hygiene2  hygiene3  hygiene4  hygiene5  psupport1  psupport2  psupport3  psupport4  psupport5  political_ideology  happy  slf_ladder  riskperc1  riskperc2  self_esteem  sex1  tested_positive  optim1  optim2  slfcont1  slfcont2  slfcont3  slfcont4  urban
##   1   2  cnarc1  cnarc2  cnarc3  ctheory1  ctheory2  ctheory3  ctheory4  CRT1  CRT2  CRT3  generosity1  generosity2  generosity3  health_cond  know_tested_positive  ladder  marital1  moralid1  moralid10  moralid2  moralid3  moralid4  moralid5  moralid6  moralid7  moralid8  moralid9  mcoop1  mcoop2  mcoop3  mcoop4  mcoop5  mcoop6  mcoop7  narc1  narc2  narc3  narc4  narc5  narc6  nidentity1  nidentity2  omind1  omind2  omind3  omind4  omind5  contact4  contact5  hygiene1  hygiene2  hygiene3  hygiene4  hygiene5  psupport1  psupport2  psupport3  psupport4  psupport5  political_ideology  happy  slf_ladder  riskperc1  riskperc2  self_esteem  sex1  tested_positive  optim1  optim2  slfcont1  slfcont2  slfcont3  slfcont4  urban
##   1   3  cnarc1  cnarc2  cnarc3  ctheory1  ctheory2  ctheory3  ctheory4  CRT1  CRT2  CRT3  generosity1  generosity2  generosity3  health_cond  know_tested_positive  ladder  marital1  moralid1  moralid10  moralid2  moralid3  moralid4  moralid5  moralid6  moralid7  moralid8  moralid9  mcoop1  mcoop2  mcoop3  mcoop4  mcoop5  mcoop6  mcoop7  narc1  narc2  narc3  narc4  narc5  narc6  nidentity1  nidentity2  omind1  omind2  omind3  omind4  omind5  contact4  contact5  hygiene1  hygiene2  hygiene3  hygiene4  hygiene5  psupport1  psupport2  psupport3  psupport4  psupport5  political_ideology  happy  slf_ladder  riskperc1  riskperc2  self_esteem  sex1  tested_positive  optim1  optim2  slfcont1  slfcont2  slfcont3  slfcont4  urban
##   1   4  cnarc1  cnarc2  cnarc3  ctheory1  ctheory2  ctheory3  ctheory4  CRT1  CRT2  CRT3  generosity1  generosity2  generosity3  health_cond  know_tested_positive  ladder  marital1  moralid1  moralid10  moralid2  moralid3  moralid4  moralid5  moralid6  moralid7  moralid8  moralid9  mcoop1  mcoop2  mcoop3  mcoop4  mcoop5  mcoop6  mcoop7  narc1  narc2  narc3  narc4  narc5  narc6  nidentity1  nidentity2  omind1  omind2  omind3  omind4  omind5  contact4  contact5  hygiene1  hygiene2  hygiene3  hygiene4  hygiene5  psupport1  psupport2  psupport3  psupport4  psupport5  political_ideology  happy  slf_ladder  riskperc1  riskperc2  self_esteem  sex1  tested_positive  optim1  optim2  slfcont1  slfcont2  slfcont3  slfcont4  urban
##   1   5  cnarc1  cnarc2  cnarc3  ctheory1  ctheory2  ctheory3  ctheory4  CRT1  CRT2  CRT3  generosity1  generosity2  generosity3  health_cond  know_tested_positive  ladder  marital1  moralid1  moralid10  moralid2  moralid3  moralid4  moralid5  moralid6  moralid7  moralid8  moralid9  mcoop1  mcoop2  mcoop3  mcoop4  mcoop5  mcoop6  mcoop7  narc1  narc2  narc3  narc4  narc5  narc6  nidentity1  nidentity2  omind1  omind2  omind3  omind4  omind5  contact4  contact5  hygiene1  hygiene2  hygiene3  hygiene4  hygiene5  psupport1  psupport2  psupport3  psupport4  psupport5  political_ideology  happy  slf_ladder  riskperc1  riskperc2  self_esteem  sex1  tested_positive  optim1  optim2  slfcont1  slfcont2  slfcont3  slfcont4  urban
##   1   6  cnarc1  cnarc2  cnarc3  ctheory1  ctheory2  ctheory3  ctheory4  CRT1  CRT2  CRT3  generosity1  generosity2  generosity3  health_cond  know_tested_positive  ladder  marital1  moralid1  moralid10  moralid2  moralid3  moralid4  moralid5  moralid6  moralid7  moralid8  moralid9  mcoop1  mcoop2  mcoop3  mcoop4  mcoop5  mcoop6  mcoop7  narc1  narc2  narc3  narc4  narc5  narc6  nidentity1  nidentity2  omind1  omind2  omind3  omind4  omind5  contact4  contact5  hygiene1  hygiene2  hygiene3  hygiene4  hygiene5  psupport1  psupport2  psupport3  psupport4  psupport5  political_ideology  happy  slf_ladder  riskperc1  riskperc2  self_esteem  sex1  tested_positive  optim1  optim2  slfcont1  slfcont2  slfcont3  slfcont4  urban
##   1   7  cnarc1  cnarc2  cnarc3  ctheory1  ctheory2  ctheory3  ctheory4  CRT1  CRT2  CRT3  generosity1  generosity2  generosity3  health_cond  know_tested_positive  ladder  marital1  moralid1  moralid10  moralid2  moralid3  moralid4  moralid5  moralid6  moralid7  moralid8  moralid9  mcoop1  mcoop2  mcoop3  mcoop4  mcoop5  mcoop6  mcoop7  narc1  narc2  narc3  narc4  narc5  narc6  nidentity1  nidentity2  omind1  omind2  omind3  omind4  omind5  contact4  contact5  hygiene1  hygiene2  hygiene3  hygiene4  hygiene5  psupport1  psupport2  psupport3  psupport4  psupport5  political_ideology  happy  slf_ladder  riskperc1  riskperc2  self_esteem  sex1  tested_positive  optim1  optim2  slfcont1  slfcont2  slfcont3  slfcont4  urban
##   1   8  cnarc1  cnarc2  cnarc3  ctheory1  ctheory2  ctheory3  ctheory4  CRT1  CRT2  CRT3  generosity1  generosity2  generosity3  health_cond  know_tested_positive  ladder  marital1  moralid1  moralid10  moralid2  moralid3  moralid4  moralid5  moralid6  moralid7  moralid8  moralid9  mcoop1  mcoop2  mcoop3  mcoop4  mcoop5  mcoop6  mcoop7  narc1  narc2  narc3  narc4  narc5  narc6  nidentity1  nidentity2  omind1  omind2  omind3  omind4  omind5  contact4  contact5  hygiene1  hygiene2  hygiene3  hygiene4  hygiene5  psupport1  psupport2  psupport3  psupport4  psupport5  political_ideology  happy  slf_ladder  riskperc1  riskperc2  self_esteem  sex1  tested_positive  optim1  optim2  slfcont1  slfcont2  slfcont3  slfcont4  urban
##   1   9  cnarc1  cnarc2  cnarc3  ctheory1  ctheory2  ctheory3  ctheory4  CRT1  CRT2  CRT3  generosity1  generosity2  generosity3  health_cond  know_tested_positive  ladder  marital1  moralid1  moralid10  moralid2  moralid3  moralid4  moralid5  moralid6  moralid7  moralid8  moralid9  mcoop1  mcoop2  mcoop3  mcoop4  mcoop5  mcoop6  mcoop7  narc1  narc2  narc3  narc4  narc5  narc6  nidentity1  nidentity2  omind1  omind2  omind3  omind4  omind5  contact4  contact5  hygiene1  hygiene2  hygiene3  hygiene4  hygiene5  psupport1  psupport2  psupport3  psupport4  psupport5  political_ideology  happy  slf_ladder  riskperc1  riskperc2  self_esteem  sex1  tested_positive  optim1  optim2  slfcont1  slfcont2  slfcont3  slfcont4  urban
##   1   10  cnarc1  cnarc2  cnarc3  ctheory1  ctheory2  ctheory3  ctheory4  CRT1  CRT2  CRT3  generosity1  generosity2  generosity3  health_cond  know_tested_positive  ladder  marital1  moralid1  moralid10  moralid2  moralid3  moralid4  moralid5  moralid6  moralid7  moralid8  moralid9  mcoop1  mcoop2  mcoop3  mcoop4  mcoop5  mcoop6  mcoop7  narc1  narc2  narc3  narc4  narc5  narc6  nidentity1  nidentity2  omind1  omind2  omind3  omind4  omind5  contact4  contact5  hygiene1  hygiene2  hygiene3  hygiene4  hygiene5  psupport1  psupport2  psupport3  psupport4  psupport5  political_ideology  happy  slf_ladder  riskperc1  riskperc2  self_esteem  sex1  tested_positive  optim1  optim2  slfcont1  slfcont2  slfcont3  slfcont4  urban
##   2   1  cnarc1  cnarc2  cnarc3  ctheory1  ctheory2  ctheory3  ctheory4  CRT1  CRT2  CRT3  generosity1  generosity2  generosity3  health_cond  know_tested_positive  ladder  marital1  moralid1  moralid10  moralid2  moralid3  moralid4  moralid5  moralid6  moralid7  moralid8  moralid9  mcoop1  mcoop2  mcoop3  mcoop4  mcoop5  mcoop6  mcoop7  narc1  narc2  narc3  narc4  narc5  narc6  nidentity1  nidentity2  omind1  omind2  omind3  omind4  omind5  contact4  contact5  hygiene1  hygiene2  hygiene3  hygiene4  hygiene5  psupport1  psupport2  psupport3  psupport4  psupport5  political_ideology  happy  slf_ladder  riskperc1  riskperc2  self_esteem  sex1  tested_positive  optim1  optim2  slfcont1  slfcont2  slfcont3  slfcont4  urban
##   2   2  cnarc1  cnarc2  cnarc3  ctheory1  ctheory2  ctheory3  ctheory4  CRT1  CRT2  CRT3  generosity1  generosity2  generosity3  health_cond  know_tested_positive  ladder  marital1  moralid1  moralid10  moralid2  moralid3  moralid4  moralid5  moralid6  moralid7  moralid8  moralid9  mcoop1  mcoop2  mcoop3  mcoop4  mcoop5  mcoop6  mcoop7  narc1  narc2  narc3  narc4  narc5  narc6  nidentity1  nidentity2  omind1  omind2  omind3  omind4  omind5  contact4  contact5  hygiene1  hygiene2  hygiene3  hygiene4  hygiene5  psupport1  psupport2  psupport3  psupport4  psupport5  political_ideology  happy  slf_ladder  riskperc1  riskperc2  self_esteem  sex1  tested_positive  optim1  optim2  slfcont1  slfcont2  slfcont3  slfcont4  urban
##   2   3  cnarc1  cnarc2  cnarc3  ctheory1  ctheory2  ctheory3  ctheory4  CRT1  CRT2  CRT3  generosity1  generosity2  generosity3  health_cond  know_tested_positive  ladder  marital1  moralid1  moralid10  moralid2  moralid3  moralid4  moralid5  moralid6  moralid7  moralid8  moralid9  mcoop1  mcoop2  mcoop3  mcoop4  mcoop5  mcoop6  mcoop7  narc1  narc2  narc3  narc4  narc5  narc6  nidentity1  nidentity2  omind1  omind2  omind3  omind4  omind5  contact4  contact5  hygiene1  hygiene2  hygiene3  hygiene4  hygiene5  psupport1  psupport2  psupport3  psupport4  psupport5  political_ideology  happy  slf_ladder  riskperc1  riskperc2  self_esteem  sex1  tested_positive  optim1  optim2  slfcont1  slfcont2  slfcont3  slfcont4  urban
##   2   4  cnarc1  cnarc2  cnarc3  ctheory1  ctheory2  ctheory3  ctheory4  CRT1  CRT2  CRT3  generosity1  generosity2  generosity3  health_cond  know_tested_positive  ladder  marital1  moralid1  moralid10  moralid2  moralid3  moralid4  moralid5  moralid6  moralid7  moralid8  moralid9  mcoop1  mcoop2  mcoop3  mcoop4  mcoop5  mcoop6  mcoop7  narc1  narc2  narc3  narc4  narc5  narc6  nidentity1  nidentity2  omind1  omind2  omind3  omind4  omind5  contact4  contact5  hygiene1  hygiene2  hygiene3  hygiene4  hygiene5  psupport1  psupport2  psupport3  psupport4  psupport5  political_ideology  happy  slf_ladder  riskperc1  riskperc2  self_esteem  sex1  tested_positive  optim1  optim2  slfcont1  slfcont2  slfcont3  slfcont4  urban
##   2   5  cnarc1  cnarc2  cnarc3  ctheory1  ctheory2  ctheory3  ctheory4  CRT1  CRT2  CRT3  generosity1  generosity2  generosity3  health_cond  know_tested_positive  ladder  marital1  moralid1  moralid10  moralid2  moralid3  moralid4  moralid5  moralid6  moralid7  moralid8  moralid9  mcoop1  mcoop2  mcoop3  mcoop4  mcoop5  mcoop6  mcoop7  narc1  narc2  narc3  narc4  narc5  narc6  nidentity1  nidentity2  omind1  omind2  omind3  omind4  omind5  contact4  contact5  hygiene1  hygiene2  hygiene3  hygiene4  hygiene5  psupport1  psupport2  psupport3  psupport4  psupport5  political_ideology  happy  slf_ladder  riskperc1  riskperc2  self_esteem  sex1  tested_positive  optim1  optim2  slfcont1  slfcont2  slfcont3  slfcont4  urban
##   2   6  cnarc1  cnarc2  cnarc3  ctheory1  ctheory2  ctheory3  ctheory4  CRT1  CRT2  CRT3  generosity1  generosity2  generosity3  health_cond  know_tested_positive  ladder  marital1  moralid1  moralid10  moralid2  moralid3  moralid4  moralid5  moralid6  moralid7  moralid8  moralid9  mcoop1  mcoop2  mcoop3  mcoop4  mcoop5  mcoop6  mcoop7  narc1  narc2  narc3  narc4  narc5  narc6  nidentity1  nidentity2  omind1  omind2  omind3  omind4  omind5  contact4  contact5  hygiene1  hygiene2  hygiene3  hygiene4  hygiene5  psupport1  psupport2  psupport3  psupport4  psupport5  political_ideology  happy  slf_ladder  riskperc1  riskperc2  self_esteem  sex1  tested_positive  optim1  optim2  slfcont1  slfcont2  slfcont3  slfcont4  urban
##   2   7  cnarc1  cnarc2  cnarc3  ctheory1  ctheory2  ctheory3  ctheory4  CRT1  CRT2  CRT3  generosity1  generosity2  generosity3  health_cond  know_tested_positive  ladder  marital1  moralid1  moralid10  moralid2  moralid3  moralid4  moralid5  moralid6  moralid7  moralid8  moralid9  mcoop1  mcoop2  mcoop3  mcoop4  mcoop5  mcoop6  mcoop7  narc1  narc2  narc3  narc4  narc5  narc6  nidentity1  nidentity2  omind1  omind2  omind3  omind4  omind5  contact4  contact5  hygiene1  hygiene2  hygiene3  hygiene4  hygiene5  psupport1  psupport2  psupport3  psupport4  psupport5  political_ideology  happy  slf_ladder  riskperc1  riskperc2  self_esteem  sex1  tested_positive  optim1  optim2  slfcont1  slfcont2  slfcont3  slfcont4  urban
##   2   8  cnarc1  cnarc2  cnarc3  ctheory1  ctheory2  ctheory3  ctheory4  CRT1  CRT2  CRT3  generosity1  generosity2  generosity3  health_cond  know_tested_positive  ladder  marital1  moralid1  moralid10  moralid2  moralid3  moralid4  moralid5  moralid6  moralid7  moralid8  moralid9  mcoop1  mcoop2  mcoop3  mcoop4  mcoop5  mcoop6  mcoop7  narc1  narc2  narc3  narc4  narc5  narc6  nidentity1  nidentity2  omind1  omind2  omind3  omind4  omind5  contact4  contact5  hygiene1  hygiene2  hygiene3  hygiene4  hygiene5  psupport1  psupport2  psupport3  psupport4  psupport5  political_ideology  happy  slf_ladder  riskperc1  riskperc2  self_esteem  sex1  tested_positive  optim1  optim2  slfcont1  slfcont2  slfcont3  slfcont4  urban
##   2   9  cnarc1  cnarc2  cnarc3  ctheory1  ctheory2  ctheory3  ctheory4  CRT1  CRT2  CRT3  generosity1  generosity2  generosity3  health_cond  know_tested_positive  ladder  marital1  moralid1  moralid10  moralid2  moralid3  moralid4  moralid5  moralid6  moralid7  moralid8  moralid9  mcoop1  mcoop2  mcoop3  mcoop4  mcoop5  mcoop6  mcoop7  narc1  narc2  narc3  narc4  narc5  narc6  nidentity1  nidentity2  omind1  omind2  omind3  omind4  omind5  contact4  contact5  hygiene1  hygiene2  hygiene3  hygiene4  hygiene5  psupport1  psupport2  psupport3  psupport4  psupport5  political_ideology  happy  slf_ladder  riskperc1  riskperc2  self_esteem  sex1  tested_positive  optim1  optim2  slfcont1  slfcont2  slfcont3  slfcont4  urban
##   2   10  cnarc1  cnarc2  cnarc3  ctheory1  ctheory2  ctheory3  ctheory4  CRT1  CRT2  CRT3  generosity1  generosity2  generosity3  health_cond  know_tested_positive  ladder  marital1  moralid1  moralid10  moralid2  moralid3  moralid4  moralid5  moralid6  moralid7  moralid8  moralid9  mcoop1  mcoop2  mcoop3  mcoop4  mcoop5  mcoop6  mcoop7  narc1  narc2  narc3  narc4  narc5  narc6  nidentity1  nidentity2  omind1  omind2  omind3  omind4  omind5  contact4  contact5  hygiene1  hygiene2  hygiene3  hygiene4  hygiene5  psupport1  psupport2  psupport3  psupport4  psupport5  political_ideology  happy  slf_ladder  riskperc1  riskperc2  self_esteem  sex1  tested_positive  optim1  optim2  slfcont1  slfcont2  slfcont3  slfcont4  urban
##   3   1  cnarc1  cnarc2  cnarc3  ctheory1  ctheory2  ctheory3  ctheory4  CRT1  CRT2  CRT3  generosity1  generosity2  generosity3  health_cond  know_tested_positive  ladder  marital1  moralid1  moralid10  moralid2  moralid3  moralid4  moralid5  moralid6  moralid7  moralid8  moralid9  mcoop1  mcoop2  mcoop3  mcoop4  mcoop5  mcoop6  mcoop7  narc1  narc2  narc3  narc4  narc5  narc6  nidentity1  nidentity2  omind1  omind2  omind3  omind4  omind5  contact4  contact5  hygiene1  hygiene2  hygiene3  hygiene4  hygiene5  psupport1  psupport2  psupport3  psupport4  psupport5  political_ideology  happy  slf_ladder  riskperc1  riskperc2  self_esteem  sex1  tested_positive  optim1  optim2  slfcont1  slfcont2  slfcont3  slfcont4  urban
##   3   2  cnarc1  cnarc2  cnarc3  ctheory1  ctheory2  ctheory3  ctheory4  CRT1  CRT2  CRT3  generosity1  generosity2  generosity3  health_cond  know_tested_positive  ladder  marital1  moralid1  moralid10  moralid2  moralid3  moralid4  moralid5  moralid6  moralid7  moralid8  moralid9  mcoop1  mcoop2  mcoop3  mcoop4  mcoop5  mcoop6  mcoop7  narc1  narc2  narc3  narc4  narc5  narc6  nidentity1  nidentity2  omind1  omind2  omind3  omind4  omind5  contact4  contact5  hygiene1  hygiene2  hygiene3  hygiene4  hygiene5  psupport1  psupport2  psupport3  psupport4  psupport5  political_ideology  happy  slf_ladder  riskperc1  riskperc2  self_esteem  sex1  tested_positive  optim1  optim2  slfcont1  slfcont2  slfcont3  slfcont4  urban
##   3   3  cnarc1  cnarc2  cnarc3  ctheory1  ctheory2  ctheory3  ctheory4  CRT1  CRT2  CRT3  generosity1  generosity2  generosity3  health_cond  know_tested_positive  ladder  marital1  moralid1  moralid10  moralid2  moralid3  moralid4  moralid5  moralid6  moralid7  moralid8  moralid9  mcoop1  mcoop2  mcoop3  mcoop4  mcoop5  mcoop6  mcoop7  narc1  narc2  narc3  narc4  narc5  narc6  nidentity1  nidentity2  omind1  omind2  omind3  omind4  omind5  contact4  contact5  hygiene1  hygiene2  hygiene3  hygiene4  hygiene5  psupport1  psupport2  psupport3  psupport4  psupport5  political_ideology  happy  slf_ladder  riskperc1  riskperc2  self_esteem  sex1  tested_positive  optim1  optim2  slfcont1  slfcont2  slfcont3  slfcont4  urban
##   3   4  cnarc1  cnarc2  cnarc3  ctheory1  ctheory2  ctheory3  ctheory4  CRT1  CRT2  CRT3  generosity1  generosity2  generosity3  health_cond  know_tested_positive  ladder  marital1  moralid1  moralid10  moralid2  moralid3  moralid4  moralid5  moralid6  moralid7  moralid8  moralid9  mcoop1  mcoop2  mcoop3  mcoop4  mcoop5  mcoop6  mcoop7  narc1  narc2  narc3  narc4  narc5  narc6  nidentity1  nidentity2  omind1  omind2  omind3  omind4  omind5  contact4  contact5  hygiene1  hygiene2  hygiene3  hygiene4  hygiene5  psupport1  psupport2  psupport3  psupport4  psupport5  political_ideology  happy  slf_ladder  riskperc1  riskperc2  self_esteem  sex1  tested_positive  optim1  optim2  slfcont1  slfcont2  slfcont3  slfcont4  urban
##   3   5  cnarc1  cnarc2  cnarc3  ctheory1  ctheory2  ctheory3  ctheory4  CRT1  CRT2  CRT3  generosity1  generosity2  generosity3  health_cond  know_tested_positive  ladder  marital1  moralid1  moralid10  moralid2  moralid3  moralid4  moralid5  moralid6  moralid7  moralid8  moralid9  mcoop1  mcoop2  mcoop3  mcoop4  mcoop5  mcoop6  mcoop7  narc1  narc2  narc3  narc4  narc5  narc6  nidentity1  nidentity2  omind1  omind2  omind3  omind4  omind5  contact4  contact5  hygiene1  hygiene2  hygiene3  hygiene4  hygiene5  psupport1  psupport2  psupport3  psupport4  psupport5  political_ideology  happy  slf_ladder  riskperc1  riskperc2  self_esteem  sex1  tested_positive  optim1  optim2  slfcont1  slfcont2  slfcont3  slfcont4  urban
##   3   6  cnarc1  cnarc2  cnarc3  ctheory1  ctheory2  ctheory3  ctheory4  CRT1  CRT2  CRT3  generosity1  generosity2  generosity3  health_cond  know_tested_positive  ladder  marital1  moralid1  moralid10  moralid2  moralid3  moralid4  moralid5  moralid6  moralid7  moralid8  moralid9  mcoop1  mcoop2  mcoop3  mcoop4  mcoop5  mcoop6  mcoop7  narc1  narc2  narc3  narc4  narc5  narc6  nidentity1  nidentity2  omind1  omind2  omind3  omind4  omind5  contact4  contact5  hygiene1  hygiene2  hygiene3  hygiene4  hygiene5  psupport1  psupport2  psupport3  psupport4  psupport5  political_ideology  happy  slf_ladder  riskperc1  riskperc2  self_esteem  sex1  tested_positive  optim1  optim2  slfcont1  slfcont2  slfcont3  slfcont4  urban
##   3   7  cnarc1  cnarc2  cnarc3  ctheory1  ctheory2  ctheory3  ctheory4  CRT1  CRT2  CRT3  generosity1  generosity2  generosity3  health_cond  know_tested_positive  ladder  marital1  moralid1  moralid10  moralid2  moralid3  moralid4  moralid5  moralid6  moralid7  moralid8  moralid9  mcoop1  mcoop2  mcoop3  mcoop4  mcoop5  mcoop6  mcoop7  narc1  narc2  narc3  narc4  narc5  narc6  nidentity1  nidentity2  omind1  omind2  omind3  omind4  omind5  contact4  contact5  hygiene1  hygiene2  hygiene3  hygiene4  hygiene5  psupport1  psupport2  psupport3  psupport4  psupport5  political_ideology  happy  slf_ladder  riskperc1  riskperc2  self_esteem  sex1  tested_positive  optim1  optim2  slfcont1  slfcont2  slfcont3  slfcont4  urban
##   3   8  cnarc1  cnarc2  cnarc3  ctheory1  ctheory2  ctheory3  ctheory4  CRT1  CRT2  CRT3  generosity1  generosity2  generosity3  health_cond  know_tested_positive  ladder  marital1  moralid1  moralid10  moralid2  moralid3  moralid4  moralid5  moralid6  moralid7  moralid8  moralid9  mcoop1  mcoop2  mcoop3  mcoop4  mcoop5  mcoop6  mcoop7  narc1  narc2  narc3  narc4  narc5  narc6  nidentity1  nidentity2  omind1  omind2  omind3  omind4  omind5  contact4  contact5  hygiene1  hygiene2  hygiene3  hygiene4  hygiene5  psupport1  psupport2  psupport3  psupport4  psupport5  political_ideology  happy  slf_ladder  riskperc1  riskperc2  self_esteem  sex1  tested_positive  optim1  optim2  slfcont1  slfcont2  slfcont3  slfcont4  urban
##   3   9  cnarc1  cnarc2  cnarc3  ctheory1  ctheory2  ctheory3  ctheory4  CRT1  CRT2  CRT3  generosity1  generosity2  generosity3  health_cond  know_tested_positive  ladder  marital1  moralid1  moralid10  moralid2  moralid3  moralid4  moralid5  moralid6  moralid7  moralid8  moralid9  mcoop1  mcoop2  mcoop3  mcoop4  mcoop5  mcoop6  mcoop7  narc1  narc2  narc3  narc4  narc5  narc6  nidentity1  nidentity2  omind1  omind2  omind3  omind4  omind5  contact4  contact5  hygiene1  hygiene2  hygiene3  hygiene4  hygiene5  psupport1  psupport2  psupport3  psupport4  psupport5  political_ideology  happy  slf_ladder  riskperc1  riskperc2  self_esteem  sex1  tested_positive  optim1  optim2  slfcont1  slfcont2  slfcont3  slfcont4  urban
##   3   10  cnarc1  cnarc2  cnarc3  ctheory1  ctheory2  ctheory3  ctheory4  CRT1  CRT2  CRT3  generosity1  generosity2  generosity3  health_cond  know_tested_positive  ladder  marital1  moralid1  moralid10  moralid2  moralid3  moralid4  moralid5  moralid6  moralid7  moralid8  moralid9  mcoop1  mcoop2  mcoop3  mcoop4  mcoop5  mcoop6  mcoop7  narc1  narc2  narc3  narc4  narc5  narc6  nidentity1  nidentity2  omind1  omind2  omind3  omind4  omind5  contact4  contact5  hygiene1  hygiene2  hygiene3  hygiene4  hygiene5  psupport1  psupport2  psupport3  psupport4  psupport5  political_ideology  happy  slf_ladder  riskperc1  riskperc2  self_esteem  sex1  tested_positive  optim1  optim2  slfcont1  slfcont2  slfcont3  slfcont4  urban
##   4   1  cnarc1  cnarc2  cnarc3  ctheory1  ctheory2  ctheory3  ctheory4  CRT1  CRT2  CRT3  generosity1  generosity2  generosity3  health_cond  know_tested_positive  ladder  marital1  moralid1  moralid10  moralid2  moralid3  moralid4  moralid5  moralid6  moralid7  moralid8  moralid9  mcoop1  mcoop2  mcoop3  mcoop4  mcoop5  mcoop6  mcoop7  narc1  narc2  narc3  narc4  narc5  narc6  nidentity1  nidentity2  omind1  omind2  omind3  omind4  omind5  contact4  contact5  hygiene1  hygiene2  hygiene3  hygiene4  hygiene5  psupport1  psupport2  psupport3  psupport4  psupport5  political_ideology  happy  slf_ladder  riskperc1  riskperc2  self_esteem  sex1  tested_positive  optim1  optim2  slfcont1  slfcont2  slfcont3  slfcont4  urban
##   4   2  cnarc1  cnarc2  cnarc3  ctheory1  ctheory2  ctheory3  ctheory4  CRT1  CRT2  CRT3  generosity1  generosity2  generosity3  health_cond  know_tested_positive  ladder  marital1  moralid1  moralid10  moralid2  moralid3  moralid4  moralid5  moralid6  moralid7  moralid8  moralid9  mcoop1  mcoop2  mcoop3  mcoop4  mcoop5  mcoop6  mcoop7  narc1  narc2  narc3  narc4  narc5  narc6  nidentity1  nidentity2  omind1  omind2  omind3  omind4  omind5  contact4  contact5  hygiene1  hygiene2  hygiene3  hygiene4  hygiene5  psupport1  psupport2  psupport3  psupport4  psupport5  political_ideology  happy  slf_ladder  riskperc1  riskperc2  self_esteem  sex1  tested_positive  optim1  optim2  slfcont1  slfcont2  slfcont3  slfcont4  urban
##   4   3  cnarc1  cnarc2  cnarc3  ctheory1  ctheory2  ctheory3  ctheory4  CRT1  CRT2  CRT3  generosity1  generosity2  generosity3  health_cond  know_tested_positive  ladder  marital1  moralid1  moralid10  moralid2  moralid3  moralid4  moralid5  moralid6  moralid7  moralid8  moralid9  mcoop1  mcoop2  mcoop3  mcoop4  mcoop5  mcoop6  mcoop7  narc1  narc2  narc3  narc4  narc5  narc6  nidentity1  nidentity2  omind1  omind2  omind3  omind4  omind5  contact4  contact5  hygiene1  hygiene2  hygiene3  hygiene4  hygiene5  psupport1  psupport2  psupport3  psupport4  psupport5  political_ideology  happy  slf_ladder  riskperc1  riskperc2  self_esteem  sex1  tested_positive  optim1  optim2  slfcont1  slfcont2  slfcont3  slfcont4  urban
##   4   4  cnarc1  cnarc2  cnarc3  ctheory1  ctheory2  ctheory3  ctheory4  CRT1  CRT2  CRT3  generosity1  generosity2  generosity3  health_cond  know_tested_positive  ladder  marital1  moralid1  moralid10  moralid2  moralid3  moralid4  moralid5  moralid6  moralid7  moralid8  moralid9  mcoop1  mcoop2  mcoop3  mcoop4  mcoop5  mcoop6  mcoop7  narc1  narc2  narc3  narc4  narc5  narc6  nidentity1  nidentity2  omind1  omind2  omind3  omind4  omind5  contact4  contact5  hygiene1  hygiene2  hygiene3  hygiene4  hygiene5  psupport1  psupport2  psupport3  psupport4  psupport5  political_ideology  happy  slf_ladder  riskperc1  riskperc2  self_esteem  sex1  tested_positive  optim1  optim2  slfcont1  slfcont2  slfcont3  slfcont4  urban
##   4   5  cnarc1  cnarc2  cnarc3  ctheory1  ctheory2  ctheory3  ctheory4  CRT1  CRT2  CRT3  generosity1  generosity2  generosity3  health_cond  know_tested_positive  ladder  marital1  moralid1  moralid10  moralid2  moralid3  moralid4  moralid5  moralid6  moralid7  moralid8  moralid9  mcoop1  mcoop2  mcoop3  mcoop4  mcoop5  mcoop6  mcoop7  narc1  narc2  narc3  narc4  narc5  narc6  nidentity1  nidentity2  omind1  omind2  omind3  omind4  omind5  contact4  contact5  hygiene1  hygiene2  hygiene3  hygiene4  hygiene5  psupport1  psupport2  psupport3  psupport4  psupport5  political_ideology  happy  slf_ladder  riskperc1  riskperc2  self_esteem  sex1  tested_positive  optim1  optim2  slfcont1  slfcont2  slfcont3  slfcont4  urban
##   4   6  cnarc1  cnarc2  cnarc3  ctheory1  ctheory2  ctheory3  ctheory4  CRT1  CRT2  CRT3  generosity1  generosity2  generosity3  health_cond  know_tested_positive  ladder  marital1  moralid1  moralid10  moralid2  moralid3  moralid4  moralid5  moralid6  moralid7  moralid8  moralid9  mcoop1  mcoop2  mcoop3  mcoop4  mcoop5  mcoop6  mcoop7  narc1  narc2  narc3  narc4  narc5  narc6  nidentity1  nidentity2  omind1  omind2  omind3  omind4  omind5  contact4  contact5  hygiene1  hygiene2  hygiene3  hygiene4  hygiene5  psupport1  psupport2  psupport3  psupport4  psupport5  political_ideology  happy  slf_ladder  riskperc1  riskperc2  self_esteem  sex1  tested_positive  optim1  optim2  slfcont1  slfcont2  slfcont3  slfcont4  urban
##   4   7  cnarc1  cnarc2  cnarc3  ctheory1  ctheory2  ctheory3  ctheory4  CRT1  CRT2  CRT3  generosity1  generosity2  generosity3  health_cond  know_tested_positive  ladder  marital1  moralid1  moralid10  moralid2  moralid3  moralid4  moralid5  moralid6  moralid7  moralid8  moralid9  mcoop1  mcoop2  mcoop3  mcoop4  mcoop5  mcoop6  mcoop7  narc1  narc2  narc3  narc4  narc5  narc6  nidentity1  nidentity2  omind1  omind2  omind3  omind4  omind5  contact4  contact5  hygiene1  hygiene2  hygiene3  hygiene4  hygiene5  psupport1  psupport2  psupport3  psupport4  psupport5  political_ideology  happy  slf_ladder  riskperc1  riskperc2  self_esteem  sex1  tested_positive  optim1  optim2  slfcont1  slfcont2  slfcont3  slfcont4  urban
##   4   8  cnarc1  cnarc2  cnarc3  ctheory1  ctheory2  ctheory3  ctheory4  CRT1  CRT2  CRT3  generosity1  generosity2  generosity3  health_cond  know_tested_positive  ladder  marital1  moralid1  moralid10  moralid2  moralid3  moralid4  moralid5  moralid6  moralid7  moralid8  moralid9  mcoop1  mcoop2  mcoop3  mcoop4  mcoop5  mcoop6  mcoop7  narc1  narc2  narc3  narc4  narc5  narc6  nidentity1  nidentity2  omind1  omind2  omind3  omind4  omind5  contact4  contact5  hygiene1  hygiene2  hygiene3  hygiene4  hygiene5  psupport1  psupport2  psupport3  psupport4  psupport5  political_ideology  happy  slf_ladder  riskperc1  riskperc2  self_esteem  sex1  tested_positive  optim1  optim2  slfcont1  slfcont2  slfcont3  slfcont4  urban
##   4   9  cnarc1  cnarc2  cnarc3  ctheory1  ctheory2  ctheory3  ctheory4  CRT1  CRT2  CRT3  generosity1  generosity2  generosity3  health_cond  know_tested_positive  ladder  marital1  moralid1  moralid10  moralid2  moralid3  moralid4  moralid5  moralid6  moralid7  moralid8  moralid9  mcoop1  mcoop2  mcoop3  mcoop4  mcoop5  mcoop6  mcoop7  narc1  narc2  narc3  narc4  narc5  narc6  nidentity1  nidentity2  omind1  omind2  omind3  omind4  omind5  contact4  contact5  hygiene1  hygiene2  hygiene3  hygiene4  hygiene5  psupport1  psupport2  psupport3  psupport4  psupport5  political_ideology  happy  slf_ladder  riskperc1  riskperc2  self_esteem  sex1  tested_positive  optim1  optim2  slfcont1  slfcont2  slfcont3  slfcont4  urban
##   4   10  cnarc1  cnarc2  cnarc3  ctheory1  ctheory2  ctheory3  ctheory4  CRT1  CRT2  CRT3  generosity1  generosity2  generosity3  health_cond  know_tested_positive  ladder  marital1  moralid1  moralid10  moralid2  moralid3  moralid4  moralid5  moralid6  moralid7  moralid8  moralid9  mcoop1  mcoop2  mcoop3  mcoop4  mcoop5  mcoop6  mcoop7  narc1  narc2  narc3  narc4  narc5  narc6  nidentity1  nidentity2  omind1  omind2  omind3  omind4  omind5  contact4  contact5  hygiene1  hygiene2  hygiene3  hygiene4  hygiene5  psupport1  psupport2  psupport3  psupport4  psupport5  political_ideology  happy  slf_ladder  riskperc1  riskperc2  self_esteem  sex1  tested_positive  optim1  optim2  slfcont1  slfcont2  slfcont3  slfcont4  urban
##   5   1  cnarc1  cnarc2  cnarc3  ctheory1  ctheory2  ctheory3  ctheory4  CRT1  CRT2  CRT3  generosity1  generosity2  generosity3  health_cond  know_tested_positive  ladder  marital1  moralid1  moralid10  moralid2  moralid3  moralid4  moralid5  moralid6  moralid7  moralid8  moralid9  mcoop1  mcoop2  mcoop3  mcoop4  mcoop5  mcoop6  mcoop7  narc1  narc2  narc3  narc4  narc5  narc6  nidentity1  nidentity2  omind1  omind2  omind3  omind4  omind5  contact4  contact5  hygiene1  hygiene2  hygiene3  hygiene4  hygiene5  psupport1  psupport2  psupport3  psupport4  psupport5  political_ideology  happy  slf_ladder  riskperc1  riskperc2  self_esteem  sex1  tested_positive  optim1  optim2  slfcont1  slfcont2  slfcont3  slfcont4  urban
##   5   2  cnarc1  cnarc2  cnarc3  ctheory1  ctheory2  ctheory3  ctheory4  CRT1  CRT2  CRT3  generosity1  generosity2  generosity3  health_cond  know_tested_positive  ladder  marital1  moralid1  moralid10  moralid2  moralid3  moralid4  moralid5  moralid6  moralid7  moralid8  moralid9  mcoop1  mcoop2  mcoop3  mcoop4  mcoop5  mcoop6  mcoop7  narc1  narc2  narc3  narc4  narc5  narc6  nidentity1  nidentity2  omind1  omind2  omind3  omind4  omind5  contact4  contact5  hygiene1  hygiene2  hygiene3  hygiene4  hygiene5  psupport1  psupport2  psupport3  psupport4  psupport5  political_ideology  happy  slf_ladder  riskperc1  riskperc2  self_esteem  sex1  tested_positive  optim1  optim2  slfcont1  slfcont2  slfcont3  slfcont4  urban
##   5   3  cnarc1  cnarc2  cnarc3  ctheory1  ctheory2  ctheory3  ctheory4  CRT1  CRT2  CRT3  generosity1  generosity2  generosity3  health_cond  know_tested_positive  ladder  marital1  moralid1  moralid10  moralid2  moralid3  moralid4  moralid5  moralid6  moralid7  moralid8  moralid9  mcoop1  mcoop2  mcoop3  mcoop4  mcoop5  mcoop6  mcoop7  narc1  narc2  narc3  narc4  narc5  narc6  nidentity1  nidentity2  omind1  omind2  omind3  omind4  omind5  contact4  contact5  hygiene1  hygiene2  hygiene3  hygiene4  hygiene5  psupport1  psupport2  psupport3  psupport4  psupport5  political_ideology  happy  slf_ladder  riskperc1  riskperc2  self_esteem  sex1  tested_positive  optim1  optim2  slfcont1  slfcont2  slfcont3  slfcont4  urban
##   5   4  cnarc1  cnarc2  cnarc3  ctheory1  ctheory2  ctheory3  ctheory4  CRT1  CRT2  CRT3  generosity1  generosity2  generosity3  health_cond  know_tested_positive  ladder  marital1  moralid1  moralid10  moralid2  moralid3  moralid4  moralid5  moralid6  moralid7  moralid8  moralid9  mcoop1  mcoop2  mcoop3  mcoop4  mcoop5  mcoop6  mcoop7  narc1  narc2  narc3  narc4  narc5  narc6  nidentity1  nidentity2  omind1  omind2  omind3  omind4  omind5  contact4  contact5  hygiene1  hygiene2  hygiene3  hygiene4  hygiene5  psupport1  psupport2  psupport3  psupport4  psupport5  political_ideology  happy  slf_ladder  riskperc1  riskperc2  self_esteem  sex1  tested_positive  optim1  optim2  slfcont1  slfcont2  slfcont3  slfcont4  urban
##   5   5  cnarc1  cnarc2  cnarc3  ctheory1  ctheory2  ctheory3  ctheory4  CRT1  CRT2  CRT3  generosity1  generosity2  generosity3  health_cond  know_tested_positive  ladder  marital1  moralid1  moralid10  moralid2  moralid3  moralid4  moralid5  moralid6  moralid7  moralid8  moralid9  mcoop1  mcoop2  mcoop3  mcoop4  mcoop5  mcoop6  mcoop7  narc1  narc2  narc3  narc4  narc5  narc6  nidentity1  nidentity2  omind1  omind2  omind3  omind4  omind5  contact4  contact5  hygiene1  hygiene2  hygiene3  hygiene4  hygiene5  psupport1  psupport2  psupport3  psupport4  psupport5  political_ideology  happy  slf_ladder  riskperc1  riskperc2  self_esteem  sex1  tested_positive  optim1  optim2  slfcont1  slfcont2  slfcont3  slfcont4  urban
##   5   6  cnarc1  cnarc2  cnarc3  ctheory1  ctheory2  ctheory3  ctheory4  CRT1  CRT2  CRT3  generosity1  generosity2  generosity3  health_cond  know_tested_positive  ladder  marital1  moralid1  moralid10  moralid2  moralid3  moralid4  moralid5  moralid6  moralid7  moralid8  moralid9  mcoop1  mcoop2  mcoop3  mcoop4  mcoop5  mcoop6  mcoop7  narc1  narc2  narc3  narc4  narc5  narc6  nidentity1  nidentity2  omind1  omind2  omind3  omind4  omind5  contact4  contact5  hygiene1  hygiene2  hygiene3  hygiene4  hygiene5  psupport1  psupport2  psupport3  psupport4  psupport5  political_ideology  happy  slf_ladder  riskperc1  riskperc2  self_esteem  sex1  tested_positive  optim1  optim2  slfcont1  slfcont2  slfcont3  slfcont4  urban
##   5   7  cnarc1  cnarc2  cnarc3  ctheory1  ctheory2  ctheory3  ctheory4  CRT1  CRT2  CRT3  generosity1  generosity2  generosity3  health_cond  know_tested_positive  ladder  marital1  moralid1  moralid10  moralid2  moralid3  moralid4  moralid5  moralid6  moralid7  moralid8  moralid9  mcoop1  mcoop2  mcoop3  mcoop4  mcoop5  mcoop6  mcoop7  narc1  narc2  narc3  narc4  narc5  narc6  nidentity1  nidentity2  omind1  omind2  omind3  omind4  omind5  contact4  contact5  hygiene1  hygiene2  hygiene3  hygiene4  hygiene5  psupport1  psupport2  psupport3  psupport4  psupport5  political_ideology  happy  slf_ladder  riskperc1  riskperc2  self_esteem  sex1  tested_positive  optim1  optim2  slfcont1  slfcont2  slfcont3  slfcont4  urban
##   5   8  cnarc1  cnarc2  cnarc3  ctheory1  ctheory2  ctheory3  ctheory4  CRT1  CRT2  CRT3  generosity1  generosity2  generosity3  health_cond  know_tested_positive  ladder  marital1  moralid1  moralid10  moralid2  moralid3  moralid4  moralid5  moralid6  moralid7  moralid8  moralid9  mcoop1  mcoop2  mcoop3  mcoop4  mcoop5  mcoop6  mcoop7  narc1  narc2  narc3  narc4  narc5  narc6  nidentity1  nidentity2  omind1  omind2  omind3  omind4  omind5  contact4  contact5  hygiene1  hygiene2  hygiene3  hygiene4  hygiene5  psupport1  psupport2  psupport3  psupport4  psupport5  political_ideology  happy  slf_ladder  riskperc1  riskperc2  self_esteem  sex1  tested_positive  optim1  optim2  slfcont1  slfcont2  slfcont3  slfcont4  urban
##   5   9  cnarc1  cnarc2  cnarc3  ctheory1  ctheory2  ctheory3  ctheory4  CRT1  CRT2  CRT3  generosity1  generosity2  generosity3  health_cond  know_tested_positive  ladder  marital1  moralid1  moralid10  moralid2  moralid3  moralid4  moralid5  moralid6  moralid7  moralid8  moralid9  mcoop1  mcoop2  mcoop3  mcoop4  mcoop5  mcoop6  mcoop7  narc1  narc2  narc3  narc4  narc5  narc6  nidentity1  nidentity2  omind1  omind2  omind3  omind4  omind5  contact4  contact5  hygiene1  hygiene2  hygiene3  hygiene4  hygiene5  psupport1  psupport2  psupport3  psupport4  psupport5  political_ideology  happy  slf_ladder  riskperc1  riskperc2  self_esteem  sex1  tested_positive  optim1  optim2  slfcont1  slfcont2  slfcont3  slfcont4  urban
##   5   10  cnarc1  cnarc2  cnarc3  ctheory1  ctheory2  ctheory3  ctheory4  CRT1  CRT2  CRT3  generosity1  generosity2  generosity3  health_cond  know_tested_positive  ladder  marital1  moralid1  moralid10  moralid2  moralid3  moralid4  moralid5  moralid6  moralid7  moralid8  moralid9  mcoop1  mcoop2  mcoop3  mcoop4  mcoop5  mcoop6  mcoop7  narc1  narc2  narc3  narc4  narc5  narc6  nidentity1  nidentity2  omind1  omind2  omind3  omind4  omind5  contact4  contact5  hygiene1  hygiene2  hygiene3  hygiene4  hygiene5  psupport1  psupport2  psupport3  psupport4  psupport5  political_ideology  happy  slf_ladder  riskperc1  riskperc2  self_esteem  sex1  tested_positive  optim1  optim2  slfcont1  slfcont2  slfcont3  slfcont4  urban
##   6   1  cnarc1  cnarc2  cnarc3  ctheory1  ctheory2  ctheory3  ctheory4  CRT1  CRT2  CRT3  generosity1  generosity2  generosity3  health_cond  know_tested_positive  ladder  marital1  moralid1  moralid10  moralid2  moralid3  moralid4  moralid5  moralid6  moralid7  moralid8  moralid9  mcoop1  mcoop2  mcoop3  mcoop4  mcoop5  mcoop6  mcoop7  narc1  narc2  narc3  narc4  narc5  narc6  nidentity1  nidentity2  omind1  omind2  omind3  omind4  omind5  contact4  contact5  hygiene1  hygiene2  hygiene3  hygiene4  hygiene5  psupport1  psupport2  psupport3  psupport4  psupport5  political_ideology  happy  slf_ladder  riskperc1  riskperc2  self_esteem  sex1  tested_positive  optim1  optim2  slfcont1  slfcont2  slfcont3  slfcont4  urban
##   6   2  cnarc1  cnarc2  cnarc3  ctheory1  ctheory2  ctheory3  ctheory4  CRT1  CRT2  CRT3  generosity1  generosity2  generosity3  health_cond  know_tested_positive  ladder  marital1  moralid1  moralid10  moralid2  moralid3  moralid4  moralid5  moralid6  moralid7  moralid8  moralid9  mcoop1  mcoop2  mcoop3  mcoop4  mcoop5  mcoop6  mcoop7  narc1  narc2  narc3  narc4  narc5  narc6  nidentity1  nidentity2  omind1  omind2  omind3  omind4  omind5  contact4  contact5  hygiene1  hygiene2  hygiene3  hygiene4  hygiene5  psupport1  psupport2  psupport3  psupport4  psupport5  political_ideology  happy  slf_ladder  riskperc1  riskperc2  self_esteem  sex1  tested_positive  optim1  optim2  slfcont1  slfcont2  slfcont3  slfcont4  urban
##   6   3  cnarc1  cnarc2  cnarc3  ctheory1  ctheory2  ctheory3  ctheory4  CRT1  CRT2  CRT3  generosity1  generosity2  generosity3  health_cond  know_tested_positive  ladder  marital1  moralid1  moralid10  moralid2  moralid3  moralid4  moralid5  moralid6  moralid7  moralid8  moralid9  mcoop1  mcoop2  mcoop3  mcoop4  mcoop5  mcoop6  mcoop7  narc1  narc2  narc3  narc4  narc5  narc6  nidentity1  nidentity2  omind1  omind2  omind3  omind4  omind5  contact4  contact5  hygiene1  hygiene2  hygiene3  hygiene4  hygiene5  psupport1  psupport2  psupport3  psupport4  psupport5  political_ideology  happy  slf_ladder  riskperc1  riskperc2  self_esteem  sex1  tested_positive  optim1  optim2  slfcont1  slfcont2  slfcont3  slfcont4  urban
##   6   4  cnarc1  cnarc2  cnarc3  ctheory1  ctheory2  ctheory3  ctheory4  CRT1  CRT2  CRT3  generosity1  generosity2  generosity3  health_cond  know_tested_positive  ladder  marital1  moralid1  moralid10  moralid2  moralid3  moralid4  moralid5  moralid6  moralid7  moralid8  moralid9  mcoop1  mcoop2  mcoop3  mcoop4  mcoop5  mcoop6  mcoop7  narc1  narc2  narc3  narc4  narc5  narc6  nidentity1  nidentity2  omind1  omind2  omind3  omind4  omind5  contact4  contact5  hygiene1  hygiene2  hygiene3  hygiene4  hygiene5  psupport1  psupport2  psupport3  psupport4  psupport5  political_ideology  happy  slf_ladder  riskperc1  riskperc2  self_esteem  sex1  tested_positive  optim1  optim2  slfcont1  slfcont2  slfcont3  slfcont4  urban
##   6   5  cnarc1  cnarc2  cnarc3  ctheory1  ctheory2  ctheory3  ctheory4  CRT1  CRT2  CRT3  generosity1  generosity2  generosity3  health_cond  know_tested_positive  ladder  marital1  moralid1  moralid10  moralid2  moralid3  moralid4  moralid5  moralid6  moralid7  moralid8  moralid9  mcoop1  mcoop2  mcoop3  mcoop4  mcoop5  mcoop6  mcoop7  narc1  narc2  narc3  narc4  narc5  narc6  nidentity1  nidentity2  omind1  omind2  omind3  omind4  omind5  contact4  contact5  hygiene1  hygiene2  hygiene3  hygiene4  hygiene5  psupport1  psupport2  psupport3  psupport4  psupport5  political_ideology  happy  slf_ladder  riskperc1  riskperc2  self_esteem  sex1  tested_positive  optim1  optim2  slfcont1  slfcont2  slfcont3  slfcont4  urban
##   6   6  cnarc1  cnarc2  cnarc3  ctheory1  ctheory2  ctheory3  ctheory4  CRT1  CRT2  CRT3  generosity1  generosity2  generosity3  health_cond  know_tested_positive  ladder  marital1  moralid1  moralid10  moralid2  moralid3  moralid4  moralid5  moralid6  moralid7  moralid8  moralid9  mcoop1  mcoop2  mcoop3  mcoop4  mcoop5  mcoop6  mcoop7  narc1  narc2  narc3  narc4  narc5  narc6  nidentity1  nidentity2  omind1  omind2  omind3  omind4  omind5  contact4  contact5  hygiene1  hygiene2  hygiene3  hygiene4  hygiene5  psupport1  psupport2  psupport3  psupport4  psupport5  political_ideology  happy  slf_ladder  riskperc1  riskperc2  self_esteem  sex1  tested_positive  optim1  optim2  slfcont1  slfcont2  slfcont3  slfcont4  urban
##   6   7  cnarc1  cnarc2  cnarc3  ctheory1  ctheory2  ctheory3  ctheory4  CRT1  CRT2  CRT3  generosity1  generosity2  generosity3  health_cond  know_tested_positive  ladder  marital1  moralid1  moralid10  moralid2  moralid3  moralid4  moralid5  moralid6  moralid7  moralid8  moralid9  mcoop1  mcoop2  mcoop3  mcoop4  mcoop5  mcoop6  mcoop7  narc1  narc2  narc3  narc4  narc5  narc6  nidentity1  nidentity2  omind1  omind2  omind3  omind4  omind5  contact4  contact5  hygiene1  hygiene2  hygiene3  hygiene4  hygiene5  psupport1  psupport2  psupport3  psupport4  psupport5  political_ideology  happy  slf_ladder  riskperc1  riskperc2  self_esteem  sex1  tested_positive  optim1  optim2  slfcont1  slfcont2  slfcont3  slfcont4  urban
##   6   8  cnarc1  cnarc2  cnarc3  ctheory1  ctheory2  ctheory3  ctheory4  CRT1  CRT2  CRT3  generosity1  generosity2  generosity3  health_cond  know_tested_positive  ladder  marital1  moralid1  moralid10  moralid2  moralid3  moralid4  moralid5  moralid6  moralid7  moralid8  moralid9  mcoop1  mcoop2  mcoop3  mcoop4  mcoop5  mcoop6  mcoop7  narc1  narc2  narc3  narc4  narc5  narc6  nidentity1  nidentity2  omind1  omind2  omind3  omind4  omind5  contact4  contact5  hygiene1  hygiene2  hygiene3  hygiene4  hygiene5  psupport1  psupport2  psupport3  psupport4  psupport5  political_ideology  happy  slf_ladder  riskperc1  riskperc2  self_esteem  sex1  tested_positive  optim1  optim2  slfcont1  slfcont2  slfcont3  slfcont4  urban
##   6   9  cnarc1  cnarc2  cnarc3  ctheory1  ctheory2  ctheory3  ctheory4  CRT1  CRT2  CRT3  generosity1  generosity2  generosity3  health_cond  know_tested_positive  ladder  marital1  moralid1  moralid10  moralid2  moralid3  moralid4  moralid5  moralid6  moralid7  moralid8  moralid9  mcoop1  mcoop2  mcoop3  mcoop4  mcoop5  mcoop6  mcoop7  narc1  narc2  narc3  narc4  narc5  narc6  nidentity1  nidentity2  omind1  omind2  omind3  omind4  omind5  contact4  contact5  hygiene1  hygiene2  hygiene3  hygiene4  hygiene5  psupport1  psupport2  psupport3  psupport4  psupport5  political_ideology  happy  slf_ladder  riskperc1  riskperc2  self_esteem  sex1  tested_positive  optim1  optim2  slfcont1  slfcont2  slfcont3  slfcont4  urban
##   6   10  cnarc1  cnarc2  cnarc3  ctheory1  ctheory2  ctheory3  ctheory4  CRT1  CRT2  CRT3  generosity1  generosity2  generosity3  health_cond  know_tested_positive  ladder  marital1  moralid1  moralid10  moralid2  moralid3  moralid4  moralid5  moralid6  moralid7  moralid8  moralid9  mcoop1  mcoop2  mcoop3  mcoop4  mcoop5  mcoop6  mcoop7  narc1  narc2  narc3  narc4  narc5  narc6  nidentity1  nidentity2  omind1  omind2  omind3  omind4  omind5  contact4  contact5  hygiene1  hygiene2  hygiene3  hygiene4  hygiene5  psupport1  psupport2  psupport3  psupport4  psupport5  political_ideology  happy  slf_ladder  riskperc1  riskperc2  self_esteem  sex1  tested_positive  optim1  optim2  slfcont1  slfcont2  slfcont3  slfcont4  urban
##   7   1  cnarc1  cnarc2  cnarc3  ctheory1  ctheory2  ctheory3  ctheory4  CRT1  CRT2  CRT3  generosity1  generosity2  generosity3  health_cond  know_tested_positive  ladder  marital1  moralid1  moralid10  moralid2  moralid3  moralid4  moralid5  moralid6  moralid7  moralid8  moralid9  mcoop1  mcoop2  mcoop3  mcoop4  mcoop5  mcoop6  mcoop7  narc1  narc2  narc3  narc4  narc5  narc6  nidentity1  nidentity2  omind1  omind2  omind3  omind4  omind5  contact4  contact5  hygiene1  hygiene2  hygiene3  hygiene4  hygiene5  psupport1  psupport2  psupport3  psupport4  psupport5  political_ideology  happy  slf_ladder  riskperc1  riskperc2  self_esteem  sex1  tested_positive  optim1  optim2  slfcont1  slfcont2  slfcont3  slfcont4  urban
##   7   2  cnarc1  cnarc2  cnarc3  ctheory1  ctheory2  ctheory3  ctheory4  CRT1  CRT2  CRT3  generosity1  generosity2  generosity3  health_cond  know_tested_positive  ladder  marital1  moralid1  moralid10  moralid2  moralid3  moralid4  moralid5  moralid6  moralid7  moralid8  moralid9  mcoop1  mcoop2  mcoop3  mcoop4  mcoop5  mcoop6  mcoop7  narc1  narc2  narc3  narc4  narc5  narc6  nidentity1  nidentity2  omind1  omind2  omind3  omind4  omind5  contact4  contact5  hygiene1  hygiene2  hygiene3  hygiene4  hygiene5  psupport1  psupport2  psupport3  psupport4  psupport5  political_ideology  happy  slf_ladder  riskperc1  riskperc2  self_esteem  sex1  tested_positive  optim1  optim2  slfcont1  slfcont2  slfcont3  slfcont4  urban
##   7   3  cnarc1  cnarc2  cnarc3  ctheory1  ctheory2  ctheory3  ctheory4  CRT1  CRT2  CRT3  generosity1  generosity2  generosity3  health_cond  know_tested_positive  ladder  marital1  moralid1  moralid10  moralid2  moralid3  moralid4  moralid5  moralid6  moralid7  moralid8  moralid9  mcoop1  mcoop2  mcoop3  mcoop4  mcoop5  mcoop6  mcoop7  narc1  narc2  narc3  narc4  narc5  narc6  nidentity1  nidentity2  omind1  omind2  omind3  omind4  omind5  contact4  contact5  hygiene1  hygiene2  hygiene3  hygiene4  hygiene5  psupport1  psupport2  psupport3  psupport4  psupport5  political_ideology  happy  slf_ladder  riskperc1  riskperc2  self_esteem  sex1  tested_positive  optim1  optim2  slfcont1  slfcont2  slfcont3  slfcont4  urban
##   7   4  cnarc1  cnarc2  cnarc3  ctheory1  ctheory2  ctheory3  ctheory4  CRT1  CRT2  CRT3  generosity1  generosity2  generosity3  health_cond  know_tested_positive  ladder  marital1  moralid1  moralid10  moralid2  moralid3  moralid4  moralid5  moralid6  moralid7  moralid8  moralid9  mcoop1  mcoop2  mcoop3  mcoop4  mcoop5  mcoop6  mcoop7  narc1  narc2  narc3  narc4  narc5  narc6  nidentity1  nidentity2  omind1  omind2  omind3  omind4  omind5  contact4  contact5  hygiene1  hygiene2  hygiene3  hygiene4  hygiene5  psupport1  psupport2  psupport3  psupport4  psupport5  political_ideology  happy  slf_ladder  riskperc1  riskperc2  self_esteem  sex1  tested_positive  optim1  optim2  slfcont1  slfcont2  slfcont3  slfcont4  urban
##   7   5  cnarc1  cnarc2  cnarc3  ctheory1  ctheory2  ctheory3  ctheory4  CRT1  CRT2  CRT3  generosity1  generosity2  generosity3  health_cond  know_tested_positive  ladder  marital1  moralid1  moralid10  moralid2  moralid3  moralid4  moralid5  moralid6  moralid7  moralid8  moralid9  mcoop1  mcoop2  mcoop3  mcoop4  mcoop5  mcoop6  mcoop7  narc1  narc2  narc3  narc4  narc5  narc6  nidentity1  nidentity2  omind1  omind2  omind3  omind4  omind5  contact4  contact5  hygiene1  hygiene2  hygiene3  hygiene4  hygiene5  psupport1  psupport2  psupport3  psupport4  psupport5  political_ideology  happy  slf_ladder  riskperc1  riskperc2  self_esteem  sex1  tested_positive  optim1  optim2  slfcont1  slfcont2  slfcont3  slfcont4  urban
##   7   6  cnarc1  cnarc2  cnarc3  ctheory1  ctheory2  ctheory3  ctheory4  CRT1  CRT2  CRT3  generosity1  generosity2  generosity3  health_cond  know_tested_positive  ladder  marital1  moralid1  moralid10  moralid2  moralid3  moralid4  moralid5  moralid6  moralid7  moralid8  moralid9  mcoop1  mcoop2  mcoop3  mcoop4  mcoop5  mcoop6  mcoop7  narc1  narc2  narc3  narc4  narc5  narc6  nidentity1  nidentity2  omind1  omind2  omind3  omind4  omind5  contact4  contact5  hygiene1  hygiene2  hygiene3  hygiene4  hygiene5  psupport1  psupport2  psupport3  psupport4  psupport5  political_ideology  happy  slf_ladder  riskperc1  riskperc2  self_esteem  sex1  tested_positive  optim1  optim2  slfcont1  slfcont2  slfcont3  slfcont4  urban
##   7   7  cnarc1  cnarc2  cnarc3  ctheory1  ctheory2  ctheory3  ctheory4  CRT1  CRT2  CRT3  generosity1  generosity2  generosity3  health_cond  know_tested_positive  ladder  marital1  moralid1  moralid10  moralid2  moralid3  moralid4  moralid5  moralid6  moralid7  moralid8  moralid9  mcoop1  mcoop2  mcoop3  mcoop4  mcoop5  mcoop6  mcoop7  narc1  narc2  narc3  narc4  narc5  narc6  nidentity1  nidentity2  omind1  omind2  omind3  omind4  omind5  contact4  contact5  hygiene1  hygiene2  hygiene3  hygiene4  hygiene5  psupport1  psupport2  psupport3  psupport4  psupport5  political_ideology  happy  slf_ladder  riskperc1  riskperc2  self_esteem  sex1  tested_positive  optim1  optim2  slfcont1  slfcont2  slfcont3  slfcont4  urban
##   7   8  cnarc1  cnarc2  cnarc3  ctheory1  ctheory2  ctheory3  ctheory4  CRT1  CRT2  CRT3  generosity1  generosity2  generosity3  health_cond  know_tested_positive  ladder  marital1  moralid1  moralid10  moralid2  moralid3  moralid4  moralid5  moralid6  moralid7  moralid8  moralid9  mcoop1  mcoop2  mcoop3  mcoop4  mcoop5  mcoop6  mcoop7  narc1  narc2  narc3  narc4  narc5  narc6  nidentity1  nidentity2  omind1  omind2  omind3  omind4  omind5  contact4  contact5  hygiene1  hygiene2  hygiene3  hygiene4  hygiene5  psupport1  psupport2  psupport3  psupport4  psupport5  political_ideology  happy  slf_ladder  riskperc1  riskperc2  self_esteem  sex1  tested_positive  optim1  optim2  slfcont1  slfcont2  slfcont3  slfcont4  urban
##   7   9  cnarc1  cnarc2  cnarc3  ctheory1  ctheory2  ctheory3  ctheory4  CRT1  CRT2  CRT3  generosity1  generosity2  generosity3  health_cond  know_tested_positive  ladder  marital1  moralid1  moralid10  moralid2  moralid3  moralid4  moralid5  moralid6  moralid7  moralid8  moralid9  mcoop1  mcoop2  mcoop3  mcoop4  mcoop5  mcoop6  mcoop7  narc1  narc2  narc3  narc4  narc5  narc6  nidentity1  nidentity2  omind1  omind2  omind3  omind4  omind5  contact4  contact5  hygiene1  hygiene2  hygiene3  hygiene4  hygiene5  psupport1  psupport2  psupport3  psupport4  psupport5  political_ideology  happy  slf_ladder  riskperc1  riskperc2  self_esteem  sex1  tested_positive  optim1  optim2  slfcont1  slfcont2  slfcont3  slfcont4  urban
##   7   10  cnarc1  cnarc2  cnarc3  ctheory1  ctheory2  ctheory3  ctheory4  CRT1  CRT2  CRT3  generosity1  generosity2  generosity3  health_cond  know_tested_positive  ladder  marital1  moralid1  moralid10  moralid2  moralid3  moralid4  moralid5  moralid6  moralid7  moralid8  moralid9  mcoop1  mcoop2  mcoop3  mcoop4  mcoop5  mcoop6  mcoop7  narc1  narc2  narc3  narc4  narc5  narc6  nidentity1  nidentity2  omind1  omind2  omind3  omind4  omind5  contact4  contact5  hygiene1  hygiene2  hygiene3  hygiene4  hygiene5  psupport1  psupport2  psupport3  psupport4  psupport5  political_ideology  happy  slf_ladder  riskperc1  riskperc2  self_esteem  sex1  tested_positive  optim1  optim2  slfcont1  slfcont2  slfcont3  slfcont4  urban
##   8   1  cnarc1  cnarc2  cnarc3  ctheory1  ctheory2  ctheory3  ctheory4  CRT1  CRT2  CRT3  generosity1  generosity2  generosity3  health_cond  know_tested_positive  ladder  marital1  moralid1  moralid10  moralid2  moralid3  moralid4  moralid5  moralid6  moralid7  moralid8  moralid9  mcoop1  mcoop2  mcoop3  mcoop4  mcoop5  mcoop6  mcoop7  narc1  narc2  narc3  narc4  narc5  narc6  nidentity1  nidentity2  omind1  omind2  omind3  omind4  omind5  contact4  contact5  hygiene1  hygiene2  hygiene3  hygiene4  hygiene5  psupport1  psupport2  psupport3  psupport4  psupport5  political_ideology  happy  slf_ladder  riskperc1  riskperc2  self_esteem  sex1  tested_positive  optim1  optim2  slfcont1  slfcont2  slfcont3  slfcont4  urban
##   8   2  cnarc1  cnarc2  cnarc3  ctheory1  ctheory2  ctheory3  ctheory4  CRT1  CRT2  CRT3  generosity1  generosity2  generosity3  health_cond  know_tested_positive  ladder  marital1  moralid1  moralid10  moralid2  moralid3  moralid4  moralid5  moralid6  moralid7  moralid8  moralid9  mcoop1  mcoop2  mcoop3  mcoop4  mcoop5  mcoop6  mcoop7  narc1  narc2  narc3  narc4  narc5  narc6  nidentity1  nidentity2  omind1  omind2  omind3  omind4  omind5  contact4  contact5  hygiene1  hygiene2  hygiene3  hygiene4  hygiene5  psupport1  psupport2  psupport3  psupport4  psupport5  political_ideology  happy  slf_ladder  riskperc1  riskperc2  self_esteem  sex1  tested_positive  optim1  optim2  slfcont1  slfcont2  slfcont3  slfcont4  urban
##   8   3  cnarc1  cnarc2  cnarc3  ctheory1  ctheory2  ctheory3  ctheory4  CRT1  CRT2  CRT3  generosity1  generosity2  generosity3  health_cond  know_tested_positive  ladder  marital1  moralid1  moralid10  moralid2  moralid3  moralid4  moralid5  moralid6  moralid7  moralid8  moralid9  mcoop1  mcoop2  mcoop3  mcoop4  mcoop5  mcoop6  mcoop7  narc1  narc2  narc3  narc4  narc5  narc6  nidentity1  nidentity2  omind1  omind2  omind3  omind4  omind5  contact4  contact5  hygiene1  hygiene2  hygiene3  hygiene4  hygiene5  psupport1  psupport2  psupport3  psupport4  psupport5  political_ideology  happy  slf_ladder  riskperc1  riskperc2  self_esteem  sex1  tested_positive  optim1  optim2  slfcont1  slfcont2  slfcont3  slfcont4  urban
##   8   4  cnarc1  cnarc2  cnarc3  ctheory1  ctheory2  ctheory3  ctheory4  CRT1  CRT2  CRT3  generosity1  generosity2  generosity3  health_cond  know_tested_positive  ladder  marital1  moralid1  moralid10  moralid2  moralid3  moralid4  moralid5  moralid6  moralid7  moralid8  moralid9  mcoop1  mcoop2  mcoop3  mcoop4  mcoop5  mcoop6  mcoop7  narc1  narc2  narc3  narc4  narc5  narc6  nidentity1  nidentity2  omind1  omind2  omind3  omind4  omind5  contact4  contact5  hygiene1  hygiene2  hygiene3  hygiene4  hygiene5  psupport1  psupport2  psupport3  psupport4  psupport5  political_ideology  happy  slf_ladder  riskperc1  riskperc2  self_esteem  sex1  tested_positive  optim1  optim2  slfcont1  slfcont2  slfcont3  slfcont4  urban
##   8   5  cnarc1  cnarc2  cnarc3  ctheory1  ctheory2  ctheory3  ctheory4  CRT1  CRT2  CRT3  generosity1  generosity2  generosity3  health_cond  know_tested_positive  ladder  marital1  moralid1  moralid10  moralid2  moralid3  moralid4  moralid5  moralid6  moralid7  moralid8  moralid9  mcoop1  mcoop2  mcoop3  mcoop4  mcoop5  mcoop6  mcoop7  narc1  narc2  narc3  narc4  narc5  narc6  nidentity1  nidentity2  omind1  omind2  omind3  omind4  omind5  contact4  contact5  hygiene1  hygiene2  hygiene3  hygiene4  hygiene5  psupport1  psupport2  psupport3  psupport4  psupport5  political_ideology  happy  slf_ladder  riskperc1  riskperc2  self_esteem  sex1  tested_positive  optim1  optim2  slfcont1  slfcont2  slfcont3  slfcont4  urban
##   8   6  cnarc1  cnarc2  cnarc3  ctheory1  ctheory2  ctheory3  ctheory4  CRT1  CRT2  CRT3  generosity1  generosity2  generosity3  health_cond  know_tested_positive  ladder  marital1  moralid1  moralid10  moralid2  moralid3  moralid4  moralid5  moralid6  moralid7  moralid8  moralid9  mcoop1  mcoop2  mcoop3  mcoop4  mcoop5  mcoop6  mcoop7  narc1  narc2  narc3  narc4  narc5  narc6  nidentity1  nidentity2  omind1  omind2  omind3  omind4  omind5  contact4  contact5  hygiene1  hygiene2  hygiene3  hygiene4  hygiene5  psupport1  psupport2  psupport3  psupport4  psupport5  political_ideology  happy  slf_ladder  riskperc1  riskperc2  self_esteem  sex1  tested_positive  optim1  optim2  slfcont1  slfcont2  slfcont3  slfcont4  urban
##   8   7  cnarc1  cnarc2  cnarc3  ctheory1  ctheory2  ctheory3  ctheory4  CRT1  CRT2  CRT3  generosity1  generosity2  generosity3  health_cond  know_tested_positive  ladder  marital1  moralid1  moralid10  moralid2  moralid3  moralid4  moralid5  moralid6  moralid7  moralid8  moralid9  mcoop1  mcoop2  mcoop3  mcoop4  mcoop5  mcoop6  mcoop7  narc1  narc2  narc3  narc4  narc5  narc6  nidentity1  nidentity2  omind1  omind2  omind3  omind4  omind5  contact4  contact5  hygiene1  hygiene2  hygiene3  hygiene4  hygiene5  psupport1  psupport2  psupport3  psupport4  psupport5  political_ideology  happy  slf_ladder  riskperc1  riskperc2  self_esteem  sex1  tested_positive  optim1  optim2  slfcont1  slfcont2  slfcont3  slfcont4  urban
##   8   8  cnarc1  cnarc2  cnarc3  ctheory1  ctheory2  ctheory3  ctheory4  CRT1  CRT2  CRT3  generosity1  generosity2  generosity3  health_cond  know_tested_positive  ladder  marital1  moralid1  moralid10  moralid2  moralid3  moralid4  moralid5  moralid6  moralid7  moralid8  moralid9  mcoop1  mcoop2  mcoop3  mcoop4  mcoop5  mcoop6  mcoop7  narc1  narc2  narc3  narc4  narc5  narc6  nidentity1  nidentity2  omind1  omind2  omind3  omind4  omind5  contact4  contact5  hygiene1  hygiene2  hygiene3  hygiene4  hygiene5  psupport1  psupport2  psupport3  psupport4  psupport5  political_ideology  happy  slf_ladder  riskperc1  riskperc2  self_esteem  sex1  tested_positive  optim1  optim2  slfcont1  slfcont2  slfcont3  slfcont4  urban
##   8   9  cnarc1  cnarc2  cnarc3  ctheory1  ctheory2  ctheory3  ctheory4  CRT1  CRT2  CRT3  generosity1  generosity2  generosity3  health_cond  know_tested_positive  ladder  marital1  moralid1  moralid10  moralid2  moralid3  moralid4  moralid5  moralid6  moralid7  moralid8  moralid9  mcoop1  mcoop2  mcoop3  mcoop4  mcoop5  mcoop6  mcoop7  narc1  narc2  narc3  narc4  narc5  narc6  nidentity1  nidentity2  omind1  omind2  omind3  omind4  omind5  contact4  contact5  hygiene1  hygiene2  hygiene3  hygiene4  hygiene5  psupport1  psupport2  psupport3  psupport4  psupport5  political_ideology  happy  slf_ladder  riskperc1  riskperc2  self_esteem  sex1  tested_positive  optim1  optim2  slfcont1  slfcont2  slfcont3  slfcont4  urban
##   8   10  cnarc1  cnarc2  cnarc3  ctheory1  ctheory2  ctheory3  ctheory4  CRT1  CRT2  CRT3  generosity1  generosity2  generosity3  health_cond  know_tested_positive  ladder  marital1  moralid1  moralid10  moralid2  moralid3  moralid4  moralid5  moralid6  moralid7  moralid8  moralid9  mcoop1  mcoop2  mcoop3  mcoop4  mcoop5  mcoop6  mcoop7  narc1  narc2  narc3  narc4  narc5  narc6  nidentity1  nidentity2  omind1  omind2  omind3  omind4  omind5  contact4  contact5  hygiene1  hygiene2  hygiene3  hygiene4  hygiene5  psupport1  psupport2  psupport3  psupport4  psupport5  political_ideology  happy  slf_ladder  riskperc1  riskperc2  self_esteem  sex1  tested_positive  optim1  optim2  slfcont1  slfcont2  slfcont3  slfcont4  urban
##   9   1  cnarc1  cnarc2  cnarc3  ctheory1  ctheory2  ctheory3  ctheory4  CRT1  CRT2  CRT3  generosity1  generosity2  generosity3  health_cond  know_tested_positive  ladder  marital1  moralid1  moralid10  moralid2  moralid3  moralid4  moralid5  moralid6  moralid7  moralid8  moralid9  mcoop1  mcoop2  mcoop3  mcoop4  mcoop5  mcoop6  mcoop7  narc1  narc2  narc3  narc4  narc5  narc6  nidentity1  nidentity2  omind1  omind2  omind3  omind4  omind5  contact4  contact5  hygiene1  hygiene2  hygiene3  hygiene4  hygiene5  psupport1  psupport2  psupport3  psupport4  psupport5  political_ideology  happy  slf_ladder  riskperc1  riskperc2  self_esteem  sex1  tested_positive  optim1  optim2  slfcont1  slfcont2  slfcont3  slfcont4  urban
##   9   2  cnarc1  cnarc2  cnarc3  ctheory1  ctheory2  ctheory3  ctheory4  CRT1  CRT2  CRT3  generosity1  generosity2  generosity3  health_cond  know_tested_positive  ladder  marital1  moralid1  moralid10  moralid2  moralid3  moralid4  moralid5  moralid6  moralid7  moralid8  moralid9  mcoop1  mcoop2  mcoop3  mcoop4  mcoop5  mcoop6  mcoop7  narc1  narc2  narc3  narc4  narc5  narc6  nidentity1  nidentity2  omind1  omind2  omind3  omind4  omind5  contact4  contact5  hygiene1  hygiene2  hygiene3  hygiene4  hygiene5  psupport1  psupport2  psupport3  psupport4  psupport5  political_ideology  happy  slf_ladder  riskperc1  riskperc2  self_esteem  sex1  tested_positive  optim1  optim2  slfcont1  slfcont2  slfcont3  slfcont4  urban
##   9   3  cnarc1  cnarc2  cnarc3  ctheory1  ctheory2  ctheory3  ctheory4  CRT1  CRT2  CRT3  generosity1  generosity2  generosity3  health_cond  know_tested_positive  ladder  marital1  moralid1  moralid10  moralid2  moralid3  moralid4  moralid5  moralid6  moralid7  moralid8  moralid9  mcoop1  mcoop2  mcoop3  mcoop4  mcoop5  mcoop6  mcoop7  narc1  narc2  narc3  narc4  narc5  narc6  nidentity1  nidentity2  omind1  omind2  omind3  omind4  omind5  contact4  contact5  hygiene1  hygiene2  hygiene3  hygiene4  hygiene5  psupport1  psupport2  psupport3  psupport4  psupport5  political_ideology  happy  slf_ladder  riskperc1  riskperc2  self_esteem  sex1  tested_positive  optim1  optim2  slfcont1  slfcont2  slfcont3  slfcont4  urban
##   9   4  cnarc1  cnarc2  cnarc3  ctheory1  ctheory2  ctheory3  ctheory4  CRT1  CRT2  CRT3  generosity1  generosity2  generosity3  health_cond  know_tested_positive  ladder  marital1  moralid1  moralid10  moralid2  moralid3  moralid4  moralid5  moralid6  moralid7  moralid8  moralid9  mcoop1  mcoop2  mcoop3  mcoop4  mcoop5  mcoop6  mcoop7  narc1  narc2  narc3  narc4  narc5  narc6  nidentity1  nidentity2  omind1  omind2  omind3  omind4  omind5  contact4  contact5  hygiene1  hygiene2  hygiene3  hygiene4  hygiene5  psupport1  psupport2  psupport3  psupport4  psupport5  political_ideology  happy  slf_ladder  riskperc1  riskperc2  self_esteem  sex1  tested_positive  optim1  optim2  slfcont1  slfcont2  slfcont3  slfcont4  urban
##   9   5  cnarc1  cnarc2  cnarc3  ctheory1  ctheory2  ctheory3  ctheory4  CRT1  CRT2  CRT3  generosity1  generosity2  generosity3  health_cond  know_tested_positive  ladder  marital1  moralid1  moralid10  moralid2  moralid3  moralid4  moralid5  moralid6  moralid7  moralid8  moralid9  mcoop1  mcoop2  mcoop3  mcoop4  mcoop5  mcoop6  mcoop7  narc1  narc2  narc3  narc4  narc5  narc6  nidentity1  nidentity2  omind1  omind2  omind3  omind4  omind5  contact4  contact5  hygiene1  hygiene2  hygiene3  hygiene4  hygiene5  psupport1  psupport2  psupport3  psupport4  psupport5  political_ideology  happy  slf_ladder  riskperc1  riskperc2  self_esteem  sex1  tested_positive  optim1  optim2  slfcont1  slfcont2  slfcont3  slfcont4  urban
##   9   6  cnarc1  cnarc2  cnarc3  ctheory1  ctheory2  ctheory3  ctheory4  CRT1  CRT2  CRT3  generosity1  generosity2  generosity3  health_cond  know_tested_positive  ladder  marital1  moralid1  moralid10  moralid2  moralid3  moralid4  moralid5  moralid6  moralid7  moralid8  moralid9  mcoop1  mcoop2  mcoop3  mcoop4  mcoop5  mcoop6  mcoop7  narc1  narc2  narc3  narc4  narc5  narc6  nidentity1  nidentity2  omind1  omind2  omind3  omind4  omind5  contact4  contact5  hygiene1  hygiene2  hygiene3  hygiene4  hygiene5  psupport1  psupport2  psupport3  psupport4  psupport5  political_ideology  happy  slf_ladder  riskperc1  riskperc2  self_esteem  sex1  tested_positive  optim1  optim2  slfcont1  slfcont2  slfcont3  slfcont4  urban
##   9   7  cnarc1  cnarc2  cnarc3  ctheory1  ctheory2  ctheory3  ctheory4  CRT1  CRT2  CRT3  generosity1  generosity2  generosity3  health_cond  know_tested_positive  ladder  marital1  moralid1  moralid10  moralid2  moralid3  moralid4  moralid5  moralid6  moralid7  moralid8  moralid9  mcoop1  mcoop2  mcoop3  mcoop4  mcoop5  mcoop6  mcoop7  narc1  narc2  narc3  narc4  narc5  narc6  nidentity1  nidentity2  omind1  omind2  omind3  omind4  omind5  contact4  contact5  hygiene1  hygiene2  hygiene3  hygiene4  hygiene5  psupport1  psupport2  psupport3  psupport4  psupport5  political_ideology  happy  slf_ladder  riskperc1  riskperc2  self_esteem  sex1  tested_positive  optim1  optim2  slfcont1  slfcont2  slfcont3  slfcont4  urban
##   9   8  cnarc1  cnarc2  cnarc3  ctheory1  ctheory2  ctheory3  ctheory4  CRT1  CRT2  CRT3  generosity1  generosity2  generosity3  health_cond  know_tested_positive  ladder  marital1  moralid1  moralid10  moralid2  moralid3  moralid4  moralid5  moralid6  moralid7  moralid8  moralid9  mcoop1  mcoop2  mcoop3  mcoop4  mcoop5  mcoop6  mcoop7  narc1  narc2  narc3  narc4  narc5  narc6  nidentity1  nidentity2  omind1  omind2  omind3  omind4  omind5  contact4  contact5  hygiene1  hygiene2  hygiene3  hygiene4  hygiene5  psupport1  psupport2  psupport3  psupport4  psupport5  political_ideology  happy  slf_ladder  riskperc1  riskperc2  self_esteem  sex1  tested_positive  optim1  optim2  slfcont1  slfcont2  slfcont3  slfcont4  urban
##   9   9  cnarc1  cnarc2  cnarc3  ctheory1  ctheory2  ctheory3  ctheory4  CRT1  CRT2  CRT3  generosity1  generosity2  generosity3  health_cond  know_tested_positive  ladder  marital1  moralid1  moralid10  moralid2  moralid3  moralid4  moralid5  moralid6  moralid7  moralid8  moralid9  mcoop1  mcoop2  mcoop3  mcoop4  mcoop5  mcoop6  mcoop7  narc1  narc2  narc3  narc4  narc5  narc6  nidentity1  nidentity2  omind1  omind2  omind3  omind4  omind5  contact4  contact5  hygiene1  hygiene2  hygiene3  hygiene4  hygiene5  psupport1  psupport2  psupport3  psupport4  psupport5  political_ideology  happy  slf_ladder  riskperc1  riskperc2  self_esteem  sex1  tested_positive  optim1  optim2  slfcont1  slfcont2  slfcont3  slfcont4  urban
##   9   10  cnarc1  cnarc2  cnarc3  ctheory1  ctheory2  ctheory3  ctheory4  CRT1  CRT2  CRT3  generosity1  generosity2  generosity3  health_cond  know_tested_positive  ladder  marital1  moralid1  moralid10  moralid2  moralid3  moralid4  moralid5  moralid6  moralid7  moralid8  moralid9  mcoop1  mcoop2  mcoop3  mcoop4  mcoop5  mcoop6  mcoop7  narc1  narc2  narc3  narc4  narc5  narc6  nidentity1  nidentity2  omind1  omind2  omind3  omind4  omind5  contact4  contact5  hygiene1  hygiene2  hygiene3  hygiene4  hygiene5  psupport1  psupport2  psupport3  psupport4  psupport5  political_ideology  happy  slf_ladder  riskperc1  riskperc2  self_esteem  sex1  tested_positive  optim1  optim2  slfcont1  slfcont2  slfcont3  slfcont4  urban
##   10   1  cnarc1  cnarc2  cnarc3  ctheory1  ctheory2  ctheory3  ctheory4  CRT1  CRT2  CRT3  generosity1  generosity2  generosity3  health_cond  know_tested_positive  ladder  marital1  moralid1  moralid10  moralid2  moralid3  moralid4  moralid5  moralid6  moralid7  moralid8  moralid9  mcoop1  mcoop2  mcoop3  mcoop4  mcoop5  mcoop6  mcoop7  narc1  narc2  narc3  narc4  narc5  narc6  nidentity1  nidentity2  omind1  omind2  omind3  omind4  omind5  contact4  contact5  hygiene1  hygiene2  hygiene3  hygiene4  hygiene5  psupport1  psupport2  psupport3  psupport4  psupport5  political_ideology  happy  slf_ladder  riskperc1  riskperc2  self_esteem  sex1  tested_positive  optim1  optim2  slfcont1  slfcont2  slfcont3  slfcont4  urban
##   10   2  cnarc1  cnarc2  cnarc3  ctheory1  ctheory2  ctheory3  ctheory4  CRT1  CRT2  CRT3  generosity1  generosity2  generosity3  health_cond  know_tested_positive  ladder  marital1  moralid1  moralid10  moralid2  moralid3  moralid4  moralid5  moralid6  moralid7  moralid8  moralid9  mcoop1  mcoop2  mcoop3  mcoop4  mcoop5  mcoop6  mcoop7  narc1  narc2  narc3  narc4  narc5  narc6  nidentity1  nidentity2  omind1  omind2  omind3  omind4  omind5  contact4  contact5  hygiene1  hygiene2  hygiene3  hygiene4  hygiene5  psupport1  psupport2  psupport3  psupport4  psupport5  political_ideology  happy  slf_ladder  riskperc1  riskperc2  self_esteem  sex1  tested_positive  optim1  optim2  slfcont1  slfcont2  slfcont3  slfcont4  urban
##   10   3  cnarc1  cnarc2  cnarc3  ctheory1  ctheory2  ctheory3  ctheory4  CRT1  CRT2  CRT3  generosity1  generosity2  generosity3  health_cond  know_tested_positive  ladder  marital1  moralid1  moralid10  moralid2  moralid3  moralid4  moralid5  moralid6  moralid7  moralid8  moralid9  mcoop1  mcoop2  mcoop3  mcoop4  mcoop5  mcoop6  mcoop7  narc1  narc2  narc3  narc4  narc5  narc6  nidentity1  nidentity2  omind1  omind2  omind3  omind4  omind5  contact4  contact5  hygiene1  hygiene2  hygiene3  hygiene4  hygiene5  psupport1  psupport2  psupport3  psupport4  psupport5  political_ideology  happy  slf_ladder  riskperc1  riskperc2  self_esteem  sex1  tested_positive  optim1  optim2  slfcont1  slfcont2  slfcont3  slfcont4  urban
##   10   4  cnarc1  cnarc2  cnarc3  ctheory1  ctheory2  ctheory3  ctheory4  CRT1  CRT2  CRT3  generosity1  generosity2  generosity3  health_cond  know_tested_positive  ladder  marital1  moralid1  moralid10  moralid2  moralid3  moralid4  moralid5  moralid6  moralid7  moralid8  moralid9  mcoop1  mcoop2  mcoop3  mcoop4  mcoop5  mcoop6  mcoop7  narc1  narc2  narc3  narc4  narc5  narc6  nidentity1  nidentity2  omind1  omind2  omind3  omind4  omind5  contact4  contact5  hygiene1  hygiene2  hygiene3  hygiene4  hygiene5  psupport1  psupport2  psupport3  psupport4  psupport5  political_ideology  happy  slf_ladder  riskperc1  riskperc2  self_esteem  sex1  tested_positive  optim1  optim2  slfcont1  slfcont2  slfcont3  slfcont4  urban
##   10   5  cnarc1  cnarc2  cnarc3  ctheory1  ctheory2  ctheory3  ctheory4  CRT1  CRT2  CRT3  generosity1  generosity2  generosity3  health_cond  know_tested_positive  ladder  marital1  moralid1  moralid10  moralid2  moralid3  moralid4  moralid5  moralid6  moralid7  moralid8  moralid9  mcoop1  mcoop2  mcoop3  mcoop4  mcoop5  mcoop6  mcoop7  narc1  narc2  narc3  narc4  narc5  narc6  nidentity1  nidentity2  omind1  omind2  omind3  omind4  omind5  contact4  contact5  hygiene1  hygiene2  hygiene3  hygiene4  hygiene5  psupport1  psupport2  psupport3  psupport4  psupport5  political_ideology  happy  slf_ladder  riskperc1  riskperc2  self_esteem  sex1  tested_positive  optim1  optim2  slfcont1  slfcont2  slfcont3  slfcont4  urban
##   10   6  cnarc1  cnarc2  cnarc3  ctheory1  ctheory2  ctheory3  ctheory4  CRT1  CRT2  CRT3  generosity1  generosity2  generosity3  health_cond  know_tested_positive  ladder  marital1  moralid1  moralid10  moralid2  moralid3  moralid4  moralid5  moralid6  moralid7  moralid8  moralid9  mcoop1  mcoop2  mcoop3  mcoop4  mcoop5  mcoop6  mcoop7  narc1  narc2  narc3  narc4  narc5  narc6  nidentity1  nidentity2  omind1  omind2  omind3  omind4  omind5  contact4  contact5  hygiene1  hygiene2  hygiene3  hygiene4  hygiene5  psupport1  psupport2  psupport3  psupport4  psupport5  political_ideology  happy  slf_ladder  riskperc1  riskperc2  self_esteem  sex1  tested_positive  optim1  optim2  slfcont1  slfcont2  slfcont3  slfcont4  urban
##   10   7  cnarc1  cnarc2  cnarc3  ctheory1  ctheory2  ctheory3  ctheory4  CRT1  CRT2  CRT3  generosity1  generosity2  generosity3  health_cond  know_tested_positive  ladder  marital1  moralid1  moralid10  moralid2  moralid3  moralid4  moralid5  moralid6  moralid7  moralid8  moralid9  mcoop1  mcoop2  mcoop3  mcoop4  mcoop5  mcoop6  mcoop7  narc1  narc2  narc3  narc4  narc5  narc6  nidentity1  nidentity2  omind1  omind2  omind3  omind4  omind5  contact4  contact5  hygiene1  hygiene2  hygiene3  hygiene4  hygiene5  psupport1  psupport2  psupport3  psupport4  psupport5  political_ideology  happy  slf_ladder  riskperc1  riskperc2  self_esteem  sex1  tested_positive  optim1  optim2  slfcont1  slfcont2  slfcont3  slfcont4  urban
##   10   8  cnarc1  cnarc2  cnarc3  ctheory1  ctheory2  ctheory3  ctheory4  CRT1  CRT2  CRT3  generosity1  generosity2  generosity3  health_cond  know_tested_positive  ladder  marital1  moralid1  moralid10  moralid2  moralid3  moralid4  moralid5  moralid6  moralid7  moralid8  moralid9  mcoop1  mcoop2  mcoop3  mcoop4  mcoop5  mcoop6  mcoop7  narc1  narc2  narc3  narc4  narc5  narc6  nidentity1  nidentity2  omind1  omind2  omind3  omind4  omind5  contact4  contact5  hygiene1  hygiene2  hygiene3  hygiene4  hygiene5  psupport1  psupport2  psupport3  psupport4  psupport5  political_ideology  happy  slf_ladder  riskperc1  riskperc2  self_esteem  sex1  tested_positive  optim1  optim2  slfcont1  slfcont2  slfcont3  slfcont4  urban
##   10   9  cnarc1  cnarc2  cnarc3  ctheory1  ctheory2  ctheory3  ctheory4  CRT1  CRT2  CRT3  generosity1  generosity2  generosity3  health_cond  know_tested_positive  ladder  marital1  moralid1  moralid10  moralid2  moralid3  moralid4  moralid5  moralid6  moralid7  moralid8  moralid9  mcoop1  mcoop2  mcoop3  mcoop4  mcoop5  mcoop6  mcoop7  narc1  narc2  narc3  narc4  narc5  narc6  nidentity1  nidentity2  omind1  omind2  omind3  omind4  omind5  contact4  contact5  hygiene1  hygiene2  hygiene3  hygiene4  hygiene5  psupport1  psupport2  psupport3  psupport4  psupport5  political_ideology  happy  slf_ladder  riskperc1  riskperc2  self_esteem  sex1  tested_positive  optim1  optim2  slfcont1  slfcont2  slfcont3  slfcont4  urban
##   10   10  cnarc1  cnarc2  cnarc3  ctheory1  ctheory2  ctheory3  ctheory4  CRT1  CRT2  CRT3  generosity1  generosity2  generosity3  health_cond  know_tested_positive  ladder  marital1  moralid1  moralid10  moralid2  moralid3  moralid4  moralid5  moralid6  moralid7  moralid8  moralid9  mcoop1  mcoop2  mcoop3  mcoop4  mcoop5  mcoop6  mcoop7  narc1  narc2  narc3  narc4  narc5  narc6  nidentity1  nidentity2  omind1  omind2  omind3  omind4  omind5  contact4  contact5  hygiene1  hygiene2  hygiene3  hygiene4  hygiene5  psupport1  psupport2  psupport3  psupport4  psupport5  political_ideology  happy  slf_ladder  riskperc1  riskperc2  self_esteem  sex1  tested_positive  optim1  optim2  slfcont1  slfcont2  slfcont3  slfcont4  urban
```

```
## Warning: Number of logged events: 7082
```

```
## 
##  iter imp variable
##   1   1
##   1   2
##   1   3
##   1   4
##   1   5
##   1   6
##   1   7
##   1   8
##   1   9
##   1   10
##   2   1
##   2   2
##   2   3
##   2   4
##   2   5
##   2   6
##   2   7
##   2   8
##   2   9
##   2   10
##   3   1
##   3   2
##   3   3
##   3   4
##   3   5
##   3   6
##   3   7
##   3   8
##   3   9
##   3   10
##   4   1
##   4   2
##   4   3
##   4   4
##   4   5
##   4   6
##   4   7
##   4   8
##   4   9
##   4   10
##   5   1
##   5   2
##   5   3
##   5   4
##   5   5
##   5   6
##   5   7
##   5   8
##   5   9
##   5   10
##   6   1
##   6   2
##   6   3
##   6   4
##   6   5
##   6   6
##   6   7
##   6   8
##   6   9
##   6   10
##   7   1
##   7   2
##   7   3
##   7   4
##   7   5
##   7   6
##   7   7
##   7   8
##   7   9
##   7   10
##   8   1
##   8   2
##   8   3
##   8   4
##   8   5
##   8   6
##   8   7
##   8   8
##   8   9
##   8   10
##   9   1
##   9   2
##   9   3
##   9   4
##   9   5
##   9   6
##   9   7
##   9   8
##   9   9
##   9   10
##   10   1
##   10   2
##   10   3
##   10   4
##   10   5
##   10   6
##   10   7
##   10   8
##   10   9
##   10   10
```

```
## Warning: Number of logged events: 4
```

```
## 
##  iter imp variable
##   1   1  marital1
##   1   2  marital1
##   1   3  marital1
##   1   4  marital1
##   1   5  marital1
##   1   6  marital1
##   1   7  marital1
##   1   8  marital1
##   1   9  marital1
##   1   10  marital1
##   2   1  marital1
##   2   2  marital1
##   2   3  marital1
##   2   4  marital1
##   2   5  marital1
##   2   6  marital1
##   2   7  marital1
##   2   8  marital1
##   2   9  marital1
##   2   10  marital1
##   3   1  marital1
##   3   2  marital1
##   3   3  marital1
##   3   4  marital1
##   3   5  marital1
##   3   6  marital1
##   3   7  marital1
##   3   8  marital1
##   3   9  marital1
##   3   10  marital1
##   4   1  marital1
##   4   2  marital1
##   4   3  marital1
##   4   4  marital1
##   4   5  marital1
##   4   6  marital1
##   4   7  marital1
##   4   8  marital1
##   4   9  marital1
##   4   10  marital1
##   5   1  marital1
##   5   2  marital1
##   5   3  marital1
##   5   4  marital1
##   5   5  marital1
##   5   6  marital1
##   5   7  marital1
##   5   8  marital1
##   5   9  marital1
##   5   10  marital1
##   6   1  marital1
##   6   2  marital1
##   6   3  marital1
##   6   4  marital1
##   6   5  marital1
##   6   6  marital1
##   6   7  marital1
##   6   8  marital1
##   6   9  marital1
##   6   10  marital1
##   7   1  marital1
##   7   2  marital1
##   7   3  marital1
##   7   4  marital1
##   7   5  marital1
##   7   6  marital1
##   7   7  marital1
##   7   8  marital1
##   7   9  marital1
##   7   10  marital1
##   8   1  marital1
##   8   2  marital1
##   8   3  marital1
##   8   4  marital1
##   8   5  marital1
##   8   6  marital1
##   8   7  marital1
##   8   8  marital1
##   8   9  marital1
##   8   10  marital1
##   9   1  marital1
##   9   2  marital1
##   9   3  marital1
##   9   4  marital1
##   9   5  marital1
##   9   6  marital1
##   9   7  marital1
##   9   8  marital1
##   9   9  marital1
##   9   10  marital1
##   10   1  marital1
##   10   2  marital1
##   10   3  marital1
##   10   4  marital1
##   10   5  marital1
##   10   6  marital1
##   10   7  marital1
##   10   8  marital1
##   10   9  marital1
##   10   10  marital1
```

```
## Warning: Number of logged events: 104
```

```
## 
##  iter imp variable
##   1   1  age  children  cnarc1  cnarc2  cnarc3  ctheory1  ctheory2  ctheory3  ctheory4  CRT1  CRT2  CRT3  employ_status1  health_cond  know_tested_positive  ladder  marital1  mor_circle  moralid4  moralid6  mcoop1  mcoop2  mcoop3  mcoop4  mcoop5  mcoop6  mcoop7  narc1  narc3  narc4  narc5  narc6  nidentity1  nidentity2  omind2  omind4  omind5  omind6  contact1  contact2  hygiene1  hygiene2  hygiene3  hygiene4  hygiene5  psupport2  psupport4  political_ideology  happy  slf_ladder  riskperc1  riskperc2  self_esteem  sex1  sbelong1  sbelong2  sbelong3  sbelong4  tested_positive  optim1  optim2  slfcont2
##   1   2  age  children  cnarc1  cnarc2  cnarc3  ctheory1  ctheory2  ctheory3  ctheory4  CRT1  CRT2  CRT3  employ_status1  health_cond  know_tested_positive  ladder  marital1  mor_circle  moralid4  moralid6  mcoop1  mcoop2  mcoop3  mcoop4  mcoop5  mcoop6  mcoop7  narc1  narc3  narc4  narc5  narc6  nidentity1  nidentity2  omind2  omind4  omind5  omind6  contact1  contact2  hygiene1  hygiene2  hygiene3  hygiene4  hygiene5  psupport2  psupport4  political_ideology  happy  slf_ladder  riskperc1  riskperc2  self_esteem  sex1  sbelong1  sbelong2  sbelong3  sbelong4  tested_positive  optim1  optim2  slfcont2
##   1   3  age  children  cnarc1  cnarc2  cnarc3  ctheory1  ctheory2  ctheory3  ctheory4  CRT1  CRT2  CRT3  employ_status1  health_cond  know_tested_positive  ladder  marital1  mor_circle  moralid4  moralid6  mcoop1  mcoop2  mcoop3  mcoop4  mcoop5  mcoop6  mcoop7  narc1  narc3  narc4  narc5  narc6  nidentity1  nidentity2  omind2  omind4  omind5  omind6  contact1  contact2  hygiene1  hygiene2  hygiene3  hygiene4  hygiene5  psupport2  psupport4  political_ideology  happy  slf_ladder  riskperc1  riskperc2  self_esteem  sex1  sbelong1  sbelong2  sbelong3  sbelong4  tested_positive  optim1  optim2  slfcont2
##   1   4  age  children  cnarc1  cnarc2  cnarc3  ctheory1  ctheory2  ctheory3  ctheory4  CRT1  CRT2  CRT3  employ_status1  health_cond  know_tested_positive  ladder  marital1  mor_circle  moralid4  moralid6  mcoop1  mcoop2  mcoop3  mcoop4  mcoop5  mcoop6  mcoop7  narc1  narc3  narc4  narc5  narc6  nidentity1  nidentity2  omind2  omind4  omind5  omind6  contact1  contact2  hygiene1  hygiene2  hygiene3  hygiene4  hygiene5  psupport2  psupport4  political_ideology  happy  slf_ladder  riskperc1  riskperc2  self_esteem  sex1  sbelong1  sbelong2  sbelong3  sbelong4  tested_positive  optim1  optim2  slfcont2
##   1   5  age  children  cnarc1  cnarc2  cnarc3  ctheory1  ctheory2  ctheory3  ctheory4  CRT1  CRT2  CRT3  employ_status1  health_cond  know_tested_positive  ladder  marital1  mor_circle  moralid4  moralid6  mcoop1  mcoop2  mcoop3  mcoop4  mcoop5  mcoop6  mcoop7  narc1  narc3  narc4  narc5  narc6  nidentity1  nidentity2  omind2  omind4  omind5  omind6  contact1  contact2  hygiene1  hygiene2  hygiene3  hygiene4  hygiene5  psupport2  psupport4  political_ideology  happy  slf_ladder  riskperc1  riskperc2  self_esteem  sex1  sbelong1  sbelong2  sbelong3  sbelong4  tested_positive  optim1  optim2  slfcont2
##   1   6  age  children  cnarc1  cnarc2  cnarc3  ctheory1  ctheory2  ctheory3  ctheory4  CRT1  CRT2  CRT3  employ_status1  health_cond  know_tested_positive  ladder  marital1  mor_circle  moralid4  moralid6  mcoop1  mcoop2  mcoop3  mcoop4  mcoop5  mcoop6  mcoop7  narc1  narc3  narc4  narc5  narc6  nidentity1  nidentity2  omind2  omind4  omind5  omind6  contact1  contact2  hygiene1  hygiene2  hygiene3  hygiene4  hygiene5  psupport2  psupport4  political_ideology  happy  slf_ladder  riskperc1  riskperc2  self_esteem  sex1  sbelong1  sbelong2  sbelong3  sbelong4  tested_positive  optim1  optim2  slfcont2
##   1   7  age  children  cnarc1  cnarc2  cnarc3  ctheory1  ctheory2  ctheory3  ctheory4  CRT1  CRT2  CRT3  employ_status1  health_cond  know_tested_positive  ladder  marital1  mor_circle  moralid4  moralid6  mcoop1  mcoop2  mcoop3  mcoop4  mcoop5  mcoop6  mcoop7  narc1  narc3  narc4  narc5  narc6  nidentity1  nidentity2  omind2  omind4  omind5  omind6  contact1  contact2  hygiene1  hygiene2  hygiene3  hygiene4  hygiene5  psupport2  psupport4  political_ideology  happy  slf_ladder  riskperc1  riskperc2  self_esteem  sex1  sbelong1  sbelong2  sbelong3  sbelong4  tested_positive  optim1  optim2  slfcont2
##   1   8  age  children  cnarc1  cnarc2  cnarc3  ctheory1  ctheory2  ctheory3  ctheory4  CRT1  CRT2  CRT3  employ_status1  health_cond  know_tested_positive  ladder  marital1  mor_circle  moralid4  moralid6  mcoop1  mcoop2  mcoop3  mcoop4  mcoop5  mcoop6  mcoop7  narc1  narc3  narc4  narc5  narc6  nidentity1  nidentity2  omind2  omind4  omind5  omind6  contact1  contact2  hygiene1  hygiene2  hygiene3  hygiene4  hygiene5  psupport2  psupport4  political_ideology  happy  slf_ladder  riskperc1  riskperc2  self_esteem  sex1  sbelong1  sbelong2  sbelong3  sbelong4  tested_positive  optim1  optim2  slfcont2
##   1   9  age  children  cnarc1  cnarc2  cnarc3  ctheory1  ctheory2  ctheory3  ctheory4  CRT1  CRT2  CRT3  employ_status1  health_cond  know_tested_positive  ladder  marital1  mor_circle  moralid4  moralid6  mcoop1  mcoop2  mcoop3  mcoop4  mcoop5  mcoop6  mcoop7  narc1  narc3  narc4  narc5  narc6  nidentity1  nidentity2  omind2  omind4  omind5  omind6  contact1  contact2  hygiene1  hygiene2  hygiene3  hygiene4  hygiene5  psupport2  psupport4  political_ideology  happy  slf_ladder  riskperc1  riskperc2  self_esteem  sex1  sbelong1  sbelong2  sbelong3  sbelong4  tested_positive  optim1  optim2  slfcont2
##   1   10  age  children  cnarc1  cnarc2  cnarc3  ctheory1  ctheory2  ctheory3  ctheory4  CRT1  CRT2  CRT3  employ_status1  health_cond  know_tested_positive  ladder  marital1  mor_circle  moralid4  moralid6  mcoop1  mcoop2  mcoop3  mcoop4  mcoop5  mcoop6  mcoop7  narc1  narc3  narc4  narc5  narc6  nidentity1  nidentity2  omind2  omind4  omind5  omind6  contact1  contact2  hygiene1  hygiene2  hygiene3  hygiene4  hygiene5  psupport2  psupport4  political_ideology  happy  slf_ladder  riskperc1  riskperc2  self_esteem  sex1  sbelong1  sbelong2  sbelong3  sbelong4  tested_positive  optim1  optim2  slfcont2
##   2   1  age  children  cnarc1  cnarc2  cnarc3  ctheory1  ctheory2  ctheory3  ctheory4  CRT1  CRT2  CRT3  employ_status1  health_cond  know_tested_positive  ladder  marital1  mor_circle  moralid4  moralid6  mcoop1  mcoop2  mcoop3  mcoop4  mcoop5  mcoop6  mcoop7  narc1  narc3  narc4  narc5  narc6  nidentity1  nidentity2  omind2  omind4  omind5  omind6  contact1  contact2  hygiene1  hygiene2  hygiene3  hygiene4  hygiene5  psupport2  psupport4  political_ideology  happy  slf_ladder  riskperc1  riskperc2  self_esteem  sex1  sbelong1  sbelong2  sbelong3  sbelong4  tested_positive  optim1  optim2  slfcont2
##   2   2  age  children  cnarc1  cnarc2  cnarc3  ctheory1  ctheory2  ctheory3  ctheory4  CRT1  CRT2  CRT3  employ_status1  health_cond  know_tested_positive  ladder  marital1  mor_circle  moralid4  moralid6  mcoop1  mcoop2  mcoop3  mcoop4  mcoop5  mcoop6  mcoop7  narc1  narc3  narc4  narc5  narc6  nidentity1  nidentity2  omind2  omind4  omind5  omind6  contact1  contact2  hygiene1  hygiene2  hygiene3  hygiene4  hygiene5  psupport2  psupport4  political_ideology  happy  slf_ladder  riskperc1  riskperc2  self_esteem  sex1  sbelong1  sbelong2  sbelong3  sbelong4  tested_positive  optim1  optim2  slfcont2
##   2   3  age  children  cnarc1  cnarc2  cnarc3  ctheory1  ctheory2  ctheory3  ctheory4  CRT1  CRT2  CRT3  employ_status1  health_cond  know_tested_positive  ladder  marital1  mor_circle  moralid4  moralid6  mcoop1  mcoop2  mcoop3  mcoop4  mcoop5  mcoop6  mcoop7  narc1  narc3  narc4  narc5  narc6  nidentity1  nidentity2  omind2  omind4  omind5  omind6  contact1  contact2  hygiene1  hygiene2  hygiene3  hygiene4  hygiene5  psupport2  psupport4  political_ideology  happy  slf_ladder  riskperc1  riskperc2  self_esteem  sex1  sbelong1  sbelong2  sbelong3  sbelong4  tested_positive  optim1  optim2  slfcont2
##   2   4  age  children  cnarc1  cnarc2  cnarc3  ctheory1  ctheory2  ctheory3  ctheory4  CRT1  CRT2  CRT3  employ_status1  health_cond  know_tested_positive  ladder  marital1  mor_circle  moralid4  moralid6  mcoop1  mcoop2  mcoop3  mcoop4  mcoop5  mcoop6  mcoop7  narc1  narc3  narc4  narc5  narc6  nidentity1  nidentity2  omind2  omind4  omind5  omind6  contact1  contact2  hygiene1  hygiene2  hygiene3  hygiene4  hygiene5  psupport2  psupport4  political_ideology  happy  slf_ladder  riskperc1  riskperc2  self_esteem  sex1  sbelong1  sbelong2  sbelong3  sbelong4  tested_positive  optim1  optim2  slfcont2
##   2   5  age  children  cnarc1  cnarc2  cnarc3  ctheory1  ctheory2  ctheory3  ctheory4  CRT1  CRT2  CRT3  employ_status1  health_cond  know_tested_positive  ladder  marital1  mor_circle  moralid4  moralid6  mcoop1  mcoop2  mcoop3  mcoop4  mcoop5  mcoop6  mcoop7  narc1  narc3  narc4  narc5  narc6  nidentity1  nidentity2  omind2  omind4  omind5  omind6  contact1  contact2  hygiene1  hygiene2  hygiene3  hygiene4  hygiene5  psupport2  psupport4  political_ideology  happy  slf_ladder  riskperc1  riskperc2  self_esteem  sex1  sbelong1  sbelong2  sbelong3  sbelong4  tested_positive  optim1  optim2  slfcont2
##   2   6  age  children  cnarc1  cnarc2  cnarc3  ctheory1  ctheory2  ctheory3  ctheory4  CRT1  CRT2  CRT3  employ_status1  health_cond  know_tested_positive  ladder  marital1  mor_circle  moralid4  moralid6  mcoop1  mcoop2  mcoop3  mcoop4  mcoop5  mcoop6  mcoop7  narc1  narc3  narc4  narc5  narc6  nidentity1  nidentity2  omind2  omind4  omind5  omind6  contact1  contact2  hygiene1  hygiene2  hygiene3  hygiene4  hygiene5  psupport2  psupport4  political_ideology  happy  slf_ladder  riskperc1  riskperc2  self_esteem  sex1  sbelong1  sbelong2  sbelong3  sbelong4  tested_positive  optim1  optim2  slfcont2
##   2   7  age  children  cnarc1  cnarc2  cnarc3  ctheory1  ctheory2  ctheory3  ctheory4  CRT1  CRT2  CRT3  employ_status1  health_cond  know_tested_positive  ladder  marital1  mor_circle  moralid4  moralid6  mcoop1  mcoop2  mcoop3  mcoop4  mcoop5  mcoop6  mcoop7  narc1  narc3  narc4  narc5  narc6  nidentity1  nidentity2  omind2  omind4  omind5  omind6  contact1  contact2  hygiene1  hygiene2  hygiene3  hygiene4  hygiene5  psupport2  psupport4  political_ideology  happy  slf_ladder  riskperc1  riskperc2  self_esteem  sex1  sbelong1  sbelong2  sbelong3  sbelong4  tested_positive  optim1  optim2  slfcont2
##   2   8  age  children  cnarc1  cnarc2  cnarc3  ctheory1  ctheory2  ctheory3  ctheory4  CRT1  CRT2  CRT3  employ_status1  health_cond  know_tested_positive  ladder  marital1  mor_circle  moralid4  moralid6  mcoop1  mcoop2  mcoop3  mcoop4  mcoop5  mcoop6  mcoop7  narc1  narc3  narc4  narc5  narc6  nidentity1  nidentity2  omind2  omind4  omind5  omind6  contact1  contact2  hygiene1  hygiene2  hygiene3  hygiene4  hygiene5  psupport2  psupport4  political_ideology  happy  slf_ladder  riskperc1  riskperc2  self_esteem  sex1  sbelong1  sbelong2  sbelong3  sbelong4  tested_positive  optim1  optim2  slfcont2
##   2   9  age  children  cnarc1  cnarc2  cnarc3  ctheory1  ctheory2  ctheory3  ctheory4  CRT1  CRT2  CRT3  employ_status1  health_cond  know_tested_positive  ladder  marital1  mor_circle  moralid4  moralid6  mcoop1  mcoop2  mcoop3  mcoop4  mcoop5  mcoop6  mcoop7  narc1  narc3  narc4  narc5  narc6  nidentity1  nidentity2  omind2  omind4  omind5  omind6  contact1  contact2  hygiene1  hygiene2  hygiene3  hygiene4  hygiene5  psupport2  psupport4  political_ideology  happy  slf_ladder  riskperc1  riskperc2  self_esteem  sex1  sbelong1  sbelong2  sbelong3  sbelong4  tested_positive  optim1  optim2  slfcont2
##   2   10  age  children  cnarc1  cnarc2  cnarc3  ctheory1  ctheory2  ctheory3  ctheory4  CRT1  CRT2  CRT3  employ_status1  health_cond  know_tested_positive  ladder  marital1  mor_circle  moralid4  moralid6  mcoop1  mcoop2  mcoop3  mcoop4  mcoop5  mcoop6  mcoop7  narc1  narc3  narc4  narc5  narc6  nidentity1  nidentity2  omind2  omind4  omind5  omind6  contact1  contact2  hygiene1  hygiene2  hygiene3  hygiene4  hygiene5  psupport2  psupport4  political_ideology  happy  slf_ladder  riskperc1  riskperc2  self_esteem  sex1  sbelong1  sbelong2  sbelong3  sbelong4  tested_positive  optim1  optim2  slfcont2
##   3   1  age  children  cnarc1  cnarc2  cnarc3  ctheory1  ctheory2  ctheory3  ctheory4  CRT1  CRT2  CRT3  employ_status1  health_cond  know_tested_positive  ladder  marital1  mor_circle  moralid4  moralid6  mcoop1  mcoop2  mcoop3  mcoop4  mcoop5  mcoop6  mcoop7  narc1  narc3  narc4  narc5  narc6  nidentity1  nidentity2  omind2  omind4  omind5  omind6  contact1  contact2  hygiene1  hygiene2  hygiene3  hygiene4  hygiene5  psupport2  psupport4  political_ideology  happy  slf_ladder  riskperc1  riskperc2  self_esteem  sex1  sbelong1  sbelong2  sbelong3  sbelong4  tested_positive  optim1  optim2  slfcont2
##   3   2  age  children  cnarc1  cnarc2  cnarc3  ctheory1  ctheory2  ctheory3  ctheory4  CRT1  CRT2  CRT3  employ_status1  health_cond  know_tested_positive  ladder  marital1  mor_circle  moralid4  moralid6  mcoop1  mcoop2  mcoop3  mcoop4  mcoop5  mcoop6  mcoop7  narc1  narc3  narc4  narc5  narc6  nidentity1  nidentity2  omind2  omind4  omind5  omind6  contact1  contact2  hygiene1  hygiene2  hygiene3  hygiene4  hygiene5  psupport2  psupport4  political_ideology  happy  slf_ladder  riskperc1  riskperc2  self_esteem  sex1  sbelong1  sbelong2  sbelong3  sbelong4  tested_positive  optim1  optim2  slfcont2
##   3   3  age  children  cnarc1  cnarc2  cnarc3  ctheory1  ctheory2  ctheory3  ctheory4  CRT1  CRT2  CRT3  employ_status1  health_cond  know_tested_positive  ladder  marital1  mor_circle  moralid4  moralid6  mcoop1  mcoop2  mcoop3  mcoop4  mcoop5  mcoop6  mcoop7  narc1  narc3  narc4  narc5  narc6  nidentity1  nidentity2  omind2  omind4  omind5  omind6  contact1  contact2  hygiene1  hygiene2  hygiene3  hygiene4  hygiene5  psupport2  psupport4  political_ideology  happy  slf_ladder  riskperc1  riskperc2  self_esteem  sex1  sbelong1  sbelong2  sbelong3  sbelong4  tested_positive  optim1  optim2  slfcont2
##   3   4  age  children  cnarc1  cnarc2  cnarc3  ctheory1  ctheory2  ctheory3  ctheory4  CRT1  CRT2  CRT3  employ_status1  health_cond  know_tested_positive  ladder  marital1  mor_circle  moralid4  moralid6  mcoop1  mcoop2  mcoop3  mcoop4  mcoop5  mcoop6  mcoop7  narc1  narc3  narc4  narc5  narc6  nidentity1  nidentity2  omind2  omind4  omind5  omind6  contact1  contact2  hygiene1  hygiene2  hygiene3  hygiene4  hygiene5  psupport2  psupport4  political_ideology  happy  slf_ladder  riskperc1  riskperc2  self_esteem  sex1  sbelong1  sbelong2  sbelong3  sbelong4  tested_positive  optim1  optim2  slfcont2
##   3   5  age  children  cnarc1  cnarc2  cnarc3  ctheory1  ctheory2  ctheory3  ctheory4  CRT1  CRT2  CRT3  employ_status1  health_cond  know_tested_positive  ladder  marital1  mor_circle  moralid4  moralid6  mcoop1  mcoop2  mcoop3  mcoop4  mcoop5  mcoop6  mcoop7  narc1  narc3  narc4  narc5  narc6  nidentity1  nidentity2  omind2  omind4  omind5  omind6  contact1  contact2  hygiene1  hygiene2  hygiene3  hygiene4  hygiene5  psupport2  psupport4  political_ideology  happy  slf_ladder  riskperc1  riskperc2  self_esteem  sex1  sbelong1  sbelong2  sbelong3  sbelong4  tested_positive  optim1  optim2  slfcont2
##   3   6  age  children  cnarc1  cnarc2  cnarc3  ctheory1  ctheory2  ctheory3  ctheory4  CRT1  CRT2  CRT3  employ_status1  health_cond  know_tested_positive  ladder  marital1  mor_circle  moralid4  moralid6  mcoop1  mcoop2  mcoop3  mcoop4  mcoop5  mcoop6  mcoop7  narc1  narc3  narc4  narc5  narc6  nidentity1  nidentity2  omind2  omind4  omind5  omind6  contact1  contact2  hygiene1  hygiene2  hygiene3  hygiene4  hygiene5  psupport2  psupport4  political_ideology  happy  slf_ladder  riskperc1  riskperc2  self_esteem  sex1  sbelong1  sbelong2  sbelong3  sbelong4  tested_positive  optim1  optim2  slfcont2
##   3   7  age  children  cnarc1  cnarc2  cnarc3  ctheory1  ctheory2  ctheory3  ctheory4  CRT1  CRT2  CRT3  employ_status1  health_cond  know_tested_positive  ladder  marital1  mor_circle  moralid4  moralid6  mcoop1  mcoop2  mcoop3  mcoop4  mcoop5  mcoop6  mcoop7  narc1  narc3  narc4  narc5  narc6  nidentity1  nidentity2  omind2  omind4  omind5  omind6  contact1  contact2  hygiene1  hygiene2  hygiene3  hygiene4  hygiene5  psupport2  psupport4  political_ideology  happy  slf_ladder  riskperc1  riskperc2  self_esteem  sex1  sbelong1  sbelong2  sbelong3  sbelong4  tested_positive  optim1  optim2  slfcont2
##   3   8  age  children  cnarc1  cnarc2  cnarc3  ctheory1  ctheory2  ctheory3  ctheory4  CRT1  CRT2  CRT3  employ_status1  health_cond  know_tested_positive  ladder  marital1  mor_circle  moralid4  moralid6  mcoop1  mcoop2  mcoop3  mcoop4  mcoop5  mcoop6  mcoop7  narc1  narc3  narc4  narc5  narc6  nidentity1  nidentity2  omind2  omind4  omind5  omind6  contact1  contact2  hygiene1  hygiene2  hygiene3  hygiene4  hygiene5  psupport2  psupport4  political_ideology  happy  slf_ladder  riskperc1  riskperc2  self_esteem  sex1  sbelong1  sbelong2  sbelong3  sbelong4  tested_positive  optim1  optim2  slfcont2
##   3   9  age  children  cnarc1  cnarc2  cnarc3  ctheory1  ctheory2  ctheory3  ctheory4  CRT1  CRT2  CRT3  employ_status1  health_cond  know_tested_positive  ladder  marital1  mor_circle  moralid4  moralid6  mcoop1  mcoop2  mcoop3  mcoop4  mcoop5  mcoop6  mcoop7  narc1  narc3  narc4  narc5  narc6  nidentity1  nidentity2  omind2  omind4  omind5  omind6  contact1  contact2  hygiene1  hygiene2  hygiene3  hygiene4  hygiene5  psupport2  psupport4  political_ideology  happy  slf_ladder  riskperc1  riskperc2  self_esteem  sex1  sbelong1  sbelong2  sbelong3  sbelong4  tested_positive  optim1  optim2  slfcont2
##   3   10  age  children  cnarc1  cnarc2  cnarc3  ctheory1  ctheory2  ctheory3  ctheory4  CRT1  CRT2  CRT3  employ_status1  health_cond  know_tested_positive  ladder  marital1  mor_circle  moralid4  moralid6  mcoop1  mcoop2  mcoop3  mcoop4  mcoop5  mcoop6  mcoop7  narc1  narc3  narc4  narc5  narc6  nidentity1  nidentity2  omind2  omind4  omind5  omind6  contact1  contact2  hygiene1  hygiene2  hygiene3  hygiene4  hygiene5  psupport2  psupport4  political_ideology  happy  slf_ladder  riskperc1  riskperc2  self_esteem  sex1  sbelong1  sbelong2  sbelong3  sbelong4  tested_positive  optim1  optim2  slfcont2
##   4   1  age  children  cnarc1  cnarc2  cnarc3  ctheory1  ctheory2  ctheory3  ctheory4  CRT1  CRT2  CRT3  employ_status1  health_cond  know_tested_positive  ladder  marital1  mor_circle  moralid4  moralid6  mcoop1  mcoop2  mcoop3  mcoop4  mcoop5  mcoop6  mcoop7  narc1  narc3  narc4  narc5  narc6  nidentity1  nidentity2  omind2  omind4  omind5  omind6  contact1  contact2  hygiene1  hygiene2  hygiene3  hygiene4  hygiene5  psupport2  psupport4  political_ideology  happy  slf_ladder  riskperc1  riskperc2  self_esteem  sex1  sbelong1  sbelong2  sbelong3  sbelong4  tested_positive  optim1  optim2  slfcont2
##   4   2  age  children  cnarc1  cnarc2  cnarc3  ctheory1  ctheory2  ctheory3  ctheory4  CRT1  CRT2  CRT3  employ_status1  health_cond  know_tested_positive  ladder  marital1  mor_circle  moralid4  moralid6  mcoop1  mcoop2  mcoop3  mcoop4  mcoop5  mcoop6  mcoop7  narc1  narc3  narc4  narc5  narc6  nidentity1  nidentity2  omind2  omind4  omind5  omind6  contact1  contact2  hygiene1  hygiene2  hygiene3  hygiene4  hygiene5  psupport2  psupport4  political_ideology  happy  slf_ladder  riskperc1  riskperc2  self_esteem  sex1  sbelong1  sbelong2  sbelong3  sbelong4  tested_positive  optim1  optim2  slfcont2
##   4   3  age  children  cnarc1  cnarc2  cnarc3  ctheory1  ctheory2  ctheory3  ctheory4  CRT1  CRT2  CRT3  employ_status1  health_cond  know_tested_positive  ladder  marital1  mor_circle  moralid4  moralid6  mcoop1  mcoop2  mcoop3  mcoop4  mcoop5  mcoop6  mcoop7  narc1  narc3  narc4  narc5  narc6  nidentity1  nidentity2  omind2  omind4  omind5  omind6  contact1  contact2  hygiene1  hygiene2  hygiene3  hygiene4  hygiene5  psupport2  psupport4  political_ideology  happy  slf_ladder  riskperc1  riskperc2  self_esteem  sex1  sbelong1  sbelong2  sbelong3  sbelong4  tested_positive  optim1  optim2  slfcont2
##   4   4  age  children  cnarc1  cnarc2  cnarc3  ctheory1  ctheory2  ctheory3  ctheory4  CRT1  CRT2  CRT3  employ_status1  health_cond  know_tested_positive  ladder  marital1  mor_circle  moralid4  moralid6  mcoop1  mcoop2  mcoop3  mcoop4  mcoop5  mcoop6  mcoop7  narc1  narc3  narc4  narc5  narc6  nidentity1  nidentity2  omind2  omind4  omind5  omind6  contact1  contact2  hygiene1  hygiene2  hygiene3  hygiene4  hygiene5  psupport2  psupport4  political_ideology  happy  slf_ladder  riskperc1  riskperc2  self_esteem  sex1  sbelong1  sbelong2  sbelong3  sbelong4  tested_positive  optim1  optim2  slfcont2
##   4   5  age  children  cnarc1  cnarc2  cnarc3  ctheory1  ctheory2  ctheory3  ctheory4  CRT1  CRT2  CRT3  employ_status1  health_cond  know_tested_positive  ladder  marital1  mor_circle  moralid4  moralid6  mcoop1  mcoop2  mcoop3  mcoop4  mcoop5  mcoop6  mcoop7  narc1  narc3  narc4  narc5  narc6  nidentity1  nidentity2  omind2  omind4  omind5  omind6  contact1  contact2  hygiene1  hygiene2  hygiene3  hygiene4  hygiene5  psupport2  psupport4  political_ideology  happy  slf_ladder  riskperc1  riskperc2  self_esteem  sex1  sbelong1  sbelong2  sbelong3  sbelong4  tested_positive  optim1  optim2  slfcont2
##   4   6  age  children  cnarc1  cnarc2  cnarc3  ctheory1  ctheory2  ctheory3  ctheory4  CRT1  CRT2  CRT3  employ_status1  health_cond  know_tested_positive  ladder  marital1  mor_circle  moralid4  moralid6  mcoop1  mcoop2  mcoop3  mcoop4  mcoop5  mcoop6  mcoop7  narc1  narc3  narc4  narc5  narc6  nidentity1  nidentity2  omind2  omind4  omind5  omind6  contact1  contact2  hygiene1  hygiene2  hygiene3  hygiene4  hygiene5  psupport2  psupport4  political_ideology  happy  slf_ladder  riskperc1  riskperc2  self_esteem  sex1  sbelong1  sbelong2  sbelong3  sbelong4  tested_positive  optim1  optim2  slfcont2
##   4   7  age  children  cnarc1  cnarc2  cnarc3  ctheory1  ctheory2  ctheory3  ctheory4  CRT1  CRT2  CRT3  employ_status1  health_cond  know_tested_positive  ladder  marital1  mor_circle  moralid4  moralid6  mcoop1  mcoop2  mcoop3  mcoop4  mcoop5  mcoop6  mcoop7  narc1  narc3  narc4  narc5  narc6  nidentity1  nidentity2  omind2  omind4  omind5  omind6  contact1  contact2  hygiene1  hygiene2  hygiene3  hygiene4  hygiene5  psupport2  psupport4  political_ideology  happy  slf_ladder  riskperc1  riskperc2  self_esteem  sex1  sbelong1  sbelong2  sbelong3  sbelong4  tested_positive  optim1  optim2  slfcont2
##   4   8  age  children  cnarc1  cnarc2  cnarc3  ctheory1  ctheory2  ctheory3  ctheory4  CRT1  CRT2  CRT3  employ_status1  health_cond  know_tested_positive  ladder  marital1  mor_circle  moralid4  moralid6  mcoop1  mcoop2  mcoop3  mcoop4  mcoop5  mcoop6  mcoop7  narc1  narc3  narc4  narc5  narc6  nidentity1  nidentity2  omind2  omind4  omind5  omind6  contact1  contact2  hygiene1  hygiene2  hygiene3  hygiene4  hygiene5  psupport2  psupport4  political_ideology  happy  slf_ladder  riskperc1  riskperc2  self_esteem  sex1  sbelong1  sbelong2  sbelong3  sbelong4  tested_positive  optim1  optim2  slfcont2
##   4   9  age  children  cnarc1  cnarc2  cnarc3  ctheory1  ctheory2  ctheory3  ctheory4  CRT1  CRT2  CRT3  employ_status1  health_cond  know_tested_positive  ladder  marital1  mor_circle  moralid4  moralid6  mcoop1  mcoop2  mcoop3  mcoop4  mcoop5  mcoop6  mcoop7  narc1  narc3  narc4  narc5  narc6  nidentity1  nidentity2  omind2  omind4  omind5  omind6  contact1  contact2  hygiene1  hygiene2  hygiene3  hygiene4  hygiene5  psupport2  psupport4  political_ideology  happy  slf_ladder  riskperc1  riskperc2  self_esteem  sex1  sbelong1  sbelong2  sbelong3  sbelong4  tested_positive  optim1  optim2  slfcont2
##   4   10  age  children  cnarc1  cnarc2  cnarc3  ctheory1  ctheory2  ctheory3  ctheory4  CRT1  CRT2  CRT3  employ_status1  health_cond  know_tested_positive  ladder  marital1  mor_circle  moralid4  moralid6  mcoop1  mcoop2  mcoop3  mcoop4  mcoop5  mcoop6  mcoop7  narc1  narc3  narc4  narc5  narc6  nidentity1  nidentity2  omind2  omind4  omind5  omind6  contact1  contact2  hygiene1  hygiene2  hygiene3  hygiene4  hygiene5  psupport2  psupport4  political_ideology  happy  slf_ladder  riskperc1  riskperc2  self_esteem  sex1  sbelong1  sbelong2  sbelong3  sbelong4  tested_positive  optim1  optim2  slfcont2
##   5   1  age  children  cnarc1  cnarc2  cnarc3  ctheory1  ctheory2  ctheory3  ctheory4  CRT1  CRT2  CRT3  employ_status1  health_cond  know_tested_positive  ladder  marital1  mor_circle  moralid4  moralid6  mcoop1  mcoop2  mcoop3  mcoop4  mcoop5  mcoop6  mcoop7  narc1  narc3  narc4  narc5  narc6  nidentity1  nidentity2  omind2  omind4  omind5  omind6  contact1  contact2  hygiene1  hygiene2  hygiene3  hygiene4  hygiene5  psupport2  psupport4  political_ideology  happy  slf_ladder  riskperc1  riskperc2  self_esteem  sex1  sbelong1  sbelong2  sbelong3  sbelong4  tested_positive  optim1  optim2  slfcont2
##   5   2  age  children  cnarc1  cnarc2  cnarc3  ctheory1  ctheory2  ctheory3  ctheory4  CRT1  CRT2  CRT3  employ_status1  health_cond  know_tested_positive  ladder  marital1  mor_circle  moralid4  moralid6  mcoop1  mcoop2  mcoop3  mcoop4  mcoop5  mcoop6  mcoop7  narc1  narc3  narc4  narc5  narc6  nidentity1  nidentity2  omind2  omind4  omind5  omind6  contact1  contact2  hygiene1  hygiene2  hygiene3  hygiene4  hygiene5  psupport2  psupport4  political_ideology  happy  slf_ladder  riskperc1  riskperc2  self_esteem  sex1  sbelong1  sbelong2  sbelong3  sbelong4  tested_positive  optim1  optim2  slfcont2
##   5   3  age  children  cnarc1  cnarc2  cnarc3  ctheory1  ctheory2  ctheory3  ctheory4  CRT1  CRT2  CRT3  employ_status1  health_cond  know_tested_positive  ladder  marital1  mor_circle  moralid4  moralid6  mcoop1  mcoop2  mcoop3  mcoop4  mcoop5  mcoop6  mcoop7  narc1  narc3  narc4  narc5  narc6  nidentity1  nidentity2  omind2  omind4  omind5  omind6  contact1  contact2  hygiene1  hygiene2  hygiene3  hygiene4  hygiene5  psupport2  psupport4  political_ideology  happy  slf_ladder  riskperc1  riskperc2  self_esteem  sex1  sbelong1  sbelong2  sbelong3  sbelong4  tested_positive  optim1  optim2  slfcont2
##   5   4  age  children  cnarc1  cnarc2  cnarc3  ctheory1  ctheory2  ctheory3  ctheory4  CRT1  CRT2  CRT3  employ_status1  health_cond  know_tested_positive  ladder  marital1  mor_circle  moralid4  moralid6  mcoop1  mcoop2  mcoop3  mcoop4  mcoop5  mcoop6  mcoop7  narc1  narc3  narc4  narc5  narc6  nidentity1  nidentity2  omind2  omind4  omind5  omind6  contact1  contact2  hygiene1  hygiene2  hygiene3  hygiene4  hygiene5  psupport2  psupport4  political_ideology  happy  slf_ladder  riskperc1  riskperc2  self_esteem  sex1  sbelong1  sbelong2  sbelong3  sbelong4  tested_positive  optim1  optim2  slfcont2
##   5   5  age  children  cnarc1  cnarc2  cnarc3  ctheory1  ctheory2  ctheory3  ctheory4  CRT1  CRT2  CRT3  employ_status1  health_cond  know_tested_positive  ladder  marital1  mor_circle  moralid4  moralid6  mcoop1  mcoop2  mcoop3  mcoop4  mcoop5  mcoop6  mcoop7  narc1  narc3  narc4  narc5  narc6  nidentity1  nidentity2  omind2  omind4  omind5  omind6  contact1  contact2  hygiene1  hygiene2  hygiene3  hygiene4  hygiene5  psupport2  psupport4  political_ideology  happy  slf_ladder  riskperc1  riskperc2  self_esteem  sex1  sbelong1  sbelong2  sbelong3  sbelong4  tested_positive  optim1  optim2  slfcont2
##   5   6  age  children  cnarc1  cnarc2  cnarc3  ctheory1  ctheory2  ctheory3  ctheory4  CRT1  CRT2  CRT3  employ_status1  health_cond  know_tested_positive  ladder  marital1  mor_circle  moralid4  moralid6  mcoop1  mcoop2  mcoop3  mcoop4  mcoop5  mcoop6  mcoop7  narc1  narc3  narc4  narc5  narc6  nidentity1  nidentity2  omind2  omind4  omind5  omind6  contact1  contact2  hygiene1  hygiene2  hygiene3  hygiene4  hygiene5  psupport2  psupport4  political_ideology  happy  slf_ladder  riskperc1  riskperc2  self_esteem  sex1  sbelong1  sbelong2  sbelong3  sbelong4  tested_positive  optim1  optim2  slfcont2
##   5   7  age  children  cnarc1  cnarc2  cnarc3  ctheory1  ctheory2  ctheory3  ctheory4  CRT1  CRT2  CRT3  employ_status1  health_cond  know_tested_positive  ladder  marital1  mor_circle  moralid4  moralid6  mcoop1  mcoop2  mcoop3  mcoop4  mcoop5  mcoop6  mcoop7  narc1  narc3  narc4  narc5  narc6  nidentity1  nidentity2  omind2  omind4  omind5  omind6  contact1  contact2  hygiene1  hygiene2  hygiene3  hygiene4  hygiene5  psupport2  psupport4  political_ideology  happy  slf_ladder  riskperc1  riskperc2  self_esteem  sex1  sbelong1  sbelong2  sbelong3  sbelong4  tested_positive  optim1  optim2  slfcont2
##   5   8  age  children  cnarc1  cnarc2  cnarc3  ctheory1  ctheory2  ctheory3  ctheory4  CRT1  CRT2  CRT3  employ_status1  health_cond  know_tested_positive  ladder  marital1  mor_circle  moralid4  moralid6  mcoop1  mcoop2  mcoop3  mcoop4  mcoop5  mcoop6  mcoop7  narc1  narc3  narc4  narc5  narc6  nidentity1  nidentity2  omind2  omind4  omind5  omind6  contact1  contact2  hygiene1  hygiene2  hygiene3  hygiene4  hygiene5  psupport2  psupport4  political_ideology  happy  slf_ladder  riskperc1  riskperc2  self_esteem  sex1  sbelong1  sbelong2  sbelong3  sbelong4  tested_positive  optim1  optim2  slfcont2
##   5   9  age  children  cnarc1  cnarc2  cnarc3  ctheory1  ctheory2  ctheory3  ctheory4  CRT1  CRT2  CRT3  employ_status1  health_cond  know_tested_positive  ladder  marital1  mor_circle  moralid4  moralid6  mcoop1  mcoop2  mcoop3  mcoop4  mcoop5  mcoop6  mcoop7  narc1  narc3  narc4  narc5  narc6  nidentity1  nidentity2  omind2  omind4  omind5  omind6  contact1  contact2  hygiene1  hygiene2  hygiene3  hygiene4  hygiene5  psupport2  psupport4  political_ideology  happy  slf_ladder  riskperc1  riskperc2  self_esteem  sex1  sbelong1  sbelong2  sbelong3  sbelong4  tested_positive  optim1  optim2  slfcont2
##   5   10  age  children  cnarc1  cnarc2  cnarc3  ctheory1  ctheory2  ctheory3  ctheory4  CRT1  CRT2  CRT3  employ_status1  health_cond  know_tested_positive  ladder  marital1  mor_circle  moralid4  moralid6  mcoop1  mcoop2  mcoop3  mcoop4  mcoop5  mcoop6  mcoop7  narc1  narc3  narc4  narc5  narc6  nidentity1  nidentity2  omind2  omind4  omind5  omind6  contact1  contact2  hygiene1  hygiene2  hygiene3  hygiene4  hygiene5  psupport2  psupport4  political_ideology  happy  slf_ladder  riskperc1  riskperc2  self_esteem  sex1  sbelong1  sbelong2  sbelong3  sbelong4  tested_positive  optim1  optim2  slfcont2
##   6   1  age  children  cnarc1  cnarc2  cnarc3  ctheory1  ctheory2  ctheory3  ctheory4  CRT1  CRT2  CRT3  employ_status1  health_cond  know_tested_positive  ladder  marital1  mor_circle  moralid4  moralid6  mcoop1  mcoop2  mcoop3  mcoop4  mcoop5  mcoop6  mcoop7  narc1  narc3  narc4  narc5  narc6  nidentity1  nidentity2  omind2  omind4  omind5  omind6  contact1  contact2  hygiene1  hygiene2  hygiene3  hygiene4  hygiene5  psupport2  psupport4  political_ideology  happy  slf_ladder  riskperc1  riskperc2  self_esteem  sex1  sbelong1  sbelong2  sbelong3  sbelong4  tested_positive  optim1  optim2  slfcont2
##   6   2  age  children  cnarc1  cnarc2  cnarc3  ctheory1  ctheory2  ctheory3  ctheory4  CRT1  CRT2  CRT3  employ_status1  health_cond  know_tested_positive  ladder  marital1  mor_circle  moralid4  moralid6  mcoop1  mcoop2  mcoop3  mcoop4  mcoop5  mcoop6  mcoop7  narc1  narc3  narc4  narc5  narc6  nidentity1  nidentity2  omind2  omind4  omind5  omind6  contact1  contact2  hygiene1  hygiene2  hygiene3  hygiene4  hygiene5  psupport2  psupport4  political_ideology  happy  slf_ladder  riskperc1  riskperc2  self_esteem  sex1  sbelong1  sbelong2  sbelong3  sbelong4  tested_positive  optim1  optim2  slfcont2
##   6   3  age  children  cnarc1  cnarc2  cnarc3  ctheory1  ctheory2  ctheory3  ctheory4  CRT1  CRT2  CRT3  employ_status1  health_cond  know_tested_positive  ladder  marital1  mor_circle  moralid4  moralid6  mcoop1  mcoop2  mcoop3  mcoop4  mcoop5  mcoop6  mcoop7  narc1  narc3  narc4  narc5  narc6  nidentity1  nidentity2  omind2  omind4  omind5  omind6  contact1  contact2  hygiene1  hygiene2  hygiene3  hygiene4  hygiene5  psupport2  psupport4  political_ideology  happy  slf_ladder  riskperc1  riskperc2  self_esteem  sex1  sbelong1  sbelong2  sbelong3  sbelong4  tested_positive  optim1  optim2  slfcont2
##   6   4  age  children  cnarc1  cnarc2  cnarc3  ctheory1  ctheory2  ctheory3  ctheory4  CRT1  CRT2  CRT3  employ_status1  health_cond  know_tested_positive  ladder  marital1  mor_circle  moralid4  moralid6  mcoop1  mcoop2  mcoop3  mcoop4  mcoop5  mcoop6  mcoop7  narc1  narc3  narc4  narc5  narc6  nidentity1  nidentity2  omind2  omind4  omind5  omind6  contact1  contact2  hygiene1  hygiene2  hygiene3  hygiene4  hygiene5  psupport2  psupport4  political_ideology  happy  slf_ladder  riskperc1  riskperc2  self_esteem  sex1  sbelong1  sbelong2  sbelong3  sbelong4  tested_positive  optim1  optim2  slfcont2
##   6   5  age  children  cnarc1  cnarc2  cnarc3  ctheory1  ctheory2  ctheory3  ctheory4  CRT1  CRT2  CRT3  employ_status1  health_cond  know_tested_positive  ladder  marital1  mor_circle  moralid4  moralid6  mcoop1  mcoop2  mcoop3  mcoop4  mcoop5  mcoop6  mcoop7  narc1  narc3  narc4  narc5  narc6  nidentity1  nidentity2  omind2  omind4  omind5  omind6  contact1  contact2  hygiene1  hygiene2  hygiene3  hygiene4  hygiene5  psupport2  psupport4  political_ideology  happy  slf_ladder  riskperc1  riskperc2  self_esteem  sex1  sbelong1  sbelong2  sbelong3  sbelong4  tested_positive  optim1  optim2  slfcont2
##   6   6  age  children  cnarc1  cnarc2  cnarc3  ctheory1  ctheory2  ctheory3  ctheory4  CRT1  CRT2  CRT3  employ_status1  health_cond  know_tested_positive  ladder  marital1  mor_circle  moralid4  moralid6  mcoop1  mcoop2  mcoop3  mcoop4  mcoop5  mcoop6  mcoop7  narc1  narc3  narc4  narc5  narc6  nidentity1  nidentity2  omind2  omind4  omind5  omind6  contact1  contact2  hygiene1  hygiene2  hygiene3  hygiene4  hygiene5  psupport2  psupport4  political_ideology  happy  slf_ladder  riskperc1  riskperc2  self_esteem  sex1  sbelong1  sbelong2  sbelong3  sbelong4  tested_positive  optim1  optim2  slfcont2
##   6   7  age  children  cnarc1  cnarc2  cnarc3  ctheory1  ctheory2  ctheory3  ctheory4  CRT1  CRT2  CRT3  employ_status1  health_cond  know_tested_positive  ladder  marital1  mor_circle  moralid4  moralid6  mcoop1  mcoop2  mcoop3  mcoop4  mcoop5  mcoop6  mcoop7  narc1  narc3  narc4  narc5  narc6  nidentity1  nidentity2  omind2  omind4  omind5  omind6  contact1  contact2  hygiene1  hygiene2  hygiene3  hygiene4  hygiene5  psupport2  psupport4  political_ideology  happy  slf_ladder  riskperc1  riskperc2  self_esteem  sex1  sbelong1  sbelong2  sbelong3  sbelong4  tested_positive  optim1  optim2  slfcont2
##   6   8  age  children  cnarc1  cnarc2  cnarc3  ctheory1  ctheory2  ctheory3  ctheory4  CRT1  CRT2  CRT3  employ_status1  health_cond  know_tested_positive  ladder  marital1  mor_circle  moralid4  moralid6  mcoop1  mcoop2  mcoop3  mcoop4  mcoop5  mcoop6  mcoop7  narc1  narc3  narc4  narc5  narc6  nidentity1  nidentity2  omind2  omind4  omind5  omind6  contact1  contact2  hygiene1  hygiene2  hygiene3  hygiene4  hygiene5  psupport2  psupport4  political_ideology  happy  slf_ladder  riskperc1  riskperc2  self_esteem  sex1  sbelong1  sbelong2  sbelong3  sbelong4  tested_positive  optim1  optim2  slfcont2
##   6   9  age  children  cnarc1  cnarc2  cnarc3  ctheory1  ctheory2  ctheory3  ctheory4  CRT1  CRT2  CRT3  employ_status1  health_cond  know_tested_positive  ladder  marital1  mor_circle  moralid4  moralid6  mcoop1  mcoop2  mcoop3  mcoop4  mcoop5  mcoop6  mcoop7  narc1  narc3  narc4  narc5  narc6  nidentity1  nidentity2  omind2  omind4  omind5  omind6  contact1  contact2  hygiene1  hygiene2  hygiene3  hygiene4  hygiene5  psupport2  psupport4  political_ideology  happy  slf_ladder  riskperc1  riskperc2  self_esteem  sex1  sbelong1  sbelong2  sbelong3  sbelong4  tested_positive  optim1  optim2  slfcont2
##   6   10  age  children  cnarc1  cnarc2  cnarc3  ctheory1  ctheory2  ctheory3  ctheory4  CRT1  CRT2  CRT3  employ_status1  health_cond  know_tested_positive  ladder  marital1  mor_circle  moralid4  moralid6  mcoop1  mcoop2  mcoop3  mcoop4  mcoop5  mcoop6  mcoop7  narc1  narc3  narc4  narc5  narc6  nidentity1  nidentity2  omind2  omind4  omind5  omind6  contact1  contact2  hygiene1  hygiene2  hygiene3  hygiene4  hygiene5  psupport2  psupport4  political_ideology  happy  slf_ladder  riskperc1  riskperc2  self_esteem  sex1  sbelong1  sbelong2  sbelong3  sbelong4  tested_positive  optim1  optim2  slfcont2
##   7   1  age  children  cnarc1  cnarc2  cnarc3  ctheory1  ctheory2  ctheory3  ctheory4  CRT1  CRT2  CRT3  employ_status1  health_cond  know_tested_positive  ladder  marital1  mor_circle  moralid4  moralid6  mcoop1  mcoop2  mcoop3  mcoop4  mcoop5  mcoop6  mcoop7  narc1  narc3  narc4  narc5  narc6  nidentity1  nidentity2  omind2  omind4  omind5  omind6  contact1  contact2  hygiene1  hygiene2  hygiene3  hygiene4  hygiene5  psupport2  psupport4  political_ideology  happy  slf_ladder  riskperc1  riskperc2  self_esteem  sex1  sbelong1  sbelong2  sbelong3  sbelong4  tested_positive  optim1  optim2  slfcont2
##   7   2  age  children  cnarc1  cnarc2  cnarc3  ctheory1  ctheory2  ctheory3  ctheory4  CRT1  CRT2  CRT3  employ_status1  health_cond  know_tested_positive  ladder  marital1  mor_circle  moralid4  moralid6  mcoop1  mcoop2  mcoop3  mcoop4  mcoop5  mcoop6  mcoop7  narc1  narc3  narc4  narc5  narc6  nidentity1  nidentity2  omind2  omind4  omind5  omind6  contact1  contact2  hygiene1  hygiene2  hygiene3  hygiene4  hygiene5  psupport2  psupport4  political_ideology  happy  slf_ladder  riskperc1  riskperc2  self_esteem  sex1  sbelong1  sbelong2  sbelong3  sbelong4  tested_positive  optim1  optim2  slfcont2
##   7   3  age  children  cnarc1  cnarc2  cnarc3  ctheory1  ctheory2  ctheory3  ctheory4  CRT1  CRT2  CRT3  employ_status1  health_cond  know_tested_positive  ladder  marital1  mor_circle  moralid4  moralid6  mcoop1  mcoop2  mcoop3  mcoop4  mcoop5  mcoop6  mcoop7  narc1  narc3  narc4  narc5  narc6  nidentity1  nidentity2  omind2  omind4  omind5  omind6  contact1  contact2  hygiene1  hygiene2  hygiene3  hygiene4  hygiene5  psupport2  psupport4  political_ideology  happy  slf_ladder  riskperc1  riskperc2  self_esteem  sex1  sbelong1  sbelong2  sbelong3  sbelong4  tested_positive  optim1  optim2  slfcont2
##   7   4  age  children  cnarc1  cnarc2  cnarc3  ctheory1  ctheory2  ctheory3  ctheory4  CRT1  CRT2  CRT3  employ_status1  health_cond  know_tested_positive  ladder  marital1  mor_circle  moralid4  moralid6  mcoop1  mcoop2  mcoop3  mcoop4  mcoop5  mcoop6  mcoop7  narc1  narc3  narc4  narc5  narc6  nidentity1  nidentity2  omind2  omind4  omind5  omind6  contact1  contact2  hygiene1  hygiene2  hygiene3  hygiene4  hygiene5  psupport2  psupport4  political_ideology  happy  slf_ladder  riskperc1  riskperc2  self_esteem  sex1  sbelong1  sbelong2  sbelong3  sbelong4  tested_positive  optim1  optim2  slfcont2
##   7   5  age  children  cnarc1  cnarc2  cnarc3  ctheory1  ctheory2  ctheory3  ctheory4  CRT1  CRT2  CRT3  employ_status1  health_cond  know_tested_positive  ladder  marital1  mor_circle  moralid4  moralid6  mcoop1  mcoop2  mcoop3  mcoop4  mcoop5  mcoop6  mcoop7  narc1  narc3  narc4  narc5  narc6  nidentity1  nidentity2  omind2  omind4  omind5  omind6  contact1  contact2  hygiene1  hygiene2  hygiene3  hygiene4  hygiene5  psupport2  psupport4  political_ideology  happy  slf_ladder  riskperc1  riskperc2  self_esteem  sex1  sbelong1  sbelong2  sbelong3  sbelong4  tested_positive  optim1  optim2  slfcont2
##   7   6  age  children  cnarc1  cnarc2  cnarc3  ctheory1  ctheory2  ctheory3  ctheory4  CRT1  CRT2  CRT3  employ_status1  health_cond  know_tested_positive  ladder  marital1  mor_circle  moralid4  moralid6  mcoop1  mcoop2  mcoop3  mcoop4  mcoop5  mcoop6  mcoop7  narc1  narc3  narc4  narc5  narc6  nidentity1  nidentity2  omind2  omind4  omind5  omind6  contact1  contact2  hygiene1  hygiene2  hygiene3  hygiene4  hygiene5  psupport2  psupport4  political_ideology  happy  slf_ladder  riskperc1  riskperc2  self_esteem  sex1  sbelong1  sbelong2  sbelong3  sbelong4  tested_positive  optim1  optim2  slfcont2
##   7   7  age  children  cnarc1  cnarc2  cnarc3  ctheory1  ctheory2  ctheory3  ctheory4  CRT1  CRT2  CRT3  employ_status1  health_cond  know_tested_positive  ladder  marital1  mor_circle  moralid4  moralid6  mcoop1  mcoop2  mcoop3  mcoop4  mcoop5  mcoop6  mcoop7  narc1  narc3  narc4  narc5  narc6  nidentity1  nidentity2  omind2  omind4  omind5  omind6  contact1  contact2  hygiene1  hygiene2  hygiene3  hygiene4  hygiene5  psupport2  psupport4  political_ideology  happy  slf_ladder  riskperc1  riskperc2  self_esteem  sex1  sbelong1  sbelong2  sbelong3  sbelong4  tested_positive  optim1  optim2  slfcont2
##   7   8  age  children  cnarc1  cnarc2  cnarc3  ctheory1  ctheory2  ctheory3  ctheory4  CRT1  CRT2  CRT3  employ_status1  health_cond  know_tested_positive  ladder  marital1  mor_circle  moralid4  moralid6  mcoop1  mcoop2  mcoop3  mcoop4  mcoop5  mcoop6  mcoop7  narc1  narc3  narc4  narc5  narc6  nidentity1  nidentity2  omind2  omind4  omind5  omind6  contact1  contact2  hygiene1  hygiene2  hygiene3  hygiene4  hygiene5  psupport2  psupport4  political_ideology  happy  slf_ladder  riskperc1  riskperc2  self_esteem  sex1  sbelong1  sbelong2  sbelong3  sbelong4  tested_positive  optim1  optim2  slfcont2
##   7   9  age  children  cnarc1  cnarc2  cnarc3  ctheory1  ctheory2  ctheory3  ctheory4  CRT1  CRT2  CRT3  employ_status1  health_cond  know_tested_positive  ladder  marital1  mor_circle  moralid4  moralid6  mcoop1  mcoop2  mcoop3  mcoop4  mcoop5  mcoop6  mcoop7  narc1  narc3  narc4  narc5  narc6  nidentity1  nidentity2  omind2  omind4  omind5  omind6  contact1  contact2  hygiene1  hygiene2  hygiene3  hygiene4  hygiene5  psupport2  psupport4  political_ideology  happy  slf_ladder  riskperc1  riskperc2  self_esteem  sex1  sbelong1  sbelong2  sbelong3  sbelong4  tested_positive  optim1  optim2  slfcont2
##   7   10  age  children  cnarc1  cnarc2  cnarc3  ctheory1  ctheory2  ctheory3  ctheory4  CRT1  CRT2  CRT3  employ_status1  health_cond  know_tested_positive  ladder  marital1  mor_circle  moralid4  moralid6  mcoop1  mcoop2  mcoop3  mcoop4  mcoop5  mcoop6  mcoop7  narc1  narc3  narc4  narc5  narc6  nidentity1  nidentity2  omind2  omind4  omind5  omind6  contact1  contact2  hygiene1  hygiene2  hygiene3  hygiene4  hygiene5  psupport2  psupport4  political_ideology  happy  slf_ladder  riskperc1  riskperc2  self_esteem  sex1  sbelong1  sbelong2  sbelong3  sbelong4  tested_positive  optim1  optim2  slfcont2
##   8   1  age  children  cnarc1  cnarc2  cnarc3  ctheory1  ctheory2  ctheory3  ctheory4  CRT1  CRT2  CRT3  employ_status1  health_cond  know_tested_positive  ladder  marital1  mor_circle  moralid4  moralid6  mcoop1  mcoop2  mcoop3  mcoop4  mcoop5  mcoop6  mcoop7  narc1  narc3  narc4  narc5  narc6  nidentity1  nidentity2  omind2  omind4  omind5  omind6  contact1  contact2  hygiene1  hygiene2  hygiene3  hygiene4  hygiene5  psupport2  psupport4  political_ideology  happy  slf_ladder  riskperc1  riskperc2  self_esteem  sex1  sbelong1  sbelong2  sbelong3  sbelong4  tested_positive  optim1  optim2  slfcont2
##   8   2  age  children  cnarc1  cnarc2  cnarc3  ctheory1  ctheory2  ctheory3  ctheory4  CRT1  CRT2  CRT3  employ_status1  health_cond  know_tested_positive  ladder  marital1  mor_circle  moralid4  moralid6  mcoop1  mcoop2  mcoop3  mcoop4  mcoop5  mcoop6  mcoop7  narc1  narc3  narc4  narc5  narc6  nidentity1  nidentity2  omind2  omind4  omind5  omind6  contact1  contact2  hygiene1  hygiene2  hygiene3  hygiene4  hygiene5  psupport2  psupport4  political_ideology  happy  slf_ladder  riskperc1  riskperc2  self_esteem  sex1  sbelong1  sbelong2  sbelong3  sbelong4  tested_positive  optim1  optim2  slfcont2
##   8   3  age  children  cnarc1  cnarc2  cnarc3  ctheory1  ctheory2  ctheory3  ctheory4  CRT1  CRT2  CRT3  employ_status1  health_cond  know_tested_positive  ladder  marital1  mor_circle  moralid4  moralid6  mcoop1  mcoop2  mcoop3  mcoop4  mcoop5  mcoop6  mcoop7  narc1  narc3  narc4  narc5  narc6  nidentity1  nidentity2  omind2  omind4  omind5  omind6  contact1  contact2  hygiene1  hygiene2  hygiene3  hygiene4  hygiene5  psupport2  psupport4  political_ideology  happy  slf_ladder  riskperc1  riskperc2  self_esteem  sex1  sbelong1  sbelong2  sbelong3  sbelong4  tested_positive  optim1  optim2  slfcont2
##   8   4  age  children  cnarc1  cnarc2  cnarc3  ctheory1  ctheory2  ctheory3  ctheory4  CRT1  CRT2  CRT3  employ_status1  health_cond  know_tested_positive  ladder  marital1  mor_circle  moralid4  moralid6  mcoop1  mcoop2  mcoop3  mcoop4  mcoop5  mcoop6  mcoop7  narc1  narc3  narc4  narc5  narc6  nidentity1  nidentity2  omind2  omind4  omind5  omind6  contact1  contact2  hygiene1  hygiene2  hygiene3  hygiene4  hygiene5  psupport2  psupport4  political_ideology  happy  slf_ladder  riskperc1  riskperc2  self_esteem  sex1  sbelong1  sbelong2  sbelong3  sbelong4  tested_positive  optim1  optim2  slfcont2
##   8   5  age  children  cnarc1  cnarc2  cnarc3  ctheory1  ctheory2  ctheory3  ctheory4  CRT1  CRT2  CRT3  employ_status1  health_cond  know_tested_positive  ladder  marital1  mor_circle  moralid4  moralid6  mcoop1  mcoop2  mcoop3  mcoop4  mcoop5  mcoop6  mcoop7  narc1  narc3  narc4  narc5  narc6  nidentity1  nidentity2  omind2  omind4  omind5  omind6  contact1  contact2  hygiene1  hygiene2  hygiene3  hygiene4  hygiene5  psupport2  psupport4  political_ideology  happy  slf_ladder  riskperc1  riskperc2  self_esteem  sex1  sbelong1  sbelong2  sbelong3  sbelong4  tested_positive  optim1  optim2  slfcont2
##   8   6  age  children  cnarc1  cnarc2  cnarc3  ctheory1  ctheory2  ctheory3  ctheory4  CRT1  CRT2  CRT3  employ_status1  health_cond  know_tested_positive  ladder  marital1  mor_circle  moralid4  moralid6  mcoop1  mcoop2  mcoop3  mcoop4  mcoop5  mcoop6  mcoop7  narc1  narc3  narc4  narc5  narc6  nidentity1  nidentity2  omind2  omind4  omind5  omind6  contact1  contact2  hygiene1  hygiene2  hygiene3  hygiene4  hygiene5  psupport2  psupport4  political_ideology  happy  slf_ladder  riskperc1  riskperc2  self_esteem  sex1  sbelong1  sbelong2  sbelong3  sbelong4  tested_positive  optim1  optim2  slfcont2
##   8   7  age  children  cnarc1  cnarc2  cnarc3  ctheory1  ctheory2  ctheory3  ctheory4  CRT1  CRT2  CRT3  employ_status1  health_cond  know_tested_positive  ladder  marital1  mor_circle  moralid4  moralid6  mcoop1  mcoop2  mcoop3  mcoop4  mcoop5  mcoop6  mcoop7  narc1  narc3  narc4  narc5  narc6  nidentity1  nidentity2  omind2  omind4  omind5  omind6  contact1  contact2  hygiene1  hygiene2  hygiene3  hygiene4  hygiene5  psupport2  psupport4  political_ideology  happy  slf_ladder  riskperc1  riskperc2  self_esteem  sex1  sbelong1  sbelong2  sbelong3  sbelong4  tested_positive  optim1  optim2  slfcont2
##   8   8  age  children  cnarc1  cnarc2  cnarc3  ctheory1  ctheory2  ctheory3  ctheory4  CRT1  CRT2  CRT3  employ_status1  health_cond  know_tested_positive  ladder  marital1  mor_circle  moralid4  moralid6  mcoop1  mcoop2  mcoop3  mcoop4  mcoop5  mcoop6  mcoop7  narc1  narc3  narc4  narc5  narc6  nidentity1  nidentity2  omind2  omind4  omind5  omind6  contact1  contact2  hygiene1  hygiene2  hygiene3  hygiene4  hygiene5  psupport2  psupport4  political_ideology  happy  slf_ladder  riskperc1  riskperc2  self_esteem  sex1  sbelong1  sbelong2  sbelong3  sbelong4  tested_positive  optim1  optim2  slfcont2
##   8   9  age  children  cnarc1  cnarc2  cnarc3  ctheory1  ctheory2  ctheory3  ctheory4  CRT1  CRT2  CRT3  employ_status1  health_cond  know_tested_positive  ladder  marital1  mor_circle  moralid4  moralid6  mcoop1  mcoop2  mcoop3  mcoop4  mcoop5  mcoop6  mcoop7  narc1  narc3  narc4  narc5  narc6  nidentity1  nidentity2  omind2  omind4  omind5  omind6  contact1  contact2  hygiene1  hygiene2  hygiene3  hygiene4  hygiene5  psupport2  psupport4  political_ideology  happy  slf_ladder  riskperc1  riskperc2  self_esteem  sex1  sbelong1  sbelong2  sbelong3  sbelong4  tested_positive  optim1  optim2  slfcont2
##   8   10  age  children  cnarc1  cnarc2  cnarc3  ctheory1  ctheory2  ctheory3  ctheory4  CRT1  CRT2  CRT3  employ_status1  health_cond  know_tested_positive  ladder  marital1  mor_circle  moralid4  moralid6  mcoop1  mcoop2  mcoop3  mcoop4  mcoop5  mcoop6  mcoop7  narc1  narc3  narc4  narc5  narc6  nidentity1  nidentity2  omind2  omind4  omind5  omind6  contact1  contact2  hygiene1  hygiene2  hygiene3  hygiene4  hygiene5  psupport2  psupport4  political_ideology  happy  slf_ladder  riskperc1  riskperc2  self_esteem  sex1  sbelong1  sbelong2  sbelong3  sbelong4  tested_positive  optim1  optim2  slfcont2
##   9   1  age  children  cnarc1  cnarc2  cnarc3  ctheory1  ctheory2  ctheory3  ctheory4  CRT1  CRT2  CRT3  employ_status1  health_cond  know_tested_positive  ladder  marital1  mor_circle  moralid4  moralid6  mcoop1  mcoop2  mcoop3  mcoop4  mcoop5  mcoop6  mcoop7  narc1  narc3  narc4  narc5  narc6  nidentity1  nidentity2  omind2  omind4  omind5  omind6  contact1  contact2  hygiene1  hygiene2  hygiene3  hygiene4  hygiene5  psupport2  psupport4  political_ideology  happy  slf_ladder  riskperc1  riskperc2  self_esteem  sex1  sbelong1  sbelong2  sbelong3  sbelong4  tested_positive  optim1  optim2  slfcont2
##   9   2  age  children  cnarc1  cnarc2  cnarc3  ctheory1  ctheory2  ctheory3  ctheory4  CRT1  CRT2  CRT3  employ_status1  health_cond  know_tested_positive  ladder  marital1  mor_circle  moralid4  moralid6  mcoop1  mcoop2  mcoop3  mcoop4  mcoop5  mcoop6  mcoop7  narc1  narc3  narc4  narc5  narc6  nidentity1  nidentity2  omind2  omind4  omind5  omind6  contact1  contact2  hygiene1  hygiene2  hygiene3  hygiene4  hygiene5  psupport2  psupport4  political_ideology  happy  slf_ladder  riskperc1  riskperc2  self_esteem  sex1  sbelong1  sbelong2  sbelong3  sbelong4  tested_positive  optim1  optim2  slfcont2
##   9   3  age  children  cnarc1  cnarc2  cnarc3  ctheory1  ctheory2  ctheory3  ctheory4  CRT1  CRT2  CRT3  employ_status1  health_cond  know_tested_positive  ladder  marital1  mor_circle  moralid4  moralid6  mcoop1  mcoop2  mcoop3  mcoop4  mcoop5  mcoop6  mcoop7  narc1  narc3  narc4  narc5  narc6  nidentity1  nidentity2  omind2  omind4  omind5  omind6  contact1  contact2  hygiene1  hygiene2  hygiene3  hygiene4  hygiene5  psupport2  psupport4  political_ideology  happy  slf_ladder  riskperc1  riskperc2  self_esteem  sex1  sbelong1  sbelong2  sbelong3  sbelong4  tested_positive  optim1  optim2  slfcont2
##   9   4  age  children  cnarc1  cnarc2  cnarc3  ctheory1  ctheory2  ctheory3  ctheory4  CRT1  CRT2  CRT3  employ_status1  health_cond  know_tested_positive  ladder  marital1  mor_circle  moralid4  moralid6  mcoop1  mcoop2  mcoop3  mcoop4  mcoop5  mcoop6  mcoop7  narc1  narc3  narc4  narc5  narc6  nidentity1  nidentity2  omind2  omind4  omind5  omind6  contact1  contact2  hygiene1  hygiene2  hygiene3  hygiene4  hygiene5  psupport2  psupport4  political_ideology  happy  slf_ladder  riskperc1  riskperc2  self_esteem  sex1  sbelong1  sbelong2  sbelong3  sbelong4  tested_positive  optim1  optim2  slfcont2
##   9   5  age  children  cnarc1  cnarc2  cnarc3  ctheory1  ctheory2  ctheory3  ctheory4  CRT1  CRT2  CRT3  employ_status1  health_cond  know_tested_positive  ladder  marital1  mor_circle  moralid4  moralid6  mcoop1  mcoop2  mcoop3  mcoop4  mcoop5  mcoop6  mcoop7  narc1  narc3  narc4  narc5  narc6  nidentity1  nidentity2  omind2  omind4  omind5  omind6  contact1  contact2  hygiene1  hygiene2  hygiene3  hygiene4  hygiene5  psupport2  psupport4  political_ideology  happy  slf_ladder  riskperc1  riskperc2  self_esteem  sex1  sbelong1  sbelong2  sbelong3  sbelong4  tested_positive  optim1  optim2  slfcont2
##   9   6  age  children  cnarc1  cnarc2  cnarc3  ctheory1  ctheory2  ctheory3  ctheory4  CRT1  CRT2  CRT3  employ_status1  health_cond  know_tested_positive  ladder  marital1  mor_circle  moralid4  moralid6  mcoop1  mcoop2  mcoop3  mcoop4  mcoop5  mcoop6  mcoop7  narc1  narc3  narc4  narc5  narc6  nidentity1  nidentity2  omind2  omind4  omind5  omind6  contact1  contact2  hygiene1  hygiene2  hygiene3  hygiene4  hygiene5  psupport2  psupport4  political_ideology  happy  slf_ladder  riskperc1  riskperc2  self_esteem  sex1  sbelong1  sbelong2  sbelong3  sbelong4  tested_positive  optim1  optim2  slfcont2
##   9   7  age  children  cnarc1  cnarc2  cnarc3  ctheory1  ctheory2  ctheory3  ctheory4  CRT1  CRT2  CRT3  employ_status1  health_cond  know_tested_positive  ladder  marital1  mor_circle  moralid4  moralid6  mcoop1  mcoop2  mcoop3  mcoop4  mcoop5  mcoop6  mcoop7  narc1  narc3  narc4  narc5  narc6  nidentity1  nidentity2  omind2  omind4  omind5  omind6  contact1  contact2  hygiene1  hygiene2  hygiene3  hygiene4  hygiene5  psupport2  psupport4  political_ideology  happy  slf_ladder  riskperc1  riskperc2  self_esteem  sex1  sbelong1  sbelong2  sbelong3  sbelong4  tested_positive  optim1  optim2  slfcont2
##   9   8  age  children  cnarc1  cnarc2  cnarc3  ctheory1  ctheory2  ctheory3  ctheory4  CRT1  CRT2  CRT3  employ_status1  health_cond  know_tested_positive  ladder  marital1  mor_circle  moralid4  moralid6  mcoop1  mcoop2  mcoop3  mcoop4  mcoop5  mcoop6  mcoop7  narc1  narc3  narc4  narc5  narc6  nidentity1  nidentity2  omind2  omind4  omind5  omind6  contact1  contact2  hygiene1  hygiene2  hygiene3  hygiene4  hygiene5  psupport2  psupport4  political_ideology  happy  slf_ladder  riskperc1  riskperc2  self_esteem  sex1  sbelong1  sbelong2  sbelong3  sbelong4  tested_positive  optim1  optim2  slfcont2
##   9   9  age  children  cnarc1  cnarc2  cnarc3  ctheory1  ctheory2  ctheory3  ctheory4  CRT1  CRT2  CRT3  employ_status1  health_cond  know_tested_positive  ladder  marital1  mor_circle  moralid4  moralid6  mcoop1  mcoop2  mcoop3  mcoop4  mcoop5  mcoop6  mcoop7  narc1  narc3  narc4  narc5  narc6  nidentity1  nidentity2  omind2  omind4  omind5  omind6  contact1  contact2  hygiene1  hygiene2  hygiene3  hygiene4  hygiene5  psupport2  psupport4  political_ideology  happy  slf_ladder  riskperc1  riskperc2  self_esteem  sex1  sbelong1  sbelong2  sbelong3  sbelong4  tested_positive  optim1  optim2  slfcont2
##   9   10  age  children  cnarc1  cnarc2  cnarc3  ctheory1  ctheory2  ctheory3  ctheory4  CRT1  CRT2  CRT3  employ_status1  health_cond  know_tested_positive  ladder  marital1  mor_circle  moralid4  moralid6  mcoop1  mcoop2  mcoop3  mcoop4  mcoop5  mcoop6  mcoop7  narc1  narc3  narc4  narc5  narc6  nidentity1  nidentity2  omind2  omind4  omind5  omind6  contact1  contact2  hygiene1  hygiene2  hygiene3  hygiene4  hygiene5  psupport2  psupport4  political_ideology  happy  slf_ladder  riskperc1  riskperc2  self_esteem  sex1  sbelong1  sbelong2  sbelong3  sbelong4  tested_positive  optim1  optim2  slfcont2
##   10   1  age  children  cnarc1  cnarc2  cnarc3  ctheory1  ctheory2  ctheory3  ctheory4  CRT1  CRT2  CRT3  employ_status1  health_cond  know_tested_positive  ladder  marital1  mor_circle  moralid4  moralid6  mcoop1  mcoop2  mcoop3  mcoop4  mcoop5  mcoop6  mcoop7  narc1  narc3  narc4  narc5  narc6  nidentity1  nidentity2  omind2  omind4  omind5  omind6  contact1  contact2  hygiene1  hygiene2  hygiene3  hygiene4  hygiene5  psupport2  psupport4  political_ideology  happy  slf_ladder  riskperc1  riskperc2  self_esteem  sex1  sbelong1  sbelong2  sbelong3  sbelong4  tested_positive  optim1  optim2  slfcont2
##   10   2  age  children  cnarc1  cnarc2  cnarc3  ctheory1  ctheory2  ctheory3  ctheory4  CRT1  CRT2  CRT3  employ_status1  health_cond  know_tested_positive  ladder  marital1  mor_circle  moralid4  moralid6  mcoop1  mcoop2  mcoop3  mcoop4  mcoop5  mcoop6  mcoop7  narc1  narc3  narc4  narc5  narc6  nidentity1  nidentity2  omind2  omind4  omind5  omind6  contact1  contact2  hygiene1  hygiene2  hygiene3  hygiene4  hygiene5  psupport2  psupport4  political_ideology  happy  slf_ladder  riskperc1  riskperc2  self_esteem  sex1  sbelong1  sbelong2  sbelong3  sbelong4  tested_positive  optim1  optim2  slfcont2
##   10   3  age  children  cnarc1  cnarc2  cnarc3  ctheory1  ctheory2  ctheory3  ctheory4  CRT1  CRT2  CRT3  employ_status1  health_cond  know_tested_positive  ladder  marital1  mor_circle  moralid4  moralid6  mcoop1  mcoop2  mcoop3  mcoop4  mcoop5  mcoop6  mcoop7  narc1  narc3  narc4  narc5  narc6  nidentity1  nidentity2  omind2  omind4  omind5  omind6  contact1  contact2  hygiene1  hygiene2  hygiene3  hygiene4  hygiene5  psupport2  psupport4  political_ideology  happy  slf_ladder  riskperc1  riskperc2  self_esteem  sex1  sbelong1  sbelong2  sbelong3  sbelong4  tested_positive  optim1  optim2  slfcont2
##   10   4  age  children  cnarc1  cnarc2  cnarc3  ctheory1  ctheory2  ctheory3  ctheory4  CRT1  CRT2  CRT3  employ_status1  health_cond  know_tested_positive  ladder  marital1  mor_circle  moralid4  moralid6  mcoop1  mcoop2  mcoop3  mcoop4  mcoop5  mcoop6  mcoop7  narc1  narc3  narc4  narc5  narc6  nidentity1  nidentity2  omind2  omind4  omind5  omind6  contact1  contact2  hygiene1  hygiene2  hygiene3  hygiene4  hygiene5  psupport2  psupport4  political_ideology  happy  slf_ladder  riskperc1  riskperc2  self_esteem  sex1  sbelong1  sbelong2  sbelong3  sbelong4  tested_positive  optim1  optim2  slfcont2
##   10   5  age  children  cnarc1  cnarc2  cnarc3  ctheory1  ctheory2  ctheory3  ctheory4  CRT1  CRT2  CRT3  employ_status1  health_cond  know_tested_positive  ladder  marital1  mor_circle  moralid4  moralid6  mcoop1  mcoop2  mcoop3  mcoop4  mcoop5  mcoop6  mcoop7  narc1  narc3  narc4  narc5  narc6  nidentity1  nidentity2  omind2  omind4  omind5  omind6  contact1  contact2  hygiene1  hygiene2  hygiene3  hygiene4  hygiene5  psupport2  psupport4  political_ideology  happy  slf_ladder  riskperc1  riskperc2  self_esteem  sex1  sbelong1  sbelong2  sbelong3  sbelong4  tested_positive  optim1  optim2  slfcont2
##   10   6  age  children  cnarc1  cnarc2  cnarc3  ctheory1  ctheory2  ctheory3  ctheory4  CRT1  CRT2  CRT3  employ_status1  health_cond  know_tested_positive  ladder  marital1  mor_circle  moralid4  moralid6  mcoop1  mcoop2  mcoop3  mcoop4  mcoop5  mcoop6  mcoop7  narc1  narc3  narc4  narc5  narc6  nidentity1  nidentity2  omind2  omind4  omind5  omind6  contact1  contact2  hygiene1  hygiene2  hygiene3  hygiene4  hygiene5  psupport2  psupport4  political_ideology  happy  slf_ladder  riskperc1  riskperc2  self_esteem  sex1  sbelong1  sbelong2  sbelong3  sbelong4  tested_positive  optim1  optim2  slfcont2
##   10   7  age  children  cnarc1  cnarc2  cnarc3  ctheory1  ctheory2  ctheory3  ctheory4  CRT1  CRT2  CRT3  employ_status1  health_cond  know_tested_positive  ladder  marital1  mor_circle  moralid4  moralid6  mcoop1  mcoop2  mcoop3  mcoop4  mcoop5  mcoop6  mcoop7  narc1  narc3  narc4  narc5  narc6  nidentity1  nidentity2  omind2  omind4  omind5  omind6  contact1  contact2  hygiene1  hygiene2  hygiene3  hygiene4  hygiene5  psupport2  psupport4  political_ideology  happy  slf_ladder  riskperc1  riskperc2  self_esteem  sex1  sbelong1  sbelong2  sbelong3  sbelong4  tested_positive  optim1  optim2  slfcont2
##   10   8  age  children  cnarc1  cnarc2  cnarc3  ctheory1  ctheory2  ctheory3  ctheory4  CRT1  CRT2  CRT3  employ_status1  health_cond  know_tested_positive  ladder  marital1  mor_circle  moralid4  moralid6  mcoop1  mcoop2  mcoop3  mcoop4  mcoop5  mcoop6  mcoop7  narc1  narc3  narc4  narc5  narc6  nidentity1  nidentity2  omind2  omind4  omind5  omind6  contact1  contact2  hygiene1  hygiene2  hygiene3  hygiene4  hygiene5  psupport2  psupport4  political_ideology  happy  slf_ladder  riskperc1  riskperc2  self_esteem  sex1  sbelong1  sbelong2  sbelong3  sbelong4  tested_positive  optim1  optim2  slfcont2
##   10   9  age  children  cnarc1  cnarc2  cnarc3  ctheory1  ctheory2  ctheory3  ctheory4  CRT1  CRT2  CRT3  employ_status1  health_cond  know_tested_positive  ladder  marital1  mor_circle  moralid4  moralid6  mcoop1  mcoop2  mcoop3  mcoop4  mcoop5  mcoop6  mcoop7  narc1  narc3  narc4  narc5  narc6  nidentity1  nidentity2  omind2  omind4  omind5  omind6  contact1  contact2  hygiene1  hygiene2  hygiene3  hygiene4  hygiene5  psupport2  psupport4  political_ideology  happy  slf_ladder  riskperc1  riskperc2  self_esteem  sex1  sbelong1  sbelong2  sbelong3  sbelong4  tested_positive  optim1  optim2  slfcont2
##   10   10  age  children  cnarc1  cnarc2  cnarc3  ctheory1  ctheory2  ctheory3  ctheory4  CRT1  CRT2  CRT3  employ_status1  health_cond  know_tested_positive  ladder  marital1  mor_circle  moralid4  moralid6  mcoop1  mcoop2  mcoop3  mcoop4  mcoop5  mcoop6  mcoop7  narc1  narc3  narc4  narc5  narc6  nidentity1  nidentity2  omind2  omind4  omind5  omind6  contact1  contact2  hygiene1  hygiene2  hygiene3  hygiene4  hygiene5  psupport2  psupport4  political_ideology  happy  slf_ladder  riskperc1  riskperc2  self_esteem  sex1  sbelong1  sbelong2  sbelong3  sbelong4  tested_positive  optim1  optim2  slfcont2
```

```
## Warning: Number of logged events: 6203
```

```
## 
##  iter imp variable
##   1   1  age  children  cnarc1  cnarc2  cnarc3  ctheory1  ctheory2  ctheory3  ctheory4  CRT1  CRT2  CRT3  employ_status1  generosity1  generosity2  generosity3  health_cond  know_tested_positive  ladder  marital1  mor_circle  moralid1  moralid10  moralid2  moralid3  moralid4  moralid5  moralid6  moralid7  moralid8  moralid9  mcoop1  mcoop2  mcoop3  mcoop4  mcoop5  mcoop6  mcoop7  narc1  narc2  narc3  narc4  narc5  narc6  nidentity1  nidentity2  omind1  omind4  omind5  omind6  contact1  contact2  contact3  contact4  contact5  hygiene1  hygiene2  hygiene3  hygiene4  hygiene5  psupport1  psupport2  psupport3  psupport4  psupport5  political_ideology  happy  slf_ladder  riskperc1  riskperc2  self_esteem  sex1  sbelong1  sbelong2  sbelong3  sbelong4  tested_positive  optim1  optim2  slfcont1  slfcont2  slfcont3  slfcont4  urban
##   1   2  age  children  cnarc1  cnarc2  cnarc3  ctheory1  ctheory2  ctheory3  ctheory4  CRT1  CRT2  CRT3  employ_status1  generosity1  generosity2  generosity3  health_cond  know_tested_positive  ladder  marital1  mor_circle  moralid1  moralid10  moralid2  moralid3  moralid4  moralid5  moralid6  moralid7  moralid8  moralid9  mcoop1  mcoop2  mcoop3  mcoop4  mcoop5  mcoop6  mcoop7  narc1  narc2  narc3  narc4  narc5  narc6  nidentity1  nidentity2  omind1  omind4  omind5  omind6  contact1  contact2  contact3  contact4  contact5  hygiene1  hygiene2  hygiene3  hygiene4  hygiene5  psupport1  psupport2  psupport3  psupport4  psupport5  political_ideology  happy  slf_ladder  riskperc1  riskperc2  self_esteem  sex1  sbelong1  sbelong2  sbelong3  sbelong4  tested_positive  optim1  optim2  slfcont1  slfcont2  slfcont3  slfcont4  urban
##   1   3  age  children  cnarc1  cnarc2  cnarc3  ctheory1  ctheory2  ctheory3  ctheory4  CRT1  CRT2  CRT3  employ_status1  generosity1  generosity2  generosity3  health_cond  know_tested_positive  ladder  marital1  mor_circle  moralid1  moralid10  moralid2  moralid3  moralid4  moralid5  moralid6  moralid7  moralid8  moralid9  mcoop1  mcoop2  mcoop3  mcoop4  mcoop5  mcoop6  mcoop7  narc1  narc2  narc3  narc4  narc5  narc6  nidentity1  nidentity2  omind1  omind4  omind5  omind6  contact1  contact2  contact3  contact4  contact5  hygiene1  hygiene2  hygiene3  hygiene4  hygiene5  psupport1  psupport2  psupport3  psupport4  psupport5  political_ideology  happy  slf_ladder  riskperc1  riskperc2  self_esteem  sex1  sbelong1  sbelong2  sbelong3  sbelong4  tested_positive  optim1  optim2  slfcont1  slfcont2  slfcont3  slfcont4  urban
##   1   4  age  children  cnarc1  cnarc2  cnarc3  ctheory1  ctheory2  ctheory3  ctheory4  CRT1  CRT2  CRT3  employ_status1  generosity1  generosity2  generosity3  health_cond  know_tested_positive  ladder  marital1  mor_circle  moralid1  moralid10  moralid2  moralid3  moralid4  moralid5  moralid6  moralid7  moralid8  moralid9  mcoop1  mcoop2  mcoop3  mcoop4  mcoop5  mcoop6  mcoop7  narc1  narc2  narc3  narc4  narc5  narc6  nidentity1  nidentity2  omind1  omind4  omind5  omind6  contact1  contact2  contact3  contact4  contact5  hygiene1  hygiene2  hygiene3  hygiene4  hygiene5  psupport1  psupport2  psupport3  psupport4  psupport5  political_ideology  happy  slf_ladder  riskperc1  riskperc2  self_esteem  sex1  sbelong1  sbelong2  sbelong3  sbelong4  tested_positive  optim1  optim2  slfcont1  slfcont2  slfcont3  slfcont4  urban
##   1   5  age  children  cnarc1  cnarc2  cnarc3  ctheory1  ctheory2  ctheory3  ctheory4  CRT1  CRT2  CRT3  employ_status1  generosity1  generosity2  generosity3  health_cond  know_tested_positive  ladder  marital1  mor_circle  moralid1  moralid10  moralid2  moralid3  moralid4  moralid5  moralid6  moralid7  moralid8  moralid9  mcoop1  mcoop2  mcoop3  mcoop4  mcoop5  mcoop6  mcoop7  narc1  narc2  narc3  narc4  narc5  narc6  nidentity1  nidentity2  omind1  omind4  omind5  omind6  contact1  contact2  contact3  contact4  contact5  hygiene1  hygiene2  hygiene3  hygiene4  hygiene5  psupport1  psupport2  psupport3  psupport4  psupport5  political_ideology  happy  slf_ladder  riskperc1  riskperc2  self_esteem  sex1  sbelong1  sbelong2  sbelong3  sbelong4  tested_positive  optim1  optim2  slfcont1  slfcont2  slfcont3  slfcont4  urban
##   1   6  age  children  cnarc1  cnarc2  cnarc3  ctheory1  ctheory2  ctheory3  ctheory4  CRT1  CRT2  CRT3  employ_status1  generosity1  generosity2  generosity3  health_cond  know_tested_positive  ladder  marital1  mor_circle  moralid1  moralid10  moralid2  moralid3  moralid4  moralid5  moralid6  moralid7  moralid8  moralid9  mcoop1  mcoop2  mcoop3  mcoop4  mcoop5  mcoop6  mcoop7  narc1  narc2  narc3  narc4  narc5  narc6  nidentity1  nidentity2  omind1  omind4  omind5  omind6  contact1  contact2  contact3  contact4  contact5  hygiene1  hygiene2  hygiene3  hygiene4  hygiene5  psupport1  psupport2  psupport3  psupport4  psupport5  political_ideology  happy  slf_ladder  riskperc1  riskperc2  self_esteem  sex1  sbelong1  sbelong2  sbelong3  sbelong4  tested_positive  optim1  optim2  slfcont1  slfcont2  slfcont3  slfcont4  urban
##   1   7  age  children  cnarc1  cnarc2  cnarc3  ctheory1  ctheory2  ctheory3  ctheory4  CRT1  CRT2  CRT3  employ_status1  generosity1  generosity2  generosity3  health_cond  know_tested_positive  ladder  marital1  mor_circle  moralid1  moralid10  moralid2  moralid3  moralid4  moralid5  moralid6  moralid7  moralid8  moralid9  mcoop1  mcoop2  mcoop3  mcoop4  mcoop5  mcoop6  mcoop7  narc1  narc2  narc3  narc4  narc5  narc6  nidentity1  nidentity2  omind1  omind4  omind5  omind6  contact1  contact2  contact3  contact4  contact5  hygiene1  hygiene2  hygiene3  hygiene4  hygiene5  psupport1  psupport2  psupport3  psupport4  psupport5  political_ideology  happy  slf_ladder  riskperc1  riskperc2  self_esteem  sex1  sbelong1  sbelong2  sbelong3  sbelong4  tested_positive  optim1  optim2  slfcont1  slfcont2  slfcont3  slfcont4  urban
##   1   8  age  children  cnarc1  cnarc2  cnarc3  ctheory1  ctheory2  ctheory3  ctheory4  CRT1  CRT2  CRT3  employ_status1  generosity1  generosity2  generosity3  health_cond  know_tested_positive  ladder  marital1  mor_circle  moralid1  moralid10  moralid2  moralid3  moralid4  moralid5  moralid6  moralid7  moralid8  moralid9  mcoop1  mcoop2  mcoop3  mcoop4  mcoop5  mcoop6  mcoop7  narc1  narc2  narc3  narc4  narc5  narc6  nidentity1  nidentity2  omind1  omind4  omind5  omind6  contact1  contact2  contact3  contact4  contact5  hygiene1  hygiene2  hygiene3  hygiene4  hygiene5  psupport1  psupport2  psupport3  psupport4  psupport5  political_ideology  happy  slf_ladder  riskperc1  riskperc2  self_esteem  sex1  sbelong1  sbelong2  sbelong3  sbelong4  tested_positive  optim1  optim2  slfcont1  slfcont2  slfcont3  slfcont4  urban
##   1   9  age  children  cnarc1  cnarc2  cnarc3  ctheory1  ctheory2  ctheory3  ctheory4  CRT1  CRT2  CRT3  employ_status1  generosity1  generosity2  generosity3  health_cond  know_tested_positive  ladder  marital1  mor_circle  moralid1  moralid10  moralid2  moralid3  moralid4  moralid5  moralid6  moralid7  moralid8  moralid9  mcoop1  mcoop2  mcoop3  mcoop4  mcoop5  mcoop6  mcoop7  narc1  narc2  narc3  narc4  narc5  narc6  nidentity1  nidentity2  omind1  omind4  omind5  omind6  contact1  contact2  contact3  contact4  contact5  hygiene1  hygiene2  hygiene3  hygiene4  hygiene5  psupport1  psupport2  psupport3  psupport4  psupport5  political_ideology  happy  slf_ladder  riskperc1  riskperc2  self_esteem  sex1  sbelong1  sbelong2  sbelong3  sbelong4  tested_positive  optim1  optim2  slfcont1  slfcont2  slfcont3  slfcont4  urban
##   1   10  age  children  cnarc1  cnarc2  cnarc3  ctheory1  ctheory2  ctheory3  ctheory4  CRT1  CRT2  CRT3  employ_status1  generosity1  generosity2  generosity3  health_cond  know_tested_positive  ladder  marital1  mor_circle  moralid1  moralid10  moralid2  moralid3  moralid4  moralid5  moralid6  moralid7  moralid8  moralid9  mcoop1  mcoop2  mcoop3  mcoop4  mcoop5  mcoop6  mcoop7  narc1  narc2  narc3  narc4  narc5  narc6  nidentity1  nidentity2  omind1  omind4  omind5  omind6  contact1  contact2  contact3  contact4  contact5  hygiene1  hygiene2  hygiene3  hygiene4  hygiene5  psupport1  psupport2  psupport3  psupport4  psupport5  political_ideology  happy  slf_ladder  riskperc1  riskperc2  self_esteem  sex1  sbelong1  sbelong2  sbelong3  sbelong4  tested_positive  optim1  optim2  slfcont1  slfcont2  slfcont3  slfcont4  urban
##   2   1  age  children  cnarc1  cnarc2  cnarc3  ctheory1  ctheory2  ctheory3  ctheory4  CRT1  CRT2  CRT3  employ_status1  generosity1  generosity2  generosity3  health_cond  know_tested_positive  ladder  marital1  mor_circle  moralid1  moralid10  moralid2  moralid3  moralid4  moralid5  moralid6  moralid7  moralid8  moralid9  mcoop1  mcoop2  mcoop3  mcoop4  mcoop5  mcoop6  mcoop7  narc1  narc2  narc3  narc4  narc5  narc6  nidentity1  nidentity2  omind1  omind4  omind5  omind6  contact1  contact2  contact3  contact4  contact5  hygiene1  hygiene2  hygiene3  hygiene4  hygiene5  psupport1  psupport2  psupport3  psupport4  psupport5  political_ideology  happy  slf_ladder  riskperc1  riskperc2  self_esteem  sex1  sbelong1  sbelong2  sbelong3  sbelong4  tested_positive  optim1  optim2  slfcont1  slfcont2  slfcont3  slfcont4  urban
##   2   2  age  children  cnarc1  cnarc2  cnarc3  ctheory1  ctheory2  ctheory3  ctheory4  CRT1  CRT2  CRT3  employ_status1  generosity1  generosity2  generosity3  health_cond  know_tested_positive  ladder  marital1  mor_circle  moralid1  moralid10  moralid2  moralid3  moralid4  moralid5  moralid6  moralid7  moralid8  moralid9  mcoop1  mcoop2  mcoop3  mcoop4  mcoop5  mcoop6  mcoop7  narc1  narc2  narc3  narc4  narc5  narc6  nidentity1  nidentity2  omind1  omind4  omind5  omind6  contact1  contact2  contact3  contact4  contact5  hygiene1  hygiene2  hygiene3  hygiene4  hygiene5  psupport1  psupport2  psupport3  psupport4  psupport5  political_ideology  happy  slf_ladder  riskperc1  riskperc2  self_esteem  sex1  sbelong1  sbelong2  sbelong3  sbelong4  tested_positive  optim1  optim2  slfcont1  slfcont2  slfcont3  slfcont4  urban
##   2   3  age  children  cnarc1  cnarc2  cnarc3  ctheory1  ctheory2  ctheory3  ctheory4  CRT1  CRT2  CRT3  employ_status1  generosity1  generosity2  generosity3  health_cond  know_tested_positive  ladder  marital1  mor_circle  moralid1  moralid10  moralid2  moralid3  moralid4  moralid5  moralid6  moralid7  moralid8  moralid9  mcoop1  mcoop2  mcoop3  mcoop4  mcoop5  mcoop6  mcoop7  narc1  narc2  narc3  narc4  narc5  narc6  nidentity1  nidentity2  omind1  omind4  omind5  omind6  contact1  contact2  contact3  contact4  contact5  hygiene1  hygiene2  hygiene3  hygiene4  hygiene5  psupport1  psupport2  psupport3  psupport4  psupport5  political_ideology  happy  slf_ladder  riskperc1  riskperc2  self_esteem  sex1  sbelong1  sbelong2  sbelong3  sbelong4  tested_positive  optim1  optim2  slfcont1  slfcont2  slfcont3  slfcont4  urban
##   2   4  age  children  cnarc1  cnarc2  cnarc3  ctheory1  ctheory2  ctheory3  ctheory4  CRT1  CRT2  CRT3  employ_status1  generosity1  generosity2  generosity3  health_cond  know_tested_positive  ladder  marital1  mor_circle  moralid1  moralid10  moralid2  moralid3  moralid4  moralid5  moralid6  moralid7  moralid8  moralid9  mcoop1  mcoop2  mcoop3  mcoop4  mcoop5  mcoop6  mcoop7  narc1  narc2  narc3  narc4  narc5  narc6  nidentity1  nidentity2  omind1  omind4  omind5  omind6  contact1  contact2  contact3  contact4  contact5  hygiene1  hygiene2  hygiene3  hygiene4  hygiene5  psupport1  psupport2  psupport3  psupport4  psupport5  political_ideology  happy  slf_ladder  riskperc1  riskperc2  self_esteem  sex1  sbelong1  sbelong2  sbelong3  sbelong4  tested_positive  optim1  optim2  slfcont1  slfcont2  slfcont3  slfcont4  urban
##   2   5  age  children  cnarc1  cnarc2  cnarc3  ctheory1  ctheory2  ctheory3  ctheory4  CRT1  CRT2  CRT3  employ_status1  generosity1  generosity2  generosity3  health_cond  know_tested_positive  ladder  marital1  mor_circle  moralid1  moralid10  moralid2  moralid3  moralid4  moralid5  moralid6  moralid7  moralid8  moralid9  mcoop1  mcoop2  mcoop3  mcoop4  mcoop5  mcoop6  mcoop7  narc1  narc2  narc3  narc4  narc5  narc6  nidentity1  nidentity2  omind1  omind4  omind5  omind6  contact1  contact2  contact3  contact4  contact5  hygiene1  hygiene2  hygiene3  hygiene4  hygiene5  psupport1  psupport2  psupport3  psupport4  psupport5  political_ideology  happy  slf_ladder  riskperc1  riskperc2  self_esteem  sex1  sbelong1  sbelong2  sbelong3  sbelong4  tested_positive  optim1  optim2  slfcont1  slfcont2  slfcont3  slfcont4  urban
##   2   6  age  children  cnarc1  cnarc2  cnarc3  ctheory1  ctheory2  ctheory3  ctheory4  CRT1  CRT2  CRT3  employ_status1  generosity1  generosity2  generosity3  health_cond  know_tested_positive  ladder  marital1  mor_circle  moralid1  moralid10  moralid2  moralid3  moralid4  moralid5  moralid6  moralid7  moralid8  moralid9  mcoop1  mcoop2  mcoop3  mcoop4  mcoop5  mcoop6  mcoop7  narc1  narc2  narc3  narc4  narc5  narc6  nidentity1  nidentity2  omind1  omind4  omind5  omind6  contact1  contact2  contact3  contact4  contact5  hygiene1  hygiene2  hygiene3  hygiene4  hygiene5  psupport1  psupport2  psupport3  psupport4  psupport5  political_ideology  happy  slf_ladder  riskperc1  riskperc2  self_esteem  sex1  sbelong1  sbelong2  sbelong3  sbelong4  tested_positive  optim1  optim2  slfcont1  slfcont2  slfcont3  slfcont4  urban
##   2   7  age  children  cnarc1  cnarc2  cnarc3  ctheory1  ctheory2  ctheory3  ctheory4  CRT1  CRT2  CRT3  employ_status1  generosity1  generosity2  generosity3  health_cond  know_tested_positive  ladder  marital1  mor_circle  moralid1  moralid10  moralid2  moralid3  moralid4  moralid5  moralid6  moralid7  moralid8  moralid9  mcoop1  mcoop2  mcoop3  mcoop4  mcoop5  mcoop6  mcoop7  narc1  narc2  narc3  narc4  narc5  narc6  nidentity1  nidentity2  omind1  omind4  omind5  omind6  contact1  contact2  contact3  contact4  contact5  hygiene1  hygiene2  hygiene3  hygiene4  hygiene5  psupport1  psupport2  psupport3  psupport4  psupport5  political_ideology  happy  slf_ladder  riskperc1  riskperc2  self_esteem  sex1  sbelong1  sbelong2  sbelong3  sbelong4  tested_positive  optim1  optim2  slfcont1  slfcont2  slfcont3  slfcont4  urban
##   2   8  age  children  cnarc1  cnarc2  cnarc3  ctheory1  ctheory2  ctheory3  ctheory4  CRT1  CRT2  CRT3  employ_status1  generosity1  generosity2  generosity3  health_cond  know_tested_positive  ladder  marital1  mor_circle  moralid1  moralid10  moralid2  moralid3  moralid4  moralid5  moralid6  moralid7  moralid8  moralid9  mcoop1  mcoop2  mcoop3  mcoop4  mcoop5  mcoop6  mcoop7  narc1  narc2  narc3  narc4  narc5  narc6  nidentity1  nidentity2  omind1  omind4  omind5  omind6  contact1  contact2  contact3  contact4  contact5  hygiene1  hygiene2  hygiene3  hygiene4  hygiene5  psupport1  psupport2  psupport3  psupport4  psupport5  political_ideology  happy  slf_ladder  riskperc1  riskperc2  self_esteem  sex1  sbelong1  sbelong2  sbelong3  sbelong4  tested_positive  optim1  optim2  slfcont1  slfcont2  slfcont3  slfcont4  urban
##   2   9  age  children  cnarc1  cnarc2  cnarc3  ctheory1  ctheory2  ctheory3  ctheory4  CRT1  CRT2  CRT3  employ_status1  generosity1  generosity2  generosity3  health_cond  know_tested_positive  ladder  marital1  mor_circle  moralid1  moralid10  moralid2  moralid3  moralid4  moralid5  moralid6  moralid7  moralid8  moralid9  mcoop1  mcoop2  mcoop3  mcoop4  mcoop5  mcoop6  mcoop7  narc1  narc2  narc3  narc4  narc5  narc6  nidentity1  nidentity2  omind1  omind4  omind5  omind6  contact1  contact2  contact3  contact4  contact5  hygiene1  hygiene2  hygiene3  hygiene4  hygiene5  psupport1  psupport2  psupport3  psupport4  psupport5  political_ideology  happy  slf_ladder  riskperc1  riskperc2  self_esteem  sex1  sbelong1  sbelong2  sbelong3  sbelong4  tested_positive  optim1  optim2  slfcont1  slfcont2  slfcont3  slfcont4  urban
##   2   10  age  children  cnarc1  cnarc2  cnarc3  ctheory1  ctheory2  ctheory3  ctheory4  CRT1  CRT2  CRT3  employ_status1  generosity1  generosity2  generosity3  health_cond  know_tested_positive  ladder  marital1  mor_circle  moralid1  moralid10  moralid2  moralid3  moralid4  moralid5  moralid6  moralid7  moralid8  moralid9  mcoop1  mcoop2  mcoop3  mcoop4  mcoop5  mcoop6  mcoop7  narc1  narc2  narc3  narc4  narc5  narc6  nidentity1  nidentity2  omind1  omind4  omind5  omind6  contact1  contact2  contact3  contact4  contact5  hygiene1  hygiene2  hygiene3  hygiene4  hygiene5  psupport1  psupport2  psupport3  psupport4  psupport5  political_ideology  happy  slf_ladder  riskperc1  riskperc2  self_esteem  sex1  sbelong1  sbelong2  sbelong3  sbelong4  tested_positive  optim1  optim2  slfcont1  slfcont2  slfcont3  slfcont4  urban
##   3   1  age  children  cnarc1  cnarc2  cnarc3  ctheory1  ctheory2  ctheory3  ctheory4  CRT1  CRT2  CRT3  employ_status1  generosity1  generosity2  generosity3  health_cond  know_tested_positive  ladder  marital1  mor_circle  moralid1  moralid10  moralid2  moralid3  moralid4  moralid5  moralid6  moralid7  moralid8  moralid9  mcoop1  mcoop2  mcoop3  mcoop4  mcoop5  mcoop6  mcoop7  narc1  narc2  narc3  narc4  narc5  narc6  nidentity1  nidentity2  omind1  omind4  omind5  omind6  contact1  contact2  contact3  contact4  contact5  hygiene1  hygiene2  hygiene3  hygiene4  hygiene5  psupport1  psupport2  psupport3  psupport4  psupport5  political_ideology  happy  slf_ladder  riskperc1  riskperc2  self_esteem  sex1  sbelong1  sbelong2  sbelong3  sbelong4  tested_positive  optim1  optim2  slfcont1  slfcont2  slfcont3  slfcont4  urban
##   3   2  age  children  cnarc1  cnarc2  cnarc3  ctheory1  ctheory2  ctheory3  ctheory4  CRT1  CRT2  CRT3  employ_status1  generosity1  generosity2  generosity3  health_cond  know_tested_positive  ladder  marital1  mor_circle  moralid1  moralid10  moralid2  moralid3  moralid4  moralid5  moralid6  moralid7  moralid8  moralid9  mcoop1  mcoop2  mcoop3  mcoop4  mcoop5  mcoop6  mcoop7  narc1  narc2  narc3  narc4  narc5  narc6  nidentity1  nidentity2  omind1  omind4  omind5  omind6  contact1  contact2  contact3  contact4  contact5  hygiene1  hygiene2  hygiene3  hygiene4  hygiene5  psupport1  psupport2  psupport3  psupport4  psupport5  political_ideology  happy  slf_ladder  riskperc1  riskperc2  self_esteem  sex1  sbelong1  sbelong2  sbelong3  sbelong4  tested_positive  optim1  optim2  slfcont1  slfcont2  slfcont3  slfcont4  urban
##   3   3  age  children  cnarc1  cnarc2  cnarc3  ctheory1  ctheory2  ctheory3  ctheory4  CRT1  CRT2  CRT3  employ_status1  generosity1  generosity2  generosity3  health_cond  know_tested_positive  ladder  marital1  mor_circle  moralid1  moralid10  moralid2  moralid3  moralid4  moralid5  moralid6  moralid7  moralid8  moralid9  mcoop1  mcoop2  mcoop3  mcoop4  mcoop5  mcoop6  mcoop7  narc1  narc2  narc3  narc4  narc5  narc6  nidentity1  nidentity2  omind1  omind4  omind5  omind6  contact1  contact2  contact3  contact4  contact5  hygiene1  hygiene2  hygiene3  hygiene4  hygiene5  psupport1  psupport2  psupport3  psupport4  psupport5  political_ideology  happy  slf_ladder  riskperc1  riskperc2  self_esteem  sex1  sbelong1  sbelong2  sbelong3  sbelong4  tested_positive  optim1  optim2  slfcont1  slfcont2  slfcont3  slfcont4  urban
##   3   4  age  children  cnarc1  cnarc2  cnarc3  ctheory1  ctheory2  ctheory3  ctheory4  CRT1  CRT2  CRT3  employ_status1  generosity1  generosity2  generosity3  health_cond  know_tested_positive  ladder  marital1  mor_circle  moralid1  moralid10  moralid2  moralid3  moralid4  moralid5  moralid6  moralid7  moralid8  moralid9  mcoop1  mcoop2  mcoop3  mcoop4  mcoop5  mcoop6  mcoop7  narc1  narc2  narc3  narc4  narc5  narc6  nidentity1  nidentity2  omind1  omind4  omind5  omind6  contact1  contact2  contact3  contact4  contact5  hygiene1  hygiene2  hygiene3  hygiene4  hygiene5  psupport1  psupport2  psupport3  psupport4  psupport5  political_ideology  happy  slf_ladder  riskperc1  riskperc2  self_esteem  sex1  sbelong1  sbelong2  sbelong3  sbelong4  tested_positive  optim1  optim2  slfcont1  slfcont2  slfcont3  slfcont4  urban
##   3   5  age  children  cnarc1  cnarc2  cnarc3  ctheory1  ctheory2  ctheory3  ctheory4  CRT1  CRT2  CRT3  employ_status1  generosity1  generosity2  generosity3  health_cond  know_tested_positive  ladder  marital1  mor_circle  moralid1  moralid10  moralid2  moralid3  moralid4  moralid5  moralid6  moralid7  moralid8  moralid9  mcoop1  mcoop2  mcoop3  mcoop4  mcoop5  mcoop6  mcoop7  narc1  narc2  narc3  narc4  narc5  narc6  nidentity1  nidentity2  omind1  omind4  omind5  omind6  contact1  contact2  contact3  contact4  contact5  hygiene1  hygiene2  hygiene3  hygiene4  hygiene5  psupport1  psupport2  psupport3  psupport4  psupport5  political_ideology  happy  slf_ladder  riskperc1  riskperc2  self_esteem  sex1  sbelong1  sbelong2  sbelong3  sbelong4  tested_positive  optim1  optim2  slfcont1  slfcont2  slfcont3  slfcont4  urban
##   3   6  age  children  cnarc1  cnarc2  cnarc3  ctheory1  ctheory2  ctheory3  ctheory4  CRT1  CRT2  CRT3  employ_status1  generosity1  generosity2  generosity3  health_cond  know_tested_positive  ladder  marital1  mor_circle  moralid1  moralid10  moralid2  moralid3  moralid4  moralid5  moralid6  moralid7  moralid8  moralid9  mcoop1  mcoop2  mcoop3  mcoop4  mcoop5  mcoop6  mcoop7  narc1  narc2  narc3  narc4  narc5  narc6  nidentity1  nidentity2  omind1  omind4  omind5  omind6  contact1  contact2  contact3  contact4  contact5  hygiene1  hygiene2  hygiene3  hygiene4  hygiene5  psupport1  psupport2  psupport3  psupport4  psupport5  political_ideology  happy  slf_ladder  riskperc1  riskperc2  self_esteem  sex1  sbelong1  sbelong2  sbelong3  sbelong4  tested_positive  optim1  optim2  slfcont1  slfcont2  slfcont3  slfcont4  urban
##   3   7  age  children  cnarc1  cnarc2  cnarc3  ctheory1  ctheory2  ctheory3  ctheory4  CRT1  CRT2  CRT3  employ_status1  generosity1  generosity2  generosity3  health_cond  know_tested_positive  ladder  marital1  mor_circle  moralid1  moralid10  moralid2  moralid3  moralid4  moralid5  moralid6  moralid7  moralid8  moralid9  mcoop1  mcoop2  mcoop3  mcoop4  mcoop5  mcoop6  mcoop7  narc1  narc2  narc3  narc4  narc5  narc6  nidentity1  nidentity2  omind1  omind4  omind5  omind6  contact1  contact2  contact3  contact4  contact5  hygiene1  hygiene2  hygiene3  hygiene4  hygiene5  psupport1  psupport2  psupport3  psupport4  psupport5  political_ideology  happy  slf_ladder  riskperc1  riskperc2  self_esteem  sex1  sbelong1  sbelong2  sbelong3  sbelong4  tested_positive  optim1  optim2  slfcont1  slfcont2  slfcont3  slfcont4  urban
##   3   8  age  children  cnarc1  cnarc2  cnarc3  ctheory1  ctheory2  ctheory3  ctheory4  CRT1  CRT2  CRT3  employ_status1  generosity1  generosity2  generosity3  health_cond  know_tested_positive  ladder  marital1  mor_circle  moralid1  moralid10  moralid2  moralid3  moralid4  moralid5  moralid6  moralid7  moralid8  moralid9  mcoop1  mcoop2  mcoop3  mcoop4  mcoop5  mcoop6  mcoop7  narc1  narc2  narc3  narc4  narc5  narc6  nidentity1  nidentity2  omind1  omind4  omind5  omind6  contact1  contact2  contact3  contact4  contact5  hygiene1  hygiene2  hygiene3  hygiene4  hygiene5  psupport1  psupport2  psupport3  psupport4  psupport5  political_ideology  happy  slf_ladder  riskperc1  riskperc2  self_esteem  sex1  sbelong1  sbelong2  sbelong3  sbelong4  tested_positive  optim1  optim2  slfcont1  slfcont2  slfcont3  slfcont4  urban
##   3   9  age  children  cnarc1  cnarc2  cnarc3  ctheory1  ctheory2  ctheory3  ctheory4  CRT1  CRT2  CRT3  employ_status1  generosity1  generosity2  generosity3  health_cond  know_tested_positive  ladder  marital1  mor_circle  moralid1  moralid10  moralid2  moralid3  moralid4  moralid5  moralid6  moralid7  moralid8  moralid9  mcoop1  mcoop2  mcoop3  mcoop4  mcoop5  mcoop6  mcoop7  narc1  narc2  narc3  narc4  narc5  narc6  nidentity1  nidentity2  omind1  omind4  omind5  omind6  contact1  contact2  contact3  contact4  contact5  hygiene1  hygiene2  hygiene3  hygiene4  hygiene5  psupport1  psupport2  psupport3  psupport4  psupport5  political_ideology  happy  slf_ladder  riskperc1  riskperc2  self_esteem  sex1  sbelong1  sbelong2  sbelong3  sbelong4  tested_positive  optim1  optim2  slfcont1  slfcont2  slfcont3  slfcont4  urban
##   3   10  age  children  cnarc1  cnarc2  cnarc3  ctheory1  ctheory2  ctheory3  ctheory4  CRT1  CRT2  CRT3  employ_status1  generosity1  generosity2  generosity3  health_cond  know_tested_positive  ladder  marital1  mor_circle  moralid1  moralid10  moralid2  moralid3  moralid4  moralid5  moralid6  moralid7  moralid8  moralid9  mcoop1  mcoop2  mcoop3  mcoop4  mcoop5  mcoop6  mcoop7  narc1  narc2  narc3  narc4  narc5  narc6  nidentity1  nidentity2  omind1  omind4  omind5  omind6  contact1  contact2  contact3  contact4  contact5  hygiene1  hygiene2  hygiene3  hygiene4  hygiene5  psupport1  psupport2  psupport3  psupport4  psupport5  political_ideology  happy  slf_ladder  riskperc1  riskperc2  self_esteem  sex1  sbelong1  sbelong2  sbelong3  sbelong4  tested_positive  optim1  optim2  slfcont1  slfcont2  slfcont3  slfcont4  urban
##   4   1  age  children  cnarc1  cnarc2  cnarc3  ctheory1  ctheory2  ctheory3  ctheory4  CRT1  CRT2  CRT3  employ_status1  generosity1  generosity2  generosity3  health_cond  know_tested_positive  ladder  marital1  mor_circle  moralid1  moralid10  moralid2  moralid3  moralid4  moralid5  moralid6  moralid7  moralid8  moralid9  mcoop1  mcoop2  mcoop3  mcoop4  mcoop5  mcoop6  mcoop7  narc1  narc2  narc3  narc4  narc5  narc6  nidentity1  nidentity2  omind1  omind4  omind5  omind6  contact1  contact2  contact3  contact4  contact5  hygiene1  hygiene2  hygiene3  hygiene4  hygiene5  psupport1  psupport2  psupport3  psupport4  psupport5  political_ideology  happy  slf_ladder  riskperc1  riskperc2  self_esteem  sex1  sbelong1  sbelong2  sbelong3  sbelong4  tested_positive  optim1  optim2  slfcont1  slfcont2  slfcont3  slfcont4  urban
##   4   2  age  children  cnarc1  cnarc2  cnarc3  ctheory1  ctheory2  ctheory3  ctheory4  CRT1  CRT2  CRT3  employ_status1  generosity1  generosity2  generosity3  health_cond  know_tested_positive  ladder  marital1  mor_circle  moralid1  moralid10  moralid2  moralid3  moralid4  moralid5  moralid6  moralid7  moralid8  moralid9  mcoop1  mcoop2  mcoop3  mcoop4  mcoop5  mcoop6  mcoop7  narc1  narc2  narc3  narc4  narc5  narc6  nidentity1  nidentity2  omind1  omind4  omind5  omind6  contact1  contact2  contact3  contact4  contact5  hygiene1  hygiene2  hygiene3  hygiene4  hygiene5  psupport1  psupport2  psupport3  psupport4  psupport5  political_ideology  happy  slf_ladder  riskperc1  riskperc2  self_esteem  sex1  sbelong1  sbelong2  sbelong3  sbelong4  tested_positive  optim1  optim2  slfcont1  slfcont2  slfcont3  slfcont4  urban
##   4   3  age  children  cnarc1  cnarc2  cnarc3  ctheory1  ctheory2  ctheory3  ctheory4  CRT1  CRT2  CRT3  employ_status1  generosity1  generosity2  generosity3  health_cond  know_tested_positive  ladder  marital1  mor_circle  moralid1  moralid10  moralid2  moralid3  moralid4  moralid5  moralid6  moralid7  moralid8  moralid9  mcoop1  mcoop2  mcoop3  mcoop4  mcoop5  mcoop6  mcoop7  narc1  narc2  narc3  narc4  narc5  narc6  nidentity1  nidentity2  omind1  omind4  omind5  omind6  contact1  contact2  contact3  contact4  contact5  hygiene1  hygiene2  hygiene3  hygiene4  hygiene5  psupport1  psupport2  psupport3  psupport4  psupport5  political_ideology  happy  slf_ladder  riskperc1  riskperc2  self_esteem  sex1  sbelong1  sbelong2  sbelong3  sbelong4  tested_positive  optim1  optim2  slfcont1  slfcont2  slfcont3  slfcont4  urban
##   4   4  age  children  cnarc1  cnarc2  cnarc3  ctheory1  ctheory2  ctheory3  ctheory4  CRT1  CRT2  CRT3  employ_status1  generosity1  generosity2  generosity3  health_cond  know_tested_positive  ladder  marital1  mor_circle  moralid1  moralid10  moralid2  moralid3  moralid4  moralid5  moralid6  moralid7  moralid8  moralid9  mcoop1  mcoop2  mcoop3  mcoop4  mcoop5  mcoop6  mcoop7  narc1  narc2  narc3  narc4  narc5  narc6  nidentity1  nidentity2  omind1  omind4  omind5  omind6  contact1  contact2  contact3  contact4  contact5  hygiene1  hygiene2  hygiene3  hygiene4  hygiene5  psupport1  psupport2  psupport3  psupport4  psupport5  political_ideology  happy  slf_ladder  riskperc1  riskperc2  self_esteem  sex1  sbelong1  sbelong2  sbelong3  sbelong4  tested_positive  optim1  optim2  slfcont1  slfcont2  slfcont3  slfcont4  urban
##   4   5  age  children  cnarc1  cnarc2  cnarc3  ctheory1  ctheory2  ctheory3  ctheory4  CRT1  CRT2  CRT3  employ_status1  generosity1  generosity2  generosity3  health_cond  know_tested_positive  ladder  marital1  mor_circle  moralid1  moralid10  moralid2  moralid3  moralid4  moralid5  moralid6  moralid7  moralid8  moralid9  mcoop1  mcoop2  mcoop3  mcoop4  mcoop5  mcoop6  mcoop7  narc1  narc2  narc3  narc4  narc5  narc6  nidentity1  nidentity2  omind1  omind4  omind5  omind6  contact1  contact2  contact3  contact4  contact5  hygiene1  hygiene2  hygiene3  hygiene4  hygiene5  psupport1  psupport2  psupport3  psupport4  psupport5  political_ideology  happy  slf_ladder  riskperc1  riskperc2  self_esteem  sex1  sbelong1  sbelong2  sbelong3  sbelong4  tested_positive  optim1  optim2  slfcont1  slfcont2  slfcont3  slfcont4  urban
##   4   6  age  children  cnarc1  cnarc2  cnarc3  ctheory1  ctheory2  ctheory3  ctheory4  CRT1  CRT2  CRT3  employ_status1  generosity1  generosity2  generosity3  health_cond  know_tested_positive  ladder  marital1  mor_circle  moralid1  moralid10  moralid2  moralid3  moralid4  moralid5  moralid6  moralid7  moralid8  moralid9  mcoop1  mcoop2  mcoop3  mcoop4  mcoop5  mcoop6  mcoop7  narc1  narc2  narc3  narc4  narc5  narc6  nidentity1  nidentity2  omind1  omind4  omind5  omind6  contact1  contact2  contact3  contact4  contact5  hygiene1  hygiene2  hygiene3  hygiene4  hygiene5  psupport1  psupport2  psupport3  psupport4  psupport5  political_ideology  happy  slf_ladder  riskperc1  riskperc2  self_esteem  sex1  sbelong1  sbelong2  sbelong3  sbelong4  tested_positive  optim1  optim2  slfcont1  slfcont2  slfcont3  slfcont4  urban
##   4   7  age  children  cnarc1  cnarc2  cnarc3  ctheory1  ctheory2  ctheory3  ctheory4  CRT1  CRT2  CRT3  employ_status1  generosity1  generosity2  generosity3  health_cond  know_tested_positive  ladder  marital1  mor_circle  moralid1  moralid10  moralid2  moralid3  moralid4  moralid5  moralid6  moralid7  moralid8  moralid9  mcoop1  mcoop2  mcoop3  mcoop4  mcoop5  mcoop6  mcoop7  narc1  narc2  narc3  narc4  narc5  narc6  nidentity1  nidentity2  omind1  omind4  omind5  omind6  contact1  contact2  contact3  contact4  contact5  hygiene1  hygiene2  hygiene3  hygiene4  hygiene5  psupport1  psupport2  psupport3  psupport4  psupport5  political_ideology  happy  slf_ladder  riskperc1  riskperc2  self_esteem  sex1  sbelong1  sbelong2  sbelong3  sbelong4  tested_positive  optim1  optim2  slfcont1  slfcont2  slfcont3  slfcont4  urban
##   4   8  age  children  cnarc1  cnarc2  cnarc3  ctheory1  ctheory2  ctheory3  ctheory4  CRT1  CRT2  CRT3  employ_status1  generosity1  generosity2  generosity3  health_cond  know_tested_positive  ladder  marital1  mor_circle  moralid1  moralid10  moralid2  moralid3  moralid4  moralid5  moralid6  moralid7  moralid8  moralid9  mcoop1  mcoop2  mcoop3  mcoop4  mcoop5  mcoop6  mcoop7  narc1  narc2  narc3  narc4  narc5  narc6  nidentity1  nidentity2  omind1  omind4  omind5  omind6  contact1  contact2  contact3  contact4  contact5  hygiene1  hygiene2  hygiene3  hygiene4  hygiene5  psupport1  psupport2  psupport3  psupport4  psupport5  political_ideology  happy  slf_ladder  riskperc1  riskperc2  self_esteem  sex1  sbelong1  sbelong2  sbelong3  sbelong4  tested_positive  optim1  optim2  slfcont1  slfcont2  slfcont3  slfcont4  urban
##   4   9  age  children  cnarc1  cnarc2  cnarc3  ctheory1  ctheory2  ctheory3  ctheory4  CRT1  CRT2  CRT3  employ_status1  generosity1  generosity2  generosity3  health_cond  know_tested_positive  ladder  marital1  mor_circle  moralid1  moralid10  moralid2  moralid3  moralid4  moralid5  moralid6  moralid7  moralid8  moralid9  mcoop1  mcoop2  mcoop3  mcoop4  mcoop5  mcoop6  mcoop7  narc1  narc2  narc3  narc4  narc5  narc6  nidentity1  nidentity2  omind1  omind4  omind5  omind6  contact1  contact2  contact3  contact4  contact5  hygiene1  hygiene2  hygiene3  hygiene4  hygiene5  psupport1  psupport2  psupport3  psupport4  psupport5  political_ideology  happy  slf_ladder  riskperc1  riskperc2  self_esteem  sex1  sbelong1  sbelong2  sbelong3  sbelong4  tested_positive  optim1  optim2  slfcont1  slfcont2  slfcont3  slfcont4  urban
##   4   10  age  children  cnarc1  cnarc2  cnarc3  ctheory1  ctheory2  ctheory3  ctheory4  CRT1  CRT2  CRT3  employ_status1  generosity1  generosity2  generosity3  health_cond  know_tested_positive  ladder  marital1  mor_circle  moralid1  moralid10  moralid2  moralid3  moralid4  moralid5  moralid6  moralid7  moralid8  moralid9  mcoop1  mcoop2  mcoop3  mcoop4  mcoop5  mcoop6  mcoop7  narc1  narc2  narc3  narc4  narc5  narc6  nidentity1  nidentity2  omind1  omind4  omind5  omind6  contact1  contact2  contact3  contact4  contact5  hygiene1  hygiene2  hygiene3  hygiene4  hygiene5  psupport1  psupport2  psupport3  psupport4  psupport5  political_ideology  happy  slf_ladder  riskperc1  riskperc2  self_esteem  sex1  sbelong1  sbelong2  sbelong3  sbelong4  tested_positive  optim1  optim2  slfcont1  slfcont2  slfcont3  slfcont4  urban
##   5   1  age  children  cnarc1  cnarc2  cnarc3  ctheory1  ctheory2  ctheory3  ctheory4  CRT1  CRT2  CRT3  employ_status1  generosity1  generosity2  generosity3  health_cond  know_tested_positive  ladder  marital1  mor_circle  moralid1  moralid10  moralid2  moralid3  moralid4  moralid5  moralid6  moralid7  moralid8  moralid9  mcoop1  mcoop2  mcoop3  mcoop4  mcoop5  mcoop6  mcoop7  narc1  narc2  narc3  narc4  narc5  narc6  nidentity1  nidentity2  omind1  omind4  omind5  omind6  contact1  contact2  contact3  contact4  contact5  hygiene1  hygiene2  hygiene3  hygiene4  hygiene5  psupport1  psupport2  psupport3  psupport4  psupport5  political_ideology  happy  slf_ladder  riskperc1  riskperc2  self_esteem  sex1  sbelong1  sbelong2  sbelong3  sbelong4  tested_positive  optim1  optim2  slfcont1  slfcont2  slfcont3  slfcont4  urban
##   5   2  age  children  cnarc1  cnarc2  cnarc3  ctheory1  ctheory2  ctheory3  ctheory4  CRT1  CRT2  CRT3  employ_status1  generosity1  generosity2  generosity3  health_cond  know_tested_positive  ladder  marital1  mor_circle  moralid1  moralid10  moralid2  moralid3  moralid4  moralid5  moralid6  moralid7  moralid8  moralid9  mcoop1  mcoop2  mcoop3  mcoop4  mcoop5  mcoop6  mcoop7  narc1  narc2  narc3  narc4  narc5  narc6  nidentity1  nidentity2  omind1  omind4  omind5  omind6  contact1  contact2  contact3  contact4  contact5  hygiene1  hygiene2  hygiene3  hygiene4  hygiene5  psupport1  psupport2  psupport3  psupport4  psupport5  political_ideology  happy  slf_ladder  riskperc1  riskperc2  self_esteem  sex1  sbelong1  sbelong2  sbelong3  sbelong4  tested_positive  optim1  optim2  slfcont1  slfcont2  slfcont3  slfcont4  urban
##   5   3  age  children  cnarc1  cnarc2  cnarc3  ctheory1  ctheory2  ctheory3  ctheory4  CRT1  CRT2  CRT3  employ_status1  generosity1  generosity2  generosity3  health_cond  know_tested_positive  ladder  marital1  mor_circle  moralid1  moralid10  moralid2  moralid3  moralid4  moralid5  moralid6  moralid7  moralid8  moralid9  mcoop1  mcoop2  mcoop3  mcoop4  mcoop5  mcoop6  mcoop7  narc1  narc2  narc3  narc4  narc5  narc6  nidentity1  nidentity2  omind1  omind4  omind5  omind6  contact1  contact2  contact3  contact4  contact5  hygiene1  hygiene2  hygiene3  hygiene4  hygiene5  psupport1  psupport2  psupport3  psupport4  psupport5  political_ideology  happy  slf_ladder  riskperc1  riskperc2  self_esteem  sex1  sbelong1  sbelong2  sbelong3  sbelong4  tested_positive  optim1  optim2  slfcont1  slfcont2  slfcont3  slfcont4  urban
##   5   4  age  children  cnarc1  cnarc2  cnarc3  ctheory1  ctheory2  ctheory3  ctheory4  CRT1  CRT2  CRT3  employ_status1  generosity1  generosity2  generosity3  health_cond  know_tested_positive  ladder  marital1  mor_circle  moralid1  moralid10  moralid2  moralid3  moralid4  moralid5  moralid6  moralid7  moralid8  moralid9  mcoop1  mcoop2  mcoop3  mcoop4  mcoop5  mcoop6  mcoop7  narc1  narc2  narc3  narc4  narc5  narc6  nidentity1  nidentity2  omind1  omind4  omind5  omind6  contact1  contact2  contact3  contact4  contact5  hygiene1  hygiene2  hygiene3  hygiene4  hygiene5  psupport1  psupport2  psupport3  psupport4  psupport5  political_ideology  happy  slf_ladder  riskperc1  riskperc2  self_esteem  sex1  sbelong1  sbelong2  sbelong3  sbelong4  tested_positive  optim1  optim2  slfcont1  slfcont2  slfcont3  slfcont4  urban
##   5   5  age  children  cnarc1  cnarc2  cnarc3  ctheory1  ctheory2  ctheory3  ctheory4  CRT1  CRT2  CRT3  employ_status1  generosity1  generosity2  generosity3  health_cond  know_tested_positive  ladder  marital1  mor_circle  moralid1  moralid10  moralid2  moralid3  moralid4  moralid5  moralid6  moralid7  moralid8  moralid9  mcoop1  mcoop2  mcoop3  mcoop4  mcoop5  mcoop6  mcoop7  narc1  narc2  narc3  narc4  narc5  narc6  nidentity1  nidentity2  omind1  omind4  omind5  omind6  contact1  contact2  contact3  contact4  contact5  hygiene1  hygiene2  hygiene3  hygiene4  hygiene5  psupport1  psupport2  psupport3  psupport4  psupport5  political_ideology  happy  slf_ladder  riskperc1  riskperc2  self_esteem  sex1  sbelong1  sbelong2  sbelong3  sbelong4  tested_positive  optim1  optim2  slfcont1  slfcont2  slfcont3  slfcont4  urban
##   5   6  age  children  cnarc1  cnarc2  cnarc3  ctheory1  ctheory2  ctheory3  ctheory4  CRT1  CRT2  CRT3  employ_status1  generosity1  generosity2  generosity3  health_cond  know_tested_positive  ladder  marital1  mor_circle  moralid1  moralid10  moralid2  moralid3  moralid4  moralid5  moralid6  moralid7  moralid8  moralid9  mcoop1  mcoop2  mcoop3  mcoop4  mcoop5  mcoop6  mcoop7  narc1  narc2  narc3  narc4  narc5  narc6  nidentity1  nidentity2  omind1  omind4  omind5  omind6  contact1  contact2  contact3  contact4  contact5  hygiene1  hygiene2  hygiene3  hygiene4  hygiene5  psupport1  psupport2  psupport3  psupport4  psupport5  political_ideology  happy  slf_ladder  riskperc1  riskperc2  self_esteem  sex1  sbelong1  sbelong2  sbelong3  sbelong4  tested_positive  optim1  optim2  slfcont1  slfcont2  slfcont3  slfcont4  urban
##   5   7  age  children  cnarc1  cnarc2  cnarc3  ctheory1  ctheory2  ctheory3  ctheory4  CRT1  CRT2  CRT3  employ_status1  generosity1  generosity2  generosity3  health_cond  know_tested_positive  ladder  marital1  mor_circle  moralid1  moralid10  moralid2  moralid3  moralid4  moralid5  moralid6  moralid7  moralid8  moralid9  mcoop1  mcoop2  mcoop3  mcoop4  mcoop5  mcoop6  mcoop7  narc1  narc2  narc3  narc4  narc5  narc6  nidentity1  nidentity2  omind1  omind4  omind5  omind6  contact1  contact2  contact3  contact4  contact5  hygiene1  hygiene2  hygiene3  hygiene4  hygiene5  psupport1  psupport2  psupport3  psupport4  psupport5  political_ideology  happy  slf_ladder  riskperc1  riskperc2  self_esteem  sex1  sbelong1  sbelong2  sbelong3  sbelong4  tested_positive  optim1  optim2  slfcont1  slfcont2  slfcont3  slfcont4  urban
##   5   8  age  children  cnarc1  cnarc2  cnarc3  ctheory1  ctheory2  ctheory3  ctheory4  CRT1  CRT2  CRT3  employ_status1  generosity1  generosity2  generosity3  health_cond  know_tested_positive  ladder  marital1  mor_circle  moralid1  moralid10  moralid2  moralid3  moralid4  moralid5  moralid6  moralid7  moralid8  moralid9  mcoop1  mcoop2  mcoop3  mcoop4  mcoop5  mcoop6  mcoop7  narc1  narc2  narc3  narc4  narc5  narc6  nidentity1  nidentity2  omind1  omind4  omind5  omind6  contact1  contact2  contact3  contact4  contact5  hygiene1  hygiene2  hygiene3  hygiene4  hygiene5  psupport1  psupport2  psupport3  psupport4  psupport5  political_ideology  happy  slf_ladder  riskperc1  riskperc2  self_esteem  sex1  sbelong1  sbelong2  sbelong3  sbelong4  tested_positive  optim1  optim2  slfcont1  slfcont2  slfcont3  slfcont4  urban
##   5   9  age  children  cnarc1  cnarc2  cnarc3  ctheory1  ctheory2  ctheory3  ctheory4  CRT1  CRT2  CRT3  employ_status1  generosity1  generosity2  generosity3  health_cond  know_tested_positive  ladder  marital1  mor_circle  moralid1  moralid10  moralid2  moralid3  moralid4  moralid5  moralid6  moralid7  moralid8  moralid9  mcoop1  mcoop2  mcoop3  mcoop4  mcoop5  mcoop6  mcoop7  narc1  narc2  narc3  narc4  narc5  narc6  nidentity1  nidentity2  omind1  omind4  omind5  omind6  contact1  contact2  contact3  contact4  contact5  hygiene1  hygiene2  hygiene3  hygiene4  hygiene5  psupport1  psupport2  psupport3  psupport4  psupport5  political_ideology  happy  slf_ladder  riskperc1  riskperc2  self_esteem  sex1  sbelong1  sbelong2  sbelong3  sbelong4  tested_positive  optim1  optim2  slfcont1  slfcont2  slfcont3  slfcont4  urban
##   5   10  age  children  cnarc1  cnarc2  cnarc3  ctheory1  ctheory2  ctheory3  ctheory4  CRT1  CRT2  CRT3  employ_status1  generosity1  generosity2  generosity3  health_cond  know_tested_positive  ladder  marital1  mor_circle  moralid1  moralid10  moralid2  moralid3  moralid4  moralid5  moralid6  moralid7  moralid8  moralid9  mcoop1  mcoop2  mcoop3  mcoop4  mcoop5  mcoop6  mcoop7  narc1  narc2  narc3  narc4  narc5  narc6  nidentity1  nidentity2  omind1  omind4  omind5  omind6  contact1  contact2  contact3  contact4  contact5  hygiene1  hygiene2  hygiene3  hygiene4  hygiene5  psupport1  psupport2  psupport3  psupport4  psupport5  political_ideology  happy  slf_ladder  riskperc1  riskperc2  self_esteem  sex1  sbelong1  sbelong2  sbelong3  sbelong4  tested_positive  optim1  optim2  slfcont1  slfcont2  slfcont3  slfcont4  urban
##   6   1  age  children  cnarc1  cnarc2  cnarc3  ctheory1  ctheory2  ctheory3  ctheory4  CRT1  CRT2  CRT3  employ_status1  generosity1  generosity2  generosity3  health_cond  know_tested_positive  ladder  marital1  mor_circle  moralid1  moralid10  moralid2  moralid3  moralid4  moralid5  moralid6  moralid7  moralid8  moralid9  mcoop1  mcoop2  mcoop3  mcoop4  mcoop5  mcoop6  mcoop7  narc1  narc2  narc3  narc4  narc5  narc6  nidentity1  nidentity2  omind1  omind4  omind5  omind6  contact1  contact2  contact3  contact4  contact5  hygiene1  hygiene2  hygiene3  hygiene4  hygiene5  psupport1  psupport2  psupport3  psupport4  psupport5  political_ideology  happy  slf_ladder  riskperc1  riskperc2  self_esteem  sex1  sbelong1  sbelong2  sbelong3  sbelong4  tested_positive  optim1  optim2  slfcont1  slfcont2  slfcont3  slfcont4  urban
##   6   2  age  children  cnarc1  cnarc2  cnarc3  ctheory1  ctheory2  ctheory3  ctheory4  CRT1  CRT2  CRT3  employ_status1  generosity1  generosity2  generosity3  health_cond  know_tested_positive  ladder  marital1  mor_circle  moralid1  moralid10  moralid2  moralid3  moralid4  moralid5  moralid6  moralid7  moralid8  moralid9  mcoop1  mcoop2  mcoop3  mcoop4  mcoop5  mcoop6  mcoop7  narc1  narc2  narc3  narc4  narc5  narc6  nidentity1  nidentity2  omind1  omind4  omind5  omind6  contact1  contact2  contact3  contact4  contact5  hygiene1  hygiene2  hygiene3  hygiene4  hygiene5  psupport1  psupport2  psupport3  psupport4  psupport5  political_ideology  happy  slf_ladder  riskperc1  riskperc2  self_esteem  sex1  sbelong1  sbelong2  sbelong3  sbelong4  tested_positive  optim1  optim2  slfcont1  slfcont2  slfcont3  slfcont4  urban
##   6   3  age  children  cnarc1  cnarc2  cnarc3  ctheory1  ctheory2  ctheory3  ctheory4  CRT1  CRT2  CRT3  employ_status1  generosity1  generosity2  generosity3  health_cond  know_tested_positive  ladder  marital1  mor_circle  moralid1  moralid10  moralid2  moralid3  moralid4  moralid5  moralid6  moralid7  moralid8  moralid9  mcoop1  mcoop2  mcoop3  mcoop4  mcoop5  mcoop6  mcoop7  narc1  narc2  narc3  narc4  narc5  narc6  nidentity1  nidentity2  omind1  omind4  omind5  omind6  contact1  contact2  contact3  contact4  contact5  hygiene1  hygiene2  hygiene3  hygiene4  hygiene5  psupport1  psupport2  psupport3  psupport4  psupport5  political_ideology  happy  slf_ladder  riskperc1  riskperc2  self_esteem  sex1  sbelong1  sbelong2  sbelong3  sbelong4  tested_positive  optim1  optim2  slfcont1  slfcont2  slfcont3  slfcont4  urban
##   6   4  age  children  cnarc1  cnarc2  cnarc3  ctheory1  ctheory2  ctheory3  ctheory4  CRT1  CRT2  CRT3  employ_status1  generosity1  generosity2  generosity3  health_cond  know_tested_positive  ladder  marital1  mor_circle  moralid1  moralid10  moralid2  moralid3  moralid4  moralid5  moralid6  moralid7  moralid8  moralid9  mcoop1  mcoop2  mcoop3  mcoop4  mcoop5  mcoop6  mcoop7  narc1  narc2  narc3  narc4  narc5  narc6  nidentity1  nidentity2  omind1  omind4  omind5  omind6  contact1  contact2  contact3  contact4  contact5  hygiene1  hygiene2  hygiene3  hygiene4  hygiene5  psupport1  psupport2  psupport3  psupport4  psupport5  political_ideology  happy  slf_ladder  riskperc1  riskperc2  self_esteem  sex1  sbelong1  sbelong2  sbelong3  sbelong4  tested_positive  optim1  optim2  slfcont1  slfcont2  slfcont3  slfcont4  urban
##   6   5  age  children  cnarc1  cnarc2  cnarc3  ctheory1  ctheory2  ctheory3  ctheory4  CRT1  CRT2  CRT3  employ_status1  generosity1  generosity2  generosity3  health_cond  know_tested_positive  ladder  marital1  mor_circle  moralid1  moralid10  moralid2  moralid3  moralid4  moralid5  moralid6  moralid7  moralid8  moralid9  mcoop1  mcoop2  mcoop3  mcoop4  mcoop5  mcoop6  mcoop7  narc1  narc2  narc3  narc4  narc5  narc6  nidentity1  nidentity2  omind1  omind4  omind5  omind6  contact1  contact2  contact3  contact4  contact5  hygiene1  hygiene2  hygiene3  hygiene4  hygiene5  psupport1  psupport2  psupport3  psupport4  psupport5  political_ideology  happy  slf_ladder  riskperc1  riskperc2  self_esteem  sex1  sbelong1  sbelong2  sbelong3  sbelong4  tested_positive  optim1  optim2  slfcont1  slfcont2  slfcont3  slfcont4  urban
##   6   6  age  children  cnarc1  cnarc2  cnarc3  ctheory1  ctheory2  ctheory3  ctheory4  CRT1  CRT2  CRT3  employ_status1  generosity1  generosity2  generosity3  health_cond  know_tested_positive  ladder  marital1  mor_circle  moralid1  moralid10  moralid2  moralid3  moralid4  moralid5  moralid6  moralid7  moralid8  moralid9  mcoop1  mcoop2  mcoop3  mcoop4  mcoop5  mcoop6  mcoop7  narc1  narc2  narc3  narc4  narc5  narc6  nidentity1  nidentity2  omind1  omind4  omind5  omind6  contact1  contact2  contact3  contact4  contact5  hygiene1  hygiene2  hygiene3  hygiene4  hygiene5  psupport1  psupport2  psupport3  psupport4  psupport5  political_ideology  happy  slf_ladder  riskperc1  riskperc2  self_esteem  sex1  sbelong1  sbelong2  sbelong3  sbelong4  tested_positive  optim1  optim2  slfcont1  slfcont2  slfcont3  slfcont4  urban
##   6   7  age  children  cnarc1  cnarc2  cnarc3  ctheory1  ctheory2  ctheory3  ctheory4  CRT1  CRT2  CRT3  employ_status1  generosity1  generosity2  generosity3  health_cond  know_tested_positive  ladder  marital1  mor_circle  moralid1  moralid10  moralid2  moralid3  moralid4  moralid5  moralid6  moralid7  moralid8  moralid9  mcoop1  mcoop2  mcoop3  mcoop4  mcoop5  mcoop6  mcoop7  narc1  narc2  narc3  narc4  narc5  narc6  nidentity1  nidentity2  omind1  omind4  omind5  omind6  contact1  contact2  contact3  contact4  contact5  hygiene1  hygiene2  hygiene3  hygiene4  hygiene5  psupport1  psupport2  psupport3  psupport4  psupport5  political_ideology  happy  slf_ladder  riskperc1  riskperc2  self_esteem  sex1  sbelong1  sbelong2  sbelong3  sbelong4  tested_positive  optim1  optim2  slfcont1  slfcont2  slfcont3  slfcont4  urban
##   6   8  age  children  cnarc1  cnarc2  cnarc3  ctheory1  ctheory2  ctheory3  ctheory4  CRT1  CRT2  CRT3  employ_status1  generosity1  generosity2  generosity3  health_cond  know_tested_positive  ladder  marital1  mor_circle  moralid1  moralid10  moralid2  moralid3  moralid4  moralid5  moralid6  moralid7  moralid8  moralid9  mcoop1  mcoop2  mcoop3  mcoop4  mcoop5  mcoop6  mcoop7  narc1  narc2  narc3  narc4  narc5  narc6  nidentity1  nidentity2  omind1  omind4  omind5  omind6  contact1  contact2  contact3  contact4  contact5  hygiene1  hygiene2  hygiene3  hygiene4  hygiene5  psupport1  psupport2  psupport3  psupport4  psupport5  political_ideology  happy  slf_ladder  riskperc1  riskperc2  self_esteem  sex1  sbelong1  sbelong2  sbelong3  sbelong4  tested_positive  optim1  optim2  slfcont1  slfcont2  slfcont3  slfcont4  urban
##   6   9  age  children  cnarc1  cnarc2  cnarc3  ctheory1  ctheory2  ctheory3  ctheory4  CRT1  CRT2  CRT3  employ_status1  generosity1  generosity2  generosity3  health_cond  know_tested_positive  ladder  marital1  mor_circle  moralid1  moralid10  moralid2  moralid3  moralid4  moralid5  moralid6  moralid7  moralid8  moralid9  mcoop1  mcoop2  mcoop3  mcoop4  mcoop5  mcoop6  mcoop7  narc1  narc2  narc3  narc4  narc5  narc6  nidentity1  nidentity2  omind1  omind4  omind5  omind6  contact1  contact2  contact3  contact4  contact5  hygiene1  hygiene2  hygiene3  hygiene4  hygiene5  psupport1  psupport2  psupport3  psupport4  psupport5  political_ideology  happy  slf_ladder  riskperc1  riskperc2  self_esteem  sex1  sbelong1  sbelong2  sbelong3  sbelong4  tested_positive  optim1  optim2  slfcont1  slfcont2  slfcont3  slfcont4  urban
##   6   10  age  children  cnarc1  cnarc2  cnarc3  ctheory1  ctheory2  ctheory3  ctheory4  CRT1  CRT2  CRT3  employ_status1  generosity1  generosity2  generosity3  health_cond  know_tested_positive  ladder  marital1  mor_circle  moralid1  moralid10  moralid2  moralid3  moralid4  moralid5  moralid6  moralid7  moralid8  moralid9  mcoop1  mcoop2  mcoop3  mcoop4  mcoop5  mcoop6  mcoop7  narc1  narc2  narc3  narc4  narc5  narc6  nidentity1  nidentity2  omind1  omind4  omind5  omind6  contact1  contact2  contact3  contact4  contact5  hygiene1  hygiene2  hygiene3  hygiene4  hygiene5  psupport1  psupport2  psupport3  psupport4  psupport5  political_ideology  happy  slf_ladder  riskperc1  riskperc2  self_esteem  sex1  sbelong1  sbelong2  sbelong3  sbelong4  tested_positive  optim1  optim2  slfcont1  slfcont2  slfcont3  slfcont4  urban
##   7   1  age  children  cnarc1  cnarc2  cnarc3  ctheory1  ctheory2  ctheory3  ctheory4  CRT1  CRT2  CRT3  employ_status1  generosity1  generosity2  generosity3  health_cond  know_tested_positive  ladder  marital1  mor_circle  moralid1  moralid10  moralid2  moralid3  moralid4  moralid5  moralid6  moralid7  moralid8  moralid9  mcoop1  mcoop2  mcoop3  mcoop4  mcoop5  mcoop6  mcoop7  narc1  narc2  narc3  narc4  narc5  narc6  nidentity1  nidentity2  omind1  omind4  omind5  omind6  contact1  contact2  contact3  contact4  contact5  hygiene1  hygiene2  hygiene3  hygiene4  hygiene5  psupport1  psupport2  psupport3  psupport4  psupport5  political_ideology  happy  slf_ladder  riskperc1  riskperc2  self_esteem  sex1  sbelong1  sbelong2  sbelong3  sbelong4  tested_positive  optim1  optim2  slfcont1  slfcont2  slfcont3  slfcont4  urban
##   7   2  age  children  cnarc1  cnarc2  cnarc3  ctheory1  ctheory2  ctheory3  ctheory4  CRT1  CRT2  CRT3  employ_status1  generosity1  generosity2  generosity3  health_cond  know_tested_positive  ladder  marital1  mor_circle  moralid1  moralid10  moralid2  moralid3  moralid4  moralid5  moralid6  moralid7  moralid8  moralid9  mcoop1  mcoop2  mcoop3  mcoop4  mcoop5  mcoop6  mcoop7  narc1  narc2  narc3  narc4  narc5  narc6  nidentity1  nidentity2  omind1  omind4  omind5  omind6  contact1  contact2  contact3  contact4  contact5  hygiene1  hygiene2  hygiene3  hygiene4  hygiene5  psupport1  psupport2  psupport3  psupport4  psupport5  political_ideology  happy  slf_ladder  riskperc1  riskperc2  self_esteem  sex1  sbelong1  sbelong2  sbelong3  sbelong4  tested_positive  optim1  optim2  slfcont1  slfcont2  slfcont3  slfcont4  urban
##   7   3  age  children  cnarc1  cnarc2  cnarc3  ctheory1  ctheory2  ctheory3  ctheory4  CRT1  CRT2  CRT3  employ_status1  generosity1  generosity2  generosity3  health_cond  know_tested_positive  ladder  marital1  mor_circle  moralid1  moralid10  moralid2  moralid3  moralid4  moralid5  moralid6  moralid7  moralid8  moralid9  mcoop1  mcoop2  mcoop3  mcoop4  mcoop5  mcoop6  mcoop7  narc1  narc2  narc3  narc4  narc5  narc6  nidentity1  nidentity2  omind1  omind4  omind5  omind6  contact1  contact2  contact3  contact4  contact5  hygiene1  hygiene2  hygiene3  hygiene4  hygiene5  psupport1  psupport2  psupport3  psupport4  psupport5  political_ideology  happy  slf_ladder  riskperc1  riskperc2  self_esteem  sex1  sbelong1  sbelong2  sbelong3  sbelong4  tested_positive  optim1  optim2  slfcont1  slfcont2  slfcont3  slfcont4  urban
##   7   4  age  children  cnarc1  cnarc2  cnarc3  ctheory1  ctheory2  ctheory3  ctheory4  CRT1  CRT2  CRT3  employ_status1  generosity1  generosity2  generosity3  health_cond  know_tested_positive  ladder  marital1  mor_circle  moralid1  moralid10  moralid2  moralid3  moralid4  moralid5  moralid6  moralid7  moralid8  moralid9  mcoop1  mcoop2  mcoop3  mcoop4  mcoop5  mcoop6  mcoop7  narc1  narc2  narc3  narc4  narc5  narc6  nidentity1  nidentity2  omind1  omind4  omind5  omind6  contact1  contact2  contact3  contact4  contact5  hygiene1  hygiene2  hygiene3  hygiene4  hygiene5  psupport1  psupport2  psupport3  psupport4  psupport5  political_ideology  happy  slf_ladder  riskperc1  riskperc2  self_esteem  sex1  sbelong1  sbelong2  sbelong3  sbelong4  tested_positive  optim1  optim2  slfcont1  slfcont2  slfcont3  slfcont4  urban
##   7   5  age  children  cnarc1  cnarc2  cnarc3  ctheory1  ctheory2  ctheory3  ctheory4  CRT1  CRT2  CRT3  employ_status1  generosity1  generosity2  generosity3  health_cond  know_tested_positive  ladder  marital1  mor_circle  moralid1  moralid10  moralid2  moralid3  moralid4  moralid5  moralid6  moralid7  moralid8  moralid9  mcoop1  mcoop2  mcoop3  mcoop4  mcoop5  mcoop6  mcoop7  narc1  narc2  narc3  narc4  narc5  narc6  nidentity1  nidentity2  omind1  omind4  omind5  omind6  contact1  contact2  contact3  contact4  contact5  hygiene1  hygiene2  hygiene3  hygiene4  hygiene5  psupport1  psupport2  psupport3  psupport4  psupport5  political_ideology  happy  slf_ladder  riskperc1  riskperc2  self_esteem  sex1  sbelong1  sbelong2  sbelong3  sbelong4  tested_positive  optim1  optim2  slfcont1  slfcont2  slfcont3  slfcont4  urban
##   7   6  age  children  cnarc1  cnarc2  cnarc3  ctheory1  ctheory2  ctheory3  ctheory4  CRT1  CRT2  CRT3  employ_status1  generosity1  generosity2  generosity3  health_cond  know_tested_positive  ladder  marital1  mor_circle  moralid1  moralid10  moralid2  moralid3  moralid4  moralid5  moralid6  moralid7  moralid8  moralid9  mcoop1  mcoop2  mcoop3  mcoop4  mcoop5  mcoop6  mcoop7  narc1  narc2  narc3  narc4  narc5  narc6  nidentity1  nidentity2  omind1  omind4  omind5  omind6  contact1  contact2  contact3  contact4  contact5  hygiene1  hygiene2  hygiene3  hygiene4  hygiene5  psupport1  psupport2  psupport3  psupport4  psupport5  political_ideology  happy  slf_ladder  riskperc1  riskperc2  self_esteem  sex1  sbelong1  sbelong2  sbelong3  sbelong4  tested_positive  optim1  optim2  slfcont1  slfcont2  slfcont3  slfcont4  urban
##   7   7  age  children  cnarc1  cnarc2  cnarc3  ctheory1  ctheory2  ctheory3  ctheory4  CRT1  CRT2  CRT3  employ_status1  generosity1  generosity2  generosity3  health_cond  know_tested_positive  ladder  marital1  mor_circle  moralid1  moralid10  moralid2  moralid3  moralid4  moralid5  moralid6  moralid7  moralid8  moralid9  mcoop1  mcoop2  mcoop3  mcoop4  mcoop5  mcoop6  mcoop7  narc1  narc2  narc3  narc4  narc5  narc6  nidentity1  nidentity2  omind1  omind4  omind5  omind6  contact1  contact2  contact3  contact4  contact5  hygiene1  hygiene2  hygiene3  hygiene4  hygiene5  psupport1  psupport2  psupport3  psupport4  psupport5  political_ideology  happy  slf_ladder  riskperc1  riskperc2  self_esteem  sex1  sbelong1  sbelong2  sbelong3  sbelong4  tested_positive  optim1  optim2  slfcont1  slfcont2  slfcont3  slfcont4  urban
##   7   8  age  children  cnarc1  cnarc2  cnarc3  ctheory1  ctheory2  ctheory3  ctheory4  CRT1  CRT2  CRT3  employ_status1  generosity1  generosity2  generosity3  health_cond  know_tested_positive  ladder  marital1  mor_circle  moralid1  moralid10  moralid2  moralid3  moralid4  moralid5  moralid6  moralid7  moralid8  moralid9  mcoop1  mcoop2  mcoop3  mcoop4  mcoop5  mcoop6  mcoop7  narc1  narc2  narc3  narc4  narc5  narc6  nidentity1  nidentity2  omind1  omind4  omind5  omind6  contact1  contact2  contact3  contact4  contact5  hygiene1  hygiene2  hygiene3  hygiene4  hygiene5  psupport1  psupport2  psupport3  psupport4  psupport5  political_ideology  happy  slf_ladder  riskperc1  riskperc2  self_esteem  sex1  sbelong1  sbelong2  sbelong3  sbelong4  tested_positive  optim1  optim2  slfcont1  slfcont2  slfcont3  slfcont4  urban
##   7   9  age  children  cnarc1  cnarc2  cnarc3  ctheory1  ctheory2  ctheory3  ctheory4  CRT1  CRT2  CRT3  employ_status1  generosity1  generosity2  generosity3  health_cond  know_tested_positive  ladder  marital1  mor_circle  moralid1  moralid10  moralid2  moralid3  moralid4  moralid5  moralid6  moralid7  moralid8  moralid9  mcoop1  mcoop2  mcoop3  mcoop4  mcoop5  mcoop6  mcoop7  narc1  narc2  narc3  narc4  narc5  narc6  nidentity1  nidentity2  omind1  omind4  omind5  omind6  contact1  contact2  contact3  contact4  contact5  hygiene1  hygiene2  hygiene3  hygiene4  hygiene5  psupport1  psupport2  psupport3  psupport4  psupport5  political_ideology  happy  slf_ladder  riskperc1  riskperc2  self_esteem  sex1  sbelong1  sbelong2  sbelong3  sbelong4  tested_positive  optim1  optim2  slfcont1  slfcont2  slfcont3  slfcont4  urban
##   7   10  age  children  cnarc1  cnarc2  cnarc3  ctheory1  ctheory2  ctheory3  ctheory4  CRT1  CRT2  CRT3  employ_status1  generosity1  generosity2  generosity3  health_cond  know_tested_positive  ladder  marital1  mor_circle  moralid1  moralid10  moralid2  moralid3  moralid4  moralid5  moralid6  moralid7  moralid8  moralid9  mcoop1  mcoop2  mcoop3  mcoop4  mcoop5  mcoop6  mcoop7  narc1  narc2  narc3  narc4  narc5  narc6  nidentity1  nidentity2  omind1  omind4  omind5  omind6  contact1  contact2  contact3  contact4  contact5  hygiene1  hygiene2  hygiene3  hygiene4  hygiene5  psupport1  psupport2  psupport3  psupport4  psupport5  political_ideology  happy  slf_ladder  riskperc1  riskperc2  self_esteem  sex1  sbelong1  sbelong2  sbelong3  sbelong4  tested_positive  optim1  optim2  slfcont1  slfcont2  slfcont3  slfcont4  urban
##   8   1  age  children  cnarc1  cnarc2  cnarc3  ctheory1  ctheory2  ctheory3  ctheory4  CRT1  CRT2  CRT3  employ_status1  generosity1  generosity2  generosity3  health_cond  know_tested_positive  ladder  marital1  mor_circle  moralid1  moralid10  moralid2  moralid3  moralid4  moralid5  moralid6  moralid7  moralid8  moralid9  mcoop1  mcoop2  mcoop3  mcoop4  mcoop5  mcoop6  mcoop7  narc1  narc2  narc3  narc4  narc5  narc6  nidentity1  nidentity2  omind1  omind4  omind5  omind6  contact1  contact2  contact3  contact4  contact5  hygiene1  hygiene2  hygiene3  hygiene4  hygiene5  psupport1  psupport2  psupport3  psupport4  psupport5  political_ideology  happy  slf_ladder  riskperc1  riskperc2  self_esteem  sex1  sbelong1  sbelong2  sbelong3  sbelong4  tested_positive  optim1  optim2  slfcont1  slfcont2  slfcont3  slfcont4  urban
##   8   2  age  children  cnarc1  cnarc2  cnarc3  ctheory1  ctheory2  ctheory3  ctheory4  CRT1  CRT2  CRT3  employ_status1  generosity1  generosity2  generosity3  health_cond  know_tested_positive  ladder  marital1  mor_circle  moralid1  moralid10  moralid2  moralid3  moralid4  moralid5  moralid6  moralid7  moralid8  moralid9  mcoop1  mcoop2  mcoop3  mcoop4  mcoop5  mcoop6  mcoop7  narc1  narc2  narc3  narc4  narc5  narc6  nidentity1  nidentity2  omind1  omind4  omind5  omind6  contact1  contact2  contact3  contact4  contact5  hygiene1  hygiene2  hygiene3  hygiene4  hygiene5  psupport1  psupport2  psupport3  psupport4  psupport5  political_ideology  happy  slf_ladder  riskperc1  riskperc2  self_esteem  sex1  sbelong1  sbelong2  sbelong3  sbelong4  tested_positive  optim1  optim2  slfcont1  slfcont2  slfcont3  slfcont4  urban
##   8   3  age  children  cnarc1  cnarc2  cnarc3  ctheory1  ctheory2  ctheory3  ctheory4  CRT1  CRT2  CRT3  employ_status1  generosity1  generosity2  generosity3  health_cond  know_tested_positive  ladder  marital1  mor_circle  moralid1  moralid10  moralid2  moralid3  moralid4  moralid5  moralid6  moralid7  moralid8  moralid9  mcoop1  mcoop2  mcoop3  mcoop4  mcoop5  mcoop6  mcoop7  narc1  narc2  narc3  narc4  narc5  narc6  nidentity1  nidentity2  omind1  omind4  omind5  omind6  contact1  contact2  contact3  contact4  contact5  hygiene1  hygiene2  hygiene3  hygiene4  hygiene5  psupport1  psupport2  psupport3  psupport4  psupport5  political_ideology  happy  slf_ladder  riskperc1  riskperc2  self_esteem  sex1  sbelong1  sbelong2  sbelong3  sbelong4  tested_positive  optim1  optim2  slfcont1  slfcont2  slfcont3  slfcont4  urban
##   8   4  age  children  cnarc1  cnarc2  cnarc3  ctheory1  ctheory2  ctheory3  ctheory4  CRT1  CRT2  CRT3  employ_status1  generosity1  generosity2  generosity3  health_cond  know_tested_positive  ladder  marital1  mor_circle  moralid1  moralid10  moralid2  moralid3  moralid4  moralid5  moralid6  moralid7  moralid8  moralid9  mcoop1  mcoop2  mcoop3  mcoop4  mcoop5  mcoop6  mcoop7  narc1  narc2  narc3  narc4  narc5  narc6  nidentity1  nidentity2  omind1  omind4  omind5  omind6  contact1  contact2  contact3  contact4  contact5  hygiene1  hygiene2  hygiene3  hygiene4  hygiene5  psupport1  psupport2  psupport3  psupport4  psupport5  political_ideology  happy  slf_ladder  riskperc1  riskperc2  self_esteem  sex1  sbelong1  sbelong2  sbelong3  sbelong4  tested_positive  optim1  optim2  slfcont1  slfcont2  slfcont3  slfcont4  urban
##   8   5  age  children  cnarc1  cnarc2  cnarc3  ctheory1  ctheory2  ctheory3  ctheory4  CRT1  CRT2  CRT3  employ_status1  generosity1  generosity2  generosity3  health_cond  know_tested_positive  ladder  marital1  mor_circle  moralid1  moralid10  moralid2  moralid3  moralid4  moralid5  moralid6  moralid7  moralid8  moralid9  mcoop1  mcoop2  mcoop3  mcoop4  mcoop5  mcoop6  mcoop7  narc1  narc2  narc3  narc4  narc5  narc6  nidentity1  nidentity2  omind1  omind4  omind5  omind6  contact1  contact2  contact3  contact4  contact5  hygiene1  hygiene2  hygiene3  hygiene4  hygiene5  psupport1  psupport2  psupport3  psupport4  psupport5  political_ideology  happy  slf_ladder  riskperc1  riskperc2  self_esteem  sex1  sbelong1  sbelong2  sbelong3  sbelong4  tested_positive  optim1  optim2  slfcont1  slfcont2  slfcont3  slfcont4  urban
##   8   6  age  children  cnarc1  cnarc2  cnarc3  ctheory1  ctheory2  ctheory3  ctheory4  CRT1  CRT2  CRT3  employ_status1  generosity1  generosity2  generosity3  health_cond  know_tested_positive  ladder  marital1  mor_circle  moralid1  moralid10  moralid2  moralid3  moralid4  moralid5  moralid6  moralid7  moralid8  moralid9  mcoop1  mcoop2  mcoop3  mcoop4  mcoop5  mcoop6  mcoop7  narc1  narc2  narc3  narc4  narc5  narc6  nidentity1  nidentity2  omind1  omind4  omind5  omind6  contact1  contact2  contact3  contact4  contact5  hygiene1  hygiene2  hygiene3  hygiene4  hygiene5  psupport1  psupport2  psupport3  psupport4  psupport5  political_ideology  happy  slf_ladder  riskperc1  riskperc2  self_esteem  sex1  sbelong1  sbelong2  sbelong3  sbelong4  tested_positive  optim1  optim2  slfcont1  slfcont2  slfcont3  slfcont4  urban
##   8   7  age  children  cnarc1  cnarc2  cnarc3  ctheory1  ctheory2  ctheory3  ctheory4  CRT1  CRT2  CRT3  employ_status1  generosity1  generosity2  generosity3  health_cond  know_tested_positive  ladder  marital1  mor_circle  moralid1  moralid10  moralid2  moralid3  moralid4  moralid5  moralid6  moralid7  moralid8  moralid9  mcoop1  mcoop2  mcoop3  mcoop4  mcoop5  mcoop6  mcoop7  narc1  narc2  narc3  narc4  narc5  narc6  nidentity1  nidentity2  omind1  omind4  omind5  omind6  contact1  contact2  contact3  contact4  contact5  hygiene1  hygiene2  hygiene3  hygiene4  hygiene5  psupport1  psupport2  psupport3  psupport4  psupport5  political_ideology  happy  slf_ladder  riskperc1  riskperc2  self_esteem  sex1  sbelong1  sbelong2  sbelong3  sbelong4  tested_positive  optim1  optim2  slfcont1  slfcont2  slfcont3  slfcont4  urban
##   8   8  age  children  cnarc1  cnarc2  cnarc3  ctheory1  ctheory2  ctheory3  ctheory4  CRT1  CRT2  CRT3  employ_status1  generosity1  generosity2  generosity3  health_cond  know_tested_positive  ladder  marital1  mor_circle  moralid1  moralid10  moralid2  moralid3  moralid4  moralid5  moralid6  moralid7  moralid8  moralid9  mcoop1  mcoop2  mcoop3  mcoop4  mcoop5  mcoop6  mcoop7  narc1  narc2  narc3  narc4  narc5  narc6  nidentity1  nidentity2  omind1  omind4  omind5  omind6  contact1  contact2  contact3  contact4  contact5  hygiene1  hygiene2  hygiene3  hygiene4  hygiene5  psupport1  psupport2  psupport3  psupport4  psupport5  political_ideology  happy  slf_ladder  riskperc1  riskperc2  self_esteem  sex1  sbelong1  sbelong2  sbelong3  sbelong4  tested_positive  optim1  optim2  slfcont1  slfcont2  slfcont3  slfcont4  urban
##   8   9  age  children  cnarc1  cnarc2  cnarc3  ctheory1  ctheory2  ctheory3  ctheory4  CRT1  CRT2  CRT3  employ_status1  generosity1  generosity2  generosity3  health_cond  know_tested_positive  ladder  marital1  mor_circle  moralid1  moralid10  moralid2  moralid3  moralid4  moralid5  moralid6  moralid7  moralid8  moralid9  mcoop1  mcoop2  mcoop3  mcoop4  mcoop5  mcoop6  mcoop7  narc1  narc2  narc3  narc4  narc5  narc6  nidentity1  nidentity2  omind1  omind4  omind5  omind6  contact1  contact2  contact3  contact4  contact5  hygiene1  hygiene2  hygiene3  hygiene4  hygiene5  psupport1  psupport2  psupport3  psupport4  psupport5  political_ideology  happy  slf_ladder  riskperc1  riskperc2  self_esteem  sex1  sbelong1  sbelong2  sbelong3  sbelong4  tested_positive  optim1  optim2  slfcont1  slfcont2  slfcont3  slfcont4  urban
##   8   10  age  children  cnarc1  cnarc2  cnarc3  ctheory1  ctheory2  ctheory3  ctheory4  CRT1  CRT2  CRT3  employ_status1  generosity1  generosity2  generosity3  health_cond  know_tested_positive  ladder  marital1  mor_circle  moralid1  moralid10  moralid2  moralid3  moralid4  moralid5  moralid6  moralid7  moralid8  moralid9  mcoop1  mcoop2  mcoop3  mcoop4  mcoop5  mcoop6  mcoop7  narc1  narc2  narc3  narc4  narc5  narc6  nidentity1  nidentity2  omind1  omind4  omind5  omind6  contact1  contact2  contact3  contact4  contact5  hygiene1  hygiene2  hygiene3  hygiene4  hygiene5  psupport1  psupport2  psupport3  psupport4  psupport5  political_ideology  happy  slf_ladder  riskperc1  riskperc2  self_esteem  sex1  sbelong1  sbelong2  sbelong3  sbelong4  tested_positive  optim1  optim2  slfcont1  slfcont2  slfcont3  slfcont4  urban
##   9   1  age  children  cnarc1  cnarc2  cnarc3  ctheory1  ctheory2  ctheory3  ctheory4  CRT1  CRT2  CRT3  employ_status1  generosity1  generosity2  generosity3  health_cond  know_tested_positive  ladder  marital1  mor_circle  moralid1  moralid10  moralid2  moralid3  moralid4  moralid5  moralid6  moralid7  moralid8  moralid9  mcoop1  mcoop2  mcoop3  mcoop4  mcoop5  mcoop6  mcoop7  narc1  narc2  narc3  narc4  narc5  narc6  nidentity1  nidentity2  omind1  omind4  omind5  omind6  contact1  contact2  contact3  contact4  contact5  hygiene1  hygiene2  hygiene3  hygiene4  hygiene5  psupport1  psupport2  psupport3  psupport4  psupport5  political_ideology  happy  slf_ladder  riskperc1  riskperc2  self_esteem  sex1  sbelong1  sbelong2  sbelong3  sbelong4  tested_positive  optim1  optim2  slfcont1  slfcont2  slfcont3  slfcont4  urban
##   9   2  age  children  cnarc1  cnarc2  cnarc3  ctheory1  ctheory2  ctheory3  ctheory4  CRT1  CRT2  CRT3  employ_status1  generosity1  generosity2  generosity3  health_cond  know_tested_positive  ladder  marital1  mor_circle  moralid1  moralid10  moralid2  moralid3  moralid4  moralid5  moralid6  moralid7  moralid8  moralid9  mcoop1  mcoop2  mcoop3  mcoop4  mcoop5  mcoop6  mcoop7  narc1  narc2  narc3  narc4  narc5  narc6  nidentity1  nidentity2  omind1  omind4  omind5  omind6  contact1  contact2  contact3  contact4  contact5  hygiene1  hygiene2  hygiene3  hygiene4  hygiene5  psupport1  psupport2  psupport3  psupport4  psupport5  political_ideology  happy  slf_ladder  riskperc1  riskperc2  self_esteem  sex1  sbelong1  sbelong2  sbelong3  sbelong4  tested_positive  optim1  optim2  slfcont1  slfcont2  slfcont3  slfcont4  urban
##   9   3  age  children  cnarc1  cnarc2  cnarc3  ctheory1  ctheory2  ctheory3  ctheory4  CRT1  CRT2  CRT3  employ_status1  generosity1  generosity2  generosity3  health_cond  know_tested_positive  ladder  marital1  mor_circle  moralid1  moralid10  moralid2  moralid3  moralid4  moralid5  moralid6  moralid7  moralid8  moralid9  mcoop1  mcoop2  mcoop3  mcoop4  mcoop5  mcoop6  mcoop7  narc1  narc2  narc3  narc4  narc5  narc6  nidentity1  nidentity2  omind1  omind4  omind5  omind6  contact1  contact2  contact3  contact4  contact5  hygiene1  hygiene2  hygiene3  hygiene4  hygiene5  psupport1  psupport2  psupport3  psupport4  psupport5  political_ideology  happy  slf_ladder  riskperc1  riskperc2  self_esteem  sex1  sbelong1  sbelong2  sbelong3  sbelong4  tested_positive  optim1  optim2  slfcont1  slfcont2  slfcont3  slfcont4  urban
##   9   4  age  children  cnarc1  cnarc2  cnarc3  ctheory1  ctheory2  ctheory3  ctheory4  CRT1  CRT2  CRT3  employ_status1  generosity1  generosity2  generosity3  health_cond  know_tested_positive  ladder  marital1  mor_circle  moralid1  moralid10  moralid2  moralid3  moralid4  moralid5  moralid6  moralid7  moralid8  moralid9  mcoop1  mcoop2  mcoop3  mcoop4  mcoop5  mcoop6  mcoop7  narc1  narc2  narc3  narc4  narc5  narc6  nidentity1  nidentity2  omind1  omind4  omind5  omind6  contact1  contact2  contact3  contact4  contact5  hygiene1  hygiene2  hygiene3  hygiene4  hygiene5  psupport1  psupport2  psupport3  psupport4  psupport5  political_ideology  happy  slf_ladder  riskperc1  riskperc2  self_esteem  sex1  sbelong1  sbelong2  sbelong3  sbelong4  tested_positive  optim1  optim2  slfcont1  slfcont2  slfcont3  slfcont4  urban
##   9   5  age  children  cnarc1  cnarc2  cnarc3  ctheory1  ctheory2  ctheory3  ctheory4  CRT1  CRT2  CRT3  employ_status1  generosity1  generosity2  generosity3  health_cond  know_tested_positive  ladder  marital1  mor_circle  moralid1  moralid10  moralid2  moralid3  moralid4  moralid5  moralid6  moralid7  moralid8  moralid9  mcoop1  mcoop2  mcoop3  mcoop4  mcoop5  mcoop6  mcoop7  narc1  narc2  narc3  narc4  narc5  narc6  nidentity1  nidentity2  omind1  omind4  omind5  omind6  contact1  contact2  contact3  contact4  contact5  hygiene1  hygiene2  hygiene3  hygiene4  hygiene5  psupport1  psupport2  psupport3  psupport4  psupport5  political_ideology  happy  slf_ladder  riskperc1  riskperc2  self_esteem  sex1  sbelong1  sbelong2  sbelong3  sbelong4  tested_positive  optim1  optim2  slfcont1  slfcont2  slfcont3  slfcont4  urban
##   9   6  age  children  cnarc1  cnarc2  cnarc3  ctheory1  ctheory2  ctheory3  ctheory4  CRT1  CRT2  CRT3  employ_status1  generosity1  generosity2  generosity3  health_cond  know_tested_positive  ladder  marital1  mor_circle  moralid1  moralid10  moralid2  moralid3  moralid4  moralid5  moralid6  moralid7  moralid8  moralid9  mcoop1  mcoop2  mcoop3  mcoop4  mcoop5  mcoop6  mcoop7  narc1  narc2  narc3  narc4  narc5  narc6  nidentity1  nidentity2  omind1  omind4  omind5  omind6  contact1  contact2  contact3  contact4  contact5  hygiene1  hygiene2  hygiene3  hygiene4  hygiene5  psupport1  psupport2  psupport3  psupport4  psupport5  political_ideology  happy  slf_ladder  riskperc1  riskperc2  self_esteem  sex1  sbelong1  sbelong2  sbelong3  sbelong4  tested_positive  optim1  optim2  slfcont1  slfcont2  slfcont3  slfcont4  urban
##   9   7  age  children  cnarc1  cnarc2  cnarc3  ctheory1  ctheory2  ctheory3  ctheory4  CRT1  CRT2  CRT3  employ_status1  generosity1  generosity2  generosity3  health_cond  know_tested_positive  ladder  marital1  mor_circle  moralid1  moralid10  moralid2  moralid3  moralid4  moralid5  moralid6  moralid7  moralid8  moralid9  mcoop1  mcoop2  mcoop3  mcoop4  mcoop5  mcoop6  mcoop7  narc1  narc2  narc3  narc4  narc5  narc6  nidentity1  nidentity2  omind1  omind4  omind5  omind6  contact1  contact2  contact3  contact4  contact5  hygiene1  hygiene2  hygiene3  hygiene4  hygiene5  psupport1  psupport2  psupport3  psupport4  psupport5  political_ideology  happy  slf_ladder  riskperc1  riskperc2  self_esteem  sex1  sbelong1  sbelong2  sbelong3  sbelong4  tested_positive  optim1  optim2  slfcont1  slfcont2  slfcont3  slfcont4  urban
##   9   8  age  children  cnarc1  cnarc2  cnarc3  ctheory1  ctheory2  ctheory3  ctheory4  CRT1  CRT2  CRT3  employ_status1  generosity1  generosity2  generosity3  health_cond  know_tested_positive  ladder  marital1  mor_circle  moralid1  moralid10  moralid2  moralid3  moralid4  moralid5  moralid6  moralid7  moralid8  moralid9  mcoop1  mcoop2  mcoop3  mcoop4  mcoop5  mcoop6  mcoop7  narc1  narc2  narc3  narc4  narc5  narc6  nidentity1  nidentity2  omind1  omind4  omind5  omind6  contact1  contact2  contact3  contact4  contact5  hygiene1  hygiene2  hygiene3  hygiene4  hygiene5  psupport1  psupport2  psupport3  psupport4  psupport5  political_ideology  happy  slf_ladder  riskperc1  riskperc2  self_esteem  sex1  sbelong1  sbelong2  sbelong3  sbelong4  tested_positive  optim1  optim2  slfcont1  slfcont2  slfcont3  slfcont4  urban
##   9   9  age  children  cnarc1  cnarc2  cnarc3  ctheory1  ctheory2  ctheory3  ctheory4  CRT1  CRT2  CRT3  employ_status1  generosity1  generosity2  generosity3  health_cond  know_tested_positive  ladder  marital1  mor_circle  moralid1  moralid10  moralid2  moralid3  moralid4  moralid5  moralid6  moralid7  moralid8  moralid9  mcoop1  mcoop2  mcoop3  mcoop4  mcoop5  mcoop6  mcoop7  narc1  narc2  narc3  narc4  narc5  narc6  nidentity1  nidentity2  omind1  omind4  omind5  omind6  contact1  contact2  contact3  contact4  contact5  hygiene1  hygiene2  hygiene3  hygiene4  hygiene5  psupport1  psupport2  psupport3  psupport4  psupport5  political_ideology  happy  slf_ladder  riskperc1  riskperc2  self_esteem  sex1  sbelong1  sbelong2  sbelong3  sbelong4  tested_positive  optim1  optim2  slfcont1  slfcont2  slfcont3  slfcont4  urban
##   9   10  age  children  cnarc1  cnarc2  cnarc3  ctheory1  ctheory2  ctheory3  ctheory4  CRT1  CRT2  CRT3  employ_status1  generosity1  generosity2  generosity3  health_cond  know_tested_positive  ladder  marital1  mor_circle  moralid1  moralid10  moralid2  moralid3  moralid4  moralid5  moralid6  moralid7  moralid8  moralid9  mcoop1  mcoop2  mcoop3  mcoop4  mcoop5  mcoop6  mcoop7  narc1  narc2  narc3  narc4  narc5  narc6  nidentity1  nidentity2  omind1  omind4  omind5  omind6  contact1  contact2  contact3  contact4  contact5  hygiene1  hygiene2  hygiene3  hygiene4  hygiene5  psupport1  psupport2  psupport3  psupport4  psupport5  political_ideology  happy  slf_ladder  riskperc1  riskperc2  self_esteem  sex1  sbelong1  sbelong2  sbelong3  sbelong4  tested_positive  optim1  optim2  slfcont1  slfcont2  slfcont3  slfcont4  urban
##   10   1  age  children  cnarc1  cnarc2  cnarc3  ctheory1  ctheory2  ctheory3  ctheory4  CRT1  CRT2  CRT3  employ_status1  generosity1  generosity2  generosity3  health_cond  know_tested_positive  ladder  marital1  mor_circle  moralid1  moralid10  moralid2  moralid3  moralid4  moralid5  moralid6  moralid7  moralid8  moralid9  mcoop1  mcoop2  mcoop3  mcoop4  mcoop5  mcoop6  mcoop7  narc1  narc2  narc3  narc4  narc5  narc6  nidentity1  nidentity2  omind1  omind4  omind5  omind6  contact1  contact2  contact3  contact4  contact5  hygiene1  hygiene2  hygiene3  hygiene4  hygiene5  psupport1  psupport2  psupport3  psupport4  psupport5  political_ideology  happy  slf_ladder  riskperc1  riskperc2  self_esteem  sex1  sbelong1  sbelong2  sbelong3  sbelong4  tested_positive  optim1  optim2  slfcont1  slfcont2  slfcont3  slfcont4  urban
##   10   2  age  children  cnarc1  cnarc2  cnarc3  ctheory1  ctheory2  ctheory3  ctheory4  CRT1  CRT2  CRT3  employ_status1  generosity1  generosity2  generosity3  health_cond  know_tested_positive  ladder  marital1  mor_circle  moralid1  moralid10  moralid2  moralid3  moralid4  moralid5  moralid6  moralid7  moralid8  moralid9  mcoop1  mcoop2  mcoop3  mcoop4  mcoop5  mcoop6  mcoop7  narc1  narc2  narc3  narc4  narc5  narc6  nidentity1  nidentity2  omind1  omind4  omind5  omind6  contact1  contact2  contact3  contact4  contact5  hygiene1  hygiene2  hygiene3  hygiene4  hygiene5  psupport1  psupport2  psupport3  psupport4  psupport5  political_ideology  happy  slf_ladder  riskperc1  riskperc2  self_esteem  sex1  sbelong1  sbelong2  sbelong3  sbelong4  tested_positive  optim1  optim2  slfcont1  slfcont2  slfcont3  slfcont4  urban
##   10   3  age  children  cnarc1  cnarc2  cnarc3  ctheory1  ctheory2  ctheory3  ctheory4  CRT1  CRT2  CRT3  employ_status1  generosity1  generosity2  generosity3  health_cond  know_tested_positive  ladder  marital1  mor_circle  moralid1  moralid10  moralid2  moralid3  moralid4  moralid5  moralid6  moralid7  moralid8  moralid9  mcoop1  mcoop2  mcoop3  mcoop4  mcoop5  mcoop6  mcoop7  narc1  narc2  narc3  narc4  narc5  narc6  nidentity1  nidentity2  omind1  omind4  omind5  omind6  contact1  contact2  contact3  contact4  contact5  hygiene1  hygiene2  hygiene3  hygiene4  hygiene5  psupport1  psupport2  psupport3  psupport4  psupport5  political_ideology  happy  slf_ladder  riskperc1  riskperc2  self_esteem  sex1  sbelong1  sbelong2  sbelong3  sbelong4  tested_positive  optim1  optim2  slfcont1  slfcont2  slfcont3  slfcont4  urban
##   10   4  age  children  cnarc1  cnarc2  cnarc3  ctheory1  ctheory2  ctheory3  ctheory4  CRT1  CRT2  CRT3  employ_status1  generosity1  generosity2  generosity3  health_cond  know_tested_positive  ladder  marital1  mor_circle  moralid1  moralid10  moralid2  moralid3  moralid4  moralid5  moralid6  moralid7  moralid8  moralid9  mcoop1  mcoop2  mcoop3  mcoop4  mcoop5  mcoop6  mcoop7  narc1  narc2  narc3  narc4  narc5  narc6  nidentity1  nidentity2  omind1  omind4  omind5  omind6  contact1  contact2  contact3  contact4  contact5  hygiene1  hygiene2  hygiene3  hygiene4  hygiene5  psupport1  psupport2  psupport3  psupport4  psupport5  political_ideology  happy  slf_ladder  riskperc1  riskperc2  self_esteem  sex1  sbelong1  sbelong2  sbelong3  sbelong4  tested_positive  optim1  optim2  slfcont1  slfcont2  slfcont3  slfcont4  urban
##   10   5  age  children  cnarc1  cnarc2  cnarc3  ctheory1  ctheory2  ctheory3  ctheory4  CRT1  CRT2  CRT3  employ_status1  generosity1  generosity2  generosity3  health_cond  know_tested_positive  ladder  marital1  mor_circle  moralid1  moralid10  moralid2  moralid3  moralid4  moralid5  moralid6  moralid7  moralid8  moralid9  mcoop1  mcoop2  mcoop3  mcoop4  mcoop5  mcoop6  mcoop7  narc1  narc2  narc3  narc4  narc5  narc6  nidentity1  nidentity2  omind1  omind4  omind5  omind6  contact1  contact2  contact3  contact4  contact5  hygiene1  hygiene2  hygiene3  hygiene4  hygiene5  psupport1  psupport2  psupport3  psupport4  psupport5  political_ideology  happy  slf_ladder  riskperc1  riskperc2  self_esteem  sex1  sbelong1  sbelong2  sbelong3  sbelong4  tested_positive  optim1  optim2  slfcont1  slfcont2  slfcont3  slfcont4  urban
##   10   6  age  children  cnarc1  cnarc2  cnarc3  ctheory1  ctheory2  ctheory3  ctheory4  CRT1  CRT2  CRT3  employ_status1  generosity1  generosity2  generosity3  health_cond  know_tested_positive  ladder  marital1  mor_circle  moralid1  moralid10  moralid2  moralid3  moralid4  moralid5  moralid6  moralid7  moralid8  moralid9  mcoop1  mcoop2  mcoop3  mcoop4  mcoop5  mcoop6  mcoop7  narc1  narc2  narc3  narc4  narc5  narc6  nidentity1  nidentity2  omind1  omind4  omind5  omind6  contact1  contact2  contact3  contact4  contact5  hygiene1  hygiene2  hygiene3  hygiene4  hygiene5  psupport1  psupport2  psupport3  psupport4  psupport5  political_ideology  happy  slf_ladder  riskperc1  riskperc2  self_esteem  sex1  sbelong1  sbelong2  sbelong3  sbelong4  tested_positive  optim1  optim2  slfcont1  slfcont2  slfcont3  slfcont4  urban
##   10   7  age  children  cnarc1  cnarc2  cnarc3  ctheory1  ctheory2  ctheory3  ctheory4  CRT1  CRT2  CRT3  employ_status1  generosity1  generosity2  generosity3  health_cond  know_tested_positive  ladder  marital1  mor_circle  moralid1  moralid10  moralid2  moralid3  moralid4  moralid5  moralid6  moralid7  moralid8  moralid9  mcoop1  mcoop2  mcoop3  mcoop4  mcoop5  mcoop6  mcoop7  narc1  narc2  narc3  narc4  narc5  narc6  nidentity1  nidentity2  omind1  omind4  omind5  omind6  contact1  contact2  contact3  contact4  contact5  hygiene1  hygiene2  hygiene3  hygiene4  hygiene5  psupport1  psupport2  psupport3  psupport4  psupport5  political_ideology  happy  slf_ladder  riskperc1  riskperc2  self_esteem  sex1  sbelong1  sbelong2  sbelong3  sbelong4  tested_positive  optim1  optim2  slfcont1  slfcont2  slfcont3  slfcont4  urban
##   10   8  age  children  cnarc1  cnarc2  cnarc3  ctheory1  ctheory2  ctheory3  ctheory4  CRT1  CRT2  CRT3  employ_status1  generosity1  generosity2  generosity3  health_cond  know_tested_positive  ladder  marital1  mor_circle  moralid1  moralid10  moralid2  moralid3  moralid4  moralid5  moralid6  moralid7  moralid8  moralid9  mcoop1  mcoop2  mcoop3  mcoop4  mcoop5  mcoop6  mcoop7  narc1  narc2  narc3  narc4  narc5  narc6  nidentity1  nidentity2  omind1  omind4  omind5  omind6  contact1  contact2  contact3  contact4  contact5  hygiene1  hygiene2  hygiene3  hygiene4  hygiene5  psupport1  psupport2  psupport3  psupport4  psupport5  political_ideology  happy  slf_ladder  riskperc1  riskperc2  self_esteem  sex1  sbelong1  sbelong2  sbelong3  sbelong4  tested_positive  optim1  optim2  slfcont1  slfcont2  slfcont3  slfcont4  urban
##   10   9  age  children  cnarc1  cnarc2  cnarc3  ctheory1  ctheory2  ctheory3  ctheory4  CRT1  CRT2  CRT3  employ_status1  generosity1  generosity2  generosity3  health_cond  know_tested_positive  ladder  marital1  mor_circle  moralid1  moralid10  moralid2  moralid3  moralid4  moralid5  moralid6  moralid7  moralid8  moralid9  mcoop1  mcoop2  mcoop3  mcoop4  mcoop5  mcoop6  mcoop7  narc1  narc2  narc3  narc4  narc5  narc6  nidentity1  nidentity2  omind1  omind4  omind5  omind6  contact1  contact2  contact3  contact4  contact5  hygiene1  hygiene2  hygiene3  hygiene4  hygiene5  psupport1  psupport2  psupport3  psupport4  psupport5  political_ideology  happy  slf_ladder  riskperc1  riskperc2  self_esteem  sex1  sbelong1  sbelong2  sbelong3  sbelong4  tested_positive  optim1  optim2  slfcont1  slfcont2  slfcont3  slfcont4  urban
##   10   10  age  children  cnarc1  cnarc2  cnarc3  ctheory1  ctheory2  ctheory3  ctheory4  CRT1  CRT2  CRT3  employ_status1  generosity1  generosity2  generosity3  health_cond  know_tested_positive  ladder  marital1  mor_circle  moralid1  moralid10  moralid2  moralid3  moralid4  moralid5  moralid6  moralid7  moralid8  moralid9  mcoop1  mcoop2  mcoop3  mcoop4  mcoop5  mcoop6  mcoop7  narc1  narc2  narc3  narc4  narc5  narc6  nidentity1  nidentity2  omind1  omind4  omind5  omind6  contact1  contact2  contact3  contact4  contact5  hygiene1  hygiene2  hygiene3  hygiene4  hygiene5  psupport1  psupport2  psupport3  psupport4  psupport5  political_ideology  happy  slf_ladder  riskperc1  riskperc2  self_esteem  sex1  sbelong1  sbelong2  sbelong3  sbelong4  tested_positive  optim1  optim2  slfcont1  slfcont2  slfcont3  slfcont4  urban
```

```
## Warning: Number of logged events: 4
```

```
f8 <- bind_rows(outloops) #binding country-based imputed datasets into the original one
write.csv(f8, "finaldata_marina.csv")
```
